# Alcohol use and burden for 195 countries and territories, 1990–2016: a systematic analysis for the Global Burden of Disease Study 2016

**DOI:** 10.1016/S0140-6736(18)31310-2

**Published:** 2018-09-22

**Authors:** GBD 2016 Alcohol Collaborators

## Abstract

**Background:**

Alcohol use is a leading risk factor for death and disability, but its overall association with health remains complex given the possible protective effects of moderate alcohol consumption on some conditions. With our comprehensive approach to health accounting within the Global Burden of Diseases, Injuries, and Risk Factors Study 2016, we generated improved estimates of alcohol use and alcohol-attributable deaths and disability-adjusted life-years (DALYs) for 195 locations from 1990 to 2016, for both sexes and for 5-year age groups between the ages of 15 years and 95 years and older.

**Methods:**

Using 694 data sources of individual and population-level alcohol consumption, along with 592 prospective and retrospective studies on the risk of alcohol use, we produced estimates of the prevalence of current drinking, abstention, the distribution of alcohol consumption among current drinkers in standard drinks daily (defined as 10 g of pure ethyl alcohol), and alcohol-attributable deaths and DALYs. We made several methodological improvements compared with previous estimates: first, we adjusted alcohol sales estimates to take into account tourist and unrecorded consumption; second, we did a new meta-analysis of relative risks for 23 health outcomes associated with alcohol use; and third, we developed a new method to quantify the level of alcohol consumption that minimises the overall risk to individual health.

**Findings:**

Globally, alcohol use was the seventh leading risk factor for both deaths and DALYs in 2016, accounting for 2·2% (95% uncertainty interval [UI] 1·5–3·0) of age-standardised female deaths and 6·8% (5·8–8·0) of age-standardised male deaths. Among the population aged 15–49 years, alcohol use was the leading risk factor globally in 2016, with 3·8% (95% UI 3·2–4·3) of female deaths and 12·2% (10·8–13·6) of male deaths attributable to alcohol use. For the population aged 15–49 years, female attributable DALYs were 2·3% (95% UI 2·0–2·6) and male attributable DALYs were 8·9% (7·8–9·9). The three leading causes of attributable deaths in this age group were tuberculosis (1·4% [95% UI 1·0–1·7] of total deaths), road injuries (1·2% [0·7–1·9]), and self-harm (1·1% [0·6–1·5]). For populations aged 50 years and older, cancers accounted for a large proportion of total alcohol-attributable deaths in 2016, constituting 27·1% (95% UI 21·2–33·3) of total alcohol-attributable female deaths and 18·9% (15·3–22·6) of male deaths. The level of alcohol consumption that minimised harm across health outcomes was zero (95% UI 0·0–0·8) standard drinks per week.

**Interpretation:**

Alcohol use is a leading risk factor for global disease burden and causes substantial health loss. We found that the risk of all-cause mortality, and of cancers specifically, rises with increasing levels of consumption, and the level of consumption that minimises health loss is zero. These results suggest that alcohol control policies might need to be revised worldwide, refocusing on efforts to lower overall population-level consumption.

**Funding:**

Bill & Melinda Gates Foundation.

## Introduction

Alcohol use has a complex association with health. Researchers have recognised alcohol use as a leading risk factor for disease burden, and studies link its consumption to 60 acute and chronic diseases.^1^, ^2^, ^3^ Additionally, some research suggests that low levels of alcohol consumption can have a protective effect on ischaemic heart disease, diabetes, and several other outcomes.^4^, ^5^, ^6^ This finding remains an open question, and recent studies have challenged this view by use of mendelian randomisation and meta-analyses.^7^, ^8^, ^9^, ^10^

Determination of harm due to alcohol use is complicated further by the multiple mechanisms through which alcohol use affects health: through cumulative consumption leading to adverse effects on organs and tissues; by acute intoxication leading to injuries or poisoning; and by dependent drinking leading to impairments and potentially self-harm or violence. These effects are also influenced by an individual's consumption volume and pattern of drinking.^2^ Measuring the health effects of alcohol use requires careful consideration of all these factors.

**Research in context**

**Evidence before this study**

Although researchers recognise alcohol use as a leading risk factor for premature death and disability, some evidence suggests that low intake might have a protective effect on specific conditions such as ischaemic heart disease and diabetes. Monitoring of consumption behaviour is required to analyse the health effects of alcohol use. Historically, researchers have relied on self-reported survey data to estimate consumption levels and trends. However, these data have systematic biases that make cross-country comparisons unreliable. The Global Status Report on Alcohol and Health, as well as previous iterations of the Global Burden of Diseases, Injuries, and Risk Factors Study, have sought to produce harmonised, cross-country comparisons of alcohol consumption and its harms, by leveraging data on alcohol sales, the prevalence of current drinking and abstention, and self-reports of consumption amounts.

**Added value of this study**

In this analysis we improved available estimates of alcohol use and its associated health burden in five ways. First, we consolidated 694 individual and population-level data sources to estimate alcohol consumption levels among current drinkers. Second, we developed a method to adjust population-level consumption for alcohol consumed by tourists. Third, we improved pre-existing methods that account for unrecorded population-level consumption. Fourth, we did a new systematic review and meta-analysis of alcohol use and 23 associated health outcomes, which we used to estimate new dose–response curves of relative risk. Fifth, using the new relative risk curves and a new analytical method, we estimated the exposure of alcohol consumption that minimises an individual's total attributable risk.

**Implications of all the available evidence**

The total attributable burden of alcohol use was larger than previous evidence has indicated and increases monotonically with consumption. Based on weighted relative risk curves for each health outcome associated with alcohol use, the level of consumption that minimises health loss due to alcohol use is zero. These findings strongly suggest that alcohol control policies should aim to reduce total population-level consumption. To potentially reduce the effects of alcohol use on future health loss, there is a need for countries to revisit their alcohol control policies and assess how they can be modified to further lower population-level consumption.

Several studies have attempted to address these factors to provide global estimates of alcohol consumption and its associated health effects. The most comprehensive among these studies have been WHO's Global Status Report on Alcohol and Health, as well as previous iterations of the Global Burden of Diseases, Injuries, and Risk Factors Study (GBD).^11^, ^12^, ^13^ The present study aims to build upon pre-existing work and to address several limitations found in earlier research.

First, the available studies have assessed the risk of alcohol use by relying on external meta-analyses, which do not control for confounding in the selection of the reference category within constituent studies. This approach is problematic because of the so-called sick quitter hypothesis, which emphasises the importance of reference category selection in correctly assessing risk among drinkers, along with other confounding study characteristics such as survival bias.^8^, ^14^, ^15^, ^16^, ^17^ Until recently, most meta-analyses of alcohol consumption have not controlled for the composition of the reference category. Subsequently, assessments of harm relying on these studies have been biased. We sought to resolve this issue within our meta-analyses by including controls for different reference categories and the average age of participants.

Second, previous studies have used sales data to estimate population-level alcohol stock. Researchers have noted the benefit of using sales data instead of survey data for quantifying alcohol stock available within a location.^18^, ^19^ However, sales data still have bias because of consumption by tourists and unrecorded consumption from illicit sales, home brewing, and local beverages. Without correction for these factors, estimates relying on sales data can be biased and lead to inaccurate cross-national comparisons. In the current study, we adjusted the estimates of population-level alcohol stock to account for the effects of tourism and unrecorded consumption.

Third, previous studies have assumed zero as the counterfactual exposure level that minimises harm. Within a comparative risk assessment approach, a counterfactual level of consumption that minimises harm is required to estimate population attributable fractions (PAFs).^1^ However, this counterfactual level needs to be estimated, rather than assumed, given the complexities involved in estimating the risk of alcohol use across outcomes. Relying on this assumption can fail to capture any potential non-linear effects between alcohol use and health. Our study proposes a new method for the use of available evidence to establish a counterfactual level of exposure across varied relative risks, which provides tangible evidence for low-risk drinking recommendations.

In the present study, we aimed to address these limitations and provide the best available estimates of alcohol use and the associated health burden. We estimated the prevalence of current drinking (having one or more drinks in the past year); abstention from alcohol (having no alcohol in the past year); the distribution of alcohol consumption among current drinkers in standard drinks daily; and the disease burden attributable to alcohol use, in terms of deaths and disability-adjusted life-years (DALYs). We produced these estimates for 195 locations from 1990 to 2016, for both sexes and for 5-year age groups between the ages of 15 years and 95 years and older. We also did a new meta-analysis to assess the dose–response risk of alcohol consumption for 23 outcomes. Lastly, we estimated the level of alcohol consumption that minimises an individual's total attributable risk of any health loss.

## Methods

### Study design

This study follows the comparative risk assessment framework developed in previous iterations of GBD.^20^ In the following sections, we summarise our methods and briefly present innovations. A full explanation is available in appendix 1. This study fully adheres to the Guidelines for Accurate and Transparent Health Estimates Reporting (GATHER) statement.^21^

We estimated alcohol use exposure as grams of pure ethanol consumed daily by current drinkers (which we present here in terms of standard drinks daily, defined as 10 g of pure ethyl alcohol). We estimated relative risks by dose in grams of pure ethyl alcohol, for each included risk–outcome pair. We ascertained which cause and injury outcomes to include by reviewing prospective and observational studies of alcohol use, and then assessing the causal association using Bradford-Hill's criteria for causation.^22^ We included 23 outcomes, and the full list of risk–outcome pairs, as well as the corresponding data sources, are provided in appendix 1 (pp 52–140).

### Data sources

We found sources that included indicators of current drinking prevalence and alcohol consumed in grams per day using the Global Health Data Exchange (GHDx) and PubMed.^23^ For the meta-analysis, we searched PubMed, the GHDx, and references of previously published meta-analyses. For our exposure estimates, we extracted 121 029 data points from 694 sources across all exposure indicators. For our relative risk estimates, we extracted 3992 relative risk estimates across 592 studies. These relative risk estimates corresponded to a combined study population of 28 million individuals and 649 000 registered cases of respective outcomes. We list all the included data sources in appendix 1 (pp 52–140).

To estimate standard drinks consumed daily by current drinkers, we followed the general approach used by Rehm and colleagues.^18^ We briefly explain this method here, along with two methodological innovations to account for bias in the sales model: an adjustment to account for tourist consumption and an updated adjustment for unrecorded consumption. A full explanation of this approach is available in appendix 1 (pp 18–49).

To estimate exposure, we combined estimates of population-level alcohol stock and individual-level alcohol consumption to produce standard drinks consumed daily among current drinkers and current drinker prevalence, within a specific location, year, age group, and sex. We started by estimating population-level alcohol stock in litres per capita from sales data, individual-level estimates of the prevalence of current drinkers and abstainers from survey data, and individual-level estimates of the amount of alcohol consumed in grams per day from survey data. Then, for a given location and year, we rescaled age-specific and sex-specific estimates of individual-level consumption so that they aggregated to the estimates of population-level consumption. When surveys reported amount consumed in terms of beverage types, we converted these data into grams of pure ethanol using density equations and assumptions of the average alcohol content by drink type (appendix 1, p 50). Finally, we rescaled estimates of current drinking and abstention so that, within a given location, year, age group, and sex, the two estimates summed to one.

After we derived our model of population-level alcohol stock from sales data, we controlled for sources of bias that could arise from tourism and unrecorded consumption not recorded in formal sales. To account for tourist consumption, we computed an additive measure for alcohol consumed abroad by domestic citizens and subtractive measures for alcohol consumed domestically by tourists. We extracted data on the number of tourists by country of origin and destination from the World Tourism Organization and used these data to obtain estimates of total tourists, percentage of tourists by location, and average duration of stay using a spatiotemporal Gaussian process regression.^24^ We combined these estimates with measures of alcohol in litres per capita by location, to calculate net amounts of total population-level alcohol stock consumed by tourists or domestic citizens travelling abroad.

To account for alcohol stock not captured within formal alcohol sales data (ie, unrecorded consumption from illicit production, home brewing, local beverages, or alcohol sold as a non-alcohol product), we collated estimates across published studies of the percentage of total alcohol stock due to unrecorded consumption. We sampled 1000 times from a uniform distribution with a range between zero and the average of these collated studies by location (sampling from the uncertainty interval from each study, then averaging the draws) to generate a conservative estimate of the total stock likely to be unrecorded. We used a conservative approach because of the wide heterogeneity in both the methods and estimates within included data sources. We provide estimates of these percentages in appendix 1 (pp 46–49).

### Systematic review and meta-analysis

We did a new systematic review for each associated outcome to incorporate new findings on risk and to improve upon existing approaches. This strategy allowed us to systematically control for reference category confounding in constituent studies across associated outcomes. We provide the search strategy, search diagrams, dose–response curves for each included outcome, and references for each outcome in appendix 1 (pp 57–146).

Drawing from our systematic review, we did a meta-analysis of risk outcomes for alcohol use. For each outcome, we estimated the dose–response relative risk curve using mixed-effects logistic regression with non-linear splines for doses between 0 and 12·5 standard drinks daily. We selected 12·5 standard drinks daily as a cutoff point given the absence of available data beyond this range. We present additional details of the model in appendix 1 (pp 51–138). We tested the significance of including a study-level confounding variable on the composition of the reference category (eg, whether former drinkers were included in the abstainer category or not). When found to be significant, this variable was included as a predictor within the model, which was the case for ischaemic heart disease, ischaemic stroke, and diabetes.

Using our dose–response curves, we estimated the consumption level that minimises harm, which is defined in the comparative risk assessment approach as the theoretical minimum risk exposure level (TMREL). We chose a theoretical minimum on the basis of a weighted average relative risk curve across all attributable outcomes. We constructed weights for each risk outcome based on the respective global, age-standardised DALY rate per 100 000 in 2016 for both sexes. Our TMREL was the minimum of this weighted all-attributable outcome dose–response curve.

### Attributable burden due to alcohol use

We calculated PAFs using our estimates of exposure, relative risks, and TMREL, following the same approach taken within the GBD studies.^20^ For alcohol-use disorders, which are by definition fully attributable, we assumed a PAF of 1.^24^ Following this calculation, we multiplied PAFs by outcome-specific estimates of deaths and DALYs and summed these across outcomes to calculate the total attributable burden in specific locations. We aggregated both exposure and burden results at the global level and have presented them by quintile of the Socio-demographic Index (SDI). SDI is a summary measure of overall development, based on educational attainment, fertility, and income per capita within a location. Locations categorised by SDI quintile are found in appendix 1 (pp 8–12).^25^ We also constructed age-standardised values of all estimates, using the same age weights as those used in the GBD standard population.

We made one adjustment to road injury PAFs to estimate how much burden occurred to others because of alcohol use by another individual. We based this adjustment on data from the US Fatality Analysis Reporting System (FARS), which includes the average number of deaths in automobile accidents involving alcohol and the percentage of those deaths distributed by age and sex. We multiplied age-specific and sex-specific alcohol-attributable and road-injury-attributable DALYs by the average number of fatalities, given the driver's age and sex. We then redistributed these attributable DALYs according to the FARS-derived probabilities that a population by age and sex would be involved in a road injury, given the exposed driver's age and sex. Because of data availability, we assumed that locations outside the USA would follow a similar pattern to what we estimated with FARS. After redistributing the attributable DALYs, we derived PAFs again by dividing the redistributed attributable DALYs by total DALYs within specific demographics.

### Uncertainty analysis

For all steps, we calculated uncertainty for estimation of exposure, attributable deaths, and DALYs by taking 1000 draws from the data's uncertainty due to sampling error and modelling uncertainty arising from hyper-parameter selection and parameter estimation. We then used these draws throughout the entire modelling process. When reporting uncertainty intervals, we present the 2·5th and 97·5th percentiles of the draws.

### Role of the funding source

The funders of the study had no role in study design, data collection, data analysis, data interpretation, or writing of the report. The corresponding author had full access to all the data in the study and had final responsibility for the decision to submit for publication.

## Results

### Global, regional, and national trends in alcohol consumption

In 2016, 32·5% (95% uncertainty interval [UI] 30·0–35·2) of people globally were current drinkers. 25% (95% UI 23–27) of females were current drinkers, as were 39% (36–43) of males (appendix 2). These percentages corresponded to 2·4 billion (95% UI 2·2–2·6) people globally who were current drinkers, with 1·5 billion (1·4–1·6) male current drinkers and 0·9 billion (0·8–1·0) female current drinkers (appendix 2, pp 2–1994). Globally, the mean amount of alcohol consumed was 0·73 (95% UI 0·68–0·78) standard drinks daily for females and 1·7 (1·5–1·9) standard drinks daily for males.

**Figure 1:**
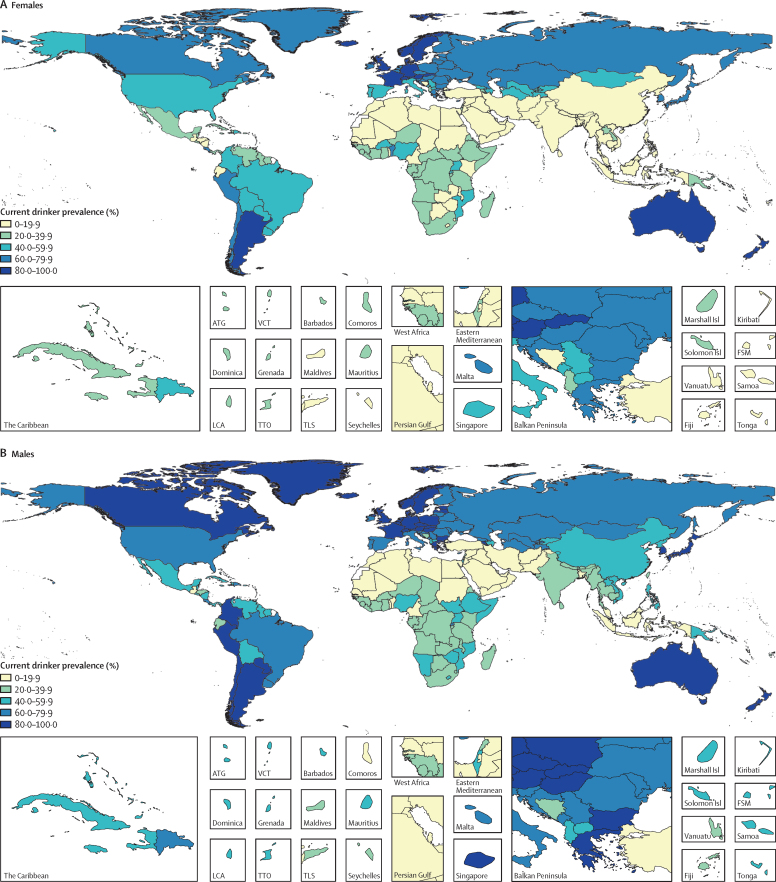
Age-standardised prevalence of current drinking for females (A) and males (B) in 2016, in 195 locations Current drinkers are defined as individuals who reported having consumed alcohol within the past 12 months. ATG=Antigua and Barbuda. VCT=Saint Vincent and the Grenadines. Isl=Islands. FSM=Federated States of Micronesia. LCA=Saint Lucia. TTO=Trinidad and Tobago. TLS=Timor-Leste.

The prevalence of current drinking varied considerably by location (figure 1). Prevalence was highest for high SDI locations, where 72% (95% UI 69–75) of females and 83% (80–85) of males were current drinkers (locations comprising each SDI quintile are provided in appendix 2, pp 8–12). Drinking prevalence was lowest in low-to-middle SDI locations, where 8·9% (95% UI 6·6–9·7) of females and 20% (17–22) of males were current drinkers. Across SDI quintiles, females consumed less alcohol than males, with the size of this disparity decreasing with higher levels of SDI. For example, we found large differences between females and males in Nepal, with only 1·5% (95% UI 1·2–1·9) of females being current drinkers in 2016, compared with 21% (17–25) of males. Conversely, many high SDI locations had similar prevalence between sexes. For example, we found minimal differences in Sweden, where 86% (95% UI 84–88) of females and 87% (85–89) of males were current drinkers.

**Figure 2:**
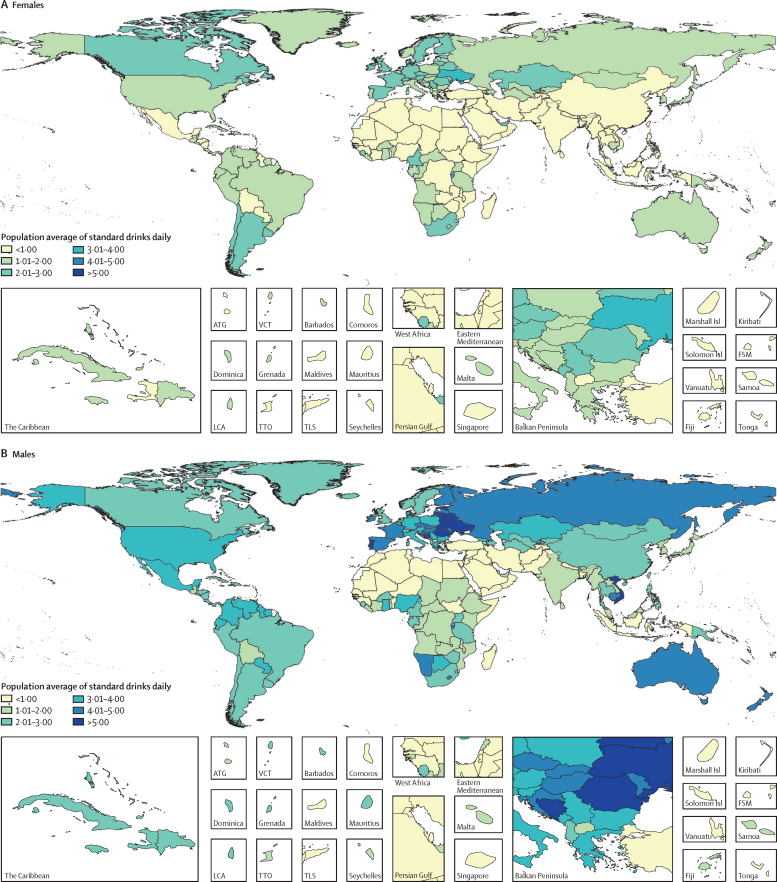
Average standard drinks (10 g of pure ethanol per serving) consumed per day, age-standardised, for females (A) and males (B) in 2016, in 195 locations ATG=Antigua and Barbuda. VCT=Saint Vincent and the Grenadines. Isl=Islands. FSM=Federated States of Micronesia. LCA=Saint Lucia. TTO=Trinidad and Tobago. TLS=Timor-Leste.

The population average of standard drinks consumed daily among current drinkers in 2016 also differed widely by location and sex (figure 2). High SDI locations had the highest average of standard drinks consumed daily, with 1·9 (95% UI 1·3–2·7) standard drinks consumed daily among females and 2·9 (2·0–4·1) among males. Low SDI locations had the smallest average for males, with 1·4 (0·6–2·4) standard drinks consumed daily, while low-to-middle SDI locations had the lowest average for females, with 0·3 (0·1–0·6) standard drinks consumed daily.

### Global patterns in alcohol-attributable deaths and disease burden

In 2016, 2·8 million deaths (95% UI 2·4–3·3) were attributed to alcohol use. This corresponds to 2·2% (95% UI 1·5–3·0) of total age-standardised deaths among females and 6·8% (5·8–8·0) among males. In terms of overall disease burden, alcohol use led to 1·6% (95% UI 1·4–2·0) of total DALYs globally in 2016 among females and 6·0% (5·4–6·7) among males. Globally, alcohol use was ranked as the seventh leading risk factor for premature death and disability in 2016, compared with other risk factors in the GBD studies. Among the population aged 15–49 years, alcohol use was the leading global risk factor for risk-attributable disease burden, causing 8·9% (95% UI 7·8–9·9) of attributable DALYs for men and 2·3% (2·0–2·6) for women. Among the population aged 15–49 years, 3·8% (95% UI 3·2–4·3) of female deaths and 12·2% (10·8–13·6) of male deaths were attributable to alcohol use.

**Figure 3:**
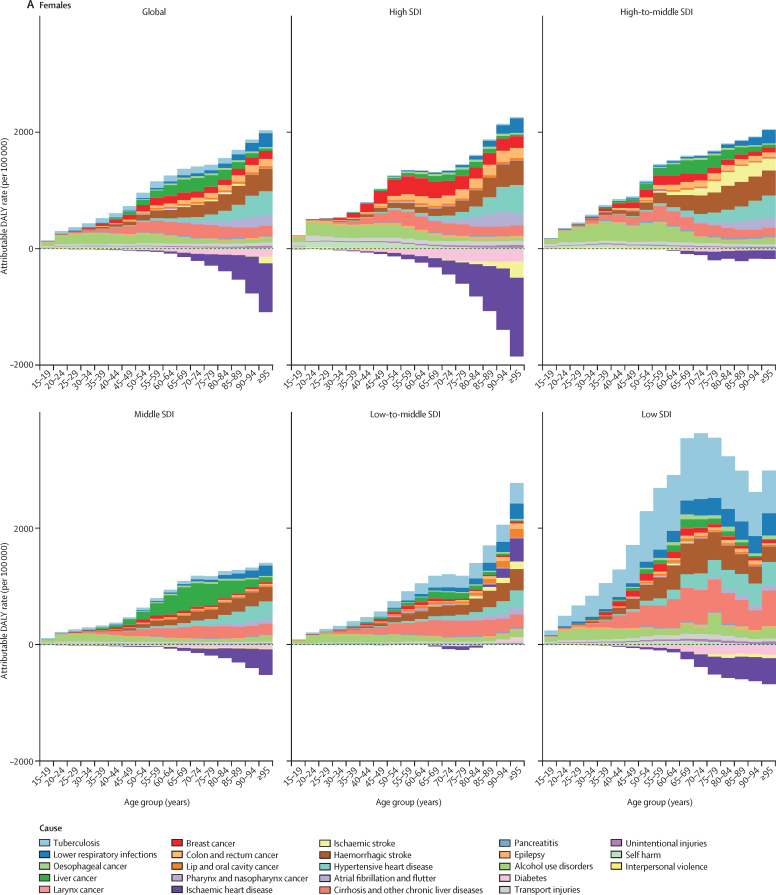

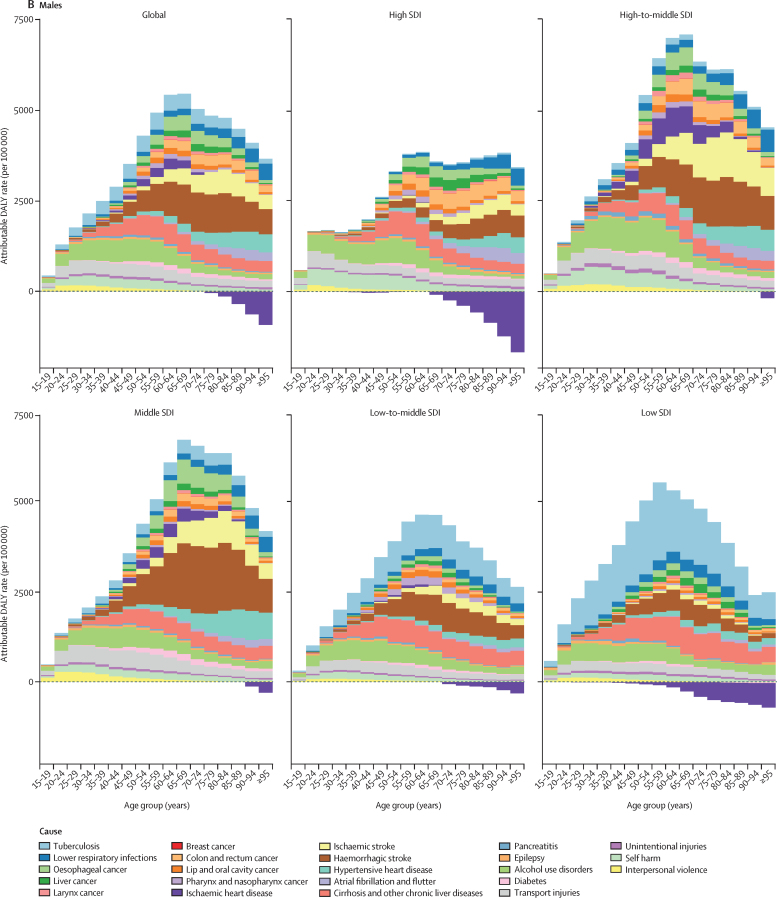
Attributable DALY rate disaggregated by outcome, shown globally and for each region, by age and sex, in 2016 (A) Females. (B) Males. DALY=disability-adjusted life-year. SDI=Socio-demographic Index.

Both total burden attributable to alcohol use and the proportion of causes associated with alcohol use varied by sex, age, and SDI quintile (figure 3; appendix 2, pp 1997–2186). In absolute terms, the alcohol-attributable burden by age was smaller for females than for males (figure 3). For females, the alcohol-attributable burden increased with age, while for males the burden increased until between 55–65 years of age, after which attributable burden decreased. Females, particularly in high SDI locations, experienced some protective effects for ischaemic heart disease and diabetes beyond 60 years of age. For males, only high SDI and low SDI locations had noticeable protective effects for ischaemic heart disease, but the effect was small compared with the total attributable burden in those locations.

For both males and females, health outcomes comprising the attributable burden changed across the lifespan (figure 3). The three leading causes of attributable deaths in this age group were tuberculosis (1·4% [95% UI 1·0–1·7] of total deaths), road injuries (1·2% [0·7–1·9]), and self-harm (1·1% [0·6–1·5]). For females aged 15–49 years, alcohol use disorders constituted the largest proportion of the attributable burden across SDI quintiles; the primary exception was tuberculosis, which accounted for the largest proportion of attributable burden in low SDI settings. In this age range, transport injuries and alcohol use disorders were the predominant causes of attributable burden for males in high-to-middle SDI quintiles; for low-to-middle SDI and low SDI quintiles, tuberculosis was the primary cause of the attributable burden for both sexes.

Beyond 50 years of age, the causes of total attributable burden became more complex by SDI quintile. For populations aged 50 years and older, cancers accounted for a large proportion of total alcohol-attributable deaths in 2016, constituting 27·1% (95% UI 21·2–33·3) of total alcohol-attributable female deaths and 18·9% (15·3–22·6) of alcohol-attributable male deaths. In high SDI countries, cancers were the predominant source of attributable burden among both sexes. In low SDI countries, tuberculosis was the primary cause of burden for both sexes, followed by cirrhosis and other chronic liver diseases. The profile of attributable burden in high-to-middle SDI and middle SDI countries for females and males was largely composed of ischaemic stroke and haemorrhagic stroke, followed by liver cancer for females. In all SDI quintiles, haemorrhagic stroke and hypertensive heart disease were the largest sources of burden for females aged 80 years and older. For men in this age group, the composition of the burden was similar to that of males aged 50 years or older.

### Health risks associated with alcohol consumption

**Figure 4:**
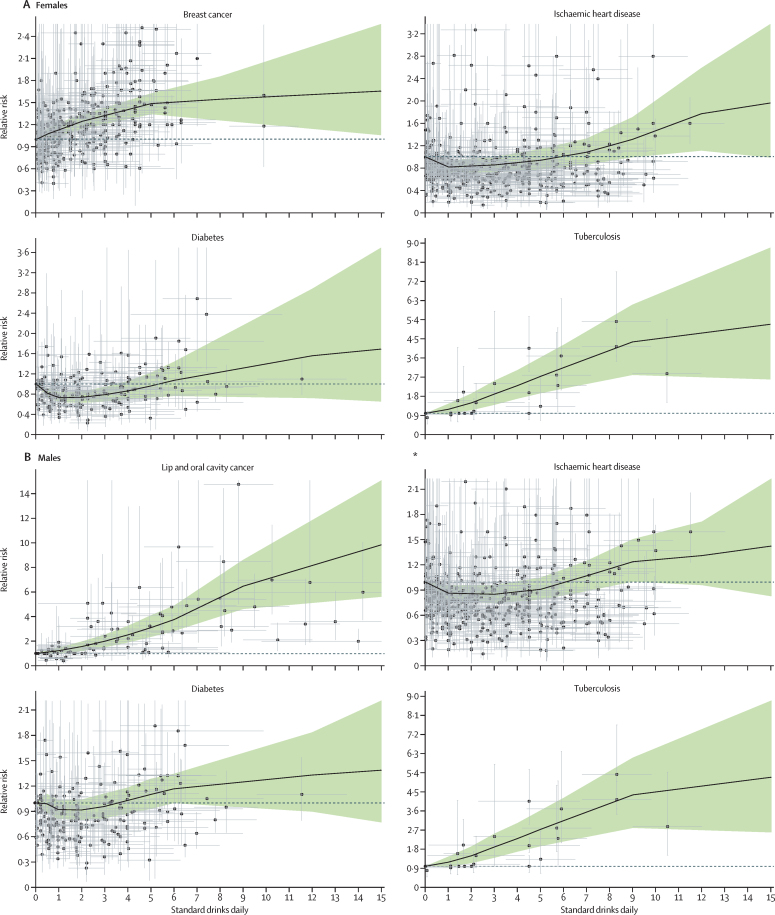
Relative risk curves for selected conditions by number of standard drinks consumed daily (A) Relative risk curves for breast cancer, ischaemic heart disease, diabetes, and tuberculosis for females. (B) Relative risk curves for lip and oral cavity cancer, ischaemic heart disease, diabetes, and tuberculosis for males. Points are relative risk estimates from studies. The vertical and horizontal bars capture the uncertainty in each study, related to the sample size and number of drinks consumed by individuals in the study. The black line represents the estimated relative risk for each condition at each level of consumption. The shaded green areas represent the 95% uncertainty interval associated with the estimated relative risk. The dotted line is a reference line for a relative risk of 1. The relative risk curves for all other health outcomes associated with alcohol use are presented in appendix 2 (pp 57–146).

Figure 4 shows the relative risk curves for selected health outcomes, separately for females and males. Estimated relative risk curves for each health outcome are presented in appendix 2 (pp 52–140). With this analysis, we only found statistically significant evidence for the J-shaped curve for ischaemic heart disease; non-significant J-shaped curves were observed for diabetes and ischaemic stroke. For ischaemic heart disease, we found a minimum relative risk of 0·86 (0·80–0·96) for men and 0·82 (0·72–0·95) for women, occurring at 0·83 standard drinks daily for men and 0·92 standard drinks daily for women. We found no significant difference in relative risk curves for ischaemic heart disease or diabetes when estimating the curves by age. For all other outcomes, including all cancers, we found that relative risk monotonically increased with alcohol consumption (appendix 2, pp 57–146).

**Figure 5:**
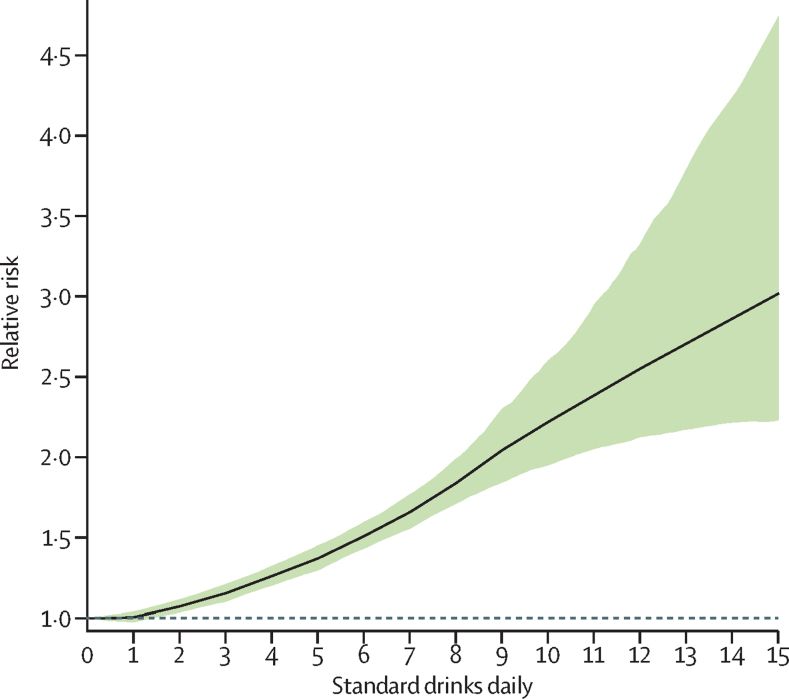
Weighted relative risk of alcohol for all attributable causes, by standard drinks consumed per day Age-standardised weights determined by the DALY rate in 2016, for both sexes. The dotted line is a reference line for a relative risk of 1. DALY=disability-adjusted life-year.

In estimating the weighted relative risk curve, we found that consuming zero (95% UI 0·0–0·8) standard drinks daily minimised the overall risk of all health loss (figure 5). The risk rose monotonically with increasing amounts of daily drinking. This weighted relative risk curve took into account the protective effects of alcohol use associated with ischaemic heart disease and diabetes in females. However, these protective effects were offset by the risks associated with cancers, which increased monotonically with consumption. In a sensitivity analysis, where we explored how the weighted relative risk curve changed on the basis of the choice of weights for various health outcomes, the curve changed significantly only in settings where diabetes and ischaemic heart disease comprised more than 60% of total deaths in a population.

## Discussion

In 2016, alcohol use led to 2·8 million deaths and was the leading risk factor for premature death and disability among people aged 15–49 years, with nearly 9% of all attributable DALYs for men and more than 2% for women. Our findings indicate that alcohol use was associated with far more health loss for males than for females, with the attributable burden for men around three times higher than that for women in 2016. By evaluating all associated relative risks for alcohol use, we found that consuming zero standard drinks daily minimises the overall risk to health.

Previous research has analysed all-cause risk due to alcohol use by either investigating all-cause risk in particular cohorts and survey series, or through meta-analyses of those studies.^26^, ^27^ Past findings subsequently suggested a persistent protective effect for some low or moderate levels of alcohol consumption on all-cause mortality. However, these studies were limited by small sample sizes, inadequate control for confounders, and non-optimal choices of a reference category for calculating relative risks. More recent research, which has used methodologies such as mendelian randomisation, pooling cohort studies, and multivariable adjusted meta-analyses, increasingly shows either a non-significant or no protective effect of drinking on all-cause mortality or cardiovascular outcomes.^7^, ^14^, ^28^ Our results on the weighted attributable risk are consistent with this body of work. Taken together, these findings emphasise that alcohol use, regardless of amount, leads to health loss across populations. Although we found some protective effects for ischaemic heart disease and diabetes among women, these effects were offset when overall health risks were considered—especially because of the strong association between alcohol consumption and the risk of cancer, injuries, and communicable disease. These findings stress the importance of assessing how alcohol use affects population health across the lifespan.

Evaluating attributable burden across SDI quintiles revealed the magnitude by which outcomes of alcohol use differ and how total attributable burden relates to increasing SDI. Our results indicate that alcohol use and its harmful effects on health could become an increasing challenge amid gains in SDI. Given that most low and low-to-middle SDI settings currently have lower average alcohol consumption than high-to-middle SDI settings, it is crucial for decision makers and government agencies to enact or maintain strong alcohol control policies today to prevent the potential for rising alcohol use in the future. Effective policies now could yield substantial population health benefits for years to come.

Our results point to a need to revisit alcohol control policies and health programmes, and to consider recommendations for abstention. In terms of reducing population-level alcohol use, WHO provides a set of best buys—policies that provide an individual year of healthy life at less than the cost of the average individual income.^29^ Governments should consider how these recommendations can be implemented within their local contexts and broader policy platforms, including excise taxes on alcohol, controlling the physical availability of alcohol and the hours of sale, and controlling alcohol advertising. Any of these policy actions would contribute to reductions in population-level consumption—an important step toward decreasing the health loss associated with alcohol use.

Failing to address harms from alcohol use, particularly at high levels of consumption, can have dire effects on population health. The mortality crisis in Russia is a striking example, where alcohol use was the primary culprit of increases in mortality starting in the 1980s and led to 75% of deaths among men aged 15–55 years.^30^ Current global trends—namely, population ageing— portend a growing toll of the alcohol-attributable burden in the absence of policies, particularly since many cancers disproportionately affect older individuals. Consequently, low-to-middle SDI countries could benefit from policy action today to keep alcohol consumption low and prevent greater health loss in the future. High and high-to-middle SDI locations need to consider stronger alcohol reduction policies, such as those recommended by WHO, in an effort to reduce population-level consumption.

Our results should be interpreted within the context of the study's limitations. First, our consumption estimates might not fully capture illicit production or unrecorded consumption given our use of sales data in estimation. We have sought to adjust for consumption beyond sales data, but given the heterogeneity of these estimates it is likely that additional methodological refinements are necessary to improve the quantification of unrecorded consumption. Second, drinking patterns within a year are assumed to be consistent; however, past work shows that drinking patterns, rather than average levels of consumption such as standard daily drinks, might be related to different levels of risk and harm. Unfortunately, the data requirements for assessment of such drinking patterns by age, sex, and location far exceed what is currently available. For instance, few prospective studies quantify the effects of drinking patterns and average levels of consumption in tandem, a requirement for correctly assessing the risk of alcohol-attributable outcomes. Third, the data used to estimate motor vehicle harm caused to others from alcohol use were only available for the USA (ie, FARS data). Although it is unlikely that the patterns observed in FARS are drastically different from those of other locations (appendix 1, pp 141–144), this assumption needs to be tested with more location-specific estimates. Fourth, we were unable to find robust data about the harm caused to others from alcohol-attributable interpersonal violence, a major potential source of health loss. More retrospective studies are needed to assess the harm to others caused through an individual's alcohol use.^30^ Fifth, consumption for populations younger than 15 years was not assessed because of data sparseness on alcohol use for these age groups. In the absence of such data, potential approaches to address this limitation, such as assuming consumption patterns of older age groups or trying to extrapolate past levels of alcohol consumption, are likely to introduce additional bias or error. More research on youth drinking and the associated risk is required to estimate alcohol-attributable burden for this age group. Last, we sought to quantify the risk of alcohol use only for outcomes with evidence meeting the criteria for the comparative risk assessment approach of GBD studies. However, there are additional outcomes, such as dementia and psoriasis, for which accumulating evidence suggests that alcohol use might be a risk factor.^31^, ^32^, ^33^ In combination, these limitations suggest that our results are likely to underestimate both the health risks and overall attributable burden of alcohol use.

## Conclusion

Alcohol use is a leading risk factor for disease burden worldwide, accounting for nearly 10% of global deaths among populations aged 15–49 years, and poses dire ramifications for future population health in the absence of policy action today. The widely held view of the health benefits of alcohol needs revising, particularly as improved methods and analyses continue to show how much alcohol use contributes to global death and disability. Our results show that the safest level of drinking is none. This level is in conflict with most health guidelines, which espouse health benefits associated with consuming up to two drinks per day. Alcohol use contributes to health loss from many causes and exacts its toll across the lifespan, particularly among men. Policies that focus on reducing population-level consumption will be most effective in reducing the health loss from alcohol use.

Correspondence to: Prof Emmanuela Gakidou, Institute for Health Metrics and Evaluation, University of Washington, Seattle, WA 98121, USA **gakidou@uw.edu**
