# Department of Error

**DOI:** 10.1016/S0140-6736(18)32338-9

**Published:** 2018-09-29

*GBD 2016 Alcohol Collaborators. Alcohol use and burden for 195 countries and territories, 1990–2016: a systematic analysis for the Global Burden of Disease Study 2016.* Lancet *2018;* 392: *1015–35—*Alan D Lopez has been added to the list of GBD 2016 Alcohol Collaborators; the names of Masood Ali Shaikh and Dhirendra Narain Sinha have been corrected; and affiliations have been updated for Karzan Abdulmuhsin Mohammad. Raw data underlying figures and relative risk curves have been made publicly available on Mendeley Data, a secure online repository for research data, as of Sept 19, 2018 (DOI:10.17632/5thy2mcwn7.1). A data sharing statement containing a link to this dataset has been added at the end of the Article. These corrections have been made to the online version as of Sept 27, 2018.

