# Department of Error

**DOI:** 10.1016/S0140-6736(19)31050-5

**Published:** 2019-06-22

*GBD 2016 Alcohol Collaborators. Alcohol use and burden for 195 countries and territories, 1990–2016: a systematic analysis for the Global Burden of Disease Study 2016.* Lancet *2018;* 392: *1015–35—*In this Global Health Metrics paper, Stefan Lorkowski has been added to the collaborators list; affiliation details and a declaration of interests statement for Stefan Lorkowski have also been added. These corrections have been made to the online version as of June 20, 2019.

