# Supplementary material for: Alcohol use and burden for 195 countries and territories, 1990–2016: a systematic analysis for the Global Burden of Disease Study 2016
Source: Lancet. 2018 Sep 22;392(10152):1015–35. doi: 10.1016/S0140-6736(18)31310-2 (PMC6148333; doi:10.1016/S0140-6736(18)31310-2)
Supplement: Supplementary appendix 2 [file mmc2.pdf]

THE LANCET

Supplementary appendix 2

This appendix formed part of the original submission and has been peer reviewed. We post it as supplied by the authors.

Supplement to: GBD 2016 Alcohol Collaborators. Alcohol use and burden for 195 countries and territories, 1990–2016: a systematic analysis for the Global Burden of Disease Study 2016. *Lancet* 2018; published online Aug 23. http://dx.doi.org/10.1016/S0140-6736(18)31310-2.

Supplemental Results Appendix for Alcohol use and burden: a systematic analysis from the Global Burden of Disease Study 2016 for 195 countries and territories, 1990–2016

Contents

Supplemental Results Table 1: Prevalence of current drinkers and abstainers, Population average of standard drinks daily, Percent of total attributable deaths, Percent of total attributable DALYs, Attributable deaths, and DALYs to alcohol use by location, year, sex, and age for GBD 2016 2

Supplemental Results Figure 1: Attributable DALY rate disaggregated by cause by location, sex, and age in 2016 3

Supplemental Results Table 1: Prevalence of current drinkers and abstainers, Population average of standard drinks daily, Percent of total attributable deaths, Percent of total attributable DALYs, Attributable deaths, and DALYs to alcohol use by location, year, sex, and age for GBD 2016

Location	Year	Sex	Age	Prevalence of current drinkers	Prevalence of abstainers	Population average of standard drinks daily	Percent of total attributable deaths	Percent of total attributable DALYs	Attributable deaths	Attributable DALYs
Global	1990	Female	All Ages	26% (24-29%)	74% (71-76%)	0.76 (0.74-0.8)	1.3% (1.1-1.6%)	2% (1.4-2.8%)	450000 (300000-610000)	15000000 (12000000-18000000)
Global	1990	Female	15 to 19	21% (16-28%)	79% (72-84%)	0.56 (0.38-0.77)	1.1% (0.77-1.4%)	1.3% (0.84-2%)	4200 (2700-6300)	470000 (330000-640000)
Global	1990	Female	20 to 24	27% (20-34%)	73% (66-80%)	0.88 (0.64-1.2)	1.8% (1.4-2.4%)	2% (1.3-2.7%)	7700 (5200-11000)	890000 (650000-1200000)
Global	1990	Female	25 to 29	31% (23-38%)	69% (62-77%)	1 (0.77-1.3)	2.3% (1.8-2.8%)	2.7% (1.9-3.6%)	10000 (7100-13000)	1000000 (760000-1300000)
Global	1990	Female	30 to 34	34% (26-42%)	66% (58-74%)	1.1 (0.84-1.5)	2.6% (2-3.2%)	3.5% (2.6-4.4%)	13000 (9900-17000)	1100000 (850000-1300000)
Global	1990	Female	35 to 39	36% (28-43%)	64% (57-72%)	1.2 (0.9-1.5)	2.8% (2.3-3.5%)	4.2% (3.2-5.3%)	18000 (14000-23000)	1200000 (950000-1500000)
Global	1990	Female	40 to 44	38% (30-47%)	62% (53-70%)	1.3 (1-1.7)	3.2% (2.5-4%)	5% (3.8-6.2%)	22000 (17000-28000)	1200000 (980000-1500000)
Global	1990	Female	45 to 49	38% (28-45%)	62% (55-72%)	1.4 (1-1.8)	3.3% (2.5-4.3%)	5% (3.9-6.4%)	26000 (20000-34000)	1200000 (960000-1600000)
Global	1990	Female	50 to 54	39% (30-46%)	61% (54-70%)	1.4 (1.1-1.9)	3.5% (2.7-4.6%)	5.1% (3.8-6.5%)	37000 (28000-48000)	1500000 (1100000-1900000)
Global	1990	Female	55 to 59	38% (29-46%)	62% (54-71%)	1.5 (1.1-1.9)	3.4% (2.4-4.5%)	4.6% (3.4-6.1%)	44000 (32000-59000)	1500000 (1100000-2000000)
Global	1990	Female	60 to 64	40% (31-49%)	60% (51-69%)	1.5 (1.1-2.1)	3% (1.9-4.2%)	3.9% (2.5-5.5%)	50000 (32000-71000)	1500000 (940000-2000000)
Global	1990	Female	65 to 69	41% (32-49%)	59% (51-68%)	1.5 (1.1-1.9)	2.6% (1.6-3.6%)	3.3% (2.1-4.7%)	53000 (33000-75000)	1300000 (800000-1800000)
Global	1990	Female	70 to 74	38% (29-46%)	62% (54-71%)	1.2 (0.88-1.6)	1.9% (0.88-3%)	2.4% (1.1-3.7%)	44000 (20000-69000)	830000 (370000-1300000)
Global	1990	Female	75 to 79	39% (29-49%)	61% (51-71%)	1.3 (0.92-1.8)	1.7% (0.39-3.1%)	2% (0.42-3.6%)	43000 (9300-80000)	650000 (150000-1200000)
Global	1990	Female	80 to 84	40% (30-50%)	60% (50-70%)	1.4 (0.99-1.8)	1.6% (0.27-3%)	1.8% (0.26-3.5%)	37000 (5300-73000)	420000 (72000-800000)

Location	Year	Sex	Age	Prevalence of current drinkers	Prevalence of abstainers	Population average of standard drinks daily	Percent of total attributable deaths	Percent of total attributable DALYs	Attributable deaths	Attributable DALYs
Global	1990	Female	85 to 89	41% (31-51%)	59% (49-69%)	1.4 (0.99-2)	1.4% (0.11-3%)	1.6% (-0.016-3.4%)	24000 (-250-52000)	200000 (15000-410000)
Global	1990	Female	90 to 94	44% (33-54%)	56% (46-67%)	1.5 (0.98-2.1)	1.3% (-0.35-3.2%)	1.3% (-0.62-3.7%)	9700 (-4500-27000)	57000 (-16000-150000)
Global	1990	Female	95 plus	47% (33-59%)	53% (41-67%)	1.5 (0.85-2.3)	1% (-1.1-3.5%)	1% (-1.5-4%)	2500 (-3700-10000)	10000 (-11000-35000)
Global	1990	Male	All Ages	39% (35-43%)	61% (57-65%)	1.4 (1.4-1.5)	4.5% (4-4.9%)	6.3% (5.4-7.2%)	1600000 (1300000-1800000)	58000000 (52000000-64000000)
Global	1990	Male	15 to 19	28% (21-36%)	72% (64-79%)	0.77 (0.54-1)	3.1% (2.3-4.2%)	4% (2.8-5.7%)	16000 (11000-23000)	1500000 (1000000-2000000)
Global	1990	Male	20 to 24	43% (33-52%)	57% (48-67%)	1.6 (1.2-2)	7.2% (5.6-8.9%)	8.6% (6.5-11%)	47000 (35000-60000)	3900000 (3100000-4900000)
Global	1990	Male	25 to 29	51% (40-60%)	49% (40-60%)	2.1 (1.6-2.6)	9% (7.3-11%)	11% (8.6-14%)	62000 (49000-76000)	4800000 (3800000-5700000)
Global	1990	Male	30 to 34	55% (44-64%)	45% (36-56%)	2.4 (1.9-3)	10% (8.3-12%)	13% (10-15%)	77000 (62000-91000)	5200000 (4300000-6100000)
Global	1990	Male	35 to 39	58% (48-66%)	42% (34-52%)	2.5 (1.9-3.2)	11% (8.8-13%)	14% (11-16%)	93000 (76000-110000)	5500000 (4500000-6500000)
Global	1990	Male	40 to 44	60% (50-68%)	40% (32-50%)	2.7 (2.1-3.4)	11% (9.1-13%)	14% (12-16%)	100000 (85000-120000)	5300000 (4400000-6200000)
Global	1990	Male	45 to 49	58% (48-67%)	42% (33-52%)	2.8 (2.1-3.6)	11% (9-13%)	14% (11-16%)	120000 (96000-140000)	5300000 (4400000-6200000)
Global	1990	Male	50 to 54	59% (49-67%)	41% (33-51%)	3 (2.3-3.8)	11% (8.9-13%)	13% (11-16%)	160000 (130000-190000)	6100000 (5000000-7200000)
Global	1990	Male	55 to 59	58% (48-67%)	42% (33-52%)	3 (2.3-3.7)	9.9% (8-12%)	12% (9.6-14%)	170000 (140000-210000)	5900000 (4800000-7000000)
Global	1990	Male	60 to 64	58% (48-67%)	42% (33-52%)	2.9 (2.3-3.7)	8.6% (6.7-11%)	9.9% (7.7-12%)	190000 (150000-240000)	5400000 (4200000-6700000)
Global	1990	Male	65 to 69	57% (47-66%)	43% (34-53%)	2.7 (2.2-3.3)	7.1% (5.5-8.9%)	8.2% (6.2-10%)	170000 (130000-210000)	4000000 (3100000-5000000)

Location	Year	Sex	Age	Prevalence of current drinkers	Prevalence of abstainers	Population average of standard drinks daily	Percent of total attributable deaths	Percent of total attributable DALYs	Attributable deaths	Attributable DALYs
Global	1990	Male	70 to 74	54% (44-63%)	46% (37-56%)	2.1 (1.7-2.7)	5.6% (3.9-7.4%)	6.3% (4.4-8.4%)	130000 (94000-180000)	2600000 (1800000-3400000)
Global	1990	Male	75 to 79	54% (43-65%)	46% (35-57%)	2.3 (1.7-2.9)	4.7% (3.1-6.6%)	5.2% (3.3-7.4%)	110000 (70000-160000)	1600000 (1100000-2300000)
Global	1990	Male	80 to 84	54% (42-64%)	46% (36-58%)	2.3 (1.8-2.9)	4.1% (2.6-5.7%)	4.4% (2.8-6.3%)	69000 (43000-98000)	770000 (500000-1100000)
Global	1990	Male	85 to 89	53% (41-63%)	47% (37-59%)	2.1 (1.6-2.8)	3.3% (1.9-4.8%)	3.5% (1.9-5.3%)	32000 (18000-48000)	260000 (150000-390000)
Global	1990	Male	90 to 94	55% (43-65%)	45% (35-57%)	2 (1.4-2.7)	2.6% (1.1-4.2%)	2.7% (0.95-4.6%)	9200 (3300-16000)	53000 (22000-88000)
Global	1990	Male	95 plus	58% (44-69%)	42% (31-56%)	1.9 (1.2-2.7)	2% (0.19-3.9%)	1.9% (-0.18-4.1%)	1700 (-160-3800)	7100 (680-14000)
Global	1995	Female	All Ages	26% (24-28%)	74% (72-76%)	0.73 (0.7-0.75)	1.4% (1.1-1.7%)	2% (1.3-2.8%)	470000 (300000-650000)	16000000 (13000000-19000000)
Global	1995	Female	15 to 19	21% (16-27%)	79% (73-84%)	0.55 (0.38-0.76)	1.1% (0.8-1.5%)	1.4% (0.91-2.1%)	4300 (2800-6300)	480000 (330000-640000)
Global	1995	Female	20 to 24	27% (21-33%)	73% (67-79%)	0.85 (0.61-1.1)	1.8% (1.4-2.3%)	1.9% (1.3-2.7%)	7900 (5300-11000)	900000 (660000-1200000)
Global	1995	Female	25 to 29	29% (23-36%)	71% (64-77%)	0.9 (0.68-1.2)	2.1% (1.6-2.5%)	2.4% (1.7-3.1%)	10000 (7500-14000)	1000000 (780000-1300000)
Global	1995	Female	30 to 34	33% (26-40%)	67% (60-74%)	1 (0.77-1.3)	2.4% (1.9-2.9%)	3.2% (2.4-4%)	14000 (11000-18000)	1200000 (910000-1400000)
Global	1995	Female	35 to 39	35% (29-42%)	65% (58-71%)	1.1 (0.88-1.5)	2.9% (2.3-3.5%)	4.3% (3.4-5.2%)	20000 (16000-25000)	1300000 (1100000-1600000)
Global	1995	Female	40 to 44	36% (30-44%)	64% (56-70%)	1.2 (0.89-1.5)	3.2% (2.5-3.9%)	4.9% (3.9-6%)	27000 (22000-33000)	1500000 (1200000-1800000)
Global	1995	Female	45 to 49	38% (31-45%)	62% (55-69%)	1.3 (0.99-1.7)	3.4% (2.7-4.3%)	5.2% (4.1-6.6%)	33000 (26000-41000)	1500000 (1200000-1900000)
Global	1995	Female	50 to 54	37% (30-44%)	63% (56-70%)	1.3 (1-1.7)	3.4% (2.5-4.4%)	5% (3.7-6.3%)	37000 (28000-48000)	1500000 (1100000-1900000)
Global	1995	Female	55 to 59	38% (31-45%)	62% (55-69%)	1.4 (1-1.9)	3.4% (2.5-4.6%)	4.8% (3.5-6.3%)	49000 (36000-65000)	1700000 (1200000-2200000)

Location	Year	Sex	Age	Prevalence of current drinkers	Prevalence of abstainers	Population average of standard drinks daily	Percent of total attributable deaths	Percent of total attributable DALYs	Attributable deaths	Attributable DALYs
Global	1995	Female	60 to 64	37% (30-45%)	63% (55-70%)	1.4 (1-1.8)	2.8% (1.8-4%)	3.7% (2.4-5.2%)	51000 (33000-72000)	1500000 (950000-2100000)
Global	1995	Female	65 to 69	39% (32-47%)	61% (53-68%)	1.3 (1-1.7)	2.5% (1.5-3.5%)	3.1% (1.9-4.5%)	56000 (34000-79000)	1300000 (800000-1900000)
Global	1995	Female	70 to 74	40% (32-48%)	60% (52-68%)	1.3 (0.93-1.7)	2% (0.85-3.2%)	2.4% (1-3.9%)	53000 (23000-84000)	1000000 (420000-1600000)
Global	1995	Female	75 to 79	37% (28-47%)	63% (53-72%)	1.1 (0.78-1.5)	1.4% (0.31-2.7%)	1.7% (0.32-3.2%)	37000 (7100-70000)	550000 (120000-1000000)
Global	1995	Female	80 to 84	39% (29-49%)	61% (51-71%)	1.3 (0.91-1.7)	1.3% (0.1-2.7%)	1.5% (0.066-3.1%)	34000 (1600-72000)	390000 (30000-790000)
Global	1995	Female	85 to 89	42% (32-52%)	58% (48-68%)	1.4 (0.94-1.9)	1.2% (-0.11-2.7%)	1.3% (-0.25-3%)	24000 (-4600-55000)	200000 (-18000-440000)
Global	1995	Female	90 to 94	45% (34-55%)	55% (45-66%)	1.4 (0.94-2)	1% (-0.57-2.9%)	1% (-0.85-3.3%)	9700 (-7900-30000)	58000 (-33000-170000)
Global	1995	Female	95 plus	49% (35-61%)	51% (39-65%)	1.5 (0.85-2.2)	0.84% (-1.2-3.3%)	0.78% (-1.6-3.7%)	2500 (-5300-12000)	11000 (-16000-42000)
Global	1995	Male	All Ages	39% (36-43%)	61% (57-64%)	1.5 (1.4-1.5)	5.2% (4.6-5.7%)	7% (6-8%)	1900000 (1600000-2100000)	69000000 (62000000-77000000)
Global	1995	Male	15 to 19	28% (21-35%)	72% (65-79%)	0.77 (0.54-1)	3.3% (2.4-4.3%)	4.3% (2.9-6%)	17000 (12000-24000)	1500000 (1100000-2000000)
Global	1995	Male	20 to 24	42% (33-50%)	58% (50-67%)	1.6 (1.2-2)	7.4% (5.8-9.2%)	8.9% (6.7-12%)	52000 (39000-67000)	4300000 (3300000-5400000)
Global	1995	Male	25 to 29	50% (41-59%)	50% (41-59%)	2 (1.5-2.5)	9.1% (7.3-11%)	11% (8.7-14%)	73000 (57000-90000)	5500000 (4300000-6600000)
Global	1995	Male	30 to 34	55% (45-63%)	45% (37-55%)	2.4 (1.9-3)	10% (8.5-12%)	13% (10-15%)	94000 (77000-110000)	6300000 (5200000-7300000)
Global	1995	Male	35 to 39	58% (49-65%)	42% (35-51%)	2.6 (2-3.3)	11% (9.4-13%)	14% (12-17%)	120000 (96000-140000)	6800000 (5600000-8000000)
Global	1995	Male	40 to 44	59% (51-67%)	41% (33-49%)	2.7 (2-3.3)	12% (10-14%)	15% (13-18%)	150000 (120000-170000)	7500000 (6400000-8600000)
Global	1995	Male	45 to 49	60% (51-68%)	40% (32-49%)	2.9 (2.2-3.8)	12% (10-14%)	15% (13-18%)	160000 (130000-190000)	7200000 (6000000-8400000)

Location	Year	Sex	Age	Prevalence of current drinkers	Prevalence of abstainers	Population average of standard drinks daily	Percent of total attributable deaths	Percent of total attributable DALYs	Attributable deaths	Attributable DALYs
Global	1995	Male	50 to 54	59% (50-66%)	41% (34-50%)	2.9 (2.3-3.7)	11% (9.2-13%)	14% (11-16%)	160000 (130000-190000)	6400000 (5300000-7600000)
Global	1995	Male	55 to 59	59% (50-67%)	41% (33-50%)	3 (2.3-3.8)	11% (8.5-13%)	13% (10-15%)	210000 (170000-250000)	6900000 (5600000-8200000)
Global	1995	Male	60 to 64	57% (48-66%)	43% (34-52%)	2.8 (2.2-3.6)	8.9% (7-11%)	10% (8.1-13%)	210000 (170000-260000)	6100000 (4700000-7500000)
Global	1995	Male	65 to 69	57% (48-65%)	43% (35-52%)	2.6 (2.1-3.2)	7.4% (5.6-9.3%)	8.5% (6.5-11%)	200000 (160000-260000)	4800000 (3700000-6000000)
Global	1995	Male	70 to 74	55% (46-63%)	45% (37-54%)	2.3 (1.8-2.9)	5.9% (4.2-7.8%)	6.7% (4.8-8.9%)	160000 (120000-220000)	3100000 (2300000-4100000)
Global	1995	Male	75 to 79	52% (41-62%)	48% (38-59%)	1.9 (1.4-2.4)	4.6% (3.1-6.4%)	5.1% (3.3-7.2%)	110000 (73000-160000)	1700000 (1100000-2300000)
Global	1995	Male	80 to 84	53% (42-63%)	47% (37-58%)	2 (1.6-2.5)	4% (2.6-5.6%)	4.4% (2.8-6.2%)	80000 (51000-110000)	890000 (580000-1300000)
Global	1995	Male	85 to 89	54% (43-64%)	46% (36-57%)	2 (1.5-2.6)	3.3% (1.9-4.8%)	3.5% (1.9-5.3%)	39000 (21000-59000)	310000 (180000-470000)
Global	1995	Male	90 to 94	56% (44-66%)	44% (34-56%)	1.9 (1.4-2.6)	2.5% (1-4.2%)	2.6% (0.88-4.5%)	12000 (3900-20000)	67000 (27000-110000)
Global	1995	Male	95 plus	58% (45-70%)	42% (30-55%)	1.8 (1.2-2.6)	2% (0.19-4%)	2% (-0.11-4.3%)	2200 (-120-4800)	9000 (840-18000)
Global	2000	Female	All Ages	25% (23-27%)	75% (73-77%)	0.71 (0.68-0.74)	1.4% (1.2-1.7%)	2% (1.3-2.8%)	480000 (310000-660000)	17000000 (14000000-20000000)
Global	2000	Female	15 to 19	21% (16-27%)	79% (73-84%)	0.53 (0.37-0.74)	1.1% (0.82-1.5%)	1.5% (0.96-2.1%)	4600 (2900-6700)	510000 (360000-690000)
Global	2000	Female	20 to 24	27% (21-33%)	73% (67-79%)	0.87 (0.63-1.2)	1.9% (1.4-2.4%)	2.1% (1.4-2.8%)	8100 (5500-11000)	930000 (680000-1200000)
Global	2000	Female	25 to 29	28% (22-34%)	72% (66-78%)	0.89 (0.67-1.1)	2% (1.6-2.5%)	2.3% (1.7-3%)	11000 (7900-14000)	1100000 (820000-1300000)
Global	2000	Female	30 to 34	29% (23-36%)	71% (64-77%)	0.93 (0.69-1.2)	2.2% (1.7-2.6%)	2.8% (2.2-3.5%)	14000 (11000-18000)	1200000 (910000-1400000)
Global	2000	Female	35 to 39	32% (26-39%)	68% (61-74%)	1.1 (0.8-1.4)	2.6% (2.1-3.2%)	3.7% (3-4.6%)	20000 (16000-25000)	1400000 (1100000-1600000)

Location	Year	Sex	Age	Prevalence of current drinkers	Prevalence of abstainers	Population average of standard drinks daily	Percent of total attributable deaths	Percent of total attributable DALYs	Attributable deaths	Attributable DALYs
Global	2000	Female	40 to 44	34% (28-42%)	66% (58-72%)	1.2 (0.89-1.5)	3% (2.4-3.7%)	4.6% (3.7-5.6%)	28000 (22000-33000)	1500000 (1300000-1900000)
Global	2000	Female	45 to 49	35% (27-42%)	65% (58-73%)	1.2 (0.88-1.5)	3.2% (2.5-4%)	4.9% (3.9-6.2%)	36000 (28000-45000)	1700000 (1300000-2100000)
Global	2000	Female	50 to 54	36% (30-44%)	64% (56-70%)	1.3 (0.97-1.7)	3.4% (2.6-4.3%)	5% (3.8-6.4%)	42000 (32000-54000)	1700000 (1300000-2100000)
Global	2000	Female	55 to 59	35% (29-42%)	65% (58-71%)	1.3 (0.97-1.7)	3.2% (2.3-4.2%)	4.5% (3.3-5.9%)	46000 (34000-60000)	1600000 (1100000-2000000)
Global	2000	Female	60 to 64	36% (28-44%)	64% (56-72%)	1.3 (0.94-1.8)	2.8% (1.8-4%)	3.8% (2.4-5.3%)	54000 (35000-75000)	1500000 (1000000-2200000)
Global	2000	Female	65 to 69	35% (28-44%)	65% (56-72%)	1.2 (0.88-1.5)	2.3% (1.5-3.3%)	3% (1.9-4.2%)	56000 (36000-78000)	1300000 (830000-1800000)
Global	2000	Female	70 to 74	37% (29-45%)	63% (55-71%)	1.1 (0.81-1.5)	1.9% (0.83-3%)	2.3% (1-3.7%)	54000 (24000-86000)	1000000 (440000-1600000)
Global	2000	Female	75 to 79	37% (29-47%)	63% (53-71%)	1.1 (0.78-1.5)	1.4% (0.32-2.7%)	1.7% (0.36-3.2%)	43000 (9000-81000)	650000 (140000-1200000)
Global	2000	Female	80 to 84	37% (27-46%)	63% (54-73%)	1.1 (0.76-1.5)	1.1% (0.16-2.3%)	1.3% (0.16-2.7%)	30000 (3700-62000)	340000 (47000-680000)
Global	2000	Female	85 to 89	41% (30-51%)	59% (49-70%)	1.2 (0.86-1.7)	1% (-0.14-2.4%)	1.1% (-0.29-2.8%)	23000 (-5900-56000)	190000 (-26000-450000)
Global	2000	Female	90 to 94	46% (35-55%)	54% (45-65%)	1.4 (0.91-1.9)	0.9% (-0.63-2.8%)	0.93% (-0.91-3.2%)	10000 (-10000-36000)	64000 (-44000-200000)
Global	2000	Female	95 plus	50% (36-62%)	50% (38-64%)	1.4 (0.84-2.2)	0.74% (-1.3-3.2%)	0.67% (-1.7-3.5%)	2800 (-7000-14000)	12000 (-20000-52000)
Global	2000	Male	All Ages	40% (37-43%)	60% (57-63%)	1.5 (1.4-1.6)	5.5% (5-6.1%)	7.2% (6.2-8.2%)	2000000 (1700000-2300000)	75000000 (67000000-83000000)
Global	2000	Male	15 to 19	29% (23-36%)	71% (64-77%)	0.84 (0.59-1.1)	3.4% (2.5-4.5%)	4.6% (3.1-6.4%)	19000 (13000-26000)	1700000 (1200000-2300000)
Global	2000	Male	20 to 24	43% (34-50%)	57% (50-66%)	1.8 (1.3-2.4)	7.8% (6.2-9.7%)	9.5% (7.2-12%)	55000 (42000-71000)	4500000 (3500000-5600000)
Global	2000	Male	25 to 29	50% (41-58%)	50% (42-59%)	2.2 (1.7-2.7)	9.2% (7.4-11%)	11% (8.7-14%)	77000 (60000-94000)	5800000 (4600000-7000000)

Location	Year	Sex	Age	Prevalence of current drinkers	Prevalence of abstainers	Population average of standard drinks daily	Percent of total attributable deaths	Percent of total attributable DALYs	Attributable deaths	Attributable DALYs
Global	2000	Male	30 to 34	54% (44-62%)	46% (38-56%)	2.3 (1.8-3)	9.8% (8.2-12%)	12% (9.9-14%)	97000 (79000-120000)	6500000 (5400000-7600000)
Global	2000	Male	35 to 39	57% (49-64%)	43% (36-51%)	2.5 (1.9-3.3)	11% (9-13%)	14% (11-16%)	120000 (100000-140000)	7200000 (6000000-8400000)
Global	2000	Male	40 to 44	58% (51-66%)	42% (34-49%)	2.7 (2.1-3.4)	12% (9.8-14%)	15% (12-17%)	150000 (130000-180000)	7900000 (6700000-9100000)
Global	2000	Male	45 to 49	59% (50-67%)	41% (33-50%)	2.8 (2.1-3.6)	12% (10-14%)	15% (13-18%)	190000 (160000-220000)	8600000 (7300000-10000000)
Global	2000	Male	50 to 54	60% (51-67%)	40% (33-49%)	2.9 (2.2-3.7)	12% (9.5-14%)	14% (12-17%)	190000 (160000-230000)	7600000 (6300000-9000000)
Global	2000	Male	55 to 59	57% (49-65%)	43% (35-51%)	2.8 (2.2-3.6)	10% (8.2-12%)	12% (10-15%)	190000 (150000-220000)	6400000 (5200000-7600000)
Global	2000	Male	60 to 64	57% (48-65%)	43% (35-52%)	2.7 (2.1-3.5)	8.9% (6.9-11%)	10% (8.1-13%)	220000 (170000-280000)	6400000 (5000000-7900000)
Global	2000	Male	65 to 69	55% (46-64%)	45% (36-54%)	2.4 (1.9-2.9)	7.4% (5.7-9.3%)	8.6% (6.7-11%)	210000 (160000-260000)	5000000 (3900000-6300000)
Global	2000	Male	70 to 74	55% (46-63%)	45% (37-54%)	2.2 (1.7-2.7)	5.9% (4.2-7.9%)	6.7% (4.7-9%)	190000 (130000-250000)	3600000 (2500000-4700000)
Global	2000	Male	75 to 79	53% (43-63%)	47% (37-57%)	2 (1.5-2.5)	4.7% (3.1-6.5%)	5.2% (3.4-7.4%)	130000 (85000-180000)	2000000 (1300000-2700000)
Global	2000	Male	80 to 84	51% (39-61%)	49% (39-61%)	1.7 (1.3-2.1)	3.8% (2.5-5.3%)	4.2% (2.6-5.9%)	78000 (49000-110000)	870000 (580000-1200000)
Global	2000	Male	85 to 89	53% (41-62%)	47% (38-59%)	1.8 (1.3-2.3)	3.1% (1.8-4.7%)	3.4% (1.9-5.2%)	44000 (24000-66000)	350000 (210000-530000)
Global	2000	Male	90 to 94	56% (45-66%)	44% (34-55%)	1.8 (1.3-2.4)	2.4% (0.93-4.1%)	2.5% (0.75-4.4%)	14000 (4100-24000)	80000 (30000-130000)
Global	2000	Male	95 plus	58% (45-70%)	42% (30-55%)	1.8 (1.1-2.5)	2% (0.11-4%)	1.9% (-0.2-4.3%)	2700 (-290-6000)	11000 (610-23000)
Global	2005	Female	All Ages	24% (23-27%)	76% (73-77%)	0.71 (0.69-0.74)	1.5% (1.3-1.8%)	2.1% (1.4-2.8%)	510000 (350000-670000)	17000000 (14000000-20000000)
Global	2005	Female	15 to 19	20% (16-25%)	80% (75-84%)	0.51 (0.36-0.7)	1.1% (0.8-1.5%)	1.5% (0.97-2.1%)	4400 (2900-6400)	510000 (360000-700000)

Location	Year	Sex	Age	Prevalence of current drinkers	Prevalence of abstainers	Population average of standard drinks daily	Percent of total attributable deaths	Percent of total attributable DALYs	Attributable deaths	Attributable DALYs
Global	2005	Female	20 to 24	26% (21-31%)	74% (69-79%)	0.87 (0.64-1.2)	1.9% (1.5-2.4%)	2.2% (1.6-3%)	8400 (5900-11000)	970000 (730000-1300000)
Global	2005	Female	25 to 29	28% (22-33%)	72% (67-78%)	0.94 (0.71-1.2)	2.1% (1.7-2.5%)	2.5% (1.8-3.1%)	11000 (8200-14000)	1100000 (840000-1400000)
Global	2005	Female	30 to 34	29% (23-34%)	71% (66-77%)	0.94 (0.71-1.2)	2.2% (1.8-2.6%)	2.8% (2.2-3.5%)	15000 (12000-18000)	1200000 (960000-1500000)
Global	2005	Female	35 to 39	30% (25-36%)	70% (64-75%)	0.98 (0.75-1.3)	2.4% (1.9-2.9%)	3.5% (2.8-4.3%)	20000 (16000-24000)	1300000 (1100000-1600000)
Global	2005	Female	40 to 44	33% (28-39%)	67% (61-72%)	1.1 (0.85-1.4)	2.8% (2.3-3.4%)	4.4% (3.6-5.3%)	28000 (23000-34000)	1600000 (1300000-1900000)
Global	2005	Female	45 to 49	34% (28-40%)	66% (60-72%)	1.2 (0.91-1.5)	3.2% (2.5-4%)	5% (4-6.2%)	38000 (30000-47000)	1800000 (1400000-2200000)
Global	2005	Female	50 to 54	34% (28-40%)	66% (60-72%)	1.2 (0.89-1.5)	3.3% (2.5-4.2%)	4.9% (3.8-6.2%)	48000 (37000-59000)	1900000 (1500000-2400000)
Global	2005	Female	55 to 59	35% (30-41%)	65% (59-70%)	1.3 (0.96-1.7)	3.2% (2.3-4.1%)	4.6% (3.4-6%)	52000 (39000-67000)	1800000 (1300000-2300000)
Global	2005	Female	60 to 64	34% (28-40%)	66% (60-72%)	1.2 (0.89-1.7)	2.6% (1.8-3.6%)	3.6% (2.5-4.9%)	49000 (34000-66000)	1400000 (950000-1900000)
Global	2005	Female	65 to 69	34% (27-41%)	66% (59-73%)	1.1 (0.86-1.5)	2.3% (1.5-3.2%)	3.1% (2-4.2%)	57000 (38000-78000)	1300000 (880000-1900000)
Global	2005	Female	70 to 74	33% (27-40%)	67% (60-73%)	1 (0.74-1.4)	1.8% (0.93-2.8%)	2.3% (1.2-3.5%)	53000 (28000-80000)	1000000 (510000-1500000)
Global	2005	Female	75 to 79	34% (27-42%)	66% (58-73%)	1 (0.7-1.4)	1.4% (0.41-2.5%)	1.7% (0.55-3.1%)	46000 (14000-81000)	690000 (200000-1200000)
Global	2005	Female	80 to 84	36% (27-45%)	64% (55-73%)	1.1 (0.78-1.5)	1.3% (0.32-2.4%)	1.5% (0.38-2.8%)	40000 (10000-76000)	440000 (110000-830000)
Global	2005	Female	85 to 89	37% (28-46%)	63% (54-72%)	1.1 (0.75-1.5)	1% (0.026-2.2%)	1.2% (-0.015-2.5%)	23000 (-310-51000)	190000 (4800-410000)
Global	2005	Female	90 to 94	43% (33-53%)	57% (47-67%)	1.3 (0.87-1.8)	0.93% (-0.46-2.6%)	0.99% (-0.67-3%)	13000 (-8700-39000)	76000 (-37000-210000)
Global	2005	Female	95 plus	49% (36-62%)	51% (38-64%)	1.4 (0.85-2.2)	0.86% (-1-3.2%)	0.83% (-1.4-3.6%)	4300 (-7300-18000)	18000 (-21000-66000)

Location	Year	Sex	Age	Prevalence of current drinkers	Prevalence of abstainers	Population average of standard drinks daily	Percent of total attributable deaths	Percent of total attributable DALYs	Attributable deaths	Attributable DALYs
Global	2005	Male	All Ages	41% (38-43%)	59% (57-62%)	1.6 (1.5-1.7)	5.9% (5.3-6.5%)	7.4% (6.5-8.4%)	2100000 (1800000-2400000)	79000000 (72000000-88000000)
Global	2005	Male	15 to 19	30% (25-37%)	70% (63-75%)	0.81 (0.58-1.1)	3.4% (2.5-4.4%)	4.6% (3.2-6.3%)	19000 (13000-26000)	1800000 (1300000-2300000)
Global	2005	Male	20 to 24	43% (37-49%)	57% (51-63%)	1.9 (1.4-2.4)	7.9% (6.3-9.7%)	9.8% (7.5-12%)	56000 (43000-71000)	4700000 (3700000-5800000)
Global	2005	Male	25 to 29	49% (43-56%)	51% (44-57%)	2.4 (1.8-3)	9.3% (7.6-11%)	11% (9.1-14%)	77000 (61000-93000)	5800000 (4700000-6900000)
Global	2005	Male	30 to 34	53% (46-60%)	47% (40-54%)	2.5 (1.9-3.1)	9.9% (8.3-12%)	12% (10-15%)	100000 (82000-120000)	6700000 (5600000-7800000)
Global	2005	Male	35 to 39	56% (50-62%)	44% (38-50%)	2.5 (1.8-3.2)	10% (8.7-12%)	13% (11-15%)	120000 (100000-150000)	7400000 (6200000-8600000)
Global	2005	Male	40 to 44	57% (51-64%)	43% (36-49%)	2.7 (2.1-3.4)	11% (9.7-13%)	15% (12-17%)	160000 (140000-190000)	8400000 (7200000-9600000)
Global	2005	Male	45 to 49	58% (52-65%)	42% (35-48%)	2.9 (2.2-3.7)	12% (10-14%)	15% (13-18%)	200000 (170000-230000)	9100000 (7800000-10000000)
Global	2005	Male	50 to 54	59% (52-65%)	41% (35-48%)	2.8 (2.1-3.5)	12% (9.9-14%)	15% (12-17%)	240000 (200000-280000)	9400000 (7900000-11000000)
Global	2005	Male	55 to 59	58% (52-65%)	42% (35-48%)	2.8 (2.2-3.6)	11% (8.6-12%)	13% (11-15%)	220000 (190000-270000)	7700000 (6400000-9000000)
Global	2005	Male	60 to 64	56% (49-63%)	44% (37-51%)	2.6 (2-3.4)	8.6% (6.7-11%)	10% (8-13%)	200000 (160000-240000)	5800000 (4500000-7100000)
Global	2005	Male	65 to 69	55% (48-62%)	45% (38-52%)	2.4 (1.9-2.9)	7.4% (5.7-9.2%)	8.7% (6.6-11%)	220000 (170000-270000)	5300000 (4100000-6500000)
Global	2005	Male	70 to 74	53% (45-60%)	47% (40-55%)	2.1 (1.6-2.6)	5.8% (4.1-7.7%)	6.7% (4.6-8.8%)	190000 (130000-250000)	3600000 (2600000-4800000)
Global	2005	Male	75 to 79	52% (44-61%)	48% (39-56%)	1.9 (1.5-2.4)	4.6% (2.9-6.4%)	5.1% (3.2-7.3%)	140000 (91000-200000)	2200000 (1400000-3000000)

Location	Year	Sex	Age	Prevalence of current drinkers	Prevalence of abstainers	Population average of standard drinks daily	Percent of total attributable deaths	Percent of total attributable DALYs	Attributable deaths	Attributable DALYs
Global	2005	Male	80 to 84	51% (40-61%)	49% (39-60%)	1.8 (1.4-2.2)	3.8% (2.5-5.3%)	4.2% (2.7-6%)	94000 (61000-130000)	1100000 (710000-1500000)
Global	2005	Male	85 to 89	50% (40-60%)	50% (40-60%)	1.6 (1.2-2)	2.9% (1.7-4.2%)	3.1% (1.7-4.7%)	42000 (23000-64000)	350000 (200000-520000)
Global	2005	Male	90 to 94	55% (44-64%)	45% (36-56%)	1.7 (1.3-2.3)	2.4% (0.87-3.9%)	2.4% (0.71-4.3%)	16000 (4700-28000)	93000 (35000-160000)
Global	2005	Male	95 plus	58% (46-70%)	42% (30-54%)	1.8 (1.1-2.5)	2% (0.12-4.2%)	1.9% (-0.22-4.3%)	3400 (-390-7700)	14000 (830-29000)
Global	2010	Female	All Ages	24% (23-26%)	76% (74-77%)	0.71 (0.68-0.74)	1.5% (1.3-1.8%)	2.1% (1.5-2.8%)	520000 (360000-680000)	17000000 (14000000-20000000)
Global	2010	Female	15 to 19	19% (15-24%)	81% (76-85%)	0.49 (0.34-0.7)	1% (0.75-1.4%)	1.4% (0.86-2%)	3600 (2300-5400)	450000 (320000-610000)
Global	2010	Female	20 to 24	25% (20-31%)	75% (69-80%)	0.82 (0.59-1.1)	1.9% (1.5-2.4%)	2.2% (1.5-3%)	7600 (5300-10000)	960000 (710000-1300000)
Global	2010	Female	25 to 29	27% (21-33%)	73% (67-79%)	0.93 (0.7-1.2)	2.1% (1.7-2.5%)	2.5% (1.8-3.2%)	10000 (7500-13000)	1100000 (830000-1400000)
Global	2010	Female	30 to 34	28% (22-34%)	72% (66-78%)	0.98 (0.73-1.3)	2.1% (1.7-2.6%)	2.8% (2.2-3.5%)	13000 (10000-16000)	1100000 (900000-1400000)
Global	2010	Female	35 to 39	30% (24-36%)	70% (64-76%)	0.98 (0.75-1.3)	2.3% (1.9-2.8%)	3.4% (2.7-4.2%)	18000 (14000-22000)	1300000 (1000000-1500000)
Global	2010	Female	40 to 44	31% (26-37%)	69% (63-74%)	1 (0.76-1.3)	2.5% (2-3%)	4% (3.2-4.9%)	24000 (20000-30000)	1400000 (1200000-1700000)
Global	2010	Female	45 to 49	33% (26-39%)	67% (61-74%)	1.1 (0.85-1.4)	2.9% (2.3-3.6%)	4.7% (3.7-5.9%)	35000 (28000-43000)	1700000 (1400000-2100000)
Global	2010	Female	50 to 54	33% (28-40%)	67% (60-72%)	1.2 (0.9-1.5)	3.2% (2.4-4%)	4.9% (3.8-6.1%)	46000 (36000-58000)	1900000 (1500000-2400000)
Global	2010	Female	55 to 59	33% (27-39%)	67% (61-73%)	1.2 (0.88-1.6)	3.1% (2.3-4%)	4.7% (3.5-5.9%)	56000 (42000-71000)	1900000 (1500000-2500000)
Global	2010	Female	60 to 64	34% (28-41%)	66% (59-72%)	1.2 (0.89-1.6)	2.7% (1.8-3.6%)	3.8% (2.6-5%)	55000 (38000-73000)	1600000 (1100000-2100000)
Global	2010	Female	65 to 69	34% (28-40%)	66% (60-72%)	1.1 (0.85-1.4)	2.2% (1.5-3%)	3.1% (2.1-4.1%)	52000 (36000-70000)	1200000 (830000-1700000)

Location	Year	Sex	Age	Prevalence of current drinkers	Prevalence of abstainers	Population average of standard drinks daily	Percent of total attributable deaths	Percent of total attributable DALYs	Attributable deaths	Attributable DALYs
Global	2010	Female	70 to 74	34% (27-41%)	66% (59-73%)	1 (0.75-1.4)	1.9% (0.97-2.8%)	2.4% (1.3-3.6%)	56000 (31000-84000)	1100000 (570000-1600000)
Global	2010	Female	75 to 79	33% (25-41%)	67% (59-75%)	0.95 (0.66-1.3)	1.4% (0.54-2.5%)	1.8% (0.71-3%)	47000 (18000-79000)	710000 (270000-1200000)
Global	2010	Female	80 to 84	35% (26-43%)	65% (57-74%)	1 (0.72-1.4)	1.3% (0.38-2.3%)	1.5% (0.48-2.8%)	44000 (14000-82000)	490000 (150000-910000)
Global	2010	Female	85 to 89	38% (29-48%)	62% (52-71%)	1.1 (0.77-1.5)	1.1% (0.16-2.3%)	1.3% (0.15-2.6%)	32000 (3700-65000)	260000 (36000-520000)
Global	2010	Female	90 to 94	42% (32-51%)	58% (49-68%)	1.2 (0.77-1.6)	0.85% (-0.31-2.3%)	0.93% (-0.46-2.7%)	13000 (-6300-36000)	74000 (-26000-200000)
Global	2010	Female	95 plus	49% (36-61%)	51% (39-64%)	1.4 (0.79-2.1)	0.84% (-0.93-3.1%)	0.83% (-1.3-3.6%)	5300 (-8400-23000)	22000 (-23000-82000)
Global	2010	Male	All Ages	40% (38-43%)	60% (57-62%)	1.6 (1.5-1.8)	5.9% (5.3-6.5%)	7.4% (6.4-8.4%)	2100000 (1900000-2400000)	79000000 (71000000-88000000)
Global	2010	Male	15 to 19	28% (22-34%)	72% (66-78%)	0.82 (0.56-1.1)	3.1% (2.3-4.1%)	4.1% (2.7-5.7%)	16000 (11000-23000)	1500000 (1100000-2100000)
Global	2010	Male	20 to 24	42% (36-49%)	58% (51-64%)	1.8 (1.3-2.4)	7.5% (5.9-9.2%)	9.4% (7-12%)	54000 (40000-68000)	4600000 (3600000-5700000)
Global	2010	Male	25 to 29	48% (41-54%)	52% (46-59%)	2.4 (1.8-3.1)	9.1% (7.4-11%)	11% (8.9-14%)	74000 (59000-90000)	5700000 (4600000-6800000)
Global	2010	Male	30 to 34	51% (44-58%)	49% (42-56%)	2.6 (2-3.4)	9.6% (8.1-11%)	12% (9.9-14%)	91000 (75000-110000)	6200000 (5200000-7200000)
Global	2010	Male	35 to 39	54% (48-61%)	46% (39-52%)	2.5 (1.9-3.3)	10% (8.4-12%)	13% (11-15%)	120000 (96000-140000)	7000000 (5900000-8100000)
Global	2010	Male	40 to 44	56% (49-63%)	44% (37-51%)	2.5 (1.9-3.2)	11% (8.9-12%)	14% (12-16%)	150000 (120000-170000)	7800000 (6600000-9000000)
Global	2010	Male	45 to 49	57% (50-63%)	43% (37-50%)	2.8 (2.1-3.5)	11% (9.5-13%)	15% (12-17%)	190000 (160000-220000)	8700000 (7400000-10000000)
Global	2010	Male	50 to 54	57% (50-63%)	43% (37-50%)	2.9 (2.2-3.6)	11% (9.3-13%)	14% (12-16%)	220000 (190000-260000)	9000000 (7600000-10000000)
Global	2010	Male	55 to 59	56% (49-63%)	44% (37-51%)	2.7 (2.1-3.5)	10% (8.5-12%)	13% (11-15%)	250000 (210000-300000)	8700000 (7300000-10000000)

Location	Year	Sex	Age	Prevalence of current drinkers	Prevalence of abstainers	Population average of standard drinks daily	Percent of total attributable deaths	Percent of total attributable DALYs	Attributable deaths	Attributable DALYs
Global	2010	Male	60 to 64	56% (49-64%)	44% (36-51%)	2.7 (2-3.4)	8.7% (6.9-11%)	11% (8.3-13%)	230000 (180000-280000)	6700000 (5400000-8100000)
Global	2010	Male	65 to 69	54% (46-61%)	46% (39-54%)	2.4 (1.9-2.9)	7.1% (5.6-8.7%)	8.4% (6.6-10%)	200000 (160000-240000)	4800000 (3800000-5900000)
Global	2010	Male	70 to 74	53% (45-60%)	47% (40-55%)	2.1 (1.6-2.7)	5.8% (4.2-7.7%)	6.8% (4.7-8.9%)	200000 (140000-260000)	3800000 (2700000-5000000)
Global	2010	Male	75 to 79	51% (43-60%)	49% (40-57%)	1.9 (1.4-2.4)	4.7% (3.1-6.4%)	5.3% (3.5-7.4%)	150000 (100000-210000)	2300000 (1600000-3200000)
Global	2010	Male	80 to 84	52% (41-61%)	48% (39-59%)	1.8 (1.4-2.2)	3.9% (2.6-5.4%)	4.3% (2.8-6.2%)	110000 (73000-160000)	1300000 (860000-1800000)
Global	2010	Male	85 to 89	53% (43-62%)	47% (38-57%)	1.8 (1.3-2.3)	3.1% (1.9-4.5%)	3.4% (1.9-5.1%)	58000 (32000-86000)	470000 (280000-690000)
Global	2010	Male	90 to 94	54% (44-63%)	46% (37-56%)	1.6 (1.2-2.2)	2.3% (0.92-3.8%)	2.4% (0.79-4.1%)	18000 (5800-31000)	100000 (42000-170000)
Global	2010	Male	95 plus	59% (47-70%)	41% (30-53%)	1.7 (1.1-2.5)	2.1% (0.25-4.1%)	2% (-0.076-4.4%)	4600 (-170-9900)	19000 (2300-38000)
Global	2016	Female	All Ages	25% (23-27%)	75% (73-77%)	0.74 (0.69-0.8)	1.7% (1.4-2%)	2.3% (1.6-3%)	560000 (390000-750000)	18000000 (15000000-22000000)
Global	2016	Female	15 to 19	19% (14-25%)	81% (75-86%)	0.52 (0.34-0.75)	1% (0.74-1.4%)	1.4% (0.84-2.1%)	3100 (1900-4700)	410000 (290000-560000)
Global	2016	Female	20 to 24	25% (19-32%)	75% (68-81%)	0.87 (0.62-1.2)	1.9% (1.5-2.5%)	2.3% (1.6-3.1%)	6400 (4400-8900)	870000 (640000-1200000)
Global	2016	Female	25 to 29	27% (21-34%)	73% (66-79%)	0.92 (0.67-1.2)	2.2% (1.7-2.7%)	2.8% (2-3.7%)	9700 (7000-13000)	1100000 (830000-1400000)
Global	2016	Female	30 to 34	29% (21-36%)	71% (64-79%)	0.99 (0.73-1.3)	2.3% (1.9-2.9%)	3.4% (2.6-4.4%)	14000 (10000-17000)	1200000 (960000-1500000)
Global	2016	Female	35 to 39	31% (23-38%)	69% (62-77%)	1.1 (0.78-1.4)	2.5% (2-3.2%)	4.2% (3.2-5.3%)	18000 (14000-24000)	1300000 (1000000-1600000)
Global	2016	Female	40 to 44	31% (24-39%)	69% (61-76%)	1.1 (0.78-1.4)	2.6% (2.1-3.3%)	4.7% (3.6-5.8%)	25000 (19000-31000)	1500000 (1200000-1800000)
Global	2016	Female	45 to 49	31% (23-38%)	69% (62-77%)	1.1 (0.76-1.4)	2.7% (2.1-3.5%)	4.8% (3.7-6%)	34000 (27000-43000)	1700000 (1300000-2100000)

Location	Year	Sex	Age	Prevalence of current drinkers	Prevalence of abstainers	Population average of standard drinks daily	Percent of total attributable deaths	Percent of total attributable DALYs	Attributable deaths	Attributable DALYs
Global	2016	Female	50 to 54	32% (25-39%)	68% (61-75%)	1.1 (0.84-1.5)	3% (2.3-3.9%)	5% (3.8-6.2%)	48000 (37000-60000)	2000000 (1500000-2500000)
Global	2016	Female	55 to 59	33% (26-41%)	67% (59-74%)	1.2 (0.9-1.6)	3.1% (2.3-4%)	4.8% (3.6-6.2%)	58000 (44000-75000)	2000000 (1500000-2600000)
Global	2016	Female	60 to 64	33% (26-41%)	67% (59-74%)	1.2 (0.85-1.6)	2.7% (1.9-3.6%)	3.9% (2.8-5.3%)	65000 (46000-86000)	1900000 (1300000-2500000)
Global	2016	Female	65 to 69	35% (28-43%)	65% (57-72%)	1.1 (0.84-1.5)	2.3% (1.5-3.1%)	3.2% (2.3-4.3%)	63000 (43000-83000)	1500000 (1000000-2000000)
Global	2016	Female	70 to 74	34% (27-42%)	66% (58-73%)	1 (0.74-1.4)	1.8% (1-2.8%)	2.5% (1.4-3.7%)	54000 (31000-80000)	1000000 (580000-1500000)
Global	2016	Female	75 to 79	34% (26-44%)	66% (56-74%)	1 (0.68-1.4)	1.5% (0.55-2.6%)	1.9% (0.75-3.3%)	52000 (20000-89000)	780000 (290000-1300000)
Global	2016	Female	80 to 84	35% (26-45%)	65% (55-74%)	1 (0.71-1.4)	1.3% (0.44-2.3%)	1.6% (0.55-2.9%)	46000 (16000-84000)	510000 (180000-930000)
Global	2016	Female	85 to 89	38% (28-47%)	62% (53-72%)	1.1 (0.73-1.5)	1.1% (0.17-2.2%)	1.3% (0.15-2.7%)	37000 (4200-79000)	300000 (47000-620000)
Global	2016	Female	90 to 94	44% (33-53%)	56% (47-67%)	1.3 (0.81-1.8)	0.95% (-0.37-2.5%)	1% (-0.49-2.9%)	20000 (-9300-56000)	120000 (-45000-310000)
Global	2016	Female	95 plus	48% (35-61%)	52% (39-65%)	1.3 (0.72-1.9)	0.78% (-0.81-2.8%)	0.77% (-1.2-3.2%)	5900 (-9000-24000)	25000 (-26000-89000)
Global	2016	Male	All Ages	39% (36-43%)	61% (57-64%)	1.7 (1.5-1.9)	6.2% (5.6-6.9%)	7.5% (6.5-8.6%)	2300000 (1900000-2600000)	81000000 (72000000-91000000)
Global	2016	Male	15 to 19	25% (19-32%)	75% (68-81%)	0.79 (0.53-1.1)	2.9% (2.1-3.9%)	3.8% (2.6-5.4%)	14000 (9200-19000)	1400000 (990000-1800000)
Global	2016	Male	20 to 24	38% (30-46%)	62% (54-70%)	1.7 (1.3-2.3)	7% (5.5-8.8%)	8.7% (6.5-11%)	46000 (34000-59000)	4000000 (3100000-5100000)
Global	2016	Male	25 to 29	45% (36-54%)	55% (46-64%)	2.2 (1.6-2.9)	8.8% (7.2-11%)	11% (8.8-14%)	72000 (56000-89000)	5600000 (4600000-6800000)
Global	2016	Male	30 to 34	48% (38-57%)	52% (43-62%)	2.6 (1.9-3.5)	9.6% (8-11%)	13% (10-15%)	90000 (74000-110000)	6300000 (5200000-7400000)
Global	2016	Male	35 to 39	51% (42-59%)	49% (41-58%)	2.7 (2-3.7)	9.9% (8.3-12%)	13% (11-16%)	110000 (88000-130000)	6600000 (5500000-7700000)

Location	Year	Sex	Age	Prevalence of current drinkers	Prevalence of abstainers	Population average of standard drinks daily	Percent of total attributable deaths	Percent of total attributable DALYs	Attributable deaths	Attributable DALYs
Global	2016	Male	40 to 44	53% (44-62%)	47% (38-56%)	2.7 (1.9-3.5)	10% (8.7-12%)	14% (12-16%)	140000 (110000-160000)	7400000 (6200000-8600000)
Global	2016	Male	45 to 49	55% (45-64%)	45% (36-55%)	2.6 (1.9-3.5)	11% (9.1-13%)	14% (12-17%)	180000 (150000-210000)	8500000 (7200000-9900000)
Global	2016	Male	50 to 54	56% (46-64%)	44% (36-54%)	2.7 (2-3.6)	11% (9.1-13%)	14% (12-16%)	230000 (190000-270000)	9200000 (7700000-11000000)
Global	2016	Male	55 to 59	55% (47-64%)	45% (36-53%)	2.8 (2.1-3.6)	10% (8.3-12%)	13% (10-15%)	250000 (210000-300000)	8800000 (7400000-10000000)
Global	2016	Male	60 to 64	55% (46-65%)	45% (35-54%)	2.6 (1.9-3.4)	8.9% (7.1-11%)	11% (8.7-13%)	280000 (230000-340000)	8300000 (6700000-10000000)
Global	2016	Male	65 to 69	55% (46-64%)	45% (36-54%)	2.4 (1.8-3)	7.4% (5.9-9.1%)	8.9% (7.1-11%)	250000 (200000-310000)	6100000 (4900000-7400000)
Global	2016	Male	70 to 74	53% (43-61%)	47% (39-57%)	2.1 (1.6-2.7)	5.7% (4.2-7.5%)	6.7% (4.8-8.7%)	190000 (140000-250000)	3800000 (2800000-4900000)
Global	2016	Male	75 to 79	51% (41-62%)	49% (38-59%)	1.9 (1.4-2.5)	4.6% (3.1-6.4%)	5.3% (3.4-7.4%)	170000 (110000-230000)	2500000 (1700000-3500000)
Global	2016	Male	80 to 84	51% (40-61%)	49% (39-60%)	1.7 (1.3-2.2)	3.9% (2.6-5.3%)	4.4% (2.8-6.1%)	130000 (83000-180000)	1400000 (970000-2000000)
Global	2016	Male	85 to 89	52% (41-62%)	48% (38-59%)	1.7 (1.2-2.2)	3.1% (1.9-4.5%)	3.4% (1.9-5%)	76000 (43000-110000)	620000 (370000-900000)
Global	2016	Male	90 to 94	56% (45-66%)	44% (34-55%)	1.8 (1.3-2.4)	2.5% (1-4%)	2.6% (0.91-4.4%)	28000 (9700-47000)	160000 (68000-260000)
Global	2016	Male	95 plus	58% (45-69%)	42% (31-55%)	1.6 (0.98-2.3)	2% (0.29-3.8%)	1.9% (-0.027-4.1%)	5500 (-77-12000)	23000 (3400-45000)
High SDI	1990	Female	All Ages	73% (69-76%)	27% (24-31%)	2.3 (2-2.7)	3.3% (2-4.8%)	3.2% (0.62-6.1%)	140000 (33000-260000)	4100000 (2500000-5900000)
High SDI	1990	Female	15 to 19	69% (59-78%)	31% (22-41%)	2 (1.4-2.8)	3% (1.9-4.4%)	9.3% (5-14%)	1100 (580-1600)	110000 (71000-160000)
High SDI	1990	Female	20 to 24	80% (72-86%)	20% (14-28%)	3.1 (2.2-4.1)	4.6% (3.3-6.1%)	12% (7.4-17%)	1800 (1100-2500)	210000 (150000-280000)
High SDI	1990	Female	25 to 29	83% (76-88%)	17% (12-24%)	2.9 (2.2-3.8)	4.2% (3-5.4%)	11% (7-15%)	2000 (1300-2800)	220000 (150000-290000)
High SDI	1990	Female	30 to 34	84% (76-89%)	16% (11-24%)	2.9 (2.1-3.7)	4% (2.9-5.3%)	11% (7.4-14%)	2600 (1800-3500)	230000 (160000-290000)

Location	Year	Sex	Age	Prevalence of current drinkers	Prevalence of abstainers	Population average of standard drinks daily	Percent of total attributable deaths	Percent of total attributable DALYs	Attributable deaths	Attributable DALYs
High SDI	1990	Female	35 to 39	84% (77-89%)	16% (11-23%)	2.9 (2.2-3.9)	4.4% (3.2-5.8%)	12% (8.5-15%)	3800 (2800-4900)	260000 (190000-340000)
High SDI	1990	Female	40 to 44	83% (77-89%)	17% (11-23%)	3.1 (2.3-4)	4.9% (3.5-6.4%)	12% (8.9-15%)	5600 (4100-7100)	320000 (230000-410000)
High SDI	1990	Female	45 to 49	82% (75-88%)	18% (12-25%)	3.2 (2.3-4.3)	5.3% (3.7-7.2%)	12% (8.4-15%)	7000 (5100-9100)	330000 (240000-440000)
High SDI	1990	Female	50 to 54	81% (74-86%)	19% (14-26%)	3.3 (2.4-4.4)	5.6% (3.7-7.7%)	11% (7.6-14%)	9200 (6500-12000)	380000 (260000-510000)
High SDI	1990	Female	55 to 59	79% (71-85%)	21% (15-29%)	3.3 (2.4-4.4)	5.3% (3.3-7.5%)	9.2% (6.1-13%)	11000 (7400-16000)	400000 (250000-560000)
High SDI	1990	Female	60 to 64	76% (68-82%)	24% (18-32%)	3.1 (2.2-4.3)	4.5% (2.3-7.1%)	7.2% (3.9-11%)	13000 (7000-21000)	400000 (200000-630000)
High SDI	1990	Female	65 to 69	72% (63-80%)	28% (20-37%)	2.9 (2.1-3.8)	3.8% (1.6-6.2%)	5.5% (2.4-8.8%)	16000 (6200-26000)	390000 (150000-640000)
High SDI	1990	Female	70 to 74	68% (58-76%)	32% (24-42%)	2.4 (1.6-3.3)	2.5% (0.14-5.2%)	3.4% (0.23-6.9%)	12000 (140-25000)	240000 (5700-500000)
High SDI	1990	Female	75 to 79	66% (56-76%)	34% (24-44%)	2.4 (1.6-3.3)	2.3% (-0.54-5.5%)	2.8% (-0.79-6.9%)	17000 (-5300-41000)	260000 (-65000-630000)
High SDI	1990	Female	80 to 84	64% (52-73%)	36% (27-48%)	2.3 (1.6-3.2)	2.1% (-0.68-5.2%)	2.4% (-1.1-6.3%)	18000 (-7700-47000)	210000 (-66000-530000)
High SDI	1990	Female	85 to 89	61% (50-71%)	39% (29-50%)	2.2 (1.5-3.1)	1.7% (-1.1-4.9%)	1.8% (-1.6-5.8%)	13000 (-9700-39000)	110000 (-61000-310000)
High SDI	1990	Female	90 to 94	60% (49-70%)	40% (30-51%)	2.1 (1.3-3.1)	1.3% (-1.5-4.7%)	1.3% (-2.2-5.4%)	5300 (-7600-21000)	33000 (-34000-110000)
High SDI	1990	Female	95 plus	59% (44-72%)	41% (28-56%)	1.9 (1.1-3)	0.73% (-2.3-4.3%)	0.57% (-3.2-4.9%)	1100 (-4300-7500)	5400 (-13000-27000)
High SDI	1990	Male	All Ages	82% (79-85%)	18% (15-21%)	3.6 (3.1-4.1)	8.4% (6.5-10%)	8.2% (5.5-11%)	350000 (230000-460000)	12000000 (9200000-15000000)
High SDI	1990	Male	15 to 19	73% (64-81%)	27% (19-36%)	2.4 (1.7-3.2)	7.2% (4.7-10%)	13% (7.3-18%)	4000 (2400-5800)	350000 (230000-480000)
High SDI	1990	Male	20 to 24	86% (78-91%)	14% (9.3-22%)	4.5 (3.5-5.8)	14% (10-18%)	21% (14-28%)	9900 (6900-13000)	840000 (620000-1100000)
High SDI	1990	Male	25 to 29	89% (83-93%)	11% (7.3-17%)	4.7 (3.6-5.9)	13% (9.7-16%)	20% (14-26%)	10000 (7400-14000)	850000 (640000-1100000)
High SDI	1990	Male	30 to 34	91% (86-94%)	9.2% (5.6-14%)	4.6 (3.6-5.9)	12% (8.8-15%)	18% (13-23%)	11000 (8200-14000)	820000 (630000-1000000)
High SDI	1990	Male	35 to 39	92% (87-95%)	8.3% (5.1-13%)	4.4 (3.4-5.7)	11% (8.5-14%)	17% (12-21%)	13000 (9700-16000)	830000 (640000-1000000)

Location	Year	Sex	Age	Prevalence of current drinkers	Prevalence of abstainers	Population average of standard drinks daily	Percent of total attributable deaths	Percent of total attributable DALYs	Attributable deaths	Attributable DALYs
High SDI	1990	Male	40 to 44	92%<br>(88-95%)	7.7%<br>(4.7-12%)	4.6<br>(3.5-5.8)	11%<br>(8.4-14%)	16%<br>(12-20%)	16000<br>(12000-21000)	910000<br>(700000-1100000)
High SDI	1990	Male	45 to 49	92%<br>(88-95%)	7.8%<br>(4.8-12%)	5<br>(3.8-6.5)	12%<br>(8.8-15%)	16%<br>(12-20%)	21000<br>(15000-26000)	970000<br>(740000-1200000)
High SDI	1990	Male	50 to 54	92%<br>(88-95%)	8.1%<br>(5.1-12%)	5.5<br>(4.2-7)	12%<br>(8.8-15%)	15%<br>(11-19%)	28000<br>(21000-36000)	1100000<br>(870000-1400000)
High SDI	1990	Male	55 to 59	91%<br>(86-94%)	9.3%<br>(6-14%)	5.7<br>(4.4-7.2)	11%<br>(7.9-14%)	13%<br>(9.6-17%)	35000<br>(26000-45000)	1200000<br>(900000-1600000)
High SDI	1990	Male	60 to 64	89%<br>(84-93%)	11%<br>(6.9-16%)	5.5<br>(4.2-7.1)	9.1%<br>(6.2-12%)	11%<br>(7.1-15%)	41000<br>(27000-56000)	1200000<br>(820000-1600000)
High SDI	1990	Male	65 to 69	88%<br>(83-92%)	12%<br>(7.9-17%)	5.1<br>(3.9-6.4)	7.6%<br>(4.7-11%)	8.7%<br>(5.3-12%)	41000<br>(25000-58000)	1000000<br>(630000-1400000)
High SDI	1990	Male	70 to 74	86%<br>(80-91%)	14%<br>(9.4-20%)	4.2<br>(3.1-5.4)	5.5%<br>(2.7-8.4%)	6.1%<br>(2.7-9.6%)	29000<br>(13000-47000)	590000<br>(280000-920000)
High SDI	1990	Male	75 to 79	85%<br>(79-91%)	15%<br>(9.4-21%)	4.1<br>(3-5.4)	5.2%<br>(2.2-8.4%)	5.7%<br>(2.1-9.4%)	35000<br>(13000-59000)	550000<br>(230000-900000)
High SDI	1990	Male	80 to 84	84%<br>(76-90%)	16%<br>(10-24%)	3.8<br>(2.8-5)	4.7%<br>(1.7-7.8%)	5%<br>(1.6-8.5%)	29000<br>(8900-50000)	330000<br>(120000-560000)
High SDI	1990	Male	85 to 89	82%<br>(74-88%)	18%<br>(12-26%)	3.5<br>(2.5-4.6)	3.8%<br>(0.98-6.8%)	4%<br>(0.66-7.4%)	15000<br>(2600-28000)	120000<br>(32000-220000)
High SDI	1990	Male	90 to 94	82%<br>(74-88%)	18%<br>(12-26%)	3.2<br>(2.2-4.4)	2.8%<br>(-0.18-5.8%)	2.7%<br>(-0.73-6.3%)	4200<br>(-900-9500)	25000<br>(-830-52000)
High SDI	1990	Male	95 plus	81%<br>(71-89%)	19%<br>(11-29%)	2.8<br>(1.7-4.1)	1.7%<br>(-1.4-5%)	1.4%<br>(-2.3-5.3%)	650<br>(-890-2200)	3000<br>(-2200-8500)
High SDI	1995	Female	All Ages	73%<br>(70-76%)	27%<br>(24-30%)	2.2<br>(1.9-2.6)	3.1%<br>(1.8-4.4%)	2.8%<br>(0.36-5.5%)	120000<br>(21000-240000)	3800000<br>(2300000-5500000)
High SDI	1995	Female	15 to 19	68%<br>(59-77%)	32%<br>(23-41%)	1.9<br>(1.3-2.7)	2.9%<br>(1.8-4.2%)	8.9%<br>(4.8-14%)	930<br>(510-1400)	100000<br>(65000-150000)
High SDI	1995	Female	20 to 24	80%<br>(72-86%)	20%<br>(14-28%)	3<br>(2.1-4)	4.4%<br>(3.1-5.8%)	12%<br>(7-17%)	1500<br>(940-2200)	190000<br>(130000-250000)
High SDI	1995	Female	25 to 29	83%<br>(76-88%)	17%<br>(12-24%)	2.8<br>(2.1-3.7)	3.9%<br>(2.8-5.1%)	10%<br>(6.4-14%)	1700<br>(1100-2400)	190000<br>(140000-250000)
High SDI	1995	Female	30 to 34	84%<br>(77-89%)	16%<br>(11-23%)	2.8<br>(2.1-3.7)	3.7%<br>(2.7-4.9%)	10%<br>(6.8-13%)	2400<br>(1600-3200)	210000<br>(160000-280000)
High SDI	1995	Female	35 to 39	83%<br>(77-89%)	17%<br>(11-23%)	2.8<br>(2.1-3.7)	4.2%<br>(3-5.4%)	11%<br>(7.9-14%)	3800<br>(2800-4800)	260000<br>(200000-340000)

Location	Year	Sex	Age	Prevalence of current drinkers	Prevalence of abstainers	Population average of standard drinks daily	Percent of total attributable deaths	Percent of total attributable DALYs	Attributable deaths	Attributable DALYs
High SDI	1995	Female	40 to 44	83% (77-88%)	17% (12-23%)	2.9 (2.2-3.8)	4.7% (3.3-6.1%)	11% (8.5-14%)	5600 (4200-7000)	320000 (240000-410000)
High SDI	1995	Female	45 to 49	83% (76-88%)	17% (12-24%)	3.1 (2.2-4.1)	5% (3.5-6.7%)	11% (8.3-14%)	7700 (5700-9900)	370000 (270000-480000)
High SDI	1995	Female	50 to 54	81% (74-86%)	19% (14-26%)	3.1 (2.2-4.1)	5.2% (3.5-7.1%)	10% (7.4-13%)	8900 (6400-12000)	360000 (250000-490000)
High SDI	1995	Female	55 to 59	79% (72-85%)	21% (15-28%)	3.2 (2.3-4.2)	5% (3.2-7%)	9% (6.2-12%)	11000 (7200-15000)	380000 (240000-520000)
High SDI	1995	Female	60 to 64	76% (68-82%)	24% (18-32%)	3 (2.1-4.1)	4.2% (2.2-6.5%)	6.8% (3.9-10%)	12000 (6400-18000)	350000 (180000-540000)
High SDI	1995	Female	65 to 69	72% (64-80%)	28% (20-36%)	2.7 (2-3.5)	3.3% (1.3-5.5%)	5% (2.2-8%)	13000 (5400-22000)	330000 (130000-550000)
High SDI	1995	Female	70 to 74	69% (60-77%)	31% (23-40%)	2.4 (1.7-3.3)	2.6% (0.33-5.2%)	3.7% (0.56-7%)	15000 (1300-30000)	300000 (26000-590000)
High SDI	1995	Female	75 to 79	65% (55-75%)	35% (25-45%)	2.1 (1.4-3)	1.6% (-0.81-4.3%)	2% (-1.1-5.4%)	9700 (-5900-28000)	160000 (-82000-430000)
High SDI	1995	Female	80 to 84	64% (52-73%)	36% (27-48%)	2.1 (1.5-2.9)	1.6% (-0.89-4.3%)	1.8% (-1.3-5.3%)	13000 (-10000-39000)	160000 (-92000-450000)
High SDI	1995	Female	85 to 89	62% (51-71%)	38% (29-49%)	2.1 (1.4-2.9)	1.3% (-1.2-4.2%)	1.4% (-1.7-4.9%)	10000 (-13000-38000)	94000 (-84000-300000)
High SDI	1995	Female	90 to 94	61% (50-71%)	39% (29-50%)	2 (1.3-2.9)	0.9% (-1.7-4.1%)	0.83% (-2.3-4.7%)	4300 (-10000-22000)	29000 (-48000-120000)
High SDI	1995	Female	95 plus	60% (46-72%)	40% (28-54%)	1.9 (1-2.9)	0.5% (-2.4-4%)	0.3% (-3.3-4.5%)	810 (-5900-8800)	4700 (-18000-32000)
High SDI	1995	Male	All Ages	83% (80-85%)	17% (15-20%)	3.4 (3-3.9)	8.1% (6.4-9.9%)	7.8% (5.2-10%)	330000 (220000-450000)	12000000 (9100000-14000000)
High SDI	1995	Male	15 to 19	73% (64-80%)	27% (20-36%)	2.3 (1.7-3)	6.9% (4.5-9.6%)	12% (7-18%)	3500 (2100-5100)	310000 (210000-430000)
High SDI	1995	Male	20 to 24	86% (79-91%)	14% (9.1-21%)	4.3 (3.3-5.4)	13% (9.7-17%)	20% (13-27%)	8600 (5900-11000)	750000 (550000-950000)
High SDI	1995	Male	25 to 29	89% (84-93%)	11% (7.1-16%)	4.4 (3.4-5.6)	12% (9.1-15%)	19% (12-25%)	8800 (6000-12000)	740000 (550000-940000)
High SDI	1995	Male	30 to 34	91% (86-94%)	8.9% (5.5-14%)	4.4 (3.4-5.6)	11% (8-13%)	16% (11-21%)	10000 (7200-13000)	760000 (570000-960000)
High SDI	1995	Male	35 to 39	92% (88-95%)	8.2% (5.2-12%)	4.2 (3.3-5.4)	10% (8-13%)	16% (11-20%)	13000 (9600-16000)	840000 (650000-1000000)
High SDI	1995	Male	40 to 44	92% (88-95%)	7.8% (4.9-12%)	4.3 (3.3-5.5)	11% (8.3-13%)	16% (12-20%)	17000 (13000-22000)	960000 (750000-1200000)
High SDI	1995	Male	45 to 49	92% (88-95%)	7.6% (4.7-12%)	4.8 (3.6-6.1)	11% (8.5-14%)	16% (12-20%)	23000 (18000-29000)	1100000 (860000-1400000)

Location	Year	Sex	Age	Prevalence of current drinkers	Prevalence of abstainers	Population average of standard drinks daily	Percent of total attributable deaths	Percent of total attributable DALYs	Attributable deaths	Attributable DALYs
High SDI	1995	Male	50 to 54	92% (88-95%)	8% (5.2-12%)	5 (3.8-6.4)	11% (8.3-14%)	15% (11-18%)	27000 (20000-33000)	1100000 (830000-1400000)
High SDI	1995	Male	55 to 59	91% (87-94%)	8.8% (5.8-13%)	5.4 (4.1-6.9)	11% (8-13%)	13% (10-17%)	34000 (26000-43000)	1200000 (910000-1500000)
High SDI	1995	Male	60 to 64	90% (85-93%)	10% (6.7-15%)	5.2 (3.9-6.8)	9% (6.3-12%)	11% (7.5-15%)	39000 (27000-52000)	1100000 (810000-1500000)
High SDI	1995	Male	65 to 69	88% (83-92%)	12% (7.8-17%)	4.7 (3.7-5.9)	7.4% (4.9-10%)	8.6% (5.5-12%)	42000 (26000-58000)	1000000 (670000-1400000)
High SDI	1995	Male	70 to 74	87% (81-91%)	13% (9.1-19%)	4.2 (3.1-5.4)	5.9% (3.2-8.7%)	6.6% (3.4-10%)	38000 (19000-58000)	760000 (400000-1100000)
High SDI	1995	Male	75 to 79	85% (78-90%)	15% (9.9-22%)	3.5 (2.5-4.6)	4% (1.4-6.9%)	4.3% (1.2-7.7%)	24000 (6200-42000)	380000 (120000-650000)
High SDI	1995	Male	80 to 84	84% (76-89%)	16% (11-24%)	3.5 (2.6-4.5)	4% (1.4-6.9%)	4.3% (1.2-7.6%)	26000 (6500-46000)	300000 (99000-520000)
High SDI	1995	Male	85 to 89	83% (75-88%)	17% (12-25%)	3.2 (2.3-4.3)	3.4% (0.7-6.2%)	3.5% (0.35-6.8%)	15000 (1500-29000)	130000 (26000-240000)
High SDI	1995	Male	90 to 94	83% (75-88%)	17% (12-25%)	3 (2-4.1)	2.5% (-0.41-5.5%)	2.4% (-1-6%)	4700 (-1800-11000)	29000 (-4300-64000)
High SDI	1995	Male	95 plus	82% (72-89%)	18% (11-28%)	2.7 (1.7-4)	1.7% (-1.4-5%)	1.5% (-2.2-5.3%)	790 (-1100-2700)	3600 (-2700-10000)
High SDI	2000	Female	All Ages	73% (69-76%)	27% (24-31%)	2.2 (1.9-2.5)	2.9% (1.7-4.1%)	2.5% (0.29-5%)	110000 (18000-220000)	3600000 (2100000-5100000)
High SDI	2000	Female	15 to 19	68% (58-76%)	32% (24-42%)	1.9 (1.3-2.6)	2.7% (1.7-3.9%)	8.9% (4.7-14%)	840 (450-1300)	95000 (62000-130000)
High SDI	2000	Female	20 to 24	79% (71-86%)	21% (14-29%)	2.9 (2.1-3.9)	4.1% (2.9-5.4%)	12% (7-17%)	1400 (830-1900)	160000 (120000-220000)
High SDI	2000	Female	25 to 29	83% (76-88%)	17% (12-24%)	2.8 (2.1-3.7)	3.7% (2.6-4.8%)	11% (6.6-15%)	1500 (930-2100)	170000 (120000-220000)
High SDI	2000	Female	30 to 34	84% (77-89%)	16% (11-23%)	2.7 (2-3.6)	3.5% (2.5-4.6%)	10% (7-14%)	2000 (1400-2700)	190000 (130000-250000)
High SDI	2000	Female	35 to 39	84% (78-89%)	16% (11-22%)	2.8 (2.1-3.7)	4% (2.9-5.2%)	11% (8.1-14%)	3500 (2600-4500)	250000 (190000-320000)
High SDI	2000	Female	40 to 44	83% (77-88%)	17% (12-23%)	2.9 (2.1-3.7)	4.5% (3.3-5.9%)	11% (8.6-14%)	5500 (4200-7000)	320000 (240000-410000)
High SDI	2000	Female	45 to 49	82% (75-87%)	18% (13-25%)	3 (2.2-3.9)	4.9% (3.5-6.5%)	11% (8.3-14%)	7700 (5800-9700)	370000 (270000-480000)
High SDI	2000	Female	50 to 54	81% (74-86%)	19% (14-26%)	3 (2.2-3.9)	4.9% (3.3-6.6%)	9.9% (7.2-13%)	9600 (7100-12000)	400000 (280000-530000)

Location	Year	Sex	Age	Prevalence of current drinkers	Prevalence of abstainers	Population average of standard drinks daily	Percent of total attributable deaths	Percent of total attributable DALYs	Attributable deaths	Attributable DALYs
High SDI	2000	Female	55 to 59	79% (72-85%)	21% (15-28%)	3 (2.2-3.9)	4.6% (2.9-6.4%)	8.5% (6-11%)	10000 (7000-13000)	350000 (230000-490000)
High SDI	2000	Female	60 to 64	76% (68-82%)	24% (18-32%)	2.8 (2-3.9)	3.9% (2.1-6%)	6.7% (4-9.8%)	11000 (6400-16000)	330000 (180000-500000)
High SDI	2000	Female	65 to 69	71% (62-79%)	29% (21-38%)	2.5 (1.8-3.3)	3% (1.3-4.9%)	4.8% (2.3-7.4%)	11000 (5200-18000)	280000 (120000-460000)
High SDI	2000	Female	70 to 74	68% (59-76%)	32% (24-41%)	2.2 (1.5-3.1)	2.3% (0.27-4.5%)	3.3% (0.64-6.3%)	12000 (1800-24000)	240000 (23000-490000)
High SDI	2000	Female	75 to 79	66% (56-75%)	34% (25-44%)	2.1 (1.4-2.9)	1.6% (-0.55-4.1%)	2.1% (-0.71-5.3%)	12000 (-4700-31000)	190000 (-67000-490000)
High SDI	2000	Female	80 to 84	63% (51-73%)	37% (27-49%)	1.9 (1.3-2.6)	1% (-1-3.3%)	1.2% (-1.4-4.1%)	7800 (-8900-27000)	95000 (-91000-310000)
High SDI	2000	Female	85 to 89	62% (51-72%)	38% (28-49%)	1.9 (1.3-2.8)	1% (-1.2-3.6%)	1% (-1.7-4.2%)	8700 (-14000-34000)	79000 (-92000-280000)
High SDI	2000	Female	90 to 94	62% (51-72%)	38% (28-49%)	1.9 (1.2-2.9)	0.7% (-1.7-3.6%)	0.61% (-2.4-4.1%)	3800 (-13000-24000)	27000 (-61000-130000)
High SDI	2000	Female	95 plus	63% (49-75%)	37% (25-51%)	1.9 (1-2.9)	0.35% (-2.5-3.8%)	0.13% (-3.3-4.3%)	520 (-7800-11000)	4200 (-24000-38000)
High SDI	2000	Male	All Ages	83% (81-86%)	17% (14-19%)	3.4 (2.9-3.9)	8% (6.3-9.7%)	7.5% (5.1-10%)	320000 (220000-430000)	11000000 (8800000-14000000)
High SDI	2000	Male	15 to 19	72% (64-79%)	28% (21-36%)	2.2 (1.6-3)	6.5% (4.3-8.9%)	12% (6.9-18%)	2900 (1700-4200)	270000 (180000-370000)
High SDI	2000	Male	20 to 24	86% (79-91%)	14% (9.5-21%)	4.2 (3.3-5.4)	13% (9.2-16%)	20% (13-27%)	7300 (5000-9600)	640000 (470000-810000)
High SDI	2000	Male	25 to 29	89% (85-93%)	11% (7-15%)	4.3 (3.3-5.4)	12% (8.7-15%)	20% (13-26%)	7400 (5100-9800)	630000 (470000-800000)
High SDI	2000	Male	30 to 34	91% (87-95%)	8.7% (5.4-13%)	4.2 (3.3-5.4)	11% (8-14%)	18% (12-24%)	8300 (5800-11000)	640000 (480000-810000)
High SDI	2000	Male	35 to 39	92% (88-95%)	8% (5.1-12%)	4.1 (3.1-5.3)	11% (8-13%)	17% (12-22%)	12000 (8500-15000)	770000 (590000-950000)
High SDI	2000	Male	40 to 44	92% (89-95%)	7.7% (4.9-11%)	4.2 (3.3-5.4)	11% (8.6-14%)	17% (13-21%)	17000 (13000-21000)	960000 (750000-1200000)
High SDI	2000	Male	45 to 49	92% (88-95%)	7.6% (4.9-12%)	4.6 (3.5-5.9)	12% (8.9-14%)	17% (13-21%)	24000 (19000-30000)	1200000 (900000-1400000)
High SDI	2000	Male	50 to 54	92% (89-95%)	7.8% (5.1-11%)	4.8 (3.7-6.1)	11% (8.3-13%)	15% (11-18%)	30000 (23000-37000)	1200000 (940000-1500000)
High SDI	2000	Male	55 to 59	91% (88-94%)	8.6% (5.7-12%)	4.9 (3.8-6.3)	9.9% (7.5-12%)	13% (9.7-16%)	31000 (23000-39000)	1100000 (840000-1400000)

Location	Year	Sex	Age	Prevalence of current drinkers	Prevalence of abstainers	Population average of standard drinks daily	Percent of total attributable deaths	Percent of total attributable DALYs	Attributable deaths	Attributable DALYs
High SDI	2000	Male	60 to 64	90% (86-94%)	9.7% (6.5-14%)	4.9 (3.7-6.4)	9% (6.4-12%)	11% (7.9-15%)	37000 (26000-48000)	1100000 (790000-1400000)
High SDI	2000	Male	65 to 69	89% (84-93%)	11% (7.5-16%)	4.4 (3.5-5.6)	7.3% (4.9-9.8%)	8.7% (5.8-12%)	38000 (25000-52000)	960000 (650000-1300000)
High SDI	2000	Male	70 to 74	87% (82-91%)	13% (8.8-18%)	4 (3-5.1)	5.7% (3.1-8.4%)	6.6% (3.5-9.8%)	38000 (20000-57000)	760000 (420000-1100000)
High SDI	2000	Male	75 to 79	86% (80-91%)	14% (9.4-20%)	3.5 (2.6-4.7)	4.4% (1.8-7.2%)	4.9% (1.7-8.2%)	31000 (11000-53000)	500000 (200000-820000)
High SDI	2000	Male	80 to 84	84% (76-89%)	16% (11-24%)	3 (2.2-4)	3.2% (0.79-5.7%)	3.3% (0.51-6.3%)	18000 (2700-34000)	220000 (53000-390000)
High SDI	2000	Male	85 to 89	83% (75-89%)	17% (11-25%)	3 (2.2-4)	3% (0.47-5.7%)	3% (0.076-6.2%)	14000 (390-29000)	130000 (19000-240000)
High SDI	2000	Male	90 to 94	83% (76-89%)	17% (11-24%)	2.9 (2-4)	2.3% (-0.47-5.3%)	2.2% (-1.1-5.7%)	5100 (-2400-13000)	32000 (-6600-74000)
High SDI	2000	Male	95 plus	83% (74-90%)	17% (9.9-26%)	2.6 (1.6-3.9)	1.6% (-1.4-4.9%)	1.3% (-2.3-5.2%)	870 (-1400-3400)	4100 (-3600-13000)
High SDI	2005	Female	All Ages	72% (69-75%)	28% (25-31%)	2.2 (1.9-2.5)	2.8% (1.8-3.9%)	2.5% (0.56-4.7%)	110000 (27000-200000)	3500000 (2300000-4900000)
High SDI	2005	Female	15 to 19	67% (58-75%)	33% (25-42%)	1.9 (1.3-2.6)	2.6% (1.7-3.6%)	8.8% (4.6-13%)	730 (400-1100)	88000 (58000-120000)
High SDI	2005	Female	20 to 24	79% (71-85%)	21% (15-29%)	3 (2.2-4)	4.1% (3-5.4%)	12% (7-17%)	1300 (830-1900)	170000 (120000-220000)
High SDI	2005	Female	25 to 29	82% (75-87%)	18% (13-25%)	2.8 (2.1-3.7)	3.7% (2.7-4.7%)	11% (6.6-15%)	1400 (880-1900)	160000 (120000-210000)
High SDI	2005	Female	30 to 34	83% (77-88%)	17% (12-23%)	2.7 (2-3.6)	3.4% (2.4-4.5%)	11% (6.8-14%)	1900 (1200-2500)	170000 (120000-230000)
High SDI	2005	Female	35 to 39	83% (78-88%)	17% (12-22%)	2.8 (2.1-3.7)	3.8% (2.7-4.9%)	11% (8-14%)	3100 (2300-4000)	230000 (170000-290000)
High SDI	2005	Female	40 to 44	83% (77-88%)	17% (12-23%)	2.9 (2.2-3.8)	4.3% (3.1-5.7%)	12% (8.6-15%)	5300 (4000-6700)	310000 (230000-400000)
High SDI	2005	Female	45 to 49	82% (75-87%)	18% (13-25%)	3 (2.2-3.9)	4.8% (3.5-6.4%)	11% (8.4-14%)	7900 (6100-9900)	390000 (290000-490000)
High SDI	2005	Female	50 to 54	80% (74-85%)	20% (15-26%)	2.9 (2.2-3.8)	4.8% (3.4-6.5%)	10% (7.5-13%)	9900 (7500-13000)	410000 (300000-540000)
High SDI	2005	Female	55 to 59	78% (72-84%)	22% (16-28%)	2.9 (2.1-3.8)	4.4% (2.9-6.1%)	8.4% (6.2-11%)	11000 (8300-15000)	400000 (280000-540000)
High SDI	2005	Female	60 to 64	75% (68-81%)	25% (19-32%)	2.7 (2-3.7)	3.7% (2.1-5.5%)	6.6% (4.3-9.1%)	11000 (6800-15000)	320000 (180000-460000)
High SDI	2005	Female	65 to 69	71% (62-78%)	29% (22-38%)	2.4 (1.8-3.2)	3% (1.5-4.6%)	4.9% (2.8-7.2%)	11000 (6200-16000)	270000 (130000-420000)

Location	Year	Sex	Age	Prevalence of current drinkers	Prevalence of abstainers	Population average of standard drinks daily	Percent of total attributable deaths	Percent of total attributable DALYs	Attributable deaths	Attributable DALYs
High SDI	2005	Female	70 to 74	67% (59-74%)	33% (26-41%)	2.1 (1.5-2.9)	2.2% (0.5-4%)	3.4% (1.1-5.8%)	11000 (3400-20000)	220000 (48000-410000)
High SDI	2005	Female	75 to 79	65% (55-73%)	35% (27-45%)	1.9 (1.3-2.7)	1.5% (-0.27-3.6%)	2.1% (-0.23-4.7%)	11000 (-1500-26000)	170000 (-30000-410000)
High SDI	2005	Female	80 to 84	63% (51-72%)	37% (28-49%)	1.9 (1.3-2.6)	1.2% (-0.66-3.2%)	1.4% (-0.9-4%)	11000 (-6600-32000)	130000 (-68000-370000)
High SDI	2005	Female	85 to 89	61% (50-71%)	39% (29-50%)	1.8 (1.2-2.5)	0.76% (-1.1-2.9%)	0.81% (-1.5-3.4%)	6300 (-11000-25000)	57000 (-76000-210000)
High SDI	2005	Female	90 to 94	62% (52-72%)	38% (28-48%)	1.9 (1.2-2.7)	0.67% (-1.5-3.3%)	0.61% (-2.1-3.8%)	4200 (-13000-25000)	29000 (-61000-140000)
High SDI	2005	Female	95 plus	64% (50-76%)	36% (24-50%)	1.9 (1.1-3)	0.48% (-2.2-3.7%)	0.32% (-2.9-4.2%)	1200 (-8700-13000)	6800 (-27000-48000)
High SDI	2005	Male	All Ages	83% (81-86%)	17% (14-19%)	3.4 (2.9-3.9)	7.9% (6.3-9.6%)	7.6% (5.2-10%)	330000 (230000-430000)	11000000 (8900000-13000000)
High SDI	2005	Male	15 to 19	71% (63-78%)	29% (22-37%)	2.3 (1.6-3)	6.1% (4.2-8.3%)	12% (7.1-17%)	2600 (1600-3600)	250000 (170000-330000)
High SDI	2005	Male	20 to 24	86% (80-90%)	14% (9.6-20%)	4.4 (3.4-5.6)	12% (9.1-16%)	20% (13-27%)	7100 (5000-9300)	630000 (470000-800000)
High SDI	2005	Male	25 to 29	89% (85-93%)	11% (7.1-15%)	4.4 (3.4-5.5)	12% (8.7-15%)	20% (13-26%)	6900 (4800-9000)	590000 (450000-750000)
High SDI	2005	Male	30 to 34	91% (87-95%)	8.6% (5.5-13%)	4.3 (3.3-5.5)	10% (7.8-13%)	18% (12-24%)	7600 (5300-9900)	590000 (440000-740000)
High SDI	2005	Male	35 to 39	92% (88-95%)	7.9% (5-12%)	4.1 (3.1-5.3)	10% (7.6-13%)	17% (12-22%)	10000 (7300-13000)	680000 (520000-850000)
High SDI	2005	Male	40 to 44	92% (89-95%)	7.7% (4.9-11%)	4.2 (3.3-5.4)	11% (8.3-13%)	17% (13-21%)	16000 (12000-20000)	910000 (710000-1100000)
High SDI	2005	Male	45 to 49	92% (88-95%)	7.7% (5-12%)	4.7 (3.6-6)	12% (9-14%)	17% (13-21%)	25000 (19000-30000)	1200000 (930000-1400000)
High SDI	2005	Male	50 to 54	92% (88-95%)	8% (5.3-12%)	4.8 (3.7-6.1)	11% (8.8-14%)	16% (12-19%)	32000 (25000-39000)	1300000 (1000000-1600000)
High SDI	2005	Male	55 to 59	91% (88-94%)	8.7% (5.8-12%)	4.8 (3.7-6.1)	9.9% (7.7-12%)	13% (10-16%)	36000 (28000-44000)	1300000 (1000000-1600000)
High SDI	2005	Male	60 to 64	90% (86-93%)	9.8% (6.6-14%)	4.7 (3.5-6)	8.5% (6.2-11%)	11% (7.8-14%)	34000 (25000-44000)	1000000 (770000-1300000)
High SDI	2005	Male	65 to 69	89% (84-93%)	11% (7.4-16%)	4.4 (3.4-5.5)	7.3% (5.1-9.6%)	9% (6.2-12%)	37000 (26000-49000)	940000 (660000-1200000)

Location	Year	Sex	Age	Prevalence of current drinkers	Prevalence of abstainers	Population average of standard drinks daily	Percent of total attributable deaths	Percent of total attributable DALYs	Attributable deaths	Attributable DALYs
High SDI	2005	Male	70 to 74	88% (83-91%)	12% (8.9-17%)	3.8 (2.9-5)	5.7% (3.3-8.2%)	6.7% (3.8-9.7%)	36000 (20000-52000)	730000 (430000-1000000)
High SDI	2005	Male	75 to 79	86% (80-91%)	14% (9.4-20%)	3.4 (2.5-4.5)	4.4% (1.9-7%)	4.9% (2-8%)	32000 (13000-53000)	520000 (230000-830000)
High SDI	2005	Male	80 to 84	84% (77-90%)	16% (10-23%)	3.1 (2.3-4.1)	3.5% (1.2-6%)	3.8% (1-6.7%)	25000 (7100-45000)	300000 (110000-520000)
High SDI	2005	Male	85 to 89	83% (76-88%)	17% (12-24%)	2.8 (2-3.7)	2.4% (0.26-4.9%)	2.5% (-0.15-5.3%)	11000 (-550-24000)	100000 (11000-200000)
High SDI	2005	Male	90 to 94	84% (77-89%)	16% (11-23%)	2.8 (1.9-3.8)	2.1% (-0.4-5%)	2% (-0.97-5.4%)	5600 (-2600-15000)	36000 (-6700-83000)
High SDI	2005	Male	95 plus	84% (75-91%)	16% (9.4-25%)	2.6 (1.6-3.9)	1.7% (-1.2-5%)	1.4% (-2-5.4%)	1200 (-1600-4500)	5600 (-3900-17000)
High SDI	2010	Female	All Ages	72% (68-75%)	28% (25-32%)	2.2 (1.8-2.6)	2.7% (1.8-3.8%)	2.5% (0.65-4.6%)	110000 (30000-200000)	3500000 (2300000-4900000)
High SDI	2010	Female	15 to 19	61% (53-69%)	39% (31-47%)	1.6 (1.1-2.3)	2.2% (1.5-3.1%)	7.9% (4.1-12%)	540 (290-830)	73000 (48000-100000)
High SDI	2010	Female	20 to 24	79% (71-85%)	21% (15-29%)	3 (2.2-4)	4% (2.9-5.3%)	12% (7-17%)	1200 (770-1700)	160000 (110000-210000)
High SDI	2010	Female	25 to 29	82% (75-87%)	18% (13-25%)	2.8 (2.1-3.7)	3.7% (2.7-4.8%)	11% (6.5-15%)	1400 (900-2000)	170000 (120000-220000)
High SDI	2010	Female	30 to 34	83% (76-88%)	17% (12-24%)	2.7 (2-3.6)	3.4% (2.4-4.5%)	11% (6.9-14%)	1800 (1200-2400)	170000 (120000-220000)
High SDI	2010	Female	35 to 39	83% (77-88%)	17% (12-23%)	2.8 (2-3.7)	3.6% (2.6-4.8%)	11% (7.8-15%)	2800 (2000-3700)	210000 (150000-270000)
High SDI	2010	Female	40 to 44	82% (76-87%)	18% (13-24%)	2.9 (2.1-3.8)	4.1% (2.9-5.4%)	12% (8.5-15%)	4700 (3500-5900)	280000 (210000-360000)
High SDI	2010	Female	45 to 49	82% (75-87%)	18% (13-25%)	3 (2.2-3.9)	4.6% (3.3-6.1%)	11% (8.6-14%)	7600 (5900-9500)	380000 (280000-480000)
High SDI	2010	Female	50 to 54	80% (74-85%)	20% (15-26%)	2.9 (2.1-3.8)	4.8% (3.4-6.4%)	10% (7.6-13%)	10000 (7700-13000)	430000 (310000-550000)
High SDI	2010	Female	55 to 59	78% (71-84%)	22% (16-29%)	2.8 (2.1-3.8)	4.3% (2.9-5.9%)	8.5% (6.2-11%)	11000 (8500-15000)	410000 (280000-550000)
High SDI	2010	Female	60 to 64	75% (68-81%)	25% (19-32%)	2.6 (1.9-3.6)	3.5% (2-5.2%)	6.5% (4.3-8.9%)	12000 (8000-16000)	360000 (210000-520000)
High SDI	2010	Female	65 to 69	71% (63-78%)	29% (22-37%)	2.4 (1.7-3.1)	2.8% (1.4-4.3%)	4.8% (2.9-6.9%)	11000 (6500-16000)	260000 (140000-400000)
High SDI	2010	Female	70 to 74	68% (60-74%)	32% (26-40%)	2.1 (1.5-2.9)	2.2% (0.62-3.9%)	3.5% (1.4-5.8%)	11000 (4400-19000)	220000 (63000-390000)
High SDI	2010	Female	75 to 79	65% (55-73%)	35% (27-45%)	1.9 (1.3-2.7)	1.5% (-0.11-3.4%)	2.2% (0.097-4.6%)	11000 (280-22000)	160000 (-11000-360000)
High SDI	2010	Female	80 to 84	63% (51-72%)	37% (28-49%)	1.8 (1.2-2.5)	1.1% (-0.54-3%)	1.4% (-0.69-3.8%)	11000 (-4900-28000)	130000 (-56000-340000)

Location	Year	Sex	Age	Prevalence of current drinkers	Prevalence of abstainers	Population average of standard drinks daily	Percent of total attributable deaths	Percent of total attributable DALYs	Attributable deaths	Attributable DALYs
High SDI	2010	Female	85 to 89	62%<br>(51-71%)	38%<br>(29-49%)	1.8<br>(1.2-2.6)	0.91%<br>(-0.89-3%)	1%<br>(-1.2-3.6%)	9900<br>(-11000-33000)	87000<br>(-77000-270000)
High SDI	2010	Female	90 to 94	62%<br>(51-71%)	38%<br>(29-49%)	1.7<br>(1.1-2.5)	0.5%<br>(-1.4-2.8%)	0.44%<br>(-1.9-3.2%)	3200<br>(-11000-21000)	22000<br>(-57000-120000)
High SDI	2010	Female	95 plus	64%<br>(50-76%)	36%<br>(24-50%)	1.8<br>(1-2.9)	0.47%<br>(-2-3.5%)	0.33%<br>(-2.7-4%)	1600<br>(-10000-16000)	8500<br>(-31000-58000)
High SDI	2010	Male	All Ages	83%<br>(81-85%)	17%<br>(15-19%)	3.4<br>(2.9-4)	7.7%<br>(6-9.4%)	7.3%<br>(5-9.7%)	320000<br>(220000-430000)	11000000<br>(8500000-13000000)
High SDI	2010	Male	15 to 19	68%<br>(60-76%)	32%<br>(24-40%)	2.2<br>(1.5-2.9)	5.5%<br>(3.8-7.4%)	11%<br>(6.6-17%)	2000<br>(1200-2800)	200000<br>(140000-270000)
High SDI	2010	Male	20 to 24	86%<br>(79-90%)	14%<br>(9.6-21%)	4.4<br>(3.4-5.7)	12%<br>(8.8-15%)	20%<br>(13-27%)	6200<br>(4300-8100)	570000<br>(430000-720000)
High SDI	2010	Male	25 to 29	89%<br>(84-93%)	11%<br>(7.3-16%)	4.4<br>(3.4-5.7)	11%<br>(8.4-14%)	19%<br>(13-26%)	6500<br>(4500-8500)	570000<br>(430000-720000)
High SDI	2010	Male	30 to 34	91%<br>(86-94%)	9.1%<br>(5.7-14%)	4.2<br>(3.2-5.5)	10%<br>(7.6-13%)	18%<br>(12-24%)	6900<br>(4800-9000)	550000<br>(410000-690000)
High SDI	2010	Male	35 to 39	92%<br>(88-95%)	8.2%<br>(5.2-12%)	4<br>(3-5.3)	9.8%<br>(7.3-12%)	17%<br>(12-23%)	9000<br>(6400-12000)	610000<br>(460000-770000)
High SDI	2010	Male	40 to 44	92%<br>(88-95%)	7.8%<br>(4.9-12%)	4.2<br>(3.1-5.4)	10%<br>(7.7-13%)	17%<br>(13-22%)	14000<br>(10000-17000)	780000<br>(600000-970000)
High SDI	2010	Male	45 to 49	92%<br>(88-95%)	7.9%<br>(5-12%)	4.6<br>(3.4-5.9)	11%<br>(8.6-14%)	17%<br>(13-21%)	22000<br>(17000-28000)	1100000<br>(850000-1300000)
High SDI	2010	Male	50 to 54	92%<br>(88-94%)	8.3%<br>(5.5-12%)	4.8<br>(3.6-6.1)	11%<br>(8.7-14%)	16%<br>(12-20%)	32000<br>(25000-39000)	1300000<br>(1000000-1600000)
High SDI	2010	Male	55 to 59	91%<br>(87-94%)	9.3%<br>(6.2-13%)	4.8<br>(3.6-6.1)	10%<br>(7.9-13%)	14%<br>(11-17%)	38000<br>(29000-46000)	1300000<br>(1100000-1600000)
High SDI	2010	Male	60 to 64	90%<br>(85-93%)	10%<br>(6.9-15%)	4.5<br>(3.4-5.9)	8.4%<br>(6.1-11%)	11%<br>(8-14%)	39000<br>(28000-49000)	1200000<br>(880000-1500000)
High SDI	2010	Male	65 to 69	88%<br>(84-92%)	12%<br>(7.8-16%)	4.1<br>(3.2-5.2)	6.8%<br>(4.8-9%)	8.6%<br>(6-11%)	35000<br>(24000-45000)	880000<br>(620000-1200000)
High SDI	2010	Male	70 to 74	88%<br>(83-91%)	12%<br>(8.7-17%)	3.8<br>(2.8-4.9)	5.6%<br>(3.4-8%)	6.8%<br>(4-9.7%)	34000<br>(20000-49000)	710000<br>(430000-1000000)
High SDI	2010	Male	75 to 79	86%<br>(81-91%)	14%<br>(9.1-19%)	3.3<br>(2.4-4.5)	4.3%<br>(2-6.7%)	4.9%<br>(2.2-7.8%)	31000<br>(13000-49000)	500000<br>(230000-790000)
High SDI	2010	Male	80 to 84	85%<br>(78-90%)	15%<br>(9.9-22%)	3.1<br>(2.2-4)	3.4%<br>(1.2-5.8%)	3.7%<br>(1.1-6.5%)	27000<br>(7900-47000)	320000<br>(120000-550000)

Location	Year	Sex	Age	Prevalence of current drinkers	Prevalence of abstainers	Population average of standard drinks daily	Percent of total attributable deaths	Percent of total attributable DALYs	Attributable deaths	Attributable DALYs
High SDI	2010	Male	85 to 89	84% (77-89%)	16% (11-23%)	2.9 (2.1-3.9)	2.7% (0.52-5.1%)	2.8% (0.16-5.6%)	17000 (1200-34000)	150000 (29000-280000)
High SDI	2010	Male	90 to 94	83% (76-89%)	17% (11-24%)	2.6 (1.8-3.6)	1.8% (-0.45-4.4%)	1.7% (-1-4.7%)	4900 (-2800-14000)	32000 (-8200-78000)
High SDI	2010	Male	95 plus	84% (75-91%)	16% (9.5-25%)	2.5 (1.5-3.8)	1.7% (-1-4.9%)	1.5% (-1.8-5.2%)	1600 (-1900-5700)	7400 (-4500-21000)
High SDI	2016	Female	All Ages	72% (69-75%)	28% (25-31%)	2.2 (1.6-2.8)	2.6% (1.5-4%)	2.4% (0.29-4.8%)	110000 (14000-240000)	3500000 (2000000-5400000)
High SDI	2016	Female	15 to 19	61% (52-69%)	39% (31-48%)	1.6 (1-2.4)	2.1% (1.4-3%)	7.8% (4-12%)	470 (240-740)	65000 (43000-92000)
High SDI	2016	Female	20 to 24	79% (70-85%)	21% (15-30%)	3 (2-4.3)	3.9% (2.8-5.2%)	12% (6.8-17%)	1200 (720-1700)	160000 (110000-210000)
High SDI	2016	Female	25 to 29	83% (76-88%)	17% (12-24%)	2.9 (2-4)	3.6% (2.6-4.8%)	11% (6.4-16%)	1400 (850-2000)	160000 (120000-220000)
High SDI	2016	Female	30 to 34	83% (76-88%)	17% (12-24%)	2.7 (1.8-3.9)	3.4% (2.4-4.6%)	11% (6.7-15%)	1800 (1200-2500)	170000 (120000-230000)
High SDI	2016	Female	35 to 39	83% (76-88%)	17% (12-24%)	2.7 (1.8-3.9)	3.6% (2.4-4.9%)	11% (7.6-15%)	2600 (1800-3500)	200000 (140000-260000)
High SDI	2016	Female	40 to 44	83% (76-88%)	17% (12-24%)	2.8 (1.9-4)	3.9% (2.7-5.4%)	12% (8.2-15%)	4400 (3100-5800)	260000 (180000-350000)
High SDI	2016	Female	45 to 49	82% (75-87%)	18% (13-25%)	2.9 (2-4.2)	4.4% (3-6.1%)	11% (8.3-15%)	7000 (5100-9100)	350000 (240000-470000)
High SDI	2016	Female	50 to 54	80% (74-86%)	20% (14-26%)	2.9 (2-4.1)	4.6% (3.1-6.5%)	10% (7.4-13%)	10000 (7200-13000)	430000 (290000-580000)
High SDI	2016	Female	55 to 59	78% (71-84%)	22% (16-29%)	2.8 (1.9-3.9)	4.2% (2.7-6.1%)	8.4% (5.9-11%)	12000 (8100-16000)	420000 (270000-600000)
High SDI	2016	Female	60 to 64	75% (68-82%)	25% (18-32%)	2.6 (1.8-3.8)	3.5% (1.9-5.4%)	6.5% (4.1-9.3%)	13000 (7700-18000)	380000 (200000-580000)
High SDI	2016	Female	65 to 69	72% (63-79%)	28% (21-37%)	2.4 (1.6-3.3)	2.7% (1.2-4.4%)	4.8% (2.7-7.1%)	12000 (6800-19000)	310000 (140000-500000)
High SDI	2016	Female	70 to 74	68% (59-76%)	32% (24-41%)	2.1 (1.4-3.1)	2% (0.44-3.9%)	3.4% (1.2-5.9%)	11000 (3900-20000)	220000 (48000-430000)
High SDI	2016	Female	75 to 79	67% (57-76%)	33% (24-43%)	2 (1.2-2.9)	1.5% (-0.23-3.7%)	2.2% (-0.064-5%)	11000 (-490-25000)	170000 (-25000-410000)
High SDI	2016	Female	80 to 84	65% (53-74%)	35% (26-47%)	1.8 (1.2-2.7)	1.1% (-0.71-3.2%)	1.4% (-0.89-4.1%)	10000 (-6300-30000)	120000 (-76000-360000)
High SDI	2016	Female	85 to 89	63% (52-73%)	37% (27-48%)	1.8 (1.1-2.7)	0.84% (-1.1-3.1%)	0.95% (-1.4-3.7%)	9700 (-13000-37000)	86000 (-100000-310000)
High SDI	2016	Female	90 to 94	64% (53-73%)	36% (27-47%)	1.8 (1.1-2.8)	0.64% (-1.5-3.3%)	0.61% (-2-3.8%)	5900 (-18000-35000)	40000 (-87000-200000)
High SDI	2016	Female	95 plus	64% (50-76%)	36% (24-50%)	1.7 (0.91-2.8)	0.29% (-2.1-3.4%)	0.13% (-2.8-3.8%)	780 (-12000-16000)	5900 (-36000-59000)

Location	Year	Sex	Age	Prevalence of current drinkers	Prevalence of abstainers	Population average of standard drinks daily	Percent of total attributable deaths	Percent of total attributable DALYs	Attributable deaths	Attributable DALYs
High SDI	2016	Male	All Ages	83% (80-85%)	17% (15-20%)	3.3 (2.5-4.4)	7.3% (5.4-9.4%)	6.8% (4.1-9.7%)	330000 (200000-480000)	11000000 (7800000-14000000)
High SDI	2016	Male	15 to 19	68% (59-76%)	32% (24-41%)	2.1 (1.4-3.1)	5.2% (3.5-7.3%)	11% (6.3-17%)	1700 (980-2500)	180000 (120000-240000)
High SDI	2016	Male	20 to 24	85% (78-90%)	15% (10-22%)	4.4 (3.1-6.1)	12% (8.5-15%)	20% (12-27%)	6000 (4000-8100)	550000 (410000-710000)
High SDI	2016	Male	25 to 29	89% (83-93%)	11% (7.4-17%)	4.4 (3.1-6.1)	11% (8.1-14%)	19% (12-27%)	6400 (4300-8700)	570000 (420000-730000)
High SDI	2016	Male	30 to 34	91% (85-94%)	9.4% (5.8-15%)	4.2 (2.9-5.9)	10% (7.4-13%)	18% (12-25%)	7000 (4800-9500)	560000 (410000-720000)
High SDI	2016	Male	35 to 39	91% (87-95%)	8.8% (5.4-13%)	4 (2.7-5.6)	9.7% (7.1-13%)	17% (12-23%)	8400 (5800-11000)	580000 (420000-740000)
High SDI	2016	Male	40 to 44	92% (88-95%)	8.1% (5-12%)	4 (2.8-5.6)	9.8% (7.2-13%)	17% (12-22%)	12000 (8600-16000)	720000 (530000-920000)
High SDI	2016	Male	45 to 49	92% (87-95%)	8.1% (5-13%)	4.4 (3-6.2)	11% (7.9-14%)	17% (12-22%)	20000 (14000-26000)	970000 (720000-1200000)
High SDI	2016	Male	50 to 54	91% (87-94%)	8.6% (5.6-13%)	4.6 (3.2-6.4)	11% (8.1-14%)	16% (12-20%)	30000 (22000-38000)	1300000 (940000-1600000)
High SDI	2016	Male	55 to 59	90% (86-94%)	9.6% (6.3-14%)	4.6 (3.2-6.4)	10% (7.4-13%)	14% (10-18%)	37000 (27000-48000)	1300000 (990000-1700000)
High SDI	2016	Male	60 to 64	89% (85-93%)	11% (7.1-15%)	4.5 (3.1-6.2)	8.5% (5.9-11%)	11% (7.8-15%)	41000 (28000-54000)	1200000 (870000-1700000)
High SDI	2016	Male	65 to 69	88% (83-92%)	12% (7.9-17%)	4 (2.8-5.4)	6.6% (4.3-9%)	8.4% (5.5-11%)	39000 (25000-54000)	1000000 (650000-1400000)
High SDI	2016	Male	70 to 74	87% (82-91%)	13% (8.9-18%)	3.6 (2.4-5)	5.2% (2.8-7.8%)	6.4% (3.4-9.5%)	34000 (18000-52000)	710000 (380000-1100000)
High SDI	2016	Male	75 to 79	87% (80-91%)	13% (8.7-20%)	3.3 (2.2-4.7)	4.1% (1.7-6.8%)	4.7% (1.9-8.1%)	31000 (12000-53000)	510000 (210000-860000)
High SDI	2016	Male	80 to 84	85% (77-90%)	15% (9.5-23%)	3 (2-4.2)	3.2% (0.91-5.9%)	3.5% (0.76-6.7%)	27000 (5800-51000)	330000 (92000-610000)
High SDI	2016	Male	85 to 89	83% (76-89%)	17% (11-24%)	2.7 (1.8-3.9)	2.5% (0.2-5.2%)	2.6% (-0.16-5.8%)	19000 (-960-43000)	170000 (13000-360000)
High SDI	2016	Male	90 to 94	83% (76-89%)	17% (11-24%)	2.6 (1.7-3.9)	2% (-0.5-4.9%)	1.9% (-1-5.4%)	8600 (-4500-24000)	55000 (-14000-140000)
High SDI	2016	Male	95 plus	83% (73-90%)	17% (10-27%)	2.3 (1.3-3.6)	1.3% (-1.2-4.4%)	1% (-2-4.7%)	1400 (-2500-6100)	6800 (-6500-23000)

Location	Year	Sex	Age	Prevalence of current drinkers	Prevalence of abstainers	Population average of standard drinks daily	Percent of total attributable deaths	Percent of total attributable DALYs	Attributable deaths	Attributable DALYs
High-middle SDI	1990	Female	All Ages	49% (45-54%)	51% (46-55%)	1.6 (1.3-1.9)	3.6% (2.2-5.2%)	3.7% (1.4-6.5%)	99000 (34000-170000)	3300000 (2000000-4700000)
High-middle SDI	1990	Female	15 to 19	46% (36-57%)	54% (43-64%)	1.5 (0.93-2.3)	3% (1.9-4.5%)	6.3% (3.3-10%)	680 (350-1100)	75000 (46000-110000)
High-middle SDI	1990	Female	20 to 24	54% (44-63%)	46% (37-56%)	2.1 (1.3-3.1)	4.6% (3.2-6.2%)	7.9% (4.6-12%)	1100 (630-1600)	130000 (89000-190000)
High-middle SDI	1990	Female	25 to 29	59% (49-67%)	41% (33-51%)	2.2 (1.5-3.1)	5.3% (3.9-6.8%)	8.8% (5.8-12%)	1500 (970-2100)	170000 (120000-230000)
High-middle SDI	1990	Female	30 to 34	63% (53-71%)	37% (29-47%)	2.4 (1.6-3.3)	5.9% (4.5-7.5%)	10% (7.3-14%)	2300 (1600-3100)	220000 (160000-280000)
High-middle SDI	1990	Female	35 to 39	64% (55-72%)	36% (28-45%)	2.6 (1.7-3.6)	6.7% (5-8.5%)	12% (8.6-16%)	3500 (2400-4700)	260000 (190000-340000)
High-middle SDI	1990	Female	40 to 44	65% (57-73%)	35% (27-43%)	2.7 (1.8-3.8)	7.3% (5.4-9.5%)	13% (9.2-17%)	4600 (3300-6200)	280000 (200000-370000)
High-middle SDI	1990	Female	45 to 49	64% (54-73%)	36% (27-46%)	2.8 (1.8-3.9)	7.3% (5.1-9.9%)	12% (8.5-17%)	5100 (3500-7100)	260000 (180000-350000)
High-middle SDI	1990	Female	50 to 54	65% (56-73%)	35% (27-44%)	2.5 (1.7-3.6)	7.4% (5-10%)	11% (7.5-16%)	9000 (5900-13000)	400000 (270000-540000)
High-middle SDI	1990	Female	55 to 59	60% (49-69%)	40% (31-51%)	2.4 (1.6-3.5)	6.5% (3.8-9.5%)	9.6% (5.6-14%)	9500 (5400-14000)	350000 (200000-510000)
High-middle SDI	1990	Female	60 to 64	56% (45-67%)	44% (33-55%)	2.2 (1.4-3.3)	5.1% (2.2-8.5%)	7% (3-12%)	12000 (4500-20000)	350000 (150000-600000)
High-middle SDI	1990	Female	65 to 69	53% (42-64%)	47% (36-58%)	2 (1.3-3)	4.3% (1.4-7.6%)	5.7% (1.8-10%)	11000 (2800-21000)	280000 (79000-510000)
High-middle SDI	1990	Female	70 to 74	48% (37-58%)	52% (42-63%)	1.8 (1.1-2.7)	3.3% (0.35-6.9%)	4.2% (0.45-8.7%)	8800 (-93-19000)	180000 (4800-380000)
High-middle SDI	1990	Female	75 to 79	40% (29-52%)	60% (48-71%)	1.5 (0.84-2.3)	2.4% (-0.41-6%)	2.9% (-0.6-7.3%)	11000 (-3500-28000)	160000 (-43000-420000)
High-middle SDI	1990	Female	80 to 84	36% (25-48%)	64% (52-75%)	1.4 (0.8-2.1)	2.2% (-0.4-5.4%)	2.5% (-0.57-6.4%)	9500 (-3200-25000)	110000 (-28000-280000)
High-middle SDI	1990	Female	85 to 89	35% (24-47%)	65% (53-76%)	1.4 (0.77-2.2)	2% (-0.58-5.3%)	2.3% (-0.82-6.3%)	6100 (-2900-17000)	49000 (-18000-130000)
High-middle SDI	1990	Female	90 to 94	37% (26-48%)	63% (52-74%)	1.4 (0.76-2.4)	2% (-0.8-5.7%)	2.3% (-1.1-6.7%)	2700 (-1500-8200)	15000 (-6300-43000)
High-middle SDI	1990	Female	95 plus	38% (24-54%)	62% (46-76%)	1.4 (0.65-2.6)	1.8% (-1.4-6.1%)	2% (-1.8-7.1%)	910 (-820-3200)	3400 (-2400-11000)
High-middle SDI	1990	Male	All Ages	57% (53-61%)	43% (39-47%)	3.1 (2.6-3.7)	9.3% (6.9-12%)	9.5% (6.3-13%)	290000 (190000-390000)	11000000 (8000000-14000000)
High-middle SDI	1990	Male	15 to 19	52% (41-63%)	48% (37-59%)	1.9 (1.2-2.9)	6.2% (3.9-9.1%)	9.4% (5.4-15%)	2400 (1400-3700)	210000 (130000-310000)
High-middle SDI	1990	Male	20 to 24	59% (50-67%)	41% (33-50%)	3.3 (2.2-4.7)	11% (7.8-16%)	15% (9.5-21%)	6600 (4200-9300)	540000 (370000-730000)

Location	Year	Sex	Age	Prevalence of current drinkers	Prevalence of abstainers	Population average of standard drinks daily	Percent of total attributable deaths	Percent of total attributable DALYs	Attributable deaths	Attributable DALYs
High-middle SDI	1990	Male	25 to 29	65% (58-72%)	35% (28-42%)	4.1 (2.8-5.6)	14% (10-18%)	18% (13-24%)	10000 (7200-14000)	770000 (570000-1000000)
High-middle SDI	1990	Male	30 to 34	69% (62-76%)	31% (24-38%)	4.7 (3.3-6.4)	15% (12-19%)	20% (15-26%)	15000 (11000-19000)	990000 (750000-1300000)
High-middle SDI	1990	Male	35 to 39	71% (64-78%)	29% (22-36%)	5.1 (3.4-7.1)	16% (12-21%)	21% (15-27%)	19000 (14000-25000)	1100000 (820000-1400000)
High-middle SDI	1990	Male	40 to 44	73% (66-79%)	27% (21-34%)	5.5 (3.8-7.6)	17% (12-21%)	22% (16-28%)	22000 (16000-28000)	1100000 (820000-1400000)
High-middle SDI	1990	Male	45 to 49	72% (64-78%)	28% (22-36%)	5.9 (4.1-8.3)	16% (12-21%)	20% (15-26%)	22000 (15000-29000)	980000 (710000-1300000)
High-middle SDI	1990	Male	50 to 54	76% (69-81%)	24% (19-31%)	6 (4-8.2)	16% (11-20%)	19% (13-25%)	38000 (25000-50000)	1500000 (1000000-1900000)
High-middle SDI	1990	Male	55 to 59	73% (65-80%)	27% (20-35%)	5.6 (3.9-7.7)	13% (8.4-18%)	15% (9.9-21%)	34000 (21000-47000)	1200000 (740000-1600000)
High-middle SDI	1990	Male	60 to 64	74% (65-81%)	26% (19-35%)	5.2 (3.5-7.3)	11% (6-16%)	12% (6.8-19%)	38000 (20000-57000)	1100000 (590000-1600000)
High-middle SDI	1990	Male	65 to 69	71% (62-78%)	29% (22-38%)	4.9 (3.5-6.6)	9% (4.6-14%)	10% (5.1-16%)	26000 (12000-42000)	640000 (310000-990000)
High-middle SDI	1990	Male	70 to 74	69% (60-76%)	31% (24-40%)	4.2 (2.8-5.8)	7% (2.9-11%)	8% (3.2-13%)	17000 (5900-29000)	330000 (120000-550000)
High-middle SDI	1990	Male	75 to 79	65% (55-74%)	35% (26-45%)	3.6 (2.3-5.2)	5.4% (1.4-10%)	6% (1.4-11%)	17000 (2800-33000)	260000 (53000-500000)
High-middle SDI	1990	Male	80 to 84	63% (52-73%)	37% (27-48%)	3.4 (2.2-4.7)	4.9% (1-9.3%)	5.4% (0.93-10%)	12000 (1300-24000)	140000 (22000-270000)
High-middle SDI	1990	Male	85 to 89	60% (47-70%)	40% (30-53%)	3 (1.9-4.4)	4.2% (0.7-8.2%)	4.6% (0.48-9.2%)	5800 (340-12000)	46000 (5900-93000)
High-middle SDI	1990	Male	90 to 94	58% (46-69%)	42% (31-54%)	2.7 (1.6-4.1)	3.4% (0.22-7.3%)	3.6% (-0.1-8.1%)	1700 (-87-3900)	9600 (440-21000)
High-middle SDI	1990	Male	95 plus	58% (43-72%)	42% (28-57%)	2.4 (1.2-4)	2.6% (-0.74-6.7%)	2.6% (-1.3-7.4%)	340 (-180-960)	1300 (-390-3500)
High-middle SDI	1995	Female	All Ages	49% (45-54%)	51% (46-55%)	1.5 (1.2-1.8)	3.7% (2.3-5.2%)	3.5% (1.2-6.1%)	110000 (34000-190000)	3800000 (2300000-5400000)
High-middle SDI	1995	Female	15 to 19	47% (38-57%)	53% (43-62%)	1.4 (0.89-2.2)	3.1% (1.9-4.5%)	6.2% (3.2-9.8%)	790 (430-1200)	83000 (53000-120000)

Location	Year	Sex	Age	Prevalence of current drinkers	Prevalence of abstainers	Population average of standard drinks daily	Percent of total attributable deaths	Percent of total attributable DALYs	Attributable deaths	Attributable DALYs
High-middle SDI	1995	Female	20 to 24	54% (45-63%)	46% (37-55%)	2 (1.3-3)	4.7% (3.3-6.4%)	7.8% (4.6-12%)	1400 (850-2100)	160000 (110000-220000)
High-middle SDI	1995	Female	25 to 29	57% (48-66%)	43% (34-52%)	2.1 (1.3-2.9)	5.2% (3.9-6.8%)	8.4% (5.4-12%)	1800 (1200-2600)	180000 (130000-240000)
High-middle SDI	1995	Female	30 to 34	61% (52-69%)	39% (31-48%)	2.2 (1.5-3.1)	6.1% (4.6-7.7%)	10% (7.1-13%)	3100 (2200-4100)	260000 (190000-330000)
High-middle SDI	1995	Female	35 to 39	63% (55-71%)	37% (29-45%)	2.4 (1.6-3.4)	7.3% (5.6-9.2%)	12% (9-15%)	5400 (4000-7000)	360000 (270000-450000)
High-middle SDI	1995	Female	40 to 44	64% (56-72%)	36% (28-44%)	2.5 (1.7-3.4)	7.9% (6-10%)	13% (9.6-17%)	7700 (5700-10000)	430000 (320000-540000)
High-middle SDI	1995	Female	45 to 49	65% (56-73%)	35% (27-44%)	2.5 (1.7-3.6)	7.8% (5.6-10%)	12% (8.7-16%)	8700 (6100-12000)	420000 (300000-550000)
High-middle SDI	1995	Female	50 to 54	63% (54-71%)	37% (29-46%)	2.5 (1.6-3.5)	7.2% (4.8-10%)	11% (7.3-15%)	8200 (5400-11000)	340000 (230000-470000)
High-middle SDI	1995	Female	55 to 59	61% (51-70%)	39% (30-49%)	2.2 (1.4-3.3)	6.7% (3.9-9.8%)	9.4% (5.5-14%)	13000 (7800-20000)	470000 (280000-700000)
High-middle SDI	1995	Female	60 to 64	56% (46-67%)	44% (33-54%)	2.1 (1.3-3.2)	4.9% (2-8.4%)	6.8% (2.8-12%)	11000 (3900-19000)	330000 (120000-570000)
High-middle SDI	1995	Female	65 to 69	54% (43-64%)	46% (36-57%)	1.9 (1.2-2.7)	3.7% (0.81-7%)	4.8% (1.1-9.1%)	12000 (1500-25000)	310000 (49000-610000)
High-middle SDI	1995	Female	70 to 74	49% (38-59%)	51% (41-62%)	1.7 (1-2.6)	2.9% (-0.15-6.5%)	3.7% (-0.14-8.2%)	11000 (-2700-26000)	210000 (-41000-510000)
High-middle SDI	1995	Female	75 to 79	42% (31-54%)	58% (46-69%)	1.4 (0.82-2.3)	2.1% (-0.58-5.5%)	2.6% (-0.77-6.7%)	7100 (-4200-21000)	110000 (-54000-320000)
High-middle SDI	1995	Female	80 to 84	37% (27-49%)	63% (51-73%)	1.3 (0.75-2)	1.7% (-0.77-4.7%)	2% (-1-5.6%)	7500 (-6400-24000)	87000 (-61000-270000)
High-middle SDI	1995	Female	85 to 89	37% (26-48%)	63% (52-74%)	1.3 (0.74-2.1)	1.7% (-0.87-4.9%)	1.9% (-1.1-5.7%)	5500 (-5200-19000)	46000 (-35000-150000)
High-middle SDI	1995	Female	90 to 94	38% (27-50%)	62% (50-73%)	1.4 (0.74-2.3)	1.7% (-1-5.3%)	1.9% (-1.3-6.2%)	2600 (-2600-9700)	15000 (-12000-51000)
High-middle SDI	1995	Female	95 plus	40% (26-56%)	60% (44-74%)	1.5 (0.68-2.6)	1.8% (-1.5-6.2%)	2% (-1.9-7.2%)	930 (-1000-3500)	3400 (-3100-12000)
High-middle SDI	1995	Male	All Ages	57% (53-61%)	43% (39-47%)	3 (2.5-3.6)	10% (7.6-13%)	9.9% (6.7-13%)	390000 (260000-530000)	15000000 (11000000-19000000)
High-middle SDI	1995	Male	15 to 19	53% (43-63%)	47% (37-57%)	1.9 (1.1-2.8)	6.3% (4-9.3%)	9.2% (5.3-14%)	3000 (1700-4600)	260000 (160000-370000)
High-middle SDI	1995	Male	20 to 24	60% (51-67%)	40% (33-49%)	3.2 (2.1-4.5)	12% (8.1-16%)	15% (9.5-21%)	9400 (6100-13000)	730000 (510000-990000)
High-middle SDI	1995	Male	25 to 29	63% (55-70%)	37% (30-45%)	3.9 (2.6-5.4)	14% (10-18%)	17% (12-23%)	13000 (9200-18000)	950000 (690000-1200000)

Location	Year	Sex	Age	Prevalence of current drinkers	Prevalence of abstainers	Population average of standard drinks daily	Percent of total attributable deaths	Percent of total attributable DALYs	Attributable deaths	Attributable DALYs
High-middle SDI	1995	Male	30 to 34	67% (59-74%)	33% (26-41%)	4.4 (3-6)	15% (12-19%)	19% (14-24%)	21000 (16000-27000)	1300000 (1000000-1700000)
High-middle SDI	1995	Male	35 to 39	70% (63-76%)	30% (24-37%)	4.8 (3.3-6.6)	17% (13-21%)	21% (15-26%)	32000 (23000-41000)	1800000 (1300000-2300000)
High-middle SDI	1995	Male	40 to 44	72% (65-78%)	28% (22-35%)	5.2 (3.6-7.1)	18% (13-22%)	21% (16-27%)	41000 (30000-54000)	2000000 (1500000-2600000)
High-middle SDI	1995	Male	45 to 49	74% (66-80%)	26% (20-34%)	5.7 (4-7.9)	18% (13-22%)	21% (15-27%)	44000 (31000-57000)	1900000 (1400000-2500000)
High-middle SDI	1995	Male	50 to 54	72% (65-78%)	28% (22-35%)	5.7 (3.9-7.8)	15% (11-20%)	18% (12-24%)	37000 (25000-49000)	1400000 (960000-1800000)
High-middle SDI	1995	Male	55 to 59	74% (66-80%)	26% (20-34%)	5.2 (3.6-7.1)	14% (8.8-19%)	16% (10-22%)	54000 (33000-75000)	1800000 (1100000-2500000)
High-middle SDI	1995	Male	60 to 64	71% (63-79%)	29% (21-37%)	4.9 (3.4-7)	11% (5.9-16%)	12% (6.7-18%)	42000 (22000-64000)	1200000 (630000-1800000)
High-middle SDI	1995	Male	65 to 69	73% (64-80%)	27% (20-36%)	4.4 (3.1-6)	8.6% (4-13%)	9.7% (4.4-15%)	39000 (16000-65000)	940000 (400000-1500000)
High-middle SDI	1995	Male	70 to 74	69% (60-76%)	31% (24-40%)	4 (2.8-5.6)	7% (2.7-12%)	7.9% (3-13%)	24000 (7300-42000)	470000 (150000-810000)
High-middle SDI	1995	Male	75 to 79	65% (55-73%)	35% (27-45%)	3.4 (2.2-4.9)	5.2% (1.6-9.5%)	5.8% (1.6-11%)	14000 (2300-27000)	210000 (45000-400000)
High-middle SDI	1995	Male	80 to 84	64% (52-73%)	36% (27-48%)	3.1 (2.1-4.4)	4.5% (0.9-8.6%)	4.9% (0.79-9.6%)	12000 (410-24000)	130000 (13000-270000)
High-middle SDI	1995	Male	85 to 89	59% (48-69%)	41% (31-52%)	2.8 (1.8-4.1)	3.9% (0.56-7.8%)	4.2% (0.37-8.7%)	6300 (-180-14000)	51000 (2700-110000)
High-middle SDI	1995	Male	90 to 94	58% (47-69%)	42% (31-53%)	2.6 (1.6-4)	3.3% (0.21-7%)	3.5% (-0.048-7.9%)	2000 (-230-4700)	11000 (-35-25000)
High-middle SDI	1995	Male	95 plus	58% (43-71%)	42% (29-57%)	2.3 (1.2-3.9)	2.7% (-0.57-6.7%)	2.8% (-1.1-7.4%)	400 (-190-1100)	1600 (-430-4000)
High-middle SDI	2000	Female	All Ages	48% (44-52%)	52% (48-56%)	1.5 (1.2-1.8)	3.7% (2.3-5.1%)	3.4% (1.2-5.9%)	110000 (35000-190000)	3900000 (2400000-5400000)
High-middle SDI	2000	Female	15 to 19	47% (37-57%)	53% (43-63%)	1.4 (0.87-2.2)	3.1% (2-4.5%)	6.4% (3.4-10%)	800 (450-1200)	85000 (54000-120000)
High-middle SDI	2000	Female	20 to 24	52% (43-61%)	48% (39-57%)	2 (1.3-2.9)	4.8% (3.4-6.4%)	8.1% (4.9-12%)	1600 (980-2300)	170000 (120000-230000)

Location	Year	Sex	Age	Prevalence of current drinkers	Prevalence of abstainers	Population average of standard drinks daily	Percent of total attributable deaths	Percent of total attributable DALYs	Attributable deaths	Attributable DALYs
High-middle SDI	2000	Female	25 to 29	56% (47-64%)	44% (36-53%)	2 (1.3-2.9)	5.3% (4-6.8%)	8.8% (5.8-12%)	2000 (1400-2800)	200000 (150000-270000)
High-middle SDI	2000	Female	30 to 34	57% (48-66%)	43% (34-52%)	2.1 (1.4-3)	5.7% (4.4-7.2%)	9.8% (7-13%)	2800 (2000-3700)	230000 (180000-300000)
High-middle SDI	2000	Female	35 to 39	60% (51-68%)	40% (32-49%)	2.3 (1.6-3.3)	6.9% (5.4-8.6%)	12% (9-15%)	4900 (3700-6300)	330000 (260000-420000)
High-middle SDI	2000	Female	40 to 44	62% (54-70%)	38% (30-46%)	2.5 (1.7-3.4)	7.9% (6.2-9.9%)	13% (10-17%)	7800 (5900-9900)	440000 (340000-560000)
High-middle SDI	2000	Female	45 to 49	63% (54-71%)	37% (29-46%)	2.4 (1.7-3.4)	7.8% (5.8-10%)	12% (9.1-16%)	9900 (7200-13000)	480000 (360000-620000)
High-middle SDI	2000	Female	50 to 54	61% (52-70%)	39% (30-48%)	2.3 (1.5-3.3)	7.4% (5.1-10%)	11% (7.7-15%)	10000 (7200-14000)	430000 (300000-590000)
High-middle SDI	2000	Female	55 to 59	56% (46-66%)	44% (34-54%)	2.2 (1.4-3.2)	6.3% (3.8-9.3%)	9.3% (5.6-14%)	9200 (5500-14000)	330000 (200000-480000)
High-middle SDI	2000	Female	60 to 64	55% (44-66%)	45% (34-56%)	1.9 (1.2-3)	4.9% (2.1-8.4%)	6.8% (2.8-12%)	13000 (4800-22000)	380000 (150000-660000)
High-middle SDI	2000	Female	65 to 69	51% (40-62%)	49% (38-60%)	1.8 (1.1-2.6)	3.7% (0.96-6.8%)	4.9% (1.3-9.1%)	10000 (1700-21000)	260000 (54000-510000)
High-middle SDI	2000	Female	70 to 74	46% (35-57%)	54% (43-65%)	1.5 (0.88-2.3)	2.6% (-0.21-5.9%)	3.3% (-0.28-7.5%)	11000 (-3000-28000)	220000 (-43000-540000)
High-middle SDI	2000	Female	75 to 79	39% (28-51%)	61% (49-72%)	1.3 (0.71-2)	2% (-0.65-5.3%)	2.4% (-0.82-6.5%)	9000 (-5300-27000)	140000 (-68000-410000)
High-middle SDI	2000	Female	80 to 84	37% (26-49%)	63% (51-74%)	1.2 (0.71-1.9)	1.8% (-0.6-4.7%)	2.1% (-0.79-5.7%)	6400 (-4200-20000)	74000 (-39000-220000)
High-middle SDI	2000	Female	85 to 89	37% (26-48%)	63% (52-74%)	1.2 (0.69-1.9)	1.5% (-0.9-4.6%)	1.6% (-1.2-5.3%)	5200 (-5700-19000)	44000 (-38000-150000)
High-middle SDI	2000	Female	90 to 94	38% (27-49%)	62% (51-73%)	1.3 (0.7-2.1)	1.5% (-1.1-5%)	1.7% (-1.4-5.9%)	2800 (-3300-11000)	16000 (-15000-57000)
High-middle SDI	2000	Female	95 plus	40% (26-55%)	60% (45-74%)	1.4 (0.65-2.5)	1.7% (-1.4-6.1%)	1.9% (-1.8-7.1%)	1100 (-1200-4200)	3900 (-3600-14000)
High-middle SDI	2000	Male	All Ages	57% (53-60%)	43% (40-47%)	3 (2.5-3.6)	10% (7.9-13%)	10% (6.9-13%)	410000 (280000-540000)	16000000 (12000000-19000000)
High-middle SDI	2000	Male	15 to 19	55% (45-64%)	45% (36-55%)	1.8 (1.1-2.7)	6.5% (4.1-9.4%)	9.5% (5.3-14%)	3000 (1700-4600)	260000 (160000-370000)
High-middle SDI	2000	Male	20 to 24	58% (50-65%)	42% (35-50%)	3 (2-4.3)	12% (8.1-15%)	15% (9.6-21%)	10000 (6800-14000)	790000 (560000-1100000)
High-middle SDI	2000	Male	25 to 29	61% (54-68%)	39% (32-46%)	3.7 (2.6-5.2)	14% (10-17%)	17% (12-23%)	15000 (10000-19000)	1000000 (760000-1300000)
High-middle SDI	2000	Male	30 to 34	64% (56-70%)	36% (30-44%)	4.2 (3-5.8)	14% (11-18%)	18% (13-23%)	18000 (13000-23000)	1200000 (880000-1400000)

Location	Year	Sex	Age	Prevalence of current drinkers	Prevalence of abstainers	Population average of standard drinks daily	Percent of total attributable deaths	Percent of total attributable DALYs	Attributable deaths	Attributable DALYs
High-middle SDI	2000	Male	35 to 39	68% (61-74%)	32% (26-39%)	4.7 (3.2-6.4)	16% (13-20%)	20% (15-25%)	28000 (21000-36000)	1600000 (1200000-2000000)
High-middle SDI	2000	Male	40 to 44	70% (64-76%)	30% (24-36%)	5.2 (3.6-7)	18% (14-22%)	22% (17-27%)	42000 (32000-53000)	2100000 (1600000-2600000)
High-middle SDI	2000	Male	45 to 49	72% (64-77%)	28% (23-36%)	5.6 (4-7.6)	18% (14-22%)	21% (16-26%)	51000 (38000-64000)	2200000 (1700000-2800000)
High-middle SDI	2000	Male	50 to 54	73% (66-79%)	27% (21-34%)	5.7 (4-7.8)	16% (12-21%)	20% (14-25%)	49000 (35000-64000)	1900000 (1300000-2400000)
High-middle SDI	2000	Male	55 to 59	70% (63-77%)	30% (23-37%)	5.2 (3.7-7)	13% (8.9-18%)	16% (11-21%)	36000 (24000-49000)	1200000 (790000-1600000)
High-middle SDI	2000	Male	60 to 64	72% (64-79%)	28% (21-36%)	4.7 (3.2-6.7)	11% (6.5-16%)	13% (7.3-19%)	51000 (27000-75000)	1400000 (800000-2100000)
High-middle SDI	2000	Male	65 to 69	70% (61-77%)	30% (23-39%)	4.3 (3.1-5.8)	8.9% (4.6-13%)	10% (5.2-16%)	37000 (17000-58000)	890000 (420000-1400000)
High-middle SDI	2000	Male	70 to 74	70% (61-77%)	30% (23-39%)	3.7 (2.5-5.1)	6.8% (2.6-11%)	7.6% (2.8-13%)	32000 (9100-57000)	610000 (200000-1100000)
High-middle SDI	2000	Male	75 to 79	63% (54-72%)	37% (28-46%)	3.2 (2.1-4.6)	5.4% (1.7-9.6%)	6% (1.8-11%)	19000 (3700-36000)	280000 (66000-530000)
High-middle SDI	2000	Male	80 to 84	61% (50-71%)	39% (29-50%)	2.9 (1.9-4.1)	4.6% (1.3-8.3%)	5.1% (1.3-9.3%)	10000 (1500-20000)	110000 (23000-220000)
High-middle SDI	2000	Male	85 to 89	60% (48-70%)	40% (30-52%)	2.6 (1.7-3.8)	3.7% (0.66-7.3%)	4% (0.51-8.1%)	6100 (120-13000)	50000 (4600-100000)
High-middle SDI	2000	Male	90 to 94	58% (46-69%)	42% (31-54%)	2.4 (1.5-3.7)	3.2% (0.19-6.7%)	3.4% (-0.065-7.5%)	2200 (-270-5200)	13000 (-79-28000)
High-middle SDI	2000	Male	95 plus	58% (44-71%)	42% (29-56%)	2.2 (1.1-3.7)	2.7% (-0.46-6.6%)	2.8% (-0.95-7.3%)	490 (-190-1300)	1900 (-400-4700)
High-middle SDI	2005	Female	All Ages	47% (43-51%)	53% (49-57%)	1.6 (1.3-1.9)	4% (2.7-5.4%)	3.7% (1.6-6.1%)	130000 (58000-220000)	4500000 (3000000-6100000)
High-middle SDI	2005	Female	15 to 19	46% (37-55%)	54% (45-63%)	1.5 (0.91-2.2)	3.1% (2.1-4.5%)	6.7% (3.7-10%)	760 (430-1200)	83000 (54000-120000)
High-middle SDI	2005	Female	20 to 24	50% (41-59%)	50% (41-59%)	2 (1.3-2.9)	4.8% (3.5-6.3%)	8.3% (5.3-12%)	1700 (1100-2400)	180000 (130000-250000)
High-middle SDI	2005	Female	25 to 29	54% (45-62%)	46% (38-55%)	2 (1.4-2.9)	5.5% (4.2-6.9%)	9% (6.2-12%)	2600 (1800-3400)	240000 (180000-310000)

Location	Year	Sex	Age	Prevalence of current drinkers	Prevalence of abstainers	Population average of standard drinks daily	Percent of total attributable deaths	Percent of total attributable DALYs	Attributable deaths	Attributable DALYs
High-middle SDI	2005	Female	30 to 34	56% (47-65%)	44% (35-53%)	2.2 (1.5-3.1)	6.1% (4.8-7.6%)	10% (7.5-13%)	3800 (2800-4900)	290000 (230000-370000)
High-middle SDI	2005	Female	35 to 39	57% (49-66%)	43% (34-51%)	2.4 (1.7-3.3)	6.9% (5.4-8.6%)	12% (8.9-15%)	5400 (4100-7000)	350000 (270000-440000)
High-middle SDI	2005	Female	40 to 44	60% (53-68%)	40% (32-47%)	2.6 (1.8-3.5)	8.1% (6.3-10%)	13% (10-17%)	8800 (6800-11000)	480000 (380000-610000)
High-middle SDI	2005	Female	45 to 49	62% (53-69%)	38% (31-47%)	2.5 (1.8-3.5)	8.3% (6.4-11%)	13% (9.9-17%)	12000 (9100-15000)	570000 (440000-720000)
High-middle SDI	2005	Female	50 to 54	59% (50-68%)	41% (32-50%)	2.3 (1.6-3.3)	7.9% (5.6-10%)	12% (8.4-16%)	14000 (9900-18000)	570000 (410000-750000)
High-middle SDI	2005	Female	55 to 59	56% (46-65%)	44% (35-54%)	2.2 (1.4-3.2)	6.9% (4.4-9.8%)	10% (6.4-14%)	14000 (8700-19000)	470000 (310000-670000)
High-middle SDI	2005	Female	60 to 64	51% (41-61%)	49% (39-59%)	2 (1.2-3)	5.1% (2.4-8.2%)	7.2% (3.6-12%)	9800 (4600-16000)	300000 (140000-480000)
High-middle SDI	2005	Female	65 to 69	49% (39-59%)	51% (41-61%)	1.7 (1.1-2.5)	4% (1.3-7%)	5.3% (1.8-9.3%)	14000 (3900-25000)	340000 (110000-600000)
High-middle SDI	2005	Female	70 to 74	42% (32-53%)	58% (47-68%)	1.4 (0.87-2.2)	2.9% (0.28-6%)	3.8% (0.4-7.7%)	11000 (-180-25000)	220000 (5200-480000)
High-middle SDI	2005	Female	75 to 79	36% (26-48%)	64% (52-74%)	1.2 (0.69-1.9)	2.1% (-0.3-5.2%)	2.6% (-0.41-6.4%)	12000 (-3600-32000)	190000 (-42000-480000)
High-middle SDI	2005	Female	80 to 84	35% (25-47%)	65% (53-75%)	1.2 (0.7-1.9)	2% (-0.33-5%)	2.4% (-0.46-5.9%)	10000 (-3500-28000)	120000 (-30000-310000)
High-middle SDI	2005	Female	85 to 89	36% (25-47%)	64% (53-75%)	1.2 (0.69-1.9)	1.8% (-0.41-4.8%)	2.1% (-0.57-5.7%)	6200 (-2500-18000)	50000 (-16000-140000)
High-middle SDI	2005	Female	90 to 94	37% (26-48%)	63% (52-74%)	1.3 (0.68-2.1)	1.7% (-0.8-5.1%)	1.9% (-1.1-6%)	3800 (-2600-13000)	21000 (-12000-66000)
High-middle SDI	2005	Female	95 plus	39% (26-54%)	61% (46-74%)	1.4 (0.64-2.4)	1.9% (-1.1-6.2%)	2.1% (-1.5-7.1%)	1500 (-1100-5100)	5500 (-3300-18000)
High-middle SDI	2005	Male	All Ages	56% (52-60%)	44% (40-48%)	3.2 (2.7-3.8)	11% (8.5-13%)	10% (7.5-13%)	470000 (340000-600000)	17000000 (14000000-21000000)
High-middle SDI	2005	Male	15 to 19	53% (44-62%)	47% (38-56%)	1.9 (1.2-2.8)	6.5% (4.2-9.2%)	9.9% (5.6-15%)	2800 (1600-4200)	240000 (150000-350000)
High-middle SDI	2005	Male	20 to 24	55% (48-62%)	45% (38-52%)	3.1 (2.1-4.3)	11% (8.2-15%)	15% (10-21%)	10000 (7000-14000)	790000 (570000-1000000)
High-middle SDI	2005	Male	25 to 29	59% (53-66%)	41% (34-47%)	3.9 (2.7-5.3)	13% (10-17%)	17% (12-23%)	17000 (12000-22000)	1200000 (880000-1500000)
High-middle SDI	2005	Male	30 to 34	61% (55-68%)	39% (32-45%)	4.4 (3.1-5.9)	14% (11-17%)	18% (14-23%)	22000 (17000-28000)	1400000 (1100000-1700000)

Location	Year	Sex	Age	Prevalence of current drinkers	Prevalence of abstainers	Population average of standard drinks daily	Percent of total attributable deaths	Percent of total attributable DALYs	Attributable deaths	Attributable DALYs
High-middle SDI	2005	Male	35 to 39	63% (56-70%)	37% (30-44%)	4.7 (3.2-6.4)	15% (12-18%)	19% (14-24%)	28000 (21000-34000)	1500000 (1200000-1900000)
High-middle SDI	2005	Male	40 to 44	67% (61-73%)	33% (27-39%)	5.2 (3.6-7)	17% (14-20%)	21% (16-25%)	44000 (34000-54000)	2100000 (1700000-2600000)
High-middle SDI	2005	Male	45 to 49	70% (63-76%)	30% (24-37%)	5.8 (4.2-7.9)	18% (14-22%)	22% (17-26%)	60000 (46000-74000)	2600000 (2000000-3100000)
High-middle SDI	2005	Male	50 to 54	71% (64-76%)	29% (24-36%)	5.8 (4.1-7.9)	17% (13-21%)	20% (15-25%)	65000 (48000-81000)	2400000 (1800000-3100000)
High-middle SDI	2005	Male	55 to 59	71% (64-77%)	29% (23-36%)	5.5 (3.9-7.4)	15% (10-19%)	17% (12-22%)	54000 (37000-70000)	1800000 (1200000-2300000)
High-middle SDI	2005	Male	60 to 64	68% (61-75%)	32% (25-39%)	4.9 (3.3-6.9)	11% (6.8-16%)	13% (8-18%)	37000 (21000-53000)	1100000 (620000-1500000)
High-middle SDI	2005	Male	65 to 69	70% (62-77%)	30% (23-38%)	4.4 (3.1-5.9)	9.7% (5.2-14%)	11% (5.8-16%)	48000 (23000-72000)	1100000 (570000-1700000)
High-middle SDI	2005	Male	70 to 74	66% (58-73%)	34% (27-42%)	3.7 (2.6-5.1)	7.2% (3.2-11%)	8.2% (3.6-13%)	32000 (11000-54000)	620000 (230000-1000000)
High-middle SDI	2005	Male	75 to 79	64% (54-73%)	36% (27-46%)	3.2 (2.1-4.6)	5.5% (1.6-9.9%)	6.2% (1.7-11%)	25000 (4000-49000)	380000 (80000-730000)
High-middle SDI	2005	Male	80 to 84	61% (50-71%)	39% (29-50%)	2.9 (1.9-4.2)	4.9% (1.6-8.7%)	5.5% (1.6-9.9%)	14000 (3000-27000)	160000 (40000-300000)
High-middle SDI	2005	Male	85 to 89	57% (46-67%)	43% (33-54%)	2.5 (1.6-3.7)	3.9% (1.1-7.2%)	4.3% (1-8.1%)	6000 (950-12000)	48000 (11000-92000)
High-middle SDI	2005	Male	90 to 94	58% (47-68%)	42% (32-53%)	2.4 (1.4-3.6)	3.2% (0.36-6.5%)	3.4% (0.1-7.3%)	2400 (-100-5400)	14000 (790-29000)
High-middle SDI	2005	Male	95 plus	58% (43-71%)	42% (29-57%)	2.2 (1.1-3.7)	2.8% (-0.32-6.6%)	2.9% (-0.77-7.3%)	630 (-180-1600)	2400 (-300-5800)
High-middle SDI	2010	Female	All Ages	47% (43-51%)	53% (49-57%)	1.6 (1.3-2)	3.8% (2.5-5.2%)	3.6% (1.5-6.1%)	120000 (51000-210000)	4100000 (2700000-5600000)
High-middle SDI	2010	Female	15 to 19	44% (34-53%)	56% (47-66%)	1.4 (0.84-2.1)	2.7% (1.8-4%)	6.3% (3.3-9.9%)	520 (280-820)	61000 (38000-89000)
High-middle SDI	2010	Female	20 to 24	48% (39-58%)	52% (42-61%)	1.9 (1.2-2.8)	4.5% (3.3-6%)	7.9% (5-11%)	1300 (820-1800)	160000 (110000-220000)
High-middle SDI	2010	Female	25 to 29	51% (41-60%)	49% (40-59%)	2 (1.3-2.8)	5% (3.8-6.2%)	8.1% (5.6-11%)	2100 (1400-2700)	220000 (160000-280000)

Location	Year	Sex	Age	Prevalence of current drinkers	Prevalence of abstainers	Population average of standard drinks daily	Percent of total attributable deaths	Percent of total attributable DALYs	Attributable deaths	Attributable DALYs
High-middle SDI	2010	Female	30 to 34	54% (45-63%)	46% (37-55%)	2.1 (1.4-3)	5.5% (4.4-6.9%)	9.4% (6.8-12%)	3300 (2400-4200)	280000 (210000-350000)
High-middle SDI	2010	Female	35 to 39	56% (48-65%)	44% (35-52%)	2.4 (1.6-3.4)	6.4% (5-7.9%)	11% (8.5-14%)	4800 (3600-6000)	330000 (250000-410000)
High-middle SDI	2010	Female	40 to 44	57% (49-66%)	43% (34-51%)	2.5 (1.7-3.5)	6.8% (5.3-8.6%)	12% (9.2-15%)	6200 (4700-7900)	360000 (280000-450000)
High-middle SDI	2010	Female	45 to 49	59% (50-67%)	41% (33-50%)	2.5 (1.7-3.6)	7.2% (5.5-9.3%)	12% (9.2-16%)	8800 (6600-11000)	440000 (330000-560000)
High-middle SDI	2010	Female	50 to 54	59% (50-67%)	41% (33-50%)	2.4 (1.6-3.4)	7.3% (5.3-9.7%)	11% (8.3-15%)	12000 (8500-16000)	500000 (360000-670000)
High-middle SDI	2010	Female	55 to 59	56% (45-66%)	44% (34-55%)	2.2 (1.4-3.3)	6.6% (4.3-9.3%)	9.9% (6.5-14%)	14000 (8800-19000)	490000 (320000-680000)
High-middle SDI	2010	Female	60 to 64	53% (42-64%)	47% (36-58%)	2.1 (1.3-3.2)	5.2% (2.7-8.3%)	7.5% (3.9-12%)	12000 (5700-19000)	360000 (180000-570000)
High-middle SDI	2010	Female	65 to 69	50% (39-61%)	50% (39-61%)	1.9 (1.2-2.7)	4% (1.6-7%)	5.7% (2.2-9.7%)	9400 (3300-17000)	240000 (86000-420000)
High-middle SDI	2010	Female	70 to 74	46% (35-57%)	54% (43-65%)	1.6 (0.95-2.5)	3.2% (0.39-6.6%)	4.1% (0.48-8.5%)	13000 (610-29000)	270000 (23000-580000)
High-middle SDI	2010	Female	75 to 79	39% (28-51%)	61% (49-72%)	1.3 (0.76-2.1)	2.4% (-0.15-5.6%)	3% (-0.25-7%)	11000 (-2100-28000)	170000 (-22000-430000)
High-middle SDI	2010	Female	80 to 84	36% (25-48%)	64% (52-75%)	1.2 (0.72-1.9)	2% (-0.3-5.1%)	2.4% (-0.45-6.2%)	13000 (-3400-33000)	140000 (-27000-370000)
High-middle SDI	2010	Female	85 to 89	36% (25-47%)	64% (53-75%)	1.2 (0.69-2)	1.9% (-0.4-5%)	2.2% (-0.59-5.9%)	8800 (-3300-25000)	71000 (-19000-190000)
High-middle SDI	2010	Female	90 to 94	37% (27-49%)	63% (51-73%)	1.3 (0.68-2.1)	1.7% (-0.59-4.9%)	2% (-0.83-5.8%)	3600 (-1900-11000)	20000 (-8300-60000)
High-middle SDI	2010	Female	95 plus	40% (26-54%)	60% (46-74%)	1.4 (0.64-2.4)	1.9% (-0.95-5.8%)	2.1% (-1.3-6.8%)	1700 (-1100-5700)	6300 (-3300-20000)
High-middle SDI	2010	Male	All Ages	54% (50-58%)	46% (42-50%)	3.3 (2.7-3.9)	9.9% (7.9-12%)	9.7% (7-12%)	400000 (290000-530000)	15000000 (11000000-18000000)
High-middle SDI	2010	Male	15 to 19	49% (39-58%)	51% (42-61%)	1.8 (1.1-2.7)	5.5% (3.5-8%)	9.2% (5.2-14%)	1700 (960-2600)	160000 (99000-230000)
High-middle SDI	2010	Male	20 to 24	53% (45-60%)	47% (40-55%)	3 (2-4.3)	11% (7.6-14%)	15% (9.6-20%)	7500 (5000-10000)	610000 (440000-810000)
High-middle SDI	2010	Male	25 to 29	56% (49-62%)	44% (38-51%)	3.8 (2.5-5.3)	12% (9.3-15%)	16% (12-22%)	13000 (9600-17000)	950000 (720000-1200000)
High-middle SDI	2010	Male	30 to 34	59% (52-66%)	41% (34-48%)	4.3 (3-6)	13% (10-16%)	17% (13-22%)	19000 (14000-23000)	1200000 (940000-1500000)
High-middle SDI	2010	Male	35 to 39	60% (53-67%)	40% (33-47%)	4.6 (3.1-6.5)	14% (11-17%)	18% (14-23%)	23000 (18000-29000)	1300000 (1000000-1600000)

Location	Year	Sex	Age	Prevalence of current drinkers	Prevalence of abstainers	Population average of standard drinks daily	Percent of total attributable deaths	Percent of total attributable DALYs	Attributable deaths	Attributable DALYs
High-middle SDI	2010	Male	40 to 44	62% (55-69%)	38% (31-45%)	4.9 (3.4-6.8)	14% (11-17%)	19% (15-23%)	28000 (22000-35000)	1400000 (1100000-1700000)
High-middle SDI	2010	Male	45 to 49	66% (58-72%)	34% (28-42%)	5.6 (3.9-7.7)	16% (13-19%)	20% (16-25%)	41000 (32000-51000)	1800000 (1400000-2200000)
High-middle SDI	2010	Male	50 to 54	68% (61-74%)	32% (26-39%)	5.8 (3.9-8)	16% (12-20%)	20% (15-24%)	53000 (40000-66000)	2000000 (1500000-2500000)
High-middle SDI	2010	Male	55 to 59	68% (60-75%)	32% (25-40%)	5.3 (3.7-7.3)	14% (10-18%)	17% (12-22%)	53000 (38000-69000)	1800000 (1300000-2300000)
High-middle SDI	2010	Male	60 to 64	69% (61-76%)	31% (24-39%)	5 (3.4-7.1)	12% (7.5-16%)	14% (8.8-19%)	45000 (28000-62000)	1300000 (800000-1800000)
High-middle SDI	2010	Male	65 to 69	66% (58-73%)	34% (27-42%)	4.4 (3.1-5.9)	9.3% (5.5-13%)	11% (6.4-16%)	30000 (16000-44000)	730000 (410000-1100000)
High-middle SDI	2010	Male	70 to 74	66% (58-73%)	34% (27-42%)	3.7 (2.5-5.2)	7.4% (3.4-12%)	8.5% (3.8-14%)	35000 (14000-58000)	690000 (280000-1100000)
High-middle SDI	2010	Male	75 to 79	61% (51-70%)	39% (30-49%)	3.2 (2.1-4.7)	5.7% (2.1-9.8%)	6.5% (2.3-11%)	24000 (6400-43000)	360000 (110000-650000)
High-middle SDI	2010	Male	80 to 84	62% (50-71%)	38% (29-50%)	2.9 (1.9-4.2)	4.9% (1.5-8.9%)	5.5% (1.4-10%)	18000 (3300-35000)	210000 (48000-390000)
High-middle SDI	2010	Male	85 to 89	58% (47-68%)	42% (32-53%)	2.7 (1.7-3.9)	4.2% (1.2-7.7%)	4.7% (1.2-8.7%)	8800 (1600-17000)	71000 (17000-130000)
High-middle SDI	2010	Male	90 to 94	56% (46-67%)	44% (33-54%)	2.4 (1.4-3.7)	3.3% (0.57-6.5%)	3.6% (0.36-7.3%)	2600 (110-5500)	15000 (1900-30000)
High-middle SDI	2010	Male	95 plus	58% (44-71%)	42% (29-56%)	2.3 (1.2-3.8)	2.9% (-0.13-6.7%)	3.1% (-0.56-7.4%)	730 (-150-1800)	2800 (-170-6400)
High-middle SDI	2016	Female	All Ages	46% (42-51%)	54% (49-58%)	1.7 (1.2-2.2)	3.7% (2.3-5.4%)	3.5% (1.2-6.4%)	130000 (41000-250000)	4000000 (2200000-6400000)
High-middle SDI	2016	Female	15 to 19	42% (32-52%)	58% (48-68%)	1.4 (0.75-2.2)	2.4% (1.5-3.6%)	6% (3-9.8%)	380 (180-670)	47000 (28000-73000)
High-middle SDI	2016	Female	20 to 24	47% (38-57%)	53% (43-62%)	1.9 (1.1-3.1)	4.1% (2.9-5.6%)	7.7% (4.5-11%)	870 (450-1500)	120000 (75000-170000)
High-middle SDI	2016	Female	25 to 29	51% (41-60%)	49% (40-59%)	2.1 (1.2-3.1)	4.9% (3.7-6.4%)	8.3% (5.5-12%)	1900 (970-3100)	210000 (140000-300000)
High-middle SDI	2016	Female	30 to 34	51% (41-61%)	49% (39-59%)	2.1 (1.3-3.1)	5.2% (3.9-6.8%)	8.9% (6.3-12%)	3100 (1700-5300)	270000 (170000-410000)
High-middle SDI	2016	Female	35 to 39	53% (44-62%)	47% (38-56%)	2.3 (1.4-3.5)	5.7% (4.2-7.6%)	10% (7.6-14%)	4600 (2400-7900)	320000 (200000-500000)

Location	Year	Sex	Age	Prevalence of current drinkers	Prevalence of abstainers	Population average of standard drinks daily	Percent of total attributable deaths	Percent of total attributable DALYs	Attributable deaths	Attributable DALYs
High-middle SDI	2016	Female	40 to 44	55% (46-64%)	45% (36-54%)	2.5 (1.5-3.7)	6.3% (4.5-8.4%)	12% (8.5-15%)	6000 (3300-9800)	350000 (220000-540000)
High-middle SDI	2016	Female	45 to 49	56% (46-65%)	44% (35-54%)	2.5 (1.5-3.7)	6.1% (4.3-8.5%)	11% (8-15%)	6800 (4000-11000)	350000 (220000-530000)
High-middle SDI	2016	Female	50 to 54	57% (47-66%)	43% (34-53%)	2.4 (1.5-3.7)	6.6% (4.5-9.2%)	11% (7.6-15%)	10000 (5900-16000)	440000 (270000-690000)
High-middle SDI	2016	Female	55 to 59	55% (45-66%)	45% (34-55%)	2.3 (1.4-3.6)	6.3% (3.9-9.2%)	9.8% (6.2-14%)	13000 (7300-22000)	480000 (280000-760000)
High-middle SDI	2016	Female	60 to 64	54% (42-65%)	46% (35-58%)	2.2 (1.2-3.4)	5.1% (2.5-8.4%)	7.6% (3.7-12%)	14000 (6100-24000)	420000 (200000-730000)
High-middle SDI	2016	Female	65 to 69	52% (41-63%)	48% (37-59%)	2 (1.2-3)	4.1% (1.4-7.3%)	5.8% (2.1-10%)	12000 (3700-23000)	300000 (100000-560000)
High-middle SDI	2016	Female	70 to 74	47% (36-58%)	53% (42-64%)	1.8 (0.98-2.8)	3.3% (0.44-6.9%)	4.5% (0.67-9.4%)	9800 (730-22000)	200000 (20000-450000)
High-middle SDI	2016	Female	75 to 79	42% (30-55%)	58% (45-70%)	1.5 (0.78-2.5)	2.5% (-0.32-6.3%)	3.1% (-0.51-8%)	13000 (-3300-38000)	210000 (-40000-580000)
High-middle SDI	2016	Female	80 to 84	38% (27-50%)	62% (50-73%)	1.4 (0.74-2.3)	2.3% (-0.21-5.7%)	2.8% (-0.31-7%)	12000 (-2700-33000)	140000 (-23000-370000)
High-middle SDI	2016	Female	85 to 89	37% (25-49%)	63% (51-75%)	1.3 (0.7-2.2)	1.9% (-0.5-5.4%)	2.2% (-0.72-6.5%)	11000 (-5200-34000)	91000 (-32000-260000)
High-middle SDI	2016	Female	90 to 94	38% (27-50%)	62% (50-73%)	1.4 (0.72-2.5)	2% (-0.62-5.8%)	2.2% (-0.9-6.9%)	6000 (-3100-19000)	34000 (-13000-100000)
High-middle SDI	2016	Female	95 plus	41% (27-56%)	59% (44-73%)	1.5 (0.67-2.8)	2.1% (-0.84-6.4%)	2.4% (-1.2-7.6%)	2300 (-1200-7400)	8400 (-3500-26000)
High-middle SDI	2016	Male	All Ages	52% (48-56%)	48% (44-52%)	3.1 (2.3-4.2)	9.3% (7-12%)	9.1% (6.1-12%)	380000 (220000-570000)	13000000 (8800000-19000000)
High-middle SDI	2016	Male	15 to 19	46% (36-55%)	54% (45-64%)	1.6 (0.91-2.6)	5% (3.1-7.5%)	8.6% (4.6-14%)	1300 (640-2200)	120000 (72000-190000)
High-middle SDI	2016	Male	20 to 24	51% (42-58%)	49% (42-58%)	2.8 (1.7-4.3)	9.8% (6.8-13%)	14% (8.6-20%)	5100 (2900-7900)	430000 (270000-630000)
High-middle SDI	2016	Male	25 to 29	55% (47-62%)	45% (38-53%)	3.7 (2.3-5.4)	12% (8.9-15%)	16% (11-22%)	12000 (7100-18000)	880000 (580000-1200000)
High-middle SDI	2016	Male	30 to 34	56% (47-63%)	44% (37-53%)	4 (2.6-5.9)	12% (9.3-15%)	16% (12-21%)	18000 (11000-27000)	1200000 (750000-1700000)
High-middle SDI	2016	Male	35 to 39	58% (50-65%)	42% (35-50%)	4.4 (2.7-6.5)	13% (9.5-16%)	17% (12-22%)	21000 (13000-32000)	1200000 (780000-1800000)
High-middle SDI	2016	Male	40 to 44	60% (52-67%)	40% (33-48%)	4.7 (3-6.9)	13% (10-17%)	18% (14-23%)	26000 (16000-39000)	1300000 (860000-1900000)

Location	Year	Sex	Age	Prevalence of current drinkers	Prevalence of abstainers	Population average of standard drinks daily	Percent of total attributable deaths	Percent of total attributable DALYs	Attributable deaths	Attributable DALYs
High-middle SDI	2016	Male	45 to 49	60% (52-67%)	40% (33-48%)	5 (3.2-7.3)	13% (10-17%)	18% (14-22%)	30000 (19000-45000)	1400000 (880000-1900000)
High-middle SDI	2016	Male	50 to 54	64% (56-71%)	36% (29-44%)	5.3 (3.3-7.8)	14% (11-18%)	18% (14-22%)	44000 (27000-65000)	1700000 (1100000-2500000)
High-middle SDI	2016	Male	55 to 59	65% (57-72%)	35% (28-43%)	5 (3.2-7.3)	13% (9.3-17%)	16% (12-21%)	50000 (31000-75000)	1700000 (1100000-2500000)
High-middle SDI	2016	Male	60 to 64	66% (57-73%)	34% (27-43%)	4.7 (2.9-7.1)	11% (7-15%)	13% (8.3-18%)	50000 (28000-76000)	1400000 (810000-2200000)
High-middle SDI	2016	Male	65 to 69	67% (58-74%)	33% (26-42%)	4.3 (2.8-6.3)	9.3% (5.3-13%)	11% (6.3-16%)	38000 (19000-61000)	930000 (480000-1400000)
High-middle SDI	2016	Male	70 to 74	63% (55-71%)	37% (29-45%)	3.7 (2.3-5.5)	7% (3.3-11%)	8.3% (3.9-13%)	24000 (9300-41000)	470000 (190000-780000)
High-middle SDI	2016	Male	75 to 79	63% (53-72%)	37% (28-47%)	3.2 (1.9-4.9)	5.6% (1.7-10%)	6.4% (1.8-11%)	26000 (5300-50000)	400000 (97000-750000)
High-middle SDI	2016	Male	80 to 84	61% (49-70%)	39% (30-51%)	3 (1.8-4.6)	5% (1.5-8.9%)	5.6% (1.5-10%)	18000 (3400-35000)	210000 (48000-390000)
High-middle SDI	2016	Male	85 to 89	58% (46-68%)	42% (32-54%)	2.6 (1.5-4.1)	3.9% (0.6-7.7%)	4.3% (0.44-8.8%)	11000 (360-24000)	94000 (10000-190000)
High-middle SDI	2016	Male	90 to 94	57% (46-68%)	43% (32-54%)	2.5 (1.4-4.1)	3.5% (0.42-7.1%)	3.8% (0.22-8.1%)	4200 (-26-9200)	24000 (1800-50000)
High-middle SDI	2016	Male	95 plus	57% (43-69%)	43% (31-57%)	2.2 (1.1-3.9)	2.9% (-0.15-6.6%)	3.1% (-0.57-7.4%)	930 (-190-2300)	3500 (-250-8100)
Middle SDI	1990	Female	All Ages	16% (14-19%)	84% (81-86%)	0.33 (0.26-0.4)	1.2% (0.84-1.5%)	1.8% (1.1-2.5%)	110000 (67000-160000)	3800000 (2800000-5000000)
Middle SDI	1990	Female	15 to 19	14% (9.1-20%)	86% (80-91%)	0.26 (0.14-0.45)	0.97% (0.59-1.5%)	1.4% (0.71-2.4%)	1300 (630-2200)	140000 (87000-220000)
Middle SDI	1990	Female	20 to 24	17% (11-25%)	83% (75-89%)	0.37 (0.2-0.62)	1.5% (0.99-2.2%)	1.8% (0.87-3%)	2300 (1100-3900)	280000 (180000-410000)
Middle SDI	1990	Female	25 to 29	19% (13-27%)	81% (73-87%)	0.45 (0.25-0.72)	1.8% (1.2-2.5%)	2.2% (1.1-3.5%)	2900 (1600-4600)	300000 (200000-420000)
Middle SDI	1990	Female	30 to 34	21% (13-30%)	79% (70-87%)	0.51 (0.28-0.82)	1.9% (1.3-2.6%)	2.5% (1.5-3.9%)	3500 (2100-5200)	290000 (200000-410000)
Middle SDI	1990	Female	35 to 39	22% (14-31%)	78% (69-86%)	0.52 (0.28-0.84)	1.9% (1.3-2.7%)	2.9% (1.8-4.3%)	4500 (2800-6600)	310000 (210000-430000)
Middle SDI	1990	Female	40 to 44	23% (15-32%)	77% (68-85%)	0.58 (0.31-0.93)	2.2% (1.5-3.1%)	3.5% (2.2-5%)	5200 (3300-7600)	290000 (200000-410000)
Middle SDI	1990	Female	45 to 49	22% (14-31%)	78% (69-86%)	0.62 (0.32-1)	2.4% (1.5-3.5%)	3.7% (2.3-5.5%)	6400 (4000-9600)	300000 (190000-430000)

Location	Year	Sex	Age	Prevalence of current drinkers	Prevalence of abstainers	Population average of standard drinks daily	Percent of total attributable deaths	Percent of total attributable DALYs	Attributable deaths	Attributable DALYs
Middle SDI	1990	Female	50 to 54	22% (14-31%)	78% (69-86%)	0.63 (0.33-1.1)	2.5% (1.5-3.7%)	3.8% (2.3-5.6%)	8900 (5400-13000)	350000 (210000-510000)
Middle SDI	1990	Female	55 to 59	21% (13-30%)	79% (70-87%)	0.62 (0.33-1.1)	2.5% (1.5-3.8%)	3.6% (2.1-5.5%)	11000 (6800-17000)	370000 (220000-570000)
Middle SDI	1990	Female	60 to 64	21% (13-31%)	79% (69-87%)	0.63 (0.32-1.1)	2.3% (1.2-3.7%)	3.1% (1.7-5%)	13000 (7100-20000)	350000 (190000-570000)
Middle SDI	1990	Female	65 to 69	21% (13-29%)	79% (71-87%)	0.59 (0.32-0.98)	2.1% (1.1-3.3%)	2.7% (1.5-4.3%)	14000 (7600-22000)	310000 (170000-490000)
Middle SDI	1990	Female	70 to 74	20% (13-28%)	80% (72-87%)	0.5 (0.25-0.87)	1.6% (0.7-2.8%)	2% (0.89-3.5%)	13000 (6100-22000)	240000 (110000-410000)
Middle SDI	1990	Female	75 to 79	18% (11-27%)	82% (73-89%)	0.46 (0.22-0.82)	1.2% (0.35-2.4%)	1.5% (0.45-2.9%)	9900 (3100-19000)	140000 (42000-270000)
Middle SDI	1990	Female	80 to 84	17% (11-25%)	83% (75-89%)	0.46 (0.22-0.8)	1% (0.17-2.1%)	1.2% (0.21-2.5%)	6500 (1000-14000)	69000 (11000-140000)
Middle SDI	1990	Female	85 to 89	18% (11-27%)	82% (73-89%)	0.47 (0.23-0.85)	0.84% (0.019-2%)	0.99% (0.0038-2.4%)	3700 (-31-8800)	28000 (500-65000)
Middle SDI	1990	Female	90 to 94	20% (13-29%)	80% (71-87%)	0.54 (0.25-1)	0.71% (-0.19-2%)	0.81% (-0.26-2.4%)	1300 (-440-3800)	6900 (-1900-20000)
Middle SDI	1990	Female	95 plus	23% (13-36%)	77% (64-87%)	0.64 (0.24-1.3)	0.72% (-0.44-2.5%)	0.77% (-0.6-2.9%)	350 (-270-1300)	1300 (-790-4500)
Middle SDI	1990	Male	All Ages	40% (36-45%)	60% (55-64%)	1.6 (1.4-1.9)	5.7% (4.7-6.6%)	7.7% (6.3-9.3%)	610000 (490000-740000)	22000000 (18000000-26000000)
Middle SDI	1990	Male	15 to 19	29% (20-39%)	71% (61-80%)	0.63 (0.37-0.99)	2.9% (1.9-4.3%)	3.8% (2.1-5.9%)	6200 (3500-9700)	550000 (340000-810000)
Middle SDI	1990	Male	20 to 24	45% (33-56%)	55% (44-67%)	1.6 (1-2.2)	6.8% (4.8-9.2%)	8.3% (5.4-12%)	19000 (13000-27000)	1600000 (1100000-2100000)
Middle SDI	1990	Male	25 to 29	52% (40-63%)	48% (37-60%)	2.2 (1.5-3)	8.8% (6.4-11%)	11% (7.6-15%)	24000 (17000-33000)	1800000 (1300000-2300000)
Middle SDI	1990	Male	30 to 34	55% (43-66%)	45% (34-57%)	2.7 (1.9-3.6)	9.7% (7.4-12%)	12% (9.2-16%)	28000 (20000-35000)	1800000 (1400000-2300000)
Middle SDI	1990	Male	35 to 39	58% (46-68%)	42% (32-54%)	3.1 (2.2-4.3)	10% (8.1-13%)	13% (10-17%)	34000 (25000-43000)	2000000 (1500000-2500000)
Middle SDI	1990	Male	40 to 44	58% (47-69%)	42% (31-53%)	3.5 (2.5-4.6)	11% (8.7-14%)	14% (11-17%)	35000 (27000-43000)	1800000 (1400000-2200000)
Middle SDI	1990	Male	45 to 49	58% (46-69%)	42% (31-54%)	3.7 (2.6-5)	12% (9.2-14%)	15% (11-18%)	42000 (32000-52000)	1900000 (1500000-2300000)

Location	Year	Sex	Age	Prevalence of current drinkers	Prevalence of abstainers	Population average of standard drinks daily	Percent of total attributable deaths	Percent of total attributable DALYs	Attributable deaths	Attributable DALYs
Middle SDI	1990	Male	50 to 54	57% (46-68%)	43% (32-54%)	3.8 (2.6-5.1)	12% (8.9-14%)	14% (11-17%)	55000 (42000-68000)	2200000 (1700000-2700000)
Middle SDI	1990	Male	55 to 59	56% (44-67%)	44% (33-56%)	3.7 (2.6-5.1)	11% (8.4-14%)	13% (9.9-16%)	69000 (52000-86000)	2300000 (1800000-2900000)
Middle SDI	1990	Male	60 to 64	55% (43-66%)	45% (34-57%)	3.4 (2.3-4.8)	10% (7.3-13%)	12% (8.4-15%)	77000 (56000-98000)	2200000 (1600000-2800000)
Middle SDI	1990	Male	65 to 69	52% (40-63%)	48% (37-60%)	2.9 (2-4)	8.6% (6.2-11%)	9.8% (7.1-13%)	73000 (53000-95000)	1700000 (1200000-2200000)
Middle SDI	1990	Male	70 to 74	48% (37-59%)	52% (41-63%)	2.3 (1.5-3.3)	7% (4.7-9.6%)	7.8% (5.2-11%)	68000 (46000-94000)	1300000 (860000-1700000)
Middle SDI	1990	Male	75 to 79	45% (33-57%)	55% (43-67%)	1.9 (1.2-2.9)	5.5% (3.4-8.1%)	6.1% (3.6-9.1%)	45000 (27000-66000)	650000 (400000-950000)
Middle SDI	1990	Male	80 to 84	41% (29-54%)	59% (46-71%)	1.7 (1-2.4)	4.4% (2.5-6.6%)	4.9% (2.8-7.4%)	23000 (13000-34000)	240000 (140000-360000)
Middle SDI	1990	Male	85 to 89	39% (28-51%)	61% (49-72%)	1.5 (0.88-2.3)	3.5% (1.8-5.5%)	3.8% (1.9-6.1%)	9600 (4900-15000)	74000 (39000-120000)
Middle SDI	1990	Male	90 to 94	40% (29-51%)	60% (49-71%)	1.5 (0.85-2.3)	2.8% (1.2-4.7%)	3% (1.2-5.2%)	2900 (1200-4900)	16000 (6800-26000)
Middle SDI	1990	Male	95 plus	43% (29-57%)	57% (43-71%)	1.5 (0.77-2.5)	2.6% (0.78-4.8%)	2.7% (0.62-5.2%)	620 (140-1200)	2300 (690-4300)
Middle SDI	1995	Female	All Ages	17% (14-20%)	83% (80-86%)	0.37 (0.3-0.45)	1.4% (1-1.8%)	2% (1.3-2.9%)	130000 (83000-190000)	4400000 (3200000-5700000)
Middle SDI	1995	Female	15 to 19	15% (10-22%)	85% (78-90%)	0.33 (0.17-0.56)	1.1% (0.66-1.6%)	1.7% (0.83-2.8%)	1200 (600-2000)	140000 (83000-210000)
Middle SDI	1995	Female	20 to 24	18% (12-26%)	82% (74-88%)	0.43 (0.23-0.71)	1.6% (1-2.3%)	1.9% (0.96-3.2%)	2300 (1100-3800)	280000 (170000-410000)
Middle SDI	1995	Female	25 to 29	19% (13-27%)	81% (73-87%)	0.46 (0.26-0.75)	1.7% (1.1-2.4%)	2.1% (1.1-3.3%)	3100 (1600-4900)	320000 (210000-460000)
Middle SDI	1995	Female	30 to 34	21% (14-30%)	79% (70-86%)	0.54 (0.29-0.86)	1.8% (1.3-2.5%)	2.5% (1.5-3.8%)	3700 (2300-5600)	320000 (220000-450000)
Middle SDI	1995	Female	35 to 39	22% (15-31%)	78% (69-85%)	0.59 (0.32-0.96)	2% (1.4-2.8%)	3.1% (2-4.6%)	4700 (3000-6900)	320000 (220000-450000)
Middle SDI	1995	Female	40 to 44	22% (15-31%)	78% (69-85%)	0.59 (0.32-0.95)	2.2% (1.5-3%)	3.5% (2.3-5.1%)	6700 (4400-9700)	370000 (250000-520000)
Middle SDI	1995	Female	45 to 49	22% (13-30%)	78% (70-87%)	0.65 (0.34-1.1)	2.4% (1.6-3.5%)	3.9% (2.4-5.7%)	8000 (5000-12000)	370000 (240000-530000)
Middle SDI	1995	Female	50 to 54	21% (14-30%)	79% (70-86%)	0.68 (0.36-1.2)	2.7% (1.7-3.9%)	4.1% (2.5-5.9%)	9600 (6000-14000)	380000 (240000-550000)

Location	Year	Sex	Age	Prevalence of current drinkers	Prevalence of abstainers	Population average of standard drinks daily	Percent of total attributable deaths	Percent of total attributable DALYs	Attributable deaths	Attributable DALYs
Middle SDI	1995	Female	55 to 59	21% (14-30%)	79% (70-86%)	0.7 (0.36-1.2)	2.7% (1.7-4.1%)	4% (2.5-6%)	13000 (7900-19000)	420000 (260000-630000)
Middle SDI	1995	Female	60 to 64	20% (13-30%)	80% (70-87%)	0.67 (0.35-1.2)	2.5% (1.4-3.9%)	3.4% (2-5.3%)	16000 (9100-24000)	430000 (250000-670000)
Middle SDI	1995	Female	65 to 69	21% (13-29%)	79% (71-87%)	0.64 (0.35-1.1)	2.3% (1.3-3.6%)	3.1% (1.7-4.7%)	17000 (9700-26000)	390000 (220000-600000)
Middle SDI	1995	Female	70 to 74	20% (13-28%)	80% (72-87%)	0.57 (0.3-1)	1.8% (0.84-3.2%)	2.4% (1.1-4%)	16000 (7800-27000)	290000 (140000-500000)
Middle SDI	1995	Female	75 to 79	19% (12-28%)	81% (72-88%)	0.52 (0.26-0.92)	1.4% (0.46-2.7%)	1.8% (0.59-3.3%)	13000 (4600-24000)	180000 (62000-350000)
Middle SDI	1995	Female	80 to 84	19% (12-27%)	81% (73-88%)	0.53 (0.27-0.9)	1.2% (0.29-2.4%)	1.4% (0.35-2.9%)	9600 (2300-19000)	100000 (24000-200000)
Middle SDI	1995	Female	85 to 89	19% (12-29%)	81% (71-88%)	0.56 (0.27-0.99)	1% (0.13-2.3%)	1.2% (0.14-2.8%)	5700 (580-13000)	43000 (4800-97000)
Middle SDI	1995	Female	90 to 94	21% (14-31%)	79% (69-86%)	0.63 (0.3-1.2)	0.9% (-0.12-2.4%)	1% (-0.17-2.8%)	2200 (-390-6000)	12000 (-1600-31000)
Middle SDI	1995	Female	95 plus	25% (14-38%)	75% (62-86%)	0.74 (0.29-1.5)	0.93% (-0.39-2.9%)	1% (-0.55-3.4%)	620 (-340-2100)	2300 (-980-7200)
Middle SDI	1995	Male	All Ages	41% (37-46%)	59% (54-63%)	1.9 (1.7-2.2)	6.8% (5.7-7.8%)	8.7% (7.1-10%)	740000 (610000-880000)	27000000 (22000000-31000000)
Middle SDI	1995	Male	15 to 19	30% (22-40%)	70% (60-78%)	0.73 (0.42-1.1)	3.3% (2.1-4.8%)	4.3% (2.5-6.7%)	6500 (3700-10000)	570000 (350000-840000)
Middle SDI	1995	Male	20 to 24	45% (34-56%)	55% (44-66%)	1.8 (1.2-2.5)	7.4% (5.3-10%)	9.1% (6-13%)	22000 (14000-30000)	1700000 (1200000-2400000)
Middle SDI	1995	Male	25 to 29	53% (41-63%)	47% (37-59%)	2.5 (1.7-3.4)	9.3% (6.8-12%)	12% (8.1-15%)	31000 (22000-42000)	2300000 (1600000-2900000)
Middle SDI	1995	Male	30 to 34	56% (43-66%)	44% (34-57%)	3 (2.2-4)	10% (7.9-13%)	13% (9.7-16%)	35000 (26000-45000)	2300000 (1800000-2900000)
Middle SDI	1995	Male	35 to 39	57% (47-67%)	43% (33-53%)	3.5 (2.5-4.7)	11% (8.7-14%)	14% (11-18%)	39000 (30000-49000)	2300000 (1800000-2800000)
Middle SDI	1995	Male	40 to 44	59% (48-69%)	41% (31-52%)	3.9 (2.9-5.2)	12% (9.4-14%)	15% (12-18%)	50000 (39000-61000)	2600000 (2100000-3100000)
Middle SDI	1995	Male	45 to 49	59% (46-69%)	41% (31-54%)	4.2 (3-5.7)	12% (10-15%)	16% (12-19%)	54000 (42000-66000)	2400000 (1900000-3000000)
Middle SDI	1995	Male	50 to 54	58% (46-68%)	42% (32-54%)	4.3 (3-5.7)	12% (9.7-15%)	15% (12-18%)	59000 (45000-72000)	2300000 (1800000-2800000)

Location	Year	Sex	Age	Prevalence of current drinkers	Prevalence of abstainers	Population average of standard drinks daily	Percent of total attributable deaths	Percent of total attributable DALYs	Attributable deaths	Attributable DALYs
Middle SDI	1995	Male	55 to 59	56% (44-67%)	44% (33-56%)	4.1 (2.9-5.6)	12% (9.1-14%)	14% (11-17%)	74000 (57000-91000)	2500000 (1900000-3100000)
Middle SDI	1995	Male	60 to 64	54% (42-65%)	46% (35-58%)	3.7 (2.6-5.3)	11% (8.1-14%)	12% (9.2-16%)	91000 (68000-110000)	2600000 (1900000-3200000)
Middle SDI	1995	Male	65 to 69	51% (40-62%)	49% (38-60%)	3.2 (2.2-4.3)	9.2% (6.8-12%)	11% (7.6-14%)	88000 (64000-110000)	2100000 (1500000-2600000)
Middle SDI	1995	Male	70 to 74	48% (37-59%)	52% (41-63%)	2.6 (1.7-3.7)	7.6% (5.2-10%)	8.4% (5.8-11%)	77000 (53000-100000)	1400000 (1000000-1900000)
Middle SDI	1995	Male	75 to 79	45% (33-58%)	55% (42-67%)	2.2 (1.4-3.3)	6.3% (4.1-9%)	7% (4.5-10%)	60000 (38000-85000)	860000 (560000-1200000)
Middle SDI	1995	Male	80 to 84	43% (31-55%)	57% (45-69%)	2 (1.3-2.9)	5.3% (3.2-7.6%)	5.8% (3.5-8.4%)	36000 (22000-51000)	380000 (240000-550000)
Middle SDI	1995	Male	85 to 89	40% (29-52%)	60% (48-71%)	1.7 (1-2.6)	4.1% (2.3-6.2%)	4.5% (2.5-6.9%)	15000 (8200-22000)	110000 (64000-170000)
Middle SDI	1995	Male	90 to 94	41% (30-52%)	59% (48-70%)	1.7 (0.98-2.6)	3.3% (1.6-5.3%)	3.5% (1.6-5.9%)	4300 (2000-7100)	23000 (11000-37000)
Middle SDI	1995	Male	95 plus	44% (30-57%)	56% (43-70%)	1.6 (0.85-2.7)	2.9% (0.93-5.3%)	3% (0.76-5.7%)	870 (220-1700)	3300 (1000-6000)
Middle SDI	2000	Female	All Ages	17% (15-20%)	83% (80-85%)	0.4 (0.33-0.48)	1.5% (1.1-1.9%)	2.1% (1.3-3%)	140000 (89000-210000)	4700000 (3500000-6100000)
Middle SDI	2000	Female	15 to 19	17% (12-24%)	83% (76-88%)	0.38 (0.2-0.63)	1.1% (0.71-1.7%)	1.9% (0.95-3.2%)	1400 (690-2300)	150000 (93000-230000)
Middle SDI	2000	Female	20 to 24	20% (14-28%)	80% (72-86%)	0.52 (0.28-0.85)	1.7% (1.1-2.5%)	2.2% (1.2-3.6%)	2300 (1200-3800)	270000 (170000-400000)
Middle SDI	2000	Female	25 to 29	20% (13-27%)	80% (73-87%)	0.5 (0.28-0.8)	1.7% (1.2-2.4%)	2.1% (1.2-3.3%)	3300 (1800-5200)	340000 (220000-480000)
Middle SDI	2000	Female	30 to 34	20% (13-28%)	80% (72-87%)	0.51 (0.28-0.82)	1.7% (1.2-2.3%)	2.3% (1.4-3.5%)	4000 (2500-6100)	350000 (240000-500000)
Middle SDI	2000	Female	35 to 39	21% (14-29%)	79% (71-86%)	0.58 (0.32-0.94)	1.9% (1.3-2.6%)	2.9% (1.9-4.2%)	5000 (3200-7400)	360000 (240000-500000)
Middle SDI	2000	Female	40 to 44	21% (14-29%)	79% (71-86%)	0.62 (0.34-1)	2.1% (1.4-2.9%)	3.4% (2.2-4.9%)	6500 (4200-9400)	370000 (250000-520000)
Middle SDI	2000	Female	45 to 49	20% (13-28%)	80% (72-87%)	0.62 (0.32-1)	2.2% (1.4-3.1%)	3.6% (2.2-5.2%)	8900 (5600-13000)	420000 (270000-610000)
Middle SDI	2000	Female	50 to 54	20% (13-29%)	80% (71-87%)	0.68 (0.36-1.1)	2.5% (1.5-3.6%)	3.8% (2.4-5.6%)	11000 (6700-16000)	430000 (270000-620000)
Middle SDI	2000	Female	55 to 59	20% (14-29%)	80% (71-86%)	0.72 (0.38-1.2)	2.6% (1.6-4%)	3.9% (2.4-5.8%)	13000 (7800-19000)	420000 (260000-630000)

Location	Year	Sex	Age	Prevalence of current drinkers	Prevalence of abstainers	Population average of standard drinks daily	Percent of total attributable deaths	Percent of total attributable DALYs	Attributable deaths	Attributable DALYs
Middle SDI	2000	Female	60 to 64	20% (13-29%)	80% (71-87%)	0.7 (0.37-1.2)	2.4% (1.4-3.9%)	3.4% (1.9-5.3%)	16000 (9100-25000)	440000 (250000-710000)
Middle SDI	2000	Female	65 to 69	19% (12-27%)	81% (73-88%)	0.62 (0.34-1)	2.2% (1.2-3.5%)	3% (1.7-4.6%)	19000 (11000-29000)	430000 (240000-660000)
Middle SDI	2000	Female	70 to 74	19% (13-27%)	81% (73-87%)	0.57 (0.29-1)	1.8% (0.83-3.1%)	2.4% (1.1-4%)	18000 (8500-30000)	330000 (150000-560000)
Middle SDI	2000	Female	75 to 79	18% (12-27%)	82% (73-88%)	0.53 (0.26-0.94)	1.4% (0.44-2.6%)	1.8% (0.58-3.3%)	14000 (4600-26000)	200000 (63000-370000)
Middle SDI	2000	Female	80 to 84	18% (12-26%)	82% (74-88%)	0.53 (0.27-0.91)	1.2% (0.29-2.4%)	1.5% (0.38-2.9%)	11000 (2700-22000)	120000 (27000-230000)
Middle SDI	2000	Female	85 to 89	19% (12-28%)	81% (72-88%)	0.57 (0.28-0.99)	1.1% (0.15-2.4%)	1.3% (0.17-2.8%)	7200 (790-16000)	54000 (6700-120000)
Middle SDI	2000	Female	90 to 94	21% (14-30%)	79% (70-86%)	0.65 (0.31-1.2)	1% (-0.04-2.6%)	1.2% (-0.08-3%)	3200 (-270-8200)	17000 (-780-43000)
Middle SDI	2000	Female	95 plus	24% (14-37%)	76% (63-86%)	0.77 (0.3-1.6)	1.1% (-0.3-3.1%)	1.2% (-0.44-3.6%)	990 (-370-3000)	3600 (-1000-10000)
Middle SDI	2000	Male	All Ages	43% (39-48%)	57% (52-61%)	2.1 (1.8-2.4)	7.4% (6.3-8.6%)	9.1% (7.4-11%)	820000 (660000-980000)	29000000 (25000000-34000000)
Middle SDI	2000	Male	15 to 19	34% (26-43%)	66% (57-74%)	0.87 (0.52-1.3)	3.7% (2.4-5.4%)	5% (2.9-7.6%)	7600 (4300-12000)	650000 (400000-960000)
Middle SDI	2000	Male	20 to 24	46% (36-56%)	54% (44-64%)	1.9 (1.3-2.7)	8% (5.7-11%)	9.9% (6.5-14%)	21000 (14000-30000)	1700000 (1200000-2300000)
Middle SDI	2000	Male	25 to 29	52% (41-62%)	48% (38-59%)	2.5 (1.7-3.5)	9.4% (6.9-12%)	12% (8.2-16%)	30000 (20000-40000)	2200000 (1600000-2900000)
Middle SDI	2000	Male	30 to 34	56% (43-66%)	44% (34-57%)	3.1 (2.2-4.1)	10% (7.9-13%)	13% (9.7-17%)	38000 (28000-49000)	2500000 (1900000-3200000)
Middle SDI	2000	Male	35 to 39	58% (47-68%)	42% (32-53%)	3.6 (2.6-4.8)	11% (8.7-14%)	14% (11-18%)	44000 (33000-56000)	2600000 (2000000-3300000)
Middle SDI	2000	Male	40 to 44	58% (48-68%)	42% (32-52%)	4 (2.9-5.2)	12% (9.5-15%)	15% (12-19%)	52000 (40000-64000)	2700000 (2100000-3300000)
Middle SDI	2000	Male	45 to 49	59% (47-69%)	41% (31-53%)	4.3 (3.1-5.8)	13% (10-15%)	16% (12-19%)	69000 (54000-84000)	3200000 (2500000-3800000)
Middle SDI	2000	Male	50 to 54	58% (47-68%)	42% (32-53%)	4.4 (3.1-5.8)	12% (9.7-15%)	15% (12-18%)	69000 (53000-84000)	2800000 (2200000-3400000)

Location	Year	Sex	Age	Prevalence of current drinkers	Prevalence of abstainers	Population average of standard drinks daily	Percent of total attributable deaths	Percent of total attributable DALYs	Attributable deaths	Attributable DALYs
Middle SDI	2000	Male	55 to 59	56% (45-66%)	44% (34-55%)	4.2 (2.9-5.7)	12% (9-14%)	14% (11-17%)	75000 (57000-92000)	2600000 (2000000-3200000)
Middle SDI	2000	Male	60 to 64	53% (42-65%)	47% (35-58%)	3.7 (2.6-5.3)	11% (7.9-13%)	12% (9.2-16%)	92000 (68000-120000)	2600000 (2000000-3300000)
Middle SDI	2000	Male	65 to 69	51% (39-61%)	49% (39-61%)	3.2 (2.2-4.3)	9.3% (6.8-12%)	11% (7.7-14%)	100000 (73000-130000)	2400000 (1700000-3000000)
Middle SDI	2000	Male	70 to 74	48% (37-58%)	52% (42-63%)	2.6 (1.7-3.7)	7.7% (5.3-10%)	8.6% (5.8-12%)	90000 (61000-120000)	1700000 (1200000-2300000)
Middle SDI	2000	Male	75 to 79	45% (34-57%)	55% (43-66%)	2.2 (1.5-3.3)	6.3% (4-9%)	7% (4.4-10%)	64000 (41000-92000)	930000 (600000-1300000)
Middle SDI	2000	Male	80 to 84	43% (31-55%)	57% (45-69%)	2 (1.3-2.9)	5.4% (3.3-7.8%)	5.9% (3.6-8.7%)	42000 (26000-61000)	460000 (280000-660000)
Middle SDI	2000	Male	85 to 89	41% (30-52%)	59% (48-70%)	1.8 (1.1-2.7)	4.3% (2.5-6.5%)	4.8% (2.7-7.2%)	20000 (11000-30000)	150000 (88000-230000)
Middle SDI	2000	Male	90 to 94	41% (30-52%)	59% (48-70%)	1.7 (1-2.6)	3.4% (1.7-5.6%)	3.7% (1.7-6.1%)	5600 (2600-9400)	30000 (15000-50000)
Middle SDI	2000	Male	95 plus	44% (30-57%)	56% (43-70%)	1.7 (0.88-2.8)	3% (0.99-5.6%)	3.1% (0.83-6%)	1100 (290-2200)	4300 (1400-8100)
Middle SDI	2005	Female	All Ages	18% (16-20%)	82% (80-84%)	0.43 (0.35-0.52)	1.5% (1.1-1.9%)	2% (1.3-2.9%)	150000 (93000-210000)	4800000 (3500000-6200000)
Middle SDI	2005	Female	15 to 19	18% (13-24%)	82% (76-87%)	0.38 (0.21-0.62)	1.1% (0.72-1.7%)	1.9% (1-3.2%)	1500 (770-2400)	170000 (100000-240000)
Middle SDI	2005	Female	20 to 24	22% (16-28%)	78% (72-84%)	0.57 (0.32-0.9)	1.8% (1.2-2.5%)	2.4% (1.3-3.8%)	2600 (1400-4000)	300000 (190000-430000)
Middle SDI	2005	Female	25 to 29	21% (16-28%)	79% (72-84%)	0.59 (0.36-0.91)	1.8% (1.3-2.4%)	2.4% (1.4-3.6%)	3100 (1800-4900)	320000 (210000-460000)
Middle SDI	2005	Female	30 to 34	20% (15-27%)	80% (73-85%)	0.54 (0.32-0.82)	1.6% (1.2-2.2%)	2.3% (1.5-3.4%)	4100 (2500-6100)	350000 (240000-500000)
Middle SDI	2005	Female	35 to 39	21% (16-28%)	79% (72-84%)	0.55 (0.33-0.85)	1.7% (1.2-2.3%)	2.7% (1.7-3.8%)	4900 (3000-7400)	360000 (240000-500000)
Middle SDI	2005	Female	40 to 44	22% (16-29%)	78% (71-84%)	0.62 (0.37-0.95)	1.9% (1.3-2.6%)	3.2% (2.1-4.5%)	6600 (4300-9500)	390000 (260000-540000)
Middle SDI	2005	Female	45 to 49	21% (15-27%)	79% (73-85%)	0.67 (0.39-1.1)	2% (1.3-3%)	3.5% (2.2-5.1%)	8400 (5300-13000)	410000 (270000-590000)
Middle SDI	2005	Female	50 to 54	20% (15-27%)	80% (73-85%)	0.64 (0.37-1)	2.2% (1.4-3.2%)	3.5% (2.2-5.1%)	12000 (7700-18000)	480000 (310000-700000)
Middle SDI	2005	Female	55 to 59	19% (14-25%)	81% (75-86%)	0.66 (0.38-1.1)	2.3% (1.4-3.5%)	3.5% (2.2-5.3%)	14000 (8400-20000)	460000 (280000-690000)

Location	Year	Sex	Age	Prevalence of current drinkers	Prevalence of abstainers	Population average of standard drinks daily	Percent of total attributable deaths	Percent of total attributable DALYs	Attributable deaths	Attributable DALYs
Middle SDI	2005	Female	60 to 64	18% (12-25%)	82% (75-88%)	0.65 (0.35-1.1)	2.2% (1.3-3.5%)	3.2% (1.9-4.9%)	14000 (8400-22000)	400000 (230000-620000)
Middle SDI	2005	Female	65 to 69	17% (12-24%)	83% (76-88%)	0.58 (0.32-0.94)	2% (1.2-3.2%)	2.8% (1.6-4.3%)	17000 (9900-27000)	400000 (220000-620000)
Middle SDI	2005	Female	70 to 74	16% (11-23%)	84% (77-89%)	0.52 (0.27-0.9)	1.7% (0.83-2.8%)	2.2% (1.1-3.6%)	18000 (8900-30000)	330000 (160000-550000)
Middle SDI	2005	Female	75 to 79	16% (10-23%)	84% (77-90%)	0.49 (0.24-0.87)	1.3% (0.48-2.4%)	1.7% (0.64-3%)	14000 (5300-26000)	210000 (72000-380000)
Middle SDI	2005	Female	80 to 84	15% (9.2-22%)	85% (78-91%)	0.49 (0.24-0.85)	1.2% (0.36-2.3%)	1.5% (0.46-2.8%)	12000 (3600-24000)	130000 (38000-250000)
Middle SDI	2005	Female	85 to 89	15% (9.3-22%)	85% (78-91%)	0.51 (0.25-0.9)	1.1% (0.22-2.2%)	1.3% (0.27-2.6%)	8100 (1500-17000)	62000 (12000-130000)
Middle SDI	2005	Female	90 to 94	17% (11-25%)	83% (75-89%)	0.58 (0.28-1.1)	1% (0.05-2.3%)	1.2% (0.032-2.8%)	3800 (86-9200)	20000 (1200-48000)
Middle SDI	2005	Female	95 plus	20% (12-31%)	80% (69-88%)	0.73 (0.28-1.5)	1.2% (-0.15-3%)	1.3% (-0.27-3.5%)	1400 (-280-3700)	5000 (-580-13000)
Middle SDI	2005	Male	All Ages	44% (41-48%)	56% (52-59%)	2.3 (2-2.6)	7.8% (6.6-9.1%)	9.1% (7.4-11%)	850000 (690000-1000000)	30000000 (26000000-36000000)
Middle SDI	2005	Male	15 to 19	36% (29-45%)	64% (55-71%)	0.93 (0.58-1.4)	3.8% (2.5-5.4%)	5.3% (3.2-7.9%)	8300 (4800-13000)	730000 (470000-1000000)
Middle SDI	2005	Male	20 to 24	49% (41-57%)	51% (43-59%)	2.1 (1.4-2.9)	8.4% (6.1-11%)	11% (7.1-15%)	21000 (14000-30000)	1700000 (1200000-2300000)
Middle SDI	2005	Male	25 to 29	53% (44-61%)	47% (39-56%)	2.7 (1.9-3.7)	9.7% (7.1-12%)	13% (8.8-17%)	26000 (17000-35000)	1900000 (1400000-2600000)
Middle SDI	2005	Male	30 to 34	56% (46-65%)	44% (35-54%)	3.2 (2.4-4.2)	10% (7.9-13%)	13% (9.9-17%)	35000 (26000-46000)	2400000 (1800000-3000000)
Middle SDI	2005	Male	35 to 39	58% (49-66%)	42% (34-51%)	3.7 (2.7-4.8)	11% (8.6-14%)	14% (11-18%)	46000 (35000-59000)	2800000 (2200000-3500000)
Middle SDI	2005	Male	40 to 44	58% (49-67%)	42% (33-51%)	4 (3-5.3)	12% (9.5-14%)	15% (12-19%)	58000 (45000-72000)	3100000 (2400000-3700000)
Middle SDI	2005	Male	45 to 49	58% (49-67%)	42% (33-51%)	4.3 (3.2-5.7)	12% (9.9-15%)	16% (13-19%)	66000 (52000-81000)	3100000 (2400000-3700000)
Middle SDI	2005	Male	50 to 54	59% (49-68%)	41% (32-51%)	4.5 (3.3-5.9)	12% (9.8-15%)	15% (12-18%)	89000 (70000-110000)	3600000 (2800000-4300000)

Location	Year	Sex	Age	Prevalence of current drinkers	Prevalence of abstainers	Population average of standard drinks daily	Percent of total attributable deaths	Percent of total attributable DALYs	Attributable deaths	Attributable DALYs
Middle SDI	2005	Male	55 to 59	56% (47-65%)	44% (35-53%)	4.2 (3.1-5.7)	12% (9-14%)	14% (11-17%)	86000 (67000-110000)	3000000 (2300000-3600000)
Middle SDI	2005	Male	60 to 64	53% (44-63%)	47% (37-56%)	3.8 (2.7-5.3)	10% (7.7-13%)	12% (9.1-15%)	84000 (62000-110000)	2400000 (1800000-3000000)
Middle SDI	2005	Male	65 to 69	50% (40-59%)	50% (41-60%)	3.1 (2.2-4.2)	8.8% (6.5-11%)	10% (7.5-13%)	96000 (70000-120000)	2300000 (1700000-2900000)
Middle SDI	2005	Male	70 to 74	46% (36-56%)	54% (44-64%)	2.5 (1.7-3.6)	7.3% (5-9.6%)	8.2% (5.6-11%)	91000 (62000-120000)	1700000 (1200000-2300000)
Middle SDI	2005	Male	75 to 79	43% (33-54%)	57% (46-67%)	2.1 (1.4-3.2)	5.8% (3.7-8.3%)	6.5% (4.1-9.3%)	67000 (42000-96000)	990000 (620000-1400000)
Middle SDI	2005	Male	80 to 84	40% (29-52%)	60% (48-71%)	1.9 (1.2-2.8)	5% (2.9-7.3%)	5.5% (3.2-8.1%)	45000 (26000-67000)	490000 (290000-730000)
Middle SDI	2005	Male	85 to 89	39% (28-50%)	61% (50-72%)	1.7 (1.1-2.6)	4% (2.3-6.2%)	4.4% (2.4-6.9%)	21000 (12000-33000)	170000 (94000-260000)
Middle SDI	2005	Male	90 to 94	40% (29-51%)	60% (49-71%)	1.6 (1-2.5)	3.2% (1.6-5.4%)	3.5% (1.6-6%)	6900 (3100-12000)	38000 (18000-64000)
Middle SDI	2005	Male	95 plus	42% (29-56%)	58% (44-71%)	1.6 (0.86-2.7)	2.9% (0.88-5.4%)	3% (0.72-5.9%)	1400 (320-2800)	5200 (1500-10000)
Middle SDI	2010	Female	All Ages	18% (16-20%)	82% (80-84%)	0.47 (0.38-0.57)	1.6% (1.2-2%)	2.1% (1.4-3%)	160000 (100000-220000)	5000000 (3700000-6400000)
Middle SDI	2010	Female	15 to 19	18% (13-24%)	82% (76-87%)	0.41 (0.22-0.68)	1.1% (0.73-1.7%)	2% (1-3.3%)	1200 (640-2000)	140000 (91000-220000)
Middle SDI	2010	Female	20 to 24	21% (15-28%)	79% (72-85%)	0.54 (0.3-0.87)	1.8% (1.2-2.5%)	2.3% (1.2-3.7%)	2500 (1400-3800)	310000 (200000-440000)
Middle SDI	2010	Female	25 to 29	22% (16-28%)	78% (72-84%)	0.63 (0.37-0.99)	1.9% (1.3-2.5%)	2.5% (1.4-3.8%)	2900 (1700-4500)	320000 (210000-450000)
Middle SDI	2010	Female	30 to 34	22% (16-29%)	78% (71-84%)	0.63 (0.37-0.97)	1.8% (1.2-2.4%)	2.5% (1.6-3.7%)	3100 (1900-4700)	300000 (200000-420000)
Middle SDI	2010	Female	35 to 39	22% (16-28%)	78% (72-84%)	0.59 (0.35-0.93)	1.7% (1.2-2.3%)	2.8% (1.8-4%)	4300 (2700-6400)	330000 (220000-460000)
Middle SDI	2010	Female	40 to 44	21% (16-28%)	79% (72-84%)	0.61 (0.36-0.95)	1.8% (1.2-2.4%)	3.2% (2.1-4.4%)	6300 (4100-9100)	390000 (260000-540000)
Middle SDI	2010	Female	45 to 49	21% (15-28%)	79% (72-85%)	0.68 (0.39-1.1)	2% (1.3-2.9%)	3.6% (2.3-5.2%)	9100 (5800-13000)	450000 (290000-640000)
Middle SDI	2010	Female	50 to 54	20% (15-27%)	80% (73-85%)	0.72 (0.41-1.2)	2.3% (1.4-3.3%)	3.9% (2.5-5.6%)	12000 (7800-18000)	500000 (320000-730000)
Middle SDI	2010	Female	55 to 59	19% (14-25%)	81% (75-86%)	0.67 (0.38-1.1)	2.3% (1.4-3.5%)	3.8% (2.4-5.5%)	16000 (10000-24000)	550000 (340000-820000)

Location	Year	Sex	Age	Prevalence of current drinkers	Prevalence of abstainers	Population average of standard drinks daily	Percent of total attributable deaths	Percent of total attributable DALYs	Attributable deaths	Attributable DALYs
Middle SDI	2010	Female	60 to 64	17% (12-24%)	83% (76-88%)	0.64 (0.34-1.1)	2.2% (1.3-3.4%)	3.4% (2.1-5%)	17000 (10000-25000)	470000 (280000-720000)
Middle SDI	2010	Female	65 to 69	17% (12-24%)	83% (76-88%)	0.59 (0.32-0.95)	2.1% (1.2-3.1%)	3% (1.8-4.4%)	17000 (10000-26000)	390000 (230000-600000)
Middle SDI	2010	Female	70 to 74	16% (11-23%)	84% (77-89%)	0.54 (0.27-0.95)	1.7% (0.87-2.8%)	2.3% (1.2-3.8%)	18000 (9300-29000)	340000 (170000-550000)
Middle SDI	2010	Female	75 to 79	15% (9.8-22%)	85% (78-90%)	0.5 (0.24-0.89)	1.4% (0.53-2.4%)	1.8% (0.72-3.1%)	16000 (6300-28000)	230000 (88000-410000)
Middle SDI	2010	Female	80 to 84	15% (9-22%)	85% (78-91%)	0.52 (0.25-0.9)	1.2% (0.4-2.3%)	1.5% (0.51-2.8%)	14000 (4600-27000)	150000 (49000-290000)
Middle SDI	2010	Female	85 to 89	15% (9.4-23%)	85% (77-91%)	0.56 (0.27-1)	1.1% (0.25-2.3%)	1.4% (0.3-2.8%)	9900 (2000-20000)	76000 (17000-150000)
Middle SDI	2010	Female	90 to 94	17% (11-26%)	83% (74-89%)	0.64 (0.3-1.2)	1% (0.087-2.4%)	1.2% (0.04-2.9%)	4800 (180-11000)	26000 (2300-59000)
Middle SDI	2010	Female	95 plus	21% (12-32%)	79% (68-88%)	0.78 (0.3-1.6)	1.1% (-0.15-2.9%)	1.3% (-0.31-3.4%)	1800 (-370-4700)	6500 (-710-17000)
Middle SDI	2010	Male	All Ages	44% (41-48%)	56% (52-59%)	2.5 (2.2-2.9)	8.2% (6.9-9.5%)	9.3% (7.6-11%)	910000 (730000-1100000)	32000000 (27000000-37000000)
Middle SDI	2010	Male	15 to 19	34% (27-42%)	66% (58-73%)	0.93 (0.56-1.4)	3.8% (2.5-5.4%)	5.3% (3.2-8%)	7200 (4100-11000)	640000 (400000-930000)
Middle SDI	2010	Male	20 to 24	48% (39-57%)	52% (43-61%)	2.1 (1.5-3)	8.3% (5.9-11%)	11% (7.1-15%)	22000 (14000-31000)	1800000 (1300000-2500000)
Middle SDI	2010	Male	25 to 29	52% (43-60%)	48% (40-57%)	2.8 (2-3.9)	9.7% (7.2-13%)	13% (9-17%)	26000 (17000-35000)	2000000 (1400000-2600000)
Middle SDI	2010	Male	30 to 34	55% (45-63%)	45% (37-55%)	3.4 (2.4-4.5)	10% (7.9-13%)	14% (10-18%)	30000 (22000-40000)	2100000 (1600000-2700000)
Middle SDI	2010	Male	35 to 39	57% (48-65%)	43% (35-52%)	3.9 (2.8-5.2)	11% (8.7-14%)	15% (11-18%)	42000 (31000-54000)	2600000 (2000000-3300000)
Middle SDI	2010	Male	40 to 44	57% (48-67%)	43% (33-52%)	4.3 (3.2-5.6)	12% (9.5-14%)	15% (12-19%)	59000 (46000-74000)	3200000 (2600000-3900000)
Middle SDI	2010	Male	45 to 49	58% (48-67%)	42% (33-52%)	4.6 (3.4-6.1)	12% (10-15%)	16% (13-19%)	73000 (57000-89000)	3400000 (2700000-4200000)
Middle SDI	2010	Male	50 to 54	57% (47-66%)	43% (34-53%)	4.7 (3.4-6.1)	12% (9.7-15%)	15% (12-18%)	85000 (66000-100000)	3500000 (2700000-4200000)

Location	Year	Sex	Age	Prevalence of current drinkers	Prevalence of abstainers	Population average of standard drinks daily	Percent of total attributable deaths	Percent of total attributable DALYs	Attributable deaths	Attributable DALYs
Middle SDI	2010	Male	55 to 59	56% (46-65%)	44% (35-54%)	4.6 (3.4-6.1)	12% (9.2-14%)	14% (11-17%)	110000 (83000-130000)	3700000 (2900000-4500000)
Middle SDI	2010	Male	60 to 64	53% (43-63%)	47% (37-57%)	4 (2.8-5.6)	10% (7.9-13%)	12% (9.4-15%)	98000 (75000-120000)	2900000 (2200000-3600000)
Middle SDI	2010	Male	65 to 69	49% (40-59%)	51% (41-60%)	3.3 (2.4-4.5)	8.9% (6.8-11%)	10% (7.9-13%)	92000 (70000-120000)	2200000 (1700000-2800000)
Middle SDI	2010	Male	70 to 74	46% (36-55%)	54% (45-64%)	2.7 (1.9-3.8)	7.4% (5.2-9.7%)	8.5% (5.9-11%)	93000 (65000-120000)	1800000 (1300000-2400000)
Middle SDI	2010	Male	75 to 79	43% (33-54%)	57% (46-67%)	2.3 (1.5-3.4)	6.1% (4-8.5%)	6.9% (4.4-9.7%)	78000 (50000-110000)	1200000 (760000-1600000)
Middle SDI	2010	Male	80 to 84	41% (30-53%)	59% (47-70%)	2.1 (1.3-3)	5.3% (3.3-7.6%)	5.9% (3.6-8.6%)	58000 (35000-84000)	640000 (400000-920000)
Middle SDI	2010	Male	85 to 89	39% (28-50%)	61% (50-72%)	1.9 (1.1-2.8)	4.3% (2.5-6.5%)	4.8% (2.7-7.2%)	28000 (16000-42000)	220000 (120000-330000)
Middle SDI	2010	Male	90 to 94	40% (29-51%)	60% (49-71%)	1.8 (1.1-2.8)	3.5% (1.7-5.7%)	3.8% (1.7-6.4%)	8900 (4000-15000)	49000 (24000-80000)
Middle SDI	2010	Male	95 plus	43% (29-57%)	57% (43-71%)	1.8 (0.97-3)	3.2% (1.1-5.9%)	3.3% (0.88-6.4%)	2000 (540-3900)	7800 (2600-14000)
Middle SDI	2016	Female	All Ages	19% (16-22%)	81% (78-84%)	0.52 (0.39-0.69)	1.7% (1.2-2.2%)	2.3% (1.4-3.4%)	170000 (110000-250000)	5300000 (3800000-7100000)
Middle SDI	2016	Female	15 to 19	18% (12-26%)	82% (74-88%)	0.46 (0.23-0.79)	1.1% (0.72-1.7%)	2.1% (1.1-3.6%)	880 (440-1500)	110000 (70000-170000)
Middle SDI	2016	Female	20 to 24	22% (15-30%)	78% (70-85%)	0.61 (0.32-1)	1.9% (1.3-2.6%)	2.5% (1.3-4.1%)	1700 (910-2800)	240000 (160000-360000)
Middle SDI	2016	Female	25 to 29	23% (16-31%)	77% (69-84%)	0.62 (0.34-1)	1.8% (1.3-2.5%)	2.4% (1.4-3.9%)	2500 (1400-3900)	310000 (210000-440000)
Middle SDI	2016	Female	30 to 34	24% (16-33%)	76% (67-84%)	0.67 (0.37-1.1)	1.8% (1.3-2.5%)	2.7% (1.7-4.1%)	3100 (1900-4700)	310000 (210000-440000)
Middle SDI	2016	Female	35 to 39	25% (17-34%)	75% (66-83%)	0.72 (0.38-1.2)	1.9% (1.3-2.6%)	3.3% (2.1-4.8%)	4100 (2500-6100)	320000 (210000-450000)
Middle SDI	2016	Female	40 to 44	23% (16-32%)	77% (68-84%)	0.69 (0.37-1.1)	1.9% (1.3-2.6%)	3.6% (2.3-5.1%)	6200 (4000-9200)	380000 (250000-550000)
Middle SDI	2016	Female	45 to 49	21% (13-30%)	79% (70-87%)	0.68 (0.36-1.1)	2% (1.3-2.8%)	3.8% (2.4-5.5%)	9700 (6100-14000)	480000 (310000-700000)
Middle SDI	2016	Female	50 to 54	20% (13-29%)	80% (71-87%)	0.73 (0.39-1.2)	2.3% (1.4-3.3%)	4.1% (2.6-5.8%)	14000 (8700-20000)	570000 (360000-830000)
Middle SDI	2016	Female	55 to 59	20% (13-29%)	80% (71-87%)	0.77 (0.4-1.3)	2.5% (1.6-3.7%)	4.3% (2.8-6.1%)	17000 (11000-26000)	590000 (360000-880000)

Location	Year	Sex	Age	Prevalence of current drinkers	Prevalence of abstainers	Population average of standard drinks daily	Percent of total attributable deaths	Percent of total attributable DALYs	Attributable deaths	Attributable DALYs
Middle SDI	2016	Female	60 to 64	19% (11-29%)	81% (71-89%)	0.69 (0.34-1.2)	2.3% (1.4-3.6%)	3.7% (2.3-5.5%)	21000 (13000-33000)	610000 (360000-940000)
Middle SDI	2016	Female	65 to 69	19% (12-27%)	81% (73-88%)	0.64 (0.33-1.1)	2.1% (1.2-3.3%)	3.2% (2-4.8%)	21000 (13000-32000)	490000 (280000-760000)
Middle SDI	2016	Female	70 to 74	18% (11-26%)	82% (74-89%)	0.59 (0.29-1.1)	1.8% (0.9-2.9%)	2.6% (1.4-4.1%)	18000 (9600-30000)	340000 (170000-580000)
Middle SDI	2016	Female	75 to 79	17% (10-26%)	83% (74-90%)	0.55 (0.25-1)	1.4% (0.55-2.6%)	1.9% (0.78-3.4%)	17000 (6600-30000)	240000 (91000-440000)
Middle SDI	2016	Female	80 to 84	16% (9.5-24%)	84% (76-90%)	0.57 (0.27-1)	1.3% (0.39-2.4%)	1.6% (0.51-3%)	16000 (4800-30000)	170000 (52000-320000)
Middle SDI	2016	Female	85 to 89	17% (9.9-26%)	83% (74-90%)	0.61 (0.28-1.1)	1.1% (0.22-2.3%)	1.4% (0.25-2.8%)	12000 (2200-25000)	95000 (19000-200000)
Middle SDI	2016	Female	90 to 94	19% (12-29%)	81% (71-88%)	0.71 (0.32-1.4)	1% (-0.0074-2.4%)	1.2% (-0.08-2.9%)	6300 (-320-15000)	35000 (350-81000)
Middle SDI	2016	Female	95 plus	23% (12-35%)	77% (65-88%)	0.85 (0.32-1.8)	1.1% (-0.27-3%)	1.2% (-0.46-3.5%)	2400 (-770-6600)	8800 (-1800-23000)
Middle SDI	2016	Male	All Ages	45% (41-49%)	55% (51-59%)	2.8 (2.3-3.3)	8.5% (7-10%)	9.4% (7.6-11%)	1000000 (800000-1200000)	34000000 (28000000-40000000)
Middle SDI	2016	Male	15 to 19	32% (24-42%)	68% (58-76%)	0.9 (0.51-1.5)	3.7% (2.3-5.3%)	5.2% (2.9-8.1%)	5800 (3100-9300)	520000 (320000-780000)
Middle SDI	2016	Male	20 to 24	45% (35-55%)	55% (45-65%)	2.1 (1.3-3.1)	8.2% (5.7-11%)	11% (6.8-15%)	18000 (11000-27000)	1500000 (1000000-2100000)
Middle SDI	2016	Male	25 to 29	50% (39-60%)	50% (40-61%)	2.8 (1.9-4.1)	9.4% (6.9-12%)	13% (8.6-17%)	26000 (17000-36000)	2100000 (1500000-2700000)
Middle SDI	2016	Male	30 to 34	53% (41-63%)	47% (37-59%)	3.4 (2.3-4.8)	10% (7.7-13%)	14% (10-18%)	30000 (21000-40000)	2100000 (1600000-2700000)
Middle SDI	2016	Male	35 to 39	55% (45-65%)	45% (35-55%)	3.9 (2.6-5.5)	11% (8.2-13%)	15% (11-19%)	35000 (25000-46000)	2200000 (1600000-2800000)
Middle SDI	2016	Male	40 to 44	57% (47-67%)	43% (33-53%)	4.4 (3.1-6)	11% (9.1-14%)	15% (12-19%)	51000 (39000-65000)	2800000 (2200000-3500000)
Middle SDI	2016	Male	45 to 49	58% (47-68%)	42% (32-53%)	4.9 (3.5-6.6)	12% (9.8-15%)	16% (13-19%)	76000 (59000-94000)	3700000 (2900000-4500000)
Middle SDI	2016	Male	50 to 54	58% (47-68%)	42% (32-53%)	5 (3.6-6.7)	12% (9.6-15%)	15% (12-19%)	94000 (73000-120000)	3900000 (3100000-4800000)

Location	Year	Sex	Age	Prevalence of current drinkers	Prevalence of abstainers	Population average of standard drinks daily	Percent of total attributable deaths	Percent of total attributable DALYs	Attributable deaths	Attributable DALYs
Middle SDI	2016	Male	55 to 59	57% (46-67%)	43% (33-54%)	4.8 (3.5-6.6)	12% (9.1-14%)	14% (11-17%)	100000 (80000-130000)	3700000 (2900000-4600000)
Middle SDI	2016	Male	60 to 64	55% (44-66%)	45% (34-56%)	4.5 (3.1-6.3)	11% (8.3-13%)	13% (10-16%)	130000 (100000-170000)	3900000 (3000000-4900000)
Middle SDI	2016	Male	65 to 69	52% (41-63%)	48% (37-59%)	3.7 (2.6-5.1)	9.5% (7.2-12%)	11% (8.4-14%)	120000 (92000-160000)	3000000 (2300000-3800000)
Middle SDI	2016	Male	70 to 74	48% (37-58%)	52% (42-63%)	2.9 (1.9-4.3)	7.6% (5.3-10%)	8.8% (6-12%)	97000 (67000-130000)	1900000 (1300000-2500000)
Middle SDI	2016	Male	75 to 79	44% (32-56%)	56% (44-68%)	2.5 (1.5-3.7)	6.3% (4-8.8%)	7.1% (4.5-10%)	84000 (53000-120000)	1300000 (810000-1800000)
Middle SDI	2016	Male	80 to 84	42% (30-54%)	58% (46-70%)	2.2 (1.4-3.3)	5.5% (3.4-7.8%)	6.1% (3.7-8.9%)	68000 (41000-98000)	750000 (460000-1100000)
Middle SDI	2016	Male	85 to 89	40% (29-51%)	60% (49-71%)	2 (1.2-3.1)	4.6% (2.7-6.9%)	5.1% (2.9-7.8%)	39000 (22000-59000)	310000 (180000-460000)
Middle SDI	2016	Male	90 to 94	41% (31-53%)	59% (47-69%)	1.9 (1.2-3)	3.7% (1.8-6%)	4.1% (1.8-6.7%)	13000 (5900-22000)	72000 (35000-120000)
Middle SDI	2016	Male	95 plus	44% (31-58%)	56% (42-69%)	1.9 (1-3.2)	3.3% (1.1-6.2%)	3.5% (0.87-6.7%)	2900 (720-5500)	11000 (3600-20000)
Low-middle SDI	1990	Female	All Ages	7.6% (6-9.5%)	92% (90-94%)	0.2 (0.15-0.26)	0.54% (0.38-0.73%)	0.88% (0.53-1.3%)	61000 (37000-93000)	2400000 (1700000-3300000)
Low-middle SDI	1990	Female	15 to 19	6.7% (4-10%)	93% (90-96%)	0.15 (0.074-0.28)	0.47% (0.25-0.79%)	0.49% (0.17-0.99%)	570 (180-1200)	74000 (39000-130000)
Low-middle SDI	1990	Female	20 to 24	7.8% (4.7-12%)	92% (88-95%)	0.24 (0.11-0.42)	0.91% (0.54-1.4%)	0.84% (0.35-1.6%)	1300 (530-2500)	160000 (90000-250000)
Low-middle SDI	1990	Female	25 to 29	8.5% (5.1-13%)	92% (87-95%)	0.3 (0.14-0.53)	1.2% (0.73-1.9%)	1.2% (0.57-2.3%)	2000 (890-3600)	190000 (110000-300000)
Low-middle SDI	1990	Female	30 to 34	9.5% (5.7-15%)	91% (85-94%)	0.36 (0.17-0.62)	1.4% (0.89-2.2%)	1.7% (0.91-2.9%)	2700 (1400-4500)	210000 (130000-320000)
Low-middle SDI	1990	Female	35 to 39	10% (6-15%)	90% (85-94%)	0.4 (0.19-0.69)	1.7% (1.1-2.6%)	2.2% (1.3-3.7%)	3600 (2000-5900)	230000 (140000-350000)
Low-middle SDI	1990	Female	40 to 44	11% (7-17%)	89% (83-93%)	0.42 (0.2-0.73)	1.8% (1.1-2.7%)	2.5% (1.5-4%)	4000 (2300-6400)	220000 (140000-330000)
Low-middle SDI	1990	Female	45 to 49	11% (6.7-17%)	89% (83-93%)	0.39 (0.17-0.71)	1.8% (1.1-2.8%)	2.4% (1.4-4%)	4700 (2600-7700)	220000 (130000-340000)
Low-middle SDI	1990	Female	50 to 54	12% (7.1-18%)	88% (82-93%)	0.41 (0.19-0.76)	1.8% (1.1-2.9%)	2.4% (1.4-4%)	6100 (3400-10000)	240000 (140000-390000)

Location	Year	Sex	Age	Prevalence of current drinkers	Prevalence of abstainers	Population average of standard drinks daily	Percent of total attributable deaths	Percent of total attributable DALYs	Attributable deaths	Attributable DALYs
Low-middle SDI	1990	Female	55 to 59	12% (7-19%)	88% (81-93%)	0.48 (0.22-0.91)	1.9% (1-3.1%)	2.4% (1.3-4.1%)	7600 (3900-13000)	250000 (140000-420000)
Low-middle SDI	1990	Female	60 to 64	13% (7.3-20%)	87% (80-93%)	0.51 (0.23-0.99)	1.7% (0.8-2.9%)	2.1% (0.97-3.7%)	7800 (3700-14000)	220000 (110000-380000)
Low-middle SDI	1990	Female	65 to 69	13% (7.6-20%)	87% (80-92%)	0.48 (0.22-0.87)	1.4% (0.66-2.5%)	1.7% (0.78-3.1%)	7500 (3400-13000)	170000 (81000-310000)
Low-middle SDI	1990	Female	70 to 74	13% (7.5-19%)	87% (81-93%)	0.41 (0.18-0.78)	1.1% (0.4-2.2%)	1.3% (0.46-2.7%)	6100 (2200-12000)	110000 (42000-220000)
Low-middle SDI	1990	Female	75 to 79	12% (6.5-20%)	88% (80-93%)	0.35 (0.14-0.72)	0.79% (0.17-1.8%)	0.92% (0.18-2.1%)	3700 (830-8300)	53000 (13000-120000)
Low-middle SDI	1990	Female	80 to 84	11% (5.9-20%)	89% (80-94%)	0.33 (0.13-0.72)	0.61% (0.088-1.5%)	0.71% (0.086-1.7%)	2200 (330-5300)	23000 (4000-55000)
Low-middle SDI	1990	Female	85 to 89	12% (6.5-22%)	88% (78-93%)	0.36 (0.15-0.76)	0.5% (-0.01-1.3%)	0.56% (-0.042-1.5%)	990 (-41-2600)	7600 (71-20000)
Low-middle SDI	1990	Female	90 to 94	15% (7.8-26%)	85% (74-92%)	0.42 (0.16-0.9)	0.41% (-0.15-1.3%)	0.45% (-0.22-1.5%)	290 (-130-950)	1600 (-520-5000)
Low-middle SDI	1990	Female	95 plus	18% (8.5-31%)	82% (69-91%)	0.5 (0.17-1.1)	0.41% (-0.3-1.6%)	0.41% (-0.42-1.8%)	67 (-64-280)	260 (-180-980)
Low-middle SDI	1990	Male	All Ages	17% (15-20%)	83% (80-85%)	0.76 (0.6-0.96)	2.2% (1.7-2.7%)	3.1% (2.2-4.1%)	240000 (170000-320000)	10000000 (7900000-13000000)
Low-middle SDI	1990	Male	15 to 19	10% (6.9-15%)	90% (85-93%)	0.36 (0.19-0.61)	1.5% (0.93-2.2%)	1.6% (0.84-2.6%)	2100 (1100-3500)	220000 (140000-340000)
Low-middle SDI	1990	Male	20 to 24	18% (12-24%)	82% (76-88%)	0.85 (0.47-1.4)	4% (2.7-5.7%)	4.2% (2.5-6.5%)	7500 (4300-11000)	680000 (440000-970000)
Low-middle SDI	1990	Male	25 to 29	25% (18-33%)	75% (67-82%)	1.3 (0.79-2)	6% (4.2-8.2%)	6.7% (4.2-9.7%)	13000 (7800-18000)	1000000 (690000-1400000)
Low-middle SDI	1990	Male	30 to 34	31% (22-40%)	69% (60-78%)	1.6 (1-2.4)	7.5% (5.4-9.9%)	8.8% (5.9-12%)	18000 (12000-25000)	1200000 (870000-1600000)
Low-middle SDI	1990	Male	35 to 39	34% (25-42%)	66% (58-75%)	1.8 (1.1-2.7)	7.9% (5.7-11%)	9.6% (6.5-13%)	21000 (14000-29000)	1200000 (890000-1600000)
Low-middle SDI	1990	Male	40 to 44	34% (26-42%)	66% (58-74%)	1.9 (1.1-2.8)	8% (5.7-11%)	9.8% (6.7-13%)	22000 (15000-30000)	1100000 (800000-1500000)
Low-middle SDI	1990	Male	45 to 49	32% (23-40%)	68% (60-77%)	1.8 (1.1-2.9)	7.7% (5.3-11%)	9.5% (6.4-13%)	25000 (17000-35000)	1100000 (780000-1600000)
Low-middle SDI	1990	Male	50 to 54	30% (23-39%)	70% (61-77%)	1.7 (1-2.7)	6.9% (4.5-9.6%)	8.4% (5.4-12%)	27000 (17000-39000)	1100000 (690000-1500000)

Location	Year	Sex	Age	Prevalence of current drinkers	Prevalence of abstainers	Population average of standard drinks daily	Percent of total attributable deaths	Percent of total attributable DALYs	Attributable deaths	Attributable DALYs
Low-middle SDI	1990	Male	55 to 59	29%<br>(21-37%)	71%<br>(63-79%)	1.6<br>(0.94-2.5)	6%<br>(4-8.4%)	7.2%<br>(4.8-10%)	28000<br>(18000-40000)	940000<br>(620000-1300000)
Low-middle SDI	1990	Male	60 to 64	27%<br>(20-36%)	73%<br>(64-80%)	1.4<br>(0.79-2.3)	4.8%<br>(2.9-7.1%)	5.6%<br>(3.4-8.5%)	26000<br>(15000-40000)	740000<br>(450000-1100000)
Low-middle SDI	1990	Male	65 to 69	26%<br>(19-33%)	74%<br>(67-81%)	1.2<br>(0.73-1.9)	3.8%<br>(2.2-5.7%)	4.5%<br>(2.6-6.7%)	22000<br>(12000-33000)	510000<br>(290000-760000)
Low-middle SDI	1990	Male	70 to 74	24%<br>(17-31%)	76%<br>(69-83%)	1<br>(0.57-1.6)	2.9%<br>(1.5-4.6%)	3.3%<br>(1.7-5.4%)	16000<br>(7900-26000)	300000<br>(150000-480000)
Low-middle SDI	1990	Male	75 to 79	21%<br>(14-28%)	79%<br>(72-86%)	0.77<br>(0.4-1.3)	2%<br>(0.91-3.4%)	2.3%<br>(0.98-3.9%)	8900<br>(3800-16000)	130000<br>(58000-220000)
Low-middle SDI	1990	Male	80 to 84	18%<br>(12-25%)	82%<br>(75-88%)	0.6<br>(0.33-0.98)	1.3%<br>(0.55-2.4%)	1.5%<br>(0.58-2.7%)	3800<br>(1500-7000)	42000<br>(17000-75000)
Low-middle SDI	1990	Male	85 to 89	18%<br>(12-25%)	82%<br>(75-88%)	0.52<br>(0.28-0.87)	0.95%<br>(0.33-1.8%)	1%<br>(0.3-2%)	1500<br>(430-2900)	12000<br>(4100-23000)
Low-middle SDI	1990	Male	90 to 94	19%<br>(13-26%)	81%<br>(74-87%)	0.5<br>(0.25-0.87)	0.67%<br>(0.12-1.4%)	0.68%<br>(0.042-1.6%)	360<br>(20-830)	2100<br>(380-4500)
Low-middle SDI	1990	Male	95 plus	21%<br>(13-31%)	79%<br>(69-87%)	0.49<br>(0.2-0.93)	0.59%<br>(0.0079-1.4%)	0.54%<br>(-0.14-1.5%)	66<br>(-17-190)	290<br>(3.4-710)
Low-middle SDI	1995	Female	All Ages	6.4%<br>(5.4-7.6%)	94%<br>(92-95%)	0.18<br>(0.14-0.23)	0.53%<br>(0.39-0.7%)	0.86%<br>(0.54-1.2%)	64000<br>(40000-93000)	2500000<br>(1800000-3300000)
Low-middle SDI	1995	Female	15 to 19	5.6%<br>(3.7-8%)	94%<br>(92-96%)	0.15<br>(0.073-0.26)	0.46%<br>(0.26-0.75%)	0.46%<br>(0.18-0.92%)	590<br>(220-1200)	80000<br>(43000-130000)
Low-middle SDI	1995	Female	20 to 24	6.9%<br>(4.5-9.6%)	93%<br>(90-95%)	0.23<br>(0.12-0.39)	0.84%<br>(0.51-1.3%)	0.74%<br>(0.32-1.3%)	1300<br>(560-2300)	160000<br>(95000-250000)
Low-middle SDI	1995	Female	25 to 29	7.4%<br>(4.9-10%)	93%<br>(90-95%)	0.27<br>(0.14-0.46)	1%<br>(0.65-1.5%)	1%<br>(0.5-1.7%)	1900<br>(930-3300)	190000<br>(120000-290000)
Low-middle SDI	1995	Female	30 to 34	8%<br>(5.3-11%)	92%<br>(89-95%)	0.31<br>(0.17-0.52)	1.2%<br>(0.81-1.7%)	1.4%<br>(0.8-2.2%)	2600<br>(1500-4100)	210000<br>(130000-300000)
Low-middle SDI	1995	Female	35 to 39	8.7%<br>(5.9-12%)	91%<br>(88-94%)	0.34<br>(0.18-0.56)	1.4%<br>(0.97-2.1%)	1.8%<br>(1.1-2.8%)	3500<br>(2100-5500)	230000<br>(150000-330000)
Low-middle SDI	1995	Female	40 to 44	9.1%<br>(6.4-12%)	91%<br>(88-94%)	0.35<br>(0.19-0.59)	1.6%<br>(1-2.2%)	2.1%<br>(1.3-3.1%)	4200<br>(2600-6400)	230000<br>(150000-340000)
Low-middle SDI	1995	Female	45 to 49	9.5%<br>(6.4-13%)	90%<br>(87-94%)	0.35<br>(0.17-0.61)	1.6%<br>(1.1-2.4%)	2.2%<br>(1.3-3.3%)	4700<br>(2800-7300)	230000<br>(140000-330000)
Low-middle SDI	1995	Female	50 to 54	9.9%<br>(7-14%)	90%<br>(86-93%)	0.37<br>(0.19-0.64)	1.7%<br>(1.1-2.6%)	2.3%<br>(1.4-3.5%)	6100<br>(3600-9500)	250000<br>(150000-370000)
Low-middle SDI	1995	Female	55 to 59	9.9%<br>(6.9-14%)	90%<br>(86-93%)	0.43<br>(0.21-0.74)	1.7%<br>(1-2.7%)	2.2%<br>(1.3-3.5%)	7600<br>(4300-12000)	260000<br>(150000-410000)
Low-middle SDI	1995	Female	60 to 64	9.8%<br>(6.6-14%)	90%<br>(86-93%)	0.43<br>(0.21-0.77)	1.5%<br>(0.8-2.4%)	1.9%<br>(0.97-3.1%)	7800<br>(4000-13000)	220000<br>(120000-370000)

Location	Year	Sex	Age	Prevalence of current drinkers	Prevalence of abstainers	Population average of standard drinks daily	Percent of total attributable deaths	Percent of total attributable DALYs	Attributable deaths	Attributable DALYs
Low-middle SDI	1995	Female	65 to 69	10% (6.7-14%)	90% (86-93%)	0.4 (0.2-0.69)	1.3% (0.66-2.2%)	1.6% (0.78-2.7%)	7800 (3900-13000)	180000 (92000-300000)
Low-middle SDI	1995	Female	70 to 74	10% (6.9-15%)	90% (85-93%)	0.36 (0.18-0.65)	1.1% (0.42-2%)	1.3% (0.5-2.4%)	6600 (2600-12000)	120000 (49000-230000)
Low-middle SDI	1995	Female	75 to 79	10% (6.5-15%)	90% (85-93%)	0.34 (0.15-0.66)	0.8% (0.23-1.6%)	0.94% (0.26-2%)	4400 (1200-9100)	63000 (18000-130000)
Low-middle SDI	1995	Female	80 to 84	11% (6.5-16%)	89% (84-94%)	0.36 (0.16-0.7)	0.65% (0.14-1.4%)	0.75% (0.15-1.7%)	2700 (550-6000)	29000 (6500-63000)
Low-middle SDI	1995	Female	85 to 89	12% (7.3-19%)	88% (81-93%)	0.41 (0.18-0.81)	0.55% (0.049-1.3%)	0.63% (0.032-1.5%)	1300 (66-3300)	10000 (920-24000)
Low-middle SDI	1995	Female	90 to 94	14% (8.7-23%)	86% (77-91%)	0.49 (0.2-0.99)	0.48% (-0.067-1.4%)	0.53% (-0.12-1.6%)	430 (-99-1300)	2300 (-320-6600)
Low-middle SDI	1995	Female	95 plus	17% (9.6-28%)	83% (72-90%)	0.58 (0.2-1.3)	0.49% (-0.21-1.6%)	0.5% (-0.32-1.9%)	110 (-69-390)	410 (-180-1400)
Low-middle SDI	1995	Male	All Ages	17% (15-19%)	83% (81-85%)	0.91 (0.72-1.1)	2.6% (2.1-3.2%)	3.6% (2.7-4.6%)	300000 (220000-390000)	13000000 (10000000-16000000)
Low-middle SDI	1995	Male	15 to 19	8.5% (5.7-12%)	92% (88-94%)	0.34 (0.18-0.57)	1.4% (0.92-2.1%)	1.5% (0.85-2.5%)	2200 (1200-3500)	240000 (150000-360000)
Low-middle SDI	1995	Male	20 to 24	16% (12-21%)	84% (79-88%)	0.89 (0.51-1.4)	4% (2.7-5.5%)	4.2% (2.6-6.2%)	8300 (5000-13000)	760000 (500000-1100000)
Low-middle SDI	1995	Male	25 to 29	24% (18-30%)	76% (70-82%)	1.5 (0.92-2.2)	6% (4.3-8%)	6.7% (4.3-9.5%)	15000 (9400-21000)	1100000 (810000-1500000)
Low-middle SDI	1995	Male	30 to 34	29% (23-36%)	71% (64-77%)	1.9 (1.2-2.8)	7.6% (5.6-10%)	9% (6.2-12%)	21000 (15000-29000)	1500000 (1100000-1900000)
Low-middle SDI	1995	Male	35 to 39	33% (26-40%)	67% (60-74%)	2.2 (1.4-3.1)	8.2% (6.1-11%)	10% (7.1-13%)	26000 (18000-34000)	1500000 (1100000-2000000)
Low-middle SDI	1995	Male	40 to 44	34% (28-41%)	66% (59-72%)	2.3 (1.4-3.3)	8.5% (6.3-11%)	10% (7.5-14%)	30000 (21000-39000)	1500000 (1100000-2000000)
Low-middle SDI	1995	Male	45 to 49	33% (26-39%)	67% (61-74%)	2.3 (1.4-3.4)	8.3% (6-11%)	10% (7.1-14%)	31000 (22000-42000)	1400000 (1000000-1800000)
Low-middle SDI	1995	Male	50 to 54	31% (25-37%)	69% (63-75%)	2.2 (1.3-3.3)	7.5% (5.2-10%)	9.2% (6.2-12%)	33000 (22000-45000)	1300000 (870000-1700000)
Low-middle SDI	1995	Male	55 to 59	29% (23-36%)	71% (64-77%)	2 (1.2-3)	6.7% (4.6-9.1%)	8.1% (5.5-11%)	35000 (23000-49000)	1200000 (810000-1600000)

Location	Year	Sex	Age	Prevalence of current drinkers	Prevalence of abstainers	Population average of standard drinks daily	Percent of total attributable deaths	Percent of total attributable DALYs	Attributable deaths	Attributable DALYs
Low-middle SDI	1995	Male	60 to 64	27% (21-34%)	73% (66-79%)	1.7 (1-2.7)	5.3% (3.5-7.6%)	6.2% (4-9%)	32000 (20000-47000)	920000 (600000-1300000)
Low-middle SDI	1995	Male	65 to 69	25% (19-31%)	75% (69-81%)	1.5 (0.88-2.2)	4.3% (2.7-6.1%)	5% (3.1-7.2%)	28000 (17000-40000)	650000 (410000-940000)
Low-middle SDI	1995	Male	70 to 74	23% (18-29%)	77% (71-82%)	1.2 (0.7-1.8)	3.2% (1.8-5%)	3.7% (2.1-5.8%)	20000 (11000-32000)	380000 (220000-590000)
Low-middle SDI	1995	Male	75 to 79	20% (15-26%)	80% (74-85%)	0.91 (0.51-1.5)	2.3% (1.1-3.7%)	2.6% (1.2-4.3%)	12000 (5600-20000)	170000 (86000-280000)
Low-middle SDI	1995	Male	80 to 84	18% (13-23%)	82% (77-87%)	0.72 (0.42-1.2)	1.6% (0.74-2.6%)	1.8% (0.8-3%)	5400 (2400-9100)	59000 (28000-98000)
Low-middle SDI	1995	Male	85 to 89	18% (13-24%)	82% (76-87%)	0.65 (0.37-1)	1.1% (0.48-2.1%)	1.3% (0.48-2.3%)	2200 (830-4100)	18000 (7300-31000)
Low-middle SDI	1995	Male	90 to 94	19% (13-25%)	81% (75-87%)	0.61 (0.33-1)	0.81% (0.24-1.6%)	0.84% (0.16-1.8%)	560 (110-1200)	3300 (970-6500)
Low-middle SDI	1995	Male	95 plus	21% (14-29%)	79% (71-86%)	0.59 (0.26-1.1)	0.7% (0.087-1.6%)	0.65% (-0.054-1.7%)	100 (-9.1-270)	450 (54-1000)
Low-middle SDI	2000	Female	All Ages	6.4% (5.4-7.5%)	94% (92-95%)	0.19 (0.14-0.24)	0.56% (0.41-0.73%)	0.87% (0.55-1.3%)	68000 (42000-100000)	2600000 (1900000-3600000)
Low-middle SDI	2000	Female	15 to 19	5.7% (3.9-8.1%)	94% (92-96%)	0.15 (0.079-0.27)	0.48% (0.27-0.76%)	0.47% (0.19-0.91%)	640 (250-1300)	89000 (50000-150000)
Low-middle SDI	2000	Female	20 to 24	6.9% (4.7-9.5%)	93% (91-95%)	0.23 (0.12-0.39)	0.84% (0.52-1.3%)	0.7% (0.32-1.3%)	1300 (600-2400)	180000 (100000-270000)
Low-middle SDI	2000	Female	25 to 29	7.4% (5.2-10%)	93% (90-95%)	0.28 (0.15-0.45)	0.98% (0.64-1.4%)	0.91% (0.46-1.5%)	1900 (990-3300)	200000 (130000-300000)
Low-middle SDI	2000	Female	30 to 34	7.8% (5.4-11%)	92% (89-95%)	0.31 (0.17-0.5)	1.1% (0.78-1.6%)	1.2% (0.75-1.9%)	2600 (1500-4200)	220000 (140000-320000)
Low-middle SDI	2000	Female	35 to 39	8.3% (5.8-11%)	92% (89-94%)	0.33 (0.18-0.53)	1.3% (0.93-1.9%)	1.7% (1.1-2.5%)	3700 (2300-5600)	250000 (170000-350000)
Low-middle SDI	2000	Female	40 to 44	8.7% (6.3-11%)	91% (89-94%)	0.34 (0.18-0.56)	1.4% (0.98-2%)	1.9% (1.2-2.8%)	4400 (2700-6600)	250000 (170000-360000)
Low-middle SDI	2000	Female	45 to 49	8.8% (6.1-12%)	91% (88-94%)	0.33 (0.17-0.57)	1.5% (0.99-2.1%)	2% (1.3-3%)	5200 (3100-8000)	250000 (160000-370000)
Low-middle SDI	2000	Female	50 to 54	9.4% (6.6-13%)	91% (87-93%)	0.36 (0.18-0.62)	1.6% (1-2.4%)	2.1% (1.3-3.2%)	6300 (3800-9900)	260000 (160000-390000)
Low-middle SDI	2000	Female	55 to 59	9.4% (6.4-13%)	91% (87-94%)	0.42 (0.21-0.73)	1.6% (0.98-2.5%)	2.1% (1.2-3.3%)	7800 (4300-12000)	260000 (150000-410000)
Low-middle SDI	2000	Female	60 to 64	9.3% (6.2-13%)	91% (87-94%)	0.42 (0.2-0.75)	1.4% (0.76-2.3%)	1.8% (0.92-2.9%)	8200 (4200-14000)	240000 (120000-390000)
Low-middle SDI	2000	Female	65 to 69	9.3% (6.2-13%)	91% (87-94%)	0.38 (0.19-0.66)	1.2% (0.65-2.1%)	1.5% (0.78-2.5%)	8500 (4300-14000)	200000 (100000-330000)
Low-middle SDI	2000	Female	70 to 74	9.6% (6.4-14%)	90% (86-94%)	0.35 (0.17-0.64)	1% (0.41-1.8%)	1.2% (0.48-2.2%)	7200 (2800-13000)	130000 (54000-250000)

Location	Year	Sex	Age	Prevalence of current drinkers	Prevalence of abstainers	Population average of standard drinks daily	Percent of total attributable deaths	Percent of total attributable DALYs	Attributable deaths	Attributable DALYs
Low-middle SDI	2000	Female	75 to 79	9.7% (6.1-15%)	90% (85-94%)	0.33 (0.15-0.64)	0.76% (0.22-1.5%)	0.9% (0.25-1.8%)	4800 (1300-9800)	69000 (20000-140000)
Low-middle SDI	2000	Female	80 to 84	9.9% (5.9-15%)	90% (85-94%)	0.34 (0.15-0.68)	0.62% (0.15-1.3%)	0.72% (0.16-1.6%)	3000 (660-6500)	32000 (7800-69000)
Low-middle SDI	2000	Female	85 to 89	11% (6.6-18%)	89% (82-93%)	0.39 (0.17-0.8)	0.53% (0.061-1.2%)	0.6% (0.051-1.4%)	1500 (120-3600)	12000 (1300-27000)
Low-middle SDI	2000	Female	90 to 94	13% (7.8-21%)	87% (79-92%)	0.47 (0.2-0.94)	0.46% (-0.048-1.3%)	0.51% (-0.099-1.5%)	500 (-100-1400)	2800 (-290-7600)
Low-middle SDI	2000	Female	95 plus	16% (8.6-27%)	84% (73-91%)	0.56 (0.2-1.2)	0.46% (-0.17-1.5%)	0.47% (-0.27-1.7%)	130 (-76-460)	490 (-190-1600)
Low-middle SDI	2000	Male	All Ages	18% (16-20%)	82% (80-84%)	1 (0.83-1.3)	2.9% (2.4-3.5%)	3.8% (2.9-4.8%)	340000 (250000-430000)	15000000 (12000000-18000000)
Low-middle SDI	2000	Male	15 to 19	11% (8.1-15%)	89% (85-92%)	0.48 (0.27-0.78)	1.8% (1.2-2.6%)	2% (1.1-3.1%)	3200 (1800-5100)	340000 (220000-500000)
Low-middle SDI	2000	Male	20 to 24	21% (16-26%)	79% (74-84%)	1.3 (0.77-1.9)	4.9% (3.5-6.7%)	5.3% (3.4-7.7%)	12000 (7400-17000)	1000000 (720000-1400000)
Low-middle SDI	2000	Male	25 to 29	28% (22-33%)	72% (67-78%)	1.9 (1.2-2.7)	6.7% (4.9-8.8%)	7.4% (5-10%)	19000 (13000-26000)	1500000 (1100000-1900000)
Low-middle SDI	2000	Male	30 to 34	31% (25-37%)	69% (63-75%)	2.1 (1.4-3.1)	7.7% (5.9-9.9%)	8.9% (6.4-12%)	25000 (18000-34000)	1700000 (1300000-2200000)
Low-middle SDI	2000	Male	35 to 39	33% (27-38%)	67% (62-73%)	2.3 (1.5-3.3)	8% (6-10%)	9.5% (6.9-12%)	30000 (21000-39000)	1800000 (1300000-2200000)
Low-middle SDI	2000	Male	40 to 44	33% (27-39%)	67% (61-73%)	2.4 (1.5-3.4)	8.1% (6.1-10%)	9.9% (7.2-13%)	34000 (24000-44000)	1800000 (1300000-2200000)
Low-middle SDI	2000	Male	45 to 49	32% (26-38%)	68% (62-74%)	2.4 (1.5-3.5)	8% (5.9-11%)	9.8% (7-13%)	38000 (27000-50000)	1700000 (1200000-2200000)
Low-middle SDI	2000	Male	50 to 54	30% (24-36%)	70% (64-76%)	2.2 (1.3-3.3)	7.3% (5.1-9.7%)	8.9% (6-12%)	36000 (24000-49000)	1400000 (970000-1900000)
Low-middle SDI	2000	Male	55 to 59	27% (21-33%)	73% (67-79%)	1.9 (1.2-3)	6.3% (4.4-8.5%)	7.6% (5.2-10%)	35000 (23000-49000)	1200000 (820000-1600000)
Low-middle SDI	2000	Male	60 to 64	24% (18-31%)	76% (69-82%)	1.6 (0.94-2.6)	5% (3.3-7.1%)	5.8% (3.8-8.4%)	33000 (21000-48000)	950000 (620000-1400000)
Low-middle SDI	2000	Male	65 to 69	22% (16-28%)	78% (72-84%)	1.4 (0.81-2.1)	4% (2.6-5.7%)	4.7% (3-6.7%)	29000 (18000-42000)	680000 (440000-980000)

Location	Year	Sex	Age	Prevalence of current drinkers	Prevalence of abstainers	Population average of standard drinks daily	Percent of total attributable deaths	Percent of total attributable DALYs	Attributable deaths	Attributable DALYs
Low-middle SDI	2000	Male	70 to 74	20% (15-26%)	80% (74-85%)	1.1 (0.64-1.8)	3% (1.8-4.6%)	3.5% (2-5.4%)	21000 (12000-34000)	410000 (240000-630000)
Low-middle SDI	2000	Male	75 to 79	17% (12-23%)	83% (77-88%)	0.82 (0.44-1.4)	2.1% (1.1-3.4%)	2.4% (1.2-3.9%)	13000 (6300-21000)	190000 (97000-300000)
Low-middle SDI	2000	Male	80 to 84	15% (9.9-20%)	85% (80-90%)	0.63 (0.35-1)	1.4% (0.73-2.4%)	1.6% (0.77-2.7%)	5900 (2800-10000)	65000 (32000-110000)
Low-middle SDI	2000	Male	85 to 89	15% (9.8-21%)	85% (79-90%)	0.57 (0.31-0.94)	1.1% (0.48-1.9%)	1.2% (0.48-2.1%)	2500 (990-4500)	20000 (8700-35000)
Low-middle SDI	2000	Male	90 to 94	16% (11-22%)	84% (78-89%)	0.54 (0.28-0.94)	0.77% (0.26-1.5%)	0.8% (0.2-1.7%)	650 (160-1400)	3800 (1300-7400)
Low-middle SDI	2000	Male	95 plus	18% (11-26%)	82% (74-89%)	0.53 (0.22-0.99)	0.67% (0.12-1.5%)	0.62% (-0.0097-1.6%)	130 (-3-320)	540 (95-1200)
Low-middle SDI	2005	Female	All Ages	6.5% (5.5-7.7%)	93% (92-95%)	0.2 (0.15-0.25)	0.57% (0.42-0.75%)	0.86% (0.54-1.2%)	67000 (41000-100000)	2600000 (1900000-3500000)
Low-middle SDI	2005	Female	15 to 19	5.8% (3.9-8.2%)	94% (92-96%)	0.16 (0.079-0.27)	0.45% (0.27-0.71%)	0.44% (0.18-0.83%)	570 (230-1100)	86000 (49000-140000)
Low-middle SDI	2005	Female	20 to 24	6.9% (4.8-9.6%)	93% (90-95%)	0.24 (0.12-0.41)	0.82% (0.52-1.2%)	0.68% (0.32-1.2%)	1200 (580-2200)	180000 (110000-270000)
Low-middle SDI	2005	Female	25 to 29	7.5% (5.3-10%)	92% (90-95%)	0.29 (0.16-0.47)	0.92% (0.62-1.3%)	0.85% (0.45-1.4%)	1800 (940-3100)	200000 (130000-290000)
Low-middle SDI	2005	Female	30 to 34	8.1% (5.6-11%)	92% (89-94%)	0.32 (0.18-0.52)	1% (0.71-1.4%)	1.1% (0.71-1.8%)	2500 (1400-3900)	210000 (140000-310000)
Low-middle SDI	2005	Female	35 to 39	8.4% (5.9-11%)	92% (89-94%)	0.34 (0.19-0.56)	1.2% (0.85-1.7%)	1.6% (1-2.3%)	3500 (2100-5400)	240000 (160000-340000)
Low-middle SDI	2005	Female	40 to 44	8.6% (6.2-11%)	91% (89-94%)	0.35 (0.18-0.57)	1.3% (0.88-1.8%)	1.7% (1.2-2.5%)	4300 (2700-6400)	250000 (160000-350000)
Low-middle SDI	2005	Female	45 to 49	8.8% (6.1-12%)	91% (88-94%)	0.33 (0.17-0.58)	1.3% (0.88-1.9%)	1.8% (1.2-2.7%)	5100 (3100-7800)	250000 (160000-360000)
Low-middle SDI	2005	Female	50 to 54	8.9% (6.3-12%)	91% (88-94%)	0.36 (0.18-0.62)	1.4% (0.92-2.1%)	1.9% (1.2-2.9%)	6300 (3700-9900)	260000 (160000-390000)
Low-middle SDI	2005	Female	55 to 59	9.3% (6.4-13%)	91% (87-94%)	0.43 (0.22-0.75)	1.5% (0.9-2.3%)	2% (1.1-3.1%)	7500 (4100-12000)	260000 (150000-410000)
Low-middle SDI	2005	Female	60 to 64	9.1% (6.1-13%)	91% (87-94%)	0.43 (0.21-0.77)	1.3% (0.71-2.1%)	1.7% (0.87-2.7%)	7800 (3900-13000)	230000 (120000-370000)
Low-middle SDI	2005	Female	65 to 69	9.2% (6.2-13%)	91% (87-94%)	0.39 (0.2-0.67)	1.1% (0.59-1.9%)	1.4% (0.71-2.4%)	8400 (4100-14000)	200000 (100000-330000)
Low-middle SDI	2005	Female	70 to 74	9.2% (6.2-13%)	91% (87-94%)	0.35 (0.17-0.64)	0.93% (0.37-1.7%)	1.1% (0.44-2.1%)	7400 (2800-14000)	140000 (55000-250000)
Low-middle SDI	2005	Female	75 to 79	9.2% (5.8-14%)	91% (86-94%)	0.33 (0.15-0.64)	0.71% (0.2-1.4%)	0.85% (0.23-1.7%)	5100 (1300-10000)	74000 (20000-150000)
Low-middle SDI	2005	Female	80 to 84	9.4% (5.6-15%)	91% (85-94%)	0.34 (0.15-0.66)	0.59% (0.14-1.2%)	0.69% (0.15-1.5%)	3300 (650-7200)	36000 (7800-77000)

Location	Year	Sex	Age	Prevalence of current drinkers	Prevalence of abstainers	Population average of standard drinks daily	Percent of total attributable deaths	Percent of total attributable DALYs	Attributable deaths	Attributable DALYs
Low-middle SDI	2005	Female	85 to 89	10% (6.2-17%)	90% (83-94%)	0.38 (0.17-0.78)	0.52% (0.066-1.2%)	0.59% (0.053-1.4%)	1800 (130-4200)	14000 (1600-32000)
Low-middle SDI	2005	Female	90 to 94	12% (7.3-20%)	88% (80-93%)	0.46 (0.19-0.91)	0.46% (-0.029-1.2%)	0.5% (-0.078-1.4%)	600 (-100-1700)	3300 (-210-8800)
Low-middle SDI	2005	Female	95 plus	15% (8-25%)	85% (75-92%)	0.55 (0.2-1.2)	0.47% (-0.15-1.5%)	0.48% (-0.26-1.7%)	150 (-85-540)	600 (-200-1900)
Low-middle SDI	2005	Male	All Ages	19% (17-21%)	81% (79-83%)	1.1 (0.89-1.4)	3.2% (2.6-3.9%)	4% (3.1-5%)	370000 (280000-460000)	16000000 (13000000-19000000)
Low-middle SDI	2005	Male	15 to 19	11% (8.1-15%)	89% (85-92%)	0.48 (0.27-0.79)	1.8% (1.2-2.5%)	1.9% (1.1-3%)	3200 (1800-5200)	350000 (230000-510000)
Low-middle SDI	2005	Male	20 to 24	21% (16-26%)	79% (74-84%)	1.3 (0.79-1.9)	5% (3.6-6.7%)	5.4% (3.5-7.8%)	13000 (8100-18000)	1100000 (780000-1500000)
Low-middle SDI	2005	Male	25 to 29	28% (22-33%)	72% (67-78%)	1.9 (1.2-2.7)	6.6% (4.9-8.6%)	7.3% (5-10%)	20000 (14000-27000)	1600000 (1100000-2000000)
Low-middle SDI	2005	Male	30 to 34	31% (25-38%)	69% (62-75%)	2.2 (1.5-3.2)	7.5% (5.7-9.5%)	8.6% (6.3-11%)	27000 (19000-35000)	1800000 (1400000-2300000)
Low-middle SDI	2005	Male	35 to 39	33% (27-39%)	67% (61-73%)	2.4 (1.6-3.4)	7.8% (6-9.9%)	9.2% (6.8-12%)	32000 (24000-42000)	1900000 (1500000-2400000)
Low-middle SDI	2005	Male	40 to 44	33% (27-39%)	67% (61-73%)	2.4 (1.6-3.5)	7.9% (6-10%)	9.5% (7.1-12%)	36000 (27000-47000)	1900000 (1400000-2400000)
Low-middle SDI	2005	Male	45 to 49	32% (26-38%)	68% (62-74%)	2.4 (1.5-3.6)	7.8% (5.8-10%)	9.5% (6.9-12%)	40000 (29000-54000)	1800000 (1400000-2400000)
Low-middle SDI	2005	Male	50 to 54	30% (24-36%)	70% (64-76%)	2.3 (1.4-3.4)	7.2% (5.2-9.5%)	8.7% (6.1-12%)	42000 (29000-57000)	1700000 (1200000-2200000)
Low-middle SDI	2005	Male	55 to 59	27% (22-34%)	73% (66-78%)	2 (1.3-3.1)	6.4% (4.5-8.5%)	7.7% (5.4-10%)	38000 (26000-52000)	1300000 (910000-1700000)
Low-middle SDI	2005	Male	60 to 64	25% (19-32%)	75% (68-81%)	1.7 (1-2.7)	5.1% (3.4-7.1%)	6% (3.9-8.4%)	35000 (22000-50000)	1000000 (660000-1400000)
Low-middle SDI	2005	Male	65 to 69	23% (17-29%)	77% (71-83%)	1.5 (0.88-2.2)	4% (2.6-5.7%)	4.7% (3-6.7%)	31000 (20000-46000)	740000 (480000-1100000)
Low-middle SDI	2005	Male	70 to 74	21% (16-27%)	79% (73-84%)	1.2 (0.69-1.9)	3% (1.8-4.6%)	3.5% (2-5.4%)	24000 (13000-36000)	450000 (260000-690000)

Location	Year	Sex	Age	Prevalence of current drinkers	Prevalence of abstainers	Population average of standard drinks daily	Percent of total attributable deaths	Percent of total attributable DALYs	Attributable deaths	Attributable DALYs
Low-middle SDI	2005	Male	75 to 79	18% (13-24%)	82% (76-87%)	0.89 (0.48-1.4)	2.1% (1.1-3.4%)	2.4% (1.2-3.9%)	14000 (7100-24000)	210000 (110000-350000)
Low-middle SDI	2005	Male	80 to 84	15% (10-21%)	85% (79-90%)	0.66 (0.37-1.1)	1.5% (0.73-2.4%)	1.6% (0.77-2.8%)	7100 (3300-12000)	79000 (39000-130000)
Low-middle SDI	2005	Male	85 to 89	15% (9.7-21%)	85% (79-90%)	0.59 (0.32-0.99)	1.1% (0.49-1.9%)	1.2% (0.5-2.1%)	3000 (1200-5500)	24000 (11000-42000)
Low-middle SDI	2005	Male	90 to 94	16% (11-22%)	84% (78-89%)	0.57 (0.29-0.97)	0.8% (0.27-1.6%)	0.82% (0.2-1.7%)	800 (190-1700)	4700 (1600-9300)
Low-middle SDI	2005	Male	95 plus	18% (11-26%)	82% (74-89%)	0.56 (0.24-1)	0.69% (0.14-1.6%)	0.63% (-0.011-1.6%)	150 (-3.8-400)	670 (130-1500)
Low-middle SDI	2010	Female	All Ages	7.3% (6.1-8.7%)	93% (91-94%)	0.22 (0.16-0.29)	0.63% (0.47-0.83%)	0.91% (0.57-1.3%)	69000 (42000-100000)	2700000 (1900000-3600000)
Low-middle SDI	2010	Female	15 to 19	6.2% (4.1-8.9%)	94% (91-96%)	0.17 (0.078-0.31)	0.47% (0.28-0.73%)	0.43% (0.17-0.82%)	510 (200-1000)	85000 (49000-140000)
Low-middle SDI	2010	Female	20 to 24	7.2% (4.8-10%)	93% (90-95%)	0.25 (0.12-0.45)	0.88% (0.57-1.3%)	0.69% (0.33-1.2%)	1100 (510-2000)	180000 (110000-270000)
Low-middle SDI	2010	Female	25 to 29	7.8% (5.2-11%)	92% (89-95%)	0.31 (0.16-0.53)	0.99% (0.67-1.4%)	0.9% (0.48-1.5%)	1700 (890-2900)	210000 (140000-310000)
Low-middle SDI	2010	Female	30 to 34	8.2% (5.6-11%)	92% (89-94%)	0.34 (0.18-0.57)	1.1% (0.73-1.5%)	1.2% (0.7-1.8%)	2300 (1300-3700)	220000 (140000-310000)
Low-middle SDI	2010	Female	35 to 39	8.7% (5.9-12%)	91% (88-94%)	0.36 (0.19-0.62)	1.2% (0.83-1.6%)	1.5% (0.99-2.2%)	3300 (2000-5000)	240000 (160000-330000)
Low-middle SDI	2010	Female	40 to 44	8.7% (6.1-12%)	91% (88-94%)	0.36 (0.18-0.62)	1.2% (0.86-1.7%)	1.7% (1.1-2.5%)	4000 (2500-6100)	240000 (160000-350000)
Low-middle SDI	2010	Female	45 to 49	8.7% (5.8-12%)	91% (88-94%)	0.35 (0.17-0.62)	1.3% (0.87-1.8%)	1.8% (1.1-2.7%)	5000 (3100-7700)	250000 (160000-370000)
Low-middle SDI	2010	Female	50 to 54	9.1% (6.2-13%)	91% (87-94%)	0.38 (0.18-0.69)	1.4% (0.9-2.1%)	1.9% (1.2-2.9%)	6600 (3900-10000)	270000 (170000-410000)
Low-middle SDI	2010	Female	55 to 59	9.2% (6.2-13%)	91% (87-94%)	0.46 (0.22-0.83)	1.5% (0.86-2.3%)	2% (1.1-3.1%)	8000 (4400-13000)	280000 (160000-440000)
Low-middle SDI	2010	Female	60 to 64	9.6% (6.3-14%)	90% (86-94%)	0.48 (0.23-0.9)	1.3% (0.7-2.2%)	1.7% (0.87-2.9%)	8200 (4100-14000)	240000 (120000-400000)
Low-middle SDI	2010	Female	65 to 69	9.5% (6.2-14%)	90% (86-94%)	0.43 (0.21-0.77)	1.2% (0.57-1.9%)	1.5% (0.7-2.5%)	8500 (4100-15000)	200000 (100000-340000)
Low-middle SDI	2010	Female	70 to 74	9.1% (6-13%)	91% (87-94%)	0.37 (0.17-0.7)	0.91% (0.37-1.7%)	1.1% (0.44-2.1%)	7700 (3000-14000)	150000 (58000-270000)
Low-middle SDI	2010	Female	75 to 79	8.3% (5.1-13%)	92% (87-95%)	0.32 (0.14-0.64)	0.69% (0.21-1.4%)	0.83% (0.24-1.7%)	5500 (1600-11000)	81000 (24000-160000)
Low-middle SDI	2010	Female	80 to 84	7.8% (4.5-13%)	92% (87-96%)	0.3 (0.13-0.61)	0.57% (0.15-1.2%)	0.67% (0.16-1.4%)	3800 (850-8200)	41000 (10000-87000)
Low-middle SDI	2010	Female	85 to 89	8.4% (4.8-14%)	92% (86-95%)	0.32 (0.14-0.65)	0.47% (0.067-1.1%)	0.54% (0.052-1.3%)	2000 (160-4700)	16000 (2000-36000)

Location	Year	Sex	Age	Prevalence of current drinkers	Prevalence of abstainers	Population average of standard drinks daily	Percent of total attributable deaths	Percent of total attributable DALYs	Attributable deaths	Attributable DALYs
Low-middle SDI	2010	Female	90 to 94	9.5% (5.5-16%)	90% (84-95%)	0.37 (0.15-0.75)	0.42% (-0.0035-1.1%)	0.46% (-0.048-1.3%)	700 (-77-1900)	3900 (-41-10000)
Low-middle SDI	2010	Female	95 plus	11% (5.9-20%)	89% (80-94%)	0.43 (0.15-0.98)	0.43% (-0.098-1.3%)	0.44% (-0.19-1.4%)	180 (-79-590)	720 (-160-2100)
Low-middle SDI	2010	Male	All Ages	20% (18-22%)	80% (78-82%)	1.2 (0.93-1.6)	3.5% (2.8-4.2%)	4.2% (3.3-5.3%)	390000 (290000-490000)	17000000 (13000000-20000000)
Low-middle SDI	2010	Male	15 to 19	12% (8.5-16%)	88% (84-91%)	0.51 (0.28-0.85)	1.7% (1.1-2.5%)	1.9% (1.1-3.1%)	3100 (1700-5100)	340000 (220000-500000)
Low-middle SDI	2010	Male	20 to 24	22% (18-27%)	78% (73-82%)	1.4 (0.83-2.1)	5% (3.5-6.7%)	5.6% (3.6-8%)	12000 (7900-18000)	1100000 (770000-1500000)
Low-middle SDI	2010	Male	25 to 29	29% (24-34%)	71% (66-76%)	2 (1.3-3)	6.8% (5-8.8%)	7.8% (5.3-11%)	21000 (14000-28000)	1600000 (1200000-2100000)
Low-middle SDI	2010	Male	30 to 34	32% (26-37%)	68% (63-74%)	2.3 (1.5-3.4)	7.5% (5.8-9.5%)	8.7% (6.4-11%)	26000 (19000-35000)	1800000 (1400000-2300000)
Low-middle SDI	2010	Male	35 to 39	33% (28-39%)	67% (61-72%)	2.4 (1.6-3.5)	7.7% (5.9-9.7%)	9.2% (6.8-12%)	32000 (24000-42000)	1900000 (1500000-2500000)
Low-middle SDI	2010	Male	40 to 44	33% (28-38%)	67% (62-72%)	2.5 (1.6-3.6)	7.6% (5.8-9.6%)	9.3% (6.9-12%)	36000 (26000-47000)	1900000 (1400000-2400000)
Low-middle SDI	2010	Male	45 to 49	32% (26-37%)	68% (63-74%)	2.4 (1.5-3.7)	7.5% (5.6-9.7%)	9.2% (6.7-12%)	41000 (30000-55000)	1900000 (1400000-2500000)
Low-middle SDI	2010	Male	50 to 54	30% (25-36%)	70% (64-75%)	2.3 (1.4-3.6)	7% (5-9.1%)	8.5% (6-11%)	44000 (31000-59000)	1700000 (1200000-2300000)
Low-middle SDI	2010	Male	55 to 59	29% (23-35%)	71% (65-77%)	2.2 (1.3-3.4)	6.3% (4.4-8.3%)	7.6% (5.3-10%)	44000 (30000-60000)	1500000 (1100000-2000000)
Low-middle SDI	2010	Male	60 to 64	28% (21-35%)	72% (65-79%)	1.9 (1.2-3.1)	5.1% (3.4-7.2%)	6.1% (3.9-8.6%)	38000 (24000-54000)	1100000 (710000-1500000)
Low-middle SDI	2010	Male	65 to 69	26% (20-33%)	74% (67-80%)	1.7 (1-2.6)	4.1% (2.6-5.9%)	4.8% (3-6.9%)	33000 (20000-48000)	790000 (500000-1100000)
Low-middle SDI	2010	Male	70 to 74	24% (18-31%)	76% (69-82%)	1.4 (0.82-2.2)	3.1% (1.7-4.7%)	3.6% (2-5.6%)	27000 (14000-42000)	510000 (280000-790000)
Low-middle SDI	2010	Male	75 to 79	21% (15-28%)	79% (72-85%)	1.1 (0.6-1.8)	2.2% (1.1-3.6%)	2.5% (1.2-4.1%)	17000 (7700-28000)	250000 (120000-410000)

Location	Year	Sex	Age	Prevalence of current drinkers	Prevalence of abstainers	Population average of standard drinks daily	Percent of total attributable deaths	Percent of total attributable DALYs	Attributable deaths	Attributable DALYs
Low-middle SDI	2010	Male	80 to 84	18% (12-24%)	82% (76-88%)	0.83 (0.45-1.3)	1.5% (0.69-2.5%)	1.7% (0.73-2.9%)	8800 (3800-15000)	98000 (44000-170000)
Low-middle SDI	2010	Male	85 to 89	17% (11-25%)	83% (75-89%)	0.7 (0.37-1.2)	1.1% (0.45-2%)	1.2% (0.42-2.2%)	3800 (1300-7100)	31000 (12000-55000)
Low-middle SDI	2010	Male	90 to 94	18% (12-26%)	82% (74-88%)	0.68 (0.34-1.2)	0.79% (0.2-1.6%)	0.79% (0.1-1.8%)	990 (120-2200)	6000 (1500-12000)
Low-middle SDI	2010	Male	95 plus	22% (13-32%)	78% (68-87%)	0.69 (0.29-1.3)	0.68% (0.035-1.6%)	0.6% (-0.15-1.7%)	180 (-49-530)	830 (37-2000)
Low-middle SDI	2016	Female	All Ages	8% (6.6-9.7%)	92% (90-93%)	0.26 (0.17-0.4)	0.82% (0.59-1.1%)	1.1% (0.69-1.7%)	83000 (49000-130000)	3200000 (2300000-4400000)
Low-middle SDI	2016	Female	15 to 19	7% (4.5-10%)	93% (90-96%)	0.19 (0.075-0.38)	0.51% (0.3-0.83%)	0.47% (0.17-0.99%)	520 (190-1100)	90000 (51000-150000)
Low-middle SDI	2016	Female	20 to 24	8.1% (5.1-12%)	92% (88-95%)	0.29 (0.12-0.57)	1% (0.64-1.5%)	0.82% (0.37-1.6%)	1200 (490-2200)	190000 (120000-300000)
Low-middle SDI	2016	Female	25 to 29	8.6% (5.5-12%)	91% (88-94%)	0.36 (0.16-0.68)	1.2% (0.78-1.7%)	1.1% (0.57-2%)	1800 (900-3400)	230000 (150000-350000)
Low-middle SDI	2016	Female	30 to 34	9% (5.8-13%)	91% (87-94%)	0.42 (0.19-0.78)	1.3% (0.86-1.9%)	1.5% (0.87-2.5%)	2700 (1500-4600)	260000 (170000-390000)
Low-middle SDI	2016	Female	35 to 39	9.4% (6.1-13%)	91% (87-94%)	0.45 (0.2-0.82)	1.4% (0.97-2%)	2% (1.2-3%)	3900 (2200-6200)	290000 (180000-420000)
Low-middle SDI	2016	Female	40 to 44	9.6% (6.5-13%)	90% (87-94%)	0.45 (0.2-0.82)	1.5% (0.97-2.1%)	2.1% (1.3-3.2%)	4800 (2800-7500)	290000 (190000-430000)
Low-middle SDI	2016	Female	45 to 49	9.5% (6.1-13%)	90% (87-94%)	0.42 (0.19-0.81)	1.5% (0.95-2.2%)	2.1% (1.3-3.3%)	5800 (3400-9300)	300000 (190000-440000)
Low-middle SDI	2016	Female	50 to 54	9.4% (6.2-13%)	91% (87-94%)	0.44 (0.19-0.84)	1.6% (0.98-2.4%)	2.2% (1.3-3.5%)	7800 (4600-13000)	330000 (200000-510000)
Low-middle SDI	2016	Female	55 to 59	9.6% (6.2-14%)	90% (86-94%)	0.51 (0.23-0.99)	1.6% (0.91-2.6%)	2.2% (1.2-3.6%)	9500 (5100-16000)	330000 (190000-540000)
Low-middle SDI	2016	Female	60 to 64	9.7% (6.1-15%)	90% (85-94%)	0.54 (0.24-1.1)	1.4% (0.72-2.4%)	1.9% (0.94-3.3%)	10000 (5000-18000)	300000 (150000-510000)
Low-middle SDI	2016	Female	65 to 69	9.8% (6.3-14%)	90% (86-94%)	0.49 (0.22-0.94)	1.3% (0.61-2.2%)	1.6% (0.77-2.8%)	10000 (4800-18000)	240000 (120000-420000)
Low-middle SDI	2016	Female	70 to 74	9.3% (6-14%)	91% (86-94%)	0.41 (0.18-0.83)	0.99% (0.4-1.9%)	1.2% (0.48-2.4%)	8700 (3400-17000)	170000 (66000-310000)
Low-middle SDI	2016	Female	75 to 79	8.4% (5.1-13%)	92% (87-95%)	0.35 (0.14-0.73)	0.74% (0.23-1.5%)	0.9% (0.27-1.9%)	6600 (1900-14000)	96000 (29000-200000)
Low-middle SDI	2016	Female	80 to 84	7.7% (4.3-13%)	92% (87-96%)	0.34 (0.14-0.7)	0.63% (0.18-1.3%)	0.75% (0.2-1.6%)	5000 (1300-11000)	54000 (15000-120000)
Low-middle SDI	2016	Female	85 to 89	8.2% (4.5-14%)	92% (86-95%)	0.36 (0.14-0.75)	0.55% (0.12-1.2%)	0.64% (0.11-1.5%)	3000 (480-6900)	23000 (4700-53000)
Low-middle SDI	2016	Female	90 to 94	9.3% (5.2-16%)	91% (84-95%)	0.4 (0.15-0.86)	0.49% (0.035-1.3%)	0.54% (-0.0036-1.5%)	1100 (-13-3100)	6300 (450-16000)

Location	Year	Sex	Age	Prevalence of current drinkers	Prevalence of abstainers	Population average of standard drinks daily	Percent of total attributable deaths	Percent of total attributable DALYs	Attributable deaths	Attributable DALYs
Low-middle SDI	2016	Female	95 plus	11% (5.5-19%)	89% (81-95%)	0.47 (0.15-1.1)	0.52% (-0.048-1.4%)	0.54% (-0.13-1.6%)	320 (-78-960)	1200 (-110-3400)
Low-middle SDI	2016	Male	All Ages	20% (17-22%)	80% (78-83%)	1.3 (0.94-1.7)	3.9% (3.2-4.7%)	4.6% (3.5-5.9%)	430000 (320000-550000)	18000000 (14000000-22000000)
Low-middle SDI	2016	Male	15 to 19	10% (6.9-15%)	90% (85-93%)	0.46 (0.22-0.85)	1.6% (0.99-2.4%)	1.7% (0.89-2.9%)	2700 (1300-4700)	300000 (190000-470000)
Low-middle SDI	2016	Male	20 to 24	18% (13-24%)	82% (76-87%)	1.2 (0.64-2)	4.4% (3-6.2%)	4.9% (2.9-7.4%)	11000 (6200-16000)	990000 (650000-1400000)
Low-middle SDI	2016	Male	25 to 29	26% (19-32%)	74% (68-81%)	1.9 (1.1-3)	6.3% (4.5-8.3%)	7.4% (4.9-10%)	19000 (12000-26000)	1500000 (1100000-2000000)
Low-middle SDI	2016	Male	30 to 34	29% (22-37%)	71% (63-78%)	2.3 (1.4-3.6)	7.3% (5.5-9.4%)	8.9% (6.4-12%)	26000 (18000-36000)	1800000 (1400000-2400000)
Low-middle SDI	2016	Male	35 to 39	32% (24-39%)	68% (61-76%)	2.5 (1.5-3.9)	7.7% (5.9-9.9%)	9.6% (7-12%)	33000 (24000-44000)	2000000 (1500000-2600000)
Low-middle SDI	2016	Male	40 to 44	32% (25-39%)	68% (61-75%)	2.6 (1.6-4)	7.8% (5.9-9.9%)	9.8% (7.2-13%)	37000 (27000-49000)	2000000 (1500000-2600000)
Low-middle SDI	2016	Male	45 to 49	31% (24-38%)	69% (62-76%)	2.6 (1.5-4.1)	7.7% (5.7-10%)	9.7% (7-13%)	44000 (31000-58000)	2000000 (1500000-2600000)
Low-middle SDI	2016	Male	50 to 54	30% (24-37%)	70% (63-76%)	2.5 (1.4-4)	7.1% (5.1-9.3%)	8.9% (6.2-12%)	48000 (33000-64000)	1900000 (1400000-2500000)
Low-middle SDI	2016	Male	55 to 59	29% (23-36%)	71% (64-77%)	2.4 (1.4-3.8)	6.5% (4.5-8.6%)	8% (5.5-11%)	51000 (35000-69000)	1800000 (1200000-2300000)
Low-middle SDI	2016	Male	60 to 64	28% (22-36%)	72% (64-78%)	2.2 (1.3-3.5)	5.3% (3.5-7.5%)	6.4% (4.1-9.1%)	47000 (30000-68000)	1400000 (890000-1900000)
Low-middle SDI	2016	Male	65 to 69	27% (21-34%)	73% (66-79%)	1.9 (1.2-3)	4.3% (2.7-6.2%)	5.1% (3.1-7.4%)	39000 (24000-58000)	940000 (580000-1400000)
Low-middle SDI	2016	Male	70 to 74	25% (19-32%)	75% (68-81%)	1.6 (0.89-2.5)	3.3% (1.8-5.1%)	3.8% (2-6%)	30000 (16000-48000)	580000 (320000-900000)
Low-middle SDI	2016	Male	75 to 79	22% (16-29%)	78% (71-84%)	1.2 (0.66-2)	2.3% (1.1-3.9%)	2.7% (1.2-4.5%)	20000 (9300-34000)	300000 (150000-500000)
Low-middle SDI	2016	Male	80 to 84	18% (12-25%)	82% (75-88%)	0.95 (0.52-1.6)	1.6% (0.75-2.8%)	1.8% (0.78-3.2%)	12000 (5000-21000)	130000 (60000-220000)

Location	Year	Sex	Age	Prevalence of current drinkers	Prevalence of abstainers	Population average of standard drinks daily	Percent of total attributable deaths	Percent of total attributable DALYs	Attributable deaths	Attributable DALYs
Low-middle SDI	2016	Male	85 to 89	18% (11-25%)	82% (75-89%)	0.81 (0.43-1.4)	1.2% (0.5-2.2%)	1.3% (0.48-2.4%)	5300 (2000-9900)	43000 (18000-77000)
Low-middle SDI	2016	Male	90 to 94	19% (13-26%)	81% (74-87%)	0.77 (0.39-1.4)	0.88% (0.22-1.8%)	0.91% (0.12-2%)	1500 (210-3400)	9100 (2200-19000)
Low-middle SDI	2016	Male	95 plus	22% (13-31%)	78% (69-87%)	0.77 (0.33-1.5)	0.77% (0.054-1.8%)	0.7% (-0.14-1.9%)	290 (-60-810)	1300 (87-3100)
Low SDI	1990	Female	All Ages	24% (20-29%)	76% (71-80%)	0.62 (0.47-0.81)	1% (0.59-1.5%)	1.9% (0.96-2.9%)	41000 (21000-64000)	1500000 (870000-2200000)
Low SDI	1990	Female	15 to 19	24% (15-35%)	76% (65-85%)	0.68 (0.35-1.2)	1.5% (0.65-2.5%)	1.8% (0.6-3.4%)	670 (210-1300)	61000 (27000-110000)
Low SDI	1990	Female	20 to 24	30% (19-43%)	70% (57-81%)	1 (0.53-1.7)	2.3% (1.1-3.9%)	2.5% (0.89-4.7%)	1200 (420-2300)	110000 (51000-180000)
Low SDI	1990	Female	25 to 29	33% (21-46%)	67% (54-79%)	1.2 (0.69-2)	3% (1.5-4.8%)	3.4% (1.4-5.9%)	1700 (720-3000)	130000 (66000-210000)
Low SDI	1990	Female	30 to 34	35% (23-49%)	65% (51-77%)	1.4 (0.75-2.2)	3.4% (1.8-5.4%)	4.1% (1.9-6.9%)	2100 (960-3600)	140000 (71000-220000)
Low SDI	1990	Female	35 to 39	38% (25-52%)	62% (48-75%)	1.3 (0.74-2.2)	3.6% (1.8-5.8%)	4.5% (2-7.5%)	2500 (1100-4100)	140000 (68000-220000)
Low SDI	1990	Female	40 to 44	39% (27-53%)	61% (47-73%)	1.3 (0.7-2.2)	3.7% (1.8-5.9%)	4.7% (2.2-7.7%)	2600 (1200-4300)	130000 (63000-210000)
Low SDI	1990	Female	45 to 49	39% (25-53%)	61% (47-75%)	1.3 (0.68-2.3)	4.1% (1.9-6.7%)	5.3% (2.4-8.8%)	3100 (1300-5300)	130000 (60000-220000)
Low SDI	1990	Female	50 to 54	37% (25-52%)	63% (48-75%)	1.3 (0.67-2.3)	4.6% (2.1-7.6%)	5.8% (2.5-9.7%)	3900 (1700-6700)	140000 (64000-250000)
Low SDI	1990	Female	55 to 59	36% (24-51%)	64% (49-76%)	1.3 (0.67-2.3)	4.7% (2-7.9%)	5.8% (2.4-9.7%)	4600 (1900-8000)	150000 (60000-250000)
Low SDI	1990	Female	60 to 64	37% (23-52%)	63% (48-77%)	1.3 (0.65-2.4)	4.2% (1.7-7.4%)	5.1% (2.1-8.9%)	4700 (1900-8300)	130000 (51000-220000)
Low SDI	1990	Female	65 to 69	37% (25-51%)	63% (49-75%)	1.2 (0.63-2.1)	3.9% (1.4-6.9%)	4.6% (1.7-8.1%)	5000 (1800-8900)	110000 (41000-200000)
Low SDI	1990	Female	70 to 74	36% (24-50%)	64% (50-76%)	1 (0.51-1.9)	3.2% (0.75-6.2%)	3.7% (0.84-7.2%)	4000 (870-7800)	71000 (16000-140000)
Low SDI	1990	Female	75 to 79	33% (21-48%)	67% (52-79%)	0.87 (0.39-1.6)	2.4% (0.28-5.1%)	2.8% (0.31-5.9%)	2600 (290-5500)	36000 (4100-76000)
Low SDI	1990	Female	80 to 84	32% (19-47%)	68% (53-81%)	0.79 (0.37-1.4)	1.9% (0.11-4.1%)	2.2% (0.11-4.8%)	1300 (59-2800)	13000 (690-29000)
Low SDI	1990	Female	85 to 89	31% (19-46%)	69% (54-81%)	0.76 (0.35-1.4)	1.4% (-0.095-3.5%)	1.7% (-0.15-4.1%)	510 (-43-1200)	3700 (-260-9100)
Low SDI	1990	Female	90 to 94	33% (21-48%)	67% (52-79%)	0.77 (0.34-1.5)	1.1% (-0.32-3.1%)	1.3% (-0.43-3.6%)	140 (-45-380)	710 (-200-1900)
Low SDI	1990	Female	95 plus	36% (21-55%)	64% (45-79%)	0.8 (0.3-1.7)	1.2% (-0.36-3.3%)	1.3% (-0.52-3.9%)	36 (-14-110)	130 (-39-360)

Location	Year	Sex	Age	Prevalence of current drinkers	Prevalence of abstainers	Population average of standard drinks daily	Percent of total attributable deaths	Percent of total attributable DALYs	Attributable deaths	Attributable DALYs
Low SDI	1990	Male	All Ages	29% (24-35%)	71% (65-76%)	0.89 (0.68-1.1)	1.8% (1.2-2.5%)	3% (1.9-4.1%)	73000 (46000-100000)	3100000 (2100000-4100000)
Low SDI	1990	Male	15 to 19	26% (17-37%)	74% (63-83%)	0.81 (0.44-1.4)	2.6% (1.4-4.2%)	3% (1.4-5.2%)	1400 (590-2400)	120000 (64000-200000)
Low SDI	1990	Male	20 to 24	36% (24-49%)	64% (51-76%)	1.4 (0.76-2.2)	5% (2.8-7.6%)	5.3% (2.6-8.6%)	3300 (1600-5500)	270000 (150000-410000)
Low SDI	1990	Male	25 to 29	42% (28-55%)	58% (45-72%)	1.8 (1.1-2.8)	6.2% (3.7-9.3%)	6.8% (3.5-11%)	4700 (2500-7300)	330000 (200000-490000)
Low SDI	1990	Male	30 to 34	45% (31-59%)	55% (41-69%)	2.1 (1.2-3.2)	7.1% (4.3-10%)	8% (4.4-12%)	5600 (3100-8400)	350000 (210000-500000)
Low SDI	1990	Male	35 to 39	46% (33-60%)	54% (40-67%)	2.2 (1.3-3.5)	7.8% (4.7-11%)	8.9% (5.1-13%)	6400 (3600-9500)	350000 (210000-510000)
Low SDI	1990	Male	40 to 44	47% (34-61%)	53% (39-66%)	2.2 (1.2-3.5)	8% (4.8-12%)	9.4% (5.4-14%)	7000 (4000-10000)	340000 (200000-490000)
Low SDI	1990	Male	45 to 49	48% (34-62%)	52% (38-66%)	2.2 (1.2-3.5)	8.2% (4.9-12%)	9.7% (5.6-14%)	7400 (4200-11000)	310000 (180000-470000)
Low SDI	1990	Male	50 to 54	49% (35-62%)	51% (38-65%)	2.1 (1.2-3.5)	8.3% (4.7-12%)	9.9% (5.5-15%)	7800 (4200-12000)	290000 (160000-440000)
Low SDI	1990	Male	55 to 59	49% (35-62%)	51% (38-65%)	2.1 (1.1-3.4)	8% (4.5-12%)	9.4% (5.2-14%)	8100 (4400-12000)	260000 (140000-400000)
Low SDI	1990	Male	60 to 64	46% (32-61%)	54% (39-68%)	1.8 (0.97-3.2)	6.6% (3.5-10%)	7.6% (4-12%)	7100 (3700-11000)	190000 (100000-310000)
Low SDI	1990	Male	65 to 69	44% (30-58%)	56% (42-70%)	1.5 (0.82-2.5)	5.1% (2.5-8.1%)	5.8% (2.8-9.4%)	5600 (2700-9200)	130000 (63000-210000)
Low SDI	1990	Male	70 to 74	42% (28-56%)	58% (44-72%)	1.2 (0.64-2.1)	3.8% (1.5-6.7%)	4.4% (1.7-7.8%)	4200 (1600-7500)	76000 (30000-130000)
Low SDI	1990	Male	75 to 79	40% (27-55%)	60% (45-73%)	1 (0.5-1.8)	3% (0.92-5.7%)	3.4% (1-6.5%)	2600 (770-5100)	37000 (11000-70000)
Low SDI	1990	Male	80 to 84	38% (24-53%)	62% (47-76%)	0.86 (0.43-1.5)	2.3% (0.67-4.5%)	2.6% (0.71-5.1%)	1100 (300-2200)	12000 (3400-23000)
Low SDI	1990	Male	85 to 89	35% (22-49%)	65% (51-78%)	0.71 (0.35-1.3)	1.7% (0.46-3.3%)	1.9% (0.44-3.8%)	390 (91-790)	3000 (800-5900)
Low SDI	1990	Male	90 to 94	35% (22-49%)	65% (51-78%)	0.64 (0.3-1.2)	1.1% (0.12-2.5%)	1.2% (0.0068-2.8%)	83 (0.39-200)	460 (46-1000)
Low SDI	1990	Male	95 plus	37% (21-55%)	63% (45-79%)	0.62 (0.23-1.3)	1.1% (-0.038-2.7%)	1.1% (-0.24-3%)	18 (-3.9-48)	70 (-2.7-170)
Low SDI	1995	Female	All Ages	24% (20-29%)	76% (71-80%)	0.6 (0.46-0.78)	1% (0.57-1.5%)	1.8% (0.86-2.8%)	45000 (22000-71000)	1700000 (950000-2500000)
Low SDI	1995	Female	15 to 19	24% (16-35%)	76% (65-84%)	0.68 (0.36-1.1)	1.5% (0.67-2.6%)	1.8% (0.61-3.5%)	790 (260-1500)	72000 (33000-120000)
Low SDI	1995	Female	20 to 24	31% (20-43%)	69% (57-80%)	1 (0.54-1.6)	2.1% (1-3.6%)	2.2% (0.75-4.3%)	1400 (480-2600)	120000 (58000-200000)

Location	Year	Sex	Age	Prevalence of current drinkers	Prevalence of abstainers	Population average of standard drinks daily	Percent of total attributable deaths	Percent of total attributable DALYs	Attributable deaths	Attributable DALYs
Low SDI	1995	Female	25 to 29	33% (21-46%)	67% (54-79%)	1.2 (0.68-1.9)	2.5% (1.2-4%)	2.7% (1-4.8%)	1900 (820-3300)	140000 (74000-230000)
Low SDI	1995	Female	30 to 34	35% (23-50%)	65% (50-77%)	1.3 (0.73-2.1)	2.7% (1.4-4.4%)	3.1% (1.3-5.4%)	2300 (1000-4000)	150000 (78000-240000)
Low SDI	1995	Female	35 to 39	38% (25-52%)	62% (48-75%)	1.3 (0.71-2.1)	3% (1.5-4.9%)	3.6% (1.5-6.2%)	2700 (1200-4600)	150000 (75000-250000)
Low SDI	1995	Female	40 to 44	39% (27-53%)	61% (47-73%)	1.3 (0.68-2.1)	3.2% (1.6-5.3%)	4% (1.8-6.7%)	2900 (1400-4900)	140000 (71000-240000)
Low SDI	1995	Female	45 to 49	39% (25-53%)	61% (47-75%)	1.3 (0.66-2.2)	3.6% (1.6-6%)	4.5% (1.9-7.7%)	3500 (1500-6000)	150000 (67000-250000)
Low SDI	1995	Female	50 to 54	37% (25-52%)	63% (48-75%)	1.3 (0.65-2.2)	4.2% (1.8-7%)	5.1% (2.2-8.7%)	4500 (1800-7700)	160000 (69000-280000)
Low SDI	1995	Female	55 to 59	36% (23-51%)	64% (49-77%)	1.3 (0.64-2.2)	4.3% (1.7-7.3%)	5.2% (2.1-8.9%)	4900 (1900-8600)	160000 (62000-270000)
Low SDI	1995	Female	60 to 64	37% (23-52%)	63% (48-77%)	1.3 (0.62-2.3)	4% (1.5-7%)	4.7% (1.8-8.4%)	5200 (2000-9400)	140000 (54000-250000)
Low SDI	1995	Female	65 to 69	37% (25-51%)	63% (49-75%)	1.2 (0.61-2)	3.8% (1.3-6.7%)	4.4% (1.5-7.8%)	5400 (1900-9800)	120000 (43000-220000)
Low SDI	1995	Female	70 to 74	36% (24-50%)	64% (50-76%)	1 (0.48-1.8)	3% (0.62-5.8%)	3.4% (0.7-6.7%)	4400 (850-8800)	79000 (16000-160000)
Low SDI	1995	Female	75 to 79	33% (21-48%)	67% (52-79%)	0.83 (0.37-1.5)	2.2% (0.2-4.8%)	2.6% (0.21-5.5%)	2800 (250-6100)	39000 (3700-84000)
Low SDI	1995	Female	80 to 84	31% (19-46%)	69% (54-81%)	0.75 (0.35-1.4)	1.7% (0.0058-3.8%)	1.9% (-0.0058-4.4%)	1400 (-2.8-3100)	14000 (110-32000)
Low SDI	1995	Female	85 to 89	31% (19-45%)	69% (55-81%)	0.73 (0.34-1.3)	1.3% (-0.17-3.3%)	1.5% (-0.23-3.8%)	560 (-80-1400)	4200 (-520-10000)
Low SDI	1995	Female	90 to 94	33% (21-48%)	67% (52-79%)	0.74 (0.33-1.4)	1% (-0.37-2.9%)	1.2% (-0.49-3.5%)	150 (-62-450)	790 (-280-2300)
Low SDI	1995	Female	95 plus	36% (21-55%)	64% (45-79%)	0.75 (0.29-1.6)	1.1% (-0.4-3.1%)	1.2% (-0.57-3.6%)	41 (-19-130)	150 (-55-430)
Low SDI	1995	Male	All Ages	29% (24-35%)	71% (65-76%)	0.87 (0.67-1.1)	1.9% (1.3-2.5%)	2.9% (1.9-4.1%)	81000 (52000-110000)	3500000 (2300000-4700000)
Low SDI	1995	Male	15 to 19	26% (18-38%)	74% (62-82%)	0.83 (0.45-1.4)	2.8% (1.5-4.4%)	3.2% (1.5-5.5%)	1700 (770-2900)	150000 (80000-240000)
Low SDI	1995	Male	20 to 24	37% (25-49%)	63% (51-75%)	1.3 (0.77-2.1)	5.1% (2.9-7.9%)	5.4% (2.5-8.9%)	3900 (1900-6400)	320000 (180000-480000)
Low SDI	1995	Male	25 to 29	42% (29-55%)	58% (45-71%)	1.8 (1.1-2.7)	5.9% (3.4-8.9%)	6.2% (3.2-10%)	5400 (2900-8300)	380000 (230000-560000)
Low SDI	1995	Male	30 to 34	45% (31-59%)	55% (41-69%)	2 (1.2-3.1)	6.1% (3.6-9.1%)	6.7% (3.6-10%)	6300 (3600-9500)	390000 (240000-570000)
Low SDI	1995	Male	35 to 39	46% (33-60%)	54% (40-67%)	2.1 (1.2-3.3)	6.6% (3.9-9.8%)	7.4% (4.1-11%)	7100 (4100-11000)	390000 (240000-570000)

Location	Year	Sex	Age	Prevalence of current drinkers	Prevalence of abstainers	Population average of standard drinks daily	Percent of total attributable deaths	Percent of total attributable DALYs	Attributable deaths	Attributable DALYs
Low SDI	1995	Male	40 to 44	47% (34-60%)	53% (40-66%)	2.1 (1.2-3.4)	6.9% (4-10%)	7.9% (4.4-12%)	7800 (4500-12000)	380000 (230000-550000)
Low SDI	1995	Male	45 to 49	48% (34-61%)	52% (39-66%)	2.1 (1.2-3.4)	7.1% (4.1-11%)	8.3% (4.6-12%)	8200 (4600-12000)	350000 (200000-520000)
Low SDI	1995	Male	50 to 54	48% (35-62%)	52% (38-65%)	2 (1.1-3.3)	7.5% (4.1-11%)	8.7% (4.7-13%)	8700 (4600-13000)	320000 (180000-490000)
Low SDI	1995	Male	55 to 59	48% (34-62%)	52% (38-66%)	2 (1.1-3.2)	7.3% (4.1-11%)	8.6% (4.7-13%)	8600 (4600-13000)	280000 (150000-420000)
Low SDI	1995	Male	60 to 64	46% (32-61%)	54% (39-68%)	1.8 (0.94-3)	6.1% (3.3-9.5%)	7.1% (3.7-11%)	7900 (4100-13000)	220000 (110000-340000)
Low SDI	1995	Male	65 to 69	44% (30-58%)	56% (42-70%)	1.5 (0.79-2.4)	4.7% (2.3-7.6%)	5.4% (2.6-8.8%)	6100 (2800-10000)	140000 (66000-220000)
Low SDI	1995	Male	70 to 74	41% (28-55%)	59% (45-72%)	1.2 (0.61-2)	3.6% (1.4-6.3%)	4.1% (1.5-7.3%)	4600 (1800-8200)	83000 (33000-150000)
Low SDI	1995	Male	75 to 79	39% (26-54%)	61% (46-74%)	0.96 (0.47-1.7)	2.7% (0.83-5.2%)	3.1% (0.9-6%)	3000 (880-5700)	42000 (13000-80000)
Low SDI	1995	Male	80 to 84	37% (23-51%)	63% (49-77%)	0.8 (0.4-1.4)	2.1% (0.58-4.1%)	2.4% (0.6-4.6%)	1300 (340-2600)	14000 (3900-27000)
Low SDI	1995	Male	85 to 89	34% (22-48%)	66% (52-78%)	0.67 (0.33-1.2)	1.6% (0.43-3.2%)	1.8% (0.41-3.6%)	450 (110-930)	3500 (950-7000)
Low SDI	1995	Male	90 to 94	34% (22-49%)	66% (51-78%)	0.6 (0.28-1.1)	1% (0.081-2.3%)	1.1% (-0.032-2.6%)	94 (-2.3-230)	530 (43-1200)
Low SDI	1995	Male	95 plus	37% (20-55%)	63% (45-80%)	0.57 (0.22-1.2)	1% (-0.066-2.6%)	1% (-0.27-2.8%)	22 (-5.6-59)	86 (-5.3-210)
Low SDI	2000	Female	All Ages	23% (19-28%)	77% (72-81%)	0.59 (0.45-0.76)	1.1% (0.63-1.6%)	1.9% (0.96-3%)	52000 (27000-82000)	1900000 (1100000-2800000)
Low SDI	2000	Female	15 to 19	24% (16-34%)	76% (66-84%)	0.68 (0.37-1.1)	1.5% (0.74-2.6%)	1.9% (0.73-3.6%)	910 (340-1700)	83000 (41000-140000)
Low SDI	2000	Female	20 to 24	29% (20-41%)	71% (59-80%)	0.98 (0.54-1.6)	2.2% (1.1-3.6%)	2.4% (0.88-4.4%)	1500 (590-2800)	140000 (70000-230000)
Low SDI	2000	Female	25 to 29	31% (21-43%)	69% (57-79%)	1.2 (0.67-1.8)	2.3% (1.2-3.8%)	2.4% (1-4.4%)	2100 (960-3700)	160000 (86000-260000)
Low SDI	2000	Female	30 to 34	33% (22-46%)	67% (54-78%)	1.2 (0.71-2)	2.4% (1.3-3.9%)	2.6% (1.2-4.6%)	2500 (1200-4200)	160000 (88000-260000)
Low SDI	2000	Female	35 to 39	36% (25-49%)	64% (51-75%)	1.2 (0.7-2)	2.6% (1.3-4.3%)	3% (1.4-5.3%)	2900 (1400-4900)	160000 (84000-260000)
Low SDI	2000	Female	40 to 44	37% (26-50%)	63% (50-74%)	1.2 (0.67-2)	2.9% (1.5-4.7%)	3.5% (1.6-5.9%)	3200 (1600-5400)	160000 (81000-260000)
Low SDI	2000	Female	45 to 49	37% (25-50%)	63% (50-75%)	1.3 (0.68-2.2)	3.5% (1.7-5.8%)	4.3% (1.9-7.3%)	4000 (1800-6900)	170000 (82000-290000)
Low SDI	2000	Female	50 to 54	36% (25-50%)	64% (50-75%)	1.2 (0.66-2.1)	4.2% (1.9-6.9%)	5.1% (2.3-8.6%)	5200 (2300-8900)	190000 (86000-320000)

Location	Year	Sex	Age	Prevalence of current drinkers	Prevalence of abstainers	Population average of standard drinks daily	Percent of total attributable deaths	Percent of total attributable DALYs	Attributable deaths	Attributable DALYs
Low SDI	2000	Female	55 to 59	35% (24-49%)	65% (51-76%)	1.3 (0.66-2.2)	4.5% (2-7.6%)	5.5% (2.4-9.3%)	6000 (2500-10000)	190000 (82000-320000)
Low SDI	2000	Female	60 to 64	36% (23-50%)	64% (50-77%)	1.2 (0.63-2.2)	4% (1.7-7%)	4.8% (2-8.5%)	5800 (2300-10000)	150000 (62000-280000)
Low SDI	2000	Female	65 to 69	37% (25-50%)	63% (50-75%)	1.2 (0.63-2)	4% (1.5-7%)	4.7% (1.8-8.2%)	6500 (2500-12000)	140000 (56000-260000)
Low SDI	2000	Female	70 to 74	35% (23-48%)	65% (52-77%)	1 (0.49-1.8)	3.1% (0.78-6.1%)	3.6% (0.87-7%)	5000 (1200-10000)	91000 (23000-180000)
Low SDI	2000	Female	75 to 79	32% (20-47%)	68% (53-80%)	0.85 (0.39-1.6)	2.5% (0.39-5.2%)	2.9% (0.43-6%)	3700 (580-7700)	51000 (8300-110000)
Low SDI	2000	Female	80 to 84	31% (19-46%)	69% (54-81%)	0.77 (0.36-1.4)	1.9% (0.15-4.1%)	2.2% (0.16-4.8%)	1900 (150-4100)	19000 (1500-42000)
Low SDI	2000	Female	85 to 89	31% (19-45%)	69% (55-81%)	0.72 (0.34-1.3)	1.4% (-0.11-3.4%)	1.6% (-0.16-3.9%)	690 (-57-1700)	5100 (-360-12000)
Low SDI	2000	Female	90 to 94	33% (21-47%)	67% (53-79%)	0.74 (0.34-1.4)	1.1% (-0.31-3.1%)	1.3% (-0.41-3.6%)	190 (-59-540)	1000 (-260-2700)
Low SDI	2000	Female	95 plus	36% (21-54%)	64% (46-79%)	0.77 (0.3-1.6)	1.2% (-0.34-3.4%)	1.3% (-0.51-3.9%)	53 (-20-160)	190 (-53-540)
Low SDI	2000	Male	All Ages	29% (24-34%)	71% (66-76%)	0.89 (0.69-1.1)	2% (1.4-2.7%)	3.1% (2.1-4.3%)	94000 (62000-130000)	4000000 (2800000-5300000)
Low SDI	2000	Male	15 to 19	26% (18-36%)	74% (64-82%)	0.85 (0.47-1.4)	2.9% (1.7-4.5%)	3.4% (1.7-5.7%)	2000 (970-3400)	180000 (99000-280000)
Low SDI	2000	Male	20 to 24	36% (26-48%)	64% (52-74%)	1.4 (0.8-2.1)	5.5% (3.2-8.1%)	5.9% (3.1-9.3%)	4700 (2500-7600)	380000 (220000-570000)
Low SDI	2000	Male	25 to 29	42% (30-54%)	58% (46-70%)	1.8 (1.1-2.7)	6% (3.6-9%)	6.4% (3.4-10%)	6400 (3600-9700)	450000 (280000-650000)
Low SDI	2000	Male	30 to 34	45% (32-57%)	55% (43-68%)	2.1 (1.3-3.1)	6% (3.7-8.8%)	6.5% (3.7-9.9%)	7300 (4300-11000)	450000 (290000-650000)
Low SDI	2000	Male	35 to 39	46% (34-59%)	54% (41-66%)	2.2 (1.3-3.4)	6.2% (3.7-9%)	6.9% (3.9-10%)	8100 (4800-12000)	440000 (280000-640000)
Low SDI	2000	Male	40 to 44	47% (35-60%)	53% (40-65%)	2.2 (1.3-3.4)	6.6% (4-9.5%)	7.5% (4.3-11%)	8800 (5200-13000)	420000 (260000-610000)
Low SDI	2000	Male	45 to 49	48% (35-60%)	52% (40-65%)	2.2 (1.2-3.4)	7% (4.2-10%)	8.1% (4.7-12%)	9400 (5500-14000)	400000 (240000-590000)
Low SDI	2000	Male	50 to 54	49% (36-61%)	51% (39-64%)	2.1 (1.2-3.4)	7.6% (4.4-11%)	8.8% (5-13%)	9800 (5500-15000)	370000 (210000-550000)
Low SDI	2000	Male	55 to 59	48% (36-61%)	52% (39-64%)	2.1 (1.2-3.3)	7.7% (4.5-11%)	9% (5.1-13%)	10000 (5600-15000)	320000 (180000-480000)
Low SDI	2000	Male	60 to 64	46% (33-60%)	54% (40-67%)	1.8 (1-3.1)	6.3% (3.4-9.8%)	7.4% (3.9-11%)	8500 (4400-13000)	230000 (120000-360000)
Low SDI	2000	Male	65 to 69	44% (31-57%)	56% (43-69%)	1.5 (0.85-2.4)	5% (2.5-8.1%)	5.8% (2.9-9.4%)	7200 (3500-12000)	160000 (81000-260000)

Location	Year	Sex	Age	Prevalence of current drinkers	Prevalence of abstainers	Population average of standard drinks daily	Percent of total attributable deaths	Percent of total attributable DALYs	Attributable deaths	Attributable DALYs
Low SDI	2000	Male	70 to 74	42% (29-55%)	58% (45-71%)	1.2 (0.67-2)	3.8% (1.5-6.7%)	4.4% (1.7-7.7%)	5200 (2100-9200)	94000 (38000-170000)
Low SDI	2000	Male	75 to 79	40% (27-55%)	60% (45-73%)	1 (0.53-1.8)	3.1% (1-5.8%)	3.6% (1.1-6.7%)	3600 (1200-6900)	51000 (17000-96000)
Low SDI	2000	Male	80 to 84	37% (23-52%)	63% (48-77%)	0.85 (0.44-1.4)	2.3% (0.74-4.5%)	2.7% (0.79-5.1%)	1700 (520-3300)	18000 (5800-34000)
Low SDI	2000	Male	85 to 89	34% (22-48%)	66% (52-78%)	0.69 (0.35-1.2)	1.7% (0.49-3.3%)	1.9% (0.47-3.8%)	600 (150-1200)	4600 (1300-8900)
Low SDI	2000	Male	90 to 94	35% (23-49%)	65% (51-77%)	0.63 (0.3-1.2)	1.2% (0.17-2.5%)	1.3% (0.07-2.9%)	130 (7.6-300)	710 (100-1500)
Low SDI	2000	Male	95 plus	38% (21-56%)	62% (44-79%)	0.6 (0.23-1.2)	1.2% (0.027-2.9%)	1.3% (-0.16-3.2%)	31 (-3.9-79)	120 (2.8-280)
Low SDI	2005	Female	All Ages	23% (19-28%)	77% (72-81%)	0.6 (0.45-0.78)	1.1% (0.65-1.6%)	1.9% (0.96-3%)	53000 (27000-85000)	1900000 (1100000-2900000)
Low SDI	2005	Female	15 to 19	24% (16-34%)	76% (66-84%)	0.69 (0.38-1.1)	1.4% (0.71-2.4%)	1.7% (0.65-3.3%)	900 (340-1700)	86000 (42000-150000)
Low SDI	2005	Female	20 to 24	30% (20-41%)	70% (59-80%)	1 (0.55-1.6)	2.3% (1.2-3.7%)	2.4% (0.93-4.5%)	1600 (610-2900)	150000 (76000-240000)
Low SDI	2005	Female	25 to 29	31% (21-43%)	69% (57-79%)	1.2 (0.68-1.9)	2.3% (1.3-3.8%)	2.5% (1.1-4.6%)	2100 (980-3700)	170000 (93000-260000)
Low SDI	2005	Female	30 to 34	33% (23-46%)	67% (54-77%)	1.3 (0.73-2)	2.3% (1.2-3.7%)	2.5% (1.2-4.4%)	2500 (1200-4200)	170000 (93000-260000)
Low SDI	2005	Female	35 to 39	36% (25-49%)	64% (51-75%)	1.3 (0.71-2)	2.4% (1.3-3.9%)	2.8% (1.3-4.8%)	2900 (1400-4800)	160000 (87000-270000)
Low SDI	2005	Female	40 to 44	37% (26-50%)	63% (50-74%)	1.3 (0.69-2.1)	2.7% (1.4-4.3%)	3.2% (1.5-5.4%)	3200 (1500-5400)	160000 (82000-260000)
Low SDI	2005	Female	45 to 49	37% (25-50%)	63% (50-75%)	1.3 (0.68-2.2)	3.3% (1.6-5.5%)	4.1% (1.9-6.9%)	4100 (1900-7100)	180000 (85000-300000)
Low SDI	2005	Female	50 to 54	36% (24-50%)	64% (50-76%)	1.3 (0.66-2.2)	4.1% (1.9-6.7%)	5% (2.3-8.4%)	5300 (2300-9200)	200000 (88000-340000)
Low SDI	2005	Female	55 to 59	35% (23-49%)	65% (51-77%)	1.3 (0.65-2.2)	4.3% (1.9-7.3%)	5.3% (2.3-9.1%)	6100 (2500-11000)	190000 (81000-340000)
Low SDI	2005	Female	60 to 64	35% (23-50%)	65% (50-77%)	1.3 (0.63-2.3)	4% (1.6-7%)	4.8% (2-8.4%)	6100 (2400-11000)	160000 (64000-290000)
Low SDI	2005	Female	65 to 69	36% (24-50%)	64% (50-76%)	1.2 (0.61-2)	3.8% (1.4-6.7%)	4.5% (1.7-8%)	6300 (2200-11000)	140000 (50000-250000)
Low SDI	2005	Female	70 to 74	35% (23-48%)	65% (52-77%)	1 (0.5-1.8)	3.1% (0.77-6.1%)	3.7% (0.87-7.2%)	5400 (1200-11000)	98000 (23000-190000)
Low SDI	2005	Female	75 to 79	32% (20-47%)	68% (53-80%)	0.82 (0.38-1.5)	2.4% (0.31-5%)	2.7% (0.35-5.8%)	3500 (420-7500)	49000 (6000-100000)
Low SDI	2005	Female	80 to 84	31% (19-46%)	69% (54-81%)	0.78 (0.37-1.4)	1.9% (0.16-4.2%)	2.2% (0.19-4.8%)	2100 (160-4600)	22000 (1700-47000)

Location	Year	Sex	Age	Prevalence of current drinkers	Prevalence of abstainers	Population average of standard drinks daily	Percent of total attributable deaths	Percent of total attributable DALYs	Attributable deaths	Attributable DALYs
Low SDI	2005	Female	85 to 89	31% (19-45%)	69% (55-81%)	0.75 (0.35-1.4)	1.5% (-0.06-3.5%)	1.7% (-0.094-4.1%)	860 (-48-2100)	6400 (-270-15000)
Low SDI	2005	Female	90 to 94	32% (20-47%)	68% (53-80%)	0.74 (0.33-1.4)	1.2% (-0.28-3.1%)	1.3% (-0.39-3.7%)	230 (-66-630)	1200 (-280-3200)
Low SDI	2005	Female	95 plus	36% (21-54%)	64% (46-79%)	0.78 (0.3-1.6)	1.3% (-0.31-3.5%)	1.4% (-0.46-4.1%)	64 (-20-180)	220 (-54-620)
Low SDI	2005	Male	All Ages	29% (24-34%)	71% (66-76%)	0.9 (0.69-1.1)	2.2% (1.6-3%)	3.4% (2.2-4.7%)	99000 (65000-140000)	4300000 (3000000-5700000)
Low SDI	2005	Male	15 to 19	27% (19-37%)	73% (63-81%)	0.88 (0.5-1.4)	3% (1.8-4.7%)	3.6% (1.8-5.9%)	2200 (1100-3600)	190000 (110000-300000)
Low SDI	2005	Male	20 to 24	37% (27-48%)	63% (52-73%)	1.4 (0.82-2.2)	6.1% (3.7-8.9%)	6.7% (3.6-11%)	5200 (2700-8300)	420000 (250000-630000)
Low SDI	2005	Male	25 to 29	41% (30-53%)	59% (47-70%)	1.8 (1.1-2.7)	6.8% (4.2-9.9%)	7.5% (4.1-12%)	7000 (3900-11000)	500000 (310000-720000)
Low SDI	2005	Male	30 to 34	44% (32-56%)	56% (44-68%)	2.1 (1.3-3.1)	6.5% (4.1-9.3%)	7.2% (4.1-11%)	7900 (4700-12000)	500000 (320000-710000)
Low SDI	2005	Male	35 to 39	45% (34-58%)	55% (42-66%)	2.2 (1.3-3.4)	6.3% (4-9.2%)	7.1% (4.2-11%)	8400 (5000-12000)	460000 (290000-660000)
Low SDI	2005	Male	40 to 44	46% (35-59%)	54% (41-65%)	2.2 (1.3-3.4)	6.5% (4-9.3%)	7.4% (4.4-11%)	9100 (5400-13000)	440000 (270000-640000)
Low SDI	2005	Male	45 to 49	48% (35-60%)	52% (40-65%)	2.2 (1.2-3.5)	7% (4.3-10%)	8.1% (4.8-12%)	9700 (5700-14000)	420000 (250000-610000)
Low SDI	2005	Male	50 to 54	48% (36-61%)	52% (39-64%)	2.1 (1.2-3.4)	7.5% (4.4-11%)	8.9% (5.1-13%)	10000 (5800-16000)	390000 (220000-570000)
Low SDI	2005	Male	55 to 59	48% (36-61%)	52% (39-64%)	2.1 (1.2-3.4)	7.7% (4.5-11%)	9.2% (5.2-14%)	10000 (5800-16000)	340000 (190000-510000)
Low SDI	2005	Male	60 to 64	46% (33-60%)	54% (40-67%)	1.8 (0.99-3.1)	6.4% (3.5-9.9%)	7.5% (4-12%)	9300 (4800-15000)	250000 (140000-400000)
Low SDI	2005	Male	65 to 69	43% (30-57%)	57% (43-70%)	1.5 (0.84-2.5)	5% (2.5-8.1%)	5.8% (2.8-9.5%)	7300 (3500-12000)	170000 (82000-270000)
Low SDI	2005	Male	70 to 74	42% (29-55%)	58% (45-71%)	1.3 (0.68-2.1)	3.9% (1.6-6.9%)	4.6% (1.8-8%)	5900 (2400-10000)	110000 (44000-190000)
Low SDI	2005	Male	75 to 79	40% (27-54%)	60% (46-73%)	1.1 (0.52-1.9)	3.1% (1-5.8%)	3.6% (1.1-6.8%)	3800 (1200-7200)	54000 (18000-100000)
Low SDI	2005	Male	80 to 84	38% (24-53%)	62% (47-76%)	0.88 (0.45-1.5)	2.4% (0.79-4.6%)	2.8% (0.84-5.3%)	1900 (590-3700)	20000 (6600-39000)
Low SDI	2005	Male	85 to 89	34% (22-48%)	66% (52-78%)	0.69 (0.35-1.2)	1.7% (0.52-3.3%)	1.9% (0.51-3.8%)	730 (190-1400)	5600 (1700-11000)
Low SDI	2005	Male	90 to 94	34% (22-49%)	66% (51-78%)	0.6 (0.29-1.1)	1.1% (0.16-2.5%)	1.2% (0.059-2.8%)	160 (7.7-360)	860 (120-1900)
Low SDI	2005	Male	95 plus	38% (21-56%)	62% (44-79%)	0.59 (0.23-1.2)	1.3% (0.061-3%)	1.3% (-0.12-3.4%)	38 (-3.6-97)	150 (6.6-340)

Location	Year	Sex	Age	Prevalence of current drinkers	Prevalence of abstainers	Population average of standard drinks daily	Percent of total attributable deaths	Percent of total attributable DALYs	Attributable deaths	Attributable DALYs
Low SDI	2010	Female	All Ages	23% (19-28%)	77% (72-81%)	0.61 (0.44-0.84)	1.2% (0.65-1.8%)	2% (0.94-3.3%)	55000 (25000-90000)	2000000 (1100000-3000000)
Low SDI	2010	Female	15 to 19	24% (16-34%)	76% (66-84%)	0.67 (0.34-1.2)	1.3% (0.62-2.2%)	1.5% (0.51-2.9%)	850 (300-1600)	86000 (42000-150000)
Low SDI	2010	Female	20 to 24	30% (20-42%)	70% (58-80%)	1 (0.53-1.7)	2.2% (1.2-3.6%)	2.3% (0.87-4.4%)	1500 (580-2800)	150000 (79000-240000)
Low SDI	2010	Female	25 to 29	32% (22-44%)	68% (56-78%)	1.2 (0.67-2)	2.4% (1.3-3.9%)	2.7% (1.1-4.8%)	2100 (960-3700)	170000 (96000-270000)
Low SDI	2010	Female	30 to 34	34% (23-47%)	66% (53-77%)	1.3 (0.73-2.2)	2.4% (1.3-3.9%)	2.8% (1.3-4.8%)	2600 (1200-4400)	180000 (97000-280000)
Low SDI	2010	Female	35 to 39	37% (25-50%)	63% (50-75%)	1.3 (0.7-2.2)	2.5% (1.3-4.1%)	3% (1.4-5.2%)	3000 (1400-5000)	170000 (90000-280000)
Low SDI	2010	Female	40 to 44	38% (27-51%)	62% (49-73%)	1.3 (0.67-2.2)	2.8% (1.4-4.5%)	3.4% (1.6-5.7%)	3300 (1500-5600)	170000 (83000-270000)
Low SDI	2010	Female	45 to 49	38% (25-51%)	62% (49-75%)	1.3 (0.66-2.3)	3.4% (1.6-5.6%)	4.3% (1.9-7.3%)	4200 (1800-7300)	180000 (82000-310000)
Low SDI	2010	Female	50 to 54	37% (25-52%)	63% (48-75%)	1.3 (0.64-2.3)	4.1% (1.8-6.8%)	5.2% (2.3-8.7%)	5400 (2300-9500)	200000 (87000-350000)
Low SDI	2010	Female	55 to 59	36% (23-51%)	64% (49-77%)	1.3 (0.64-2.3)	4.4% (1.8-7.5%)	5.5% (2.2-9.4%)	6300 (2400-11000)	200000 (78000-360000)
Low SDI	2010	Female	60 to 64	36% (23-51%)	64% (49-77%)	1.3 (0.6-2.3)	3.9% (1.4-6.9%)	4.7% (1.8-8.5%)	6200 (2100-11000)	170000 (58000-310000)
Low SDI	2010	Female	65 to 69	37% (25-51%)	63% (49-75%)	1.2 (0.59-2.1)	3.7% (1.3-6.7%)	4.5% (1.5-8.1%)	6700 (2100-12000)	150000 (48000-280000)
Low SDI	2010	Female	70 to 74	35% (24-49%)	65% (51-76%)	1 (0.48-1.8)	2.9% (0.56-6%)	3.4% (0.64-7.1%)	5300 (910-11000)	96000 (17000-200000)
Low SDI	2010	Female	75 to 79	33% (21-48%)	67% (52-79%)	0.86 (0.38-1.6)	2.4% (0.23-5.1%)	2.8% (0.26-6%)	4000 (310-8800)	56000 (4600-120000)
Low SDI	2010	Female	80 to 84	32% (19-47%)	68% (53-81%)	0.77 (0.35-1.4)	1.8% (0.04-4.1%)	2.1% (0.043-4.8%)	2200 (3.3-5200)	23000 (150-53000)
Low SDI	2010	Female	85 to 89	31% (19-45%)	69% (55-81%)	0.74 (0.34-1.4)	1.4% (-0.13-3.5%)	1.7% (-0.16-4.1%)	970 (-100-2400)	7200 (-700-18000)
Low SDI	2010	Female	90 to 94	33% (20-47%)	67% (53-80%)	0.75 (0.33-1.4)	1.1% (-0.34-3.2%)	1.3% (-0.47-3.8%)	280 (-99-790)	1400 (-450-4000)
Low SDI	2010	Female	95 plus	36% (20-53%)	64% (47-80%)	0.76 (0.29-1.6)	1.2% (-0.37-3.5%)	1.3% (-0.54-4%)	71 (-29-220)	250 (-79-740)
Low SDI	2010	Male	All Ages	28% (23-34%)	72% (66-77%)	0.89 (0.65-1.2)	2.4% (1.7-3.4%)	3.6% (2.3-5.1%)	110000 (66000-150000)	4600000 (3100000-6300000)
Low SDI	2010	Male	15 to 19	26% (18-37%)	74% (63-82%)	0.82 (0.44-1.4)	2.7% (1.6-4.3%)	3% (1.4-5.2%)	2100 (990-3700)	200000 (110000-320000)
Low SDI	2010	Male	20 to 24	36% (26-48%)	64% (52-74%)	1.4 (0.76-2.2)	6% (3.6-8.8%)	6.4% (3.3-10%)	5400 (2700-8900)	460000 (270000-690000)

Location	Year	Sex	Age	Prevalence of current drinkers	Prevalence of abstainers	Population average of standard drinks daily	Percent of total attributable deaths	Percent of total attributable DALYs	Attributable deaths	Attributable DALYs
Low SDI	2010	Male	25 to 29	41% (29-53%)	59% (47-71%)	1.8 (1.1-2.8)	7.1% (4.4-10%)	7.9% (4.3-12%)	7600 (4100-12000)	550000 (340000-810000)
Low SDI	2010	Male	30 to 34	43% (31-57%)	57% (43-69%)	2 (1.2-3.2)	7% (4.4-10%)	8% (4.6-12%)	8600 (4900-13000)	550000 (340000-790000)
Low SDI	2010	Male	35 to 39	45% (33-58%)	55% (42-67%)	2.1 (1.2-3.5)	6.9% (4.3-9.9%)	7.9% (4.6-12%)	9100 (5300-14000)	510000 (320000-750000)
Low SDI	2010	Male	40 to 44	46% (34-59%)	54% (41-66%)	2.2 (1.2-3.6)	7% (4.3-10%)	8.2% (4.9-12%)	9700 (5600-15000)	480000 (290000-700000)
Low SDI	2010	Male	45 to 49	47% (34-60%)	53% (40-66%)	2.2 (1.2-3.6)	7.4% (4.5-11%)	8.7% (5.1-13%)	10000 (5900-16000)	440000 (260000-660000)
Low SDI	2010	Male	50 to 54	48% (35-61%)	52% (39-65%)	2.1 (1.1-3.6)	7.8% (4.5-12%)	9.3% (5.2-14%)	11000 (6000-17000)	410000 (230000-620000)
Low SDI	2010	Male	55 to 59	47% (35-61%)	53% (39-65%)	2 (1.1-3.4)	7.7% (4.3-11%)	9.2% (5.1-14%)	11000 (6000-17000)	360000 (200000-550000)
Low SDI	2010	Male	60 to 64	46% (32-61%)	54% (39-68%)	1.8 (0.94-3.2)	6.3% (3.3-9.9%)	7.5% (3.8-12%)	9800 (4900-16000)	270000 (140000-430000)
Low SDI	2010	Male	65 to 69	44% (30-58%)	56% (42-70%)	1.5 (0.79-2.5)	4.9% (2.3-8.1%)	5.8% (2.7-9.5%)	8000 (3600-14000)	180000 (85000-310000)
Low SDI	2010	Male	70 to 74	42% (28-55%)	58% (45-72%)	1.2 (0.63-2.2)	3.7% (1.4-6.7%)	4.3% (1.6-7.8%)	6000 (2200-11000)	110000 (41000-200000)
Low SDI	2010	Male	75 to 79	40% (26-55%)	60% (45-74%)	1 (0.5-1.9)	3% (0.9-5.8%)	3.4% (1-6.7%)	4200 (1200-8300)	59000 (18000-120000)
Low SDI	2010	Male	80 to 84	38% (24-53%)	62% (47-76%)	0.88 (0.42-1.6)	2.3% (0.69-4.5%)	2.7% (0.74-5.3%)	2200 (600-4400)	23000 (6800-46000)
Low SDI	2010	Male	85 to 89	34% (22-49%)	66% (51-78%)	0.7 (0.34-1.3)	1.7% (0.47-3.4%)	1.9% (0.45-3.9%)	820 (200-1700)	6300 (1700-13000)
Low SDI	2010	Male	90 to 94	34% (22-49%)	66% (51-78%)	0.6 (0.28-1.1)	1.1% (0.12-2.4%)	1.1% (0.0095-2.7%)	180 (1.8-440)	1000 (120-2300)
Low SDI	2010	Male	95 plus	37% (21-55%)	63% (45-79%)	0.56 (0.22-1.1)	1.2% (0.03-2.8%)	1.2% (-0.16-3.1%)	45 (-5.8-120)	170 (4.5-420)
Low SDI	2016	Female	All Ages	24% (20-29%)	76% (71-80%)	0.63 (0.36-1)	1.4% (0.68-2.3%)	2.4% (0.88-4.2%)	60000 (22000-110000)	2200000 (1100000-3600000)
Low SDI	2016	Female	15 to 19	24% (17-35%)	76% (65-83%)	0.67 (0.3-1.3)	1.3% (0.61-2.3%)	1.6% (0.5-3.3%)	860 (270-1800)	92000 (42000-160000)
Low SDI	2016	Female	20 to 24	30% (20-42%)	70% (58-80%)	1 (0.45-1.9)	2.3% (1.2-3.8%)	2.5% (0.87-4.8%)	1500 (520-3000)	160000 (82000-270000)
Low SDI	2016	Female	25 to 29	32% (21-44%)	68% (56-79%)	1.2 (0.57-2.3)	2.7% (1.5-4.3%)	3.1% (1.3-5.7%)	2100 (870-3900)	190000 (99000-300000)
Low SDI	2016	Female	30 to 34	34% (23-47%)	66% (53-77%)	1.3 (0.63-2.5)	2.9% (1.5-4.6%)	3.5% (1.5-6.2%)	2700 (1200-4900)	200000 (100000-320000)
Low SDI	2016	Female	35 to 39	37% (25-49%)	63% (51-75%)	1.3 (0.61-2.5)	3.1% (1.6-5%)	3.9% (1.8-6.8%)	3300 (1400-5800)	200000 (96000-330000)

Location	Year	Sex	Age	Prevalence of current drinkers	Prevalence of abstainers	Population average of standard drinks daily	Percent of total attributable deaths	Percent of total attributable DALYs	Attributable deaths	Attributable DALYs
Low SDI	2016	Female	40 to 44	38% (27-51%)	62% (49-73%)	1.3 (0.6-2.5)	3.3% (1.6-5.4%)	4.4% (2-7.4%)	3700 (1600-6500)	190000 (88000-320000)
Low SDI	2016	Female	45 to 49	38% (26-51%)	62% (49-74%)	1.3 (0.57-2.6)	3.8% (1.7-6.7%)	5.1% (2.1-9%)	4600 (1800-8300)	200000 (82000-360000)
Low SDI	2016	Female	50 to 54	37% (25-52%)	63% (48-75%)	1.3 (0.55-2.6)	4.3% (1.7-7.6%)	5.7% (2.2-10%)	5800 (2100-11000)	220000 (81000-390000)
Low SDI	2016	Female	55 to 59	36% (23-50%)	64% (50-77%)	1.3 (0.54-2.6)	4.4% (1.5-8.1%)	5.7% (2-10%)	6700 (2100-13000)	210000 (69000-400000)
Low SDI	2016	Female	60 to 64	36% (23-51%)	64% (49-77%)	1.3 (0.53-2.6)	3.9% (1.2-7.5%)	4.9% (1.5-9.3%)	6700 (1900-13000)	180000 (50000-360000)
Low SDI	2016	Female	65 to 69	37% (24-51%)	63% (49-76%)	1.2 (0.51-2.3)	3.6% (0.91-7.1%)	4.4% (1.1-8.7%)	7100 (1700-14000)	160000 (37000-320000)
Low SDI	2016	Female	70 to 74	36% (24-49%)	64% (51-76%)	1 (0.42-2.1)	2.9% (0.29-6.5%)	3.4% (0.33-7.7%)	5900 (510-14000)	110000 (9900-240000)
Low SDI	2016	Female	75 to 79	33% (21-48%)	67% (52-79%)	0.85 (0.33-1.8)	2.3% (0.0096-5.5%)	2.7% (0.017-6.5%)	4300 (-37-11000)	60000 (-580-150000)
Low SDI	2016	Female	80 to 84	32% (19-47%)	68% (53-81%)	0.78 (0.31-1.6)	1.8% (-0.15-4.5%)	2.1% (-0.17-5.2%)	2600 (-270-6600)	27000 (-2800-68000)
Low SDI	2016	Female	85 to 89	32% (20-46%)	68% (54-80%)	0.76 (0.3-1.6)	1.5% (-0.27-4%)	1.7% (-0.33-4.7%)	1200 (-250-3400)	9100 (-1800-25000)
Low SDI	2016	Female	90 to 94	33% (21-48%)	67% (52-79%)	0.76 (0.29-1.6)	1.1% (-0.47-3.6%)	1.3% (-0.6-4.2%)	350 (-160-1100)	1800 (-760-5800)
Low SDI	2016	Female	95 plus	36% (21-54%)	64% (46-79%)	0.81 (0.27-1.8)	1.3% (-0.41-4%)	1.5% (-0.58-4.7%)	100 (-42-330)	370 (-120-1100)
Low SDI	2016	Male	All Ages	29% (24-34%)	71% (66-76%)	0.91 (0.53-1.5)	3% (1.8-4.3%)	4.2% (2.3-6.4%)	120000 (62000-180000)	5200000 (3100000-7600000)
Low SDI	2016	Male	15 to 19	26% (18-37%)	74% (63-82%)	0.82 (0.38-1.5)	2.8% (1.5-4.6%)	3.1% (1.3-5.7%)	2400 (950-4400)	230000 (120000-380000)
Low SDI	2016	Male	20 to 24	36% (25-48%)	64% (52-75%)	1.3 (0.64-2.4)	6.1% (3.5-9.4%)	6.5% (3-11%)	5900 (2600-10000)	520000 (280000-820000)
Low SDI	2016	Male	25 to 29	41% (29-53%)	59% (47-71%)	1.8 (0.89-3.2)	7.5% (4.5-11%)	8.4% (4.3-14%)	8300 (4000-14000)	620000 (350000-950000)
Low SDI	2016	Male	30 to 34	44% (31-57%)	56% (43-69%)	2.1 (1-3.7)	7.8% (4.6-11%)	9% (4.8-14%)	9500 (4800-15000)	620000 (350000-930000)
Low SDI	2016	Male	35 to 39	45% (33-57%)	55% (43-67%)	2.2 (1-3.9)	7.7% (4.5-11%)	9.1% (5-14%)	10000 (5300-16000)	580000 (330000-880000)
Low SDI	2016	Male	40 to 44	46% (34-59%)	54% (41-66%)	2.2 (1-4)	7.9% (4.5-12%)	9.5% (5.2-14%)	11000 (5600-17000)	540000 (300000-830000)
Low SDI	2016	Male	45 to 49	47% (34-60%)	53% (40-66%)	2.2 (1-4.1)	8.2% (4.5-12%)	9.9% (5.3-15%)	11000 (5700-18000)	500000 (260000-770000)
Low SDI	2016	Male	50 to 54	48% (35-61%)	52% (39-65%)	2.2 (0.98-4.1)	8.2% (4.3-13%)	10% (5-16%)	12000 (5700-19000)	450000 (220000-720000)

Location	Year	Sex	Age	Prevalence of current drinkers	Prevalence of abstainers	Population average of standard drinks daily	Percent of total attributable deaths	Percent of total attributable DALYs	Attributable deaths	Attributable DALYs
Low SDI	2016	Male	55 to 59	47% (35-61%)	53% (39-65%)	2.1 (0.95-3.9)	7.8% (3.9-12%)	9.5% (4.7-15%)	12000 (5600-19000)	390000 (190000-630000)
Low SDI	2016	Male	60 to 64	46% (32-60%)	54% (40-68%)	1.8 (0.81-3.5)	6.3% (2.9-10%)	7.5% (3.3-13%)	11000 (4600-18000)	290000 (130000-500000)
Low SDI	2016	Male	65 to 69	43% (30-57%)	57% (43-70%)	1.5 (0.68-2.8)	4.8% (1.9-8.6%)	5.7% (2.2-10%)	8600 (3200-16000)	200000 (76000-360000)
Low SDI	2016	Male	70 to 74	42% (28-55%)	58% (45-72%)	1.2 (0.54-2.4)	3.6% (1.1-7.1%)	4.2% (1.3-8.4%)	6600 (1900-13000)	120000 (36000-240000)
Low SDI	2016	Male	75 to 79	40% (26-55%)	60% (45-74%)	1 (0.43-2.1)	2.9% (0.72-6.1%)	3.3% (0.8-7.2%)	4500 (1000-9900)	64000 (16000-140000)
Low SDI	2016	Male	80 to 84	38% (24-53%)	62% (47-76%)	0.88 (0.38-1.7)	2.3% (0.52-4.9%)	2.6% (0.54-5.7%)	2500 (500-5500)	27000 (5900-58000)
Low SDI	2016	Male	85 to 89	35% (22-50%)	65% (50-78%)	0.73 (0.31-1.5)	1.7% (0.37-3.9%)	1.9% (0.34-4.5%)	1000 (180-2400)	8000 (1700-18000)
Low SDI	2016	Male	90 to 94	35% (22-49%)	65% (51-78%)	0.63 (0.25-1.3)	1.2% (0.088-2.9%)	1.2% (-0.025-3.3%)	240 (-6.1-650)	1300 (97-3400)
Low SDI	2016	Male	95 plus	38% (21-55%)	62% (45-79%)	0.58 (0.2-1.3)	1.2% (0.021-3.1%)	1.2% (-0.17-3.5%)	60 (-8.7-170)	230 (2.7-610)
China	1990	Female	All Ages	12% (9.9-14%)	88% (86-90%)	0.14 (0.11-0.17)	0.85% (0.61-1.1%)	1.3% (0.81-1.8%)	49000 (31000-68000)	1500000 (1100000-2000000)
China	1990	Female	15 to 19	8.7% (5.4-13%)	91% (87-95%)	0.086 (0.046-0.15)	0.63% (0.42-0.92%)	0.72% (0.38-1.2%)	410 (220-710)	51000 (34000-74000)
China	1990	Female	20 to 24	11% (6.4-17%)	89% (83-94%)	0.095 (0.049-0.16)	0.91% (0.59-1.3%)	0.67% (0.23-1.3%)	560 (200-1100)	97000 (61000-140000)
China	1990	Female	25 to 29	12% (7.3-19%)	88% (81-93%)	0.12 (0.061-0.2)	1% (0.68-1.4%)	0.79% (0.3-1.5%)	540 (210-1000)	87000 (58000-120000)
China	1990	Female	30 to 34	14% (8.3-23%)	86% (77-92%)	0.16 (0.081-0.26)	1% (0.7-1.4%)	1% (0.54-1.7%)	720 (370-1200)	82000 (55000-120000)
China	1990	Female	35 to 39	17% (9.7-25%)	83% (75-90%)	0.21 (0.11-0.34)	1.2% (0.84-1.6%)	1.6% (0.99-2.3%)	1400 (870-2100)	110000 (75000-150000)
China	1990	Female	40 to 44	18% (11-28%)	82% (72-89%)	0.26 (0.15-0.41)	1.4% (0.98-1.9%)	2.1% (1.4-3%)	1800 (1200-2600)	100000 (76000-140000)
China	1990	Female	45 to 49	18% (11-27%)	82% (73-89%)	0.29 (0.16-0.44)	1.6% (1.1-2.2%)	2.5% (1.6-3.5%)	2400 (1600-3500)	110000 (75000-150000)
China	1990	Female	50 to 54	18% (11-27%)	82% (73-89%)	0.3 (0.17-0.47)	1.7% (1.2-2.5%)	2.6% (1.7-3.7%)	3700 (2400-5100)	140000 (92000-190000)
China	1990	Female	55 to 59	18% (11-26%)	82% (74-89%)	0.31 (0.18-0.49)	1.9% (1.2-2.7%)	2.7% (1.7-3.8%)	5400 (3500-7700)	170000 (110000-240000)
China	1990	Female	60 to 64	18% (11-27%)	82% (73-89%)	0.32 (0.18-0.54)	1.7% (1.1-2.6%)	2.3% (1.5-3.4%)	6400 (4000-9300)	170000 (110000-250000)
China	1990	Female	65 to 69	18% (12-26%)	82% (74-88%)	0.34 (0.2-0.52)	1.6% (1-2.4%)	2.1% (1.3-3%)	7500 (4700-11000)	160000 (100000-240000)

Location	Year	Sex	Age	Prevalence of current drinkers	Prevalence of abstainers	Population average of standard drinks daily	Percent of total attributable deaths	Percent of total attributable DALYs	Attributable deaths	Attributable DALYs
China	1990	Female	70 to 74	18% (11-25%)	82% (75-89%)	0.33 (0.19-0.54)	1.3% (0.75-2.1%)	1.7% (0.93-2.6%)	7900 (4500-12000)	140000 (77000-220000)
China	1990	Female	75 to 79	16% (10-24%)	84% (76-90%)	0.3 (0.16-0.51)	0.97% (0.42-1.7%)	1.2% (0.54-2%)	5600 (2500-9500)	76000 (32000-130000)
China	1990	Female	80 to 84	16% (9.7-23%)	84% (77-90%)	0.3 (0.17-0.48)	0.62% (0.12-1.2%)	0.73% (0.16-1.5%)	2700 (580-5400)	28000 (5600-55000)
China	1990	Female	85 to 89	16% (10-24%)	84% (76-90%)	0.31 (0.16-0.52)	0.43% (-0.011-1%)	0.51% (-0.019-1.2%)	1200 (-45-2800)	8800 (-230-21000)
China	1990	Female	90 to 94	18% (12-27%)	82% (73-88%)	0.35 (0.19-0.6)	0.3% (-0.19-1%)	0.33% (-0.23-1.1%)	310 (-210-1000)	1600 (-1000-5200)
China	1990	Female	95 plus	21% (12-32%)	79% (68-88%)	0.41 (0.18-0.76)	0.25% (-0.39-1.2%)	0.24% (-0.49-1.3%)	55 (-110-300)	220 (-350-1000)
China	1990	Male	All Ages	41% (37-47%)	59% (53-63%)	1.8 (1.6-2.1)	6.3% (5.4-7.2%)	8.8% (7.4-10%)	420000 (340000-500000)	14000000 (12000000-16000000)
China	1990	Male	15 to 19	24% (16-36%)	76% (64-84%)	0.46 (0.28-0.72)	2.3% (1.5-3.2%)	2.8% (1.7-4.4%)	2300 (1400-3600)	210000 (140000-310000)
China	1990	Male	20 to 24	43% (30-56%)	57% (44-70%)	1.4 (0.92-1.9)	5.8% (4.3-7.8%)	7.1% (4.8-9.7%)	8200 (5500-11000)	690000 (500000-910000)
China	1990	Male	25 to 29	52% (38-65%)	48% (35-62%)	2.1 (1.5-2.8)	7.8% (5.8-9.8%)	10% (7-13%)	9800 (6800-13000)	750000 (550000-960000)
China	1990	Male	30 to 34	57% (41-69%)	43% (31-59%)	2.7 (2-3.4)	9% (7-11%)	12% (8.9-15%)	12000 (9000-15000)	810000 (630000-990000)
China	1990	Male	35 to 39	59% (45-71%)	41% (29-55%)	3.3 (2.4-4.3)	9.9% (7.9-12%)	13% (10-16%)	17000 (13000-21000)	1100000 (840000-1300000)
China	1990	Male	40 to 44	61% (48-73%)	39% (27-52%)	3.8 (2.8-4.8)	11% (8.9-13%)	14% (11-17%)	19000 (15000-23000)	1000000 (820000-1200000)
China	1990	Male	45 to 49	62% (47-74%)	38% (26-53%)	4.2 (3.1-5.3)	12% (10-14%)	15% (12-18%)	25000 (20000-30000)	1100000 (930000-1400000)
China	1990	Male	50 to 54	62% (48-74%)	38% (26-52%)	4.3 (3.1-5.4)	13% (10-15%)	15% (12-18%)	37000 (29000-44000)	1400000 (1200000-1700000)
China	1990	Male	55 to 59	60% (47-72%)	40% (28-53%)	4.1 (3-5.4)	12% (9.8-15%)	15% (11-17%)	50000 (39000-60000)	1700000 (1300000-2000000)
China	1990	Male	60 to 64	59% (45-71%)	41% (29-55%)	3.8 (2.7-5.1)	12% (8.9-14%)	13% (10-16%)	59000 (45000-73000)	1700000 (1300000-2000000)
China	1990	Male	65 to 69	55% (42-67%)	45% (33-58%)	3.2 (2.4-4.3)	10% (7.8-13%)	11% (8.7-14%)	58000 (44000-73000)	1300000 (1000000-1700000)

Location	Year	Sex	Age	Prevalence of current drinkers	Prevalence of abstainers	Population average of standard drinks daily	Percent of total attributable deaths	Percent of total attributable DALYs	Attributable deaths	Attributable DALYs
China	1990	Male	70 to 74	51% (39-63%)	49% (37-61%)	2.7 (1.9-3.7)	8.3% (6-11%)	9.1% (6.5-12%)	57000 (41000-76000)	1100000 (760000-1400000)
China	1990	Male	75 to 79	48% (34-61%)	52% (39-66%)	2.3 (1.5-3.3)	6.9% (4.6-9.7%)	7.5% (4.9-11%)	37000 (25000-53000)	540000 (360000-750000)
China	1990	Male	80 to 84	44% (31-57%)	56% (43-69%)	2 (1.3-2.8)	5.7% (3.6-8%)	6.2% (3.9-8.7%)	18000 (11000-25000)	190000 (120000-270000)
China	1990	Male	85 to 89	42% (30-54%)	58% (46-70%)	1.8 (1.1-2.6)	4.6% (2.8-6.8%)	5% (3-7.4%)	7100 (4300-10000)	54000 (33000-79000)
China	1990	Male	90 to 94	43% (31-55%)	57% (45-69%)	1.8 (1.1-2.6)	3.8% (2.1-5.8%)	4.1% (2.2-6.3%)	2000 (1100-3000)	11000 (5800-16000)
China	1990	Male	95 plus	46% (31-60%)	54% (40-69%)	1.8 (0.99-2.8)	3.4% (1.5-5.7%)	3.5% (1.4-6.1%)	390 (160-680)	1400 (620-2400)
China	1995	Female	All Ages	12% (10-15%)	88% (85-90%)	0.16 (0.13-0.2)	1.1% (0.79-1.4%)	1.6% (1-2.2%)	62000 (40000-85000)	1900000 (1400000-2400000)
China	1995	Female	15 to 19	8.7% (5.5-13%)	91% (87-94%)	0.099 (0.052-0.17)	0.68% (0.46-0.96%)	0.79% (0.43-1.4%)	290 (150-490)	38000 (25000-55000)
China	1995	Female	20 to 24	11% (6.8-18%)	89% (82-93%)	0.11 (0.058-0.2)	0.98% (0.64-1.4%)	0.77% (0.33-1.5%)	570 (240-1100)	94000 (60000-140000)
China	1995	Female	25 to 29	13% (8.1-20%)	87% (80-92%)	0.14 (0.076-0.24)	1% (0.71-1.4%)	0.9% (0.37-1.7%)	800 (330-1500)	110000 (74000-160000)
China	1995	Female	30 to 34	15% (9-24%)	85% (76-91%)	0.18 (0.097-0.29)	1.1% (0.78-1.5%)	1.2% (0.69-2%)	980 (560-1600)	100000 (71000-150000)
China	1995	Female	35 to 39	17% (9.6-25%)	83% (75-90%)	0.23 (0.13-0.38)	1.3% (0.92-1.7%)	1.8% (1.1-2.6%)	1400 (910-2100)	110000 (75000-140000)
China	1995	Female	40 to 44	17% (11-26%)	83% (74-89%)	0.27 (0.16-0.44)	1.5% (1.1-2%)	2.4% (1.6-3.3%)	2700 (1900-3900)	150000 (110000-210000)
China	1995	Female	45 to 49	17% (10-26%)	83% (74-90%)	0.3 (0.17-0.47)	1.7% (1.2-2.4%)	2.8% (1.8-3.9%)	3400 (2200-4800)	150000 (100000-210000)
China	1995	Female	50 to 54	17% (11-26%)	83% (74-89%)	0.32 (0.19-0.51)	1.9% (1.3-2.7%)	3% (2-4.2%)	4000 (2700-5600)	150000 (100000-210000)
China	1995	Female	55 to 59	17% (11-25%)	83% (75-89%)	0.33 (0.19-0.52)	2.1% (1.4-2.9%)	3.1% (2.1-4.3%)	5800 (3900-8000)	190000 (130000-260000)
China	1995	Female	60 to 64	17% (10-26%)	83% (74-90%)	0.34 (0.19-0.57)	2% (1.3-2.8%)	2.7% (1.8-3.9%)	7800 (5300-11000)	210000 (140000-300000)
China	1995	Female	65 to 69	17% (11-25%)	83% (75-89%)	0.36 (0.21-0.56)	1.9% (1.2-2.6%)	2.5% (1.7-3.4%)	9000 (6100-12000)	200000 (130000-270000)
China	1995	Female	70 to 74	17% (11-25%)	83% (75-89%)	0.36 (0.21-0.6)	1.6% (0.92-2.4%)	2% (1.2-3%)	9400 (5600-15000)	170000 (97000-260000)
China	1995	Female	75 to 79	17% (11-26%)	83% (74-89%)	0.37 (0.2-0.61)	1.2% (0.54-2.1%)	1.5% (0.68-2.5%)	7800 (3700-13000)	110000 (48000-180000)
China	1995	Female	80 to 84	18% (11-26%)	82% (74-89%)	0.38 (0.22-0.61)	0.83% (0.25-1.6%)	0.99% (0.31-1.9%)	4600 (1400-8700)	47000 (14000-89000)

Location	Year	Sex	Age	Prevalence of current drinkers	Prevalence of abstainers	Population average of standard drinks daily	Percent of total attributable deaths	Percent of total attributable DALYs	Attributable deaths	Attributable DALYs
China	1995	Female	85 to 89	19% (12-28%)	81% (72-88%)	0.41 (0.22-0.67)	0.64% (0.074-1.4%)	0.75% (0.092-1.7%)	2200 (270-4900)	16000 (1900-36000)
China	1995	Female	90 to 94	21% (14-30%)	79% (70-86%)	0.46 (0.25-0.78)	0.51% (-0.13-1.4%)	0.57% (-0.16-1.6%)	700 (-200-2000)	3700 (-1000-10000)
China	1995	Female	95 plus	24% (14-36%)	76% (64-86%)	0.53 (0.24-0.96)	0.47% (-0.37-1.7%)	0.49% (-0.46-1.9%)	150 (-140-610)	570 (-450-2100)
China	1995	Male	All Ages	43% (38-48%)	57% (52-62%)	2.2 (2-2.5)	7.7% (6.6-8.8%)	10% (8.5-12%)	510000 (430000-600000)	17000000 (14000000-19000000)
China	1995	Male	15 to 19	23% (15-34%)	77% (66-85%)	0.51 (0.31-0.79)	2.5% (1.7-3.5%)	3.2% (2-4.9%)	1800 (1100-2800)	170000 (110000-250000)
China	1995	Male	20 to 24	42% (30-55%)	58% (45-70%)	1.6 (1.1-2.1)	6.6% (4.9-8.8%)	8.1% (5.5-11%)	8800 (5900-12000)	730000 (530000-980000)
China	1995	Male	25 to 29	53% (39-65%)	47% (35-61%)	2.4 (1.7-3.2)	8.8% (6.6-11%)	11% (8.1-15%)	15000 (11000-19000)	1100000 (810000-1400000)
China	1995	Male	30 to 34	57% (42-69%)	43% (31-58%)	3.1 (2.3-3.9)	9.9% (7.8-12%)	13% (9.8-16%)	17000 (13000-21000)	1100000 (880000-1400000)
China	1995	Male	35 to 39	59% (47-71%)	41% (29-53%)	3.8 (2.8-4.9)	11% (8.9-13%)	14% (11-18%)	19000 (15000-23000)	1100000 (920000-1400000)
China	1995	Male	40 to 44	61% (49-73%)	39% (27-51%)	4.4 (3.3-5.5)	12% (10-15%)	15% (13-18%)	30000 (25000-36000)	1600000 (1300000-1900000)
China	1995	Male	45 to 49	62% (48-74%)	38% (26-52%)	4.8 (3.6-6.1)	13% (11-16%)	16% (13-19%)	33000 (27000-39000)	1500000 (1300000-1800000)
China	1995	Male	50 to 54	62% (48-73%)	38% (27-52%)	4.9 (3.7-6.3)	14% (11-16%)	16% (13-19%)	38000 (31000-45000)	1500000 (1200000-1800000)
China	1995	Male	55 to 59	60% (46-72%)	40% (28-54%)	4.7 (3.4-6.1)	13% (11-16%)	16% (12-18%)	52000 (41000-61000)	1800000 (1400000-2100000)
China	1995	Male	60 to 64	57% (43-70%)	43% (30-57%)	4.2 (3-5.7)	12% (9.8-15%)	14% (11-17%)	69000 (54000-84000)	2000000 (1500000-2400000)
China	1995	Male	65 to 69	54% (41-66%)	46% (34-59%)	3.6 (2.6-4.8)	11% (8.5-14%)	12% (9.4-15%)	69000 (53000-86000)	1600000 (1200000-2000000)
China	1995	Male	70 to 74	51% (38-62%)	49% (38-62%)	3 (2.1-4.2)	9.2% (6.8-12%)	10% (7.4-13%)	63000 (46000-83000)	1200000 (860000-1500000)

Location	Year	Sex	Age	Prevalence of current drinkers	Prevalence of abstainers	Population average of standard drinks daily	Percent of total attributable deaths	Percent of total attributable DALYs	Attributable deaths	Attributable DALYs
China	1995	Male	75 to 79	48% (35-62%)	52% (38-65%)	2.7 (1.8-3.8)	7.9% (5.4-11%)	8.6% (5.9-12%)	51000 (35000-70000)	730000 (510000-1000000)
China	1995	Male	80 to 84	46% (33-59%)	54% (41-67%)	2.5 (1.6-3.4)	6.8% (4.5-9.3%)	7.4% (4.9-10%)	30000 (20000-41000)	320000 (210000-430000)
China	1995	Male	85 to 89	45% (32-57%)	55% (43-68%)	2.2 (1.4-3.2)	5.7% (3.6-8%)	6.1% (3.9-8.7%)	11000 (7200-16000)	87000 (55000-120000)
China	1995	Male	90 to 94	45% (34-57%)	55% (43-66%)	2.1 (1.4-3.2)	4.7% (2.8-6.9%)	5% (2.9-7.6%)	3100 (1800-4700)	17000 (9700-25000)
China	1995	Male	95 plus	48% (34-62%)	52% (38-66%)	2.1 (1.2-3.3)	4.2% (1.9-6.8%)	4.4% (1.9-7.2%)	590 (250-980)	2200 (950-3500)
China	2000	Female	All Ages	13% (11-16%)	87% (84-89%)	0.16 (0.14-0.2)	1.1% (0.77-1.4%)	1.4% (0.88-2%)	56000 (34000-80000)	1700000 (1300000-2200000)
China	2000	Female	15 to 19	12% (8-18%)	88% (82-92%)	0.14 (0.074-0.23)	0.76% (0.51-1.1%)	0.99% (0.54-1.7%)	320 (170-540)	42000 (28000-59000)
China	2000	Female	20 to 24	13% (8.4-20%)	87% (80-92%)	0.13 (0.068-0.22)	1.1% (0.74-1.5%)	0.87% (0.4-1.6%)	400 (190-720)	71000 (47000-100000)
China	2000	Female	25 to 29	14% (8.7-21%)	86% (79-91%)	0.15 (0.081-0.24)	1.1% (0.77-1.5%)	0.93% (0.46-1.6%)	620 (310-1100)	99000 (67000-140000)
China	2000	Female	30 to 34	15% (9-23%)	85% (77-91%)	0.18 (0.096-0.29)	1.1% (0.78-1.5%)	1.2% (0.68-1.8%)	900 (530-1400)	110000 (77000-150000)
China	2000	Female	35 to 39	16% (9.4-24%)	84% (76-91%)	0.22 (0.12-0.35)	1.2% (0.85-1.5%)	1.6% (1-2.3%)	1300 (830-1800)	110000 (77000-140000)
China	2000	Female	40 to 44	16% (9.8-24%)	84% (76-90%)	0.25 (0.14-0.39)	1.3% (0.94-1.7%)	2% (1.4-2.9%)	1900 (1300-2700)	120000 (85000-160000)
China	2000	Female	45 to 49	16% (9.2-23%)	84% (77-91%)	0.27 (0.15-0.42)	1.5% (0.98-2%)	2.4% (1.5-3.4%)	3400 (2200-4800)	160000 (110000-220000)
China	2000	Female	50 to 54	16% (10-24%)	84% (76-90%)	0.3 (0.18-0.47)	1.7% (1.1-2.3%)	2.7% (1.7-3.8%)	4300 (2700-6000)	160000 (110000-230000)
China	2000	Female	55 to 59	16% (10-24%)	84% (76-90%)	0.31 (0.18-0.49)	1.8% (1.2-2.7%)	2.8% (1.8-4%)	5100 (3300-7400)	160000 (110000-230000)
China	2000	Female	60 to 64	16% (9.6-24%)	84% (76-90%)	0.31 (0.18-0.53)	1.7% (1.1-2.5%)	2.4% (1.5-3.5%)	6700 (4300-9900)	180000 (120000-260000)
China	2000	Female	65 to 69	16% (9.8-23%)	84% (77-90%)	0.32 (0.19-0.5)	1.7% (1-2.4%)	2.2% (1.5-3.2%)	8900 (5700-13000)	200000 (120000-280000)
China	2000	Female	70 to 74	16% (10-24%)	84% (76-90%)	0.33 (0.19-0.54)	1.4% (0.74-2.1%)	1.7% (0.98-2.7%)	8800 (4900-14000)	150000 (84000-240000)
China	2000	Female	75 to 79	16% (10-24%)	84% (76-90%)	0.34 (0.18-0.56)	1% (0.38-1.7%)	1.3% (0.52-2.1%)	6600 (2800-11000)	90000 (35000-160000)
China	2000	Female	80 to 84	17% (11-24%)	83% (76-89%)	0.36 (0.2-0.57)	0.66% (0.089-1.4%)	0.81% (0.14-1.6%)	4000 (710-8200)	41000 (5600-84000)
China	2000	Female	85 to 89	18% (12-26%)	82% (74-88%)	0.39 (0.21-0.63)	0.5% (-0.091-1.3%)	0.58% (-0.1-1.5%)	2100 (-370-5400)	15000 (-2700-40000)

Location	Year	Sex	Age	Prevalence of current drinkers	Prevalence of abstainers	Population average of standard drinks daily	Percent of total attributable deaths	Percent of total attributable DALYs	Attributable deaths	Attributable DALYs
China	2000	Female	90 to 94	20% (14-29%)	80% (71-86%)	0.44 (0.24-0.75)	0.38% (-0.27-1.3%)	0.42% (-0.32-1.5%)	650 (-490-2300)	3400 (-2400-12000)
China	2000	Female	95 plus	23% (14-34%)	77% (66-86%)	0.52 (0.23-0.93)	0.36% (-0.51-1.6%)	0.36% (-0.62-1.8%)	150 (-270-750)	600 (-850-2700)
China	2000	Male	All Ages	45% (40-50%)	55% (50-60%)	2.3 (2.1-2.6)	8.4% (7.2-9.6%)	10% (8.6-12%)	550000 (450000-640000)	18000000 (15000000-20000000)
China	2000	Male	15 to 19	29% (20-40%)	71% (60-80%)	0.64 (0.39-0.97)	3% (2.1-4.1%)	3.9% (2.4-5.8%)	2100 (1300-3100)	200000 (130000-280000)
China	2000	Male	20 to 24	43% (30-55%)	57% (45-70%)	1.6 (1.1-2.1)	7% (5.1-9.4%)	8.6% (5.8-12%)	6900 (4600-9600)	580000 (420000-790000)
China	2000	Male	25 to 29	52% (38-64%)	48% (36-62%)	2.3 (1.7-3.1)	8.8% (6.6-11%)	11% (8-15%)	13000 (8900-17000)	980000 (720000-1200000)
China	2000	Male	30 to 34	56% (41-69%)	44% (31-59%)	3 (2.2-3.8)	9.9% (7.8-12%)	13% (9.7-16%)	19000 (14000-23000)	1300000 (990000-1600000)
China	2000	Male	35 to 39	59% (47-71%)	41% (29-53%)	3.7 (2.8-4.8)	11% (8.9-14%)	14% (11-18%)	22000 (17000-27000)	1300000 (1100000-1600000)
China	2000	Male	40 to 44	61% (49-73%)	39% (27-51%)	4.4 (3.4-5.5)	12% (10-15%)	16% (13-19%)	28000 (23000-33000)	1500000 (1200000-1800000)
China	2000	Male	45 to 49	62% (48-74%)	38% (26-52%)	4.8 (3.6-6.1)	13% (11-16%)	16% (13-19%)	43000 (35000-51000)	2000000 (1600000-2300000)
China	2000	Male	50 to 54	62% (48-73%)	38% (27-52%)	4.9 (3.7-6.2)	13% (11-16%)	16% (13-19%)	44000 (35000-52000)	1800000 (1400000-2100000)
China	2000	Male	55 to 59	60% (46-71%)	40% (29-54%)	4.6 (3.4-6)	13% (10-15%)	15% (12-18%)	50000 (40000-59000)	1700000 (1400000-2000000)
China	2000	Male	60 to 64	56% (43-69%)	44% (31-57%)	4.1 (3-5.5)	12% (9.4-15%)	14% (11-17%)	67000 (52000-81000)	1900000 (1500000-2300000)
China	2000	Male	65 to 69	53% (41-65%)	47% (35-59%)	3.5 (2.5-4.6)	11% (8.2-13%)	12% (9.2-15%)	77000 (59000-97000)	1800000 (1400000-2300000)
China	2000	Male	70 to 74	51% (39-62%)	49% (38-61%)	3 (2.1-4.1)	9.1% (6.6-12%)	10% (7.2-13%)	72000 (52000-93000)	1300000 (960000-1700000)
China	2000	Male	75 to 79	48% (36-61%)	52% (39-64%)	2.7 (1.8-3.8)	7.8% (5.3-11%)	8.5% (5.8-12%)	52000 (35000-71000)	750000 (510000-1000000)

Location	Year	Sex	Age	Prevalence of current drinkers	Prevalence of abstainers	Population average of standard drinks daily	Percent of total attributable deaths	Percent of total attributable DALYs	Attributable deaths	Attributable DALYs
China	2000	Male	80 to 84	46% (33-58%)	54% (42-67%)	2.4 (1.6-3.4)	6.6% (4.4-9.2%)	7.2% (4.8-10%)	34000 (22000-47000)	370000 (240000-510000)
China	2000	Male	85 to 89	44% (32-56%)	56% (44-68%)	2.2 (1.4-3.1)	5.5% (3.4-7.8%)	5.9% (3.7-8.5%)	16000 (9600-22000)	120000 (75000-170000)
China	2000	Male	90 to 94	45% (33-56%)	55% (44-67%)	2.1 (1.3-3.1)	4.5% (2.6-6.9%)	4.9% (2.7-7.5%)	4000 (2200-6200)	21000 (12000-33000)
China	2000	Male	95 plus	48% (34-61%)	52% (39-66%)	2.1 (1.2-3.1)	4.1% (1.7-6.9%)	4.2% (1.6-7.3%)	750 (280-1300)	2800 (1100-4700)
China	2005	Female	All Ages	15% (13-16%)	85% (84-87%)	0.18 (0.15-0.21)	0.98% (0.73-1.3%)	1.2% (0.76-1.7%)	47000 (29000-66000)	1500000 (1100000-1900000)
China	2005	Female	15 to 19	15% (11-20%)	85% (80-89%)	0.16 (0.09-0.25)	0.77% (0.52-1.1%)	1.1% (0.59-1.8%)	350 (190-570)	48000 (31000-67000)
China	2005	Female	20 to 24	17% (12-23%)	83% (77-88%)	0.16 (0.091-0.25)	1.1% (0.78-1.5%)	0.98% (0.44-1.7%)	300 (140-510)	63000 (42000-91000)
China	2005	Female	25 to 29	16% (11-21%)	84% (79-89%)	0.16 (0.096-0.24)	1.1% (0.81-1.5%)	0.99% (0.51-1.6%)	330 (170-540)	65000 (45000-91000)
China	2005	Female	30 to 34	16% (12-22%)	84% (78-88%)	0.18 (0.12-0.26)	1.1% (0.76-1.4%)	1.1% (0.68-1.7%)	620 (380-930)	88000 (62000-120000)
China	2005	Female	35 to 39	18% (13-24%)	82% (76-87%)	0.23 (0.15-0.34)	1.1% (0.8-1.4%)	1.5% (0.98-2.1%)	1200 (800-1700)	110000 (81000-150000)
China	2005	Female	40 to 44	18% (14-25%)	82% (75-86%)	0.27 (0.17-0.38)	1.1% (0.81-1.5%)	1.9% (1.3-2.6%)	1700 (1200-2400)	110000 (80000-150000)
China	2005	Female	45 to 49	17% (13-23%)	83% (77-87%)	0.28 (0.19-0.41)	1.2% (0.82-1.7%)	2.1% (1.4-3%)	2400 (1600-3500)	120000 (82000-160000)
China	2005	Female	50 to 54	17% (12-23%)	83% (77-88%)	0.3 (0.2-0.42)	1.4% (0.89-2%)	2.3% (1.5-3.3%)	4600 (3000-6500)	180000 (120000-250000)
China	2005	Female	55 to 59	16% (12-21%)	84% (79-88%)	0.29 (0.19-0.41)	1.5% (0.95-2.2%)	2.4% (1.6-3.5%)	5200 (3400-7700)	170000 (110000-240000)
China	2005	Female	60 to 64	14% (10-19%)	86% (81-90%)	0.27 (0.17-0.41)	1.4% (0.92-2.1%)	2.2% (1.4-3.1%)	5400 (3500-7600)	140000 (91000-200000)
China	2005	Female	65 to 69	14% (9.7-19%)	86% (81-90%)	0.27 (0.18-0.38)	1.4% (0.89-2%)	1.9% (1.3-2.7%)	7200 (4600-10000)	160000 (98000-220000)
China	2005	Female	70 to 74	13% (9.1-19%)	87% (81-91%)	0.26 (0.16-0.42)	1.1% (0.63-1.7%)	1.5% (0.87-2.2%)	7600 (4400-11000)	130000 (76000-200000)
China	2005	Female	75 to 79	13% (8.2-19%)	87% (81-92%)	0.25 (0.14-0.41)	0.76% (0.3-1.3%)	0.98% (0.42-1.6%)	5300 (2300-8700)	72000 (28000-120000)
China	2005	Female	80 to 84	12% (7.3-18%)	88% (82-93%)	0.25 (0.14-0.4)	0.44% (0.018-0.93%)	0.55% (0.044-1.1%)	2900 (230-5900)	29000 (1100-61000)
China	2005	Female	85 to 89	13% (7.8-19%)	87% (81-92%)	0.27 (0.14-0.44)	0.27% (-0.15-0.78%)	0.32% (-0.17-0.9%)	1300 (-690-3600)	9500 (-4900-27000)
China	2005	Female	90 to 94	14% (8.9-21%)	86% (79-91%)	0.31 (0.16-0.52)	0.16% (-0.32-0.77%)	0.15% (-0.38-0.84%)	290 (-720-1600)	1800 (-3500-8800)

Location	Year	Sex	Age	Prevalence of current drinkers	Prevalence of abstainers	Population average of standard drinks daily	Percent of total attributable deaths	Percent of total attributable DALYs	Attributable deaths	Attributable DALYs
China	2005	Female	95 plus	17% (9.6-26%)	83% (74-90%)	0.37 (0.16-0.67)	0.13% (-0.51-0.95%)	0.087% (-0.65-1%)	46 (-350-550)	270 (-1100-2000)
China	2005	Male	All Ages	47% (44-51%)	53% (49-56%)	2.5 (2.2-2.8)	8.8% (7.5-10%)	10% (8.3-12%)	540000 (450000-640000)	18000000 (15000000-20000000)
China	2005	Male	15 to 19	35% (27-44%)	65% (56-73%)	0.75 (0.49-1.1)	3.2% (2.2-4.4%)	4.5% (2.9-6.6%)	2800 (1800-4200)	260000 (180000-360000)
China	2005	Male	20 to 24	48% (40-58%)	52% (42-60%)	1.8 (1.3-2.4)	7.4% (5.5-9.7%)	9.7% (6.6-14%)	6500 (4400-9000)	550000 (400000-730000)
China	2005	Male	25 to 29	54% (44-64%)	46% (36-56%)	2.4 (1.8-3.2)	9.1% (6.8-11%)	12% (8.8-16%)	8900 (6300-12000)	700000 (520000-890000)
China	2005	Male	30 to 34	57% (47-67%)	43% (33-53%)	3.1 (2.4-3.8)	9.9% (7.7-12%)	13% (9.8-16%)	16000 (12000-20000)	1100000 (860000-1400000)
China	2005	Male	35 to 39	59% (50-69%)	41% (31-50%)	3.7 (2.9-4.7)	11% (8.9-13%)	14% (11-18%)	24000 (19000-30000)	1500000 (1200000-1800000)
China	2005	Male	40 to 44	61% (50-71%)	39% (29-50%)	4.3 (3.4-5.3)	12% (10-15%)	16% (13-19%)	32000 (26000-38000)	1700000 (1400000-2000000)
China	2005	Male	45 to 49	62% (52-72%)	38% (28-48%)	4.7 (3.7-5.9)	13% (11-15%)	16% (13-19%)	37000 (30000-43000)	1700000 (1400000-2000000)
China	2005	Male	50 to 54	62% (52-72%)	38% (28-48%)	4.9 (3.8-6.1)	13% (11-15%)	16% (13-18%)	57000 (46000-67000)	2300000 (1900000-2700000)
China	2005	Male	55 to 59	60% (51-69%)	40% (31-49%)	4.6 (3.5-5.9)	13% (10-15%)	15% (12-18%)	57000 (46000-67000)	2000000 (1600000-2300000)
China	2005	Male	60 to 64	56% (46-66%)	44% (34-54%)	4 (3-5.3)	11% (9-14%)	13% (10-16%)	57000 (44000-69000)	1700000 (1300000-2000000)
China	2005	Male	65 to 69	52% (42-61%)	48% (39-58%)	3.3 (2.5-4.3)	9.9% (7.7-12%)	11% (8.6-14%)	70000 (54000-86000)	1700000 (1300000-2000000)
China	2005	Male	70 to 74	48% (38-58%)	52% (42-62%)	2.8 (2-3.7)	8.2% (5.9-10%)	9.1% (6.5-12%)	69000 (49000-89000)	1300000 (950000-1700000)
China	2005	Male	75 to 79	44% (34-55%)	56% (45-66%)	2.4 (1.6-3.3)	6.8% (4.6-9.2%)	7.4% (5-10%)	51000 (34000-71000)	750000 (510000-1000000)
China	2005	Male	80 to 84	42% (30-55%)	58% (45-70%)	2.1 (1.4-3)	5.7% (3.6-8.1%)	6.3% (3.9-8.8%)	34000 (21000-48000)	370000 (230000-520000)

Location	Year	Sex	Age	Prevalence of current drinkers	Prevalence of abstainers	Population average of standard drinks daily	Percent of total attributable deaths	Percent of total attributable DALYs	Attributable deaths	Attributable DALYs
China	2005	Male	85 to 89	41% (29-52%)	59% (48-71%)	1.9 (1.3-2.8)	4.7% (2.9-6.9%)	5.1% (3.1-7.6%)	16000 (9400-23000)	120000 (74000-180000)
China	2005	Male	90 to 94	42% (31-53%)	58% (47-69%)	1.9 (1.2-2.7)	3.8% (2-6%)	4% (2.1-6.5%)	4700 (2400-7500)	25000 (13000-41000)
China	2005	Male	95 plus	45% (31-59%)	55% (41-69%)	1.8 (1.1-2.8)	3.4% (1.2-6.1%)	3.5% (1.1-6.5%)	810 (260-1500)	3000 (1100-5500)
China	2010	Female	All Ages	14% (13-16%)	86% (84-87%)	0.21 (0.17-0.24)	1.1% (0.8-1.3%)	1.3% (0.87-1.9%)	50000 (32000-70000)	1600000 (1200000-2000000)
China	2010	Female	15 to 19	14% (10-19%)	86% (81-90%)	0.16 (0.092-0.26)	0.74% (0.51-1%)	1.1% (0.62-1.8%)	210 (120-340)	34000 (23000-48000)
China	2010	Female	20 to 24	16% (12-22%)	84% (78-88%)	0.17 (0.096-0.26)	1.1% (0.8-1.6%)	0.99% (0.48-1.7%)	310 (150-530)	75000 (50000-110000)
China	2010	Female	25 to 29	16% (11-22%)	84% (78-89%)	0.17 (0.1-0.27)	1.1% (0.86-1.5%)	1% (0.58-1.6%)	290 (170-470)	65000 (45000-91000)
China	2010	Female	30 to 34	16% (12-22%)	84% (78-88%)	0.2 (0.13-0.29)	1.1% (0.79-1.4%)	1.2% (0.78-1.8%)	420 (280-630)	67000 (46000-92000)
China	2010	Female	35 to 39	18% (13-23%)	82% (77-87%)	0.25 (0.16-0.37)	1.1% (0.81-1.4%)	1.6% (1.1-2.3%)	960 (650-1300)	96000 (70000-130000)
China	2010	Female	40 to 44	18% (13-24%)	82% (76-87%)	0.28 (0.19-0.41)	1.1% (0.82-1.5%)	2.1% (1.4-2.8%)	1900 (1300-2600)	130000 (92000-170000)
China	2010	Female	45 to 49	17% (12-23%)	83% (77-88%)	0.3 (0.2-0.43)	1.2% (0.86-1.7%)	2.4% (1.6-3.4%)	2700 (1800-3800)	140000 (95000-180000)
China	2010	Female	50 to 54	16% (12-22%)	84% (78-88%)	0.32 (0.21-0.45)	1.4% (0.96-2%)	2.7% (1.8-3.7%)	4000 (2600-5500)	160000 (110000-210000)
China	2010	Female	55 to 59	15% (11-20%)	85% (80-89%)	0.31 (0.21-0.44)	1.6% (1.1-2.3%)	2.9% (1.9-4%)	6600 (4300-9200)	210000 (140000-290000)
China	2010	Female	60 to 64	14% (9.8-19%)	86% (81-90%)	0.29 (0.19-0.44)	1.6% (1-2.2%)	2.5% (1.8-3.5%)	6900 (4700-9400)	190000 (130000-250000)
China	2010	Female	65 to 69	13% (9.5-18%)	87% (82-90%)	0.28 (0.18-0.41)	1.5% (0.99-2%)	2.2% (1.6-3%)	6900 (4800-9300)	150000 (100000-210000)
China	2010	Female	70 to 74	13% (8.8-18%)	87% (82-91%)	0.28 (0.17-0.45)	1.2% (0.69-1.7%)	1.6% (1-2.4%)	7300 (4500-11000)	130000 (78000-190000)
China	2010	Female	75 to 79	12% (8.1-18%)	88% (82-92%)	0.28 (0.16-0.44)	0.81% (0.32-1.4%)	1.1% (0.46-1.8%)	5900 (2500-9700)	82000 (32000-140000)
China	2010	Female	80 to 84	12% (7.3-18%)	88% (82-93%)	0.28 (0.15-0.44)	0.49% (0.049-0.97%)	0.61% (0.073-1.2%)	3400 (410-6800)	36000 (3600-72000)
China	2010	Female	85 to 89	13% (7.7-19%)	87% (81-92%)	0.29 (0.15-0.49)	0.31% (-0.15-0.84%)	0.35% (-0.19-1%)	1500 (-830-4300)	12000 (-5700-32000)
China	2010	Female	90 to 94	14% (8.7-21%)	86% (79-91%)	0.33 (0.18-0.57)	0.18% (-0.33-0.86%)	0.16% (-0.44-0.95%)	360 (-990-2100)	2400 (-4600-12000)
China	2010	Female	95 plus	16% (9.2-25%)	84% (75-91%)	0.4 (0.17-0.73)	0.16% (-0.53-1.1%)	0.1% (-0.73-1.2%)	72 (-510-850)	450 (-1500-3200)

Location	Year	Sex	Age	Prevalence of current drinkers	Prevalence of abstainers	Population average of standard drinks daily	Percent of total attributable deaths	Percent of total attributable DALYs	Attributable deaths	Attributable DALYs
China	2010	Male	All Ages	47% (44-52%)	53% (48-56%)	3 (2.6-3.3)	9.4% (8-11%)	11% (8.8-12%)	580000 (480000-680000)	19000000 (16000000-21000000)
China	2010	Male	15 to 19	32% (25-41%)	68% (59-75%)	0.76 (0.5-1.1)	3.1% (2.2-4.3%)	4.7% (3.1-6.8%)	2000 (1300-2900)	190000 (130000-260000)
China	2010	Male	20 to 24	48% (39-58%)	52% (42-61%)	2 (1.4-2.6)	7.4% (5.5-9.7%)	10% (6.8-14%)	7400 (4900-10000)	650000 (480000-860000)
China	2010	Male	25 to 29	53% (44-63%)	47% (37-56%)	2.7 (2-3.5)	9.3% (7-12%)	13% (9.2-17%)	8400 (5900-11000)	680000 (500000-870000)
China	2010	Male	30 to 34	57% (47-67%)	43% (33-53%)	3.4 (2.6-4.3)	10% (8-13%)	14% (11-18%)	11000 (8600-14000)	830000 (650000-1000000)
China	2010	Male	35 to 39	59% (50-68%)	41% (32-50%)	4.1 (3.2-5.2)	11% (9.2-14%)	15% (12-19%)	21000 (16000-26000)	1300000 (1100000-1600000)
China	2010	Male	40 to 44	60% (50-71%)	40% (29-50%)	4.7 (3.7-5.8)	12% (10-15%)	16% (13-20%)	34000 (28000-41000)	1900000 (1600000-2300000)
China	2010	Male	45 to 49	61% (51-71%)	39% (29-49%)	5.2 (4.1-6.5)	13% (11-15%)	17% (14-20%)	41000 (34000-49000)	2000000 (1700000-2400000)
China	2010	Male	50 to 54	61% (51-71%)	39% (29-49%)	5.4 (4.2-6.6)	13% (11-16%)	16% (14-19%)	50000 (41000-58000)	2100000 (1700000-2400000)
China	2010	Male	55 to 59	59% (50-69%)	41% (31-50%)	5.1 (3.9-6.4)	13% (10-15%)	15% (13-18%)	69000 (57000-82000)	2500000 (2000000-2900000)
China	2010	Male	60 to 64	56% (46-66%)	44% (34-54%)	4.4 (3.3-5.8)	12% (9.3-14%)	14% (11-16%)	67000 (54000-81000)	2000000 (1600000-2400000)
China	2010	Male	65 to 69	51% (42-62%)	49% (38-58%)	3.6 (2.8-4.7)	10% (8.1-12%)	12% (9.3-14%)	66000 (53000-81000)	1600000 (1300000-2000000)
China	2010	Male	70 to 74	47% (38-57%)	53% (43-62%)	3 (2.2-4.1)	8.5% (6.2-11%)	9.6% (7-12%)	71000 (51000-90000)	1400000 (1000000-1700000)
China	2010	Male	75 to 79	44% (35-55%)	56% (45-65%)	2.6 (1.8-3.6)	7.1% (4.9-9.5%)	7.9% (5.4-11%)	60000 (41000-81000)	900000 (620000-1200000)
China	2010	Male	80 to 84	42% (31-55%)	58% (45-69%)	2.4 (1.6-3.3)	6.2% (4.1-8.6%)	6.8% (4.5-9.5%)	45000 (29000-62000)	490000 (330000-680000)
China	2010	Male	85 to 89	41% (30-52%)	59% (48-70%)	2.2 (1.4-3.1)	5.2% (3.2-7.6%)	5.7% (3.5-8.2%)	20000 (12000-29000)	160000 (96000-230000)

Location	Year	Sex	Age	Prevalence of current drinkers	Prevalence of abstainers	Population average of standard drinks daily	Percent of total attributable deaths	Percent of total attributable DALYs	Attributable deaths	Attributable DALYs
China	2010	Male	90 to 94	42% (31-53%)	58% (47-69%)	2.1 (1.4-3)	4.3% (2.3-6.6%)	4.6% (2.3-7.2%)	6000 (3100-9500)	33000 (18000-51000)
China	2010	Male	95 plus	45% (31-59%)	55% (41-69%)	2.1 (1.2-3.2)	3.8% (1.5-6.6%)	3.9% (1.3-7%)	1300 (430-2300)	4800 (1800-8400)
China	2016	Female	All Ages	16% (13-19%)	84% (81-87%)	0.24 (0.2-0.3)	1.2% (0.9-1.5%)	1.6% (1-2.2%)	59000 (39000-81000)	1700000 (1300000-2200000)
China	2016	Female	15 to 19	14% (8.4-21%)	86% (79-92%)	0.17 (0.085-0.29)	0.72% (0.5-1%)	1.2% (0.66-2%)	120 (67-200)	21000 (15000-31000)
China	2016	Female	20 to 24	16% (9.4-25%)	84% (75-91%)	0.18 (0.086-0.3)	1.1% (0.79-1.6%)	1.1% (0.55-1.9%)	190 (97-340)	52000 (34000-77000)
China	2016	Female	25 to 29	18% (10-27%)	82% (73-90%)	0.2 (0.1-0.35)	1.2% (0.86-1.5%)	1.2% (0.65-1.9%)	340 (190-560)	78000 (54000-110000)
China	2016	Female	30 to 34	19% (11-30%)	81% (70-89%)	0.25 (0.13-0.41)	1.1% (0.83-1.5%)	1.4% (0.9-2.2%)	440 (280-680)	70000 (48000-100000)
China	2016	Female	35 to 39	20% (12-31%)	80% (69-88%)	0.3 (0.16-0.5)	1.1% (0.83-1.5%)	2% (1.3-2.9%)	680 (440-1000)	70000 (51000-96000)
China	2016	Female	40 to 44	19% (11-29%)	81% (71-89%)	0.33 (0.18-0.53)	1.2% (0.86-1.6%)	2.5% (1.7-3.5%)	1600 (1100-2300)	110000 (79000-150000)
China	2016	Female	45 to 49	18% (9.6-27%)	82% (73-90%)	0.33 (0.18-0.52)	1.3% (0.92-1.8%)	2.9% (1.9-4%)	3400 (2200-4600)	170000 (120000-220000)
China	2016	Female	50 to 54	16% (9.4-26%)	84% (74-91%)	0.33 (0.18-0.53)	1.5% (1.1-2.1%)	3.1% (2.1-4.2%)	4900 (3400-6600)	190000 (140000-260000)
China	2016	Female	55 to 59	15% (9.1-24%)	85% (76-91%)	0.33 (0.19-0.54)	1.8% (1.2-2.4%)	3.4% (2.4-4.5%)	6200 (4400-8300)	200000 (140000-270000)
China	2016	Female	60 to 64	15% (8.5-25%)	85% (75-92%)	0.34 (0.18-0.58)	1.8% (1.2-2.5%)	3.1% (2.2-4.2%)	9700 (6900-13000)	260000 (180000-360000)
China	2016	Female	65 to 69	15% (8.8-24%)	85% (76-91%)	0.35 (0.19-0.56)	1.6% (1.1-2.3%)	2.6% (1.8-3.6%)	9700 (6800-13000)	220000 (140000-300000)
China	2016	Female	70 to 74	15% (8.9-23%)	85% (77-91%)	0.34 (0.19-0.58)	1.3% (0.77-1.9%)	2% (1.2-2.9%)	7800 (4900-11000)	140000 (81000-210000)
China	2016	Female	75 to 79	14% (8-22%)	86% (78-92%)	0.33 (0.17-0.56)	0.96% (0.42-1.6%)	1.3% (0.63-2.1%)	6600 (3100-11000)	92000 (40000-150000)
China	2016	Female	80 to 84	14% (7.9-21%)	86% (79-92%)	0.33 (0.17-0.56)	0.6% (0.11-1.2%)	0.79% (0.16-1.5%)	4300 (860-8200)	45000 (8300-87000)
China	2016	Female	85 to 89	14% (8.4-23%)	86% (77-92%)	0.35 (0.18-0.6)	0.39% (-0.11-0.99%)	0.46% (-0.15-1.2%)	2400 (-800-6300)	19000 (-5300-48000)
China	2016	Female	90 to 94	16% (9.7-25%)	84% (75-90%)	0.4 (0.21-0.72)	0.23% (-0.38-1%)	0.22% (-0.53-1.1%)	620 (-1500-3200)	4100 (-6700-18000)
China	2016	Female	95 plus	19% (11-31%)	81% (69-89%)	0.49 (0.2-0.92)	0.18% (-0.69-1.3%)	0.11% (-0.91-1.4%)	110 (-900-1400)	740 (-2700-5300)
China	2016	Male	All Ages	48% (44-53%)	52% (47-56%)	3.3 (2.9-3.7)	9.8% (8.4-11%)	11% (9.1-13%)	650000 (540000-760000)	20000000 (17000000-23000000)

Location	Year	Sex	Age	Prevalence of current drinkers	Prevalence of abstainers	Population average of standard drinks daily	Percent of total attributable deaths	Percent of total attributable DALYs	Attributable deaths	Attributable DALYs
China	2016	Male	15 to 19	28% (20-40%)	72% (60-80%)	0.72 (0.45-1.1)	3% (2.1-4.1%)	4.6% (2.9-6.8%)	1200 (770-1800)	120000 (87000-170000)
China	2016	Male	20 to 24	42% (30-54%)	58% (46-70%)	1.8 (1.2-2.5)	7.1% (5.2-9.5%)	9.9% (6.5-14%)	4900 (3200-7000)	460000 (330000-610000)
China	2016	Male	25 to 29	49% (36-62%)	51% (38-64%)	2.6 (1.9-3.6)	8.7% (6.5-11%)	12% (8.6-17%)	9500 (6600-13000)	790000 (590000-1000000)
China	2016	Male	30 to 34	54% (39-67%)	46% (33-61%)	3.4 (2.4-4.5)	9.8% (7.6-12%)	14% (10-18%)	11000 (8000-14000)	810000 (620000-1000000)
China	2016	Male	35 to 39	57% (45-70%)	43% (30-55%)	4.2 (3.1-5.6)	11% (8.6-13%)	15% (12-19%)	13000 (10000-16000)	880000 (690000-1100000)
China	2016	Male	40 to 44	60% (48-73%)	40% (27-52%)	4.9 (3.8-6.3)	12% (9.8-14%)	16% (13-20%)	26000 (21000-31000)	1500000 (1200000-1800000)
China	2016	Male	45 to 49	62% (49-74%)	38% (26-51%)	5.6 (4.3-7.1)	13% (11-15%)	17% (14-20%)	45000 (37000-53000)	2200000 (1800000-2600000)
China	2016	Male	50 to 54	63% (50-74%)	37% (26-50%)	5.9 (4.5-7.4)	13% (11-15%)	17% (14-20%)	57000 (47000-66000)	2400000 (2000000-2800000)
China	2016	Male	55 to 59	62% (50-73%)	38% (27-50%)	5.6 (4.3-7.2)	13% (11-15%)	16% (13-19%)	63000 (52000-74000)	2200000 (1900000-2700000)
China	2016	Male	60 to 64	59% (46-71%)	41% (29-54%)	5.1 (3.7-6.7)	12% (9.9-15%)	14% (12-17%)	94000 (76000-110000)	2800000 (2300000-3400000)
China	2016	Male	65 to 69	55% (43-67%)	45% (33-57%)	4.2 (3.1-5.5)	11% (8.7-13%)	13% (10-16%)	93000 (73000-110000)	2300000 (1800000-2800000)
China	2016	Male	70 to 74	50% (38-61%)	50% (39-62%)	3.4 (2.4-4.7)	9.1% (6.6-12%)	10% (7.5-13%)	74000 (54000-94000)	1400000 (1100000-1800000)
China	2016	Male	75 to 79	46% (33-59%)	54% (41-67%)	2.9 (1.9-4)	7.6% (5.1-10%)	8.4% (5.7-11%)	66000 (45000-89000)	1000000 (680000-1300000)
China	2016	Male	80 to 84	43% (31-56%)	57% (44-69%)	2.6 (1.7-3.6)	6.5% (4.3-9%)	7.2% (4.7-10%)	53000 (34000-73000)	580000 (390000-810000)
China	2016	Male	85 to 89	42% (31-53%)	58% (47-69%)	2.4 (1.6-3.4)	5.6% (3.7-8.1%)	6.1% (3.9-8.9%)	30000 (19000-43000)	230000 (150000-340000)
China	2016	Male	90 to 94	44% (33-55%)	56% (45-67%)	2.3 (1.5-3.4)	4.7% (2.6-7.1%)	5.1% (2.7-7.8%)	9300 (4900-14000)	51000 (28000-77000)

Location	Year	Sex	Age	Prevalence of current drinkers	Prevalence of abstainers	Population average of standard drinks daily	Percent of total attributable deaths	Percent of total attributable DALYs	Attributable deaths	Attributable DALYs
China	2016	Male	95 plus	47% (33-60%)	53% (40-67%)	2.3 (1.3-3.5)	4.2% (1.7-7.2%)	4.4% (1.5-7.6%)	1800 (640-3200)	6900 (2800-12000)
North Korea	1990	Female	All Ages	16% (13-20%)	84% (80-87%)	0.26 (0.22-0.31)	0.85% (0.5-1.2%)	1.2% (0.47-2.1%)	680 (270-1200)	24000 (15000-35000)
North Korea	1990	Female	15 to 19	16% (9.8-25%)	84% (75-90%)	0.25 (0.13-0.43)	0.92% (0.56-1.5%)	1.4% (0.63-2.6%)	8.6 (3.7-17)	1100 (680-1800)
North Korea	1990	Female	20 to 24	19% (12-29%)	81% (71-88%)	0.27 (0.13-0.47)	1.3% (0.8-1.9%)	1.3% (0.47-2.7%)	10 (3.6-20)	1800 (1100-2800)
North Korea	1990	Female	25 to 29	20% (11-31%)	80% (69-89%)	0.28 (0.15-0.48)	1.3% (0.81-1.8%)	1.3% (0.51-2.4%)	8.7 (3.4-16)	1400 (890-2100)
North Korea	1990	Female	30 to 34	21% (12-33%)	79% (67-88%)	0.32 (0.17-0.54)	1.2% (0.77-1.7%)	1.4% (0.66-2.5%)	13 (6.1-24)	1600 (1000-2400)
North Korea	1990	Female	35 to 39	22% (13-35%)	78% (65-87%)	0.4 (0.21-0.68)	1.2% (0.8-1.9%)	1.8% (0.91-3.1%)	13 (6.6-23)	1100 (660-1600)
North Korea	1990	Female	40 to 44	23% (14-36%)	77% (64-86%)	0.46 (0.25-0.75)	1.3% (0.77-2%)	2.1% (1.1-3.4%)	31 (16-51)	1900 (1100-2900)
North Korea	1990	Female	45 to 49	22% (12-35%)	78% (65-88%)	0.48 (0.25-0.81)	1.3% (0.69-2.2%)	2.1% (1-3.7%)	50 (24-86)	2400 (1300-4000)
North Korea	1990	Female	50 to 54	22% (13-34%)	78% (66-87%)	0.5 (0.29-0.82)	1.4% (0.68-2.5%)	2.2% (1-3.8%)	74 (33-130)	2900 (1400-5000)
North Korea	1990	Female	55 to 59	21% (12-32%)	79% (68-88%)	0.51 (0.25-0.85)	1.5% (0.52-2.7%)	2.2% (0.79-3.9%)	82 (30-150)	2700 (980-4900)
North Korea	1990	Female	60 to 64	19% (11-31%)	81% (69-89%)	0.49 (0.24-0.87)	1.3% (0.41-2.7%)	1.9% (0.65-3.7%)	86 (30-170)	2400 (750-4800)
North Korea	1990	Female	65 to 69	19% (11-29%)	81% (71-89%)	0.49 (0.25-0.83)	1.3% (0.34-2.5%)	1.7% (0.51-3.3%)	91 (27-180)	2100 (590-4100)
North Korea	1990	Female	70 to 74	18% (11-28%)	82% (72-89%)	0.47 (0.23-0.85)	1.1% (0.2-2.4%)	1.4% (0.29-3%)	85 (17-180)	1500 (280-3400)
North Korea	1990	Female	75 to 79	16% (9.9-27%)	84% (73-90%)	0.45 (0.22-0.8)	0.83% (-0.018-2%)	1% (0.0095-2.5%)	63 (0.59-150)	890 (-20-2200)
North Korea	1990	Female	80 to 84	16% (9.3-26%)	84% (74-91%)	0.43 (0.22-0.73)	0.58% (-0.18-1.7%)	0.69% (-0.2-2%)	36 (-10-110)	380 (-110-1100)
North Korea	1990	Female	85 to 89	16% (9.3-25%)	84% (75-91%)	0.44 (0.22-0.78)	0.45% (-0.3-1.5%)	0.51% (-0.38-1.8%)	22 (-16-74)	170 (-110-560)
North Korea	1990	Female	90 to 94	18% (11-27%)	82% (73-89%)	0.48 (0.23-0.86)	0.36% (-0.44-1.6%)	0.38% (-0.55-1.8%)	7.8 (-11-38)	45 (-55-200)
North Korea	1990	Female	95 plus	20% (11-33%)	80% (67-89%)	0.54 (0.23-1)	0.36% (-0.62-1.9%)	0.32% (-0.8-2.1%)	2.2 (-5.4-14)	9.3 (-16-50)
North Korea	1990	Male	All Ages	37% (31-45%)	63% (55-69%)	1.5 (1.4-1.7)	5.6% (4.4-6.8%)	7.9% (6-9.9%)	4100 (3000-5200)	170000 (130000-200000)
North Korea	1990	Male	15 to 19	30% (19-45%)	70% (55-81%)	0.54 (0.27-0.97)	2.6% (1.6-4.1%)	3.3% (1.7-5.6%)	44 (22-76)	4100 (2400-6600)
North Korea	1990	Male	20 to 24	42% (28-58%)	58% (42-72%)	1.3 (0.73-2)	5.8% (3.7-8.5%)	6.9% (3.7-11%)	130 (71-220)	12000 (7100-17000)

Location	Year	Sex	Age	Prevalence of current drinkers	Prevalence of abstainers	Population average of standard drinks daily	Percent of total attributable deaths	Percent of total attributable DALYs	Attributable deaths	Attributable DALYs
North Korea	1990	Male	25 to 29	48% (31-65%)	52% (35-69%)	1.9 (1.1-2.9)	7.8% (4.9-11%)	9.8% (5.5-15%)	150 (81-220)	11000 (6900-17000)
North Korea	1990	Male	30 to 34	51% (34-68%)	49% (32-66%)	2.4 (1.5-3.5)	8.7% (5.8-12%)	11% (7.1-16%)	210 (130-310)	15000 (9600-20000)
North Korea	1990	Male	35 to 39	54% (37-70%)	46% (30-63%)	2.9 (1.8-4.4)	10% (6.7-14%)	13% (8.1-18%)	160 (98-230)	9600 (6200-13000)
North Korea	1990	Male	40 to 44	55% (39-72%)	45% (28-61%)	3.4 (2.1-4.9)	11% (7.4-14%)	14% (9.1-19%)	310 (200-430)	17000 (11000-23000)
North Korea	1990	Male	45 to 49	56% (37-73%)	44% (27-63%)	3.8 (2.4-5.5)	11% (7.9-15%)	14% (9.4-19%)	490 (330-660)	23000 (16000-30000)
North Korea	1990	Male	50 to 54	55% (37-73%)	45% (27-63%)	3.9 (2.5-5.6)	11% (7.5-15%)	14% (8.9-18%)	630 (390-860)	25000 (16000-34000)
North Korea	1990	Male	55 to 59	54% (36-71%)	46% (29-64%)	3.6 (2.2-5.6)	11% (6.8-15%)	13% (7.8-17%)	550 (320-780)	19000 (11000-26000)
North Korea	1990	Male	60 to 64	51% (33-69%)	49% (31-67%)	3.2 (1.8-5.2)	9.2% (5.1-14%)	11% (5.7-16%)	430 (220-670)	13000 (6700-19000)
North Korea	1990	Male	65 to 69	48% (32-65%)	52% (35-68%)	2.7 (1.5-4.1)	7.9% (4.2-12%)	9% (4.6-14%)	370 (190-600)	8700 (4600-14000)
North Korea	1990	Male	70 to 74	44% (29-61%)	56% (39-71%)	2.3 (1.2-3.6)	6.3% (2.9-10%)	7% (3.1-12%)	280 (130-490)	5500 (2600-9200)
North Korea	1990	Male	75 to 79	41% (27-59%)	59% (41-73%)	1.9 (0.99-3.3)	5.2% (2.1-9.4%)	5.8% (2.2-11%)	190 (74-360)	2900 (1100-5300)
North Korea	1990	Male	80 to 84	39% (24-56%)	61% (44-76%)	1.8 (0.9-2.9)	4.6% (1.9-8.3%)	5% (2-9.1%)	77 (29-140)	850 (340-1500)
North Korea	1990	Male	85 to 89	38% (24-53%)	62% (47-76%)	1.6 (0.85-2.7)	3.8% (1.4-7.1%)	4% (1.4-7.8%)	35 (12-68)	280 (100-530)
North Korea	1990	Male	90 to 94	39% (27-55%)	61% (45-73%)	1.6 (0.82-2.7)	3.1% (0.88-6.6%)	3.3% (0.77-7.2%)	12 (2.8-27)	66 (19-140)
North Korea	1990	Male	95 plus	43% (26-62%)	57% (38-74%)	1.7 (0.74-3.1)	2.8% (0.24-6.9%)	2.9% (-0.041-7.3%)	2.8 (-0.042-7.2)	11 (0.92-26)
North Korea	1995	Female	All Ages	16% (13-20%)	84% (80-87%)	0.28 (0.24-0.34)	0.55% (0.3-0.91%)	0.87% (0.37-1.6%)	930 (400-1600)	31000 (18000-46000)
North Korea	1995	Female	15 to 19	16% (10-25%)	84% (75-90%)	0.26 (0.14-0.47)	0.76% (0.46-1.2%)	0.97% (0.39-1.9%)	6.2 (2.7-12)	790 (490-1300)
North Korea	1995	Female	20 to 24	20% (12-30%)	80% (70-88%)	0.29 (0.14-0.51)	1.1% (0.65-1.7%)	0.92% (0.33-1.9%)	11 (4.1-22)	1900 (1100-3000)
North Korea	1995	Female	25 to 29	20% (12-31%)	80% (69-88%)	0.3 (0.16-0.49)	1% (0.64-1.5%)	0.93% (0.35-1.8%)	13 (5.1-24)	2000 (1200-2900)
North Korea	1995	Female	30 to 34	21% (12-33%)	79% (67-88%)	0.34 (0.19-0.56)	1% (0.64-1.5%)	1.1% (0.48-1.9%)	13 (6.4-24)	1600 (990-2300)
North Korea	1995	Female	35 to 39	23% (13-35%)	77% (65-87%)	0.42 (0.23-0.71)	1.1% (0.65-1.6%)	1.4% (0.69-2.5%)	26 (13-46)	2100 (1300-3200)
North Korea	1995	Female	40 to 44	23% (14-36%)	77% (64-86%)	0.48 (0.26-0.79)	1.2% (0.69-1.9%)	1.7% (0.86-3%)	24 (13-41)	1400 (840-2200)

Location	Year	Sex	Age	Prevalence of current drinkers	Prevalence of abstainers	Population average of standard drinks daily	Percent of total attributable deaths	Percent of total attributable DALYs	Attributable deaths	Attributable DALYs
North Korea	1995	Female	45 to 49	22% (13-35%)	78% (65-87%)	0.52 (0.26-0.88)	1.2% (0.66-2.2%)	1.9% (0.87-3.4%)	53 (25-95)	2500 (1300-4300)
North Korea	1995	Female	50 to 54	22% (13-34%)	78% (66-87%)	0.54 (0.31-0.9)	1.4% (0.66-2.4%)	2% (0.96-3.5%)	89 (41-150)	3500 (1700-5900)
North Korea	1995	Female	55 to 59	21% (13-32%)	79% (68-87%)	0.54 (0.29-0.9)	1.4% (0.55-2.6%)	2% (0.82-3.6%)	120 (45-210)	3900 (1500-7000)
North Korea	1995	Female	60 to 64	20% (11-32%)	80% (68-89%)	0.53 (0.27-0.91)	1.3% (0.45-2.5%)	1.8% (0.64-3.3%)	120 (44-230)	3300 (1100-6400)
North Korea	1995	Female	65 to 69	19% (11-30%)	81% (70-89%)	0.52 (0.27-0.87)	1.3% (0.43-2.4%)	1.6% (0.61-3.1%)	130 (47-250)	2900 (1000-5700)
North Korea	1995	Female	70 to 74	18% (11-28%)	82% (72-89%)	0.5 (0.26-0.88)	1.1% (0.24-2.3%)	1.3% (0.33-2.8%)	130 (30-260)	2300 (490-4800)
North Korea	1995	Female	75 to 79	17% (10-28%)	83% (72-90%)	0.48 (0.23-0.85)	0.81% (0.042-2%)	0.96% (0.077-2.3%)	96 (8.2-230)	1400 (72-3300)
North Korea	1995	Female	80 to 84	16% (9.6-26%)	84% (74-90%)	0.46 (0.24-0.78)	0.57% (-0.097-1.5%)	0.64% (-0.1-1.7%)	54 (-9.5-140)	570 (-100-1500)
North Korea	1995	Female	85 to 89	17% (9.9-25%)	83% (75-90%)	0.48 (0.24-0.82)	0.44% (-0.17-1.3%)	0.47% (-0.2-1.5%)	32 (-14-99)	250 (-93-750)
North Korea	1995	Female	90 to 94	18% (11-27%)	82% (73-89%)	0.52 (0.25-0.95)	0.34% (-0.27-1.3%)	0.33% (-0.32-1.4%)	14 (-12-55)	76 (-58-290)
North Korea	1995	Female	95 plus	21% (11-34%)	79% (66-89%)	0.59 (0.24-1.2)	0.3% (-0.34-1.4%)	0.27% (-0.41-1.4%)	4.2 (-6.8-22)	18 (-21-81)
North Korea	1995	Male	All Ages	38% (31-46%)	62% (54-69%)	1.7 (1.5-2)	3.5% (2.3-5.4%)	5.4% (3.5-7.9%)	5400 (4000-6800)	210000 (170000-250000)
North Korea	1995	Male	15 to 19	30% (19-45%)	70% (55-81%)	0.57 (0.29-1)	2.3% (1.4-3.7%)	2.7% (1.4-4.9%)	32 (16-58)	3000 (1800-4900)
North Korea	1995	Male	20 to 24	42% (28-58%)	58% (42-72%)	1.4 (0.77-2.2)	5.3% (3.3-7.9%)	6% (3.2-9.6%)	150 (80-240)	12000 (7600-19000)
North Korea	1995	Male	25 to 29	48% (31-65%)	52% (35-69%)	2 (1.2-3.1)	6.8% (4.3-10%)	8% (4.3-13%)	220 (130-340)	17000 (10000-24000)
North Korea	1995	Male	30 to 34	51% (33-69%)	49% (31-67%)	2.6 (1.6-3.8)	7.9% (5.2-11%)	9.4% (5.7-14%)	220 (130-320)	15000 (9800-21000)
North Korea	1995	Male	35 to 39	54% (37-71%)	46% (29-63%)	3.1 (1.9-4.8)	8.6% (5.6-12%)	10% (6.3-15%)	330 (200-480)	20000 (13000-28000)
North Korea	1995	Male	40 to 44	55% (39-73%)	45% (27-61%)	3.6 (2.2-5.3)	9.6% (6.3-13%)	11% (7.1-16%)	250 (160-350)	13000 (9200-18000)
North Korea	1995	Male	45 to 49	56% (37-72%)	44% (28-63%)	4 (2.5-5.9)	10% (6.8-14%)	12% (7.7-16%)	510 (330-690)	23000 (16000-31000)
North Korea	1995	Male	50 to 54	55% (38-73%)	45% (27-62%)	4.1 (2.6-6.1)	10% (6.8-14%)	12% (7.7-16%)	750 (490-1000)	30000 (20000-40000)
North Korea	1995	Male	55 to 59	54% (36-71%)	46% (29-64%)	3.9 (2.3-5.7)	9.7% (6.3-13%)	11% (7-15%)	910 (550-1300)	31000 (20000-43000)
North Korea	1995	Male	60 to 64	51% (33-69%)	49% (31-67%)	3.4 (1.9-5.5)	8.5% (4.8-12%)	9.5% (5.1-14%)	710 (380-1100)	20000 (11000-31000)

Location	Year	Sex	Age	Prevalence of current drinkers	Prevalence of abstainers	Population average of standard drinks daily	Percent of total attributable deaths	Percent of total attributable DALYs	Attributable deaths	Attributable DALYs
North Korea	1995	Male	65 to 69	48% (31-65%)	52% (35-69%)	2.9 (1.6-4.3)	7.3% (4.1-11%)	8% (4.4-12%)	500 (270-770)	12000 (6400-18000)
North Korea	1995	Male	70 to 74	44% (29-61%)	56% (39-71%)	2.4 (1.3-3.8)	5.6% (2.7-9.2%)	6.1% (2.8-10%)	350 (170-590)	6800 (3300-11000)
North Korea	1995	Male	75 to 79	41% (26-59%)	59% (41-74%)	2 (1-3.5)	4.6% (1.9-8.1%)	4.9% (1.9-8.8%)	240 (95-440)	3600 (1500-6400)
North Korea	1995	Male	80 to 84	39% (24-56%)	61% (44-76%)	1.8 (0.97-3.1)	3.8% (1.5-7%)	3.9% (1.5-7.5%)	110 (43-190)	1200 (510-2100)
North Korea	1995	Male	85 to 89	38% (24-53%)	62% (47-76%)	1.7 (0.89-2.8)	3% (1.1-5.8%)	3.1% (1-6.3%)	57 (20-110)	450 (170-820)
North Korea	1995	Male	90 to 94	40% (26-55%)	60% (45-74%)	1.7 (0.87-2.9)	2.4% (0.62-5.2%)	2.4% (0.52-5.4%)	17 (4.2-38)	95 (26-200)
North Korea	1995	Male	95 plus	43% (26-62%)	57% (38-74%)	1.8 (0.78-3.3)	1.8% (0.16-4.6%)	1.7% (0.048-4.7%)	4.2 (0.16-10)	17 (1.9-39)
North Korea	2000	Female	All Ages	16% (13-20%)	84% (80-87%)	0.27 (0.22-0.32)	0.52% (0.27-0.89%)	0.77% (0.26-1.5%)	970 (320-1800)	32000 (17000-50000)
North Korea	2000	Female	15 to 19	16% (9.9-25%)	84% (75-90%)	0.25 (0.13-0.44)	0.74% (0.44-1.2%)	0.94% (0.39-1.9%)	8 (3.4-16)	990 (580-1600)
North Korea	2000	Female	20 to 24	20% (12-30%)	80% (70-88%)	0.27 (0.13-0.49)	1% (0.63-1.6%)	0.91% (0.33-1.9%)	7.9 (2.9-16)	1300 (780-2000)
North Korea	2000	Female	25 to 29	20% (12-32%)	80% (68-88%)	0.28 (0.14-0.46)	0.99% (0.63-1.4%)	0.87% (0.32-1.7%)	13 (5-25)	2000 (1200-3000)
North Korea	2000	Female	30 to 34	21% (12-34%)	79% (66-88%)	0.31 (0.17-0.52)	0.94% (0.61-1.4%)	0.96% (0.43-1.8%)	19 (8.5-34)	2200 (1400-3300)
North Korea	2000	Female	35 to 39	23% (14-35%)	77% (65-86%)	0.39 (0.21-0.65)	1% (0.62-1.5%)	1.3% (0.61-2.2%)	24 (12-43)	1900 (1200-2900)
North Korea	2000	Female	40 to 44	23% (14-36%)	77% (64-86%)	0.45 (0.25-0.73)	1.1% (0.59-1.7%)	1.5% (0.73-2.6%)	44 (22-75)	2600 (1500-4100)
North Korea	2000	Female	45 to 49	23% (13-36%)	77% (64-87%)	0.48 (0.24-0.82)	1.1% (0.53-2.1%)	1.7% (0.73-3.1%)	37 (17-68)	1800 (870-3100)
North Korea	2000	Female	50 to 54	22% (13-35%)	78% (65-87%)	0.5 (0.27-0.85)	1.3% (0.55-2.3%)	1.8% (0.77-3.3%)	82 (35-150)	3200 (1400-5800)
North Korea	2000	Female	55 to 59	21% (13-32%)	79% (68-87%)	0.5 (0.26-0.83)	1.3% (0.38-2.4%)	1.7% (0.54-3.3%)	120 (36-230)	3900 (1200-7400)
North Korea	2000	Female	60 to 64	20% (11-32%)	80% (68-89%)	0.48 (0.25-0.81)	1.1% (0.28-2.2%)	1.5% (0.39-3%)	140 (34-280)	3900 (860-7600)
North Korea	2000	Female	65 to 69	19% (11-30%)	81% (70-89%)	0.48 (0.26-0.8)	1.1% (0.24-2.2%)	1.4% (0.36-2.8%)	140 (36-290)	3200 (730-6600)
North Korea	2000	Female	70 to 74	18% (11-29%)	82% (71-89%)	0.47 (0.25-0.84)	0.9% (0.07-2.1%)	1.1% (0.13-2.6%)	140 (15-300)	2400 (170-5600)
North Korea	2000	Female	75 to 79	17% (10-27%)	83% (73-90%)	0.44 (0.22-0.81)	0.67% (-0.098-1.8%)	0.79% (-0.096-2.2%)	100 (-12-270)	1400 (-210-3800)
North Korea	2000	Female	80 to 84	17% (9.7-26%)	83% (74-90%)	0.43 (0.22-0.75)	0.43% (-0.24-1.4%)	0.49% (-0.28-1.6%)	51 (-30-170)	540 (-300-1800)

Location	Year	Sex	Age	Prevalence of current drinkers	Prevalence of abstainers	Population average of standard drinks daily	Percent of total attributable deaths	Percent of total attributable DALYs	Attributable deaths	Attributable DALYs
North Korea	2000	Female	85 to 89	17%<br>(10-26%)	83%<br>(74-90%)	0.44<br>(0.23-0.77)	0.31%<br>(-0.3-1.2%)	0.33%<br>(-0.35-1.3%)	28<br>(-29-110)	210<br>(-200-830)
North Korea	2000	Female	90 to 94	18%<br>(11-28%)	82%<br>(72-89%)	0.48<br>(0.23-0.85)	0.21%<br>(-0.37-1.1%)	0.2%<br>(-0.44-1.2%)	9<br>(-19-52)	53<br>(-93-270)
North Korea	2000	Female	95 plus	21%<br>(11-35%)	79%<br>(65-89%)	0.54<br>(0.22-1.1)	0.17%<br>(-0.47-1.2%)	0.14%<br>(-0.56-1.2%)	2.6<br>(-10-23)	12<br>(-35-87)
North Korea	2000	Male	All Ages	38%<br>(32-46%)	62%<br>(54-68%)	1.6<br>(1.4-1.9)	3.7%<br>(2.5-5.5%)	5.5%<br>(3.5-8%)	6300<br>(4500-8100)	240000<br>(190000-300000)
North Korea	2000	Male	15 to 19	30%<br>(19-44%)	70%<br>(56-81%)	0.52<br>(0.26-0.93)	2.3%<br>(1.3-3.6%)	2.7%<br>(1.3-4.7%)	41<br>(20-73)	3700<br>(2200-6100)
North Korea	2000	Male	20 to 24	42%<br>(28-58%)	58%<br>(42-72%)	1.2<br>(0.69-2)	5.4%<br>(3.4-8.2%)	6.1%<br>(3.3-9.8%)	110<br>(60-180)	9200<br>(5600-14000)
North Korea	2000	Male	25 to 29	48%<br>(32-65%)	52%<br>(35-68%)	1.9<br>(1.1-2.9)	6.8%<br>(4.2-10%)	7.8%<br>(4.4-12%)	240<br>(130-380)	18000<br>(11000-26000)
North Korea	2000	Male	30 to 34	51%<br>(34-68%)	49%<br>(32-66%)	2.4<br>(1.5-3.5)	7.6%<br>(5.1-11%)	8.9%<br>(5.5-13%)	330<br>(200-490)	22000<br>(14000-31000)
North Korea	2000	Male	35 to 39	54%<br>(38-70%)	46%<br>(30-62%)	2.8<br>(1.7-4.2)	8.5%<br>(5.5-12%)	10%<br>(6-15%)	330<br>(200-500)	20000<br>(12000-28000)
North Korea	2000	Male	40 to 44	55%<br>(39-73%)	45%<br>(27-61%)	3.3<br>(2-4.8)	9.2%<br>(6-13%)	11%<br>(6.6-15%)	510<br>(320-730)	27000<br>(17000-37000)
North Korea	2000	Male	45 to 49	56%<br>(37-72%)	44%<br>(28-63%)	3.6<br>(2.2-5.4)	10%<br>(6.5-14%)	12%<br>(7.2-16%)	390<br>(250-540)	18000<br>(12000-24000)
North Korea	2000	Male	50 to 54	56%<br>(37-73%)	44%<br>(27-63%)	3.7<br>(2.3-5.6)	10%<br>(6.3-14%)	11%<br>(7-16%)	750<br>(450-1000)	30000<br>(19000-40000)
North Korea	2000	Male	55 to 59	54%<br>(36-73%)	46%<br>(27-64%)	3.5<br>(2.1-5.3)	9.4%<br>(5.6-13%)	11%<br>(6.1-15%)	1000<br>(590-1500)	35000<br>(21000-49000)
North Korea	2000	Male	60 to 64	51%<br>(33-70%)	49%<br>(30-67%)	3.1<br>(1.7-4.9)	8.2%<br>(4.5-12%)	9%<br>(4.8-14%)	1100<br>(560-1600)	31000<br>(17000-46000)
North Korea	2000	Male	65 to 69	48%<br>(32-65%)	52%<br>(35-68%)	2.6<br>(1.6-3.9)	7%<br>(3.7-10%)	7.6%<br>(3.9-12%)	690<br>(360-1100)	16000<br>(8900-25000)
North Korea	2000	Male	70 to 74	45%<br>(29-62%)	55%<br>(38-71%)	2.2<br>(1.2-3.6)	5.3%<br>(2.4-8.9%)	5.7%<br>(2.5-9.7%)	390<br>(180-670)	7500<br>(3400-13000)
North Korea	2000	Male	75 to 79	42%<br>(27-60%)	58%<br>(40-73%)	1.9<br>(0.97-3.2)	4.3%<br>(1.6-7.9%)	4.5%<br>(1.7-8.5%)	240<br>(88-450)	3500<br>(1300-6500)
North Korea	2000	Male	80 to 84	39%<br>(24-56%)	61%<br>(44-76%)	1.7<br>(0.9-2.8)	3.4%<br>(1.3-6.6%)	3.6%<br>(1.2-7%)	100<br>(39-200)	1100<br>(450-2100)
North Korea	2000	Male	85 to 89	38%<br>(25-54%)	62%<br>(46-75%)	1.6<br>(0.81-2.6)	2.7%<br>(0.9-5.5%)	2.7%<br>(0.8-5.8%)	65<br>(21-130)	510<br>(180-1000)
North Korea	2000	Male	90 to 94	40%<br>(26-55%)	60%<br>(45-74%)	1.5<br>(0.79-2.7)	2%<br>(0.43-4.7%)	2%<br>(0.33-4.9%)	21<br>(4.1-49)	120<br>(29-270)
North Korea	2000	Male	95 plus	43%<br>(26-62%)	57%<br>(38-74%)	1.6<br>(0.7-3)	1.6%<br>(0.093-4.5%)	1.5%<br>(-0.042-4.5%)	4.4<br>(-0.14-12)	18<br>(1.2-45)
North Korea	2005	Female	All Ages	17%<br>(14-20%)	83%<br>(80-86%)	0.24<br>(0.2-0.28)	0.63%<br>(0.27-1.1%)	0.73%<br>(0.043-1.6%)	730<br>(45-1600)	26000<br>(11000-45000)

Location	Year	Sex	Age	Prevalence of current drinkers	Prevalence of abstainers	Population average of standard drinks daily	Percent of total attributable deaths	Percent of total attributable DALYs	Attributable deaths	Attributable DALYs
North Korea	2005	Female	15 to 19	16%<br>(10-26%)	84%<br>(74-90%)	0.21<br>(0.11-0.36)	0.83%<br>(0.5-1.3%)	1.2%<br>(0.5-2.2%)	7.4<br>(2.9-14)	940<br>(570-1500)
North Korea	2005	Female	20 to 24	20%<br>(12-30%)	80%<br>(70-88%)	0.23<br>(0.11-0.42)	1.1%<br>(0.7-1.8%)	1.1%<br>(0.39-2.2%)	8.2<br>(2.8-17)	1500<br>(870-2300)
North Korea	2005	Female	25 to 29	20%<br>(12-31%)	80%<br>(69-88%)	0.24<br>(0.12-0.41)	1.1%<br>(0.72-1.5%)	1%<br>(0.4-2%)	7.9<br>(2.9-16)	1300<br>(790-1900)
North Korea	2005	Female	30 to 34	21%<br>(12-34%)	79%<br>(66-88%)	0.27<br>(0.14-0.45)	1%<br>(0.64-1.5%)	1.1%<br>(0.45-1.9%)	16<br>(6.6-29)	2000<br>(1200-3000)
North Korea	2005	Female	35 to 39	23%<br>(13-35%)	77%<br>(65-87%)	0.33<br>(0.18-0.54)	1%<br>(0.62-1.5%)	1.3%<br>(0.58-2.3%)	28<br>(12-49)	2400<br>(1400-3600)
North Korea	2005	Female	40 to 44	23%<br>(14-36%)	77%<br>(64-86%)	0.38<br>(0.21-0.61)	1%<br>(0.57-1.6%)	1.5%<br>(0.68-2.6%)	34<br>(15-57)	2100<br>(1200-3200)
North Korea	2005	Female	45 to 49	23%<br>(12-35%)	77%<br>(65-88%)	0.41<br>(0.21-0.69)	1%<br>(0.48-1.8%)	1.6%<br>(0.64-2.9%)	56<br>(22-100)	2700<br>(1300-4800)
North Korea	2005	Female	50 to 54	22%<br>(13-35%)	78%<br>(65-87%)	0.43<br>(0.23-0.7)	1.1%<br>(0.41-2%)	1.6%<br>(0.6-3.1%)	46<br>(16-86)	1800<br>(670-3300)
North Korea	2005	Female	55 to 59	21%<br>(13-33%)	79%<br>(67-87%)	0.43<br>(0.22-0.71)	1%<br>(0.2-2.1%)	1.5%<br>(0.3-3%)	86<br>(18-180)	2800<br>(560-5800)
North Korea	2005	Female	60 to 64	20%<br>(11-32%)	80%<br>(68-89%)	0.41<br>(0.21-0.71)	0.88%<br>(0.04-2%)	1.2%<br>(0.12-2.7%)	110<br>(9.6-240)	2900<br>(130-6600)
North Korea	2005	Female	65 to 69	19%<br>(11-30%)	81%<br>(70-89%)	0.41<br>(0.22-0.67)	0.8%<br>(-0.023-1.9%)	1.1%<br>(0.031-2.5%)	130<br>(3.7-290)	2800<br>(-75-6600)
North Korea	2005	Female	70 to 74	18%<br>(11-29%)	82%<br>(71-89%)	0.4<br>(0.21-0.72)	0.65%<br>(-0.16-1.7%)	0.84%<br>(-0.17-2.2%)	110<br>(-21-280)	1900<br>(-460-5200)
North Korea	2005	Female	75 to 79	17%<br>(11-27%)	83%<br>(73-89%)	0.38<br>(0.19-0.68)	0.42%<br>(-0.35-1.5%)	0.53%<br>(-0.39-1.9%)	70<br>(-51-250)	960<br>(-790-3600)
North Korea	2005	Female	80 to 84	17%<br>(9.6-26%)	83%<br>(74-90%)	0.37<br>(0.19-0.63)	0.2%<br>(-0.53-1.2%)	0.24%<br>(-0.65-1.4%)	25<br>(-66-150)	260<br>(-690-1500)
North Korea	2005	Female	85 to 89	17%<br>(10-26%)	83%<br>(74-90%)	0.38<br>(0.19-0.67)	0.089%<br>(-0.62-1.1%)	0.083%<br>(-0.75-1.3%)	5.9<br>(-54-90)	55<br>(-390-680)
North Korea	2005	Female	90 to 94	18%<br>(11-28%)	82%<br>(72-89%)	0.41<br>(0.2-0.73)	-0.0014%<br>(-0.75-1.1%)	-0.047%<br>(-0.96-1.2%)	-1.7<br>(-33-43)	-0.27<br>(-160-230)
North Korea	2005	Female	95 plus	21%<br>(11-34%)	79%<br>(66-89%)	0.47<br>(0.19-0.94)	-0.054%<br>(-1-1.4%)	-0.14%<br>(-1.3-1.5%)	-1.6<br>(-14-17)	-2.3<br>(-45-60)
North Korea	2005	Male	All Ages	39%<br>(33-47%)	61%<br>(53-67%)	1.4<br>(1.3-1.6)	5.4%<br>(4-6.7%)	6.9%<br>(4.8-9.1%)	6200<br>(4200-8200)	240000<br>(180000-300000)
North Korea	2005	Male	15 to 19	30%<br>(19-45%)	70%<br>(55-81%)	0.44<br>(0.21-0.81)	2.5%<br>(1.4-4%)	3%<br>(1.4-5.4%)	40<br>(19-72)	3700<br>(2100-5900)
North Korea	2005	Male	20 to 24	42%<br>(28-59%)	58%<br>(41-72%)	1.1<br>(0.57-1.7)	5.5%<br>(3.4-8.3%)	6.2%<br>(3.4-10%)	120<br>(66-200)	10000<br>(6200-16000)
North Korea	2005	Male	25 to 29	48%<br>(31-64%)	52%<br>(36-69%)	1.6<br>(0.88-2.5)	7.2%<br>(4.4-10%)	8.6%<br>(4.7-13%)	160<br>(81-240)	12000<br>(6900-17000)
North Korea	2005	Male	30 to 34	51%<br>(33-68%)	49%<br>(32-67%)	2<br>(1.2-2.9)	7.8%<br>(5.1-11%)	9.3%<br>(5.7-14%)	290<br>(170-440)	20000<br>(13000-28000)

Location	Year	Sex	Age	Prevalence of current drinkers	Prevalence of abstainers	Population average of standard drinks daily	Percent of total attributable deaths	Percent of total attributable DALYs	Attributable deaths	Attributable DALYs
North Korea	2005	Male	35 to 39	54% (37-71%)	46% (29-63%)	2.4 (1.5-3.5)	8.6% (5.7-12%)	10% (6.5-15%)	420 (240-620)	25000 (16000-36000)
North Korea	2005	Male	40 to 44	55% (39-73%)	45% (27-61%)	2.8 (1.8-4)	9.4% (6.5-13%)	11% (7.5-16%)	430 (270-600)	23000 (15000-31000)
North Korea	2005	Male	45 to 49	56% (37-72%)	44% (28-63%)	3.1 (1.8-4.5)	10% (6.4-14%)	12% (7.4-17%)	680 (420-990)	31000 (20000-45000)
North Korea	2005	Male	50 to 54	55% (37-72%)	45% (28-63%)	3.2 (1.9-4.7)	10% (5.9-14%)	12% (6.8-17%)	490 (280-700)	19000 (12000-27000)
North Korea	2005	Male	55 to 59	53% (36-71%)	47% (29-64%)	3 (1.7-4.5)	9.2% (5-14%)	11% (5.4-16%)	850 (440-1300)	29000 (16000-43000)
North Korea	2005	Male	60 to 64	51% (33-70%)	49% (30-67%)	2.6 (1.5-4.3)	7.9% (3.8-13%)	8.9% (4.2-14%)	1000 (470-1700)	29000 (14000-47000)
North Korea	2005	Male	65 to 69	48% (32-66%)	52% (34-68%)	2.2 (1.3-3.3)	6.5% (3.2-10%)	7.3% (3.4-12%)	880 (420-1400)	21000 (10000-34000)
North Korea	2005	Male	70 to 74	45% (29-62%)	55% (38-71%)	1.8 (1-3)	5% (2-8.9%)	5.5% (2.2-10%)	460 (180-830)	8700 (3500-16000)
North Korea	2005	Male	75 to 79	41% (27-58%)	59% (42-73%)	1.6 (0.8-2.7)	3.9% (1.2-7.6%)	4.2% (1.3-8.4%)	220 (67-440)	3200 (1000-6400)
North Korea	2005	Male	80 to 84	39% (24-56%)	61% (44-76%)	1.4 (0.73-2.4)	3.3% (1.1-6.7%)	3.5% (1.1-7.4%)	87 (26-180)	970 (320-2000)
North Korea	2005	Male	85 to 89	38% (24-53%)	62% (47-76%)	1.3 (0.7-2.2)	2.6% (0.69-5.4%)	2.7% (0.61-5.8%)	43 (9.4-93)	350 (94-730)
North Korea	2005	Male	90 to 94	39% (26-54%)	61% (46-74%)	1.3 (0.68-2.2)	2.1% (0.26-5.2%)	2.1% (0.08-5.6%)	17 (0.66-46)	98 (12-250)
North Korea	2005	Male	95 plus	43% (26-61%)	57% (39-74%)	1.3 (0.59-2.5)	1.9% (-0.19-5.4%)	1.8% (-0.54-5.7%)	3.8 (-1.1-12)	15 (-1.5-44)
North Korea	2010	Female	All Ages	17% (14-21%)	83% (79-86%)	0.24 (0.19-0.29)	0.6% (0.22-1.1%)	0.63% (-0.094-1.5%)	700 (-100-1700)	26000 (8900-46000)
North Korea	2010	Female	15 to 19	16% (10-26%)	84% (74-90%)	0.2 (0.1-0.36)	0.82% (0.49-1.3%)	1.2% (0.49-2.1%)	7.4 (3-14)	960 (540-1500)
North Korea	2010	Female	20 to 24	20% (12-30%)	80% (70-88%)	0.22 (0.1-0.4)	1.1% (0.72-1.7%)	1% (0.33-2.1%)	7.7 (2.3-15)	1500 (880-2300)
North Korea	2010	Female	25 to 29	20% (12-32%)	80% (68-88%)	0.23 (0.11-0.4)	1.1% (0.71-1.6%)	0.99% (0.35-1.9%)	8.8 (3.1-17)	1500 (940-2200)
North Korea	2010	Female	30 to 34	21% (12-33%)	79% (67-88%)	0.26 (0.13-0.43)	1% (0.62-1.5%)	1% (0.43-1.9%)	9.9 (3.9-18)	1300 (780-1900)
North Korea	2010	Female	35 to 39	23% (14-35%)	77% (65-86%)	0.32 (0.17-0.52)	0.98% (0.61-1.5%)	1.3% (0.56-2.3%)	26 (11-45)	2200 (1300-3300)
North Korea	2010	Female	40 to 44	23% (14-37%)	77% (63-86%)	0.36 (0.2-0.59)	0.98% (0.55-1.5%)	1.4% (0.64-2.5%)	43 (19-75)	2700 (1500-4200)
North Korea	2010	Female	45 to 49	23% (12-36%)	77% (64-88%)	0.39 (0.2-0.65)	0.98% (0.44-1.7%)	1.5% (0.59-2.7%)	49 (18-93)	2400 (1100-4100)
North Korea	2010	Female	50 to 54	22% (14-35%)	78% (65-86%)	0.41 (0.22-0.67)	1% (0.35-1.9%)	1.5% (0.51-2.8%)	80 (27-150)	3100 (1100-5700)

Location	Year	Sex	Age	Prevalence of current drinkers	Prevalence of abstainers	Population average of standard drinks daily	Percent of total attributable deaths	Percent of total attributable DALYs	Attributable deaths	Attributable DALYs
North Korea	2010	Female	55 to 59	21% (13-33%)	79% (67-87%)	0.41 (0.22-0.68)	0.95% (0.18-2%)	1.4% (0.28-2.9%)	57 (12-120)	1800 (370-3900)
North Korea	2010	Female	60 to 64	20% (11-32%)	80% (68-89%)	0.4 (0.2-0.7)	0.81% (0.024-1.9%)	1.1% (0.083-2.6%)	96 (7.1-210)	2600 (78-6000)
North Korea	2010	Female	65 to 69	19% (12-29%)	81% (71-88%)	0.39 (0.21-0.66)	0.7% (-0.075-1.6%)	0.97% (-0.055-2.2%)	120 (-7.5-270)	2600 (-280-6100)
North Korea	2010	Female	70 to 74	18% (12-29%)	82% (71-88%)	0.38 (0.2-0.69)	0.56% (-0.22-1.7%)	0.74% (-0.24-2.1%)	120 (-40-350)	2100 (-840-6300)
North Korea	2010	Female	75 to 79	17% (10-27%)	83% (73-90%)	0.37 (0.18-0.66)	0.33% (-0.42-1.4%)	0.42% (-0.5-1.7%)	67 (-81-280)	910 (-1200-3900)
North Korea	2010	Female	80 to 84	17% (10-26%)	83% (74-90%)	0.36 (0.18-0.61)	0.11% (-0.63-1.1%)	0.13% (-0.75-1.3%)	17 (-98-170)	180 (-1000-1700)
North Korea	2010	Female	85 to 89	17% (10-26%)	83% (74-90%)	0.37 (0.19-0.64)	0.0026% (-0.74-0.98%)	-0.018% (-0.88-1.1%)	-1.7 (-79-98)	1.9 (-560-740)
North Korea	2010	Female	90 to 94	19% (11-28%)	81% (72-89%)	0.4 (0.19-0.72)	-0.087% (-0.86-1%)	-0.15% (-1-1.1%)	-6 (-42-45)	-21 (-200-230)
North Korea	2010	Female	95 plus	21% (12-35%)	79% (65-88%)	0.45 (0.18-0.9)	-0.14% (-1.2-1.3%)	-0.24% (-1.4-1.4%)	-3.2 (-19-19)	-7.1 (-59-65)
North Korea	2010	Male	All Ages	40% (34-47%)	60% (53-66%)	1.4 (1.2-1.7)	5.4% (4-6.9%)	6.6% (4.4-9%)	6500 (4300-9100)	250000 (180000-320000)
North Korea	2010	Male	15 to 19	30% (19-45%)	70% (55-81%)	0.42 (0.2-0.77)	2.4% (1.4-3.9%)	3% (1.4-5.3%)	42 (20-73)	3900 (2200-6100)
North Korea	2010	Male	20 to 24	43% (28-58%)	57% (42-72%)	1 (0.55-1.7)	5.4% (3.3-8.1%)	6.1% (3.2-10%)	120 (65-210)	10000 (6100-16000)
North Korea	2010	Male	25 to 29	48% (31-64%)	52% (36-69%)	1.5 (0.82-2.4)	6.9% (4.2-10%)	8.1% (4.3-13%)	180 (91-280)	13000 (7900-20000)
North Korea	2010	Male	30 to 34	51% (33-68%)	49% (32-67%)	1.9 (1.2-2.9)	7.7% (5-11%)	9.2% (5.4-14%)	190 (110-300)	13000 (8400-19000)
North Korea	2010	Male	35 to 39	53% (37-70%)	47% (30-63%)	2.3 (1.4-3.3)	8.2% (5.3-12%)	9.9% (5.9-15%)	390 (220-600)	24000 (15000-35000)
North Korea	2010	Male	40 to 44	55% (38-72%)	45% (28-62%)	2.6 (1.7-3.8)	8.8% (5.8-12%)	11% (6.6-15%)	550 (330-820)	29000 (19000-42000)
North Korea	2010	Male	45 to 49	56% (37-72%)	44% (28-63%)	2.9 (1.8-4.4)	9.4% (6-13%)	11% (6.8-16%)	590 (350-880)	27000 (17000-40000)
North Korea	2010	Male	50 to 54	56% (38-72%)	44% (28-62%)	3 (1.9-4.3)	9.4% (5.5-13%)	11% (6.1-16%)	900 (500-1300)	36000 (21000-52000)
North Korea	2010	Male	55 to 59	54% (36-72%)	46% (28-64%)	2.8 (1.7-4.4)	8.7% (4.9-13%)	9.9% (5.4-15%)	580 (310-880)	20000 (11000-30000)
North Korea	2010	Male	60 to 64	51% (33-70%)	49% (30-67%)	2.5 (1.4-4.1)	7.5% (3.6-12%)	8.4% (3.9-14%)	920 (430-1500)	27000 (13000-44000)
North Korea	2010	Male	65 to 69	48% (32-66%)	52% (34-68%)	2.1 (1.2-3.3)	6.1% (2.9-10%)	6.8% (3.2-12%)	930 (430-1600)	22000 (11000-37000)
North Korea	2010	Male	70 to 74	45% (29-62%)	55% (38-71%)	1.7 (0.95-2.8)	4.7% (1.8-8.8%)	5.2% (1.9-9.8%)	690 (250-1300)	13000 (4900-24000)

Location	Year	Sex	Age	Prevalence of current drinkers	Prevalence of abstainers	Population average of standard drinks daily	Percent of total attributable deaths	Percent of total attributable DALYs	Attributable deaths	Attributable DALYs
North Korea	2010	Male	75 to 79	41% (27-59%)	59% (41-73%)	1.5 (0.74-2.5)	3.6% (1-7.5%)	3.9% (1.1-8.2%)	300 (80-620)	4400 (1300-9000)
North Korea	2010	Male	80 to 84	39% (24-56%)	61% (44-76%)	1.3 (0.7-2.3)	3% (0.91-6.3%)	3.2% (0.85-7%)	100 (28-230)	1200 (350-2500)
North Korea	2010	Male	85 to 89	38% (24-53%)	62% (47-76%)	1.3 (0.65-2.1)	2.4% (0.51-5.1%)	2.5% (0.39-5.6%)	43 (6.7-100)	340 (74-770)
North Korea	2010	Male	90 to 94	40% (26-55%)	60% (45-74%)	1.2 (0.65-2.1)	1.8% (0.11-4.8%)	1.9% (-0.069-5.2%)	14 (-0.51-38)	78 (4.8-200)
North Korea	2010	Male	95 plus	43% (26-62%)	57% (38-74%)	1.3 (0.56-2.4)	1.7% (-0.34-5.2%)	1.5% (-0.69-5.5%)	3.7 (-1.7-13)	15 (-3.2-47)
North Korea	2016	Female	All Ages	18% (15-21%)	82% (79-85%)	0.26 (0.17-0.36)	0.68% (0.19-1.3%)	0.71% (-0.13-1.8%)	870 (-150-2300)	30000 (8000-57000)
North Korea	2016	Female	15 to 19	17% (11-26%)	83% (74-89%)	0.21 (0.096-0.4)	0.87% (0.52-1.3%)	1.2% (0.5-2.3%)	6.9 (2.8-13)	920 (540-1400)
North Korea	2016	Female	20 to 24	20% (12-30%)	80% (70-88%)	0.23 (0.098-0.44)	1.2% (0.74-1.8%)	1.1% (0.36-2.1%)	7.9 (2.5-16)	1500 (880-2300)
North Korea	2016	Female	25 to 29	20% (12-32%)	80% (68-88%)	0.23 (0.11-0.43)	1.1% (0.72-1.6%)	0.99% (0.33-2%)	8.6 (2.8-18)	1500 (910-2400)
North Korea	2016	Female	30 to 34	21% (13-33%)	79% (67-87%)	0.26 (0.13-0.49)	1% (0.65-1.5%)	1% (0.41-2%)	12 (4.6-23)	1600 (940-2300)
North Korea	2016	Female	35 to 39	23% (13-36%)	77% (64-87%)	0.33 (0.16-0.59)	1% (0.62-1.6%)	1.3% (0.59-2.4%)	17 (7.1-31)	1500 (840-2300)
North Korea	2016	Female	40 to 44	23% (14-37%)	77% (63-86%)	0.38 (0.19-0.67)	1% (0.56-1.8%)	1.5% (0.69-2.8%)	40 (18-79)	2500 (1300-4400)
North Korea	2016	Female	45 to 49	23% (13-37%)	77% (63-87%)	0.4 (0.2-0.72)	1% (0.46-2%)	1.6% (0.62-3.1%)	71 (27-140)	3500 (1500-6600)
North Korea	2016	Female	50 to 54	22% (14-35%)	78% (65-86%)	0.42 (0.21-0.76)	1.1% (0.39-2.1%)	1.6% (0.56-3.2%)	81 (29-160)	3200 (1200-6000)
North Korea	2016	Female	55 to 59	22% (13-33%)	78% (67-87%)	0.42 (0.21-0.72)	1% (0.2-2.2%)	1.5% (0.31-3.3%)	120 (24-250)	3800 (720-8200)
North Korea	2016	Female	60 to 64	20% (12-32%)	80% (68-88%)	0.41 (0.21-0.72)	0.9% (0.06-2.2%)	1.3% (0.14-3%)	84 (8.7-190)	2200 (150-5300)
North Korea	2016	Female	65 to 69	19% (12-30%)	81% (70-88%)	0.4 (0.21-0.7)	0.82% (-0.074-2%)	1.1% (-0.035-2.7%)	120 (-3.6-290)	2700 (-240-6500)
North Korea	2016	Female	70 to 74	18% (11-29%)	82% (71-89%)	0.39 (0.19-0.73)	0.67% (-0.24-2%)	0.88% (-0.23-2.5%)	150 (-40-420)	2600 (-890-7600)
North Korea	2016	Female	75 to 79	17% (11-27%)	83% (73-89%)	0.38 (0.18-0.7)	0.43% (-0.38-1.7%)	0.53% (-0.44-2.1%)	110 (-90-440)	1500 (-1300-6300)
North Korea	2016	Female	80 to 84	17% (10-27%)	83% (73-90%)	0.37 (0.17-0.66)	0.2% (-0.63-1.4%)	0.23% (-0.75-1.7%)	39 (-120-280)	410 (-1300-3000)
North Korea	2016	Female	85 to 89	18% (10-27%)	82% (73-90%)	0.39 (0.18-0.71)	0.069% (-0.75-1.3%)	0.058% (-0.9-1.5%)	6.7 (-100-170)	69 (-740-1300)
North Korea	2016	Female	90 to 94	19% (12-29%)	81% (71-88%)	0.42 (0.18-0.79)	-0.027% (-0.85-1.3%)	-0.079% (-1.1-1.5%)	-4.2 (-55-81)	-8.1 (-270-410)

Location	Year	Sex	Age	Prevalence of current drinkers	Prevalence of abstainers	Population average of standard drinks daily	Percent of total attributable deaths	Percent of total attributable DALYs	Attributable deaths	Attributable DALYs
North Korea	2016	Female	95 plus	22% (12-35%)	78% (65-88%)	0.48 (0.18-0.99)	-0.068% (-1.2-1.7%)	-0.16% (-1.4-1.9%)	-2.5 (-22-30)	-4 (-70-110)
North Korea	2016	Male	All Ages	41% (35-49%)	59% (51-65%)	1.5 (1.1-2.1)	5.7% (3.7-7.9%)	6.8% (3.7-10%)	7500 (4200-11000)	270000 (170000-380000)
North Korea	2016	Male	15 to 19	30% (19-45%)	70% (55-81%)	0.44 (0.19-0.84)	2.5% (1.3-4.1%)	3% (1.3-5.6%)	42 (18-78)	3900 (2100-6400)
North Korea	2016	Male	20 to 24	43% (28-59%)	57% (41-72%)	1.1 (0.51-1.8)	5.4% (3.2-8.6%)	6.2% (3.1-11%)	140 (64-230)	11000 (6300-18000)
North Korea	2016	Male	25 to 29	49% (31-65%)	51% (35-69%)	1.6 (0.77-2.7)	6.9% (4.1-11%)	8.2% (4.3-13%)	180 (89-300)	14000 (8000-22000)
North Korea	2016	Male	30 to 34	52% (34-69%)	48% (31-66%)	2 (1-3.2)	7.6% (4.7-11%)	9.1% (5.1-14%)	230 (130-380)	16000 (9600-24000)
North Korea	2016	Male	35 to 39	54% (38-71%)	46% (29-62%)	2.3 (1.2-3.9)	8.5% (5-13%)	10% (5.5-16%)	260 (140-440)	16000 (9400-25000)
North Korea	2016	Male	40 to 44	55% (39-73%)	45% (27-61%)	2.7 (1.5-4.3)	9% (5.5-13%)	11% (6.2-17%)	520 (290-850)	28000 (16000-44000)
North Korea	2016	Male	45 to 49	56% (37-73%)	44% (27-63%)	3 (1.6-4.7)	9.3% (5.4-14%)	11% (6-17%)	830 (440-1300)	39000 (22000-59000)
North Korea	2016	Male	50 to 54	56% (38-73%)	44% (27-62%)	3.1 (1.8-4.8)	9.4% (5.1-14%)	11% (5.7-17%)	870 (440-1300)	35000 (19000-52000)
North Korea	2016	Male	55 to 59	54% (36-72%)	46% (28-64%)	2.9 (1.6-4.8)	8.7% (4.6-13%)	10% (5.1-16%)	1200 (580-1800)	40000 (21000-62000)
North Korea	2016	Male	60 to 64	52% (34-70%)	48% (30-66%)	2.6 (1.4-4.3)	7.5% (3.2-12%)	8.4% (3.5-14%)	750 (310-1300)	22000 (9400-36000)
North Korea	2016	Male	65 to 69	48% (33-66%)	52% (34-67%)	2.2 (1.2-3.5)	6.3% (2.8-11%)	7.1% (3-12%)	870 (360-1500)	21000 (9000-35000)
North Korea	2016	Male	70 to 74	45% (29-62%)	55% (38-71%)	1.8 (0.91-3.2)	4.9% (1.6-9.7%)	5.4% (1.6-11%)	830 (240-1700)	16000 (4900-32000)
North Korea	2016	Male	75 to 79	42% (27-60%)	58% (40-73%)	1.5 (0.72-2.7)	3.9% (0.95-7.9%)	4.2% (0.94-8.7%)	540 (120-1100)	8000 (2000-16000)
North Korea	2016	Male	80 to 84	40% (25-57%)	60% (43-75%)	1.4 (0.68-2.4)	3.2% (0.64-7%)	3.4% (0.58-7.7%)	190 (32-420)	2100 (420-4500)
North Korea	2016	Male	85 to 89	39% (25-54%)	61% (46-75%)	1.3 (0.63-2.3)	2.5% (0.33-6%)	2.6% (0.21-6.6%)	64 (5.2-160)	510 (69-1200)
North Korea	2016	Male	90 to 94	40% (26-56%)	60% (44-74%)	1.3 (0.62-2.4)	1.9% (-0.064-5.6%)	2% (-0.27-6.2%)	17 (-2.3-53)	96 (-3.2-280)
North Korea	2016	Male	95 plus	43% (26-62%)	57% (38-74%)	1.3 (0.54-2.6)	1.7% (-0.42-5.8%)	1.6% (-0.77-6.3%)	3.5 (-1.7-13)	14 (-3.4-47)
Taiwan	1990	Female	All Ages	16% (13-20%)	84% (80-87%)	0.3 (0.17-0.5)	1.2% (0.55-2.2%)	1.6% (0.31-3.6%)	630 (120-1500)	23000 (11000-43000)
Taiwan	1990	Female	15 to 19	15% (9.4-24%)	85% (76-91%)	0.28 (0.12-0.58)	0.97% (0.52-1.6%)	1.6% (0.56-3.4%)	7.5 (2.5-16)	930 (490-1600)
Taiwan	1990	Female	20 to 24	19% (11-29%)	81% (71-89%)	0.3 (0.12-0.61)	1.4% (0.87-2.1%)	1.8% (0.69-3.5%)	9.8 (3.9-19)	1600 (950-2500)

Location	Year	Sex	Age	Prevalence of current drinkers	Prevalence of abstainers	Population average of standard drinks daily	Percent of total attributable deaths	Percent of total attributable DALYs	Attributable deaths	Attributable DALYs
Taiwan	1990	Female	25 to 29	19% (11-30%)	81% (70-89%)	0.31 (0.13-0.63)	1.4% (0.91-2.1%)	1.9% (0.83-3.5%)	13 (5.6-24)	1800 (1100-2800)
Taiwan	1990	Female	30 to 34	20% (12-32%)	80% (68-88%)	0.36 (0.16-0.71)	1.4% (0.94-2.2%)	2.3% (1.2-4.2%)	18 (9.7-33)	1900 (1200-3000)
Taiwan	1990	Female	35 to 39	22% (13-34%)	78% (66-87%)	0.45 (0.19-0.86)	1.6% (1.1-2.6%)	3.2% (1.9-5.3%)	30 (18-50)	2200 (1500-3400)
Taiwan	1990	Female	40 to 44	22% (13-35%)	78% (65-87%)	0.51 (0.22-0.99)	1.8% (1.1-2.9%)	3.6% (2.1-6%)	32 (19-54)	1800 (1200-3000)
Taiwan	1990	Female	45 to 49	22% (12-34%)	78% (66-88%)	0.55 (0.22-1.1)	1.8% (0.92-3.2%)	3.5% (1.9-6.3%)	41 (22-76)	1900 (1000-3500)
Taiwan	1990	Female	50 to 54	21% (13-33%)	79% (67-87%)	0.57 (0.24-1.2)	1.8% (0.77-3.5%)	3.3% (1.6-6.2%)	57 (28-110)	2200 (980-4300)
Taiwan	1990	Female	55 to 59	20% (12-31%)	80% (69-88%)	0.57 (0.23-1.1)	1.6% (0.5-3.5%)	2.8% (1.2-5.6%)	66 (29-130)	2100 (670-4500)
Taiwan	1990	Female	60 to 64	19% (11-30%)	81% (70-89%)	0.55 (0.21-1.1)	1.4% (0.18-3.3%)	2.2% (0.61-4.9%)	72 (20-160)	1900 (260-4600)
Taiwan	1990	Female	65 to 69	18% (11-28%)	82% (72-89%)	0.54 (0.23-1)	1.2% (-0.075-3.3%)	1.8% (0.13-4.5%)	77 (5.5-190)	1700 (-99-4600)
Taiwan	1990	Female	70 to 74	17% (11-27%)	83% (73-89%)	0.52 (0.21-1)	1% (-0.29-3.3%)	1.4% (-0.23-4.2%)	70 (-11-210)	1300 (-350-4100)
Taiwan	1990	Female	75 to 79	16% (9.6-26%)	84% (74-90%)	0.5 (0.2-1)	0.83% (-0.57-3.2%)	1% (-0.58-3.7%)	66 (-38-240)	940 (-590-3500)
Taiwan	1990	Female	80 to 84	15% (8.8-25%)	85% (75-91%)	0.48 (0.19-0.92)	0.67% (-0.59-2.8%)	0.8% (-0.66-3.3%)	42 (-35-170)	450 (-380-1900)
Taiwan	1990	Female	85 to 89	16% (9.1-24%)	84% (76-91%)	0.49 (0.19-0.98)	0.58% (-0.61-2.6%)	0.66% (-0.72-3%)	21 (-23-95)	160 (-180-730)
Taiwan	1990	Female	90 to 94	17% (10-26%)	83% (74-90%)	0.52 (0.21-1.1)	0.49% (-0.68-2.5%)	0.52% (-0.82-2.8%)	6.8 (-11-37)	39 (-53-200)
Taiwan	1990	Female	95 plus	19% (10-32%)	81% (68-90%)	0.6 (0.19-1.4)	0.55% (-0.81-3.3%)	0.54% (-1-3.6%)	2 (-3.7-13)	7.9 (-12-47)
Taiwan	1990	Male	All Ages	39% (32-46%)	61% (54-68%)	2 (1.1-3.2)	8.6% (5.4-12%)	10% (5.6-15%)	6800 (3700-9600)	240000 (150000-320000)
Taiwan	1990	Male	15 to 19	29% (18-43%)	71% (57-82%)	0.61 (0.25-1.3)	3.1% (1.5-5.5%)	4.1% (1.6-7.8%)	53 (21-100)	4500 (2200-8200)
Taiwan	1990	Male	20 to 24	41% (27-57%)	59% (43-73%)	1.5 (0.67-2.8)	6.7% (3.7-11%)	8.7% (4-16%)	130 (59-230)	11000 (5700-18000)
Taiwan	1990	Male	25 to 29	47% (31-64%)	53% (36-69%)	2.2 (0.99-4)	8.7% (4.6-14%)	12% (5.4-20%)	210 (97-350)	16000 (8100-25000)
Taiwan	1990	Male	30 to 34	50% (33-67%)	50% (33-67%)	2.8 (1.3-5)	10% (5.7-15%)	14% (7.2-22%)	270 (140-420)	18000 (9900-27000)
Taiwan	1990	Male	35 to 39	53% (36-69%)	47% (31-64%)	3.3 (1.5-6.2)	11% (6.9-16%)	16% (9-23%)	360 (200-530)	21000 (13000-30000)
Taiwan	1990	Male	40 to 44	54% (37-72%)	46% (28-63%)	3.9 (1.8-7)	13% (8.2-18%)	17% (10-24%)	380 (230-530)	20000 (12000-27000)

Location	Year	Sex	Age	Prevalence of current drinkers	Prevalence of abstainers	Population average of standard drinks daily	Percent of total attributable deaths	Percent of total attributable DALYs	Attributable deaths	Attributable DALYs
Taiwan	1990	Male	45 to 49	55% (36-72%)	45% (28-64%)	4.3 (2-8.3)	14% (8.8-19%)	18% (11-24%)	480 (300-640)	22000 (14000-29000)
Taiwan	1990	Male	50 to 54	54% (36-72%)	46% (28-64%)	4.4 (2-8.3)	14% (8.2-18%)	17% (10-23%)	600 (360-810)	24000 (15000-32000)
Taiwan	1990	Male	55 to 59	53% (35-70%)	47% (30-65%)	4.1 (2-7.7)	12% (7.1-17%)	15% (8.5-20%)	740 (430-1000)	26000 (15000-35000)
Taiwan	1990	Male	60 to 64	50% (33-68%)	50% (32-67%)	3.7 (1.7-6.9)	10% (5.3-15%)	12% (6.3-18%)	930 (470-1300)	27000 (14000-40000)
Taiwan	1990	Male	65 to 69	47% (31-64%)	53% (36-69%)	3.1 (1.4-5.7)	9.1% (4-14%)	11% (4.6-16%)	890 (390-1400)	22000 (9500-33000)
Taiwan	1990	Male	70 to 74	43% (28-60%)	57% (40-72%)	2.5 (1.1-4.9)	7.4% (2.7-12%)	8.5% (3-14%)	680 (240-1100)	13000 (4900-22000)
Taiwan	1990	Male	75 to 79	40% (25-58%)	60% (42-75%)	2.2 (0.91-4.2)	6.3% (1.8-11%)	7% (2-13%)	580 (160-1000)	8500 (2500-15000)
Taiwan	1990	Male	80 to 84	38% (23-54%)	62% (46-77%)	2 (0.79-3.7)	5.7% (1.4-11%)	6.3% (1.4-12%)	310 (71-590)	3400 (850-6500)
Taiwan	1990	Male	85 to 89	37% (24-52%)	63% (48-76%)	1.8 (0.75-3.6)	4.8% (1.1-9.8%)	5.3% (1.1-11%)	130 (25-260)	990 (220-2000)
Taiwan	1990	Male	90 to 94	39% (25-54%)	61% (46-75%)	1.8 (0.76-3.6)	4% (0.52-8.5%)	4.3% (0.45-9.4%)	32 (3.4-70)	170 (22-370)
Taiwan	1990	Male	95 plus	42% (26-61%)	58% (39-74%)	1.9 (0.65-4)	3.6% (0.072-8.6%)	3.7% (-0.19-9.3%)	6.5 (-0.33-16)	24 (0.46-57)
Taiwan	1995	Female	All Ages	16% (13-20%)	84% (80-87%)	0.34 (0.19-0.55)	1.4% (0.71-2.5%)	2% (0.58-4.1%)	880 (260-1800)	29000 (15000-50000)
Taiwan	1995	Female	15 to 19	15% (9.4-24%)	85% (76-91%)	0.3 (0.12-0.61)	1% (0.57-1.8%)	1.8% (0.63-3.7%)	8.2 (2.9-17)	1000 (540-1800)
Taiwan	1995	Female	20 to 24	19% (11-28%)	81% (72-89%)	0.32 (0.13-0.69)	1.5% (0.92-2.3%)	1.9% (0.75-3.7%)	9.2 (3.7-18)	1500 (890-2400)
Taiwan	1995	Female	25 to 29	19% (11-29%)	81% (71-89%)	0.33 (0.14-0.69)	1.5% (1-2.3%)	2% (0.93-3.6%)	12 (5.3-21)	1700 (1100-2600)
Taiwan	1995	Female	30 to 34	20% (12-31%)	80% (69-88%)	0.38 (0.15-0.77)	1.5% (1-2.3%)	2.5% (1.4-4.3%)	19 (11-34)	2000 (1300-3100)
Taiwan	1995	Female	35 to 39	22% (13-34%)	78% (66-87%)	0.47 (0.2-0.9)	1.7% (1.2-2.6%)	3.4% (2.1-5.5%)	34 (21-55)	2500 (1700-3800)
Taiwan	1995	Female	40 to 44	22% (13-35%)	78% (65-87%)	0.54 (0.24-1)	1.9% (1.1-3%)	3.8% (2.3-6.3%)	49 (29-81)	2900 (1800-4600)
Taiwan	1995	Female	45 to 49	21% (12-34%)	79% (66-88%)	0.58 (0.23-1.2)	1.9% (1-3.3%)	3.8% (2.2-6.6%)	49 (27-85)	2300 (1300-4000)
Taiwan	1995	Female	50 to 54	21% (12-33%)	79% (67-88%)	0.6 (0.25-1.2)	2% (0.95-3.7%)	3.8% (2.1-6.7%)	62 (33-110)	2400 (1200-4400)
Taiwan	1995	Female	55 to 59	20% (12-31%)	80% (69-88%)	0.6 (0.25-1.2)	2% (0.81-3.9%)	3.5% (1.8-6.4%)	85 (42-160)	2700 (1200-5300)
Taiwan	1995	Female	60 to 64	19% (11-30%)	81% (70-89%)	0.58 (0.23-1.2)	1.8% (0.56-3.8%)	3% (1.2-5.6%)	100 (42-190)	2700 (860-5600)

Location	Year	Sex	Age	Prevalence of current drinkers	Prevalence of abstainers	Population average of standard drinks daily	Percent of total attributable deaths	Percent of total attributable DALYs	Attributable deaths	Attributable DALYs
Taiwan	1995	Female	65 to 69	18% (11-29%)	82% (71-89%)	0.57 (0.24-1.1)	1.7% (0.33-3.8%)	2.6% (0.8-5.3%)	120 (37-240)	2700 (500-5900)
Taiwan	1995	Female	70 to 74	17% (11-27%)	83% (73-89%)	0.55 (0.22-1.1)	1.5% (0.077-3.8%)	2% (0.27-4.9%)	120 (15-290)	2200 (120-5500)
Taiwan	1995	Female	75 to 79	16% (9.6-26%)	84% (74-90%)	0.53 (0.2-1)	1.1% (-0.22-3.5%)	1.5% (-0.16-4.3%)	91 (-11-270)	1300 (-250-4000)
Taiwan	1995	Female	80 to 84	15% (9-25%)	85% (75-91%)	0.51 (0.2-1)	0.88% (-0.39-3%)	1.1% (-0.42-3.6%)	69 (-27-230)	740 (-330-2500)
Taiwan	1995	Female	85 to 89	16% (9.5-24%)	84% (76-91%)	0.53 (0.21-1.1)	0.77% (-0.49-2.8%)	0.89% (-0.53-3.3%)	39 (-23-140)	300 (-180-1100)
Taiwan	1995	Female	90 to 94	17% (10-26%)	83% (74-90%)	0.57 (0.22-1.2)	0.65% (-0.56-2.6%)	0.71% (-0.68-3%)	13 (-12-55)	74 (-63-300)
Taiwan	1995	Female	95 plus	20% (11-33%)	80% (67-89%)	0.65 (0.21-1.5)	0.73% (-0.79-3.5%)	0.74% (-0.99-3.9%)	4.1 (-5.4-21)	16 (-17-75)
Taiwan	1995	Male	All Ages	39% (33-47%)	61% (53-67%)	2.2 (1.2-3.5)	9.1% (5.8-12%)	11% (5.7-14%)	7800 (4400-11000)	270000 (170000-360000)
Taiwan	1995	Male	15 to 19	28% (18-43%)	72% (57-82%)	0.65 (0.25-1.4)	3.2% (1.6-5.8%)	4.3% (1.7-8.5%)	56 (22-110)	4800 (2400-8700)
Taiwan	1995	Male	20 to 24	41% (27-57%)	59% (43-73%)	1.5 (0.71-3)	7.2% (3.9-12%)	9.3% (4.3-17%)	120 (56-220)	10000 (5500-17000)
Taiwan	1995	Male	25 to 29	47% (30-64%)	53% (36-70%)	2.3 (1-4.4)	9.3% (5-14%)	13% (5.8-21%)	200 (90-320)	15000 (7800-23000)
Taiwan	1995	Male	30 to 34	50% (32-68%)	50% (32-68%)	3 (1.3-5.4)	11% (5.9-16%)	15% (7.3-22%)	300 (150-460)	20000 (11000-30000)
Taiwan	1995	Male	35 to 39	53% (36-70%)	47% (30-64%)	3.6 (1.6-6.5)	12% (7.4-17%)	16% (9.5-24%)	430 (250-620)	25000 (15000-36000)
Taiwan	1995	Male	40 to 44	54% (37-72%)	46% (28-63%)	4.1 (1.9-7.4)	14% (8.8-19%)	18% (11-25%)	590 (360-800)	31000 (19000-41000)
Taiwan	1995	Male	45 to 49	54% (36-72%)	46% (28-64%)	4.6 (2.1-8.6)	15% (9.4-19%)	19% (12-25%)	580 (360-760)	26000 (17000-35000)
Taiwan	1995	Male	50 to 54	54% (36-72%)	46% (28-64%)	4.7 (2.1-8.9)	14% (9.1-19%)	18% (11-23%)	630 (390-830)	25000 (16000-33000)
Taiwan	1995	Male	55 to 59	52% (34-70%)	48% (30-66%)	4.4 (2.1-7.8)	13% (7.9-17%)	16% (9.4-21%)	740 (450-970)	26000 (15000-34000)
Taiwan	1995	Male	60 to 64	50% (32-68%)	50% (32-68%)	3.9 (1.8-7.2)	11% (6-15%)	13% (7.1-18%)	870 (470-1200)	26000 (14000-36000)
Taiwan	1995	Male	65 to 69	47% (30-65%)	53% (35-70%)	3.3 (1.5-6.1)	9.3% (4.4-14%)	11% (5-16%)	1100 (490-1600)	26000 (12000-39000)
Taiwan	1995	Male	70 to 74	43% (27-60%)	57% (40-73%)	2.7 (1.1-5.1)	7.6% (2.9-12%)	8.7% (3.3-14%)	910 (350-1500)	18000 (6900-28000)
Taiwan	1995	Male	75 to 79	40% (25-57%)	60% (43-75%)	2.3 (0.94-4.4)	6.2% (2-11%)	7% (2.3-12%)	640 (200-1100)	9700 (3100-16000)
Taiwan	1995	Male	80 to 84	37% (23-54%)	63% (46-77%)	2.1 (0.86-4)	5.7% (1.6-10%)	6.3% (1.7-11%)	470 (120-830)	5100 (1400-9200)

Location	Year	Sex	Age	Prevalence of current drinkers	Prevalence of abstainers	Population average of standard drinks daily	Percent of total attributable deaths	Percent of total attributable DALYs	Attributable deaths	Attributable DALYs
Taiwan	1995	Male	85 to 89	37% (23-51%)	63% (49-77%)	1.9 (0.82-3.8)	4.9% (1.2-9.5%)	5.4% (1.2-11%)	190 (43-370)	1500 (360-2900)
Taiwan	1995	Male	90 to 94	38% (25-54%)	62% (46-75%)	1.9 (0.8-3.7)	4.2% (0.67-8.6%)	4.5% (0.57-9.5%)	52 (6.4-110)	280 (46-570)
Taiwan	1995	Male	95 plus	42% (25-60%)	58% (40-75%)	2 (0.66-4.2)	3.8% (0.078-8.7%)	3.9% (-0.14-9.5%)	10 (-0.37-25)	38 (0.81-89)
Taiwan	2000	Female	All Ages	17% (14-20%)	83% (80-86%)	0.38 (0.21-0.62)	1.6% (0.81-2.7%)	2.2% (0.84-4.4%)	1100 (400-2100)	34000 (17000-58000)
Taiwan	2000	Female	15 to 19	15% (9.5-24%)	85% (76-91%)	0.31 (0.13-0.65)	1% (0.59-1.7%)	1.8% (0.67-3.7%)	6.7 (2.5-14)	910 (490-1500)
Taiwan	2000	Female	20 to 24	19% (11-29%)	81% (71-89%)	0.34 (0.14-0.71)	1.5% (0.96-2.3%)	2% (0.82-3.9%)	8.8 (3.7-18)	1600 (980-2500)
Taiwan	2000	Female	25 to 29	19% (11-30%)	81% (70-89%)	0.35 (0.14-0.72)	1.6% (1-2.3%)	2.2% (0.99-4.1%)	9.9 (4.5-18)	1600 (1000-2400)
Taiwan	2000	Female	30 to 34	20% (12-32%)	80% (68-88%)	0.4 (0.16-0.82)	1.6% (1-2.3%)	2.6% (1.4-4.5%)	17 (9.3-30)	1900 (1200-3000)
Taiwan	2000	Female	35 to 39	21% (13-34%)	79% (66-87%)	0.5 (0.21-0.98)	1.7% (1.2-2.7%)	3.5% (2.2-5.8%)	34 (21-55)	2600 (1800-3900)
Taiwan	2000	Female	40 to 44	22% (13-35%)	78% (65-87%)	0.57 (0.25-1.1)	1.9% (1.1-3.1%)	3.9% (2.3-6.6%)	52 (30-88)	3100 (1900-5100)
Taiwan	2000	Female	45 to 49	21% (12-34%)	79% (66-88%)	0.61 (0.25-1.2)	1.9% (1-3.2%)	3.9% (2.3-6.7%)	68 (38-120)	3300 (1900-5600)
Taiwan	2000	Female	50 to 54	21% (12-33%)	79% (67-88%)	0.63 (0.26-1.3)	2% (0.97-3.6%)	3.9% (2.2-6.7%)	68 (37-120)	2700 (1300-4800)
Taiwan	2000	Female	55 to 59	20% (12-31%)	80% (69-88%)	0.63 (0.26-1.3)	2.1% (0.9-4.1%)	3.8% (2-6.8%)	86 (44-160)	2800 (1200-5500)
Taiwan	2000	Female	60 to 64	19% (11-30%)	81% (70-89%)	0.62 (0.23-1.2)	2% (0.73-4.2%)	3.5% (1.6-6.4%)	120 (53-220)	3200 (1100-6300)
Taiwan	2000	Female	65 to 69	18% (11-29%)	82% (71-89%)	0.61 (0.26-1.2)	2% (0.6-4.1%)	3.2% (1.2-5.9%)	150 (57-270)	3300 (990-6700)
Taiwan	2000	Female	70 to 74	17% (10-27%)	83% (73-90%)	0.6 (0.24-1.2)	1.8% (0.3-4.2%)	2.6% (0.65-5.6%)	160 (40-350)	3000 (480-7000)
Taiwan	2000	Female	75 to 79	16% (9.6-26%)	84% (74-90%)	0.56 (0.22-1.1)	1.4% (0.014-3.5%)	1.9% (0.16-4.4%)	130 (12-310)	1900 (19-4800)
Taiwan	2000	Female	80 to 84	15% (9-24%)	85% (76-91%)	0.54 (0.22-1)	1.1% (-0.19-3.2%)	1.3% (-0.18-3.9%)	89 (-11-260)	970 (-170-2900)
Taiwan	2000	Female	85 to 89	16% (9.2-25%)	84% (75-91%)	0.56 (0.23-1.1)	0.93% (-0.33-3%)	1.1% (-0.35-3.5%)	61 (-20-190)	480 (-170-1500)
Taiwan	2000	Female	90 to 94	17% (10-27%)	83% (73-90%)	0.6 (0.23-1.2)	0.8% (-0.47-3%)	0.88% (-0.57-3.4%)	23 (-15-88)	130 (-74-490)
Taiwan	2000	Female	95 plus	20% (11-32%)	80% (68-89%)	0.69 (0.22-1.6)	0.88% (-0.67-3.8%)	0.91% (-0.85-4.2%)	7.8 (-7.3-37)	30 (-22-130)
Taiwan	2000	Male	All Ages	40% (34-48%)	60% (52-66%)	2.5 (1.4-4)	9.7% (6.2-13%)	11% (6.3-15%)	8500 (5000-12000)	290000 (190000-380000)

Location	Year	Sex	Age	Prevalence of current drinkers	Prevalence of abstainers	Population average of standard drinks daily	Percent of total attributable deaths	Percent of total attributable DALYs	Attributable deaths	Attributable DALYs
Taiwan	2000	Male	15 to 19	28% (18-43%)	72% (57-82%)	0.67 (0.27-1.4)	3.1% (1.6-5.5%)	4.5% (1.9-8.7%)	40 (17-77)	3700 (1900-6500)
Taiwan	2000	Male	20 to 24	41% (26-56%)	59% (44-74%)	1.6 (0.72-3.2)	7.1% (3.9-12%)	9.6% (4.5-18%)	110 (49-200)	9400 (5100-16000)
Taiwan	2000	Male	25 to 29	47% (30-64%)	53% (36-70%)	2.5 (1.1-4.6)	9.5% (5.2-15%)	14% (6.4-22%)	160 (77-270)	13000 (6700-20000)
Taiwan	2000	Male	30 to 34	50% (33-67%)	50% (33-67%)	3.2 (1.4-5.7)	11% (6.5-16%)	16% (8.1-24%)	280 (140-420)	19000 (11000-27000)
Taiwan	2000	Male	35 to 39	52% (36-69%)	48% (31-64%)	3.7 (1.7-7)	13% (7.9-18%)	17% (10-24%)	450 (270-650)	27000 (16000-37000)
Taiwan	2000	Male	40 to 44	53% (37-71%)	47% (29-63%)	4.3 (2-7.9)	15% (9.5-19%)	19% (12-26%)	680 (420-910)	35000 (23000-47000)
Taiwan	2000	Male	45 to 49	54% (35-72%)	46% (28-65%)	4.8 (2.2-8.8)	15% (10-20%)	20% (13-26%)	850 (560-1100)	39000 (26000-50000)
Taiwan	2000	Male	50 to 54	54% (36-72%)	46% (28-64%)	4.9 (2.3-9.1)	15% (9.9-19%)	19% (12-24%)	730 (500-960)	30000 (20000-39000)
Taiwan	2000	Male	55 to 59	52% (35-71%)	48% (29-65%)	4.6 (2.1-8.5)	13% (8.3-17%)	16% (10-21%)	740 (460-970)	26000 (16000-34000)
Taiwan	2000	Male	60 to 64	49% (31-68%)	51% (32-69%)	4.1 (1.7-7.6)	11% (6.1-15%)	13% (7.4-18%)	800 (450-1100)	24000 (13000-33000)
Taiwan	2000	Male	65 to 69	46% (31-63%)	54% (37-69%)	3.4 (1.5-6.4)	9.3% (4.7-13%)	11% (5.5-16%)	900 (460-1300)	22000 (11000-32000)
Taiwan	2000	Male	70 to 74	43% (27-60%)	57% (40-73%)	2.8 (1.2-5.4)	7.5% (3.1-12%)	8.7% (3.6-14%)	1000 (440-1600)	20000 (8800-32000)
Taiwan	2000	Male	75 to 79	40% (25-58%)	60% (42-75%)	2.4 (0.98-4.7)	6.2% (2.3-10%)	7.1% (2.5-12%)	820 (290-1400)	13000 (4500-21000)
Taiwan	2000	Male	80 to 84	37% (23-54%)	63% (46-77%)	2.2 (0.94-4.2)	5.7% (1.9-9.8%)	6.4% (2-11%)	530 (170-920)	5900 (1900-10000)
Taiwan	2000	Male	85 to 89	36% (23-52%)	64% (48-77%)	2 (0.87-3.9)	5% (1.3-9.3%)	5.6% (1.4-10%)	290 (73-540)	2300 (610-4200)
Taiwan	2000	Male	90 to 94	38% (25-54%)	62% (46-75%)	2 (0.83-4)	4.3% (0.8-8.4%)	4.7% (0.75-9.4%)	80 (13-160)	440 (81-850)
Taiwan	2000	Male	95 plus	42% (25-60%)	58% (40-75%)	2.1 (0.71-4.5)	4.1% (0.27-8.9%)	4.2% (0.025-9.8%)	18 (0.11-41)	67 (4.6-150)
Taiwan	2005	Female	All Ages	17% (14-21%)	83% (79-86%)	0.41 (0.22-0.69)	1.5% (0.76-2.7%)	2.1% (0.8-4.2%)	1100 (420-2200)	34000 (17000-60000)
Taiwan	2005	Female	15 to 19	15% (9.4-25%)	85% (75-91%)	0.31 (0.13-0.66)	1% (0.59-1.7%)	1.9% (0.71-4.1%)	4.6 (1.7-9.7)	690 (400-1100)
Taiwan	2005	Female	20 to 24	19% (11-29%)	81% (71-89%)	0.34 (0.14-0.74)	1.6% (1-2.4%)	2.1% (0.84-4.4%)	8 (3.1-17)	1500 (930-2400)
Taiwan	2005	Female	25 to 29	19% (11-30%)	81% (70-89%)	0.35 (0.14-0.73)	1.6% (1-2.4%)	2.3% (0.91-4.6%)	11 (4.3-22)	1800 (1100-2700)
Taiwan	2005	Female	30 to 34	20% (12-31%)	80% (69-88%)	0.4 (0.16-0.81)	1.6% (1-2.4%)	2.7% (1.3-4.9%)	15 (7.7-28)	1800 (1100-2700)

Location	Year	Sex	Age	Prevalence of current drinkers	Prevalence of abstainers	Population average of standard drinks daily	Percent of total attributable deaths	Percent of total attributable DALYs	Attributable deaths	Attributable DALYs
Taiwan	2005	Female	35 to 39	21% (13-34%)	79% (66-87%)	0.5 (0.21-0.99)	1.7% (1.1-2.7%)	3.5% (2-5.9%)	29 (16-49)	2300 (1500-3700)
Taiwan	2005	Female	40 to 44	22% (13-35%)	78% (65-87%)	0.57 (0.24-1.2)	1.8% (1.1-3%)	3.8% (2.1-6.5%)	47 (27-80)	2900 (1800-4900)
Taiwan	2005	Female	45 to 49	21% (12-33%)	79% (67-88%)	0.61 (0.25-1.2)	1.7% (0.87-3%)	3.7% (2.1-6.5%)	64 (35-110)	3200 (1700-5600)
Taiwan	2005	Female	50 to 54	21% (13-33%)	79% (67-87%)	0.64 (0.26-1.2)	1.8% (0.8-3.2%)	3.7% (1.9-6.4%)	86 (44-150)	3500 (1600-6300)
Taiwan	2005	Female	55 to 59	20% (12-31%)	80% (69-88%)	0.64 (0.25-1.3)	1.8% (0.76-3.6%)	3.5% (1.8-6.4%)	83 (42-150)	2800 (1200-5400)
Taiwan	2005	Female	60 to 64	19% (11-30%)	81% (70-89%)	0.62 (0.24-1.2)	1.9% (0.67-3.9%)	3.4% (1.6-6.3%)	100 (49-180)	2800 (1000-5600)
Taiwan	2005	Female	65 to 69	18% (11-28%)	82% (72-89%)	0.61 (0.26-1.2)	1.9% (0.67-3.9%)	3.2% (1.4-5.9%)	150 (65-270)	3300 (1100-6700)
Taiwan	2005	Female	70 to 74	17% (11-27%)	83% (73-89%)	0.6 (0.24-1.3)	1.8% (0.39-4%)	2.6% (0.77-5.5%)	170 (46-340)	3100 (670-7100)
Taiwan	2005	Female	75 to 79	16% (9.6-26%)	84% (74-90%)	0.56 (0.22-1.2)	1.3% (0.11-3.4%)	1.8% (0.32-4.3%)	140 (26-350)	2100 (180-5400)
Taiwan	2005	Female	80 to 84	15% (9-25%)	85% (75-91%)	0.55 (0.23-1.1)	1% (-0.11-2.9%)	1.3% (-0.056-3.5%)	110 (-4.6-290)	1200 (-120-3300)
Taiwan	2005	Female	85 to 89	16% (9.5-25%)	84% (75-91%)	0.57 (0.23-1.1)	0.88% (-0.23-2.8%)	1% (-0.23-3.2%)	66 (-14-210)	530 (-140-1600)
Taiwan	2005	Female	90 to 94	17% (10-27%)	83% (73-90%)	0.62 (0.24-1.3)	0.77% (-0.37-2.9%)	0.85% (-0.46-3.3%)	31 (-17-120)	180 (-87-680)
Taiwan	2005	Female	95 plus	20% (11-33%)	80% (67-89%)	0.71 (0.23-1.6)	0.88% (-0.58-3.8%)	0.91% (-0.76-4.2%)	12 (-10-56)	46 (-31-200)
Taiwan	2005	Male	All Ages	41% (35-49%)	59% (51-65%)	2.6 (1.4-4.3)	10% (6.6-13%)	11% (6.3-15%)	9100 (5300-13000)	310000 (200000-410000)
Taiwan	2005	Male	15 to 19	28% (17-43%)	72% (57-83%)	0.67 (0.27-1.4)	3% (1.6-5.2%)	4.6% (1.9-9%)	26 (11-51)	2600 (1400-4400)
Taiwan	2005	Male	20 to 24	40% (26-57%)	60% (43-74%)	1.6 (0.68-3.3)	6.9% (3.9-11%)	9.8% (4.3-18%)	86 (37-160)	7900 (4500-13000)
Taiwan	2005	Male	25 to 29	46% (29-63%)	54% (37-71%)	2.4 (1-4.7)	9.4% (5.2-14%)	14% (6.4-22%)	170 (81-290)	14000 (7300-21000)
Taiwan	2005	Male	30 to 34	50% (32-67%)	50% (33-68%)	3.1 (1.4-5.8)	11% (6.7-17%)	16% (8.7-24%)	260 (140-400)	18000 (10000-25000)
Taiwan	2005	Male	35 to 39	52% (36-70%)	48% (30-64%)	3.7 (1.7-7)	13% (8.2-18%)	18% (11-26%)	450 (260-630)	27000 (16000-37000)
Taiwan	2005	Male	40 to 44	54% (37-71%)	46% (29-63%)	4.3 (2-8)	15% (10-20%)	20% (13-27%)	750 (460-990)	39000 (25000-51000)
Taiwan	2005	Male	45 to 49	54% (36-71%)	46% (29-64%)	4.8 (2.2-9)	16% (10-20%)	21% (13-27%)	970 (620-1300)	44000 (29000-57000)
Taiwan	2005	Male	50 to 54	54% (35-71%)	46% (29-65%)	4.9 (2.2-9)	15% (9.6-19%)	19% (12-25%)	1100 (690-1400)	43000 (28000-56000)

Location	Year	Sex	Age	Prevalence of current drinkers	Prevalence of abstainers	Population average of standard drinks daily	Percent of total attributable deaths	Percent of total attributable DALYs	Attributable deaths	Attributable DALYs
Taiwan	2005	Male	55 to 59	52% (34-70%)	48% (30-66%)	4.6 (2.1-8.3)	13% (8.2-17%)	16% (10-21%)	830 (510-1100)	29000 (18000-38000)
Taiwan	2005	Male	60 to 64	49% (31-68%)	51% (32-69%)	4.1 (1.8-7.8)	11% (6.1-15%)	13% (7.3-18%)	770 (430-1100)	23000 (13000-32000)
Taiwan	2005	Male	65 to 69	46% (30-65%)	54% (35-70%)	3.4 (1.5-6.6)	9% (4.7-13%)	11% (5.5-16%)	810 (410-1200)	20000 (10000-29000)
Taiwan	2005	Male	70 to 74	43% (28-61%)	57% (39-72%)	2.8 (1.2-5.5)	7.1% (3.2-11%)	8.4% (3.6-13%)	840 (370-1300)	17000 (7400-27000)
Taiwan	2005	Male	75 to 79	39% (25-56%)	61% (44-75%)	2.4 (0.96-4.8)	5.8% (2.1-9.8%)	6.6% (2.3-11%)	910 (320-1600)	14000 (5100-24000)
Taiwan	2005	Male	80 to 84	37% (22-54%)	63% (46-78%)	2.1 (0.92-4.1)	5.1% (1.7-9.1%)	5.8% (1.9-10%)	640 (210-1200)	7400 (2500-13000)
Taiwan	2005	Male	85 to 89	36% (22-52%)	64% (48-78%)	2 (0.87-3.8)	4.6% (1.2-8.7%)	5.1% (1.3-9.8%)	330 (82-640)	2700 (730-5100)
Taiwan	2005	Male	90 to 94	38% (25-53%)	62% (47-75%)	2 (0.81-4)	4.1% (0.83-8.1%)	4.4% (0.77-9.1%)	130 (22-270)	730 (150-1400)
Taiwan	2005	Male	95 plus	41% (25-59%)	59% (41-75%)	2 (0.71-4.6)	3.9% (0.43-8.7%)	4.1% (0.26-9.7%)	30 (1.8-70)	110 (12-250)
Taiwan	2010	Female	All Ages	18% (15-22%)	82% (78-85%)	0.44 (0.24-0.73)	1.5% (0.76-2.6%)	2.1% (0.85-4.2%)	1200 (500-2400)	35000 (18000-61000)
Taiwan	2010	Female	15 to 19	16% (9.7-25%)	84% (75-90%)	0.33 (0.13-0.69)	0.98% (0.57-1.6%)	2% (0.8-4.2%)	3.7 (1.4-7.5)	630 (360-1000)
Taiwan	2010	Female	20 to 24	19% (12-29%)	81% (71-88%)	0.35 (0.14-0.8)	1.6% (0.98-2.4%)	2.4% (0.94-5%)	5.6 (2.2-12)	1200 (710-1800)
Taiwan	2010	Female	25 to 29	19% (11-30%)	81% (70-89%)	0.37 (0.15-0.74)	1.6% (1.1-2.4%)	2.5% (0.89-5.1%)	9.7 (3.6-20)	1700 (1000-2500)
Taiwan	2010	Female	30 to 34	20% (12-31%)	80% (69-88%)	0.42 (0.17-0.88)	1.6% (1-2.4%)	2.8% (1.3-5.4%)	16 (7.4-31)	1900 (1200-3000)
Taiwan	2010	Female	35 to 39	22% (13-34%)	78% (66-87%)	0.52 (0.22-1)	1.7% (1-2.6%)	3.6% (2-6.3%)	25 (14-45)	2100 (1400-3300)
Taiwan	2010	Female	40 to 44	22% (14-35%)	78% (65-86%)	0.59 (0.25-1.1)	1.7% (1-2.8%)	3.9% (2.2-6.6%)	43 (24-74)	2700 (1700-4600)
Taiwan	2010	Female	45 to 49	22% (12-34%)	78% (66-88%)	0.62 (0.26-1.2)	1.6% (0.88-2.8%)	3.8% (2.1-6.4%)	63 (35-110)	3200 (1700-5500)
Taiwan	2010	Female	50 to 54	21% (13-33%)	79% (67-87%)	0.65 (0.28-1.3)	1.7% (0.77-3%)	3.7% (2-6.2%)	85 (46-140)	3500 (1700-6400)
Taiwan	2010	Female	55 to 59	20% (12-32%)	80% (68-88%)	0.66 (0.27-1.3)	1.7% (0.7-3.4%)	3.6% (1.9-6.3%)	110 (58-190)	3700 (1500-7500)
Taiwan	2010	Female	60 to 64	19% (11-31%)	81% (69-89%)	0.64 (0.25-1.3)	1.8% (0.68-3.6%)	3.4% (1.8-6.1%)	110 (54-190)	3000 (1100-6100)
Taiwan	2010	Female	65 to 69	18% (11-29%)	82% (71-89%)	0.63 (0.27-1.3)	1.9% (0.75-3.6%)	3.4% (1.6-5.9%)	130 (62-230)	3100 (1200-6100)
Taiwan	2010	Female	70 to 74	17% (11-28%)	83% (72-89%)	0.62 (0.27-1.3)	1.8% (0.52-3.8%)	2.8% (1.1-5.6%)	170 (67-340)	3300 (1000-7200)

Location	Year	Sex	Age	Prevalence of current drinkers	Prevalence of abstainers	Population average of standard drinks daily	Percent of total attributable deaths	Percent of total attributable DALYs	Attributable deaths	Attributable DALYs
Taiwan	2010	Female	75 to 79	16% (9.8-27%)	84% (73-90%)	0.59 (0.24-1.3)	1.4% (0.23-3.4%)	2% (0.48-4.5%)	160 (39-380)	2400 (410-6100)
Taiwan	2010	Female	80 to 84	16% (9.5-25%)	84% (75-91%)	0.57 (0.25-1.1)	1.1% (-0.022-3%)	1.4% (0.069-3.7%)	130 (6.7-370)	1500 (-29-4300)
Taiwan	2010	Female	85 to 89	16% (9.7-25%)	84% (75-90%)	0.59 (0.25-1.2)	0.92% (-0.13-2.8%)	1.1% (-0.13-3.4%)	95 (-11-300)	780 (-110-2500)
Taiwan	2010	Female	90 to 94	18% (11-27%)	82% (73-89%)	0.64 (0.24-1.3)	0.82% (-0.32-2.8%)	0.91% (-0.4-3.3%)	44 (-19-160)	260 (-100-900)
Taiwan	2010	Female	95 plus	20% (11-33%)	80% (67-89%)	0.73 (0.26-1.6)	0.94% (-0.46-3.9%)	0.99% (-0.63-4.4%)	21 (-13-94)	79 (-40-330)
Taiwan	2010	Male	All Ages	43% (36-51%)	57% (49-64%)	2.8 (1.5-4.7)	10% (6.9-13%)	11% (6.3-14%)	9500 (5600-13000)	320000 (210000-410000)
Taiwan	2010	Male	15 to 19	28% (18-43%)	72% (57-82%)	0.68 (0.27-1.5)	2.8% (1.6-4.8%)	4.6% (2-9%)	20 (8.7-39)	2200 (1300-3700)
Taiwan	2010	Male	20 to 24	41% (27-57%)	59% (43-73%)	1.7 (0.7-3.4)	6.8% (4-11%)	10% (4.6-20%)	60 (26-110)	5900 (3400-9700)
Taiwan	2010	Male	25 to 29	47% (30-64%)	53% (36-70%)	2.5 (1.1-4.8)	9% (5.2-13%)	14% (6.5-22%)	130 (59-210)	11000 (6100-16000)
Taiwan	2010	Male	30 to 34	50% (32-67%)	50% (33-68%)	3.2 (1.5-6)	11% (6.5-16%)	16% (9-24%)	230 (130-340)	17000 (9800-24000)
Taiwan	2010	Male	35 to 39	52% (36-69%)	48% (31-64%)	3.8 (1.7-7.3)	13% (8.2-18%)	19% (11-26%)	380 (230-530)	23000 (14000-32000)
Taiwan	2010	Male	40 to 44	53% (37-71%)	47% (29-63%)	4.3 (2-8)	15% (10-20%)	21% (13-27%)	680 (430-900)	36000 (23000-47000)
Taiwan	2010	Male	45 to 49	54% (36-71%)	46% (29-64%)	4.8 (2.3-8.5)	16% (10-20%)	21% (14-27%)	980 (650-1300)	46000 (30000-58000)
Taiwan	2010	Male	50 to 54	54% (36-71%)	46% (29-64%)	4.9 (2.3-8.8)	15% (9.9-19%)	20% (13-24%)	1100 (740-1400)	46000 (30000-59000)
Taiwan	2010	Male	55 to 59	52% (35-71%)	48% (29-65%)	4.7 (2.2-8.1)	13% (8.5-17%)	17% (11-22%)	1200 (750-1500)	41000 (27000-54000)
Taiwan	2010	Male	60 to 64	50% (32-69%)	50% (31-68%)	4.1 (1.8-7.6)	11% (6.2-15%)	14% (7.6-19%)	840 (470-1100)	26000 (14000-35000)
Taiwan	2010	Male	65 to 69	47% (31-64%)	53% (36-69%)	3.5 (1.6-6.4)	9% (4.7-13%)	11% (5.8-16%)	770 (400-1100)	20000 (10000-28000)
Taiwan	2010	Male	70 to 74	43% (27-60%)	57% (40-73%)	2.9 (1.3-5.6)	7.1% (3.2-11%)	8.4% (3.8-13%)	770 (340-1200)	16000 (7000-24000)
Taiwan	2010	Male	75 to 79	40% (25-58%)	60% (42-75%)	2.4 (1.1-4.9)	5.7% (2.2-9.8%)	6.6% (2.5-11%)	780 (290-1300)	12000 (4600-21000)
Taiwan	2010	Male	80 to 84	38% (23-54%)	62% (46-77%)	2.2 (0.97-4.4)	5% (1.7-9%)	5.7% (1.9-10%)	820 (270-1500)	9500 (3300-17000)
Taiwan	2010	Male	85 to 89	37% (23-52%)	63% (48-77%)	2.1 (0.89-4.1)	4.5% (1.4-8.5%)	5% (1.5-9.7%)	480 (140-920)	4000 (1300-7600)
Taiwan	2010	Male	90 to 94	38% (25-53%)	62% (47-75%)	2 (0.84-4.1)	4% (1-8%)	4.4% (0.96-9.2%)	180 (38-370)	1000 (260-2100)

Location	Year	Sex	Age	Prevalence of current drinkers	Prevalence of abstainers	Population average of standard drinks daily	Percent of total attributable deaths	Percent of total attributable DALYs	Attributable deaths	Attributable DALYs
Taiwan	2010	Male	95 plus	42% (25-61%)	58% (39-75%)	2.1 (0.8-4.5)	4% (0.71-8.9%)	4.2% (0.49-9.9%)	61 (7-140)	220 (40-500)
Taiwan	2016	Female	All Ages	18% (15-22%)	82% (78-85%)	0.47 (0.26-0.79)	1.6% (0.79-2.7%)	2.2% (0.89-4.2%)	1600 (610-3000)	40000 (20000-71000)
Taiwan	2016	Female	15 to 19	16% (9.8-25%)	84% (75-90%)	0.34 (0.14-0.7)	0.96% (0.58-1.6%)	2% (0.77-4.1%)	3.1 (1.2-6.6)	560 (320-930)
Taiwan	2016	Female	20 to 24	19% (12-29%)	81% (71-88%)	0.36 (0.14-0.79)	1.6% (1-2.4%)	2.4% (0.95-4.9%)	5.2 (2-11)	1100 (700-1800)
Taiwan	2016	Female	25 to 29	19% (12-30%)	81% (70-88%)	0.37 (0.15-0.78)	1.6% (1-2.4%)	2.5% (0.98-5%)	6.9 (2.6-14)	1300 (800-1900)
Taiwan	2016	Female	30 to 34	20% (12-32%)	80% (68-88%)	0.42 (0.18-0.86)	1.5% (0.97-2.4%)	2.8% (1.3-5.5%)	15 (6.3-28)	1800 (1100-2900)
Taiwan	2016	Female	35 to 39	22% (13-34%)	78% (66-87%)	0.52 (0.22-1)	1.7% (1.1-2.7%)	3.7% (2-6.6%)	28 (14-51)	2400 (1500-3900)
Taiwan	2016	Female	40 to 44	22% (13-35%)	78% (65-87%)	0.59 (0.26-1.2)	1.7% (1-2.9%)	3.9% (2.2-6.6%)	40 (21-70)	2600 (1500-4300)
Taiwan	2016	Female	45 to 49	22% (12-34%)	78% (66-88%)	0.64 (0.27-1.2)	1.7% (0.91-2.9%)	3.9% (2.1-6.5%)	60 (32-110)	3100 (1600-5500)
Taiwan	2016	Female	50 to 54	21% (13-33%)	79% (67-87%)	0.67 (0.3-1.3)	1.7% (0.83-3.2%)	3.8% (2.2-6.4%)	93 (50-150)	3900 (1800-7300)
Taiwan	2016	Female	55 to 59	20% (12-32%)	80% (68-88%)	0.67 (0.29-1.3)	1.7% (0.72-3.3%)	3.7% (2-6.2%)	120 (64-220)	4200 (1800-8200)
Taiwan	2016	Female	60 to 64	19% (11-31%)	81% (69-89%)	0.65 (0.26-1.3)	1.8% (0.72-3.6%)	3.6% (1.9-6.3%)	170 (89-320)	4900 (1900-9900)
Taiwan	2016	Female	65 to 69	18% (11-29%)	82% (71-89%)	0.64 (0.28-1.2)	2% (0.84-3.7%)	3.5% (1.8-6%)	160 (79-270)	3700 (1600-7100)
Taiwan	2016	Female	70 to 74	17% (11-27%)	83% (73-89%)	0.62 (0.26-1.3)	1.9% (0.66-3.9%)	3.1% (1.3-5.8%)	200 (84-390)	3800 (1300-7900)
Taiwan	2016	Female	75 to 79	16% (9.9-26%)	84% (74-90%)	0.6 (0.24-1.2)	1.5% (0.34-3.5%)	2.2% (0.68-4.7%)	210 (63-440)	3100 (680-7200)
Taiwan	2016	Female	80 to 84	16% (9.3-26%)	84% (74-91%)	0.59 (0.25-1.2)	1.2% (0.095-3.1%)	1.5% (0.2-3.8%)	180 (23-450)	2000 (160-5300)
Taiwan	2016	Female	85 to 89	17% (9.7-26%)	83% (74-90%)	0.61 (0.26-1.2)	0.99% (-0.085-2.9%)	1.2% (-0.11-3.5%)	140 (-13-420)	1200 (-95-3500)
Taiwan	2016	Female	90 to 94	18% (11-29%)	82% (71-89%)	0.66 (0.27-1.4)	0.9% (-0.24-3.1%)	1% (-0.32-3.6%)	81 (-26-290)	470 (-120-1600)
Taiwan	2016	Female	95 plus	21% (11-34%)	79% (66-89%)	0.76 (0.27-1.7)	1.1% (-0.45-4%)	1.1% (-0.61-4.5%)	38 (-21-160)	140 (-60-540)
Taiwan	2016	Male	All Ages	44% (37-52%)	56% (48-63%)	3 (1.7-5)	10% (6.9-13%)	10% (6.1-14%)	11000 (6100-15000)	340000 (220000-450000)
Taiwan	2016	Male	15 to 19	29% (18-43%)	71% (57-82%)	0.7 (0.28-1.5)	2.7% (1.6-4.5%)	4.6% (2-8.7%)	17 (7.3-33)	1900 (1100-3300)
Taiwan	2016	Male	20 to 24	41% (27-57%)	59% (43-73%)	1.7 (0.72-3.4)	6.7% (4-11%)	10% (4.7-19%)	56 (25-110)	5800 (3400-9400)

Location	Year	Sex	Age	Prevalence of current drinkers	Prevalence of abstainers	Population average of standard drinks daily	Percent of total attributable deaths	Percent of total attributable DALYs	Attributable deaths	Attributable DALYs
Taiwan	2016	Male	25 to 29	47% (30-64%)	53% (36-70%)	2.5 (1.1-4.9)	8.8% (5.2-13%)	14% (7-23%)	94 (45-160)	8300 (4800-13000)
Taiwan	2016	Male	30 to 34	50% (33-68%)	50% (32-67%)	3.2 (1.5-6.2)	11% (6.6-15%)	16% (9-24%)	210 (110-320)	15000 (9300-22000)
Taiwan	2016	Male	35 to 39	53% (37-70%)	47% (30-63%)	3.9 (1.7-7.6)	13% (8.1-18%)	19% (11-25%)	410 (240-590)	25000 (15000-35000)
Taiwan	2016	Male	40 to 44	54% (38-72%)	46% (28-62%)	4.4 (2-8.2)	15% (9.6-20%)	21% (13-27%)	590 (360-820)	31000 (20000-43000)
Taiwan	2016	Male	45 to 49	55% (36-71%)	45% (29-64%)	4.9 (2.3-8.8)	16% (11-20%)	21% (14-27%)	910 (610-1200)	43000 (29000-57000)
Taiwan	2016	Male	50 to 54	54% (36-72%)	46% (28-64%)	5 (2.5-9.2)	15% (10-19%)	20% (14-25%)	1200 (790-1600)	49000 (33000-66000)
Taiwan	2016	Male	55 to 59	53% (35-71%)	47% (29-65%)	4.7 (2.3-8.6)	13% (8.8-17%)	17% (11-22%)	1300 (840-1800)	46000 (30000-63000)
Taiwan	2016	Male	60 to 64	50% (33-69%)	50% (31-67%)	4.2 (1.9-7.8)	11% (6.5-15%)	14% (8.1-19%)	1300 (770-1900)	41000 (24000-57000)
Taiwan	2016	Male	65 to 69	47% (32-65%)	53% (35-68%)	3.6 (1.6-6.4)	9.1% (5-13%)	11% (6.1-16%)	900 (480-1300)	23000 (12000-33000)
Taiwan	2016	Male	70 to 74	43% (28-61%)	57% (39-72%)	2.9 (1.3-5.7)	7.3% (3.3-11%)	8.7% (3.8-13%)	850 (390-1300)	17000 (8000-27000)
Taiwan	2016	Male	75 to 79	40% (26-58%)	60% (42-74%)	2.5 (1.1-5.1)	5.9% (2.3-9.8%)	6.8% (2.5-11%)	780 (290-1300)	12000 (4700-21000)
Taiwan	2016	Male	80 to 84	38% (24-55%)	62% (45-76%)	2.3 (1-4.3)	5.2% (1.7-9.1%)	5.9% (1.9-11%)	780 (260-1400)	9100 (3100-16000)
Taiwan	2016	Male	85 to 89	37% (23-52%)	63% (48-77%)	2.1 (0.93-4.1)	4.6% (1.4-8.5%)	5.1% (1.5-9.6%)	750 (230-1400)	6200 (2000-11000)
Taiwan	2016	Male	90 to 94	38% (25-54%)	62% (46-75%)	2.1 (0.91-4.2)	4.1% (1.1-8.1%)	4.5% (1.1-9.1%)	330 (80-670)	1900 (520-3800)
Taiwan	2016	Male	95 plus	42% (25-61%)	58% (39-75%)	2.2 (0.8-4.6)	4.1% (0.76-8.9%)	4.4% (0.58-9.9%)	110 (15-260)	420 (80-910)
Cambodia	1990	Female	All Ages	8.5% (5.9-12%)	92% (88-94%)	0.24 (0.15-0.36)	1.1% (0.63-1.6%)	2.1% (1.1-3.3%)	890 (450-1500)	28000 (17000-42000)
Cambodia	1990	Female	15 to 19	8.3% (3.7-16%)	92% (84-96%)	0.22 (0.069-0.49)	0.72% (0.34-1.4%)	0.9% (0.31-1.9%)	6.9 (2.4-15)	700 (320-1400)
Cambodia	1990	Female	20 to 24	10% (4.1-20%)	90% (80-96%)	0.29 (0.099-0.64)	1.4% (0.63-2.7%)	1.6% (0.5-3.5%)	16 (5.1-36)	1500 (670-2900)
Cambodia	1990	Female	25 to 29	11% (4.6-21%)	89% (79-95%)	0.34 (0.11-0.72)	1.6% (0.75-3.2%)	2% (0.65-4.4%)	20 (6.7-46)	1700 (730-3200)
Cambodia	1990	Female	30 to 34	12% (4.8-22%)	88% (78-95%)	0.39 (0.13-0.81)	1.9% (0.92-3.5%)	2.4% (1-4.9%)	27 (11-55)	1900 (900-3500)
Cambodia	1990	Female	35 to 39	13% (5.6-24%)	87% (76-94%)	0.43 (0.15-0.91)	2.4% (1.2-4.4%)	3.3% (1.5-6%)	42 (19-77)	2400 (1200-4300)
Cambodia	1990	Female	40 to 44	13% (5.9-24%)	87% (76-94%)	0.48 (0.17-1)	2.6% (1.3-4.8%)	3.6% (1.7-6.5%)	43 (20-78)	2200 (1100-3900)

Location	Year	Sex	Age	Prevalence of current drinkers	Prevalence of abstainers	Population average of standard drinks daily	Percent of total attributable deaths	Percent of total attributable DALYs	Attributable deaths	Attributable DALYs
Cambodia	1990	Female	45 to 49	13% (5.7-24%)	87% (76-94%)	0.54 (0.18-1.2)	2.9% (1.4-5.5%)	3.9% (1.8-7.5%)	53 (24-100)	2300 (1100-4400)
Cambodia	1990	Female	50 to 54	14% (6.1-26%)	86% (74-94%)	0.61 (0.2-1.4)	3.6% (1.6-6.8%)	4.6% (2-8.7%)	78 (34-150)	2900 (1300-5500)
Cambodia	1990	Female	55 to 59	14% (6.5-26%)	86% (74-94%)	0.66 (0.21-1.5)	3.7% (1.5-7.1%)	4.5% (1.8-8.7%)	94 (38-190)	3100 (1200-6000)
Cambodia	1990	Female	60 to 64	14% (6.2-26%)	86% (74-94%)	0.66 (0.21-1.6)	3.7% (1.3-7.4%)	4.4% (1.5-9%)	110 (39-230)	3100 (1100-6300)
Cambodia	1990	Female	65 to 69	13% (6.2-24%)	87% (76-94%)	0.61 (0.19-1.4)	3.4% (1.1-6.8%)	4% (1.3-8%)	120 (40-240)	2800 (920-5500)
Cambodia	1990	Female	70 to 74	13% (6-23%)	87% (77-94%)	0.55 (0.17-1.3)	3% (0.89-6.7%)	3.5% (1-7.7%)	110 (32-250)	2000 (600-4500)
Cambodia	1990	Female	75 to 79	12% (5.5-23%)	88% (77-94%)	0.51 (0.14-1.3)	2.5% (0.6-5.7%)	2.8% (0.68-6.5%)	77 (18-180)	1100 (260-2500)
Cambodia	1990	Female	80 to 84	12% (5.4-23%)	88% (77-95%)	0.51 (0.15-1.2)	2.5% (0.67-5.6%)	2.9% (0.77-6.4%)	52 (14-120)	540 (150-1200)
Cambodia	1990	Female	85 to 89	13% (5.7-24%)	87% (76-94%)	0.55 (0.16-1.4)	2.3% (0.58-5.6%)	2.7% (0.67-6.5%)	25 (6.2-62)	190 (47-450)
Cambodia	1990	Female	90 to 94	15% (6.5-28%)	85% (72-94%)	0.62 (0.16-1.6)	2.1% (0.42-5.3%)	2.5% (0.48-6.3%)	8.5 (1.6-21)	44 (8.6-110)
Cambodia	1990	Female	95 plus	19% (7.1-37%)	81% (63-93%)	0.76 (0.15-2.1)	2.3% (0.3-6%)	2.6% (0.33-7%)	2.4 (0.31-6.6)	8.4 (1.1-22)
Cambodia	1990	Male	All Ages	25% (19-32%)	75% (68-81%)	0.72 (0.49-0.98)	2.5% (1.7-3.6%)	4.1% (2.6-6%)	1800 (1100-2700)	76000 (49000-110000)
Cambodia	1990	Male	15 to 19	23% (13-38%)	77% (62-87%)	0.39 (0.16-0.74)	1.5% (0.75-2.6%)	1.8% (0.71-3.3%)	19 (7.6-35)	1700 (810-2900)
Cambodia	1990	Male	20 to 24	35% (20-53%)	65% (47-80%)	0.92 (0.43-1.6)	4.4% (2.2-6.9%)	5.1% (2.3-8.5%)	72 (32-120)	5500 (2700-8800)
Cambodia	1990	Male	25 to 29	41% (22-59%)	59% (41-78%)	1.3 (0.62-2.1)	6.6% (3.4-11%)	8% (3.7-13%)	100 (47-170)	6900 (3600-11000)
Cambodia	1990	Male	30 to 34	44% (24-63%)	56% (37-76%)	1.6 (0.83-2.6)	8.1% (4.7-12%)	10% (5.6-16%)	130 (73-210)	8100 (4700-12000)
Cambodia	1990	Male	35 to 39	46% (28-66%)	54% (34-72%)	1.8 (0.88-3.1)	9.4% (5.5-14%)	12% (6.6-18%)	180 (97-270)	9500 (5500-15000)
Cambodia	1990	Male	40 to 44	46% (28-67%)	54% (33-72%)	1.9 (0.96-3.2)	9.6% (5.7-14%)	12% (6.8-18%)	170 (96-260)	8200 (4700-13000)
Cambodia	1990	Male	45 to 49	44% (25-64%)	56% (36-75%)	1.9 (0.89-3.3)	9.5% (5.4-15%)	11% (6.5-18%)	190 (110-300)	8300 (4700-13000)
Cambodia	1990	Male	50 to 54	42% (24-62%)	58% (38-76%)	1.8 (0.83-3.1)	8.7% (4.6-14%)	10% (5.4-17%)	220 (110-350)	8200 (4400-13000)
Cambodia	1990	Male	55 to 59	40% (23-60%)	60% (40-77%)	1.6 (0.74-2.9)	7.4% (3.7-13%)	8.7% (4.2-15%)	220 (100-370)	7100 (3400-12000)
Cambodia	1990	Male	60 to 64	37% (21-57%)	63% (43-79%)	1.4 (0.62-2.7)	6.5% (2.8-11%)	7.4% (3.2-13%)	200 (87-360)	5600 (2400-10000)

Location	Year	Sex	Age	Prevalence of current drinkers	Prevalence of abstainers	Population average of standard drinks daily	Percent of total attributable deaths	Percent of total attributable DALYs	Attributable deaths	Attributable DALYs
Cambodia	1990	Male	65 to 69	33% (19-52%)	67% (48-81%)	1.2 (0.5-2.1)	5.1% (2.3-9.3%)	5.9% (2.6-11%)	150 (65-280)	3500 (1500-6400)
Cambodia	1990	Male	70 to 74	30% (17-47%)	70% (53-83%)	0.95 (0.41-1.8)	4.3% (1.6-8.7%)	4.9% (1.7-9.9%)	110 (39-220)	2000 (740-4100)
Cambodia	1990	Male	75 to 79	27% (15-44%)	73% (56-85%)	0.81 (0.33-1.6)	3.3% (1.1-6.8%)	3.7% (1.2-7.7%)	54 (17-110)	770 (250-1600)
Cambodia	1990	Male	80 to 84	25% (13-42%)	75% (58-87%)	0.7 (0.27-1.4)	2.9% (0.96-5.9%)	3.3% (1.1-6.7%)	23 (7.4-46)	240 (80-490)
Cambodia	1990	Male	85 to 89	24% (13-39%)	76% (61-87%)	0.62 (0.25-1.3)	2.1% (0.69-4.5%)	2.4% (0.75-5.2%)	6.9 (2.2-15)	53 (17-110)
Cambodia	1990	Male	90 to 94	25% (13-42%)	75% (58-87%)	0.6 (0.23-1.2)	1.6% (0.38-3.7%)	1.8% (0.4-4.3%)	2.3 (0.49-5.5)	12 (2.9-28)
Cambodia	1990	Male	95 plus	29% (13-48%)	71% (52-87%)	0.63 (0.21-1.4)	1.4% (0.27-3.7%)	1.5% (0.22-4.1%)	0.55 (0.077-1.5)	2 (0.37-5.2)
Cambodia	1995	Female	All Ages	8.5% (6.1-12%)	92% (88-94%)	0.41 (0.26-0.6)	1.7% (1.2-2.3%)	3.3% (2.1-4.6%)	1600 (1000-2200)	49000 (35000-67000)
Cambodia	1995	Female	15 to 19	8.3% (3.9-15%)	92% (85-96%)	0.38 (0.13-0.85)	1.2% (0.52-2.2%)	1.6% (0.53-3.4%)	11 (3.7-23)	1000 (440-2000)
Cambodia	1995	Female	20 to 24	10% (4.4-19%)	90% (81-96%)	0.52 (0.18-1.1)	2.1% (0.96-4%)	2.8% (0.96-5.6%)	30 (11-62)	2500 (1100-4700)
Cambodia	1995	Female	25 to 29	11% (4.8-21%)	89% (79-95%)	0.6 (0.19-1.3)	2.4% (1.1-4.4%)	3.2% (1.2-6.2%)	37 (14-72)	2800 (1300-5100)
Cambodia	1995	Female	30 to 34	12% (4.9-23%)	88% (77-95%)	0.67 (0.23-1.4)	2.8% (1.4-4.9%)	3.8% (1.7-6.9%)	48 (21-86)	3200 (1500-5500)
Cambodia	1995	Female	35 to 39	13% (5.3-24%)	87% (76-95%)	0.74 (0.25-1.5)	3.5% (1.8-6%)	4.9% (2.4-8.5%)	70 (34-120)	3900 (2000-6600)
Cambodia	1995	Female	40 to 44	13% (6-24%)	87% (76-94%)	0.83 (0.29-1.8)	3.9% (2-6.5%)	5.3% (2.7-9%)	86 (43-140)	4300 (2200-7200)
Cambodia	1995	Female	45 to 49	13% (5.8-24%)	87% (76-94%)	0.94 (0.31-2.1)	4.3% (2.2-7.1%)	5.7% (2.9-9.8%)	92 (46-160)	4000 (2000-6800)
Cambodia	1995	Female	50 to 54	14% (6.1-25%)	86% (75-94%)	1.1 (0.34-2.4)	5.2% (2.7-8.7%)	6.7% (3.3-11%)	130 (65-220)	4900 (2500-8100)
Cambodia	1995	Female	55 to 59	14% (6.2-25%)	86% (75-94%)	1.2 (0.36-2.6)	5.3% (2.6-9.1%)	6.6% (3.2-11%)	160 (74-270)	5100 (2500-8800)
Cambodia	1995	Female	60 to 64	14% (5.9-26%)	86% (74-94%)	1.2 (0.34-2.7)	5.3% (2.4-9.4%)	6.4% (3-12%)	180 (83-320)	5100 (2300-8900)
Cambodia	1995	Female	65 to 69	13% (6-24%)	87% (76-94%)	1.1 (0.33-2.4)	5.3% (2.2-9.1%)	6.2% (2.7-11%)	210 (90-360)	4800 (2100-8200)
Cambodia	1995	Female	70 to 74	13% (5.8-23%)	87% (77-94%)	0.97 (0.29-2.3)	4.7% (1.8-8.7%)	5.5% (2.1-10%)	200 (78-370)	3700 (1400-6800)
Cambodia	1995	Female	75 to 79	12% (5.3-23%)	88% (77-95%)	0.9 (0.25-2.3)	4% (1.3-7.6%)	4.6% (1.5-8.7%)	150 (49-290)	2200 (710-4100)
Cambodia	1995	Female	80 to 84	12% (5.1-23%)	88% (77-95%)	0.89 (0.26-2.1)	4.1% (1.5-7.6%)	4.7% (1.7-8.8%)	100 (36-190)	1100 (370-2000)

Location	Year	Sex	Age	Prevalence of current drinkers	Prevalence of abstainers	Population average of standard drinks daily	Percent of total attributable deaths	Percent of total attributable DALYs	Attributable deaths	Attributable DALYs
Cambodia	1995	Female	85 to 89	13% (5.9-24%)	87% (76-94%)	0.96 (0.28-2.3)	3.8% (1.4-7.6%)	4.4% (1.6-8.9%)	47 (16-95)	350 (120-700)
Cambodia	1995	Female	90 to 94	15% (6.6-28%)	85% (72-93%)	1.1 (0.27-2.8)	3.5% (1.1-7.1%)	4.1% (1.3-8.3%)	15 (4.8-31)	78 (25-160)
Cambodia	1995	Female	95 plus	19% (7.8-36%)	81% (64-92%)	1.3 (0.26-3.6)	3.6% (0.84-7.8%)	4.1% (0.95-9%)	4.1 (0.92-9)	14 (3.3-31)
Cambodia	1995	Male	All Ages	25% (19-32%)	75% (68-81%)	1.2 (0.83-1.6)	4.1% (2.9-5.2%)	6.7% (4.6-8.8%)	3400 (2300-4500)	130000 (97000-170000)
Cambodia	1995	Male	15 to 19	23% (13-38%)	77% (62-87%)	0.67 (0.29-1.3)	2.3% (1.2-3.9%)	2.8% (1.3-5.1%)	27 (12-49)	2200 (1200-3900)
Cambodia	1995	Male	20 to 24	35% (19-54%)	65% (46-81%)	1.6 (0.76-2.7)	6.4% (3.6-9.9%)	7.6% (3.9-12%)	130 (69-210)	9700 (5300-15000)
Cambodia	1995	Male	25 to 29	41% (23-59%)	59% (41-77%)	2.2 (1.1-3.6)	9.4% (5.5-15%)	12% (6.4-18%)	200 (110-310)	13000 (7700-20000)
Cambodia	1995	Male	30 to 34	44% (25-63%)	56% (37-75%)	2.7 (1.4-4.5)	11% (7.1-17%)	14% (8.5-21%)	230 (140-330)	14000 (8500-20000)
Cambodia	1995	Male	35 to 39	46% (29-66%)	54% (34-71%)	3.1 (1.6-5.3)	13% (8.4-19%)	16% (10-23%)	290 (170-400)	15000 (9400-21000)
Cambodia	1995	Male	40 to 44	46% (29-66%)	54% (34-71%)	3.3 (1.6-5.5)	14% (9-19%)	17% (11-23%)	340 (220-480)	16000 (11000-23000)
Cambodia	1995	Male	45 to 49	44% (25-64%)	56% (36-75%)	3.2 (1.6-5.6)	14% (8.7-19%)	17% (11-23%)	320 (190-440)	14000 (8300-18000)
Cambodia	1995	Male	50 to 54	42% (24-62%)	58% (38-76%)	3.1 (1.4-5.3)	13% (7.6-18%)	16% (9.2-21%)	340 (200-470)	13000 (7500-17000)
Cambodia	1995	Male	55 to 59	40% (23-59%)	60% (41-77%)	2.8 (1.3-5)	12% (6.4-16%)	14% (7.6-20%)	370 (210-540)	12000 (6700-18000)
Cambodia	1995	Male	60 to 64	37% (21-57%)	63% (43-79%)	2.4 (1.1-4.6)	10% (5.4-16%)	12% (6.2-18%)	380 (190-580)	11000 (5400-16000)
Cambodia	1995	Male	65 to 69	34% (19-52%)	66% (48-81%)	2 (0.9-3.7)	8.8% (4.4-14%)	10% (5.1-16%)	320 (160-510)	7300 (3600-12000)
Cambodia	1995	Male	70 to 74	30% (17-46%)	70% (54-83%)	1.7 (0.73-3.2)	7.6% (3.4-13%)	8.7% (3.8-15%)	250 (110-430)	4600 (2000-7900)
Cambodia	1995	Male	75 to 79	27% (15-44%)	73% (56-85%)	1.4 (0.57-2.8)	6% (2.5-11%)	6.9% (2.8-12%)	140 (57-250)	2000 (820-3600)
Cambodia	1995	Male	80 to 84	25% (13-41%)	75% (59-87%)	1.2 (0.49-2.5)	5.4% (2.2-10%)	6.2% (2.5-12%)	62 (24-110)	650 (250-1200)
Cambodia	1995	Male	85 to 89	24% (13-39%)	76% (61-87%)	1.1 (0.45-2.2)	4.2% (1.7-8%)	4.8% (1.9-9.3%)	21 (8.1-40)	160 (62-300)
Cambodia	1995	Male	90 to 94	25% (14-41%)	75% (59-86%)	1.1 (0.43-2.2)	3.3% (1.2-6.8%)	3.8% (1.3-7.9%)	4.7 (1.6-9.8)	25 (8.8-51)
Cambodia	1995	Male	95 plus	29% (14-50%)	71% (50-86%)	1.1 (0.37-2.4)	3% (0.88-6.6%)	3.3% (0.88-7.5%)	1.2 (0.31-2.6)	4.2 (1.2-9.3)
Cambodia	2000	Female	All Ages	8.5% (6.1-12%)	92% (88-94%)	0.81 (0.53-1.2)	2.4% (1.8-3.1%)	4.6% (3.3-6.2%)	2300 (1600-3100)	72000 (54000-92000)

Location	Year	Sex	Age	Prevalence of current drinkers	Prevalence of abstainers	Population average of standard drinks daily	Percent of total attributable deaths	Percent of total attributable DALYs	Attributable deaths	Attributable DALYs
Cambodia	2000	Female	15 to 19	8.2% (3.9-15%)	92% (85-96%)	0.73 (0.26-1.6)	1.8% (0.85-3.2%)	2.7% (1.1-5.1%)	33 (13-61)	2800 (1300-5000)
Cambodia	2000	Female	20 to 24	9.9% (4.2-18%)	90% (82-96%)	0.98 (0.34-2.1)	2.9% (1.4-4.9%)	4% (1.7-7.3%)	34 (15-62)	2800 (1400-4700)
Cambodia	2000	Female	25 to 29	11% (4.5-20%)	89% (80-95%)	1.1 (0.38-2.5)	3.1% (1.6-5.1%)	4.2% (1.8-7.3%)	52 (24-91)	3900 (2000-6400)
Cambodia	2000	Female	30 to 34	12% (4.7-22%)	88% (78-95%)	1.3 (0.43-2.7)	3.6% (1.9-5.7%)	5% (2.4-8.1%)	72 (36-120)	4700 (2500-7400)
Cambodia	2000	Female	35 to 39	13% (5.4-23%)	87% (77-95%)	1.4 (0.48-2.9)	4.4% (2.4-6.9%)	6.2% (3.3-9.8%)	100 (56-160)	5900 (3300-9000)
Cambodia	2000	Female	40 to 44	13% (6.2-23%)	87% (77-94%)	1.6 (0.57-3.3)	4.9% (2.9-7.5%)	6.8% (3.9-10%)	120 (71-190)	6100 (3600-9100)
Cambodia	2000	Female	45 to 49	13% (5.9-24%)	87% (76-94%)	1.8 (0.6-4)	5.4% (3.1-8.3%)	7.3% (4.2-12%)	160 (91-250)	7000 (4100-11000)
Cambodia	2000	Female	50 to 54	14% (6.1-26%)	86% (74-94%)	2 (0.66-4.5)	6.5% (3.8-10%)	8.4% (5-13%)	190 (110-300)	7200 (4300-11000)
Cambodia	2000	Female	55 to 59	14% (6.4-26%)	86% (74-94%)	2.2 (0.73-4.9)	6.6% (3.7-11%)	8.2% (4.7-13%)	220 (130-360)	7200 (4100-12000)
Cambodia	2000	Female	60 to 64	14% (5.9-26%)	86% (74-94%)	2.2 (0.65-5.3)	6.5% (3.7-11%)	8% (4.6-13%)	260 (150-430)	7100 (4000-12000)
Cambodia	2000	Female	65 to 69	13% (6.2-24%)	87% (76-94%)	2.1 (0.64-4.5)	6.5% (3.5-11%)	7.8% (4.2-13%)	300 (160-490)	6900 (3700-11000)
Cambodia	2000	Female	70 to 74	13% (6.1-23%)	87% (77-94%)	1.8 (0.56-4.3)	6% (3.1-10%)	7.1% (3.7-12%)	270 (140-450)	5000 (2700-8400)
Cambodia	2000	Female	75 to 79	12% (5.4-23%)	88% (77-95%)	1.7 (0.47-4.3)	5.3% (2.6-9%)	6.1% (3-10%)	200 (99-350)	2900 (1400-4900)
Cambodia	2000	Female	80 to 84	12% (5.7-24%)	88% (76-94%)	1.8 (0.53-4)	5.4% (2.6-9.1%)	6.3% (3-11%)	150 (70-250)	1500 (730-2600)
Cambodia	2000	Female	85 to 89	13% (5.9-25%)	87% (75-94%)	1.9 (0.56-4.7)	5% (2.4-9.1%)	5.9% (2.8-11%)	73 (34-130)	540 (250-960)
Cambodia	2000	Female	90 to 94	16% (6.7-28%)	84% (72-93%)	2.2 (0.53-5.5)	4.7% (2.1-8.4%)	5.5% (2.5-9.7%)	22 (10-40)	120 (51-200)
Cambodia	2000	Female	95 plus	19% (7.1-38%)	81% (62-93%)	2.6 (0.54-7.4)	4.9% (2-9.4%)	5.6% (2.2-11%)	5.8 (2.3-11)	20 (7.9-38)
Cambodia	2000	Male	All Ages	25% (19-32%)	75% (68-81%)	2.3 (1.6-3.1)	6% (5-7.1%)	9.7% (7.9-12%)	5300 (4300-6400)	210000 (170000-250000)
Cambodia	2000	Male	15 to 19	23% (13-38%)	77% (62-87%)	1.3 (0.56-2.4)	3.8% (1.9-6.7%)	5% (2.4-9.1%)	88 (42-160)	7100 (3600-12000)
Cambodia	2000	Male	20 to 24	35% (20-53%)	65% (47-80%)	3.1 (1.5-5.2)	10% (6.3-15%)	13% (7.4-19%)	170 (100-260)	12000 (7400-18000)
Cambodia	2000	Male	25 to 29	41% (23-59%)	59% (41-77%)	4.3 (2.2-7.1)	12% (8.2-17%)	15% (9.5-21%)	330 (220-450)	21000 (15000-29000)
Cambodia	2000	Male	30 to 34	44% (25-63%)	56% (37-75%)	5.3 (2.8-8.7)	14% (9.4-18%)	17% (11-23%)	410 (290-530)	24000 (17000-32000)

Location	Year	Sex	Age	Prevalence of current drinkers	Prevalence of abstainers	Population average of standard drinks daily	Percent of total attributable deaths	Percent of total attributable DALYs	Attributable deaths	Attributable DALYs
Cambodia	2000	Male	35 to 39	46% (28-66%)	54% (34-72%)	6 (3-10)	15% (11-20%)	19% (13-25%)	470 (350-600)	25000 (19000-32000)
Cambodia	2000	Male	40 to 44	45% (28-65%)	55% (35-72%)	6.2 (3.1-11)	16% (12-20%)	19% (14-25%)	440 (320-550)	21000 (16000-26000)
Cambodia	2000	Male	45 to 49	44% (25-64%)	56% (36-75%)	6.2 (3-11)	16% (12-21%)	20% (14-25%)	520 (380-670)	22000 (16000-28000)
Cambodia	2000	Male	50 to 54	42% (23-62%)	58% (38-77%)	5.9 (2.8-10)	16% (11-20%)	19% (14-24%)	460 (330-590)	17000 (13000-22000)
Cambodia	2000	Male	55 to 59	39% (23-60%)	61% (40-77%)	5.3 (2.5-9.6)	15% (11-20%)	18% (13-23%)	480 (340-630)	15000 (11000-20000)
Cambodia	2000	Male	60 to 64	37% (20-56%)	63% (44-80%)	4.6 (2-8.7)	14% (9.4-18%)	16% (11-21%)	520 (350-710)	14000 (9800-20000)
Cambodia	2000	Male	65 to 69	33% (19-52%)	67% (48-81%)	3.9 (1.7-7.3)	12% (8.1-17%)	14% (9.5-20%)	510 (330-710)	12000 (7700-16000)
Cambodia	2000	Male	70 to 74	30% (17-46%)	70% (54-83%)	3.2 (1.4-6.1)	11% (6.9-16%)	13% (7.9-19%)	430 (260-630)	7900 (4800-11000)
Cambodia	2000	Male	75 to 79	27% (15-44%)	73% (56-85%)	2.7 (1.1-5.4)	9.3% (5.2-14%)	11% (6-16%)	260 (150-390)	3800 (2100-5600)
Cambodia	2000	Male	80 to 84	25% (13-41%)	75% (59-87%)	2.3 (0.92-4.6)	8.8% (4.6-13%)	10% (5.2-15%)	140 (70-200)	1400 (740-2200)
Cambodia	2000	Male	85 to 89	23% (12-38%)	77% (62-88%)	2 (0.83-4.2)	7.3% (3.7-11%)	8.4% (4.2-13%)	54 (27-85)	410 (210-640)
Cambodia	2000	Male	90 to 94	25% (13-40%)	75% (60-87%)	2 (0.8-4)	6.2% (2.9-9.8%)	7.1% (3.2-11%)	14 (6.1-22)	70 (32-110)
Cambodia	2000	Male	95 plus	28% (13-48%)	72% (52-87%)	2 (0.69-4.5)	5.7% (2.3-9.8%)	6.4% (2.5-11%)	2.3 (0.89-4)	8.1 (3.3-14)
Cambodia	2005	Female	All Ages	8.6% (6.2-12%)	91% (88-94%)	1.1 (0.73-1.6)	2.8% (2.2-3.7%)	5.3% (3.8-7.1%)	2500 (1800-3400)	78000 (60000-100000)
Cambodia	2005	Female	15 to 19	8.1% (3.6-15%)	92% (85-96%)	0.93 (0.31-2)	1.9% (0.95-3.2%)	3.2% (1.4-5.7%)	28 (12-50)	2500 (1300-4200)
Cambodia	2005	Female	20 to 24	9.8% (4.2-18%)	90% (82-96%)	1.3 (0.44-2.6)	2.9% (1.6-4.7%)	4.2% (1.9-7.3%)	53 (24-91)	4600 (2500-7300)
Cambodia	2005	Female	25 to 29	11% (4.4-19%)	89% (81-96%)	1.5 (0.49-3.2)	3.2% (1.7-5%)	4.6% (2.2-7.8%)	35 (17-59)	2700 (1500-4300)
Cambodia	2005	Female	30 to 34	12% (4.8-22%)	88% (78-95%)	1.6 (0.56-3.5)	3.6% (2.1-5.7%)	5.2% (2.7-8.4%)	64 (34-100)	4300 (2500-6700)
Cambodia	2005	Female	35 to 39	12% (5.2-23%)	88% (77-95%)	1.8 (0.62-3.8)	4.4% (2.5-6.7%)	6.4% (3.5-9.7%)	98 (54-150)	5700 (3300-8400)
Cambodia	2005	Female	40 to 44	13% (5.8-23%)	87% (77-94%)	2 (0.7-4.1)	4.9% (3-7.3%)	7.2% (4.4-11%)	130 (80-200)	6800 (4100-10000)
Cambodia	2005	Female	45 to 49	13% (5.6-24%)	87% (76-94%)	2.3 (0.76-5.2)	5.5% (3.3-8.3%)	7.8% (4.6-12%)	160 (95-250)	7300 (4300-11000)
Cambodia	2005	Female	50 to 54	13% (6.1-25%)	87% (75-94%)	2.6 (0.86-5.9)	6.5% (3.9-10%)	8.7% (5.3-13%)	240 (150-380)	9200 (5600-14000)

Location	Year	Sex	Age	Prevalence of current drinkers	Prevalence of abstainers	Population average of standard drinks daily	Percent of total attributable deaths	Percent of total attributable DALYs	Attributable deaths	Attributable DALYs
Cambodia	2005	Female	55 to 59	14% (6.3-26%)	86% (74-94%)	2.9 (0.93-6.6)	6.7% (3.9-11%)	8.6% (5-14%)	250 (140-410)	8200 (4700-13000)
Cambodia	2005	Female	60 to 64	14% (6.1-26%)	86% (74-94%)	3 (0.88-6.7)	6.6% (3.8-11%)	8.3% (4.8-14%)	280 (160-460)	7800 (4500-13000)
Cambodia	2005	Female	65 to 69	13% (6-23%)	87% (77-94%)	2.7 (0.85-5.8)	6.6% (3.6-11%)	8.1% (4.5-13%)	320 (180-530)	7400 (4100-12000)
Cambodia	2005	Female	70 to 74	13% (5.8-23%)	87% (77-94%)	2.4 (0.71-5.6)	6.1% (3.2-10%)	7.4% (3.9-12%)	320 (170-540)	6000 (3100-9900)
Cambodia	2005	Female	75 to 79	12% (5.2-23%)	88% (77-95%)	2.3 (0.62-5.6)	5.4% (2.7-9.1%)	6.4% (3.1-11%)	240 (120-400)	3400 (1700-5600)
Cambodia	2005	Female	80 to 84	12% (5.3-23%)	88% (77-95%)	2.3 (0.69-5.3)	5.5% (2.6-9%)	6.4% (3.1-11%)	160 (79-280)	1700 (840-2900)
Cambodia	2005	Female	85 to 89	13% (5.9-25%)	87% (75-94%)	2.5 (0.72-6)	5.2% (2.5-9.2%)	6.1% (2.9-11%)	89 (43-160)	660 (320-1200)
Cambodia	2005	Female	90 to 94	15% (6.8-28%)	85% (72-93%)	2.8 (0.7-7.1)	4.8% (2.4-8.4%)	5.7% (2.8-9.9%)	29 (15-51)	150 (76-260)
Cambodia	2005	Female	95 plus	19% (7.4-36%)	81% (64-93%)	3.3 (0.67-9.1)	5% (2.1-9.1%)	5.8% (2.5-11%)	7 (3-13)	24 (11-44)
Cambodia	2005	Male	All Ages	25% (20-33%)	75% (67-80%)	3.1 (2.3-4.2)	7.2% (5.9-8.5%)	11% (9.1-13%)	5900 (4900-7200)	230000 (190000-270000)
Cambodia	2005	Male	15 to 19	23% (13-38%)	77% (62-87%)	1.6 (0.7-3)	4.7% (2.4-7.9%)	6.4% (3.1-11%)	96 (45-170)	7700 (4000-13000)
Cambodia	2005	Male	20 to 24	34% (19-52%)	66% (48-81%)	3.9 (1.9-6.5)	12% (7.2-16%)	15% (8.9-21%)	350 (210-500)	25000 (16000-35000)
Cambodia	2005	Male	25 to 29	39% (22-57%)	61% (43-78%)	5.5 (2.7-9.4)	14% (9.5-18%)	17% (12-23%)	240 (160-330)	16000 (11000-21000)
Cambodia	2005	Male	30 to 34	43% (24-62%)	57% (38-76%)	6.8 (3.6-11)	14% (9.5-18%)	17% (11-22%)	390 (280-510)	24000 (17000-30000)
Cambodia	2005	Male	35 to 39	45% (27-65%)	55% (35-73%)	7.7 (3.9-13)	15% (11-19%)	18% (13-24%)	510 (380-650)	27000 (21000-35000)
Cambodia	2005	Male	40 to 44	45% (27-65%)	55% (35-73%)	8 (4-13)	15% (12-19%)	19% (14-23%)	560 (420-700)	27000 (20000-34000)
Cambodia	2005	Male	45 to 49	43% (24-63%)	57% (37-76%)	8 (3.9-13)	16% (12-20%)	20% (15-25%)	510 (380-640)	22000 (16000-27000)
Cambodia	2005	Male	50 to 54	41% (23-61%)	59% (39-77%)	7.5 (3.6-13)	16% (12-20%)	19% (15-24%)	570 (430-730)	22000 (16000-27000)
Cambodia	2005	Male	55 to 59	39% (23-59%)	61% (41-77%)	6.7 (3.3-12)	15% (11-20%)	18% (13-24%)	520 (380-680)	17000 (12000-22000)
Cambodia	2005	Male	60 to 64	36% (20-55%)	64% (45-80%)	5.8 (2.6-11)	14% (9.8-18%)	17% (12-22%)	520 (360-700)	15000 (10000-19000)
Cambodia	2005	Male	65 to 69	33% (19-51%)	67% (49-81%)	4.9 (2.2-9.2)	13% (8.8-17%)	15% (10-20%)	530 (360-730)	12000 (8400-17000)
Cambodia	2005	Male	70 to 74	29% (16-46%)	71% (54-84%)	4.1 (1.8-7.8)	12% (7.6-16%)	13% (8.9-19%)	500 (330-720)	9300 (6000-13000)

Location	Year	Sex	Age	Prevalence of current drinkers	Prevalence of abstainers	Population average of standard drinks daily	Percent of total attributable deaths	Percent of total attributable DALYs	Attributable deaths	Attributable DALYs
Cambodia	2005	Male	75 to 79	27% (15-43%)	73% (57-85%)	3.4 (1.4-6.9)	9.8% (6.3-14%)	11% (7.1-16%)	330 (200-480)	4700 (2900-6900)
Cambodia	2005	Male	80 to 84	24% (12-40%)	76% (60-88%)	3 (1.2-5.9)	9.3% (5.5-14%)	11% (6.3-16%)	180 (100-260)	1900 (1100-2800)
Cambodia	2005	Male	85 to 89	23% (12-37%)	77% (63-88%)	2.6 (1-5.3)	7.9% (4.5-12%)	9% (5.1-14%)	81 (46-120)	610 (350-940)
Cambodia	2005	Male	90 to 94	24% (13-40%)	76% (60-87%)	2.5 (1-5.1)	6.8% (3.7-10%)	7.8% (4.2-12%)	23 (12-34)	120 (66-180)
Cambodia	2005	Male	95 plus	27% (13-47%)	73% (53-87%)	2.6 (0.84-5.7)	6.4% (3-11%)	7.2% (3.3-12%)	4.1 (1.9-6.9)	15 (6.7-24)
Cambodia	2010	Female	All Ages	8.7% (6.2-12%)	91% (88-94%)	1.3 (0.83-2)	3.1% (2.3-4.1%)	5.7% (4.1-7.6%)	2600 (1800-3400)	77000 (59000-100000)
Cambodia	2010	Female	15 to 19	8% (3.5-16%)	92% (84-96%)	1 (0.34-2.3)	1.7% (0.91-3%)	3% (1.3-5.6%)	21 (9.2-40)	2000 (1100-3400)
Cambodia	2010	Female	20 to 24	9.6% (3.8-18%)	90% (82-96%)	1.4 (0.47-3.1)	2.7% (1.5-4.4%)	4.1% (1.9-7.1%)	31 (14-56)	2900 (1700-4700)
Cambodia	2010	Female	25 to 29	10% (4.4-20%)	90% (80-96%)	1.6 (0.52-3.6)	3.1% (1.8-4.9%)	4.9% (2.3-8%)	61 (29-100)	5000 (2800-7900)
Cambodia	2010	Female	30 to 34	11% (4.6-22%)	89% (78-95%)	1.8 (0.63-4)	3.7% (2.2-5.7%)	5.8% (3.2-9.3%)	40 (22-66)	2700 (1600-4300)
Cambodia	2010	Female	35 to 39	12% (5.2-23%)	88% (77-95%)	2 (0.66-4.3)	4.4% (2.6-6.6%)	6.9% (4.1-11%)	82 (47-130)	4900 (2900-7400)
Cambodia	2010	Female	40 to 44	13% (5.8-23%)	87% (77-94%)	2.3 (0.77-4.8)	4.9% (3.1-7.3%)	7.6% (4.8-11%)	120 (74-180)	6100 (3900-8900)
Cambodia	2010	Female	45 to 49	13% (5.2-24%)	87% (76-95%)	2.6 (0.8-5.7)	5.4% (3.2-8.2%)	8% (4.8-12%)	170 (100-260)	7500 (4600-11000)
Cambodia	2010	Female	50 to 54	13% (5.6-25%)	87% (75-94%)	2.8 (0.9-6.4)	6.3% (3.8-9.8%)	8.7% (5.3-13%)	230 (140-360)	8800 (5400-14000)
Cambodia	2010	Female	55 to 59	13% (6.2-25%)	87% (75-94%)	3.1 (0.94-7)	6.4% (3.7-10%)	8.4% (5-13%)	280 (170-450)	9400 (5600-15000)
Cambodia	2010	Female	60 to 64	13% (5.8-25%)	87% (75-94%)	3.2 (0.96-7.4)	6.3% (3.7-10%)	8% (4.8-13%)	280 (160-450)	7900 (4700-13000)
Cambodia	2010	Female	65 to 69	13% (5.8-24%)	87% (76-94%)	3 (0.94-6.5)	6.3% (3.4-10%)	7.9% (4.4-13%)	320 (180-520)	7400 (4100-12000)
Cambodia	2010	Female	70 to 74	12% (5.5-22%)	88% (78-95%)	2.7 (0.78-6.5)	5.8% (3.1-9.6%)	7.1% (3.8-12%)	310 (160-520)	5800 (3100-9700)
Cambodia	2010	Female	75 to 79	12% (5.1-22%)	88% (78-95%)	2.5 (0.66-6.2)	5.1% (2.6-8.5%)	6.1% (3.1-10%)	270 (140-440)	3900 (2000-6300)
Cambodia	2010	Female	80 to 84	12% (5.6-23%)	88% (77-94%)	2.5 (0.75-5.9)	5.2% (2.7-8.6%)	6.2% (3.2-10%)	190 (100-320)	2000 (1100-3400)
Cambodia	2010	Female	85 to 89	13% (5.6-24%)	87% (76-94%)	2.7 (0.79-6.7)	5% (2.5-8.7%)	5.9% (2.9-10%)	100 (50-180)	760 (380-1300)
Cambodia	2010	Female	90 to 94	15% (6.4-28%)	85% (72-94%)	3.2 (0.75-8.3)	4.8% (2.3-8.4%)	5.7% (2.7-9.9%)	37 (18-65)	190 (92-330)

Location	Year	Sex	Age	Prevalence of current drinkers	Prevalence of abstainers	Population average of standard drinks daily	Percent of total attributable deaths	Percent of total attributable DALYs	Attributable deaths	Attributable DALYs
Cambodia	2010	Female	95 plus	19% (7.2-36%)	81% (64-93%)	3.9 (0.75-11)	5.1% (2.2-9.3%)	5.9% (2.5-11%)	9.5 (4-18)	33 (14-60)
Cambodia	2010	Male	All Ages	26% (20-33%)	74% (67-80%)	3.7 (2.6-5.1)	8.3% (6.8-9.7%)	12% (10-14%)	6400 (5200-7600)	250000 (200000-290000)
Cambodia	2010	Male	15 to 19	22% (12-37%)	78% (63-88%)	1.7 (0.72-3.3)	4.7% (2.4-7.8%)	6.4% (3-11%)	98 (46-170)	7900 (4000-13000)
Cambodia	2010	Male	20 to 24	34% (19-51%)	66% (49-81%)	4.2 (2-7.2)	12% (7.4-16%)	15% (9-21%)	270 (160-390)	19000 (12000-28000)
Cambodia	2010	Male	25 to 29	39% (22-58%)	61% (42-78%)	5.9 (2.9-10)	14% (9.8-18%)	18% (12-23%)	510 (340-690)	34000 (23000-45000)
Cambodia	2010	Male	30 to 34	42% (23-62%)	58% (38-77%)	7.4 (3.8-13)	15% (11-19%)	19% (13-24%)	270 (190-350)	16000 (12000-21000)
Cambodia	2010	Male	35 to 39	45% (27-63%)	55% (37-73%)	8.4 (4.2-15)	15% (12-19%)	19% (14-24%)	470 (350-590)	25000 (19000-32000)
Cambodia	2010	Male	40 to 44	44% (27-64%)	56% (36-73%)	8.8 (4.3-15)	16% (12-19%)	19% (15-24%)	550 (420-690)	27000 (21000-34000)
Cambodia	2010	Male	45 to 49	43% (25-62%)	57% (38-75%)	8.7 (4.2-15)	16% (12-20%)	20% (15-25%)	660 (500-840)	29000 (21000-36000)
Cambodia	2010	Male	50 to 54	41% (22-60%)	59% (40-78%)	8.2 (3.9-14)	16% (12-20%)	20% (15-24%)	540 (400-680)	21000 (15000-26000)
Cambodia	2010	Male	55 to 59	38% (23-58%)	62% (42-77%)	7.4 (3.5-14)	15% (11-19%)	18% (13-24%)	610 (440-800)	20000 (15000-26000)
Cambodia	2010	Male	60 to 64	36% (20-56%)	64% (44-80%)	6.4 (2.8-12)	14% (9.7-18%)	16% (12-22%)	560 (390-750)	16000 (11000-21000)
Cambodia	2010	Male	65 to 69	32% (18-50%)	68% (50-82%)	5.4 (2.4-10)	12% (8.7-17%)	15% (10-20%)	530 (370-740)	12000 (8600-17000)
Cambodia	2010	Male	70 to 74	29% (16-45%)	71% (55-84%)	4.4 (1.9-8.8)	11% (7.5-16%)	13% (8.8-18%)	520 (350-720)	9700 (6500-13000)
Cambodia	2010	Male	75 to 79	26% (15-42%)	74% (58-85%)	3.8 (1.5-7.5)	9.6% (6.3-14%)	11% (7.2-16%)	400 (250-560)	5700 (3600-8100)
Cambodia	2010	Male	80 to 84	24% (12-40%)	76% (60-88%)	3.2 (1.3-6.5)	9.2% (5.6-13%)	11% (6.4-15%)	230 (140-340)	2500 (1400-3600)
Cambodia	2010	Male	85 to 89	22% (12-36%)	78% (64-88%)	2.8 (1.1-5.8)	7.8% (4.6-11%)	8.9% (5.2-13%)	100 (59-160)	790 (450-1200)
Cambodia	2010	Male	90 to 94	24% (13-39%)	76% (61-87%)	2.7 (1.1-5.9)	6.7% (3.9-10%)	7.7% (4.5-12%)	32 (18-49)	170 (97-260)
Cambodia	2010	Male	95 plus	27% (12-47%)	73% (53-88%)	2.8 (0.92-6.4)	6.6% (3.2-11%)	7.3% (3.4-12%)	6.6 (3.1-11)	23 (11-38)
Cambodia	2016	Female	All Ages	8.8% (6.3-12%)	91% (88-94%)	1.4 (0.79-2.2)	3.3% (2.4-4.3%)	5.8% (4.1-7.7%)	2600 (1900-3500)	77000 (58000-100000)
Cambodia	2016	Female	15 to 19	7.9% (3.5-15%)	92% (85-96%)	1 (0.32-2.4)	1.5% (0.8-2.5%)	2.7% (1.2-4.8%)	14 (5.9-26)	1500 (770-2400)
Cambodia	2016	Female	20 to 24	9.4% (3.9-18%)	91% (82-96%)	1.4 (0.45-3.2)	2.3% (1.4-3.7%)	3.6% (1.7-6.1%)	25 (12-44)	2700 (1600-4200)

Location	Year	Sex	Age	Prevalence of current drinkers	Prevalence of abstainers	Population average of standard drinks daily	Percent of total attributable deaths	Percent of total attributable DALYs	Attributable deaths	Attributable DALYs
Cambodia	2016	Female	25 to 29	10% (4.4-19%)	90% (81-96%)	1.6 (0.53-3.5)	2.8% (1.7-4.3%)	4.5% (2.3-7.6%)	33 (16-55)	2900 (1700-4500)
Cambodia	2016	Female	30 to 34	11% (4.7-21%)	89% (79-95%)	1.8 (0.58-4.1)	3.3% (2-5.1%)	5.5% (3.1-8.5%)	73 (40-120)	5400 (3300-8100)
Cambodia	2016	Female	35 to 39	12% (5.4-22%)	88% (78-95%)	2 (0.71-4.5)	4% (2.5-6%)	6.7% (4-9.9%)	62 (36-94)	3800 (2400-5600)
Cambodia	2016	Female	40 to 44	12% (5.7-22%)	88% (78-94%)	2.3 (0.75-4.9)	4.6% (2.9-6.7%)	7.4% (4.7-11%)	98 (61-150)	5100 (3300-7500)
Cambodia	2016	Female	45 to 49	13% (5.6-24%)	87% (76-94%)	2.6 (0.8-6.1)	5% (3-7.6%)	7.7% (4.6-12%)	150 (91-230)	6900 (4100-10000)
Cambodia	2016	Female	50 to 54	13% (5.6-25%)	87% (75-94%)	2.9 (0.87-6.4)	5.8% (3.5-8.9%)	8.3% (5.1-13%)	230 (140-370)	9100 (5500-14000)
Cambodia	2016	Female	55 to 59	13% (5.9-25%)	87% (75-94%)	3.1 (0.94-7.3)	5.9% (3.4-9.4%)	8.1% (4.7-13%)	280 (160-440)	9200 (5200-15000)
Cambodia	2016	Female	60 to 64	13% (5.6-24%)	87% (76-94%)	3.2 (0.92-7.3)	5.8% (3.5-9.2%)	7.6% (4.6-12%)	320 (190-520)	9200 (5500-15000)
Cambodia	2016	Female	65 to 69	13% (5.7-24%)	87% (76-94%)	3 (0.87-6.8)	5.8% (3.2-9.2%)	7.4% (4.1-12%)	330 (190-540)	7800 (4400-13000)
Cambodia	2016	Female	70 to 74	12% (5.9-22%)	88% (78-94%)	2.7 (0.77-6.9)	5.4% (2.9-8.8%)	6.8% (3.7-11%)	320 (170-530)	6000 (3200-9900)
Cambodia	2016	Female	75 to 79	12% (5.2-23%)	88% (77-95%)	2.6 (0.68-6.6)	4.8% (2.5-8.1%)	5.9% (3-9.8%)	270 (140-460)	3900 (2000-6600)
Cambodia	2016	Female	80 to 84	12% (5.2-23%)	88% (77-95%)	2.6 (0.74-6.1)	4.9% (2.5-8.1%)	5.9% (3-9.7%)	220 (110-370)	2300 (1200-3900)
Cambodia	2016	Female	85 to 89	13% (5.7-24%)	87% (76-94%)	2.8 (0.76-7.1)	4.7% (2.3-8.3%)	5.6% (2.7-9.9%)	140 (69-250)	1100 (520-1900)
Cambodia	2016	Female	90 to 94	15% (6.3-27%)	85% (73-94%)	3.2 (0.75-8.7)	4.4% (2.1-7.8%)	5.3% (2.5-9.3%)	49 (23-86)	250 (120-440)
Cambodia	2016	Female	95 plus	18% (7-35%)	82% (65-93%)	3.8 (0.71-11)	4.7% (2-8.6%)	5.4% (2.3-10%)	13 (5.5-24)	44 (19-82)
Cambodia	2016	Male	All Ages	27% (21-34%)	73% (66-79%)	4 (2.5-6.1)	9% (7.4-11%)	13% (10-15%)	6700 (5400-8000)	250000 (210000-300000)
Cambodia	2016	Male	15 to 19	22% (12-35%)	78% (65-88%)	1.7 (0.67-3.5)	4.5% (2.3-7.7%)	6.3% (2.9-11%)	81 (36-140)	6600 (3200-11000)
Cambodia	2016	Male	20 to 24	33% (18-51%)	67% (49-82%)	4.2 (1.9-7.7)	11% (6.8-15%)	14% (8.4-20%)	300 (180-450)	22000 (13000-32000)
Cambodia	2016	Male	25 to 29	39% (21-57%)	61% (43-79%)	5.9 (2.8-11)	14% (9.7-18%)	18% (12-23%)	320 (210-450)	22000 (15000-29000)
Cambodia	2016	Male	30 to 34	42% (23-62%)	58% (38-77%)	7.3 (3.5-13)	14% (11-19%)	19% (14-25%)	550 (380-740)	34000 (24000-45000)
Cambodia	2016	Male	35 to 39	44% (27-63%)	56% (37-73%)	8.4 (3.8-16)	16% (12-20%)	20% (15-26%)	350 (260-450)	19000 (14000-24000)
Cambodia	2016	Male	40 to 44	44% (27-64%)	56% (36-73%)	8.8 (3.9-16)	15% (12-19%)	20% (16-25%)	450 (330-570)	22000 (16000-28000)

Location	Year	Sex	Age	Prevalence of current drinkers	Prevalence of abstainers	Population average of standard drinks daily	Percent of total attributable deaths	Percent of total attributable DALYs	Attributable deaths	Attributable DALYs
Cambodia	2016	Male	45 to 49	42% (24-61%)	58% (39-76%)	8.6 (3.8-16)	16% (12-20%)	20% (15-25%)	610 (460-780)	27000 (20000-34000)
Cambodia	2016	Male	50 to 54	40% (23-61%)	60% (39-77%)	8.1 (3.5-15)	15% (11-19%)	19% (14-24%)	730 (530-930)	28000 (20000-35000)
Cambodia	2016	Male	55 to 59	38% (22-59%)	62% (41-78%)	7.4 (3.3-14)	14% (10-18%)	17% (13-22%)	610 (440-810)	20000 (15000-27000)
Cambodia	2016	Male	60 to 64	35% (19-55%)	65% (45-81%)	6.4 (2.7-13)	13% (9.1-17%)	16% (11-20%)	650 (460-870)	18000 (13000-24000)
Cambodia	2016	Male	65 to 69	32% (18-51%)	68% (49-82%)	5.3 (2.2-11)	12% (8.1-16%)	14% (9.7-19%)	600 (420-810)	14000 (9600-19000)
Cambodia	2016	Male	70 to 74	28% (16-45%)	72% (55-84%)	4.4 (1.8-9)	10% (6.8-14%)	12% (7.9-17%)	520 (340-720)	9600 (6300-13000)
Cambodia	2016	Male	75 to 79	26% (14-42%)	74% (58-86%)	3.7 (1.4-7.8)	8.8% (5.6-12%)	10% (6.4-14%)	420 (260-590)	6000 (3800-8500)
Cambodia	2016	Male	80 to 84	24% (12-40%)	76% (60-88%)	3.2 (1.2-6.7)	8.3% (5-12%)	9.5% (5.8-14%)	280 (170-410)	3000 (1800-4400)
Cambodia	2016	Male	85 to 89	22% (11-37%)	78% (63-89%)	2.8 (1.1-6.1)	7% (4-10%)	8% (4.6-12%)	130 (75-200)	1000 (570-1500)
Cambodia	2016	Male	90 to 94	23% (12-38%)	77% (62-88%)	2.7 (1-5.7)	6.1% (3.3-9.3%)	7% (3.7-11%)	40 (22-62)	210 (120-320)
Cambodia	2016	Male	95 plus	27% (12-45%)	73% (55-88%)	2.8 (0.88-6.9)	6% (2.8-9.6%)	6.6% (3-11%)	8.8 (4.1-15)	31 (15-51)
Indonesia	1990	Female	All Ages	2.5% (2-3.1%)	98% (97-98%)	0.11 (0.1-0.12)	0.76% (0.61-0.93%)	1.3% (0.92-1.7%)	8400 (6200-11000)	290000 (230000-350000)
Indonesia	1990	Female	15 to 19	2.3% (1.3-3.8%)	98% (96-99%)	0.092 (0.046-0.16)	0.54% (0.35-0.83%)	0.61% (0.32-1.1%)	80 (41-140)	9000 (5700-14000)
Indonesia	1990	Female	20 to 24	2.8% (1.5-4.7%)	97% (95-99%)	0.12 (0.06-0.21)	0.96% (0.62-1.4%)	0.92% (0.46-1.5%)	150 (76-250)	17000 (11000-25000)
Indonesia	1990	Female	25 to 29	3.1% (1.7-4.9%)	97% (95-98%)	0.14 (0.073-0.23)	1.1% (0.78-1.6%)	1.2% (0.66-1.9%)	190 (100-300)	19000 (12000-27000)
Indonesia	1990	Female	30 to 34	3.4% (1.8-5.5%)	97% (94-98%)	0.16 (0.084-0.26)	1.2% (0.88-1.8%)	1.4% (0.92-2.2%)	240 (150-360)	19000 (13000-28000)
Indonesia	1990	Female	35 to 39	3.7% (1.9-5.9%)	96% (94-98%)	0.19 (0.096-0.3)	1.5% (1.1-2.2%)	2% (1.3-3%)	330 (210-480)	21000 (14000-29000)
Indonesia	1990	Female	40 to 44	3.7% (2.1-6.1%)	96% (94-98%)	0.22 (0.11-0.36)	1.7% (1.2-2.4%)	2.3% (1.6-3.4%)	400 (270-590)	22000 (15000-31000)
Indonesia	1990	Female	45 to 49	3.7% (1.9-5.8%)	96% (94-98%)	0.25 (0.12-0.42)	1.9% (1.3-2.8%)	2.5% (1.7-3.8%)	570 (370-860)	26000 (18000-39000)
Indonesia	1990	Female	50 to 54	3.7% (2.1-6.2%)	96% (94-98%)	0.25 (0.13-0.43)	2.1% (1.4-3%)	2.7% (1.8-4%)	830 (550-1200)	32000 (22000-48000)
Indonesia	1990	Female	55 to 59	3.7% (2.1-6%)	96% (94-98%)	0.24 (0.12-0.41)	1.8% (1.2-2.8%)	2.4% (1.5-3.6%)	900 (570-1400)	30000 (19000-45000)
Indonesia	1990	Female	60 to 64	3.7% (2-6.3%)	96% (94-98%)	0.23 (0.11-0.43)	1.8% (1.1-2.8%)	2.2% (1.3-3.5%)	1000 (600-1600)	28000 (17000-44000)

Location	Year	Sex	Age	Prevalence of current drinkers	Prevalence of abstainers	Population average of standard drinks daily	Percent of total attributable deaths	Percent of total attributable DALYs	Attributable deaths	Attributable DALYs
Indonesia	1990	Female	65 to 69	3.7% (2.1-5.8%)	96% (94-98%)	0.22 (0.11-0.39)	1.6% (0.95-2.6%)	2% (1.1-3.1%)	970 (580-1500)	22000 (13000-36000)
Indonesia	1990	Female	70 to 74	3.6% (2.1-5.8%)	96% (94-98%)	0.21 (0.098-0.37)	1.5% (0.82-2.4%)	1.7% (0.96-2.8%)	960 (540-1600)	18000 (10000-29000)
Indonesia	1990	Female	75 to 79	3.5% (2-5.9%)	96% (94-98%)	0.2 (0.087-0.37)	1.2% (0.68-2.1%)	1.4% (0.79-2.4%)	810 (440-1300)	12000 (6400-19000)
Indonesia	1990	Female	80 to 84	3.5% (1.9-5.8%)	96% (94-98%)	0.19 (0.091-0.34)	1.2% (0.65-2%)	1.4% (0.75-2.3%)	570 (300-930)	6000 (3200-9700)
Indonesia	1990	Female	85 to 89	3.8% (2.1-6.4%)	96% (94-98%)	0.2 (0.093-0.37)	1.1% (0.55-1.9%)	1.3% (0.65-2.2%)	300 (150-520)	2200 (1200-3900)
Indonesia	1990	Female	90 to 94	4.4% (2.5-7.2%)	96% (93-97%)	0.23 (0.1-0.43)	0.96% (0.46-1.8%)	1.1% (0.54-2.1%)	96 (46-180)	500 (240-900)
Indonesia	1990	Female	95 plus	5.3% (2.6-9.3%)	95% (91-97%)	0.26 (0.098-0.52)	1% (0.42-1.9%)	1.1% (0.46-2.2%)	24 (9.9-46)	84 (36-160)
Indonesia	1990	Male	All Ages	6.6% (5.4-8.1%)	93% (92-95%)	0.061 (0.055-0.068)	0.64% (0.44-0.81%)	0.87% (0.56-1.2%)	6200 (4000-8400)	270000 (190000-350000)
Indonesia	1990	Male	15 to 19	5.6% (3.3-9%)	94% (91-97%)	0.027 (0.013-0.047)	0.44% (0.3-0.61%)	0.32% (0.11-0.55%)	47 (16-81)	7600 (4800-11000)
Indonesia	1990	Male	20 to 24	8.8% (4.9-14%)	91% (86-95%)	0.068 (0.037-0.11)	0.94% (0.55-1.4%)	0.64% (0.18-1.2%)	130 (36-240)	19000 (11000-28000)
Indonesia	1990	Male	25 to 29	10% (5.8-16%)	90% (84-94%)	0.1 (0.054-0.16)	1.2% (0.72-1.8%)	1% (0.3-1.8%)	210 (60-360)	23000 (13000-35000)
Indonesia	1990	Male	30 to 34	11% (6.3-18%)	89% (82-94%)	0.13 (0.073-0.2)	1.7% (1-2.3%)	1.7% (0.84-2.6%)	360 (180-540)	29000 (17000-40000)
Indonesia	1990	Male	35 to 39	12% (6.9-19%)	88% (81-93%)	0.15 (0.082-0.24)	2.1% (1.4-2.9%)	2.5% (1.4-3.6%)	510 (290-750)	32000 (20000-44000)
Indonesia	1990	Male	40 to 44	12% (7-19%)	88% (81-93%)	0.15 (0.08-0.26)	2.3% (1.5-3%)	2.8% (1.8-3.9%)	570 (370-790)	30000 (20000-40000)
Indonesia	1990	Male	45 to 49	11% (6-17%)	89% (83-94%)	0.14 (0.076-0.24)	2.2% (1.5-3%)	2.8% (1.8-3.8%)	720 (460-990)	32000 (22000-43000)
Indonesia	1990	Male	50 to 54	10% (6.3-16%)	90% (84-94%)	0.13 (0.066-0.21)	1.9% (1.3-2.6%)	2.4% (1.6-3.3%)	820 (530-1100)	31000 (21000-42000)
Indonesia	1990	Male	55 to 59	9.8% (5.8-15%)	90% (85-94%)	0.11 (0.056-0.18)	1.5% (0.97-2%)	1.8% (1.1-2.6%)	730 (450-1000)	24000 (15000-33000)
Indonesia	1990	Male	60 to 64	9.2% (5.4-15%)	91% (85-95%)	0.091 (0.044-0.16)	1.2% (0.7-1.7%)	1.5% (0.81-2.1%)	690 (390-1000)	19000 (11000-28000)
Indonesia	1990	Male	65 to 69	8.6% (5.2-13%)	91% (87-95%)	0.08 (0.042-0.13)	0.88% (0.48-1.3%)	1% (0.54-1.5%)	510 (270-760)	12000 (6600-17000)
Indonesia	1990	Male	70 to 74	7.5% (4.7-12%)	92% (88-95%)	0.066 (0.033-0.12)	0.68% (0.35-1%)	0.78% (0.38-1.2%)	390 (190-600)	7300 (3800-11000)
Indonesia	1990	Male	75 to 79	6.5% (3.8-10%)	94% (90-96%)	0.054 (0.024-0.097)	0.57% (0.27-0.84%)	0.65% (0.29-0.99%)	300 (140-460)	4400 (2100-6500)
Indonesia	1990	Male	80 to 84	5.8% (3.3-9%)	94% (91-97%)	0.046 (0.022-0.08)	0.46% (0.2-0.69%)	0.52% (0.21-0.8%)	150 (61-230)	1600 (710-2500)

Location	Year	Sex	Age	Prevalence of current drinkers	Prevalence of abstainers	Population average of standard drinks daily	Percent of total attributable deaths	Percent of total attributable DALYs	Attributable deaths	Attributable DALYs
Indonesia	1990	Male	85 to 89	5.6% (3.2-8.9%)	94% (91-97%)	0.043 (0.021-0.075)	0.33% (0.13-0.52%)	0.37% (0.13-0.6%)	63 (22-100)	480 (190-760)
Indonesia	1990	Male	90 to 94	6% (3.6-9.6%)	94% (90-96%)	0.043 (0.02-0.08)	0.25% (0.083-0.42%)	0.28% (0.072-0.48%)	17 (4.5-30)	91 (30-160)
Indonesia	1990	Male	95 plus	7.1% (3.8-12%)	93% (88-96%)	0.048 (0.019-0.092)	0.22% (0.048-0.4%)	0.23% (0.021-0.44%)	3.5 (0.33-6.8)	14 (2.9-24)
Indonesia	1995	Female	All Ages	2.2% (1.8-2.8%)	98% (97-98%)	0.11 (0.1-0.12)	0.84% (0.69-1%)	1.4% (1-1.8%)	9400 (7000-13000)	300000 (250000-380000)
Indonesia	1995	Female	15 to 19	2.2% (1.2-3.6%)	98% (96-99%)	0.1 (0.05-0.18)	0.57% (0.37-0.84%)	0.66% (0.35-1.1%)	75 (40-130)	9000 (5800-13000)
Indonesia	1995	Female	20 to 24	2.5% (1.3-4.2%)	97% (96-99%)	0.13 (0.061-0.22)	0.98% (0.66-1.4%)	0.95% (0.51-1.5%)	130 (72-220)	17000 (11000-25000)
Indonesia	1995	Female	25 to 29	2.6% (1.4-4.3%)	97% (96-99%)	0.14 (0.072-0.22)	1.1% (0.77-1.5%)	1.2% (0.67-1.8%)	170 (100-270)	18000 (13000-26000)
Indonesia	1995	Female	30 to 34	2.7% (1.4-4.5%)	97% (96-99%)	0.15 (0.076-0.24)	1.2% (0.85-1.6%)	1.4% (0.9-2%)	220 (140-320)	19000 (14000-26000)
Indonesia	1995	Female	35 to 39	2.8% (1.4-4.6%)	97% (95-99%)	0.16 (0.082-0.27)	1.4% (1-1.9%)	1.9% (1.2-2.7%)	330 (220-480)	22000 (16000-30000)
Indonesia	1995	Female	40 to 44	2.9% (1.6-4.9%)	97% (95-98%)	0.2 (0.1-0.33)	1.6% (1.1-2.2%)	2.2% (1.5-3.1%)	410 (280-580)	23000 (16000-31000)
Indonesia	1995	Female	45 to 49	3.1% (1.5-4.9%)	97% (95-98%)	0.24 (0.12-0.42)	1.8% (1.2-2.6%)	2.4% (1.6-3.6%)	530 (350-790)	25000 (17000-36000)
Indonesia	1995	Female	50 to 54	3.1% (1.7-5.2%)	97% (95-98%)	0.25 (0.13-0.43)	2% (1.3-2.9%)	2.6% (1.8-3.8%)	800 (540-1200)	32000 (22000-46000)
Indonesia	1995	Female	55 to 59	3.1% (1.7-5.1%)	97% (95-98%)	0.24 (0.11-0.41)	1.8% (1.2-2.6%)	2.3% (1.5-3.4%)	950 (620-1400)	32000 (21000-47000)
Indonesia	1995	Female	60 to 64	3.2% (1.8-5.4%)	97% (95-98%)	0.23 (0.11-0.43)	1.7% (1.1-2.7%)	2.2% (1.4-3.4%)	1100 (720-1700)	32000 (20000-49000)
Indonesia	1995	Female	65 to 69	3.3% (1.9-5.2%)	97% (95-98%)	0.23 (0.11-0.41)	1.7% (1-2.6%)	2% (1.2-3.1%)	1200 (750-1900)	28000 (17000-44000)
Indonesia	1995	Female	70 to 74	3.4% (2-5.4%)	97% (95-98%)	0.23 (0.11-0.4)	1.5% (0.9-2.5%)	1.8% (1.1-2.9%)	1100 (670-1900)	21000 (12000-35000)
Indonesia	1995	Female	75 to 79	3.4% (1.9-5.8%)	97% (94-98%)	0.23 (0.1-0.42)	1.4% (0.77-2.2%)	1.6% (0.9-2.6%)	970 (540-1600)	14000 (7700-23000)
Indonesia	1995	Female	80 to 84	3.5% (1.9-5.8%)	96% (94-98%)	0.23 (0.1-0.4)	1.3% (0.74-2.2%)	1.6% (0.87-2.6%)	730 (400-1200)	7600 (4200-12000)
Indonesia	1995	Female	85 to 89	3.8% (2.1-6.5%)	96% (94-98%)	0.24 (0.11-0.43)	1.2% (0.63-2.1%)	1.4% (0.74-2.4%)	430 (230-730)	3200 (1700-5500)
Indonesia	1995	Female	90 to 94	4.4% (2.5-7%)	96% (93-98%)	0.26 (0.11-0.5)	1.1% (0.55-1.9%)	1.3% (0.64-2.3%)	150 (76-280)	790 (400-1400)
Indonesia	1995	Female	95 plus	5.3% (2.6-9.2%)	95% (91-97%)	0.3 (0.11-0.6)	1.1% (0.49-1.9%)	1.3% (0.56-2.3%)	39 (17-68)	130 (59-240)
Indonesia	1995	Male	All Ages	7.5% (6-9.3%)	93% (91-94%)	0.078 (0.07-0.086)	0.81% (0.61-0.99%)	1.1% (0.76-1.4%)	7700 (5300-9700)	330000 (250000-410000)

Location	Year	Sex	Age	Prevalence of current drinkers	Prevalence of abstainers	Population average of standard drinks daily	Percent of total attributable deaths	Percent of total attributable DALYs	Attributable deaths	Attributable DALYs
Indonesia	1995	Male	15 to 19	8% (4.8-13%)	92% (87-95%)	0.045 (0.022-0.078)	0.5% (0.33-0.72%)	0.42% (0.16-0.72%)	61 (22-100)	8700 (5400-13000)
Indonesia	1995	Male	20 to 24	13% (7.4-19%)	87% (81-93%)	0.11 (0.063-0.18)	1.1% (0.62-1.7%)	0.9% (0.27-1.6%)	180 (51-320)	22000 (12000-34000)
Indonesia	1995	Male	25 to 29	13% (7.5-19%)	87% (81-92%)	0.15 (0.081-0.23)	1.5% (0.83-2.2%)	1.4% (0.47-2.3%)	280 (96-480)	28000 (15000-42000)
Indonesia	1995	Male	30 to 34	12% (6.9-19%)	88% (81-93%)	0.16 (0.092-0.25)	1.9% (1.3-2.6%)	2.1% (1.2-3%)	450 (250-650)	35000 (23000-47000)
Indonesia	1995	Male	35 to 39	12% (6.8-18%)	88% (82-93%)	0.17 (0.095-0.28)	2.3% (1.6-3.1%)	2.9% (1.9-4%)	680 (440-950)	42000 (29000-56000)
Indonesia	1995	Male	40 to 44	11% (6.3-17%)	89% (83-94%)	0.16 (0.083-0.27)	2.5% (1.8-3.2%)	3.2% (2.2-4.2%)	760 (530-1000)	39000 (28000-51000)
Indonesia	1995	Male	45 to 49	9.3% (4.9-15%)	91% (85-95%)	0.14 (0.071-0.24)	2.4% (1.7-3.1%)	3.1% (2.2-4.1%)	790 (550-1000)	35000 (25000-45000)
Indonesia	1995	Male	50 to 54	8.2% (4.9-13%)	92% (87-95%)	0.12 (0.059-0.2)	2.1% (1.5-2.7%)	2.7% (1.9-3.4%)	870 (630-1200)	34000 (24000-43000)
Indonesia	1995	Male	55 to 59	7.5% (4.4-12%)	93% (88-96%)	0.094 (0.047-0.16)	1.6% (1.2-2.1%)	2% (1.4-2.7%)	870 (600-1100)	29000 (20000-38000)
Indonesia	1995	Male	60 to 64	7.3% (4.2-12%)	93% (88-96%)	0.083 (0.04-0.15)	1.3% (0.88-1.8%)	1.6% (1-2.2%)	850 (550-1200)	24000 (16000-33000)
Indonesia	1995	Male	65 to 69	7.3% (4.3-11%)	93% (89-96%)	0.078 (0.039-0.13)	1% (0.64-1.4%)	1.2% (0.73-1.6%)	680 (420-950)	16000 (10000-22000)
Indonesia	1995	Male	70 to 74	6.7% (4.1-10%)	93% (90-96%)	0.067 (0.034-0.12)	0.78% (0.48-1.1%)	0.91% (0.54-1.3%)	510 (310-750)	9600 (5800-14000)
Indonesia	1995	Male	75 to 79	5.9% (3.5-9.5%)	94% (90-97%)	0.056 (0.025-0.1)	0.66% (0.36-0.92%)	0.76% (0.39-1.1%)	360 (190-520)	5200 (2900-7400)
Indonesia	1995	Male	80 to 84	5.4% (3.1-8.3%)	95% (92-97%)	0.049 (0.023-0.085)	0.53% (0.26-0.76%)	0.61% (0.29-0.89%)	200 (93-290)	2100 (1000-3000)
Indonesia	1995	Male	85 to 89	5.5% (3-8.6%)	95% (91-97%)	0.048 (0.023-0.084)	0.38% (0.18-0.56%)	0.43% (0.19-0.66%)	91 (40-140)	690 (330-1000)
Indonesia	1995	Male	90 to 94	6% (3.5-9.3%)	94% (91-96%)	0.049 (0.023-0.094)	0.28% (0.12-0.45%)	0.32% (0.11-0.52%)	26 (9.4-43)	140 (56-220)
Indonesia	1995	Male	95 plus	7% (3.6-11%)	93% (89-96%)	0.053 (0.021-0.1)	0.27% (0.092-0.44%)	0.28% (0.065-0.49%)	5.3 (1.3-9.5)	20 (7.1-34)
Indonesia	2000	Female	All Ages	2.3% (1.9-2.8%)	98% (97-98%)	0.12 (0.12-0.13)	0.99% (0.81-1.2%)	1.6% (1.2-2.1%)	12000 (8600-15000)	360000 (290000-440000)
Indonesia	2000	Female	15 to 19	2.2% (1.2-3.6%)	98% (96-99%)	0.11 (0.053-0.19)	0.59% (0.38-0.86%)	0.73% (0.39-1.2%)	73 (39-120)	9000 (5900-13000)
Indonesia	2000	Female	20 to 24	2.6% (1.3-4.3%)	97% (96-99%)	0.14 (0.066-0.24)	1% (0.68-1.4%)	1% (0.56-1.7%)	130 (70-220)	17000 (11000-24000)
Indonesia	2000	Female	25 to 29	2.7% (1.5-4.4%)	97% (96-99%)	0.15 (0.076-0.24)	1.2% (0.82-1.6%)	1.3% (0.73-2%)	180 (100-270)	19000 (13000-27000)
Indonesia	2000	Female	30 to 34	2.7% (1.4-4.5%)	97% (96-99%)	0.16 (0.082-0.26)	1.2% (0.9-1.7%)	1.5% (0.97-2.2%)	240 (160-360)	21000 (16000-29000)

Location	Year	Sex	Age	Prevalence of current drinkers	Prevalence of abstainers	Population average of standard drinks daily	Percent of total attributable deaths	Percent of total attributable DALYs	Attributable deaths	Attributable DALYs
Indonesia	2000	Female	35 to 39	2.8% (1.4-4.6%)	97% (95-99%)	0.18 (0.092-0.29)	1.4% (1-1.9%)	1.9% (1.3-2.8%)	370 (250-540)	25000 (18000-34000)
Indonesia	2000	Female	40 to 44	3% (1.6-4.9%)	97% (95-98%)	0.21 (0.11-0.35)	1.6% (1.2-2.2%)	2.3% (1.6-3.2%)	520 (370-730)	29000 (21000-39000)
Indonesia	2000	Female	45 to 49	3.1% (1.5-4.9%)	97% (95-98%)	0.26 (0.13-0.45)	1.8% (1.3-2.6%)	2.5% (1.7-3.7%)	640 (440-940)	30000 (21000-43000)
Indonesia	2000	Female	50 to 54	3.2% (1.8-5.3%)	97% (95-98%)	0.27 (0.13-0.47)	2% (1.4-2.9%)	2.7% (1.8-3.9%)	850 (580-1200)	33000 (23000-48000)
Indonesia	2000	Female	55 to 59	3.2% (1.8-5.2%)	97% (95-98%)	0.26 (0.12-0.45)	1.9% (1.3-2.7%)	2.4% (1.6-3.6%)	1100 (700-1500)	35000 (24000-51000)
Indonesia	2000	Female	60 to 64	3.3% (1.7-5.6%)	97% (94-98%)	0.25 (0.12-0.47)	1.8% (1.2-2.8%)	2.3% (1.5-3.5%)	1300 (860-2000)	38000 (24000-58000)
Indonesia	2000	Female	65 to 69	3.4% (1.8-5.4%)	97% (95-98%)	0.25 (0.12-0.44)	1.8% (1.1-2.7%)	2.1% (1.3-3.3%)	1500 (960-2400)	36000 (22000-55000)
Indonesia	2000	Female	70 to 74	3.4% (2-5.5%)	97% (95-98%)	0.25 (0.12-0.45)	1.6% (0.98-2.6%)	2% (1.2-3.1%)	1500 (920-2400)	29000 (17000-45000)
Indonesia	2000	Female	75 to 79	3.5% (1.9-5.9%)	97% (94-98%)	0.24 (0.11-0.45)	1.5% (0.83-2.4%)	1.7% (0.97-2.8%)	1300 (720-2100)	18000 (10000-29000)
Indonesia	2000	Female	80 to 84	3.6% (1.9-6.1%)	96% (94-98%)	0.25 (0.11-0.44)	1.4% (0.82-2.3%)	1.7% (0.96-2.7%)	950 (540-1500)	9900 (5600-16000)
Indonesia	2000	Female	85 to 89	3.9% (2.2-6.6%)	96% (93-98%)	0.26 (0.12-0.47)	1.3% (0.71-2.2%)	1.5% (0.84-2.6%)	550 (300-910)	4100 (2300-6700)
Indonesia	2000	Female	90 to 94	4.5% (2.6-7.2%)	95% (93-97%)	0.29 (0.13-0.55)	1.2% (0.63-2%)	1.4% (0.74-2.4%)	210 (110-370)	1100 (580-1900)
Indonesia	2000	Female	95 plus	5.4% (2.7-9.7%)	95% (90-97%)	0.33 (0.13-0.66)	1.2% (0.57-2.1%)	1.4% (0.64-2.5%)	58 (27-110)	200 (97-360)
Indonesia	2000	Male	All Ages	7.7% (6.2-9.6%)	92% (90-94%)	0.085 (0.075-0.094)	0.97% (0.75-1.2%)	1.2% (0.92-1.5%)	9100 (6700-11000)	390000 (300000-470000)
Indonesia	2000	Male	15 to 19	8% (4.8-12%)	92% (88-95%)	0.046 (0.023-0.081)	0.53% (0.35-0.74%)	0.46% (0.19-0.77%)	68 (28-110)	9500 (6100-14000)
Indonesia	2000	Male	20 to 24	13% (7.3-19%)	87% (81-93%)	0.12 (0.065-0.19)	1.2% (0.69-1.8%)	0.99% (0.35-1.7%)	200 (76-360)	25000 (14000-37000)
Indonesia	2000	Male	25 to 29	13% (7.4-19%)	87% (81-93%)	0.15 (0.082-0.24)	1.5% (0.94-2.2%)	1.5% (0.58-2.4%)	320 (130-530)	31000 (18000-46000)
Indonesia	2000	Male	30 to 34	12% (6.8-19%)	88% (81-93%)	0.17 (0.094-0.26)	2% (1.4-2.6%)	2.2% (1.4-3.1%)	530 (330-750)	41000 (28000-54000)
Indonesia	2000	Male	35 to 39	12% (7-18%)	88% (82-93%)	0.18 (0.098-0.28)	2.5% (1.8-3.2%)	3.1% (2.1-4.1%)	810 (550-1100)	49000 (35000-65000)
Indonesia	2000	Male	40 to 44	11% (6.4-17%)	89% (83-94%)	0.17 (0.088-0.27)	2.6% (2-3.3%)	3.4% (2.5-4.4%)	990 (730-1300)	51000 (39000-66000)
Indonesia	2000	Male	45 to 49	9.3% (5-14%)	91% (86-95%)	0.14 (0.074-0.24)	2.5% (1.9-3.3%)	3.3% (2.3-4.3%)	1000 (750-1400)	46000 (34000-60000)
Indonesia	2000	Male	50 to 54	8.1% (4.8-13%)	92% (87-95%)	0.12 (0.06-0.21)	2.2% (1.6-2.8%)	2.8% (2.1-3.6%)	960 (700-1200)	37000 (27000-46000)

Location	Year	Sex	Age	Prevalence of current drinkers	Prevalence of abstainers	Population average of standard drinks daily	Percent of total attributable deaths	Percent of total attributable DALYs	Attributable deaths	Attributable DALYs
Indonesia	2000	Male	55 to 59	7.5% (4.4-11%)	93% (89-96%)	0.097 (0.049-0.17)	1.7% (1.3-2.2%)	2.2% (1.5-2.8%)	940 (680-1200)	31000 (23000-40000)
Indonesia	2000	Male	60 to 64	7.3% (4.2-12%)	93% (88-96%)	0.086 (0.041-0.16)	1.4% (0.97-1.9%)	1.7% (1.1-2.3%)	990 (660-1300)	28000 (19000-37000)
Indonesia	2000	Male	65 to 69	7.3% (4.3-11%)	93% (89-96%)	0.08 (0.042-0.13)	1% (0.68-1.4%)	1.3% (0.78-1.7%)	800 (510-1100)	19000 (12000-25000)
Indonesia	2000	Male	70 to 74	6.8% (4.1-11%)	93% (89-96%)	0.07 (0.035-0.12)	0.82% (0.53-1.2%)	0.96% (0.61-1.4%)	630 (390-890)	12000 (7600-17000)
Indonesia	2000	Male	75 to 79	5.9% (3.4-9.6%)	94% (90-97%)	0.058 (0.026-0.1)	0.69% (0.41-0.94%)	0.81% (0.47-1.1%)	430 (250-600)	6200 (3700-8500)
Indonesia	2000	Male	80 to 84	5.5% (3.1-8.6%)	94% (91-97%)	0.052 (0.024-0.089)	0.56% (0.3-0.78%)	0.64% (0.33-0.91%)	220 (110-320)	2400 (1300-3300)
Indonesia	2000	Male	85 to 89	5.5% (3.1-8.6%)	95% (91-97%)	0.05 (0.024-0.088)	0.41% (0.21-0.58%)	0.46% (0.23-0.69%)	110 (52-160)	810 (420-1200)
Indonesia	2000	Male	90 to 94	6% (3.5-9.4%)	94% (91-96%)	0.052 (0.023-0.096)	0.3% (0.14-0.45%)	0.34% (0.14-0.53%)	34 (14-53)	180 (84-280)
Indonesia	2000	Male	95 plus	7.1% (3.8-12%)	93% (88-96%)	0.056 (0.022-0.11)	0.28% (0.11-0.45%)	0.29% (0.078-0.5%)	7.2 (2-12)	28 (10-45)
Indonesia	2005	Female	All Ages	2.4% (1.9-3%)	98% (97-98%)	0.14 (0.13-0.15)	1.1% (0.89-1.3%)	1.7% (1.3-2.2%)	12000 (9000-16000)	370000 (300000-450000)
Indonesia	2005	Female	15 to 19	2.3% (1.3-3.7%)	98% (96-99%)	0.12 (0.056-0.2)	0.59% (0.39-0.85%)	0.75% (0.4-1.3%)	63 (34-110)	8300 (5500-12000)
Indonesia	2005	Female	20 to 24	2.6% (1.4-4.3%)	97% (96-99%)	0.15 (0.069-0.25)	1% (0.7-1.4%)	1% (0.55-1.7%)	110 (59-180)	16000 (10000-22000)
Indonesia	2005	Female	25 to 29	2.7% (1.4-4.4%)	97% (96-99%)	0.16 (0.083-0.26)	1.1% (0.83-1.5%)	1.2% (0.73-1.9%)	160 (94-250)	19000 (13000-26000)
Indonesia	2005	Female	30 to 34	2.8% (1.5-4.7%)	97% (95-99%)	0.17 (0.088-0.28)	1.2% (0.88-1.6%)	1.4% (0.96-2.1%)	230 (150-330)	21000 (15000-28000)
Indonesia	2005	Female	35 to 39	2.9% (1.5-4.8%)	97% (95-99%)	0.19 (0.098-0.31)	1.4% (1-1.8%)	1.9% (1.3-2.6%)	370 (260-520)	26000 (19000-34000)
Indonesia	2005	Female	40 to 44	3.1% (1.8-5%)	97% (95-98%)	0.23 (0.12-0.37)	1.6% (1.2-2.1%)	2.2% (1.6-3.1%)	550 (390-760)	31000 (23000-42000)
Indonesia	2005	Female	45 to 49	3.2% (1.6-5.1%)	97% (95-98%)	0.27 (0.13-0.47)	1.8% (1.3-2.5%)	2.5% (1.7-3.6%)	730 (500-1100)	35000 (24000-48000)
Indonesia	2005	Female	50 to 54	3.3% (1.8-5.5%)	97% (95-98%)	0.29 (0.14-0.49)	2% (1.4-2.8%)	2.6% (1.8-3.7%)	960 (660-1400)	38000 (26000-54000)
Indonesia	2005	Female	55 to 59	3.3% (1.8-5.4%)	97% (95-98%)	0.27 (0.13-0.48)	1.8% (1.2-2.7%)	2.4% (1.6-3.5%)	1100 (720-1500)	36000 (25000-52000)
Indonesia	2005	Female	60 to 64	3.4% (1.8-5.8%)	97% (94-98%)	0.27 (0.13-0.5)	1.8% (1.1-2.7%)	2.2% (1.4-3.4%)	1300 (810-1900)	36000 (23000-54000)
Indonesia	2005	Female	65 to 69	3.5% (2-5.6%)	96% (94-98%)	0.27 (0.13-0.47)	1.7% (1.1-2.7%)	2.1% (1.3-3.3%)	1500 (930-2300)	35000 (22000-54000)
Indonesia	2005	Female	70 to 74	3.6% (2.1-5.8%)	96% (94-98%)	0.27 (0.13-0.48)	1.6% (0.97-2.6%)	2% (1.2-3.1%)	1600 (960-2500)	30000 (18000-47000)

Location	Year	Sex	Age	Prevalence of current drinkers	Prevalence of abstainers	Population average of standard drinks daily	Percent of total attributable deaths	Percent of total attributable DALYs	Attributable deaths	Attributable DALYs
Indonesia	2005	Female	75 to 79	3.6% (2-6.1%)	96% (94-98%)	0.26 (0.12-0.48)	1.5% (0.84-2.4%)	1.7% (0.98-2.8%)	1300 (750-2100)	19000 (11000-31000)
Indonesia	2005	Female	80 to 84	3.7% (2-6.1%)	96% (94-98%)	0.26 (0.12-0.46)	1.4% (0.85-2.3%)	1.7% (1-2.7%)	1100 (650-1700)	12000 (6800-18000)
Indonesia	2005	Female	85 to 89	4% (2.2-6.7%)	96% (93-98%)	0.28 (0.13-0.51)	1.3% (0.75-2.2%)	1.6% (0.88-2.6%)	630 (360-1100)	4700 (2700-7900)
Indonesia	2005	Female	90 to 94	4.7% (2.7-7.6%)	95% (92-97%)	0.31 (0.14-0.59)	1.2% (0.66-2.1%)	1.4% (0.78-2.5%)	240 (130-420)	1200 (680-2100)
Indonesia	2005	Female	95 plus	5.6% (2.7-9.9%)	94% (90-97%)	0.35 (0.14-0.7)	1.2% (0.6-2.2%)	1.4% (0.67-2.5%)	72 (34-130)	250 (120-440)
Indonesia	2005	Male	All Ages	7.6% (6.2-9.4%)	92% (91-94%)	0.084 (0.074-0.094)	1.1% (0.88-1.3%)	1.4% (1-1.6%)	10000 (7900-12000)	440000 (350000-520000)
Indonesia	2005	Male	15 to 19	7.8% (4.6-12%)	92% (88-95%)	0.044 (0.021-0.078)	0.55% (0.38-0.76%)	0.5% (0.23-0.8%)	70 (32-110)	9600 (6400-14000)
Indonesia	2005	Male	20 to 24	12% (7.3-19%)	88% (81-93%)	0.11 (0.06-0.18)	1.2% (0.77-1.7%)	1% (0.45-1.7%)	210 (91-350)	25000 (15000-36000)
Indonesia	2005	Male	25 to 29	13% (7.4-19%)	87% (81-93%)	0.15 (0.08-0.24)	1.6% (1-2.1%)	1.5% (0.67-2.3%)	340 (150-530)	33000 (21000-47000)
Indonesia	2005	Male	30 to 34	12% (6.8-19%)	88% (81-93%)	0.16 (0.091-0.26)	2% (1.5-2.6%)	2.3% (1.5-3.1%)	580 (380-790)	44000 (31000-57000)
Indonesia	2005	Male	35 to 39	12% (6.8-18%)	88% (82-93%)	0.17 (0.095-0.27)	2.5% (1.9-3.2%)	3.1% (2.2-4.1%)	910 (650-1200)	56000 (41000-72000)
Indonesia	2005	Male	40 to 44	11% (6.2-17%)	89% (83-94%)	0.16 (0.085-0.26)	2.6% (2.1-3.3%)	3.4% (2.6-4.3%)	1100 (860-1400)	59000 (45000-73000)
Indonesia	2005	Male	45 to 49	9.1% (4.8-14%)	91% (86-95%)	0.14 (0.072-0.23)	2.6% (1.9-3.3%)	3.4% (2.5-4.2%)	1300 (940-1600)	56000 (42000-71000)
Indonesia	2005	Male	50 to 54	8% (4.7-13%)	92% (87-95%)	0.11 (0.059-0.19)	2.2% (1.7-2.8%)	2.9% (2.2-3.6%)	1200 (920-1500)	47000 (36000-59000)
Indonesia	2005	Male	55 to 59	7.4% (4.4-12%)	93% (88-96%)	0.095 (0.048-0.16)	1.7% (1.3-2.2%)	2.2% (1.6-2.8%)	1100 (820-1400)	37000 (28000-46000)
Indonesia	2005	Male	60 to 64	7.3% (4.2-12%)	93% (88-96%)	0.084 (0.04-0.15)	1.4% (1-1.8%)	1.7% (1.2-2.3%)	1000 (720-1300)	29000 (20000-37000)
Indonesia	2005	Male	65 to 69	7.2% (4.3-11%)	93% (89-96%)	0.078 (0.041-0.13)	1% (0.71-1.4%)	1.3% (0.84-1.6%)	830 (570-1100)	19000 (14000-26000)
Indonesia	2005	Male	70 to 74	6.6% (4.1-10%)	93% (90-96%)	0.067 (0.032-0.12)	0.81% (0.54-1.1%)	0.97% (0.63-1.3%)	670 (440-920)	12000 (8400-17000)
Indonesia	2005	Male	75 to 79	5.8% (3.3-9.4%)	94% (91-97%)	0.055 (0.025-0.1)	0.71% (0.44-0.94%)	0.84% (0.5-1.1%)	480 (280-640)	6800 (4200-9100)
Indonesia	2005	Male	80 to 84	5.3% (3-8.3%)	95% (92-97%)	0.048 (0.023-0.082)	0.59% (0.35-0.8%)	0.7% (0.39-0.94%)	280 (160-390)	3000 (1700-4100)
Indonesia	2005	Male	85 to 89	5.2% (3-8.2%)	95% (92-97%)	0.046 (0.022-0.081)	0.43% (0.23-0.58%)	0.49% (0.27-0.68%)	130 (69-180)	990 (540-1300)
Indonesia	2005	Male	90 to 94	5.7% (3.3-9%)	94% (91-97%)	0.047 (0.022-0.088)	0.32% (0.16-0.46%)	0.36% (0.17-0.53%)	41 (19-61)	220 (110-320)

Location	Year	Sex	Age	Prevalence of current drinkers	Prevalence of abstainers	Population average of standard drinks daily	Percent of total attributable deaths	Percent of total attributable DALYs	Attributable deaths	Attributable DALYs
Indonesia	2005	Male	95 plus	6.8% (3.6-11%)	93% (89-96%)	0.052 (0.02-0.1)	0.29% (0.13-0.44%)	0.3% (0.1-0.47%)	9.7 (3.3-15)	37 (16-55)
Indonesia	2010	Female	All Ages	3% (2.4-3.7%)	97% (96-98%)	0.17 (0.15-0.18)	1.2% (0.95-1.4%)	1.7% (1.3-2.2%)	12000 (9300-16000)	390000 (320000-480000)
Indonesia	2010	Female	15 to 19	2.6% (1.5-4.3%)	97% (96-99%)	0.12 (0.06-0.23)	0.59% (0.39-0.86%)	0.76% (0.4-1.3%)	54 (29-96)	7600 (4900-11000)
Indonesia	2010	Female	20 to 24	3.2% (1.7-5.2%)	97% (95-98%)	0.17 (0.078-0.3)	1.1% (0.73-1.5%)	1.1% (0.58-1.9%)	98 (51-170)	15000 (9900-22000)
Indonesia	2010	Female	25 to 29	3.5% (1.9-5.5%)	97% (94-98%)	0.19 (0.095-0.31)	1.2% (0.86-1.6%)	1.3% (0.76-2.1%)	160 (90-250)	20000 (14000-28000)
Indonesia	2010	Female	30 to 34	3.7% (2-6.1%)	96% (94-98%)	0.21 (0.11-0.34)	1.3% (0.93-1.7%)	1.6% (1-2.3%)	230 (150-330)	23000 (16000-31000)
Indonesia	2010	Female	35 to 39	4.1% (2.1-6.6%)	96% (93-98%)	0.24 (0.13-0.39)	1.5% (1.1-2%)	2.1% (1.4-2.9%)	410 (280-580)	29000 (21000-40000)
Indonesia	2010	Female	40 to 44	4.1% (2.4-6.8%)	96% (93-98%)	0.28 (0.15-0.45)	1.7% (1.2-2.3%)	2.4% (1.7-3.3%)	630 (440-870)	36000 (26000-49000)
Indonesia	2010	Female	45 to 49	4.1% (2.1-6.4%)	96% (94-98%)	0.32 (0.16-0.53)	1.9% (1.3-2.6%)	2.6% (1.8-3.8%)	880 (600-1300)	42000 (29000-59000)
Indonesia	2010	Female	50 to 54	4.1% (2.3-6.8%)	96% (93-98%)	0.33 (0.17-0.55)	2% (1.4-2.8%)	2.7% (1.9-3.9%)	1100 (770-1600)	46000 (32000-65000)
Indonesia	2010	Female	55 to 59	4.1% (2.3-6.6%)	96% (93-98%)	0.32 (0.16-0.54)	1.9% (1.2-2.7%)	2.4% (1.6-3.6%)	1100 (730-1600)	38000 (25000-55000)
Indonesia	2010	Female	60 to 64	4.1% (2.3-7%)	96% (93-98%)	0.3 (0.14-0.55)	1.8% (1.1-2.7%)	2.3% (1.4-3.4%)	1200 (730-1900)	35000 (21000-53000)
Indonesia	2010	Female	65 to 69	4.1% (2.3-6.4%)	96% (94-98%)	0.29 (0.15-0.49)	1.7% (1-2.6%)	2.1% (1.3-3.2%)	1400 (840-2100)	32000 (20000-50000)
Indonesia	2010	Female	70 to 74	4% (2.4-6.4%)	96% (94-98%)	0.27 (0.13-0.48)	1.6% (0.92-2.4%)	1.9% (1.1-2.9%)	1600 (930-2400)	29000 (18000-45000)
Indonesia	2010	Female	75 to 79	3.9% (2.2-6.5%)	96% (94-98%)	0.26 (0.12-0.46)	1.3% (0.77-2.1%)	1.6% (0.91-2.6%)	1300 (720-2000)	18000 (10000-30000)
Indonesia	2010	Female	80 to 84	3.9% (2.1-6.3%)	96% (94-98%)	0.26 (0.12-0.44)	1.4% (0.78-2.1%)	1.6% (0.92-2.5%)	1100 (640-1800)	12000 (6700-19000)
Indonesia	2010	Female	85 to 89	4.2% (2.3-7%)	96% (93-98%)	0.27 (0.12-0.49)	1.2% (0.71-2.1%)	1.5% (0.83-2.5%)	710 (400-1200)	5300 (3000-9000)
Indonesia	2010	Female	90 to 94	4.9% (2.8-7.9%)	95% (92-97%)	0.3 (0.13-0.58)	1.1% (0.61-2%)	1.3% (0.72-2.4%)	270 (140-470)	1400 (750-2400)
Indonesia	2010	Female	95 plus	5.9% (2.9-11%)	94% (89-97%)	0.35 (0.13-0.7)	1.2% (0.55-2.1%)	1.4% (0.62-2.5%)	78 (35-140)	270 (120-470)
Indonesia	2010	Male	All Ages	7.1% (5.8-8.6%)	93% (91-94%)	0.073 (0.065-0.082)	1.2% (0.96-1.4%)	1.4% (1.1-1.7%)	11000 (8600-13000)	470000 (380000-550000)
Indonesia	2010	Male	15 to 19	5.3% (3.1-8.4%)	95% (92-97%)	0.026 (0.012-0.049)	0.55% (0.38-0.72%)	0.47% (0.21-0.7%)	60 (27-90)	8800 (5900-12000)
Indonesia	2010	Male	20 to 24	8.4% (4.7-13%)	92% (87-95%)	0.066 (0.034-0.11)	1.1% (0.81-1.5%)	0.85% (0.45-1.3%)	150 (83-240)	21000 (15000-30000)

Location	Year	Sex	Age	Prevalence of current drinkers	Prevalence of abstainers	Population average of standard drinks daily	Percent of total attributable deaths	Percent of total attributable DALYs	Attributable deaths	Attributable DALYs
Indonesia	2010	Male	25 to 29	9.9% (5.5-15%)	90% (85-94%)	0.1 (0.052-0.17)	1.4% (1-1.9%)	1.3% (0.66-1.8%)	290 (150-420)	32000 (22000-43000)
Indonesia	2010	Male	30 to 34	11% (6.2-17%)	89% (83-94%)	0.13 (0.072-0.2)	1.9% (1.4-2.4%)	2.1% (1.4-2.8%)	530 (360-700)	43000 (32000-55000)
Indonesia	2010	Male	35 to 39	12% (6.6-18%)	88% (82-93%)	0.15 (0.082-0.23)	2.4% (1.9-3%)	3% (2.2-3.8%)	920 (670-1200)	58000 (45000-72000)
Indonesia	2010	Male	40 to 44	11% (6.8-18%)	89% (82-93%)	0.15 (0.08-0.24)	2.5% (2-3%)	3.3% (2.6-4.1%)	1200 (930-1500)	63000 (50000-77000)
Indonesia	2010	Male	45 to 49	11% (5.8-16%)	89% (84-94%)	0.14 (0.076-0.23)	2.5% (1.9-3.1%)	3.3% (2.5-4.1%)	1500 (1100-1800)	66000 (49000-82000)
Indonesia	2010	Male	50 to 54	10% (6-15%)	90% (85-94%)	0.13 (0.068-0.21)	2.2% (1.6-2.7%)	2.8% (2.1-3.6%)	1500 (1100-2000)	59000 (45000-76000)
Indonesia	2010	Male	55 to 59	9.4% (5.6-15%)	91% (85-94%)	0.11 (0.056-0.17)	1.7% (1.3-2.2%)	2.2% (1.6-2.7%)	1300 (960-1700)	44000 (33000-55000)
Indonesia	2010	Male	60 to 64	8.9% (5.2-14%)	91% (86-95%)	0.09 (0.045-0.16)	1.4% (0.95-1.8%)	1.7% (1.2-2.2%)	1100 (720-1400)	30000 (20000-38000)
Indonesia	2010	Male	65 to 69	8.3% (4.9-13%)	92% (87-95%)	0.079 (0.04-0.13)	0.99% (0.7-1.3%)	1.2% (0.83-1.6%)	850 (590-1100)	20000 (14000-26000)
Indonesia	2010	Male	70 to 74	7.2% (4.5-11%)	93% (89-95%)	0.064 (0.031-0.11)	0.76% (0.52-1%)	0.91% (0.61-1.2%)	690 (460-920)	13000 (8800-17000)
Indonesia	2010	Male	75 to 79	6.1% (3.6-9.9%)	94% (90-96%)	0.051 (0.023-0.092)	0.67% (0.43-0.89%)	0.8% (0.5-1.1%)	500 (310-670)	7200 (4500-9600)
Indonesia	2010	Male	80 to 84	5.4% (3.1-8.4%)	95% (92-97%)	0.044 (0.02-0.075)	0.57% (0.34-0.74%)	0.67% (0.38-0.88%)	310 (180-420)	3300 (2000-4400)
Indonesia	2010	Male	85 to 89	5.3% (3-8.3%)	95% (92-97%)	0.041 (0.02-0.072)	0.41% (0.24-0.54%)	0.47% (0.27-0.64%)	160 (88-210)	1200 (700-1600)
Indonesia	2010	Male	90 to 94	5.7% (3.4-9%)	94% (91-97%)	0.041 (0.019-0.077)	0.3% (0.16-0.42%)	0.34% (0.17-0.49%)	48 (24-69)	250 (140-360)
Indonesia	2010	Male	95 plus	6.7% (3.5-11%)	93% (89-96%)	0.045 (0.018-0.091)	0.28% (0.13-0.41%)	0.29% (0.11-0.45%)	11 (4.2-17)	42 (19-60)
Indonesia	2016	Female	All Ages	3.1% (2.5-3.7%)	97% (96-97%)	0.2 (0.15-0.24)	1.3% (1-1.6%)	1.8% (1.4-2.5%)	13000 (9700-18000)	420000 (330000-540000)
Indonesia	2016	Female	15 to 19	2.6% (1.5-4.3%)	97% (96-98%)	0.14 (0.064-0.27)	0.6% (0.39-0.88%)	0.81% (0.41-1.5%)	54 (27-97)	7900 (5100-12000)
Indonesia	2016	Female	20 to 24	3.2% (1.7-5.3%)	97% (95-98%)	0.19 (0.082-0.37)	1.1% (0.76-1.6%)	1.1% (0.58-2%)	89 (44-150)	15000 (9900-21000)
Indonesia	2016	Female	25 to 29	3.5% (1.9-5.5%)	97% (95-98%)	0.21 (0.097-0.36)	1.2% (0.88-1.7%)	1.4% (0.74-2.2%)	130 (70-210)	18000 (12000-25000)
Indonesia	2016	Female	30 to 34	3.7% (2-6.2%)	96% (94-98%)	0.23 (0.11-0.4)	1.3% (0.92-1.7%)	1.6% (1-2.4%)	210 (130-320)	23000 (16000-31000)
Indonesia	2016	Female	35 to 39	4.1% (2.2-6.5%)	96% (93-98%)	0.27 (0.13-0.45)	1.5% (1-2%)	2.1% (1.4-3%)	380 (250-550)	29000 (21000-40000)
Indonesia	2016	Female	40 to 44	4.1% (2.4-6.8%)	96% (93-98%)	0.31 (0.16-0.52)	1.6% (1.2-2.2%)	2.4% (1.7-3.4%)	610 (420-870)	37000 (26000-50000)

Location	Year	Sex	Age	Prevalence of current drinkers	Prevalence of abstainers	Population average of standard drinks daily	Percent of total attributable deaths	Percent of total attributable DALYs	Attributable deaths	Attributable DALYs
Indonesia	2016	Female	45 to 49	4.1% (2.1-6.5%)	96% (93-98%)	0.35 (0.18-0.6)	1.8% (1.3-2.6%)	2.6% (1.8-3.9%)	910 (610-1300)	45000 (31000-64000)
Indonesia	2016	Female	50 to 54	4.1% (2.3-6.8%)	96% (93-98%)	0.37 (0.2-0.63)	1.9% (1.3-2.8%)	2.7% (1.8-3.9%)	1200 (820-1800)	51000 (35000-73000)
Indonesia	2016	Female	55 to 59	4.2% (2.3-6.7%)	96% (93-98%)	0.35 (0.18-0.6)	1.8% (1.2-2.7%)	2.5% (1.6-3.7%)	1400 (890-2000)	48000 (32000-71000)
Indonesia	2016	Female	60 to 64	4.2% (2.3-7%)	96% (93-98%)	0.34 (0.16-0.64)	1.8% (1.1-2.7%)	2.3% (1.4-3.6%)	1400 (860-2200)	41000 (25000-63000)
Indonesia	2016	Female	65 to 69	4.2% (2.4-6.5%)	96% (94-98%)	0.33 (0.16-0.6)	1.7% (1-2.7%)	2.2% (1.3-3.5%)	1500 (890-2300)	35000 (21000-55000)
Indonesia	2016	Female	70 to 74	4% (2.4-6.3%)	96% (94-98%)	0.31 (0.14-0.59)	1.6% (0.94-2.5%)	2% (1.2-3.2%)	1500 (910-2400)	29000 (17000-46000)
Indonesia	2016	Female	75 to 79	3.9% (2.2-6.6%)	96% (93-98%)	0.29 (0.13-0.56)	1.4% (0.76-2.3%)	1.7% (0.92-2.8%)	1500 (810-2500)	22000 (12000-36000)
Indonesia	2016	Female	80 to 84	4% (2.1-6.6%)	96% (93-98%)	0.29 (0.13-0.53)	1.4% (0.76-2.2%)	1.6% (0.92-2.6%)	1200 (660-1900)	13000 (7100-20000)
Indonesia	2016	Female	85 to 89	4.3% (2.4-7.1%)	96% (93-98%)	0.31 (0.14-0.58)	1.3% (0.7-2.1%)	1.5% (0.83-2.5%)	770 (420-1300)	5800 (3200-9700)
Indonesia	2016	Female	90 to 94	5% (2.9-7.9%)	95% (92-97%)	0.34 (0.15-0.65)	1.2% (0.6-2%)	1.4% (0.71-2.4%)	330 (170-560)	1700 (900-2900)
Indonesia	2016	Female	95 plus	6% (3-10%)	94% (90-97%)	0.4 (0.15-0.85)	1.2% (0.54-2.2%)	1.4% (0.61-2.5%)	97 (42-180)	340 (150-610)
Indonesia	2016	Male	All Ages	7.3% (6-8.8%)	93% (91-94%)	0.082 (0.062-0.1)	1.3% (1.1-1.5%)	1.5% (1.2-1.8%)	12000 (10000-15000)	520000 (420000-620000)
Indonesia	2016	Male	15 to 19	5.2% (3.1-8.6%)	95% (91-97%)	0.028 (0.013-0.057)	0.59% (0.4-0.79%)	0.54% (0.23-0.78%)	70 (30-100)	10000 (6700-14000)
Indonesia	2016	Male	20 to 24	8.4% (4.7-13%)	92% (87-95%)	0.073 (0.034-0.14)	1.2% (0.89-1.7%)	0.96% (0.54-1.5%)	170 (98-270)	24000 (17000-33000)
Indonesia	2016	Male	25 to 29	9.9% (5.7-15%)	90% (85-94%)	0.11 (0.052-0.19)	1.6% (1.2-2.1%)	1.4% (0.85-2.1%)	260 (160-380)	29000 (21000-40000)
Indonesia	2016	Male	30 to 34	11% (6-17%)	89% (83-94%)	0.14 (0.07-0.23)	2% (1.6-2.6%)	2.3% (1.6-3%)	530 (370-700)	44000 (33000-57000)
Indonesia	2016	Male	35 to 39	12% (6.7-18%)	88% (82-93%)	0.16 (0.082-0.27)	2.5% (1.9-3.1%)	3.2% (2.4-4%)	910 (670-1200)	59000 (45000-73000)
Indonesia	2016	Male	40 to 44	11% (6.7-18%)	89% (82-93%)	0.16 (0.084-0.27)	2.5% (2-3.1%)	3.4% (2.6-4.2%)	1200 (960-1500)	66000 (52000-81000)
Indonesia	2016	Male	45 to 49	11% (5.8-16%)	89% (84-94%)	0.15 (0.079-0.27)	2.5% (1.9-3.1%)	3.3% (2.5-4.1%)	1600 (1200-2000)	71000 (55000-90000)
Indonesia	2016	Male	50 to 54	10% (6-16%)	90% (84-94%)	0.14 (0.072-0.23)	2.1% (1.7-2.7%)	2.8% (2.1-3.6%)	1700 (1200-2200)	65000 (50000-84000)
Indonesia	2016	Male	55 to 59	9.4% (5.6-15%)	91% (85-94%)	0.11 (0.058-0.19)	1.7% (1.3-2.2%)	2.2% (1.6-2.8%)	1600 (1200-2100)	53000 (40000-68000)
Indonesia	2016	Male	60 to 64	8.8% (5.2-14%)	91% (86-95%)	0.097 (0.046-0.18)	1.4% (1-1.8%)	1.7% (1.2-2.3%)	1400 (990-1900)	41000 (28000-54000)

Location	Year	Sex	Age	Prevalence of current drinkers	Prevalence of abstainers	Population average of standard drinks daily	Percent of total attributable deaths	Percent of total attributable DALYs	Attributable deaths	Attributable DALYs
Indonesia	2016	Male	65 to 69	8.3% (5.1-13%)	92% (87-95%)	0.085 (0.042-0.15)	1% (0.75-1.3%)	1.2% (0.89-1.6%)	980 (700-1300)	23000 (17000-30000)
Indonesia	2016	Male	70 to 74	7.2% (4.5-11%)	93% (89-95%)	0.07 (0.033-0.13)	0.8% (0.58-1.1%)	0.97% (0.68-1.3%)	780 (540-1100)	15000 (10000-20000)
Indonesia	2016	Male	75 to 79	6.1% (3.5-9.8%)	94% (90-96%)	0.056 (0.024-0.11)	0.71% (0.49-0.94%)	0.85% (0.58-1.1%)	630 (410-870)	9100 (6100-12000)
Indonesia	2016	Male	80 to 84	5.4% (3-8.4%)	95% (92-97%)	0.047 (0.021-0.085)	0.61% (0.39-0.79%)	0.72% (0.44-0.95%)	360 (220-480)	3800 (2500-5000)
Indonesia	2016	Male	85 to 89	5.2% (3-8.3%)	95% (92-97%)	0.044 (0.021-0.081)	0.44% (0.28-0.58%)	0.51% (0.32-0.68%)	170 (110-230)	1300 (840-1700)
Indonesia	2016	Male	90 to 94	5.6% (3.4-8.9%)	94% (91-97%)	0.045 (0.021-0.084)	0.33% (0.19-0.45%)	0.38% (0.21-0.53%)	59 (33-83)	310 (190-430)
Indonesia	2016	Male	95 plus	6.6% (3.6-11%)	93% (89-96%)	0.049 (0.018-0.1)	0.31% (0.15-0.44%)	0.33% (0.14-0.49%)	14 (5.8-21)	52 (26-75)
Laos	1990	Female	All Ages	24% (20-28%)	76% (72-80%)	0.63 (0.52-0.76)	1.2% (0.68-1.8%)	2.5% (1.3-4%)	710 (370-1100)	22000 (13000-32000)
Laos	1990	Female	15 to 19	24% (17-33%)	76% (67-83%)	0.77 (0.46-1.2)	1.9% (1-3.3%)	2.7% (1.3-4.6%)	13 (6.2-23)	1000 (550-1700)
Laos	1990	Female	20 to 24	31% (21-42%)	69% (58-79%)	1.1 (0.62-1.6)	3% (1.6-4.9%)	3.7% (1.8-6.4%)	22 (10-37)	1700 (890-2700)
Laos	1990	Female	25 to 29	33% (23-43%)	67% (57-77%)	1.1 (0.69-1.6)	3.3% (1.7-5.4%)	4.2% (2-7.4%)	24 (11-41)	1600 (840-2700)
Laos	1990	Female	30 to 34	34% (23-46%)	66% (54-77%)	1.1 (0.69-1.7)	3.2% (1.7-5.1%)	4.1% (2-6.9%)	22 (11-36)	1400 (720-2200)
Laos	1990	Female	35 to 39	36% (24-46%)	64% (54-76%)	1.2 (0.75-1.9)	4.3% (2.2-7.1%)	5.8% (2.9-9.5%)	31 (16-52)	1700 (870-2700)
Laos	1990	Female	40 to 44	35% (25-46%)	65% (54-75%)	1.3 (0.77-1.9)	4.5% (2.2-7.5%)	6.1% (2.9-10%)	34 (16-55)	1600 (790-2600)
Laos	1990	Female	45 to 49	34% (23-45%)	66% (55-77%)	1.2 (0.7-2)	4.9% (2.3-8.7%)	6.5% (2.9-11%)	40 (18-70)	1700 (770-2900)
Laos	1990	Female	50 to 54	34% (24-45%)	66% (55-76%)	1.2 (0.72-2)	4.9% (1.9-8.9%)	6.3% (2.4-11%)	51 (20-91)	1900 (730-3400)
Laos	1990	Female	55 to 59	34% (25-46%)	66% (54-75%)	1.3 (0.7-2.1)	4.7% (1.6-8.8%)	5.8% (1.9-11%)	61 (20-110)	1900 (660-3600)
Laos	1990	Female	60 to 64	32% (22-44%)	68% (56-78%)	1.2 (0.7-2.1)	4.3% (1.3-8.6%)	5.2% (1.5-10%)	73 (22-140)	2000 (590-3900)
Laos	1990	Female	65 to 69	28% (19-37%)	72% (63-81%)	1.2 (0.72-1.8)	4.3% (1.7-8.1%)	5.1% (2-9.6%)	85 (32-160)	1900 (730-3500)
Laos	1990	Female	70 to 74	27% (19-36%)	73% (64-81%)	1.2 (0.7-1.9)	4.6% (1.6-8.8%)	5.3% (1.9-10%)	96 (34-190)	1700 (620-3400)
Laos	1990	Female	75 to 79	28% (19-38%)	72% (62-81%)	1.3 (0.71-2)	4.3% (1.4-8.3%)	4.9% (1.6-9.7%)	78 (25-150)	1100 (360-2100)
Laos	1990	Female	80 to 84	29% (20-38%)	71% (62-80%)	1.2 (0.75-1.9)	4.2% (1.4-7.8%)	4.9% (1.7-9%)	53 (18-98)	550 (190-1000)

Location	Year	Sex	Age	Prevalence of current drinkers	Prevalence of abstainers	Population average of standard drinks daily	Percent of total attributable deaths	Percent of total attributable DALYs	Attributable deaths	Attributable DALYs
Laos	1990	Female	85 to 89	29%<br>(20-40%)	71%<br>(60-80%)	1.2<br>(0.73-1.9)	3.6%<br>(1.2-6.7%)	4.1%<br>(1.4-7.8%)	20<br>(6.8-38)	150<br>(52-280)
Laos	1990	Female	90 to 94	31%<br>(21-42%)	69%<br>(58-79%)	1.3<br>(0.72-2.1)	3.1%<br>(0.74-6.4%)	3.7%<br>(0.81-7.7%)	5.3<br>(1.2-11)	27<br>(6.4-56)
Laos	1990	Female	95 plus	34%<br>(22-47%)	66%<br>(53-78%)	1.4<br>(0.64-2.5)	3.3%<br>(0.33-7.4%)	3.7%<br>(0.29-8.5%)	1.2<br>(0.095-2.8)	4.2<br>(0.41-9.5)
Laos	1990	Male	All Ages	48%<br>(41-54%)	52%<br>(46-59%)	1.4<br>(1.2-1.6)	2.9%<br>(2-3.8%)	4.9%<br>(3.3-6.7%)	1500<br>(1000-2100)	61000<br>(45000-80000)
Laos	1990	Male	15 to 19	46%<br>(34-58%)	54%<br>(42-66%)	1<br>(0.62-1.5)	3.5%<br>(1.9-5.5%)	4.4%<br>(2.4-7.1%)	26<br>(14-42)	2000<br>(1100-3200)
Laos	1990	Male	20 to 24	66%<br>(53-76%)	34%<br>(24-47%)	2.4<br>(1.6-3.1)	9.9%<br>(6.5-14%)	12%<br>(7.8-17%)	89<br>(56-130)	6300<br>(4100-8700)
Laos	1990	Male	25 to 29	71%<br>(60-79%)	29%<br>(21-40%)	2.9<br>(2-3.7)	14%<br>(8.7-19%)	17%<br>(10-23%)	120<br>(72-160)	7500<br>(4800-10000)
Laos	1990	Male	30 to 34	75%<br>(65-82%)	25%<br>(18-35%)	3.2<br>(2.4-4.2)	15%<br>(10-20%)	18%<br>(12-25%)	120<br>(80-170)	7200<br>(4800-9800)
Laos	1990	Male	35 to 39	76%<br>(67-83%)	24%<br>(17-33%)	3.3<br>(2.4-4.3)	16%<br>(11-21%)	19%<br>(13-26%)	130<br>(88-180)	6900<br>(4700-9300)
Laos	1990	Male	40 to 44	75%<br>(65-82%)	25%<br>(18-35%)	3.2<br>(2.3-4.3)	15%<br>(9.9-21%)	18%<br>(12-26%)	120<br>(77-170)	5700<br>(3700-7900)
Laos	1990	Male	45 to 49	72%<br>(60-79%)	28%<br>(21-40%)	3.2<br>(2.2-4.3)	15%<br>(9.6-21%)	18%<br>(11-26%)	140<br>(83-190)	5700<br>(3500-7900)
Laos	1990	Male	50 to 54	69%<br>(58-77%)	31%<br>(23-42%)	2.9<br>(1.9-4)	14%<br>(7.9-20%)	16%<br>(9.2-24%)	150<br>(87-220)	5600<br>(3300-8200)
Laos	1990	Male	55 to 59	67%<br>(55-77%)	33%<br>(23-45%)	2.5<br>(1.7-3.5)	11%<br>(5.9-17%)	13%<br>(7-20%)	140<br>(77-220)	4500<br>(2500-7000)
Laos	1990	Male	60 to 64	63%<br>(50-74%)	37%<br>(26-50%)	2.1<br>(1.4-3.2)	9%<br>(4.4-15%)	11%<br>(5-17%)	140<br>(65-230)	3800<br>(1800-6300)
Laos	1990	Male	65 to 69	56%<br>(43-67%)	44%<br>(33-57%)	1.8<br>(1.2-2.6)	7.5%<br>(3.6-13%)	8.7%<br>(4.1-15%)	120<br>(57-200)	2700<br>(1300-4500)
Laos	1990	Male	70 to 74	52%<br>(39-64%)	48%<br>(36-61%)	1.7<br>(1-2.4)	6.7%<br>(2.7-11%)	7.7%<br>(3.1-13%)	110<br>(43-190)	1900<br>(810-3300)
Laos	1990	Male	75 to 79	52%<br>(38-67%)	48%<br>(33-62%)	1.6<br>(0.92-2.4)	5%<br>(1.6-9.2%)	5.8%<br>(1.8-11%)	64<br>(19-120)	890<br>(290-1600)
Laos	1990	Male	80 to 84	50%<br>(34-65%)	50%<br>(35-66%)	1.3<br>(0.8-1.9)	4.1%<br>(1.2-7.9%)	4.8%<br>(1.3-9.2%)	31<br>(8.4-59)	320<br>(93-610)
Laos	1990	Male	85 to 89	45%<br>(31-59%)	55%<br>(41-69%)	1.1<br>(0.62-1.7)	2.6%<br>(0.52-5.3%)	2.9%<br>(0.51-6.1%)	9.2<br>(1.6-19)	69<br>(14-140)
Laos	1990	Male	90 to 94	44%<br>(30-58%)	56%<br>(42-70%)	0.95<br>(0.54-1.5)	1.6%<br>(-0.03-3.8%)	1.8%<br>(-0.13-4.5%)	1.9<br>(-0.14-4.7)	9.9<br>(-0.19-24)
Laos	1990	Male	95 plus	45%<br>(29-64%)	55%<br>(36-71%)	0.86<br>(0.39-1.6)	1.1%<br>(-0.49-3.5%)	1.1%<br>(-0.72-3.9%)	0.26<br>(-0.17-0.88)	1<br>(-0.43-3.1)
Laos	1995	Female	All Ages	24%<br>(20-28%)	76%<br>(72-80%)	0.62<br>(0.52-0.75)	1.2%<br>(0.73-1.8%)	2.5%<br>(1.3-4%)	730<br>(390-1100)	23000<br>(14000-33000)

Location	Year	Sex	Age	Prevalence of current drinkers	Prevalence of abstainers	Population average of standard drinks daily	Percent of total attributable deaths	Percent of total attributable DALYs	Attributable deaths	Attributable DALYs
Laos	1995	Female	15 to 19	24% (16-33%)	76% (67-84%)	0.76 (0.47-1.2)	2.1% (1.1-3.3%)	2.9% (1.5-4.9%)	15 (7.5-25)	1200 (640-1900)
Laos	1995	Female	20 to 24	31% (21-42%)	69% (58-79%)	1.1 (0.62-1.6)	3% (1.6-5%)	3.9% (1.9-6.6%)	24 (12-41)	1900 (990-3000)
Laos	1995	Female	25 to 29	33% (23-44%)	67% (56-77%)	1.1 (0.68-1.6)	3.3% (1.8-5.2%)	4.3% (2.1-7.2%)	25 (12-43)	1800 (930-2900)
Laos	1995	Female	30 to 34	34% (23-46%)	66% (54-77%)	1.1 (0.66-1.7)	3.2% (1.7-5.1%)	4.2% (2.1-7%)	24 (12-40)	1500 (840-2400)
Laos	1995	Female	35 to 39	36% (24-46%)	64% (54-76%)	1.2 (0.73-1.9)	4.2% (2.2-6.9%)	5.8% (2.9-9.6%)	34 (17-57)	1900 (1000-3000)
Laos	1995	Female	40 to 44	35% (25-46%)	65% (54-75%)	1.3 (0.76-1.9)	4.5% (2.3-7.1%)	6.1% (3.1-9.6%)	37 (19-59)	1800 (930-2800)
Laos	1995	Female	45 to 49	34% (23-44%)	66% (56-77%)	1.2 (0.72-2)	4.7% (2.1-8.1%)	6.3% (2.8-11%)	42 (19-72)	1800 (820-3000)
Laos	1995	Female	50 to 54	34% (24-45%)	66% (55-76%)	1.2 (0.71-2)	4.7% (1.8-8.5%)	6.1% (2.3-11%)	49 (18-88)	1800 (680-3200)
Laos	1995	Female	55 to 59	34% (25-45%)	66% (55-75%)	1.2 (0.68-2.1)	4.4% (1.4-8.6%)	5.5% (1.8-11%)	58 (18-110)	1800 (590-3600)
Laos	1995	Female	60 to 64	32% (22-44%)	68% (56-78%)	1.2 (0.68-2)	4.1% (1.2-8.4%)	4.9% (1.4-9.8%)	67 (19-130)	1800 (510-3700)
Laos	1995	Female	65 to 69	29% (20-37%)	71% (63-80%)	1.2 (0.72-1.8)	4.1% (1.5-7.5%)	4.9% (1.8-8.9%)	85 (31-160)	1900 (700-3500)
Laos	1995	Female	70 to 74	27% (19-37%)	73% (63-81%)	1.2 (0.71-1.9)	4.4% (1.6-8.4%)	5.1% (1.8-9.8%)	97 (34-190)	1800 (630-3500)
Laos	1995	Female	75 to 79	28% (19-39%)	72% (61-81%)	1.2 (0.69-2)	4.1% (1.3-8.3%)	4.7% (1.5-9.6%)	82 (25-170)	1200 (350-2300)
Laos	1995	Female	80 to 84	29% (19-39%)	71% (61-81%)	1.2 (0.73-2)	4.1% (1.5-7.5%)	4.7% (1.7-8.7%)	58 (21-110)	600 (220-1100)
Laos	1995	Female	85 to 89	29% (20-40%)	71% (60-80%)	1.2 (0.72-2)	3.5% (1.2-6.9%)	4% (1.4-8%)	24 (8.1-47)	170 (61-340)
Laos	1995	Female	90 to 94	31% (22-42%)	69% (58-78%)	1.3 (0.7-2.1)	3% (0.74-6.3%)	3.5% (0.82-7.4%)	6.3 (1.5-13)	32 (8.2-67)
Laos	1995	Female	95 plus	34% (22-46%)	66% (54-78%)	1.3 (0.61-2.5)	3.2% (0.25-7.4%)	3.6% (0.2-8.5%)	1.5 (0.085-3.5)	5.1 (0.41-12)
Laos	1995	Male	All Ages	48% (41-54%)	52% (46-59%)	1.3 (1.2-1.5)	3% (2.1-4%)	4.9% (3.3-6.8%)	1600 (1100-2100)	65000 (48000-84000)
Laos	1995	Male	15 to 19	46% (34-58%)	54% (42-66%)	1 (0.63-1.4)	3.6% (2-5.5%)	4.7% (2.5-7.3%)	27 (15-43)	2200 (1200-3300)
Laos	1995	Male	20 to 24	66% (54-76%)	34% (24-46%)	2.3 (1.6-3.1)	10% (6.7-14%)	13% (8.1-18%)	97 (61-140)	6900 (4500-9400)
Laos	1995	Male	25 to 29	72% (61-79%)	28% (21-39%)	2.8 (2.1-3.7)	14% (9.2-19%)	17% (11-23%)	120 (80-180)	8100 (5400-11000)
Laos	1995	Male	30 to 34	75% (65-82%)	25% (18-35%)	3.2 (2.4-4.1)	15% (10-20%)	18% (12-25%)	130 (89-190)	8000 (5400-11000)

Location	Year	Sex	Age	Prevalence of current drinkers	Prevalence of abstainers	Population average of standard drinks daily	Percent of total attributable deaths	Percent of total attributable DALYs	Attributable deaths	Attributable DALYs
Laos	1995	Male	35 to 39	76% (67-83%)	24% (17-33%)	3.3 (2.4-4.2)	16% (11-21%)	19% (13-26%)	140 (95-200)	7600 (5100-10000)
Laos	1995	Male	40 to 44	75% (66-82%)	25% (18-34%)	3.2 (2.3-4.2)	15% (10-20%)	18% (12-25%)	140 (94-200)	6800 (4600-9400)
Laos	1995	Male	45 to 49	72% (60-79%)	28% (21-40%)	3.1 (2.2-4.2)	15% (9.6-21%)	18% (11-25%)	140 (88-200)	5900 (3700-8200)
Laos	1995	Male	50 to 54	69% (58-77%)	31% (23-42%)	2.9 (1.9-4)	13% (7.9-19%)	16% (9.2-23%)	140 (84-210)	5300 (3200-7600)
Laos	1995	Male	55 to 59	67% (56-77%)	33% (23-44%)	2.5 (1.7-3.5)	10% (5.8-16%)	12% (6.8-19%)	140 (77-210)	4500 (2500-6800)
Laos	1995	Male	60 to 64	63% (50-74%)	37% (26-50%)	2.1 (1.4-3.2)	8.6% (4.3-14%)	10% (5-17%)	140 (67-230)	3800 (1900-6200)
Laos	1995	Male	65 to 69	56% (43-67%)	44% (33-57%)	1.8 (1.3-2.6)	7.1% (3.4-12%)	8.2% (3.9-14%)	120 (59-210)	2800 (1400-4600)
Laos	1995	Male	70 to 74	52% (40-64%)	48% (36-60%)	1.6 (1-2.4)	6.3% (2.6-11%)	7.3% (3-13%)	110 (45-190)	2000 (840-3500)
Laos	1995	Male	75 to 79	53% (37-67%)	47% (33-63%)	1.5 (0.92-2.3)	4.7% (1.4-8.7%)	5.4% (1.5-10%)	68 (19-120)	960 (290-1700)
Laos	1995	Male	80 to 84	50% (34-65%)	50% (35-66%)	1.3 (0.76-1.9)	3.8% (1-7.4%)	4.4% (1.1-8.7%)	33 (8.3-65)	350 (92-680)
Laos	1995	Male	85 to 89	45% (31-60%)	55% (40-69%)	1 (0.6-1.7)	2.4% (0.43-5%)	2.7% (0.39-5.7%)	10 (1.5-22)	77 (14-160)
Laos	1995	Male	90 to 94	45% (31-59%)	55% (41-69%)	0.93 (0.53-1.5)	1.4% (-0.11-3.5%)	1.6% (-0.25-4.1%)	2 (-0.32-5.1)	11 (-0.83-26)
Laos	1995	Male	95 plus	46% (29-65%)	54% (35-71%)	0.85 (0.4-1.6)	1% (-0.53-3.2%)	1% (-0.8-3.6%)	0.29 (-0.23-1)	1.1 (-0.61-3.6)
Laos	2000	Female	All Ages	22% (19-25%)	78% (75-81%)	0.54 (0.45-0.65)	1.1% (0.69-1.7%)	2.2% (1.2-3.6%)	630 (330-1000)	20000 (12000-29000)
Laos	2000	Female	15 to 19	22% (16-29%)	78% (71-84%)	0.66 (0.41-1)	1.9% (1-3%)	2.7% (1.4-4.4%)	14 (7.1-23)	1100 (620-1800)
Laos	2000	Female	20 to 24	29% (22-37%)	71% (63-78%)	0.96 (0.6-1.5)	2.8% (1.5-4.5%)	3.5% (1.7-6.1%)	22 (11-36)	1700 (940-2700)
Laos	2000	Female	25 to 29	30% (23-38%)	70% (62-77%)	0.95 (0.65-1.4)	3% (1.7-4.6%)	3.9% (1.9-6.4%)	23 (11-38)	1600 (900-2600)
Laos	2000	Female	30 to 34	31% (24-41%)	69% (59-76%)	0.97 (0.64-1.4)	2.8% (1.6-4.4%)	3.8% (1.9-6.1%)	22 (11-35)	1400 (790-2200)
Laos	2000	Female	35 to 39	34% (26-42%)	66% (58-74%)	1.1 (0.73-1.6)	3.7% (2.1-6.1%)	5.2% (2.8-8.4%)	31 (17-52)	1700 (980-2800)
Laos	2000	Female	40 to 44	34% (26-42%)	66% (58-74%)	1.2 (0.76-1.7)	3.9% (2.1-6.3%)	5.5% (2.9-8.8%)	34 (18-55)	1700 (900-2600)
Laos	2000	Female	45 to 49	31% (23-38%)	69% (62-77%)	1.1 (0.67-1.7)	3.9% (1.7-6.7%)	5.4% (2.3-9.1%)	37 (16-63)	1600 (720-2700)
Laos	2000	Female	50 to 54	28% (20-36%)	72% (64-80%)	0.96 (0.57-1.6)	3.6% (1.3-6.6%)	4.7% (1.7-8.5%)	39 (14-71)	1500 (510-2600)

Location	Year	Sex	Age	Prevalence of current drinkers	Prevalence of abstainers	Population average of standard drinks daily	Percent of total attributable deaths	Percent of total attributable DALYs	Attributable deaths	Attributable DALYs
Laos	2000	Female	55 to 59	27% (19-36%)	73% (64-81%)	0.95 (0.54-1.6)	3.3% (1.1-6.4%)	4.2% (1.3-8.1%)	42 (13-81)	1300 (430-2600)
Laos	2000	Female	60 to 64	26% (18-35%)	74% (65-82%)	0.95 (0.54-1.6)	3.1% (0.87-6.5%)	3.8% (1.1-8%)	51 (14-110)	1400 (380-2900)
Laos	2000	Female	65 to 69	24% (17-32%)	76% (68-83%)	0.95 (0.58-1.5)	3.2% (1.2-6%)	3.8% (1.5-7.1%)	63 (23-120)	1400 (540-2700)
Laos	2000	Female	70 to 74	24% (16-32%)	76% (68-84%)	1 (0.59-1.7)	3.5% (1.2-7.1%)	4.2% (1.4-8.3%)	82 (28-160)	1500 (500-3000)
Laos	2000	Female	75 to 79	26% (18-36%)	74% (64-82%)	1.2 (0.68-1.9)	3.6% (1-7.4%)	4.2% (1.2-8.5%)	76 (21-160)	1100 (300-2200)
Laos	2000	Female	80 to 84	28% (19-38%)	72% (62-81%)	1.2 (0.71-1.9)	3.7% (1.2-7%)	4.3% (1.4-8.3%)	59 (20-110)	610 (210-1200)
Laos	2000	Female	85 to 89	28% (19-38%)	72% (62-81%)	1.2 (0.67-1.8)	3.1% (1-6.2%)	3.7% (1.1-7.3%)	25 (7.8-50)	190 (59-360)
Laos	2000	Female	90 to 94	30% (21-40%)	70% (60-79%)	1.2 (0.65-2)	2.7% (0.58-5.8%)	3.2% (0.64-6.8%)	7 (1.4-15)	36 (7.6-76)
Laos	2000	Female	95 plus	32% (21-45%)	68% (55-79%)	1.3 (0.59-2.3)	2.9% (0.15-6.8%)	3.2% (0.08-7.8%)	1.6 (0.04-3.9)	5.5 (0.3-13)
Laos	2000	Male	All Ages	51% (45-57%)	49% (43-55%)	1.4 (1.2-1.6)	3.3% (2.4-4.4%)	5.3% (3.7-7.3%)	1600 (1100-2200)	69000 (52000-88000)
Laos	2000	Male	15 to 19	49% (40-59%)	51% (41-60%)	1 (0.68-1.4)	3.7% (2.2-5.4%)	5% (2.9-7.5%)	30 (17-47)	2400 (1400-3600)
Laos	2000	Male	20 to 24	70% (61-77%)	30% (23-39%)	2.4 (1.7-3.1)	10% (6.9-14%)	13% (8.5-18%)	100 (67-140)	7200 (4900-9900)
Laos	2000	Male	25 to 29	76% (69-81%)	24% (19-31%)	3 (2.2-3.8)	14% (9.4-19%)	17% (11-24%)	130 (87-180)	8700 (5800-12000)
Laos	2000	Male	30 to 34	79% (73-83%)	21% (17-27%)	3.3 (2.5-4.2)	15% (11-20%)	19% (13-25%)	140 (97-190)	8400 (5900-11000)
Laos	2000	Male	35 to 39	80% (75-84%)	20% (16-25%)	3.4 (2.6-4.3)	15% (11-21%)	19% (13-26%)	150 (100-210)	8100 (5600-11000)
Laos	2000	Male	40 to 44	78% (73-83%)	22% (17-27%)	3.3 (2.5-4.3)	15% (10-20%)	18% (12-25%)	150 (100-210)	7300 (5000-10000)
Laos	2000	Male	45 to 49	76% (69-81%)	24% (19-31%)	3.3 (2.4-4.4)	15% (9.7-20%)	18% (12-25%)	170 (110-230)	7000 (4600-9700)
Laos	2000	Male	50 to 54	73% (66-78%)	27% (22-34%)	3 (2.1-4.1)	13% (8-18%)	16% (9.4-22%)	150 (90-220)	5500 (3400-8000)
Laos	2000	Male	55 to 59	71% (62-78%)	29% (22-38%)	2.5 (1.8-3.5)	10% (5.9-15%)	12% (6.8-18%)	130 (75-200)	4200 (2500-6200)
Laos	2000	Male	60 to 64	64% (54-73%)	36% (27-46%)	2.1 (1.4-3)	8% (4-13%)	9.5% (4.6-15%)	130 (64-220)	3600 (1800-5900)
Laos	2000	Male	65 to 69	53% (42-64%)	47% (36-58%)	1.7 (1.2-2.3)	6.3% (3-11%)	7.4% (3.5-13%)	110 (55-190)	2600 (1300-4400)
Laos	2000	Male	70 to 74	54% (43-64%)	46% (36-57%)	1.7 (1.1-2.4)	6% (2.4-10%)	7% (2.8-12%)	120 (47-200)	2100 (850-3600)

Location	Year	Sex	Age	Prevalence of current drinkers	Prevalence of abstainers	Population average of standard drinks daily	Percent of total attributable deaths	Percent of total attributable DALYs	Attributable deaths	Attributable DALYs
Laos	2000	Male	75 to 79	56% (43-68%)	44% (32-57%)	1.6 (1-2.4)	4.6% (1.3-8.6%)	5.3% (1.4-9.9%)	74 (20-140)	1100 (300-2000)
Laos	2000	Male	80 to 84	50% (34-65%)	50% (35-66%)	1.3 (0.75-1.9)	3.6% (0.84-6.9%)	4.1% (0.91-8%)	36 (8-71)	380 (89-730)
Laos	2000	Male	85 to 89	43% (30-58%)	57% (42-70%)	0.97 (0.57-1.5)	2.1% (0.36-4.6%)	2.4% (0.33-5.3%)	11 (1.5-24)	84 (14-180)
Laos	2000	Male	90 to 94	42% (29-57%)	58% (43-71%)	0.85 (0.47-1.4)	1.3% (-0.11-3.1%)	1.4% (-0.28-3.6%)	2.2 (-0.44-5.6)	12 (-1-29)
Laos	2000	Male	95 plus	44% (26-62%)	56% (38-74%)	0.77 (0.36-1.4)	0.92% (-0.53-3%)	0.9% (-0.76-3.4%)	0.31 (-0.26-1.2)	1.2 (-0.71-4.1)
Laos	2005	Female	All Ages	22% (19-25%)	78% (75-81%)	0.53 (0.45-0.64)	1.1% (0.67-1.6%)	2.1% (1.1-3.4%)	540 (280-880)	17000 (11000-25000)
Laos	2005	Female	15 to 19	21% (15-28%)	79% (72-85%)	0.62 (0.39-0.93)	1.7% (0.95-2.6%)	2.5% (1.3-4%)	13 (6.6-21)	1100 (620-1700)
Laos	2005	Female	20 to 24	28% (21-36%)	72% (64-79%)	0.91 (0.56-1.4)	2.5% (1.4-3.9%)	3.2% (1.6-5.5%)	19 (9.2-32)	1500 (890-2400)
Laos	2005	Female	25 to 29	30% (23-37%)	70% (63-77%)	0.91 (0.61-1.3)	2.6% (1.5-4.1%)	3.5% (1.7-5.9%)	19 (9.4-32)	1400 (790-2200)
Laos	2005	Female	30 to 34	30% (23-39%)	70% (61-77%)	0.93 (0.6-1.4)	2.5% (1.5-3.9%)	3.4% (1.8-5.4%)	18 (9.7-29)	1200 (740-1900)
Laos	2005	Female	35 to 39	33% (25-41%)	67% (59-75%)	1.1 (0.67-1.6)	3.2% (1.8-5%)	4.5% (2.4-7.2%)	26 (14-42)	1500 (830-2300)
Laos	2005	Female	40 to 44	33% (26-40%)	67% (60-74%)	1.1 (0.73-1.6)	3.4% (1.8-5.3%)	4.8% (2.5-7.7%)	30 (15-48)	1500 (800-2400)
Laos	2005	Female	45 to 49	30% (23-38%)	70% (62-77%)	1 (0.64-1.6)	3.3% (1.4-5.6%)	4.7% (2-7.9%)	32 (14-56)	1400 (610-2400)
Laos	2005	Female	50 to 54	27% (19-36%)	73% (64-81%)	0.92 (0.53-1.5)	3% (0.99-5.6%)	4% (1.3-7.4%)	34 (11-63)	1300 (420-2400)
Laos	2005	Female	55 to 59	27% (19-36%)	73% (64-81%)	0.91 (0.52-1.5)	2.7% (0.75-5.4%)	3.5% (1-7.1%)	36 (10-71)	1200 (320-2300)
Laos	2005	Female	60 to 64	25% (17-34%)	75% (66-83%)	0.91 (0.5-1.5)	2.6% (0.66-5.7%)	3.2% (0.82-7.2%)	40 (9.9-88)	1100 (270-2500)
Laos	2005	Female	65 to 69	23% (16-31%)	77% (69-84%)	0.9 (0.54-1.4)	2.7% (0.85-5.2%)	3.3% (1.1-6.3%)	51 (17-100)	1200 (370-2300)
Laos	2005	Female	70 to 74	23% (16-31%)	77% (69-84%)	0.97 (0.56-1.6)	3% (0.92-6.3%)	3.6% (1.1-7.4%)	68 (20-140)	1200 (370-2500)
Laos	2005	Female	75 to 79	26% (18-36%)	74% (64-82%)	1.1 (0.63-1.8)	3.1% (0.82-6.6%)	3.6% (0.92-7.7%)	68 (18-150)	960 (260-2100)
Laos	2005	Female	80 to 84	27% (19-38%)	73% (62-81%)	1.1 (0.68-1.8)	3.2% (1-6.1%)	3.8% (1.2-7.2%)	54 (17-110)	570 (180-1100)
Laos	2005	Female	85 to 89	28% (19-38%)	72% (62-81%)	1.1 (0.64-1.8)	2.7% (0.83-5.5%)	3.2% (0.95-6.5%)	25 (7.5-51)	190 (57-380)
Laos	2005	Female	90 to 94	29% (20-40%)	71% (60-80%)	1.1 (0.64-1.9)	2.4% (0.4-5.3%)	2.8% (0.39-6.2%)	7.4 (1-17)	38 (6.3-84)

Location	Year	Sex	Age	Prevalence of current drinkers	Prevalence of abstainers	Population average of standard drinks daily	Percent of total attributable deaths	Percent of total attributable DALYs	Attributable deaths	Attributable DALYs
Laos	2005	Female	95 plus	32% (21-44%)	68% (56-79%)	1.2 (0.56-2.1)	2.5% (-0.053-6.1%)	2.8% (-0.17-7%)	1.7 (-0.11-4.3)	5.9 (-0.13-15)
Laos	2005	Male	All Ages	51% (46-58%)	49% (42-54%)	1.4 (1.2-1.6)	3.4% (2.5-4.6%)	5.2% (3.6-7.1%)	1500 (1100-2000)	64000 (48000-82000)
Laos	2005	Male	15 to 19	48% (39-58%)	52% (42-61%)	0.99 (0.65-1.3)	3.6% (2.2-5.3%)	4.9% (2.8-7.3%)	31 (18-47)	2500 (1500-3700)
Laos	2005	Male	20 to 24	69% (60-76%)	31% (24-40%)	2.3 (1.7-3)	9.7% (6.4-13%)	12% (8-17%)	97 (61-130)	6900 (4600-9400)
Laos	2005	Male	25 to 29	75% (69-80%)	25% (20-31%)	2.9 (2.1-3.7)	13% (8.2-17%)	16% (10-22%)	120 (75-160)	7800 (5000-10000)
Laos	2005	Male	30 to 34	79% (73-83%)	21% (17-27%)	3.3 (2.5-4.1)	14% (9.5-18%)	17% (12-23%)	130 (85-170)	7600 (5300-10000)
Laos	2005	Male	35 to 39	79% (74-84%)	21% (16-26%)	3.3 (2.5-4.2)	14% (9.9-19%)	17% (12-24%)	130 (92-180)	7200 (5000-9800)
Laos	2005	Male	40 to 44	78% (73-83%)	22% (17-27%)	3.2 (2.4-4.2)	13% (9.2-18%)	16% (11-23%)	140 (94-200)	6800 (4700-9300)
Laos	2005	Male	45 to 49	75% (69-81%)	25% (19-31%)	3.2 (2.3-4.1)	13% (8.8-18%)	16% (11-22%)	160 (100-220)	6800 (4500-9300)
Laos	2005	Male	50 to 54	72% (65-78%)	28% (22-35%)	2.9 (2-3.9)	11% (7.1-17%)	14% (8.5-20%)	160 (95-230)	5800 (3600-8400)
Laos	2005	Male	55 to 59	70% (61-78%)	30% (22-39%)	2.5 (1.7-3.4)	8.9% (5-13%)	11% (5.8-16%)	120 (66-180)	4000 (2200-5900)
Laos	2005	Male	60 to 64	63% (53-72%)	37% (28-47%)	2 (1.4-3)	6.9% (3.1-12%)	8.2% (3.7-14%)	110 (49-190)	3000 (1400-5100)
Laos	2005	Male	65 to 69	53% (42-63%)	47% (37-58%)	1.6 (1.1-2.2)	5.5% (2.6-9%)	6.4% (3-11%)	100 (49-170)	2300 (1100-3800)
Laos	2005	Male	70 to 74	53% (42-63%)	47% (37-58%)	1.6 (1-2.2)	5.1% (2.1-8.6%)	6% (2.4-9.9%)	100 (43-170)	1900 (780-3100)
Laos	2005	Male	75 to 79	55% (43-67%)	45% (33-57%)	1.5 (0.99-2.3)	3.9% (0.98-7.3%)	4.5% (1-8.4%)	69 (16-130)	990 (250-1800)
Laos	2005	Male	80 to 84	50% (35-63%)	50% (37-65%)	1.2 (0.74-1.8)	3% (0.67-5.7%)	3.5% (0.69-6.5%)	35 (6.8-66)	370 (81-690)
Laos	2005	Male	85 to 89	42% (28-57%)	58% (43-72%)	0.92 (0.53-1.4)	1.7% (0.18-3.8%)	2% (0.1-4.4%)	11 (0.55-24)	82 (8.8-180)
Laos	2005	Male	90 to 94	41% (28-55%)	59% (45-72%)	0.8 (0.45-1.3)	1% (-0.25-2.6%)	1.1% (-0.46-2.9%)	2.1 (-0.88-5.6)	12 (-2.9-30)
Laos	2005	Male	95 plus	42% (26-61%)	58% (39-74%)	0.72 (0.34-1.4)	0.71% (-0.61-2.5%)	0.64% (-0.92-2.7%)	0.28 (-0.4-1.2)	1.2 (-1-4.1)
Laos	2010	Female	All Ages	24% (20-27%)	76% (73-80%)	0.63 (0.51-0.76)	1.2% (0.71-1.9%)	2.4% (1.1-4%)	560 (280-920)	18000 (11000-26000)
Laos	2010	Female	15 to 19	23% (15-31%)	77% (69-85%)	0.71 (0.42-1.1)	1.7% (0.98-2.8%)	2.7% (1.4-4.5%)	13 (6.5-21)	1100 (630-1800)
Laos	2010	Female	20 to 24	30% (20-40%)	70% (60-80%)	0.98 (0.59-1.5)	2.4% (1.4-3.9%)	3.2% (1.5-5.5%)	18 (8.1-31)	1600 (860-2500)

Location	Year	Sex	Age	Prevalence of current drinkers	Prevalence of abstainers	Population average of standard drinks daily	Percent of total attributable deaths	Percent of total attributable DALYs	Attributable deaths	Attributable DALYs
Laos	2010	Female	25 to 29	31% (21-41%)	69% (59-79%)	0.98 (0.62-1.5)	2.5% (1.5-4%)	3.4% (1.7-5.8%)	17 (8.2-28)	1300 (750-2100)
Laos	2010	Female	30 to 34	32% (22-44%)	68% (56-78%)	1 (0.61-1.6)	2.4% (1.4-3.8%)	3.4% (1.7-5.6%)	16 (8.2-27)	1100 (670-1800)
Laos	2010	Female	35 to 39	34% (23-45%)	66% (55-77%)	1.1 (0.67-1.8)	3% (1.6-4.7%)	4.4% (2.3-7%)	23 (12-38)	1400 (740-2100)
Laos	2010	Female	40 to 44	33% (24-44%)	67% (56-76%)	1.2 (0.71-1.8)	3.1% (1.6-5.1%)	4.7% (2.4-7.6%)	27 (14-44)	1400 (740-2200)
Laos	2010	Female	45 to 49	32% (21-42%)	68% (58-79%)	1.1 (0.65-1.9)	3.1% (1.4-5.5%)	4.6% (2-8%)	32 (14-56)	1400 (610-2500)
Laos	2010	Female	50 to 54	32% (22-42%)	68% (58-78%)	1.1 (0.63-1.9)	3.2% (0.92-6.2%)	4.4% (1.3-8.4%)	37 (11-72)	1400 (420-2800)
Laos	2010	Female	55 to 59	32% (23-43%)	68% (57-77%)	1.2 (0.63-1.9)	2.9% (0.73-6.3%)	3.9% (0.92-8.4%)	41 (9.9-87)	1300 (340-2900)
Laos	2010	Female	60 to 64	30% (20-41%)	70% (59-80%)	1.1 (0.61-2)	2.7% (0.51-6.4%)	3.5% (0.67-8%)	44 (8.4-100)	1200 (230-2800)
Laos	2010	Female	65 to 69	27% (18-36%)	73% (64-82%)	1.1 (0.63-1.8)	2.9% (0.75-6%)	3.6% (0.93-7.4%)	53 (14-110)	1200 (320-2500)
Laos	2010	Female	70 to 74	25% (18-35%)	75% (65-82%)	1.1 (0.64-1.9)	3.2% (0.82-6.5%)	3.8% (1-7.9%)	70 (19-140)	1300 (330-2600)
Laos	2010	Female	75 to 79	26% (18-37%)	74% (63-82%)	1.2 (0.65-2)	3.1% (0.59-6.4%)	3.6% (0.67-7.8%)	66 (12-140)	950 (180-2000)
Laos	2010	Female	80 to 84	27% (18-37%)	73% (63-82%)	1.2 (0.68-1.9)	3.2% (0.99-6.3%)	3.8% (1.2-7.5%)	56 (17-110)	590 (180-1200)
Laos	2010	Female	85 to 89	28% (19-38%)	72% (62-81%)	1.2 (0.68-1.9)	2.9% (0.78-5.8%)	3.4% (0.89-6.8%)	32 (8.4-64)	240 (65-480)
Laos	2010	Female	90 to 94	30% (20-40%)	70% (60-80%)	1.2 (0.67-2.1)	2.5% (0.38-5.7%)	3% (0.39-6.7%)	10 (1.4-23)	53 (8.3-120)
Laos	2010	Female	95 plus	32% (20-45%)	68% (55-80%)	1.3 (0.58-2.4)	2.7% (-0.077-6.6%)	3% (-0.18-7.5%)	2.4 (-0.15-6.2)	8.5 (-0.24-21)
Laos	2010	Male	All Ages	48% (41-54%)	52% (46-59%)	1.4 (1.2-1.7)	3.6% (2.3-4.9%)	5.2% (3.2-7.1%)	1400 (940-1900)	61000 (42000-78000)
Laos	2010	Male	15 to 19	44% (32-56%)	56% (44-68%)	0.93 (0.57-1.3)	3.3% (2-5.1%)	4.7% (2.6-7.2%)	30 (17-47)	2500 (1500-3700)
Laos	2010	Male	20 to 24	64% (51-74%)	36% (26-49%)	2.2 (1.5-3)	8.8% (5.7-12%)	11% (7-16%)	96 (59-140)	7000 (4500-9700)
Laos	2010	Male	25 to 29	70% (58-79%)	30% (21-42%)	2.7 (1.9-3.6)	11% (6.1-15%)	14% (7.2-20%)	110 (56-150)	7300 (4000-10000)
Laos	2010	Male	30 to 34	73% (62-81%)	27% (19-38%)	3.1 (2.2-4)	12% (6.8-16%)	15% (8.3-21%)	110 (61-160)	6800 (3900-9400)
Laos	2010	Male	35 to 39	75% (64-82%)	25% (18-36%)	3.2 (2.3-4.3)	12% (7.8-17%)	16% (9.5-22%)	120 (72-170)	6600 (4100-9300)
Laos	2010	Male	40 to 44	74% (64-81%)	26% (19-36%)	3.1 (2.2-4.3)	12% (7.8-17%)	15% (9.6-21%)	120 (78-180)	6100 (4000-8600)

Location	Year	Sex	Age	Prevalence of current drinkers	Prevalence of abstainers	Population average of standard drinks daily	Percent of total attributable deaths	Percent of total attributable DALYs	Attributable deaths	Attributable DALYs
Laos	2010	Male	45 to 49	70% (57-79%)	30% (21-43%)	3 (2.1-4.2)	12% (7.5-17%)	15% (9.3-21%)	150 (92-210)	6300 (4100-9000)
Laos	2010	Male	50 to 54	68% (57-76%)	32% (24-43%)	2.8 (1.8-3.9)	10% (6.1-15%)	13% (7.4-19%)	150 (87-220)	5600 (3300-8300)
Laos	2010	Male	55 to 59	66% (53-76%)	34% (24-47%)	2.4 (1.5-3.4)	8% (4.1-12%)	9.6% (4.9-15%)	130 (66-200)	4300 (2200-6700)
Laos	2010	Male	60 to 64	61% (47-73%)	39% (27-53%)	2 (1.3-3.1)	6.3% (2.9-11%)	7.4% (3.4-13%)	100 (48-180)	2900 (1400-5100)
Laos	2010	Male	65 to 69	54% (41-66%)	46% (34-59%)	1.7 (1.1-2.5)	5.2% (2.5-8.6%)	6.1% (2.9-10%)	95 (45-160)	2200 (1100-3700)
Laos	2010	Male	70 to 74	50% (37-62%)	50% (38-63%)	1.5 (0.97-2.4)	4.6% (1.9-7.7%)	5.3% (2.2-8.9%)	93 (37-160)	1700 (730-2900)
Laos	2010	Male	75 to 79	50% (35-65%)	50% (35-65%)	1.4 (0.83-2.2)	3.3% (0.9-6.2%)	3.8% (0.92-7.2%)	61 (15-120)	890 (230-1700)
Laos	2010	Male	80 to 84	47% (31-62%)	53% (38-69%)	1.2 (0.71-1.8)	2.7% (0.67-5.1%)	3% (0.68-6%)	33 (7.4-65)	360 (90-690)
Laos	2010	Male	85 to 89	43% (29-57%)	57% (43-71%)	0.97 (0.56-1.5)	1.6% (0.15-3.4%)	1.8% (0.026-3.7%)	11 (0.17-24)	89 (8.6-190)
Laos	2010	Male	90 to 94	42% (28-57%)	58% (43-72%)	0.85 (0.47-1.4)	0.89% (-0.34-2.4%)	0.93% (-0.56-2.7%)	2.2 (-1.3-6.5)	13 (-4.8-35)
Laos	2010	Male	95 plus	43% (26-61%)	57% (39-74%)	0.76 (0.34-1.4)	0.59% (-0.66-2.4%)	0.49% (-1-2.6%)	0.27 (-0.55-1.4)	1.3 (-1.4-5.2)
Laos	2016	Female	All Ages	23% (20-27%)	77% (73-80%)	0.66 (0.45-0.92)	1.4% (0.59-2.4%)	2.6% (0.92-4.9%)	560 (200-1000)	18000 (8300-29000)
Laos	2016	Female	15 to 19	22% (15-30%)	78% (70-85%)	0.73 (0.36-1.2)	1.7% (0.88-2.7%)	2.8% (1.3-4.8%)	9 (3.9-16)	850 (450-1400)
Laos	2016	Female	20 to 24	29% (19-39%)	71% (61-81%)	1 (0.52-1.6)	2.3% (1.3-3.8%)	3.3% (1.4-5.8%)	15 (6.3-27)	1400 (770-2300)
Laos	2016	Female	25 to 29	30% (21-41%)	70% (59-79%)	1 (0.58-1.6)	2.3% (1.3-3.7%)	3.3% (1.5-5.8%)	15 (6.7-26)	1300 (710-2100)
Laos	2016	Female	30 to 34	31% (21-43%)	69% (57-79%)	1 (0.58-1.7)	2.3% (1.2-3.6%)	3.3% (1.5-5.8%)	14 (6.3-25)	1100 (580-1800)
Laos	2016	Female	35 to 39	33% (22-44%)	67% (56-78%)	1.2 (0.63-1.9)	2.7% (1.4-4.5%)	4.3% (1.9-7.2%)	20 (9-35)	1300 (630-2100)
Laos	2016	Female	40 to 44	33% (23-43%)	67% (57-77%)	1.2 (0.64-2)	3% (1.4-5.1%)	4.7% (2.1-8%)	26 (12-45)	1400 (640-2400)
Laos	2016	Female	45 to 49	31% (21-42%)	69% (58-79%)	1.2 (0.64-2)	3% (1.2-5.4%)	4.5% (1.8-8.4%)	31 (12-58)	1400 (570-2600)
Laos	2016	Female	50 to 54	31% (22-42%)	69% (58-78%)	1.2 (0.61-2.1)	3% (0.85-6.1%)	4.4% (1.2-8.8%)	39 (11-77)	1500 (410-3000)
Laos	2016	Female	55 to 59	32% (23-43%)	68% (57-77%)	1.2 (0.62-2.2)	2.9% (0.5-6.5%)	4% (0.74-9%)	44 (8.4-97)	1500 (250-3300)
Laos	2016	Female	60 to 64	30% (20-41%)	70% (59-80%)	1.2 (0.61-2.2)	2.7% (0.35-6.6%)	3.5% (0.47-8.5%)	47 (6.6-110)	1300 (170-3200)

Location	Year	Sex	Age	Prevalence of current drinkers	Prevalence of abstainers	Population average of standard drinks daily	Percent of total attributable deaths	Percent of total attributable DALYs	Attributable deaths	Attributable DALYs
Laos	2016	Female	65 to 69	26% (18-35%)	74% (65-82%)	1.1 (0.6-2)	2.8% (0.54-6.2%)	3.6% (0.72-7.9%)	56 (11-120)	1300 (260-2800)
Laos	2016	Female	70 to 74	25% (18-35%)	75% (65-82%)	1.2 (0.62-2.1)	3.1% (0.47-6.6%)	3.8% (0.57-8%)	66 (10-140)	1200 (180-2700)
Laos	2016	Female	75 to 79	26% (17-37%)	74% (63-83%)	1.2 (0.62-2.1)	3% (0.23-6.7%)	3.6% (0.2-8.1%)	65 (3.6-150)	950 (71-2200)
Laos	2016	Female	80 to 84	27% (18-37%)	73% (63-82%)	1.2 (0.67-2)	3.2% (0.65-6.7%)	3.8% (0.74-8.1%)	56 (11-120)	600 (120-1300)
Laos	2016	Female	85 to 89	28% (18-38%)	72% (62-82%)	1.2 (0.65-2.1)	2.9% (0.48-6.5%)	3.4% (0.49-7.6%)	37 (5.1-82)	280 (46-610)
Laos	2016	Female	90 to 94	29% (20-40%)	71% (60-80%)	1.3 (0.67-2.2)	2.6% (0.17-6.5%)	3.1% (0.15-7.7%)	15 (0.74-38)	78 (5.3-190)
Laos	2016	Female	95 plus	32% (20-45%)	68% (55-80%)	1.4 (0.57-2.6)	2.8% (-0.14-7.2%)	3.1% (-0.28-8%)	3.9 (-0.34-10)	14 (-0.67-35)
Laos	2016	Male	All Ages	49% (42-55%)	51% (45-58%)	1.5 (1.1-2.1)	3.9% (2.2-5.9%)	5.4% (2.9-8.3%)	1400 (800-2100)	62000 (38000-87000)
Laos	2016	Male	15 to 19	43% (31-56%)	57% (44-69%)	0.96 (0.52-1.5)	3.3% (1.9-5.2%)	4.8% (2.5-7.8%)	26 (14-42)	2100 (1200-3300)
Laos	2016	Male	20 to 24	64% (50-74%)	36% (26-50%)	2.3 (1.4-3.4)	8.5% (5.3-12%)	11% (6.6-17%)	94 (54-140)	7100 (4400-10000)
Laos	2016	Male	25 to 29	70% (58-79%)	30% (21-42%)	2.8 (1.9-4.3)	10% (5.8-15%)	14% (6.8-20%)	110 (57-170)	7900 (4300-11000)
Laos	2016	Male	30 to 34	73% (63-81%)	27% (19-37%)	3.2 (2.1-4.7)	11% (5.1-16%)	14% (5.8-21%)	110 (43-160)	7000 (3200-10000)
Laos	2016	Male	35 to 39	75% (63-82%)	25% (18-37%)	3.3 (2.1-4.9)	11% (5.9-17%)	15% (6.9-22%)	120 (55-180)	6600 (3400-9600)
Laos	2016	Male	40 to 44	74% (63-81%)	26% (19-37%)	3.3 (2-4.8)	11% (6.3-17%)	14% (7.5-21%)	120 (63-180)	6000 (3300-9100)
Laos	2016	Male	45 to 49	70% (57-79%)	30% (21-43%)	3.2 (2-4.8)	11% (6.4-16%)	14% (7.8-21%)	140 (76-210)	6100 (3400-9200)
Laos	2016	Male	50 to 54	68% (56-77%)	32% (23-44%)	2.9 (1.7-4.5)	9.7% (5.2-15%)	12% (6.4-19%)	150 (77-230)	5700 (3000-8900)
Laos	2016	Male	55 to 59	66% (54-76%)	34% (24-46%)	2.5 (1.5-3.9)	7.7% (3.5-13%)	9.3% (4.1-16%)	130 (60-230)	4400 (2000-7700)
Laos	2016	Male	60 to 64	61% (47-73%)	39% (27-53%)	2.1 (1.2-3.6)	6% (2.4-12%)	7.2% (2.7-14%)	120 (46-230)	3400 (1300-6600)
Laos	2016	Male	65 to 69	53% (40-65%)	47% (35-60%)	1.8 (1-3)	4.9% (2-9.6%)	5.8% (2.3-11%)	98 (39-190)	2300 (950-4400)
Laos	2016	Male	70 to 74	49% (37-62%)	51% (38-63%)	1.6 (0.93-2.6)	4.2% (1.5-8%)	4.9% (1.7-9.3%)	84 (30-160)	1600 (570-3100)
Laos	2016	Male	75 to 79	50% (34-64%)	50% (36-66%)	1.5 (0.79-2.5)	3.1% (0.67-6.7%)	3.5% (0.69-7.8%)	58 (11-130)	860 (180-1900)
Laos	2016	Male	80 to 84	47% (31-61%)	53% (39-69%)	1.2 (0.67-2)	2.4% (0.44-5.3%)	2.7% (0.41-6.2%)	32 (5-73)	350 (66-780)

Location	Year	Sex	Age	Prevalence of current drinkers	Prevalence of abstainers	Population average of standard drinks daily	Percent of total attributable deaths	Percent of total attributable DALYs	Attributable deaths	Attributable DALYs
Laos	2016	Male	85 to 89	42% (28-57%)	58% (43-72%)	1 (0.53-1.6)	1.5% (-0.0076-3.6%)	1.6% (-0.15-4.2%)	12 (-1.2-31)	96 (-0.5-240)
Laos	2016	Male	90 to 94	41% (28-55%)	59% (45-72%)	0.88 (0.46-1.5)	0.8% (-0.48-2.6%)	0.8% (-0.76-2.9%)	2.5 (-2.3-9)	15 (-9-49)
Laos	2016	Male	95 plus	42% (26-60%)	58% (40-74%)	0.79 (0.34-1.5)	0.53% (-0.78-2.4%)	0.39% (-1.1-2.5%)	0.29 (-0.82-1.8)	1.5 (-2.3-6.8)
Malaysia	1990	Female	All Ages	2.5% (2.1-3.1%)	97% (97-98%)	0.46 (0.32-0.62)	1.4% (1.1-1.9%)	2.6% (1.8-3.5%)	910 (640-1300)	27000 (21000-35000)
Malaysia	1990	Female	15 to 19	2.4% (1.4-3.7%)	98% (96-99%)	0.42 (0.2-0.77)	0.93% (0.62-1.4%)	1.9% (1.1-3%)	8.8 (5.2-14)	950 (630-1400)
Malaysia	1990	Female	20 to 24	2.9% (1.6-4.4%)	97% (96-98%)	0.56 (0.27-1)	1.3% (0.9-1.9%)	2.2% (1.3-3.4%)	12 (7.1-19)	1400 (930-2000)
Malaysia	1990	Female	25 to 29	3.1% (1.8-4.9%)	97% (95-98%)	0.66 (0.33-1.1)	1.5% (1-2.1%)	2.5% (1.5-3.8%)	15 (9-24)	1600 (1100-2300)
Malaysia	1990	Female	30 to 34	3.3% (1.8-5.5%)	97% (95-98%)	0.72 (0.37-1.3)	1.6% (1.1-2.3%)	2.7% (1.6-4.2%)	19 (12-29)	1600 (1100-2400)
Malaysia	1990	Female	35 to 39	3.5% (1.9-5.4%)	96% (95-98%)	0.73 (0.35-1.2)	1.7% (1.1-2.4%)	3% (1.8-4.4%)	25 (15-37)	1800 (1100-2500)
Malaysia	1990	Female	40 to 44	3.6% (2.1-5.5%)	96% (95-98%)	0.81 (0.39-1.4)	1.9% (1.2-2.7%)	3.2% (2.1-4.7%)	32 (20-47)	1800 (1200-2600)
Malaysia	1990	Female	45 to 49	3.5% (2-5.4%)	96% (95-98%)	0.97 (0.44-1.7)	2.1% (1.4-3.1%)	3.5% (2.2-5.2%)	39 (25-58)	1900 (1200-2700)
Malaysia	1990	Female	50 to 54	3.6% (2.2-5.5%)	96% (94-98%)	1.1 (0.49-2)	2.5% (1.6-3.7%)	3.7% (2.3-5.5%)	63 (39-95)	2600 (1600-3800)
Malaysia	1990	Female	55 to 59	3.7% (2.2-5.7%)	96% (94-98%)	1.1 (0.52-2)	2.6% (1.6-4.1%)	3.6% (2.3-5.7%)	76 (47-120)	2600 (1600-4000)
Malaysia	1990	Female	60 to 64	3.6% (2.1-5.8%)	96% (94-98%)	1.1 (0.48-2)	2.5% (1.6-3.9%)	3.3% (2-5.1%)	97 (60-150)	2800 (1700-4400)
Malaysia	1990	Female	65 to 69	3.4% (1.9-5%)	97% (95-98%)	1 (0.46-1.8)	2.5% (1.5-3.8%)	3.1% (1.8-4.7%)	110 (65-170)	2600 (1500-3900)
Malaysia	1990	Female	70 to 74	3.2% (2-5%)	97% (95-98%)	0.99 (0.43-1.9)	2.4% (1.4-3.9%)	2.8% (1.7-4.7%)	130 (75-210)	2400 (1400-3900)
Malaysia	1990	Female	75 to 79	3.3% (1.9-5.2%)	97% (95-98%)	1 (0.41-2)	2.2% (1.2-3.6%)	2.6% (1.5-4.3%)	110 (60-180)	1600 (870-2500)
Malaysia	1990	Female	80 to 84	3.4% (1.8-5.1%)	97% (95-98%)	1 (0.42-1.9)	2.2% (1.2-3.6%)	2.6% (1.4-4.3%)	88 (45-140)	930 (480-1500)
Malaysia	1990	Female	85 to 89	3.5% (2-5.5%)	96% (94-98%)	1 (0.47-1.8)	2.2% (1.2-3.6%)	2.5% (1.4-4.3%)	58 (30-97)	430 (230-730)
Malaysia	1990	Female	90 to 94	3.8% (2.3-6%)	96% (94-98%)	1.1 (0.45-2.1)	2.1% (1.2-3.6%)	2.5% (1.3-4.3%)	23 (12-39)	120 (65-200)
Malaysia	1990	Female	95 plus	4.3% (2.5-7.1%)	96% (93-98%)	1.2 (0.45-2.6)	2.3% (1.2-3.9%)	2.7% (1.4-4.6%)	8.4 (4.4-15)	29 (15-49)
Malaysia	1990	Male	All Ages	7.9% (6.5-9.5%)	92% (90-94%)	1.8 (1.3-2.3)	4.9% (3.9-6%)	6.5% (5.1-8%)	3100 (2400-3800)	120000 (92000-140000)

Location	Year	Sex	Age	Prevalence of current drinkers	Prevalence of abstainers	Population average of standard drinks daily	Percent of total attributable deaths	Percent of total attributable DALYs	Attributable deaths	Attributable DALYs
Malaysia	1990	Male	15 to 19	7% (4.3-10%)	93% (90-96%)	0.87 (0.4-1.6)	3.5% (2.2-5.6%)	5.4% (3.1-8.8%)	61 (36-98)	5100 (3100-7900)
Malaysia	1990	Male	20 to 24	11% (6.5-16%)	89% (84-94%)	2.1 (1.1-3.6)	7% (4.3-10%)	9.5% (5.3-15%)	130 (74-200)	10000 (6400-15000)
Malaysia	1990	Male	25 to 29	12% (7.5-18%)	88% (82-93%)	2.8 (1.5-4.7)	7.7% (5.1-11%)	11% (6.8-16%)	150 (91-210)	11000 (7300-15000)
Malaysia	1990	Male	30 to 34	13% (7.8-20%)	87% (80-92%)	3.4 (1.9-5.5)	7.8% (5.3-11%)	11% (7.5-16%)	150 (100-220)	10000 (7200-14000)
Malaysia	1990	Male	35 to 39	13% (8.2-19%)	87% (81-92%)	4 (2.1-6.5)	8% (5.7-11%)	12% (8.2-16%)	170 (110-230)	9900 (7100-13000)
Malaysia	1990	Male	40 to 44	13% (8.4-19%)	87% (81-92%)	4.1 (2.1-6.6)	8.3% (5.9-11%)	12% (8.2-15%)	190 (130-250)	10000 (7100-13000)
Malaysia	1990	Male	45 to 49	13% (7.6-19%)	87% (81-92%)	3.7 (1.9-6.4)	8.4% (6-11%)	11% (8.1-15%)	220 (160-290)	9900 (7100-13000)
Malaysia	1990	Male	50 to 54	13% (8-18%)	87% (82-92%)	3.7 (1.8-6.4)	8% (5.7-11%)	10% (7.2-14%)	290 (210-390)	11000 (8100-15000)
Malaysia	1990	Male	55 to 59	12% (7.6-18%)	88% (82-92%)	3.9 (1.9-6.8)	7.7% (5.3-11%)	9.3% (6.4-13%)	310 (210-440)	10000 (7200-14000)
Malaysia	1990	Male	60 to 64	11% (7-17%)	89% (83-93%)	3.4 (1.6-6.2)	6.8% (4.6-9.5%)	8% (5.4-11%)	340 (230-480)	9700 (6600-13000)
Malaysia	1990	Male	65 to 69	9.6% (6.1-14%)	90% (86-94%)	2.6 (1.2-4.3)	5.9% (3.9-8.4%)	6.8% (4.5-9.8%)	310 (210-440)	7200 (4800-10000)
Malaysia	1990	Male	70 to 74	8.2% (5.3-12%)	92% (88-95%)	1.9 (0.9-3.4)	4.9% (3.3-7.1%)	5.7% (3.7-8.1%)	290 (190-420)	5500 (3600-7900)
Malaysia	1990	Male	75 to 79	7.4% (4.6-12%)	93% (88-95%)	1.6 (0.67-3.1)	4% (2.6-6%)	4.6% (2.9-6.9%)	210 (130-320)	3000 (1900-4600)
Malaysia	1990	Male	80 to 84	6.7% (3.9-9.9%)	93% (90-96%)	1.3 (0.62-2.5)	3.6% (2.3-5.3%)	4.2% (2.6-6.2%)	140 (86-210)	1500 (930-2200)
Malaysia	1990	Male	85 to 89	6.4% (3.8-9.9%)	94% (90-96%)	1.2 (0.55-2.2)	3.2% (1.9-4.8%)	3.7% (2.2-5.6%)	79 (47-120)	600 (370-900)
Malaysia	1990	Male	90 to 94	6.8% (4.1-11%)	93% (89-96%)	1.2 (0.52-2.3)	2.9% (1.8-4.3%)	3.4% (2-5.1%)	27 (16-41)	140 (85-210)
Malaysia	1990	Male	95 plus	7.9% (4.3-13%)	92% (87-96%)	1.3 (0.49-2.7)	3.1% (1.7-4.9%)	3.4% (1.9-5.6%)	7.2 (3.9-12)	26 (14-42)
Malaysia	1995	Female	All Ages	2.6% (2.1-3.1%)	97% (97-98%)	0.38 (0.26-0.5)	1.4% (1.1-1.8%)	2.5% (1.8-3.5%)	980 (700-1400)	29000 (22000-37000)
Malaysia	1995	Female	15 to 19	2.4% (1.4-3.6%)	98% (96-99%)	0.34 (0.16-0.61)	0.87% (0.59-1.3%)	1.8% (1-2.9%)	8.4 (4.9-13)	950 (620-1400)
Malaysia	1995	Female	20 to 24	2.9% (1.6-4.5%)	97% (96-98%)	0.45 (0.21-0.81)	1.3% (0.84-1.8%)	2.1% (1.3-3.2%)	11 (6.6-17)	1400 (920-2000)
Malaysia	1995	Female	25 to 29	3.2% (1.8-4.9%)	97% (95-98%)	0.53 (0.27-0.91)	1.4% (0.97-2%)	2.4% (1.4-3.6%)	14 (8.2-21)	1500 (1000-2200)
Malaysia	1995	Female	30 to 34	3.3% (1.8-5.5%)	97% (94-98%)	0.57 (0.3-1)	1.5% (1-2.1%)	2.5% (1.6-3.9%)	18 (11-28)	1700 (1200-2500)

Location	Year	Sex	Age	Prevalence of current drinkers	Prevalence of abstainers	Population average of standard drinks daily	Percent of total attributable deaths	Percent of total attributable DALYs	Attributable deaths	Attributable DALYs
Malaysia	1995	Female	35 to 39	3.5% (1.8-5.4%)	96% (95-98%)	0.57 (0.28-0.98)	1.5% (0.98-2.1%)	2.7% (1.7-4.1%)	24 (15-36)	1800 (1200-2500)
Malaysia	1995	Female	40 to 44	3.5% (2.1-5.5%)	96% (95-98%)	0.63 (0.31-1.1)	1.6% (1.1-2.4%)	2.9% (1.9-4.2%)	32 (20-47)	2000 (1300-2800)
Malaysia	1995	Female	45 to 49	3.5% (2-5.3%)	96% (95-98%)	0.76 (0.35-1.3)	1.9% (1.2-2.8%)	3.1% (2-4.7%)	44 (28-67)	2200 (1400-3300)
Malaysia	1995	Female	50 to 54	3.6% (2.2-5.4%)	96% (95-98%)	0.86 (0.41-1.6)	2.3% (1.4-3.3%)	3.4% (2.1-5%)	57 (36-85)	2400 (1500-3400)
Malaysia	1995	Female	55 to 59	3.7% (2.2-5.6%)	96% (94-98%)	0.87 (0.41-1.6)	2.4% (1.5-3.6%)	3.4% (2.1-5.1%)	81 (51-120)	2800 (1800-4300)
Malaysia	1995	Female	60 to 64	3.6% (2.1-5.7%)	96% (94-98%)	0.84 (0.39-1.6)	2.4% (1.5-3.7%)	3.1% (1.9-4.8%)	98 (60-150)	2800 (1800-4400)
Malaysia	1995	Female	65 to 69	3.4% (1.9-5%)	97% (95-98%)	0.8 (0.37-1.4)	2.3% (1.4-3.5%)	2.9% (1.7-4.5%)	120 (71-180)	2800 (1700-4200)
Malaysia	1995	Female	70 to 74	3.2% (2-5%)	97% (95-98%)	0.78 (0.34-1.5)	2.2% (1.3-3.7%)	2.7% (1.6-4.5%)	130 (78-220)	2500 (1500-4000)
Malaysia	1995	Female	75 to 79	3.3% (1.9-5.2%)	97% (95-98%)	0.8 (0.33-1.6)	2.1% (1.1-3.5%)	2.5% (1.4-4.2%)	120 (67-200)	1800 (970-2900)
Malaysia	1995	Female	80 to 84	3.4% (1.8-4.9%)	97% (95-98%)	0.79 (0.33-1.4)	2.2% (1.1-3.5%)	2.6% (1.3-4.1%)	100 (51-160)	1100 (540-1700)
Malaysia	1995	Female	85 to 89	3.5% (2-5.5%)	97% (94-98%)	0.79 (0.37-1.4)	2.1% (1.1-3.6%)	2.5% (1.3-4.2%)	72 (38-120)	530 (290-900)
Malaysia	1995	Female	90 to 94	3.8% (2.3-5.9%)	96% (94-98%)	0.84 (0.36-1.6)	2.1% (1.1-3.6%)	2.5% (1.3-4.2%)	32 (17-54)	160 (88-280)
Malaysia	1995	Female	95 plus	4.4% (2.5-6.9%)	96% (93-98%)	0.94 (0.36-2.1)	2.3% (1.2-3.9%)	2.7% (1.4-4.5%)	9 (4.6-15)	31 (16-51)
Malaysia	1995	Male	All Ages	8.1% (6.7-9.7%)	92% (90-93%)	1.5 (1.1-1.9)	4.9% (3.9-5.9%)	6.2% (4.9-7.6%)	3300 (2600-4000)	130000 (100000-150000)
Malaysia	1995	Male	15 to 19	6.9% (4.2-10%)	93% (90-96%)	0.68 (0.31-1.2)	3.3% (2-5.4%)	4.8% (2.7-7.9%)	68 (37-110)	5700 (3300-8800)
Malaysia	1995	Male	20 to 24	11% (6.3-16%)	89% (84-94%)	1.7 (0.86-2.8)	6.7% (4.2-10%)	8.8% (5.1-14%)	150 (85-230)	12000 (7300-17000)
Malaysia	1995	Male	25 to 29	12% (7.4-18%)	88% (82-93%)	2.2 (1.1-3.7)	7.2% (4.8-10%)	9.9% (6.2-15%)	150 (95-220)	11000 (7600-16000)
Malaysia	1995	Male	30 to 34	13% (7.8-20%)	87% (80-92%)	2.7 (1.5-4.5)	7.2% (4.9-9.8%)	10% (6.7-14%)	170 (110-240)	12000 (8000-16000)
Malaysia	1995	Male	35 to 39	13% (8.3-20%)	87% (80-92%)	3.2 (1.7-5.1)	7.5% (5.4-10%)	11% (7.4-14%)	190 (130-260)	12000 (8500-16000)
Malaysia	1995	Male	40 to 44	13% (8.5-19%)	87% (81-92%)	3.2 (1.7-5.3)	7.6% (5.5-10%)	11% (7.6-14%)	220 (160-290)	12000 (8500-15000)
Malaysia	1995	Male	45 to 49	13% (7.6-18%)	87% (82-92%)	2.9 (1.5-4.9)	7.7% (5.5-10%)	10% (7.4-14%)	250 (180-340)	12000 (8300-15000)
Malaysia	1995	Male	50 to 54	13% (8.2-18%)	87% (82-92%)	2.9 (1.5-4.9)	7.4% (5.3-10%)	9.5% (6.7-13%)	290 (200-390)	11000 (8100-15000)

Location	Year	Sex	Age	Prevalence of current drinkers	Prevalence of abstainers	Population average of standard drinks daily	Percent of total attributable deaths	Percent of total attributable DALYs	Attributable deaths	Attributable DALYs
Malaysia	1995	Male	55 to 59	12% (8.1-18%)	88% (82-92%)	3.1 (1.5-5.4)	7.1% (4.9-9.7%)	8.7% (6.1-12%)	350 (240-490)	12000 (8300-16000)
Malaysia	1995	Male	60 to 64	11% (7-17%)	89% (83-93%)	2.7 (1.3-4.8)	6.3% (4.3-8.9%)	7.5% (5.1-11%)	350 (240-490)	9900 (6800-14000)
Malaysia	1995	Male	65 to 69	9.7% (6-14%)	90% (86-94%)	2 (1-3.5)	5.4% (3.6-7.6%)	6.3% (4.2-8.9%)	340 (230-480)	7900 (5300-11000)
Malaysia	1995	Male	70 to 74	8.2% (5.4-12%)	92% (88-95%)	1.5 (0.7-2.6)	4.5% (3-6.4%)	5.1% (3.4-7.4%)	280 (190-410)	5300 (3500-7600)
Malaysia	1995	Male	75 to 79	7.3% (4.5-11%)	93% (89-96%)	1.2 (0.53-2.4)	3.6% (2.2-5.4%)	4.1% (2.5-6.2%)	200 (130-310)	3000 (1900-4500)
Malaysia	1995	Male	80 to 84	6.6% (3.9-9.8%)	93% (90-96%)	1 (0.47-1.9)	3.3% (2-4.9%)	3.7% (2.3-5.6%)	140 (87-220)	1600 (950-2300)
Malaysia	1995	Male	85 to 89	6.4% (3.7-9.9%)	94% (90-96%)	0.93 (0.44-1.7)	3% (1.8-4.6%)	3.4% (2-5.3%)	86 (50-130)	650 (390-1000)
Malaysia	1995	Male	90 to 94	6.9% (4.2-11%)	93% (89-96%)	0.95 (0.42-1.9)	2.8% (1.6-4.3%)	3.2% (1.8-5%)	35 (20-55)	180 (110-280)
Malaysia	1995	Male	95 plus	8% (4.4-13%)	92% (87-96%)	1 (0.38-2.3)	3% (1.5-4.9%)	3.3% (1.6-5.6%)	8.3 (4.1-14)	30 (15-50)
Malaysia	2000	Female	All Ages	2.3% (1.9-2.8%)	98% (97-98%)	0.23 (0.16-0.3)	1.3% (0.97-1.7%)	2.3% (1.6-3.1%)	970 (680-1300)	27000 (21000-35000)
Malaysia	2000	Female	15 to 19	2.3% (1.4-3.4%)	98% (97-99%)	0.22 (0.11-0.39)	0.73% (0.48-1.1%)	1.4% (0.84-2.3%)	6.8 (3.9-11)	860 (560-1200)
Malaysia	2000	Female	20 to 24	2.5% (1.5-3.9%)	97% (96-99%)	0.26 (0.13-0.48)	1.1% (0.72-1.5%)	1.6% (0.93-2.4%)	8.1 (4.8-13)	1200 (810-1800)
Malaysia	2000	Female	25 to 29	2.5% (1.4-3.9%)	97% (96-99%)	0.29 (0.14-0.5)	1.1% (0.77-1.5%)	1.6% (0.98-2.5%)	8.8 (5.2-13)	1300 (840-1800)
Malaysia	2000	Female	30 to 34	2.5% (1.4-4.1%)	98% (96-99%)	0.29 (0.15-0.51)	1.1% (0.74-1.6%)	1.7% (1.1-2.6%)	12 (7.2-18)	1300 (900-1900)
Malaysia	2000	Female	35 to 39	2.7% (1.4-4.1%)	97% (96-99%)	0.3 (0.14-0.51)	1.1% (0.74-1.5%)	1.9% (1.1-2.8%)	17 (10-26)	1500 (990-2100)
Malaysia	2000	Female	40 to 44	3% (1.8-4.5%)	97% (96-98%)	0.36 (0.18-0.6)	1.3% (0.85-1.8%)	2.2% (1.4-3.2%)	27 (17-41)	1800 (1200-2600)
Malaysia	2000	Female	45 to 49	2.9% (1.7-4.4%)	97% (96-98%)	0.43 (0.2-0.77)	1.5% (0.99-2.2%)	2.4% (1.5-3.7%)	40 (25-61)	2100 (1400-3200)
Malaysia	2000	Female	50 to 54	3% (1.9-4.6%)	97% (95-98%)	0.48 (0.23-0.85)	1.8% (1.2-2.6%)	2.7% (1.7-4%)	58 (37-85)	2500 (1600-3600)
Malaysia	2000	Female	55 to 59	3.5% (2.2-5.1%)	97% (95-98%)	0.54 (0.27-0.98)	2% (1.3-3%)	2.9% (1.8-4.3%)	71 (45-110)	2500 (1600-3700)
Malaysia	2000	Female	60 to 64	3.5% (2.1-5.3%)	97% (95-98%)	0.55 (0.27-0.98)	2.1% (1.3-3.2%)	2.7% (1.7-4.2%)	100 (62-150)	2900 (1900-4500)
Malaysia	2000	Female	65 to 69	3.2% (1.9-4.8%)	97% (95-98%)	0.52 (0.25-0.94)	2.1% (1.3-3.1%)	2.6% (1.6-4%)	120 (71-180)	2800 (1700-4300)
Malaysia	2000	Female	70 to 74	3.3% (2.1-5%)	97% (95-98%)	0.54 (0.25-0.98)	2% (1.2-3.4%)	2.5% (1.5-4.1%)	140 (81-230)	2600 (1500-4200)

Location	Year	Sex	Age	Prevalence of current drinkers	Prevalence of abstainers	Population average of standard drinks daily	Percent of total attributable deaths	Percent of total attributable DALYs	Attributable deaths	Attributable DALYs
Malaysia	2000	Female	75 to 79	3.5% (2.1-5.4%)	97% (95-98%)	0.57 (0.25-1.1)	2% (1.1-3.4%)	2.4% (1.3-4%)	140 (74-230)	2000 (1100-3200)
Malaysia	2000	Female	80 to 84	3.4% (1.9-5.1%)	97% (95-98%)	0.55 (0.24-0.98)	2% (1-3.3%)	2.4% (1.2-3.9%)	100 (53-170)	1100 (570-1800)
Malaysia	2000	Female	85 to 89	3.5% (2-5.4%)	97% (95-98%)	0.54 (0.25-0.96)	1.9% (1-3.2%)	2.3% (1.2-3.9%)	74 (39-130)	560 (300-950)
Malaysia	2000	Female	90 to 94	3.8% (2.3-6%)	96% (94-98%)	0.57 (0.25-1.1)	1.9% (0.96-3.3%)	2.2% (1.1-4%)	35 (18-62)	180 (92-320)
Malaysia	2000	Female	95 plus	4.3% (2.5-6.8%)	96% (93-98%)	0.63 (0.26-1.3)	2% (0.94-3.6%)	2.3% (1.1-4.2%)	11 (5.1-20)	39 (18-68)
Malaysia	2000	Male	All Ages	8.2% (7.1-9.5%)	92% (90-93%)	1 (0.74-1.3)	4.8% (3.9-5.8%)	6% (4.7-7.3%)	3500 (2800-4400)	140000 (110000-170000)
Malaysia	2000	Male	15 to 19	6.5% (4.1-9.6%)	94% (90-96%)	0.42 (0.2-0.76)	2.6% (1.5-4%)	3.7% (1.9-6.1%)	53 (27-87)	4700 (2700-7200)
Malaysia	2000	Male	20 to 24	10% (6.8-14%)	90% (86-93%)	1.1 (0.58-1.8)	5.6% (3.7-8.2%)	7.4% (4.4-12%)	120 (72-200)	10000 (6500-15000)
Malaysia	2000	Male	25 to 29	11% (7.8-15%)	89% (85-92%)	1.4 (0.78-2.3)	5.9% (4.2-8.4%)	8% (5.3-12%)	130 (88-200)	10000 (7200-14000)
Malaysia	2000	Male	30 to 34	12% (8.2-17%)	88% (83-92%)	1.7 (0.97-2.7)	6.1% (4.4-8.3%)	8.2% (5.7-11%)	160 (110-220)	11000 (8100-15000)
Malaysia	2000	Male	35 to 39	12% (8.1-17%)	88% (83-92%)	1.9 (1.1-3.1)	6.3% (4.7-8.6%)	8.7% (6.1-12%)	200 (140-270)	12000 (9100-17000)
Malaysia	2000	Male	40 to 44	13% (9.1-17%)	87% (83-91%)	2.1 (1.1-3.3)	6.7% (5-8.8%)	9.2% (6.8-12%)	250 (190-330)	13000 (10000-17000)
Malaysia	2000	Male	45 to 49	14% (9.5-18%)	86% (82-91%)	2.1 (1.2-3.4)	7.3% (5.5-9.2%)	9.8% (7.3-12%)	310 (230-390)	14000 (11000-18000)
Malaysia	2000	Male	50 to 54	14% (9.5-19%)	86% (81-90%)	2.1 (1.1-3.5)	7.2% (5.3-9.5%)	9.2% (6.7-12%)	350 (260-470)	14000 (10000-18000)
Malaysia	2000	Male	55 to 59	15% (10-20%)	85% (80-90%)	2.5 (1.3-4.2)	7.5% (5.2-9.9%)	9.2% (6.5-12%)	410 (280-540)	14000 (9800-18000)
Malaysia	2000	Male	60 to 64	14% (9.5-20%)	86% (80-91%)	2.3 (1.2-3.8)	6.7% (4.7-9%)	8% (5.6-11%)	470 (330-640)	13000 (9600-18000)
Malaysia	2000	Male	65 to 69	10% (6.9-14%)	90% (86-93%)	1.4 (0.78-2.4)	5.2% (3.5-7.1%)	6% (4.1-8.3%)	360 (250-500)	8500 (5900-12000)
Malaysia	2000	Male	70 to 74	8.4% (5.6-12%)	92% (88-94%)	1 (0.51-1.8)	3.9% (2.4-5.7%)	4.5% (2.8-6.5%)	290 (180-420)	5500 (3400-7900)
Malaysia	2000	Male	75 to 79	7.6% (4.9-11%)	92% (89-95%)	0.84 (0.39-1.6)	3.1% (1.7-4.8%)	3.5% (1.8-5.5%)	190 (100-300)	2900 (1600-4500)
Malaysia	2000	Male	80 to 84	6.7% (4-9.7%)	93% (90-96%)	0.7 (0.33-1.3)	2.7% (1.5-4.1%)	3.1% (1.6-4.8%)	120 (64-190)	1300 (720-2000)
Malaysia	2000	Male	85 to 89	6.3% (3.6-9.8%)	94% (90-96%)	0.61 (0.28-1.1)	2.3% (1.2-3.8%)	2.7% (1.3-4.4%)	78 (39-130)	610 (310-1000)
Malaysia	2000	Male	90 to 94	6.7% (4.1-11%)	93% (89-96%)	0.62 (0.27-1.2)	2.2% (0.9-3.7%)	2.5% (0.99-4.3%)	32 (13-57)	170 (73-300)

Location	Year	Sex	Age	Prevalence of current drinkers	Prevalence of abstainers	Population average of standard drinks daily	Percent of total attributable deaths	Percent of total attributable DALYs	Attributable deaths	Attributable DALYs
Malaysia	2000	Male	95 plus	7.8% (4.3-13%)	92% (87-96%)	0.67 (0.25-1.4)	2.3% (0.83-4.1%)	2.5% (0.82-4.7%)	8.9 (3-17)	32 (12-60)
Malaysia	2005	Female	All Ages	2.3% (1.9-2.8%)	98% (97-98%)	0.2 (0.14-0.27)	1.2% (0.89-1.6%)	2.1% (1.5-3%)	1000 (700-1500)	29000 (21000-37000)
Malaysia	2005	Female	15 to 19	2.2% (1.4-3.4%)	98% (97-99%)	0.18 (0.085-0.34)	0.63% (0.4-0.94%)	1.3% (0.73-2.2%)	6.1 (3.5-10)	810 (520-1200)
Malaysia	2005	Female	20 to 24	2.5% (1.5-3.8%)	97% (96-99%)	0.22 (0.11-0.4)	0.95% (0.64-1.4%)	1.4% (0.79-2.2%)	7.5 (4.3-12)	1200 (790-1800)
Malaysia	2005	Female	25 to 29	2.5% (1.4-3.9%)	98% (96-99%)	0.24 (0.12-0.42)	0.99% (0.69-1.4%)	1.4% (0.84-2.2%)	8.2 (4.8-13)	1300 (850-1800)
Malaysia	2005	Female	30 to 34	2.4% (1.3-4.1%)	98% (96-99%)	0.24 (0.12-0.43)	0.98% (0.66-1.4%)	1.5% (0.92-2.4%)	10 (6.3-16)	1300 (830-1800)
Malaysia	2005	Female	35 to 39	2.6% (1.4-4.2%)	97% (96-99%)	0.25 (0.11-0.45)	0.96% (0.64-1.4%)	1.6% (0.92-2.5%)	15 (8.6-23)	1400 (910-2000)
Malaysia	2005	Female	40 to 44	2.9% (1.8-4.3%)	97% (96-98%)	0.3 (0.15-0.5)	1.1% (0.73-1.6%)	1.9% (1.2-2.9%)	26 (15-39)	1700 (1100-2500)
Malaysia	2005	Female	45 to 49	2.9% (1.7-4.3%)	97% (96-98%)	0.36 (0.18-0.63)	1.4% (0.88-2%)	2.2% (1.4-3.4%)	43 (27-66)	2300 (1500-3500)
Malaysia	2005	Female	50 to 54	3% (1.9-4.6%)	97% (95-98%)	0.41 (0.19-0.72)	1.6% (1-2.4%)	2.5% (1.5-3.7%)	63 (39-96)	2700 (1700-4100)
Malaysia	2005	Female	55 to 59	3.4% (2.1-5%)	97% (95-98%)	0.46 (0.23-0.82)	1.8% (1.1-2.7%)	2.6% (1.6-3.9%)	82 (50-120)	2900 (1800-4400)
Malaysia	2005	Female	60 to 64	3.4% (2.1-5.2%)	97% (95-98%)	0.46 (0.22-0.83)	1.8% (1.1-2.8%)	2.4% (1.5-3.8%)	95 (56-150)	2900 (1700-4500)
Malaysia	2005	Female	65 to 69	3.2% (2-4.8%)	97% (95-98%)	0.44 (0.22-0.78)	1.9% (1.1-2.8%)	2.4% (1.4-3.6%)	120 (72-190)	2900 (1700-4400)
Malaysia	2005	Female	70 to 74	3.3% (2.1-5%)	97% (95-98%)	0.46 (0.21-0.83)	1.8% (1-3%)	2.3% (1.3-3.7%)	140 (78-230)	2700 (1500-4400)
Malaysia	2005	Female	75 to 79	3.5% (2.1-5.4%)	97% (95-98%)	0.49 (0.21-0.93)	1.9% (0.94-3.2%)	2.2% (1.1-3.8%)	140 (71-240)	2100 (1100-3500)
Malaysia	2005	Female	80 to 84	3.5% (1.9-5.2%)	97% (95-98%)	0.47 (0.21-0.84)	1.9% (0.94-3.1%)	2.2% (1.1-3.6%)	120 (60-200)	1300 (640-2100)
Malaysia	2005	Female	85 to 89	3.4% (2-5.4%)	97% (95-98%)	0.45 (0.21-0.82)	1.8% (0.93-3.2%)	2.1% (1-3.7%)	89 (44-150)	660 (340-1200)
Malaysia	2005	Female	90 to 94	3.8% (2.2-5.8%)	96% (94-98%)	0.48 (0.21-0.93)	1.9% (0.87-3.3%)	2.1% (0.97-3.9%)	49 (22-88)	250 (120-450)
Malaysia	2005	Female	95 plus	4.3% (2.4-7%)	96% (93-98%)	0.54 (0.21-1.1)	2% (0.76-3.7%)	2.2% (0.84-4.1%)	15 (5.5-28)	49 (19-91)
Malaysia	2005	Male	All Ages	8.3% (7.1-9.6%)	92% (90-93%)	0.89 (0.63-1.2)	4.4% (3.5-5.5%)	5.4% (4.1-6.8%)	3600 (2800-4600)	140000 (110000-170000)
Malaysia	2005	Male	15 to 19	6.3% (4-9.2%)	94% (91-96%)	0.35 (0.16-0.63)	2.2% (1.3-3.3%)	3.3% (1.7-5.4%)	47 (24-76)	4200 (2500-6300)
Malaysia	2005	Male	20 to 24	9.9% (6.6-14%)	90% (86-93%)	0.89 (0.47-1.5)	4.8% (3.1-7.3%)	6.5% (3.7-10%)	110 (64-190)	9700 (6200-15000)

Location	Year	Sex	Age	Prevalence of current drinkers	Prevalence of abstainers	Population average of standard drinks daily	Percent of total attributable deaths	Percent of total attributable DALYs	Attributable deaths	Attributable DALYs
Malaysia	2005	Male	25 to 29	11% (7.5-16%)	89% (84-92%)	1.2 (0.64-2)	5.1% (3.5-7.3%)	7% (4.5-10%)	120 (79-180)	9900 (6800-14000)
Malaysia	2005	Male	30 to 34	12% (8-17%)	88% (83-92%)	1.4 (0.81-2.2)	5.4% (3.8-7.4%)	7.3% (4.9-10%)	150 (99-210)	10000 (7500-14000)
Malaysia	2005	Male	35 to 39	12% (7.9-17%)	88% (83-92%)	1.6 (0.88-2.6)	5.8% (4.1-7.9%)	7.9% (5.3-11%)	190 (120-260)	11000 (8000-15000)
Malaysia	2005	Male	40 to 44	12% (8.7-17%)	88% (83-91%)	1.7 (0.91-2.8)	6.1% (4.4-8.2%)	8.4% (5.9-11%)	240 (170-330)	13000 (9400-17000)
Malaysia	2005	Male	45 to 49	14% (9.2-18%)	86% (82-91%)	1.8 (0.98-2.9)	6.6% (4.8-8.7%)	8.9% (6.3-11%)	330 (230-420)	15000 (11000-19000)
Malaysia	2005	Male	50 to 54	13% (9.1-18%)	87% (82-91%)	1.8 (0.92-2.8)	6.5% (4.5-8.7%)	8.4% (5.9-11%)	380 (260-510)	15000 (11000-20000)
Malaysia	2005	Male	55 to 59	15% (10-19%)	85% (81-90%)	2 (1.1-3.5)	6.9% (4.7-9.3%)	8.5% (5.8-12%)	460 (310-620)	16000 (11000-21000)
Malaysia	2005	Male	60 to 64	14% (9.1-19%)	86% (81-91%)	1.9 (0.95-3.1)	6.1% (4.1-8.2%)	7.4% (5-10%)	470 (320-650)	14000 (9000-19000)
Malaysia	2005	Male	65 to 69	10% (6.8-14%)	90% (86-93%)	1.2 (0.63-2)	4.7% (2.9-6.5%)	5.5% (3.4-7.7%)	400 (250-560)	9500 (6000-13000)
Malaysia	2005	Male	70 to 74	8.2% (5.4-12%)	92% (88-95%)	0.85 (0.42-1.5)	3.4% (2-5.1%)	4% (2.2-6%)	300 (160-450)	5700 (3200-8500)
Malaysia	2005	Male	75 to 79	7.4% (4.7-11%)	93% (89-95%)	0.7 (0.31-1.3)	2.6% (1.3-4.3%)	3% (1.4-5%)	210 (93-330)	3100 (1400-4900)
Malaysia	2005	Male	80 to 84	6.5% (4-9.5%)	93% (90-96%)	0.58 (0.27-1)	2.3% (1.1-3.6%)	2.6% (1.2-4.2%)	120 (56-190)	1300 (640-2100)
Malaysia	2005	Male	85 to 89	6.1% (3.5-9.6%)	94% (90-97%)	0.5 (0.24-0.91)	2% (0.85-3.4%)	2.2% (0.93-3.9%)	75 (31-130)	580 (250-1000)
Malaysia	2005	Male	90 to 94	6.5% (3.9-9.8%)	94% (90-96%)	0.5 (0.22-0.95)	1.8% (0.69-3.4%)	2% (0.73-3.9%)	38 (14-72)	200 (78-380)
Malaysia	2005	Male	95 plus	7.5% (4.1-12%)	93% (88-96%)	0.54 (0.2-1.1)	1.9% (0.63-3.8%)	2% (0.61-4.1%)	9.9 (3-20)	36 (12-70)
Malaysia	2010	Female	All Ages	2.7% (2.2-3.2%)	97% (97-98%)	0.28 (0.19-0.39)	1.3% (0.99-1.7%)	2.2% (1.5-3.2%)	1200 (840-1700)	34000 (26000-45000)
Malaysia	2010	Female	15 to 19	2.3% (1.4-3.6%)	98% (96-99%)	0.22 (0.095-0.41)	0.64% (0.41-0.97%)	1.4% (0.77-2.4%)	6.7 (3.7-11)	930 (590-1400)
Malaysia	2010	Female	20 to 24	2.8% (1.6-4.4%)	97% (96-98%)	0.29 (0.13-0.52)	1% (0.7-1.5%)	1.6% (0.93-2.6%)	9.3 (5.3-15)	1500 (970-2200)
Malaysia	2010	Female	25 to 29	3.1% (1.7-4.8%)	97% (95-98%)	0.34 (0.17-0.61)	1.1% (0.77-1.6%)	1.8% (1.1-2.8%)	12 (7-18)	1700 (1100-2500)
Malaysia	2010	Female	30 to 34	3.2% (1.8-5.4%)	97% (95-98%)	0.37 (0.19-0.65)	1.2% (0.81-1.6%)	2% (1.3-3.1%)	14 (8.5-21)	1600 (1000-2300)
Malaysia	2010	Female	35 to 39	3.4% (1.9-5.3%)	97% (95-98%)	0.37 (0.18-0.65)	1.2% (0.76-1.7%)	2.1% (1.3-3.2%)	19 (11-29)	1700 (1100-2400)
Malaysia	2010	Female	40 to 44	3.4% (2.1-5.3%)	97% (95-98%)	0.41 (0.2-0.71)	1.3% (0.84-1.9%)	2.3% (1.5-3.6%)	31 (19-48)	2100 (1400-3100)

Location	Year	Sex	Age	Prevalence of current drinkers	Prevalence of abstainers	Population average of standard drinks daily	Percent of total attributable deaths	Percent of total attributable DALYs	Attributable deaths	Attributable DALYs
Malaysia	2010	Female	45 to 49	3.4% (1.9-5.4%)	97% (95-98%)	0.49 (0.22-0.88)	1.5% (0.97-2.3%)	2.6% (1.6-4.1%)	54 (33-85)	2900 (1800-4400)
Malaysia	2010	Female	50 to 54	3.5% (2.1-5.5%)	96% (95-98%)	0.55 (0.25-0.99)	1.8% (1.1-2.7%)	2.8% (1.7-4.2%)	82 (51-120)	3600 (2300-5400)
Malaysia	2010	Female	55 to 59	3.7% (2.2-5.6%)	96% (94-98%)	0.56 (0.26-1)	1.9% (1.2-2.9%)	2.8% (1.7-4.4%)	100 (63-160)	3800 (2400-5700)
Malaysia	2010	Female	60 to 64	3.5% (2-5.6%)	96% (94-98%)	0.54 (0.25-1)	1.8% (1.1-2.9%)	2.6% (1.5-4%)	110 (70-180)	3500 (2100-5500)
Malaysia	2010	Female	65 to 69	3.3% (1.9-5%)	97% (95-98%)	0.52 (0.23-0.93)	1.9% (1.1-2.9%)	2.5% (1.4-3.8%)	130 (74-200)	3100 (1800-4800)
Malaysia	2010	Female	70 to 74	3.2% (2-4.8%)	97% (95-98%)	0.51 (0.22-0.97)	1.8% (1-3.1%)	2.3% (1.3-3.9%)	160 (91-280)	3100 (1700-5200)
Malaysia	2010	Female	75 to 79	3.2% (1.8-5.1%)	97% (95-98%)	0.51 (0.21-1)	1.8% (0.93-3.1%)	2.1% (1.1-3.6%)	150 (76-250)	2200 (1100-3700)
Malaysia	2010	Female	80 to 84	3.3% (1.8-4.9%)	97% (95-98%)	0.51 (0.22-0.97)	1.9% (0.95-3%)	2.2% (1.1-3.6%)	140 (68-220)	1400 (730-2400)
Malaysia	2010	Female	85 to 89	3.5% (2-5.6%)	97% (94-98%)	0.53 (0.25-0.98)	1.9% (1-3.4%)	2.2% (1.2-3.9%)	110 (57-200)	840 (440-1400)
Malaysia	2010	Female	90 to 94	3.8% (2.3-6.1%)	96% (94-98%)	0.56 (0.24-1.1)	2% (1-3.5%)	2.3% (1.1-4%)	52 (26-93)	270 (140-470)
Malaysia	2010	Female	95 plus	4.3% (2.5-6.8%)	96% (93-98%)	0.62 (0.24-1.3)	2.1% (0.92-3.7%)	2.3% (1-4.2%)	13 (5.6-23)	44 (19-78)
Malaysia	2010	Male	All Ages	8.6% (7.2-10%)	91% (90-93%)	1 (0.72-1.4)	4.4% (3.5-5.4%)	5.3% (4-6.5%)	4100 (3100-5100)	150000 (120000-190000)
Malaysia	2010	Male	15 to 19	6.7% (4.1-9.8%)	93% (90-96%)	0.42 (0.19-0.78)	2.4% (1.4-3.7%)	3.7% (1.9-6.2%)	52 (27-87)	4600 (2700-7200)
Malaysia	2010	Male	20 to 24	10% (6.3-15%)	90% (85-94%)	1.1 (0.53-1.8)	5.1% (3.3-7.7%)	7.2% (4-12%)	130 (74-220)	11000 (7200-17000)
Malaysia	2010	Male	25 to 29	12% (7.2-17%)	88% (83-93%)	1.4 (0.73-2.4)	5.6% (3.8-7.9%)	8% (5-12%)	150 (93-220)	12000 (8100-17000)
Malaysia	2010	Male	30 to 34	13% (7.7-19%)	87% (81-92%)	1.7 (0.94-2.8)	5.8% (4-8%)	8% (5.3-12%)	170 (110-240)	12000 (8400-16000)
Malaysia	2010	Male	35 to 39	13% (7.9-19%)	87% (81-92%)	2 (1-3.4)	6.2% (4.3-8.5%)	8.6% (5.7-12%)	210 (140-290)	13000 (8800-18000)
Malaysia	2010	Male	40 to 44	13% (8.1-19%)	87% (81-92%)	2 (1-3.4)	6.3% (4.4-8.4%)	8.7% (5.9-12%)	260 (180-350)	14000 (9900-19000)
Malaysia	2010	Male	45 to 49	13% (7.4-18%)	87% (82-93%)	1.9 (0.94-3.1)	6.4% (4.5-8.7%)	8.6% (5.9-11%)	350 (240-460)	16000 (11000-21000)
Malaysia	2010	Male	50 to 54	12% (7.7-18%)	88% (82-92%)	1.8 (0.91-3.1)	6.2% (4.2-8.5%)	8% (5.4-11%)	420 (290-590)	17000 (12000-23000)
Malaysia	2010	Male	55 to 59	12% (7.5-17%)	88% (83-92%)	1.9 (0.91-3.5)	6% (4-8.3%)	7.5% (4.9-10%)	470 (320-670)	16000 (11000-23000)
Malaysia	2010	Male	60 to 64	11% (6.8-16%)	89% (84-93%)	1.7 (0.79-3)	5.3% (3.4-7.4%)	6.4% (4.1-9%)	490 (320-690)	14000 (9200-20000)

Location	Year	Sex	Age	Prevalence of current drinkers	Prevalence of abstainers	Population average of standard drinks daily	Percent of total attributable deaths	Percent of total attributable DALYs	Attributable deaths	Attributable DALYs
Malaysia	2010	Male	65 to 69	9.3% (5.8-13%)	91% (87-94%)	1.3 (0.6-2.1)	4.5% (2.9-6.4%)	5.3% (3.4-7.6%)	420 (270-610)	10000 (6500-14000)
Malaysia	2010	Male	70 to 74	8% (5.1-12%)	92% (88-95%)	0.94 (0.44-1.7)	3.6% (2.1-5.2%)	4.2% (2.5-6.1%)	390 (230-580)	7500 (4500-11000)
Malaysia	2010	Male	75 to 79	7.1% (4.3-11%)	93% (89-96%)	0.76 (0.33-1.5)	2.7% (1.4-4.3%)	3.2% (1.5-5.2%)	250 (120-410)	3800 (1900-6100)
Malaysia	2010	Male	80 to 84	6.4% (3.7-9.3%)	94% (91-96%)	0.65 (0.3-1.3)	2.4% (1.2-3.7%)	2.8% (1.4-4.4%)	170 (82-260)	1900 (960-2900)
Malaysia	2010	Male	85 to 89	6.1% (3.4-9.5%)	94% (91-97%)	0.57 (0.26-1.1)	2.1% (0.99-3.4%)	2.4% (1.1-4%)	100 (47-170)	800 (380-1300)
Malaysia	2010	Male	90 to 94	6.4% (3.9-9.9%)	94% (90-96%)	0.57 (0.24-1.1)	2% (0.8-3.5%)	2.2% (0.84-4%)	45 (17-82)	240 (98-420)
Malaysia	2010	Male	95 plus	7.4% (4-12%)	93% (88-96%)	0.61 (0.23-1.3)	2% (0.75-3.8%)	2.2% (0.71-4.2%)	12 (3.9-23)	43 (16-82)
Malaysia	2016	Female	All Ages	2.7% (2.2-3.3%)	97% (97-98%)	0.36 (0.22-0.56)	1.3% (1-1.8%)	2.3% (1.6-3.1%)	1300 (930-1900)	39000 (29000-51000)
Malaysia	2016	Female	15 to 19	2.3% (1.4-3.5%)	98% (96-99%)	0.27 (0.11-0.51)	0.66% (0.42-0.98%)	1.5% (0.87-2.4%)	6.3 (3.6-10)	920 (590-1300)
Malaysia	2016	Female	20 to 24	2.8% (1.6-4.3%)	97% (96-98%)	0.35 (0.15-0.66)	1% (0.67-1.4%)	1.7% (1-2.6%)	9 (5.4-14)	1500 (1000-2200)
Malaysia	2016	Female	25 to 29	3.1% (1.7-4.8%)	97% (95-98%)	0.41 (0.18-0.76)	1.1% (0.78-1.6%)	1.9% (1.1-3%)	11 (6.7-18)	1800 (1200-2600)
Malaysia	2016	Female	30 to 34	3.2% (1.8-5.3%)	97% (95-98%)	0.44 (0.2-0.84)	1.1% (0.79-1.6%)	2.1% (1.3-3.2%)	17 (9.9-26)	2000 (1300-2900)
Malaysia	2016	Female	35 to 39	3.4% (1.8-5.2%)	97% (95-98%)	0.44 (0.19-0.85)	1.1% (0.76-1.6%)	2.2% (1.3-3.2%)	20 (12-31)	1900 (1200-2700)
Malaysia	2016	Female	40 to 44	3.4% (2-5.3%)	97% (95-98%)	0.49 (0.23-0.9)	1.3% (0.83-1.8%)	2.4% (1.5-3.6%)	30 (19-46)	2200 (1400-3100)
Malaysia	2016	Female	45 to 49	3.4% (1.9-5.3%)	97% (95-98%)	0.6 (0.24-1.2)	1.5% (0.94-2.3%)	2.6% (1.6-4%)	52 (31-81)	2900 (1800-4300)
Malaysia	2016	Female	50 to 54	3.5% (2.1-5.3%)	96% (95-98%)	0.67 (0.28-1.3)	1.7% (1.1-2.6%)	2.8% (1.8-4.2%)	85 (54-130)	3900 (2500-5700)
Malaysia	2016	Female	55 to 59	3.6% (2.2-5.7%)	96% (94-98%)	0.68 (0.29-1.4)	1.8% (1.1-2.8%)	2.8% (1.7-4.2%)	120 (74-190)	4400 (2800-6800)
Malaysia	2016	Female	60 to 64	3.5% (2-5.5%)	97% (94-98%)	0.65 (0.28-1.3)	1.8% (1.1-2.8%)	2.5% (1.5-4%)	130 (81-210)	4200 (2500-6600)
Malaysia	2016	Female	65 to 69	3.2% (1.9-4.9%)	97% (95-98%)	0.62 (0.26-1.2)	1.8% (1.1-2.8%)	2.4% (1.5-3.8%)	170 (100-270)	4300 (2600-6500)
Malaysia	2016	Female	70 to 74	3.1% (1.9-4.8%)	97% (95-98%)	0.61 (0.24-1.3)	1.8% (1-2.9%)	2.3% (1.3-3.7%)	180 (100-290)	3400 (1900-5500)
Malaysia	2016	Female	75 to 79	3.2% (1.9-5%)	97% (95-98%)	0.62 (0.24-1.3)	1.7% (0.92-2.9%)	2.1% (1.1-3.5%)	180 (94-310)	2700 (1500-4600)
Malaysia	2016	Female	80 to 84	3.3% (1.8-4.9%)	97% (95-98%)	0.62 (0.25-1.2)	1.8% (0.95-2.9%)	2.2% (1.1-3.5%)	150 (77-240)	1600 (860-2600)

Location	Year	Sex	Age	Prevalence of current drinkers	Prevalence of abstainers	Population average of standard drinks daily	Percent of total attributable deaths	Percent of total attributable DALYs	Attributable deaths	Attributable DALYs
Malaysia	2016	Female	85 to 89	3.5% (2-5.6%)	97% (94-98%)	0.64 (0.27-1.2)	1.8% (0.96-3.2%)	2.2% (1.1-3.7%)	110 (56-190)	840 (430-1400)
Malaysia	2016	Female	90 to 94	3.8% (2.3-6%)	96% (94-98%)	0.69 (0.28-1.4)	1.9% (0.99-3.2%)	2.2% (1.1-3.7%)	58 (30-100)	300 (160-510)
Malaysia	2016	Female	95 plus	4.4% (2.5-7%)	96% (93-98%)	0.77 (0.28-1.7)	2% (0.98-3.5%)	2.3% (1.1-4.1%)	17 (7.7-29)	57 (27-97)
Malaysia	2016	Male	All Ages	8.9% (7.3-11%)	91% (89-93%)	1.3 (0.82-2.1)	4.6% (3.6-5.7%)	5.3% (4.1-6.6%)	4800 (3700-6000)	180000 (140000-220000)
Malaysia	2016	Male	15 to 19	6.5% (4-9.7%)	93% (90-96%)	0.5 (0.21-0.98)	2.5% (1.5-3.9%)	4.1% (2.1-6.8%)	49 (25-81)	4400 (2700-7000)
Malaysia	2016	Male	20 to 24	10% (6.3-15%)	90% (85-94%)	1.3 (0.59-2.4)	5.3% (3.5-7.9%)	7.6% (4.3-12%)	140 (79-210)	11000 (7200-17000)
Malaysia	2016	Male	25 to 29	12% (7-17%)	88% (83-93%)	1.7 (0.81-3.1)	5.9% (4-8.2%)	8.6% (5.4-12%)	170 (110-250)	14000 (9500-19000)
Malaysia	2016	Male	30 to 34	12% (7.5-19%)	88% (81-92%)	2.1 (1-3.7)	6.1% (4.2-8.3%)	8.7% (5.7-12%)	220 (140-310)	15000 (11000-21000)
Malaysia	2016	Male	35 to 39	13% (7.8-19%)	87% (81-92%)	2.4 (1.1-4.5)	6.3% (4.3-8.6%)	9% (6-13%)	230 (150-330)	14000 (10000-20000)
Malaysia	2016	Male	40 to 44	13% (8-18%)	87% (82-92%)	2.5 (1.2-4.5)	6.3% (4.4-8.4%)	8.9% (6.1-12%)	280 (190-370)	15000 (11000-20000)
Malaysia	2016	Male	45 to 49	12% (7.3-18%)	88% (82-93%)	2.3 (1-4.2)	6.4% (4.4-8.6%)	8.7% (5.9-12%)	360 (250-490)	17000 (12000-23000)
Malaysia	2016	Male	50 to 54	12% (7.7-18%)	88% (82-92%)	2.2 (0.99-4.1)	6.1% (4.2-8.3%)	8% (5.6-11%)	450 (320-620)	18000 (13000-25000)
Malaysia	2016	Male	55 to 59	12% (7.3-17%)	88% (83-93%)	2.3 (1-4.4)	5.8% (4-8%)	7.3% (5.1-10%)	550 (380-760)	19000 (13000-26000)
Malaysia	2016	Male	60 to 64	11% (6.6-17%)	89% (83-93%)	2 (0.9-4.2)	5.2% (3.5-7.3%)	6.3% (4.2-9%)	570 (380-790)	16000 (11000-23000)
Malaysia	2016	Male	65 to 69	9.2% (5.7-13%)	91% (87-94%)	1.5 (0.7-2.9)	4.4% (2.9-6.3%)	5.3% (3.4-7.6%)	580 (380-820)	14000 (9100-20000)
Malaysia	2016	Male	70 to 74	7.9% (5.1-12%)	92% (88-95%)	1.1 (0.5-2.2)	3.6% (2.2-5.3%)	4.2% (2.6-6.2%)	430 (270-640)	8200 (5200-12000)
Malaysia	2016	Male	75 to 79	7% (4.3-11%)	93% (89-96%)	0.92 (0.37-1.9)	2.8% (1.5-4.4%)	3.3% (1.8-5.3%)	320 (170-520)	4800 (2700-7600)
Malaysia	2016	Male	80 to 84	6.2% (3.7-9.3%)	94% (91-96%)	0.77 (0.32-1.5)	2.5% (1.4-3.7%)	2.9% (1.5-4.4%)	240 (130-370)	2600 (1400-4100)
Malaysia	2016	Male	85 to 89	5.9% (3.3-9.3%)	94% (91-97%)	0.68 (0.27-1.3)	2.2% (1.1-3.6%)	2.5% (1.2-4.2%)	160 (77-260)	1200 (620-2000)
Malaysia	2016	Male	90 to 94	6.4% (3.9-10%)	94% (90-96%)	0.69 (0.28-1.5)	2.1% (0.94-3.5%)	2.4% (1-4%)	72 (31-120)	380 (170-640)
Malaysia	2016	Male	95 plus	7.4% (4.1-12%)	93% (88-96%)	0.74 (0.27-1.6)	2.2% (0.91-3.9%)	2.4% (0.89-4.4%)	18 (6.7-33)	65 (26-120)
Maldives	1990	Female	All Ages	14% (9.8-18%)	86% (82-90%)	0.1 (0.061-0.15)	0.14% (-0.024-0.31%)	0.027% (-0.34-0.45%)	0.17 (-2.2-3)	61 (-10-140)

Location	Year	Sex	Age	Prevalence of current drinkers	Prevalence of abstainers	Population average of standard drinks daily	Percent of total attributable deaths	Percent of total attributable DALYs	Attributable deaths	Attributable DALYs
Maldives	1990	Female	15 to 19	13% (6.7-23%)	87% (77-93%)	0.099 (0.034-0.21)	0.36% (0.2-0.58%)	0.27% (0.034-0.63%)	0.039 (0.005-0.091)	7.4 (3.9-12)
Maldives	1990	Female	20 to 24	16% (7.5-29%)	84% (71-93%)	0.13 (0.049-0.28)	0.54% (0.29-0.88%)	0.24% (-0.044-0.72%)	0.045 (-0.0081-0.13)	12 (6.4-20)
Maldives	1990	Female	25 to 29	18% (8.1-31%)	82% (69-92%)	0.16 (0.052-0.32)	0.49% (0.23-0.83%)	0.21% (-0.13-0.67%)	0.049 (-0.029-0.15)	11 (5.1-19)
Maldives	1990	Female	30 to 34	19% (8.5-35%)	81% (65-92%)	0.18 (0.062-0.37)	0.46% (0.16-0.8%)	0.25% (-0.16-0.74%)	0.053 (-0.033-0.15)	8.6 (3-15)
Maldives	1990	Female	35 to 39	21% (9.4-35%)	79% (65-91%)	0.19 (0.065-0.39)	0.47% (0.043-0.92%)	0.39% (-0.21-1.1%)	0.068 (-0.041-0.19)	6.5 (0.59-13)
Maldives	1990	Female	40 to 44	21% (10-35%)	79% (65-90%)	0.22 (0.074-0.47)	0.57% (0.13-1.1%)	0.69% (0.014-1.5%)	0.08 (0.0015-0.18)	5.5 (1.2-11)
Maldives	1990	Female	45 to 49	21% (9.8-35%)	79% (65-90%)	0.24 (0.079-0.53)	0.55% (0.016-1.3%)	0.71% (-0.094-1.9%)	0.14 (-0.02-0.39)	7.5 (0.21-18)
Maldives	1990	Female	50 to 54	21% (10-36%)	79% (64-90%)	0.27 (0.092-0.57)	0.43% (-0.26-1.3%)	0.55% (-0.41-1.7%)	0.17 (-0.13-0.55)	7.2 (-4.2-21)
Maldives	1990	Female	55 to 59	21% (11-37%)	79% (63-89%)	0.29 (0.1-0.63)	0.24% (-0.6-1.3%)	0.31% (-0.77-1.7%)	0.12 (-0.3-0.63)	3.9 (-9.7-21)
Maldives	1990	Female	60 to 64	21% (11-36%)	79% (64-89%)	0.29 (0.1-0.67)	-0.065% (-1-1%)	-0.087% (-1.2-1.3%)	-0.037 (-0.52-0.53)	-0.93 (-14-15)
Maldives	1990	Female	65 to 69	21% (10-34%)	79% (66-90%)	0.27 (0.094-0.55)	-0.27% (-1.2-0.64%)	-0.33% (-1.4-0.81%)	-0.15 (-0.68-0.38)	-3.4 (-15-8.5)
Maldives	1990	Female	70 to 74	20% (9.9-34%)	80% (66-90%)	0.25 (0.08-0.54)	-0.24% (-1.1-0.71%)	-0.29% (-1.2-0.86%)	-0.12 (-0.51-0.34)	-2.2 (-9.4-6.2)
Maldives	1990	Female	75 to 79	19% (9.2-34%)	81% (66-91%)	0.23 (0.071-0.58)	-0.34% (-1.1-0.48%)	-0.41% (-1.4-0.59%)	-0.11 (-0.38-0.16)	-1.6 (-5.4-2.2)
Maldives	1990	Female	80 to 84	19% (9.1-34%)	81% (66-91%)	0.24 (0.071-0.55)	-0.49% (-1.4-0.29%)	-0.58% (-1.7-0.33%)	-0.1 (-0.29-0.059)	-1 (-2.9-0.63)
Maldives	1990	Female	85 to 89	21% (11-36%)	79% (64-89%)	0.25 (0.077-0.6)	-0.59% (-1.5-0.14%)	-0.72% (-1.8-0.14%)	-0.054 (-0.14-0.01)	-0.38 (-1-0.092)
Maldives	1990	Female	90 to 94	24% (12-41%)	76% (59-88%)	0.29 (0.079-0.7)	-0.55% (-1.5-0.31%)	-0.69% (-1.8-0.31%)	-0.015 (-0.039-0.0067)	-0.07 (-0.19-0.04)
Maldives	1990	Female	95 plus	29% (13-49%)	71% (51-87%)	0.34 (0.069-0.91)	-0.59% (-1.9-0.45%)	-0.77% (-2.3-0.42%)	-0.0039 (-0.011-0.0022)	-0.012 (-0.037-0.009)
Maldives	1990	Male	All Ages	35% (29-42%)	65% (58-71%)	0.26 (0.17-0.38)	0.29% (-0.2-0.77%)	0.018% (-1-0.95%)	0.13 (-7.5-7.1)	140 (-94-360)
Maldives	1990	Male	15 to 19	34% (21-49%)	66% (51-79%)	0.16 (0.071-0.3)	0.83% (0.47-1.4%)	0.82% (0.23-1.7%)	0.095 (0.028-0.2)	14 (7.7-23)
Maldives	1990	Male	20 to 24	47% (31-63%)	53% (37-69%)	0.35 (0.18-0.59)	1.8% (0.95-3%)	1.7% (0.24-3.5%)	0.21 (0.03-0.44)	29 (14-47)
Maldives	1990	Male	25 to 29	51% (34-68%)	49% (32-66%)	0.47 (0.24-0.77)	2% (0.87-3.3%)	1.8% (-0.16-4.1%)	0.22 (-0.019-0.5)	27 (12-47)
Maldives	1990	Male	30 to 34	53% (35-70%)	47% (30-65%)	0.55 (0.29-0.91)	1.7% (0.37-3.3%)	1.5% (-0.71-4%)	0.21 (-0.1-0.56)	23 (4.5-43)

Location	Year	Sex	Age	Prevalence of current drinkers	Prevalence of abstainers	Population average of standard drinks daily	Percent of total attributable deaths	Percent of total attributable DALYs	Attributable deaths	Attributable DALYs
Maldives	1990	Male	35 to 39	55% (39-71%)	45% (29-61%)	0.62 (0.31-1)	1.7% (0.019-3.5%)	1.7% (-0.89-4.4%)	0.22 (-0.12-0.58)	18 (0.21-37)
Maldives	1990	Male	40 to 44	55% (40-73%)	45% (27-60%)	0.65 (0.32-1.1)	1.3% (-0.44-3.1%)	1.3% (-1.3-4%)	0.19 (-0.18-0.56)	13 (-4.4-31)
Maldives	1990	Male	45 to 49	54% (36-71%)	46% (29-64%)	0.65 (0.32-1.1)	1.1% (-0.71-2.9%)	1.1% (-1.6-3.7%)	0.23 (-0.32-0.79)	14 (-9.2-38)
Maldives	1990	Male	50 to 54	52% (34-70%)	48% (30-66%)	0.63 (0.31-1.1)	0.49% (-1.3-2.3%)	0.35% (-2.1-2.8%)	0.13 (-0.79-1)	9.5 (-25-43)
Maldives	1990	Male	55 to 59	51% (34-68%)	49% (32-66%)	0.58 (0.29-1)	0.33% (-1.4-2%)	0.22% (-2.1-2.4%)	0.1 (-1.1-1.1)	6.9 (-29-40)
Maldives	1990	Male	60 to 64	48% (31-67%)	52% (33-69%)	0.52 (0.25-0.95)	0.034% (-1.7-1.6%)	-0.1% (-2.3-1.9%)	-0.063 (-1.4-1.1)	0.71 (-36-33)
Maldives	1990	Male	65 to 69	45% (29-63%)	55% (37-71%)	0.45 (0.22-0.77)	-0.26% (-1.7-1.1%)	-0.44% (-2.3-1.3%)	-0.27 (-1.4-0.73)	-4.5 (-29-18)
Maldives	1990	Male	70 to 74	41% (26-59%)	59% (41-74%)	0.38 (0.18-0.69)	-0.49% (-1.8-0.57%)	-0.7% (-2.4-0.62%)	-0.39 (-1.4-0.36)	-6.1 (-23-7.3)
Maldives	1990	Male	75 to 79	38% (23-56%)	62% (44-77%)	0.33 (0.15-0.62)	-0.59% (-1.9-0.32%)	-0.81% (-2.4-0.32%)	-0.36 (-1.1-0.14)	-4.5 (-15-2.4)
Maldives	1990	Male	80 to 84	35% (20-53%)	65% (47-80%)	0.29 (0.13-0.53)	-0.65% (-1.9-0.24%)	-0.81% (-2.2-0.22%)	-0.25 (-0.69-0.069)	-2.4 (-6.8-0.85)
Maldives	1990	Male	85 to 89	34% (20-51%)	66% (49-80%)	0.26 (0.11-0.48)	-0.54% (-1.5-0.18%)	-0.68% (-1.8-0.17%)	-0.11 (-0.29-0.026)	-0.72 (-2-0.25)
Maldives	1990	Male	90 to 94	36% (22-51%)	64% (49-78%)	0.25 (0.11-0.48)	-0.46% (-1.2-0.14%)	-0.61% (-1.5-0.099%)	-0.034 (-0.086-0.0057)	-0.15 (-0.42-0.043)
Maldives	1990	Male	95 plus	39% (22-58%)	61% (42-78%)	0.25 (0.087-0.51)	-0.54% (-1.5-0.1%)	-0.74% (-1.9-0.012%)	-0.012 (-0.03-0.00019)	-0.033 (-0.093-0.0064)
Maldives	1995	Female	All Ages	13% (9.8-18%)	87% (82-90%)	0.15 (0.099-0.21)	0.29% (0.045-0.59%)	0.25% (-0.36-1%)	1.3 (-1.8-5.2)	100 (15-210)
Maldives	1995	Female	15 to 19	13% (6.6-23%)	87% (77-93%)	0.14 (0.049-0.3)	0.47% (0.27-0.74%)	0.46% (0.13-1.1%)	0.041 (0.012-0.09)	8.5 (4.8-14)
Maldives	1995	Female	20 to 24	16% (7.5-28%)	84% (72-92%)	0.19 (0.072-0.4)	0.77% (0.47-1.2%)	0.45% (0.062-1.2%)	0.053 (0.0073-0.14)	14 (8.3-22)
Maldives	1995	Female	25 to 29	17% (8.1-31%)	83% (69-92%)	0.22 (0.081-0.44)	0.74% (0.46-1.1%)	0.41% (0.027-0.98%)	0.056 (0.0035-0.14)	13 (7.7-21)
Maldives	1995	Female	30 to 34	19% (8.6-33%)	81% (67-91%)	0.25 (0.096-0.53)	0.67% (0.39-1%)	0.47% (0.019-1.2%)	0.068 (0.0028-0.17)	11 (6.2-18)
Maldives	1995	Female	35 to 39	20% (8.9-35%)	80% (65-91%)	0.28 (0.1-0.56)	0.66% (0.25-1.2%)	0.66% (-0.015-1.6%)	0.11 (-0.0026-0.27)	11 (4-20)
Maldives	1995	Female	40 to 44	21% (10-35%)	79% (65-90%)	0.31 (0.12-0.67)	0.77% (0.31-1.5%)	1.1% (0.27-2.4%)	0.12 (0.029-0.26)	7.9 (3.2-15)
Maldives	1995	Female	45 to 49	21% (10-34%)	79% (66-90%)	0.36 (0.12-0.74)	0.82% (0.21-1.8%)	1.2% (0.21-3%)	0.15 (0.026-0.36)	7.7 (1.9-17)
Maldives	1995	Female	50 to 54	21% (10-37%)	79% (63-90%)	0.39 (0.14-0.81)	0.78% (-0.1-2%)	1.1% (-0.17-2.8%)	0.29 (-0.048-0.77)	12 (-1.6-31)

Location	Year	Sex	Age	Prevalence of current drinkers	Prevalence of abstainers	Population average of standard drinks daily	Percent of total attributable deaths	Percent of total attributable DALYs	Attributable deaths	Attributable DALYs
Maldives	1995	Female	55 to 59	21% (11-37%)	79% (63-89%)	0.43 (0.16-0.88)	0.64% (-0.32-1.9%)	0.88% (-0.45-2.7%)	0.33 (-0.18-1)	11 (-5.9-34)
Maldives	1995	Female	60 to 64	21% (10-37%)	79% (63-90%)	0.43 (0.15-0.9)	0.32% (-0.84-1.8%)	0.4% (-1.1-2.3%)	0.2 (-0.55-1.1)	5.7 (-15-32)
Maldives	1995	Female	65 to 69	20% (9.9-34%)	80% (66-90%)	0.4 (0.14-0.79)	0.076% (-1-1.4%)	0.089% (-1.3-1.7%)	0.041 (-0.61-0.77)	1 (-14-18)
Maldives	1995	Female	70 to 74	20% (9.6-33%)	80% (67-90%)	0.36 (0.12-0.79)	0.067% (-0.89-1.5%)	0.081% (-1.1-1.8%)	0.037 (-0.51-0.79)	0.7 (-9.4-15)
Maldives	1995	Female	75 to 79	19% (8.9-32%)	81% (68-91%)	0.34 (0.1-0.81)	-0.11% (-1.1-1.1%)	-0.14% (-1.2-1.3%)	-0.05 (-0.45-0.44)	-0.64 (-6.1-6.4)
Maldives	1995	Female	80 to 84	19% (9.1-33%)	81% (67-91%)	0.34 (0.11-0.76)	-0.25% (-1.3-0.84%)	-0.32% (-1.5-0.91%)	-0.067 (-0.32-0.2)	-0.65 (-3.3-2.1)
Maldives	1995	Female	85 to 89	21% (9.8-36%)	79% (64-90%)	0.37 (0.11-0.82)	-0.3% (-1.2-0.73%)	-0.41% (-1.6-0.83%)	-0.035 (-0.13-0.07)	-0.23 (-0.95-0.57)
Maldives	1995	Female	90 to 94	24% (12-42%)	76% (58-88%)	0.42 (0.12-1)	-0.31% (-1.4-1%)	-0.42% (-1.6-1.1%)	-0.014 (-0.056-0.038)	-0.062 (-0.27-0.21)
Maldives	1995	Female	95 plus	28% (13-49%)	72% (51-87%)	0.49 (0.11-1.3)	-0.34% (-1.7-1.6%)	-0.48% (-2.2-1.7%)	-0.0033 (-0.014-0.012)	-0.0089 (-0.046-0.043)
Maldives	1995	Male	All Ages	34% (28-42%)	66% (58-72%)	0.36 (0.26-0.5)	0.52% (-0.1-1.1%)	0.18% (-1-1.4%)	1.2 (-7-9.4)	210 (-45-450)
Maldives	1995	Male	15 to 19	33% (21-49%)	67% (51-79%)	0.23 (0.11-0.41)	0.99% (0.57-1.6%)	1.1% (0.4-2.2%)	0.12 (0.041-0.24)	17 (9.6-28)
Maldives	1995	Male	20 to 24	46% (31-62%)	54% (38-69%)	0.5 (0.27-0.82)	2.3% (1.3-3.5%)	2.4% (0.8-4.5%)	0.28 (0.092-0.53)	35 (19-54)
Maldives	1995	Male	25 to 29	51% (33-68%)	49% (32-67%)	0.67 (0.37-1)	2.5% (1.4-3.8%)	2.8% (0.7-5.2%)	0.26 (0.068-0.5)	32 (17-49)
Maldives	1995	Male	30 to 34	53% (35-70%)	47% (30-65%)	0.8 (0.46-1.2)	2.3% (1.1-3.8%)	2.5% (0.31-5.1%)	0.28 (0.039-0.57)	30 (13-49)
Maldives	1995	Male	35 to 39	55% (39-72%)	45% (28-61%)	0.89 (0.48-1.4)	2.3% (0.81-3.9%)	2.7% (0.13-5.5%)	0.35 (0.017-0.72)	28 (9.2-48)
Maldives	1995	Male	40 to 44	55% (39-72%)	45% (28-61%)	0.94 (0.5-1.5)	2% (0.41-3.8%)	2.4% (-0.29-5.3%)	0.28 (-0.033-0.64)	19 (3.7-37)
Maldives	1995	Male	45 to 49	53% (36-70%)	47% (30-64%)	0.94 (0.48-1.6)	1.7% (-0.11-3.7%)	2.1% (-0.81-5%)	0.3 (-0.12-0.74)	17 (-0.95-36)
Maldives	1995	Male	50 to 54	52% (35-69%)	48% (31-65%)	0.91 (0.47-1.5)	1% (-0.89-3.1%)	1.1% (-1.7-3.9%)	0.33 (-0.5-1.2)	17 (-14-49)
Maldives	1995	Male	55 to 59	50% (34-67%)	50% (33-66%)	0.84 (0.43-1.4)	0.76% (-1.1-2.7%)	0.75% (-1.7-3.3%)	0.39 (-0.92-1.7)	17 (-24-61)
Maldives	1995	Male	60 to 64	48% (30-66%)	52% (34-70%)	0.75 (0.37-1.3)	0.39% (-1.3-2.5%)	0.32% (-1.9-2.9%)	0.24 (-1.4-2.1)	10 (-34-63)
Maldives	1995	Male	65 to 69	44% (29-62%)	56% (38-71%)	0.65 (0.34-1.1)	-0.039% (-1.5-1.5%)	-0.18% (-2.1-1.8%)	-0.13 (-1.5-1.3)	-0.74 (-32-31)
Maldives	1995	Male	70 to 74	41% (26-58%)	59% (42-74%)	0.56 (0.27-1)	-0.45% (-1.9-0.87%)	-0.66% (-2.4-0.93%)	-0.47 (-1.7-0.66)	-7 (-30-14)

Location	Year	Sex	Age	Prevalence of current drinkers	Prevalence of abstainers	Population average of standard drinks daily	Percent of total attributable deaths	Percent of total attributable DALYs	Attributable deaths	Attributable DALYs
Maldives	1995	Male	75 to 79	38% (23-56%)	62% (44-77%)	0.48 (0.21-0.89)	-0.64% (-2.1-0.42%)	-0.88% (-2.7-0.44%)	-0.49 (-1.5-0.24)	-6 (-19-4.1)
Maldives	1995	Male	80 to 84	35% (20-53%)	65% (47-80%)	0.42 (0.19-0.76)	-0.67% (-1.9-0.29%)	-0.87% (-2.4-0.28%)	-0.31 (-0.85-0.096)	-2.9 (-8.4-1.3)
Maldives	1995	Male	85 to 89	34% (19-51%)	66% (49-81%)	0.37 (0.17-0.7)	-0.54% (-1.5-0.19%)	-0.74% (-1.9-0.16%)	-0.13 (-0.35-0.027)	-0.87 (-2.5-0.29)
Maldives	1995	Male	90 to 94	35% (21-52%)	65% (48-79%)	0.36 (0.16-0.67)	-0.46% (-1.2-0.15%)	-0.67% (-1.7-0.098%)	-0.041 (-0.1-0.0059)	-0.18 (-0.47-0.057)
Maldives	1995	Male	95 plus	39% (22-59%)	61% (41-78%)	0.36 (0.13-0.75)	-0.58% (-1.5-0.12%)	-0.86% (-2--0.014%)	-0.013 (-0.031--0.00021)	-0.036 (-0.096-0.0073)
Maldives	2000	Female	All Ages	13% (9.7-18%)	87% (82-90%)	0.19 (0.13-0.27)	0.48% (0.15-0.87%)	0.53% (-0.29-1.6%)	2.4 (-1.3-7.1)	140 (44-260)
Maldives	2000	Female	15 to 19	13% (6.3-22%)	87% (78-94%)	0.18 (0.064-0.36)	0.53% (0.31-0.84%)	0.63% (0.21-1.4%)	0.041 (0.014-0.09)	11 (5.9-17)
Maldives	2000	Female	20 to 24	16% (7.1-28%)	84% (72-93%)	0.24 (0.087-0.5)	0.95% (0.6-1.4%)	0.68% (0.19-1.6%)	0.046 (0.012-0.11)	15 (9.2-24)
Maldives	2000	Female	25 to 29	17% (7.4-30%)	83% (70-93%)	0.28 (0.097-0.57)	0.95% (0.6-1.3%)	0.63% (0.18-1.3%)	0.046 (0.013-0.099)	14 (8.6-21)
Maldives	2000	Female	30 to 34	18% (8.1-33%)	82% (67-92%)	0.31 (0.12-0.66)	0.85% (0.54-1.3%)	0.72% (0.25-1.5%)	0.061 (0.02-0.13)	13 (7.5-20)
Maldives	2000	Female	35 to 39	20% (8.7-34%)	80% (66-91%)	0.34 (0.12-0.68)	0.82% (0.45-1.4%)	0.95% (0.24-2.1%)	0.12 (0.031-0.27)	13 (7-22)
Maldives	2000	Female	40 to 44	20% (9.9-33%)	80% (67-90%)	0.38 (0.14-0.79)	0.9% (0.44-1.6%)	1.4% (0.53-2.8%)	0.18 (0.065-0.35)	13 (6.1-22)
Maldives	2000	Female	45 to 49	20% (9.6-34%)	80% (66-90%)	0.43 (0.15-0.89)	0.97% (0.38-2%)	1.6% (0.49-3.6%)	0.18 (0.054-0.41)	9.9 (3.7-21)
Maldives	2000	Female	50 to 54	21% (10-36%)	79% (64-90%)	0.48 (0.18-0.99)	1.1% (0.098-2.6%)	1.5% (0.11-3.6%)	0.29 (0.021-0.71)	12 (1.1-28)
Maldives	2000	Female	55 to 59	21% (10-35%)	79% (65-90%)	0.53 (0.19-1.1)	0.96% (-0.049-2.5%)	1.4% (-0.1-3.7%)	0.39 (-0.027-1)	14 (-0.77-35)
Maldives	2000	Female	60 to 64	21% (9.9-35%)	79% (65-90%)	0.53 (0.18-1.1)	0.7% (-0.56-2.4%)	0.89% (-0.74-3.1%)	0.49 (-0.4-1.7)	14 (-11-49)
Maldives	2000	Female	65 to 69	20% (9.6-34%)	80% (66-90%)	0.5 (0.18-1)	0.42% (-0.77-2%)	0.52% (-0.97-2.4%)	0.28 (-0.51-1.3)	6.7 (-12-31)
Maldives	2000	Female	70 to 74	19% (9.5-32%)	81% (68-91%)	0.45 (0.16-1)	0.35% (-0.68-2%)	0.43% (-0.84-2.5%)	0.21 (-0.41-1.2)	4 (-7.6-22)
Maldives	2000	Female	75 to 79	19% (8.6-33%)	81% (67-91%)	0.43 (0.13-0.96)	0.13% (-0.92-1.6%)	0.14% (-1.1-1.9%)	0.062 (-0.51-0.87)	1 (-7-13)
Maldives	2000	Female	80 to 84	19% (9-34%)	81% (66-91%)	0.43 (0.13-0.94)	0.0018% (-1-1.4%)	-0.026% (-1.3-1.6%)	-0.0071 (-0.36-0.44)	0.01 (-3.6-4.8)
Maldives	2000	Female	85 to 89	20% (9.6-36%)	80% (64-90%)	0.45 (0.14-1)	-0.062% (-0.99-1.2%)	-0.13% (-1.3-1.4%)	-0.016 (-0.16-0.17)	-0.068 (-1.1-1.4)
Maldives	2000	Female	90 to 94	23% (11-41%)	77% (59-89%)	0.51 (0.14-1.2)	-0.036% (-1.2-1.7%)	-0.11% (-1.5-1.9%)	-0.0062 (-0.084-0.11)	-0.012 (-0.39-0.58)

Location	Year	Sex	Age	Prevalence of current drinkers	Prevalence of abstainers	Population average of standard drinks daily	Percent of total attributable deaths	Percent of total attributable DALYs	Attributable deaths	Attributable DALYs
Maldives	2000	Female	95 plus	28% (12-49%)	72% (51-88%)	0.61 (0.14-1.6)	-0.033% (-1.5-2.3%)	-0.15% (-1.9-2.5%)	-0.0018 (-0.023-0.03)	-0.0015 (-0.072-0.11)
Maldives	2000	Male	All Ages	35% (28-42%)	65% (58-72%)	0.47 (0.35-0.61)	0.77% (0.17-1.4%)	0.32% (-0.94-1.6%)	2 (-5.9-10)	260 (54-490)
Maldives	2000	Male	15 to 19	33% (20-49%)	67% (51-80%)	0.28 (0.13-0.5)	1.1% (0.64-1.7%)	1.4% (0.51-2.6%)	0.16 (0.06-0.31)	23 (13-37)
Maldives	2000	Male	20 to 24	46% (30-62%)	54% (38-70%)	0.62 (0.33-1)	2.6% (1.6-3.8%)	3% (1.2-5.1%)	0.32 (0.13-0.57)	41 (25-63)
Maldives	2000	Male	25 to 29	50% (33-67%)	50% (33-67%)	0.83 (0.45-1.3)	2.9% (1.9-4.1%)	3.6% (1.5-6.1%)	0.26 (0.11-0.45)	35 (21-51)
Maldives	2000	Male	30 to 34	53% (35-70%)	47% (30-65%)	0.99 (0.56-1.5)	2.7% (1.6-3.9%)	3.3% (1.1-5.8%)	0.27 (0.092-0.48)	33 (19-48)
Maldives	2000	Male	35 to 39	54% (38-71%)	46% (29-62%)	1.1 (0.59-1.7)	2.7% (1.4-4.1%)	3.5% (1.1-6.1%)	0.39 (0.12-0.69)	34 (17-52)
Maldives	2000	Male	40 to 44	54% (38-72%)	46% (28-62%)	1.2 (0.62-1.8)	2.3% (0.98-3.9%)	3.1% (0.66-5.7%)	0.41 (0.083-0.76)	29 (11-48)
Maldives	2000	Male	45 to 49	53% (35-71%)	47% (29-65%)	1.2 (0.61-1.9)	2.1% (0.46-3.9%)	2.7% (-0.0068-5.6%)	0.36 (-0.00094-0.76)	21 (4.4-39)
Maldives	2000	Male	50 to 54	51% (35-69%)	49% (31-65%)	1.1 (0.6-1.9)	1.4% (-0.58-3.4%)	1.5% (-1.3-4.4%)	0.32 (-0.27-0.93)	16 (-6.9-40)
Maldives	2000	Male	55 to 59	50% (33-67%)	50% (33-67%)	1 (0.55-1.7)	1.1% (-0.69-3.1%)	1.2% (-1.2-3.8%)	0.49 (-0.5-1.6)	20 (-12-58)
Maldives	2000	Male	60 to 64	47% (30-67%)	53% (33-70%)	0.93 (0.47-1.6)	0.7% (-0.99-2.8%)	0.68% (-1.5-3.3%)	0.46 (-1-2.2)	17 (-23-66)
Maldives	2000	Male	65 to 69	44% (28-62%)	56% (38-72%)	0.81 (0.41-1.4)	0.22% (-1.3-1.9%)	0.11% (-1.8-2.2%)	0.093 (-1.6-1.8)	5.5 (-31-45)
Maldives	2000	Male	70 to 74	40% (26-58%)	60% (42-74%)	0.68 (0.34-1.2)	-0.29% (-1.7-1.1%)	-0.5% (-2.3-1.2%)	-0.41 (-1.8-0.98)	-5.3 (-31-21)
Maldives	2000	Male	75 to 79	37% (22-55%)	63% (45-78%)	0.59 (0.26-1.1)	-0.55% (-1.9-0.64%)	-0.79% (-2.5-0.62%)	-0.51 (-1.7-0.39)	-6 (-22-7.3)
Maldives	2000	Male	80 to 84	35% (20-52%)	65% (48-80%)	0.51 (0.22-0.92)	-0.58% (-1.8-0.43%)	-0.8% (-2.2-0.41%)	-0.36 (-1-0.19)	-3.3 (-9.9-2.4)
Maldives	2000	Male	85 to 89	33% (19-50%)	67% (50-81%)	0.46 (0.22-0.85)	-0.45% (-1.4-0.29%)	-0.67% (-1.8-0.27%)	-0.15 (-0.4-0.06)	-0.92 (-2.8-0.6)
Maldives	2000	Male	90 to 94	35% (21-52%)	65% (48-79%)	0.44 (0.2-0.81)	-0.47% (-1.3-0.18%)	-0.68% (-1.7-0.14%)	-0.068 (-0.17-0.013)	-0.29 (-0.78-0.11)
Maldives	2000	Male	95 plus	38% (20-58%)	62% (42-80%)	0.45 (0.16-0.9)	-0.64% (-1.6-0.12%)	-0.95% (-2.2-0.002%)	-0.023 (-0.054-0.000047)	-0.064 (-0.16-0.012)
Maldives	2005	Female	All Ages	14% (10-18%)	86% (82-90%)	0.24 (0.16-0.33)	0.61% (0.28-1.1%)	0.73% (-0.17-2.1%)	3 (-0.69-8.7)	160 (72-300)
Maldives	2005	Female	15 to 19	12% (6.1-22%)	88% (78-94%)	0.2 (0.07-0.41)	0.55% (0.33-0.86%)	0.71% (0.27-1.5%)	0.037 (0.014-0.081)	11 (6.5-18)

Location	Year	Sex	Age	Prevalence of current drinkers	Prevalence of abstainers	Population average of standard drinks daily	Percent of total attributable deaths	Percent of total attributable DALYs	Attributable deaths	Attributable DALYs
Maldives	2005	Female	20 to 24	15% (7.1-26%)	85% (74-93%)	0.27 (0.1-0.55)	1% (0.66-1.6%)	0.8% (0.27-1.8%)	0.043 (0.014-0.097)	20 (12-31)
Maldives	2005	Female	25 to 29	16% (7.5-29%)	84% (71-92%)	0.31 (0.1-0.61)	1.1% (0.7-1.5%)	0.78% (0.28-1.6%)	0.036 (0.013-0.075)	16 (9.9-24)
Maldives	2005	Female	30 to 34	18% (7.9-31%)	82% (69-92%)	0.34 (0.12-0.71)	0.93% (0.59-1.4%)	0.88% (0.35-1.8%)	0.053 (0.021-0.11)	14 (8.1-21)
Maldives	2005	Female	35 to 39	19% (8.5-33%)	81% (67-91%)	0.38 (0.14-0.77)	0.88% (0.53-1.4%)	1.2% (0.45-2.4%)	0.094 (0.035-0.19)	13 (7.6-21)
Maldives	2005	Female	40 to 44	19% (9.4-32%)	81% (68-91%)	0.42 (0.15-0.9)	0.93% (0.49-1.6%)	1.6% (0.69-3.3%)	0.17 (0.072-0.34)	14 (7.2-24)
Maldives	2005	Female	45 to 49	20% (8.9-34%)	80% (66-91%)	0.48 (0.17-1)	1% (0.44-2.1%)	1.9% (0.71-4.2%)	0.23 (0.085-0.5)	14 (6-28)
Maldives	2005	Female	50 to 54	20% (9.7-35%)	80% (65-90%)	0.54 (0.19-1.1)	1.1% (0.27-2.5%)	1.8% (0.4-4.2%)	0.3 (0.067-0.69)	13 (3.1-29)
Maldives	2005	Female	55 to 59	20% (10-35%)	80% (65-90%)	0.59 (0.2-1.2)	1.1% (0.13-2.7%)	1.7% (0.19-4.2%)	0.29 (0.033-0.71)	11 (1.2-25)
Maldives	2005	Female	60 to 64	20% (10-35%)	80% (65-90%)	0.6 (0.2-1.2)	0.92% (-0.3-2.9%)	1.3% (-0.41-4%)	0.49 (-0.16-1.5)	15 (-5.1-45)
Maldives	2005	Female	65 to 69	20% (9.5-32%)	80% (68-90%)	0.55 (0.2-1.2)	0.66% (-0.45-2.4%)	0.88% (-0.64-3.1%)	0.47 (-0.33-1.7)	11 (-7.6-41)
Maldives	2005	Female	70 to 74	19% (9.6-32%)	81% (68-90%)	0.5 (0.17-1.1)	0.56% (-0.5-2.5%)	0.72% (-0.62-3.2%)	0.41 (-0.36-1.8)	7.9 (-6.7-35)
Maldives	2005	Female	75 to 79	18% (8.5-32%)	82% (68-91%)	0.47 (0.14-1.1)	0.35% (-0.68-2%)	0.4% (-0.85-2.4%)	0.21 (-0.44-1.2)	3.1 (-6.3-18)
Maldives	2005	Female	80 to 84	18% (8.7-32%)	82% (68-91%)	0.47 (0.15-1)	0.23% (-0.76-1.9%)	0.25% (-0.99-2.2%)	0.089 (-0.35-0.83)	1.1 (-3.5-9)
Maldives	2005	Female	85 to 89	20% (9.8-34%)	80% (66-90%)	0.51 (0.16-1.1)	0.17% (-0.74-1.8%)	0.14% (-1-2.1%)	0.026 (-0.18-0.38)	0.3 (-1.3-3)
Maldives	2005	Female	90 to 94	23% (11-40%)	77% (60-89%)	0.58 (0.16-1.4)	0.23% (-0.83-2.3%)	0.2% (-1.1-2.7%)	0.017 (-0.096-0.23)	0.12 (-0.46-1.2)
Maldives	2005	Female	95 plus	27% (12-47%)	73% (53-88%)	0.68 (0.15-1.8)	0.26% (-1.2-2.7%)	0.18% (-1.5-3%)	0.0043 (-0.035-0.07)	0.025 (-0.11-0.26)
Maldives	2005	Male	All Ages	36% (29-44%)	64% (56-71%)	0.57 (0.42-0.76)	0.96% (0.31-1.7%)	0.39% (-0.82-1.8%)	2.3 (-5-11)	300 (91-520)
Maldives	2005	Male	15 to 19	32% (20-48%)	68% (52-80%)	0.3 (0.14-0.55)	1.1% (0.65-1.7%)	1.5% (0.58-2.9%)	0.15 (0.06-0.31)	25 (14-39)
Maldives	2005	Male	20 to 24	45% (28-62%)	55% (38-72%)	0.69 (0.36-1.1)	2.6% (1.7-3.8%)	3.2% (1.5-5.5%)	0.35 (0.17-0.61)	51 (32-76)
Maldives	2005	Male	25 to 29	50% (32-67%)	50% (33-68%)	0.92 (0.48-1.4)	3% (2-4.1%)	3.8% (1.7-6.2%)	0.29 (0.13-0.49)	41 (26-59)
Maldives	2005	Male	30 to 34	52% (34-70%)	48% (30-66%)	1.1 (0.61-1.7)	2.8% (1.8-3.9%)	3.7% (1.6-6.2%)	0.26 (0.11-0.44)	35 (22-50)
Maldives	2005	Male	35 to 39	54% (37-70%)	46% (30-63%)	1.2 (0.69-1.9)	2.8% (1.7-4%)	3.9% (1.6-6.4%)	0.37 (0.16-0.61)	35 (20-51)

Location	Year	Sex	Age	Prevalence of current drinkers	Prevalence of abstainers	Population average of standard drinks daily	Percent of total attributable deaths	Percent of total attributable DALYs	Attributable deaths	Attributable DALYs
Maldives	2005	Male	40 to 44	54% (37-71%)	46% (29-63%)	1.3 (0.69-2.1)	2.4% (1.2-3.9%)	3.4% (0.99-6%)	0.42 (0.13-0.73)	32 (15-51)
Maldives	2005	Male	45 to 49	52% (33-69%)	48% (31-67%)	1.3 (0.69-2.2)	2.2% (0.74-4%)	3% (0.39-6.1%)	0.5 (0.063-0.99)	30 (9.5-57)
Maldives	2005	Male	50 to 54	51% (32-68%)	49% (32-68%)	1.2 (0.65-2)	1.5% (-0.18-3.6%)	1.8% (-0.96-4.9%)	0.36 (-0.19-0.95)	19 (-1.9-44)
Maldives	2005	Male	55 to 59	49% (32-66%)	51% (34-68%)	1.1 (0.59-2)	1.3% (-0.35-3.3%)	1.5% (-0.95-4.3%)	0.39 (-0.25-1.1)	16 (-4.2-41)
Maldives	2005	Male	60 to 64	46% (30-64%)	54% (36-70%)	1 (0.5-1.8)	0.9% (-0.73-2.9%)	0.93% (-1.2-3.5%)	0.46 (-0.61-1.7)	17 (-13-53)
Maldives	2005	Male	65 to 69	43% (27-60%)	57% (40-73%)	0.88 (0.46-1.5)	0.41% (-0.96-2.1%)	0.33% (-1.5-2.5%)	0.26 (-1.2-1.9)	9.6 (-23-49)
Maldives	2005	Male	70 to 74	39% (25-57%)	61% (43-75%)	0.74 (0.35-1.4)	-0.13% (-1.5-1.3%)	-0.33% (-2.1-1.3%)	-0.32 (-2-1.3)	-2.9 (-34-28)
Maldives	2005	Male	75 to 79	36% (22-53%)	64% (47-78%)	0.64 (0.28-1.2)	-0.42% (-1.7-0.78%)	-0.66% (-2.3-0.79%)	-0.51 (-1.8-0.59)	-5.6 (-23-11)
Maldives	2005	Male	80 to 84	34% (19-51%)	66% (49-81%)	0.56 (0.25-1)	-0.46% (-1.6-0.53%)	-0.68% (-2.1-0.53%)	-0.37 (-1.1-0.28)	-3.2 (-11-3.5)
Maldives	2005	Male	85 to 89	32% (18-48%)	68% (52-82%)	0.49 (0.22-0.93)	-0.35% (-1.1-0.34%)	-0.56% (-1.6-0.33%)	-0.16 (-0.47-0.093)	-0.94 (-3.1-0.97)
Maldives	2005	Male	90 to 94	33% (20-49%)	67% (51-80%)	0.48 (0.21-0.94)	-0.37% (-1.1-0.26%)	-0.59% (-1.5-0.21%)	-0.08 (-0.2-0.028)	-0.33 (-0.95-0.22)
Maldives	2005	Male	95 plus	37% (20-58%)	63% (42-80%)	0.48 (0.18-1)	-0.56% (-1.5-0.19%)	-0.89% (-2.2-0.055%)	-0.033 (-0.079-0.0019)	-0.087 (-0.24-0.03)
Maldives	2010	Female	All Ages	14% (10-19%)	86% (81-90%)	0.24 (0.14-0.37)	0.61% (0.29-1.1%)	0.63% (-0.25-2.1%)	2.6 (-1-8.4)	170 (74-300)
Maldives	2010	Female	15 to 19	12% (6.2-22%)	88% (78-94%)	0.18 (0.06-0.41)	0.56% (0.34-0.9%)	0.69% (0.26-1.5%)	0.031 (0.012-0.066)	10 (6-17)
Maldives	2010	Female	20 to 24	15% (7-27%)	85% (73-93%)	0.25 (0.088-0.53)	1.1% (0.65-1.6%)	0.75% (0.25-1.6%)	0.041 (0.013-0.089)	23 (13-35)
Maldives	2010	Female	25 to 29	16% (7.2-29%)	84% (71-93%)	0.28 (0.091-0.59)	1.1% (0.75-1.5%)	0.74% (0.26-1.6%)	0.036 (0.013-0.076)	21 (13-31)
Maldives	2010	Female	30 to 34	18% (7.9-33%)	82% (67-92%)	0.32 (0.11-0.67)	0.97% (0.62-1.4%)	0.86% (0.36-1.7%)	0.046 (0.019-0.094)	16 (9.4-25)
Maldives	2010	Female	35 to 39	19% (8.3-33%)	81% (67-92%)	0.35 (0.12-0.73)	0.87% (0.54-1.3%)	1.2% (0.49-2.3%)	0.073 (0.03-0.15)	13 (7.6-20)
Maldives	2010	Female	40 to 44	19% (9.5-33%)	81% (67-91%)	0.39 (0.14-0.84)	0.85% (0.48-1.4%)	1.6% (0.74-3.3%)	0.14 (0.061-0.28)	13 (7.1-23)
Maldives	2010	Female	45 to 49	19% (8.8-32%)	81% (68-91%)	0.44 (0.15-1)	0.88% (0.41-1.8%)	1.8% (0.75-4.1%)	0.2 (0.08-0.44)	13 (6.4-29)
Maldives	2010	Female	50 to 54	19% (9.4-33%)	81% (67-91%)	0.49 (0.16-1.1)	0.96% (0.3-2.1%)	1.8% (0.58-4%)	0.3 (0.096-0.7)	15 (4.6-32)
Maldives	2010	Female	55 to 59	20% (9.8-34%)	80% (66-90%)	0.55 (0.17-1.2)	0.94% (0.15-2.3%)	1.7% (0.31-4.1%)	0.28 (0.052-0.66)	11 (1.8-26)

Location	Year	Sex	Age	Prevalence of current drinkers	Prevalence of abstainers	Population average of standard drinks daily	Percent of total attributable deaths	Percent of total attributable DALYs	Attributable deaths	Attributable DALYs
Maldives	2010	Female	60 to 64	20%<br>(9.3-34%)	80%<br>(66-91%)	0.55<br>(0.17-1.2)	0.81%<br>(-0.21-2.5%)	1.3%<br>(-0.24-3.8%)	0.28<br>(-0.054-0.85)	8.6<br>(-2.2-27)
Maldives	2010	Female	65 to 69	19%<br>(9.4-32%)	81%<br>(68-91%)	0.51<br>(0.17-1.1)	0.57%<br>(-0.4-2.1%)	0.81%<br>(-0.56-2.9%)	0.36<br>(-0.24-1.3)	8.9<br>(-6.3-33)
Maldives	2010	Female	70 to 74	19%<br>(9-31%)	81%<br>(69-91%)	0.47<br>(0.14-1.1)	0.49%<br>(-0.45-2.3%)	0.64%<br>(-0.56-2.9%)	0.45<br>(-0.39-2)	8.6<br>(-8-40)
Maldives	2010	Female	75 to 79	18%<br>(8.2-31%)	82%<br>(69-92%)	0.44<br>(0.12-1.1)	0.29%<br>(-0.64-1.9%)	0.34%<br>(-0.83-2.3%)	0.24<br>(-0.57-1.6)	3.7<br>(-8.2-24)
Maldives	2010	Female	80 to 84	18%<br>(8.9-33%)	82%<br>(67-91%)	0.44<br>(0.13-1)	0.17%<br>(-0.79-1.6%)	0.18%<br>(-1-1.9%)	0.082<br>(-0.47-0.87)	1.1<br>(-4.9-9.8)
Maldives	2010	Female	85 to 89	20%<br>(9.3-34%)	80%<br>(66-91%)	0.48<br>(0.14-1.2)	0.12%<br>(-0.69-1.5%)	0.09%<br>(-1-1.9%)	0.021<br>(-0.22-0.41)	0.29<br>(-1.6-3.5)
Maldives	2010	Female	90 to 94	23%<br>(10-40%)	77%<br>(60-90%)	0.55<br>(0.14-1.4)	0.18%<br>(-0.68-1.8%)	0.15%<br>(-0.95-2.2%)	0.017<br>(-0.11-0.25)	0.15<br>(-0.55-1.5)
Maldives	2010	Female	95 plus	27%<br>(11-48%)	73%<br>(52-89%)	0.64<br>(0.14-1.7)	0.22%<br>(-1-2.4%)	0.12%<br>(-1.4-2.7%)	0.0042<br>(-0.049-0.094)	0.033<br>(-0.15-0.37)
Maldives	2010	Male	All Ages	37%<br>(30-45%)	63%<br>(55-70%)	0.58<br>(0.37-0.85)	0.98%<br>(0.27-1.8%)	0.24%<br>(-0.99-1.6%)	1.5<br>(-6.2-10)	310<br>(81-550)
Maldives	2010	Male	15 to 19	31%<br>(19-47%)	69%<br>(53-81%)	0.28<br>(0.12-0.51)	1.1%<br>(0.66-1.7%)	1.4%<br>(0.55-2.8%)	0.13<br>(0.05-0.25)	22<br>(13-34)
Maldives	2010	Male	20 to 24	44%<br>(27-61%)	56%<br>(39-73%)	0.63<br>(0.31-1.1)	2.5%<br>(1.7-3.7%)	3%<br>(1.3-5.5%)	0.37<br>(0.16-0.68)	57<br>(36-86)
Maldives	2010	Male	25 to 29	49%<br>(31-66%)	51%<br>(34-69%)	0.85<br>(0.44-1.4)	2.8%<br>(1.9-4%)	3.5%<br>(1.5-5.9%)	0.34<br>(0.14-0.58)	53<br>(33-77)
Maldives	2010	Male	30 to 34	52%<br>(33-68%)	48%<br>(32-67%)	1<br>(0.56-1.7)	2.7%<br>(1.7-3.7%)	3.4%<br>(1.3-5.8%)	0.28<br>(0.11-0.49)	39<br>(24-56)
Maldives	2010	Male	35 to 39	53%<br>(36-70%)	47%<br>(30-64%)	1.1<br>(0.6-2)	2.6%<br>(1.6-3.9%)	3.8%<br>(1.6-6.3%)	0.3<br>(0.12-0.5)	34<br>(20-50)
Maldives	2010	Male	40 to 44	53%<br>(36-72%)	47%<br>(28-64%)	1.2<br>(0.61-2.1)	2.3%<br>(1.1-3.6%)	3.3%<br>(0.93-6.1%)	0.37<br>(0.1-0.68)	32<br>(15-51)
Maldives	2010	Male	45 to 49	52%<br>(33-69%)	48%<br>(31-67%)	1.2<br>(0.59-2.1)	2%<br>(0.66-3.7%)	2.8%<br>(0.26-6%)	0.49<br>(0.042-1.1)	32<br>(10-60)
Maldives	2010	Male	50 to 54	50%<br>(32-68%)	50%<br>(32-68%)	1.2<br>(0.56-2)	1.4%<br>(-0.16-3.2%)	1.6%<br>(-1-4.5%)	0.42<br>(-0.28-1.2)	24<br>(-2.8-58)
Maldives	2010	Male	55 to 59	48%<br>(32-66%)	52%<br>(34-68%)	1.1<br>(0.5-1.9)	1.2%<br>(-0.25-3.2%)	1.4%<br>(-0.84-4.2%)	0.41<br>(-0.26-1.2)	18<br>(-3.7-47)
Maldives	2010	Male	60 to 64	46%<br>(29-64%)	54%<br>(36-71%)	0.95<br>(0.42-1.7)	0.82%<br>(-0.7-2.6%)	0.84%<br>(-1.2-3.3%)	0.31<br>(-0.41-1.2)	12<br>(-9.5-38)
Maldives	2010	Male	65 to 69	42%<br>(27-60%)	58%<br>(40-73%)	0.82<br>(0.39-1.5)	0.32%<br>(-1-2%)	0.22%<br>(-1.5-2.4%)	0.15<br>(-1-1.5)	6.5<br>(-20-40)
Maldives	2010	Male	70 to 74	39%<br>(24-57%)	61%<br>(43-76%)	0.69<br>(0.31-1.4)	-0.2%<br>(-1.5-1.2%)	-0.42%<br>(-2-1.3%)	-0.44<br>(-2.1-1.4)	-4.8<br>(-36-30)
Maldives	2010	Male	75 to 79	35%<br>(21-55%)	65%<br>(45-79%)	0.59<br>(0.24-1.2)	-0.41%<br>(-1.7-0.65%)	-0.67%<br>(-2.3-0.63%)	-0.72<br>(-2.5-0.7)	-7.8<br>(-31-13)

Location	Year	Sex	Age	Prevalence of current drinkers	Prevalence of abstainers	Population average of standard drinks daily	Percent of total attributable deaths	Percent of total attributable DALYs	Attributable deaths	Attributable DALYs
Maldives	2010	Male	80 to 84	33%<br>(19-50%)	67%<br>(50-81%)	0.51<br>(0.2-1)	-0.48%<br>(-1.6-0.43%)	-0.71%<br>(-2.1-0.4%)	-0.56<br>(-1.7-0.31)	-4.8<br>(-16-4.3)
Maldives	2010	Male	85 to 89	31%<br>(18-47%)	69%<br>(53-82%)	0.46<br>(0.2-0.91)	-0.35%<br>(-1.1-0.29%)	-0.56%<br>(-1.6-0.25%)	-0.22<br>(-0.61-0.099)	-1.3<br>(-4.1-1.1)
Maldives	2010	Male	90 to 94	33%<br>(19-49%)	67%<br>(51-81%)	0.44<br>(0.18-0.89)	-0.29%<br>(-0.91-0.23%)	-0.51%<br>(-1.4-0.16%)	-0.08<br>(-0.21-0.025)	-0.31<br>(-0.99-0.25)
Maldives	2010	Male	95 plus	37%<br>(20-57%)	63%<br>(43-80%)	0.45<br>(0.16-0.98)	-0.42%<br>(-1.3-0.24%)	-0.78%<br>(-1.9-0.091%)	-0.035<br>(-0.087-0.0042)	-0.086<br>(-0.26-0.047)
Maldives	2016	Female	All Ages	14%<br>(11-19%)	86%<br>(81-89%)	0.23<br>(0.098-0.41)	0.62%<br>(0.3-1.2%)	0.54%<br>(-0.26-2.1%)	2.4<br>(-1.1-9.6)	180<br>(80-350)
Maldives	2016	Female	15 to 19	12%<br>(6.2-21%)	88%<br>(79-94%)	0.16<br>(0.045-0.39)	0.56%<br>(0.33-0.89%)	0.66%<br>(0.25-1.4%)	0.02<br>(0.0072-0.044)	8.1<br>(4.5-13)
Maldives	2016	Female	20 to 24	15%<br>(6.9-26%)	85%<br>(74-93%)	0.22<br>(0.062-0.52)	1.1%<br>(0.69-1.6%)	0.72%<br>(0.23-1.8%)	0.031<br>(0.0093-0.08)	20<br>(12-31)
Maldives	2016	Female	25 to 29	16%<br>(7.1-28%)	84%<br>(72-93%)	0.25<br>(0.07-0.58)	1.1%<br>(0.73-1.6%)	0.69%<br>(0.24-1.5%)	0.038<br>(0.012-0.088)	24<br>(15-36)
Maldives	2016	Female	30 to 34	17%<br>(7.5-31%)	83%<br>(69-92%)	0.27<br>(0.078-0.6)	0.97%<br>(0.61-1.4%)	0.82%<br>(0.35-1.7%)	0.059<br>(0.022-0.13)	22<br>(13-34)
Maldives	2016	Female	35 to 39	19%<br>(8.3-33%)	81%<br>(67-92%)	0.31<br>(0.086-0.72)	0.86%<br>(0.52-1.3%)	1.1%<br>(0.49-2.3%)	0.09<br>(0.033-0.2)	17<br>(9.6-27)
Maldives	2016	Female	40 to 44	19%<br>(9.4-33%)	81%<br>(67-91%)	0.34<br>(0.1-0.83)	0.82%<br>(0.45-1.4%)	1.5%<br>(0.7-3.2%)	0.14<br>(0.057-0.29)	15<br>(7.7-26)
Maldives	2016	Female	45 to 49	19%<br>(8.7-33%)	81%<br>(67-91%)	0.39<br>(0.11-0.95)	0.81%<br>(0.37-1.6%)	1.7%<br>(0.67-3.9%)	0.2<br>(0.077-0.47)	14<br>(6.4-29)
Maldives	2016	Female	50 to 54	20%<br>(9.2-34%)	80%<br>(66-91%)	0.44<br>(0.13-1.1)	0.86%<br>(0.27-2%)	1.6%<br>(0.48-3.9%)	0.33<br>(0.091-0.85)	16<br>(4.7-39)
Maldives	2016	Female	55 to 59	20%<br>(9.9-34%)	80%<br>(66-90%)	0.48<br>(0.13-1.2)	0.81%<br>(0.11-2.2%)	1.5%<br>(0.26-4%)	0.37<br>(0.065-1)	15<br>(1.8-40)
Maldives	2016	Female	60 to 64	20%<br>(9.5-34%)	80%<br>(66-90%)	0.48<br>(0.12-1.2)	0.68%<br>(-0.15-2.4%)	1.1%<br>(-0.17-3.7%)	0.34<br>(-0.051-1.2)	11<br>(-2.4-39)
Maldives	2016	Female	65 to 69	20%<br>(9.5-33%)	80%<br>(67-90%)	0.45<br>(0.12-1.1)	0.45%<br>(-0.41-2%)	0.68%<br>(-0.54-2.9%)	0.22<br>(-0.18-0.99)	5.5<br>(-5.1-26)
Maldives	2016	Female	70 to 74	19%<br>(9.4-31%)	81%<br>(69-91%)	0.41<br>(0.11-1)	0.39%<br>(-0.39-1.9%)	0.55%<br>(-0.49-2.7%)	0.3<br>(-0.27-1.5)	5.9<br>(-5.9-29)
Maldives	2016	Female	75 to 79	18%<br>(8.8-32%)	82%<br>(68-91%)	0.39<br>(0.093-0.99)	0.21%<br>(-0.59-1.7%)	0.27%<br>(-0.76-2.3%)	0.21<br>(-0.64-1.7)	3.4<br>(-9.5-27)
Maldives	2016	Female	80 to 84	18%<br>(8.5-32%)	82%<br>(68-91%)	0.39<br>(0.094-0.94)	0.083%<br>(-0.74-1.5%)	0.076%<br>(-0.94-1.9%)	0.047<br>(-0.64-1.2)	0.73<br>(-6.6-13)
Maldives	2016	Female	85 to 89	20%<br>(9.5-34%)	80%<br>(66-91%)	0.42<br>(0.1-1.1)	0.038%<br>(-0.7-1.3%)	-0.012%<br>(-0.97-1.7%)	-0.0051<br>(-0.36-0.59)	0.14<br>(-2.7-5.1)
Maldives	2016	Female	90 to 94	22%<br>(11-39%)	78%<br>(61-89%)	0.47<br>(0.11-1.3)	0.059%<br>(-0.7-1.6%)	-0.004%<br>(-0.97-2%)	-0.0012<br>(-0.2-0.38)	0.079<br>(-0.99-2.2)
Maldives	2016	Female	95 plus	27%<br>(12-47%)	73%<br>(53-88%)	0.56<br>(0.11-1.6)	0.09%<br>(-0.94-2.3%)	-0.019%<br>(-1.3-2.5%)	-0.0015<br>(-0.095-0.18)	0.028<br>(-0.3-0.69)

Location	Year	Sex	Age	Prevalence of current drinkers	Prevalence of abstainers	Population average of standard drinks daily	Percent of total attributable deaths	Percent of total attributable DALYs	Attributable deaths	Attributable DALYs
Maldives	2016	Male	All Ages	38% (32-46%)	62% (54-68%)	0.55 (0.25-0.93)	1.1% (0.4-2%)	0.28% (-0.84-1.6%)	1.9 (-5.7-11)	350 (120-670)
Maldives	2016	Male	15 to 19	31% (18-46%)	69% (54-82%)	0.24 (0.083-0.51)	1% (0.63-1.6%)	1.3% (0.48-2.6%)	0.075 (0.026-0.16)	16 (9.3-25)
Maldives	2016	Male	20 to 24	44% (28-60%)	56% (40-72%)	0.55 (0.21-1.1)	2.5% (1.6-3.7%)	2.7% (0.97-5.4%)	0.28 (0.091-0.57)	50 (30-78)
Maldives	2016	Male	25 to 29	49% (31-66%)	51% (34-69%)	0.74 (0.28-1.4)	2.8% (1.8-4.1%)	3.1% (1.1-5.8%)	0.35 (0.11-0.7)	61 (38-92)
Maldives	2016	Male	30 to 34	51% (33-68%)	49% (32-67%)	0.88 (0.36-1.7)	2.6% (1.6-3.9%)	2.9% (1-5.3%)	0.36 (0.12-0.72)	56 (33-86)
Maldives	2016	Male	35 to 39	53% (35-70%)	47% (30-65%)	0.98 (0.41-1.9)	2.5% (1.5-4%)	3.3% (1.2-6.5%)	0.37 (0.12-0.79)	43 (25-71)
Maldives	2016	Male	40 to 44	53% (37-71%)	47% (29-63%)	1 (0.43-2)	2.2% (0.98-3.7%)	2.9% (0.55-5.9%)	0.4 (0.074-0.93)	36 (16-66)
Maldives	2016	Male	45 to 49	52% (34-71%)	48% (29-66%)	1 (0.43-2.1)	1.9% (0.62-3.7%)	2.4% (0.019-5.8%)	0.49 (0.0033-1.2)	34 (11-68)
Maldives	2016	Male	50 to 54	50% (32-69%)	50% (31-68%)	1 (0.4-2)	1.2% (-0.13-3.2%)	1.4% (-0.91-4.3%)	0.44 (-0.29-1.4)	26 (-2.9-68)
Maldives	2016	Male	55 to 59	48% (31-67%)	52% (33-69%)	0.92 (0.34-1.9)	1.1% (-0.21-3.1%)	1.2% (-0.81-4.1%)	0.53 (-0.35-1.9)	25 (-5.1-73)
Maldives	2016	Male	60 to 64	45% (29-64%)	55% (36-71%)	0.82 (0.28-1.7)	0.76% (-0.55-2.7%)	0.78% (-1-3.4%)	0.39 (-0.52-1.7)	15 (-10-53)
Maldives	2016	Male	65 to 69	42% (26-59%)	58% (41-74%)	0.7 (0.26-1.4)	0.33% (-0.8-1.9%)	0.23% (-1.3-2.3%)	0.12 (-0.65-1.2)	5.3 (-12-31)
Maldives	2016	Male	70 to 74	38% (24-55%)	62% (45-76%)	0.59 (0.2-1.3)	-0.082% (-1.2-1.1%)	-0.28% (-1.8-1.3%)	-0.22 (-1.3-0.98)	-1.5 (-22-21)
Maldives	2016	Male	75 to 79	35% (21-53%)	65% (47-79%)	0.51 (0.16-1.1)	-0.28% (-1.4-0.62%)	-0.52% (-2-0.63%)	-0.55 (-2.1-0.67)	-5.5 (-26-12)
Maldives	2016	Male	80 to 84	32% (18-50%)	68% (50-82%)	0.44 (0.15-0.92)	-0.37% (-1.3-0.42%)	-0.57% (-1.8-0.4%)	-0.59 (-1.9-0.46)	-4.9 (-18-5.8)
Maldives	2016	Male	85 to 89	31% (17-47%)	69% (53-83%)	0.39 (0.13-0.88)	-0.28% (-0.99-0.27%)	-0.48% (-1.4-0.27%)	-0.32 (-0.95-0.16)	-1.8 (-6.4-1.7)
Maldives	2016	Male	90 to 94	32% (19-49%)	68% (51-81%)	0.38 (0.13-0.81)	-0.28% (-0.91-0.21%)	-0.48% (-1.4-0.16%)	-0.13 (-0.39-0.045)	-0.52 (-1.8-0.4)
Maldives	2016	Male	95 plus	36% (19-57%)	64% (43-81%)	0.38 (0.11-0.94)	-0.41% (-1.3-0.19%)	-0.72% (-1.9-0.049%)	-0.061 (-0.16-0.0041)	-0.15 (-0.47-0.074)
Myanmar	1990	Female	All Ages	2.1% (1.8-2.7%)	98% (97-98%)	0.025 (0.016-0.035)	0.41% (0.32-0.52%)	0.58% (0.43-0.77%)	1200 (880-1600)	45000 (36000-56000)
Myanmar	1990	Female	15 to 19	2% (1.2-3.1%)	98% (97-99%)	0.024 (0.011-0.045)	0.38% (0.24-0.55%)	0.3% (0.15-0.5%)	10 (5.1-18)	1700 (1000-2500)
Myanmar	1990	Female	20 to 24	2.5% (1.3-3.8%)	98% (96-99%)	0.032 (0.014-0.062)	0.64% (0.42-0.9%)	0.38% (0.18-0.67%)	17 (8.1-30)	3100 (2000-4500)
Myanmar	1990	Female	25 to 29	2.7% (1.5-4.3%)	97% (96-99%)	0.035 (0.015-0.067)	0.7% (0.47-0.97%)	0.46% (0.22-0.77%)	21 (9.8-36)	3100 (2000-4500)

Location	Year	Sex	Age	Prevalence of current drinkers	Prevalence of abstainers	Population average of standard drinks daily	Percent of total attributable deaths	Percent of total attributable DALYs	Attributable deaths	Attributable DALYs
Myanmar	1990	Female	30 to 34	2.9% (1.6-4.8%)	97% (95-98%)	0.038 (0.018-0.072)	0.81% (0.57-1.1%)	0.74% (0.44-1.1%)	37 (22-56)	3600 (2500-5000)
Myanmar	1990	Female	35 to 39	3% (1.7-4.7%)	97% (95-98%)	0.042 (0.019-0.076)	1% (0.72-1.4%)	1.1% (0.71-1.6%)	65 (41-97)	4500 (3200-6200)
Myanmar	1990	Female	40 to 44	3% (1.8-4.7%)	97% (95-98%)	0.044 (0.021-0.08)	1.2% (0.84-1.6%)	1.4% (0.96-2%)	74 (49-110)	4100 (2900-5600)
Myanmar	1990	Female	45 to 49	3.1% (1.7-4.6%)	97% (95-98%)	0.047 (0.021-0.088)	1.2% (0.81-1.6%)	1.4% (0.91-2%)	88 (57-130)	4100 (2800-5700)
Myanmar	1990	Female	50 to 54	3.1% (1.8-4.9%)	97% (95-98%)	0.052 (0.024-0.096)	1.1% (0.78-1.6%)	1.3% (0.86-2%)	110 (73-170)	4500 (3000-6600)
Myanmar	1990	Female	55 to 59	3.2% (1.8-4.8%)	97% (95-98%)	0.057 (0.026-0.11)	0.96% (0.66-1.4%)	1.1% (0.76-1.7%)	130 (84-190)	4400 (2900-6300)
Myanmar	1990	Female	60 to 64	3% (1.8-4.9%)	97% (95-98%)	0.057 (0.026-0.11)	0.82% (0.51-1.2%)	0.97% (0.6-1.5%)	150 (92-220)	4200 (2600-6100)
Myanmar	1990	Female	65 to 69	2.8% (1.6-4.3%)	97% (96-98%)	0.054 (0.025-0.097)	0.69% (0.44-1%)	0.81% (0.51-1.2%)	140 (87-210)	3200 (2000-4800)
Myanmar	1990	Female	70 to 74	2.7% (1.6-4.2%)	97% (96-98%)	0.053 (0.023-0.1)	0.6% (0.36-0.96%)	0.7% (0.41-1.1%)	140 (80-220)	2500 (1500-4100)
Myanmar	1990	Female	75 to 79	2.8% (1.6-4.5%)	97% (96-98%)	0.054 (0.024-0.11)	0.47% (0.26-0.78%)	0.55% (0.29-0.91%)	97 (52-160)	1400 (740-2300)
Myanmar	1990	Female	80 to 84	3% (1.7-4.5%)	97% (96-98%)	0.056 (0.025-0.1)	0.42% (0.22-0.7%)	0.49% (0.25-0.83%)	63 (32-110)	650 (330-1100)
Myanmar	1990	Female	85 to 89	3.4% (1.8-5.5%)	97% (95-98%)	0.062 (0.027-0.12)	0.35% (0.16-0.6%)	0.41% (0.19-0.71%)	28 (13-50)	210 (98-360)
Myanmar	1990	Female	90 to 94	4% (2.2-6.5%)	96% (93-98%)	0.07 (0.029-0.14)	0.29% (0.12-0.55%)	0.34% (0.13-0.66%)	7.9 (3-15)	40 (16-76)
Myanmar	1990	Female	95 plus	5% (2.3-9.4%)	95% (91-98%)	0.084 (0.027-0.18)	0.34% (0.11-0.7%)	0.37% (0.11-0.81%)	2 (0.6-4.4)	7 (2.3-15)
Myanmar	1990	Male	All Ages	19% (16-22%)	81% (78-84%)	0.48 (0.34-0.63)	3.3% (2-4.7%)	4.8% (2.8-7.1%)	11000 (6400-16000)	420000 (270000-590000)
Myanmar	1990	Male	15 to 19	16% (10-22%)	84% (78-90%)	0.21 (0.11-0.38)	1.2% (0.56-2.2%)	1.4% (0.4-2.7%)	71 (20-140)	6600 (2900-11000)
Myanmar	1990	Male	20 to 24	25% (17-34%)	75% (66-83%)	0.55 (0.3-0.9)	4% (1.8-7%)	4.6% (1.8-8.4%)	320 (130-610)	25000 (11000-44000)
Myanmar	1990	Male	25 to 29	30% (21-41%)	70% (59-79%)	0.79 (0.46-1.2)	6.7% (3.3-11%)	8.1% (3.6-14%)	560 (250-990)	38000 (19000-64000)
Myanmar	1990	Male	30 to 34	32% (22-44%)	68% (56-78%)	0.98 (0.55-1.5)	8.6% (4.7-13%)	11% (5.5-17%)	780 (400-1200)	47000 (25000-73000)
Myanmar	1990	Male	35 to 39	34% (22-44%)	66% (56-78%)	1.1 (0.62-1.8)	10% (5.6-15%)	12% (6.9-19%)	1000 (550-1500)	54000 (31000-81000)
Myanmar	1990	Male	40 to 44	33% (23-43%)	67% (57-77%)	1.2 (0.68-1.9)	11% (6.5-16%)	13% (7.8-19%)	1000 (590-1500)	47000 (28000-70000)
Myanmar	1990	Male	45 to 49	32% (21-42%)	68% (58-79%)	1.3 (0.68-2.1)	11% (6.5-17%)	14% (7.7-20%)	1200 (670-1800)	49000 (28000-75000)

Location	Year	Sex	Age	Prevalence of current drinkers	Prevalence of abstainers	Population average of standard drinks daily	Percent of total attributable deaths	Percent of total attributable DALYs	Attributable deaths	Attributable DALYs
Myanmar	1990	Male	50 to 54	29% (21-39%)	71% (61-79%)	1.2 (0.63-1.9)	9.7% (5.3-15%)	12% (6.4-18%)	1200 (660-1900)	45000 (24000-70000)
Myanmar	1990	Male	55 to 59	26% (18-35%)	74% (65-82%)	0.96 (0.51-1.6)	7.3% (4-12%)	8.7% (4.7-14%)	1100 (600-1900)	37000 (19000-59000)
Myanmar	1990	Male	60 to 64	24% (16-33%)	76% (67-84%)	0.81 (0.42-1.4)	5.8% (2.7-10%)	6.8% (3.2-12%)	1100 (510-1900)	30000 (14000-52000)
Myanmar	1990	Male	65 to 69	21% (14-29%)	79% (71-86%)	0.7 (0.36-1.2)	4.5% (2.1-8.1%)	5.3% (2.4-9.2%)	880 (400-1500)	20000 (9200-34000)
Myanmar	1990	Male	70 to 74	19% (13-26%)	81% (74-87%)	0.59 (0.28-1)	3.7% (1.5-7%)	4.3% (1.8-8%)	730 (300-1400)	13000 (5500-25000)
Myanmar	1990	Male	75 to 79	17% (11-24%)	83% (76-89%)	0.5 (0.24-0.93)	2.8% (1.1-5.3%)	3.2% (1.2-6.1%)	410 (150-810)	5800 (2100-11000)
Myanmar	1990	Male	80 to 84	15% (9.8-21%)	85% (79-90%)	0.42 (0.2-0.72)	2.3% (0.88-4.3%)	2.7% (0.97-5%)	210 (77-390)	2200 (810-4100)
Myanmar	1990	Male	85 to 89	15% (9.2-23%)	85% (77-91%)	0.37 (0.17-0.69)	1.7% (0.6-3.4%)	2% (0.67-3.9%)	80 (27-160)	590 (200-1100)
Myanmar	1990	Male	90 to 94	17% (11-24%)	83% (76-89%)	0.36 (0.16-0.67)	1.3% (0.31-2.8%)	1.5% (0.34-3.3%)	21 (4.6-44)	110 (24-220)
Myanmar	1990	Male	95 plus	19% (11-30%)	81% (70-89%)	0.37 (0.14-0.7)	1.2% (0.18-2.7%)	1.3% (0.13-3.1%)	3.8 (0.38-9.1)	13 (2.1-31)
Myanmar	1995	Female	All Ages	2.2% (1.8-2.7%)	98% (97-98%)	0.028 (0.018-0.04)	0.47% (0.37-0.59%)	0.64% (0.47-0.84%)	1400 (1000-1800)	52000 (41000-64000)
Myanmar	1995	Female	15 to 19	2% (1.2-3.1%)	98% (97-99%)	0.026 (0.012-0.049)	0.4% (0.25-0.58%)	0.32% (0.16-0.51%)	11 (5.8-19)	1800 (1100-2700)
Myanmar	1995	Female	20 to 24	2.5% (1.4-3.8%)	98% (96-99%)	0.034 (0.015-0.066)	0.68% (0.46-0.95%)	0.4% (0.21-0.7%)	18 (9.4-32)	3300 (2100-4800)
Myanmar	1995	Female	25 to 29	2.7% (1.5-4.2%)	97% (96-99%)	0.038 (0.017-0.072)	0.73% (0.5-1%)	0.49% (0.26-0.81%)	23 (12-38)	3500 (2300-4900)
Myanmar	1995	Female	30 to 34	2.9% (1.5-4.7%)	97% (95-98%)	0.041 (0.018-0.077)	0.85% (0.61-1.1%)	0.78% (0.5-1.1%)	40 (26-60)	4000 (2700-5400)
Myanmar	1995	Female	35 to 39	3% (1.6-4.6%)	97% (95-98%)	0.045 (0.021-0.081)	1% (0.76-1.4%)	1.2% (0.77-1.7%)	71 (47-100)	5000 (3500-6900)
Myanmar	1995	Female	40 to 44	3% (1.8-4.7%)	97% (95-98%)	0.048 (0.023-0.087)	1.2% (0.86-1.6%)	1.5% (1-2.1%)	100 (70-150)	5800 (4200-8100)
Myanmar	1995	Female	45 to 49	3.1% (1.7-4.7%)	97% (95-98%)	0.051 (0.023-0.095)	1.2% (0.85-1.6%)	1.5% (1-2.1%)	100 (68-150)	4700 (3300-6600)
Myanmar	1995	Female	50 to 54	3.1% (1.8-4.7%)	97% (95-98%)	0.056 (0.026-0.1)	1.2% (0.8-1.7%)	1.4% (0.94-2%)	120 (79-180)	4800 (3200-6700)
Myanmar	1995	Female	55 to 59	3.1% (1.8-4.8%)	97% (95-98%)	0.062 (0.029-0.11)	1% (0.67-1.4%)	1.2% (0.78-1.7%)	140 (87-190)	4600 (3000-6300)
Myanmar	1995	Female	60 to 64	3% (1.8-4.9%)	97% (95-98%)	0.061 (0.028-0.12)	0.87% (0.54-1.3%)	1% (0.62-1.6%)	160 (95-240)	4400 (2700-6600)
Myanmar	1995	Female	65 to 69	2.8% (1.6-4.2%)	97% (96-98%)	0.058 (0.028-0.1)	0.73% (0.48-1.1%)	0.87% (0.55-1.3%)	170 (110-250)	3900 (2500-5700)

Location	Year	Sex	Age	Prevalence of current drinkers	Prevalence of abstainers	Population average of standard drinks daily	Percent of total attributable deaths	Percent of total attributable DALYs	Attributable deaths	Attributable DALYs
Myanmar	1995	Female	70 to 74	2.7% (1.7-4.2%)	97% (96-98%)	0.057 (0.025-0.11)	0.65% (0.38-1%)	0.76% (0.43-1.2%)	160 (91-250)	2900 (1700-4600)
Myanmar	1995	Female	75 to 79	2.8% (1.7-4.5%)	97% (96-98%)	0.059 (0.026-0.12)	0.51% (0.28-0.83%)	0.6% (0.32-0.97%)	120 (64-200)	1700 (920-2700)
Myanmar	1995	Female	80 to 84	3% (1.6-4.5%)	97% (95-98%)	0.061 (0.027-0.11)	0.46% (0.25-0.77%)	0.53% (0.28-0.9%)	79 (42-130)	810 (440-1400)
Myanmar	1995	Female	85 to 89	3.4% (1.8-5.6%)	97% (94-98%)	0.067 (0.029-0.13)	0.38% (0.19-0.65%)	0.45% (0.22-0.76%)	35 (17-59)	250 (120-430)
Myanmar	1995	Female	90 to 94	4% (2.2-6.6%)	96% (93-98%)	0.077 (0.032-0.14)	0.32% (0.14-0.6%)	0.38% (0.16-0.71%)	10 (4.5-19)	53 (23-99)
Myanmar	1995	Female	95 plus	4.9% (2.3-9.2%)	95% (91-98%)	0.092 (0.03-0.2)	0.37% (0.13-0.72%)	0.41% (0.13-0.83%)	2.8 (0.92-5.7)	9.9 (3.5-19)
Myanmar	1995	Male	All Ages	19% (16-23%)	81% (77-84%)	0.55 (0.39-0.73)	3.8% (2.4-5.3%)	5.3% (3.3-7.6%)	12000 (7600-18000)	490000 (310000-680000)
Myanmar	1995	Male	15 to 19	16% (10-23%)	84% (77-90%)	0.23 (0.12-0.42)	1.3% (0.65-2.3%)	1.5% (0.53-2.8%)	82 (29-160)	7500 (3600-13000)
Myanmar	1995	Male	20 to 24	26% (17-34%)	74% (66-83%)	0.6 (0.32-1)	4.2% (2-6.9%)	4.8% (2-8.2%)	370 (150-640)	28000 (13000-47000)
Myanmar	1995	Male	25 to 29	30% (21-41%)	70% (59-79%)	0.87 (0.5-1.4)	6.9% (3.6-11%)	8.2% (4-14%)	660 (310-1100)	44000 (23000-72000)
Myanmar	1995	Male	30 to 34	33% (22-45%)	67% (55-78%)	1.1 (0.61-1.7)	8.8% (5-13%)	11% (5.9-17%)	900 (490-1400)	54000 (31000-82000)
Myanmar	1995	Male	35 to 39	34% (23-45%)	66% (55-77%)	1.2 (0.68-1.9)	10% (6.1-15%)	13% (7.4-19%)	1200 (680-1800)	63000 (37000-94000)
Myanmar	1995	Male	40 to 44	33% (23-44%)	67% (56-77%)	1.3 (0.75-2.1)	11% (6.7-16%)	13% (8.2-19%)	1400 (830-2000)	66000 (40000-95000)
Myanmar	1995	Male	45 to 49	32% (21-42%)	68% (58-79%)	1.4 (0.73-2.3)	11% (6.9-17%)	14% (8.4-20%)	1300 (790-2000)	56000 (33000-84000)
Myanmar	1995	Male	50 to 54	29% (21-40%)	71% (60-79%)	1.3 (0.67-2.1)	10% (5.6-15%)	12% (6.7-18%)	1300 (710-2100)	49000 (26000-75000)
Myanmar	1995	Male	55 to 59	27% (18-36%)	73% (64-82%)	1.1 (0.56-1.7)	7.7% (4.3-12%)	9.2% (5.1-14%)	1200 (650-1900)	39000 (21000-61000)
Myanmar	1995	Male	60 to 64	24% (16-34%)	76% (66-84%)	0.89 (0.46-1.6)	6.1% (3.1-10%)	7.1% (3.6-12%)	1200 (570-2000)	32000 (16000-55000)
Myanmar	1995	Male	65 to 69	21% (14-29%)	79% (71-86%)	0.77 (0.4-1.3)	4.8% (2.3-8.1%)	5.6% (2.7-9.4%)	1100 (510-1800)	24000 (11000-40000)
Myanmar	1995	Male	70 to 74	19% (13-26%)	81% (74-87%)	0.64 (0.31-1.1)	4% (1.7-7.3%)	4.6% (2-8.3%)	840 (350-1500)	15000 (6500-28000)
Myanmar	1995	Male	75 to 79	17% (11-24%)	83% (76-89%)	0.55 (0.26-0.99)	3% (1.2-5.5%)	3.4% (1.3-6.4%)	520 (200-980)	7300 (2900-14000)
Myanmar	1995	Male	80 to 84	15% (9.8-21%)	85% (79-90%)	0.46 (0.22-0.78)	2.5% (0.96-4.5%)	2.8% (1.1-5.2%)	260 (99-490)	2800 (1100-5100)
Myanmar	1995	Male	85 to 89	15% (9.1-22%)	85% (78-91%)	0.41 (0.18-0.72)	1.9% (0.68-3.6%)	2.1% (0.75-4.1%)	99 (36-190)	730 (270-1400)

Location	Year	Sex	Age	Prevalence of current drinkers	Prevalence of abstainers	Population average of standard drinks daily	Percent of total attributable deaths	Percent of total attributable DALYs	Attributable deaths	Attributable DALYs
Myanmar	1995	Male	90 to 94	17% (11-24%)	83% (76-89%)	0.4 (0.17-0.7)	1.4% (0.43-2.9%)	1.7% (0.48-3.4%)	27 (7.7-56)	140 (42-280)
Myanmar	1995	Male	95 plus	20% (11-30%)	80% (70-89%)	0.4 (0.15-0.76)	1.3% (0.29-2.9%)	1.4% (0.27-3.3%)	5.4 (1-12)	19 (4.3-43)
Myanmar	2000	Female	All Ages	2% (1.6-2.4%)	98% (98-98%)	0.029 (0.019-0.04)	0.52% (0.41-0.64%)	0.7% (0.53-0.92%)	1500 (1100-1900)	56000 (45000-68000)
Myanmar	2000	Female	15 to 19	1.9% (1.1-2.9%)	98% (97-99%)	0.027 (0.012-0.052)	0.4% (0.26-0.58%)	0.31% (0.17-0.5%)	11 (5.8-18)	1800 (1200-2700)
Myanmar	2000	Female	20 to 24	2.2% (1.2-3.3%)	98% (97-99%)	0.034 (0.015-0.066)	0.68% (0.46-0.97%)	0.39% (0.21-0.66%)	17 (9.3-29)	3400 (2200-5000)
Myanmar	2000	Female	25 to 29	2.2% (1.3-3.4%)	98% (97-99%)	0.034 (0.016-0.065)	0.74% (0.53-0.99%)	0.46% (0.28-0.73%)	21 (13-34)	3500 (2400-4900)
Myanmar	2000	Female	30 to 34	2.2% (1.2-3.5%)	98% (96-99%)	0.036 (0.017-0.066)	0.84% (0.63-1.1%)	0.74% (0.5-1.1%)	39 (26-57)	4100 (2900-5400)
Myanmar	2000	Female	35 to 39	2.4% (1.4-3.8%)	98% (96-99%)	0.041 (0.019-0.073)	1% (0.76-1.4%)	1.1% (0.77-1.7%)	70 (46-100)	5000 (3700-6900)
Myanmar	2000	Female	40 to 44	2.6% (1.5-3.8%)	97% (96-99%)	0.045 (0.023-0.08)	1.2% (0.87-1.6%)	1.5% (1-2%)	110 (73-150)	6000 (4300-8100)
Myanmar	2000	Female	45 to 49	2.6% (1.5-3.9%)	97% (96-99%)	0.049 (0.022-0.091)	1.2% (0.89-1.6%)	1.5% (1.1-2.1%)	130 (94-190)	6400 (4500-8600)
Myanmar	2000	Female	50 to 54	2.9% (1.8-4.2%)	97% (96-98%)	0.059 (0.027-0.1)	1.2% (0.84-1.7%)	1.5% (0.98-2.1%)	130 (88-190)	5200 (3600-7400)
Myanmar	2000	Female	55 to 59	3.1% (1.9-4.5%)	97% (95-98%)	0.068 (0.032-0.12)	1.1% (0.71-1.5%)	1.3% (0.81-1.8%)	140 (89-200)	4600 (3100-6700)
Myanmar	2000	Female	60 to 64	2.8% (1.7-4.5%)	97% (96-98%)	0.065 (0.03-0.12)	0.93% (0.6-1.3%)	1.1% (0.71-1.7%)	160 (100-240)	4500 (2900-6500)
Myanmar	2000	Female	65 to 69	2.6% (1.5-3.9%)	97% (96-98%)	0.061 (0.03-0.11)	0.78% (0.52-1.1%)	0.93% (0.61-1.4%)	180 (120-260)	4100 (2700-5900)
Myanmar	2000	Female	70 to 74	2.6% (1.6-4%)	97% (96-98%)	0.062 (0.028-0.12)	0.69% (0.44-1.1%)	0.82% (0.52-1.3%)	190 (120-290)	3400 (2200-5300)
Myanmar	2000	Female	75 to 79	2.8% (1.6-4.4%)	97% (96-98%)	0.065 (0.028-0.13)	0.56% (0.33-0.89%)	0.66% (0.38-1.1%)	140 (78-220)	1900 (1100-3100)
Myanmar	2000	Female	80 to 84	3% (1.6-4.4%)	97% (96-98%)	0.068 (0.03-0.13)	0.5% (0.27-0.8%)	0.59% (0.31-0.96%)	95 (51-150)	980 (540-1600)
Myanmar	2000	Female	85 to 89	3.3% (1.8-5.4%)	97% (95-98%)	0.074 (0.033-0.14)	0.42% (0.23-0.69%)	0.49% (0.26-0.82%)	45 (24-75)	330 (180-540)
Myanmar	2000	Female	90 to 94	3.9% (2.2-6.5%)	96% (93-98%)	0.084 (0.036-0.16)	0.35% (0.17-0.62%)	0.41% (0.19-0.73%)	13 (6.1-23)	67 (32-120)
Myanmar	2000	Female	95 plus	4.9% (2.4-9.2%)	95% (91-98%)	0.1 (0.034-0.22)	0.4% (0.16-0.76%)	0.45% (0.17-0.88%)	3.7 (1.4-7.2)	13 (5.3-25)
Myanmar	2000	Male	All Ages	21% (19-23%)	79% (77-81%)	0.69 (0.49-0.91)	4.3% (2.9-5.9%)	5.8% (3.8-7.9%)	14000 (8800-19000)	560000 (380000-760000)
Myanmar	2000	Male	15 to 19	15% (11-20%)	85% (80-89%)	0.25 (0.13-0.41)	1.4% (0.75-2.2%)	1.6% (0.7-2.8%)	87 (38-150)	8000 (4300-13000)

Location	Year	Sex	Age	Prevalence of current drinkers	Prevalence of abstainers	Population average of standard drinks daily	Percent of total attributable deaths	Percent of total attributable DALYs	Attributable deaths	Attributable DALYs
Myanmar	2000	Male	20 to 24	27% (21-33%)	73% (67-79%)	0.7 (0.41-1.1)	4.6% (2.6-7.2%)	5.2% (2.7-8.6%)	420 (200-690)	32000 (17000-50000)
Myanmar	2000	Male	25 to 29	34% (28-40%)	66% (60-72%)	1.1 (0.7-1.6)	7.6% (4.2-11%)	9% (4.7-14%)	780 (410-1200)	52000 (29000-79000)
Myanmar	2000	Male	30 to 34	37% (30-43%)	63% (57-70%)	1.3 (0.84-1.9)	9.3% (5.7-13%)	11% (6.6-17%)	1100 (640-1500)	64000 (39000-91000)
Myanmar	2000	Male	35 to 39	37% (30-43%)	63% (57-70%)	1.5 (0.89-2.2)	11% (6.6-15%)	13% (7.9-18%)	1300 (810-1900)	72000 (44000-100000)
Myanmar	2000	Male	40 to 44	37% (31-43%)	63% (57-69%)	1.6 (1-2.4)	11% (7.5-16%)	14% (9.1-19%)	1600 (1000-2300)	77000 (50000-110000)
Myanmar	2000	Male	45 to 49	35% (29-41%)	65% (59-71%)	1.7 (1-2.6)	12% (7.9-17%)	15% (9.5-21%)	1900 (1200-2700)	80000 (51000-110000)
Myanmar	2000	Male	50 to 54	32% (26-38%)	68% (62-74%)	1.6 (0.9-2.4)	11% (6.5-16%)	13% (7.8-19%)	1600 (900-2300)	57000 (34000-85000)
Myanmar	2000	Male	55 to 59	26% (21-33%)	74% (67-79%)	1.2 (0.68-1.8)	7.9% (4.6-12%)	9.4% (5.5-14%)	1200 (700-1900)	40000 (23000-62000)
Myanmar	2000	Male	60 to 64	20% (15-27%)	80% (73-85%)	0.85 (0.47-1.5)	5.8% (3.1-9.3%)	6.7% (3.6-11%)	1100 (570-1800)	30000 (16000-49000)
Myanmar	2000	Male	65 to 69	17% (12-22%)	83% (78-88%)	0.68 (0.38-1.1)	4.3% (2.4-7.1%)	5% (2.7-8.3%)	940 (500-1500)	21000 (11000-35000)
Myanmar	2000	Male	70 to 74	15% (11-21%)	85% (79-89%)	0.57 (0.28-0.98)	3.6% (1.8-6.3%)	4.1% (2-7.3%)	850 (410-1500)	15000 (7500-27000)
Myanmar	2000	Male	75 to 79	15% (9.6-21%)	85% (79-90%)	0.53 (0.25-0.93)	2.8% (1.3-5.1%)	3.2% (1.4-5.8%)	540 (230-1000)	7600 (3300-14000)
Myanmar	2000	Male	80 to 84	15% (9.5-20%)	85% (80-90%)	0.49 (0.24-0.83)	2.5% (1.1-4.4%)	2.9% (1.2-5.2%)	310 (130-560)	3200 (1400-5700)
Myanmar	2000	Male	85 to 89	15% (9.5-22%)	85% (78-91%)	0.46 (0.22-0.79)	2% (0.82-3.6%)	2.3% (0.93-4.2%)	130 (52-230)	950 (390-1700)
Myanmar	2000	Male	90 to 94	17% (11-25%)	83% (75-89%)	0.45 (0.21-0.81)	1.6% (0.54-3.1%)	1.8% (0.62-3.7%)	35 (11-69)	180 (61-360)
Myanmar	2000	Male	95 plus	20% (11-30%)	80% (70-89%)	0.45 (0.17-0.85)	1.4% (0.38-3%)	1.6% (0.37-3.4%)	7.2 (1.7-16)	25 (6.7-54)
Myanmar	2005	Female	All Ages	2% (1.6-2.4%)	98% (98-98%)	0.032 (0.021-0.045)	0.57% (0.45-0.69%)	0.76% (0.58-0.96%)	1500 (1200-1900)	55000 (45000-67000)
Myanmar	2005	Female	15 to 19	1.8% (1.1-2.9%)	98% (97-99%)	0.029 (0.013-0.055)	0.41% (0.26-0.58%)	0.3% (0.17-0.48%)	8 (4.4-13)	1600 (990-2300)
Myanmar	2005	Female	20 to 24	2.1% (1.1-3.2%)	98% (97-99%)	0.036 (0.016-0.071)	0.72% (0.49-1%)	0.38% (0.22-0.61%)	14 (8.2-23)	3300 (2200-4800)
Myanmar	2005	Female	25 to 29	2.1% (1.2-3.3%)	98% (97-99%)	0.037 (0.016-0.069)	0.76% (0.54-1%)	0.45% (0.29-0.66%)	18 (12-28)	3400 (2300-4800)
Myanmar	2005	Female	30 to 34	2.2% (1.2-3.5%)	98% (96-99%)	0.038 (0.018-0.073)	0.84% (0.63-1.1%)	0.72% (0.49-1%)	34 (23-49)	3800 (2700-5200)
Myanmar	2005	Female	35 to 39	2.4% (1.4-3.6%)	98% (96-99%)	0.044 (0.021-0.078)	1% (0.74-1.3%)	1.1% (0.75-1.6%)	64 (42-92)	4800 (3500-6400)

Location	Year	Sex	Age	Prevalence of current drinkers	Prevalence of abstainers	Population average of standard drinks daily	Percent of total attributable deaths	Percent of total attributable DALYs	Attributable deaths	Attributable DALYs
Myanmar	2005	Female	40 to 44	2.5% (1.5-3.7%)	97% (96-98%)	0.049 (0.025-0.085)	1.1% (0.84-1.5%)	1.4% (1-2%)	96 (67-140)	5600 (4100-7500)
Myanmar	2005	Female	45 to 49	2.6% (1.5-3.8%)	97% (96-99%)	0.053 (0.024-0.097)	1.2% (0.86-1.6%)	1.5% (1-2.1%)	130 (87-180)	6100 (4400-8200)
Myanmar	2005	Female	50 to 54	2.8% (1.8-4.1%)	97% (96-98%)	0.063 (0.03-0.11)	1.2% (0.83-1.6%)	1.5% (1-2.1%)	160 (110-240)	6600 (4600-9200)
Myanmar	2005	Female	55 to 59	3% (1.8-4.5%)	97% (95-98%)	0.073 (0.035-0.13)	1% (0.71-1.5%)	1.3% (0.85-1.9%)	140 (93-210)	4800 (3200-6900)
Myanmar	2005	Female	60 to 64	2.8% (1.7-4.3%)	97% (96-98%)	0.07 (0.032-0.13)	0.94% (0.62-1.4%)	1.2% (0.76-1.7%)	150 (99-220)	4300 (2800-6100)
Myanmar	2005	Female	65 to 69	2.6% (1.5-3.8%)	97% (96-99%)	0.065 (0.032-0.12)	0.8% (0.54-1.1%)	0.98% (0.64-1.4%)	170 (110-240)	3900 (2600-5500)
Myanmar	2005	Female	70 to 74	2.6% (1.6-4.1%)	97% (96-98%)	0.067 (0.03-0.13)	0.72% (0.46-1.1%)	0.87% (0.55-1.3%)	190 (120-280)	3400 (2200-5200)
Myanmar	2005	Female	75 to 79	2.7% (1.6-4.4%)	97% (96-98%)	0.071 (0.032-0.14)	0.57% (0.35-0.9%)	0.69% (0.41-1.1%)	160 (93-240)	2200 (1300-3400)
Myanmar	2005	Female	80 to 84	2.9% (1.6-4.4%)	97% (96-98%)	0.074 (0.032-0.14)	0.52% (0.3-0.81%)	0.62% (0.36-0.97%)	110 (62-170)	1100 (650-1700)
Myanmar	2005	Female	85 to 89	3.3% (1.8-5.3%)	97% (95-98%)	0.081 (0.036-0.16)	0.44% (0.24-0.72%)	0.52% (0.28-0.85%)	52 (28-87)	380 (210-630)
Myanmar	2005	Female	90 to 94	3.9% (2.2-6.4%)	96% (94-98%)	0.092 (0.039-0.17)	0.37% (0.19-0.64%)	0.43% (0.22-0.75%)	16 (8.1-28)	84 (43-150)
Myanmar	2005	Female	95 plus	4.8% (2.4-9%)	95% (91-98%)	0.11 (0.037-0.24)	0.42% (0.18-0.78%)	0.47% (0.18-0.9%)	4.4 (1.7-8.5)	16 (6.7-29)
Myanmar	2005	Male	All Ages	21% (19-24%)	79% (76-81%)	0.78 (0.56-1)	4.5% (3.2-6%)	5.9% (4-7.8%)	14000 (9100-20000)	550000 (380000-770000)
Myanmar	2005	Male	15 to 19	15% (11-20%)	85% (80-89%)	0.26 (0.14-0.44)	1.4% (0.84-2.2%)	1.7% (0.8-2.8%)	74 (35-120)	7000 (4100-11000)
Myanmar	2005	Male	20 to 24	26% (21-32%)	74% (68-79%)	0.75 (0.44-1.2)	4.4% (2.7-6.6%)	5.1% (2.8-8%)	380 (200-610)	30000 (17000-46000)
Myanmar	2005	Male	25 to 29	34% (28-39%)	66% (61-72%)	1.2 (0.74-1.8)	7% (4.3-10%)	8.3% (4.7-13%)	720 (400-1100)	49000 (29000-74000)
Myanmar	2005	Male	30 to 34	36% (30-42%)	64% (58-70%)	1.4 (0.91-2.1)	8.4% (5.4-12%)	10% (6.1-15%)	980 (590-1400)	60000 (37000-85000)
Myanmar	2005	Male	35 to 39	36% (30-42%)	64% (58-70%)	1.6 (0.98-2.4)	9.7% (6.3-14%)	12% (7.6-16%)	1300 (820-1900)	70000 (45000-100000)
Myanmar	2005	Male	40 to 44	36% (30-42%)	64% (58-70%)	1.7 (1.1-2.6)	11% (7.1-14%)	13% (8.6-18%)	1600 (1000-2300)	74000 (49000-110000)
Myanmar	2005	Male	45 to 49	35% (28-40%)	65% (60-72%)	1.8 (1.1-2.8)	11% (7.5-16%)	14% (9-19%)	1900 (1200-2700)	79000 (51000-110000)
Myanmar	2005	Male	50 to 54	32% (26-37%)	68% (63-74%)	1.7 (0.96-2.6)	10% (6.3-15%)	12% (7.6-18%)	1800 (1100-2800)	69000 (41000-100000)
Myanmar	2005	Male	55 to 59	26% (20-32%)	74% (68-80%)	1.2 (0.73-2)	7.5% (4.7-11%)	9.1% (5.5-13%)	1200 (730-2000)	40000 (24000-63000)

Location	Year	Sex	Age	Prevalence of current drinkers	Prevalence of abstainers	Population average of standard drinks daily	Percent of total attributable deaths	Percent of total attributable DALYs	Attributable deaths	Attributable DALYs
Myanmar	2005	Male	60 to 64	20% (15-26%)	80% (74-85%)	0.9 (0.49-1.5)	5.6% (3.1-8.9%)	6.6% (3.6-10%)	1000 (550-1700)	28000 (15000-46000)
Myanmar	2005	Male	65 to 69	17% (12-22%)	83% (78-88%)	0.72 (0.4-1.2)	4.2% (2.4-6.7%)	4.9% (2.8-8%)	870 (480-1400)	20000 (11000-32000)
Myanmar	2005	Male	70 to 74	15% (10-20%)	85% (80-90%)	0.6 (0.3-1)	3.5% (1.8-5.9%)	4% (2.1-6.9%)	800 (400-1400)	15000 (7400-25000)
Myanmar	2005	Male	75 to 79	14% (9.4-20%)	86% (80-91%)	0.56 (0.27-1)	2.7% (1.3-4.9%)	3.2% (1.5-5.6%)	580 (260-1100)	8200 (3800-15000)
Myanmar	2005	Male	80 to 84	14% (9.4-20%)	86% (80-91%)	0.52 (0.26-0.88)	2.5% (1.1-4.2%)	2.9% (1.3-5%)	340 (150-600)	3600 (1600-6300)
Myanmar	2005	Male	85 to 89	15% (9.3-22%)	85% (78-91%)	0.48 (0.22-0.85)	1.9% (0.88-3.5%)	2.2% (1-4%)	140 (62-260)	1100 (470-1900)
Myanmar	2005	Male	90 to 94	16% (11-24%)	84% (76-89%)	0.47 (0.22-0.84)	1.5% (0.58-2.9%)	1.8% (0.65-3.4%)	40 (15-78)	210 (78-400)
Myanmar	2005	Male	95 plus	19% (11-29%)	81% (71-89%)	0.47 (0.18-0.89)	1.4% (0.42-2.8%)	1.5% (0.41-3.2%)	8 (2.1-17)	28 (9-58)
Myanmar	2010	Female	All Ages	2.2% (1.9-2.7%)	98% (97-98%)	0.045 (0.029-0.064)	0.67% (0.54-0.82%)	0.88% (0.67-1.1%)	1600 (1200-2000)	57000 (45000-69000)
Myanmar	2010	Female	15 to 19	1.9% (1.1-3%)	98% (97-99%)	0.037 (0.017-0.073)	0.44% (0.28-0.65%)	0.32% (0.18-0.49%)	6 (3.5-9.1)	1400 (880-2100)
Myanmar	2010	Female	20 to 24	2.4% (1.3-3.7%)	98% (96-99%)	0.049 (0.021-0.097)	0.82% (0.54-1.2%)	0.41% (0.24-0.66%)	9.9 (5.6-16)	2800 (1800-4200)
Myanmar	2010	Female	25 to 29	2.6% (1.4-4.2%)	97% (96-99%)	0.054 (0.023-0.11)	0.85% (0.61-1.2%)	0.5% (0.32-0.76%)	15 (9.6-23)	3200 (2200-4600)
Myanmar	2010	Female	30 to 34	2.8% (1.5-4.5%)	97% (95-98%)	0.058 (0.026-0.11)	0.89% (0.66-1.2%)	0.78% (0.54-1.1%)	30 (20-43)	3800 (2700-5100)
Myanmar	2010	Female	35 to 39	2.9% (1.6-4.5%)	97% (95-98%)	0.063 (0.03-0.12)	1% (0.77-1.3%)	1.2% (0.83-1.7%)	58 (39-82)	4600 (3300-6200)
Myanmar	2010	Female	40 to 44	3% (1.8-4.5%)	97% (96-98%)	0.068 (0.033-0.12)	1.2% (0.88-1.5%)	1.5% (1.1-2.1%)	93 (65-130)	5600 (4200-7400)
Myanmar	2010	Female	45 to 49	3% (1.6-4.5%)	97% (95-98%)	0.072 (0.032-0.13)	1.2% (0.87-1.6%)	1.6% (1.1-2.2%)	120 (84-180)	6100 (4500-8200)
Myanmar	2010	Female	50 to 54	3% (1.8-4.7%)	97% (95-98%)	0.08 (0.036-0.15)	1.2% (0.84-1.7%)	1.6% (1.1-2.2%)	160 (110-240)	6600 (4700-9200)
Myanmar	2010	Female	55 to 59	3.1% (1.7-4.8%)	97% (95-98%)	0.087 (0.041-0.17)	1.1% (0.73-1.5%)	1.4% (0.91-2%)	180 (120-270)	6300 (4200-8900)
Myanmar	2010	Female	60 to 64	2.9% (1.7-4.8%)	97% (95-98%)	0.087 (0.039-0.16)	0.98% (0.64-1.4%)	1.3% (0.81-1.8%)	160 (100-230)	4600 (3000-6600)
Myanmar	2010	Female	65 to 69	2.7% (1.5-4.1%)	97% (96-98%)	0.083 (0.04-0.15)	0.86% (0.58-1.2%)	1.1% (0.72-1.6%)	170 (110-240)	3800 (2600-5600)
Myanmar	2010	Female	70 to 74	2.6% (1.6-4.1%)	97% (96-98%)	0.082 (0.035-0.16)	0.78% (0.49-1.2%)	0.96% (0.59-1.5%)	180 (120-270)	3300 (2100-5100)
Myanmar	2010	Female	75 to 79	2.7% (1.6-4.5%)	97% (96-98%)	0.084 (0.037-0.17)	0.62% (0.37-0.99%)	0.76% (0.45-1.2%)	160 (93-250)	2200 (1300-3600)

Location	Year	Sex	Age	Prevalence of current drinkers	Prevalence of abstainers	Population average of standard drinks daily	Percent of total attributable deaths	Percent of total attributable DALYs	Attributable deaths	Attributable DALYs
Myanmar	2010	Female	80 to 84	3% (1.6-4.5%)	97% (95-98%)	0.088 (0.038-0.16)	0.57% (0.34-0.91%)	0.69% (0.41-1.1%)	120 (72-190)	1300 (740-2000)
Myanmar	2010	Female	85 to 89	3.3% (1.8-5.5%)	97% (94-98%)	0.097 (0.041-0.19)	0.49% (0.28-0.81%)	0.58% (0.33-0.97%)	68 (38-110)	500 (290-840)
Myanmar	2010	Female	90 to 94	3.9% (2.2-6.5%)	96% (94-98%)	0.11 (0.046-0.21)	0.42% (0.22-0.69%)	0.49% (0.24-0.83%)	22 (11-37)	110 (58-190)
Myanmar	2010	Female	95 plus	4.8% (2.3-9.2%)	95% (91-98%)	0.13 (0.043-0.28)	0.47% (0.21-0.87%)	0.52% (0.22-0.97%)	6.2 (2.6-11)	22 (9.7-39)
Myanmar	2010	Male	All Ages	20% (17-23%)	80% (77-83%)	0.86 (0.61-1.2)	5% (3.5-6.6%)	6.4% (4.3-8.4%)	14000 (9300-20000)	540000 (380000-740000)
Myanmar	2010	Male	15 to 19	15% (9.9-21%)	85% (79-90%)	0.32 (0.16-0.56)	1.5% (0.95-2.4%)	1.9% (0.95-3.1%)	71 (34-120)	6700 (4000-10000)
Myanmar	2010	Male	20 to 24	25% (16-33%)	75% (67-84%)	0.83 (0.43-1.4)	4.5% (2.7-6.8%)	5.2% (2.9-8.3%)	300 (160-510)	24000 (15000-39000)
Myanmar	2010	Male	25 to 29	29% (19-39%)	71% (61-81%)	1.2 (0.69-1.9)	6.5% (4.1-9.5%)	7.8% (4.5-12%)	580 (340-930)	41000 (26000-63000)
Myanmar	2010	Male	30 to 34	31% (21-43%)	69% (57-79%)	1.5 (0.84-2.4)	7.7% (4.9-11%)	9.3% (5.6-14%)	840 (510-1300)	53000 (34000-76000)
Myanmar	2010	Male	35 to 39	33% (22-43%)	67% (57-78%)	1.7 (0.93-2.7)	8.9% (6-13%)	11% (7.2-16%)	1100 (720-1700)	63000 (41000-91000)
Myanmar	2010	Male	40 to 44	32% (22-42%)	68% (58-78%)	1.8 (1-2.9)	9.6% (6.5-13%)	12% (8.1-16%)	1400 (880-2100)	69000 (44000-100000)
Myanmar	2010	Male	45 to 49	30% (20-40%)	70% (60-80%)	1.9 (1-3.1)	10% (6.8-14%)	13% (8.6-18%)	1700 (1100-2600)	74000 (47000-110000)
Myanmar	2010	Male	50 to 54	28% (19-38%)	72% (62-81%)	1.7 (0.93-2.8)	9.7% (5.8-14%)	12% (7.3-17%)	1800 (1100-2700)	68000 (41000-100000)
Myanmar	2010	Male	55 to 59	25% (17-34%)	75% (66-83%)	1.4 (0.74-2.4)	7.8% (4.7-11%)	9.5% (5.8-14%)	1600 (950-2500)	52000 (32000-81000)
Myanmar	2010	Male	60 to 64	23% (15-32%)	77% (68-85%)	1.2 (0.62-2.1)	6.3% (3.5-9.9%)	7.5% (4.2-12%)	1200 (640-1900)	33000 (18000-53000)
Myanmar	2010	Male	65 to 69	20% (14-27%)	80% (73-86%)	1 (0.55-1.8)	5.1% (2.8-8.1%)	6% (3.3-9.8%)	1000 (550-1700)	24000 (13000-39000)
Myanmar	2010	Male	70 to 74	18% (12-24%)	82% (76-88%)	0.87 (0.42-1.5)	4.2% (2.1-7%)	4.9% (2.4-8.3%)	930 (450-1600)	17000 (8400-30000)
Myanmar	2010	Male	75 to 79	16% (10-23%)	84% (77-90%)	0.74 (0.34-1.4)	3.2% (1.5-5.7%)	3.7% (1.8-6.5%)	680 (310-1200)	9800 (4500-17000)
Myanmar	2010	Male	80 to 84	15% (9.1-21%)	85% (79-91%)	0.62 (0.29-1.1)	2.7% (1.3-4.7%)	3.1% (1.4-5.4%)	410 (190-720)	4300 (2000-7600)
Myanmar	2010	Male	85 to 89	14% (8.5-21%)	86% (79-91%)	0.54 (0.25-0.97)	2.1% (0.99-3.7%)	2.4% (1.1-4.3%)	180 (85-330)	1400 (660-2500)
Myanmar	2010	Male	90 to 94	15% (9.8-23%)	85% (77-90%)	0.52 (0.24-0.95)	1.6% (0.69-3.1%)	1.9% (0.77-3.6%)	51 (21-99)	270 (110-510)
Myanmar	2010	Male	95 plus	18% (9.8-28%)	82% (72-90%)	0.53 (0.2-1)	1.5% (0.53-3%)	1.6% (0.52-3.4%)	11 (3.4-23)	39 (14-80)

Location	Year	Sex	Age	Prevalence of current drinkers	Prevalence of abstainers	Population average of standard drinks daily	Percent of total attributable deaths	Percent of total attributable DALYs	Attributable deaths	Attributable DALYs
Myanmar	2016	Female	All Ages	2.3% (1.9-2.7%)	98% (97-98%)	0.054 (0.032-0.082)	0.78% (0.63-0.96%)	0.99% (0.76-1.3%)	1600 (1200-2100)	58000 (46000-71000)
Myanmar	2016	Female	15 to 19	1.9% (1.1-3%)	98% (97-99%)	0.043 (0.018-0.088)	0.48% (0.3-0.69%)	0.35% (0.21-0.57%)	5.4 (3.3-8.7)	1400 (850-2100)
Myanmar	2016	Female	20 to 24	2.4% (1.3-3.6%)	98% (96-99%)	0.057 (0.023-0.12)	0.92% (0.62-1.3%)	0.45% (0.27-0.75%)	8.2 (4.8-13)	2800 (1800-4200)
Myanmar	2016	Female	25 to 29	2.6% (1.4-4.1%)	97% (96-99%)	0.062 (0.027-0.12)	0.96% (0.67-1.3%)	0.56% (0.36-0.85%)	12 (7.6-18)	3100 (2000-4300)
Myanmar	2016	Female	30 to 34	2.7% (1.4-4.6%)	97% (95-99%)	0.067 (0.028-0.13)	0.98% (0.72-1.3%)	0.87% (0.6-1.2%)	24 (16-34)	3500 (2400-4900)
Myanmar	2016	Female	35 to 39	2.9% (1.5-4.4%)	97% (96-98%)	0.072 (0.032-0.14)	1.1% (0.8-1.4%)	1.3% (0.92-1.8%)	50 (34-70)	4400 (3100-5700)
Myanmar	2016	Female	40 to 44	2.9% (1.7-4.5%)	97% (96-98%)	0.078 (0.036-0.15)	1.2% (0.87-1.6%)	1.6% (1.2-2.3%)	85 (60-120)	5400 (4000-7200)
Myanmar	2016	Female	45 to 49	2.9% (1.6-4.5%)	97% (96-98%)	0.084 (0.035-0.16)	1.2% (0.88-1.6%)	1.7% (1.2-2.3%)	120 (82-170)	6100 (4500-8300)
Myanmar	2016	Female	50 to 54	3% (1.8-4.6%)	97% (95-98%)	0.092 (0.041-0.18)	1.2% (0.83-1.7%)	1.6% (1.1-2.3%)	150 (100-220)	6500 (4500-8900)
Myanmar	2016	Female	55 to 59	3% (1.7-4.6%)	97% (95-98%)	0.1 (0.042-0.19)	1.1% (0.74-1.5%)	1.5% (0.95-2.1%)	180 (110-260)	6300 (4300-8900)
Myanmar	2016	Female	60 to 64	2.9% (1.7-4.7%)	97% (95-98%)	0.1 (0.044-0.19)	1% (0.64-1.4%)	1.3% (0.85-1.9%)	210 (130-310)	6100 (4000-8800)
Myanmar	2016	Female	65 to 69	2.7% (1.6-4%)	97% (96-98%)	0.096 (0.043-0.18)	0.9% (0.6-1.3%)	1.2% (0.77-1.7%)	190 (120-280)	4300 (2800-6400)
Myanmar	2016	Female	70 to 74	2.6% (1.6-4%)	97% (96-98%)	0.095 (0.039-0.19)	0.82% (0.51-1.3%)	1.1% (0.65-1.6%)	180 (110-270)	3300 (2000-5000)
Myanmar	2016	Female	75 to 79	2.7% (1.6-4.3%)	97% (96-98%)	0.098 (0.039-0.2)	0.67% (0.39-1.1%)	0.83% (0.49-1.3%)	160 (91-250)	2200 (1300-3600)
Myanmar	2016	Female	80 to 84	2.9% (1.6-4.4%)	97% (96-98%)	0.1 (0.041-0.19)	0.62% (0.36-1%)	0.76% (0.45-1.3%)	130 (73-210)	1300 (770-2200)
Myanmar	2016	Female	85 to 89	3.3% (1.8-5.3%)	97% (95-98%)	0.11 (0.046-0.22)	0.53% (0.29-0.93%)	0.64% (0.35-1.1%)	83 (45-140)	620 (330-1100)
Myanmar	2016	Female	90 to 94	3.9% (2.2-6.5%)	96% (93-98%)	0.13 (0.051-0.26)	0.47% (0.23-0.8%)	0.55% (0.26-0.94%)	32 (15-56)	170 (83-290)
Myanmar	2016	Female	95 plus	4.9% (2.4-9.2%)	95% (91-98%)	0.16 (0.05-0.34)	0.52% (0.22-1%)	0.59% (0.24-1.1%)	9.2 (3.7-18)	33 (14-63)
Myanmar	2016	Male	All Ages	20% (17-23%)	80% (77-83%)	1.1 (0.67-1.6)	5.8% (4.1-7.7%)	7.1% (4.7-9.5%)	15000 (9700-20000)	560000 (390000-750000)
Myanmar	2016	Male	15 to 19	15% (9.8-21%)	85% (79-90%)	0.37 (0.17-0.68)	1.7% (1-2.7%)	2.1% (1.1-3.7%)	78 (40-140)	7400 (4400-12000)
Myanmar	2016	Male	20 to 24	24% (16-34%)	76% (66-84%)	0.97 (0.47-1.7)	4.9% (2.9-7.7%)	5.7% (3-9.5%)	310 (160-520)	25000 (15000-40000)
Myanmar	2016	Male	25 to 29	29% (20-39%)	71% (61-80%)	1.4 (0.72-2.4)	7% (4.4-10%)	8.6% (5.1-13%)	500 (280-790)	36000 (22000-55000)

Location	Year	Sex	Age	Prevalence of current drinkers	Prevalence of abstainers	Population average of standard drinks daily	Percent of total attributable deaths	Percent of total attributable DALYs	Attributable deaths	Attributable DALYs
Myanmar	2016	Male	30 to 34	31% (21-43%)	69% (57-79%)	1.7 (0.93-2.9)	8.3% (5.4-12%)	10% (6.4-15%)	740 (440-1100)	47000 (30000-69000)
Myanmar	2016	Male	35 to 39	32% (22-43%)	68% (57-78%)	1.9 (1-3.4)	9.5% (6.3-13%)	12% (8-17%)	1000 (660-1500)	58000 (39000-83000)
Myanmar	2016	Male	40 to 44	32% (22-42%)	68% (58-78%)	2.1 (1.1-3.6)	10% (6.9-14%)	13% (9-17%)	1300 (860-1800)	65000 (44000-89000)
Myanmar	2016	Male	45 to 49	30% (19-39%)	70% (61-81%)	2.2 (1.1-3.8)	11% (7.2-15%)	14% (9.3-19%)	1700 (1100-2400)	74000 (48000-100000)
Myanmar	2016	Male	50 to 54	28% (19-38%)	72% (62-81%)	2 (1-3.6)	10% (6.3-14%)	13% (8-17%)	1800 (1100-2600)	70000 (44000-97000)
Myanmar	2016	Male	55 to 59	25% (17-35%)	75% (65-83%)	1.7 (0.82-3)	8.3% (5-12%)	10% (6.1-15%)	1800 (1100-2600)	59000 (36000-86000)
Myanmar	2016	Male	60 to 64	23% (15-33%)	77% (67-85%)	1.4 (0.67-2.5)	6.7% (3.6-10%)	8% (4.4-12%)	1700 (920-2600)	47000 (26000-73000)
Myanmar	2016	Male	65 to 69	20% (13-27%)	80% (73-87%)	1.2 (0.6-2.2)	5.4% (2.9-8.6%)	6.5% (3.4-10%)	1300 (680-2100)	30000 (16000-48000)
Myanmar	2016	Male	70 to 74	18% (12-24%)	82% (76-88%)	1 (0.46-1.8)	4.5% (2.1-7.4%)	5.3% (2.4-8.8%)	1000 (480-1700)	19000 (9200-32000)
Myanmar	2016	Male	75 to 79	16% (10-23%)	84% (77-90%)	0.85 (0.38-1.6)	3.5% (1.6-6.1%)	4% (1.8-7.1%)	760 (340-1300)	11000 (4900-19000)
Myanmar	2016	Male	80 to 84	14% (9.1-20%)	86% (80-91%)	0.71 (0.31-1.3)	3% (1.3-5.1%)	3.4% (1.5-5.9%)	470 (210-810)	5000 (2200-8600)
Myanmar	2016	Male	85 to 89	14% (8.4-21%)	86% (79-92%)	0.62 (0.26-1.2)	2.3% (1-4.1%)	2.6% (1.2-4.7%)	230 (110-430)	1800 (810-3200)
Myanmar	2016	Male	90 to 94	15% (9.5-23%)	85% (77-90%)	0.6 (0.25-1.2)	1.8% (0.73-3.5%)	2.1% (0.84-4%)	76 (31-150)	400 (160-780)
Myanmar	2016	Male	95 plus	18% (10-28%)	82% (72-90%)	0.62 (0.22-1.3)	1.7% (0.57-3.4%)	1.8% (0.56-3.8%)	16 (4.9-34)	57 (20-120)
Philippines	1990	Female	All Ages	16% (13-19%)	84% (81-87%)	0.38 (0.32-0.47)	1.2% (0.78-1.7%)	2.1% (1.2-3.3%)	3300 (1800-5100)	120000 (79000-170000)
Philippines	1990	Female	15 to 19	16% (11-23%)	84% (77-89%)	0.4 (0.23-0.64)	1.1% (0.66-1.8%)	1.8% (0.88-3.1%)	52 (26-91)	5300 (3100-8400)
Philippines	1990	Female	20 to 24	20% (13-28%)	80% (72-87%)	0.55 (0.31-0.89)	1.8% (1.1-2.7%)	2.3% (1.1-4%)	77 (35-130)	8300 (4800-13000)
Philippines	1990	Female	25 to 29	22% (14-31%)	78% (69-86%)	0.62 (0.36-0.97)	2.1% (1.2-3.3%)	2.9% (1.4-5%)	110 (50-180)	9500 (5600-15000)
Philippines	1990	Female	30 to 34	23% (15-33%)	77% (67-85%)	0.68 (0.39-1)	2.3% (1.4-3.6%)	3.5% (1.7-5.7%)	130 (67-220)	10000 (5800-15000)
Philippines	1990	Female	35 to 39	24% (15-32%)	76% (68-85%)	0.74 (0.42-1.1)	2.7% (1.5-4.4%)	4.3% (2.1-7.1%)	180 (86-290)	11000 (6100-17000)
Philippines	1990	Female	40 to 44	23% (16-31%)	77% (69-84%)	0.74 (0.43-1.1)	2.8% (1.5-4.5%)	4.6% (2.4-7.5%)	180 (90-290)	9400 (5000-15000)
Philippines	1990	Female	45 to 49	22% (14-30%)	78% (70-86%)	0.71 (0.4-1.2)	2.9% (1.4-4.9%)	4.6% (2.1-7.8%)	200 (94-350)	9200 (4500-15000)

Location	Year	Sex	Age	Prevalence of current drinkers	Prevalence of abstainers	Population average of standard drinks daily	Percent of total attributable deaths	Percent of total attributable DALYs	Attributable deaths	Attributable DALYs
Philippines	1990	Female	50 to 54	22% (15-31%)	78% (69-85%)	0.79 (0.45-1.3)	3.3% (1.6-5.8%)	5% (2.4-8.7%)	270 (130-480)	11000 (5100-18000)
Philippines	1990	Female	55 to 59	23% (15-31%)	77% (69-85%)	0.97 (0.56-1.5)	4.2% (2-7%)	6.2% (3-10%)	370 (180-620)	12000 (6000-21000)
Philippines	1990	Female	60 to 64	21% (14-30%)	79% (70-86%)	0.93 (0.54-1.5)	3.9% (1.9-6.8%)	5.5% (2.6-9.7%)	390 (180-690)	11000 (5300-19000)
Philippines	1990	Female	65 to 69	19% (13-27%)	81% (73-87%)	0.76 (0.44-1.2)	3.1% (1.3-5.4%)	4.3% (1.9-7.5%)	360 (160-640)	8400 (3800-15000)
Philippines	1990	Female	70 to 74	18% (13-26%)	82% (74-87%)	0.65 (0.35-1.1)	2.3% (0.64-4.7%)	3.1% (0.88-6.2%)	300 (86-610)	5700 (1500-11000)
Philippines	1990	Female	75 to 79	19% (12-27%)	81% (73-88%)	0.62 (0.32-1.1)	1.8% (0.19-4.2%)	2.2% (0.23-5.3%)	250 (27-600)	3700 (380-8800)
Philippines	1990	Female	80 to 84	20% (13-27%)	80% (73-87%)	0.65 (0.36-1.1)	1.6% (0.18-3.7%)	1.9% (0.19-4.3%)	210 (22-500)	2300 (250-5300)
Philippines	1990	Female	85 to 89	22% (14-31%)	78% (69-86%)	0.7 (0.37-1.2)	1.4% (0.039-3.3%)	1.6% (0.019-3.8%)	130 (1.5-310)	960 (28-2300)
Philippines	1990	Female	90 to 94	24% (16-34%)	76% (66-84%)	0.8 (0.42-1.4)	1.2% (-0.14-3.3%)	1.3% (-0.24-3.8%)	46 (-8.3-130)	250 (-30-690)
Philippines	1990	Female	95 plus	28% (16-42%)	72% (58-84%)	0.93 (0.42-1.7)	1.3% (-0.34-4.2%)	1.4% (-0.51-4.8%)	18 (-6.5-59)	65 (-16-200)
Philippines	1990	Male	All Ages	56% (50-62%)	44% (38-50%)	1.7 (1.5-1.9)	5.8% (4.7-6.9%)	8.2% (6.5-10%)	18000 (14000-23000)	780000 (630000-930000)
Philippines	1990	Male	15 to 19	54% (43-65%)	46% (35-57%)	1.3 (0.79-1.8)	4.3% (2.8-6%)	6.1% (3.8-8.6%)	320 (200-450)	27000 (17000-37000)
Philippines	1990	Male	20 to 24	74% (64-81%)	26% (19-36%)	2.8 (2-3.7)	11% (7.8-14%)	14% (9.7-18%)	1000 (730-1400)	79000 (57000-100000)
Philippines	1990	Male	25 to 29	77% (69-84%)	23% (16-31%)	3.1 (2.3-4)	12% (9.3-16%)	16% (12-20%)	1300 (940-1700)	89000 (67000-110000)
Philippines	1990	Male	30 to 34	79% (71-85%)	21% (15-29%)	3.4 (2.7-4.3)	13% (10-16%)	17% (13-22%)	1400 (1000-1800)	87000 (66000-110000)
Philippines	1990	Male	35 to 39	79% (71-85%)	21% (15-29%)	3.8 (2.7-4.9)	15% (11-18%)	19% (14-24%)	1600 (1200-2100)	90000 (67000-110000)
Philippines	1990	Male	40 to 44	78% (71-84%)	22% (16-29%)	3.9 (2.8-5.1)	15% (11-19%)	20% (15-25%)	1600 (1200-2100)	79000 (58000-100000)
Philippines	1990	Male	45 to 49	75% (66-82%)	25% (18-34%)	3.9 (2.7-5.3)	16% (11-21%)	20% (14-26%)	1800 (1300-2400)	80000 (55000-110000)
Philippines	1990	Male	50 to 54	71% (62-78%)	29% (22-38%)	3.6 (2.5-5)	15% (10-20%)	19% (13-25%)	1900 (1300-2600)	73000 (48000-97000)
Philippines	1990	Male	55 to 59	66% (55-75%)	34% (25-45%)	3.3 (2.3-4.6)	14% (9.3-19%)	17% (12-23%)	1900 (1300-2700)	63000 (42000-88000)
Philippines	1990	Male	60 to 64	62% (49-72%)	38% (28-51%)	2.8 (1.9-4.2)	12% (7.2-17%)	14% (8.5-21%)	1700 (1000-2500)	47000 (28000-69000)
Philippines	1990	Male	65 to 69	58% (45-70%)	42% (30-55%)	2.2 (1.5-3.2)	8.9% (5.2-14%)	11% (6.3-16%)	1300 (750-2000)	30000 (18000-46000)

Location	Year	Sex	Age	Prevalence of current drinkers	Prevalence of abstainers	Population average of standard drinks daily	Percent of total attributable deaths	Percent of total attributable DALYs	Attributable deaths	Attributable DALYs
Philippines	1990	Male	70 to 74	53% (40-65%)	47% (35-60%)	1.7 (1.1-2.6)	6.3% (3.1-10%)	7.4% (3.6-12%)	920 (440-1500)	17000 (8400-29000)
Philippines	1990	Male	75 to 79	47% (33-62%)	53% (38-67%)	1.4 (0.81-2.3)	4.3% (1.5-8%)	4.9% (1.6-9.4%)	610 (190-1200)	8800 (2900-17000)
Philippines	1990	Male	80 to 84	42% (29-56%)	58% (44-71%)	1.2 (0.69-1.8)	3% (0.77-5.7%)	3.2% (0.76-6.4%)	350 (80-690)	3700 (950-7400)
Philippines	1990	Male	85 to 89	41% (28-55%)	59% (45-72%)	1 (0.58-1.6)	1.9% (0.29-3.9%)	2% (0.2-4.4%)	140 (14-310)	1100 (170-2400)
Philippines	1990	Male	90 to 94	43% (29-57%)	57% (43-71%)	0.99 (0.57-1.6)	1.2% (-0.21-3.1%)	1.2% (-0.42-3.4%)	29 (-10-83)	170 (-31-440)
Philippines	1990	Male	95 plus	46% (28-64%)	54% (36-72%)	0.99 (0.48-1.7)	0.99% (-0.51-3.3%)	0.9% (-0.84-3.4%)	5.3 (-4.9-20)	23 (-12-76)
Philippines	1995	Female	All Ages	16% (14-19%)	84% (81-86%)	0.39 (0.32-0.48)	1.2% (0.85-1.7%)	2.1% (1.2-3.3%)	3400 (1900-5300)	120000 (84000-170000)
Philippines	1995	Female	15 to 19	16% (11-22%)	84% (78-89%)	0.4 (0.23-0.62)	1.1% (0.66-1.6%)	1.7% (0.89-2.9%)	46 (24-78)	5100 (3100-7900)
Philippines	1995	Female	20 to 24	20% (13-29%)	80% (71-87%)	0.54 (0.29-0.88)	1.7% (1-2.6%)	2.3% (1-3.9%)	71 (33-120)	8200 (4900-12000)
Philippines	1995	Female	25 to 29	22% (14-30%)	78% (70-86%)	0.61 (0.36-0.97)	2% (1.2-3.1%)	2.8% (1.4-4.9%)	100 (50-170)	9500 (5800-15000)
Philippines	1995	Female	30 to 34	22% (15-33%)	78% (67-85%)	0.67 (0.38-1.1)	2.2% (1.3-3.2%)	3.3% (1.8-5.2%)	130 (68-200)	10000 (6100-15000)
Philippines	1995	Female	35 to 39	23% (15-32%)	77% (68-85%)	0.73 (0.42-1.1)	2.5% (1.5-3.8%)	4% (2.1-6.5%)	170 (90-270)	11000 (6500-17000)
Philippines	1995	Female	40 to 44	23% (16-31%)	77% (69-84%)	0.74 (0.44-1.1)	2.5% (1.4-3.9%)	4.3% (2.4-6.6%)	200 (110-310)	11000 (6300-17000)
Philippines	1995	Female	45 to 49	22% (14-30%)	78% (70-86%)	0.71 (0.39-1.1)	2.6% (1.4-4.4%)	4.2% (2.1-6.9%)	210 (100-340)	9600 (5000-16000)
Philippines	1995	Female	50 to 54	22% (15-30%)	78% (70-85%)	0.79 (0.45-1.3)	3% (1.5-5%)	4.6% (2.3-7.6%)	270 (140-450)	11000 (5400-18000)
Philippines	1995	Female	55 to 59	23% (15-31%)	77% (69-85%)	0.98 (0.55-1.6)	3.8% (1.9-6.2%)	5.6% (2.9-9.2%)	380 (200-620)	13000 (6800-21000)
Philippines	1995	Female	60 to 64	21% (14-31%)	79% (69-86%)	0.94 (0.53-1.5)	3.7% (1.7-6.3%)	5.1% (2.4-8.7%)	450 (200-760)	13000 (5800-22000)
Philippines	1995	Female	65 to 69	19% (13-27%)	81% (73-87%)	0.77 (0.46-1.2)	2.9% (1.3-4.9%)	3.9% (1.9-6.7%)	370 (180-640)	8700 (4100-15000)
Philippines	1995	Female	70 to 74	18% (13-25%)	82% (75-87%)	0.65 (0.36-1.1)	2.2% (0.63-4.2%)	2.9% (0.89-5.5%)	340 (100-650)	6300 (1800-12000)
Philippines	1995	Female	75 to 79	19% (12-26%)	81% (74-88%)	0.62 (0.32-1.1)	1.6% (0.16-3.8%)	2.1% (0.25-4.8%)	260 (30-600)	3700 (370-8700)
Philippines	1995	Female	80 to 84	20% (13-27%)	80% (73-87%)	0.65 (0.37-1.1)	1.4% (0.056-3.4%)	1.7% (0.021-3.9%)	230 (2.9-540)	2400 (94-5700)
Philippines	1995	Female	85 to 89	21% (14-30%)	79% (70-86%)	0.7 (0.39-1.2)	1.2% (-0.096-2.9%)	1.3% (-0.15-3.4%)	150 (-16-360)	1100 (-88-2700)

Location	Year	Sex	Age	Prevalence of current drinkers	Prevalence of abstainers	Population average of standard drinks daily	Percent of total attributable deaths	Percent of total attributable DALYs	Attributable deaths	Attributable DALYs
Philippines	1995	Female	90 to 94	24%<br>(16-34%)	76%<br>(66-84%)	0.79<br>(0.42-1.4)	1%<br>(-0.27-3.1%)	1.1%<br>(-0.4-3.6%)	51<br>(-18-160)	280<br>(-73-850)
Philippines	1995	Female	95 plus	28%<br>(16-42%)	72%<br>(58-84%)	0.92<br>(0.41-1.7)	1.2%<br>(-0.49-4.1%)	1.3%<br>(-0.67-4.6%)	18<br>(-9.5-66)	68<br>(-27-230)
Philippines	1995	Male	All Ages	56%<br>(50-62%)	44%<br>(38-50%)	1.8<br>(1.6-2)	6%<br>(4.9-7.1%)	8.2%<br>(6.4-10%)	19000<br>(15000-24000)	810000<br>(660000-970000)
Philippines	1995	Male	15 to 19	54%<br>(43-65%)	46%<br>(35-57%)	1.3<br>(0.81-1.8)	4.1%<br>(2.8-5.7%)	6.2%<br>(4.1-8.6%)	300<br>(200-430)	26000<br>(18000-36000)
Philippines	1995	Male	20 to 24	74%<br>(63-81%)	26%<br>(19-37%)	2.8<br>(2-3.6)	10%<br>(7.4-13%)	13%<br>(9.7-17%)	900<br>(650-1200)	71000<br>(53000-90000)
Philippines	1995	Male	25 to 29	77%<br>(69-84%)	23%<br>(16-31%)	3.1<br>(2.3-3.9)	11%<br>(8.6-14%)	15%<br>(11-19%)	1200<br>(840-1500)	82000<br>(60000-100000)
Philippines	1995	Male	30 to 34	79%<br>(71-85%)	21%<br>(15-29%)	3.4<br>(2.6-4.3)	12%<br>(9.1-15%)	16%<br>(12-20%)	1300<br>(970-1700)	84000<br>(63000-110000)
Philippines	1995	Male	35 to 39	79%<br>(71-85%)	21%<br>(15-29%)	3.8<br>(2.7-4.9)	13%<br>(10-17%)	18%<br>(13-23%)	1600<br>(1200-2100)	90000<br>(67000-110000)
Philippines	1995	Male	40 to 44	78%<br>(70-84%)	22%<br>(16-30%)	3.9<br>(2.9-5)	14%<br>(10-18%)	19%<br>(14-24%)	1800<br>(1300-2400)	92000<br>(67000-120000)
Philippines	1995	Male	45 to 49	75%<br>(65-82%)	25%<br>(18-35%)	3.9<br>(2.7-5.3)	15%<br>(10-19%)	19%<br>(13-24%)	2000<br>(1400-2600)	87000<br>(61000-110000)
Philippines	1995	Male	50 to 54	71%<br>(62-78%)	29%<br>(22-38%)	3.6<br>(2.5-5)	14%<br>(9.4-19%)	17%<br>(11-23%)	2100<br>(1400-2900)	81000<br>(53000-110000)
Philippines	1995	Male	55 to 59	66%<br>(55-75%)	34%<br>(25-45%)	3.3<br>(2.3-4.6)	13%<br>(8.6-18%)	16%<br>(11-22%)	2200<br>(1400-3000)	71000<br>(47000-98000)
Philippines	1995	Male	60 to 64	62%<br>(49-73%)	38%<br>(27-51%)	2.9<br>(1.9-4.2)	11%<br>(7-17%)	13%<br>(8.2-20%)	2100<br>(1300-3100)	59000<br>(37000-86000)
Philippines	1995	Male	65 to 69	58%<br>(45-70%)	42%<br>(30-55%)	2.2<br>(1.5-3.2)	8.4%<br>(4.9-13%)	9.8%<br>(5.9-15%)	1500<br>(870-2200)	34000<br>(20000-51000)
Philippines	1995	Male	70 to 74	53%<br>(41-65%)	47%<br>(35-59%)	1.8<br>(1.1-2.6)	5.9%<br>(2.9-9.7%)	6.8%<br>(3.3-11%)	1100<br>(500-1700)	20000<br>(9500-33000)
Philippines	1995	Male	75 to 79	46%<br>(32-61%)	54%<br>(39-68%)	1.4<br>(0.81-2.3)	4%<br>(1.3-7.6%)	4.5%<br>(1.4-8.7%)	630<br>(190-1200)	9100<br>(3100-17000)
Philippines	1995	Male	80 to 84	42%<br>(29-56%)	58%<br>(44-71%)	1.2<br>(0.69-1.8)	2.6%<br>(0.55-5.3%)	2.8%<br>(0.45-5.9%)	350<br>(56-730)	3800<br>(810-7700)
Philippines	1995	Male	85 to 89	41%<br>(28-56%)	59%<br>(44-72%)	1<br>(0.59-1.6)	1.6%<br>(-0.056-3.7%)	1.7%<br>(-0.17-4%)	140<br>(-14-330)	1100<br>(-39-2600)
Philippines	1995	Male	90 to 94	43%<br>(30-57%)	57%<br>(43-70%)	1<br>(0.57-1.6)	0.92%<br>(-0.49-2.8%)	0.9%<br>(-0.76-3.1%)	29<br>(-25-100)	180<br>(-96-540)
Philippines	1995	Male	95 plus	47%<br>(29-65%)	53%<br>(35-71%)	1<br>(0.48-1.8)	0.81%<br>(-0.78-2.9%)	0.66%<br>(-1.2-3.1%)	5.3<br>(-9.5-25)	25<br>(-23-92)
Philippines	2000	Female	All Ages	13%<br>(11-15%)	87%<br>(85-89%)	0.33<br>(0.28-0.4)	1.2%<br>(0.86-1.7%)	2.1%<br>(1.2-3.2%)	3800<br>(2200-5700)	130000<br>(91000-180000)
Philippines	2000	Female	15 to 19	13%<br>(9.1-17%)	87%<br>(83-91%)	0.34<br>(0.2-0.51)	0.9%<br>(0.57-1.3%)	1.3%<br>(0.72-2.2%)	37<br>(20-61)	4700<br>(2800-7000)

Location	Year	Sex	Age	Prevalence of current drinkers	Prevalence of abstainers	Population average of standard drinks daily	Percent of total attributable deaths	Percent of total attributable DALYs	Attributable deaths	Attributable DALYs
Philippines	2000	Female	20 to 24	15% (11-20%)	85% (80-89%)	0.41 (0.25-0.64)	1.5% (0.93-2.1%)	1.7% (0.87-2.9%)	59 (29-98)	7800 (4800-11000)
Philippines	2000	Female	25 to 29	15% (11-21%)	85% (79-89%)	0.46 (0.29-0.7)	1.7% (1.1-2.5%)	2.2% (1.2-3.6%)	82 (44-140)	8600 (5500-13000)
Philippines	2000	Female	30 to 34	18% (14-23%)	82% (77-86%)	0.55 (0.35-0.81)	1.9% (1.2-2.7%)	2.7% (1.5-4.2%)	120 (65-180)	9600 (6100-14000)
Philippines	2000	Female	35 to 39	19% (14-24%)	81% (76-86%)	0.61 (0.39-0.9)	2.1% (1.3-3.1%)	3.3% (1.9-5.2%)	160 (95-250)	11000 (6800-16000)
Philippines	2000	Female	40 to 44	18% (14-23%)	82% (77-86%)	0.6 (0.39-0.86)	2.2% (1.3-3.2%)	3.5% (2.2-5.4%)	200 (120-300)	11000 (6900-16000)
Philippines	2000	Female	45 to 49	18% (13-23%)	82% (77-87%)	0.59 (0.37-0.91)	2.2% (1.3-3.5%)	3.5% (2-5.6%)	240 (130-370)	11000 (6600-17000)
Philippines	2000	Female	50 to 54	18% (14-24%)	82% (76-86%)	0.68 (0.41-1)	2.6% (1.4-4.2%)	3.9% (2.1-6.2%)	290 (160-460)	11000 (6100-18000)
Philippines	2000	Female	55 to 59	20% (16-26%)	80% (74-84%)	0.92 (0.59-1.4)	3.5% (2-5.7%)	5.2% (2.9-8.3%)	430 (240-700)	15000 (8100-24000)
Philippines	2000	Female	60 to 64	20% (15-26%)	80% (74-85%)	0.91 (0.57-1.4)	3.5% (1.8-5.8%)	4.8% (2.4-7.9%)	510 (260-840)	15000 (7400-25000)
Philippines	2000	Female	65 to 69	16% (12-22%)	84% (78-88%)	0.67 (0.42-1)	2.6% (1.3-4.4%)	3.5% (1.7-5.8%)	440 (220-740)	10000 (5100-18000)
Philippines	2000	Female	70 to 74	14% (10-20%)	86% (80-90%)	0.53 (0.3-0.88)	1.9% (0.68-3.6%)	2.4% (0.87-4.7%)	340 (120-650)	6300 (2300-12000)
Philippines	2000	Female	75 to 79	15% (9.8-22%)	85% (78-90%)	0.52 (0.28-0.91)	1.5% (0.25-3.3%)	1.8% (0.33-4%)	290 (51-640)	4200 (720-9500)
Philippines	2000	Female	80 to 84	18% (11-25%)	82% (75-89%)	0.61 (0.34-1)	1.4% (0.24-3.3%)	1.7% (0.26-3.8%)	260 (40-590)	2700 (450-6200)
Philippines	2000	Female	85 to 89	20% (13-29%)	80% (71-87%)	0.7 (0.39-1.1)	1.3% (0.077-3.1%)	1.5% (0.035-3.5%)	200 (4.5-460)	1500 (86-3400)
Philippines	2000	Female	90 to 94	23% (15-33%)	77% (67-85%)	0.79 (0.42-1.4)	1.2% (-0.13-3.3%)	1.3% (-0.23-3.8%)	88 (-15-250)	470 (-49-1300)
Philippines	2000	Female	95 plus	27% (15-40%)	73% (60-85%)	0.93 (0.42-1.7)	1.5% (-0.28-4.5%)	1.6% (-0.41-5.1%)	34 (-8.6-110)	120 (-22-360)
Philippines	2000	Male	All Ages	60% (56-65%)	40% (35-44%)	2 (1.8-2.2)	6.7% (5.4-7.9%)	8.9% (7-11%)	23000 (18000-29000)	970000 (800000-1200000)
Philippines	2000	Male	15 to 19	63% (56-69%)	37% (31-44%)	1.6 (1.1-2.2)	4.5% (2.8-6.1%)	6.9% (4.3-9.4%)	360 (230-500)	31000 (20000-42000)
Philippines	2000	Male	20 to 24	79% (74-82%)	21% (18-26%)	3.3 (2.4-4.1)	10% (7.5-13%)	14% (9.9-18%)	1000 (740-1300)	79000 (59000-100000)
Philippines	2000	Male	25 to 29	80% (76-84%)	20% (16-24%)	3.4 (2.6-4.3)	11% (8.3-14%)	15% (11-19%)	1200 (870-1600)	86000 (64000-110000)
Philippines	2000	Male	30 to 34	82% (78-85%)	18% (15-22%)	3.8 (3-4.8)	12% (9.1-15%)	16% (12-21%)	1500 (1100-1900)	93000 (69000-120000)

Location	Year	Sex	Age	Prevalence of current drinkers	Prevalence of abstainers	Population average of standard drinks daily	Percent of total attributable deaths	Percent of total attributable DALYs	Attributable deaths	Attributable DALYs
Philippines	2000	Male	35 to 39	81% (78-85%)	19% (15-22%)	4.2 (3.1-5.4)	14% (9.8-18%)	18% (13-24%)	1900 (1300-2400)	110000 (76000-140000)
Philippines	2000	Male	40 to 44	80% (76-84%)	20% (16-24%)	4.3 (3.3-5.5)	14% (11-18%)	19% (14-24%)	2200 (1600-2800)	110000 (82000-140000)
Philippines	2000	Male	45 to 49	78% (73-82%)	22% (18-27%)	4.3 (3.2-5.7)	15% (11-19%)	19% (14-25%)	2700 (1900-3600)	120000 (84000-150000)
Philippines	2000	Male	50 to 54	73% (68-78%)	27% (22-32%)	3.9 (2.8-5.2)	14% (9.8-19%)	18% (12-23%)	2700 (1800-3700)	100000 (69000-140000)
Philippines	2000	Male	55 to 59	66% (58-74%)	34% (26-42%)	3.5 (2.5-4.7)	13% (8.7-18%)	16% (10-22%)	2700 (1800-3700)	89000 (60000-120000)
Philippines	2000	Male	60 to 64	60% (50-70%)	40% (30-50%)	2.9 (2-4.1)	11% (6.9-16%)	13% (8-19%)	2500 (1600-3700)	70000 (44000-100000)
Philippines	2000	Male	65 to 69	55% (44-66%)	45% (34-56%)	2.2 (1.5-3)	8.1% (5-12%)	9.4% (5.9-14%)	1900 (1200-2900)	45000 (28000-66000)
Philippines	2000	Male	70 to 74	51% (39-61%)	49% (39-61%)	1.7 (1.2-2.5)	5.8% (3-9.3%)	6.6% (3.4-11%)	1200 (610-2000)	23000 (12000-37000)
Philippines	2000	Male	75 to 79	44% (31-57%)	56% (43-69%)	1.4 (0.82-2.2)	4% (1.6-7.4%)	4.4% (1.7-8.3%)	770 (280-1400)	11000 (4400-21000)
Philippines	2000	Male	80 to 84	39% (26-53%)	61% (47-74%)	1.1 (0.67-1.8)	2.7% (0.87-5.3%)	3% (0.86-5.8%)	400 (120-780)	4400 (1400-8400)
Philippines	2000	Male	85 to 89	38% (26-52%)	62% (48-74%)	1 (0.57-1.6)	1.8% (0.21-3.8%)	1.8% (0.11-4.1%)	180 (10-400)	1400 (170-3000)
Philippines	2000	Male	90 to 94	40% (27-55%)	60% (45-73%)	0.96 (0.53-1.6)	1.1% (-0.34-3.2%)	1.1% (-0.54-3.4%)	46 (-22-140)	260 (-78-750)
Philippines	2000	Male	95 plus	44% (27-62%)	56% (38-73%)	0.95 (0.45-1.7)	1% (-0.58-3.6%)	0.93% (-0.85-3.7%)	11 (-9.8-43)	45 (-26-150)
Philippines	2005	Female	All Ages	13% (11-15%)	87% (85-89%)	0.34 (0.29-0.42)	1.3% (0.92-1.8%)	2.2% (1.3-3.4%)	4500 (2500-6800)	150000 (100000-210000)
Philippines	2005	Female	15 to 19	13% (8.8-18%)	87% (82-91%)	0.34 (0.2-0.52)	0.88% (0.57-1.3%)	1.3% (0.7-2.2%)	40 (21-68)	5100 (3200-7500)
Philippines	2005	Female	20 to 24	15% (10-20%)	85% (80-90%)	0.42 (0.25-0.66)	1.5% (0.95-2.1%)	1.7% (0.83-2.8%)	65 (31-110)	8400 (5400-13000)
Philippines	2005	Female	25 to 29	15% (11-20%)	85% (80-89%)	0.46 (0.29-0.71)	1.6% (1.1-2.4%)	2.1% (1.1-3.4%)	89 (48-150)	9400 (5900-14000)
Philippines	2005	Female	30 to 34	17% (13-23%)	83% (77-87%)	0.56 (0.36-0.82)	1.8% (1.2-2.6%)	2.6% (1.4-4.1%)	120 (68-200)	10000 (6500-15000)
Philippines	2005	Female	35 to 39	18% (14-23%)	82% (77-86%)	0.61 (0.39-0.91)	2% (1.3-3%)	3.2% (1.8-4.9%)	180 (100-280)	12000 (7300-18000)
Philippines	2005	Female	40 to 44	18% (13-23%)	82% (77-87%)	0.6 (0.39-0.89)	2.1% (1.2-3.1%)	3.4% (2-5.2%)	220 (130-350)	12000 (7600-19000)
Philippines	2005	Female	45 to 49	18% (13-22%)	82% (78-87%)	0.6 (0.38-0.93)	2.2% (1.2-3.5%)	3.4% (1.8-5.5%)	280 (150-460)	13000 (7000-21000)
Philippines	2005	Female	50 to 54	18% (13-23%)	82% (77-87%)	0.68 (0.42-1)	2.5% (1.3-4.1%)	3.8% (1.9-6.2%)	390 (190-630)	16000 (7600-25000)

Location	Year	Sex	Age	Prevalence of current drinkers	Prevalence of abstainers	Population average of standard drinks daily	Percent of total attributable deaths	Percent of total attributable DALYs	Attributable deaths	Attributable DALYs
Philippines	2005	Female	55 to 59	20% (15-25%)	80% (75-85%)	0.92 (0.6-1.4)	3.5% (1.9-5.8%)	5.1% (2.8-8.2%)	530 (290-890)	18000 (9600-30000)
Philippines	2005	Female	60 to 64	19% (14-25%)	81% (75-86%)	0.91 (0.58-1.4)	3.4% (1.7-5.8%)	4.7% (2.3-7.9%)	590 (290-980)	17000 (8200-28000)
Philippines	2005	Female	65 to 69	16% (11-21%)	84% (79-89%)	0.67 (0.43-1)	2.5% (1.2-4.4%)	3.4% (1.7-5.9%)	520 (250-910)	12000 (5900-22000)
Philippines	2005	Female	70 to 74	14% (10-19%)	86% (81-90%)	0.54 (0.3-0.9)	1.9% (0.55-3.8%)	2.4% (0.74-4.8%)	420 (130-830)	7900 (2400-16000)
Philippines	2005	Female	75 to 79	15% (9.9-21%)	85% (79-90%)	0.53 (0.28-0.92)	1.5% (0.22-3.4%)	1.8% (0.3-4.2%)	330 (54-760)	4800 (710-11000)
Philippines	2005	Female	80 to 84	17% (11-25%)	83% (75-89%)	0.61 (0.35-1)	1.4% (0.18-3.5%)	1.7% (0.2-4%)	310 (37-750)	3300 (400-7800)
Philippines	2005	Female	85 to 89	20% (13-29%)	80% (71-87%)	0.72 (0.38-1.2)	1.4% (0.1-3.3%)	1.6% (0.082-3.7%)	250 (13-600)	1900 (140-4500)
Philippines	2005	Female	90 to 94	23% (15-32%)	77% (68-85%)	0.81 (0.42-1.4)	1.3% (-0.093-3.6%)	1.5% (-0.19-4.1%)	120 (-15-330)	610 (-44-1700)
Philippines	2005	Female	95 plus	27% (16-40%)	73% (60-84%)	0.95 (0.43-1.7)	1.6% (-0.17-4.7%)	1.7% (-0.29-5.2%)	50 (-8.4-150)	180 (-18-520)
Philippines	2005	Male	All Ages	61% (56-65%)	39% (35-44%)	2.1 (1.9-2.3)	7.3% (6-8.7%)	9.5% (7.5-12%)	28000 (22000-34000)	1100000 (930000-1400000)
Philippines	2005	Male	15 to 19	63% (56-69%)	37% (31-44%)	1.6 (1.1-2.2)	4.5% (3-6.2%)	7.1% (4.5-9.7%)	410 (260-580)	34000 (22000-47000)
Philippines	2005	Male	20 to 24	78% (74-82%)	22% (18-26%)	3.3 (2.4-4.2)	10% (7.7-13%)	14% (10-19%)	1100 (810-1500)	87000 (63000-110000)
Philippines	2005	Male	25 to 29	80% (76-83%)	20% (17-24%)	3.5 (2.6-4.4)	11% (8.3-14%)	15% (11-19%)	1300 (960-1700)	93000 (68000-120000)
Philippines	2005	Male	30 to 34	82% (78-85%)	18% (15-22%)	3.9 (3-4.9)	12% (9.1-16%)	16% (12-21%)	1600 (1100-2000)	99000 (73000-130000)
Philippines	2005	Male	35 to 39	81% (77-85%)	19% (15-23%)	4.2 (3.2-5.4)	14% (10-18%)	18% (13-24%)	2100 (1500-2700)	120000 (84000-150000)
Philippines	2005	Male	40 to 44	80% (76-84%)	20% (16-24%)	4.4 (3.3-5.6)	15% (11-19%)	19% (14-25%)	2600 (1900-3400)	130000 (95000-160000)
Philippines	2005	Male	45 to 49	78% (73-82%)	22% (18-27%)	4.4 (3.2-5.8)	15% (11-20%)	19% (14-25%)	3200 (2300-4300)	140000 (100000-190000)
Philippines	2005	Male	50 to 54	73% (67-77%)	27% (23-33%)	4 (2.8-5.3)	15% (9.9-19%)	18% (12-24%)	3700 (2500-4900)	140000 (96000-190000)
Philippines	2005	Male	55 to 59	66% (57-74%)	34% (26-43%)	3.5 (2.6-4.8)	13% (8.7-18%)	16% (10-22%)	3400 (2200-4700)	110000 (73000-160000)
Philippines	2005	Male	60 to 64	59% (50-68%)	41% (32-50%)	2.9 (2-4.1)	11% (6.7-16%)	13% (7.8-19%)	3000 (1800-4300)	83000 (51000-120000)
Philippines	2005	Male	65 to 69	55% (44-65%)	45% (35-56%)	2.2 (1.5-3.1)	8.1% (4.9-12%)	9.4% (5.8-14%)	2300 (1400-3400)	53000 (33000-78000)

Location	Year	Sex	Age	Prevalence of current drinkers	Prevalence of abstainers	Population average of standard drinks daily	Percent of total attributable deaths	Percent of total attributable DALYs	Attributable deaths	Attributable DALYs
Philippines	2005	Male	70 to 74	50% (39-61%)	50% (39-61%)	1.8 (1.2-2.6)	5.8% (3-9.5%)	6.6% (3.4-11%)	1600 (800-2600)	29000 (15000-48000)
Philippines	2005	Male	75 to 79	43% (31-57%)	57% (43-69%)	1.4 (0.82-2.2)	3.9% (1.5-7.3%)	4.3% (1.6-8.2%)	850 (320-1600)	12000 (4700-23000)
Philippines	2005	Male	80 to 84	38% (25-52%)	62% (48-75%)	1.1 (0.67-1.7)	2.6% (0.9-5.1%)	2.9% (0.87-5.6%)	440 (140-880)	4800 (1600-9300)
Philippines	2005	Male	85 to 89	37% (25-51%)	63% (49-75%)	0.99 (0.56-1.5)	1.7% (0.28-3.7%)	1.8% (0.17-4%)	200 (18-440)	1600 (250-3300)
Philippines	2005	Male	90 to 94	39% (26-53%)	61% (47-74%)	0.95 (0.54-1.6)	1.2% (-0.22-3.1%)	1.2% (-0.43-3.3%)	51 (-19-140)	290 (-55-760)
Philippines	2005	Male	95 plus	43% (26-62%)	57% (38-74%)	0.95 (0.45-1.7)	1.1% (-0.52-3.4%)	1% (-0.78-3.5%)	13 (-9.8-46)	53 (-25-170)
Philippines	2010	Female	All Ages	15% (13-18%)	85% (82-87%)	0.41 (0.33-0.51)	1.5% (0.97-2%)	2.2% (1.1-3.6%)	5300 (2700-8400)	180000 (120000-250000)
Philippines	2010	Female	15 to 19	14% (9.4-21%)	86% (79-91%)	0.37 (0.21-0.6)	0.94% (0.58-1.5%)	1.4% (0.64-2.4%)	45 (22-82)	5700 (3500-9200)
Philippines	2010	Female	20 to 24	18% (12-27%)	82% (73-88%)	0.51 (0.27-0.83)	1.6% (1-2.4%)	1.9% (0.86-3.3%)	76 (34-130)	9600 (5800-14000)
Philippines	2010	Female	25 to 29	20% (13-28%)	80% (72-87%)	0.57 (0.33-0.92)	1.8% (1.1-2.7%)	2.3% (1.1-4%)	110 (50-180)	11000 (6500-17000)
Philippines	2010	Female	30 to 34	20% (13-29%)	80% (71-87%)	0.63 (0.36-0.99)	1.9% (1.2-2.8%)	2.6% (1.3-4.3%)	140 (71-230)	12000 (7000-18000)
Philippines	2010	Female	35 to 39	21% (14-29%)	79% (71-86%)	0.68 (0.39-1.1)	2.1% (1.3-3.3%)	3.2% (1.7-5.2%)	200 (110-330)	13000 (7900-21000)
Philippines	2010	Female	40 to 44	21% (14-29%)	79% (71-86%)	0.69 (0.42-1.1)	2.2% (1.2-3.5%)	3.5% (1.9-5.6%)	270 (150-440)	15000 (8900-23000)
Philippines	2010	Female	45 to 49	20% (12-28%)	80% (72-88%)	0.66 (0.36-1.1)	2.2% (1.1-3.7%)	3.4% (1.7-5.9%)	330 (160-560)	16000 (8000-26000)
Philippines	2010	Female	50 to 54	20% (14-28%)	80% (72-86%)	0.74 (0.43-1.2)	2.5% (1.2-4.5%)	3.8% (1.8-6.7%)	450 (210-780)	18000 (8400-31000)
Philippines	2010	Female	55 to 59	21% (14-29%)	79% (71-86%)	0.92 (0.53-1.4)	3.3% (1.7-5.6%)	4.7% (2.4-7.9%)	650 (330-1100)	22000 (11000-38000)
Philippines	2010	Female	60 to 64	20% (13-28%)	80% (72-87%)	0.88 (0.5-1.4)	3.1% (1.5-5.5%)	4.2% (2-7.5%)	700 (340-1300)	20000 (9500-36000)
Philippines	2010	Female	65 to 69	18% (12-25%)	82% (75-88%)	0.72 (0.43-1.1)	2.4% (1-4.4%)	3.2% (1.4-5.8%)	580 (260-1000)	14000 (6000-25000)
Philippines	2010	Female	70 to 74	17% (11-24%)	83% (76-89%)	0.61 (0.33-1)	1.9% (0.42-3.9%)	2.4% (0.62-5%)	560 (140-1200)	10000 (2400-22000)
Philippines	2010	Female	75 to 79	17% (11-25%)	83% (75-89%)	0.59 (0.31-1)	1.4% (0.046-3.5%)	1.8% (0.09-4.3%)	450 (23-1100)	6400 (190-16000)
Philippines	2010	Female	80 to 84	18% (12-25%)	82% (75-88%)	0.62 (0.34-1)	1.3% (0.0026-3.2%)	1.5% (-0.023-3.7%)	350 (-5.3-890)	3700 (7.6-9400)
Philippines	2010	Female	85 to 89	20% (13-28%)	80% (72-87%)	0.67 (0.35-1.1)	1.2% (-0.039-2.9%)	1.3% (-0.076-3.3%)	240 (-15-600)	1800 (-62-4500)

Location	Year	Sex	Age	Prevalence of current drinkers	Prevalence of abstainers	Population average of standard drinks daily	Percent of total attributable deaths	Percent of total attributable DALYs	Attributable deaths	Attributable DALYs
Philippines	2010	Female	90 to 94	22% (14-32%)	78% (68-86%)	0.76 (0.4-1.3)	1.1% (-0.25-3.2%)	1.2% (-0.38-3.6%)	120 (-36-350)	620 (-140-1800)
Philippines	2010	Female	95 plus	26% (15-39%)	74% (61-85%)	0.88 (0.4-1.6)	1.4% (-0.37-4.3%)	1.4% (-0.5-4.7%)	48 (-17-160)	180 (-47-550)
Philippines	2010	Male	All Ages	56% (50-62%)	44% (38-50%)	1.9 (1.7-2.1)	6.9% (5.5-8.3%)	8.5% (6.6-11%)	29000 (21000-36000)	1100000 (920000-1400000)
Philippines	2010	Male	15 to 19	51% (40-63%)	49% (37-60%)	1.2 (0.74-1.8)	3.6% (2.3-5.2%)	5.4% (3.2-7.8%)	330 (200-490)	29000 (18000-41000)
Philippines	2010	Male	20 to 24	72% (61-80%)	28% (20-39%)	2.7 (1.9-3.7)	9.2% (6.5-12%)	12% (8.5-17%)	1000 (690-1400)	78000 (55000-100000)
Philippines	2010	Male	25 to 29	76% (66-83%)	24% (17-34%)	3 (2.2-4)	10% (7.3-13%)	13% (9.3-18%)	1200 (840-1600)	87000 (62000-110000)
Philippines	2010	Male	30 to 34	78% (69-84%)	22% (16-31%)	3.4 (2.6-4.4)	11% (7.9-14%)	14% (10-19%)	1500 (1000-2000)	95000 (68000-120000)
Philippines	2010	Male	35 to 39	78% (69-84%)	22% (16-31%)	3.8 (2.7-5)	12% (8.6-16%)	16% (11-21%)	2000 (1300-2600)	110000 (77000-150000)
Philippines	2010	Male	40 to 44	77% (68-83%)	23% (17-32%)	3.9 (2.8-5.1)	13% (9.3-17%)	17% (12-22%)	2600 (1800-3400)	130000 (92000-170000)
Philippines	2010	Male	45 to 49	74% (62-81%)	26% (19-38%)	3.8 (2.7-5.2)	13% (8.9-18%)	17% (11-23%)	3200 (2100-4400)	140000 (96000-190000)
Philippines	2010	Male	50 to 54	69% (59-77%)	31% (23-41%)	3.5 (2.4-4.9)	13% (8.2-18%)	16% (9.9-21%)	3700 (2300-5000)	140000 (91000-190000)
Philippines	2010	Male	55 to 59	64% (52-74%)	36% (26-48%)	3.2 (2.2-4.4)	12% (7.5-16%)	14% (8.9-20%)	3900 (2400-5500)	130000 (82000-180000)
Philippines	2010	Male	60 to 64	59% (46-70%)	41% (30-54%)	2.8 (1.8-4.1)	9.7% (5.6-15%)	11% (6.6-17%)	3400 (2000-5200)	96000 (56000-140000)
Philippines	2010	Male	65 to 69	55% (41-67%)	45% (33-59%)	2.1 (1.4-3.1)	7.2% (4.3-11%)	8.3% (4.8-13%)	2400 (1400-3700)	56000 (34000-85000)
Philippines	2010	Male	70 to 74	49% (36-62%)	51% (38-64%)	1.6 (1-2.5)	5% (2.5-8.5%)	5.7% (2.8-9.7%)	1700 (850-2900)	32000 (16000-54000)
Philippines	2010	Male	75 to 79	43% (30-58%)	57% (42-70%)	1.3 (0.76-2.1)	3.3% (1.2-6.3%)	3.7% (1.3-7.1%)	990 (340-1900)	15000 (5000-28000)
Philippines	2010	Male	80 to 84	39% (26-53%)	61% (47-74%)	1.1 (0.64-1.7)	2.3% (0.64-4.5%)	2.4% (0.61-5%)	470 (120-960)	5200 (1500-11000)
Philippines	2010	Male	85 to 89	38% (25-52%)	62% (48-75%)	0.95 (0.54-1.5)	1.4% (0.068-3.4%)	1.5% (-0.045-3.6%)	170 (-5.2-420)	1400 (66-3200)
Philippines	2010	Male	90 to 94	39% (27-54%)	61% (46-73%)	0.92 (0.51-1.5)	0.92% (-0.4-2.8%)	0.91% (-0.56-3%)	43 (-26-140)	250 (-110-730)
Philippines	2010	Male	95 plus	43% (26-62%)	57% (38-74%)	0.92 (0.44-1.7)	0.87% (-0.66-3.2%)	0.76% (-0.93-3.3%)	9.6 (-12-41)	41 (-31-150)
Philippines	2016	Female	All Ages	16% (13-19%)	84% (81-87%)	0.41 (0.3-0.58)	1.4% (0.78-2.2%)	2.1% (0.81-3.8%)	5400 (2000-10000)	180000 (98000-300000)

Location	Year	Sex	Age	Prevalence of current drinkers	Prevalence of abstainers	Population average of standard drinks daily	Percent of total attributable deaths	Percent of total attributable DALYs	Attributable deaths	Attributable DALYs
Philippines	2016	Female	15 to 19	15% (9.8-21%)	85% (79-90%)	0.36 (0.19-0.61)	0.88% (0.52-1.4%)	1.2% (0.56-2.3%)	38 (16-73)	5200 (3000-8400)
Philippines	2016	Female	20 to 24	18% (12-26%)	82% (74-88%)	0.49 (0.25-0.81)	1.5% (0.9-2.3%)	1.7% (0.7-3.1%)	71 (27-140)	10000 (5600-16000)
Philippines	2016	Female	25 to 29	20% (13-28%)	80% (72-87%)	0.55 (0.29-0.92)	1.7% (1-2.6%)	2.2% (0.9-3.9%)	99 (40-180)	11000 (6000-17000)
Philippines	2016	Female	30 to 34	21% (14-30%)	79% (70-86%)	0.61 (0.32-1)	1.7% (0.98-2.7%)	2.3% (1.1-4.1%)	120 (52-220)	11000 (6000-18000)
Philippines	2016	Female	35 to 39	22% (14-30%)	78% (70-86%)	0.67 (0.36-1.1)	1.9% (1.1-3.2%)	3% (1.4-5.2%)	190 (86-340)	13000 (7100-22000)
Philippines	2016	Female	40 to 44	21% (14-29%)	79% (71-86%)	0.67 (0.38-1.1)	2% (1.1-3.5%)	3.3% (1.7-5.7%)	250 (120-460)	14000 (7400-24000)
Philippines	2016	Female	45 to 49	21% (13-28%)	79% (72-87%)	0.64 (0.33-1.1)	2% (0.96-3.6%)	3.2% (1.5-5.8%)	330 (150-620)	16000 (7300-29000)
Philippines	2016	Female	50 to 54	21% (14-29%)	79% (71-86%)	0.72 (0.39-1.2)	2.3% (0.96-4.2%)	3.5% (1.5-6.5%)	450 (190-850)	18000 (7700-34000)
Philippines	2016	Female	55 to 59	21% (14-29%)	79% (71-86%)	0.89 (0.49-1.5)	2.9% (1.2-5.4%)	4.3% (1.9-7.7%)	700 (300-1300)	24000 (10000-46000)
Philippines	2016	Female	60 to 64	20% (13-28%)	80% (72-87%)	0.85 (0.47-1.5)	2.7% (0.99-5.4%)	3.8% (1.5-7.4%)	710 (270-1400)	21000 (7500-40000)
Philippines	2016	Female	65 to 69	18% (12-25%)	82% (75-88%)	0.7 (0.36-1.1)	2.2% (0.72-4.2%)	2.9% (1-5.6%)	650 (220-1300)	15000 (5000-31000)
Philippines	2016	Female	70 to 74	17% (11-24%)	83% (76-89%)	0.6 (0.3-1.1)	1.7% (0.27-3.9%)	2.2% (0.44-5%)	510 (99-1200)	9700 (1600-24000)
Philippines	2016	Female	75 to 79	17% (11-26%)	83% (74-89%)	0.58 (0.26-1.1)	1.3% (-0.081-3.5%)	1.6% (-0.078-4.3%)	450 (-20-1200)	6500 (-360-18000)
Philippines	2016	Female	80 to 84	18% (12-26%)	82% (74-88%)	0.6 (0.3-1)	1.1% (-0.1-3.1%)	1.3% (-0.12-3.7%)	400 (-37-1100)	4300 (-400-12000)
Philippines	2016	Female	85 to 89	20% (13-29%)	80% (71-87%)	0.66 (0.34-1.1)	1% (-0.26-2.9%)	1.1% (-0.33-3.3%)	270 (-78-790)	2000 (-510-5800)
Philippines	2016	Female	90 to 94	23% (15-32%)	77% (68-85%)	0.74 (0.38-1.3)	0.97% (-0.44-3.2%)	1.1% (-0.56-3.6%)	100 (-56-350)	550 (-250-1800)
Philippines	2016	Female	95 plus	27% (15-40%)	73% (60-85%)	0.86 (0.4-1.6)	1.2% (-0.54-4%)	1.3% (-0.72-4.4%)	45 (-26-160)	170 (-75-560)
Philippines	2016	Male	All Ages	57% (51-63%)	43% (37-49%)	1.9 (1.4-2.5)	6.7% (4.6-9.1%)	7.9% (5-11%)	29000 (18000-43000)	1200000 (780000-1600000)
Philippines	2016	Male	15 to 19	51% (40-63%)	49% (37-60%)	1.2 (0.64-1.8)	3.3% (2-5.1%)	5% (2.7-7.8%)	300 (160-500)	26000 (15000-42000)
Philippines	2016	Male	20 to 24	72% (61-80%)	28% (20-39%)	2.6 (1.6-3.8)	8.6% (5.7-12%)	12% (7.3-17%)	1000 (630-1600)	81000 (51000-120000)
Philippines	2016	Male	25 to 29	76% (67-83%)	24% (17-33%)	2.9 (1.9-4.2)	9.6% (6.5-13%)	13% (8.1-18%)	1300 (780-1800)	90000 (59000-130000)

Location	Year	Sex	Age	Prevalence of current drinkers	Prevalence of abstainers	Population average of standard drinks daily	Percent of total attributable deaths	Percent of total attributable DALYs	Attributable deaths	Attributable DALYs
Philippines	2016	Male	30 to 34	78% (69-84%)	22% (16-31%)	3.3 (2.2-4.6)	10% (6.8-14%)	14% (8.6-19%)	1400 (860-2100)	92000 (59000-130000)
Philippines	2016	Male	35 to 39	78% (69-84%)	22% (16-31%)	3.6 (2.5-5.2)	11% (7.4-16%)	15% (9.4-21%)	1900 (1100-2900)	110000 (69000-160000)
Philippines	2016	Male	40 to 44	77% (68-83%)	23% (17-32%)	3.7 (2.5-5.3)	12% (7.9-17%)	16% (10-22%)	2400 (1500-3600)	120000 (77000-180000)
Philippines	2016	Male	45 to 49	74% (63-81%)	26% (19-37%)	3.7 (2.4-5.3)	12% (7.4-18%)	15% (9.3-22%)	3200 (1900-4700)	140000 (84000-210000)
Philippines	2016	Male	50 to 54	70% (60-77%)	30% (23-40%)	3.4 (2.2-5.1)	12% (6.8-17%)	14% (8.3-21%)	3700 (2100-5600)	140000 (82000-210000)
Philippines	2016	Male	55 to 59	64% (52-75%)	36% (25-48%)	3.1 (2-4.7)	11% (6.2-16%)	13% (7.3-20%)	4100 (2300-6500)	140000 (77000-210000)
Philippines	2016	Male	60 to 64	59% (47-71%)	41% (29-53%)	2.7 (1.6-4.1)	8.9% (4.7-14%)	10% (5.6-17%)	3700 (1900-6200)	110000 (55000-170000)
Philippines	2016	Male	65 to 69	56% (42-67%)	44% (33-58%)	2.1 (1.2-3.1)	6.5% (3.2-11%)	7.5% (3.6-12%)	2800 (1300-4600)	65000 (31000-110000)
Philippines	2016	Male	70 to 74	50% (37-62%)	50% (38-63%)	1.6 (0.9-2.5)	4.5% (1.8-8.2%)	5.1% (2-9.4%)	1600 (570-3000)	31000 (11000-57000)
Philippines	2016	Male	75 to 79	43% (30-58%)	57% (42-70%)	1.3 (0.68-2.2)	2.9% (0.65-6.3%)	3.2% (0.65-7%)	980 (190-2200)	15000 (3300-32000)
Philippines	2016	Male	80 to 84	39% (26-53%)	61% (47-74%)	1 (0.56-1.7)	2% (0.33-4.4%)	2.1% (0.24-4.9%)	540 (54-1300)	6000 (1000-14000)
Philippines	2016	Male	85 to 89	38% (25-52%)	62% (48-75%)	0.91 (0.48-1.5)	1.2% (-0.17-3.2%)	1.3% (-0.28-3.5%)	210 (-46-590)	1700 (-230-4500)
Philippines	2016	Male	90 to 94	40% (27-53%)	60% (47-73%)	0.89 (0.49-1.5)	0.78% (-0.63-2.8%)	0.74% (-0.85-3.1%)	37 (-42-150)	220 (-180-770)
Philippines	2016	Male	95 plus	44% (26-62%)	56% (38-74%)	0.89 (0.42-1.7)	0.74% (-0.72-3%)	0.61% (-1-3%)	8.1 (-14-40)	37 (-36-150)
Sri Lanka	1990	Female	All Ages	5% (4.2-6%)	95% (94-96%)	0.11 (0.08-0.13)	0.51% (0.38-0.67%)	0.56% (0.27-0.91%)	210 (100-340)	11000 (7800-14000)
Sri Lanka	1990	Female	15 to 19	4.5% (2.9-6.7%)	95% (93-97%)	0.088 (0.046-0.15)	0.5% (0.29-0.8%)	0.47% (0.13-1%)	3.9 (1-8.6)	630 (350-1000)
Sri Lanka	1990	Female	20 to 24	5.7% (3.4-8.4%)	94% (92-97%)	0.12 (0.06-0.21)	0.9% (0.55-1.3%)	0.65% (0.17-1.4%)	6.3 (1.6-14)	1200 (710-1800)
Sri Lanka	1990	Female	25 to 29	6.2% (3.7-9.4%)	94% (91-96%)	0.14 (0.075-0.24)	0.98% (0.63-1.4%)	0.76% (0.32-1.5%)	6.6 (2.7-13)	1200 (740-1700)
Sri Lanka	1990	Female	30 to 34	6.6% (3.9-10%)	93% (90-96%)	0.15 (0.083-0.26)	0.99% (0.69-1.3%)	0.97% (0.56-1.6%)	8.5 (4.9-14)	1200 (770-1600)
Sri Lanka	1990	Female	35 to 39	6.9% (3.9-10%)	93% (90-96%)	0.16 (0.079-0.27)	0.93% (0.66-1.3%)	1.1% (0.69-1.7%)	9.1 (5.7-14)	950 (660-1300)
Sri Lanka	1990	Female	40 to 44	6.8% (4.1-10%)	93% (90-96%)	0.16 (0.085-0.29)	0.91% (0.64-1.2%)	1.3% (0.86-1.9%)	12 (7.5-17)	900 (630-1200)
Sri Lanka	1990	Female	45 to 49	6.7% (3.8-9.9%)	93% (90-96%)	0.18 (0.091-0.32)	0.95% (0.65-1.4%)	1.4% (0.9-2.2%)	15 (9.1-23)	890 (610-1300)

Location	Year	Sex	Age	Prevalence of current drinkers	Prevalence of abstainers	Population average of standard drinks daily	Percent of total attributable deaths	Percent of total attributable DALYs	Attributable deaths	Attributable DALYs
Sri Lanka	1990	Female	50 to 54	6.8% (4.2-10%)	93% (90-96%)	0.21 (0.11-0.36)	0.93% (0.62-1.4%)	1.4% (0.84-2.2%)	18 (11-28)	860 (570-1300)
Sri Lanka	1990	Female	55 to 59	6.9% (4.3-10%)	93% (90-96%)	0.23 (0.12-0.4)	0.87% (0.51-1.4%)	1.3% (0.7-2.1%)	20 (11-34)	790 (460-1300)
Sri Lanka	1990	Female	60 to 64	6.7% (4-10%)	93% (90-96%)	0.23 (0.12-0.4)	0.75% (0.36-1.3%)	1.1% (0.47-1.8%)	22 (10-39)	710 (350-1200)
Sri Lanka	1990	Female	65 to 69	6.1% (3.7-9%)	94% (91-96%)	0.21 (0.11-0.35)	0.6% (0.25-1.1%)	0.81% (0.32-1.4%)	23 (9-41)	590 (250-1000)
Sri Lanka	1990	Female	70 to 74	5.9% (3.8-8.9%)	94% (91-96%)	0.2 (0.1-0.34)	0.52% (0.16-1%)	0.67% (0.19-1.3%)	23 (6.2-45)	460 (140-900)
Sri Lanka	1990	Female	75 to 79	6.3% (3.8-9.5%)	94% (90-96%)	0.2 (0.097-0.37)	0.38% (0.0096-0.86%)	0.46% (-0.017-1.1%)	17 (-0.64-41)	270 (6.8-620)
Sri Lanka	1990	Female	80 to 84	6.7% (4-9.6%)	93% (90-96%)	0.21 (0.11-0.37)	0.31% (-0.04-0.79%)	0.35% (-0.074-0.92%)	14 (-3-37)	160 (-20-410)
Sri Lanka	1990	Female	85 to 89	7.5% (4.4-12%)	93% (88-96%)	0.23 (0.11-0.4)	0.24% (-0.11-0.71%)	0.25% (-0.15-0.8%)	9.3 (-5.3-30)	76 (-34-240)
Sri Lanka	1990	Female	90 to 94	8.6% (5.1-13%)	91% (87-95%)	0.25 (0.12-0.46)	0.17% (-0.2-0.71%)	0.16% (-0.27-0.79%)	2.7 (-4.6-13)	17 (-21-72)
Sri Lanka	1990	Female	95 plus	10% (5.2-17%)	90% (83-95%)	0.28 (0.12-0.57)	0.15% (-0.32-0.89%)	0.11% (-0.42-0.93%)	0.56 (-2.2-4.7)	3 (-6.2-18)
Sri Lanka	1990	Male	All Ages	33% (28-39%)	67% (61-72%)	0.48 (0.38-0.58)	2% (1.3-2.8%)	2% (1.1-2.9%)	1300 (660-1900)	63000 (38000-87000)
Sri Lanka	1990	Male	15 to 19	26% (16-37%)	74% (63-84%)	0.19 (0.095-0.33)	1% (0.39-2%)	1.1% (0.15-2.5%)	19 (2.5-43)	1900 (710-3700)
Sri Lanka	1990	Male	20 to 24	39% (25-53%)	61% (47-75%)	0.46 (0.27-0.73)	2.5% (0.99-4.6%)	2.6% (0.62-5.3%)	79 (19-160)	6500 (2500-12000)
Sri Lanka	1990	Male	25 to 29	46% (31-60%)	54% (40-69%)	0.66 (0.39-0.98)	3.4% (1.6-5.4%)	3.8% (1.4-6.5%)	100 (38-180)	7800 (3700-12000)
Sri Lanka	1990	Male	30 to 34	51% (34-66%)	49% (34-66%)	0.85 (0.52-1.2)	4.1% (2.3-6.2%)	4.7% (2.3-7.8%)	120 (59-200)	8300 (4600-12000)
Sri Lanka	1990	Male	35 to 39	54% (39-68%)	46% (32-61%)	1 (0.64-1.5)	4.7% (3-6.9%)	5.8% (3.4-8.5%)	130 (78-200)	8000 (5100-12000)
Sri Lanka	1990	Male	40 to 44	55% (41-70%)	45% (30-59%)	1.1 (0.69-1.6)	4.8% (3-6.6%)	5.9% (3.5-8.6%)	140 (83-210)	7700 (4800-11000)
Sri Lanka	1990	Male	45 to 49	54% (37-68%)	46% (32-63%)	1.1 (0.64-1.6)	4.5% (2.7-6.5%)	5.6% (3.2-8.1%)	140 (82-220)	6700 (4100-9700)
Sri Lanka	1990	Male	50 to 54	52% (37-67%)	48% (33-63%)	1 (0.58-1.5)	3.6% (2-5.4%)	4.4% (2.2-6.8%)	130 (63-200)	5300 (2900-8000)
Sri Lanka	1990	Male	55 to 59	50% (35-65%)	50% (35-65%)	0.87 (0.5-1.4)	2.6% (1.2-4.1%)	3.2% (1.3-5.2%)	110 (43-180)	3900 (1800-6200)
Sri Lanka	1990	Male	60 to 64	46% (31-63%)	54% (37-69%)	0.74 (0.4-1.2)	1.9% (0.68-3.1%)	2.2% (0.52-3.8%)	90 (21-160)	2800 (1000-4700)
Sri Lanka	1990	Male	65 to 69	41% (27-55%)	59% (45-73%)	0.6 (0.33-0.95)	1.4% (0.42-2.3%)	1.5% (0.34-2.7%)	76 (17-130)	2000 (610-3300)

Location	Year	Sex	Age	Prevalence of current drinkers	Prevalence of abstainers	Population average of standard drinks daily	Percent of total attributable deaths	Percent of total attributable DALYs	Attributable deaths	Attributable DALYs
Sri Lanka	1990	Male	70 to 74	36% (24-50%)	64% (50-76%)	0.49 (0.26-0.8)	0.98% (0.21-1.9%)	1.1% (0.12-2.2%)	59 (6.5-120)	1200 (260-2400)
Sri Lanka	1990	Male	75 to 79	33% (20-49%)	67% (51-80%)	0.43 (0.23-0.75)	0.51% (-0.17-1.2%)	0.49% (-0.32-1.4%)	27 (-17-75)	480 (-150-1200)
Sri Lanka	1990	Male	80 to 84	29% (17-44%)	71% (56-83%)	0.38 (0.2-0.63)	0.32% (-0.26-0.95%)	0.29% (-0.41-1%)	16 (-22-54)	220 (-180-630)
Sri Lanka	1990	Male	85 to 89	27% (16-40%)	73% (60-84%)	0.32 (0.17-0.57)	0.14% (-0.42-0.62%)	0.086% (-0.57-0.64%)	3.6 (-24-27)	50 (-150-220)
Sri Lanka	1990	Male	90 to 94	27% (17-39%)	73% (61-83%)	0.3 (0.15-0.54)	0.0016% (-0.51-0.46%)	-0.078% (-0.7-0.46%)	-1.2 (-11-7.4)	0.16 (-48-44)
Sri Lanka	1990	Male	95 plus	28% (16-44%)	72% (56-84%)	0.3 (0.13-0.58)	-0.066% (-0.68-0.42%)	-0.2% (-0.93-0.36%)	-0.73 (-3.3-1.3)	-0.9 (-9.4-5.8)
Sri Lanka	1995	Female	All Ages	5.4% (4.6-6.4%)	95% (94-95%)	0.11 (0.085-0.15)	0.59% (0.43-0.78%)	0.6% (0.29-0.99%)	250 (120-410)	13000 (8900-17000)
Sri Lanka	1995	Female	15 to 19	4.8% (3-7.2%)	95% (93-97%)	0.091 (0.046-0.16)	0.52% (0.29-0.86%)	0.51% (0.13-1.1%)	4.9 (1.3-11)	730 (400-1200)
Sri Lanka	1995	Female	20 to 24	6% (3.6-9%)	94% (91-96%)	0.12 (0.062-0.22)	0.92% (0.57-1.4%)	0.7% (0.19-1.5%)	7 (1.9-15)	1300 (730-2000)
Sri Lanka	1995	Female	25 to 29	6.5% (3.9-10%)	93% (90-96%)	0.14 (0.076-0.25)	0.99% (0.68-1.4%)	0.79% (0.32-1.5%)	7.3 (2.9-14)	1300 (810-1800)
Sri Lanka	1995	Female	30 to 34	6.9% (4-11%)	93% (89-96%)	0.15 (0.082-0.27)	1% (0.69-1.4%)	1% (0.58-1.7%)	9.1 (5.2-15)	1200 (820-1700)
Sri Lanka	1995	Female	35 to 39	7.2% (4.2-11%)	93% (89-96%)	0.16 (0.081-0.27)	0.95% (0.7-1.3%)	1.1% (0.71-1.8%)	11 (6.9-18)	1200 (820-1600)
Sri Lanka	1995	Female	40 to 44	7.2% (4.4-10%)	93% (90-96%)	0.17 (0.086-0.28)	0.94% (0.67-1.3%)	1.4% (0.9-2%)	14 (9.2-20)	1100 (740-1400)
Sri Lanka	1995	Female	45 to 49	7% (4-10%)	93% (90-96%)	0.19 (0.092-0.33)	1% (0.72-1.5%)	1.5% (0.98-2.3%)	21 (14-31)	1200 (830-1700)
Sri Lanka	1995	Female	50 to 54	7.1% (4.5-11%)	93% (89-95%)	0.21 (0.11-0.37)	1% (0.66-1.5%)	1.5% (0.91-2.2%)	24 (15-37)	1100 (730-1600)
Sri Lanka	1995	Female	55 to 59	7.2% (4.5-11%)	93% (89-95%)	0.23 (0.12-0.42)	0.9% (0.51-1.5%)	1.3% (0.72-2.2%)	25 (14-41)	970 (560-1500)
Sri Lanka	1995	Female	60 to 64	6.9% (4.1-10%)	93% (90-96%)	0.23 (0.11-0.4)	0.75% (0.34-1.3%)	1% (0.46-1.8%)	27 (12-48)	860 (390-1500)
Sri Lanka	1995	Female	65 to 69	6.4% (3.8-9.4%)	94% (91-96%)	0.21 (0.11-0.35)	0.6% (0.25-1.1%)	0.79% (0.31-1.5%)	29 (11-54)	740 (310-1300)
Sri Lanka	1995	Female	70 to 74	6.2% (3.9-9.3%)	94% (91-96%)	0.2 (0.1-0.35)	0.5% (0.13-1%)	0.62% (0.13-1.3%)	29 (6.7-59)	580 (150-1200)
Sri Lanka	1995	Female	75 to 79	6.6% (4-9.9%)	93% (90-96%)	0.21 (0.1-0.37)	0.36% (-0.038-0.87%)	0.43% (-0.066-1.1%)	19 (-2.8-46)	300 (-30-710)
Sri Lanka	1995	Female	80 to 84	7.1% (4.3-10%)	93% (90-96%)	0.22 (0.11-0.38)	0.25% (-0.11-0.77%)	0.28% (-0.17-0.9%)	12 (-7.1-37)	140 (-64-420)
Sri Lanka	1995	Female	85 to 89	7.9% (4.6-12%)	92% (88-95%)	0.23 (0.12-0.4)	0.17% (-0.18-0.65%)	0.17% (-0.25-0.71%)	6.3 (-9.7-28)	58 (-64-220)

Location	Year	Sex	Age	Prevalence of current drinkers	Prevalence of abstainers	Population average of standard drinks daily	Percent of total attributable deaths	Percent of total attributable DALYs	Attributable deaths	Attributable DALYs
Sri Lanka	1995	Female	90 to 94	9%<br>(5.4-14%)	91%<br>(86-95%)	0.25<br>(0.12-0.46)	0.1%<br>(-0.27-0.58%)	0.071%<br>(-0.39-0.65%)	1.4<br>(-7.4-12)	12<br>(-33-69)
Sri Lanka	1995	Female	95 plus	11%<br>(5.8-18%)	89%<br>(82-94%)	0.29<br>(0.12-0.58)	0.086%<br>(-0.4-0.79%)	0.018%<br>(-0.57-0.86%)	0.11<br>(-3.5-5.2)	2.1<br>(-9.8-20)
Sri Lanka	1995	Male	All Ages	35%<br>(30-42%)	65%<br>(58-70%)	0.5<br>(0.39-0.61)	2.6%<br>(1.7-3.6%)	2.5%<br>(1.4-3.6%)	1700<br>(990-2500)	83000<br>(54000-110000)
Sri Lanka	1995	Male	15 to 19	27%<br>(17-39%)	73%<br>(61-83%)	0.19<br>(0.096-0.33)	1%<br>(0.4-2%)	1.1%<br>(0.19-2.5%)	21<br>(3.7-46)	2000<br>(760-3800)
Sri Lanka	1995	Male	20 to 24	40%<br>(27-55%)	60%<br>(45-73%)	0.46<br>(0.27-0.74)	2.6%<br>(1-4.8%)	2.8%<br>(0.74-5.5%)	91<br>(25-180)	7300<br>(2800-13000)
Sri Lanka	1995	Male	25 to 29	47%<br>(32-62%)	53%<br>(38-68%)	0.65<br>(0.39-0.98)	3.7%<br>(1.8-5.9%)	4%<br>(1.6-6.9%)	130<br>(50-220)	9400<br>(4600-15000)
Sri Lanka	1995	Male	30 to 34	52%<br>(35-67%)	48%<br>(33-65%)	0.85<br>(0.53-1.3)	4.5%<br>(2.7-6.8%)	5.3%<br>(2.8-8.4%)	150<br>(79-240)	9900<br>(5800-15000)
Sri Lanka	1995	Male	35 to 39	56%<br>(40-70%)	44%<br>(30-60%)	1<br>(0.64-1.5)	5.5%<br>(3.6-7.8%)	6.8%<br>(4.2-9.8%)	200<br>(120-290)	12000<br>(7500-16000)
Sri Lanka	1995	Male	40 to 44	57%<br>(42-70%)	43%<br>(30-58%)	1.1<br>(0.68-1.6)	5.6%<br>(3.7-7.6%)	7%<br>(4.4-9.7%)	200<br>(130-290)	11000<br>(6900-14000)
Sri Lanka	1995	Male	45 to 49	55%<br>(38-69%)	45%<br>(31-62%)	1.1<br>(0.66-1.6)	5.4%<br>(3.3-7.6%)	6.7%<br>(3.8-9.5%)	230<br>(130-340)	11000<br>(6400-15000)
Sri Lanka	1995	Male	50 to 54	53%<br>(38-68%)	47%<br>(32-62%)	0.99<br>(0.58-1.5)	4.3%<br>(2.4-6.2%)	5.2%<br>(2.7-7.7%)	190<br>(98-290)	7700<br>(4100-11000)
Sri Lanka	1995	Male	55 to 59	51%<br>(35-67%)	49%<br>(33-65%)	0.87<br>(0.49-1.4)	3.1%<br>(1.5-4.7%)	3.7%<br>(1.7-5.7%)	150<br>(68-240)	5300<br>(2700-8100)
Sri Lanka	1995	Male	60 to 64	48%<br>(32-63%)	52%<br>(37-68%)	0.74<br>(0.4-1.2)	2.1%<br>(0.83-3.4%)	2.5%<br>(0.78-4.1%)	120<br>(39-210)	3600<br>(1400-6000)
Sri Lanka	1995	Male	65 to 69	42%<br>(28-57%)	58%<br>(43-72%)	0.6<br>(0.33-0.96)	1.5%<br>(0.52-2.5%)	1.7%<br>(0.48-3%)	100<br>(29-180)	2600<br>(900-4300)
Sri Lanka	1995	Male	70 to 74	37%<br>(24-51%)	63%<br>(49-76%)	0.49<br>(0.26-0.8)	1.1%<br>(0.26-1.9%)	1.2%<br>(0.22-2.2%)	79<br>(14-150)	1600<br>(390-3000)
Sri Lanka	1995	Male	75 to 79	34%<br>(21-50%)	66%<br>(50-79%)	0.44<br>(0.23-0.76)	0.52%<br>(-0.11-1.3%)	0.51%<br>(-0.26-1.4%)	29<br>(-14-77)	520<br>(-110-1200)
Sri Lanka	1995	Male	80 to 84	30%<br>(18-45%)	70%<br>(55-82%)	0.38<br>(0.2-0.63)	0.33%<br>(-0.24-0.91%)	0.3%<br>(-0.42-0.99%)	15<br>(-21-50)	220<br>(-160-590)
Sri Lanka	1995	Male	85 to 89	28%<br>(17-41%)	72%<br>(59-83%)	0.33<br>(0.17-0.56)	0.15%<br>(-0.38-0.61%)	0.082%<br>(-0.56-0.62%)	3.1<br>(-21-24)	51<br>(-130-210)
Sri Lanka	1995	Male	90 to 94	28%<br>(17-41%)	72%<br>(59-83%)	0.31<br>(0.15-0.56)	0.014%<br>(-0.49-0.42%)	-0.089%<br>(-0.71-0.4%)	-1.5<br>(-12-6.7)	1.4<br>(-50-44)
Sri Lanka	1995	Male	95 plus	30%<br>(17-46%)	70%<br>(54-83%)	0.31<br>(0.13-0.59)	-0.00028%<br>(-0.58-0.43%)	-0.18%<br>(-0.9-0.35%)	-0.72<br>(-3.7-1.5)	0.0039<br>(-9.6-7.1)
Sri Lanka	2000	Female	All Ages	5%<br>(4.3-5.9%)	95%<br>(94-96%)	0.14<br>(0.1-0.18)	0.74%<br>(0.54-1%)	0.87%<br>(0.45-1.4%)	380<br>(190-620)	16000<br>(11000-22000)
Sri Lanka	2000	Female	15 to 19	4.5%<br>(2.9-6.7%)	95%<br>(93-97%)	0.11<br>(0.057-0.2)	0.6%<br>(0.35-1%)	0.65%<br>(0.2-1.4%)	5.6<br>(1.7-12)	790<br>(440-1300)

Location	Year	Sex	Age	Prevalence of current drinkers	Prevalence of abstainers	Population average of standard drinks daily	Percent of total attributable deaths	Percent of total attributable DALYs	Attributable deaths	Attributable DALYs
Sri Lanka	2000	Female	20 to 24	5.6% (3.5-8.1%)	94% (92-97%)	0.15 (0.073-0.25)	1% (0.66-1.6%)	0.86% (0.28-1.8%)	7.6 (2.5-16)	1300 (800-2100)
Sri Lanka	2000	Female	25 to 29	6.3% (3.9-9.3%)	94% (91-96%)	0.18 (0.092-0.3)	1.1% (0.76-1.6%)	0.99% (0.42-1.9%)	7.3 (3.1-14)	1200 (800-1800)
Sri Lanka	2000	Female	30 to 34	6.4% (4-9.7%)	94% (90-96%)	0.18 (0.097-0.33)	1.1% (0.76-1.6%)	1.2% (0.7-2%)	9.4 (5.3-17)	1300 (840-1800)
Sri Lanka	2000	Female	35 to 39	6.2% (3.7-9%)	94% (91-96%)	0.17 (0.091-0.3)	1% (0.73-1.4%)	1.3% (0.82-2.1%)	12 (7.3-19)	1200 (850-1700)
Sri Lanka	2000	Female	40 to 44	6.1% (3.9-8.7%)	94% (91-96%)	0.18 (0.096-0.3)	1% (0.72-1.4%)	1.5% (1-2.2%)	16 (11-23)	1300 (900-1700)
Sri Lanka	2000	Female	45 to 49	6% (3.6-8.6%)	94% (91-96%)	0.2 (0.1-0.35)	1.1% (0.75-1.6%)	1.7% (1.1-2.5%)	24 (16-35)	1400 (940-1900)
Sri Lanka	2000	Female	50 to 54	5.8% (3.8-8.5%)	94% (91-96%)	0.22 (0.11-0.37)	1.1% (0.72-1.6%)	1.6% (1-2.5%)	32 (20-50)	1500 (1000-2200)
Sri Lanka	2000	Female	55 to 59	6.2% (3.9-8.9%)	94% (91-96%)	0.25 (0.13-0.44)	1.1% (0.63-1.7%)	1.6% (0.92-2.6%)	34 (19-55)	1300 (790-2200)
Sri Lanka	2000	Female	60 to 64	6.2% (3.9-9.3%)	94% (91-96%)	0.27 (0.13-0.47)	0.96% (0.46-1.7%)	1.3% (0.65-2.3%)	38 (18-67)	1200 (590-2100)
Sri Lanka	2000	Female	65 to 69	5.9% (3.7-8.5%)	94% (92-96%)	0.25 (0.13-0.41)	0.84% (0.39-1.5%)	1.1% (0.51-2%)	44 (21-80)	1100 (530-2000)
Sri Lanka	2000	Female	70 to 74	6% (3.9-8.7%)	94% (91-96%)	0.25 (0.12-0.44)	0.75% (0.26-1.4%)	0.94% (0.31-1.9%)	50 (16-99)	980 (320-1900)
Sri Lanka	2000	Female	75 to 79	6.4% (3.9-9.7%)	94% (90-96%)	0.26 (0.12-0.47)	0.59% (0.089-1.3%)	0.72% (0.074-1.6%)	36 (3.8-82)	570 (80-1300)
Sri Lanka	2000	Female	80 to 84	6.8% (4.2-9.9%)	93% (90-96%)	0.27 (0.13-0.46)	0.5% (0.025-1.2%)	0.58% (0.0084-1.4%)	30 (0.45-74)	340 (17-820)
Sri Lanka	2000	Female	85 to 89	7.7% (4.4-12%)	92% (88-96%)	0.29 (0.14-0.5)	0.42% (-0.0014-1.1%)	0.48% (-0.032-1.3%)	18 (-1.2-47)	150 (-0.52-390)
Sri Lanka	2000	Female	90 to 94	8.8% (5.3-14%)	91% (86-95%)	0.32 (0.16-0.58)	0.36% (-0.099-1.1%)	0.39% (-0.17-1.3%)	8.4 (-3.9-28)	50 (-14-150)
Sri Lanka	2000	Female	95 plus	11% (5.6-18%)	89% (82-94%)	0.36 (0.15-0.73)	0.39% (-0.25-1.5%)	0.37% (-0.37-1.7%)	2.8 (-2.8-13)	12 (-7.6-47)
Sri Lanka	2000	Male	All Ages	38% (33-43%)	62% (57-67%)	0.72 (0.55-0.91)	3.5% (2.4-4.8%)	3.4% (2.1-4.8%)	2500 (1600-3600)	110000 (76000-150000)
Sri Lanka	2000	Male	15 to 19	27% (19-38%)	73% (62-81%)	0.25 (0.13-0.43)	1.3% (0.55-2.3%)	1.5% (0.36-3.2%)	23 (5.2-47)	2200 (930-4000)
Sri Lanka	2000	Male	20 to 24	40% (29-52%)	60% (48-71%)	0.6 (0.34-0.95)	3.3% (1.6-5.7%)	3.7% (1.3-6.8%)	100 (37-190)	8200 (3900-14000)
Sri Lanka	2000	Male	25 to 29	48% (36-60%)	52% (40-64%)	0.85 (0.52-1.3)	4.5% (2.5-7.2%)	5.3% (2.4-8.8%)	130 (59-220)	9500 (5200-15000)
Sri Lanka	2000	Male	30 to 34	54% (40-66%)	46% (34-60%)	1.1 (0.72-1.7)	5.6% (3.4-8.2%)	6.9% (3.8-11%)	170 (97-270)	11000 (6700-16000)
Sri Lanka	2000	Male	35 to 39	59% (47-71%)	41% (29-53%)	1.4 (0.89-2.1)	6.9% (4.6-9.7%)	8.7% (5.5-12%)	260 (170-370)	15000 (9900-21000)

Location	Year	Sex	Age	Prevalence of current drinkers	Prevalence of abstainers	Population average of standard drinks daily	Percent of total attributable deaths	Percent of total attributable DALYs	Attributable deaths	Attributable DALYs
Sri Lanka	2000	Male	40 to 44	60% (48-72%)	40% (28-52%)	1.5 (0.96-2.2)	6.9% (4.8-9.3%)	8.8% (5.8-12%)	310 (200-430)	16000 (11000-21000)
Sri Lanka	2000	Male	45 to 49	58% (44-70%)	42% (30-56%)	1.5 (0.95-2.2)	6.6% (4.2-9.1%)	8.2% (5.1-11%)	350 (210-490)	15000 (9700-21000)
Sri Lanka	2000	Male	50 to 54	57% (44-69%)	43% (31-56%)	1.4 (0.84-2.1)	5.4% (3.2-7.8%)	6.5% (3.7-9.5%)	340 (190-510)	13000 (8000-20000)
Sri Lanka	2000	Male	55 to 59	55% (42-68%)	45% (32-58%)	1.2 (0.75-2)	3.9% (2.1-6.2%)	4.8% (2.4-7.5%)	250 (120-390)	8500 (4600-13000)
Sri Lanka	2000	Male	60 to 64	50% (37-64%)	50% (36-63%)	1 (0.59-1.7)	2.8% (1.2-4.7%)	3.3% (1.3-5.7%)	190 (74-320)	5500 (2400-9400)
Sri Lanka	2000	Male	65 to 69	43% (30-56%)	57% (44-70%)	0.8 (0.46-1.2)	2% (0.87-3.4%)	2.3% (0.93-3.9%)	150 (60-260)	3800 (1600-6200)
Sri Lanka	2000	Male	70 to 74	37% (25-50%)	63% (50-75%)	0.65 (0.35-1)	1.5% (0.46-2.8%)	1.6% (0.44-3.2%)	130 (34-240)	2500 (790-4700)
Sri Lanka	2000	Male	75 to 79	36% (23-50%)	64% (50-77%)	0.6 (0.32-1)	0.8% (-0.022-1.9%)	0.84% (-0.17-2.1%)	54 (-11-140)	910 (-24-2100)
Sri Lanka	2000	Male	80 to 84	31% (18-46%)	69% (54-82%)	0.5 (0.26-0.85)	0.57% (-0.14-1.5%)	0.58% (-0.28-1.6%)	33 (-16-93)	420 (-100-1100)
Sri Lanka	2000	Male	85 to 89	26% (16-39%)	74% (61-84%)	0.41 (0.21-0.7)	0.39% (-0.11-0.99%)	0.37% (-0.26-1.1%)	14 (-9.4-41)	140 (-41-350)
Sri Lanka	2000	Male	90 to 94	26% (16-39%)	74% (61-84%)	0.38 (0.19-0.68)	0.22% (-0.29-0.74%)	0.14% (-0.5-0.8%)	2.7 (-8.9-15)	26 (-34-86)
Sri Lanka	2000	Male	95 plus	28% (15-43%)	72% (57-85%)	0.37 (0.16-0.73)	0.21% (-0.37-0.77%)	0.044% (-0.67-0.67%)	0.24 (-3.6-3.6)	4.6 (-7.9-17)
Sri Lanka	2005	Female	All Ages	4.8% (4.1-5.6%)	95% (94-96%)	0.21 (0.15-0.28)	1% (0.71-1.4%)	1.5% (0.81-2.3%)	680 (370-1100)	22000 (16000-32000)
Sri Lanka	2005	Female	15 to 19	4.2% (2.7-6.3%)	96% (94-97%)	0.16 (0.081-0.28)	0.75% (0.42-1.2%)	0.98% (0.37-2.1%)	6.2 (2.3-13)	810 (450-1400)
Sri Lanka	2005	Female	20 to 24	5.2% (3.1-7.6%)	95% (92-97%)	0.21 (0.1-0.38)	1.3% (0.8-2%)	1.3% (0.5-2.6%)	9.3 (3.5-19)	1500 (910-2400)
Sri Lanka	2005	Female	25 to 29	5.8% (3.7-8.6%)	94% (91-96%)	0.26 (0.13-0.45)	1.4% (0.88-2%)	1.5% (0.68-2.8%)	9.8 (4.4-19)	1500 (960-2300)
Sri Lanka	2005	Female	30 to 34	6% (3.7-9.2%)	94% (91-96%)	0.27 (0.14-0.47)	1.3% (0.91-1.9%)	1.7% (0.91-3%)	11 (5.8-19)	1400 (880-2000)
Sri Lanka	2005	Female	35 to 39	5.8% (3.5-8.4%)	94% (92-97%)	0.25 (0.13-0.44)	1.2% (0.82-1.7%)	1.7% (1-2.8%)	13 (7.6-22)	1400 (910-2000)
Sri Lanka	2005	Female	40 to 44	5.7% (3.6-8.2%)	94% (92-96%)	0.26 (0.14-0.46)	1.2% (0.82-1.7%)	1.9% (1.2-2.9%)	18 (11-28)	1400 (980-2000)
Sri Lanka	2005	Female	45 to 49	5.6% (3.3-8.1%)	94% (92-97%)	0.3 (0.15-0.53)	1.3% (0.83-1.9%)	2.1% (1.3-3.3%)	29 (18-45)	1700 (1100-2600)
Sri Lanka	2005	Female	50 to 54	5.5% (3.5-8%)	95% (92-97%)	0.32 (0.17-0.55)	1.4% (0.87-2.2%)	2.2% (1.3-3.5%)	40 (24-65)	1900 (1200-3000)
Sri Lanka	2005	Female	55 to 59	5.8% (3.7-8.3%)	94% (92-96%)	0.37 (0.19-0.64)	1.4% (0.82-2.4%)	2.3% (1.3-3.8%)	53 (30-88)	2100 (1200-3400)

Location	Year	Sex	Age	Prevalence of current drinkers	Prevalence of abstainers	Population average of standard drinks daily	Percent of total attributable deaths	Percent of total attributable DALYs	Attributable deaths	Attributable DALYs
Sri Lanka	2005	Female	60 to 64	5.8% (3.7-8.7%)	94% (91-96%)	0.39 (0.19-0.66)	1.4% (0.71-2.4%)	2.1% (1-3.6%)	61 (29-100)	2000 (970-3300)
Sri Lanka	2005	Female	65 to 69	5.5% (3.5-7.7%)	94% (92-96%)	0.36 (0.19-0.59)	1.4% (0.67-2.3%)	1.9% (0.93-3.1%)	74 (37-120)	1900 (950-3100)
Sri Lanka	2005	Female	70 to 74	5.6% (3.5-8.3%)	94% (92-97%)	0.36 (0.18-0.64)	1.3% (0.58-2.5%)	1.7% (0.73-3.2%)	95 (41-180)	1900 (810-3600)
Sri Lanka	2005	Female	75 to 79	6.1% (3.7-9.3%)	94% (91-96%)	0.38 (0.18-0.68)	1.2% (0.41-2.3%)	1.5% (0.49-2.8%)	83 (28-160)	1300 (440-2600)
Sri Lanka	2005	Female	80 to 84	6.6% (3.9-9.5%)	93% (91-96%)	0.4 (0.2-0.69)	1.1% (0.37-2.3%)	1.4% (0.43-2.8%)	82 (26-170)	920 (300-1800)
Sri Lanka	2005	Female	85 to 89	7.3% (4.2-11%)	93% (89-96%)	0.43 (0.21-0.77)	1% (0.3-2.1%)	1.2% (0.33-2.6%)	52 (14-110)	420 (130-880)
Sri Lanka	2005	Female	90 to 94	8.4% (5-13%)	92% (87-95%)	0.47 (0.23-0.89)	0.97% (0.21-2.3%)	1.1% (0.22-2.8%)	28 (5.4-70)	160 (36-380)
Sri Lanka	2005	Female	95 plus	10% (5.1-16%)	90% (84-95%)	0.54 (0.21-1.1)	1.1% (0.096-2.7%)	1.2% (0.039-3.2%)	12 (0.36-31)	43 (4-110)
Sri Lanka	2005	Male	All Ages	37% (32-43%)	63% (57-68%)	1.1 (0.86-1.4)	4.6% (3.3-6.2%)	4.8% (3.1-6.9%)	3500 (2300-5000)	140000 (100000-190000)
Sri Lanka	2005	Male	15 to 19	25% (17-36%)	75% (64-83%)	0.37 (0.19-0.64)	1.7% (0.92-2.9%)	2.3% (0.92-4.3%)	22 (8.6-43)	2200 (1100-3700)
Sri Lanka	2005	Male	20 to 24	39% (27-51%)	61% (49-73%)	0.9 (0.51-1.4)	4.5% (2.5-7.3%)	5.7% (2.7-9.7%)	94 (43-160)	7700 (4100-12000)
Sri Lanka	2005	Male	25 to 29	46% (34-58%)	54% (42-66%)	1.3 (0.76-2)	6% (3.4-8.9%)	7.8% (4-12%)	130 (66-210)	9700 (5600-14000)
Sri Lanka	2005	Male	30 to 34	53% (39-66%)	47% (34-61%)	1.7 (1.1-2.5)	7.2% (4.6-10%)	9.8% (5.9-15%)	160 (95-250)	11000 (6900-16000)
Sri Lanka	2005	Male	35 to 39	58% (46-69%)	42% (31-54%)	2.2 (1.4-3.2)	8.7% (5.8-12%)	12% (7.5-17%)	270 (170-380)	16000 (10000-22000)
Sri Lanka	2005	Male	40 to 44	58% (47-71%)	42% (29-53%)	2.3 (1.5-3.3)	9.1% (6.3-12%)	12% (8.1-16%)	370 (240-510)	19000 (13000-26000)
Sri Lanka	2005	Male	45 to 49	57% (43-69%)	43% (31-57%)	2.3 (1.5-3.3)	8.8% (5.8-12%)	11% (7.4-16%)	460 (290-670)	21000 (13000-30000)
Sri Lanka	2005	Male	50 to 54	56% (43-68%)	44% (32-57%)	2.1 (1.3-3.1)	7.5% (4.6-11%)	9.4% (5.6-14%)	470 (280-720)	19000 (11000-28000)
Sri Lanka	2005	Male	55 to 59	54% (41-66%)	46% (34-59%)	1.9 (1.1-3)	5.9% (3.3-9.2%)	7.2% (4.1-12%)	420 (230-670)	15000 (8200-23000)
Sri Lanka	2005	Male	60 to 64	49% (36-63%)	51% (37-64%)	1.6 (0.89-2.5)	4.4% (2.1-7.3%)	5.3% (2.4-8.8%)	320 (140-550)	9400 (4400-16000)
Sri Lanka	2005	Male	65 to 69	41% (29-55%)	59% (45-71%)	1.2 (0.69-1.9)	3.3% (1.5-5.5%)	3.8% (1.6-6.5%)	260 (120-450)	6400 (3100-11000)
Sri Lanka	2005	Male	70 to 74	36% (24-49%)	64% (51-76%)	0.98 (0.52-1.6)	2.5% (1-4.6%)	2.9% (1.1-5.3%)	230 (88-420)	4500 (1800-8100)
Sri Lanka	2005	Male	75 to 79	34% (22-49%)	66% (51-78%)	0.91 (0.47-1.6)	1.7% (0.4-3.7%)	1.9% (0.36-4.2%)	140 (26-310)	2200 (530-4700)

Location	Year	Sex	Age	Prevalence of current drinkers	Prevalence of abstainers	Population average of standard drinks daily	Percent of total attributable deaths	Percent of total attributable DALYs	Attributable deaths	Attributable DALYs
Sri Lanka	2005	Male	80 to 84	29% (17-44%)	71% (56-83%)	0.75 (0.38-1.3)	1.3% (0.24-2.8%)	1.4% (0.18-3.2%)	90 (11-200)	1100 (190-2300)
Sri Lanka	2005	Male	85 to 89	25% (15-37%)	75% (63-85%)	0.6 (0.3-1.1)	0.88% (0.15-1.9%)	0.94% (0.072-2.2%)	39 (3-91)	340 (61-750)
Sri Lanka	2005	Male	90 to 94	25% (15-37%)	75% (63-85%)	0.56 (0.28-1)	0.63% (0.0096-1.5%)	0.62% (-0.14-1.7%)	14 (-3.2-38)	90 (1.4-220)
Sri Lanka	2005	Male	95 plus	26% (14-42%)	74% (58-86%)	0.55 (0.22-1.1)	0.61% (-0.089-1.7%)	0.49% (-0.36-1.7%)	3.4 (-2.6-12)	18 (-2.6-48)
Sri Lanka	2010	Female	All Ages	5.2% (4.4-6.1%)	95% (94-96%)	0.34 (0.25-0.46)	1.5% (1-2%)	2.3% (1.4-3.6%)	1200 (690-1900)	34000 (23000-48000)
Sri Lanka	2010	Female	15 to 19	4.4% (2.7-6.5%)	96% (93-97%)	0.24 (0.12-0.45)	0.82% (0.46-1.4%)	1% (0.38-2.2%)	7.2 (2.6-15)	920 (500-1600)
Sri Lanka	2010	Female	20 to 24	5.5% (3.3-8.2%)	95% (92-97%)	0.33 (0.16-0.61)	1.4% (0.85-2.2%)	1.5% (0.54-2.9%)	10 (3.9-21)	1600 (940-2500)
Sri Lanka	2010	Female	25 to 29	6% (3.6-9.3%)	94% (91-96%)	0.38 (0.19-0.68)	1.5% (0.97-2.2%)	1.7% (0.77-3.2%)	12 (5.4-24)	1700 (1100-2700)
Sri Lanka	2010	Female	30 to 34	6.4% (3.7-10%)	94% (90-96%)	0.41 (0.21-0.75)	1.5% (0.98-2.1%)	1.9% (0.97-3.4%)	14 (7.4-27)	1800 (1100-2600)
Sri Lanka	2010	Female	35 to 39	6.6% (3.7-10%)	93% (90-96%)	0.42 (0.21-0.76)	1.5% (0.93-2.2%)	2.1% (1.2-3.7%)	17 (9.1-30)	1700 (1000-2500)
Sri Lanka	2010	Female	40 to 44	6.6% (4-9.9%)	93% (90-96%)	0.45 (0.22-0.78)	1.5% (0.93-2.2%)	2.4% (1.4-3.9%)	24 (14-38)	1800 (1100-2800)
Sri Lanka	2010	Female	45 to 49	6.4% (3.7-9.5%)	94% (90-96%)	0.49 (0.25-0.89)	1.7% (1-2.6%)	2.8% (1.6-4.6%)	40 (23-66)	2400 (1500-3700)
Sri Lanka	2010	Female	50 to 54	6.5% (4.1-9.7%)	93% (90-96%)	0.56 (0.29-0.99)	1.9% (1.2-3.1%)	3.1% (1.8-5%)	66 (38-110)	3100 (1900-4900)
Sri Lanka	2010	Female	55 to 59	6.7% (4.1-10%)	93% (90-96%)	0.62 (0.31-1.1)	2.1% (1.2-3.4%)	3.3% (1.8-5.3%)	88 (46-140)	3500 (1900-5600)
Sri Lanka	2010	Female	60 to 64	6.4% (3.9-9.9%)	94% (90-96%)	0.61 (0.31-1)	2.1% (1.1-3.5%)	3.1% (1.6-5.2%)	110 (59-190)	3700 (2000-6400)
Sri Lanka	2010	Female	65 to 69	5.9% (3.6-8.5%)	94% (92-96%)	0.56 (0.29-0.95)	2% (1.1-3.4%)	2.8% (1.4-4.7%)	130 (69-230)	3400 (1800-5800)
Sri Lanka	2010	Female	70 to 74	5.7% (3.7-8.6%)	94% (91-96%)	0.54 (0.28-0.95)	2% (0.97-3.7%)	2.6% (1.2-4.6%)	150 (70-270)	2900 (1400-5200)
Sri Lanka	2010	Female	75 to 79	6.1% (3.7-9.3%)	94% (91-96%)	0.56 (0.26-0.99)	1.8% (0.78-3.4%)	2.3% (0.96-4.3%)	140 (60-280)	2200 (960-4300)
Sri Lanka	2010	Female	80 to 84	6.6% (4-9.8%)	93% (90-96%)	0.59 (0.29-1)	1.9% (0.84-3.5%)	2.3% (1-4.3%)	160 (70-320)	1800 (800-3400)
Sri Lanka	2010	Female	85 to 89	7.3% (4.3-11%)	93% (89-96%)	0.63 (0.3-1.1)	1.8% (0.72-3.4%)	2.2% (0.85-4.2%)	120 (47-240)	950 (380-1800)
Sri Lanka	2010	Female	90 to 94	8.4% (5.1-13%)	92% (87-95%)	0.7 (0.34-1.4)	1.8% (0.64-3.7%)	2.1% (0.72-4.6%)	66 (22-140)	360 (130-770)
Sri Lanka	2010	Female	95 plus	10% (5.3-17%)	90% (83-95%)	0.81 (0.33-1.6)	1.9% (0.48-3.9%)	2.3% (0.5-4.8%)	28 (6.1-59)	100 (26-210)

Location	Year	Sex	Age	Prevalence of current drinkers	Prevalence of abstainers	Population average of standard drinks daily	Percent of total attributable deaths	Percent of total attributable DALYs	Attributable deaths	Attributable DALYs
Sri Lanka	2010	Male	All Ages	35% (30-41%)	65% (59-70%)	1.6 (1.2-2)	5.6% (4-7.5%)	6.3% (4.1-9%)	4700 (3000-6900)	180000 (120000-240000)
Sri Lanka	2010	Male	15 to 19	25% (16-37%)	75% (63-84%)	0.52 (0.25-0.94)	1.5% (0.79-2.6%)	1.7% (0.71-3.4%)	22 (9.7-42)	2200 (1200-3600)
Sri Lanka	2010	Male	20 to 24	38% (25-52%)	62% (48-75%)	1.3 (0.68-2.2)	3.7% (2-6.2%)	4.1% (1.9-7.6%)	91 (43-160)	7400 (4000-12000)
Sri Lanka	2010	Male	25 to 29	45% (30-59%)	55% (41-70%)	1.9 (1-3)	5.4% (3.1-8.6%)	6.5% (3.3-11%)	140 (74-230)	10000 (6000-16000)
Sri Lanka	2010	Male	30 to 34	50% (33-65%)	50% (35-67%)	2.4 (1.4-3.7)	6.8% (4.3-10%)	8.7% (5-13%)	190 (110-290)	13000 (7900-19000)
Sri Lanka	2010	Male	35 to 39	53% (38-67%)	47% (33-62%)	2.9 (1.7-4.4)	8.8% (5.8-12%)	12% (7.3-17%)	270 (170-400)	16000 (10000-23000)
Sri Lanka	2010	Male	40 to 44	54% (39-68%)	46% (32-61%)	3.1 (1.8-4.6)	10% (6.8-14%)	13% (8.9-18%)	380 (250-540)	20000 (13000-27000)
Sri Lanka	2010	Male	45 to 49	53% (36-67%)	47% (33-64%)	3.1 (1.9-4.7)	10% (7.1-14%)	14% (9.1-19%)	530 (340-750)	24000 (16000-34000)
Sri Lanka	2010	Male	50 to 54	51% (36-65%)	49% (35-64%)	2.8 (1.7-4.4)	9.4% (5.9-13%)	12% (7.5-17%)	600 (360-860)	24000 (14000-34000)
Sri Lanka	2010	Male	55 to 59	49% (34-65%)	51% (35-66%)	2.5 (1.4-4)	7.9% (4.5-12%)	9.8% (5.7-15%)	580 (320-890)	20000 (12000-31000)
Sri Lanka	2010	Male	60 to 64	45% (31-62%)	55% (38-69%)	2.1 (1.1-3.5)	6.2% (3-9.8%)	7.7% (3.6-12%)	540 (250-890)	16000 (7600-26000)
Sri Lanka	2010	Male	65 to 69	40% (26-55%)	60% (45-74%)	1.7 (0.91-2.7)	5.1% (2.5-8.5%)	6.1% (2.9-10%)	450 (210-760)	11000 (5400-18000)
Sri Lanka	2010	Male	70 to 74	35% (22-49%)	65% (51-78%)	1.4 (0.73-2.4)	4.1% (1.8-7.4%)	4.8% (2-8.6%)	360 (150-650)	6900 (3000-12000)
Sri Lanka	2010	Male	75 to 79	32% (19-48%)	68% (52-81%)	1.2 (0.62-2.3)	3.1% (1.1-6.2%)	3.5% (1.2-7.2%)	250 (80-510)	3800 (1300-7600)
Sri Lanka	2010	Male	80 to 84	28% (16-43%)	72% (57-84%)	1.1 (0.51-1.9)	2.5% (0.86-5.2%)	2.8% (0.86-6%)	180 (54-380)	2000 (700-4100)
Sri Lanka	2010	Male	85 to 89	25% (15-38%)	75% (62-85%)	0.9 (0.46-1.6)	1.8% (0.54-3.9%)	2.1% (0.52-4.5%)	92 (23-200)	760 (230-1600)
Sri Lanka	2010	Male	90 to 94	25% (15-37%)	75% (63-85%)	0.84 (0.41-1.5)	1.4% (0.28-3.2%)	1.5% (0.18-3.6%)	37 (4.4-91)	220 (45-510)
Sri Lanka	2010	Male	95 plus	27% (15-43%)	73% (57-85%)	0.82 (0.32-1.7)	1.3% (0.16-3.4%)	1.3% (-0.049-3.7%)	13 (-0.47-37)	55 (6.4-140)
Sri Lanka	2016	Female	All Ages	5.3% (4.4-6.1%)	95% (94-96%)	0.43 (0.28-0.62)	1.8% (1.2-2.5%)	2.8% (1.7-4.3%)	1400 (800-2300)	39000 (25000-57000)
Sri Lanka	2016	Female	15 to 19	4.3% (2.7-6.6%)	96% (93-97%)	0.3 (0.14-0.54)	1.1% (0.58-1.8%)	1.9% (0.72-3.9%)	7.5 (2.6-16)	950 (500-1600)
Sri Lanka	2016	Female	20 to 24	5.4% (3.2-8.1%)	95% (92-97%)	0.4 (0.19-0.75)	1.8% (1-2.8%)	2.6% (0.97-5%)	10 (3.7-21)	1600 (890-2500)
Sri Lanka	2016	Female	25 to 29	6% (3.5-9.2%)	94% (91-96%)	0.47 (0.21-0.86)	1.8% (1.2-2.7%)	2.7% (1.2-4.8%)	11 (4.3-22)	1600 (960-2500)

Location	Year	Sex	Age	Prevalence of current drinkers	Prevalence of abstainers	Population average of standard drinks daily	Percent of total attributable deaths	Percent of total attributable DALYs	Attributable deaths	Attributable DALYs
Sri Lanka	2016	Female	30 to 34	6.3% (3.7-10%)	94% (90-96%)	0.5 (0.23-0.98)	1.8% (1.1-2.6%)	2.9% (1.5-5%)	14 (6.3-26)	1800 (1100-2700)
Sri Lanka	2016	Female	35 to 39	6.5% (3.6-9.6%)	93% (90-96%)	0.5 (0.24-0.92)	1.7% (1.1-2.5%)	3% (1.6-5%)	19 (9.1-35)	1900 (1200-3000)
Sri Lanka	2016	Female	40 to 44	6.5% (3.9-9.6%)	94% (90-96%)	0.54 (0.25-0.97)	1.7% (1.1-2.5%)	3.2% (1.8-5.1%)	24 (13-42)	1900 (1200-3000)
Sri Lanka	2016	Female	45 to 49	6.4% (3.6-9.5%)	94% (91-96%)	0.6 (0.29-1.1)	1.9% (1.1-3%)	3.5% (2-5.4%)	40 (20-69)	2500 (1400-3900)
Sri Lanka	2016	Female	50 to 54	6.5% (4-9.6%)	94% (90-96%)	0.68 (0.33-1.2)	2.2% (1.3-3.4%)	3.8% (2.2-5.8%)	67 (35-120)	3300 (1900-5400)
Sri Lanka	2016	Female	55 to 59	6.6% (4.1-9.6%)	93% (90-96%)	0.77 (0.36-1.3)	2.3% (1.3-3.6%)	3.8% (2.2-5.8%)	95 (49-160)	3900 (2100-6300)
Sri Lanka	2016	Female	60 to 64	6.3% (3.8-9.5%)	94% (91-96%)	0.76 (0.36-1.3)	2.3% (1.3-3.7%)	3.5% (2-5.6%)	120 (62-200)	4100 (2100-6600)
Sri Lanka	2016	Female	65 to 69	5.8% (3.5-8.5%)	94% (91-97%)	0.69 (0.34-1.2)	2.2% (1.2-3.7%)	3.2% (1.8-5.1%)	180 (92-310)	4800 (2500-8100)
Sri Lanka	2016	Female	70 to 74	5.7% (3.6-8.6%)	94% (91-96%)	0.66 (0.32-1.2)	2.2% (1.1-3.6%)	2.9% (1.5-4.9%)	220 (110-390)	4400 (2200-7700)
Sri Lanka	2016	Female	75 to 79	6.1% (3.7-9.2%)	94% (91-96%)	0.69 (0.32-1.3)	2% (0.97-3.7%)	2.6% (1.2-4.8%)	160 (67-290)	2500 (1100-4500)
Sri Lanka	2016	Female	80 to 84	6.6% (3.9-9.8%)	93% (90-96%)	0.72 (0.34-1.3)	2.2% (1-3.9%)	2.7% (1.3-4.8%)	190 (82-350)	2200 (960-3900)
Sri Lanka	2016	Female	85 to 89	7.4% (4.3-11%)	93% (89-96%)	0.78 (0.35-1.5)	2.1% (0.89-3.8%)	2.6% (1.1-4.7%)	150 (62-280)	1200 (530-2200)
Sri Lanka	2016	Female	90 to 94	8.4% (5.1-13%)	92% (87-95%)	0.87 (0.39-1.7)	2.1% (0.79-3.8%)	2.5% (0.93-4.8%)	89 (31-170)	500 (190-930)
Sri Lanka	2016	Female	95 plus	10% (5.3-17%)	90% (83-95%)	1 (0.38-2.1)	2.3% (0.69-4.3%)	2.7% (0.73-5.3%)	38 (10-75)	140 (41-260)
Sri Lanka	2016	Male	All Ages	35% (30-41%)	65% (59-70%)	1.9 (1.3-2.7)	7.7% (5.3-10%)	8.5% (5.4-12%)	6100 (3500-9400)	220000 (140000-310000)
Sri Lanka	2016	Male	15 to 19	24% (15-36%)	76% (64-85%)	0.64 (0.28-1.2)	2.3% (1.2-4.1%)	3.8% (1.6-7%)	23 (8.7-46)	2300 (1200-4000)
Sri Lanka	2016	Male	20 to 24	38% (25-51%)	62% (49-75%)	1.6 (0.78-2.7)	6.7% (3.6-11%)	9.6% (4.5-16%)	100 (44-190)	8100 (4200-14000)
Sri Lanka	2016	Male	25 to 29	45% (29-59%)	55% (41-71%)	2.3 (1.1-3.7)	8.9% (5-14%)	13% (6.8-21%)	140 (63-240)	10000 (5300-16000)
Sri Lanka	2016	Male	30 to 34	50% (33-65%)	50% (35-67%)	2.9 (1.6-4.8)	10% (6.3-16%)	16% (9-23%)	200 (100-310)	13000 (7400-20000)
Sri Lanka	2016	Male	35 to 39	53% (38-68%)	47% (32-62%)	3.5 (1.9-5.7)	12% (7.5-17%)	17% (11-24%)	330 (180-500)	19000 (11000-28000)
Sri Lanka	2016	Male	40 to 44	54% (39-68%)	46% (32-61%)	3.8 (2.1-5.9)	12% (8.3-17%)	18% (12-24%)	430 (260-660)	22000 (14000-33000)
Sri Lanka	2016	Male	45 to 49	53% (36-67%)	47% (33-64%)	3.7 (2.1-6)	13% (8.5-17%)	17% (12-23%)	590 (350-890)	27000 (17000-39000)

Location	Year	Sex	Age	Prevalence of current drinkers	Prevalence of abstainers	Population average of standard drinks daily	Percent of total attributable deaths	Percent of total attributable DALYs	Attributable deaths	Attributable DALYs
Sri Lanka	2016	Male	50 to 54	51% (36-65%)	49% (35-64%)	3.4 (1.9-5.5)	11% (6.9-16%)	15% (9.5-20%)	710 (390-1100)	28000 (16000-42000)
Sri Lanka	2016	Male	55 to 59	49% (34-64%)	51% (36-66%)	3 (1.6-4.9)	9.6% (5.6-14%)	12% (7.1-18%)	730 (390-1100)	25000 (14000-39000)
Sri Lanka	2016	Male	60 to 64	45% (30-62%)	55% (38-70%)	2.6 (1.4-4.3)	7.8% (4-12%)	9.8% (5-15%)	680 (330-1100)	20000 (9900-32000)
Sri Lanka	2016	Male	65 to 69	40% (26-54%)	60% (46-74%)	2.1 (1.1-3.5)	6.5% (3.4-10%)	7.9% (4-12%)	770 (350-1300)	19000 (8600-30000)
Sri Lanka	2016	Male	70 to 74	35% (23-48%)	65% (52-77%)	1.7 (0.85-3)	5.3% (2.4-8.9%)	6.3% (2.8-10%)	640 (270-1100)	12000 (5300-22000)
Sri Lanka	2016	Male	75 to 79	32% (19-47%)	68% (53-81%)	1.5 (0.7-2.9)	4.1% (1.6-7.6%)	4.8% (1.8-8.9%)	350 (120-670)	5300 (2000-10000)
Sri Lanka	2016	Male	80 to 84	28% (16-42%)	72% (58-84%)	1.3 (0.61-2.4)	3.4% (1.3-6.6%)	4% (1.4-7.7%)	250 (81-500)	2800 (960-5500)
Sri Lanka	2016	Male	85 to 89	25% (15-38%)	75% (62-85%)	1.1 (0.51-2.1)	2.6% (0.9-5.2%)	3% (0.97-6.3%)	140 (42-290)	1100 (360-2300)
Sri Lanka	2016	Male	90 to 94	25% (16-37%)	75% (63-84%)	1 (0.47-2)	2% (0.56-4.4%)	2.3% (0.55-5.2%)	60 (14-140)	350 (96-780)
Sri Lanka	2016	Male	95 plus	27% (15-43%)	73% (57-85%)	1 (0.39-2.1)	2% (0.4-4.6%)	2% (0.23-5.1%)	21 (2.5-54)	87 (18-200)
Thailand	1990	Female	All Ages	10% (7.2-14%)	90% (86-93%)	0.62 (0.46-0.82)	3% (2.4-3.8%)	5.8% (4.2-7.8%)	7900 (5600-11000)	220000 (180000-280000)
Thailand	1990	Female	15 to 19	9% (4-17%)	91% (83-96%)	0.47 (0.16-1)	1.5% (0.76-2.8%)	2.9% (1.3-5.7%)	69 (30-140)	6300 (3100-12000)
Thailand	1990	Female	20 to 24	11% (4.5-21%)	89% (79-95%)	0.63 (0.22-1.3)	2.3% (1.2-3.8%)	3.9% (1.6-7.3%)	100 (43-190)	9400 (5000-16000)
Thailand	1990	Female	25 to 29	12% (5.1-22%)	88% (78-95%)	0.72 (0.26-1.5)	2.7% (1.5-4.5%)	4.8% (2.2-8.8%)	120 (56-230)	11000 (5900-18000)
Thailand	1990	Female	30 to 34	13% (5.5-24%)	87% (76-94%)	0.83 (0.32-1.7)	3% (1.8-4.8%)	5.5% (3.1-8.9%)	160 (88-260)	12000 (7200-18000)
Thailand	1990	Female	35 to 39	14% (6.4-26%)	86% (74-94%)	0.93 (0.37-1.8)	3.5% (2.2-5.4%)	6.5% (3.8-9.9%)	220 (130-340)	14000 (8500-20000)
Thailand	1990	Female	40 to 44	15% (6.7-26%)	85% (74-93%)	1 (0.38-2)	4.3% (2.7-6.5%)	7.4% (4.6-11%)	300 (180-460)	16000 (10000-24000)
Thailand	1990	Female	45 to 49	15% (6.5-27%)	85% (73-93%)	1.2 (0.39-2.5)	5.2% (3.2-7.8%)	8.5% (5.3-13%)	420 (260-640)	19000 (12000-29000)
Thailand	1990	Female	50 to 54	15% (6.8-28%)	85% (72-93%)	1.3 (0.48-2.8)	6% (3.7-8.9%)	9.2% (5.7-13%)	630 (390-920)	25000 (16000-36000)
Thailand	1990	Female	55 to 59	15% (7.2-28%)	85% (72-93%)	1.4 (0.51-2.9)	6% (3.7-9.1%)	8.9% (5.5-14%)	720 (440-1100)	24000 (15000-37000)
Thailand	1990	Female	60 to 64	15% (6.7-28%)	85% (72-93%)	1.4 (0.51-3.2)	6% (3.6-9.5%)	8.5% (5.1-13%)	820 (480-1300)	23000 (14000-37000)
Thailand	1990	Female	65 to 69	15% (6.8-26%)	85% (74-93%)	1.3 (0.49-2.8)	6.2% (3.5-9.5%)	8.5% (4.9-13%)	870 (510-1300)	20000 (12000-32000)

Location	Year	Sex	Age	Prevalence of current drinkers	Prevalence of abstainers	Population average of standard drinks daily	Percent of total attributable deaths	Percent of total attributable DALYs	Attributable deaths	Attributable DALYs
Thailand	1990	Female	70 to 74	14% (6.7-25%)	86% (75-93%)	1.2 (0.4-2.8)	5.5% (2.8-9%)	7.2% (3.7-12%)	860 (440-1400)	16000 (8200-26000)
Thailand	1990	Female	75 to 79	14% (6.2-25%)	86% (75-94%)	1.1 (0.34-2.6)	4.7% (2.1-8.3%)	6.1% (2.7-11%)	760 (350-1300)	11000 (5000-19000)
Thailand	1990	Female	80 to 84	14% (6-26%)	86% (74-94%)	1.1 (0.34-2.6)	5% (2.2-8.6%)	6.4% (2.8-11%)	830 (360-1400)	8700 (3800-15000)
Thailand	1990	Female	85 to 89	15% (6.3-27%)	85% (73-94%)	1.2 (0.36-2.8)	4.7% (1.9-8.9%)	5.8% (2.4-11%)	620 (250-1200)	4600 (1900-8700)
Thailand	1990	Female	90 to 94	17% (7.3-31%)	83% (69-93%)	1.4 (0.35-3.4)	4.2% (1.5-8.1%)	5.1% (1.8-9.9%)	280 (98-550)	1400 (510-2800)
Thailand	1990	Female	95 plus	21% (8.2-40%)	79% (60-92%)	1.7 (0.34-4.4)	4.2% (1.1-8.9%)	4.9% (1.2-11%)	83 (20-180)	290 (72-600)
Thailand	1990	Male	All Ages	31% (24-39%)	69% (61-76%)	1.8 (1.5-2.1)	8.1% (6.7-9.7%)	11% (8.9-14%)	21000 (17000-26000)	800000 (670000-950000)
Thailand	1990	Male	15 to 19	25% (15-41%)	75% (59-85%)	0.82 (0.37-1.5)	3.7% (1.9-6.3%)	5.1% (2.5-9.2%)	300 (150-540)	23000 (12000-41000)
Thailand	1990	Male	20 to 24	37% (21-56%)	63% (44-79%)	1.9 (1-3.1)	8.8% (5.1-14%)	11% (6.2-18%)	920 (500-1500)	65000 (38000-100000)
Thailand	1990	Male	25 to 29	43% (24-61%)	57% (39-76%)	2.7 (1.5-4.1)	12% (7.6-17%)	16% (9.4-23%)	1200 (750-1800)	81000 (50000-120000)
Thailand	1990	Male	30 to 34	46% (26-65%)	54% (35-74%)	3.3 (1.9-4.9)	14% (9.4-18%)	18% (12-24%)	1400 (930-1900)	84000 (58000-110000)
Thailand	1990	Male	35 to 39	49% (30-68%)	51% (32-70%)	3.8 (2.1-5.8)	15% (11-20%)	20% (14-26%)	1500 (1100-2000)	84000 (59000-110000)
Thailand	1990	Male	40 to 44	48% (30-69%)	52% (31-70%)	3.9 (2.3-6.2)	15% (11-20%)	20% (14-26%)	1600 (1100-2000)	78000 (56000-99000)
Thailand	1990	Male	45 to 49	47% (27-66%)	53% (34-73%)	3.9 (2.1-6.1)	15% (11-20%)	20% (14-26%)	1700 (1200-2200)	76000 (53000-97000)
Thailand	1990	Male	50 to 54	45% (26-64%)	55% (36-74%)	3.7 (2-5.9)	15% (10-19%)	19% (13-24%)	2100 (1400-2700)	80000 (56000-100000)
Thailand	1990	Male	55 to 59	42% (25-62%)	58% (38-75%)	3.3 (1.7-5.5)	13% (8.9-17%)	17% (11-22%)	2100 (1500-2800)	70000 (48000-94000)
Thailand	1990	Male	60 to 64	39% (23-59%)	61% (41-77%)	2.9 (1.5-5.1)	12% (7.3-16%)	14% (9.2-20%)	2100 (1300-2900)	59000 (38000-81000)
Thailand	1990	Male	65 to 69	36% (21-54%)	64% (46-79%)	2.5 (1.2-4.2)	10% (6.1-15%)	13% (7.7-18%)	1800 (1100-2600)	42000 (25000-60000)
Thailand	1990	Male	70 to 74	32% (18-50%)	68% (50-82%)	2 (0.94-3.7)	9.1% (5-14%)	11% (6-17%)	1600 (860-2400)	29000 (16000-45000)
Thailand	1990	Male	75 to 79	29% (17-47%)	71% (53-83%)	1.7 (0.75-3.2)	7.1% (3.4-11%)	8.5% (4.1-14%)	1100 (540-1800)	16000 (7800-26000)
Thailand	1990	Male	80 to 84	27% (14-44%)	73% (56-86%)	1.5 (0.65-2.8)	6.6% (3.3-11%)	7.8% (3.8-13%)	860 (430-1400)	9100 (4500-15000)
Thailand	1990	Male	85 to 89	26% (13-41%)	74% (59-87%)	1.3 (0.59-2.5)	5.1% (2.3-9%)	5.9% (2.7-11%)	430 (200-780)	3300 (1500-5900)

Location	Year	Sex	Age	Prevalence of current drinkers	Prevalence of abstainers	Population average of standard drinks daily	Percent of total attributable deaths	Percent of total attributable DALYs	Attributable deaths	Attributable DALYs
Thailand	1990	Male	90 to 94	27% (15-44%)	73% (56-85%)	1.3 (0.53-2.5)	3.9% (1.5-7.5%)	4.5% (1.7-8.7%)	150 (56-290)	770 (300-1500)
Thailand	1990	Male	95 plus	31% (15-50%)	69% (50-85%)	1.3 (0.46-2.9)	3.6% (1.1-7.6%)	3.9% (1.1-8.5%)	35 (9.7-76)	120 (39-270)
Thailand	1995	Female	All Ages	11% (7.9-14%)	89% (86-92%)	0.74 (0.56-0.98)	3.4% (2.7-4.3%)	6.1% (4.4-8%)	9000 (6600-12000)	260000 (210000-320000)
Thailand	1995	Female	15 to 19	9.2% (4.3-17%)	91% (83-96%)	0.53 (0.18-1.1)	1.6% (0.82-3%)	3.1% (1.4-5.9%)	71 (31-140)	6500 (3200-12000)
Thailand	1995	Female	20 to 24	11% (4.9-21%)	89% (79-95%)	0.72 (0.24-1.5)	2.4% (1.3-4.1%)	3.6% (1.5-6.7%)	120 (51-230)	11000 (5600-19000)
Thailand	1995	Female	25 to 29	12% (5.5-22%)	88% (78-94%)	0.83 (0.3-1.6)	2.8% (1.5-4.5%)	4.2% (1.8-7.5%)	160 (70-290)	13000 (7100-22000)
Thailand	1995	Female	30 to 34	13% (5.6-25%)	87% (75-94%)	0.94 (0.34-1.9)	3.1% (1.9-4.9%)	5.1% (2.8-8.3%)	200 (110-320)	14000 (8600-22000)
Thailand	1995	Female	35 to 39	14% (6.2-26%)	86% (74-94%)	1 (0.41-2)	3.6% (2.2-5.5%)	6.2% (3.7-9.5%)	260 (150-410)	17000 (10000-25000)
Thailand	1995	Female	40 to 44	15% (6.9-27%)	85% (73-93%)	1.2 (0.43-2.3)	4.2% (2.7-6.3%)	7.2% (4.5-11%)	370 (220-550)	20000 (12000-29000)
Thailand	1995	Female	45 to 49	15% (6.3-27%)	85% (73-94%)	1.3 (0.45-2.7)	5.1% (3.2-7.4%)	8.3% (5.3-12%)	480 (300-710)	22000 (14000-33000)
Thailand	1995	Female	50 to 54	15% (6.7-28%)	85% (72-93%)	1.4 (0.54-3)	5.9% (3.8-8.6%)	9.1% (5.9-13%)	620 (400-900)	25000 (16000-36000)
Thailand	1995	Female	55 to 59	15% (7.1-28%)	85% (72-93%)	1.6 (0.55-3.2)	5.9% (3.6-8.8%)	8.7% (5.5-13%)	780 (490-1200)	27000 (17000-41000)
Thailand	1995	Female	60 to 64	15% (6.8-28%)	85% (72-93%)	1.6 (0.55-3.5)	5.9% (3.6-9.3%)	8.4% (5-13%)	970 (590-1500)	28000 (17000-44000)
Thailand	1995	Female	65 to 69	15% (6.7-26%)	85% (74-93%)	1.5 (0.55-3)	5.9% (3.6-9%)	8.2% (5.1-12%)	1100 (650-1600)	25000 (15000-39000)
Thailand	1995	Female	70 to 74	14% (6.7-25%)	86% (75-93%)	1.3 (0.46-3.1)	5.3% (2.9-8.5%)	7.1% (4-11%)	970 (540-1500)	18000 (10000-29000)
Thailand	1995	Female	75 to 79	14% (5.9-25%)	86% (75-94%)	1.3 (0.38-2.8)	4.6% (2.2-7.8%)	5.9% (2.9-9.9%)	830 (400-1400)	12000 (5900-20000)
Thailand	1995	Female	80 to 84	14% (6-25%)	86% (75-94%)	1.2 (0.39-2.8)	4.9% (2.3-8.1%)	6.1% (2.8-10%)	910 (410-1500)	9700 (4400-16000)
Thailand	1995	Female	85 to 89	15% (6.7-27%)	85% (73-93%)	1.3 (0.42-3)	4.6% (2-8.5%)	5.6% (2.4-10%)	740 (320-1400)	5600 (2400-10000)
Thailand	1995	Female	90 to 94	17% (7.6-31%)	83% (69-92%)	1.5 (0.41-3.6)	4.1% (1.6-7.9%)	4.9% (1.9-9.5%)	350 (130-680)	1800 (720-3500)
Thailand	1995	Female	95 plus	21% (8.1-39%)	79% (61-92%)	1.8 (0.39-4.7)	4.1% (1.2-8.4%)	4.7% (1.3-9.9%)	110 (30-230)	390 (110-790)
Thailand	1995	Male	All Ages	33% (26-41%)	67% (59-74%)	2.2 (1.9-2.5)	9.6% (7.9-11%)	12% (9.9-14%)	27000 (22000-32000)	1100000 (890000-1300000)

Location	Year	Sex	Age	Prevalence of current drinkers	Prevalence of abstainers	Population average of standard drinks daily	Percent of total attributable deaths	Percent of total attributable DALYs	Attributable deaths	Attributable DALYs
Thailand	1995	Male	15 to 19	26% (14-41%)	74% (59-86%)	0.91 (0.42-1.7)	4.3% (2.2-7.7%)	5.6% (2.7-10%)	440 (210-790)	33000 (16000-59000)
Thailand	1995	Male	20 to 24	38% (22-56%)	62% (44-78%)	2.2 (1.1-3.6)	9.6% (5.4-15%)	12% (6.4-19%)	1400 (740-2200)	95000 (53000-150000)
Thailand	1995	Male	25 to 29	44% (25-62%)	56% (38-75%)	3 (1.7-4.6)	12% (7.6-18%)	14% (8.6-21%)	2200 (1300-3300)	140000 (87000-210000)
Thailand	1995	Male	30 to 34	47% (28-66%)	53% (34-72%)	3.7 (2.2-5.4)	14% (9.6-19%)	16% (11-22%)	2400 (1600-3200)	140000 (96000-190000)
Thailand	1995	Male	35 to 39	49% (32-69%)	51% (31-68%)	4.2 (2.4-6.3)	15% (11-20%)	18% (13-24%)	2400 (1700-3100)	130000 (90000-170000)
Thailand	1995	Male	40 to 44	49% (31-68%)	51% (32-69%)	4.4 (2.6-6.6)	15% (11-19%)	19% (13-24%)	2200 (1500-2800)	110000 (77000-140000)
Thailand	1995	Male	45 to 49	47% (28-67%)	53% (33-72%)	4.3 (2.3-6.8)	15% (11-19%)	19% (14-24%)	2100 (1500-2800)	93000 (67000-120000)
Thailand	1995	Male	50 to 54	45% (26-64%)	55% (36-74%)	4.1 (2.2-6.5)	14% (10-18%)	18% (13-23%)	2100 (1500-2600)	80000 (57000-100000)
Thailand	1995	Male	55 to 59	43% (26-63%)	57% (37-74%)	3.7 (2-6.1)	13% (8.9-17%)	16% (11-21%)	2200 (1500-2900)	75000 (52000-99000)
Thailand	1995	Male	60 to 64	40% (23-60%)	60% (40-77%)	3.3 (1.6-5.6)	11% (7.5-15%)	14% (9.3-19%)	2300 (1500-3100)	66000 (43000-89000)
Thailand	1995	Male	65 to 69	37% (21-55%)	63% (45-79%)	2.8 (1.4-4.6)	10% (6.4-14%)	13% (8-18%)	2200 (1400-3000)	51000 (32000-70000)
Thailand	1995	Male	70 to 74	33% (19-50%)	67% (50-81%)	2.3 (1.1-4.1)	8.9% (5.2-13%)	11% (6.5-16%)	1800 (1000-2600)	33000 (19000-49000)
Thailand	1995	Male	75 to 79	30% (17-47%)	70% (53-83%)	1.9 (0.87-3.6)	7% (3.6-11%)	8.3% (4.3-13%)	1200 (610-1900)	18000 (9100-27000)
Thailand	1995	Male	80 to 84	27% (15-44%)	73% (56-85%)	1.7 (0.72-3.1)	6.3% (3.2-10%)	7.4% (3.7-12%)	940 (470-1500)	10000 (5100-16000)
Thailand	1995	Male	85 to 89	26% (14-41%)	74% (59-86%)	1.5 (0.69-2.7)	5% (2.5-8.5%)	5.7% (2.8-10%)	520 (250-910)	4000 (2000-6900)
Thailand	1995	Male	90 to 94	28% (15-45%)	72% (55-85%)	1.5 (0.65-2.8)	3.9% (1.6-7.1%)	4.4% (1.8-8.4%)	180 (73-340)	960 (400-1800)
Thailand	1995	Male	95 plus	31% (15-52%)	69% (48-85%)	1.5 (0.55-3.1)	3.5% (1.2-7.4%)	3.8% (1.1-8.1%)	42 (12-89)	150 (49-320)
Thailand	2000	Female	All Ages	11% (8.1-15%)	89% (85-92%)	0.89 (0.66-1.2)	3.6% (2.8-4.5%)	6.1% (4.4-8.1%)	10000 (7400-14000)	290000 (230000-370000)
Thailand	2000	Female	15 to 19	9.4% (4.4-17%)	91% (83-96%)	0.59 (0.2-1.3)	1.7% (0.83-3.1%)	3.4% (1.5-6.8%)	61 (26-120)	5700 (2800-10000)
Thailand	2000	Female	20 to 24	11% (5-21%)	89% (79-95%)	0.8 (0.27-1.7)	2.3% (1.2-3.8%)	3.5% (1.5-6.4%)	110 (45-190)	9600 (4900-16000)
Thailand	2000	Female	25 to 29	12% (5.1-22%)	88% (78-95%)	0.9 (0.32-1.9)	2.7% (1.4-4.4%)	3.6% (1.6-6.4%)	210 (95-370)	16000 (8300-27000)
Thailand	2000	Female	30 to 34	13% (5.4-24%)	87% (76-95%)	1 (0.36-2)	3% (1.8-4.8%)	4.2% (2.3-6.7%)	280 (150-460)	20000 (12000-31000)

Location	Year	Sex	Age	Prevalence of current drinkers	Prevalence of abstainers	Population average of standard drinks daily	Percent of total attributable deaths	Percent of total attributable DALYs	Attributable deaths	Attributable DALYs
Thailand	2000	Female	35 to 39	14% (6-26%)	86% (74-94%)	1.1 (0.43-2.2)	3.3% (2-5%)	5.2% (3-7.9%)	320 (190-490)	20000 (12000-30000)
Thailand	2000	Female	40 to 44	15% (6.8-26%)	85% (74-93%)	1.3 (0.49-2.5)	3.9% (2.5-5.8%)	6.5% (4.1-9.5%)	430 (270-630)	23000 (15000-34000)
Thailand	2000	Female	45 to 49	15% (6.5-27%)	85% (73-94%)	1.4 (0.49-2.9)	4.6% (3-7%)	7.7% (4.9-11%)	550 (350-810)	26000 (17000-39000)
Thailand	2000	Female	50 to 54	15% (6.7-29%)	85% (71-93%)	1.6 (0.62-3.3)	5.6% (3.6-8.1%)	8.7% (5.7-13%)	670 (430-990)	27000 (18000-40000)
Thailand	2000	Female	55 to 59	16% (7.3-29%)	84% (71-93%)	1.8 (0.65-3.8)	5.8% (3.6-8.8%)	8.6% (5.4-13%)	770 (480-1100)	27000 (17000-41000)
Thailand	2000	Female	60 to 64	16% (7-29%)	84% (71-93%)	1.8 (0.63-4)	5.8% (3.6-9.1%)	8.3% (5.3-13%)	1000 (650-1600)	30000 (18000-49000)
Thailand	2000	Female	65 to 69	15% (6.9-27%)	85% (73-93%)	1.7 (0.61-3.3)	5.8% (3.6-8.9%)	8.2% (5-12%)	1200 (730-1900)	29000 (18000-45000)
Thailand	2000	Female	70 to 74	14% (6.8-26%)	86% (74-93%)	1.5 (0.51-3.4)	5.2% (2.9-8.4%)	7.1% (4-11%)	1200 (670-1900)	23000 (13000-37000)
Thailand	2000	Female	75 to 79	14% (6.2-26%)	86% (74-94%)	1.4 (0.4-3.2)	4.5% (2.1-7.7%)	5.9% (2.8-9.7%)	980 (460-1600)	15000 (6900-24000)
Thailand	2000	Female	80 to 84	14% (6.3-26%)	86% (74-94%)	1.4 (0.42-3.1)	4.8% (2.1-8.1%)	6% (2.7-10%)	1000 (460-1800)	11000 (4900-19000)
Thailand	2000	Female	85 to 89	15% (6.7-28%)	85% (72-93%)	1.5 (0.47-3.4)	4.6% (2-8.3%)	5.6% (2.5-10%)	800 (350-1500)	6100 (2600-11000)
Thailand	2000	Female	90 to 94	17% (7.7-31%)	83% (69-92%)	1.7 (0.47-4.1)	4.2% (1.6-8.2%)	5.1% (2-9.9%)	440 (170-840)	2300 (910-4400)
Thailand	2000	Female	95 plus	21% (8.3-40%)	79% (60-92%)	2 (0.42-5.6)	4.3% (1.2-8.7%)	4.9% (1.3-10%)	150 (40-310)	510 (150-1000)
Thailand	2000	Male	All Ages	34% (27-42%)	66% (58-73%)	2.6 (2.2-3)	9.9% (8.3-12%)	12% (10-14%)	29000 (24000-35000)	1100000 (950000-1400000)
Thailand	2000	Male	15 to 19	26% (15-41%)	74% (59-85%)	1 (0.45-1.9)	4.7% (2.4-8.3%)	6.3% (3.1-12%)	390 (190-710)	29000 (15000-52000)
Thailand	2000	Male	20 to 24	38% (22-56%)	62% (44-78%)	2.4 (1.2-4.1)	10% (5.8-16%)	13% (7.1-21%)	1100 (600-1800)	80000 (44000-130000)
Thailand	2000	Male	25 to 29	44% (25-62%)	56% (38-75%)	3.3 (1.8-5.2)	12% (7.2-17%)	14% (8.1-20%)	1800 (1100-2700)	120000 (72000-170000)
Thailand	2000	Male	30 to 34	47% (27-66%)	53% (34-73%)	4 (2.3-6)	12% (8.4-16%)	14% (9.5-19%)	2400 (1700-3300)	140000 (100000-200000)
Thailand	2000	Male	35 to 39	49% (31-69%)	51% (31-69%)	4.5 (2.7-6.8)	13% (9.3-17%)	15% (11-20%)	2500 (1800-3400)	140000 (99000-180000)
Thailand	2000	Male	40 to 44	49% (30-69%)	51% (31-70%)	4.7 (2.8-7)	13% (10-17%)	17% (12-21%)	2600 (1900-3400)	130000 (95000-170000)
Thailand	2000	Male	45 to 49	47% (27-67%)	53% (33-73%)	4.6 (2.6-7.2)	14% (11-18%)	18% (14-22%)	2600 (1900-3300)	110000 (86000-150000)

Location	Year	Sex	Age	Prevalence of current drinkers	Prevalence of abstainers	Population average of standard drinks daily	Percent of total attributable deaths	Percent of total attributable DALYs	Attributable deaths	Attributable DALYs
Thailand	2000	Male	50 to 54	45% (26-65%)	55% (35-74%)	4.4 (2.4-6.9)	14% (10-17%)	18% (13-22%)	2400 (1800-3100)	94000 (69000-120000)
Thailand	2000	Male	55 to 59	43% (25-63%)	57% (37-75%)	4 (2.2-6.7)	13% (9.2-17%)	17% (12-21%)	2300 (1600-2900)	77000 (54000-99000)
Thailand	2000	Male	60 to 64	40% (23-61%)	60% (39-77%)	3.6 (1.7-6.1)	12% (7.7-15%)	15% (9.9-19%)	2600 (1700-3400)	73000 (48000-97000)
Thailand	2000	Male	65 to 69	36% (21-56%)	64% (44-79%)	3 (1.5-5.1)	10% (6.9-14%)	13% (8.7-18%)	2500 (1600-3400)	59000 (39000-81000)
Thailand	2000	Male	70 to 74	33% (19-49%)	67% (51-81%)	2.5 (1.2-4.4)	9% (5.5-13%)	11% (6.8-16%)	2300 (1400-3300)	43000 (26000-63000)
Thailand	2000	Male	75 to 79	30% (17-47%)	70% (53-83%)	2.1 (0.91-3.9)	7.1% (4-11%)	8.7% (4.9-13%)	1500 (830-2200)	22000 (12000-33000)
Thailand	2000	Male	80 to 84	28% (14-44%)	72% (56-86%)	1.8 (0.82-3.4)	6.4% (3.2-9.9%)	7.6% (3.8-12%)	1100 (570-1700)	12000 (6200-19000)
Thailand	2000	Male	85 to 89	26% (14-41%)	74% (59-86%)	1.6 (0.73-3)	5.2% (2.4-8.7%)	6.1% (2.8-10%)	620 (280-1000)	4700 (2200-8000)
Thailand	2000	Male	90 to 94	28% (16-44%)	72% (56-84%)	1.6 (0.73-3)	4.2% (1.8-7.6%)	4.8% (2-8.9%)	240 (98-450)	1300 (540-2400)
Thailand	2000	Male	95 plus	31% (15-52%)	69% (48-85%)	1.6 (0.57-3.4)	3.8% (1.2-8%)	4.1% (1.2-8.8%)	56 (16-120)	200 (68-420)
Thailand	2005	Female	All Ages	11% (8.2-15%)	89% (85-92%)	0.97 (0.73-1.3)	3.6% (2.8-4.6%)	6.1% (4.4-8.2%)	11000 (7700-14000)	280000 (220000-360000)
Thailand	2005	Female	15 to 19	9.1% (4.2-17%)	91% (83-96%)	0.61 (0.21-1.4)	1.5% (0.77-2.7%)	3.2% (1.4-6.4%)	46 (20-93)	4500 (2200-8100)
Thailand	2005	Female	20 to 24	11% (4.7-20%)	89% (80-95%)	0.82 (0.27-1.7)	2% (1.1-3.3%)	3.4% (1.5-6.3%)	69 (30-130)	6900 (3800-11000)
Thailand	2005	Female	25 to 29	12% (5.1-22%)	88% (78-95%)	0.94 (0.32-2)	2.2% (1.2-3.6%)	3.2% (1.4-5.7%)	120 (55-210)	10000 (5600-17000)
Thailand	2005	Female	30 to 34	13% (5.2-24%)	87% (76-95%)	1.1 (0.36-2.2)	2.5% (1.4-4%)	3.7% (1.9-6.2%)	180 (90-300)	14000 (7700-21000)
Thailand	2005	Female	35 to 39	14% (5.7-26%)	86% (74-94%)	1.2 (0.44-2.3)	2.8% (1.7-4.3%)	4.7% (2.6-7.2%)	250 (140-390)	16000 (9500-24000)
Thailand	2005	Female	40 to 44	14% (6.5-26%)	86% (74-93%)	1.3 (0.49-2.5)	3.5% (2.2-5.1%)	6% (3.7-8.8%)	370 (230-540)	21000 (13000-31000)
Thailand	2005	Female	45 to 49	14% (6.4-25%)	86% (75-94%)	1.5 (0.53-2.9)	4.1% (2.6-6.1%)	7.2% (4.5-11%)	520 (330-790)	25000 (16000-38000)
Thailand	2005	Female	50 to 54	15% (6.9-27%)	85% (73-93%)	1.7 (0.67-3.5)	5% (3.2-7.4%)	8.2% (5.3-12%)	720 (460-1100)	30000 (19000-44000)
Thailand	2005	Female	55 to 59	15% (7.2-29%)	85% (71-93%)	1.9 (0.69-3.8)	5.5% (3.3-8.5%)	8.4% (5.2-13%)	840 (530-1300)	29000 (18000-46000)
Thailand	2005	Female	60 to 64	15% (6.9-28%)	85% (72-93%)	1.9 (0.68-4)	5.5% (3.4-8.7%)	8% (5-13%)	950 (590-1500)	28000 (17000-45000)
Thailand	2005	Female	65 to 69	15% (6.9-27%)	85% (73-93%)	1.7 (0.64-3.4)	5.6% (3.4-8.5%)	8% (4.9-12%)	1300 (780-1900)	31000 (19000-48000)

Location	Year	Sex	Age	Prevalence of current drinkers	Prevalence of abstainers	Population average of standard drinks daily	Percent of total attributable deaths	Percent of total attributable DALYs	Attributable deaths	Attributable DALYs
Thailand	2005	Female	70 to 74	14% (6.5-25%)	86% (75-93%)	1.6 (0.52-3.5)	5% (2.8-7.9%)	6.8% (3.9-11%)	1300 (760-2100)	26000 (15000-40000)
Thailand	2005	Female	75 to 79	13% (6.1-25%)	87% (75-94%)	1.4 (0.43-3.3)	4.3% (2.1-7.3%)	5.7% (2.9-9.4%)	1200 (610-2000)	18000 (9000-30000)
Thailand	2005	Female	80 to 84	14% (6.3-26%)	86% (74-94%)	1.4 (0.45-3.2)	4.5% (2-7.7%)	5.7% (2.6-9.8%)	1300 (570-2200)	14000 (6100-23000)
Thailand	2005	Female	85 to 89	15% (6.6-27%)	85% (73-93%)	1.6 (0.49-3.5)	4.4% (2-8.1%)	5.5% (2.4-10%)	920 (400-1700)	7000 (3100-13000)
Thailand	2005	Female	90 to 94	17% (7.5-32%)	83% (68-93%)	1.8 (0.48-4.3)	4.2% (1.7-7.7%)	5.1% (2-9.6%)	480 (190-910)	2500 (1000-4700)
Thailand	2005	Female	95 plus	21% (8.1-40%)	79% (60-92%)	2.1 (0.44-5.8)	4.2% (1.3-8.7%)	4.9% (1.4-10%)	180 (50-360)	610 (180-1200)
Thailand	2005	Male	All Ages	34% (28-43%)	66% (57-72%)	2.8 (2.4-3.2)	10% (8.6-12%)	12% (10-15%)	29000 (24000-35000)	1100000 (920000-1300000)
Thailand	2005	Male	15 to 19	25% (14-41%)	75% (59-86%)	1 (0.46-1.9)	4.6% (2.5-8.2%)	6.5% (3.3-12%)	330 (170-600)	25000 (14000-45000)
Thailand	2005	Male	20 to 24	37% (21-56%)	63% (44-79%)	2.4 (1.2-4.2)	10% (5.9-16%)	14% (7.5-22%)	890 (480-1400)	64000 (37000-100000)
Thailand	2005	Male	25 to 29	43% (25-61%)	57% (39-75%)	3.4 (1.8-5.5)	12% (7.6-17%)	15% (9.2-22%)	1300 (790-1900)	89000 (56000-130000)
Thailand	2005	Male	30 to 34	46% (26-65%)	54% (35-74%)	4.1 (2.4-6.3)	12% (8.4-16%)	15% (10-19%)	1700 (1200-2300)	110000 (74000-140000)
Thailand	2005	Male	35 to 39	48% (29-68%)	52% (32-71%)	4.7 (2.6-7)	12% (8.4-16%)	15% (10-19%)	2000 (1300-2600)	110000 (78000-140000)
Thailand	2005	Male	40 to 44	48% (31-67%)	52% (33-69%)	4.9 (3-7.3)	13% (10-17%)	17% (13-21%)	2400 (1800-3100)	120000 (92000-150000)
Thailand	2005	Male	45 to 49	47% (27-66%)	53% (34-73%)	4.9 (2.8-7.3)	14% (11-17%)	19% (14-23%)	2800 (2100-3400)	120000 (95000-150000)
Thailand	2005	Male	50 to 54	45% (26-64%)	55% (36-74%)	4.6 (2.6-7)	14% (11-18%)	19% (14-23%)	2900 (2200-3600)	110000 (85000-140000)
Thailand	2005	Male	55 to 59	42% (26-62%)	58% (38-74%)	4.2 (2.2-6.9)	13% (9.6-17%)	18% (13-22%)	2700 (1900-3500)	92000 (67000-120000)
Thailand	2005	Male	60 to 64	39% (22-59%)	61% (41-78%)	3.7 (1.8-6.3)	12% (8.1-16%)	15% (10-20%)	2500 (1700-3300)	73000 (50000-96000)
Thailand	2005	Male	65 to 69	36% (20-54%)	64% (46-80%)	3.1 (1.5-5.1)	10% (7.1-14%)	13% (9.1-18%)	2700 (1800-3600)	64000 (44000-87000)
Thailand	2005	Male	70 to 74	32% (18-50%)	68% (50-82%)	2.5 (1.2-4.7)	8.8% (5.5-13%)	11% (6.9-16%)	2500 (1500-3600)	47000 (29000-67000)
Thailand	2005	Male	75 to 79	29% (17-46%)	71% (54-83%)	2.1 (0.95-4.1)	7% (4-11%)	8.7% (5-13%)	1900 (1100-2800)	28000 (16000-41000)
Thailand	2005	Male	80 to 84	27% (15-43%)	73% (57-85%)	1.9 (0.84-3.5)	6.2% (3.1-9.7%)	7.4% (3.8-12%)	1400 (730-2300)	16000 (8000-25000)

Location	Year	Sex	Age	Prevalence of current drinkers	Prevalence of abstainers	Population average of standard drinks daily	Percent of total attributable deaths	Percent of total attributable DALYs	Attributable deaths	Attributable DALYs
Thailand	2005	Male	85 to 89	25%<br>(14-40%)	75%<br>(60-86%)	1.6<br>(0.73-3)	5.1%<br>(2.3-8.3%)	6%<br>(2.7-9.9%)	750<br>(340-1300)	5800<br>(2600-9500)
Thailand	2005	Male	90 to 94	27%<br>(15-43%)	73%<br>(57-85%)	1.6<br>(0.72-3)	4.1%<br>(1.7-7.5%)	4.8%<br>(1.9-8.7%)	280<br>(110-520)	1500<br>(620-2700)
Thailand	2005	Male	95 plus	30%<br>(15-50%)	70%<br>(50-85%)	1.6<br>(0.58-3.5)	3.9%<br>(1.2-7.9%)	4.2%<br>(1.2-8.7%)	73<br>(20-150)	260<br>(80-530)
Thailand	2010	Female	All Ages	11%<br>(8.3-15%)	89%<br>(85-92%)	1<br>(0.76-1.4)	3.6%<br>(2.8-4.6%)	6.1%<br>(4.4-8.2%)	11000<br>(7600-14000)	270000<br>(220000-350000)
Thailand	2010	Female	15 to 19	9%<br>(4.2-17%)	91%<br>(83-96%)	0.63<br>(0.21-1.4)	1.5%<br>(0.78-2.8%)	3.3%<br>(1.4-6.5%)	41<br>(18-82)	4000<br>(2000-7400)
Thailand	2010	Female	20 to 24	11%<br>(4.6-20%)	89%<br>(80-95%)	0.84<br>(0.26-1.9)	1.9%<br>(1.1-3.1%)	3.5%<br>(1.5-6.4%)	48<br>(21-88)	5400<br>(3000-8900)
Thailand	2010	Female	25 to 29	12%<br>(5.2-23%)	88%<br>(77-95%)	0.95<br>(0.31-2)	2.1%<br>(1.2-3.3%)	3.4%<br>(1.5-6.2%)	71<br>(31-130)	7000<br>(3900-12000)
Thailand	2010	Female	30 to 34	13%<br>(5.3-25%)	87%<br>(75-95%)	1.1<br>(0.38-2.2)	2.3%<br>(1.3-3.6%)	3.9%<br>(1.8-6.6%)	120<br>(56-210)	9900<br>(5500-16000)
Thailand	2010	Female	35 to 39	14%<br>(5.8-26%)	86%<br>(74-94%)	1.2<br>(0.45-2.3)	2.7%<br>(1.6-4.3%)	4.9%<br>(2.6-8%)	200<br>(110-330)	14000<br>(7700-21000)
Thailand	2010	Female	40 to 44	14%<br>(6.5-25%)	86%<br>(75-94%)	1.3<br>(0.49-2.5)	3.3%<br>(2.1-5%)	6.1%<br>(3.8-9.2%)	340<br>(210-520)	19000<br>(13000-29000)
Thailand	2010	Female	45 to 49	14%<br>(5.8-26%)	86%<br>(74-94%)	1.4<br>(0.54-2.9)	3.9%<br>(2.4-6%)	7.2%<br>(4.6-11%)	510<br>(320-760)	25000<br>(16000-37000)
Thailand	2010	Female	50 to 54	15%<br>(6.4-28%)	85%<br>(72-94%)	1.6<br>(0.64-3.4)	4.7%<br>(2.9-7%)	8.1%<br>(5.1-12%)	740<br>(460-1100)	31000<br>(19000-46000)
Thailand	2010	Female	55 to 59	15%<br>(6.8-28%)	85%<br>(72-93%)	1.8<br>(0.65-3.7)	5%<br>(3.1-7.8%)	8.2%<br>(5.2-13%)	880<br>(550-1400)	31000<br>(20000-49000)
Thailand	2010	Female	60 to 64	15%<br>(6.4-28%)	85%<br>(72-94%)	1.8<br>(0.63-4)	5.1%<br>(3.2-8.1%)	7.9%<br>(5-12%)	970<br>(610-1500)	29000<br>(18000-46000)
Thailand	2010	Female	65 to 69	14%<br>(6.1-26%)	86%<br>(74-94%)	1.7<br>(0.62-3.3)	5.3%<br>(3.3-7.9%)	7.8%<br>(5-11%)	1100<br>(700-1700)	27000<br>(17000-42000)
Thailand	2010	Female	70 to 74	14%<br>(6.3-25%)	86%<br>(75-94%)	1.5<br>(0.52-3.6)	4.7%<br>(2.7-7.4%)	6.7%<br>(4-10%)	1400<br>(790-2100)	26000<br>(15000-41000)
Thailand	2010	Female	75 to 79	13%<br>(5.9-25%)	87%<br>(75-94%)	1.5<br>(0.44-3.4)	4.2%<br>(2.1-6.9%)	5.7%<br>(3-9.2%)	1300<br>(680-2200)	20000<br>(10000-33000)
Thailand	2010	Female	80 to 84	13%<br>(5.9-26%)	87%<br>(74-94%)	1.5<br>(0.43-3.2)	4.2%<br>(1.9-7%)	5.5%<br>(2.6-9.5%)	1300<br>(620-2300)	15000<br>(6800-25000)
Thailand	2010	Female	85 to 89	15%<br>(6.4-28%)	85%<br>(72-94%)	1.6<br>(0.45-3.6)	4.1%<br>(1.8-7.6%)	5.2%<br>(2.3-9.6%)	1000<br>(440-1900)	8000<br>(3400-15000)
Thailand	2010	Female	90 to 94	17%<br>(7.8-32%)	83%<br>(68-92%)	1.8<br>(0.47-4.3)	3.8%<br>(1.4-7.1%)	4.8%<br>(1.7-9.2%)	470<br>(180-900)	2500<br>(940-4800)
Thailand	2010	Female	95 plus	21%<br>(8.1-39%)	79%<br>(61-92%)	2.2<br>(0.45-5.8)	4%<br>(1.2-8.1%)	4.8%<br>(1.3-9.8%)	170<br>(46-340)	590<br>(170-1200)

Location	Year	Sex	Age	Prevalence of current drinkers	Prevalence of abstainers	Population average of standard drinks daily	Percent of total attributable deaths	Percent of total attributable DALYs	Attributable deaths	Attributable DALYs
Thailand	2010	Male	All Ages	35% (28-43%)	65% (57-72%)	2.9 (2.4-3.5)	11% (9-13%)	13% (11-15%)	30000 (24000-35000)	1100000 (910000-1200000)
Thailand	2010	Male	15 to 19	24% (14-39%)	76% (61-86%)	1 (0.45-1.9)	4.6% (2.6-7.9%)	6.6% (3.6-12%)	260 (140-450)	20000 (11000-34000)
Thailand	2010	Male	20 to 24	36% (20-54%)	64% (46-80%)	2.4 (1.2-4.1)	9.9% (5.7-15%)	14% (7.5-22%)	610 (330-950)	45000 (26000-69000)
Thailand	2010	Male	25 to 29	42% (24-60%)	58% (40-76%)	3.3 (1.7-5.6)	12% (7.5-17%)	16% (9.7-23%)	920 (560-1300)	63000 (39000-89000)
Thailand	2010	Male	30 to 34	45% (26-64%)	55% (36-74%)	4.1 (2.2-6.5)	13% (8.8-17%)	17% (11-22%)	1400 (950-1900)	88000 (61000-120000)
Thailand	2010	Male	35 to 39	48% (30-67%)	52% (33-70%)	4.7 (2.7-7.2)	13% (9.4-17%)	17% (12-22%)	1900 (1300-2500)	110000 (75000-140000)
Thailand	2010	Male	40 to 44	48% (30-67%)	52% (33-70%)	4.9 (2.9-7.2)	14% (10-17%)	18% (13-23%)	2400 (1800-3100)	120000 (92000-160000)
Thailand	2010	Male	45 to 49	46% (27-65%)	54% (35-73%)	4.8 (2.6-7.2)	15% (11-18%)	20% (15-24%)	3000 (2300-3800)	140000 (100000-170000)
Thailand	2010	Male	50 to 54	44% (25-63%)	56% (37-75%)	4.5 (2.5-7)	14% (11-18%)	19% (15-24%)	3300 (2500-4100)	130000 (100000-160000)
Thailand	2010	Male	55 to 59	42% (25-62%)	58% (38-75%)	4.1 (2.2-6.6)	13% (9.6-17%)	18% (13-22%)	3200 (2300-4000)	110000 (80000-140000)
Thailand	2010	Male	60 to 64	39% (22-58%)	61% (42-78%)	3.6 (1.7-6.1)	12% (8.2-15%)	15% (11-20%)	2800 (2000-3700)	82000 (57000-110000)
Thailand	2010	Male	65 to 69	35% (20-53%)	65% (47-80%)	3 (1.5-5)	10% (7-14%)	13% (9.1-18%)	2500 (1700-3400)	61000 (41000-81000)
Thailand	2010	Male	70 to 74	31% (18-49%)	69% (51-82%)	2.5 (1.2-4.5)	8.6% (5.4-12%)	11% (6.9-15%)	2600 (1600-3600)	49000 (31000-68000)
Thailand	2010	Male	75 to 79	29% (16-46%)	71% (54-84%)	2.1 (0.93-4)	6.8% (3.8-10%)	8.6% (4.8-13%)	2000 (1100-3000)	30000 (17000-44000)
Thailand	2010	Male	80 to 84	26% (14-44%)	74% (56-86%)	1.8 (0.79-3.4)	5.7% (2.9-9.1%)	7.1% (3.6-11%)	1500 (740-2400)	17000 (8300-26000)
Thailand	2010	Male	85 to 89	25% (13-40%)	75% (60-87%)	1.6 (0.69-3.1)	4.5% (2.1-7.6%)	5.5% (2.5-9.5%)	790 (350-1400)	6300 (2900-11000)
Thailand	2010	Male	90 to 94	26% (14-44%)	74% (56-86%)	1.6 (0.66-2.9)	3.6% (1.5-6.9%)	4.4% (1.7-8.4%)	260 (100-510)	1500 (610-2700)
Thailand	2010	Male	95 plus	30% (14-51%)	70% (49-86%)	1.6 (0.56-3.4)	3.5% (1.1-7.3%)	3.9% (1-8.5%)	64 (17-140)	250 (76-520)
Thailand	2016	Female	All Ages	12% (8.6-15%)	88% (85-91%)	1.1 (0.73-1.6)	3.5% (2.7-4.7%)	5.9% (4.1-7.9%)	12000 (7900-16000)	280000 (210000-370000)
Thailand	2016	Female	15 to 19	8.9% (4.2-16%)	91% (84-96%)	0.62 (0.2-1.4)	1.5% (0.75-2.6%)	3.3% (1.4-6.6%)	33 (14-63)	3400 (1700-6200)
Thailand	2016	Female	20 to 24	11% (4.5-20%)	89% (80-95%)	0.82 (0.25-1.9)	1.9% (1.1-3%)	3.5% (1.6-6.4%)	39 (18-73)	4800 (2800-7800)

Location	Year	Sex	Age	Prevalence of current drinkers	Prevalence of abstainers	Population average of standard drinks daily	Percent of total attributable deaths	Percent of total attributable DALYs	Attributable deaths	Attributable DALYs
Thailand	2016	Female	25 to 29	11% (4.8-21%)	89% (79-95%)	0.93 (0.29-2)	1.9% (1.1-3%)	3.3% (1.4-6%)	51 (21-95)	5700 (3300-9100)
Thailand	2016	Female	30 to 34	12% (5.2-23%)	88% (77-95%)	1 (0.34-2.2)	2.1% (1.2-3.3%)	3.6% (1.7-6.3%)	83 (37-150)	7600 (4300-12000)
Thailand	2016	Female	35 to 39	14% (5.7-25%)	86% (75-94%)	1.2 (0.41-2.4)	2.4% (1.4-3.8%)	4.5% (2.1-7.3%)	150 (71-260)	11000 (6100-18000)
Thailand	2016	Female	40 to 44	14% (6.4-24%)	86% (76-94%)	1.3 (0.47-2.7)	3.1% (1.9-4.7%)	5.7% (3.4-8.7%)	290 (170-450)	17000 (10000-26000)
Thailand	2016	Female	45 to 49	14% (6-26%)	86% (74-94%)	1.5 (0.5-3.1)	3.7% (2.3-5.6%)	6.8% (4.2-10%)	480 (290-740)	24000 (15000-36000)
Thailand	2016	Female	50 to 54	15% (6.3-28%)	85% (72-94%)	1.6 (0.63-3.6)	4.4% (2.7-6.7%)	7.9% (4.8-12%)	730 (440-1100)	31000 (19000-46000)
Thailand	2016	Female	55 to 59	15% (6.9-28%)	85% (72-93%)	1.8 (0.64-3.7)	4.7% (2.9-7.4%)	8.1% (5.1-12%)	940 (590-1500)	34000 (21000-54000)
Thailand	2016	Female	60 to 64	15% (6.5-28%)	85% (72-93%)	1.8 (0.63-3.9)	4.8% (3-7.8%)	7.7% (4.9-12%)	1200 (730-1800)	36000 (22000-57000)
Thailand	2016	Female	65 to 69	14% (6.6-27%)	86% (73-93%)	1.7 (0.61-3.5)	5% (3.1-7.6%)	7.6% (4.8-11%)	1300 (820-1900)	32000 (20000-48000)
Thailand	2016	Female	70 to 74	14% (6.6-25%)	86% (75-93%)	1.5 (0.51-3.5)	4.4% (2.6-6.9%)	6.5% (3.8-9.8%)	1300 (720-2000)	25000 (14000-39000)
Thailand	2016	Female	75 to 79	13% (5.9-25%)	87% (75-94%)	1.4 (0.4-3.3)	3.8% (2-6.4%)	5.3% (2.8-8.6%)	1400 (700-2200)	21000 (10000-34000)
Thailand	2016	Female	80 to 84	13% (6-25%)	87% (75-94%)	1.4 (0.43-3.4)	3.8% (1.7-6.4%)	5.1% (2.3-8.8%)	1500 (680-2500)	16000 (7500-28000)
Thailand	2016	Female	85 to 89	14% (6.8-27%)	86% (73-93%)	1.6 (0.46-3.7)	3.7% (1.5-6.7%)	4.9% (2-8.8%)	1300 (510-2300)	9800 (4000-18000)
Thailand	2016	Female	90 to 94	17% (7.3-30%)	83% (70-93%)	1.8 (0.46-4.6)	3.5% (1.2-6.6%)	4.5% (1.6-8.5%)	660 (230-1300)	3500 (1200-6700)
Thailand	2016	Female	95 plus	20% (7.8-39%)	80% (61-92%)	2.2 (0.43-6)	3.7% (1.1-7.5%)	4.5% (1.2-9.3%)	220 (57-450)	770 (210-1500)
Thailand	2016	Male	All Ages	35% (29-44%)	65% (56-71%)	3 (2.2-4.1)	11% (8.7-13%)	13% (9.7-15%)	32000 (24000-40000)	1100000 (880000-1400000)
Thailand	2016	Male	15 to 19	24% (14-38%)	76% (62-86%)	0.99 (0.41-2)	4.5% (2.4-7.7%)	6.8% (3.4-12%)	220 (110-390)	17000 (9100-29000)
Thailand	2016	Male	20 to 24	36% (20-55%)	64% (45-80%)	2.4 (1.1-4.5)	9.9% (5.7-15%)	14% (7.5-23%)	550 (290-910)	42000 (24000-65000)
Thailand	2016	Male	25 to 29	42% (24-60%)	58% (40-76%)	3.4 (1.5-6)	12% (7.2-17%)	17% (9.5-24%)	800 (450-1200)	56000 (33000-80000)
Thailand	2016	Male	30 to 34	45% (26-65%)	55% (35-74%)	4.1 (2.1-7)	13% (8.6-17%)	17% (11-24%)	1200 (760-1700)	76000 (50000-110000)
Thailand	2016	Male	35 to 39	47% (30-66%)	53% (34-70%)	4.6 (2.4-7.7)	14% (9.7-18%)	19% (13-24%)	1800 (1200-2500)	100000 (71000-140000)

Location	Year	Sex	Age	Prevalence of current drinkers	Prevalence of abstainers	Population average of standard drinks daily	Percent of total attributable deaths	Percent of total attributable DALYs	Attributable deaths	Attributable DALYs
Thailand	2016	Male	40 to 44	47% (30-67%)	53% (33-70%)	4.8 (2.5-8)	14% (11-19%)	19% (14-24%)	2500 (1800-3300)	120000 (90000-160000)
Thailand	2016	Male	45 to 49	46% (27-65%)	54% (35-73%)	4.8 (2.4-7.8)	15% (11-19%)	20% (15-25%)	3200 (2300-4100)	140000 (100000-180000)
Thailand	2016	Male	50 to 54	44% (25-64%)	56% (36-75%)	4.5 (2.3-7.4)	14% (11-18%)	19% (14-24%)	3600 (2600-4700)	140000 (100000-180000)
Thailand	2016	Male	55 to 59	42% (24-62%)	58% (38-76%)	4.1 (2.1-6.8)	13% (9.2-17%)	17% (13-22%)	3700 (2700-4800)	130000 (90000-160000)
Thailand	2016	Male	60 to 64	38% (22-59%)	62% (41-78%)	3.6 (1.7-6.3)	11% (7.7-15%)	15% (11-20%)	3600 (2400-4900)	110000 (72000-140000)
Thailand	2016	Male	65 to 69	35% (20-54%)	65% (46-80%)	3 (1.4-5.4)	9.9% (6.6-13%)	13% (8.9-17%)	3200 (2100-4300)	76000 (50000-100000)
Thailand	2016	Male	70 to 74	31% (18-49%)	69% (51-82%)	2.4 (1.1-4.6)	8.2% (5-12%)	11% (6.3-15%)	2600 (1500-3800)	51000 (29000-73000)
Thailand	2016	Male	75 to 79	28% (16-45%)	72% (55-84%)	2.1 (0.84-4)	6.4% (3.4-9.8%)	8.1% (4.5-12%)	2200 (1100-3300)	33000 (17000-49000)
Thailand	2016	Male	80 to 84	26% (14-43%)	74% (57-86%)	1.8 (0.73-3.4)	5.3% (2.5-8.4%)	6.6% (3.2-11%)	1700 (800-2700)	19000 (9000-30000)
Thailand	2016	Male	85 to 89	24% (13-39%)	76% (61-87%)	1.6 (0.65-3.1)	4.2% (1.8-7.2%)	5.2% (2.1-9.1%)	920 (380-1700)	7400 (3100-13000)
Thailand	2016	Male	90 to 94	26% (14-42%)	74% (58-86%)	1.5 (0.62-3)	3.4% (1.2-6.5%)	4.2% (1.4-8.4%)	380 (130-750)	2100 (750-4100)
Thailand	2016	Male	95 plus	29% (14-50%)	71% (50-86%)	1.6 (0.51-3.5)	3.4% (0.92-7.2%)	3.7% (0.82-8.5%)	88 (19-200)	340 (93-730)
Timor-Leste	1990	Female	All Ages	8.4% (5.9-12%)	92% (88-94%)	0.024 (0.018-0.032)	0.12% (0.055-0.19%)	0.091% (-0.031-0.22%)	3.2 (-1.1-8)	300 (130-480)
Timor-Leste	1990	Female	15 to 19	8.3% (3.8-16%)	92% (84-96%)	0.022 (0.0077-0.047)	0.27% (0.16-0.4%)	0.16% (0.037-0.33%)	0.13 (0.03-0.28)	26 (15-40)
Timor-Leste	1990	Female	20 to 24	10% (4.2-19%)	90% (81-96%)	0.029 (0.01-0.061)	0.44% (0.25-0.66%)	0.15% (-0.028-0.37%)	0.15 (-0.029-0.37)	44 (24-68)
Timor-Leste	1990	Female	25 to 29	11% (4.7-21%)	89% (79-95%)	0.034 (0.013-0.068)	0.47% (0.27-0.72%)	0.17% (-0.048-0.47%)	0.17 (-0.049-0.46)	43 (23-68)
Timor-Leste	1990	Female	30 to 34	12% (4.7-23%)	88% (77-95%)	0.038 (0.014-0.076)	0.47% (0.25-0.7%)	0.26% (0.012-0.54%)	0.25 (0.012-0.53)	39 (20-61)
Timor-Leste	1990	Female	35 to 39	13% (5.3-24%)	87% (76-95%)	0.043 (0.016-0.082)	0.46% (0.24-0.69%)	0.34% (0.066-0.66%)	0.33 (0.064-0.67)	34 (17-53)
Timor-Leste	1990	Female	40 to 44	13% (5.9-24%)	87% (76-94%)	0.048 (0.018-0.099)	0.46% (0.23-0.71%)	0.43% (0.11-0.8%)	0.43 (0.11-0.81)	32 (15-51)
Timor-Leste	1990	Female	45 to 49	14% (6-25%)	86% (75-94%)	0.055 (0.019-0.12)	0.43% (0.16-0.71%)	0.42% (0.06-0.84%)	0.45 (0.061-0.92)	26 (9.4-46)
Timor-Leste	1990	Female	50 to 54	14% (6.1-26%)	86% (74-94%)	0.061 (0.021-0.14)	0.44% (0.086-0.82%)	0.48% (0.0077-1%)	0.56 (0.0082-1.2)	25 (5-48)
Timor-Leste	1990	Female	55 to 59	14% (6.3-26%)	86% (74-94%)	0.067 (0.022-0.14)	0.33% (-0.055-0.64%)	0.36% (-0.14-0.78%)	0.42 (-0.15-0.98)	16 (-2.8-34)

Location	Year	Sex	Age	Prevalence of current drinkers	Prevalence of abstainers	Population average of standard drinks daily	Percent of total attributable deaths	Percent of total attributable DALYs	Attributable deaths	Attributable DALYs
Timor-Leste	1990	Female	60 to 64	14% (6.1-27%)	86% (73-94%)	0.068 (0.021-0.16)	0.19% (-0.24-0.54%)	0.2% (-0.33-0.64%)	0.28 (-0.47-0.95)	8.8 (-12-27)
Timor-Leste	1990	Female	65 to 69	14% (6-25%)	86% (75-94%)	0.064 (0.021-0.13)	0.091% (-0.36-0.42%)	0.096% (-0.44-0.52%)	0.13 (-0.59-0.66)	3.3 (-13-15)
Timor-Leste	1990	Female	70 to 74	13% (6.1-24%)	87% (76-94%)	0.058 (0.018-0.14)	0.054% (-0.38-0.35%)	0.056% (-0.46-0.41%)	0.073 (-0.59-0.57)	1.5 (-11-10)
Timor-Leste	1990	Female	75 to 79	12% (5.4-24%)	88% (76-95%)	0.054 (0.015-0.13)	-0.028% (-0.47-0.23%)	-0.039% (-0.56-0.26%)	-0.036 (-0.51-0.26)	-0.41 (-7-3.6)
Timor-Leste	1990	Female	80 to 84	12% (5.5-24%)	88% (76-94%)	0.053 (0.016-0.12)	-0.057% (-0.46-0.19%)	-0.074% (-0.55-0.22%)	-0.044 (-0.32-0.13)	-0.41 (-3.3-1.4)
Timor-Leste	1990	Female	85 to 89	13% (5.7-25%)	87% (75-94%)	0.056 (0.017-0.14)	-0.093% (-0.49-0.13%)	-0.12% (-0.59-0.14%)	-0.052 (-0.26-0.065)	-0.35 (-1.9-0.49)
Timor-Leste	1990	Female	90 to 94	15% (6.3-29%)	85% (71-94%)	0.065 (0.017-0.16)	-0.12% (-0.54-0.11%)	-0.16% (-0.67-0.12%)	-0.036 (-0.14-0.027)	-0.17 (-0.71-0.14)
Timor-Leste	1990	Female	95 plus	19% (7.1-37%)	81% (63-93%)	0.08 (0.016-0.22)	-0.13% (-0.67-0.15%)	-0.18% (-0.84-0.16%)	-0.0099 (-0.045-0.0088)	-0.029 (-0.14-0.033)
Timor-Leste	1990	Male	All Ages	26% (20-34%)	74% (66-80%)	0.079 (0.07-0.089)	0.31% (0.094-0.54%)	0.35% (-0.041-0.76%)	12 (-1.5-26)	850 (280-1400)
Timor-Leste	1990	Male	15 to 19	23% (14-38%)	77% (62-86%)	0.04 (0.019-0.074)	0.52% (0.34-0.79%)	0.4% (0.19-0.7%)	0.32 (0.15-0.58)	48 (30-73)
Timor-Leste	1990	Male	20 to 24	35% (19-53%)	65% (47-81%)	0.094 (0.049-0.15)	0.98% (0.56-1.5%)	0.64% (0.15-1.3%)	0.72 (0.16-1.4)	100 (59-160)
Timor-Leste	1990	Male	25 to 29	40% (22-59%)	60% (41-78%)	0.13 (0.074-0.2)	1.2% (0.45-2.1%)	0.89% (-0.19-2.1%)	0.92 (-0.19-2.2)	120 (42-200)
Timor-Leste	1990	Male	30 to 34	44% (25-63%)	56% (37-75%)	0.16 (0.098-0.24)	1.5% (0.48-2.5%)	1.3% (-0.11-2.9%)	1.2 (-0.11-2.7)	120 (40-210)
Timor-Leste	1990	Male	35 to 39	46% (28-66%)	54% (34-72%)	0.19 (0.1-0.29)	1.6% (0.49-2.8%)	1.7% (-0.049-3.6%)	1.5 (-0.039-3.2)	120 (33-210)
Timor-Leste	1990	Male	40 to 44	46% (28-66%)	54% (34-72%)	0.2 (0.11-0.31)	1.6% (0.36-2.9%)	1.8% (-0.068-3.9%)	1.7 (-0.052-3.6)	110 (24-190)
Timor-Leste	1990	Male	45 to 49	44% (25-64%)	56% (36-75%)	0.19 (0.1-0.32)	1.5% (0.2-2.9%)	1.8% (-0.21-3.8%)	1.7 (-0.2-3.7)	88 (11-170)
Timor-Leste	1990	Male	50 to 54	42% (24-62%)	58% (38-76%)	0.18 (0.094-0.3)	1.2% (-0.15-2.5%)	1.4% (-0.53-3.2%)	1.3 (-0.49-3.1)	60 (-7.1-120)
Timor-Leste	1990	Male	55 to 59	40% (23-60%)	60% (40-77%)	0.16 (0.079-0.29)	0.96% (-0.12-2%)	1.1% (-0.4-2.6%)	0.96 (-0.33-2.3)	37 (-4.2-79)
Timor-Leste	1990	Male	60 to 64	37% (21-56%)	63% (44-79%)	0.14 (0.067-0.26)	0.75% (-0.22-1.7%)	0.82% (-0.46-2.1%)	0.83 (-0.43-2.1)	27 (-7.3-62)
Timor-Leste	1990	Male	65 to 69	34% (19-52%)	66% (48-81%)	0.12 (0.057-0.21)	0.5% (-0.39-1.3%)	0.52% (-0.63-1.6%)	0.45 (-0.54-1.4)	12 (-9.5-33)
Timor-Leste	1990	Male	70 to 74	30% (17-47%)	70% (53-83%)	0.096 (0.043-0.18)	0.35% (-0.4-1.1%)	0.35% (-0.58-1.2%)	0.3 (-0.49-1.1)	6.6 (-7.2-20)
Timor-Leste	1990	Male	75 to 79	27% (15-45%)	73% (55-85%)	0.081 (0.033-0.16)	0.21% (-0.44-0.72%)	0.19% (-0.61-0.82%)	0.11 (-0.36-0.5)	2.2 (-4.5-7.3)

Location	Year	Sex	Age	Prevalence of current drinkers	Prevalence of abstainers	Population average of standard drinks daily	Percent of total attributable deaths	Percent of total attributable DALYs	Attributable deaths	Attributable DALYs
Timor-Leste	1990	Male	80 to 84	25%<br>(13-42%)	75%<br>(58-87%)	0.07<br>(0.029-0.13)	0.16%<br>(-0.37-0.6%)	0.13%<br>(-0.52-0.67%)	0.044<br>(-0.17-0.23)	0.67<br>(-1.5-2.5)
Timor-Leste	1990	Male	85 to 89	24%<br>(12-39%)	76%<br>(61-88%)	0.062<br>(0.026-0.12)	0.11%<br>(-0.28-0.42%)	0.079%<br>(-0.39-0.48%)	0.02<br>(-0.1-0.12)	0.24<br>(-0.61-0.95)
Timor-Leste	1990	Male	90 to 94	25%<br>(14-41%)	75%<br>(59-86%)	0.06<br>(0.024-0.12)	0.05%<br>(-0.29-0.31%)	0.015%<br>(-0.41-0.33%)	0.0018<br>(-0.049-0.04)	0.038<br>(-0.22-0.23)
Timor-Leste	1990	Male	95 plus	29%<br>(13-48%)	71%<br>(52-87%)	0.063<br>(0.021-0.13)	0.069%<br>(-0.33-0.34%)	0.0032%<br>(-0.46-0.34%)	0.00011<br>(-0.016-0.012)	0.0095<br>(-0.044-0.047)
Timor-Leste	1995	Female	All Ages	8.4%<br>(6-11%)	92%<br>(89-94%)	0.027<br>(0.02-0.036)	0.14%<br>(0.076-0.21%)	0.11%<br>(-0.021-0.24%)	3.7<br>(-0.68-8.2)	340<br>(180-500)
Timor-Leste	1995	Female	15 to 19	8.2%<br>(3.6-15%)	92%<br>(85-96%)	0.025<br>(0.0085-0.055)	0.29%<br>(0.17-0.44%)	0.17%<br>(0.049-0.33%)	0.11<br>(0.03-0.21)	21<br>(12-34)
Timor-Leste	1995	Female	20 to 24	10%<br>(4.3-18%)	90%<br>(82-96%)	0.033<br>(0.011-0.069)	0.48%<br>(0.28-0.71%)	0.15%<br>(0.0066-0.34%)	0.15<br>(0.0065-0.34)	48<br>(27-75)
Timor-Leste	1995	Female	25 to 29	11%<br>(4.7-20%)	89%<br>(80-95%)	0.038<br>(0.013-0.075)	0.51%<br>(0.31-0.74%)	0.18%<br>(0.0035-0.41%)	0.17<br>(0.0036-0.41)	46<br>(27-71)
Timor-Leste	1995	Female	30 to 34	12%<br>(4.8-22%)	88%<br>(78-95%)	0.043<br>(0.017-0.087)	0.5%<br>(0.31-0.72%)	0.27%<br>(0.056-0.52%)	0.27<br>(0.056-0.54)	44<br>(26-66)
Timor-Leste	1995	Female	35 to 39	13%<br>(5.3-24%)	87%<br>(76-95%)	0.048<br>(0.019-0.093)	0.48%<br>(0.29-0.71%)	0.35%<br>(0.088-0.66%)	0.37<br>(0.089-0.7)	39<br>(22-60)
Timor-Leste	1995	Female	40 to 44	13%<br>(6.1-24%)	87%<br>(76-94%)	0.055<br>(0.02-0.11)	0.49%<br>(0.28-0.71%)	0.45%<br>(0.17-0.78%)	0.47<br>(0.18-0.82)	35<br>(20-53)
Timor-Leste	1995	Female	45 to 49	13%<br>(5.7-25%)	87%<br>(75-94%)	0.063<br>(0.021-0.14)	0.45%<br>(0.22-0.69%)	0.45%<br>(0.12-0.8%)	0.52<br>(0.14-0.95)	31<br>(15-49)
Timor-Leste	1995	Female	50 to 54	13%<br>(5.8-26%)	87%<br>(74-94%)	0.069<br>(0.024-0.15)	0.47%<br>(0.14-0.82%)	0.52%<br>(0.09-1%)	0.67<br>(0.12-1.3)	30<br>(9.6-55)
Timor-Leste	1995	Female	55 to 59	14%<br>(6-25%)	86%<br>(75-94%)	0.076<br>(0.025-0.16)	0.35%<br>(-0.017-0.68%)	0.4%<br>(-0.075-0.84%)	0.56<br>(-0.1-1.2)	21<br>(-0.99-42)
Timor-Leste	1995	Female	60 to 64	13%<br>(6-25%)	87%<br>(75-94%)	0.076<br>(0.024-0.18)	0.21%<br>(-0.2-0.52%)	0.23%<br>(-0.29-0.64%)	0.33<br>(-0.39-0.93)	10<br>(-9.7-27)
Timor-Leste	1995	Female	65 to 69	13%<br>(5.8-24%)	87%<br>(76-94%)	0.07<br>(0.024-0.15)	0.1%<br>(-0.31-0.41%)	0.11%<br>(-0.37-0.51%)	0.19<br>(-0.68-0.83)	4.8<br>(-15-19)
Timor-Leste	1995	Female	70 to 74	12%<br>(5.9-23%)	88%<br>(77-94%)	0.063<br>(0.02-0.15)	0.07%<br>(-0.31-0.36%)	0.078%<br>(-0.38-0.42%)	0.11<br>(-0.56-0.63)	2.2<br>(-9.8-11)
Timor-Leste	1995	Female	75 to 79	12%<br>(5.2-23%)	88%<br>(77-95%)	0.059<br>(0.017-0.14)	-0.024%<br>(-0.44-0.23%)	-0.035%<br>(-0.54-0.27%)	-0.041<br>(-0.63-0.31)	-0.47<br>(-8.8-4.4)
Timor-Leste	1995	Female	80 to 84	12%<br>(5.4-23%)	88%<br>(77-95%)	0.059<br>(0.018-0.13)	-0.061%<br>(-0.44-0.17%)	-0.078%<br>(-0.52-0.2%)	-0.05<br>(-0.34-0.13)	-0.48<br>(-3.5-1.4)
Timor-Leste	1995	Female	85 to 89	13%<br>(5.9-24%)	87%<br>(76-94%)	0.064<br>(0.02-0.15)	-0.1%<br>(-0.5-0.13%)	-0.13%<br>(-0.59-0.14%)	-0.051<br>(-0.24-0.054)	-0.35<br>(-1.7-0.41)
Timor-Leste	1995	Female	90 to 94	15%<br>(6.8-28%)	85%<br>(72-93%)	0.073<br>(0.02-0.17)	-0.14%<br>(-0.57-0.1%)	-0.18%<br>(-0.72-0.1%)	-0.034<br>(-0.14-0.02)	-0.16<br>(-0.66-0.12)
Timor-Leste	1995	Female	95 plus	18%<br>(7.2-36%)	82%<br>(64-93%)	0.09<br>(0.019-0.24)	-0.14%<br>(-0.68-0.15%)	-0.2%<br>(-0.85-0.16%)	-0.014<br>(-0.061-0.011)	-0.04<br>(-0.2-0.041)

Location	Year	Sex	Age	Prevalence of current drinkers	Prevalence of abstainers	Population average of standard drinks daily	Percent of total attributable deaths	Percent of total attributable DALYs	Attributable deaths	Attributable DALYs
Timor-Leste	1995	Male	All Ages	26% (21-34%)	74% (66-79%)	0.092 (0.081-0.1)	0.39% (0.16-0.65%)	0.45% (0.023-0.93%)	15 (0.86-31)	1000 (410-1600)
Timor-Leste	1995	Male	15 to 19	23% (13-38%)	77% (62-87%)	0.045 (0.021-0.086)	0.52% (0.33-0.76%)	0.39% (0.18-0.7%)	0.27 (0.13-0.47)	39 (25-57)
Timor-Leste	1995	Male	20 to 24	35% (19-53%)	65% (47-81%)	0.11 (0.054-0.17)	0.92% (0.51-1.4%)	0.59% (0.14-1.1%)	0.82 (0.21-1.6)	120 (63-180)
Timor-Leste	1995	Male	25 to 29	40% (22-59%)	60% (41-78%)	0.15 (0.083-0.22)	1.2% (0.57-1.9%)	0.83% (0.051-1.7%)	1 (0.059-2.2)	130 (62-200)
Timor-Leste	1995	Male	30 to 34	44% (24-63%)	56% (37-76%)	0.18 (0.11-0.27)	1.4% (0.61-2.3%)	1.3% (0.1-2.5%)	1.5 (0.11-3.1)	140 (61-240)
Timor-Leste	1995	Male	35 to 39	46% (28-65%)	54% (35-72%)	0.21 (0.12-0.32)	1.6% (0.67-2.8%)	1.7% (0.19-3.5%)	1.9 (0.19-4)	140 (54-250)
Timor-Leste	1995	Male	40 to 44	46% (28-65%)	54% (35-72%)	0.22 (0.12-0.34)	1.7% (0.55-2.8%)	1.9% (0.16-3.7%)	2 (0.14-3.9)	120 (41-220)
Timor-Leste	1995	Male	45 to 49	44% (26-64%)	56% (36-74%)	0.22 (0.11-0.35)	1.6% (0.38-2.8%)	1.9% (0.16-3.7%)	2.2 (0.17-4.3)	110 (26-200)
Timor-Leste	1995	Male	50 to 54	42% (24-61%)	58% (39-76%)	0.21 (0.11-0.34)	1.3% (0.098-2.5%)	1.6% (-0.19-3.2%)	1.8 (-0.23-3.8)	80 (5.8-150)
Timor-Leste	1995	Male	55 to 59	39% (23-60%)	61% (40-77%)	0.19 (0.091-0.32)	1% (0.088-2%)	1.2% (-0.11-2.6%)	1.4 (-0.13-3)	53 (4.4-100)
Timor-Leste	1995	Male	60 to 64	37% (20-57%)	63% (43-80%)	0.16 (0.076-0.3)	0.81% (-0.036-1.6%)	0.91% (-0.24-2.1%)	1 (-0.28-2.4)	34 (-1.5-69)
Timor-Leste	1995	Male	65 to 69	33% (19-51%)	67% (49-81%)	0.13 (0.064-0.24)	0.54% (-0.25-1.3%)	0.58% (-0.46-1.6%)	0.69 (-0.51-1.9)	19 (-8.1-44)
Timor-Leste	1995	Male	70 to 74	30% (17-46%)	70% (54-83%)	0.11 (0.049-0.2)	0.38% (-0.31-1%)	0.39% (-0.47-1.2%)	0.39 (-0.47-1.2)	8.4 (-6.7-23)
Timor-Leste	1995	Male	75 to 79	27% (15-44%)	73% (56-85%)	0.092 (0.038-0.18)	0.23% (-0.34-0.73%)	0.21% (-0.5-0.84%)	0.17 (-0.39-0.68)	3.2 (-4.6-10)
Timor-Leste	1995	Male	80 to 84	25% (13-41%)	75% (59-87%)	0.079 (0.033-0.15)	0.17% (-0.3-0.59%)	0.15% (-0.44-0.69%)	0.057 (-0.17-0.26)	0.83 (-1.5-2.9)
Timor-Leste	1995	Male	85 to 89	23% (13-39%)	77% (61-87%)	0.07 (0.03-0.13)	0.11% (-0.26-0.44%)	0.09% (-0.37-0.49%)	0.026 (-0.1-0.14)	0.29 (-0.63-1.1)
Timor-Leste	1995	Male	90 to 94	25% (14-41%)	75% (59-86%)	0.068 (0.028-0.13)	0.05% (-0.28-0.32%)	0.014% (-0.39-0.35%)	0.0021 (-0.057-0.052)	0.045 (-0.24-0.29)
Timor-Leste	1995	Male	95 plus	28% (14-48%)	72% (52-86%)	0.07 (0.024-0.15)	0.074% (-0.3-0.34%)	0.0071% (-0.44-0.32%)	0.00032 (-0.019-0.014)	0.013 (-0.053-0.061)
Timor-Leste	2000	Female	All Ages	7.7% (5.5-11%)	92% (89-94%)	0.03 (0.023-0.04)	0.16% (0.085-0.24%)	0.13% (-0.043-0.3%)	3.3 (-1.1-7.5)	280 (150-410)
Timor-Leste	2000	Female	15 to 19	7.8% (3.4-15%)	92% (85-97%)	0.029 (0.01-0.064)	0.33% (0.19-0.5%)	0.2% (0.066-0.38%)	0.13 (0.039-0.25)	27 (16-41)
Timor-Leste	2000	Female	20 to 24	9.5% (3.9-18%)	91% (82-96%)	0.04 (0.013-0.086)	0.54% (0.32-0.79%)	0.18% (0.015-0.4%)	0.088 (0.0071-0.2)	29 (17-45)
Timor-Leste	2000	Female	25 to 29	10% (4.3-20%)	90% (80-96%)	0.046 (0.016-0.097)	0.57% (0.36-0.82%)	0.2% (0.016-0.45%)	0.11 (0.0085-0.25)	32 (19-48)

Location	Year	Sex	Age	Prevalence of current drinkers	Prevalence of abstainers	Population average of standard drinks daily	Percent of total attributable deaths	Percent of total attributable DALYs	Attributable deaths	Attributable DALYs
Timor-Leste	2000	Female	30 to 34	11% (4.5-22%)	89% (78-95%)	0.052 (0.019-0.11)	0.55% (0.35-0.78%)	0.3% (0.075-0.55%)	0.17 (0.041-0.31)	29 (17-43)
Timor-Leste	2000	Female	35 to 39	12% (5.3-23%)	88% (77-95%)	0.058 (0.022-0.11)	0.52% (0.31-0.74%)	0.38% (0.1-0.69%)	0.27 (0.07-0.5)	31 (18-45)
Timor-Leste	2000	Female	40 to 44	13% (5.9-22%)	87% (78-94%)	0.066 (0.024-0.13)	0.51% (0.31-0.74%)	0.48% (0.17-0.8%)	0.37 (0.13-0.64)	29 (17-43)
Timor-Leste	2000	Female	45 to 49	13% (5.6-23%)	87% (77-94%)	0.075 (0.025-0.16)	0.47% (0.24-0.72%)	0.49% (0.17-0.85%)	0.44 (0.14-0.8)	27 (13-41)
Timor-Leste	2000	Female	50 to 54	13% (5.6-25%)	87% (75-94%)	0.083 (0.03-0.17)	0.49% (0.17-0.82%)	0.57% (0.14-1%)	0.65 (0.16-1.2)	29 (10-49)
Timor-Leste	2000	Female	55 to 59	13% (6.1-25%)	87% (75-94%)	0.091 (0.032-0.19)	0.38% (0.0095-0.73%)	0.45% (-0.043-0.92%)	0.6 (-0.049-1.3)	22 (0.53-43)
Timor-Leste	2000	Female	60 to 64	13% (5.8-25%)	87% (75-94%)	0.093 (0.031-0.2)	0.23% (-0.2-0.59%)	0.27% (-0.25-0.74%)	0.41 (-0.43-1.2)	12 (-11-33)
Timor-Leste	2000	Female	65 to 69	13% (5.8-23%)	87% (77-94%)	0.086 (0.029-0.18)	0.11% (-0.33-0.42%)	0.12% (-0.42-0.54%)	0.19 (-0.63-0.81)	4.8 (-13-19)
Timor-Leste	2000	Female	70 to 74	12% (5.7-23%)	88% (77-94%)	0.078 (0.025-0.18)	0.074% (-0.32-0.37%)	0.086% (-0.38-0.46%)	0.15 (-0.65-0.8)	2.8 (-12-14)
Timor-Leste	2000	Female	75 to 79	12% (5.4-22%)	88% (78-95%)	0.073 (0.022-0.18)	-0.028% (-0.47-0.24%)	-0.038% (-0.55-0.29%)	-0.047 (-0.68-0.36)	-0.57 (-9.6-5.1)
Timor-Leste	2000	Female	80 to 84	12% (5.5-23%)	88% (77-95%)	0.072 (0.022-0.16)	-0.066% (-0.47-0.18%)	-0.085% (-0.56-0.22%)	-0.065 (-0.46-0.17)	-0.63 (-4.5-1.8)
Timor-Leste	2000	Female	85 to 89	13% (5.7-23%)	87% (77-94%)	0.077 (0.024-0.18)	-0.11% (-0.52-0.13%)	-0.14% (-0.63-0.15%)	-0.067 (-0.3-0.071)	-0.46 (-2.2-0.53)
Timor-Leste	2000	Female	90 to 94	15% (6.3-27%)	85% (73-94%)	0.089 (0.024-0.21)	-0.15% (-0.61-0.11%)	-0.19% (-0.74-0.12%)	-0.036 (-0.14-0.023)	-0.16 (-0.67-0.12)
Timor-Leste	2000	Female	95 plus	18% (6.7-34%)	82% (66-93%)	0.11 (0.022-0.29)	-0.15% (-0.7-0.16%)	-0.21% (-0.88-0.17%)	-0.014 (-0.056-0.011)	-0.039 (-0.18-0.042)
Timor-Leste	2000	Male	All Ages	23% (18-30%)	77% (70-82%)	0.097 (0.085-0.11)	0.46% (0.19-0.74%)	0.56% (0.056-1.1%)	14 (1.4-28)	860 (360-1400)
Timor-Leste	2000	Male	15 to 19	22% (12-37%)	78% (63-88%)	0.054 (0.024-0.1)	0.65% (0.42-0.97%)	0.57% (0.28-1%)	0.36 (0.17-0.65)	53 (33-78)
Timor-Leste	2000	Male	20 to 24	34% (19-52%)	66% (48-81%)	0.13 (0.063-0.22)	1.3% (0.74-1.9%)	0.99% (0.29-1.8%)	0.53 (0.14-1)	71 (39-110)
Timor-Leste	2000	Male	25 to 29	39% (22-58%)	61% (42-78%)	0.18 (0.098-0.29)	1.5% (0.73-2.3%)	1.2% (0.087-2.5%)	0.75 (0.051-1.5)	92 (45-140)
Timor-Leste	2000	Male	30 to 34	43% (24-62%)	57% (38-76%)	0.22 (0.13-0.34)	1.7% (0.74-2.7%)	1.6% (0.12-3.2%)	1 (0.077-2)	97 (41-160)
Timor-Leste	2000	Male	35 to 39	45% (27-65%)	55% (35-73%)	0.26 (0.14-0.39)	1.8% (0.79-3%)	2% (0.21-4%)	1.5 (0.14-3.2)	120 (48-200)
Timor-Leste	2000	Male	40 to 44	44% (27-64%)	56% (36-73%)	0.27 (0.15-0.4)	1.8% (0.66-3%)	2.1% (0.23-4%)	1.7 (0.18-3.4)	110 (38-190)
Timor-Leste	2000	Male	45 to 49	43% (24-63%)	57% (37-76%)	0.26 (0.14-0.42)	1.7% (0.49-2.9%)	2.1% (0.24-4%)	1.9 (0.2-3.7)	99 (28-170)

Location	Year	Sex	Age	Prevalence of current drinkers	Prevalence of abstainers	Population average of standard drinks daily	Percent of total attributable deaths	Percent of total attributable DALYs	Attributable deaths	Attributable DALYs
Timor-Leste	2000	Male	50 to 54	41% (23-61%)	59% (39-77%)	0.25 (0.13-0.4)	1.4% (0.27-2.5%)	1.7% (0.061-3.4%)	1.9 (0.065-3.8)	84 (16-160)
Timor-Leste	2000	Male	55 to 59	39% (22-59%)	61% (41-78%)	0.22 (0.11-0.37)	1.1% (0.21-2.1%)	1.3% (0.034-2.7%)	1.6 (0.035-3.3)	59 (9.7-110)
Timor-Leste	2000	Male	60 to 64	36% (20-56%)	64% (44-80%)	0.19 (0.094-0.34)	0.86% (0.023-1.7%)	0.98% (-0.12-2.1%)	1.3 (-0.18-2.8)	41 (1.2-82)
Timor-Leste	2000	Male	65 to 69	32% (18-51%)	68% (49-82%)	0.16 (0.079-0.27)	0.57% (-0.2-1.3%)	0.63% (-0.41-1.6%)	0.74 (-0.44-1.9)	20 (-6.1-45)
Timor-Leste	2000	Male	70 to 74	29% (16-45%)	71% (55-84%)	0.13 (0.06-0.24)	0.41% (-0.29-1%)	0.43% (-0.45-1.2%)	0.52 (-0.56-1.5)	11 (-8.1-29)
Timor-Leste	2000	Male	75 to 79	26% (15-43%)	74% (57-85%)	0.11 (0.048-0.21)	0.24% (-0.32-0.71%)	0.22% (-0.5-0.8%)	0.18 (-0.41-0.67)	3.4 (-4.8-10)
Timor-Leste	2000	Male	80 to 84	24% (12-40%)	76% (60-88%)	0.095 (0.041-0.17)	0.18% (-0.33-0.62%)	0.16% (-0.46-0.71%)	0.074 (-0.22-0.35)	1.1 (-2-3.9)
Timor-Leste	2000	Male	85 to 89	23% (12-37%)	77% (63-88%)	0.084 (0.036-0.16)	0.11% (-0.27-0.45%)	0.082% (-0.39-0.5%)	0.026 (-0.12-0.16)	0.31 (-0.78-1.3)
Timor-Leste	2000	Male	90 to 94	24% (13-41%)	76% (59-87%)	0.082 (0.034-0.16)	0.042% (-0.3-0.33%)	0.0032% (-0.43-0.35%)	0.00049 (-0.069-0.057)	0.041 (-0.3-0.32)
Timor-Leste	2000	Male	95 plus	28% (13-48%)	72% (52-87%)	0.084 (0.029-0.18)	0.064% (-0.34-0.35%)	-0.0078% (-0.51-0.34%)	-0.00042 (-0.027-0.018)	0.014 (-0.073-0.073)
Timor-Leste	2005	Female	All Ages	7.6% (5.4-10%)	92% (90-95%)	0.033 (0.023-0.046)	0.19% (0.11-0.27%)	0.14% (-0.03-0.32%)	3.6 (-0.73-7.9)	310 (180-440)
Timor-Leste	2005	Female	15 to 19	7.7% (3.5-15%)	92% (85-97%)	0.033 (0.011-0.071)	0.36% (0.22-0.52%)	0.21% (0.088-0.4%)	0.12 (0.046-0.23)	30 (17-44)
Timor-Leste	2005	Female	20 to 24	9.3% (4.1-18%)	91% (82-96%)	0.044 (0.015-0.096)	0.62% (0.38-0.94%)	0.19% (0.046-0.4%)	0.12 (0.028-0.27)	49 (29-77)
Timor-Leste	2005	Female	25 to 29	10% (4.3-19%)	90% (81-96%)	0.05 (0.017-0.11)	0.64% (0.43-0.93%)	0.22% (0.029-0.45%)	0.087 (0.012-0.18)	29 (18-44)
Timor-Leste	2005	Female	30 to 34	11% (4.4-21%)	89% (79-96%)	0.057 (0.02-0.12)	0.6% (0.39-0.84%)	0.3% (0.049-0.54%)	0.15 (0.026-0.27)	31 (19-46)
Timor-Leste	2005	Female	35 to 39	12% (5-22%)	88% (78-95%)	0.063 (0.022-0.12)	0.55% (0.37-0.74%)	0.39% (0.11-0.67%)	0.22 (0.066-0.39)	28 (18-40)
Timor-Leste	2005	Female	40 to 44	12% (5.4-22%)	88% (78-95%)	0.071 (0.025-0.15)	0.52% (0.32-0.73%)	0.49% (0.2-0.8%)	0.36 (0.13-0.59)	31 (18-44)
Timor-Leste	2005	Female	45 to 49	12% (5.4-22%)	88% (78-95%)	0.081 (0.027-0.18)	0.48% (0.27-0.71%)	0.51% (0.18-0.87%)	0.44 (0.16-0.79)	28 (15-42)
Timor-Leste	2005	Female	50 to 54	13% (5.5-23%)	87% (77-94%)	0.089 (0.032-0.19)	0.52% (0.22-0.85%)	0.62% (0.2-1.1%)	0.67 (0.21-1.3)	31 (12-52)
Timor-Leste	2005	Female	55 to 59	13% (5.9-24%)	87% (76-94%)	0.098 (0.033-0.22)	0.41% (0.052-0.73%)	0.5% (0.012-0.97%)	0.7 (0.017-1.3)	26 (3.3-46)
Timor-Leste	2005	Female	60 to 64	13% (5.7-25%)	87% (75-94%)	0.099 (0.032-0.22)	0.26% (-0.16-0.65%)	0.31% (-0.21-0.83%)	0.53 (-0.36-1.4)	16 (-9.8-39)
Timor-Leste	2005	Female	65 to 69	13% (5.6-23%)	87% (77-94%)	0.092 (0.03-0.19)	0.13% (-0.27-0.44%)	0.15% (-0.36-0.55%)	0.28 (-0.7-1)	7 (-16-24)

Location	Year	Sex	Age	Prevalence of current drinkers	Prevalence of abstainers	Population average of standard drinks daily	Percent of total attributable deaths	Percent of total attributable DALYs	Attributable deaths	Attributable DALYs
Timor-Leste	2005	Female	70 to 74	12% (5.4-22%)	88% (78-95%)	0.083 (0.026-0.2)	0.09% (-0.27-0.37%)	0.11% (-0.34-0.46%)	0.19 (-0.62-0.83)	3.7 (-11-15)
Timor-Leste	2005	Female	75 to 79	11% (5.1-22%)	89% (78-95%)	0.077 (0.023-0.19)	-0.014% (-0.44-0.27%)	-0.023% (-0.55-0.32%)	-0.039 (-0.87-0.55)	-0.4 (-12-7.8)
Timor-Leste	2005	Female	80 to 84	11% (5.2-22%)	89% (78-95%)	0.078 (0.024-0.18)	-0.056% (-0.47-0.19%)	-0.074% (-0.57-0.22%)	-0.08 (-0.61-0.24)	-0.76 (-6.2-2.6)
Timor-Leste	2005	Female	85 to 89	12% (5.5-23%)	88% (77-95%)	0.084 (0.025-0.2)	-0.11% (-0.52-0.13%)	-0.14% (-0.64-0.15%)	-0.089 (-0.39-0.094)	-0.61 (-2.8-0.72)
Timor-Leste	2005	Female	90 to 94	14% (6.3-27%)	86% (73-94%)	0.099 (0.026-0.24)	-0.15% (-0.57-0.11%)	-0.2% (-0.71-0.13%)	-0.05 (-0.18-0.033)	-0.23 (-0.87-0.18)
Timor-Leste	2005	Female	95 plus	18% (6.9-36%)	82% (64-93%)	0.12 (0.024-0.35)	-0.16% (-0.69-0.22%)	-0.22% (-0.88-0.23%)	-0.015 (-0.059-0.015)	-0.042 (-0.19-0.06)
Timor-Leste	2005	Male	All Ages	23% (18-30%)	77% (70-82%)	0.1 (0.083-0.13)	0.56% (0.27-0.87%)	0.66% (0.15-1.2%)	17 (3.8-31)	1000 (510-1600)
Timor-Leste	2005	Male	15 to 19	22% (12-36%)	78% (64-88%)	0.058 (0.027-0.11)	0.74% (0.47-1.1%)	0.7% (0.35-1.2%)	0.43 (0.21-0.77)	62 (38-94)
Timor-Leste	2005	Male	20 to 24	33% (18-51%)	67% (49-82%)	0.14 (0.067-0.23)	1.5% (0.88-2.2%)	1.3% (0.38-2.3%)	0.92 (0.28-1.7)	130 (70-190)
Timor-Leste	2005	Male	25 to 29	38% (21-56%)	62% (44-79%)	0.19 (0.1-0.33)	1.7% (0.94-2.6%)	1.5% (0.18-3%)	0.71 (0.081-1.4)	86 (46-130)
Timor-Leste	2005	Male	30 to 34	42% (23-61%)	58% (39-77%)	0.24 (0.13-0.4)	1.9% (0.92-2.9%)	1.9% (0.21-3.6%)	1.1 (0.13-2.2)	110 (52-170)
Timor-Leste	2005	Male	35 to 39	44% (26-64%)	56% (36-74%)	0.28 (0.15-0.43)	2% (0.83-3.2%)	2.3% (0.29-4.4%)	1.5 (0.19-3)	110 (49-190)
Timor-Leste	2005	Male	40 to 44	44% (26-64%)	56% (36-74%)	0.29 (0.15-0.47)	1.9% (0.73-3%)	2.3% (0.3-4.2%)	2 (0.25-3.7)	120 (47-210)
Timor-Leste	2005	Male	45 to 49	42% (24-62%)	58% (38-76%)	0.29 (0.14-0.48)	1.8% (0.65-3%)	2.3% (0.43-4.2%)	2.2 (0.4-4.2)	120 (40-200)
Timor-Leste	2005	Male	50 to 54	40% (22-60%)	60% (40-78%)	0.27 (0.14-0.44)	1.5% (0.43-2.5%)	1.9% (0.27-3.4%)	2.2 (0.34-4.1)	94 (27-160)
Timor-Leste	2005	Male	55 to 59	38% (21-57%)	62% (43-79%)	0.24 (0.11-0.41)	1.1% (0.33-2.1%)	1.4% (0.19-2.7%)	2 (0.26-3.9)	75 (21-140)
Timor-Leste	2005	Male	60 to 64	35% (19-54%)	65% (46-81%)	0.21 (0.096-0.37)	0.9% (0.12-1.6%)	1% (0.0027-2.1%)	1.7 (0.0044-3.5)	54 (6.6-100)
Timor-Leste	2005	Male	65 to 69	32% (17-49%)	68% (51-83%)	0.17 (0.083-0.3)	0.59% (-0.037-1.2%)	0.66% (-0.18-1.5%)	1.1 (-0.3-2.4)	28 (-1.8-57)
Timor-Leste	2005	Male	70 to 74	28% (15-45%)	72% (55-85%)	0.14 (0.063-0.26)	0.43% (-0.18-0.95%)	0.47% (-0.33-1.2%)	0.66 (-0.47-1.6)	14 (-6.1-31)
Timor-Leste	2005	Male	75 to 79	26% (14-42%)	74% (58-86%)	0.12 (0.05-0.23)	0.25% (-0.27-0.67%)	0.24% (-0.41-0.76%)	0.3 (-0.49-0.92)	5.4 (-5.7-14)
Timor-Leste	2005	Male	80 to 84	24% (13-39%)	76% (61-87%)	0.1 (0.043-0.2)	0.19% (-0.24-0.59%)	0.17% (-0.36-0.66%)	0.12 (-0.27-0.48)	1.7 (-2.2-5.4)
Timor-Leste	2005	Male	85 to 89	22% (11-36%)	78% (64-89%)	0.089 (0.037-0.17)	0.11% (-0.24-0.41%)	0.086% (-0.34-0.45%)	0.038 (-0.15-0.2)	0.44 (-0.95-1.6)

Location	Year	Sex	Age	Prevalence of current drinkers	Prevalence of abstainers	Population average of standard drinks daily	Percent of total attributable deaths	Percent of total attributable DALYs	Attributable deaths	Attributable DALYs
Timor-Leste	2005	Male	90 to 94	23%<br>(12-39%)	77%<br>(61-88%)	0.086<br>(0.035-0.18)	0.044%<br>(-0.3-0.31%)	0.0062%<br>(-0.42-0.32%)	0.0012<br>(-0.085-0.067)	0.056<br>(-0.38-0.38)
Timor-Leste	2005	Male	95 plus	27%<br>(12-46%)	73%<br>(54-88%)	0.088<br>(0.029-0.2)	0.065%<br>(-0.34-0.33%)	-0.0099%<br>(-0.51-0.32%)	-0.00061<br>(-0.032-0.019)	0.016<br>(-0.085-0.081)
Timor-Leste	2010	Female	All Ages	7.6%<br>(5.5-10%)	92%<br>(90-94%)	0.037<br>(0.024-0.056)	0.21%<br>(0.12-0.31%)	0.14%<br>(-0.051-0.32%)	3.8<br>(-1.3-8.8)	340<br>(200-500)
Timor-Leste	2010	Female	15 to 19	7.5%<br>(3.4-14%)	92%<br>(86-97%)	0.034<br>(0.011-0.081)	0.4%<br>(0.25-0.61%)	0.22%<br>(0.1-0.41%)	0.09<br>(0.037-0.17)	30<br>(17-47)
Timor-Leste	2010	Female	20 to 24	9.1%<br>(3.8-17%)	91%<br>(83-96%)	0.046<br>(0.015-0.1)	0.76%<br>(0.47-1.1%)	0.21%<br>(0.083-0.42%)	0.093<br>(0.035-0.2)	51<br>(31-79)
Timor-Leste	2010	Female	25 to 29	9.9%<br>(4.2-19%)	90%<br>(81-96%)	0.052<br>(0.018-0.12)	0.75%<br>(0.5-1.1%)	0.23%<br>(0.039-0.43%)	0.099<br>(0.017-0.19)	45<br>(28-68)
Timor-Leste	2010	Female	30 to 34	11%<br>(4.4-21%)	89%<br>(79-96%)	0.06<br>(0.02-0.13)	0.69%<br>(0.46-0.97%)	0.33%<br>(0.053-0.54%)	0.12<br>(0.02-0.21)	31<br>(20-48)
Timor-Leste	2010	Female	35 to 39	12%<br>(4.9-22%)	88%<br>(78-95%)	0.065<br>(0.023-0.13)	0.6%<br>(0.4-0.83%)	0.42%<br>(0.071-0.73%)	0.22<br>(0.037-0.4)	34<br>(21-49)
Timor-Leste	2010	Female	40 to 44	12%<br>(5.5-22%)	88%<br>(78-95%)	0.073<br>(0.025-0.15)	0.56%<br>(0.37-0.77%)	0.53%<br>(0.24-0.86%)	0.33<br>(0.14-0.56)	32<br>(20-46)
Timor-Leste	2010	Female	45 to 49	12%<br>(5.2-23%)	88%<br>(77-95%)	0.083<br>(0.026-0.18)	0.51%<br>(0.3-0.74%)	0.55%<br>(0.23-0.93%)	0.41<br>(0.16-0.72)	28<br>(16-42)
Timor-Leste	2010	Female	50 to 54	12%<br>(5.3-24%)	88%<br>(76-95%)	0.093<br>(0.031-0.21)	0.52%<br>(0.24-0.83%)	0.65%<br>(0.24-1.2%)	0.56<br>(0.19-1)	27<br>(12-46)
Timor-Leste	2010	Female	55 to 59	13%<br>(5.7-23%)	87%<br>(77-94%)	0.1<br>(0.034-0.23)	0.41%<br>(0.098-0.71%)	0.52%<br>(0.086-1%)	0.49<br>(0.085-0.94)	19<br>(4.4-34)
Timor-Leste	2010	Female	60 to 64	13%<br>(5.4-23%)	87%<br>(77-95%)	0.1<br>(0.033-0.23)	0.27%<br>(-0.1-0.63%)	0.34%<br>(-0.15-0.83%)	0.93<br>(-0.43-2.3)	29<br>(-11-69)
Timor-Leste	2010	Female	65 to 69	12%<br>(5.3-22%)	88%<br>(78-95%)	0.095<br>(0.031-0.2)	0.14%<br>(-0.24-0.44%)	0.18%<br>(-0.34-0.59%)	0.44<br>(-0.83-1.6)	11<br>(-18-37)
Timor-Leste	2010	Female	70 to 74	12%<br>(5.5-21%)	88%<br>(79-95%)	0.086<br>(0.028-0.2)	0.11%<br>(-0.24-0.38%)	0.13%<br>(-0.31-0.5%)	0.31<br>(-0.73-1.2)	6<br>(-14-22)
Timor-Leste	2010	Female	75 to 79	11%<br>(5-22%)	89%<br>(78-95%)	0.082<br>(0.023-0.2)	0.0032%<br>(-0.39-0.29%)	-0.0022%<br>(-0.48-0.36%)	-0.0044<br>(-1-0.78)	0.14<br>(-15-12)
Timor-Leste	2010	Female	80 to 84	11%<br>(5-22%)	89%<br>(78-95%)	0.082<br>(0.024-0.2)	-0.042%<br>(-0.42-0.21%)	-0.058%<br>(-0.52-0.24%)	-0.09<br>(-0.8-0.38)	-0.84<br>(-8.5-4.2)
Timor-Leste	2010	Female	85 to 89	12%<br>(5.3-23%)	88%<br>(77-95%)	0.089<br>(0.026-0.22)	-0.1%<br>(-0.5-0.15%)	-0.14%<br>(-0.62-0.17%)	-0.12<br>(-0.56-0.15)	-0.82<br>(-4.1-1.1)
Timor-Leste	2010	Female	90 to 94	14%<br>(6-27%)	86%<br>(73-94%)	0.1<br>(0.025-0.26)	-0.15%<br>(-0.56-0.16%)	-0.2%<br>(-0.72-0.17%)	-0.069<br>(-0.26-0.057)	-0.31<br>(-1.2-0.34)
Timor-Leste	2010	Female	95 plus	18%<br>(6.9-36%)	82%<br>(64-93%)	0.13<br>(0.024-0.37)	-0.16%<br>(-0.68-0.29%)	-0.23%<br>(-0.89-0.28%)	-0.022<br>(-0.088-0.027)	-0.061<br>(-0.28-0.11)
Timor-Leste	2010	Male	All Ages	23%<br>(18-29%)	77%<br>(71-82%)	0.11<br>(0.078-0.16)	0.62%<br>(0.35-0.94%)	0.71%<br>(0.24-1.2%)	20<br>(6.9-37)	1200<br>(670-1800)
Timor-Leste	2010	Male	15 to 19	21%<br>(12-34%)	79%<br>(66-88%)	0.059<br>(0.026-0.12)	0.79%<br>(0.52-1.1%)	0.74%<br>(0.36-1.3%)	0.4<br>(0.2-0.74)	64<br>(39-96)

Location	Year	Sex	Age	Prevalence of current drinkers	Prevalence of abstainers	Population average of standard drinks daily	Percent of total attributable deaths	Percent of total attributable DALYs	Attributable deaths	Attributable DALYs
Timor-Leste	2010	Male	20 to 24	32%<br>(17-49%)	68%<br>(51-83%)	0.14<br>(0.066-0.25)	1.7%<br>(1.1-2.3%)	1.4%<br>(0.54-2.5%)	0.87<br>(0.33-1.6)	130<br>(82-190)
Timor-Leste	2010	Male	25 to 29	37%<br>(21-55%)	63%<br>(45-79%)	0.2<br>(0.1-0.36)	1.9%<br>(1.1-2.7%)	1.6%<br>(0.22-3%)	0.98<br>(0.13-1.9)	130<br>(76-200)
Timor-Leste	2010	Male	30 to 34	41%<br>(23-61%)	59%<br>(39-77%)	0.25<br>(0.13-0.44)	2%<br>(1.1-3%)	2.1%<br>(0.27-4%)	1<br>(0.13-2)	110<br>(54-160)
Timor-Leste	2010	Male	35 to 39	43%<br>(26-62%)	57%<br>(38-74%)	0.29<br>(0.14-0.48)	2.1%<br>(0.98-3.4%)	2.6%<br>(0.29-4.8%)	1.7<br>(0.18-3.5)	140<br>(62-230)
Timor-Leste	2010	Male	40 to 44	43%<br>(26-63%)	57%<br>(37-74%)	0.3<br>(0.15-0.51)	2%<br>(0.88-3.2%)	2.7%<br>(0.38-4.7%)	2<br>(0.29-3.9)	130<br>(55-220)
Timor-Leste	2010	Male	45 to 49	42%<br>(23-61%)	58%<br>(39-77%)	0.3<br>(0.14-0.53)	1.9%<br>(0.75-3.2%)	2.6%<br>(0.52-4.8%)	2.3<br>(0.46-4.4)	120<br>(46-210)
Timor-Leste	2010	Male	50 to 54	39%<br>(22-58%)	61%<br>(42-78%)	0.28<br>(0.13-0.49)	1.6%<br>(0.61-2.6%)	2.1%<br>(0.56-3.7%)	2.1<br>(0.53-3.9)	92<br>(35-160)
Timor-Leste	2010	Male	55 to 59	37%<br>(21-56%)	63%<br>(44-79%)	0.25<br>(0.11-0.44)	1.2%<br>(0.55-2.1%)	1.6%<br>(0.46-2.8%)	1.6<br>(0.45-3)	62<br>(26-110)
Timor-Leste	2010	Male	60 to 64	34%<br>(18-54%)	66%<br>(46-82%)	0.21<br>(0.096-0.4)	0.97%<br>(0.32-1.6%)	1.2%<br>(0.24-2.1%)	3.5<br>(0.73-6.8)	110<br>(35-200)
Timor-Leste	2010	Male	65 to 69	31%<br>(17-48%)	69%<br>(52-83%)	0.17<br>(0.079-0.32)	0.66%<br>(0.14-1.2%)	0.76%<br>(0.011-1.5%)	1.9<br>(0.024-4)	52<br>(11-99)
Timor-Leste	2010	Male	70 to 74	27%<br>(15-44%)	73%<br>(56-85%)	0.14<br>(0.059-0.29)	0.49%<br>(0.046-0.95%)	0.56%<br>(-0.04-1.2%)	1.2<br>(-0.083-2.6)	25<br>(2.3-51)
Timor-Leste	2010	Male	75 to 79	25%<br>(14-40%)	75%<br>(60-86%)	0.12<br>(0.048-0.24)	0.29%<br>(-0.14-0.64%)	0.29%<br>(-0.27-0.75%)	0.54<br>(-0.47-1.4)	9.8<br>(-4.5-22)
Timor-Leste	2010	Male	80 to 84	23%<br>(12-38%)	77%<br>(62-88%)	0.1<br>(0.042-0.21)	0.22%<br>(-0.17-0.53%)	0.2%<br>(-0.27-0.62%)	0.24<br>(-0.33-0.74)	3.3<br>(-2.7-8.3)
Timor-Leste	2010	Male	85 to 89	21%<br>(11-35%)	79%<br>(65-89%)	0.09<br>(0.036-0.18)	0.13%<br>(-0.18-0.38%)	0.1%<br>(-0.28-0.42%)	0.067<br>(-0.18-0.27)	0.75<br>(-1.1-2.3)
Timor-Leste	2010	Male	90 to 94	22%<br>(12-38%)	78%<br>(62-88%)	0.087<br>(0.034-0.18)	0.054%<br>(-0.27-0.28%)	0.016%<br>(-0.38-0.3%)	0.0044<br>(-0.11-0.079)	0.093<br>(-0.46-0.48)
Timor-Leste	2010	Male	95 plus	26%<br>(11-45%)	74%<br>(55-89%)	0.09<br>(0.026-0.2)	0.075%<br>(-0.3-0.31%)	-0.0043%<br>(-0.48-0.29%)	-0.00033<br>(-0.039-0.024)	0.025<br>(-0.099-0.1)
Timor-Leste	2016	Female	All Ages	7.8%<br>(5.6-10%)	92%<br>(90-94%)	0.04<br>(0.023-0.064)	0.26%<br>(0.15-0.38%)	0.15%<br>(-0.078-0.38%)	4.1<br>(-2.1-11)	380<br>(220-570)
Timor-Leste	2016	Female	15 to 19	7.6%<br>(3.2-14%)	92%<br>(86-97%)	0.036<br>(0.01-0.083)	0.44%<br>(0.26-0.68%)	0.25%<br>(0.11-0.46%)	0.091<br>(0.034-0.18)	32<br>(18-53)
Timor-Leste	2016	Female	20 to 24	9.1%<br>(3.8-17%)	91%<br>(83-96%)	0.047<br>(0.015-0.11)	0.84%<br>(0.52-1.3%)	0.23%<br>(0.089-0.44%)	0.091<br>(0.032-0.19)	58<br>(34-92)
Timor-Leste	2016	Female	25 to 29	9.8%<br>(4.1-18%)	90%<br>(82-96%)	0.054<br>(0.018-0.12)	0.83%<br>(0.55-1.1%)	0.25%<br>(0.033-0.47%)	0.097<br>(0.013-0.21)	50<br>(32-76)
Timor-Leste	2016	Female	30 to 34	11%<br>(4.4-20%)	89%<br>(80-96%)	0.061<br>(0.019-0.14)	0.75%<br>(0.5-1.1%)	0.35%<br>(0.045-0.6%)	0.15<br>(0.019-0.28)	44<br>(27-65)
Timor-Leste	2016	Female	35 to 39	12%<br>(5-22%)	88%<br>(78-95%)	0.069<br>(0.021-0.15)	0.66%<br>(0.45-0.9%)	0.47%<br>(0.11-0.79%)	0.19<br>(0.053-0.36)	30<br>(20-44)

Location	Year	Sex	Age	Prevalence of current drinkers	Prevalence of abstainers	Population average of standard drinks daily	Percent of total attributable deaths	Percent of total attributable DALYs	Attributable deaths	Attributable DALYs
Timor-Leste	2016	Female	40 to 44	12% (5.5-22%)	88% (78-94%)	0.078 (0.025-0.18)	0.6% (0.39-0.83%)	0.59% (0.21-0.96%)	0.36 (0.11-0.62)	35 (21-51)
Timor-Leste	2016	Female	45 to 49	12% (5.3-22%)	88% (78-95%)	0.088 (0.028-0.2)	0.55% (0.35-0.79%)	0.62% (0.28-1%)	0.51 (0.2-0.91)	34 (20-52)
Timor-Leste	2016	Female	50 to 54	13% (5.6-25%)	87% (75-94%)	0.099 (0.031-0.23)	0.55% (0.27-0.88%)	0.69% (0.28-1.2%)	0.7 (0.26-1.3)	34 (15-57)
Timor-Leste	2016	Female	55 to 59	13% (5.5-25%)	87% (75-95%)	0.11 (0.033-0.26)	0.42% (0.093-0.78%)	0.54% (0.063-1.1%)	0.65 (0.061-1.4)	25 (5-49)
Timor-Leste	2016	Female	60 to 64	13% (5.6-25%)	87% (75-94%)	0.11 (0.032-0.27)	0.28% (-0.095-0.65%)	0.35% (-0.14-0.87%)	0.42 (-0.16-1.1)	13 (-4.8-32)
Timor-Leste	2016	Female	65 to 69	13% (5.6-23%)	87% (77-94%)	0.1 (0.031-0.23)	0.15% (-0.25-0.51%)	0.19% (-0.35-0.65%)	0.66 (-1.3-2.3)	17 (-28-55)
Timor-Leste	2016	Female	70 to 74	12% (5.6-22%)	88% (78-94%)	0.095 (0.027-0.23)	0.12% (-0.24-0.42%)	0.15% (-0.31-0.54%)	0.53 (-1.2-2.1)	10 (-22-39)
Timor-Leste	2016	Female	75 to 79	12% (5-22%)	88% (78-95%)	0.089 (0.024-0.22)	0.0087% (-0.37-0.3%)	0.0034% (-0.48-0.39%)	0.0094 (-1.2-1)	0.44 (-17-15)
Timor-Leste	2016	Female	80 to 84	12% (5-22%)	88% (78-95%)	0.089 (0.024-0.23)	-0.035% (-0.42-0.23%)	-0.051% (-0.53-0.29%)	-0.1 (-1.1-0.57)	-0.93 (-11-6.1)
Timor-Leste	2016	Female	85 to 89	13% (5.4-23%)	87% (77-95%)	0.096 (0.026-0.26)	-0.097% (-0.5-0.21%)	-0.13% (-0.63-0.22%)	-0.18 (-0.89-0.32)	-1.2 (-6.5-2.6)
Timor-Leste	2016	Female	90 to 94	15% (6-27%)	85% (73-94%)	0.11 (0.025-0.29)	-0.14% (-0.57-0.21%)	-0.19% (-0.73-0.22%)	-0.1 (-0.39-0.13)	-0.46 (-1.9-0.7)
Timor-Leste	2016	Female	95 plus	18% (6.8-35%)	82% (65-93%)	0.14 (0.026-0.41)	-0.15% (-0.69-0.32%)	-0.22% (-0.9-0.33%)	-0.029 (-0.12-0.047)	-0.079 (-0.38-0.18)
Timor-Leste	2016	Male	All Ages	23% (18-30%)	77% (70-82%)	0.12 (0.07-0.18)	0.79% (0.45-1.2%)	0.83% (0.31-1.4%)	24 (8.8-43)	1400 (780-2000)
Timor-Leste	2016	Male	15 to 19	21% (12-34%)	79% (66-88%)	0.062 (0.025-0.13)	0.83% (0.53-1.2%)	0.81% (0.41-1.5%)	0.46 (0.21-0.85)	73 (45-110)
Timor-Leste	2016	Male	20 to 24	32% (18-50%)	68% (50-82%)	0.15 (0.063-0.29)	1.7% (1.1-2.5%)	1.5% (0.62-2.8%)	1.1 (0.4-2)	150 (94-230)
Timor-Leste	2016	Male	25 to 29	38% (21-56%)	62% (44-79%)	0.21 (0.093-0.38)	2% (1.2-2.9%)	1.8% (0.25-3.3%)	1.2 (0.15-2.4)	150 (83-230)
Timor-Leste	2016	Male	30 to 34	41% (22-61%)	59% (39-78%)	0.26 (0.12-0.47)	2.2% (1.2-3.2%)	2.2% (0.27-4.2%)	1.5 (0.17-3)	150 (76-240)
Timor-Leste	2016	Male	35 to 39	43% (26-62%)	57% (38-74%)	0.3 (0.13-0.54)	2.3% (1.1-3.6%)	2.8% (0.35-5%)	1.6 (0.17-3.2)	120 (57-200)
Timor-Leste	2016	Male	40 to 44	43% (25-63%)	57% (37-75%)	0.31 (0.14-0.58)	2.2% (0.91-3.5%)	2.8% (0.34-4.9%)	2.3 (0.26-4.3)	140 (58-240)
Timor-Leste	2016	Male	45 to 49	42% (24-60%)	58% (40-76%)	0.31 (0.14-0.57)	2.1% (0.93-3.3%)	2.8% (0.77-4.9%)	2.9 (0.74-5.4)	150 (64-250)
Timor-Leste	2016	Male	50 to 54	40% (22-60%)	60% (40-78%)	0.29 (0.12-0.55)	1.7% (0.77-2.7%)	2.3% (0.8-3.8%)	2.7 (0.84-5)	120 (54-210)
Timor-Leste	2016	Male	55 to 59	37% (21-57%)	63% (43-79%)	0.26 (0.11-0.51)	1.3% (0.59-2.1%)	1.7% (0.56-2.9%)	2.4 (0.76-4.4)	90 (39-160)

Location	Year	Sex	Age	Prevalence of current drinkers	Prevalence of abstainers	Population average of standard drinks daily	Percent of total attributable deaths	Percent of total attributable DALYs	Attributable deaths	Attributable DALYs
Timor-Leste	2016	Male	60 to 64	34% (18-54%)	66% (46-82%)	0.22 (0.09-0.44)	1% (0.41-1.7%)	1.3% (0.36-2.2%)	1.7 (0.48-3.3)	55 (21-97)
Timor-Leste	2016	Male	65 to 69	31% (17-49%)	69% (51-83%)	0.19 (0.074-0.36)	0.71% (0.18-1.3%)	0.83% (0.085-1.6%)	3 (0.34-6.1)	80 (20-150)
Timor-Leste	2016	Male	70 to 74	28% (15-45%)	72% (55-85%)	0.15 (0.06-0.31)	0.53% (0.083-1%)	0.61% (-0.015-1.2%)	2.1 (-0.048-4.4)	45 (6.9-86)
Timor-Leste	2016	Male	75 to 79	25% (13-41%)	75% (59-87%)	0.13 (0.046-0.26)	0.33% (-0.12-0.68%)	0.34% (-0.23-0.8%)	0.76 (-0.53-1.8)	13 (-5-29)
Timor-Leste	2016	Male	80 to 84	23% (11-38%)	77% (62-89%)	0.11 (0.039-0.24)	0.24% (-0.14-0.55%)	0.23% (-0.25-0.62%)	0.35 (-0.37-0.96)	4.7 (-2.7-11)
Timor-Leste	2016	Male	85 to 89	21% (11-35%)	79% (65-89%)	0.094 (0.034-0.2)	0.14% (-0.18-0.38%)	0.12% (-0.27-0.41%)	0.13 (-0.3-0.45)	1.4 (-1.8-3.7)
Timor-Leste	2016	Male	90 to 94	22% (12-37%)	78% (63-88%)	0.092 (0.033-0.2)	0.061% (-0.27-0.29%)	0.023% (-0.39-0.3%)	0.0091 (-0.15-0.12)	0.15 (-0.67-0.68)
Timor-Leste	2016	Male	95 plus	26% (12-45%)	74% (55-88%)	0.095 (0.027-0.21)	0.084% (-0.32-0.32%)	0.0064% (-0.5-0.3%)	0.00064 (-0.047-0.029)	0.033 (-0.12-0.13)
Vietnam	1990	Female	All Ages	9.3% (7.8-11%)	91% (89-92%)	0.13 (0.084-0.18)	0.86% (0.5-1.3%)	0.8% (0.074-1.9%)	2000 (180-4700)	90000 (51000-140000)
Vietnam	1990	Female	15 to 19	9.3% (5.8-14%)	91% (86-94%)	0.13 (0.062-0.23)	1.1% (0.7-1.5%)	0.8% (0.26-1.6%)	22 (6.9-42)	5200 (3200-7800)
Vietnam	1990	Female	20 to 24	11% (6.6-15%)	89% (85-93%)	0.15 (0.075-0.28)	2.2% (1.4-3.2%)	1% (0.23-2.3%)	33 (6.9-74)	11000 (6400-17000)
Vietnam	1990	Female	25 to 29	11% (7-17%)	89% (83-93%)	0.16 (0.081-0.29)	2.2% (1.5-3.2%)	1.1% (0.15-2.6%)	40 (5-97)	11000 (6600-17000)
Vietnam	1990	Female	30 to 34	12% (7.4-18%)	88% (82-93%)	0.18 (0.089-0.31)	1.9% (1.2-2.7%)	1.1% (0.31-2.3%)	44 (12-93)	8900 (5400-14000)
Vietnam	1990	Female	35 to 39	12% (7.6-18%)	88% (82-92%)	0.2 (0.098-0.35)	1.7% (1.1-2.6%)	1.4% (0.44-2.8%)	56 (18-120)	7300 (4300-11000)
Vietnam	1990	Female	40 to 44	12% (7.7-17%)	88% (83-92%)	0.21 (0.11-0.37)	1.6% (1-2.5%)	1.6% (0.7-2.9%)	60 (27-110)	5400 (3200-8200)
Vietnam	1990	Female	45 to 49	12% (7.1-17%)	88% (83-93%)	0.23 (0.11-0.44)	1.5% (0.95-2.5%)	1.6% (0.7-3.2%)	76 (32-150)	5000 (2900-8400)
Vietnam	1990	Female	50 to 54	12% (8.1-18%)	88% (82-92%)	0.26 (0.13-0.47)	1.4% (0.78-2.4%)	1.6% (0.63-3.1%)	110 (44-230)	5700 (3000-10000)
Vietnam	1990	Female	55 to 59	13% (8.3-19%)	87% (81-92%)	0.29 (0.15-0.52)	1.3% (0.59-2.4%)	1.5% (0.51-3.1%)	170 (53-360)	6700 (2900-13000)
Vietnam	1990	Female	60 to 64	13% (7.9-19%)	87% (81-92%)	0.29 (0.14-0.51)	1.2% (0.4-2.4%)	1.5% (0.37-3.1%)	210 (50-460)	6600 (2100-14000)
Vietnam	1990	Female	65 to 69	11% (7.2-16%)	89% (84-93%)	0.26 (0.13-0.44)	0.92% (0.16-2.1%)	1.1% (0.11-2.6%)	220 (23-540)	5500 (970-12000)
Vietnam	1990	Female	70 to 74	11% (7.1-16%)	89% (84-93%)	0.25 (0.13-0.45)	0.78% (0.007-2.1%)	0.93% (-0.06-2.5%)	230 (-12-640)	4400 (30-12000)
Vietnam	1990	Female	75 to 79	11% (7.2-17%)	89% (83-93%)	0.25 (0.12-0.46)	0.61% (-0.19-2%)	0.71% (-0.27-2.4%)	220 (-78-750)	3300 (-950-11000)

Location	Year	Sex	Age	Prevalence of current drinkers	Prevalence of abstainers	Population average of standard drinks daily	Percent of total attributable deaths	Percent of total attributable DALYs	Attributable deaths	Attributable DALYs
Vietnam	1990	Female	80 to 84	12% (7.5-17%)	88% (83-93%)	0.27 (0.13-0.46)	0.65% (-0.2-2%)	0.77% (-0.28-2.4%)	260 (-94-820)	2800 (-840-8400)
Vietnam	1990	Female	85 to 89	13% (8.2-20%)	87% (80-92%)	0.3 (0.14-0.53)	0.55% (-0.27-1.8%)	0.64% (-0.34-2.2%)	170 (-90-600)	1300 (-590-4300)
Vietnam	1990	Female	90 to 94	16% (9.7-24%)	84% (76-90%)	0.34 (0.16-0.62)	0.41% (-0.44-1.8%)	0.44% (-0.58-2.1%)	60 (-77-280)	330 (-350-1400)
Vietnam	1990	Female	95 plus	19% (10-31%)	81% (69-90%)	0.41 (0.16-0.84)	0.4% (-0.66-2.1%)	0.38% (-0.88-2.4%)	16 (-37-100)	67 (-110-360)
Vietnam	1990	Male	All Ages	43% (37-49%)	57% (51-63%)	0.75 (0.52-1)	3.4% (2-5%)	4.2% (1.9-6.6%)	11000 (4900-17000)	420000 (250000-640000)
Vietnam	1990	Male	15 to 19	35% (25-46%)	65% (54-75%)	0.39 (0.2-0.61)	2.2% (1.1-3.7%)	2.8% (1.1-5%)	150 (54-280)	14000 (6900-23000)
Vietnam	1990	Male	20 to 24	52% (40-63%)	48% (37-60%)	0.89 (0.53-1.4)	5.2% (3-7.9%)	6% (2.9-9.7%)	450 (200-770)	38000 (21000-60000)
Vietnam	1990	Male	25 to 29	62% (51-71%)	38% (29-49%)	1.2 (0.73-1.7)	6.6% (3.6-10%)	8% (3.6-13%)	630 (280-1100)	46000 (24000-73000)
Vietnam	1990	Male	30 to 34	70% (58-78%)	30% (22-42%)	1.5 (0.97-2.1)	7.7% (4.4-12%)	9.7% (5-15%)	770 (370-1200)	50000 (27000-78000)
Vietnam	1990	Male	35 to 39	72% (62-79%)	28% (21-38%)	1.8 (1.2-2.7)	8.8% (5.2-13%)	11% (6.3-17%)	880 (460-1400)	50000 (28000-76000)
Vietnam	1990	Male	40 to 44	71% (61-79%)	29% (21-39%)	1.9 (1.2-2.8)	8.7% (5.3-12%)	11% (6.5-16%)	700 (390-1100)	35000 (20000-53000)
Vietnam	1990	Male	45 to 49	66% (53-75%)	34% (25-47%)	1.7 (1.1-2.5)	8% (4.8-12%)	10% (5.7-15%)	730 (410-1100)	32000 (18000-49000)
Vietnam	1990	Male	50 to 54	63% (51-72%)	37% (28-49%)	1.6 (0.99-2.4)	7.2% (4.1-11%)	8.9% (4.9-13%)	900 (480-1400)	34000 (19000-53000)
Vietnam	1990	Male	55 to 59	61% (48-71%)	39% (29-52%)	1.6 (1-2.4)	6.6% (3.3-10%)	8.1% (4-13%)	1200 (580-1900)	39000 (20000-63000)
Vietnam	1990	Male	60 to 64	57% (44-69%)	43% (31-56%)	1.5 (0.87-2.3)	5.2% (2.2-8.7%)	6.2% (2.5-10%)	1100 (440-1900)	31000 (13000-53000)
Vietnam	1990	Male	65 to 69	53% (41-65%)	47% (35-59%)	1.3 (0.74-1.9)	3.9% (1.4-6.9%)	4.5% (1.5-8.1%)	990 (320-1800)	23000 (8000-41000)
Vietnam	1990	Male	70 to 74	50% (38-63%)	50% (37-62%)	1.1 (0.61-1.8)	3.3% (0.67-6.5%)	3.8% (0.72-7.7%)	890 (150-1800)	16000 (3000-33000)
Vietnam	1990	Male	75 to 79	49% (35-64%)	51% (36-65%)	0.98 (0.51-1.7)	2.3% (-0.14-5.4%)	2.6% (-0.25-6.3%)	600 (-58-1500)	8700 (-500-21000)
Vietnam	1990	Male	80 to 84	46% (30-61%)	54% (39-70%)	0.82 (0.44-1.4)	1.9% (-0.36-4.8%)	2.2% (-0.5-5.6%)	400 (-91-1000)	4200 (-840-11000)
Vietnam	1990	Male	85 to 89	42% (28-57%)	58% (43-72%)	0.67 (0.35-1.2)	1% (-0.68-3.1%)	1.1% (-0.88-3.6%)	130 (-99-430)	1000 (-660-3200)
Vietnam	1990	Male	90 to 94	41% (28-56%)	59% (44-72%)	0.58 (0.29-1)	0.36% (-0.98-2.1%)	0.33% (-1.3-2.3%)	16 (-62-120)	100 (-280-610)
Vietnam	1990	Male	95 plus	43% (27-61%)	57% (39-73%)	0.52 (0.22-1)	0.075% (-1.2-1.5%)	-0.084% (-1.6-1.7%)	-0.99 (-19-20)	3.6 (-55-75)

Location	Year	Sex	Age	Prevalence of current drinkers	Prevalence of abstainers	Population average of standard drinks daily	Percent of total attributable deaths	Percent of total attributable DALYs	Attributable deaths	Attributable DALYs
Vietnam	1995	Female	All Ages	9.6% (8-11%)	90% (89-92%)	0.19 (0.13-0.26)	1.3% (0.79-2%)	1.5% (0.47-3%)	3900 (1200-7900)	130000 (78000-200000)
Vietnam	1995	Female	15 to 19	9.3% (5.8-14%)	91% (86-94%)	0.19 (0.088-0.33)	1.2% (0.82-1.8%)	1.1% (0.45-2.1%)	26 (11-50)	5800 (3700-8600)
Vietnam	1995	Female	20 to 24	11% (6.5-15%)	89% (85-93%)	0.22 (0.11-0.39)	2.6% (1.7-3.7%)	1.5% (0.52-3.1%)	43 (14-88)	12000 (7700-19000)
Vietnam	1995	Female	25 to 29	11% (7.2-17%)	89% (83-93%)	0.23 (0.12-0.41)	2.6% (1.8-3.7%)	1.8% (0.6-3.6%)	56 (17-120)	13000 (7800-19000)
Vietnam	1995	Female	30 to 34	12% (7.3-19%)	88% (81-93%)	0.25 (0.13-0.44)	2.3% (1.5-3.2%)	1.7% (0.74-3.3%)	64 (25-120)	11000 (7000-16000)
Vietnam	1995	Female	35 to 39	12% (7.5-18%)	88% (82-93%)	0.28 (0.14-0.47)	2.1% (1.4-3.1%)	2.1% (0.91-3.8%)	97 (40-190)	11000 (6600-16000)
Vietnam	1995	Female	40 to 44	12% (7.9-17%)	88% (83-92%)	0.3 (0.16-0.5)	2% (1.3-3%)	2.2% (1.1-4%)	120 (55-210)	9500 (5800-14000)
Vietnam	1995	Female	45 to 49	12% (7-17%)	88% (83-93%)	0.33 (0.16-0.59)	2% (1.2-3.2%)	2.3% (1.1-4.3%)	120 (51-230)	7400 (4200-12000)
Vietnam	1995	Female	50 to 54	13% (8.2-19%)	87% (81-92%)	0.37 (0.19-0.65)	2% (1.1-3.4%)	2.4% (1-4.6%)	160 (60-290)	7400 (3900-13000)
Vietnam	1995	Female	55 to 59	13% (8.3-19%)	87% (81-92%)	0.42 (0.22-0.72)	1.9% (0.93-3.5%)	2.4% (0.97-4.7%)	230 (86-450)	8600 (4000-16000)
Vietnam	1995	Female	60 to 64	13% (8.1-19%)	87% (81-92%)	0.41 (0.2-0.74)	1.9% (0.79-3.6%)	2.4% (0.86-4.6%)	370 (130-740)	11000 (4500-21000)
Vietnam	1995	Female	65 to 69	11% (7.2-16%)	89% (84-93%)	0.37 (0.19-0.61)	1.6% (0.56-3.1%)	1.9% (0.6-4%)	390 (110-810)	9500 (3100-19000)
Vietnam	1995	Female	70 to 74	11% (7-15%)	89% (85-93%)	0.35 (0.18-0.62)	1.4% (0.36-3.1%)	1.7% (0.41-3.9%)	470 (100-1100)	9000 (2200-21000)
Vietnam	1995	Female	75 to 79	11% (7.2-17%)	89% (83-93%)	0.36 (0.18-0.65)	1.2% (0.15-3%)	1.5% (0.16-3.6%)	470 (49-1200)	6800 (860-17000)
Vietnam	1995	Female	80 to 84	12% (7.7-17%)	88% (83-92%)	0.38 (0.19-0.64)	1.3% (0.17-3.1%)	1.6% (0.19-3.7%)	610 (69-1500)	6400 (780-15000)
Vietnam	1995	Female	85 to 89	13% (8.1-20%)	87% (80-92%)	0.42 (0.21-0.73)	1.2% (0.069-2.8%)	1.4% (0.047-3.4%)	460 (15-1100)	3400 (190-8300)
Vietnam	1995	Female	90 to 94	16% (9.7-23%)	84% (77-90%)	0.48 (0.23-0.89)	1% (-0.14-2.9%)	1.2% (-0.21-3.5%)	200 (-37-580)	1100 (-140-3000)
Vietnam	1995	Female	95 plus	19% (10-31%)	81% (69-90%)	0.57 (0.22-1.2)	1.1% (-0.33-3.4%)	1.2% (-0.49-3.9%)	70 (-30-240)	250 (-76-820)
Vietnam	1995	Male	All Ages	44% (39-50%)	56% (50-61%)	1.1 (0.81-1.5)	5.1% (3.4-7%)	6.4% (3.8-9.2%)	17000 (9700-25000)	640000 (420000-900000)
Vietnam	1995	Male	15 to 19	36% (26-46%)	64% (54-74%)	0.55 (0.3-0.87)	2.8% (1.6-4.4%)	3.6% (1.7-6.2%)	200 (85-370)	18000 (9900-30000)
Vietnam	1995	Male	20 to 24	52% (39-63%)	48% (37-61%)	1.3 (0.78-1.8)	6.5% (4.2-9.5%)	7.8% (4.4-12%)	620 (320-1000)	50000 (30000-79000)
Vietnam	1995	Male	25 to 29	62% (51-72%)	38% (28-49%)	1.7 (1.1-2.4)	8.4% (5.2-12%)	11% (6.1-16%)	870 (460-1400)	62000 (38000-94000)

Location	Year	Sex	Age	Prevalence of current drinkers	Prevalence of abstainers	Population average of standard drinks daily	Percent of total attributable deaths	Percent of total attributable DALYs	Attributable deaths	Attributable DALYs
Vietnam	1995	Male	30 to 34	69% (58-78%)	31% (22-42%)	2.1 (1.4-3)	9.8% (6.4-14%)	13% (7.9-18%)	1100 (640-1700)	70000 (43000-100000)
Vietnam	1995	Male	35 to 39	72% (61-80%)	28% (20-39%)	2.6 (1.7-3.7)	11% (7.4-15%)	14% (9.4-20%)	1400 (830-2000)	77000 (49000-110000)
Vietnam	1995	Male	40 to 44	71% (61-79%)	29% (21-39%)	2.7 (1.7-3.8)	11% (7.3-15%)	14% (9.4-19%)	1300 (830-2000)	67000 (43000-96000)
Vietnam	1995	Male	45 to 49	66% (54-76%)	34% (24-46%)	2.4 (1.5-3.5)	10% (6.8-15%)	13% (8.5-19%)	1100 (670-1600)	48000 (31000-70000)
Vietnam	1995	Male	50 to 54	63% (52-72%)	37% (28-48%)	2.3 (1.4-3.4)	9.7% (5.9-14%)	12% (7.3-17%)	1200 (680-1700)	44000 (26000-66000)
Vietnam	1995	Male	55 to 59	61% (48-70%)	39% (30-52%)	2.3 (1.4-3.4)	9.2% (5.5-14%)	11% (6.7-17%)	1500 (870-2300)	50000 (29000-76000)
Vietnam	1995	Male	60 to 64	57% (44-68%)	43% (32-56%)	2.1 (1.3-3.1)	7.6% (4.2-12%)	9% (4.9-14%)	1900 (960-3000)	52000 (27000-82000)
Vietnam	1995	Male	65 to 69	53% (40-65%)	47% (35-60%)	1.8 (1.1-2.7)	5.9% (2.8-9.7%)	6.9% (3.2-11%)	1600 (750-2700)	38000 (18000-62000)
Vietnam	1995	Male	70 to 74	50% (38-62%)	50% (38-62%)	1.6 (0.88-2.4)	5.2% (2-9%)	6.1% (2.3-11%)	1700 (630-2900)	30000 (12000-53000)
Vietnam	1995	Male	75 to 79	49% (34-64%)	51% (36-66%)	1.4 (0.75-2.2)	3.9% (0.85-7.4%)	4.4% (0.86-8.5%)	1200 (220-2300)	17000 (3600-33000)
Vietnam	1995	Male	80 to 84	46% (30-61%)	54% (39-70%)	1.2 (0.63-1.8)	3.3% (0.54-6.7%)	3.8% (0.51-7.8%)	880 (120-1800)	9300 (1500-18000)
Vietnam	1995	Male	85 to 89	42% (28-58%)	58% (42-72%)	0.94 (0.51-1.5)	2% (-0.048-4.6%)	2.3% (-0.15-5.3%)	350 (-23-810)	2700 (-64-6000)
Vietnam	1995	Male	90 to 94	42% (28-57%)	58% (43-72%)	0.82 (0.42-1.4)	1% (-0.57-3.2%)	1.1% (-0.84-3.6%)	72 (-55-240)	400 (-220-1200)
Vietnam	1995	Male	95 plus	43% (26-62%)	57% (38-74%)	0.72 (0.31-1.4)	0.5% (-1-2.5%)	0.39% (-1.4-2.7%)	6.8 (-24-48)	34 (-69-170)
Vietnam	2000	Female	All Ages	8.8% (7.3-11%)	91% (89-93%)	0.29 (0.2-0.4)	2.1% (1.4-3.1%)	3.1% (1.5-5.1%)	8500 (3900-14000)	220000 (130000-310000)
Vietnam	2000	Female	15 to 19	9% (5.7-13%)	91% (87-94%)	0.3 (0.15-0.53)	1.5% (1-2.2%)	1.7% (0.74-3.2%)	39 (17-73)	7300 (4600-11000)
Vietnam	2000	Female	20 to 24	9.8% (6.3-14%)	90% (86-94%)	0.34 (0.17-0.6)	3% (2-4.2%)	2.3% (1-4.3%)	60 (25-120)	14000 (8900-22000)
Vietnam	2000	Female	25 to 29	10% (6.6-15%)	90% (85-93%)	0.35 (0.18-0.6)	3.1% (2.2-4.3%)	2.7% (1.3-5%)	80 (34-160)	15000 (9700-22000)
Vietnam	2000	Female	30 to 34	10% (6.9-16%)	90% (84-93%)	0.37 (0.2-0.61)	2.7% (1.8-3.7%)	2.6% (1.4-4.4%)	91 (45-170)	14000 (8600-20000)
Vietnam	2000	Female	35 to 39	10% (6.3-15%)	90% (85-94%)	0.38 (0.2-0.64)	2.7% (1.8-3.8%)	3% (1.6-5.3%)	140 (68-250)	14000 (9100-21000)
Vietnam	2000	Female	40 to 44	9.7% (6.3-14%)	90% (86-94%)	0.4 (0.21-0.65)	2.5% (1.7-3.7%)	3.1% (1.7-5.1%)	190 (100-320)	14000 (9000-21000)
Vietnam	2000	Female	45 to 49	9.9% (6.1-14%)	90% (86-94%)	0.44 (0.22-0.81)	2.6% (1.6-4.1%)	3.4% (1.8-5.8%)	240 (120-420)	14000 (8200-22000)

Location	Year	Sex	Age	Prevalence of current drinkers	Prevalence of abstainers	Population average of standard drinks daily	Percent of total attributable deaths	Percent of total attributable DALYs	Attributable deaths	Attributable DALYs
Vietnam	2000	Female	50 to 54	11% (7.6-16%)	89% (84-92%)	0.53 (0.28-0.93)	2.9% (1.7-4.6%)	3.8% (1.8-6.5%)	270 (130-470)	12000 (6800-20000)
Vietnam	2000	Female	55 to 59	12% (8.1-17%)	88% (83-92%)	0.64 (0.34-1.1)	3.1% (1.6-5.2%)	4.1% (2-7.1%)	360 (170-630)	13000 (7000-22000)
Vietnam	2000	Female	60 to 64	12% (7.7-17%)	88% (83-92%)	0.64 (0.32-1.1)	3.3% (1.6-5.6%)	4.2% (2-7.4%)	570 (260-990)	17000 (8300-29000)
Vietnam	2000	Female	65 to 69	11% (6.9-15%)	89% (85-93%)	0.57 (0.3-0.93)	2.9% (1.3-4.9%)	3.7% (1.7-6.3%)	800 (340-1400)	19000 (8700-34000)
Vietnam	2000	Female	70 to 74	9.9% (6.6-14%)	90% (86-93%)	0.53 (0.27-0.93)	2.6% (1.1-4.9%)	3.3% (1.4-6.1%)	920 (360-1800)	17000 (7000-33000)
Vietnam	2000	Female	75 to 79	11% (6.7-16%)	89% (84-93%)	0.56 (0.28-1)	2.5% (0.87-4.8%)	3.1% (1.1-5.9%)	1100 (340-2200)	16000 (5100-31000)
Vietnam	2000	Female	80 to 84	12% (7.7-17%)	88% (83-92%)	0.62 (0.32-1.1)	2.8% (1.1-5.1%)	3.4% (1.3-6.2%)	1500 (540-2800)	16000 (5900-29000)
Vietnam	2000	Female	85 to 89	14% (8.3-20%)	86% (80-92%)	0.71 (0.35-1.2)	2.7% (0.97-5.3%)	3.3% (1.1-6.3%)	1200 (420-2400)	9200 (3200-18000)
Vietnam	2000	Female	90 to 94	16% (9.8-24%)	84% (76-90%)	0.82 (0.39-1.5)	2.6% (0.71-5.4%)	3% (0.78-6.5%)	640 (160-1400)	3300 (900-7000)
Vietnam	2000	Female	95 plus	20% (10-32%)	80% (68-90%)	1 (0.39-2)	2.8% (0.43-6.5%)	3.2% (0.41-7.7%)	250 (33-590)	850 (130-2000)
Vietnam	2000	Male	All Ages	49% (45-54%)	51% (46-55%)	2.1 (1.5-2.7)	8.8% (6.4-11%)	11% (7.3-15%)	32000 (20000-45000)	1100000 (760000-1500000)
Vietnam	2000	Male	15 to 19	34% (26-44%)	66% (56-74%)	0.87 (0.5-1.3)	3.7% (2.3-5.7%)	5.1% (2.8-8.2%)	300 (150-510)	26000 (15000-41000)
Vietnam	2000	Male	20 to 24	53% (43-62%)	47% (38-57%)	2.1 (1.4-3.1)	9.1% (6-13%)	11% (7.1-17%)	930 (550-1400)	73000 (45000-110000)
Vietnam	2000	Male	25 to 29	67% (59-73%)	33% (27-41%)	3 (2-4.3)	12% (8.1-16%)	16% (10-22%)	1300 (790-1900)	90000 (59000-130000)
Vietnam	2000	Male	30 to 34	74% (67-80%)	26% (20-33%)	3.8 (2.6-5.3)	14% (9.7-18%)	18% (13-25%)	1600 (980-2400)	100000 (67000-150000)
Vietnam	2000	Male	35 to 39	76% (70-81%)	24% (19-30%)	4.6 (3.1-6.5)	15% (11-20%)	21% (15-27%)	2000 (1300-2900)	110000 (75000-160000)
Vietnam	2000	Male	40 to 44	75% (69-81%)	25% (19-31%)	4.8 (3.2-6.7)	16% (11-20%)	21% (15-26%)	2300 (1500-3300)	120000 (78000-160000)
Vietnam	2000	Male	45 to 49	71% (63-77%)	29% (23-37%)	4.2 (2.8-6)	15% (11-20%)	20% (14-26%)	2500 (1600-3400)	110000 (72000-150000)
Vietnam	2000	Male	50 to 54	68% (59-75%)	32% (25-41%)	4 (2.6-5.8)	15% (9.9-20%)	19% (12-25%)	2100 (1300-2900)	80000 (51000-110000)
Vietnam	2000	Male	55 to 59	66% (56-73%)	34% (27-44%)	4.1 (2.7-6)	15% (10-21%)	19% (12-25%)	2500 (1600-3400)	81000 (53000-110000)
Vietnam	2000	Male	60 to 64	62% (52-70%)	38% (30-48%)	3.8 (2.4-5.6)	13% (8.1-19%)	16% (9.5-22%)	3000 (1800-4300)	84000 (51000-120000)

Location	Year	Sex	Age	Prevalence of current drinkers	Prevalence of abstainers	Population average of standard drinks daily	Percent of total attributable deaths	Percent of total attributable DALYs	Attributable deaths	Attributable DALYs
Vietnam	2000	Male	65 to 69	59% (49-68%)	41% (32-51%)	3.4 (2.1-4.9)	11% (6.2-16%)	13% (7.2-19%)	3700 (2000-5400)	84000 (45000-120000)
Vietnam	2000	Male	70 to 74	57% (46-67%)	43% (33-54%)	2.9 (1.7-4.4)	10% (5-16%)	12% (5.9-18%)	3600 (1700-5500)	66000 (32000-100000)
Vietnam	2000	Male	75 to 79	53% (40-67%)	47% (33-60%)	2.5 (1.4-4)	7.9% (3.2-13%)	9% (3.6-15%)	2900 (1100-4900)	41000 (17000-69000)
Vietnam	2000	Male	80 to 84	48% (32-63%)	52% (37-68%)	2 (1.1-3.1)	6.5% (2.6-11%)	7.5% (2.9-13%)	2100 (820-3700)	22000 (8900-39000)
Vietnam	2000	Male	85 to 89	43% (29-59%)	57% (41-71%)	1.6 (0.87-2.6)	4.5% (1.5-8.2%)	5.1% (1.6-9.3%)	960 (300-1800)	7300 (2300-13000)
Vietnam	2000	Male	90 to 94	43% (29-58%)	57% (42-71%)	1.4 (0.74-2.4)	2.8% (0.51-6%)	3.2% (0.43-6.8%)	270 (36-580)	1400 (250-3000)
Vietnam	2000	Male	95 plus	44% (28-62%)	56% (38-72%)	1.2 (0.53-2.4)	1.9% (-0.26-5.5%)	1.9% (-0.58-5.9%)	44 (-13-130)	170 (-24-480)
Vietnam	2005	Female	All Ages	9.1% (7.7-11%)	91% (89-92%)	0.47 (0.32-0.66)	3.1% (2.2-4.1%)	4.8% (2.8-7.1%)	14000 (7800-21000)	320000 (210000-440000)
Vietnam	2005	Female	15 to 19	9.2% (5.8-13%)	91% (87-94%)	0.47 (0.23-0.83)	1.8% (1.2-2.8%)	2.6% (1.2-4.8%)	58 (26-110)	9100 (5700-14000)
Vietnam	2005	Female	20 to 24	9.9% (6.3-14%)	90% (86-94%)	0.51 (0.25-0.94)	3.4% (2.3-4.8%)	3.3% (1.5-5.9%)	82 (38-150)	17000 (11000-25000)
Vietnam	2005	Female	25 to 29	10% (6.8-15%)	90% (85-93%)	0.53 (0.28-0.93)	3.5% (2.5-4.8%)	3.5% (1.7-6.1%)	98 (45-180)	17000 (11000-25000)
Vietnam	2005	Female	30 to 34	10% (6.8-16%)	90% (84-93%)	0.55 (0.3-0.92)	3.1% (2.2-4.4%)	3.4% (1.9-5.6%)	120 (57-210)	16000 (10000-24000)
Vietnam	2005	Female	35 to 39	10% (6.4-15%)	90% (85-94%)	0.58 (0.31-0.99)	3.2% (2.2-4.5%)	4.1% (2.3-6.6%)	180 (91-320)	17000 (11000-26000)
Vietnam	2005	Female	40 to 44	9.8% (6.2-14%)	90% (86-94%)	0.6 (0.32-1)	3.2% (2.1-4.6%)	4.3% (2.5-6.7%)	270 (150-440)	19000 (12000-29000)
Vietnam	2005	Female	45 to 49	9.9% (6.1-14%)	90% (86-94%)	0.67 (0.34-1.3)	3.5% (2.2-5.3%)	4.8% (2.7-7.8%)	410 (220-690)	23000 (14000-35000)
Vietnam	2005	Female	50 to 54	11% (7.7-16%)	89% (84-92%)	0.82 (0.44-1.5)	4% (2.4-6%)	5.5% (3.1-8.7%)	550 (310-910)	24000 (15000-38000)
Vietnam	2005	Female	55 to 59	12% (8.1-18%)	88% (82-92%)	0.99 (0.53-1.7)	4.4% (2.6-6.8%)	6% (3.3-9.3%)	590 (320-950)	21000 (12000-33000)
Vietnam	2005	Female	60 to 64	12% (7.8-17%)	88% (83-92%)	0.98 (0.49-1.7)	4.6% (2.5-7.2%)	6.1% (3.3-9.7%)	760 (400-1200)	22000 (12000-35000)
Vietnam	2005	Female	65 to 69	11% (6.9-15%)	89% (85-93%)	0.86 (0.45-1.5)	4.2% (2.3-6.5%)	5.4% (2.9-8.4%)	1000 (520-1700)	25000 (13000-39000)
Vietnam	2005	Female	70 to 74	9.9% (6.7-14%)	90% (86-93%)	0.8 (0.42-1.4)	3.9% (2-6.4%)	4.9% (2.4-8.1%)	1500 (710-2600)	28000 (14000-48000)
Vietnam	2005	Female	75 to 79	11% (6.9-16%)	89% (84-93%)	0.85 (0.43-1.5)	3.8% (1.8-6.4%)	4.7% (2.2-7.9%)	1700 (790-2900)	26000 (12000-43000)
Vietnam	2005	Female	80 to 84	12% (7.5-17%)	88% (83-93%)	0.97 (0.49-1.7)	4.3% (2.1-7%)	5.3% (2.6-8.6%)	2600 (1300-4200)	28000 (13000-45000)

Location	Year	Sex	Age	Prevalence of current drinkers	Prevalence of abstainers	Population average of standard drinks daily	Percent of total attributable deaths	Percent of total attributable DALYs	Attributable deaths	Attributable DALYs
Vietnam	2005	Female	85 to 89	14%<br>(8.5-21%)	86%<br>(79-92%)	1.1<br>(0.54-1.9)	4.4%<br>(2-7.3%)	5.3%<br>(2.4-8.9%)	2300<br>(1000-3900)	17000<br>(7800-29000)
Vietnam	2005	Female	90 to 94	17%<br>(10-24%)	83%<br>(76-90%)	1.3<br>(0.63-2.4)	4.3%<br>(1.8-7.7%)	5.1%<br>(2.1-9.4%)	1300<br>(530-2300)	6600<br>(2800-12000)
Vietnam	2005	Female	95 plus	20%<br>(11-33%)	80%<br>(67-89%)	1.6<br>(0.62-3.2)	4.7%<br>(1.5-9.2%)	5.4%<br>(1.7-11%)	540<br>(160-1100)	1900<br>(600-3600)
Vietnam	2005	Male	All Ages	51%<br>(47-56%)	49%<br>(44-53%)	3.5<br>(2.5-4.5)	12%<br>(9.5-15%)	16%<br>(12-19%)	47000<br>(33000-62000)	1500000<br>(1200000-2000000)
Vietnam	2005	Male	15 to 19	34%<br>(26-44%)	66%<br>(56-74%)	1.3<br>(0.75-2)	4.9%<br>(3-7.5%)	6.9%<br>(3.8-11%)	430<br>(230-700)	36000<br>(21000-56000)
Vietnam	2005	Male	20 to 24	53%<br>(44-62%)	47%<br>(38-56%)	3.3<br>(2.1-4.7)	12%<br>(8-16%)	15%<br>(10-22%)	1300<br>(760-2000)	98000<br>(64000-150000)
Vietnam	2005	Male	25 to 29	67%<br>(58-73%)	33%<br>(27-42%)	4.5<br>(3-6.5)	14%<br>(9.9-19%)	19%<br>(13-26%)	1600<br>(910-2300)	110000<br>(69000-160000)
Vietnam	2005	Male	30 to 34	74%<br>(67-80%)	26%<br>(20-33%)	5.7<br>(3.9-8)	16%<br>(12-20%)	22%<br>(16-28%)	1900<br>(1200-2800)	120000<br>(79000-170000)
Vietnam	2005	Male	35 to 39	76%<br>(70-82%)	24%<br>(18-30%)	7<br>(4.6-9.9)	18%<br>(13-22%)	25%<br>(19-30%)	2500<br>(1600-3500)	140000<br>(98000-190000)
Vietnam	2005	Male	40 to 44	75%<br>(69-81%)	25%<br>(19-31%)	7.3<br>(4.9-10)	19%<br>(14-22%)	25%<br>(20-29%)	3100<br>(2200-4300)	160000<br>(110000-210000)
Vietnam	2005	Male	45 to 49	71%<br>(63-78%)	29%<br>(22-37%)	6.5<br>(4.4-9.1)	19%<br>(15-23%)	25%<br>(20-30%)	3900<br>(2800-5300)	170000<br>(130000-230000)
Vietnam	2005	Male	50 to 54	68%<br>(59-75%)	32%<br>(25-41%)	6.1<br>(4-8.8)	19%<br>(14-23%)	24%<br>(18-29%)	4200<br>(3000-5500)	160000<br>(120000-210000)
Vietnam	2005	Male	55 to 59	66%<br>(56-73%)	34%<br>(27-44%)	6.2<br>(4.1-8.9)	20%<br>(15-24%)	24%<br>(19-29%)	3600<br>(2600-4700)	120000<br>(88000-150000)
Vietnam	2005	Male	60 to 64	62%<br>(52-71%)	38%<br>(29-48%)	5.8<br>(3.7-8.6)	18%<br>(13-22%)	21%<br>(15-26%)	3700<br>(2500-4800)	100000<br>(72000-130000)
Vietnam	2005	Male	65 to 69	59%<br>(49-68%)	41%<br>(32-51%)	5.1<br>(3.1-7.4)	16%<br>(10-20%)	19%<br>(12-23%)	4400<br>(2900-5800)	100000<br>(68000-130000)
Vietnam	2005	Male	70 to 74	57%<br>(46-66%)	43%<br>(34-54%)	4.5<br>(2.7-6.7)	15%<br>(8.9-20%)	17%<br>(10-23%)	5700<br>(3400-7800)	110000<br>(62000-140000)
Vietnam	2005	Male	75 to 79	53%<br>(39-66%)	47%<br>(34-61%)	3.7<br>(2.1-6)	12%<br>(6.5-17%)	14%<br>(7.4-20%)	4600<br>(2400-6700)	65000<br>(35000-95000)
Vietnam	2005	Male	80 to 84	48%<br>(31-63%)	52%<br>(37-69%)	3<br>(1.7-4.8)	10%<br>(5.1-15%)	12%<br>(5.8-18%)	3800<br>(1900-5900)	40000<br>(20000-62000)
Vietnam	2005	Male	85 to 89	43%<br>(28-58%)	57%<br>(42-72%)	2.4<br>(1.3-4)	7.5%<br>(3.2-12%)	8.5%<br>(3.7-14%)	1900<br>(810-3300)	15000<br>(6300-24000)
Vietnam	2005	Male	90 to 94	42%<br>(29-58%)	58%<br>(42-71%)	2.1<br>(1.1-3.6)	5.2%<br>(1.8-10%)	5.8%<br>(1.9-12%)	590<br>(200-1200)	3100<br>(1100-6100)
Vietnam	2005	Male	95 plus	44%<br>(27-63%)	56%<br>(37-73%)	1.8<br>(0.78-3.7)	3.8%<br>(0.54-9%)	4%<br>(0.37-9.9%)	120<br>(11-290)	430<br>(61-1000)

Location	Year	Sex	Age	Prevalence of current drinkers	Prevalence of abstainers	Population average of standard drinks daily	Percent of total attributable deaths	Percent of total attributable DALYs	Attributable deaths	Attributable DALYs
Vietnam	2010	Female	All Ages	10% (8.7-12%)	90% (88-91%)	0.76 (0.51-1.1)	4% (2.9-5.1%)	6.1% (4-8.4%)	19000 (12000-26000)	420000 (300000-550000)
Vietnam	2010	Female	15 to 19	9.3% (5.9-14%)	91% (86-94%)	0.65 (0.31-1.1)	2.2% (1.4-3.3%)	3.6% (1.6-6.2%)	72 (31-130)	10000 (6500-16000)
Vietnam	2010	Female	20 to 24	10% (6.5-15%)	90% (85-94%)	0.76 (0.36-1.4)	4% (2.7-5.5%)	4.4% (2.1-7.4%)	110 (48-190)	20000 (13000-29000)
Vietnam	2010	Female	25 to 29	11% (7.1-16%)	89% (84-93%)	0.81 (0.4-1.5)	4.1% (2.9-5.5%)	4.7% (2.4-7.8%)	120 (59-220)	20000 (13000-29000)
Vietnam	2010	Female	30 to 34	12% (7.3-18%)	88% (82-93%)	0.87 (0.43-1.5)	3.7% (2.5-5.1%)	4.7% (2.5-7.5%)	150 (73-260)	19000 (12000-27000)
Vietnam	2010	Female	35 to 39	12% (7.4-17%)	88% (83-93%)	0.95 (0.49-1.6)	3.9% (2.7-5.3%)	5.6% (3.3-8.6%)	240 (130-400)	22000 (14000-31000)
Vietnam	2010	Female	40 to 44	12% (7.7-17%)	88% (83-92%)	1 (0.53-1.8)	4.1% (2.7-5.7%)	6.2% (3.7-9.1%)	360 (200-570)	25000 (16000-36000)
Vietnam	2010	Female	45 to 49	12% (6.9-17%)	88% (83-93%)	1.1 (0.54-2.1)	4.5% (2.9-6.6%)	6.8% (4-10%)	590 (330-920)	32000 (20000-47000)
Vietnam	2010	Female	50 to 54	12% (8.1-18%)	88% (82-92%)	1.2 (0.65-2.3)	5% (3.3-7.5%)	7.3% (4.4-11%)	870 (500-1300)	38000 (24000-56000)
Vietnam	2010	Female	55 to 59	13% (8.1-19%)	87% (81-92%)	1.4 (0.73-2.5)	5.4% (3.4-8%)	7.6% (4.7-11%)	1000 (610-1600)	38000 (24000-58000)
Vietnam	2010	Female	60 to 64	13% (7.9-19%)	87% (81-92%)	1.4 (0.69-2.6)	5.5% (3.4-8.3%)	7.5% (4.5-11%)	1000 (590-1500)	30000 (18000-45000)
Vietnam	2010	Female	65 to 69	11% (7.2-16%)	89% (84-93%)	1.3 (0.64-2.2)	5.2% (3.1-7.7%)	6.8% (4-10%)	1100 (640-1700)	28000 (16000-42000)
Vietnam	2010	Female	70 to 74	11% (7.2-16%)	89% (84-93%)	1.2 (0.59-2.2)	4.9% (2.8-7.7%)	6.3% (3.7-9.8%)	1600 (860-2600)	30000 (17000-49000)
Vietnam	2010	Female	75 to 79	11% (7.2-17%)	89% (83-93%)	1.2 (0.59-2.3)	4.7% (2.6-7.2%)	5.9% (3.3-9.2%)	2300 (1200-3600)	34000 (18000-53000)
Vietnam	2010	Female	80 to 84	12% (7.5-17%)	88% (83-92%)	1.3 (0.67-2.3)	5% (2.9-7.6%)	6.2% (3.6-9.5%)	3300 (1800-5000)	35000 (20000-53000)
Vietnam	2010	Female	85 to 89	14% (8.3-20%)	86% (80-92%)	1.5 (0.74-2.6)	5.2% (2.8-8.2%)	6.4% (3.4-10%)	3100 (1600-4800)	23000 (12000-36000)
Vietnam	2010	Female	90 to 94	16% (9.6-24%)	84% (76-90%)	1.7 (0.81-3.2)	5.2% (2.7-8.7%)	6.3% (3.1-10%)	1900 (950-3100)	9800 (5100-16000)
Vietnam	2010	Female	95 plus	19% (10-31%)	81% (69-90%)	2 (0.81-4.2)	5.7% (2.3-10%)	6.6% (2.6-12%)	800 (310-1500)	2800 (1100-5000)
Vietnam	2010	Male	All Ages	49% (44-54%)	51% (46-56%)	4.8 (3.4-6.4)	14% (11-17%)	18% (14-21%)	56000 (41000-72000)	1800000 (1400000-2300000)
Vietnam	2010	Male	15 to 19	35% (25-46%)	65% (54-75%)	1.9 (1-2.9)	6.4% (3.8-9.9%)	9.2% (5.1-15%)	550 (280-910)	46000 (25000-72000)
Vietnam	2010	Male	20 to 24	51% (39-62%)	49% (38-61%)	4.4 (2.6-6.6)	14% (9.7-20%)	19% (12-28%)	1600 (950-2500)	120000 (78000-180000)

Location	Year	Sex	Age	Prevalence of current drinkers	Prevalence of abstainers	Population average of standard drinks daily	Percent of total attributable deaths	Percent of total attributable DALYs	Attributable deaths	Attributable DALYs
Vietnam	2010	Male	25 to 29	62% (51-71%)	38% (29-49%)	5.8 (3.6-8.7)	16% (12-21%)	22% (16-29%)	1800 (1100-2600)	130000 (82000-170000)
Vietnam	2010	Male	30 to 34	69% (58-78%)	31% (22-42%)	7.4 (4.7-11)	17% (13-21%)	24% (19-30%)	2000 (1300-2800)	130000 (91000-170000)
Vietnam	2010	Male	35 to 39	72% (61-79%)	28% (21-39%)	9 (5.7-13)	19% (14-22%)	26% (21-31%)	2500 (1800-3500)	150000 (110000-200000)
Vietnam	2010	Male	40 to 44	71% (61-78%)	29% (22-39%)	9.4 (5.9-14)	20% (16-23%)	27% (22-31%)	3400 (2500-4500)	170000 (130000-230000)
Vietnam	2010	Male	45 to 49	66% (53-75%)	34% (25-47%)	8.3 (5.4-12)	21% (17-24%)	27% (23-32%)	4600 (3400-6100)	200000 (150000-260000)
Vietnam	2010	Male	50 to 54	63% (52-72%)	37% (28-48%)	7.8 (5-12)	20% (17-24%)	26% (22-30%)	5600 (4200-7200)	210000 (160000-270000)
Vietnam	2010	Male	55 to 59	60% (48-70%)	40% (30-52%)	7.8 (5-12)	21% (17-24%)	25% (21-30%)	5900 (4400-7500)	190000 (150000-240000)
Vietnam	2010	Male	60 to 64	57% (44-68%)	43% (32-56%)	7.3 (4.3-11)	19% (15-22%)	22% (18-26%)	4300 (3200-5500)	120000 (90000-150000)
Vietnam	2010	Male	65 to 69	53% (40-65%)	47% (35-60%)	6.3 (3.7-9.6)	17% (13-20%)	20% (15-24%)	4200 (3000-5400)	98000 (72000-130000)
Vietnam	2010	Male	70 to 74	50% (37-63%)	50% (37-63%)	5.4 (3-8.4)	16% (11-20%)	18% (13-23%)	5000 (3500-6600)	94000 (66000-120000)
Vietnam	2010	Male	75 to 79	49% (34-64%)	51% (36-66%)	4.7 (2.6-8)	14% (8.7-18%)	16% (9.9-21%)	5500 (3400-7600)	80000 (49000-110000)
Vietnam	2010	Male	80 to 84	46% (30-61%)	54% (39-70%)	4 (2.1-6.5)	12% (7.4-17%)	14% (8.3-20%)	5100 (3000-7100)	54000 (32000-76000)
Vietnam	2010	Male	85 to 89	41% (28-56%)	59% (44-72%)	3.2 (1.7-5.4)	9.7% (5.2-14%)	11% (5.9-16%)	2900 (1500-4300)	22000 (11000-33000)
Vietnam	2010	Male	90 to 94	40% (27-56%)	60% (44-73%)	2.8 (1.4-4.7)	7.2% (3.2-12%)	8.2% (3.5-14%)	1000 (430-1700)	5400 (2400-8700)
Vietnam	2010	Male	95 plus	42% (25-61%)	58% (39-75%)	2.4 (1-4.8)	5.6% (1.5-11%)	6.1% (1.4-12%)	220 (50-440)	790 (210-1500)
Vietnam	2016	Female	All Ages	10% (8.8-12%)	90% (88-91%)	0.85 (0.51-1.3)	4% (2.9-5.1%)	5.9% (3.8-8.1%)	19000 (12000-27000)	430000 (310000-560000)
Vietnam	2016	Female	15 to 19	9.3% (5.8-14%)	91% (86-94%)	0.71 (0.31-1.3)	2.3% (1.5-3.3%)	3.7% (1.6-6.5%)	51 (22-93)	7700 (4800-11000)
Vietnam	2016	Female	20 to 24	10% (6.5-15%)	90% (85-93%)	0.82 (0.36-1.6)	4.1% (2.8-5.5%)	4.4% (2.1-7.5%)	91 (42-160)	19000 (12000-27000)
Vietnam	2016	Female	25 to 29	11% (6.8-17%)	89% (83-93%)	0.86 (0.41-1.7)	4.1% (2.9-5.5%)	4.4% (2.1-7.5%)	110 (50-200)	21000 (14000-30000)
Vietnam	2016	Female	30 to 34	12% (7.2-18%)	88% (82-93%)	0.92 (0.42-1.8)	3.7% (2.6-5.1%)	4.4% (2.3-7.4%)	140 (67-250)	20000 (13000-29000)
Vietnam	2016	Female	35 to 39	12% (7.3-17%)	88% (83-93%)	1 (0.49-1.9)	3.8% (2.6-5.3%)	5.4% (2.9-8.2%)	220 (110-370)	22000 (14000-31000)
Vietnam	2016	Female	40 to 44	12% (7.6-17%)	88% (83-92%)	1.1 (0.53-2)	3.9% (2.6-5.5%)	5.9% (3.4-8.8%)	330 (180-540)	25000 (16000-36000)

Location	Year	Sex	Age	Prevalence of current drinkers	Prevalence of abstainers	Population average of standard drinks daily	Percent of total attributable deaths	Percent of total attributable DALYs	Attributable deaths	Attributable DALYs
Vietnam	2016	Female	45 to 49	12% (7-17%)	88% (83-93%)	1.2 (0.54-2.1)	4.3% (2.8-6.2%)	6.6% (3.8-9.7%)	530 (300-840)	31000 (19000-45000)
Vietnam	2016	Female	50 to 54	12% (7.8-18%)	88% (82-92%)	1.3 (0.64-2.4)	4.8% (3.1-6.9%)	7% (4.3-10%)	830 (480-1300)	38000 (23000-56000)
Vietnam	2016	Female	55 to 59	13% (8.4-18%)	87% (82-92%)	1.5 (0.73-2.7)	5.1% (3.3-7.4%)	7.2% (4.4-11%)	1200 (710-1800)	45000 (28000-66000)
Vietnam	2016	Female	60 to 64	12% (8-18%)	88% (82-92%)	1.5 (0.71-2.8)	5.1% (3.1-7.6%)	7% (4.2-10%)	1400 (810-2100)	44000 (26000-65000)
Vietnam	2016	Female	65 to 69	11% (7.3-16%)	89% (84-93%)	1.4 (0.67-2.6)	4.9% (2.9-7.2%)	6.5% (3.8-9.7%)	1300 (750-1900)	32000 (19000-47000)
Vietnam	2016	Female	70 to 74	11% (7.2-16%)	89% (84-93%)	1.3 (0.6-2.4)	4.6% (2.6-7.1%)	5.9% (3.4-9.1%)	1300 (720-2000)	26000 (15000-40000)
Vietnam	2016	Female	75 to 79	11% (7.2-17%)	89% (83-93%)	1.3 (0.62-2.7)	4.4% (2.4-6.9%)	5.6% (3.1-8.6%)	1700 (940-2700)	26000 (14000-40000)
Vietnam	2016	Female	80 to 84	12% (7.7-17%)	88% (83-92%)	1.4 (0.69-2.5)	4.7% (2.6-7.1%)	5.8% (3.2-9%)	3000 (1700-4600)	33000 (19000-50000)
Vietnam	2016	Female	85 to 89	14% (8.3-20%)	86% (80-92%)	1.6 (0.7-2.9)	4.9% (2.6-7.6%)	5.9% (3.1-9.4%)	3500 (1800-5500)	26000 (14000-41000)
Vietnam	2016	Female	90 to 94	16% (9.7-24%)	84% (76-90%)	1.8 (0.81-3.5)	5% (2.4-8.1%)	5.9% (2.8-9.8%)	2200 (1100-3700)	12000 (5600-19000)
Vietnam	2016	Female	95 plus	19% (9.9-31%)	81% (69-90%)	2.2 (0.79-4.7)	5.5% (2.2-9.8%)	6.3% (2.4-12%)	1000 (390-1900)	3600 (1400-6600)
Vietnam	2016	Male	All Ages	50% (45-55%)	50% (45-55%)	5.4 (3.4-7.9)	14% (12-17%)	18% (14-21%)	60000 (44000-76000)	1900000 (1500000-2400000)
Vietnam	2016	Male	15 to 19	35% (24-46%)	65% (54-76%)	2 (1-3.3)	6.6% (3.8-11%)	9.7% (5.3-16%)	410 (210-710)	34000 (19000-56000)
Vietnam	2016	Male	20 to 24	51% (39-62%)	49% (38-61%)	4.7 (2.6-7.5)	14% (9.6-20%)	19% (12-28%)	1400 (800-2200)	110000 (70000-160000)
Vietnam	2016	Male	25 to 29	62% (50-71%)	38% (29-50%)	6.2 (3.5-9.7)	16% (11-21%)	22% (15-29%)	1800 (1000-2600)	130000 (82000-180000)
Vietnam	2016	Male	30 to 34	69% (57-78%)	31% (22-43%)	7.9 (4.6-12)	17% (13-22%)	24% (18-30%)	2000 (1200-3000)	130000 (87000-190000)
Vietnam	2016	Male	35 to 39	72% (61-79%)	28% (21-39%)	9.7 (5.7-16)	18% (14-22%)	26% (20-31%)	2400 (1600-3500)	140000 (97000-200000)
Vietnam	2016	Male	40 to 44	70% (61-79%)	30% (21-39%)	10 (5.9-16)	19% (15-22%)	26% (21-30%)	3100 (2200-4300)	160000 (120000-220000)
Vietnam	2016	Male	45 to 49	66% (53-75%)	34% (25-47%)	8.9 (5.3-14)	20% (16-24%)	27% (22-31%)	4400 (3200-6000)	200000 (150000-260000)
Vietnam	2016	Male	50 to 54	63% (51-72%)	37% (28-49%)	8.3 (4.8-13)	20% (16-23%)	25% (21-29%)	5800 (4300-7700)	230000 (170000-290000)
Vietnam	2016	Male	55 to 59	60% (48-71%)	40% (29-52%)	8.4 (4.9-13)	20% (16-24%)	25% (20-29%)	7400 (5500-9400)	250000 (190000-310000)

Location	Year	Sex	Age	Prevalence of current drinkers	Prevalence of abstainers	Population average of standard drinks daily	Percent of total attributable deaths	Percent of total attributable DALYs	Attributable deaths	Attributable DALYs
Vietnam	2016	Male	60 to 64	57% (44-68%)	43% (32-56%)	7.8 (4.3-12)	18% (14-21%)	22% (17-26%)	7100 (5400-9000)	200000 (150000-250000)
Vietnam	2016	Male	65 to 69	53% (39-64%)	47% (36-61%)	6.6 (3.6-11)	16% (12-19%)	19% (14-23%)	5100 (3800-6500)	120000 (89000-150000)
Vietnam	2016	Male	70 to 74	50% (37-62%)	50% (38-63%)	5.7 (2.9-9.5)	14% (9.9-18%)	17% (12-21%)	4400 (3000-5800)	83000 (57000-110000)
Vietnam	2016	Male	75 to 79	49% (34-64%)	51% (36-66%)	5 (2.5-8.8)	13% (7.8-17%)	15% (9-20%)	4400 (2700-6100)	64000 (40000-88000)
Vietnam	2016	Male	80 to 84	45% (30-60%)	55% (40-70%)	4.2 (2.1-7.3)	12% (6.7-16%)	13% (7.6-19%)	5000 (2900-7100)	54000 (31000-75000)
Vietnam	2016	Male	85 to 89	41% (26-56%)	59% (44-74%)	3.4 (1.6-5.9)	9.2% (4.7-14%)	11% (5.2-16%)	3400 (1700-5100)	26000 (13000-39000)
Vietnam	2016	Male	90 to 94	40% (27-56%)	60% (44-73%)	2.9 (1.4-5.4)	7.1% (2.8-12%)	8.1% (3-13%)	1300 (480-2100)	6700 (2700-11000)
Vietnam	2016	Male	95 plus	42% (25-61%)	58% (39-75%)	2.6 (1-5.1)	5.7% (1.3-11%)	6.1% (1.1-12%)	300 (56-620)	1100 (260-2200)
Fiji	1990	Female	All Ages	7.9% (6.1-10%)	92% (90-94%)	0.19 (0.14-0.25)	0.42% (0.22-0.66%)	0.24% (-0.051-0.62%)	4.5 (-0.9-13)	440 (220-730)
Fiji	1990	Female	15 to 19	9.2% (5.2-16%)	91% (84-95%)	0.25 (0.12-0.45)	0.77% (0.44-1.3%)	1% (0.4-2.1%)	0.31 (0.11-0.7)	39 (21-67)
Fiji	1990	Female	20 to 24	12% (6.5-20%)	88% (80-94%)	0.41 (0.21-0.69)	1.4% (0.88-2.2%)	1.6% (0.7-3%)	0.69 (0.26-1.4)	83 (47-140)
Fiji	1990	Female	25 to 29	12% (6.7-21%)	88% (79-93%)	0.36 (0.2-0.59)	1.4% (0.89-1.9%)	1.5% (0.77-2.5%)	0.89 (0.37-1.7)	94 (56-150)
Fiji	1990	Female	30 to 34	12% (6-20%)	88% (80-94%)	0.33 (0.17-0.57)	1.1% (0.68-1.7%)	1.1% (0.58-2%)	0.85 (0.36-1.6)	80 (45-130)
Fiji	1990	Female	35 to 39	11% (6-19%)	89% (81-94%)	0.34 (0.16-0.59)	1.1% (0.59-1.7%)	1.2% (0.57-2.1%)	0.98 (0.38-1.9)	71 (36-120)
Fiji	1990	Female	40 to 44	10% (5.6-19%)	90% (81-94%)	0.29 (0.14-0.53)	0.79% (0.35-1.4%)	0.92% (0.33-1.8%)	0.86 (0.27-1.9)	52 (22-100)
Fiji	1990	Female	45 to 49	10% (5-18%)	90% (82-95%)	0.22 (0.094-0.4)	0.37% (-0.04-0.92%)	0.42% (-0.088-1.2%)	0.47 (-0.11-1.4)	25 (-3.2-66)
Fiji	1990	Female	50 to 54	10% (5.4-18%)	90% (82-95%)	0.19 (0.077-0.37)	0.071% (-0.41-0.58%)	0.092% (-0.47-0.73%)	0.12 (-0.62-1)	4.7 (-27-40)
Fiji	1990	Female	55 to 59	9.3% (5.1-16%)	91% (84-95%)	0.18 (0.079-0.35)	0.036% (-0.43-0.59%)	0.079% (-0.46-0.76%)	0.12 (-0.72-1.2)	2.4 (-28-39)
Fiji	1990	Female	60 to 64	8.5% (4.3-15%)	91% (85-96%)	0.16 (0.07-0.33)	-0.05% (-0.52-0.5%)	-0.026% (-0.55-0.63%)	-0.037 (-0.86-1.1)	-2.6 (-29-30)
Fiji	1990	Female	65 to 69	8.9% (4.8-15%)	91% (85-95%)	0.16 (0.076-0.31)	-0.072% (-0.56-0.42%)	-0.044% (-0.59-0.54%)	-0.067 (-0.98-0.89)	-3.1 (-25-20)
Fiji	1990	Female	70 to 74	9.1% (5.2-15%)	91% (85-95%)	0.16 (0.074-0.31)	-0.15% (-0.64-0.36%)	-0.16% (-0.7-0.43%)	-0.27 (-1.2-0.84)	-6 (-25-15)
Fiji	1990	Female	75 to 79	8.9% (4.9-16%)	91% (84-95%)	0.15 (0.064-0.3)	-0.082% (-0.51-0.4%)	-0.073% (-0.55-0.47%)	-0.11 (-0.9-0.81)	-2.2 (-14-11)

Location	Year	Sex	Age	Prevalence of current drinkers	Prevalence of abstainers	Population average of standard drinks daily	Percent of total attributable deaths	Percent of total attributable DALYs	Attributable deaths	Attributable DALYs
Fiji	1990	Female	80 to 84	8.9% (4.8-15%)	91% (85-95%)	0.15 (0.067-0.28)	-0.13% (-0.52-0.26%)	-0.15% (-0.58-0.3%)	-0.16 (-0.67-0.33)	-1.8 (-7.2-3.6)
Fiji	1990	Female	85 to 89	9.1% (4.9-15%)	91% (85-95%)	0.15 (0.07-0.27)	-0.14% (-0.49-0.24%)	-0.16% (-0.56-0.26%)	-0.11 (-0.37-0.17)	-0.8 (-2.8-1.3)
Fiji	1990	Female	90 to 94	9.8% (5.5-16%)	90% (84-95%)	0.16 (0.07-0.3)	-0.1% (-0.4-0.26%)	-0.12% (-0.47-0.28%)	-0.031 (-0.12-0.068)	-0.15 (-0.6-0.39)
Fiji	1990	Female	95 plus	11% (5.6-20%)	89% (80-94%)	0.18 (0.07-0.37)	-0.089% (-0.53-0.41%)	-0.14% (-0.64-0.42%)	-0.0082 (-0.037-0.024)	-0.02 (-0.12-0.091)
Fiji	1990	Male	All Ages	37% (29-47%)	63% (53-71%)	1.2 (1-1.4)	3.1% (1.9-4.2%)	2.8% (1.4-4.4%)	76 (37-130)	4200 (2600-6300)
Fiji	1990	Male	15 to 19	34% (19-51%)	66% (49-81%)	0.79 (0.37-1.4)	2.3% (1.2-3.8%)	3% (1.4-5.4%)	1.8 (0.81-3.3)	160 (82-270)
Fiji	1990	Male	20 to 24	52% (33-70%)	48% (30-67%)	2.1 (1.2-3.2)	7.1% (4.2-10%)	8.8% (5-13%)	8.1 (4.2-13)	610 (360-950)
Fiji	1990	Male	25 to 29	59% (39-76%)	41% (24-61%)	2.8 (1.8-4)	8.6% (5.5-12%)	11% (6.4-15%)	12 (6.8-19)	820 (490-1200)
Fiji	1990	Male	30 to 34	60% (39-77%)	40% (23-61%)	3 (1.8-4.3)	7.6% (4.7-11%)	9.2% (5.3-14%)	12 (6-19)	760 (420-1200)
Fiji	1990	Male	35 to 39	58% (39-75%)	42% (25-61%)	2.7 (1.6-4.1)	6.5% (3.2-10%)	7.6% (3.4-12%)	10 (4-18)	580 (260-990)
Fiji	1990	Male	40 to 44	56% (37-73%)	44% (27-63%)	2.3 (1.2-3.6)	4.7% (1.5-8.7%)	5.2% (1.3-10%)	8 (1.9-17)	420 (130-830)
Fiji	1990	Male	45 to 49	52% (31-71%)	48% (29-69%)	1.7 (0.88-2.9)	2.8% (0.23-6.4%)	3% (-0.27-7.2%)	5.4 (-0.52-14)	270 (23-630)
Fiji	1990	Male	50 to 54	49% (30-68%)	51% (32-70%)	1.4 (0.68-2.3)	2.1% (-0.28-5.5%)	2.2% (-0.56-6.1%)	4.5 (-1.2-13)	190 (-24-530)
Fiji	1990	Male	55 to 59	47% (29-67%)	53% (33-71%)	1.2 (0.59-2.2)	1.8% (-0.24-4.9%)	1.8% (-0.46-5.5%)	4.1 (-1.1-12)	150 (-21-420)
Fiji	1990	Male	60 to 64	44% (26-64%)	56% (36-74%)	1 (0.5-1.9)	1.5% (-0.22-4.7%)	1.6% (-0.38-5.1%)	3.7 (-0.88-12)	120 (-17-350)
Fiji	1990	Male	65 to 69	43% (24-62%)	57% (38-76%)	0.93 (0.46-1.6)	1.2% (-0.28-3.5%)	1.3% (-0.36-3.8%)	2.8 (-0.77-8.5)	72 (-16-210)
Fiji	1990	Male	70 to 74	40% (23-57%)	60% (43-77%)	0.79 (0.38-1.4)	0.9% (-0.42-3%)	0.93% (-0.49-3.2%)	2.1 (-1.2-7.2)	42 (-20-140)
Fiji	1990	Male	75 to 79	36% (21-55%)	64% (45-79%)	0.66 (0.3-1.3)	0.68% (-0.4-2.4%)	0.7% (-0.48-2.5%)	1.3 (-0.9-4.8)	20 (-12-73)
Fiji	1990	Male	80 to 84	33% (18-52%)	67% (48-82%)	0.58 (0.27-1.1)	0.54% (-0.29-1.8%)	0.53% (-0.39-1.9%)	0.6 (-0.41-2.1)	7.2 (-3.8-24)
Fiji	1990	Male	85 to 89	32% (19-48%)	68% (52-81%)	0.55 (0.26-0.97)	0.38% (-0.37-1.5%)	0.35% (-0.47-1.6%)	0.19 (-0.25-0.84)	1.7 (-1.7-6.9)
Fiji	1990	Male	90 to 94	33% (20-49%)	67% (51-80%)	0.56 (0.25-1)	0.21% (-0.46-1.2%)	0.15% (-0.61-1.3%)	0.026 (-0.11-0.23)	0.23 (-0.49-1.3)
Fiji	1990	Male	95 plus	36% (19-57%)	64% (43-81%)	0.61 (0.25-1.2)	0.11% (-0.77-1.3%)	-0.024% (-1-1.4%)	-0.0012 (-0.05-0.067)	0.021 (-0.14-0.25)

Location	Year	Sex	Age	Prevalence of current drinkers	Prevalence of abstainers	Population average of standard drinks daily	Percent of total attributable deaths	Percent of total attributable DALYs	Attributable deaths	Attributable DALYs
Fiji	1995	Female	All Ages	8.2% (6.4-11%)	92% (89-94%)	0.19 (0.14-0.25)	0.35% (0.13-0.6%)	0.12% (-0.19-0.52%)	2.8 (-4-12)	410 (160-710)
Fiji	1995	Female	15 to 19	9.4% (5.4-16%)	91% (84-95%)	0.25 (0.12-0.45)	0.79% (0.42-1.3%)	1% (0.4-2.1%)	0.39 (0.14-0.81)	46 (25-80)
Fiji	1995	Female	20 to 24	12% (6.5-20%)	88% (80-93%)	0.41 (0.21-0.7)	1.5% (0.87-2.3%)	1.6% (0.71-2.9%)	0.75 (0.31-1.5)	89 (51-140)
Fiji	1995	Female	25 to 29	12% (6.7-21%)	88% (79-93%)	0.35 (0.19-0.6)	1.4% (0.91-2%)	1.4% (0.78-2.5%)	0.89 (0.41-1.6)	91 (56-140)
Fiji	1995	Female	30 to 34	12% (6.1-20%)	88% (80-94%)	0.33 (0.17-0.56)	1.1% (0.64-1.7%)	1.1% (0.52-1.9%)	0.9 (0.4-1.7)	84 (48-140)
Fiji	1995	Female	35 to 39	11% (5.8-20%)	89% (80-94%)	0.33 (0.16-0.57)	0.99% (0.55-1.6%)	1.1% (0.48-2%)	1.2 (0.48-2.2)	85 (45-140)
Fiji	1995	Female	40 to 44	11% (5.8-18%)	89% (82-94%)	0.28 (0.14-0.51)	0.7% (0.24-1.4%)	0.79% (0.16-1.7%)	0.91 (0.19-2)	56 (20-110)
Fiji	1995	Female	45 to 49	10% (5.3-19%)	90% (81-95%)	0.22 (0.098-0.4)	0.24% (-0.2-0.8%)	0.27% (-0.27-0.98%)	0.38 (-0.4-1.4)	20 (-18-66)
Fiji	1995	Female	50 to 54	10% (5.6-18%)	90% (82-94%)	0.19 (0.081-0.36)	-0.067% (-0.57-0.48%)	-0.068% (-0.65-0.58%)	-0.12 (-1.1-0.95)	-5.8 (-49-39)
Fiji	1995	Female	55 to 59	9.3% (5.1-16%)	91% (84-95%)	0.17 (0.076-0.34)	-0.092% (-0.6-0.46%)	-0.071% (-0.64-0.59%)	-0.13 (-1.2-1.1)	-7.1 (-47-36)
Fiji	1995	Female	60 to 64	8.6% (4.5-15%)	91% (85-96%)	0.16 (0.07-0.32)	-0.18% (-0.7-0.36%)	-0.18% (-0.76-0.44%)	-0.35 (-1.5-0.84)	-12 (-48-24)
Fiji	1995	Female	65 to 69	9.1% (5.1-16%)	91% (84-95%)	0.16 (0.077-0.31)	-0.2% (-0.7-0.3%)	-0.19% (-0.78-0.38%)	-0.37 (-1.5-0.69)	-10 (-38-15)
Fiji	1995	Female	70 to 74	9.4% (5.3-16%)	91% (84-95%)	0.16 (0.074-0.31)	-0.28% (-0.78-0.22%)	-0.3% (-0.86-0.28%)	-0.57 (-1.7-0.54)	-12 (-34-9.8)
Fiji	1995	Female	75 to 79	9.2% (5.1-16%)	91% (84-95%)	0.15 (0.065-0.28)	-0.18% (-0.65-0.27%)	-0.19% (-0.7-0.34%)	-0.4 (-1.5-0.74)	-6.4 (-23-10)
Fiji	1995	Female	80 to 84	9% (4.9-16%)	91% (84-95%)	0.14 (0.068-0.27)	-0.23% (-0.64-0.15%)	-0.25% (-0.71-0.17%)	-0.36 (-1.1-0.25)	-4 (-12-2.6)
Fiji	1995	Female	85 to 89	9.1% (5.1-15%)	91% (85-95%)	0.15 (0.066-0.27)	-0.22% (-0.61-0.14%)	-0.25% (-0.7-0.15%)	-0.2 (-0.58-0.13)	-1.5 (-4.3-1.1)
Fiji	1995	Female	90 to 94	9.9% (5.4-16%)	90% (84-95%)	0.16 (0.067-0.29)	-0.17% (-0.51-0.21%)	-0.21% (-0.59-0.21%)	-0.062 (-0.18-0.064)	-0.31 (-0.93-0.37)
Fiji	1995	Female	95 plus	11% (5.7-20%)	89% (80-94%)	0.18 (0.067-0.35)	-0.17% (-0.64-0.36%)	-0.23% (-0.79-0.35%)	-0.018 (-0.059-0.028)	-0.05 (-0.18-0.11)
Fiji	1995	Male	All Ages	39% (30-48%)	61% (52-70%)	1.2 (1.1-1.4)	3% (1.9-4.3%)	2.7% (1.3-4.4%)	83 (40-140)	4600 (2800-6900)
Fiji	1995	Male	15 to 19	33% (19-51%)	67% (49-81%)	0.76 (0.35-1.3)	2.3% (1.2-3.9%)	3% (1.3-5.5%)	2.1 (0.93-3.9)	180 (94-320)
Fiji	1995	Male	20 to 24	51% (33-69%)	49% (31-67%)	2.1 (1.2-3.1)	7.2% (4.2-11%)	8.8% (4.9-14%)	8.8 (4.7-14)	660 (380-1000)
Fiji	1995	Male	25 to 29	59% (39-76%)	41% (24-61%)	2.8 (1.8-4)	8.6% (5.4-12%)	10% (6.3-15%)	12 (6.8-18)	810 (500-1200)

Location	Year	Sex	Age	Prevalence of current drinkers	Prevalence of abstainers	Population average of standard drinks daily	Percent of total attributable deaths	Percent of total attributable DALYs	Attributable deaths	Attributable DALYs
Fiji	1995	Male	30 to 34	60% (39-77%)	40% (23-61%)	2.9 (1.8-4.2)	7.6% (4.7-11%)	9% (5.2-14%)	12 (6.5-20)	800 (460-1200)
Fiji	1995	Male	35 to 39	59% (40-75%)	41% (25-60%)	2.7 (1.6-4)	6.3% (3.1-9.9%)	7.2% (3.1-12%)	12 (4.8-22)	710 (330-1200)
Fiji	1995	Male	40 to 44	56% (38-74%)	44% (26-62%)	2.2 (1.3-3.4)	4.4% (1.3-8.4%)	4.9% (1.1-9.7%)	9.2 (1.7-20)	490 (140-1000)
Fiji	1995	Male	45 to 49	52% (31-70%)	48% (30-69%)	1.7 (0.89-2.8)	2.7% (0.063-6%)	2.7% (-0.41-6.7%)	6.1 (-0.74-16)	300 (8.3-740)
Fiji	1995	Male	50 to 54	49% (30-68%)	51% (32-70%)	1.3 (0.66-2.3)	1.9% (-0.37-5.1%)	2% (-0.67-5.6%)	5 (-1.7-14)	220 (-40-570)
Fiji	1995	Male	55 to 59	47% (28-66%)	53% (34-72%)	1.2 (0.58-2.1)	1.6% (-0.33-4.6%)	1.6% (-0.54-5%)	4.4 (-1.4-13)	160 (-34-460)
Fiji	1995	Male	60 to 64	45% (26-65%)	55% (35-74%)	1 (0.5-1.9)	1.4% (-0.32-4.4%)	1.4% (-0.44-4.8%)	3.9 (-1.3-14)	120 (-28-390)
Fiji	1995	Male	65 to 69	43% (25-62%)	57% (38-75%)	0.9 (0.45-1.6)	1.1% (-0.35-3.3%)	1.1% (-0.45-3.5%)	3 (-1.3-9.3)	76 (-25-220)
Fiji	1995	Male	70 to 74	40% (23-58%)	60% (42-77%)	0.77 (0.37-1.4)	0.8% (-0.5-2.8%)	0.82% (-0.58-3%)	1.9 (-1.3-6.7)	38 (-24-130)
Fiji	1995	Male	75 to 79	36% (21-55%)	64% (45-79%)	0.65 (0.29-1.2)	0.6% (-0.47-2.3%)	0.61% (-0.58-2.4%)	1.3 (-1.2-5.4)	21 (-17-81)
Fiji	1995	Male	80 to 84	33% (18-51%)	67% (49-82%)	0.57 (0.28-1)	0.47% (-0.34-1.7%)	0.46% (-0.45-1.8%)	0.56 (-0.56-2.2)	6.9 (-5-25)
Fiji	1995	Male	85 to 89	32% (18-49%)	68% (51-82%)	0.54 (0.25-0.96)	0.32% (-0.42-1.5%)	0.28% (-0.54-1.5%)	0.19 (-0.36-1)	1.8 (-2.5-8.3)
Fiji	1995	Male	90 to 94	33% (20-50%)	67% (50-80%)	0.55 (0.25-1)	0.17% (-0.5-1.2%)	0.093% (-0.67-1.2%)	0.02 (-0.15-0.26)	0.22 (-0.65-1.5)
Fiji	1995	Male	95 plus	36% (20-57%)	64% (43-80%)	0.6 (0.25-1.1)	0.065% (-0.8-1.3%)	-0.076% (-1.1-1.3%)	-0.0039 (-0.057-0.07)	0.013 (-0.17-0.27)
Fiji	2000	Female	All Ages	7.6% (6-9.7%)	92% (90-94%)	0.17 (0.13-0.23)	0.28% (0.07-0.51%)	0.03% (-0.26-0.38%)	0.81 (-6.8-9.9)	360 (86-660)
Fiji	2000	Female	15 to 19	9.3% (5.8-15%)	91% (85-94%)	0.24 (0.13-0.41)	0.78% (0.44-1.3%)	0.99% (0.41-2%)	0.42 (0.17-0.83)	50 (29-84)
Fiji	2000	Female	20 to 24	11% (6.6-18%)	89% (82-93%)	0.37 (0.21-0.63)	1.4% (0.84-2.1%)	1.4% (0.68-2.7%)	0.77 (0.34-1.5)	94 (56-150)
Fiji	2000	Female	25 to 29	10% (5.9-18%)	90% (82-94%)	0.3 (0.16-0.49)	1.2% (0.85-1.8%)	1.3% (0.69-2.1%)	0.75 (0.39-1.3)	82 (53-120)
Fiji	2000	Female	30 to 34	9.9% (5.5-17%)	90% (83-94%)	0.27 (0.14-0.46)	0.96% (0.62-1.5%)	0.91% (0.47-1.6%)	0.66 (0.31-1.2)	67 (42-110)
Fiji	2000	Female	35 to 39	9.8% (5.2-18%)	90% (82-95%)	0.28 (0.14-0.51)	0.88% (0.48-1.4%)	0.93% (0.41-1.7%)	1 (0.42-1.9)	77 (41-130)
Fiji	2000	Female	40 to 44	9.3% (5.1-16%)	91% (84-95%)	0.25 (0.12-0.45)	0.62% (0.21-1.2%)	0.67% (0.14-1.4%)	0.93 (0.17-2.1)	59 (20-120)
Fiji	2000	Female	45 to 49	9.2% (4.7-16%)	91% (84-95%)	0.19 (0.082-0.35)	0.19% (-0.21-0.65%)	0.19% (-0.29-0.79%)	0.29 (-0.45-1.2)	17 (-20-60)

Location	Year	Sex	Age	Prevalence of current drinkers	Prevalence of abstainers	Population average of standard drinks daily	Percent of total attributable deaths	Percent of total attributable DALYs	Attributable deaths	Attributable DALYs
Fiji	2000	Female	50 to 54	9.1% (5.1-16%)	91% (84-95%)	0.16 (0.067-0.32)	-0.11% (-0.58-0.35%)	-0.13% (-0.66-0.43%)	-0.25 (-1.3-0.83)	-10 (-56-34)
Fiji	2000	Female	55 to 59	8.4% (4.5-15%)	92% (85-95%)	0.15 (0.067-0.3)	-0.11% (-0.57-0.37%)	-0.11% (-0.63-0.46%)	-0.25 (-1.4-1.1)	-11 (-56-36)
Fiji	2000	Female	60 to 64	8% (4.1-15%)	92% (85-96%)	0.14 (0.062-0.29)	-0.2% (-0.71-0.29%)	-0.21% (-0.79-0.34%)	-0.53 (-1.9-0.79)	-17 (-59-24)
Fiji	2000	Female	65 to 69	8.8% (4.8-15%)	91% (85-95%)	0.15 (0.074-0.28)	-0.24% (-0.74-0.22%)	-0.25% (-0.82-0.28%)	-0.61 (-2-0.72)	-16 (-51-15)
Fiji	2000	Female	70 to 74	9.3% (5.2-16%)	91% (84-95%)	0.15 (0.071-0.29)	-0.33% (-0.86-0.16%)	-0.36% (-0.96-0.18%)	-0.87 (-2.4-0.43)	-17 (-47-8)
Fiji	2000	Female	75 to 79	9.2% (5-16%)	91% (84-95%)	0.15 (0.062-0.28)	-0.24% (-0.7-0.2%)	-0.25% (-0.77-0.24%)	-0.54 (-1.7-0.49)	-8.4 (-26-7.1)
Fiji	2000	Female	80 to 84	9% (4.9-16%)	91% (84-95%)	0.14 (0.067-0.26)	-0.28% (-0.7-0.098%)	-0.31% (-0.77-0.11%)	-0.55 (-1.4-0.2)	-6 (-15-2)
Fiji	2000	Female	85 to 89	9.2% (4.9-15%)	91% (85-95%)	0.14 (0.068-0.27)	-0.26% (-0.67-0.11%)	-0.3% (-0.76-0.11%)	-0.3 (-0.78-0.11)	-2.3 (-5.9-0.94)
Fiji	2000	Female	90 to 94	10% (5.4-17%)	90% (83-95%)	0.16 (0.068-0.29)	-0.21% (-0.57-0.17%)	-0.25% (-0.66-0.18%)	-0.092 (-0.24-0.068)	-0.46 (-1.2-0.36)
Fiji	2000	Female	95 plus	12% (5.6-21%)	88% (79-94%)	0.18 (0.068-0.36)	-0.21% (-0.71-0.31%)	-0.28% (-0.86-0.31%)	-0.025 (-0.078-0.028)	-0.074 (-0.25-0.11)
Fiji	2000	Male	All Ages	42% (34-50%)	58% (50-66%)	1.3 (1.1-1.5)	3% (1.9-4.2%)	2.6% (1.2-4.2%)	87 (42-140)	4900 (3200-7000)
Fiji	2000	Male	15 to 19	40% (25-56%)	60% (44-75%)	0.92 (0.47-1.5)	2.7% (1.5-4.4%)	3.6% (1.8-6.3%)	2.8 (1.3-4.9)	240 (130-400)
Fiji	2000	Male	20 to 24	59% (42-73%)	41% (27-58%)	2.5 (1.7-3.4)	8.2% (5.2-12%)	10% (6.2-15%)	11 (7-17)	850 (540-1200)
Fiji	2000	Male	25 to 29	64% (48-78%)	36% (22-52%)	3 (2-4.2)	9.2% (6-13%)	11% (7.1-16%)	12 (7.4-18)	850 (540-1200)
Fiji	2000	Male	30 to 34	62% (45-77%)	38% (23-55%)	3 (2-4.2)	7.6% (4.7-11%)	9.1% (5.3-14%)	11 (6.4-17)	730 (440-1100)
Fiji	2000	Male	35 to 39	60% (44-75%)	40% (25-56%)	2.7 (1.7-3.8)	6.3% (3.1-9.9%)	7.3% (3.2-12%)	12 (5.1-20)	710 (350-1200)
Fiji	2000	Male	40 to 44	56% (40-72%)	44% (28-60%)	2.2 (1.3-3.3)	4.3% (1.1-8%)	4.7% (0.87-9.2%)	10 (1.9-21)	550 (150-1000)
Fiji	2000	Male	45 to 49	51% (33-67%)	49% (33-67%)	1.6 (0.94-2.5)	2.4% (0.1-5.5%)	2.5% (-0.23-6%)	6.1 (-0.62-16)	310 (13-720)
Fiji	2000	Male	50 to 54	47% (30-64%)	53% (36-70%)	1.2 (0.63-2)	1.7% (-0.37-4.5%)	1.7% (-0.7-4.9%)	4.9 (-2-15)	220 (-49-590)
Fiji	2000	Male	55 to 59	44% (28-61%)	56% (39-72%)	1.1 (0.55-1.8)	1.4% (-0.27-4.1%)	1.4% (-0.46-4.5%)	4.6 (-1.5-14)	180 (-34-510)
Fiji	2000	Male	60 to 64	42% (25-60%)	58% (40-75%)	0.91 (0.46-1.7)	1.2% (-0.32-3.8%)	1.2% (-0.43-4.1%)	4.1 (-1.4-14)	130 (-33-410)
Fiji	2000	Male	65 to 69	41% (24-60%)	59% (40-76%)	0.84 (0.43-1.5)	0.97% (-0.39-3%)	0.99% (-0.5-3.2%)	3.1 (-1.6-9.8)	78 (-33-240)

Location	Year	Sex	Age	Prevalence of current drinkers	Prevalence of abstainers	Population average of standard drinks daily	Percent of total attributable deaths	Percent of total attributable DALYs	Attributable deaths	Attributable DALYs
Fiji	2000	Male	70 to 74	40%<br>(23-57%)	60%<br>(43-77%)	0.74<br>(0.35-1.3)	0.69%<br>(-0.55-2.4%)	0.69%<br>(-0.66-2.6%)	1.9<br>(-1.9-7.6)	40<br>(-34-150)
Fiji	2000	Male	75 to 79	36%<br>(21-55%)	64%<br>(45-79%)	0.63<br>(0.29-1.2)	0.52%<br>(-0.51-2.1%)	0.51%<br>(-0.62-2.2%)	1.1<br>(-1.3-4.7)	17<br>(-17-72)
Fiji	2000	Male	80 to 84	34%<br>(18-52%)	66%<br>(48-82%)	0.56<br>(0.27-1)	0.41%<br>(-0.38-1.6%)	0.39%<br>(-0.47-1.6%)	0.54<br>(-0.66-2.3)	6.7<br>(-6.3-26)
Fiji	2000	Male	85 to 89	32%<br>(18-49%)	68%<br>(51-82%)	0.53<br>(0.25-0.95)	0.27%<br>(-0.46-1.4%)	0.22%<br>(-0.6-1.5%)	0.17<br>(-0.48-1.1)	1.8<br>(-3.2-9.1)
Fiji	2000	Male	90 to 94	34%<br>(20-51%)	66%<br>(49-80%)	0.55<br>(0.25-1)	0.13%<br>(-0.56-1.2%)	0.055%<br>(-0.74-1.2%)	0.015<br>(-0.21-0.34)	0.22<br>(-0.95-2)
Fiji	2000	Male	95 plus	37%<br>(20-57%)	63%<br>(43-80%)	0.6<br>(0.24-1.2)	0.039%<br>(-0.85-1.3%)	-0.1%<br>(-1.1-1.3%)	-0.007<br>(-0.078-0.086)	0.01<br>(-0.23-0.35)
Fiji	2005	Female	All Ages	7.6%<br>(6.1-9.7%)	92%<br>(90-94%)	0.19<br>(0.14-0.25)	0.27%<br>(0.045-0.53%)	0.028%<br>(-0.3-0.41%)	0.83<br>(-8.7-12)	370<br>(58-740)
Fiji	2005	Female	15 to 19	9%<br>(5.6-15%)	91%<br>(85-94%)	0.25<br>(0.14-0.44)	0.81%<br>(0.45-1.3%)	1.1%<br>(0.44-2.1%)	0.38<br>(0.16-0.78)	45<br>(25-75)
Fiji	2005	Female	20 to 24	11%<br>(6.6-18%)	89%<br>(82-93%)	0.4<br>(0.21-0.65)	1.4%<br>(0.89-2.2%)	1.6%<br>(0.73-2.8%)	0.75<br>(0.33-1.4)	94<br>(56-150)
Fiji	2005	Female	25 to 29	10%<br>(5.8-18%)	90%<br>(82-94%)	0.32<br>(0.18-0.53)	1.3%<br>(0.86-1.8%)	1.3%<br>(0.71-2.2%)	0.75<br>(0.38-1.3)	86<br>(56-130)
Fiji	2005	Female	30 to 34	9.7%<br>(5.4-17%)	90%<br>(83-95%)	0.29<br>(0.15-0.5)	1%<br>(0.64-1.5%)	0.95%<br>(0.5-1.7%)	0.61<br>(0.3-1.1)	65<br>(40-100)
Fiji	2005	Female	35 to 39	9.6%<br>(5-17%)	90%<br>(83-95%)	0.3<br>(0.15-0.54)	0.91%<br>(0.51-1.5%)	0.98%<br>(0.45-1.8%)	0.89<br>(0.37-1.7)	72<br>(38-120)
Fiji	2005	Female	40 to 44	9%<br>(5-15%)	91%<br>(85-95%)	0.26<br>(0.13-0.47)	0.66%<br>(0.23-1.2%)	0.73%<br>(0.18-1.5%)	1.1<br>(0.26-2.3)	70<br>(24-140)
Fiji	2005	Female	45 to 49	9%<br>(4.6-16%)	91%<br>(84-95%)	0.2<br>(0.091-0.37)	0.23%<br>(-0.21-0.72%)	0.24%<br>(-0.27-0.84%)	0.46<br>(-0.51-1.6)	26<br>(-24-83)
Fiji	2005	Female	50 to 54	9%<br>(5-16%)	91%<br>(84-95%)	0.17<br>(0.073-0.35)	-0.074%<br>(-0.58-0.45%)	-0.093%<br>(-0.67-0.52%)	-0.21<br>(-1.6-1.2)	-8.2<br>(-66-49)
Fiji	2005	Female	55 to 59	8.3%<br>(4.5-15%)	92%<br>(85-95%)	0.17<br>(0.074-0.32)	-0.086%<br>(-0.56-0.43%)	-0.08%<br>(-0.63-0.51%)	-0.21<br>(-1.7-1.4)	-9.3<br>(-63-47)
Fiji	2005	Female	60 to 64	7.9%<br>(4.1-15%)	92%<br>(85-96%)	0.16<br>(0.068-0.31)	-0.18%<br>(-0.72-0.37%)	-0.19%<br>(-0.79-0.43%)	-0.6<br>(-2.5-1.4)	-19<br>(-76-41)
Fiji	2005	Female	65 to 69	8.8%<br>(4.8-16%)	91%<br>(84-95%)	0.17<br>(0.079-0.31)	-0.22%<br>(-0.75-0.29%)	-0.23%<br>(-0.82-0.34%)	-0.67<br>(-2.4-1)	-18<br>(-61-24)
Fiji	2005	Female	70 to 74	9.2%<br>(5.2-16%)	91%<br>(84-95%)	0.17<br>(0.078-0.33)	-0.3%<br>(-0.84-0.24%)	-0.33%<br>(-0.94-0.28%)	-0.96<br>(-2.7-0.81)	-19<br>(-52-15)
Fiji	2005	Female	75 to 79	9%<br>(4.9-16%)	91%<br>(84-95%)	0.16<br>(0.069-0.3)	-0.2%<br>(-0.7-0.3%)	-0.21%<br>(-0.77-0.35%)	-0.5<br>(-1.8-0.83)	-8<br>(-28-12)
Fiji	2005	Female	80 to 84	8.9%<br>(4.8-16%)	91%<br>(84-95%)	0.15<br>(0.072-0.28)	-0.25%<br>(-0.66-0.19%)	-0.28%<br>(-0.75-0.2%)	-0.52<br>(-1.4-0.37)	-5.6<br>(-15-4.4)
Fiji	2005	Female	85 to 89	9.1%<br>(4.9-15%)	91%<br>(85-95%)	0.16<br>(0.073-0.29)	-0.23%<br>(-0.64-0.19%)	-0.27%<br>(-0.72-0.2%)	-0.3<br>(-0.82-0.24)	-2.2<br>(-6.2-1.9)

Location	Year	Sex	Age	Prevalence of current drinkers	Prevalence of abstainers	Population average of standard drinks daily	Percent of total attributable deaths	Percent of total attributable DALYs	Attributable deaths	Attributable DALYs
Fiji	2005	Female	90 to 94	9.9% (5.5-16%)	90% (84-94%)	0.17 (0.074-0.33)	-0.19% (-0.58-0.25%)	-0.23% (-0.67-0.27%)	-0.098 (-0.29-0.11)	-0.48 (-1.5-0.61)
Fiji	2005	Female	95 plus	11% (5.6-20%)	89% (80-94%)	0.19 (0.076-0.39)	-0.19% (-0.74-0.37%)	-0.26% (-0.89-0.37%)	-0.027 (-0.09-0.038)	-0.075 (-0.29-0.15)
Fiji	2005	Male	All Ages	43% (36-51%)	57% (49-64%)	1.5 (1.3-1.8)	3.2% (2.1-4.5%)	2.7% (1.4-4.4%)	100 (48-170)	5500 (3400-8100)
Fiji	2005	Male	15 to 19	40% (25-56%)	60% (44-75%)	1 (0.52-1.7)	2.9% (1.6-4.8%)	4% (2-6.7%)	2.4 (1.2-4.3)	200 (110-340)
Fiji	2005	Male	20 to 24	59% (42-73%)	41% (27-58%)	2.7 (1.8-3.8)	8.7% (5.4-12%)	11% (6.4-16%)	11 (6.4-17)	830 (500-1200)
Fiji	2005	Male	25 to 29	64% (48-78%)	36% (22-52%)	3.3 (2.2-4.5)	9.8% (6.4-14%)	12% (7.8-18%)	12 (7.4-18)	880 (550-1300)
Fiji	2005	Male	30 to 34	62% (44-77%)	38% (23-56%)	3.3 (2.1-4.6)	8.4% (5.3-12%)	10% (6.2-15%)	11 (6.2-16)	710 (440-1000)
Fiji	2005	Male	35 to 39	60% (44-75%)	40% (25-56%)	2.9 (1.9-4.2)	7.1% (4-10%)	8.5% (4.5-13%)	12 (5.7-19)	700 (390-1100)
Fiji	2005	Male	40 to 44	56% (40-72%)	44% (28-60%)	2.4 (1.5-3.5)	5.1% (1.8-8.7%)	5.7% (1.5-10%)	12 (3.4-22)	660 (240-1100)
Fiji	2005	Male	45 to 49	50% (33-66%)	50% (34-67%)	1.7 (1-2.6)	3.1% (0.58-6.2%)	3.2% (0.25-6.9%)	9.5 (0.7-22)	480 (87-1000)
Fiji	2005	Male	50 to 54	46% (30-64%)	54% (36-70%)	1.3 (0.68-2.1)	2.2% (-0.045-5.1%)	2.2% (-0.3-5.7%)	7.3 (-1-20)	320 (-6.8-810)
Fiji	2005	Male	55 to 59	44% (28-61%)	56% (39-72%)	1.1 (0.6-1.9)	1.8% (-0.1-4.6%)	1.9% (-0.25-5%)	6.9 (-0.97-19)	260 (-15-680)
Fiji	2005	Male	60 to 64	42% (26-61%)	58% (39-74%)	0.99 (0.5-1.8)	1.6% (-0.16-4.1%)	1.6% (-0.27-4.5%)	6.7 (-1.2-20)	210 (-23-580)
Fiji	2005	Male	65 to 69	41% (24-60%)	59% (40-76%)	0.92 (0.47-1.6)	1.3% (-0.18-3.6%)	1.3% (-0.3-3.8%)	5.2 (-1.1-15)	130 (-18-360)
Fiji	2005	Male	70 to 74	39% (22-57%)	61% (43-78%)	0.8 (0.37-1.5)	0.94% (-0.45-3%)	0.96% (-0.56-3.1%)	3.3 (-2-11)	67 (-35-210)
Fiji	2005	Male	75 to 79	36% (21-54%)	64% (46-79%)	0.67 (0.3-1.3)	0.71% (-0.43-2.5%)	0.72% (-0.5-2.6%)	2 (-1.5-7.4)	32 (-19-110)
Fiji	2005	Male	80 to 84	33% (18-51%)	67% (49-82%)	0.6 (0.29-1.1)	0.56% (-0.31-1.9%)	0.55% (-0.38-1.9%)	0.9 (-0.61-3.2)	11 (-5.9-36)
Fiji	2005	Male	85 to 89	31% (18-48%)	69% (52-82%)	0.56 (0.26-1)	0.42% (-0.37-1.6%)	0.4% (-0.48-1.7%)	0.34 (-0.41-1.5)	3.1 (-2.7-12)
Fiji	2005	Male	90 to 94	32% (20-49%)	68% (51-80%)	0.57 (0.27-1)	0.27% (-0.44-1.4%)	0.2% (-0.6-1.5%)	0.066 (-0.19-0.47)	0.51 (-0.86-2.7)
Fiji	2005	Male	95 plus	36% (19-56%)	64% (44-81%)	0.62 (0.25-1.2)	0.2% (-0.71-1.6%)	0.078% (-0.94-1.6%)	0.0066 (-0.079-0.14)	0.066 (-0.23-0.53)
Fiji	2010	Female	All Ages	8.4% (6.7-11%)	92% (89-93%)	0.26 (0.19-0.35)	0.39% (0.082-0.79%)	0.18% (-0.26-0.79%)	5.6 (-8.2-25)	560 (120-1100)
Fiji	2010	Female	15 to 19	9% (5.2-15%)	91% (85-95%)	0.31 (0.15-0.56)	0.95% (0.52-1.7%)	1.3% (0.52-2.7%)	0.5 (0.2-1.1)	56 (30-100)

Location	Year	Sex	Age	Prevalence of current drinkers	Prevalence of abstainers	Population average of standard drinks daily	Percent of total attributable deaths	Percent of total attributable DALYs	Attributable deaths	Attributable DALYs
Fiji	2010	Female	20 to 24	12% (6.2-19%)	88% (81-94%)	0.51 (0.25-0.9)	1.7% (1-2.6%)	2.1% (0.92-3.9%)	0.84 (0.36-1.6)	100 (60-160)
Fiji	2010	Female	25 to 29	12% (6.4-20%)	88% (80-94%)	0.45 (0.24-0.74)	1.6% (1-2.3%)	1.8% (0.94-3%)	0.96 (0.5-1.7)	110 (67-160)
Fiji	2010	Female	30 to 34	11% (5.8-19%)	89% (81-94%)	0.41 (0.21-0.71)	1.3% (0.78-1.9%)	1.4% (0.69-2.5%)	0.87 (0.43-1.6)	88 (53-140)
Fiji	2010	Female	35 to 39	11% (5.5-19%)	89% (81-94%)	0.41 (0.2-0.74)	1.2% (0.68-1.9%)	1.4% (0.65-2.6%)	1 (0.46-2)	83 (45-140)
Fiji	2010	Female	40 to 44	10% (5.5-18%)	90% (82-95%)	0.36 (0.17-0.64)	0.93% (0.36-1.8%)	1.1% (0.33-2.3%)	1.3 (0.4-2.9)	84 (34-170)
Fiji	2010	Female	45 to 49	10% (4.9-18%)	90% (82-95%)	0.27 (0.12-0.52)	0.42% (-0.11-1.2%)	0.51% (-0.2-1.4%)	0.9 (-0.36-2.5)	48 (-13-130)
Fiji	2010	Female	50 to 54	10% (5.4-18%)	90% (82-95%)	0.24 (0.097-0.46)	0.072% (-0.56-0.88%)	0.093% (-0.64-1.1%)	0.23 (-1.8-3)	9.3 (-75-120)
Fiji	2010	Female	55 to 59	9.1% (5.1-16%)	91% (84-95%)	0.22 (0.098-0.43)	0.046% (-0.56-0.83%)	0.077% (-0.63-1%)	0.22 (-1.9-2.9)	5.6 (-72-100)
Fiji	2010	Female	60 to 64	8.4% (4.2-15%)	92% (85-96%)	0.2 (0.089-0.4)	-0.041% (-0.67-0.73%)	-0.035% (-0.75-0.85%)	-0.13 (-2.8-3)	-5.2 (-82-88)
Fiji	2010	Female	65 to 69	8.7% (4.7-15%)	91% (85-95%)	0.2 (0.092-0.38)	-0.058% (-0.66-0.64%)	-0.047% (-0.73-0.75%)	-0.18 (-2.7-2.8)	-6.1 (-68-67)
Fiji	2010	Female	70 to 74	9% (5.2-15%)	91% (85-95%)	0.2 (0.091-0.4)	-0.12% (-0.71-0.63%)	-0.13% (-0.8-0.73%)	-0.49 (-3-2.6)	-10 (-57-52)
Fiji	2010	Female	75 to 79	8.9% (4.9-15%)	91% (85-95%)	0.19 (0.08-0.37)	-0.028% (-0.56-0.71%)	-0.021% (-0.64-0.84%)	-0.069 (-2-2.4)	-1.5 (-30-37)
Fiji	2010	Female	80 to 84	8.7% (4.7-15%)	91% (85-95%)	0.19 (0.084-0.35)	-0.084% (-0.55-0.52%)	-0.098% (-0.63-0.58%)	-0.22 (-1.4-1.4)	-2.4 (-15-15)
Fiji	2010	Female	85 to 89	8.9% (4.7-15%)	91% (85-95%)	0.19 (0.085-0.36)	-0.079% (-0.52-0.46%)	-0.1% (-0.6-0.49%)	-0.12 (-0.73-0.61)	-0.83 (-5.5-4.8)
Fiji	2010	Female	90 to 94	9.7% (5.4-16%)	90% (84-95%)	0.2 (0.09-0.38)	-0.062% (-0.47-0.5%)	-0.093% (-0.55-0.53%)	-0.043 (-0.26-0.25)	-0.17 (-1.3-1.4)
Fiji	2010	Female	95 plus	11% (5.5-20%)	89% (80-95%)	0.23 (0.087-0.46)	-0.057% (-0.6-0.61%)	-0.12% (-0.73-0.62%)	-0.015 (-0.088-0.076)	-0.027 (-0.28-0.29)
Fiji	2010	Male	All Ages	41% (33-50%)	59% (50-67%)	1.7 (1.4-2.1)	3.7% (2.4-5.2%)	3.4% (1.6-5.5%)	140 (63-220)	6500 (4000-9300)
Fiji	2010	Male	15 to 19	32% (18-50%)	68% (50-82%)	0.96 (0.47-1.7)	2.7% (1.5-4.8%)	3.9% (1.8-7.4%)	2 (0.88-3.8)	180 (94-320)
Fiji	2010	Male	20 to 24	50% (32-68%)	50% (32-68%)	2.6 (1.5-4)	8.5% (5.1-12%)	11% (6.3-17%)	9.1 (4.8-15)	700 (400-1100)
Fiji	2010	Male	25 to 29	58% (38-75%)	42% (25-62%)	3.5 (2.1-5.1)	9.9% (6.6-13%)	13% (8.4-18%)	12 (7.1-17)	850 (550-1200)
Fiji	2010	Male	30 to 34	59% (37-77%)	41% (23-63%)	3.7 (2.2-5.4)	9.1% (5.9-12%)	12% (7.3-16%)	11 (6.6-17)	760 (480-1100)
Fiji	2010	Male	35 to 39	58% (39-76%)	42% (24-61%)	3.4 (1.9-5.1)	8.1% (4.7-12%)	10% (5.5-15%)	12 (6.1-18)	700 (410-1000)

Location	Year	Sex	Age	Prevalence of current drinkers	Prevalence of abstainers	Population average of standard drinks daily	Percent of total attributable deaths	Percent of total attributable DALYs	Attributable deaths	Attributable DALYs
Fiji	2010	Male	40 to 44	55%<br>(37-73%)	45%<br>(27-63%)	2.8<br>(1.6-4.4)	6.4%<br>(2.9-10%)	7.5%<br>(3-13%)	13<br>(5-22)	690<br>(320-1100)
Fiji	2010	Male	45 to 49	51%<br>(31-71%)	49%<br>(29-69%)	2.1<br>(1.1-3.4)	4.5%<br>(1.4-8.2%)	4.9%<br>(1.2-9.4%)	13<br>(3.3-27)	650<br>(200-1200)
Fiji	2010	Male	50 to 54	48%<br>(29-68%)	52%<br>(32-71%)	1.7<br>(0.85-2.9)	3.4%<br>(0.51-7.3%)	3.7%<br>(0.28-8.4%)	14<br>(1.1-31)	580<br>(88-1200)
Fiji	2010	Male	55 to 59	46%<br>(28-66%)	54%<br>(34-72%)	1.5<br>(0.74-2.7)	3%<br>(0.4-6.6%)	3.2%<br>(0.26-7.3%)	14<br>(1.1-30)	480<br>(63-1000)
Fiji	2010	Male	60 to 64	43%<br>(25-63%)	57%<br>(37-75%)	1.3<br>(0.61-2.4)	2.5%<br>(0.25-5.9%)	2.7%<br>(0.17-6.5%)	13<br>(0.77-32)	390<br>(39-930)
Fiji	2010	Male	65 to 69	42%<br>(24-61%)	58%<br>(39-76%)	1.1<br>(0.56-1.9)	2.1%<br>(0.15-4.9%)	2.2%<br>(0.11-5.3%)	10<br>(0.49-24)	260<br>(17-580)
Fiji	2010	Male	70 to 74	39%<br>(23-56%)	61%<br>(44-77%)	0.96<br>(0.45-1.8)	1.5%<br>(-0.18-4.3%)	1.6%<br>(-0.26-4.6%)	6.8<br>(-1.1-19)	130<br>(-15-370)
Fiji	2010	Male	75 to 79	35%<br>(21-53%)	65%<br>(47-79%)	0.8<br>(0.35-1.5)	1.2%<br>(-0.25-3.5%)	1.2%<br>(-0.32-3.8%)	4.1<br>(-1.1-12)	63<br>(-14-180)
Fiji	2010	Male	80 to 84	33%<br>(18-51%)	67%<br>(49-82%)	0.72<br>(0.33-1.3)	0.97%<br>(-0.12-2.8%)	1%<br>(-0.19-3%)	2<br>(-0.37-5.9)	23<br>(-2.7-66)
Fiji	2010	Male	85 to 89	31%<br>(18-47%)	69%<br>(53-82%)	0.68<br>(0.31-1.3)	0.82%<br>(-0.18-2.5%)	0.84%<br>(-0.27-2.7%)	0.79<br>(-0.25-2.4)	6.6<br>(-1.5-19)
Fiji	2010	Male	90 to 94	32%<br>(19-49%)	68%<br>(51-81%)	0.69<br>(0.32-1.3)	0.59%<br>(-0.27-2%)	0.57%<br>(-0.43-2.2%)	0.19<br>(-0.13-0.7)	1.2<br>(-0.54-4)
Fiji	2010	Male	95 plus	35%<br>(18-56%)	65%<br>(44-82%)	0.75<br>(0.3-1.4)	0.55%<br>(-0.5-2.4%)	0.47%<br>(-0.7-2.5%)	0.042<br>(-0.063-0.21)	0.19<br>(-0.18-0.79)
Fiji	2016	Female	All Ages	8.4%<br>(6.7-11%)	92%<br>(89-93%)	0.28<br>(0.18-0.43)	0.47%<br>(0.046-1.1%)	0.28%<br>(-0.27-1.1%)	10<br>(-10-41)	730<br>(76-1700)
Fiji	2016	Female	15 to 19	9%<br>(5.2-15%)	91%<br>(85-95%)	0.34<br>(0.15-0.67)	1.1%<br>(0.53-2%)	1.5%<br>(0.54-3.3%)	0.53<br>(0.18-1.2)	57<br>(27-100)
Fiji	2016	Female	20 to 24	12%<br>(6.4-19%)	88%<br>(81-94%)	0.57<br>(0.26-1)	1.9%<br>(1.1-3.1%)	2.4%<br>(0.93-4.6%)	0.98<br>(0.34-2.1)	110<br>(58-190)
Fiji	2016	Female	25 to 29	12%<br>(6.2-20%)	88%<br>(80-94%)	0.49<br>(0.24-0.86)	1.7%<br>(1-2.7%)	2%<br>(0.98-3.7%)	1<br>(0.42-2.1)	110<br>(63-180)
Fiji	2016	Female	30 to 34	12%<br>(5.9-20%)	88%<br>(80-94%)	0.45<br>(0.21-0.82)	1.4%<br>(0.81-2.3%)	1.6%<br>(0.71-3%)	1.1<br>(0.41-2.4)	110<br>(57-190)
Fiji	2016	Female	35 to 39	11%<br>(5.8-19%)	89%<br>(81-94%)	0.45<br>(0.21-0.82)	1.4%<br>(0.71-2.4%)	1.6%<br>(0.69-3.2%)	1.5<br>(0.5-3.2)	110<br>(53-210)
Fiji	2016	Female	40 to 44	10%<br>(5.5-18%)	90%<br>(82-94%)	0.39<br>(0.17-0.78)	1.1%<br>(0.38-2.3%)	1.3%<br>(0.34-2.9%)	1.6<br>(0.35-3.7)	96<br>(33-210)
Fiji	2016	Female	45 to 49	10%<br>(5.1-17%)	90%<br>(83-95%)	0.3<br>(0.12-0.59)	0.55%<br>(-0.075-1.6%)	0.66%<br>(-0.16-2%)	1.1<br>(-0.24-3.5)	59<br>(-7.5-170)
Fiji	2016	Female	50 to 54	10%<br>(5.5-18%)	90%<br>(82-94%)	0.25<br>(0.1-0.51)	0.2%<br>(-0.48-1.2%)	0.25%<br>(-0.57-1.5%)	0.66<br>(-1.7-3.7)	28<br>(-71-160)
Fiji	2016	Female	55 to 59	9.1%<br>(5.1-16%)	91%<br>(84-95%)	0.24<br>(0.099-0.48)	0.17%<br>(-0.52-1.1%)	0.22%<br>(-0.57-1.4%)	0.81<br>(-2.2-5.1)	26<br>(-86-180)

Location	Year	Sex	Age	Prevalence of current drinkers	Prevalence of abstainers	Population average of standard drinks daily	Percent of total attributable deaths	Percent of total attributable DALYs	Attributable deaths	Attributable DALYs
Fiji	2016	Female	60 to 64	8.4% (4.3-15%)	92% (85-96%)	0.22 (0.087-0.46)	0.083% (-0.61-1%)	0.11% (-0.67-1.2%)	0.43 (-3.1-5.3)	12 (-92-160)
Fiji	2016	Female	65 to 69	8.9% (4.6-16%)	91% (84-95%)	0.22 (0.092-0.43)	0.058% (-0.61-0.96%)	0.085% (-0.67-1.1%)	0.35 (-3.1-4.6)	6.9 (-77-110)
Fiji	2016	Female	70 to 74	9.2% (5.1-16%)	91% (84-95%)	0.22 (0.089-0.46)	-0.016% (-0.66-0.97%)	-0.013% (-0.75-1.1%)	-0.088 (-3.9-5.3)	-2.1 (-76-100)
Fiji	2016	Female	75 to 79	8.9% (5-16%)	91% (84-95%)	0.21 (0.082-0.44)	0.072% (-0.56-0.94%)	0.09% (-0.6-1.1%)	0.36 (-2.6-4.6)	4.8 (-39-67)
Fiji	2016	Female	80 to 84	8.8% (4.7-15%)	91% (85-95%)	0.21 (0.089-0.4)	0.0092% (-0.53-0.84%)	0.005% (-0.61-0.95%)	0.0095 (-2-2.9)	0.3 (-22-32)
Fiji	2016	Female	85 to 89	9% (4.9-15%)	91% (85-95%)	0.21 (0.089-0.4)	0.0038% (-0.51-0.76%)	-0.012% (-0.58-0.84%)	-0.023 (-1.1-1.5)	0.041 (-7.7-11)
Fiji	2016	Female	90 to 94	9.7% (5.5-16%)	90% (84-95%)	0.22 (0.093-0.44)	0.017% (-0.45-0.78%)	-0.007% (-0.55-0.84%)	-0.0045 (-0.32-0.51)	0.058 (-1.6-2.7)
Fiji	2016	Female	95 plus	11% (5.7-20%)	89% (80-94%)	0.25 (0.093-0.54)	0.021% (-0.59-0.98%)	-0.033% (-0.76-1%)	-0.0051 (-0.12-0.16)	0.012 (-0.36-0.57)
Fiji	2016	Male	All Ages	41% (33-50%)	59% (50-67%)	1.9 (1.3-2.7)	4.3% (2.3-6.5%)	3.9% (1.6-6.6%)	180 (67-340)	8100 (4100-14000)
Fiji	2016	Male	15 to 19	33% (19-50%)	67% (50-81%)	1.1 (0.47-2)	3.2% (1.5-6%)	4.4% (1.9-8.8%)	2.4 (0.91-4.9)	210 (95-390)
Fiji	2016	Male	20 to 24	50% (32-69%)	50% (31-68%)	2.9 (1.5-4.8)	9.5% (5.3-14%)	12% (6.5-19%)	11 (5.2-19)	840 (440-1400)
Fiji	2016	Male	25 to 29	58% (37-74%)	42% (26-63%)	3.8 (2.2-6.1)	11% (6.8-15%)	14% (8.5-20%)	13 (6.7-21)	950 (510-1400)
Fiji	2016	Male	30 to 34	59% (38-76%)	41% (24-62%)	4.1 (2.3-6.4)	10% (6.3-14%)	13% (7.5-18%)	15 (7.6-25)	980 (540-1500)
Fiji	2016	Male	35 to 39	58% (40-76%)	42% (24-60%)	3.7 (2.1-6.2)	8.9% (5-13%)	11% (5.7-16%)	16 (6.8-28)	950 (450-1600)
Fiji	2016	Male	40 to 44	55% (37-73%)	45% (27-63%)	3.1 (1.6-5.4)	7.1% (3.3-11%)	8.3% (3.4-14%)	15 (5.4-28)	800 (340-1400)
Fiji	2016	Male	45 to 49	51% (29-70%)	49% (30-71%)	2.4 (1.1-4.2)	5.2% (1.6-9.5%)	5.8% (1.3-11%)	15 (2.9-31)	710 (200-1400)
Fiji	2016	Male	50 to 54	48% (28-68%)	52% (32-72%)	1.9 (0.87-3.4)	4.1% (0.71-8.5%)	4.4% (0.45-9.3%)	18 (1.9-41)	740 (140-1600)
Fiji	2016	Male	55 to 59	46% (28-66%)	54% (34-72%)	1.7 (0.77-3.1)	3.6% (0.55-7.5%)	3.8% (0.41-8.4%)	19 (1.7-47)	700 (100-1600)
Fiji	2016	Male	60 to 64	44% (26-64%)	56% (36-74%)	1.4 (0.61-2.7)	3% (0.27-6.7%)	3.2% (0.19-7.4%)	17 (1-40)	510 (47-1200)
Fiji	2016	Male	65 to 69	42% (24-61%)	58% (39-76%)	1.2 (0.56-2.2)	2.6% (0.28-5.9%)	2.8% (0.24-6.4%)	15 (1.2-36)	360 (39-850)
Fiji	2016	Male	70 to 74	39% (23-57%)	61% (43-77%)	1.1 (0.46-2)	2% (-0.058-5.2%)	2.1% (-0.11-5.6%)	11 (-0.65-29)	220 (-6.4-570)
Fiji	2016	Male	75 to 79	35% (20-54%)	65% (46-80%)	0.89 (0.36-1.8)	1.6% (-0.12-4.4%)	1.6% (-0.19-4.7%)	6.8 (-0.84-20)	100 (-8.5-300)

Location	Year	Sex	Age	Prevalence of current drinkers	Prevalence of abstainers	Population average of standard drinks daily	Percent of total attributable deaths	Percent of total attributable DALYs	Attributable deaths	Attributable DALYs
Fiji	2016	Male	80 to 84	32% (17-50%)	68% (50-83%)	0.79 (0.34-1.5)	1.3% (-0.031-3.4%)	1.3% (-0.086-3.7%)	3.5 (-0.22-9.8)	40 (-0.92-110)
Fiji	2016	Male	85 to 89	31% (17-47%)	69% (53-83%)	0.75 (0.34-1.4)	1.1% (-0.1-3.2%)	1.2% (-0.17-3.4%)	1.4 (-0.2-4.1)	12 (-1-31)
Fiji	2016	Male	90 to 94	32% (19-50%)	68% (50-81%)	0.77 (0.34-1.4)	0.86% (-0.2-2.7%)	0.86% (-0.32-2.9%)	0.33 (-0.12-1.1)	2 (-0.49-6.2)
Fiji	2016	Male	95 plus	36% (18-56%)	64% (44-82%)	0.84 (0.32-1.8)	0.85% (-0.47-3.1%)	0.79% (-0.65-3.3%)	0.069 (-0.055-0.29)	0.29 (-0.15-1.1)
Kiribati	1990	Female	All Ages	7.4% (5.8-9.6%)	93% (90-94%)	0.14 (0.11-0.18)	0.39% (0.24-0.57%)	0.41% (0.16-0.71%)	1.5 (0.59-2.6)	84 (52-120)
Kiribati	1990	Female	15 to 19	9.1% (5.1-16%)	91% (84-95%)	0.18 (0.092-0.32)	0.95% (0.44-1.9%)	1.3% (0.42-2.7%)	0.06 (0.019-0.13)	5.7 (2.6-11)
Kiribati	1990	Female	20 to 24	12% (6.4-19%)	88% (81-94%)	0.31 (0.16-0.5)	1.7% (0.87-2.7%)	2% (0.8-3.6%)	0.16 (0.066-0.3)	15 (7.8-25)
Kiribati	1990	Female	25 to 29	12% (6.5-21%)	88% (79-94%)	0.3 (0.17-0.47)	1.5% (0.93-2.3%)	1.7% (0.9-2.9%)	0.15 (0.076-0.25)	13 (7.9-19)
Kiribati	1990	Female	30 to 34	12% (6.1-20%)	88% (80-94%)	0.29 (0.15-0.49)	1.4% (0.81-2.2%)	1.6% (0.79-2.7%)	0.16 (0.078-0.29)	12 (6.9-19)
Kiribati	1990	Female	35 to 39	11% (5.9-19%)	89% (81-94%)	0.27 (0.14-0.49)	1.3% (0.74-2.2%)	1.6% (0.76-2.8%)	0.16 (0.079-0.3)	10 (5.7-17)
Kiribati	1990	Female	40 to 44	10% (5.6-18%)	90% (82-94%)	0.24 (0.12-0.42)	1.3% (0.58-2.3%)	1.6% (0.64-2.9%)	0.18 (0.076-0.34)	9.7 (4.5-17)
Kiribati	1990	Female	45 to 49	10% (5-18%)	90% (82-95%)	0.21 (0.095-0.39)	0.72% (0.22-1.4%)	0.9% (0.23-1.9%)	0.11 (0.03-0.24)	5.2 (1.5-10)
Kiribati	1990	Female	50 to 54	10% (5.4-18%)	90% (82-95%)	0.19 (0.088-0.35)	0.55% (-0.045-1.4%)	0.71% (0.01-1.8%)	0.12 (0.0017-0.31)	4.5 (-0.34-12)
Kiribati	1990	Female	55 to 59	9.2% (5.1-16%)	91% (84-95%)	0.16 (0.073-0.3)	0.4% (-0.11-1.1%)	0.53% (-0.098-1.4%)	0.093 (-0.019-0.24)	2.8 (-0.81-7.5)
Kiribati	1990	Female	60 to 64	8.4% (4.3-15%)	92% (85-96%)	0.14 (0.062-0.27)	0.25% (-0.19-0.78%)	0.34% (-0.16-0.98%)	0.09 (-0.042-0.27)	2.2 (-1.6-7.1)
Kiribati	1990	Female	65 to 69	8.8% (4.8-15%)	91% (85-95%)	0.14 (0.064-0.25)	0.22% (-0.21-0.78%)	0.3% (-0.2-0.92%)	0.079 (-0.05-0.25)	1.5 (-1.4-5.4)
Kiribati	1990	Female	70 to 74	9.1% (5.3-15%)	91% (85-95%)	0.14 (0.063-0.26)	0.16% (-0.3-0.81%)	0.22% (-0.32-0.98%)	0.069 (-0.1-0.32)	1.1 (-2-5.4)
Kiribati	1990	Female	75 to 79	9% (4.9-16%)	91% (84-95%)	0.13 (0.059-0.24)	0.21% (-0.21-0.71%)	0.26% (-0.21-0.84%)	0.075 (-0.064-0.24)	0.96 (-0.96-3.3)
Kiribati	1990	Female	80 to 84	8.8% (4.8-15%)	91% (85-95%)	0.12 (0.06-0.22)	0.07% (-0.2-0.37%)	0.089% (-0.21-0.42%)	0.018 (-0.044-0.085)	0.17 (-0.5-0.87)
Kiribati	1990	Female	85 to 89	9% (4.8-15%)	91% (85-95%)	0.13 (0.058-0.23)	-0.0034% (-0.24-0.23%)	-0.00054% (-0.25-0.26%)	-0.00005 (-0.026-0.026)	-0.0028 (-0.2-0.2)
Kiribati	1990	Female	90 to 94	9.7% (5.4-16%)	90% (84-95%)	0.14 (0.06-0.25)	0.03% (-0.17-0.26%)	0.034% (-0.19-0.29%)	0.0014 (-0.0075-0.012)	0.007 (-0.041-0.062)
Kiribati	1990	Female	95 plus	11% (5.4-20%)	89% (80-95%)	0.15 (0.06-0.31)	0.11% (-0.18-0.44%)	0.098% (-0.23-0.46%)	0.0012 (-0.0029-0.0057)	0.0055 (-0.0084-0.021)

Location	Year	Sex	Age	Prevalence of current drinkers	Prevalence of abstainers	Population average of standard drinks daily	Percent of total attributable deaths	Percent of total attributable DALYs	Attributable deaths	Attributable DALYs
Kiribati	1990	Male	All Ages	39%<br>(31-48%)	61%<br>(52-69%)	0.76<br>(0.68-0.84)	2.3%<br>(1.6-3%)	2.7%<br>(1.7-3.8%)	12<br>(7.5-17)	580<br>(400-750)
Kiribati	1990	Male	15 to 19	37%<br>(22-55%)	63%<br>(45-78%)	0.51<br>(0.25-0.83)	2.1%<br>(0.84-4%)	2.5%<br>(0.85-5.2%)	0.2<br>(0.069-0.42)	17<br>(6.8-32)
Kiribati	1990	Male	20 to 24	56%<br>(37-73%)	44%<br>(27-63%)	1.4<br>(0.84-2)	7.2%<br>(4-11%)	8.5%<br>(4.4-13%)	1.2<br>(0.61-1.8)	85<br>(47-130)
Kiribati	1990	Male	25 to 29	63%<br>(44-79%)	37%<br>(21-56%)	1.9<br>(1.3-2.6)	9.1%<br>(5.8-13%)	11%<br>(6.7-15%)	1.5<br>(0.91-2.2)	97<br>(61-140)
Kiribati	1990	Male	30 to 34	64%<br>(43-80%)	36%<br>(20-57%)	2<br>(1.2-2.9)	8.8%<br>(5.5-13%)	10%<br>(6.4-15%)	1.5<br>(0.89-2.2)	90<br>(55-130)
Kiribati	1990	Male	35 to 39	62%<br>(44-79%)	38%<br>(21-56%)	1.8<br>(1.1-2.6)	6.3%<br>(3.5-9.6%)	7.2%<br>(3.9-11%)	1.1<br>(0.57-1.7)	60<br>(33-93)
Kiribati	1990	Male	40 to 44	60%<br>(41-75%)	40%<br>(25-59%)	1.5<br>(0.85-2.4)	5.8%<br>(2.8-9.4%)	6.6%<br>(3.1-11%)	1.2<br>(0.54-2)	58<br>(28-95)
Kiribati	1990	Male	45 to 49	56%<br>(34-74%)	44%<br>(26-66%)	1.3<br>(0.71-2.2)	5%<br>(2.2-8.6%)	5.6%<br>(2.4-9.7%)	1.2<br>(0.5-2.1)	52<br>(23-91)
Kiribati	1990	Male	50 to 54	53%<br>(34-72%)	47%<br>(28-66%)	1.1<br>(0.56-1.9)	3.8%<br>(1.2-7.3%)	4.2%<br>(1.3-8.1%)	1.1<br>(0.32-2.1)	41<br>(13-79)
Kiribati	1990	Male	55 to 59	51%<br>(33-71%)	49%<br>(29-67%)	0.9<br>(0.47-1.6)	2.6%<br>(0.61-5.2%)	2.8%<br>(0.6-5.8%)	0.76<br>(0.16-1.6)	25<br>(5.8-52)
Kiribati	1990	Male	60 to 64	48%<br>(29-69%)	52%<br>(31-71%)	0.75<br>(0.37-1.4)	1.9%<br>(0.29-4.5%)	2.1%<br>(0.28-4.9%)	0.68<br>(0.088-1.6)	19<br>(2.7-44)
Kiribati	1990	Male	65 to 69	46%<br>(27-65%)	54%<br>(35-73%)	0.68<br>(0.36-1.1)	2.2%<br>(0.52-4.7%)	2.4%<br>(0.58-5.2%)	0.73<br>(0.17-1.6)	16<br>(3.8-35)
Kiribati	1990	Male	70 to 74	44%<br>(26-61%)	56%<br>(39-74%)	0.6<br>(0.3-1)	1.5%<br>(0.17-3.6%)	1.7%<br>(0.17-4%)	0.49<br>(0.05-1.1)	8.9<br>(1-21)
Kiribati	1990	Male	75 to 79	40%<br>(24-59%)	60%<br>(41-76%)	0.51<br>(0.25-0.92)	1.3%<br>(0.17-3%)	1.4%<br>(0.18-3.3%)	0.32<br>(0.039-0.74)	4.5<br>(0.57-10)
Kiribati	1990	Male	80 to 84	37%<br>(21-56%)	63%<br>(44-79%)	0.46<br>(0.24-0.77)	0.84%<br>(0.12-1.9%)	0.92%<br>(0.13-2%)	0.12<br>(0.017-0.27)	1.3<br>(0.19-2.9)
Kiribati	1990	Male	85 to 89	36%<br>(21-53%)	64%<br>(47-79%)	0.43<br>(0.21-0.76)	0.6%<br>(-0.018-1.5%)	0.65%<br>(-0.044-1.7%)	0.04<br>(-0.0027-0.1)	0.31<br>(-0.0094-0.79)
Kiribati	1990	Male	90 to 94	37%<br>(23-54%)	63%<br>(46-77%)	0.44<br>(0.21-0.79)	0.36%<br>(-0.089-1%)	0.37%<br>(-0.13-1.1%)	0.0079<br>(-0.0029-0.023)	0.045<br>(-0.011-0.13)
Kiribati	1990	Male	95 plus	40%<br>(22-61%)	60%<br>(39-78%)	0.48<br>(0.2-0.91)	0.016%<br>(-0.54-0.68%)	-0.08%<br>(-0.71-0.65%)	-0.00048<br>(-0.0042-0.0038)	0.00037<br>(-0.012-0.016)
Kiribati	1995	Female	All Ages	7.4%<br>(5.8-9.5%)	93%<br>(91-94%)	0.14<br>(0.11-0.17)	0.4%<br>(0.24-0.58%)	0.4%<br>(0.14-0.7%)	1.5<br>(0.52-2.6)	83<br>(50-120)
Kiribati	1995	Female	15 to 19	9.2%<br>(5.1-16%)	91%<br>(84-95%)	0.18<br>(0.093-0.33)	0.92%<br>(0.42-1.7%)	1.2%<br>(0.39-2.5%)	0.064<br>(0.02-0.14)	6.3<br>(2.9-12)
Kiribati	1995	Female	20 to 24	12%<br>(6.3-20%)	88%<br>(80-94%)	0.3<br>(0.16-0.51)	1.6%<br>(0.87-2.6%)	1.8%<br>(0.73-3.5%)	0.11<br>(0.047-0.22)	11<br>(5.8-18)
Kiribati	1995	Female	25 to 29	12%<br>(6.6-20%)	88%<br>(80-93%)	0.3<br>(0.17-0.46)	1.5%<br>(0.94-2.2%)	1.6%<br>(0.86-2.7%)	0.16<br>(0.082-0.27)	14<br>(8.6-21)

Location	Year	Sex	Age	Prevalence of current drinkers	Prevalence of abstainers	Population average of standard drinks daily	Percent of total attributable deaths	Percent of total attributable DALYs	Attributable deaths	Attributable DALYs
Kiribati	1995	Female	30 to 34	12% (6.2-20%)	88% (80-94%)	0.29 (0.15-0.48)	1.3% (0.82-2.1%)	1.5% (0.79-2.7%)	0.16 (0.082-0.28)	12 (7.3-19)
Kiribati	1995	Female	35 to 39	11% (5.9-19%)	89% (81-94%)	0.28 (0.15-0.49)	1.3% (0.75-2.1%)	1.6% (0.77-2.7%)	0.19 (0.095-0.33)	12 (7-20)
Kiribati	1995	Female	40 to 44	10% (5.6-18%)	90% (82-94%)	0.24 (0.12-0.42)	1.2% (0.59-2.2%)	1.6% (0.67-2.8%)	0.19 (0.08-0.34)	9.9 (4.7-17)
Kiribati	1995	Female	45 to 49	10% (5.1-18%)	90% (82-95%)	0.21 (0.097-0.38)	0.68% (0.17-1.3%)	0.85% (0.21-1.7%)	0.12 (0.027-0.24)	5.5 (1.4-10)
Kiribati	1995	Female	50 to 54	10% (5.3-18%)	90% (82-95%)	0.19 (0.086-0.36)	0.49% (-0.11-1.3%)	0.64% (-0.092-1.6%)	0.11 (-0.016-0.29)	4 (-0.85-11)
Kiribati	1995	Female	55 to 59	9.1% (4.9-16%)	91% (84-95%)	0.16 (0.072-0.3)	0.35% (-0.14-0.97%)	0.48% (-0.11-1.3%)	0.098 (-0.022-0.27)	2.9 (-1.2-8.3)
Kiribati	1995	Female	60 to 64	8.3% (4.3-15%)	92% (85-96%)	0.13 (0.061-0.26)	0.21% (-0.21-0.74%)	0.3% (-0.18-0.92%)	0.073 (-0.045-0.22)	1.7 (-1.7-5.9)
Kiribati	1995	Female	65 to 69	8.9% (4.9-15%)	91% (85-95%)	0.14 (0.065-0.25)	0.17% (-0.27-0.68%)	0.25% (-0.27-0.83%)	0.07 (-0.073-0.24)	1.3 (-2-5)
Kiribati	1995	Female	70 to 74	9.3% (5.3-16%)	91% (84-95%)	0.14 (0.062-0.25)	0.12% (-0.34-0.7%)	0.18% (-0.35-0.87%)	0.058 (-0.12-0.28)	0.84 (-2.3-4.9)
Kiribati	1995	Female	75 to 79	9.1% (4.9-16%)	91% (84-95%)	0.13 (0.057-0.25)	0.18% (-0.23-0.65%)	0.23% (-0.23-0.76%)	0.069 (-0.071-0.23)	0.87 (-1.1-3.1)
Kiribati	1995	Female	80 to 84	8.8% (4.7-15%)	91% (85-95%)	0.12 (0.058-0.21)	0.049% (-0.22-0.33%)	0.066% (-0.23-0.38%)	0.014 (-0.049-0.083)	0.13 (-0.56-0.84)
Kiribati	1995	Female	85 to 89	8.9% (4.9-15%)	91% (85-95%)	0.12 (0.056-0.23)	-0.021% (-0.26-0.21%)	-0.02% (-0.3-0.23%)	-0.0024 (-0.034-0.027)	-0.02 (-0.26-0.2)
Kiribati	1995	Female	90 to 94	9.7% (5.3-16%)	90% (84-95%)	0.13 (0.059-0.25)	0.014% (-0.19-0.23%)	0.016% (-0.21-0.26%)	0.0006 (-0.008-0.01)	0.0031 (-0.043-0.052)
Kiribati	1995	Female	95 plus	11% (5.5-20%)	89% (80-95%)	0.15 (0.059-0.3)	0.1% (-0.22-0.43%)	0.079% (-0.27-0.44%)	0.00087 (-0.003-0.0049)	0.0043 (-0.0089-0.018)
Kiribati	1995	Male	All Ages	38% (30-48%)	62% (52-70%)	0.74 (0.67-0.81)	2.4% (1.7-3.2%)	2.8% (1.8-3.9%)	13 (8-18)	610 (430-800)
Kiribati	1995	Male	15 to 19	37% (22-55%)	63% (45-78%)	0.5 (0.24-0.84)	2% (0.76-3.9%)	2.3% (0.7-4.8%)	0.23 (0.073-0.49)	19 (7.7-38)
Kiribati	1995	Male	20 to 24	55% (36-72%)	45% (28-64%)	1.3 (0.81-2)	6.6% (3.5-10%)	7.7% (3.8-12%)	0.94 (0.46-1.5)	67 (35-110)
Kiribati	1995	Male	25 to 29	62% (43-78%)	38% (22-57%)	1.9 (1.2-2.6)	8.5% (5.4-12%)	9.9% (6.1-14%)	1.6 (1-2.4)	110 (68-150)
Kiribati	1995	Male	30 to 34	63% (42-79%)	37% (21-58%)	2 (1.3-2.8)	8.2% (5.1-12%)	9.5% (5.7-14%)	1.7 (1-2.5)	100 (63-150)
Kiribati	1995	Male	35 to 39	62% (44-78%)	38% (22-56%)	1.8 (1.1-2.6)	6% (3.2-9.1%)	6.8% (3.5-10%)	1.3 (0.72-2.1)	74 (41-110)
Kiribati	1995	Male	40 to 44	59% (42-76%)	41% (24-58%)	1.5 (0.86-2.4)	5.6% (2.6-8.9%)	6.3% (2.8-10%)	1.3 (0.59-2.2)	65 (30-100)
Kiribati	1995	Male	45 to 49	55% (34-73%)	45% (27-66%)	1.3 (0.71-2.1)	4.7% (2-8.1%)	5.3% (2.2-9.1%)	1.4 (0.56-2.4)	59 (25-100)

Location	Year	Sex	Age	Prevalence of current drinkers	Prevalence of abstainers	Population average of standard drinks daily	Percent of total attributable deaths	Percent of total attributable DALYs	Attributable deaths	Attributable DALYs
Kiribati	1995	Male	50 to 54	52% (33-71%)	48% (29-67%)	1.1 (0.55-1.9)	3.6% (1.1-6.9%)	4% (1.1-7.6%)	1.1 (0.31-2.1)	41 (12-79)
Kiribati	1995	Male	55 to 59	50% (32-70%)	50% (30-68%)	0.88 (0.44-1.5)	2.4% (0.55-5.1%)	2.7% (0.58-5.6%)	0.77 (0.16-1.6)	25 (5.9-53)
Kiribati	1995	Male	60 to 64	48% (29-68%)	52% (32-71%)	0.74 (0.37-1.3)	1.8% (0.16-4.3%)	2% (0.17-4.7%)	0.65 (0.054-1.5)	18 (1.5-42)
Kiribati	1995	Male	65 to 69	47% (28-65%)	53% (35-72%)	0.68 (0.36-1.1)	2.1% (0.4-4.5%)	2.3% (0.45-5%)	0.78 (0.15-1.7)	18 (3.2-37)
Kiribati	1995	Male	70 to 74	44% (27-63%)	56% (37-73%)	0.6 (0.3-1.1)	1.5% (0.093-3.5%)	1.6% (0.12-3.9%)	0.52 (0.038-1.3)	9.5 (0.62-23)
Kiribati	1995	Male	75 to 79	40% (24-59%)	60% (41-76%)	0.51 (0.24-0.91)	1.3% (0.13-3%)	1.4% (0.14-3.4%)	0.33 (0.034-0.79)	4.7 (0.51-11)
Kiribati	1995	Male	80 to 84	37% (21-55%)	63% (45-79%)	0.45 (0.23-0.76)	0.8% (0.077-1.8%)	0.88% (0.075-2%)	0.13 (0.011-0.29)	1.4 (0.13-3)
Kiribati	1995	Male	85 to 89	35% (20-52%)	65% (48-80%)	0.43 (0.2-0.76)	0.55% (-0.047-1.4%)	0.6% (-0.071-1.6%)	0.04 (-0.0048-0.1)	0.31 (-0.026-0.79)
Kiribati	1995	Male	90 to 94	37% (23-54%)	63% (46-77%)	0.43 (0.2-0.79)	0.33% (-0.13-0.95%)	0.33% (-0.19-1%)	0.0068 (-0.0039-0.021)	0.039 (-0.015-0.12)
Kiribati	1995	Male	95 plus	40% (22-60%)	60% (40-78%)	0.47 (0.19-0.89)	-0.016% (-0.59-0.64%)	-0.12% (-0.77-0.59%)	-0.00066 (-0.0043-0.0033)	-0.00034 (-0.013-0.014)
Kiribati	2000	Female	All Ages	7.3% (5.8-9.3%)	93% (91-94%)	0.13 (0.11-0.17)	0.38% (0.24-0.55%)	0.35% (0.11-0.63%)	1.3 (0.41-2.4)	78 (47-110)
Kiribati	2000	Female	15 to 19	9.5% (5.4-16%)	91% (84-95%)	0.19 (0.097-0.33)	0.91% (0.44-1.7%)	1.2% (0.42-2.5%)	0.071 (0.024-0.15)	7.1 (3.3-13)
Kiribati	2000	Female	20 to 24	12% (6.5-20%)	88% (80-93%)	0.3 (0.16-0.51)	1.6% (0.86-2.5%)	1.8% (0.73-3.4%)	0.12 (0.047-0.22)	11 (6.3-19)
Kiribati	2000	Female	25 to 29	12% (6.7-19%)	88% (81-93%)	0.29 (0.17-0.45)	1.4% (0.94-2.1%)	1.6% (0.87-2.6%)	0.11 (0.063-0.19)	10 (6.7-16)
Kiribati	2000	Female	30 to 34	11% (6-18%)	89% (82-94%)	0.26 (0.15-0.44)	1.2% (0.79-1.8%)	1.3% (0.72-2.2%)	0.15 (0.077-0.25)	12 (7.5-18)
Kiribati	2000	Female	35 to 39	9.1% (4.8-17%)	91% (83-95%)	0.22 (0.11-0.4)	1.1% (0.68-1.8%)	1.3% (0.66-2.3%)	0.17 (0.084-0.3)	11 (6.7-18)
Kiribati	2000	Female	40 to 44	8.6% (4.7-15%)	91% (85-95%)	0.2 (0.097-0.35)	1.1% (0.58-1.7%)	1.3% (0.64-2.3%)	0.19 (0.092-0.34)	10 (5.6-18)
Kiribati	2000	Female	45 to 49	10% (5.5-17%)	90% (83-95%)	0.2 (0.1-0.36)	0.61% (0.13-1.2%)	0.76% (0.16-1.6%)	0.12 (0.024-0.24)	5.4 (1.2-10)
Kiribati	2000	Female	50 to 54	10% (6.1-17%)	90% (83-94%)	0.19 (0.095-0.34)	0.39% (-0.18-1.2%)	0.53% (-0.16-1.5%)	0.1 (-0.031-0.28)	3.7 (-1.6-10)
Kiribati	2000	Female	55 to 59	8.9% (5.1-16%)	91% (84-95%)	0.15 (0.071-0.28)	0.28% (-0.18-0.83%)	0.39% (-0.14-1.1%)	0.081 (-0.029-0.23)	2.4 (-1.6-7.1)
Kiribati	2000	Female	60 to 64	8.1% (4.2-14%)	92% (86-96%)	0.13 (0.059-0.24)	0.16% (-0.24-0.64%)	0.24% (-0.22-0.81%)	0.062 (-0.058-0.21)	1.4 (-2-5.4)
Kiribati	2000	Female	65 to 69	8.9% (4.8-15%)	91% (85-95%)	0.13 (0.064-0.24)	0.12% (-0.3-0.58%)	0.19% (-0.29-0.74%)	0.055 (-0.084-0.21)	0.92 (-2.4-4.5)

Location	Year	Sex	Age	Prevalence of current drinkers	Prevalence of abstainers	Population average of standard drinks daily	Percent of total attributable deaths	Percent of total attributable DALYs	Attributable deaths	Attributable DALYs
Kiribati	2000	Female	70 to 74	9.4%<br>(5.4-16%)	91%<br>(84-95%)	0.14<br>(0.062-0.25)	0.046%<br>(-0.39-0.54%)	0.094%<br>(-0.41-0.69%)	0.032<br>(-0.14-0.24)	0.34<br>(-2.9-4.1)
Kiribati	2000	Female	75 to 79	9.3%<br>(5.3-16%)	91%<br>(84-95%)	0.13<br>(0.058-0.24)	0.12%<br>(-0.27-0.54%)	0.17%<br>(-0.27-0.65%)	0.052<br>(-0.084-0.21)	0.61<br>(-1.4-2.8)
Kiribati	2000	Female	80 to 84	9.2%<br>(4.9-16%)	91%<br>(84-95%)	0.13<br>(0.061-0.22)	0.019%<br>(-0.26-0.3%)	0.034%<br>(-0.28-0.35%)	0.0077<br>(-0.062-0.08)	0.052<br>(-0.71-0.82)
Kiribati	2000	Female	85 to 89	9.4%<br>(5-15%)	91%<br>(85-95%)	0.13<br>(0.06-0.24)	-0.046%<br>(-0.32-0.21%)	-0.047%<br>(-0.35-0.24%)	-0.0059<br>(-0.045-0.03)	-0.049<br>(-0.35-0.22)
Kiribati	2000	Female	90 to 94	10%<br>(5.7-17%)	90%<br>(83-94%)	0.14<br>(0.063-0.26)	-0.0019%<br>(-0.22-0.22%)	-0.00095%<br>(-0.24-0.25%)	-0.000044<br>(-0.01-0.011)	-0.00052<br>(-0.056-0.057)
Kiribati	2000	Female	95 plus	12%<br>(5.7-21%)	88%<br>(79-94%)	0.16<br>(0.063-0.31)	0.084%<br>(-0.26-0.45%)	0.062%<br>(-0.33-0.46%)	0.00065<br>(-0.0035-0.0049)	0.0035<br>(-0.011-0.018)
Kiribati	2000	Male	All Ages	40%<br>(32-48%)	60%<br>(52-68%)	0.76<br>(0.69-0.83)	2.5%<br>(1.7-3.4%)	2.9%<br>(1.7-4%)	13<br>(7.9-19)	640<br>(440-850)
Kiribati	2000	Male	15 to 19	38%<br>(22-56%)	62%<br>(44-78%)	0.51<br>(0.25-0.84)	2.1%<br>(0.79-4%)	2.5%<br>(0.75-5.1%)	0.28<br>(0.085-0.57)	23<br>(9-44)
Kiribati	2000	Male	20 to 24	56%<br>(38-74%)	44%<br>(26-62%)	1.4<br>(0.86-2)	6.9%<br>(3.6-11%)	8.1%<br>(4-13%)	1.1<br>(0.54-1.8)	79<br>(41-120)
Kiribati	2000	Male	25 to 29	65%<br>(50-79%)	35%<br>(21-50%)	2<br>(1.4-2.7)	8.9%<br>(5.5-13%)	10%<br>(6.3-15%)	1.3<br>(0.81-2)	88<br>(54-130)
Kiribati	2000	Male	30 to 34	66%<br>(49-80%)	34%<br>(20-51%)	2.1<br>(1.4-2.8)	8.1%<br>(4.9-12%)	9.4%<br>(5.4-14%)	1.9<br>(1.1-2.7)	110<br>(67-160)
Kiribati	2000	Male	35 to 39	62%<br>(47-77%)	38%<br>(23-53%)	1.7<br>(1.1-2.5)	5.7%<br>(3.2-8.6%)	6.4%<br>(3.5-9.9%)	1.5<br>(0.8-2.4)	82<br>(46-130)
Kiribati	2000	Male	40 to 44	59%<br>(43-74%)	41%<br>(26-57%)	1.5<br>(0.89-2.2)	5.1%<br>(2.2-8.3%)	5.7%<br>(2.4-9.3%)	1.5<br>(0.63-2.6)	74<br>(33-120)
Kiribati	2000	Male	45 to 49	57%<br>(38-72%)	43%<br>(28-62%)	1.4<br>(0.81-2.1)	4.4%<br>(1.7-7.7%)	4.9%<br>(1.9-8.7%)	1.4<br>(0.54-2.5)	63<br>(24-110)
Kiribati	2000	Male	50 to 54	55%<br>(36-70%)	45%<br>(30-64%)	1.1<br>(0.63-1.9)	3.4%<br>(0.86-6.6%)	3.7%<br>(0.84-7.4%)	1.3<br>(0.31-2.5)	48<br>(13-95)
Kiribati	2000	Male	55 to 59	51%<br>(34-67%)	49%<br>(33-66%)	0.88<br>(0.47-1.4)	2.2%<br>(0.3-4.7%)	2.4%<br>(0.27-5.2%)	0.76<br>(0.088-1.7)	25<br>(3.4-57)
Kiribati	2000	Male	60 to 64	48%<br>(30-66%)	52%<br>(34-70%)	0.73<br>(0.38-1.3)	1.6%<br>(0.0088-4%)	1.8%<br>(-0.0011-4.4%)	0.61<br>(-0.00037-1.5)	17<br>(0.091-42)
Kiribati	2000	Male	65 to 69	47%<br>(29-65%)	53%<br>(35-71%)	0.69<br>(0.37-1.2)	1.8%<br>(0.24-4.2%)	2%<br>(0.27-4.6%)	0.66<br>(0.088-1.5)	15<br>(1.9-34)
Kiribati	2000	Male	70 to 74	44%<br>(27-62%)	56%<br>(38-73%)	0.6<br>(0.31-1.1)	1.3%<br>(0.0028-3.3%)	1.4%<br>(0.016-3.6%)	0.44<br>(0.0049-1.1)	8.1<br>(0.017-21)
Kiribati	2000	Male	75 to 79	41%<br>(24-59%)	59%<br>(41-76%)	0.52<br>(0.25-0.91)	1.1%<br>(0.077-2.9%)	1.3%<br>(0.083-3.2%)	0.29<br>(0.019-0.73)	4.1<br>(0.28-10)
Kiribati	2000	Male	80 to 84	38%<br>(21-57%)	62%<br>(43-79%)	0.46<br>(0.24-0.78)	0.75%<br>(0.022-1.8%)	0.83%<br>(0.02-2%)	0.12<br>(0.0029-0.29)	1.3<br>(0.037-3.1)
Kiribati	2000	Male	85 to 89	37%<br>(22-54%)	63%<br>(46-78%)	0.44<br>(0.21-0.77)	0.48%<br>(-0.13-1.4%)	0.52%<br>(-0.16-1.6%)	0.036<br>(-0.011-0.11)	0.28<br>(-0.074-0.84)

Location	Year	Sex	Age	Prevalence of current drinkers	Prevalence of abstainers	Population average of standard drinks daily	Percent of total attributable deaths	Percent of total attributable DALYs	Attributable deaths	Attributable DALYs
Kiribati	2000	Male	90 to 94	38% (23-55%)	62% (45-77%)	0.45 (0.21-0.81)	0.28% (-0.2-0.98%)	0.27% (-0.28-1.1%)	0.0058 (-0.0059-0.023)	0.035 (-0.026-0.12)
Kiribati	2000	Male	95 plus	41% (23-62%)	59% (38-77%)	0.49 (0.2-0.91)	-0.059% (-0.69-0.64%)	-0.17% (-0.89-0.6%)	-0.00092 (-0.0047-0.0032)	-0.0012 (-0.014-0.013)
Kiribati	2005	Female	All Ages	7.5% (6-9.7%)	92% (90-94%)	0.14 (0.12-0.18)	0.38% (0.23-0.55%)	0.34% (0.086-0.6%)	1.3 (0.35-2.4)	82 (49-120)
Kiribati	2005	Female	15 to 19	9.5% (5.4-16%)	90% (84-95%)	0.19 (0.099-0.33)	0.89% (0.45-1.6%)	1.2% (0.42-2.4%)	0.079 (0.028-0.17)	8 (3.9-15)
Kiribati	2005	Female	20 to 24	12% (6.7-20%)	88% (80-93%)	0.31 (0.16-0.52)	1.5% (0.87-2.5%)	1.8% (0.75-3.3%)	0.14 (0.057-0.26)	14 (7.6-23)
Kiribati	2005	Female	25 to 29	12% (6.9-20%)	88% (80-93%)	0.29 (0.17-0.46)	1.4% (0.91-2%)	1.5% (0.83-2.5%)	0.12 (0.066-0.21)	12 (7.3-17)
Kiribati	2005	Female	30 to 34	11% (6.1-18%)	89% (82-94%)	0.26 (0.15-0.45)	1.2% (0.77-1.8%)	1.3% (0.71-2.2%)	0.12 (0.061-0.2)	9.7 (6-15)
Kiribati	2005	Female	35 to 39	9.2% (5-17%)	91% (83-95%)	0.23 (0.12-0.41)	1.1% (0.69-1.7%)	1.3% (0.69-2.3%)	0.18 (0.092-0.32)	12 (7.6-20)
Kiribati	2005	Female	40 to 44	8.7% (4.8-15%)	91% (85-95%)	0.2 (0.098-0.36)	1% (0.58-1.7%)	1.3% (0.62-2.2%)	0.19 (0.097-0.34)	11 (5.9-18)
Kiribati	2005	Female	45 to 49	10% (5.6-16%)	90% (84-94%)	0.21 (0.1-0.35)	0.57% (0.12-1.1%)	0.73% (0.12-1.5%)	0.13 (0.02-0.29)	6.3 (1.2-13)
Kiribati	2005	Female	50 to 54	11% (6.3-17%)	89% (83-94%)	0.19 (0.096-0.33)	0.33% (-0.23-0.99%)	0.47% (-0.2-1.3%)	0.098 (-0.042-0.27)	3.4 (-2.4-10)
Kiribati	2005	Female	55 to 59	9.1% (4.9-16%)	91% (84-95%)	0.15 (0.073-0.29)	0.24% (-0.24-0.76%)	0.36% (-0.2-1%)	0.086 (-0.046-0.24)	2.4 (-2.3-7.4)
Kiribati	2005	Female	60 to 64	8.2% (4.2-15%)	92% (85-96%)	0.13 (0.061-0.25)	0.14% (-0.28-0.59%)	0.22% (-0.27-0.76%)	0.055 (-0.07-0.19)	1.2 (-2.4-5)
Kiribati	2005	Female	65 to 69	9% (4.8-16%)	91% (84-95%)	0.14 (0.066-0.25)	0.096% (-0.35-0.56%)	0.17% (-0.35-0.69%)	0.051 (-0.1-0.21)	0.81 (-3-4.6)
Kiribati	2005	Female	70 to 74	9.6% (5.4-16%)	90% (84-95%)	0.14 (0.065-0.27)	0.021% (-0.44-0.48%)	0.067% (-0.46-0.63%)	0.029 (-0.2-0.27)	0.2 (-4.1-4.6)
Kiribati	2005	Female	75 to 79	9.4% (5.2-16%)	91% (84-95%)	0.13 (0.06-0.25)	0.1% (-0.3-0.53%)	0.15% (-0.29-0.65%)	0.049 (-0.094-0.22)	0.55 (-1.6-2.9)
Kiribati	2005	Female	80 to 84	9.2% (4.8-16%)	91% (84-95%)	0.13 (0.062-0.23)	0.0058% (-0.3-0.29%)	0.019% (-0.32-0.35%)	0.0045 (-0.077-0.082)	0.016 (-0.88-0.82)
Kiribati	2005	Female	85 to 89	9.4% (5.1-15%)	91% (85-95%)	0.13 (0.061-0.24)	-0.059% (-0.36-0.21%)	-0.063% (-0.39-0.23%)	-0.0088 (-0.056-0.031)	-0.07 (-0.43-0.24)
Kiribati	2005	Female	90 to 94	10% (5.7-17%)	90% (83-94%)	0.14 (0.066-0.26)	-0.012% (-0.24-0.23%)	-0.014% (-0.27-0.24%)	-0.00072 (-0.013-0.012)	-0.0037 (-0.071-0.067)
Kiribati	2005	Female	95 plus	12% (5.8-21%)	88% (79-94%)	0.16 (0.066-0.32)	0.072% (-0.29-0.43%)	0.043% (-0.38-0.46%)	0.00054 (-0.0048-0.0058)	0.0035 (-0.014-0.021)
Kiribati	2005	Male	All Ages	41% (33-50%)	59% (50-67%)	0.79 (0.72-0.86)	2.6% (1.7-3.5%)	2.8% (1.7-4%)	14 (8.3-20)	690 (470-940)
Kiribati	2005	Male	15 to 19	38% (22-56%)	62% (44-78%)	0.51 (0.26-0.84)	2.1% (0.83-4%)	2.5% (0.83-5.1%)	0.33 (0.11-0.7)	28 (11-53)

Location	Year	Sex	Age	Prevalence of current drinkers	Prevalence of abstainers	Population average of standard drinks daily	Percent of total attributable deaths	Percent of total attributable DALYs	Attributable deaths	Attributable DALYs
Kiribati	2005	Male	20 to 24	58% (39-74%)	42% (26-61%)	1.4 (0.89-2)	6.8% (3.7-10%)	8.1% (4-13%)	1.3 (0.64-2.1)	96 (50-150)
Kiribati	2005	Male	25 to 29	66% (50-80%)	34% (20-50%)	2 (1.4-2.7)	8.7% (5.5-12%)	10% (6.2-15%)	1.6 (0.97-2.3)	110 (65-150)
Kiribati	2005	Male	30 to 34	66% (50-80%)	34% (20-50%)	2.1 (1.4-3)	7.9% (4.9-12%)	9.2% (5.5-14%)	1.5 (0.86-2.2)	90 (54-130)
Kiribati	2005	Male	35 to 39	62% (46-77%)	38% (23-54%)	1.7 (1.1-2.5)	5.7% (3.1-8.6%)	6.4% (3.3-9.9%)	1.6 (0.85-2.6)	91 (51-140)
Kiribati	2005	Male	40 to 44	59% (44-75%)	41% (25-56%)	1.5 (0.9-2.2)	5% (2.2-8.3%)	5.6% (2.3-9.3%)	1.7 (0.68-2.9)	84 (35-140)
Kiribati	2005	Male	45 to 49	57% (39-73%)	43% (27-61%)	1.4 (0.8-2.1)	4.2% (1.5-7.6%)	4.7% (1.6-8.5%)	1.6 (0.56-2.9)	71 (25-120)
Kiribati	2005	Male	50 to 54	55% (37-71%)	45% (29-63%)	1.1 (0.63-1.9)	3.2% (0.65-6.3%)	3.5% (0.76-7%)	1.2 (0.26-2.5)	47 (11-96)
Kiribati	2005	Male	55 to 59	51% (34-68%)	49% (32-66%)	0.89 (0.49-1.5)	2% (0.21-4.6%)	2.2% (0.2-5.1%)	0.82 (0.074-1.9)	28 (3-61)
Kiribati	2005	Male	60 to 64	48% (30-67%)	52% (33-70%)	0.73 (0.38-1.3)	1.5% (0.016-3.9%)	1.7% (0.014-4.3%)	0.61 (0.0056-1.6)	17 (0.18-43)
Kiribati	2005	Male	65 to 69	47% (28-66%)	53% (34-72%)	0.69 (0.37-1.1)	1.7% (0.079-3.9%)	1.9% (0.094-4.3%)	0.63 (0.032-1.4)	14 (0.68-33)
Kiribati	2005	Male	70 to 74	45% (27-62%)	55% (38-73%)	0.61 (0.3-1.1)	1.2% (-0.066-3%)	1.3% (-0.1-3.4%)	0.52 (-0.039-1.3)	9.5 (-0.54-25)
Kiribati	2005	Male	75 to 79	41% (25-60%)	59% (40-75%)	0.52 (0.25-0.94)	1.1% (0.024-2.7%)	1.2% (0.047-2.9%)	0.29 (0.012-0.72)	4.2 (0.091-10)
Kiribati	2005	Male	80 to 84	38% (21-56%)	62% (44-79%)	0.46 (0.24-0.77)	0.72% (0.019-1.8%)	0.78% (0.011-1.9%)	0.12 (0.0017-0.29)	1.3 (0.034-3.1)
Kiribati	2005	Male	85 to 89	36% (21-54%)	64% (46-79%)	0.44 (0.21-0.76)	0.44% (-0.2-1.4%)	0.47% (-0.24-1.5%)	0.034 (-0.018-0.11)	0.27 (-0.12-0.84)
Kiribati	2005	Male	90 to 94	37% (23-54%)	63% (46-77%)	0.44 (0.21-0.8)	0.24% (-0.26-0.94%)	0.23% (-0.36-1%)	0.0054 (-0.0084-0.024)	0.034 (-0.036-0.13)
Kiribati	2005	Male	95 plus	41% (22-61%)	59% (39-78%)	0.48 (0.2-0.93)	-0.083% (-0.71-0.65%)	-0.2% (-0.91-0.6%)	-0.0012 (-0.0054-0.0034)	-0.0019 (-0.016-0.015)
Kiribati	2010	Female	All Ages	7.8% (6.1-9.9%)	92% (90-94%)	0.15 (0.12-0.19)	0.41% (0.24-0.61%)	0.36% (0.11-0.65%)	1.5 (0.45-2.7)	92 (54-130)
Kiribati	2010	Female	15 to 19	9.6% (5.5-16%)	90% (84-94%)	0.19 (0.098-0.33)	0.88% (0.46-1.6%)	1.2% (0.43-2.3%)	0.075 (0.028-0.16)	7.8 (4-14)
Kiribati	2010	Female	20 to 24	12% (6.8-20%)	88% (80-93%)	0.31 (0.17-0.51)	1.5% (0.91-2.4%)	1.7% (0.78-3.2%)	0.15 (0.064-0.28)	16 (9-25)
Kiribati	2010	Female	25 to 29	12% (6.9-20%)	88% (80-93%)	0.29 (0.17-0.45)	1.4% (0.92-2%)	1.5% (0.83-2.5%)	0.14 (0.074-0.24)	14 (8.5-21)
Kiribati	2010	Female	30 to 34	11% (6-18%)	89% (82-94%)	0.26 (0.15-0.44)	1.2% (0.77-1.8%)	1.3% (0.72-2.1%)	0.13 (0.069-0.23)	11 (7-17)
Kiribati	2010	Female	35 to 39	9.2% (4.8-17%)	91% (83-95%)	0.23 (0.11-0.43)	1.1% (0.7-1.8%)	1.3% (0.7-2.3%)	0.16 (0.08-0.29)	11 (6.6-18)

Location	Year	Sex	Age	Prevalence of current drinkers	Prevalence of abstainers	Population average of standard drinks daily	Percent of total attributable deaths	Percent of total attributable DALYs	Attributable deaths	Attributable DALYs
Kiribati	2010	Female	40 to 44	8.7% (4.8-16%)	91% (84-95%)	0.21 (0.099-0.38)	1% (0.58-1.7%)	1.3% (0.66-2.2%)	0.23 (0.11-0.4)	13 (7-21)
Kiribati	2010	Female	45 to 49	10% (5.4-17%)	90% (83-95%)	0.21 (0.11-0.36)	0.6% (0.14-1.2%)	0.76% (0.14-1.5%)	0.17 (0.032-0.33)	7.8 (1.8-15)
Kiribati	2010	Female	50 to 54	11% (6.2-18%)	89% (82-94%)	0.2 (0.096-0.35)	0.35% (-0.2-0.94%)	0.48% (-0.21-1.3%)	0.12 (-0.057-0.33)	4.4 (-2.6-12)
Kiribati	2010	Female	55 to 59	9.2% (5-16%)	91% (84-95%)	0.16 (0.075-0.3)	0.27% (-0.25-0.77%)	0.38% (-0.21-1%)	0.11 (-0.06-0.29)	3.1 (-2.8-9.2)
Kiribati	2010	Female	60 to 64	8.3% (4.4-15%)	92% (85-96%)	0.13 (0.062-0.26)	0.15% (-0.23-0.62%)	0.23% (-0.21-0.79%)	0.065 (-0.061-0.22)	1.5 (-2.2-5.9)
Kiribati	2010	Female	65 to 69	9% (5-15%)	91% (85-95%)	0.14 (0.067-0.24)	0.11% (-0.33-0.56%)	0.19% (-0.32-0.73%)	0.066 (-0.11-0.25)	1.1 (-3.3-5.4)
Kiribati	2010	Female	70 to 74	9.5% (5.5-16%)	90% (84-94%)	0.14 (0.065-0.27)	0.022% (-0.44-0.49%)	0.069% (-0.45-0.6%)	0.029 (-0.19-0.27)	0.21 (-4-4.5)
Kiribati	2010	Female	75 to 79	9.3% (5.1-16%)	91% (84-95%)	0.13 (0.059-0.26)	0.11% (-0.29-0.52%)	0.16% (-0.29-0.63%)	0.06 (-0.11-0.24)	0.69 (-1.8-3.2)
Kiribati	2010	Female	80 to 84	9.2% (4.9-16%)	91% (84-95%)	0.13 (0.061-0.23)	0.011% (-0.29-0.3%)	0.024% (-0.31-0.36%)	0.0068 (-0.086-0.097)	0.037 (-0.99-1)
Kiribati	2010	Female	85 to 89	9.5% (5.1-15%)	91% (85-95%)	0.13 (0.062-0.23)	-0.053% (-0.37-0.18%)	-0.057% (-0.41-0.21%)	-0.0086 (-0.063-0.03)	-0.069 (-0.48-0.22)
Kiribati	2010	Female	90 to 94	10% (5.7-17%)	90% (83-94%)	0.14 (0.065-0.27)	-0.0089% (-0.24-0.23%)	-0.012% (-0.27-0.25%)	-0.00066 (-0.015-0.014)	-0.003 (-0.08-0.078)
Kiribati	2010	Female	95 plus	12% (5.8-20%)	88% (80-94%)	0.16 (0.068-0.31)	0.081% (-0.29-0.44%)	0.049% (-0.37-0.45%)	0.00071 (-0.0053-0.0065)	0.0045 (-0.016-0.024)
Kiribati	2010	Male	All Ages	42% (35-51%)	58% (49-65%)	0.84 (0.75-0.94)	2.7% (1.8-3.7%)	2.9% (1.7-4.1%)	15 (8.8-23)	760 (500-1000)
Kiribati	2010	Male	15 to 19	38% (22-56%)	62% (44-78%)	0.51 (0.26-0.84)	2.1% (0.8-3.9%)	2.5% (0.71-5.1%)	0.33 (0.094-0.67)	28 (10-52)
Kiribati	2010	Male	20 to 24	57% (39-74%)	43% (26-61%)	1.4 (0.88-2)	6.7% (3.6-11%)	8% (3.9-13%)	1.6 (0.76-2.5)	110 (60-180)
Kiribati	2010	Male	25 to 29	66% (51-79%)	34% (21-49%)	2 (1.4-2.7)	8.6% (5.2-12%)	10% (5.9-15%)	1.9 (1.1-2.8)	130 (78-180)
Kiribati	2010	Male	30 to 34	66% (49-81%)	34% (19-51%)	2.1 (1.4-2.9)	7.7% (4.8-11%)	8.9% (5.3-13%)	1.7 (1-2.6)	110 (65-160)
Kiribati	2010	Male	35 to 39	62% (46-78%)	38% (22-54%)	1.7 (1.1-2.5)	5.6% (3-8.6%)	6.3% (3.3-9.9%)	1.4 (0.71-2.3)	78 (41-120)
Kiribati	2010	Male	40 to 44	60% (43-75%)	40% (25-57%)	1.5 (0.9-2.3)	4.9% (2-8.1%)	5.5% (2.1-9.1%)	1.9 (0.68-3.2)	92 (36-160)
Kiribati	2010	Male	45 to 49	58% (39-73%)	42% (27-61%)	1.4 (0.83-2.2)	4.1% (1.4-7.5%)	4.6% (1.5-8.3%)	1.9 (0.62-3.6)	84 (29-150)
Kiribati	2010	Male	50 to 54	55% (37-71%)	45% (29-63%)	1.1 (0.64-1.9)	3.1% (0.55-6%)	3.4% (0.51-6.8%)	1.4 (0.22-2.9)	54 (11-110)
Kiribati	2010	Male	55 to 59	51% (34-68%)	49% (32-66%)	0.89 (0.5-1.4)	2% (0.24-4.3%)	2.1% (0.2-4.8%)	0.93 (0.085-2)	31 (3.6-68)

Location	Year	Sex	Age	Prevalence of current drinkers	Prevalence of abstainers	Population average of standard drinks daily	Percent of total attributable deaths	Percent of total attributable DALYs	Attributable deaths	Attributable DALYs
Kiribati	2010	Male	60 to 64	48% (30-66%)	52% (34-70%)	0.74 (0.4-1.3)	1.5% (0.0019-3.8%)	1.6% (-0.013-4.1%)	0.65 (-0.0055-1.7)	18 (0.022-48)
Kiribati	2010	Male	65 to 69	47% (28-66%)	53% (34-72%)	0.69 (0.37-1.2)	1.6% (0.13-3.7%)	1.8% (0.17-4.1%)	0.7 (0.067-1.7)	16 (1.3-37)
Kiribati	2010	Male	70 to 74	45% (28-63%)	55% (37-72%)	0.61 (0.3-1.1)	1.2% (-0.058-3%)	1.3% (-0.066-3.4%)	0.48 (-0.026-1.3)	8.8 (-0.45-23)
Kiribati	2010	Male	75 to 79	41% (25-60%)	59% (40-75%)	0.52 (0.26-0.94)	1.1% (0.016-2.6%)	1.2% (0.024-2.9%)	0.32 (0.0065-0.79)	4.5 (0.067-11)
Kiribati	2010	Male	80 to 84	38% (22-57%)	62% (43-78%)	0.46 (0.24-0.77)	0.71% (-0.01-1.7%)	0.78% (-0.024-1.8%)	0.14 (-0.0041-0.32)	1.4 (-0.021-3.4)
Kiribati	2010	Male	85 to 89	37% (22-53%)	63% (47-78%)	0.44 (0.21-0.76)	0.43% (-0.21-1.3%)	0.45% (-0.26-1.4%)	0.033 (-0.019-0.1)	0.26 (-0.13-0.8)
Kiribati	2010	Male	90 to 94	37% (23-55%)	63% (45-77%)	0.44 (0.21-0.79)	0.23% (-0.26-0.89%)	0.21% (-0.35-0.94%)	0.005 (-0.0085-0.023)	0.032 (-0.038-0.13)
Kiribati	2010	Male	95 plus	41% (22-61%)	59% (39-78%)	0.47 (0.2-0.91)	-0.091% (-0.71-0.62%)	-0.21% (-0.91-0.54%)	-0.0013 (-0.0057-0.0033)	-0.0022 (-0.017-0.015)
Kiribati	2016	Female	All Ages	8.4% (6.6-11%)	92% (89-93%)	0.17 (0.12-0.23)	0.47% (0.23-0.77%)	0.39% (0.076-0.82%)	1.8 (0.35-3.9)	110 (52-170)
Kiribati	2016	Female	15 to 19	9.5% (5.4-16%)	91% (84-95%)	0.19 (0.093-0.36)	0.88% (0.44-1.6%)	1.2% (0.4-2.5%)	0.074 (0.025-0.16)	8 (3.9-15)
Kiribati	2016	Female	20 to 24	12% (6.7-20%)	88% (80-93%)	0.32 (0.15-0.58)	1.5% (0.86-2.5%)	1.8% (0.72-3.6%)	0.15 (0.055-0.3)	16 (8.3-26)
Kiribati	2016	Female	25 to 29	13% (6.7-21%)	87% (79-93%)	0.32 (0.15-0.53)	1.5% (0.89-2.2%)	1.6% (0.81-2.8%)	0.16 (0.075-0.3)	16 (9.3-25)
Kiribati	2016	Female	30 to 34	12% (6.3-21%)	88% (79-94%)	0.31 (0.15-0.54)	1.3% (0.78-2%)	1.5% (0.71-2.5%)	0.17 (0.079-0.32)	15 (8.4-23)
Kiribati	2016	Female	35 to 39	12% (6.1-21%)	88% (79-94%)	0.29 (0.14-0.53)	1.3% (0.74-2.1%)	1.5% (0.79-2.8%)	0.21 (0.1-0.39)	14 (7.9-24)
Kiribati	2016	Female	40 to 44	11% (6-19%)	89% (81-94%)	0.26 (0.12-0.49)	1.1% (0.56-2.1%)	1.5% (0.64-2.8%)	0.23 (0.094-0.45)	13 (5.9-23)
Kiribati	2016	Female	45 to 49	11% (5.3-19%)	89% (81-95%)	0.22 (0.099-0.43)	0.63% (0.086-1.4%)	0.8% (0.14-1.8%)	0.19 (0.033-0.42)	9.1 (1.3-20)
Kiribati	2016	Female	50 to 54	11% (5.7-19%)	89% (81-94%)	0.2 (0.09-0.38)	0.38% (-0.17-1.1%)	0.53% (-0.18-1.5%)	0.17 (-0.06-0.48)	6.2 (-2.9-18)
Kiribati	2016	Female	55 to 59	9.6% (5.2-17%)	90% (83-95%)	0.16 (0.072-0.33)	0.28% (-0.25-0.94%)	0.41% (-0.21-1.2%)	0.14 (-0.076-0.44)	4.2 (-3.7-14)
Kiribati	2016	Female	60 to 64	8.8% (4.6-16%)	91% (84-95%)	0.14 (0.061-0.28)	0.16% (-0.27-0.67%)	0.25% (-0.24-0.87%)	0.099 (-0.096-0.34)	2.2 (-3.8-9)
Kiribati	2016	Female	65 to 69	9.4% (5-16%)	91% (84-95%)	0.15 (0.067-0.28)	0.13% (-0.34-0.67%)	0.21% (-0.33-0.83%)	0.079 (-0.13-0.31)	1.3 (-3.4-6.9)
Kiribati	2016	Female	70 to 74	9.7% (5.6-16%)	90% (84-94%)	0.15 (0.063-0.29)	0.039% (-0.44-0.61%)	0.088% (-0.45-0.76%)	0.038 (-0.19-0.33)	0.38 (-4.1-5.7)
Kiribati	2016	Female	75 to 79	9.5% (5.3-17%)	90% (83-95%)	0.14 (0.059-0.27)	0.13% (-0.29-0.6%)	0.18% (-0.29-0.72%)	0.086 (-0.14-0.35)	1 (-2.3-4.6)

Location	Year	Sex	Age	Prevalence of current drinkers	Prevalence of abstainers	Population average of standard drinks daily	Percent of total attributable deaths	Percent of total attributable DALYs	Attributable deaths	Attributable DALYs
Kiribati	2016	Female	80 to 84	9.3% (4.9-16%)	91% (84-95%)	0.13 (0.061-0.25)	0.019% (-0.29-0.35%)	0.034% (-0.31-0.41%)	0.012 (-0.11-0.14)	0.081 (-1.3-1.5)
Kiribati	2016	Female	85 to 89	9.5% (5.1-16%)	91% (84-95%)	0.14 (0.061-0.26)	-0.038% (-0.34-0.24%)	-0.041% (-0.38-0.27%)	-0.0082 (-0.076-0.053)	-0.066 (-0.58-0.42)
Kiribati	2016	Female	90 to 94	10% (5.6-16%)	90% (84-94%)	0.15 (0.064-0.28)	0.0032% (-0.24-0.26%)	0.00096% (-0.27-0.29%)	0.000058 (-0.019-0.02)	0.0013 (-0.099-0.11)
Kiribati	2016	Female	95 plus	12% (5.9-21%)	88% (79-94%)	0.17 (0.065-0.34)	0.087% (-0.31-0.48%)	0.061% (-0.39-0.51%)	0.0011 (-0.0072-0.0092)	0.0061 (-0.022-0.033)
Kiribati	2016	Male	All Ages	43% (35-52%)	57% (48-65%)	0.88 (0.65-1.1)	3% (1.6-4.6%)	3.1% (1.5-5.1%)	18 (8.1-30)	870 (470-1400)
Kiribati	2016	Male	15 to 19	38% (23-56%)	62% (44-77%)	0.53 (0.25-0.95)	2.1% (0.79-4.3%)	2.6% (0.71-5.8%)	0.34 (0.09-0.74)	29 (11-58)
Kiribati	2016	Male	20 to 24	56% (37-73%)	44% (27-63%)	1.4 (0.81-2.3)	6.8% (3.3-11%)	8% (3.5-14%)	1.6 (0.67-2.8)	110 (54-190)
Kiribati	2016	Male	25 to 29	63% (43-78%)	37% (22-57%)	1.9 (1.2-2.9)	8.3% (4.7-13%)	9.8% (5.2-15%)	2.2 (1.1-3.5)	150 (81-230)
Kiribati	2016	Male	30 to 34	64% (43-80%)	36% (20-57%)	2.1 (1.2-3.2)	7.7% (4.3-12%)	8.9% (4.5-14%)	2.1 (1-3.4)	130 (66-200)
Kiribati	2016	Male	35 to 39	63% (46-80%)	37% (20-54%)	1.8 (1.1-2.9)	5.9% (2.9-9.7%)	6.6% (3-11%)	1.7 (0.71-2.9)	93 (43-160)
Kiribati	2016	Male	40 to 44	61% (43-78%)	39% (22-57%)	1.6 (0.86-2.7)	5.1% (2-9.2%)	5.7% (2.1-10%)	1.7 (0.53-3.1)	83 (29-150)
Kiribati	2016	Male	45 to 49	57% (34-75%)	43% (25-66%)	1.4 (0.7-2.4)	4.2% (1.3-7.9%)	4.6% (1.3-9%)	2 (0.59-4.1)	90 (28-180)
Kiribati	2016	Male	50 to 54	54% (34-73%)	46% (27-66%)	1.2 (0.58-2.1)	3.2% (0.56-6.7%)	3.5% (0.54-7.5%)	2 (0.29-4.3)	76 (13-170)
Kiribati	2016	Male	55 to 59	52% (33-71%)	48% (29-67%)	0.95 (0.46-1.7)	2.1% (0.15-5.3%)	2.4% (0.083-5.9%)	1.3 (0.046-3.2)	43 (3-110)
Kiribati	2016	Male	60 to 64	49% (30-69%)	51% (31-70%)	0.79 (0.36-1.5)	1.6% (-0.049-4.5%)	1.8% (-0.053-5%)	0.95 (-0.028-2.8)	27 (-0.79-77)
Kiribati	2016	Male	65 to 69	48% (30-67%)	52% (33-70%)	0.71 (0.35-1.2)	1.7% (0.093-4.3%)	1.9% (0.13-4.7%)	0.9 (0.062-2.3)	21 (1.1-54)
Kiribati	2016	Male	70 to 74	45% (28-62%)	55% (38-72%)	0.62 (0.3-1.1)	1.2% (-0.094-3.2%)	1.4% (-0.091-3.6%)	0.57 (-0.037-1.6)	11 (-0.81-29)
Kiribati	2016	Male	75 to 79	40% (24-59%)	60% (41-76%)	0.52 (0.24-0.97)	1.1% (-0.0042-2.7%)	1.2% (-0.00046-3.1%)	0.43 (-0.00017-1.1)	6.2 (-0.022-16)
Kiribati	2016	Male	80 to 84	37% (21-56%)	63% (44-79%)	0.47 (0.23-0.87)	0.77% (-0.019-2%)	0.84% (-0.019-2.1%)	0.18 (-0.0036-0.44)	1.9 (-0.046-4.8)
Kiribati	2016	Male	85 to 89	36% (22-52%)	64% (48-78%)	0.45 (0.21-0.82)	0.49% (-0.19-1.6%)	0.52% (-0.23-1.7%)	0.047 (-0.021-0.16)	0.37 (-0.15-1.2)
Kiribati	2016	Male	90 to 94	38% (23-55%)	62% (45-77%)	0.46 (0.21-0.85)	0.27% (-0.26-1%)	0.25% (-0.35-1.1%)	0.0067 (-0.0092-0.028)	0.042 (-0.041-0.16)
Kiribati	2016	Male	95 plus	41% (22-62%)	59% (38-78%)	0.5 (0.2-0.97)	-0.036% (-0.7-0.8%)	-0.16% (-0.91-0.78%)	-0.001 (-0.0059-0.0051)	-0.00091 (-0.018-0.021)

Location	Year	Sex	Age	Prevalence of current drinkers	Prevalence of abstainers	Population average of standard drinks daily	Percent of total attributable deaths	Percent of total attributable DALYs	Attributable deaths	Attributable DALYs
Marshall Islands	1990	Female	All Ages	18%<br>(13-25%)	82%<br>(75-87%)	0.28<br>(0.15-0.48)	0.061%<br>(-0.39-0.7%)	-0.39%<br>(-1.2-0.7%)	-0.46<br>(-1.4-0.79)	4.5<br>(-27-53)
Marshall Islands	1990	Female	15 to 19	20%<br>(9-36%)	80%<br>(64-91%)	0.35<br>(0.13-0.82)	1%<br>(0.4-2.1%)	1.5%<br>(0.33-3.7%)	0.034<br>(0.0076-0.088)	3.6<br>(1.4-7.5)
Marshall Islands	1990	Female	20 to 24	24%<br>(10-43%)	76%<br>(57-90%)	0.55<br>(0.19-1.2)	1.7%<br>(0.81-3.2%)	2.2%<br>(0.63-5.2%)	0.056<br>(0.017-0.13)	5.8<br>(2.8-11)
Marshall Islands	1990	Female	25 to 29	26%<br>(10-46%)	74%<br>(54-90%)	0.57<br>(0.21-1.2)	1.6%<br>(0.75-2.9%)	2%<br>(0.58-4.2%)	0.055<br>(0.016-0.12)	5.2<br>(2.5-9.7)
Marshall Islands	1990	Female	30 to 34	27%<br>(11-47%)	73%<br>(53-89%)	0.59<br>(0.22-1.2)	1.3%<br>(0.45-2.7%)	1.6%<br>(0.24-3.9%)	0.05<br>(0.008-0.12)	4.2<br>(1.5-9)
Marshall Islands	1990	Female	35 to 39	27%<br>(11-46%)	73%<br>(54-89%)	0.6<br>(0.22-1.2)	0.97%<br>(-0.034-2.6%)	1.3%<br>(-0.24-3.8%)	0.049<br>(-0.0096-0.15)	3.3<br>(-0.13-8.9)
Marshall Islands	1990	Female	40 to 44	26%<br>(13-44%)	74%<br>(56-87%)	0.55<br>(0.21-1.1)	0.46%<br>(-0.89-2.5%)	0.65%<br>(-1.3-3.5%)	0.028<br>(-0.054-0.16)	1.5<br>(-2.9-8.2)
Marshall Islands	1990	Female	45 to 49	26%<br>(12-44%)	74%<br>(56-88%)	0.48<br>(0.16-1)	-0.22%<br>(-1.6-1.6%)	-0.15%<br>(-2-2.3%)	-0.008<br>(-0.1-0.12)	-0.74<br>(-5.6-5.4)
Marshall Islands	1990	Female	50 to 54	26%<br>(12-44%)	74%<br>(56-88%)	0.43<br>(0.13-0.95)	-0.78%<br>(-2.6-0.91%)	-0.8%<br>(-2.9-1.5%)	-0.05<br>(-0.19-0.093)	-2.6<br>(-8.6-3.1)
Marshall Islands	1990	Female	55 to 59	25%<br>(12-45%)	75%<br>(55-88%)	0.39<br>(0.12-0.95)	-1%<br>(-3-0.6%)	-1%<br>(-3.3-0.98%)	-0.074<br>(-0.24-0.069)	-3.2<br>(-9.7-1.9)
Marshall Islands	1990	Female	60 to 64	25%<br>(12-46%)	75%<br>(54-88%)	0.37<br>(0.11-0.87)	-1.3%<br>(-3.4-0.27%)	-1.3%<br>(-3.8-0.51%)	-0.11<br>(-0.31-0.043)	-3.8<br>(-10-0.8)
Marshall Islands	1990	Female	65 to 69	26%<br>(12-43%)	74%<br>(57-88%)	0.37<br>(0.12-0.82)	-1.3%<br>(-3.1-0.23%)	-1.4%<br>(-3.4-0.39%)	-0.13<br>(-0.32-0.034)	-3.5<br>(-8.1-0.58)
Marshall Islands	1990	Female	70 to 74	26%<br>(12-43%)	74%<br>(57-88%)	0.36<br>(0.11-0.83)	-1.3%<br>(-2.9-0.2%)	-1.4%<br>(-3.3-0.35%)	-0.13<br>(-0.32-0.035)	-2.7<br>(-6.4-0.43)
Marshall Islands	1990	Female	75 to 79	25%<br>(11-44%)	75%<br>(56-89%)	0.34<br>(0.093-0.85)	-1%<br>(-2.7-0.29%)	-1.1%<br>(-3-0.4%)	-0.094<br>(-0.26-0.035)	-1.5<br>(-4-0.43)
Marshall Islands	1990	Female	80 to 84	24%<br>(10-43%)	76%<br>(57-90%)	0.32<br>(0.086-0.77)	-1%<br>(-2.5-0.1%)	-1.1%<br>(-2.9-0.16%)	-0.07<br>(-0.18-0.0099)	-0.79<br>(-2-0.073)
Marshall Islands	1990	Female	85 to 89	25%<br>(11-43%)	75%<br>(57-89%)	0.33<br>(0.11-0.85)	-1%<br>(-2.5-0.1%)	-1.2%<br>(-2.9-0.12%)	-0.041<br>(-0.1-0.004)	-0.32<br>(-0.76-0.031)
Marshall Islands	1990	Female	90 to 94	28%<br>(13-48%)	72%<br>(52-87%)	0.37<br>(0.12-0.86)	-0.97%<br>(-2.2-0.17%)	-1.1%<br>(-2.5-0.19%)	-0.015<br>(-0.033-0.0025)	-0.077<br>(-0.17-0.013)
Marshall Islands	1990	Female	95 plus	33%<br>(15-56%)	67%<br>(44-85%)	0.43<br>(0.12-1.1)	-1.3%<br>(-3-0.31%)	-1.5%<br>(-3.4-0.33%)	-0.0048<br>(-0.011-0.0011)	-0.016<br>(-0.038-0.004)
Marshall Islands	1990	Male	All Ages	39%<br>(31-49%)	61%<br>(51-69%)	0.64<br>(0.37-1.1)	1.9%<br>(0.86-3.5%)	2%<br>(0.65-4.3%)	3.4<br>(1.1-7.2)	190<br>(85-350)
Marshall Islands	1990	Male	15 to 19	37%<br>(22-55%)	63%<br>(45-78%)	0.43<br>(0.16-0.87)	1.9%<br>(0.79-3.6%)	2.4%<br>(0.76-4.9%)	0.11<br>(0.035-0.23)	9.8<br>(4-18)
Marshall Islands	1990	Male	20 to 24	55%<br>(37-73%)	45%<br>(27-63%)	1.2<br>(0.55-2.1)	5.4%<br>(2.5-9.6%)	6.5%<br>(2.7-12%)	0.4<br>(0.16-0.73)	30<br>(14-53)
Marshall Islands	1990	Male	25 to 29	63%<br>(43-78%)	37%<br>(22-57%)	1.6<br>(0.8-2.9)	6.8%<br>(3.5-12%)	8.2%<br>(3.9-15%)	0.51<br>(0.24-0.93)	35<br>(18-61)

Location	Year	Sex	Age	Prevalence of current drinkers	Prevalence of abstainers	Population average of standard drinks daily	Percent of total attributable deaths	Percent of total attributable DALYs	Attributable deaths	Attributable DALYs
Marshall Islands	1990	Male	30 to 34	64%<br>(43-80%)	36%<br>(20-57%)	1.7<br>(0.84-3.2)	6.2%<br>(3.2-11%)	7.3%<br>(3.4-14%)	0.47<br>(0.22-0.89)	30<br>(16-54)
Marshall Islands	1990	Male	35 to 39	62%<br>(44-79%)	38%<br>(21-56%)	1.5<br>(0.71-2.9)	4.8%<br>(2.1-9.3%)	5.6%<br>(2.2-11%)	0.4<br>(0.16-0.8)	23<br>(10-45)
Marshall Islands	1990	Male	40 to 44	59%<br>(42-76%)	41%<br>(24-58%)	1.3<br>(0.58-2.6)	3.7%<br>(1.1-8%)	4.1%<br>(0.98-9.3%)	0.33<br>(0.079-0.76)	17<br>(5.3-38)
Marshall Islands	1990	Male	45 to 49	56%<br>(34-74%)	44%<br>(26-66%)	1.1<br>(0.48-2.3)	2.9%<br>(0.61-6.7%)	3.2%<br>(0.44-7.8%)	0.3<br>(0.039-0.74)	14<br>(2.9-33)
Marshall Islands	1990	Male	50 to 54	53%<br>(34-71%)	47%<br>(29-66%)	0.94<br>(0.38-1.9)	2.2%<br>(0.14-6%)	2.4%<br>(0.0055-6.8%)	0.27<br>(0.00058-0.75)	11<br>(0.75-29)
Marshall Islands	1990	Male	55 to 59	51%<br>(33-70%)	49%<br>(30-67%)	0.77<br>(0.31-1.6)	1.4%<br>(-0.16-4.2%)	1.5%<br>(-0.29-4.6%)	0.18<br>(-0.036-0.58)	6.5<br>(-0.81-20)
Marshall Islands	1990	Male	60 to 64	48%<br>(29-68%)	52%<br>(32-71%)	0.64<br>(0.26-1.4)	0.97%<br>(-0.4-3.6%)	1%<br>(-0.46-3.9%)	0.14<br>(-0.062-0.53)	4.2<br>(-1.7-16)
Marshall Islands	1990	Male	65 to 69	46%<br>(28-65%)	54%<br>(35-72%)	0.58<br>(0.23-1.2)	0.85%<br>(-0.36-3.1%)	0.94%<br>(-0.39-3.5%)	0.12<br>(-0.05-0.46)	2.9<br>(-1.3-11)
Marshall Islands	1990	Male	70 to 74	44%<br>(26-61%)	56%<br>(39-74%)	0.52<br>(0.2-1.1)	0.58%<br>(-0.42-2.7%)	0.63%<br>(-0.48-3%)	0.072<br>(-0.055-0.33)	1.4<br>(-1-6.3)
Marshall Islands	1990	Male	75 to 79	40%<br>(24-59%)	60%<br>(41-76%)	0.44<br>(0.16-0.98)	0.43%<br>(-0.41-2.2%)	0.45%<br>(-0.47-2.4%)	0.038<br>(-0.04-0.21)	0.59<br>(-0.55-3)
Marshall Islands	1990	Male	80 to 84	37%<br>(21-55%)	63%<br>(45-79%)	0.39<br>(0.15-0.86)	0.34%<br>(-0.34-1.6%)	0.35%<br>(-0.4-1.7%)	0.018<br>(-0.021-0.092)	0.21<br>(-0.21-1)
Marshall Islands	1990	Male	85 to 89	35%<br>(21-52%)	65%<br>(48-79%)	0.37<br>(0.15-0.82)	0.17%<br>(-0.48-1.3%)	0.15%<br>(-0.59-1.4%)	0.0034<br>(-0.013-0.03)	0.033<br>(-0.093-0.24)
Marshall Islands	1990	Male	90 to 94	37%<br>(23-54%)	63%<br>(46-77%)	0.38<br>(0.15-0.82)	0.12%<br>(-0.44-1.1%)	0.074%<br>(-0.57-1.1%)	0.00051<br>(-0.0039-0.0076)	0.0047<br>(-0.018-0.044)
Marshall Islands	1990	Male	95 plus	40%<br>(22-62%)	60%<br>(38-78%)	0.41<br>(0.14-0.94)	-0.093%<br>(-0.79-1%)	-0.22%<br>(-1-0.98%)	-0.00034<br>(-0.0016-0.0015)	-0.00056<br>(-0.0049-0.0062)
Marshall Islands	1995	Female	All Ages	19%<br>(13-26%)	81%<br>(74-87%)	0.29<br>(0.15-0.49)	-0.091%<br>(-0.65-0.71%)	-0.69%<br>(-1.6-0.59%)	-0.95<br>(-2.2-0.84)	-7.3<br>(-51-55)
Marshall Islands	1995	Female	15 to 19	20%<br>(9-35%)	80%<br>(65-91%)	0.35<br>(0.12-0.78)	0.98%<br>(0.38-2.1%)	1.5%<br>(0.28-3.6%)	0.036<br>(0.0068-0.089)	3.7<br>(1.4-7.6)
Marshall Islands	1995	Female	20 to 24	24%<br>(10-43%)	76%<br>(57-90%)	0.55<br>(0.19-1.2)	1.7%<br>(0.79-3.2%)	2.1%<br>(0.59-4.8%)	0.068<br>(0.018-0.16)	7<br>(3.2-14)
Marshall Islands	1995	Female	25 to 29	26%<br>(10-45%)	74%<br>(55-90%)	0.56<br>(0.2-1.2)	1.4%<br>(0.59-2.8%)	1.7%<br>(0.41-4%)	0.062<br>(0.015-0.15)	5.8<br>(2.3-11)
Marshall Islands	1995	Female	30 to 34	27%<br>(10-48%)	73%<br>(52-90%)	0.59<br>(0.21-1.2)	1.1%<br>(0.083-2.7%)	1.2%<br>(-0.15-3.6%)	0.052<br>(-0.0065-0.16)	4.4<br>(0.36-11)
Marshall Islands	1995	Female	35 to 39	28%<br>(11-47%)	72%<br>(53-89%)	0.61<br>(0.22-1.2)	0.69%<br>(-0.55-2.6%)	0.85%<br>(-0.82-3.5%)	0.045<br>(-0.043-0.19)	3<br>(-2.4-11)
Marshall Islands	1995	Female	40 to 44	27%<br>(13-44%)	73%<br>(56-87%)	0.56<br>(0.2-1.2)	0.095%<br>(-1.5-2.3%)	0.15%<br>(-2.1-3.1%)	0.0091<br>(-0.12-0.18)	0.4<br>(-6.6-9.7)
Marshall Islands	1995	Female	45 to 49	26%<br>(11-45%)	74%<br>(55-89%)	0.48<br>(0.16-1)	-0.59%<br>(-2.3-1.4%)	-0.63%<br>(-2.8-2%)	-0.043<br>(-0.19-0.13)	-2.5<br>(-10-6.1)

Location	Year	Sex	Age	Prevalence of current drinkers	Prevalence of abstainers	Population average of standard drinks daily	Percent of total attributable deaths	Percent of total attributable DALYs	Attributable deaths	Attributable DALYs
Marshall Islands	1995	Female	50 to 54	25% (11-46%)	75% (54-89%)	0.42 (0.13-0.95)	-1.1% (-3.3-0.54%)	-1.3% (-3.8-0.81%)	-0.1 (-0.3-0.067)	-4.8 (-14-2.3)
Marshall Islands	1995	Female	55 to 59	25% (11-45%)	75% (55-89%)	0.38 (0.12-0.93)	-1.3% (-3.5-0.25%)	-1.4% (-3.9-0.42%)	-0.13 (-0.35-0.037)	-5.1 (-14-1)
Marshall Islands	1995	Female	60 to 64	25% (11-46%)	75% (54-89%)	0.36 (0.11-0.88)	-1.6% (-3.9-0.13%)	-1.7% (-4.4-0.24%)	-0.18 (-0.45-0.024)	-5.7 (-14-0.49)
Marshall Islands	1995	Female	65 to 69	27% (12-47%)	73% (53-88%)	0.38 (0.12-0.86)	-1.7% (-3.8--0.054%)	-1.8% (-4.2-0.096%)	-0.21 (-0.47-0.011)	-5.3 (-12--0.17)
Marshall Islands	1995	Female	70 to 74	27% (13-45%)	73% (55-87%)	0.37 (0.11-0.84)	-1.6% (-3.5--0.058%)	-1.8% (-3.9--0.011%)	-0.21 (-0.45--0.0012)	-4.2 (-8.9--0.14)
Marshall Islands	1995	Female	75 to 79	26% (11-45%)	74% (55-89%)	0.34 (0.093-0.85)	-1.3% (-3.2-0.14%)	-1.4% (-3.5-0.26%)	-0.15 (-0.38-0.029)	-2.3 (-5.7-0.26)
Marshall Islands	1995	Female	80 to 84	25% (11-44%)	75% (56-89%)	0.32 (0.088-0.77)	-1.3% (-2.9--0.039%)	-1.4% (-3.2--0.037%)	-0.11 (-0.25--0.0029)	-1.2 (-2.7--0.039)
Marshall Islands	1995	Female	85 to 89	26% (11-44%)	74% (56-89%)	0.33 (0.1-0.81)	-1.3% (-3--0.078%)	-1.4% (-3.3--0.096%)	-0.065 (-0.15--0.0045)	-0.5 (-1.1--0.033)
Marshall Islands	1995	Female	90 to 94	29% (13-48%)	71% (52-87%)	0.37 (0.12-0.86)	-1.2% (-2.7-0.046%)	-1.4% (-3-0.045%)	-0.022 (-0.047-0.0007)	-0.11 (-0.25-0.0042)
Marshall Islands	1995	Female	95 plus	34% (14-57%)	66% (43-86%)	0.43 (0.11-1)	-1.6% (-3.6-0.31%)	-1.8% (-4.2-0.29%)	-0.0073 (-0.016-0.0011)	-0.024 (-0.055-0.0048)
Marshall Islands	1995	Male	All Ages	39% (31-48%)	61% (52-69%)	0.64 (0.36-1.1)	1.9% (0.82-3.6%)	1.9% (0.58-4.3%)	3.4 (1.1-7.8)	200 (89-390)
Marshall Islands	1995	Male	15 to 19	37% (22-55%)	63% (45-78%)	0.43 (0.16-0.88)	1.8% (0.74-3.5%)	2.3% (0.69-4.6%)	0.11 (0.033-0.23)	9.8 (4-18)
Marshall Islands	1995	Male	20 to 24	55% (36-72%)	45% (28-64%)	1.1 (0.54-2.1)	5.2% (2.5-9.1%)	6.2% (2.6-11%)	0.44 (0.18-0.82)	34 (16-60)
Marshall Islands	1995	Male	25 to 29	62% (43-78%)	38% (22-57%)	1.6 (0.78-2.9)	6.5% (3.3-12%)	7.7% (3.6-14%)	0.54 (0.25-1)	38 (19-67)
Marshall Islands	1995	Male	30 to 34	63% (42-79%)	37% (21-58%)	1.7 (0.81-3.2)	5.8% (2.9-11%)	6.7% (3.1-13%)	0.5 (0.23-0.98)	32 (16-60)
Marshall Islands	1995	Male	35 to 39	62% (44-78%)	38% (22-56%)	1.5 (0.71-2.8)	4.5% (1.9-9%)	5.2% (1.9-11%)	0.43 (0.16-0.88)	25 (11-50)
Marshall Islands	1995	Male	40 to 44	59% (42-76%)	41% (24-58%)	1.3 (0.56-2.5)	3.3% (0.91-7.7%)	3.7% (0.78-9%)	0.36 (0.075-0.85)	19 (5.2-43)
Marshall Islands	1995	Male	45 to 49	55% (34-73%)	45% (27-66%)	1.1 (0.45-2.2)	2.5% (0.34-6.2%)	2.8% (0.19-7.2%)	0.3 (0.02-0.77)	14 (2-36)
Marshall Islands	1995	Male	50 to 54	52% (32-71%)	48% (29-68%)	0.91 (0.36-1.8)	1.9% (-0.093-5.4%)	2% (-0.26-6.2%)	0.26 (-0.033-0.78)	11 (-0.53-31)
Marshall Islands	1995	Male	55 to 59	50% (32-70%)	50% (30-68%)	0.75 (0.29-1.6)	1.1% (-0.37-4.3%)	1.2% (-0.44-4.6%)	0.16 (-0.064-0.62)	6 (-1.9-22)
Marshall Islands	1995	Male	60 to 64	48% (28-68%)	52% (32-72%)	0.62 (0.25-1.4)	0.78% (-0.51-3.5%)	0.83% (-0.62-3.9%)	0.12 (-0.094-0.56)	3.7 (-2.5-16)
Marshall Islands	1995	Male	65 to 69	47% (28-65%)	53% (35-72%)	0.57 (0.23-1.2)	0.66% (-0.51-2.9%)	0.72% (-0.59-3.1%)	0.1 (-0.084-0.45)	2.4 (-2-11)

Location	Year	Sex	Age	Prevalence of current drinkers	Prevalence of abstainers	Population average of standard drinks daily	Percent of total attributable deaths	Percent of total attributable DALYs	Attributable deaths	Attributable DALYs
Marshall Islands	1995	Male	70 to 74	44% (27-62%)	56% (38-73%)	0.51 (0.19-1.1)	0.43% (-0.59-2.4%)	0.46% (-0.66-2.5%)	0.057 (-0.08-0.31)	1.1 (-1.5-6)
Marshall Islands	1995	Male	75 to 79	40% (24-58%)	60% (42-76%)	0.43 (0.16-0.97)	0.3% (-0.56-2%)	0.3% (-0.63-2.1%)	0.029 (-0.061-0.2)	0.46 (-0.86-2.9)
Marshall Islands	1995	Male	80 to 84	37% (21-55%)	63% (45-79%)	0.38 (0.15-0.84)	0.25% (-0.43-1.5%)	0.25% (-0.49-1.6%)	0.013 (-0.026-0.093)	0.16 (-0.28-1)
Marshall Islands	1995	Male	85 to 89	35% (21-52%)	65% (48-79%)	0.36 (0.14-0.77)	0.097% (-0.58-1.2%)	0.063% (-0.68-1.2%)	0.0017 (-0.018-0.033)	0.022 (-0.13-0.26)
Marshall Islands	1995	Male	90 to 94	37% (23-54%)	63% (46-77%)	0.37 (0.14-0.83)	0.053% (-0.5-0.95%)	-0.0014% (-0.65-0.99%)	-0.00001 (-0.005-0.0076)	0.0024 (-0.024-0.044)
Marshall Islands	1995	Male	95 plus	41% (22-61%)	59% (39-78%)	0.41 (0.14-0.96)	-0.15% (-0.87-0.92%)	-0.29% (-1.1-0.89%)	-0.00048 (-0.0019-0.0015)	-0.00097 (-0.0057-0.006)
Marshall Islands	2000	Female	All Ages	19% (13-26%)	81% (74-87%)	0.29 (0.16-0.49)	-0.21% (-0.81-0.55%)	-0.86% (-1.8-0.35%)	-1.5 (-3.1-0.63)	-20 (-77-54)
Marshall Islands	2000	Female	15 to 19	20% (9.3-36%)	80% (64-91%)	0.35 (0.12-0.78)	0.98% (0.37-2.2%)	1.4% (0.26-3.6%)	0.041 (0.0076-0.1)	4.1 (1.5-9.1)
Marshall Islands	2000	Female	20 to 24	24% (11-43%)	76% (57-89%)	0.55 (0.2-1.2)	1.6% (0.76-3.2%)	2% (0.56-4.6%)	0.077 (0.021-0.18)	7.7 (3.5-15)
Marshall Islands	2000	Female	25 to 29	26% (11-45%)	74% (55-89%)	0.56 (0.2-1.2)	1.3% (0.38-2.7%)	1.5% (0.13-3.6%)	0.071 (0.0067-0.17)	6.6 (1.9-14)
Marshall Islands	2000	Female	30 to 34	27% (10-46%)	73% (54-90%)	0.58 (0.21-1.2)	0.88% (-0.19-2.3%)	0.95% (-0.54-3.1%)	0.05 (-0.028-0.16)	4.2 (-0.95-11)
Marshall Islands	2000	Female	35 to 39	27% (12-46%)	73% (54-88%)	0.6 (0.22-1.2)	0.43% (-1-2.6%)	0.51% (-1.3-3.4%)	0.033 (-0.086-0.22)	2.2 (-5.2-13)
Marshall Islands	2000	Female	40 to 44	26% (13-44%)	74% (56-87%)	0.55 (0.21-1.2)	-0.2% (-2-2.1%)	-0.19% (-2.4-2.7%)	-0.014 (-0.19-0.21)	-1.1 (-10-11)
Marshall Islands	2000	Female	45 to 49	26% (11-43%)	74% (57-89%)	0.47 (0.16-0.97)	-0.84% (-2.8-1.2%)	-0.92% (-3.3-1.6%)	-0.082 (-0.29-0.14)	-4.5 (-15-6.5)
Marshall Islands	2000	Female	50 to 54	25% (12-44%)	75% (56-88%)	0.41 (0.13-0.94)	-1.4% (-3.7-0.35%)	-1.5% (-4.2-0.51%)	-0.16 (-0.42-0.056)	-7 (-19-2)
Marshall Islands	2000	Female	55 to 59	25% (12-44%)	75% (56-88%)	0.37 (0.12-0.9)	-1.5% (-3.8-0.086%)	-1.7% (-4.2-0.16%)	-0.19 (-0.48-0.017)	-7.2 (-18-0.39)
Marshall Islands	2000	Female	60 to 64	25% (11-44%)	75% (56-89%)	0.36 (0.11-0.86)	-1.7% (-4--0.11%)	-1.9% (-4.5--0.095%)	-0.24 (-0.56--0.012)	-7.5 (-17--0.47)
Marshall Islands	2000	Female	65 to 69	26% (12-45%)	74% (55-88%)	0.36 (0.12-0.84)	-1.8% (-3.9--0.18%)	-2% (-4.4--0.12%)	-0.28 (-0.6--0.016)	-6.9 (-15--0.71)
Marshall Islands	2000	Female	70 to 74	26% (12-44%)	74% (56-88%)	0.35 (0.11-0.8)	-1.8% (-3.7--0.13%)	-2% (-4.3--0.12%)	-0.28 (-0.6--0.018)	-5.4 (-12--0.4)
Marshall Islands	2000	Female	75 to 79	25% (11-44%)	75% (56-89%)	0.33 (0.087-0.83)	-1.4% (-3.4--0.022%)	-1.6% (-3.8-0.026%)	-0.21 (-0.48-0.0034)	-3.1 (-7.2--0.048)
Marshall Islands	2000	Female	80 to 84	24% (10-43%)	76% (57-90%)	0.31 (0.085-0.76)	-1.4% (-3.2--0.076%)	-1.6% (-3.5--0.083%)	-0.15 (-0.36--0.008)	-1.7 (-3.8--0.086)
Marshall Islands	2000	Female	85 to 89	25% (11-43%)	75% (57-89%)	0.33 (0.098-0.78)	-1.4% (-3.2--0.093%)	-1.6% (-3.6--0.11%)	-0.093 (-0.21--0.0064)	-0.7 (-1.6--0.047)

Location	Year	Sex	Age	Prevalence of current drinkers	Prevalence of abstainers	Population average of standard drinks daily	Percent of total attributable deaths	Percent of total attributable DALYs	Attributable deaths	Attributable DALYs
Marshall Islands	2000	Female	90 to 94	29% (13-49%)	71% (51-87%)	0.38 (0.11-0.89)	-1.4% (-2.9-0.071%)	-1.6% (-3.3-0.03%)	-0.033 (-0.07-0.00063)	-0.17 (-0.36-0.0088)
Marshall Islands	2000	Female	95 plus	34% (15-56%)	66% (44-85%)	0.44 (0.12-1.1)	-1.7% (-4-0.25%)	-2% (-4.5-0.2%)	-0.01 (-0.023-0.00097)	-0.034 (-0.079-0.0047)
Marshall Islands	2000	Male	All Ages	39% (30-49%)	61% (51-70%)	0.66 (0.36-1.1)	1.8% (0.77-3.5%)	1.8% (0.48-3.9%)	3.7 (1-8.2)	220 (91-410)
Marshall Islands	2000	Male	15 to 19	37% (21-55%)	63% (45-79%)	0.43 (0.16-0.87)	1.9% (0.8-3.6%)	2.3% (0.72-4.8%)	0.12 (0.038-0.25)	11 (4.5-20)
Marshall Islands	2000	Male	20 to 24	55% (35-72%)	45% (28-65%)	1.2 (0.52-2.1)	5.2% (2.5-9.2%)	6.2% (2.6-11%)	0.48 (0.2-0.88)	36 (17-64)
Marshall Islands	2000	Male	25 to 29	62% (43-78%)	38% (22-57%)	1.6 (0.78-2.8)	6.5% (3.3-11%)	7.7% (3.5-14%)	0.65 (0.3-1.2)	45 (23-78)
Marshall Islands	2000	Male	30 to 34	63% (42-79%)	37% (21-58%)	1.7 (0.84-3.1)	5.6% (2.7-10%)	6.5% (2.8-12%)	0.55 (0.24-1)	35 (17-63)
Marshall Islands	2000	Male	35 to 39	62% (44-78%)	38% (22-56%)	1.5 (0.69-2.8)	4.4% (1.7-8.8%)	4.9% (1.7-10%)	0.47 (0.16-0.98)	28 (11-55)
Marshall Islands	2000	Male	40 to 44	59% (42-76%)	41% (24-58%)	1.3 (0.57-2.5)	3.2% (0.75-7.2%)	3.5% (0.55-8.3%)	0.39 (0.06-0.95)	21 (4.7-48)
Marshall Islands	2000	Male	45 to 49	56% (34-73%)	44% (27-66%)	1.1 (0.47-2.3)	2.4% (0.13-6.1%)	2.5% (-0.028-6.9%)	0.33 (-0.0036-0.9)	16 (0.88-41)
Marshall Islands	2000	Male	50 to 54	52% (33-70%)	48% (30-67%)	0.93 (0.37-1.9)	1.7% (-0.33-5.4%)	1.8% (-0.58-6%)	0.27 (-0.08-0.87)	11 (-2.3-34)
Marshall Islands	2000	Male	55 to 59	50% (32-68%)	50% (32-68%)	0.76 (0.3-1.6)	1% (-0.51-3.9%)	1% (-0.64-4.3%)	0.16 (-0.097-0.68)	6 (-3-24)
Marshall Islands	2000	Male	60 to 64	48% (28-67%)	52% (33-72%)	0.64 (0.26-1.3)	0.67% (-0.69-3.2%)	0.69% (-0.8-3.5%)	0.11 (-0.13-0.57)	3.5 (-3.6-17)
Marshall Islands	2000	Male	65 to 69	46% (27-65%)	54% (35-73%)	0.57 (0.24-1.2)	0.55% (-0.62-2.7%)	0.59% (-0.69-3%)	0.093 (-0.11-0.46)	2.3 (-2.6-11)
Marshall Islands	2000	Male	70 to 74	43% (25-61%)	57% (39-75%)	0.51 (0.19-1.1)	0.33% (-0.7-2.1%)	0.34% (-0.79-2.4%)	0.047 (-0.11-0.31)	0.95 (-1.9-6.1)
Marshall Islands	2000	Male	75 to 79	40% (23-59%)	60% (41-77%)	0.43 (0.17-0.97)	0.22% (-0.66-1.7%)	0.21% (-0.75-1.9%)	0.022 (-0.08-0.2)	0.38 (-1.2-2.9)
Marshall Islands	2000	Male	80 to 84	37% (20-55%)	63% (45-80%)	0.39 (0.15-0.82)	0.18% (-0.54-1.4%)	0.17% (-0.63-1.4%)	0.011 (-0.04-0.094)	0.14 (-0.41-1)
Marshall Islands	2000	Male	85 to 89	35% (21-53%)	65% (47-79%)	0.37 (0.15-0.75)	0.035% (-0.68-1.1%)	-0.013% (-0.82-1.1%)	-0.00041 (-0.027-0.037)	0.0098 (-0.19-0.3)
Marshall Islands	2000	Male	90 to 94	37% (23-55%)	63% (45-77%)	0.38 (0.14-0.8)	-0.0041% (-0.6-0.9%)	-0.071% (-0.74-0.93%)	-0.00076 (-0.008-0.0099)	-0.00027 (-0.038-0.058)
Marshall Islands	2000	Male	95 plus	41% (22-62%)	59% (38-78%)	0.42 (0.14-0.94)	-0.2% (-0.95-0.94%)	-0.35% (-1.2-0.9%)	-0.00077 (-0.0027-0.0019)	-0.0017 (-0.0082-0.0083)
Marshall Islands	2005	Female	All Ages	19% (13-25%)	81% (75-87%)	0.31 (0.17-0.53)	-0.31% (-1.1-0.68%)	-1% (-2.2-0.39%)	-2 (-4.4-0.85)	-32 (-110-73)
Marshall Islands	2005	Female	15 to 19	20% (9.6-35%)	80% (65-90%)	0.37 (0.13-0.8)	1% (0.38-2.2%)	1.5% (0.31-3.6%)	0.046 (0.0099-0.11)	4.6 (1.7-9.6)

Location	Year	Sex	Age	Prevalence of current drinkers	Prevalence of abstainers	Population average of standard drinks daily	Percent of total attributable deaths	Percent of total attributable DALYs	Attributable deaths	Attributable DALYs
Marshall Islands	2005	Female	20 to 24	24%<br>(11-42%)	76%<br>(58-89%)	0.58<br>(0.22-1.2)	1.7%<br>(0.74-3.3%)	2.1%<br>(0.58-4.8%)	0.092<br>(0.026-0.22)	8.9<br>(3.8-17)
Marshall Islands	2005	Female	25 to 29	26%<br>(11-44%)	74%<br>(56-89%)	0.59<br>(0.2-1.2)	1.3%<br>(0.36-2.9%)	1.5%<br>(0.084-3.9%)	0.083<br>(0.005-0.23)	7.5<br>(2-17)
Marshall Islands	2005	Female	30 to 34	27%<br>(10-45%)	73%<br>(55-90%)	0.6<br>(0.21-1.3)	0.83%<br>(-0.35-2.6%)	0.88%<br>(-0.74-3.3%)	0.061<br>(-0.052-0.23)	5.1<br>(-2.3-16)
Marshall Islands	2005	Female	35 to 39	27%<br>(11-46%)	73%<br>(54-89%)	0.63<br>(0.23-1.3)	0.38%<br>(-1.2-2.5%)	0.44%<br>(-1.6-3.2%)	0.037<br>(-0.13-0.28)	2.4<br>(-8-16)
Marshall Islands	2005	Female	40 to 44	26%<br>(13-44%)	74%<br>(56-87%)	0.57<br>(0.22-1.2)	-0.26%<br>(-2.1-2.3%)	-0.27%<br>(-2.6-2.9%)	-0.026<br>(-0.25-0.28)	-1.7<br>(-14-15)
Marshall Islands	2005	Female	45 to 49	26%<br>(11-44%)	74%<br>(56-89%)	0.49<br>(0.17-1.1)	-0.91%<br>(-3.1-1.2%)	-1%<br>(-3.6-1.5%)	-0.11<br>(-0.41-0.17)	-6.1<br>(-21-7.6)
Marshall Islands	2005	Female	50 to 54	26%<br>(12-44%)	74%<br>(56-88%)	0.44<br>(0.13-1)	-1.5%<br>(-3.9-0.5%)	-1.7%<br>(-4.5-0.73%)	-0.22<br>(-0.6-0.092)	-9.7<br>(-25-3.2)
Marshall Islands	2005	Female	55 to 59	25%<br>(11-45%)	75%<br>(55-89%)	0.4<br>(0.12-0.94)	-1.6%<br>(-4-0.23%)	-1.8%<br>(-4.5-0.36%)	-0.26<br>(-0.67-0.058)	-9.6<br>(-24-1.5)
Marshall Islands	2005	Female	60 to 64	25%<br>(11-45%)	75%<br>(55-89%)	0.38<br>(0.11-0.89)	-1.8%<br>(-4.2--0.1%)	-2%<br>(-4.7--0.071%)	-0.32<br>(-0.76--0.012)	-9.7<br>(-23--0.56)
Marshall Islands	2005	Female	65 to 69	27%<br>(12-46%)	73%<br>(54-88%)	0.39<br>(0.13-0.88)	-1.9%<br>(-4.2--0.24%)	-2.1%<br>(-4.7--0.23%)	-0.36<br>(-0.82--0.039)	-8.9<br>(-20--1.1)
Marshall Islands	2005	Female	70 to 74	27%<br>(12-44%)	73%<br>(56-88%)	0.38<br>(0.11-0.85)	-1.9%<br>(-4--0.22%)	-2.1%<br>(-4.5--0.23%)	-0.38<br>(-0.82--0.043)	-7.4<br>(-16--0.81)
Marshall Islands	2005	Female	75 to 79	25%<br>(11-45%)	75%<br>(55-89%)	0.35<br>(0.097-0.9)	-1.5%<br>(-3.6-0.07%)	-1.7%<br>(-4-0.09%)	-0.28<br>(-0.65-0.015)	-4.1<br>(-9.5-0.17)
Marshall Islands	2005	Female	80 to 84	24%<br>(11-44%)	76%<br>(56-89%)	0.33<br>(0.094-0.81)	-1.5%<br>(-3.5--0.1%)	-1.7%<br>(-3.9--0.12%)	-0.21<br>(-0.49--0.015)	-2.2<br>(-5.3--0.16)
Marshall Islands	2005	Female	85 to 89	25%<br>(11-44%)	75%<br>(56-89%)	0.34<br>(0.11-0.81)	-1.5%<br>(-3.4--0.00053%)	-1.7%<br>(-3.8--0.05%)	-0.12<br>(-0.27--0.0036)	-0.9<br>(-2--0.00033)
Marshall Islands	2005	Female	90 to 94	28%<br>(13-48%)	72%<br>(52-87%)	0.39<br>(0.12-0.94)	-1.4%<br>(-3.1-0.14%)	-1.7%<br>(-3.5-0.098%)	-0.044<br>(-0.093-0.0026)	-0.23<br>(-0.49-0.021)
Marshall Islands	2005	Female	95 plus	33%<br>(14-57%)	67%<br>(43-86%)	0.46<br>(0.12-1.1)	-1.8%<br>(-4.2-0.2%)	-2.1%<br>(-4.9-0.17%)	-0.014<br>(-0.033-0.0011)	-0.047<br>(-0.11-0.0052)
Marshall Islands	2005	Male	All Ages	40%<br>(32-49%)	60%<br>(51-68%)	0.7<br>(0.39-1.2)	2%<br>(0.77-4%)	1.9%<br>(0.41-4.3%)	4.5<br>(1-9.9)	260<br>(97-510)
Marshall Islands	2005	Male	15 to 19	36%<br>(21-54%)	64%<br>(46-79%)	0.44<br>(0.17-0.9)	1.9%<br>(0.85-3.7%)	2.4%<br>(0.81-4.9%)	0.13<br>(0.045-0.29)	12<br>(5-22)
Marshall Islands	2005	Male	20 to 24	55%<br>(36-72%)	45%<br>(28-64%)	1.2<br>(0.55-2.2)	5.4%<br>(2.6-9.4%)	6.4%<br>(2.7-12%)	0.54<br>(0.22-1)	41<br>(19-74)
Marshall Islands	2005	Male	25 to 29	62%<br>(43-78%)	38%<br>(22-57%)	1.6<br>(0.79-2.9)	6.6%<br>(3.4-12%)	7.8%<br>(3.5-14%)	0.73<br>(0.33-1.3)	51<br>(26-88)
Marshall Islands	2005	Male	30 to 34	63%<br>(42-80%)	37%<br>(20-58%)	1.8<br>(0.88-3.1)	5.7%<br>(2.6-10%)	6.5%<br>(2.7-12%)	0.71<br>(0.29-1.3)	46<br>(21-80)
Marshall Islands	2005	Male	35 to 39	62%<br>(43-78%)	38%<br>(22-57%)	1.6<br>(0.73-2.9)	4.5%<br>(1.6-8.9%)	5%<br>(1.6-10%)	0.59<br>(0.18-1.2)	35<br>(12-68)

Location	Year	Sex	Age	Prevalence of current drinkers	Prevalence of abstainers	Population average of standard drinks daily	Percent of total attributable deaths	Percent of total attributable DALYs	Attributable deaths	Attributable DALYs
Marshall Islands	2005	Male	40 to 44	59% (41-76%)	41% (24-59%)	1.4 (0.58-2.7)	3.2% (0.64-7.4%)	3.5% (0.5-8.5%)	0.49 (0.071-1.2)	26 (5.6-61)
Marshall Islands	2005	Male	45 to 49	55% (34-73%)	45% (27-66%)	1.2 (0.48-2.3)	2.4% (0.014-6.5%)	2.5% (-0.2-7.2%)	0.42 (-0.029-1.2)	20 (0.12-56)
Marshall Islands	2005	Male	50 to 54	52% (33-71%)	48% (29-67%)	0.97 (0.39-1.9)	1.7% (-0.44-5.5%)	1.8% (-0.67-6.1%)	0.34 (-0.13-1.2)	14 (-3.9-46)
Marshall Islands	2005	Male	55 to 59	50% (32-69%)	50% (31-68%)	0.79 (0.31-1.6)	0.98% (-0.7-4%)	1% (-0.81-4.2%)	0.19 (-0.16-0.81)	7.2 (-5.1-29)
Marshall Islands	2005	Male	60 to 64	48% (28-68%)	52% (32-72%)	0.66 (0.25-1.4)	0.65% (-0.76-3.2%)	0.65% (-0.9-3.5%)	0.13 (-0.18-0.68)	4 (-4.8-20)
Marshall Islands	2005	Male	65 to 69	46% (28-64%)	54% (36-72%)	0.6 (0.24-1.2)	0.51% (-0.72-2.6%)	0.53% (-0.82-2.8%)	0.1 (-0.16-0.55)	2.5 (-3.6-13)
Marshall Islands	2005	Male	70 to 74	43% (26-61%)	57% (39-74%)	0.53 (0.2-1.1)	0.3% (-0.77-2%)	0.29% (-0.86-2.2%)	0.049 (-0.14-0.37)	1 (-2.6-7.3)
Marshall Islands	2005	Male	75 to 79	39% (24-58%)	61% (42-76%)	0.44 (0.17-0.97)	0.19% (-0.74-1.6%)	0.17% (-0.85-1.8%)	0.022 (-0.11-0.22)	0.39 (-1.5-3.3)
Marshall Islands	2005	Male	80 to 84	36% (21-55%)	64% (45-79%)	0.39 (0.16-0.82)	0.16% (-0.59-1.3%)	0.13% (-0.69-1.4%)	0.01 (-0.053-0.1)	0.15 (-0.55-1.2)
Marshall Islands	2005	Male	85 to 89	35% (20-52%)	65% (48-80%)	0.37 (0.15-0.79)	0.014% (-0.71-1%)	-0.044% (-0.85-1.1%)	-0.0017 (-0.034-0.043)	0.0047 (-0.24-0.36)
Marshall Islands	2005	Male	90 to 94	36% (22-53%)	64% (47-78%)	0.38 (0.14-0.8)	-0.029% (-0.66-0.84%)	-0.11% (-0.83-0.87%)	-0.0015 (-0.011-0.012)	-0.0024 (-0.054-0.067)
Marshall Islands	2005	Male	95 plus	40% (22-61%)	60% (39-78%)	0.41 (0.13-0.93)	-0.21% (-0.98-0.83%)	-0.37% (-1.3-0.74%)	-0.0012 (-0.004-0.0023)	-0.0026 (-0.012-0.01)
Marshall Islands	2010	Female	All Ages	19% (13-26%)	81% (74-87%)	0.33 (0.17-0.57)	-0.36% (-1.2-0.75%)	-1.1% (-2.4-0.43%)	-2.4 (-5.2-0.92)	-40 (-130-84)
Marshall Islands	2010	Female	15 to 19	20% (8.9-35%)	80% (65-91%)	0.38 (0.13-0.84)	1% (0.38-2.2%)	1.5% (0.33-3.7%)	0.05 (0.011-0.13)	5 (2-11)
Marshall Islands	2010	Female	20 to 24	24% (10-42%)	76% (58-90%)	0.59 (0.21-1.3)	1.7% (0.76-3.3%)	2.1% (0.55-4.9%)	0.093 (0.025-0.22)	9.2 (4-18)
Marshall Islands	2010	Female	25 to 29	25% (11-44%)	75% (56-89%)	0.61 (0.21-1.3)	1.4% (0.35-3%)	1.5% (0.051-4.2%)	0.09 (0.0029-0.25)	8.3 (2-19)
Marshall Islands	2010	Female	30 to 34	26% (10-46%)	74% (54-90%)	0.63 (0.22-1.3)	0.88% (-0.4-2.7%)	0.93% (-0.79-3.6%)	0.068 (-0.057-0.24)	5.8 (-2.6-17)
Marshall Islands	2010	Female	35 to 39	27% (11-45%)	73% (55-89%)	0.65 (0.24-1.4)	0.38% (-1.4-2.6%)	0.44% (-1.8-3.3%)	0.044 (-0.19-0.33)	2.9 (-11-20)
Marshall Islands	2010	Female	40 to 44	26% (13-44%)	74% (56-87%)	0.61 (0.23-1.3)	-0.29% (-2.3-2.3%)	-0.29% (-2.8-2.8%)	-0.031 (-0.31-0.32)	-2.2 (-18-17)
Marshall Islands	2010	Female	45 to 49	26% (12-44%)	74% (56-88%)	0.53 (0.18-1.1)	-0.94% (-3.2-1.2%)	-1% (-3.7-1.7%)	-0.13 (-0.49-0.22)	-7.2 (-25-9.8)
Marshall Islands	2010	Female	50 to 54	26% (12-46%)	74% (54-88%)	0.46 (0.15-1)	-1.5% (-4-0.77%)	-1.7% (-4.5-1%)	-0.26 (-0.71-0.16)	-11 (-31-6.2)
Marshall Islands	2010	Female	55 to 59	25% (11-44%)	75% (56-89%)	0.42 (0.13-0.97)	-1.6% (-4-0.31%)	-1.8% (-4.6-0.44%)	-0.32 (-0.85-0.079)	-12 (-30-2.2)

Location	Year	Sex	Age	Prevalence of current drinkers	Prevalence of abstainers	Population average of standard drinks daily	Percent of total attributable deaths	Percent of total attributable DALYs	Attributable deaths	Attributable DALYs
Marshall Islands	2010	Female	60 to 64	25% (11-44%)	75% (56-89%)	0.4 (0.11-0.94)	-1.8% (-4.2-0.014%)	-2% (-4.7-0.094%)	-0.38 (-0.9-0.018)	-12 (-27-0.092)
Marshall Islands	2010	Female	65 to 69	26% (11-45%)	74% (55-89%)	0.41 (0.13-0.93)	-1.9% (-4.3--0.16%)	-2.1% (-4.8--0.14%)	-0.43 (-0.98--0.028)	-11 (-24--0.88)
Marshall Islands	2010	Female	70 to 74	26% (11-43%)	74% (57-89%)	0.39 (0.11-0.92)	-1.9% (-4--0.21%)	-2.1% (-4.5--0.23%)	-0.44 (-0.96--0.049)	-8.5 (-18--0.96)
Marshall Islands	2010	Female	75 to 79	25% (11-43%)	75% (57-89%)	0.36 (0.096-0.9)	-1.5% (-3.6-0.0049%)	-1.7% (-4-0.0012%)	-0.34 (-0.8-0.00027)	-4.9 (-12-0.018)
Marshall Islands	2010	Female	80 to 84	24% (10-43%)	76% (57-90%)	0.35 (0.1-0.86)	-1.5% (-3.5--0.15%)	-1.7% (-3.9--0.2%)	-0.26 (-0.6--0.032)	-2.8 (-6.5--0.28)
Marshall Islands	2010	Female	85 to 89	25% (11-43%)	75% (57-89%)	0.36 (0.12-0.89)	-1.5% (-3.4--0.12%)	-1.7% (-3.9--0.16%)	-0.14 (-0.31--0.013)	-1.1 (-2.4--0.077)
Marshall Islands	2010	Female	90 to 94	28% (13-48%)	72% (52-87%)	0.41 (0.13-0.99)	-1.5% (-3.2-0.13%)	-1.7% (-3.7-0.094%)	-0.051 (-0.11-0.0028)	-0.26 (-0.57-0.022)
Marshall Islands	2010	Female	95 plus	33% (14-56%)	67% (44-86%)	0.48 (0.14-1.1)	-1.8% (-4.2-0.27%)	-2.1% (-4.9-0.19%)	-0.017 (-0.039-0.0015)	-0.055 (-0.13-0.008)
Marshall Islands	2010	Male	All Ages	40% (32-50%)	60% (50-68%)	0.74 (0.41-1.3)	2.2% (0.8-4.3%)	2% (0.38-4.5%)	5.2 (1-12)	300 (110-590)
Marshall Islands	2010	Male	15 to 19	36% (21-55%)	64% (45-79%)	0.46 (0.17-0.93)	2% (0.9-3.8%)	2.5% (0.83-5.2%)	0.15 (0.048-0.32)	13 (5.5-25)
Marshall Islands	2010	Male	20 to 24	54% (36-72%)	46% (28-64%)	1.2 (0.58-2.2)	5.5% (2.7-9.8%)	6.6% (2.9-13%)	0.58 (0.24-1.1)	43 (20-81)
Marshall Islands	2010	Male	25 to 29	61% (42-77%)	39% (23-58%)	1.7 (0.82-3.1)	6.8% (3.4-12%)	8.1% (3.6-15%)	0.81 (0.36-1.5)	56 (28-98)
Marshall Islands	2010	Male	30 to 34	63% (41-79%)	37% (21-59%)	1.8 (0.88-3.3)	5.9% (2.7-11%)	6.7% (2.8-13%)	0.79 (0.32-1.5)	51 (23-92)
Marshall Islands	2010	Male	35 to 39	62% (43-78%)	38% (22-57%)	1.6 (0.75-3.1)	4.6% (1.6-9.1%)	5.2% (1.6-11%)	0.75 (0.22-1.5)	44 (15-86)
Marshall Islands	2010	Male	40 to 44	59% (41-76%)	41% (24-59%)	1.4 (0.61-2.7)	3.3% (0.57-7.9%)	3.6% (0.46-9%)	0.58 (0.076-1.4)	31 (5.5-72)
Marshall Islands	2010	Male	45 to 49	55% (34-73%)	45% (27-66%)	1.2 (0.51-2.4)	2.5% (-0.1-6.6%)	2.6% (-0.39-7.3%)	0.51 (-0.068-1.4)	24 (-1-63)
Marshall Islands	2010	Male	50 to 54	52% (33-71%)	48% (29-67%)	1 (0.4-2)	1.8% (-0.44-5.5%)	1.9% (-0.61-6%)	0.42 (-0.14-1.3)	18 (-4.5-53)
Marshall Islands	2010	Male	55 to 59	50% (32-69%)	50% (31-68%)	0.82 (0.33-1.7)	1.1% (-0.65-4.1%)	1.1% (-0.84-4.5%)	0.25 (-0.2-1)	9.3 (-6-36)
Marshall Islands	2010	Male	60 to 64	47% (28-67%)	53% (33-72%)	0.68 (0.25-1.5)	0.7% (-0.75-3.3%)	0.71% (-0.9-3.6%)	0.16 (-0.21-0.84)	5.1 (-5.6-25)
Marshall Islands	2010	Male	65 to 69	45% (27-64%)	55% (36-73%)	0.62 (0.24-1.3)	0.54% (-0.73-2.8%)	0.56% (-0.85-3%)	0.12 (-0.19-0.67)	3.1 (-4.2-16)
Marshall Islands	2010	Male	70 to 74	43% (26-61%)	57% (39-74%)	0.55 (0.22-1.2)	0.32% (-0.78-2.3%)	0.32% (-0.89-2.5%)	0.061 (-0.17-0.49)	1.3 (-3.1-9.4)
Marshall Islands	2010	Male	75 to 79	39% (24-58%)	61% (42-76%)	0.47 (0.18-1)	0.21% (-0.76-2%)	0.19% (-0.85-2.1%)	0.028 (-0.13-0.32)	0.51 (-1.7-4.8)

Location	Year	Sex	Age	Prevalence of current drinkers	Prevalence of abstainers	Population average of standard drinks daily	Percent of total attributable deaths	Percent of total attributable DALYs	Attributable deaths	Attributable DALYs
Marshall Islands	2010	Male	80 to 84	37% (21-56%)	63% (44-79%)	0.42 (0.17-0.89)	0.17% (-0.6-1.5%)	0.14% (-0.72-1.6%)	0.013 (-0.062-0.15)	0.19 (-0.65-1.7)
Marshall Islands	2010	Male	85 to 89	35% (20-52%)	65% (48-80%)	0.39 (0.16-0.85)	0.029% (-0.69-1.1%)	-0.032% (-0.85-1.2%)	-0.0014 (-0.038-0.053)	0.011 (-0.26-0.43)
Marshall Islands	2010	Male	90 to 94	36% (22-54%)	64% (46-78%)	0.4 (0.16-0.87)	-0.021% (-0.68-0.9%)	-0.1% (-0.85-0.92%)	-0.0016 (-0.013-0.014)	-0.0019 (-0.061-0.082)
Marshall Islands	2010	Male	95 plus	40% (21-60%)	60% (40-79%)	0.43 (0.15-0.97)	-0.19% (-1-0.99%)	-0.35% (-1.3-0.98%)	-0.0013 (-0.0049-0.0037)	-0.0028 (-0.015-0.015)
Marshall Islands	2016	Female	All Ages	19% (14-26%)	81% (74-86%)	0.36 (0.2-0.61)	-0.4% (-1.3-0.89%)	-1.2% (-2.6-0.62%)	-2.9 (-6.5-1.6)	-48 (-170-110)
Marshall Islands	2016	Female	15 to 19	20% (8.8-35%)	80% (65-91%)	0.41 (0.13-0.94)	1% (0.41-2.4%)	1.6% (0.38-4%)	0.054 (0.013-0.15)	5.6 (2.2-13)
Marshall Islands	2016	Female	20 to 24	24% (9.9-42%)	76% (58-90%)	0.64 (0.23-1.4)	1.8% (0.78-3.5%)	2.2% (0.64-5.4%)	0.1 (0.027-0.25)	10 (4.6-20)
Marshall Islands	2016	Female	25 to 29	25% (11-44%)	75% (56-89%)	0.65 (0.23-1.3)	1.4% (0.29-3.2%)	1.6% (-0.087-4.3%)	0.091 (-0.0053-0.26)	8.7 (1.9-20)
Marshall Islands	2016	Female	30 to 34	26% (9.8-46%)	74% (54-90%)	0.67 (0.23-1.3)	0.92% (-0.39-2.9%)	0.97% (-0.84-4%)	0.072 (-0.062-0.27)	6.4 (-2.8-20)
Marshall Islands	2016	Female	35 to 39	27% (11-45%)	73% (55-89%)	0.69 (0.25-1.5)	0.41% (-1.4-2.8%)	0.45% (-1.9-3.6%)	0.046 (-0.2-0.38)	3.4 (-11-24)
Marshall Islands	2016	Female	40 to 44	26% (12-43%)	74% (57-88%)	0.63 (0.24-1.3)	-0.23% (-2.3-2.2%)	-0.28% (-3-2.9%)	-0.035 (-0.37-0.38)	-2 (-20-20)
Marshall Islands	2016	Female	45 to 49	26% (12-44%)	74% (56-88%)	0.54 (0.19-1.2)	-0.88% (-3.1-1.4%)	-1% (-3.7-1.7%)	-0.15 (-0.55-0.26)	-7.6 (-27-12)
Marshall Islands	2016	Female	50 to 54	25% (11-44%)	75% (56-89%)	0.48 (0.15-1)	-1.4% (-3.8-1.1%)	-1.6% (-4.4-1.2%)	-0.28 (-0.81-0.22)	-12 (-35-9.1)
Marshall Islands	2016	Female	55 to 59	25% (11-45%)	75% (55-89%)	0.44 (0.15-1)	-1.5% (-4.1-0.63%)	-1.8% (-4.6-0.78%)	-0.36 (-0.99-0.15)	-13 (-36-5.3)
Marshall Islands	2016	Female	60 to 64	25% (12-45%)	75% (55-88%)	0.43 (0.13-1)	-1.8% (-4.3-0.27%)	-2% (-4.8-0.36%)	-0.46 (-1.1-0.091)	-14 (-33-2.3)
Marshall Islands	2016	Female	65 to 69	26% (12-45%)	74% (55-88%)	0.44 (0.14-1)	-1.9% (-4.3-0.16%)	-2.2% (-4.8-0.19%)	-0.51 (-1.2-0.048)	-13 (-29-1.2)
Marshall Islands	2016	Female	70 to 74	26% (12-44%)	74% (56-88%)	0.42 (0.13-1)	-1.9% (-4.1-0.17%)	-2.1% (-4.7-0.19%)	-0.53 (-1.2-0.043)	-10 (-23-0.91)
Marshall Islands	2016	Female	75 to 79	25% (11-44%)	75% (56-89%)	0.39 (0.11-0.99)	-1.5% (-3.7-0.35%)	-1.7% (-4.2-0.4%)	-0.41 (-1-0.095)	-6 (-15-1.3)
Marshall Islands	2016	Female	80 to 84	24% (11-43%)	76% (57-89%)	0.37 (0.11-0.94)	-1.5% (-3.6-0.14%)	-1.7% (-4.1-0.14%)	-0.31 (-0.78-0.026)	-3.3 (-8.2-0.32)
Marshall Islands	2016	Female	85 to 89	25% (11-44%)	75% (56-89%)	0.39 (0.13-0.93)	-1.5% (-3.6-0.099%)	-1.7% (-4.1-0.071%)	-0.17 (-0.41-0.0065)	-1.3 (-3.1-0.078)
Marshall Islands	2016	Female	90 to 94	28% (13-48%)	72% (52-87%)	0.43 (0.13-1)	-1.5% (-3.4-0.25%)	-1.7% (-4-0.18%)	-0.061 (-0.14-0.0061)	-0.31 (-0.72-0.047)
Marshall Islands	2016	Female	95 plus	33% (14-56%)	67% (44-86%)	0.51 (0.14-1.2)	-1.8% (-4.5-0.33%)	-2.2% (-5.2-0.27%)	-0.019 (-0.045-0.0023)	-0.063 (-0.15-0.011)

Location	Year	Sex	Age	Prevalence of current drinkers	Prevalence of abstainers	Population average of standard drinks daily	Percent of total attributable deaths	Percent of total attributable DALYs	Attributable deaths	Attributable DALYs
Marshall Islands	2016	Male	All Ages	41%<br>(32-50%)	59%<br>(50-68%)	0.82<br>(0.45-1.4)	2.4%<br>(0.83-4.9%)	2.1%<br>(0.41-5.2%)	6.3<br>(1.2-15)	360<br>(120-730)
Marshall Islands	2016	Male	15 to 19	36%<br>(21-55%)	64%<br>(45-79%)	0.5<br>(0.19-0.99)	2.1%<br>(0.93-4%)	2.7%<br>(0.92-5.5%)	0.17<br>(0.058-0.37)	15<br>(6.6-30)
Marshall Islands	2016	Male	20 to 24	55%<br>(36-72%)	45%<br>(28-64%)	1.3<br>(0.63-2.5)	5.8%<br>(2.8-11%)	7%<br>(3-13%)	0.68<br>(0.28-1.3)	51<br>(25-94)
Marshall Islands	2016	Male	25 to 29	62%<br>(42-77%)	38%<br>(23-58%)	1.9<br>(0.86-3.4)	7.1%<br>(3.5-12%)	8.5%<br>(3.8-16%)	0.88<br>(0.39-1.7)	62<br>(29-110)
Marshall Islands	2016	Male	30 to 34	63%<br>(42-79%)	37%<br>(21-58%)	2<br>(0.96-3.7)	6.1%<br>(2.8-11%)	7.1%<br>(2.8-14%)	0.89<br>(0.35-1.7)	58<br>(26-110)
Marshall Islands	2016	Male	35 to 39	62%<br>(44-79%)	38%<br>(21-56%)	1.7<br>(0.85-3.2)	4.9%<br>(1.7-9.5%)	5.6%<br>(1.7-12%)	0.86<br>(0.27-1.7)	51<br>(18-98)
Marshall Islands	2016	Male	40 to 44	59%<br>(41-76%)	41%<br>(24-59%)	1.5<br>(0.65-2.9)	3.5%<br>(0.67-7.9%)	3.8%<br>(0.47-9.2%)	0.75<br>(0.095-1.8)	41<br>(7.4-94)
Marshall Islands	2016	Male	45 to 49	55%<br>(33-74%)	45%<br>(26-67%)	1.3<br>(0.56-2.5)	2.6%<br>(-0.039-6.9%)	2.8%<br>(-0.29-7.9%)	0.63<br>(-0.062-1.8)	31<br>(-0.45-81)
Marshall Islands	2016	Male	50 to 54	52%<br>(32-72%)	48%<br>(28-68%)	1.1<br>(0.46-2.2)	2%<br>(-0.49-6%)	2.1%<br>(-0.67-6.5%)	0.53<br>(-0.17-1.7)	23<br>(-5.6-67)
Marshall Islands	2016	Male	55 to 59	50%<br>(32-69%)	50%<br>(31-68%)	0.89<br>(0.34-1.9)	1.2%<br>(-0.57-4.8%)	1.2%<br>(-0.75-5.2%)	0.34<br>(-0.21-1.5)	13<br>(-6.2-51)
Marshall Islands	2016	Male	60 to 64	47%<br>(28-67%)	53%<br>(33-72%)	0.73<br>(0.27-1.6)	0.83%<br>(-0.72-3.9%)	0.85%<br>(-0.88-4.3%)	0.24<br>(-0.25-1.2)	7.5<br>(-6.8-37)
Marshall Islands	2016	Male	65 to 69	46%<br>(27-65%)	54%<br>(35-73%)	0.66<br>(0.26-1.3)	0.65%<br>(-0.7-3.3%)	0.67%<br>(-0.81-3.6%)	0.17<br>(-0.2-0.91)	4.5<br>(-4.8-22)
Marshall Islands	2016	Male	70 to 74	43%<br>(26-60%)	57%<br>(40-74%)	0.58<br>(0.23-1.2)	0.39%<br>(-0.73-2.6%)	0.39%<br>(-0.84-2.8%)	0.089<br>(-0.2-0.64)	1.9<br>(-3.6-12)
Marshall Islands	2016	Male	75 to 79	39%<br>(23-58%)	61%<br>(42-77%)	0.5<br>(0.19-1.1)	0.27%<br>(-0.74-2.1%)	0.24%<br>(-0.86-2.3%)	0.043<br>(-0.16-0.39)	0.78<br>(-2.2-5.9)
Marshall Islands	2016	Male	80 to 84	36%<br>(20-54%)	64%<br>(46-80%)	0.44<br>(0.18-0.94)	0.23%<br>(-0.54-1.7%)	0.2%<br>(-0.67-1.8%)	0.021<br>(-0.07-0.19)	0.29<br>(-0.68-2.2)
Marshall Islands	2016	Male	85 to 89	34%<br>(21-51%)	66%<br>(49-79%)	0.42<br>(0.17-0.89)	0.078%<br>(-0.71-1.4%)	0.017%<br>(-0.88-1.4%)	0.00088<br>(-0.047-0.079)	0.036<br>(-0.33-0.64)
Marshall Islands	2016	Male	90 to 94	36%<br>(22-54%)	64%<br>(46-78%)	0.44<br>(0.16-0.94)	0.015%<br>(-0.75-1.1%)	-0.072%<br>(-0.94-1.2%)	-0.0013<br>(-0.016-0.02)	0.0015<br>(-0.076-0.11)
Marshall Islands	2016	Male	95 plus	40%<br>(21-61%)	60%<br>(39-79%)	0.48<br>(0.17-1.1)	-0.15%<br>(-1.1-1.2%)	-0.32%<br>(-1.4-1.2%)	-0.0014<br>(-0.0057-0.005)	-0.0025<br>(-0.018-0.02)
Federated States of Micronesia	1990	Female	All Ages	15%<br>(12-19%)	85%<br>(81-88%)	0.23<br>(0.15-0.33)	0.4%<br>(0.14-0.76%)	0.29%<br>(-0.13-0.9%)	0.96<br>(-0.42-3)	72<br>(26-140)
Federated States of Micronesia	1990	Female	15 to 19	19%<br>(12-30%)	81%<br>(70-88%)	0.33<br>(0.15-0.58)	0.97%<br>(0.44-1.7%)	1.4%<br>(0.43-2.9%)	0.081<br>(0.024-0.17)	8.2<br>(3.6-15)
Federated States of Micronesia	1990	Female	20 to 24	24%<br>(14-36%)	76%<br>(64-86%)	0.52<br>(0.26-0.9)	1.7%<br>(0.91-2.8%)	2.1%<br>(0.82-4.1%)	0.14<br>(0.057-0.28)	14<br>(7.5-24)
Federated States of Micronesia	1990	Female	25 to 29	24%<br>(14-37%)	76%<br>(63-86%)	0.48<br>(0.24-0.81)	1.5%<br>(0.9-2.5%)	1.8%<br>(0.82-3.4%)	0.13<br>(0.059-0.26)	12<br>(6.6-20)

Location	Year	Sex	Age	Prevalence of current drinkers	Prevalence of abstainers	Population average of standard drinks daily	Percent of total attributable deaths	Percent of total attributable DALYs	Attributable deaths	Attributable DALYs
Federated States of Micronesia	1990	Female	30 to 34	23%<br>(13-37%)	77%<br>(63-87%)	0.47<br>(0.23-0.81)	1.3%<br>(0.66-2.2%)	1.5%<br>(0.63-2.9%)	0.13<br>(0.051-0.27)	11<br>(5.3-18)
Federated States of Micronesia	1990	Female	35 to 39	22%<br>(13-36%)	78%<br>(64-87%)	0.49<br>(0.24-0.89)	1.4%<br>(0.67-2.5%)	1.7%<br>(0.75-3.4%)	0.19<br>(0.079-0.39)	12<br>(5.8-23)
Federated States of Micronesia	1990	Female	40 to 44	21%<br>(12-34%)	79%<br>(66-88%)	0.45<br>(0.21-0.84)	1.3%<br>(0.52-2.5%)	1.7%<br>(0.61-3.4%)	0.16<br>(0.056-0.34)	8.3<br>(3.3-17)
Federated States of Micronesia	1990	Female	45 to 49	21%<br>(11-33%)	79%<br>(67-89%)	0.39<br>(0.18-0.72)	0.78%<br>(0.046-1.7%)	1%<br>(0.084-2.4%)	0.12<br>(0.0098-0.27)	5.1<br>(0.32-12)
Federated States of Micronesia	1990	Female	50 to 54	21%<br>(12-33%)	79%<br>(67-88%)	0.35<br>(0.16-0.68)	0.47%<br>(-0.22-1.5%)	0.68%<br>(-0.23-2.2%)	0.086<br>(-0.027-0.27)	2.9<br>(-1.4-9.7)
Federated States of Micronesia	1990	Female	55 to 59	19%<br>(11-30%)	81%<br>(70-89%)	0.28<br>(0.13-0.54)	0.27%<br>(-0.43-1.2%)	0.43%<br>(-0.43-1.6%)	0.075<br>(-0.071-0.29)	1.9<br>(-2.9-8.6)
Federated States of Micronesia	1990	Female	60 to 64	17%<br>(9.8-29%)	83%<br>(71-90%)	0.24<br>(0.11-0.47)	0.0092%<br>(-0.63-0.79%)	0.095%<br>(-0.66-1%)	0.021<br>(-0.14-0.23)	0.074<br>(-4.6-5.8)
Federated States of Micronesia	1990	Female	65 to 69	18%<br>(10-28%)	82%<br>(72-90%)	0.24<br>(0.11-0.44)	-0.019%<br>(-0.7-0.75%)	0.063%<br>(-0.73-0.95%)	0.018<br>(-0.2-0.28)	-0.15<br>(-5.3-5.8)
Federated States of Micronesia	1990	Female	70 to 74	18%<br>(11-29%)	82%<br>(71-89%)	0.24<br>(0.11-0.46)	-0.17%<br>(-0.8-0.61%)	-0.13%<br>(-0.86-0.78%)	-0.045<br>(-0.29-0.25)	-1.2<br>(-5.7-4.4)
Federated States of Micronesia	1990	Female	75 to 79	18%<br>(11-30%)	82%<br>(70-89%)	0.23<br>(0.11-0.44)	-0.13%<br>(-0.74-0.62%)	-0.095%<br>(-0.79-0.73%)	-0.03<br>(-0.25-0.23)	-0.65<br>(-3.8-3.1)
Federated States of Micronesia	1990	Female	80 to 84	18%<br>(10-29%)	82%<br>(71-90%)	0.23<br>(0.1-0.41)	-0.28%<br>(-0.88-0.3%)	-0.3%<br>(-0.98-0.37%)	-0.069<br>(-0.22-0.086)	-0.78<br>(-2.4-0.84)
Federated States of Micronesia	1990	Female	85 to 89	18%<br>(11-29%)	82%<br>(71-89%)	0.23<br>(0.11-0.42)	-0.32%<br>(-0.9-0.24%)	-0.36%<br>(-1-0.29%)	-0.041<br>(-0.12-0.033)	-0.31<br>(-0.87-0.23)
Federated States of Micronesia	1990	Female	90 to 94	20%<br>(12-30%)	80%<br>(70-88%)	0.24<br>(0.1-0.46)	-0.26%<br>(-0.76-0.28%)	-0.29%<br>(-0.86-0.31%)	-0.013<br>(-0.037-0.014)	-0.067<br>(-0.2-0.071)
Federated States of Micronesia	1990	Female	95 plus	22%<br>(12-36%)	78%<br>(64-88%)	0.27<br>(0.1-0.53)	-0.29%<br>(-0.98-0.4%)	-0.36%<br>(-1.2-0.41%)	-0.0044<br>(-0.014-0.0049)	-0.013<br>(-0.046-0.018)
Federated States of Micronesia	1990	Male	All Ages	36%<br>(27-45%)	64%<br>(55-73%)	0.71<br>(0.5-0.98)	2.1%<br>(1.3-3.2%)	2.2%<br>(1.1-3.6%)	9<br>(4.5-15)	470<br>(290-700)
Federated States of Micronesia	1990	Male	15 to 19	36%<br>(21-53%)	64%<br>(47-79%)	0.46<br>(0.21-0.82)	1.9%<br>(0.86-3.5%)	2.4%<br>(0.83-4.8%)	0.25<br>(0.087-0.5)	22<br>(9.6-40)
Federated States of Micronesia	1990	Male	20 to 24	54%<br>(35-72%)	46%<br>(28-65%)	1.3<br>(0.75-2.1)	5.9%<br>(3.3-9.4%)	7.1%<br>(3.7-12%)	0.96<br>(0.48-1.6)	72<br>(40-110)
Federated States of Micronesia	1990	Male	25 to 29	61%<br>(42-77%)	39%<br>(23-58%)	2<br>(1.2-3.1)	8.2%<br>(5-12%)	9.9%<br>(5.9-15%)	1.3<br>(0.72-2)	86<br>(54-130)
Federated States of Micronesia	1990	Male	30 to 34	62%<br>(41-79%)	38%<br>(21-59%)	2.2<br>(1.2-3.4)	7.6%<br>(4.5-12%)	9.2%<br>(5.2-14%)	1.3<br>(0.67-2.1)	80<br>(46-120)
Federated States of Micronesia	1990	Male	35 to 39	61%<br>(43-78%)	39%<br>(22-57%)	1.8<br>(1-2.9)	5.8%<br>(3.2-9.4%)	6.8%<br>(3.5-11%)	1.1<br>(0.55-1.8)	62<br>(34-100)
Federated States of Micronesia	1990	Male	40 to 44	58%<br>(40-75%)	42%<br>(25-60%)	1.6<br>(0.89-2.6)	4.6%<br>(2.2-7.9%)	5.2%<br>(2.2-9.2%)	0.78<br>(0.34-1.4)	40<br>(20-71)
Federated States of Micronesia	1990	Male	45 to 49	54%<br>(33-72%)	46%<br>(28-67%)	1.4<br>(0.72-2.5)	4.1%<br>(1.7-7.4%)	4.6%<br>(1.7-8.5%)	0.81<br>(0.3-1.5)	37<br>(15-67)

Location	Year	Sex	Age	Prevalence of current drinkers	Prevalence of abstainers	Population average of standard drinks daily	Percent of total attributable deaths	Percent of total attributable DALYs	Attributable deaths	Attributable DALYs
Federated States of Micronesia	1990	Male	50 to 54	51% (32-70%)	49% (30-68%)	1.2 (0.58-2.1)	3.3% (1.1-6.4%)	3.6% (0.94-7.3%)	0.62 (0.16-1.3)	25 (7.9-50)
Federated States of Micronesia	1990	Male	55 to 59	49% (31-69%)	51% (31-69%)	0.88 (0.44-1.6)	1.9% (0.35-4.3%)	2% (0.25-4.9%)	0.48 (0.061-1.1)	17 (3.1-38)
Federated States of Micronesia	1990	Male	60 to 64	46% (27-66%)	54% (34-73%)	0.69 (0.32-1.3)	1.3% (0.012-3.6%)	1.4% (-0.1-3.9%)	0.41 (-0.029-1.2)	12 (0.12-33)
Federated States of Micronesia	1990	Male	65 to 69	45% (26-64%)	55% (36-74%)	0.62 (0.3-1.2)	1.2% (-0.02-2.8%)	1.3% (-0.061-3.2%)	0.42 (-0.023-1.1)	9.9 (-0.17-25)
Federated States of Micronesia	1990	Male	70 to 74	42% (25-59%)	58% (41-75%)	0.55 (0.26-1)	0.81% (-0.16-2.2%)	0.88% (-0.22-2.5%)	0.33 (-0.082-0.94)	6.3 (-1.3-17)
Federated States of Micronesia	1990	Male	75 to 79	38% (23-57%)	62% (43-77%)	0.46 (0.21-0.91)	0.58% (-0.22-1.9%)	0.61% (-0.27-2.1%)	0.21 (-0.092-0.72)	3.1 (-1.2-10)
Federated States of Micronesia	1990	Male	80 to 84	36% (20-54%)	64% (46-80%)	0.41 (0.19-0.75)	0.43% (-0.2-1.3%)	0.44% (-0.26-1.5%)	0.096 (-0.058-0.31)	1.1 (-0.54-3.4)
Federated States of Micronesia	1990	Male	85 to 89	34% (21-51%)	66% (49-79%)	0.39 (0.18-0.72)	0.26% (-0.32-1%)	0.25% (-0.42-1.1%)	0.025 (-0.042-0.11)	0.22 (-0.27-0.88)
Federated States of Micronesia	1990	Male	90 to 94	35% (21-52%)	65% (48-79%)	0.4 (0.17-0.75)	0.17% (-0.32-0.81%)	0.13% (-0.44-0.85%)	0.0057 (-0.019-0.038)	0.042 (-0.081-0.21)
Federated States of Micronesia	1990	Male	95 plus	38% (21-59%)	62% (41-79%)	0.43 (0.16-0.84)	-0.081% (-0.75-0.69%)	-0.21% (-0.97-0.66%)	-0.0022 (-0.011-0.0072)	-0.0034 (-0.032-0.029)
Federated States of Micronesia	1995	Female	All Ages	16% (13-20%)	84% (80-87%)	0.24 (0.18-0.31)	0.43% (0.17-0.75%)	0.26% (-0.17-0.79%)	0.92 (-0.59-2.7)	77 (30-130)
Federated States of Micronesia	1995	Female	15 to 19	19% (12-30%)	81% (70-88%)	0.34 (0.18-0.57)	0.99% (0.49-1.8%)	1.4% (0.52-3%)	0.089 (0.029-0.18)	9.1 (4.3-16)
Federated States of Micronesia	1995	Female	20 to 24	24% (14-36%)	76% (64-86%)	0.54 (0.29-0.87)	1.7% (0.96-2.7%)	2.1% (0.89-3.9%)	0.14 (0.055-0.27)	14 (7.7-23)
Federated States of Micronesia	1995	Female	25 to 29	24% (14-37%)	76% (63-86%)	0.49 (0.28-0.8)	1.6% (0.97-2.4%)	1.9% (0.96-3.2%)	0.14 (0.066-0.25)	12 (7.3-20)
Federated States of Micronesia	1995	Female	30 to 34	23% (13-37%)	77% (63-87%)	0.48 (0.26-0.79)	1.3% (0.75-2%)	1.5% (0.72-2.6%)	0.14 (0.065-0.26)	11 (6.5-19)
Federated States of Micronesia	1995	Female	35 to 39	22% (12-35%)	78% (65-88%)	0.51 (0.26-0.85)	1.4% (0.71-2.3%)	1.7% (0.81-3.1%)	0.22 (0.095-0.42)	14 (7.2-24)
Federated States of Micronesia	1995	Female	40 to 44	21% (12-34%)	79% (66-88%)	0.47 (0.24-0.8)	1.2% (0.51-2.2%)	1.6% (0.63-3.1%)	0.23 (0.083-0.45)	12 (5-22)
Federated States of Micronesia	1995	Female	45 to 49	21% (11-34%)	79% (66-89%)	0.41 (0.2-0.72)	0.74% (0.054-1.6%)	1% (0.078-2.3%)	0.14 (0.011-0.31)	5.9 (0.39-13)
Federated States of Micronesia	1995	Female	50 to 54	21% (12-33%)	79% (67-88%)	0.36 (0.17-0.67)	0.41% (-0.27-1.4%)	0.62% (-0.23-1.9%)	0.089 (-0.034-0.27)	2.9 (-1.9-9.9)
Federated States of Micronesia	1995	Female	55 to 59	19% (11-30%)	81% (70-89%)	0.29 (0.14-0.54)	0.21% (-0.46-1%)	0.38% (-0.43-1.4%)	0.064 (-0.072-0.24)	1.5 (-3.3-7.3)
Federated States of Micronesia	1995	Female	60 to 64	17% (9.7-29%)	83% (71-90%)	0.25 (0.12-0.46)	-0.053% (-0.71-0.62%)	0.034% (-0.73-0.83%)	0.0098 (-0.2-0.23)	-0.48 (-6.8-5.8)
Federated States of Micronesia	1995	Female	65 to 69	18% (11-30%)	82% (70-89%)	0.25 (0.13-0.46)	-0.098% (-0.77-0.61%)	-0.017% (-0.79-0.83%)	-0.0045 (-0.21-0.22)	-0.7 (-5.6-4.4)

Location	Year	Sex	Age	Prevalence of current drinkers	Prevalence of abstainers	Population average of standard drinks daily	Percent of total attributable deaths	Percent of total attributable DALYs	Attributable deaths	Attributable DALYs
Federated States of Micronesia	1995	Female	70 to 74	19% (11-30%)	81% (70-89%)	0.25 (0.12-0.47)	-0.26% (-0.96-0.4%)	-0.23% (-1-0.53%)	-0.092 (-0.4-0.21)	-2.2 (-8.1-3.2)
Federated States of Micronesia	1995	Female	75 to 79	19% (12-30%)	81% (70-88%)	0.24 (0.12-0.44)	-0.18% (-0.82-0.5%)	-0.16% (-0.87-0.62%)	-0.063 (-0.36-0.24)	-1.2 (-5.5-3.2)
Federated States of Micronesia	1995	Female	80 to 84	18% (11-29%)	82% (71-89%)	0.23 (0.11-0.41)	-0.35% (-0.97-0.23%)	-0.37% (-1.1-0.29%)	-0.11 (-0.31-0.083)	-1.2 (-3.4-0.83)
Federated States of Micronesia	1995	Female	85 to 89	19% (11-29%)	81% (71-89%)	0.24 (0.11-0.42)	-0.38% (-1-0.15%)	-0.42% (-1.1-0.19%)	-0.058 (-0.16-0.027)	-0.45 (-1.2-0.17)
Federated States of Micronesia	1995	Female	90 to 94	20% (12-30%)	80% (70-88%)	0.25 (0.12-0.45)	-0.31% (-0.82-0.23%)	-0.35% (-0.95-0.27%)	-0.016 (-0.042-0.012)	-0.081 (-0.22-0.064)
Federated States of Micronesia	1995	Female	95 plus	22% (12-37%)	78% (63-88%)	0.28 (0.11-0.54)	-0.34% (-1.1-0.32%)	-0.42% (-1.3-0.31%)	-0.0052 (-0.016-0.0038)	-0.016 (-0.051-0.015)
Federated States of Micronesia	1995	Male	All Ages	36% (28-46%)	64% (54-72%)	0.75 (0.6-0.91)	2.4% (1.5-3.2%)	2.3% (1.3-3.4%)	9.9 (5.5-15)	520 (340-730)
Federated States of Micronesia	1995	Male	15 to 19	36% (20-54%)	64% (46-80%)	0.47 (0.23-0.83)	2% (0.94-3.5%)	2.5% (0.94-4.9%)	0.3 (0.11-0.57)	26 (12-45)
Federated States of Micronesia	1995	Male	20 to 24	53% (34-71%)	47% (29-66%)	1.4 (0.84-2)	6% (3.4-9.3%)	7.3% (3.8-12%)	0.93 (0.47-1.5)	70 (39-110)
Federated States of Micronesia	1995	Male	25 to 29	61% (41-77%)	39% (23-59%)	2.1 (1.3-3)	8.3% (5.4-12%)	10% (6.4-15%)	1.1 (0.68-1.7)	77 (48-110)
Federated States of Micronesia	1995	Male	30 to 34	62% (41-78%)	38% (22-59%)	2.2 (1.4-3.3)	7.7% (4.9-11%)	9.2% (5.6-14%)	1.4 (0.84-2.3)	92 (55-140)
Federated States of Micronesia	1995	Male	35 to 39	61% (42-77%)	39% (23-58%)	1.9 (1.1-2.8)	5.9% (3.5-8.6%)	6.9% (3.9-11%)	1.3 (0.69-2)	72 (43-110)
Federated States of Micronesia	1995	Male	40 to 44	58% (40-75%)	42% (25-60%)	1.6 (0.97-2.5)	4.5% (2.5-7.2%)	5.1% (2.6-8.5%)	1.1 (0.54-1.9)	60 (31-99)
Federated States of Micronesia	1995	Male	45 to 49	54% (33-72%)	46% (28-67%)	1.5 (0.78-2.4)	4% (1.7-6.4%)	4.4% (1.8-7.4%)	1 (0.43-1.8)	48 (21-80)
Federated States of Micronesia	1995	Male	50 to 54	51% (32-70%)	49% (30-68%)	1.2 (0.62-2)	3.2% (1.1-5.9%)	3.5% (1-6.6%)	0.68 (0.19-1.3)	28 (9.4-51)
Federated States of Micronesia	1995	Male	55 to 59	49% (31-68%)	51% (32-69%)	0.9 (0.48-1.6)	1.8% (0.42-4%)	1.9% (0.31-4.5%)	0.41 (0.062-0.96)	14 (3.1-33)
Federated States of Micronesia	1995	Male	60 to 64	47% (27-66%)	53% (34-73%)	0.72 (0.35-1.3)	1.2% (0.05-3.2%)	1.3% (-0.063-3.5%)	0.54 (-0.025-1.5)	16 (0.6-42)
Federated States of Micronesia	1995	Male	65 to 69	45% (27-63%)	55% (37-73%)	0.65 (0.33-1.1)	1.1% (-0.0012-2.6%)	1.2% (-0.034-3%)	0.39 (-0.0096-0.94)	9.1 (-0.0093-22)
Federated States of Micronesia	1995	Male	70 to 74	43% (25-61%)	57% (39-75%)	0.57 (0.29-1.1)	0.77% (-0.19-2.1%)	0.83% (-0.24-2.3%)	0.33 (-0.095-0.92)	6.3 (-1.7-17)
Federated States of Micronesia	1995	Male	75 to 79	39% (23-58%)	61% (42-77%)	0.49 (0.24-0.89)	0.55% (-0.23-1.7%)	0.58% (-0.31-1.9%)	0.23 (-0.12-0.75)	3.5 (-1.4-11)
Federated States of Micronesia	1995	Male	80 to 84	36% (20-53%)	64% (47-80%)	0.43 (0.22-0.76)	0.41% (-0.2-1.2%)	0.42% (-0.29-1.3%)	0.098 (-0.067-0.31)	1.1 (-0.54-3.4)
Federated States of Micronesia	1995	Male	85 to 89	34% (20-51%)	66% (49-80%)	0.41 (0.19-0.7)	0.24% (-0.33-1%)	0.23% (-0.44-1.1%)	0.026 (-0.049-0.12)	0.24 (-0.32-0.97)

Location	Year	Sex	Age	Prevalence of current drinkers	Prevalence of abstainers	Population average of standard drinks daily	Percent of total attributable deaths	Percent of total attributable DALYs	Attributable deaths	Attributable DALYs
Federated States of Micronesia	1995	Male	90 to 94	35% (22-52%)	65% (48-78%)	0.41 (0.19-0.76)	0.15% (-0.33-0.76%)	0.11% (-0.47-0.78%)	0.0038 (-0.016-0.028)	0.032 (-0.071-0.16)
Federated States of Micronesia	1995	Male	95 plus	39% (21-59%)	61% (41-79%)	0.45 (0.18-0.86)	-0.093% (-0.73-0.66%)	-0.23% (-0.97-0.62%)	-0.0025 (-0.011-0.0067)	-0.004 (-0.031-0.029)
Federated States of Micronesia	2000	Female	All Ages	15% (12-19%)	85% (81-88%)	0.21 (0.17-0.27)	0.33% (0.1-0.61%)	0.082% (-0.3-0.5%)	0.29 (-1.1-1.8)	58 (17-100)
Federated States of Micronesia	2000	Female	15 to 19	19% (11-30%)	81% (70-89%)	0.3 (0.16-0.51)	0.86% (0.45-1.5%)	1.2% (0.42-2.5%)	0.075 (0.025-0.16)	8.3 (4.1-15)
Federated States of Micronesia	2000	Female	20 to 24	22% (13-34%)	78% (66-87%)	0.45 (0.24-0.72)	1.4% (0.81-2.2%)	1.6% (0.71-3%)	0.11 (0.044-0.21)	12 (6.9-20)
Federated States of Micronesia	2000	Female	25 to 29	21% (13-33%)	79% (67-87%)	0.38 (0.23-0.6)	1.3% (0.85-1.9%)	1.4% (0.77-2.3%)	0.11 (0.05-0.19)	11 (6.6-16)
Federated States of Micronesia	2000	Female	30 to 34	20% (11-32%)	80% (68-89%)	0.37 (0.2-0.6)	1.1% (0.63-1.6%)	1.1% (0.55-1.9%)	0.11 (0.051-0.2)	9.9 (5.6-15)
Federated States of Micronesia	2000	Female	35 to 39	20% (11-33%)	80% (67-89%)	0.41 (0.21-0.71)	1.1% (0.59-1.9%)	1.3% (0.61-2.4%)	0.17 (0.07-0.31)	11 (5.6-18)
Federated States of Micronesia	2000	Female	40 to 44	20% (12-32%)	80% (68-88%)	0.4 (0.2-0.69)	0.94% (0.35-1.7%)	1.2% (0.41-2.3%)	0.19 (0.059-0.36)	10 (3.6-18)
Federated States of Micronesia	2000	Female	45 to 49	20% (10-32%)	80% (68-90%)	0.35 (0.17-0.6)	0.49% (-0.11-1.2%)	0.69% (-0.12-1.7%)	0.12 (-0.02-0.31)	5.2 (-1.3-13)
Federated States of Micronesia	2000	Female	50 to 54	20% (11-31%)	80% (69-89%)	0.31 (0.15-0.58)	0.16% (-0.55-0.85%)	0.3% (-0.57-1.2%)	0.057 (-0.11-0.23)	1.5 (-5-8.1)
Federated States of Micronesia	2000	Female	55 to 59	18% (11-30%)	82% (70-89%)	0.25 (0.12-0.46)	0.012% (-0.64-0.6%)	0.13% (-0.66-0.87%)	0.024 (-0.12-0.16)	0.091 (-4.9-4.6)
Federated States of Micronesia	2000	Female	60 to 64	17% (9.3-28%)	83% (72-91%)	0.22 (0.1-0.4)	-0.2% (-0.89-0.35%)	-0.14% (-0.92-0.52%)	-0.029 (-0.19-0.099)	-1.4 (-6.1-2.3)
Federated States of Micronesia	2000	Female	65 to 69	18% (11-28%)	82% (72-89%)	0.22 (0.11-0.38)	-0.25% (-0.9-0.31%)	-0.2% (-0.93-0.47%)	-0.063 (-0.29-0.14)	-2.2 (-7.8-2.6)
Federated States of Micronesia	2000	Female	70 to 74	19% (11-30%)	81% (70-89%)	0.22 (0.11-0.4)	-0.42% (-1-0.11%)	-0.42% (-1.1-0.23%)	-0.12 (-0.31-0.059)	-2.5 (-6.3-0.7)
Federated States of Micronesia	2000	Female	75 to 79	18% (11-29%)	82% (71-89%)	0.21 (0.1-0.39)	-0.33% (-0.95-0.18%)	-0.32% (-1-0.28%)	-0.14 (-0.46-0.12)	-2.3 (-7-1.3)
Federated States of Micronesia	2000	Female	80 to 84	18% (10-30%)	82% (70-90%)	0.21 (0.1-0.36)	-0.47% (-1.1--0.015%)	-0.51% (-1.2-0.017%)	-0.19 (-0.46-0.0066)	-2.1 (-5--0.068)
Federated States of Micronesia	2000	Female	85 to 89	18% (11-28%)	82% (72-89%)	0.21 (0.11-0.37)	-0.48% (-1.1--0.043%)	-0.54% (-1.2--0.036%)	-0.1 (-0.24--0.007)	-0.79 (-1.8--0.071)
Federated States of Micronesia	2000	Female	90 to 94	20% (12-31%)	80% (69-88%)	0.23 (0.11-0.41)	-0.41% (-0.97-0.0079%)	-0.47% (-1.1-0.012%)	-0.028 (-0.067-0.00069)	-0.15 (-0.35-0.0026)
Federated States of Micronesia	2000	Female	95 plus	22% (12-36%)	78% (64-88%)	0.25 (0.1-0.48)	-0.45% (-1.2-0.12%)	-0.55% (-1.4-0.12%)	-0.0074 (-0.019-0.0017)	-0.023 (-0.063-0.0064)
Federated States of Micronesia	2000	Male	All Ages	39% (31-47%)	61% (53-69%)	0.76 (0.65-0.89)	2.3% (1.5-3.1%)	2.1% (1.2-3%)	8.6 (4.8-13)	480 (320-650)
Federated States of Micronesia	2000	Male	15 to 19	35% (21-53%)	65% (47-79%)	0.43 (0.22-0.73)	1.9% (0.91-3.2%)	2.4% (0.91-4.5%)	0.28 (0.11-0.53)	24 (12-42)

Location	Year	Sex	Age	Prevalence of current drinkers	Prevalence of abstainers	Population average of standard drinks daily	Percent of total attributable deaths	Percent of total attributable DALYs	Attributable deaths	Attributable DALYs
Federated States of Micronesia	2000	Male	20 to 24	54% (36-72%)	46% (28-64%)	1.3 (0.84-1.9)	5.6% (3.3-8.6%)	6.8% (3.6-11%)	0.93 (0.5-1.5)	70 (41-110)
Federated States of Micronesia	2000	Male	25 to 29	64% (47-78%)	36% (22-53%)	2.1 (1.4-2.8)	7.9% (5.2-11%)	9.6% (6.1-14%)	1.1 (0.69-1.7)	79 (51-110)
Federated States of Micronesia	2000	Male	30 to 34	66% (47-81%)	34% (19-53%)	2.3 (1.4-3.1)	7.2% (4.8-10%)	8.6% (5.4-13%)	1.2 (0.71-1.9)	78 (48-110)
Federated States of Micronesia	2000	Male	35 to 39	64% (47-79%)	36% (21-53%)	1.9 (1.2-2.6)	5.5% (3.3-8.1%)	6.4% (3.6-9.8%)	1.1 (0.6-1.8)	67 (37-100)
Federated States of Micronesia	2000	Male	40 to 44	60% (43-75%)	40% (25-57%)	1.6 (0.96-2.3)	4% (2-6.5%)	4.5% (1.9-7.5%)	0.92 (0.39-1.6)	49 (24-82)
Federated States of Micronesia	2000	Male	45 to 49	54% (35-70%)	46% (30-65%)	1.4 (0.83-2)	3.3% (1.5-5.4%)	3.6% (1.3-6.1%)	1 (0.38-1.8)	48 (21-81)
Federated States of Micronesia	2000	Male	50 to 54	50% (32-67%)	50% (33-68%)	1.1 (0.6-1.7)	2.5% (0.82-4.7%)	2.7% (0.76-5.2%)	0.74 (0.2-1.4)	31 (9.8-55)
Federated States of Micronesia	2000	Male	55 to 59	47% (30-66%)	53% (34-70%)	0.79 (0.43-1.4)	1.4% (0.19-3%)	1.4% (0.046-3.3%)	0.3 (0.0075-0.7)	11 (1.5-24)
Federated States of Micronesia	2000	Male	60 to 64	45% (26-65%)	55% (35-74%)	0.63 (0.31-1.1)	0.88% (-0.17-2.4%)	0.91% (-0.31-2.6%)	0.23 (-0.075-0.66)	7.1 (-1.3-20)
Federated States of Micronesia	2000	Male	65 to 69	44% (26-62%)	56% (38-74%)	0.58 (0.3-1)	0.78% (-0.14-2%)	0.85% (-0.21-2.2%)	0.32 (-0.083-0.88)	7.8 (-1.3-21)
Federated States of Micronesia	2000	Male	70 to 74	42% (24-59%)	58% (41-76%)	0.52 (0.26-0.92)	0.5% (-0.33-1.6%)	0.53% (-0.41-1.8%)	0.13 (-0.1-0.43)	2.5 (-1.6-8.1)
Federated States of Micronesia	2000	Male	75 to 79	38% (22-57%)	62% (43-78%)	0.44 (0.22-0.8)	0.35% (-0.35-1.3%)	0.36% (-0.42-1.5%)	0.15 (-0.18-0.61)	2.4 (-2.4-9.1)
Federated States of Micronesia	2000	Male	80 to 84	35% (19-54%)	65% (46-81%)	0.39 (0.2-0.66)	0.27% (-0.3-0.91%)	0.27% (-0.38-0.99%)	0.08 (-0.12-0.3)	0.98 (-1.1-3.3)
Federated States of Micronesia	2000	Male	85 to 89	34% (19-51%)	66% (49-81%)	0.37 (0.19-0.63)	0.12% (-0.4-0.68%)	0.088% (-0.52-0.71%)	0.013 (-0.074-0.1)	0.15 (-0.5-0.85)
Federated States of Micronesia	2000	Male	90 to 94	35% (21-53%)	65% (47-79%)	0.37 (0.18-0.68)	0.052% (-0.44-0.54%)	-0.0023% (-0.55-0.54%)	-0.00009 (-0.027-0.027)	0.015 (-0.13-0.16)
Federated States of Micronesia	2000	Male	95 plus	39% (21-59%)	61% (41-79%)	0.41 (0.16-0.77)	-0.18% (-0.84-0.42%)	-0.33% (-1.1-0.35%)	-0.0036 (-0.012-0.0038)	-0.0078 (-0.035-0.018)
Federated States of Micronesia	2005	Female	All Ages	15% (12-19%)	85% (81-88%)	0.2 (0.15-0.25)	0.24% (-0.053-0.52%)	-0.099% (-0.54-0.32%)	-0.36 (-2-1.1)	38 (-8.2-82)
Federated States of Micronesia	2005	Female	15 to 19	19% (11-29%)	81% (71-89%)	0.27 (0.15-0.45)	0.77% (0.38-1.3%)	1% (0.32-2%)	0.061 (0.018-0.13)	7 (3.5-12)
Federated States of Micronesia	2005	Female	20 to 24	22% (13-34%)	78% (66-87%)	0.4 (0.22-0.65)	1.3% (0.76-2%)	1.4% (0.57-2.6%)	0.11 (0.043-0.22)	13 (7.6-21)
Federated States of Micronesia	2005	Female	25 to 29	20% (12-33%)	80% (67-88%)	0.34 (0.2-0.56)	1.2% (0.74-1.7%)	1.2% (0.56-2%)	0.089 (0.038-0.16)	9.2 (5.5-13)
Federated States of Micronesia	2005	Female	30 to 34	20% (12-32%)	80% (68-88%)	0.33 (0.18-0.56)	0.95% (0.53-1.5%)	0.98% (0.4-1.7%)	0.077 (0.031-0.14)	6.9 (3.7-11)
Federated States of Micronesia	2005	Female	35 to 39	20% (12-33%)	80% (67-88%)	0.38 (0.2-0.65)	0.95% (0.44-1.6%)	1.1% (0.4-2.1%)	0.14 (0.047-0.26)	8.8 (4.1-15)

Location	Year	Sex	Age	Prevalence of current drinkers	Prevalence of abstainers	Population average of standard drinks daily	Percent of total attributable deaths	Percent of total attributable DALYs	Attributable deaths	Attributable DALYs
Federated States of Micronesia	2005	Female	40 to 44	20% (12-32%)	80% (68-88%)	0.36 (0.18-0.63)	0.77% (0.17-1.5%)	0.98% (0.14-2%)	0.14 (0.021-0.3)	7.7 (1.8-15)
Federated States of Micronesia	2005	Female	45 to 49	19% (10-31%)	81% (69-90%)	0.31 (0.15-0.53)	0.32% (-0.27-0.91%)	0.47% (-0.33-1.3%)	0.093 (-0.063-0.26)	3.8 (-3.2-11)
Federated States of Micronesia	2005	Female	50 to 54	20% (12-32%)	80% (68-88%)	0.28 (0.14-0.5)	-0.016% (-0.72-0.58%)	0.083% (-0.72-0.85%)	0.02 (-0.19-0.21)	-0.2 (-8.4-7.4)
Federated States of Micronesia	2005	Female	55 to 59	18% (11-30%)	82% (70-89%)	0.23 (0.12-0.42)	-0.14% (-0.85-0.4%)	-0.044% (-0.86-0.62%)	-0.01 (-0.21-0.16)	-1.4 (-8.4-4.2)
Federated States of Micronesia	2005	Female	60 to 64	17% (9.4-29%)	83% (71-91%)	0.2 (0.091-0.36)	-0.32% (-1-0.19%)	-0.28% (-1.1-0.33%)	-0.063 (-0.25-0.079)	-2.4 (-7.9-1.6)
Federated States of Micronesia	2005	Female	65 to 69	18% (11-29%)	82% (71-89%)	0.2 (0.098-0.35)	-0.37% (-1.1-0.13%)	-0.34% (-1.2-0.28%)	-0.092 (-0.31-0.071)	-2.8 (-7.9-0.93)
Federated States of Micronesia	2005	Female	70 to 74	19% (11-30%)	81% (70-89%)	0.2 (0.096-0.36)	-0.55% (-1.2--0.023%)	-0.56% (-1.3-0.03%)	-0.24 (-0.56-0.013)	-4.9 (-11--0.2)
Federated States of Micronesia	2005	Female	75 to 79	18% (11-29%)	82% (71-89%)	0.19 (0.092-0.35)	-0.44% (-1-0.044%)	-0.45% (-1.1-0.088%)	-0.17 (-0.45-0.034)	-2.8 (-6.8-0.24)
Federated States of Micronesia	2005	Female	80 to 84	18% (10-29%)	82% (71-90%)	0.19 (0.095-0.32)	-0.56% (-1.2--0.12%)	-0.62% (-1.3--0.12%)	-0.26 (-0.58--0.051)	-2.9 (-6.2--0.61)
Federated States of Micronesia	2005	Female	85 to 89	18% (11-28%)	82% (72-89%)	0.19 (0.098-0.35)	-0.56% (-1.2--0.12%)	-0.62% (-1.3--0.13%)	-0.19 (-0.39--0.038)	-1.4 (-3--0.3)
Federated States of Micronesia	2005	Female	90 to 94	19% (12-30%)	81% (70-88%)	0.21 (0.1-0.38)	-0.49% (-1--0.074%)	-0.55% (-1.2--0.082%)	-0.054 (-0.12--0.0079)	-0.28 (-0.6--0.044)
Federated States of Micronesia	2005	Female	95 plus	22% (12-36%)	78% (64-88%)	0.23 (0.092-0.43)	-0.53% (-1.3-0.046%)	-0.63% (-1.5-0.029%)	-0.014 (-0.032-0.00063)	-0.043 (-0.1-0.0038)
Federated States of Micronesia	2005	Male	All Ages	39% (31-48%)	61% (52-69%)	0.68 (0.57-0.79)	2.2% (1.4-3%)	1.7% (0.86-2.6%)	6.7 (3.4-11)	400 (250-570)
Federated States of Micronesia	2005	Male	15 to 19	35% (20-53%)	65% (47-80%)	0.37 (0.19-0.62)	1.7% (0.82-2.9%)	2.1% (0.82-4.1%)	0.24 (0.089-0.48)	22 (11-38)
Federated States of Micronesia	2005	Male	20 to 24	55% (37-72%)	45% (28-63%)	1.1 (0.74-1.6)	5% (3-7.6%)	6% (3.2-9.7%)	0.94 (0.49-1.6)	73 (42-120)
Federated States of Micronesia	2005	Male	25 to 29	65% (48-79%)	35% (21-52%)	1.8 (1.3-2.6)	7% (4.4-10%)	8.5% (5-12%)	1.1 (0.62-1.6)	75 (46-110)
Federated States of Micronesia	2005	Male	30 to 34	66% (48-81%)	34% (19-52%)	2 (1.3-2.8)	6.3% (3.9-9.3%)	7.5% (4.3-11%)	0.81 (0.44-1.3)	53 (31-80)
Federated States of Micronesia	2005	Male	35 to 39	64% (48-79%)	36% (21-52%)	1.7 (1.1-2.5)	4.8% (2.7-7.2%)	5.5% (2.8-8.7%)	0.75 (0.37-1.2)	45 (24-72)
Federated States of Micronesia	2005	Male	40 to 44	60% (43-75%)	40% (25-57%)	1.4 (0.86-2.1)	3.3% (1.5-5.6%)	3.7% (1.4-6.5%)	0.73 (0.28-1.3)	40 (18-69)
Federated States of Micronesia	2005	Male	45 to 49	54% (35-70%)	46% (30-65%)	1.2 (0.71-1.8)	2.6% (1-4.5%)	2.8% (0.82-5%)	0.71 (0.21-1.3)	35 (13-63)
Federated States of Micronesia	2005	Male	50 to 54	50% (32-68%)	50% (32-68%)	0.95 (0.54-1.5)	2% (0.48-3.6%)	2.1% (0.37-4%)	0.71 (0.13-1.4)	30 (7.5-59)
Federated States of Micronesia	2005	Male	55 to 59	48% (30-66%)	52% (34-70%)	0.7 (0.38-1.2)	1% (-0.059-2.4%)	1% (-0.22-2.7%)	0.32 (-0.066-0.83)	12 (-0.77-30)

Location	Year	Sex	Age	Prevalence of current drinkers	Prevalence of abstainers	Population average of standard drinks daily	Percent of total attributable deaths	Percent of total attributable DALYs	Attributable deaths	Attributable DALYs
Federated States of Micronesia	2005	Male	60 to 64	45% (26-65%)	55% (35-74%)	0.56 (0.28-1)	0.59% (-0.34-1.9%)	0.59% (-0.48-2.1%)	0.14 (-0.12-0.51)	4.5 (-2.6-15)
Federated States of Micronesia	2005	Male	65 to 69	44% (25-63%)	56% (37-75%)	0.52 (0.26-0.89)	0.51% (-0.32-1.5%)	0.53% (-0.39-1.7%)	0.15 (-0.11-0.49)	3.7 (-2.3-12)
Federated States of Micronesia	2005	Male	70 to 74	41% (24-60%)	59% (40-76%)	0.45 (0.22-0.8)	0.27% (-0.45-1.2%)	0.27% (-0.56-1.3%)	0.11 (-0.22-0.53)	2.3 (-4-11)
Federated States of Micronesia	2005	Male	75 to 79	37% (22-57%)	63% (43-78%)	0.38 (0.19-0.69)	0.18% (-0.49-0.93%)	0.17% (-0.57-0.99%)	0.047 (-0.16-0.28)	0.84 (-2.2-4.3)
Federated States of Micronesia	2005	Male	80 to 84	35% (19-53%)	65% (47-81%)	0.34 (0.17-0.6)	0.15% (-0.38-0.68%)	0.12% (-0.47-0.71%)	0.037 (-0.14-0.22)	0.53 (-1.4-2.4)
Federated States of Micronesia	2005	Male	85 to 89	33% (19-50%)	67% (50-81%)	0.32 (0.16-0.56)	0.011% (-0.5-0.48%)	-0.037% (-0.61-0.5%)	-0.0084 (-0.14-0.11)	0.022 (-0.96-0.93)
Federated States of Micronesia	2005	Male	90 to 94	34% (21-51%)	66% (49-79%)	0.33 (0.15-0.59)	-0.031% (-0.48-0.36%)	-0.097% (-0.66-0.35%)	-0.0072 (-0.049-0.026)	-0.013 (-0.22-0.16)
Federated States of Micronesia	2005	Male	95 plus	37% (20-58%)	63% (42-80%)	0.35 (0.14-0.67)	-0.24% (-0.84-0.28%)	-0.39% (-1.1-0.19%)	-0.0069 (-0.02-0.0034)	-0.017 (-0.06-0.019)
Federated States of Micronesia	2010	Female	All Ages	16% (13-20%)	84% (80-87%)	0.2 (0.15-0.27)	0.18% (-0.13-0.46%)	-0.17% (-0.62-0.24%)	-0.66 (-2.4-0.94)	28 (-19-74)
Federated States of Micronesia	2010	Female	15 to 19	18% (11-29%)	82% (71-89%)	0.25 (0.12-0.43)	0.74% (0.37-1.2%)	0.93% (0.26-1.9%)	0.056 (0.015-0.12)	6.8 (3.3-12)
Federated States of Micronesia	2010	Female	20 to 24	23% (14-35%)	77% (65-86%)	0.4 (0.2-0.65)	1.3% (0.73-2%)	1.3% (0.51-2.7%)	0.098 (0.035-0.2)	12 (6.4-20)
Federated States of Micronesia	2010	Female	25 to 29	23% (13-36%)	77% (64-87%)	0.37 (0.21-0.61)	1.2% (0.73-1.8%)	1.3% (0.55-2.2%)	0.11 (0.047-0.21)	11 (6.7-18)
Federated States of Micronesia	2010	Female	30 to 34	23% (13-36%)	77% (64-87%)	0.36 (0.18-0.61)	0.8% (0.35-1.3%)	0.75% (0.073-1.6%)	0.055 (0.0051-0.12)	5.4 (2.3-9.5)
Federated States of Micronesia	2010	Female	35 to 39	22% (12-35%)	78% (65-88%)	0.38 (0.2-0.66)	0.91% (0.39-1.6%)	1.1% (0.32-2%)	0.096 (0.027-0.19)	6.3 (2.5-11)
Federated States of Micronesia	2010	Female	40 to 44	20% (12-33%)	80% (67-88%)	0.35 (0.17-0.62)	0.7% (0.08-1.4%)	0.86% (0.027-1.9%)	0.12 (0.004-0.26)	6.4 (0.78-13)
Federated States of Micronesia	2010	Female	45 to 49	20% (11-32%)	80% (68-89%)	0.3 (0.15-0.54)	0.27% (-0.38-0.86%)	0.37% (-0.42-1.2%)	0.068 (-0.077-0.22)	2.8 (-3.8-9.4)
Federated States of Micronesia	2010	Female	50 to 54	20% (11-33%)	80% (67-89%)	0.27 (0.13-0.47)	-0.088% (-0.83-0.49%)	-0.026% (-0.9-0.69%)	-0.0067 (-0.25-0.19)	-1.1 (-11-6.6)
Federated States of Micronesia	2010	Female	55 to 59	18% (11-30%)	82% (70-89%)	0.22 (0.11-0.4)	-0.19% (-0.94-0.33%)	-0.13% (-0.98-0.52%)	-0.041 (-0.31-0.18)	-2.6 (-12-4.6)
Federated States of Micronesia	2010	Female	60 to 64	17% (9.5-28%)	83% (72-91%)	0.19 (0.086-0.35)	-0.38% (-1.1-0.16%)	-0.36% (-1.2-0.26%)	-0.11 (-0.38-0.08)	-4 (-12-1.6)
Federated States of Micronesia	2010	Female	65 to 69	18% (11-28%)	82% (72-89%)	0.19 (0.09-0.33)	-0.41% (-1.1-0.089%)	-0.4% (-1.2-0.18%)	-0.11 (-0.35-0.052)	-3.3 (-8.9-0.7)
Federated States of Micronesia	2010	Female	70 to 74	18% (11-29%)	82% (71-89%)	0.19 (0.087-0.34)	-0.59% (-1.2--0.068%)	-0.62% (-1.3--0.036%)	-0.21 (-0.47--0.012)	-4.3 (-9.2--0.54)
Federated States of Micronesia	2010	Female	75 to 79	18% (11-29%)	82% (71-89%)	0.18 (0.081-0.32)	-0.48% (-1.1--0.037%)	-0.5% (-1.2--0.0021%)	-0.23 (-0.58--0.00094)	-3.6 (-8.7--0.29)

Location	Year	Sex	Age	Prevalence of current drinkers	Prevalence of abstainers	Population average of standard drinks daily	Percent of total attributable deaths	Percent of total attributable DALYs	Attributable deaths	Attributable DALYs
Federated States of Micronesia	2010	Female	80 to 84	18% (10-29%)	82% (71-90%)	0.17 (0.083-0.3)	-0.58% (-1.2--0.15%)	-0.64% (-1.3--0.14%)	-0.28 (-0.62--0.06)	-3.1 (-6.6--0.76)
Federated States of Micronesia	2010	Female	85 to 89	18% (11-28%)	82% (72-89%)	0.17 (0.087-0.3)	-0.58% (-1.2--0.13%)	-0.65% (-1.3--0.14%)	-0.17 (-0.35--0.037)	-1.3 (-2.7--0.3)
Federated States of Micronesia	2010	Female	90 to 94	19% (12-29%)	81% (71-88%)	0.19 (0.088-0.35)	-0.52% (-1--0.13%)	-0.59% (-1.2--0.15%)	-0.072 (-0.15--0.018)	-0.37 (-0.76--0.086)
Federated States of Micronesia	2010	Female	95 plus	22% (12-35%)	78% (65-88%)	0.21 (0.084-0.39)	-0.55% (-1.3-0.025%)	-0.67% (-1.5--0.011%)	-0.019 (-0.043--0.00029)	-0.06 (-0.14-0.0026)
Federated States of Micronesia	2010	Male	All Ages	38% (30-48%)	62% (52-70%)	0.62 (0.49-0.76)	1.9% (1.1-2.8%)	1.4% (0.56-2.4%)	5.9 (2.3-10)	360 (200-550)
Federated States of Micronesia	2010	Male	15 to 19	34% (20-52%)	66% (48-80%)	0.34 (0.16-0.58)	1.6% (0.81-2.8%)	2% (0.73-3.9%)	0.21 (0.076-0.44)	20 (10-36)
Federated States of Micronesia	2010	Male	20 to 24	52% (34-70%)	48% (30-66%)	1 (0.62-1.5)	4.6% (2.7-7.2%)	5.4% (2.8-8.8%)	0.89 (0.45-1.5)	69 (40-110)
Federated States of Micronesia	2010	Male	25 to 29	59% (40-76%)	41% (24-60%)	1.5 (0.99-2.2)	6.1% (3.7-8.8%)	7.2% (4-11%)	1.2 (0.64-1.8)	82 (50-120)
Federated States of Micronesia	2010	Male	30 to 34	61% (39-78%)	39% (22-61%)	1.6 (0.96-2.5)	5.1% (2.5-8%)	5.7% (2.3-9.4%)	0.72 (0.28-1.2)	48 (22-78)
Federated States of Micronesia	2010	Male	35 to 39	59% (41-77%)	41% (23-59%)	1.4 (0.8-2.3)	4.3% (2.2-7%)	4.8% (2.2-8.1%)	0.53 (0.24-0.96)	32 (16-54)
Federated States of Micronesia	2010	Male	40 to 44	57% (39-75%)	43% (25-61%)	1.2 (0.66-1.9)	3% (1.2-5.3%)	3.2% (0.99-5.8%)	0.52 (0.15-1.1)	28 (11-52)
Federated States of Micronesia	2010	Male	45 to 49	53% (32-72%)	47% (28-68%)	1.1 (0.6-1.8)	2.4% (0.76-4.2%)	2.4% (0.54-4.6%)	0.64 (0.14-1.3)	31 (10-59)
Federated States of Micronesia	2010	Male	50 to 54	50% (31-69%)	50% (31-69%)	0.89 (0.47-1.5)	1.7% (0.24-3.5%)	1.8% (0.027-3.8%)	0.6 (0.0097-1.4)	26 (3.5-55)
Federated States of Micronesia	2010	Male	55 to 59	48% (30-67%)	52% (33-70%)	0.66 (0.35-1.1)	0.81% (-0.25-2.2%)	0.78% (-0.44-2.3%)	0.36 (-0.21-1)	14 (-4.1-37)
Federated States of Micronesia	2010	Male	60 to 64	45% (26-65%)	55% (35-74%)	0.52 (0.25-0.96)	0.43% (-0.5-1.7%)	0.41% (-0.68-1.8%)	0.16 (-0.28-0.72)	5.5 (-6.8-21)
Federated States of Micronesia	2010	Male	65 to 69	43% (25-62%)	57% (38-75%)	0.47 (0.23-0.82)	0.35% (-0.41-1.3%)	0.35% (-0.52-1.4%)	0.098 (-0.14-0.39)	2.6 (-3-9.4)
Federated States of Micronesia	2010	Male	70 to 74	41% (24-58%)	59% (42-76%)	0.4 (0.19-0.73)	0.17% (-0.5-0.96%)	0.15% (-0.58-1.1%)	0.047 (-0.19-0.32)	1.1 (-3.3-6.2)
Federated States of Micronesia	2010	Male	75 to 79	37% (22-56%)	63% (44-78%)	0.34 (0.16-0.63)	0.099% (-0.52-0.74%)	0.069% (-0.61-0.77%)	0.027 (-0.24-0.31)	0.63 (-3.2-4.8)
Federated States of Micronesia	2010	Male	80 to 84	35% (19-53%)	65% (47-81%)	0.31 (0.15-0.54)	0.088% (-0.43-0.59%)	0.057% (-0.53-0.6%)	0.017 (-0.16-0.19)	0.32 (-1.5-2.2)
Federated States of Micronesia	2010	Male	85 to 89	33% (19-50%)	67% (50-81%)	0.29 (0.15-0.51)	-0.041% (-0.53-0.39%)	-0.098% (-0.66-0.39%)	-0.017 (-0.12-0.066)	-0.061 (-0.82-0.58)
Federated States of Micronesia	2010	Male	90 to 94	34% (21-51%)	66% (49-79%)	0.29 (0.14-0.52)	-0.066% (-0.5-0.28%)	-0.14% (-0.66-0.26%)	-0.012 (-0.058-0.023)	-0.035 (-0.26-0.14)
Federated States of Micronesia	2010	Male	95 plus	37% (20-57%)	63% (43-80%)	0.31 (0.13-0.61)	-0.26% (-0.86-0.19%)	-0.41% (-1.1-0.11%)	-0.0085 (-0.023-0.0023)	-0.021 (-0.069-0.015)

Location	Year	Sex	Age	Prevalence of current drinkers	Prevalence of abstainers	Population average of standard drinks daily	Percent of total attributable deaths	Percent of total attributable DALYs	Attributable deaths	Attributable DALYs
Federated States of Micronesia	2016	Female	All Ages	16% (13-21%)	84% (79-87%)	0.2 (0.13-0.3)	0.15% (-0.17-0.5%)	-0.21% (-0.67-0.25%)	-0.82 (-2.6-0.96)	24 (-26-81)
Federated States of Micronesia	2016	Female	15 to 19	18% (11-28%)	82% (72-89%)	0.24 (0.11-0.44)	0.72% (0.36-1.3%)	0.9% (0.22-2.1%)	0.051 (0.011-0.12)	6.4 (3-11)
Federated States of Micronesia	2016	Female	20 to 24	23% (14-35%)	77% (65-86%)	0.39 (0.18-0.7)	1.2% (0.69-2.1%)	1.3% (0.39-2.9%)	0.1 (0.03-0.23)	13 (6.8-22)
Federated States of Micronesia	2016	Female	25 to 29	23% (13-36%)	77% (64-87%)	0.36 (0.18-0.62)	1.2% (0.68-1.8%)	1.2% (0.48-2.3%)	0.11 (0.04-0.21)	11 (6.3-18)
Federated States of Micronesia	2016	Female	30 to 34	22% (12-36%)	78% (64-88%)	0.34 (0.17-0.61)	0.89% (0.46-1.5%)	0.87% (0.24-1.7%)	0.094 (0.024-0.2)	8.9 (4.4-15)
Federated States of Micronesia	2016	Female	35 to 39	22% (12-35%)	78% (65-88%)	0.36 (0.17-0.7)	0.74% (0.22-1.6%)	0.8% (0.068-1.9%)	0.08 (0.0061-0.21)	5.7 (1.7-13)
Federated States of Micronesia	2016	Female	40 to 44	20% (12-33%)	80% (67-88%)	0.34 (0.15-0.66)	0.53% (0.045-1.4%)	0.61% (0.016-1.8%)	0.06 (0.0013-0.17)	3.5 (0.28-8.8)
Federated States of Micronesia	2016	Female	45 to 49	20% (11-32%)	80% (68-89%)	0.29 (0.12-0.54)	0.23% (-0.45-0.92%)	0.32% (-0.54-1.3%)	0.053 (-0.086-0.22)	2.2 (-4.2-9)
Federated States of Micronesia	2016	Female	50 to 54	20% (11-33%)	80% (67-89%)	0.26 (0.11-0.49)	-0.12% (-0.86-0.55%)	-0.067% (-0.93-0.73%)	-0.015 (-0.23-0.18)	-1.4 (-9.7-5.8)
Federated States of Micronesia	2016	Female	55 to 59	18% (11-30%)	82% (70-89%)	0.21 (0.094-0.41)	-0.22% (-0.92-0.37%)	-0.16% (-1-0.52%)	-0.056 (-0.34-0.19)	-3.1 (-13-4.8)
Federated States of Micronesia	2016	Female	60 to 64	17% (9.5-28%)	83% (72-91%)	0.18 (0.076-0.35)	-0.38% (-1.1-0.13%)	-0.37% (-1.2-0.22%)	-0.16 (-0.52-0.096)	-5.6 (-16-1.9)
Federated States of Micronesia	2016	Female	65 to 69	17% (10-28%)	83% (72-90%)	0.18 (0.079-0.33)	-0.44% (-1.1-0.12%)	-0.43% (-1.2-0.17%)	-0.18 (-0.53-0.076)	-5.1 (-13-1.4)
Federated States of Micronesia	2016	Female	70 to 74	18% (11-29%)	82% (71-89%)	0.18 (0.079-0.35)	-0.6% (-1.2--0.086%)	-0.64% (-1.4--0.058%)	-0.24 (-0.51--0.022)	-4.9 (-10--0.72)
Federated States of Micronesia	2016	Female	75 to 79	18% (11-28%)	82% (72-89%)	0.17 (0.07-0.32)	-0.49% (-1.1--0.028%)	-0.51% (-1.2-0.0093%)	-0.19 (-0.46-0.0037)	-2.9 (-6.9--0.18)
Federated States of Micronesia	2016	Female	80 to 84	18% (10-28%)	82% (72-90%)	0.17 (0.074-0.31)	-0.59% (-1.2--0.15%)	-0.65% (-1.3--0.15%)	-0.26 (-0.54--0.058)	-2.8 (-5.8--0.71)
Federated States of Micronesia	2016	Female	85 to 89	18% (11-27%)	82% (73-89%)	0.17 (0.077-0.32)	-0.58% (-1.2--0.15%)	-0.66% (-1.3--0.16%)	-0.19 (-0.38--0.044)	-1.4 (-2.9--0.33)
Federated States of Micronesia	2016	Female	90 to 94	19% (12-29%)	81% (71-88%)	0.18 (0.074-0.36)	-0.52% (-1--0.15%)	-0.6% (-1.2--0.17%)	-0.063 (-0.13--0.017)	-0.32 (-0.67--0.087)
Federated States of Micronesia	2016	Female	95 plus	22% (12-35%)	78% (65-88%)	0.2 (0.077-0.43)	-0.56% (-1.3--0.0013%)	-0.67% (-1.5--0.025%)	-0.023 (-0.053--0.00081)	-0.074 (-0.17--0.00016)
Federated States of Micronesia	2016	Male	All Ages	39% (31-49%)	61% (51-69%)	0.65 (0.43-0.92)	1.9% (0.98-3.2%)	1.4% (0.41-2.7%)	6.2 (1.7-13)	380 (190-660)
Federated States of Micronesia	2016	Male	15 to 19	34% (20-51%)	66% (49-80%)	0.33 (0.14-0.61)	1.6% (0.77-2.8%)	1.9% (0.69-3.9%)	0.19 (0.067-0.4)	18 (8.8-33)
Federated States of Micronesia	2016	Male	20 to 24	52% (33-70%)	48% (30-67%)	0.97 (0.52-1.6)	4.4% (2.4-7.3%)	5.2% (2.5-9.1%)	0.85 (0.38-1.5)	67 (36-110)
Federated States of Micronesia	2016	Male	25 to 29	59% (39-76%)	41% (24-61%)	1.5 (0.86-2.3)	5.8% (3.3-9%)	6.9% (3.5-11%)	1.2 (0.61-2.1)	89 (49-140)

Location	Year	Sex	Age	Prevalence of current drinkers	Prevalence of abstainers	Population average of standard drinks daily	Percent of total attributable deaths	Percent of total attributable DALYs	Attributable deaths	Attributable DALYs
Federated States of Micronesia	2016	Male	30 to 34	60% (39-78%)	40% (22-61%)	1.6 (0.86-2.6)	5.1% (2.7-8.3%)	5.8% (2.7-10%)	1.1 (0.51-2)	73 (38-120)
Federated States of Micronesia	2016	Male	35 to 39	59% (40-77%)	41% (23-60%)	1.4 (0.74-2.3)	3.3% (0.92-6.3%)	3.6% (0.43-7.3%)	0.57 (0.063-1.2)	35 (9.8-69)
Federated States of Micronesia	2016	Male	40 to 44	56% (38-74%)	44% (26-62%)	1.2 (0.59-2)	2.7% (0.83-5.5%)	2.9% (0.43-6.2%)	0.39 (0.058-0.86)	21 (6.4-44)
Federated States of Micronesia	2016	Male	45 to 49	52% (31-71%)	48% (29-69%)	1.1 (0.51-1.9)	2.3% (0.49-4.8%)	2.4% (0.11-5.1%)	0.47 (0.019-1.1)	23 (4.4-49)
Federated States of Micronesia	2016	Male	50 to 54	49% (29-69%)	51% (31-71%)	0.86 (0.42-1.6)	1.7% (0.00082-3.8%)	1.7% (-0.23-4.2%)	0.58 (-0.075-1.5)	25 (0.013-59)
Federated States of Micronesia	2016	Male	55 to 59	47% (29-67%)	53% (33-71%)	0.64 (0.3-1.2)	0.79% (-0.37-2.4%)	0.77% (-0.56-2.5%)	0.33 (-0.25-1.1)	13 (-5.8-39)
Federated States of Micronesia	2016	Male	60 to 64	45% (26-66%)	55% (34-74%)	0.5 (0.22-0.98)	0.42% (-0.51-1.7%)	0.39% (-0.71-1.8%)	0.23 (-0.42-1.1)	7.8 (-10-33)
Federated States of Micronesia	2016	Male	65 to 69	43% (25-62%)	57% (38-75%)	0.45 (0.21-0.82)	0.32% (-0.46-1.3%)	0.32% (-0.57-1.5%)	0.17 (-0.28-0.78)	4.4 (-6.2-19)
Federated States of Micronesia	2016	Male	70 to 74	40% (24-58%)	60% (42-76%)	0.39 (0.18-0.74)	0.16% (-0.53-1%)	0.14% (-0.64-1.1%)	0.046 (-0.22-0.37)	1.1 (-3.7-7.2)
Federated States of Micronesia	2016	Male	75 to 79	36% (21-55%)	64% (45-79%)	0.33 (0.14-0.64)	0.11% (-0.49-0.81%)	0.073% (-0.59-0.87%)	0.021 (-0.17-0.25)	0.49 (-2.3-3.7)
Federated States of Micronesia	2016	Male	80 to 84	33% (18-51%)	67% (49-82%)	0.29 (0.13-0.56)	0.11% (-0.39-0.63%)	0.076% (-0.48-0.68%)	0.021 (-0.13-0.19)	0.35 (-1.3-2.1)
Federated States of Micronesia	2016	Male	85 to 89	32% (19-48%)	68% (52-81%)	0.28 (0.12-0.54)	-0.03% (-0.52-0.43%)	-0.087% (-0.66-0.42%)	-0.017 (-0.13-0.08)	-0.05 (-0.87-0.7)
Federated States of Micronesia	2016	Male	90 to 94	33% (20-51%)	67% (49-80%)	0.29 (0.12-0.57)	-0.061% (-0.51-0.33%)	-0.13% (-0.66-0.3%)	-0.0087 (-0.043-0.02)	-0.023 (-0.2-0.13)
Federated States of Micronesia	2016	Male	95 plus	37% (19-57%)	63% (43-81%)	0.32 (0.12-0.67)	-0.26% (-0.87-0.23%)	-0.41% (-1.1-0.16%)	-0.01 (-0.029-0.004)	-0.025 (-0.086-0.023)
Papua New Guinea	1990	Female	All Ages	20% (14-27%)	80% (73-86%)	1 (0.66-1.5)	2% (1.2-2.9%)	3.1% (1.6-4.9%)	630 (320-1000)	25000 (15000-37000)
Papua New Guinea	1990	Female	15 to 19	21% (9.7-37%)	79% (63-90%)	1.3 (0.5-2.5)	2.6% (1.1-4.9%)	3.9% (1.4-7.8%)	17 (6.1-36)	1400 (560-2700)
Papua New Guinea	1990	Female	20 to 24	26% (12-45%)	74% (55-88%)	2 (0.86-4)	4% (2.1-6.9%)	5.4% (2.5-9.4%)	28 (13-51)	2100 (1100-3700)
Papua New Guinea	1990	Female	25 to 29	27% (11-47%)	73% (53-89%)	2.1 (0.89-3.8)	4.7% (2.4-7.7%)	6.1% (3-10%)	36 (16-61)	2400 (1200-4000)
Papua New Guinea	1990	Female	30 to 34	28% (11-49%)	72% (51-89%)	2.1 (0.78-4.1)	4.5% (2.3-7.4%)	5.8% (2.7-9.5%)	38 (17-66)	2300 (1200-3900)
Papua New Guinea	1990	Female	35 to 39	28% (12-48%)	72% (52-88%)	2.2 (0.85-4.1)	5% (2.6-8.1%)	6.3% (3.2-10%)	51 (24-88)	2800 (1400-4700)
Papua New Guinea	1990	Female	40 to 44	28% (14-46%)	72% (54-86%)	2 (0.81-3.9)	5.3% (2.5-8.9%)	6.6% (3.1-11%)	55 (24-97)	2700 (1200-4600)
Papua New Guinea	1990	Female	45 to 49	27% (12-46%)	73% (54-88%)	1.7 (0.66-3.4)	4.9% (2.1-9%)	6.1% (2.6-11%)	55 (22-100)	2400 (960-4300)

Location	Year	Sex	Age	Prevalence of current drinkers	Prevalence of abstainers	Population average of standard drinks daily	Percent of total attributable deaths	Percent of total attributable DALYs	Attributable deaths	Attributable DALYs
Papua New Guinea	1990	Female	50 to 54	27% (13-46%)	73% (54-87%)	1.5 (0.58-3.2)	4.6% (1.5-9%)	5.6% (1.9-11%)	59 (20-120)	2200 (730-4400)
Papua New Guinea	1990	Female	55 to 59	27% (13-47%)	73% (53-87%)	1.4 (0.53-3.1)	4% (1.2-8.4%)	4.7% (1.4-9.9%)	56 (16-120)	1800 (500-3800)
Papua New Guinea	1990	Female	60 to 64	27% (13-47%)	73% (53-87%)	1.3 (0.48-3.1)	3.3% (0.62-7.4%)	3.7% (0.74-8.4%)	48 (9.5-110)	1300 (250-3100)
Papua New Guinea	1990	Female	65 to 69	28% (13-46%)	72% (54-87%)	1.4 (0.48-2.9)	3.6% (0.79-7.6%)	4% (0.9-8.6%)	57 (14-120)	1300 (300-2800)
Papua New Guinea	1990	Female	70 to 74	27% (12-45%)	73% (55-88%)	1.3 (0.44-2.8)	3.1% (0.54-7.2%)	3.5% (0.62-8.1%)	49 (8.7-120)	890 (150-2200)
Papua New Guinea	1990	Female	75 to 79	26% (11-44%)	74% (56-89%)	1.2 (0.39-2.8)	3.1% (0.48-7.2%)	3.4% (0.5-8%)	40 (6.2-95)	560 (86-1300)
Papua New Guinea	1990	Female	80 to 84	26% (11-45%)	74% (55-89%)	1.2 (0.36-2.6)	2.6% (0.45-6.5%)	2.9% (0.47-7.2%)	24 (3.9-60)	250 (43-630)
Papua New Guinea	1990	Female	85 to 89	27% (12-47%)	73% (53-88%)	1.2 (0.44-2.7)	2.5% (0.42-6.4%)	2.8% (0.43-7.1%)	9.3 (1.5-24)	70 (12-180)
Papua New Guinea	1990	Female	90 to 94	30% (15-50%)	70% (50-85%)	1.4 (0.47-3)	2.3% (0.32-5.8%)	2.6% (0.32-6.4%)	2.5 (0.31-6.2)	13 (1.8-32)
Papua New Guinea	1990	Female	95 plus	35% (16-57%)	65% (43-84%)	1.6 (0.5-3.6)	2.5% (0.042-6.6%)	2.7% (-0.039-7.2%)	0.6 (-0.0085-1.6)	2.1 (0.035-5.4)
Papua New Guinea	1990	Male	All Ages	40% (32-50%)	60% (50-68%)	2.3 (1.6-3.1)	4.9% (3.4-6.2%)	6.6% (4.5-8.6%)	1500 (1000-2100)	70000 (49000-92000)
Papua New Guinea	1990	Male	15 to 19	39% (23-57%)	61% (43-77%)	1.6 (0.71-2.9)	4.7% (2.2-8.7%)	6.2% (2.7-12%)	41 (18-78)	3100 (1400-5800)
Papua New Guinea	1990	Male	20 to 24	57% (38-74%)	43% (26-62%)	4.2 (2.3-6.7)	14% (8.4-19%)	17% (10-24%)	130 (82-200)	9300 (5800-13000)
Papua New Guinea	1990	Male	25 to 29	64% (45-79%)	36% (21-55%)	5.8 (3.3-9.1)	16% (11-20%)	19% (13-26%)	150 (100-210)	9600 (6500-13000)
Papua New Guinea	1990	Male	30 to 34	65% (45-81%)	35% (19-55%)	6.2 (3.5-9.9)	15% (11-20%)	18% (13-24%)	150 (100-210)	8900 (6200-12000)
Papua New Guinea	1990	Male	35 to 39	64% (46-79%)	36% (21-54%)	5.5 (2.9-9.4)	14% (9.2-18%)	17% (11-22%)	160 (99-220)	8400 (5400-12000)
Papua New Guinea	1990	Male	40 to 44	61% (44-77%)	39% (23-56%)	4.8 (2.3-8.5)	12% (7.7-17%)	15% (8.7-20%)	160 (92-230)	7500 (4500-11000)
Papua New Guinea	1990	Male	45 to 49	58% (36-75%)	42% (25-64%)	4.2 (2-7.4)	11% (6.5-15%)	13% (7.3-18%)	160 (90-240)	6800 (3900-10000)
Papua New Guinea	1990	Male	50 to 54	55% (35-74%)	45% (26-65%)	3.5 (1.6-6.4)	9.4% (4.8-14%)	11% (5.4-16%)	150 (78-230)	5800 (3000-8600)
Papua New Guinea	1990	Male	55 to 59	53% (34-73%)	47% (27-66%)	2.8 (1.3-5.3)	7.3% (3.4-11%)	8% (3.7-13%)	120 (55-200)	4100 (1800-6400)
Papua New Guinea	1990	Male	60 to 64	50% (30-70%)	50% (30-70%)	2.4 (1.1-4.7)	6% (2.4-9.9%)	6.5% (2.6-11%)	100 (41-180)	2900 (1200-4900)
Papua New Guinea	1990	Male	65 to 69	48% (29-68%)	52% (32-71%)	2.2 (0.98-4.1)	5.5% (2.3-9.2%)	6% (2.5-10%)	84 (33-140)	1900 (780-3300)

Location	Year	Sex	Age	Prevalence of current drinkers	Prevalence of abstainers	Population average of standard drinks daily	Percent of total attributable deaths	Percent of total attributable DALYs	Attributable deaths	Attributable DALYs
Papua New Guinea	1990	Male	70 to 74	46% (27-63%)	54% (37-73%)	1.9 (0.86-3.7)	4.6% (1.7-8.1%)	5% (1.8-8.9%)	56 (20-100)	1000 (380-1800)
Papua New Guinea	1990	Male	75 to 79	42% (26-60%)	58% (40-74%)	1.6 (0.7-3.1)	3.8% (1.3-7%)	4.1% (1.4-7.6%)	30 (9.9-57)	440 (150-810)
Papua New Guinea	1990	Male	80 to 84	39% (22-57%)	61% (43-78%)	1.4 (0.64-2.7)	3.4% (1.3-6.1%)	3.6% (1.3-6.6%)	14 (5.2-25)	150 (58-270)
Papua New Guinea	1990	Male	85 to 89	37% (22-54%)	63% (46-78%)	1.4 (0.6-2.6)	3% (0.99-5.7%)	3.2% (0.99-6.2%)	5.3 (1.6-10)	41 (14-79)
Papua New Guinea	1990	Male	90 to 94	39% (24-56%)	61% (44-76%)	1.4 (0.59-2.8)	2.4% (0.75-4.8%)	2.6% (0.72-5.3%)	1.3 (0.35-2.7)	7.1 (2.2-14)
Papua New Guinea	1990	Male	95 plus	42% (24-63%)	58% (37-76%)	1.5 (0.55-3.2)	2.4% (0.44-5.3%)	2.5% (0.3-5.7%)	0.29 (0.035-0.67)	1.1 (0.19-2.5)
Papua New Guinea	1995	Female	All Ages	20% (14-27%)	80% (73-86%)	1.1 (0.73-1.7)	2.3% (1.4-3.3%)	3.4% (1.8-5.4%)	780 (410-1300)	30000 (18000-45000)
Papua New Guinea	1995	Female	15 to 19	21% (9.8-38%)	79% (62-90%)	1.4 (0.52-2.7)	2.8% (1.2-5.2%)	4.1% (1.5-8.2%)	19 (7-39)	1500 (640-2900)
Papua New Guinea	1995	Female	20 to 24	26% (11-44%)	74% (56-89%)	2.1 (0.87-4.1)	4.1% (2.1-7%)	5.6% (2.6-9.8%)	36 (16-64)	2700 (1400-4600)
Papua New Guinea	1995	Female	25 to 29	27% (12-46%)	73% (54-88%)	2.2 (0.95-4)	4.9% (2.6-8%)	6.4% (3.2-11%)	43 (21-73)	2900 (1500-4800)
Papua New Guinea	1995	Female	30 to 34	28% (11-49%)	72% (51-89%)	2.3 (0.87-4.4)	4.8% (2.4-7.7%)	6.1% (2.9-10%)	45 (21-78)	2800 (1400-4700)
Papua New Guinea	1995	Female	35 to 39	29% (13-48%)	71% (52-87%)	2.4 (0.98-4.5)	5.5% (2.9-8.6%)	6.9% (3.6-11%)	62 (30-100)	3400 (1700-5600)
Papua New Guinea	1995	Female	40 to 44	28% (14-47%)	72% (53-86%)	2.2 (0.91-4.4)	5.7% (2.8-9.4%)	7.2% (3.4-12%)	72 (33-120)	3500 (1700-5900)
Papua New Guinea	1995	Female	45 to 49	28% (12-47%)	72% (53-88%)	1.9 (0.71-3.7)	5.3% (2.2-9.5%)	6.6% (2.8-12%)	70 (29-130)	3000 (1200-5500)
Papua New Guinea	1995	Female	50 to 54	27% (12-47%)	73% (53-88%)	1.7 (0.62-3.3)	5% (1.7-9.6%)	6% (2.1-12%)	73 (26-140)	2700 (960-5400)
Papua New Guinea	1995	Female	55 to 59	26% (12-46%)	74% (54-88%)	1.5 (0.55-3.3)	4.3% (1.3-8.9%)	5.1% (1.6-10%)	69 (21-140)	2200 (670-4700)
Papua New Guinea	1995	Female	60 to 64	27% (12-47%)	73% (53-88%)	1.4 (0.5-3.3)	3.5% (0.66-7.7%)	4% (0.77-8.6%)	59 (12-130)	1600 (300-3600)
Papua New Guinea	1995	Female	65 to 69	28% (13-47%)	72% (53-87%)	1.5 (0.53-3)	3.9% (0.9-8.1%)	4.4% (1-9.2%)	71 (17-150)	1600 (360-3400)
Papua New Guinea	1995	Female	70 to 74	28% (14-47%)	72% (53-86%)	1.4 (0.51-2.9)	3.4% (0.6-7.7%)	3.8% (0.67-8.5%)	62 (11-150)	1100 (200-2700)
Papua New Guinea	1995	Female	75 to 79	26% (12-47%)	74% (53-88%)	1.3 (0.43-2.9)	3.4% (0.56-7.5%)	3.7% (0.62-8.5%)	51 (8.9-120)	720 (120-1600)
Papua New Guinea	1995	Female	80 to 84	26% (12-46%)	74% (54-88%)	1.3 (0.41-2.8)	2.9% (0.56-6.9%)	3.2% (0.59-7.7%)	32 (5.9-78)	340 (64-820)
Papua New Guinea	1995	Female	85 to 89	27% (12-45%)	73% (55-88%)	1.3 (0.48-2.9)	2.8% (0.52-6.9%)	3.1% (0.54-7.7%)	13 (2.3-32)	96 (19-240)

Location	Year	Sex	Age	Prevalence of current drinkers	Prevalence of abstainers	Population average of standard drinks daily	Percent of total attributable deaths	Percent of total attributable DALYs	Attributable deaths	Attributable DALYs
Papua New Guinea	1995	Female	90 to 94	30% (15-50%)	70% (50-85%)	1.5 (0.5-3.2)	2.5% (0.46-6.2%)	2.8% (0.49-6.9%)	3.2 (0.58-8)	17 (3.1-42)
Papua New Guinea	1995	Female	95 plus	35% (16-57%)	65% (43-84%)	1.7 (0.53-3.8)	2.8% (0.22-7%)	3% (0.16-7.8%)	0.82 (0.043-2.1)	2.9 (0.23-7.2)
Papua New Guinea	1995	Male	All Ages	41% (33-50%)	59% (50-67%)	2.5 (1.7-3.4)	5.2% (3.8-6.8%)	7% (4.9-9.1%)	1800 (1200-2400)	81000 (58000-110000)
Papua New Guinea	1995	Male	15 to 19	39% (23-57%)	61% (43-77%)	1.7 (0.75-3.1)	4.9% (2.3-8.8%)	6.4% (2.8-12%)	44 (19-84)	3400 (1600-6200)
Papua New Guinea	1995	Male	20 to 24	56% (38-73%)	44% (27-62%)	4.5 (2.5-7.1)	14% (8.7-19%)	17% (11-24%)	170 (100-240)	12000 (7400-16000)
Papua New Guinea	1995	Male	25 to 29	63% (44-79%)	37% (21-56%)	6.2 (3.5-9.4)	16% (11-21%)	19% (14-26%)	180 (120-240)	11000 (7800-15000)
Papua New Guinea	1995	Male	30 to 34	65% (44-80%)	35% (20-56%)	6.6 (3.7-10)	15% (11-20%)	18% (13-24%)	170 (120-230)	10000 (7100-13000)
Papua New Guinea	1995	Male	35 to 39	64% (45-79%)	36% (21-55%)	5.8 (3.2-10)	14% (9.9-18%)	17% (12-22%)	180 (110-240)	9400 (6200-13000)
Papua New Guinea	1995	Male	40 to 44	61% (43-77%)	39% (23-57%)	5.1 (2.5-9.1)	13% (8-17%)	15% (9.3-20%)	180 (110-260)	8800 (5400-12000)
Papua New Guinea	1995	Male	45 to 49	57% (36-74%)	43% (26-64%)	4.4 (2.1-8)	11% (6.9-15%)	13% (7.7-18%)	180 (100-260)	7500 (4500-11000)
Papua New Guinea	1995	Male	50 to 54	54% (34-72%)	46% (28-66%)	3.7 (1.7-6.7)	9.6% (5.3-14%)	11% (5.8-16%)	170 (92-260)	6400 (3500-9500)
Papua New Guinea	1995	Male	55 to 59	52% (34-71%)	48% (29-66%)	3 (1.4-5.7)	7.5% (3.6-11%)	8.3% (3.9-13%)	140 (65-230)	4600 (2200-7300)
Papua New Guinea	1995	Male	60 to 64	50% (30-70%)	50% (30-70%)	2.5 (1.1-4.9)	6.2% (2.7-10%)	6.8% (2.9-11%)	120 (50-190)	3300 (1400-5400)
Papua New Guinea	1995	Male	65 to 69	48% (29-67%)	52% (33-71%)	2.3 (1-4.3)	5.8% (2.6-9.4%)	6.4% (2.7-10%)	100 (41-170)	2300 (960-3800)
Papua New Guinea	1995	Male	70 to 74	46% (28-64%)	54% (36-72%)	2.1 (0.94-4)	4.9% (1.9-8.4%)	5.3% (2-9.2%)	68 (25-120)	1300 (480-2200)
Papua New Guinea	1995	Male	75 to 79	42% (26-60%)	58% (40-74%)	1.8 (0.77-3.3)	4.1% (1.4-7.1%)	4.4% (1.5-7.8%)	38 (13-68)	540 (190-970)
Papua New Guinea	1995	Male	80 to 84	39% (22-57%)	61% (43-78%)	1.6 (0.67-2.9)	3.6% (1.4-6.3%)	3.9% (1.5-6.9%)	18 (6.9-31)	200 (77-340)
Papua New Guinea	1995	Male	85 to 89	37% (22-54%)	63% (46-78%)	1.5 (0.63-2.8)	3.2% (1.1-5.9%)	3.4% (1.1-6.4%)	6.8 (2.3-13)	53 (19-100)
Papua New Guinea	1995	Male	90 to 94	39% (24-56%)	61% (44-76%)	1.5 (0.63-3)	2.6% (0.83-5.1%)	2.8% (0.77-5.7%)	1.6 (0.46-3.3)	8.9 (2.9-18)
Papua New Guinea	1995	Male	95 plus	42% (24-63%)	58% (37-76%)	1.6 (0.58-3.4)	2.6% (0.54-5.8%)	2.7% (0.41-6.1%)	0.38 (0.057-0.85)	1.4 (0.28-3.1)
Papua New Guinea	2000	Female	All Ages	20% (13-27%)	80% (73-87%)	1.1 (0.68-1.5)	2.2% (1.2-3.3%)	3.2% (1.6-5.2%)	810 (390-1300)	32000 (18000-48000)
Papua New Guinea	2000	Female	15 to 19	21% (9.3-36%)	79% (64-91%)	1.3 (0.48-2.5)	2.2% (0.91-4.3%)	3.3% (1.1-6.7%)	16 (5.3-32)	1300 (540-2500)

Location	Year	Sex	Age	Prevalence of current drinkers	Prevalence of abstainers	Population average of standard drinks daily	Percent of total attributable deaths	Percent of total attributable DALYs	Attributable deaths	Attributable DALYs
Papua New Guinea	2000	Female	20 to 24	25% (11-45%)	75% (55-89%)	2 (0.81-3.9)	3.7% (1.9-6.1%)	4.9% (2.2-8.8%)	33 (14-58)	2500 (1300-4300)
Papua New Guinea	2000	Female	25 to 29	27% (11-46%)	73% (54-89%)	2 (0.9-3.7)	4.4% (2.3-7%)	5.7% (2.8-9.5%)	49 (23-83)	3300 (1700-5500)
Papua New Guinea	2000	Female	30 to 34	27% (12-47%)	73% (53-88%)	2.1 (0.82-4)	4.2% (2.2-7.1%)	5.4% (2.5-9.2%)	47 (22-82)	2900 (1500-5000)
Papua New Guinea	2000	Female	35 to 39	28% (11-47%)	72% (53-89%)	2.2 (0.84-4.1)	4.9% (2.4-7.9%)	6.1% (3-10%)	64 (29-110)	3500 (1600-6000)
Papua New Guinea	2000	Female	40 to 44	27% (13-45%)	73% (55-87%)	2 (0.8-4)	5.2% (2.4-8.8%)	6.5% (2.9-11%)	74 (32-130)	3600 (1600-6400)
Papua New Guinea	2000	Female	45 to 49	27% (12-44%)	73% (56-88%)	1.7 (0.66-3.4)	4.8% (1.9-9%)	5.9% (2.4-11%)	78 (31-150)	3400 (1300-6400)
Papua New Guinea	2000	Female	50 to 54	26% (12-46%)	74% (54-88%)	1.5 (0.57-3)	4.4% (1.4-8.9%)	5.3% (1.7-11%)	78 (25-160)	2900 (930-5900)
Papua New Guinea	2000	Female	55 to 59	26% (12-45%)	74% (55-88%)	1.4 (0.49-3.1)	3.8% (1-8.2%)	4.5% (1.2-9.7%)	72 (19-160)	2300 (600-5200)
Papua New Guinea	2000	Female	60 to 64	26% (12-45%)	74% (55-88%)	1.3 (0.47-3)	3.1% (0.48-7.1%)	3.5% (0.58-8.2%)	61 (10-140)	1700 (250-4000)
Papua New Guinea	2000	Female	65 to 69	27% (13-46%)	73% (54-87%)	1.3 (0.49-2.8)	3.4% (0.61-7.5%)	3.9% (0.71-8.6%)	71 (14-160)	1600 (310-3700)
Papua New Guinea	2000	Female	70 to 74	27% (13-45%)	73% (55-87%)	1.3 (0.46-2.7)	3% (0.34-7.2%)	3.3% (0.41-8%)	62 (7.3-150)	1100 (120-2800)
Papua New Guinea	2000	Female	75 to 79	26% (11-44%)	74% (56-89%)	1.2 (0.39-2.7)	2.9% (0.4-7.1%)	3.2% (0.45-7.9%)	52 (7.1-130)	740 (99-1800)
Papua New Guinea	2000	Female	80 to 84	25% (11-45%)	75% (55-89%)	1.1 (0.35-2.6)	2.5% (0.4-6.6%)	2.8% (0.42-7.2%)	34 (5.1-89)	360 (56-940)
Papua New Guinea	2000	Female	85 to 89	26% (12-46%)	74% (54-88%)	1.2 (0.41-2.7)	2.4% (0.38-6.3%)	2.7% (0.39-7%)	14 (2-36)	100 (17-270)
Papua New Guinea	2000	Female	90 to 94	29% (13-49%)	71% (51-87%)	1.4 (0.46-3)	2.3% (0.33-5.8%)	2.5% (0.34-6.5%)	3.6 (0.49-9.3)	19 (2.8-49)
Papua New Guinea	2000	Female	95 plus	34% (15-58%)	66% (42-85%)	1.6 (0.47-3.6)	2.5% (0.05-6.7%)	2.7% (-0.011-7.6%)	0.89 (-0.0036-2.4)	3.2 (0.061-8.3)
Papua New Guinea	2000	Male	All Ages	41% (32-50%)	59% (50-68%)	2.4 (1.7-3.3)	5.1% (3.5-6.7%)	6.6% (4.5-8.8%)	1800 (1200-2500)	84000 (61000-110000)
Papua New Guinea	2000	Male	15 to 19	38% (22-56%)	62% (44-78%)	1.6 (0.69-2.9)	4.3% (1.9-7.8%)	5.7% (2.3-11%)	39 (16-76)	3100 (1400-5600)
Papua New Guinea	2000	Male	20 to 24	56% (37-73%)	44% (27-63%)	4.2 (2.3-6.6)	12% (7.7-18%)	16% (9.3-22%)	160 (95-240)	11000 (6700-16000)
Papua New Guinea	2000	Male	25 to 29	63% (43-79%)	37% (21-57%)	5.8 (3.3-8.9)	14% (9.5-19%)	17% (12-23%)	200 (130-280)	13000 (8700-17000)
Papua New Guinea	2000	Male	30 to 34	64% (43-80%)	36% (20-57%)	6.1 (3.4-9.7)	14% (9.3-18%)	16% (11-22%)	180 (120-250)	11000 (7100-15000)
Papua New Guinea	2000	Male	35 to 39	63% (45-79%)	37% (21-55%)	5.4 (2.9-9.4)	13% (8.4-17%)	15% (10-20%)	180 (110-260)	9700 (6300-14000)

Location	Year	Sex	Age	Prevalence of current drinkers	Prevalence of abstainers	Population average of standard drinks daily	Percent of total attributable deaths	Percent of total attributable DALYs	Attributable deaths	Attributable DALYs
Papua New Guinea	2000	Male	40 to 44	61% (43-77%)	39% (23-57%)	4.8 (2.3-8.4)	12% (7.1-16%)	14% (8.1-19%)	190 (110-270)	9000 (5400-13000)
Papua New Guinea	2000	Male	45 to 49	57% (35-74%)	43% (26-65%)	4.1 (1.9-7.5)	10% (6.2-14%)	12% (6.9-17%)	190 (110-290)	8200 (4800-12000)
Papua New Guinea	2000	Male	50 to 54	54% (34-72%)	46% (28-66%)	3.5 (1.6-6.3)	8.9% (4.7-13%)	10% (5.1-15%)	180 (87-270)	6600 (3400-10000)
Papua New Guinea	2000	Male	55 to 59	52% (34-70%)	48% (30-66%)	2.8 (1.3-5.4)	6.9% (3.2-11%)	7.7% (3.5-12%)	140 (62-230)	4700 (2100-7500)
Papua New Guinea	2000	Male	60 to 64	49% (30-69%)	51% (31-70%)	2.4 (1.1-4.6)	5.7% (2.4-9.5%)	6.3% (2.5-11%)	120 (49-210)	3400 (1400-5700)
Papua New Guinea	2000	Male	65 to 69	47% (28-66%)	53% (34-72%)	2.1 (0.98-4)	5.3% (2.3-8.9%)	5.8% (2.4-9.8%)	100 (41-180)	2400 (980-4000)
Papua New Guinea	2000	Male	70 to 74	45% (27-63%)	55% (37-73%)	1.9 (0.84-3.6)	4.4% (1.5-7.8%)	4.8% (1.6-8.6%)	71 (25-130)	1300 (470-2400)
Papua New Guinea	2000	Male	75 to 79	41% (24-60%)	59% (40-76%)	1.6 (0.69-3)	3.6% (1.2-6.7%)	3.9% (1.2-7.3%)	40 (13-75)	580 (190-1100)
Papua New Guinea	2000	Male	80 to 84	38% (21-57%)	62% (43-79%)	1.4 (0.63-2.7)	3.3% (1.2-5.8%)	3.5% (1.2-6.3%)	20 (7-35)	210 (80-380)
Papua New Guinea	2000	Male	85 to 89	37% (21-54%)	63% (46-79%)	1.4 (0.62-2.7)	2.9% (0.97-5.4%)	3.1% (0.98-6%)	7.4 (2.4-15)	58 (20-110)
Papua New Guinea	2000	Male	90 to 94	38% (23-57%)	62% (43-77%)	1.4 (0.61-2.8)	2.3% (0.72-4.7%)	2.5% (0.67-5.2%)	1.8 (0.49-3.8)	9.9 (3-20)
Papua New Guinea	2000	Male	95 plus	42% (23-63%)	58% (37-77%)	1.5 (0.55-3.3)	2.4% (0.44-5.2%)	2.4% (0.3-5.6%)	0.4 (0.048-0.93)	1.5 (0.29-3.3)
Papua New Guinea	2005	Female	All Ages	20% (14-27%)	80% (73-86%)	0.97 (0.63-1.4)	2.1% (1.1-3.3%)	2.9% (1.3-5%)	850 (370-1500)	34000 (18000-53000)
Papua New Guinea	2005	Female	15 to 19	21% (9.7-37%)	79% (63-90%)	1.1 (0.45-2.3)	2.1% (0.86-4.2%)	3.1% (1.1-6.4%)	16 (5.4-33)	1300 (540-2600)
Papua New Guinea	2005	Female	20 to 24	25% (11-45%)	75% (55-89%)	1.8 (0.72-3.5)	3.5% (1.7-5.9%)	4.6% (2-8.3%)	34 (14-62)	2600 (1300-4500)
Papua New Guinea	2005	Female	25 to 29	27% (11-47%)	73% (53-89%)	1.8 (0.77-3.4)	4.1% (2.1-6.7%)	5.2% (2.4-8.9%)	51 (23-88)	3500 (1700-5800)
Papua New Guinea	2005	Female	30 to 34	28% (11-48%)	72% (52-89%)	1.9 (0.71-3.6)	4% (2-6.6%)	5% (2.3-8.4%)	58 (27-100)	3600 (1800-6200)
Papua New Guinea	2005	Female	35 to 39	28% (12-47%)	72% (53-88%)	2 (0.79-3.6)	4.6% (2.2-7.6%)	5.7% (2.6-9.5%)	73 (32-130)	4000 (1800-6900)
Papua New Guinea	2005	Female	40 to 44	27% (14-46%)	73% (54-86%)	1.8 (0.72-3.5)	4.7% (2-8.4%)	5.9% (2.5-11%)	80 (33-150)	3900 (1600-7100)
Papua New Guinea	2005	Female	45 to 49	27% (12-46%)	73% (54-88%)	1.5 (0.59-3)	4.3% (1.6-8.4%)	5.3% (1.9-10%)	82 (29-160)	3500 (1300-6900)
Papua New Guinea	2005	Female	50 to 54	27% (12-45%)	73% (55-88%)	1.4 (0.52-2.7)	3.8% (1-8.1%)	4.6% (1.2-9.8%)	85 (22-190)	3200 (810-7000)
Papua New Guinea	2005	Female	55 to 59	26% (12-46%)	74% (54-88%)	1.2 (0.46-2.7)	3.2% (0.58-7.5%)	3.8% (0.74-8.8%)	75 (14-180)	2400 (440-5700)

Location	Year	Sex	Age	Prevalence of current drinkers	Prevalence of abstainers	Population average of standard drinks daily	Percent of total attributable deaths	Percent of total attributable DALYs	Attributable deaths	Attributable DALYs
Papua New Guinea	2005	Female	60 to 64	26% (12-46%)	74% (54-88%)	1.2 (0.43-2.7)	2.6% (0.11-6.6%)	3% (0.14-7.5%)	61 (2.7-150)	1700 (65-4300)
Papua New Guinea	2005	Female	65 to 69	27% (13-46%)	73% (54-87%)	1.2 (0.43-2.5)	2.8% (0.22-6.8%)	3.2% (0.32-7.7%)	70 (6.3-170)	1600 (130-3900)
Papua New Guinea	2005	Female	70 to 74	27% (13-44%)	73% (56-87%)	1.1 (0.41-2.3)	2.4% (-0.053-6.5%)	2.7% (-0.018-7.2%)	60 (-0.45-160)	1100 (-25-3000)
Papua New Guinea	2005	Female	75 to 79	26% (11-44%)	74% (56-89%)	1.1 (0.34-2.4)	2.4% (0.12-6.3%)	2.7% (0.15-7%)	51 (2.7-140)	720 (34-2000)
Papua New Guinea	2005	Female	80 to 84	25% (11-44%)	75% (56-89%)	1 (0.33-2.3)	2.1% (0.17-5.8%)	2.3% (0.17-6.4%)	34 (2.7-99)	360 (29-1000)
Papua New Guinea	2005	Female	85 to 89	26% (12-45%)	74% (55-88%)	1.1 (0.39-2.4)	2% (0.078-5.7%)	2.2% (0.051-6.4%)	14 (0.32-40)	110 (4.2-300)
Papua New Guinea	2005	Female	90 to 94	30% (14-49%)	70% (51-86%)	1.2 (0.43-2.8)	1.9% (0.07-5.3%)	2.1% (0.03-6%)	3.7 (0.053-11)	20 (0.73-56)
Papua New Guinea	2005	Female	95 plus	34% (15-57%)	66% (43-85%)	1.4 (0.44-3.2)	2.1% (-0.22-6.2%)	2.2% (-0.32-6.9%)	0.89 (-0.13-2.8)	3.2 (-0.35-9.5)
Papua New Guinea	2005	Male	All Ages	41% (33-50%)	59% (50-67%)	2.2 (1.5-2.9)	5% (3.3-6.7%)	6.3% (4-8.6%)	2000 (1200-2700)	91000 (62000-120000)
Papua New Guinea	2005	Male	15 to 19	38% (22-56%)	62% (44-78%)	1.4 (0.59-2.5)	3.9% (1.8-7.1%)	5.1% (2.2-9.6%)	38 (15-75)	3000 (1300-5600)
Papua New Guinea	2005	Male	20 to 24	56% (37-73%)	44% (27-63%)	3.7 (2-5.9)	12% (6.9-17%)	14% (8.3-21%)	160 (91-240)	11000 (6600-16000)
Papua New Guinea	2005	Male	25 to 29	63% (44-79%)	37% (21-56%)	5.1 (2.9-7.7)	13% (9-18%)	16% (11-22%)	210 (130-290)	13000 (8700-18000)
Papua New Guinea	2005	Male	30 to 34	64% (44-80%)	36% (20-56%)	5.4 (3.1-8.5)	13% (8.5-17%)	15% (9.9-21%)	220 (140-320)	13000 (8700-18000)
Papua New Guinea	2005	Male	35 to 39	63% (45-79%)	37% (21-55%)	4.8 (2.6-8.1)	12% (7.5-16%)	14% (8.8-19%)	210 (120-310)	11000 (6800-16000)
Papua New Guinea	2005	Male	40 to 44	60% (42-77%)	40% (23-58%)	4.2 (2-7.3)	11% (6.1-15%)	12% (6.8-18%)	210 (110-310)	9900 (5400-15000)
Papua New Guinea	2005	Male	45 to 49	56% (35-74%)	44% (26-65%)	3.6 (1.7-6.5)	9.4% (5.2-14%)	11% (5.8-16%)	200 (100-310)	8700 (4600-13000)
Papua New Guinea	2005	Male	50 to 54	53% (34-73%)	47% (27-66%)	3 (1.4-5.6)	8% (3.8-12%)	9% (4.1-14%)	190 (85-310)	7300 (3300-11000)
Papua New Guinea	2005	Male	55 to 59	51% (33-71%)	49% (29-67%)	2.4 (1.1-4.6)	6.1% (2.6-10%)	6.8% (2.7-11%)	150 (57-250)	4800 (2000-8200)
Papua New Guinea	2005	Male	60 to 64	49% (30-69%)	51% (31-70%)	2.1 (0.93-4.1)	5% (1.9-9%)	5.5% (2-9.9%)	120 (43-220)	3400 (1300-6100)
Papua New Guinea	2005	Male	65 to 69	47% (28-66%)	53% (34-72%)	1.9 (0.87-3.5)	4.6% (1.7-8.2%)	5.1% (1.9-9.1%)	100 (37-190)	2400 (880-4400)
Papua New Guinea	2005	Male	70 to 74	44% (27-62%)	56% (38-73%)	1.6 (0.73-3.1)	3.8% (1.2-7.3%)	4.1% (1.3-8%)	70 (22-140)	1300 (420-2500)
Papua New Guinea	2005	Male	75 to 79	40% (25-60%)	60% (40-75%)	1.4 (0.59-2.6)	3.1% (0.92-6.1%)	3.3% (0.94-6.6%)	40 (11-81)	590 (170-1200)

Location	Year	Sex	Age	Prevalence of current drinkers	Prevalence of abstainers	Population average of standard drinks daily	Percent of total attributable deaths	Percent of total attributable DALYs	Attributable deaths	Attributable DALYs
Papua New Guinea	2005	Male	80 to 84	38% (21-56%)	62% (44-79%)	1.2 (0.53-2.3)	2.7% (0.91-5.2%)	2.9% (0.9-5.5%)	20 (6.3-39)	220 (74-420)
Papua New Guinea	2005	Male	85 to 89	36% (22-53%)	64% (47-78%)	1.2 (0.52-2.3)	2.3% (0.7-4.8%)	2.5% (0.69-5.2%)	7.6 (2.1-16)	60 (18-120)
Papua New Guinea	2005	Male	90 to 94	37% (23-55%)	63% (45-77%)	1.2 (0.52-2.4)	1.9% (0.48-4.2%)	2% (0.42-4.6%)	1.8 (0.38-4.2)	10 (2.5-23)
Papua New Guinea	2005	Male	95 plus	41% (22-62%)	59% (38-78%)	1.3 (0.46-2.7)	1.9% (0.22-4.5%)	1.9% (0.047-4.8%)	0.4 (0.0094-0.99)	1.5 (0.18-3.6)
Papua New Guinea	2010	Female	All Ages	20% (14-27%)	80% (73-86%)	0.9 (0.57-1.3)	2% (0.96-3.4%)	2.7% (1-5%)	870 (310-1600)	35000 (16000-57000)
Papua New Guinea	2010	Female	15 to 19	20% (9.7-36%)	80% (64-90%)	1 (0.39-2.1)	2.1% (0.83-4.1%)	3.1% (1-6.5%)	17 (5.2-36)	1400 (550-2800)
Papua New Guinea	2010	Female	20 to 24	25% (11-44%)	75% (56-89%)	1.6 (0.65-3.2)	3.4% (1.7-5.9%)	4.5% (2-8.3%)	33 (13-60)	2600 (1200-4500)
Papua New Guinea	2010	Female	25 to 29	26% (11-46%)	74% (54-89%)	1.7 (0.71-3.2)	3.9% (2-6.6%)	5.1% (2.3-8.7%)	49 (21-88)	3400 (1700-5800)
Papua New Guinea	2010	Female	30 to 34	27% (11-47%)	73% (53-89%)	1.7 (0.65-3.3)	3.8% (1.9-6.4%)	4.8% (2.2-8.4%)	57 (25-100)	3600 (1700-6300)
Papua New Guinea	2010	Female	35 to 39	28% (12-47%)	72% (53-88%)	1.8 (0.73-3.3)	4.4% (2.1-7.4%)	5.5% (2.5-9.5%)	84 (35-150)	4600 (2000-8200)
Papua New Guinea	2010	Female	40 to 44	27% (13-46%)	73% (54-87%)	1.7 (0.69-3.3)	4.4% (1.9-8.2%)	5.6% (2.3-10%)	86 (33-170)	4200 (1600-7900)
Papua New Guinea	2010	Female	45 to 49	27% (12-45%)	73% (55-88%)	1.4 (0.54-2.9)	3.9% (1.2-8%)	4.9% (1.5-10%)	83 (26-170)	3600 (1100-7500)
Papua New Guinea	2010	Female	50 to 54	27% (12-47%)	73% (53-88%)	1.3 (0.49-2.6)	3.4% (0.72-7.8%)	4.2% (0.95-9.3%)	85 (19-200)	3200 (680-7400)
Papua New Guinea	2010	Female	55 to 59	26% (12-47%)	74% (53-88%)	1.2 (0.43-2.6)	2.9% (0.31-7%)	3.4% (0.42-8.2%)	80 (9.1-200)	2600 (270-6600)
Papua New Guinea	2010	Female	60 to 64	26% (12-46%)	74% (54-88%)	1.1 (0.38-2.5)	2.2% (-0.21-6.1%)	2.6% (-0.19-7.1%)	62 (-4.5-170)	1700 (-160-4700)
Papua New Guinea	2010	Female	65 to 69	27% (13-46%)	73% (54-87%)	1.1 (0.41-2.3)	2.3% (-0.074-6.3%)	2.7% (-0.063-7.2%)	68 (-1.4-180)	1500 (-45-4100)
Papua New Guinea	2010	Female	70 to 74	27% (12-45%)	73% (55-88%)	1.1 (0.36-2.3)	2.1% (-0.28-6%)	2.4% (-0.26-6.6%)	61 (-6.9-170)	1100 (-150-3200)
Papua New Guinea	2010	Female	75 to 79	26% (11-45%)	74% (55-89%)	0.99 (0.32-2.3)	2.1% (-0.13-6.2%)	2.3% (-0.12-6.7%)	51 (-2.5-150)	730 (-43-2200)
Papua New Guinea	2010	Female	80 to 84	25% (11-45%)	75% (55-89%)	0.95 (0.3-2.1)	1.8% (-0.023-5.3%)	2% (-0.037-6%)	35 (-0.7-100)	380 (-4.7-1100)
Papua New Guinea	2010	Female	85 to 89	26% (12-45%)	74% (55-88%)	0.99 (0.35-2.2)	1.7% (-0.15-5.6%)	1.9% (-0.21-6.1%)	14 (-1.5-46)	110 (-10-340)
Papua New Guinea	2010	Female	90 to 94	29% (14-49%)	71% (51-86%)	1.1 (0.38-2.4)	1.6% (-0.16-4.9%)	1.8% (-0.23-5.4%)	3.8 (-0.49-12)	20 (-2.1-60)
Papua New Guinea	2010	Female	95 plus	34% (15-56%)	66% (44-85%)	1.3 (0.4-2.9)	1.7% (-0.52-6%)	1.8% (-0.7-6.6%)	0.88 (-0.34-3.3)	3.2 (-0.98-11)

Location	Year	Sex	Age	Prevalence of current drinkers	Prevalence of abstainers	Population average of standard drinks daily	Percent of total attributable deaths	Percent of total attributable DALYs	Attributable deaths	Attributable DALYs
Papua New Guinea	2010	Male	All Ages	41% (33-51%)	59% (49-67%)	2 (1.4-2.8)	5% (3.2-7%)	6.2% (3.7-8.7%)	2100 (1200-3000)	97000 (62000-130000)
Papua New Guinea	2010	Male	15 to 19	38% (23-56%)	62% (44-77%)	1.3 (0.55-2.4)	3.8% (1.8-7%)	5.1% (2.2-9.6%)	41 (17-81)	3200 (1400-6100)
Papua New Guinea	2010	Male	20 to 24	56% (38-73%)	44% (27-62%)	3.4 (1.8-5.6)	11% (6.6-16%)	14% (7.8-21%)	160 (88-240)	11000 (6500-17000)
Papua New Guinea	2010	Male	25 to 29	63% (43-79%)	37% (21-57%)	4.7 (2.7-7.3)	13% (8.4-18%)	16% (10-22%)	210 (130-300)	14000 (8500-19000)
Papua New Guinea	2010	Male	30 to 34	64% (43-80%)	36% (20-57%)	5 (2.7-7.9)	12% (8.1-17%)	15% (9.4-21%)	230 (140-330)	14000 (8700-19000)
Papua New Guinea	2010	Male	35 to 39	63% (44-79%)	37% (21-56%)	4.4 (2.3-7.4)	11% (6.8-16%)	14% (7.9-19%)	250 (130-380)	13000 (7600-20000)
Papua New Guinea	2010	Male	40 to 44	61% (43-77%)	39% (23-57%)	3.9 (1.9-6.9)	10% (5.5-15%)	12% (6.2-18%)	230 (120-360)	11000 (5800-17000)
Papua New Guinea	2010	Male	45 to 49	57% (35-75%)	43% (25-65%)	3.4 (1.6-6.1)	8.9% (4.5-14%)	10% (5.1-16%)	220 (110-350)	9300 (4700-15000)
Papua New Guinea	2010	Male	50 to 54	54% (34-72%)	46% (28-66%)	2.8 (1.3-5.1)	7.5% (3.4-12%)	8.5% (3.7-14%)	200 (85-340)	7600 (3400-13000)
Papua New Guinea	2010	Male	55 to 59	52% (33-71%)	48% (29-67%)	2.3 (1.1-4.3)	5.7% (2.3-9.7%)	6.3% (2.5-11%)	160 (60-290)	5300 (2100-9500)
Papua New Guinea	2010	Male	60 to 64	49% (29-68%)	51% (32-71%)	1.9 (0.89-3.7)	4.6% (1.6-8.6%)	5% (1.6-9.6%)	130 (40-240)	3500 (1200-6700)
Papua New Guinea	2010	Male	65 to 69	47% (28-66%)	53% (34-72%)	1.7 (0.78-3.2)	4.2% (1.3-7.7%)	4.5% (1.4-8.6%)	110 (31-210)	2500 (770-4800)
Papua New Guinea	2010	Male	70 to 74	44% (27-62%)	56% (38-73%)	1.5 (0.67-2.9)	3.4% (0.95-6.9%)	3.6% (0.98-7.6%)	73 (20-150)	1400 (400-2800)
Papua New Guinea	2010	Male	75 to 79	41% (25-59%)	59% (41-75%)	1.3 (0.56-2.5)	2.7% (0.73-5.6%)	2.9% (0.74-6.1%)	42 (10-89)	620 (160-1300)
Papua New Guinea	2010	Male	80 to 84	38% (22-57%)	62% (43-78%)	1.2 (0.51-2.2)	2.4% (0.72-4.9%)	2.6% (0.72-5.4%)	22 (5.9-46)	240 (70-500)
Papua New Guinea	2010	Male	85 to 89	36% (22-53%)	64% (47-78%)	1.1 (0.46-2.2)	2% (0.53-4.5%)	2.2% (0.5-4.9%)	8.2 (1.9-19)	65 (16-140)
Papua New Guinea	2010	Male	90 to 94	38% (23-56%)	62% (44-77%)	1.1 (0.46-2.2)	1.7% (0.34-3.8%)	1.7% (0.25-4.2%)	2 (0.28-4.7)	11 (2.2-26)
Papua New Guinea	2010	Male	95 plus	41% (22-62%)	59% (38-78%)	1.2 (0.44-2.5)	1.7% (0.082-4.2%)	1.7% (-0.097-4.5%)	0.42 (-0.025-1.2)	1.7 (0.082-4.2)
Papua New Guinea	2016	Female	All Ages	20% (14-27%)	80% (73-86%)	0.94 (0.54-1.5)	2.1% (0.93-3.7%)	2.8% (0.79-5.4%)	940 (250-1900)	37000 (15000-65000)
Papua New Guinea	2016	Female	15 to 19	21% (9.6-36%)	79% (64-90%)	1.1 (0.38-2.2)	2.1% (0.84-4.2%)	3.3% (1.1-6.9%)	18 (5.8-38)	1600 (620-3100)
Papua New Guinea	2016	Female	20 to 24	25% (11-44%)	75% (56-89%)	1.7 (0.66-3.3)	3.4% (1.7-6.1%)	4.8% (2.1-8.9%)	34 (15-64)	2800 (1400-4800)
Papua New Guinea	2016	Female	25 to 29	27% (11-46%)	73% (54-89%)	1.7 (0.69-3.6)	4% (2-6.8%)	5.4% (2.4-9.7%)	47 (20-86)	3400 (1600-5900)

Location	Year	Sex	Age	Prevalence of current drinkers	Prevalence of abstainers	Population average of standard drinks daily	Percent of total attributable deaths	Percent of total attributable DALYs	Attributable deaths	Attributable DALYs
Papua New Guinea	2016	Female	30 to 34	27% (10-48%)	73% (52-90%)	1.8 (0.61-3.6)	3.8% (1.7-6.7%)	5% (2.1-9%)	55 (21-100)	3500 (1600-6400)
Papua New Guinea	2016	Female	35 to 39	28% (11-47%)	72% (53-89%)	1.8 (0.69-3.8)	4.3% (1.9-7.5%)	5.5% (2.3-9.7%)	81 (32-150)	4500 (1900-8200)
Papua New Guinea	2016	Female	40 to 44	28% (13-46%)	72% (54-87%)	1.7 (0.66-3.8)	4.4% (1.6-8.1%)	5.6% (2.1-10%)	100 (36-200)	4900 (1800-9400)
Papua New Guinea	2016	Female	45 to 49	27% (12-45%)	73% (55-88%)	1.4 (0.53-2.9)	3.8% (1.1-7.7%)	4.9% (1.4-9.8%)	94 (26-190)	4100 (1100-8500)
Papua New Guinea	2016	Female	50 to 54	27% (13-46%)	73% (54-87%)	1.3 (0.45-2.6)	3.3% (0.46-7.6%)	4.1% (0.67-9.3%)	89 (15-210)	3400 (520-7800)
Papua New Guinea	2016	Female	55 to 59	26% (12-45%)	74% (55-88%)	1.1 (0.41-2.5)	2.7% (0.29-7%)	3.3% (0.42-8.2%)	83 (11-210)	2700 (280-6900)
Papua New Guinea	2016	Female	60 to 64	26% (12-46%)	74% (54-88%)	1.1 (0.36-2.6)	2.1% (-0.29-6.2%)	2.5% (-0.3-7.1%)	71 (-8.1-210)	2000 (-280-5600)
Papua New Guinea	2016	Female	65 to 69	27% (12-47%)	73% (53-88%)	1.1 (0.38-2.4)	2.2% (-0.19-6.4%)	2.6% (-0.17-7.3%)	77 (-4.9-210)	1700 (-150-5000)
Papua New Guinea	2016	Female	70 to 74	27% (13-46%)	73% (54-87%)	1.1 (0.37-2.4)	2% (-0.43-6.6%)	2.3% (-0.42-7.4%)	68 (-12-220)	1200 (-250-4000)
Papua New Guinea	2016	Female	75 to 79	26% (11-45%)	74% (55-89%)	1 (0.31-2.4)	2% (-0.32-6.2%)	2.3% (-0.34-6.9%)	59 (-9.1-180)	840 (-130-2600)
Papua New Guinea	2016	Female	80 to 84	25% (11-45%)	75% (55-89%)	0.97 (0.29-2.3)	1.8% (-0.26-5.9%)	2% (-0.27-6.6%)	43 (-5.8-140)	460 (-68-1500)
Papua New Guinea	2016	Female	85 to 89	26% (12-46%)	74% (54-88%)	1 (0.35-2.5)	1.8% (-0.32-5.8%)	2% (-0.4-6.4%)	18 (-3.6-60)	130 (-24-450)
Papua New Guinea	2016	Female	90 to 94	30% (13-49%)	70% (51-87%)	1.2 (0.36-2.7)	1.7% (-0.28-5.5%)	1.9% (-0.34-6.1%)	4.8 (-0.88-15)	26 (-4.1-82)
Papua New Guinea	2016	Female	95 plus	34% (14-57%)	66% (43-86%)	1.3 (0.4-3.2)	1.7% (-0.69-6.3%)	1.8% (-0.86-6.9%)	1.1 (-0.51-3.9)	3.8 (-1.5-14)
Papua New Guinea	2016	Male	All Ages	42% (34-51%)	58% (49-66%)	2.1 (1.3-3.2)	5.3% (3.2-7.5%)	6.3% (3.5-9.2%)	2300 (1200-3300)	100000 (62000-150000)
Papua New Guinea	2016	Male	15 to 19	38% (22-56%)	62% (44-78%)	1.3 (0.52-2.6)	3.9% (1.8-7.6%)	5.3% (2.2-10%)	47 (20-97)	3700 (1700-7300)
Papua New Guinea	2016	Male	20 to 24	56% (37-73%)	44% (27-63%)	3.5 (1.7-6)	11% (6.4-16%)	14% (7.6-21%)	180 (92-270)	12000 (6800-19000)
Papua New Guinea	2016	Male	25 to 29	63% (43-79%)	37% (21-57%)	4.8 (2.5-8.2)	13% (8.1-18%)	16% (10-23%)	210 (120-300)	14000 (8400-20000)
Papua New Guinea	2016	Male	30 to 34	64% (43-81%)	36% (19-57%)	5.1 (2.7-8.7)	12% (7.8-17%)	15% (9.2-21%)	230 (130-320)	14000 (8200-19000)
Papua New Guinea	2016	Male	35 to 39	63% (45-80%)	37% (20-55%)	4.5 (2.2-8.2)	11% (6.5-16%)	14% (7.7-20%)	250 (130-370)	14000 (7400-20000)
Papua New Guinea	2016	Male	40 to 44	60% (42-77%)	40% (23-58%)	4 (1.8-7.5)	10% (5.1-15%)	12% (5.6-17%)	270 (130-420)	13000 (6600-20000)
Papua New Guinea	2016	Male	45 to 49	56% (35-74%)	44% (26-65%)	3.4 (1.6-6.3)	8.7% (4.2-13%)	10% (4.8-15%)	250 (110-400)	11000 (5100-17000)

Location	Year	Sex	Age	Prevalence of current drinkers	Prevalence of abstainers	Population average of standard drinks daily	Percent of total attributable deaths	Percent of total attributable DALYs	Attributable deaths	Attributable DALYs
Papua New Guinea	2016	Male	50 to 54	53% (33-73%)	47% (27-67%)	2.8 (1.2-5.3)	7.4% (3.1-12%)	8.4% (3.4-14%)	220 (88-370)	8400 (3600-14000)
Papua New Guinea	2016	Male	55 to 59	51% (33-71%)	49% (29-67%)	2.3 (1-4.5)	5.6% (2-9.7%)	6.2% (2.2-11%)	170 (61-330)	5800 (2100-11000)
Papua New Guinea	2016	Male	60 to 64	49% (29-69%)	51% (31-71%)	1.9 (0.8-3.8)	4.4% (1.4-8.4%)	4.9% (1.4-9.3%)	140 (40-280)	4000 (1200-7700)
Papua New Guinea	2016	Male	65 to 69	47% (29-66%)	53% (34-71%)	1.7 (0.73-3.5)	4% (1.2-7.7%)	4.4% (1.2-8.6%)	120 (32-230)	2800 (790-5400)
Papua New Guinea	2016	Male	70 to 74	45% (27-62%)	55% (38-73%)	1.5 (0.63-3.1)	3.3% (0.87-6.7%)	3.6% (0.9-7.3%)	82 (20-170)	1500 (390-3200)
Papua New Guinea	2016	Male	75 to 79	40% (24-59%)	60% (41-76%)	1.3 (0.53-2.7)	2.7% (0.61-5.5%)	2.9% (0.61-6%)	50 (10-110)	740 (170-1500)
Papua New Guinea	2016	Male	80 to 84	37% (21-56%)	63% (44-79%)	1.2 (0.49-2.3)	2.4% (0.62-5.2%)	2.6% (0.61-5.6%)	27 (6.4-59)	300 (75-640)
Papua New Guinea	2016	Male	85 to 89	36% (21-52%)	64% (48-79%)	1.1 (0.46-2.4)	2.1% (0.43-4.7%)	2.2% (0.39-5.1%)	10 (1.9-24)	82 (17-190)
Papua New Guinea	2016	Male	90 to 94	38% (23-55%)	62% (45-77%)	1.2 (0.46-2.4)	1.7% (0.3-4.1%)	1.8% (0.19-4.4%)	2.6 (0.27-6.4)	15 (2.5-34)
Papua New Guinea	2016	Male	95 plus	42% (22-62%)	58% (38-78%)	1.3 (0.43-2.8)	1.8% (0.1-4.6%)	1.8% (-0.14-5%)	0.57 (-0.047-1.6)	2.2 (0.13-5.8)
Samoa	1990	Female	All Ages	18% (12-24%)	82% (76-88%)	0.42 (0.31-0.53)	0.8% (0.39-1.3%)	0.84% (0.039-1.9%)	3.1 (0.13-7)	160 (79-260)
Samoa	1990	Female	15 to 19	19% (7.9-34%)	81% (66-92%)	0.51 (0.22-0.96)	1.2% (0.62-2.2%)	2.2% (0.81-4.4%)	0.13 (0.049-0.27)	15 (7.3-26)
Samoa	1990	Female	20 to 24	23% (9.3-41%)	77% (59-91%)	0.81 (0.35-1.4)	2.1% (1.3-3.4%)	3.2% (1.3-6%)	0.2 (0.079-0.37)	22 (12-35)
Samoa	1990	Female	25 to 29	24% (10-43%)	76% (57-90%)	0.83 (0.37-1.5)	2.2% (1.3-3.3%)	3.2% (1.5-5.5%)	0.22 (0.1-0.4)	22 (13-35)
Samoa	1990	Female	30 to 34	25% (9.9-45%)	75% (55-90%)	0.87 (0.35-1.5)	2% (1.2-3.3%)	3% (1.4-5.3%)	0.2 (0.088-0.37)	17 (9.3-27)
Samoa	1990	Female	35 to 39	26% (11-44%)	74% (56-89%)	0.9 (0.38-1.6)	2.1% (1.1-3.5%)	3.1% (1.4-5.4%)	0.3 (0.13-0.55)	20 (10-33)
Samoa	1990	Female	40 to 44	25% (12-43%)	75% (57-88%)	0.83 (0.35-1.5)	2% (0.85-3.6%)	3% (1.2-5.7%)	0.3 (0.11-0.58)	16 (6.7-31)
Samoa	1990	Female	45 to 49	25% (11-43%)	75% (57-89%)	0.72 (0.28-1.4)	1.4% (0.39-3.1%)	2.1% (0.64-4.7%)	0.33 (0.093-0.73)	15 (3.9-33)
Samoa	1990	Female	50 to 54	25% (11-44%)	75% (56-89%)	0.63 (0.24-1.3)	1.1% (-0.0077-3%)	1.6% (0.15-4.3%)	0.28 (0.027-0.73)	10 (-0.07-28)
Samoa	1990	Female	55 to 59	24% (11-42%)	76% (58-89%)	0.56 (0.21-1.1)	0.8% (-0.27-2.3%)	1.3% (-0.13-3.3%)	0.32 (-0.035-0.86)	9.6 (-3.1-28)
Samoa	1990	Female	60 to 64	24% (11-44%)	76% (56-89%)	0.54 (0.2-1.1)	0.42% (-0.74-1.8%)	0.7% (-0.67-2.5%)	0.2 (-0.2-0.74)	4.6 (-8.1-20)
Samoa	1990	Female	65 to 69	25% (11-42%)	75% (58-89%)	0.54 (0.21-1.1)	0.38% (-0.78-1.7%)	0.65% (-0.73-2.4%)	0.23 (-0.28-0.86)	4.2 (-8.5-19)

Location	Year	Sex	Age	Prevalence of current drinkers	Prevalence of abstainers	Population average of standard drinks daily	Percent of total attributable deaths	Percent of total attributable DALYs	Attributable deaths	Attributable DALYs
Samoa	1990	Female	70 to 74	24%<br>(11-40%)	76%<br>(60-89%)	0.52<br>(0.19-1)	0.23%<br>(-0.86-1.8%)	0.39%<br>(-0.9-2.4%)	0.13<br>(-0.31-0.79)	1.8<br>(-6.7-14)
Samoa	1990	Female	75 to 79	23%<br>(10-42%)	77%<br>(58-90%)	0.48<br>(0.16-1)	0.27%<br>(-0.8-1.8%)	0.4%<br>(-0.87-2.2%)	0.15<br>(-0.35-0.85)	1.8<br>(-5.6-12)
Samoa	1990	Female	80 to 84	23%<br>(9.3-42%)	77%<br>(58-91%)	0.46<br>(0.16-0.99)	0.12%<br>(-0.81-1.4%)	0.19%<br>(-0.89-1.7%)	0.06<br>(-0.29-0.53)	0.5<br>(-3.3-5.6)
Samoa	1990	Female	85 to 89	24%<br>(11-42%)	76%<br>(58-89%)	0.48<br>(0.18-1)	0.03%<br>(-0.87-1.2%)	0.049%<br>(-1-1.4%)	0.0098<br>(-0.2-0.27)	0.054<br>(-1.5-2)
Samoa	1990	Female	90 to 94	27%<br>(12-46%)	73%<br>(54-88%)	0.56<br>(0.2-1.2)	0.088%<br>(-0.82-1.5%)	0.11%<br>(-0.94-1.7%)	0.011<br>(-0.095-0.17)	0.054<br>(-0.51-0.87)
Samoa	1990	Female	95 plus	32%<br>(13-55%)	68%<br>(45-87%)	0.65<br>(0.21-1.5)	0.039%<br>(-1.3-1.8%)	-0.0092%<br>(-1.5-2%)	-0.00024<br>(-0.048-0.062)	0.0047<br>(-0.15-0.22)
Samoa	1990	Male	All Ages	36%<br>(28-46%)	64%<br>(54-72%)	0.91<br>(0.76-1.1)	3.1%<br>(2-4.3%)	3.3%<br>(2-4.9%)	17<br>(11-26)	860<br>(590-1200)
Samoa	1990	Male	15 to 19	35%<br>(20-53%)	65%<br>(47-80%)	0.64<br>(0.32-1.1)	2.4%<br>(1.2-4.2%)	3.3%<br>(1.3-6.2%)	0.55<br>(0.22-1)	47<br>(23-82)
Samoa	1990	Male	20 to 24	53%<br>(35-71%)	47%<br>(29-65%)	1.7<br>(1.1-2.5)	7%<br>(4.2-11%)	9.1%<br>(5.1-14%)	1.9<br>(1-3.1)	140<br>(82-220)
Samoa	1990	Male	25 to 29	61%<br>(41-77%)	39%<br>(23-59%)	2.4<br>(1.5-3.4)	8.9%<br>(5.6-13%)	12%<br>(7-17%)	2.3<br>(1.3-3.5)	160<br>(98-230)
Samoa	1990	Male	30 to 34	62%<br>(41-79%)	38%<br>(21-59%)	2.6<br>(1.6-3.8)	8.3%<br>(5.1-12%)	11%<br>(6.2-16%)	1.6<br>(0.9-2.6)	100<br>(61-160)
Samoa	1990	Male	35 to 39	60%<br>(42-77%)	40%<br>(23-58%)	2.3<br>(1.3-3.5)	7%<br>(4-11%)	8.7%<br>(4.7-14%)	1.6<br>(0.85-2.6)	91<br>(52-150)
Samoa	1990	Male	40 to 44	57%<br>(39-74%)	43%<br>(26-61%)	2<br>(1-3.2)	5.7%<br>(2.9-9.5%)	6.9%<br>(3.3-12%)	1.1<br>(0.51-2)	59<br>(28-100)
Samoa	1990	Male	45 to 49	53%<br>(32-72%)	47%<br>(28-68%)	1.7<br>(0.83-3)	4.8%<br>(2.2-8.4%)	5.6%<br>(2.4-10%)	1.4<br>(0.58-2.5)	63<br>(28-110)
Samoa	1990	Male	50 to 54	51%<br>(31-69%)	49%<br>(31-69%)	1.4<br>(0.71-2.4)	4%<br>(1.6-7.4%)	4.6%<br>(1.7-8.9%)	1.5<br>(0.52-2.9)	59<br>(23-110)
Samoa	1990	Male	55 to 59	48%<br>(30-69%)	52%<br>(31-70%)	1.1<br>(0.57-2)	2.9%<br>(0.94-6.1%)	3.3%<br>(0.97-7.1%)	1.3<br>(0.36-2.8)	44<br>(14-94)
Samoa	1990	Male	60 to 64	46%<br>(27-66%)	54%<br>(34-73%)	0.95<br>(0.45-1.7)	2.3%<br>(0.58-5.3%)	2.6%<br>(0.6-6.1%)	1.1<br>(0.26-2.7)	33<br>(8.6-78)
Samoa	1990	Male	65 to 69	44%<br>(26-64%)	56%<br>(36-74%)	0.87<br>(0.43-1.5)	2%<br>(0.48-4.1%)	2.2%<br>(0.53-4.8%)	1.3<br>(0.31-2.9)	31<br>(8-68)
Samoa	1990	Male	70 to 74	42%<br>(25-59%)	58%<br>(41-75%)	0.76<br>(0.37-1.4)	1.5%<br>(0.21-3.6%)	1.7%<br>(0.18-4%)	0.85<br>(0.092-2.1)	16<br>(2.1-39)
Samoa	1990	Male	75 to 79	38%<br>(22-57%)	62%<br>(43-78%)	0.64<br>(0.31-1.2)	1.2%<br>(0.11-3%)	1.3%<br>(0.056-3.4%)	0.55<br>(0.027-1.5)	8.2<br>(0.73-22)
Samoa	1990	Male	80 to 84	35%<br>(19-53%)	65%<br>(47-81%)	0.57<br>(0.28-1)	0.97%<br>(0.13-2.2%)	1.1%<br>(0.11-2.5%)	0.28<br>(0.031-0.67)	3.1<br>(0.44-7.2)
Samoa	1990	Male	85 to 89	34%<br>(20-50%)	66%<br>(50-80%)	0.54<br>(0.26-0.99)	0.74%<br>(-0.0085-1.8%)	0.8%<br>(-0.062-2.1%)	0.1<br>(-0.0079-0.28)	0.84<br>(-0.0096-2.1)

Location	Year	Sex	Age	Prevalence of current drinkers	Prevalence of abstainers	Population average of standard drinks daily	Percent of total attributable deaths	Percent of total attributable DALYs	Attributable deaths	Attributable DALYs
Samoa	1990	Male	90 to 94	35% (21-52%)	65% (48-79%)	0.55 (0.25-1.1)	0.55% (-0.067-1.5%)	0.56% (-0.15-1.7%)	0.026 (-0.0066-0.078)	0.15 (-0.018-0.43)
Samoa	1990	Male	95 plus	38% (21-59%)	62% (41-79%)	0.61 (0.24-1.2)	0.36% (-0.47-1.6%)	0.28% (-0.68-1.7%)	0.0025 (-0.0061-0.016)	0.013 (-0.016-0.057)
Samoa	1995	Female	All Ages	18% (12-24%)	82% (76-88%)	0.43 (0.32-0.56)	0.85% (0.4-1.4%)	0.84% (-0.022-2%)	3.3 (-0.087-7.8)	170 (81-290)
Samoa	1995	Female	15 to 19	19% (8.5-33%)	81% (67-91%)	0.53 (0.23-0.98)	1.3% (0.65-2.2%)	2.3% (0.85-4.6%)	0.12 (0.045-0.24)	13 (6.6-23)
Samoa	1995	Female	20 to 24	23% (9.4-39%)	77% (61-91%)	0.83 (0.36-1.5)	2.2% (1.3-3.5%)	3.3% (1.4-6.2%)	0.2 (0.083-0.39)	22 (13-37)
Samoa	1995	Female	25 to 29	24% (9.7-42%)	76% (58-90%)	0.85 (0.37-1.5)	2.2% (1.4-3.4%)	3.3% (1.6-5.6%)	0.24 (0.11-0.43)	23 (14-37)
Samoa	1995	Female	30 to 34	25% (9.4-44%)	75% (56-91%)	0.89 (0.35-1.6)	2.1% (1.2-3.3%)	3% (1.4-5.3%)	0.28 (0.12-0.49)	22 (13-36)
Samoa	1995	Female	35 to 39	26% (11-44%)	74% (56-89%)	0.93 (0.38-1.6)	2.1% (1.1-3.6%)	3.2% (1.5-5.6%)	0.29 (0.12-0.54)	19 (9.8-33)
Samoa	1995	Female	40 to 44	25% (12-42%)	75% (58-88%)	0.86 (0.37-1.6)	2% (0.87-3.7%)	3% (1.2-5.6%)	0.41 (0.16-0.8)	22 (9.6-42)
Samoa	1995	Female	45 to 49	24% (11-42%)	76% (58-89%)	0.73 (0.29-1.4)	1.4% (0.42-3.1%)	2.2% (0.65-4.6%)	0.31 (0.089-0.69)	14 (3.7-30)
Samoa	1995	Female	50 to 54	24% (11-43%)	76% (57-89%)	0.64 (0.24-1.3)	1% (-0.067-2.8%)	1.6% (0.1-4.1%)	0.35 (0.023-0.94)	13 (-0.73-36)
Samoa	1995	Female	55 to 59	23% (11-41%)	77% (59-89%)	0.57 (0.22-1.1)	0.77% (-0.31-2.3%)	1.2% (-0.16-3.2%)	0.26 (-0.035-0.7)	7.6 (-3.2-23)
Samoa	1995	Female	60 to 64	23% (10-42%)	77% (58-90%)	0.55 (0.2-1.2)	0.4% (-0.73-1.8%)	0.69% (-0.7-2.6%)	0.23 (-0.24-0.89)	5.1 (-8.9-24)
Samoa	1995	Female	65 to 69	25% (11-43%)	75% (57-89%)	0.56 (0.22-1.1)	0.36% (-0.87-1.9%)	0.63% (-0.85-2.6%)	0.24 (-0.32-0.93)	4.2 (-9.8-21)
Samoa	1995	Female	70 to 74	25% (11-42%)	75% (58-89%)	0.54 (0.2-1.1)	0.2% (-0.94-1.7%)	0.36% (-0.99-2.3%)	0.15 (-0.42-0.98)	2 (-9.3-17)
Samoa	1995	Female	75 to 79	23% (10-42%)	77% (58-90%)	0.5 (0.16-1.1)	0.25% (-0.9-1.8%)	0.38% (-0.92-2.3%)	0.16 (-0.38-0.91)	1.8 (-6.3-13)
Samoa	1995	Female	80 to 84	23% (9.7-42%)	77% (58-90%)	0.47 (0.17-0.99)	0.12% (-0.84-1.4%)	0.18% (-0.95-1.6%)	0.072 (-0.37-0.67)	0.6 (-4.3-7.1)
Samoa	1995	Female	85 to 89	24% (11-42%)	76% (58-89%)	0.49 (0.18-1)	0.033% (-0.88-1.2%)	0.051% (-1-1.5%)	0.014 (-0.27-0.4)	0.083 (-2.1-3)
Samoa	1995	Female	90 to 94	27% (13-47%)	73% (53-87%)	0.56 (0.22-1.2)	0.091% (-0.87-1.5%)	0.11% (-1-1.7%)	0.012 (-0.11-0.19)	0.06 (-0.57-0.96)
Samoa	1995	Female	95 plus	32% (14-55%)	68% (45-86%)	0.66 (0.22-1.5)	0.05% (-1.3-1.8%)	-0.0024% (-1.6-2.1%)	-0.000039 (-0.056-0.074)	0.0074 (-0.18-0.26)
Samoa	1995	Male	All Ages	36% (28-46%)	64% (54-72%)	0.98 (0.81-1.2)	3.3% (2.3-4.6%)	3.4% (2-5%)	18 (11-27)	890 (620-1200)
Samoa	1995	Male	15 to 19	35% (20-53%)	65% (47-80%)	0.65 (0.32-1.1)	2.4% (1.2-4.2%)	3.4% (1.4-6.3%)	0.5 (0.21-0.96)	43 (21-76)

Location	Year	Sex	Age	Prevalence of current drinkers	Prevalence of abstainers	Population average of standard drinks daily	Percent of total attributable deaths	Percent of total attributable DALYs	Attributable deaths	Attributable DALYs
Samoa	1995	Male	20 to 24	53% (34-70%)	47% (30-66%)	1.8 (1.1-2.7)	7.2% (4.3-11%)	9.4% (5.3-15%)	1.8 (0.91-2.8)	130 (75-210)
Samoa	1995	Male	25 to 29	60% (40-76%)	40% (24-60%)	2.5 (1.6-3.5)	9% (5.9-13%)	12% (7.3-17%)	2.2 (1.3-3.3)	150 (96-220)
Samoa	1995	Male	30 to 34	61% (40-78%)	39% (22-60%)	2.7 (1.7-3.9)	8.2% (5.2-12%)	11% (6.4-16%)	2.1 (1.2-3.4)	140 (83-210)
Samoa	1995	Male	35 to 39	60% (42-77%)	40% (23-58%)	2.3 (1.4-3.7)	7% (4-11%)	8.9% (4.9-14%)	1.5 (0.78-2.4)	86 (48-140)
Samoa	1995	Male	40 to 44	57% (40-75%)	43% (25-60%)	2 (1.1-3.3)	5.7% (3-9.4%)	7% (3.3-12%)	1.6 (0.75-2.8)	83 (41-140)
Samoa	1995	Male	45 to 49	53% (32-71%)	47% (29-68%)	1.7 (0.91-3)	4.8% (2.2-8.7%)	5.7% (2.4-11%)	1.2 (0.51-2.3)	58 (26-100)
Samoa	1995	Male	50 to 54	50% (30-69%)	50% (31-70%)	1.4 (0.74-2.5)	4% (1.6-7.7%)	4.6% (1.7-9.2%)	1.5 (0.53-3)	60 (24-120)
Samoa	1995	Male	55 to 59	48% (30-68%)	52% (32-70%)	1.2 (0.58-2)	2.8% (0.92-5.9%)	3.2% (0.88-6.9%)	1.2 (0.34-2.7)	43 (15-92)
Samoa	1995	Male	60 to 64	46% (27-66%)	54% (34-73%)	0.97 (0.45-1.8)	2.2% (0.51-5.1%)	2.5% (0.52-5.9%)	1.1 (0.24-2.7)	34 (7.9-78)
Samoa	1995	Male	65 to 69	44% (26-63%)	56% (37-74%)	0.88 (0.44-1.5)	1.9% (0.41-4.2%)	2.2% (0.4-4.8%)	1.2 (0.22-2.6)	28 (5.6-61)
Samoa	1995	Male	70 to 74	42% (25-59%)	58% (41-75%)	0.78 (0.38-1.4)	1.5% (0.19-3.6%)	1.7% (0.17-4.1%)	1 (0.1-2.5)	20 (2.5-48)
Samoa	1995	Male	75 to 79	38% (22-57%)	62% (43-78%)	0.67 (0.32-1.3)	1.2% (0.095-3%)	1.3% (0.07-3.3%)	0.6 (0.036-1.6)	9 (0.78-23)
Samoa	1995	Male	80 to 84	35% (20-53%)	65% (47-80%)	0.59 (0.29-1.1)	0.98% (0.14-2.3%)	1.1% (0.11-2.6%)	0.34 (0.033-0.82)	3.7 (0.53-8.9)
Samoa	1995	Male	85 to 89	33% (19-50%)	67% (50-81%)	0.56 (0.27-1)	0.75% (0.002-1.9%)	0.8% (-0.055-2.1%)	0.14 (-0.01-0.38)	1.2 (0.0031-3)
Samoa	1995	Male	90 to 94	35% (21-52%)	65% (48-79%)	0.57 (0.26-1.1)	0.55% (-0.083-1.6%)	0.57% (-0.17-1.7%)	0.033 (-0.01-0.1)	0.2 (-0.03-0.54)
Samoa	1995	Male	95 plus	38% (20-59%)	62% (41-80%)	0.63 (0.25-1.3)	0.37% (-0.49-1.7%)	0.29% (-0.69-1.9%)	0.0041 (-0.0097-0.026)	0.021 (-0.028-0.097)
Samoa	2000	Female	All Ages	17% (12-24%)	83% (76-88%)	0.44 (0.32-0.56)	0.83% (0.37-1.5%)	0.74% (-0.13-1.9%)	3.1 (-0.53-8.1)	170 (70-290)
Samoa	2000	Female	15 to 19	18% (8-34%)	82% (66-92%)	0.54 (0.22-1)	1.2% (0.63-2.3%)	2.3% (0.83-4.9%)	0.11 (0.04-0.24)	12 (6.1-22)
Samoa	2000	Female	20 to 24	22% (9.4-41%)	78% (59-91%)	0.84 (0.35-1.5)	2.2% (1.2-3.5%)	3.3% (1.3-6.3%)	0.19 (0.072-0.36)	21 (11-34)
Samoa	2000	Female	25 to 29	24% (10-43%)	76% (57-90%)	0.87 (0.38-1.5)	2.3% (1.4-3.5%)	3.3% (1.6-5.7%)	0.24 (0.11-0.43)	23 (13-36)
Samoa	2000	Female	30 to 34	25% (9.6-44%)	75% (56-90%)	0.9 (0.35-1.6)	2.1% (1.2-3.4%)	3% (1.3-5.2%)	0.28 (0.12-0.5)	23 (13-37)
Samoa	2000	Female	35 to 39	25% (9.7-44%)	75% (56-90%)	0.93 (0.39-1.6)	2.1% (1.1-3.5%)	3.1% (1.4-5.5%)	0.36 (0.16-0.67)	24 (12-42)

Location	Year	Sex	Age	Prevalence of current drinkers	Prevalence of abstainers	Population average of standard drinks daily	Percent of total attributable deaths	Percent of total attributable DALYs	Attributable deaths	Attributable DALYs
Samoa	2000	Female	40 to 44	25% (12-43%)	75% (57-88%)	0.86 (0.36-1.6)	1.9% (0.79-3.5%)	2.8% (1.1-5.3%)	0.4 (0.15-0.81)	22 (9.2-42)
Samoa	2000	Female	45 to 49	24% (10-41%)	76% (59-90%)	0.73 (0.29-1.4)	1.3% (0.28-3%)	2% (0.51-4.6%)	0.33 (0.082-0.74)	15 (3.1-34)
Samoa	2000	Female	50 to 54	24% (10-42%)	76% (58-90%)	0.64 (0.25-1.3)	0.94% (-0.26-2.7%)	1.5% (-0.053-3.8%)	0.28 (-0.01-0.76)	10 (-2.8-29)
Samoa	2000	Female	55 to 59	23% (10-42%)	77% (58-90%)	0.57 (0.22-1.2)	0.66% (-0.45-2.3%)	1.1% (-0.33-3.2%)	0.26 (-0.076-0.76)	7.4 (-5.5-24)
Samoa	2000	Female	60 to 64	23% (10-40%)	77% (60-90%)	0.54 (0.2-1.2)	0.3% (-0.87-1.7%)	0.55% (-0.85-2.4%)	0.16 (-0.25-0.68)	3.3 (-9.6-18)
Samoa	2000	Female	65 to 69	24% (11-41%)	76% (59-89%)	0.56 (0.22-1.1)	0.25% (-1-1.8%)	0.48% (-1-2.4%)	0.17 (-0.38-0.83)	2.8 (-12-19)
Samoa	2000	Female	70 to 74	24% (11-40%)	76% (60-89%)	0.54 (0.2-1.1)	0.1% (-1-1.5%)	0.23% (-1.1-2%)	0.11 (-0.53-0.94)	1.1 (-12-17)
Samoa	2000	Female	75 to 79	23% (10-41%)	77% (59-90%)	0.51 (0.17-1.1)	0.17% (-0.99-1.7%)	0.29% (-1.1-2.1%)	0.15 (-0.54-1.1)	1.6 (-8.9-15)
Samoa	2000	Female	80 to 84	23% (9.1-41%)	77% (59-91%)	0.49 (0.17-1)	0.069% (-0.91-1.5%)	0.12% (-1-1.8%)	0.058 (-0.51-0.85)	0.43 (-5.7-9.1)
Samoa	2000	Female	85 to 89	23% (10-43%)	77% (57-90%)	0.51 (0.19-1.1)	0.008% (-0.99-1.3%)	0.017% (-1.1-1.5%)	0.006 (-0.38-0.53)	0.027 (-2.9-4)
Samoa	2000	Female	90 to 94	26% (12-46%)	74% (54-88%)	0.57 (0.21-1.2)	0.078% (-0.92-1.5%)	0.085% (-1.1-1.8%)	0.013 (-0.16-0.28)	0.073 (-0.83-1.4)
Samoa	2000	Female	95 plus	31% (13-55%)	69% (45-87%)	0.68 (0.22-1.5)	0.037% (-1.4-1.9%)	-0.025% (-1.7-2.1%)	-0.00092 (-0.065-0.085)	0.0059 (-0.21-0.29)
Samoa	2000	Male	All Ages	36% (28-46%)	64% (54-72%)	1 (0.84-1.2)	3.3% (2.2-4.6%)	3.3% (1.9-5%)	18 (10-27)	880 (590-1200)
Samoa	2000	Male	15 to 19	34% (19-52%)	66% (48-81%)	0.67 (0.31-1.2)	2.5% (1.3-4.4%)	3.4% (1.5-6.6%)	0.45 (0.19-0.88)	39 (20-70)
Samoa	2000	Male	20 to 24	52% (33-69%)	48% (31-67%)	1.8 (1.1-2.8)	7.2% (4.4-11%)	9.5% (5.3-15%)	1.5 (0.81-2.5)	120 (66-180)
Samoa	2000	Male	25 to 29	59% (39-76%)	41% (24-61%)	2.5 (1.6-3.7)	9% (5.6-13%)	12% (7.1-18%)	2 (1.1-3.1)	140 (84-210)
Samoa	2000	Male	30 to 34	61% (40-77%)	39% (23-60%)	2.7 (1.7-3.9)	8% (5.1-11%)	10% (6.3-16%)	2 (1.1-3.1)	130 (78-200)
Samoa	2000	Male	35 to 39	59% (41-76%)	41% (24-59%)	2.4 (1.4-3.6)	6.9% (4-10%)	8.8% (4.7-14%)	1.8 (0.89-3)	110 (58-170)
Samoa	2000	Male	40 to 44	57% (39-74%)	43% (26-61%)	2.1 (1.1-3.3)	5.6% (2.9-9%)	6.9% (3.3-11%)	1.7 (0.74-3)	89 (43-150)
Samoa	2000	Male	45 to 49	53% (32-70%)	47% (30-68%)	1.8 (0.89-3)	4.7% (2.2-8.4%)	5.6% (2.4-10%)	1.5 (0.62-2.9)	72 (32-130)
Samoa	2000	Male	50 to 54	50% (30-68%)	50% (32-70%)	1.5 (0.74-2.6)	3.9% (1.5-7.4%)	4.6% (1.5-9.1%)	1.3 (0.46-2.7)	56 (22-110)
Samoa	2000	Male	55 to 59	48% (30-67%)	52% (33-70%)	1.2 (0.59-2.1)	2.7% (0.86-5.7%)	3.1% (0.81-6.8%)	1.2 (0.3-2.7)	42 (13-91)

Location	Year	Sex	Age	Prevalence of current drinkers	Prevalence of abstainers	Population average of standard drinks daily	Percent of total attributable deaths	Percent of total attributable DALYs	Attributable deaths	Attributable DALYs
Samoa	2000	Male	60 to 64	45%<br>(26-65%)	55%<br>(35-74%)	1<br>(0.44-1.9)	2.2%<br>(0.42-5%)	2.4%<br>(0.38-5.8%)	1.1<br>(0.17-2.7)	33<br>(6.4-80)
Samoa	2000	Male	65 to 69	43%<br>(25-62%)	57%<br>(38-75%)	0.91<br>(0.44-1.6)	1.9%<br>(0.29-4.1%)	2.1%<br>(0.28-4.7%)	1.1<br>(0.15-2.5)	28<br>(4.5-61)
Samoa	2000	Male	70 to 74	41%<br>(23-59%)	59%<br>(41-77%)	0.79<br>(0.38-1.4)	1.4%<br>(0.1-3.7%)	1.6%<br>(0.074-4.1%)	0.89<br>(0.041-2.3)	17<br>(1.3-44)
Samoa	2000	Male	75 to 79	37%<br>(21-55%)	63%<br>(45-79%)	0.67<br>(0.32-1.3)	1.1%<br>(0.038-3%)	1.2%<br>(-0.019-3.4%)	0.69<br>(-0.01-1.9)	11<br>(0.37-29)
Samoa	2000	Male	80 to 84	34%<br>(18-53%)	66%<br>(47-82%)	0.6<br>(0.29-1.1)	0.94%<br>(0.075-2.4%)	1%<br>(0.05-2.6%)	0.39<br>(0.02-1)	4.4<br>(0.36-11)
Samoa	2000	Male	85 to 89	33%<br>(18-50%)	67%<br>(50-82%)	0.57<br>(0.28-1)	0.71%<br>(-0.055-2%)	0.75%<br>(-0.11-2.2%)	0.16<br>(-0.023-0.46)	1.3<br>(-0.095-3.6)
Samoa	2000	Male	90 to 94	34%<br>(21-52%)	66%<br>(48-79%)	0.58<br>(0.26-1.1)	0.53%<br>(-0.16-1.5%)	0.53%<br>(-0.28-1.7%)	0.043<br>(-0.023-0.14)	0.25<br>(-0.081-0.75)
Samoa	2000	Male	95 plus	38%<br>(20-59%)	62%<br>(41-80%)	0.64<br>(0.26-1.3)	0.35%<br>(-0.53-1.8%)	0.27%<br>(-0.76-1.9%)	0.0046<br>(-0.013-0.033)	0.024<br>(-0.037-0.12)
Samoa	2005	Female	All Ages	17%<br>(12-23%)	83%<br>(77-88%)	0.47<br>(0.35-0.61)	0.88%<br>(0.35-1.5%)	0.78%<br>(-0.17-2%)	3.6<br>(-0.9-9.7)	180<br>(73-330)
Samoa	2005	Female	15 to 19	18%<br>(8.4-32%)	82%<br>(68-92%)	0.58<br>(0.25-1.1)	1.3%<br>(0.67-2.4%)	2.4%<br>(0.92-5.2%)	0.12<br>(0.043-0.26)	13<br>(6.5-24)
Samoa	2005	Female	20 to 24	22%<br>(9.3-41%)	78%<br>(59-91%)	0.91<br>(0.38-1.7)	2.3%<br>(1.3-3.8%)	3.6%<br>(1.5-7.1%)	0.19<br>(0.075-0.37)	21<br>(12-35)
Samoa	2005	Female	25 to 29	24%<br>(9.9-42%)	76%<br>(58-90%)	0.92<br>(0.39-1.7)	2.4%<br>(1.4-3.7%)	3.6%<br>(1.6-6.3%)	0.24<br>(0.11-0.44)	23<br>(13-37)
Samoa	2005	Female	30 to 34	24%<br>(9.5-43%)	76%<br>(57-91%)	0.95<br>(0.38-1.7)	2.2%<br>(1.2-3.5%)	3.2%<br>(1.5-5.5%)	0.29<br>(0.13-0.51)	24<br>(13-39)
Samoa	2005	Female	35 to 39	25%<br>(10-43%)	75%<br>(57-90%)	0.98<br>(0.41-1.7)	2.2%<br>(1.1-3.6%)	3.2%<br>(1.5-5.6%)	0.4<br>(0.18-0.71)	26<br>(14-46)
Samoa	2005	Female	40 to 44	24%<br>(12-41%)	76%<br>(59-88%)	0.9<br>(0.39-1.6)	1.9%<br>(0.84-3.5%)	2.9%<br>(1.2-5.5%)	0.45<br>(0.18-0.89)	25<br>(10-47)
Samoa	2005	Female	45 to 49	24%<br>(10-41%)	76%<br>(59-90%)	0.77<br>(0.31-1.5)	1.4%<br>(0.3-3.1%)	2.1%<br>(0.6-4.6%)	0.39<br>(0.11-0.88)	18<br>(4-41)
Samoa	2005	Female	50 to 54	24%<br>(11-41%)	76%<br>(59-89%)	0.68<br>(0.27-1.3)	0.97%<br>(-0.26-2.8%)	1.5%<br>(-0.011-4%)	0.35<br>(-0.0025-0.93)	13<br>(-3.5-37)
Samoa	2005	Female	55 to 59	23%<br>(10-42%)	77%<br>(58-90%)	0.62<br>(0.23-1.3)	0.71%<br>(-0.47-2.3%)	1.2%<br>(-0.3-3.3%)	0.29<br>(-0.078-0.84)	8.3<br>(-5.5-27)
Samoa	2005	Female	60 to 64	23%<br>(10-42%)	77%<br>(58-90%)	0.6<br>(0.22-1.3)	0.35%<br>(-0.99-2%)	0.64%<br>(-0.96-2.6%)	0.19<br>(-0.29-0.83)	4<br>(-12-23)
Samoa	2005	Female	65 to 69	25%<br>(11-43%)	75%<br>(57-89%)	0.62<br>(0.23-1.2)	0.29%<br>(-1-2%)	0.54%<br>(-1.1-2.7%)	0.21<br>(-0.43-1)	3.5<br>(-13-23)
Samoa	2005	Female	70 to 74	25%<br>(11-42%)	75%<br>(58-89%)	0.59<br>(0.21-1.2)	0.14%<br>(-1.1-1.7%)	0.28%<br>(-1.2-2.2%)	0.13<br>(-0.55-1.1)	1.5<br>(-12-20)
Samoa	2005	Female	75 to 79	23%<br>(10-41%)	77%<br>(59-90%)	0.55<br>(0.19-1.2)	0.22%<br>(-1-1.9%)	0.34%<br>(-1.1-2.3%)	0.21<br>(-0.69-1.4)	2.5<br>(-11-20)

Location	Year	Sex	Age	Prevalence of current drinkers	Prevalence of abstainers	Population average of standard drinks daily	Percent of total attributable deaths	Percent of total attributable DALYs	Attributable deaths	Attributable DALYs
Samoa	2005	Female	80 to 84	22% (9.8-40%)	78% (60-90%)	0.52 (0.18-1.1)	0.13% (-0.9-1.5%)	0.19% (-1-1.9%)	0.11 (-0.6-1.1)	1 (-7.1-12)
Samoa	2005	Female	85 to 89	23% (11-41%)	77% (59-89%)	0.54 (0.21-1.1)	0.068% (-0.91-1.4%)	0.083% (-1.1-1.7%)	0.035 (-0.46-0.7)	0.26 (-3.5-5.3)
Samoa	2005	Female	90 to 94	27% (12-46%)	73% (54-88%)	0.62 (0.23-1.3)	0.14% (-0.93-1.7%)	0.15% (-1.1-2%)	0.028 (-0.21-0.38)	0.16 (-1-2)
Samoa	2005	Female	95 plus	31% (12-54%)	69% (46-88%)	0.74 (0.24-1.6)	0.094% (-1.4-2.3%)	0.032% (-1.7-2.6%)	0.0018 (-0.095-0.15)	0.021 (-0.31-0.51)
Samoa	2005	Male	All Ages	36% (29-45%)	64% (55-71%)	1.1 (0.91-1.3)	3.4% (2.3-4.7%)	3.3% (1.9-5.1%)	19 (11-29)	910 (610-1300)
Samoa	2005	Male	15 to 19	34% (19-51%)	66% (49-81%)	0.7 (0.33-1.2)	2.6% (1.3-4.5%)	3.6% (1.5-6.8%)	0.44 (0.18-0.87)	38 (19-70)
Samoa	2005	Male	20 to 24	52% (33-70%)	48% (30-67%)	1.9 (1.1-2.9)	7.5% (4.5-11%)	9.9% (5.3-16%)	1.5 (0.77-2.4)	110 (64-180)
Samoa	2005	Male	25 to 29	60% (40-76%)	40% (24-60%)	2.7 (1.7-3.9)	9.2% (5.9-13%)	12% (7.6-19%)	1.8 (1-2.8)	130 (78-190)
Samoa	2005	Male	30 to 34	61% (40-78%)	39% (22-60%)	2.9 (1.8-4.2)	8.3% (5.3-12%)	11% (6.5-16%)	1.8 (1-2.8)	120 (72-180)
Samoa	2005	Male	35 to 39	59% (41-76%)	41% (24-59%)	2.5 (1.5-3.8)	7.2% (4.2-11%)	9.3% (5.1-14%)	1.9 (0.95-3.1)	110 (62-180)
Samoa	2005	Male	40 to 44	57% (39-74%)	43% (26-61%)	2.2 (1.2-3.4)	5.8% (3.1-9.1%)	7.3% (3.5-12%)	1.8 (0.81-3)	95 (48-160)
Samoa	2005	Male	45 to 49	52% (32-71%)	48% (29-68%)	1.9 (0.96-3.2)	4.9% (2.3-8.5%)	5.9% (2.5-11%)	1.7 (0.74-3.2)	84 (39-150)
Samoa	2005	Male	50 to 54	49% (30-69%)	51% (31-70%)	1.5 (0.8-2.6)	4.1% (1.5-7.6%)	4.7% (1.6-9.3%)	1.6 (0.54-3.2)	69 (26-130)
Samoa	2005	Male	55 to 59	47% (30-66%)	53% (34-70%)	1.3 (0.63-2.2)	2.9% (0.92-5.9%)	3.3% (0.9-7.1%)	1.3 (0.35-2.8)	46 (15-99)
Samoa	2005	Male	60 to 64	45% (26-65%)	55% (35-74%)	1.1 (0.49-1.9)	2.3% (0.52-5.3%)	2.6% (0.5-6.2%)	1.2 (0.23-2.8)	35 (7.9-81)
Samoa	2005	Male	65 to 69	43% (25-62%)	57% (38-75%)	0.95 (0.48-1.7)	1.9% (0.4-4.2%)	2.2% (0.41-4.8%)	1.2 (0.22-2.6)	29 (6-63)
Samoa	2005	Male	70 to 74	40% (23-58%)	60% (42-77%)	0.83 (0.4-1.5)	1.5% (0.14-3.8%)	1.7% (0.13-4.3%)	0.95 (0.076-2.4)	19 (2-47)
Samoa	2005	Male	75 to 79	36% (21-55%)	64% (45-79%)	0.7 (0.33-1.3)	1.2% (0.053-3.1%)	1.3% (-0.0023-3.5%)	0.7 (-0.0014-1.9)	11 (0.54-29)
Samoa	2005	Male	80 to 84	34% (19-52%)	66% (48-81%)	0.62 (0.3-1.1)	0.97% (0.14-2.5%)	1% (0.096-2.7%)	0.45 (0.041-1.2)	5.2 (0.74-13)
Samoa	2005	Male	85 to 89	32% (18-49%)	68% (51-82%)	0.59 (0.29-1.1)	0.74% (-0.02-1.9%)	0.78% (-0.085-2.1%)	0.22 (-0.024-0.62)	1.8 (-0.049-4.9)
Samoa	2005	Male	90 to 94	34% (21-51%)	66% (49-79%)	0.61 (0.28-1.2)	0.55% (-0.17-1.7%)	0.55% (-0.29-1.8%)	0.056 (-0.03-0.18)	0.34 (-0.1-1)
Samoa	2005	Male	95 plus	37% (19-58%)	63% (42-81%)	0.66 (0.26-1.4)	0.4% (-0.55-1.9%)	0.31% (-0.78-2.1%)	0.008 (-0.02-0.052)	0.041 (-0.055-0.19)

Location	Year	Sex	Age	Prevalence of current drinkers	Prevalence of abstainers	Population average of standard drinks daily	Percent of total attributable deaths	Percent of total attributable DALYs	Attributable deaths	Attributable DALYs
Samoa	2010	Female	All Ages	17% (12-23%)	83% (77-88%)	0.48 (0.35-0.64)	0.92% (0.36-1.7%)	0.81% (-0.2-2.1%)	4.2 (-1-11)	200 (82-380)
Samoa	2010	Female	15 to 19	18% (8.2-33%)	82% (67-92%)	0.58 (0.24-1.1)	1.3% (0.65-2.4%)	2.4% (0.9-5.2%)	0.13 (0.047-0.27)	14 (6.8-26)
Samoa	2010	Female	20 to 24	22% (8.9-39%)	78% (61-91%)	0.91 (0.37-1.7)	2.3% (1.2-3.8%)	3.5% (1.4-6.9%)	0.19 (0.075-0.38)	22 (12-37)
Samoa	2010	Female	25 to 29	23% (9.7-41%)	77% (59-90%)	0.92 (0.39-1.7)	2.4% (1.4-3.9%)	3.5% (1.6-6.4%)	0.24 (0.11-0.45)	23 (13-38)
Samoa	2010	Female	30 to 34	24% (9.2-43%)	76% (57-91%)	0.96 (0.36-1.8)	2.2% (1.2-3.6%)	3.2% (1.5-5.7%)	0.29 (0.13-0.53)	24 (13-40)
Samoa	2010	Female	35 to 39	25% (9.6-43%)	75% (57-90%)	1 (0.4-1.8)	2.2% (1.1-3.7%)	3.3% (1.5-5.6%)	0.4 (0.17-0.74)	27 (14-46)
Samoa	2010	Female	40 to 44	24% (12-41%)	76% (59-88%)	0.91 (0.4-1.6)	2% (0.87-3.7%)	3% (1.2-5.7%)	0.5 (0.19-0.96)	27 (12-51)
Samoa	2010	Female	45 to 49	24% (9.9-41%)	76% (59-90%)	0.78 (0.32-1.5)	1.4% (0.38-3.2%)	2.2% (0.6-4.5%)	0.46 (0.13-1)	21 (5.6-47)
Samoa	2010	Female	50 to 54	23% (10-42%)	77% (58-90%)	0.69 (0.26-1.4)	1% (-0.21-3%)	1.6% (0.048-4.1%)	0.42 (0.014-1.1)	16 (-3.4-44)
Samoa	2010	Female	55 to 59	23% (10-41%)	77% (59-90%)	0.63 (0.24-1.3)	0.78% (-0.46-2.5%)	1.2% (-0.28-3.5%)	0.37 (-0.081-1.1)	11 (-6.8-36)
Samoa	2010	Female	60 to 64	23% (10-41%)	77% (59-90%)	0.6 (0.22-1.3)	0.42% (-0.86-2.3%)	0.71% (-0.89-3.1%)	0.22 (-0.26-0.94)	5.1 (-11-28)
Samoa	2010	Female	65 to 69	24% (11-42%)	76% (58-89%)	0.61 (0.23-1.2)	0.36% (-1-2.1%)	0.62% (-1-2.8%)	0.26 (-0.43-1.2)	4.7 (-13-28)
Samoa	2010	Female	70 to 74	24% (10-41%)	76% (59-90%)	0.58 (0.21-1.2)	0.2% (-1-1.9%)	0.34% (-1.1-2.5%)	0.17 (-0.58-1.3)	2.5 (-13-24)
Samoa	2010	Female	75 to 79	23% (9.5-41%)	77% (59-90%)	0.55 (0.17-1.2)	0.27% (-0.94-2%)	0.4% (-1.1-2.5%)	0.24 (-0.67-1.5)	3 (-11-22)
Samoa	2010	Female	80 to 84	22% (8.8-42%)	78% (58-91%)	0.53 (0.17-1.1)	0.17% (-0.89-1.6%)	0.24% (-1-1.9%)	0.17 (-0.74-1.4)	1.6 (-8.6-15)
Samoa	2010	Female	85 to 89	23% (11-42%)	77% (58-89%)	0.56 (0.21-1.2)	0.1% (-0.9-1.5%)	0.12% (-1-1.8%)	0.069 (-0.59-1)	0.53 (-4.6-7.7)
Samoa	2010	Female	90 to 94	27% (12-47%)	73% (53-88%)	0.64 (0.23-1.3)	0.16% (-0.93-1.8%)	0.17% (-1.1-2%)	0.044 (-0.28-0.51)	0.25 (-1.4-2.7)
Samoa	2010	Female	95 plus	31% (13-55%)	69% (45-87%)	0.75 (0.25-1.6)	0.11% (-1.4-2.3%)	0.044% (-1.8-2.5%)	0.0033 (-0.13-0.19)	0.033 (-0.42-0.68)
Samoa	2010	Male	All Ages	37% (29-46%)	63% (54-71%)	1.1 (0.89-1.4)	3.5% (2.3-4.8%)	3.3% (1.9-5.2%)	19 (11-31)	930 (620-1300)
Samoa	2010	Male	15 to 19	34% (19-52%)	66% (48-81%)	0.71 (0.35-1.2)	2.5% (1.3-4.5%)	3.6% (1.6-6.9%)	0.46 (0.18-0.87)	40 (20-70)
Samoa	2010	Male	20 to 24	51% (33-69%)	49% (31-67%)	1.9 (1.1-3)	7.4% (4.5-11%)	10% (5.5-16%)	1.4 (0.71-2.4)	110 (60-180)
Samoa	2010	Male	25 to 29	59% (39-76%)	41% (24-61%)	2.7 (1.6-4)	9.2% (5.6-13%)	12% (7.2-18%)	1.7 (0.94-2.7)	120 (72-180)

Location	Year	Sex	Age	Prevalence of current drinkers	Prevalence of abstainers	Population average of standard drinks daily	Percent of total attributable deaths	Percent of total attributable DALYs	Attributable deaths	Attributable DALYs
Samoa	2010	Male	30 to 34	60% (39-77%)	40% (23-61%)	2.9 (1.7-4.3)	8.3% (5.2-12%)	11% (6.4-17%)	1.7 (0.91-2.6)	110 (67-170)
Samoa	2010	Male	35 to 39	59% (40-76%)	41% (24-60%)	2.5 (1.5-3.9)	7.1% (4.1-11%)	9.4% (5-15%)	1.8 (0.92-2.9)	110 (60-170)
Samoa	2010	Male	40 to 44	56% (39-74%)	44% (26-61%)	2.2 (1.2-3.5)	5.8% (2.9-9.2%)	7.3% (3.4-12%)	1.9 (0.85-3.3)	100 (50-170)
Samoa	2010	Male	45 to 49	53% (32-71%)	47% (29-68%)	1.9 (1-3.3)	4.9% (2.4-8.4%)	6% (2.6-11%)	1.9 (0.75-3.5)	91 (41-160)
Samoa	2010	Male	50 to 54	49% (31-68%)	51% (32-69%)	1.6 (0.8-2.7)	4.1% (1.6-7.8%)	4.9% (1.7-9.5%)	1.9 (0.65-3.9)	82 (30-160)
Samoa	2010	Male	55 to 59	47% (30-67%)	53% (33-70%)	1.3 (0.65-2.2)	3% (0.97-6.2%)	3.4% (0.97-7.3%)	1.5 (0.43-3.4)	55 (17-120)
Samoa	2010	Male	60 to 64	45% (26-64%)	55% (36-74%)	1.1 (0.51-2)	2.3% (0.55-5.4%)	2.6% (0.51-6.4%)	1.2 (0.23-3)	36 (8.7-86)
Samoa	2010	Male	65 to 69	43% (25-61%)	57% (39-75%)	0.96 (0.48-1.7)	1.9% (0.47-4.4%)	2.2% (0.49-5.1%)	1.2 (0.27-2.8)	29 (7.3-67)
Samoa	2010	Male	70 to 74	40% (24-58%)	60% (42-76%)	0.83 (0.41-1.5)	1.5% (0.18-3.8%)	1.7% (0.13-4.3%)	0.99 (0.079-2.5)	20 (2.5-49)
Samoa	2010	Male	75 to 79	37% (22-55%)	63% (45-78%)	0.71 (0.34-1.3)	1.2% (0.097-3.1%)	1.3% (0.03-3.5%)	0.7 (0.017-1.9)	11 (0.94-30)
Samoa	2010	Male	80 to 84	34% (19-53%)	66% (47-81%)	0.63 (0.31-1.1)	0.99% (0.16-2.4%)	1.1% (0.13-2.7%)	0.5 (0.056-1.3)	5.7 (0.9-14)
Samoa	2010	Male	85 to 89	32% (19-49%)	68% (51-81%)	0.6 (0.29-1.1)	0.75% (-0.014-2%)	0.8% (-0.074-2.3%)	0.27 (-0.024-0.73)	2.2 (-0.042-5.9)
Samoa	2010	Male	90 to 94	34% (20-50%)	66% (50-80%)	0.61 (0.28-1.2)	0.56% (-0.15-1.8%)	0.56% (-0.26-1.9%)	0.083 (-0.039-0.28)	0.5 (-0.13-1.6)
Samoa	2010	Male	95 plus	37% (20-58%)	63% (42-80%)	0.67 (0.26-1.3)	0.43% (-0.49-2.1%)	0.34% (-0.72-2.2%)	0.011 (-0.024-0.074)	0.057 (-0.065-0.27)
Samoa	2016	Female	All Ages	18% (13-24%)	82% (76-87%)	0.49 (0.31-0.71)	0.91% (0.2-2%)	0.79% (-0.37-2.5%)	4.6 (-2-16)	210 (44-450)
Samoa	2016	Female	15 to 19	18% (7.7-33%)	82% (67-92%)	0.58 (0.22-1.2)	1.3% (0.62-2.4%)	2.4% (0.76-5.3%)	0.13 (0.041-0.3)	14 (6.9-27)
Samoa	2016	Female	20 to 24	22% (8.9-39%)	78% (61-91%)	0.91 (0.35-1.7)	2.2% (1.3-3.7%)	3.4% (1.2-7%)	0.21 (0.074-0.43)	24 (13-42)
Samoa	2016	Female	25 to 29	23% (9.6-41%)	77% (59-90%)	0.93 (0.36-1.9)	2.3% (1.3-3.9%)	3.4% (1.3-6.9%)	0.22 (0.081-0.45)	22 (12-39)
Samoa	2016	Female	30 to 34	24% (9.1-44%)	76% (56-91%)	0.96 (0.35-1.9)	2.2% (1.1-3.8%)	3.2% (1.2-6.3%)	0.26 (0.097-0.51)	21 (11-38)
Samoa	2016	Female	35 to 39	25% (9.7-43%)	75% (57-90%)	1 (0.38-1.9)	2.2% (1.1-4%)	3.3% (1.4-6.1%)	0.38 (0.15-0.72)	25 (12-47)
Samoa	2016	Female	40 to 44	24% (11-41%)	76% (59-89%)	0.93 (0.38-1.8)	2% (0.77-3.9%)	3% (1.1-5.9%)	0.48 (0.16-0.99)	27 (10-53)
Samoa	2016	Female	45 to 49	24% (10-42%)	76% (58-90%)	0.79 (0.3-1.6)	1.5% (0.28-3.4%)	2.2% (0.54-5%)	0.49 (0.11-1.2)	23 (4.2-53)

Location	Year	Sex	Age	Prevalence of current drinkers	Prevalence of abstainers	Population average of standard drinks daily	Percent of total attributable deaths	Percent of total attributable DALYs	Attributable deaths	Attributable DALYs
Samoa	2016	Female	50 to 54	24% (11-42%)	76% (58-89%)	0.7 (0.27-1.4)	1.1% (-0.25-3.1%)	1.6% (-0.064-4.3%)	0.48 (-0.017-1.4)	18 (-4.2-59)
Samoa	2016	Female	55 to 59	23% (11-41%)	77% (59-89%)	0.64 (0.23-1.4)	0.79% (-0.53-2.9%)	1.2% (-0.43-4%)	0.45 (-0.16-1.5)	14 (-9.4-52)
Samoa	2016	Female	60 to 64	24% (11-43%)	76% (57-89%)	0.62 (0.21-1.3)	0.43% (-0.97-2.5%)	0.72% (-0.98-3.4%)	0.3 (-0.41-1.4)	6.9 (-16-41)
Samoa	2016	Female	65 to 69	25% (11-43%)	75% (57-89%)	0.63 (0.22-1.3)	0.4% (-1.1-2.5%)	0.66% (-1.1-3.3%)	0.28 (-0.46-1.4)	5.3 (-14-33)
Samoa	2016	Female	70 to 74	24% (11-41%)	76% (59-89%)	0.6 (0.21-1.3)	0.23% (-1.1-2.2%)	0.37% (-1.2-2.8%)	0.21 (-0.73-1.6)	3.2 (-16-31)
Samoa	2016	Female	75 to 79	23% (9.8-41%)	77% (59-90%)	0.56 (0.17-1.3)	0.3% (-1-2.2%)	0.42% (-1.1-2.7%)	0.29 (-0.73-1.8)	3.7 (-12-27)
Samoa	2016	Female	80 to 84	22% (8.9-41%)	78% (59-91%)	0.54 (0.16-1.2)	0.2% (-0.98-1.9%)	0.26% (-1.1-2.2%)	0.21 (-0.96-1.9)	2.1 (-11-21)
Samoa	2016	Female	85 to 89	24% (10-42%)	76% (58-90%)	0.56 (0.19-1.2)	0.14% (-0.97-1.7%)	0.15% (-1.1-2.1%)	0.11 (-0.8-1.4)	0.84 (-6.2-11)
Samoa	2016	Female	90 to 94	27% (12-46%)	73% (54-88%)	0.65 (0.22-1.4)	0.18% (-0.92-2%)	0.19% (-1.1-2.3%)	0.072 (-0.41-0.86)	0.42 (-2.1-4.6)
Samoa	2016	Female	95 plus	32% (12-54%)	68% (46-88%)	0.76 (0.24-1.7)	0.12% (-1.5-2.4%)	0.054% (-1.8-2.7%)	0.0058 (-0.19-0.29)	0.051 (-0.62-1)
Samoa	2016	Male	All Ages	37% (29-45%)	63% (55-71%)	1.1 (0.77-1.5)	3.5% (2.1-5.5%)	3.3% (1.5-5.8%)	21 (9.3-38)	980 (550-1500)
Samoa	2016	Male	15 to 19	34% (19-51%)	66% (49-81%)	0.72 (0.33-1.3)	2.5% (1.3-4.4%)	3.7% (1.6-7.2%)	0.45 (0.17-0.88)	41 (19-73)
Samoa	2016	Male	20 to 24	52% (34-69%)	48% (31-66%)	2 (1-3.2)	7.5% (4.2-12%)	10% (5.1-17%)	1.6 (0.78-2.9)	130 (66-210)
Samoa	2016	Male	25 to 29	59% (39-75%)	41% (25-61%)	2.7 (1.5-4.5)	9.1% (5.3-14%)	12% (6.7-20%)	1.7 (0.83-2.7)	120 (67-190)
Samoa	2016	Male	30 to 34	60% (39-78%)	40% (22-61%)	2.9 (1.5-4.8)	8.3% (4.9-13%)	11% (5.9-17%)	1.5 (0.76-2.6)	100 (57-160)
Samoa	2016	Male	35 to 39	59% (40-77%)	41% (23-60%)	2.6 (1.3-4.3)	7.3% (4-12%)	9.5% (4.7-16%)	1.6 (0.78-2.7)	98 (51-160)
Samoa	2016	Male	40 to 44	56% (38-74%)	44% (26-62%)	2.2 (1.1-3.7)	5.9% (2.7-9.8%)	7.4% (3-13%)	1.8 (0.72-3.3)	98 (42-170)
Samoa	2016	Male	45 to 49	52% (30-71%)	48% (29-70%)	1.9 (0.93-3.4)	5% (2.2-8.8%)	6% (2.3-11%)	2.1 (0.76-4)	100 (44-190)
Samoa	2016	Male	50 to 54	49% (30-69%)	51% (31-70%)	1.6 (0.74-2.8)	4.2% (1.5-7.9%)	5% (1.5-9.4%)	2.2 (0.65-4.4)	93 (30-180)
Samoa	2016	Male	55 to 59	47% (29-67%)	53% (33-71%)	1.3 (0.6-2.4)	3.1% (0.9-6.7%)	3.5% (0.89-7.8%)	1.9 (0.5-4.5)	70 (21-160)
Samoa	2016	Male	60 to 64	45% (26-65%)	55% (35-74%)	1.1 (0.47-2.1)	2.4% (0.47-5.7%)	2.7% (0.41-6.7%)	1.6 (0.23-4.2)	49 (9.6-120)
Samoa	2016	Male	65 to 69	43% (25-61%)	57% (39-75%)	0.96 (0.43-1.7)	2% (0.36-4.7%)	2.3% (0.38-5.5%)	1.3 (0.2-3.2)	32 (5.6-76)

Location	Year	Sex	Age	Prevalence of current drinkers	Prevalence of abstainers	Population average of standard drinks daily	Percent of total attributable deaths	Percent of total attributable DALYs	Attributable deaths	Attributable DALYs
Samoa	2016	Male	70 to 74	40% (24-58%)	60% (42-76%)	0.85 (0.39-1.6)	1.5% (0.18-4.2%)	1.7% (0.15-4.7%)	1 (0.093-2.8)	20 (2.3-55)
Samoa	2016	Male	75 to 79	36% (21-55%)	64% (45-79%)	0.72 (0.3-1.4)	1.2% (0.068-3.4%)	1.3% (0.013-3.8%)	0.83 (0.0066-2.4)	13 (0.69-37)
Samoa	2016	Male	80 to 84	33% (18-51%)	67% (49-82%)	0.63 (0.29-1.2)	1% (0.12-2.7%)	1.1% (0.084-3%)	0.57 (0.04-1.6)	6.6 (0.69-18)
Samoa	2016	Male	85 to 89	32% (19-48%)	68% (52-81%)	0.6 (0.27-1.1)	0.8% (-0.051-2.1%)	0.85% (-0.14-2.4%)	0.31 (-0.054-0.86)	2.6 (-0.16-6.8)
Samoa	2016	Male	90 to 94	34% (20-51%)	66% (49-80%)	0.63 (0.26-1.2)	0.62% (-0.13-1.9%)	0.62% (-0.23-2.1%)	0.11 (-0.041-0.38)	0.66 (-0.13-2.1)
Samoa	2016	Male	95 plus	38% (19-59%)	62% (41-81%)	0.69 (0.25-1.5)	0.49% (-0.48-2.2%)	0.4% (-0.72-2.3%)	0.021 (-0.038-0.12)	0.099 (-0.097-0.44)
Solomon Islands	1990	Female	All Ages	18% (13-25%)	82% (75-87%)	0.14 (0.11-0.18)	0.064% (-0.2-0.3%)	-0.2% (-0.65-0.21%)	-2.2 (-7.3-2.4)	41 (-130-190)
Solomon Islands	1990	Female	15 to 19	20% (8.9-36%)	80% (64-91%)	0.19 (0.079-0.35)	0.51% (0.18-1%)	0.5% (-0.023-1.3%)	0.13 (-0.0057-0.34)	17 (5.8-33)
Solomon Islands	1990	Female	20 to 24	24% (10-43%)	76% (57-90%)	0.29 (0.13-0.53)	0.88% (0.43-1.5%)	0.76% (0.15-1.8%)	0.22 (0.045-0.52)	27 (13-49)
Solomon Islands	1990	Female	25 to 29	25% (11-45%)	75% (55-89%)	0.29 (0.13-0.51)	0.86% (0.47-1.4%)	0.77% (0.23-1.5%)	0.26 (0.075-0.53)	27 (14-45)
Solomon Islands	1990	Female	30 to 34	27% (11-47%)	73% (53-89%)	0.31 (0.12-0.55)	0.64% (0.22-1.1%)	0.55% (0.016-1.3%)	0.22 (0.0063-0.5)	20 (6.8-37)
Solomon Islands	1990	Female	35 to 39	27% (11-47%)	73% (53-89%)	0.32 (0.13-0.56)	0.57% (0.052-1.2%)	0.58% (-0.11-1.4%)	0.28 (-0.05-0.7)	19 (1.8-40)
Solomon Islands	1990	Female	40 to 44	27% (13-45%)	73% (55-87%)	0.3 (0.13-0.55)	0.33% (-0.31-0.99%)	0.36% (-0.49-1.2%)	0.2 (-0.28-0.71)	12 (-11-35)
Solomon Islands	1990	Female	45 to 49	26% (11-45%)	74% (55-89%)	0.26 (0.11-0.48)	-0.056% (-0.77-0.47%)	-0.062% (-0.91-0.6%)	-0.042 (-0.64-0.42)	-2 (-28-17)
Solomon Islands	1990	Female	50 to 54	26% (12-46%)	74% (54-88%)	0.23 (0.087-0.45)	-0.3% (-1.2-0.32%)	-0.3% (-1.3-0.45%)	-0.19 (-0.86-0.27)	-8.5 (-35-9.2)
Solomon Islands	1990	Female	55 to 59	25% (12-45%)	75% (55-88%)	0.2 (0.075-0.42)	-0.42% (-1.5-0.19%)	-0.41% (-1.6-0.28%)	-0.33 (-1.3-0.23)	-13 (-46-5.8)
Solomon Islands	1990	Female	60 to 64	25% (11-45%)	75% (55-89%)	0.19 (0.069-0.41)	-0.63% (-1.8-0.029%)	-0.65% (-1.9-0.076%)	-0.5 (-1.4-0.057)	-16 (-43-0.67)
Solomon Islands	1990	Female	65 to 69	26% (12-43%)	74% (57-88%)	0.2 (0.073-0.38)	-0.7% (-1.7--0.0095%)	-0.72% (-1.9-0.039%)	-0.68 (-1.7-0.034)	-17 (-43--0.25)
Solomon Islands	1990	Female	70 to 74	26% (12-44%)	74% (56-88%)	0.19 (0.07-0.37)	-0.73% (-1.6--0.058%)	-0.76% (-1.8--0.039%)	-0.65 (-1.5--0.032)	-13 (-30--1.1)
Solomon Islands	1990	Female	75 to 79	25% (11-43%)	75% (57-89%)	0.17 (0.055-0.39)	-0.62% (-1.7--0.0024%)	-0.65% (-1.8-0.025%)	-0.51 (-1.4-0.018)	-7.6 (-20--0.029)
Solomon Islands	1990	Female	80 to 84	24% (10-43%)	76% (57-90%)	0.16 (0.053-0.35)	-0.6% (-1.5--0.068%)	-0.65% (-1.7--0.068%)	-0.38 (-0.95--0.039)	-4.1 (-10--0.47)
Solomon Islands	1990	Female	85 to 89	25% (11-44%)	75% (56-89%)	0.17 (0.063-0.36)	-0.65% (-1.6--0.11%)	-0.72% (-1.8--0.12%)	-0.17 (-0.43--0.031)	-1.3 (-3.2--0.22)

Location	Year	Sex	Age	Prevalence of current drinkers	Prevalence of abstainers	Population average of standard drinks daily	Percent of total attributable deaths	Percent of total attributable DALYs	Attributable deaths	Attributable DALYs
Solomon Islands	1990	Female	90 to 94	29% (13-48%)	71% (52-87%)	0.2 (0.068-0.41)	-0.56% (-1.3--0.1%)	-0.63% (-1.4--0.12%)	-0.047 (-0.11--0.0088)	-0.25 (-0.56--0.046)
Solomon Islands	1990	Female	95 plus	34% (15-55%)	66% (45-85%)	0.23 (0.075-0.47)	-0.77% (-1.9--0.06%)	-0.9% (-2.1--0.091%)	-0.017 (-0.039--0.0017)	-0.055 (-0.13--0.0044)
Solomon Islands	1990	Male	All Ages	37% (30-47%)	63% (53-70%)	0.3 (0.25-0.37)	0.87% (0.39-1.4%)	0.72% (0.085-1.4%)	11 (1.3-21)	680 (310-1100)
Solomon Islands	1990	Male	15 to 19	37% (22-55%)	63% (45-78%)	0.23 (0.11-0.38)	1% (0.44-1.9%)	1% (0.26-2.3%)	0.43 (0.1-0.94)	42 (18-79)
Solomon Islands	1990	Male	20 to 24	56% (37-73%)	44% (27-63%)	0.6 (0.36-0.89)	2.8% (1.4-4.5%)	3% (1.2-5.2%)	1.5 (0.58-2.6)	120 (59-200)
Solomon Islands	1990	Male	25 to 29	63% (44-78%)	37% (22-56%)	0.83 (0.5-1.2)	3.4% (1.8-5.4%)	3.6% (1.6-6.2%)	1.9 (0.82-3.2)	140 (71-220)
Solomon Islands	1990	Male	30 to 34	64% (43-80%)	36% (20-57%)	0.89 (0.53-1.3)	3% (1.5-4.9%)	3.2% (1.3-5.5%)	1.7 (0.63-3)	110 (53-190)
Solomon Islands	1990	Male	35 to 39	62% (44-79%)	38% (21-56%)	0.78 (0.45-1.2)	2.2% (1-3.8%)	2.3% (0.72-4.2%)	1.2 (0.38-2.4)	76 (34-130)
Solomon Islands	1990	Male	40 to 44	60% (41-76%)	40% (24-59%)	0.68 (0.37-1.1)	1.6% (0.44-3.1%)	1.6% (0.14-3.3%)	1.1 (0.097-2.3)	60 (16-120)
Solomon Islands	1990	Male	45 to 49	56% (34-74%)	44% (26-66%)	0.58 (0.3-0.99)	1.2% (0.0075-2.6%)	1.2% (-0.29-2.8%)	1.1 (-0.27-2.5)	55 (0.34-120)
Solomon Islands	1990	Male	50 to 54	53% (34-72%)	47% (28-66%)	0.48 (0.24-0.83)	0.93% (-0.19-2.1%)	0.88% (-0.38-2.2%)	0.84 (-0.37-2.2)	37 (-7.5-88)
Solomon Islands	1990	Male	55 to 59	51% (33-70%)	49% (30-67%)	0.39 (0.19-0.7)	0.47% (-0.42-1.4%)	0.42% (-0.62-1.5%)	0.46 (-0.67-1.7)	19 (-17-57)
Solomon Islands	1990	Male	60 to 64	48% (29-68%)	52% (32-71%)	0.33 (0.16-0.59)	0.29% (-0.49-1.1%)	0.27% (-0.62-1.2%)	0.28 (-0.65-1.3)	9.3 (-16-36)
Solomon Islands	1990	Male	65 to 69	47% (28-66%)	53% (34-72%)	0.3 (0.15-0.51)	0.25% (-0.46-1%)	0.24% (-0.56-1.1%)	0.28 (-0.63-1.3)	7.1 (-13-30)
Solomon Islands	1990	Male	70 to 74	44% (27-62%)	56% (38-73%)	0.26 (0.13-0.47)	0.12% (-0.46-0.76%)	0.11% (-0.53-0.83%)	0.13 (-0.61-0.97)	2.8 (-11-18)
Solomon Islands	1990	Male	75 to 79	40% (25-59%)	60% (41-75%)	0.23 (0.1-0.43)	0.061% (-0.43-0.56%)	0.04% (-0.5-0.59%)	0.04 (-0.5-0.61)	0.96 (-6.6-8.9)
Solomon Islands	1990	Male	80 to 84	37% (21-56%)	63% (44-79%)	0.2 (0.099-0.35)	0.055% (-0.32-0.4%)	0.032% (-0.39-0.43%)	0.024 (-0.29-0.31)	0.48 (-2.7-3.6)
Solomon Islands	1990	Male	85 to 89	35% (21-52%)	65% (48-79%)	0.19 (0.088-0.33)	-0.034% (-0.42-0.29%)	-0.073% (-0.52-0.3%)	-0.028 (-0.2-0.12)	-0.11 (-1.4-0.97)
Solomon Islands	1990	Male	90 to 94	37% (22-54%)	63% (46-78%)	0.19 (0.089-0.36)	-0.04% (-0.4-0.24%)	-0.087% (-0.51-0.23%)	-0.012 (-0.072-0.033)	-0.033 (-0.32-0.2)
Solomon Islands	1990	Male	95 plus	41% (22-62%)	59% (38-78%)	0.22 (0.088-0.43)	-0.22% (-0.72-0.11%)	-0.34% (-0.94-0.055%)	-0.013 (-0.037-0.0022)	-0.034 (-0.11-0.017)
Solomon Islands	1995	Female	All Ages	19% (13-25%)	81% (75-87%)	0.13 (0.096-0.17)	0.015% (-0.25-0.24%)	-0.28% (-0.75-0.12%)	-3.6 (-9.9-1.4)	11 (-180-170)
Solomon Islands	1995	Female	15 to 19	20% (9.4-36%)	80% (64-91%)	0.16 (0.07-0.31)	0.49% (0.18-0.96%)	0.47% (-0.03-1.3%)	0.13 (-0.0084-0.35)	18 (6.8-35)

Location	Year	Sex	Age	Prevalence of current drinkers	Prevalence of abstainers	Population average of standard drinks daily	Percent of total attributable deaths	Percent of total attributable DALYs	Attributable deaths	Attributable DALYs
Solomon Islands	1995	Female	20 to 24	25% (10-42%)	75% (58-90%)	0.26 (0.12-0.46)	0.82% (0.41-1.3%)	0.66% (0.11-1.5%)	0.24 (0.042-0.58)	33 (16-56)
Solomon Islands	1995	Female	25 to 29	26% (11-45%)	74% (55-89%)	0.26 (0.11-0.45)	0.8% (0.44-1.2%)	0.67% (0.19-1.3%)	0.26 (0.073-0.54)	29 (15-46)
Solomon Islands	1995	Female	30 to 34	27% (11-48%)	73% (52-89%)	0.27 (0.11-0.49)	0.55% (0.18-0.99%)	0.42% (-0.054-1%)	0.19 (-0.025-0.47)	20 (6.6-37)
Solomon Islands	1995	Female	35 to 39	27% (11-46%)	73% (54-89%)	0.29 (0.12-0.5)	0.44% (-0.069-0.91%)	0.4% (-0.21-1.1%)	0.23 (-0.11-0.63)	17 (-2.4-37)
Solomon Islands	1995	Female	40 to 44	27% (13-44%)	73% (56-87%)	0.26 (0.12-0.47)	0.2% (-0.45-0.73%)	0.18% (-0.57-0.89%)	0.11 (-0.39-0.55)	7.2 (-18-27)
Solomon Islands	1995	Female	45 to 49	26% (12-44%)	74% (56-88%)	0.23 (0.09-0.44)	-0.15% (-0.88-0.34%)	-0.18% (-1-0.44%)	-0.13 (-0.78-0.3)	-6 (-35-13)
Solomon Islands	1995	Female	50 to 54	26% (12-44%)	74% (56-88%)	0.2 (0.076-0.4)	-0.4% (-1.3-0.16%)	-0.42% (-1.4-0.23%)	-0.38 (-1.3-0.2)	-16 (-54-7)
Solomon Islands	1995	Female	55 to 59	25% (11-44%)	75% (56-89%)	0.18 (0.067-0.37)	-0.49% (-1.6-0.1%)	-0.5% (-1.7-0.18%)	-0.41 (-1.4-0.14)	-16 (-50-3.4)
Solomon Islands	1995	Female	60 to 64	25% (11-45%)	75% (55-89%)	0.17 (0.058-0.38)	-0.7% (-1.9--0.046%)	-0.72% (-2-0.0047%)	-0.73 (-2.1-0.0047)	-23 (-62--1.4)
Solomon Islands	1995	Female	65 to 69	27% (12-45%)	73% (55-88%)	0.18 (0.061-0.34)	-0.78% (-1.8--0.054%)	-0.81% (-2--0.024%)	-0.79 (-2--0.022)	-20 (-49--1.4)
Solomon Islands	1995	Female	70 to 74	27% (12-45%)	73% (55-88%)	0.17 (0.06-0.33)	-0.8% (-1.8--0.12%)	-0.85% (-1.9--0.1%)	-0.97 (-2.3--0.12)	-19 (-43--2.7)
Solomon Islands	1995	Female	75 to 79	25% (11-46%)	75% (54-89%)	0.15 (0.048-0.34)	-0.66% (-1.7--0.034%)	-0.69% (-1.8--0.02%)	-0.6 (-1.6--0.017)	-9 (-23--0.43)
Solomon Islands	1995	Female	80 to 84	24% (11-45%)	76% (55-89%)	0.14 (0.047-0.31)	-0.64% (-1.6--0.088%)	-0.7% (-1.7--0.1%)	-0.45 (-1.1--0.066)	-4.8 (-12--0.65)
Solomon Islands	1995	Female	85 to 89	25% (11-44%)	75% (56-89%)	0.15 (0.054-0.32)	-0.68% (-1.6--0.12%)	-0.75% (-1.8--0.13%)	-0.22 (-0.52--0.039)	-1.7 (-4--0.29)
Solomon Islands	1995	Female	90 to 94	29% (14-49%)	71% (51-86%)	0.17 (0.06-0.36)	-0.6% (-1.3--0.13%)	-0.68% (-1.5--0.14%)	-0.056 (-0.12--0.012)	-0.29 (-0.65--0.062)
Solomon Islands	1995	Female	95 plus	34% (15-56%)	66% (44-85%)	0.2 (0.067-0.43)	-0.81% (-1.9--0.088%)	-0.94% (-2.2--0.13%)	-0.018 (-0.043--0.0026)	-0.061 (-0.14--0.0066)
Solomon Islands	1995	Male	All Ages	38% (30-47%)	62% (53-70%)	0.28 (0.22-0.34)	0.81% (0.34-1.3%)	0.63% (0.022-1.2%)	10 (0.35-20)	690 (280-1100)
Solomon Islands	1995	Male	15 to 19	37% (22-56%)	63% (44-78%)	0.2 (0.095-0.33)	0.96% (0.45-1.8%)	0.99% (0.24-2.2%)	0.44 (0.11-0.99)	46 (21-85)
Solomon Islands	1995	Male	20 to 24	55% (37-72%)	45% (28-63%)	0.52 (0.31-0.77)	2.5% (1.3-4.2%)	2.6% (0.94-4.8%)	1.7 (0.59-3)	140 (66-240)
Solomon Islands	1995	Male	25 to 29	62% (43-78%)	38% (22-57%)	0.73 (0.46-1)	3% (1.6-4.8%)	3.2% (1.4-5.5%)	1.8 (0.79-3.3)	140 (72-230)
Solomon Islands	1995	Male	30 to 34	63% (43-79%)	37% (21-57%)	0.78 (0.47-1.2)	2.6% (1.3-4.2%)	2.6% (0.98-4.7%)	1.6 (0.59-3)	110 (55-190)
Solomon Islands	1995	Male	35 to 39	62% (44-78%)	38% (22-56%)	0.68 (0.39-1.1)	2% (0.87-3.3%)	1.9% (0.47-3.6%)	1.3 (0.32-2.5)	83 (34-140)

Location	Year	Sex	Age	Prevalence of current drinkers	Prevalence of abstainers	Population average of standard drinks daily	Percent of total attributable deaths	Percent of total attributable DALYs	Attributable deaths	Attributable DALYs
Solomon Islands	1995	Male	40 to 44	60% (42-77%)	40% (23-58%)	0.59 (0.31-0.97)	1.4% (0.25-2.7%)	1.3% (-0.12-2.9%)	0.91 (-0.074-2)	54 (10-110)
Solomon Islands	1995	Male	45 to 49	56% (34-73%)	44% (27-66%)	0.51 (0.26-0.88)	1% (-0.1-2.2%)	0.95% (-0.42-2.3%)	0.83 (-0.38-2.1)	44 (-4.1-96)
Solomon Islands	1995	Male	50 to 54	53% (32-72%)	47% (28-68%)	0.42 (0.2-0.73)	0.75% (-0.29-1.7%)	0.67% (-0.55-1.8%)	0.77 (-0.61-2.2)	37 (-14-86)
Solomon Islands	1995	Male	55 to 59	51% (33-70%)	49% (30-67%)	0.34 (0.16-0.61)	0.36% (-0.5-1.1%)	0.29% (-0.7-1.2%)	0.33 (-0.81-1.4)	15 (-21-49)
Solomon Islands	1995	Male	60 to 64	48% (29-68%)	52% (32-71%)	0.29 (0.14-0.52)	0.22% (-0.51-0.88%)	0.18% (-0.67-0.95%)	0.23 (-0.84-1.2)	8.6 (-20-35)
Solomon Islands	1995	Male	65 to 69	47% (28-66%)	53% (34-72%)	0.26 (0.13-0.47)	0.16% (-0.46-0.81%)	0.14% (-0.59-0.88%)	0.16 (-0.66-1)	4.7 (-14-24)
Solomon Islands	1995	Male	70 to 74	44% (27-63%)	56% (37-73%)	0.23 (0.11-0.42)	0.055% (-0.47-0.61%)	0.035% (-0.55-0.66%)	0.041 (-0.67-0.79)	1.3 (-11-15)
Solomon Islands	1995	Male	75 to 79	40% (24-59%)	60% (41-76%)	0.2 (0.093-0.38)	0.011% (-0.47-0.42%)	-0.02% (-0.57-0.43%)	-0.02 (-0.57-0.44)	0.18 (-7.4-6.6)
Solomon Islands	1995	Male	80 to 84	37% (21-55%)	63% (45-79%)	0.18 (0.085-0.32)	0.032% (-0.33-0.34%)	0.004% (-0.39-0.34%)	0.0029 (-0.29-0.24)	0.27 (-2.8-2.9)
Solomon Islands	1995	Male	85 to 89	36% (21-52%)	64% (48-79%)	0.17 (0.077-0.3)	-0.057% (-0.45-0.23%)	-0.1% (-0.55-0.22%)	-0.039 (-0.21-0.085)	-0.18 (-1.5-0.74)
Solomon Islands	1995	Male	90 to 94	37% (23-55%)	63% (45-77%)	0.17 (0.076-0.32)	-0.053% (-0.42-0.2%)	-0.1% (-0.54-0.19%)	-0.014 (-0.072-0.026)	-0.042 (-0.33-0.16)
Solomon Islands	1995	Male	95 plus	41% (22-62%)	59% (38-78%)	0.19 (0.078-0.38)	-0.23% (-0.73-0.1%)	-0.35% (-0.96-0.043%)	-0.013 (-0.036-0.0017)	-0.034 (-0.11-0.015)
Solomon Islands	2000	Female	All Ages	19% (13-26%)	81% (74-87%)	0.14 (0.11-0.19)	0.013% (-0.29-0.25%)	-0.31% (-0.83-0.11%)	-4.6 (-13-1.6)	11 (-230-200)
Solomon Islands	2000	Female	15 to 19	20% (9.2-35%)	80% (65-91%)	0.18 (0.074-0.34)	0.53% (0.21-1%)	0.56% (0.034-1.4%)	0.17 (0.009-0.42)	21 (8.1-40)
Solomon Islands	2000	Female	20 to 24	24% (11-43%)	76% (57-89%)	0.28 (0.12-0.5)	0.9% (0.46-1.5%)	0.79% (0.2-1.7%)	0.34 (0.082-0.73)	42 (21-72)
Solomon Islands	2000	Female	25 to 29	26% (11-45%)	74% (55-89%)	0.29 (0.13-0.49)	0.88% (0.48-1.3%)	0.79% (0.27-1.5%)	0.41 (0.12-0.81)	43 (23-66)
Solomon Islands	2000	Female	30 to 34	27% (11-48%)	73% (52-89%)	0.3 (0.12-0.55)	0.59% (0.2-1%)	0.49% (-0.052-1.1%)	0.25 (-0.028-0.6)	25 (8.3-45)
Solomon Islands	2000	Female	35 to 39	28% (11-47%)	72% (53-89%)	0.31 (0.12-0.55)	0.45% (-0.076-0.94%)	0.44% (-0.18-1.1%)	0.29 (-0.13-0.74)	21 (-3.7-45)
Solomon Islands	2000	Female	40 to 44	27% (13-45%)	73% (55-87%)	0.28 (0.12-0.52)	0.18% (-0.52-0.71%)	0.17% (-0.66-0.87%)	0.13 (-0.49-0.64)	8.2 (-23-32)
Solomon Islands	2000	Female	45 to 49	26% (11-44%)	74% (56-89%)	0.24 (0.098-0.47)	-0.18% (-0.95-0.37%)	-0.2% (-1.1-0.46%)	-0.16 (-0.89-0.36)	-7.6 (-40-16)
Solomon Islands	2000	Female	50 to 54	26% (12-44%)	74% (56-88%)	0.22 (0.079-0.44)	-0.43% (-1.4-0.16%)	-0.45% (-1.5-0.23%)	-0.45 (-1.6-0.23)	-20 (-65-7.5)
Solomon Islands	2000	Female	55 to 59	25% (11-44%)	75% (56-89%)	0.19 (0.072-0.4)	-0.54% (-1.7-0.095%)	-0.53% (-1.8-0.18%)	-0.65 (-2.2-0.22)	-25 (-80-4.4)

Location	Year	Sex	Age	Prevalence of current drinkers	Prevalence of abstainers	Population average of standard drinks daily	Percent of total attributable deaths	Percent of total attributable DALYs	Attributable deaths	Attributable DALYs
Solomon Islands	2000	Female	60 to 64	25% (11-44%)	75% (56-89%)	0.18 (0.062-0.39)	-0.74% (-2--0.047%)	-0.76% (-2.1--0.018%)	-0.81 (-2.3--0.017)	-25 (-68--1.5)
Solomon Islands	2000	Female	65 to 69	26% (12-44%)	74% (56-88%)	0.19 (0.065-0.37)	-0.82% (-1.9--0.077%)	-0.85% (-2.1--0.045%)	-1.1 (-2.8--0.052)	-28 (-68--2.7)
Solomon Islands	2000	Female	70 to 74	26% (12-44%)	74% (56-88%)	0.18 (0.063-0.35)	-0.86% (-1.9--0.15%)	-0.91% (-2.1--0.15%)	-1.1 (-2.5--0.17)	-22 (-47--3.6)
Solomon Islands	2000	Female	75 to 79	25% (11-44%)	75% (56-89%)	0.16 (0.053-0.35)	-0.71% (-1.8--0.047%)	-0.75% (-1.9--0.037%)	-0.89 (-2.2--0.044)	-13 (-33--0.82)
Solomon Islands	2000	Female	80 to 84	24% (10-44%)	76% (56-90%)	0.15 (0.05-0.33)	-0.7% (-1.7--0.12%)	-0.76% (-1.8--0.14%)	-0.63 (-1.5--0.11)	-6.8 (-16--1.2)
Solomon Islands	2000	Female	85 to 89	25% (12-44%)	75% (56-88%)	0.16 (0.057-0.34)	-0.74% (-1.8--0.16%)	-0.83% (-2--0.17%)	-0.27 (-0.63--0.057)	-2 (-4.8--0.44)
Solomon Islands	2000	Female	90 to 94	29% (13-48%)	71% (52-87%)	0.19 (0.065-0.39)	-0.67% (-1.5--0.14%)	-0.75% (-1.7--0.16%)	-0.074 (-0.16--0.016)	-0.38 (-0.87--0.079)
Solomon Islands	2000	Female	95 plus	34% (14-56%)	66% (44-86%)	0.22 (0.07-0.47)	-0.9% (-2.1--0.11%)	-1% (-2.4--0.15%)	-0.022 (-0.051--0.0033)	-0.073 (-0.17--0.0089)
Solomon Islands	2000	Male	All Ages	39% (30-48%)	61% (52-70%)	0.31 (0.25-0.39)	0.93% (0.43-1.5%)	0.72% (0.087-1.4%)	13 (1.6-25)	880 (410-1400)
Solomon Islands	2000	Male	15 to 19	37% (22-55%)	63% (45-78%)	0.22 (0.1-0.38)	1.1% (0.51-1.9%)	1.2% (0.34-2.5%)	0.54 (0.15-1.1)	53 (24-97)
Solomon Islands	2000	Male	20 to 24	55% (36-72%)	45% (28-64%)	0.58 (0.34-0.86)	2.9% (1.5-4.6%)	3.1% (1.3-5.5%)	2.2 (0.89-3.9)	180 (89-290)
Solomon Islands	2000	Male	25 to 29	62% (43-78%)	38% (22-57%)	0.79 (0.51-1.1)	3.4% (1.9-5.1%)	3.6% (1.8-5.9%)	2.7 (1.3-4.6)	200 (110-320)
Solomon Islands	2000	Male	30 to 34	63% (42-79%)	37% (21-58%)	0.83 (0.5-1.3)	2.8% (1.5-4.4%)	2.9% (1.2-4.9%)	2 (0.86-3.7)	140 (72-240)
Solomon Islands	2000	Male	35 to 39	62% (44-78%)	38% (22-56%)	0.73 (0.42-1.2)	2.1% (0.89-3.5%)	2.1% (0.56-3.9%)	1.7 (0.41-3.1)	110 (44-180)
Solomon Islands	2000	Male	40 to 44	59% (42-76%)	41% (24-58%)	0.64 (0.34-1.1)	1.4% (0.32-2.8%)	1.3% (-0.049-3%)	1.2 (-0.042-2.7)	71 (16-140)
Solomon Islands	2000	Male	45 to 49	56% (35-73%)	44% (27-65%)	0.55 (0.27-0.96)	1.1% (-0.058-2.3%)	1% (-0.39-2.5%)	0.94 (-0.37-2.4)	51 (-2.7-110)
Solomon Islands	2000	Male	50 to 54	53% (33-71%)	47% (29-67%)	0.46 (0.22-0.81)	0.8% (-0.24-1.8%)	0.72% (-0.49-2%)	0.81 (-0.57-2.3)	38 (-12-90)
Solomon Islands	2000	Male	55 to 59	50% (32-69%)	50% (31-68%)	0.38 (0.18-0.68)	0.38% (-0.49-1.3%)	0.3% (-0.68-1.3%)	0.42 (-0.97-1.9)	19 (-26-64)
Solomon Islands	2000	Male	60 to 64	48% (28-68%)	52% (32-72%)	0.31 (0.15-0.58)	0.21% (-0.5-0.95%)	0.17% (-0.66-0.98%)	0.23 (-0.93-1.4)	9 (-22-40)
Solomon Islands	2000	Male	65 to 69	46% (27-65%)	54% (35-73%)	0.29 (0.14-0.5)	0.15% (-0.43-0.78%)	0.14% (-0.54-0.85%)	0.2 (-0.83-1.3)	5.7 (-16-29)
Solomon Islands	2000	Male	70 to 74	44% (26-62%)	56% (38-74%)	0.25 (0.12-0.47)	0.042% (-0.51-0.58%)	0.019% (-0.61-0.65%)	0.023 (-0.74-0.77)	1.1 (-13-15)
Solomon Islands	2000	Male	75 to 79	40% (24-59%)	60% (41-76%)	0.22 (0.1-0.41)	-0.01% (-0.5-0.43%)	-0.045% (-0.6-0.45%)	-0.049 (-0.67-0.5)	-0.17 (-8.7-7.6)

Location	Year	Sex	Age	Prevalence of current drinkers	Prevalence of abstainers	Population average of standard drinks daily	Percent of total attributable deaths	Percent of total attributable DALYs	Attributable deaths	Attributable DALYs
Solomon Islands	2000	Male	80 to 84	37% (20-56%)	63% (44-80%)	0.19 (0.093-0.35)	0.021% (-0.37-0.36%)	-0.01% (-0.45-0.37%)	-0.0082 (-0.37-0.3)	0.2 (-3.6-3.4)
Solomon Islands	2000	Male	85 to 89	36% (20-53%)	64% (47-80%)	0.18 (0.082-0.33)	-0.076% (-0.49-0.23%)	-0.13% (-0.61-0.22%)	-0.051 (-0.24-0.089)	-0.26 (-1.7-0.77)
Solomon Islands	2000	Male	90 to 94	37% (23-55%)	63% (45-77%)	0.19 (0.083-0.36)	-0.075% (-0.46-0.2%)	-0.13% (-0.59-0.19%)	-0.019 (-0.084-0.026)	-0.062 (-0.39-0.17)
Solomon Islands	2000	Male	95 plus	41% (23-61%)	59% (39-77%)	0.21 (0.083-0.41)	-0.26% (-0.78-0.12%)	-0.39% (-0.99-0.046%)	-0.015 (-0.038-0.0017)	-0.039 (-0.12-0.018)
Solomon Islands	2005	Female	All Ages	19% (13-26%)	81% (74-87%)	0.19 (0.14-0.24)	0.024% (-0.33-0.34%)	-0.34% (-0.95-0.16%)	-6.2 (-18-2.9)	23 (-310-320)
Solomon Islands	2005	Female	15 to 19	20% (9.1-36%)	80% (64-91%)	0.23 (0.092-0.42)	0.64% (0.26-1.2%)	0.75% (0.13-1.8%)	0.25 (0.046-0.61)	28 (11-54)
Solomon Islands	2005	Female	20 to 24	25% (10-42%)	75% (58-90%)	0.35 (0.16-0.63)	1.1% (0.56-1.8%)	1.1% (0.35-2.3%)	0.51 (0.15-1.1)	55 (28-98)
Solomon Islands	2005	Female	25 to 29	26% (11-44%)	74% (56-89%)	0.36 (0.16-0.63)	1% (0.55-1.7%)	1% (0.36-1.9%)	0.66 (0.23-1.3)	60 (32-99)
Solomon Islands	2005	Female	30 to 34	27% (11-47%)	73% (53-89%)	0.38 (0.16-0.68)	0.73% (0.25-1.3%)	0.69% (0.054-1.5%)	0.5 (0.038-1.1)	42 (14-78)
Solomon Islands	2005	Female	35 to 39	28% (11-47%)	72% (53-89%)	0.39 (0.17-0.69)	0.61% (-0.016-1.3%)	0.65% (-0.14-1.6%)	0.55 (-0.13-1.3)	35 (-0.8-76)
Solomon Islands	2005	Female	40 to 44	27% (13-44%)	73% (56-87%)	0.36 (0.15-0.67)	0.27% (-0.53-1%)	0.3% (-0.7-1.3%)	0.28 (-0.65-1.3)	16 (-30-62)
Solomon Islands	2005	Female	45 to 49	26% (12-45%)	74% (55-88%)	0.31 (0.12-0.58)	-0.19% (-1.1-0.52%)	-0.19% (-1.3-0.65%)	-0.19 (-1.3-0.67)	-10 (-61-27)
Solomon Islands	2005	Female	50 to 54	26% (12-45%)	74% (55-88%)	0.27 (0.1-0.56)	-0.51% (-1.7-0.25%)	-0.51% (-1.8-0.38%)	-0.59 (-2.2-0.42)	-27 (-93-13)
Solomon Islands	2005	Female	55 to 59	25% (12-46%)	75% (54-88%)	0.25 (0.091-0.51)	-0.63% (-1.9-0.12%)	-0.62% (-2-0.22%)	-0.85 (-2.8-0.29)	-33 (-99-6)
Solomon Islands	2005	Female	60 to 64	26% (12-45%)	74% (55-88%)	0.24 (0.084-0.52)	-0.87% (-2.2--0.091%)	-0.89% (-2.3--0.041%)	-1.4 (-3.6--0.065)	-42 (-110--4)
Solomon Islands	2005	Female	65 to 69	27% (12-45%)	73% (55-88%)	0.24 (0.086-0.48)	-0.97% (-2.2--0.1%)	-1% (-2.3--0.048%)	-1.4 (-3.3--0.06)	-35 (-80--3.3)
Solomon Islands	2005	Female	70 to 74	27% (12-44%)	73% (56-88%)	0.23 (0.081-0.44)	-1% (-2.2--0.17%)	-1.1% (-2.3--0.16%)	-1.8 (-4.2--0.27)	-36 (-80--6.1)
Solomon Islands	2005	Female	75 to 79	25% (11-45%)	75% (55-89%)	0.21 (0.069-0.46)	-0.82% (-2--0.071%)	-0.87% (-2.2--0.055%)	-1.2 (-3--0.073)	-18 (-45--1.5)
Solomon Islands	2005	Female	80 to 84	24% (11-44%)	76% (56-89%)	0.2 (0.066-0.42)	-0.81% (-2--0.17%)	-0.88% (-2.2--0.16%)	-0.96 (-2.3--0.18)	-10 (-24--2.2)
Solomon Islands	2005	Female	85 to 89	25% (12-43%)	75% (57-88%)	0.21 (0.075-0.44)	-0.86% (-2--0.19%)	-0.95% (-2.2--0.21%)	-0.44 (-1--0.097)	-3.3 (-7.7--0.72)
Solomon Islands	2005	Female	90 to 94	29% (14-48%)	71% (52-86%)	0.24 (0.084-0.49)	-0.76% (-1.6--0.18%)	-0.86% (-1.8--0.2%)	-0.1 (-0.22--0.025)	-0.53 (-1.1--0.13)
Solomon Islands	2005	Female	95 plus	34% (15-57%)	66% (43-85%)	0.28 (0.094-0.59)	-1% (-2.3--0.15%)	-1.2% (-2.6--0.21%)	-0.033 (-0.073--0.0059)	-0.11 (-0.24--0.016)

Location	Year	Sex	Age	Prevalence of current drinkers	Prevalence of abstainers	Population average of standard drinks daily	Percent of total attributable deaths	Percent of total attributable DALYs	Attributable deaths	Attributable DALYs
Solomon Islands	2005	Male	All Ages	39% (31-48%)	61% (52-69%)	0.4 (0.32-0.49)	1.1% (0.59-1.8%)	0.92% (0.24-1.7%)	20 (5.5-36)	1200 (640-1900)
Solomon Islands	2005	Male	15 to 19	37% (22-56%)	63% (44-78%)	0.27 (0.12-0.46)	1.2% (0.59-2.2%)	1.4% (0.44-2.8%)	0.72 (0.23-1.5)	68 (32-120)
Solomon Islands	2005	Male	20 to 24	55% (37-72%)	45% (28-63%)	0.71 (0.42-1.1)	3.4% (1.9-5.4%)	3.8% (1.8-6.4%)	2.8 (1.3-4.7)	220 (120-350)
Solomon Islands	2005	Male	25 to 29	63% (43-79%)	37% (21-57%)	1 (0.63-1.4)	4.1% (2.3-6.1%)	4.6% (2.4-7.1%)	3.7 (1.9-6.1)	270 (150-410)
Solomon Islands	2005	Male	30 to 34	64% (43-80%)	36% (20-57%)	1.1 (0.65-1.6)	3.5% (1.9-5.4%)	3.7% (1.7-6.2%)	3.3 (1.5-5.6)	220 (120-350)
Solomon Islands	2005	Male	35 to 39	62% (43-78%)	38% (22-57%)	0.93 (0.55-1.4)	2.6% (1.2-4.2%)	2.6% (0.97-4.7%)	2.5 (0.9-4.7)	150 (72-270)
Solomon Islands	2005	Male	40 to 44	59% (41-76%)	41% (24-59%)	0.81 (0.44-1.3)	1.8% (0.5-3.4%)	1.7% (0.24-3.7%)	2 (0.27-4.3)	110 (32-220)
Solomon Islands	2005	Male	45 to 49	56% (34-74%)	44% (26-66%)	0.69 (0.34-1.2)	1.3% (0.019-2.9%)	1.3% (-0.28-3.1%)	1.6 (-0.35-3.9)	80 (1.5-170)
Solomon Islands	2005	Male	50 to 54	53% (33-72%)	47% (28-67%)	0.57 (0.29-0.99)	1% (-0.21-2.3%)	0.96% (-0.47-2.5%)	1.3 (-0.58-3.5)	58 (-13-140)
Solomon Islands	2005	Male	55 to 59	51% (33-70%)	49% (30-67%)	0.47 (0.22-0.83)	0.5% (-0.44-1.6%)	0.45% (-0.65-1.7%)	0.69 (-0.99-2.7)	28 (-25-90)
Solomon Islands	2005	Male	60 to 64	48% (29-68%)	52% (32-71%)	0.39 (0.19-0.71)	0.3% (-0.46-1.2%)	0.27% (-0.58-1.3%)	0.48 (-1.1-2.4)	17 (-26-69)
Solomon Islands	2005	Male	65 to 69	46% (27-65%)	54% (35-73%)	0.36 (0.18-0.62)	0.25% (-0.44-1.1%)	0.24% (-0.53-1.1%)	0.4 (-0.89-1.9)	10 (-19-45)
Solomon Islands	2005	Male	70 to 74	43% (26-61%)	57% (39-74%)	0.31 (0.15-0.56)	0.1% (-0.49-0.8%)	0.087% (-0.57-0.87%)	0.15 (-0.99-1.5)	3.6 (-17-29)
Solomon Islands	2005	Male	75 to 79	39% (23-59%)	61% (41-77%)	0.27 (0.12-0.49)	0.032% (-0.51-0.63%)	0.0023% (-0.62-0.65%)	0.0032 (-0.84-0.91)	0.71 (-11-13)
Solomon Islands	2005	Male	80 to 84	37% (21-55%)	63% (45-79%)	0.24 (0.12-0.42)	0.035% (-0.41-0.44%)	0.0037% (-0.5-0.45%)	0.0038 (-0.5-0.47)	0.42 (-4.9-5.3)
Solomon Islands	2005	Male	85 to 89	35% (20-52%)	65% (48-80%)	0.22 (0.11-0.4)	-0.065% (-0.5-0.31%)	-0.12% (-0.61-0.3%)	-0.058 (-0.31-0.15)	-0.27 (-2.1-1.3)
Solomon Islands	2005	Male	90 to 94	36% (22-54%)	64% (46-78%)	0.23 (0.11-0.43)	-0.081% (-0.49-0.25%)	-0.15% (-0.64-0.23%)	-0.024 (-0.11-0.038)	-0.08 (-0.49-0.23)
Solomon Islands	2005	Male	95 plus	40% (21-61%)	60% (39-79%)	0.25 (0.1-0.51)	-0.27% (-0.83-0.13%)	-0.41% (-1.1-0.052%)	-0.018 (-0.048-0.0023)	-0.047 (-0.14-0.023)
Solomon Islands	2010	Female	All Ages	19% (13-26%)	81% (74-87%)	0.23 (0.17-0.3)	0.11% (-0.3-0.51%)	-0.29% (-0.97-0.34%)	-5.9 (-20-6.8)	110 (-300-530)
Solomon Islands	2010	Female	15 to 19	20% (9-36%)	80% (64-91%)	0.27 (0.11-0.53)	0.78% (0.32-1.5%)	1% (0.25-2.3%)	0.36 (0.086-0.88)	37 (15-75)
Solomon Islands	2010	Female	20 to 24	24% (10-42%)	76% (58-90%)	0.42 (0.18-0.78)	1.3% (0.68-2.2%)	1.4% (0.5-3%)	0.68 (0.23-1.5)	69 (35-120)
Solomon Islands	2010	Female	25 to 29	25% (11-44%)	75% (56-89%)	0.43 (0.19-0.76)	1.3% (0.68-2.1%)	1.4% (0.55-2.5%)	0.89 (0.34-1.7)	77 (41-130)

Location	Year	Sex	Age	Prevalence of current drinkers	Prevalence of abstainers	Population average of standard drinks daily	Percent of total attributable deaths	Percent of total attributable DALYs	Attributable deaths	Attributable DALYs
Solomon Islands	2010	Female	30 to 34	26% (10-46%)	74% (54-90%)	0.45 (0.19-0.81)	0.96% (0.36-1.8%)	1% (0.19-2.2%)	0.85 (0.15-1.9)	66 (24-130)
Solomon Islands	2010	Female	35 to 39	27% (12-46%)	73% (54-88%)	0.47 (0.21-0.82)	0.86% (0.12-1.8%)	0.99% (0.043-2.2%)	1.1 (0.041-2.4)	65 (7.9-130)
Solomon Islands	2010	Female	40 to 44	26% (13-44%)	74% (56-87%)	0.44 (0.19-0.8)	0.49% (-0.41-1.5%)	0.58% (-0.52-1.9%)	0.6 (-0.54-1.9)	31 (-27-93)
Solomon Islands	2010	Female	45 to 49	26% (11-45%)	74% (55-89%)	0.38 (0.15-0.73)	-0.049% (-1.1-0.85%)	-0.022% (-1.2-1.2%)	-0.027 (-1.5-1.3)	-3 (-68-53)
Solomon Islands	2010	Female	50 to 54	26% (12-46%)	74% (54-88%)	0.34 (0.13-0.67)	-0.45% (-1.7-0.49%)	-0.45% (-1.9-0.66%)	-0.57 (-2.5-0.84)	-26 (-100-28)
Solomon Islands	2010	Female	55 to 59	25% (11-45%)	75% (55-89%)	0.3 (0.11-0.61)	-0.61% (-2.1-0.29%)	-0.6% (-2.2-0.48%)	-0.82 (-3.1-0.66)	-32 (-110-15)
Solomon Islands	2010	Female	60 to 64	25% (11-44%)	75% (56-89%)	0.29 (0.1-0.6)	-0.89% (-2.3-0.022%)	-0.92% (-2.5-0.11%)	-1.5 (-4.2-0.18)	-46 (-120-1.3)
Solomon Islands	2010	Female	65 to 69	26% (12-44%)	74% (56-88%)	0.29 (0.11-0.56)	-0.98% (-2.3--0.049%)	-1% (-2.5-0.027%)	-1.9 (-4.7-0.05)	-48 (-110--2.1)
Solomon Islands	2010	Female	70 to 74	26% (12-44%)	74% (56-88%)	0.28 (0.099-0.56)	-1% (-2.3--0.11%)	-1.1% (-2.5--0.099%)	-1.8 (-4.3--0.16)	-36 (-83--4.3)
Solomon Islands	2010	Female	75 to 79	25% (11-43%)	75% (57-89%)	0.26 (0.085-0.54)	-0.83% (-2.1--0.041%)	-0.88% (-2.2-0.011%)	-1.7 (-4.2-0.022)	-25 (-61--1.1)
Solomon Islands	2010	Female	80 to 84	24% (11-44%)	76% (56-89%)	0.25 (0.084-0.53)	-0.83% (-2--0.12%)	-0.91% (-2.2--0.13%)	-1.3 (-3.1--0.17)	-14 (-33--2.1)
Solomon Islands	2010	Female	85 to 89	25% (12-44%)	75% (56-88%)	0.26 (0.095-0.56)	-0.88% (-2--0.17%)	-0.98% (-2.3--0.2%)	-0.54 (-1.2--0.11)	-4 (-9.4--0.79)
Solomon Islands	2010	Female	90 to 94	29% (14-48%)	71% (52-86%)	0.29 (0.1-0.62)	-0.78% (-1.7--0.14%)	-0.88% (-2--0.15%)	-0.14 (-0.31--0.024)	-0.72 (-1.6--0.13)
Solomon Islands	2010	Female	95 plus	34% (15-56%)	66% (44-85%)	0.35 (0.11-0.73)	-1% (-2.4--0.099%)	-1.2% (-2.7--0.15%)	-0.038 (-0.085--0.0045)	-0.12 (-0.28--0.012)
Solomon Islands	2010	Male	All Ages	39% (31-49%)	61% (51-69%)	0.5 (0.4-0.62)	1.5% (0.83-2.2%)	1.2% (0.45-2.1%)	29 (11-50)	1700 (980-2600)
Solomon Islands	2010	Male	15 to 19	37% (22-55%)	63% (45-78%)	0.33 (0.16-0.56)	1.5% (0.71-2.6%)	1.8% (0.65-3.5%)	0.95 (0.34-1.9)	86 (40-150)
Solomon Islands	2010	Male	20 to 24	55% (36-72%)	45% (28-64%)	0.89 (0.53-1.3)	4.1% (2.4-6.3%)	4.7% (2.4-7.6%)	3.4 (1.7-5.7)	260 (150-410)
Solomon Islands	2010	Male	25 to 29	62% (43-78%)	38% (22-57%)	1.2 (0.77-1.8)	5% (3-7.4%)	5.7% (3.1-8.8%)	4.6 (2.5-7.2)	320 (190-490)
Solomon Islands	2010	Male	30 to 34	63% (42-79%)	37% (21-58%)	1.3 (0.8-2)	4.2% (2.4-6.4%)	4.7% (2.4-7.4%)	4.6 (2.3-7.7)	300 (170-480)
Solomon Islands	2010	Male	35 to 39	62% (44-79%)	38% (21-56%)	1.1 (0.66-1.8)	3.2% (1.7-5.2%)	3.4% (1.5-5.9%)	4.1 (1.8-7.1)	240 (120-400)
Solomon Islands	2010	Male	40 to 44	59% (41-76%)	41% (24-59%)	1 (0.54-1.6)	2.3% (0.76-4.2%)	2.4% (0.49-4.6%)	3 (0.62-6.1)	160 (53-300)
Solomon Islands	2010	Male	45 to 49	56% (34-73%)	44% (27-66%)	0.86 (0.44-1.5)	1.8% (0.23-3.5%)	1.8% (-0.047-3.9%)	2.6 (-0.073-5.7)	130 (16-260)

Location	Year	Sex	Age	Prevalence of current drinkers	Prevalence of abstainers	Population average of standard drinks daily	Percent of total attributable deaths	Percent of total attributable DALYs	Attributable deaths	Attributable DALYs
Solomon Islands	2010	Male	50 to 54	53% (34-71%)	47% (29-66%)	0.71 (0.36-1.3)	1.4% (0.005-3.1%)	1.4% (-0.27-3.3%)	2.2 (-0.45-5.4)	93 (0.35-220)
Solomon Islands	2010	Male	55 to 59	50% (32-70%)	50% (30-68%)	0.58 (0.28-1)	0.75% (-0.33-2%)	0.72% (-0.55-2.2%)	1.2 (-0.95-3.7)	45 (-21-130)
Solomon Islands	2010	Male	60 to 64	48% (28-67%)	52% (33-72%)	0.49 (0.23-0.9)	0.5% (-0.41-1.8%)	0.5% (-0.56-1.9%)	0.9 (-0.99-3.6)	28 (-23-110)
Solomon Islands	2010	Male	65 to 69	46% (28-64%)	54% (36-72%)	0.44 (0.21-0.77)	0.42% (-0.36-1.5%)	0.43% (-0.45-1.6%)	0.87 (-0.95-3.3)	22 (-19-77)
Solomon Islands	2010	Male	70 to 74	43% (27-61%)	57% (39-73%)	0.38 (0.18-0.7)	0.22% (-0.44-1%)	0.21% (-0.53-1.1%)	0.4 (-0.97-2.1)	8.4 (-17-41)
Solomon Islands	2010	Male	75 to 79	40% (24-58%)	60% (42-76%)	0.33 (0.15-0.61)	0.11% (-0.48-0.83%)	0.089% (-0.58-0.86%)	0.16 (-1.1-1.6)	3.3 (-14-24)
Solomon Islands	2010	Male	80 to 84	37% (21-55%)	63% (45-79%)	0.29 (0.14-0.51)	0.093% (-0.4-0.61%)	0.064% (-0.49-0.65%)	0.084 (-0.66-0.84)	1.4 (-6.3-9.4)
Solomon Islands	2010	Male	85 to 89	35% (20-52%)	65% (48-80%)	0.28 (0.13-0.5)	-0.017% (-0.46-0.43%)	-0.067% (-0.58-0.45%)	-0.037 (-0.32-0.25)	-0.081 (-2.2-2.1)
Solomon Islands	2010	Male	90 to 94	37% (22-54%)	63% (46-78%)	0.28 (0.13-0.53)	-0.058% (-0.51-0.31%)	-0.13% (-0.65-0.3%)	-0.023 (-0.12-0.056)	-0.063 (-0.55-0.35)
Solomon Islands	2010	Male	95 plus	40% (21-61%)	60% (39-79%)	0.31 (0.12-0.64)	-0.27% (-0.84-0.19%)	-0.41% (-1.1-0.12%)	-0.019 (-0.052-0.0053)	-0.048 (-0.15-0.036)
Solomon Islands	2016	Female	All Ages	19% (13-26%)	81% (74-87%)	0.27 (0.18-0.4)	0.16% (-0.35-0.72%)	-0.25% (-1.1-0.64%)	-5.5 (-23-13)	170 (-380-760)
Solomon Islands	2016	Female	15 to 19	20% (9-35%)	80% (65-91%)	0.31 (0.13-0.63)	0.87% (0.39-1.8%)	1.2% (0.33-2.8%)	0.44 (0.12-1.1)	45 (20-91)
Solomon Islands	2016	Female	20 to 24	24% (9.9-43%)	76% (57-90%)	0.49 (0.21-0.96)	1.5% (0.77-2.6%)	1.7% (0.58-3.6%)	0.81 (0.27-1.8)	82 (41-150)
Solomon Islands	2016	Female	25 to 29	26% (11-45%)	74% (55-89%)	0.51 (0.2-0.95)	1.5% (0.78-2.5%)	1.6% (0.64-3.2%)	0.98 (0.33-2.1)	84 (44-150)
Solomon Islands	2016	Female	30 to 34	27% (10-47%)	73% (53-90%)	0.53 (0.21-1)	1.1% (0.43-2.2%)	1.3% (0.29-2.9%)	0.98 (0.21-2.3)	74 (26-150)
Solomon Islands	2016	Female	35 to 39	27% (11-47%)	73% (53-89%)	0.54 (0.23-0.99)	1% (0.17-2.2%)	1.2% (0.083-2.7%)	1.4 (0.091-3.3)	84 (14-180)
Solomon Islands	2016	Female	40 to 44	26% (13-44%)	74% (56-87%)	0.5 (0.21-0.96)	0.63% (-0.42-1.9%)	0.78% (-0.52-2.5%)	1 (-0.72-3.3)	52 (-37-170)
Solomon Islands	2016	Female	45 to 49	26% (11-45%)	74% (55-89%)	0.43 (0.17-0.84)	0.06% (-1.1-1.2%)	0.13% (-1.3-1.6%)	0.17 (-1.7-2.2)	4.4 (-80-88)
Solomon Islands	2016	Female	50 to 54	26% (12-44%)	74% (56-88%)	0.38 (0.14-0.77)	-0.37% (-1.6-0.82%)	-0.34% (-1.8-1.1%)	-0.5 (-2.7-1.8)	-25 (-120-59)
Solomon Islands	2016	Female	55 to 59	25% (12-45%)	75% (55-88%)	0.34 (0.13-0.71)	-0.56% (-2-0.5%)	-0.53% (-2.2-0.74%)	-0.88 (-3.6-1.2)	-36 (-130-32)
Solomon Islands	2016	Female	60 to 64	25% (11-46%)	75% (54-89%)	0.33 (0.11-0.71)	-0.88% (-2.4-0.19%)	-0.9% (-2.6-0.31%)	-1.5 (-4.4-0.51)	-48 (-130-11)
Solomon Islands	2016	Female	65 to 69	27% (12-45%)	73% (55-88%)	0.34 (0.12-0.68)	-0.97% (-2.4-0.1%)	-1% (-2.7-0.22%)	-2 (-5.4-0.4)	-51 (-130-5.6)

Location	Year	Sex	Age	Prevalence of current drinkers	Prevalence of abstainers	Population average of standard drinks daily	Percent of total attributable deaths	Percent of total attributable DALYs	Attributable deaths	Attributable DALYs
Solomon Islands	2016	Female	70 to 74	26% (12-44%)	74% (56-88%)	0.32 (0.11-0.67)	-1% (-2.3-0.11%)	-1.1% (-2.5-0.18%)	-2.4 (-5.9-0.37)	-48 (-110-4.6)
Solomon Islands	2016	Female	75 to 79	25% (11-44%)	75% (56-89%)	0.3 (0.093-0.67)	-0.79% (-2.1-0.17%)	-0.84% (-2.2-0.25%)	-1.5 (-4.2-0.43)	-23 (-62-5.2)
Solomon Islands	2016	Female	80 to 84	24% (11-43%)	76% (57-89%)	0.28 (0.091-0.62)	-0.8% (-2-0.03%)	-0.88% (-2.2-0.072%)	-1.4 (-3.5-0.11)	-15 (-37-0.57)
Solomon Islands	2016	Female	85 to 89	25% (12-45%)	75% (55-88%)	0.3 (0.1-0.64)	-0.85% (-2.1--0.066%)	-0.95% (-2.3--0.069%)	-0.79 (-1.9--0.058)	-5.9 (-14--0.47)
Solomon Islands	2016	Female	90 to 94	29% (13-48%)	71% (52-87%)	0.34 (0.12-0.71)	-0.76% (-1.7-0.005%)	-0.86% (-2-0.02%)	-0.18 (-0.41-0.0041)	-0.91 (-2.1-0.006)
Solomon Islands	2016	Female	95 plus	34% (14-56%)	66% (44-86%)	0.4 (0.12-0.87)	-1% (-2.5-0.03%)	-1.2% (-2.8--0.027%)	-0.056 (-0.13--0.0013)	-0.18 (-0.42-0.0051)
Solomon Islands	2016	Male	All Ages	39% (31-48%)	61% (52-69%)	0.58 (0.41-0.8)	1.8% (0.92-2.9%)	1.5% (0.51-2.8%)	38 (13-71)	2200 (1100-3500)
Solomon Islands	2016	Male	15 to 19	37% (22-55%)	63% (45-78%)	0.38 (0.17-0.67)	1.6% (0.79-2.9%)	2% (0.73-3.9%)	1.2 (0.42-2.3)	110 (50-180)
Solomon Islands	2016	Male	20 to 24	55% (36-72%)	45% (28-64%)	1 (0.56-1.7)	4.5% (2.5-7.1%)	5.2% (2.6-8.8%)	4.3 (2.1-7.5)	340 (190-560)
Solomon Islands	2016	Male	25 to 29	62% (42-77%)	38% (23-58%)	1.4 (0.8-2.2)	5.5% (3.1-8.5%)	6.4% (3.2-10%)	5.2 (2.6-8.8)	370 (200-590)
Solomon Islands	2016	Male	30 to 34	63% (42-79%)	37% (21-58%)	1.5 (0.85-2.4)	4.7% (2.6-7.8%)	5.3% (2.6-9.2%)	5 (2.5-8.5)	330 (170-540)
Solomon Islands	2016	Male	35 to 39	62% (44-79%)	38% (21-56%)	1.3 (0.72-2.1)	3.7% (1.8-6.2%)	4.1% (1.7-7.2%)	4.9 (2-9.1)	290 (140-510)
Solomon Islands	2016	Male	40 to 44	59% (41-76%)	41% (24-59%)	1.1 (0.61-2)	2.7% (0.94-5.4%)	2.8% (0.72-6.1%)	4.5 (1.1-9.7)	240 (83-490)
Solomon Islands	2016	Male	45 to 49	55% (33-73%)	45% (27-67%)	0.98 (0.47-1.8)	2.1% (0.38-4.4%)	2.2% (0.12-4.9%)	3.7 (0.19-8.6)	180 (33-390)
Solomon Islands	2016	Male	50 to 54	52% (32-71%)	48% (29-68%)	0.82 (0.38-1.5)	1.6% (0.12-3.8%)	1.7% (-0.16-4.1%)	3.1 (-0.29-8.1)	130 (9.1-320)
Solomon Islands	2016	Male	55 to 59	50% (31-69%)	50% (31-69%)	0.66 (0.31-1.2)	0.93% (-0.31-2.6%)	0.92% (-0.5-2.8%)	1.8 (-0.95-6)	69 (-21-210)
Solomon Islands	2016	Male	60 to 64	48% (28-68%)	52% (32-72%)	0.54 (0.25-1.1)	0.65% (-0.33-2.1%)	0.65% (-0.45-2.3%)	1.3 (-0.88-4.8)	41 (-21-140)
Solomon Islands	2016	Male	65 to 69	46% (27-65%)	54% (35-73%)	0.5 (0.23-0.91)	0.57% (-0.29-1.8%)	0.59% (-0.38-2%)	1.2 (-0.79-4.2)	30 (-15-100)
Solomon Islands	2016	Male	70 to 74	43% (26-61%)	57% (39-74%)	0.44 (0.2-0.84)	0.34% (-0.43-1.5%)	0.35% (-0.52-1.6%)	0.76 (-1.1-3.4)	16 (-19-66)
Solomon Islands	2016	Male	75 to 79	39% (23-58%)	61% (42-77%)	0.38 (0.17-0.74)	0.22% (-0.41-1.1%)	0.2% (-0.51-1.2%)	0.36 (-0.9-2.2)	6.3 (-12-32)
Solomon Islands	2016	Male	80 to 84	36% (20-54%)	64% (46-80%)	0.33 (0.15-0.63)	0.17% (-0.35-0.8%)	0.15% (-0.43-0.85%)	0.21 (-0.59-1.2)	2.9 (-5.8-14)
Solomon Islands	2016	Male	85 to 89	35% (20-51%)	65% (49-80%)	0.32 (0.14-0.61)	0.048% (-0.45-0.63%)	0.0019% (-0.56-0.66%)	0.0014 (-0.43-0.5)	0.31 (-2.9-4.2)

Location	Year	Sex	Age	Prevalence of current drinkers	Prevalence of abstainers	Population average of standard drinks daily	Percent of total attributable deaths	Percent of total attributable DALYs	Attributable deaths	Attributable DALYs
Solomon Islands	2016	Male	90 to 94	37% (23-54%)	63% (46-77%)	0.33 (0.14-0.64)	-0.019% (-0.51-0.45%)	-0.089% (-0.66-0.46%)	-0.019 (-0.14-0.097)	-0.024 (-0.65-0.58)
Solomon Islands	2016	Male	95 plus	40% (22-61%)	60% (39-78%)	0.37 (0.14-0.74)	-0.23% (-0.87-0.37%)	-0.38% (-1.2-0.28%)	-0.019 (-0.058-0.015)	-0.046 (-0.17-0.074)
Tonga	1990	Female	All Ages	17% (12-24%)	83% (76-88%)	0.14 (0.099-0.19)	-0.044% (-0.32-0.19%)	-0.42% (-0.91--0.014%)	-1.2 (-2.8--0.047)	-6.2 (-46-27)
Tonga	1990	Female	15 to 19	18% (8.2-33%)	82% (67-92%)	0.17 (0.072-0.34)	0.48% (0.25-0.77%)	0.4% (0.075-1%)	0.016 (0.0027-0.039)	3.3 (1.7-5.4)
Tonga	1990	Female	20 to 24	23% (9.7-41%)	77% (59-90%)	0.27 (0.12-0.49)	0.87% (0.53-1.3%)	0.58% (0.19-1.2%)	0.034 (0.011-0.074)	7.6 (4.5-12)
Tonga	1990	Female	25 to 29	24% (10-44%)	76% (56-90%)	0.28 (0.12-0.51)	0.84% (0.54-1.2%)	0.59% (0.21-1.2%)	0.03 (0.011-0.066)	5.4 (3.2-8.1)
Tonga	1990	Female	30 to 34	25% (9.9-45%)	75% (55-90%)	0.29 (0.11-0.53)	0.7% (0.41-1.1%)	0.58% (0.21-1.1%)	0.03 (0.011-0.06)	3.9 (2.1-5.9)
Tonga	1990	Female	35 to 39	25% (10-43%)	75% (57-90%)	0.3 (0.12-0.56)	0.58% (0.25-0.97%)	0.62% (0.19-1.2%)	0.046 (0.014-0.097)	3.8 (1.6-6.5)
Tonga	1990	Female	40 to 44	25% (12-42%)	75% (58-88%)	0.27 (0.11-0.52)	0.39% (-0.067-0.84%)	0.52% (-0.048-1.2%)	0.045 (-0.0037-0.11)	2.6 (-0.41-5.8)
Tonga	1990	Female	45 to 49	24% (11-42%)	76% (58-89%)	0.24 (0.088-0.47)	0.023% (-0.63-0.49%)	0.13% (-0.62-0.76%)	0.013 (-0.067-0.076)	0.15 (-4.1-3.3)
Tonga	1990	Female	50 to 54	24% (11-43%)	76% (57-89%)	0.21 (0.076-0.42)	-0.33% (-1.2-0.26%)	-0.27% (-1.3-0.4%)	-0.038 (-0.19-0.055)	-2.5 (-9.7-1.9)
Tonga	1990	Female	55 to 59	23% (10-42%)	77% (58-90%)	0.19 (0.067-0.41)	-0.52% (-1.6-0.12%)	-0.46% (-1.7-0.25%)	-0.091 (-0.35-0.051)	-4.5 (-14-1.1)
Tonga	1990	Female	60 to 64	24% (11-43%)	76% (57-89%)	0.18 (0.063-0.38)	-0.72% (-2-0.019%)	-0.71% (-2.1-0.09%)	-0.18 (-0.53-0.023)	-6.7 (-18-0.16)
Tonga	1990	Female	65 to 69	24% (11-41%)	76% (59-89%)	0.18 (0.065-0.37)	-0.87% (-2--0.11%)	-0.91% (-2.2--0.059%)	-0.25 (-0.63--0.017)	-7 (-17--0.9)
Tonga	1990	Female	70 to 74	24% (12-40%)	76% (60-88%)	0.17 (0.058-0.36)	-0.86% (-1.8--0.16%)	-0.92% (-2--0.15%)	-0.28 (-0.64--0.045)	-5.9 (-13--1.1)
Tonga	1990	Female	75 to 79	23% (10-42%)	77% (58-90%)	0.16 (0.051-0.35)	-0.6% (-1.6--0.056%)	-0.62% (-1.7--0.027%)	-0.18 (-0.51--0.0074)	-3 (-8.1--0.28)
Tonga	1990	Female	80 to 84	22% (9.2-42%)	78% (58-91%)	0.15 (0.045-0.34)	-0.6% (-1.6--0.079%)	-0.64% (-1.7--0.076%)	-0.19 (-0.5--0.021)	-2.2 (-5.7--0.28)
Tonga	1990	Female	85 to 89	23% (10-42%)	77% (58-90%)	0.16 (0.053-0.35)	-0.63% (-1.5--0.13%)	-0.68% (-1.7--0.13%)	-0.13 (-0.32--0.027)	-1.1 (-2.5--0.22)
Tonga	1990	Female	90 to 94	27% (12-47%)	73% (53-88%)	0.18 (0.059-0.39)	-0.68% (-1.5--0.15%)	-0.75% (-1.7--0.16%)	-0.064 (-0.14--0.014)	-0.34 (-0.76--0.075)
Tonga	1990	Female	95 plus	32% (13-53%)	68% (47-87%)	0.22 (0.067-0.49)	-0.83% (-2--0.052%)	-0.97% (-2.3--0.08%)	-0.021 (-0.049--0.0017)	-0.069 (-0.17--0.0043)
Tonga	1990	Male	All Ages	36% (28-45%)	64% (55-72%)	0.3 (0.22-0.39)	0.88% (0.51-1.3%)	0.62% (0.18-1.1%)	2.2 (0.67-3.8)	150 (89-230)
Tonga	1990	Male	15 to 19	35% (20-52%)	65% (48-80%)	0.2 (0.099-0.35)	1.1% (0.59-1.8%)	1.1% (0.43-2.2%)	0.096 (0.037-0.19)	10 (5.7-17)

Location	Year	Sex	Age	Prevalence of current drinkers	Prevalence of abstainers	Population average of standard drinks daily	Percent of total attributable deaths	Percent of total attributable DALYs	Attributable deaths	Attributable DALYs
Tonga	1990	Male	20 to 24	53% (34-71%)	47% (29-66%)	0.55 (0.33-0.84)	2.7% (1.6-4%)	2.8% (1.4-4.8%)	0.4 (0.18-0.68)	36 (21-55)
Tonga	1990	Male	25 to 29	60% (40-77%)	40% (23-60%)	0.77 (0.46-1.2)	3.3% (2-4.8%)	3.6% (1.9-5.6%)	0.38 (0.18-0.61)	31 (18-45)
Tonga	1990	Male	30 to 34	61% (40-78%)	39% (22-60%)	0.83 (0.48-1.3)	2.7% (1.6-3.9%)	2.8% (1.4-4.5%)	0.22 (0.1-0.38)	17 (10-27)
Tonga	1990	Male	35 to 39	60% (42-76%)	40% (24-58%)	0.73 (0.38-1.2)	2.2% (1.2-3.4%)	2.3% (0.98-3.9%)	0.23 (0.097-0.4)	16 (8.6-25)
Tonga	1990	Male	40 to 44	57% (39-74%)	43% (26-61%)	0.63 (0.3-1.1)	1.6% (0.85-2.6%)	1.7% (0.67-2.9%)	0.18 (0.062-0.33)	11 (5.7-19)
Tonga	1990	Male	45 to 49	53% (32-72%)	47% (28-68%)	0.54 (0.24-0.97)	1.5% (0.69-2.2%)	1.5% (0.53-2.6%)	0.18 (0.06-0.32)	9.7 (4.5-16)
Tonga	1990	Male	50 to 54	50% (31-69%)	50% (31-69%)	0.44 (0.2-0.79)	1.1% (0.35-1.9%)	1.1% (0.19-2.1%)	0.19 (0.03-0.37)	8.8 (2.7-16)
Tonga	1990	Male	55 to 59	48% (29-68%)	52% (32-71%)	0.36 (0.17-0.66)	0.63% (-0.091-1.3%)	0.61% (-0.27-1.4%)	0.14 (-0.06-0.33)	5.6 (-0.8-11)
Tonga	1990	Male	60 to 64	45% (27-65%)	55% (35-73%)	0.3 (0.13-0.57)	0.34% (-0.26-0.89%)	0.33% (-0.37-0.95%)	0.1 (-0.12-0.31)	3.5 (-2.7-9.4)
Tonga	1990	Male	65 to 69	44% (26-62%)	56% (38-74%)	0.27 (0.13-0.49)	0.23% (-0.26-0.69%)	0.23% (-0.36-0.76%)	0.076 (-0.12-0.25)	2 (-2.2-6.1)
Tonga	1990	Male	70 to 74	41% (24-58%)	59% (42-76%)	0.24 (0.1-0.44)	0.084% (-0.35-0.48%)	0.067% (-0.43-0.53%)	0.023 (-0.15-0.18)	0.6 (-2.6-3.6)
Tonga	1990	Male	75 to 79	37% (22-56%)	63% (44-78%)	0.21 (0.089-0.39)	-0.025% (-0.45-0.29%)	-0.068% (-0.55-0.28%)	-0.021 (-0.17-0.089)	-0.13 (-2.2-1.5)
Tonga	1990	Male	80 to 84	34% (19-53%)	66% (47-81%)	0.18 (0.082-0.33)	-0.001% (-0.36-0.26%)	-0.038% (-0.45-0.26%)	-0.0093 (-0.11-0.065)	-0.004 (-1.1-0.77)
Tonga	1990	Male	85 to 89	33% (19-49%)	67% (51-81%)	0.17 (0.081-0.31)	-0.081% (-0.45-0.17%)	-0.13% (-0.56-0.16%)	-0.02 (-0.082-0.024)	-0.1 (-0.57-0.21)
Tonga	1990	Male	90 to 94	34% (21-52%)	66% (48-79%)	0.18 (0.077-0.33)	-0.076% (-0.44-0.18%)	-0.13% (-0.57-0.17%)	-0.0077 (-0.032-0.0094)	-0.026 (-0.15-0.061)
Tonga	1990	Male	95 plus	38% (20-60%)	62% (40-80%)	0.2 (0.077-0.41)	-0.24% (-0.72-0.071%)	-0.38% (-0.96-0.00049%)	-0.0049 (-0.012-0.0000063)	-0.012 (-0.036-0.0035)
Tonga	1995	Female	All Ages	18% (12-24%)	82% (76-88%)	0.17 (0.12-0.22)	-0.029% (-0.33-0.22%)	-0.4% (-0.96-0.042%)	-1.2 (-3-0.13)	-4.1 (-45-32)
Tonga	1995	Female	15 to 19	18% (8.3-33%)	82% (67-92%)	0.2 (0.086-0.38)	0.55% (0.3-0.89%)	0.56% (0.13-1.3%)	0.02 (0.0047-0.046)	3.5 (1.9-5.8)
Tonga	1995	Female	20 to 24	23% (9.7-40%)	77% (60-90%)	0.32 (0.14-0.58)	0.96% (0.58-1.4%)	0.74% (0.26-1.5%)	0.031 (0.011-0.067)	6.2 (3.6-9.5)
Tonga	1995	Female	25 to 29	24% (9.9-42%)	76% (58-90%)	0.33 (0.13-0.59)	0.94% (0.6-1.4%)	0.75% (0.31-1.4%)	0.042 (0.017-0.08)	6.7 (4.1-10)
Tonga	1995	Female	30 to 34	25% (8.9-44%)	75% (56-91%)	0.34 (0.14-0.61)	0.8% (0.45-1.2%)	0.74% (0.33-1.4%)	0.039 (0.017-0.073)	4.6 (2.5-7.1)

Location	Year	Sex	Age	Prevalence of current drinkers	Prevalence of abstainers	Population average of standard drinks daily	Percent of total attributable deaths	Percent of total attributable DALYs	Attributable deaths	Attributable DALYs
Tonga	1995	Female	35 to 39	25%<br>(11-43%)	75%<br>(57-89%)	0.35<br>(0.15-0.63)	0.7%<br>(0.34-1.2%)	0.82%<br>(0.35-1.5%)	0.059<br>(0.025-0.12)	4.5<br>(2.3-7.5)
Tonga	1995	Female	40 to 44	24%<br>(11-41%)	76%<br>(59-89%)	0.32<br>(0.13-0.6)	0.54%<br>(0.082-1%)	0.76%<br>(0.14-1.6%)	0.072<br>(0.012-0.15)	4<br>(0.57-7.7)
Tonga	1995	Female	45 to 49	24%<br>(10-41%)	76%<br>(59-90%)	0.28<br>(0.1-0.55)	0.16%<br>(-0.46-0.7%)	0.35%<br>(-0.41-1%)	0.037<br>(-0.044-0.11)	1.1<br>(-3.1-4.9)
Tonga	1995	Female	50 to 54	24%<br>(11-42%)	76%<br>(58-89%)	0.25<br>(0.091-0.48)	-0.21%<br>(-1.1-0.37%)	-0.078%<br>(-1.1-0.61%)	-0.011<br>(-0.16-0.084)	-1.6<br>(-8.2-2.8)
Tonga	1995	Female	55 to 59	23%<br>(10-41%)	77%<br>(59-90%)	0.22<br>(0.083-0.46)	-0.41%<br>(-1.5-0.22%)	-0.3%<br>(-1.5-0.42%)	-0.06<br>(-0.3-0.083)	-3.6<br>(-13-1.8)
Tonga	1995	Female	60 to 64	23%<br>(11-42%)	77%<br>(58-89%)	0.21<br>(0.077-0.44)	-0.67%<br>(-1.9-0.079%)	-0.63%<br>(-2-0.18%)	-0.16<br>(-0.51-0.045)	-6.4<br>(-18-0.7)
Tonga	1995	Female	65 to 69	25%<br>(11-43%)	75%<br>(57-89%)	0.22<br>(0.081-0.43)	-0.86%<br>(-2.1--0.068%)	-0.88%<br>(-2.2--0.021%)	-0.29<br>(-0.73--0.0073)	-8.3<br>(-20--0.64)
Tonga	1995	Female	70 to 74	25%<br>(11-42%)	75%<br>(58-89%)	0.21<br>(0.073-0.42)	-0.9%<br>(-2--0.14%)	-0.97%<br>(-2.2--0.13%)	-0.33<br>(-0.76--0.04)	-7.2<br>(-16--1.1)
Tonga	1995	Female	75 to 79	23%<br>(10-42%)	77%<br>(58-90%)	0.19<br>(0.063-0.43)	-0.62%<br>(-1.6--0.028%)	-0.64%<br>(-1.7-0.011%)	-0.22<br>(-0.61-0.0039)	-3.8<br>(-9.7--0.17)
Tonga	1995	Female	80 to 84	22%<br>(9.8-42%)	78%<br>(58-90%)	0.18<br>(0.059-0.38)	-0.61%<br>(-1.5--0.091%)	-0.65%<br>(-1.7--0.072%)	-0.23<br>(-0.58--0.024)	-2.7<br>(-6.5--0.39)
Tonga	1995	Female	85 to 89	24%<br>(10-41%)	76%<br>(59-90%)	0.19<br>(0.066-0.4)	-0.65%<br>(-1.5--0.12%)	-0.71%<br>(-1.7--0.13%)	-0.15<br>(-0.36--0.027)	-1.2<br>(-2.8--0.22)
Tonga	1995	Female	90 to 94	27%<br>(12-46%)	73%<br>(54-88%)	0.21<br>(0.075-0.46)	-0.67%<br>(-1.4--0.14%)	-0.74%<br>(-1.6--0.15%)	-0.065<br>(-0.14--0.013)	-0.35<br>(-0.75--0.075)
Tonga	1995	Female	95 plus	32%<br>(13-55%)	68%<br>(45-87%)	0.26<br>(0.082-0.55)	-0.82%<br>(-1.9--0.051%)	-0.96%<br>(-2.3--0.09%)	-0.025<br>(-0.06--0.0023)	-0.084<br>(-0.2--0.0051)
Tonga	1995	Male	All Ages	36%<br>(28-45%)	64%<br>(55-72%)	0.35<br>(0.27-0.45)	0.98%<br>(0.6-1.4%)	0.69%<br>(0.23-1.1%)	2.5<br>(0.86-4.3)	170<br>(100-240)
Tonga	1995	Male	15 to 19	34%<br>(20-52%)	66%<br>(48-80%)	0.24<br>(0.11-0.42)	1.2%<br>(0.7-2%)	1.4%<br>(0.59-2.6%)	0.11<br>(0.048-0.21)	11<br>(6.6-19)
Tonga	1995	Male	20 to 24	52%<br>(33-70%)	48%<br>(30-67%)	0.66<br>(0.39-1)	3.1%<br>(1.9-4.6%)	3.5%<br>(1.8-5.6%)	0.36<br>(0.19-0.6)	31<br>(18-47)
Tonga	1995	Male	25 to 29	60%<br>(40-76%)	40%<br>(24-60%)	0.92<br>(0.57-1.4)	3.7%<br>(2.4-5.2%)	4.1%<br>(2.4-6.3%)	0.46<br>(0.25-0.72)	36<br>(22-53)
Tonga	1995	Male	30 to 34	61%<br>(40-78%)	39%<br>(22-60%)	0.99<br>(0.57-1.5)	3%<br>(1.9-4.4%)	3.2%<br>(1.7-5.2%)	0.28<br>(0.14-0.47)	21<br>(13-32)
Tonga	1995	Male	35 to 39	59%<br>(41-77%)	41%<br>(23-59%)	0.87<br>(0.49-1.4)	2.5%<br>(1.5-3.7%)	2.7%<br>(1.3-4.3%)	0.26<br>(0.12-0.45)	18<br>(10-28)
Tonga	1995	Male	40 to 44	57%<br>(39-74%)	43%<br>(26-61%)	0.76<br>(0.39-1.3)	1.9%<br>(1-2.8%)	1.9%<br>(0.83-3.2%)	0.24<br>(0.1-0.42)	15<br>(8.2-24)
Tonga	1995	Male	45 to 49	53%<br>(32-71%)	47%<br>(29-68%)	0.64<br>(0.31-1.1)	1.7%<br>(0.78-2.6%)	1.8%<br>(0.66-2.9%)	0.22<br>(0.078-0.38)	12<br>(5.4-19)
Tonga	1995	Male	50 to 54	50%<br>(30-69%)	50%<br>(31-70%)	0.52<br>(0.25-0.92)	1.2%<br>(0.38-2.1%)	1.3%<br>(0.24-2.3%)	0.21<br>(0.038-0.39)	9.7<br>(2.9-17)

Location	Year	Sex	Age	Prevalence of current drinkers	Prevalence of abstainers	Population average of standard drinks daily	Percent of total attributable deaths	Percent of total attributable DALYs	Attributable deaths	Attributable DALYs
Tonga	1995	Male	55 to 59	48% (30-67%)	52% (33-70%)	0.42 (0.21-0.73)	0.71% (-0.066-1.4%)	0.71% (-0.23-1.6%)	0.17 (-0.059-0.37)	6.7 (-0.63-14)
Tonga	1995	Male	60 to 64	45% (27-66%)	55% (34-73%)	0.35 (0.17-0.65)	0.41% (-0.27-0.98%)	0.4% (-0.39-1.1%)	0.12 (-0.11-0.33)	4.1 (-2.6-11)
Tonga	1995	Male	65 to 69	44% (26-63%)	56% (37-74%)	0.32 (0.16-0.56)	0.28% (-0.26-0.78%)	0.29% (-0.32-0.87%)	0.12 (-0.13-0.36)	3 (-2.9-8.6)
Tonga	1995	Male	70 to 74	41% (25-60%)	59% (40-75%)	0.29 (0.14-0.52)	0.11% (-0.39-0.55%)	0.096% (-0.46-0.6%)	0.037 (-0.19-0.23)	0.9 (-3.4-4.4)
Tonga	1995	Male	75 to 79	37% (22-55%)	63% (45-78%)	0.24 (0.11-0.45)	-0.015% (-0.43-0.34%)	-0.056% (-0.54-0.33%)	-0.019 (-0.19-0.12)	-0.082 (-2.5-1.9)
Tonga	1995	Male	80 to 84	34% (19-52%)	66% (48-81%)	0.22 (0.1-0.39)	0.0045% (-0.37-0.3%)	-0.032% (-0.45-0.3%)	-0.0089 (-0.12-0.081)	0.014 (-1.2-0.96)
Tonga	1995	Male	85 to 89	33% (19-50%)	67% (50-81%)	0.2 (0.096-0.37)	-0.081% (-0.48-0.19%)	-0.13% (-0.6-0.18%)	-0.023 (-0.1-0.03)	-0.12 (-0.69-0.28)
Tonga	1995	Male	90 to 94	34% (21-52%)	66% (48-79%)	0.21 (0.093-0.39)	-0.081% (-0.47-0.2%)	-0.14% (-0.62-0.18%)	-0.0096 (-0.041-0.012)	-0.032 (-0.19-0.077)
Tonga	1995	Male	95 plus	38% (21-59%)	62% (41-79%)	0.24 (0.092-0.48)	-0.25% (-0.74-0.087%)	-0.4% (-0.99--0.0034%)	-0.0069 (-0.017--0.000061)	-0.017 (-0.051-0.006)
Tonga	2000	Female	All Ages	18% (12-24%)	82% (76-88%)	0.22 (0.16-0.29)	0.021% (-0.3-0.33%)	-0.36% (-0.97-0.15%)	-1.2 (-3.2-0.51)	3 (-46-46)
Tonga	2000	Female	15 to 19	18% (8.5-33%)	82% (67-91%)	0.27 (0.11-0.52)	0.62% (0.34-0.98%)	0.71% (0.19-1.6%)	0.027 (0.0073-0.059)	4.3 (2.4-6.9)
Tonga	2000	Female	20 to 24	22% (9.7-39%)	78% (61-90%)	0.42 (0.18-0.78)	1.1% (0.68-1.6%)	0.94% (0.34-1.9%)	0.041 (0.015-0.088)	7.2 (4.4-11)
Tonga	2000	Female	25 to 29	24% (9.5-42%)	76% (58-91%)	0.43 (0.18-0.76)	1.1% (0.69-1.5%)	0.96% (0.4-1.8%)	0.044 (0.018-0.084)	6.3 (3.9-9.5)
Tonga	2000	Female	30 to 34	25% (9.2-44%)	75% (56-91%)	0.45 (0.17-0.81)	0.93% (0.55-1.4%)	0.95% (0.45-1.8%)	0.058 (0.026-0.11)	6.3 (3.5-9.6)
Tonga	2000	Female	35 to 39	25% (10-43%)	75% (57-90%)	0.46 (0.19-0.83)	0.89% (0.44-1.5%)	1.1% (0.46-2.1%)	0.072 (0.028-0.14)	5.2 (2.6-9)
Tonga	2000	Female	40 to 44	24% (11-42%)	76% (58-89%)	0.43 (0.18-0.8)	0.73% (0.15-1.4%)	1.1% (0.31-2.2%)	0.097 (0.026-0.2)	5.2 (1.1-10)
Tonga	2000	Female	45 to 49	24% (10-41%)	76% (59-90%)	0.37 (0.14-0.7)	0.33% (-0.36-1%)	0.62% (-0.24-1.6%)	0.074 (-0.029-0.19)	2.6 (-2.9-7.9)
Tonga	2000	Female	50 to 54	24% (11-41%)	76% (59-89%)	0.32 (0.12-0.62)	-0.086% (-1-0.62%)	0.1% (-0.93-1%)	0.013 (-0.13-0.13)	-0.65 (-7.9-4.7)
Tonga	2000	Female	55 to 59	23% (10-42%)	77% (58-90%)	0.29 (0.11-0.59)	-0.33% (-1.4-0.37%)	-0.18% (-1.3-0.67%)	-0.033 (-0.25-0.11)	-2.7 (-12-3.1)
Tonga	2000	Female	60 to 64	23% (11-43%)	77% (57-89%)	0.28 (0.099-0.6)	-0.63% (-1.9-0.16%)	-0.57% (-2-0.32%)	-0.14 (-0.51-0.081)	-5.7 (-18-1.4)
Tonga	2000	Female	65 to 69	25% (11-43%)	75% (57-89%)	0.28 (0.1-0.56)	-0.83% (-2.1-0.019%)	-0.84% (-2.3-0.12%)	-0.27 (-0.76-0.039)	-7.9 (-21-0.17)

Location	Year	Sex	Age	Prevalence of current drinkers	Prevalence of abstainers	Population average of standard drinks daily	Percent of total attributable deaths	Percent of total attributable DALYs	Attributable deaths	Attributable DALYs
Tonga	2000	Female	70 to 74	24% (11-42%)	76% (58-89%)	0.27 (0.098-0.53)	-0.88% (-2--0.11%)	-0.95% (-2.2--0.071%)	-0.39 (-0.94--0.034)	-8.4 (-20--1)
Tonga	2000	Female	75 to 79	23% (10-42%)	77% (58-90%)	0.25 (0.079-0.52)	-0.59% (-1.5-0.028%)	-0.6% (-1.7-0.078%)	-0.24 (-0.68-0.031)	-4.2 (-11-0.21)
Tonga	2000	Female	80 to 84	22% (8.6-42%)	78% (58-91%)	0.23 (0.072-0.48)	-0.58% (-1.5--0.014%)	-0.62% (-1.6-0.013%)	-0.25 (-0.64-0.0058)	-3 (-7.6--0.073)
Tonga	2000	Female	85 to 89	23% (10-41%)	77% (59-90%)	0.25 (0.087-0.53)	-0.63% (-1.6--0.046%)	-0.69% (-1.7--0.039%)	-0.18 (-0.46--0.01)	-1.5 (-3.6--0.11)
Tonga	2000	Female	90 to 94	27% (11-46%)	73% (54-89%)	0.29 (0.094-0.62)	-0.63% (-1.5--0.049%)	-0.7% (-1.7--0.05%)	-0.074 (-0.18--0.0054)	-0.4 (-0.94--0.029)
Tonga	2000	Female	95 plus	32% (13-55%)	68% (45-87%)	0.34 (0.11-0.74)	-0.78% (-2-0.061%)	-0.92% (-2.3-0.049%)	-0.027 (-0.068-0.0013)	-0.089 (-0.23-0.0069)
Tonga	2000	Male	All Ages	36% (28-45%)	64% (55-72%)	0.48 (0.38-0.6)	1.2% (0.79-1.7%)	0.86% (0.39-1.5%)	3.2 (1.4-5.3)	200 (130-290)
Tonga	2000	Male	15 to 19	34% (19-52%)	66% (48-81%)	0.33 (0.16-0.56)	1.4% (0.79-2.3%)	1.7% (0.74-3%)	0.15 (0.068-0.29)	15 (8.5-25)
Tonga	2000	Male	20 to 24	52% (33-70%)	48% (30-67%)	0.88 (0.53-1.3)	3.6% (2.2-5.4%)	4.1% (2.2-6.6%)	0.45 (0.24-0.74)	38 (23-57)
Tonga	2000	Male	25 to 29	59% (39-75%)	41% (25-61%)	1.2 (0.76-1.8)	4.2% (2.7-5.9%)	4.8% (2.8-7.2%)	0.45 (0.24-0.71)	35 (22-52)
Tonga	2000	Male	30 to 34	61% (39-77%)	39% (23-61%)	1.3 (0.75-2)	3.3% (2.1-4.9%)	3.6% (2-5.7%)	0.39 (0.2-0.65)	29 (17-46)
Tonga	2000	Male	35 to 39	59% (41-76%)	41% (24-59%)	1.1 (0.64-1.8)	2.9% (1.8-4.3%)	3.1% (1.7-5%)	0.29 (0.14-0.48)	19 (11-30)
Tonga	2000	Male	40 to 44	57% (39-74%)	43% (26-61%)	1 (0.52-1.7)	2.2% (1.2-3.5%)	2.4% (1.1-4%)	0.28 (0.12-0.49)	17 (8.5-28)
Tonga	2000	Male	45 to 49	53% (32-71%)	47% (29-68%)	0.86 (0.43-1.6)	2% (0.99-3.2%)	2.2% (0.88-3.6%)	0.34 (0.14-0.6)	18 (8.6-30)
Tonga	2000	Male	50 to 54	50% (31-68%)	50% (32-69%)	0.71 (0.34-1.2)	1.5% (0.5-2.7%)	1.6% (0.33-3%)	0.25 (0.049-0.48)	11 (3.6-20)
Tonga	2000	Male	55 to 59	48% (30-66%)	52% (34-70%)	0.57 (0.28-0.99)	0.91% (0.11-1.8%)	0.93% (-0.072-2%)	0.2 (-0.015-0.45)	7.9 (0.9-16)
Tonga	2000	Male	60 to 64	45% (26-66%)	55% (34-74%)	0.47 (0.22-0.87)	0.56% (-0.2-1.3%)	0.57% (-0.31-1.5%)	0.18 (-0.092-0.47)	5.7 (-1.8-14)
Tonga	2000	Male	65 to 69	43% (25-62%)	57% (38-75%)	0.43 (0.22-0.74)	0.42% (-0.16-1.1%)	0.45% (-0.23-1.2%)	0.17 (-0.083-0.45)	4.2 (-1.7-11)
Tonga	2000	Male	70 to 74	41% (24-59%)	59% (41-76%)	0.38 (0.19-0.69)	0.21% (-0.35-0.81%)	0.21% (-0.39-0.87%)	0.096 (-0.18-0.39)	2 (-3.1-7.6)
Tonga	2000	Male	75 to 79	37% (21-56%)	63% (44-79%)	0.33 (0.15-0.61)	0.056% (-0.42-0.53%)	0.02% (-0.5-0.56%)	0.0077 (-0.19-0.2)	0.34 (-2.5-3.2)
Tonga	2000	Male	80 to 84	35% (19-53%)	65% (47-81%)	0.29 (0.13-0.51)	0.058% (-0.36-0.44%)	0.024% (-0.44-0.44%)	0.0071 (-0.13-0.13)	0.21 (-1.3-1.6)
Tonga	2000	Male	85 to 89	33% (19-50%)	67% (50-81%)	0.27 (0.13-0.51)	-0.04% (-0.46-0.3%)	-0.094% (-0.58-0.29%)	-0.018 (-0.11-0.056)	-0.065 (-0.75-0.49)

Location	Year	Sex	Age	Prevalence of current drinkers	Prevalence of abstainers	Population average of standard drinks daily	Percent of total attributable deaths	Percent of total attributable DALYs	Attributable deaths	Attributable DALYs
Tonga	2000	Male	90 to 94	35%<br>(21-52%)	65%<br>(48-79%)	0.28<br>(0.12-0.52)	-0.054%<br>(-0.45-0.25%)	-0.12%<br>(-0.6-0.23%)	-0.0092<br>(-0.045-0.017)	-0.025<br>(-0.21-0.11)
Tonga	2000	Male	95 plus	38%<br>(21-59%)	62%<br>(41-79%)	0.32<br>(0.13-0.61)	-0.23%<br>(-0.74-0.16%)	-0.39%<br>(-1-0.076%)	-0.0076<br>(-0.02-0.0015)	-0.018<br>(-0.057-0.013)
Tonga	2005	Female	All Ages	18%<br>(13-24%)	82%<br>(76-87%)	0.26<br>(0.19-0.34)	0.057%<br>(-0.31-0.39%)	-0.34%<br>(-1-0.22%)	-1.2<br>(-3.3-0.76)	8.2<br>(-44-57)
Tonga	2005	Female	15 to 19	18%<br>(8.3-33%)	82%<br>(67-92%)	0.3<br>(0.12-0.59)	0.66%<br>(0.36-1.1%)	0.82%<br>(0.24-1.9%)	0.025<br>(0.0072-0.056)	3.9<br>(2-6.7)
Tonga	2005	Female	20 to 24	22%<br>(9.1-41%)	78%<br>(59-91%)	0.47<br>(0.19-0.9)	1.2%<br>(0.7-1.7%)	1.1%<br>(0.39-2.2%)	0.045<br>(0.017-0.093)	8<br>(4.7-12)
Tonga	2005	Female	25 to 29	24%<br>(9.6-43%)	76%<br>(57-90%)	0.48<br>(0.2-0.88)	1.1%<br>(0.72-1.7%)	1.1%<br>(0.44-2.2%)	0.048<br>(0.019-0.095)	6.9<br>(4.2-11)
Tonga	2005	Female	30 to 34	25%<br>(9.1-44%)	75%<br>(56-91%)	0.5<br>(0.2-0.91)	1%<br>(0.59-1.5%)	1.1%<br>(0.49-2%)	0.062<br>(0.026-0.12)	6.7<br>(3.7-10)
Tonga	2005	Female	35 to 39	25%<br>(10-43%)	75%<br>(57-90%)	0.52<br>(0.21-0.91)	0.98%<br>(0.51-1.6%)	1.3%<br>(0.59-2.4%)	0.1<br>(0.045-0.2)	7.6<br>(3.9-13)
Tonga	2005	Female	40 to 44	24%<br>(12-41%)	76%<br>(59-88%)	0.48<br>(0.2-0.88)	0.82%<br>(0.21-1.6%)	1.2%<br>(0.41-2.4%)	0.11<br>(0.034-0.21)	5.7<br>(1.5-11)
Tonga	2005	Female	45 to 49	24%<br>(10-41%)	76%<br>(59-90%)	0.42<br>(0.15-0.79)	0.38%<br>(-0.42-1.1%)	0.75%<br>(-0.2-1.9%)	0.09<br>(-0.023-0.22)	3.1<br>(-3.5-9.5)
Tonga	2005	Female	50 to 54	24%<br>(11-41%)	76%<br>(59-89%)	0.36<br>(0.14-0.72)	-0.078%<br>(-1.1-0.78%)	0.17%<br>(-1.1-1.2%)	0.022<br>(-0.14-0.16)	-0.6<br>(-8.8-5.7)
Tonga	2005	Female	55 to 59	23%<br>(10-42%)	77%<br>(58-90%)	0.33<br>(0.12-0.68)	-0.31%<br>(-1.4-0.49%)	-0.12%<br>(-1.3-0.91%)	-0.019<br>(-0.21-0.13)	-2.3<br>(-10-3.4)
Tonga	2005	Female	60 to 64	23%<br>(10-41%)	77%<br>(59-90%)	0.32<br>(0.11-0.67)	-0.61%<br>(-2-0.24%)	-0.53%<br>(-2-0.47%)	-0.11<br>(-0.41-0.099)	-4.8<br>(-15-2)
Tonga	2005	Female	65 to 69	25%<br>(11-43%)	75%<br>(57-89%)	0.32<br>(0.12-0.64)	-0.82%<br>(-2.2-0.085%)	-0.82%<br>(-2.4-0.24%)	-0.23<br>(-0.66-0.072)	-7.1<br>(-18-0.84)
Tonga	2005	Female	70 to 74	25%<br>(11-41%)	75%<br>(59-89%)	0.31<br>(0.11-0.61)	-0.88%<br>(-2.1--0.071%)	-0.94%<br>(-2.3--0.011%)	-0.37<br>(-0.9--0.0048)	-8.2<br>(-19--0.65)
Tonga	2005	Female	75 to 79	23%<br>(10-42%)	77%<br>(58-90%)	0.29<br>(0.095-0.62)	-0.59%<br>(-1.6-0.07%)	-0.6%<br>(-1.8-0.14%)	-0.29<br>(-0.85-0.068)	-5.1<br>(-14-0.67)
Tonga	2005	Female	80 to 84	23%<br>(9.7-41%)	77%<br>(59-90%)	0.27<br>(0.094-0.56)	-0.58%<br>(-1.5--0.0038%)	-0.62%<br>(-1.7-0.052%)	-0.3<br>(-0.83-0.023)	-3.6<br>(-9.7--0.024)
Tonga	2005	Female	85 to 89	23%<br>(11-41%)	77%<br>(59-89%)	0.28<br>(0.11-0.59)	-0.62%<br>(-1.6--0.033%)	-0.69%<br>(-1.8--0.028%)	-0.22<br>(-0.57--0.0091)	-1.8<br>(-4.5--0.097)
Tonga	2005	Female	90 to 94	27%<br>(12-46%)	73%<br>(54-88%)	0.32<br>(0.11-0.7)	-0.63%<br>(-1.5--0.015%)	-0.7%<br>(-1.7--0.012%)	-0.096<br>(-0.23--0.0015)	-0.52<br>(-1.2--0.013)
Tonga	2005	Female	95 plus	32%<br>(13-54%)	68%<br>(46-87%)	0.38<br>(0.13-0.84)	-0.79%<br>(-2-0.12%)	-0.95%<br>(-2.4-0.1%)	-0.034<br>(-0.088-0.0039)	-0.11<br>(-0.3-0.017)
Tonga	2005	Male	All Ages	37%<br>(29-46%)	63%<br>(54-71%)	0.56<br>(0.44-0.71)	1.3%<br>(0.87-1.9%)	0.99%<br>(0.46-1.6%)	3.9<br>(1.7-6.7)	230<br>(150-340)
Tonga	2005	Male	15 to 19	34%<br>(19-52%)	66%<br>(48-81%)	0.36<br>(0.16-0.63)	1.5%<br>(0.85-2.5%)	1.8%<br>(0.82-3.3%)	0.14<br>(0.063-0.26)	14<br>(7.4-22)

Location	Year	Sex	Age	Prevalence of current drinkers	Prevalence of abstainers	Population average of standard drinks daily	Percent of total attributable deaths	Percent of total attributable DALYs	Attributable deaths	Attributable DALYs
Tonga	2005	Male	20 to 24	52%<br>(33-71%)	48%<br>(29-67%)	0.99<br>(0.56-1.5)	3.8%<br>(2.3-5.7%)	4.4%<br>(2.4-7%)	0.5<br>(0.26-0.83)	41<br>(25-63)
Tonga	2005	Male	25 to 29	60%<br>(40-76%)	40%<br>(24-60%)	1.4<br>(0.86-2.1)	4.6%<br>(2.9-6.5%)	5.2%<br>(3-7.9%)	0.48<br>(0.25-0.76)	37<br>(22-55)
Tonga	2005	Male	30 to 34	61%<br>(40-78%)	39%<br>(22-60%)	1.5<br>(0.87-2.2)	3.7%<br>(2.3-5.7%)	4%<br>(2.3-6.6%)	0.41<br>(0.21-0.69)	31<br>(19-49)
Tonga	2005	Male	35 to 39	59%<br>(41-76%)	41%<br>(24-59%)	1.3<br>(0.73-2)	3.1%<br>(1.9-4.8%)	3.5%<br>(1.8-5.6%)	0.45<br>(0.22-0.75)	30<br>(17-47)
Tonga	2005	Male	40 to 44	57%<br>(39-74%)	43%<br>(26-61%)	1.1<br>(0.6-1.9)	2.4%<br>(1.3-3.9%)	2.6%<br>(1.2-4.4%)	0.34<br>(0.14-0.6)	20<br>(10-34)
Tonga	2005	Male	45 to 49	53%<br>(32-71%)	47%<br>(29-68%)	0.97<br>(0.47-1.7)	2.2%<br>(1.1-3.6%)	2.4%<br>(1-4.1%)	0.42<br>(0.17-0.74)	22<br>(10-36)
Tonga	2005	Male	50 to 54	50%<br>(31-69%)	50%<br>(31-69%)	0.78<br>(0.38-1.4)	1.7%<br>(0.6-3.1%)	1.9%<br>(0.51-3.5%)	0.33<br>(0.088-0.66)	14<br>(5-28)
Tonga	2005	Male	55 to 59	48%<br>(29-67%)	52%<br>(33-71%)	0.63<br>(0.31-1.1)	1.1%<br>(0.19-2.2%)	1.1%<br>(0.083-2.4%)	0.21<br>(0.015-0.47)	8.2<br>(1.5-17)
Tonga	2005	Male	60 to 64	45%<br>(27-66%)	55%<br>(34-73%)	0.53<br>(0.25-1)	0.68%<br>(-0.13-1.7%)	0.72%<br>(-0.18-1.9%)	0.2<br>(-0.05-0.54)	6.4<br>(-1.1-16)
Tonga	2005	Male	65 to 69	44%<br>(25-62%)	56%<br>(38-75%)	0.49<br>(0.24-0.85)	0.52%<br>(-0.11-1.3%)	0.57%<br>(-0.16-1.5%)	0.22<br>(-0.062-0.58)	5.4<br>(-1.2-14)
Tonga	2005	Male	70 to 74	41%<br>(24-58%)	59%<br>(42-76%)	0.43<br>(0.21-0.77)	0.29%<br>(-0.32-1%)	0.3%<br>(-0.39-1.1%)	0.13<br>(-0.17-0.48)	2.7<br>(-2.8-9.4)
Tonga	2005	Male	75 to 79	37%<br>(21-56%)	63%<br>(44-79%)	0.36<br>(0.16-0.68)	0.11%<br>(-0.37-0.71%)	0.084%<br>(-0.47-0.72%)	0.039<br>(-0.21-0.33)	0.86<br>(-2.8-5.2)
Tonga	2005	Male	80 to 84	34%<br>(19-53%)	66%<br>(47-81%)	0.32<br>(0.15-0.58)	0.1%<br>(-0.33-0.53%)	0.069%<br>(-0.43-0.56%)	0.024<br>(-0.15-0.2)	0.42<br>(-1.4-2.2)
Tonga	2005	Male	85 to 89	33%<br>(19-49%)	67%<br>(51-81%)	0.3<br>(0.14-0.55)	-0.0051%<br>(-0.43-0.4%)	-0.056%<br>(-0.56-0.39%)	-0.012<br>(-0.11-0.08)	-0.0085<br>(-0.78-0.7)
Tonga	2005	Male	90 to 94	34%<br>(20-52%)	66%<br>(48-80%)	0.31<br>(0.14-0.56)	-0.029%<br>(-0.41-0.33%)	-0.096%<br>(-0.55-0.3%)	-0.0083<br>(-0.048-0.027)	-0.015<br>(-0.21-0.17)
Tonga	2005	Male	95 plus	37%<br>(20-58%)	63%<br>(42-80%)	0.34<br>(0.13-0.66)	-0.2%<br>(-0.71-0.22%)	-0.36%<br>(-0.97-0.15%)	-0.0083<br>(-0.022-0.0033)	-0.019<br>(-0.065-0.019)
Tonga	2010	Female	All Ages	18%<br>(13-24%)	82%<br>(76-87%)	0.28<br>(0.19-0.39)	0.097%<br>(-0.29-0.48%)	-0.31%<br>(-0.95-0.31%)	-1.1<br>(-3.5-1.1)	14<br>(-40-67)
Tonga	2010	Female	15 to 19	18%<br>(8.3-32%)	82%<br>(68-92%)	0.32<br>(0.13-0.64)	0.68%<br>(0.35-1.1%)	0.89%<br>(0.26-2%)	0.029<br>(0.0082-0.067)	4.5<br>(2.3-7.6)
Tonga	2010	Female	20 to 24	22%<br>(8.9-40%)	78%<br>(60-91%)	0.49<br>(0.2-0.97)	1.2%<br>(0.72-1.9%)	1.2%<br>(0.41-2.5%)	0.041<br>(0.014-0.089)	7.2<br>(4.2-12)
Tonga	2010	Female	25 to 29	23%<br>(10-42%)	77%<br>(58-90%)	0.51<br>(0.22-0.95)	1.2%<br>(0.74-1.8%)	1.2%<br>(0.47-2.3%)	0.054<br>(0.021-0.11)	7.8<br>(4.6-12)
Tonga	2010	Female	30 to 34	25%<br>(9.6-44%)	75%<br>(56-90%)	0.54<br>(0.22-0.99)	1.1%<br>(0.61-1.7%)	1.2%<br>(0.53-2.2%)	0.07<br>(0.029-0.13)	7.5<br>(4.1-12)
Tonga	2010	Female	35 to 39	25%<br>(9.6-43%)	75%<br>(57-90%)	0.56<br>(0.23-1)	1.1%<br>(0.54-1.7%)	1.5%<br>(0.66-2.6%)	0.11<br>(0.045-0.21)	8.3<br>(4.3-14)

Location	Year	Sex	Age	Prevalence of current drinkers	Prevalence of abstainers	Population average of standard drinks daily	Percent of total attributable deaths	Percent of total attributable DALYs	Attributable deaths	Attributable DALYs
Tonga	2010	Female	40 to 44	25% (12-42%)	75% (58-88%)	0.52 (0.22-0.99)	0.89% (0.23-1.7%)	1.4% (0.42-2.7%)	0.13 (0.037-0.26)	6.9 (1.7-14)
Tonga	2010	Female	45 to 49	24% (10-42%)	76% (58-90%)	0.45 (0.17-0.87)	0.44% (-0.42-1.3%)	0.83% (-0.22-2.2%)	0.091 (-0.026-0.24)	3.4 (-3.3-11)
Tonga	2010	Female	50 to 54	24% (10-42%)	76% (58-90%)	0.39 (0.14-0.77)	-0.032% (-1.3-0.96%)	0.21% (-1.2-1.5%)	0.032 (-0.19-0.23)	-0.3 (-12-8.5)
Tonga	2010	Female	55 to 59	23% (11-42%)	77% (58-89%)	0.35 (0.13-0.75)	-0.29% (-1.5-0.6%)	-0.11% (-1.4-1.1%)	-0.017 (-0.24-0.16)	-2.2 (-11-4.3)
Tonga	2010	Female	60 to 64	23% (10-42%)	77% (58-90%)	0.34 (0.12-0.72)	-0.61% (-2-0.31%)	-0.53% (-2.2-0.6%)	-0.11 (-0.45-0.11)	-4.6 (-16-2.3)
Tonga	2010	Female	65 to 69	24% (11-42%)	76% (58-89%)	0.34 (0.12-0.69)	-0.81% (-2.2-0.15%)	-0.82% (-2.4-0.31%)	-0.2 (-0.6-0.072)	-6.1 (-17-1)
Tonga	2010	Female	70 to 74	24% (11-40%)	76% (60-89%)	0.32 (0.12-0.65)	-0.83% (-2-0.0067%)	-0.9% (-2.3-0.079%)	-0.31 (-0.78-0.023)	-6.9 (-17-0.061)
Tonga	2010	Female	75 to 79	23% (10-40%)	77% (60-90%)	0.29 (0.095-0.64)	-0.54% (-1.6-0.14%)	-0.55% (-1.7-0.24%)	-0.26 (-0.81-0.11)	-4.6 (-13-1.3)
Tonga	2010	Female	80 to 84	22% (9.2-41%)	78% (59-91%)	0.28 (0.094-0.6)	-0.54% (-1.4-0.057%)	-0.58% (-1.6-0.089%)	-0.33 (-0.92-0.052)	-4 (-11-0.43)
Tonga	2010	Female	85 to 89	23% (10-41%)	77% (59-90%)	0.3 (0.11-0.64)	-0.6% (-1.6-0.01%)	-0.66% (-1.8-0.029%)	-0.28 (-0.77-0.012)	-2.2 (-6.1-0.04)
Tonga	2010	Female	90 to 94	27% (12-46%)	73% (54-88%)	0.35 (0.11-0.75)	-0.62% (-1.5-0.093%)	-0.7% (-1.7-0.1%)	-0.13 (-0.3-0.017)	-0.67 (-1.6-0.11)
Tonga	2010	Female	95 plus	31% (14-55%)	69% (45-86%)	0.41 (0.13-0.94)	-0.78% (-2.1-0.19%)	-0.94% (-2.5-0.16%)	-0.049 (-0.13-0.0082)	-0.16 (-0.44-0.039)
Tonga	2010	Male	All Ages	37% (29-46%)	63% (54-71%)	0.59 (0.44-0.8)	1.4% (0.89-2.1%)	1.1% (0.48-1.9%)	4.3 (1.8-7.8)	250 (150-380)
Tonga	2010	Male	15 to 19	34% (20-52%)	66% (48-80%)	0.38 (0.17-0.69)	1.5% (0.86-2.6%)	1.9% (0.84-3.5%)	0.16 (0.071-0.29)	15 (8.2-24)
Tonga	2010	Male	20 to 24	52% (33-69%)	48% (31-67%)	1 (0.56-1.7)	4% (2.3-6.2%)	4.7% (2.4-7.8%)	0.45 (0.22-0.78)	38 (21-59)
Tonga	2010	Male	25 to 29	59% (40-76%)	41% (24-60%)	1.5 (0.86-2.3)	4.7% (2.9-6.9%)	5.5% (3-8.5%)	0.55 (0.29-0.89)	42 (25-65)
Tonga	2010	Male	30 to 34	60% (39-77%)	40% (23-61%)	1.6 (0.88-2.4)	3.8% (2.4-5.9%)	4.3% (2.3-6.9%)	0.44 (0.22-0.76)	33 (19-53)
Tonga	2010	Male	35 to 39	59% (40-77%)	41% (23-60%)	1.4 (0.77-2.2)	3.3% (1.9-5.1%)	3.7% (2-6.2%)	0.47 (0.23-0.85)	32 (17-52)
Tonga	2010	Male	40 to 44	57% (39-74%)	43% (26-61%)	1.2 (0.6-2)	2.6% (1.3-4.3%)	2.8% (1.3-5%)	0.39 (0.16-0.72)	24 (11-41)
Tonga	2010	Male	45 to 49	53% (32-72%)	47% (28-68%)	1 (0.49-1.8)	2.3% (1.1-4%)	2.5% (1.1-4.5%)	0.41 (0.17-0.75)	21 (9.7-37)
Tonga	2010	Male	50 to 54	50% (31-68%)	50% (32-69%)	0.84 (0.4-1.5)	1.8% (0.68-3.4%)	2% (0.67-3.8%)	0.46 (0.15-0.95)	20 (7.3-41)
Tonga	2010	Male	55 to 59	48% (30-67%)	52% (33-70%)	0.68 (0.32-1.2)	1.2% (0.24-2.5%)	1.3% (0.16-2.8%)	0.27 (0.034-0.63)	10 (2.2-23)

Location	Year	Sex	Age	Prevalence of current drinkers	Prevalence of abstainers	Population average of standard drinks daily	Percent of total attributable deaths	Percent of total attributable DALYs	Attributable deaths	Attributable DALYs
Tonga	2010	Male	60 to 64	45% (26-65%)	55% (35-74%)	0.57 (0.25-1.1)	0.78% (-0.051-2%)	0.83% (-0.12-2.2%)	0.21 (-0.032-0.6)	6.8 (-0.39-18)
Tonga	2010	Male	65 to 69	43% (25-63%)	57% (37-75%)	0.51 (0.25-0.91)	0.6% (-0.087-1.6%)	0.65% (-0.12-1.8%)	0.23 (-0.043-0.63)	5.6 (-0.83-15)
Tonga	2010	Male	70 to 74	40% (25-58%)	60% (42-75%)	0.44 (0.22-0.84)	0.34% (-0.27-1.2%)	0.36% (-0.33-1.3%)	0.15 (-0.14-0.57)	3.2 (-2.4-11)
Tonga	2010	Male	75 to 79	37% (22-55%)	63% (45-78%)	0.38 (0.17-0.71)	0.16% (-0.34-0.81%)	0.13% (-0.43-0.84%)	0.056 (-0.19-0.37)	1.1 (-2.4-6.1)
Tonga	2010	Male	80 to 84	34% (19-52%)	66% (48-81%)	0.34 (0.15-0.61)	0.14% (-0.29-0.62%)	0.11% (-0.38-0.65%)	0.041 (-0.15-0.25)	0.64 (-1.4-2.9)
Tonga	2010	Male	85 to 89	33% (19-49%)	67% (51-81%)	0.32 (0.15-0.58)	0.028% (-0.39-0.46%)	-0.021% (-0.5-0.47%)	-0.0051 (-0.12-0.11)	0.06 (-0.84-0.99)
Tonga	2010	Male	90 to 94	34% (21-51%)	66% (49-79%)	0.33 (0.14-0.61)	0.00034% (-0.4-0.41%)	-0.063% (-0.54-0.4%)	-0.0061 (-0.052-0.039)	0.00021 (-0.23-0.24)
Tonga	2010	Male	95 plus	37% (19-58%)	63% (42-81%)	0.36 (0.13-0.73)	-0.17% (-0.7-0.32%)	-0.32% (-0.96-0.25%)	-0.0087 (-0.026-0.0068)	-0.019 (-0.076-0.035)
Tonga	2016	Female	All Ages	18% (13-24%)	82% (76-87%)	0.28 (0.16-0.44)	0.12% (-0.3-0.64%)	-0.27% (-0.96-0.55%)	-1 (-3.7-2)	18 (-42-93)
Tonga	2016	Female	15 to 19	18% (8.3-32%)	82% (68-92%)	0.32 (0.11-0.67)	0.71% (0.37-1.2%)	0.92% (0.25-2.1%)	0.03 (0.0078-0.07)	4.7 (2.4-8.3)
Tonga	2016	Female	20 to 24	22% (9.3-39%)	78% (61-91%)	0.5 (0.19-0.98)	1.2% (0.72-1.9%)	1.2% (0.39-2.7%)	0.047 (0.015-0.1)	8.3 (4.8-13)
Tonga	2016	Female	25 to 29	24% (10-42%)	76% (58-90%)	0.51 (0.2-0.99)	1.2% (0.74-1.9%)	1.2% (0.44-2.5%)	0.046 (0.015-0.099)	6.7 (3.9-11)
Tonga	2016	Female	30 to 34	25% (9-45%)	75% (55-91%)	0.53 (0.2-1.1)	1.1% (0.61-1.8%)	1.2% (0.52-2.5%)	0.063 (0.023-0.13)	6.8 (3.5-12)
Tonga	2016	Female	35 to 39	26% (9.7-44%)	74% (56-90%)	0.56 (0.2-1.1)	1.1% (0.5-2%)	1.5% (0.61-3%)	0.11 (0.039-0.22)	7.8 (3.5-15)
Tonga	2016	Female	40 to 44	25% (12-43%)	75% (57-88%)	0.52 (0.18-1.1)	0.94% (0.2-2%)	1.5% (0.42-3.1%)	0.15 (0.038-0.31)	8 (1.7-17)
Tonga	2016	Female	45 to 49	24% (11-42%)	76% (58-89%)	0.44 (0.15-0.91)	0.5% (-0.32-1.6%)	0.93% (-0.13-2.4%)	0.11 (-0.015-0.29)	4.3 (-2.9-13)
Tonga	2016	Female	50 to 54	24% (11-42%)	76% (58-89%)	0.38 (0.13-0.82)	0.052% (-1-1.2%)	0.34% (-0.99-1.8%)	0.049 (-0.15-0.28)	0.45 (-9.1-10)
Tonga	2016	Female	55 to 59	23% (10-42%)	77% (58-90%)	0.35 (0.12-0.77)	-0.2% (-1.4-0.76%)	0.018% (-1.3-1.3%)	0.0031 (-0.27-0.26)	-2 (-14-7.9)
Tonga	2016	Female	60 to 64	23% (10-41%)	77% (59-90%)	0.33 (0.11-0.74)	-0.48% (-1.8-0.46%)	-0.38% (-1.9-0.8%)	-0.084 (-0.43-0.16)	-4.2 (-16-4.2)
Tonga	2016	Female	65 to 69	24% (11-42%)	76% (58-89%)	0.34 (0.12-0.7)	-0.7% (-2-0.3%)	-0.69% (-2.3-0.45%)	-0.18 (-0.59-0.12)	-5.8 (-17-2.4)
Tonga	2016	Female	70 to 74	24% (11-42%)	76% (58-89%)	0.32 (0.11-0.72)	-0.76% (-1.9-0.18%)	-0.8% (-2.1-0.29%)	-0.27 (-0.68-0.099)	-6 (-15-1.4)
Tonga	2016	Female	75 to 79	23% (10-41%)	77% (59-90%)	0.3 (0.088-0.66)	-0.49% (-1.5-0.35%)	-0.48% (-1.6-0.46%)	-0.21 (-0.71-0.21)	-3.8 (-12-2.7)

Location	Year	Sex	Age	Prevalence of current drinkers	Prevalence of abstainers	Population average of standard drinks daily	Percent of total attributable deaths	Percent of total attributable DALYs	Attributable deaths	Attributable DALYs
Tonga	2016	Female	80 to 84	23% (9.5-41%)	77% (59-91%)	0.29 (0.088-0.62)	-0.51% (-1.5-0.24%)	-0.53% (-1.6-0.33%)	-0.31 (-0.96-0.19)	-3.8 (-11-1.7)
Tonga	2016	Female	85 to 89	24% (10-42%)	76% (58-90%)	0.3 (0.095-0.68)	-0.55% (-1.5-0.19%)	-0.61% (-1.7-0.25%)	-0.33 (-0.92-0.13)	-2.6 (-7.3-0.89)
Tonga	2016	Female	90 to 94	27% (12-46%)	73% (54-88%)	0.35 (0.11-0.78)	-0.56% (-1.4-0.2%)	-0.64% (-1.6-0.22%)	-0.18 (-0.46-0.066)	-0.96 (-2.4-0.34)
Tonga	2016	Female	95 plus	31% (13-54%)	69% (46-87%)	0.41 (0.12-0.98)	-0.72% (-2.1-0.27%)	-0.87% (-2.5-0.24%)	-0.069 (-0.2-0.019)	-0.22 (-0.65-0.089)
Tonga	2016	Male	All Ages	37% (29-46%)	63% (54-71%)	0.6 (0.37-0.9)	1.6% (0.86-2.5%)	1.2% (0.46-2.2%)	4.6 (1.8-9)	270 (150-440)
Tonga	2016	Male	15 to 19	34% (20-52%)	66% (48-80%)	0.39 (0.16-0.74)	1.5% (0.83-2.7%)	1.9% (0.8-3.7%)	0.17 (0.069-0.32)	16 (8.4-28)
Tonga	2016	Male	20 to 24	52% (34-69%)	48% (31-66%)	1.1 (0.51-1.8)	4% (2.3-6.4%)	4.7% (2.3-8.1%)	0.54 (0.25-0.97)	45 (25-74)
Tonga	2016	Male	25 to 29	59% (39-76%)	41% (24-61%)	1.5 (0.76-2.4)	4.7% (2.7-7.5%)	5.6% (2.8-9.6%)	0.51 (0.25-0.88)	39 (22-65)
Tonga	2016	Male	30 to 34	61% (40-78%)	39% (22-60%)	1.6 (0.78-2.7)	3.9% (2.2-6.5%)	4.4% (2.1-8.1%)	0.42 (0.19-0.81)	32 (17-55)
Tonga	2016	Male	35 to 39	59% (40-77%)	41% (23-60%)	1.4 (0.69-2.4)	3.4% (1.9-5.9%)	3.9% (1.9-7.1%)	0.43 (0.2-0.83)	29 (15-51)
Tonga	2016	Male	40 to 44	57% (38-75%)	43% (25-62%)	1.2 (0.54-2.1)	2.6% (1.3-4.8%)	2.9% (1.2-5.6%)	0.44 (0.18-0.93)	27 (12-51)
Tonga	2016	Male	45 to 49	52% (31-71%)	48% (29-69%)	1 (0.43-1.9)	2.4% (1-4.3%)	2.6% (1-4.8%)	0.48 (0.18-0.92)	25 (10-45)
Tonga	2016	Male	50 to 54	49% (30-69%)	51% (31-70%)	0.83 (0.36-1.6)	1.9% (0.71-3.8%)	2.1% (0.69-4.3%)	0.45 (0.14-0.96)	20 (7.2-41)
Tonga	2016	Male	55 to 59	47% (29-67%)	53% (33-71%)	0.68 (0.3-1.3)	1.2% (0.27-2.8%)	1.3% (0.16-3.2%)	0.41 (0.048-0.97)	16 (3.4-36)
Tonga	2016	Male	60 to 64	45% (26-66%)	55% (34-74%)	0.56 (0.22-1.1)	0.85% (-0.022-2.3%)	0.91% (-0.066-2.6%)	0.27 (-0.021-0.75)	8.5 (-0.21-23)
Tonga	2016	Male	65 to 69	43% (25-62%)	57% (38-75%)	0.51 (0.23-0.96)	0.67% (-0.05-1.8%)	0.73% (-0.091-2%)	0.23 (-0.026-0.62)	5.7 (-0.43-15)
Tonga	2016	Male	70 to 74	41% (24-58%)	59% (42-76%)	0.45 (0.2-0.9)	0.4% (-0.2-1.4%)	0.43% (-0.26-1.5%)	0.17 (-0.098-0.56)	3.4 (-1.6-11)
Tonga	2016	Male	75 to 79	37% (21-55%)	63% (45-79%)	0.38 (0.16-0.77)	0.2% (-0.33-1%)	0.18% (-0.42-1%)	0.079 (-0.19-0.45)	1.5 (-2.2-7.2)
Tonga	2016	Male	80 to 84	34% (19-52%)	66% (48-81%)	0.34 (0.15-0.65)	0.17% (-0.27-0.72%)	0.15% (-0.33-0.76%)	0.06 (-0.13-0.31)	0.85 (-1.3-3.6)
Tonga	2016	Male	85 to 89	32% (18-48%)	68% (52-82%)	0.32 (0.14-0.61)	0.061% (-0.39-0.56%)	0.016% (-0.5-0.58%)	0.0044 (-0.14-0.16)	0.15 (-0.91-1.4)
Tonga	2016	Male	90 to 94	34% (21-52%)	66% (48-79%)	0.33 (0.14-0.66)	0.026% (-0.39-0.52%)	-0.035% (-0.51-0.49%)	-0.0043 (-0.062-0.06)	0.019 (-0.28-0.38)
Tonga	2016	Male	95 plus	38% (19-59%)	62% (41-81%)	0.37 (0.13-0.82)	-0.15% (-0.68-0.48%)	-0.29% (-0.92-0.38%)	-0.009 (-0.028-0.012)	-0.018 (-0.085-0.058)

Location	Year	Sex	Age	Prevalence of current drinkers	Prevalence of abstainers	Population average of standard drinks daily	Percent of total attributable deaths	Percent of total attributable DALYs	Attributable deaths	Attributable DALYs
Vanuatu	1990	Female	All Ages	9.9% (7.8-12%)	90% (88-92%)	0.28 (0.22-0.35)	0.79% (0.48-1.2%)	0.88% (0.39-1.5%)	4.7 (2.1-8.1)	230 (140-340)
Vanuatu	1990	Female	15 to 19	13% (7.3-21%)	87% (79-93%)	0.39 (0.19-0.66)	1.1% (0.54-2.1%)	1.7% (0.6-3.4%)	0.15 (0.05-0.31)	14 (6.7-26)
Vanuatu	1990	Female	20 to 24	16% (9.3-26%)	84% (74-91%)	0.64 (0.33-1.1)	2.1% (1.2-3.4%)	2.7% (1.2-4.9%)	0.32 (0.14-0.6)	28 (16-48)
Vanuatu	1990	Female	25 to 29	16% (9.9-24%)	84% (76-90%)	0.63 (0.37-0.94)	2.1% (1.3-3.2%)	2.7% (1.4-4.2%)	0.42 (0.21-0.69)	33 (19-50)
Vanuatu	1990	Female	30 to 34	16% (9.4-24%)	84% (76-91%)	0.63 (0.36-1)	2% (1.2-3%)	2.4% (1.2-4.1%)	0.44 (0.21-0.75)	31 (18-49)
Vanuatu	1990	Female	35 to 39	14% (8.5-22%)	86% (78-91%)	0.6 (0.33-0.99)	2.1% (1.2-3.5%)	2.6% (1.3-4.5%)	0.56 (0.27-0.97)	33 (17-54)
Vanuatu	1990	Female	40 to 44	13% (7.9-20%)	87% (80-92%)	0.51 (0.25-0.89)	2% (0.97-3.4%)	2.5% (1.1-4.6%)	0.55 (0.24-1)	28 (13-49)
Vanuatu	1990	Female	45 to 49	13% (7.4-19%)	87% (81-93%)	0.42 (0.21-0.75)	1.4% (0.56-2.7%)	1.7% (0.65-3.4%)	0.43 (0.15-0.84)	19 (7.4-37)
Vanuatu	1990	Female	50 to 54	12% (7.7-19%)	88% (81-92%)	0.36 (0.18-0.66)	1.1% (0.3-2.5%)	1.4% (0.32-3.1%)	0.38 (0.089-0.85)	14 (3.8-32)
Vanuatu	1990	Female	55 to 59	11% (6.7-17%)	89% (83-93%)	0.31 (0.14-0.58)	0.94% (0.2-2.1%)	1.1% (0.26-2.5%)	0.32 (0.073-0.74)	10 (2.2-24)
Vanuatu	1990	Female	60 to 64	10% (6-16%)	90% (84-94%)	0.27 (0.13-0.51)	0.56% (-0.06-1.6%)	0.68% (-0.062-1.9%)	0.23 (-0.019-0.63)	6.3 (-0.67-17)
Vanuatu	1990	Female	65 to 69	12% (6.8-19%)	88% (81-93%)	0.28 (0.14-0.49)	0.56% (-0.1-1.5%)	0.68% (-0.12-1.7%)	0.28 (-0.05-0.73)	6.2 (-1-16)
Vanuatu	1990	Female	70 to 74	12% (7.5-20%)	88% (80-93%)	0.28 (0.13-0.51)	0.44% (-0.28-1.4%)	0.51% (-0.3-1.6%)	0.22 (-0.13-0.72)	3.9 (-2.5-13)
Vanuatu	1990	Female	75 to 79	12% (7-21%)	88% (79-93%)	0.26 (0.12-0.48)	0.43% (-0.22-1.3%)	0.49% (-0.23-1.5%)	0.23 (-0.11-0.74)	3.2 (-1.7-10)
Vanuatu	1990	Female	80 to 84	12% (6.6-21%)	88% (79-93%)	0.26 (0.11-0.46)	0.27% (-0.26-1%)	0.31% (-0.29-1.2%)	0.13 (-0.12-0.49)	1.4 (-1.3-5.2)
Vanuatu	1990	Female	85 to 89	13% (6.9-20%)	87% (80-93%)	0.26 (0.12-0.49)	0.19% (-0.26-0.9%)	0.21% (-0.32-0.99%)	0.04 (-0.059-0.19)	0.31 (-0.42-1.5)
Vanuatu	1990	Female	90 to 94	14% (7.9-22%)	86% (78-92%)	0.28 (0.12-0.52)	0.21% (-0.25-0.96%)	0.22% (-0.3-1.1%)	0.012 (-0.016-0.061)	0.067 (-0.082-0.32)
Vanuatu	1990	Female	95 plus	16% (7.7-27%)	84% (73-92%)	0.32 (0.12-0.65)	0.26% (-0.3-1.2%)	0.25% (-0.39-1.3%)	0.003 (-0.0048-0.016)	0.012 (-0.014-0.055)
Vanuatu	1990	Male	All Ages	29% (21-36%)	71% (64-79%)	0.69 (0.55-0.83)	2.1% (1.4-3%)	2.1% (1.2-3.1%)	16 (8.8-25)	800 (510-1100)
Vanuatu	1990	Male	15 to 19	28% (16-45%)	72% (55-84%)	0.4 (0.18-0.71)	1.5% (0.78-2.6%)	1.8% (0.72-3.4%)	0.27 (0.1-0.54)	25 (12-43)
Vanuatu	1990	Male	20 to 24	48% (29-67%)	52% (33-71%)	1.2 (0.72-1.9)	5.1% (2.9-8.1%)	6% (3.1-10%)	1.2 (0.6-2)	91 (51-150)
Vanuatu	1990	Male	25 to 29	58% (42-71%)	42% (29-58%)	2 (1.3-2.9)	7.5% (4.8-11%)	9% (5.3-13%)	2 (1.2-3.1)	140 (83-200)

Location	Year	Sex	Age	Prevalence of current drinkers	Prevalence of abstainers	Population average of standard drinks daily	Percent of total attributable deaths	Percent of total attributable DALYs	Attributable deaths	Attributable DALYs
Vanuatu	1990	Male	30 to 34	60% (43-74%)	40% (26-57%)	2.3 (1.4-3.3)	7.4% (4.4-11%)	8.8% (4.9-13%)	2.1 (1.1-3.3)	130 (74-190)
Vanuatu	1990	Male	35 to 39	57% (41-70%)	43% (30-59%)	1.8 (1.1-2.8)	5.6% (3.1-8.7%)	6.4% (3.3-10%)	1.7 (0.87-2.8)	98 (54-160)
Vanuatu	1990	Male	40 to 44	52% (37-65%)	48% (35-63%)	1.5 (0.78-2.4)	4.5% (2.2-7.5%)	5% (2.2-8.9%)	1.7 (0.71-3)	85 (40-150)
Vanuatu	1990	Male	45 to 49	46% (30-60%)	54% (40-70%)	1.2 (0.62-2.1)	3.6% (1.5-6.5%)	3.9% (1.4-7.5%)	1.5 (0.56-3.1)	69 (29-130)
Vanuatu	1990	Male	50 to 54	43% (28-57%)	57% (43-72%)	0.97 (0.5-1.7)	2.9% (1.1-5.8%)	3.1% (0.96-6.6%)	1.4 (0.43-2.9)	55 (19-110)
Vanuatu	1990	Male	55 to 59	44% (29-57%)	56% (43-71%)	0.84 (0.44-1.4)	2.1% (0.59-4.4%)	2.2% (0.49-4.9%)	0.97 (0.22-2.1)	33 (8.9-70)
Vanuatu	1990	Male	60 to 64	41% (26-56%)	59% (44-74%)	0.68 (0.35-1.2)	1.7% (0.38-3.7%)	1.8% (0.3-4.1%)	0.96 (0.17-2.3)	28 (6.5-63)
Vanuatu	1990	Male	65 to 69	38% (22-56%)	62% (44-78%)	0.57 (0.29-1)	1.4% (0.34-2.9%)	1.5% (0.32-3.2%)	0.94 (0.19-2)	22 (5.4-46)
Vanuatu	1990	Male	70 to 74	34% (20-51%)	66% (49-80%)	0.46 (0.23-0.81)	0.91% (0.047-2.1%)	0.98% (0.019-2.3%)	0.6 (0.011-1.4)	11 (0.55-27)
Vanuatu	1990	Male	75 to 79	30% (16-47%)	70% (53-84%)	0.38 (0.17-0.71)	0.64% (-0.034-1.7%)	0.68% (-0.08-1.8%)	0.46 (-0.058-1.2)	6.9 (-0.36-18)
Vanuatu	1990	Male	80 to 84	27% (14-43%)	73% (57-86%)	0.33 (0.16-0.6)	0.49% (0.012-1.2%)	0.51% (-0.025-1.3%)	0.3 (-0.015-0.76)	3.4 (0.078-8.4)
Vanuatu	1990	Male	85 to 89	26% (14-41%)	74% (59-86%)	0.32 (0.14-0.59)	0.36% (-0.052-0.94%)	0.36% (-0.11-1%)	0.1 (-0.03-0.28)	0.83 (-0.12-2.2)
Vanuatu	1990	Male	90 to 94	27% (15-43%)	73% (57-85%)	0.33 (0.15-0.61)	0.26% (-0.12-0.75%)	0.25% (-0.19-0.8%)	0.021 (-0.016-0.07)	0.13 (-0.06-0.39)
Vanuatu	1990	Male	95 plus	30% (15-49%)	70% (51-85%)	0.36 (0.15-0.71)	0.07% (-0.41-0.8%)	-0.015% (-0.58-0.8%)	-0.0003 (-0.011-0.015)	0.0055 (-0.032-0.061)
Vanuatu	1995	Female	All Ages	10% (7.9-13%)	90% (87-92%)	0.25 (0.2-0.32)	0.72% (0.43-1.1%)	0.73% (0.26-1.3%)	4.4 (1.5-7.9)	240 (140-360)
Vanuatu	1995	Female	15 to 19	13% (7.4-22%)	87% (78-93%)	0.35 (0.17-0.59)	1.1% (0.56-2%)	1.7% (0.62-3.3%)	0.17 (0.062-0.35)	16 (7.9-29)
Vanuatu	1995	Female	20 to 24	16% (9.4-26%)	84% (74-91%)	0.57 (0.3-0.95)	1.9% (1.1-3.2%)	2.5% (1.1-4.5%)	0.33 (0.15-0.61)	30 (17-49)
Vanuatu	1995	Female	25 to 29	16% (9.7-25%)	84% (75-90%)	0.56 (0.33-0.85)	2% (1.2-3%)	2.5% (1.3-3.9%)	0.44 (0.23-0.71)	35 (21-53)
Vanuatu	1995	Female	30 to 34	16% (9.1-23%)	84% (77-91%)	0.55 (0.33-0.87)	1.8% (1-2.7%)	2.1% (1.1-3.6%)	0.46 (0.22-0.78)	33 (19-52)
Vanuatu	1995	Female	35 to 39	14% (8.5-22%)	86% (78-92%)	0.53 (0.29-0.89)	1.9% (1-3.1%)	2.3% (1.2-4%)	0.59 (0.28-1)	35 (19-59)
Vanuatu	1995	Female	40 to 44	13% (8.1-20%)	87% (80-92%)	0.45 (0.23-0.78)	1.7% (0.85-3%)	2.1% (0.96-3.9%)	0.57 (0.25-1)	29 (14-51)
Vanuatu	1995	Female	45 to 49	13% (7.3-19%)	87% (81-93%)	0.37 (0.19-0.67)	1.2% (0.43-2.3%)	1.4% (0.47-3%)	0.41 (0.13-0.86)	18 (6.8-37)

Location	Year	Sex	Age	Prevalence of current drinkers	Prevalence of abstainers	Population average of standard drinks daily	Percent of total attributable deaths	Percent of total attributable DALYs	Attributable deaths	Attributable DALYs
Vanuatu	1995	Female	50 to 54	12%<br>(7.5-18%)	88%<br>(82-93%)	0.32<br>(0.16-0.59)	0.91%<br>(0.16-2.1%)	1.1%<br>(0.18-2.6%)	0.37<br>(0.057-0.9)	14<br>(2.5-34)
Vanuatu	1995	Female	55 to 59	11%<br>(6.7-16%)	89%<br>(84-93%)	0.28<br>(0.13-0.51)	0.73%<br>(0.052-1.8%)	0.9%<br>(0.064-2.2%)	0.32<br>(0.023-0.81)	10<br>(0.7-26)
Vanuatu	1995	Female	60 to 64	10%<br>(6-16%)	90%<br>(84-94%)	0.25<br>(0.11-0.47)	0.39%<br>(-0.2-1.3%)	0.46%<br>(-0.23-1.5%)	0.18<br>(-0.084-0.61)	5<br>(-2.5-17)
Vanuatu	1995	Female	65 to 69	12%<br>(6.6-19%)	88%<br>(81-93%)	0.25<br>(0.12-0.43)	0.36%<br>(-0.25-1.2%)	0.44%<br>(-0.29-1.4%)	0.2<br>(-0.12-0.63)	4.4<br>(-3.1-14)
Vanuatu	1995	Female	70 to 74	13%<br>(7.3-20%)	87%<br>(80-93%)	0.25<br>(0.12-0.45)	0.22%<br>(-0.43-1.1%)	0.26%<br>(-0.48-1.2%)	0.13<br>(-0.23-0.59)	2.2<br>(-4.3-11)
Vanuatu	1995	Female	75 to 79	12%<br>(7.1-21%)	88%<br>(79-93%)	0.23<br>(0.1-0.43)	0.24%<br>(-0.34-1%)	0.28%<br>(-0.39-1.2%)	0.14<br>(-0.19-0.6)	1.9<br>(-2.8-8.3)
Vanuatu	1995	Female	80 to 84	12%<br>(6.7-21%)	88%<br>(79-93%)	0.23<br>(0.1-0.41)	0.12%<br>(-0.37-0.79%)	0.13%<br>(-0.42-0.9%)	0.068<br>(-0.21-0.44)	0.73<br>(-2.2-4.7)
Vanuatu	1995	Female	85 to 89	13%<br>(7-20%)	87%<br>(80-93%)	0.23<br>(0.11-0.43)	0.067%<br>(-0.38-0.71%)	0.065%<br>(-0.44-0.78%)	0.016<br>(-0.11-0.18)	0.14<br>(-0.8-1.4)
Vanuatu	1995	Female	90 to 94	13%<br>(7.6-22%)	87%<br>(78-92%)	0.25<br>(0.11-0.47)	0.092%<br>(-0.31-0.75%)	0.087%<br>(-0.38-0.84%)	0.0062<br>(-0.027-0.058)	0.038<br>(-0.13-0.31)
Vanuatu	1995	Female	95 plus	15%<br>(7.9-26%)	85%<br>(74-92%)	0.28<br>(0.11-0.57)	0.13%<br>(-0.39-0.97%)	0.1%<br>(-0.48-1.1%)	0.0016<br>(-0.0074-0.017)	0.0078<br>(-0.023-0.058)
Vanuatu	1995	Male	All Ages	30%<br>(22-37%)	70%<br>(63-78%)	0.62<br>(0.5-0.77)	1.9%<br>(1.2-2.7%)	1.8%<br>(0.94-2.7%)	15<br>(7.9-24)	790<br>(490-1100)
Vanuatu	1995	Male	15 to 19	29%<br>(15-45%)	71%<br>(55-85%)	0.35<br>(0.16-0.63)	1.5%<br>(0.75-2.6%)	1.7%<br>(0.7-3.4%)	0.31<br>(0.12-0.62)	28<br>(14-49)
Vanuatu	1995	Male	20 to 24	49%<br>(30-66%)	51%<br>(34-70%)	1.1<br>(0.63-1.7)	4.8%<br>(2.8-7.4%)	5.7%<br>(2.9-9.1%)	1.3<br>(0.65-2.1)	97<br>(54-150)
Vanuatu	1995	Male	25 to 29	59%<br>(41-72%)	41%<br>(28-59%)	1.8<br>(1.2-2.5)	6.9%<br>(4.3-9.9%)	8.2%<br>(4.8-12%)	2<br>(1.2-3.1)	140<br>(86-210)
Vanuatu	1995	Male	30 to 34	60%<br>(43-75%)	40%<br>(25-57%)	2<br>(1.3-2.9)	6.7%<br>(4.1-9.7%)	7.8%<br>(4.4-12%)	2.2<br>(1.2-3.4)	140<br>(79-210)
Vanuatu	1995	Male	35 to 39	57%<br>(41-70%)	43%<br>(30-59%)	1.6<br>(0.97-2.5)	4.9%<br>(2.7-7.7%)	5.5%<br>(2.8-9%)	1.8<br>(0.9-2.9)	100<br>(57-160)
Vanuatu	1995	Male	40 to 44	52%<br>(38-65%)	48%<br>(35-62%)	1.3<br>(0.71-2)	3.8%<br>(1.8-6.7%)	4.2%<br>(1.7-7.6%)	1.5<br>(0.61-2.9)	80<br>(36-140)
Vanuatu	1995	Male	45 to 49	45%<br>(30-60%)	55%<br>(40-70%)	1.1<br>(0.56-1.9)	3%<br>(1.1-5.7%)	3.2%<br>(0.99-6.4%)	1.4<br>(0.43-3)	66<br>(23-130)
Vanuatu	1995	Male	50 to 54	43%<br>(29-56%)	57%<br>(44-71%)	0.85<br>(0.43-1.5)	2.4%<br>(0.72-4.7%)	2.5%<br>(0.55-5.2%)	1.3<br>(0.29-2.8)	51<br>(16-110)
Vanuatu	1995	Male	55 to 59	44%<br>(29-58%)	56%<br>(42-71%)	0.74<br>(0.38-1.3)	1.6%<br>(0.3-3.5%)	1.7%<br>(0.19-3.9%)	0.89<br>(0.096-2)	31<br>(5.8-69)
Vanuatu	1995	Male	60 to 64	41%<br>(26-57%)	59%<br>(43-74%)	0.6<br>(0.31-1.1)	1.3%<br>(0.21-3.1%)	1.4%<br>(0.12-3.4%)	0.81<br>(0.072-2)	24<br>(3.6-57)
Vanuatu	1995	Male	65 to 69	38%<br>(22-55%)	62%<br>(45-78%)	0.5<br>(0.25-0.86)	1.1%<br>(0.13-2.4%)	1.1%<br>(0.076-2.7%)	0.75<br>(0.049-1.8)	18<br>(2.2-41)

Location	Year	Sex	Age	Prevalence of current drinkers	Prevalence of abstainers	Population average of standard drinks daily	Percent of total attributable deaths	Percent of total attributable DALYs	Attributable deaths	Attributable DALYs
Vanuatu	1995	Male	70 to 74	34% (20-50%)	66% (50-80%)	0.41 (0.21-0.72)	0.66% (-0.08-1.8%)	0.7% (-0.13-2%)	0.47 (-0.09-1.3)	9.1 (-1.1-24)
Vanuatu	1995	Male	75 to 79	30% (17-47%)	70% (53-83%)	0.34 (0.16-0.62)	0.47% (-0.12-1.4%)	0.48% (-0.18-1.5%)	0.33 (-0.13-1)	5 (-1.3-15)
Vanuatu	1995	Male	80 to 84	27% (15-44%)	73% (56-85%)	0.3 (0.14-0.54)	0.37% (-0.064-0.96%)	0.37% (-0.12-1%)	0.24 (-0.079-0.68)	2.8 (-0.48-7.5)
Vanuatu	1995	Male	85 to 89	26% (14-41%)	74% (59-86%)	0.28 (0.13-0.51)	0.24% (-0.15-0.79%)	0.23% (-0.21-0.84%)	0.076 (-0.068-0.27)	0.68 (-0.42-2.2)
Vanuatu	1995	Male	90 to 94	27% (16-43%)	73% (57-84%)	0.29 (0.13-0.55)	0.17% (-0.16-0.64%)	0.15% (-0.25-0.67%)	0.015 (-0.026-0.069)	0.11 (-0.1-0.39)
Vanuatu	1995	Male	95 plus	30% (15-50%)	70% (50-85%)	0.32 (0.13-0.64)	-0.014% (-0.46-0.55%)	-0.11% (-0.65-0.49%)	-0.0027 (-0.016-0.012)	-0.0013 (-0.044-0.051)
Vanuatu	2000	Female	All Ages	10% (7.9-13%)	90% (87-92%)	0.22 (0.17-0.28)	0.56% (0.3-0.88%)	0.49% (0.089-0.99%)	3.2 (0.6-6.7)	200 (110-320)
Vanuatu	2000	Female	15 to 19	13% (7.3-21%)	87% (79-93%)	0.29 (0.14-0.49)	0.95% (0.48-1.7%)	1.3% (0.49-2.7%)	0.16 (0.06-0.34)	16 (8.3-30)
Vanuatu	2000	Female	20 to 24	16% (9.4-26%)	84% (74-91%)	0.49 (0.26-0.81)	1.7% (0.95-2.8%)	2% (0.88-3.8%)	0.3 (0.13-0.56)	29 (16-48)
Vanuatu	2000	Female	25 to 29	16% (9.9-24%)	84% (76-90%)	0.48 (0.27-0.73)	1.7% (1.1-2.6%)	2.1% (1.1-3.4%)	0.38 (0.19-0.65)	31 (19-49)
Vanuatu	2000	Female	30 to 34	16% (9.4-24%)	84% (76-91%)	0.47 (0.27-0.75)	1.5% (0.86-2.2%)	1.7% (0.85-2.9%)	0.39 (0.18-0.7)	29 (16-48)
Vanuatu	2000	Female	35 to 39	14% (8.6-22%)	86% (78-91%)	0.45 (0.24-0.78)	1.5% (0.82-2.6%)	1.8% (0.87-3.3%)	0.53 (0.24-1)	33 (17-57)
Vanuatu	2000	Female	40 to 44	13% (7.9-20%)	87% (80-92%)	0.39 (0.19-0.69)	1.3% (0.62-2.5%)	1.6% (0.67-3.2%)	0.5 (0.21-0.99)	27 (13-49)
Vanuatu	2000	Female	45 to 49	13% (7.1-19%)	87% (81-93%)	0.32 (0.16-0.58)	0.86% (0.26-1.8%)	1% (0.24-2.3%)	0.34 (0.078-0.79)	16 (4.7-35)
Vanuatu	2000	Female	50 to 54	12% (7.8-19%)	88% (81-92%)	0.28 (0.13-0.5)	0.6% (-0.048-1.5%)	0.72% (-0.077-1.9%)	0.26 (-0.029-0.72)	10 (-0.85-27)
Vanuatu	2000	Female	55 to 59	11% (6.7-17%)	89% (83-93%)	0.23 (0.11-0.42)	0.45% (-0.15-1.3%)	0.56% (-0.17-1.6%)	0.24 (-0.071-0.66)	7.6 (-2.5-21)
Vanuatu	2000	Female	60 to 64	10% (5.9-16%)	90% (84-94%)	0.21 (0.095-0.38)	0.16% (-0.36-0.84%)	0.2% (-0.41-1%)	0.089 (-0.18-0.44)	2.4 (-5.2-12)
Vanuatu	2000	Female	65 to 69	12% (6.5-19%)	88% (81-93%)	0.21 (0.1-0.37)	0.13% (-0.43-0.77%)	0.17% (-0.5-0.91%)	0.08 (-0.23-0.44)	1.6 (-5.6-9.8)
Vanuatu	2000	Female	70 to 74	13% (7.3-20%)	87% (80-93%)	0.21 (0.097-0.38)	-0.011% (-0.57-0.67%)	0.00039% (-0.65-0.76%)	0.00022 (-0.34-0.39)	-0.11 (-6.3-7.1)
Vanuatu	2000	Female	75 to 79	12% (7-21%)	88% (79-93%)	0.2 (0.087-0.37)	0.032% (-0.5-0.64%)	0.044% (-0.55-0.73%)	0.023 (-0.29-0.38)	0.27 (-4.2-5.4)
Vanuatu	2000	Female	80 to 84	12% (6.8-21%)	88% (79-93%)	0.19 (0.089-0.34)	-0.052% (-0.5-0.49%)	-0.061% (-0.57-0.54%)	-0.032 (-0.3-0.29)	-0.32 (-3.2-3)
Vanuatu	2000	Female	85 to 89	13% (7.1-20%)	87% (80-93%)	0.2 (0.088-0.36)	-0.078% (-0.48-0.45%)	-0.098% (-0.57-0.49%)	-0.028 (-0.16-0.14)	-0.19 (-1.2-1.1)

Location	Year	Sex	Age	Prevalence of current drinkers	Prevalence of abstainers	Population average of standard drinks daily	Percent of total attributable deaths	Percent of total attributable DALYs	Attributable deaths	Attributable DALYs
Vanuatu	2000	Female	90 to 94	13% (7.9-22%)	87% (78-92%)	0.21 (0.09-0.41)	-0.045% (-0.41-0.48%)	-0.068% (-0.48-0.53%)	-0.0061 (-0.045-0.047)	-0.024 (-0.22-0.24)
Vanuatu	2000	Female	95 plus	16% (7.9-27%)	84% (73-92%)	0.24 (0.096-0.49)	-0.028% (-0.55-0.62%)	-0.077% (-0.68-0.66%)	-0.0015 (-0.013-0.012)	-0.002 (-0.04-0.044)
Vanuatu	2000	Male	All Ages	30% (23-37%)	70% (63-77%)	0.55 (0.43-0.67)	1.6% (0.95-2.2%)	1.4% (0.63-2.2%)	13 (5.8-20)	710 (420-1000)
Vanuatu	2000	Male	15 to 19	29% (16-47%)	71% (53-84%)	0.3 (0.14-0.53)	1.4% (0.72-2.4%)	1.6% (0.64-3.1%)	0.32 (0.12-0.63)	30 (15-53)
Vanuatu	2000	Male	20 to 24	50% (29-68%)	50% (32-71%)	0.97 (0.55-1.5)	4.3% (2.5-6.7%)	5% (2.6-8.1%)	1.3 (0.67-2.2)	100 (58-160)
Vanuatu	2000	Male	25 to 29	59% (41-72%)	41% (28-59%)	1.6 (1-2.2)	6.1% (3.8-8.7%)	7.1% (4.2-10%)	1.8 (1-2.9)	130 (76-190)
Vanuatu	2000	Male	30 to 34	60% (43-73%)	40% (27-57%)	1.7 (1.1-2.5)	5.6% (3.3-8.3%)	6.4% (3.5-9.8%)	1.9 (0.99-3)	120 (70-190)
Vanuatu	2000	Male	35 to 39	56% (41-70%)	44% (30-59%)	1.4 (0.83-2.1)	4.1% (2.2-6.6%)	4.5% (2.1-7.6%)	1.6 (0.72-2.8)	95 (48-160)
Vanuatu	2000	Male	40 to 44	52% (38-65%)	48% (35-62%)	1.1 (0.6-1.8)	3% (1.3-5.3%)	3.2% (1.1-6%)	1.3 (0.44-2.5)	70 (29-130)
Vanuatu	2000	Male	45 to 49	46% (29-60%)	54% (40-71%)	0.91 (0.47-1.6)	2.3% (0.74-4.3%)	2.4% (0.55-4.8%)	1.1 (0.23-2.3)	53 (16-100)
Vanuatu	2000	Male	50 to 54	43% (28-57%)	57% (43-72%)	0.74 (0.38-1.3)	1.8% (0.4-3.6%)	1.8% (0.2-3.9%)	1 (0.095-2.2)	42 (9-87)
Vanuatu	2000	Male	55 to 59	44% (29-58%)	56% (42-71%)	0.63 (0.33-1.1)	1.1% (0.036-2.6%)	1.1% (-0.14-2.8%)	0.67 (-0.089-1.6)	25 (0.81-55)
Vanuatu	2000	Male	60 to 64	41% (26-56%)	59% (44-74%)	0.52 (0.27-0.92)	0.86% (-0.059-2.2%)	0.87% (-0.15-2.4%)	0.55 (-0.098-1.5)	17 (-1.2-44)
Vanuatu	2000	Male	65 to 69	38% (23-54%)	62% (46-77%)	0.44 (0.22-0.77)	0.7% (-0.065-1.8%)	0.72% (-0.13-1.9%)	0.48 (-0.085-1.3)	12 (-1.1-30)
Vanuatu	2000	Male	70 to 74	34% (20-49%)	66% (51-80%)	0.35 (0.18-0.63)	0.41% (-0.23-1.3%)	0.42% (-0.3-1.4%)	0.3 (-0.22-1)	6 (-3.4-19)
Vanuatu	2000	Male	75 to 79	30% (17-47%)	70% (53-83%)	0.29 (0.13-0.54)	0.27% (-0.25-1%)	0.26% (-0.34-1.1%)	0.19 (-0.24-0.78)	3.2 (-2.9-12)
Vanuatu	2000	Male	80 to 84	27% (14-43%)	73% (57-86%)	0.25 (0.12-0.45)	0.23% (-0.16-0.71%)	0.21% (-0.22-0.75%)	0.14 (-0.14-0.49)	1.8 (-1.2-5.6)
Vanuatu	2000	Male	85 to 89	26% (14-40%)	74% (60-86%)	0.24 (0.11-0.43)	0.13% (-0.23-0.55%)	0.098% (-0.31-0.57%)	0.035 (-0.11-0.2)	0.38 (-0.68-1.6)
Vanuatu	2000	Male	90 to 94	27% (16-42%)	73% (58-84%)	0.25 (0.11-0.48)	0.082% (-0.24-0.44%)	0.04% (-0.34-0.45%)	0.0048 (-0.041-0.055)	0.058 (-0.17-0.31)
Vanuatu	2000	Male	95 plus	30% (15-50%)	70% (50-85%)	0.28 (0.11-0.56)	-0.095% (-0.54-0.33%)	-0.2% (-0.76-0.28%)	-0.0058 (-0.021-0.0077)	-0.01 (-0.06-0.037)
Vanuatu	2005	Female	All Ages	10% (8.2-13%)	90% (87-92%)	0.18 (0.14-0.24)	0.4% (0.17-0.67%)	0.27% (-0.075-0.68%)	2.1 (-0.55-5.4)	170 (70-290)
Vanuatu	2005	Female	15 to 19	13% (7.3-21%)	87% (79-93%)	0.24 (0.11-0.4)	0.75% (0.39-1.3%)	0.98% (0.33-2.1%)	0.13 (0.043-0.28)	14 (7.2-26)

Location	Year	Sex	Age	Prevalence of current drinkers	Prevalence of abstainers	Population average of standard drinks daily	Percent of total attributable deaths	Percent of total attributable DALYs	Attributable deaths	Attributable DALYs
Vanuatu	2005	Female	20 to 24	16%<br>(8.9-26%)	84%<br>(74-91%)	0.38<br>(0.2-0.63)	1.3%<br>(0.74-2.2%)	1.5%<br>(0.59-2.8%)	0.27<br>(0.11-0.51)	28<br>(16-47)
Vanuatu	2005	Female	25 to 29	16%<br>(9.9-24%)	84%<br>(76-90%)	0.38<br>(0.22-0.59)	1.4%<br>(0.87-2.2%)	1.6%<br>(0.8-2.8%)	0.34<br>(0.16-0.62)	30<br>(17-48)
Vanuatu	2005	Female	30 to 34	16%<br>(9.5-24%)	84%<br>(76-91%)	0.39<br>(0.22-0.6)	1.2%<br>(0.65-1.8%)	1.3%<br>(0.54-2.2%)	0.31<br>(0.12-0.58)	25<br>(13-42)
Vanuatu	2005	Female	35 to 39	14%<br>(8.6-22%)	86%<br>(78-91%)	0.37<br>(0.2-0.63)	1.2%<br>(0.61-2%)	1.4%<br>(0.6-2.5%)	0.46<br>(0.19-0.89)	29<br>(15-52)
Vanuatu	2005	Female	40 to 44	13%<br>(8.1-20%)	87%<br>(80-92%)	0.31<br>(0.16-0.54)	0.97%<br>(0.42-1.8%)	1.2%<br>(0.41-2.3%)	0.44<br>(0.15-0.88)	24<br>(9.9-44)
Vanuatu	2005	Female	45 to 49	13%<br>(7.1-19%)	87%<br>(81-93%)	0.26<br>(0.13-0.45)	0.56%<br>(0.045-1.3%)	0.66%<br>(-0.02-1.6%)	0.28<br>(-0.0074-0.7)	13<br>(1.1-30)
Vanuatu	2005	Female	50 to 54	12%<br>(7.8-18%)	88%<br>(82-92%)	0.22<br>(0.11-0.39)	0.31%<br>(-0.25-1%)	0.37%<br>(-0.31-1.3%)	0.17<br>(-0.14-0.6)	6.7<br>(-5.1-23)
Vanuatu	2005	Female	55 to 59	11%<br>(6.6-17%)	89%<br>(83-93%)	0.19<br>(0.091-0.36)	0.22%<br>(-0.33-0.86%)	0.28%<br>(-0.38-1%)	0.14<br>(-0.19-0.54)	4.4<br>(-6.8-17)
Vanuatu	2005	Female	60 to 64	10%<br>(6-16%)	90%<br>(84-94%)	0.17<br>(0.078-0.32)	-0.0038%<br>(-0.5-0.55%)	0.012%<br>(-0.56-0.69%)	0.0069<br>(-0.32-0.39)	-0.06<br>(-9.4-10)
Vanuatu	2005	Female	65 to 69	12%<br>(6.8-19%)	88%<br>(81-93%)	0.17<br>(0.082-0.31)	-0.051%<br>(-0.59-0.49%)	-0.033%<br>(-0.65-0.58%)	-0.02<br>(-0.39-0.36)	-0.83<br>(-9.4-7.9)
Vanuatu	2005	Female	70 to 74	13%<br>(7.6-21%)	87%<br>(79-92%)	0.17<br>(0.08-0.32)	-0.19%<br>(-0.71-0.33%)	-0.21%<br>(-0.79-0.4%)	-0.13<br>(-0.49-0.26)	-2.6<br>(-9.4-4.5)
Vanuatu	2005	Female	75 to 79	13%<br>(7.1-21%)	87%<br>(79-93%)	0.16<br>(0.07-0.3)	-0.13%<br>(-0.62-0.34%)	-0.14%<br>(-0.7-0.4%)	-0.082<br>(-0.41-0.24)	-1.2<br>(-5.9-3.3)
Vanuatu	2005	Female	80 to 84	12%<br>(6.8-21%)	88%<br>(79-93%)	0.16<br>(0.071-0.28)	-0.19%<br>(-0.61-0.2%)	-0.22%<br>(-0.69-0.23%)	-0.12<br>(-0.38-0.13)	-1.2<br>(-4-1.3)
Vanuatu	2005	Female	85 to 89	13%<br>(7.1-21%)	87%<br>(79-93%)	0.16<br>(0.074-0.3)	-0.2%<br>(-0.62-0.2%)	-0.24%<br>(-0.71-0.22%)	-0.067<br>(-0.21-0.065)	-0.48<br>(-1.5-0.51)
Vanuatu	2005	Female	90 to 94	14%<br>(7.8-22%)	86%<br>(78-92%)	0.17<br>(0.075-0.35)	-0.16%<br>(-0.52-0.24%)	-0.2%<br>(-0.61-0.26%)	-0.02<br>(-0.061-0.026)	-0.097<br>(-0.3-0.14)
Vanuatu	2005	Female	95 plus	16%<br>(7.9-27%)	84%<br>(73-92%)	0.2<br>(0.079-0.42)	-0.16%<br>(-0.67-0.38%)	-0.23%<br>(-0.81-0.4%)	-0.0054<br>(-0.019-0.0094)	-0.015<br>(-0.06-0.034)
Vanuatu	2005	Male	All Ages	31%<br>(23-38%)	69%<br>(62-77%)	0.45<br>(0.34-0.56)	1.2%<br>(0.72-1.8%)	1%<br>(0.36-1.7%)	10<br>(3.8-18)	630<br>(360-930)
Vanuatu	2005	Male	15 to 19	28%<br>(15-45%)	72%<br>(55-85%)	0.24<br>(0.11-0.42)	1.2%<br>(0.61-2%)	1.3%<br>(0.49-2.6%)	0.29<br>(0.11-0.57)	28<br>(15-47)
Vanuatu	2005	Male	20 to 24	48%<br>(30-66%)	52%<br>(34-70%)	0.74<br>(0.42-1.1)	3.5%<br>(2.1-5.5%)	3.9%<br>(2-6.5%)	1.2<br>(0.61-2.2)	100<br>(59-160)
Vanuatu	2005	Male	25 to 29	58%<br>(42-72%)	42%<br>(28-58%)	1.2<br>(0.76-1.8)	4.9%<br>(2.9-7.2%)	5.5%<br>(3-8.5%)	1.7<br>(0.91-2.8)	120<br>(70-190)
Vanuatu	2005	Male	30 to 34	60%<br>(43-74%)	40%<br>(26-57%)	1.4<br>(0.79-2)	4.4%<br>(2.5-6.9%)	4.9%<br>(2.5-8%)	1.6<br>(0.77-2.7)	110<br>(59-170)
Vanuatu	2005	Male	35 to 39	56%<br>(42-70%)	44%<br>(30-58%)	1.1<br>(0.64-1.7)	3.2%<br>(1.7-5%)	3.4%<br>(1.4-5.7%)	1.3<br>(0.56-2.3)	82<br>(43-140)

Location	Year	Sex	Age	Prevalence of current drinkers	Prevalence of abstainers	Population average of standard drinks daily	Percent of total attributable deaths	Percent of total attributable DALYs	Attributable deaths	Attributable DALYs
Vanuatu	2005	Male	40 to 44	52%<br>(37-65%)	48%<br>(35-63%)	0.9<br>(0.5-1.5)	2.3%<br>(0.92-4%)	2.3%<br>(0.62-4.4%)	1.1<br>(0.29-2.2)	62<br>(23-110)
Vanuatu	2005	Male	45 to 49	46%<br>(29-60%)	54%<br>(40-71%)	0.72<br>(0.38-1.2)	1.7%<br>(0.45-3.3%)	1.7%<br>(0.16-3.6%)	0.94<br>(0.081-2)	48<br>(12-94)
Vanuatu	2005	Male	50 to 54	43%<br>(28-57%)	57%<br>(43-72%)	0.57<br>(0.29-0.97)	1.3%<br>(0.12-2.6%)	1.2%<br>(-0.15-2.9%)	0.78<br>(-0.085-1.8)	35<br>(3.2-72)
Vanuatu	2005	Male	55 to 59	44%<br>(29-57%)	56%<br>(43-71%)	0.49<br>(0.25-0.85)	0.7%<br>(-0.18-1.8%)	0.64%<br>(-0.41-1.9%)	0.45<br>(-0.29-1.4)	18<br>(-5.1-48)
Vanuatu	2005	Male	60 to 64	41%<br>(25-56%)	59%<br>(44-75%)	0.41<br>(0.2-0.74)	0.47%<br>(-0.27-1.5%)	0.44%<br>(-0.41-1.6%)	0.33<br>(-0.31-1.2)	11<br>(-6.3-36)
Vanuatu	2005	Male	65 to 69	38%<br>(21-55%)	62%<br>(45-79%)	0.34<br>(0.17-0.61)	0.38%<br>(-0.24-1.2%)	0.37%<br>(-0.34-1.3%)	0.29<br>(-0.26-1)	7.7<br>(-4.7-24)
Vanuatu	2005	Male	70 to 74	34%<br>(20-50%)	66%<br>(50-80%)	0.28<br>(0.14-0.51)	0.19%<br>(-0.33-0.81%)	0.17%<br>(-0.41-0.86%)	0.13<br>(-0.32-0.67)	3<br>(-5.3-13)
Vanuatu	2005	Male	75 to 79	30%<br>(17-47%)	70%<br>(53-83%)	0.22<br>(0.1-0.41)	0.11%<br>(-0.34-0.61%)	0.083%<br>(-0.42-0.62%)	0.06<br>(-0.31-0.45)	1.3<br>(-3.9-6.9)
Vanuatu	2005	Male	80 to 84	27%<br>(14-43%)	73%<br>(57-86%)	0.2<br>(0.094-0.35)	0.11%<br>(-0.25-0.48%)	0.083%<br>(-0.32-0.49%)	0.052<br>(-0.21-0.31)	0.83<br>(-1.9-3.5)
Vanuatu	2005	Male	85 to 89	26%<br>(14-40%)	74%<br>(60-86%)	0.19<br>(0.087-0.34)	0.017%<br>(-0.32-0.32%)	-0.025%<br>(-0.42-0.32%)	-0.0088<br>(-0.14-0.11)	0.051<br>(-0.95-0.94)
Vanuatu	2005	Male	90 to 94	27%<br>(15-41%)	73%<br>(59-85%)	0.19<br>(0.09-0.37)	-0.0014%<br>(-0.33-0.27%)	-0.053%<br>(-0.43-0.26%)	-0.0067<br>(-0.054-0.032)	-0.00078<br>(-0.24-0.2)
Vanuatu	2005	Male	95 plus	29%<br>(14-49%)	71%<br>(51-86%)	0.21<br>(0.086-0.42)	-0.16%<br>(-0.55-0.15%)	-0.28%<br>(-0.76-0.091%)	-0.0088<br>(-0.023-0.0028)	-0.02<br>(-0.069-0.019)
Vanuatu	2010	Female	All Ages	10%<br>(8-12%)	90%<br>(88-92%)	0.14<br>(0.097-0.19)	0.28%<br>(0.081-0.5%)	0.089%<br>(-0.2-0.41%)	0.8<br>(-1.7-3.7)	130<br>(38-240)
Vanuatu	2010	Female	15 to 19	13%<br>(7.5-21%)	87%<br>(79-92%)	0.18<br>(0.085-0.32)	0.6%<br>(0.3-1%)	0.7%<br>(0.19-1.5%)	0.092<br>(0.025-0.2)	12<br>(5.8-20)
Vanuatu	2010	Female	20 to 24	16%<br>(9.2-25%)	84%<br>(75-91%)	0.29<br>(0.15-0.5)	1%<br>(0.6-1.7%)	1%<br>(0.35-2.1%)	0.21<br>(0.074-0.43)	26<br>(14-42)
Vanuatu	2010	Female	25 to 29	16%<br>(10-23%)	84%<br>(77-90%)	0.29<br>(0.16-0.46)	1.1%<br>(0.69-1.7%)	1.2%<br>(0.53-2.2%)	0.28<br>(0.12-0.51)	28<br>(16-44)
Vanuatu	2010	Female	30 to 34	15%<br>(9.4-23%)	85%<br>(77-91%)	0.29<br>(0.16-0.47)	0.88%<br>(0.46-1.4%)	0.85%<br>(0.3-1.6%)	0.23<br>(0.077-0.44)	21<br>(11-36)
Vanuatu	2010	Female	35 to 39	13%<br>(8.3-21%)	87%<br>(79-92%)	0.27<br>(0.14-0.45)	0.85%<br>(0.44-1.5%)	0.92%<br>(0.35-1.8%)	0.32<br>(0.12-0.67)	23<br>(11-40)
Vanuatu	2010	Female	40 to 44	12%<br>(7.6-18%)	88%<br>(82-92%)	0.22<br>(0.11-0.4)	0.67%<br>(0.26-1.3%)	0.74%<br>(0.2-1.6%)	0.3<br>(0.079-0.67)	18<br>(6.6-35)
Vanuatu	2010	Female	45 to 49	11%<br>(6.7-16%)	89%<br>(84-93%)	0.17<br>(0.088-0.31)	0.35%<br>(-0.047-0.84%)	0.38%<br>(-0.14-1%)	0.21<br>(-0.072-0.59)	11<br>(-1.4-26)
Vanuatu	2010	Female	50 to 54	11%<br>(6.8-16%)	89%<br>(84-93%)	0.15<br>(0.07-0.27)	0.14%<br>(-0.27-0.62%)	0.15%<br>(-0.36-0.76%)	0.079<br>(-0.19-0.37)	3.6<br>(-6.6-15)
Vanuatu	2010	Female	55 to 59	9.3%<br>(5.8-14%)	91%<br>(86-94%)	0.13<br>(0.059-0.24)	0.09%<br>(-0.33-0.51%)	0.11%<br>(-0.37-0.62%)	0.077<br>(-0.26-0.42)	2.5<br>(-9.1-14)

Location	Year	Sex	Age	Prevalence of current drinkers	Prevalence of abstainers	Population average of standard drinks daily	Percent of total attributable deaths	Percent of total attributable DALYs	Attributable deaths	Attributable DALYs
Vanuatu	2010	Female	60 to 64	9% (5.3-15%)	91% (85-95%)	0.12 (0.055-0.22)	-0.076% (-0.48-0.3%)	-0.081% (-0.57-0.38%)	-0.049 (-0.34-0.23)	-1.5 (-9.7-6.1)
Vanuatu	2010	Female	65 to 69	11% (6.2-17%)	89% (83-94%)	0.13 (0.06-0.23)	-0.14% (-0.61-0.26%)	-0.14% (-0.69-0.32%)	-0.12 (-0.58-0.28)	-3.3 (-14-5.9)
Vanuatu	2010	Female	70 to 74	12% (7.5-20%)	88% (80-93%)	0.13 (0.059-0.24)	-0.3% (-0.74-0.1%)	-0.33% (-0.84-0.14%)	-0.33 (-0.86-0.14)	-6.3 (-16-2.2)
Vanuatu	2010	Female	75 to 79	12% (7-21%)	88% (79-93%)	0.12 (0.054-0.23)	-0.23% (-0.67-0.11%)	-0.25% (-0.76-0.14%)	-0.18 (-0.53-0.094)	-2.6 (-7.6-1.2)
Vanuatu	2010	Female	80 to 84	12% (6.8-21%)	88% (79-93%)	0.12 (0.053-0.22)	-0.29% (-0.67-0.006%)	-0.33% (-0.74-0.018%)	-0.19 (-0.45-0.01)	-2 (-4.7-0.037)
Vanuatu	2010	Female	85 to 89	13% (7.1-20%)	87% (80-93%)	0.12 (0.055-0.24)	-0.29% (-0.72-0.012%)	-0.34% (-0.83-0.011%)	-0.096 (-0.24-0.0032)	-0.7 (-1.8-0.031)
Vanuatu	2010	Female	90 to 94	14% (7.8-22%)	86% (78-92%)	0.13 (0.054-0.26)	-0.26% (-0.63-0.0092%)	-0.31% (-0.72-0.0013%)	-0.031 (-0.072-0.00013)	-0.15 (-0.36-0.0054)
Vanuatu	2010	Female	95 plus	16% (8-27%)	84% (73-92%)	0.15 (0.057-0.31)	-0.26% (-0.77-0.16%)	-0.34% (-0.93-0.14%)	-0.0089 (-0.024-0.0036)	-0.026 (-0.076-0.016)
Vanuatu	2010	Male	All Ages	34% (26-41%)	66% (59-74%)	0.39 (0.27-0.53)	1% (0.54-1.7%)	0.77% (0.17-1.4%)	8.6 (2.1-16)	570 (300-930)
Vanuatu	2010	Male	15 to 19	29% (15-47%)	71% (53-85%)	0.19 (0.078-0.34)	1% (0.54-1.7%)	1.1% (0.39-2.2%)	0.22 (0.079-0.45)	23 (12-39)
Vanuatu	2010	Male	20 to 24	51% (31-69%)	49% (31-69%)	0.62 (0.32-1)	3% (1.6-4.8%)	3.3% (1.4-5.7%)	1.1 (0.49-2)	93 (49-150)
Vanuatu	2010	Male	25 to 29	63% (46-74%)	37% (26-54%)	1.1 (0.64-1.6)	4.2% (2.5-6.3%)	4.7% (2.4-7.4%)	1.8 (0.88-2.9)	130 (75-210)
Vanuatu	2010	Male	30 to 34	65% (50-76%)	35% (24-50%)	1.2 (0.69-1.8)	3.6% (2-5.7%)	3.9% (1.8-6.5%)	1.5 (0.66-2.6)	100 (54-170)
Vanuatu	2010	Male	35 to 39	60% (47-71%)	40% (29-53%)	0.92 (0.54-1.5)	2.5% (1.2-4.3%)	2.6% (0.92-4.9%)	1.1 (0.39-2.2)	73 (34-130)
Vanuatu	2010	Male	40 to 44	53% (41-66%)	47% (34-59%)	0.72 (0.38-1.2)	1.7% (0.52-3.2%)	1.6% (0.2-3.4%)	0.83 (0.092-1.8)	51 (15-97)
Vanuatu	2010	Male	45 to 49	47% (32-59%)	53% (41-68%)	0.56 (0.31-0.99)	1.3% (0.2-2.5%)	1.1% (-0.16-2.7%)	0.77 (-0.11-1.8)	43 (6.4-87)
Vanuatu	2010	Male	50 to 54	42% (30-55%)	58% (45-70%)	0.42 (0.21-0.76)	0.94% (-0.02-2%)	0.84% (-0.33-2.1%)	0.58 (-0.21-1.5)	28 (-0.56-61)
Vanuatu	2010	Male	55 to 59	41% (27-54%)	59% (46-73%)	0.34 (0.17-0.6)	0.47% (-0.22-1.2%)	0.38% (-0.45-1.2%)	0.32 (-0.38-1.1)	15 (-6.9-40)
Vanuatu	2010	Male	60 to 64	39% (25-53%)	61% (47-75%)	0.28 (0.14-0.51)	0.26% (-0.34-0.94%)	0.2% (-0.49-0.94%)	0.16 (-0.39-0.76)	6.4 (-8.2-23)
Vanuatu	2010	Male	65 to 69	37% (22-52%)	63% (48-78%)	0.25 (0.12-0.46)	0.19% (-0.32-0.75%)	0.15% (-0.42-0.79%)	0.17 (-0.51-0.88)	5.5 (-9.3-22)
Vanuatu	2010	Male	70 to 74	34% (20-49%)	66% (51-80%)	0.21 (0.099-0.39)	0.061% (-0.38-0.53%)	0.026% (-0.48-0.57%)	0.024 (-0.45-0.54)	1.2 (-7.3-10)
Vanuatu	2010	Male	75 to 79	30% (17-46%)	70% (54-83%)	0.17 (0.076-0.33)	0.025% (-0.36-0.37%)	-0.016% (-0.45-0.37%)	-0.012 (-0.36-0.29)	0.31 (-4.6-4.4)

Location	Year	Sex	Age	Prevalence of current drinkers	Prevalence of abstainers	Population average of standard drinks daily	Percent of total attributable deaths	Percent of total attributable DALYs	Attributable deaths	Attributable DALYs
Vanuatu	2010	Male	80 to 84	27% (14-42%)	73% (58-86%)	0.15 (0.068-0.28)	0.055% (-0.27-0.32%)	0.019% (-0.34-0.32%)	0.012 (-0.21-0.19)	0.39 (-1.9-2.3)
Vanuatu	2010	Male	85 to 89	26% (14-40%)	74% (60-86%)	0.14 (0.067-0.28)	-0.033% (-0.35-0.2%)	-0.083% (-0.45-0.19%)	-0.028 (-0.15-0.063)	-0.092 (-0.99-0.59)
Vanuatu	2010	Male	90 to 94	27% (15-42%)	73% (58-85%)	0.15 (0.066-0.29)	-0.03% (-0.33-0.2%)	-0.085% (-0.44-0.18%)	-0.011 (-0.056-0.023)	-0.022 (-0.25-0.15)
Vanuatu	2010	Male	95 plus	29% (14-49%)	71% (51-86%)	0.16 (0.064-0.33)	-0.18% (-0.57-0.095%)	-0.29% (-0.77-0.044%)	-0.0096 (-0.025-0.0015)	-0.023 (-0.075-0.012)
Vanuatu	2016	Female	All Ages	10% (8.2-13%)	90% (87-92%)	0.14 (0.072-0.23)	0.28% (0.084-0.57%)	0.089% (-0.22-0.51%)	0.95 (-2.3-5.4)	150 (42-310)
Vanuatu	2016	Female	15 to 19	13% (7.4-21%)	87% (79-93%)	0.18 (0.067-0.36)	0.59% (0.28-1.1%)	0.68% (0.14-1.7%)	0.092 (0.02-0.23)	12 (5.7-23)
Vanuatu	2016	Female	20 to 24	16% (9-26%)	84% (74-91%)	0.29 (0.12-0.55)	1.1% (0.58-1.8%)	1% (0.28-2.3%)	0.19 (0.052-0.43)	25 (13-45)
Vanuatu	2016	Female	25 to 29	16% (10-23%)	84% (77-90%)	0.28 (0.13-0.5)	1.1% (0.67-1.8%)	1.1% (0.44-2.2%)	0.31 (0.12-0.61)	33 (18-52)
Vanuatu	2016	Female	30 to 34	15% (9.4-22%)	85% (78-91%)	0.27 (0.12-0.51)	0.87% (0.43-1.4%)	0.82% (0.24-1.8%)	0.26 (0.069-0.56)	26 (12-45)
Vanuatu	2016	Female	35 to 39	13% (8.2-21%)	87% (79-92%)	0.25 (0.11-0.49)	0.84% (0.42-1.6%)	0.91% (0.3-2%)	0.35 (0.11-0.79)	25 (12-49)
Vanuatu	2016	Female	40 to 44	12% (7.4-18%)	88% (82-93%)	0.21 (0.089-0.43)	0.67% (0.25-1.4%)	0.74% (0.18-1.8%)	0.34 (0.075-0.8)	20 (7.2-42)
Vanuatu	2016	Female	45 to 49	11% (6.5-17%)	89% (83-93%)	0.17 (0.072-0.34)	0.38% (-0.02-0.99%)	0.42% (-0.1-1.2%)	0.23 (-0.061-0.69)	12 (-0.59-32)
Vanuatu	2016	Female	50 to 54	11% (6.8-16%)	89% (84-93%)	0.14 (0.059-0.3)	0.17% (-0.24-0.72%)	0.19% (-0.32-0.86%)	0.14 (-0.24-0.66)	6.1 (-8-27)
Vanuatu	2016	Female	55 to 59	9.3% (5.8-14%)	91% (86-94%)	0.12 (0.048-0.25)	0.12% (-0.27-0.58%)	0.15% (-0.33-0.72%)	0.12 (-0.25-0.54)	3.8 (-8.3-18)
Vanuatu	2016	Female	60 to 64	9% (5.2-14%)	91% (86-95%)	0.11 (0.045-0.23)	-0.041% (-0.43-0.36%)	-0.039% (-0.49-0.45%)	-0.035 (-0.43-0.38)	-1.2 (-13-11)
Vanuatu	2016	Female	65 to 69	11% (6.5-17%)	89% (83-94%)	0.12 (0.05-0.25)	-0.1% (-0.53-0.33%)	-0.095% (-0.62-0.42%)	-0.085 (-0.54-0.35)	-2.5 (-13-7.7)
Vanuatu	2016	Female	70 to 74	13% (7.5-20%)	87% (80-93%)	0.13 (0.048-0.28)	-0.26% (-0.72-0.15%)	-0.29% (-0.8-0.2%)	-0.34 (-0.97-0.24)	-6.6 (-18-4.2)
Vanuatu	2016	Female	75 to 79	12% (7.2-21%)	88% (79-93%)	0.12 (0.046-0.25)	-0.2% (-0.65-0.21%)	-0.21% (-0.72-0.24%)	-0.25 (-0.89-0.29)	-3.8 (-13-3.9)
Vanuatu	2016	Female	80 to 84	12% (6.7-21%)	88% (79-93%)	0.12 (0.044-0.24)	-0.27% (-0.64-0.063%)	-0.3% (-0.75-0.074%)	-0.25 (-0.6-0.059)	-2.6 (-6.4-0.61)
Vanuatu	2016	Female	85 to 89	13% (6.9-20%)	87% (80-93%)	0.12 (0.048-0.25)	-0.26% (-0.7-0.08%)	-0.31% (-0.8-0.089%)	-0.093 (-0.24-0.026)	-0.68 (-1.8-0.2)
Vanuatu	2016	Female	90 to 94	14% (7.8-23%)	86% (77-92%)	0.13 (0.046-0.27)	-0.24% (-0.6-0.062%)	-0.29% (-0.71-0.056%)	-0.028 (-0.068-0.0057)	-0.14 (-0.34-0.036)
Vanuatu	2016	Female	95 plus	16% (7.8-27%)	84% (73-92%)	0.14 (0.048-0.33)	-0.25% (-0.76-0.17%)	-0.32% (-0.92-0.15%)	-0.0076 (-0.022-0.0037)	-0.023 (-0.07-0.016)

Location	Year	Sex	Age	Prevalence of current drinkers	Prevalence of abstainers	Population average of standard drinks daily	Percent of total attributable deaths	Percent of total attributable DALYs	Attributable deaths	Attributable DALYs
Vanuatu	2016	Male	All Ages	34% (27-41%)	66% (59-73%)	0.39 (0.2-0.63)	1.1% (0.49-2%)	0.78% (0.13-1.7%)	9.9 (1.7-22)	650 (290-1200)
Vanuatu	2016	Male	15 to 19	29% (16-45%)	71% (55-84%)	0.18 (0.065-0.36)	1% (0.51-1.8%)	1.1% (0.34-2.2%)	0.26 (0.085-0.55)	27 (14-48)
Vanuatu	2016	Male	20 to 24	50% (32-67%)	50% (33-68%)	0.58 (0.26-1.1)	2.9% (1.5-5.1%)	3.2% (1.2-6%)	1 (0.38-2)	87 (42-150)
Vanuatu	2016	Male	25 to 29	63% (48-73%)	37% (27-52%)	1 (0.51-1.7)	4.1% (2.1-6.7%)	4.5% (1.9-7.9%)	1.8 (0.73-3.3)	140 (66-230)
Vanuatu	2016	Male	30 to 34	66% (50-76%)	34% (24-50%)	1.2 (0.55-2.1)	3.6% (1.7-6.3%)	3.8% (1.3-7.4%)	1.8 (0.61-3.7)	130 (57-230)
Vanuatu	2016	Male	35 to 39	60% (47-72%)	40% (28-53%)	0.92 (0.44-1.7)	2.6% (1.1-4.9%)	2.6% (0.7-5.6%)	1.4 (0.34-3)	90 (37-180)
Vanuatu	2016	Male	40 to 44	53% (41-65%)	47% (35-59%)	0.69 (0.31-1.3)	1.7% (0.45-3.7%)	1.6% (0.099-4%)	0.97 (0.061-2.4)	60 (16-130)
Vanuatu	2016	Male	45 to 49	46% (32-58%)	54% (42-68%)	0.55 (0.24-1.1)	1.3% (0.2-2.9%)	1.2% (-0.14-3%)	0.85 (-0.11-2.2)	47 (7.3-110)
Vanuatu	2016	Male	50 to 54	42% (29-54%)	58% (46-71%)	0.42 (0.18-0.85)	0.96% (0.0078-2.4%)	0.87% (-0.27-2.5%)	0.79 (-0.25-2.4)	38 (0.37-95)
Vanuatu	2016	Male	55 to 59	41% (27-54%)	59% (46-73%)	0.33 (0.14-0.64)	0.49% (-0.22-1.3%)	0.39% (-0.44-1.3%)	0.37 (-0.42-1.3)	17 (-8-49)
Vanuatu	2016	Male	60 to 64	38% (24-52%)	62% (48-76%)	0.27 (0.12-0.51)	0.28% (-0.32-0.98%)	0.22% (-0.49-1%)	0.24 (-0.51-1.1)	9.6 (-10-33)
Vanuatu	2016	Male	65 to 69	36% (21-54%)	64% (46-79%)	0.24 (0.1-0.48)	0.23% (-0.26-0.79%)	0.19% (-0.35-0.86%)	0.21 (-0.39-0.95)	6.3 (-7.5-24)
Vanuatu	2016	Male	70 to 74	33% (20-50%)	67% (50-80%)	0.2 (0.081-0.42)	0.095% (-0.31-0.64%)	0.061% (-0.42-0.68%)	0.08 (-0.55-0.91)	2.6 (-8.7-18)
Vanuatu	2016	Male	75 to 79	29% (17-46%)	71% (54-83%)	0.17 (0.061-0.35)	0.052% (-0.33-0.47%)	0.012% (-0.44-0.47%)	0.013 (-0.46-0.5)	0.88 (-5.9-7.9)
Vanuatu	2016	Male	80 to 84	26% (14-42%)	74% (58-86%)	0.15 (0.055-0.29)	0.084% (-0.25-0.37%)	0.051% (-0.33-0.38%)	0.034 (-0.22-0.25)	0.67 (-1.9-3)
Vanuatu	2016	Male	85 to 89	25% (14-40%)	75% (60-86%)	0.14 (0.057-0.3)	-0.012% (-0.34-0.26%)	-0.061% (-0.43-0.23%)	-0.02 (-0.14-0.076)	-0.033 (-0.93-0.72)
Vanuatu	2016	Male	90 to 94	26% (15-42%)	74% (58-85%)	0.15 (0.055-0.31)	-0.013% (-0.31-0.22%)	-0.067% (-0.41-0.21%)	-0.0079 (-0.049-0.025)	-0.0088 (-0.22-0.16)
Vanuatu	2016	Male	95 plus	30% (15-49%)	70% (51-85%)	0.16 (0.055-0.35)	-0.17% (-0.56-0.12%)	-0.28% (-0.76-0.056%)	-0.0092 (-0.025-0.0018)	-0.021 (-0.072-0.016)
Armenia	1990	Female	All Ages	30% (26-35%)	70% (65-74%)	1 (0.78-1.3)	2.4% (1.6-3.4%)	3.8% (2-6.1%)	450 (230-730)	13000 (8200-18000)
Armenia	1990	Female	15 to 19	25% (18-34%)	75% (66-82%)	0.78 (0.45-1.2)	1.6% (1-2.3%)	3.2% (1.9-5%)	1.7 (0.97-2.6)	220 (140-320)
Armenia	1990	Female	20 to 24	36% (25-47%)	64% (53-75%)	1.2 (0.73-1.9)	2.8% (2-3.8%)	4% (2.4-6.2%)	3.5 (2.1-5.4)	470 (310-680)
Armenia	1990	Female	25 to 29	41% (29-52%)	59% (48-71%)	1.5 (0.89-2.2)	3.1% (2.2-4.1%)	4.6% (3-6.7%)	5.7 (3.6-8.3)	700 (480-960)

Location	Year	Sex	Age	Prevalence of current drinkers	Prevalence of abstainers	Population average of standard drinks daily	Percent of total attributable deaths	Percent of total attributable DALYs	Attributable deaths	Attributable DALYs
Armenia	1990	Female	30 to 34	44% (31-56%)	56% (44-69%)	1.6 (0.98-2.4)	3.2% (2.4-4.3%)	5.3% (3.5-7.6%)	8.3 (5.5-12)	820 (600-1100)
Armenia	1990	Female	35 to 39	44% (32-55%)	56% (45-68%)	1.7 (1-2.6)	3.4% (2.4-4.6%)	6% (3.9-8.3%)	9.9 (6.5-14)	770 (540-1000)
Armenia	1990	Female	40 to 44	43% (32-54%)	57% (46-68%)	1.8 (1.1-2.7)	3.7% (2.5-5.1%)	6.4% (4.1-9.1%)	11 (6.9-15)	660 (450-930)
Armenia	1990	Female	45 to 49	42% (28-53%)	58% (47-72%)	1.8 (0.99-2.8)	4% (2.5-5.9%)	6.6% (4.1-10%)	12 (7-18)	590 (380-880)
Armenia	1990	Female	50 to 54	39% (28-52%)	61% (48-72%)	1.7 (1-2.7)	4.2% (2.6-6.4%)	6.4% (3.9-9.9%)	31 (18-49)	1300 (820-2100)
Armenia	1990	Female	55 to 59	37% (22-53%)	63% (47-78%)	1.7 (0.91-2.8)	4.3% (2.3-7%)	6.2% (3.2-10%)	38 (19-64)	1400 (710-2200)
Armenia	1990	Female	60 to 64	34% (20-51%)	66% (49-80%)	1.6 (0.83-2.7)	4.3% (1.9-7.4%)	5.8% (2.5-10%)	62 (27-110)	1800 (810-3200)
Armenia	1990	Female	65 to 69	32% (20-47%)	68% (53-80%)	1.5 (0.8-2.4)	4.3% (2-7.4%)	5.6% (2.6-9.7%)	56 (26-95)	1400 (640-2300)
Armenia	1990	Female	70 to 74	28% (18-42%)	72% (58-82%)	1.3 (0.69-2.2)	3.8% (1.3-7.2%)	4.8% (1.6-9.1%)	43 (14-82)	820 (280-1500)
Armenia	1990	Female	75 to 79	24% (15-38%)	76% (62-85%)	1.1 (0.54-1.9)	3.2% (0.77-6.7%)	3.8% (0.87-8%)	59 (13-130)	880 (210-1800)
Armenia	1990	Female	80 to 84	22% (13-33%)	78% (67-87%)	0.99 (0.52-1.6)	2.8% (0.73-5.9%)	3.3% (0.77-7%)	51 (12-110)	550 (140-1200)
Armenia	1990	Female	85 to 89	21% (13-32%)	79% (68-87%)	0.98 (0.5-1.7)	2.5% (0.46-6%)	2.9% (0.4-7.2%)	37 (5.1-93)	280 (53-690)
Armenia	1990	Female	90 to 94	23% (14-34%)	77% (66-86%)	1 (0.52-2)	2.2% (0.13-6.2%)	2.6% (-0.034-7.4%)	14 (-0.19-41)	77 (4.3-210)
Armenia	1990	Female	95 plus	25% (14-40%)	75% (60-86%)	1.1 (0.46-2.2)	2.2% (-0.32-6.8%)	2.5% (-0.6-8.1%)	4.1 (-0.95-13)	15 (-2.1-46)
Armenia	1990	Male	All Ages	79% (76-83%)	21% (17-24%)	1.2 (0.95-1.6)	2.5% (1.4-3.7%)	1.2% (-0.63-3.2%)	160 (-83-420)	16000 (8900-25000)
Armenia	1990	Male	15 to 19	68% (58-76%)	32% (24-42%)	0.57 (0.31-0.95)	3.8% (2.2-5.8%)	4.4% (1.9-7.9%)	8.4 (3.5-15)	870 (500-1400)
Armenia	1990	Male	20 to 24	90% (84-94%)	10% (6.4-16%)	1.3 (0.8-2)	7.9% (5.3-11%)	8.4% (4.5-13%)	22 (11-34)	2100 (1400-3000)
Armenia	1990	Male	25 to 29	93% (89-95%)	7.4% (4.9-11%)	2 (1.2-3)	9.1% (6.4-12%)	10% (6-15%)	32 (19-50)	3000 (2000-4100)
Armenia	1990	Male	30 to 34	94% (91-96%)	5.8% (3.6-9.3%)	2.3 (1.5-3.3)	8.3% (5.6-11%)	9.3% (5.3-14%)	37 (21-56)	3000 (2000-4200)
Armenia	1990	Male	35 to 39	95% (93-97%)	4.9% (3.1-7.5%)	2.3 (1.4-3.4)	6.6% (4.1-9.5%)	7.3% (3.5-12%)	28 (13-44)	2100 (1200-3000)
Armenia	1990	Male	40 to 44	95% (93-97%)	4.8% (2.9-7.4%)	2.3 (1.4-3.5)	5.2% (2.8-8%)	5.5% (2.1-9.4%)	20 (7.5-35)	1300 (700-2100)
Armenia	1990	Male	45 to 49	95% (91-97%)	5.4% (3.3-8.8%)	2.5 (1.5-3.8)	4.7% (1.8-8%)	4.9% (0.96-9.4%)	18 (3.4-35)	990 (390-1700)

Location	Year	Sex	Age	Prevalence of current drinkers	Prevalence of abstainers	Population average of standard drinks daily	Percent of total attributable deaths	Percent of total attributable DALYs	Attributable deaths	Attributable DALYs
Armenia	1990	Male	50 to 54	93% (90-96%)	6.5% (4-10%)	2.4 (1.5-3.5)	3.6% (0.87-6.9%)	3.6% (-0.089-7.8%)	35 (-0.85-77)	1700 (430-3300)
Armenia	1990	Male	55 to 59	92% (85-96%)	8.2% (4.1-15%)	2.1 (1.3-3.1)	2.4% (0.13-5.1%)	2.3% (-0.66-5.6%)	25 (-7.1-60)	1100 (57-2300)
Armenia	1990	Male	60 to 64	90% (82-95%)	9.9% (4.7-18%)	1.8 (1.1-2.7)	1.3% (-0.97-3.9%)	1% (-1.7-4.2%)	17 (-29-72)	720 (-580-2300)
Armenia	1990	Male	65 to 69	89% (78-95%)	11% (5-22%)	1.6 (0.99-2.3)	0.4% (-1.7-2.6%)	0.11% (-2.5-2.8%)	1.2 (-27-31)	120 (-510-800)
Armenia	1990	Male	70 to 74	87% (77-93%)	13% (6.9-23%)	1.3 (0.78-2.1)	-0.4% (-2.5-1.8%)	-0.77% (-3.3-1.9%)	-5.4 (-23-13)	-61 (-390-280)
Armenia	1990	Male	75 to 79	85% (74-92%)	15% (7.7-26%)	1.1 (0.64-1.8)	-1.1% (-3.1-0.93%)	-1.5% (-4-0.96%)	-17 (-44-10)	-190 (-580-180)
Armenia	1990	Male	80 to 84	83% (71-91%)	17% (9.1-29%)	1 (0.6-1.6)	-1.7% (-3.9-0.33%)	-2.3% (-5-0.2%)	-23 (-49-1.9)	-210 (-470-39)
Armenia	1990	Male	85 to 89	81% (70-90%)	19% (10-30%)	0.95 (0.56-1.5)	-2.1% (-4.3--0.17%)	-2.8% (-5.6--0.49%)	-22 (-43--3.7)	-140 (-300--12)
Armenia	1990	Male	90 to 94	81% (70-90%)	19% (10-30%)	0.89 (0.51-1.5)	-2.2% (-4.6--0.27%)	-3.1% (-6--0.64%)	-9.2 (-18--1.9)	-41 (-84--4.9)
Armenia	1990	Male	95 plus	80% (66-91%)	20% (8.9-34%)	0.81 (0.4-1.5)	-2.1% (-4.4--0.15%)	-3% (-6--0.65%)	-2.5 (-4.9--0.53)	-7 (-15--0.52)
Armenia	1995	Female	All Ages	27% (24-30%)	73% (70-76%)	0.94 (0.75-1.1)	2.8% (1.9-4%)	4.2% (2.3-6.8%)	510 (280-830)	13000 (8900-19000)
Armenia	1995	Female	15 to 19	21% (18-26%)	79% (74-82%)	0.65 (0.42-0.94)	1.4% (0.98-1.9%)	2.8% (1.8-4.1%)	1.3 (0.84-1.9)	190 (130-270)
Armenia	1995	Female	20 to 24	29% (23-35%)	71% (65-77%)	0.98 (0.65-1.4)	2.6% (1.9-3.5%)	3.6% (2.3-5.4%)	2.4 (1.6-3.7)	340 (240-480)
Armenia	1995	Female	25 to 29	35% (29-42%)	65% (58-71%)	1.2 (0.85-1.7)	3% (2.3-3.9%)	4.4% (3-6.2%)	3.6 (2.4-5.1)	450 (320-610)
Armenia	1995	Female	30 to 34	38% (32-44%)	62% (56-68%)	1.4 (0.95-1.9)	3.1% (2.4-4%)	5% (3.6-6.8%)	7 (4.8-9.4)	690 (510-910)
Armenia	1995	Female	35 to 39	37% (31-44%)	63% (56-69%)	1.4 (0.95-1.9)	3.2% (2.4-4.1%)	5.5% (4-7.4%)	11 (7.5-14)	810 (610-1100)
Armenia	1995	Female	40 to 44	37% (31-44%)	63% (56-69%)	1.5 (1-2)	3.5% (2.5-4.5%)	5.9% (4.3-7.9%)	13 (9.5-18)	800 (580-1100)
Armenia	1995	Female	45 to 49	37% (31-45%)	63% (55-69%)	1.6 (1-2.3)	3.8% (2.6-5.4%)	6.2% (4.1-9.1%)	14 (9.4-21)	720 (480-1000)
Armenia	1995	Female	50 to 54	37% (27-49%)	63% (51-73%)	1.6 (0.99-2.5)	4.3% (2.6-6.4%)	6.5% (4-9.9%)	16 (9.7-25)	680 (430-1000)
Armenia	1995	Female	55 to 59	37% (23-54%)	63% (46-77%)	1.7 (0.93-2.7)	4.6% (2.3-7.4%)	6.5% (3.3-11%)	49 (25-81)	1700 (900-2800)
Armenia	1995	Female	60 to 64	34% (21-53%)	66% (47-79%)	1.6 (0.87-2.7)	4.4% (2-7.6%)	5.9% (2.6-10%)	57 (25-96)	1700 (740-2800)
Armenia	1995	Female	65 to 69	32% (20-48%)	68% (52-80%)	1.5 (0.85-2.4)	4.5% (2.2-7.6%)	5.7% (2.8-9.6%)	96 (46-160)	2300 (1100-3800)

Location	Year	Sex	Age	Prevalence of current drinkers	Prevalence of abstainers	Population average of standard drinks daily	Percent of total attributable deaths	Percent of total attributable DALYs	Attributable deaths	Attributable DALYs
Armenia	1995	Female	70 to 74	28%<br>(18-42%)	72%<br>(58-82%)	1.3<br>(0.72-2.1)	4%<br>(1.5-7.6%)	4.9%<br>(1.9-9.5%)	72<br>(27-140)	1400<br>(530-2600)
Armenia	1995	Female	75 to 79	24%<br>(15-37%)	76%<br>(63-85%)	1.1<br>(0.57-1.9)	3.3%<br>(0.9-6.9%)	3.9%<br>(0.98-8.4%)	48<br>(12-100)	710<br>(190-1500)
Armenia	1995	Female	80 to 84	22%<br>(13-33%)	78%<br>(67-87%)	1<br>(0.53-1.7)	2.9%<br>(0.79-5.8%)	3.4%<br>(0.82-7%)	48<br>(12-100)	520<br>(140-1100)
Armenia	1995	Female	85 to 89	22%<br>(13-33%)	78%<br>(67-87%)	1<br>(0.51-1.7)	2.6%<br>(0.56-5.9%)	3%<br>(0.53-7%)	45<br>(8.2-100)	340<br>(73-790)
Armenia	1995	Female	90 to 94	23%<br>(14-33%)	77%<br>(67-86%)	1<br>(0.53-1.9)	2.3%<br>(0.21-6%)	2.6%<br>(0.059-7.4%)	20<br>(0.44-55)	110<br>(9.9-280)
Armenia	1995	Female	95 plus	25%<br>(14-40%)	75%<br>(60-86%)	1.1<br>(0.5-2.2)	2.3%<br>(-0.3-7.1%)	2.6%<br>(-0.6-8.4%)	6.6<br>(-1.5-21)	24<br>(-3-72)
Armenia	1995	Male	All Ages	82%<br>(79-84%)	18%<br>(16-21%)	1.3<br>(1.1-1.6)	2.4%<br>(1.1-3.7%)	0.93%<br>(-1.1-3%)	140<br>(-160-440)	15000<br>(6700-23000)
Armenia	1995	Male	15 to 19	74%<br>(67-79%)	26%<br>(21-33%)	0.66<br>(0.38-1.1)	3.8%<br>(2.2-5.8%)	4.4%<br>(2.1-7.4%)	9.4<br>(4.3-16)	910<br>(520-1400)
Armenia	1995	Male	20 to 24	92%<br>(88-94%)	8.1%<br>(5.6-12%)	1.4<br>(0.87-2.1)	7.7%<br>(5.1-11%)	8.2%<br>(4.6-12%)	19<br>(11-29)	1800<br>(1200-2500)
Armenia	1995	Male	25 to 29	94%<br>(91-96%)	6.3%<br>(4.3-9.1%)	2.1<br>(1.3-3.1)	9%<br>(6.5-12%)	9.9%<br>(6.3-14%)	23<br>(15-34)	2000<br>(1400-2700)
Armenia	1995	Male	30 to 34	95%<br>(93-97%)	5%<br>(3.2-7.4%)	2.4<br>(1.7-3.4)	8.3%<br>(5.7-11%)	9.2%<br>(5.6-13%)	34<br>(21-51)	2700<br>(1800-3600)
Armenia	1995	Male	35 to 39	96%<br>(94-97%)	4.2%<br>(2.8-6%)	2.4<br>(1.5-3.4)	6.5%<br>(3.9-9.2%)	7.1%<br>(3.6-11%)	34<br>(16-53)	2400<br>(1400-3400)
Armenia	1995	Male	40 to 44	96%<br>(94-97%)	4.1%<br>(2.8-6%)	2.4<br>(1.5-3.4)	4.9%<br>(2.4-7.7%)	5.2%<br>(1.6-8.9%)	28<br>(8.4-47)	1700<br>(830-2700)
Armenia	1995	Male	45 to 49	95%<br>(93-97%)	4.6%<br>(2.9-6.9%)	2.6<br>(1.7-3.9)	4.4%<br>(1.4-8.1%)	4.5%<br>(0.69-9.4%)	24<br>(3.8-50)	1300<br>(420-2400)
Armenia	1995	Male	50 to 54	94%<br>(92-96%)	5.7%<br>(3.6-8.2%)	2.5<br>(1.6-3.7)	3.5%<br>(0.53-7.1%)	3.5%<br>(-0.27-8.1%)	19<br>(-1.4-43)	880<br>(130-1800)
Armenia	1995	Male	55 to 59	92%<br>(86-96%)	7.7%<br>(4.1-14%)	2.1<br>(1.4-2.9)	2.1%<br>(-0.32-4.8%)	1.9%<br>(-1-5.3%)	28<br>(-15-76)	1200<br>(-190-2800)
Armenia	1995	Male	60 to 64	90%<br>(82-95%)	9.8%<br>(4.7-18%)	1.8<br>(1.1-2.6)	1%<br>(-1.3-3.6%)	0.8%<br>(-1.9-3.9%)	14<br>(-34-68)	590<br>(-730-2100)
Armenia	1995	Male	65 to 69	89%<br>(79-95%)	11%<br>(5-21%)	1.6<br>(1-2.2)	0.12%<br>(-2.1-2.4%)	-0.16%<br>(-2.8-2.6%)	-3.9<br>(-68-63)	79<br>(-1400-1600)
Armenia	1995	Male	70 to 74	87%<br>(77-93%)	13%<br>(6.8-23%)	1.3<br>(0.82-2)	-0.68%<br>(-2.9-1.6%)	-1%<br>(-3.6-1.6%)	-15<br>(-53-23)	-210<br>(-870-460)
Armenia	1995	Male	75 to 79	85%<br>(75-93%)	15%<br>(7.3-25%)	1.1<br>(0.66-1.8)	-1.3%<br>(-3.5-0.78%)	-1.7%<br>(-4.3-0.71%)	-14<br>(-35-5.9)	-180<br>(-470-100)
Armenia	1995	Male	80 to 84	83%<br>(71-91%)	17%<br>(8.9-29%)	1<br>(0.62-1.6)	-1.8%<br>(-4.2-0.25%)	-2.5%<br>(-5.3-0.13%)	-20<br>(-43-1.1)	-190<br>(-420-25)
Armenia	1995	Male	85 to 89	82%<br>(71-90%)	18%<br>(10-29%)	0.95<br>(0.58-1.4)	-2.3%<br>(-4.6--0.31%)	-3%<br>(-5.9--0.54%)	-27<br>(-52--4.9)	-180<br>(-360--24)

Location	Year	Sex	Age	Prevalence of current drinkers	Prevalence of abstainers	Population average of standard drinks daily	Percent of total attributable deaths	Percent of total attributable DALYs	Attributable deaths	Attributable DALYs
Armenia	1995	Male	90 to 94	81%<br>(70-90%)	19%<br>(10-30%)	0.89<br>(0.52-1.4)	-2.3%<br>(-4.7--0.35%)	-3.2%<br>(-6.1--0.74%)	-12<br>(-24--2.9)	-56<br>(-110--8.1)
Armenia	1995	Male	95 plus	80%<br>(66-91%)	20%<br>(8.7-34%)	0.81<br>(0.4-1.4)	-2.2%<br>(-4.7--0.22%)	-3.2%<br>(-6.2--0.68%)	-3.4<br>(-6.5--0.73)	-9.7<br>(-20--0.93)
Armenia	2000	Female	All Ages	27%<br>(24-31%)	73%<br>(69-76%)	0.97<br>(0.78-1.1)	3%<br>(1.9-4.3%)	4.1%<br>(2.2-6.9%)	500<br>(260-830)	13000<br>(8500-19000)
Armenia	2000	Female	15 to 19	21%<br>(17-26%)	79%<br>(74-83%)	0.63<br>(0.42-0.91)	1.3%<br>(0.92-1.8%)	2.8%<br>(1.8-4%)	1.1<br>(0.73-1.6)	190<br>(120-260)
Armenia	2000	Female	20 to 24	29%<br>(24-35%)	71%<br>(65-76%)	0.97<br>(0.66-1.4)	2.4%<br>(1.8-3.3%)	3.7%<br>(2.4-5.4%)	1.8<br>(1.2-2.7)	320<br>(220-460)
Armenia	2000	Female	25 to 29	35%<br>(30-41%)	65%<br>(59-70%)	1.2<br>(0.85-1.7)	2.9%<br>(2.2-3.8%)	4.5%<br>(3.1-6.2%)	2.8<br>(1.9-3.9)	370<br>(260-490)
Armenia	2000	Female	30 to 34	38%<br>(32-44%)	62%<br>(56-68%)	1.4<br>(0.97-1.8)	3%<br>(2.3-3.9%)	5.1%<br>(3.7-6.9%)	4.1<br>(2.9-5.5)	430<br>(320-580)
Armenia	2000	Female	35 to 39	37%<br>(32-43%)	63%<br>(57-68%)	1.4<br>(0.97-1.9)	3.1%<br>(2.3-3.9%)	5.5%<br>(4.1-7.2%)	8.4<br>(6.2-11)	680<br>(520-880)
Armenia	2000	Female	40 to 44	37%<br>(32-43%)	63%<br>(57-68%)	1.4<br>(1-1.9)	3.3%<br>(2.5-4.3%)	5.9%<br>(4.5-7.7%)	14<br>(10-18)	870<br>(670-1100)
Armenia	2000	Female	45 to 49	37%<br>(31-44%)	63%<br>(56-69%)	1.5<br>(1-2.2)	3.7%<br>(2.5-5.1%)	6.3%<br>(4.3-8.7%)	18<br>(12-26)	930<br>(630-1300)
Armenia	2000	Female	50 to 54	37%<br>(27-49%)	63%<br>(51-73%)	1.6<br>(0.96-2.5)	4.1%<br>(2.6-6.2%)	6.5%<br>(4.1-9.7%)	20<br>(12-30)	830<br>(530-1200)
Armenia	2000	Female	55 to 59	36%<br>(22-54%)	64%<br>(46-78%)	1.6<br>(0.89-2.7)	4.4%<br>(2.3-7.3%)	6.4%<br>(3.3-10%)	22<br>(11-36)	770<br>(400-1300)
Armenia	2000	Female	60 to 64	34%<br>(20-52%)	66%<br>(48-80%)	1.6<br>(0.83-2.6)	4.2%<br>(1.7-7.5%)	5.7%<br>(2.4-10%)	61<br>(26-110)	1800<br>(790-3300)
Armenia	2000	Female	65 to 69	31%<br>(19-48%)	69%<br>(52-81%)	1.4<br>(0.84-2.3)	4.2%<br>(2-7.2%)	5.5%<br>(2.7-9.4%)	71<br>(34-120)	1700<br>(840-2900)
Armenia	2000	Female	70 to 74	27%<br>(17-42%)	73%<br>(58-83%)	1.2<br>(0.69-2.1)	3.9%<br>(1.5-7.4%)	4.8%<br>(1.8-9.2%)	110<br>(40-210)	2100<br>(800-4000)
Armenia	2000	Female	75 to 79	23%<br>(15-37%)	77%<br>(63-85%)	1.1<br>(0.56-1.8)	3.2%<br>(0.84-6.6%)	3.8%<br>(1-7.9%)	69<br>(17-150)	1000<br>(260-2100)
Armenia	2000	Female	80 to 84	21%<br>(13-33%)	79%<br>(67-87%)	0.97<br>(0.52-1.6)	2.7%<br>(0.85-5.4%)	3.2%<br>(0.94-6.6%)	43<br>(13-89)	480<br>(150-960)
Armenia	2000	Female	85 to 89	21%<br>(13-32%)	79%<br>(68-87%)	0.97<br>(0.5-1.7)	2.4%<br>(0.52-5.5%)	2.8%<br>(0.53-6.7%)	33<br>(6.3-78)	260<br>(57-590)
Armenia	2000	Female	90 to 94	22%<br>(14-33%)	78%<br>(67-86%)	1<br>(0.51-1.8)	2.1%<br>(0.14-5.7%)	2.4%<br>(-0.025-6.8%)	19<br>(-0.2-55)	100<br>(6.9-280)
Armenia	2000	Female	95 plus	25%<br>(14-40%)	75%<br>(60-86%)	1.1<br>(0.48-2.1)	2.1%<br>(-0.47-6.8%)	2.4%<br>(-0.78-8.1%)	7.2<br>(-2.4-25)	26<br>(-5.8-84)
Armenia	2000	Male	All Ages	83%<br>(80-86%)	17%<br>(14-20%)	1.3<br>(1.1-1.6)	2.1%<br>(0.76-3.4%)	0.48%<br>(-1.5-2.5%)	63<br>(-200-330)	10000<br>(3900-17000)
Armenia	2000	Male	15 to 19	76%<br>(71-80%)	24%<br>(20-29%)	0.68<br>(0.4-1.1)	3.5%<br>(2.3-5%)	4.1%<br>(2.1-6.9%)	6.1<br>(3.2-10)	700<br>(450-1000)

Location	Year	Sex	Age	Prevalence of current drinkers	Prevalence of abstainers	Population average of standard drinks daily	Percent of total attributable deaths	Percent of total attributable DALYs	Attributable deaths	Attributable DALYs
Armenia	2000	Male	20 to 24	92% (89-95%)	7.5% (5.3-11%)	1.4 (0.9-2.1)	7.2% (5.1-9.4%)	8% (4.7-12%)	11 (6.3-16)	1200 (840-1600)
Armenia	2000	Male	25 to 29	94% (92-96%)	5.8% (4.2-8%)	2.1 (1.3-3.1)	8.4% (6.2-11%)	9.8% (6.1-14%)	13 (7.9-19)	1300 (920-1700)
Armenia	2000	Male	30 to 34	95% (93-97%)	4.6% (3.1-6.7%)	2.4 (1.7-3.5)	8.1% (5.8-11%)	9.6% (6-14%)	17 (11-26)	1400 (1000-1900)
Armenia	2000	Male	35 to 39	96% (95-97%)	4% (2.7-5.3%)	2.4 (1.5-3.4)	6.6% (4.2-9.1%)	7.5% (3.9-11%)	23 (12-36)	1700 (1100-2500)
Armenia	2000	Male	40 to 44	96% (95-97%)	3.8% (2.6-5.4%)	2.4 (1.5-3.4)	4.9% (2.5-7.6%)	5.4% (1.8-8.9%)	26 (8.5-44)	1700 (820-2600)
Armenia	2000	Male	45 to 49	96% (94-97%)	4.2% (2.8-6.2%)	2.6 (1.8-3.8)	4% (1.4-7.5%)	4.1% (0.54-8.8%)	24 (3-51)	1300 (460-2500)
Armenia	2000	Male	50 to 54	95% (92-97%)	5.2% (3.5-7.6%)	2.5 (1.6-3.6)	3.2% (0.4-6.6%)	3.1% (-0.51-7.5%)	18 (-3-45)	900 (120-1900)
Armenia	2000	Male	55 to 59	93% (87-96%)	7.3% (3.8-13%)	2.1 (1.4-2.9)	1.8% (-0.62-4.4%)	1.6% (-1.4-4.8%)	9.1 (-8.2-28)	420 (-150-1000)
Armenia	2000	Male	60 to 64	90% (82-95%)	9.8% (4.6-18%)	1.7 (1.1-2.6)	0.92% (-1.3-3.4%)	0.69% (-2-3.7%)	12 (-35-62)	520 (-710-1900)
Armenia	2000	Male	65 to 69	89% (79-95%)	11% (5.2-21%)	1.5 (1-2.2)	0.072% (-2-2.3%)	-0.21% (-2.7-2.4%)	-4 (-52-47)	38 (-1000-1200)
Armenia	2000	Male	70 to 74	87% (77-93%)	13% (6.6-23%)	1.3 (0.8-2)	-0.63% (-2.7-1.5%)	-0.97% (-3.5-1.4%)	-26 (-93-39)	-360 (-1600-830)
Armenia	2000	Male	75 to 79	85% (73-92%)	15% (7.7-27%)	1.1 (0.67-1.8)	-1.2% (-3.2-0.76%)	-1.6% (-4-0.73%)	-21 (-54-9.8)	-260 (-720-170)
Armenia	2000	Male	80 to 84	83% (71-91%)	17% (9-29%)	1 (0.61-1.5)	-1.6% (-3.8-0.31%)	-2.3% (-5.1-0.13%)	-15 (-33-0.86)	-140 (-320-27)
Armenia	2000	Male	85 to 89	82% (70-90%)	18% (10-30%)	0.94 (0.59-1.4)	-2.2% (-4.4--0.22%)	-3% (-5.8--0.54%)	-15 (-30--2.7)	-100 (-210--10)
Armenia	2000	Male	90 to 94	81% (70-90%)	19% (10-30%)	0.88 (0.51-1.4)	-2.4% (-4.7--0.41%)	-3.3% (-6.1--0.8%)	-12 (-22--2.8)	-52 (-100--8.9)
Armenia	2000	Male	95 plus	80% (66-91%)	20% (8.7-34%)	0.8 (0.4-1.4)	-2.3% (-4.7--0.33%)	-3.3% (-6.3--0.74%)	-3.8 (-7.3--0.86)	-11 (-22--1.6)
Armenia	2005	Female	All Ages	28% (26-32%)	72% (68-74%)	1.1 (0.86-1.3)	3.3% (2.2-4.7%)	4.5% (2.5-7.4%)	570 (310-940)	14000 (9200-20000)
Armenia	2005	Female	15 to 19	22% (18-27%)	78% (73-82%)	0.67 (0.43-0.94)	1.4% (0.98-1.9%)	2.9% (1.9-4.1%)	1.2 (0.76-1.7)	190 (130-270)
Armenia	2005	Female	20 to 24	29% (24-36%)	71% (64-76%)	1 (0.67-1.5)	2.5% (1.8-3.4%)	3.8% (2.5-5.5%)	1.9 (1.2-2.8)	350 (240-500)
Armenia	2005	Female	25 to 29	36% (31-42%)	64% (58-69%)	1.3 (0.9-1.7)	2.9% (2.2-3.7%)	4.6% (3.2-6.5%)	2.7 (1.8-3.8)	390 (280-530)
Armenia	2005	Female	30 to 34	38% (33-45%)	62% (55-67%)	1.4 (1-2)	3% (2.3-3.9%)	5.3% (3.9-7.2%)	3.6 (2.6-5)	390 (300-530)
Armenia	2005	Female	35 to 39	37% (32-43%)	63% (57-68%)	1.4 (1-2)	3.1% (2.4-4%)	5.7% (4.2-7.6%)	5.8 (4.3-7.8)	480 (370-630)

Location	Year	Sex	Age	Prevalence of current drinkers	Prevalence of abstainers	Population average of standard drinks daily	Percent of total attributable deaths	Percent of total attributable DALYs	Attributable deaths	Attributable DALYs
Armenia	2005	Female	40 to 44	38% (32-44%)	62% (56-68%)	1.5 (1.1-2)	3.4% (2.6-4.4%)	6.1% (4.6-7.9%)	13 (9.6-17)	820 (620-1100)
Armenia	2005	Female	45 to 49	38% (31-44%)	62% (56-69%)	1.6 (1.1-2.2)	3.7% (2.6-5.1%)	6.4% (4.5-9%)	21 (14-30)	1100 (760-1500)
Armenia	2005	Female	50 to 54	38% (27-50%)	62% (50-73%)	1.7 (1-2.5)	4.3% (2.8-6.3%)	6.7% (4.4-9.9%)	28 (18-42)	1200 (770-1800)
Armenia	2005	Female	55 to 59	37% (23-54%)	63% (46-77%)	1.7 (0.92-2.9)	4.7% (2.4-7.8%)	6.8% (3.6-11%)	29 (16-49)	1000 (560-1700)
Armenia	2005	Female	60 to 64	34% (21-52%)	66% (48-79%)	1.6 (0.87-2.8)	4.5% (2-7.9%)	6.2% (2.8-11%)	32 (15-56)	960 (440-1700)
Armenia	2005	Female	65 to 69	32% (20-48%)	68% (52-80%)	1.5 (0.87-2.4)	4.5% (2.2-7.6%)	5.9% (3-9.9%)	88 (45-150)	2100 (1100-3600)
Armenia	2005	Female	70 to 74	28% (17-42%)	72% (58-83%)	1.3 (0.71-2.2)	4.2% (1.8-7.8%)	5.2% (2.2-9.8%)	96 (40-180)	1800 (760-3400)
Armenia	2005	Female	75 to 79	24% (15-37%)	76% (63-85%)	1.1 (0.58-1.9)	3.5% (1.1-6.8%)	4.2% (1.4-8.3%)	110 (36-220)	1600 (540-3200)
Armenia	2005	Female	80 to 84	22% (13-33%)	78% (67-87%)	1 (0.55-1.7)	3.1% (1.2-5.9%)	3.7% (1.4-7.1%)	80 (29-150)	870 (330-1600)
Armenia	2005	Female	85 to 89	22% (13-33%)	78% (67-87%)	1 (0.54-1.8)	2.7% (0.89-5.8%)	3.2% (0.95-7.1%)	33 (10-75)	260 (85-580)
Armenia	2005	Female	90 to 94	23% (14-34%)	77% (66-86%)	1.1 (0.54-1.9)	2.4% (0.4-6.1%)	2.8% (0.34-7.6%)	17 (2-44)	89 (15-230)
Armenia	2005	Female	95 plus	26% (15-41%)	74% (59-85%)	1.2 (0.5-2.2)	2.4% (-0.2-6.9%)	2.8% (-0.47-8.2%)	7.5 (-1.3-22)	27 (-2.3-77)
Armenia	2005	Male	All Ages	84% (82-87%)	16% (13-18%)	1.5 (1.2-1.7)	2.3% (1-3.7%)	0.77% (-1.3-2.9%)	110 (-180-400)	12000 (5200-19000)
Armenia	2005	Male	15 to 19	76% (69-80%)	24% (20-31%)	0.69 (0.4-1.1)	3.3% (2.2-4.6%)	4.1% (2.2-6.4%)	5 (2.6-8.1)	640 (420-910)
Armenia	2005	Male	20 to 24	92% (89-95%)	7.6% (5.2-11%)	1.5 (0.94-2.2)	7.1% (5.1-9.2%)	8.1% (4.9-12%)	11 (6.6-16)	1300 (910-1800)
Armenia	2005	Male	25 to 29	94% (92-96%)	5.9% (4.2-8.3%)	2.2 (1.4-3.2)	8.3% (6-11%)	9.9% (6-14%)	14 (8.3-20)	1400 (1000-1900)
Armenia	2005	Male	30 to 34	95% (93-97%)	4.7% (3.1-6.7%)	2.5 (1.7-3.6)	8.2% (5.9-11%)	10% (6.1-14%)	17 (10-25)	1400 (990-1900)
Armenia	2005	Male	35 to 39	96% (94-97%)	4% (2.7-5.8%)	2.4 (1.6-3.6)	7.1% (4.6-10%)	8.6% (4.5-13%)	20 (10-30)	1400 (900-2000)
Armenia	2005	Male	40 to 44	96% (94-97%)	3.9% (2.6-5.7%)	2.5 (1.6-3.5)	5.5% (3-8.2%)	6.2% (2.5-10%)	28 (11-46)	1800 (930-2600)
Armenia	2005	Male	45 to 49	96% (94-97%)	4.3% (2.9-6.4%)	2.7 (1.8-3.8)	4.6% (2-7.8%)	4.9% (1.3-9.5%)	34 (8.6-66)	1900 (780-3200)
Armenia	2005	Male	50 to 54	95% (92-97%)	5.3% (3.5-7.8%)	2.6 (1.7-3.6)	3.5% (0.7-6.9%)	3.6% (-0.15-8%)	30 (-1.2-68)	1400 (300-2900)
Armenia	2005	Male	55 to 59	93% (87-96%)	7.4% (4-13%)	2.2 (1.4-3)	2.1% (-0.33-5%)	2% (-1.1-5.5%)	16 (-8.5-45)	700 (-100-1700)

Location	Year	Sex	Age	Prevalence of current drinkers	Prevalence of abstainers	Population average of standard drinks daily	Percent of total attributable deaths	Percent of total attributable DALYs	Attributable deaths	Attributable DALYs
Armenia	2005	Male	60 to 64	90% (82-95%)	9.8% (4.7-18%)	1.8 (1.1-2.7)	1.3% (-0.9-3.8%)	1.1% (-1.6-4.2%)	9.5 (-14-36)	360 (-260-1100)
Armenia	2005	Male	65 to 69	89% (79-95%)	11% (5.1-21%)	1.6 (1.1-2.3)	0.44% (-1.6-2.7%)	0.24% (-2.2-2.9%)	5.2 (-47-63)	260 (-910-1600)
Armenia	2005	Male	70 to 74	87% (77-93%)	13% (6.9-23%)	1.4 (0.85-2.1)	-0.25% (-2.3-1.9%)	-0.51% (-3-2%)	-12 (-69-48)	-120 (-1100-970)
Armenia	2005	Male	75 to 79	85% (74-92%)	15% (7.6-26%)	1.2 (0.7-1.8)	-0.79% (-2.7-1.2%)	-1.1% (-3.5-1.2%)	-29 (-90-32)	-340 (-1200-520)
Armenia	2005	Male	80 to 84	83% (71-91%)	17% (8.9-29%)	1 (0.65-1.6)	-1.3% (-3.3-0.59%)	-1.8% (-4.4-0.52%)	-23 (-56-6.9)	-200 (-540-93)
Armenia	2005	Male	85 to 89	82% (70-90%)	18% (10-30%)	0.97 (0.6-1.4)	-1.6% (-3.8-0.16%)	-2.3% (-5--0.027%)	-9.1 (-20--0.1)	-59 (-130-5.9)
Armenia	2005	Male	90 to 94	81% (70-90%)	19% (10-30%)	0.9 (0.55-1.4)	-2.1% (-4.3--0.22%)	-2.9% (-5.6--0.53%)	-5.7 (-11--1.1)	-25 (-52--2.6)
Armenia	2005	Male	95 plus	80% (66-91%)	20% (9.4-34%)	0.82 (0.43-1.4)	-2.1% (-4.5--0.18%)	-3% (-5.8--0.57%)	-2.6 (-5.1--0.49)	-7.4 (-16--0.62)
Armenia	2010	Female	All Ages	32% (28-37%)	68% (63-72%)	1.3 (1.1-1.7)	3.7% (2.5-5.3%)	5.1% (2.9-8.2%)	670 (390-1100)	15000 (10000-22000)
Armenia	2010	Female	15 to 19	25% (17-35%)	75% (65-83%)	0.87 (0.51-1.3)	1.6% (1.1-2.2%)	3.4% (2.1-5.1%)	1.1 (0.71-1.7)	170 (110-240)
Armenia	2010	Female	20 to 24	36% (25-47%)	64% (53-75%)	1.4 (0.84-2.1)	2.9% (2.1-4%)	4.7% (2.9-7.2%)	2.3 (1.4-3.6)	390 (260-550)
Armenia	2010	Female	25 to 29	41% (29-53%)	59% (47-71%)	1.6 (0.99-2.4)	3.1% (2.3-4%)	5.3% (3.4-7.5%)	2.8 (1.8-4)	420 (290-580)
Armenia	2010	Female	30 to 34	43% (31-57%)	57% (43-69%)	1.8 (1.1-2.7)	3.2% (2.4-4.3%)	6.1% (4.1-8.5%)	4 (2.7-5.7)	450 (320-610)
Armenia	2010	Female	35 to 39	44% (31-56%)	56% (44-69%)	1.9 (1.2-2.9)	3.5% (2.5-4.8%)	6.7% (4.6-9.4%)	5.8 (3.9-8.2)	470 (340-650)
Armenia	2010	Female	40 to 44	43% (32-54%)	57% (46-68%)	2 (1.2-2.9)	3.9% (2.7-5.3%)	7% (4.7-9.6%)	11 (7.3-15)	680 (480-940)
Armenia	2010	Female	45 to 49	42% (29-54%)	58% (46-71%)	2 (1.2-3)	4.3% (2.9-6.2%)	7.5% (4.9-11%)	22 (14-32)	1100 (770-1600)
Armenia	2010	Female	50 to 54	40% (27-53%)	60% (47-73%)	1.9 (1.1-3)	4.7% (3-7%)	7.5% (4.8-11%)	34 (22-51)	1500 (970-2200)
Armenia	2010	Female	55 to 59	37% (23-54%)	63% (46-77%)	1.9 (1-3.2)	5% (2.8-8.1%)	7.3% (4.2-12%)	40 (23-64)	1400 (820-2300)
Armenia	2010	Female	60 to 64	34% (20-52%)	66% (48-80%)	1.8 (0.97-3)	4.9% (2.4-8.2%)	6.7% (3.4-11%)	40 (19-67)	1200 (580-2000)
Armenia	2010	Female	65 to 69	32% (20-49%)	68% (51-80%)	1.7 (0.94-2.7)	5% (2.7-8.2%)	6.6% (3.5-11%)	50 (26-83)	1200 (640-2000)
Armenia	2010	Female	70 to 74	28% (18-43%)	72% (57-82%)	1.4 (0.78-2.4)	4.7% (2.1-8.7%)	5.9% (2.7-11%)	120 (55-230)	2300 (1100-4300)
Armenia	2010	Female	75 to 79	24% (15-37%)	76% (63-85%)	1.2 (0.61-2.1)	4.1% (1.6-7.5%)	4.9% (1.9-9.1%)	110 (43-210)	1600 (640-3000)

Location	Year	Sex	Age	Prevalence of current drinkers	Prevalence of abstainers	Population average of standard drinks daily	Percent of total attributable deaths	Percent of total attributable DALYs	Attributable deaths	Attributable DALYs
Armenia	2010	Female	80 to 84	22% (14-33%)	78% (67-86%)	1.1 (0.59-1.9)	3.9% (1.7-6.9%)	4.5% (2-8.2%)	120 (53-220)	1300 (590-2400)
Armenia	2010	Female	85 to 89	21% (13-32%)	79% (68-87%)	1.1 (0.57-1.9)	3.4% (1.3-6.6%)	4.1% (1.6-8.1%)	73 (28-150)	570 (230-1100)
Armenia	2010	Female	90 to 94	22% (14-34%)	78% (66-86%)	1.2 (0.57-2.2)	2.9% (0.78-6.6%)	3.5% (0.85-8.3%)	22 (5.3-52)	120 (32-270)
Armenia	2010	Female	95 plus	25% (15-40%)	75% (60-85%)	1.3 (0.56-2.4)	2.9% (0.27-7.6%)	3.4% (0.086-9.2%)	7.8 (0.2-21)	29 (2.5-74)
Armenia	2010	Male	All Ages	83% (80-85%)	17% (15-20%)	1.6 (1.3-1.9)	2.9% (1.5-4.4%)	1.3% (-0.78-3.5%)	190 (-110-490)	14000 (7300-22000)
Armenia	2010	Male	15 to 19	69% (59-76%)	31% (24-41%)	0.64 (0.35-1)	3.2% (2.2-4.5%)	3.9% (2-6.3%)	4.1 (2.1-6.6)	540 (350-770)
Armenia	2010	Male	20 to 24	90% (84-94%)	10% (6.5-16%)	1.5 (0.92-2.2)	7% (5.1-9.2%)	8% (4.7-12%)	12 (7.3-18)	1500 (1000-2000)
Armenia	2010	Male	25 to 29	93% (88-95%)	7.4% (4.8-12%)	2.2 (1.4-3.3)	8.3% (6.1-11%)	10% (6.1-15%)	16 (9.6-24)	1700 (1200-2300)
Armenia	2010	Male	30 to 34	94% (91-96%)	5.8% (3.5-9.5%)	2.6 (1.7-3.8)	8.3% (6-11%)	10% (6.5-15%)	18 (11-26)	1600 (1100-2100)
Armenia	2010	Male	35 to 39	95% (92-97%)	4.9% (3.1-7.5%)	2.5 (1.5-3.8)	7.4% (4.9-10%)	9.1% (4.9-14%)	20 (10-29)	1400 (920-2000)
Armenia	2010	Male	40 to 44	95% (93-97%)	4.8% (2.8-7.2%)	2.6 (1.5-3.8)	6.1% (3.5-9.2%)	7.2% (3.3-12%)	25 (12-41)	1500 (890-2300)
Armenia	2010	Male	45 to 49	95% (91-97%)	5.4% (3.2-8.5%)	2.7 (1.8-4)	5.5% (2.8-8.9%)	6.3% (2.4-11%)	42 (16-73)	2200 (1100-3500)
Armenia	2010	Male	50 to 54	94% (90-96%)	6.5% (3.9-10%)	2.7 (1.7-3.8)	4.2% (1.3-7.7%)	4.5% (0.69-9%)	44 (6.5-89)	2000 (640-3800)
Armenia	2010	Male	55 to 59	92% (85-96%)	8.2% (4.2-15%)	2.3 (1.5-3.3)	2.8% (0.3-5.8%)	2.8% (-0.35-6.7%)	31 (-3.8-73)	1200 (140-2700)
Armenia	2010	Male	60 to 64	90% (82-95%)	9.8% (4.6-18%)	2 (1.2-3)	1.7% (-0.67-4.7%)	1.6% (-1.3-5.1%)	17 (-14-54)	610 (-240-1700)
Armenia	2010	Male	65 to 69	89% (79-95%)	11% (5.2-21%)	1.8 (1.2-2.6)	1% (-1-3.3%)	0.89% (-1.6-3.7%)	10 (-17-41)	310 (-320-1000)
Armenia	2010	Male	70 to 74	87% (77-93%)	13% (6.8-23%)	1.5 (0.92-2.3)	0.26% (-1.8-2.5%)	0.078% (-2.4-2.8%)	1.9 (-60-69)	140 (-960-1400)
Armenia	2010	Male	75 to 79	85% (73-93%)	15% (7.5-27%)	1.3 (0.75-2)	-0.26% (-2.2-1.7%)	-0.52% (-2.9-1.8%)	-12 (-67-43)	-100 (-850-680)
Armenia	2010	Male	80 to 84	83% (71-91%)	17% (8.7-29%)	1.2 (0.7-1.7)	-0.86% (-3-0.98%)	-1.2% (-3.7-1%)	-24 (-73-19)	-210 (-710-230)
Armenia	2010	Male	85 to 89	82% (70-90%)	18% (10-30%)	1.1 (0.66-1.6)	-1% (-2.9-0.65%)	-1.6% (-4.1-0.54%)	-13 (-35-4.6)	-80 (-230-51)
Armenia	2010	Male	90 to 94	81% (69-90%)	19% (10-31%)	1 (0.59-1.6)	-1.4% (-3.4-0.22%)	-2.2% (-4.8--0.052%)	-3.9 (-8.6--0.09)	-16 (-39-2.6)
Armenia	2010	Male	95 plus	80% (66-91%)	20% (9.1-34%)	0.91 (0.45-1.6)	-1.5% (-3.5-0.27%)	-2.4% (-5--0.15%)	-1.1 (-2.4--0.071)	-3 (-7.2-0.56)

Location	Year	Sex	Age	Prevalence of current drinkers	Prevalence of abstainers	Population average of standard drinks daily	Percent of total attributable deaths	Percent of total attributable DALYs	Attributable deaths	Attributable DALYs
Armenia	2016	Female	All Ages	32% (28-37%)	68% (63-72%)	1.6 (1.2-2.2)	4.1% (2.7-5.9%)	5.9% (3.3-9.5%)	780 (430-1300)	17000 (11000-25000)
Armenia	2016	Female	15 to 19	25% (17-34%)	75% (66-83%)	1 (0.55-1.6)	1.7% (1.1-2.5%)	4% (2.3-6.2%)	0.72 (0.42-1.1)	120 (74-180)
Armenia	2016	Female	20 to 24	36% (25-47%)	64% (53-75%)	1.6 (0.95-2.5)	3.1% (2.1-4.3%)	5.4% (3.3-8.4%)	2 (1.2-3.1)	370 (240-530)
Armenia	2016	Female	25 to 29	41% (29-52%)	59% (48-71%)	1.9 (1.1-3)	3.2% (2.4-4.3%)	5.9% (3.7-8.7%)	2.9 (1.8-4.4)	510 (350-720)
Armenia	2016	Female	30 to 34	43% (31-56%)	57% (44-69%)	2.1 (1.2-3.3)	3.4% (2.4-4.5%)	6.8% (4.4-10%)	4.5 (2.9-6.8)	570 (400-780)
Armenia	2016	Female	35 to 39	44% (31-55%)	56% (45-69%)	2.2 (1.3-3.4)	3.5% (2.5-4.9%)	7.5% (4.8-11%)	6.3 (3.9-9.2)	580 (400-810)
Armenia	2016	Female	40 to 44	43% (31-55%)	57% (45-69%)	2.3 (1.3-3.4)	3.9% (2.7-5.5%)	7.8% (5.1-11%)	9.4 (5.9-14)	650 (440-930)
Armenia	2016	Female	45 to 49	42% (28-53%)	58% (47-72%)	2.4 (1.3-3.7)	4.4% (2.9-6.6%)	8.3% (5.4-13%)	15 (9.1-23)	820 (540-1200)
Armenia	2016	Female	50 to 54	39% (27-52%)	61% (48-73%)	2.3 (1.3-3.6)	4.8% (3.1-7.3%)	8.3% (5.3-13%)	32 (20-50)	1500 (940-2300)
Armenia	2016	Female	55 to 59	36% (22-54%)	64% (46-78%)	2.2 (1.2-3.6)	5.1% (3-8.1%)	8.2% (4.8-13%)	52 (29-84)	2000 (1200-3200)
Armenia	2016	Female	60 to 64	34% (21-53%)	66% (47-79%)	2.1 (1.1-3.5)	5.2% (2.8-8.5%)	7.6% (4.2-12%)	68 (36-110)	2200 (1200-3600)
Armenia	2016	Female	65 to 69	32% (20-47%)	68% (53-80%)	1.9 (1.1-3.2)	5.4% (2.9-9%)	7.6% (4.2-13%)	68 (36-120)	1700 (920-2900)
Armenia	2016	Female	70 to 74	28% (17-42%)	72% (58-83%)	1.7 (0.87-2.9)	5.1% (2.4-9.3%)	6.7% (3.1-12%)	56 (26-100)	1100 (510-2000)
Armenia	2016	Female	75 to 79	23% (15-37%)	77% (63-85%)	1.4 (0.7-2.6)	4.4% (1.8-8.3%)	5.6% (2.3-10%)	130 (51-240)	1900 (810-3600)
Armenia	2016	Female	80 to 84	21% (13-33%)	79% (67-87%)	1.3 (0.68-2.2)	4.4% (1.9-8.2%)	5.4% (2.3-10%)	170 (71-320)	1800 (790-3400)
Armenia	2016	Female	85 to 89	21% (13-32%)	79% (68-87%)	1.3 (0.67-2.3)	4.1% (1.6-8.1%)	5.1% (1.8-10%)	95 (35-190)	740 (290-1500)
Armenia	2016	Female	90 to 94	23% (14-33%)	77% (67-86%)	1.4 (0.68-2.6)	3.8% (1.1-8.2%)	4.6% (1.2-10%)	57 (15-130)	310 (89-670)
Armenia	2016	Female	95 plus	25% (14-40%)	75% (60-86%)	1.5 (0.63-3)	3.8% (0.64-8.9%)	4.5% (0.56-11%)	16 (1.9-39)	57 (9.6-140)
Armenia	2016	Male	All Ages	84% (81-86%)	16% (14-19%)	1.9 (1.4-2.6)	3.5% (1.9-5.5%)	1.9% (-0.34-4.5%)	260 (-46-630)	17000 (8500-26000)
Armenia	2016	Male	15 to 19	68% (58-76%)	32% (24-42%)	0.76 (0.4-1.3)	3.5% (2.4-5.1%)	4.5% (2.4-7.4%)	2.7 (1.4-4.5)	340 (230-500)
Armenia	2016	Male	20 to 24	90% (84-94%)	10% (6.4-16%)	1.7 (1-2.8)	7.8% (5.6-11%)	9.3% (5.3-14%)	10 (5.8-16)	1200 (810-1700)
Armenia	2016	Male	25 to 29	93% (88-95%)	7.5% (4.8-12%)	2.6 (1.6-4.1)	9.2% (6.7-12%)	11% (6.6-17%)	17 (9.1-26)	1700 (1200-2400)

Location	Year	Sex	Age	Prevalence of current drinkers	Prevalence of abstainers	Population average of standard drinks daily	Percent of total attributable deaths	Percent of total attributable DALYs	Attributable deaths	Attributable DALYs
Armenia	2016	Male	30 to 34	94% (90-96%)	5.9% (3.6-9.7%)	3.1 (2-4.5)	9.1% (6.3-12%)	12% (6.8-17%)	21 (12-33)	1900 (1300-2600)
Armenia	2016	Male	35 to 39	95% (92-97%)	5% (3.1-7.6%)	3 (1.7-4.6)	8% (5.1-11%)	10% (5.3-16%)	21 (10-34)	1600 (980-2300)
Armenia	2016	Male	40 to 44	95% (93-97%)	4.8% (2.9-7.3%)	3 (1.7-4.5)	7% (4-11%)	8.6% (4.1-14%)	23 (10-39)	1400 (800-2200)
Armenia	2016	Male	45 to 49	95% (91-97%)	5.4% (3.2-8.8%)	3.2 (2-5)	6.9% (3.7-11%)	8.2% (3.6-15%)	32 (14-58)	1700 (840-2800)
Armenia	2016	Male	50 to 54	93% (90-96%)	6.6% (3.9-10%)	3.2 (2-4.8)	5.7% (2.2-10%)	6.5% (1.9-12%)	54 (16-100)	2400 (950-4400)
Armenia	2016	Male	55 to 59	92% (85-96%)	8.3% (4.2-15%)	2.7 (1.8-4)	3.8% (0.99-7.8%)	4.2% (0.45-9.1%)	54 (5.8-120)	2100 (560-4100)
Armenia	2016	Male	60 to 64	90% (82-95%)	9.9% (4.6-18%)	2.3 (1.4-3.6)	2.6% (-0.067-6.5%)	2.7% (-0.52-7.2%)	42 (-8.4-110)	1400 (-37-3300)
Armenia	2016	Male	65 to 69	89% (79-95%)	11% (5.3-21%)	2.1 (1.3-3.1)	1.7% (-0.49-4.5%)	1.7% (-1-5.1%)	23 (-14-70)	670 (-200-1800)
Armenia	2016	Male	70 to 74	87% (76-93%)	13% (7-24%)	1.8 (1-2.7)	0.89% (-1.3-3.7%)	0.78% (-1.8-4.1%)	7.8 (-18-40)	200 (-290-800)
Armenia	2016	Male	75 to 79	85% (73-92%)	15% (7.6-27%)	1.5 (0.82-2.4)	0.25% (-1.8-2.7%)	0.033% (-2.4-2.9%)	0.66 (-51-62)	92 (-630-990)
Armenia	2016	Male	80 to 84	83% (71-91%)	17% (9.1-29%)	1.3 (0.76-2.1)	-0.47% (-2.7-1.7%)	-0.82% (-3.6-1.7%)	-18 (-80-37)	-130 (-760-470)
Armenia	2016	Male	85 to 89	81% (69-90%)	19% (10-31%)	1.2 (0.73-1.9)	-0.81% (-2.8-1.1%)	-1.3% (-3.9-1.1%)	-15 (-45-12)	-85 (-310-110)
Armenia	2016	Male	90 to 94	81% (69-89%)	19% (11-31%)	1.2 (0.66-1.9)	-1.3% (-3.4-0.74%)	-2% (-4.8-0.49%)	-9.9 (-24-2.6)	-40 (-110-24)
Armenia	2016	Male	95 plus	80% (65-91%)	20% (9.1-35%)	1 (0.5-1.9)	-1.3% (-3.5-0.65%)	-2.2% (-5-0.23%)	-1.7 (-3.8-0.18)	-4.3 (-11-2.1)
Azerbaijan	1990	Female	All Ages	55% (48-62%)	45% (38-52%)	0.98 (0.73-1.3)	0.94% (0.021-2%)	0.47% (-2-3.2%)	120 (-510-810)	13000 (260-27000)
Azerbaijan	1990	Female	15 to 19	55% (43-70%)	45% (30-57%)	0.94 (0.54-1.5)	1.7% (1-2.7%)	3% (1.4-5.2%)	5.1 (2.4-8.8)	630 (370-980)
Azerbaijan	1990	Female	20 to 24	62% (49-75%)	38% (25-51%)	1.3 (0.75-2.1)	3% (1.8-4.4%)	4.3% (1.9-7.4%)	11 (4.9-19)	1300 (810-2000)
Azerbaijan	1990	Female	25 to 29	65% (51-77%)	35% (23-49%)	1.5 (0.91-2.2)	3.3% (2.1-4.7%)	4.8% (2.1-7.8%)	16 (7-27)	1700 (1000-2500)
Azerbaijan	1990	Female	30 to 34	66% (52-79%)	34% (21-48%)	1.6 (0.99-2.3)	3.4% (2.1-4.9%)	5.5% (2.6-8.4%)	19 (9.1-31)	1700 (1000-2400)
Azerbaijan	1990	Female	35 to 39	66% (53-80%)	34% (20-47%)	1.6 (0.97-2.5)	3.4% (2-5.1%)	5.7% (2.8-9.1%)	20 (9.7-32)	1400 (810-2100)
Azerbaijan	1990	Female	40 to 44	66% (52-79%)	34% (21-48%)	1.6 (0.99-2.6)	3.3% (1.7-5.3%)	5.8% (2.5-9.6%)	17 (7.3-29)	970 (480-1600)
Azerbaijan	1990	Female	45 to 49	66% (52-79%)	34% (21-48%)	1.7 (0.98-2.6)	3% (0.95-5.6%)	5.1% (1.4-9.7%)	24 (6.2-46)	1100 (370-2200)

Location	Year	Sex	Age	Prevalence of current drinkers	Prevalence of abstainers	Population average of standard drinks daily	Percent of total attributable deaths	Percent of total attributable DALYs	Attributable deaths	Attributable DALYs
Azerbaijan	1990	Female	50 to 54	65% (50-77%)	35% (23-50%)	1.6 (0.96-2.6)	2.6% (0.16-5.9%)	3.9% (0.067-8.8%)	37 (0.58-82)	1500 (94-3400)
Azerbaijan	1990	Female	55 to 59	63% (49-76%)	37% (24-51%)	1.6 (0.95-2.6)	2.2% (-0.73-5.7%)	3% (-1.4-8%)	36 (-17-99)	1300 (-460-3500)
Azerbaijan	1990	Female	60 to 64	61% (47-76%)	39% (24-53%)	1.5 (0.89-2.6)	1.7% (-1.4-5.8%)	2.1% (-2.3-7.6%)	35 (-39-130)	1100 (-970-3800)
Azerbaijan	1990	Female	65 to 69	59% (47-74%)	41% (26-53%)	1.5 (0.93-2.3)	1.2% (-2-4.9%)	1.6% (-2.7-6.5%)	25 (-42-100)	600 (-930-2400)
Azerbaijan	1990	Female	70 to 74	57% (45-69%)	43% (31-55%)	1.4 (0.85-2.2)	0.16% (-3.5-4.2%)	0.15% (-4.4-5.3%)	2.9 (-83-100)	72 (-1500-1900)
Azerbaijan	1990	Female	75 to 79	54% (44-67%)	46% (33-56%)	1.4 (0.77-2.1)	-0.63% (-4.1-3.6%)	-0.87% (-5.2-4.2%)	-25 (-150-120)	-310 (-2000-1800)
Azerbaijan	1990	Female	80 to 84	52% (40-66%)	48% (34-60%)	1.3 (0.78-2)	-0.83% (-4.3-3%)	-1.1% (-5.4-3.4%)	-24 (-120-74)	-220 (-1200-810)
Azerbaijan	1990	Female	85 to 89	52% (41-63%)	48% (37-59%)	1.3 (0.77-1.9)	-1.6% (-5-2%)	-2% (-6-2.3%)	-42 (-130-48)	-290 (-910-360)
Azerbaijan	1990	Female	90 to 94	53% (42-64%)	47% (36-58%)	1.3 (0.76-2.1)	-2.2% (-5.6-1.8%)	-2.7% (-6.8-2%)	-28 (-70-20)	-130 (-340-110)
Azerbaijan	1990	Female	95 plus	53% (40-68%)	47% (32-60%)	1.2 (0.62-2.2)	-2.5% (-6-1.9%)	-3.1% (-7.4-2.1%)	-9.6 (-23-6.5)	-30 (-74-23)
Azerbaijan	1990	Male	All Ages	48% (39-58%)	52% (42-61%)	1.9 (1.5-2.5)	5.3% (3.8-7%)	6.2% (3.7-9.1%)	1800 (1100-2700)	88000 (62000-120000)
Azerbaijan	1990	Male	15 to 19	36% (22-53%)	64% (47-78%)	0.47 (0.24-0.8)	4.2% (2.7-5.8%)	4.8% (2.3-7.3%)	20 (9.1-30)	2300 (1400-3200)
Azerbaijan	1990	Male	20 to 24	57% (38-74%)	43% (26-62%)	1.6 (0.98-2.4)	11% (8-14%)	11% (7.3-16%)	65 (41-93)	6400 (4600-8500)
Azerbaijan	1990	Male	25 to 29	68% (50-82%)	32% (18-50%)	3.5 (2.4-4.9)	16% (12-20%)	19% (14-25%)	140 (95-190)	11000 (8300-14000)
Azerbaijan	1990	Male	30 to 34	75% (56-87%)	25% (13-44%)	4.5 (3.1-6.3)	17% (13-22%)	21% (16-28%)	170 (120-230)	12000 (8800-15000)
Azerbaijan	1990	Male	35 to 39	77% (61-89%)	23% (11-39%)	4.1 (2.8-5.8)	15% (11-20%)	19% (13-26%)	150 (100-210)	9400 (6600-12000)
Azerbaijan	1990	Male	40 to 44	77% (62-89%)	23% (11-38%)	4.1 (2.7-5.9)	14% (9.9-20%)	17% (11-25%)	120 (74-170)	6300 (4300-8700)
Azerbaijan	1990	Male	45 to 49	76% (58-88%)	24% (12-42%)	4.6 (2.9-6.7)	15% (9.9-20%)	18% (11-25%)	180 (110-270)	8600 (5600-12000)
Azerbaijan	1990	Male	50 to 54	75% (58-87%)	25% (13-42%)	4.4 (2.8-6.3)	13% (8.1-18%)	15% (8.8-22%)	290 (160-420)	12000 (7100-16000)
Azerbaijan	1990	Male	55 to 59	72% (54-86%)	28% (14-46%)	3.4 (2.2-5.1)	9.4% (4.7-15%)	11% (4.7-17%)	260 (110-420)	8900 (4300-14000)
Azerbaijan	1990	Male	60 to 64	69% (49-85%)	31% (15-51%)	2.7 (1.6-4.4)	6.5% (2.3-12%)	7.1% (2.1-14%)	200 (59-400)	6000 (2100-11000)
Azerbaijan	1990	Male	65 to 69	66% (47-82%)	34% (18-53%)	2.4 (1.4-3.7)	4.9% (1.2-9.6%)	5.2% (0.95-11%)	96 (18-200)	2400 (580-4700)

Location	Year	Sex	Age	Prevalence of current drinkers	Prevalence of abstainers	Population average of standard drinks daily	Percent of total attributable deaths	Percent of total attributable DALYs	Attributable deaths	Attributable DALYs
Azerbaijan	1990	Male	70 to 74	62% (43-78%)	38% (22-57%)	1.9 (1.1-3.2)	3.4% (0.082-8.2%)	3.5% (-0.26-9.2%)	59 (-4.3-150)	1200 (27-2900)
Azerbaijan	1990	Male	75 to 79	57% (38-76%)	43% (24-62%)	1.6 (0.82-2.7)	2.6% (-0.33-6.9%)	2.7% (-0.66-7.6%)	58 (-14-160)	900 (-110-2300)
Azerbaijan	1990	Male	80 to 84	54% (35-71%)	46% (29-65%)	1.4 (0.76-2.3)	2% (-0.84-5.4%)	1.9% (-1.4-5.8%)	26 (-19-78)	310 (-140-860)
Azerbaijan	1990	Male	85 to 89	52% (34-69%)	48% (31-66%)	1.3 (0.68-2.1)	1.3% (-1.6-4.2%)	1.2% (-2.2-4.6%)	10 (-20-41)	100 (-120-320)
Azerbaijan	1990	Male	90 to 94	51% (34-68%)	49% (32-66%)	1.2 (0.61-1.9)	0.68% (-2.1-3.5%)	0.38% (-2.9-3.7%)	1.4 (-11-14)	15 (-47-79)
Azerbaijan	1990	Male	95 plus	52% (30-72%)	48% (28-70%)	1.1 (0.46-2)	0.68% (-2.1-3.5%)	0.28% (-3.1-3.4%)	0.31 (-3.5-3.8)	2.9 (-9.2-15)
Azerbaijan	1995	Female	All Ages	55% (49-62%)	45% (38-51%)	1 (0.78-1.3)	1.2% (0.075-2.3%)	0.81% (-1.8-3.6%)	210 (-470-920)	16000 (1000-32000)
Azerbaijan	1995	Female	15 to 19	55% (43-70%)	45% (30-57%)	0.96 (0.57-1.5)	1.8% (1-2.9%)	3.2% (1.3-5.4%)	6.7 (2.7-12)	740 (400-1200)
Azerbaijan	1995	Female	20 to 24	62% (49-74%)	38% (26-51%)	1.3 (0.78-2.1)	3.2% (2-5%)	4.7% (2-8.1%)	14 (6-24)	1500 (860-2300)
Azerbaijan	1995	Female	25 to 29	64% (51-77%)	36% (23-49%)	1.5 (0.91-2.2)	3.6% (2.2-5.2%)	5.2% (2.2-8.5%)	20 (8.8-34)	1900 (1100-2900)
Azerbaijan	1995	Female	30 to 34	66% (53-79%)	34% (21-47%)	1.6 (1-2.3)	3.7% (2.2-5.3%)	5.8% (2.9-9%)	30 (15-46)	2400 (1400-3400)
Azerbaijan	1995	Female	35 to 39	66% (53-80%)	34% (20-47%)	1.6 (1-2.4)	3.6% (2.1-5.5%)	6% (3-9.4%)	34 (17-54)	2200 (1300-3300)
Azerbaijan	1995	Female	40 to 44	66% (52-80%)	34% (20-48%)	1.6 (1-2.4)	3.6% (1.9-5.7%)	6.1% (3-9.7%)	33 (15-54)	1800 (930-2800)
Azerbaijan	1995	Female	45 to 49	66% (51-79%)	34% (21-49%)	1.7 (1-2.6)	3.2% (0.97-6%)	5.1% (1.3-9.7%)	26 (6.8-49)	1200 (360-2200)
Azerbaijan	1995	Female	50 to 54	65% (51-78%)	35% (22-49%)	1.6 (0.98-2.6)	2.7% (0.16-5.7%)	4% (0.064-9%)	28 (0.44-62)	1100 (60-2500)
Azerbaijan	1995	Female	55 to 59	63% (49-76%)	37% (24-51%)	1.6 (0.98-2.6)	2.2% (-0.8-5.8%)	2.9% (-1.4-8.1%)	46 (-23-130)	1600 (-610-4400)
Azerbaijan	1995	Female	60 to 64	61% (48-76%)	39% (24-52%)	1.6 (0.92-2.5)	1.7% (-1.7-5.9%)	2% (-2.5-7.6%)	44 (-53-160)	1300 (-1400-4700)
Azerbaijan	1995	Female	65 to 69	59% (47-74%)	41% (26-53%)	1.5 (0.96-2.3)	1.1% (-2.4-4.9%)	1.4% (-3.1-6.3%)	39 (-87-170)	920 (-1900-4000)
Azerbaijan	1995	Female	70 to 74	57% (45-70%)	43% (30-55%)	1.4 (0.85-2.2)	0.028% (-3.8-4.3%)	-0.0021% (-4.8-5.2%)	-0.096 (-100-120)	14 (-1900-2200)
Azerbaijan	1995	Female	75 to 79	54% (44-67%)	46% (33-56%)	1.4 (0.78-2.2)	-0.69% (-4.2-3.6%)	-0.92% (-5.2-4.1%)	-22 (-120-96)	-270 (-1700-1400)
Azerbaijan	1995	Female	80 to 84	53% (41-66%)	47% (34-59%)	1.3 (0.81-2)	-0.83% (-4.3-2.8%)	-1.1% (-5.2-3.3%)	-22 (-110-66)	-210 (-1100-700)
Azerbaijan	1995	Female	85 to 89	52% (41-64%)	48% (36-59%)	1.3 (0.77-1.9)	-1.6% (-5.1-1.9%)	-2% (-6.1-2.3%)	-31 (-97-35)	-220 (-690-260)

Location	Year	Sex	Age	Prevalence of current drinkers	Prevalence of abstainers	Population average of standard drinks daily	Percent of total attributable deaths	Percent of total attributable DALYs	Attributable deaths	Attributable DALYs
Azerbaijan	1995	Female	90 to 94	53% (42-64%)	47% (36-58%)	1.3 (0.75-2.1)	-2.2% (-5.8-1.8%)	-2.8% (-7-2.1%)	-27 (-68-20)	-130 (-330-100)
Azerbaijan	1995	Female	95 plus	53% (41-68%)	47% (32-59%)	1.3 (0.62-2.2)	-2.5% (-6.2-1.6%)	-3.1% (-7.4-1.8%)	-10 (-24-5.7)	-32 (-78-20)
Azerbaijan	1995	Male	All Ages	50% (41-59%)	50% (41-59%)	2 (1.6-2.5)	6.6% (4.9-8.6%)	7.5% (4.9-11%)	2500 (1600-3500)	120000 (86000-150000)
Azerbaijan	1995	Male	15 to 19	36% (22-53%)	64% (47-78%)	0.48 (0.26-0.84)	4.3% (2.7-6.2%)	4.8% (2.4-7.4%)	25 (12-39)	2600 (1600-3700)
Azerbaijan	1995	Male	20 to 24	58% (38-74%)	42% (26-62%)	1.6 (1-2.4)	11% (8.3-15%)	12% (8-17%)	99 (63-140)	8800 (6300-12000)
Azerbaijan	1995	Male	25 to 29	68% (50-82%)	32% (18-50%)	3.5 (2.4-4.9)	18% (14-22%)	21% (15-26%)	190 (130-250)	14000 (10000-18000)
Azerbaijan	1995	Male	30 to 34	75% (56-87%)	25% (13-44%)	4.5 (3.1-6.2)	19% (15-24%)	23% (17-29%)	280 (200-360)	19000 (14000-23000)
Azerbaijan	1995	Male	35 to 39	77% (62-88%)	23% (12-38%)	4.1 (3-5.7)	17% (12-21%)	20% (14-26%)	270 (190-360)	16000 (12000-21000)
Azerbaijan	1995	Male	40 to 44	78% (62-89%)	22% (11-38%)	4.1 (2.8-5.7)	15% (10-20%)	18% (12-24%)	240 (150-340)	13000 (8600-17000)
Azerbaijan	1995	Male	45 to 49	77% (58-88%)	23% (12-42%)	4.6 (3-6.7)	16% (11-21%)	18% (12-25%)	200 (130-290)	9300 (6300-13000)
Azerbaijan	1995	Male	50 to 54	75% (58-87%)	25% (13-42%)	4.4 (2.9-6.3)	14% (8.8-19%)	16% (9.6-22%)	250 (150-360)	10000 (6300-14000)
Azerbaijan	1995	Male	55 to 59	73% (55-86%)	27% (14-45%)	3.4 (2.2-5)	9.7% (5-15%)	11% (5.2-17%)	300 (140-490)	10000 (5400-16000)
Azerbaijan	1995	Male	60 to 64	69% (50-84%)	31% (16-50%)	2.8 (1.6-4.3)	6.6% (2.2-12%)	7.1% (2.1-14%)	250 (75-480)	7400 (2600-14000)
Azerbaijan	1995	Male	65 to 69	66% (48-82%)	34% (18-52%)	2.4 (1.5-3.7)	4.9% (1.3-9.7%)	5.3% (1.1-11%)	190 (40-410)	4700 (1300-9500)
Azerbaijan	1995	Male	70 to 74	62% (44-78%)	38% (22-56%)	2 (1.1-3.3)	3.3% (0.024-8.2%)	3.5% (-0.39-9.1%)	74 (-7.8-190)	1500 (10-3600)
Azerbaijan	1995	Male	75 to 79	58% (40-75%)	42% (25-60%)	1.6 (0.86-2.7)	2.6% (-0.37-6.8%)	2.7% (-0.79-7.5%)	40 (-11-120)	630 (-89-1700)
Azerbaijan	1995	Male	80 to 84	54% (35-71%)	46% (29-65%)	1.4 (0.79-2.3)	2% (-0.98-5.4%)	1.9% (-1.6-5.8%)	21 (-16-64)	250 (-120-700)
Azerbaijan	1995	Male	85 to 89	52% (34-68%)	48% (32-66%)	1.3 (0.69-2.1)	1.3% (-1.6-4.3%)	1.1% (-2.3-4.6%)	7.7 (-16-31)	74 (-91-250)
Azerbaijan	1995	Male	90 to 94	51% (35-68%)	49% (32-65%)	1.2 (0.64-2)	0.65% (-2.2-3.5%)	0.34% (-2.9-3.6%)	1.1 (-9.2-11)	12 (-39-64)
Azerbaijan	1995	Male	95 plus	52% (31-72%)	48% (28-69%)	1.1 (0.48-2)	0.62% (-2.3-3.6%)	0.2% (-3.4-3.6%)	0.17 (-2.9-3.1)	2.1 (-7.6-12)
Azerbaijan	2000	Female	All Ages	56% (50-63%)	44% (37-50%)	1.1 (0.85-1.4)	1.5% (0.14-2.9%)	1.1% (-2-4.3%)	300 (-510-1100)	18000 (1700-36000)
Azerbaijan	2000	Female	15 to 19	55% (43-69%)	45% (31-57%)	1 (0.59-1.6)	1.7% (0.94-2.7%)	3.3% (1.2-5.6%)	6.2 (2.3-10)	740 (390-1200)

Location	Year	Sex	Age	Prevalence of current drinkers	Prevalence of abstainers	Population average of standard drinks daily	Percent of total attributable deaths	Percent of total attributable DALYs	Attributable deaths	Attributable DALYs
Azerbaijan	2000	Female	20 to 24	62% (49-74%)	38% (26-51%)	1.4 (0.8-2.2)	3.3% (1.9-5%)	5.2% (1.9-9%)	13 (4.7-22)	1400 (790-2200)
Azerbaijan	2000	Female	25 to 29	65% (51-77%)	35% (23-49%)	1.6 (0.94-2.4)	3.7% (2.2-5.4%)	5.9% (2.5-9.6%)	18 (7.6-29)	1700 (1000-2600)
Azerbaijan	2000	Female	30 to 34	66% (52-78%)	34% (22-48%)	1.7 (1.1-2.5)	3.8% (2.4-5.5%)	6.6% (3.3-10%)	27 (13-42)	2200 (1300-3100)
Azerbaijan	2000	Female	35 to 39	66% (52-79%)	34% (21-48%)	1.7 (1.1-2.5)	3.7% (2.2-5.5%)	6.7% (3.6-10%)	38 (20-60)	2600 (1500-3800)
Azerbaijan	2000	Female	40 to 44	66% (52-79%)	34% (21-48%)	1.7 (1.1-2.5)	3.7% (2.1-5.7%)	6.7% (3.6-10%)	43 (23-68)	2400 (1300-3700)
Azerbaijan	2000	Female	45 to 49	66% (51-79%)	34% (21-49%)	1.7 (1.1-2.7)	3.4% (1.4-5.9%)	5.9% (2.3-10%)	41 (15-70)	1900 (730-3200)
Azerbaijan	2000	Female	50 to 54	65% (51-78%)	35% (22-49%)	1.7 (1-2.7)	3.1% (0.39-6.3%)	4.6% (0.48-9.4%)	30 (3.1-63)	1200 (160-2400)
Azerbaijan	2000	Female	55 to 59	63% (49-77%)	37% (23-51%)	1.7 (1-2.7)	2.6% (-0.52-6.1%)	3.5% (-0.98-8.5%)	31 (-8.5-75)	1100 (-220-2600)
Azerbaijan	2000	Female	60 to 64	61% (48-76%)	39% (24-52%)	1.6 (0.93-2.6)	2.1% (-1.5-6.5%)	2.5% (-2.3-8.1%)	62 (-57-200)	1900 (-1400-5700)
Azerbaijan	2000	Female	65 to 69	59% (47-73%)	41% (27-53%)	1.6 (1-2.4)	1.6% (-2.1-5.6%)	2% (-2.7-6.9%)	62 (-85-230)	1500 (-1900-5300)
Azerbaijan	2000	Female	70 to 74	57% (45-70%)	43% (30-55%)	1.5 (0.89-2.3)	0.49% (-3.4-4.9%)	0.58% (-4.2-6%)	21 (-160-220)	400 (-2800-4000)
Azerbaijan	2000	Female	75 to 79	54% (44-67%)	46% (33-56%)	1.4 (0.81-2.2)	-0.3% (-4.1-4.1%)	-0.44% (-5-4.8%)	-12 (-130-130)	-140 (-1800-1900)
Azerbaijan	2000	Female	80 to 84	53% (41-66%)	47% (34-59%)	1.4 (0.82-2.1)	-0.53% (-4.1-3.4%)	-0.72% (-4.9-3.9%)	-14 (-96-76)	-130 (-960-840)
Azerbaijan	2000	Female	85 to 89	52% (40-64%)	48% (36-60%)	1.3 (0.81-2)	-1.3% (-4.9-2.6%)	-1.6% (-5.7-3%)	-32 (-120-59)	-220 (-830-450)
Azerbaijan	2000	Female	90 to 94	53% (42-64%)	47% (36-58%)	1.3 (0.77-2.1)	-2% (-5.6-2%)	-2.4% (-6.8-2.4%)	-24 (-68-23)	-110 (-330-120)
Azerbaijan	2000	Female	95 plus	53% (40-67%)	47% (33-60%)	1.3 (0.64-2.3)	-2.3% (-6.1-2.2%)	-2.9% (-7.4-2.5%)	-12 (-31-10)	-38 (-99-35)
Azerbaijan	2000	Male	All Ages	51% (42-61%)	49% (39-58%)	2.2 (1.7-2.7)	7% (5.1-9%)	7.7% (4.6-11%)	2400 (1400-3600)	110000 (79000-140000)
Azerbaijan	2000	Male	15 to 19	36% (22-54%)	64% (46-78%)	0.49 (0.27-0.85)	4.2% (2.9-5.8%)	4.8% (2.7-7.2%)	20 (11-31)	2300 (1600-3300)
Azerbaijan	2000	Male	20 to 24	57% (38-74%)	43% (26-62%)	1.6 (1-2.5)	12% (8.7-15%)	13% (9.1-18%)	67 (44-94)	6500 (4700-8600)
Azerbaijan	2000	Male	25 to 29	68% (51-82%)	32% (18-49%)	3.7 (2.5-5.1)	18% (14-23%)	23% (17-30%)	130 (98-170)	11000 (8200-13000)
Azerbaijan	2000	Male	30 to 34	75% (56-88%)	25% (12-44%)	4.7 (3.2-6.5)	20% (16-24%)	26% (20-32%)	190 (140-250)	13000 (10000-17000)
Azerbaijan	2000	Male	35 to 39	77% (62-89%)	23% (11-38%)	4.3 (3-5.7)	17% (13-21%)	22% (16-28%)	240 (170-320)	15000 (11000-19000)

Location	Year	Sex	Age	Prevalence of current drinkers	Prevalence of abstainers	Population average of standard drinks daily	Percent of total attributable deaths	Percent of total attributable DALYs	Attributable deaths	Attributable DALYs
Azerbaijan	2000	Male	40 to 44	78% (61-89%)	22% (11-39%)	4.3 (3-5.8)	15% (11-20%)	19% (13-25%)	260 (170-360)	14000 (10000-19000)
Azerbaijan	2000	Male	45 to 49	77% (58-89%)	23% (11-42%)	4.8 (3.2-6.8)	16% (11-21%)	19% (13-26%)	270 (180-380)	13000 (8900-17000)
Azerbaijan	2000	Male	50 to 54	75% (57-87%)	25% (13-43%)	4.6 (3-6.5)	14% (9.3-20%)	16% (10-23%)	220 (130-320)	8900 (5600-12000)
Azerbaijan	2000	Male	55 to 59	73% (55-86%)	27% (14-45%)	3.6 (2.3-5.2)	10% (5.2-16%)	12% (5.4-18%)	190 (88-300)	6500 (3400-9800)
Azerbaijan	2000	Male	60 to 64	69% (50-85%)	31% (15-50%)	2.9 (1.7-4.5)	7.2% (2.4-13%)	7.8% (2.2-15%)	290 (85-560)	8400 (2900-15000)
Azerbaijan	2000	Male	65 to 69	67% (49-83%)	33% (17-51%)	2.5 (1.5-3.8)	5.5% (1.6-11%)	6% (1.4-12%)	260 (61-520)	6200 (1700-12000)
Azerbaijan	2000	Male	70 to 74	62% (44-78%)	38% (22-56%)	2.1 (1.2-3.4)	3.8% (0.24-9.3%)	4.1% (-0.046-10%)	170 (-1.8-400)	3200 (200-7500)
Azerbaijan	2000	Male	75 to 79	58% (39-76%)	42% (24-61%)	1.7 (0.9-2.9)	3% (-0.37-7.8%)	3.2% (-0.65-8.6%)	60 (-12-160)	910 (-110-2400)
Azerbaijan	2000	Male	80 to 84	55% (35-72%)	45% (28-65%)	1.5 (0.82-2.4)	2.2% (-0.95-5.9%)	2.2% (-1.5-6.4%)	21 (-14-62)	250 (-110-670)
Azerbaijan	2000	Male	85 to 89	52% (35-69%)	48% (31-65%)	1.3 (0.74-2.2)	1.5% (-1.5-4.5%)	1.4% (-2.1-4.9%)	10 (-16-37)	95 (-93-290)
Azerbaijan	2000	Male	90 to 94	51% (35-69%)	49% (31-65%)	1.2 (0.66-2.1)	0.78% (-2.2-3.7%)	0.5% (-3-4%)	1.6 (-9.7-13)	15 (-41-70)
Azerbaijan	2000	Male	95 plus	52% (31-72%)	48% (28-69%)	1.1 (0.5-2.1)	0.72% (-2.3-3.9%)	0.33% (-3.3-4%)	0.32 (-3.1-3.9)	2.7 (-8.6-15)
Azerbaijan	2005	Female	All Ages	57% (51-65%)	43% (35-49%)	1.2 (0.93-1.6)	1.8% (0.39-3.4%)	1.8% (-1.5-5.2%)	520 (-430-1500)	24000 (5200-44000)
Azerbaijan	2005	Female	15 to 19	55% (43-69%)	45% (31-57%)	1.1 (0.6-1.7)	1.8% (0.99-2.8%)	3.3% (1.3-5.7%)	7.6 (3.1-13)	900 (480-1400)
Azerbaijan	2005	Female	20 to 24	62% (49-74%)	38% (26-51%)	1.5 (0.85-2.4)	3.3% (1.9-4.9%)	5.1% (2.1-8.7%)	14 (5.6-26)	1700 (940-2600)
Azerbaijan	2005	Female	25 to 29	65% (51-77%)	35% (23-49%)	1.7 (1-2.6)	3.6% (2.2-5.3%)	5.8% (2.7-9.4%)	17 (7.9-29)	1700 (1000-2600)
Azerbaijan	2005	Female	30 to 34	66% (52-79%)	34% (21-48%)	1.8 (1.1-2.8)	3.8% (2.4-5.5%)	6.4% (3.3-10%)	23 (12-38)	2000 (1200-2800)
Azerbaijan	2005	Female	35 to 39	66% (53-81%)	34% (19-47%)	1.8 (1.1-2.7)	3.9% (2.2-5.8%)	6.8% (3.5-11%)	37 (19-58)	2500 (1400-3700)
Azerbaijan	2005	Female	40 to 44	66% (52-79%)	34% (21-48%)	1.8 (1.1-2.7)	3.8% (2.1-5.9%)	6.9% (3.6-11%)	57 (28-90)	3100 (1700-4800)
Azerbaijan	2005	Female	45 to 49	66% (51-79%)	34% (21-49%)	1.9 (1.1-2.8)	3.7% (1.6-6.3%)	6.4% (2.7-11%)	66 (27-110)	3000 (1300-5100)
Azerbaijan	2005	Female	50 to 54	64% (50-78%)	36% (22-50%)	1.8 (1.1-2.8)	3.5% (0.69-6.8%)	5.3% (1.1-10%)	61 (12-120)	2400 (470-4600)
Azerbaijan	2005	Female	55 to 59	63% (50-76%)	37% (24-50%)	1.8 (1.1-2.8)	3.1% (-0.21-6.8%)	4.3% (-0.38-9.3%)	44 (-3.8-97)	1500 (-91-3300)

Location	Year	Sex	Age	Prevalence of current drinkers	Prevalence of abstainers	Population average of standard drinks daily	Percent of total attributable deaths	Percent of total attributable DALYs	Attributable deaths	Attributable DALYs
Azerbaijan	2005	Female	60 to 64	61% (48-75%)	39% (25-52%)	1.7 (0.99-2.8)	2.8% (-0.93-7%)	3.5% (-1.4-9.1%)	52 (-20-140)	1500 (-490-4000)
Azerbaijan	2005	Female	65 to 69	59% (48-73%)	41% (27-52%)	1.7 (1.1-2.5)	2.3% (-1.6-6.4%)	2.9% (-1.9-8%)	100 (-65-280)	2300 (-1500-6400)
Azerbaijan	2005	Female	70 to 74	57% (46-70%)	43% (30-54%)	1.6 (0.96-2.5)	1.2% (-3.1-5.9%)	1.5% (-3.8-7.1%)	66 (-160-330)	1200 (-2900-6000)
Azerbaijan	2005	Female	75 to 79	54% (44-67%)	46% (33-56%)	1.5 (0.88-2.4)	0.38% (-3.6-5.1%)	0.39% (-4.3-5.9%)	15 (-160-230)	240 (-2300-3300)
Azerbaijan	2005	Female	80 to 84	53% (40-66%)	47% (34-60%)	1.5 (0.9-2.3)	0.084% (-3.6-4.2%)	0.017% (-4.4-4.8%)	0.43 (-130-140)	30 (-1300-1500)
Azerbaijan	2005	Female	85 to 89	52% (41-63%)	48% (37-59%)	1.4 (0.86-2.2)	-0.61% (-4.3-3.6%)	-0.81% (-5.1-4.3%)	-13 (-85-70)	-84 (-600-520)
Azerbaijan	2005	Female	90 to 94	53% (42-64%)	47% (36-58%)	1.4 (0.82-2.3)	-1.3% (-5.1-3.2%)	-1.7% (-6.2-3.7%)	-18 (-66-39)	-82 (-320-200)
Azerbaijan	2005	Female	95 plus	53% (40-68%)	47% (32-60%)	1.4 (0.71-2.4)	-1.7% (-5.6-3.1%)	-2.2% (-6.7-3.3%)	-7.9 (-25-13)	-24 (-79-43)
Azerbaijan	2005	Male	All Ages	53% (45-62%)	47% (38-55%)	2.5 (2-3.2)	8.2% (5.9-11%)	8.8% (5.4-13%)	3100 (1900-4600)	130000 (96000-180000)
Azerbaijan	2005	Male	15 to 19	29% (18-45%)	71% (55-82%)	0.42 (0.23-0.74)	4% (3-5.2%)	4.5% (2.9-6.4%)	22 (14-33)	2600 (1900-3600)
Azerbaijan	2005	Male	20 to 24	54% (37-69%)	46% (31-63%)	1.6 (1.1-2.4)	11% (8.8-14%)	13% (8.9-17%)	82 (54-120)	8100 (5900-11000)
Azerbaijan	2005	Male	25 to 29	71% (57-83%)	29% (17-43%)	4.2 (2.9-5.7)	19% (15-23%)	24% (18-30%)	160 (110-210)	12000 (9300-16000)
Azerbaijan	2005	Male	30 to 34	78% (64-88%)	22% (12-36%)	5.4 (3.8-7.2)	21% (16-25%)	27% (20-33%)	210 (150-270)	14000 (11000-18000)
Azerbaijan	2005	Male	35 to 39	79% (68-89%)	21% (11-32%)	4.8 (3.5-6.4)	18% (13-23%)	23% (16-30%)	230 (160-330)	14000 (10000-19000)
Azerbaijan	2005	Male	40 to 44	79% (66-89%)	21% (11-34%)	4.8 (3.5-6.4)	16% (11-21%)	20% (13-27%)	340 (220-490)	18000 (13000-25000)
Azerbaijan	2005	Male	45 to 49	80% (66-89%)	20% (11-34%)	5.5 (3.9-7.4)	17% (12-22%)	20% (14-28%)	420 (270-580)	19000 (13000-26000)
Azerbaijan	2005	Male	50 to 54	79% (64-88%)	21% (12-36%)	5.3 (3.6-7.3)	15% (10-21%)	18% (11-25%)	410 (250-570)	16000 (10000-22000)
Azerbaijan	2005	Male	55 to 59	75% (59-86%)	25% (14-41%)	4 (2.7-5.8)	12% (6-18%)	13% (6.3-21%)	230 (110-370)	8000 (4100-12000)
Azerbaijan	2005	Male	60 to 64	70% (51-84%)	30% (16-49%)	3.2 (1.9-4.8)	8.3% (3.1-14%)	9.2% (3-16%)	200 (62-370)	5900 (2100-10000)
Azerbaijan	2005	Male	65 to 69	67% (47-82%)	33% (18-53%)	2.7 (1.6-4.1)	6.6% (2-12%)	7.2% (2-13%)	310 (83-590)	7400 (2200-14000)
Azerbaijan	2005	Male	70 to 74	63% (44-78%)	37% (22-56%)	2.3 (1.3-3.7)	4.8% (0.75-10%)	5.2% (0.61-12%)	270 (31-590)	5100 (790-11000)
Azerbaijan	2005	Male	75 to 79	58% (39-75%)	42% (25-61%)	1.9 (0.99-3)	3.8% (0.24-8.8%)	4.1% (-0.059-9.7%)	150 (-2.1-360)	2200 (140-5100)

Location	Year	Sex	Age	Prevalence of current drinkers	Prevalence of abstainers	Population average of standard drinks daily	Percent of total attributable deaths	Percent of total attributable DALYs	Attributable deaths	Attributable DALYs
Azerbaijan	2005	Male	80 to 84	55% (36-72%)	45% (28-64%)	1.6 (0.9-2.6)	2.9% (-0.38-7.1%)	3% (-0.87-7.9%)	56 (-17-150)	640 (-89-1600)
Azerbaijan	2005	Male	85 to 89	52% (34-69%)	48% (31-66%)	1.5 (0.8-2.4)	2.1% (-0.7-5.7%)	2.1% (-1.3-6.2%)	13 (-7.8-39)	110 (-39-300)
Azerbaijan	2005	Male	90 to 94	51% (35-69%)	49% (31-65%)	1.3 (0.72-2.3)	1.2% (-1.7-4.1%)	1.1% (-2.4-4.4%)	3.5 (-8-14)	24 (-32-78)
Azerbaijan	2005	Male	95 plus	52% (31-72%)	48% (28-69%)	1.2 (0.56-2.3)	1% (-2-4.4%)	0.72% (-2.9-4.6%)	0.67 (-2.7-4.3)	3.7 (-7.1-16)
Azerbaijan	2010	Female	All Ages	57% (51-64%)	43% (36-49%)	1.4 (1-1.9)	2.2% (0.47-4.1%)	2.4% (-1.4-6.5%)	760 (-440-2100)	30000 (6200-55000)
Azerbaijan	2010	Female	15 to 19	55% (43-70%)	45% (30-57%)	1.2 (0.65-1.9)	1.9% (1-3.1%)	3.5% (1.5-6.2%)	6.7 (2.9-12)	860 (470-1400)
Azerbaijan	2010	Female	20 to 24	62% (49-74%)	38% (26-51%)	1.7 (0.94-2.7)	3.3% (2-4.9%)	5.3% (2.3-9.1%)	15 (6.2-26)	1900 (1100-2900)
Azerbaijan	2010	Female	25 to 29	64% (51-77%)	36% (23-49%)	1.9 (1.1-2.9)	3.6% (2.3-5.2%)	6% (3-9.6%)	18 (8.7-29)	2000 (1200-2800)
Azerbaijan	2010	Female	30 to 34	66% (52-78%)	34% (22-48%)	2 (1.2-3.1)	3.8% (2.6-5.5%)	6.7% (3.8-10%)	22 (12-35)	2000 (1300-2800)
Azerbaijan	2010	Female	35 to 39	66% (53-79%)	34% (21-47%)	2 (1.2-3.1)	3.9% (2.4-5.8%)	7.3% (4-11%)	31 (17-50)	2200 (1300-3300)
Azerbaijan	2010	Female	40 to 44	66% (52-79%)	34% (21-48%)	2 (1.2-3.2)	4% (2.2-6.2%)	7.4% (3.9-12%)	50 (26-81)	2800 (1600-4500)
Azerbaijan	2010	Female	45 to 49	66% (51-79%)	34% (21-49%)	2.1 (1.2-3.1)	4% (1.7-6.7%)	7.1% (3.1-12%)	79 (34-130)	3700 (1700-6300)
Azerbaijan	2010	Female	50 to 54	64% (51-78%)	36% (22-49%)	2 (1.2-3.1)	4% (1.2-7.4%)	6.2% (1.9-11%)	93 (28-180)	3700 (1100-6900)
Azerbaijan	2010	Female	55 to 59	63% (50-76%)	37% (24-50%)	2 (1.2-3.2)	3.7% (0.35-7.5%)	5.2% (0.41-11%)	85 (6.5-180)	3000 (240-6200)
Azerbaijan	2010	Female	60 to 64	61% (47-76%)	39% (24-53%)	1.9 (1.1-3.2)	3.6% (-0.55-8.6%)	4.5% (-0.96-11%)	72 (-15-180)	2100 (-320-5100)
Azerbaijan	2010	Female	65 to 69	59% (48-74%)	41% (26-52%)	1.9 (1.1-2.9)	3.1% (-0.97-7.5%)	4% (-1.2-9.7%)	77 (-23-190)	1800 (-490-4400)
Azerbaijan	2010	Female	70 to 74	57% (45-70%)	43% (30-55%)	1.8 (1-2.8)	2% (-2.7-7.3%)	2.4% (-3.3-8.9%)	120 (-160-440)	2300 (-2900-8200)
Azerbaijan	2010	Female	75 to 79	54% (44-67%)	46% (33-56%)	1.7 (0.94-2.7)	1.2% (-3.3-6.5%)	1.3% (-4-7.5%)	68 (-210-390)	1000 (-2800-5600)
Azerbaijan	2010	Female	80 to 84	53% (40-66%)	47% (34-60%)	1.6 (0.95-2.6)	0.81% (-3.4-5.6%)	0.86% (-4-6.5%)	36 (-170-280)	420 (-1700-2900)
Azerbaijan	2010	Female	85 to 89	52% (40-64%)	48% (36-60%)	1.6 (0.95-2.5)	0.063% (-3.9-4.8%)	-0.057% (-4.8-5.5%)	-1.3 (-120-140)	14 (-830-1000)
Azerbaijan	2010	Female	90 to 94	53% (42-65%)	47% (35-58%)	1.6 (0.91-2.6)	-0.65% (-4.9-4.7%)	-0.89% (-5.8-5.3%)	-9.2 (-62-56)	-39 (-300-290)
Azerbaijan	2010	Female	95 plus	53% (40-68%)	47% (32-60%)	1.6 (0.78-2.7)	-1.1% (-5.4-5%)	-1.4% (-6.6-5.5%)	-6.1 (-27-23)	-17 (-87-81)

Location	Year	Sex	Age	Prevalence of current drinkers	Prevalence of abstainers	Population average of standard drinks daily	Percent of total attributable deaths	Percent of total attributable DALYs	Attributable deaths	Attributable DALYs
Azerbaijan	2010	Male	All Ages	55% (46-63%)	45% (37-54%)	2.9 (2.2-3.8)	8.8% (6.1-12%)	9.4% (5.5-14%)	3600 (2100-5400)	150000 (100000-200000)
Azerbaijan	2010	Male	15 to 19	29% (18-45%)	71% (55-82%)	0.46 (0.24-0.82)	4.2% (3.1-5.5%)	4.7% (3.1-6.8%)	20 (13-30)	2500 (1800-3500)
Azerbaijan	2010	Male	20 to 24	53% (37-69%)	47% (31-63%)	1.8 (1.1-2.7)	11% (8.8-14%)	13% (8.8-17%)	73 (49-100)	8200 (6000-11000)
Azerbaijan	2010	Male	25 to 29	71% (57-82%)	29% (18-43%)	4.6 (3-6.4)	18% (14-22%)	23% (17-30%)	150 (100-200)	13000 (9700-16000)
Azerbaijan	2010	Male	30 to 34	78% (63-88%)	22% (12-37%)	5.9 (4.1-8.1)	20% (16-25%)	26% (20-35%)	190 (140-260)	14000 (11000-17000)
Azerbaijan	2010	Male	35 to 39	79% (67-88%)	21% (12-33%)	5.3 (3.7-7.5)	18% (13-24%)	23% (16-32%)	210 (140-290)	13000 (9200-18000)
Azerbaijan	2010	Male	40 to 44	79% (67-89%)	21% (11-33%)	5.3 (3.7-7.3)	17% (12-23%)	21% (14-30%)	280 (180-400)	15000 (10000-21000)
Azerbaijan	2010	Male	45 to 49	80% (64-89%)	20% (11-36%)	6.1 (4.2-8.5)	17% (12-23%)	21% (14-29%)	460 (310-660)	22000 (15000-30000)
Azerbaijan	2010	Male	50 to 54	78% (64-88%)	22% (12-36%)	5.9 (4-8.2)	16% (11-22%)	19% (13-27%)	560 (360-800)	22000 (15000-31000)
Azerbaijan	2010	Male	55 to 59	74% (60-86%)	26% (14-40%)	4.5 (2.9-6.6)	13% (6.9-19%)	15% (7.4-22%)	450 (220-700)	15000 (8000-23000)
Azerbaijan	2010	Male	60 to 64	70% (52-85%)	30% (15-48%)	3.5 (2.1-5.4)	9.4% (3.8-16%)	10% (3.7-18%)	260 (90-450)	7500 (2800-13000)
Azerbaijan	2010	Male	65 to 69	66% (48-82%)	34% (18-52%)	3 (1.8-4.6)	7.7% (2.8-13%)	8.5% (2.8-15%)	230 (72-410)	5400 (1900-9400)
Azerbaijan	2010	Male	70 to 74	62% (44-78%)	38% (22-56%)	2.5 (1.4-4.2)	5.8% (1.1-12%)	6.4% (1-14%)	340 (53-730)	6500 (1200-13000)
Azerbaijan	2010	Male	75 to 79	58% (39-76%)	42% (24-61%)	2.1 (1-3.4)	4.7% (0.6-10%)	5% (0.46-12%)	240 (21-560)	3600 (490-8200)
Azerbaijan	2010	Male	80 to 84	55% (36-72%)	45% (28-64%)	1.8 (0.97-2.9)	3.5% (-0.016-8.6%)	3.8% (-0.31-9.4%)	120 (-11-310)	1400 (-6.5-3300)
Azerbaijan	2010	Male	85 to 89	52% (34-69%)	48% (31-66%)	1.6 (0.86-2.7)	2.7% (-0.44-7%)	2.8% (-0.87-7.7%)	39 (-13-110)	330 (-50-840)
Azerbaijan	2010	Male	90 to 94	51% (35-69%)	49% (31-65%)	1.5 (0.77-2.6)	1.6% (-1.6-5.7%)	1.5% (-2.2-6%)	6.5 (-9.2-26)	39 (-40-140)
Azerbaijan	2010	Male	95 plus	52% (31-72%)	48% (28-69%)	1.4 (0.61-2.6)	1.2% (-2.2-5.8%)	0.98% (-2.9-6.1%)	0.95 (-2.8-5.9)	4.3 (-8-21)
Azerbaijan	2016	Female	All Ages	58% (51-65%)	42% (35-49%)	1.5 (0.99-2.1)	2.3% (0.42-4.3%)	2.4% (-1.4-6.9%)	710 (-400-2100)	29000 (5200-59000)
Azerbaijan	2016	Female	15 to 19	55% (43-69%)	45% (31-57%)	1.2 (0.62-2)	1.8% (1-2.9%)	3.5% (1.6-6.5%)	4.3 (1.9-7.8)	570 (310-930)
Azerbaijan	2016	Female	20 to 24	62% (49-74%)	38% (26-51%)	1.7 (0.89-2.8)	3.2% (2-4.8%)	5.2% (2.3-9.1%)	11 (4.8-20)	1500 (870-2300)
Azerbaijan	2016	Female	25 to 29	64% (51-78%)	36% (22-49%)	1.9 (1-3)	3.5% (2.2-5%)	5.7% (2.7-9.6%)	17 (7.6-31)	2100 (1300-3100)

Location	Year	Sex	Age	Prevalence of current drinkers	Prevalence of abstainers	Population average of standard drinks daily	Percent of total attributable deaths	Percent of total attributable DALYs	Attributable deaths	Attributable DALYs
Azerbaijan	2016	Female	30 to 34	66% (52-79%)	34% (21-48%)	2 (1.2-3.1)	3.7% (2.4-5.3%)	6.7% (3.7-11%)	25 (13-42)	2400 (1500-3600)
Azerbaijan	2016	Female	35 to 39	66% (53-80%)	34% (20-47%)	2 (1.1-3.4)	3.7% (2.2-5.7%)	7.3% (4-12%)	28 (14-47)	2100 (1200-3300)
Azerbaijan	2016	Female	40 to 44	66% (52-79%)	34% (21-48%)	2.1 (1.2-3.4)	3.8% (2-6.1%)	7.4% (3.5-12%)	41 (17-70)	2400 (1200-4000)
Azerbaijan	2016	Female	45 to 49	66% (50-79%)	34% (21-50%)	2.1 (1.2-3.4)	3.8% (1.6-6.7%)	7% (3-12%)	59 (24-110)	2900 (1200-5200)
Azerbaijan	2016	Female	50 to 54	64% (50-77%)	36% (23-50%)	2 (1.1-3.2)	3.7% (0.93-7.3%)	6.1% (1.5-12%)	100 (24-210)	4100 (1000-8400)
Azerbaijan	2016	Female	55 to 59	62% (50-75%)	38% (25-50%)	2 (1.1-3.3)	3.4% (0.18-7.6%)	5.1% (0.12-12%)	100 (2.4-230)	3600 (200-8200)
Azerbaijan	2016	Female	60 to 64	61% (47-75%)	39% (25-53%)	2 (1.1-3.3)	3.3% (-0.5-8.1%)	4.5% (-0.77-11%)	130 (-21-320)	3800 (-560-9600)
Azerbaijan	2016	Female	65 to 69	59% (47-74%)	41% (26-53%)	1.9 (1.1-3.1)	2.9% (-0.96-7.6%)	4% (-1.4-10%)	67 (-24-180)	1600 (-540-4300)
Azerbaijan	2016	Female	70 to 74	57% (46-70%)	43% (30-54%)	1.8 (0.95-3)	1.9% (-2.4-7.2%)	2.5% (-3.1-9.3%)	53 (-70-200)	1000 (-1300-3800)
Azerbaijan	2016	Female	75 to 79	54% (44-67%)	46% (33-56%)	1.7 (0.89-2.9)	1.2% (-2.9-6.9%)	1.4% (-3.9-8.5%)	64 (-180-410)	1000 (-2600-6000)
Azerbaijan	2016	Female	80 to 84	52% (40-66%)	48% (34-60%)	1.6 (0.9-2.7)	0.8% (-3.1-6%)	0.87% (-3.9-7.2%)	38 (-180-310)	460 (-1900-3400)
Azerbaijan	2016	Female	85 to 89	52% (40-63%)	48% (37-60%)	1.6 (0.89-2.6)	0.051% (-4-5.2%)	-0.094% (-5.1-6.1%)	-3 (-150-180)	12 (-1100-1400)
Azerbaijan	2016	Female	90 to 94	53% (42-64%)	47% (36-58%)	1.6 (0.87-2.7)	-0.61% (-4.8-5%)	-0.92% (-5.8-5.6%)	-13 (-87-80)	-54 (-420-420)
Azerbaijan	2016	Female	95 plus	53% (40-68%)	47% (32-60%)	1.6 (0.73-2.8)	-1% (-5.2-5.6%)	-1.4% (-6.5-6.2%)	-5 (-23-21)	-14 (-72-74)
Azerbaijan	2016	Male	All Ages	56% (47-64%)	44% (36-53%)	3.1 (2.1-4.2)	9.3% (6.2-13%)	9.4% (5.1-14%)	3800 (2000-6200)	160000 (100000-230000)
Azerbaijan	2016	Male	15 to 19	36% (22-54%)	64% (46-78%)	0.59 (0.29-1.1)	4.5% (3.2-6.1%)	5.2% (3.2-7.8%)	19 (11-31)	2200 (1500-3200)
Azerbaijan	2016	Male	20 to 24	57% (37-74%)	43% (26-63%)	2 (1.1-3.2)	12% (8.5-15%)	13% (8.5-18%)	90 (52-140)	9000 (6000-13000)
Azerbaijan	2016	Male	25 to 29	68% (49-82%)	32% (18-51%)	4.4 (2.8-6.7)	17% (13-22%)	22% (15-29%)	190 (120-290)	16000 (11000-21000)
Azerbaijan	2016	Male	30 to 34	75% (56-87%)	25% (13-44%)	5.7 (3.6-8.4)	19% (14-24%)	25% (18-32%)	250 (160-370)	18000 (13000-25000)
Azerbaijan	2016	Male	35 to 39	77% (62-88%)	23% (12-38%)	5.2 (3.3-7.9)	17% (12-23%)	22% (14-31%)	210 (130-330)	14000 (9000-20000)
Azerbaijan	2016	Male	40 to 44	77% (62-89%)	23% (11-38%)	5.2 (3.3-7.7)	16% (11-22%)	20% (12-29%)	250 (140-400)	14000 (8700-21000)
Azerbaijan	2016	Male	45 to 49	76% (57-88%)	24% (12-43%)	5.7 (3.6-8.8)	16% (11-22%)	20% (13-28%)	340 (200-520)	16000 (10000-24000)

Location	Year	Sex	Age	Prevalence of current drinkers	Prevalence of abstainers	Population average of standard drinks daily	Percent of total attributable deaths	Percent of total attributable DALYs	Attributable deaths	Attributable DALYs
Azerbaijan	2016	Male	50 to 54	74% (57-87%)	26% (13-43%)	5.5 (3.4-8.3)	15% (9.6-21%)	18% (11-26%)	600 (340-930)	25000 (15000-36000)
Azerbaijan	2016	Male	55 to 59	72% (55-86%)	28% (14-45%)	4.3 (2.6-6.7)	12% (6.1-18%)	14% (6.6-22%)	530 (240-890)	19000 (9600-30000)
Azerbaijan	2016	Male	60 to 64	69% (49-85%)	31% (15-51%)	3.5 (1.9-5.6)	9% (2.8-15%)	10% (2.9-18%)	500 (150-910)	15000 (4900-26000)
Azerbaijan	2016	Male	65 to 69	66% (47-82%)	34% (18-53%)	3 (1.7-4.8)	7.3% (2.5-13%)	8.3% (2.5-15%)	220 (61-420)	5300 (1700-9900)
Azerbaijan	2016	Male	70 to 74	61% (43-78%)	39% (22-57%)	2.5 (1.3-4.2)	5.6% (1-12%)	6.2% (0.72-14%)	170 (21-370)	3200 (570-7200)
Azerbaijan	2016	Male	75 to 79	57% (37-74%)	43% (26-63%)	2 (0.98-3.5)	4.5% (0.52-10%)	4.9% (0.15-12%)	220 (6.5-540)	3400 (350-7800)
Azerbaijan	2016	Male	80 to 84	53% (34-70%)	47% (30-66%)	1.7 (0.9-3)	3.4% (-0.19-8.7%)	3.7% (-0.61-9.7%)	160 (-28-440)	1900 (-110-4800)
Azerbaijan	2016	Male	85 to 89	51% (33-68%)	49% (32-67%)	1.6 (0.8-2.7)	2.6% (-0.55-7.5%)	2.7% (-1.1-8.3%)	68 (-26-210)	570 (-120-1600)
Azerbaijan	2016	Male	90 to 94	50% (34-67%)	50% (33-66%)	1.5 (0.72-2.6)	1.7% (-1.4-5.9%)	1.6% (-2.1-6.5%)	15 (-18-57)	92 (-74-300)
Azerbaijan	2016	Male	95 plus	51% (30-70%)	49% (30-70%)	1.4 (0.57-2.6)	1.5% (-1.8-5.6%)	1.2% (-2.7-5.9%)	1.3 (-2.8-6.4)	6 (-7.2-23)
Georgia	1990	Female	All Ages	53% (49-58%)	47% (42-51%)	0.41 (0.31-0.55)	-0.63% (-1.7-0.35%)	-2.4% (-4.5--0.46%)	-650 (-1200--130)	-5800 (-16000-3200)
Georgia	1990	Female	15 to 19	45% (33-57%)	55% (43-67%)	0.33 (0.19-0.52)	0.89% (0.5-1.4%)	0.99% (0.13-2.1%)	0.81 (0.11-1.7)	180 (99-290)
Georgia	1990	Female	20 to 24	61% (48-71%)	39% (29-52%)	0.52 (0.31-0.81)	1.6% (0.94-2.4%)	1.1% (-0.2-2.6%)	1.4 (-0.28-3.3)	380 (210-590)
Georgia	1990	Female	25 to 29	65% (54-73%)	35% (27-46%)	0.58 (0.36-0.85)	1.7% (1-2.5%)	1.1% (-0.23-2.5%)	2 (-0.41-4.5)	540 (300-810)
Georgia	1990	Female	30 to 34	66% (55-75%)	34% (25-45%)	0.6 (0.38-0.89)	1.6% (0.94-2.3%)	1.2% (-0.24-2.6%)	2.6 (-0.53-5.7)	540 (290-830)
Georgia	1990	Female	35 to 39	66% (55-74%)	34% (26-45%)	0.58 (0.37-0.91)	1.4% (0.68-2.1%)	1.4% (-0.12-2.9%)	3.4 (-0.28-7.5)	460 (220-730)
Georgia	1990	Female	40 to 44	65% (55-74%)	35% (26-45%)	0.58 (0.37-0.94)	1.2% (0.41-2%)	1.4% (-0.22-3.1%)	4.4 (-0.69-10)	390 (140-690)
Georgia	1990	Female	45 to 49	65% (53-74%)	35% (26-47%)	0.59 (0.35-0.92)	0.72% (-0.25-1.7%)	0.73% (-1.2-2.6%)	2.9 (-4.5-10)	240 (-74-540)
Georgia	1990	Female	50 to 54	63% (53-72%)	37% (28-47%)	0.57 (0.35-0.89)	0.39% (-0.83-1.5%)	0.33% (-1.7-2.2%)	3.1 (-16-21)	230 (-500-900)
Georgia	1990	Female	55 to 59	60% (48-70%)	40% (30-52%)	0.54 (0.32-0.83)	-0.19% (-1.6-1%)	-0.43% (-2.6-1.5%)	-5.1 (-31-17)	-120 (-970-620)
Georgia	1990	Female	60 to 64	57% (45-67%)	43% (33-55%)	0.54 (0.32-0.82)	-1.1% (-2.8-0.33%)	-1.7% (-4.1-0.49%)	-36 (-89-11)	-970 (-2400-280)
Georgia	1990	Female	65 to 69	55% (44-65%)	45% (35-56%)	0.55 (0.35-0.79)	-1.8% (-3.8--0.054%)	-2.4% (-5.1-0.016%)	-58 (-120-0.36)	-1400 (-2900--41)

Location	Year	Sex	Age	Prevalence of current drinkers	Prevalence of abstainers	Population average of standard drinks daily	Percent of total attributable deaths	Percent of total attributable DALYs	Attributable deaths	Attributable DALYs
Georgia	1990	Female	70 to 74	52% (40-62%)	48% (38-60%)	0.54 (0.33-0.8)	-2.3% (-4.5--0.38%)	-2.9% (-5.8--0.39%)	-80 (-160--11)	-1500 (-3000--250)
Georgia	1990	Female	75 to 79	49% (36-62%)	51% (38-64%)	0.5 (0.29-0.76)	-2.7% (-4.9--0.84%)	-3.4% (-6.2--1%)	-150 (-270--44)	-2100 (-3800--620)
Georgia	1990	Female	80 to 84	47% (33-59%)	53% (41-67%)	0.46 (0.27-0.68)	-2.9% (-5.2--0.96%)	-3.6% (-6.5--1.2%)	-150 (-260--48)	-1500 (-2700--500)
Georgia	1990	Female	85 to 89	45% (32-58%)	55% (42-68%)	0.44 (0.25-0.68)	-3% (-5.3--1.2%)	-3.7% (-6.5--1.4%)	-120 (-210--46)	-870 (-1500--340)
Georgia	1990	Female	90 to 94	45% (33-58%)	55% (42-67%)	0.44 (0.24-0.71)	-3.2% (-5.5--1.2%)	-3.8% (-6.6--1.5%)	-62 (-110--24)	-310 (-520--120)
Georgia	1990	Female	95 plus	47% (31-63%)	53% (37-69%)	0.46 (0.22-0.8)	-3.4% (-5.9--1.1%)	-4.1% (-7.2--1.3%)	-21 (-36--6.5)	-66 (-110--21)
Georgia	1990	Male	All Ages	80% (76-83%)	20% (17-24%)	3 (2.4-3.8)	8.5% (5.7-12%)	8.9% (4.8-14%)	2400 (1300-3800)	94000 (61000-130000)
Georgia	1990	Male	15 to 19	70% (59-80%)	30% (20-41%)	1.4 (0.94-2)	5% (3.6-7%)	7% (4.5-10%)	15 (9.4-22)	1500 (1000-2000)
Georgia	1990	Male	20 to 24	85% (78-90%)	15% (10-22%)	3 (2.2-4)	11% (8.1-15%)	14% (9.4-19%)	49 (33-69)	4200 (3000-5600)
Georgia	1990	Male	25 to 29	88% (83-92%)	12% (8.3-17%)	4.5 (3.2-5.9)	15% (11-20%)	19% (14-26%)	99 (68-130)	7300 (5300-9700)
Georgia	1990	Male	30 to 34	90% (85-93%)	10% (7-15%)	5.2 (3.8-6.9)	16% (12-21%)	21% (15-28%)	130 (90-170)	8500 (6300-11000)
Georgia	1990	Male	35 to 39	91% (87-94%)	9.3% (6.4-13%)	4.9 (3.5-6.5)	15% (10-19%)	19% (12-25%)	130 (84-180)	7700 (5300-10000)
Georgia	1990	Male	40 to 44	91% (87-94%)	9.1% (6.3-13%)	4.8 (3.6-6.5)	14% (9.8-19%)	18% (12-24%)	140 (91-200)	7400 (5000-9800)
Georgia	1990	Male	45 to 49	90% (85-93%)	9.5% (6.6-15%)	5.4 (3.9-7.3)	15% (11-20%)	19% (13-25%)	180 (120-240)	7900 (5400-11000)
Georgia	1990	Male	50 to 54	91% (87-93%)	9.4% (6.6-13%)	5.4 (4-7.2)	14% (9.4-19%)	17% (11-23%)	360 (220-490)	14000 (9000-19000)
Georgia	1990	Male	55 to 59	91% (87-94%)	8.7% (5.8-13%)	4.6 (3.4-6.1)	12% (7-17%)	14% (8.1-20%)	340 (190-500)	11000 (6600-16000)
Georgia	1990	Male	60 to 64	90% (85-93%)	10% (7-15%)	4.1 (2.9-5.6)	10% (4.8-16%)	12% (5.3-18%)	420 (190-670)	12000 (5900-19000)
Georgia	1990	Male	65 to 69	86% (80-90%)	14% (9.6-20%)	3.8 (2.7-5)	8.5% (3.4-14%)	9.7% (3.6-16%)	260 (98-450)	6200 (2500-10000)
Georgia	1990	Male	70 to 74	83% (77-88%)	17% (12-23%)	3.3 (2.4-4.4)	6.6% (1.7-12%)	7.3% (1.6-14%)	160 (35-300)	3000 (770-5600)
Georgia	1990	Male	75 to 79	82% (73-88%)	18% (12-27%)	2.9 (2-3.9)	4.3% (-0.099-9.8%)	4.6% (-0.53-11%)	130 (-16-330)	2100 (-52-4800)
Georgia	1990	Male	80 to 84	80% (70-87%)	20% (13-30%)	2.4 (1.6-3.2)	2.1% (-1.5-6.7%)	2% (-2.2-7.5%)	49 (-54-180)	610 (-450-2000)
Georgia	1990	Male	85 to 89	79% (69-86%)	21% (14-31%)	2 (1.3-2.8)	0.48% (-2.7-4.2%)	0.16% (-3.6-4.6%)	2.5 (-56-71)	64 (-360-580)

Location	Year	Sex	Age	Prevalence of current drinkers	Prevalence of abstainers	Population average of standard drinks daily	Percent of total attributable deaths	Percent of total attributable DALYs	Attributable deaths	Attributable DALYs
Georgia	1990	Male	90 to 94	79%<br>(69-86%)	21%<br>(14-31%)	1.8<br>(1.1-2.7)	-0.89%<br>(-4-2.7%)	-1.5%<br>(-5.3-2.7%)	-9.9<br>(-35-19)	-35<br>(-160-110)
Georgia	1990	Male	95 plus	78%<br>(65-88%)	22%<br>(12-35%)	1.5<br>(0.77-2.5)	-1.7%<br>(-4.8-2%)	-2.5%<br>(-6.3-1.8%)	-4.5<br>(-11-3.2)	-12<br>(-34-14)
Georgia	1995	Female	All Ages	55%<br>(51-58%)	45%<br>(42-49%)	0.37<br>(0.29-0.47)	-0.92%<br>(-2.1-0.097%)	-3%<br>(-5.3--0.86%)	-730<br>(-1300--210)	-7200<br>(-16000-760)
Georgia	1995	Female	15 to 19	53%<br>(45-61%)	47%<br>(39-55%)	0.34<br>(0.22-0.49)	0.78%<br>(0.42-1.2%)	0.62%<br>(-0.27-1.6%)	0.41<br>(-0.18-1)	140<br>(74-230)
Georgia	1995	Female	20 to 24	65%<br>(58-71%)	35%<br>(29-42%)	0.48<br>(0.33-0.7)	1.4%<br>(0.77-2.2%)	0.5%<br>(-0.8-1.9%)	0.47<br>(-0.76-1.8)	280<br>(140-450)
Georgia	1995	Female	25 to 29	67%<br>(61-73%)	33%<br>(27-39%)	0.52<br>(0.36-0.72)	1.3%<br>(0.63-2.1%)	0.23%<br>(-1.2-1.7%)	0.31<br>(-1.7-2.3)	310<br>(140-500)
Georgia	1995	Female	30 to 34	68%<br>(62-73%)	32%<br>(27-38%)	0.53<br>(0.37-0.74)	1.3%<br>(0.55-2%)	0.4%<br>(-1.2-2%)	0.77<br>(-2.3-3.8)	400<br>(160-670)
Georgia	1995	Female	35 to 39	66%<br>(60-72%)	34%<br>(28-40%)	0.5<br>(0.34-0.74)	1.1%<br>(0.36-1.7%)	0.6%<br>(-0.96-2.1%)	1.4<br>(-2.2-5.2)	350<br>(110-590)
Georgia	1995	Female	40 to 44	65%<br>(59-72%)	35%<br>(28-41%)	0.5<br>(0.34-0.74)	0.93%<br>(0.22-1.7%)	0.9%<br>(-0.65-2.5%)	2.8<br>(-2-7.7)	310<br>(78-570)
Georgia	1995	Female	45 to 49	65%<br>(59-72%)	35%<br>(28-41%)	0.51<br>(0.34-0.75)	0.42%<br>(-0.49-1.3%)	0.15%<br>(-1.6-2%)	0.57<br>(-6.1-7.2)	140<br>(-150-420)
Georgia	1995	Female	50 to 54	64%<br>(57-70%)	36%<br>(30-43%)	0.49<br>(0.32-0.72)	0.073%<br>(-1-1.1%)	-0.21%<br>(-2.2-1.6%)	-0.99<br>(-10-7.8)	24<br>(-330-360)
Georgia	1995	Female	55 to 59	59%<br>(52-65%)	41%<br>(35-48%)	0.44<br>(0.29-0.65)	-0.39%<br>(-1.7-0.73%)	-0.78%<br>(-2.8-1.1%)	-8.9<br>(-32-12)	-240<br>(-1000-450)
Georgia	1995	Female	60 to 64	55%<br>(49-62%)	45%<br>(38-51%)	0.44<br>(0.29-0.63)	-1.1%<br>(-2.6-0.17%)	-1.7%<br>(-3.9-0.25%)	-26<br>(-58-3.7)	-700<br>(-1600-120)
Georgia	1995	Female	65 to 69	55%<br>(49-61%)	45%<br>(39-51%)	0.47<br>(0.34-0.65)	-1.9%<br>(-3.8--0.28%)	-2.6%<br>(-5.3--0.3%)	-68<br>(-140--8.3)	-1600<br>(-3200--250)
Georgia	1995	Female	70 to 74	53%<br>(46-60%)	47%<br>(40-54%)	0.47<br>(0.33-0.67)	-2.3%<br>(-4.4--0.59%)	-3%<br>(-5.7--0.66%)	-89<br>(-170--19)	-1700<br>(-3100--430)
Georgia	1995	Female	75 to 79	53%<br>(44-61%)	47%<br>(39-56%)	0.47<br>(0.31-0.66)	-3.1%<br>(-5.2--1.2%)	-3.9%<br>(-6.6--1.4%)	-120<br>(-200--43)	-1700<br>(-2800--620)
Georgia	1995	Female	80 to 84	53%<br>(40-65%)	47%<br>(35-60%)	0.45<br>(0.29-0.67)	-3.5%<br>(-5.9--1.3%)	-4.3%<br>(-7.4--1.6%)	-160<br>(-270--60)	-1600<br>(-2800--630)
Georgia	1995	Female	85 to 89	51%<br>(39-62%)	49%<br>(38-61%)	0.43<br>(0.28-0.62)	-3.5%<br>(-6.1--1.5%)	-4.3%<br>(-7.4--1.7%)	-150<br>(-270--63)	-1100<br>(-1900--460)
Georgia	1995	Female	90 to 94	51%<br>(39-62%)	49%<br>(38-61%)	0.43<br>(0.25-0.66)	-3.6%<br>(-6.1--1.4%)	-4.4%<br>(-7.4--1.7%)	-85<br>(-140--32)	-420<br>(-700--160)
Georgia	1995	Female	95 plus	52%<br>(36-68%)	48%<br>(32-64%)	0.44<br>(0.22-0.73)	-3.8%<br>(-6.4--1.2%)	-4.6%<br>(-7.6--1.5%)	-29<br>(-49--9.6)	-95<br>(-160--32)
Georgia	1995	Male	All Ages	84%<br>(82-87%)	16%<br>(13-18%)	2.9<br>(2.4-3.4)	7.2%<br>(4.5-9.9%)	6.6%<br>(2.7-11%)	1600<br>(650-2600)	68000<br>(42000-97000)
Georgia	1995	Male	15 to 19	80%<br>(74-85%)	20%<br>(15-26%)	1.5<br>(1.1-2)	4.8%<br>(3.4-6.4%)	6.9%<br>(4.4-9.6%)	12<br>(7.4-17)	1200<br>(810-1600)

Location	Year	Sex	Age	Prevalence of current drinkers	Prevalence of abstainers	Population average of standard drinks daily	Percent of total attributable deaths	Percent of total attributable DALYs	Attributable deaths	Attributable DALYs
Georgia	1995	Male	20 to 24	89% (86-92%)	11% (8.5-14%)	2.9 (2.3-3.6)	10% (7.6-13%)	12% (8.7-17%)	41 (28-56)	3500 (2500-4600)
Georgia	1995	Male	25 to 29	91% (89-93%)	9.1% (7.3-11%)	4.2 (3.3-5.3)	14% (10-17%)	17% (12-23%)	70 (49-94)	5200 (3800-6700)
Georgia	1995	Male	30 to 34	92% (90-94%)	8% (6.2-10%)	4.8 (3.8-6)	14% (11-18%)	19% (13-24%)	100 (72-140)	6900 (5100-8900)
Georgia	1995	Male	35 to 39	93% (91-94%)	7.4% (5.7-9.3%)	4.5 (3.5-5.6)	13% (9-17%)	16% (10-21%)	110 (72-150)	6700 (4700-8900)
Georgia	1995	Male	40 to 44	93% (91-95%)	7.1% (5.5-9%)	4.5 (3.6-5.6)	12% (7.9-16%)	15% (9.1-21%)	120 (73-170)	6500 (4200-8900)
Georgia	1995	Male	45 to 49	93% (91-94%)	7.4% (5.8-9.2%)	5.1 (3.9-6.4)	13% (8.6-18%)	16% (10-22%)	150 (91-210)	6800 (4500-9300)
Georgia	1995	Male	50 to 54	93% (91-94%)	7.2% (5.6-8.9%)	5.1 (3.9-6.4)	13% (7.6-17%)	15% (8.6-21%)	160 (89-230)	6300 (3800-9000)
Georgia	1995	Male	55 to 59	93% (91-95%)	6.5% (4.8-9%)	4.4 (3.4-5.4)	10% (5.2-15%)	12% (5.7-18%)	270 (130-410)	9300 (4800-14000)
Georgia	1995	Male	60 to 64	92% (90-94%)	7.8% (6-10%)	3.9 (3-4.9)	7.6% (2.7-13%)	8.7% (2.5-15%)	230 (66-400)	6700 (2300-12000)
Georgia	1995	Male	65 to 69	89% (86-92%)	11% (8.5-14%)	3.5 (2.8-4.4)	6% (1.4-11%)	6.8% (1.1-13%)	250 (42-470)	6100 (1500-11000)
Georgia	1995	Male	70 to 74	87% (83-89%)	13% (11-17%)	3.1 (2.4-4)	4.3% (-0.11-9%)	4.7% (-0.66-10%)	120 (-16-270)	2500 (-58-5200)
Georgia	1995	Male	75 to 79	86% (81-90%)	14% (10-19%)	2.8 (2.1-3.6)	2.3% (-1.9-7%)	2.2% (-2.8-7.7%)	41 (-51-140)	670 (-590-2000)
Georgia	1995	Male	80 to 84	84% (78-89%)	16% (11-22%)	2.3 (1.7-2.9)	0.38% (-3.2-4.2%)	0.059% (-4.2-4.6%)	1.1 (-84-93)	91 (-780-1000)
Georgia	1995	Male	85 to 89	83% (77-88%)	17% (12-23%)	1.9 (1.4-2.6)	-0.89% (-4.1-2.6%)	-1.4% (-5.4-2.7%)	-25 (-94-48)	-130 (-620-390)
Georgia	1995	Male	90 to 94	82% (75-88%)	18% (12-25%)	1.7 (1.1-2.4)	-2% (-5.2-1.5%)	-2.8% (-6.7-1.3%)	-21 (-50-9.6)	-88 (-230-66)
Georgia	1995	Male	95 plus	81% (71-90%)	19% (10-29%)	1.4 (0.78-2.3)	-2.5% (-5.6-0.81%)	-3.4% (-7.1-0.51%)	-7.1 (-15-1)	-20 (-46-6.6)
Georgia	2000	Female	All Ages	51% (47-55%)	49% (45-53%)	0.33 (0.26-0.42)	-0.74% (-1.7-0.13%)	-2.5% (-4.4--0.72%)	-580 (-1000--170)	-5300 (-12000-970)
Georgia	2000	Female	15 to 19	44% (33-54%)	56% (46-67%)	0.27 (0.17-0.4)	0.78% (0.46-1.2%)	0.66% (0.037-1.4%)	0.41 (0.02-0.88)	140 (78-220)
Georgia	2000	Female	20 to 24	58% (47-69%)	42% (31-53%)	0.41 (0.26-0.62)	1.5% (0.93-2.2%)	0.77% (-0.33-1.9%)	0.59 (-0.26-1.5)	280 (160-450)
Georgia	2000	Female	25 to 29	62% (52-71%)	38% (29-48%)	0.46 (0.3-0.65)	1.6% (0.99-2.3%)	0.69% (-0.51-1.9%)	0.64 (-0.48-1.8)	310 (170-470)
Georgia	2000	Female	30 to 34	63% (52-73%)	37% (27-48%)	0.47 (0.31-0.67)	1.4% (0.71-2.1%)	0.63% (-0.87-2%)	0.79 (-1-2.6)	310 (150-480)
Georgia	2000	Female	35 to 39	63% (52-72%)	37% (28-48%)	0.45 (0.29-0.67)	1.1% (0.47-1.7%)	0.64% (-0.8-2%)	1.3 (-1.6-4.1)	350 (140-550)

Location	Year	Sex	Age	Prevalence of current drinkers	Prevalence of abstainers	Population average of standard drinks daily	Percent of total attributable deaths	Percent of total attributable DALYs	Attributable deaths	Attributable DALYs
Georgia	2000	Female	40 to 44	62% (52-71%)	38% (29-48%)	0.45 (0.29-0.68)	1% (0.41-1.6%)	1.1% (-0.26-2.4%)	2.9 (-0.68-6.6)	340 (130-550)
Georgia	2000	Female	45 to 49	62% (51-71%)	38% (29-49%)	0.46 (0.29-0.68)	0.6% (-0.12-1.3%)	0.52% (-1.1-2%)	1.8 (-3.5-6.7)	200 (-36-420)
Georgia	2000	Female	50 to 54	60% (50-69%)	40% (31-50%)	0.44 (0.27-0.67)	0.27% (-0.66-1.1%)	0.1% (-1.6-1.7%)	0.44 (-7.4-7.1)	91 (-210-350)
Georgia	2000	Female	55 to 59	56% (46-66%)	44% (34-54%)	0.41 (0.25-0.65)	-0.22% (-1.3-0.75%)	-0.54% (-2.3-1.1%)	-3.2 (-14-6.7)	-72 (-420-240)
Georgia	2000	Female	60 to 64	53% (41-64%)	47% (36-59%)	0.4 (0.26-0.61)	-0.99% (-2.4-0.18%)	-1.5% (-3.7-0.32%)	-25 (-60-4.9)	-660 (-1600-140)
Georgia	2000	Female	65 to 69	50% (39-60%)	50% (40-61%)	0.41 (0.28-0.58)	-1.6% (-3.3--0.2%)	-2.2% (-4.5--0.2%)	-46 (-97--3.9)	-1100 (-2300--150)
Georgia	2000	Female	70 to 74	47% (37-57%)	53% (43-63%)	0.4 (0.26-0.59)	-1.9% (-3.7--0.38%)	-2.4% (-4.8--0.41%)	-87 (-170--14)	-1600 (-3200--360)
Georgia	2000	Female	75 to 79	46% (35-58%)	54% (42-65%)	0.38 (0.23-0.57)	-2.3% (-4.1--0.74%)	-2.9% (-5.4--0.88%)	-110 (-190--33)	-1500 (-2700--490)
Georgia	2000	Female	80 to 84	45% (32-57%)	55% (43-68%)	0.36 (0.22-0.54)	-2.6% (-4.7--0.93%)	-3.3% (-5.9--1.2%)	-100 (-190--37)	-1100 (-1900--390)
Georgia	2000	Female	85 to 89	44% (32-57%)	56% (43-68%)	0.35 (0.21-0.53)	-2.8% (-5.1--1.1%)	-3.5% (-6.3--1.4%)	-110 (-200--44)	-810 (-1500--320)
Georgia	2000	Female	90 to 94	45% (32-58%)	55% (42-68%)	0.36 (0.19-0.57)	-2.9% (-5--1.1%)	-3.6% (-6.1--1.3%)	-78 (-130--30)	-380 (-650--150)
Georgia	2000	Female	95 plus	47% (30-63%)	53% (37-70%)	0.37 (0.18-0.66)	-3.1% (-5.3--0.97%)	-3.7% (-6.5--1.2%)	-29 (-50--9.1)	-92 (-160--29)
Georgia	2000	Male	All Ages	83% (81-86%)	17% (14-19%)	2.8 (2.3-3.2)	6.6% (4-9.1%)	5.7% (2-9.7%)	1400 (490-2300)	56000 (34000-80000)
Georgia	2000	Male	15 to 19	77% (70-83%)	23% (17-30%)	1.4 (1-1.8)	4.3% (3.1-5.5%)	6.3% (4.1-8.8%)	7.9 (5.1-11)	890 (630-1200)
Georgia	2000	Male	20 to 24	88% (85-91%)	12% (9-15%)	2.7 (2.1-3.4)	9.8% (7.6-12%)	13% (9.1-17%)	28 (20-39)	2600 (1900-3400)
Georgia	2000	Male	25 to 29	90% (87-92%)	10% (7.7-13%)	3.9 (3.1-4.9)	13% (10-16%)	17% (13-22%)	52 (37-68)	4000 (3100-5100)
Georgia	2000	Male	30 to 34	92% (89-94%)	8.5% (6.4-11%)	4.6 (3.6-5.6)	14% (10-18%)	18% (13-24%)	69 (49-92)	4700 (3500-6100)
Georgia	2000	Male	35 to 39	92% (90-94%)	8% (6.1-10%)	4.2 (3.3-5.2)	12% (8.8-16%)	15% (10-21%)	92 (61-130)	5600 (4000-7400)
Georgia	2000	Male	40 to 44	92% (90-94%)	7.8% (5.7-9.8%)	4.2 (3.3-5.2)	12% (7.8-15%)	14% (8.9-20%)	110 (68-160)	6100 (3900-8100)
Georgia	2000	Male	45 to 49	92% (90-94%)	8.1% (6.1-10%)	4.7 (3.7-5.9)	12% (8.3-17%)	15% (9.6-22%)	150 (89-210)	6800 (4400-9300)
Georgia	2000	Male	50 to 54	92% (90-94%)	7.8% (5.9-10%)	4.7 (3.7-5.9)	12% (6.9-16%)	14% (8-20%)	140 (80-210)	5900 (3500-8300)
Georgia	2000	Male	55 to 59	93% (90-95%)	7.2% (5.1-9.6%)	4.1 (3.2-5)	9.2% (4.7-14%)	11% (5-17%)	130 (59-200)	4500 (2300-6800)

Location	Year	Sex	Age	Prevalence of current drinkers	Prevalence of abstainers	Population average of standard drinks daily	Percent of total attributable deaths	Percent of total attributable DALYs	Attributable deaths	Attributable DALYs
Georgia	2000	Male	60 to 64	91% (89-94%)	8.6% (6.4-11%)	3.6 (2.8-4.6)	6.9% (2.4-12%)	7.9% (2.4-14%)	210 (60-370)	6100 (2100-11000)
Georgia	2000	Male	65 to 69	88% (84-91%)	12% (9-16%)	3.3 (2.6-4.1)	5.4% (1.2-9.9%)	6% (0.96-11%)	200 (31-370)	4700 (1100-8700)
Georgia	2000	Male	70 to 74	86% (82-89%)	14% (11-18%)	2.9 (2.3-3.6)	3.7% (-0.3-8.1%)	4.1% (-0.77-9.2%)	180 (-34-400)	3500 (-290-7600)
Georgia	2000	Male	75 to 79	84% (78-89%)	16% (11-22%)	2.5 (1.9-3.2)	1.9% (-1.7-6.1%)	1.9% (-2.3-6.7%)	50 (-63-180)	840 (-790-2700)
Georgia	2000	Male	80 to 84	82% (73-88%)	18% (12-27%)	2.1 (1.5-2.7)	0.21% (-3.1-3.6%)	-0.1% (-4-3.9%)	-1.7 (-67-65)	42 (-610-730)
Georgia	2000	Male	85 to 89	81% (72-87%)	19% (13-28%)	1.7 (1.2-2.4)	-1% (-4-2.1%)	-1.5% (-5.1-2.1%)	-20 (-64-28)	-110 (-430-230)
Georgia	2000	Male	90 to 94	80% (71-87%)	20% (13-29%)	1.5 (0.99-2.2)	-1.8% (-4.7-1.2%)	-2.5% (-6.1-1%)	-20 (-49-7.9)	-85 (-230-56)
Georgia	2000	Male	95 plus	79% (67-89%)	21% (11-33%)	1.3 (0.68-2.1)	-2.2% (-4.9-0.77%)	-3% (-6.3-0.42%)	-6.3 (-14-0.87)	-18 (-42-6.5)
Georgia	2005	Female	All Ages	51% (47-55%)	49% (45-53%)	0.36 (0.28-0.46)	-0.56% (-1.5-0.3%)	-2% (-3.8--0.34%)	-480 (-930--82)	-4000 (-11000-2200)
Georgia	2005	Female	15 to 19	43% (33-54%)	57% (46-67%)	0.28 (0.17-0.43)	0.73% (0.43-1.1%)	0.5% (-0.087-1.2%)	0.32 (-0.057-0.78)	140 (74-220)
Georgia	2005	Female	20 to 24	58% (47-68%)	42% (32-53%)	0.44 (0.28-0.65)	1.5% (0.85-2.2%)	0.66% (-0.4-1.8%)	0.52 (-0.33-1.4)	280 (150-430)
Georgia	2005	Female	25 to 29	62% (51-70%)	38% (30-49%)	0.48 (0.32-0.69)	1.6% (1-2.1%)	0.88% (-0.21-2%)	0.82 (-0.21-1.9)	310 (190-450)
Georgia	2005	Female	30 to 34	63% (51-72%)	37% (28-49%)	0.49 (0.33-0.71)	1.4% (0.84-2%)	0.94% (-0.34-2.1%)	1.2 (-0.44-2.8)	310 (170-470)
Georgia	2005	Female	35 to 39	62% (51-72%)	38% (28-49%)	0.48 (0.3-0.72)	1.2% (0.64-1.8%)	1.1% (-0.089-2.3%)	1.8 (-0.15-3.8)	300 (150-440)
Georgia	2005	Female	40 to 44	61% (52-71%)	39% (29-48%)	0.47 (0.31-0.71)	1.1% (0.5-1.7%)	1.3% (0.11-2.5%)	4 (0.33-7.7)	390 (170-600)
Georgia	2005	Female	45 to 49	61% (50-71%)	39% (29-50%)	0.48 (0.3-0.72)	0.7% (0.0079-1.4%)	0.81% (-0.63-2.2%)	3.5 (-2.7-9.3)	280 (3.2-540)
Georgia	2005	Female	50 to 54	60% (50-69%)	40% (31-50%)	0.46 (0.29-0.72)	0.37% (-0.51-1.1%)	0.39% (-1.2-1.8%)	2.2 (-6.7-10)	150 (-210-450)
Georgia	2005	Female	55 to 59	56% (45-65%)	44% (35-55%)	0.43 (0.27-0.67)	-0.062% (-1-0.81%)	-0.19% (-1.8-1.3%)	-1.4 (-13-9.5)	-25 (-420-320)
Georgia	2005	Female	60 to 64	52% (41-62%)	48% (38-59%)	0.42 (0.27-0.65)	-0.74% (-2-0.33%)	-1.1% (-3.1-0.55%)	-11 (-30-4.9)	-300 (-830-130)
Georgia	2005	Female	65 to 69	50% (39-59%)	50% (41-61%)	0.43 (0.3-0.62)	-1.2% (-2.8-0.06%)	-1.6% (-3.7-0.21%)	-40 (-91-5.1)	-980 (-2100-45)
Georgia	2005	Female	70 to 74	47% (36-57%)	53% (43-64%)	0.42 (0.27-0.63)	-1.5% (-3.1--0.12%)	-1.9% (-3.9-0.014%)	-56 (-120-0.42)	-1100 (-2300--83)
Georgia	2005	Female	75 to 79	45% (34-57%)	55% (43-66%)	0.4 (0.25-0.62)	-1.9% (-3.6--0.47%)	-2.4% (-4.5--0.54%)	-110 (-200--25)	-1600 (-2900--400)

Location	Year	Sex	Age	Prevalence of current drinkers	Prevalence of abstainers	Population average of standard drinks daily	Percent of total attributable deaths	Percent of total attributable DALYs	Attributable deaths	Attributable DALYs
Georgia	2005	Female	80 to 84	44% (31-57%)	56% (43-69%)	0.38 (0.23-0.58)	-2.2% (-4--0.65%)	-2.7% (-5--0.77%)	-110 (-200--31)	-1200 (-2100--360)
Georgia	2005	Female	85 to 89	44% (31-56%)	56% (44-69%)	0.37 (0.22-0.57)	-2.4% (-4.4--0.81%)	-2.9% (-5.4--0.97%)	-84 (-160--28)	-610 (-1100--210)
Georgia	2005	Female	90 to 94	44% (31-57%)	56% (43-69%)	0.38 (0.21-0.61)	-2.5% (-4.4--0.86%)	-3.1% (-5.3--1%)	-59 (-100--20)	-290 (-510--100)
Georgia	2005	Female	95 plus	46% (30-63%)	54% (37-70%)	0.39 (0.19-0.7)	-2.7% (-4.7--0.81%)	-3.3% (-5.8--1%)	-29 (-51--9)	-94 (-170--29)
Georgia	2005	Male	All Ages	84% (81-86%)	16% (14-19%)	3.1 (2.6-3.6)	7.7% (5.1-10%)	7% (3.3-11%)	1900 (890-2900)	68000 (45000-92000)
Georgia	2005	Male	15 to 19	77% (70-83%)	23% (17-30%)	1.5 (1.1-1.9)	4.4% (3.1-5.8%)	6.1% (3.7-8.6%)	9.8 (5.9-14)	1100 (740-1400)
Georgia	2005	Male	20 to 24	88% (84-91%)	12% (9.3-16%)	2.9 (2.3-3.6)	10% (7.7-13%)	13% (9.1-17%)	32 (22-45)	2900 (2100-3800)
Georgia	2005	Male	25 to 29	90% (87-92%)	10% (7.9-13%)	4.2 (3.3-5.3)	14% (10-17%)	17% (12-23%)	51 (35-67)	3900 (2900-5000)
Georgia	2005	Male	30 to 34	91% (89-93%)	8.7% (6.6-11%)	4.9 (3.9-6.2)	15% (11-18%)	19% (13-24%)	73 (52-96)	5000 (3700-6400)
Georgia	2005	Male	35 to 39	92% (90-94%)	8.2% (6.2-10%)	4.5 (3.5-5.7)	13% (9.8-17%)	17% (12-22%)	81 (54-110)	4900 (3500-6500)
Georgia	2005	Male	40 to 44	92% (90-94%)	8% (5.9-10%)	4.5 (3.5-5.6)	13% (9.1-16%)	16% (11-21%)	120 (82-170)	6600 (4600-8700)
Georgia	2005	Male	45 to 49	92% (89-94%)	8.3% (6.3-11%)	5.1 (4-6.3)	14% (9.8-18%)	17% (11-23%)	190 (130-250)	8600 (6100-11000)
Georgia	2005	Male	50 to 54	92% (90-94%)	8% (6-10%)	5.1 (4-6.3)	13% (8.8-17%)	16% (10-21%)	220 (140-290)	8600 (5700-11000)
Georgia	2005	Male	55 to 59	93% (90-95%)	7.3% (5.2-10%)	4.4 (3.4-5.4)	11% (6.2-15%)	12% (6.9-18%)	190 (110-280)	6600 (3800-9400)
Georgia	2005	Male	60 to 64	91% (88-93%)	8.8% (6.5-12%)	3.9 (2.9-5)	8.4% (3.7-13%)	9.6% (3.9-16%)	160 (66-270)	4700 (2100-7600)
Georgia	2005	Male	65 to 69	88% (84-91%)	12% (9.2-16%)	3.5 (2.8-4.4)	6.9% (2.4-11%)	7.8% (2.5-13%)	290 (93-490)	6900 (2500-12000)
Georgia	2005	Male	70 to 74	85% (81-88%)	15% (12-19%)	3.1 (2.4-3.9)	5.3% (1-9.7%)	5.9% (0.85-11%)	230 (34-440)	4500 (890-8400)
Georgia	2005	Male	75 to 79	84% (78-88%)	16% (12-22%)	2.7 (2.1-3.5)	3.4% (-0.45-8%)	3.7% (-0.87-9%)	180 (-43-440)	2700 (-370-6400)
Georgia	2005	Male	80 to 84	82% (73-88%)	18% (12-27%)	2.2 (1.6-2.9)	1.8% (-1.6-5.5%)	1.7% (-2.1-6.2%)	52 (-60-180)	620 (-550-1900)
Georgia	2005	Male	85 to 89	80% (72-87%)	20% (13-28%)	1.9 (1.3-2.6)	0.32% (-2.6-3.6%)	0.077% (-3.3-3.9%)	0.85 (-38-43)	30 (-250-350)
Georgia	2005	Male	90 to 94	80% (71-87%)	20% (13-29%)	1.7 (1.1-2.4)	-0.75% (-3.6-2.3%)	-1.2% (-4.6-2.3%)	-6.7 (-26-13)	-24 (-120-72)
Georgia	2005	Male	95 plus	79% (66-89%)	21% (11-34%)	1.4 (0.75-2.3)	-1.4% (-4.2-1.6%)	-2% (-5.3-1.5%)	-4.4 (-12-3.3)	-12 (-36-14)

Location	Year	Sex	Age	Prevalence of current drinkers	Prevalence of abstainers	Population average of standard drinks daily	Percent of total attributable deaths	Percent of total attributable DALYs	Attributable deaths	Attributable DALYs
Georgia	2010	Female	All Ages	54% (50-58%)	46% (42-50%)	0.48 (0.37-0.61)	-0.46% (-1.4-0.4%)	-2% (-3.8--0.25%)	-450 (-870--55)	-3000 (-9100-2700)
Georgia	2010	Female	15 to 19	44% (33-55%)	56% (45-67%)	0.35 (0.21-0.54)	0.81% (0.45-1.2%)	0.74% (0.023-1.7%)	0.43 (0.013-0.99)	130 (70-210)
Georgia	2010	Female	20 to 24	60% (48-70%)	40% (30-52%)	0.55 (0.33-0.86)	1.7% (1.1-2.5%)	1.2% (0.13-2.5%)	0.94 (0.1-2)	310 (190-490)
Georgia	2010	Female	25 to 29	64% (52-73%)	36% (27-48%)	0.62 (0.4-0.91)	1.8% (1.2-2.5%)	1.4% (0.27-2.7%)	1.2 (0.23-2.3)	340 (220-510)
Georgia	2010	Female	30 to 34	66% (54-74%)	34% (26-46%)	0.63 (0.41-0.93)	1.8% (1.2-2.4%)	1.8% (0.7-3.1%)	2 (0.82-3.4)	380 (240-530)
Georgia	2010	Female	35 to 39	65% (53-74%)	35% (26-47%)	0.62 (0.39-0.93)	1.5% (0.99-2.1%)	1.8% (0.6-3%)	2.6 (0.89-4.6)	340 (210-490)
Georgia	2010	Female	40 to 44	65% (55-73%)	35% (27-45%)	0.62 (0.39-0.93)	1.3% (0.69-2%)	1.8% (0.6-3.2%)	4.2 (1.3-7.5)	350 (180-560)
Georgia	2010	Female	45 to 49	64% (52-74%)	36% (26-48%)	0.63 (0.4-0.98)	0.91% (0.18-1.7%)	1.2% (-0.13-2.8%)	5.3 (-0.58-12)	350 (72-670)
Georgia	2010	Female	50 to 54	63% (51-71%)	37% (29-49%)	0.61 (0.37-0.94)	0.6% (-0.27-1.5%)	0.82% (-0.73-2.5%)	5.1 (-4.7-15)	260 (-120-680)
Georgia	2010	Female	55 to 59	59% (48-69%)	41% (31-52%)	0.57 (0.35-0.87)	0.056% (-0.99-1.1%)	0.027% (-1.7-1.7%)	0.22 (-14-14)	25 (-440-470)
Georgia	2010	Female	60 to 64	57% (45-67%)	43% (33-55%)	0.57 (0.36-0.89)	-0.73% (-2.1-0.47%)	-1.1% (-3.2-0.67%)	-12 (-35-7.5)	-340 (-980-230)
Georgia	2010	Female	65 to 69	54% (43-64%)	46% (36-57%)	0.59 (0.39-0.86)	-1.3% (-2.8-0.066%)	-1.7% (-3.9-0.27%)	-24 (-55-3.7)	-600 (-1300-31)
Georgia	2010	Female	70 to 74	51% (39-62%)	49% (38-61%)	0.57 (0.36-0.84)	-1.5% (-3.2--0.027%)	-1.9% (-4.2-0.11%)	-60 (-130-4)	-1200 (-2500--23)
Georgia	2010	Female	75 to 79	48% (35-61%)	52% (39-65%)	0.52 (0.32-0.82)	-1.8% (-3.6--0.37%)	-2.4% (-4.7--0.44%)	-75 (-150--13)	-1100 (-2200--220)
Georgia	2010	Female	80 to 84	46% (33-59%)	54% (41-67%)	0.48 (0.29-0.73)	-2% (-3.8--0.54%)	-2.6% (-4.9--0.65%)	-100 (-200--26)	-1100 (-2100--300)
Georgia	2010	Female	85 to 89	44% (32-58%)	56% (42-68%)	0.47 (0.27-0.71)	-2.2% (-4.2--0.74%)	-2.8% (-5.3--0.9%)	-110 (-210--35)	-810 (-1500--270)
Georgia	2010	Female	90 to 94	45% (32-58%)	55% (42-68%)	0.47 (0.26-0.76)	-2.4% (-4.3--0.79%)	-3% (-5.3--0.95%)	-58 (-100--19)	-290 (-520--95)
Georgia	2010	Female	95 plus	47% (31-64%)	53% (36-69%)	0.49 (0.22-0.86)	-2.6% (-4.7--0.77%)	-3.2% (-5.9--0.95%)	-27 (-49--8)	-87 (-160--27)
Georgia	2010	Male	All Ages	81% (78-84%)	19% (16-22%)	3.5 (2.9-4.2)	9.7% (6.9-13%)	9.4% (5.4-14%)	2500 (1400-3700)	84000 (59000-110000)
Georgia	2010	Male	15 to 19	69% (58-79%)	31% (21-42%)	1.5 (1-2.1)	4.6% (3.3-6.2%)	6.5% (4.1-9.4%)	8.9 (5.5-13)	940 (660-1300)
Georgia	2010	Male	20 to 24	85% (77-90%)	15% (10-23%)	3.2 (2.4-4.3)	11% (8.3-14%)	14% (9.6-19%)	38 (26-53)	3400 (2500-4500)
Georgia	2010	Male	25 to 29	88% (82-92%)	12% (8.2-18%)	4.8 (3.6-6.2)	15% (11-19%)	19% (14-25%)	59 (41-79)	4500 (3400-5800)

Location	Year	Sex	Age	Prevalence of current drinkers	Prevalence of abstainers	Population average of standard drinks daily	Percent of total attributable deaths	Percent of total attributable DALYs	Attributable deaths	Attributable DALYs
Georgia	2010	Male	30 to 34	90%<br>(84-93%)	10%<br>(7-16%)	5.6<br>(4.2-7.4)	16%<br>(12-19%)	21%<br>(16-26%)	75<br>(55-96)	5100<br>(3900-6400)
Georgia	2010	Male	35 to 39	91%<br>(86-93%)	9.5%<br>(6.5-14%)	5.2<br>(3.9-6.7)	15%<br>(11-18%)	19%<br>(14-24%)	92<br>(68-120)	5500<br>(4200-7000)
Georgia	2010	Male	40 to 44	91%<br>(87-94%)	9.3%<br>(6.4-13%)	5.2<br>(3.9-6.6)	15%<br>(11-18%)	18%<br>(14-23%)	130<br>(91-160)	6500<br>(4800-8300)
Georgia	2010	Male	45 to 49	90%<br>(86-93%)	9.6%<br>(6.7-14%)	5.8<br>(4.4-7.6)	16%<br>(12-20%)	19%<br>(14-24%)	230<br>(170-290)	10000<br>(7700-13000)
Georgia	2010	Male	50 to 54	91%<br>(87-94%)	9.5%<br>(6.5-13%)	5.8<br>(4.3-7.5)	15%<br>(11-19%)	18%<br>(13-23%)	290<br>(200-380)	11000<br>(8100-15000)
Georgia	2010	Male	55 to 59	91%<br>(87-94%)	8.8%<br>(5.8-13%)	4.9<br>(3.7-6.5)	13%<br>(8.5-17%)	15%<br>(9.7-21%)	290<br>(180-400)	9700<br>(6300-13000)
Georgia	2010	Male	60 to 64	90%<br>(84-93%)	10%<br>(7-16%)	4.4<br>(3.2-5.9)	11%<br>(6-16%)	12%<br>(6.7-19%)	270<br>(140-400)	7600<br>(4200-11000)
Georgia	2010	Male	65 to 69	86%<br>(80-90%)	14%<br>(9.7-20%)	4<br>(3-5.2)	9.4%<br>(5-14%)	11%<br>(5.4-17%)	230<br>(120-360)	5500<br>(2900-8600)
Georgia	2010	Male	70 to 74	83%<br>(77-88%)	17%<br>(12-23%)	3.5<br>(2.6-4.6)	7.7%<br>(2.9-12%)	8.7%<br>(3-14%)	390<br>(140-660)	7500<br>(2800-12000)
Georgia	2010	Male	75 to 79	81%<br>(73-88%)	19%<br>(12-27%)	3<br>(2.2-4.1)	5.8%<br>(1.3-11%)	6.4%<br>(1.1-12%)	260<br>(45-480)	3800<br>(820-7000)
Georgia	2010	Male	80 to 84	80%<br>(69-87%)	20%<br>(13-31%)	2.5<br>(1.7-3.4)	3.8%<br>(0.14-8.1%)	4.1%<br>(-0.13-9.1%)	150<br>(-4.6-330)	1600<br>(58-3500)
Georgia	2010	Male	85 to 89	79%<br>(69-86%)	21%<br>(14-31%)	2.1<br>(1.4-3)	2%<br>(-1.3-5.9%)	2%<br>(-1.9-6.7%)	44<br>(-41-140)	370<br>(-250-1100)
Georgia	2010	Male	90 to 94	78%<br>(68-86%)	22%<br>(14-32%)	1.9<br>(1.2-2.7)	0.63%<br>(-2.3-4.3%)	0.39%<br>(-3.2-4.7%)	2.2<br>(-19-27)	21<br>(-81-150)
Georgia	2010	Male	95 plus	78%<br>(63-88%)	22%<br>(12-37%)	1.6<br>(0.84-2.6)	-0.28%<br>(-3.3-3.4%)	-0.76%<br>(-4.5-3.5%)	-1.3<br>(-7.7-6.1)	-1.9<br>(-23-23)
Georgia	2016	Female	All Ages	54%<br>(50-58%)	46%<br>(42-50%)	0.52<br>(0.36-0.7)	-0.35%<br>(-1.3-0.51%)	-1.8%<br>(-3.6--0.16%)	-410<br>(-800--37)	-2100<br>(-7300-3200)
Georgia	2016	Female	15 to 19	44%<br>(33-56%)	56%<br>(44-67%)	0.38<br>(0.21-0.61)	0.91%<br>(0.52-1.4%)	1%<br>(0.16-2.2%)	0.37<br>(0.058-0.81)	94<br>(51-150)
Georgia	2016	Female	20 to 24	60%<br>(48-70%)	40%<br>(30-52%)	0.6<br>(0.35-0.98)	1.8%<br>(1.1-2.7%)	1.5%<br>(0.31-3%)	0.9<br>(0.18-1.9)	270<br>(160-400)
Georgia	2016	Female	25 to 29	64%<br>(53-73%)	36%<br>(27-47%)	0.67<br>(0.41-1)	2%<br>(1.4-2.7%)	1.7%<br>(0.56-3.2%)	1.4<br>(0.43-2.6)	360<br>(230-530)
Georgia	2016	Female	30 to 34	66%<br>(54-74%)	34%<br>(26-46%)	0.69<br>(0.42-1)	2%<br>(1.4-2.7%)	2.3%<br>(1-3.7%)	2.2<br>(1-3.8)	380<br>(250-560)
Georgia	2016	Female	35 to 39	65%<br>(54-73%)	35%<br>(27-46%)	0.67<br>(0.41-1)	1.7%<br>(1.1-2.3%)	2.2%<br>(1.1-3.5%)	2.9<br>(1.3-5)	360<br>(230-520)
Georgia	2016	Female	40 to 44	65%<br>(55-73%)	35%<br>(27-45%)	0.68<br>(0.41-1)	1.5%<br>(0.87-2.2%)	2.3%<br>(1-3.8%)	4.4<br>(1.9-7.8)	360<br>(210-570)
Georgia	2016	Female	45 to 49	64%<br>(52-74%)	36%<br>(26-48%)	0.69<br>(0.41-1.1)	1.1%<br>(0.41-2%)	1.8%<br>(0.39-3.4%)	5.3<br>(1-11)	330<br>(120-590)

Location	Year	Sex	Age	Prevalence of current drinkers	Prevalence of abstainers	Population average of standard drinks daily	Percent of total attributable deaths	Percent of total attributable DALYs	Attributable deaths	Attributable DALYs
Georgia	2016	Female	50 to 54	63% (51-71%)	37% (29-49%)	0.66 (0.39-1)	0.85% (0.041-1.8%)	1.4% (-0.12-3.1%)	7.3 (-0.61-17)	340 (17-760)
Georgia	2016	Female	55 to 59	59% (48-69%)	41% (31-52%)	0.62 (0.36-0.98)	0.28% (-0.67-1.2%)	0.44% (-1.1-2.1%)	3.5 (-9.6-17)	130 (-330-590)
Georgia	2016	Female	60 to 64	57% (45-68%)	43% (32-55%)	0.62 (0.37-0.96)	-0.51% (-1.8-0.6%)	-0.73% (-2.7-0.99%)	-8.1 (-30-11)	-250 (-880-300)
Georgia	2016	Female	65 to 69	54% (43-64%)	46% (36-57%)	0.64 (0.39-0.94)	-1% (-2.4-0.27%)	-1.3% (-3.4-0.53%)	-17 (-45-7.2)	-460 (-1100-120)
Georgia	2016	Female	70 to 74	51% (39-62%)	49% (38-61%)	0.61 (0.36-0.94)	-1.3% (-2.9-0.12%)	-1.7% (-3.9-0.34%)	-31 (-73-5.8)	-640 (-1400-62)
Georgia	2016	Female	75 to 79	48% (35-61%)	52% (39-65%)	0.56 (0.32-0.87)	-1.6% (-3.3--0.15%)	-2.1% (-4.4--0.13%)	-75 (-150--4.4)	-1100 (-2300--110)
Georgia	2016	Female	80 to 84	46% (33-58%)	54% (42-67%)	0.52 (0.31-0.8)	-1.9% (-3.6--0.42%)	-2.4% (-4.6--0.49%)	-94 (-180--20)	-1000 (-1900--230)
Georgia	2016	Female	85 to 89	45% (32-58%)	55% (42-68%)	0.51 (0.29-0.8)	-2.1% (-4--0.57%)	-2.6% (-5--0.67%)	-100 (-200--27)	-750 (-1400--200)
Georgia	2016	Female	90 to 94	45% (32-59%)	55% (41-68%)	0.52 (0.27-0.87)	-2.2% (-4.1--0.58%)	-2.8% (-5.1--0.73%)	-82 (-150--22)	-410 (-760--110)
Georgia	2016	Female	95 plus	47% (31-64%)	53% (36-69%)	0.54 (0.23-0.99)	-2.4% (-4.6--0.51%)	-3% (-5.6--0.68%)	-29 (-55--6.6)	-92 (-170--19)
Georgia	2016	Male	All Ages	82% (78-84%)	18% (16-22%)	3.9 (2.9-5.3)	11% (7.2-14%)	10% (5.5-15%)	2700 (1400-4200)	85000 (54000-120000)
Georgia	2016	Male	15 to 19	69% (58-79%)	31% (21-42%)	1.6 (1-2.4)	5.4% (3.6-7.5%)	7.4% (4.6-11%)	9.1 (5.3-14)	880 (570-1300)
Georgia	2016	Male	20 to 24	85% (78-90%)	15% (10-22%)	3.6 (2.4-5.1)	12% (9-17%)	16% (10-22%)	39 (23-58)	3300 (2200-4600)
Georgia	2016	Male	25 to 29	88% (82-92%)	12% (8.3-18%)	5.3 (3.7-7.5)	16% (12-21%)	21% (15-28%)	67 (43-94)	5100 (3500-6900)
Georgia	2016	Male	30 to 34	90% (85-93%)	10% (7.1-15%)	6.2 (4.3-8.8)	17% (13-21%)	22% (16-28%)	82 (54-120)	5600 (3900-7700)
Georgia	2016	Male	35 to 39	91% (87-94%)	9.4% (6.5-13%)	5.8 (4-8)	16% (12-20%)	21% (15-26%)	95 (60-140)	5700 (3900-8200)
Georgia	2016	Male	40 to 44	91% (87-94%)	9.3% (6.4-13%)	5.8 (4-8)	15% (11-19%)	20% (14-25%)	120 (76-180)	6300 (4200-9000)
Georgia	2016	Male	45 to 49	90% (85-93%)	9.7% (6.8-15%)	6.4 (4.6-8.7)	16% (12-20%)	20% (15-25%)	180 (120-260)	8000 (5500-11000)
Georgia	2016	Male	50 to 54	90% (86-93%)	9.5% (6.6-14%)	6.4 (4.5-8.8)	16% (11-20%)	19% (14-24%)	270 (170-380)	11000 (7000-15000)
Georgia	2016	Male	55 to 59	91% (87-94%)	8.8% (5.9-13%)	5.5 (3.7-7.6)	14% (8.7-18%)	16% (10-22%)	310 (180-450)	11000 (6500-15000)
Georgia	2016	Male	60 to 64	89% (84-93%)	11% (7.2-16%)	4.9 (3.2-6.8)	12% (6.4-17%)	14% (7.2-20%)	320 (160-480)	9100 (4800-14000)
Georgia	2016	Male	65 to 69	86% (79-90%)	14% (10-21%)	4.4 (3-6.2)	11% (5.6-16%)	13% (6.2-19%)	280 (130-440)	6700 (3400-10000)

Location	Year	Sex	Age	Prevalence of current drinkers	Prevalence of abstainers	Population average of standard drinks daily	Percent of total attributable deaths	Percent of total attributable DALYs	Attributable deaths	Attributable DALYs
Georgia	2016	Male	70 to 74	83% (77-88%)	17% (12-23%)	3.9 (2.6-5.5)	9.3% (4-15%)	11% (4.2-17%)	260 (100-430)	4900 (2000-8000)
Georgia	2016	Male	75 to 79	81% (73-88%)	19% (12-27%)	3.4 (2.1-4.9)	7.4% (2.2-13%)	8.4% (2.3-15%)	330 (85-620)	4900 (1400-9100)
Georgia	2016	Male	80 to 84	80% (69-87%)	20% (13-31%)	2.8 (1.8-4)	5.5% (1-11%)	6.1% (0.91-13%)	240 (34-510)	2600 (520-5400)
Georgia	2016	Male	85 to 89	79% (68-86%)	21% (14-32%)	2.3 (1.4-3.5)	3.3% (-0.39-8.4%)	3.6% (-0.79-9.4%)	92 (-19-250)	740 (-85-1900)
Georgia	2016	Male	90 to 94	78% (68-86%)	22% (14-32%)	2 (1.2-3.2)	1.8% (-1.9-6.3%)	1.7% (-2.7-7.2%)	21 (-31-87)	130 (-140-460)
Georgia	2016	Male	95 plus	77% (63-88%)	23% (12-37%)	1.7 (0.85-2.9)	0.61% (-2.9-4.9%)	0.28% (-3.9-5.5%)	0.59 (-8-11)	5 (-24-42)
Kazakhstan	1990	Female	All Ages	57% (51-63%)	43% (37-49%)	2.3 (1.7-3)	5.6% (3.3-7.9%)	8% (3.9-13%)	5100 (2400-8400)	150000 (90000-210000)
Kazakhstan	1990	Female	15 to 19	47% (34-59%)	53% (41-66%)	1.8 (0.96-2.9)	4% (2.2-6.4%)	8.1% (4.3-14%)	41 (21-67)	3600 (2000-5700)
Kazakhstan	1990	Female	20 to 24	67% (54-78%)	33% (22-46%)	3 (1.8-4.8)	6.8% (4.3-9.9%)	13% (7.6-19%)	78 (45-120)	6500 (4100-9200)
Kazakhstan	1990	Female	25 to 29	74% (62-82%)	26% (18-38%)	3.7 (2.4-5.6)	7.6% (5.3-11%)	15% (9.9-20%)	110 (75-160)	8800 (6100-12000)
Kazakhstan	1990	Female	30 to 34	76% (66-85%)	24% (15-34%)	4.1 (2.6-5.9)	7.8% (5.5-10%)	15% (10-20%)	140 (93-190)	9700 (6700-13000)
Kazakhstan	1990	Female	35 to 39	77% (67-84%)	23% (16-33%)	4.1 (2.6-5.9)	8.1% (5.5-11%)	15% (10-20%)	160 (100-220)	9700 (6500-13000)
Kazakhstan	1990	Female	40 to 44	76% (65-84%)	24% (16-35%)	4.1 (2.6-5.9)	8.7% (5.7-12%)	15% (10-21%)	180 (110-250)	9500 (6300-13000)
Kazakhstan	1990	Female	45 to 49	74% (61-83%)	26% (17-39%)	3.9 (2.4-6)	9.6% (6.1-13%)	15% (9.9-22%)	200 (120-280)	8900 (5700-13000)
Kazakhstan	1990	Female	50 to 54	72% (60-82%)	28% (18-40%)	3.7 (2.2-5.8)	9.9% (5.7-14%)	15% (8.3-21%)	460 (260-690)	18000 (10000-27000)
Kazakhstan	1990	Female	55 to 59	69% (56-81%)	31% (19-44%)	3.4 (2.1-5.3)	9.5% (5-14%)	13% (7.1-20%)	390 (210-600)	13000 (7200-20000)
Kazakhstan	1990	Female	60 to 64	67% (52-80%)	33% (20-48%)	3.1 (1.8-5)	8.5% (3.3-14%)	11% (4.5-19%)	640 (250-1100)	18000 (7500-31000)
Kazakhstan	1990	Female	65 to 69	66% (50-79%)	34% (21-50%)	2.9 (1.7-4.5)	7.9% (3.1-13%)	10% (4-17%)	590 (220-1000)	14000 (5400-24000)
Kazakhstan	1990	Female	70 to 74	58% (43-71%)	42% (29-57%)	2.4 (1.3-4)	6.4% (1.3-13%)	8% (1.6-16%)	470 (93-950)	9000 (1800-18000)
Kazakhstan	1990	Female	75 to 79	47% (33-63%)	53% (37-67%)	2 (1-3.5)	5.1% (0.65-12%)	6.2% (0.7-14%)	570 (65-1300)	8400 (1000-19000)
Kazakhstan	1990	Female	80 to 84	42% (28-57%)	58% (43-72%)	2 (1-3.3)	5.1% (0.97-12%)	6% (1-14%)	510 (85-1200)	5600 (1100-12000)
Kazakhstan	1990	Female	85 to 89	41% (27-56%)	59% (44-73%)	2.1 (1.1-3.5)	5.1% (0.79-12%)	6.1% (0.78-14%)	370 (47-860)	2900 (430-6400)

Location	Year	Sex	Age	Prevalence of current drinkers	Prevalence of abstainers	Population average of standard drinks daily	Percent of total attributable deaths	Percent of total attributable DALYs	Attributable deaths	Attributable DALYs
Kazakhstan	1990	Female	90 to 94	42% (28-57%)	58% (43-72%)	2.3 (1.1-3.9)	5.1% (0.57-12%)	6.1% (0.45-15%)	160 (11-390)	850 (96-2000)
Kazakhstan	1990	Female	95 plus	45% (28-64%)	55% (36-72%)	2.5 (1.1-4.8)	5.6% (-0.14-14%)	6.5% (-0.5-17%)	45 (-3.4-120)	160 (-3.9-390)
Kazakhstan	1990	Male	All Ages	60% (54-68%)	40% (32-46%)	3 (2.2-3.9)	10% (7.3-13%)	12% (7.6-16%)	8300 (5300-12000)	340000 (240000-450000)
Kazakhstan	1990	Male	15 to 19	47% (34-60%)	53% (40-66%)	1.3 (0.66-2.2)	5.5% (3.2-8.6%)	7.4% (3.7-12%)	79 (39-130)	7000 (4000-11000)
Kazakhstan	1990	Male	20 to 24	74% (61-84%)	26% (16-39%)	3.5 (2.1-5.3)	14% (9.5-20%)	18% (11-26%)	290 (180-430)	22000 (15000-31000)
Kazakhstan	1990	Male	25 to 29	82% (72-88%)	18% (12-28%)	5.3 (3.4-7.9)	19% (13-25%)	24% (16-33%)	560 (360-770)	38000 (26000-51000)
Kazakhstan	1990	Male	30 to 34	86% (77-91%)	14% (8.6-23%)	6.1 (4-8.6)	20% (14-25%)	26% (18-33%)	700 (480-930)	44000 (31000-57000)
Kazakhstan	1990	Male	35 to 39	85% (77-91%)	15% (8.9-23%)	5.6 (3.5-8)	18% (12-23%)	23% (15-30%)	660 (410-900)	37000 (25000-50000)
Kazakhstan	1990	Male	40 to 44	85% (77-91%)	15% (9.2-23%)	5.2 (3.2-7.6)	17% (11-22%)	20% (13-27%)	610 (370-850)	31000 (19000-42000)
Kazakhstan	1990	Male	45 to 49	84% (75-90%)	16% (9.7-25%)	5.4 (3.4-8.3)	17% (10-22%)	20% (12-26%)	600 (360-830)	26000 (17000-36000)
Kazakhstan	1990	Male	50 to 54	83% (74-89%)	17% (11-26%)	5.5 (3.2-8.5)	16% (9-21%)	18% (10-24%)	1300 (690-1800)	49000 (28000-68000)
Kazakhstan	1990	Male	55 to 59	81% (70-88%)	19% (12-30%)	4.7 (2.9-7.1)	13% (7.4-18%)	15% (8.1-21%)	910 (470-1300)	30000 (16000-43000)
Kazakhstan	1990	Male	60 to 64	79% (67-88%)	21% (12-33%)	4.1 (2.3-6.4)	11% (5-16%)	12% (5.5-19%)	1100 (470-1700)	30000 (14000-48000)
Kazakhstan	1990	Male	65 to 69	78% (65-88%)	22% (12-35%)	3.8 (2.3-5.8)	9.6% (4.2-15%)	11% (4.6-18%)	610 (260-1000)	14000 (6200-23000)
Kazakhstan	1990	Male	70 to 74	74% (61-84%)	26% (16-39%)	3.3 (1.8-5.1)	7.8% (2.4-13%)	8.7% (2.5-15%)	400 (120-700)	7500 (2300-13000)
Kazakhstan	1990	Male	75 to 79	68% (52-81%)	32% (19-48%)	2.6 (1.4-4.3)	5.6% (0.77-11%)	6.2% (0.69-12%)	330 (38-670)	4900 (710-9800)
Kazakhstan	1990	Male	80 to 84	64% (47-78%)	36% (22-53%)	2.3 (1.2-3.6)	4% (-0.22-9.1%)	4.3% (-0.53-10%)	170 (-23-420)	2000 (-120-4600)
Kazakhstan	1990	Male	85 to 89	61% (45-75%)	39% (25-55%)	2.1 (1.1-3.4)	2.4% (-1.1-7.6%)	2.4% (-1.7-8.5%)	57 (-40-200)	490 (-220-1600)
Kazakhstan	1990	Male	90 to 94	60% (44-75%)	40% (25-56%)	1.9 (0.98-3.2)	1.1% (-2-5.9%)	0.82% (-2.9-6.4%)	7.3 (-26-59)	57 (-110-320)
Kazakhstan	1990	Male	95 plus	60% (40-77%)	40% (23-60%)	1.6 (0.71-3.2)	0.24% (-2.7-5.4%)	-0.24% (-3.8-5.9%)	-0.56 (-8.8-14)	2.2 (-25-51)
Kazakhstan	1995	Female	All Ages	60% (54-65%)	40% (35-46%)	2.2 (1.7-2.7)	5.9% (3.5-8.5%)	7.4% (3.1-12%)	5700 (2400-9600)	170000 (100000-250000)
Kazakhstan	1995	Female	15 to 19	51% (42-60%)	49% (40-58%)	1.7 (1-2.7)	4.3% (2.4-6.6%)	8.1% (4.4-12%)	49 (27-78)	4200 (2400-6500)

Location	Year	Sex	Age	Prevalence of current drinkers	Prevalence of abstainers	Population average of standard drinks daily	Percent of total attributable deaths	Percent of total attributable DALYs	Attributable deaths	Attributable DALYs
Kazakhstan	1995	Female	20 to 24	69% (60-76%)	31% (24-40%)	2.8 (1.7-4.3)	7.6% (4.8-11%)	13% (7.8-19%)	110 (62-150)	8300 (5300-12000)
Kazakhstan	1995	Female	25 to 29	75% (68-81%)	25% (19-32%)	3.4 (2.3-4.9)	8.9% (6.2-12%)	15% (10-20%)	140 (90-190)	9800 (6800-13000)
Kazakhstan	1995	Female	30 to 34	77% (71-83%)	23% (17-29%)	3.7 (2.5-5.1)	9% (6.5-12%)	15% (11-20%)	190 (130-250)	12000 (8400-16000)
Kazakhstan	1995	Female	35 to 39	77% (71-83%)	23% (17-29%)	3.7 (2.5-5.2)	8.8% (6.1-12%)	15% (10-19%)	230 (150-310)	13000 (9200-18000)
Kazakhstan	1995	Female	40 to 44	76% (70-82%)	24% (18-30%)	3.6 (2.5-5.1)	9.2% (6.2-13%)	14% (9.8-20%)	280 (190-390)	14000 (9600-20000)
Kazakhstan	1995	Female	45 to 49	76% (67-82%)	24% (18-33%)	3.6 (2.3-5.4)	9.7% (6.1-14%)	14% (8.9-20%)	310 (190-460)	14000 (8600-20000)
Kazakhstan	1995	Female	50 to 54	74% (64-82%)	26% (18-36%)	3.4 (2.1-5.1)	10% (5.5-15%)	14% (7.7-21%)	330 (180-500)	12000 (7000-19000)
Kazakhstan	1995	Female	55 to 59	71% (59-82%)	29% (18-41%)	3.1 (1.9-4.8)	9.3% (4.6-14%)	12% (6-19%)	680 (330-1100)	23000 (11000-36000)
Kazakhstan	1995	Female	60 to 64	70% (56-82%)	30% (18-44%)	2.9 (1.7-4.6)	7.8% (2.5-14%)	9.8% (3.2-17%)	480 (160-860)	14000 (4400-24000)
Kazakhstan	1995	Female	65 to 69	68% (54-80%)	32% (20-46%)	2.7 (1.6-4)	6.9% (2-13%)	8.4% (2.3-15%)	800 (220-1500)	19000 (5100-34000)
Kazakhstan	1995	Female	70 to 74	61% (47-73%)	39% (27-53%)	2.2 (1.2-3.6)	5.4% (0.26-12%)	6.5% (0.25-14%)	590 (21-1300)	11000 (470-24000)
Kazakhstan	1995	Female	75 to 79	51% (35-66%)	49% (34-65%)	1.9 (0.97-3.2)	4.3% (-0.26-11%)	5% (-0.44-13%)	410 (-32-1100)	6000 (-350-15000)
Kazakhstan	1995	Female	80 to 84	45% (31-60%)	55% (40-69%)	1.9 (1-3.1)	4.2% (0.058-10%)	4.8% (-0.16-12%)	480 (-15-1200)	5200 (72-13000)
Kazakhstan	1995	Female	85 to 89	44% (30-58%)	56% (42-70%)	2 (1.1-3.2)	4.2% (-0.13-11%)	4.9% (-0.4-12%)	380 (-30-1000)	3000 (-85-7400)
Kazakhstan	1995	Female	90 to 94	45% (31-59%)	55% (41-69%)	2.1 (1.1-3.6)	4.3% (-0.43-11%)	5.1% (-0.79-14%)	170 (-27-460)	920 (-90-2400)
Kazakhstan	1995	Female	95 plus	47% (30-65%)	53% (35-70%)	2.4 (1.1-4.3)	4.8% (-0.91-13%)	5.6% (-1.3-16%)	61 (-15-170)	210 (-42-580)
Kazakhstan	1995	Male	All Ages	67% (61-73%)	33% (27-39%)	2.9 (2.3-3.6)	12% (8.5-15%)	12% (7.9-16%)	11000 (7000-15000)	470000 (340000-600000)
Kazakhstan	1995	Male	15 to 19	50% (40-60%)	50% (40-60%)	1.2 (0.67-2)	5.8% (3.4-9.1%)	7.6% (3.9-12%)	97 (49-160)	8200 (4400-13000)
Kazakhstan	1995	Male	20 to 24	79% (71-85%)	21% (15-29%)	3.4 (2.2-5)	16% (11-22%)	20% (13-28%)	450 (290-640)	33000 (22000-45000)
Kazakhstan	1995	Male	25 to 29	87% (82-90%)	13% (9.6-18%)	5.1 (3.4-7.3)	22% (16-28%)	26% (19-34%)	690 (470-910)	45000 (32000-59000)
Kazakhstan	1995	Male	30 to 34	89% (85-92%)	11% (7.5-15%)	5.7 (3.9-7.9)	22% (16-28%)	27% (20-34%)	960 (670-1200)	58000 (42000-74000)
Kazakhstan	1995	Male	35 to 39	89% (84-93%)	11% (7.4-16%)	5.3 (3.6-7.3)	20% (14-26%)	24% (16-31%)	1100 (720-1500)	60000 (40000-80000)

Location	Year	Sex	Age	Prevalence of current drinkers	Prevalence of abstainers	Population average of standard drinks daily	Percent of total attributable deaths	Percent of total attributable DALYs	Attributable deaths	Attributable DALYs
Kazakhstan	1995	Male	40 to 44	88% (84-92%)	12% (7.9-16%)	5.1 (3.3-7.1)	19% (12-24%)	21% (14-29%)	1100 (710-1600)	56000 (36000-76000)
Kazakhstan	1995	Male	45 to 49	88% (82-92%)	12% (8.1-18%)	5.3 (3.5-7.7)	18% (11-24%)	20% (12-27%)	1100 (680-1600)	48000 (30000-67000)
Kazakhstan	1995	Male	50 to 54	87% (82-91%)	13% (9.2-18%)	5.3 (3.3-7.8)	16% (9.1-23%)	18% (9.8-25%)	950 (510-1400)	36000 (19000-50000)
Kazakhstan	1995	Male	55 to 59	85% (78-90%)	15% (10-22%)	4.6 (2.9-6.6)	13% (6.7-19%)	14% (7.1-21%)	1700 (810-2600)	55000 (28000-84000)
Kazakhstan	1995	Male	60 to 64	81% (72-88%)	19% (12-28%)	3.8 (2.3-5.8)	10% (4.2-16%)	11% (4.4-18%)	980 (400-1600)	27000 (12000-45000)
Kazakhstan	1995	Male	65 to 69	79% (68-88%)	21% (12-32%)	3.4 (2.2-5)	8.3% (3.3-14%)	9% (3.4-15%)	1100 (410-1900)	26000 (10000-43000)
Kazakhstan	1995	Male	70 to 74	76% (63-85%)	24% (15-37%)	3 (1.7-4.4)	6.5% (1.6-12%)	7.1% (1.5-14%)	480 (100-940)	9100 (2300-17000)
Kazakhstan	1995	Male	75 to 79	70% (55-82%)	30% (18-45%)	2.4 (1.3-3.8)	4.3% (0.091-9.6%)	4.6% (-0.18-11%)	220 (-7.7-510)	3300 (66-7300)
Kazakhstan	1995	Male	80 to 84	66% (50-79%)	34% (21-50%)	2.1 (1.2-3.2)	2.6% (-0.97-7.5%)	2.7% (-1.4-8.2%)	120 (-61-360)	1400 (-490-3800)
Kazakhstan	1995	Male	85 to 89	63% (47-77%)	37% (23-53%)	1.9 (1.1-3)	1.2% (-2-5.6%)	1% (-2.8-6.1%)	28 (-78-170)	290 (-480-1300)
Kazakhstan	1995	Male	90 to 94	62% (47-76%)	38% (24-53%)	1.7 (0.93-2.8)	0.19% (-2.6-4.4%)	-0.21% (-3.6-4.7%)	-2 (-36-46)	12 (-160-260)
Kazakhstan	1995	Male	95 plus	62% (42-79%)	38% (21-58%)	1.5 (0.69-2.8)	-0.4% (-3.2-3.9%)	-1% (-4.4-4.2%)	-2.6 (-11-11)	-4.2 (-33-40)
Kazakhstan	2000	Female	All Ages	60% (55-65%)	40% (35-45%)	2.3 (1.8-2.8)	6.3% (4.1-8.8%)	7.4% (3.5-12%)	5300 (2500-8800)	170000 (110000-240000)
Kazakhstan	2000	Female	15 to 19	50% (41-60%)	50% (40-59%)	1.7 (1.1-2.6)	4.5% (2.6-6.8%)	8.7% (4.9-14%)	50 (28-78)	4200 (2500-6300)
Kazakhstan	2000	Female	20 to 24	69% (60-76%)	31% (24-40%)	2.9 (1.8-4.3)	8.4% (5.5-12%)	15% (9.4-21%)	120 (73-170)	9100 (6000-13000)
Kazakhstan	2000	Female	25 to 29	76% (69-81%)	24% (19-31%)	3.6 (2.4-5)	10% (7.3-14%)	17% (12-23%)	170 (120-230)	12000 (8400-16000)
Kazakhstan	2000	Female	30 to 34	78% (72-84%)	22% (16-28%)	3.9 (2.7-5.3)	10% (7.7-14%)	17% (13-23%)	190 (140-260)	12000 (8900-16000)
Kazakhstan	2000	Female	35 to 39	78% (72-83%)	22% (17-28%)	3.8 (2.6-5.1)	9.9% (7.1-13%)	16% (12-21%)	250 (170-340)	14000 (10000-19000)
Kazakhstan	2000	Female	40 to 44	77% (71-82%)	23% (18-29%)	3.7 (2.6-5.1)	10% (7.2-13%)	16% (11-21%)	320 (220-440)	16000 (11000-22000)
Kazakhstan	2000	Female	45 to 49	75% (67-82%)	25% (18-33%)	3.6 (2.4-5.3)	10% (6.5-14%)	15% (9.8-21%)	400 (250-560)	18000 (11000-25000)
Kazakhstan	2000	Female	50 to 54	71% (62-79%)	29% (21-38%)	3.2 (2.1-4.7)	9.8% (5.8-14%)	14% (8-20%)	390 (220-580)	15000 (8800-22000)
Kazakhstan	2000	Female	55 to 59	70% (59-80%)	30% (20-41%)	3.1 (1.9-4.5)	9.4% (4.9-14%)	12% (6.4-19%)	370 (190-560)	12000 (6300-19000)

Location	Year	Sex	Age	Prevalence of current drinkers	Prevalence of abstainers	Population average of standard drinks daily	Percent of total attributable deaths	Percent of total attributable DALYs	Attributable deaths	Attributable DALYs
Kazakhstan	2000	Female	60 to 64	69% (57-80%)	31% (20-43%)	2.9 (1.7-4.4)	7.8% (2.6-14%)	9.9% (3.3-17%)	700 (230-1200)	20000 (6600-35000)
Kazakhstan	2000	Female	65 to 69	65% (53-77%)	35% (23-47%)	2.5 (1.6-3.7)	6.6% (1.8-12%)	8.1% (2.1-15%)	470 (120-880)	11000 (3000-20000)
Kazakhstan	2000	Female	70 to 74	54% (42-65%)	46% (35-58%)	1.9 (1.1-3)	4.8% (0.64-10%)	5.7% (0.65-12%)	620 (72-1300)	12000 (1600-25000)
Kazakhstan	2000	Female	75 to 79	42% (29-58%)	58% (42-71%)	1.5 (0.81-2.7)	3.6% (0.088-9%)	4.2% (0.0076-11%)	380 (0.74-980)	5600 (140-14000)
Kazakhstan	2000	Female	80 to 84	42% (28-56%)	58% (44-72%)	1.7 (0.97-2.8)	3.8% (0.15-9.2%)	4.4% (-0.08-11%)	330 (-6-800)	3600 (150-8500)
Kazakhstan	2000	Female	85 to 89	43% (30-58%)	57% (42-70%)	2 (1.1-3.2)	4.1% (-0.058-10%)	4.7% (-0.26-12%)	300 (-17-760)	2300 (-34-5700)
Kazakhstan	2000	Female	90 to 94	44% (30-59%)	56% (41-70%)	2.1 (1.2-3.6)	4.3% (-0.31-11%)	4.9% (-0.85-13%)	180 (-30-480)	950 (-70-2500)
Kazakhstan	2000	Female	95 plus	46% (29-65%)	54% (35-71%)	2.4 (1.1-4.4)	4.7% (-0.87-13%)	5.5% (-1.5-16%)	64 (-17-190)	220 (-42-620)
Kazakhstan	2000	Male	All Ages	68% (62-73%)	32% (27-38%)	3.1 (2.5-3.7)	13% (10-16%)	13% (9.3-17%)	12000 (7900-16000)	500000 (370000-640000)
Kazakhstan	2000	Male	15 to 19	52% (43-62%)	48% (38-57%)	1.3 (0.74-2.1)	6.2% (3.6-9.6%)	8.3% (4.3-13%)	100 (54-170)	8700 (4900-14000)
Kazakhstan	2000	Male	20 to 24	80% (72-85%)	20% (15-28%)	3.6 (2.3-5.3)	17% (12-23%)	21% (14-29%)	480 (310-670)	34000 (23000-47000)
Kazakhstan	2000	Male	25 to 29	87% (82-91%)	13% (9.4-18%)	5.3 (3.6-7.4)	24% (17-30%)	28% (21-36%)	820 (580-1100)	53000 (38000-69000)
Kazakhstan	2000	Male	30 to 34	89% (85-93%)	11% (7.3-15%)	6 (4.2-8.1)	24% (18-30%)	29% (21-36%)	930 (660-1200)	56000 (40000-71000)
Kazakhstan	2000	Male	35 to 39	89% (85-92%)	11% (7.6-15%)	5.5 (3.8-7.4)	22% (16-28%)	26% (18-33%)	1100 (790-1500)	62000 (44000-81000)
Kazakhstan	2000	Male	40 to 44	88% (84-92%)	12% (8.2-16%)	5.2 (3.5-7.1)	20% (14-26%)	23% (15-30%)	1300 (860-1800)	64000 (43000-86000)
Kazakhstan	2000	Male	45 to 49	87% (82-92%)	13% (8.4-18%)	5.4 (3.7-7.7)	19% (13-25%)	22% (14-28%)	1500 (910-2000)	62000 (40000-85000)
Kazakhstan	2000	Male	50 to 54	87% (82-91%)	13% (9.3-18%)	5.5 (3.5-8)	18% (10-24%)	20% (11-27%)	1300 (700-1800)	49000 (28000-69000)
Kazakhstan	2000	Male	55 to 59	84% (78-89%)	16% (11-22%)	4.7 (3.1-6.8)	14% (7.6-20%)	16% (8-23%)	900 (450-1300)	29000 (16000-44000)
Kazakhstan	2000	Male	60 to 64	80% (72-88%)	20% (12-28%)	3.8 (2.3-5.8)	10% (4.5-17%)	11% (4.7-19%)	1400 (580-2300)	39000 (17000-64000)
Kazakhstan	2000	Male	65 to 69	79% (69-87%)	21% (13-31%)	3.5 (2.4-5)	8.6% (3.4-14%)	9.4% (3.5-16%)	790 (300-1300)	18000 (7200-30000)
Kazakhstan	2000	Male	70 to 74	74% (63-83%)	26% (17-37%)	2.9 (1.8-4.3)	6.5% (1.7-12%)	7.1% (1.7-13%)	740 (180-1400)	14000 (3600-26000)
Kazakhstan	2000	Male	75 to 79	66% (50-78%)	34% (22-50%)	2.2 (1.2-3.5)	4.1% (0.24-9.3%)	4.5% (0.0021-10%)	230 (0.11-550)	3500 (200-7900)

Location	Year	Sex	Age	Prevalence of current drinkers	Prevalence of abstainers	Population average of standard drinks daily	Percent of total attributable deaths	Percent of total attributable DALYs	Attributable deaths	Attributable DALYs
Kazakhstan	2000	Male	80 to 84	64% (48-78%)	36% (22-52%)	2 (1.2-3.1)	2.6% (-0.9-7.1%)	2.6% (-1.4-7.9%)	82 (-45-250)	970 (-330-2800)
Kazakhstan	2000	Male	85 to 89	64% (48-76%)	36% (24-52%)	2 (1.2-3)	1.1% (-2.2-5.5%)	0.9% (-3-6%)	18 (-61-120)	200 (-390-960)
Kazakhstan	2000	Male	90 to 94	63% (47-76%)	37% (24-53%)	1.8 (0.98-2.8)	0.063% (-3-4.2%)	-0.38% (-4.1-4.6%)	-3.9 (-42-46)	3.8 (-180-260)
Kazakhstan	2000	Male	95 plus	62% (42-78%)	38% (22-58%)	1.5 (0.73-2.8)	-0.53% (-3.5-3.8%)	-1.2% (-4.8-3.9%)	-3.1 (-13-10)	-5.5 (-37-40)
Kazakhstan	2005	Female	All Ages	61% (57-66%)	39% (34-43%)	2.5 (2.1-3.1)	7.2% (5-9.8%)	8.5% (4.5-13%)	6300 (3300-10000)	200000 (140000-270000)
Kazakhstan	2005	Female	15 to 19	48% (38-59%)	52% (41-62%)	1.8 (1.1-2.7)	4.7% (2.7-7.4%)	9.6% (5.4-15%)	59 (33-93)	5000 (2900-7500)
Kazakhstan	2005	Female	20 to 24	69% (59-77%)	31% (23-41%)	3.1 (2-4.5)	8.9% (6-13%)	16% (10-22%)	130 (83-190)	10000 (6900-14000)
Kazakhstan	2005	Female	25 to 29	77% (70-83%)	23% (17-30%)	3.9 (2.6-5.3)	11% (7.9-14%)	18% (13-24%)	190 (130-260)	13000 (9600-18000)
Kazakhstan	2005	Female	30 to 34	79% (73-85%)	21% (15-27%)	4.2 (3-5.7)	12% (8.6-15%)	19% (14-24%)	250 (180-330)	16000 (11000-20000)
Kazakhstan	2005	Female	35 to 39	78% (72-84%)	22% (16-28%)	4.1 (2.8-5.7)	11% (8.2-15%)	18% (13-23%)	280 (190-380)	16000 (11000-21000)
Kazakhstan	2005	Female	40 to 44	77% (70-83%)	23% (17-30%)	4 (2.7-5.6)	12% (8.2-15%)	18% (13-23%)	390 (270-530)	20000 (14000-27000)
Kazakhstan	2005	Female	45 to 49	75% (66-82%)	25% (18-34%)	3.9 (2.6-5.6)	11% (7.8-15%)	17% (11-23%)	490 (330-680)	22000 (15000-30000)
Kazakhstan	2005	Female	50 to 54	71% (62-79%)	29% (21-38%)	3.5 (2.3-5.3)	11% (7-16%)	15% (9.9-22%)	550 (350-800)	21000 (14000-31000)
Kazakhstan	2005	Female	55 to 59	71% (60-81%)	29% (19-40%)	3.4 (2.1-5)	10% (5.9-15%)	14% (7.9-21%)	540 (300-800)	18000 (10000-27000)
Kazakhstan	2005	Female	60 to 64	69% (57-80%)	31% (20-43%)	3.1 (1.9-4.8)	8.9% (3.7-15%)	11% (4.7-19%)	460 (190-770)	13000 (5500-22000)
Kazakhstan	2005	Female	65 to 69	66% (53-77%)	34% (23-47%)	2.7 (1.7-4.1)	7.5% (2.7-13%)	9.3% (3.5-16%)	810 (300-1400)	19000 (7200-33000)
Kazakhstan	2005	Female	70 to 74	55% (42-67%)	45% (33-58%)	2.1 (1.2-3.4)	5.5% (0.9-11%)	6.6% (1.1-13%)	510 (82-1100)	9700 (1600-20000)
Kazakhstan	2005	Female	75 to 79	44% (30-59%)	56% (41-70%)	1.7 (0.92-2.9)	4.2% (0.31-10%)	4.9% (0.26-12%)	590 (33-1400)	8600 (650-21000)
Kazakhstan	2005	Female	80 to 84	43% (29-58%)	57% (42-71%)	1.9 (1.1-3.1)	4.4% (0.33-10%)	5.1% (0.24-12%)	470 (24-1200)	5200 (410-12000)
Kazakhstan	2005	Female	85 to 89	44% (30-59%)	56% (41-70%)	2.2 (1.2-3.5)	4.8% (0.37-12%)	5.5% (0.14-14%)	270 (7.2-710)	2100 (170-5200)
Kazakhstan	2005	Female	90 to 94	45% (32-60%)	55% (40-68%)	2.4 (1.3-4.1)	5% (-0.22-13%)	5.8% (-0.61-16%)	170 (-19-470)	900 (-41-2400)
Kazakhstan	2005	Female	95 plus	47% (30-65%)	53% (35-70%)	2.6 (1.2-4.8)	5.6% (-0.95-15%)	6.4% (-1.4-18%)	78 (-18-210)	270 (-46-710)

Location	Year	Sex	Age	Prevalence of current drinkers	Prevalence of abstainers	Population average of standard drinks daily	Percent of total attributable deaths	Percent of total attributable DALYs	Attributable deaths	Attributable DALYs
Kazakhstan	2005	Male	All Ages	69% (64-75%)	31% (25-36%)	3.4 (2.8-4.1)	14% (11-18%)	15% (11-19%)	14000 (9700-18000)	580000 (450000-730000)
Kazakhstan	2005	Male	15 to 19	54% (44-64%)	46% (36-56%)	1.5 (0.84-2.3)	6.8% (4-10%)	9.3% (4.9-15%)	130 (65-200)	10000 (5900-16000)
Kazakhstan	2005	Male	20 to 24	80% (72-86%)	20% (14-28%)	3.8 (2.5-5.5)	18% (12-24%)	21% (14-29%)	530 (340-760)	38000 (26000-53000)
Kazakhstan	2005	Male	25 to 29	87% (83-91%)	13% (9.4-17%)	5.7 (3.8-7.8)	24% (18-31%)	28% (20-36%)	950 (670-1300)	62000 (45000-81000)
Kazakhstan	2005	Male	30 to 34	90% (86-93%)	10% (7.1-14%)	6.4 (4.5-8.7)	25% (19-31%)	29% (22-36%)	1200 (850-1500)	70000 (51000-89000)
Kazakhstan	2005	Male	35 to 39	89% (84-93%)	11% (7.5-16%)	5.8 (4-8)	23% (17-29%)	27% (19-34%)	1200 (820-1600)	65000 (46000-85000)
Kazakhstan	2005	Male	40 to 44	87% (82-91%)	13% (8.6-18%)	5.4 (3.7-7.3)	21% (15-27%)	24% (17-31%)	1500 (1000-2100)	74000 (50000-98000)
Kazakhstan	2005	Male	45 to 49	87% (81-91%)	13% (8.5-19%)	5.6 (3.9-8)	21% (14-26%)	23% (16-29%)	1800 (1200-2400)	77000 (51000-100000)
Kazakhstan	2005	Male	50 to 54	87% (81-91%)	13% (9.4-19%)	5.8 (3.7-8.3)	19% (12-25%)	21% (13-27%)	1800 (1100-2400)	68000 (41000-89000)
Kazakhstan	2005	Male	55 to 59	83% (76-89%)	17% (11-24%)	4.9 (3.2-7.1)	15% (8.7-21%)	17% (9.1-24%)	1300 (720-1900)	44000 (25000-62000)
Kazakhstan	2005	Male	60 to 64	81% (72-88%)	19% (12-28%)	4.1 (2.5-6.2)	12% (5.4-18%)	13% (5.6-20%)	860 (380-1400)	24000 (11000-38000)
Kazakhstan	2005	Male	65 to 69	80% (70-88%)	20% (12-30%)	3.8 (2.6-5.3)	9.6% (4.1-15%)	10% (4.3-17%)	1300 (540-2100)	30000 (13000-49000)
Kazakhstan	2005	Male	70 to 74	75% (63-84%)	25% (16-37%)	3.2 (2-4.7)	7.3% (2.3-13%)	7.9% (2.3-14%)	660 (180-1200)	12000 (3800-22000)
Kazakhstan	2005	Male	75 to 79	66% (50-80%)	34% (20-50%)	2.4 (1.3-3.8)	4.7% (0.55-10%)	5.1% (0.37-11%)	430 (32-970)	6500 (730-14000)
Kazakhstan	2005	Male	80 to 84	65% (48-78%)	35% (22-52%)	2.2 (1.3-3.4)	3.1% (-0.55-8%)	3.2% (-1.1-8.8%)	130 (-42-350)	1500 (-260-3900)
Kazakhstan	2005	Male	85 to 89	64% (49-77%)	36% (23-51%)	2.2 (1.3-3.3)	1.5% (-2.1-6.4%)	1.3% (-2.9-6.9%)	19 (-43-100)	190 (-270-800)
Kazakhstan	2005	Male	90 to 94	63% (48-77%)	37% (23-52%)	1.9 (1.1-3.1)	0.28% (-3-5.1%)	-0.13% (-4.1-5.5%)	-0.93 (-29-39)	12 (-130-220)
Kazakhstan	2005	Male	95 plus	62% (44-79%)	38% (21-56%)	1.7 (0.77-3.1)	-0.37% (-3.8-4.9%)	-0.97% (-5-5.2%)	-2.3 (-12-13)	-3.4 (-36-47)
Kazakhstan	2010	Female	All Ages	64% (59-69%)	36% (31-41%)	2.7 (2.2-3.4)	7.5% (5.1-10%)	9.5% (4.9-15%)	6200 (3300-9900)	190000 (130000-260000)
Kazakhstan	2010	Female	15 to 19	52% (39-64%)	48% (36-61%)	2 (1.2-3.1)	4.7% (2.7-7.3%)	11% (5.9-17%)	48 (25-76)	4200 (2400-6400)
Kazakhstan	2010	Female	20 to 24	71% (59-80%)	29% (20-41%)	3.3 (2.1-4.9)	7.8% (5.2-11%)	16% (10-22%)	110 (70-160)	9100 (6000-13000)
Kazakhstan	2010	Female	25 to 29	76% (66-83%)	24% (17-34%)	4 (2.6-5.6)	9.5% (6.9-13%)	18% (13-23%)	150 (100-200)	11000 (7800-14000)

Location	Year	Sex	Age	Prevalence of current drinkers	Prevalence of abstainers	Population average of standard drinks daily	Percent of total attributable deaths	Percent of total attributable DALYs	Attributable deaths	Attributable DALYs
Kazakhstan	2010	Female	30 to 34	79% (69-85%)	21% (15-31%)	4.3 (2.9-6)	11% (7.8-13%)	19% (14-24%)	200 (140-260)	13000 (9600-17000)
Kazakhstan	2010	Female	35 to 39	79% (70-85%)	21% (15-30%)	4.2 (2.8-6.1)	11% (8-14%)	19% (14-24%)	250 (180-330)	15000 (11000-19000)
Kazakhstan	2010	Female	40 to 44	78% (69-85%)	22% (15-31%)	4.2 (2.8-6)	11% (8-15%)	19% (14-24%)	310 (220-400)	16000 (12000-21000)
Kazakhstan	2010	Female	45 to 49	76% (65-84%)	24% (16-35%)	4.2 (2.7-6.1)	11% (8-15%)	18% (13-24%)	440 (310-600)	20000 (14000-27000)
Kazakhstan	2010	Female	50 to 54	75% (64-83%)	25% (17-36%)	3.9 (2.5-5.9)	11% (7.6-16%)	17% (11-23%)	530 (350-740)	21000 (14000-29000)
Kazakhstan	2010	Female	55 to 59	72% (60-83%)	28% (17-40%)	3.6 (2.2-5.5)	11% (6.3-15%)	15% (8.9-22%)	580 (350-860)	20000 (12000-29000)
Kazakhstan	2010	Female	60 to 64	71% (57-83%)	29% (17-43%)	3.3 (2-5.2)	9.3% (4.3-15%)	12% (5.8-20%)	520 (240-850)	15000 (7000-25000)
Kazakhstan	2010	Female	65 to 69	70% (56-82%)	30% (18-44%)	3.1 (1.9-4.7)	8.3% (3.4-14%)	11% (4.3-18%)	500 (200-840)	12000 (4900-20000)
Kazakhstan	2010	Female	70 to 74	63% (49-75%)	37% (25-51%)	2.6 (1.5-4.3)	6.6% (1.3-13%)	8.3% (1.6-16%)	790 (140-1600)	15000 (2800-30000)
Kazakhstan	2010	Female	75 to 79	53% (38-68%)	47% (32-62%)	2.2 (1.2-3.9)	5.4% (0.58-12%)	6.4% (0.55-14%)	480 (39-1100)	7100 (740-16000)
Kazakhstan	2010	Female	80 to 84	48% (33-63%)	52% (37-67%)	2.3 (1.2-3.6)	5.5% (0.68-12%)	6.2% (0.53-14%)	610 (50-1400)	6600 (800-15000)
Kazakhstan	2010	Female	85 to 89	46% (32-61%)	54% (39-68%)	2.4 (1.3-3.8)	5.3% (0.66-12%)	6.2% (0.41-15%)	420 (27-1000)	3300 (400-7500)
Kazakhstan	2010	Female	90 to 94	47% (33-61%)	53% (39-67%)	2.5 (1.4-4.4)	5.4% (0.26-13%)	6.4% (-0.088-16%)	150 (-2.1-390)	830 (41-2000)
Kazakhstan	2010	Female	95 plus	49% (32-67%)	51% (33-68%)	2.8 (1.3-5)	5.9% (-0.59-15%)	6.8% (-1.2-18%)	64 (-11-170)	220 (-21-560)
Kazakhstan	2010	Male	All Ages	68% (61-73%)	32% (27-39%)	3.4 (2.7-4.2)	14% (11-17%)	15% (10-19%)	12000 (8100-16000)	490000 (380000-620000)
Kazakhstan	2010	Male	15 to 19	52% (39-64%)	48% (36-61%)	1.4 (0.78-2.4)	6.2% (3.7-9.4%)	9.1% (4.7-15%)	81 (41-130)	7100 (4200-11000)
Kazakhstan	2010	Male	20 to 24	77% (65-85%)	23% (15-35%)	3.6 (2.4-5.5)	16% (11-21%)	20% (13-29%)	390 (240-570)	30000 (20000-42000)
Kazakhstan	2010	Male	25 to 29	84% (75-89%)	16% (11-25%)	5.5 (3.6-7.9)	22% (16-28%)	27% (19-35%)	690 (480-920)	47000 (34000-61000)
Kazakhstan	2010	Male	30 to 34	87% (80-92%)	13% (7.8-20%)	6.2 (4.2-8.8)	24% (18-29%)	28% (21-35%)	930 (660-1200)	57000 (42000-72000)
Kazakhstan	2010	Male	35 to 39	87% (80-92%)	13% (8.2-20%)	5.7 (3.8-8)	22% (17-28%)	27% (19-33%)	1000 (710-1300)	56000 (41000-72000)
Kazakhstan	2010	Male	40 to 44	86% (80-91%)	14% (8.5-20%)	5.4 (3.5-7.4)	21% (15-26%)	25% (18-31%)	1100 (770-1400)	54000 (39000-70000)
Kazakhstan	2010	Male	45 to 49	86% (78-91%)	14% (8.9-22%)	5.6 (3.8-8.3)	20% (15-25%)	24% (17-30%)	1500 (1000-2000)	65000 (48000-85000)

Location	Year	Sex	Age	Prevalence of current drinkers	Prevalence of abstainers	Population average of standard drinks daily	Percent of total attributable deaths	Percent of total attributable DALYs	Attributable deaths	Attributable DALYs
Kazakhstan	2010	Male	50 to 54	85% (77-90%)	15% (10-23%)	5.7 (3.6-8.5)	19% (12-24%)	21% (14-28%)	1600 (1000-2100)	60000 (40000-80000)
Kazakhstan	2010	Male	55 to 59	82% (73-89%)	18% (11-27%)	4.9 (3.1-7.2)	15% (9.5-21%)	17% (10-24%)	1300 (800-1900)	45000 (28000-63000)
Kazakhstan	2010	Male	60 to 64	81% (71-89%)	19% (11-29%)	4.2 (2.5-6.4)	12% (6.2-18%)	13% (6.5-20%)	970 (470-1500)	28000 (14000-42000)
Kazakhstan	2010	Male	65 to 69	80% (69-89%)	20% (11-31%)	3.9 (2.5-5.6)	10% (5-16%)	11% (5.3-18%)	740 (330-1200)	17000 (8100-27000)
Kazakhstan	2010	Male	70 to 74	77% (65-86%)	23% (14-35%)	3.4 (2.1-5.1)	8% (2.9-13%)	8.9% (3-15%)	940 (300-1600)	18000 (6300-30000)
Kazakhstan	2010	Male	75 to 79	71% (57-83%)	29% (17-43%)	2.8 (1.6-4.4)	5.7% (0.96-11%)	6.2% (0.75-13%)	360 (44-740)	5400 (890-11000)
Kazakhstan	2010	Male	80 to 84	68% (52-80%)	32% (20-48%)	2.4 (1.4-3.7)	3.7% (-0.42-9.1%)	3.9% (-0.92-10%)	200 (-47-510)	2200 (-250-5500)
Kazakhstan	2010	Male	85 to 89	65% (49-78%)	35% (22-51%)	2.2 (1.3-3.5)	2% (-1.6-7%)	1.8% (-2.5-7.8%)	42 (-57-180)	410 (-330-1400)
Kazakhstan	2010	Male	90 to 94	64% (50-78%)	36% (22-50%)	2 (1.1-3.3)	0.72% (-2.4-5.4%)	0.27% (-3.6-5.9%)	1.5 (-20-33)	25 (-83-190)
Kazakhstan	2010	Male	95 plus	63% (44-80%)	37% (20-56%)	1.7 (0.81-3.2)	0.057% (-3.1-4.9%)	-0.65% (-4.6-5.2%)	-1.1 (-7.2-8.6)	0.4 (-20-34)
Kazakhstan	2016	Female	All Ages	64% (58-69%)	36% (31-42%)	2.7 (2-3.5)	7.1% (4.4-10%)	9.4% (4.4-15%)	5700 (2400-9800)	170000 (100000-250000)
Kazakhstan	2016	Female	15 to 19	52% (40-64%)	48% (36-60%)	2 (1.1-3.3)	4.1% (2.3-6.7%)	11% (5.5-18%)	26 (13-45)	2400 (1300-3900)
Kazakhstan	2016	Female	20 to 24	71% (59-80%)	29% (20-41%)	3.3 (1.9-5.2)	6.7% (4.3-9.8%)	15% (9.5-23%)	64 (37-99)	5800 (3700-8400)
Kazakhstan	2016	Female	25 to 29	76% (67-83%)	24% (17-33%)	3.9 (2.5-5.8)	8.2% (5.8-11%)	17% (12-24%)	120 (78-180)	9900 (6900-14000)
Kazakhstan	2016	Female	30 to 34	79% (69-85%)	21% (15-31%)	4.3 (2.7-6.3)	9.4% (6.8-13%)	19% (14-25%)	170 (110-240)	12000 (8100-16000)
Kazakhstan	2016	Female	35 to 39	79% (70-86%)	21% (14-30%)	4.2 (2.6-6.3)	10% (7-13%)	19% (14-24%)	200 (130-290)	12000 (8400-17000)
Kazakhstan	2016	Female	40 to 44	78% (70-85%)	22% (15-30%)	4.2 (2.6-6.3)	10% (6.9-14%)	19% (13-24%)	260 (170-380)	14000 (9500-20000)
Kazakhstan	2016	Female	45 to 49	77% (66-84%)	23% (16-34%)	4.1 (2.5-6.4)	10% (6.9-14%)	18% (12-24%)	310 (190-450)	14000 (9500-21000)
Kazakhstan	2016	Female	50 to 54	75% (64-83%)	25% (17-36%)	3.9 (2.4-6.1)	10% (6.7-15%)	17% (11-23%)	480 (300-720)	20000 (12000-29000)
Kazakhstan	2016	Female	55 to 59	73% (60-83%)	27% (17-40%)	3.6 (2.1-5.8)	9.9% (5.6-15%)	15% (8.7-22%)	560 (300-850)	19000 (11000-29000)
Kazakhstan	2016	Female	60 to 64	71% (57-82%)	29% (18-43%)	3.4 (1.9-5.5)	8.8% (3.8-15%)	12% (5.4-20%)	630 (270-1100)	19000 (8000-31000)
Kazakhstan	2016	Female	65 to 69	70% (55-82%)	30% (18-45%)	3.1 (1.8-4.8)	7.9% (2.7-13%)	11% (3.7-18%)	480 (160-860)	12000 (3900-21000)

Location	Year	Sex	Age	Prevalence of current drinkers	Prevalence of abstainers	Population average of standard drinks daily	Percent of total attributable deaths	Percent of total attributable DALYs	Attributable deaths	Attributable DALYs
Kazakhstan	2016	Female	70 to 74	63% (49-75%)	37% (25-51%)	2.6 (1.4-4.3)	6.5% (1.3-13%)	8.3% (1.6-16%)	470 (81-980)	9100 (1700-19000)
Kazakhstan	2016	Female	75 to 79	53% (37-68%)	47% (32-63%)	2.2 (1.2-4.1)	5.3% (0.55-12%)	6.5% (0.6-15%)	670 (56-1600)	10000 (1100-23000)
Kazakhstan	2016	Female	80 to 84	48% (33-63%)	52% (37-67%)	2.3 (1.2-3.8)	5.5% (0.42-13%)	6.4% (0.29-15%)	670 (30-1600)	7400 (550-17000)
Kazakhstan	2016	Female	85 to 89	47% (33-61%)	53% (39-67%)	2.4 (1.3-4.1)	5.5% (0.33-13%)	6.5% (0.095-16%)	340 (5.6-820)	2600 (170-6200)
Kazakhstan	2016	Female	90 to 94	48% (34-62%)	52% (38-66%)	2.6 (1.3-4.6)	5.6% (-0.093-14%)	6.6% (-0.47-17%)	200 (-16-520)	1100 (-18-2700)
Kazakhstan	2016	Female	95 plus	50% (33-68%)	50% (32-67%)	2.8 (1.4-5.2)	6% (-0.54-15%)	7.1% (-1.1-19%)	58 (-9-160)	200 (-18-520)
Kazakhstan	2016	Male	All Ages	67% (61-73%)	33% (27-39%)	3.3 (2.4-4.5)	14% (10-18%)	14% (9.3-19%)	11000 (6400-16000)	440000 (300000-600000)
Kazakhstan	2016	Male	15 to 19	52% (39-65%)	48% (35-61%)	1.4 (0.75-2.5)	5.9% (3.6-9.3%)	9.4% (5-16%)	45 (22-77)	4300 (2500-6600)
Kazakhstan	2016	Male	20 to 24	77% (65-85%)	23% (15-35%)	3.6 (2.2-5.5)	15% (10-20%)	20% (13-29%)	250 (140-380)	20000 (12000-29000)
Kazakhstan	2016	Male	25 to 29	84% (75-89%)	16% (11-25%)	5.4 (3.4-8.2)	21% (15-27%)	27% (19-35%)	580 (360-840)	42000 (29000-57000)
Kazakhstan	2016	Male	30 to 34	87% (80-92%)	13% (7.9-20%)	6.1 (3.9-9.1)	23% (17-28%)	28% (21-36%)	800 (540-1100)	51000 (36000-69000)
Kazakhstan	2016	Male	35 to 39	87% (80-92%)	13% (8.2-20%)	5.7 (3.6-8.4)	22% (16-27%)	27% (20-34%)	840 (550-1200)	48000 (33000-67000)
Kazakhstan	2016	Male	40 to 44	86% (80-91%)	14% (8.6-20%)	5.4 (3.3-7.8)	21% (15-26%)	26% (18-32%)	930 (590-1300)	47000 (32000-66000)
Kazakhstan	2016	Male	45 to 49	86% (78-91%)	14% (8.9-22%)	5.6 (3.5-8.8)	20% (14-25%)	24% (17-31%)	1000 (650-1500)	46000 (31000-65000)
Kazakhstan	2016	Male	50 to 54	85% (77-90%)	15% (10-23%)	5.6 (3.3-8.8)	19% (12-24%)	22% (14-29%)	1400 (850-2100)	57000 (35000-82000)
Kazakhstan	2016	Male	55 to 59	83% (74-89%)	17% (11-26%)	4.8 (2.9-7.3)	15% (9.3-21%)	18% (11-25%)	1300 (720-2000)	46000 (26000-68000)
Kazakhstan	2016	Male	60 to 64	81% (71-88%)	19% (12-29%)	4.2 (2.4-6.6)	12% (5.9-18%)	13% (6.5-21%)	1200 (540-1900)	34000 (16000-54000)
Kazakhstan	2016	Male	65 to 69	80% (70-89%)	20% (11-30%)	3.9 (2.4-5.8)	10% (4.9-16%)	11% (5.3-18%)	760 (320-1300)	18000 (8100-29000)
Kazakhstan	2016	Male	70 to 74	77% (65-85%)	23% (15-35%)	3.4 (1.9-5.4)	8% (2.7-14%)	9% (2.8-16%)	530 (160-980)	10000 (3300-18000)
Kazakhstan	2016	Male	75 to 79	72% (57-83%)	28% (17-43%)	2.8 (1.5-4.6)	5.7% (0.96-12%)	6.4% (0.87-13%)	510 (70-1100)	7800 (1200-16000)
Kazakhstan	2016	Male	80 to 84	68% (53-80%)	32% (20-47%)	2.4 (1.3-3.9)	3.9% (-0.33-9.9%)	4.1% (-0.71-11%)	260 (-45-700)	2900 (-250-7500)
Kazakhstan	2016	Male	85 to 89	65% (50-78%)	35% (22-50%)	2.2 (1.2-3.6)	2.1% (-1.5-7.5%)	2% (-2.3-8.3%)	40 (-47-170)	380 (-270-1400)

Location	Year	Sex	Age	Prevalence of current drinkers	Prevalence of abstainers	Population average of standard drinks daily	Percent of total attributable deaths	Percent of total attributable DALYs	Attributable deaths	Attributable DALYs
Kazakhstan	2016	Male	90 to 94	64% (50-78%)	36% (22-50%)	2 (1-3.4)	0.87% (-2.2-5.4%)	0.46% (-3.4-6.1%)	4.1 (-30-55)	48 (-120-310)
Kazakhstan	2016	Male	95 plus	63% (44-80%)	37% (20-56%)	1.7 (0.76-3.2)	0.21% (-2.8-5.3%)	-0.47% (-4.3-5.4%)	-0.65 (-6-7.9)	1.3 (-16-31)
Kyrgyzstan	1990	Female	All Ages	53% (47-61%)	47% (39-53%)	1 (0.78-1.3)	1.9% (0.96-3%)	1.8% (-0.63-4.6%)	300 (-110-790)	16000 (7900-26000)
Kyrgyzstan	1990	Female	15 to 19	56% (43-70%)	44% (30-57%)	1.1 (0.6-1.7)	2.2% (1.3-3.6%)	4.3% (2.1-7%)	6 (2.9-9.9)	590 (340-910)
Kyrgyzstan	1990	Female	20 to 24	62% (49-75%)	38% (25-51%)	1.5 (0.86-2.4)	3.6% (2.3-5.4%)	5.5% (2.9-8.9%)	9.9 (5.2-16)	980 (610-1500)
Kyrgyzstan	1990	Female	25 to 29	65% (51-77%)	35% (23-49%)	1.7 (1-2.5)	4.5% (3.1-6.3%)	6.9% (4.2-10%)	16 (9.8-24)	1500 (970-2000)
Kyrgyzstan	1990	Female	30 to 34	66% (52-79%)	34% (21-48%)	1.8 (1.1-2.7)	5.2% (3.8-7%)	8.5% (5.7-12%)	22 (15-30)	1700 (1200-2300)
Kyrgyzstan	1990	Female	35 to 39	66% (53-80%)	34% (20-47%)	1.8 (1.1-2.8)	5.7% (4.2-7.7%)	9.4% (6.5-13%)	25 (17-34)	1600 (1200-2200)
Kyrgyzstan	1990	Female	40 to 44	66% (53-79%)	34% (21-47%)	1.8 (1.1-2.8)	6.4% (4.5-8.8%)	10% (7.1-14%)	27 (18-38)	1500 (1000-2000)
Kyrgyzstan	1990	Female	45 to 49	66% (51-79%)	34% (21-49%)	1.9 (1.1-2.9)	6.4% (4-9.3%)	9.8% (5.8-15%)	24 (15-36)	1100 (710-1700)
Kyrgyzstan	1990	Female	50 to 54	65% (51-77%)	35% (23-49%)	1.9 (1.1-2.8)	6.4% (3.5-9.7%)	9.1% (4.7-14%)	57 (30-91)	2300 (1300-3600)
Kyrgyzstan	1990	Female	55 to 59	63% (50-76%)	37% (24-50%)	1.8 (1.1-2.8)	5.3% (2.2-8.9%)	7.4% (3-13%)	55 (22-94)	1900 (820-3200)
Kyrgyzstan	1990	Female	60 to 64	61% (48-76%)	39% (24-52%)	1.8 (1-2.8)	3.8% (0.51-7.6%)	5% (0.57-10%)	61 (7-130)	1800 (240-3600)
Kyrgyzstan	1990	Female	65 to 69	60% (48-74%)	40% (26-52%)	1.7 (1.1-2.5)	2.4% (-0.64-6.1%)	3.2% (-0.97-8.1%)	40 (-12-100)	940 (-250-2300)
Kyrgyzstan	1990	Female	70 to 74	58% (46-70%)	42% (30-54%)	1.6 (0.95-2.5)	1.3% (-2.2-5.4%)	1.6% (-2.8-6.9%)	20 (-35-84)	380 (-650-1600)
Kyrgyzstan	1990	Female	75 to 79	55% (44-67%)	45% (33-56%)	1.5 (0.87-2.4)	0.23% (-3.2-4.5%)	0.21% (-4-5.5%)	3.8 (-73-98)	74 (-1000-1400)
Kyrgyzstan	1990	Female	80 to 84	53% (41-66%)	47% (34-59%)	1.5 (0.9-2.2)	-0.63% (-3.8-3.4%)	-0.87% (-4.7-4%)	-16 (-89-75)	-150 (-900-800)
Kyrgyzstan	1990	Female	85 to 89	52% (41-63%)	48% (37-59%)	1.4 (0.87-2.2)	-1.2% (-4.4-2.7%)	-1.6% (-5.5-3%)	-26 (-92-50)	-180 (-650-400)
Kyrgyzstan	1990	Female	90 to 94	53% (42-64%)	47% (36-58%)	1.5 (0.88-2.4)	-1.6% (-4.8-3%)	-2% (-5.9-3.3%)	-17 (-50-28)	-79 (-240-150)
Kyrgyzstan	1990	Female	95 plus	53% (41-68%)	47% (32-59%)	1.4 (0.75-2.5)	-1.7% (-5.1-3.1%)	-2.3% (-6.2-3.5%)	-5.5 (-15-8.5)	-17 (-49-30)
Kyrgyzstan	1990	Male	All Ages	36% (31-41%)	64% (59-69%)	2.8 (2.2-3.4)	8.6% (7.2-10%)	11% (9-14%)	2200 (1700-2700)	90000 (75000-110000)
Kyrgyzstan	1990	Male	15 to 19	26% (18-35%)	74% (65-82%)	0.91 (0.52-1.5)	4.1% (2.8-6.1%)	5.4% (3.2-8.7%)	13 (7.6-21)	1300 (870-1900)

Location	Year	Sex	Age	Prevalence of current drinkers	Prevalence of abstainers	Population average of standard drinks daily	Percent of total attributable deaths	Percent of total attributable DALYs	Attributable deaths	Attributable DALYs
Kyrgyzstan	1990	Male	20 to 24	51% (38-64%)	49% (36-62%)	3 (1.9-4.3)	13% (8.9-17%)	16% (11-23%)	60 (39-85)	4900 (3500-6700)
Kyrgyzstan	1990	Male	25 to 29	64% (51-74%)	36% (26-49%)	5.2 (3.5-7.1)	19% (15-24%)	24% (18-31%)	130 (95-170)	9500 (7300-12000)
Kyrgyzstan	1990	Male	30 to 34	72% (60-82%)	28% (18-40%)	6.5 (4.5-8.7)	22% (18-26%)	27% (22-33%)	170 (140-220)	11000 (9000-14000)
Kyrgyzstan	1990	Male	35 to 39	75% (64-84%)	25% (16-36%)	6.3 (4.3-8.9)	22% (18-26%)	27% (22-33%)	170 (130-210)	9800 (7800-12000)
Kyrgyzstan	1990	Male	40 to 44	75% (64-84%)	25% (16-36%)	6.6 (4.5-9.1)	23% (19-27%)	28% (23-33%)	170 (140-200)	8600 (7100-10000)
Kyrgyzstan	1990	Male	45 to 49	72% (59-82%)	28% (18-41%)	7.4 (4.6-11)	23% (20-27%)	28% (23-33%)	160 (130-190)	7200 (6000-8500)
Kyrgyzstan	1990	Male	50 to 54	68% (54-80%)	32% (20-46%)	6.9 (4.4-9.9)	22% (18-25%)	25% (21-30%)	310 (260-380)	12000 (10000-15000)
Kyrgyzstan	1990	Male	55 to 59	64% (45-80%)	36% (20-55%)	5.1 (3-7.7)	18% (14-22%)	21% (16-25%)	270 (200-340)	9200 (7100-11000)
Kyrgyzstan	1990	Male	60 to 64	59% (39-77%)	41% (23-61%)	4 (2.2-6.2)	14% (9-18%)	15% (10-21%)	290 (180-380)	8200 (5300-11000)
Kyrgyzstan	1990	Male	65 to 69	55% (35-73%)	45% (27-65%)	3.4 (2-5.3)	11% (6.7-16%)	13% (7.5-18%)	160 (98-230)	3800 (2300-5400)
Kyrgyzstan	1990	Male	70 to 74	50% (32-68%)	50% (32-68%)	2.9 (1.6-4.8)	8.4% (3.9-13%)	9.4% (4.3-15%)	96 (44-150)	1800 (860-2800)
Kyrgyzstan	1990	Male	75 to 79	46% (29-65%)	54% (35-71%)	2.3 (1.2-4.1)	6% (1.9-10%)	6.6% (2.1-12%)	81 (25-150)	1200 (390-2100)
Kyrgyzstan	1990	Male	80 to 84	43% (26-60%)	57% (40-74%)	2 (1.1-3.4)	4.5% (1.1-8.9%)	5% (1-9.8%)	56 (11-110)	620 (140-1200)
Kyrgyzstan	1990	Male	85 to 89	41% (26-57%)	59% (43-74%)	1.8 (0.96-3)	3.3% (0.35-7.6%)	3.6% (0.13-8.4%)	27 (0.97-64)	220 (23-490)
Kyrgyzstan	1990	Male	90 to 94	40% (26-57%)	60% (43-74%)	1.7 (0.9-2.9)	2.2% (-0.39-6.4%)	2.3% (-0.77-7.1%)	8 (-2.7-25)	47 (-8.3-130)
Kyrgyzstan	1990	Male	95 plus	42% (24-62%)	58% (38-76%)	1.6 (0.68-3)	1.5% (-1.2-5.8%)	1.3% (-1.8-6.2%)	1.3 (-1.8-6.1)	5.9 (-4.5-22)
Kyrgyzstan	1995	Female	All Ages	54% (48-61%)	46% (39-52%)	0.92 (0.71-1.2)	2% (0.81-3.2%)	1.3% (-1.3-4.1%)	230 (-230-750)	16000 (6600-26000)
Kyrgyzstan	1995	Female	15 to 19	56% (44-70%)	44% (30-56%)	0.96 (0.55-1.5)	2.1% (1.2-3.2%)	3.8% (1.8-6.4%)	5.7 (2.8-9.8)	580 (320-910)
Kyrgyzstan	1995	Female	20 to 24	62% (49-75%)	38% (25-51%)	1.3 (0.77-2.1)	3.5% (2.1-5.4%)	5.2% (2.7-8.5%)	11 (5.7-19)	1100 (660-1700)
Kyrgyzstan	1995	Female	25 to 29	65% (51-77%)	35% (23-49%)	1.5 (0.91-2.3)	4.4% (3-6.3%)	6.6% (3.8-9.9%)	15 (8.6-23)	1300 (860-1800)
Kyrgyzstan	1995	Female	30 to 34	66% (52-79%)	34% (21-48%)	1.6 (0.97-2.4)	5.2% (3.7-7%)	7.9% (5.2-11%)	27 (17-38)	2000 (1400-2700)
Kyrgyzstan	1995	Female	35 to 39	66% (53-80%)	34% (20-47%)	1.6 (0.97-2.4)	5.7% (4-7.8%)	8.7% (5.9-12%)	35 (23-48)	2200 (1600-2900)

Location	Year	Sex	Age	Prevalence of current drinkers	Prevalence of abstainers	Population average of standard drinks daily	Percent of total attributable deaths	Percent of total attributable DALYs	Attributable deaths	Attributable DALYs
Kyrgyzstan	1995	Female	40 to 44	66% (53-80%)	34% (20-47%)	1.6 (0.99-2.4)	6.3% (4.3-8.6%)	9.6% (6.4-13%)	41 (27-58)	2200 (1500-2900)
Kyrgyzstan	1995	Female	45 to 49	66% (52-79%)	34% (21-48%)	1.7 (0.98-2.6)	6.1% (3.7-9.2%)	8.8% (5-13%)	40 (22-60)	1800 (1100-2700)
Kyrgyzstan	1995	Female	50 to 54	65% (51-78%)	35% (22-49%)	1.6 (1-2.6)	6% (3-9.5%)	7.9% (3.8-13%)	35 (16-57)	1400 (690-2100)
Kyrgyzstan	1995	Female	55 to 59	63% (50-76%)	37% (24-50%)	1.6 (0.96-2.4)	4.7% (1.6-8.4%)	6.1% (1.9-11%)	64 (20-120)	2100 (720-3900)
Kyrgyzstan	1995	Female	60 to 64	61% (48-76%)	39% (24-52%)	1.6 (0.91-2.5)	3% (-0.1-7%)	3.8% (-0.3-9%)	47 (-3.8-110)	1300 (-49-3100)
Kyrgyzstan	1995	Female	65 to 69	60% (48-74%)	40% (26-52%)	1.5 (0.95-2.2)	1.6% (-1.5-5.4%)	2% (-2-6.7%)	38 (-38-130)	890 (-840-3000)
Kyrgyzstan	1995	Female	70 to 74	58% (45-70%)	42% (30-55%)	1.5 (0.85-2.1)	0.43% (-2.9-4.3%)	0.52% (-3.6-5.4%)	9.7 (-67-99)	180 (-1200-1800)
Kyrgyzstan	1995	Female	75 to 79	55% (44-68%)	45% (32-56%)	1.4 (0.79-2.1)	-0.64% (-3.9-3.4%)	-0.82% (-4.7-3.9%)	-13 (-78-64)	-180 (-1100-920)
Kyrgyzstan	1995	Female	80 to 84	53% (41-66%)	47% (34-59%)	1.3 (0.82-2)	-1.6% (-4.8-2.1%)	-1.9% (-5.7-2.5%)	-37 (-110-48)	-370 (-1100-500)
Kyrgyzstan	1995	Female	85 to 89	53% (41-64%)	47% (36-59%)	1.3 (0.78-1.9)	-2.2% (-5.3-1.7%)	-2.7% (-6.3-1.9%)	-47 (-110-33)	-330 (-800-250)
Kyrgyzstan	1995	Female	90 to 94	53% (43-64%)	47% (36-57%)	1.3 (0.78-2.1)	-2.5% (-5.6-1.5%)	-3.1% (-6.7-1.7%)	-29 (-65-16)	-140 (-320-83)
Kyrgyzstan	1995	Female	95 plus	53% (41-68%)	47% (32-59%)	1.3 (0.66-2.2)	-2.7% (-6-1.6%)	-3.3% (-7.1-1.8%)	-11 (-24-6)	-35 (-77-21)
Kyrgyzstan	1995	Male	All Ages	36% (31-42%)	64% (58-69%)	2.5 (2-3)	10% (8.5-12%)	12% (9.6-15%)	2700 (2100-3300)	110000 (91000-130000)
Kyrgyzstan	1995	Male	15 to 19	26% (18-35%)	74% (65-82%)	0.81 (0.46-1.3)	3.9% (2.6-5.7%)	4.9% (2.9-7.9%)	14 (8.3-22)	1400 (920-2100)
Kyrgyzstan	1995	Male	20 to 24	52% (39-64%)	48% (36-61%)	2.7 (1.7-3.8)	12% (8.7-16%)	15% (10-20%)	68 (46-94)	5500 (3900-7300)
Kyrgyzstan	1995	Male	25 to 29	64% (52-75%)	36% (25-48%)	4.6 (3.2-6.3)	19% (15-23%)	23% (17-28%)	140 (100-170)	9600 (7400-12000)
Kyrgyzstan	1995	Male	30 to 34	73% (61-82%)	27% (18-39%)	5.8 (4-7.7)	22% (17-26%)	26% (20-31%)	210 (170-260)	13000 (11000-16000)
Kyrgyzstan	1995	Male	35 to 39	76% (65-84%)	24% (16-35%)	5.7 (3.9-7.7)	22% (17-26%)	25% (20-31%)	250 (190-300)	14000 (11000-17000)
Kyrgyzstan	1995	Male	40 to 44	75% (65-83%)	25% (17-35%)	5.9 (4.1-8)	23% (18-27%)	26% (21-31%)	290 (230-350)	14000 (12000-17000)
Kyrgyzstan	1995	Male	45 to 49	72% (59-82%)	28% (18-41%)	6.6 (4.2-9.3)	23% (19-28%)	27% (22-32%)	280 (230-340)	12000 (9900-14000)
Kyrgyzstan	1995	Male	50 to 54	69% (55-81%)	31% (19-45%)	6.1 (3.9-9)	22% (18-27%)	25% (20-30%)	230 (180-280)	8800 (7000-11000)
Kyrgyzstan	1995	Male	55 to 59	64% (46-81%)	36% (19-54%)	4.6 (2.8-6.9)	18% (13-22%)	20% (14-25%)	370 (260-460)	12000 (8800-15000)

Location	Year	Sex	Age	Prevalence of current drinkers	Prevalence of abstainers	Population average of standard drinks daily	Percent of total attributable deaths	Percent of total attributable DALYs	Attributable deaths	Attributable DALYs
Kyrgyzstan	1995	Male	60 to 64	59% (39-77%)	41% (23-61%)	3.5 (1.9-5.6)	13% (7.9-18%)	14% (8.6-20%)	300 (180-410)	8300 (5100-11000)
Kyrgyzstan	1995	Male	65 to 69	55% (35-73%)	45% (27-65%)	3.1 (1.8-4.7)	10% (5.7-15%)	11% (6.2-17%)	290 (160-430)	6700 (3700-9900)
Kyrgyzstan	1995	Male	70 to 74	51% (32-68%)	49% (32-68%)	2.5 (1.4-4.1)	7.5% (3.2-12%)	8.2% (3.4-13%)	130 (54-210)	2400 (1000-3900)
Kyrgyzstan	1995	Male	75 to 79	46% (29-65%)	54% (35-71%)	2.1 (1.1-3.5)	5% (1.3-9.6%)	5.4% (1.2-11%)	55 (13-110)	800 (210-1600)
Kyrgyzstan	1995	Male	80 to 84	43% (26-61%)	57% (39-74%)	1.8 (0.95-3)	3.5% (0.46-7.8%)	3.8% (0.3-8.5%)	39 (3.2-87)	430 (57-930)
Kyrgyzstan	1995	Male	85 to 89	41% (26-57%)	59% (43-74%)	1.6 (0.87-2.7)	2.4% (-0.23-6.1%)	2.5% (-0.53-6.7%)	19 (-4.1-51)	160 (-14-390)
Kyrgyzstan	1995	Male	90 to 94	41% (27-57%)	59% (43-73%)	1.5 (0.83-2.7)	1.5% (-0.96-5.3%)	1.4% (-1.4-5.8%)	4.6 (-4.7-19)	28 (-18-100)
Kyrgyzstan	1995	Male	95 plus	42% (25-62%)	58% (38-75%)	1.4 (0.62-2.8)	0.89% (-1.6-5%)	0.63% (-2.2-5.3%)	0.65 (-2.3-5.5)	3.6 (-6.5-20)
Kyrgyzstan	2000	Female	All Ages	55% (48-62%)	45% (38-52%)	0.95 (0.73-1.2)	2% (0.96-3.3%)	1.2% (-1.3-4.2%)	210 (-220-700)	16000 (7100-26000)
Kyrgyzstan	2000	Female	15 to 19	56% (44-70%)	44% (30-56%)	0.95 (0.54-1.5)	1.9% (1.1-3.1%)	3.8% (1.8-6.4%)	5.4 (2.5-9.2)	580 (320-910)
Kyrgyzstan	2000	Female	20 to 24	62% (50-74%)	38% (26-50%)	1.3 (0.76-2.1)	3.6% (2.2-5.5%)	5.8% (2.8-9.4%)	11 (5.6-19)	1100 (670-1700)
Kyrgyzstan	2000	Female	25 to 29	65% (51-77%)	35% (23-49%)	1.5 (0.91-2.3)	4.6% (3.1-6.3%)	7.3% (4.2-11%)	17 (9.6-25)	1500 (980-2100)
Kyrgyzstan	2000	Female	30 to 34	66% (52-79%)	34% (21-48%)	1.6 (0.95-2.4)	5.3% (3.9-7%)	8.6% (5.9-12%)	23 (16-32)	1700 (1300-2300)
Kyrgyzstan	2000	Female	35 to 39	66% (53-79%)	34% (21-47%)	1.6 (0.95-2.4)	5.7% (4.2-7.7%)	9.4% (6.5-13%)	35 (24-47)	2200 (1600-2900)
Kyrgyzstan	2000	Female	40 to 44	66% (52-79%)	34% (21-48%)	1.6 (1-2.4)	6.2% (4.3-8.2%)	10% (7.1-14%)	45 (31-61)	2400 (1700-3200)
Kyrgyzstan	2000	Female	45 to 49	66% (51-79%)	34% (21-49%)	1.6 (0.97-2.5)	6% (3.6-8.7%)	9.3% (5.4-14%)	47 (27-69)	2200 (1400-3100)
Kyrgyzstan	2000	Female	50 to 54	65% (51-78%)	35% (22-49%)	1.6 (0.97-2.5)	5.7% (3-8.9%)	8.2% (4-13%)	43 (21-67)	1700 (880-2700)
Kyrgyzstan	2000	Female	55 to 59	63% (50-77%)	37% (23-50%)	1.6 (0.95-2.4)	4.7% (1.6-8.3%)	6.3% (1.9-11%)	33 (9.9-59)	1100 (380-2000)
Kyrgyzstan	2000	Female	60 to 64	61% (48-76%)	39% (24-52%)	1.5 (0.91-2.5)	3% (-0.24-7%)	3.8% (-0.38-8.8%)	48 (-4.9-110)	1400 (-100-3200)
Kyrgyzstan	2000	Female	65 to 69	60% (47-73%)	40% (27-53%)	1.5 (0.93-2.2)	1.6% (-1.6-5.3%)	2% (-2-6.7%)	30 (-30-100)	700 (-670-2400)
Kyrgyzstan	2000	Female	70 to 74	57% (45-70%)	43% (30-55%)	1.4 (0.85-2.1)	0.35% (-3-4.3%)	0.42% (-3.7-5.3%)	9.6 (-85-120)	190 (-1600-2300)
Kyrgyzstan	2000	Female	75 to 79	55% (44-67%)	45% (33-56%)	1.4 (0.79-2)	-0.82% (-4-3%)	-1.1% (-5-3.6%)	-22 (-100-71)	-290 (-1400-1000)

Location	Year	Sex	Age	Prevalence of current drinkers	Prevalence of abstainers	Population average of standard drinks daily	Percent of total attributable deaths	Percent of total attributable DALYs	Attributable deaths	Attributable DALYs
Kyrgyzstan	2000	Female	80 to 84	53% (41-66%)	47% (34-59%)	1.3 (0.8-1.9)	-1.9% (-5.1-1.7%)	-2.4% (-6.3-2%)	-38 (-100-33)	-380 (-1000-360)
Kyrgyzstan	2000	Female	85 to 89	52% (41-64%)	48% (36-59%)	1.3 (0.77-1.9)	-2.5% (-5.6-1.1%)	-3.1% (-6.8-1.1%)	-40 (-88-15)	-280 (-620-130)
Kyrgyzstan	2000	Female	90 to 94	53% (43-64%)	47% (36-57%)	1.3 (0.77-2)	-2.8% (-5.9-0.94%)	-3.4% (-7.2-0.94%)	-28 (-59-8)	-140 (-290-48)
Kyrgyzstan	2000	Female	95 plus	53% (41-68%)	47% (32-59%)	1.2 (0.65-2.1)	-2.9% (-6.2-1.1%)	-3.6% (-7.6-1.1%)	-11 (-23-3.5)	-35 (-74-13)
Kyrgyzstan	2000	Male	All Ages	37% (33-43%)	63% (57-67%)	2.6 (2.1-3.1)	11% (9.8-13%)	13% (11-16%)	2700 (2200-3300)	110000 (96000-130000)
Kyrgyzstan	2000	Male	15 to 19	26% (18-34%)	74% (66-82%)	0.79 (0.46-1.3)	3.8% (2.6-5.5%)	5.1% (3.1-8.2%)	13 (7.7-21)	1400 (910-2000)
Kyrgyzstan	2000	Male	20 to 24	51% (38-63%)	49% (37-62%)	2.6 (1.7-3.8)	13% (9.5-17%)	17% (12-22%)	73 (51-98)	5800 (4200-7700)
Kyrgyzstan	2000	Male	25 to 29	64% (52-74%)	36% (26-48%)	4.5 (3.1-6.2)	20% (16-24%)	25% (19-30%)	150 (120-190)	11000 (8600-13000)
Kyrgyzstan	2000	Male	30 to 34	72% (60-82%)	28% (18-40%)	5.6 (3.9-7.5)	22% (18-26%)	27% (22-32%)	200 (160-230)	13000 (10000-15000)
Kyrgyzstan	2000	Male	35 to 39	75% (64-84%)	25% (16-36%)	5.5 (3.9-7.6)	23% (19-27%)	27% (22-32%)	270 (210-320)	15000 (12000-18000)
Kyrgyzstan	2000	Male	40 to 44	75% (64-83%)	25% (17-36%)	5.8 (4.1-7.6)	24% (20-28%)	29% (24-33%)	330 (270-390)	17000 (14000-19000)
Kyrgyzstan	2000	Male	45 to 49	72% (58-82%)	28% (18-42%)	6.4 (4.1-9)	24% (20-28%)	29% (24-33%)	340 (280-400)	15000 (12000-18000)
Kyrgyzstan	2000	Male	50 to 54	68% (54-80%)	32% (20-46%)	6 (3.8-8.7)	23% (19-27%)	27% (21-32%)	290 (230-350)	11000 (9100-14000)
Kyrgyzstan	2000	Male	55 to 59	63% (44-80%)	37% (20-56%)	4.4 (2.6-6.9)	18% (13-23%)	21% (15-27%)	210 (150-260)	6800 (4900-8700)
Kyrgyzstan	2000	Male	60 to 64	59% (39-77%)	41% (23-61%)	3.5 (1.9-5.5)	13% (8.3-18%)	15% (9.2-21%)	300 (190-430)	8600 (5300-12000)
Kyrgyzstan	2000	Male	65 to 69	55% (35-74%)	45% (26-65%)	3 (1.8-4.7)	10% (5.8-15%)	12% (6.3-18%)	240 (130-370)	5700 (3200-8400)
Kyrgyzstan	2000	Male	70 to 74	51% (32-68%)	49% (32-68%)	2.5 (1.4-4)	7.4% (3.2-12%)	8.3% (3.4-14%)	200 (82-330)	3700 (1600-6200)
Kyrgyzstan	2000	Male	75 to 79	46% (29-65%)	54% (35-71%)	2.1 (1.1-3.5)	4.8% (1.2-9.3%)	5.3% (1.1-11%)	70 (15-140)	1000 (260-2000)
Kyrgyzstan	2000	Male	80 to 84	43% (26-60%)	57% (40-74%)	1.8 (0.96-3)	3.2% (0.28-7.5%)	3.4% (0.061-8.3%)	28 (0.5-67)	310 (27-720)
Kyrgyzstan	2000	Male	85 to 89	41% (26-57%)	59% (43-74%)	1.6 (0.86-2.6)	2% (-0.49-5.7%)	2% (-0.88-6.3%)	10 (-4.6-32)	86 (-21-250)
Kyrgyzstan	2000	Male	90 to 94	40% (26-57%)	60% (43-74%)	1.5 (0.79-2.6)	1.1% (-1.2-4.7%)	1% (-1.7-5.1%)	2.7 (-4.8-14)	18 (-19-74)
Kyrgyzstan	2000	Male	95 plus	42% (24-61%)	58% (39-76%)	1.4 (0.61-2.7)	0.69% (-1.7-4.7%)	0.36% (-2.4-5%)	0.29 (-1.9-4)	2.1 (-5.4-15)

Location	Year	Sex	Age	Prevalence of current drinkers	Prevalence of abstainers	Population average of standard drinks daily	Percent of total attributable deaths	Percent of total attributable DALYs	Attributable deaths	Attributable DALYs
Kyrgyzstan	2005	Female	All Ages	55% (49-63%)	45% (37-51%)	1 (0.78-1.3)	2.4% (1.2-3.8%)	1.5% (-1.4-4.6%)	260 (-250-810)	19000 (9300-30000)
Kyrgyzstan	2005	Female	15 to 19	55% (43-69%)	45% (31-57%)	0.97 (0.55-1.5)	2% (1.1-3.1%)	4.2% (1.9-7.1%)	6 (2.8-10)	650 (370-1000)
Kyrgyzstan	2005	Female	20 to 24	62% (49-74%)	38% (26-51%)	1.4 (0.77-2.1)	3.7% (2.3-5.5%)	6.4% (3.3-10%)	13 (6.6-20)	1300 (780-1900)
Kyrgyzstan	2005	Female	25 to 29	65% (51-78%)	35% (22-49%)	1.5 (0.93-2.3)	4.9% (3.4-6.8%)	8.2% (5.1-12%)	20 (12-28)	1700 (1100-2300)
Kyrgyzstan	2005	Female	30 to 34	66% (52-80%)	34% (20-48%)	1.6 (0.96-2.4)	5.8% (4.4-7.6%)	9.7% (7-13%)	30 (21-40)	2100 (1600-2800)
Kyrgyzstan	2005	Female	35 to 39	66% (53-80%)	34% (20-47%)	1.6 (0.99-2.5)	6.3% (4.7-8.3%)	11% (7.6-14%)	40 (29-52)	2400 (1800-3100)
Kyrgyzstan	2005	Female	40 to 44	66% (52-79%)	34% (21-48%)	1.7 (1.1-2.4)	6.8% (5-9%)	11% (8.2-15%)	57 (41-76)	3000 (2200-3900)
Kyrgyzstan	2005	Female	45 to 49	66% (51-79%)	34% (21-49%)	1.7 (1-2.5)	6.5% (4.3-9.2%)	10% (6.7-14%)	65 (42-93)	2900 (2000-4100)
Kyrgyzstan	2005	Female	50 to 54	64% (50-78%)	36% (22-50%)	1.7 (1-2.5)	6.4% (3.6-9.5%)	9.2% (5-14%)	67 (37-100)	2600 (1500-3900)
Kyrgyzstan	2005	Female	55 to 59	63% (50-76%)	37% (24-50%)	1.6 (0.99-2.5)	5.4% (2.3-9.1%)	7.4% (3-13%)	57 (23-96)	1900 (810-3200)
Kyrgyzstan	2005	Female	60 to 64	61% (48-75%)	39% (25-52%)	1.6 (0.92-2.5)	3.3% (-0.099-7.6%)	4.2% (-0.23-9.6%)	31 (-1.7-71)	870 (-26-2000)
Kyrgyzstan	2005	Female	65 to 69	59% (48-74%)	41% (26-52%)	1.5 (0.97-2.3)	1.6% (-1.6-5.3%)	2% (-2.2-6.7%)	34 (-37-110)	800 (-800-2600)
Kyrgyzstan	2005	Female	70 to 74	57% (46-70%)	43% (30-54%)	1.5 (0.88-2.2)	0.26% (-3.3-4.5%)	0.29% (-4.1-5.5%)	5.6 (-81-110)	120 (-1400-1900)
Kyrgyzstan	2005	Female	75 to 79	55% (44-67%)	45% (33-56%)	1.4 (0.8-2.1)	-0.96% (-4.4-2.9%)	-1.2% (-5.4-3.4%)	-34 (-150-94)	-450 (-2100-1400)
Kyrgyzstan	2005	Female	80 to 84	53% (41-66%)	47% (34-59%)	1.3 (0.84-2)	-2% (-5.5-1.8%)	-2.5% (-6.6-1.9%)	-59 (-160-46)	-590 (-1600-500)
Kyrgyzstan	2005	Female	85 to 89	52% (42-63%)	48% (37-58%)	1.3 (0.79-2)	-2.5% (-6-1.1%)	-3.1% (-7.2-1.1%)	-41 (-94-15)	-280 (-660-120)
Kyrgyzstan	2005	Female	90 to 94	53% (43-64%)	47% (36-57%)	1.3 (0.79-2.1)	-2.8% (-6-1.1%)	-3.4% (-7.3-1.2%)	-22 (-47-7.9)	-110 (-230-44)
Kyrgyzstan	2005	Female	95 plus	53% (41-68%)	47% (32-59%)	1.3 (0.67-2.2)	-2.8% (-6.4-1.7%)	-3.5% (-7.7-1.8%)	-9.7 (-22-5.1)	-30 (-69-18)
Kyrgyzstan	2005	Male	All Ages	39% (34-44%)	61% (56-66%)	2.8 (2.3-3.4)	13% (11-15%)	14% (12-17%)	3200 (2500-3800)	130000 (110000-150000)
Kyrgyzstan	2005	Male	15 to 19	26% (18-34%)	74% (66-82%)	0.81 (0.47-1.3)	3.9% (2.7-5.6%)	5.6% (3.4-8.7%)	13 (8.2-21)	1400 (990-2100)
Kyrgyzstan	2005	Male	20 to 24	51% (38-63%)	49% (37-62%)	2.7 (1.7-3.9)	13% (9.4-17%)	17% (12-23%)	70 (48-94)	5800 (4200-7500)
Kyrgyzstan	2005	Male	25 to 29	64% (51-75%)	36% (25-49%)	4.6 (3.2-6.3)	20% (16-24%)	26% (20-32%)	150 (120-190)	11000 (8600-13000)

Location	Year	Sex	Age	Prevalence of current drinkers	Prevalence of abstainers	Population average of standard drinks daily	Percent of total attributable deaths	Percent of total attributable DALYs	Attributable deaths	Attributable DALYs
Kyrgyzstan	2005	Male	30 to 34	72% (60-82%)	28% (18-40%)	5.8 (4.1-7.7)	23% (19-27%)	28% (23-33%)	240 (190-280)	15000 (12000-18000)
Kyrgyzstan	2005	Male	35 to 39	75% (64-84%)	25% (16-36%)	5.7 (4-7.8)	24% (20-28%)	29% (23-33%)	280 (230-330)	16000 (13000-18000)
Kyrgyzstan	2005	Male	40 to 44	75% (64-83%)	25% (17-36%)	5.9 (4.2-7.8)	25% (21-29%)	30% (25-34%)	390 (320-460)	19000 (16000-22000)
Kyrgyzstan	2005	Male	45 to 49	72% (58-81%)	28% (19-42%)	6.6 (4.4-9.3)	25% (21-29%)	30% (25-34%)	440 (370-520)	19000 (16000-23000)
Kyrgyzstan	2005	Male	50 to 54	68% (55-80%)	32% (20-45%)	6.2 (4-8.8)	24% (19-28%)	27% (22-32%)	420 (340-510)	16000 (13000-19000)
Kyrgyzstan	2005	Male	55 to 59	63% (44-80%)	37% (20-56%)	4.6 (2.7-7)	19% (14-24%)	22% (16-27%)	300 (220-380)	9900 (7400-12000)
Kyrgyzstan	2005	Male	60 to 64	59% (39-77%)	41% (23-61%)	3.5 (1.9-5.7)	14% (8.7-19%)	16% (9.5-21%)	190 (120-270)	5300 (3300-7400)
Kyrgyzstan	2005	Male	65 to 69	55% (35-73%)	45% (27-65%)	3.1 (1.8-4.9)	11% (6.2-16%)	12% (6.8-18%)	290 (160-420)	6600 (3800-9500)
Kyrgyzstan	2005	Male	70 to 74	50% (32-68%)	50% (32-68%)	2.6 (1.4-4.1)	7.6% (3.3-13%)	8.4% (3.5-14%)	190 (79-320)	3500 (1500-5900)
Kyrgyzstan	2005	Male	75 to 79	45% (28-64%)	55% (36-72%)	2.1 (1.1-3.5)	4.9% (1.2-9.4%)	5.4% (1.1-11%)	130 (27-250)	1900 (460-3700)
Kyrgyzstan	2005	Male	80 to 84	42% (26-60%)	58% (40-74%)	1.8 (1-3.1)	3.2% (0.12-7.3%)	3.4% (-0.071-8.2%)	51 (-1-120)	570 (22-1300)
Kyrgyzstan	2005	Male	85 to 89	40% (26-57%)	60% (43-74%)	1.6 (0.89-2.6)	2% (-0.72-5.8%)	2% (-1.1-6.4%)	8.9 (-5.2-29)	75 (-28-220)
Kyrgyzstan	2005	Male	90 to 94	40% (26-56%)	60% (44-74%)	1.5 (0.81-2.6)	1.2% (-1.3-4.9%)	1.1% (-1.8-5.4%)	2.1 (-3.4-10)	13 (-14-54)
Kyrgyzstan	2005	Male	95 plus	41% (24-61%)	59% (39-76%)	1.4 (0.62-2.7)	0.8% (-1.8-5%)	0.52% (-2.5-5.4%)	0.34 (-1.6-3.5)	2 (-4.5-13)
Kyrgyzstan	2010	Female	All Ages	55% (49-63%)	45% (37-51%)	1 (0.78-1.3)	2.2% (1.2-3.5%)	1.1% (-1.6-4%)	180 (-280-690)	17000 (8900-27000)
Kyrgyzstan	2010	Female	15 to 19	55% (42-69%)	45% (31-58%)	0.95 (0.54-1.5)	1.9% (1-3%)	3.8% (1.6-6.5%)	5.8 (2.5-10)	630 (350-980)
Kyrgyzstan	2010	Female	20 to 24	62% (48-74%)	38% (26-52%)	1.3 (0.77-2.1)	3.5% (2.2-5.1%)	5.9% (3.1-9.4%)	13 (6.9-22)	1400 (880-2100)
Kyrgyzstan	2010	Female	25 to 29	64% (51-77%)	36% (23-49%)	1.5 (0.91-2.3)	4.5% (3.1-6.1%)	7.7% (4.7-11%)	17 (10-25)	1600 (1000-2200)
Kyrgyzstan	2010	Female	30 to 34	66% (52-78%)	34% (22-48%)	1.6 (0.95-2.4)	5.6% (4.1-7.3%)	9.6% (6.8-13%)	27 (19-36)	2000 (1500-2600)
Kyrgyzstan	2010	Female	35 to 39	66% (53-80%)	34% (20-47%)	1.6 (0.98-2.5)	5.9% (4.4-7.8%)	10% (7.4-14%)	35 (25-47)	2200 (1600-2900)
Kyrgyzstan	2010	Female	40 to 44	66% (52-79%)	34% (21-48%)	1.6 (1-2.5)	6.5% (4.7-8.5%)	11% (8.1-14%)	48 (34-62)	2500 (1900-3300)
Kyrgyzstan	2010	Female	45 to 49	66% (51-79%)	34% (21-49%)	1.7 (1-2.6)	5.9% (3.9-8.5%)	9.8% (6.4-14%)	59 (38-83)	2800 (1800-3800)

Location	Year	Sex	Age	Prevalence of current drinkers	Prevalence of abstainers	Population average of standard drinks daily	Percent of total attributable deaths	Percent of total attributable DALYs	Attributable deaths	Attributable DALYs
Kyrgyzstan	2010	Female	50 to 54	64% (50-78%)	36% (22-50%)	1.7 (1-2.5)	5.7% (3.4-8.7%)	8.7% (4.9-13%)	68 (38-100)	2700 (1600-4100)
Kyrgyzstan	2010	Female	55 to 59	62% (50-76%)	38% (24-50%)	1.6 (0.98-2.5)	4.8% (2.1-8%)	6.9% (2.8-12%)	60 (25-100)	2000 (900-3400)
Kyrgyzstan	2010	Female	60 to 64	61% (47-76%)	39% (24-53%)	1.6 (0.92-2.5)	2.9% (-0.063-6.3%)	3.8% (-0.3-8.4%)	33 (-2.6-74)	950 (-22-2100)
Kyrgyzstan	2010	Female	65 to 69	59% (47-74%)	41% (26-53%)	1.5 (0.96-2.2)	1.3% (-1.7-4.8%)	1.7% (-2.4-6.3%)	14 (-21-53)	340 (-450-1200)
Kyrgyzstan	2010	Female	70 to 74	57% (45-70%)	43% (30-55%)	1.5 (0.85-2.2)	-0.02% (-3.5-3.9%)	-0.097% (-4.6-4.7%)	-1.7 (-85-88)	-6.8 (-1500-1600)
Kyrgyzstan	2010	Female	75 to 79	54% (44-67%)	46% (33-56%)	1.4 (0.78-2.1)	-1.3% (-4.7-2.6%)	-1.7% (-5.8-3%)	-34 (-120-65)	-450 (-1700-930)
Kyrgyzstan	2010	Female	80 to 84	53% (40-66%)	47% (34-60%)	1.3 (0.81-2)	-2.3% (-5.7-1.4%)	-2.9% (-7-1.5%)	-63 (-150-33)	-610 (-1500-380)
Kyrgyzstan	2010	Female	85 to 89	52% (41-63%)	48% (37-59%)	1.3 (0.78-1.9)	-2.8% (-6.3-0.63%)	-3.5% (-7.7-0.55%)	-69 (-150-11)	-480 (-1100-110)
Kyrgyzstan	2010	Female	90 to 94	53% (42-64%)	47% (36-58%)	1.3 (0.76-2.1)	-3% (-6.4-0.78%)	-3.7% (-7.8-0.65%)	-26 (-54-4.6)	-120 (-260-32)
Kyrgyzstan	2010	Female	95 plus	53% (40-68%)	47% (32-60%)	1.3 (0.65-2.2)	-3% (-6.6-1.2%)	-3.8% (-8-1.2%)	-8.7 (-19-3)	-27 (-60-11)
Kyrgyzstan	2010	Male	All Ages	39% (35-43%)	61% (57-65%)	2.8 (2.3-3.4)	12% (10-14%)	13% (11-16%)	2900 (2300-3600)	120000 (100000-150000)
Kyrgyzstan	2010	Male	15 to 19	19% (13-28%)	81% (72-87%)	0.6 (0.34-0.99)	3.1% (2.2-4.4%)	3.9% (2.3-6.4%)	10 (6.1-16)	1200 (820-1700)
Kyrgyzstan	2010	Male	20 to 24	48% (39-58%)	52% (42-61%)	2.5 (1.6-3.5)	10% (7.7-14%)	13% (9.3-19%)	63 (43-87)	5500 (4100-7300)
Kyrgyzstan	2010	Male	25 to 29	65% (55-72%)	35% (28-45%)	4.7 (3.3-6.3)	18% (14-22%)	23% (18-29%)	130 (100-170)	9800 (7800-12000)
Kyrgyzstan	2010	Male	30 to 34	73% (64-81%)	27% (19-36%)	5.9 (4.2-7.9)	22% (19-26%)	27% (22-32%)	210 (170-250)	13000 (11000-16000)
Kyrgyzstan	2010	Male	35 to 39	77% (69-83%)	23% (17-31%)	5.8 (4.1-7.8)	23% (19-27%)	28% (23-33%)	260 (210-300)	15000 (12000-17000)
Kyrgyzstan	2010	Male	40 to 44	77% (70-83%)	23% (17-30%)	6.1 (4.4-7.9)	24% (20-28%)	29% (25-34%)	320 (270-380)	16000 (14000-19000)
Kyrgyzstan	2010	Male	45 to 49	74% (64-82%)	26% (18-36%)	6.8 (4.8-9.3)	24% (21-28%)	29% (25-34%)	420 (350-490)	18000 (16000-22000)
Kyrgyzstan	2010	Male	50 to 54	69% (56-80%)	31% (20-44%)	6.1 (4-8.6)	23% (19-27%)	27% (22-32%)	450 (370-540)	17000 (14000-21000)
Kyrgyzstan	2010	Male	55 to 59	62% (43-79%)	38% (21-57%)	4.4 (2.6-6.9)	18% (13-23%)	21% (15-27%)	360 (260-460)	12000 (8600-15000)
Kyrgyzstan	2010	Male	60 to 64	57% (37-75%)	43% (25-63%)	3.4 (1.9-5.5)	13% (8.1-18%)	15% (8.8-21%)	220 (130-310)	6200 (3800-8700)
Kyrgyzstan	2010	Male	65 to 69	54% (34-72%)	46% (28-66%)	3 (1.7-4.8)	10% (5.7-15%)	12% (6.3-18%)	140 (76-210)	3300 (1800-4900)

Location	Year	Sex	Age	Prevalence of current drinkers	Prevalence of abstainers	Population average of standard drinks daily	Percent of total attributable deaths	Percent of total attributable DALYs	Attributable deaths	Attributable DALYs
Kyrgyzstan	2010	Male	70 to 74	49% (31-67%)	51% (33-69%)	2.5 (1.3-4)	7.1% (2.7-12%)	7.9% (3-14%)	170 (63-300)	3200 (1300-5500)
Kyrgyzstan	2010	Male	75 to 79	44% (28-63%)	56% (37-72%)	2 (1-3.4)	4.5% (0.82-9.2%)	4.9% (0.67-10%)	92 (13-190)	1400 (240-2800)
Kyrgyzstan	2010	Male	80 to 84	42% (25-59%)	58% (41-75%)	1.7 (0.92-2.9)	2.8% (-0.32-7.1%)	2.9% (-0.62-7.8%)	49 (-10-130)	550 (-64-1400)
Kyrgyzstan	2010	Male	85 to 89	40% (24-55%)	60% (45-76%)	1.6 (0.8-2.6)	1.6% (-1.1-5.3%)	1.5% (-1.6-5.7%)	17 (-18-64)	150 (-100-500)
Kyrgyzstan	2010	Male	90 to 94	39% (25-55%)	61% (45-75%)	1.4 (0.77-2.5)	0.92% (-1.5-4.6%)	0.75% (-2-5%)	1.7 (-4.6-11)	12 (-19-60)
Kyrgyzstan	2010	Male	95 plus	40% (23-61%)	60% (39-77%)	1.3 (0.59-2.6)	0.56% (-2-4.7%)	0.24% (-2.7-5%)	0.14 (-1.6-3)	1.3 (-4.6-11)
Kyrgyzstan	2016	Female	All Ages	55% (49-62%)	45% (38-51%)	0.98 (0.69-1.3)	2.1% (1-3.4%)	0.93% (-1.7-4.1%)	150 (-260-630)	16000 (7700-26000)
Kyrgyzstan	2016	Female	15 to 19	55% (42-69%)	45% (31-58%)	0.91 (0.51-1.4)	1.8% (0.99-2.8%)	4.1% (1.6-7.2%)	3.8 (1.4-6.7)	450 (250-710)
Kyrgyzstan	2016	Female	20 to 24	62% (49-74%)	38% (26-51%)	1.3 (0.72-2.1)	3.2% (2-4.7%)	5.9% (3-9.7%)	8.9 (4.5-15)	1000 (640-1600)
Kyrgyzstan	2016	Female	25 to 29	65% (51-77%)	35% (23-49%)	1.4 (0.8-2.3)	4% (2.8-5.5%)	7.4% (4.6-11%)	16 (9.7-24)	1600 (1100-2200)
Kyrgyzstan	2016	Female	30 to 34	66% (52-79%)	34% (21-48%)	1.5 (0.89-2.4)	4.9% (3.7-6.6%)	9.4% (6.7-13%)	25 (17-34)	1900 (1400-2500)
Kyrgyzstan	2016	Female	35 to 39	66% (53-80%)	34% (20-47%)	1.5 (0.88-2.4)	5.4% (4-7.1%)	10% (7.5-14%)	30 (21-41)	1900 (1400-2600)
Kyrgyzstan	2016	Female	40 to 44	66% (52-79%)	34% (21-48%)	1.6 (0.91-2.4)	5.8% (4.2-7.8%)	11% (7.9-15%)	41 (29-57)	2200 (1600-3000)
Kyrgyzstan	2016	Female	45 to 49	66% (51-79%)	34% (21-49%)	1.6 (0.92-2.5)	5.3% (3.5-7.5%)	9.6% (6.2-14%)	45 (29-65)	2100 (1400-3000)
Kyrgyzstan	2016	Female	50 to 54	64% (50-77%)	36% (23-50%)	1.6 (0.88-2.5)	5.1% (3-7.9%)	8.3% (4.6-13%)	66 (36-100)	2600 (1500-4000)
Kyrgyzstan	2016	Female	55 to 59	63% (50-75%)	37% (25-50%)	1.5 (0.88-2.4)	4.2% (1.8-7.3%)	6.4% (2.5-11%)	64 (25-110)	2200 (910-3800)
Kyrgyzstan	2016	Female	60 to 64	61% (48-75%)	39% (25-52%)	1.5 (0.83-2.4)	2.5% (-0.28-5.8%)	3.5% (-0.54-8.1%)	43 (-6.4-100)	1300 (-130-3000)
Kyrgyzstan	2016	Female	65 to 69	59% (47-74%)	41% (26-53%)	1.4 (0.84-2.2)	1.1% (-1.8-4.4%)	1.5% (-2.6-6.1%)	15 (-27-64)	380 (-580-1500)
Kyrgyzstan	2016	Female	70 to 74	57% (46-70%)	43% (30-54%)	1.4 (0.75-2.2)	-0.22% (-3.4-3.7%)	-0.36% (-4.7-4.7%)	-3.9 (-49-48)	-57 (-900-920)
Kyrgyzstan	2016	Female	75 to 79	54% (43-67%)	46% (33-57%)	1.3 (0.69-2.1)	-1.3% (-4.4-2.5%)	-1.8% (-5.8-3%)	-35 (-110-54)	-460 (-1500-820)
Kyrgyzstan	2016	Female	80 to 84	52% (40-66%)	48% (34-60%)	1.2 (0.7-2)	-2.3% (-5.5-1.3%)	-3% (-7-1.4%)	-64 (-150-29)	-630 (-1500-340)
Kyrgyzstan	2016	Female	85 to 89	52% (41-63%)	48% (37-59%)	1.2 (0.7-1.9)	-2.9% (-6.3-0.58%)	-3.8% (-8-0.46%)	-57 (-120-6.9)	-400 (-840-75)

Location	Year	Sex	Age	Prevalence of current drinkers	Prevalence of abstainers	Population average of standard drinks daily	Percent of total attributable deaths	Percent of total attributable DALYs	Attributable deaths	Attributable DALYs
Kyrgyzstan	2016	Female	90 to 94	53% (42-64%)	47% (36-58%)	1.2 (0.7-2)	-3.1% (-6.3-0.47%)	-4% (-7.9-0.31%)	-39 (-78-3.1)	-190 (-370-28)
Kyrgyzstan	2016	Female	95 plus	53% (40-68%)	47% (32-60%)	1.2 (0.6-2.2)	-3.2% (-6.8-0.72%)	-4% (-8.4-0.62%)	-11 (-22-1.6)	-33 (-70-7.3)
Kyrgyzstan	2016	Male	All Ages	39% (35-44%)	61% (56-65%)	2.7 (2-3.6)	11% (9.3-14%)	13% (9.6-16%)	2600 (1900-3400)	110000 (90000-140000)
Kyrgyzstan	2016	Male	15 to 19	19% (13-27%)	81% (73-87%)	0.56 (0.3-0.94)	3.3% (2.3-4.6%)	4.6% (2.7-7.3%)	6.9 (4-11)	920 (640-1300)
Kyrgyzstan	2016	Male	20 to 24	48% (38-58%)	52% (42-62%)	2.3 (1.4-3.4)	11% (7.9-14%)	15% (10-22%)	45 (29-64)	4300 (3100-5800)
Kyrgyzstan	2016	Male	25 to 29	65% (55-72%)	35% (28-45%)	4.5 (3-6.3)	18% (14-22%)	24% (18-31%)	130 (90-170)	9900 (7400-13000)
Kyrgyzstan	2016	Male	30 to 34	73% (63-80%)	27% (20-37%)	5.6 (3.8-7.8)	21% (17-26%)	28% (22-33%)	190 (150-240)	13000 (10000-16000)
Kyrgyzstan	2016	Male	35 to 39	76% (68-83%)	24% (17-32%)	5.5 (3.6-8)	22% (18-26%)	28% (22-34%)	210 (160-270)	13000 (9800-15000)
Kyrgyzstan	2016	Male	40 to 44	77% (69-83%)	23% (17-31%)	5.8 (3.8-8.3)	23% (19-27%)	29% (24-34%)	270 (210-330)	14000 (11000-17000)
Kyrgyzstan	2016	Male	45 to 49	74% (63-82%)	26% (18-37%)	6.5 (4.3-9.4)	23% (19-26%)	29% (24-33%)	310 (250-380)	14000 (11000-17000)
Kyrgyzstan	2016	Male	50 to 54	68% (55-80%)	32% (20-45%)	5.8 (3.6-8.8)	21% (17-25%)	26% (21-31%)	430 (330-530)	17000 (13000-21000)
Kyrgyzstan	2016	Male	55 to 59	62% (42-79%)	38% (21-58%)	4.2 (2.4-6.8)	17% (12-21%)	20% (14-26%)	380 (250-500)	13000 (8700-17000)
Kyrgyzstan	2016	Male	60 to 64	57% (36-75%)	43% (25-64%)	3.2 (1.6-5.2)	12% (7.1-17%)	14% (7.8-20%)	290 (170-430)	8400 (4900-12000)
Kyrgyzstan	2016	Male	65 to 69	53% (33-73%)	47% (27-67%)	2.8 (1.5-4.5)	9.4% (4.7-14%)	11% (5.3-17%)	150 (73-230)	3500 (1800-5400)
Kyrgyzstan	2016	Male	70 to 74	48% (30-67%)	52% (33-70%)	2.3 (1.2-3.9)	6.4% (2.3-11%)	7.2% (2.4-13%)	87 (29-160)	1700 (580-3000)
Kyrgyzstan	2016	Male	75 to 79	44% (27-63%)	56% (37-73%)	1.9 (0.93-3.3)	3.9% (0.43-8.5%)	4.3% (0.22-9.6%)	70 (3.3-160)	1100 (110-2300)
Kyrgyzstan	2016	Male	80 to 84	41% (24-58%)	59% (42-76%)	1.6 (0.83-2.8)	2.3% (-0.72-6.5%)	2.4% (-1.2-7.4%)	39 (-19-120)	450 (-150-1300)
Kyrgyzstan	2016	Male	85 to 89	39% (24-55%)	61% (45-76%)	1.4 (0.72-2.5)	1.2% (-1.4-5.2%)	1.1% (-1.9-5.7%)	9.9 (-18-53)	95 (-100-410)
Kyrgyzstan	2016	Male	90 to 94	38% (24-55%)	62% (45-76%)	1.3 (0.66-2.4)	0.62% (-1.7-4.1%)	0.39% (-2.3-4.6%)	1.9 (-12-23)	19 (-50-120)
Kyrgyzstan	2016	Male	95 plus	40% (23-60%)	60% (40-77%)	1.2 (0.52-2.4)	0.29% (-2.2-4.1%)	-0.098% (-3-4.4%)	-0.073 (-2.2-3.2)	0.83 (-6.1-12)
Mongolia	1990	Female	All Ages	43% (35-51%)	57% (49-65%)	0.72 (0.54-0.95)	1.6% (0.96-2.4%)	2.6% (1.1-4.6%)	220 (93-410)	8100 (5000-12000)
Mongolia	1990	Female	15 to 19	41% (26-57%)	59% (43-74%)	0.65 (0.27-1.2)	1.6% (0.56-3.2%)	2.4% (0.37-5.2%)	3.1 (0.48-7)	300 (100-600)

Location	Year	Sex	Age	Prevalence of current drinkers	Prevalence of abstainers	Population average of standard drinks daily	Percent of total attributable deaths	Percent of total attributable DALYs	Attributable deaths	Attributable DALYs
Mongolia	1990	Female	20 to 24	54% (37-70%)	46% (30-63%)	1.1 (0.45-1.9)	3% (1.2-5.4%)	3.8% (0.98-7.7%)	6.4 (1.6-14)	600 (240-1100)
Mongolia	1990	Female	25 to 29	59% (39-75%)	41% (25-61%)	1.3 (0.57-2.3)	3.8% (1.8-6.4%)	5.1% (1.8-9.5%)	9 (3.2-17)	730 (340-1200)
Mongolia	1990	Female	30 to 34	61% (41-77%)	39% (23-59%)	1.4 (0.68-2.4)	4.2% (2.4-6.8%)	6% (2.8-11%)	9.7 (4.5-17)	690 (380-1100)
Mongolia	1990	Female	35 to 39	62% (45-78%)	38% (22-55%)	1.5 (0.66-2.8)	4.4% (2.2-7.5%)	6.3% (2.8-11%)	10 (4.6-19)	630 (320-1100)
Mongolia	1990	Female	40 to 44	62% (46-79%)	38% (21-54%)	1.5 (0.64-2.7)	4.9% (2.5-8.3%)	7.2% (3.2-12%)	12 (5.3-21)	610 (300-1000)
Mongolia	1990	Female	45 to 49	61% (41-77%)	39% (23-59%)	1.6 (0.7-3)	4.9% (2.2-9.4%)	7% (2.9-13%)	15 (6.3-29)	670 (290-1300)
Mongolia	1990	Female	50 to 54	59% (41-75%)	41% (25-59%)	1.5 (0.66-3)	5.2% (2-10%)	7.1% (2.6-14%)	21 (7.7-42)	820 (310-1600)
Mongolia	1990	Female	55 to 59	56% (39-74%)	44% (26-61%)	1.5 (0.6-3.1)	5.1% (1.7-9.9%)	6.7% (2.2-13%)	26 (8.2-51)	850 (280-1700)
Mongolia	1990	Female	60 to 64	53% (35-71%)	47% (29-65%)	1.4 (0.56-3.1)	4.4% (1.2-9.5%)	5.6% (1.5-12%)	28 (8-60)	770 (220-1700)
Mongolia	1990	Female	65 to 69	50% (34-68%)	50% (32-66%)	1.3 (0.56-2.5)	3.8% (0.95-8.2%)	4.8% (1.2-10%)	28 (7.4-61)	640 (160-1400)
Mongolia	1990	Female	70 to 74	45% (30-62%)	55% (38-70%)	1.1 (0.42-2.2)	2.9% (0.34-7.4%)	3.6% (0.45-8.7%)	27 (3.5-67)	480 (58-1200)
Mongolia	1990	Female	75 to 79	40% (26-57%)	60% (43-74%)	0.92 (0.34-1.9)	2% (-0.49-5.9%)	2.3% (-0.58-7.1%)	15 (-3.5-45)	210 (-49-640)
Mongolia	1990	Female	80 to 84	37% (23-54%)	63% (46-77%)	0.92 (0.34-1.9)	1.3% (-1.1-5.2%)	1.5% (-1.4-6%)	7.6 (-6.6-30)	82 (-67-310)
Mongolia	1990	Female	85 to 89	36% (24-52%)	64% (48-76%)	0.98 (0.37-2.1)	0.91% (-1.5-5%)	0.98% (-1.9-5.8%)	3.7 (-6.9-22)	29 (-49-160)
Mongolia	1990	Female	90 to 94	38% (25-53%)	62% (47-75%)	1.1 (0.34-2.4)	0.73% (-2-5.8%)	0.75% (-2.4-6.5%)	1.1 (-3.4-9.3)	6.2 (-16-48)
Mongolia	1990	Female	95 plus	41% (25-60%)	59% (40-75%)	1.2 (0.29-3)	0.76% (-2.6-7%)	0.75% (-3.1-8.1%)	0.31 (-1.3-3.3)	1.2 (-4.1-11)
Mongolia	1990	Male	All Ages	51% (41-61%)	49% (39-59%)	1.1 (0.83-1.3)	3.2% (2.4-4.2%)	4.2% (2.9-5.9%)	430 (290-630)	19000 (14000-26000)
Mongolia	1990	Male	15 to 19	42% (26-60%)	58% (40-74%)	0.48 (0.24-0.83)	2.9% (1.6-4.6%)	3.1% (1.1-6%)	5.8 (2.1-11)	610 (330-990)
Mongolia	1990	Male	20 to 24	63% (44-79%)	37% (21-56%)	1.2 (0.74-1.9)	7.1% (4.7-10%)	7.8% (4.2-13%)	17 (9.1-28)	1600 (990-2400)
Mongolia	1990	Male	25 to 29	73% (56-85%)	27% (15-44%)	2.1 (1.3-3.1)	11% (7.4-15%)	12% (7.7-18%)	32 (19-46)	2500 (1600-3400)
Mongolia	1990	Male	30 to 34	79% (62-90%)	21% (10-38%)	2.6 (1.6-3.7)	12% (8.4-16%)	14% (9-20%)	32 (21-48)	2200 (1600-3100)
Mongolia	1990	Male	35 to 39	81% (68-91%)	19% (9.1-32%)	2.6 (1.6-4)	10% (7.2-15%)	12% (7.8-18%)	29 (18-45)	1800 (1200-2600)

Location	Year	Sex	Age	Prevalence of current drinkers	Prevalence of abstainers	Population average of standard drinks daily	Percent of total attributable deaths	Percent of total attributable DALYs	Attributable deaths	Attributable DALYs
Mongolia	1990	Male	40 to 44	81% (67-91%)	19% (8.8-33%)	2.6 (1.5-4)	9.9% (6.6-15%)	11% (7.1-18%)	29 (18-45)	1500 (1000-2300)
Mongolia	1990	Male	45 to 49	80% (63-91%)	20% (9.4-37%)	2.7 (1.5-4.2)	10% (6.7-14%)	12% (7.3-17%)	40 (24-61)	1800 (1200-2700)
Mongolia	1990	Male	50 to 54	79% (63-90%)	21% (10-37%)	2.7 (1.5-4.1)	9.6% (5.9-14%)	11% (6.5-16%)	53 (31-80)	2100 (1300-3100)
Mongolia	1990	Male	55 to 59	77% (61-89%)	23% (11-39%)	2.3 (1.2-3.6)	7.9% (4.9-12%)	8.9% (5.3-14%)	52 (31-82)	1800 (1100-2700)
Mongolia	1990	Male	60 to 64	74% (56-87%)	26% (13-44%)	1.9 (0.99-3.1)	6.3% (3.7-10%)	7.1% (4-11%)	51 (28-85)	1400 (820-2400)
Mongolia	1990	Male	65 to 69	71% (53-85%)	29% (15-47%)	1.6 (0.91-2.7)	4.8% (2.6-7.9%)	5.5% (2.8-9.1%)	42 (22-70)	970 (510-1600)
Mongolia	1990	Male	70 to 74	67% (49-82%)	33% (18-51%)	1.3 (0.73-2.3)	3.3% (1.4-5.9%)	3.7% (1.5-6.6%)	30 (12-56)	560 (230-1000)
Mongolia	1990	Male	75 to 79	63% (45-80%)	37% (20-55%)	1.1 (0.54-1.9)	2% (0.27-4.3%)	2.2% (0.19-4.8%)	14 (1.3-32)	210 (30-470)
Mongolia	1990	Male	80 to 84	60% (41-77%)	40% (23-59%)	0.96 (0.48-1.6)	1% (-0.53-2.9%)	1.1% (-0.76-3.2%)	4.4 (-3.3-14)	52 (-26-150)
Mongolia	1990	Male	85 to 89	58% (40-74%)	42% (26-60%)	0.9 (0.45-1.6)	0.4% (-1-2.1%)	0.29% (-1.4-2.2%)	0.71 (-3.5-5.4)	8.4 (-22-44)
Mongolia	1990	Male	90 to 94	57% (40-74%)	43% (26-60%)	0.85 (0.41-1.6)	-0.087% (-1.6-1.5%)	-0.32% (-2.2-1.5%)	-0.29 (-2-1.3)	-0.46 (-8.6-8)
Mongolia	1990	Male	95 plus	58% (36-77%)	42% (23-64%)	0.82 (0.33-1.6)	-0.32% (-2-1.3%)	-0.68% (-2.7-1.2%)	-0.16 (-0.61-0.28)	-0.3 (-1.8-1.2)
Mongolia	1995	Female	All Ages	44% (37-53%)	56% (47-63%)	0.71 (0.54-0.93)	2.1% (1.2-3.1%)	2.9% (1.2-5.4%)	250 (100-460)	9300 (5600-14000)
Mongolia	1995	Female	15 to 19	41% (27-59%)	59% (41-73%)	0.61 (0.25-1.1)	1.6% (0.56-3.1%)	2.4% (0.34-5.3%)	2.7 (0.38-6.2)	280 (96-530)
Mongolia	1995	Female	20 to 24	54% (37-70%)	46% (30-63%)	1 (0.43-1.8)	3% (1.3-5.3%)	3.9% (0.89-7.9%)	6.7 (1.5-14)	630 (260-1100)
Mongolia	1995	Female	25 to 29	59% (41-75%)	41% (25-59%)	1.2 (0.57-2.1)	3.8% (1.9-6.5%)	5.1% (1.7-9.6%)	9.5 (3.3-18)	790 (370-1300)
Mongolia	1995	Female	30 to 34	61% (42-77%)	39% (23-58%)	1.3 (0.67-2.3)	4.3% (2.4-7%)	6% (2.9-10%)	13 (6.1-23)	920 (520-1500)
Mongolia	1995	Female	35 to 39	62% (45-78%)	38% (22-55%)	1.4 (0.66-2.6)	4.4% (2.3-7.4%)	6.1% (2.7-11%)	14 (5.9-24)	820 (420-1400)
Mongolia	1995	Female	40 to 44	62% (45-78%)	38% (22-55%)	1.4 (0.61-2.6)	4.9% (2.4-8.3%)	6.9% (3.1-12%)	16 (7.3-29)	830 (400-1400)
Mongolia	1995	Female	45 to 49	61% (43-76%)	39% (24-57%)	1.5 (0.65-3)	5% (2-10%)	6.6% (2.4-13%)	18 (6.5-38)	780 (310-1600)
Mongolia	1995	Female	50 to 54	59% (42-75%)	41% (25-58%)	1.5 (0.62-2.9)	5.2% (1.8-11%)	6.6% (2.2-14%)	25 (8.2-52)	940 (330-1900)
Mongolia	1995	Female	55 to 59	56% (39-73%)	44% (27-61%)	1.4 (0.57-2.9)	5.1% (1.5-10%)	6.3% (1.8-13%)	29 (8.1-60)	930 (270-1900)

Location	Year	Sex	Age	Prevalence of current drinkers	Prevalence of abstainers	Population average of standard drinks daily	Percent of total attributable deaths	Percent of total attributable DALYs	Attributable deaths	Attributable DALYs
Mongolia	1995	Female	60 to 64	53% (35-71%)	47% (29-65%)	1.4 (0.53-2.9)	4.3% (1.1-9.5%)	5.2% (1.3-12%)	32 (8-71)	880 (220-2000)
Mongolia	1995	Female	65 to 69	51% (34-68%)	49% (32-66%)	1.2 (0.52-2.3)	3.6% (0.64-8.2%)	4.3% (0.83-9.8%)	32 (5.9-73)	710 (120-1700)
Mongolia	1995	Female	70 to 74	46% (30-62%)	54% (38-70%)	1 (0.38-2.1)	2.7% (0.091-7.1%)	3.3% (0.16-8.3%)	25 (1.1-63)	440 (15-1200)
Mongolia	1995	Female	75 to 79	40% (27-57%)	60% (43-73%)	0.87 (0.31-1.9)	1.7% (-0.59-5.7%)	2% (-0.7-6.6%)	18 (-6.3-58)	250 (-86-820)
Mongolia	1995	Female	80 to 84	37% (24-54%)	63% (46-76%)	0.86 (0.32-1.8)	1.1% (-1.3-5%)	1.3% (-1.6-5.7%)	7.3 (-9.1-32)	78 (-91-340)
Mongolia	1995	Female	85 to 89	37% (24-51%)	63% (49-76%)	0.93 (0.35-2)	0.71% (-1.7-4.9%)	0.75% (-2.1-5.5%)	2.9 (-8.2-22)	24 (-58-160)
Mongolia	1995	Female	90 to 94	38% (25-53%)	62% (47-75%)	1 (0.32-2.4)	0.54% (-2.1-5.8%)	0.53% (-2.6-6.6%)	1 (-4.8-12)	6 (-23-63)
Mongolia	1995	Female	95 plus	41% (25-60%)	59% (40-75%)	1.2 (0.28-2.9)	0.58% (-2.7-6.8%)	0.54% (-3.3-7.8%)	0.26 (-1.6-3.8)	1.1 (-5.2-13)
Mongolia	1995	Male	All Ages	52% (42-63%)	48% (37-58%)	1 (0.81-1.3)	4.3% (3.3-5.6%)	4.9% (3.4-6.9%)	520 (350-720)	24000 (18000-31000)
Mongolia	1995	Male	15 to 19	42% (26-61%)	58% (39-74%)	0.46 (0.23-0.81)	2.9% (1.5-4.5%)	3.2% (1.1-5.9%)	5.9 (2.2-11)	620 (330-990)
Mongolia	1995	Male	20 to 24	63% (44-79%)	37% (21-56%)	1.2 (0.68-1.8)	7.1% (4.5-11%)	7.6% (4-13%)	23 (12-38)	2000 (1200-3000)
Mongolia	1995	Male	25 to 29	74% (57-85%)	26% (15-43%)	2 (1.2-2.9)	11% (7.4-15%)	12% (7.5-18%)	42 (26-61)	3200 (2200-4300)
Mongolia	1995	Male	30 to 34	79% (63-90%)	21% (9.6-37%)	2.4 (1.6-3.5)	12% (8.5-16%)	13% (9-19%)	53 (35-75)	3600 (2500-4900)
Mongolia	1995	Male	35 to 39	81% (68-91%)	19% (9-32%)	2.4 (1.4-3.6)	10% (7.4-15%)	12% (7.6-17%)	43 (27-64)	2600 (1800-3700)
Mongolia	1995	Male	40 to 44	82% (69-91%)	18% (8.6-31%)	2.4 (1.4-3.7)	9.8% (6.6-15%)	11% (6.9-17%)	43 (26-68)	2300 (1500-3400)
Mongolia	1995	Male	45 to 49	81% (64-91%)	19% (9.1-36%)	2.6 (1.4-4)	10% (6.4-15%)	11% (6.7-17%)	48 (28-72)	2200 (1300-3100)
Mongolia	1995	Male	50 to 54	79% (64-90%)	21% (10-36%)	2.5 (1.4-3.9)	9.4% (5.7-14%)	10% (6.1-16%)	57 (33-88)	2200 (1300-3300)
Mongolia	1995	Male	55 to 59	77% (61-89%)	23% (11-39%)	2.1 (1.1-3.4)	7.5% (4.7-12%)	8.3% (4.9-13%)	55 (32-87)	1900 (1100-2800)
Mongolia	1995	Male	60 to 64	74% (57-87%)	26% (13-43%)	1.8 (0.91-3)	6% (3.2-9.8%)	6.5% (3.4-11%)	56 (28-95)	1600 (830-2600)
Mongolia	1995	Male	65 to 69	72% (54-86%)	28% (14-46%)	1.5 (0.84-2.5)	4.5% (2.3-7.5%)	5% (2.5-8.4%)	44 (22-76)	1000 (510-1700)
Mongolia	1995	Male	70 to 74	68% (50-82%)	32% (18-50%)	1.3 (0.69-2.2)	3% (1.2-5.6%)	3.3% (1.2-6.2%)	28 (9.6-52)	520 (190-960)
Mongolia	1995	Male	75 to 79	64% (46-79%)	36% (21-54%)	1 (0.52-1.8)	1.8% (0.13-4%)	1.9% (0.033-4.4%)	15 (0.26-35)	230 (16-500)

Location	Year	Sex	Age	Prevalence of current drinkers	Prevalence of abstainers	Population average of standard drinks daily	Percent of total attributable deaths	Percent of total attributable DALYs	Attributable deaths	Attributable DALYs
Mongolia	1995	Male	80 to 84	61% (42-77%)	39% (23-58%)	0.92 (0.46-1.5)	0.91% (-0.69-2.7%)	0.89% (-0.98-3%)	4.1 (-4.5-14)	49 (-38-150)
Mongolia	1995	Male	85 to 89	58% (40-75%)	42% (25-60%)	0.85 (0.43-1.5)	0.3% (-1.1-1.9%)	0.18% (-1.5-2%)	0.47 (-4.1-5.2)	6.8 (-26-42)
Mongolia	1995	Male	90 to 94	58% (40-74%)	42% (26-60%)	0.8 (0.38-1.4)	-0.14% (-1.7-1.4%)	-0.38% (-2.2-1.4%)	-0.39 (-2.3-1.4)	-0.87 (-10-8.3)
Mongolia	1995	Male	95 plus	59% (36-78%)	41% (22-64%)	0.78 (0.32-1.5)	-0.38% (-1.9-1.2%)	-0.73% (-2.6-1.1%)	-0.19 (-0.66-0.28)	-0.38 (-1.9-1.2)
Mongolia	2000	Female	All Ages	47% (39-56%)	53% (44-61%)	0.82 (0.62-1.1)	3.1% (1.9-4.5%)	4.2% (1.9-7.3%)	340 (160-600)	13000 (8000-18000)
Mongolia	2000	Female	15 to 19	43% (28-60%)	57% (40-72%)	0.67 (0.27-1.3)	1.8% (0.64-3.4%)	2.9% (0.43-6.3%)	3.1 (0.45-7)	310 (110-600)
Mongolia	2000	Female	20 to 24	56% (39-71%)	44% (29-61%)	1.1 (0.46-1.9)	3.5% (1.5-6.4%)	4.8% (1.3-9.6%)	8 (2.1-16)	730 (310-1300)
Mongolia	2000	Female	25 to 29	60% (42-76%)	40% (24-58%)	1.3 (0.58-2.3)	4.7% (2.4-7.5%)	6.3% (2.4-11%)	12 (4.5-21)	1000 (490-1600)
Mongolia	2000	Female	30 to 34	63% (42-78%)	37% (22-58%)	1.4 (0.75-2.4)	5.4% (3.3-8.2%)	7.5% (3.9-12%)	16 (8.5-27)	1200 (700-1800)
Mongolia	2000	Female	35 to 39	63% (47-79%)	37% (21-53%)	1.5 (0.71-2.7)	5.4% (3-8.8%)	7.5% (3.6-12%)	23 (11-38)	1400 (720-2200)
Mongolia	2000	Female	40 to 44	63% (47-79%)	37% (21-53%)	1.6 (0.67-2.7)	5.9% (3-9.7%)	8.1% (3.8-14%)	27 (12-46)	1400 (690-2300)
Mongolia	2000	Female	45 to 49	62% (44-78%)	38% (22-56%)	1.6 (0.7-3.2)	6% (2.5-11%)	7.8% (2.9-15%)	28 (10-54)	1200 (480-2300)
Mongolia	2000	Female	50 to 54	60% (42-75%)	40% (25-58%)	1.6 (0.68-3.2)	6.4% (2.3-13%)	8% (2.8-16%)	30 (11-60)	1100 (420-2200)
Mongolia	2000	Female	55 to 59	57% (40-74%)	43% (26-60%)	1.6 (0.61-3.2)	6.3% (1.9-12%)	7.8% (2.4-15%)	38 (11-76)	1200 (380-2400)
Mongolia	2000	Female	60 to 64	55% (37-72%)	45% (28-63%)	1.5 (0.57-3.1)	5.5% (1.6-12%)	6.6% (1.9-14%)	40 (11-85)	1100 (300-2300)
Mongolia	2000	Female	65 to 69	52% (36-70%)	48% (30-64%)	1.3 (0.58-2.6)	4.5% (1.1-9.9%)	5.4% (1.4-12%)	42 (11-92)	940 (240-2100)
Mongolia	2000	Female	70 to 74	47% (32-63%)	53% (37-68%)	1.1 (0.42-2.3)	3.5% (0.28-8.6%)	4.1% (0.4-10%)	34 (3.3-84)	600 (50-1500)
Mongolia	2000	Female	75 to 79	42% (28-59%)	58% (41-72%)	0.97 (0.34-2.1)	2.4% (-0.42-7.2%)	2.8% (-0.49-8.3%)	21 (-3.6-63)	290 (-52-880)
Mongolia	2000	Female	80 to 84	39% (25-55%)	61% (45-75%)	0.97 (0.37-2)	1.8% (-1.1-6.5%)	2% (-1.3-7.4%)	12 (-7.7-45)	130 (-78-460)
Mongolia	2000	Female	85 to 89	38% (26-54%)	62% (46-74%)	1 (0.39-2.2)	1.3% (-1.5-6.2%)	1.5% (-1.8-7.1%)	6 (-7.4-29)	46 (-53-210)
Mongolia	2000	Female	90 to 94	40% (26-55%)	60% (45-74%)	1.1 (0.37-2.7)	1.1% (-2-7.2%)	1.2% (-2.5-8.3%)	2.1 (-4.4-15)	11 (-21-75)
Mongolia	2000	Female	95 plus	43% (26-61%)	57% (39-74%)	1.3 (0.31-3.3)	1.1% (-2.7-7.9%)	1.1% (-3.3-8.9%)	0.66 (-2-5.4)	2.5 (-6.4-19)

Location	Year	Sex	Age	Prevalence of current drinkers	Prevalence of abstainers	Population average of standard drinks daily	Percent of total attributable deaths	Percent of total attributable DALYs	Attributable deaths	Attributable DALYs
Mongolia	2000	Male	All Ages	56% (46-66%)	44% (34-54%)	1.2 (0.98-1.5)	6% (4.7-7.5%)	6.5% (4.6-8.7%)	660 (460-890)	31000 (24000-39000)
Mongolia	2000	Male	15 to 19	44% (28-63%)	56% (37-72%)	0.51 (0.26-0.91)	3.2% (1.9-5%)	3.7% (1.5-6.8%)	6.7 (2.7-12)	730 (420-1100)
Mongolia	2000	Male	20 to 24	65% (47-80%)	35% (20-53%)	1.3 (0.75-2)	8.1% (5.2-12%)	8.7% (4.7-14%)	25 (13-40)	2300 (1400-3300)
Mongolia	2000	Male	25 to 29	75% (59-86%)	25% (14-41%)	2.2 (1.3-3.2)	13% (8.5-17%)	14% (8.4-20%)	51 (31-73)	3900 (2700-5300)
Mongolia	2000	Male	30 to 34	80% (64-91%)	20% (9.1-36%)	2.7 (1.7-3.8)	14% (10-18%)	15% (11-21%)	63 (43-87)	4300 (3200-5700)
Mongolia	2000	Male	35 to 39	82% (69-92%)	18% (8.4-31%)	2.6 (1.6-3.8)	12% (8.9-16%)	14% (9.4-19%)	71 (48-100)	4300 (3100-5800)
Mongolia	2000	Male	40 to 44	82% (69-92%)	18% (8.3-31%)	2.6 (1.5-3.9)	12% (8-16%)	13% (8.3-18%)	69 (44-100)	3600 (2400-5000)
Mongolia	2000	Male	45 to 49	82% (65-91%)	18% (9-35%)	2.8 (1.6-4.4)	12% (7.6-16%)	13% (8-19%)	73 (46-110)	3300 (2100-4700)
Mongolia	2000	Male	50 to 54	80% (64-90%)	20% (9.8-36%)	2.8 (1.5-4.3)	11% (6.5-15%)	12% (6.8-17%)	67 (39-100)	2600 (1600-3800)
Mongolia	2000	Male	55 to 59	79% (63-90%)	21% (9.9-37%)	2.4 (1.2-3.8)	8.5% (5.1-13%)	9.3% (5.4-14%)	65 (37-100)	2200 (1300-3300)
Mongolia	2000	Male	60 to 64	76% (57-88%)	24% (12-43%)	2 (1-3.3)	6.7% (3.6-11%)	7.3% (3.7-13%)	64 (32-110)	1800 (920-3000)
Mongolia	2000	Male	65 to 69	73% (55-87%)	27% (13-45%)	1.7 (0.96-2.8)	5.1% (2.5-8.7%)	5.6% (2.6-9.8%)	53 (25-91)	1200 (600-2100)
Mongolia	2000	Male	70 to 74	70% (52-83%)	30% (17-48%)	1.4 (0.75-2.4)	3.5% (1.2-6.7%)	3.8% (1.2-7.4%)	33 (10-63)	610 (200-1200)
Mongolia	2000	Male	75 to 79	66% (47-81%)	34% (19-53%)	1.2 (0.58-2)	2.2% (0.2-5%)	2.4% (0.11-5.4%)	17 (0.82-38)	250 (22-550)
Mongolia	2000	Male	80 to 84	63% (44-78%)	37% (22-56%)	1 (0.51-1.7)	1.3% (-0.63-3.4%)	1.3% (-0.88-3.6%)	5.7 (-4-16)	67 (-33-180)
Mongolia	2000	Male	85 to 89	60% (42-76%)	40% (24-58%)	0.95 (0.48-1.6)	0.59% (-1-2.5%)	0.49% (-1.5-2.7%)	1.4 (-4-7.4)	14 (-25-58)
Mongolia	2000	Male	90 to 94	59% (41-76%)	41% (24-59%)	0.89 (0.42-1.6)	0.032% (-1.6-1.9%)	-0.21% (-2.2-1.9%)	-0.22 (-2.4-2.1)	0.2 (-11-12)
Mongolia	2000	Male	95 plus	60% (38-78%)	40% (22-62%)	0.85 (0.35-1.6)	-0.27% (-2-1.6%)	-0.64% (-2.6-1.5%)	-0.19 (-0.76-0.42)	-0.3 (-2.2-1.8)
Mongolia	2005	Female	All Ages	51% (44-60%)	49% (40-56%)	1.1 (0.83-1.4)	5% (3.4-6.8%)	7.2% (4-11%)	570 (310-890)	19000 (13000-27000)
Mongolia	2005	Female	15 to 19	46% (31-62%)	54% (38-69%)	0.83 (0.35-1.5)	2.2% (0.86-4%)	4.2% (0.94-8.8%)	4 (0.87-8.3)	400 (160-750)
Mongolia	2005	Female	20 to 24	58% (42-73%)	42% (27-58%)	1.3 (0.56-2.3)	4.5% (2.2-7.6%)	7.3% (2.6-14%)	9.3 (3.3-17)	860 (430-1500)
Mongolia	2005	Female	25 to 29	62% (44-77%)	38% (23-56%)	1.6 (0.69-2.8)	6.4% (3.6-10%)	9.8% (4.4-16%)	16 (7.1-27)	1300 (700-2000)

Location	Year	Sex	Age	Prevalence of current drinkers	Prevalence of abstainers	Population average of standard drinks daily	Percent of total attributable deaths	Percent of total attributable DALYs	Attributable deaths	Attributable DALYs
Mongolia	2005	Female	30 to 34	64% (45-79%)	36% (21-55%)	1.7 (0.89-2.9)	7.4% (4.8-11%)	12% (6.8-18%)	22 (13-34)	1600 (1000-2300)
Mongolia	2005	Female	35 to 39	65% (49-80%)	35% (20-51%)	1.8 (0.89-3.2)	7.6% (4.7-11%)	12% (6.7-18%)	30 (16-46)	1800 (1100-2700)
Mongolia	2005	Female	40 to 44	65% (49-80%)	35% (20-51%)	1.9 (0.86-3.2)	8.3% (4.8-13%)	12% (6.6-19%)	45 (25-72)	2300 (1300-3600)
Mongolia	2005	Female	45 to 49	64% (46-79%)	36% (21-54%)	1.9 (0.88-3.7)	8.4% (3.8-15%)	12% (4.9-20%)	50 (22-90)	2200 (1000-3900)
Mongolia	2005	Female	50 to 54	62% (45-77%)	38% (23-55%)	1.9 (0.86-3.8)	9.2% (3.9-16%)	12% (5-21%)	55 (22-94)	2000 (850-3500)
Mongolia	2005	Female	55 to 59	59% (43-75%)	41% (25-57%)	1.9 (0.77-3.8)	9.2% (3.5-16%)	12% (4.4-20%)	55 (21-95)	1800 (670-3100)
Mongolia	2005	Female	60 to 64	57% (41-73%)	43% (27-59%)	1.8 (0.71-3.9)	8.4% (3.3-15%)	10% (4.1-19%)	61 (24-110)	1700 (620-2900)
Mongolia	2005	Female	65 to 69	55% (39-71%)	45% (29-61%)	1.7 (0.73-3.3)	7% (2.6-13%)	8.6% (3.2-16%)	61 (23-110)	1400 (490-2600)
Mongolia	2005	Female	70 to 74	51% (35-67%)	49% (33-65%)	1.4 (0.53-3)	5.8% (1.5-12%)	7.1% (1.9-15%)	60 (16-120)	1100 (290-2200)
Mongolia	2005	Female	75 to 79	45% (31-63%)	55% (37-69%)	1.3 (0.44-2.7)	4.5% (0.67-11%)	5.4% (0.8-12%)	42 (6.6-97)	590 (86-1400)
Mongolia	2005	Female	80 to 84	43% (27-60%)	57% (40-73%)	1.3 (0.47-2.5)	4.1% (0.21-10%)	4.8% (0.26-12%)	29 (1.6-69)	300 (15-720)
Mongolia	2005	Female	85 to 89	42% (28-57%)	58% (43-72%)	1.3 (0.5-2.8)	3.6% (-0.48-10%)	4.2% (-0.57-12%)	18 (-2.5-51)	130 (-17-370)
Mongolia	2005	Female	90 to 94	43% (30-58%)	57% (42-70%)	1.5 (0.46-3.4)	3% (-1.4-10%)	3.4% (-1.6-12%)	6.5 (-3.1-23)	34 (-15-120)
Mongolia	2005	Female	95 plus	46% (30-63%)	54% (37-70%)	1.6 (0.4-4.1)	2.7% (-2.2-11%)	3% (-2.7-12%)	1.7 (-1.5-7.1)	6 (-4.9-24)
Mongolia	2005	Male	All Ages	62% (53-71%)	38% (29-47%)	1.7 (1.3-2)	10% (7.8-12%)	11% (8-14%)	1300 (890-1600)	57000 (43000-70000)
Mongolia	2005	Male	15 to 19	48% (31-65%)	52% (35-69%)	0.67 (0.33-1.1)	4.6% (2.8-6.9%)	5.8% (2.7-9.6%)	12 (5.4-20)	1200 (700-1800)
Mongolia	2005	Male	20 to 24	68% (50-83%)	32% (17-50%)	1.6 (0.97-2.5)	11% (7.3-15%)	12% (7.3-18%)	38 (22-57)	3300 (2200-4600)
Mongolia	2005	Male	25 to 29	77% (62-88%)	23% (12-38%)	2.7 (1.6-3.9)	16% (11-21%)	19% (12-25%)	76 (50-110)	5600 (4000-7400)
Mongolia	2005	Male	30 to 34	82% (68-91%)	18% (8.5-32%)	3.3 (2.1-4.6)	19% (14-24%)	21% (15-28%)	110 (77-150)	7400 (5300-9600)
Mongolia	2005	Male	35 to 39	84% (72-92%)	16% (7.9-28%)	3.2 (2-4.6)	18% (13-24%)	21% (14-27%)	140 (88-190)	7700 (5300-10000)
Mongolia	2005	Male	40 to 44	84% (72-92%)	16% (7.5-28%)	3.2 (1.9-4.7)	17% (12-22%)	19% (13-25%)	170 (110-230)	8300 (5600-11000)
Mongolia	2005	Male	45 to 49	83% (69-92%)	17% (7.8-31%)	3.5 (2-5.2)	17% (11-22%)	18% (12-25%)	170 (110-240)	7600 (5000-10000)

Location	Year	Sex	Age	Prevalence of current drinkers	Prevalence of abstainers	Population average of standard drinks daily	Percent of total attributable deaths	Percent of total attributable DALYs	Attributable deaths	Attributable DALYs
Mongolia	2005	Male	50 to 54	82% (68-91%)	18% (8.9-32%)	3.4 (1.9-5.2)	15% (9.2-21%)	16% (9.8-23%)	150 (87-210)	5500 (3400-7700)
Mongolia	2005	Male	55 to 59	81% (66-91%)	19% (9.2-34%)	2.9 (1.6-4.5)	12% (7.2-18%)	13% (7.6-20%)	110 (61-160)	3500 (2100-5200)
Mongolia	2005	Male	60 to 64	78% (62-90%)	22% (10-38%)	2.4 (1.3-4.1)	9.4% (4.9-15%)	10% (5.2-17%)	99 (51-160)	2800 (1500-4500)
Mongolia	2005	Male	65 to 69	75% (59-88%)	25% (12-41%)	2.1 (1.2-3.4)	7.2% (3.6-12%)	7.9% (3.8-13%)	82 (40-140)	1900 (940-3100)
Mongolia	2005	Male	70 to 74	72% (55-85%)	28% (15-45%)	1.8 (0.98-2.9)	5.4% (2.1-10%)	5.9% (2.3-11%)	57 (23-110)	1100 (430-2000)
Mongolia	2005	Male	75 to 79	68% (51-83%)	32% (17-49%)	1.5 (0.76-2.5)	4% (1.2-8%)	4.4% (1.2-8.8%)	32 (8.7-65)	470 (140-930)
Mongolia	2005	Male	80 to 84	65% (47-80%)	35% (20-53%)	1.3 (0.66-2.1)	3% (0.48-6.2%)	3.2% (0.33-6.8%)	14 (1.5-30)	160 (27-320)
Mongolia	2005	Male	85 to 89	63% (45-78%)	37% (22-55%)	1.2 (0.61-2)	2% (-0.31-5.1%)	2% (-0.58-5.6%)	5.6 (-1.6-15)	46 (-7.1-120)
Mongolia	2005	Male	90 to 94	62% (45-78%)	38% (22-55%)	1.1 (0.54-2)	1% (-1-3.7%)	0.89% (-1.5-3.9%)	0.99 (-1.6-4.4)	6.5 (-6.8-24)
Mongolia	2005	Male	95 plus	63% (41-80%)	37% (20-59%)	1.1 (0.44-2)	0.44% (-1.8-3.3%)	0.14% (-2.4-3.4%)	0.042 (-0.75-1.1)	0.53 (-2.1-4)
Mongolia	2010	Female	All Ages	53% (45-60%)	47% (40-55%)	1.3 (0.97-1.7)	6.2% (4.3-8.3%)	9.2% (5.6-13%)	730 (440-1100)	25000 (17000-33000)
Mongolia	2010	Female	15 to 19	47% (32-63%)	53% (37-68%)	0.97 (0.38-1.8)	2.5% (1-4.6%)	5.1% (1.3-10%)	4 (1.1-8.2)	390 (150-750)
Mongolia	2010	Female	20 to 24	59% (44-74%)	41% (26-56%)	1.5 (0.64-2.8)	5% (2.6-8.3%)	8.7% (3.4-15%)	12 (4.8-22)	1100 (590-1900)
Mongolia	2010	Female	25 to 29	63% (45-77%)	37% (23-55%)	1.8 (0.83-3.3)	7% (4.2-10%)	12% (5.9-19%)	17 (8.6-28)	1400 (840-2200)
Mongolia	2010	Female	30 to 34	65% (47-79%)	35% (21-53%)	2 (1-3.4)	8.2% (5.6-12%)	14% (8.7-20%)	25 (15-38)	1800 (1200-2600)
Mongolia	2010	Female	35 to 39	66% (50-80%)	34% (20-50%)	2 (0.97-3.6)	8.8% (5.6-13%)	14% (8.2-20%)	36 (21-55)	2200 (1300-3200)
Mongolia	2010	Female	40 to 44	65% (50-80%)	35% (20-50%)	2.1 (0.96-3.8)	9.8% (5.9-14%)	15% (8.6-22%)	52 (30-81)	2700 (1500-4000)
Mongolia	2010	Female	45 to 49	64% (46-79%)	36% (21-54%)	2.2 (1-4.2)	9.7% (5-15%)	14% (6.9-22%)	69 (33-120)	3100 (1600-5000)
Mongolia	2010	Female	50 to 54	62% (46-77%)	38% (23-54%)	2.2 (1-4.2)	11% (4.9-17%)	14% (6.5-23%)	80 (36-130)	3000 (1400-5000)
Mongolia	2010	Female	55 to 59	60% (43-75%)	40% (25-57%)	2.2 (0.85-4.3)	10% (4.4-17%)	14% (6-22%)	72 (31-120)	2300 (1000-3800)
Mongolia	2010	Female	60 to 64	58% (41-75%)	42% (25-59%)	2.1 (0.82-4.5)	9.8% (4.4-16%)	13% (5.8-21%)	68 (31-110)	1800 (830-3100)
Mongolia	2010	Female	65 to 69	56% (40-72%)	44% (28-60%)	1.9 (0.83-3.8)	8.6% (3.7-15%)	10% (4.6-19%)	84 (36-150)	1900 (800-3400)

Location	Year	Sex	Age	Prevalence of current drinkers	Prevalence of abstainers	Population average of standard drinks daily	Percent of total attributable deaths	Percent of total attributable DALYs	Attributable deaths	Attributable DALYs
Mongolia	2010	Female	70 to 74	51% (36-67%)	49% (33-64%)	1.6 (0.61-3.4)	7.2% (2.7-14%)	9% (3.3-17%)	71 (27-130)	1300 (460-2500)
Mongolia	2010	Female	75 to 79	46% (32-63%)	54% (37-68%)	1.4 (0.52-3.1)	5.8% (1.5-12%)	7.1% (1.9-15%)	57 (15-120)	800 (200-1600)
Mongolia	2010	Female	80 to 84	43% (29-61%)	57% (39-71%)	1.5 (0.54-3)	5.5% (1.2-12%)	6.7% (1.4-14%)	45 (9.6-95)	470 (97-990)
Mongolia	2010	Female	85 to 89	43% (29-57%)	57% (43-71%)	1.5 (0.56-3.3)	5% (0.44-13%)	5.9% (0.41-15%)	25 (1.7-63)	180 (16-460)
Mongolia	2010	Female	90 to 94	44% (30-59%)	56% (41-70%)	1.7 (0.53-3.9)	4.2% (-0.67-12%)	4.9% (-0.95-14%)	8.7 (-1.6-24)	44 (-7-120)
Mongolia	2010	Female	95 plus	47% (30-64%)	53% (36-70%)	1.9 (0.47-4.6)	3.7% (-1.9-12%)	4.2% (-2.3-14%)	2.4 (-1.2-7.9)	8.3 (-4-26)
Mongolia	2010	Male	All Ages	64% (56-73%)	36% (27-44%)	2 (1.6-2.5)	12% (9.2-15%)	14% (9.8-17%)	1600 (1100-2100)	71000 (53000-90000)
Mongolia	2010	Male	15 to 19	49% (32-67%)	51% (33-68%)	0.75 (0.37-1.3)	5.3% (3.2-7.9%)	6.9% (3.4-11%)	12 (5.6-20)	1200 (700-1800)
Mongolia	2010	Male	20 to 24	69% (51-83%)	31% (17-49%)	1.8 (1.1-2.9)	12% (8.3-16%)	14% (8.4-20%)	47 (27-70)	4100 (2700-5700)
Mongolia	2010	Male	25 to 29	78% (63-88%)	22% (12-37%)	3.1 (1.9-4.6)	18% (13-24%)	21% (14-29%)	84 (54-120)	6400 (4400-8400)
Mongolia	2010	Male	30 to 34	83% (68-92%)	17% (8.3-32%)	3.7 (2.4-5.2)	21% (15-27%)	25% (17-33%)	130 (84-180)	8500 (5900-11000)
Mongolia	2010	Male	35 to 39	84% (72-92%)	16% (7.7-28%)	3.6 (2.3-5.3)	22% (14-29%)	25% (16-34%)	170 (100-230)	9700 (6400-13000)
Mongolia	2010	Male	40 to 44	85% (73-92%)	15% (7.5-27%)	3.7 (2.2-5.4)	20% (14-26%)	23% (15-30%)	190 (120-260)	9600 (6500-13000)
Mongolia	2010	Male	45 to 49	84% (69-92%)	16% (8-31%)	3.9 (2.3-6)	20% (14-26%)	22% (15-29%)	240 (160-340)	11000 (7300-15000)
Mongolia	2010	Male	50 to 54	83% (69-92%)	17% (8.4-31%)	3.9 (2.1-5.8)	17% (11-23%)	19% (12-26%)	220 (140-310)	8400 (5400-12000)
Mongolia	2010	Male	55 to 59	81% (67-91%)	19% (9-33%)	3.3 (1.8-5.1)	14% (9.2-20%)	16% (10-22%)	150 (93-220)	5000 (3100-7200)
Mongolia	2010	Male	60 to 64	79% (62-90%)	21% (9.9-38%)	2.8 (1.5-4.6)	11% (6.3-17%)	13% (6.8-19%)	110 (58-170)	3000 (1700-4600)
Mongolia	2010	Male	65 to 69	76% (60-89%)	24% (11-40%)	2.5 (1.4-3.9)	8.8% (4.5-14%)	9.8% (4.9-16%)	100 (52-170)	2400 (1200-3900)
Mongolia	2010	Male	70 to 74	73% (57-85%)	27% (15-43%)	2 (1.1-3.4)	6.8% (3.1-12%)	7.7% (3.4-13%)	73 (33-130)	1300 (610-2300)
Mongolia	2010	Male	75 to 79	69% (52-83%)	31% (17-48%)	1.7 (0.85-2.9)	5.4% (2.2-9.9%)	6.1% (2.3-11%)	43 (16-79)	620 (250-1100)
Mongolia	2010	Male	80 to 84	67% (48-81%)	33% (19-52%)	1.5 (0.76-2.5)	4.3% (1.5-8.3%)	4.8% (1.6-9.3%)	22 (7.4-43)	240 (88-460)
Mongolia	2010	Male	85 to 89	64% (46-79%)	36% (21-54%)	1.4 (0.7-2.3)	3% (0.56-6.5%)	3.3% (0.37-7.4%)	8.4 (0.94-18)	66 (12-140)

Location	Year	Sex	Age	Prevalence of current drinkers	Prevalence of abstainers	Population average of standard drinks daily	Percent of total attributable deaths	Percent of total attributable DALYs	Attributable deaths	Attributable DALYs
Mongolia	2010	Male	90 to 94	63% (46-78%)	37% (22-54%)	1.3 (0.61-2.3)	1.8% (-0.53-5.1%)	1.8% (-1.1-5.6%)	1.5 (-0.9-4.9)	9.1 (-2.7-26)
Mongolia	2010	Male	95 plus	63% (42-81%)	37% (19-58%)	1.2 (0.5-2.2)	1.1% (-1.4-4.6%)	0.82% (-2.1-4.9%)	0.22 (-0.56-1.3)	1.1 (-1.5-4.8)
Mongolia	2016	Female	All Ages	52% (45-60%)	48% (40-55%)	1.4 (0.93-1.9)	6.5% (4.4-8.8%)	9.7% (5.7-14%)	810 (460-1200)	26000 (17000-36000)
Mongolia	2016	Female	15 to 19	46% (31-62%)	54% (38-69%)	1 (0.38-2)	2.5% (1.1-4.9%)	5.3% (1.6-11%)	3.2 (0.95-6.7)	320 (130-590)
Mongolia	2016	Female	20 to 24	59% (42-73%)	41% (27-58%)	1.6 (0.65-3.1)	4.9% (2.6-8%)	8.9% (3.6-16%)	8.4 (3.2-15)	820 (420-1300)
Mongolia	2016	Female	25 to 29	63% (45-77%)	37% (23-55%)	1.9 (0.82-3.5)	6.6% (4-10%)	12% (5.8-19%)	17 (8-29)	1500 (850-2300)
Mongolia	2016	Female	30 to 34	65% (46-79%)	35% (21-54%)	2.1 (1-3.6)	7.8% (5.2-11%)	14% (8.4-21%)	24 (14-38)	1800 (1200-2700)
Mongolia	2016	Female	35 to 39	65% (50-79%)	35% (21-50%)	2.2 (0.99-4)	8.5% (5.4-12%)	14% (8.4-21%)	34 (18-54)	2100 (1300-3200)
Mongolia	2016	Female	40 to 44	65% (49-80%)	35% (20-51%)	2.2 (0.93-4.1)	9.4% (5.7-14%)	15% (8.7-22%)	50 (27-79)	2600 (1500-4000)
Mongolia	2016	Female	45 to 49	64% (46-79%)	36% (21-54%)	2.3 (1-4.5)	9.3% (4.9-15%)	14% (7.1-22%)	63 (31-100)	2800 (1500-4500)
Mongolia	2016	Female	50 to 54	62% (45-76%)	38% (24-55%)	2.3 (1-4.5)	10% (5-16%)	14% (6.9-23%)	92 (43-150)	3500 (1600-5600)
Mongolia	2016	Female	55 to 59	60% (43-76%)	40% (24-57%)	2.3 (0.9-4.4)	10% (4.7-16%)	14% (6.5-22%)	97 (43-160)	3200 (1400-5100)
Mongolia	2016	Female	60 to 64	58% (40-74%)	42% (26-60%)	2.2 (0.84-4.5)	9.8% (4.7-16%)	13% (6.5-21%)	88 (41-140)	2400 (1100-3900)
Mongolia	2016	Female	65 to 69	55% (39-72%)	45% (28-61%)	2 (0.85-4)	8.7% (4.2-14%)	12% (5.7-19%)	74 (36-120)	1700 (790-2800)
Mongolia	2016	Female	70 to 74	51% (36-67%)	49% (33-64%)	1.7 (0.63-3.7)	7.6% (3-14%)	9.8% (4.2-18%)	74 (31-130)	1300 (540-2500)
Mongolia	2016	Female	75 to 79	46% (31-63%)	54% (37-69%)	1.5 (0.55-3.4)	6.1% (1.7-12%)	7.7% (2.3-15%)	63 (19-130)	890 (270-1800)
Mongolia	2016	Female	80 to 84	43% (28-59%)	57% (41-72%)	1.6 (0.57-3.2)	5.8% (1.2-12%)	7.1% (1.6-15%)	61 (13-130)	640 (140-1300)
Mongolia	2016	Female	85 to 89	42% (28-56%)	58% (44-72%)	1.6 (0.59-3.6)	5.3% (0.58-12%)	6.3% (0.71-15%)	39 (4.6-92)	290 (33-680)
Mongolia	2016	Female	90 to 94	43% (30-59%)	57% (41-70%)	1.8 (0.56-4.3)	4.6% (-0.42-12%)	5.3% (-0.56-14%)	15 (-1.5-40)	76 (-6.9-200)
Mongolia	2016	Female	95 plus	46% (30-64%)	54% (36-70%)	2 (0.49-4.9)	4.1% (-1.7-12%)	4.6% (-2.1-14%)	3.4 (-1.5-10)	12 (-4.7-34)
Mongolia	2016	Male	All Ages	64% (56-73%)	36% (27-44%)	2.2 (1.6-3)	13% (9.9-16%)	14% (10-19%)	1900 (1300-2500)	81000 (58000-110000)
Mongolia	2016	Male	15 to 19	48% (31-66%)	52% (34-69%)	0.81 (0.36-1.5)	5.5% (3.4-8.4%)	7.2% (3.6-12%)	10 (4.9-17)	990 (590-1500)

Location	Year	Sex	Age	Prevalence of current drinkers	Prevalence of abstainers	Population average of standard drinks daily	Percent of total attributable deaths	Percent of total attributable DALYs	Attributable deaths	Attributable DALYs
Mongolia	2016	Male	20 to 24	69% (50-83%)	31% (17-50%)	2 (1.1-3.1)	13% (8.3-18%)	14% (8.4-21%)	42 (24-64)	3700 (2300-5300)
Mongolia	2016	Male	25 to 29	78% (63-88%)	22% (12-37%)	3.3 (1.9-5.1)	19% (13-25%)	22% (14-31%)	100 (62-140)	7600 (5200-10000)
Mongolia	2016	Male	30 to 34	83% (69-92%)	17% (8.3-31%)	4 (2.4-6)	22% (15-28%)	26% (17-34%)	140 (89-200)	9500 (6500-13000)
Mongolia	2016	Male	35 to 39	84% (72-92%)	16% (8-28%)	3.9 (2.3-6.1)	22% (15-29%)	26% (16-35%)	170 (110-250)	10000 (6700-14000)
Mongolia	2016	Male	40 to 44	84% (73-92%)	16% (7.6-27%)	3.9 (2.3-6.2)	21% (14-27%)	24% (16-32%)	210 (130-300)	11000 (7100-15000)
Mongolia	2016	Male	45 to 49	84% (69-92%)	16% (7.8-31%)	4.2 (2.4-6.7)	20% (14-26%)	23% (15-30%)	250 (150-360)	11000 (7300-15000)
Mongolia	2016	Male	50 to 54	82% (69-91%)	18% (8.7-31%)	4.1 (2.2-6.5)	18% (12-24%)	20% (13-27%)	270 (160-390)	11000 (6500-15000)
Mongolia	2016	Male	55 to 59	81% (67-91%)	19% (9.4-33%)	3.5 (1.9-5.6)	15% (9.6-21%)	17% (11-23%)	220 (130-330)	7300 (4500-11000)
Mongolia	2016	Male	60 to 64	78% (61-90%)	22% (10-39%)	3 (1.5-5)	12% (6.9-18%)	13% (7.6-21%)	160 (86-250)	4400 (2500-6800)
Mongolia	2016	Male	65 to 69	76% (59-89%)	24% (11-41%)	2.6 (1.4-4.3)	9.7% (5.3-15%)	11% (5.9-18%)	100 (55-170)	2400 (1300-4000)
Mongolia	2016	Male	70 to 74	72% (55-85%)	28% (15-45%)	2.2 (1.2-3.7)	7.6% (3.7-13%)	8.6% (4.3-15%)	84 (40-140)	1600 (760-2700)
Mongolia	2016	Male	75 to 79	68% (50-83%)	32% (17-50%)	1.8 (0.84-3.2)	6% (2.6-11%)	6.8% (2.9-13%)	61 (26-110)	880 (380-1600)
Mongolia	2016	Male	80 to 84	66% (47-80%)	34% (20-53%)	1.6 (0.77-2.8)	4.7% (1.7-9.3%)	5.3% (1.9-11%)	37 (12-74)	400 (150-790)
Mongolia	2016	Male	85 to 89	63% (45-78%)	37% (22-55%)	1.5 (0.7-2.6)	3.4% (0.82-7.5%)	3.8% (0.73-8.6%)	14 (2.6-31)	110 (24-230)
Mongolia	2016	Male	90 to 94	62% (45-78%)	38% (22-55%)	1.4 (0.63-2.5)	2.1% (-0.38-6%)	2.1% (-0.75-6.6%)	2.6 (-0.93-8.1)	15 (-2.7-44)
Mongolia	2016	Male	95 plus	63% (41-80%)	37% (20-59%)	1.3 (0.5-2.5)	1.4% (-1.1-5.7%)	1.2% (-1.8-6.1%)	0.33 (-0.52-1.7)	1.6 (-1.3-6.5)
Tajikistan	1990	Female	All Ages	49% (43-57%)	51% (43-57%)	0.53 (0.4-0.69)	0.37% (-0.055-0.82%)	-0.2% (-1.5-1.4%)	-41 (-320-280)	4800 (-720-11000)
Tajikistan	1990	Female	15 to 19	52% (39-68%)	48% (32-61%)	0.54 (0.23-1)	1.3% (0.65-2.2%)	1.8% (0.51-3.6%)	3.4 (1-6.9)	420 (210-740)
Tajikistan	1990	Female	20 to 24	61% (48-74%)	39% (26-52%)	0.79 (0.36-1.4)	2.1% (1.2-3.3%)	2.2% (0.69-4.4%)	6.3 (1.9-13)	790 (420-1300)
Tajikistan	1990	Female	25 to 29	64% (50-77%)	36% (23-50%)	0.92 (0.44-1.6)	2.5% (1.5-3.9%)	3% (1.2-5.4%)	9.3 (3.8-17)	990 (560-1600)
Tajikistan	1990	Female	30 to 34	65% (51-78%)	35% (22-49%)	1 (0.54-1.7)	2.7% (1.7-4.1%)	3.6% (1.8-6%)	11 (5.6-19)	950 (580-1500)
Tajikistan	1990	Female	35 to 39	65% (52-79%)	35% (21-48%)	1 (0.5-1.9)	2.7% (1.6-4.5%)	3.9% (1.8-6.9%)	12 (5.5-22)	800 (440-1400)

Location	Year	Sex	Age	Prevalence of current drinkers	Prevalence of abstainers	Population average of standard drinks daily	Percent of total attributable deaths	Percent of total attributable DALYs	Attributable deaths	Attributable DALYs
Tajikistan	1990	Female	40 to 44	65% (51-78%)	35% (22-49%)	1.1 (0.48-1.9)	2.7% (1.3-4.8%)	4.1% (1.6-7.6%)	11 (4.4-20)	610 (290-1100)
Tajikistan	1990	Female	45 to 49	65% (50-78%)	35% (22-50%)	1.1 (0.51-2.1)	2.5% (0.73-5.3%)	3.7% (0.85-8.2%)	9.6 (2.4-22)	450 (140-1000)
Tajikistan	1990	Female	50 to 54	64% (49-77%)	36% (23-51%)	1.2 (0.53-2.1)	2.2% (0.11-5.5%)	3.1% (-0.025-8%)	17 (-0.15-45)	700 (34-1700)
Tajikistan	1990	Female	55 to 59	62% (47-76%)	38% (24-53%)	1.2 (0.5-2.1)	1.5% (-0.93-5%)	2.1% (-1.4-7.2%)	15 (-9.7-50)	510 (-300-1700)
Tajikistan	1990	Female	60 to 64	60% (45-75%)	40% (25-55%)	1.1 (0.47-2.2)	0.45% (-2.2-4.5%)	0.57% (-3.1-5.8%)	6 (-31-61)	180 (-830-1700)
Tajikistan	1990	Female	65 to 69	58% (45-73%)	42% (27-55%)	1 (0.49-1.9)	-0.47% (-3-2.7%)	-0.59% (-3.9-3.6%)	-6.3 (-43-42)	-150 (-990-960)
Tajikistan	1990	Female	70 to 74	55% (43-68%)	45% (32-57%)	0.93 (0.37-1.8)	-1.1% (-3.7-2.3%)	-1.4% (-4.7-2.8%)	-16 (-54-33)	-300 (-990-610)
Tajikistan	1990	Female	75 to 79	52% (40-65%)	48% (35-60%)	0.87 (0.32-1.7)	-1.7% (-4.3-1.7%)	-2.1% (-5.3-2.1%)	-29 (-75-30)	-410 (-1100-430)
Tajikistan	1990	Female	80 to 84	50% (37-64%)	50% (36-63%)	0.89 (0.37-1.8)	-2% (-4.8-1.4%)	-2.5% (-6-1.5%)	-37 (-88-23)	-380 (-900-260)
Tajikistan	1990	Female	85 to 89	49% (37-61%)	51% (39-63%)	0.94 (0.37-1.9)	-2.2% (-5-2%)	-2.7% (-6-2.2%)	-34 (-77-27)	-240 (-550-230)
Tajikistan	1990	Female	90 to 94	50% (38-62%)	50% (38-62%)	1 (0.34-2.3)	-2% (-5-2.9%)	-2.6% (-6.1-3.2%)	-16 (-37-20)	-74 (-180-100)
Tajikistan	1990	Female	95 plus	51% (38-66%)	49% (34-62%)	1.1 (0.28-2.7)	-1.7% (-4.9-4.7%)	-2.2% (-6-5.3%)	-3.7 (-10-8.9)	-11 (-32-30)
Tajikistan	1990	Male	All Ages	60% (51-69%)	40% (31-49%)	0.83 (0.66-1)	1.4% (0.85-1.9%)	0.98% (-0.14-2.2%)	230 (-31-520)	20000 (13000-29000)
Tajikistan	1990	Male	15 to 19	55% (39-71%)	45% (29-61%)	0.46 (0.26-0.76)	2.6% (1.7-3.6%)	2.6% (1.3-4.3%)	6.9 (3.4-12)	950 (610-1400)
Tajikistan	1990	Male	20 to 24	73% (57-85%)	27% (15-43%)	1 (0.65-1.5)	6% (4.2-7.9%)	6.1% (3.4-9%)	21 (12-32)	2300 (1600-3200)
Tajikistan	1990	Male	25 to 29	80% (68-89%)	20% (11-32%)	1.7 (1.1-2.4)	7.9% (5.6-10%)	8.6% (5.3-12%)	42 (25-59)	3700 (2600-4800)
Tajikistan	1990	Male	30 to 34	84% (73-92%)	16% (8-27%)	2 (1.4-2.7)	8.3% (6.1-11%)	9.4% (6.1-13%)	45 (29-63)	3500 (2400-4600)
Tajikistan	1990	Male	35 to 39	86% (76-93%)	14% (7.4-24%)	2 (1.3-2.9)	7.4% (5.2-10%)	8.5% (5.2-12%)	37 (22-55)	2500 (1700-3500)
Tajikistan	1990	Male	40 to 44	86% (76-93%)	14% (7.1-24%)	2 (1.3-3)	6.6% (4.3-9.3%)	7.4% (4.4-11%)	30 (17-46)	1800 (1200-2600)
Tajikistan	1990	Male	45 to 49	85% (73-93%)	15% (7.3-27%)	2.2 (1.3-3.2)	6.1% (3.4-8.8%)	6.8% (3.3-11%)	32 (15-51)	1700 (910-2500)
Tajikistan	1990	Male	50 to 54	84% (73-92%)	16% (8-27%)	2.2 (1.3-3.2)	5% (2.3-7.9%)	5.5% (1.9-9.2%)	49 (16-86)	2200 (990-3600)
Tajikistan	1990	Male	55 to 59	83% (70-91%)	17% (8.7-30%)	1.9 (1.1-2.9)	3.4% (0.81-6.3%)	3.6% (0.35-7.3%)	37 (3.9-78)	1500 (370-2800)

Location	Year	Sex	Age	Prevalence of current drinkers	Prevalence of abstainers	Population average of standard drinks daily	Percent of total attributable deaths	Percent of total attributable DALYs	Attributable deaths	Attributable DALYs
Tajikistan	1990	Male	60 to 64	81% (67-91%)	19% (9.4-33%)	1.6 (0.95-2.6)	1.9% (-0.69-4.9%)	1.8% (-1.3-5.4%)	27 (-19-77)	920 (-330-2300)
Tajikistan	1990	Male	65 to 69	79% (64-90%)	21% (10-36%)	1.5 (0.9-2.2)	0.68% (-1.7-3.3%)	0.51% (-2.4-3.6%)	5.3 (-25-38)	200 (-480-950)
Tajikistan	1990	Male	70 to 74	76% (62-86%)	24% (14-38%)	1.2 (0.74-1.9)	-0.45% (-2.8-2.2%)	-0.76% (-3.6-2.4%)	-6.7 (-33-21)	-86 (-560-430)
Tajikistan	1990	Male	75 to 79	73% (57-85%)	27% (15-43%)	1.1 (0.57-1.7)	-1.3% (-3.5-0.97%)	-1.7% (-4.4-0.95%)	-20 (-51-11)	-250 (-680-180)
Tajikistan	1990	Male	80 to 84	70% (53-83%)	30% (17-47%)	0.96 (0.54-1.5)	-1.9% (-4.2-0.16%)	-2.5% (-5.2-0.091%)	-30 (-64-1)	-280 (-630-23)
Tajikistan	1990	Male	85 to 89	68% (52-81%)	32% (19-48%)	0.89 (0.51-1.4)	-2.3% (-4.5--0.27%)	-2.9% (-5.6--0.46%)	-28 (-54--4.3)	-190 (-370--22)
Tajikistan	1990	Male	90 to 94	67% (52-80%)	33% (20-48%)	0.82 (0.46-1.4)	-2.4% (-4.7--0.53%)	-3.1% (-5.9--0.76%)	-14 (-27--3.5)	-66 (-130--14)
Tajikistan	1990	Male	95 plus	66% (46-82%)	34% (18-54%)	0.75 (0.33-1.4)	-2.3% (-4.5--0.39%)	-3% (-5.7--0.71%)	-3.4 (-6.4--0.81)	-10 (-20--1.7)
Tajikistan	1995	Female	All Ages	49% (43-57%)	51% (43-57%)	0.51 (0.4-0.64)	0.42% (-0.076-0.93%)	-0.24% (-1.8-1.4%)	-50 (-370-290)	5300 (-890-12000)
Tajikistan	1995	Female	15 to 19	53% (40-68%)	47% (32-60%)	0.54 (0.23-0.98)	1.2% (0.58-2.1%)	1.6% (0.46-3.3%)	4 (1.1-8.2)	480 (220-830)
Tajikistan	1995	Female	20 to 24	61% (48-73%)	39% (27-52%)	0.78 (0.34-1.4)	2.1% (1.1-3.5%)	2.2% (0.6-4.4%)	7.4 (2-15)	880 (430-1500)
Tajikistan	1995	Female	25 to 29	64% (50-76%)	36% (24-50%)	0.9 (0.44-1.5)	2.6% (1.4-4.1%)	3% (1-5.5%)	11 (3.7-21)	1100 (570-1700)
Tajikistan	1995	Female	30 to 34	65% (51-79%)	35% (21-49%)	0.98 (0.53-1.6)	2.7% (1.6-4.2%)	3.6% (1.7-6%)	16 (7.5-27)	1200 (740-1900)
Tajikistan	1995	Female	35 to 39	65% (52-80%)	35% (20-48%)	1 (0.51-1.8)	2.7% (1.5-4.5%)	3.8% (1.7-6.7%)	16 (7.1-29)	1100 (580-1800)
Tajikistan	1995	Female	40 to 44	65% (51-79%)	35% (21-49%)	1.1 (0.48-1.8)	2.7% (1.2-4.9%)	4% (1.5-7.6%)	16 (5.4-31)	850 (370-1600)
Tajikistan	1995	Female	45 to 49	65% (50-78%)	35% (22-50%)	1.1 (0.51-2.2)	2.3% (0.47-5.4%)	3.4% (0.39-8.1%)	12 (1.5-29)	570 (120-1300)
Tajikistan	1995	Female	50 to 54	64% (49-77%)	36% (23-51%)	1.2 (0.53-2.2)	2% (-0.25-5.7%)	2.8% (-0.56-7.8%)	10 (-2.1-29)	410 (-54-1100)
Tajikistan	1995	Female	55 to 59	62% (48-75%)	38% (25-52%)	1.2 (0.49-2.1)	1.3% (-1.3-5.2%)	1.7% (-1.9-6.9%)	14 (-15-59)	480 (-480-1900)
Tajikistan	1995	Female	60 to 64	60% (45-75%)	40% (25-55%)	1.1 (0.46-2.2)	0.27% (-2.6-4.8%)	0.31% (-3.4-6.2%)	3.2 (-36-65)	100 (-980-1800)
Tajikistan	1995	Female	65 to 69	58% (45-73%)	42% (27-55%)	1 (0.48-1.9)	-0.73% (-3.6-3%)	-0.92% (-4.5-3.7%)	-13 (-61-53)	-300 (-1400-1200)
Tajikistan	1995	Female	70 to 74	56% (43-69%)	44% (31-57%)	0.92 (0.36-1.8)	-1.3% (-4.1-2.5%)	-1.6% (-5.1-3.1%)	-23 (-73-44)	-420 (-1300-800)
Tajikistan	1995	Female	75 to 79	52% (41-66%)	48% (34-59%)	0.86 (0.32-1.7)	-1.8% (-4.6-1.5%)	-2.2% (-5.6-1.7%)	-28 (-70-22)	-400 (-980-310)

Location	Year	Sex	Age	Prevalence of current drinkers	Prevalence of abstainers	Population average of standard drinks daily	Percent of total attributable deaths	Percent of total attributable DALYs	Attributable deaths	Attributable DALYs
Tajikistan	1995	Female	80 to 84	50% (37-65%)	50% (35-63%)	0.88 (0.35-1.7)	-2.2% (-5.1-1.3%)	-2.7% (-6.1-1.4%)	-36 (-84-20)	-370 (-860-210)
Tajikistan	1995	Female	85 to 89	50% (38-62%)	50% (38-62%)	0.93 (0.36-1.9)	-2.3% (-5.2-1.7%)	-2.8% (-6.2-1.8%)	-36 (-80-24)	-260 (-580-190)
Tajikistan	1995	Female	90 to 94	51% (39-62%)	49% (38-61%)	1 (0.35-2.3)	-2.2% (-5.1-2.8%)	-2.7% (-6.2-3.3%)	-19 (-43-22)	-89 (-210-120)
Tajikistan	1995	Female	95 plus	51% (38-67%)	49% (33-62%)	1 (0.28-2.6)	-1.8% (-5-4.5%)	-2.3% (-6.1-5.3%)	-5.1 (-14-12)	-16 (-44-41)
Tajikistan	1995	Male	All Ages	61% (52-70%)	39% (30-48%)	0.82 (0.68-0.95)	1.7% (1.1-2.4%)	1.4% (0.11-2.7%)	330 (25-640)	26000 (17000-37000)
Tajikistan	1995	Male	15 to 19	56% (40-72%)	44% (28-60%)	0.46 (0.26-0.74)	2.4% (1.5-3.4%)	2.3% (1.1-3.8%)	9.4 (4.5-16)	1200 (710-1700)
Tajikistan	1995	Male	20 to 24	73% (58-85%)	27% (15-42%)	1 (0.66-1.4)	5.6% (3.7-7.6%)	5.5% (3-8%)	33 (17-50)	3200 (2100-4600)
Tajikistan	1995	Male	25 to 29	80% (69-89%)	20% (11-31%)	1.7 (1.1-2.3)	8% (5.6-10%)	8.4% (5.4-12%)	57 (35-81)	4600 (3100-6100)
Tajikistan	1995	Male	30 to 34	84% (73-92%)	16% (8-27%)	2 (1.4-2.6)	8.6% (6.3-11%)	9.5% (6.4-13%)	73 (48-100)	5200 (3700-6800)
Tajikistan	1995	Male	35 to 39	86% (75-93%)	14% (7.2-25%)	2 (1.3-2.7)	7.8% (5.5-10%)	8.8% (5.5-12%)	64 (39-89)	4100 (2800-5500)
Tajikistan	1995	Male	40 to 44	86% (77-93%)	14% (7-23%)	2 (1.3-2.9)	6.7% (4.4-9.1%)	7.4% (4.4-11%)	50 (28-73)	2800 (1800-3900)
Tajikistan	1995	Male	45 to 49	86% (74-93%)	14% (7.2-26%)	2.2 (1.4-3.2)	6.1% (3.3-9.1%)	6.7% (3.1-11%)	41 (19-66)	2000 (1100-3100)
Tajikistan	1995	Male	50 to 54	85% (73-92%)	15% (8-27%)	2.1 (1.3-3.1)	4.9% (2.1-8.1%)	5.3% (1.6-9.3%)	35 (11-65)	1600 (660-2700)
Tajikistan	1995	Male	55 to 59	83% (71-91%)	17% (8.8-29%)	1.9 (1.1-2.7)	3.1% (0.32-6.1%)	3.2% (-0.23-7%)	40 (-2.9-87)	1600 (160-3000)
Tajikistan	1995	Male	60 to 64	81% (67-91%)	19% (9.4-33%)	1.6 (0.94-2.5)	1.5% (-1.2-4.6%)	1.4% (-1.9-5.1%)	20 (-27-74)	720 (-540-2200)
Tajikistan	1995	Male	65 to 69	79% (65-89%)	21% (11-35%)	1.4 (0.9-2.1)	0.36% (-2.2-3.1%)	0.12% (-3-3.4%)	2.1 (-54-61)	170 (-1100-1500)
Tajikistan	1995	Male	70 to 74	76% (62-86%)	24% (14-38%)	1.2 (0.71-1.9)	-0.64% (-3.1-1.9%)	-0.98% (-3.9-2%)	-12 (-48-25)	-170 (-810-510)
Tajikistan	1995	Male	75 to 79	73% (58-85%)	27% (15-42%)	1.1 (0.58-1.7)	-1.5% (-3.8-0.77%)	-1.9% (-4.6-0.8%)	-17 (-41-7.2)	-210 (-550-120)
Tajikistan	1995	Male	80 to 84	71% (54-83%)	29% (17-46%)	0.95 (0.54-1.4)	-2% (-4.4-0.14%)	-2.6% (-5.3-0.047%)	-24 (-51-0.45)	-230 (-510-17)
Tajikistan	1995	Male	85 to 89	69% (53-80%)	31% (20-47%)	0.87 (0.51-1.4)	-2.3% (-4.5--0.34%)	-2.9% (-5.5--0.54%)	-24 (-45--4.4)	-160 (-310--24)
Tajikistan	1995	Male	90 to 94	68% (53-80%)	32% (20-47%)	0.81 (0.45-1.3)	-2.4% (-4.7--0.5%)	-3.1% (-5.8--0.74%)	-13 (-25--3.1)	-62 (-120--13)
Tajikistan	1995	Male	95 plus	67% (48-82%)	33% (18-52%)	0.73 (0.33-1.3)	-2.3% (-4.4--0.4%)	-2.9% (-5.6--0.71%)	-4 (-7.7--0.98)	-12 (-24--2.2)

Location	Year	Sex	Age	Prevalence of current drinkers	Prevalence of abstainers	Population average of standard drinks daily	Percent of total attributable deaths	Percent of total attributable DALYs	Attributable deaths	Attributable DALYs
Tajikistan	2000	Female	All Ages	50% (44-59%)	50% (41-56%)	0.46 (0.37-0.58)	0.4% (-0.15-0.95%)	-0.47% (-2.1-1.2%)	-91 (-400-240)	4500 (-1700-11000)
Tajikistan	2000	Female	15 to 19	53% (40-68%)	47% (32-60%)	0.48 (0.22-0.85)	1.1% (0.55-1.9%)	1.5% (0.41-3.1%)	3.5 (0.91-7.1)	480 (230-820)
Tajikistan	2000	Female	20 to 24	61% (49-73%)	39% (27-51%)	0.69 (0.31-1.2)	2% (1-3.3%)	2.2% (0.5-4.6%)	6.7 (1.6-14)	840 (420-1400)
Tajikistan	2000	Female	25 to 29	64% (49-76%)	36% (24-51%)	0.8 (0.38-1.4)	2.4% (1.2-3.9%)	2.9% (0.64-5.5%)	10 (2.3-20)	1000 (490-1700)
Tajikistan	2000	Female	30 to 34	65% (50-78%)	35% (22-50%)	0.87 (0.46-1.5)	2.7% (1.5-4.1%)	3.5% (1.4-6%)	14 (5.7-24)	1200 (630-1800)
Tajikistan	2000	Female	35 to 39	65% (52-79%)	35% (21-48%)	0.9 (0.45-1.5)	2.6% (1.4-4.3%)	3.8% (1.7-6.5%)	18 (7.7-31)	1200 (630-1900)
Tajikistan	2000	Female	40 to 44	65% (51-79%)	35% (21-49%)	0.95 (0.43-1.6)	2.5% (1.1-4.4%)	3.8% (1.5-7%)	18 (6.7-34)	980 (440-1700)
Tajikistan	2000	Female	45 to 49	65% (49-79%)	35% (21-51%)	0.99 (0.45-1.9)	2% (0.26-4.5%)	3% (0.091-7%)	13 (0.42-32)	620 (80-1400)
Tajikistan	2000	Female	50 to 54	64% (49-78%)	36% (22-51%)	1 (0.46-1.9)	1.6% (-0.4-4.8%)	2.3% (-0.78-6.9%)	10 (-3.4-30)	400 (-110-1200)
Tajikistan	2000	Female	55 to 59	62% (48-76%)	38% (24-52%)	1 (0.44-1.9)	0.85% (-1.6-4.4%)	1.1% (-2.2-5.9%)	5.5 (-11-29)	190 (-340-920)
Tajikistan	2000	Female	60 to 64	60% (46-75%)	40% (25-54%)	0.99 (0.41-1.9)	-0.19% (-2.9-3.8%)	-0.28% (-3.8-4.8%)	-3.2 (-43-53)	-80 (-1100-1500)
Tajikistan	2000	Female	65 to 69	58% (45-73%)	42% (27-55%)	0.91 (0.42-1.7)	-1.2% (-3.9-2.4%)	-1.4% (-4.8-2.8%)	-19 (-67-38)	-450 (-1500-890)
Tajikistan	2000	Female	70 to 74	55% (43-68%)	45% (32-57%)	0.81 (0.32-1.6)	-1.6% (-4.4-1.8%)	-1.9% (-5.2-2.1%)	-34 (-94-36)	-620 (-1700-640)
Tajikistan	2000	Female	75 to 79	52% (41-66%)	48% (34-59%)	0.76 (0.28-1.5)	-2% (-4.6-0.89%)	-2.4% (-5.6-0.98%)	-38 (-88-15)	-540 (-1200-210)
Tajikistan	2000	Female	80 to 84	50% (37-65%)	50% (35-63%)	0.78 (0.31-1.5)	-2.4% (-5.1-0.69%)	-2.8% (-6.2-0.74%)	-36 (-79-9)	-370 (-810-110)
Tajikistan	2000	Female	85 to 89	50% (37-62%)	50% (38-63%)	0.83 (0.32-1.8)	-2.5% (-5.3-0.97%)	-3% (-6.2-1%)	-33 (-69-10)	-240 (-490-84)
Tajikistan	2000	Female	90 to 94	51% (39-62%)	49% (38-61%)	0.89 (0.3-2.1)	-2.4% (-5.1-2.3%)	-2.9% (-6.2-2.5%)	-19 (-41-16)	-93 (-200-86)
Tajikistan	2000	Female	95 plus	51% (38-66%)	49% (34-62%)	0.93 (0.24-2.4)	-2% (-4.9-4.1%)	-2.5% (-5.9-4.6%)	-6.1 (-14-11)	-19 (-47-38)
Tajikistan	2000	Male	All Ages	61% (52-70%)	39% (30-48%)	0.75 (0.62-0.85)	1.8% (1.1-2.5%)	1.1% (-0.24-2.5%)	250 (-53-570)	24000 (15000-33000)
Tajikistan	2000	Male	15 to 19	56% (40-72%)	44% (28-60%)	0.41 (0.23-0.64)	2.3% (1.5-3.2%)	2.2% (1.1-3.7%)	8.1 (4.1-14)	1100 (740-1700)
Tajikistan	2000	Male	20 to 24	73% (57-85%)	27% (15-43%)	0.91 (0.59-1.3)	5.4% (3.6-7.3%)	5.3% (2.7-8.1%)	26 (13-42)	2800 (1800-4000)
Tajikistan	2000	Male	25 to 29	80% (69-89%)	20% (11-31%)	1.5 (0.98-2)	7.6% (5.4-10%)	8.2% (5-12%)	48 (28-71)	4100 (2800-5700)

Location	Year	Sex	Age	Prevalence of current drinkers	Prevalence of abstainers	Population average of standard drinks daily	Percent of total attributable deaths	Percent of total attributable DALYs	Attributable deaths	Attributable DALYs
Tajikistan	2000	Male	30 to 34	85%<br>(73-92%)	15%<br>(7.7-27%)	1.8<br>(1.3-2.4)	8.2%<br>(6.1-11%)	9.2%<br>(6.1-13%)	59<br>(39-82)	4400<br>(3100-5800)
Tajikistan	2000	Male	35 to 39	86%<br>(75-93%)	14%<br>(7.1-25%)	1.8<br>(1.2-2.4)	7.5%<br>(5.3-9.9%)	8.6%<br>(5.4-12%)	61<br>(38-88)	4100<br>(2800-5600)
Tajikistan	2000	Male	40 to 44	86%<br>(76-93%)	14%<br>(7-24%)	1.8<br>(1.2-2.5)	6.4%<br>(4.1-8.6%)	7.2%<br>(4-10%)	51<br>(28-76)	3100<br>(1900-4200)
Tajikistan	2000	Male	45 to 49	86%<br>(73-93%)	14%<br>(7.1-27%)	1.9<br>(1.2-2.8)	5.6%<br>(3-8.2%)	6.1%<br>(2.5-9.7%)	42<br>(17-69)	2200<br>(1100-3400)
Tajikistan	2000	Male	50 to 54	85%<br>(73-92%)	15%<br>(8-27%)	1.9<br>(1.1-2.8)	4.3%<br>(1.6-7.4%)	4.6%<br>(1.1-8.5%)	31<br>(6.9-58)	1400<br>(520-2400)
Tajikistan	2000	Male	55 to 59	83%<br>(71-92%)	17%<br>(8.1-29%)	1.7<br>(1-2.5)	2.8%<br>(0.19-5.6%)	2.8%<br>(-0.63-6.4%)	22<br>(-4.8-52)	890<br>(58-1800)
Tajikistan	2000	Male	60 to 64	81%<br>(68-91%)	19%<br>(9.4-32%)	1.4<br>(0.83-2.3)	1.1%<br>(-1.5-3.9%)	0.9%<br>(-2.2-4.2%)	14<br>(-35-67)	560<br>(-770-1900)
Tajikistan	2000	Male	65 to 69	79%<br>(65-89%)	21%<br>(11-35%)	1.3<br>(0.79-1.9)	-0.075%<br>(-2.6-2.6%)	-0.35%<br>(-3.3-2.8%)	-6<br>(-58-48)	-34<br>(-1200-1200)
Tajikistan	2000	Male	70 to 74	76%<br>(62-86%)	24%<br>(14-38%)	1.1<br>(0.63-1.7)	-0.95%<br>(-3.4-1.3%)	-1.3%<br>(-4.2-1.4%)	-28<br>(-89-29)	-430<br>(-1500-620)
Tajikistan	2000	Male	75 to 79	73%<br>(57-85%)	27%<br>(15-43%)	0.94<br>(0.5-1.5)	-1.6%<br>(-3.8-0.46%)	-2%<br>(-4.7-0.4%)	-26<br>(-60-5.4)	-330<br>(-800-100)
Tajikistan	2000	Male	80 to 84	71%<br>(54-84%)	29%<br>(16-46%)	0.85<br>(0.46-1.3)	-2.1%<br>(-4.5-0.033%)	-2.6%<br>(-5.4--0.13%)	-20<br>(-41--0.94)	-190<br>(-400-3)
Tajikistan	2000	Male	85 to 89	69%<br>(53-80%)	31%<br>(20-47%)	0.78<br>(0.44-1.2)	-2.4%<br>(-4.6--0.42%)	-2.9%<br>(-5.6--0.58%)	-17<br>(-33--3.4)	-120<br>(-230--21)
Tajikistan	2000	Male	90 to 94	68%<br>(53-80%)	32%<br>(20-47%)	0.72<br>(0.39-1.2)	-2.4%<br>(-4.7--0.53%)	-3.1%<br>(-5.8--0.8%)	-10<br>(-20--2.7)	-48<br>(-94--11)
Tajikistan	2000	Male	95 plus	67%<br>(48-82%)	33%<br>(18-52%)	0.65<br>(0.3-1.2)	-2.2%<br>(-4.5--0.38%)	-2.9%<br>(-5.5--0.7%)	-3.3<br>(-6.5--0.77)	-9.8<br>(-20--1.7)
Tajikistan	2005	Female	All Ages	51%<br>(45-60%)	49%<br>(40-55%)	0.44<br>(0.35-0.56)	0.41%<br>(-0.26-1%)	-0.74%<br>(-2.4-1%)	-130<br>(-440-190)	4100<br>(-2300-11000)
Tajikistan	2005	Female	15 to 19	52%<br>(40-67%)	48%<br>(33-60%)	0.43<br>(0.18-0.76)	1.1%<br>(0.54-1.8%)	1.5%<br>(0.3-3.1%)	3.5<br>(0.68-7.1)	520<br>(260-870)
Tajikistan	2005	Female	20 to 24	61%<br>(48-73%)	39%<br>(27-52%)	0.62<br>(0.27-1.1)	2%<br>(1-3.1%)	2.2%<br>(0.35-4.6%)	6.1<br>(0.92-13)	890<br>(440-1400)
Tajikistan	2005	Female	25 to 29	64%<br>(49-77%)	36%<br>(23-51%)	0.72<br>(0.34-1.3)	2.4%<br>(1.2-3.9%)	2.9%<br>(0.52-5.6%)	8.2<br>(1.4-17)	960<br>(480-1500)
Tajikistan	2005	Female	30 to 34	65%<br>(51-79%)	35%<br>(21-49%)	0.79<br>(0.4-1.3)	2.6%<br>(1.6-3.9%)	3.6%<br>(1.5-6.1%)	12<br>(5.2-20)	1100<br>(630-1600)
Tajikistan	2005	Female	35 to 39	65%<br>(52-80%)	35%<br>(20-48%)	0.81<br>(0.39-1.4)	2.5%<br>(1.5-3.9%)	4%<br>(2-6.7%)	15<br>(7.3-25)	1100<br>(630-1600)
Tajikistan	2005	Female	40 to 44	65%<br>(51-79%)	35%<br>(21-49%)	0.84<br>(0.38-1.5)	2.4%<br>(1.2-4%)	3.9%<br>(1.6-6.9%)	19<br>(7.9-33)	1100<br>(540-1800)
Tajikistan	2005	Female	45 to 49	65%<br>(49-78%)	35%<br>(22-51%)	0.89<br>(0.41-1.6)	1.9%<br>(0.47-3.8%)	2.9%<br>(0.4-6.4%)	17<br>(2.3-37)	800<br>(190-1700)

Location	Year	Sex	Age	Prevalence of current drinkers	Prevalence of abstainers	Population average of standard drinks daily	Percent of total attributable deaths	Percent of total attributable DALYs	Attributable deaths	Attributable DALYs
Tajikistan	2005	Female	50 to 54	63% (49-77%)	37% (23-51%)	0.91 (0.42-1.7)	1.4% (-0.33-3.9%)	2% (-0.58-5.8%)	12 (-3.5-34)	480 (-120-1300)
Tajikistan	2005	Female	55 to 59	62% (48-75%)	38% (25-52%)	0.92 (0.39-1.7)	0.64% (-1.5-3.8%)	0.86% (-2.2-5.1%)	5.7 (-14-35)	190 (-440-1100)
Tajikistan	2005	Female	60 to 64	60% (45-74%)	40% (26-55%)	0.89 (0.36-1.8)	-0.45% (-3-3.3%)	-0.6% (-3.8-4.2%)	-4.2 (-27-29)	-110 (-730-800)
Tajikistan	2005	Female	65 to 69	58% (46-73%)	42% (27-54%)	0.82 (0.37-1.5)	-1.3% (-3.7-1.6%)	-1.6% (-4.6-2.1%)	-24 (-71-32)	-560 (-1600-730)
Tajikistan	2005	Female	70 to 74	56% (43-69%)	44% (31-57%)	0.73 (0.29-1.4)	-1.8% (-4.4-1.1%)	-2.1% (-5.2-1.2%)	-40 (-99-26)	-740 (-1800-460)
Tajikistan	2005	Female	75 to 79	52% (41-65%)	48% (35-59%)	0.68 (0.25-1.3)	-2.2% (-4.7-0.25%)	-2.6% (-5.5-0.29%)	-49 (-110-5.5)	-690 (-1500-72)
Tajikistan	2005	Female	80 to 84	50% (37-64%)	50% (36-63%)	0.7 (0.28-1.3)	-2.5% (-5.3-0.072%)	-3% (-6.2-0.044%)	-52 (-110-0.76)	-540 (-1100-15)
Tajikistan	2005	Female	85 to 89	50% (38-62%)	50% (38-62%)	0.74 (0.27-1.5)	-2.7% (-5.3-0.08%)	-3.3% (-6.3-0.021%)	-37 (-71-0.23)	-260 (-510-7.9)
Tajikistan	2005	Female	90 to 94	51% (39-62%)	49% (38-61%)	0.79 (0.27-1.8)	-2.6% (-5.2-1.1%)	-3.2% (-6.3-1.1%)	-20 (-39-6.9)	-96 (-190-41)
Tajikistan	2005	Female	95 plus	51% (38-67%)	49% (33-62%)	0.83 (0.22-2.1)	-2.3% (-4.9-2.6%)	-2.8% (-5.8-2.9%)	-7.2 (-15-7.3)	-23 (-49-26)
Tajikistan	2005	Male	All Ages	63% (55-72%)	37% (28-45%)	0.71 (0.6-0.82)	1.9% (1.1-2.7%)	0.96% (-0.67-2.4%)	210 (-140-540)	24000 (14000-33000)
Tajikistan	2005	Male	15 to 19	56% (40-72%)	44% (28-60%)	0.37 (0.21-0.58)	2.3% (1.6-3.1%)	2.1% (0.99-3.5%)	7.6 (3.7-13)	1200 (790-1800)
Tajikistan	2005	Male	20 to 24	73% (58-85%)	27% (15-42%)	0.82 (0.55-1.1)	5.3% (3.5-7.2%)	5.1% (2.3-7.8%)	23 (10-37)	2900 (1800-4100)
Tajikistan	2005	Male	25 to 29	80% (69-89%)	20% (11-31%)	1.3 (0.89-1.8)	7.4% (5.3-9.7%)	7.9% (4.6-11%)	38 (22-56)	3700 (2600-5000)
Tajikistan	2005	Male	30 to 34	85% (73-92%)	15% (7.9-27%)	1.6 (1.1-2.1)	8% (5.9-10%)	9% (5.8-13%)	52 (33-74)	4200 (3000-5700)
Tajikistan	2005	Male	35 to 39	86% (76-93%)	14% (7.3-24%)	1.6 (1-2.2)	7.3% (5.2-9.5%)	8.5% (5.4-12%)	54 (33-79)	3800 (2600-5100)
Tajikistan	2005	Male	40 to 44	86% (77-93%)	14% (7-23%)	1.6 (1.1-2.2)	6.3% (4.2-8.4%)	7.2% (4.2-10%)	58 (33-86)	3500 (2400-4900)
Tajikistan	2005	Male	45 to 49	86% (74-93%)	14% (7.3-26%)	1.7 (1.1-2.5)	5.4% (2.9-7.8%)	6% (2.6-9.4%)	52 (23-85)	2800 (1500-4200)
Tajikistan	2005	Male	50 to 54	85% (73-92%)	15% (7.7-27%)	1.7 (1-2.5)	4.1% (1.5-6.8%)	4.3% (0.82-7.9%)	40 (7.9-76)	1900 (670-3200)
Tajikistan	2005	Male	55 to 59	83% (71-92%)	17% (8.4-29%)	1.5 (0.9-2.2)	2.6% (0.079-5.3%)	2.6% (-0.68-6%)	23 (-6-54)	960 (29-2000)
Tajikistan	2005	Male	60 to 64	81% (68-91%)	19% (9.4-32%)	1.3 (0.71-2.1)	0.9% (-1.6-3.4%)	0.67% (-2.4-3.8%)	7.8 (-28-43)	340 (-610-1300)
Tajikistan	2005	Male	65 to 69	79% (64-89%)	21% (11-36%)	1.1 (0.7-1.7)	-0.17% (-2.7-2.3%)	-0.44% (-3.4-2.6%)	-9.1 (-71-51)	-92 (-1500-1200)

Location	Year	Sex	Age	Prevalence of current drinkers	Prevalence of abstainers	Population average of standard drinks daily	Percent of total attributable deaths	Percent of total attributable DALYs	Attributable deaths	Attributable DALYs
Tajikistan	2005	Male	70 to 74	76% (62-87%)	24% (13-38%)	0.98 (0.57-1.5)	-1.1% (-3.7-1.1%)	-1.5% (-4.3-1.1%)	-33 (-100-27)	-540 (-1700-550)
Tajikistan	2005	Male	75 to 79	73% (58-85%)	27% (15-42%)	0.84 (0.45-1.3)	-1.6% (-3.9-0.38%)	-2.1% (-4.7-0.33%)	-47 (-110-6.9)	-620 (-1500-150)
Tajikistan	2005	Male	80 to 84	71% (55-83%)	29% (17-45%)	0.75 (0.42-1.1)	-2.1% (-4.5-0.011%)	-2.6% (-5.4--0.09%)	-32 (-66--1)	-310 (-660-1.6)
Tajikistan	2005	Male	85 to 89	69% (53-80%)	31% (20-47%)	0.69 (0.4-1.1)	-2.4% (-4.6--0.35%)	-2.9% (-5.6--0.52%)	-14 (-27--2.3)	-93 (-190--15)
Tajikistan	2005	Male	90 to 94	68% (53-80%)	32% (20-47%)	0.64 (0.36-1.1)	-2.4% (-4.6--0.5%)	-3% (-5.7--0.73%)	-7 (-13--1.7)	-32 (-63--6.6)
Tajikistan	2005	Male	95 plus	66% (48-82%)	34% (18-52%)	0.58 (0.26-1)	-2.2% (-4.4--0.38%)	-2.8% (-5.5--0.64%)	-2.5 (-4.9--0.59)	-7.5 (-15--1.4)
Tajikistan	2010	Female	All Ages	52% (45-60%)	48% (40-55%)	0.41 (0.32-0.52)	0.38% (-0.16-0.91%)	-0.86% (-2.5-0.65%)	-150 (-420-110)	3800 (-1600-9300)
Tajikistan	2010	Female	15 to 19	52% (40-67%)	48% (33-60%)	0.38 (0.17-0.68)	0.98% (0.55-1.5%)	1.3% (0.31-2.5%)	2.9 (0.7-6.1)	470 (250-800)
Tajikistan	2010	Female	20 to 24	61% (48-74%)	39% (26-52%)	0.55 (0.25-0.97)	1.8% (1-2.8%)	1.8% (0.31-3.6%)	5.9 (1.1-12)	1000 (540-1600)
Tajikistan	2010	Female	25 to 29	64% (49-76%)	36% (24-51%)	0.64 (0.3-1.1)	2.1% (1.3-3.2%)	2.3% (0.66-4.5%)	7.5 (2.1-14)	1000 (590-1600)
Tajikistan	2010	Female	30 to 34	65% (51-77%)	35% (23-49%)	0.69 (0.37-1.2)	2.3% (1.5-3.4%)	3.1% (1.5-5%)	10 (5-17)	1000 (650-1500)
Tajikistan	2010	Female	35 to 39	65% (52-79%)	35% (21-48%)	0.71 (0.35-1.3)	2.2% (1.4-3.4%)	3.4% (1.7-5.6%)	13 (6.6-22)	980 (610-1500)
Tajikistan	2010	Female	40 to 44	65% (51-79%)	35% (21-49%)	0.74 (0.34-1.3)	2% (1.1-3.3%)	3.3% (1.4-5.8%)	16 (6.4-28)	940 (480-1500)
Tajikistan	2010	Female	45 to 49	65% (49-78%)	35% (22-51%)	0.78 (0.36-1.5)	1.5% (0.33-3.3%)	2.3% (0.25-5.3%)	15 (1.6-37)	780 (170-1700)
Tajikistan	2010	Female	50 to 54	63% (49-78%)	37% (22-51%)	0.8 (0.37-1.5)	1% (-0.37-3%)	1.4% (-0.8-4.7%)	11 (-6.3-35)	490 (-180-1400)
Tajikistan	2010	Female	55 to 59	61% (48-76%)	39% (24-52%)	0.81 (0.33-1.6)	0.27% (-1.6-2.6%)	0.34% (-2.3-3.6%)	2.8 (-19-30)	110 (-580-1000)
Tajikistan	2010	Female	60 to 64	60% (46-75%)	40% (25-54%)	0.78 (0.31-1.6)	-0.72% (-2.8-2.1%)	-1% (-3.9-2.7%)	-8.3 (-32-23)	-230 (-870-630)
Tajikistan	2010	Female	65 to 69	58% (45-73%)	42% (27-55%)	0.72 (0.33-1.4)	-1.5% (-3.7-0.67%)	-1.9% (-4.8-0.89%)	-16 (-41-8)	-380 (-930-170)
Tajikistan	2010	Female	70 to 74	56% (43-69%)	44% (31-57%)	0.64 (0.25-1.3)	-1.9% (-4.1-0.47%)	-2.3% (-5.2-0.63%)	-39 (-89-10)	-730 (-1600-170)
Tajikistan	2010	Female	75 to 79	52% (40-66%)	48% (34-60%)	0.6 (0.22-1.2)	-2.3% (-4.5--0.25%)	-2.8% (-5.6--0.28%)	-49 (-98--5.2)	-690 (-1400--82)
Tajikistan	2010	Female	80 to 84	50% (37-64%)	50% (36-63%)	0.61 (0.24-1.2)	-2.6% (-5.1--0.53%)	-3.2% (-6.3--0.63%)	-51 (-98--10)	-520 (-1000--110)
Tajikistan	2010	Female	85 to 89	50% (37-62%)	50% (38-63%)	0.65 (0.25-1.3)	-2.8% (-5.2--0.55%)	-3.4% (-6.3--0.71%)	-47 (-86--9.3)	-340 (-620--65)

Location	Year	Sex	Age	Prevalence of current drinkers	Prevalence of abstainers	Population average of standard drinks daily	Percent of total attributable deaths	Percent of total attributable DALYs	Attributable deaths	Attributable DALYs
Tajikistan	2010	Female	90 to 94	51% (38-63%)	49% (37-62%)	0.7 (0.24-1.6)	-2.7% (-5-0.041%)	-3.4% (-6.2--0.056%)	-20 (-36--0.35)	-97 (-180-1.5)
Tajikistan	2010	Female	95 plus	51% (38-66%)	49% (34-62%)	0.73 (0.19-1.9)	-2.4% (-4.8-1%)	-3% (-5.9-0.95%)	-7.1 (-14-2.3)	-23 (-45-9.9)
Tajikistan	2010	Male	All Ages	64% (55-73%)	36% (27-45%)	0.66 (0.55-0.77)	1.9% (1.2-2.7%)	0.77% (-0.79-2.2%)	180 (-180-500)	25000 (15000-35000)
Tajikistan	2010	Male	15 to 19	56% (40-71%)	44% (29-60%)	0.32 (0.18-0.5)	2.2% (1.5-3%)	1.9% (0.99-3.1%)	7.5 (3.8-12)	1200 (820-1800)
Tajikistan	2010	Male	20 to 24	73% (58-85%)	27% (15-42%)	0.72 (0.48-0.99)	4.9% (3.4-6.6%)	4.4% (2.2-6.6%)	26 (13-40)	3400 (2300-4800)
Tajikistan	2010	Male	25 to 29	80% (69-89%)	20% (11-31%)	1.2 (0.78-1.6)	6.7% (5-8.5%)	6.7% (4.2-9.3%)	41 (24-58)	4200 (3000-5600)
Tajikistan	2010	Male	30 to 34	85% (73-92%)	15% (7.7-27%)	1.4 (0.98-1.9)	7.3% (5.6-9.4%)	7.9% (5.1-11%)	48 (31-69)	4100 (3000-5500)
Tajikistan	2010	Male	35 to 39	86% (76-93%)	14% (7.4-24%)	1.4 (0.9-2)	6.7% (4.9-8.7%)	7.4% (4.6-10%)	50 (31-72)	3700 (2500-4900)
Tajikistan	2010	Male	40 to 44	86% (76-93%)	14% (7-24%)	1.4 (0.92-2)	5.7% (3.7-7.7%)	6.4% (3.4-9.6%)	49 (25-74)	3200 (2000-4500)
Tajikistan	2010	Male	45 to 49	85% (73-93%)	15% (7.4-27%)	1.5 (0.95-2.2)	4.8% (2.7-7.1%)	5.2% (1.9-8.5%)	52 (19-85)	2900 (1600-4500)
Tajikistan	2010	Male	50 to 54	85% (72-92%)	15% (8.1-28%)	1.5 (0.87-2.2)	3.5% (1.2-6%)	3.6% (0.24-6.8%)	45 (3.2-87)	2200 (730-3800)
Tajikistan	2010	Male	55 to 59	83% (71-91%)	17% (8.5-29%)	1.3 (0.78-2)	2.2% (-0.13-4.4%)	2.1% (-0.95-5%)	25 (-12-60)	1100 (-66-2200)
Tajikistan	2010	Male	60 to 64	81% (68-91%)	19% (9.3-32%)	1.1 (0.64-1.8)	0.71% (-1.5-2.9%)	0.42% (-2.4-3.3%)	5.2 (-29-41)	300 (-630-1300)
Tajikistan	2010	Male	65 to 69	79% (65-89%)	21% (11-35%)	1 (0.62-1.5)	-0.26% (-2.5-1.9%)	-0.59% (-3.4-2.1%)	-7.7 (-44-26)	-91 (-890-660)
Tajikistan	2010	Male	70 to 74	76% (63-86%)	24% (14-37%)	0.86 (0.5-1.3)	-1.2% (-3.5-0.99%)	-1.6% (-4.3-1.1%)	-35 (-100-24)	-570 (-1700-490)
Tajikistan	2010	Male	75 to 79	73% (58-85%)	27% (15-42%)	0.73 (0.39-1.2)	-1.7% (-4-0.29%)	-2.2% (-4.8-0.18%)	-43 (-99-3.7)	-560 (-1300-95)
Tajikistan	2010	Male	80 to 84	71% (54-83%)	29% (17-46%)	0.66 (0.37-1)	-2.2% (-4.4--0.062%)	-2.7% (-5.4--0.19%)	-44 (-89--3)	-430 (-890--12)
Tajikistan	2010	Male	85 to 89	69% (53-80%)	31% (20-47%)	0.6 (0.35-0.94)	-2.4% (-4.7--0.39%)	-2.9% (-5.6--0.55%)	-30 (-59--5.8)	-210 (-410--34)
Tajikistan	2010	Male	90 to 94	67% (53-80%)	33% (20-47%)	0.56 (0.3-0.93)	-2.4% (-4.7--0.49%)	-3% (-5.7--0.7%)	-8.4 (-16--1.9)	-40 (-77--7.8)
Tajikistan	2010	Male	95 plus	66% (47-82%)	34% (18-53%)	0.5 (0.22-0.91)	-2.1% (-4.5--0.33%)	-2.7% (-5.5--0.59%)	-2.3 (-4.6--0.49)	-6.9 (-14--1)
Tajikistan	2016	Female	All Ages	52% (46-60%)	48% (40-54%)	0.4 (0.29-0.54)	0.4% (-0.14-0.92%)	-0.74% (-2.3-0.63%)	-130 (-380-110)	4300 (-1400-9800)
Tajikistan	2016	Female	15 to 19	52% (39-67%)	48% (33-61%)	0.36 (0.14-0.65)	0.97% (0.53-1.6%)	1.2% (0.34-2.5%)	2.5 (0.68-5.4)	440 (230-730)

Location	Year	Sex	Age	Prevalence of current drinkers	Prevalence of abstainers	Population average of standard drinks daily	Percent of total attributable deaths	Percent of total attributable DALYs	Attributable deaths	Attributable DALYs
Tajikistan	2016	Female	20 to 24	61% (48-74%)	39% (26-52%)	0.52 (0.23-0.95)	1.8% (1.1-2.7%)	1.7% (0.47-3.4%)	4.8 (1.2-9.4)	940 (520-1400)
Tajikistan	2016	Female	25 to 29	64% (50-77%)	36% (23-50%)	0.6 (0.28-1.1)	2.1% (1.4-3%)	2.2% (0.78-4.1%)	7.9 (2.9-15)	1200 (760-1800)
Tajikistan	2016	Female	30 to 34	65% (50-78%)	35% (22-50%)	0.65 (0.33-1.1)	2.2% (1.6-3.2%)	3% (1.6-4.8%)	11 (5.4-18)	1200 (770-1700)
Tajikistan	2016	Female	35 to 39	65% (52-79%)	35% (21-48%)	0.67 (0.33-1.2)	2.2% (1.4-3.2%)	3.4% (1.8-5.3%)	12 (6.4-20)	980 (610-1400)
Tajikistan	2016	Female	40 to 44	65% (52-79%)	35% (21-48%)	0.7 (0.31-1.3)	2% (1.1-3.1%)	3.2% (1.5-5.6%)	15 (7.2-27)	930 (510-1500)
Tajikistan	2016	Female	45 to 49	65% (49-78%)	35% (22-51%)	0.73 (0.33-1.4)	1.4% (0.35-2.9%)	2.2% (0.27-4.9%)	14 (1.7-31)	730 (180-1500)
Tajikistan	2016	Female	50 to 54	63% (49-77%)	37% (23-51%)	0.74 (0.32-1.4)	0.88% (-0.37-2.6%)	1.3% (-0.87-4.1%)	12 (-8.3-38)	540 (-240-1500)
Tajikistan	2016	Female	55 to 59	62% (48-75%)	38% (25-52%)	0.75 (0.31-1.4)	0.12% (-1.5-1.9%)	0.13% (-2.3-2.8%)	1.4 (-27-33)	67 (-860-1100)
Tajikistan	2016	Female	60 to 64	60% (46-74%)	40% (26-54%)	0.73 (0.29-1.5)	-0.79% (-2.7-1.5%)	-1.1% (-3.8-2%)	-14 (-45-23)	-380 (-1300-720)
Tajikistan	2016	Female	65 to 69	58% (46-73%)	42% (27-54%)	0.68 (0.3-1.3)	-1.4% (-3.5-0.49%)	-1.9% (-4.7-0.72%)	-18 (-45-6.9)	-430 (-1000-140)
Tajikistan	2016	Female	70 to 74	55% (43-69%)	45% (31-57%)	0.61 (0.23-1.2)	-1.7% (-3.9-0.32%)	-2.2% (-5-0.41%)	-22 (-50-3.9)	-420 (-930-78)
Tajikistan	2016	Female	75 to 79	52% (40-66%)	48% (34-60%)	0.57 (0.21-1.2)	-2.1% (-4.2--0.23%)	-2.6% (-5.4--0.25%)	-39 (-80--3.5)	-570 (-1100--61)
Tajikistan	2016	Female	80 to 84	50% (37-65%)	50% (35-63%)	0.58 (0.23-1.2)	-2.5% (-4.9--0.54%)	-3.2% (-6.1--0.64%)	-46 (-88--9.7)	-480 (-920--100)
Tajikistan	2016	Female	85 to 89	50% (37-62%)	50% (38-63%)	0.61 (0.24-1.3)	-2.8% (-5--0.7%)	-3.4% (-6.2--0.88%)	-40 (-72--10)	-290 (-520--73)
Tajikistan	2016	Female	90 to 94	51% (39-63%)	49% (37-61%)	0.66 (0.22-1.5)	-2.8% (-5--0.4%)	-3.4% (-6.2--0.61%)	-25 (-44--4.3)	-120 (-220--18)
Tajikistan	2016	Female	95 plus	51% (38-66%)	49% (34-62%)	0.69 (0.18-1.7)	-2.5% (-4.9-0.38%)	-3.2% (-6-0.26%)	-7.1 (-13-0.6)	-23 (-43-3.5)
Tajikistan	2016	Male	All Ages	65% (57-73%)	35% (27-43%)	0.65 (0.5-0.82)	2.1% (1.2-3%)	0.95% (-0.56-2.5%)	230 (-150-590)	29000 (18000-41000)
Tajikistan	2016	Male	15 to 19	56% (40-72%)	44% (28-60%)	0.3 (0.16-0.49)	2.2% (1.5-3.1%)	1.9% (0.99-3.1%)	7.4 (3.8-12)	1300 (810-1800)
Tajikistan	2016	Male	20 to 24	73% (56-85%)	27% (15-44%)	0.67 (0.42-0.99)	5.1% (3.5-6.8%)	4.4% (2.2-6.6%)	26 (13-42)	3500 (2300-5000)
Tajikistan	2016	Male	25 to 29	80% (69-89%)	20% (11-31%)	1.1 (0.7-1.6)	6.7% (5.1-8.7%)	6.5% (4.1-9.5%)	50 (30-75)	5400 (3800-7300)
Tajikistan	2016	Male	30 to 34	85% (73-92%)	15% (8-27%)	1.3 (0.89-1.8)	7.3% (5.5-9.4%)	7.7% (5-11%)	57 (35-85)	5100 (3700-6800)
Tajikistan	2016	Male	35 to 39	86% (76-93%)	14% (7.3-24%)	1.3 (0.81-1.9)	6.7% (4.8-8.8%)	7.3% (4.4-11%)	51 (30-76)	4000 (2700-5400)

Location	Year	Sex	Age	Prevalence of current drinkers	Prevalence of abstainers	Population average of standard drinks daily	Percent of total attributable deaths	Percent of total attributable DALYs	Attributable deaths	Attributable DALYs
Tajikistan	2016	Male	40 to 44	86% (76-93%)	14% (7-24%)	1.3 (0.82-2)	5.6% (3.6-7.8%)	6.2% (3.3-9.5%)	50 (26-76)	3300 (2100-4700)
Tajikistan	2016	Male	45 to 49	85% (72-93%)	15% (7.5-28%)	1.4 (0.87-2.1)	4.7% (2.4-6.9%)	5% (1.7-8.3%)	50 (16-88)	2900 (1500-4500)
Tajikistan	2016	Male	50 to 54	84% (72-92%)	16% (8.1-28%)	1.4 (0.82-2.1)	3.5% (1.3-5.9%)	3.6% (0.6-6.9%)	51 (8-100)	2700 (1000-4500)
Tajikistan	2016	Male	55 to 59	83% (71-91%)	17% (8.6-29%)	1.2 (0.71-1.9)	2.2% (0.081-4.3%)	2.1% (-0.8-5.1%)	33 (-13-83)	1500 (54-3100)
Tajikistan	2016	Male	60 to 64	81% (67-91%)	19% (9.4-33%)	1.1 (0.58-1.7)	0.8% (-1.3-2.9%)	0.51% (-2.2-3.3%)	8.4 (-36-55)	470 (-750-1800)
Tajikistan	2016	Male	65 to 69	79% (64-90%)	21% (10-36%)	0.93 (0.54-1.4)	-0.13% (-2.3-1.9%)	-0.49% (-3.3-2.1%)	-6 (-40-25)	-46 (-810-660)
Tajikistan	2016	Male	70 to 74	76% (62-87%)	24% (13-38%)	0.8 (0.44-1.3)	-0.94% (-3.2-1%)	-1.4% (-4.1-1.1%)	-20 (-62-16)	-310 (-1000-330)
Tajikistan	2016	Male	75 to 79	73% (57-84%)	27% (16-43%)	0.68 (0.35-1.1)	-1.5% (-3.6-0.35%)	-2% (-4.6-0.33%)	-35 (-85-5.7)	-460 (-1200-120)
Tajikistan	2016	Male	80 to 84	70% (53-82%)	30% (18-47%)	0.61 (0.32-0.98)	-2% (-4.2--0.013%)	-2.5% (-5.3--0.083%)	-43 (-93--1.5)	-420 (-920--3.1)
Tajikistan	2016	Male	85 to 89	68% (52-80%)	32% (20-48%)	0.56 (0.31-0.89)	-2.2% (-4.4--0.27%)	-2.8% (-5.5--0.47%)	-34 (-67--5.7)	-230 (-470--28)
Tajikistan	2016	Male	90 to 94	67% (52-80%)	33% (20-48%)	0.52 (0.28-0.85)	-2.2% (-4.4--0.36%)	-2.8% (-5.5--0.56%)	-17 (-33--3.4)	-78 (-160--13)
Tajikistan	2016	Male	95 plus	66% (47-82%)	34% (18-53%)	0.47 (0.21-0.86)	-1.9% (-4.1--0.24%)	-2.6% (-5.2--0.5%)	-2.2 (-4.5--0.41)	-6.4 (-14--0.75)
Turkmenistan	1990	Female	All Ages	51% (44-59%)	49% (41-56%)	0.69 (0.48-0.95)	1% (0.24-2%)	1% (-1.1-4.1%)	150 (-160-580)	8500 (2000-17000)
Turkmenistan	1990	Female	15 to 19	53% (39-68%)	47% (32-61%)	0.68 (0.27-1.3)	1.8% (0.93-3.1%)	2.9% (1.2-5.7%)	4.4 (1.7-8.4)	450 (220-780)
Turkmenistan	1990	Female	20 to 24	61% (48-74%)	39% (26-52%)	0.99 (0.4-1.8)	2.9% (1.5-4.8%)	3.8% (1.3-7.4%)	7.6 (2.6-15)	770 (390-1300)
Turkmenistan	1990	Female	25 to 29	64% (49-77%)	36% (23-51%)	1.1 (0.51-2.1)	3.4% (2-5.5%)	4.5% (2.1-8.1%)	11 (5.2-20)	1000 (590-1600)
Turkmenistan	1990	Female	30 to 34	65% (51-78%)	35% (22-49%)	1.2 (0.62-2.2)	3.7% (2.3-5.6%)	5.3% (2.7-8.9%)	13 (6.7-23)	1000 (600-1600)
Turkmenistan	1990	Female	35 to 39	65% (51-79%)	35% (21-49%)	1.3 (0.6-2.4)	3.8% (2.2-6.1%)	5.7% (2.9-10%)	13 (6.6-24)	860 (500-1400)
Turkmenistan	1990	Female	40 to 44	65% (51-78%)	35% (22-49%)	1.3 (0.59-2.4)	3.8% (1.9-6.8%)	5.9% (2.5-11%)	13 (5.3-24)	680 (330-1200)
Turkmenistan	1990	Female	45 to 49	65% (50-78%)	35% (22-50%)	1.4 (0.61-2.8)	3.7% (1.1-8.3%)	5.4% (1.2-13%)	12 (2.7-28)	540 (150-1200)
Turkmenistan	1990	Female	50 to 54	64% (49-77%)	36% (23-51%)	1.4 (0.62-2.8)	4% (0.73-9.8%)	5.7% (0.94-14%)	27 (4.4-65)	1000 (200-2500)
Turkmenistan	1990	Female	55 to 59	62% (47-75%)	38% (25-53%)	1.4 (0.6-2.9)	3.7% (-0.033-10%)	4.9% (-0.22-14%)	29 (-1.3-80)	950 (-8.4-2600)

Location	Year	Sex	Age	Prevalence of current drinkers	Prevalence of abstainers	Population average of standard drinks daily	Percent of total attributable deaths	Percent of total attributable DALYs	Attributable deaths	Attributable DALYs
Turkmenistan	1990	Female	60 to 64	60% (45-75%)	40% (25-55%)	1.4 (0.55-3)	2.7% (-1.3-9.9%)	3.4% (-1.6-13%)	31 (-15-120)	870 (-400-3200)
Turkmenistan	1990	Female	65 to 69	58% (45-73%)	42% (27-55%)	1.3 (0.55-2.6)	1.6% (-2.2-7.8%)	2% (-2.9-9.8%)	20 (-27-95)	450 (-620-2200)
Turkmenistan	1990	Female	70 to 74	55% (42-69%)	45% (31-58%)	1.2 (0.43-2.3)	0.67% (-2.9-6.2%)	0.81% (-3.7-7.8%)	8 (-37-77)	150 (-660-1400)
Turkmenistan	1990	Female	75 to 79	52% (40-66%)	48% (34-60%)	1.1 (0.39-2.2)	-0.25% (-3.7-5.2%)	-0.38% (-4.6-6.2%)	-4.5 (-55-74)	-52 (-760-1000)
Turkmenistan	1990	Female	80 to 84	50% (37-64%)	50% (36-63%)	1.1 (0.42-2.3)	-1.1% (-4.6-4.2%)	-1.5% (-5.8-4.7%)	-15 (-59-49)	-140 (-590-540)
Turkmenistan	1990	Female	85 to 89	49% (37-62%)	51% (38-63%)	1.2 (0.45-2.4)	-1.4% (-4.9-4.2%)	-1.8% (-6.1-4.8%)	-13 (-46-37)	-91 (-320-280)
Turkmenistan	1990	Female	90 to 94	50% (39-63%)	50% (37-61%)	1.2 (0.41-2.9)	-1.4% (-5-5.3%)	-1.9% (-6.3-6.3%)	-6 (-20-20)	-27 (-98-110)
Turkmenistan	1990	Female	95 plus	51% (38-66%)	49% (34-62%)	1.3 (0.34-3.2)	-1.2% (-5.1-6.6%)	-1.6% (-6.3-7.6%)	-1.5 (-5.8-7)	-4.3 (-19-24)
Turkmenistan	1990	Male	All Ages	61% (53-70%)	39% (30-47%)	1.1 (0.77-1.4)	2.5% (1.5-3.6%)	2.7% (0.74-4.7%)	430 (120-770)	25000 (15000-36000)
Turkmenistan	1990	Male	15 to 19	56% (40-71%)	44% (29-60%)	0.57 (0.29-0.98)	3.3% (2.1-4.9%)	3.7% (1.9-6.2%)	8.9 (4.4-15)	970 (610-1400)
Turkmenistan	1990	Male	20 to 24	73% (57-85%)	27% (15-43%)	1.3 (0.76-2)	7.8% (5.4-10%)	8.4% (5-12%)	27 (16-40)	2600 (1800-3500)
Turkmenistan	1990	Male	25 to 29	80% (68-89%)	20% (11-32%)	2 (1.3-3.1)	10% (7-13%)	11% (7.2-16%)	43 (28-61)	3500 (2400-4700)
Turkmenistan	1990	Male	30 to 34	84% (73-92%)	16% (7.9-27%)	2.5 (1.6-3.6)	9.8% (6.9-13%)	11% (7.4-16%)	47 (30-66)	3400 (2400-4600)
Turkmenistan	1990	Male	35 to 39	86% (76-93%)	14% (7.4-24%)	2.5 (1.4-3.8)	9.4% (6.2-13%)	11% (6.4-16%)	46 (27-70)	2900 (1900-4200)
Turkmenistan	1990	Male	40 to 44	86% (76-93%)	14% (7.1-24%)	2.5 (1.5-3.9)	8.9% (5.3-13%)	10% (5.2-16%)	41 (21-65)	2200 (1300-3400)
Turkmenistan	1990	Male	45 to 49	85% (73-93%)	15% (7.4-27%)	2.7 (1.5-4.2)	8.6% (4.2-14%)	9.5% (4.1-16%)	42 (17-71)	2000 (980-3200)
Turkmenistan	1990	Male	50 to 54	84% (73-92%)	16% (8.1-27%)	2.6 (1.5-4.2)	7.7% (3-13%)	8.5% (2.7-15%)	73 (23-130)	3000 (1200-5100)
Turkmenistan	1990	Male	55 to 59	83% (70-91%)	17% (8.8-30%)	2.3 (1.4-3.6)	6% (1.8-11%)	6.6% (1.5-13%)	64 (14-130)	2200 (640-4300)
Turkmenistan	1990	Male	60 to 64	81% (67-91%)	19% (9.4-33%)	2 (1.1-3.3)	4.2% (0.055-9.4%)	4.5% (-0.3-11%)	58 (-3.9-140)	1700 (22-3900)
Turkmenistan	1990	Male	65 to 69	79% (64-89%)	21% (11-36%)	1.8 (1-2.8)	2.5% (-1.2-6.6%)	2.6% (-1.7-7.3%)	24 (-16-68)	600 (-300-1600)
Turkmenistan	1990	Male	70 to 74	76% (62-86%)	24% (14-38%)	1.5 (0.83-2.4)	0.86% (-2.4-4.6%)	0.78% (-3-5.1%)	5.8 (-23-38)	130 (-370-710)
Turkmenistan	1990	Male	75 to 79	73% (57-85%)	27% (15-43%)	1.3 (0.65-2.2)	-0.74% (-3.7-2.4%)	-1.1% (-4.5-2.7%)	-9.2 (-39-23)	-100 (-520-350)

Location	Year	Sex	Age	Prevalence of current drinkers	Prevalence of abstainers	Population average of standard drinks daily	Percent of total attributable deaths	Percent of total attributable DALYs	Attributable deaths	Attributable DALYs
Turkmenistan	1990	Male	80 to 84	70%<br>(54-83%)	30%<br>(17-46%)	1.2<br>(0.64-1.9)	-1.8%<br>(-4.6-0.93%)	-2.4%<br>(-5.6-0.91%)	-15<br>(-36-5.7)	-140<br>(-350-74)
Turkmenistan	1990	Male	85 to 89	68%<br>(53-81%)	32%<br>(19-47%)	1.1<br>(0.57-1.7)	-2.4%<br>(-5.1-0.0088%)	-3.2%<br>(-6.3--0.2%)	-13<br>(-26--0.82)	-88<br>(-180-0.31)
Turkmenistan	1990	Male	90 to 94	67%<br>(52-81%)	33%<br>(19-48%)	0.99<br>(0.51-1.7)	-2.7%<br>(-5.2--0.26%)	-3.5%<br>(-6.6--0.51%)	-5.8<br>(-11--0.85)	-26<br>(-51--2.6)
Turkmenistan	1990	Male	95 plus	66%<br>(46-82%)	34%<br>(18-54%)	0.9<br>(0.39-1.7)	-2.6%<br>(-5.2--0.3%)	-3.5%<br>(-6.7--0.62%)	-1.4<br>(-2.7--0.25)	-4.1<br>(-8.3--0.48)
Turkmenistan	1995	Female	All Ages	51%<br>(45-59%)	49%<br>(41-55%)	0.84<br>(0.6-1.1)	1.4%<br>(0.36-2.8%)	1.7%<br>(-1.1-5.5%)	260<br>(-170-850)	12000<br>(3200-24000)
Turkmenistan	1995	Female	15 to 19	52%<br>(40-67%)	48%<br>(33-60%)	0.81<br>(0.34-1.5)	2.1%<br>(1-3.6%)	3.3%<br>(1.2-6.4%)	6.2<br>(2.3-12)	600<br>(290-1100)
Turkmenistan	1995	Female	20 to 24	61%<br>(48-73%)	39%<br>(27-52%)	1.2<br>(0.51-2.2)	3.4%<br>(1.6-5.8%)	4.6%<br>(1.6-8.8%)	11<br>(3.8-22)	1100<br>(490-1900)
Turkmenistan	1995	Female	25 to 29	64%<br>(50-76%)	36%<br>(24-50%)	1.4<br>(0.62-2.5)	3.9%<br>(2.1-6.2%)	5.2%<br>(2.3-9.4%)	14<br>(6.2-26)	1200<br>(650-2000)
Turkmenistan	1995	Female	30 to 34	65%<br>(50-79%)	35%<br>(21-50%)	1.5<br>(0.75-2.6)	4.1%<br>(2.4-6.7%)	5.9%<br>(2.8-10%)	19<br>(9.1-33)	1400<br>(790-2300)
Turkmenistan	1995	Female	35 to 39	65%<br>(52-80%)	35%<br>(20-48%)	1.5<br>(0.73-2.7)	4.2%<br>(2.3-7%)	6.5%<br>(3-11%)	21<br>(9.9-37)	1300<br>(710-2200)
Turkmenistan	1995	Female	40 to 44	65%<br>(51-79%)	35%<br>(21-49%)	1.6<br>(0.71-2.8)	4.4%<br>(2-7.8%)	6.9%<br>(2.8-13%)	23<br>(9.1-42)	1200<br>(530-2100)
Turkmenistan	1995	Female	45 to 49	65%<br>(49-79%)	35%<br>(21-51%)	1.7<br>(0.72-3.2)	4.2%<br>(0.97-9.4%)	6.3%<br>(1.1-14%)	20<br>(3.7-46)	920<br>(220-2000)
Turkmenistan	1995	Female	50 to 54	63%<br>(48-77%)	37%<br>(23-52%)	1.7<br>(0.76-3.3)	4.6%<br>(0.53-12%)	6.4%<br>(0.54-16%)	23<br>(1.9-57)	870<br>(110-2100)
Turkmenistan	1995	Female	55 to 59	61%<br>(48-75%)	39%<br>(25-52%)	1.7<br>(0.7-3.5)	4.4%<br>(-0.39-12%)	5.7%<br>(-0.65-16%)	44<br>(-5-120)	1400<br>(-130-4000)
Turkmenistan	1995	Female	60 to 64	60%<br>(45-75%)	40%<br>(25-55%)	1.7<br>(0.67-3.5)	3.3%<br>(-1.5-12%)	4.2%<br>(-2.1-15%)	39<br>(-19-140)	1100<br>(-510-3800)
Turkmenistan	1995	Female	65 to 69	58%<br>(45-73%)	42%<br>(27-55%)	1.5<br>(0.69-3)	2.3%<br>(-2.5-9.5%)	2.8%<br>(-3-12%)	38<br>(-42-160)	870<br>(-930-3700)
Turkmenistan	1995	Female	70 to 74	55%<br>(43-69%)	45%<br>(31-57%)	1.4<br>(0.54-2.7)	1.2%<br>(-3.3-7.9%)	1.4%<br>(-4-9.5%)	19<br>(-55-130)	360<br>(-980-2400)
Turkmenistan	1995	Female	75 to 79	52%<br>(40-66%)	48%<br>(34-60%)	1.3<br>(0.48-2.6)	0.31%<br>(-3.7-6.8%)	0.29%<br>(-4.5-8%)	3.5<br>(-55-100)	64<br>(-760-1400)
Turkmenistan	1995	Female	80 to 84	50%<br>(37-65%)	50%<br>(35-63%)	1.3<br>(0.54-2.6)	-0.44%<br>(-4.5-5.5%)	-0.68%<br>(-5.6-6.4%)	-7.5<br>(-62-72)	-61<br>(-620-760)
Turkmenistan	1995	Female	85 to 89	50%<br>(38-62%)	50%<br>(38-62%)	1.4<br>(0.53-2.9)	-0.64%<br>(-4.9-6.1%)	-0.9%<br>(-6-7.2%)	-8.4<br>(-55-66)	-51<br>(-390-500)
Turkmenistan	1995	Female	90 to 94	51%<br>(39-63%)	49%<br>(37-61%)	1.5<br>(0.5-3.4)	-0.61%<br>(-4.9-7.5%)	-0.91%<br>(-6.1-8.8%)	-3.6<br>(-24-36)	-14<br>(-120-190)
Turkmenistan	1995	Female	95 plus	51%<br>(38-67%)	49%<br>(33-62%)	1.6<br>(0.4-3.9)	-0.37%<br>(-5.2-8.6%)	-0.64%<br>(-6.5-10%)	-0.81<br>(-8.2-13)	-1.8<br>(-26-43)

Location	Year	Sex	Age	Prevalence of current drinkers	Prevalence of abstainers	Population average of standard drinks daily	Percent of total attributable deaths	Percent of total attributable DALYs	Attributable deaths	Attributable DALYs
Turkmenistan	1995	Male	All Ages	62% (54-71%)	38% (29-46%)	1.3 (0.99-1.7)	3.4% (2.1-4.9%)	3.7% (1.2-6.6%)	680 (230-1200)	37000 (22000-52000)
Turkmenistan	1995	Male	15 to 19	56% (40-72%)	44% (28-60%)	0.69 (0.36-1.2)	3.6% (2.2-5.3%)	4% (2-6.7%)	13 (6.3-21)	1300 (800-1900)
Turkmenistan	1995	Male	20 to 24	73% (57-85%)	27% (15-43%)	1.6 (0.95-2.3)	8.8% (6.1-12%)	9.6% (5.8-14%)	40 (24-57)	3600 (2400-4800)
Turkmenistan	1995	Male	25 to 29	80% (68-89%)	20% (11-32%)	2.5 (1.5-3.6)	11% (8-15%)	13% (8.3-18%)	61 (39-85)	4700 (3300-6200)
Turkmenistan	1995	Male	30 to 34	84% (72-92%)	16% (7.9-28%)	3 (2-4.2)	11% (8-15%)	13% (8.5-18%)	71 (47-98)	4900 (3500-6600)
Turkmenistan	1995	Male	35 to 39	86% (75-93%)	14% (7.3-25%)	3 (1.9-4.4)	11% (7.2-16%)	13% (7.8-19%)	83 (50-120)	5000 (3300-7000)
Turkmenistan	1995	Male	40 to 44	86% (76-93%)	14% (7.1-24%)	3.1 (1.8-4.6)	11% (6.1-16%)	12% (6.2-19%)	82 (43-130)	4300 (2600-6600)
Turkmenistan	1995	Male	45 to 49	86% (74-93%)	14% (7.4-26%)	3.3 (2-5.1)	10% (5.2-17%)	12% (5.3-20%)	77 (35-130)	3600 (1800-5800)
Turkmenistan	1995	Male	50 to 54	85% (73-92%)	15% (7.9-27%)	3.2 (1.8-5)	9.1% (3.5-16%)	10% (3.3-18%)	68 (23-120)	2700 (1000-4800)
Turkmenistan	1995	Male	55 to 59	83% (71-91%)	17% (8.8-29%)	2.8 (1.6-4.3)	6.8% (1.8-13%)	7.4% (1.5-15%)	93 (19-190)	3200 (850-6300)
Turkmenistan	1995	Male	60 to 64	81% (67-90%)	19% (9.5-33%)	2.4 (1.3-3.9)	4.8% (-0.13-11%)	5.2% (-0.58-13%)	71 (-7.8-170)	2100 (-58-4800)
Turkmenistan	1995	Male	65 to 69	79% (65-89%)	21% (11-35%)	2.2 (1.2-3.3)	2.9% (-1.4-8.3%)	3.1% (-1.9-9.2%)	52 (-30-160)	1300 (-570-3600)
Turkmenistan	1995	Male	70 to 74	76% (62-86%)	24% (14-38%)	1.8 (1-3)	1.2% (-2.8-6.2%)	1.1% (-3.4-6.8%)	12 (-37-74)	260 (-620-1400)
Turkmenistan	1995	Male	75 to 79	73% (58-85%)	27% (15-42%)	1.6 (0.84-2.6)	-0.5% (-4.1-3.5%)	-0.82% (-5-3.8%)	-6.1 (-37-28)	-59 (-480-410)
Turkmenistan	1995	Male	80 to 84	71% (54-83%)	29% (17-46%)	1.4 (0.79-2.2)	-1.7% (-5-1.7%)	-2.3% (-6.3-1.7%)	-14 (-39-11)	-130 (-370-130)
Turkmenistan	1995	Male	85 to 89	68% (52-80%)	32% (20-48%)	1.3 (0.71-2.1)	-2.4% (-5.5-0.56%)	-3.1% (-6.7-0.36%)	-15 (-32-1.7)	-97 (-220-22)
Turkmenistan	1995	Male	90 to 94	67% (53-80%)	33% (20-47%)	1.2 (0.63-2)	-2.7% (-5.5-0.042%)	-3.5% (-6.9--0.15%)	-6.7 (-13--0.29)	-30 (-62-0.48)
Turkmenistan	1995	Male	95 plus	66% (47-82%)	34% (18-53%)	1.1 (0.47-2)	-2.7% (-5.5--0.016%)	-3.5% (-7--0.38%)	-1.9 (-3.7--0.2)	-5.6 (-12--0.032)
Turkmenistan	2000	Female	All Ages	52% (46-60%)	48% (40-54%)	1 (0.75-1.4)	2.2% (0.82-4%)	2.8% (-0.57-7.7%)	430 (-82-1200)	18000 (6700-33000)
Turkmenistan	2000	Female	15 to 19	52% (40-67%)	48% (33-60%)	0.97 (0.41-1.8)	2.5% (1.1-4.5%)	4.1% (1.5-7.8%)	9.1 (3.3-18)	830 (380-1500)
Turkmenistan	2000	Female	20 to 24	61% (49-73%)	39% (27-51%)	1.4 (0.61-2.6)	4.2% (2-7.2%)	6% (2.2-11%)	17 (6.2-31)	1500 (680-2600)
Turkmenistan	2000	Female	25 to 29	64% (49-76%)	36% (24-51%)	1.6 (0.72-3)	4.9% (2.6-8.2%)	7% (3.1-13%)	21 (9.3-38)	1700 (860-2800)

Location	Year	Sex	Age	Prevalence of current drinkers	Prevalence of abstainers	Population average of standard drinks daily	Percent of total attributable deaths	Percent of total attributable DALYs	Attributable deaths	Attributable DALYs
Turkmenistan	2000	Female	30 to 34	65% (50-78%)	35% (22-50%)	1.8 (0.89-3.1)	5.1% (2.9-8.2%)	7.7% (3.8-13%)	25 (12-42)	1800 (940-2800)
Turkmenistan	2000	Female	35 to 39	65% (52-79%)	35% (21-48%)	1.8 (0.85-3.2)	5% (2.6-8.2%)	8% (3.7-14%)	30 (14-52)	1800 (940-3000)
Turkmenistan	2000	Female	40 to 44	65% (51-79%)	35% (21-49%)	1.9 (0.87-3.4)	5.3% (2.4-9.2%)	8.5% (3.6-15%)	36 (15-62)	1900 (850-3200)
Turkmenistan	2000	Female	45 to 49	65% (49-79%)	35% (21-51%)	2 (0.87-3.8)	5.3% (1.5-11%)	7.9% (1.9-17%)	37 (9-77)	1700 (490-3400)
Turkmenistan	2000	Female	50 to 54	64% (49-77%)	36% (23-51%)	2 (0.87-3.9)	5.8% (0.95-13%)	8.1% (1.2-18%)	39 (6.2-86)	1500 (250-3300)
Turkmenistan	2000	Female	55 to 59	62% (48-76%)	38% (24-52%)	2.1 (0.85-4.2)	5.7% (-0.039-14%)	7.6% (-0.16-19%)	39 (-0.8-96)	1300 (-8.5-3100)
Turkmenistan	2000	Female	60 to 64	60% (46-75%)	40% (25-54%)	2 (0.8-4.2)	4.6% (-1.5-14%)	5.7% (-2-17%)	62 (-21-190)	1700 (-530-5100)
Turkmenistan	2000	Female	65 to 69	58% (45-73%)	42% (27-55%)	1.9 (0.81-3.7)	3.4% (-2.2-12%)	4.2% (-2.7-15%)	51 (-34-180)	1200 (-750-4100)
Turkmenistan	2000	Female	70 to 74	55% (43-68%)	45% (32-57%)	1.6 (0.65-3.3)	2.1% (-3.2-10%)	2.4% (-4-12%)	42 (-68-210)	790 (-1200-3900)
Turkmenistan	2000	Female	75 to 79	52% (40-65%)	48% (35-60%)	1.5 (0.56-3.1)	1.1% (-3.7-8.8%)	1.2% (-4.7-10%)	18 (-69-150)	280 (-950-2100)
Turkmenistan	2000	Female	80 to 84	50% (37-65%)	50% (35-63%)	1.6 (0.62-3.2)	0.4% (-4.2-7.8%)	0.3% (-5.3-9.3%)	3.2 (-57-100)	55 (-550-1100)
Turkmenistan	2000	Female	85 to 89	50% (37-62%)	50% (38-63%)	1.7 (0.62-3.5)	0.3% (-4.5-8.6%)	0.17% (-5.7-10%)	1.4 (-47-85)	22 (-330-640)
Turkmenistan	2000	Female	90 to 94	50% (39-62%)	50% (38-61%)	1.8 (0.59-4.1)	0.35% (-4.8-9.7%)	0.21% (-6-12%)	0.89 (-26-49)	9.1 (-120-250)
Turkmenistan	2000	Female	95 plus	51% (37-66%)	49% (34-63%)	1.9 (0.45-4.8)	0.56% (-5-10%)	0.44% (-6.3-12%)	0.58 (-8.2-16)	3 (-26-54)
Turkmenistan	2000	Male	All Ages	64% (55-72%)	36% (28-45%)	1.7 (1.3-2.1)	5.1% (3.2-7.3%)	5.6% (2.5-9.4%)	1100 (470-1800)	55000 (35000-76000)
Turkmenistan	2000	Male	15 to 19	56% (40-72%)	44% (28-60%)	0.83 (0.43-1.4)	4% (2.3-6.1%)	4.5% (2.2-7.8%)	17 (8.4-30)	1700 (990-2600)
Turkmenistan	2000	Male	20 to 24	73% (57-85%)	27% (15-43%)	1.8 (1.1-2.8)	10% (6.8-14%)	11% (6.9-16%)	58 (35-85)	4900 (3300-6700)
Turkmenistan	2000	Male	25 to 29	80% (69-89%)	20% (11-31%)	2.9 (1.8-4.4)	14% (9.5-18%)	16% (10-23%)	98 (62-140)	7100 (4800-9600)
Turkmenistan	2000	Male	30 to 34	84% (72-92%)	16% (7.7-28%)	3.5 (2.3-5.1)	14% (9.7-19%)	16% (11-23%)	110 (73-160)	7300 (5100-10000)
Turkmenistan	2000	Male	35 to 39	86% (75-93%)	14% (7.2-25%)	3.5 (2.2-5.3)	13% (9.1-19%)	16% (10-23%)	130 (81-180)	7400 (5000-10000)
Turkmenistan	2000	Male	40 to 44	86% (76-93%)	14% (7.1-24%)	3.6 (2.2-5.4)	13% (7.9-19%)	15% (8.6-23%)	140 (78-220)	7300 (4300-11000)
Turkmenistan	2000	Male	45 to 49	86% (73-93%)	14% (7.3-27%)	3.9 (2.4-6)	13% (7-20%)	15% (7.5-23%)	140 (71-220)	6400 (3600-9800)

Location	Year	Sex	Age	Prevalence of current drinkers	Prevalence of abstainers	Population average of standard drinks daily	Percent of total attributable deaths	Percent of total attributable DALYs	Attributable deaths	Attributable DALYs
Turkmenistan	2000	Male	50 to 54	85% (73-92%)	15% (8-27%)	3.8 (2.2-5.9)	11% (4.6-19%)	13% (4.5-22%)	110 (42-200)	4500 (1800-7600)
Turkmenistan	2000	Male	55 to 59	83% (71-92%)	17% (8.3-29%)	3.3 (1.9-5.2)	8.5% (2.6-16%)	9.4% (2.4-18%)	81 (20-160)	2800 (820-5300)
Turkmenistan	2000	Male	60 to 64	81% (68-91%)	19% (9.3-32%)	2.9 (1.6-4.8)	6.3% (0.49-14%)	6.8% (0.12-16%)	110 (2-260)	3100 (240-7100)
Turkmenistan	2000	Male	65 to 69	79% (65-90%)	21% (10-35%)	2.6 (1.5-4)	4.1% (-1.1-11%)	4.4% (-1.6-12%)	69 (-25-190)	1600 (-440-4300)
Turkmenistan	2000	Male	70 to 74	76% (61-86%)	24% (14-39%)	2.2 (1.2-3.6)	1.8% (-2.9-7.8%)	1.9% (-3.5-8.7%)	33 (-61-160)	670 (-1000-2900)
Turkmenistan	2000	Male	75 to 79	73% (57-85%)	27% (15-43%)	1.9 (1-3.2)	0.21% (-4.1-5.2%)	-0.038% (-4.9-5.8%)	-0.34 (-47-56)	32 (-610-830)
Turkmenistan	2000	Male	80 to 84	71% (54-84%)	29% (16-46%)	1.7 (0.94-2.7)	-1.2% (-4.9-3.2%)	-1.8% (-6.2-3.5%)	-9.2 (-33-18)	-75 (-320-210)
Turkmenistan	2000	Male	85 to 89	69% (53-81%)	31% (19-47%)	1.6 (0.85-2.5)	-2% (-5.5-1.5%)	-2.7% (-6.7-1.5%)	-11 (-26-5.8)	-67 (-180-52)
Turkmenistan	2000	Male	90 to 94	68% (53-80%)	32% (20-47%)	1.4 (0.75-2.5)	-2.5% (-5.6-0.75%)	-3.3% (-7.1-0.54%)	-6.2 (-13-1)	-27 (-61-8.3)
Turkmenistan	2000	Male	95 plus	67% (48-82%)	33% (18-52%)	1.3 (0.58-2.4)	-2.5% (-5.6-0.57%)	-3.4% (-7.1-0.22%)	-1.8 (-3.7-0.11)	-5.1 (-11-1.2)
Turkmenistan	2005	Female	All Ages	53% (47-61%)	47% (39-53%)	1.2 (0.83-1.6)	2.7% (1.1-4.6%)	3.5% (-0.14-8.8%)	520 (-20-1300)	21000 (8800-36000)
Turkmenistan	2005	Female	15 to 19	52% (40-67%)	48% (33-60%)	1 (0.42-1.9)	2.6% (1.2-4.6%)	4.2% (1.5-8%)	10 (3.7-20)	950 (420-1700)
Turkmenistan	2005	Female	20 to 24	61% (48-73%)	39% (27-52%)	1.5 (0.61-2.6)	4.4% (2.1-7.7%)	6.5% (2.3-12%)	19 (6.7-36)	1700 (770-2900)
Turkmenistan	2005	Female	25 to 29	64% (49-77%)	36% (23-51%)	1.7 (0.76-3.2)	5.3% (2.9-8.7%)	7.8% (3.6-14%)	24 (11-43)	1900 (1000-3100)
Turkmenistan	2005	Female	30 to 34	65% (50-78%)	35% (22-50%)	1.8 (0.92-3.4)	5.5% (3.3-8.8%)	8.5% (4.4-15%)	28 (15-48)	2000 (1100-3100)
Turkmenistan	2005	Female	35 to 39	65% (52-80%)	35% (20-48%)	1.9 (0.89-3.3)	5.5% (2.9-8.9%)	9% (4.4-15%)	30 (15-51)	1900 (1000-3000)
Turkmenistan	2005	Female	40 to 44	65% (51-79%)	35% (21-49%)	2 (0.89-3.6)	5.8% (2.7-9.6%)	9.5% (4.3-16%)	43 (20-71)	2200 (1100-3600)
Turkmenistan	2005	Female	45 to 49	65% (49-78%)	35% (22-51%)	2.1 (0.92-4)	5.7% (1.9-12%)	8.9% (2.5-18%)	51 (15-100)	2300 (750-4500)
Turkmenistan	2005	Female	50 to 54	63% (48-77%)	37% (23-52%)	2.1 (0.93-4.1)	6.1% (1.4-13%)	8.9% (2.1-18%)	57 (13-120)	2200 (520-4500)
Turkmenistan	2005	Female	55 to 59	61% (48-75%)	39% (25-52%)	2.2 (0.9-4.3)	6.2% (0.45-14%)	8.4% (0.52-20%)	55 (3.5-130)	1800 (130-4300)
Turkmenistan	2005	Female	60 to 64	60% (45-74%)	40% (26-55%)	2.1 (0.82-4.3)	5.2% (-1.2-15%)	6.5% (-1.6-18%)	45 (-11-120)	1200 (-270-3400)
Turkmenistan	2005	Female	65 to 69	58% (46-73%)	42% (27-54%)	1.9 (0.87-3.8)	4% (-1.9-13%)	5% (-2.3-16%)	69 (-32-220)	1600 (-710-5100)

Location	Year	Sex	Age	Prevalence of current drinkers	Prevalence of abstainers	Population average of standard drinks daily	Percent of total attributable deaths	Percent of total attributable DALYs	Attributable deaths	Attributable DALYs
Turkmenistan	2005	Female	70 to 74	55% (43-69%)	45% (31-57%)	1.7 (0.69-3.4)	2.6% (-3.1-11%)	3.1% (-3.8-14%)	46 (-57-200)	860 (-1000-3700)
Turkmenistan	2005	Female	75 to 79	52% (40-65%)	48% (35-60%)	1.6 (0.62-3.2)	1.5% (-3.8-9.4%)	1.7% (-4.8-11%)	29 (-85-200)	440 (-1200-2800)
Turkmenistan	2005	Female	80 to 84	50% (37-64%)	50% (36-63%)	1.7 (0.66-3.3)	0.78% (-3.8-8.3%)	0.78% (-5-10%)	9.5 (-60-120)	120 (-590-1300)
Turkmenistan	2005	Female	85 to 89	49% (38-61%)	51% (39-62%)	1.7 (0.66-3.7)	0.66% (-4.3-8.8%)	0.6% (-5.5-11%)	4.7 (-43-85)	46 (-300-630)
Turkmenistan	2005	Female	90 to 94	50% (39-62%)	50% (38-61%)	1.9 (0.63-4.2)	0.65% (-4.6-10%)	0.57% (-5.9-12%)	2.1 (-21-46)	15 (-100-230)
Turkmenistan	2005	Female	95 plus	51% (38-66%)	49% (34-62%)	1.9 (0.49-4.9)	0.8% (-4.9-11%)	0.72% (-6.2-13%)	0.96 (-8.1-17)	4.3 (-26-56)
Turkmenistan	2005	Male	All Ages	65% (57-74%)	35% (26-43%)	1.9 (1.4-2.4)	6.1% (4-8.5%)	6.7% (3.2-11%)	1300 (630-2100)	64000 (42000-88000)
Turkmenistan	2005	Male	15 to 19	56% (40-72%)	44% (28-60%)	0.87 (0.46-1.5)	4.1% (2.5-6.2%)	4.8% (2.5-7.9%)	20 (10-33)	2000 (1200-3000)
Turkmenistan	2005	Male	20 to 24	73% (58-85%)	27% (15-42%)	1.9 (1.2-2.9)	10% (7-14%)	12% (7.2-17%)	61 (37-88)	5200 (3600-7100)
Turkmenistan	2005	Male	25 to 29	80% (69-89%)	20% (11-31%)	3.1 (1.9-4.6)	14% (9.9-19%)	16% (11-23%)	110 (68-150)	7700 (5300-10000)
Turkmenistan	2005	Male	30 to 34	85% (73-92%)	15% (7.8-27%)	3.7 (2.4-5.3)	14% (10-19%)	17% (11-23%)	130 (87-180)	8200 (5900-11000)
Turkmenistan	2005	Male	35 to 39	86% (76-93%)	14% (7.3-24%)	3.7 (2.3-5.4)	14% (10-20%)	17% (11-24%)	140 (92-200)	8100 (5600-11000)
Turkmenistan	2005	Male	40 to 44	86% (76-93%)	14% (6.9-24%)	3.8 (2.3-5.5)	14% (8.8-20%)	16% (9.7-24%)	160 (96-240)	8300 (5300-12000)
Turkmenistan	2005	Male	45 to 49	86% (73-93%)	14% (7.4-27%)	4.1 (2.5-6.1)	14% (7.9-21%)	16% (8.5-25%)	190 (100-290)	8500 (4800-13000)
Turkmenistan	2005	Male	50 to 54	85% (73-92%)	15% (7.7-27%)	4 (2.3-6)	13% (5.8-20%)	14% (5.9-23%)	170 (70-280)	6600 (3000-11000)
Turkmenistan	2005	Male	55 to 59	83% (70-91%)	17% (8.5-30%)	3.5 (2.1-5.3)	9.7% (3.6-18%)	11% (3.5-21%)	120 (38-220)	4000 (1500-7300)
Turkmenistan	2005	Male	60 to 64	81% (67-90%)	19% (9.5-33%)	3 (1.6-5)	7.4% (1.3-16%)	8.2% (1.1-18%)	86 (11-190)	2400 (430-5200)
Turkmenistan	2005	Male	65 to 69	79% (64-89%)	21% (11-36%)	2.7 (1.6-4.2)	4.9% (-0.71-12%)	5.3% (-1.2-14%)	93 (-20-240)	2200 (-320-5600)
Turkmenistan	2005	Male	70 to 74	76% (62-86%)	24% (14-38%)	2.3 (1.3-3.8)	2.5% (-2.6-9.1%)	2.6% (-3.2-10%)	42 (-53-170)	840 (-870-3100)
Turkmenistan	2005	Male	75 to 79	73% (58-84%)	27% (16-42%)	2 (1.1-3.3)	0.69% (-3.6-6.5%)	0.52% (-4.5-7.4%)	7.9 (-69-110)	170 (-890-1600)
Turkmenistan	2005	Male	80 to 84	71% (55-83%)	29% (17-45%)	1.8 (0.98-2.8)	-0.77% (-4.7-3.6%)	-1.3% (-6.1-3.9%)	-9.4 (-44-28)	-68 (-420-320)
Turkmenistan	2005	Male	85 to 89	68% (53-80%)	32% (20-47%)	1.6 (0.91-2.6)	-1.7% (-5.1-2.3%)	-2.4% (-6.5-2.4%)	-7.1 (-19-7.1)	-43 (-130-59)

Location	Year	Sex	Age	Prevalence of current drinkers	Prevalence of abstainers	Population average of standard drinks daily	Percent of total attributable deaths	Percent of total attributable DALYs	Attributable deaths	Attributable DALYs
Turkmenistan	2005	Male	90 to 94	67% (52-80%)	33% (20-48%)	1.5 (0.79-2.6)	-2.3% (-5.5-1.2%)	-3.1% (-6.9-1%)	-4.1 (-9.1-1.4)	-18 (-42-9)
Turkmenistan	2005	Male	95 plus	66% (47-82%)	34% (18-53%)	1.4 (0.61-2.6)	-2.3% (-5.5-1.2%)	-3.2% (-7.2-0.88%)	-1.4 (-3.1-0.37)	-3.9 (-9.3-1.9)
Turkmenistan	2010	Female	All Ages	54% (47-61%)	46% (39-53%)	1.2 (0.84-1.6)	2.8% (1.2-4.8%)	3.6% (-0.033-8.6%)	520 (-4.8-1200)	21000 (9400-36000)
Turkmenistan	2010	Female	15 to 19	52% (39-68%)	48% (32-61%)	0.96 (0.39-1.8)	2.3% (1-4.1%)	3.7% (1.2-7.5%)	8.4 (2.7-17)	810 (360-1500)
Turkmenistan	2010	Female	20 to 24	61% (47-74%)	39% (26-53%)	1.4 (0.58-2.6)	4.1% (2-7%)	6% (2.2-11%)	17 (6.1-32)	1600 (760-2800)
Turkmenistan	2010	Female	25 to 29	64% (49-77%)	36% (23-51%)	1.6 (0.76-3.1)	4.9% (2.8-7.8%)	7.6% (3.7-13%)	22 (11-38)	1800 (1000-2900)
Turkmenistan	2010	Female	30 to 34	65% (50-77%)	35% (23-50%)	1.8 (0.91-3.3)	5.3% (3.3-8.4%)	8.7% (4.9-14%)	27 (15-43)	1900 (1200-2900)
Turkmenistan	2010	Female	35 to 39	65% (52-79%)	35% (21-48%)	1.8 (0.85-3.3)	5.4% (3-8.5%)	9.2% (4.7-15%)	31 (16-49)	1900 (1100-2900)
Turkmenistan	2010	Female	40 to 44	65% (52-78%)	35% (22-48%)	1.9 (0.87-3.5)	5.6% (3-9.2%)	9.6% (4.7-16%)	39 (19-64)	2000 (1100-3300)
Turkmenistan	2010	Female	45 to 49	65% (49-79%)	35% (21-51%)	2 (0.9-3.8)	5.4% (2-10%)	8.7% (3-17%)	52 (18-100)	2400 (860-4500)
Turkmenistan	2010	Female	50 to 54	63% (49-77%)	37% (23-51%)	2.1 (0.91-3.9)	5.7% (1.4-12%)	8.5% (2-17%)	65 (15-130)	2500 (610-5100)
Turkmenistan	2010	Female	55 to 59	61% (48-75%)	39% (25-52%)	2.1 (0.88-4.1)	5.7% (0.35-13%)	7.9% (0.36-18%)	70 (3.1-160)	2300 (140-5300)
Turkmenistan	2010	Female	60 to 64	60% (46-75%)	40% (25-54%)	2 (0.8-4.1)	4.8% (-0.92-13%)	6.2% (-1.3-17%)	55 (-11-150)	1500 (-300-4200)
Turkmenistan	2010	Female	65 to 69	58% (45-73%)	42% (27-55%)	1.9 (0.85-3.6)	3.8% (-1.7-12%)	4.8% (-2.2-15%)	41 (-19-130)	950 (-410-3100)
Turkmenistan	2010	Female	70 to 74	55% (42-69%)	45% (31-58%)	1.7 (0.65-3.2)	2.6% (-2.9-10%)	3.1% (-3.6-13%)	51 (-59-210)	940 (-1100-3800)
Turkmenistan	2010	Female	75 to 79	52% (40-65%)	48% (35-60%)	1.5 (0.57-3.2)	1.4% (-3.5-8.7%)	1.6% (-4.5-11%)	24 (-67-160)	370 (-930-2200)
Turkmenistan	2010	Female	80 to 84	50% (37-64%)	50% (36-63%)	1.6 (0.62-3.1)	0.73% (-3.7-7.6%)	0.73% (-4.9-9.6%)	8.8 (-58-110)	120 (-590-1200)
Turkmenistan	2010	Female	85 to 89	49% (37-62%)	51% (38-63%)	1.7 (0.63-3.5)	0.57% (-4.1-8.3%)	0.5% (-5.3-10%)	5 (-53-100)	53 (-380-810)
Turkmenistan	2010	Female	90 to 94	50% (38-62%)	50% (38-62%)	1.8 (0.61-4)	0.51% (-4.4-9.3%)	0.41% (-5.8-11%)	1.6 (-23-45)	13 (-110-240)
Turkmenistan	2010	Female	95 plus	51% (36-66%)	49% (34-64%)	1.9 (0.48-4.7)	0.63% (-4.7-9.7%)	0.51% (-6.1-12%)	0.63 (-7.6-15)	3.3 (-24-50)
Turkmenistan	2010	Male	All Ages	66% (58-74%)	34% (26-42%)	1.9 (1.4-2.4)	6.4% (4.2-8.8%)	6.8% (3.4-11%)	1200 (620-2000)	62000 (42000-85000)
Turkmenistan	2010	Male	15 to 19	55% (40-72%)	45% (28-60%)	0.81 (0.42-1.4)	3.8% (2.5-5.5%)	4.4% (2.3-7.1%)	15 (7.6-24)	1600 (990-2300)

Location	Year	Sex	Age	Prevalence of current drinkers	Prevalence of abstainers	Population average of standard drinks daily	Percent of total attributable deaths	Percent of total attributable DALYs	Attributable deaths	Attributable DALYs
Turkmenistan	2010	Male	20 to 24	73% (57-85%)	27% (15-43%)	1.8 (1.1-2.8)	9.6% (6.8-13%)	11% (7-15%)	55 (35-79)	5100 (3500-6900)
Turkmenistan	2010	Male	25 to 29	80% (69-89%)	20% (11-31%)	3 (1.8-4.4)	13% (9.3-17%)	15% (10-21%)	88 (59-120)	6800 (4800-8800)
Turkmenistan	2010	Male	30 to 34	84% (72-92%)	16% (7.9-28%)	3.6 (2.3-5.2)	13% (9.7-18%)	16% (11-22%)	110 (73-150)	7200 (5200-9600)
Turkmenistan	2010	Male	35 to 39	86% (75-93%)	14% (7.3-25%)	3.5 (2.2-5.3)	14% (9.9-20%)	17% (11-24%)	130 (88-190)	7700 (5500-10000)
Turkmenistan	2010	Male	40 to 44	86% (76-93%)	14% (7.1-24%)	3.6 (2.2-5.4)	14% (9.1-19%)	16% (11-24%)	150 (95-210)	7700 (5200-11000)
Turkmenistan	2010	Male	45 to 49	85% (72-93%)	15% (7.5-28%)	3.9 (2.4-5.8)	13% (7.8-20%)	16% (8.7-24%)	180 (100-280)	8400 (5000-12000)
Turkmenistan	2010	Male	50 to 54	84% (73-92%)	16% (8.2-27%)	3.8 (2.2-5.7)	12% (5.9-19%)	14% (6.1-22%)	190 (87-320)	7800 (3800-12000)
Turkmenistan	2010	Male	55 to 59	83% (71-91%)	17% (8.6-29%)	3.3 (2-5.1)	9.3% (3.5-17%)	11% (3.5-20%)	150 (50-280)	5100 (1900-9200)
Turkmenistan	2010	Male	60 to 64	81% (67-91%)	19% (9.3-33%)	2.9 (1.6-4.8)	7.1% (1.4-15%)	7.9% (1.1-18%)	100 (14-220)	2900 (570-6100)
Turkmenistan	2010	Male	65 to 69	79% (65-89%)	21% (11-35%)	2.6 (1.4-4)	4.7% (-0.43-11%)	5.1% (-0.87-13%)	59 (-9.8-150)	1400 (-130-3400)
Turkmenistan	2010	Male	70 to 74	76% (62-86%)	24% (14-38%)	2.2 (1.3-3.6)	2.4% (-2.4-8.8%)	2.5% (-3.1-10%)	43 (-54-170)	870 (-860-3200)
Turkmenistan	2010	Male	75 to 79	73% (58-85%)	27% (15-42%)	1.9 (1-3.2)	0.75% (-3.4-6.2%)	0.59% (-4.3-7%)	8.1 (-60-97)	170 (-780-1400)
Turkmenistan	2010	Male	80 to 84	71% (54-83%)	29% (17-46%)	1.7 (0.94-2.7)	-0.79% (-4.5-3.3%)	-1.3% (-5.8-3.7%)	-11 (-51-32)	-86 (-490-370)
Turkmenistan	2010	Male	85 to 89	69% (53-80%)	31% (20-47%)	1.6 (0.87-2.5)	-1.7% (-5-2%)	-2.4% (-6.3-2%)	-12 (-32-10)	-74 (-220-86)
Turkmenistan	2010	Male	90 to 94	67% (52-80%)	33% (20-48%)	1.4 (0.76-2.4)	-2.2% (-5.1-1.1%)	-3% (-6.6-0.93%)	-4 (-8.7-1.2)	-17 (-40-8.5)
Turkmenistan	2010	Male	95 plus	66% (47-82%)	34% (18-53%)	1.3 (0.57-2.5)	-2.2% (-5.2-0.98%)	-3.2% (-6.8-0.73%)	-1.1 (-2.4-0.25)	-3.2 (-7.5-1.4)
Turkmenistan	2016	Female	All Ages	54% (48-62%)	46% (38-52%)	1.2 (0.8-1.7)	2.9% (1.2-5.1%)	3.6% (-0.26-8.9%)	520 (-36-1300)	21000 (8800-37000)
Turkmenistan	2016	Female	15 to 19	52% (39-67%)	48% (33-61%)	0.95 (0.36-1.9)	2.1% (0.98-3.7%)	3.6% (1.2-7.3%)	5.3 (1.8-11)	550 (260-1000)
Turkmenistan	2016	Female	20 to 24	61% (47-73%)	39% (27-53%)	1.4 (0.56-2.7)	3.7% (1.9-6.2%)	5.8% (2.1-11%)	13 (4.5-24)	1300 (680-2200)
Turkmenistan	2016	Female	25 to 29	64% (50-77%)	36% (23-50%)	1.6 (0.7-3)	4.5% (2.6-7%)	7.3% (3.6-13%)	20 (9.3-34)	1800 (1000-2800)
Turkmenistan	2016	Female	30 to 34	65% (51-78%)	35% (22-49%)	1.8 (0.85-3.2)	5% (3.2-7.6%)	8.6% (4.8-14%)	25 (14-40)	1900 (1200-2900)
Turkmenistan	2016	Female	35 to 39	65% (52-79%)	35% (21-48%)	1.8 (0.8-3.3)	5.1% (3-8%)	9.3% (5-15%)	30 (16-48)	1900 (1100-3000)

Location	Year	Sex	Age	Prevalence of current drinkers	Prevalence of abstainers	Population average of standard drinks daily	Percent of total attributable deaths	Percent of total attributable DALYs	Attributable deaths	Attributable DALYs
Turkmenistan	2016	Female	40 to 44	65% (51-79%)	35% (21-49%)	1.9 (0.79-3.5)	5.4% (2.9-8.8%)	9.6% (4.8-16%)	38 (19-64)	2000 (1100-3300)
Turkmenistan	2016	Female	45 to 49	65% (49-79%)	35% (21-51%)	2 (0.84-3.7)	5.1% (1.9-9.9%)	8.5% (3.1-16%)	45 (16-86)	2000 (800-3900)
Turkmenistan	2016	Female	50 to 54	63% (49-76%)	37% (24-51%)	2 (0.9-3.9)	5.2% (1.3-11%)	8.1% (2-17%)	65 (16-140)	2600 (610-5300)
Turkmenistan	2016	Female	55 to 59	61% (48-75%)	39% (25-52%)	2 (0.81-3.9)	5.2% (0.4-12%)	7.5% (0.54-17%)	81 (5.7-180)	2700 (210-6200)
Turkmenistan	2016	Female	60 to 64	60% (46-75%)	40% (25-54%)	2 (0.77-4)	4.3% (-1-12%)	5.8% (-1.4-16%)	72 (-18-200)	2000 (-470-5600)
Turkmenistan	2016	Female	65 to 69	58% (45-73%)	42% (27-55%)	1.8 (0.81-3.5)	3.4% (-1.7-11%)	4.4% (-2.2-15%)	52 (-27-170)	1200 (-600-4000)
Turkmenistan	2016	Female	70 to 74	55% (43-69%)	45% (31-57%)	1.6 (0.62-3.2)	2.3% (-2.7-10%)	2.9% (-3.5-13%)	29 (-36-130)	550 (-630-2400)
Turkmenistan	2016	Female	75 to 79	52% (40-65%)	48% (35-60%)	1.5 (0.56-3.2)	1.3% (-3.4-8.6%)	1.5% (-4.4-11%)	23 (-66-160)	350 (-900-2300)
Turkmenistan	2016	Female	80 to 84	50% (37-64%)	50% (36-63%)	1.6 (0.61-3.2)	0.69% (-3.7-7.4%)	0.69% (-5-9.4%)	9 (-67-130)	120 (-680-1400)
Turkmenistan	2016	Female	85 to 89	49% (36-61%)	51% (39-64%)	1.7 (0.63-3.6)	0.56% (-4.2-8.5%)	0.5% (-5.4-11%)	5.2 (-57-110)	55 (-400-840)
Turkmenistan	2016	Female	90 to 94	50% (39-62%)	50% (38-61%)	1.8 (0.6-4.2)	0.54% (-4.4-9.4%)	0.44% (-5.8-11%)	2.7 (-35-69)	21 (-170-350)
Turkmenistan	2016	Female	95 plus	51% (37-66%)	49% (34-63%)	1.9 (0.47-4.9)	0.67% (-4.8-9.7%)	0.55% (-6.1-12%)	0.94 (-10-20)	4.8 (-33-69)
Turkmenistan	2016	Male	All Ages	67% (60-75%)	33% (25-40%)	2 (1.4-2.7)	6.9% (4.3-9.9%)	7% (3.1-12%)	1300 (590-2200)	65000 (42000-93000)
Turkmenistan	2016	Male	15 to 19	55% (39-71%)	45% (29-61%)	0.8 (0.38-1.5)	3.8% (2.5-5.7%)	4.4% (2.3-7.5%)	12 (6-19)	1300 (810-1900)
Turkmenistan	2016	Male	20 to 24	73% (56-85%)	27% (15-44%)	1.8 (0.99-2.9)	9.5% (6.7-13%)	11% (6.6-15%)	52 (32-77)	4900 (3400-6800)
Turkmenistan	2016	Male	25 to 29	80% (69-89%)	20% (11-31%)	2.9 (1.7-4.5)	13% (9-17%)	15% (9.6-21%)	95 (61-130)	7500 (5400-10000)
Turkmenistan	2016	Male	30 to 34	85% (73-92%)	15% (8.2-27%)	3.5 (2.2-5.3)	13% (9.6-17%)	16% (11-22%)	110 (73-150)	7600 (5400-10000)
Turkmenistan	2016	Male	35 to 39	86% (76-93%)	14% (7.3-24%)	3.5 (2.1-5.5)	14% (10-19%)	17% (11-24%)	130 (87-180)	7800 (5600-11000)
Turkmenistan	2016	Male	40 to 44	86% (76-93%)	14% (7.1-24%)	3.6 (2.1-5.6)	14% (9.2-19%)	17% (11-24%)	140 (92-210)	7700 (5100-11000)
Turkmenistan	2016	Male	45 to 49	85% (72-92%)	15% (7.6-28%)	3.8 (2.2-6)	13% (7.8-20%)	16% (8.8-24%)	170 (94-270)	8000 (4700-12000)
Turkmenistan	2016	Male	50 to 54	84% (72-92%)	16% (8.3-28%)	3.8 (2.1-6)	12% (5.9-19%)	14% (6.3-22%)	200 (89-330)	8100 (4000-13000)
Turkmenistan	2016	Male	55 to 59	83% (71-91%)	17% (8.6-29%)	3.3 (1.8-5.3)	9.1% (3.5-17%)	10% (3.6-20%)	180 (61-340)	6200 (2400-11000)

Location	Year	Sex	Age	Prevalence of current drinkers	Prevalence of abstainers	Population average of standard drinks daily	Percent of total attributable deaths	Percent of total attributable DALYs	Attributable deaths	Attributable DALYs
Turkmenistan	2016	Male	60 to 64	81% (67-90%)	19% (9.6-33%)	2.8 (1.4-4.8)	6.9% (1.1-15%)	7.8% (0.87-18%)	140 (16-310)	4000 (640-8600)
Turkmenistan	2016	Male	65 to 69	79% (64-89%)	21% (11-36%)	2.5 (1.4-4.1)	4.7% (-0.4-11%)	5.2% (-0.84-13%)	77 (-13-200)	1900 (-160-4500)
Turkmenistan	2016	Male	70 to 74	76% (62-87%)	24% (13-38%)	2.2 (1.2-3.7)	2.4% (-2.2-8.7%)	2.5% (-2.8-10%)	30 (-34-120)	600 (-550-2200)
Turkmenistan	2016	Male	75 to 79	73% (57-84%)	27% (16-43%)	1.9 (0.95-3.2)	0.72% (-3.3-6.2%)	0.54% (-4.3-7.1%)	7.7 (-62-100)	170 (-800-1500)
Turkmenistan	2016	Male	80 to 84	70% (54-83%)	30% (17-46%)	1.7 (0.86-2.7)	-0.91% (-4.6-3.2%)	-1.5% (-5.8-3.4%)	-15 (-61-36)	-120 (-590-420)
Turkmenistan	2016	Male	85 to 89	68% (52-80%)	32% (20-48%)	1.5 (0.79-2.6)	-1.8% (-5.2-1.8%)	-2.5% (-6.5-1.7%)	-16 (-42-11)	-100 (-280-100)
Turkmenistan	2016	Male	90 to 94	67% (52-80%)	33% (20-48%)	1.4 (0.7-2.4)	-2.3% (-5.2-0.9%)	-3.1% (-6.7-0.73%)	-8.5 (-18-2)	-37 (-85-15)
Turkmenistan	2016	Male	95 plus	66% (47-82%)	34% (18-53%)	1.3 (0.53-2.4)	-2.3% (-5.3-0.91%)	-3.2% (-6.9-0.57%)	-1.5 (-3.1-0.26)	-4.2 (-9.7-1.7)
Uzbekistan	1990	Female	All Ages	50% (44-58%)	50% (42-56%)	0.66 (0.46-0.92)	1% (0.06-2.2%)	0.68% (-1.8-4.1%)	440 (-1200-2700)	34000 (2100-75000)
Uzbekistan	1990	Female	15 to 19	52% (39-68%)	48% (32-61%)	0.67 (0.27-1.3)	1.3% (0.64-2.3%)	2.2% (0.6-4.4%)	15 (4-30)	1800 (830-3100)
Uzbekistan	1990	Female	20 to 24	61% (48-74%)	39% (26-52%)	0.97 (0.42-1.8)	2.2% (1.1-3.7%)	2.8% (0.72-5.6%)	26 (6.6-54)	3200 (1500-5400)
Uzbekistan	1990	Female	25 to 29	64% (49-77%)	36% (23-51%)	1.1 (0.51-2)	2.6% (1.5-4.4%)	3.6% (1.1-6.9%)	38 (12-74)	4000 (2100-6600)
Uzbekistan	1990	Female	30 to 34	65% (51-78%)	35% (22-49%)	1.2 (0.62-2.2)	2.9% (1.6-4.6%)	4.4% (1.9-7.8%)	50 (21-89)	4200 (2400-6600)
Uzbekistan	1990	Female	35 to 39	65% (52-79%)	35% (21-48%)	1.3 (0.55-2.4)	3% (1.7-5.3%)	5% (2.2-9.4%)	54 (24-100)	3600 (1900-6300)
Uzbekistan	1990	Female	40 to 44	65% (52-78%)	35% (22-48%)	1.3 (0.58-2.4)	3.2% (1.5-6.4%)	5.4% (2.2-10%)	55 (23-110)	3000 (1400-5500)
Uzbekistan	1990	Female	45 to 49	65% (50-78%)	35% (22-50%)	1.4 (0.6-2.7)	3.4% (1.2-7.2%)	5.6% (1.9-12%)	59 (19-130)	2700 (920-5600)
Uzbekistan	1990	Female	50 to 54	64% (49-77%)	36% (23-51%)	1.4 (0.61-2.7)	3.6% (0.84-8.3%)	5.5% (1.2-12%)	130 (28-290)	4900 (1200-11000)
Uzbekistan	1990	Female	55 to 59	62% (48-76%)	38% (24-52%)	1.4 (0.55-2.8)	3.3% (-0.011-8.5%)	4.7% (-0.075-12%)	130 (-2.1-350)	4400 (-15-12000)
Uzbekistan	1990	Female	60 to 64	60% (45-75%)	40% (25-55%)	1.4 (0.54-2.8)	2.1% (-1.4-8.2%)	2.9% (-1.9-11%)	130 (-87-480)	3500 (-2400-13000)
Uzbekistan	1990	Female	65 to 69	58% (45-73%)	42% (27-55%)	1.2 (0.55-2.4)	1.1% (-2.3-6.7%)	1.5% (-3-8.8%)	70 (-140-390)	1600 (-3200-9000)
Uzbekistan	1990	Female	70 to 74	55% (43-68%)	45% (32-57%)	1.1 (0.42-2.3)	0.13% (-3.1-5.6%)	0.17% (-4.1-6.8%)	8 (-190-320)	150 (-3400-6000)
Uzbekistan	1990	Female	75 to 79	52% (39-65%)	48% (35-61%)	1 (0.37-2.1)	-0.6% (-3.9-4.7%)	-0.81% (-4.9-5.7%)	-50 (-300-360)	-660 (-4100-5100)

Location	Year	Sex	Age	Prevalence of current drinkers	Prevalence of abstainers	Population average of standard drinks daily	Percent of total attributable deaths	Percent of total attributable DALYs	Attributable deaths	Attributable DALYs
Uzbekistan	1990	Female	80 to 84	50% (36-64%)	50% (36-64%)	1.1 (0.42-2.1)	-1.2% (-4.5-3.9%)	-1.5% (-5.5-4.6%)	-110 (-380-310)	-1000 (-3900-3400)
Uzbekistan	1990	Female	85 to 89	49% (37-61%)	51% (39-63%)	1.1 (0.42-2.4)	-1.3% (-4.7-4.6%)	-1.7% (-5.8-5.3%)	-110 (-370-330)	-720 (-2600-2600)
Uzbekistan	1990	Female	90 to 94	50% (38-62%)	50% (38-62%)	1.2 (0.41-2.8)	-1.1% (-4.7-5.3%)	-1.5% (-5.8-6.2%)	-48 (-180-200)	-220 (-880-1000)
Uzbekistan	1990	Female	95 plus	51% (38-67%)	49% (33-62%)	1.3 (0.34-3.2)	-0.81% (-4.8-6.8%)	-1.2% (-6-7.9%)	-9.9 (-50-68)	-27 (-160-230)
Uzbekistan	1990	Male	All Ages	61% (52-70%)	39% (30-48%)	1 (0.76-1.4)	2.8% (1.7-4.2%)	2.7% (0.57-5%)	2000 (400-3700)	120000 (70000-170000)
Uzbekistan	1990	Male	15 to 19	55% (40-71%)	45% (29-60%)	0.56 (0.3-0.93)	2.8% (1.8-4.2%)	3.2% (1.4-5.3%)	34 (15-59)	4200 (2500-6400)
Uzbekistan	1990	Male	20 to 24	73% (57-85%)	27% (15-43%)	1.3 (0.75-1.9)	6.4% (4.4-8.8%)	7% (3.9-11%)	100 (55-160)	11000 (7000-15000)
Uzbekistan	1990	Male	25 to 29	80% (68-89%)	20% (11-32%)	2 (1.3-3)	8.7% (6.1-12%)	10% (6.2-15%)	190 (120-280)	16000 (11000-22000)
Uzbekistan	1990	Male	30 to 34	84% (73-92%)	16% (7.8-27%)	2.4 (1.6-3.5)	8.9% (6.3-12%)	11% (6.7-15%)	210 (140-310)	16000 (11000-22000)
Uzbekistan	1990	Male	35 to 39	86% (75-93%)	14% (7.3-25%)	2.4 (1.4-3.7)	8.1% (5.1-12%)	9.7% (5.4-15%)	190 (100-290)	12000 (7600-18000)
Uzbekistan	1990	Male	40 to 44	86% (76-93%)	14% (7-24%)	2.5 (1.5-3.9)	7.6% (4.3-12%)	9.1% (4.4-15%)	170 (79-280)	9300 (5200-14000)
Uzbekistan	1990	Male	45 to 49	85% (74-93%)	15% (7.2-26%)	2.7 (1.6-4.3)	7.9% (4.2-13%)	9.3% (4.4-15%)	190 (92-320)	9200 (5000-15000)
Uzbekistan	1990	Male	50 to 54	84% (73-92%)	16% (7.9-27%)	2.6 (1.5-4.1)	7.6% (3.6-13%)	8.8% (3.8-15%)	370 (160-650)	15000 (7100-25000)
Uzbekistan	1990	Male	55 to 59	83% (70-91%)	17% (8.8-30%)	2.3 (1.2-3.7)	6.2% (2.4-11%)	7.1% (2.4-13%)	330 (110-610)	11000 (4200-20000)
Uzbekistan	1990	Male	60 to 64	81% (67-91%)	19% (9.3-33%)	2 (1-3.4)	4.4% (0.68-9.4%)	5% (0.5-11%)	310 (31-700)	9000 (1400-20000)
Uzbekistan	1990	Male	65 to 69	79% (64-89%)	21% (11-36%)	1.7 (0.98-2.8)	2.9% (-0.49-7%)	3.2% (-0.87-8.2%)	140 (-37-350)	3300 (-570-8200)
Uzbekistan	1990	Male	70 to 74	76% (62-86%)	24% (14-38%)	1.5 (0.82-2.5)	1.3% (-1.8-5%)	1.3% (-2.3-5.7%)	45 (-80-200)	940 (-1300-3700)
Uzbekistan	1990	Male	75 to 79	73% (57-85%)	27% (15-43%)	1.3 (0.65-2.1)	-0.16% (-3-3.1%)	-0.39% (-3.7-3.4%)	-17 (-170-150)	-120 (-2200-2200)
Uzbekistan	1990	Male	80 to 84	70% (54-83%)	30% (17-46%)	1.1 (0.62-1.8)	-1.4% (-4.1-1.4%)	-1.8% (-5-1.4%)	-92 (-250-70)	-840 (-2400-820)
Uzbekistan	1990	Male	85 to 89	68% (52-81%)	32% (19-48%)	1 (0.56-1.7)	-2% (-4.7-0.36%)	-2.6% (-5.8-0.26%)	-110 (-250-11)	-730 (-1700-130)
Uzbekistan	1990	Male	90 to 94	67% (52-81%)	33% (19-48%)	0.97 (0.49-1.7)	-2.3% (-4.8-0.046%)	-3% (-6--0.17%)	-60 (-120--3.4)	-270 (-580-5.6)
Uzbekistan	1990	Male	95 plus	66% (46-82%)	34% (18-54%)	0.87 (0.37-1.6)	-2.3% (-4.6-0.023%)	-3% (-6--0.26%)	-15 (-29--1.3)	-43 (-92-0.43)

Location	Year	Sex	Age	Prevalence of current drinkers	Prevalence of abstainers	Population average of standard drinks daily	Percent of total attributable deaths	Percent of total attributable DALYs	Attributable deaths	Attributable DALYs
Uzbekistan	1995	Female	All Ages	51% (44-58%)	49% (42-56%)	0.75 (0.54-1)	1.4% (0.23-2.9%)	1.2% (-1.6-5.3%)	980 (-1300-4200)	53000 (9000-110000)
Uzbekistan	1995	Female	15 to 19	52% (40-67%)	48% (33-60%)	0.74 (0.31-1.4)	1.4% (0.66-2.6%)	2.3% (0.58-4.9%)	19 (4.9-40)	2200 (970-3900)
Uzbekistan	1995	Female	20 to 24	61% (48-74%)	39% (26-52%)	1.1 (0.45-2)	2.5% (1.2-4.6%)	3.3% (0.82-6.7%)	39 (9.7-80)	4200 (1900-7500)
Uzbekistan	1995	Female	25 to 29	64% (50-76%)	36% (24-50%)	1.3 (0.58-2.2)	3.1% (1.5-5.4%)	4.3% (1.4-8.4%)	56 (17-110)	5100 (2400-8600)
Uzbekistan	1995	Female	30 to 34	65% (50-79%)	35% (21-50%)	1.4 (0.7-2.4)	3.5% (2-5.6%)	5.3% (2.5-9.4%)	83 (38-150)	6300 (3400-10000)
Uzbekistan	1995	Female	35 to 39	66% (52-80%)	34% (20-48%)	1.4 (0.65-2.5)	3.7% (2-6.4%)	6% (2.9-11%)	100 (48-180)	6300 (3300-11000)
Uzbekistan	1995	Female	40 to 44	65% (52-79%)	35% (21-48%)	1.5 (0.66-2.6)	3.8% (1.8-7.1%)	6.3% (2.9-11%)	110 (49-190)	5600 (2700-9800)
Uzbekistan	1995	Female	45 to 49	65% (49-79%)	35% (21-51%)	1.5 (0.68-3.1)	4% (1.4-8.4%)	6.2% (2-13%)	110 (33-220)	4600 (1700-9600)
Uzbekistan	1995	Female	50 to 54	63% (49-77%)	37% (23-51%)	1.6 (0.7-3.1)	4.2% (0.89-9.7%)	6.1% (1.3-14%)	110 (23-260)	4300 (890-9900)
Uzbekistan	1995	Female	55 to 59	61% (48-75%)	39% (25-52%)	1.6 (0.66-3.1)	4% (0.027-10%)	5.4% (0.0017-14%)	230 (0.068-560)	7400 (48-18000)
Uzbekistan	1995	Female	60 to 64	60% (45-75%)	40% (25-55%)	1.5 (0.59-3.2)	2.7% (-1.6-9.9%)	3.4% (-2.1-12%)	180 (-110-620)	5000 (-3000-17000)
Uzbekistan	1995	Female	65 to 69	58% (45-73%)	42% (27-55%)	1.4 (0.64-2.7)	1.5% (-2.4-7.7%)	1.9% (-3-9.5%)	140 (-230-710)	3200 (-5200-16000)
Uzbekistan	1995	Female	70 to 74	55% (42-69%)	45% (31-58%)	1.2 (0.49-2.4)	0.46% (-3.4-6.5%)	0.55% (-4.1-7.8%)	42 (-320-600)	780 (-5800-11000)
Uzbekistan	1995	Female	75 to 79	52% (40-66%)	48% (34-60%)	1.2 (0.42-2.3)	-0.36% (-4.2-5.6%)	-0.51% (-5-6.5%)	-35 (-350-460)	-430 (-4900-6500)
Uzbekistan	1995	Female	80 to 84	50% (37-65%)	50% (35-63%)	1.2 (0.47-2.3)	-0.74% (-4.5-5.1%)	-0.99% (-5.5-5.9%)	-78 (-450-470)	-720 (-4500-5100)
Uzbekistan	1995	Female	85 to 89	50% (37-63%)	50% (37-63%)	1.3 (0.49-2.6)	-0.81% (-4.8-5.9%)	-1.1% (-5.7-6.9%)	-77 (-400-500)	-490 (-2800-3700)
Uzbekistan	1995	Female	90 to 94	51% (39-62%)	49% (38-61%)	1.3 (0.46-3)	-0.63% (-4.8-7.2%)	-0.91% (-5.8-8.3%)	-37 (-240-340)	-150 (-1100-1700)
Uzbekistan	1995	Female	95 plus	51% (38-66%)	49% (34-62%)	1.4 (0.38-3.4)	-0.33% (-5.1-8.3%)	-0.58% (-6.1-9.5%)	-7.8 (-82-130)	-17 (-260-440)
Uzbekistan	1995	Male	All Ages	62% (54-71%)	38% (29-46%)	1.2 (0.9-1.5)	3.6% (2.3-5.1%)	3.7% (1.2-6.2%)	3100 (1000-5300)	160000 (100000-230000)
Uzbekistan	1995	Male	15 to 19	56% (40-72%)	44% (28-60%)	0.63 (0.34-1)	2.9% (1.8-4.2%)	3.2% (1.5-5.4%)	45 (21-76)	5200 (3100-7600)
Uzbekistan	1995	Male	20 to 24	73% (58-85%)	27% (15-42%)	1.4 (0.87-2.1)	6.9% (4.8-9.4%)	7.6% (4.5-11%)	140 (80-220)	14000 (9100-19000)
Uzbekistan	1995	Male	25 to 29	80% (68-89%)	20% (11-32%)	2.3 (1.4-3.4)	9.8% (6.9-13%)	11% (7.2-16%)	240 (150-350)	19000 (13000-26000)

Location	Year	Sex	Age	Prevalence of current drinkers	Prevalence of abstainers	Population average of standard drinks daily	Percent of total attributable deaths	Percent of total attributable DALYs	Attributable deaths	Attributable DALYs
Uzbekistan	1995	Male	30 to 34	84% (73-92%)	16% (7.9-27%)	2.7 (1.8-3.9)	10% (7.4-14%)	12% (8.1-17%)	320 (210-450)	23000 (16000-30000)
Uzbekistan	1995	Male	35 to 39	86% (75-93%)	14% (7.2-25%)	2.7 (1.7-3.9)	9.3% (6.2-13%)	11% (7-16%)	340 (200-490)	21000 (14000-29000)
Uzbekistan	1995	Male	40 to 44	86% (76-93%)	14% (7.1-24%)	2.8 (1.7-4.1)	8.9% (5.3-13%)	11% (5.8-16%)	340 (180-520)	18000 (10000-26000)
Uzbekistan	1995	Male	45 to 49	86% (74-93%)	14% (7.3-26%)	3 (1.8-4.7)	9.1% (5-14%)	11% (5.3-17%)	340 (170-560)	15000 (8400-25000)
Uzbekistan	1995	Male	50 to 54	85% (73-92%)	15% (8.1-27%)	2.9 (1.7-4.6)	8.7% (4.3-14%)	10% (4.4-17%)	340 (150-600)	14000 (6600-23000)
Uzbekistan	1995	Male	55 to 59	83% (71-91%)	17% (8.7-29%)	2.5 (1.4-4)	6.9% (2.6-13%)	7.8% (2.7-15%)	520 (180-980)	17000 (6600-32000)
Uzbekistan	1995	Male	60 to 64	81% (67-90%)	19% (9.6-33%)	2.2 (1.2-3.7)	4.9% (0.66-11%)	5.4% (0.48-12%)	400 (35-900)	11000 (1500-25000)
Uzbekistan	1995	Male	65 to 69	79% (65-89%)	21% (11-35%)	2 (1.1-3)	3.1% (-0.55-7.7%)	3.4% (-0.89-8.7%)	300 (-79-760)	7200 (-1300-18000)
Uzbekistan	1995	Male	70 to 74	76% (62-86%)	24% (14-38%)	1.7 (0.96-2.7)	1.5% (-1.9-5.3%)	1.5% (-2.5-6%)	88 (-140-340)	1800 (-2300-6400)
Uzbekistan	1995	Male	75 to 79	73% (58-85%)	27% (15-42%)	1.4 (0.77-2.4)	-0.018% (-3.2-3.6%)	-0.23% (-4-3.9%)	-9 (-160-150)	-11 (-2100-2300)
Uzbekistan	1995	Male	80 to 84	71% (54-83%)	29% (17-46%)	1.3 (0.72-2.1)	-1.2% (-4.1-1.9%)	-1.6% (-5.1-1.9%)	-75 (-240-86)	-660 (-2300-1000)
Uzbekistan	1995	Male	85 to 89	68% (53-80%)	32% (20-47%)	1.2 (0.66-1.9)	-1.9% (-4.8-0.95%)	-2.4% (-5.7-0.9%)	-96 (-230-36)	-620 (-1600-310)
Uzbekistan	1995	Male	90 to 94	67% (53-80%)	33% (20-47%)	1.1 (0.58-1.9)	-2.2% (-4.9-0.52%)	-2.8% (-6-0.42%)	-63 (-130-9.2)	-280 (-630-68)
Uzbekistan	1995	Male	95 plus	66% (47-82%)	34% (18-53%)	1 (0.44-1.9)	-2.2% (-4.7-0.48%)	-2.9% (-6-0.16%)	-20 (-41-1.1)	-59 (-130-13)
Uzbekistan	2000	Female	All Ages	52% (45-59%)	48% (41-55%)	0.85 (0.62-1.1)	1.8% (0.46-3.6%)	1.8% (-1.5-6.4%)	1600 (-1300-5400)	72000 (18000-140000)
Uzbekistan	2000	Female	15 to 19	52% (40-67%)	48% (33-60%)	0.8 (0.33-1.5)	1.6% (0.67-3%)	2.7% (0.67-5.9%)	26 (6.4-56)	2800 (1200-5200)
Uzbekistan	2000	Female	20 to 24	61% (48-73%)	39% (27-52%)	1.2 (0.49-2.2)	3% (1.3-5.3%)	4.2% (1-8.3%)	56 (14-110)	5500 (2400-9600)
Uzbekistan	2000	Female	25 to 29	64% (50-76%)	36% (24-50%)	1.4 (0.62-2.4)	3.7% (1.7-6.3%)	5.4% (1.6-10%)	80 (24-150)	6700 (3100-12000)
Uzbekistan	2000	Female	30 to 34	65% (50-78%)	35% (22-50%)	1.5 (0.73-2.5)	4% (2.3-6.6%)	6.3% (2.9-11%)	100 (47-170)	7300 (4000-12000)
Uzbekistan	2000	Female	35 to 39	65% (52-79%)	35% (21-48%)	1.5 (0.7-2.7)	4.3% (2.3-7.1%)	7% (3.4-12%)	140 (67-230)	8500 (4400-14000)
Uzbekistan	2000	Female	40 to 44	65% (51-79%)	35% (21-49%)	1.6 (0.73-2.8)	4.4% (2.1-7.6%)	7.2% (3.3-13%)	170 (77-290)	8600 (4100-15000)
Uzbekistan	2000	Female	45 to 49	65% (49-79%)	35% (21-51%)	1.6 (0.73-3.2)	4.4% (1.6-9%)	6.9% (2.4-14%)	170 (60-350)	7600 (2700-15000)

Location	Year	Sex	Age	Prevalence of current drinkers	Prevalence of abstainers	Population average of standard drinks daily	Percent of total attributable deaths	Percent of total attributable DALYs	Attributable deaths	Attributable DALYs
Uzbekistan	2000	Female	50 to 54	63% (48-77%)	37% (23-52%)	1.7 (0.76-3.3)	4.6% (1-10%)	6.5% (1.4-15%)	170 (37-380)	6500 (1500-15000)
Uzbekistan	2000	Female	55 to 59	62% (47-76%)	38% (24-53%)	1.7 (0.68-3.3)	4.4% (0.14-11%)	5.9% (0.14-14%)	170 (3.9-420)	5600 (180-13000)
Uzbekistan	2000	Female	60 to 64	60% (45-75%)	40% (25-55%)	1.6 (0.63-3.4)	3.1% (-1.5-11%)	4% (-1.9-13%)	250 (-120-850)	6900 (-3300-24000)
Uzbekistan	2000	Female	65 to 69	58% (45-73%)	42% (27-55%)	1.5 (0.67-2.9)	1.9% (-2.6-8.9%)	2.3% (-3.2-11%)	180 (-240-830)	4000 (-5500-19000)
Uzbekistan	2000	Female	70 to 74	55% (43-68%)	45% (32-57%)	1.3 (0.51-2.8)	0.81% (-3.4-7.6%)	0.95% (-4.2-9.1%)	99 (-430-940)	1900 (-7900-17000)
Uzbekistan	2000	Female	75 to 79	52% (40-65%)	48% (35-60%)	1.3 (0.45-2.5)	0.014% (-3.9-6.5%)	-0.065% (-4.8-7.4%)	-6.2 (-460-700)	24 (-6300-10000)
Uzbekistan	2000	Female	80 to 84	50% (37-65%)	50% (35-63%)	1.3 (0.51-2.4)	-0.13% (-4.2-6.4%)	-0.27% (-5.2-7.4%)	-24 (-480-680)	-150 (-4800-7200)
Uzbekistan	2000	Female	85 to 89	49% (37-61%)	51% (39-63%)	1.4 (0.52-2.8)	-0.16% (-4.5-7.4%)	-0.32% (-5.5-8.6%)	-22 (-370-570)	-92 (-2600-4200)
Uzbekistan	2000	Female	90 to 94	50% (39-62%)	50% (38-61%)	1.5 (0.5-3.4)	0.0077% (-4.6-8.5%)	-0.15% (-5.6-10%)	-5.9 (-220-380)	2 (-1100-1900)
Uzbekistan	2000	Female	95 plus	51% (37-66%)	49% (34-63%)	1.5 (0.4-3.7)	0.28% (-4.9-9.2%)	0.14% (-5.9-11%)	2.1 (-91-160)	17 (-290-550)
Uzbekistan	2000	Male	All Ages	63% (54-72%)	37% (28-46%)	1.3 (1-1.7)	4.6% (3-6.4%)	4.5% (1.7-7.5%)	4200 (1500-7000)	210000 (130000-300000)
Uzbekistan	2000	Male	15 to 19	55% (39-71%)	45% (29-61%)	0.67 (0.36-1.1)	3% (1.8-4.4%)	3.3% (1.5-5.6%)	57 (27-98)	6400 (3800-9500)
Uzbekistan	2000	Male	20 to 24	73% (57-85%)	27% (15-43%)	1.5 (0.91-2.2)	7.3% (4.9-10%)	8.1% (4.6-12%)	190 (100-280)	17000 (11000-24000)
Uzbekistan	2000	Male	25 to 29	80% (69-89%)	20% (11-31%)	2.4 (1.5-3.6)	11% (7.1-15%)	12% (7.6-18%)	340 (200-490)	26000 (17000-35000)
Uzbekistan	2000	Male	30 to 34	84% (72-92%)	16% (7.8-28%)	2.9 (1.9-4.2)	11% (8.1-15%)	14% (9.2-19%)	400 (260-560)	27000 (19000-36000)
Uzbekistan	2000	Male	35 to 39	86% (75-93%)	14% (7.3-25%)	2.9 (1.8-4.3)	11% (7.1-15%)	13% (8-18%)	470 (280-680)	28000 (18000-39000)
Uzbekistan	2000	Male	40 to 44	86% (76-93%)	14% (7.1-24%)	3 (1.9-4.4)	10% (6.2-15%)	12% (6.9-18%)	520 (290-790)	27000 (16000-39000)
Uzbekistan	2000	Male	45 to 49	85% (73-93%)	15% (7.2-27%)	3.2 (2-5)	10% (5.8-16%)	12% (6.4-19%)	550 (290-900)	25000 (14000-40000)
Uzbekistan	2000	Male	50 to 54	84% (73-92%)	16% (8.1-27%)	3.2 (1.8-4.9)	9.5% (4.8-16%)	11% (5-18%)	490 (220-830)	19000 (9300-32000)
Uzbekistan	2000	Male	55 to 59	83% (71-92%)	17% (8.2-29%)	2.7 (1.6-4.4)	7.4% (2.8-14%)	8.3% (2.8-16%)	390 (130-740)	13000 (5000-25000)
Uzbekistan	2000	Male	60 to 64	81% (67-91%)	19% (9.4-33%)	2.4 (1.3-3.9)	5.2% (0.64-12%)	5.7% (0.39-13%)	520 (35-1200)	15000 (1800-33000)
Uzbekistan	2000	Male	65 to 69	79% (65-89%)	21% (11-35%)	2.1 (1.2-3.3)	3.2% (-0.67-8.3%)	3.5% (-1.1-9.2%)	320 (-100-850)	7700 (-1700-20000)

Location	Year	Sex	Age	Prevalence of current drinkers	Prevalence of abstainers	Population average of standard drinks daily	Percent of total attributable deaths	Percent of total attributable DALYs	Attributable deaths	Attributable DALYs
Uzbekistan	2000	Male	70 to 74	76% (61-86%)	24% (14-39%)	1.8 (1-2.9)	1.5% (-2.3-5.5%)	1.5% (-3-6.1%)	160 (-320-650)	3200 (-5200-12000)
Uzbekistan	2000	Male	75 to 79	73% (57-85%)	27% (15-43%)	1.6 (0.85-2.5)	0.13% (-3.4-4.1%)	-0.071% (-4.2-4.5%)	-4.3 (-260-260)	130 (-3400-3900)
Uzbekistan	2000	Male	80 to 84	71% (54-83%)	29% (17-46%)	1.4 (0.78-2.2)	-0.9% (-4-2.4%)	-1.3% (-5-2.5%)	-59 (-230-110)	-490 (-2200-1300)
Uzbekistan	2000	Male	85 to 89	68% (53-80%)	32% (20-47%)	1.3 (0.7-2.1)	-1.6% (-4.6-1.4%)	-2.1% (-5.7-1.4%)	-61 (-160-41)	-390 (-1100-350)
Uzbekistan	2000	Male	90 to 94	67% (52-80%)	33% (20-48%)	1.2 (0.63-2)	-1.9% (-4.8-0.92%)	-2.6% (-5.9-0.79%)	-43 (-98-13)	-190 (-460-88)
Uzbekistan	2000	Male	95 plus	66% (47-82%)	34% (18-53%)	1.1 (0.47-2)	-2% (-4.8-0.81%)	-2.7% (-6-0.61%)	-17 (-37-3.7)	-48 (-110-20)
Uzbekistan	2005	Female	All Ages	52% (46-60%)	48% (40-54%)	0.97 (0.7-1.3)	2.2% (0.65-4.3%)	2.2% (-1.4-7.3%)	2100 (-1200-6700)	89000 (26000-170000)
Uzbekistan	2005	Female	15 to 19	52% (38-67%)	48% (33-62%)	0.83 (0.34-1.6)	1.7% (0.76-3%)	3% (0.78-6.3%)	28 (7.1-57)	3100 (1400-5600)
Uzbekistan	2005	Female	20 to 24	61% (47-73%)	39% (27-53%)	1.2 (0.49-2.3)	3.1% (1.5-5.4%)	4.7% (1.2-9.2%)	64 (16-130)	6400 (2900-11000)
Uzbekistan	2005	Female	25 to 29	63% (49-76%)	37% (24-51%)	1.4 (0.64-2.7)	3.8% (1.8-6.6%)	5.7% (1.9-11%)	87 (28-170)	7400 (3400-12000)
Uzbekistan	2005	Female	30 to 34	65% (50-79%)	35% (21-50%)	1.6 (0.79-2.8)	4.3% (2.3-7.1%)	6.8% (3.1-12%)	120 (52-200)	8300 (4400-14000)
Uzbekistan	2005	Female	35 to 39	65% (52-80%)	35% (20-48%)	1.6 (0.74-2.9)	4.6% (2.4-7.7%)	7.5% (3.7-13%)	150 (71-260)	9000 (4700-15000)
Uzbekistan	2005	Female	40 to 44	65% (51-79%)	35% (21-49%)	1.7 (0.77-3)	4.7% (2.3-8.1%)	7.7% (3.7-13%)	210 (96-360)	11000 (5200-18000)
Uzbekistan	2005	Female	45 to 49	65% (49-78%)	35% (22-51%)	1.7 (0.78-3.4)	4.7% (1.8-9.5%)	7.4% (2.7-15%)	250 (86-500)	11000 (3900-22000)
Uzbekistan	2005	Female	50 to 54	63% (48-77%)	37% (23-52%)	1.8 (0.83-3.4)	4.9% (1.2-11%)	7% (1.7-15%)	280 (64-600)	11000 (2400-23000)
Uzbekistan	2005	Female	55 to 59	61% (47-75%)	39% (25-53%)	1.8 (0.74-3.6)	4.7% (0.27-11%)	6.3% (0.41-15%)	260 (16-620)	8300 (470-20000)
Uzbekistan	2005	Female	60 to 64	59% (45-74%)	41% (26-55%)	1.7 (0.67-3.6)	3.5% (-1.4-12%)	4.3% (-1.9-14%)	190 (-85-640)	5300 (-2300-18000)
Uzbekistan	2005	Female	65 to 69	58% (45-73%)	42% (27-55%)	1.6 (0.71-3.1)	2.3% (-2.4-9.8%)	2.8% (-3-12%)	250 (-270-1100)	5800 (-6000-24000)
Uzbekistan	2005	Female	70 to 74	55% (42-68%)	45% (32-58%)	1.4 (0.54-2.9)	1.1% (-3.5-8.3%)	1.3% (-4.3-10%)	130 (-450-1100)	2500 (-8100-19000)
Uzbekistan	2005	Female	75 to 79	52% (39-65%)	48% (35-61%)	1.3 (0.48-2.7)	0.25% (-4.1-7.3%)	0.21% (-5-8.5%)	26 (-610-1000)	520 (-8400-15000)
Uzbekistan	2005	Female	80 to 84	50% (36-64%)	50% (36-64%)	1.4 (0.53-2.6)	0.21% (-4.2-7.6%)	0.14% (-5.1-8.7%)	17 (-600-1100)	320 (-6100-11000)
Uzbekistan	2005	Female	85 to 89	49% (37-61%)	51% (39-63%)	1.4 (0.55-3)	0.26% (-4.4-8.5%)	0.16% (-5.3-9.8%)	12 (-380-680)	160 (-2700-5200)

Location	Year	Sex	Age	Prevalence of current drinkers	Prevalence of abstainers	Population average of standard drinks daily	Percent of total attributable deaths	Percent of total attributable DALYs	Attributable deaths	Attributable DALYs
Uzbekistan	2005	Female	90 to 94	50% (38-61%)	50% (39-62%)	1.6 (0.52-3.6)	0.53% (-4.5-9.7%)	0.46% (-5.5-11%)	16 (-190-380)	110 (-890-1900)
Uzbekistan	2005	Female	95 plus	51% (36-66%)	49% (34-64%)	1.6 (0.44-4)	0.88% (-4.9-11%)	0.84% (-5.8-12%)	11 (-81-170)	47 (-260-570)
Uzbekistan	2005	Male	All Ages	64% (56-73%)	36% (27-44%)	1.5 (1.2-1.9)	5% (3.1-7.1%)	4.8% (1.5-8.2%)	5000 (1600-8500)	250000 (150000-350000)
Uzbekistan	2005	Male	15 to 19	55% (39-71%)	45% (29-61%)	0.72 (0.38-1.2)	3.2% (2-4.8%)	3.7% (1.7-6.2%)	65 (30-110)	7400 (4400-11000)
Uzbekistan	2005	Male	20 to 24	73% (58-85%)	27% (15-42%)	1.6 (0.98-2.4)	7.6% (5.2-10%)	8.7% (5.1-13%)	200 (110-300)	19000 (12000-26000)
Uzbekistan	2005	Male	25 to 29	80% (68-89%)	20% (11-32%)	2.6 (1.6-3.8)	11% (7.5-15%)	13% (7.9-18%)	350 (220-510)	27000 (18000-38000)
Uzbekistan	2005	Male	30 to 34	84% (73-92%)	16% (8.1-27%)	3.1 (2-4.4)	12% (8.3-16%)	14% (9.4-20%)	440 (290-620)	30000 (21000-41000)
Uzbekistan	2005	Male	35 to 39	86% (76-93%)	14% (7.5-24%)	3.1 (1.9-4.5)	11% (7.4-16%)	14% (8.4-20%)	480 (290-710)	28000 (18000-40000)
Uzbekistan	2005	Male	40 to 44	86% (76-93%)	14% (7-24%)	3.2 (2-4.6)	11% (6.3-16%)	13% (7.2-19%)	610 (350-930)	32000 (19000-47000)
Uzbekistan	2005	Male	45 to 49	85% (73-92%)	15% (7.5-27%)	3.4 (2.1-5.2)	10% (5.8-16%)	12% (6.4-20%)	730 (380-1200)	33000 (18000-52000)
Uzbekistan	2005	Male	50 to 54	84% (73-92%)	16% (7.8-27%)	3.3 (2-5.1)	9.8% (4.7-16%)	11% (4.9-19%)	740 (320-1300)	29000 (14000-49000)
Uzbekistan	2005	Male	55 to 59	83% (70-91%)	17% (8.6-30%)	2.9 (1.7-4.6)	7.7% (2.7-14%)	8.6% (2.8-16%)	520 (160-990)	17000 (6400-33000)
Uzbekistan	2005	Male	60 to 64	81% (67-90%)	19% (9.6-33%)	2.5 (1.3-4.2)	5.4% (0.39-12%)	5.9% (0.097-14%)	400 (6-930)	11000 (810-26000)
Uzbekistan	2005	Male	65 to 69	79% (63-89%)	21% (11-37%)	2.2 (1.3-3.5)	3.5% (-0.75-9%)	3.8% (-1.2-10%)	440 (-130-1100)	10000 (-2300-26000)
Uzbekistan	2005	Male	70 to 74	76% (61-86%)	24% (14-39%)	1.9 (1.1-3.1)	1.5% (-2.6-6%)	1.5% (-3.2-6.6%)	170 (-360-720)	3400 (-5900-14000)
Uzbekistan	2005	Male	75 to 79	73% (57-85%)	27% (15-43%)	1.6 (0.87-2.7)	0.06% (-3.7-4.3%)	-0.16% (-4.5-4.6%)	-17 (-480-510)	100 (-6400-7500)
Uzbekistan	2005	Male	80 to 84	70% (54-83%)	30% (17-46%)	1.5 (0.81-2.3)	-0.81% (-4.3-2.8%)	-1.2% (-5.2-3%)	-83 (-370-210)	-680 (-3600-2400)
Uzbekistan	2005	Male	85 to 89	68% (53-80%)	32% (20-47%)	1.4 (0.75-2.2)	-1.5% (-4.6-1.7%)	-2% (-5.6-1.6%)	-53 (-150-42)	-330 (-1000-370)
Uzbekistan	2005	Male	90 to 94	67% (52-80%)	33% (20-48%)	1.3 (0.66-2.2)	-1.8% (-4.8-1.3%)	-2.4% (-6-1.3%)	-26 (-63-13)	-110 (-300-84)
Uzbekistan	2005	Male	95 plus	66% (47-81%)	34% (19-53%)	1.1 (0.5-2.2)	-1.9% (-4.9-1.3%)	-2.6% (-6.1-1.2%)	-11 (-25-4.7)	-30 (-77-21)
Uzbekistan	2010	Female	All Ages	53% (46-60%)	47% (40-54%)	1.2 (0.85-1.6)	2.8% (1.1-5.1%)	3.5% (-0.63-9.2%)	3100 (-580-8200)	110000 (42000-210000)
Uzbekistan	2010	Female	15 to 19	51% (37-66%)	49% (34-63%)	0.96 (0.38-1.8)	1.9% (0.82-3.5%)	3.7% (0.83-7.7%)	32 (7.2-68)	3500 (1400-6400)

Location	Year	Sex	Age	Prevalence of current drinkers	Prevalence of abstainers	Population average of standard drinks daily	Percent of total attributable deaths	Percent of total attributable DALYs	Attributable deaths	Attributable DALYs
Uzbekistan	2010	Female	20 to 24	60% (46-73%)	40% (27-54%)	1.4 (0.57-2.7)	3.4% (1.6-5.9%)	5.4% (1.3-11%)	71 (18-140)	7300 (3300-13000)
Uzbekistan	2010	Female	25 to 29	63% (49-76%)	37% (24-51%)	1.7 (0.75-3.1)	3.9% (1.9-6.8%)	6.2% (2.1-12%)	89 (30-170)	8100 (3900-14000)
Uzbekistan	2010	Female	30 to 34	65% (49-78%)	35% (22-51%)	1.8 (0.91-3.3)	4.5% (2.5-7.3%)	7.7% (3.7-13%)	120 (57-210)	8800 (4900-14000)
Uzbekistan	2010	Female	35 to 39	65% (52-78%)	35% (22-48%)	1.9 (0.87-3.5)	4.9% (2.6-8.3%)	8.5% (4.2-15%)	160 (77-280)	9900 (5200-16000)
Uzbekistan	2010	Female	40 to 44	65% (51-78%)	35% (22-49%)	1.9 (0.9-3.6)	5% (2.3-8.8%)	8.4% (3.8-15%)	200 (93-360)	11000 (5000-18000)
Uzbekistan	2010	Female	45 to 49	64% (48-78%)	36% (22-52%)	2 (0.91-3.9)	5.1% (1.8-10%)	8.2% (2.9-16%)	300 (110-590)	13000 (4800-26000)
Uzbekistan	2010	Female	50 to 54	63% (47-77%)	37% (23-53%)	2.1 (0.93-4)	5.5% (1.4-11%)	8% (2-17%)	390 (100-790)	15000 (3800-30000)
Uzbekistan	2010	Female	55 to 59	61% (47-75%)	39% (25-53%)	2.1 (0.86-4.1)	5.5% (0.44-13%)	7.5% (0.61-17%)	400 (31-910)	13000 (1000-30000)
Uzbekistan	2010	Female	60 to 64	59% (45-74%)	41% (26-55%)	2 (0.79-4.3)	4.4% (-1.1-13%)	5.7% (-1.5-17%)	300 (-74-860)	8300 (-2000-24000)
Uzbekistan	2010	Female	65 to 69	57% (43-73%)	43% (27-57%)	1.9 (0.84-3.7)	3.5% (-1.8-12%)	4.4% (-2.4-15%)	220 (-130-760)	5200 (-2800-18000)
Uzbekistan	2010	Female	70 to 74	54% (41-68%)	46% (32-59%)	1.7 (0.62-3.4)	2.2% (-3.1-10%)	2.7% (-3.9-13%)	280 (-410-1300)	5300 (-7300-24000)
Uzbekistan	2010	Female	75 to 79	51% (39-64%)	49% (36-61%)	1.5 (0.55-3.2)	1.4% (-3.5-9.2%)	1.6% (-4.4-11%)	160 (-450-1100)	2500 (-6300-16000)
Uzbekistan	2010	Female	80 to 84	49% (35-64%)	51% (36-65%)	1.6 (0.6-3)	1.3% (-3.7-9.5%)	1.4% (-4.6-11%)	160 (-540-1300)	1900 (-5500-14000)
Uzbekistan	2010	Female	85 to 89	48% (35-61%)	52% (39-65%)	1.6 (0.62-3.5)	1.3% (-4.1-11%)	1.4% (-5-12%)	130 (-470-1100)	1100 (-3200-8600)
Uzbekistan	2010	Female	90 to 94	49% (36-62%)	51% (38-64%)	1.8 (0.59-4.2)	1.7% (-4-11%)	1.8% (-4.8-13%)	69 (-190-510)	380 (-900-2600)
Uzbekistan	2010	Female	95 plus	50% (36-65%)	50% (35-64%)	1.9 (0.49-4.7)	2% (-4.3-12%)	2.2% (-5.3-14%)	27 (-65-170)	98 (-210-580)
Uzbekistan	2010	Male	All Ages	65% (57-73%)	35% (27-43%)	1.9 (1.4-2.4)	6.2% (3.8-8.6%)	6.1% (2.5-10%)	6300 (2500-10000)	300000 (190000-420000)
Uzbekistan	2010	Male	15 to 19	53% (37-70%)	47% (30-63%)	0.8 (0.41-1.3)	3.5% (2-5.3%)	4.1% (1.8-6.8%)	82 (37-140)	8600 (4900-13000)
Uzbekistan	2010	Male	20 to 24	72% (55-85%)	28% (15-45%)	1.8 (1.1-2.8)	8% (5.5-11%)	9% (5.3-13%)	290 (170-440)	26000 (17000-36000)
Uzbekistan	2010	Male	25 to 29	79% (67-88%)	21% (12-33%)	3 (1.8-4.4)	11% (7.7-15%)	13% (8.3-19%)	430 (270-630)	33000 (22000-46000)
Uzbekistan	2010	Male	30 to 34	84% (71-92%)	16% (8.1-29%)	3.6 (2.3-5.2)	12% (8.9-17%)	15% (9.9-21%)	490 (320-690)	33000 (23000-45000)
Uzbekistan	2010	Male	35 to 39	85% (74-93%)	15% (7.4-26%)	3.6 (2.2-5.2)	12% (8.2-17%)	15% (9.5-22%)	550 (340-800)	33000 (21000-46000)

Location	Year	Sex	Age	Prevalence of current drinkers	Prevalence of abstainers	Population average of standard drinks daily	Percent of total attributable deaths	Percent of total attributable DALYs	Attributable deaths	Attributable DALYs
Uzbekistan	2010	Male	40 to 44	85% (75-93%)	15% (7.4-25%)	3.7 (2.3-5.3)	12% (7.2-17%)	15% (8.4-21%)	620 (350-930)	32000 (19000-47000)
Uzbekistan	2010	Male	45 to 49	85% (72-92%)	15% (7.5-28%)	3.9 (2.5-6)	12% (6.7-18%)	14% (7.6-22%)	870 (450-1400)	40000 (22000-61000)
Uzbekistan	2010	Male	50 to 54	84% (72-92%)	16% (8.5-28%)	3.8 (2.3-6)	11% (5.4-18%)	13% (5.9-21%)	1000 (450-1700)	39000 (19000-64000)
Uzbekistan	2010	Male	55 to 59	82% (70-91%)	18% (8.9-30%)	3.3 (1.9-5.3)	9% (3.4-16%)	10% (3.7-19%)	790 (270-1500)	27000 (9800-48000)
Uzbekistan	2010	Male	60 to 64	80% (67-90%)	20% (9.8-33%)	2.9 (1.6-4.8)	6.8% (1-14%)	7.6% (0.87-17%)	530 (62-1200)	15000 (2400-33000)
Uzbekistan	2010	Male	65 to 69	78% (63-89%)	22% (11-37%)	2.6 (1.5-4)	5.1% (0.12-11%)	5.6% (-0.15-13%)	370 (-10-870)	8900 (200-20000)
Uzbekistan	2010	Male	70 to 74	75% (60-86%)	25% (14-40%)	2.2 (1.2-3.6)	2.8% (-1.7-8.8%)	3% (-2.3-10%)	350 (-260-1100)	6800 (-4100-21000)
Uzbekistan	2010	Male	75 to 79	72% (56-84%)	28% (16-44%)	1.9 (1-3.1)	1% (-3.3-6.4%)	0.91% (-4.1-6.9%)	87 (-390-670)	1600 (-5000-9800)
Uzbekistan	2010	Male	80 to 84	70% (52-82%)	30% (18-48%)	1.7 (0.92-2.7)	-0.082% (-3.9-4.1%)	-0.38% (-4.8-4.4%)	-31 (-400-370)	-78 (-3800-4100)
Uzbekistan	2010	Male	85 to 89	67% (51-80%)	33% (20-49%)	1.6 (0.84-2.5)	-0.95% (-4.4-2.7%)	-1.4% (-5.4-2.8%)	-66 (-260-130)	-380 (-1700-1100)
Uzbekistan	2010	Male	90 to 94	66% (51-80%)	34% (20-49%)	1.4 (0.75-2.5)	-1.3% (-4.7-2.3%)	-1.8% (-5.8-2.2%)	-22 (-69-26)	-88 (-320-150)
Uzbekistan	2010	Male	95 plus	65% (46-81%)	35% (19-54%)	1.3 (0.57-2.4)	-1.4% (-4.7-2.5%)	-2% (-6-2.4%)	-6.2 (-19-7.4)	-16 (-57-30)
Uzbekistan	2016	Female	All Ages	53% (46-60%)	47% (40-54%)	1.3 (0.91-1.9)	3.5% (1.4-6.2%)	4.6% (-0.0042-11%)	4300 (-3.9-11000)	140000 (54000-260000)
Uzbekistan	2016	Female	15 to 19	49% (36-65%)	51% (35-64%)	1 (0.41-2)	2% (0.84-4%)	4.1% (0.91-8.9%)	29 (6.3-64)	3100 (1200-5900)
Uzbekistan	2016	Female	20 to 24	59% (46-73%)	41% (27-54%)	1.5 (0.6-3)	3.5% (1.6-6.2%)	5.8% (1.5-12%)	63 (16-130)	6700 (3100-13000)
Uzbekistan	2016	Female	25 to 29	63% (48-76%)	37% (24-52%)	1.8 (0.77-3.5)	4% (2.1-6.9%)	6.6% (2.3-13%)	98 (33-200)	9200 (4600-16000)
Uzbekistan	2016	Female	30 to 34	64% (50-78%)	36% (22-50%)	2 (0.95-3.7)	4.6% (2.7-7.6%)	8.2% (4.1-14%)	140 (66-250)	11000 (6000-18000)
Uzbekistan	2016	Female	35 to 39	65% (51-78%)	35% (22-49%)	2 (0.9-3.8)	5.1% (2.7-8.4%)	9.1% (4.6-16%)	180 (86-310)	11000 (5800-19000)
Uzbekistan	2016	Female	40 to 44	65% (50-78%)	35% (22-50%)	2.1 (0.95-3.9)	5.3% (2.5-9.3%)	9.2% (4.1-16%)	230 (100-430)	12000 (5600-22000)
Uzbekistan	2016	Female	45 to 49	64% (48-78%)	36% (22-52%)	2.2 (0.98-4.4)	5.6% (2.1-11%)	9% (3.5-18%)	300 (110-610)	14000 (5300-27000)
Uzbekistan	2016	Female	50 to 54	62% (47-76%)	38% (24-53%)	2.3 (1-4.3)	6% (1.8-12%)	9% (2.8-18%)	460 (140-970)	18000 (5100-37000)
Uzbekistan	2016	Female	55 to 59	60% (46-74%)	40% (26-54%)	2.3 (0.93-4.4)	6.1% (0.98-13%)	8.6% (1.4-18%)	580 (93-1300)	19000 (2900-43000)

Location	Year	Sex	Age	Prevalence of current drinkers	Prevalence of abstainers	Population average of standard drinks daily	Percent of total attributable deaths	Percent of total attributable DALYs	Attributable deaths	Attributable DALYs
Uzbekistan	2016	Female	60 to 64	58% (44-73%)	42% (27-56%)	2.2 (0.86-4.6)	5.1% (-0.55-13%)	6.9% (-0.84-18%)	510 (-64-1400)	14000 (-1700-39000)
Uzbekistan	2016	Female	65 to 69	57% (43-72%)	43% (28-57%)	2 (0.9-4)	4.4% (-1.2-13%)	5.7% (-1.6-17%)	400 (-110-1100)	9200 (-2500-27000)
Uzbekistan	2016	Female	70 to 74	54% (41-67%)	46% (33-59%)	1.8 (0.67-3.9)	3.2% (-2.4-12%)	4% (-3.1-15%)	250 (-190-960)	4700 (-3400-18000)
Uzbekistan	2016	Female	75 to 79	50% (37-64%)	50% (36-63%)	1.7 (0.59-3.6)	2.3% (-2.9-11%)	2.8% (-3.8-14%)	300 (-410-1500)	4400 (-5600-22000)
Uzbekistan	2016	Female	80 to 84	48% (34-62%)	52% (38-66%)	1.7 (0.63-3.5)	2.1% (-3-11%)	2.4% (-4-13%)	310 (-500-1800)	3400 (-5100-18000)
Uzbekistan	2016	Female	85 to 89	47% (34-60%)	53% (40-66%)	1.8 (0.68-3.8)	2.1% (-3.6-12%)	2.3% (-4.3-14%)	220 (-440-1400)	1800 (-3100-10000)
Uzbekistan	2016	Female	90 to 94	48% (36-61%)	52% (39-64%)	1.9 (0.67-4.4)	2.3% (-3.7-12%)	2.6% (-4.6-14%)	150 (-260-820)	780 (-1300-4200)
Uzbekistan	2016	Female	95 plus	50% (35-65%)	50% (35-65%)	2.1 (0.56-5)	2.6% (-4-13%)	2.9% (-5-15%)	46 (-80-240)	170 (-250-830)
Uzbekistan	2016	Male	All Ages	65% (57-74%)	35% (26-43%)	2.2 (1.5-2.9)	7.2% (4.5-11%)	7.2% (3.2-12%)	8000 (3500-14000)	370000 (230000-540000)
Uzbekistan	2016	Male	15 to 19	52% (35-69%)	48% (31-65%)	0.86 (0.43-1.5)	3.7% (2.1-5.9%)	4.4% (2-7.6%)	77 (32-140)	7900 (4500-13000)
Uzbekistan	2016	Male	20 to 24	71% (53-84%)	29% (16-47%)	2 (1.1-3.2)	8.6% (5.6-12%)	9.7% (5.4-15%)	300 (160-470)	27000 (17000-39000)
Uzbekistan	2016	Male	25 to 29	79% (66-88%)	21% (12-34%)	3.3 (2-5.1)	12% (8.2-17%)	14% (8.8-21%)	530 (320-810)	41000 (27000-58000)
Uzbekistan	2016	Male	30 to 34	84% (71-92%)	16% (8.4-29%)	3.9 (2.4-5.9)	13% (9.1-18%)	16% (11-23%)	600 (380-900)	41000 (28000-59000)
Uzbekistan	2016	Male	35 to 39	85% (74-92%)	15% (7.7-26%)	3.9 (2.3-5.8)	13% (8.4-18%)	16% (10-24%)	620 (360-940)	37000 (24000-54000)
Uzbekistan	2016	Male	40 to 44	85% (75-93%)	15% (7.4-25%)	4 (2.4-6)	12% (7.7-18%)	16% (9-23%)	710 (400-1100)	37000 (22000-56000)
Uzbekistan	2016	Male	45 to 49	84% (71-92%)	16% (7.9-29%)	4.3 (2.6-6.7)	13% (7.1-19%)	16% (8.4-24%)	870 (460-1400)	39000 (22000-62000)
Uzbekistan	2016	Male	50 to 54	83% (71-91%)	17% (8.6-29%)	4.2 (2.4-6.8)	12% (5.7-19%)	15% (6.6-24%)	1200 (520-2000)	46000 (22000-76000)
Uzbekistan	2016	Male	55 to 59	82% (69-91%)	18% (9-31%)	3.6 (2-5.8)	10% (3.9-18%)	12% (4.4-21%)	1200 (430-2200)	40000 (16000-73000)
Uzbekistan	2016	Male	60 to 64	80% (65-90%)	20% (9.9-35%)	3.2 (1.7-5.3)	7.9% (2-16%)	9% (1.9-18%)	940 (200-1900)	27000 (6600-54000)
Uzbekistan	2016	Male	65 to 69	77% (61-89%)	23% (11-39%)	2.8 (1.6-4.5)	6.1% (0.88-13%)	6.9% (0.7-15%)	590 (60-1300)	14000 (2100-30000)
Uzbekistan	2016	Male	70 to 74	74% (59-86%)	26% (14-41%)	2.4 (1.3-4.1)	3.9% (-1.2-11%)	4.2% (-1.7-13%)	310 (-120-930)	6100 (-1900-17000)
Uzbekistan	2016	Male	75 to 79	71% (55-83%)	29% (17-45%)	2 (1.1-3.6)	1.9% (-2.5-8%)	1.9% (-3.3-9.2%)	200 (-340-950)	3300 (-4300-14000)

Location	Year	Sex	Age	Prevalence of current drinkers	Prevalence of abstainers	Population average of standard drinks daily	Percent of total attributable deaths	Percent of total attributable DALYs	Attributable deaths	Attributable DALYs
Uzbekistan	2016	Male	80 to 84	68% (51-82%)	32% (18-49%)	1.8 (0.94-3)	0.51% (-3.4-5.4%)	0.28% (-4.3-6%)	27 (-430-610)	600 (-4000-6600)
Uzbekistan	2016	Male	85 to 89	66% (49-79%)	34% (21-51%)	1.7 (0.84-2.8)	-0.56% (-4.3-3.6%)	-1% (-5.4-3.8%)	-58 (-310-230)	-280 (-2100-1800)
Uzbekistan	2016	Male	90 to 94	65% (50-79%)	35% (21-50%)	1.5 (0.77-2.7)	-0.96% (-4.6-2.7%)	-1.5% (-5.7-2.8%)	-38 (-150-72)	-140 (-680-400)
Uzbekistan	2016	Male	95 plus	65% (44-81%)	35% (19-56%)	1.4 (0.57-2.6)	-1.1% (-4.7-3.2%)	-1.7% (-5.9-3.2%)	-7.1 (-25-14)	-18 (-76-51)
Albania	1990	Female	All Ages	25% (22-29%)	75% (71-78%)	0.51 (0.42-0.61)	1.3% (0.87-1.7%)	1.8% (0.75-3.1%)	130 (54-230)	5000 (3400-6900)
Albania	1990	Female	15 to 19	25% (18-34%)	75% (66-82%)	0.52 (0.33-0.76)	1.5% (0.98-2%)	1.7% (0.87-3%)	1 (0.52-1.8)	230 (150-320)
Albania	1990	Female	20 to 24	34% (24-44%)	66% (56-76%)	0.79 (0.52-1.1)	2.7% (1.9-3.7%)	2.4% (1.2-3.7%)	1.8 (0.93-2.8)	460 (300-660)
Albania	1990	Female	25 to 29	35% (25-46%)	65% (54-75%)	0.73 (0.49-1)	2.6% (2-3.5%)	2.1% (1.1-3.3%)	1.8 (0.95-2.8)	470 (330-670)
Albania	1990	Female	30 to 34	36% (25-48%)	64% (52-75%)	0.72 (0.45-1.1)	2.4% (1.7-3.2%)	2.2% (1.3-3.4%)	2.1 (1.2-3.2)	430 (290-600)
Albania	1990	Female	35 to 39	37% (26-48%)	63% (52-74%)	0.77 (0.49-1.1)	2.2% (1.6-2.9%)	2.6% (1.5-3.9%)	2.3 (1.3-3.5)	350 (240-470)
Albania	1990	Female	40 to 44	37% (27-47%)	63% (53-73%)	0.78 (0.51-1.1)	2.1% (1.4-2.7%)	2.8% (1.7-4.3%)	2.8 (1.7-4.2)	290 (200-390)
Albania	1990	Female	45 to 49	34% (23-44%)	66% (56-77%)	0.75 (0.44-1.1)	1.9% (1.3-2.6%)	2.8% (1.6-4.4%)	3.4 (1.9-5.4)	270 (180-370)
Albania	1990	Female	50 to 54	32% (23-43%)	68% (57-77%)	0.86 (0.54-1.3)	2.1% (1.3-3.1%)	3.3% (1.8-5.3%)	6 (3.1-9.4)	320 (200-470)
Albania	1990	Female	55 to 59	31% (20-46%)	69% (54-80%)	1.1 (0.62-1.8)	2.7% (1.5-4.4%)	4.5% (2.1-7.5%)	10 (4.7-17)	400 (210-640)
Albania	1990	Female	60 to 64	29% (19-44%)	71% (56-81%)	1.1 (0.64-1.8)	3% (1.4-4.9%)	4.6% (2.1-7.9%)	16 (7-26)	480 (230-800)
Albania	1990	Female	65 to 69	27% (18-39%)	73% (61-82%)	0.97 (0.59-1.5)	2.6% (1.1-4.4%)	3.9% (1.7-6.6%)	18 (7.8-31)	450 (200-740)
Albania	1990	Female	70 to 74	24% (16-34%)	76% (66-84%)	0.78 (0.42-1.3)	2.1% (0.59-4.1%)	3% (0.79-5.8%)	19 (5-38)	370 (100-710)
Albania	1990	Female	75 to 79	20% (13-29%)	80% (71-87%)	0.61 (0.32-1.1)	1.6% (0.19-3.6%)	2.1% (0.2-4.7%)	19 (1.8-42)	270 (32-600)
Albania	1990	Female	80 to 84	17% (11-25%)	83% (75-89%)	0.52 (0.3-0.91)	1.3% (0.087-3.2%)	1.6% (0.079-3.9%)	15 (0.71-35)	150 (10-360)
Albania	1990	Female	85 to 89	17% (10-24%)	83% (76-90%)	0.5 (0.27-0.85)	1.1% (0.067-2.7%)	1.4% (0.032-3.4%)	8.8 (0.2-22)	67 (4-160)
Albania	1990	Female	90 to 94	18% (11-26%)	82% (74-89%)	0.53 (0.27-0.91)	0.9% (-0.17-2.5%)	1.1% (-0.28-3.1%)	4.1 (-1.1-12)	22 (-4.2-62)
Albania	1990	Female	95 plus	20% (12-30%)	80% (70-88%)	0.58 (0.25-1.1)	0.81% (-0.48-2.8%)	0.9% (-0.67-3.3%)	1.5 (-1.2-5.6)	5.6 (-3.2-20)

Location	Year	Sex	Age	Prevalence of current drinkers	Prevalence of abstainers	Population average of standard drinks daily	Percent of total attributable deaths	Percent of total attributable DALYs	Attributable deaths	Attributable DALYs
Albania	1990	Male	All Ages	45% (40-51%)	55% (49-60%)	1.2 (1-1.4)	2.6% (1.9-3.4%)	2.9% (1.3-4.6%)	280 (130-450)	13000 (9200-18000)
Albania	1990	Male	15 to 19	39% (29-49%)	61% (51-71%)	0.71 (0.44-1.1)	2.9% (2-4%)	3.4% (1.9-5.3%)	4.9 (2.7-7.8)	600 (410-860)
Albania	1990	Male	20 to 24	59% (48-69%)	41% (31-52%)	1.6 (1-2.1)	6.5% (4.6-8.6%)	7.3% (4.4-11%)	16 (9.3-23)	1600 (1100-2300)
Albania	1990	Male	25 to 29	65% (53-74%)	35% (26-47%)	1.7 (1.2-2.3)	6.6% (4.8-8.5%)	7.5% (4.6-11%)	16 (9.5-22)	1600 (1200-2200)
Albania	1990	Male	30 to 34	67% (56-76%)	33% (24-44%)	1.9 (1.3-2.5)	6.2% (4.6-8%)	7.5% (4.7-11%)	14 (8.8-20)	1400 (990-1800)
Albania	1990	Male	35 to 39	67% (56-75%)	33% (25-44%)	2.1 (1.4-2.8)	5.7% (4-7.5%)	7.6% (4.7-11%)	13 (8.3-19)	1100 (770-1500)
Albania	1990	Male	40 to 44	69% (57-76%)	31% (24-43%)	2.2 (1.5-3)	5.1% (3.6-6.7%)	6.9% (4.3-9.6%)	13 (7.9-18)	900 (620-1200)
Albania	1990	Male	45 to 49	72% (60-80%)	28% (20-40%)	2.3 (1.6-3.3)	4.4% (2.8-6.3%)	5.9% (3.1-9.1%)	14 (7.5-23)	870 (530-1300)
Albania	1990	Male	50 to 54	69% (57-79%)	31% (21-43%)	2.4 (1.6-3.3)	4.2% (2.3-6.3%)	5.4% (2.6-8.8%)	21 (9.8-34)	980 (550-1500)
Albania	1990	Male	55 to 59	62% (44-79%)	38% (21-56%)	2.4 (1.4-3.5)	4.2% (2.1-7%)	5.3% (2.3-9.4%)	28 (12-49)	1100 (530-1800)
Albania	1990	Male	60 to 64	58% (39-77%)	42% (23-61%)	2.2 (1.3-3.4)	4.1% (1.8-7.2%)	5% (1.9-9%)	38 (15-70)	1200 (500-2100)
Albania	1990	Male	65 to 69	56% (38-74%)	44% (26-62%)	2 (1.2-2.8)	3.4% (1.3-6.2%)	4% (1.4-7.4%)	33 (11-61)	820 (300-1500)
Albania	1990	Male	70 to 74	53% (36-69%)	47% (31-64%)	1.7 (1-2.6)	2.6% (0.3-5.3%)	3.1% (0.19-6.3%)	33 (2-69)	650 (76-1300)
Albania	1990	Male	75 to 79	48% (33-65%)	52% (35-67%)	1.4 (0.77-2.2)	1.9% (-0.23-4.6%)	2.2% (-0.46-5.4%)	24 (-5-58)	360 (-43-860)
Albania	1990	Male	80 to 84	45% (29-62%)	55% (38-71%)	1.2 (0.67-1.9)	1.3% (-0.68-3.4%)	1.4% (-0.92-3.9%)	12 (-7.6-32)	130 (-68-340)
Albania	1990	Male	85 to 89	44% (30-59%)	56% (41-70%)	1.1 (0.63-1.7)	0.77% (-0.92-2.6%)	0.77% (-1.3-2.9%)	3.8 (-6.1-14)	33 (-39-110)
Albania	1990	Male	90 to 94	45% (31-60%)	55% (40-69%)	0.99 (0.57-1.6)	0.2% (-1.2-1.9%)	0.065% (-1.7-2.1%)	0.16 (-4.2-5)	3.1 (-18-28)
Albania	1990	Male	95 plus	46% (30-63%)	54% (37-70%)	0.9 (0.43-1.6)	-0.093% (-1.6-1.6%)	-0.37% (-2.3-1.8%)	-0.29 (-1.7-1.3)	-0.29 (-5.1-5.1)
Albania	1995	Female	All Ages	26% (22-30%)	74% (70-78%)	0.61 (0.5-0.73)	1.6% (1.1-2.2%)	2.6% (1.3-4.3%)	190 (95-310)	6200 (4300-8100)
Albania	1995	Female	15 to 19	26% (18-34%)	74% (66-82%)	0.62 (0.39-0.9)	1.7% (1.1-2.4%)	2.4% (1.2-4%)	1.6 (0.75-2.6)	250 (160-370)
Albania	1995	Female	20 to 24	34% (24-45%)	66% (55-76%)	0.92 (0.6-1.3)	3% (2.1-4.1%)	3.2% (1.6-5%)	2.3 (1.2-3.7)	470 (320-650)
Albania	1995	Female	25 to 29	35% (25-46%)	65% (54-75%)	0.84 (0.56-1.2)	2.9% (2.1-3.7%)	2.8% (1.5-4.4%)	2.1 (1.2-3.3)	440 (300-610)

Location	Year	Sex	Age	Prevalence of current drinkers	Prevalence of abstainers	Population average of standard drinks daily	Percent of total attributable deaths	Percent of total attributable DALYs	Attributable deaths	Attributable DALYs
Albania	1995	Female	30 to 34	37% (26-48%)	63% (52-74%)	0.84 (0.54-1.2)	2.6% (1.9-3.4%)	2.8% (1.6-4.2%)	2.6 (1.5-3.9)	450 (310-620)
Albania	1995	Female	35 to 39	38% (27-49%)	62% (51-73%)	0.9 (0.57-1.3)	2.4% (1.8-3.1%)	3.2% (1.9-4.6%)	3.2 (1.9-4.8)	420 (290-560)
Albania	1995	Female	40 to 44	37% (27-48%)	63% (52-73%)	0.91 (0.6-1.3)	2.3% (1.6-3%)	3.4% (2-5%)	3.9 (2.3-5.9)	370 (240-490)
Albania	1995	Female	45 to 49	35% (23-45%)	65% (55-77%)	0.89 (0.53-1.3)	2.1% (1.4-2.9%)	3.3% (1.9-5.1%)	4.2 (2.4-6.4)	300 (200-420)
Albania	1995	Female	50 to 54	33% (23-44%)	67% (56-77%)	1 (0.65-1.5)	2.4% (1.6-3.4%)	4% (2.2-6.1%)	7.1 (4-11)	370 (240-540)
Albania	1995	Female	55 to 59	32% (21-46%)	68% (54-79%)	1.3 (0.74-2.1)	3.1% (1.7-4.9%)	5.4% (2.7-8.6%)	13 (6.6-21)	530 (290-810)
Albania	1995	Female	60 to 64	30% (19-45%)	70% (55-81%)	1.3 (0.77-2.1)	3.5% (1.8-5.7%)	5.7% (2.7-9.3%)	20 (9.5-32)	610 (310-970)
Albania	1995	Female	65 to 69	28% (18-40%)	72% (60-82%)	1.1 (0.7-1.7)	3.3% (1.7-5.4%)	5.1% (2.6-8.4%)	25 (13-41)	610 (330-990)
Albania	1995	Female	70 to 74	24% (16-34%)	76% (66-84%)	0.91 (0.5-1.5)	2.8% (1-5.4%)	4% (1.4-7.6%)	29 (9.9-55)	550 (190-1000)
Albania	1995	Female	75 to 79	20% (13-30%)	80% (70-87%)	0.72 (0.38-1.3)	2.2% (0.56-4.6%)	3% (0.68-6.2%)	26 (6-54)	370 (94-780)
Albania	1995	Female	80 to 84	17% (11-25%)	83% (75-89%)	0.62 (0.35-1)	2% (0.45-4.1%)	2.4% (0.54-5.1%)	23 (4.9-48)	240 (55-510)
Albania	1995	Female	85 to 89	17% (10-25%)	83% (75-90%)	0.6 (0.32-1)	1.7% (0.36-3.6%)	2.1% (0.42-4.6%)	16 (3.1-35)	120 (25-260)
Albania	1995	Female	90 to 94	18% (12-27%)	82% (73-88%)	0.63 (0.32-1.1)	1.4% (0.022-3.6%)	1.7% (-0.018-4.4%)	7.1 (-0.072-18)	38 (0.57-94)
Albania	1995	Female	95 plus	21% (12-31%)	79% (69-88%)	0.69 (0.3-1.3)	1.3% (-0.32-3.9%)	1.5% (-0.45-4.7%)	2.6 (-0.76-7.9)	9.4 (-2.2-27)
Albania	1995	Male	All Ages	45% (40-50%)	55% (50-60%)	1.4 (1.2-1.6)	3.3% (2.4-4.3%)	3.9% (2.1-6%)	380 (200-580)	16000 (11000-21000)
Albania	1995	Male	15 to 19	40% (30-50%)	60% (50-70%)	0.85 (0.53-1.3)	3.3% (2.2-4.6%)	4% (2.2-6.2%)	6.4 (3.6-10)	690 (450-980)
Albania	1995	Male	20 to 24	60% (49-69%)	40% (31-51%)	1.8 (1.2-2.5)	7.3% (5.1-9.9%)	8.5% (5.2-12%)	20 (12-29)	1800 (1200-2400)
Albania	1995	Male	25 to 29	65% (53-74%)	35% (26-47%)	2 (1.4-2.7)	7.6% (5.3-10%)	8.9% (5.5-13%)	19 (12-27)	1600 (1100-2200)
Albania	1995	Male	30 to 34	67% (56-76%)	33% (24-44%)	2.2 (1.6-3)	7.1% (5.2-9.4%)	8.8% (5.9-12%)	19 (12-26)	1500 (1100-2000)
Albania	1995	Male	35 to 39	68% (56-75%)	32% (25-44%)	2.4 (1.6-3.3)	6.6% (4.7-8.7%)	8.7% (5.8-12%)	17 (12-25)	1300 (960-1700)
Albania	1995	Male	40 to 44	69% (59-77%)	31% (23-41%)	2.6 (1.7-3.5)	5.9% (4.2-7.9%)	8.1% (5.2-11%)	17 (11-24)	1100 (770-1500)
Albania	1995	Male	45 to 49	72% (60-80%)	28% (20-40%)	2.7 (1.9-3.8)	5.2% (3.3-7.6%)	7.2% (4-11%)	16 (9-25)	930 (590-1400)

Location	Year	Sex	Age	Prevalence of current drinkers	Prevalence of abstainers	Population average of standard drinks daily	Percent of total attributable deaths	Percent of total attributable DALYs	Attributable deaths	Attributable DALYs
Albania	1995	Male	50 to 54	69% (58-79%)	31% (21-42%)	2.8 (1.9-3.9)	5% (2.8-7.5%)	6.6% (3.3-11%)	23 (12-38)	1100 (630-1700)
Albania	1995	Male	55 to 59	63% (45-79%)	37% (21-55%)	2.8 (1.7-4)	5.1% (2.7-8.3%)	6.6% (3.1-11%)	37 (17-62)	1400 (730-2200)
Albania	1995	Male	60 to 64	59% (39-76%)	41% (24-61%)	2.6 (1.6-3.9)	5.1% (2.3-8.8%)	6.2% (2.6-11%)	49 (22-89)	1500 (680-2600)
Albania	1995	Male	65 to 69	57% (40-74%)	43% (26-60%)	2.3 (1.5-3.3)	4.3% (1.9-7.5%)	5.2% (2.1-9.3%)	51 (21-92)	1300 (550-2200)
Albania	1995	Male	70 to 74	53% (37-69%)	47% (31-63%)	2 (1.2-3)	3.5% (0.85-6.8%)	4.1% (0.89-8.1%)	43 (9.2-85)	820 (210-1600)
Albania	1995	Male	75 to 79	49% (34-66%)	51% (34-66%)	1.6 (0.9-2.6)	2.7% (0.15-6.1%)	3.1% (-0.004-7.1%)	34 (-0.045-79)	510 (28-1100)
Albania	1995	Male	80 to 84	46% (31-63%)	54% (37-69%)	1.4 (0.81-2.2)	2% (-0.24-4.6%)	2.2% (-0.46-5.3%)	20 (-4.2-49)	230 (-28-530)
Albania	1995	Male	85 to 89	45% (31-59%)	55% (41-69%)	1.3 (0.74-2)	1.4% (-0.52-3.6%)	1.5% (-0.86-4.2%)	8.2 (-4.7-23)	67 (-26-180)
Albania	1995	Male	90 to 94	45% (31-61%)	55% (39-69%)	1.2 (0.66-1.9)	0.69% (-1-2.9%)	0.63% (-1.5-3.2%)	1.5 (-3.5-7.6)	10 (-15-41)
Albania	1995	Male	95 plus	47% (30-65%)	53% (35-70%)	1.1 (0.5-2)	0.33% (-1.4-2.7%)	0.097% (-2-3%)	0.07 (-1.5-2.2)	0.97 (-4.2-8.1)
Albania	2000	Female	All Ages	26% (23-30%)	74% (70-77%)	0.78 (0.64-0.94)	2.4% (1.7-3.2%)	4.1% (2.2-6.3%)	300 (170-470)	8300 (5800-11000)
Albania	2000	Female	15 to 19	26% (18-34%)	74% (66-82%)	0.76 (0.49-1.1)	1.9% (1.3-2.9%)	3.1% (1.5-5.4%)	2.1 (1-3.6)	300 (190-440)
Albania	2000	Female	20 to 24	34% (24-44%)	66% (56-76%)	1.1 (0.72-1.6)	3.4% (2.3-4.5%)	4.1% (2.1-6.6%)	3 (1.5-4.7)	490 (330-700)
Albania	2000	Female	25 to 29	35% (25-47%)	65% (53-75%)	1 (0.69-1.5)	3.2% (2.3-4.2%)	3.6% (2-5.7%)	2.8 (1.5-4.4)	460 (320-640)
Albania	2000	Female	30 to 34	37% (25-49%)	63% (51-75%)	1 (0.66-1.5)	2.9% (2.1-3.8%)	3.6% (2.1-5.6%)	3 (1.7-4.7)	450 (310-630)
Albania	2000	Female	35 to 39	38% (27-49%)	62% (51-73%)	1.1 (0.71-1.6)	2.8% (2-3.6%)	4% (2.5-5.9%)	4.5 (2.8-6.7)	500 (360-670)
Albania	2000	Female	40 to 44	37% (27-48%)	63% (52-73%)	1.1 (0.74-1.6)	2.6% (1.9-3.5%)	4.3% (2.7-6.3%)	6.1 (3.8-8.9)	500 (340-660)
Albania	2000	Female	45 to 49	35% (24-45%)	65% (55-76%)	1.1 (0.66-1.7)	2.5% (1.7-3.5%)	4.2% (2.6-6.4%)	6.8 (4.1-10)	440 (300-600)
Albania	2000	Female	50 to 54	33% (23-44%)	67% (56-77%)	1.3 (0.8-1.9)	2.9% (2-4.3%)	5% (3.2-7.6%)	9.6 (6.1-15)	470 (320-680)
Albania	2000	Female	55 to 59	32% (21-46%)	68% (54-79%)	1.6 (0.91-2.6)	3.9% (2.2-6%)	6.7% (3.6-10%)	17 (8.9-26)	630 (370-950)
Albania	2000	Female	60 to 64	30% (19-45%)	70% (55-81%)	1.6 (0.95-2.6)	4.5% (2.4-7.1%)	7.2% (3.9-11%)	29 (15-45)	870 (480-1300)
Albania	2000	Female	65 to 69	28% (18-40%)	72% (60-82%)	1.4 (0.87-2.1)	4.4% (2.5-7%)	6.6% (3.7-11%)	36 (20-57)	870 (500-1400)

Location	Year	Sex	Age	Prevalence of current drinkers	Prevalence of abstainers	Population average of standard drinks daily	Percent of total attributable deaths	Percent of total attributable DALYs	Attributable deaths	Attributable DALYs
Albania	2000	Female	70 to 74	24%<br>(16-35%)	76%<br>(65-84%)	1.1<br>(0.61-1.8)	4%<br>(1.7-7%)	5.5%<br>(2.3-9.6%)	46<br>(20-81)	870<br>(390-1500)
Albania	2000	Female	75 to 79	20%<br>(13-31%)	80%<br>(69-87%)	0.89<br>(0.45-1.5)	3.3%<br>(1.1-6.6%)	4.3%<br>(1.4-8.7%)	44<br>(14-86)	630<br>(210-1300)
Albania	2000	Female	80 to 84	17%<br>(11-26%)	83%<br>(74-89%)	0.76<br>(0.42-1.3)	3.1%<br>(1.1-5.8%)	3.8%<br>(1.3-7.2%)	41<br>(14-78)	430<br>(150-810)
Albania	2000	Female	85 to 89	17%<br>(10-25%)	83%<br>(75-90%)	0.73<br>(0.38-1.2)	2.7%<br>(0.88-5.3%)	3.4%<br>(1-6.5%)	31<br>(9.6-60)	230<br>(73-450)
Albania	2000	Female	90 to 94	18%<br>(12-26%)	82%<br>(74-88%)	0.77<br>(0.39-1.3)	2.4%<br>(0.53-5.2%)	3%<br>(0.59-6.3%)	16<br>(3.3-35)	85<br>(18-180)
Albania	2000	Female	95 plus	20%<br>(12-30%)	80%<br>(70-88%)	0.85<br>(0.38-1.6)	2.4%<br>(0.035-5.9%)	2.8%<br>(-0.084-7.1%)	6.1<br>(-0.19-16)	22<br>(0.31-53)
Albania	2000	Male	All Ages	47%<br>(42-52%)	53%<br>(48-58%)	1.8<br>(1.5-2.2)	4.7%<br>(3.4-6.2%)	5.8%<br>(3.3-8.7%)	600<br>(340-910)	22000<br>(15000-29000)
Albania	2000	Male	15 to 19	40%<br>(30-49%)	60%<br>(51-70%)	1<br>(0.65-1.5)	3.6%<br>(2.4-5.1%)	4.5%<br>(2.5-6.9%)	7<br>(4-11)	720<br>(480-1000)
Albania	2000	Male	20 to 24	60%<br>(48-70%)	40%<br>(30-52%)	2.3<br>(1.5-3.1)	8%<br>(5.6-11%)	9.6%<br>(6-14%)	21<br>(13-29)	1800<br>(1200-2400)
Albania	2000	Male	25 to 29	65%<br>(54-74%)	35%<br>(26-46%)	2.5<br>(1.7-3.4)	8.3%<br>(5.8-11%)	10%<br>(6.3-14%)	22<br>(14-32)	1800<br>(1200-2400)
Albania	2000	Male	30 to 34	67%<br>(56-76%)	33%<br>(24-44%)	2.7<br>(1.9-3.7)	8%<br>(5.7-11%)	10%<br>(6.6-14%)	22<br>(15-31)	1700<br>(1200-2300)
Albania	2000	Male	35 to 39	68%<br>(57-75%)	32%<br>(25-43%)	3<br>(2-4.1)	7.5%<br>(5.2-10%)	10%<br>(6.6-15%)	25<br>(16-36)	1800<br>(1200-2400)
Albania	2000	Male	40 to 44	69%<br>(59-77%)	31%<br>(23-41%)	3.2<br>(2.2-4.3)	6.9%<br>(4.6-9.3%)	9.7%<br>(6-14%)	28<br>(17-40)	1800<br>(1200-2400)
Albania	2000	Male	45 to 49	72%<br>(61-80%)	28%<br>(20-39%)	3.4<br>(2.3-4.7)	6.3%<br>(3.9-9.2%)	8.8%<br>(4.9-14%)	28<br>(16-44)	1500<br>(970-2200)
Albania	2000	Male	50 to 54	69%<br>(57-80%)	31%<br>(20-43%)	3.4<br>(2.3-4.7)	6.1%<br>(3.5-9.1%)	8.3%<br>(4.1-13%)	33<br>(17-52)	1500<br>(860-2300)
Albania	2000	Male	55 to 59	63%<br>(45-79%)	37%<br>(21-55%)	3.4<br>(2.2-5)	6.5%<br>(3.7-10%)	8.6%<br>(4.6-14%)	48<br>(25-78)	1800<br>(1000-2800)
Albania	2000	Male	60 to 64	59%<br>(40-77%)	41%<br>(23-60%)	3.2<br>(2-4.8)	6.6%<br>(3.2-11%)	8.4%<br>(3.9-14%)	74<br>(34-120)	2200<br>(1100-3500)
Albania	2000	Male	65 to 69	57%<br>(39-74%)	43%<br>(26-61%)	2.9<br>(1.8-4.1)	6%<br>(2.8-9.8%)	7.3%<br>(3.4-12%)	82<br>(37-140)	2000<br>(960-3300)
Albania	2000	Male	70 to 74	53%<br>(37-69%)	47%<br>(31-63%)	2.4<br>(1.5-3.7)	5.1%<br>(1.8-9.5%)	6.1%<br>(1.9-12%)	84<br>(27-160)	1600<br>(570-3000)
Albania	2000	Male	75 to 79	49%<br>(34-65%)	51%<br>(35-66%)	2<br>(1.1-3.2)	4.2%<br>(0.87-8.8%)	4.9%<br>(0.79-10%)	58<br>(9.6-120)	850<br>(170-1800)
Albania	2000	Male	80 to 84	46%<br>(30-63%)	54%<br>(37-70%)	1.7<br>(0.98-2.8)	3.5%<br>(0.57-7.6%)	3.9%<br>(0.53-8.7%)	40<br>(5.4-90)	430<br>(74-940)
Albania	2000	Male	85 to 89	45%<br>(31-59%)	55%<br>(41-69%)	1.6<br>(0.89-2.4)	2.6%<br>(0.19-6%)	2.9%<br>(-0.029-7%)	19<br>(-0.19-45)	150<br>(11-340)

Location	Year	Sex	Age	Prevalence of current drinkers	Prevalence of abstainers	Population average of standard drinks daily	Percent of total attributable deaths	Percent of total attributable DALYs	Attributable deaths	Attributable DALYs
Albania	2000	Male	90 to 94	45%<br>(31-60%)	55%<br>(40-69%)	1.4<br>(0.79-2.4)	1.6%<br>(-0.52-4.7%)	1.7%<br>(-0.85-5.4%)	5.2<br>(-2.5-16)	30<br>(-9.3-84)
Albania	2000	Male	95 plus	46%<br>(29-65%)	54%<br>(35-71%)	1.3<br>(0.62-2.4)	1.1%<br>(-1.1-4.6%)	0.98%<br>(-1.7-5.1%)	0.83<br>(-1.5-4.4)	3.8<br>(-3.7-16)
Albania	2005	Female	All Ages	25%<br>(23-27%)	75%<br>(73-77%)	0.94<br>(0.77-1.1)	3.3%<br>(2.3-4.4%)	5.4%<br>(3.1-8.2%)	480<br>(270-720)	11000<br>(7900-15000)
Albania	2005	Female	15 to 19	22%<br>(19-27%)	78%<br>(73-81%)	0.8<br>(0.58-1.1)	2.1%<br>(1.4-2.9%)	3.6%<br>(1.9-5.7%)	2.3<br>(1.2-3.7)	330<br>(210-470)
Albania	2005	Female	20 to 24	31%<br>(26-36%)	69%<br>(64-74%)	1.2<br>(0.9-1.6)	3.7%<br>(2.7-4.9%)	5%<br>(2.8-7.6%)	3.5<br>(2-5.4)	540<br>(370-760)
Albania	2005	Female	25 to 29	33%<br>(29-38%)	67%<br>(62-71%)	1.2<br>(0.89-1.6)	3.5%<br>(2.6-4.5%)	4.5%<br>(2.6-6.6%)	2.9<br>(1.7-4.2)	440<br>(300-580)
Albania	2005	Female	30 to 34	34%<br>(29-40%)	66%<br>(60-71%)	1.1<br>(0.84-1.5)	3.1%<br>(2.3-4.1%)	4.2%<br>(2.6-6.1%)	3.2<br>(2-4.7)	430<br>(300-570)
Albania	2005	Female	35 to 39	35%<br>(31-39%)	65%<br>(61-69%)	1.2<br>(0.91-1.6)	3%<br>(2.2-3.8%)	4.6%<br>(3-6.3%)	4.8<br>(3.1-6.7)	500<br>(360-650)
Albania	2005	Female	40 to 44	33%<br>(29-38%)	67%<br>(62-71%)	1.2<br>(0.92-1.6)	2.9%<br>(2.1-3.7%)	4.8%<br>(3.2-6.6%)	7<br>(4.8-9.7)	580<br>(420-740)
Albania	2005	Female	45 to 49	32%<br>(27-36%)	68%<br>(64-73%)	1.2<br>(0.86-1.6)	2.8%<br>(2-3.7%)	4.6%<br>(3.1-6.7%)	9.4<br>(6.3-13)	600<br>(430-800)
Albania	2005	Female	50 to 54	32%<br>(24-42%)	68%<br>(58-76%)	1.5<br>(0.98-2.1)	3.4%<br>(2.4-4.7%)	5.7%<br>(3.8-8.4%)	14<br>(9.4-21)	690<br>(490-960)
Albania	2005	Female	55 to 59	32%<br>(21-47%)	68%<br>(53-79%)	2<br>(1.1-3.1)	4.7%<br>(2.7-7%)	8%<br>(4.4-12%)	23<br>(12-34)	860<br>(510-1300)
Albania	2005	Female	60 to 64	31%<br>(19-46%)	69%<br>(54-81%)	2<br>(1.1-3.2)	5.5%<br>(3.1-8.5%)	8.6%<br>(4.8-13%)	37<br>(20-57)	1100<br>(640-1700)
Albania	2005	Female	65 to 69	28%<br>(19-41%)	72%<br>(59-81%)	1.8<br>(1.1-2.6)	5.5%<br>(3.3-8.5%)	8.1%<br>(4.8-13%)	54<br>(32-83)	1300<br>(790-2000)
Albania	2005	Female	70 to 74	25%<br>(16-36%)	75%<br>(64-84%)	1.4<br>(0.79-2.3)	5.1%<br>(2.4-8.7%)	7%<br>(3.2-12%)	69<br>(32-110)	1300<br>(610-2200)
Albania	2005	Female	75 to 79	21%<br>(13-30%)	79%<br>(70-87%)	1.1<br>(0.58-1.9)	4.4%<br>(1.7-8%)	5.7%<br>(2.1-11%)	74<br>(27-140)	1100<br>(400-2000)
Albania	2005	Female	80 to 84	18%<br>(11-26%)	82%<br>(74-89%)	0.95<br>(0.52-1.6)	4.3%<br>(1.7-7.6%)	5.2%<br>(2-9.2%)	75<br>(29-130)	780<br>(310-1400)
Albania	2005	Female	85 to 89	17%<br>(11-25%)	83%<br>(75-89%)	0.91<br>(0.49-1.5)	3.9%<br>(1.5-7.5%)	4.8%<br>(1.7-9.2%)	54<br>(20-100)	400<br>(150-760)
Albania	2005	Female	90 to 94	19%<br>(12-27%)	81%<br>(73-88%)	0.95<br>(0.48-1.6)	3.5%<br>(1-7.1%)	4.3%<br>(1.2-8.7%)	31<br>(8.6-64)	160<br>(48-330)
Albania	2005	Female	95 plus	21%<br>(12-31%)	79%<br>(69-88%)	1<br>(0.46-1.9)	3.4%<br>(0.45-7.8%)	4%<br>(0.43-9.3%)	13<br>(1.4-30)	45<br>(5.8-100)
Albania	2005	Male	All Ages	51%<br>(47-54%)	49%<br>(46-53%)	2.4<br>(2-2.8)	6.1%<br>(4.3-7.9%)	7.6%<br>(4.4-11%)	850<br>(490-1200)	27000<br>(19000-35000)
Albania	2005	Male	15 to 19	38%<br>(33-44%)	62%<br>(56-67%)	1.2<br>(0.76-1.7)	3.5%<br>(2.4-4.8%)	4.5%<br>(2.4-6.8%)	6.4<br>(3.5-9.7)	720<br>(480-1000)

Location	Year	Sex	Age	Prevalence of current drinkers	Prevalence of abstainers	Population average of standard drinks daily	Percent of total attributable deaths	Percent of total attributable DALYs	Attributable deaths	Attributable DALYs
Albania	2005	Male	20 to 24	62% (56-66%)	38% (34-44%)	2.8 (1.9-3.7)	8.5% (5.9-11%)	11% (6.2-15%)	18 (11-25)	1600 (1100-2200)
Albania	2005	Male	25 to 29	70% (65-74%)	30% (26-35%)	3.2 (2.2-4.3)	9% (6.2-12%)	11% (6.8-16%)	17 (10-25)	1500 (1000-2000)
Albania	2005	Male	30 to 34	71% (66-75%)	29% (25-34%)	3.5 (2.4-4.6)	8.5% (5.9-12%)	11% (6.8-16%)	19 (11-28)	1500 (1000-2100)
Albania	2005	Male	35 to 39	70% (66-74%)	30% (26-34%)	3.7 (2.5-5.1)	8.3% (5.5-11%)	12% (6.9-17%)	22 (13-32)	1600 (1100-2200)
Albania	2005	Male	40 to 44	73% (69-77%)	27% (23-31%)	4.1 (2.9-5.4)	8% (5.1-11%)	12% (6.6-17%)	32 (18-47)	2000 (1300-2700)
Albania	2005	Male	45 to 49	77% (72-80%)	23% (20-28%)	4.4 (3.2-6)	7.8% (4.8-11%)	11% (6.1-17%)	41 (22-63)	2200 (1300-3100)
Albania	2005	Male	50 to 54	72% (62-81%)	28% (19-38%)	4.3 (3-5.9)	7.7% (4.6-11%)	11% (5.5-16%)	52 (27-78)	2300 (1300-3300)
Albania	2005	Male	55 to 59	63% (46-79%)	37% (21-54%)	4.2 (2.6-6)	7.9% (4.6-12%)	11% (5.8-16%)	62 (34-93)	2300 (1300-3300)
Albania	2005	Male	60 to 64	59% (40-77%)	41% (23-60%)	3.9 (2.4-5.8)	8.1% (4.3-12%)	10% (5.4-16%)	94 (48-140)	2800 (1500-4200)
Albania	2005	Male	65 to 69	58% (40-75%)	42% (25-60%)	3.5 (2.2-4.9)	7.7% (4-12%)	9.5% (4.7-14%)	120 (60-180)	2900 (1500-4400)
Albania	2005	Male	70 to 74	54% (37-70%)	46% (30-63%)	3 (1.8-4.5)	6.9% (2.7-12%)	8.2% (3.1-14%)	130 (49-230)	2500 (1000-4200)
Albania	2005	Male	75 to 79	49% (35-65%)	51% (35-65%)	2.4 (1.4-3.9)	5.8% (1.6-11%)	6.8% (1.8-13%)	110 (30-210)	1600 (470-3100)
Albania	2005	Male	80 to 84	47% (31-64%)	53% (36-69%)	2.1 (1.2-3.3)	5.1% (1.4-10%)	5.8% (1.6-12%)	76 (20-160)	810 (230-1700)
Albania	2005	Male	85 to 89	46% (32-60%)	54% (40-68%)	1.9 (1.1-2.9)	4.1% (0.88-8.7%)	4.7% (0.84-10%)	33 (5.9-70)	250 (55-530)
Albania	2005	Male	90 to 94	46% (32-61%)	54% (39-68%)	1.7 (0.99-2.8)	2.9% (0.11-7.2%)	3.2% (-0.13-8.2%)	12 (-0.49-31)	65 (2.6-160)
Albania	2005	Male	95 plus	47% (31-66%)	53% (34-69%)	1.6 (0.76-2.9)	2.1% (-0.84-6.8%)	2.1% (-1.4-7.8%)	2.4 (-1.5-8.8)	9.4 (-3.8-31)
Albania	2010	Female	All Ages	26% (24-29%)	74% (71-76%)	1.1 (0.88-1.3)	3.6% (2.5-5%)	5.9% (3.3-9.1%)	540 (300-840)	12000 (8400-16000)
Albania	2010	Female	15 to 19	23% (20-27%)	77% (73-80%)	0.85 (0.62-1.1)	2.1% (1.4-3%)	3.8% (2-6.1%)	1.8 (0.94-3)	270 (170-390)
Albania	2010	Female	20 to 24	31% (27-36%)	69% (64-73%)	1.3 (0.93-1.8)	3.8% (2.7-5.1%)	5.4% (3.1-8.4%)	2.9 (1.6-4.5)	470 (320-660)
Albania	2010	Female	25 to 29	34% (29-39%)	66% (61-71%)	1.3 (0.93-1.7)	3.6% (2.7-4.7%)	4.8% (2.8-7.5%)	2.5 (1.4-3.7)	400 (280-550)
Albania	2010	Female	30 to 34	34% (30-39%)	66% (61-70%)	1.2 (0.88-1.7)	3.2% (2.3-4.3%)	4.5% (2.8-6.8%)	2.6 (1.6-3.9)	370 (260-510)
Albania	2010	Female	35 to 39	35% (31-40%)	65% (60-69%)	1.3 (0.93-1.7)	3.1% (2.3-3.9%)	4.8% (3.1-6.8%)	3.9 (2.5-5.6)	430 (320-560)

Location	Year	Sex	Age	Prevalence of current drinkers	Prevalence of abstainers	Population average of standard drinks daily	Percent of total attributable deaths	Percent of total attributable DALYs	Attributable deaths	Attributable DALYs
Albania	2010	Female	40 to 44	34% (30-39%)	66% (61-70%)	1.3 (0.95-1.8)	2.9% (2.2-3.9%)	5.1% (3.5-7%)	5.8 (4-8)	490 (360-650)
Albania	2010	Female	45 to 49	32% (28-37%)	68% (63-72%)	1.3 (0.9-1.8)	2.9% (2.1-3.9%)	5% (3.3-7.3%)	9.8 (6.5-14)	630 (460-860)
Albania	2010	Female	50 to 54	33% (25-42%)	67% (58-75%)	1.6 (1.1-2.3)	3.5% (2.4-4.9%)	6.1% (4.1-8.7%)	17 (11-24)	830 (590-1100)
Albania	2010	Female	55 to 59	33% (22-47%)	67% (53-78%)	2.1 (1.2-3.4)	4.8% (2.9-7.1%)	8.4% (4.8-12%)	28 (16-43)	1100 (670-1600)
Albania	2010	Female	60 to 64	31% (20-46%)	69% (54-80%)	2.2 (1.2-3.3)	5.5% (3.2-8.3%)	9% (5.2-14%)	39 (23-62)	1200 (730-1900)
Albania	2010	Female	65 to 69	29% (19-40%)	71% (60-81%)	1.9 (1.2-2.8)	5.6% (3.2-8.6%)	8.5% (4.9-13%)	56 (32-85)	1400 (820-2100)
Albania	2010	Female	70 to 74	25% (17-35%)	75% (65-83%)	1.5 (0.83-2.4)	5.2% (2.4-8.6%)	7.2% (3.4-12%)	79 (37-130)	1500 (710-2500)
Albania	2010	Female	75 to 79	21% (13-31%)	79% (69-87%)	1.2 (0.6-2)	4.5% (1.7-8.2%)	5.9% (2.1-11%)	80 (29-150)	1200 (430-2200)
Albania	2010	Female	80 to 84	18% (12-27%)	82% (73-88%)	1 (0.55-1.7)	4.4% (1.7-7.8%)	5.5% (2.1-9.8%)	87 (33-150)	920 (360-1600)
Albania	2010	Female	85 to 89	18% (11-25%)	82% (75-89%)	0.97 (0.53-1.6)	4.2% (1.6-7.9%)	5.1% (1.9-9.7%)	68 (26-130)	510 (200-950)
Albania	2010	Female	90 to 94	19% (12-27%)	81% (73-88%)	1 (0.53-1.7)	3.9% (1.2-8%)	4.7% (1.4-9.7%)	40 (12-83)	210 (67-420)
Albania	2010	Female	95 plus	21% (12-32%)	79% (68-88%)	1.1 (0.49-2.1)	3.8% (0.63-8.8%)	4.5% (0.64-11%)	18 (2.6-42)	63 (10-140)
Albania	2010	Male	All Ages	53% (48-56%)	47% (44-52%)	2.7 (2.2-3.3)	6.6% (4.7-8.7%)	8.2% (4.7-12%)	960 (560-1400)	28000 (20000-37000)
Albania	2010	Male	15 to 19	38% (34-44%)	62% (56-66%)	1.2 (0.8-1.8)	3.5% (2.4-5%)	4.5% (2.4-7.2%)	5.4 (2.9-8.6)	640 (420-910)
Albania	2010	Male	20 to 24	62% (57-67%)	38% (33-43%)	2.9 (2-4.1)	8.6% (6-12%)	11% (6.3-16%)	18 (10-26)	1700 (1200-2400)
Albania	2010	Male	25 to 29	70% (65-74%)	30% (26-35%)	3.4 (2.3-4.6)	9.1% (6-12%)	12% (6.8-17%)	17 (9.6-25)	1500 (1000-2000)
Albania	2010	Male	30 to 34	71% (66-75%)	29% (25-34%)	3.7 (2.5-5.1)	8.8% (6.1-12%)	12% (6.9-18%)	15 (8.9-23)	1200 (820-1700)
Albania	2010	Male	35 to 39	70% (66-74%)	30% (26-34%)	3.9 (2.7-5.5)	8.5% (5.5-12%)	12% (7-18%)	19 (10-28)	1400 (890-1900)
Albania	2010	Male	40 to 44	73% (69-77%)	27% (23-31%)	4.3 (3.1-5.9)	8.4% (5.3-12%)	12% (7.1-18%)	27 (15-40)	1700 (1100-2300)
Albania	2010	Male	45 to 49	77% (72-80%)	23% (20-28%)	4.6 (3.3-6.4)	8.2% (5.1-12%)	12% (6.6-17%)	41 (23-61)	2200 (1300-3100)
Albania	2010	Male	50 to 54	72% (61-81%)	28% (19-39%)	4.5 (3.1-6.3)	8.2% (4.9-12%)	11% (6.2-17%)	59 (32-87)	2600 (1600-3700)
Albania	2010	Male	55 to 59	64% (45-80%)	36% (20-55%)	4.4 (2.9-6.4)	8.3% (4.9-12%)	11% (6.3-17%)	72 (41-110)	2700 (1600-3800)

Location	Year	Sex	Age	Prevalence of current drinkers	Prevalence of abstainers	Population average of standard drinks daily	Percent of total attributable deaths	Percent of total attributable DALYs	Attributable deaths	Attributable DALYs
Albania	2010	Male	60 to 64	59% (40-78%)	41% (22-60%)	4.2 (2.6-6.1)	8.5% (4.8-12%)	11% (5.9-16%)	95 (51-140)	2900 (1600-4200)
Albania	2010	Male	65 to 69	58% (40-75%)	42% (25-60%)	3.7 (2.4-5.3)	8.1% (4.6-12%)	10% (5.6-15%)	120 (69-190)	3100 (1700-4500)
Albania	2010	Male	70 to 74	54% (38-70%)	46% (30-62%)	3.1 (1.9-4.7)	7.3% (3.1-12%)	8.8% (3.5-15%)	160 (63-270)	3100 (1300-5100)
Albania	2010	Male	75 to 79	50% (34-67%)	50% (33-66%)	2.6 (1.4-4.1)	6.2% (1.9-12%)	7.4% (2.1-14%)	140 (41-270)	2100 (640-3900)
Albania	2010	Male	80 to 84	47% (31-63%)	53% (37-69%)	2.2 (1.3-3.6)	5.5% (1.7-11%)	6.3% (1.8-12%)	99 (29-190)	1100 (320-2100)
Albania	2010	Male	85 to 89	46% (32-60%)	54% (40-68%)	2 (1.2-3.2)	4.6% (1.2-9.5%)	5.2% (1.2-11%)	48 (11-100)	370 (93-780)
Albania	2010	Male	90 to 94	46% (32-61%)	54% (39-68%)	1.9 (1.1-3.1)	3.3% (0.2-8.2%)	3.7% (-0.0075-9.5%)	14 (-0.029-37)	78 (4.8-190)
Albania	2010	Male	95 plus	47% (31-65%)	53% (35-69%)	1.7 (0.81-3.1)	2.5% (-0.79-7.8%)	2.6% (-1.3-8.9%)	3.5 (-1.8-12)	13 (-4.4-42)
Albania	2016	Female	All Ages	29% (26-33%)	71% (67-74%)	1.2 (0.92-1.7)	3.6% (2.4-5.2%)	5.6% (2.9-9.1%)	540 (270-900)	12000 (7900-18000)
Albania	2016	Female	15 to 19	26% (19-35%)	74% (65-81%)	0.99 (0.58-1.5)	2.1% (1.4-3.1%)	4.3% (1.8-7.4%)	1.5 (0.65-2.6)	240 (140-350)
Albania	2016	Female	20 to 24	36% (25-46%)	64% (54-75%)	1.5 (0.9-2.2)	3.9% (2.7-5.3%)	5.9% (3-9.7%)	2.9 (1.5-4.9)	520 (350-740)
Albania	2016	Female	25 to 29	37% (26-48%)	63% (52-74%)	1.4 (0.85-2.2)	3.6% (2.6-4.8%)	5% (2.6-8.1%)	2.5 (1.3-4.1)	470 (310-660)
Albania	2016	Female	30 to 34	37% (26-50%)	63% (50-74%)	1.3 (0.8-2.2)	3.3% (2.3-4.5%)	4.8% (2.7-7.8%)	2.5 (1.4-4)	390 (260-560)
Albania	2016	Female	35 to 39	39% (27-50%)	61% (50-73%)	1.4 (0.82-2.2)	3.2% (2.2-4.2%)	5.2% (3-7.7%)	3.2 (1.8-5)	380 (260-520)
Albania	2016	Female	40 to 44	38% (27-49%)	62% (51-73%)	1.5 (0.88-2.2)	3.1% (2.1-4.3%)	5.6% (3.5-8.2%)	5.3 (3.2-8.2)	460 (310-640)
Albania	2016	Female	45 to 49	36% (24-46%)	64% (54-76%)	1.4 (0.81-2.2)	3% (2.1-4.3%)	5.4% (3.3-8.1%)	8.4 (5-14)	570 (380-820)
Albania	2016	Female	50 to 54	34% (24-45%)	66% (55-76%)	1.6 (1-2.5)	3.5% (2.4-5.2%)	6.2% (3.9-9.4%)	16 (10-26)	850 (560-1200)
Albania	2016	Female	55 to 59	33% (22-48%)	67% (52-78%)	2.1 (1.2-3.4)	4.6% (2.7-7%)	8.2% (4.6-13%)	31 (16-49)	1200 (720-1900)
Albania	2016	Female	60 to 64	31% (20-46%)	69% (54-80%)	2.1 (1.2-3.4)	5.3% (2.9-8%)	8.8% (4.8-13%)	46 (25-74)	1500 (830-2300)
Albania	2016	Female	65 to 69	29% (19-41%)	71% (59-81%)	1.9 (1.1-2.9)	5.2% (2.8-8.2%)	8.2% (4.3-13%)	55 (30-89)	1400 (790-2200)
Albania	2016	Female	70 to 74	25% (17-35%)	75% (65-83%)	1.5 (0.77-2.5)	4.7% (2.1-8.2%)	6.9% (2.9-12%)	67 (28-120)	1300 (560-2200)
Albania	2016	Female	75 to 79	20% (14-30%)	80% (70-86%)	1.2 (0.57-2)	4% (1.4-7.6%)	5.5% (1.8-10%)	79 (26-150)	1200 (400-2200)

Location	Year	Sex	Age	Prevalence of current drinkers	Prevalence of abstainers	Population average of standard drinks daily	Percent of total attributable deaths	Percent of total attributable DALYs	Attributable deaths	Attributable DALYs
Albania	2016	Female	80 to 84	18% (12-26%)	82% (74-88%)	0.99 (0.52-1.7)	3.9% (1.3-7.3%)	5% (1.7-9.6%)	86 (30-170)	920 (330-1800)
Albania	2016	Female	85 to 89	17% (11-25%)	83% (75-89%)	0.96 (0.49-1.7)	3.8% (1.3-7.5%)	4.8% (1.6-9.4%)	71 (24-140)	530 (190-1000)
Albania	2016	Female	90 to 94	19% (12-27%)	81% (73-88%)	1 (0.51-1.8)	3.6% (1-7.8%)	4.5% (1.2-9.7%)	44 (12-94)	230 (64-480)
Albania	2016	Female	95 plus	21% (13-32%)	79% (68-87%)	1.1 (0.48-2.2)	3.7% (0.48-8.6%)	4.4% (0.45-11%)	18 (1.8-45)	63 (8-150)
Albania	2016	Male	All Ages	53% (47-58%)	47% (42-53%)	2.8 (2.1-3.6)	6.7% (4.4-9.5%)	7.9% (4-13%)	1000 (510-1700)	29000 (18000-42000)
Albania	2016	Male	15 to 19	41% (31-50%)	59% (50-69%)	1.3 (0.79-2.1)	3.7% (2.4-5.4%)	4.9% (2.5-8.3%)	4.5 (2.3-7.6)	540 (340-810)
Albania	2016	Male	20 to 24	61% (49-70%)	39% (30-51%)	2.9 (1.8-4.3)	8.6% (5.9-12%)	11% (6.1-16%)	17 (9.4-27)	1700 (1100-2400)
Albania	2016	Male	25 to 29	66% (55-75%)	34% (25-45%)	3.2 (2-4.7)	8.8% (5.9-12%)	11% (6-17%)	18 (9.2-27)	1700 (1100-2400)
Albania	2016	Male	30 to 34	68% (58-77%)	32% (23-42%)	3.5 (2.2-5.1)	8.6% (5.7-12%)	12% (6.4-18%)	14 (7.8-22)	1300 (830-1800)
Albania	2016	Male	35 to 39	68% (58-76%)	32% (24-42%)	3.8 (2.3-5.8)	8.6% (5.5-12%)	12% (6.6-19%)	14 (7.3-22)	1100 (670-1500)
Albania	2016	Male	40 to 44	70% (60-77%)	30% (23-40%)	4.1 (2.7-6.1)	8.3% (5.2-12%)	12% (6.8-19%)	21 (11-32)	1300 (820-1900)
Albania	2016	Male	45 to 49	73% (62-80%)	27% (20-38%)	4.3 (2.8-6.5)	8% (4.8-12%)	12% (6.2-18%)	32 (16-51)	1800 (1000-2600)
Albania	2016	Male	50 to 54	70% (58-80%)	30% (20-42%)	4.4 (2.9-6.5)	8% (4.5-12%)	11% (5.8-17%)	55 (28-86)	2500 (1400-3700)
Albania	2016	Male	55 to 59	64% (46-80%)	36% (20-54%)	4.4 (2.7-6.6)	8.3% (4.8-12%)	11% (6.1-17%)	84 (43-130)	3200 (1700-4700)
Albania	2016	Male	60 to 64	59% (41-77%)	41% (23-59%)	4.1 (2.6-6.4)	8.4% (4.5-12%)	11% (5.4-16%)	110 (58-180)	3500 (1900-5400)
Albania	2016	Male	65 to 69	57% (40-75%)	43% (25-60%)	3.7 (2.3-5.6)	7.9% (4.2-12%)	9.9% (5.1-16%)	130 (64-210)	3200 (1700-5100)
Albania	2016	Male	70 to 74	54% (37-70%)	46% (30-63%)	3.1 (1.8-5)	7.1% (2.8-12%)	8.7% (3.3-15%)	150 (56-260)	2900 (1100-5100)
Albania	2016	Male	75 to 79	49% (35-66%)	51% (34-65%)	2.6 (1.3-4.4)	6% (1.6-11%)	7.2% (1.8-14%)	150 (39-300)	2300 (620-4400)
Albania	2016	Male	80 to 84	47% (31-63%)	53% (37-69%)	2.2 (1.2-3.8)	5.3% (1.4-11%)	6.2% (1.4-13%)	120 (25-240)	1300 (300-2600)
Albania	2016	Male	85 to 89	46% (32-61%)	54% (39-68%)	2 (1.1-3.4)	4.4% (0.97-9.9%)	5.1% (0.92-12%)	67 (12-160)	510 (110-1200)
Albania	2016	Male	90 to 94	46% (33-61%)	54% (39-67%)	1.9 (1-3.2)	3.2% (0.018-8.2%)	3.7% (-0.26-9.6%)	22 (-1.5-58)	120 (0.63-300)
Albania	2016	Male	95 plus	47% (31-65%)	53% (35-69%)	1.7 (0.78-3.2)	2.4% (-0.78-7.8%)	2.5% (-1.3-8.9%)	3.7 (-1.9-13)	14 (-4.7-46)

Location	Year	Sex	Age	Prevalence of current drinkers	Prevalence of abstainers	Population average of standard drinks daily	Percent of total attributable deaths	Percent of total attributable DALYs	Attributable deaths	Attributable DALYs
Bosnia and Herzegovina	1990	Female	All Ages	12% (10-14%)	88% (86-90%)	1.9 (1.4-2.5)	4.9% (3.7-6.4%)	6.6% (4.5-9.2%)	990 (650-1400)	29000 (21000-37000)
Bosnia and Herzegovina	1990	Female	15 to 19	12% (7.4-17%)	88% (83-93%)	1.3 (0.64-2.3)	3.3% (2.2-4.7%)	6.9% (3.9-11%)	4.6 (2.6-7.4)	620 (400-860)
Bosnia and Herzegovina	1990	Female	20 to 24	15% (9.8-23%)	85% (77-90%)	2.1 (1-3.7)	5.4% (4-7.1%)	8.2% (5-12%)	6.6 (3.8-9.8)	1100 (760-1500)
Bosnia and Herzegovina	1990	Female	25 to 29	16% (10-23%)	84% (77-90%)	2.1 (1.1-3.7)	5.2% (3.9-6.7%)	8.2% (5.3-11%)	8.2 (5-12)	1300 (880-1700)
Bosnia and Herzegovina	1990	Female	30 to 34	16% (10-25%)	84% (75-90%)	2.3 (1.2-4.1)	5% (3.7-6.6%)	9.3% (6.2-13%)	11 (7-16)	1300 (910-1700)
Bosnia and Herzegovina	1990	Female	35 to 39	17% (10-24%)	83% (76-90%)	2.7 (1.4-4.7)	5.2% (3.9-7%)	9.7% (6.7-13%)	17 (11-25)	1500 (1100-2000)
Bosnia and Herzegovina	1990	Female	40 to 44	16% (11-23%)	84% (77-89%)	3.1 (1.6-5)	5.6% (4.2-7.5%)	11% (7.4-14%)	25 (16-36)	1600 (1200-2200)
Bosnia and Herzegovina	1990	Female	45 to 49	16% (9.4-22%)	84% (78-91%)	3.4 (1.7-6.1)	6.7% (4.8-9.2%)	11% (7.6-16%)	38 (25-56)	1900 (1300-2700)
Bosnia and Herzegovina	1990	Female	50 to 54	15% (9.8-21%)	85% (79-90%)	3.4 (1.8-5.7)	6.7% (4.7-9.3%)	10% (7.1-15%)	69 (45-100)	2900 (2000-4100)
Bosnia and Herzegovina	1990	Female	55 to 59	14% (8.8-20%)	86% (80-91%)	3.1 (1.6-5.2)	6.6% (4.4-9.3%)	9.5% (6.2-14%)	94 (60-140)	3400 (2300-4800)
Bosnia and Herzegovina	1990	Female	60 to 64	13% (7.9-19%)	87% (81-92%)	2.8 (1.4-4.9)	6.4% (4.3-9.3%)	8.6% (5.6-13%)	130 (82-190)	3800 (2500-5400)
Bosnia and Herzegovina	1990	Female	65 to 69	12% (7.6-16%)	88% (84-92%)	2.5 (1.3-4.2)	6.3% (4.1-9.3%)	8% (5.1-12%)	150 (92-230)	3500 (2200-5300)
Bosnia and Herzegovina	1990	Female	70 to 74	10% (6.7-14%)	90% (86-93%)	2 (1-3.7)	5.5% (3.4-8.4%)	6.8% (4.1-11%)	110 (63-170)	2000 (1200-3100)
Bosnia and Herzegovina	1990	Female	75 to 79	8.8% (5.5-13%)	91% (87-95%)	1.7 (0.76-3.2)	4.6% (2.6-7.4%)	5.5% (3.1-9.1%)	130 (69-210)	1800 (1000-3000)
Bosnia and Herzegovina	1990	Female	80 to 84	7.8% (4.6-11%)	92% (89-95%)	1.5 (0.68-2.6)	4% (2.1-6.6%)	4.8% (2.4-8%)	97 (48-170)	1000 (530-1800)
Bosnia and Herzegovina	1990	Female	85 to 89	7.5% (4.4-12%)	93% (88-96%)	1.4 (0.68-2.7)	3.8% (1.8-6.7%)	4.5% (2.1-8.1%)	69 (31-120)	520 (250-910)
Bosnia and Herzegovina	1990	Female	90 to 94	8% (4.9-12%)	92% (88-95%)	1.5 (0.71-3)	3.7% (1.8-6.9%)	4.4% (2-8.1%)	31 (14-58)	160 (76-290)
Bosnia and Herzegovina	1990	Female	95 plus	9.3% (5.2-15%)	91% (85-95%)	1.8 (0.69-3.6)	4% (1.7-7.3%)	4.6% (1.9-8.5%)	9.4 (3.8-18)	32 (14-59)
Bosnia and Herzegovina	1990	Male	All Ages	37% (32-42%)	63% (58-68%)	5 (3.8-6.6)	13% (10-15%)	15% (12-19%)	2600 (2000-3400)	90000 (71000-110000)
Bosnia and Herzegovina	1990	Male	15 to 19	34% (23-48%)	66% (52-77%)	2.4 (1.2-4.3)	6.8% (4.2-10%)	11% (5.5-18%)	16 (7.8-27)	1700 (1000-2500)
Bosnia and Herzegovina	1990	Male	20 to 24	44% (30-57%)	56% (43-70%)	4.7 (2.5-8)	14% (9.6-18%)	19% (12-27%)	47 (29-68)	4300 (3000-5800)
Bosnia and Herzegovina	1990	Male	25 to 29	41% (29-53%)	59% (47-71%)	5.3 (2.8-8.6)	14% (11-17%)	20% (14-26%)	58 (40-79)	5000 (3700-6400)

Location	Year	Sex	Age	Prevalence of current drinkers	Prevalence of abstainers	Population average of standard drinks daily	Percent of total attributable deaths	Percent of total attributable DALYs	Attributable deaths	Attributable DALYs
Bosnia and Herzegovina	1990	Male	30 to 34	41% (29-54%)	59% (46-71%)	6 (3.3-9.6)	15% (12-18%)	22% (17-28%)	74 (54-96)	5500 (4200-6900)
Bosnia and Herzegovina	1990	Male	35 to 39	49% (34-62%)	51% (38-66%)	7 (3.9-11)	17% (14-21%)	25% (20-30%)	100 (78-130)	6700 (5200-8400)
Bosnia and Herzegovina	1990	Male	40 to 44	52% (38-66%)	48% (34-62%)	8.3 (4.8-13)	18% (15-22%)	26% (21-31%)	130 (100-170)	7300 (5800-9200)
Bosnia and Herzegovina	1990	Male	45 to 49	52% (37-66%)	48% (34-63%)	9.6 (5.5-15)	19% (15-23%)	24% (19-29%)	160 (120-210)	7500 (5800-9600)
Bosnia and Herzegovina	1990	Male	50 to 54	50% (36-63%)	50% (37-64%)	10 (6-15)	18% (14-22%)	22% (17-28%)	310 (230-410)	12000 (9300-16000)
Bosnia and Herzegovina	1990	Male	55 to 59	48% (34-60%)	52% (40-66%)	10 (5.7-16)	16% (12-20%)	19% (14-24%)	370 (270-500)	13000 (9300-17000)
Bosnia and Herzegovina	1990	Male	60 to 64	46% (32-60%)	54% (40-68%)	9.6 (5.1-16)	14% (10-18%)	16% (12-21%)	370 (260-500)	11000 (7700-14000)
Bosnia and Herzegovina	1990	Male	65 to 69	44% (32-56%)	56% (44-68%)	8.5 (4.9-13)	14% (9.5-18%)	15% (10-20%)	290 (200-400)	6800 (4700-9100)
Bosnia and Herzegovina	1990	Male	70 to 74	41% (30-53%)	59% (47-70%)	7 (3.7-11)	12% (8.4-17%)	14% (9.6-19%)	180 (120-250)	3400 (2300-4700)
Bosnia and Herzegovina	1990	Male	75 to 79	36% (25-48%)	64% (52-75%)	5.5 (2.7-9.8)	10% (6.6-14%)	12% (7.3-16%)	210 (130-300)	3000 (1900-4300)
Bosnia and Herzegovina	1990	Male	80 to 84	34% (24-46%)	66% (54-76%)	4.7 (2.5-7.9)	9.2% (5.5-14%)	10% (6.1-16%)	170 (100-270)	1800 (1100-2800)
Bosnia and Herzegovina	1990	Male	85 to 89	34% (23-46%)	66% (54-77%)	4.2 (2.2-7.1)	8.5% (4.8-13%)	9.6% (5.3-15%)	110 (58-170)	810 (450-1300)
Bosnia and Herzegovina	1990	Male	90 to 94	35% (24-47%)	65% (53-76%)	3.9 (2-6.9)	7.8% (4.1-12%)	8.7% (4.4-14%)	34 (17-54)	180 (95-280)
Bosnia and Herzegovina	1990	Male	95 plus	37% (24-52%)	63% (48-76%)	3.6 (1.5-6.9)	7% (2.6-12%)	7.7% (2.6-14%)	6.2 (2.2-11)	22 (8.4-37)
Bosnia and Herzegovina	1995	Female	All Ages	13% (11-15%)	87% (85-89%)	1.9 (1.4-2.4)	4.5% (3.4-5.9%)	6.1% (4.1-8.7%)	1000 (650-1500)	28000 (20000-37000)
Bosnia and Herzegovina	1995	Female	15 to 19	12% (7.7-17%)	88% (83-92%)	1.3 (0.66-2.2)	2.1% (1.4-2.9%)	2% (1.1-3.4%)	3.6 (1.9-6)	480 (310-690)
Bosnia and Herzegovina	1995	Female	20 to 24	16% (9.7-23%)	84% (77-90%)	2 (0.96-3.6)	3.7% (2.6-4.9%)	3% (1.7-4.5%)	5.8 (3.4-8.9)	930 (630-1300)
Bosnia and Herzegovina	1995	Female	25 to 29	16% (10-24%)	84% (76-90%)	2 (1-3.5)	4.1% (3.1-5.2%)	3.8% (2.3-5.7%)	6.1 (3.6-9.3)	910 (640-1200)
Bosnia and Herzegovina	1995	Female	30 to 34	17% (10-25%)	83% (75-90%)	2.2 (1.2-3.9)	4.2% (3.1-5.4%)	4.9% (3.2-7.1%)	11 (6.9-16)	1200 (850-1600)
Bosnia and Herzegovina	1995	Female	35 to 39	17% (11-24%)	83% (76-89%)	2.6 (1.4-4.3)	4.5% (3.4-6.1%)	6.3% (4.2-8.9%)	17 (11-25)	1400 (960-1800)
Bosnia and Herzegovina	1995	Female	40 to 44	17% (11-24%)	83% (76-89%)	2.9 (1.6-4.5)	5.2% (3.8-7.1%)	8.1% (5.6-12%)	28 (18-42)	1800 (1200-2400)
Bosnia and Herzegovina	1995	Female	45 to 49	16% (9.6-23%)	84% (77-90%)	3.2 (1.7-5.5)	6.1% (4.4-8.5%)	9.4% (6.4-13%)	47 (30-70)	2300 (1600-3200)

Location	Year	Sex	Age	Prevalence of current drinkers	Prevalence of abstainers	Population average of standard drinks daily	Percent of total attributable deaths	Percent of total attributable DALYs	Attributable deaths	Attributable DALYs
Bosnia and Herzegovina	1995	Female	50 to 54	15% (9.9-22%)	85% (78-90%)	3.2 (1.7-5.3)	6.3% (4.4-8.8%)	9.2% (6.3-13%)	45 (29-66)	1900 (1300-2700)
Bosnia and Herzegovina	1995	Female	55 to 59	14% (9-20%)	86% (80-91%)	3 (1.6-4.8)	6.3% (4.2-9%)	8.8% (5.8-13%)	87 (55-130)	3100 (2100-4500)
Bosnia and Herzegovina	1995	Female	60 to 64	13% (8.1-19%)	87% (81-92%)	2.7 (1.4-4.5)	6.3% (4.2-9.1%)	8.3% (5.4-12%)	130 (83-190)	3800 (2500-5600)
Bosnia and Herzegovina	1995	Female	65 to 69	12% (7.9-17%)	88% (83-92%)	2.4 (1.3-3.8)	6.2% (4-9.2%)	7.9% (4.9-12%)	170 (100-260)	4000 (2500-6000)
Bosnia and Herzegovina	1995	Female	70 to 74	10% (6.9-15%)	90% (85-93%)	2 (1-3.4)	5.5% (3.3-8.5%)	6.8% (4-11%)	150 (91-250)	2900 (1800-4500)
Bosnia and Herzegovina	1995	Female	75 to 79	8.9% (5.5-13%)	91% (87-94%)	1.6 (0.77-2.9)	4.6% (2.6-7.5%)	5.5% (3.1-9.1%)	96 (52-160)	1400 (780-2300)
Bosnia and Herzegovina	1995	Female	80 to 84	7.9% (4.7-11%)	92% (89-95%)	1.4 (0.68-2.4)	4.1% (2.1-6.7%)	4.8% (2.4-8.1%)	89 (43-150)	950 (470-1600)
Bosnia and Herzegovina	1995	Female	85 to 89	7.7% (4.4-12%)	92% (88-96%)	1.4 (0.65-2.6)	3.8% (1.8-6.6%)	4.5% (2.1-8.1%)	77 (35-140)	580 (280-1000)
Bosnia and Herzegovina	1995	Female	90 to 94	8.3% (5.1-13%)	92% (87-95%)	1.5 (0.7-2.9)	3.7% (1.8-6.8%)	4.4% (2-8.2%)	36 (16-68)	190 (90-350)
Bosnia and Herzegovina	1995	Female	95 plus	9.6% (5.4-15%)	90% (85-95%)	1.7 (0.69-3.5)	3.9% (1.7-6.9%)	4.5% (1.9-8.4%)	12 (4.8-22)	40 (18-73)
Bosnia and Herzegovina	1995	Male	All Ages	38% (33-43%)	62% (57-67%)	4.9 (3.8-6)	9.8% (7.8-12%)	13% (9.5-16%)	2600 (1900-3400)	85000 (66000-110000)
Bosnia and Herzegovina	1995	Male	15 to 19	34% (23-49%)	66% (51-77%)	2.2 (1.1-4)	3.2% (1.9-4.9%)	3% (1.5-5.2%)	14 (6.5-23)	1300 (770-2100)
Bosnia and Herzegovina	1995	Male	20 to 24	44% (30-57%)	56% (43-70%)	4.4 (2.2-7.3)	4.9% (3.2-6.6%)	4.2% (2.5-6.3%)	38 (23-57)	3400 (2300-4700)
Bosnia and Herzegovina	1995	Male	25 to 29	41% (28-53%)	59% (47-72%)	5 (2.5-8)	6.2% (4.5-7.9%)	5.7% (3.8-7.8%)	43 (28-59)	3600 (2600-4700)
Bosnia and Herzegovina	1995	Male	30 to 34	42% (29-54%)	58% (46-71%)	5.5 (3.1-8.9)	8% (6.2-10%)	8.2% (5.9-11%)	65 (46-85)	4800 (3600-6100)
Bosnia and Herzegovina	1995	Male	35 to 39	48% (35-62%)	52% (38-65%)	6.4 (3.7-9.9)	11% (8.6-14%)	12% (9.4-16%)	93 (67-120)	5900 (4600-7600)
Bosnia and Herzegovina	1995	Male	40 to 44	52% (38-66%)	48% (34-62%)	7.6 (4.5-11)	14% (11-17%)	17% (13-21%)	140 (100-180)	7500 (5800-9700)
Bosnia and Herzegovina	1995	Male	45 to 49	52% (36-65%)	48% (35-64%)	8.7 (5.1-13)	16% (13-20%)	20% (15-24%)	200 (140-260)	9200 (7000-12000)
Bosnia and Herzegovina	1995	Male	50 to 54	50% (36-63%)	50% (37-64%)	9.2 (5.6-14)	16% (13-21%)	20% (15-25%)	180 (130-250)	7400 (5500-9800)
Bosnia and Herzegovina	1995	Male	55 to 59	47% (34-60%)	53% (40-66%)	9.3 (5.5-14)	15% (11-19%)	17% (13-22%)	320 (220-440)	11000 (7800-15000)
Bosnia and Herzegovina	1995	Male	60 to 64	46% (32-59%)	54% (41-68%)	8.8 (4.8-14)	13% (9.8-18%)	15% (11-20%)	380 (260-520)	11000 (7800-15000)
Bosnia and Herzegovina	1995	Male	65 to 69	44% (32-56%)	56% (44-68%)	7.7 (4.6-12)	13% (9-17%)	15% (10-20%)	440 (290-600)	10000 (6900-14000)

Location	Year	Sex	Age	Prevalence of current drinkers	Prevalence of abstainers	Population average of standard drinks daily	Percent of total attributable deaths	Percent of total attributable DALYs	Attributable deaths	Attributable DALYs
Bosnia and Herzegovina	1995	Male	70 to 74	41% (29-53%)	59% (47-71%)	6.4 (3.6-10)	12% (8.2-16%)	14% (9.2-19%)	270 (170-370)	5000 (3300-7000)
Bosnia and Herzegovina	1995	Male	75 to 79	37% (25-50%)	63% (50-75%)	5 (2.7-8.7)	10% (6.4-14%)	11% (7.2-16%)	130 (78-190)	1900 (1200-2700)
Bosnia and Herzegovina	1995	Male	80 to 84	34% (24-47%)	66% (53-76%)	4.3 (2.3-7.1)	8.9% (5.3-13%)	10% (6-15%)	120 (71-190)	1300 (790-2000)
Bosnia and Herzegovina	1995	Male	85 to 89	34% (23-46%)	66% (54-77%)	3.9 (2.1-6.4)	8.1% (4.5-12%)	9.2% (4.9-14%)	110 (57-170)	810 (450-1300)
Bosnia and Herzegovina	1995	Male	90 to 94	35% (24-47%)	65% (53-76%)	3.5 (1.8-6.2)	7.3% (3.8-12%)	8.2% (4.1-14%)	44 (22-72)	230 (120-370)
Bosnia and Herzegovina	1995	Male	95 plus	37% (24-52%)	63% (48-76%)	3.3 (1.4-6.1)	6.4% (2.1-11%)	7.1% (2.2-13%)	8.7 (2.5-16)	31 (10-55)
Bosnia and Herzegovina	2000	Female	All Ages	11% (9.7-13%)	89% (87-90%)	1.9 (1.4-2.4)	4.6% (3.4-6.1%)	6% (3.9-8.5%)	840 (550-1200)	23000 (17000-31000)
Bosnia and Herzegovina	2000	Female	15 to 19	11% (7.1-15%)	89% (85-93%)	1.2 (0.63-2.2)	2.9% (2-4%)	6.3% (3.6-9.9%)	2.4 (1.3-4)	400 (260-560)
Bosnia and Herzegovina	2000	Female	20 to 24	13% (8.3-20%)	87% (80-92%)	1.8 (0.88-3.4)	5% (3.7-6.6%)	7.4% (4.5-11%)	3.4 (2-5.1)	680 (460-940)
Bosnia and Herzegovina	2000	Female	25 to 29	14% (8.6-20%)	86% (80-91%)	1.8 (0.89-3.3)	4.9% (3.7-6.2%)	7.1% (4.4-10%)	3.6 (2.2-5.5)	730 (510-990)
Bosnia and Herzegovina	2000	Female	30 to 34	14% (9.1-22%)	86% (78-91%)	2.1 (1.1-3.7)	4.7% (3.6-6.1%)	8.1% (5.5-12%)	5.7 (3.7-8.6)	780 (560-1000)
Bosnia and Herzegovina	2000	Female	35 to 39	15% (9.4-22%)	85% (78-91%)	2.5 (1.3-4.1)	4.7% (3.6-6.2%)	8.7% (5.9-12%)	11 (7.1-17)	1100 (760-1400)
Bosnia and Herzegovina	2000	Female	40 to 44	16% (11-22%)	84% (78-89%)	3 (1.7-4.7)	5.1% (3.9-6.9%)	9.8% (6.7-14%)	20 (13-29)	1400 (960-1900)
Bosnia and Herzegovina	2000	Female	45 to 49	15% (9.1-20%)	85% (80-91%)	3.1 (1.8-5.2)	5.6% (4.1-7.7%)	9.8% (6.8-14%)	35 (23-51)	1900 (1300-2600)
Bosnia and Herzegovina	2000	Female	50 to 54	13% (8.6-19%)	87% (81-91%)	3 (1.6-4.8)	5.6% (3.9-7.7%)	9% (6.1-13%)	43 (28-64)	1900 (1400-2700)
Bosnia and Herzegovina	2000	Female	55 to 59	12% (8-18%)	88% (82-92%)	2.7 (1.5-4.5)	5.4% (3.7-7.7%)	8.3% (5.6-12%)	41 (27-61)	1500 (1100-2200)
Bosnia and Herzegovina	2000	Female	60 to 64	11% (7.3-16%)	89% (84-93%)	2.5 (1.3-4.2)	5.4% (3.7-7.8%)	7.5% (5-11%)	96 (61-140)	2900 (1900-4100)
Bosnia and Herzegovina	2000	Female	65 to 69	10% (6.5-14%)	90% (86-93%)	2.1 (1.2-3.4)	5.3% (3.4-7.8%)	7% (4.4-10%)	140 (86-210)	3300 (2100-5000)
Bosnia and Herzegovina	2000	Female	70 to 74	9% (5.9-13%)	91% (87-94%)	1.8 (0.95-3.1)	4.8% (2.9-7.5%)	6.1% (3.6-9.5%)	150 (89-240)	2900 (1700-4500)
Bosnia and Herzegovina	2000	Female	75 to 79	8.2% (5.1-12%)	92% (88-95%)	1.6 (0.75-2.8)	4.2% (2.4-6.8%)	5.1% (2.9-8.4%)	130 (71-210)	1900 (1100-3100)
Bosnia and Herzegovina	2000	Female	80 to 84	7.5% (4.5-11%)	93% (89-96%)	1.4 (0.7-2.5)	3.8% (1.9-6.2%)	4.6% (2.3-7.8%)	82 (40-140)	890 (440-1500)
Bosnia and Herzegovina	2000	Female	85 to 89	7.2% (4.2-11%)	93% (89-96%)	1.4 (0.65-2.5)	3.3% (1.7-5.8%)	4.2% (2-7.4%)	46 (21-81)	350 (170-620)

Location	Year	Sex	Age	Prevalence of current drinkers	Prevalence of abstainers	Population average of standard drinks daily	Percent of total attributable deaths	Percent of total attributable DALYs	Attributable deaths	Attributable DALYs
Bosnia and Herzegovina	2000	Female	90 to 94	7.8% (4.9-12%)	92% (88-95%)	1.5 (0.7-2.9)	3.2% (1.5-5.9%)	4% (1.8-7.5%)	31 (14-57)	160 (79-300)
Bosnia and Herzegovina	2000	Female	95 plus	9% (5.1-14%)	91% (86-95%)	1.7 (0.66-3.5)	3.4% (1.5-6.2%)	4.2% (1.7-7.7%)	11 (4.4-20)	39 (17-71)
Bosnia and Herzegovina	2000	Male	All Ages	40% (35-44%)	60% (56-65%)	5.7 (4.5-6.9)	13% (10-16%)	15% (11-19%)	2400 (1700-3100)	76000 (58000-96000)
Bosnia and Herzegovina	2000	Male	15 to 19	36% (26-49%)	64% (51-74%)	2.5 (1.3-4.3)	7.3% (4.6-11%)	13% (6.8-20%)	12 (6.4-21)	1300 (810-2000)
Bosnia and Herzegovina	2000	Male	20 to 24	44% (32-56%)	56% (44-68%)	4.7 (2.6-7.9)	14% (10-17%)	21% (14-28%)	26 (16-37)	2600 (1800-3400)
Bosnia and Herzegovina	2000	Male	25 to 29	37% (27-48%)	63% (52-73%)	4.8 (2.6-7.8)	13% (10-16%)	20% (15-27%)	31 (21-43)	2900 (2100-3700)
Bosnia and Herzegovina	2000	Male	30 to 34	38% (26-50%)	62% (50-74%)	5.4 (3-8.5)	13% (11-17%)	22% (17-28%)	37 (26-50)	3100 (2300-3900)
Bosnia and Herzegovina	2000	Male	35 to 39	49% (38-61%)	51% (39-62%)	7 (4.1-10)	16% (13-20%)	26% (20-31%)	72 (52-97)	4900 (3800-6300)
Bosnia and Herzegovina	2000	Male	40 to 44	57% (45-69%)	43% (31-55%)	9.1 (5.8-13)	18% (15-22%)	27% (22-33%)	110 (83-150)	6500 (5100-8300)
Bosnia and Herzegovina	2000	Male	45 to 49	58% (44-70%)	42% (30-56%)	11 (6.6-16)	18% (15-22%)	26% (20-31%)	180 (130-240)	8700 (6700-11000)
Bosnia and Herzegovina	2000	Male	50 to 54	53% (39-64%)	47% (36-61%)	11 (6.4-15)	17% (14-22%)	22% (17-28%)	220 (160-290)	9100 (6900-12000)
Bosnia and Herzegovina	2000	Male	55 to 59	47% (35-59%)	53% (41-65%)	10 (6-15)	15% (11-19%)	19% (14-24%)	170 (120-230)	6100 (4400-8000)
Bosnia and Herzegovina	2000	Male	60 to 64	44% (31-57%)	56% (43-69%)	9 (5-14)	13% (9.8-17%)	16% (11-20%)	330 (230-450)	9600 (6900-13000)
Bosnia and Herzegovina	2000	Male	65 to 69	41% (31-52%)	59% (48-69%)	7.6 (4.6-11)	12% (8.5-16%)	14% (9.9-19%)	390 (260-540)	9400 (6400-13000)
Bosnia and Herzegovina	2000	Male	70 to 74	38% (28-50%)	62% (50-72%)	6.4 (3.6-10)	11% (7.7-15%)	13% (8.8-18%)	380 (240-540)	7300 (4800-10000)
Bosnia and Herzegovina	2000	Male	75 to 79	35% (25-47%)	65% (53-75%)	5.2 (2.6-8.8)	9.6% (6-13%)	11% (6.9-16%)	180 (110-270)	2700 (1700-4000)
Bosnia and Herzegovina	2000	Male	80 to 84	33% (23-45%)	67% (55-77%)	4.4 (2.4-7.3)	8.6% (5.2-13%)	9.9% (5.7-15%)	98 (56-150)	1100 (650-1700)
Bosnia and Herzegovina	2000	Male	85 to 89	33% (22-45%)	67% (55-78%)	4 (2.1-6.5)	7.7% (4.3-12%)	8.9% (4.8-14%)	57 (30-92)	450 (250-710)
Bosnia and Herzegovina	2000	Male	90 to 94	34% (23-46%)	66% (54-77%)	3.7 (1.9-6.3)	7.2% (3.9-12%)	8.2% (4.3-13%)	39 (20-65)	210 (120-340)
Bosnia and Herzegovina	2000	Male	95 plus	36% (23-51%)	64% (49-77%)	3.4 (1.5-6.6)	6.5% (2.5-11%)	7.3% (2.4-13%)	10 (3.5-18)	38 (15-65)
Bosnia and Herzegovina	2005	Female	All Ages	10% (8.9-12%)	90% (88-91%)	1.9 (1.4-2.5)	4.1% (3-5.4%)	5.2% (3.4-7.4%)	820 (530-1200)	21000 (16000-28000)
Bosnia and Herzegovina	2005	Female	15 to 19	9.8% (6.3-14%)	90% (86-94%)	1.2 (0.63-2.2)	2.6% (1.8-3.5%)	5.4% (3.2-8.5%)	1.6 (0.94-2.5)	320 (210-450)

Location	Year	Sex	Age	Prevalence of current drinkers	Prevalence of abstainers	Population average of standard drinks daily	Percent of total attributable deaths	Percent of total attributable DALYs	Attributable deaths	Attributable DALYs
Bosnia and Herzegovina	2005	Female	20 to 24	12% (7.7-18%)	88% (82-92%)	1.8 (0.86-3.3)	4.7% (3.4-6.1%)	6.2% (3.8-9.3%)	2.8 (1.7-4.3)	700 (470-980)
Bosnia and Herzegovina	2005	Female	25 to 29	13% (7.9-19%)	87% (81-92%)	1.8 (0.89-3.3)	4.6% (3.5-5.9%)	6.1% (3.9-8.6%)	2.6 (1.6-3.9)	650 (450-890)
Bosnia and Herzegovina	2005	Female	30 to 34	13% (8-20%)	87% (80-92%)	2 (1-3.6)	4.4% (3.3-5.7%)	6.9% (4.5-10%)	4.7 (3-7)	760 (540-1000)
Bosnia and Herzegovina	2005	Female	35 to 39	14% (8.5-20%)	86% (80-91%)	2.4 (1.2-4.2)	4.3% (3.3-5.7%)	7.5% (4.9-11%)	7.1 (4.5-11)	810 (570-1100)
Bosnia and Herzegovina	2005	Female	40 to 44	14% (9.8-20%)	86% (80-90%)	2.9 (1.6-4.7)	4.6% (3.4-6.2%)	8.4% (5.6-12%)	16 (9.9-23)	1200 (870-1700)
Bosnia and Herzegovina	2005	Female	45 to 49	13% (8.4-19%)	87% (81-92%)	3.1 (1.7-5.3)	4.8% (3.5-6.7%)	8.3% (5.6-12%)	28 (18-42)	1600 (1100-2200)
Bosnia and Herzegovina	2005	Female	50 to 54	12% (7.9-17%)	88% (83-92%)	2.9 (1.6-4.9)	4.7% (3.3-6.5%)	7.6% (5.1-11%)	38 (24-57)	1800 (1200-2600)
Bosnia and Herzegovina	2005	Female	55 to 59	11% (7.2-16%)	89% (84-93%)	2.7 (1.5-4.3)	4.6% (3.1-6.4%)	7.1% (4.7-10%)	46 (30-68)	1800 (1200-2600)
Bosnia and Herzegovina	2005	Female	60 to 64	10% (6.5-15%)	90% (85-93%)	2.4 (1.2-4.1)	4.7% (3.1-6.8%)	6.5% (4.2-9.5%)	57 (37-84)	1800 (1200-2500)
Bosnia and Herzegovina	2005	Female	65 to 69	9.2% (5.9-13%)	91% (87-94%)	2.1 (1.1-3.5)	4.7% (3-6.9%)	6.1% (3.8-9.1%)	120 (75-190)	3000 (1900-4600)
Bosnia and Herzegovina	2005	Female	70 to 74	8.2% (5.4-12%)	92% (88-95%)	1.8 (0.92-3.1)	4.3% (2.6-6.6%)	5.4% (3.3-8.3%)	160 (94-250)	3000 (1800-4700)
Bosnia and Herzegovina	2005	Female	75 to 79	7.5% (4.6-11%)	93% (89-95%)	1.6 (0.73-2.7)	3.7% (2.2-6%)	4.6% (2.6-7.3%)	150 (87-250)	2200 (1300-3600)
Bosnia and Herzegovina	2005	Female	80 to 84	6.8% (4.2-9.6%)	93% (90-96%)	1.4 (0.69-2.4)	3.4% (1.8-5.5%)	4.1% (2.1-6.7%)	110 (57-180)	1200 (630-1900)
Bosnia and Herzegovina	2005	Female	85 to 89	6.6% (3.8-10%)	93% (90-96%)	1.4 (0.62-2.5)	2.9% (1.4-4.9%)	3.7% (1.8-6.4%)	43 (20-75)	340 (170-570)
Bosnia and Herzegovina	2005	Female	90 to 94	7.1% (4.3-11%)	93% (89-96%)	1.5 (0.66-2.8)	2.8% (1.4-4.9%)	3.6% (1.6-6.5%)	21 (9.7-37)	110 (56-200)
Bosnia and Herzegovina	2005	Female	95 plus	8.2% (4.7-13%)	92% (87-95%)	1.7 (0.63-3.5)	3% (1.4-5.3%)	3.8% (1.6-7%)	11 (4.5-20)	41 (18-72)
Bosnia and Herzegovina	2005	Male	All Ages	38% (34-43%)	62% (57-66%)	6 (4.7-7.6)	12% (9.1-14%)	13% (9.6-17%)	2300 (1600-3000)	72000 (55000-90000)
Bosnia and Herzegovina	2005	Male	15 to 19	34% (24-46%)	66% (54-76%)	2.5 (1.3-4.4)	6.8% (4.4-9.9%)	12% (6.4-18%)	9.1 (5-15)	1100 (700-1500)
Bosnia and Herzegovina	2005	Male	20 to 24	42% (30-54%)	58% (46-70%)	4.7 (2.5-8)	13% (9.6-16%)	19% (13-26%)	22 (14-31)	2500 (1800-3300)
Bosnia and Herzegovina	2005	Male	25 to 29	34% (25-45%)	66% (55-75%)	4.8 (2.6-8)	12% (9.6-15%)	18% (13-24%)	23 (16-32)	2400 (1800-3100)
Bosnia and Herzegovina	2005	Male	30 to 34	35% (25-48%)	65% (52-75%)	5.4 (2.9-8.9)	12% (9.2-14%)	20% (15-25%)	28 (20-37)	2600 (2000-3200)
Bosnia and Herzegovina	2005	Male	35 to 39	47% (35-59%)	53% (41-65%)	7.1 (3.9-11)	14% (11-17%)	23% (18-29%)	46 (34-62)	3400 (2600-4300)

Location	Year	Sex	Age	Prevalence of current drinkers	Prevalence of abstainers	Population average of standard drinks daily	Percent of total attributable deaths	Percent of total attributable DALYs	Attributable deaths	Attributable DALYs
Bosnia and Herzegovina	2005	Male	40 to 44	55%<br>(43-67%)	45%<br>(33-57%)	9.3<br>(5.6-13)	16%<br>(13-20%)	25%<br>(20-31%)	95<br>(71-130)	5800<br>(4500-7200)
Bosnia and Herzegovina	2005	Male	45 to 49	56%<br>(42-67%)	44%<br>(33-58%)	11<br>(6.7-16)	17%<br>(13-20%)	23%<br>(18-29%)	150<br>(110-200)	7700<br>(5800-9900)
Bosnia and Herzegovina	2005	Male	50 to 54	50%<br>(37-61%)	50%<br>(39-63%)	11<br>(6.6-15)	15%<br>(12-19%)	20%<br>(15-25%)	210<br>(150-280)	9100<br>(6800-12000)
Bosnia and Herzegovina	2005	Male	55 to 59	44%<br>(33-56%)	56%<br>(44-67%)	10<br>(6-15)	13%<br>(9.9-17%)	17%<br>(12-21%)	210<br>(150-290)	7800<br>(5700-10000)
Bosnia and Herzegovina	2005	Male	60 to 64	41%<br>(30-54%)	59%<br>(46-70%)	9<br>(4.9-15)	12%<br>(8.8-16%)	14%<br>(10-19%)	210<br>(140-270)	6100<br>(4300-8100)
Bosnia and Herzegovina	2005	Male	65 to 69	39%<br>(29-49%)	61%<br>(51-71%)	7.7<br>(4.6-12)	11%<br>(7.7-15%)	13%<br>(9.1-17%)	370<br>(250-510)	9100<br>(6100-12000)
Bosnia and Herzegovina	2005	Male	70 to 74	36%<br>(26-47%)	64%<br>(53-74%)	6.4<br>(3.7-10)	10%<br>(6.9-14%)	12%<br>(7.8-16%)	390<br>(250-560)	7600<br>(4900-11000)
Bosnia and Herzegovina	2005	Male	75 to 79	32%<br>(23-44%)	68%<br>(56-77%)	5.1<br>(2.7-8.6)	8.7%<br>(5.6-12%)	10%<br>(6.3-14%)	290<br>(180-430)	4400<br>(2800-6300)
Bosnia and Herzegovina	2005	Male	80 to 84	31%<br>(21-42%)	69%<br>(58-79%)	4.3<br>(2.4-7.2)	7.9%<br>(4.7-12%)	9.2%<br>(5.4-14%)	150<br>(84-220)	1600<br>(970-2500)
Bosnia and Herzegovina	2005	Male	85 to 89	31%<br>(21-42%)	69%<br>(58-79%)	3.9<br>(2.1-6.6)	7.2%<br>(4.1-11%)	8.3%<br>(4.6-13%)	44<br>(24-70)	360<br>(200-550)
Bosnia and Herzegovina	2005	Male	90 to 94	32%<br>(21-44%)	68%<br>(56-79%)	3.7<br>(1.9-6.5)	6.8%<br>(3.8-11%)	7.8%<br>(4.1-13%)	22<br>(12-36)	120<br>(67-190)
Bosnia and Herzegovina	2005	Male	95 plus	33%<br>(21-48%)	67%<br>(52-79%)	3.4<br>(1.4-6.8)	6.3%<br>(2.8-10%)	7%<br>(2.9-12%)	9<br>(3.7-15)	34<br>(15-56)
Bosnia and Herzegovina	2010	Female	All Ages	11%<br>(9.4-13%)	89%<br>(87-91%)	2.1<br>(1.6-2.8)	4.1%<br>(3-5.4%)	5%<br>(3.3-7.2%)	850<br>(550-1300)	22000<br>(16000-29000)
Bosnia and Herzegovina	2010	Female	15 to 19	9.9%<br>(6.3-15%)	90%<br>(85-94%)	1.3<br>(0.64-2.4)	2.7%<br>(1.9-3.6%)	5.2%<br>(3.1-8.1%)	1.1<br>(0.69-1.8)	260<br>(170-370)
Bosnia and Herzegovina	2010	Female	20 to 24	13%<br>(8.1-19%)	87%<br>(81-92%)	2<br>(0.95-3.7)	5.1%<br>(3.6-6.7%)	6.2%<br>(3.7-9.2%)	2.3<br>(1.3-3.5)	670<br>(450-970)
Bosnia and Herzegovina	2010	Female	25 to 29	14%<br>(8.6-20%)	86%<br>(80-91%)	2.1<br>(1-3.8)	4.9%<br>(3.7-6.2%)	6.2%<br>(4-8.9%)	2.7<br>(1.6-4)	770<br>(540-1100)
Bosnia and Herzegovina	2010	Female	30 to 34	14%<br>(8.7-22%)	86%<br>(78-91%)	2.3<br>(1.1-4.2)	4.5%<br>(3.4-5.8%)	7.1%<br>(4.6-10%)	4.2<br>(2.6-6.5)	740<br>(510-1000)
Bosnia and Herzegovina	2010	Female	35 to 39	15%<br>(9.1-22%)	85%<br>(78-91%)	2.7<br>(1.3-4.7)	4.3%<br>(3.3-5.7%)	7.4%<br>(4.8-11%)	6.9<br>(4.2-10)	840<br>(600-1100)
Bosnia and Herzegovina	2010	Female	40 to 44	14%<br>(9.1-21%)	86%<br>(79-91%)	3<br>(1.6-5.1)	4.4%<br>(3.2-6%)	7.9%<br>(5.1-12%)	12<br>(7.1-18)	990<br>(690-1400)
Bosnia and Herzegovina	2010	Female	45 to 49	14%<br>(8.1-20%)	86%<br>(80-92%)	3.3<br>(1.7-5.8)	4.7%<br>(3.3-6.4%)	8%<br>(5.2-12%)	25<br>(16-38)	1500<br>(1100-2100)
Bosnia and Herzegovina	2010	Female	50 to 54	13%<br>(8.3-19%)	87%<br>(81-92%)	3.3<br>(1.8-5.6)	4.7%<br>(3.3-6.5%)	7.6%<br>(5-11%)	37<br>(23-56)	1800<br>(1200-2600)
Bosnia and Herzegovina	2010	Female	55 to 59	12%<br>(7.7-17%)	88%<br>(83-92%)	3<br>(1.6-5.1)	4.6%<br>(3.1-6.5%)	7.3%<br>(4.8-11%)	51<br>(32-75)	2000<br>(1400-2900)

Location	Year	Sex	Age	Prevalence of current drinkers	Prevalence of abstainers	Population average of standard drinks daily	Percent of total attributable deaths	Percent of total attributable DALYs	Attributable deaths	Attributable DALYs
Bosnia and Herzegovina	2010	Female	60 to 64	11% (6.8-17%)	89% (83-93%)	2.8 (1.3-4.7)	4.6% (3-6.7%)	6.6% (4.2-9.8%)	73 (45-110)	2300 (1500-3400)
Bosnia and Herzegovina	2010	Female	65 to 69	10% (6.5-15%)	90% (85-93%)	2.4 (1.3-4.1)	4.7% (2.9-7%)	6.4% (3.9-9.6%)	86 (52-130)	2100 (1300-3200)
Bosnia and Herzegovina	2010	Female	70 to 74	8.8% (5.7-13%)	91% (87-94%)	2 (1-3.6)	4.3% (2.6-6.7%)	5.6% (3.4-8.6%)	150 (93-240)	3000 (1800-4700)
Bosnia and Herzegovina	2010	Female	75 to 79	7.6% (4.7-12%)	92% (88-95%)	1.7 (0.76-3)	3.7% (2.1-5.9%)	4.6% (2.6-7.3%)	160 (95-270)	2400 (1400-4000)
Bosnia and Herzegovina	2010	Female	80 to 84	6.7% (4.1-9.6%)	93% (90-96%)	1.4 (0.69-2.5)	3.3% (1.8-5.3%)	4% (2.1-6.5%)	130 (68-210)	1400 (760-2300)
Bosnia and Herzegovina	2010	Female	85 to 89	6.5% (3.7-9.9%)	94% (90-96%)	1.4 (0.66-2.6)	2.8% (1.4-4.8%)	3.6% (1.8-6.2%)	68 (32-120)	530 (270-900)
Bosnia and Herzegovina	2010	Female	90 to 94	7% (4.2-11%)	93% (89-96%)	1.5 (0.68-2.9)	2.7% (1.3-4.8%)	3.5% (1.6-6.4%)	26 (12-49)	140 (73-260)
Bosnia and Herzegovina	2010	Female	95 plus	8% (4.5-13%)	92% (87-96%)	1.7 (0.62-3.7)	2.9% (1.3-5.2%)	3.7% (1.5-6.8%)	10 (4.3-19)	37 (17-65)
Bosnia and Herzegovina	2010	Male	All Ages	36% (31-40%)	64% (60-69%)	6.2 (4.7-8)	11% (8.4-13%)	12% (8.9-16%)	2200 (1600-2900)	68000 (52000-84000)
Bosnia and Herzegovina	2010	Male	15 to 19	30% (20-42%)	70% (58-80%)	2.3 (1.1-4.2)	6.3% (4.2-8.9%)	11% (5.5-17%)	6.1 (3.1-10)	770 (500-1100)
Bosnia and Herzegovina	2010	Male	20 to 24	39% (25-52%)	61% (48-75%)	4.7 (2.3-7.9)	12% (9.2-16%)	18% (12-25%)	18 (12-27)	2200 (1600-3000)
Bosnia and Herzegovina	2010	Male	25 to 29	36% (24-48%)	64% (52-76%)	5.3 (2.8-8.9)	12% (9.2-15%)	18% (13-25%)	24 (16-33)	2600 (1900-3300)
Bosnia and Herzegovina	2010	Male	30 to 34	37% (25-50%)	63% (50-75%)	6 (3.1-9.9)	12% (9.1-14%)	20% (14-26%)	25 (18-34)	2400 (1800-3000)
Bosnia and Herzegovina	2010	Male	35 to 39	44% (30-57%)	56% (43-70%)	7.1 (3.8-11)	13% (10-17%)	22% (16-29%)	42 (30-57)	3200 (2400-4100)
Bosnia and Herzegovina	2010	Male	40 to 44	47% (33-62%)	53% (38-67%)	8.4 (4.9-13)	14% (11-18%)	22% (17-29%)	67 (49-91)	4200 (3200-5500)
Bosnia and Herzegovina	2010	Male	45 to 49	47% (31-61%)	53% (39-69%)	9.6 (5.5-15)	14% (11-18%)	20% (15-26%)	130 (92-170)	6600 (5000-8500)
Bosnia and Herzegovina	2010	Male	50 to 54	45% (31-59%)	55% (41-69%)	10 (6.1-15)	14% (11-18%)	18% (14-23%)	190 (130-250)	8100 (5900-11000)
Bosnia and Herzegovina	2010	Male	55 to 59	42% (29-56%)	58% (44-71%)	10 (5.8-16)	12% (9.1-16%)	16% (12-20%)	220 (160-300)	8200 (6000-11000)
Bosnia and Herzegovina	2010	Male	60 to 64	41% (27-55%)	59% (45-73%)	9.5 (5-15)	11% (8.2-15%)	14% (9.9-18%)	270 (180-360)	8100 (5700-11000)
Bosnia and Herzegovina	2010	Male	65 to 69	40% (27-53%)	60% (47-73%)	8.4 (4.8-13)	11% (7.5-15%)	13% (8.7-17%)	240 (160-340)	6000 (4000-8200)
Bosnia and Herzegovina	2010	Male	70 to 74	36% (26-48%)	64% (52-74%)	6.9 (3.8-11)	10% (6.6-14%)	12% (7.6-16%)	380 (250-540)	7500 (4900-11000)
Bosnia and Herzegovina	2010	Male	75 to 79	32% (22-45%)	68% (55-78%)	5.3 (2.7-9.2)	8.3% (5.3-12%)	9.6% (6.1-14%)	310 (190-450)	4800 (3000-6800)

Location	Year	Sex	Age	Prevalence of current drinkers	Prevalence of abstainers	Population average of standard drinks daily	Percent of total attributable deaths	Percent of total attributable DALYs	Attributable deaths	Attributable DALYs
Bosnia and Herzegovina	2010	Male	80 to 84	29% (20-41%)	71% (59-80%)	4.4 (2.3-7.3)	7.6% (4.7-11%)	8.8% (5.3-13%)	200 (120-300)	2200 (1300-3300)
Bosnia and Herzegovina	2010	Male	85 to 89	29% (19-41%)	71% (59-81%)	4 (2.1-6.7)	6.8% (3.9-10%)	8% (4.4-13%)	85 (46-130)	690 (390-1100)
Bosnia and Herzegovina	2010	Male	90 to 94	31% (20-42%)	69% (58-80%)	3.8 (1.9-6.6)	6.6% (3.8-10%)	7.6% (4.1-12%)	25 (13-39)	130 (76-210)
Bosnia and Herzegovina	2010	Male	95 plus	33% (20-48%)	67% (52-80%)	3.5 (1.4-7)	6.2% (2.8-10%)	7% (2.9-12%)	6.5 (2.7-11)	24 (11-40)
Bosnia and Herzegovina	2016	Female	All Ages	11% (9-12%)	89% (88-91%)	2.1 (1.4-3)	3.9% (2.9-5.1%)	4.7% (3.1-6.7%)	860 (540-1300)	21000 (15000-29000)
Bosnia and Herzegovina	2016	Female	15 to 19	9.7% (6.2-15%)	90% (85-94%)	1.3 (0.59-2.4)	2.7% (1.9-3.6%)	5.2% (3-8.1%)	1 (0.57-1.7)	230 (160-330)
Bosnia and Herzegovina	2016	Female	20 to 24	13% (8.1-19%)	87% (81-92%)	2 (0.89-3.8)	5.2% (3.7-6.9%)	6.2% (3.8-9.3%)	1.7 (0.97-2.5)	530 (350-760)
Bosnia and Herzegovina	2016	Female	25 to 29	13% (8.4-20%)	87% (80-92%)	2.1 (0.93-3.7)	4.9% (3.7-6.2%)	6.2% (3.9-8.8%)	2.3 (1.3-3.5)	690 (470-940)
Bosnia and Herzegovina	2016	Female	30 to 34	14% (8.3-21%)	86% (79-92%)	2.3 (1.1-4.2)	4.5% (3.3-5.8%)	6.9% (4.4-10%)	4.4 (2.6-6.9)	830 (580-1100)
Bosnia and Herzegovina	2016	Female	35 to 39	14% (8.5-21%)	86% (79-92%)	2.7 (1.3-4.9)	4.3% (3.3-5.7%)	7.2% (4.7-11%)	6.3 (3.7-10)	830 (580-1100)
Bosnia and Herzegovina	2016	Female	40 to 44	14% (8.8-20%)	86% (80-91%)	3 (1.5-5.2)	4.4% (3.3-6%)	7.6% (5-11%)	11 (6.7-18)	1000 (690-1400)
Bosnia and Herzegovina	2016	Female	45 to 49	13% (7.6-19%)	87% (81-92%)	3.3 (1.6-5.7)	4.6% (3.3-6.4%)	7.7% (5.1-11%)	19 (12-30)	1200 (860-1700)
Bosnia and Herzegovina	2016	Female	50 to 54	12% (8-18%)	88% (82-92%)	3.2 (1.6-5.8)	4.5% (3.2-6.3%)	7.3% (4.8-11%)	33 (20-51)	1700 (1200-2400)
Bosnia and Herzegovina	2016	Female	55 to 59	11% (7.2-17%)	89% (83-93%)	2.9 (1.5-5.2)	4.3% (3-6.2%)	6.8% (4.5-10%)	47 (29-71)	1900 (1300-2900)
Bosnia and Herzegovina	2016	Female	60 to 64	11% (6.9-16%)	89% (84-93%)	2.7 (1.3-4.7)	4.3% (2.9-6.2%)	6.3% (4.1-9.3%)	73 (46-110)	2400 (1500-3600)
Bosnia and Herzegovina	2016	Female	65 to 69	9.8% (6.3-14%)	90% (86-94%)	2.4 (1.2-4.1)	4.4% (2.8-6.5%)	6.1% (3.9-9.1%)	110 (68-170)	2800 (1800-4200)
Bosnia and Herzegovina	2016	Female	70 to 74	8.5% (5.5-13%)	91% (87-94%)	2 (0.93-3.6)	4.1% (2.5-6.3%)	5.4% (3.3-8.3%)	100 (62-160)	2000 (1200-3100)
Bosnia and Herzegovina	2016	Female	75 to 79	7.3% (4.4-11%)	93% (89-96%)	1.6 (0.74-3)	3.4% (2-5.4%)	4.3% (2.6-7%)	140 (81-230)	2100 (1200-3400)
Bosnia and Herzegovina	2016	Female	80 to 84	6.4% (3.8-9.4%)	94% (91-96%)	1.4 (0.64-2.5)	3% (1.7-4.9%)	3.8% (2-6.1%)	140 (75-240)	1600 (840-2600)
Bosnia and Herzegovina	2016	Female	85 to 89	6.3% (3.6-9.7%)	94% (90-96%)	1.4 (0.66-2.6)	2.7% (1.4-4.6%)	3.5% (1.7-6%)	94 (43-170)	740 (360-1300)
Bosnia and Herzegovina	2016	Female	90 to 94	6.8% (4.2-11%)	93% (89-96%)	1.5 (0.7-3)	2.7% (1.3-4.7%)	3.4% (1.6-6.1%)	50 (22-89)	270 (130-470)
Bosnia and Herzegovina	2016	Female	95 plus	8% (4.6-13%)	92% (87-95%)	1.7 (0.64-3.7)	2.9% (1.3-5.1%)	3.6% (1.5-6.6%)	18 (7.3-33)	65 (30-120)

Location	Year	Sex	Age	Prevalence of current drinkers	Prevalence of abstainers	Population average of standard drinks daily	Percent of total attributable deaths	Percent of total attributable DALYs	Attributable deaths	Attributable DALYs
Bosnia and Herzegovina	2016	Male	All Ages	36% (31-40%)	64% (60-69%)	6.5 (4.5-9)	11% (8.4-13%)	12% (8.6-15%)	2400 (1700-3200)	70000 (53000-88000)
Bosnia and Herzegovina	2016	Male	15 to 19	30% (19-42%)	70% (58-81%)	2.4 (1.1-4.4)	6.5% (4.2-9.2%)	11% (5.6-17%)	5.5 (2.6-8.9)	710 (460-1000)
Bosnia and Herzegovina	2016	Male	20 to 24	39% (25-52%)	61% (48-75%)	4.8 (2.2-8.1)	13% (9.5-17%)	19% (12-26%)	15 (8.9-21)	1800 (1300-2400)
Bosnia and Herzegovina	2016	Male	25 to 29	35% (24-48%)	65% (52-76%)	5.4 (2.5-9)	13% (9.7-16%)	19% (13-25%)	21 (14-30)	2400 (1700-3100)
Bosnia and Herzegovina	2016	Male	30 to 34	36% (24-50%)	64% (50-76%)	6.1 (3-10)	12% (9.3-15%)	20% (14-25%)	28 (19-39)	2800 (2100-3500)
Bosnia and Herzegovina	2016	Male	35 to 39	43% (30-57%)	57% (43-70%)	7.3 (3.8-12)	13% (10-17%)	22% (17-29%)	41 (28-57)	3200 (2400-4100)
Bosnia and Herzegovina	2016	Male	40 to 44	47% (33-62%)	53% (38-67%)	8.7 (4.7-14)	14% (11-18%)	23% (17-29%)	68 (49-95)	4400 (3300-5700)
Bosnia and Herzegovina	2016	Male	45 to 49	47% (31-60%)	53% (40-69%)	9.9 (5.5-17)	14% (11-18%)	21% (16-27%)	100 (75-140)	5500 (4200-7200)
Bosnia and Herzegovina	2016	Male	50 to 54	45% (31-58%)	55% (42-69%)	10 (6.1-17)	14% (10-17%)	18% (14-23%)	180 (130-250)	7900 (5700-10000)
Bosnia and Herzegovina	2016	Male	55 to 59	42% (29-56%)	58% (44-71%)	10 (5.6-17)	12% (9-15%)	15% (12-20%)	220 (150-300)	8200 (6000-11000)
Bosnia and Herzegovina	2016	Male	60 to 64	40% (27-55%)	60% (45-73%)	9.7 (5-16)	11% (8.1-14%)	14% (9.8-18%)	300 (200-400)	9100 (6500-12000)
Bosnia and Herzegovina	2016	Male	65 to 69	39% (28-52%)	61% (48-72%)	8.6 (4.7-14)	11% (7.3-14%)	13% (8.6-17%)	360 (240-500)	9100 (6200-12000)
Bosnia and Herzegovina	2016	Male	70 to 74	36% (25-47%)	64% (53-75%)	7 (3.7-12)	9.7% (6.5-13%)	11% (7.7-16%)	270 (180-380)	5400 (3600-7600)
Bosnia and Herzegovina	2016	Male	75 to 79	32% (21-44%)	68% (56-79%)	5.4 (2.6-9.4)	8.2% (5.3-12%)	9.5% (6.1-13%)	310 (190-440)	4700 (3000-6800)
Bosnia and Herzegovina	2016	Male	80 to 84	29% (20-41%)	71% (59-80%)	4.5 (2.3-7.8)	7.4% (4.5-11%)	8.6% (5.2-13%)	250 (150-370)	2800 (1700-4200)
Bosnia and Herzegovina	2016	Male	85 to 89	30% (19-41%)	70% (59-81%)	4.1 (2.1-7.1)	6.7% (3.8-10%)	7.8% (4.3-12%)	140 (77-230)	1200 (660-1800)
Bosnia and Herzegovina	2016	Male	90 to 94	31% (21-42%)	69% (58-79%)	3.8 (1.9-7.3)	6.5% (3.7-10%)	7.5% (4.1-12%)	65 (34-100)	360 (200-550)
Bosnia and Herzegovina	2016	Male	95 plus	33% (20-47%)	67% (53-80%)	3.6 (1.4-7.4)	6.1% (2.7-10%)	6.9% (2.8-12%)	11 (4.4-18)	39 (18-65)
Bulgaria	1990	Female	All Ages	76% (70-81%)	24% (19-30%)	2 (1.7-2.3)	4% (1.9-6.5%)	4.3% (0.45-8.2%)	2100 (220-4100)	58000 (28000-89000)
Bulgaria	1990	Female	15 to 19	76% (65-85%)	24% (15-35%)	1.9 (1.3-2.6)	3.4% (2.1-4.9%)	7.3% (3.7-11%)	11 (5.3-16)	1100 (710-1600)
Bulgaria	1990	Female	20 to 24	82% (70-90%)	18% (9.7-30%)	2.3 (1.6-3.3)	4.6% (3.1-6.2%)	7.6% (4-12%)	12 (6-18)	1500 (1000-2100)
Bulgaria	1990	Female	25 to 29	83% (71-91%)	17% (8.7-29%)	2.2 (1.5-3)	4.3% (2.9-5.7%)	6.9% (3.7-10%)	12 (6.5-18)	1600 (1000-2100)

Location	Year	Sex	Age	Prevalence of current drinkers	Prevalence of abstainers	Population average of standard drinks daily	Percent of total attributable deaths	Percent of total attributable DALYs	Attributable deaths	Attributable DALYs
Bulgaria	1990	Female	30 to 34	84% (71-92%)	16% (8.3-29%)	2.3 (1.6-3.1)	4.2% (2.9-5.7%)	7.3% (4.4-10%)	19 (11-27)	1900 (1300-2600)
Bulgaria	1990	Female	35 to 39	84% (72-92%)	16% (8.4-28%)	2.6 (1.8-3.7)	4.6% (3.1-6.4%)	8.5% (5.2-12%)	33 (20-47)	2500 (1700-3400)
Bulgaria	1990	Female	40 to 44	83% (71-91%)	17% (8.7-29%)	2.8 (2-3.9)	5.1% (3.3-7.2%)	9.4% (5.9-13%)	57 (36-81)	3400 (2200-4800)
Bulgaria	1990	Female	45 to 49	82% (67-91%)	18% (9.1-33%)	2.9 (2-3.9)	5.6% (3.4-8%)	9.7% (6.1-14%)	78 (48-110)	3800 (2400-5500)
Bulgaria	1990	Female	50 to 54	80% (66-90%)	20% (10-34%)	2.9 (2-3.8)	5.8% (3.3-8.6%)	9.6% (5.5-14%)	120 (66-170)	4800 (2700-6900)
Bulgaria	1990	Female	55 to 59	79% (65-89%)	21% (11-35%)	2.8 (2-3.8)	6% (3.1-9%)	9.1% (4.9-14%)	200 (110-300)	6800 (3700-10000)
Bulgaria	1990	Female	60 to 64	76% (61-88%)	24% (12-39%)	2.7 (1.8-3.7)	5.5% (1.8-9.2%)	7.7% (2.7-13%)	270 (94-450)	7800 (2700-13000)
Bulgaria	1990	Female	65 to 69	73% (55-86%)	27% (14-45%)	2.5 (1.7-3.4)	5.1% (1.6-8.9%)	6.8% (2.3-12%)	390 (130-680)	9200 (2900-16000)
Bulgaria	1990	Female	70 to 74	68% (50-81%)	32% (19-50%)	2.3 (1.5-3.3)	4.5% (0.32-8.9%)	5.6% (0.3-11%)	290 (16-580)	5500 (420-11000)
Bulgaria	1990	Female	75 to 79	62% (44-79%)	38% (21-56%)	1.9 (1.2-3)	3.1% (-1.1-7.9%)	3.7% (-1.4-9.5%)	340 (-130-880)	5100 (-1700-13000)
Bulgaria	1990	Female	80 to 84	57% (38-74%)	43% (26-62%)	1.7 (1-2.6)	2% (-1.7-6.1%)	2.2% (-2.2-7%)	220 (-220-680)	2400 (-2000-7300)
Bulgaria	1990	Female	85 to 89	55% (37-70%)	45% (30-63%)	1.6 (0.96-2.5)	0.97% (-2.7-5%)	0.94% (-3.4-5.6%)	56 (-210-340)	500 (-1400-2600)
Bulgaria	1990	Female	90 to 94	56% (40-72%)	44% (28-60%)	1.6 (0.91-2.5)	0.48% (-3.4-5%)	0.35% (-4.4-5.6%)	7 (-87-110)	57 (-420-600)
Bulgaria	1990	Female	95 plus	59% (38-77%)	41% (23-62%)	1.6 (0.79-2.9)	0.16% (-4.3-5.9%)	-0.071% (-5.2-6.4%)	-0.4 (-30-37)	3.5 (-97-130)
Bulgaria	1990	Male	All Ages	86% (82-89%)	14% (11-18%)	5 (4.5-5.6)	15% (12-18%)	17% (13-21%)	10000 (7900-13000)	280000 (230000-330000)
Bulgaria	1990	Male	15 to 19	80% (70-87%)	20% (13-30%)	3 (2-4.1)	7.7% (5-11%)	12% (7.7-18%)	40 (25-57)	3400 (2200-4800)
Bulgaria	1990	Male	20 to 24	88% (78-94%)	12% (6.2-22%)	5.1 (3.6-6.9)	14% (10-19%)	21% (14-28%)	86 (59-120)	7000 (4900-9200)
Bulgaria	1990	Male	25 to 29	90% (82-95%)	10% (5-18%)	5.5 (3.9-7.3)	15% (11-19%)	22% (15-29%)	98 (67-130)	7600 (5500-9700)
Bulgaria	1990	Male	30 to 34	91% (83-96%)	8.8% (4-17%)	5.7 (4.1-7.4)	15% (11-19%)	22% (16-28%)	130 (96-180)	9300 (6900-12000)
Bulgaria	1990	Male	35 to 39	92% (85-96%)	8.1% (3.8-15%)	5.7 (4.1-7.6)	15% (11-20%)	21% (15-28%)	200 (140-260)	12000 (8500-15000)
Bulgaria	1990	Male	40 to 44	92% (85-96%)	7.8% (3.7-15%)	6.7 (5-8.5)	17% (13-21%)	23% (17-29%)	360 (270-440)	19000 (14000-23000)
Bulgaria	1990	Male	45 to 49	92% (83-96%)	8% (3.7-17%)	8.3 (6-11)	20% (16-24%)	26% (20-31%)	510 (400-610)	23000 (18000-27000)

Location	Year	Sex	Age	Prevalence of current drinkers	Prevalence of abstainers	Population average of standard drinks daily	Percent of total attributable deaths	Percent of total attributable DALYs	Attributable deaths	Attributable DALYs
Bulgaria	1990	Male	50 to 54	91% (83-96%)	8.6% (3.8-17%)	8.9 (6.5-11)	20% (16-24%)	25% (20-30%)	720 (570-870)	28000 (22000-34000)
Bulgaria	1990	Male	55 to 59	91% (83-96%)	9.4% (4.3-17%)	8.7 (6.6-11)	19% (15-24%)	24% (18-29%)	1100 (840-1300)	37000 (29000-45000)
Bulgaria	1990	Male	60 to 64	90% (81-95%)	10% (4.5-19%)	8.1 (5.9-11)	18% (14-23%)	22% (17-28%)	1400 (1100-1800)	41000 (31000-51000)
Bulgaria	1990	Male	65 to 69	89% (80-95%)	11% (4.7-20%)	7.2 (5.5-9)	18% (14-22%)	21% (16-26%)	1800 (1400-2200)	42000 (32000-52000)
Bulgaria	1990	Male	70 to 74	88% (78-94%)	12% (6.1-22%)	6.1 (4.4-7.9)	16% (11-21%)	18% (12-24%)	1200 (780-1500)	22000 (15000-29000)
Bulgaria	1990	Male	75 to 79	86% (76-93%)	14% (7-24%)	5.1 (3.4-7.1)	13% (6.2-19%)	14% (6.8-22%)	1400 (640-2000)	20000 (9600-29000)
Bulgaria	1990	Male	80 to 84	84% (73-92%)	16% (7.7-27%)	4.4 (3.1-6)	10% (4.2-16%)	11% (4.3-18%)	900 (350-1500)	9800 (4100-16000)
Bulgaria	1990	Male	85 to 89	83% (72-91%)	17% (9.3-28%)	3.9 (2.7-5.4)	6.9% (1-14%)	7.4% (0.53-15%)	300 (22-620)	2400 (370-4700)
Bulgaria	1990	Male	90 to 94	83% (72-91%)	17% (9-28%)	3.4 (2.2-5)	4.1% (-1.5-11%)	4.2% (-2.4-13%)	53 (-31-160)	310 (-110-870)
Bulgaria	1990	Male	95 plus	83% (70-92%)	17% (7.7-30%)	2.9 (1.5-4.8)	2.1% (-3.2-10%)	1.9% (-4.3-11%)	5.1 (-12-31)	23 (-35-110)
Bulgaria	1995	Female	All Ages	76% (71-81%)	24% (19-29%)	1.7 (1.5-2)	2.6% (0.56-4.8%)	2.1% (-1.3-5.7%)	1100 (-700-2900)	37000 (8200-68000)
Bulgaria	1995	Female	15 to 19	77% (66-85%)	23% (15-34%)	1.6 (1.1-2.2)	3% (1.9-4.3%)	6.2% (3.3-9.5%)	8.6 (4.6-13)	950 (590-1400)
Bulgaria	1995	Female	20 to 24	82% (70-90%)	18% (9.6-30%)	1.9 (1.3-2.7)	4.1% (2.7-5.7%)	6.3% (3-10%)	9.8 (4.7-16)	1400 (880-1900)
Bulgaria	1995	Female	25 to 29	83% (72-91%)	17% (8.6-28%)	1.9 (1.3-2.5)	3.9% (2.7-5.2%)	5.6% (2.8-8.6%)	10 (5-16)	1400 (910-1800)
Bulgaria	1995	Female	30 to 34	84% (71-92%)	16% (8.3-29%)	1.9 (1.3-2.6)	3.6% (2.4-5%)	5.7% (3-8.8%)	14 (7-21)	1500 (960-2000)
Bulgaria	1995	Female	35 to 39	84% (73-92%)	16% (8.3-27%)	2.2 (1.5-3)	3.9% (2.5-5.4%)	6.8% (3.8-10%)	25 (14-37)	1900 (1200-2700)
Bulgaria	1995	Female	40 to 44	83% (72-92%)	17% (8.5-28%)	2.4 (1.7-3.2)	4.3% (2.6-6.2%)	7.7% (4.3-11%)	45 (25-66)	2700 (1700-3800)
Bulgaria	1995	Female	45 to 49	82% (69-91%)	18% (8.9-31%)	2.5 (1.8-3.3)	4.5% (2.6-6.7%)	7.7% (4.3-11%)	74 (42-110)	3600 (2100-5200)
Bulgaria	1995	Female	50 to 54	81% (68-90%)	19% (10-32%)	2.4 (1.7-3.2)	4.5% (2.3-7%)	7.2% (3.6-11%)	87 (43-130)	3600 (1800-5600)
Bulgaria	1995	Female	55 to 59	79% (65-90%)	21% (10-35%)	2.3 (1.6-3.2)	4.2% (1.7-7%)	6.4% (2.6-10%)	120 (50-200)	4200 (1600-7000)
Bulgaria	1995	Female	60 to 64	77% (62-88%)	23% (12-38%)	2.2 (1.6-3)	3.4% (0.44-6.7%)	4.9% (0.57-9.5%)	160 (20-330)	4800 (610-9400)
Bulgaria	1995	Female	65 to 69	74% (57-87%)	26% (13-43%)	2.1 (1.5-2.8)	3% (-0.097-6.2%)	4% (-0.1-8.2%)	210 (-5.6-450)	5100 (-170-10000)

Location	Year	Sex	Age	Prevalence of current drinkers	Prevalence of abstainers	Population average of standard drinks daily	Percent of total attributable deaths	Percent of total attributable DALYs	Attributable deaths	Attributable DALYs
Bulgaria	1995	Female	70 to 74	69% (51-82%)	31% (18-49%)	1.9 (1.3-2.7)	2.4% (-1.4-6.3%)	3% (-1.7-7.9%)	260 (-150-660)	4900 (-2700-12000)
Bulgaria	1995	Female	75 to 79	62% (44-78%)	38% (22-56%)	1.6 (1-2.5)	1.3% (-2.4-5.1%)	1.5% (-3.1-6%)	100 (-220-420)	1600 (-2900-6200)
Bulgaria	1995	Female	80 to 84	57% (38-75%)	43% (25-62%)	1.4 (0.86-2.1)	0.4% (-3-3.9%)	0.34% (-3.6-4.3%)	35 (-380-450)	510 (-3800-4900)
Bulgaria	1995	Female	85 to 89	55% (38-71%)	45% (29-62%)	1.4 (0.77-2.1)	-0.43% (-3.5-3.2%)	-0.66% (-4.3-3.5%)	-48 (-310-250)	-270 (-2200-1900)
Bulgaria	1995	Female	90 to 94	56% (40-72%)	44% (28-60%)	1.4 (0.75-2.2)	-0.85% (-4.4-3.2%)	-1.2% (-5.2-3.4%)	-32 (-140-93)	-140 (-690-490)
Bulgaria	1995	Female	95 plus	59% (38-77%)	41% (23-62%)	1.4 (0.67-2.4)	-1.2% (-5.1-3.7%)	-1.6% (-6.1-4.1%)	-9.5 (-37-25)	-28 (-120-91)
Bulgaria	1995	Male	All Ages	86% (83-89%)	14% (11-17%)	4.4 (3.9-4.9)	14% (11-16%)	14% (10-18%)	9100 (6500-12000)	260000 (210000-310000)
Bulgaria	1995	Male	15 to 19	80% (70-88%)	20% (12-30%)	2.6 (1.7-3.5)	6.7% (4.4-9.5%)	11% (6.5-16%)	32 (19-46)	2800 (1900-3900)
Bulgaria	1995	Male	20 to 24	88% (78-94%)	12% (6.3-22%)	4.3 (3-5.8)	13% (9-17%)	18% (12-24%)	78 (51-110)	6300 (4500-8400)
Bulgaria	1995	Male	25 to 29	90% (81-95%)	10% (4.9-19%)	4.6 (3.3-6.1)	13% (9.5-17%)	19% (13-25%)	84 (57-110)	6500 (4700-8400)
Bulgaria	1995	Male	30 to 34	91% (83-96%)	8.7% (4-17%)	4.8 (3.5-6.3)	13% (9.5-17%)	18% (13-25%)	110 (75-150)	7600 (5400-9800)
Bulgaria	1995	Male	35 to 39	92% (85-96%)	8% (3.7-15%)	4.8 (3.4-6.4)	13% (9.4-18%)	18% (12-25%)	170 (110-240)	10000 (7200-14000)
Bulgaria	1995	Male	40 to 44	92% (86-96%)	7.8% (3.6-14%)	5.6 (4.2-7.2)	16% (11-20%)	20% (14-26%)	330 (230-420)	17000 (12000-21000)
Bulgaria	1995	Male	45 to 49	92% (84-96%)	8% (3.7-16%)	7 (5.2-9.2)	19% (14-23%)	23% (17-28%)	610 (450-760)	27000 (21000-34000)
Bulgaria	1995	Male	50 to 54	91% (83-96%)	8.7% (4-17%)	7.5 (5.4-9.5)	19% (15-23%)	23% (18-28%)	740 (570-910)	29000 (22000-35000)
Bulgaria	1995	Male	55 to 59	91% (82-96%)	9.5% (4.3-18%)	7.3 (5.6-9.3)	18% (14-22%)	22% (17-27%)	960 (740-1200)	32000 (25000-39000)
Bulgaria	1995	Male	60 to 64	90% (81-95%)	10% (4.6-19%)	6.8 (5-9)	17% (12-21%)	20% (14-25%)	1300 (950-1700)	38000 (27000-48000)
Bulgaria	1995	Male	65 to 69	89% (79-95%)	11% (4.9-21%)	6.1 (4.7-7.7)	16% (11-20%)	18% (13-23%)	1600 (1100-2000)	37000 (26000-47000)
Bulgaria	1995	Male	70 to 74	88% (78-94%)	12% (6.3-22%)	5.1 (3.8-6.7)	13% (7.5-18%)	15% (8.6-21%)	1600 (880-2200)	29000 (17000-41000)
Bulgaria	1995	Male	75 to 79	86% (76-93%)	14% (7-24%)	4.3 (3-6)	9.7% (3.5-16%)	11% (3.6-18%)	750 (250-1300)	11000 (4000-19000)
Bulgaria	1995	Male	80 to 84	84% (73-92%)	16% (7.6-27%)	3.7 (2.6-5)	6.8% (1.4-13%)	7.4% (1.1-15%)	620 (95-1200)	6900 (1500-13000)
Bulgaria	1995	Male	85 to 89	83% (73-91%)	17% (8.8-27%)	3.3 (2.3-4.4)	3.6% (-1.4-9.4%)	3.6% (-2-10%)	180 (-100-520)	1500 (-580-4000)

Location	Year	Sex	Age	Prevalence of current drinkers	Prevalence of abstainers	Population average of standard drinks daily	Percent of total attributable deaths	Percent of total attributable DALYs	Attributable deaths	Attributable DALYs
Bulgaria	1995	Male	90 to 94	83% (73-91%)	17% (8.7-27%)	2.8 (1.8-4.1)	1.3% (-3.3-7.5%)	1% (-4.3-8.2%)	16 (-69-130)	120 (-300-690)
Bulgaria	1995	Male	95 plus	83% (69-93%)	17% (7.4-31%)	2.4 (1.3-3.8)	-0.056% (-4.4-6.4%)	-0.61% (-5.6-6.8%)	-1.9 (-17-21)	-0.7 (-53-77)
Bulgaria	2000	Female	All Ages	76% (70-81%)	24% (19-30%)	1.5 (1.4-1.8)	1.7% (-0.14-3.7%)	0.91% (-2.2-4%)	490 (-1200-2200)	25000 (-2100-52000)
Bulgaria	2000	Female	15 to 19	76% (64-85%)	24% (15-36%)	1.4 (0.95-1.9)	2.6% (1.7-3.7%)	5.3% (2.7-8.2%)	5.7 (2.9-8.8)	690 (440-990)
Bulgaria	2000	Female	20 to 24	82% (69-90%)	18% (9.8-31%)	1.7 (1.1-2.4)	3.8% (2.6-5.1%)	5.3% (2.4-8.5%)	7.5 (3.4-12)	1200 (770-1700)
Bulgaria	2000	Female	25 to 29	83% (70-91%)	17% (8.8-30%)	1.6 (1.1-2.2)	3.5% (2.5-4.7%)	4.6% (2.2-7.2%)	7.2 (3.4-11)	1200 (780-1600)
Bulgaria	2000	Female	30 to 34	84% (71-92%)	16% (8.1-29%)	1.7 (1.2-2.3)	3.3% (2.2-4.6%)	4.7% (2.3-7.4%)	11 (5.3-17)	1300 (820-1800)
Bulgaria	2000	Female	35 to 39	84% (72-92%)	16% (8.3-28%)	1.9 (1.3-2.7)	3.4% (2.2-4.8%)	5.6% (3-8.5%)	19 (10-29)	1600 (990-2200)
Bulgaria	2000	Female	40 to 44	83% (71-91%)	17% (9-29%)	2.1 (1.5-2.8)	3.6% (2-5.1%)	6.1% (3.1-9.2%)	34 (17-52)	2100 (1200-3000)
Bulgaria	2000	Female	45 to 49	82% (67-91%)	18% (9.5-33%)	2.2 (1.6-2.9)	3.7% (1.9-5.7%)	6.2% (3-9.6%)	56 (27-85)	2800 (1400-4200)
Bulgaria	2000	Female	50 to 54	80% (67-90%)	20% (10-33%)	2.1 (1.6-2.8)	3.6% (1.6-5.8%)	5.7% (2.5-9.2%)	80 (34-130)	3300 (1500-5300)
Bulgaria	2000	Female	55 to 59	78% (64-89%)	22% (11-36%)	2 (1.4-2.7)	3.2% (0.85-5.6%)	4.8% (1.3-8.4%)	88 (24-150)	3000 (810-5300)
Bulgaria	2000	Female	60 to 64	76% (59-88%)	24% (12-41%)	1.9 (1.3-2.7)	2.3% (-0.25-5.3%)	3.3% (-0.38-7.6%)	95 (-10-210)	2700 (-300-6300)
Bulgaria	2000	Female	65 to 69	73% (56-86%)	27% (14-44%)	1.8 (1.3-2.5)	1.8% (-0.94-4.6%)	2.5% (-1.2-6.3%)	130 (-63-320)	3000 (-1400-7600)
Bulgaria	2000	Female	70 to 74	68% (50-82%)	32% (18-50%)	1.6 (1.1-2.3)	1.3% (-1.9-4.6%)	1.6% (-2.4-5.7%)	130 (-200-470)	2500 (-3700-8700)
Bulgaria	2000	Female	75 to 79	61% (42-78%)	39% (22-58%)	1.4 (0.86-2.1)	0.34% (-2.8-3.5%)	0.34% (-3.5-4.3%)	39 (-400-490)	680 (-5600-6900)
Bulgaria	2000	Female	80 to 84	56% (36-73%)	44% (27-64%)	1.2 (0.72-1.8)	-0.36% (-3.3-2.4%)	-0.52% (-3.9-2.8%)	-41 (-310-220)	-360 (-3200-2300)
Bulgaria	2000	Female	85 to 89	53% (36-69%)	47% (31-64%)	1.1 (0.66-1.8)	-1.1% (-3.9-1.9%)	-1.4% (-4.6-2%)	-100 (-350-150)	-700 (-2500-1200)
Bulgaria	2000	Female	90 to 94	55% (38-71%)	45% (29-62%)	1.1 (0.64-1.8)	-1.5% (-4.6-1.9%)	-1.8% (-5.5-2%)	-56 (-170-61)	-260 (-820-340)
Bulgaria	2000	Female	95 plus	58% (36-78%)	42% (22-64%)	1.1 (0.54-2)	-1.8% (-5.3-2.3%)	-2.3% (-6.4-2.3%)	-14 (-41-15)	-45 (-130-56)
Bulgaria	2000	Male	All Ages	86% (83-89%)	14% (11-17%)	3.9 (3.5-4.3)	11% (8.7-14%)	11% (7.5-15%)	6900 (4600-9400)	200000 (160000-250000)
Bulgaria	2000	Male	15 to 19	79% (69-87%)	21% (13-31%)	2.2 (1.5-3)	5.7% (3.8-7.9%)	9.3% (5.5-14%)	20 (12-30)	1900 (1300-2700)

Location	Year	Sex	Age	Prevalence of current drinkers	Prevalence of abstainers	Population average of standard drinks daily	Percent of total attributable deaths	Percent of total attributable DALYs	Attributable deaths	Attributable DALYs
Bulgaria	2000	Male	20 to 24	87% (77-94%)	13% (6.3-23%)	3.7 (2.7-5)	11% (7.5-14%)	15% (9.9-21%)	54 (35-75)	4800 (3300-6400)
Bulgaria	2000	Male	25 to 29	90% (81-95%)	10% (5.1-19%)	4 (2.9-5.2)	11% (8-14%)	16% (11-22%)	60 (39-82)	5100 (3600-6600)
Bulgaria	2000	Male	30 to 34	91% (82-96%)	9.1% (4.2-18%)	4.1 (3-5.4)	11% (7.9-15%)	15% (10-21%)	77 (51-110)	5700 (4000-7500)
Bulgaria	2000	Male	35 to 39	92% (84-96%)	8.2% (3.9-16%)	4.2 (3-5.7)	11% (7.5-15%)	15% (9.4-21%)	110 (69-150)	6900 (4700-9400)
Bulgaria	2000	Male	40 to 44	92% (85-96%)	7.9% (3.8-15%)	4.9 (3.7-6.3)	13% (8.8-17%)	17% (11-22%)	210 (140-290)	11000 (7800-15000)
Bulgaria	2000	Male	45 to 49	92% (84-96%)	8.2% (3.7-16%)	6.1 (4.5-7.8)	16% (11-20%)	20% (14-25%)	430 (300-560)	19000 (14000-25000)
Bulgaria	2000	Male	50 to 54	91% (83-96%)	8.9% (4.1-17%)	6.5 (4.9-8.2)	16% (12-20%)	20% (15-25%)	700 (510-880)	27000 (20000-34000)
Bulgaria	2000	Male	55 to 59	90% (81-95%)	9.8% (4.5-19%)	6.3 (4.8-8.1)	16% (12-20%)	19% (14-24%)	780 (560-990)	26000 (19000-33000)
Bulgaria	2000	Male	60 to 64	89% (80-95%)	11% (4.7-20%)	5.9 (4.3-7.8)	15% (9.7-19%)	17% (11-22%)	930 (610-1200)	27000 (18000-35000)
Bulgaria	2000	Male	65 to 69	89% (80-95%)	11% (4.8-20%)	5.2 (4.1-6.6)	13% (8.2-17%)	15% (9.4-20%)	1200 (750-1600)	28000 (18000-38000)
Bulgaria	2000	Male	70 to 74	87% (78-94%)	13% (6.5-22%)	4.5 (3.2-5.9)	10% (4.9-16%)	12% (5.4-18%)	1100 (540-1800)	22000 (11000-33000)
Bulgaria	2000	Male	75 to 79	86% (76-92%)	14% (7.5-24%)	3.7 (2.5-5.2)	7.3% (1.8-13%)	8.1% (1.9-15%)	860 (200-1600)	13000 (3300-24000)
Bulgaria	2000	Male	80 to 84	84% (72-92%)	16% (8.2-28%)	3.2 (2.3-4.3)	4.6% (0.11-10%)	4.9% (-0.28-11%)	290 (-16-660)	3300 (79-7200)
Bulgaria	2000	Male	85 to 89	83% (72-91%)	17% (9.4-28%)	2.8 (1.9-3.8)	1.9% (-2.1-6.8%)	1.8% (-2.8-7.3%)	88 (-140-350)	810 (-860-2800)
Bulgaria	2000	Male	90 to 94	83% (72-91%)	17% (9.2-28%)	2.5 (1.6-3.6)	0.19% (-3.8-5.2%)	-0.23% (-4.8-5.4%)	-4 (-81-91)	19 (-370-510)
Bulgaria	2000	Male	95 plus	82% (68-92%)	18% (8.2-32%)	2.1 (1.1-3.4)	-0.79% (-4.5-4.2%)	-1.4% (-5.8-4.4%)	-3.9 (-16-12)	-8.6 (-50-46)
Bulgaria	2005	Female	All Ages	73% (67-78%)	27% (22-33%)	1.5 (1.3-1.7)	1.6% (0.041-3.2%)	0.89% (-1.8-3.4%)	470 (-940-1800)	23000 (540-44000)
Bulgaria	2005	Female	15 to 19	75% (64-83%)	25% (17-36%)	1.3 (0.92-1.8)	2.4% (1.6-3.4%)	5.1% (2.6-8.1%)	4.3 (2.2-6.9)	580 (370-830)
Bulgaria	2005	Female	20 to 24	81% (69-89%)	19% (11-31%)	1.7 (1.1-2.3)	3.7% (2.5-4.9%)	5.1% (2.4-8.2%)	5.4 (2.5-8.7)	970 (630-1400)
Bulgaria	2005	Female	25 to 29	82% (71-90%)	18% (9.7-29%)	1.6 (1.1-2.1)	3.4% (2.4-4.4%)	4.2% (2.1-6.7%)	5.9 (2.9-9.6)	1100 (730-1500)
Bulgaria	2005	Female	30 to 34	82% (71-91%)	18% (9.3-29%)	1.6 (1.2-2.2)	3.1% (2.2-4.2%)	4.4% (2.3-6.6%)	8.6 (4.6-13)	1200 (790-1600)
Bulgaria	2005	Female	35 to 39	83% (73-91%)	17% (9.5-27%)	1.9 (1.3-2.6)	3.3% (2.1-4.5%)	5.2% (2.7-8.1%)	16 (8.5-25)	1400 (890-2000)

Location	Year	Sex	Age	Prevalence of current drinkers	Prevalence of abstainers	Population average of standard drinks daily	Percent of total attributable deaths	Percent of total attributable DALYs	Attributable deaths	Attributable DALYs
Bulgaria	2005	Female	40 to 44	83% (73-91%)	17% (9.1-27%)	2.1 (1.5-2.8)	3.4% (2-5%)	5.8% (2.9-8.9%)	28 (14-43)	1800 (1000-2600)
Bulgaria	2005	Female	45 to 49	81% (67-90%)	19% (10-33%)	2.1 (1.6-2.8)	3.6% (1.9-5.6%)	6% (2.9-9.4%)	51 (24-79)	2500 (1300-3800)
Bulgaria	2005	Female	50 to 54	79% (63-88%)	21% (12-37%)	2 (1.5-2.7)	3.5% (1.6-5.6%)	5.5% (2.4-8.9%)	71 (32-110)	3000 (1400-4600)
Bulgaria	2005	Female	55 to 59	76% (61-87%)	24% (13-39%)	1.9 (1.4-2.5)	3% (1.1-5.3%)	4.7% (1.7-7.8%)	92 (35-150)	3200 (1200-5500)
Bulgaria	2005	Female	60 to 64	73% (57-85%)	27% (15-43%)	1.8 (1.2-2.4)	2.3% (0.076-4.8%)	3.3% (0.13-7%)	85 (3.4-180)	2500 (88-5300)
Bulgaria	2005	Female	65 to 69	66% (49-82%)	34% (18-51%)	1.5 (1.1-2)	1.8% (-0.42-4.2%)	2.5% (-0.65-5.7%)	99 (-26-230)	2400 (-590-5400)
Bulgaria	2005	Female	70 to 74	58% (41-73%)	42% (27-59%)	1.3 (0.81-1.8)	1.3% (-1.1-3.7%)	1.6% (-1.4-5%)	120 (-100-360)	2200 (-1900-6800)
Bulgaria	2005	Female	75 to 79	55% (38-72%)	45% (28-62%)	1.2 (0.77-1.8)	0.43% (-2.1-3.1%)	0.48% (-2.7-3.8%)	51 (-280-410)	820 (-3900-5900)
Bulgaria	2005	Female	80 to 84	53% (34-71%)	47% (29-66%)	1.1 (0.66-1.7)	-0.21% (-2.8-2.3%)	-0.32% (-3.4-2.7%)	-39 (-420-330)	-320 (-4400-3600)
Bulgaria	2005	Female	85 to 89	51% (33-68%)	49% (32-67%)	1.1 (0.61-1.6)	-0.83% (-3.4-1.7%)	-1.1% (-4-1.9%)	-63 (-230-110)	-420 (-1700-850)
Bulgaria	2005	Female	90 to 94	52% (35-69%)	48% (31-65%)	1.1 (0.59-1.7)	-1.1% (-4-1.7%)	-1.5% (-4.8-1.9%)	-48 (-160-63)	-220 (-760-350)
Bulgaria	2005	Female	95 plus	55% (34-75%)	45% (25-66%)	1.1 (0.5-1.9)	-1.4% (-4.7-2.1%)	-1.8% (-5.6-2.2%)	-15 (-46-18)	-47 (-150-68)
Bulgaria	2005	Male	All Ages	89% (86-92%)	11% (8.5-14%)	4.1 (3.7-4.5)	11% (8.7-14%)	11% (7.5-15%)	6700 (4500-9300)	190000 (150000-240000)
Bulgaria	2005	Male	15 to 19	82% (74-89%)	18% (11-26%)	2.3 (1.5-3.1)	5.5% (3.7-7.4%)	9.5% (5.6-14%)	15 (9-22)	1600 (1100-2200)
Bulgaria	2005	Male	20 to 24	89% (81-94%)	11% (5.6-19%)	3.9 (2.7-5.1)	11% (7.6-14%)	15% (9.8-21%)	47 (30-64)	4200 (2900-5600)
Bulgaria	2005	Male	25 to 29	92% (86-96%)	8.2% (4.2-14%)	4.1 (3-5.4)	11% (7.8-14%)	16% (10-21%)	58 (37-78)	4900 (3500-6400)
Bulgaria	2005	Male	30 to 34	93% (88-97%)	6.9% (3.5-12%)	4.3 (3.2-5.6)	10% (7.3-14%)	15% (9.8-21%)	69 (44-97)	5200 (3600-7000)
Bulgaria	2005	Male	35 to 39	93% (88-97%)	6.7% (3.2-12%)	4.3 (3.1-5.7)	10% (6.9-14%)	14% (8.8-21%)	93 (57-140)	6000 (4100-8400)
Bulgaria	2005	Male	40 to 44	93% (88-97%)	6.7% (3.3-12%)	4.9 (3.7-6.3)	12% (8-16%)	16% (10-22%)	180 (110-240)	9500 (6400-13000)
Bulgaria	2005	Male	45 to 49	94% (87-97%)	6.5% (3.2-13%)	6.2 (4.7-8)	15% (11-19%)	19% (13-25%)	380 (260-490)	17000 (12000-22000)
Bulgaria	2005	Male	50 to 54	93% (87-97%)	6.9% (3.3-13%)	6.7 (5.1-8.3)	17% (12-20%)	20% (15-25%)	660 (480-820)	26000 (19000-32000)
Bulgaria	2005	Male	55 to 59	92% (86-96%)	7.6% (3.7-14%)	6.6 (5.2-8.1)	16% (12-20%)	19% (14-24%)	940 (690-1200)	31000 (23000-39000)

Location	Year	Sex	Age	Prevalence of current drinkers	Prevalence of abstainers	Population average of standard drinks daily	Percent of total attributable deaths	Percent of total attributable DALYs	Attributable deaths	Attributable DALYs
Bulgaria	2005	Male	60 to 64	92% (84-96%)	8.4% (4-16%)	6.1 (4.5-7.9)	15% (9.7-19%)	17% (11-22%)	920 (600-1200)	26000 (17000-35000)
Bulgaria	2005	Male	65 to 69	91% (83-95%)	9.5% (4.6-17%)	5.4 (4.3-6.5)	13% (8.2-17%)	15% (9.3-20%)	990 (620-1300)	23000 (15000-31000)
Bulgaria	2005	Male	70 to 74	90% (82-95%)	10% (5.4-18%)	4.6 (3.4-5.9)	10% (5.1-15%)	12% (5.6-18%)	1100 (510-1600)	20000 (10000-31000)
Bulgaria	2005	Male	75 to 79	87% (79-93%)	13% (6.6-21%)	3.8 (2.7-5.1)	7.2% (1.8-13%)	8% (1.7-15%)	810 (180-1500)	12000 (3100-22000)
Bulgaria	2005	Male	80 to 84	84% (72-91%)	16% (8.5-28%)	3.1 (2.2-4.1)	4.5% (0.3-9.6%)	4.9% (0.078-11%)	450 (7.3-990)	5100 (350-11000)
Bulgaria	2005	Male	85 to 89	82% (70-90%)	18% (10-30%)	2.7 (1.8-3.7)	2% (-1.6-6.5%)	1.9% (-2.3-7.1%)	71 (-83-260)	640 (-510-2100)
Bulgaria	2005	Male	90 to 94	82% (71-90%)	18% (9.8-29%)	2.3 (1.5-3.4)	0.43% (-3.2-4.9%)	0.066% (-4.2-5.3%)	1.2 (-76-96)	46 (-340-530)
Bulgaria	2005	Male	95 plus	81% (66-92%)	19% (8.5-34%)	2 (1.1-3.2)	-0.51% (-4.1-4.3%)	-1.1% (-5.4-4.5%)	-4.1 (-20-17)	-7.6 (-60-63)
Bulgaria	2010	Female	All Ages	72% (66-78%)	28% (22-34%)	1.5 (1.3-1.7)	1.8% (0.26-3.4%)	1.1% (-1.5-3.6%)	570 (-760-1900)	24000 (3400-43000)
Bulgaria	2010	Female	15 to 19	74% (62-83%)	26% (17-38%)	1.4 (0.94-1.9)	2.5% (1.6-3.5%)	5.1% (2.6-8%)	3.4 (1.7-5.4)	460 (300-670)
Bulgaria	2010	Female	20 to 24	81% (68-89%)	19% (11-32%)	1.7 (1.2-2.4)	3.7% (2.6-5%)	5.2% (2.5-8.4%)	4.9 (2.4-7.7)	920 (610-1300)
Bulgaria	2010	Female	25 to 29	82% (71-90%)	18% (9.8-29%)	1.7 (1.1-2.2)	3.5% (2.5-4.5%)	4.3% (2.2-6.6%)	4.8 (2.4-7.4)	940 (640-1300)
Bulgaria	2010	Female	30 to 34	82% (71-90%)	18% (9.6-29%)	1.7 (1.2-2.3)	3.3% (2.3-4.3%)	4.7% (2.7-7.1%)	8.5 (4.8-13)	1200 (780-1600)
Bulgaria	2010	Female	35 to 39	82% (71-90%)	18% (9.8-29%)	1.9 (1.4-2.6)	3.4% (2.3-4.5%)	5.5% (3.1-8.1%)	16 (9.3-24)	1500 (940-2000)
Bulgaria	2010	Female	40 to 44	82% (71-91%)	18% (9.3-29%)	2.2 (1.6-2.8)	3.5% (2.3-5%)	6% (3.4-8.7%)	27 (15-39)	1700 (1100-2400)
Bulgaria	2010	Female	45 to 49	81% (67-90%)	19% (9.9-33%)	2.2 (1.6-2.9)	3.6% (2.1-5.4%)	6.1% (3.3-9.2%)	43 (23-65)	2200 (1300-3300)
Bulgaria	2010	Female	50 to 54	78% (64-88%)	22% (12-36%)	2.1 (1.6-2.8)	3.6% (1.9-5.7%)	5.8% (2.9-8.9%)	66 (34-100)	2800 (1500-4300)
Bulgaria	2010	Female	55 to 59	75% (61-87%)	25% (13-39%)	2 (1.4-2.6)	3.2% (1.4-5.2%)	5% (2.2-7.9%)	88 (39-140)	3200 (1400-5100)
Bulgaria	2010	Female	60 to 64	72% (56-85%)	28% (15-44%)	1.8 (1.3-2.5)	2.4% (0.36-4.9%)	3.7% (0.61-7.3%)	100 (17-200)	3000 (470-6000)
Bulgaria	2010	Female	65 to 69	65% (48-80%)	35% (20-52%)	1.6 (1.1-2.1)	2% (0.02-4.2%)	2.8% (0.043-5.8%)	100 (1.6-210)	2500 (26-5300)
Bulgaria	2010	Female	70 to 74	57% (40-73%)	43% (27-60%)	1.3 (0.83-1.9)	1.5% (-0.73-4%)	2% (-0.97-5.3%)	110 (-54-300)	2200 (-1000-5700)
Bulgaria	2010	Female	75 to 79	54% (38-71%)	46% (29-62%)	1.2 (0.79-1.8)	0.77% (-1.8-3.4%)	0.9% (-2.3-4.2%)	87 (-220-400)	1400 (-3100-6000)

Location	Year	Sex	Age	Prevalence of current drinkers	Prevalence of abstainers	Population average of standard drinks daily	Percent of total attributable deaths	Percent of total attributable DALYs	Attributable deaths	Attributable DALYs
Bulgaria	2010	Female	80 to 84	52% (34-70%)	48% (30-66%)	1.1 (0.68-1.7)	0.14% (-2.3-2.6%)	0.088% (-3-3%)	10 (-350-350)	210 (-3500-3800)
Bulgaria	2010	Female	85 to 89	50% (33-66%)	50% (34-67%)	1.1 (0.63-1.6)	-0.48% (-3-2.1%)	-0.68% (-3.6-2.4%)	-64 (-340-220)	-400 (-2500-1700)
Bulgaria	2010	Female	90 to 94	51% (35-67%)	49% (33-65%)	1.1 (0.6-1.7)	-0.83% (-3.7-2.1%)	-1.1% (-4.4-2.3%)	-31 (-120-64)	-140 (-610-340)
Bulgaria	2010	Female	95 plus	54% (33-73%)	46% (27-67%)	1.1 (0.51-1.9)	-1.1% (-4.3-2.5%)	-1.5% (-5.2-2.7%)	-15 (-53-28)	-44 (-170-100)
Bulgaria	2010	Male	All Ages	89% (86-91%)	11% (8.9-14%)	4.3 (3.9-4.8)	11% (8.7-14%)	11% (7.7-15%)	6500 (4400-8800)	180000 (140000-230000)
Bulgaria	2010	Male	15 to 19	81% (73-88%)	19% (12-27%)	2.4 (1.6-3.3)	5.7% (3.9-7.9%)	9.9% (5.8-15%)	13 (7.6-19)	1300 (850-1800)
Bulgaria	2010	Male	20 to 24	89% (81-94%)	11% (6-19%)	4 (2.8-5.5)	11% (7.5-14%)	16% (9.9-22%)	40 (25-56)	3700 (2500-4900)
Bulgaria	2010	Male	25 to 29	91% (86-95%)	8.6% (4.7-14%)	4.3 (3.1-5.6)	11% (7.8-14%)	16% (10-22%)	49 (31-67)	4200 (3000-5600)
Bulgaria	2010	Male	30 to 34	93% (87-97%)	7.2% (3.4-13%)	4.4 (3.3-5.9)	10% (7.4-14%)	15% (9.8-21%)	62 (40-85)	4900 (3500-6500)
Bulgaria	2010	Male	35 to 39	93% (88-97%)	7% (3.4-12%)	4.4 (3.2-5.9)	10% (6.9-14%)	15% (9.3-21%)	85 (54-120)	5700 (3900-7800)
Bulgaria	2010	Male	40 to 44	93% (87-97%)	7.2% (3.4-13%)	5.1 (3.8-6.6)	12% (8-16%)	16% (10-22%)	160 (100-220)	8600 (5900-12000)
Bulgaria	2010	Male	45 to 49	93% (86-97%)	6.8% (3.2-14%)	6.5 (4.8-8.3)	15% (11-19%)	20% (14-25%)	330 (220-420)	15000 (11000-19000)
Bulgaria	2010	Male	50 to 54	93% (86-97%)	7.3% (3.5-14%)	6.9 (5.3-8.7)	17% (12-20%)	21% (15-26%)	570 (420-700)	22000 (17000-27000)
Bulgaria	2010	Male	55 to 59	92% (85-96%)	8% (4-15%)	6.7 (5.3-8.2)	16% (12-20%)	19% (14-24%)	830 (610-1000)	28000 (21000-35000)
Bulgaria	2010	Male	60 to 64	91% (84-96%)	8.8% (4.2-16%)	6.3 (4.7-8.1)	15% (10-19%)	17% (11-23%)	1000 (680-1300)	30000 (20000-38000)
Bulgaria	2010	Male	65 to 69	90% (82-95%)	10% (4.6-18%)	5.5 (4.4-6.7)	13% (8.6-17%)	15% (9.8-20%)	950 (610-1200)	23000 (15000-30000)
Bulgaria	2010	Male	70 to 74	89% (82-94%)	11% (5.5-18%)	4.7 (3.6-6)	11% (5.6-15%)	12% (6.3-18%)	900 (460-1300)	17000 (9100-25000)
Bulgaria	2010	Male	75 to 79	87% (77-93%)	13% (7-23%)	3.9 (2.8-5.3)	7.9% (2.5-13%)	8.9% (2.6-15%)	820 (240-1400)	12000 (4100-21000)
Bulgaria	2010	Male	80 to 84	83% (70-91%)	17% (8.9-30%)	3.2 (2.3-4.3)	5.3% (1.1-11%)	5.9% (0.86-12%)	520 (76-1000)	5800 (1300-11000)
Bulgaria	2010	Male	85 to 89	81% (69-90%)	19% (10-31%)	2.8 (1.9-3.8)	2.9% (-0.8-7.7%)	3% (-1.4-8.4%)	170 (-82-480)	1400 (-400-3800)
Bulgaria	2010	Male	90 to 94	81% (70-90%)	19% (9.9-30%)	2.4 (1.5-3.6)	1.2% (-2.6-6.2%)	0.95% (-3.4-6.7%)	14 (-51-98)	100 (-230-540)
Bulgaria	2010	Male	95 plus	81% (66-92%)	19% (8.3-34%)	2.1 (1.1-3.5)	0.12% (-3.6-5.6%)	-0.36% (-4.8-6.2%)	-1.9 (-25-32)	2.5 (-74-120)

Location	Year	Sex	Age	Prevalence of current drinkers	Prevalence of abstainers	Population average of standard drinks daily	Percent of total attributable deaths	Percent of total attributable DALYs	Attributable deaths	Attributable DALYs
Bulgaria	2016	Female	All Ages	74% (68-80%)	26% (20-32%)	1.7 (1.4-2.1)	2.1% (0.14-4.2%)	1.4% (-1.7-4.9%)	770 (-910-2600)	27000 (1700-54000)
Bulgaria	2016	Female	15 to 19	75% (62-84%)	25% (16-38%)	1.5 (0.94-2.2)	2.4% (1.6-3.5%)	5.3% (2.8-8.7%)	2 (1.1-3.4)	300 (190-440)
Bulgaria	2016	Female	20 to 24	81% (69-90%)	19% (10-31%)	1.9 (1.2-2.8)	3.7% (2.6-5%)	5.5% (2.7-9%)	4 (1.9-6.8)	840 (540-1200)
Bulgaria	2016	Female	25 to 29	83% (70-91%)	17% (9-30%)	1.8 (1.2-2.5)	3.5% (2.6-4.7%)	4.6% (2.3-7.2%)	3.9 (1.9-6.5)	820 (550-1200)
Bulgaria	2016	Female	30 to 34	83% (70-91%)	17% (8.6-30%)	1.9 (1.2-2.6)	3.4% (2.4-4.6%)	5.1% (2.9-7.7%)	7 (3.7-11)	1000 (700-1500)
Bulgaria	2016	Female	35 to 39	83% (71-91%)	17% (8.5-29%)	2.1 (1.4-3.1)	3.6% (2.4-4.9%)	6.1% (3.4-9.2%)	17 (8.8-28)	1500 (980-2200)
Bulgaria	2016	Female	40 to 44	83% (70-91%)	17% (8.9-30%)	2.3 (1.6-3.3)	3.7% (2.3-5.5%)	6.6% (3.7-9.8%)	27 (14-43)	1800 (1100-2800)
Bulgaria	2016	Female	45 to 49	81% (66-91%)	19% (9.1-34%)	2.4 (1.6-3.4)	3.9% (2.3-6%)	6.7% (3.5-10%)	38 (19-62)	2100 (1100-3200)
Bulgaria	2016	Female	50 to 54	80% (65-90%)	20% (10-35%)	2.3 (1.6-3.3)	4% (2.1-6.2%)	6.5% (3.3-10%)	62 (30-100)	2700 (1400-4300)
Bulgaria	2016	Female	55 to 59	78% (63-89%)	22% (11-37%)	2.2 (1.5-3.1)	3.6% (1.6-6%)	5.7% (2.6-9.4%)	87 (37-150)	3200 (1400-5300)
Bulgaria	2016	Female	60 to 64	75% (59-88%)	25% (12-41%)	2.1 (1.4-3.1)	2.8% (0.57-5.6%)	4.4% (0.94-8.6%)	130 (27-250)	3900 (790-7700)
Bulgaria	2016	Female	65 to 69	72% (55-85%)	28% (15-45%)	2 (1.3-2.8)	2.5% (-0.007-5.5%)	3.6% (0.023-7.7%)	130 (0.76-290)	3300 (-9.3-6900)
Bulgaria	2016	Female	70 to 74	66% (50-81%)	34% (19-50%)	1.8 (1.1-2.6)	2.1% (-0.85-5.4%)	2.9% (-1.1-7.2%)	130 (-54-350)	2600 (-1100-6700)
Bulgaria	2016	Female	75 to 79	59% (41-77%)	41% (23-59%)	1.5 (0.88-2.4)	1.2% (-1.8-4.6%)	1.5% (-2.4-5.8%)	140 (-210-530)	2200 (-3100-8000)
Bulgaria	2016	Female	80 to 84	54% (35-72%)	46% (28-65%)	1.3 (0.75-2)	0.56% (-2.2-3.6%)	0.59% (-2.8-4.3%)	65 (-310-490)	810 (-3200-5300)
Bulgaria	2016	Female	85 to 89	52% (35-69%)	48% (31-65%)	1.2 (0.66-2)	-0.087% (-2.9-3.1%)	-0.24% (-3.5-3.7%)	-29 (-420-420)	-99 (-3000-3300)
Bulgaria	2016	Female	90 to 94	53% (37-71%)	47% (29-63%)	1.2 (0.66-2)	-0.53% (-3.6-3.4%)	-0.77% (-4.5-3.8%)	-32 (-180-160)	-130 (-870-820)
Bulgaria	2016	Female	95 plus	57% (36-76%)	43% (24-64%)	1.2 (0.58-2.2)	-0.81% (-4.2-3.8%)	-1.2% (-5.2-4.2%)	-15 (-68-54)	-42 (-220-200)
Bulgaria	2016	Male	All Ages	87% (83-90%)	13% (10-17%)	4.4 (3.5-5.4)	12% (8.1-15%)	12% (6.9-16%)	6500 (3700-9700)	180000 (120000-240000)
Bulgaria	2016	Male	15 to 19	79% (68-86%)	21% (14-32%)	2.3 (1.5-3.5)	5.6% (3.7-8%)	9.9% (5.5-15%)	7.9 (4.2-13)	830 (520-1200)
Bulgaria	2016	Male	20 to 24	87% (76-93%)	13% (6.7-24%)	4 (2.6-5.8)	11% (7.3-14%)	16% (9.4-23%)	34 (20-52)	3300 (2200-4700)
Bulgaria	2016	Male	25 to 29	89% (81-95%)	11% (5.3-19%)	4.3 (2.9-6.1)	11% (7.7-14%)	16% (10-23%)	38 (23-56)	3600 (2500-5000)

Location	Year	Sex	Age	Prevalence of current drinkers	Prevalence of abstainers	Population average of standard drinks daily	Percent of total attributable deaths	Percent of total attributable DALYs	Attributable deaths	Attributable DALYs
Bulgaria	2016	Male	30 to 34	91% (82-96%)	9.3% (4.4-18%)	4.5 (3.1-6.2)	11% (7.5-14%)	16% (10-23%)	55 (33-82)	4500 (3100-6200)
Bulgaria	2016	Male	35 to 39	92% (84-96%)	8.4% (4-16%)	4.5 (3-6.4)	10% (6.9-14%)	16% (9.3-22%)	86 (49-130)	5900 (3700-8300)
Bulgaria	2016	Male	40 to 44	92% (85-96%)	8.1% (3.8-15%)	5.3 (3.8-7.2)	12% (8-17%)	17% (11-24%)	160 (93-230)	8800 (5500-12000)
Bulgaria	2016	Male	45 to 49	92% (83-96%)	8.5% (4-17%)	6.6 (4.5-9)	15% (11-19%)	20% (14-26%)	280 (180-400)	13000 (8700-18000)
Bulgaria	2016	Male	50 to 54	91% (83-96%)	9.1% (4.4-17%)	7 (4.9-9.5)	16% (12-21%)	21% (15-26%)	500 (340-680)	20000 (14000-26000)
Bulgaria	2016	Male	55 to 59	90% (82-95%)	10% (4.6-18%)	6.8 (4.8-9)	16% (12-20%)	20% (14-25%)	730 (510-1000)	25000 (18000-34000)
Bulgaria	2016	Male	60 to 64	89% (80-95%)	11% (4.8-20%)	6.3 (4.4-8.5)	15% (9.9-19%)	18% (12-23%)	1100 (710-1600)	32000 (21000-45000)
Bulgaria	2016	Male	65 to 69	88% (78-95%)	12% (4.9-22%)	5.6 (4.1-7.4)	13% (8.4-18%)	16% (9.9-21%)	1000 (620-1400)	24000 (15000-34000)
Bulgaria	2016	Male	70 to 74	87% (78-94%)	13% (6.1-22%)	4.8 (3.2-6.5)	11% (5.5-16%)	13% (6.2-19%)	770 (340-1200)	15000 (7200-23000)
Bulgaria	2016	Male	75 to 79	85% (74-93%)	15% (7.3-26%)	4 (2.6-5.8)	8.3% (2.6-14%)	9.5% (2.8-17%)	830 (240-1500)	13000 (4100-22000)
Bulgaria	2016	Male	80 to 84	83% (71-91%)	17% (8.6-29%)	3.4 (2.3-4.8)	6.2% (1.1-12%)	6.9% (0.83-14%)	590 (65-1200)	6700 (1100-13000)
Bulgaria	2016	Male	85 to 89	82% (70-90%)	18% (9.6-30%)	3 (1.9-4.3)	3.8% (-0.56-9.7%)	4% (-1.1-11%)	290 (-81-780)	2400 (-340-6100)
Bulgaria	2016	Male	90 to 94	82% (71-90%)	18% (9.7-29%)	2.7 (1.7-4)	1.8% (-2.5-7.6%)	1.7% (-3.4-8.3%)	34 (-70-170)	220 (-300-920)
Bulgaria	2016	Male	95 plus	82% (68-92%)	18% (7.9-32%)	2.2 (1.1-3.7)	0.54% (-3.5-6.3%)	0.089% (-4.8-7%)	0.63 (-33-48)	15 (-100-180)
Croatia	1990	Female	All Ages	63% (57-68%)	37% (32-43%)	1.8 (1.5-2.2)	5.6% (3.7-7.6%)	6.5% (3.5-10%)	1700 (900-2600)	42000 (28000-58000)
Croatia	1990	Female	15 to 19	65% (49-78%)	35% (22-51%)	1.3 (0.82-2)	3.1% (2-4.4%)	7.3% (4-11%)	4.4 (2.5-7)	500 (310-720)
Croatia	1990	Female	20 to 24	74% (60-84%)	26% (16-40%)	1.6 (1.1-2.3)	4.5% (3-6.1%)	8% (4.4-13%)	5.5 (3-8.6)	760 (510-1100)
Croatia	1990	Female	25 to 29	74% (61-84%)	26% (16-39%)	1.6 (1.1-2.2)	4.3% (3-5.7%)	7.4% (4.1-11%)	6.7 (3.7-10)	880 (590-1200)
Croatia	1990	Female	30 to 34	72% (59-81%)	28% (19-41%)	1.8 (1.2-2.5)	4.3% (3.1-5.7%)	8.2% (5.2-12%)	11 (6.6-15)	1100 (750-1500)
Croatia	1990	Female	35 to 39	69% (57-79%)	31% (21-43%)	2.1 (1.4-3)	5.1% (3.6-6.7%)	10% (6.9-14%)	21 (14-30)	1600 (1100-2100)
Croatia	1990	Female	40 to 44	68% (57-79%)	32% (21-43%)	2.4 (1.7-3.3)	6.3% (4.7-8.2%)	13% (9.4-16%)	37 (27-48)	2100 (1600-2700)
Croatia	1990	Female	45 to 49	68% (56-78%)	32% (22-44%)	2.8 (1.9-3.9)	8.1% (5.9-11%)	14% (10-18%)	57 (42-76)	2700 (2000-3500)

Location	Year	Sex	Age	Prevalence of current drinkers	Prevalence of abstainers	Population average of standard drinks daily	Percent of total attributable deaths	Percent of total attributable DALYs	Attributable deaths	Attributable DALYs
Croatia	1990	Female	50 to 54	68% (56-78%)	32% (22-44%)	3.1 (2.2-4.1)	8.7% (6.3-11%)	14% (10-18%)	110 (76-140)	4300 (3200-5700)
Croatia	1990	Female	55 to 59	68% (56-78%)	32% (22-44%)	3.1 (2.2-4.3)	8.8% (6.1-12%)	14% (9.6-18%)	160 (110-220)	5600 (3900-7700)
Croatia	1990	Female	60 to 64	66% (52-77%)	34% (23-48%)	3 (2.1-4.2)	7.8% (4.7-11%)	11% (6.7-16%)	190 (120-290)	5800 (3500-8700)
Croatia	1990	Female	65 to 69	62% (47-74%)	38% (26-53%)	2.7 (1.8-3.7)	6.9% (3.9-11%)	9.3% (5.3-14%)	250 (140-380)	6100 (3400-9100)
Croatia	1990	Female	70 to 74	55% (41-66%)	45% (34-59%)	2.2 (1.4-3.3)	5.7% (2-10%)	7.2% (2.5-13%)	180 (65-320)	3500 (1300-6200)
Croatia	1990	Female	75 to 79	46% (33-61%)	54% (39-67%)	1.8 (1.1-2.8)	4.1% (0.71-8.4%)	4.9% (0.69-10%)	230 (34-480)	3500 (580-7000)
Croatia	1990	Female	80 to 84	41% (27-55%)	59% (45-73%)	1.7 (0.98-2.5)	3.6% (0.7-7.4%)	4.1% (0.65-8.6%)	220 (34-470)	2400 (470-5100)
Croatia	1990	Female	85 to 89	40% (27-54%)	60% (46-73%)	1.7 (0.97-2.6)	3.3% (0.58-7.1%)	3.8% (0.45-8.3%)	130 (14-280)	1000 (180-2200)
Croatia	1990	Female	90 to 94	41% (28-56%)	59% (44-72%)	1.8 (1-2.9)	3.5% (0.39-8.1%)	4.1% (0.25-9.7%)	53 (3.3-130)	290 (30-660)
Croatia	1990	Female	95 plus	45% (28-63%)	55% (37-72%)	2 (0.99-3.5)	4.2% (0.092-10%)	4.9% (-0.17-12%)	18 (-0.6-45)	64 (1.5-160)
Croatia	1990	Male	All Ages	74% (69-79%)	26% (21-31%)	5.4 (4.6-6.4)	18% (15-21%)	21% (17-25%)	5700 (4600-7000)	170000 (140000-200000)
Croatia	1990	Male	15 to 19	66% (51-80%)	34% (20-49%)	3.1 (1.9-4.6)	8.9% (5.7-13%)	15% (8.9-22%)	23 (13-35)	2000 (1200-2800)
Croatia	1990	Male	20 to 24	78% (65-87%)	22% (13-35%)	5.2 (3.5-7.4)	18% (12-24%)	25% (17-35%)	68 (45-94)	5200 (3600-7000)
Croatia	1990	Male	25 to 29	81% (69-89%)	19% (11-31%)	5.1 (3.5-6.8)	17% (12-22%)	25% (18-33%)	72 (50-97)	5400 (3900-7000)
Croatia	1990	Male	30 to 34	82% (70-90%)	18% (9.8-30%)	5.5 (3.8-7.3)	18% (13-22%)	26% (20-33%)	100 (76-130)	6900 (5200-8800)
Croatia	1990	Male	35 to 39	82% (73-90%)	18% (9.7-27%)	6.5 (4.5-8.9)	20% (15-24%)	29% (22-35%)	160 (120-200)	9600 (7500-12000)
Croatia	1990	Male	40 to 44	83% (74-91%)	17% (9.5-26%)	7.8 (5.8-10)	22% (18-26%)	31% (26-36%)	230 (190-280)	12000 (10000-14000)
Croatia	1990	Male	45 to 49	84% (74-91%)	16% (9.1-26%)	9.5 (6.7-13)	24% (20-28%)	31% (27-36%)	310 (260-370)	14000 (11000-16000)
Croatia	1990	Male	50 to 54	84% (74-91%)	16% (8.7-26%)	9.6 (6.8-13)	23% (19-27%)	28% (24-33%)	540 (430-650)	21000 (17000-25000)
Croatia	1990	Male	55 to 59	83% (72-91%)	17% (8.8-28%)	8.8 (6.5-12)	21% (18-25%)	25% (21-30%)	720 (580-890)	24000 (20000-29000)
Croatia	1990	Male	60 to 64	82% (70-90%)	18% (9.9-30%)	8.5 (6.1-12)	19% (16-24%)	23% (18-28%)	760 (600-940)	22000 (17000-27000)
Croatia	1990	Male	65 to 69	81% (69-89%)	19% (11-31%)	8.6 (6.4-11)	19% (14-23%)	22% (17-27%)	700 (530-880)	17000 (13000-21000)

Location	Year	Sex	Age	Prevalence of current drinkers	Prevalence of abstainers	Population average of standard drinks daily	Percent of total attributable deaths	Percent of total attributable DALYs	Attributable deaths	Attributable DALYs
Croatia	1990	Male	70 to 74	79% (68-87%)	21% (13-32%)	8.3 (5.9-11)	18% (13-22%)	21% (15-26%)	520 (390-650)	9800 (7400-12000)
Croatia	1990	Male	75 to 79	76% (63-87%)	24% (13-37%)	7.4 (4.8-10)	16% (11-21%)	18% (13-24%)	680 (480-910)	10000 (7000-13000)
Croatia	1990	Male	80 to 84	74% (59-85%)	26% (15-41%)	6.1 (4.1-8.7)	14% (9.5-19%)	16% (10-22%)	550 (360-760)	5900 (4000-8100)
Croatia	1990	Male	85 to 89	72% (57-83%)	28% (17-43%)	5.1 (3.3-7.4)	11% (6.3-16%)	12% (6.9-19%)	210 (110-320)	1600 (950-2500)
Croatia	1990	Male	90 to 94	71% (56-82%)	29% (18-44%)	4.3 (2.6-6.5)	8.1% (2.9-14%)	9.2% (2.9-16%)	48 (15-82)	270 (99-450)
Croatia	1990	Male	95 plus	69% (51-83%)	31% (17-49%)	3.4 (1.7-5.8)	5.5% (0.079-12%)	5.9% (-0.71-14%)	6.5 (-0.77-15)	26 (0.37-55)
Croatia	1995	Female	All Ages	62% (57-67%)	38% (33-43%)	1.9 (1.6-2.2)	5.5% (3.9-7.4%)	6.6% (3.8-10%)	1700 (970-2600)	42000 (29000-56000)
Croatia	1995	Female	15 to 19	65% (50-79%)	35% (21-50%)	1.3 (0.83-1.9)	2.8% (1.8-4.1%)	5.2% (2.7-8.7%)	4.3 (2.3-7)	470 (300-670)
Croatia	1995	Female	20 to 24	74% (60-84%)	26% (16-40%)	1.6 (1-2.3)	4% (2.7-5.4%)	5.7% (2.8-9.5%)	4.9 (2.6-7.6)	680 (450-950)
Croatia	1995	Female	25 to 29	74% (61-84%)	26% (16-39%)	1.6 (1.1-2.2)	3.9% (2.9-5.1%)	5.8% (2.9-9.1%)	4.9 (2.6-7.4)	690 (470-940)
Croatia	1995	Female	30 to 34	72% (59-81%)	28% (19-41%)	1.8 (1.2-2.4)	3.9% (2.8-5.1%)	6.6% (4-9.7%)	8.3 (5-12)	900 (620-1200)
Croatia	1995	Female	35 to 39	69% (58-79%)	31% (21-42%)	2.1 (1.4-2.9)	4.7% (3.4-6.1%)	9% (6-12%)	19 (12-25)	1400 (960-1800)
Croatia	1995	Female	40 to 44	67% (56-79%)	33% (21-44%)	2.4 (1.7-3.2)	5.8% (4.4-7.5%)	11% (8.6-14%)	35 (26-45)	2000 (1600-2600)
Croatia	1995	Female	45 to 49	68% (55-78%)	32% (22-45%)	2.8 (1.9-3.7)	7.1% (5.3-9.3%)	13% (9.6-16%)	59 (44-76)	2800 (2100-3600)
Croatia	1995	Female	50 to 54	68% (56-78%)	32% (22-44%)	3.1 (2.2-4)	8.5% (6.3-11%)	14% (10-18%)	82 (60-110)	3300 (2400-4200)
Croatia	1995	Female	55 to 59	68% (55-78%)	32% (22-45%)	3.1 (2.2-4.2)	8.9% (6.4-12%)	14% (9.9-18%)	150 (110-190)	5100 (3600-6700)
Croatia	1995	Female	60 to 64	66% (51-77%)	34% (23-49%)	3 (2.1-4.1)	7.9% (5-11%)	11% (7.2-16%)	200 (130-280)	5900 (3800-8400)
Croatia	1995	Female	65 to 69	61% (47-73%)	39% (27-53%)	2.7 (1.9-3.6)	7% (4.2-10%)	9.5% (5.8-14%)	260 (160-380)	6200 (3800-9300)
Croatia	1995	Female	70 to 74	54% (41-66%)	46% (34-59%)	2.2 (1.4-3.2)	5.9% (2.4-9.7%)	7.5% (3.1-12%)	310 (120-510)	5900 (2400-9800)
Croatia	1995	Female	75 to 79	46% (32-60%)	54% (40-68%)	1.8 (1.1-2.7)	4.1% (0.85-8%)	4.9% (0.91-9.7%)	170 (31-340)	2600 (520-5100)
Croatia	1995	Female	80 to 84	40% (27-55%)	60% (45-73%)	1.6 (0.97-2.4)	3.5% (0.77-7.2%)	4% (0.81-8.3%)	190 (37-390)	2100 (450-4200)
Croatia	1995	Female	85 to 89	39% (26-53%)	61% (47-74%)	1.7 (0.97-2.6)	3.4% (0.62-7.1%)	3.9% (0.52-8.6%)	150 (20-320)	1200 (230-2500)

Location	Year	Sex	Age	Prevalence of current drinkers	Prevalence of abstainers	Population average of standard drinks daily	Percent of total attributable deaths	Percent of total attributable DALYs	Attributable deaths	Attributable DALYs
Croatia	1995	Female	90 to 94	41% (28-55%)	59% (45-72%)	1.8 (0.98-2.8)	3.6% (0.48-8%)	4.2% (0.25-9.8%)	73 (4.6-170)	400 (55-880)
Croatia	1995	Female	95 plus	44% (27-62%)	56% (38-73%)	2 (0.99-3.4)	4.2% (0.019-10%)	5% (-0.18-13%)	25 (-0.92-61)	89 (0.38-220)
Croatia	1995	Male	All Ages	75% (69-80%)	25% (20-31%)	5.6 (4.8-6.3)	17% (14-20%)	20% (17-24%)	5600 (4500-6900)	160000 (130000-190000)
Croatia	1995	Male	15 to 19	67% (51-80%)	33% (20-49%)	3.1 (1.9-4.6)	7.7% (4.8-11%)	11% (6.3-17%)	24 (14-36)	2000 (1300-2900)
Croatia	1995	Male	20 to 24	78% (64-88%)	22% (12-36%)	5.2 (3.5-7.2)	14% (8.9-19%)	17% (10-27%)	62 (41-86)	4700 (3300-6300)
Croatia	1995	Male	25 to 29	81% (69-89%)	19% (11-31%)	5.1 (3.6-6.9)	14% (9.7-19%)	19% (12-27%)	60 (42-80)	4400 (3200-5800)
Croatia	1995	Male	30 to 34	82% (70-90%)	18% (10-30%)	5.5 (3.9-7.4)	15% (11-19%)	21% (14-28%)	81 (59-100)	5400 (4100-6900)
Croatia	1995	Male	35 to 39	82% (73-90%)	18% (10-27%)	6.5 (4.6-8.8)	18% (14-22%)	25% (19-31%)	140 (100-170)	8100 (6200-9800)
Croatia	1995	Male	40 to 44	83% (73-91%)	17% (9.4-27%)	7.8 (5.9-9.9)	21% (17-24%)	28% (24-33%)	230 (190-280)	12000 (10000-14000)
Croatia	1995	Male	45 to 49	84% (73-91%)	16% (9-27%)	9.5 (6.9-12)	23% (19-26%)	29% (25-34%)	330 (270-400)	15000 (12000-18000)
Croatia	1995	Male	50 to 54	84% (73-91%)	16% (9.1-27%)	9.6 (6.9-12)	23% (19-27%)	28% (23-33%)	390 (310-460)	15000 (12000-18000)
Croatia	1995	Male	55 to 59	83% (72-91%)	17% (8.9-28%)	8.7 (6.4-11)	21% (17-25%)	25% (21-30%)	630 (500-760)	21000 (17000-25000)
Croatia	1995	Male	60 to 64	82% (70-90%)	18% (10-30%)	8.5 (6.2-11)	19% (15-23%)	23% (18-27%)	810 (630-1000)	23000 (18000-28000)
Croatia	1995	Male	65 to 69	80% (69-88%)	20% (12-31%)	8.5 (6.3-11)	19% (14-23%)	22% (16-26%)	880 (670-1100)	21000 (16000-26000)
Croatia	1995	Male	70 to 74	78% (67-86%)	22% (14-33%)	8.2 (5.9-11)	18% (13-23%)	21% (15-26%)	760 (560-950)	14000 (11000-18000)
Croatia	1995	Male	75 to 79	76% (63-86%)	24% (14-37%)	7.3 (4.9-10)	16% (11-21%)	18% (13-24%)	470 (330-610)	6900 (4900-9000)
Croatia	1995	Male	80 to 84	74% (59-85%)	26% (15-41%)	6.1 (4.1-8.6)	14% (9.9-20%)	16% (11-22%)	470 (310-640)	5100 (3400-6800)
Croatia	1995	Male	85 to 89	71% (57-82%)	29% (18-43%)	5.1 (3.4-7.2)	11% (6.7-16%)	13% (7.5-19%)	230 (140-350)	1900 (1100-2700)
Croatia	1995	Male	90 to 94	70% (56-82%)	30% (18-44%)	4.2 (2.6-6.4)	8.2% (3.3-13%)	9.3% (3.3-16%)	68 (24-110)	380 (150-620)
Croatia	1995	Male	95 plus	69% (50-84%)	31% (16-50%)	3.3 (1.6-5.7)	5.6% (0.12-12%)	6% (-0.56-14%)	9.8 (-0.87-23)	38 (0.79-82)
Croatia	2000	Female	All Ages	60% (54-64%)	40% (36-46%)	1.9 (1.6-2.2)	5.5% (4-7.3%)	6.7% (4-9.8%)	1700 (1000-2600)	39000 (28000-53000)
Croatia	2000	Female	15 to 19	63% (47-77%)	37% (23-53%)	1.3 (0.84-1.9)	2.9% (1.8-4.1%)	7% (3.8-11%)	3.3 (1.7-5.2)	390 (240-560)

Location	Year	Sex	Age	Prevalence of current drinkers	Prevalence of abstainers	Population average of standard drinks daily	Percent of total attributable deaths	Percent of total attributable DALYs	Attributable deaths	Attributable DALYs
Croatia	2000	Female	20 to 24	72%<br>(58-83%)	28%<br>(17-42%)	1.6<br>(1.1-2.3)	4.2%<br>(2.9-5.7%)	7.4%<br>(4.1-12%)	3.7<br>(2-5.8)	590<br>(400-830)
Croatia	2000	Female	25 to 29	71%<br>(58-82%)	29%<br>(18-42%)	1.6<br>(1.1-2.1)	3.9%<br>(2.8-5%)	6.8%<br>(3.9-10%)	3.7<br>(2.1-5.5)	610<br>(420-830)
Croatia	2000	Female	30 to 34	68%<br>(54-79%)	32%<br>(21-46%)	1.7<br>(1.1-2.3)	3.9%<br>(2.9-5.1%)	7.6%<br>(4.9-11%)	6.2<br>(4-8.7)	720<br>(510-960)
Croatia	2000	Female	35 to 39	65%<br>(54-76%)	35%<br>(24-46%)	2<br>(1.3-2.9)	4.5%<br>(3.3-5.9%)	9.6%<br>(6.6-13%)	14<br>(9.4-18)	1100<br>(780-1400)
Croatia	2000	Female	40 to 44	65%<br>(55-76%)	35%<br>(24-45%)	2.4<br>(1.8-3.2)	5.5%<br>(4.2-7.1%)	12%<br>(8.9-15%)	29<br>(22-37)	1700<br>(1300-2200)
Croatia	2000	Female	45 to 49	66%<br>(55-76%)	34%<br>(24-45%)	2.8<br>(2-3.7)	6.8%<br>(4.9-8.9%)	13%<br>(9.7-16%)	52<br>(39-67)	2600<br>(1900-3300)
Croatia	2000	Female	50 to 54	66%<br>(56-75%)	34%<br>(25-44%)	3.1<br>(2.2-4)	8%<br>(6-10%)	14%<br>(10-17%)	80<br>(60-100)	3300<br>(2500-4300)
Croatia	2000	Female	55 to 59	67%<br>(56-77%)	33%<br>(23-44%)	3.2<br>(2.3-4.2)	8.8%<br>(6.4-12%)	14%<br>(10-18%)	100<br>(76-140)	3600<br>(2600-4800)
Croatia	2000	Female	60 to 64	65%<br>(53-76%)	35%<br>(24-47%)	3.1<br>(2.2-4.2)	7.9%<br>(5.2-11%)	12%<br>(7.8-16%)	180<br>(120-240)	5200<br>(3400-7200)
Croatia	2000	Female	65 to 69	60%<br>(48-71%)	40%<br>(29-52%)	2.7<br>(2-3.5)	7.1%<br>(4.5-9.9%)	9.8%<br>(6.3-14%)	250<br>(160-350)	6000<br>(3900-8500)
Croatia	2000	Female	70 to 74	51%<br>(39-62%)	49%<br>(38-61%)	2.1<br>(1.4-3)	6%<br>(3-9.3%)	7.7%<br>(3.8-12%)	290<br>(150-460)	5700<br>(2900-8800)
Croatia	2000	Female	75 to 79	41%<br>(30-56%)	59%<br>(44-70%)	1.7<br>(1-2.5)	4.4%<br>(1.6-7.8%)	5.3%<br>(1.9-9.5%)	280<br>(100-500)	4200<br>(1500-7500)
Croatia	2000	Female	80 to 84	38%<br>(26-52%)	62%<br>(48-74%)	1.6<br>(0.98-2.3)	3.7%<br>(1.4-6.9%)	4.4%<br>(1.6-8.4%)	180<br>(65-340)	2000<br>(750-3800)
Croatia	2000	Female	85 to 89	39%<br>(26-52%)	61%<br>(48-74%)	1.7<br>(0.99-2.6)	3.5%<br>(0.94-6.9%)	4.1%<br>(0.84-8.1%)	130<br>(27-250)	1000<br>(280-2000)
Croatia	2000	Female	90 to 94	41%<br>(28-54%)	59%<br>(46-72%)	1.9<br>(1-2.9)	3.8%<br>(0.57-8.2%)	4.3%<br>(0.42-9.7%)	100<br>(9.8-230)	540<br>(81-1200)
Croatia	2000	Female	95 plus	44%<br>(28-62%)	56%<br>(38-72%)	2.1<br>(1-3.4)	4.5%<br>(0.17-10%)	5.2%<br>(0.046-12%)	40<br>(0.37-94)	140<br>(5.4-320)
Croatia	2000	Male	All Ages	77%<br>(72-82%)	23%<br>(18-28%)	6.2<br>(5.6-6.9)	18%<br>(15-21%)	21%<br>(17-25%)	5700<br>(4600-7000)	150000<br>(130000-180000)
Croatia	2000	Male	15 to 19	70%<br>(56-82%)	30%<br>(18-44%)	3.4<br>(2.2-4.8)	9.5%<br>(6.3-14%)	17%<br>(10-24%)	22<br>(13-32)	1800<br>(1200-2600)
Croatia	2000	Male	20 to 24	80%<br>(67-88%)	20%<br>(12-33%)	5.6<br>(3.9-7.6)	17%<br>(12-23%)	27%<br>(18-36%)	51<br>(35-72)	4000<br>(2900-5300)
Croatia	2000	Male	25 to 29	82%<br>(72-90%)	18%<br>(9.9-28%)	5.5<br>(4-7.2)	16%<br>(12-20%)	26%<br>(19-34%)	50<br>(36-66)	3800<br>(2800-4900)
Croatia	2000	Male	30 to 34	84%<br>(73-90%)	16%<br>(9.5-27%)	5.9<br>(4.3-7.9)	16%<br>(12-20%)	27%<br>(21-34%)	60<br>(45-77)	4200<br>(3200-5200)
Croatia	2000	Male	35 to 39	84%<br>(76-91%)	16%<br>(9.3-24%)	6.9<br>(4.9-9.3)	19%<br>(14-23%)	30%<br>(23-36%)	110<br>(80-130)	6400<br>(4900-7700)

Location	Year	Sex	Age	Prevalence of current drinkers	Prevalence of abstainers	Population average of standard drinks daily	Percent of total attributable deaths	Percent of total attributable DALYs	Attributable deaths	Attributable DALYs
Croatia	2000	Male	40 to 44	84%<br>(76-91%)	16%<br>(9.2-24%)	8.4<br>(6.5-11)	22%<br>(18-25%)	32%<br>(27-37%)	200<br>(160-240)	10000<br>(8600-12000)
Croatia	2000	Male	45 to 49	85%<br>(76-91%)	15%<br>(8.8-24%)	10<br>(7.6-13)	23%<br>(19-27%)	31%<br>(27-35%)	330<br>(280-390)	15000<br>(12000-17000)
Croatia	2000	Male	50 to 54	85%<br>(75-92%)	15%<br>(8.5-25%)	10<br>(7.6-13)	22%<br>(19-27%)	29%<br>(24-34%)	410<br>(330-490)	16000<br>(13000-19000)
Croatia	2000	Male	55 to 59	85%<br>(76-92%)	15%<br>(8.3-24%)	9.6<br>(7.4-12)	22%<br>(18-26%)	27%<br>(22-32%)	450<br>(360-550)	15000<br>(12000-18000)
Croatia	2000	Male	60 to 64	84%<br>(74-91%)	16%<br>(9.3-26%)	9.3<br>(6.8-12)	20%<br>(16-24%)	24%<br>(19-29%)	750<br>(590-920)	21000<br>(17000-26000)
Croatia	2000	Male	65 to 69	82%<br>(72-89%)	18%<br>(11-28%)	9.1<br>(7.1-11)	19%<br>(15-23%)	22%<br>(17-27%)	950<br>(720-1200)	22000<br>(17000-28000)
Croatia	2000	Male	70 to 74	80%<br>(71-87%)	20%<br>(13-29%)	8.9<br>(6.6-12)	18%<br>(13-23%)	21%<br>(15-26%)	950<br>(700-1200)	18000<br>(13000-22000)
Croatia	2000	Male	75 to 79	78%<br>(65-87%)	22%<br>(13-35%)	7.9<br>(5.5-11)	17%<br>(12-22%)	19%<br>(14-25%)	700<br>(510-910)	10000<br>(7600-13000)
Croatia	2000	Male	80 to 84	75%<br>(61-86%)	25%<br>(14-39%)	6.5<br>(4.7-8.8)	15%<br>(10-19%)	17%<br>(12-22%)	390<br>(270-530)	4300<br>(3000-5700)
Croatia	2000	Male	85 to 89	73%<br>(59-83%)	27%<br>(17-41%)	5.5<br>(3.8-7.6)	12%<br>(7.7-17%)	13%<br>(8.6-19%)	210<br>(130-300)	1600<br>(1100-2300)
Croatia	2000	Male	90 to 94	72%<br>(58-83%)	28%<br>(17-42%)	4.6<br>(3-6.8)	9.5%<br>(4.6-14%)	11%<br>(4.9-17%)	100<br>(46-160)	540<br>(270-830)
Croatia	2000	Male	95 plus	70%<br>(52-84%)	30%<br>(16-48%)	3.6<br>(1.8-6)	6.7%<br>(0.97-13%)	7.2%<br>(0.56-15%)	19<br>(1.4-40)	71<br>(10-140)
Croatia	2005	Female	All Ages	59%<br>(55-64%)	41%<br>(36-45%)	1.8<br>(1.5-2)	4.6%<br>(3.3-6%)	5.3%<br>(3-7.9%)	1400<br>(780-2100)	32000<br>(22000-42000)
Croatia	2005	Female	15 to 19	68%<br>(54-80%)	32%<br>(20-46%)	1.4<br>(0.92-1.9)	2.7%<br>(1.8-3.9%)	7.1%<br>(3.9-11%)	2.7<br>(1.5-4.2)	340<br>(220-490)
Croatia	2005	Female	20 to 24	75%<br>(62-83%)	25%<br>(17-38%)	1.6<br>(1.1-2.2)	4%<br>(2.8-5.4%)	7%<br>(3.8-11%)	3.3<br>(1.8-5.1)	560<br>(370-790)
Croatia	2005	Female	25 to 29	72%<br>(60-81%)	28%<br>(19-40%)	1.5<br>(1-2)	3.7%<br>(2.8-4.8%)	6.2%<br>(3.6-9.1%)	2.9<br>(1.6-4.3)	560<br>(390-760)
Croatia	2005	Female	30 to 34	68%<br>(57-78%)	32%<br>(22-43%)	1.6<br>(1.1-2.2)	3.6%<br>(2.7-4.8%)	7.1%<br>(4.6-9.9%)	4.7<br>(3.1-6.7)	640<br>(460-870)
Croatia	2005	Female	35 to 39	65%<br>(56-76%)	35%<br>(24-44%)	1.9<br>(1.3-2.7)	4.1%<br>(3-5.3%)	9%<br>(6.2-12%)	10<br>(7-14)	860<br>(620-1100)
Croatia	2005	Female	40 to 44	63%<br>(53-74%)	37%<br>(26-47%)	2.2<br>(1.6-2.9)	4.8%<br>(3.5-6.2%)	11%<br>(8.1-14%)	21<br>(16-27)	1300<br>(1000-1700)
Croatia	2005	Female	45 to 49	64%<br>(54-73%)	36%<br>(27-46%)	2.5<br>(1.9-3.3)	5.8%<br>(4.3-7.4%)	11%<br>(8.7-14%)	42<br>(32-54)	2100<br>(1600-2700)
Croatia	2005	Female	50 to 54	65%<br>(55-74%)	35%<br>(26-45%)	2.8<br>(2.1-3.6)	6.8%<br>(5-8.7%)	12%<br>(9.1-15%)	69<br>(52-89)	2900<br>(2200-3800)
Croatia	2005	Female	55 to 59	65%<br>(55-75%)	35%<br>(25-45%)	2.9<br>(2.2-3.7)	7.2%<br>(5.3-9.4%)	12%<br>(9.1-16%)	91<br>(68-120)	3300<br>(2500-4300)

Location	Year	Sex	Age	Prevalence of current drinkers	Prevalence of abstainers	Population average of standard drinks daily	Percent of total attributable deaths	Percent of total attributable DALYs	Attributable deaths	Attributable DALYs
Croatia	2005	Female	60 to 64	64% (54-74%)	36% (26-46%)	2.8 (2.1-3.8)	6.9% (4.6-9.6%)	10% (7.2-14%)	100 (71-150)	3100 (2100-4400)
Croatia	2005	Female	65 to 69	59% (47-69%)	41% (31-53%)	2.5 (1.8-3.2)	6% (3.9-8.5%)	8.7% (5.8-12%)	180 (120-250)	4500 (2900-6200)
Croatia	2005	Female	70 to 74	49% (39-59%)	51% (41-61%)	1.9 (1.3-2.6)	4.7% (2.1-7.4%)	6.2% (2.9-9.8%)	220 (99-350)	4300 (2000-6800)
Croatia	2005	Female	75 to 79	38% (28-51%)	62% (49-72%)	1.4 (0.9-2.1)	3.3% (1.1-6%)	4.1% (1.3-7.5%)	210 (68-390)	3200 (1100-6000)
Croatia	2005	Female	80 to 84	36% (24-49%)	64% (51-76%)	1.4 (0.86-2)	3.1% (1-5.8%)	3.6% (1.1-6.7%)	210 (66-410)	2400 (750-4500)
Croatia	2005	Female	85 to 89	37% (25-50%)	63% (50-75%)	1.5 (0.91-2.3)	2.9% (0.68-5.8%)	3.3% (0.58-6.9%)	110 (19-240)	930 (220-1900)
Croatia	2005	Female	90 to 94	39% (27-52%)	61% (48-73%)	1.7 (0.94-2.5)	2.9% (0.12-6.7%)	3.3% (-0.048-8%)	58 (-0.85-140)	330 (14-750)
Croatia	2005	Female	95 plus	42% (27-60%)	58% (40-73%)	1.9 (0.94-3)	3.4% (-0.28-8.3%)	3.8% (-0.66-9.8%)	32 (-5.4-80)	120 (-9.7-280)
Croatia	2005	Male	All Ages	79% (74-83%)	21% (17-26%)	6.1 (5.5-6.7)	17% (15-20%)	21% (17-24%)	5500 (4400-6600)	140000 (120000-170000)
Croatia	2005	Male	15 to 19	72% (59-83%)	28% (17-41%)	3.3 (2.3-4.6)	8.8% (5.9-12%)	16% (10-23%)	17 (10-24)	1500 (960-2000)
Croatia	2005	Male	20 to 24	82% (71-89%)	18% (11-29%)	5.5 (3.9-7.4)	16% (11-21%)	26% (17-35%)	44 (30-61)	3600 (2500-4700)
Croatia	2005	Male	25 to 29	84% (75-90%)	16% (9.6-25%)	5.3 (3.9-6.8)	14% (11-19%)	24% (17-32%)	42 (30-56)	3300 (2500-4300)
Croatia	2005	Male	30 to 34	85% (77-91%)	15% (9-23%)	5.7 (4.2-7.5)	14% (11-18%)	25% (19-32%)	48 (35-62)	3500 (2600-4500)
Croatia	2005	Male	35 to 39	85% (78-91%)	15% (8.7-22%)	6.7 (4.8-8.9)	17% (13-21%)	29% (22-35%)	75 (57-94)	4700 (3600-5800)
Croatia	2005	Male	40 to 44	85% (78-92%)	15% (8.4-22%)	8 (6.2-10)	20% (17-23%)	31% (26-36%)	150 (130-180)	8200 (6800-9700)
Croatia	2005	Male	45 to 49	86% (79-92%)	14% (8.2-21%)	9.7 (7.3-12)	22% (19-26%)	31% (26-35%)	280 (230-330)	13000 (11000-15000)
Croatia	2005	Male	50 to 54	86% (77-92%)	14% (8-23%)	9.8 (7.4-12)	22% (18-26%)	28% (24-33%)	420 (350-500)	17000 (14000-20000)
Croatia	2005	Male	55 to 59	86% (76-92%)	14% (7.6-24%)	9 (7-11)	21% (17-25%)	26% (22-31%)	490 (400-580)	17000 (14000-20000)
Croatia	2005	Male	60 to 64	85% (76-92%)	15% (8.4-24%)	8.9 (6.7-12)	20% (16-24%)	24% (19-28%)	500 (400-610)	14000 (12000-18000)
Croatia	2005	Male	65 to 69	82% (74-89%)	18% (11-26%)	8.7 (6.9-11)	19% (15-22%)	22% (17-27%)	810 (640-990)	19000 (15000-24000)
Croatia	2005	Male	70 to 74	80% (71-88%)	20% (12-29%)	8.4 (6.4-11)	17% (13-22%)	20% (16-25%)	960 (730-1200)	18000 (14000-23000)
Croatia	2005	Male	75 to 79	79% (67-87%)	21% (13-33%)	7.6 (5.4-10)	16% (12-20%)	18% (13-23%)	840 (610-1100)	12000 (9200-16000)

Location	Year	Sex	Age	Prevalence of current drinkers	Prevalence of abstainers	Population average of standard drinks daily	Percent of total attributable deaths	Percent of total attributable DALYs	Attributable deaths	Attributable DALYs
Croatia	2005	Male	80 to 84	77% (63-86%)	23% (14-37%)	6.3 (4.6-8.4)	14% (10-19%)	16% (11-21%)	530 (380-700)	5800 (4200-7600)
Croatia	2005	Male	85 to 89	75% (63-84%)	25% (16-37%)	5.3 (3.8-7.2)	11% (7.5-16%)	13% (8.2-18%)	180 (120-260)	1500 (960-2000)
Croatia	2005	Male	90 to 94	73% (61-84%)	27% (16-39%)	4.5 (3-6.5)	8.6% (3.8-13%)	9.5% (3.7-15%)	66 (26-110)	360 (160-570)
Croatia	2005	Male	95 plus	71% (55-85%)	29% (15-45%)	3.5 (1.8-5.7)	5.7% (0.16-12%)	6% (-0.37-14%)	15 (-0.93-35)	59 (1.7-120)
Croatia	2010	Female	All Ages	60% (55-64%)	40% (36-45%)	1.6 (1.4-1.8)	3.6% (2.4-4.8%)	3.8% (1.8-6.1%)	1000 (480-1700)	24000 (16000-33000)
Croatia	2010	Female	15 to 19	68% (53-80%)	32% (20-47%)	1.2 (0.8-1.7)	2.4% (1.6-3.3%)	6.2% (3.3-9.7%)	1.7 (0.89-2.7)	240 (150-340)
Croatia	2010	Female	20 to 24	75% (62-84%)	25% (16-38%)	1.4 (0.96-2.1)	3.8% (2.7-5%)	6.2% (3.4-9.6%)	2.2 (1.2-3.3)	460 (310-650)
Croatia	2010	Female	25 to 29	72% (61-82%)	28% (18-39%)	1.3 (0.93-1.8)	3.5% (2.6-4.7%)	5.2% (2.9-7.7%)	2.3 (1.3-3.4)	520 (360-730)
Croatia	2010	Female	30 to 34	70% (58-79%)	30% (21-42%)	1.5 (1-2)	3.4% (2.4-4.4%)	6.1% (3.8-8.6%)	3.4 (2.1-4.9)	570 (390-790)
Croatia	2010	Female	35 to 39	67% (58-77%)	33% (23-42%)	1.8 (1.2-2.5)	3.7% (2.7-4.8%)	7.9% (5.5-11%)	7.7 (5.1-10)	740 (530-960)
Croatia	2010	Female	40 to 44	64% (53-75%)	36% (25-47%)	1.9 (1.4-2.6)	4.2% (3.2-5.4%)	9.4% (7-12%)	15 (11-20)	1000 (750-1300)
Croatia	2010	Female	45 to 49	64% (54-75%)	36% (25-46%)	2.2 (1.6-3)	4.9% (3.7-6.4%)	10% (7.5-13%)	32 (24-41)	1700 (1300-2200)
Croatia	2010	Female	50 to 54	65% (54-75%)	35% (25-46%)	2.5 (1.8-3.2)	5.6% (4.2-7.4%)	10% (7.7-13%)	55 (42-71)	2400 (1800-3100)
Croatia	2010	Female	55 to 59	66% (54-76%)	34% (24-46%)	2.6 (1.9-3.4)	5.9% (4.3-7.7%)	10% (7.6-13%)	80 (60-100)	2900 (2200-3800)
Croatia	2010	Female	60 to 64	64% (52-75%)	36% (25-48%)	2.5 (1.8-3.3)	5.3% (3.5-7.3%)	8.5% (5.9-12%)	91 (63-130)	2800 (1900-4000)
Croatia	2010	Female	65 to 69	59% (47-70%)	41% (30-53%)	2.2 (1.6-2.8)	4.7% (2.9-6.6%)	7% (4.4-9.7%)	100 (64-150)	2600 (1600-3700)
Croatia	2010	Female	70 to 74	50% (39-60%)	50% (40-61%)	1.7 (1.2-2.4)	3.5% (1.3-5.8%)	4.8% (1.8-7.8%)	140 (54-230)	2900 (1100-4700)
Croatia	2010	Female	75 to 79	40% (29-54%)	60% (46-71%)	1.3 (0.85-2)	2.5% (0.51-4.8%)	3.1% (0.58-6.1%)	160 (30-300)	2400 (490-4700)
Croatia	2010	Female	80 to 84	37% (24-51%)	63% (49-76%)	1.3 (0.79-1.9)	2.2% (0.37-4.5%)	2.5% (0.32-5.3%)	160 (20-340)	1800 (310-3700)
Croatia	2010	Female	85 to 89	37% (25-51%)	63% (49-75%)	1.4 (0.79-2.1)	2% (-0.04-4.4%)	2.2% (-0.27-5.3%)	120 (-15-280)	960 (-19-2200)
Croatia	2010	Female	90 to 94	39% (27-53%)	61% (47-73%)	1.5 (0.83-2.3)	1.8% (-0.32-4.7%)	2% (-0.82-5.8%)	39 (-16-110)	230 (-43-610)
Croatia	2010	Female	95 plus	42% (26-60%)	58% (40-74%)	1.6 (0.82-2.8)	2% (-1-6.2%)	2.2% (-1.6-7.5%)	14 (-10-49)	56 (-28-170)

Location	Year	Sex	Age	Prevalence of current drinkers	Prevalence of abstainers	Population average of standard drinks daily	Percent of total attributable deaths	Percent of total attributable DALYs	Attributable deaths	Attributable DALYs
Croatia	2010	Male	All Ages	78% (74-82%)	22% (18-26%)	5.4 (4.9-6)	16% (14-18%)	18% (15-22%)	4900 (4000-5900)	120000 (110000-150000)
Croatia	2010	Male	15 to 19	72% (58-83%)	28% (17-42%)	2.9 (2-4.1)	7.5% (5-10%)	14% (8.7-21%)	10 (6.1-15)	960 (650-1300)
Croatia	2010	Male	20 to 24	82% (71-90%)	18% (10-29%)	4.9 (3.4-6.6)	14% (10-19%)	24% (16-32%)	31 (20-42)	2600 (1900-3500)
Croatia	2010	Male	25 to 29	84% (76-90%)	16% (9.6-24%)	4.7 (3.4-6)	13% (9.6-17%)	22% (16-30%)	31 (22-42)	2700 (1900-3500)
Croatia	2010	Male	30 to 34	85% (77-91%)	15% (9-23%)	5.1 (3.7-6.7)	13% (9.7-16%)	23% (17-30%)	37 (27-49)	2900 (2200-3700)
Croatia	2010	Male	35 to 39	85% (78-91%)	15% (8.9-22%)	6 (4.3-8)	15% (11-18%)	26% (20-32%)	60 (45-75)	3900 (3000-4900)
Croatia	2010	Male	40 to 44	85% (77-91%)	15% (8.9-23%)	7.1 (5.3-9)	18% (15-21%)	29% (24-33%)	110 (89-130)	6000 (4800-7200)
Croatia	2010	Male	45 to 49	86% (78-91%)	14% (8.6-22%)	8.5 (6.4-11)	20% (17-24%)	29% (25-34%)	220 (180-260)	10000 (8500-12000)
Croatia	2010	Male	50 to 54	85% (77-91%)	15% (8.7-23%)	8.6 (6.5-11)	20% (17-24%)	27% (23-31%)	350 (300-420)	14000 (12000-17000)
Croatia	2010	Male	55 to 59	84% (73-91%)	16% (8.8-27%)	7.7 (6-9.8)	19% (16-23%)	24% (21-29%)	480 (400-570)	17000 (14000-20000)
Croatia	2010	Male	60 to 64	84% (74-91%)	16% (9.4-26%)	7.7 (5.8-10)	18% (15-21%)	22% (18-26%)	530 (430-640)	15000 (13000-19000)
Croatia	2010	Male	65 to 69	82% (73-89%)	18% (11-27%)	7.7 (6.2-9.4)	17% (14-21%)	21% (17-25%)	550 (450-670)	13000 (11000-16000)
Croatia	2010	Male	70 to 74	80% (70-87%)	20% (13-30%)	7.4 (5.5-9.5)	16% (13-20%)	19% (15-23%)	790 (620-980)	15000 (12000-19000)
Croatia	2010	Male	75 to 79	79% (67-87%)	21% (13-33%)	6.6 (4.6-8.9)	14% (11-18%)	16% (12-21%)	810 (590-1100)	12000 (8900-16000)
Croatia	2010	Male	80 to 84	75% (61-85%)	25% (15-39%)	5.4 (3.8-7.2)	12% (8.6-16%)	14% (9.6-19%)	570 (390-760)	6300 (4400-8300)
Croatia	2010	Male	85 to 89	72% (59-83%)	28% (17-41%)	4.5 (3.1-6.1)	9.4% (5.4-13%)	11% (5.9-15%)	230 (130-340)	1800 (1100-2700)
Croatia	2010	Male	90 to 94	71% (57-82%)	29% (18-43%)	3.8 (2.5-5.5)	6.3% (2.1-11%)	6.9% (1.8-12%)	46 (12-84)	260 (87-450)
Croatia	2010	Male	95 plus	70% (53-83%)	30% (17-47%)	3 (1.5-5)	3.9% (-0.67-9.8%)	3.9% (-1.5-11%)	7.1 (-2.9-20)	29 (-5.1-74)
Croatia	2016	Female	All Ages	61% (56-66%)	39% (34-44%)	1.6 (1.3-2.1)	3.4% (1.9-5.1%)	3.5% (0.97-6.6%)	1000 (280-1900)	23000 (13000-34000)
Croatia	2016	Female	15 to 19	65% (49-78%)	35% (22-51%)	1.1 (0.66-1.7)	2.2% (1.4-3.1%)	5.7% (3-9.2%)	1.3 (0.68-2.2)	210 (140-300)
Croatia	2016	Female	20 to 24	74% (60-84%)	26% (16-40%)	1.3 (0.84-2.1)	3.7% (2.7-4.9%)	6% (3.2-9.6%)	1.6 (0.82-2.6)	380 (250-540)
Croatia	2016	Female	25 to 29	74% (61-84%)	26% (16-39%)	1.3 (0.88-2)	3.6% (2.7-4.7%)	5.3% (2.9-8%)	1.8 (0.95-2.9)	460 (300-650)

Location	Year	Sex	Age	Prevalence of current drinkers	Prevalence of abstainers	Population average of standard drinks daily	Percent of total attributable deaths	Percent of total attributable DALYs	Attributable deaths	Attributable DALYs
Croatia	2016	Female	30 to 34	72% (59-81%)	28% (19-41%)	1.5 (0.96-2.2)	3.4% (2.5-4.5%)	6.1% (3.8-8.9%)	3.1 (1.8-4.7)	550 (370-770)
Croatia	2016	Female	35 to 39	68% (57-79%)	32% (21-43%)	1.8 (1.1-2.6)	3.6% (2.6-4.8%)	7.9% (5.1-11%)	6.8 (4.1-10)	700 (480-960)
Croatia	2016	Female	40 to 44	67% (55-78%)	33% (22-45%)	2 (1.3-2.8)	4.1% (2.9-5.4%)	9.4% (6.8-12%)	14 (9.3-20)	980 (670-1300)
Croatia	2016	Female	45 to 49	68% (55-78%)	32% (22-45%)	2.4 (1.6-3.3)	5% (3.6-6.7%)	10% (7.5-14%)	27 (19-37)	1400 (990-1900)
Croatia	2016	Female	50 to 54	68% (55-78%)	32% (22-45%)	2.6 (1.8-3.6)	5.5% (3.9-7.6%)	10% (7.4-13%)	48 (33-67)	2100 (1500-2900)
Croatia	2016	Female	55 to 59	68% (55-78%)	32% (22-45%)	2.6 (1.8-3.7)	5.6% (3.8-7.8%)	9.8% (6.9-13%)	73 (49-100)	2700 (1800-3800)
Croatia	2016	Female	60 to 64	66% (51-77%)	34% (23-49%)	2.5 (1.7-3.5)	4.9% (2.9-7.1%)	8.1% (5.2-12%)	88 (54-130)	2800 (1600-4100)
Croatia	2016	Female	65 to 69	61% (47-73%)	39% (27-53%)	2.2 (1.4-3.1)	4.4% (2.4-6.7%)	6.6% (3.9-9.8%)	110 (63-180)	2900 (1600-4500)
Croatia	2016	Female	70 to 74	54% (40-65%)	46% (35-60%)	1.9 (1.1-2.8)	3.4% (1.1-6.4%)	4.7% (1.7-8.6%)	110 (36-190)	2100 (730-3900)
Croatia	2016	Female	75 to 79	46% (32-60%)	54% (40-68%)	1.5 (0.89-2.4)	2.6% (0.23-5.5%)	3.3% (0.25-7.3%)	140 (11-310)	2300 (170-4900)
Croatia	2016	Female	80 to 84	40% (26-55%)	60% (45-74%)	1.4 (0.8-2.1)	2.2% (0.056-5%)	2.5% (-0.05-6%)	170 (-3.4-400)	2000 (56-4500)
Croatia	2016	Female	85 to 89	39% (26-53%)	61% (47-74%)	1.4 (0.77-2.2)	1.9% (-0.42-4.7%)	2.1% (-0.73-5.7%)	130 (-48-360)	1100 (-260-2800)
Croatia	2016	Female	90 to 94	41% (28-54%)	59% (46-72%)	1.5 (0.8-2.5)	1.7% (-0.83-5.2%)	1.9% (-1.3-6.3%)	59 (-40-200)	360 (-170-1100)
Croatia	2016	Female	95 plus	44% (28-61%)	56% (39-72%)	1.7 (0.8-2.9)	2% (-1.3-6.7%)	2.2% (-2-8.3%)	23 (-22-87)	90 (-59-300)
Croatia	2016	Male	All Ages	76% (70-80%)	24% (20-30%)	5 (4-6.2)	15% (12-17%)	16% (13-20%)	4400 (3300-5600)	110000 (87000-140000)
Croatia	2016	Male	15 to 19	66% (49-80%)	34% (20-51%)	2.5 (1.5-3.8)	6.4% (4-9.2%)	13% (7.3-19%)	7.6 (4.3-12)	770 (490-1100)
Croatia	2016	Male	20 to 24	78% (64-88%)	22% (12-36%)	4.3 (2.8-6.3)	13% (9-18%)	22% (14-31%)	21 (13-31)	1900 (1300-2700)
Croatia	2016	Male	25 to 29	81% (69-89%)	19% (11-31%)	4.2 (2.8-5.9)	12% (8.8-16%)	21% (14-29%)	23 (14-33)	2100 (1500-2800)
Croatia	2016	Male	30 to 34	82% (70-90%)	18% (10-30%)	4.6 (3.1-6.4)	12% (8.8-16%)	21% (15-29%)	30 (20-41)	2500 (1800-3300)
Croatia	2016	Male	35 to 39	82% (72-90%)	18% (10-28%)	5.4 (3.6-7.7)	13% (10-17%)	24% (18-31%)	49 (33-66)	3400 (2500-4400)
Croatia	2016	Male	40 to 44	83% (73-90%)	17% (9.7-27%)	6.5 (4.6-8.9)	16% (13-20%)	28% (22-33%)	93 (69-120)	5300 (4000-6600)
Croatia	2016	Male	45 to 49	84% (73-91%)	16% (9.2-27%)	7.9 (5.4-11)	19% (16-23%)	28% (24-33%)	160 (120-210)	7800 (6000-9800)

Location	Year	Sex	Age	Prevalence of current drinkers	Prevalence of abstainers	Population average of standard drinks daily	Percent of total attributable deaths	Percent of total attributable DALYs	Attributable deaths	Attributable DALYs
Croatia	2016	Male	50 to 54	84% (73-91%)	16% (9-27%)	8 (5.5-11)	19% (16-23%)	26% (22-31%)	280 (220-360)	11000 (9000-14000)
Croatia	2016	Male	55 to 59	83% (71-91%)	17% (8.6-29%)	7.3 (5.1-9.8)	18% (15-22%)	24% (20-28%)	410 (310-510)	14000 (11000-18000)
Croatia	2016	Male	60 to 64	82% (70-90%)	18% (9.7-30%)	7.1 (4.9-9.7)	17% (13-20%)	21% (17-25%)	520 (390-650)	15000 (12000-19000)
Croatia	2016	Male	65 to 69	80% (69-89%)	20% (11-31%)	7.1 (5.2-9.5)	16% (13-19%)	20% (16-24%)	630 (480-790)	15000 (12000-19000)
Croatia	2016	Male	70 to 74	78% (66-86%)	22% (14-34%)	6.8 (4.8-9.5)	15% (11-18%)	18% (14-22%)	550 (410-710)	11000 (8100-14000)
Croatia	2016	Male	75 to 79	76% (62-86%)	24% (14-38%)	6 (3.9-8.6)	13% (9.2-17%)	15% (11-20%)	660 (450-880)	10000 (6900-13000)
Croatia	2016	Male	80 to 84	73% (58-85%)	27% (15-42%)	5 (3.2-7.2)	11% (6.9-16%)	13% (7.8-18%)	610 (370-850)	6700 (4100-9300)
Croatia	2016	Male	85 to 89	71% (55-82%)	29% (18-45%)	4.2 (2.7-6.3)	8.5% (4.2-13%)	9.7% (4.5-15%)	300 (140-470)	2400 (1200-3700)
Croatia	2016	Male	90 to 94	70% (55-82%)	30% (18-45%)	3.5 (2.1-5.5)	5.7% (1.2-11%)	6.3% (0.88-12%)	79 (11-160)	450 (99-840)
Croatia	2016	Male	95 plus	69% (50-84%)	31% (16-50%)	2.8 (1.4-5)	3.5% (-0.88-9.5%)	3.5% (-1.8-11%)	9.4 (-4.8-29)	39 (-9.6-110)
Czech Republic	1990	Female	All Ages	77% (74-80%)	23% (20-26%)	2.3 (2-2.5)	3.8% (2.1-5.8%)	3.7% (0.67-7.3%)	2300 (420-4600)	66000 (36000-100000)
Czech Republic	1990	Female	15 to 19	79% (69-88%)	21% (12-31%)	2 (1.4-2.7)	3.5% (2.3-5.1%)	9% (5-14%)	13 (7.4-21)	1400 (900-2100)
Czech Republic	1990	Female	20 to 24	88% (81-93%)	12% (7.5-19%)	2.3 (1.5-3.2)	4.6% (3.1-6.3%)	9.5% (5.1-14%)	12 (6.6-19)	1600 (1100-2300)
Czech Republic	1990	Female	25 to 29	89% (82-93%)	11% (6.8-18%)	2 (1.4-2.8)	4.1% (2.9-5.6%)	8.2% (4.4-13%)	11 (6.2-18)	1600 (1000-2200)
Czech Republic	1990	Female	30 to 34	89% (82-93%)	11% (7.1-18%)	2.3 (1.7-3.2)	4.3% (2.9-5.7%)	8.9% (5.4-13%)	21 (12-29)	2100 (1400-2800)
Czech Republic	1990	Female	35 to 39	87% (80-91%)	13% (8.5-20%)	3.1 (2.2-4.1)	5.1% (3.5-6.7%)	11% (7.4-15%)	45 (30-60)	3400 (2300-4500)
Czech Republic	1990	Female	40 to 44	85% (78-89%)	15% (11-22%)	3.5 (2.6-4.5)	5.9% (4.2-7.7%)	12% (8.4-15%)	85 (62-110)	5000 (3700-6600)
Czech Republic	1990	Female	45 to 49	83% (75-88%)	17% (12-25%)	3.6 (2.6-4.8)	6.3% (4.3-8.8%)	11% (7.7-15%)	110 (77-150)	5500 (3900-7500)
Czech Republic	1990	Female	50 to 54	82% (75-87%)	18% (13-25%)	3.7 (2.7-4.9)	6.6% (4-9.3%)	11% (6.7-15%)	130 (80-180)	5500 (3500-7700)
Czech Republic	1990	Female	55 to 59	82% (75-87%)	18% (13-25%)	3.8 (2.8-5)	6.8% (3.9-10%)	10% (5.7-15%)	220 (120-310)	7800 (4300-11000)
Czech Republic	1990	Female	60 to 64	80% (73-85%)	20% (15-27%)	3.5 (2.5-4.6)	5.9% (2.3-9.5%)	8.1% (3.3-13%)	300 (120-490)	9100 (3600-15000)
Czech Republic	1990	Female	65 to 69	74% (66-81%)	26% (19-34%)	2.9 (2.2-3.8)	4.5% (1.4-7.9%)	5.9% (1.8-10%)	380 (120-680)	9500 (3000-16000)

Location	Year	Sex	Age	Prevalence of current drinkers	Prevalence of abstainers	Population average of standard drinks daily	Percent of total attributable deaths	Percent of total attributable DALYs	Attributable deaths	Attributable DALYs
Czech Republic	1990	Female	70 to 74	66% (57-74%)	34% (26-43%)	2.4 (1.6-3.3)	3% (-0.52-6.7%)	3.7% (-0.74-8.5%)	210 (-42-490)	4200 (-740-9500)
Czech Republic	1990	Female	75 to 79	57% (45-68%)	43% (32-55%)	1.9 (1.2-2.7)	1.8% (-1.5-5.7%)	2.2% (-2-6.8%)	270 (-250-830)	4100 (-3400-12000)
Czech Republic	1990	Female	80 to 84	52% (39-63%)	48% (37-61%)	1.8 (1.2-2.5)	1.7% (-1.2-5.1%)	1.9% (-1.5-5.9%)	270 (-210-820)	3000 (-2000-8900)
Czech Republic	1990	Female	85 to 89	51% (38-63%)	49% (37-62%)	1.8 (1.2-2.8)	1.6% (-1.7-5.4%)	1.8% (-2.3-6.1%)	160 (-210-570)	1300 (-1400-4500)
Czech Republic	1990	Female	90 to 94	53% (41-66%)	47% (34-59%)	2 (1.2-3)	1.7% (-2.1-6.5%)	1.8% (-2.8-7.6%)	64 (-97-270)	370 (-470-1400)
Czech Republic	1990	Female	95 plus	57% (41-72%)	43% (28-59%)	2.2 (1.1-3.5)	1.9% (-3.2-8%)	2.1% (-4-9.4%)	14 (-27-63)	54 (-88-220)
Czech Republic	1990	Male	All Ages	88% (85-90%)	12% (10-15%)	4.7 (4.1-5.1)	13% (11-16%)	15% (11-19%)	9800 (7200-12000)	270000 (220000-330000)
Czech Republic	1990	Male	15 to 19	85% (76-92%)	15% (7.9-24%)	2.3 (1.6-3.3)	6.5% (4-9.4%)	12% (6.4-18%)	39 (22-60)	3400 (2100-5000)
Czech Republic	1990	Male	20 to 24	92% (87-95%)	8.1% (4.8-13%)	4.1 (3-5.4)	12% (8.4-17%)	20% (12-27%)	82 (51-110)	6600 (4500-8900)
Czech Republic	1990	Male	25 to 29	94% (90-96%)	6.3% (3.8-10%)	5 (3.5-6.6)	14% (9.5-18%)	23% (15-31%)	97 (64-130)	7500 (5300-9800)
Czech Republic	1990	Male	30 to 34	95% (91-97%)	5.3% (2.7-9.3%)	5.5 (4-7.3)	14% (10-18%)	23% (16-30%)	130 (91-170)	9100 (6600-12000)
Czech Republic	1990	Male	35 to 39	95% (92-97%)	4.9% (2.5-8.3%)	5.6 (4-7.7)	14% (10-19%)	21% (15-28%)	220 (150-300)	14000 (9700-18000)
Czech Republic	1990	Male	40 to 44	94% (90-96%)	6.2% (3.9-9.6%)	6.5 (4.9-8.2)	16% (12-19%)	22% (16-27%)	390 (280-480)	20000 (15000-25000)
Czech Republic	1990	Male	45 to 49	91% (87-94%)	9% (5.9-13%)	8 (6-10)	18% (14-22%)	23% (18-28%)	570 (450-690)	26000 (20000-31000)
Czech Republic	1990	Male	50 to 54	90% (85-93%)	10% (7.1-15%)	8.5 (6.4-11)	17% (13-21%)	21% (16-26%)	630 (490-780)	25000 (19000-31000)
Czech Republic	1990	Male	55 to 59	90% (86-93%)	9.7% (6.9-14%)	8.5 (6.6-11)	16% (12-21%)	19% (14-25%)	910 (670-1200)	31000 (23000-39000)
Czech Republic	1990	Male	60 to 64	90% (87-93%)	9.6% (6.9-13%)	8.4 (6.4-11)	16% (11-21%)	18% (12-24%)	1300 (900-1700)	37000 (26000-49000)
Czech Republic	1990	Male	65 to 69	90% (86-93%)	10% (7.3-14%)	7.8 (6.1-9.4)	15% (11-20%)	17% (12-23%)	1700 (1200-2200)	40000 (28000-52000)
Czech Republic	1990	Male	70 to 74	88% (84-91%)	12% (9.1-16%)	6.7 (5.1-8.6)	14% (9-19%)	16% (10-21%)	1000 (660-1400)	19000 (13000-26000)
Czech Republic	1990	Male	75 to 79	84% (77-89%)	16% (11-23%)	5.5 (4-7.5)	11% (5.8-17%)	13% (6.3-19%)	1400 (680-2000)	20000 (10000-29000)
Czech Republic	1990	Male	80 to 84	81% (73-87%)	19% (13-27%)	4.8 (3.4-6.4)	9.8% (4.6-15%)	11% (4.8-17%)	940 (420-1500)	10000 (4900-16000)
Czech Republic	1990	Male	85 to 89	79% (70-85%)	21% (15-30%)	4.3 (3-5.8)	7.8% (2.3-13%)	8.5% (2.2-15%)	340 (89-600)	2700 (790-4600)

Location	Year	Sex	Age	Prevalence of current drinkers	Prevalence of abstainers	Population average of standard drinks daily	Percent of total attributable deaths	Percent of total attributable DALYs	Attributable deaths	Attributable DALYs
Czech Republic	1990	Male	90 to 94	78% (69-85%)	22% (15-31%)	3.8 (2.6-5.4)	5.2% (-0.33-12%)	5.6% (-0.94-13%)	59 (-10-140)	330 (-20-750)
Czech Republic	1990	Male	95 plus	77% (64-86%)	23% (14-36%)	3.2 (1.9-5.2)	3.1% (-2.6-11%)	3.2% (-3.6-12%)	4.7 (-5.3-18)	19 (-16-66)
Czech Republic	1995	Female	All Ages	78% (74-81%)	22% (19-26%)	2.4 (2.1-2.7)	4% (2.3-6.1%)	4% (0.93-7.8%)	2300 (540-4500)	65000 (37000-97000)
Czech Republic	1995	Female	15 to 19	79% (69-87%)	21% (13-31%)	2 (1.4-2.8)	3.4% (2.2-4.9%)	9.3% (5.2-14%)	13 (7.1-20)	1400 (850-2000)
Czech Republic	1995	Female	20 to 24	87% (80-92%)	13% (7.7-20%)	2.3 (1.6-3.3)	4.5% (3.1-6.2%)	9.5% (5.3-14%)	14 (7.9-22)	1900 (1300-2700)
Czech Republic	1995	Female	25 to 29	89% (82-93%)	11% (6.8-18%)	2.1 (1.4-3)	4.1% (2.8-5.5%)	8.5% (5-13%)	11 (6.4-16)	1500 (1000-2100)
Czech Republic	1995	Female	30 to 34	89% (83-93%)	11% (7-17%)	2.4 (1.7-3.3)	4.1% (2.9-5.6%)	9.2% (5.9-13%)	17 (11-24)	1800 (1300-2500)
Czech Republic	1995	Female	35 to 39	87% (80-91%)	13% (8.6-20%)	3.2 (2.2-4.4)	5.1% (3.6-6.8%)	12% (8-15%)	37 (26-49)	2800 (2000-3700)
Czech Republic	1995	Female	40 to 44	84% (78-90%)	16% (10-22%)	3.6 (2.7-4.7)	6% (4.4-7.8%)	12% (9.3-16%)	77 (58-99)	4700 (3500-6200)
Czech Republic	1995	Female	45 to 49	83% (75-88%)	17% (12-25%)	3.8 (2.7-4.9)	6.3% (4.3-8.5%)	12% (8.4-15%)	120 (90-160)	6300 (4500-8400)
Czech Republic	1995	Female	50 to 54	82% (76-87%)	18% (13-24%)	3.9 (2.8-5)	6.7% (4.4-9.2%)	11% (7.5-15%)	150 (100-210)	6700 (4500-9000)
Czech Republic	1995	Female	55 to 59	82% (76-87%)	18% (13-24%)	3.9 (2.9-5.2)	6.9% (4.4-9.8%)	10% (6.5-15%)	180 (110-260)	6600 (4100-9400)
Czech Republic	1995	Female	60 to 64	80% (72-85%)	20% (15-28%)	3.6 (2.6-4.8)	6% (2.9-9.5%)	8.5% (4.1-14%)	250 (120-400)	7600 (3600-12000)
Czech Republic	1995	Female	65 to 69	74% (66-81%)	26% (19-34%)	3 (2.2-3.9)	4.7% (1.8-7.8%)	6.3% (2.2-11%)	320 (120-550)	8100 (3000-13000)
Czech Republic	1995	Female	70 to 74	66% (57-75%)	34% (25-43%)	2.5 (1.7-3.4)	3.2% (0.027-6.9%)	4.1% (-0.24-8.7%)	350 (-21-740)	6900 (58-15000)
Czech Republic	1995	Female	75 to 79	57% (45-69%)	43% (31-55%)	1.9 (1.2-2.9)	2.1% (-1.1-6.1%)	2.5% (-1.5-7.3%)	170 (-110-510)	2700 (-1400-7800)
Czech Republic	1995	Female	80 to 84	52% (39-63%)	48% (37-61%)	1.8 (1.2-2.7)	1.9% (-0.89-5.3%)	2.2% (-1.2-6.4%)	280 (-150-800)	3200 (-1500-9000)
Czech Republic	1995	Female	85 to 89	51% (38-63%)	49% (37-62%)	1.9 (1.2-2.8)	1.9% (-1.3-5.8%)	2.1% (-1.8-6.8%)	220 (-180-700)	1800 (-1200-5200)
Czech Republic	1995	Female	90 to 94	53% (41-66%)	47% (34-59%)	2.1 (1.2-3.1)	1.9% (-1.9-6.8%)	2.1% (-2.6-7.9%)	89 (-110-330)	510 (-510-1800)
Czech Republic	1995	Female	95 plus	57% (40-71%)	43% (29-60%)	2.2 (1.2-3.6)	2.1% (-3.1-8.4%)	2.3% (-3.9-9.9%)	20 (-34-88)	77 (-110-310)
Czech Republic	1995	Male	All Ages	88% (85-90%)	12% (9.8-15%)	4.9 (4.3-5.6)	14% (11-16%)	15% (11-20%)	9000 (6600-11000)	260000 (210000-310000)
Czech Republic	1995	Male	15 to 19	85% (75-92%)	15% (8.4-25%)	2.4 (1.6-3.3)	6.8% (4.1-10%)	12% (6.6-18%)	44 (24-67)	3800 (2300-5500)

Location	Year	Sex	Age	Prevalence of current drinkers	Prevalence of abstainers	Population average of standard drinks daily	Percent of total attributable deaths	Percent of total attributable DALYs	Attributable deaths	Attributable DALYs
Czech Republic	1995	Male	20 to 24	92% (87-95%)	8.2% (4.9-13%)	4.1 (3-5.5)	12% (8.4-17%)	20% (13-28%)	95 (60-130)	7700 (5200-10000)
Czech Republic	1995	Male	25 to 29	94% (89-96%)	6.4% (3.8-11%)	5.1 (3.5-6.8)	14% (9.7-18%)	24% (16-32%)	99 (64-130)	7600 (5300-9900)
Czech Republic	1995	Male	30 to 34	95% (91-97%)	5.3% (2.7-9.1%)	5.6 (4-7.5)	14% (10-18%)	24% (17-31%)	120 (84-160)	8600 (6200-11000)
Czech Republic	1995	Male	35 to 39	95% (91-97%)	5% (2.7-8.6%)	5.7 (4-7.8)	14% (10-18%)	22% (15-29%)	180 (120-230)	11000 (7700-14000)
Czech Republic	1995	Male	40 to 44	94% (90-96%)	6.3% (3.8-10%)	6.6 (4.9-8.5)	16% (12-20%)	23% (17-28%)	340 (260-420)	18000 (14000-22000)
Czech Republic	1995	Male	45 to 49	91% (86-94%)	9.1% (6.2-14%)	8.1 (6.1-10)	18% (14-22%)	24% (19-29%)	620 (490-740)	28000 (23000-34000)
Czech Republic	1995	Male	50 to 54	90% (86-93%)	10% (7.3-14%)	8.6 (6.5-11)	18% (14-21%)	22% (18-27%)	730 (580-900)	29000 (23000-35000)
Czech Republic	1995	Male	55 to 59	90% (86-93%)	9.7% (7-14%)	8.7 (6.7-11)	17% (13-21%)	20% (15-26%)	760 (570-970)	26000 (20000-33000)
Czech Republic	1995	Male	60 to 64	90% (86-93%)	9.6% (6.8-14%)	8.5 (6.4-11)	15% (11-20%)	18% (13-24%)	1000 (730-1400)	30000 (22000-40000)
Czech Republic	1995	Male	65 to 69	90% (86-93%)	10% (7.3-14%)	7.8 (6.1-9.6)	15% (11-19%)	17% (12-23%)	1400 (950-1800)	33000 (23000-43000)
Czech Republic	1995	Male	70 to 74	88% (83-91%)	12% (9.1-17%)	6.8 (5.1-8.8)	13% (8.6-18%)	15% (9.6-21%)	1500 (950-2100)	29000 (19000-39000)
Czech Republic	1995	Male	75 to 79	84% (77-89%)	16% (11-23%)	5.5 (3.9-7.6)	11% (5.8-16%)	13% (6.2-18%)	750 (370-1100)	11000 (5800-16000)
Czech Republic	1995	Male	80 to 84	81% (73-86%)	19% (14-27%)	4.8 (3.4-6.4)	9.7% (4.7-15%)	11% (4.8-17%)	860 (390-1400)	9600 (4600-15000)
Czech Republic	1995	Male	85 to 89	79% (70-85%)	21% (15-30%)	4.4 (3.1-5.9)	7.8% (2.7-13%)	8.6% (2.6-15%)	410 (120-710)	3200 (1100-5500)
Czech Republic	1995	Male	90 to 94	78% (69-84%)	22% (16-31%)	3.9 (2.5-5.5)	5.3% (-0.41-12%)	5.7% (-1.1-13%)	74 (-14-170)	410 (-32-910)
Czech Republic	1995	Male	95 plus	76% (64-85%)	24% (15-36%)	3.3 (1.9-5.2)	3.2% (-2.5-11%)	3.2% (-3.7-12%)	6.1 (-7-23)	25 (-19-83)
Czech Republic	2000	Female	All Ages	79% (76-81%)	21% (19-24%)	2.6 (2.3-2.8)	4.2% (2.8-5.9%)	4.3% (1.6-7.5%)	2300 (860-4100)	64000 (42000-90000)
Czech Republic	2000	Female	15 to 19	82% (75-89%)	18% (11-25%)	2.2 (1.5-3)	3.4% (2.1-4.9%)	10% (5.4-15%)	9.7 (5.3-15)	1100 (670-1500)
Czech Republic	2000	Female	20 to 24	89% (83-93%)	11% (7.3-17%)	2.5 (1.7-3.4)	4.4% (3.1-5.9%)	9.8% (5.6-15%)	13 (7.6-20)	1900 (1200-2600)
Czech Republic	2000	Female	25 to 29	90% (85-93%)	10% (6.7-15%)	2.2 (1.5-3)	3.9% (2.9-5.1%)	8.7% (5.3-13%)	12 (7-17)	1800 (1200-2400)
Czech Republic	2000	Female	30 to 34	90% (85-93%)	10% (6.9-15%)	2.5 (1.9-3.4)	4.1% (2.9-5.3%)	9.9% (6.8-13%)	15 (10-20)	1700 (1200-2200)
Czech Republic	2000	Female	35 to 39	88% (83-92%)	12% (8.1-17%)	3.4 (2.4-4.5)	5.1% (3.8-6.6%)	12% (9.2-16%)	31 (23-40)	2500 (1800-3200)

Location	Year	Sex	Age	Prevalence of current drinkers	Prevalence of abstainers	Population average of standard drinks daily	Percent of total attributable deaths	Percent of total attributable DALYs	Attributable deaths	Attributable DALYs
Czech Republic	2000	Female	40 to 44	86%<br>(82-90%)	14%<br>(10-18%)	3.9<br>(2.9-4.9)	6.2%<br>(4.8-8%)	14%<br>(11-17%)	64<br>(51-79)	4000<br>(3100-5000)
Czech Republic	2000	Female	45 to 49	84%<br>(80-88%)	16%<br>(12-20%)	4<br>(3-5.1)	6.7%<br>(4.8-8.9%)	13%<br>(9.6-16%)	120<br>(91-150)	6200<br>(4600-8000)
Czech Republic	2000	Female	50 to 54	83%<br>(78-87%)	17%<br>(13-22%)	4<br>(3-5.1)	7%<br>(4.9-9.3%)	12%<br>(8.7-15%)	180<br>(130-240)	8000<br>(5700-10000)
Czech Republic	2000	Female	55 to 59	82%<br>(78-86%)	18%<br>(14-22%)	4.1<br>(3.1-5.2)	7.1%<br>(4.8-9.6%)	11%<br>(7.6-15%)	210<br>(150-290)	8000<br>(5400-11000)
Czech Republic	2000	Female	60 to 64	78%<br>(73-83%)	22%<br>(17-27%)	3.5<br>(2.6-4.7)	6%<br>(3.3-8.9%)	8.8%<br>(4.9-13%)	210<br>(110-310)	6500<br>(3500-9600)
Czech Republic	2000	Female	65 to 69	71%<br>(63-79%)	29%<br>(21-37%)	3<br>(2.1-3.8)	4.8%<br>(2.4-7.4%)	6.6%<br>(3.2-10%)	260<br>(130-410)	6800<br>(3300-11000)
Czech Republic	2000	Female	70 to 74	63%<br>(54-72%)	37%<br>(28-46%)	2.3<br>(1.6-3.2)	3.4%<br>(0.57-6.6%)	4.4%<br>(0.7-8.5%)	300<br>(49-590)	6100<br>(1100-12000)
Czech Republic	2000	Female	75 to 79	53%<br>(40-64%)	47%<br>(36-60%)	1.8<br>(1.1-2.7)	2.3%<br>(-0.42-5.5%)	2.7%<br>(-0.66-6.8%)	300<br>(-70-730)	4600<br>(-870-11000)
Czech Republic	2000	Female	80 to 84	49%<br>(37-61%)	51%<br>(39-63%)	1.7<br>(1.2-2.6)	2.1%<br>(-0.46-5.1%)	2.5%<br>(-0.7-6.2%)	190<br>(-54-470)	2200<br>(-460-5300)
Czech Republic	2000	Female	85 to 89	50%<br>(37-62%)	50%<br>(38-63%)	1.9<br>(1.2-2.8)	2.2%<br>(-0.89-5.9%)	2.5%<br>(-1.3-7%)	260<br>(-140-720)	2100<br>(-820-5600)
Czech Republic	2000	Female	90 to 94	52%<br>(40-65%)	48%<br>(35-60%)	2<br>(1.2-3.1)	2.2%<br>(-1.5-6.9%)	2.4%<br>(-2-8.3%)	130<br>(-110-430)	720<br>(-480-2300)
Czech Republic	2000	Female	95 plus	56%<br>(39-71%)	44%<br>(29-61%)	2.2<br>(1.2-3.6)	2.3%<br>(-2.9-8.6%)	2.5%<br>(-3.6-9.9%)	29<br>(-42-110)	110<br>(-130-400)
Czech Republic	2000	Male	All Ages	90%<br>(88-92%)	10%<br>(8.4-12%)	5.4<br>(4.9-5.9)	14%<br>(12-17%)	16%<br>(13-20%)	8800<br>(6800-11000)	250000<br>(210000-300000)
Czech Republic	2000	Male	15 to 19	88%<br>(80-93%)	12%<br>(7.1-20%)	2.6<br>(1.8-3.6)	6.7%<br>(4.1-9.8%)	13%<br>(7.3-20%)	31<br>(17-46)	2700<br>(1700-3900)
Czech Republic	2000	Male	20 to 24	93%<br>(90-96%)	6.6%<br>(4.3-9.7%)	4.4<br>(3.1-5.9)	12%<br>(8.6-17%)	21%<br>(13-28%)	95<br>(60-130)	7800<br>(5300-10000)
Czech Republic	2000	Male	25 to 29	95%<br>(92-97%)	5.2%<br>(3.4-7.7%)	5.3<br>(3.8-7.1)	13%<br>(9.6-17%)	25%<br>(17-33%)	110<br>(73-140)	8400<br>(6100-11000)
Czech Republic	2000	Male	30 to 34	96%<br>(93-98%)	4.3%<br>(2.5-7.1%)	5.8<br>(4.3-7.7)	14%<br>(10-18%)	25%<br>(18-32%)	110<br>(78-140)	7800<br>(5800-9900)
Czech Republic	2000	Male	35 to 39	96%<br>(94-98%)	4.1%<br>(2.3-6.4%)	5.9<br>(4.3-8.1)	14%<br>(10-18%)	24%<br>(17-31%)	150<br>(100-190)	9400<br>(6900-12000)
Czech Republic	2000	Male	40 to 44	95%<br>(92-97%)	5.2%<br>(3.4-7.8%)	6.8<br>(5.2-8.8)	16%<br>(13-20%)	25%<br>(19-30%)	280<br>(220-340)	15000<br>(12000-18000)
Czech Republic	2000	Male	45 to 49	92%<br>(89-95%)	7.8%<br>(5.4-11%)	8.5<br>(6.5-11)	19%<br>(15-22%)	26%<br>(22-30%)	570<br>(470-660)	26000<br>(21000-30000)
Czech Republic	2000	Male	50 to 54	91%<br>(88-93%)	9.1%<br>(6.7-12%)	9<br>(7-11)	18%<br>(15-22%)	24%<br>(20-28%)	840<br>(690-1000)	34000<br>(28000-40000)
Czech Republic	2000	Male	55 to 59	92%<br>(89-93%)	8.5%<br>(6.6-11%)	9<br>(7.2-11)	17%<br>(14-21%)	21%<br>(17-26%)	910<br>(710-1100)	31000<br>(25000-39000)

Location	Year	Sex	Age	Prevalence of current drinkers	Prevalence of abstainers	Population average of standard drinks daily	Percent of total attributable deaths	Percent of total attributable DALYs	Attributable deaths	Attributable DALYs
Czech Republic	2000	Male	60 to 64	92% (90-94%)	8.2% (6.4-10%)	8.9 (6.9-11)	16% (12-20%)	19% (14-24%)	900 (670-1100)	26000 (20000-33000)
Czech Republic	2000	Male	65 to 69	91% (89-93%)	8.5% (6.6-11%)	8.2 (6.7-9.9)	15% (11-19%)	18% (13-23%)	1200 (850-1500)	28000 (21000-35000)
Czech Republic	2000	Male	70 to 74	90% (87-92%)	10% (8.3-13%)	7.2 (5.6-9)	14% (9.2-18%)	16% (11-21%)	1300 (890-1700)	26000 (18000-34000)
Czech Republic	2000	Male	75 to 79	86% (80-90%)	14% (10-20%)	5.9 (4.3-7.8)	11% (6.5-16%)	13% (7.1-19%)	1200 (650-1700)	18000 (10000-26000)
Czech Republic	2000	Male	80 to 84	82% (75-87%)	18% (13-25%)	5 (3.6-6.6)	10% (5.1-15%)	11% (5.4-17%)	550 (270-840)	6200 (3100-9200)
Czech Republic	2000	Male	85 to 89	80% (72-85%)	20% (15-28%)	4.5 (3.3-6)	8.2% (3.3-14%)	9.1% (3.3-15%)	450 (160-760)	3600 (1400-5900)
Czech Republic	2000	Male	90 to 94	78% (70-85%)	22% (15-30%)	4 (2.7-5.6)	5.8% (0.12-12%)	6.3% (-0.49-13%)	120 (-9.3-250)	660 (14-1400)
Czech Republic	2000	Male	95 plus	77% (66-86%)	23% (14-34%)	3.4 (2-5.3)	3.6% (-2.4-11%)	3.7% (-3.3-12%)	9.9 (-8.8-32)	40 (-25-120)
Czech Republic	2005	Female	All Ages	79% (77-81%)	21% (19-23%)	2.6 (2.4-2.9)	4.2% (2.8-5.6%)	4.2% (1.6-7%)	2200 (840-3700)	64000 (43000-85000)
Czech Republic	2005	Female	15 to 19	83% (75-89%)	17% (11-25%)	2.2 (1.5-3)	3.2% (2-4.5%)	9.9% (5.6-15%)	7.8 (4.4-12)	940 (600-1300)
Czech Republic	2005	Female	20 to 24	89% (83-93%)	11% (6.9-17%)	2.5 (1.7-3.6)	4.3% (3-5.7%)	10% (5.9-15%)	9.3 (5.4-14)	1400 (980-2000)
Czech Republic	2005	Female	25 to 29	90% (86-94%)	9.8% (6.4-14%)	2.2 (1.6-3.1)	3.9% (2.8-5.1%)	9% (5.7-13%)	11 (7.2-16)	1800 (1300-2500)
Czech Republic	2005	Female	30 to 34	90% (86-93%)	9.9% (6.7-14%)	2.6 (2-3.5)	4.1% (3-5.4%)	10% (7.2-14%)	18 (13-24)	2100 (1500-2800)
Czech Republic	2005	Female	35 to 39	88% (84-92%)	12% (8.1-16%)	3.4 (2.5-4.6)	5.1% (3.9-6.5%)	13% (9.8-16%)	31 (23-39)	2500 (1900-3200)
Czech Republic	2005	Female	40 to 44	86% (82-90%)	14% (10-18%)	3.9 (3-5)	6.3% (4.9-7.9%)	14% (11-17%)	58 (47-71)	3700 (3000-4700)
Czech Republic	2005	Female	45 to 49	84% (80-88%)	16% (12-20%)	4.1 (3-5.2)	7% (5.2-9.1%)	14% (11-17%)	100 (81-130)	5400 (4100-6800)
Czech Republic	2005	Female	50 to 54	83% (79-86%)	17% (14-21%)	4.1 (3.1-5.3)	7.3% (5.3-9.5%)	13% (9.8-16%)	170 (130-220)	7700 (5800-9900)
Czech Republic	2005	Female	55 to 59	83% (79-86%)	17% (14-21%)	4.2 (3.2-5.2)	7.2% (5-9.4%)	11% (8.4-15%)	250 (180-330)	9500 (6800-13000)
Czech Republic	2005	Female	60 to 64	79% (75-83%)	21% (17-25%)	3.7 (2.8-4.7)	6.2% (3.7-8.8%)	9.3% (5.8-13%)	250 (160-360)	8100 (4900-12000)
Czech Republic	2005	Female	65 to 69	71% (65-77%)	29% (23-35%)	3 (2.3-3.7)	4.7% (2.5-7%)	6.7% (3.7-10%)	220 (120-330)	5800 (3200-8700)
Czech Republic	2005	Female	70 to 74	60% (53-66%)	40% (34-47%)	2.2 (1.6-3)	3.2% (0.73-5.7%)	4.2% (1.1-7.5%)	230 (60-410)	4800 (1100-8600)
Czech Republic	2005	Female	75 to 79	51% (42-61%)	49% (39-58%)	1.8 (1.2-2.5)	2.2% (-0.19-5.1%)	2.7% (-0.36-6.6%)	250 (-33-590)	4000 (-330-9300)

Location	Year	Sex	Age	Prevalence of current drinkers	Prevalence of abstainers	Population average of standard drinks daily	Percent of total attributable deaths	Percent of total attributable DALYs	Attributable deaths	Attributable DALYs
Czech Republic	2005	Female	80 to 84	48% (36-58%)	52% (42-64%)	1.7 (1.1-2.4)	2% (-0.37-4.7%)	2.4% (-0.64-5.8%)	300 (-83-730)	3600 (-670-8200)
Czech Republic	2005	Female	85 to 89	47% (35-58%)	53% (42-65%)	1.8 (1.2-2.6)	2% (-0.75-5.1%)	2.2% (-1.2-6.1%)	150 (-79-420)	1300 (-470-3400)
Czech Republic	2005	Female	90 to 94	49% (38-61%)	51% (39-62%)	1.9 (1.2-2.9)	2% (-1.4-6.1%)	2.2% (-1.9-7.2%)	120 (-110-410)	710 (-510-2200)
Czech Republic	2005	Female	95 plus	53% (37-69%)	47% (31-63%)	2.1 (1.1-3.3)	2.1% (-2.7-7.6%)	2.3% (-3.4-8.9%)	37 (-55-140)	140 (-170-500)
Czech Republic	2005	Male	All Ages	91% (89-92%)	9.2% (7.9-11%)	5.6 (5.2-6)	14% (12-16%)	16% (13-20%)	8600 (6700-11000)	250000 (210000-290000)
Czech Republic	2005	Male	15 to 19	88% (82-93%)	12% (6.9-18%)	2.6 (1.8-3.6)	6.4% (3.9-9%)	13% (7.5-20%)	25 (14-38)	2300 (1400-3300)
Czech Republic	2005	Male	20 to 24	94% (91-96%)	6.2% (4.1-9.3%)	4.4 (3.2-5.9)	12% (8.6-16%)	21% (14-29%)	74 (47-100)	6200 (4300-8100)
Czech Republic	2005	Male	25 to 29	95% (93-97%)	4.9% (3.2-7.2%)	5.4 (3.8-6.9)	13% (9.4-17%)	25% (17-32%)	100 (71-140)	8200 (6100-11000)
Czech Republic	2005	Male	30 to 34	96% (93-98%)	4.1% (2.4-6.6%)	5.9 (4.3-7.5)	13% (10-17%)	25% (18-32%)	120 (89-160)	9000 (6900-11000)
Czech Republic	2005	Male	35 to 39	96% (94-98%)	3.9% (2.2-6.1%)	5.9 (4.3-7.9)	14% (10-18%)	25% (17-32%)	140 (99-180)	9100 (6700-11000)
Czech Republic	2005	Male	40 to 44	95% (93-97%)	4.9% (3.3-7.2%)	6.9 (5.3-8.6)	16% (13-20%)	26% (21-32%)	240 (190-290)	13000 (10000-15000)
Czech Republic	2005	Male	45 to 49	93% (90-95%)	7.2% (5.1-9.7%)	8.5 (6.6-11)	19% (15-22%)	28% (23-32%)	460 (380-540)	21000 (18000-25000)
Czech Republic	2005	Male	50 to 54	92% (90-94%)	8.3% (6.2-10%)	9.1 (7.2-11)	19% (16-22%)	25% (21-29%)	780 (660-920)	32000 (27000-37000)
Czech Republic	2005	Male	55 to 59	92% (91-94%)	7.8% (6.3-9.4%)	9.2 (7.4-11)	18% (14-21%)	22% (18-27%)	1100 (880-1300)	38000 (31000-45000)
Czech Republic	2005	Male	60 to 64	92% (91-94%)	7.7% (6.2-9.4%)	9 (7-11)	16% (12-20%)	19% (15-24%)	1100 (830-1300)	32000 (25000-39000)
Czech Republic	2005	Male	65 to 69	92% (90-94%)	8.2% (6.5-10%)	8.3 (6.9-9.9)	15% (11-18%)	18% (13-22%)	980 (750-1200)	24000 (19000-30000)
Czech Republic	2005	Male	70 to 74	90% (88-92%)	9.9% (8-12%)	7.3 (5.7-9.1)	13% (9-17%)	15% (10-20%)	1100 (750-1500)	22000 (15000-29000)
Czech Republic	2005	Male	75 to 79	86% (82-90%)	14% (10-18%)	6 (4.4-7.8)	11% (6.1-16%)	13% (6.5-18%)	1100 (560-1500)	17000 (9400-23000)
Czech Republic	2005	Male	80 to 84	81% (75-87%)	19% (13-25%)	5 (3.7-6.5)	9.4% (4.8-14%)	11% (5.1-16%)	880 (410-1300)	9900 (5000-15000)
Czech Republic	2005	Male	85 to 89	79% (71-85%)	21% (15-29%)	4.5 (3.2-5.9)	7.7% (2.9-13%)	8.6% (2.9-14%)	290 (98-490)	2400 (890-3900)
Czech Republic	2005	Male	90 to 94	78% (69-84%)	22% (16-31%)	4 (2.7-5.6)	5.4% (0.067-12%)	5.8% (-0.55-13%)	120 (-11-270)	690 (8.1-1500)
Czech Republic	2005	Male	95 plus	76% (66-85%)	24% (15-34%)	3.4 (1.9-5.2)	3.5% (-2.3-11%)	3.5% (-3.3-12%)	15 (-14-51)	60 (-39-180)

Location	Year	Sex	Age	Prevalence of current drinkers	Prevalence of abstainers	Population average of standard drinks daily	Percent of total attributable deaths	Percent of total attributable DALYs	Attributable deaths	Attributable DALYs
Czech Republic	2010	Female	All Ages	78% (76-81%)	22% (19-24%)	2.6 (2.3-2.8)	3.8% (2.5-5.1%)	3.4% (0.75-6.2%)	1800 (390-3300)	57000 (36000-78000)
Czech Republic	2010	Female	15 to 19	81% (72-88%)	19% (12-28%)	2 (1.4-2.8)	2.9% (1.9-4.1%)	8.8% (5-14%)	5.6 (3.2-8.6)	760 (490-1100)
Czech Republic	2010	Female	20 to 24	89% (82-93%)	11% (7.2-18%)	2.4 (1.6-3.4)	4.2% (3-5.7%)	9.2% (5.3-13%)	8.2 (4.7-12)	1400 (920-1900)
Czech Republic	2010	Female	25 to 29	90% (85-93%)	10% (6.6-15%)	2.1 (1.5-2.9)	3.9% (2.9-5.1%)	8.6% (5.6-12%)	7.9 (5.2-11)	1500 (1000-2000)
Czech Republic	2010	Female	30 to 34	89% (85-93%)	11% (7-15%)	2.5 (1.8-3.3)	4% (2.9-5.2%)	9.8% (7.1-13%)	17 (12-22)	2100 (1500-2800)
Czech Republic	2010	Female	35 to 39	88% (82-92%)	12% (8.2-18%)	3.3 (2.4-4.4)	4.7% (3.5-6%)	12% (9.4-15%)	31 (24-38)	2800 (2100-3600)
Czech Republic	2010	Female	40 to 44	85% (80-89%)	15% (11-20%)	3.7 (2.8-4.7)	5.8% (4.5-7.4%)	14% (11-16%)	51 (41-61)	3400 (2700-4300)
Czech Republic	2010	Female	45 to 49	83% (79-87%)	17% (13-21%)	3.8 (2.8-5)	6.4% (4.7-8.3%)	13% (11-17%)	84 (67-110)	4600 (3500-5900)
Czech Republic	2010	Female	50 to 54	83% (79-87%)	17% (13-21%)	4 (2.9-5.1)	6.8% (5-8.9%)	13% (9.6-16%)	130 (100-160)	6000 (4500-7800)
Czech Republic	2010	Female	55 to 59	83% (79-86%)	17% (14-21%)	4 (3.1-5)	6.6% (4.6-8.6%)	11% (8.1-14%)	210 (150-270)	8100 (5800-11000)
Czech Republic	2010	Female	60 to 64	80% (76-84%)	20% (16-24%)	3.6 (2.7-4.6)	5.6% (3.4-7.9%)	8.5% (5.5-12%)	260 (170-360)	8500 (5300-12000)
Czech Republic	2010	Female	65 to 69	73% (68-79%)	27% (21-32%)	3 (2.3-3.7)	4.3% (2.3-6.3%)	6.1% (3.4-9%)	230 (130-340)	6300 (3400-9300)
Czech Republic	2010	Female	70 to 74	63% (56-69%)	37% (31-44%)	2.3 (1.7-3.1)	2.8% (0.51-5.1%)	3.8% (0.72-6.9%)	170 (32-310)	3700 (720-7000)
Czech Republic	2010	Female	75 to 79	55% (46-63%)	45% (37-54%)	1.8 (1.2-2.6)	1.8% (-0.46-4.6%)	2.2% (-0.71-5.7%)	170 (-53-430)	2800 (-740-7100)
Czech Republic	2010	Female	80 to 84	50% (39-60%)	50% (40-61%)	1.7 (1.2-2.4)	1.5% (-0.84-4.1%)	1.7% (-1.2-4.9%)	200 (-140-550)	2500 (-1300-6600)
Czech Republic	2010	Female	85 to 89	49% (37-60%)	51% (40-63%)	1.8 (1.2-2.6)	1.4% (-1.3-4.5%)	1.5% (-1.9-5.3%)	180 (-230-640)	1600 (-1500-5100)
Czech Republic	2010	Female	90 to 94	51% (39-62%)	49% (38-61%)	1.9 (1.2-2.9)	1.3% (-2-5.3%)	1.3% (-2.8-6.4%)	55 (-120-270)	360 (-550-1500)
Czech Republic	2010	Female	95 plus	55% (39-71%)	45% (29-61%)	2.1 (1.1-3.4)	1.4% (-3.2-7%)	1.4% (-4.1-8.1%)	27 (-82-160)	120 (-260-580)
Czech Republic	2010	Male	All Ages	91% (89-92%)	9.5% (8.1-11%)	5.4 (5-5.8)	13% (11-15%)	14% (11-18%)	7900 (6100-9800)	230000 (190000-270000)
Czech Republic	2010	Male	15 to 19	87% (80-93%)	13% (7.4-20%)	2.4 (1.7-3.4)	5.7% (3.5-8%)	12% (6.9-19%)	18 (10-27)	1800 (1100-2500)
Czech Republic	2010	Male	20 to 24	93% (90-96%)	6.6% (4.4-9.9%)	4.1 (3-5.5)	11% (7.9-15%)	20% (13-28%)	59 (37-80)	5100 (3500-6800)
Czech Republic	2010	Male	25 to 29	95% (92-97%)	5.1% (3.4-7.6%)	5.1 (3.5-6.6)	12% (9.1-16%)	24% (16-32%)	76 (52-100)	6300 (4700-8200)

Location	Year	Sex	Age	Prevalence of current drinkers	Prevalence of abstainers	Population average of standard drinks daily	Percent of total attributable deaths	Percent of total attributable DALYs	Attributable deaths	Attributable DALYs
Czech Republic	2010	Male	30 to 34	96% (93-97%)	4.3% (2.5-6.7%)	5.6 (4.1-7.1)	12% (9.3-16%)	24% (17-31%)	110 (76-140)	8400 (6200-11000)
Czech Republic	2010	Male	35 to 39	96% (94-98%)	4.1% (2.3-6.3%)	5.6 (4.1-7.5)	13% (9.4-16%)	24% (17-30%)	150 (100-190)	9900 (7300-12000)
Czech Republic	2010	Male	40 to 44	95% (93-97%)	5% (3.4-7.2%)	6.5 (5-8.2)	15% (12-18%)	26% (20-31%)	210 (170-250)	12000 (9600-14000)
Czech Republic	2010	Male	45 to 49	93% (91-95%)	7.2% (5.2-9.4%)	8.1 (6.2-10)	17% (14-21%)	27% (23-32%)	360 (300-420)	18000 (15000-21000)
Czech Republic	2010	Male	50 to 54	92% (90-94%)	8.2% (6.4-10%)	8.7 (6.9-11)	18% (15-21%)	25% (21-29%)	600 (510-700)	25000 (21000-29000)
Czech Republic	2010	Male	55 to 59	92% (91-94%)	7.7% (6.3-9.2%)	8.7 (7.1-11)	17% (14-20%)	22% (19-26%)	950 (790-1100)	33000 (28000-39000)
Czech Republic	2010	Male	60 to 64	92% (90-94%)	7.8% (6.3-9.6%)	8.5 (6.7-10)	15% (12-19%)	19% (15-23%)	1200 (950-1500)	36000 (29000-44000)
Czech Republic	2010	Male	65 to 69	91% (89-93%)	8.6% (6.8-11%)	7.8 (6.5-9.2)	14% (11-17%)	17% (13-21%)	1100 (850-1400)	28000 (22000-34000)
Czech Republic	2010	Male	70 to 74	90% (88-92%)	10% (8.2-12%)	6.9 (5.4-8.5)	12% (8.2-16%)	14% (9.4-19%)	880 (580-1200)	18000 (12000-24000)
Czech Republic	2010	Male	75 to 79	86% (82-90%)	14% (10-18%)	5.7 (4.2-7.4)	9.8% (5.1-14%)	11% (5.5-16%)	870 (430-1300)	14000 (7200-20000)
Czech Republic	2010	Male	80 to 84	81% (75-86%)	19% (14-25%)	4.7 (3.5-6)	8% (3.7-12%)	9.1% (3.9-15%)	740 (310-1200)	8700 (4000-13000)
Czech Republic	2010	Male	85 to 89	78% (71-84%)	22% (16-29%)	4.2 (3.1-5.5)	6.3% (1.9-11%)	6.9% (1.7-12%)	420 (100-750)	3500 (1100-6000)
Czech Republic	2010	Male	90 to 94	77% (68-84%)	23% (16-32%)	3.7 (2.5-5.1)	4.1% (-0.59-9.6%)	4.3% (-1.6-11%)	69 (-25-180)	410 (-60-960)
Czech Republic	2010	Male	95 plus	76% (64-85%)	24% (15-36%)	3.2 (1.9-4.9)	2.4% (-2.6-9.1%)	2.2% (-3.8-10%)	12 (-20-54)	55 (-56-200)
Czech Republic	2016	Female	All Ages	78% (76-81%)	22% (19-24%)	2.5 (2-2.9)	3.2% (1.8-4.9%)	2.7% (-0.087-5.8%)	1500 (-49-3200)	48000 (26000-71000)
Czech Republic	2016	Female	15 to 19	80% (70-88%)	20% (12-30%)	1.9 (1.2-2.7)	2.6% (1.7-3.7%)	8.5% (4.9-13%)	3.2 (1.8-4.9)	480 (310-700)
Czech Republic	2016	Female	20 to 24	88% (81-93%)	12% (7.5-19%)	2.2 (1.4-3.3)	3.9% (2.7-5.2%)	8.5% (4.7-13%)	5.4 (3-8.2)	1100 (710-1600)
Czech Republic	2016	Female	25 to 29	89% (83-93%)	11% (6.6-17%)	2 (1.3-2.9)	3.8% (2.8-4.9%)	7.9% (4.9-12%)	6.5 (4-9.5)	1300 (890-1800)
Czech Republic	2016	Female	30 to 34	89% (82-93%)	11% (6.9-18%)	2.3 (1.6-3.2)	3.8% (2.8-4.9%)	9.4% (6.6-13%)	11 (7.4-14)	1600 (1100-2100)
Czech Republic	2016	Female	35 to 39	87% (80-92%)	13% (8.3-20%)	3 (2.1-4.2)	4.2% (3.1-5.5%)	11% (8.5-15%)	27 (20-35)	2600 (1900-3500)
Czech Republic	2016	Female	40 to 44	85% (80-90%)	15% (10-20%)	3.5 (2.5-4.7)	4.9% (3.6-6.5%)	12% (9.7-15%)	48 (37-61)	3500 (2600-4600)
Czech Republic	2016	Female	45 to 49	84% (78-88%)	16% (12-22%)	3.6 (2.5-4.9)	5.6% (4-7.7%)	13% (9.6-16%)	68 (51-88)	3900 (2800-5200)

Location	Year	Sex	Age	Prevalence of current drinkers	Prevalence of abstainers	Population average of standard drinks daily	Percent of total attributable deaths	Percent of total attributable DALYs	Attributable deaths	Attributable DALYs
Czech Republic	2016	Female	50 to 54	83% (78-87%)	17% (13-22%)	3.7 (2.7-4.9)	5.9% (4.2-8%)	12% (9-15%)	100 (76-130)	4800 (3400-6500)
Czech Republic	2016	Female	55 to 59	84% (81-88%)	16% (12-19%)	3.9 (2.9-5.1)	5.9% (4.1-8.2%)	10% (7.7-14%)	150 (110-190)	5900 (3900-8000)
Czech Republic	2016	Female	60 to 64	82% (79-86%)	18% (14-21%)	3.6 (2.6-4.9)	5% (2.9-7.6%)	8.1% (5-12%)	210 (130-300)	7000 (4000-10000)
Czech Republic	2016	Female	65 to 69	77% (72-81%)	23% (19-28%)	3.1 (2.3-4)	3.8% (1.9-6%)	5.7% (2.9-8.7%)	250 (130-380)	6700 (3300-11000)
Czech Republic	2016	Female	70 to 74	69% (63-74%)	31% (26-37%)	2.5 (1.7-3.3)	2.4% (0.2-4.7%)	3.5% (0.33-6.9%)	180 (16-370)	4000 (340-8100)
Czech Republic	2016	Female	75 to 79	59% (51-68%)	41% (32-49%)	1.9 (1.3-2.7)	1.4% (-0.83-4%)	1.8% (-1.2-5.2%)	110 (-71-310)	1900 (-1100-5500)
Czech Republic	2016	Female	80 to 84	53% (42-64%)	47% (36-58%)	1.7 (1.2-2.5)	1.1% (-1.2-3.8%)	1.2% (-1.7-4.6%)	120 (-170-450)	1600 (-1700-5500)
Czech Republic	2016	Female	85 to 89	53% (40-64%)	47% (36-60%)	1.8 (1.2-2.7)	0.92% (-1.8-4.4%)	0.88% (-2.5-5.3%)	100 (-310-620)	1000 (-2100-4900)
Czech Republic	2016	Female	90 to 94	55% (43-66%)	45% (34-57%)	2 (1.2-3)	0.79% (-2.7-5.1%)	0.66% (-3.6-6%)	59 (-330-540)	450 (-1500-2900)
Czech Republic	2016	Female	95 plus	59% (43-72%)	41% (28-57%)	2.1 (1.1-3.4)	0.8% (-3.8-6.7%)	0.61% (-5-7.9%)	9 (-76-120)	49 (-230-410)
Czech Republic	2016	Male	All Ages	89% (87-91%)	11% (9-13%)	5 (4.2-5.9)	12% (9-14%)	13% (8.7-17%)	6800 (4600-9000)	200000 (150000-240000)
Czech Republic	2016	Male	15 to 19	86% (77-92%)	14% (7.7-23%)	2.2 (1.4-3.2)	5.1% (3.1-7.3%)	12% (6.2-17%)	11 (6-17)	1100 (710-1600)
Czech Republic	2016	Male	20 to 24	92% (87-95%)	7.9% (4.8-13%)	3.7 (2.6-5.2)	9.9% (6.8-13%)	19% (12-27%)	39 (24-56)	3700 (2500-5000)
Czech Republic	2016	Male	25 to 29	94% (90-96%)	6.1% (3.8-10%)	4.6 (3.1-6.2)	11% (7.9-14%)	22% (14-30%)	54 (34-75)	4900 (3400-6500)
Czech Republic	2016	Male	30 to 34	95% (91-97%)	5.2% (2.9-9%)	5.1 (3.5-6.8)	11% (8.2-14%)	22% (16-30%)	68 (46-92)	5700 (4100-7400)
Czech Republic	2016	Male	35 to 39	95% (92-97%)	4.9% (2.7-8.2%)	5.1 (3.6-7.1)	11% (8.2-15%)	22% (15-29%)	120 (83-160)	8700 (6300-11000)
Czech Republic	2016	Male	40 to 44	94% (90-96%)	6.2% (3.8-9.8%)	5.9 (4.3-7.8)	13% (9.8-16%)	24% (18-30%)	200 (150-250)	12000 (8900-15000)
Czech Republic	2016	Male	45 to 49	91% (87-94%)	8.5% (5.8-13%)	7.3 (5.3-9.8)	16% (12-19%)	26% (20-30%)	290 (220-350)	14000 (11000-18000)
Czech Republic	2016	Male	50 to 54	91% (89-93%)	8.9% (6.8-11%)	7.9 (5.8-10)	16% (13-20%)	25% (20-29%)	460 (360-540)	19000 (16000-23000)
Czech Republic	2016	Male	55 to 59	92% (90-94%)	8.1% (6.4-10%)	8 (6-10)	16% (13-19%)	22% (17-26%)	660 (520-790)	24000 (19000-28000)
Czech Republic	2016	Male	60 to 64	92% (90-94%)	8% (6.4-10%)	7.9 (5.9-10)	14% (11-18%)	18% (14-22%)	970 (710-1200)	30000 (22000-37000)
Czech Republic	2016	Male	65 to 69	91% (89-93%)	8.6% (6.8-11%)	7.3 (5.7-9)	13% (8.8-16%)	16% (11-20%)	1200 (840-1500)	31000 (22000-39000)

Location	Year	Sex	Age	Prevalence of current drinkers	Prevalence of abstainers	Population average of standard drinks daily	Percent of total attributable deaths	Percent of total attributable DALYs	Attributable deaths	Attributable DALYs
Czech Republic	2016	Male	70 to 74	90% (87-92%)	10% (8.4-13%)	6.4 (4.7-8.3)	10% (6.5-14%)	13% (7.5-18%)	980 (580-1400)	20000 (13000-28000)
Czech Republic	2016	Male	75 to 79	86% (82-90%)	14% (10-18%)	5.3 (3.7-7.1)	8.4% (3.6-13%)	9.8% (3.8-16%)	650 (260-1000)	11000 (4600-17000)
Czech Republic	2016	Male	80 to 84	82% (76-87%)	18% (13-24%)	4.5 (3.2-6.1)	6.8% (2.3-12%)	7.7% (2.1-14%)	610 (160-1100)	7200 (2500-12000)
Czech Republic	2016	Male	85 to 89	80% (73-86%)	20% (14-27%)	4.1 (2.9-5.5)	5.1% (0.71-10%)	5.5% (0.2-12%)	370 (14-800)	3100 (450-6300)
Czech Republic	2016	Male	90 to 94	79% (71-85%)	21% (15-29%)	3.6 (2.4-5.1)	3.1% (-1.5-9%)	3.1% (-2.5-10%)	100 (-88-360)	660 (-330-2000)
Czech Republic	2016	Male	95 plus	77% (67-85%)	23% (15-33%)	3.1 (1.7-4.9)	1.6% (-3.3-8.3%)	1.2% (-4.7-9.4%)	4.8 (-19-39)	26 (-57-140)
Hungary	1990	Female	All Ages	73% (69-77%)	27% (23-31%)	2.7 (2.4-3)	8.9% (3.9-14%)	11% (3.7-20%)	7700 (2600-13000)	180000 (77000-280000)
Hungary	1990	Female	15 to 19	77% (64-87%)	23% (13-36%)	2.1 (1.4-3)	3.8% (1.1-6.6%)	9.2% (1.1-17%)	14 (1.7-26)	1500 (460-2600)
Hungary	1990	Female	20 to 24	85% (77-90%)	15% (9.9-23%)	2.5 (1.6-3.6)	5.4% (2.3-8.7%)	10% (1.4-20%)	18 (2.5-35)	2100 (900-3400)
Hungary	1990	Female	25 to 29	84% (76-90%)	16% (9.7-24%)	2.6 (1.7-3.6)	5.8% (2.7-8.9%)	11% (3.1-19%)	23 (6.2-39)	2300 (1100-3500)
Hungary	1990	Female	30 to 34	84% (76-90%)	16% (10-24%)	2.7 (1.8-3.7)	7.1% (3.8-10%)	13% (5.9-20%)	61 (28-92)	4600 (2500-6700)
Hungary	1990	Female	35 to 39	84% (76-90%)	16% (9.9-24%)	2.9 (2-4)	9.1% (5.3-13%)	16% (8.7-23%)	140 (73-200)	8300 (4600-12000)
Hungary	1990	Female	40 to 44	83% (75-89%)	17% (11-25%)	3.4 (2.5-4.6)	11% (6.6-15%)	18% (11-25%)	200 (120-280)	11000 (6400-15000)
Hungary	1990	Female	45 to 49	80% (72-86%)	20% (14-28%)	4.2 (3-5.7)	12% (7.6-17%)	20% (12-27%)	300 (190-420)	14000 (8500-19000)
Hungary	1990	Female	50 to 54	77% (68-83%)	23% (17-32%)	4.3 (3-5.8)	13% (7.4-18%)	19% (11-27%)	400 (230-560)	16000 (9200-23000)
Hungary	1990	Female	55 to 59	73% (64-79%)	27% (21-36%)	4.2 (2.9-5.7)	12% (6.3-18%)	17% (9.4-25%)	530 (290-800)	18000 (9600-28000)
Hungary	1990	Female	60 to 64	71% (61-78%)	29% (22-39%)	4.1 (2.9-5.7)	11% (4.6-18%)	15% (6.8-24%)	710 (320-1100)	21000 (8900-34000)
Hungary	1990	Female	65 to 69	70% (60-79%)	30% (21-40%)	4 (2.8-5.3)	11% (3.9-18%)	14% (5.4-24%)	990 (380-1700)	24000 (8900-41000)
Hungary	1990	Female	70 to 74	65% (54-74%)	35% (26-46%)	3.7 (2.4-5.1)	9.9% (2.5-18%)	13% (3.4-23%)	820 (220-1500)	16000 (4100-29000)
Hungary	1990	Female	75 to 79	55% (42-68%)	45% (32-58%)	3.1 (1.9-4.5)	8.6% (1.5-17%)	10% (1.8-21%)	1300 (230-2600)	20000 (3600-38000)
Hungary	1990	Female	80 to 84	50% (36-62%)	50% (38-64%)	2.6 (1.7-3.8)	7.2% (1.2-15%)	8.6% (1.4-18%)	1200 (190-2400)	13000 (2200-26000)
Hungary	1990	Female	85 to 89	49% (35-62%)	51% (38-65%)	2.5 (1.5-3.8)	6.5% (0.63-14%)	7.7% (0.65-17%)	680 (56-1500)	5200 (520-11000)

Location	Year	Sex	Age	Prevalence of current drinkers	Prevalence of abstainers	Population average of standard drinks daily	Percent of total attributable deaths	Percent of total attributable DALYs	Attributable deaths	Attributable DALYs
Hungary	1990	Female	90 to 94	50% (37-64%)	50% (36-63%)	2.6 (1.5-4.1)	6% (-0.29-15%)	7.1% (-0.52-18%)	260 (-20-680)	1400 (-71-3600)
Hungary	1990	Female	95 plus	54% (36-70%)	46% (30-64%)	2.6 (1.3-4.6)	5.6% (-1.6-16%)	6.6% (-2.1-19%)	54 (-17-160)	190 (-53-540)
Hungary	1990	Male	All Ages	87% (84-90%)	13% (10-16%)	6.6 (5.9-7.2)	20% (9.9-29%)	22% (9.5-33%)	17000 (7100-25000)	490000 (240000-700000)
Hungary	1990	Male	15 to 19	84% (74-91%)	16% (8.6-26%)	2.4 (1.5-3.6)	7.6% (2.1-13%)	12% (2.1-23%)	48 (8-87)	4100 (1200-7000)
Hungary	1990	Male	20 to 24	92% (87-95%)	8.3% (5.3-13%)	4.6 (3.3-6.2)	15% (7.1-24%)	23% (8.6-36%)	120 (44-190)	9200 (4300-14000)
Hungary	1990	Male	25 to 29	93% (88-96%)	7.3% (4.5-12%)	6.7 (4.6-8.9)	22% (13-30%)	32% (17-45%)	190 (100-280)	14000 (8200-19000)
Hungary	1990	Male	30 to 34	93% (88-96%)	7.2% (4.4-12%)	7.8 (5.7-10)	25% (16-33%)	34% (22-46%)	400 (250-530)	25000 (16000-33000)
Hungary	1990	Male	35 to 39	92% (88-95%)	7.8% (4.7-12%)	7.9 (5.6-11)	26% (18-35%)	34% (23-45%)	710 (480-930)	39000 (26000-51000)
Hungary	1990	Male	40 to 44	92% (88-95%)	7.9% (4.8-12%)	9.2 (6.8-12)	28% (19-36%)	35% (24-45%)	930 (650-1200)	46000 (31000-59000)
Hungary	1990	Male	45 to 49	93% (89-95%)	7.4% (4.7-11%)	11 (8.7-14)	28% (17-37%)	34% (21-44%)	1300 (780-1700)	54000 (34000-72000)
Hungary	1990	Male	50 to 54	93% (90-96%)	6.9% (4.4-10%)	12 (9.3-16)	26% (14-35%)	30% (16-42%)	1400 (770-2000)	54000 (29000-75000)
Hungary	1990	Male	55 to 59	93% (90-96%)	6.5% (4.3-9.9%)	12 (9.6-15)	24% (11-34%)	27% (13-39%)	1800 (850-2600)	59000 (27000-85000)
Hungary	1990	Male	60 to 64	91% (87-94%)	8.7% (6-13%)	12 (8.9-15)	21% (8.7-32%)	24% (10-36%)	2100 (890-3200)	60000 (25000-89000)
Hungary	1990	Male	65 to 69	87% (82-91%)	13% (9-18%)	10 (8.2-13)	19% (6.5-31%)	22% (7.5-35%)	2300 (750-3500)	53000 (17000-83000)
Hungary	1990	Male	70 to 74	85% (79-89%)	15% (11-21%)	9.1 (6.7-12)	17% (5.2-28%)	20% (6.1-32%)	1400 (430-2300)	27000 (8000-44000)
Hungary	1990	Male	75 to 79	84% (77-90%)	16% (10-23%)	7.9 (5.5-10)	16% (4.5-26%)	18% (5.1-29%)	1900 (550-3200)	29000 (8100-47000)
Hungary	1990	Male	80 to 84	84% (75-90%)	16% (10-25%)	6.9 (4.9-9.1)	14% (4.3-24%)	16% (4.7-28%)	1400 (410-2400)	16000 (4400-27000)
Hungary	1990	Male	85 to 89	84% (76-90%)	16% (10-24%)	6.2 (4.3-8.4)	12% (3.5-23%)	14% (3.8-26%)	640 (170-1200)	5000 (1400-8900)
Hungary	1990	Male	90 to 94	85% (77-91%)	15% (9.4-23%)	5.5 (3.6-7.8)	10% (2.4-20%)	12% (2.5-23%)	170 (37-350)	940 (220-1800)
Hungary	1990	Male	95 plus	85% (75-93%)	15% (7.4-25%)	4.7 (2.7-7.4)	7.8% (-0.93-18%)	8.7% (-1.7-21%)	20 (-3.8-47)	73 (-8.6-170)
Hungary	1995	Female	All Ages	74% (70-77%)	26% (23-30%)	2.5 (2.2-2.8)	8.3% (3.7-13%)	10% (3-18%)	6800 (2000-12000)	160000 (72000-250000)
Hungary	1995	Female	15 to 19	77% (66-87%)	23% (13-34%)	1.9 (1.2-2.7)	3.1% (1.1-5.2%)	8.3% (0.93-16%)	10 (1.1-19)	1200 (410-2000)

Location	Year	Sex	Age	Prevalence of current drinkers	Prevalence of abstainers	Population average of standard drinks daily	Percent of total attributable deaths	Percent of total attributable DALYs	Attributable deaths	Attributable DALYs
Hungary	1995	Female	20 to 24	85% (77-90%)	15% (9.5-23%)	2.3 (1.5-3.2)	4.6% (2.2-7.2%)	9.2% (1-17%)	14 (1.5-26)	1900 (910-3000)
Hungary	1995	Female	25 to 29	85% (77-90%)	15% (9.8-23%)	2.3 (1.6-3.2)	5.2% (2.7-7.8%)	10% (2.7-17%)	19 (5-32)	2100 (1100-3200)
Hungary	1995	Female	30 to 34	84% (76-90%)	16% (10-24%)	2.4 (1.7-3.4)	6.5% (3.7-9.4%)	13% (6.4-19%)	40 (20-60)	3100 (1800-4500)
Hungary	1995	Female	35 to 39	84% (77-90%)	16% (10-23%)	2.6 (1.8-3.7)	8.9% (5.5-13%)	16% (9.4-22%)	120 (69-170)	7100 (4200-9900)
Hungary	1995	Female	40 to 44	83% (76-88%)	17% (12-24%)	3.1 (2.3-4.2)	11% (6.8-15%)	18% (11-23%)	240 (150-320)	12000 (7700-16000)
Hungary	1995	Female	45 to 49	80% (72-86%)	20% (14-28%)	3.8 (2.7-5.2)	12% (7.4-17%)	19% (12-25%)	310 (200-430)	14000 (8900-20000)
Hungary	1995	Female	50 to 54	77% (69-83%)	23% (17-31%)	4 (2.8-5.2)	12% (7.5-17%)	18% (12-25%)	400 (250-550)	16000 (9600-22000)
Hungary	1995	Female	55 to 59	73% (64-79%)	27% (21-36%)	3.8 (2.7-5.1)	11% (6.3-17%)	16% (9.4-24%)	480 (270-710)	16000 (9000-24000)
Hungary	1995	Female	60 to 64	71% (61-78%)	29% (22-39%)	3.8 (2.6-5.2)	10% (4.4-16%)	14% (6.7-22%)	600 (280-970)	18000 (8000-29000)
Hungary	1995	Female	65 to 69	71% (61-79%)	29% (21-39%)	3.7 (2.6-4.8)	9.4% (3.2-16%)	12% (4.5-21%)	810 (290-1400)	20000 (7000-34000)
Hungary	1995	Female	70 to 74	66% (55-74%)	34% (26-45%)	3.3 (2.2-4.6)	8.7% (2-16%)	11% (2.6-21%)	1100 (270-2000)	21000 (4800-39000)
Hungary	1995	Female	75 to 79	56% (43-69%)	44% (31-57%)	2.8 (1.8-4.2)	7.4% (1-15%)	9% (1.2-18%)	740 (96-1500)	11000 (1600-23000)
Hungary	1995	Female	80 to 84	50% (36-63%)	50% (37-64%)	2.4 (1.6-3.5)	6.1% (0.34-13%)	7.3% (0.28-16%)	980 (39-2100)	11000 (630-23000)
Hungary	1995	Female	85 to 89	49% (36-63%)	51% (37-64%)	2.3 (1.4-3.6)	5.5% (0.04-12%)	6.5% (-0.063-14%)	670 (-6.3-1500)	5200 (39-12000)
Hungary	1995	Female	90 to 94	51% (38-65%)	49% (35-62%)	2.4 (1.4-3.8)	5% (-0.89-13%)	5.8% (-1.1-16%)	250 (-48-670)	1300 (-220-3600)
Hungary	1995	Female	95 plus	54% (37-71%)	46% (29-63%)	2.5 (1.2-4.2)	4.7% (-2.2-14%)	5.4% (-3-17%)	59 (-32-190)	210 (-98-640)
Hungary	1995	Male	All Ages	88% (85-90%)	12% (10-15%)	6.2 (5.6-6.8)	21% (11-29%)	22% (11-33%)	17000 (8200-25000)	510000 (270000-720000)
Hungary	1995	Male	15 to 19	84% (74-92%)	16% (8.3-26%)	2.2 (1.4-3.2)	6.5% (2-11%)	12% (1.8-21%)	36 (5.4-65)	3300 (1000-5500)
Hungary	1995	Male	20 to 24	92% (87-95%)	8.2% (5.2-13%)	4.2 (3-5.6)	14% (6.4-21%)	21% (7.6-35%)	94 (33-150)	7900 (3700-12000)
Hungary	1995	Male	25 to 29	93% (89-96%)	7.2% (4.4-11%)	6.2 (4.3-8.4)	19% (12-27%)	30% (16-43%)	160 (86-230)	12000 (7000-16000)
Hungary	1995	Male	30 to 34	93% (88-96%)	7% (4.3-12%)	7.2 (5.2-9.3)	24% (16-32%)	34% (22-44%)	290 (190-390)	18000 (13000-24000)
Hungary	1995	Male	35 to 39	92% (88-95%)	7.6% (4.7-12%)	7.3 (5.1-9.8)	27% (19-35%)	34% (24-44%)	650 (460-850)	36000 (25000-46000)

Location	Year	Sex	Age	Prevalence of current drinkers	Prevalence of abstainers	Population average of standard drinks daily	Percent of total attributable deaths	Percent of total attributable DALYs	Attributable deaths	Attributable DALYs
Hungary	1995	Male	40 to 44	92% (88-95%)	7.7% (4.6-12%)	8.4 (6.3-11)	29% (21-36%)	35% (26-44%)	1200 (880-1600)	59000 (43000-75000)
Hungary	1995	Male	45 to 49	93% (89-95%)	7.3% (4.6-11%)	11 (8-13)	29% (19-37%)	34% (23-44%)	1500 (1000-1900)	64000 (42000-83000)
Hungary	1995	Male	50 to 54	93% (90-96%)	6.9% (4.5-10%)	11 (8.5-14)	27% (16-36%)	31% (19-42%)	1700 (1000-2300)	65000 (38000-88000)
Hungary	1995	Male	55 to 59	93% (90-96%)	6.5% (4.4-9.6%)	11 (8.9-14)	24% (13-34%)	28% (15-39%)	1800 (950-2500)	58000 (31000-82000)
Hungary	1995	Male	60 to 64	91% (88-94%)	8.5% (5.9-12%)	11 (8.1-14)	21% (9.8-32%)	25% (12-36%)	2000 (930-3000)	56000 (25000-84000)
Hungary	1995	Male	65 to 69	87% (82-91%)	13% (9-18%)	9.5 (7.4-12)	19% (7.2-30%)	22% (8.4-34%)	2200 (810-3400)	51000 (19000-80000)
Hungary	1995	Male	70 to 74	85% (80-89%)	15% (11-20%)	8.4 (6.2-11)	17% (5.8-27%)	20% (6.7-31%)	2200 (750-3400)	41000 (14000-66000)
Hungary	1995	Male	75 to 79	84% (77-90%)	16% (10-23%)	7.2 (5-9.5)	15% (5.1-25%)	17% (5.8-28%)	1200 (420-2000)	18000 (6200-30000)
Hungary	1995	Male	80 to 84	84% (76-90%)	16% (10-24%)	6.3 (4.5-8.4)	14% (4.5-23%)	15% (5-26%)	1300 (440-2300)	15000 (4900-25000)
Hungary	1995	Male	85 to 89	84% (77-90%)	16% (10-23%)	5.7 (3.9-7.6)	12% (3.4-21%)	13% (3.8-24%)	670 (190-1200)	5200 (1600-9500)
Hungary	1995	Male	90 to 94	85% (78-91%)	15% (9.5-22%)	5 (3.2-7.2)	9.3% (1.4-19%)	10% (1.2-22%)	170 (19-350)	930 (140-1900)
Hungary	1995	Male	95 plus	86% (75-93%)	14% (7.2-25%)	4.3 (2.4-6.8)	6.7% (-1.9-17%)	7.3% (-2.9-19%)	22 (-9-59)	84 (-26-220)
Hungary	2000	Female	All Ages	73% (69-76%)	27% (24-31%)	2.3 (2-2.6)	7.2% (3.1-11%)	8.3% (2.3-15%)	5400 (1500-9600)	130000 (58000-210000)
Hungary	2000	Female	15 to 19	79% (67-88%)	21% (12-33%)	1.8 (1.2-2.6)	2.9% (1-4.9%)	7.8% (0.58-15%)	6.8 (0.5-13)	870 (300-1400)
Hungary	2000	Female	20 to 24	85% (77-90%)	15% (9.7-23%)	2.2 (1.4-3.1)	4.3% (2.2-6.5%)	8.3% (0.88-16%)	11 (1.1-21)	1700 (880-2700)
Hungary	2000	Female	25 to 29	84% (77-90%)	16% (10-23%)	2.2 (1.5-3)	4.5% (2.4-6.5%)	8.8% (1.9-16%)	13 (2.9-24)	1900 (1000-2800)
Hungary	2000	Female	30 to 34	84% (77-89%)	16% (11-23%)	2.3 (1.6-3.1)	5.1% (2.9-7.5%)	11% (4.5-17%)	25 (11-40)	2400 (1300-3500)
Hungary	2000	Female	35 to 39	83% (77-89%)	17% (11-23%)	2.4 (1.6-3.5)	7.1% (4.2-10%)	13% (7.3-19%)	62 (33-92)	4000 (2300-5800)
Hungary	2000	Female	40 to 44	82% (75-88%)	18% (12-25%)	2.9 (2.1-3.9)	9.3% (5.8-13%)	16% (10-21%)	160 (100-220)	8500 (5500-12000)
Hungary	2000	Female	45 to 49	80% (73-85%)	20% (15-27%)	3.5 (2.5-4.7)	11% (6.8-15%)	17% (11-23%)	310 (200-420)	14000 (9000-19000)
Hungary	2000	Female	50 to 54	75% (68-82%)	25% (18-32%)	3.6 (2.6-4.7)	11% (6.7-15%)	16% (11-22%)	350 (230-480)	14000 (8800-20000)
Hungary	2000	Female	55 to 59	70% (61-77%)	30% (23-39%)	3.3 (2.4-4.5)	10% (5.8-15%)	15% (9-21%)	410 (250-590)	14000 (8200-21000)

Location	Year	Sex	Age	Prevalence of current drinkers	Prevalence of abstainers	Population average of standard drinks daily	Percent of total attributable deaths	Percent of total attributable DALYs	Attributable deaths	Attributable DALYs
Hungary	2000	Female	60 to 64	68%<br>(59-75%)	32%<br>(25-41%)	3.3<br>(2.3-4.6)	8.8%<br>(4.1-14%)	12%<br>(6.3-20%)	460<br>(230-730)	14000<br>(6500-22000)
Hungary	2000	Female	65 to 69	68%<br>(59-76%)	32%<br>(24-41%)	3.2<br>(2.4-4.3)	8.2%<br>(2.9-14%)	11%<br>(4.2-18%)	620<br>(230-1000)	15000<br>(5500-26000)
Hungary	2000	Female	70 to 74	62%<br>(52-72%)	38%<br>(28-48%)	2.9<br>(1.9-4)	7.3%<br>(1.5-14%)	9.3%<br>(2.1-17%)	800<br>(180-1500)	16000<br>(3400-29000)
Hungary	2000	Female	75 to 79	52%<br>(41-65%)	48%<br>(35-59%)	2.4<br>(1.5-3.5)	5.9%<br>(0.44-12%)	7.2%<br>(0.4-15%)	880<br>(48-1800)	14000<br>(980-28000)
Hungary	2000	Female	80 to 84	47%<br>(34-59%)	53%<br>(41-66%)	2.1<br>(1.3-3)	4.8%<br>(-0.07-11%)	5.7%<br>(-0.19-13%)	500<br>(-17-1100)	5600<br>(-88-12000)
Hungary	2000	Female	85 to 89	46%<br>(33-59%)	54%<br>(41-67%)	2<br>(1.2-3.1)	4.3%<br>(-0.44-10%)	5%<br>(-0.63-12%)	530<br>(-67-1300)	4200<br>(-450-9800)
Hungary	2000	Female	90 to 94	48%<br>(35-61%)	52%<br>(39-65%)	2<br>(1.1-3.3)	3.9%<br>(-1.4-11%)	4.4%<br>(-1.9-14%)	230<br>(-96-700)	1300<br>(-430-3700)
Hungary	2000	Female	95 plus	51%<br>(34-69%)	49%<br>(31-66%)	2.2<br>(1-3.7)	3.7%<br>(-2.5-12%)	4.2%<br>(-3.3-15%)	54<br>(-43-190)	200<br>(-140-660)
Hungary	2000	Male	All Ages	90%<br>(88-92%)	10%<br>(8.5-12%)	6.3<br>(5.7-6.9)	20%<br>(11-28%)	22%<br>(11-32%)	16000<br>(8100-22000)	450000<br>(250000-620000)
Hungary	2000	Male	15 to 19	88%<br>(80-93%)	12%<br>(6.9-20%)	2.3<br>(1.5-3.3)	6%<br>(1.9-10%)	12%<br>(1.9-22%)	22<br>(3.4-42)	2200<br>(700-3700)
Hungary	2000	Male	20 to 24	93%<br>(90-95%)	6.7%<br>(4.5-9.7%)	4.2<br>(3-5.4)	13%<br>(6-19%)	21%<br>(7.3-33%)	81<br>(29-130)	7100<br>(3300-11000)
Hungary	2000	Male	25 to 29	94%<br>(91-96%)	5.9%<br>(3.8-8.6%)	6.1<br>(4.3-8.1)	17%<br>(10-24%)	29%<br>(15-42%)	130<br>(71-190)	10000<br>(6100-14000)
Hungary	2000	Male	30 to 34	94%<br>(91-96%)	5.8%<br>(3.8-8.5%)	7.1<br>(5.2-9.1)	21%<br>(14-28%)	33%<br>(21-43%)	220<br>(140-300)	15000<br>(9600-19000)
Hungary	2000	Male	35 to 39	94%<br>(91-96%)	6.4%<br>(4.1-8.9%)	7.1<br>(5.1-9.6)	24%<br>(17-32%)	34%<br>(24-44%)	380<br>(260-490)	21000<br>(15000-27000)
Hungary	2000	Male	40 to 44	94%<br>(91-96%)	6.4%<br>(4.2-9.1%)	8.3<br>(6.2-11)	28%<br>(20-35%)	35%<br>(26-44%)	900<br>(660-1100)	44000<br>(32000-55000)
Hungary	2000	Male	45 to 49	94%<br>(92-96%)	5.9%<br>(4-8.2%)	10<br>(8-13)	29%<br>(19-36%)	35%<br>(24-43%)	1500<br>(1000-1900)	65000<br>(44000-83000)
Hungary	2000	Male	50 to 54	94%<br>(92-96%)	5.6%<br>(3.9-7.7%)	11<br>(8.7-14)	27%<br>(17-35%)	32%<br>(20-42%)	1600<br>(1000-2200)	62000<br>(38000-82000)
Hungary	2000	Male	55 to 59	95%<br>(93-96%)	5.4%<br>(3.9-7.3%)	11<br>(8.9-14)	24%<br>(14-33%)	29%<br>(17-39%)	1700<br>(960-2400)	57000<br>(31000-78000)
Hungary	2000	Male	60 to 64	93%<br>(90-94%)	7.4%<br>(5.7-9.7%)	11<br>(8.1-13)	22%<br>(11-31%)	25%<br>(13-36%)	1700<br>(880-2500)	49000<br>(24000-70000)
Hungary	2000	Male	65 to 69	89%<br>(85-91%)	11%<br>(8.7-15%)	9.3<br>(7.4-11)	19%<br>(8-29%)	22%<br>(9.3-33%)	1900<br>(800-2800)	45000<br>(18000-67000)
Hungary	2000	Male	70 to 74	86%<br>(82-89%)	14%<br>(11-18%)	8.2<br>(6.1-10)	17%<br>(6.5-27%)	19%<br>(7.7-30%)	1900<br>(770-3000)	37000<br>(15000-58000)
Hungary	2000	Male	75 to 79	85%<br>(79-90%)	15%<br>(10-21%)	6.9<br>(5-9)	15%<br>(5.2-24%)	17%<br>(6.1-27%)	1700<br>(620-2800)	25000<br>(9300-41000)

Location	Year	Sex	Age	Prevalence of current drinkers	Prevalence of abstainers	Population average of standard drinks daily	Percent of total attributable deaths	Percent of total attributable DALYs	Attributable deaths	Attributable DALYs
Hungary	2000	Male	80 to 84	84%<br>(75-90%)	16%<br>(10-25%)	5.9<br>(4.1-7.8)	13%<br>(4.7-22%)	15%<br>(5.3-25%)	800<br>(290-1300)	8900<br>(3300-15000)
Hungary	2000	Male	85 to 89	84%<br>(76-90%)	16%<br>(10-24%)	5.3<br>(3.7-7.1)	11%<br>(3.3-20%)	12%<br>(3.6-22%)	640<br>(190-1200)	5100<br>(1500-9000)
Hungary	2000	Male	90 to 94	85%<br>(77-90%)	15%<br>(9.7-23%)	4.7<br>(3-6.7)	8.6%<br>(0.46-17%)	9.5%<br>(0.071-20%)	190<br>(1.4-400)	1000<br>(57-2100)
Hungary	2000	Male	95 plus	86%<br>(76-93%)	14%<br>(7.4-24%)	4<br>(2.2-6.4)	6%<br>(-2.5-16%)	6.4%<br>(-3.7-18%)	22<br>(-13-63)	85<br>(-37-230)
Hungary	2005	Female	All Ages	70%<br>(67-73%)	30%<br>(27-33%)	2.4<br>(2.1-2.7)	6.8%<br>(3-11%)	8%<br>(2.5-14%)	5100<br>(1600-9100)	120000<br>(55000-190000)
Hungary	2005	Female	15 to 19	79%<br>(68-87%)	21%<br>(13-32%)	2<br>(1.3-2.8)	2.9%<br>(1-4.9%)	7.9%<br>(0.86-15%)	6.1<br>(0.68-12)	800<br>(280-1300)
Hungary	2005	Female	20 to 24	85%<br>(77-90%)	15%<br>(10-23%)	2.3<br>(1.5-3.3)	4.1%<br>(2.3-6.2%)	8.4%<br>(1-16%)	7.4<br>(0.85-14)	1300<br>(710-2000)
Hungary	2005	Female	25 to 29	83%<br>(76-88%)	17%<br>(12-24%)	2.2<br>(1.5-3.1)	4.3%<br>(2.4-6.2%)	8.6%<br>(2-15%)	13<br>(3-23)	1900<br>(1000-2900)
Hungary	2005	Female	30 to 34	83%<br>(76-88%)	17%<br>(12-24%)	2.3<br>(1.6-3.2)	4.5%<br>(2.5-6.6%)	10%<br>(3.9-16%)	22<br>(8.5-34)	2300<br>(1300-3300)
Hungary	2005	Female	35 to 39	82%<br>(76-88%)	18%<br>(12-24%)	2.5<br>(1.7-3.6)	5.9%<br>(3.4-8.7%)	12%<br>(6.5-19%)	48<br>(25-73)	3400<br>(1900-4900)
Hungary	2005	Female	40 to 44	81%<br>(74-87%)	19%<br>(13-26%)	3<br>(2.1-4.1)	8%<br>(4.9-11%)	15%<br>(8.9-20%)	100<br>(62-140)	5500<br>(3300-7700)
Hungary	2005	Female	45 to 49	78%<br>(71-84%)	22%<br>(16-29%)	3.6<br>(2.6-4.9)	10%<br>(6.2-14%)	16%<br>(11-22%)	240<br>(150-320)	11000<br>(6900-15000)
Hungary	2005	Female	50 to 54	73%<br>(67-79%)	27%<br>(21-33%)	3.7<br>(2.6-4.8)	10%<br>(6.4-14%)	16%<br>(10-21%)	380<br>(250-520)	15000<br>(9500-21000)
Hungary	2005	Female	55 to 59	66%<br>(60-73%)	34%<br>(27-40%)	3.3<br>(2.4-4.5)	9.5%<br>(5.5-14%)	14%<br>(8.7-20%)	390<br>(240-570)	14000<br>(8100-20000)
Hungary	2005	Female	60 to 64	64%<br>(57-71%)	36%<br>(29-43%)	3.2<br>(2.3-4.5)	8.5%<br>(4.1-14%)	12%<br>(6.4-19%)	440<br>(230-690)	13000<br>(6400-21000)
Hungary	2005	Female	65 to 69	64%<br>(56-72%)	36%<br>(28-44%)	3.2<br>(2.3-4.1)	8%<br>(3.2-13%)	11%<br>(4.6-18%)	540<br>(230-870)	13000<br>(5300-22000)
Hungary	2005	Female	70 to 74	56%<br>(47-64%)	44%<br>(36-53%)	2.7<br>(1.8-3.7)	6.9%<br>(1.8-13%)	9%<br>(2.5-16%)	670<br>(190-1200)	13000<br>(3500-24000)
Hungary	2005	Female	75 to 79	47%<br>(37-58%)	53%<br>(42-63%)	2.2<br>(1.5-3.2)	5.8%<br>(0.81-12%)	7.1%<br>(0.94-14%)	790<br>(100-1600)	12000<br>(1800-25000)
Hungary	2005	Female	80 to 84	43%<br>(30-56%)	57%<br>(44-70%)	2<br>(1.3-2.9)	4.8%<br>(0.2-10%)	5.7%<br>(0.14-12%)	780<br>(19-1700)	8800<br>(390-19000)
Hungary	2005	Female	85 to 89	42%<br>(29-55%)	58%<br>(45-71%)	1.9<br>(1.1-2.9)	4.2%<br>(-0.11-9.8%)	5%<br>(-0.34-12%)	370<br>(-26-900)	3000<br>(-76-7100)
Hungary	2005	Female	90 to 94	43%<br>(31-57%)	57%<br>(43-69%)	1.9<br>(1.1-3.2)	4%<br>(-1-11%)	4.6%<br>(-1.4-13%)	260<br>(-84-770)	1400<br>(-370-4100)
Hungary	2005	Female	95 plus	47%<br>(30-65%)	53%<br>(35-70%)	2<br>(0.97-3.6)	3.9%<br>(-1.8-12%)	4.4%<br>(-2.5-15%)	74<br>(-42-240)	270<br>(-130-850)

Location	Year	Sex	Age	Prevalence of current drinkers	Prevalence of abstainers	Population average of standard drinks daily	Percent of total attributable deaths	Percent of total attributable DALYs	Attributable deaths	Attributable DALYs
Hungary	2005	Male	All Ages	90% (88-92%)	10% (8.4-12%)	6.8 (6.2-7.5)	20% (11-28%)	22% (10-32%)	15000 (7200-22000)	420000 (230000-600000)
Hungary	2005	Male	15 to 19	88% (81-93%)	12% (6.8-19%)	2.5 (1.7-3.5)	6.2% (2-10%)	12% (2.2-23%)	21 (3.6-39)	2100 (720-3500)
Hungary	2005	Male	20 to 24	93% (91-95%)	6.7% (4.7-9.5%)	4.4 (3.2-5.9)	13% (6.1-19%)	22% (8.1-36%)	61 (22-99)	5500 (2700-8300)
Hungary	2005	Male	25 to 29	94% (91-96%)	5.9% (4-8.6%)	6.5 (4.6-8.6)	17% (9.8-24%)	29% (15-43%)	130 (69-190)	10000 (6000-14000)
Hungary	2005	Male	30 to 34	94% (92-96%)	5.8% (3.9-8.4%)	7.5 (5.6-9.7)	19% (12-26%)	33% (20-45%)	180 (110-250)	12000 (8100-17000)
Hungary	2005	Male	35 to 39	93% (91-96%)	6.7% (4.3-9.4%)	7.5 (5.4-10)	22% (15-30%)	34% (23-44%)	310 (210-420)	18000 (12000-24000)
Hungary	2005	Male	40 to 44	93% (91-96%)	6.7% (4.4-9.5%)	8.7 (6.6-11)	26% (19-34%)	35% (25-44%)	580 (420-760)	29000 (21000-37000)
Hungary	2005	Male	45 to 49	94% (92-96%)	6% (4.2-8.3%)	11 (8.5-14)	28% (18-36%)	35% (24-44%)	1200 (810-1600)	53000 (35000-69000)
Hungary	2005	Male	50 to 54	94% (92-96%)	5.8% (4-7.8%)	12 (9.4-15)	26% (16-35%)	32% (19-42%)	1800 (1100-2400)	68000 (41000-91000)
Hungary	2005	Male	55 to 59	94% (92-96%)	5.6% (4.1-7.6%)	12 (9.4-15)	24% (13-33%)	28% (16-39%)	1800 (970-2400)	58000 (31000-81000)
Hungary	2005	Male	60 to 64	92% (90-94%)	7.6% (5.8-9.7%)	11 (8.6-14)	21% (10-31%)	25% (12-36%)	1800 (880-2600)	50000 (24000-73000)
Hungary	2005	Male	65 to 69	88% (85-91%)	12% (8.8-15%)	9.9 (8-12)	19% (7.6-29%)	22% (9.1-33%)	1700 (710-2600)	40000 (16000-61000)
Hungary	2005	Male	70 to 74	86% (83-89%)	14% (11-17%)	8.7 (6.6-11)	17% (5.8-26%)	19% (7.1-30%)	1800 (640-2800)	34000 (12000-54000)
Hungary	2005	Male	75 to 79	84% (79-89%)	16% (11-21%)	7.3 (5.3-9.5)	15% (4.5-24%)	17% (5.3-27%)	1600 (510-2700)	24000 (7600-40000)
Hungary	2005	Male	80 to 84	83% (76-89%)	17% (11-24%)	6.2 (4.4-8.3)	13% (4.2-22%)	15% (4.7-25%)	1300 (400-2100)	14000 (4600-24000)
Hungary	2005	Male	85 to 89	84% (77-90%)	16% (10-23%)	5.7 (4-7.6)	11% (3.2-20%)	13% (3.4-23%)	450 (120-810)	3600 (1000-6300)
Hungary	2005	Male	90 to 94	85% (78-90%)	15% (9.6-22%)	5.1 (3.3-7.2)	9.2% (1.4-18%)	10% (1.1-21%)	220 (23-450)	1200 (180-2400)
Hungary	2005	Male	95 plus	86% (75-92%)	14% (7.6-25%)	4.3 (2.4-6.8)	6.7% (-1.8-16%)	7.4% (-3-19%)	34 (-14-87)	130 (-37-310)
Hungary	2010	Female	All Ages	68% (65-71%)	32% (29-35%)	2 (1.8-2.3)	5.4% (2.3-8.6%)	6% (1.3-11%)	3900 (800-7300)	93000 (40000-150000)
Hungary	2010	Female	15 to 19	77% (66-87%)	23% (13-34%)	1.7 (1.1-2.4)	2.5% (0.98-4.2%)	6.5% (0.21-13%)	4.2 (0.13-8.3)	640 (250-1100)
Hungary	2010	Female	20 to 24	84% (76-89%)	16% (11-24%)	2 (1.3-2.9)	3.9% (2.2-5.7%)	7.1% (0.55-14%)	5 (0.41-9.7)	1100 (610-1700)
Hungary	2010	Female	25 to 29	81% (73-87%)	19% (13-27%)	1.9 (1.3-2.6)	3.9% (2.3-5.6%)	7.2% (1.4-13%)	7.3 (1.4-14)	1400 (770-2000)

Location	Year	Sex	Age	Prevalence of current drinkers	Prevalence of abstainers	Population average of standard drinks daily	Percent of total attributable deaths	Percent of total attributable DALYs	Attributable deaths	Attributable DALYs
Hungary	2010	Female	30 to 34	81% (74-87%)	19% (13-26%)	2 (1.4-2.8)	4% (2.3-5.7%)	8.8% (3.4-14%)	16 (6-26)	2000 (1100-2900)
Hungary	2010	Female	35 to 39	81% (74-87%)	19% (13-26%)	2.2 (1.5-3.1)	4.7% (2.6-7%)	11% (5.4-16%)	34 (17-52)	2700 (1500-4000)
Hungary	2010	Female	40 to 44	79% (72-86%)	21% (14-28%)	2.6 (1.8-3.5)	5.9% (3.3-8.7%)	12% (6.5-17%)	67 (37-96)	3900 (2100-5600)
Hungary	2010	Female	45 to 49	76% (69-83%)	24% (17-31%)	3.1 (2.2-4.2)	8.1% (4.8-12%)	14% (8.6-19%)	140 (83-190)	6500 (3900-9100)
Hungary	2010	Female	50 to 54	73% (67-78%)	27% (22-33%)	3.2 (2.3-4.3)	8.8% (5.2-12%)	14% (8.5-18%)	260 (160-360)	11000 (6500-15000)
Hungary	2010	Female	55 to 59	66% (60-73%)	34% (27-40%)	2.9 (2.2-3.9)	8.1% (4.6-12%)	12% (7.2-17%)	370 (220-530)	13000 (7400-19000)
Hungary	2010	Female	60 to 64	62% (56-69%)	38% (31-44%)	2.7 (2-3.7)	7.1% (3.4-11%)	10% (5.4-15%)	370 (190-570)	11000 (5500-18000)
Hungary	2010	Female	65 to 69	63% (56-70%)	37% (30-44%)	2.7 (2-3.5)	6.4% (2.6-11%)	9% (3.9-15%)	420 (180-680)	11000 (4300-18000)
Hungary	2010	Female	70 to 74	54% (46-61%)	46% (39-54%)	2.2 (1.5-3.1)	5.3% (1.3-9.9%)	7% (1.8-13%)	460 (120-850)	9600 (2200-18000)
Hungary	2010	Female	75 to 79	45% (37-56%)	55% (44-63%)	1.9 (1.2-2.7)	4.4% (0.36-9.3%)	5.5% (0.44-12%)	530 (40-1100)	8500 (650-18000)
Hungary	2010	Female	80 to 84	42% (30-54%)	58% (46-70%)	1.7 (1.1-2.5)	3.6% (-0.35-8.2%)	4.3% (-0.52-10%)	560 (-67-1300)	6500 (-590-15000)
Hungary	2010	Female	85 to 89	40% (28-52%)	60% (48-72%)	1.6 (0.95-2.5)	3.1% (-0.67-7.5%)	3.5% (-0.98-9%)	440 (-120-1100)	3600 (-800-9000)
Hungary	2010	Female	90 to 94	42% (30-55%)	58% (45-70%)	1.6 (0.92-2.7)	2.8% (-1.3-8.6%)	3.2% (-1.9-10%)	150 (-86-500)	860 (-420-2700)
Hungary	2010	Female	95 plus	45% (29-62%)	55% (38-71%)	1.7 (0.82-3)	2.7% (-2.2-9.6%)	3.1% (-2.9-11%)	65 (-62-240)	240 (-200-860)
Hungary	2010	Male	All Ages	89% (87-91%)	11% (9.1-13%)	6.1 (5.5-6.8)	18% (10-25%)	20% (10-29%)	13000 (6600-19000)	360000 (200000-500000)
Hungary	2010	Male	15 to 19	87% (80-92%)	13% (7.6-20%)	2.1 (1.4-3.1)	5.3% (1.8-8.9%)	11% (1.3-20%)	15 (1.8-27)	1600 (570-2600)
Hungary	2010	Male	20 to 24	93% (90-95%)	7.2% (4.9-10%)	3.9 (2.8-5.2)	11% (5.6-17%)	20% (6.9-33%)	42 (14-70)	4200 (2100-6300)
Hungary	2010	Male	25 to 29	93% (90-96%)	6.6% (4.3-9.7%)	5.7 (3.9-7.6)	14% (8.4-20%)	27% (14-41%)	75 (38-110)	6400 (3900-9200)
Hungary	2010	Male	30 to 34	94% (91-96%)	6.3% (4.3-9.2%)	6.6 (4.9-8.6)	16% (10-22%)	30% (18-42%)	140 (82-190)	10000 (6500-14000)
Hungary	2010	Male	35 to 39	93% (90-95%)	7.2% (4.6-10%)	6.6 (4.8-9)	18% (12-25%)	31% (20-41%)	220 (150-300)	14000 (8900-19000)
Hungary	2010	Male	40 to 44	92% (89-95%)	7.7% (4.8-11%)	7.6 (5.7-9.8)	22% (15-29%)	33% (23-41%)	430 (300-550)	22000 (15000-28000)
Hungary	2010	Male	45 to 49	93% (91-96%)	6.6% (4.5-9%)	9.7 (7.5-12)	26% (17-32%)	33% (23-42%)	750 (510-940)	33000 (22000-42000)

Location	Year	Sex	Age	Prevalence of current drinkers	Prevalence of abstainers	Population average of standard drinks daily	Percent of total attributable deaths	Percent of total attributable DALYs	Attributable deaths	Attributable DALYs
Hungary	2010	Male	50 to 54	94% (92-96%)	6% (4.2-8.3%)	11 (8.1-13)	25% (16-33%)	31% (19-40%)	1300 (830-1700)	51000 (31000-67000)
Hungary	2010	Male	55 to 59	94% (92-96%)	5.8% (4.2-7.7%)	11 (8.3-13)	23% (13-31%)	27% (16-37%)	1800 (1100-2500)	60000 (34000-83000)
Hungary	2010	Male	60 to 64	92% (90-94%)	7.7% (5.9-10%)	9.9 (7.7-13)	20% (10-29%)	24% (13-34%)	1700 (910-2400)	49000 (25000-69000)
Hungary	2010	Male	65 to 69	88% (85-91%)	12% (9.1-15%)	8.8 (7-11)	18% (8.7-27%)	21% (11-31%)	1600 (810-2400)	39000 (19000-58000)
Hungary	2010	Male	70 to 74	86% (82-89%)	14% (11-18%)	7.7 (5.7-9.7)	16% (6.5-24%)	18% (7.9-28%)	1500 (630-2200)	28000 (12000-43000)
Hungary	2010	Male	75 to 79	85% (79-89%)	15% (11-21%)	6.5 (4.6-8.5)	13% (5.1-22%)	16% (6-25%)	1400 (530-2200)	21000 (8100-33000)
Hungary	2010	Male	80 to 84	84% (76-90%)	16% (10-24%)	5.6 (4-7.5)	12% (4.2-20%)	14% (4.9-22%)	1100 (400-1900)	13000 (4600-21000)
Hungary	2010	Male	85 to 89	84% (77-90%)	16% (10-23%)	5.1 (3.6-6.9)	10% (2.8-18%)	11% (2.9-21%)	650 (160-1200)	5200 (1300-9200)
Hungary	2010	Male	90 to 94	85% (78-91%)	15% (9.5-22%)	4.6 (2.9-6.5)	7.8% (-0.11-16%)	8.7% (-0.58-19%)	150 (-9.9-330)	850 (-12-1800)
Hungary	2010	Male	95 plus	86% (75-93%)	14% (7.2-25%)	3.9 (2.2-6.3)	5.5% (-3-15%)	5.9% (-4.3-17%)	34 (-25-100)	130 (-73-370)
Hungary	2016	Female	All Ages	70% (67-74%)	30% (26-33%)	2 (1.6-2.5)	4.9% (1.8-8.4%)	5.2% (0.56-11%)	3500 (370-7200)	83000 (29000-140000)
Hungary	2016	Female	15 to 19	75% (61-86%)	25% (14-39%)	1.5 (0.88-2.2)	2.2% (0.91-3.7%)	5.7% (0.12-12%)	2.5 (0.052-5.2)	450 (190-750)
Hungary	2016	Female	20 to 24	84% (75-90%)	16% (10-25%)	1.8 (1.1-2.7)	3.6% (2.2-5.4%)	6.3% (0.21-13%)	3.9 (0.12-8.1)	1000 (560-1500)
Hungary	2016	Female	25 to 29	84% (74-90%)	16% (10-26%)	1.8 (1.1-2.8)	3.8% (2.2-5.5%)	6.8% (0.89-13%)	6 (0.71-12)	1200 (660-1800)
Hungary	2016	Female	30 to 34	83% (74-89%)	17% (11-26%)	1.9 (1.2-2.8)	3.8% (2.2-5.6%)	8.5% (3.1-14%)	10 (3.8-18)	1400 (770-2100)
Hungary	2016	Female	35 to 39	83% (75-90%)	17% (10-25%)	2.1 (1.4-3)	4.3% (2.3-6.5%)	10% (5-16%)	33 (16-52)	2700 (1400-4100)
Hungary	2016	Female	40 to 44	82% (74-88%)	18% (12-26%)	2.5 (1.7-3.5)	5.2% (2.7-8%)	11% (5.7-17%)	61 (31-94)	3700 (1900-5600)
Hungary	2016	Female	45 to 49	79% (70-85%)	21% (15-30%)	3 (2-4.3)	7.1% (3.8-11%)	13% (7.1-18%)	130 (72-190)	6200 (3400-9200)
Hungary	2016	Female	50 to 54	76% (68-82%)	24% (18-32%)	3.1 (2.2-4.4)	8% (4.5-12%)	13% (7.5-18%)	180 (110-260)	7500 (4200-11000)
Hungary	2016	Female	55 to 59	70% (62-77%)	30% (23-38%)	2.9 (2-4.1)	7.5% (4.1-12%)	11% (6.8-17%)	300 (170-460)	11000 (5800-17000)
Hungary	2016	Female	60 to 64	66% (57-74%)	34% (26-43%)	2.7 (1.8-3.9)	6.5% (2.9-11%)	9.5% (4.7-15%)	400 (200-660)	12000 (5400-20000)
Hungary	2016	Female	65 to 69	66% (57-74%)	34% (26-43%)	2.7 (1.8-3.6)	5.9% (2.1-10%)	8.4% (3.3-14%)	410 (150-690)	10000 (3500-18000)

Location	Year	Sex	Age	Prevalence of current drinkers	Prevalence of abstainers	Population average of standard drinks daily	Percent of total attributable deaths	Percent of total attributable DALYs	Attributable deaths	Attributable DALYs
Hungary	2016	Female	70 to 74	59%<br>(49-69%)	41%<br>(31-51%)	2.3<br>(1.5-3.4)	5%<br>(0.84-10%)	6.7%<br>(1.3-13%)	450<br>(91-910)	9300<br>(1700-19000)
Hungary	2016	Female	75 to 79	49%<br>(38-61%)	51%<br>(39-62%)	1.9<br>(1.2-2.9)	3.9%<br>(-0.2-8.9%)	4.9%<br>(-0.37-11%)	450<br>(-34-1000)	7100<br>(-340-17000)
Hungary	2016	Female	80 to 84	44%<br>(32-57%)	56%<br>(43-68%)	1.7<br>(0.99-2.6)	3%<br>(-0.71-7.7%)	3.6%<br>(-0.96-9.3%)	460<br>(-120-1200)	5300<br>(-1200-14000)
Hungary	2016	Female	85 to 89	43%<br>(30-56%)	57%<br>(44-70%)	1.6<br>(0.89-2.5)	2.5%<br>(-1.3-7.2%)	2.9%<br>(-1.7-8.5%)	360<br>(-210-1000)	3000<br>(-1500-8500)
Hungary	2016	Female	90 to 94	45%<br>(33-58%)	55%<br>(42-67%)	1.6<br>(0.89-2.7)	2.3%<br>(-1.8-8%)	2.6%<br>(-2.5-9.7%)	230<br>(-210-860)	1300<br>(-1100-4600)
Hungary	2016	Female	95 plus	49%<br>(32-66%)	51%<br>(34-68%)	1.7<br>(0.79-3.1)	2.2%<br>(-2.8-9.2%)	2.5%<br>(-3.6-11%)	46<br>(-68-200)	170<br>(-220-700)
Hungary	2016	Male	All Ages	88%<br>(86-90%)	12%<br>(9.7-14%)	5.5<br>(4.4-6.7)	17%<br>(9.7-23%)	18%<br>(9.7-27%)	12000<br>(6000-17000)	320000<br>(180000-450000)
Hungary	2016	Male	15 to 19	82%<br>(71-91%)	18%<br>(9.4-29%)	1.7<br>(1-2.6)	4.5%<br>(1.8-7.6%)	9%<br>(0.88-17%)	8.8<br>(0.81-18)	1100<br>(420-1800)
Hungary	2016	Male	20 to 24	91%<br>(86-95%)	9%<br>(5.5-14%)	3.3<br>(2.1-4.7)	9.9%<br>(5.2-15%)	17%<br>(4.9-30%)	33<br>(9.7-58)	3500<br>(1800-5500)
Hungary	2016	Male	25 to 29	92%<br>(87-95%)	7.8%<br>(4.5-13%)	4.9<br>(3.2-7)	13%<br>(7.5-18%)	25%<br>(12-38%)	54<br>(25-85)	4900<br>(2900-7200)
Hungary	2016	Male	30 to 34	92%<br>(87-95%)	7.7%<br>(4.6-13%)	5.7<br>(3.9-7.8)	14%<br>(8.8-20%)	28%<br>(16-40%)	78<br>(43-110)	6000<br>(3800-8500)
Hungary	2016	Male	35 to 39	92%<br>(87-95%)	8.3%<br>(4.8-13%)	5.8<br>(3.9-8.1)	16%<br>(10-22%)	29%<br>(18-39%)	190<br>(120-270)	12000<br>(7800-17000)
Hungary	2016	Male	40 to 44	92%<br>(87-95%)	8.4%<br>(5.1-13%)	6.7<br>(4.8-9)	20%<br>(13-26%)	31%<br>(22-40%)	360<br>(240-480)	19000<br>(13000-25000)
Hungary	2016	Male	45 to 49	93%<br>(89-95%)	7.4%<br>(4.7-11%)	8.5<br>(6-11)	23%<br>(16-30%)	32%<br>(23-40%)	680<br>(470-910)	30000<br>(21000-41000)
Hungary	2016	Male	50 to 54	94%<br>(91-96%)	6.3%<br>(4.2-9.3%)	9.5<br>(6.8-13)	24%<br>(15-31%)	30%<br>(20-39%)	930<br>(600-1200)	36000<br>(23000-48000)
Hungary	2016	Male	55 to 59	94%<br>(92-96%)	5.7%<br>(4-7.8%)	9.5<br>(7.1-13)	22%<br>(13-30%)	27%<br>(17-36%)	1500<br>(870-2000)	49000<br>(29000-68000)
Hungary	2016	Male	60 to 64	92%<br>(90-94%)	7.7%<br>(5.8-10%)	9<br>(6.5-12)	19%<br>(11-28%)	23%<br>(13-33%)	1800<br>(1100-2600)	53000<br>(30000-76000)
Hungary	2016	Male	65 to 69	88%<br>(84-91%)	12%<br>(9-16%)	7.9<br>(5.8-10)	17%<br>(9.2-25%)	20%<br>(11-29%)	1600<br>(860-2300)	38000<br>(20000-57000)
Hungary	2016	Male	70 to 74	85%<br>(81-89%)	15%<br>(11-19%)	6.9<br>(4.8-9.1)	15%<br>(7.1-22%)	18%<br>(8.6-26%)	1500<br>(720-2300)	30000<br>(14000-46000)
Hungary	2016	Male	75 to 79	85%<br>(79-90%)	15%<br>(10-21%)	5.9<br>(4-8.1)	12%<br>(4.7-20%)	15%<br>(5.6-23%)	1100<br>(430-1900)	17000<br>(6500-28000)
Hungary	2016	Male	80 to 84	84%<br>(75-90%)	16%<br>(10-25%)	5.1<br>(3.4-7)	11%<br>(3.6-18%)	12%<br>(4-21%)	1000<br>(330-1700)	11000<br>(3800-19000)
Hungary	2016	Male	85 to 89	84%<br>(76-90%)	16%<br>(10-24%)	4.5<br>(2.8-6.4)	8.4%<br>(1.2-16%)	9.5%<br>(1.1-19%)	540<br>(56-1100)	4400<br>(600-8400)

Location	Year	Sex	Age	Prevalence of current drinkers	Prevalence of abstainers	Population average of standard drinks daily	Percent of total attributable deaths	Percent of total attributable DALYs	Attributable deaths	Attributable DALYs
Hungary	2016	Male	90 to 94	85% (77-90%)	15% (9.6-23%)	4 (2.5-6)	6.2% (-1.3-14%)	6.8% (-2.1-16%)	220 (-66-540)	1300 (-260-3000)
Hungary	2016	Male	95 plus	85% (75-92%)	15% (7.5-25%)	3.4 (1.9-5.7)	4.2% (-3.6-13%)	4.4% (-5.1-15%)	22 (-26-73)	89 (-77-260)
Macedonia	1990	Female	All Ages	54% (44-63%)	46% (37-56%)	0.98 (0.73-1.3)	2.8% (1.5-4.5%)	4.2% (1.8-7.7%)	300 (130-550)	7800 (4200-12000)
Macedonia	1990	Female	15 to 19	53% (33-72%)	47% (28-67%)	0.76 (0.33-1.5)	2% (1.2-3.1%)	3.8% (1.6-7%)	1 (0.42-1.9)	150 (94-240)
Macedonia	1990	Female	20 to 24	64% (44-80%)	36% (20-56%)	1.1 (0.48-2)	3.5% (2.2-5.1%)	4.6% (1.5-9%)	1.7 (0.58-3.4)	310 (170-480)
Macedonia	1990	Female	25 to 29	65% (44-81%)	35% (19-56%)	1.2 (0.53-2.2)	3.5% (2.3-5.1%)	4.7% (1.8-8.7%)	2 (0.77-3.8)	330 (200-490)
Macedonia	1990	Female	30 to 34	66% (45-82%)	34% (18-55%)	1.3 (0.63-2.2)	3.4% (2.1-5%)	5.3% (2.3-9.6%)	3 (1.3-5.3)	360 (220-550)
Macedonia	1990	Female	35 to 39	67% (48-83%)	33% (17-52%)	1.4 (0.65-2.4)	3.5% (2.1-5.3%)	5.9% (2.5-10%)	5.1 (2.1-8.9)	450 (260-690)
Macedonia	1990	Female	40 to 44	66% (47-82%)	34% (18-53%)	1.6 (0.76-2.7)	3.6% (1.8-5.8%)	6.3% (2.5-11%)	7.1 (2.8-12)	460 (230-740)
Macedonia	1990	Female	45 to 49	64% (41-81%)	36% (19-59%)	1.7 (0.82-3.1)	4% (1.7-7.3%)	6.7% (2.4-12%)	10 (3.6-19)	520 (210-940)
Macedonia	1990	Female	50 to 54	61% (40-78%)	39% (22-60%)	1.7 (0.87-3.1)	4.4% (1.6-8.2%)	6.9% (2.5-13%)	18 (6.2-32)	750 (280-1300)
Macedonia	1990	Female	55 to 59	57% (36-76%)	43% (24-64%)	1.7 (0.79-3)	4.5% (1.2-8.6%)	6.7% (1.8-12%)	26 (7.4-49)	910 (260-1700)
Macedonia	1990	Female	60 to 64	54% (33-74%)	46% (26-67%)	1.5 (0.68-2.9)	3.9% (0.42-8.5%)	5.4% (0.75-12%)	32 (4.4-69)	920 (110-2000)
Macedonia	1990	Female	65 to 69	49% (30-70%)	51% (30-70%)	1.3 (0.65-2.3)	3.4% (0.41-7.7%)	4.6% (0.69-10%)	34 (5.2-75)	780 (95-1800)
Macedonia	1990	Female	70 to 74	43% (26-62%)	57% (38-74%)	1.1 (0.43-2.1)	3.3% (0.043-8.2%)	4.1% (0.21-9.9%)	31 (1.6-77)	570 (7.7-1400)
Macedonia	1990	Female	75 to 79	36% (22-55%)	64% (45-78%)	0.84 (0.31-1.7)	3.1% (0.081-7.5%)	3.7% (0.12-8.8%)	46 (1.4-110)	630 (16-1500)
Macedonia	1990	Female	80 to 84	31% (18-50%)	69% (50-82%)	0.75 (0.31-1.4)	3.2% (0.6-7.3%)	3.7% (0.76-8.5%)	42 (8.4-96)	430 (84-970)
Macedonia	1990	Female	85 to 89	30% (18-45%)	70% (55-82%)	0.75 (0.3-1.5)	3.5% (0.75-7.6%)	4.3% (1-9.4%)	24 (5.5-51)	170 (37-370)
Macedonia	1990	Female	90 to 94	32% (19-49%)	68% (51-81%)	0.81 (0.28-1.8)	3.9% (1.2-8.2%)	5% (1.5-11%)	12 (3.7-25)	57 (17-120)
Macedonia	1990	Female	95 plus	35% (19-55%)	65% (45-81%)	0.89 (0.25-2.1)	4.5% (1.2-9.6%)	5.6% (1.5-12%)	4.3 (1.1-9.1)	14 (3.8-31)
Macedonia	1990	Male	All Ages	61% (50-73%)	39% (27-50%)	1.5 (1.2-2)	4.7% (2.8-7%)	5.6% (2.4-9.2%)	500 (220-850)	16000 (9700-24000)
Macedonia	1990	Male	15 to 19	53% (31-76%)	47% (24-69%)	0.74 (0.34-1.3)	3.2% (2-4.8%)	4.3% (2.1-7.4%)	2.6 (1.3-4.5)	320 (200-480)

Location	Year	Sex	Age	Prevalence of current drinkers	Prevalence of abstainers	Population average of standard drinks daily	Percent of total attributable deaths	Percent of total attributable DALYs	Attributable deaths	Attributable DALYs
Macedonia	1990	Male	20 to 24	66% (43-84%)	34% (16-57%)	1.4 (0.71-2.3)	6.4% (4.4-8.7%)	7.6% (3.9-12%)	5.7 (2.9-8.8)	710 (470-1000)
Macedonia	1990	Male	25 to 29	70% (49-88%)	30% (12-51%)	1.8 (0.94-2.9)	7.1% (5.1-9.5%)	8.9% (5.1-14%)	7.5 (4.2-12)	810 (560-1200)
Macedonia	1990	Male	30 to 34	73% (51-90%)	27% (9.7-49%)	2 (1.2-3.2)	6.9% (4.9-9.7%)	9.3% (5.4-14%)	10 (5.8-16)	910 (620-1300)
Macedonia	1990	Male	35 to 39	75% (55-90%)	25% (10-45%)	2.2 (1.2-3.5)	6.6% (4.2-9.8%)	8.9% (4.5-14%)	14 (7.2-24)	1100 (660-1600)
Macedonia	1990	Male	40 to 44	76% (54-90%)	24% (9.9-46%)	2.5 (1.4-3.9)	6.7% (3.9-10%)	8.9% (4.5-15%)	19 (9.5-31)	1100 (640-1700)
Macedonia	1990	Male	45 to 49	76% (53-90%)	24% (9.9-47%)	2.9 (1.6-4.5)	7.2% (3.7-12%)	9.1% (4.2-16%)	27 (12-47)	1300 (660-2200)
Macedonia	1990	Male	50 to 54	75% (53-89%)	25% (11-47%)	3 (1.8-4.8)	7.2% (3.3-12%)	8.8% (3.7-15%)	40 (16-70)	1700 (750-2800)
Macedonia	1990	Male	55 to 59	73% (51-89%)	27% (11-49%)	3.1 (1.7-5)	7.4% (3.1-13%)	8.8% (3.4-15%)	62 (24-110)	2200 (920-3700)
Macedonia	1990	Male	60 to 64	71% (47-89%)	29% (11-53%)	3 (1.5-5)	6.8% (2.4-12%)	7.8% (2.6-15%)	70 (24-130)	2100 (730-3800)
Macedonia	1990	Male	65 to 69	70% (46-88%)	30% (12-54%)	2.7 (1.5-4.2)	5.9% (1.7-11%)	6.7% (1.7-13%)	72 (18-140)	1700 (490-3300)
Macedonia	1990	Male	70 to 74	67% (45-85%)	33% (15-55%)	2.3 (1.2-3.9)	5% (0.93-10%)	5.6% (0.92-12%)	49 (7.9-100)	930 (170-1900)
Macedonia	1990	Male	75 to 79	64% (41-83%)	36% (17-59%)	1.9 (0.87-3.3)	4.1% (0.43-9%)	4.5% (0.41-10%)	65 (5.8-150)	950 (100-2100)
Macedonia	1990	Male	80 to 84	62% (37-82%)	38% (18-63%)	1.7 (0.79-2.8)	3.4% (0.063-7.7%)	3.7% (-0.033-8.6%)	42 (-0.36-98)	460 (8.4-1000)
Macedonia	1990	Male	85 to 89	60% (37-81%)	40% (19-63%)	1.5 (0.75-2.7)	2.3% (-0.41-5.6%)	2.5% (-0.65-6.5%)	12 (-3.2-32)	99 (-18-240)
Macedonia	1990	Male	90 to 94	60% (38-82%)	40% (18-62%)	1.4 (0.67-2.7)	1.8% (-0.88-5%)	2% (-1.3-5.8%)	3.4 (-2.3-10)	19 (-9.5-53)
Macedonia	1990	Male	95 plus	62% (34-86%)	38% (14-66%)	1.3 (0.49-2.7)	1.9% (-1.3-5.5%)	2.1% (-2.1-6.3%)	0.87 (-0.87-2.6)	3.3 (-2.3-9.4)
Macedonia	1995	Female	All Ages	54% (45-63%)	46% (37-55%)	0.97 (0.77-1.2)	2.9% (1.5-4.6%)	4.1% (1.7-7.5%)	300 (130-540)	7700 (4200-12000)
Macedonia	1995	Female	15 to 19	54% (34-72%)	46% (28-66%)	0.75 (0.33-1.4)	1.9% (1.1-2.9%)	3.6% (1.4-6.5%)	0.88 (0.37-1.6)	140 (83-210)
Macedonia	1995	Female	20 to 24	64% (45-80%)	36% (20-55%)	1.1 (0.46-2)	3.4% (2.2-4.9%)	4.3% (1.4-8.7%)	1.4 (0.47-2.8)	270 (160-420)
Macedonia	1995	Female	25 to 29	65% (44-81%)	35% (19-56%)	1.1 (0.52-2)	3.4% (2.3-4.7%)	4.3% (1.6-8.1%)	1.6 (0.62-3.1)	300 (190-430)
Macedonia	1995	Female	30 to 34	66% (45-82%)	34% (18-55%)	1.2 (0.63-2.1)	3.3% (2.1-4.9%)	4.9% (2-8.8%)	2.8 (1.1-5)	340 (200-510)
Macedonia	1995	Female	35 to 39	67% (49-83%)	33% (17-51%)	1.4 (0.66-2.4)	3.3% (1.9-5.1%)	5.5% (2.3-9.7%)	4.4 (1.9-7.8)	390 (230-600)

Location	Year	Sex	Age	Prevalence of current drinkers	Prevalence of abstainers	Population average of standard drinks daily	Percent of total attributable deaths	Percent of total attributable DALYs	Attributable deaths	Attributable DALYs
Macedonia	1995	Female	40 to 44	66%<br>(48-82%)	34%<br>(18-52%)	1.5<br>(0.77-2.5)	3.4%<br>(1.7-5.5%)	5.9%<br>(2.4-10%)	7.8<br>(3.1-13)	500<br>(260-800)
Macedonia	1995	Female	45 to 49	64%<br>(41-81%)	36%<br>(19-59%)	1.7<br>(0.81-3)	3.8%<br>(1.6-7%)	6.3%<br>(2.2-12%)	11<br>(3.7-20)	540<br>(220-980)
Macedonia	1995	Female	50 to 54	61%<br>(41-79%)	39%<br>(21-59%)	1.7<br>(0.87-3)	4.2%<br>(1.5-7.8%)	6.6%<br>(2.4-12%)	16<br>(5.9-29)	670<br>(250-1200)
Macedonia	1995	Female	55 to 59	58%<br>(38-77%)	42%<br>(23-62%)	1.6<br>(0.79-2.9)	4.3%<br>(1.2-8.1%)	6.4%<br>(1.8-12%)	25<br>(7-47)	850<br>(240-1600)
Macedonia	1995	Female	60 to 64	54%<br>(33-74%)	46%<br>(26-67%)	1.5<br>(0.69-2.8)	3.7%<br>(0.36-8.1%)	5.1%<br>(0.65-11%)	31<br>(3.9-67)	870<br>(88-1900)
Macedonia	1995	Female	65 to 69	50%<br>(30-70%)	50%<br>(30-70%)	1.3<br>(0.65-2.2)	3.2%<br>(0.22-7.2%)	4.3%<br>(0.41-9.4%)	37<br>(3.5-82)	860<br>(62-1900)
Macedonia	1995	Female	70 to 74	43%<br>(26-62%)	57%<br>(38-74%)	1<br>(0.43-2)	3.1%<br>(-0.0098-7.5%)	3.9%<br>(0.1-9.3%)	45<br>(1.2-110)	810<br>(-2.6-2000)
Macedonia	1995	Female	75 to 79	36%<br>(22-56%)	64%<br>(44-78%)	0.83<br>(0.31-1.6)	2.8%<br>(0.0074-7%)	3.4%<br>(0.019-8.2%)	31<br>(0.18-76)	430<br>(1.2-1100)
Macedonia	1995	Female	80 to 84	32%<br>(18-50%)	68%<br>(50-82%)	0.73<br>(0.3-1.4)	3%<br>(0.49-7%)	3.5%<br>(0.55-8.2%)	38<br>(6-89)	390<br>(60-900)
Macedonia	1995	Female	85 to 89	30%<br>(18-46%)	70%<br>(54-82%)	0.73<br>(0.3-1.4)	3.4%<br>(0.65-7.4%)	4.1%<br>(0.84-9%)	30<br>(6-65)	210<br>(41-460)
Macedonia	1995	Female	90 to 94	32%<br>(20-49%)	68%<br>(51-80%)	0.79<br>(0.29-1.7)	3.7%<br>(1-8.1%)	4.7%<br>(1.3-10%)	13<br>(3.8-29)	66<br>(18-140)
Macedonia	1995	Female	95 plus	36%<br>(19-56%)	64%<br>(44-81%)	0.88<br>(0.24-2.1)	4.2%<br>(1.1-9.2%)	5.3%<br>(1.4-12%)	4.8<br>(1.3-11)	16<br>(4-35)
Macedonia	1995	Male	All Ages	62%<br>(50-73%)	38%<br>(27-50%)	1.5<br>(1.3-1.8)	5%<br>(3.1-7%)	5.6%<br>(2.5-8.9%)	510<br>(230-820)	16000<br>(10000-24000)
Macedonia	1995	Male	15 to 19	54%<br>(32-76%)	46%<br>(24-68%)	0.73<br>(0.35-1.3)	3.1%<br>(2-4.6%)	4.2%<br>(2-7.1%)	2.4<br>(1.1-4)	300<br>(180-440)
Macedonia	1995	Male	20 to 24	66%<br>(42-85%)	34%<br>(15-58%)	1.3<br>(0.66-2.2)	6.2%<br>(4.4-8.6%)	7.4%<br>(3.9-11%)	5.2<br>(2.7-8.2)	640<br>(430-910)
Macedonia	1995	Male	25 to 29	70%<br>(49-87%)	30%<br>(13-51%)	1.7<br>(0.97-2.8)	6.9%<br>(5-9.3%)	8.7%<br>(5.1-13%)	6.9<br>(4.1-10)	770<br>(540-1100)
Macedonia	1995	Male	30 to 34	73%<br>(50-90%)	27%<br>(10-50%)	2<br>(1.1-3.1)	6.8%<br>(4.9-9.5%)	9.2%<br>(5.7-14%)	9.1<br>(5.5-14)	830<br>(580-1200)
Macedonia	1995	Male	35 to 39	75%<br>(54-90%)	25%<br>(10-46%)	2.1<br>(1.2-3.3)	6.5%<br>(4.2-9.4%)	8.7%<br>(4.5-14%)	13<br>(6.8-20)	970<br>(600-1400)
Macedonia	1995	Male	40 to 44	76%<br>(54-91%)	24%<br>(9.3-46%)	2.4<br>(1.4-3.7)	6.6%<br>(4.1-10%)	8.7%<br>(4.6-14%)	22<br>(12-36)	1300<br>(790-1900)
Macedonia	1995	Male	45 to 49	76%<br>(51-90%)	24%<br>(10-49%)	2.8<br>(1.6-4.3)	7%<br>(3.7-11%)	8.8%<br>(4.2-15%)	29<br>(14-49)	1400<br>(740-2300)
Macedonia	1995	Male	50 to 54	75%<br>(53-89%)	25%<br>(11-47%)	3<br>(1.8-4.6)	7.1%<br>(3.2-11%)	8.5%<br>(3.7-14%)	38<br>(16-65)	1600<br>(740-2600)
Macedonia	1995	Male	55 to 59	73%<br>(51-89%)	27%<br>(11-49%)	3<br>(1.7-4.7)	7.2%<br>(3.2-12%)	8.5%<br>(3.5-15%)	59<br>(24-100)	2100<br>(900-3500)

Location	Year	Sex	Age	Prevalence of current drinkers	Prevalence of abstainers	Population average of standard drinks daily	Percent of total attributable deaths	Percent of total attributable DALYs	Attributable deaths	Attributable DALYs
Macedonia	1995	Male	60 to 64	71% (46-89%)	29% (11-54%)	2.9 (1.5-4.7)	6.5% (2-12%)	7.5% (2.2-14%)	73 (22-140)	2100 (710-3900)
Macedonia	1995	Male	65 to 69	70% (45-88%)	30% (12-55%)	2.6 (1.5-4)	5.7% (1.7-11%)	6.5% (1.8-12%)	76 (21-140)	1800 (530-3400)
Macedonia	1995	Male	70 to 74	68% (44-85%)	32% (15-56%)	2.2 (1.2-3.7)	4.8% (0.84-10%)	5.5% (0.86-11%)	73 (11-160)	1400 (230-2900)
Macedonia	1995	Male	75 to 79	65% (41-84%)	35% (16-59%)	1.8 (0.87-3.3)	4% (0.34-8.7%)	4.4% (0.31-9.7%)	40 (2.9-89)	580 (51-1300)
Macedonia	1995	Male	80 to 84	62% (37-82%)	38% (18-63%)	1.6 (0.83-2.7)	3.2% (-0.0044-7.3%)	3.5% (-0.034-8.1%)	37 (-0.37-85)	400 (-0.53-900)
Macedonia	1995	Male	85 to 89	60% (39-81%)	40% (19-61%)	1.5 (0.74-2.5)	2.2% (-0.43-5.4%)	2.4% (-0.66-6%)	17 (-4.7-42)	130 (-26-320)
Macedonia	1995	Male	90 to 94	60% (39-82%)	40% (18-61%)	1.4 (0.65-2.5)	1.7% (-0.98-4.5%)	1.9% (-1.4-5.2%)	4.3 (-3.2-12)	24 (-14-62)
Macedonia	1995	Male	95 plus	61% (34-86%)	39% (14-66%)	1.3 (0.47-2.5)	1.9% (-1.2-5%)	2.1% (-1.8-5.8%)	1.2 (-0.98-3.3)	4.4 (-2.8-12)
Macedonia	2000	Female	All Ages	55% (46-64%)	45% (36-54%)	0.92 (0.76-1.1)	2.6% (1.2-4.2%)	3.4% (1.1-6.5%)	280 (89-530)	7000 (3200-11000)
Macedonia	2000	Female	15 to 19	54% (34-72%)	46% (28-66%)	0.67 (0.3-1.3)	1.8% (1.1-2.6%)	3.1% (1.2-5.8%)	0.77 (0.31-1.4)	130 (81-200)
Macedonia	2000	Female	20 to 24	64% (44-80%)	36% (20-56%)	0.97 (0.41-1.8)	3.1% (2-4.6%)	3.6% (1.1-7.2%)	1.1 (0.34-2.1)	250 (150-380)
Macedonia	2000	Female	25 to 29	66% (45-82%)	34% (18-55%)	1 (0.49-1.8)	3.2% (2.2-4.4%)	3.7% (1.4-7%)	1.1 (0.42-2.2)	260 (170-380)
Macedonia	2000	Female	30 to 34	67% (46-82%)	33% (18-54%)	1.1 (0.57-1.9)	3% (2-4.4%)	4.2% (1.7-7.6%)	2.1 (0.86-3.9)	310 (200-460)
Macedonia	2000	Female	35 to 39	67% (49-83%)	33% (17-51%)	1.3 (0.58-2.1)	3% (1.8-4.5%)	4.7% (1.9-8.5%)	3.6 (1.5-6.5)	340 (200-520)
Macedonia	2000	Female	40 to 44	67% (48-82%)	33% (18-52%)	1.4 (0.68-2.3)	3% (1.5-5%)	5% (1.8-8.8%)	6.3 (2.3-12)	430 (210-690)
Macedonia	2000	Female	45 to 49	65% (42-81%)	35% (19-58%)	1.5 (0.76-2.7)	3.3% (1.4-5.9%)	5.4% (1.9-10%)	11 (3.9-21)	590 (240-1100)
Macedonia	2000	Female	50 to 54	62% (41-79%)	38% (21-59%)	1.6 (0.8-2.7)	3.6% (1.2-6.7%)	5.6% (1.8-10%)	15 (4.6-27)	610 (210-1100)
Macedonia	2000	Female	55 to 59	58% (38-77%)	42% (23-62%)	1.5 (0.73-2.6)	3.6% (0.85-7.1%)	5.5% (1.4-10%)	20 (5-38)	680 (160-1300)
Macedonia	2000	Female	60 to 64	55% (33-75%)	45% (25-67%)	1.4 (0.64-2.5)	3% (0.053-7%)	4.3% (0.17-9.8%)	26 (1-60)	730 (12-1700)
Macedonia	2000	Female	65 to 69	50% (30-71%)	50% (29-70%)	1.2 (0.61-1.9)	2.5% (-0.29-6%)	3.4% (-0.31-8%)	32 (-2.9-76)	730 (-81-1800)
Macedonia	2000	Female	70 to 74	43% (26-62%)	57% (38-74%)	0.94 (0.4-1.8)	2.4% (-0.36-6.3%)	3% (-0.39-7.8%)	41 (-5.6-110)	740 (-110-2000)
Macedonia	2000	Female	75 to 79	36% (22-56%)	64% (44-78%)	0.75 (0.29-1.5)	2.2% (-0.41-5.8%)	2.6% (-0.4-7%)	42 (-6-110)	580 (-100-1600)

Location	Year	Sex	Age	Prevalence of current drinkers	Prevalence of abstainers	Population average of standard drinks daily	Percent of total attributable deaths	Percent of total attributable DALYs	Attributable deaths	Attributable DALYs
Macedonia	2000	Female	80 to 84	32% (18-50%)	68% (50-82%)	0.67 (0.27-1.2)	2.4% (0.17-5.9%)	2.8% (0.2-6.9%)	32 (2.2-80)	330 (22-830)
Macedonia	2000	Female	85 to 89	31% (19-46%)	69% (54-81%)	0.67 (0.27-1.3)	2.9% (0.27-7.6%)	3.4% (0.36-9%)	25 (2.6-68)	180 (16-490)
Macedonia	2000	Female	90 to 94	32% (19-48%)	68% (52-81%)	0.72 (0.27-1.5)	3% (0.71-7%)	3.8% (0.94-9.1%)	12 (3.1-29)	61 (14-140)
Macedonia	2000	Female	95 plus	36% (19-57%)	64% (43-81%)	0.8 (0.22-1.9)	3.4% (0.79-8.1%)	4.3% (0.97-10%)	4.5 (1-11)	15 (3.5-35)
Macedonia	2000	Male	All Ages	63% (51-75%)	37% (25-49%)	1.5 (1.2-1.7)	4.7% (2.9-6.8%)	4.9% (2.1-8.1%)	480 (210-800)	16000 (9700-22000)
Macedonia	2000	Male	15 to 19	54% (32-77%)	46% (23-68%)	0.67 (0.33-1.2)	3% (1.9-4.4%)	3.9% (1.9-6.7%)	2.2 (1-3.6)	290 (170-420)
Macedonia	2000	Male	20 to 24	66% (43-84%)	34% (16-57%)	1.2 (0.62-2)	6% (4-8.3%)	6.9% (3.6-11%)	5.2 (2.7-8.5)	650 (420-920)
Macedonia	2000	Male	25 to 29	70% (48-88%)	30% (12-52%)	1.6 (0.85-2.5)	6.7% (4.8-9%)	8.2% (4.7-12%)	6.5 (3.8-10)	740 (510-1000)
Macedonia	2000	Male	30 to 34	73% (51-89%)	27% (11-49%)	1.8 (1.1-2.8)	6.5% (4.7-8.8%)	8.6% (5.2-13%)	8.1 (4.9-12)	790 (540-1100)
Macedonia	2000	Male	35 to 39	75% (54-90%)	25% (9.9-46%)	2 (1.1-3.1)	6.2% (4.1-8.9%)	8.3% (4.5-13%)	12 (6.3-18)	880 (570-1200)
Macedonia	2000	Male	40 to 44	76% (55-91%)	24% (9.3-45%)	2.2 (1.4-3.3)	6.2% (4-9%)	8.2% (4.7-13%)	20 (11-30)	1200 (730-1700)
Macedonia	2000	Male	45 to 49	76% (52-90%)	24% (10-48%)	2.5 (1.5-3.9)	6.3% (3.5-10%)	7.9% (4-13%)	32 (16-52)	1600 (880-2500)
Macedonia	2000	Male	50 to 54	75% (52-90%)	25% (10-48%)	2.7 (1.7-4)	6.2% (3-10%)	7.4% (3.1-13%)	38 (17-64)	1600 (790-2600)
Macedonia	2000	Male	55 to 59	73% (51-90%)	27% (10-49%)	2.8 (1.6-4.2)	6.4% (2.7-11%)	7.5% (3-13%)	49 (20-85)	1700 (750-2900)
Macedonia	2000	Male	60 to 64	72% (46-89%)	28% (11-54%)	2.6 (1.4-4.2)	5.8% (1.8-10%)	6.6% (1.9-12%)	65 (19-120)	1900 (600-3500)
Macedonia	2000	Male	65 to 69	70% (46-89%)	30% (11-54%)	2.4 (1.4-3.6)	4.9% (1.4-9.3%)	5.6% (1.3-11%)	76 (19-140)	1800 (510-3400)
Macedonia	2000	Male	70 to 74	68% (45-85%)	32% (15-55%)	2 (1.1-3.2)	4.1% (0.65-8.5%)	4.6% (0.65-9.7%)	68 (9.8-140)	1300 (200-2700)
Macedonia	2000	Male	75 to 79	65% (42-84%)	35% (16-58%)	1.7 (0.82-2.9)	3.3% (0.01-7.6%)	3.7% (-0.03-8.3%)	56 (-0.44-130)	820 (2.6-1900)
Macedonia	2000	Male	80 to 84	63% (39-83%)	37% (17-61%)	1.5 (0.75-2.5)	2.6% (-0.31-6.2%)	2.9% (-0.47-6.8%)	29 (-4.7-68)	310 (-36-740)
Macedonia	2000	Male	85 to 89	61% (39-82%)	39% (18-61%)	1.4 (0.68-2.4)	1.8% (-1-4.9%)	2% (-1.4-5.4%)	13 (-8.6-35)	100 (-56-260)
Macedonia	2000	Male	90 to 94	61% (38-83%)	39% (17-62%)	1.3 (0.61-2.3)	1.3% (-1.3-4.1%)	1.5% (-1.8-4.7%)	4 (-4.9-13)	22 (-22-68)
Macedonia	2000	Male	95 plus	62% (35-87%)	38% (13-65%)	1.2 (0.44-2.3)	1.7% (-1.4-4.9%)	1.9% (-1.9-5.8%)	1.3 (-1.3-4)	4.8 (-3.8-14)

Location	Year	Sex	Age	Prevalence of current drinkers	Prevalence of abstainers	Population average of standard drinks daily	Percent of total attributable deaths	Percent of total attributable DALYs	Attributable deaths	Attributable DALYs
Macedonia	2005	Female	All Ages	55% (46-64%)	45% (36-54%)	0.8 (0.65-0.96)	1.9% (0.72-3.3%)	2.3% (0.43-5.2%)	210 (41-480)	5300 (2000-9400)
Macedonia	2005	Female	15 to 19	53% (34-71%)	47% (29-66%)	0.58 (0.26-1.1)	1.6% (1-2.4%)	2.7% (0.97-5%)	0.59 (0.21-1.1)	110 (70-170)
Macedonia	2005	Female	20 to 24	63% (45-80%)	37% (20-55%)	0.82 (0.35-1.5)	3% (2-4.2%)	2.9% (0.73-6.1%)	0.72 (0.18-1.5)	230 (140-360)
Macedonia	2005	Female	25 to 29	65% (45-81%)	35% (19-55%)	0.88 (0.4-1.6)	3% (2.1-4.1%)	2.8% (0.84-5.6%)	0.81 (0.24-1.6)	250 (160-360)
Macedonia	2005	Female	30 to 34	66% (45-82%)	34% (18-55%)	0.95 (0.47-1.6)	2.7% (1.8-3.8%)	3.2% (1.2-6%)	1.3 (0.46-2.3)	260 (160-370)
Macedonia	2005	Female	35 to 39	67% (49-83%)	33% (17-51%)	1 (0.48-1.8)	2.6% (1.7-3.9%)	3.8% (1.5-6.8%)	2.6 (1-4.7)	300 (180-450)
Macedonia	2005	Female	40 to 44	66% (48-82%)	34% (18-52%)	1.2 (0.59-1.9)	2.4% (1.2-3.9%)	3.8% (1.2-6.9%)	4.1 (1.3-7.8)	320 (160-530)
Macedonia	2005	Female	45 to 49	64% (42-81%)	36% (19-58%)	1.3 (0.65-2.3)	2.5% (0.95-4.8%)	4% (1-7.9%)	7.8 (2-16)	430 (160-800)
Macedonia	2005	Female	50 to 54	61% (40-79%)	39% (21-60%)	1.3 (0.69-2.2)	2.7% (0.81-5.2%)	4.2% (1-8.3%)	13 (3.2-25)	560 (160-1100)
Macedonia	2005	Female	55 to 59	58% (37-77%)	42% (23-63%)	1.3 (0.63-2.1)	2.6% (0.43-5.3%)	4% (0.62-8.1%)	16 (2.3-32)	540 (84-1100)
Macedonia	2005	Female	60 to 64	54% (32-74%)	46% (26-68%)	1.2 (0.55-2.1)	2% (-0.5-5.3%)	2.9% (-0.59-7.4%)	16 (-3-42)	450 (-110-1200)
Macedonia	2005	Female	65 to 69	50% (30-71%)	50% (29-70%)	1 (0.51-1.6)	1.5% (-0.82-4.5%)	2.1% (-0.84-5.9%)	20 (-7.9-56)	450 (-250-1300)
Macedonia	2005	Female	70 to 74	43% (26-62%)	57% (38-74%)	0.79 (0.34-1.5)	1.4% (-0.78-4.6%)	1.9% (-0.87-6%)	27 (-12-86)	470 (-260-1600)
Macedonia	2005	Female	75 to 79	36% (22-56%)	64% (44-78%)	0.62 (0.24-1.2)	1.4% (-0.67-4.5%)	1.7% (-0.73-5.5%)	32 (-14-100)	430 (-210-1500)
Macedonia	2005	Female	80 to 84	32% (18-49%)	68% (51-82%)	0.56 (0.22-1.1)	1.6% (-0.29-5.2%)	1.9% (-0.24-6.5%)	33 (-4-110)	330 (-56-1100)
Macedonia	2005	Female	85 to 89	30% (18-46%)	70% (54-82%)	0.56 (0.22-1.1)	2.1% (0.092-6.8%)	2.5% (0.18-8.3%)	20 (1.4-66)	140 (6.2-470)
Macedonia	2005	Female	90 to 94	32% (19-48%)	68% (52-81%)	0.6 (0.22-1.3)	2.2% (0.17-6.9%)	2.7% (0.27-8.2%)	8 (0.8-25)	39 (3.1-120)
Macedonia	2005	Female	95 plus	35% (19-56%)	65% (44-81%)	0.67 (0.18-1.6)	2.5% (0.2-7.4%)	3.1% (0.31-8.8%)	4.1 (0.39-12)	13 (1-38)
Macedonia	2005	Male	All Ages	64% (52-75%)	36% (25-48%)	1.3 (1.1-1.5)	3.8% (2.3-5.4%)	3.6% (1.2-6.1%)	380 (130-640)	13000 (7600-19000)
Macedonia	2005	Male	15 to 19	54% (32-77%)	46% (23-68%)	0.57 (0.27-1)	2.8% (1.8-4%)	3.6% (1.5-6.1%)	1.6 (0.68-2.8)	240 (150-340)
Macedonia	2005	Male	20 to 24	66% (43-85%)	34% (15-57%)	1 (0.54-1.7)	5.6% (3.8-7.7%)	6% (2.8-10%)	4.2 (1.9-6.9)	590 (380-850)
Macedonia	2005	Male	25 to 29	70% (48-88%)	30% (12-52%)	1.3 (0.71-2.2)	6.1% (4.4-8.2%)	7% (3.8-11%)	5.3 (2.9-8.5)	670 (460-930)

Location	Year	Sex	Age	Prevalence of current drinkers	Prevalence of abstainers	Population average of standard drinks daily	Percent of total attributable deaths	Percent of total attributable DALYs	Attributable deaths	Attributable DALYs
Macedonia	2005	Male	30 to 34	73% (50-90%)	27% (10-50%)	1.5 (0.86-2.4)	5.7% (4-7.7%)	7.1% (3.9-11%)	5.9 (3.3-9.3)	660 (450-920)
Macedonia	2005	Male	35 to 39	75% (54-90%)	25% (10-46%)	1.7 (0.95-2.6)	5.3% (3.4-7.3%)	6.9% (3.4-11%)	8.3 (4.2-13)	720 (450-1000)
Macedonia	2005	Male	40 to 44	76% (54-90%)	24% (9.7-46%)	1.9 (1.1-2.8)	5% (3-7.4%)	6.5% (3.3-11%)	13 (6.7-21)	850 (510-1200)
Macedonia	2005	Male	45 to 49	76% (52-90%)	24% (9.7-48%)	2.1 (1.2-3.3)	5.1% (2.8-8.2%)	6.4% (2.9-11%)	24 (11-40)	1200 (680-2000)
Macedonia	2005	Male	50 to 54	75% (52-90%)	25% (10-48%)	2.3 (1.4-3.4)	5% (2.4-8.1%)	5.9% (2.4-9.9%)	37 (15-63)	1600 (750-2600)
Macedonia	2005	Male	55 to 59	73% (51-89%)	27% (11-49%)	2.3 (1.3-3.5)	5% (2.2-8.7%)	5.9% (2.3-11%)	42 (16-76)	1500 (640-2700)
Macedonia	2005	Male	60 to 64	71% (47-89%)	29% (11-53%)	2.2 (1.2-3.6)	4.4% (1.3-8.7%)	5.1% (1.3-10%)	46 (12-93)	1400 (400-2700)
Macedonia	2005	Male	65 to 69	70% (46-88%)	30% (12-54%)	2 (1.2-3)	3.6% (0.83-7.1%)	4.1% (0.87-8.1%)	54 (11-110)	1300 (310-2500)
Macedonia	2005	Male	70 to 74	67% (44-85%)	33% (15-56%)	1.7 (0.89-2.8)	3% (0.24-6.5%)	3.3% (0.17-7.3%)	56 (2.8-120)	1100 (86-2400)
Macedonia	2005	Male	75 to 79	64% (40-84%)	36% (16-60%)	1.4 (0.7-2.4)	2.3% (-0.34-5.9%)	2.5% (-0.53-6.5%)	42 (-9-110)	620 (-91-1600)
Macedonia	2005	Male	80 to 84	62% (38-81%)	38% (19-62%)	1.3 (0.63-2.1)	1.8% (-0.77-4.9%)	2% (-0.97-5.5%)	26 (-13-74)	290 (-120-780)
Macedonia	2005	Male	85 to 89	60% (37-81%)	40% (19-63%)	1.1 (0.56-2)	1.3% (-1.4-3.9%)	1.3% (-1.7-4.4%)	8.5 (-11-28)	67 (-74-210)
Macedonia	2005	Male	90 to 94	60% (38-82%)	40% (18-62%)	1.1 (0.51-1.9)	0.86% (-1.8-3.2%)	0.9% (-2.3-3.6%)	2.1 (-5.3-8.6)	12 (-25-44)
Macedonia	2005	Male	95 plus	61% (35-86%)	39% (14-65%)	0.98 (0.37-2)	1.2% (-1.9-3.9%)	1.3% (-2.5-4.4%)	1.2 (-2.3-4.2)	4.4 (-7.1-14)
Macedonia	2010	Female	All Ages	54% (45-63%)	46% (37-55%)	0.73 (0.58-0.9)	1.7% (0.66-3.1%)	2% (0.37-5.2%)	190 (35-500)	4800 (1800-8800)
Macedonia	2010	Female	15 to 19	52% (32-71%)	48% (29-68%)	0.51 (0.22-0.97)	1.6% (1-2.3%)	2.6% (0.95-4.7%)	0.46 (0.17-0.84)	100 (64-160)
Macedonia	2010	Female	20 to 24	63% (44-80%)	37% (20-56%)	0.74 (0.31-1.4)	3% (2-4.1%)	2.7% (0.8-5.4%)	0.49 (0.14-0.97)	210 (130-320)
Macedonia	2010	Female	25 to 29	65% (44-81%)	35% (19-56%)	0.79 (0.34-1.4)	2.9% (2-4%)	2.5% (0.7-4.9%)	0.64 (0.18-1.3)	250 (160-360)
Macedonia	2010	Female	30 to 34	66% (44-81%)	34% (19-56%)	0.85 (0.4-1.4)	2.6% (1.8-3.6%)	2.8% (1.1-5.2%)	1 (0.4-1.9)	250 (160-370)
Macedonia	2010	Female	35 to 39	66% (48-82%)	34% (18-52%)	0.94 (0.42-1.7)	2.5% (1.6-3.6%)	3.4% (1.5-6.3%)	2.1 (0.92-3.8)	270 (170-410)
Macedonia	2010	Female	40 to 44	65% (47-81%)	35% (19-53%)	1.1 (0.51-1.8)	2.2% (1.2-3.6%)	3.5% (1.2-6.6%)	3.4 (1.2-6.5)	300 (160-490)
Macedonia	2010	Female	45 to 49	63% (40-81%)	37% (19-60%)	1.2 (0.58-2.1)	2.3% (0.96-4.3%)	3.7% (1.1-7.5%)	6.4 (1.9-13)	380 (160-680)

Location	Year	Sex	Age	Prevalence of current drinkers	Prevalence of abstainers	Population average of standard drinks daily	Percent of total attributable deaths	Percent of total attributable DALYs	Attributable deaths	Attributable DALYs
Macedonia	2010	Female	50 to 54	60% (40-78%)	40% (22-60%)	1.2 (0.61-2)	2.3% (0.71-4.4%)	3.6% (0.9-7.2%)	10 (2.6-20)	460 (150-860)
Macedonia	2010	Female	55 to 59	57% (37-75%)	43% (25-63%)	1.1 (0.53-1.9)	2.2% (0.35-4.5%)	3.5% (0.64-7%)	16 (2.9-33)	550 (86-1100)
Macedonia	2010	Female	60 to 64	53% (32-73%)	47% (27-68%)	1 (0.48-1.9)	1.6% (-0.35-4.5%)	2.5% (-0.35-6.6%)	14 (-2-38)	390 (-83-1100)
Macedonia	2010	Female	65 to 69	49% (29-70%)	51% (30-71%)	0.88 (0.45-1.5)	1.2% (-0.71-3.8%)	1.7% (-0.73-5.3%)	15 (-6.4-44)	320 (-190-1000)
Macedonia	2010	Female	70 to 74	42% (25-61%)	58% (39-75%)	0.7 (0.29-1.4)	1.1% (-0.71-3.8%)	1.6% (-0.77-5%)	22 (-11-72)	380 (-240-1300)
Macedonia	2010	Female	75 to 79	35% (21-55%)	65% (45-79%)	0.55 (0.21-1.1)	1.1% (-0.72-4.2%)	1.4% (-0.85-5.2%)	27 (-16-99)	360 (-250-1400)
Macedonia	2010	Female	80 to 84	31% (18-48%)	69% (52-82%)	0.49 (0.2-0.93)	1.4% (-0.35-4.8%)	1.7% (-0.36-6.2%)	32 (-7.2-120)	330 (-84-1200)
Macedonia	2010	Female	85 to 89	29% (17-45%)	71% (55-83%)	0.49 (0.2-0.99)	1.8% (0.082-6%)	2.2% (0.19-7.5%)	28 (2.4-96)	190 (8.8-670)
Macedonia	2010	Female	90 to 94	31% (18-47%)	69% (53-82%)	0.53 (0.19-1.2)	1.8% (0.15-5.5%)	2.4% (0.25-7.3%)	8.3 (0.86-26)	40 (3.2-120)
Macedonia	2010	Female	95 plus	34% (18-55%)	66% (45-82%)	0.59 (0.16-1.4)	2.1% (0.22-6.2%)	2.7% (0.34-7.7%)	2.6 (0.31-7.3)	8.3 (0.84-24)
Macedonia	2010	Male	All Ages	64% (53-75%)	36% (25-47%)	1.2 (0.99-1.5)	3.4% (2-5%)	3.2% (1-5.4%)	350 (110-610)	12000 (6900-17000)
Macedonia	2010	Male	15 to 19	53% (31-75%)	47% (25-69%)	0.51 (0.24-0.91)	2.7% (1.7-3.8%)	3.3% (1.3-5.8%)	1.3 (0.51-2.3)	210 (140-310)
Macedonia	2010	Male	20 to 24	65% (42-84%)	35% (16-58%)	0.94 (0.48-1.6)	5.4% (3.7-7.5%)	5.6% (2.4-9.3%)	3.2 (1.4-5.4)	510 (330-730)
Macedonia	2010	Male	25 to 29	69% (49-87%)	31% (13-51%)	1.2 (0.65-2)	5.8% (4.3-7.7%)	6.5% (3.3-10%)	4.2 (2.2-6.9)	630 (430-870)
Macedonia	2010	Male	30 to 34	72% (50-89%)	28% (11-50%)	1.4 (0.81-2.3)	5.4% (3.8-7.2%)	6.4% (3.2-10%)	4.8 (2.4-7.8)	610 (420-850)
Macedonia	2010	Male	35 to 39	75% (54-89%)	25% (11-46%)	1.5 (0.84-2.4)	4.9% (3.2-6.8%)	6.2% (2.9-10%)	6.7 (3.2-11)	630 (400-900)
Macedonia	2010	Male	40 to 44	76% (53-90%)	24% (9.7-47%)	1.7 (0.99-2.6)	4.4% (2.6-6.6%)	5.8% (2.7-9.5%)	11 (4.9-18)	740 (430-1100)
Macedonia	2010	Male	45 to 49	75% (49-90%)	25% (10-51%)	1.9 (1.1-3)	4.6% (2.4-7.3%)	5.7% (2.6-9.6%)	20 (8.9-33)	1000 (560-1600)
Macedonia	2010	Male	50 to 54	74% (50-89%)	26% (11-50%)	2 (1.3-3.1)	4.5% (2.1-7.3%)	5.4% (2.2-9.2%)	32 (13-53)	1400 (650-2200)
Macedonia	2010	Male	55 to 59	72% (49-89%)	28% (11-51%)	2.1 (1.2-3.1)	4.4% (1.8-7.6%)	5.2% (2-9.2%)	46 (17-81)	1700 (650-2900)
Macedonia	2010	Male	60 to 64	70% (45-89%)	30% (11-55%)	2 (1.1-3.2)	3.8% (1.2-7.3%)	4.4% (1.3-8.5%)	44 (12-85)	1300 (410-2500)
Macedonia	2010	Male	65 to 69	69% (44-88%)	31% (12-56%)	1.8 (1-2.7)	3.1% (0.59-6.1%)	3.6% (0.62-7%)	44 (7.5-84)	1100 (210-2100)

Location	Year	Sex	Age	Prevalence of current drinkers	Prevalence of abstainers	Population average of standard drinks daily	Percent of total attributable deaths	Percent of total attributable DALYs	Attributable deaths	Attributable DALYs
Macedonia	2010	Male	70 to 74	67% (43-84%)	33% (16-57%)	1.5 (0.8-2.5)	2.6% (0.18-5.9%)	2.9% (0.13-6.8%)	49 (2.1-110)	930 (63-2200)
Macedonia	2010	Male	75 to 79	64% (40-84%)	36% (16-60%)	1.3 (0.6-2.2)	2% (-0.35-5.2%)	2.3% (-0.55-5.8%)	44 (-9.9-110)	640 (-110-1600)
Macedonia	2010	Male	80 to 84	61% (35-82%)	39% (18-65%)	1.1 (0.54-1.9)	1.6% (-0.78-4.1%)	1.8% (-1-4.6%)	28 (-15-73)	300 (-140-780)
Macedonia	2010	Male	85 to 89	59% (36-81%)	41% (19-64%)	1 (0.51-1.7)	1.2% (-1.4-3.5%)	1.3% (-1.7-4%)	12 (-15-35)	90 (-100-270)
Macedonia	2010	Male	90 to 94	59% (38-82%)	41% (18-62%)	0.95 (0.44-1.7)	0.88% (-1.7-3.3%)	0.97% (-2.1-3.8%)	2.5 (-5.5-9.8)	14 (-26-51)
Macedonia	2010	Male	95 plus	61% (34-85%)	39% (15-66%)	0.89 (0.32-1.8)	1.2% (-1.7-3.5%)	1.3% (-2.3-4.1%)	0.79 (-1.4-2.5)	2.9 (-4.1-8.6)
Macedonia	2016	Female	All Ages	54% (45-62%)	46% (38-55%)	0.76 (0.56-1)	1.7% (0.68-3.1%)	2% (0.44-4.9%)	200 (44-510)	5000 (2000-9300)
Macedonia	2016	Female	15 to 19	52% (32-71%)	48% (29-68%)	0.53 (0.21-1)	1.6% (0.99-2.3%)	2.7% (0.96-5.2%)	0.42 (0.15-0.79)	92 (56-150)
Macedonia	2016	Female	20 to 24	63% (44-79%)	37% (21-56%)	0.76 (0.3-1.5)	3% (2-4.2%)	2.8% (0.76-5.9%)	0.5 (0.13-1.1)	200 (120-300)
Macedonia	2016	Female	25 to 29	64% (44-80%)	36% (20-56%)	0.8 (0.35-1.5)	3% (2.1-4.1%)	2.6% (0.84-5.1%)	0.61 (0.19-1.2)	230 (150-340)
Macedonia	2016	Female	30 to 34	65% (44-81%)	35% (19-56%)	0.87 (0.41-1.6)	2.7% (1.9-3.8%)	2.9% (1.1-5.4%)	1 (0.4-2)	270 (170-400)
Macedonia	2016	Female	35 to 39	66% (47-82%)	34% (18-53%)	0.95 (0.43-1.7)	2.5% (1.7-3.7%)	3.4% (1.5-6.4%)	2 (0.86-3.8)	280 (180-420)
Macedonia	2016	Female	40 to 44	65% (47-81%)	35% (19-53%)	1.1 (0.49-1.8)	2.3% (1.3-3.7%)	3.6% (1.3-6.8%)	3.3 (1.2-6.2)	300 (160-470)
Macedonia	2016	Female	45 to 49	63% (40-80%)	37% (20-60%)	1.2 (0.54-2.1)	2.4% (1.1-4.5%)	3.9% (1.3-7.7%)	6.4 (2.1-13)	390 (170-720)
Macedonia	2016	Female	50 to 54	60% (38-78%)	40% (22-62%)	1.2 (0.58-2.1)	2.4% (0.78-4.7%)	3.8% (1.1-7.8%)	9.8 (2.8-20)	460 (150-880)
Macedonia	2016	Female	55 to 59	56% (36-76%)	44% (24-64%)	1.1 (0.52-2)	2.2% (0.42-4.4%)	3.5% (0.7-7.1%)	14 (2.7-29)	510 (95-1000)
Macedonia	2016	Female	60 to 64	53% (32-73%)	47% (27-68%)	1 (0.47-1.9)	1.7% (-0.22-4.5%)	2.6% (-0.12-6.8%)	17 (-0.82-45)	500 (-63-1300)
Macedonia	2016	Female	65 to 69	48% (29-70%)	52% (30-71%)	0.89 (0.44-1.5)	1.3% (-0.49-3.8%)	1.9% (-0.49-5.3%)	17 (-4.4-49)	370 (-150-1100)
Macedonia	2016	Female	70 to 74	42% (25-61%)	58% (39-75%)	0.7 (0.29-1.4)	1.2% (-0.66-3.9%)	1.7% (-0.69-5.2%)	21 (-8.6-64)	360 (-200-1200)
Macedonia	2016	Female	75 to 79	35% (21-53%)	65% (47-79%)	0.56 (0.22-1.2)	1.2% (-0.63-4%)	1.5% (-0.69-5.1%)	28 (-13-98)	380 (-190-1400)
Macedonia	2016	Female	80 to 84	30% (17-48%)	70% (52-83%)	0.5 (0.21-0.97)	1.3% (-0.34-4.3%)	1.6% (-0.32-5.2%)	33 (-6.3-110)	330 (-76-1100)
Macedonia	2016	Female	85 to 89	29% (18-45%)	71% (55-82%)	0.5 (0.19-1)	1.6% (-0.07-5%)	2.1% (-0.053-6.3%)	31 (-0.8-94)	220 (-10-650)

Location	Year	Sex	Age	Prevalence of current drinkers	Prevalence of abstainers	Population average of standard drinks daily	Percent of total attributable deaths	Percent of total attributable DALYs	Attributable deaths	Attributable DALYs
Macedonia	2016	Female	90 to 94	31% (19-46%)	69% (54-81%)	0.54 (0.19-1.2)	1.8% (0.092-5.1%)	2.3% (0.23-7.1%)	15 (1.4-44)	70 (3.5-210)
Macedonia	2016	Female	95 plus	34% (18-54%)	66% (46-82%)	0.61 (0.17-1.5)	2.1% (0.28-6.1%)	2.7% (0.39-7.9%)	4.4 (0.63-13)	14 (2-43)
Macedonia	2016	Male	All Ages	64% (53-75%)	36% (25-47%)	1.3 (0.96-1.7)	3.6% (2-5.5%)	3.4% (1.1-6.3%)	390 (140-720)	13000 (7200-20000)
Macedonia	2016	Male	15 to 19	53% (31-75%)	47% (25-69%)	0.52 (0.23-0.96)	2.7% (1.7-4%)	3.4% (1.4-6.1%)	1.2 (0.47-2.1)	190 (120-280)
Macedonia	2016	Male	20 to 24	65% (42-85%)	35% (15-58%)	0.96 (0.46-1.7)	5.6% (3.8-7.8%)	5.7% (2.4-9.6%)	3.2 (1.3-5.3)	510 (330-730)
Macedonia	2016	Male	25 to 29	69% (47-87%)	31% (13-53%)	1.2 (0.63-2.2)	6.2% (4.5-8.4%)	6.7% (3.1-11%)	4.2 (2-7)	630 (430-900)
Macedonia	2016	Male	30 to 34	72% (49-89%)	28% (11-51%)	1.4 (0.79-2.4)	5.7% (4-7.8%)	6.5% (3.2-11%)	5.2 (2.6-8.9)	690 (470-970)
Macedonia	2016	Male	35 to 39	74% (53-89%)	26% (11-47%)	1.5 (0.81-2.5)	5.2% (3.4-7.5%)	6.5% (3.1-11%)	7.3 (3.4-13)	700 (450-1000)
Macedonia	2016	Male	40 to 44	76% (54-90%)	24% (10-46%)	1.8 (0.99-2.7)	4.7% (2.8-7.2%)	6.1% (2.7-10%)	11 (4.7-19)	770 (450-1200)
Macedonia	2016	Male	45 to 49	75% (52-90%)	25% (10-48%)	2 (1.1-3.2)	4.8% (2.5-8.1%)	6.1% (2.6-11%)	20 (8.6-36)	1100 (560-1800)
Macedonia	2016	Male	50 to 54	74% (52-89%)	26% (11-48%)	2.1 (1.2-3.3)	4.8% (2.3-8.1%)	5.9% (2.5-10%)	31 (13-54)	1400 (670-2300)
Macedonia	2016	Male	55 to 59	72% (50-89%)	28% (11-50%)	2.1 (1.2-3.3)	4.6% (2.1-8.2%)	5.6% (2.2-10%)	45 (18-80)	1600 (740-2900)
Macedonia	2016	Male	60 to 64	70% (44-89%)	30% (11-56%)	2 (1.1-3.3)	4.1% (1.3-7.8%)	4.8% (1.4-9.2%)	58 (18-110)	1800 (590-3400)
Macedonia	2016	Male	65 to 69	69% (44-88%)	31% (12-56%)	1.8 (1-2.9)	3.3% (0.84-6.6%)	3.9% (0.89-7.9%)	53 (12-110)	1300 (330-2600)
Macedonia	2016	Male	70 to 74	67% (43-84%)	33% (16-57%)	1.6 (0.78-2.7)	2.7% (0.35-6.4%)	3.2% (0.39-7.3%)	48 (6-110)	920 (120-2100)
Macedonia	2016	Male	75 to 79	63% (39-83%)	37% (17-61%)	1.3 (0.6-2.4)	2.2% (-0.2-5.9%)	2.5% (-0.34-6.6%)	48 (-6.2-130)	700 (-64-1800)
Macedonia	2016	Male	80 to 84	61% (35-82%)	39% (18-65%)	1.1 (0.53-2)	1.8% (-0.64-4.7%)	2% (-0.95-5.3%)	34 (-16-91)	370 (-140-960)
Macedonia	2016	Male	85 to 89	59% (37-81%)	41% (19-63%)	1 (0.49-1.9)	1.2% (-1.4-3.7%)	1.3% (-1.7-4.2%)	15 (-18-46)	110 (-120-350)
Macedonia	2016	Male	90 to 94	59% (38-81%)	41% (19-62%)	0.97 (0.44-1.9)	0.92% (-1.6-3.3%)	1% (-2-3.8%)	3.9 (-7.6-14)	21 (-36-74)
Macedonia	2016	Male	95 plus	61% (34-85%)	39% (15-66%)	0.9 (0.32-1.9)	1.3% (-1.5-3.8%)	1.5% (-2-4.5%)	1.3 (-1.9-4)	4.8 (-5.6-14)
Montenegro	1990	Female	All Ages	57% (49-66%)	43% (34-51%)	2.1 (1.3-3.1)	7.1% (4.2-10%)	11% (5.5-17%)	240 (120-380)	5300 (3200-7600)
Montenegro	1990	Female	15 to 19	58% (38-75%)	42% (25-62%)	1.6 (0.64-3.3)	3.3% (1.9-5.3%)	8.6% (4-15%)	0.66 (0.31-1.2)	76 (45-130)

Location	Year	Sex	Age	Prevalence of current drinkers	Prevalence of abstainers	Population average of standard drinks daily	Percent of total attributable deaths	Percent of total attributable DALYs	Attributable deaths	Attributable DALYs
Montenegro	1990	Female	20 to 24	68% (49-82%)	32% (18-51%)	2.3 (0.92-4.5)	5.4% (3.3-8.2%)	11% (4.6-19%)	1.3 (0.56-2.3)	140 (85-220)
Montenegro	1990	Female	25 to 29	69% (50-84%)	31% (16-50%)	2.4 (1-4.6)	5.5% (3.4-8.1%)	12% (5.2-19%)	1.5 (0.69-2.6)	160 (97-240)
Montenegro	1990	Female	30 to 34	70% (49-84%)	30% (16-51%)	2.6 (1.2-4.8)	5.9% (3.6-8.8%)	13% (6.6-20%)	2.4 (1.2-3.8)	200 (120-300)
Montenegro	1990	Female	35 to 39	70% (53-84%)	30% (16-47%)	2.9 (1.3-5.4)	7% (3.8-11%)	14% (6.9-22%)	4.3 (2.1-6.7)	280 (150-430)
Montenegro	1990	Female	40 to 44	69% (51-84%)	31% (16-49%)	3.2 (1.4-6.1)	8.8% (4.4-13%)	16% (7.5-24%)	6.7 (3.2-11)	360 (180-550)
Montenegro	1990	Female	45 to 49	68% (45-83%)	32% (17-55%)	3.6 (1.5-7.2)	8.8% (4.2-14%)	16% (7.9-24%)	7.3 (3.6-11)	350 (170-520)
Montenegro	1990	Female	50 to 54	65% (44-81%)	35% (19-56%)	3.7 (1.6-7.2)	9% (4.5-13%)	16% (8.1-24%)	11 (5.4-16)	450 (230-670)
Montenegro	1990	Female	55 to 59	62% (41-79%)	38% (21-59%)	3.6 (1.6-6.7)	9.3% (4.5-14%)	16% (8-23%)	14 (7.3-22)	510 (250-790)
Montenegro	1990	Female	60 to 64	58% (37-77%)	42% (23-63%)	3.3 (1.4-6.4)	10% (4.3-16%)	14% (6.2-23%)	23 (9.8-37)	650 (280-1100)
Montenegro	1990	Female	65 to 69	54% (34-73%)	46% (27-66%)	2.8 (1.3-5.4)	9.8% (3.8-17%)	13% (5.3-22%)	22 (8.4-36)	530 (200-870)
Montenegro	1990	Female	70 to 74	47% (30-65%)	53% (35-70%)	2.3 (0.89-4.7)	8.5% (2.4-15%)	12% (3.3-20%)	19 (5.2-34)	360 (100-640)
Montenegro	1990	Female	75 to 79	40% (25-59%)	60% (41-75%)	1.8 (0.67-4.1)	7.7% (2.1-14%)	10% (2.7-19%)	31 (8.2-58)	460 (120-860)
Montenegro	1990	Female	80 to 84	36% (21-54%)	64% (46-79%)	1.7 (0.66-3.4)	7.8% (2.4-14%)	9.9% (2.9-18%)	34 (9.9-62)	370 (120-670)
Montenegro	1990	Female	85 to 89	34% (21-50%)	66% (50-79%)	1.7 (0.6-3.7)	8.1% (2.8-15%)	10% (3.4-19%)	35 (12-64)	260 (90-480)
Montenegro	1990	Female	90 to 94	36% (23-53%)	64% (47-77%)	1.8 (0.61-4.3)	8% (2.3-16%)	9.8% (2.7-19%)	20 (5.7-39)	100 (29-200)
Montenegro	1990	Female	95 plus	40% (22-60%)	60% (40-78%)	2 (0.52-5.1)	7.9% (1.6-16%)	9.7% (2.1-19%)	7.1 (1.5-14)	23 (4.6-46)
Montenegro	1990	Male	All Ages	65% (54-75%)	35% (25-46%)	3.1 (2.1-4.7)	11% (7.3-14%)	14% (8.4-20%)	310 (180-430)	9300 (6400-12000)
Montenegro	1990	Male	15 to 19	58% (36-80%)	42% (20-64%)	1.6 (0.7-2.9)	5% (3-7.9%)	7.9% (4-14%)	1.7 (0.87-3)	170 (99-270)
Montenegro	1990	Male	20 to 24	70% (48-87%)	30% (13-52%)	2.8 (1.3-5.1)	9% (6-13%)	13% (7.4-22%)	3.2 (1.8-5.3)	310 (200-470)
Montenegro	1990	Male	25 to 29	73% (54-89%)	27% (11-46%)	3.6 (1.8-6.4)	11% (6.8-15%)	16% (9-24%)	5.6 (3.1-8.7)	470 (290-670)
Montenegro	1990	Male	30 to 34	76% (55-91%)	24% (8.8-45%)	4.2 (2.1-7.1)	11% (7.4-15%)	18% (11-26%)	6.2 (3.6-8.9)	470 (300-650)
Montenegro	1990	Male	35 to 39	78% (58-91%)	22% (8.6-42%)	4.4 (2.2-7.4)	12% (7.6-17%)	19% (11-27%)	9 (5.2-13)	580 (350-810)

Location	Year	Sex	Age	Prevalence of current drinkers	Prevalence of abstainers	Population average of standard drinks daily	Percent of total attributable deaths	Percent of total attributable DALYs	Attributable deaths	Attributable DALYs
Montenegro	1990	Male	40 to 44	79% (59-91%)	21% (8.6-41%)	5 (2.5-8.4)	14% (7.9-19%)	20% (10-27%)	14 (7.3-20)	720 (410-1000)
Montenegro	1990	Male	45 to 49	79% (57-91%)	21% (8.8-43%)	5.8 (2.9-10)	14% (8.5-19%)	20% (11-27%)	15 (8.6-21)	700 (430-950)
Montenegro	1990	Male	50 to 54	77% (57-90%)	23% (9.6-43%)	6.2 (3.2-11)	15% (9.1-19%)	19% (12-25%)	26 (15-36)	1100 (660-1400)
Montenegro	1990	Male	55 to 59	76% (55-90%)	24% (9.6-45%)	6.3 (3.3-11)	15% (9.1-20%)	18% (11-24%)	39 (24-52)	1300 (830-1800)
Montenegro	1990	Male	60 to 64	74% (51-90%)	26% (9.6-49%)	6 (3-11)	14% (7.6-19%)	17% (9.2-23%)	42 (23-58)	1200 (690-1700)
Montenegro	1990	Male	65 to 69	73% (50-90%)	27% (10-50%)	5.4 (2.8-9.2)	13% (6.9-18%)	16% (8.5-23%)	36 (19-51)	860 (460-1200)
Montenegro	1990	Male	70 to 74	71% (49-86%)	29% (14-51%)	4.6 (2.2-8.8)	11% (5-17%)	14% (5.9-22%)	22 (9-33)	420 (190-640)
Montenegro	1990	Male	75 to 79	68% (45-85%)	32% (15-55%)	4 (1.7-8)	11% (3.6-18%)	13% (3.9-21%)	32 (9.9-53)	470 (150-770)
Montenegro	1990	Male	80 to 84	66% (42-84%)	34% (16-58%)	3.5 (1.6-6.7)	10% (3.3-17%)	12% (3.8-21%)	27 (8.7-49)	300 (99-520)
Montenegro	1990	Male	85 to 89	64% (43-83%)	36% (17-57%)	3.2 (1.5-6.1)	8.9% (2.2-16%)	10% (2.4-19%)	20 (4.9-38)	160 (40-290)
Montenegro	1990	Male	90 to 94	64% (42-85%)	36% (15-58%)	3 (1.3-6)	7.1% (0.86-15%)	8.3% (0.86-17%)	7.4 (0.73-15)	39 (4.7-80)
Montenegro	1990	Male	95 plus	65% (38-88%)	35% (12-62%)	2.7 (0.92-6)	5.9% (-0.32-14%)	6.8% (-0.67-16%)	1.6 (-0.16-4)	5.8 (-0.3-14)
Montenegro	1995	Female	All Ages	58% (50-67%)	42% (33-50%)	2 (1.3-3)	7.3% (4.2-11%)	11% (5.2-17%)	280 (130-440)	5900 (3400-8700)
Montenegro	1995	Female	15 to 19	58% (39-75%)	42% (25-61%)	1.6 (0.64-3)	3.2% (1.9-5.2%)	8.3% (4-15%)	0.61 (0.29-1.1)	70 (41-110)
Montenegro	1995	Female	20 to 24	68% (50-83%)	32% (17-50%)	2.2 (0.87-4.4)	5.3% (3.2-8.1%)	10% (4.5-18%)	1.2 (0.52-2.1)	140 (81-220)
Montenegro	1995	Female	25 to 29	69% (50-84%)	31% (16-50%)	2.3 (0.96-4.5)	5.5% (3.4-8.2%)	11% (5.1-19%)	1.5 (0.65-2.5)	150 (91-220)
Montenegro	1995	Female	30 to 34	70% (50-84%)	30% (16-50%)	2.5 (1.2-4.5)	5.7% (3.5-8.5%)	12% (6.4-20%)	2.3 (1.2-3.6)	190 (120-280)
Montenegro	1995	Female	35 to 39	71% (54-85%)	29% (15-46%)	2.8 (1.2-5.2)	6.6% (3.6-9.9%)	14% (6.7-21%)	3.8 (1.8-6)	260 (140-390)
Montenegro	1995	Female	40 to 44	70% (52-84%)	30% (16-48%)	3.1 (1.3-5.7)	7.6% (3.9-12%)	15% (7.4-23%)	6.3 (3.1-9.8)	350 (180-540)
Montenegro	1995	Female	45 to 49	68% (47-83%)	32% (17-53%)	3.4 (1.5-6.7)	9% (4.3-14%)	16% (7.8-24%)	8.7 (4.2-13)	410 (200-630)
Montenegro	1995	Female	50 to 54	65% (46-81%)	35% (19-54%)	3.5 (1.6-6.6)	9.5% (4.7-14%)	16% (8-24%)	11 (5.3-16)	440 (220-650)
Montenegro	1995	Female	55 to 59	62% (42-79%)	38% (21-58%)	3.4 (1.5-6.6)	9.9% (4.8-15%)	16% (7.7-24%)	18 (8.7-28)	620 (300-960)

Location	Year	Sex	Age	Prevalence of current drinkers	Prevalence of abstainers	Population average of standard drinks daily	Percent of total attributable deaths	Percent of total attributable DALYs	Attributable deaths	Attributable DALYs
Montenegro	1995	Female	60 to 64	58% (38-78%)	42% (22-62%)	3.1 (1.4-6.5)	9.4% (3.8-15%)	14% (5.7-23%)	24 (9.7-39)	690 (280-1100)
Montenegro	1995	Female	65 to 69	54% (35-74%)	46% (26-65%)	2.7 (1.3-5.1)	9.5% (3.7-16%)	13% (5.2-22%)	30 (12-50)	710 (280-1200)
Montenegro	1995	Female	70 to 74	48% (30-66%)	52% (34-70%)	2.2 (0.87-4.5)	9% (2.3-17%)	12% (3-21%)	32 (8.2-58)	590 (160-1100)
Montenegro	1995	Female	75 to 79	41% (26-60%)	59% (40-74%)	1.8 (0.62-3.7)	8% (1.9-15%)	10% (2.3-19%)	29 (6.4-55)	420 (98-790)
Montenegro	1995	Female	80 to 84	36% (21-56%)	64% (44-79%)	1.6 (0.59-3.2)	7.7% (2.3-14%)	9.7% (2.7-18%)	32 (8.7-61)	350 (100-650)
Montenegro	1995	Female	85 to 89	35% (22-51%)	65% (49-78%)	1.6 (0.57-3.4)	8% (2.5-15%)	9.9% (3.1-19%)	41 (13-78)	310 (100-580)
Montenegro	1995	Female	90 to 94	36% (23-53%)	64% (47-77%)	1.7 (0.58-4)	7.9% (2.1-15%)	9.6% (2.6-19%)	26 (7.1-51)	130 (36-250)
Montenegro	1995	Female	95 plus	40% (22-61%)	60% (39-78%)	1.9 (0.52-5)	7.8% (1.5-16%)	9.5% (1.9-20%)	9.5 (2-19)	31 (6.2-64)
Montenegro	1995	Male	All Ages	66% (55-77%)	34% (23-45%)	3.1 (2.1-4.5)	11% (7.3-14%)	14% (8.1-20%)	370 (210-530)	11000 (7100-14000)
Montenegro	1995	Male	15 to 19	59% (37-80%)	41% (20-63%)	1.5 (0.7-2.8)	5.1% (3.1-8%)	7.8% (4-13%)	1.7 (0.87-3)	160 (97-260)
Montenegro	1995	Male	20 to 24	69% (47-87%)	31% (13-53%)	2.7 (1.2-4.8)	9.4% (5.9-14%)	13% (6.9-21%)	4.2 (2.3-7.1)	370 (230-580)
Montenegro	1995	Male	25 to 29	73% (53-89%)	27% (11-47%)	3.4 (1.6-6.2)	11% (6.9-16%)	16% (8.7-24%)	5.4 (3-8.4)	440 (270-630)
Montenegro	1995	Male	30 to 34	76% (54-90%)	24% (9.5-46%)	3.9 (2-6.7)	11% (7.4-16%)	17% (10-26%)	7.1 (4.1-10)	520 (330-730)
Montenegro	1995	Male	35 to 39	78% (59-91%)	22% (8.7-41%)	4.2 (2.1-7)	12% (7.5-17%)	18% (10-26%)	9.9 (5.6-14)	620 (380-880)
Montenegro	1995	Male	40 to 44	79% (58-92%)	21% (8.2-42%)	4.7 (2.5-7.9)	13% (7.7-19%)	19% (10-27%)	16 (8.9-24)	870 (510-1200)
Montenegro	1995	Male	45 to 49	78% (55-91%)	22% (8.6-45%)	5.5 (2.7-9.4)	14% (8-20%)	19% (11-27%)	19 (10-26)	870 (500-1200)
Montenegro	1995	Male	50 to 54	77% (56-91%)	23% (9.4-44%)	5.8 (3-9.9)	15% (8.7-19%)	19% (11-25%)	22 (13-30)	900 (540-1200)
Montenegro	1995	Male	55 to 59	76% (54-91%)	24% (9.4-46%)	5.9 (3.2-10)	15% (9.1-19%)	18% (11-24%)	40 (24-55)	1400 (840-1900)
Montenegro	1995	Male	60 to 64	75% (50-91%)	25% (9.4-50%)	5.7 (2.9-10)	14% (7.6-19%)	17% (9.3-23%)	52 (28-73)	1500 (840-2100)
Montenegro	1995	Male	65 to 69	74% (49-90%)	26% (10-51%)	5.2 (2.7-8.6)	13% (6.9-19%)	16% (8.1-23%)	58 (30-84)	1400 (720-2000)
Montenegro	1995	Male	70 to 74	71% (49-87%)	29% (13-51%)	4.5 (2.2-8.2)	12% (5.1-19%)	14% (5.9-22%)	45 (19-69)	860 (370-1300)
Montenegro	1995	Male	75 to 79	68% (46-86%)	32% (14-54%)	3.8 (1.7-7.5)	11% (3.2-18%)	12% (3.6-21%)	30 (8.7-51)	440 (140-750)

Location	Year	Sex	Age	Prevalence of current drinkers	Prevalence of abstainers	Population average of standard drinks daily	Percent of total attributable deaths	Percent of total attributable DALYs	Attributable deaths	Attributable DALYs
Montenegro	1995	Male	80 to 84	66% (42-84%)	34% (16-58%)	3.4 (1.5-6.4)	10% (3.1-18%)	12% (3.5-21%)	26 (7.9-47)	280 (90-500)
Montenegro	1995	Male	85 to 89	65% (43-83%)	35% (17-57%)	3.1 (1.4-5.7)	8.7% (2.1-17%)	10% (2.3-19%)	23 (5-43)	170 (43-330)
Montenegro	1995	Male	90 to 94	64% (44-85%)	36% (15-56%)	2.8 (1.2-5.4)	6.9% (0.71-15%)	7.9% (0.66-17%)	9.9 (0.83-21)	52 (5.2-110)
Montenegro	1995	Male	95 plus	65% (39-88%)	35% (12-61%)	2.6 (0.89-5.7)	5.7% (-0.3-14%)	6.5% (-0.71-16%)	2.2 (-0.24-5.5)	7.7 (-0.41-19)
Montenegro	2000	Female	All Ages	58% (50-66%)	42% (34-50%)	2.1 (1.5-3)	7.5% (4.6-11%)	11% (5.8-17%)	300 (150-460)	6400 (3900-9200)
Montenegro	2000	Female	15 to 19	58% (38-74%)	42% (26-62%)	1.6 (0.65-3)	3.3% (1.9-5.4%)	8.3% (4.1-15%)	0.68 (0.33-1.2)	75 (44-120)
Montenegro	2000	Female	20 to 24	67% (49-83%)	33% (17-51%)	2.2 (0.91-4.3)	5.2% (3.2-8%)	10% (4.6-18%)	1.1 (0.48-1.9)	130 (76-200)
Montenegro	2000	Female	25 to 29	69% (50-84%)	31% (16-50%)	2.4 (1-4.5)	5.5% (3.4-8.1%)	11% (5.2-19%)	1.4 (0.65-2.3)	150 (89-220)
Montenegro	2000	Female	30 to 34	70% (49-84%)	30% (16-51%)	2.5 (1.2-4.5)	5.8% (3.6-8.5%)	13% (6.5-20%)	2 (1-3.1)	170 (100-250)
Montenegro	2000	Female	35 to 39	70% (52-84%)	30% (16-48%)	2.8 (1.3-5.2)	6.5% (3.7-9.8%)	14% (6.8-21%)	3.7 (1.8-5.7)	250 (140-370)
Montenegro	2000	Female	40 to 44	70% (52-84%)	30% (16-48%)	3.1 (1.5-5.6)	7.6% (4.1-11%)	15% (8-23%)	6.2 (3.1-9.5)	340 (180-520)
Montenegro	2000	Female	45 to 49	68% (46-84%)	32% (16-54%)	3.4 (1.7-6.5)	8.8% (4.5-13%)	16% (8.3-24%)	11 (5.4-16)	500 (270-760)
Montenegro	2000	Female	50 to 54	65% (45-81%)	35% (19-55%)	3.5 (1.7-6.2)	9.7% (4.9-14%)	16% (8.5-24%)	13 (6.6-19)	520 (270-770)
Montenegro	2000	Female	55 to 59	62% (42-80%)	38% (20-58%)	3.5 (1.6-6.5)	10% (5-16%)	16% (8.1-24%)	16 (7.9-24)	540 (270-830)
Montenegro	2000	Female	60 to 64	59% (38-78%)	41% (22-62%)	3.2 (1.4-6.3)	9.6% (4.1-15%)	14% (6.1-23%)	25 (11-39)	720 (310-1100)
Montenegro	2000	Female	65 to 69	54% (34-73%)	46% (27-66%)	2.7 (1.4-5.1)	9.6% (4.2-16%)	13% (5.8-22%)	35 (15-58)	840 (370-1400)
Montenegro	2000	Female	70 to 74	48% (30-66%)	52% (34-70%)	2.2 (0.9-4.3)	9% (2.7-16%)	12% (3.5-21%)	41 (12-73)	780 (240-1400)
Montenegro	2000	Female	75 to 79	41% (25-60%)	59% (40-75%)	1.8 (0.67-3.6)	8.1% (2.2-15%)	10% (2.6-19%)	37 (9.4-67)	530 (140-970)
Montenegro	2000	Female	80 to 84	36% (21-54%)	64% (46-79%)	1.6 (0.61-3.1)	8.2% (2.8-15%)	10% (3.3-18%)	33 (11-59)	350 (120-630)
Montenegro	2000	Female	85 to 89	34% (21-50%)	66% (50-79%)	1.6 (0.61-3.3)	8.5% (3-15%)	10% (3.6-19%)	38 (13-68)	280 (95-510)
Montenegro	2000	Female	90 to 94	36% (23-53%)	64% (47-77%)	1.7 (0.59-3.8)	8.3% (2.3-15%)	10% (2.8-19%)	29 (8.1-55)	150 (43-280)
Montenegro	2000	Female	95 plus	40% (23-61%)	60% (39-77%)	1.9 (0.53-4.8)	8.2% (1.7-17%)	10% (2.1-20%)	12 (2.6-24)	39 (8.3-78)

Location	Year	Sex	Age	Prevalence of current drinkers	Prevalence of abstainers	Population average of standard drinks daily	Percent of total attributable deaths	Percent of total attributable DALYs	Attributable deaths	Attributable DALYs
Montenegro	2000	Male	All Ages	66% (55-77%)	34% (23-45%)	3.3 (2.3-4.6)	11% (7.9-15%)	15% (8.7-20%)	430 (250-600)	12000 (8100-16000)
Montenegro	2000	Male	15 to 19	58% (36-79%)	42% (21-64%)	1.5 (0.71-2.7)	4.7% (2.9-7.3%)	7.7% (4-13%)	1.4 (0.71-2.3)	140 (84-210)
Montenegro	2000	Male	20 to 24	69% (47-86%)	31% (14-53%)	2.7 (1.3-4.8)	9.1% (5.8-13%)	13% (7.2-21%)	3.2 (1.7-5)	300 (190-440)
Montenegro	2000	Male	25 to 29	73% (53-89%)	27% (11-47%)	3.5 (1.7-6.3)	11% (7-15%)	16% (9.1-24%)	4.5 (2.6-6.8)	380 (240-540)
Montenegro	2000	Male	30 to 34	76% (55-91%)	24% (9.5-45%)	4 (2.1-7)	12% (7.8-16%)	18% (11-26%)	6 (3.5-8.7)	440 (280-610)
Montenegro	2000	Male	35 to 39	78% (58-91%)	22% (9.1-42%)	4.2 (2.3-7.2)	12% (7.6-17%)	18% (11-26%)	9.6 (5.5-14)	600 (380-850)
Montenegro	2000	Male	40 to 44	79% (58-92%)	21% (8.3-42%)	4.8 (2.7-7.7)	13% (8-18%)	19% (11-27%)	15 (8.8-22)	820 (500-1200)
Montenegro	2000	Male	45 to 49	79% (56-91%)	21% (8.7-44%)	5.6 (3-9.2)	14% (8.7-19%)	20% (11-26%)	26 (15-35)	1200 (710-1600)
Montenegro	2000	Male	50 to 54	77% (56-90%)	23% (9.6-44%)	5.9 (3.3-9.4)	15% (9.2-20%)	19% (12-25%)	29 (17-38)	1100 (710-1500)
Montenegro	2000	Male	55 to 59	76% (54-91%)	24% (9-46%)	6 (3.3-10)	15% (9.6-20%)	19% (12-25%)	32 (20-43)	1100 (700-1500)
Montenegro	2000	Male	60 to 64	74% (51-91%)	26% (9.4-49%)	5.8 (3-10)	14% (8.2-19%)	17% (9.9-24%)	52 (31-73)	1500 (880-2100)
Montenegro	2000	Male	65 to 69	73% (50-90%)	27% (10-50%)	5.2 (2.9-8.4)	14% (7.5-19%)	17% (8.9-23%)	67 (37-95)	1600 (900-2200)
Montenegro	2000	Male	70 to 74	71% (49-87%)	29% (13-51%)	4.5 (2.3-8)	12% (5.4-19%)	15% (6.4-22%)	67 (29-100)	1300 (560-1900)
Montenegro	2000	Male	75 to 79	68% (46-86%)	32% (14-54%)	3.8 (1.8-7.1)	11% (3.7-18%)	13% (4.1-21%)	52 (16-87)	760 (250-1300)
Montenegro	2000	Male	80 to 84	67% (43-84%)	33% (16-57%)	3.4 (1.6-6)	11% (3.6-18%)	12% (4.1-21%)	33 (11-58)	350 (120-610)
Montenegro	2000	Male	85 to 89	65% (43-83%)	35% (17-57%)	3.1 (1.5-5.6)	9.2% (2.5-17%)	11% (2.8-20%)	18 (4.6-34)	140 (37-250)
Montenegro	2000	Male	90 to 94	65% (43-85%)	35% (15-57%)	2.9 (1.3-5.4)	7.3% (1.2-15%)	8.4% (1.2-18%)	9.1 (1.3-19)	48 (7.6-99)
Montenegro	2000	Male	95 plus	65% (38-88%)	35% (12-62%)	2.6 (0.92-5.7)	6% (0.042-14%)	6.8% (-0.29-16%)	2.6 (-0.11-6.2)	9.1 (0.064-21)
Montenegro	2005	Female	All Ages	58% (50-66%)	42% (34-50%)	2.3 (1.7-3)	7.9% (5-11%)	12% (6.7-18%)	310 (170-460)	6700 (4300-9200)
Montenegro	2005	Female	15 to 19	58% (38-75%)	42% (25-62%)	1.7 (0.72-3.2)	3.4% (2-5.4%)	8.7% (4.2-15%)	0.65 (0.32-1.1)	72 (41-110)
Montenegro	2005	Female	20 to 24	67% (50-82%)	33% (18-50%)	2.3 (0.99-4.4)	5.4% (3.2-8.1%)	11% (4.8-18%)	1.2 (0.53-2)	140 (81-210)
Montenegro	2005	Female	25 to 29	69% (50-83%)	31% (17-50%)	2.5 (1.1-4.6)	5.6% (3.5-8.1%)	12% (5.5-19%)	1.4 (0.65-2.3)	150 (91-220)

Location	Year	Sex	Age	Prevalence of current drinkers	Prevalence of abstainers	Population average of standard drinks daily	Percent of total attributable deaths	Percent of total attributable DALYs	Attributable deaths	Attributable DALYs
Montenegro	2005	Female	30 to 34	70% (50-84%)	30% (16-50%)	2.7 (1.3-4.8)	5.7% (3.6-8.4%)	13% (6.9-20%)	2 (1.1-3.1)	170 (100-250)
Montenegro	2005	Female	35 to 39	70% (53-84%)	30% (16-47%)	2.9 (1.4-5.3)	6.5% (3.7-9.6%)	14% (7.2-21%)	3.1 (1.6-4.8)	210 (120-310)
Montenegro	2005	Female	40 to 44	69% (52-84%)	31% (16-48%)	3.3 (1.6-5.6)	7.6% (4.4-11%)	15% (8.3-23%)	5.7 (3.1-8.6)	320 (180-470)
Montenegro	2005	Female	45 to 49	68% (45-83%)	32% (17-55%)	3.6 (1.8-6.8)	8.8% (5-13%)	16% (9.1-24%)	9.5 (5.4-14)	460 (260-670)
Montenegro	2005	Female	50 to 54	65% (45-81%)	35% (19-55%)	3.7 (1.9-6.4)	9.5% (5.2-14%)	16% (8.9-23%)	15 (8.6-22)	630 (360-910)
Montenegro	2005	Female	55 to 59	62% (41-79%)	38% (21-59%)	3.6 (1.8-6.4)	10% (5.3-15%)	16% (8.6-23%)	17 (9.6-26)	610 (330-910)
Montenegro	2005	Female	60 to 64	58% (37-78%)	42% (22-63%)	3.3 (1.5-6.3)	9.6% (4.5-15%)	14% (6.9-22%)	19 (9.2-30)	570 (270-910)
Montenegro	2005	Female	65 to 69	54% (34-74%)	46% (26-66%)	2.9 (1.4-5.1)	9.8% (4.4-15%)	14% (6.3-22%)	36 (16-56)	860 (390-1400)
Montenegro	2005	Female	70 to 74	47% (29-66%)	53% (34-71%)	2.3 (0.97-4.3)	9.3% (3-16%)	12% (4-21%)	48 (16-84)	920 (300-1600)
Montenegro	2005	Female	75 to 79	40% (26-60%)	60% (40-74%)	1.9 (0.73-3.7)	8.5% (2.5-15%)	11% (3.1-19%)	50 (14-89)	740 (210-1300)
Montenegro	2005	Female	80 to 84	36% (21-54%)	64% (46-79%)	1.7 (0.66-3.2)	8.6% (3.1-15%)	11% (4-19%)	41 (15-71)	440 (160-760)
Montenegro	2005	Female	85 to 89	34% (21-51%)	66% (49-79%)	1.7 (0.64-3.4)	9% (3.5-16%)	11% (4.2-19%)	30 (12-52)	230 (88-390)
Montenegro	2005	Female	90 to 94	36% (23-53%)	64% (47-77%)	1.8 (0.64-3.9)	8.8% (2.9-16%)	11% (3.5-19%)	22 (7.1-39)	110 (36-200)
Montenegro	2005	Female	95 plus	40% (23-61%)	60% (39-77%)	2 (0.56-4.7)	8.7% (1.9-17%)	11% (2.5-20%)	11 (2.7-21)	37 (8.1-71)
Montenegro	2005	Male	All Ages	67% (56-78%)	33% (22-44%)	3.6 (2.7-4.7)	12% (8.9-15%)	15% (10-21%)	460 (290-620)	12000 (8900-15000)
Montenegro	2005	Male	15 to 19	59% (36-80%)	41% (20-64%)	1.6 (0.79-2.9)	4.5% (2.9-6.8%)	8.2% (4.4-14%)	0.96 (0.52-1.6)	110 (69-170)
Montenegro	2005	Male	20 to 24	69% (48-86%)	31% (14-52%)	2.9 (1.4-4.9)	9.6% (6.3-14%)	14% (7.8-22%)	3.8 (2.1-6)	350 (220-510)
Montenegro	2005	Male	25 to 29	73% (52-89%)	27% (11-48%)	3.7 (1.9-6.4)	11% (7.4-16%)	17% (10-26%)	4.7 (2.7-7.1)	390 (260-560)
Montenegro	2005	Male	30 to 34	76% (55-91%)	24% (9.3-45%)	4.2 (2.3-7)	11% (7.9-16%)	19% (12-27%)	4.8 (3-6.9)	370 (250-510)
Montenegro	2005	Male	35 to 39	78% (59-91%)	22% (9.3-41%)	4.5 (2.4-7.3)	12% (7.9-17%)	19% (12-27%)	6.9 (4.1-9.8)	450 (290-610)
Montenegro	2005	Male	40 to 44	79% (58-91%)	21% (8.5-42%)	5 (2.8-7.8)	13% (8.3-18%)	20% (12-27%)	12 (7.5-17)	680 (440-920)
Montenegro	2005	Male	45 to 49	78% (57-91%)	22% (8.6-43%)	5.8 (3.3-9)	15% (9.4-19%)	20% (13-26%)	22 (14-30)	1000 (690-1400)

Location	Year	Sex	Age	Prevalence of current drinkers	Prevalence of abstainers	Population average of standard drinks daily	Percent of total attributable deaths	Percent of total attributable DALYs	Attributable deaths	Attributable DALYs
Montenegro	2005	Male	50 to 54	77% (56-91%)	23% (9.4-44%)	6.2 (3.7-9.4)	15% (10-19%)	20% (13-25%)	34 (22-45)	1400 (940-1800)
Montenegro	2005	Male	55 to 59	76% (54-91%)	24% (9.2-46%)	6.4 (3.7-10)	16% (10-20%)	19% (13-25%)	44 (30-58)	1500 (1000-2000)
Montenegro	2005	Male	60 to 64	74% (52-91%)	26% (9.1-48%)	6.2 (3.3-10)	15% (9.1-20%)	18% (11-24%)	44 (27-59)	1300 (780-1700)
Montenegro	2005	Male	65 to 69	73% (50-90%)	27% (10-50%)	5.5 (3.2-8.8)	14% (8.4-19%)	17% (10-23%)	70 (40-95)	1700 (980-2300)
Montenegro	2005	Male	70 to 74	71% (50-86%)	29% (14-50%)	4.7 (2.5-8)	13% (6-19%)	15% (7.1-22%)	71 (34-100)	1400 (660-2000)
Montenegro	2005	Male	75 to 79	68% (44-86%)	32% (14-56%)	4 (1.9-7.2)	12% (4.4-18%)	14% (5-21%)	66 (24-110)	960 (370-1500)
Montenegro	2005	Male	80 to 84	66% (42-84%)	34% (16-58%)	3.6 (1.7-6.1)	11% (4.2-18%)	13% (4.7-21%)	42 (15-70)	450 (170-740)
Montenegro	2005	Male	85 to 89	64% (42-82%)	36% (18-58%)	3.3 (1.6-5.6)	10% (3.5-17%)	12% (3.9-20%)	20 (6.6-35)	150 (52-260)
Montenegro	2005	Male	90 to 94	64% (42-84%)	36% (16-58%)	3 (1.4-5.5)	8.1% (1.6-16%)	9.4% (1.8-18%)	6.8 (1.3-13)	35 (7.2-68)
Montenegro	2005	Male	95 plus	64% (38-87%)	36% (13-62%)	2.8 (0.99-5.7)	6.8% (0.29-14%)	7.8% (0.096-17%)	2.3 (0.028-5)	8 (0.36-17)
Montenegro	2010	Female	All Ages	58% (50-66%)	42% (34-50%)	2.4 (1.8-3.1)	8.1% (5.4-11%)	12% (7.4-18%)	310 (180-460)	6600 (4400-8900)
Montenegro	2010	Female	15 to 19	57% (37-75%)	43% (25-63%)	1.7 (0.74-3.3)	3.2% (1.9-5.1%)	9% (4.3-16%)	0.51 (0.25-0.89)	61 (36-98)
Montenegro	2010	Female	20 to 24	68% (50-83%)	32% (17-50%)	2.5 (1-4.6)	5.3% (3.2-7.9%)	11% (5-19%)	0.86 (0.39-1.5)	110 (66-170)
Montenegro	2010	Female	25 to 29	69% (49-84%)	31% (16-51%)	2.6 (1.2-4.9)	5.5% (3.5-7.8%)	12% (6-19%)	1.3 (0.61-2.1)	150 (88-210)
Montenegro	2010	Female	30 to 34	70% (50-84%)	30% (16-50%)	2.8 (1.4-5.1)	5.6% (3.7-8.1%)	13% (7.4-20%)	1.8 (0.99-2.9)	170 (110-240)
Montenegro	2010	Female	35 to 39	70% (52-85%)	30% (15-48%)	3.1 (1.5-5.5)	6.4% (3.8-9.3%)	14% (7.6-21%)	3 (1.6-4.7)	220 (130-310)
Montenegro	2010	Female	40 to 44	69% (52-84%)	31% (16-48%)	3.5 (1.6-5.9)	7.6% (4.5-11%)	16% (8.9-23%)	5.1 (2.8-7.6)	290 (170-420)
Montenegro	2010	Female	45 to 49	68% (46-83%)	32% (17-54%)	3.8 (1.9-6.9)	8.6% (5-13%)	16% (9.5-23%)	9.1 (5.4-13)	450 (270-640)
Montenegro	2010	Female	50 to 54	65% (45-81%)	35% (19-55%)	3.9 (2-6.8)	9.4% (5.5-14%)	16% (9.9-23%)	13 (7.7-19)	560 (330-790)
Montenegro	2010	Female	55 to 59	62% (42-78%)	38% (22-58%)	3.8 (1.9-6.7)	9.8% (5.5-14%)	16% (9.1-23%)	21 (12-30)	740 (410-1100)
Montenegro	2010	Female	60 to 64	58% (37-77%)	42% (23-63%)	3.5 (1.7-6.7)	9.6% (4.6-15%)	15% (6.9-22%)	22 (10-33)	650 (310-1000)
Montenegro	2010	Female	65 to 69	54% (34-75%)	46% (25-66%)	3 (1.5-5.2)	10% (4.9-16%)	15% (7-22%)	28 (14-43)	690 (330-1100)

Location	Year	Sex	Age	Prevalence of current drinkers	Prevalence of abstainers	Population average of standard drinks daily	Percent of total attributable deaths	Percent of total attributable DALYs	Attributable deaths	Attributable DALYs
Montenegro	2010	Female	70 to 74	47% (30-66%)	53% (34-70%)	2.4 (1.1-4.7)	9.4% (3.4-16%)	13% (4.7-22%)	46 (17-79)	890 (320-1500)
Montenegro	2010	Female	75 to 79	40% (26-60%)	60% (40-74%)	2 (0.8-3.9)	8.6% (2.9-15%)	11% (3.6-20%)	48 (15-85)	710 (220-1300)
Montenegro	2010	Female	80 to 84	36% (21-54%)	64% (46-79%)	1.8 (0.74-3.3)	8.8% (3.4-15%)	11% (4.3-19%)	46 (17-77)	500 (190-830)
Montenegro	2010	Female	85 to 89	34% (21-50%)	66% (50-79%)	1.8 (0.7-3.5)	9.1% (3.6-15%)	11% (4.5-19%)	40 (16-69)	300 (120-510)
Montenegro	2010	Female	90 to 94	36% (22-53%)	64% (47-78%)	1.9 (0.7-3.8)	8.9% (3.2-16%)	11% (4-19%)	19 (6.7-34)	96 (34-170)
Montenegro	2010	Female	95 plus	39% (22-60%)	61% (40-78%)	2.1 (0.61-4.6)	8.8% (2.3-16%)	11% (2.8-20%)	8.5 (2.3-16)	28 (7.3-53)
Montenegro	2010	Male	All Ages	67% (56-77%)	33% (23-44%)	3.8 (3-4.9)	12% (9.4-15%)	16% (10-21%)	460 (310-610)	12000 (9200-15000)
Montenegro	2010	Male	15 to 19	58% (36-79%)	42% (21-64%)	1.7 (0.83-3)	4.9% (3.1-7.2%)	8.7% (4.7-14%)	1.1 (0.56-1.8)	110 (71-180)
Montenegro	2010	Male	20 to 24	69% (48-87%)	31% (13-52%)	3.1 (1.6-5.3)	10% (6.6-15%)	15% (8.5-23%)	3.4 (1.9-5.4)	310 (200-460)
Montenegro	2010	Male	25 to 29	73% (53-89%)	27% (11-47%)	3.9 (2-6.6)	12% (7.7-16%)	18% (11-27%)	5.1 (3.1-7.7)	430 (280-610)
Montenegro	2010	Male	30 to 34	76% (54-91%)	24% (9.4-46%)	4.5 (2.6-7.5)	12% (8.4-16%)	20% (13-28%)	5.4 (3.5-7.6)	420 (280-560)
Montenegro	2010	Male	35 to 39	78% (59-91%)	22% (9-41%)	4.8 (2.6-7.6)	13% (8.4-17%)	21% (13-28%)	7.8 (4.8-11)	510 (330-680)
Montenegro	2010	Male	40 to 44	79% (58-92%)	21% (8.4-42%)	5.4 (3.1-8.2)	14% (9.3-18%)	21% (14-28%)	12 (7.9-17)	680 (450-890)
Montenegro	2010	Male	45 to 49	78% (54-91%)	22% (8.7-46%)	6.2 (3.6-9.6)	15% (10-19%)	21% (14-27%)	21 (14-28)	1000 (700-1300)
Montenegro	2010	Male	50 to 54	77% (55-91%)	23% (9.3-45%)	6.5 (4-9.7)	15% (11-19%)	20% (14-25%)	30 (21-39)	1200 (860-1600)
Montenegro	2010	Male	55 to 59	75% (54-91%)	25% (9.3-46%)	6.7 (3.9-10)	15% (11-20%)	20% (14-25%)	51 (36-68)	1800 (1300-2300)
Montenegro	2010	Male	60 to 64	74% (50-90%)	26% (9.5-50%)	6.5 (3.5-10)	15% (9.6-19%)	18% (12-24%)	48 (32-64)	1400 (930-1900)
Montenegro	2010	Male	65 to 69	73% (49-89%)	27% (11-51%)	5.8 (3.3-8.9)	14% (8.7-19%)	18% (11-23%)	48 (29-64)	1200 (710-1500)
Montenegro	2010	Male	70 to 74	71% (48-87%)	29% (13-52%)	5 (2.7-8.2)	13% (6.6-18%)	16% (7.8-22%)	68 (34-98)	1300 (680-1900)
Montenegro	2010	Male	75 to 79	68% (45-86%)	32% (14-55%)	4.2 (2.1-7.5)	12% (5.1-18%)	14% (5.8-21%)	66 (28-100)	980 (410-1500)
Montenegro	2010	Male	80 to 84	66% (41-85%)	34% (15-59%)	3.8 (1.8-6.3)	11% (4.9-18%)	14% (5.6-21%)	49 (20-77)	530 (220-840)
Montenegro	2010	Male	85 to 89	64% (42-83%)	36% (17-58%)	3.4 (1.7-5.9)	10% (3.6-17%)	12% (4.1-21%)	30 (10-52)	230 (82-390)

Location	Year	Sex	Age	Prevalence of current drinkers	Prevalence of abstainers	Population average of standard drinks daily	Percent of total attributable deaths	Percent of total attributable DALYs	Attributable deaths	Attributable DALYs
Montenegro	2010	Male	90 to 94	64% (44-84%)	36% (16-56%)	3.2 (1.5-5.7)	8.3% (1.9-15%)	9.7% (2.1-18%)	9 (2-17)	47 (11-88)
Montenegro	2010	Male	95 plus	65% (39-87%)	35% (13-61%)	2.9 (1.1-5.7)	7% (0.67-14%)	8.1% (0.54-17%)	1.9 (0.13-3.9)	6.7 (0.66-13)
Montenegro	2016	Female	All Ages	57% (49-66%)	43% (34-51%)	2.6 (1.8-3.5)	8.2% (5.1-11%)	12% (7.2-18%)	350 (190-520)	6900 (4300-9500)
Montenegro	2016	Female	15 to 19	57% (38-75%)	43% (25-62%)	1.8 (0.74-3.5)	3.2% (1.8-5.2%)	9.3% (4.4-16%)	0.46 (0.22-0.84)	57 (32-94)
Montenegro	2016	Female	20 to 24	67% (48-82%)	33% (18-52%)	2.6 (1-4.9)	5.1% (3.2-7.7%)	11% (5-19%)	0.74 (0.32-1.3)	100 (61-160)
Montenegro	2016	Female	25 to 29	69% (50-83%)	31% (17-50%)	2.8 (1.2-5.3)	5.3% (3.4-7.6%)	12% (5.7-20%)	0.91 (0.43-1.5)	120 (70-170)
Montenegro	2016	Female	30 to 34	70% (49-83%)	30% (17-51%)	3 (1.4-5.4)	5.4% (3.5-7.6%)	13% (7.2-20%)	1.6 (0.83-2.4)	160 (97-220)
Montenegro	2016	Female	35 to 39	70% (52-85%)	30% (15-48%)	3.2 (1.4-5.7)	6.1% (3.6-8.8%)	14% (7.6-21%)	2.7 (1.4-4.1)	200 (120-290)
Montenegro	2016	Female	40 to 44	69% (51-83%)	31% (17-49%)	3.6 (1.7-6.3)	7.3% (4.2-11%)	16% (8.4-23%)	4.8 (2.6-7.2)	290 (160-420)
Montenegro	2016	Female	45 to 49	67% (46-82%)	33% (18-54%)	4 (1.8-7.6)	8.5% (4.9-12%)	16% (9.5-23%)	7.7 (4.4-11)	390 (220-560)
Montenegro	2016	Female	50 to 54	64% (44-81%)	36% (19-56%)	4.1 (2-7.3)	9.4% (5.6-14%)	16% (9.8-23%)	13 (7.7-19)	560 (320-810)
Montenegro	2016	Female	55 to 59	61% (42-79%)	39% (21-58%)	4 (1.9-7)	9.8% (5.4-14%)	16% (9.1-23%)	18 (10-27)	660 (360-970)
Montenegro	2016	Female	60 to 64	58% (37-77%)	42% (23-63%)	3.7 (1.7-6.9)	9.5% (4.7-15%)	15% (7.5-23%)	27 (13-42)	830 (410-1300)
Montenegro	2016	Female	65 to 69	54% (34-74%)	46% (26-66%)	3.2 (1.5-5.6)	10% (4.9-15%)	15% (7.2-22%)	34 (16-53)	840 (400-1300)
Montenegro	2016	Female	70 to 74	47% (30-65%)	53% (35-70%)	2.6 (1.1-4.9)	9.4% (3.3-15%)	13% (4.9-21%)	36 (13-62)	710 (240-1200)
Montenegro	2016	Female	75 to 79	40% (25-58%)	60% (42-75%)	2.1 (0.79-4.1)	8.6% (2.7-15%)	11% (3.6-19%)	54 (17-94)	800 (250-1400)
Montenegro	2016	Female	80 to 84	35% (21-54%)	65% (46-79%)	1.9 (0.77-3.6)	8.9% (3.5-15%)	11% (4.4-19%)	52 (19-87)	570 (210-950)
Montenegro	2016	Female	85 to 89	34% (21-50%)	66% (50-79%)	1.9 (0.73-3.7)	9.2% (3.4-15%)	11% (4.2-19%)	51 (19-88)	390 (150-660)
Montenegro	2016	Female	90 to 94	36% (22-52%)	64% (48-78%)	2 (0.72-4.4)	9.1% (3.4-16%)	11% (4.1-19%)	32 (12-58)	160 (61-290)
Montenegro	2016	Female	95 plus	39% (22-60%)	61% (40-78%)	2.2 (0.62-5)	9% (2.5-16%)	11% (3.1-21%)	9.7 (2.8-18)	32 (8.9-59)
Montenegro	2016	Male	All Ages	67% (57-77%)	33% (23-43%)	4.1 (2.9-5.6)	13% (9.5-16%)	16% (11-21%)	500 (330-680)	13000 (9400-16000)
Montenegro	2016	Male	15 to 19	58% (36-79%)	42% (21-64%)	1.8 (0.84-3.3)	5% (3.1-7.8%)	9.1% (4.7-16%)	1 (0.53-1.8)	110 (69-180)

Location	Year	Sex	Age	Prevalence of current drinkers	Prevalence of abstainers	Population average of standard drinks daily	Percent of total attributable deaths	Percent of total attributable DALYs	Attributable deaths	Attributable DALYs
Montenegro	2016	Male	20 to 24	69% (47-87%)	31% (13-53%)	3.2 (1.6-5.7)	10% (6.8-15%)	16% (8.8-25%)	3.3 (1.8-5.2)	310 (190-460)
Montenegro	2016	Male	25 to 29	73% (53-89%)	27% (11-47%)	4.1 (2.1-7.4)	12% (8-17%)	19% (11-29%)	4.2 (2.5-6.5)	370 (240-520)
Montenegro	2016	Male	30 to 34	75% (54-91%)	25% (9.4-46%)	4.7 (2.5-8)	12% (8.3-16%)	21% (13-29%)	5.8 (3.6-8)	450 (300-610)
Montenegro	2016	Male	35 to 39	78% (58-90%)	22% (9.6-42%)	5 (2.6-8.4)	13% (8.6-17%)	21% (13-29%)	8.2 (5-11)	540 (360-710)
Montenegro	2016	Male	40 to 44	79% (59-92%)	21% (8.5-41%)	5.7 (3.1-9.2)	14% (9.4-19%)	22% (14-29%)	13 (8-17)	700 (460-900)
Montenegro	2016	Male	45 to 49	78% (57-91%)	22% (8.8-43%)	6.5 (3.5-11)	15% (10-19%)	22% (14-27%)	19 (12-24)	900 (600-1100)
Montenegro	2016	Male	50 to 54	77% (57-91%)	23% (9.4-43%)	7 (4-11)	15% (11-20%)	21% (15-26%)	30 (21-40)	1200 (870-1600)
Montenegro	2016	Male	55 to 59	75% (54-90%)	25% (9.7-46%)	7.1 (3.9-11)	15% (11-20%)	20% (14-26%)	44 (31-60)	1600 (1100-2100)
Montenegro	2016	Male	60 to 64	74% (50-90%)	26% (9.8-50%)	6.8 (3.6-11)	15% (9.8-19%)	18% (12-24%)	63 (41-85)	1900 (1200-2500)
Montenegro	2016	Male	65 to 69	73% (49-89%)	27% (11-51%)	6.2 (3.3-9.5)	14% (9-19%)	18% (11-24%)	61 (38-85)	1500 (970-2000)
Montenegro	2016	Male	70 to 74	70% (48-86%)	30% (14-52%)	5.3 (2.7-8.9)	13% (6.8-18%)	16% (8.3-23%)	51 (26-73)	990 (510-1400)
Montenegro	2016	Male	75 to 79	68% (44-85%)	32% (15-56%)	4.5 (2.1-7.9)	12% (4.8-18%)	14% (5.8-22%)	70 (29-110)	1100 (450-1600)
Montenegro	2016	Male	80 to 84	66% (41-84%)	34% (16-59%)	4 (2-6.8)	12% (5.1-19%)	14% (5.9-22%)	63 (27-100)	680 (300-1100)
Montenegro	2016	Male	85 to 89	64% (41-83%)	36% (17-59%)	3.7 (1.8-6.4)	11% (3.9-17%)	13% (4.3-21%)	43 (15-73)	330 (120-550)
Montenegro	2016	Male	90 to 94	64% (43-84%)	36% (16-57%)	3.4 (1.6-6.6)	8.9% (2.4-16%)	10% (2.8-19%)	18 (4.6-32)	93 (25-170)
Montenegro	2016	Male	95 plus	65% (39-87%)	35% (13-61%)	3.1 (1.1-6.3)	7.6% (0.78-15%)	8.8% (0.74-18%)	3.6 (0.3-7.1)	13 (1.3-24)
Poland	1990	Female	All Ages	76% (71-81%)	24% (19-29%)	1.5 (1.4-1.7)	2% (0.66-3.4%)	1.1% (-1.4-3.6%)	2000 (-2600-6600)	120000 (36000-190000)
Poland	1990	Female	15 to 19	76% (63-87%)	24% (13-37%)	1.4 (0.89-2.1)	2.5% (1.5-3.7%)	6.3% (3.2-9.8%)	30 (15-47)	3300 (2000-4900)
Poland	1990	Female	20 to 24	83% (71-90%)	17% (9.8-29%)	1.8 (1.1-2.5)	3.2% (2.2-4.5%)	6.3% (3.2-10%)	29 (15-46)	4000 (2700-5700)
Poland	1990	Female	25 to 29	84% (71-91%)	16% (8.7-29%)	1.7 (1.2-2.4)	3.3% (2.3-4.5%)	6% (3.4-9%)	38 (22-57)	5000 (3400-6900)
Poland	1990	Female	30 to 34	84% (71-92%)	16% (8.1-29%)	1.8 (1.2-2.5)	3.4% (2.4-4.7%)	6.5% (3.8-9.5%)	81 (47-120)	7900 (5300-11000)
Poland	1990	Female	35 to 39	84% (73-92%)	16% (8.2-27%)	1.9 (1.3-2.6)	3.9% (2.6-5.3%)	7.2% (4.5-10%)	150 (91-210)	11000 (7400-15000)

Location	Year	Sex	Age	Prevalence of current drinkers	Prevalence of abstainers	Population average of standard drinks daily	Percent of total attributable deaths	Percent of total attributable DALYs	Attributable deaths	Attributable DALYs
Poland	1990	Female	40 to 44	84%<br>(75-91%)	16%<br>(8.6-25%)	2.2<br>(1.5-3)	4.3%<br>(2.7-6%)	7.6%<br>(4.7-11%)	220<br>(140-310)	13000<br>(8300-18000)
Poland	1990	Female	45 to 49	85%<br>(77-90%)	15%<br>(9.7-23%)	2.6<br>(1.8-3.6)	4.9%<br>(3-7.1%)	8.3%<br>(5.2-12%)	250<br>(160-350)	12000<br>(7500-17000)
Poland	1990	Female	50 to 54	85%<br>(78-90%)	15%<br>(10-22%)	2.8<br>(1.9-3.7)	4.8%<br>(2.8-7%)	7.7%<br>(4.5-11%)	380<br>(230-550)	16000<br>(9200-23000)
Poland	1990	Female	55 to 59	84%<br>(78-89%)	16%<br>(11-22%)	2.7<br>(1.9-3.6)	4.2%<br>(2.1-6.5%)	6.3%<br>(3.2-9.5%)	510<br>(260-780)	18000<br>(8900-28000)
Poland	1990	Female	60 to 64	81%<br>(74-86%)	19%<br>(14-26%)	2.5<br>(1.7-3.4)	3.1%<br>(0.52-6%)	4.3%<br>(0.72-8.1%)	550<br>(90-1100)	16000<br>(2700-32000)
Poland	1990	Female	65 to 69	75%<br>(66-82%)	25%<br>(18-34%)	2.2<br>(1.5-3)	1.9%<br>(-0.64-4.4%)	2.5%<br>(-0.9-5.9%)	460<br>(-170-1100)	11000<br>(-3700-26000)
Poland	1990	Female	70 to 74	67%<br>(57-75%)	33%<br>(25-43%)	1.8<br>(1.2-2.6)	0.92%<br>(-1.8-3.7%)	1.1%<br>(-2.5-4.5%)	180<br>(-420-760)	3800<br>(-7700-15000)
Poland	1990	Female	75 to 79	58%<br>(46-70%)	42%<br>(30-54%)	1.5<br>(0.92-2.2)	0.11%<br>(-2.6-2.9%)	0.017%<br>(-3.4-3.5%)	5.4<br>(-1000-1100)	610<br>(-14000-16000)
Poland	1990	Female	80 to 84	53%<br>(40-64%)	47%<br>(36-60%)	1.3<br>(0.83-1.9)	-0.52%<br>(-3-2%)	-0.77%<br>(-3.8-2.3%)	-270<br>(-1300-800)	-2400<br>(-13000-8900)
Poland	1990	Female	85 to 89	52%<br>(39-64%)	48%<br>(36-61%)	1.2<br>(0.74-1.9)	-1.1%<br>(-3.7-1.4%)	-1.5%<br>(-4.6-1.5%)	-370<br>(-1200-400)	-2500<br>(-8400-3200)
Poland	1990	Female	90 to 94	54%<br>(41-67%)	46%<br>(33-59%)	1.3<br>(0.73-2)	-1.5%<br>(-4.2-1.4%)	-2%<br>(-5.3-1.4%)	-210<br>(-570-150)	-990<br>(-2700-880)
Poland	1990	Female	95 plus	58%<br>(41-73%)	42%<br>(27-59%)	1.3<br>(0.66-2.2)	-1.9%<br>(-5.1-1.6%)	-2.5%<br>(-6.5-1.7%)	-67<br>(-170-45)	-210<br>(-560-180)
Poland	1990	Male	All Ages	92%<br>(89-94%)	8%<br>(5.7-11%)	2.9<br>(2.6-3.2)	10%<br>(7.9-12%)	10%<br>(6.8-14%)	21000<br>(14000-29000)	760000<br>(600000-920000)
Poland	1990	Male	15 to 19	88%<br>(78-95%)	12%<br>(5.4-22%)	1.5<br>(0.99-2.3)	5.5%<br>(3.3-8.2%)	9%<br>(4.7-14%)	130<br>(69-200)	11000<br>(6700-16000)
Poland	1990	Male	20 to 24	93%<br>(86-97%)	6.9%<br>(3.1-14%)	2.5<br>(1.7-3.6)	10%<br>(6.6-14%)	14%<br>(8.3-21%)	290<br>(170-430)	23000<br>(15000-32000)
Poland	1990	Male	25 to 29	94%<br>(89-97%)	5.6%<br>(2.5-11%)	3.1<br>(2.1-4.3)	12%<br>(8.9-16%)	17%<br>(11-23%)	420<br>(280-580)	33000<br>(23000-43000)
Poland	1990	Male	30 to 34	95%<br>(90-98%)	4.8%<br>(2.1-9.7%)	3.3<br>(2.4-4.6)	13%<br>(9.6-16%)	17%<br>(12-23%)	730<br>(510-970)	51000<br>(38000-66000)
Poland	1990	Male	35 to 39	96%<br>(91-98%)	4.3%<br>(1.8-8.8%)	3.2<br>(2.1-4.3)	12%<br>(8.5-15%)	15%<br>(10-20%)	920<br>(630-1200)	57000<br>(42000-74000)
Poland	1990	Male	40 to 44	96%<br>(92-98%)	3.8%<br>(1.5-8.2%)	3.9<br>(2.8-5.1)	12%<br>(8.7-16%)	15%<br>(10-21%)	1200<br>(800-1600)	64000<br>(45000-85000)
Poland	1990	Male	45 to 49	97%<br>(93-99%)	3.1%<br>(1.4-6.6%)	5.5<br>(3.7-7.3)	16%<br>(10-21%)	19%<br>(12-25%)	1500<br>(950-2000)	69000<br>(45000-90000)
Poland	1990	Male	50 to 54	97%<br>(94-98%)	3.3%<br>(1.8-6.2%)	6.2<br>(4.4-8)	16%<br>(11-20%)	19%<br>(13-24%)	2500<br>(1700-3200)	99000<br>(69000-120000)
Poland	1990	Male	55 to 59	96%<br>(93-97%)	4.5%<br>(2.6-7.4%)	6.4<br>(4.8-8.2)	15%<br>(11-19%)	17%<br>(12-22%)	3300<br>(2300-4300)	110000<br>(80000-140000)

Location	Year	Sex	Age	Prevalence of current drinkers	Prevalence of abstainers	Population average of standard drinks daily	Percent of total attributable deaths	Percent of total attributable DALYs	Attributable deaths	Attributable DALYs
Poland	1990	Male	60 to 64	95% (92-97%)	5.1% (3-8.4%)	6.1 (4.5-8.2)	13% (8-17%)	14% (9-20%)	3600 (2200-4900)	100000 (65000-140000)
Poland	1990	Male	65 to 69	95% (91-97%)	5.3% (3.1-8.5%)	5.5 (4.1-7.1)	10% (6-15%)	12% (6.6-17%)	3100 (1700-4400)	73000 (42000-100000)
Poland	1990	Male	70 to 74	94% (91-96%)	5.8% (3.6-9.2%)	4.7 (3.3-6.3)	7.6% (2.7-12%)	8.4% (2.7-14%)	1700 (530-2800)	32000 (12000-53000)
Poland	1990	Male	75 to 79	93% (89-96%)	6.7% (3.8-11%)	3.9 (2.6-5.5)	4.9% (-0.023-10%)	5.2% (-0.5-12%)	1400 (-130-3200)	22000 (-100-47000)
Poland	1990	Male	80 to 84	93% (87-96%)	7.5% (4.1-13%)	3.4 (2.3-4.7)	2.6% (-1.7-7.8%)	2.6% (-2.5-8.7%)	610 (-590-2100)	7500 (-4800-23000)
Poland	1990	Male	85 to 89	92% (86-96%)	8% (4.5-14%)	3 (2-4.3)	0.4% (-3.6-5.3%)	-0.017% (-4.8-5.8%)	-2.2 (-580-700)	420 (-3700-5600)
Poland	1990	Male	90 to 94	92% (86-96%)	8.2% (4.3-14%)	2.7 (1.7-3.9)	-1.2% (-5.4-3.6%)	-2% (-6.9-3.8%)	-73 (-250-140)	-270 (-1200-790)
Poland	1990	Male	95 plus	92% (84-97%)	8.4% (3.5-16%)	2.3 (1.2-3.7)	-2.1% (-6-3.1%)	-3.1% (-7.9-3.1%)	-25 (-65-25)	-69 (-200-110)
Poland	1995	Female	All Ages	77% (72-81%)	23% (19-28%)	1.6 (1.4-1.8)	2.2% (0.86-3.6%)	1.2% (-1.2-3.7%)	2200 (-2300-6800)	120000 (48000-200000)
Poland	1995	Female	15 to 19	77% (63-87%)	23% (13-37%)	1.4 (0.9-2)	2.5% (1.5-3.8%)	6.6% (3.3-10%)	35 (17-55)	3800 (2300-5600)
Poland	1995	Female	20 to 24	83% (71-90%)	17% (9.7-29%)	1.7 (1.1-2.5)	3.3% (2.2-4.6%)	6.7% (3.6-10%)	32 (17-50)	4500 (3000-6400)
Poland	1995	Female	25 to 29	84% (72-91%)	16% (8.6-28%)	1.7 (1.2-2.4)	3.4% (2.4-4.5%)	6.6% (3.7-9.7%)	34 (19-50)	4500 (3100-6000)
Poland	1995	Female	30 to 34	84% (71-92%)	16% (8.4-29%)	1.8 (1.2-2.5)	3.6% (2.6-4.8%)	7.5% (4.8-11%)	64 (41-91)	6300 (4500-8400)
Poland	1995	Female	35 to 39	84% (73-92%)	16% (8.4-27%)	1.9 (1.2-2.6)	4.1% (2.9-5.5%)	8.2% (5.4-11%)	150 (98-210)	11000 (7700-15000)
Poland	1995	Female	40 to 44	84% (74-91%)	16% (8.8-26%)	2.2 (1.5-2.9)	4.6% (3-6.3%)	8.4% (5.5-11%)	270 (170-360)	15000 (11000-20000)
Poland	1995	Female	45 to 49	85% (77-91%)	15% (9.4-23%)	2.6 (1.8-3.5)	5.2% (3.4-7.2%)	8.9% (5.9-12%)	380 (250-530)	18000 (12000-25000)
Poland	1995	Female	50 to 54	85% (79-90%)	15% (10-21%)	2.8 (1.9-3.6)	5.2% (3.3-7.3%)	8.4% (5.3-12%)	350 (220-480)	14000 (9000-20000)
Poland	1995	Female	55 to 59	85% (78-89%)	15% (11-22%)	2.7 (1.9-3.6)	4.4% (2.4-6.7%)	6.8% (3.8-10%)	460 (260-690)	16000 (9000-25000)
Poland	1995	Female	60 to 64	81% (73-86%)	19% (14-27%)	2.5 (1.7-3.4)	3.2% (0.83-6%)	4.6% (1.2-8.4%)	520 (140-920)	15000 (4200-28000)
Poland	1995	Female	65 to 69	75% (66-81%)	25% (19-34%)	2.2 (1.5-2.9)	2.1% (-0.44-4.6%)	2.8% (-0.6-6.1%)	500 (-110-1100)	12000 (-2500-26000)
Poland	1995	Female	70 to 74	68% (58-75%)	32% (25-42%)	1.8 (1.2-2.6)	1% (-1.6-3.7%)	1.3% (-2.2-4.7%)	310 (-530-1200)	6300 (-10000-22000)
Poland	1995	Female	75 to 79	59% (46-70%)	41% (30-54%)	1.5 (0.93-2.2)	0.22% (-2.4-3%)	0.15% (-3.1-3.6%)	34 (-700-810)	880 (-10000-12000)

Location	Year	Sex	Age	Prevalence of current drinkers	Prevalence of abstainers	Population average of standard drinks daily	Percent of total attributable deaths	Percent of total attributable DALYs	Attributable deaths	Attributable DALYs
Poland	1995	Female	80 to 84	53% (40-64%)	47% (36-60%)	1.3 (0.82-1.9)	-0.41% (-2.8-1.9%)	-0.64% (-3.6-2.2%)	-220 (-1200-760)	-1800 (-13000-8500)
Poland	1995	Female	85 to 89	53% (40-64%)	47% (36-60%)	1.2 (0.76-1.9)	-0.98% (-3.6-1.4%)	-1.3% (-4.4-1.5%)	-400 (-1300-460)	-2600 (-9100-3700)
Poland	1995	Female	90 to 94	55% (43-67%)	45% (33-57%)	1.3 (0.75-2)	-1.4% (-4.1-1.4%)	-1.9% (-5.2-1.5%)	-250 (-700-210)	-1200 (-3400-1100)
Poland	1995	Female	95 plus	58% (42-73%)	42% (27-58%)	1.3 (0.69-2.2)	-1.8% (-5.1-1.8%)	-2.4% (-6.4-1.9%)	-81 (-220-64)	-250 (-700-250)
Poland	1995	Male	All Ages	93% (90-95%)	7.5% (5.3-10%)	3 (2.7-3.3)	11% (8.9-13%)	11% (7.7-14%)	22000 (16000-29000)	800000 (650000-960000)
Poland	1995	Male	15 to 19	89% (80-95%)	11% (5.1-20%)	1.6 (0.98-2.2)	5.5% (3.2-8%)	9.1% (4.8-14%)	140 (73-210)	12000 (7000-17000)
Poland	1995	Male	20 to 24	93% (87-97%)	6.7% (3.1-13%)	2.5 (1.7-3.6)	10% (6.6-14%)	15% (8.4-21%)	290 (170-420)	24000 (15000-33000)
Poland	1995	Male	25 to 29	95% (89-98%)	5.5% (2.4-11%)	3.1 (2.1-4.4)	13% (9.1-16%)	18% (11-24%)	360 (230-500)	28000 (20000-38000)
Poland	1995	Male	30 to 34	95% (90-98%)	4.8% (1.9-10%)	3.3 (2.4-4.6)	14% (11-18%)	19% (13-25%)	570 (410-770)	40000 (31000-52000)
Poland	1995	Male	35 to 39	96% (91-98%)	4.3% (1.7-8.6%)	3.2 (2.2-4.4)	13% (10-17%)	17% (12-22%)	1000 (720-1300)	61000 (46000-77000)
Poland	1995	Male	40 to 44	96% (92-99%)	3.7% (1.5-7.9%)	3.9 (2.8-5.1)	14% (10-18%)	18% (13-23%)	1600 (1100-2100)	84000 (62000-110000)
Poland	1995	Male	45 to 49	97% (93-99%)	3.1% (1.3-6.6%)	5.5 (3.8-7.3)	17% (11-22%)	21% (14-27%)	2300 (1500-3000)	110000 (71000-130000)
Poland	1995	Male	50 to 54	97% (94-98%)	3.3% (1.7-6.2%)	6.2 (4.4-8)	17% (12-21%)	21% (15-25%)	2200 (1600-2800)	86000 (63000-110000)
Poland	1995	Male	55 to 59	96% (93-97%)	4.4% (2.5-7.2%)	6.4 (4.8-8.3)	15% (11-20%)	18% (13-23%)	3100 (2300-3900)	100000 (77000-130000)
Poland	1995	Male	60 to 64	95% (91-97%)	5% (3-8.5%)	6.1 (4.3-8.3)	13% (8.8-17%)	15% (9.9-20%)	3500 (2300-4700)	100000 (67000-130000)
Poland	1995	Male	65 to 69	95% (91-97%)	5.2% (3.1-8.6%)	5.5 (4.1-7.1)	11% (6.4-15%)	12% (7-17%)	3400 (2000-4900)	81000 (49000-120000)
Poland	1995	Male	70 to 74	94% (91-96%)	5.8% (3.6-9.4%)	4.7 (3.3-6.3)	7.7% (3.1-13%)	8.6% (3.1-15%)	2400 (850-4000)	47000 (18000-77000)
Poland	1995	Male	75 to 79	93% (89-96%)	6.6% (3.9-11%)	3.9 (2.6-5.5)	5.1% (0.4-11%)	5.4% (-0.04-12%)	1000 (-7.7-2300)	16000 (1300-34000)
Poland	1995	Male	80 to 84	93% (87-96%)	7.3% (4.1-13%)	3.4 (2.3-4.7)	2.8% (-1.3-7.9%)	2.8% (-2-8.9%)	600 (-430-1900)	7400 (-3400-21000)
Poland	1995	Male	85 to 89	92% (87-96%)	7.8% (4.4-13%)	3 (2.1-4.2)	0.59% (-3.2-5.1%)	0.19% (-4.2-5.5%)	26 (-590-770)	720 (-3800-6200)
Poland	1995	Male	90 to 94	92% (87-96%)	8% (4.5-13%)	2.6 (1.7-3.8)	-1.1% (-5-3.7%)	-1.8% (-6.5-3.8%)	-85 (-300-180)	-310 (-1400-1100)
Poland	1995	Male	95 plus	92% (84-96%)	8.2% (3.7-16%)	2.2 (1.2-3.6)	-2% (-5.9-3%)	-3% (-7.6-3%)	-27 (-69-27)	-74 (-220-110)

Location	Year	Sex	Age	Prevalence of current drinkers	Prevalence of abstainers	Population average of standard drinks daily	Percent of total attributable deaths	Percent of total attributable DALYs	Attributable deaths	Attributable DALYs
Poland	2000	Female	All Ages	77% (73-81%)	23% (19-27%)	1.8 (1.6-2)	2.9% (1.6-4.2%)	2.1% (-0.19-4.6%)	3600 (-330-7900)	150000 (83000-220000)
Poland	2000	Female	15 to 19	77% (62-87%)	23% (13-38%)	1.5 (0.95-2.1)	2.6% (1.5-3.8%)	7.3% (3.8-11%)	35 (18-55)	3800 (2300-5600)
Poland	2000	Female	20 to 24	83% (71-91%)	17% (9.4-29%)	1.9 (1.2-2.6)	3.5% (2.3-4.8%)	7.7% (4.2-12%)	37 (20-57)	5200 (3300-7400)
Poland	2000	Female	25 to 29	84% (72-92%)	16% (8.4-28%)	1.8 (1.2-2.6)	3.6% (2.6-4.7%)	7.8% (4.6-11%)	40 (24-57)	5200 (3600-7000)
Poland	2000	Female	30 to 34	84% (72-92%)	16% (7.9-28%)	1.9 (1.3-2.7)	3.7% (2.7-5%)	8.9% (6-12%)	56 (38-77)	5700 (4000-7500)
Poland	2000	Female	35 to 39	84% (73-92%)	16% (8.2-27%)	2 (1.3-2.9)	4.5% (3.2-5.9%)	9.8% (6.7-13%)	120 (81-160)	8700 (6300-11000)
Poland	2000	Female	40 to 44	84% (74-92%)	16% (8.3-26%)	2.3 (1.6-3.1)	5.2% (3.7-6.9%)	10% (7.1-13%)	270 (190-360)	16000 (11000-21000)
Poland	2000	Female	45 to 49	85% (78-91%)	15% (9.5-22%)	2.8 (2-3.7)	5.8% (4.1-7.6%)	10% (7.4-13%)	460 (330-610)	22000 (16000-29000)
Poland	2000	Female	50 to 54	85% (79-90%)	15% (9.7-21%)	2.9 (2-3.8)	5.9% (4.1-8%)	9.7% (6.9-13%)	560 (400-730)	23000 (16000-30000)
Poland	2000	Female	55 to 59	85% (79-89%)	15% (11-21%)	2.8 (2-3.9)	5.3% (3.4-7.5%)	8.3% (5.5-12%)	470 (310-650)	16000 (11000-23000)
Poland	2000	Female	60 to 64	81% (74-86%)	19% (14-26%)	2.6 (1.8-3.7)	4% (1.8-6.8%)	6% (2.9-9.8%)	560 (260-910)	17000 (7500-28000)
Poland	2000	Female	65 to 69	75% (66-82%)	25% (18-34%)	2.3 (1.6-3.1)	2.9% (0.67-5.3%)	4.1% (1-7.4%)	610 (150-1100)	15000 (3400-27000)
Poland	2000	Female	70 to 74	67% (59-76%)	33% (24-41%)	1.9 (1.3-2.7)	1.8% (-0.7-4.5%)	2.3% (-0.95-5.8%)	540 (-220-1300)	11000 (-4400-26000)
Poland	2000	Female	75 to 79	59% (46-70%)	41% (30-54%)	1.6 (1-2.3)	0.84% (-1.7-3.5%)	0.99% (-2.3-4.3%)	300 (-700-1300)	4900 (-9900-20000)
Poland	2000	Female	80 to 84	54% (41-65%)	46% (35-59%)	1.4 (0.89-2)	0.19% (-2.1-2.4%)	0.11% (-2.7-2.9%)	26 (-660-700)	620 (-6600-7900)
Poland	2000	Female	85 to 89	53% (40-65%)	47% (35-60%)	1.3 (0.81-2)	-0.37% (-2.7-1.9%)	-0.61% (-3.4-2.2%)	-170 (-960-620)	-950 (-6800-4800)
Poland	2000	Female	90 to 94	55% (43-68%)	45% (32-57%)	1.3 (0.78-2.1)	-0.9% (-3.5-1.8%)	-1.3% (-4.4-2%)	-200 (-680-320)	-860 (-3400-1700)
Poland	2000	Female	95 plus	59% (43-73%)	41% (27-57%)	1.4 (0.7-2.3)	-1.4% (-4.6-2.4%)	-1.9% (-5.8-2.6%)	-80 (-250-110)	-240 (-800-410)
Poland	2000	Male	All Ages	93% (90-95%)	7.1% (5-9.8%)	3.4 (3-3.7)	12% (11-15%)	13% (9.7-16%)	25000 (19000-32000)	850000 (710000-1000000)
Poland	2000	Male	15 to 19	89% (80-95%)	11% (5.3-20%)	1.6 (1.1-2.4)	5.5% (3.2-8.1%)	9.7% (5.2-15%)	130 (67-200)	11000 (6700-16000)
Poland	2000	Male	20 to 24	93% (87-97%)	6.7% (3.1-13%)	2.7 (1.8-3.8)	10% (6.7-15%)	16% (8.9-22%)	310 (170-440)	25000 (16000-35000)

Location	Year	Sex	Age	Prevalence of current drinkers	Prevalence of abstainers	Population average of standard drinks daily	Percent of total attributable deaths	Percent of total attributable DALYs	Attributable deaths	Attributable DALYs
Poland	2000	Male	25 to 29	95%<br>(90-98%)	5.5%<br>(2.4-10%)	3.4<br>(2.3-4.6)	13%<br>(9.4-17%)	19%<br>(13-27%)	370<br>(240-510)	30000<br>(21000-39000)
Poland	2000	Male	30 to 34	95%<br>(90-98%)	4.7%<br>(1.9-9.8%)	3.6<br>(2.5-5)	15%<br>(11-18%)	21%<br>(16-28%)	490<br>(360-640)	35000<br>(27000-45000)
Poland	2000	Male	35 to 39	96%<br>(92-98%)	4.3%<br>(1.7-8.5%)	3.4<br>(2.2-4.7)	15%<br>(11-19%)	20%<br>(15-26%)	780<br>(580-1000)	48000<br>(37000-60000)
Poland	2000	Male	40 to 44	96%<br>(92-99%)	3.7%<br>(1.4-7.6%)	4.1<br>(2.9-5.4)	16%<br>(12-20%)	21%<br>(16-26%)	1600<br>(1200-2000)	84000<br>(65000-100000)
Poland	2000	Male	45 to 49	97%<br>(94-99%)	3%<br>(1.3-6.4%)	5.8<br>(4-7.7)	19%<br>(14-23%)	24%<br>(18-29%)	2800<br>(2000-3400)	130000<br>(93000-150000)
Poland	2000	Male	50 to 54	97%<br>(94-98%)	3.3%<br>(1.8-6.1%)	6.6<br>(4.8-8.4)	19%<br>(15-22%)	23%<br>(18-27%)	3200<br>(2500-3800)	130000<br>(97000-150000)
Poland	2000	Male	55 to 59	96%<br>(93-97%)	4.4%<br>(2.5-6.9%)	6.7<br>(5-8.8)	17%<br>(13-21%)	20%<br>(16-25%)	2600<br>(2000-3200)	87000<br>(67000-110000)
Poland	2000	Male	60 to 64	95%<br>(92-97%)	5%<br>(3-8.1%)	6.5<br>(4.6-8.7)	14%<br>(10-18%)	17%<br>(12-21%)	3300<br>(2300-4200)	94000<br>(67000-120000)
Poland	2000	Male	65 to 69	95%<br>(92-97%)	5.2%<br>(3-8.2%)	5.8<br>(4.3-7.5)	12%<br>(8-16%)	14%<br>(9.1-19%)	3500<br>(2400-4800)	85000<br>(57000-110000)
Poland	2000	Male	70 to 74	94%<br>(91-96%)	5.7%<br>(3.6-9.1%)	5<br>(3.6-6.7)	9%<br>(4.4-14%)	10%<br>(4.8-16%)	3000<br>(1400-4600)	58000<br>(28000-88000)
Poland	2000	Male	75 to 79	93%<br>(89-96%)	6.5%<br>(3.7-11%)	4.2<br>(2.8-6)	6.4%<br>(1.7-12%)	7.1%<br>(1.5-13%)	1800<br>(410-3500)	28000<br>(7300-52000)
Poland	2000	Male	80 to 84	93%<br>(87-96%)	7.2%<br>(4.1-13%)	3.6<br>(2.5-5)	4.3%<br>(0.28-9.3%)	4.6%<br>(-0.24-11%)	700<br>(-36-1600)	8200<br>(520-18000)
Poland	2000	Male	85 to 89	92%<br>(87-96%)	7.7%<br>(4.4-13%)	3.2<br>(2.2-4.5)	2.1%<br>(-1.6-6.8%)	2%<br>(-2.4-7.5%)	260<br>(-320-970)	2400<br>(-1800-7700)
Poland	2000	Male	90 to 94	92%<br>(87-96%)	7.9%<br>(4.4-13%)	2.8<br>(1.8-4.1)	0.24%<br>(-3.6-5%)	-0.29%<br>(-5-5.5%)	-16<br>(-270-300)	79<br>(-1200-1700)
Poland	2000	Male	95 plus	92%<br>(85-97%)	8.1%<br>(3.5-15%)	2.4<br>(1.2-3.8)	-0.92%<br>(-4.6-4.3%)	-1.7%<br>(-6.3-4.5%)	-20<br>(-72-52)	-43<br>(-220-200)
Poland	2005	Female	All Ages	76%<br>(72-80%)	24%<br>(20-28%)	1.9<br>(1.7-2.1)	3.2%<br>(2.3-4.3%)	2.6%<br>(0.87-4.6%)	4500<br>(1500-8000)	170000<br>(120000-220000)
Poland	2005	Female	15 to 19	76%<br>(62-86%)	24%<br>(14-38%)	1.6<br>(0.99-2.2)	2.5%<br>(1.5-3.6%)	7.7%<br>(4-12%)	27<br>(14-43)	3100<br>(1900-4500)
Poland	2005	Female	20 to 24	83%<br>(72-90%)	17%<br>(9.8-28%)	2<br>(1.3-2.9)	3.4%<br>(2.3-4.6%)	8.3%<br>(4.6-13%)	35<br>(20-54)	5100<br>(3400-7200)
Poland	2005	Female	25 to 29	84%<br>(73-91%)	16%<br>(8.8-27%)	1.9<br>(1.3-2.7)	3.5%<br>(2.5-4.5%)	8.6%<br>(5.3-12%)	41<br>(26-58)	5600<br>(3800-7400)
Poland	2005	Female	30 to 34	84%<br>(73-91%)	16%<br>(8.6-27%)	2<br>(1.4-2.8)	3.8%<br>(2.8-4.9%)	9.9%<br>(6.9-13%)	63<br>(44-85)	6300<br>(4700-8300)
Poland	2005	Female	35 to 39	85%<br>(76-92%)	15%<br>(8.5-24%)	2.1<br>(1.4-3)	4.7%<br>(3.4-6.2%)	11%<br>(8.3-15%)	110<br>(80-140)	8100<br>(6100-10000)
Poland	2005	Female	40 to 44	85%<br>(76-91%)	15%<br>(9-24%)	2.5<br>(1.7-3.3)	5.6%<br>(4.1-7.3%)	12%<br>(8.9-15%)	220<br>(160-280)	13000<br>(9800-17000)

Location	Year	Sex	Age	Prevalence of current drinkers	Prevalence of abstainers	Population average of standard drinks daily	Percent of total attributable deaths	Percent of total attributable DALYs	Attributable deaths	Attributable DALYs
Poland	2005	Female	45 to 49	86%<br>(80-90%)	14%<br>(9.8-20%)	3<br>(2.2-4)	6.5%<br>(4.8-8.4%)	12%<br>(9.2-15%)	490<br>(370-610)	24000<br>(18000-30000)
Poland	2005	Female	50 to 54	85%<br>(80-89%)	15%<br>(11-20%)	3.1<br>(2.2-4)	6.4%<br>(4.7-8.3%)	11%<br>(8.3-14%)	680<br>(520-870)	28000<br>(21000-37000)
Poland	2005	Female	55 to 59	83%<br>(79-86%)	17%<br>(14-21%)	2.9<br>(2.1-3.8)	5.8%<br>(4.1-7.8%)	9.2%<br>(6.6-12%)	720<br>(520-950)	26000<br>(18000-35000)
Poland	2005	Female	60 to 64	77%<br>(72-82%)	23%<br>(18-28%)	2.5<br>(1.8-3.5)	4.5%<br>(2.5-6.8%)	6.9%<br>(4-10%)	500<br>(290-740)	15000<br>(8700-23000)
Poland	2005	Female	65 to 69	68%<br>(62-75%)	32%<br>(25-38%)	2.1<br>(1.5-2.8)	3.1%<br>(1.5-5.1%)	4.6%<br>(2.3-7.4%)	560<br>(280-900)	14000<br>(6800-23000)
Poland	2005	Female	70 to 74	59%<br>(53-65%)	41%<br>(35-47%)	1.7<br>(1.1-2.3)	2%<br>(0.21-4.2%)	2.8%<br>(0.38-5.6%)	550<br>(74-1100)	11000<br>(1200-22000)
Poland	2005	Female	75 to 79	47%<br>(38-55%)	53%<br>(45-62%)	1.2<br>(0.8-1.8)	1.1%<br>(-0.57-3.1%)	1.4%<br>(-0.82-4%)	410<br>(-240-1200)	6600<br>(-3300-18000)
Poland	2005	Female	80 to 84	42%<br>(32-53%)	58%<br>(47-68%)	1.1<br>(0.68-1.5)	0.58%<br>(-0.96-2.3%)	0.62%<br>(-1.4-2.8%)	220<br>(-470-1000)	2800<br>(-4500-12000)
Poland	2005	Female	85 to 89	44%<br>(33-55%)	56%<br>(45-67%)	1.1<br>(0.7-1.7)	0.13%<br>(-1.6-2.1%)	0.015%<br>(-2.1-2.4%)	3.5<br>(-460-520)	270<br>(-3300-4200)
Poland	2005	Female	90 to 94	46%<br>(35-57%)	54%<br>(43-65%)	1.1<br>(0.66-1.8)	-0.29%<br>(-2.3-2%)	-0.53%<br>(-3-2.3%)	-87<br>(-500-380)	-310<br>(-2400-2100)
Poland	2005	Female	95 plus	50%<br>(34-66%)	50%<br>(34-66%)	1.2<br>(0.59-2)	-0.63%<br>(-3.2-2.3%)	-0.97%<br>(-4-2.6%)	-56<br>(-240-150)	-150<br>(-770-560)
Poland	2005	Male	All Ages	95%<br>(93-96%)	5.4%<br>(3.9-7.4%)	4<br>(3.6-4.4)	14%<br>(12-16%)	14%<br>(11-18%)	28000<br>(22000-35000)	950000<br>(810000-1100000)
Poland	2005	Male	15 to 19	91%<br>(84-95%)	9.5%<br>(5-16%)	1.8<br>(1.2-2.6)	5.6%<br>(3.4-8.1%)	11%<br>(5.7-16%)	100<br>(55-160)	9400<br>(5800-14000)
Poland	2005	Male	20 to 24	95%<br>(91-98%)	4.8%<br>(2.4-9.1%)	3.1<br>(2.1-4.3)	11%<br>(7.3-15%)	18%<br>(10-25%)	320<br>(190-450)	27000<br>(17000-37000)
Poland	2005	Male	25 to 29	96%<br>(93-98%)	4%<br>(2-7.1%)	3.8<br>(2.7-5.1)	14%<br>(9.8-17%)	22%<br>(15-29%)	420<br>(280-560)	34000<br>(24000-44000)
Poland	2005	Male	30 to 34	97%<br>(93-98%)	3.5%<br>(1.6-6.6%)	4<br>(2.9-5.5)	15%<br>(12-19%)	24%<br>(18-31%)	550<br>(410-710)	39000<br>(30000-48000)
Poland	2005	Male	35 to 39	97%<br>(94-99%)	3.2%<br>(1.4-5.6%)	3.8<br>(2.5-5.2)	16%<br>(12-20%)	24%<br>(18-30%)	750<br>(560-960)	46000<br>(35000-57000)
Poland	2005	Male	40 to 44	97%<br>(95-99%)	2.8%<br>(1.2-5.2%)	4.6<br>(3.3-6.1)	18%<br>(14-22%)	25%<br>(19-31%)	1400<br>(1100-1700)	72000<br>(56000-88000)
Poland	2005	Male	45 to 49	98%<br>(96-99%)	2.3%<br>(1.1-4.4%)	6.4<br>(4.6-8.5)	21%<br>(16-25%)	28%<br>(21-33%)	3000<br>(2300-3700)	140000<br>(110000-160000)
Poland	2005	Male	50 to 54	97%<br>(96-99%)	2.6%<br>(1.5-4.4%)	7.3<br>(5.4-9.2)	21%<br>(17-24%)	26%<br>(21-30%)	4200<br>(3400-4900)	160000<br>(130000-190000)
Poland	2005	Male	55 to 59	96%<br>(95-98%)	3.5%<br>(2.3-5.2%)	7.5<br>(5.8-9.7)	19%<br>(15-22%)	23%<br>(19-27%)	4200<br>(3400-4900)	140000<br>(110000-170000)

Location	Year	Sex	Age	Prevalence of current drinkers	Prevalence of abstainers	Population average of standard drinks daily	Percent of total attributable deaths	Percent of total attributable DALYs	Attributable deaths	Attributable DALYs
Poland	2005	Male	60 to 64	96% (94-97%)	4% (2.6-5.8%)	7.3 (5.4-9.6)	16% (12-20%)	19% (15-23%)	3000 (2300-3700)	87000 (66000-110000)
Poland	2005	Male	65 to 69	95% (93-97%)	4.7% (3-7%)	6.4 (5-8.1)	13% (9.6-17%)	16% (11-20%)	3400 (2400-4400)	83000 (60000-100000)
Poland	2005	Male	70 to 74	94% (91-96%)	6% (4-8.7%)	5.2 (3.8-7)	9.9% (5.6-14%)	11% (6.3-17%)	3100 (1700-4500)	60000 (34000-87000)
Poland	2005	Male	75 to 79	93% (89-96%)	6.8% (4.2-11%)	4.4 (3-6.1)	7.1% (2.5-12%)	8% (2.5-14%)	2200 (700-3800)	34000 (12000-58000)
Poland	2005	Male	80 to 84	93% (87-96%)	7.3% (4.1-13%)	3.8 (2.7-5.3)	5.1% (0.93-9.9%)	5.6% (0.59-11%)	1200 (130-2400)	14000 (2500-27000)
Poland	2005	Male	85 to 89	92% (87-95%)	7.9% (4.6-13%)	3.4 (2.4-4.7)	3% (-0.75-7.8%)	3% (-1.5-8.7%)	310 (-150-910)	2800 (-710-7300)
Poland	2005	Male	90 to 94	92% (87-96%)	8% (4.4-13%)	3 (2-4.2)	1% (-2.7-5.8%)	0.61% (-4.1-6.4%)	36 (-230-360)	370 (-970-2100)
Poland	2005	Male	95 plus	92% (84-96%)	8.3% (3.6-16%)	2.5 (1.3-4)	-0.27% (-3.9-5%)	-1% (-5.6-5.3%)	-17 (-91-87)	-18 (-260-350)
Poland	2010	Female	All Ages	77% (73-81%)	23% (19-27%)	1.9 (1.7-2.2)	3.2% (2.2-4.4%)	2.4% (0.58-4.6%)	4400 (1000-8200)	170000 (120000-230000)
Poland	2010	Female	15 to 19	76% (63-87%)	24% (13-37%)	1.6 (1-2.3)	2.4% (1.5-3.6%)	7.7% (4-12%)	21 (11-33)	2500 (1500-3700)
Poland	2010	Female	20 to 24	83% (73-91%)	17% (9.2-27%)	2 (1.3-2.9)	3.4% (2.3-4.5%)	8.4% (4.7-13%)	28 (15-43)	4300 (2800-6100)
Poland	2010	Female	25 to 29	84% (73-91%)	16% (8.7-27%)	1.9 (1.3-2.7)	3.5% (2.6-4.6%)	8.9% (5.7-12%)	40 (26-56)	5700 (4000-7600)
Poland	2010	Female	30 to 34	84% (74-92%)	16% (8.5-26%)	2 (1.3-2.8)	3.8% (2.8-5%)	11% (7.8-14%)	66 (48-86)	6900 (5200-9000)
Poland	2010	Female	35 to 39	85% (76-92%)	15% (8.3-24%)	2.2 (1.4-3.1)	4.7% (3.5-6.1%)	12% (9-16%)	120 (86-150)	8900 (6600-11000)
Poland	2010	Female	40 to 44	85% (77-91%)	15% (8.5-23%)	2.5 (1.7-3.4)	5.6% (4.2-7.3%)	13% (9.8-16%)	190 (140-240)	11000 (8700-15000)
Poland	2010	Female	45 to 49	86% (80-91%)	14% (9.2-20%)	3 (2.2-4.1)	6.5% (4.9-8.4%)	13% (10-16%)	360 (280-450)	18000 (14000-23000)
Poland	2010	Female	50 to 54	85% (80-89%)	15% (11-20%)	3.1 (2.2-4.1)	6.6% (4.9-8.5%)	12% (9-14%)	660 (500-830)	28000 (21000-36000)
Poland	2010	Female	55 to 59	84% (79-87%)	16% (13-21%)	2.9 (2.2-3.8)	5.9% (4.2-7.7%)	9.6% (7.2-12%)	830 (630-1100)	30000 (22000-39000)
Poland	2010	Female	60 to 64	80% (75-84%)	20% (16-25%)	2.7 (1.9-3.7)	4.6% (2.7-6.8%)	7.1% (4.4-10%)	750 (460-1100)	23000 (14000-34000)
Poland	2010	Female	65 to 69	72% (66-78%)	28% (22-34%)	2.3 (1.7-3)	3.3% (1.7-5.2%)	4.9% (2.6-7.6%)	480 (250-760)	12000 (6200-20000)
Poland	2010	Female	70 to 74	62% (55-68%)	38% (32-45%)	1.8 (1.2-2.5)	2% (0.29-4.2%)	2.9% (0.46-5.8%)	470 (76-930)	9700 (1400-19000)
Poland	2010	Female	75 to 79	50% (40-60%)	50% (40-60%)	1.3 (0.85-1.9)	1.1% (-0.56-3.2%)	1.4% (-0.87-4.1%)	370 (-220-1100)	6100 (-3100-17000)

Location	Year	Sex	Age	Prevalence of current drinkers	Prevalence of abstainers	Population average of standard drinks daily	Percent of total attributable deaths	Percent of total attributable DALYs	Attributable deaths	Attributable DALYs
Poland	2010	Female	80 to 84	46% (35-57%)	54% (43-65%)	1.2 (0.76-1.7)	0.52% (-1.1-2.4%)	0.54% (-1.5-2.9%)	200 (-570-1100)	2700 (-5600-12000)
Poland	2010	Female	85 to 89	49% (37-59%)	51% (41-63%)	1.3 (0.79-1.9)	0.028% (-1.9-2.1%)	-0.14% (-2.6-2.5%)	-49 (-890-880)	92 (-6400-7300)
Poland	2010	Female	90 to 94	51% (39-62%)	49% (38-61%)	1.3 (0.75-2)	-0.4% (-2.7-2.3%)	-0.69% (-3.5-2.6%)	-100 (-510-380)	-380 (-2500-2200)
Poland	2010	Female	95 plus	54% (38-69%)	46% (31-62%)	1.3 (0.68-2.2)	-0.72% (-3.6-2.8%)	-1.1% (-4.7-3.2%)	-80 (-340-230)	-210 (-1100-830)
Poland	2010	Male	All Ages	95% (93-96%)	5.2% (3.8-6.9%)	4.1 (3.7-4.6)	14% (12-16%)	14% (11-18%)	29000 (23000-36000)	960000 (820000-1100000)
Poland	2010	Male	15 to 19	91% (84-95%)	9.5% (4.8-16%)	1.9 (1.2-2.7)	5.6% (3.3-8.2%)	11% (5.5-17%)	89 (45-140)	8000 (4900-12000)
Poland	2010	Male	20 to 24	95% (91-98%)	4.8% (2.3-9%)	3.1 (2.1-4.4)	11% (7.2-15%)	18% (10-26%)	280 (160-400)	23000 (15000-32000)
Poland	2010	Male	25 to 29	96% (93-98%)	3.9% (1.8-6.9%)	3.8 (2.6-5.1)	13% (9.8-17%)	22% (15-29%)	410 (280-540)	33000 (24000-44000)
Poland	2010	Male	30 to 34	97% (94-98%)	3.4% (1.6-6.2%)	4 (2.9-5.4)	15% (12-19%)	25% (19-32%)	590 (440-740)	42000 (33000-52000)
Poland	2010	Male	35 to 39	97% (94-99%)	3.2% (1.4-6.1%)	3.8 (2.5-5.3)	17% (13-21%)	25% (19-32%)	810 (620-1000)	50000 (39000-61000)
Poland	2010	Male	40 to 44	97% (95-99%)	2.7% (1.2-5.4%)	4.6 (3.2-6.2)	18% (14-23%)	26% (21-32%)	1200 (930-1500)	64000 (50000-78000)
Poland	2010	Male	45 to 49	98% (96-99%)	2.2% (1.1-4.3%)	6.5 (4.5-8.6)	21% (17-26%)	29% (22-34%)	2200 (1700-2700)	100000 (79000-120000)
Poland	2010	Male	50 to 54	97% (96-98%)	2.5% (1.5-4.3%)	7.4 (5.4-9.3)	21% (17-25%)	27% (22-32%)	4000 (3300-4700)	160000 (130000-190000)
Poland	2010	Male	55 to 59	97% (95-98%)	3.5% (2.2-5%)	7.6 (5.8-9.6)	19% (16-23%)	24% (20-28%)	4900 (4000-5800)	170000 (140000-200000)
Poland	2010	Male	60 to 64	96% (94-97%)	3.9% (2.6-5.6%)	7.3 (5.5-9.6)	16% (13-20%)	20% (15-24%)	4400 (3400-5300)	130000 (99000-160000)
Poland	2010	Male	65 to 69	96% (93-97%)	4.5% (2.9-6.6%)	6.4 (5-8.2)	13% (9.9-17%)	16% (12-20%)	2900 (2100-3700)	71000 (52000-89000)
Poland	2010	Male	70 to 74	94% (92-96%)	5.8% (3.9-8.4%)	5.3 (3.8-7.1)	10% (5.6-14%)	12% (6.4-17%)	2800 (1500-4000)	55000 (32000-78000)
Poland	2010	Male	75 to 79	94% (90-96%)	6.5% (4.1-10%)	4.5 (3-6.2)	7.1% (2.8-12%)	8.1% (2.7-14%)	2200 (760-3800)	35000 (14000-59000)
Poland	2010	Male	80 to 84	93% (88-96%)	7% (4-12%)	3.9 (2.7-5.4)	5.1% (0.96-9.8%)	5.6% (0.58-11%)	1400 (150-2900)	17000 (3200-33000)
Poland	2010	Male	85 to 89	92% (87-95%)	7.9% (4.6-13%)	3.4 (2.4-4.7)	2.9% (-0.69-7.5%)	2.9% (-1.5-8.4%)	470 (-240-1400)	4300 (-1100-11000)
Poland	2010	Male	90 to 94	92% (87-95%)	8% (4.6-13%)	3 (2-4.4)	1% (-2.6-6%)	0.6% (-3.9-6.7%)	33 (-210-370)	340 (-890-2100)

Location	Year	Sex	Age	Prevalence of current drinkers	Prevalence of abstainers	Population average of standard drinks daily	Percent of total attributable deaths	Percent of total attributable DALYs	Attributable deaths	Attributable DALYs
Poland	2010	Male	95 plus	92%<br>(85-96%)	8.1%<br>(3.6-15%)	2.5<br>(1.4-4)	-0.21%<br>(-3.9-5%)	-0.92%<br>(-5.5-5.4%)	-18<br>(-110-100)	-17<br>(-310-400)
Poland	2016	Female	All Ages	78%<br>(74-81%)	22%<br>(19-26%)	2.1<br>(1.6-2.6)	3.2%<br>(1.8-4.8%)	2.4%<br>(-0.22-5.4%)	4500<br>(-400-10000)	170000<br>(92000-250000)
Poland	2016	Female	15 to 19	77%<br>(62-88%)	23%<br>(12-38%)	1.6<br>(0.97-2.5)	2.4%<br>(1.5-3.5%)	7.8%<br>(3.9-13%)	15<br>(8-25)	1900<br>(1100-2800)
Poland	2016	Female	20 to 24	83%<br>(72-91%)	17%<br>(9.4-28%)	2<br>(1.2-3.1)	3.3%<br>(2.3-4.6%)	8.4%<br>(4.6-13%)	21<br>(12-34)	3500<br>(2300-5000)
Poland	2016	Female	25 to 29	84%<br>(72-92%)	16%<br>(8.4-28%)	2<br>(1.3-3)	3.4%<br>(2.5-4.5%)	9.2%<br>(5.7-13%)	31<br>(19-45)	4700<br>(3200-6500)
Poland	2016	Female	30 to 34	84%<br>(72-92%)	16%<br>(7.9-28%)	2<br>(1.3-3)	3.7%<br>(2.7-5%)	11%<br>(7.7-14%)	62<br>(43-85)	6900<br>(5000-9200)
Poland	2016	Female	35 to 39	84%<br>(74-92%)	16%<br>(8.3-26%)	2.2<br>(1.3-3.2)	4.4%<br>(3.3-5.8%)	12%<br>(9.2-16%)	120<br>(83-160)	9400<br>(6900-12000)
Poland	2016	Female	40 to 44	85%<br>(74-92%)	15%<br>(8.4-26%)	2.5<br>(1.6-3.6)	5.3%<br>(3.8-7%)	13%<br>(10-17%)	190<br>(130-250)	12000<br>(8400-15000)
Poland	2016	Female	45 to 49	86%<br>(78-91%)	14%<br>(9.4-22%)	3.1<br>(2-4.4)	6.3%<br>(4.6-8.6%)	14%<br>(10-18%)	290<br>(220-380)	15000<br>(11000-20000)
Poland	2016	Female	50 to 54	86%<br>(80-90%)	14%<br>(9.7-20%)	3.2<br>(2.2-4.5)	6.6%<br>(4.6-8.9%)	12%<br>(9-16%)	470<br>(340-630)	21000<br>(15000-27000)
Poland	2016	Female	55 to 59	85%<br>(80-89%)	15%<br>(11-20%)	3.2<br>(2.2-4.3)	6%<br>(4.1-8.4%)	10%<br>(7.4-13%)	800<br>(560-1100)	29000<br>(20000-40000)
Poland	2016	Female	60 to 64	83%<br>(78-87%)	17%<br>(13-22%)	3<br>(2-4.2)	4.9%<br>(2.7-7.5%)	7.7%<br>(4.6-11%)	920<br>(530-1400)	28000<br>(15000-43000)
Poland	2016	Female	65 to 69	78%<br>(71-83%)	22%<br>(17-29%)	2.7<br>(1.8-3.6)	3.6%<br>(1.4-5.9%)	5.5%<br>(2.5-8.7%)	770<br>(350-1200)	20000<br>(7800-32000)
Poland	2016	Female	70 to 74	70%<br>(63-77%)	30%<br>(23-37%)	2.2<br>(1.5-3.2)	2.3%<br>(0.087-5.1%)	3.4%<br>(0.4-7.2%)	430<br>(51-910)	9000<br>(330-19000)
Poland	2016	Female	75 to 79	61%<br>(49-71%)	39%<br>(29-51%)	1.8<br>(1.1-2.7)	1.4%<br>(-0.91-4.3%)	1.8%<br>(-1.3-5.7%)	410<br>(-280-1300)	6900<br>(-4400-21000)
Poland	2016	Female	80 to 84	56%<br>(43-67%)	44%<br>(33-57%)	1.6<br>(0.95-2.3)	0.7%<br>(-1.4-3.2%)	0.75%<br>(-2.1-4%)	260<br>(-750-1400)	3700<br>(-7600-17000)
Poland	2016	Female	85 to 89	55%<br>(43-67%)	45%<br>(33-57%)	1.5<br>(0.91-2.3)	0.15%<br>(-2.1-3%)	-0.039%<br>(-3.1-3.4%)	-17<br>(-1300-1400)	570<br>(-8600-11000)
Poland	2016	Female	90 to 94	57%<br>(45-69%)	43%<br>(31-55%)	1.6<br>(0.89-2.4)	-0.34%<br>(-3.2-3.3%)	-0.67%<br>(-4.2-3.7%)	-190<br>(-1200-1000)	-620<br>(-5900-5700)
Poland	2016	Female	95 plus	61%<br>(44-75%)	39%<br>(25-56%)	1.6<br>(0.79-2.6)	-0.67%<br>(-4.1-3.9%)	-1.1%<br>(-5.2-4.5%)	-77<br>(-360-310)	-190<br>(-1100-1100)
Poland	2016	Male	All Ages	94%<br>(92-95%)	6.2%<br>(4.5-8.2%)	4.2<br>(3.2-5.1)	14%<br>(11-16%)	14%<br>(10-18%)	28000<br>(19000-36000)	890000<br>(700000-1100000)
Poland	2016	Male	15 to 19	89%<br>(80-95%)	11%<br>(5.1-20%)	1.8<br>(1.1-2.8)	5.2%<br>(3.1-7.8%)	11%<br>(5.5-17%)	56<br>(28-90)	5300<br>(3200-7900)

Location	Year	Sex	Age	Prevalence of current drinkers	Prevalence of abstainers	Population average of standard drinks daily	Percent of total attributable deaths	Percent of total attributable DALYs	Attributable deaths	Attributable DALYs
Poland	2016	Male	20 to 24	93% (88-97%)	6.6% (3-12%)	2.9 (1.9-4.3)	11% (7-15%)	18% (10-26%)	190 (110-300)	17000 (11000-24000)
Poland	2016	Male	25 to 29	95% (90-98%)	5.4% (2.4-10%)	3.6 (2.4-5.3)	13% (9.3-17%)	22% (15-30%)	310 (210-430)	26000 (19000-35000)
Poland	2016	Male	30 to 34	95% (90-98%)	4.6% (1.9-9.6%)	3.9 (2.6-5.6)	15% (11-19%)	25% (19-33%)	530 (390-720)	40000 (31000-51000)
Poland	2016	Male	35 to 39	96% (92-98%)	4.2% (1.7-8.5%)	3.7 (2.3-5.2)	16% (12-20%)	26% (20-33%)	800 (590-1000)	51000 (39000-64000)
Poland	2016	Male	40 to 44	96% (92-99%)	3.7% (1.5-7.7%)	4.5 (2.9-6.3)	18% (14-23%)	28% (22-34%)	1200 (900-1500)	64000 (49000-81000)
Poland	2016	Male	45 to 49	97% (94-99%)	3% (1.2-6.3%)	6.3 (4-8.9)	21% (16-25%)	30% (22-36%)	1700 (1300-2200)	79000 (60000-98000)
Poland	2016	Male	50 to 54	97% (94-98%)	3.3% (1.7-5.9%)	7.2 (4.8-9.7)	21% (16-25%)	28% (22-33%)	2700 (2000-3300)	110000 (83000-130000)
Poland	2016	Male	55 to 59	96% (94-98%)	4.1% (2.4-6.5%)	7.5 (5.2-10)	19% (15-23%)	24% (20-29%)	4400 (3400-5400)	150000 (120000-180000)
Poland	2016	Male	60 to 64	96% (93-97%)	4.3% (2.7-6.7%)	7.4 (5-10)	16% (12-20%)	20% (15-25%)	4900 (3600-6200)	140000 (110000-180000)
Poland	2016	Male	65 to 69	95% (93-97%)	4.6% (2.8-6.9%)	6.6 (4.8-8.6)	13% (9.4-17%)	16% (11-21%)	4200 (2900-5500)	100000 (72000-130000)
Poland	2016	Male	70 to 74	95% (92-97%)	5.2% (3.4-8.1%)	5.6 (3.8-7.8)	10% (5.6-14%)	12% (6.5-17%)	2300 (1200-3300)	46000 (25000-65000)
Poland	2016	Male	75 to 79	94% (90-97%)	6% (3.5-9.9%)	4.7 (3-6.9)	7.3% (2.8-12%)	8.5% (2.8-14%)	2100 (680-3600)	33000 (13000-55000)
Poland	2016	Male	80 to 84	93% (88-96%)	6.9% (3.8-12%)	4 (2.6-5.7)	5.2% (0.91-10%)	5.8% (0.56-12%)	1600 (150-3200)	19000 (3200-36000)
Poland	2016	Male	85 to 89	92% (87-96%)	7.7% (4.3-13%)	3.5 (2.3-5)	3.1% (-0.75-8.2%)	3.2% (-1.5-9.5%)	670 (-310-2000)	6100 (-1500-16000)
Poland	2016	Male	90 to 94	92% (87-96%)	7.8% (4.2-13%)	3.1 (1.9-4.7)	1.2% (-2.5-6.8%)	0.88% (-3.8-7.6%)	89 (-390-790)	790 (-1600-4400)
Poland	2016	Male	95 plus	92% (85-97%)	8% (3.4-15%)	2.6 (1.4-4.4)	0.0045% (-3.9-5.7%)	-0.7% (-5.6-6.3%)	-13 (-110-120)	0.29 (-310-460)
Romania	1990	Female	All Ages	64% (55-71%)	36% (29-45%)	2.6 (2.1-3.2)	8.4% (3.3-14%)	12% (3.6-22%)	15000 (4200-26000)	330000 (130000-530000)
Romania	1990	Female	15 to 19	65% (46-80%)	35% (20-54%)	2.3 (1.4-3.4)	3.7% (1.4-6.6%)	8.3% (1.8-16%)	37 (8.1-70)	3800 (1400-6800)
Romania	1990	Female	20 to 24	73% (56-85%)	27% (15-44%)	3 (1.9-4.3)	5.3% (2.6-8.4%)	9.5% (2.8-17%)	54 (15-97)	6000 (2900-9600)
Romania	1990	Female	25 to 29	74% (57-86%)	26% (14-43%)	2.9 (1.9-4.2)	5.7% (3.1-8.5%)	10% (3.9-17%)	54 (20-90)	5300 (2900-7800)
Romania	1990	Female	30 to 34	74% (57-86%)	26% (14-43%)	3 (1.9-4.4)	6.2% (3.5-9%)	11% (5.1-17%)	110 (49-170)	8600 (4800-13000)
Romania	1990	Female	35 to 39	75% (59-86%)	25% (14-41%)	3.3 (2.1-4.8)	7.3% (4.1-11%)	13% (6.8-20%)	180 (93-280)	12000 (6700-18000)

Location	Year	Sex	Age	Prevalence of current drinkers	Prevalence of abstainers	Population average of standard drinks daily	Percent of total attributable deaths	Percent of total attributable DALYs	Attributable deaths	Attributable DALYs
Romania	1990	Female	40 to 44	74% (58-86%)	26% (14-42%)	3.6 (2.4-5.1)	8.9% (4.9-13%)	16% (8.7-23%)	270 (150-400)	15000 (8700-22000)
Romania	1990	Female	45 to 49	73% (52-86%)	27% (14-48%)	3.9 (2.6-5.7)	10% (5.6-15%)	17% (9.5-25%)	370 (200-540)	17000 (9800-26000)
Romania	1990	Female	50 to 54	70% (52-84%)	30% (16-48%)	4 (2.7-5.7)	11% (6.1-17%)	17% (9.4-26%)	710 (390-1000)	29000 (16000-43000)
Romania	1990	Female	55 to 59	68% (49-83%)	32% (17-51%)	4 (2.7-5.8)	11% (5.6-18%)	17% (8.4-26%)	960 (480-1500)	33000 (16000-51000)
Romania	1990	Female	60 to 64	65% (45-82%)	35% (18-55%)	4 (2.6-5.9)	11% (4.9-19%)	16% (6.9-26%)	1400 (590-2200)	40000 (17000-65000)
Romania	1990	Female	65 to 69	61% (42-79%)	39% (21-58%)	4 (2.6-5.8)	12% (4.4-21%)	16% (6.1-27%)	2000 (730-3300)	47000 (17000-79000)
Romania	1990	Female	70 to 74	56% (38-73%)	44% (27-62%)	3.8 (2.3-5.9)	12% (3.4-22%)	15% (4.2-28%)	1700 (470-3100)	32000 (9000-60000)
Romania	1990	Female	75 to 79	49% (33-68%)	51% (32-67%)	3.3 (1.9-5.3)	10% (1.9-21%)	12% (2.4-25%)	2800 (530-5700)	40000 (7800-82000)
Romania	1990	Female	80 to 84	45% (29-63%)	55% (37-71%)	2.9 (1.7-4.5)	9.3% (1.4-20%)	11% (1.6-23%)	2400 (340-5000)	25000 (3900-54000)
Romania	1990	Female	85 to 89	43% (28-59%)	57% (41-72%)	2.8 (1.6-4.5)	8.1% (0.85-18%)	9.4% (0.88-21%)	1100 (110-2500)	8400 (870-19000)
Romania	1990	Female	90 to 94	45% (30-61%)	55% (39-70%)	2.9 (1.5-4.7)	7.7% (0.2-18%)	8.9% (0.023-22%)	440 (1.2-1100)	2300 (58-5500)
Romania	1990	Female	95 plus	48% (29-68%)	52% (32-71%)	3 (1.4-5.4)	7.6% (-0.56-20%)	8.7% (-0.87-23%)	130 (-13-350)	450 (-32-1200)
Romania	1990	Male	All Ages	70% (60-79%)	30% (21-40%)	8.1 (6.9-9.5)	16% (6.8-24%)	21% (6.8-32%)	28000 (9000-42000)	800000 (340000-1200000)
Romania	1990	Male	15 to 19	64% (43-83%)	36% (17-57%)	4.2 (2.5-6.4)	9.1% (4.1-16%)	16% (6-29%)	140 (51-260)	12000 (5300-20000)
Romania	1990	Male	20 to 24	74% (55-88%)	26% (12-45%)	7.4 (4.8-10)	17% (8.7-27%)	27% (12-43%)	340 (160-550)	27000 (14000-42000)
Romania	1990	Male	25 to 29	77% (60-91%)	23% (9.5-40%)	8.8 (6-12)	20% (11-30%)	31% (15-47%)	360 (180-540)	26000 (14000-38000)
Romania	1990	Male	30 to 34	79% (61-92%)	21% (7.8-39%)	10 (7.1-14)	22% (12-31%)	32% (17-47%)	690 (360-1000)	44000 (25000-62000)
Romania	1990	Male	35 to 39	81% (62-92%)	19% (7.6-38%)	12 (8.3-17)	23% (13-32%)	33% (18-46%)	1000 (580-1500)	59000 (34000-82000)
Romania	1990	Male	40 to 44	82% (64-92%)	18% (7.6-36%)	14 (9.7-18)	24% (13-33%)	32% (18-45%)	1300 (710-1800)	64000 (36000-88000)
Romania	1990	Male	45 to 49	81% (62-92%)	19% (7.8-38%)	14 (10-20)	23% (13-32%)	30% (16-42%)	1500 (790-2100)	65000 (35000-91000)
Romania	1990	Male	50 to 54	80% (62-92%)	20% (8.4-38%)	15 (10-20)	22% (11-32%)	27% (13-39%)	2400 (1200-3400)	91000 (45000-130000)

Location	Year	Sex	Age	Prevalence of current drinkers	Prevalence of abstainers	Population average of standard drinks daily	Percent of total attributable deaths	Percent of total attributable DALYs	Attributable deaths	Attributable DALYs
Romania	1990	Male	55 to 59	79% (60-92%)	21% (8.2-40%)	14 (10-19)	21% (8.9-30%)	25% (11-36%)	2900 (1300-4300)	98000 (43000-140000)
Romania	1990	Male	60 to 64	78% (57-92%)	22% (8.3-43%)	14 (9.2-19)	19% (6.6-29%)	23% (8.3-35%)	3400 (1200-5100)	97000 (34000-150000)
Romania	1990	Male	65 to 69	76% (55-91%)	24% (8.9-45%)	12 (8.4-17)	19% (5.4-29%)	22% (6.7-35%)	3500 (1100-5500)	83000 (25000-130000)
Romania	1990	Male	70 to 74	74% (54-88%)	26% (12-46%)	11 (7-15)	18% (4.6-28%)	20% (5.4-33%)	2300 (600-3600)	43000 (11000-69000)
Romania	1990	Male	75 to 79	72% (52-87%)	28% (13-48%)	9.1 (5.6-14)	16% (3.2-27%)	18% (3.5-31%)	3300 (640-5700)	49000 (9400-84000)
Romania	1990	Male	80 to 84	71% (48-86%)	29% (14-52%)	8.2 (5-12)	15% (2.3-26%)	17% (2.4-30%)	2800 (390-5000)	30000 (4500-53000)
Romania	1990	Male	85 to 89	69% (49-85%)	31% (15-51%)	7.5 (4.6-11)	13% (1.7-24%)	15% (1.7-27%)	1200 (130-2200)	9200 (1200-17000)
Romania	1990	Male	90 to 94	69% (49-87%)	31% (13-51%)	6.8 (4-11)	12% (1.9-22%)	13% (1.9-26%)	370 (52-700)	2000 (320-3700)
Romania	1990	Male	95 plus	69% (43-89%)	31% (11-57%)	6.1 (2.9-11)	10% (1.3-22%)	12% (0.95-24%)	76 (6.3-160)	280 (34-560)
Romania	1995	Female	All Ages	65% (57-72%)	35% (28-43%)	2.5 (2-3)	8.7% (3.5-14%)	12% (3.6-22%)	15000 (4500-27000)	340000 (130000-540000)
Romania	1995	Female	15 to 19	65% (47-80%)	35% (20-53%)	2.1 (1.3-3.1)	3.4% (1.2-6.1%)	7.9% (1.4-15%)	33 (5.9-65)	3500 (1300-6200)
Romania	1995	Female	20 to 24	73% (57-85%)	27% (15-43%)	2.7 (1.7-3.9)	5.1% (2.4-8.2%)	9.6% (2.1-18%)	48 (11-87)	5400 (2500-8800)
Romania	1995	Female	25 to 29	74% (58-86%)	26% (14-42%)	2.6 (1.7-3.9)	5.3% (2.8-8.2%)	10% (3.2-18%)	61 (19-110)	6300 (3300-9700)
Romania	1995	Female	30 to 34	75% (57-87%)	25% (13-43%)	2.8 (1.8-4)	6% (3.2-9%)	11% (4.7-18%)	73 (30-120)	5900 (3100-8900)
Romania	1995	Female	35 to 39	75% (60-87%)	25% (13-40%)	3 (1.9-4.4)	7.5% (4.1-11%)	13% (6.6-20%)	190 (93-290)	12000 (6400-18000)
Romania	1995	Female	40 to 44	74% (59-86%)	26% (14-41%)	3.3 (2.2-4.7)	8.9% (4.9-13%)	15% (8.1-22%)	340 (180-500)	18000 (10000-27000)
Romania	1995	Female	45 to 49	73% (53-85%)	27% (15-47%)	3.6 (2.3-5.1)	10% (5.7-15%)	17% (9.3-25%)	460 (260-690)	21000 (12000-32000)
Romania	1995	Female	50 to 54	71% (53-84%)	29% (16-47%)	3.7 (2.5-5.1)	11% (6.3-17%)	17% (9.4-26%)	590 (320-880)	24000 (13000-35000)
Romania	1995	Female	55 to 59	68% (49-83%)	32% (17-51%)	3.7 (2.4-5.3)	11% (5.5-18%)	17% (8.2-25%)	1100 (530-1600)	36000 (18000-56000)
Romania	1995	Female	60 to 64	65% (46-82%)	35% (18-54%)	3.7 (2.4-5.3)	11% (4.6-18%)	16% (6.6-25%)	1400 (610-2300)	40000 (17000-66000)
Romania	1995	Female	65 to 69	62% (43-80%)	38% (20-57%)	3.7 (2.4-5.2)	12% (4.1-20%)	15% (5.9-26%)	2100 (800-3500)	49000 (19000-83000)
Romania	1995	Female	70 to 74	57% (38-73%)	43% (27-62%)	3.5 (2.1-5.4)	11% (3.1-21%)	14% (4-26%)	2700 (730-5000)	51000 (14000-95000)

Location	Year	Sex	Age	Prevalence of current drinkers	Prevalence of abstainers	Population average of standard drinks daily	Percent of total attributable deaths	Percent of total attributable DALYs	Attributable deaths	Attributable DALYs
Romania	1995	Female	75 to 79	50% (34-69%)	50% (31-66%)	3.1 (1.8-4.9)	9.9% (1.8-20%)	12% (2.1-24%)	1800 (310-3900)	27000 (4800-56000)
Romania	1995	Female	80 to 84	46% (30-64%)	54% (36-70%)	2.7 (1.6-4.3)	8.8% (1.3-19%)	10% (1.2-22%)	2200 (270-4700)	23000 (3200-50000)
Romania	1995	Female	85 to 89	44% (29-61%)	56% (39-71%)	2.6 (1.4-4.1)	7.5% (0.59-17%)	8.6% (0.51-20%)	1400 (84-3200)	11000 (900-25000)
Romania	1995	Female	90 to 94	45% (30-62%)	55% (38-70%)	2.6 (1.5-4.3)	6.7% (-0.066-17%)	7.9% (-0.33-20%)	460 (-20-1200)	2400 (-24-6100)
Romania	1995	Female	95 plus	49% (30-69%)	51% (31-70%)	2.7 (1.3-5)	6.6% (-0.91-18%)	7.7% (-1.4-22%)	130 (-23-360)	450 (-63-1200)
Romania	1995	Male	All Ages	71% (60-80%)	29% (20-40%)	7.8 (6.6-9.1)	19% (8.1-28%)	22% (7.9-34%)	34000 (12000-52000)	980000 (430000-1500000)
Romania	1995	Male	15 to 19	65% (44-84%)	35% (16-56%)	3.9 (2.4-5.9)	8.8% (3.5-15%)	15% (5.4-27%)	140 (50-260)	12000 (5000-20000)
Romania	1995	Male	20 to 24	74% (53-89%)	26% (11-47%)	6.8 (4.4-9.6)	17% (8.8-26%)	26% (12-42%)	350 (160-560)	27000 (14000-42000)
Romania	1995	Male	25 to 29	77% (59-90%)	23% (9.8-41%)	8.1 (5.5-11)	20% (11-29%)	30% (16-45%)	490 (250-750)	35000 (20000-51000)
Romania	1995	Male	30 to 34	79% (60-92%)	21% (8.1-40%)	9.6 (6.5-13)	23% (13-33%)	33% (18-48%)	590 (320-850)	37000 (21000-52000)
Romania	1995	Male	35 to 39	81% (62-92%)	19% (7.7-38%)	11 (7.6-16)	26% (15-36%)	34% (19-47%)	1300 (750-1800)	71000 (42000-98000)
Romania	1995	Male	40 to 44	82% (64-93%)	18% (7-36%)	12 (8.8-17)	26% (15-36%)	34% (19-46%)	2000 (1100-2800)	98000 (55000-140000)
Romania	1995	Male	45 to 49	81% (60-93%)	19% (7.4-40%)	13 (9.1-18)	25% (14-35%)	31% (17-43%)	2300 (1200-3200)	97000 (53000-140000)
Romania	1995	Male	50 to 54	81% (62-92%)	19% (8.3-38%)	13 (9.4-18)	24% (12-35%)	29% (14-41%)	2400 (1200-3500)	91000 (45000-130000)
Romania	1995	Male	55 to 59	79% (59-92%)	21% (7.9-41%)	13 (9.3-18)	23% (9.7-33%)	27% (12-39%)	3700 (1600-5400)	120000 (52000-180000)
Romania	1995	Male	60 to 64	78% (56-92%)	22% (8.1-44%)	13 (8.5-18)	21% (7.5-31%)	24% (9.1-37%)	4200 (1500-6300)	120000 (42000-180000)
Romania	1995	Male	65 to 69	77% (55-91%)	23% (8.7-45%)	11 (7.8-15)	20% (6.1-31%)	23% (7.2-36%)	4800 (1500-7500)	110000 (34000-170000)
Romania	1995	Male	70 to 74	75% (55-89%)	25% (11-45%)	9.8 (6.6-14)	19% (5.1-30%)	22% (5.8-34%)	4500 (1200-7200)	83000 (23000-140000)
Romania	1995	Male	75 to 79	73% (52-88%)	27% (12-48%)	8.5 (5.3-12)	16% (3.7-28%)	19% (4.1-32%)	2400 (530-4000)	34000 (7900-59000)
Romania	1995	Male	80 to 84	71% (49-87%)	29% (13-51%)	7.6 (4.7-11)	16% (2.7-28%)	17% (2.9-31%)	2600 (450-4700)	28000 (5000-51000)
Romania	1995	Male	85 to 89	70% (50-85%)	30% (15-50%)	6.8 (4.3-10)	13% (2.1-24%)	15% (2.2-28%)	1600 (240-3000)	13000 (2100-23000)

Location	Year	Sex	Age	Prevalence of current drinkers	Prevalence of abstainers	Population average of standard drinks daily	Percent of total attributable deaths	Percent of total attributable DALYs	Attributable deaths	Attributable DALYs
Romania	1995	Male	90 to 94	69% (50-87%)	31% (13-50%)	6.2 (3.7-9.7)	12% (2.3-22%)	13% (2.4-26%)	480 (83-920)	2600 (520-4900)
Romania	1995	Male	95 plus	69% (45-89%)	31% (11-55%)	5.5 (2.7-9.7)	9.9% (1.1-21%)	11% (0.78-24%)	95 (6.5-210)	350 (37-720)
Romania	2000	Female	All Ages	64% (56-72%)	36% (28-44%)	2.3 (1.9-2.8)	7.8% (3.1-13%)	11% (3.1-20%)	13000 (3700-24000)	290000 (110000-480000)
Romania	2000	Female	15 to 19	64% (45-80%)	36% (20-55%)	1.9 (1.1-2.8)	3% (1-5.4%)	7.3% (1.1-14%)	24 (3.3-46)	2600 (900-4600)
Romania	2000	Female	20 to 24	72% (56-84%)	28% (16-44%)	2.4 (1.5-3.5)	4.5% (2.2-7.2%)	8.7% (1.6-17%)	35 (6.1-66)	4400 (2100-7000)
Romania	2000	Female	25 to 29	73% (57-86%)	27% (14-43%)	2.4 (1.6-3.4)	4.7% (2.4-7.3%)	9.2% (2.5-17%)	43 (12-78)	4800 (2500-7400)
Romania	2000	Female	30 to 34	74% (57-86%)	26% (14-43%)	2.5 (1.6-3.6)	5.1% (2.7-7.8%)	10% (3.9-17%)	72 (27-120)	6300 (3300-9500)
Romania	2000	Female	35 to 39	74% (59-87%)	26% (13-41%)	2.7 (1.7-4)	6.3% (3.3-9.6%)	12% (5.7-19%)	100 (48-160)	6800 (3400-10000)
Romania	2000	Female	40 to 44	74% (58-86%)	26% (14-42%)	2.9 (2-4.2)	7.7% (4.1-12%)	14% (7.3-21%)	260 (130-390)	14000 (7500-21000)
Romania	2000	Female	45 to 49	72% (52-85%)	28% (15-48%)	3.2 (2.1-4.6)	9% (5.1-14%)	15% (8.2-23%)	440 (240-680)	21000 (12000-32000)
Romania	2000	Female	50 to 54	70% (51-84%)	30% (16-49%)	3.3 (2.2-4.6)	10% (5.3-15%)	16% (8.4-24%)	570 (300-860)	23000 (12000-35000)
Romania	2000	Female	55 to 59	68% (49-83%)	32% (17-51%)	3.3 (2.1-4.8)	10% (4.9-16%)	15% (7.6-24%)	680 (330-1100)	23000 (11000-37000)
Romania	2000	Female	60 to 64	65% (45-82%)	35% (18-55%)	3.3 (2.1-4.8)	9.9% (4-16%)	14% (5.9-23%)	1200 (500-2000)	34000 (14000-57000)
Romania	2000	Female	65 to 69	61% (42-79%)	39% (21-58%)	3.3 (2.1-4.7)	10% (3.6-18%)	14% (5.5-24%)	1700 (650-2900)	41000 (15000-69000)
Romania	2000	Female	70 to 74	56% (37-72%)	44% (28-63%)	3.1 (1.9-4.8)	10% (2.6-19%)	13% (3.6-24%)	2300 (640-4400)	45000 (12000-84000)
Romania	2000	Female	75 to 79	49% (33-68%)	51% (32-67%)	2.7 (1.6-4.3)	8.7% (1.5-18%)	11% (1.8-22%)	2400 (430-5100)	35000 (6500-74000)
Romania	2000	Female	80 to 84	44% (28-62%)	56% (38-72%)	2.4 (1.4-3.7)	7.4% (0.99-16%)	8.7% (1.1-19%)	1600 (190-3400)	17000 (2300-36000)
Romania	2000	Female	85 to 89	42% (28-58%)	58% (42-72%)	2.3 (1.3-3.6)	6.5% (0.14-15%)	7.4% (-0.00032-17%)	1100 (-0.057-2600)	8400 (180-19000)
Romania	2000	Female	90 to 94	44% (29-61%)	56% (39-71%)	2.3 (1.3-3.8)	5.6% (-0.63-14%)	6.5% (-1-18%)	480 (-75-1300)	2600 (-280-6900)
Romania	2000	Female	95 plus	48% (29-68%)	52% (32-71%)	2.4 (1.1-4.4)	5.4% (-1.7-16%)	6.2% (-2.3-19%)	130 (-49-400)	450 (-150-1400)
Romania	2000	Male	All Ages	71% (61-80%)	29% (20-39%)	7.3 (6.2-8.4)	18% (8.5-27%)	22% (8.7-33%)	30000 (12000-46000)	850000 (400000-1200000)

Location	Year	Sex	Age	Prevalence of current drinkers	Prevalence of abstainers	Population average of standard drinks daily	Percent of total attributable deaths	Percent of total attributable DALYs	Attributable deaths	Attributable DALYs
Romania	2000	Male	15 to 19	63% (42-83%)	37% (17-58%)	3.5 (2.1-5.2)	7.5% (3.1-13%)	14% (4.4-25%)	88 (29-160)	7800 (3400-13000)
Romania	2000	Male	20 to 24	73% (53-88%)	27% (12-47%)	6.1 (3.9-8.6)	15% (8.2-23%)	25% (12-40%)	250 (120-410)	20000 (11000-31000)
Romania	2000	Male	25 to 29	76% (59-90%)	24% (9.9-41%)	7.3 (5-10)	18% (10-26%)	29% (16-43%)	350 (190-530)	26000 (15000-38000)
Romania	2000	Male	30 to 34	79% (60-92%)	21% (8.2-40%)	8.7 (5.9-12)	21% (13-29%)	32% (18-45%)	570 (330-810)	37000 (23000-51000)
Romania	2000	Male	35 to 39	80% (62-92%)	20% (7.9-38%)	10 (6.8-14)	24% (15-33%)	34% (20-46%)	730 (440-1000)	41000 (25000-56000)
Romania	2000	Male	40 to 44	81% (63-93%)	19% (7.5-37%)	11 (7.9-15)	26% (15-35%)	34% (20-45%)	1600 (930-2100)	77000 (46000-100000)
Romania	2000	Male	45 to 49	81% (60-92%)	19% (7.6-40%)	12 (8.3-16)	25% (14-34%)	32% (19-43%)	2200 (1300-3100)	97000 (56000-130000)
Romania	2000	Male	50 to 54	80% (60-92%)	20% (8.5-40%)	12 (8.4-16)	24% (13-33%)	29% (16-41%)	2400 (1300-3300)	91000 (47000-130000)
Romania	2000	Male	55 to 59	79% (59-92%)	21% (7.9-41%)	12 (8.3-16)	22% (10-32%)	27% (13-39%)	2500 (1200-3600)	82000 (38000-120000)
Romania	2000	Male	60 to 64	77% (55-92%)	23% (8.2-45%)	11 (7.6-16)	21% (8.4-31%)	24% (10-36%)	3600 (1500-5400)	100000 (42000-150000)
Romania	2000	Male	65 to 69	76% (54-91%)	24% (8.7-46%)	10 (6.9-14)	20% (6.9-30%)	23% (8.3-36%)	4200 (1500-6500)	98000 (35000-150000)
Romania	2000	Male	70 to 74	74% (55-89%)	26% (11-45%)	8.8 (5.8-13)	18% (6.1-29%)	21% (7.2-34%)	4500 (1500-7100)	85000 (27000-130000)
Romania	2000	Male	75 to 79	73% (52-88%)	27% (12-48%)	7.6 (4.7-11)	16% (4.4-27%)	19% (5-31%)	3500 (950-5900)	52000 (14000-88000)
Romania	2000	Male	80 to 84	71% (50-87%)	29% (13-50%)	6.8 (4.2-9.9)	15% (3.8-25%)	17% (4.3-29%)	2000 (490-3400)	22000 (5600-37000)
Romania	2000	Male	85 to 89	69% (49-86%)	31% (14-51%)	6.1 (3.8-9.1)	13% (2.9-24%)	15% (3.2-28%)	1300 (270-2300)	9700 (2100-17000)
Romania	2000	Male	90 to 94	69% (49-87%)	31% (13-51%)	5.6 (3.2-8.9)	11% (2.2-21%)	13% (2.2-25%)	520 (89-1000)	2800 (540-5500)
Romania	2000	Male	95 plus	69% (44-89%)	31% (11-56%)	5 (2.3-8.9)	9.2% (0.49-20%)	10% (-0.044-23%)	110 (-0.47-240)	390 (21-850)
Romania	2005	Female	All Ages	61% (54-68%)	39% (32-46%)	2.1 (1.7-2.5)	6.9% (2.6-11%)	9.4% (2.7-17%)	12000 (3400-21000)	250000 (98000-410000)
Romania	2005	Female	15 to 19	61% (43-77%)	39% (23-57%)	1.7 (1-2.5)	2.8% (0.96-5.1%)	6.5% (0.79-13%)	21 (2.6-42)	2400 (830-4200)
Romania	2005	Female	20 to 24	70% (54-83%)	30% (17-46%)	2.2 (1.4-3.2)	4.2% (2-6.6%)	8.2% (1.2-16%)	24 (3.6-47)	3400 (1700-5300)
Romania	2005	Female	25 to 29	72% (58-85%)	28% (15-42%)	2.2 (1.5-3.1)	4.3% (2.3-6.6%)	8.6% (1.8-16%)	34 (6.7-62)	4200 (2200-6400)
Romania	2005	Female	30 to 34	73% (58-85%)	27% (15-42%)	2.3 (1.5-3.3)	4.5% (2.3-7%)	9.4% (3.1-16%)	53 (17-91)	5000 (2400-7800)

Location	Year	Sex	Age	Prevalence of current drinkers	Prevalence of abstainers	Population average of standard drinks daily	Percent of total attributable deaths	Percent of total attributable DALYs	Attributable deaths	Attributable DALYs
Romania	2005	Female	35 to 39	73% (58-85%)	27% (15-42%)	2.4 (1.6-3.6)	5.3% (2.7-8.3%)	11% (4.6-17%)	100 (44-170)	7300 (3600-11000)
Romania	2005	Female	40 to 44	72% (57-84%)	28% (16-43%)	2.7 (1.8-3.8)	6.7% (3.5-10%)	13% (6.4-19%)	150 (75-230)	8300 (4400-13000)
Romania	2005	Female	45 to 49	71% (53-84%)	29% (16-47%)	2.9 (2-4.2)	8% (4.3-12%)	14% (7.5-21%)	360 (190-540)	17000 (8800-25000)
Romania	2005	Female	50 to 54	68% (50-81%)	32% (19-50%)	2.9 (2-4)	8.8% (4.5-13%)	14% (7.3-21%)	550 (280-830)	22000 (12000-34000)
Romania	2005	Female	55 to 59	64% (45-79%)	36% (21-55%)	2.8 (1.9-4.1)	8.9% (4.2-14%)	14% (6.7-21%)	660 (330-1000)	23000 (11000-36000)
Romania	2005	Female	60 to 64	61% (44-78%)	39% (22-56%)	2.9 (1.9-4.2)	8.8% (3.5-14%)	13% (5.4-20%)	750 (310-1200)	22000 (8900-36000)
Romania	2005	Female	65 to 69	54% (37-73%)	46% (27-63%)	2.6 (1.7-3.8)	8.8% (3-15%)	12% (4.6-20%)	1400 (530-2300)	34000 (12000-57000)
Romania	2005	Female	70 to 74	46% (30-63%)	54% (37-70%)	2.3 (1.4-3.6)	8.2% (2.2-15%)	11% (3-20%)	1800 (500-3300)	34000 (9300-64000)
Romania	2005	Female	75 to 79	45% (32-62%)	55% (38-68%)	2.3 (1.4-3.6)	7.7% (1.5-16%)	9.5% (1.8-20%)	2200 (430-4500)	33000 (6300-67000)
Romania	2005	Female	80 to 84	43% (27-61%)	57% (39-73%)	2.1 (1.3-3.3)	6.8% (0.69-14%)	8.1% (0.76-17%)	1900 (190-4000)	21000 (2200-44000)
Romania	2005	Female	85 to 89	40% (26-57%)	60% (43-74%)	2 (1.1-3.2)	5.6% (0.057-13%)	6.5% (-0.073-15%)	890 (-10-2100)	6900 (71-16000)
Romania	2005	Female	90 to 94	41% (27-58%)	59% (42-73%)	2 (1.1-3.4)	5.1% (-1-13%)	5.8% (-1.4-16%)	400 (-99-1100)	2100 (-450-5600)
Romania	2005	Female	95 plus	45% (26-66%)	55% (34-74%)	2.1 (0.98-3.8)	4.9% (-2-15%)	5.5% (-2.6-17%)	170 (-79-530)	600 (-250-1900)
Romania	2005	Male	All Ages	75% (65-83%)	25% (17-35%)	7.6 (6.4-8.8)	19% (9-27%)	22% (9.4-33%)	30000 (13000-44000)	810000 (390000-1200000)
Romania	2005	Male	15 to 19	69% (50-85%)	31% (15-50%)	3.6 (2.3-5.4)	7.6% (3-13%)	14% (4.4-25%)	89 (29-160)	7900 (3400-13000)
Romania	2005	Male	20 to 24	78% (61-90%)	22% (9.6-39%)	6.4 (4.3-8.8)	15% (8.1-23%)	26% (13-41%)	210 (100-330)	17000 (9200-25000)
Romania	2005	Male	25 to 29	80% (65-92%)	20% (8.5-35%)	7.5 (5.2-10)	17% (10-25%)	29% (15-44%)	310 (160-460)	23000 (13000-33000)
Romania	2005	Male	30 to 34	82% (67-93%)	18% (6.9-33%)	8.8 (6.2-12)	20% (12-27%)	32% (19-45%)	480 (280-660)	31000 (19000-42000)
Romania	2005	Male	35 to 39	83% (67-93%)	17% (6.9-33%)	10 (6.8-14)	22% (14-30%)	34% (21-46%)	810 (500-1100)	46000 (29000-62000)
Romania	2005	Male	40 to 44	84% (67-93%)	16% (6.7-33%)	11 (8.1-15)	25% (15-33%)	34% (20-46%)	1000 (620-1400)	50000 (30000-67000)
Romania	2005	Male	45 to 49	84% (66-93%)	16% (6.8-34%)	12 (8.6-16)	25% (14-34%)	32% (19-43%)	2000 (1200-2700)	87000 (50000-120000)

Location	Year	Sex	Age	Prevalence of current drinkers	Prevalence of abstainers	Population average of standard drinks daily	Percent of total attributable deaths	Percent of total attributable DALYs	Attributable deaths	Attributable DALYs
Romania	2005	Male	50 to 54	82% (66-93%)	18% (7.4-34%)	12 (8.7-16)	24% (13-33%)	29% (16-40%)	2700 (1500-3700)	100000 (56000-140000)
Romania	2005	Male	55 to 59	82% (64-93%)	18% (7.3-36%)	12 (8.6-16)	22% (11-32%)	27% (14-39%)	2800 (1400-4000)	94000 (46000-130000)
Romania	2005	Male	60 to 64	79% (61-92%)	21% (7.7-39%)	11 (7.5-16)	21% (8.7-30%)	25% (11-36%)	2800 (1200-4000)	78000 (33000-110000)
Romania	2005	Male	65 to 69	77% (58-92%)	23% (8.3-42%)	9.9 (7-13)	20% (7.2-30%)	23% (8.7-35%)	4000 (1500-6000)	94000 (35000-140000)
Romania	2005	Male	70 to 74	77% (60-90%)	23% (10-40%)	8.8 (6-12)	18% (6.7-29%)	22% (7.9-34%)	4400 (1600-6700)	83000 (30000-130000)
Romania	2005	Male	75 to 79	73% (53-88%)	27% (12-47%)	7.3 (4.7-11)	16% (4.8-26%)	19% (5.8-31%)	4000 (1300-6600)	59000 (19000-96000)
Romania	2005	Male	80 to 84	70% (46-86%)	30% (14-54%)	6.3 (3.9-9.1)	15% (4.3-25%)	17% (4.9-29%)	2700 (800-4600)	30000 (8700-51000)
Romania	2005	Male	85 to 89	69% (48-85%)	31% (15-52%)	5.8 (3.6-8.6)	13% (3.7-23%)	15% (4.1-26%)	1100 (300-1900)	8600 (2400-15000)
Romania	2005	Male	90 to 94	68% (48-86%)	32% (14-52%)	5.3 (3.1-8.3)	11% (2.4-22%)	13% (2.5-25%)	430 (82-820)	2300 (480-4400)
Romania	2005	Male	95 plus	68% (44-88%)	32% (12-56%)	4.7 (2.2-8.3)	9.3% (0.094-21%)	10% (-0.35-23%)	140 (-4.9-320)	510 (5-1100)
Romania	2010	Female	All Ages	60% (53-67%)	40% (33-47%)	2.1 (1.7-2.6)	6.8% (2.7-11%)	9.4% (3-17%)	11000 (3600-21000)	230000 (92000-380000)
Romania	2010	Female	15 to 19	61% (42-76%)	39% (24-58%)	1.7 (0.99-2.6)	2.8% (0.99-5.1%)	6.9% (0.72-13%)	14 (1.4-27)	1600 (520-2700)
Romania	2010	Female	20 to 24	69% (52-82%)	31% (18-48%)	2.2 (1.3-3.3)	4.3% (2.1-6.8%)	8.2% (1.1-16%)	22 (2.9-42)	3100 (1500-4900)
Romania	2010	Female	25 to 29	72% (57-84%)	28% (16-43%)	2.2 (1.5-3.2)	4.3% (2.3-6.5%)	8.5% (1.9-15%)	24 (5.2-44)	3100 (1600-4800)
Romania	2010	Female	30 to 34	73% (57-85%)	27% (15-43%)	2.3 (1.5-3.3)	4.3% (2.2-6.6%)	9.2% (2.9-15%)	44 (14-75)	4500 (2300-6900)
Romania	2010	Female	35 to 39	72% (58-85%)	28% (15-42%)	2.5 (1.6-3.6)	5% (2.6-7.7%)	11% (4.9-17%)	82 (37-130)	6100 (3100-9200)
Romania	2010	Female	40 to 44	72% (57-84%)	28% (16-43%)	2.7 (1.9-3.9)	6% (3.1-9.3%)	12% (6.2-18%)	160 (84-250)	9600 (5000-14000)
Romania	2010	Female	45 to 49	70% (53-83%)	30% (17-47%)	3 (2-4.3)	7.4% (4-11%)	13% (7.4-20%)	220 (120-330)	11000 (5800-16000)
Romania	2010	Female	50 to 54	67% (48-80%)	33% (20-52%)	3 (2-4.1)	8.2% (4.3-13%)	14% (7.4-20%)	460 (250-680)	19000 (10000-28000)
Romania	2010	Female	55 to 59	63% (45-78%)	37% (22-55%)	2.9 (1.9-4.2)	8.4% (4-13%)	13% (6.7-20%)	680 (350-1000)	24000 (12000-37000)
Romania	2010	Female	60 to 64	60% (43-77%)	40% (23-57%)	2.9 (1.9-4.2)	8.4% (3.5-14%)	13% (5.8-20%)	800 (370-1300)	24000 (10000-39000)
Romania	2010	Female	65 to 69	53% (37-72%)	47% (28-63%)	2.7 (1.7-3.8)	8.6% (3.3-14%)	12% (5-20%)	960 (390-1600)	23000 (9100-39000)

Location	Year	Sex	Age	Prevalence of current drinkers	Prevalence of abstainers	Population average of standard drinks daily	Percent of total attributable deaths	Percent of total attributable DALYs	Attributable deaths	Attributable DALYs
Romania	2010	Female	70 to 74	46% (30-63%)	54% (37-70%)	2.3 (1.4-3.7)	8.1% (2.7-15%)	11% (3.8-20%)	1700 (580-3100)	33000 (11000-61000)
Romania	2010	Female	75 to 79	44% (31-61%)	56% (39-69%)	2.3 (1.4-3.7)	7.7% (1.8-15%)	9.8% (2.3-19%)	2100 (510-4200)	31000 (7600-62000)
Romania	2010	Female	80 to 84	42% (26-60%)	58% (40-74%)	2.2 (1.3-3.4)	7.1% (1.2-14%)	8.4% (1.4-17%)	2200 (350-4400)	23000 (4000-48000)
Romania	2010	Female	85 to 89	39% (25-55%)	61% (45-75%)	2 (1.1-3.3)	5.9% (0.44-13%)	7% (0.44-15%)	1400 (84-3000)	11000 (780-23000)
Romania	2010	Female	90 to 94	40% (27-58%)	60% (42-73%)	2 (1.1-3.4)	5.1% (-0.47-13%)	6.1% (-0.8-16%)	440 (-57-1100)	2400 (-240-6000)
Romania	2010	Female	95 plus	44% (27-64%)	56% (36-73%)	2.1 (0.98-3.9)	4.9% (-1.4-14%)	5.7% (-1.9-17%)	130 (-42-380)	460 (-130-1300)
Romania	2010	Male	All Ages	75% (66-83%)	25% (17-34%)	8 (6.7-9.6)	19% (8.8-27%)	22% (9.2-32%)	30000 (13000-44000)	780000 (380000-1100000)
Romania	2010	Male	15 to 19	68% (49-84%)	32% (16-51%)	3.7 (2.3-5.6)	8.5% (3.3-14%)	16% (5.3-27%)	69 (22-120)	5900 (2400-9900)
Romania	2010	Male	20 to 24	78% (60-91%)	22% (9.5-40%)	6.6 (4.3-9.2)	16% (8.3-24%)	26% (13-41%)	210 (100-340)	17000 (9000-25000)
Romania	2010	Male	25 to 29	79% (65-91%)	21% (8.8-35%)	7.7 (5.3-11)	17% (10-25%)	30% (16-44%)	240 (120-350)	18000 (9900-25000)
Romania	2010	Male	30 to 34	82% (68-93%)	18% (7.2-32%)	9 (6.4-12)	19% (11-27%)	32% (19-45%)	410 (240-580)	27000 (16000-37000)
Romania	2010	Male	35 to 39	83% (68-93%)	17% (6.9-32%)	11 (7-14)	21% (13-29%)	34% (20-45%)	670 (400-900)	38000 (23000-52000)
Romania	2010	Male	40 to 44	84% (67-93%)	16% (6.5-33%)	12 (8.2-16)	23% (14-31%)	34% (20-45%)	1200 (710-1600)	59000 (35000-79000)
Romania	2010	Male	45 to 49	83% (64-93%)	17% (7.1-36%)	12 (8.7-17)	24% (13-33%)	31% (18-42%)	1300 (780-1900)	59000 (34000-81000)
Romania	2010	Male	50 to 54	82% (64-92%)	18% (7.8-36%)	12 (8.7-17)	23% (13-32%)	29% (16-40%)	2500 (1400-3600)	97000 (53000-140000)
Romania	2010	Male	55 to 59	81% (63-92%)	19% (7.7-37%)	12 (8.9-17)	22% (11-31%)	27% (14-38%)	3300 (1700-4700)	110000 (54000-150000)
Romania	2010	Male	60 to 64	79% (60-92%)	21% (8.1-40%)	11 (7.9-16)	20% (8.8-30%)	24% (11-35%)	3200 (1400-4700)	90000 (39000-130000)
Romania	2010	Male	65 to 69	77% (57-91%)	23% (8.8-43%)	10 (7.2-14)	19% (7.4-29%)	23% (9-35%)	3100 (1200-4600)	72000 (28000-110000)
Romania	2010	Male	70 to 74	76% (59-90%)	24% (10-41%)	9 (6.2-13)	18% (6.8-28%)	21% (8.1-33%)	4200 (1600-6500)	80000 (29000-120000)
Romania	2010	Male	75 to 79	73% (52-88%)	27% (12-48%)	7.5 (4.8-11)	16% (5.1-26%)	19% (6-30%)	4000 (1300-6500)	58000 (19000-95000)
Romania	2010	Male	80 to 84	69% (46-86%)	31% (14-54%)	6.4 (4-9.2)	15% (4.5-25%)	17% (5.3-29%)	3300 (1000-5500)	36000 (11000-60000)

Location	Year	Sex	Age	Prevalence of current drinkers	Prevalence of abstainers	Population average of standard drinks daily	Percent of total attributable deaths	Percent of total attributable DALYs	Attributable deaths	Attributable DALYs
Romania	2010	Male	85 to 89	68% (47-85%)	32% (15-53%)	5.9 (3.7-8.7)	13% (3.6-22%)	15% (4.3-26%)	1700 (470-3000)	14000 (3700-23000)
Romania	2010	Male	90 to 94	68% (49-86%)	32% (14-51%)	5.4 (3.2-8.5)	11% (2.7-21%)	13% (2.9-24%)	450 (100-850)	2500 (600-4500)
Romania	2010	Male	95 plus	68% (43-88%)	32% (12-57%)	4.8 (2.3-8.5)	9.2% (0.64-20%)	10% (0.26-23%)	84 (2.1-180)	310 (21-640)
Romania	2016	Female	All Ages	62% (54-70%)	38% (30-46%)	2.4 (1.8-3.2)	7.2% (2.8-12%)	9.9% (2.9-19%)	12000 (3400-23000)	230000 (84000-390000)
Romania	2016	Female	15 to 19	62% (44-78%)	38% (22-56%)	1.9 (1.1-3)	2.8% (1.1-5.1%)	7.4% (0.9-15%)	11 (1.2-22)	1300 (510-2400)
Romania	2016	Female	20 to 24	71% (53-84%)	29% (16-47%)	2.4 (1.4-3.8)	4.4% (2.2-7%)	8.8% (1.4-17%)	14 (2.1-27)	2100 (1000-3300)
Romania	2016	Female	25 to 29	72% (55-85%)	28% (15-45%)	2.4 (1.4-3.8)	4.4% (2.4-6.7%)	9% (2.4-16%)	22 (5.6-39)	2900 (1500-4500)
Romania	2016	Female	30 to 34	73% (55-86%)	27% (14-45%)	2.5 (1.5-3.8)	4.4% (2.4-6.7%)	9.7% (3.6-17%)	32 (11-54)	3400 (1800-5300)
Romania	2016	Female	35 to 39	73% (57-86%)	27% (14-43%)	2.7 (1.6-4.1)	4.9% (2.5-7.6%)	11% (5-18%)	69 (29-110)	5400 (2800-8400)
Romania	2016	Female	40 to 44	73% (57-85%)	27% (15-43%)	3 (1.9-4.3)	5.8% (2.9-9.1%)	13% (6.2-19%)	140 (65-220)	8200 (4100-13000)
Romania	2016	Female	45 to 49	71% (51-84%)	29% (16-49%)	3.2 (2-4.8)	7% (3.6-11%)	14% (7.3-21%)	260 (140-410)	13000 (6800-20000)
Romania	2016	Female	50 to 54	69% (50-83%)	31% (17-50%)	3.3 (2.1-4.9)	8.3% (4.3-13%)	14% (7.9-21%)	340 (190-510)	14000 (7400-21000)
Romania	2016	Female	55 to 59	66% (47-82%)	34% (18-53%)	3.2 (2-5)	8.5% (3.9-13%)	14% (6.8-21%)	560 (280-880)	20000 (9500-32000)
Romania	2016	Female	60 to 64	63% (43-80%)	37% (20-57%)	3.2 (1.9-5.1)	8.5% (3.6-14%)	13% (5.9-22%)	890 (390-1500)	27000 (11000-46000)
Romania	2016	Female	65 to 69	59% (40-78%)	41% (22-60%)	3.2 (2-4.8)	9.1% (3.6-16%)	13% (5.7-23%)	1200 (490-2000)	29000 (12000-50000)
Romania	2016	Female	70 to 74	53% (35-71%)	47% (29-65%)	3 (1.7-4.9)	9.1% (2.7-17%)	12% (4.1-23%)	1400 (440-2500)	26000 (8100-49000)
Romania	2016	Female	75 to 79	46% (31-65%)	54% (35-69%)	2.6 (1.4-4.3)	8% (1.9-17%)	10% (2.6-21%)	2100 (520-4200)	31000 (7800-63000)
Romania	2016	Female	80 to 84	41% (25-60%)	59% (40-75%)	2.2 (1.2-3.7)	7.3% (1.3-15%)	8.9% (1.6-19%)	2400 (410-5100)	26000 (4500-55000)
Romania	2016	Female	85 to 89	40% (26-57%)	60% (43-74%)	2.2 (1.2-3.6)	6.5% (0.71-15%)	7.7% (0.76-18%)	1800 (160-4000)	13000 (1500-31000)
Romania	2016	Female	90 to 94	41% (27-58%)	59% (42-73%)	2.2 (1.1-3.8)	5.7% (-0.44-15%)	6.9% (-0.64-18%)	770 (-73-2000)	4100 (-300-11000)
Romania	2016	Female	95 plus	45% (27-65%)	55% (35-73%)	2.3 (1-4.3)	5.5% (-1.5-16%)	6.5% (-2-19%)	230 (-71-670)	820 (-210-2300)

Location	Year	Sex	Age	Prevalence of current drinkers	Prevalence of abstainers	Population average of standard drinks daily	Percent of total attributable deaths	Percent of total attributable DALYs	Attributable deaths	Attributable DALYs
Romania	2016	Male	All Ages	71% (62-80%)	29% (20-38%)	8.2 (6.1-11)	18% (8.6-25%)	21% (8.7-31%)	27000 (12000-41000)	680000 (330000-1000000)
Romania	2016	Male	15 to 19	62% (40-81%)	38% (19-60%)	3.5 (1.9-5.7)	7.7% (3.1-13%)	15% (5.2-27%)	50 (17-92)	4500 (1900-7700)
Romania	2016	Male	20 to 24	72% (51-88%)	28% (12-49%)	6.3 (3.7-10)	15% (8-23%)	26% (13-41%)	120 (59-200)	10000 (5600-16000)
Romania	2016	Male	25 to 29	75% (57-90%)	25% (10-43%)	7.6 (4.7-12)	16% (9.3-23%)	29% (15-43%)	190 (96-280)	15000 (8300-21000)
Romania	2016	Male	30 to 34	78% (58-91%)	22% (8.7-42%)	8.9 (5.7-13)	18% (11-25%)	31% (17-44%)	270 (150-390)	18000 (11000-26000)
Romania	2016	Male	35 to 39	80% (61-91%)	20% (8.8-39%)	10 (6.5-16)	19% (12-27%)	32% (19-44%)	490 (300-690)	29000 (18000-41000)
Romania	2016	Male	40 to 44	81% (63-92%)	19% (7.8-37%)	12 (7.4-17)	21% (13-29%)	32% (20-44%)	880 (540-1200)	45000 (27000-62000)
Romania	2016	Male	45 to 49	81% (60-92%)	19% (8-40%)	12 (8-18)	22% (13-30%)	30% (18-41%)	1500 (880-2100)	67000 (39000-93000)
Romania	2016	Male	50 to 54	79% (60-92%)	21% (8.3-40%)	13 (8.2-18)	22% (13-31%)	28% (16-39%)	1700 (970-2400)	66000 (37000-93000)
Romania	2016	Male	55 to 59	78% (57-91%)	22% (8.7-43%)	12 (8-18)	21% (11-30%)	26% (14-37%)	2500 (1300-3600)	84000 (44000-120000)
Romania	2016	Male	60 to 64	76% (53-91%)	24% (8.9-47%)	12 (7.3-18)	19% (9.1-28%)	24% (12-34%)	3300 (1600-4900)	93000 (44000-140000)
Romania	2016	Male	65 to 69	75% (52-90%)	25% (9.7-48%)	10 (6.7-15)	18% (7.6-28%)	22% (9.4-34%)	3400 (1500-5200)	81000 (34000-120000)
Romania	2016	Male	70 to 74	73% (52-88%)	27% (12-48%)	9.1 (5.7-14)	17% (6.6-27%)	21% (8-32%)	3100 (1200-4800)	59000 (22000-92000)
Romania	2016	Male	75 to 79	71% (50-87%)	29% (13-50%)	7.7 (4.5-12)	15% (5-25%)	18% (5.9-29%)	3400 (1100-5500)	50000 (16000-81000)
Romania	2016	Male	80 to 84	69% (45-86%)	31% (14-55%)	6.8 (4-10)	14% (3.9-24%)	17% (4.4-28%)	3300 (890-5600)	37000 (9800-61000)
Romania	2016	Male	85 to 89	68% (45-85%)	32% (15-55%)	6.2 (3.7-9.6)	13% (3.4-23%)	15% (3.9-26%)	2100 (540-3600)	16000 (4300-28000)
Romania	2016	Male	90 to 94	68% (48-86%)	32% (14-52%)	5.7 (3.2-9.3)	11% (2.4-21%)	13% (2.7-25%)	740 (150-1400)	4000 (890-7500)
Romania	2016	Male	95 plus	68% (42-88%)	32% (12-58%)	5 (2.3-9.1)	9.2% (0.36-19%)	11% (-0.021-22%)	140 (-0.28-300)	520 (20-1100)
Serbia	1990	Female	All Ages	59% (50-67%)	41% (33-50%)	1.8 (1.2-2.6)	5.1% (1.2-10%)	7% (0.95-15%)	2800 (380-6200)	69000 (15000-140000)
Serbia	1990	Female	15 to 19	59% (39-76%)	41% (24-61%)	1.4 (0.56-2.8)	2.9% (0.8-5.8%)	6.7% (0.043-15%)	8.8 (0.056-21)	1000 (280-2100)
Serbia	1990	Female	20 to 24	68% (50-83%)	32% (17-50%)	2 (0.74-3.9)	4.6% (1.8-8.3%)	8.4% (-0.058-19%)	12 (-0.077-28)	1700 (630-3100)

Location	Year	Sex	Age	Prevalence of current drinkers	Prevalence of abstainers	Population average of standard drinks daily	Percent of total attributable deaths	Percent of total attributable DALYs	Attributable deaths	Attributable DALYs
Serbia	1990	Female	25 to 29	70% (50-84%)	30% (16-50%)	2.1 (0.86-3.9)	4.6% (1.8-8.1%)	8.3% (-0.16-18%)	15 (-0.28-33)	1800 (710-3200)
Serbia	1990	Female	30 to 34	70% (50-84%)	30% (16-50%)	2.2 (1-3.9)	4.5% (1.6-7.9%)	8.6% (0.63-18%)	21 (1.5-46)	2100 (720-3800)
Serbia	1990	Female	35 to 39	71% (54-85%)	29% (15-46%)	2.5 (1.1-4.5)	4.8% (1.4-9.3%)	9.3% (1.1-20%)	41 (5-88)	3100 (860-5900)
Serbia	1990	Female	40 to 44	70% (53-84%)	30% (16-47%)	2.8 (1.3-4.9)	5.4% (1.3-11%)	10% (2.2-21%)	63 (13-130)	3800 (1000-7200)
Serbia	1990	Female	45 to 49	68% (47-83%)	32% (17-53%)	3.1 (1.4-5.7)	6.4% (1.7-13%)	12% (3-23%)	79 (22-150)	3900 (1100-7500)
Serbia	1990	Female	50 to 54	66% (45-81%)	34% (19-55%)	3.1 (1.5-5.8)	7.2% (2-14%)	12% (3.2-23%)	150 (43-290)	6300 (1800-12000)
Serbia	1990	Female	55 to 59	63% (42-80%)	37% (20-58%)	3.1 (1.4-5.3)	7.5% (1.7-15%)	11% (2.7-22%)	250 (59-490)	8800 (2000-17000)
Serbia	1990	Female	60 to 64	59% (39-78%)	41% (22-61%)	2.8 (1.3-5.4)	6.9% (0.59-15%)	9.8% (0.95-21%)	340 (33-730)	9900 (820-22000)
Serbia	1990	Female	65 to 69	55% (35-74%)	45% (26-65%)	2.4 (1.1-4.5)	6.6% (0.28-15%)	8.8% (0.54-20%)	400 (24-920)	9600 (400-22000)
Serbia	1990	Female	70 to 74	48% (31-67%)	52% (33-69%)	2 (0.79-3.9)	5.6% (-0.72-14%)	7.2% (-0.68-18%)	270 (-28-690)	5100 (-700-13000)
Serbia	1990	Female	75 to 79	41% (26-61%)	59% (39-74%)	1.6 (0.58-3.4)	4.6% (-0.78-12%)	5.7% (-0.88-15%)	340 (-54-930)	5000 (-840-14000)
Serbia	1990	Female	80 to 84	37% (22-55%)	63% (45-78%)	1.4 (0.56-2.8)	4.5% (-0.44-12%)	5.4% (-0.48-14%)	340 (-32-890)	3600 (-380-9500)
Serbia	1990	Female	85 to 89	35% (22-51%)	65% (49-78%)	1.4 (0.55-3.1)	4.5% (-0.37-12%)	5.4% (-0.4-15%)	290 (-22-830)	2200 (-180-6200)
Serbia	1990	Female	90 to 94	37% (23-54%)	63% (46-77%)	1.5 (0.56-3.6)	4.6% (-0.35-13%)	5.5% (-0.33-16%)	150 (-8.8-460)	780 (-55-2300)
Serbia	1990	Female	95 plus	41% (23-62%)	59% (38-77%)	1.7 (0.46-4.2)	4.9% (-0.12-14%)	5.8% (-0.11-17%)	51 (-0.96-150)	170 (-4.5-490)
Serbia	1990	Male	All Ages	67% (56-77%)	33% (23-44%)	2.9 (2-3.9)	9.7% (4-16%)	11% (3.4-20%)	5200 (1600-9000)	160000 (64000-260000)
Serbia	1990	Male	15 to 19	59% (37-80%)	41% (20-63%)	1.3 (0.58-2.6)	5% (1.1-9.9%)	7.5% (0.32-17%)	26 (1.1-58)	2400 (540-4800)
Serbia	1990	Male	20 to 24	70% (49-87%)	30% (13-51%)	2.4 (1.1-4.4)	9.9% (3-18%)	14% (1.8-27%)	65 (9-130)	5600 (1700-10000)
Serbia	1990	Male	25 to 29	74% (54-90%)	26% (10-46%)	3.1 (1.5-5.4)	11% (4-19%)	16% (3.3-30%)	77 (15-150)	6200 (2200-11000)
Serbia	1990	Male	30 to 34	76% (56-91%)	24% (8.7-44%)	3.6 (1.8-6)	12% (4.4-19%)	17% (4.3-31%)	100 (26-190)	7400 (2700-13000)
Serbia	1990	Male	35 to 39	78% (59-91%)	22% (9-41%)	3.8 (1.9-6.5)	12% (4.4-20%)	17% (4.7-31%)	160 (41-290)	10000 (3600-17000)
Serbia	1990	Male	40 to 44	79% (59-92%)	21% (8.4-41%)	4.2 (2.3-7.1)	12% (5.2-21%)	18% (6.4-31%)	210 (78-370)	12000 (4800-19000)

Location	Year	Sex	Age	Prevalence of current drinkers	Prevalence of abstainers	Population average of standard drinks daily	Percent of total attributable deaths	Percent of total attributable DALYs	Attributable deaths	Attributable DALYs
Serbia	1990	Male	45 to 49	79% (58-91%)	21% (8.7-42%)	4.9 (2.5-8.1)	14% (6.2-22%)	18% (7.7-30%)	240 (100-390)	11000 (5000-18000)
Serbia	1990	Male	50 to 54	78% (57-91%)	22% (9.2-43%)	5.2 (2.9-8.4)	14% (6.6-22%)	18% (8.2-28%)	440 (200-680)	18000 (8400-27000)
Serbia	1990	Male	55 to 59	76% (56-91%)	24% (9.3-44%)	5.3 (2.9-8.5)	14% (5.9-22%)	17% (7.2-27%)	690 (290-1100)	24000 (10000-38000)
Serbia	1990	Male	60 to 64	75% (53-91%)	25% (9.5-47%)	5.1 (2.7-8.8)	12% (4.6-21%)	15% (5.3-25%)	820 (300-1400)	24000 (8900-41000)
Serbia	1990	Male	65 to 69	73% (51-90%)	27% (10-49%)	4.6 (2.5-7.5)	11% (3.4-20%)	13% (3.8-23%)	750 (220-1300)	18000 (5300-33000)
Serbia	1990	Male	70 to 74	71% (50-87%)	29% (13-50%)	4 (2-7)	9.5% (1.6-18%)	11% (1.9-21%)	440 (73-870)	8500 (1400-17000)
Serbia	1990	Male	75 to 79	69% (47-86%)	31% (14-53%)	3.4 (1.5-6.3)	7.7% (0.042-17%)	8.6% (-0.026-19%)	480 (-1.5-1100)	7100 (37-16000)
Serbia	1990	Male	80 to 84	67% (43-84%)	33% (16-57%)	3 (1.4-5.5)	6.4% (-0.47-15%)	7.1% (-0.67-17%)	370 (-36-870)	4100 (-320-9500)
Serbia	1990	Male	85 to 89	65% (43-84%)	35% (16-57%)	2.8 (1.3-5.1)	4.8% (-1.5-13%)	5.3% (-1.8-15%)	210 (-72-580)	1700 (-500-4500)
Serbia	1990	Male	90 to 94	65% (43-85%)	35% (15-57%)	2.6 (1.2-4.9)	3.6% (-1.9-12%)	3.9% (-2.3-13%)	65 (-38-210)	360 (-180-1100)
Serbia	1990	Male	95 plus	66% (38-88%)	34% (12-62%)	2.3 (0.82-5)	3.3% (-2-11%)	3.5% (-2.4-12%)	14 (-10-49)	53 (-31-170)
Serbia	1995	Female	All Ages	59% (51-67%)	41% (33-49%)	1.9 (1.3-2.5)	5.2% (1.2-10%)	7% (0.89-15%)	3500 (430-7700)	82000 (18000-160000)
Serbia	1995	Female	15 to 19	59% (40-75%)	41% (25-60%)	1.4 (0.59-2.6)	2.4% (0.62-4.6%)	3.7% (0.042-8.7%)	9.1 (0.11-22)	1100 (290-2100)
Serbia	1995	Female	20 to 24	68% (51-83%)	32% (17-49%)	2 (0.76-3.9)	3.9% (1.6-7%)	5.2% (-0.069-12%)	13 (-0.18-30)	1800 (710-3200)
Serbia	1995	Female	25 to 29	70% (51-84%)	30% (16-49%)	2.1 (0.84-3.9)	4.2% (1.7-7.2%)	6% (-0.12-13%)	15 (-0.3-34)	1900 (760-3300)
Serbia	1995	Female	30 to 34	71% (51-84%)	29% (16-49%)	2.2 (1-4)	4.1% (1.5-7.3%)	6.7% (0.43-14%)	21 (1.4-46)	2100 (720-3800)
Serbia	1995	Female	35 to 39	71% (55-85%)	29% (15-45%)	2.5 (1.1-4.4)	4.6% (1.3-9%)	8% (0.71-16%)	39 (3.3-79)	2800 (780-5200)
Serbia	1995	Female	40 to 44	71% (53-85%)	29% (15-47%)	2.8 (1.3-4.7)	5.4% (1.4-10%)	9.5% (1.9-19%)	80 (15-160)	4600 (1200-9000)
Serbia	1995	Female	45 to 49	69% (47-83%)	31% (17-53%)	3.1 (1.4-5.3)	6.3% (1.8-13%)	11% (2.8-21%)	120 (31-220)	5600 (1600-11000)
Serbia	1995	Female	50 to 54	66% (47-82%)	34% (18-53%)	3.1 (1.5-5.4)	7.3% (1.9-14%)	11% (3.2-22%)	150 (41-280)	5900 (1600-11000)
Serbia	1995	Female	55 to 59	63% (43-80%)	37% (20-57%)	3 (1.5-5.3)	7.7% (1.6-15%)	11% (2.4-22%)	260 (56-510)	9000 (1800-18000)
Serbia	1995	Female	60 to 64	60% (39-79%)	40% (21-61%)	2.8 (1.3-5.2)	7.1% (0.43-15%)	9.7% (0.66-21%)	390 (27-850)	11000 (650-25000)

Location	Year	Sex	Age	Prevalence of current drinkers	Prevalence of abstainers	Population average of standard drinks daily	Percent of total attributable deaths	Percent of total attributable DALYs	Attributable deaths	Attributable DALYs
Serbia	1995	Female	65 to 69	55%<br>(36-75%)	45%<br>(25-64%)	2.4<br>(1.2-4.2)	6.7%<br>(0.018-15%)	8.8%<br>(0.26-19%)	550<br>(15-1200)	13000<br>(35-30000)
Serbia	1995	Female	70 to 74	49%<br>(31-67%)	51%<br>(33-69%)	2<br>(0.81-3.8)	5.8%<br>(-1.1-15%)	7.2%<br>(-1.2-18%)	580<br>(-94-1400)	11000<br>(-2000-27000)
Serbia	1995	Female	75 to 79	42%<br>(26-62%)	58%<br>(38-74%)	1.6<br>(0.58-3.3)	4.8%<br>(-0.66-13%)	5.9%<br>(-0.74-16%)	330<br>(-42-880)	4800<br>(-700-13000)
Serbia	1995	Female	80 to 84	37%<br>(22-56%)	63%<br>(44-78%)	1.4<br>(0.57-2.7)	4.7%<br>(-0.47-12%)	5.6%<br>(-0.56-14%)	360<br>(-36-940)	3800<br>(-390-9900)
Serbia	1995	Female	85 to 89	36%<br>(22-52%)	64%<br>(48-78%)	1.4<br>(0.57-3)	4.8%<br>(-0.3-13%)	5.6%<br>(-0.34-15%)	370<br>(-22-1000)	2700<br>(-170-7600)
Serbia	1995	Female	90 to 94	37%<br>(23-54%)	63%<br>(46-77%)	1.5<br>(0.56-3.4)	4.8%<br>(-0.18-14%)	5.6%<br>(-0.16-17%)	210<br>(-5.9-620)	1000<br>(-38-3100)
Serbia	1995	Female	95 plus	41%<br>(23-62%)	59%<br>(38-77%)	1.7<br>(0.47-4.2)	5%<br>(-0.43-15%)	5.8%<br>(-0.48-17%)	74<br>(-6.1-220)	250<br>(-22-730)
Serbia	1995	Male	All Ages	67%<br>(56-77%)	33%<br>(23-44%)	2.9<br>(2.1-3.9)	9.2%<br>(3.7-15%)	11%<br>(3.2-19%)	6200<br>(1800-11000)	180000<br>(73000-300000)
Serbia	1995	Male	15 to 19	59%<br>(38-80%)	41%<br>(20-62%)	1.4<br>(0.61-2.6)	3.4%<br>(0.86-6.6%)	3.9%<br>(0.12-8.6%)	23<br>(0.69-51)	2300<br>(570-4300)
Serbia	1995	Male	20 to 24	70%<br>(47-87%)	30%<br>(13-53%)	2.4<br>(1.1-4.2)	5.7%<br>(2.1-10%)	5.8%<br>(0.97-12%)	55<br>(9.4-110)	5100<br>(1800-8900)
Serbia	1995	Male	25 to 29	74%<br>(54-89%)	26%<br>(11-46%)	3.1<br>(1.5-5.3)	7.3%<br>(2.9-12%)	8.3%<br>(1.8-16%)	68<br>(15-130)	5700<br>(2200-9700)
Serbia	1995	Male	30 to 34	76%<br>(56-91%)	24%<br>(9.4-44%)	3.5<br>(1.8-5.8)	8.6%<br>(3.3-14%)	11%<br>(2.7-20%)	88<br>(22-160)	6500<br>(2500-11000)
Serbia	1995	Male	35 to 39	78%<br>(59-91%)	22%<br>(8.5-41%)	3.7<br>(1.9-6.3)	10%<br>(3.6-17%)	13%<br>(3.5-24%)	140<br>(36-250)	8800<br>(3200-15000)
Serbia	1995	Male	40 to 44	79%<br>(59-92%)	21%<br>(7.8-41%)	4.2<br>(2.3-6.7)	12%<br>(4.8-20%)	15%<br>(5.5-27%)	260<br>(93-450)	14000<br>(5700-24000)
Serbia	1995	Male	45 to 49	79%<br>(56-92%)	21%<br>(8.4-44%)	4.8<br>(2.6-7.8)	13%<br>(5.7-21%)	17%<br>(7-28%)	360<br>(150-590)	17000<br>(7300-27000)
Serbia	1995	Male	50 to 54	78%<br>(57-91%)	22%<br>(9.3-43%)	5.1<br>(2.8-8)	14%<br>(6.4-21%)	17%<br>(7.5-27%)	400<br>(180-640)	16000<br>(7400-25000)
Serbia	1995	Male	55 to 59	76%<br>(55-91%)	24%<br>(9-45%)	5.3<br>(2.9-8.2)	14%<br>(5.8-22%)	16%<br>(6.8-26%)	700<br>(290-1100)	24000<br>(10000-38000)
Serbia	1995	Male	60 to 64	75%<br>(51-91%)	25%<br>(9.3-49%)	5.1<br>(2.6-8.5)	12%<br>(4.1-21%)	14%<br>(4.6-24%)	970<br>(320-1700)	28000<br>(9200-48000)
Serbia	1995	Male	65 to 69	74%<br>(50-90%)	26%<br>(10-50%)	4.6<br>(2.6-7.3)	11%<br>(3.2-20%)	13%<br>(3.6-23%)	1100<br>(310-2000)	26000<br>(7500-47000)
Serbia	1995	Male	70 to 74	72%<br>(50-87%)	28%<br>(13-50%)	3.9<br>(2.1-6.7)	9.6%<br>(1.4-19%)	11%<br>(1.6-22%)	880<br>(130-1700)	17000<br>(2400-33000)
Serbia	1995	Male	75 to 79	69%<br>(47-87%)	31%<br>(13-53%)	3.3<br>(1.6-6.1)	7.6%<br>(0.24-16%)	8.6%<br>(0.18-19%)	420<br>(9.1-900)	6200<br>(200-13000)
Serbia	1995	Male	80 to 84	67%<br>(44-84%)	33%<br>(16-56%)	3<br>(1.5-5.1)	6.5%<br>(-0.82-15%)	7.1%<br>(-1-16%)	360<br>(-50-800)	3900<br>(-490-8700)

Location	Year	Sex	Age	Prevalence of current drinkers	Prevalence of abstainers	Population average of standard drinks daily	Percent of total attributable deaths	Percent of total attributable DALYs	Attributable deaths	Attributable DALYs
Serbia	1995	Male	85 to 89	65% (44-83%)	35% (17-56%)	2.7 (1.3-4.8)	4.9% (-1.3-13%)	5.3% (-1.6-14%)	230 (-68-600)	1800 (-460-4700)
Serbia	1995	Male	90 to 94	65% (44-85%)	35% (15-56%)	2.5 (1.2-4.8)	3.6% (-1.7-11%)	3.9% (-2-12%)	86 (-45-280)	470 (-220-1500)
Serbia	1995	Male	95 plus	65% (39-88%)	35% (12-61%)	2.3 (0.83-4.9)	3.2% (-1.9-10%)	3.4% (-2.4-12%)	20 (-14-69)	75 (-45-250)
Serbia	2000	Female	All Ages	59% (50-67%)	41% (33-50%)	2 (1.5-2.6)	5.7% (1.3-11%)	7.5% (1.1-16%)	4000 (560-8800)	90000 (21000-180000)
Serbia	2000	Female	15 to 19	59% (39-75%)	41% (25-61%)	1.4 (0.63-2.7)	2.7% (0.85-5.1%)	6.1% (0.23-14%)	7.2 (0.28-16)	880 (270-1700)
Serbia	2000	Female	20 to 24	68% (50-83%)	32% (17-50%)	2 (0.8-3.9)	4.5% (1.9-7.7%)	7.9% (0.17-17%)	12 (0.25-26)	1600 (690-2900)
Serbia	2000	Female	25 to 29	70% (51-84%)	30% (16-49%)	2.2 (0.93-4.1)	4.5% (1.9-7.5%)	8.1% (0.48-18%)	13 (0.77-29)	1700 (730-3000)
Serbia	2000	Female	30 to 34	70% (51-84%)	30% (16-49%)	2.3 (1.1-4.2)	4.4% (1.7-7.8%)	8.5% (0.81-18%)	20 (1.8-41)	2000 (760-3400)
Serbia	2000	Female	35 to 39	71% (53-85%)	29% (15-47%)	2.5 (1.1-4.4)	4.8% (1.4-8.9%)	9.3% (1.3-19%)	33 (4.6-68)	2400 (730-4500)
Serbia	2000	Female	40 to 44	70% (53-84%)	30% (16-47%)	2.8 (1.4-4.8)	5.5% (1.5-11%)	10% (2.4-20%)	62 (14-120)	3600 (970-6800)
Serbia	2000	Female	45 to 49	69% (47-84%)	31% (16-53%)	3.1 (1.6-5.4)	6.7% (1.9-13%)	12% (3.2-22%)	140 (38-260)	6500 (1900-12000)
Serbia	2000	Female	50 to 54	66% (46-82%)	34% (18-54%)	3.2 (1.6-5.3)	7.3% (2.2-14%)	12% (3.9-22%)	180 (59-340)	7500 (2300-14000)
Serbia	2000	Female	55 to 59	63% (43-81%)	37% (19-57%)	3.2 (1.5-5.4)	8% (2-15%)	12% (3-22%)	220 (56-420)	7500 (1900-14000)
Serbia	2000	Female	60 to 64	59% (39-78%)	41% (22-61%)	2.9 (1.4-5.3)	7.4% (0.88-16%)	10% (1.4-21%)	370 (48-770)	11000 (1300-22000)
Serbia	2000	Female	65 to 69	55% (35-74%)	45% (26-65%)	2.5 (1.3-4.3)	7.1% (0.6-15%)	9.3% (0.9-20%)	590 (57-1300)	14000 (1100-31000)
Serbia	2000	Female	70 to 74	49% (31-66%)	51% (34-69%)	2 (0.89-3.8)	6.1% (-0.55-15%)	7.6% (-0.61-19%)	710 (-58-1700)	13000 (-1300-33000)
Serbia	2000	Female	75 to 79	42% (26-61%)	58% (39-74%)	1.6 (0.66-3.2)	5.2% (-0.7-13%)	6.2% (-0.73-16%)	670 (-81-1700)	9700 (-1300-26000)
Serbia	2000	Female	80 to 84	37% (22-55%)	63% (45-78%)	1.5 (0.61-2.7)	5% (-0.19-13%)	6% (-0.23-15%)	470 (-18-1200)	5000 (-180-13000)
Serbia	2000	Female	85 to 89	35% (22-51%)	65% (49-78%)	1.5 (0.6-2.9)	5.1% (0.21-13%)	6% (0.31-16%)	290 (14-760)	2200 (91-5600)
Serbia	2000	Female	90 to 94	37% (24-54%)	63% (46-76%)	1.6 (0.59-3.4)	5% (0.065-14%)	5.9% (0.097-16%)	190 (3.1-530)	960 (13-2700)
Serbia	2000	Female	95 plus	41% (24-62%)	59% (38-76%)	1.8 (0.48-4.1)	5.1% (-0.26-15%)	6% (-0.23-18%)	74 (-2.9-230)	250 (-12-750)
Serbia	2000	Male	All Ages	68% (57-78%)	32% (22-43%)	3.1 (2.4-3.9)	11% (4.7-17%)	12% (4.2-21%)	6900 (2400-12000)	190000 (82000-310000)

Location	Year	Sex	Age	Prevalence of current drinkers	Prevalence of abstainers	Population average of standard drinks daily	Percent of total attributable deaths	Percent of total attributable DALYs	Attributable deaths	Attributable DALYs
Serbia	2000	Male	15 to 19	59%<br>(36-80%)	41%<br>(20-64%)	1.4<br>(0.66-2.5)	4.5%<br>(1.2-8.6%)	6.9%<br>(0.36-15%)	19<br>(0.98-42)	1900<br>(530-3600)
Serbia	2000	Male	20 to 24	70%<br>(48-87%)	30%<br>(13-52%)	2.5<br>(1.2-4.2)	8.7%<br>(3.4-15%)	12%<br>(2.2-23%)	52<br>(9.2-100)	4700<br>(1800-8400)
Serbia	2000	Male	25 to 29	74%<br>(53-89%)	26%<br>(11-47%)	3.2<br>(1.7-5.5)	9.9%<br>(4.3-17%)	15%<br>(3.9-28%)	62<br>(15-120)	5300<br>(2300-8900)
Serbia	2000	Male	30 to 34	76%<br>(56-91%)	24%<br>(9.4-44%)	3.7<br>(2-6)	10%<br>(4.4-17%)	16%<br>(4.8-29%)	76<br>(22-140)	5800<br>(2400-9500)
Serbia	2000	Male	35 to 39	78%<br>(59-91%)	22%<br>(8.9-41%)	3.9<br>(2-6.4)	11%<br>(4.7-20%)	17%<br>(5.1-30%)	110<br>(35-200)	7300<br>(2900-12000)
Serbia	2000	Male	40 to 44	79%<br>(59-92%)	21%<br>(8.3-41%)	4.4<br>(2.5-6.9)	13%<br>(5.3-21%)	18%<br>(6.4-31%)	210<br>(77-350)	11000<br>(4800-19000)
Serbia	2000	Male	45 to 49	79%<br>(57-92%)	21%<br>(8.3-43%)	5.1<br>(2.9-7.8)	14%<br>(6.6-22%)	19%<br>(8-30%)	420<br>(180-690)	19000<br>(9100-31000)
Serbia	2000	Male	50 to 54	78%<br>(57-91%)	22%<br>(9.1-43%)	5.4<br>(3.3-8)	14%<br>(7.2-22%)	18%<br>(8.8-28%)	550<br>(270-860)	22000<br>(11000-34000)
Serbia	2000	Male	55 to 59	76%<br>(55-91%)	24%<br>(8.6-45%)	5.5<br>(3.2-8.4)	14%<br>(6.8-22%)	17%<br>(8.2-27%)	590<br>(280-930)	20000<br>(9600-31000)
Serbia	2000	Male	60 to 64	75%<br>(51-91%)	25%<br>(9.2-49%)	5.3<br>(2.8-8.4)	13%<br>(5-22%)	15%<br>(5.9-26%)	880<br>(340-1500)	25000<br>(9900-43000)
Serbia	2000	Male	65 to 69	74%<br>(50-90%)	26%<br>(9.7-50%)	4.8<br>(2.8-7.2)	12%<br>(4.2-21%)	14%<br>(4.8-24%)	1200<br>(420-2100)	29000<br>(9800-50000)
Serbia	2000	Male	70 to 74	72%<br>(50-87%)	28%<br>(13-50%)	4.1<br>(2.2-6.9)	10%<br>(2.4-19%)	12%<br>(2.7-22%)	1200<br>(270-2200)	23000<br>(5100-43000)
Serbia	2000	Male	75 to 79	69%<br>(47-86%)	31%<br>(14-53%)	3.5<br>(1.7-6)	8.3%<br>(0.89-17%)	9.4%<br>(0.96-19%)	810<br>(82-1700)	12000<br>(1300-24000)
Serbia	2000	Male	80 to 84	67%<br>(44-85%)	33%<br>(15-56%)	3.1<br>(1.6-5.2)	7.1%<br>(0.13-15%)	7.9%<br>(0.032-17%)	420<br>(1.7-900)	4600<br>(87-9800)
Serbia	2000	Male	85 to 89	66%<br>(43-83%)	34%<br>(17-57%)	2.8<br>(1.4-5)	5.5%<br>(-0.57-13%)	6.1%<br>(-0.74-14%)	170<br>(-20-390)	1300<br>(-120-3000)
Serbia	2000	Male	90 to 94	65%<br>(44-86%)	35%<br>(14-56%)	2.6<br>(1.3-4.9)	4.2%<br>(-0.99-11%)	4.6%<br>(-1.4-13%)	74<br>(-22-210)	400<br>(-98-1100)
Serbia	2000	Male	95 plus	66%<br>(39-88%)	34%<br>(12-61%)	2.4<br>(0.9-4.8)	3.6%<br>(-1.5-11%)	3.9%<br>(-1.9-12%)	22<br>(-11-69)	79<br>(-33-240)
Serbia	2005	Female	All Ages	59%<br>(51-67%)	41%<br>(33-49%)	2.2<br>(1.8-2.7)	6%<br>(1.5-11%)	7.8%<br>(1.1-16%)	4300<br>(630-8800)	91000<br>(23000-170000)
Serbia	2005	Female	15 to 19	59%<br>(39-75%)	41%<br>(25-61%)	1.6<br>(0.71-2.9)	2.7%<br>(0.96-4.9%)	7.1%<br>(0.43-16%)	5.9<br>(0.37-13)	800<br>(290-1500)
Serbia	2005	Female	20 to 24	68%<br>(51-83%)	32%<br>(17-49%)	2.2<br>(0.95-4.3)	4.6%<br>(2.1-7.7%)	9%<br>(0.67-19%)	10<br>(0.75-22)	1500<br>(690-2500)
Serbia	2005	Female	25 to 29	70%<br>(51-84%)	30%<br>(16-49%)	2.4<br>(1-4.4)	4.6%<br>(2.2-7.5%)	9%<br>(1.2-19%)	12<br>(1.6-26)	1700<br>(800-2900)
Serbia	2005	Female	30 to 34	71%<br>(51-84%)	29%<br>(16-49%)	2.6<br>(1.2-4.6)	4.6%<br>(2.1-7.9%)	9.4%<br>(1.7-19%)	19<br>(3.3-37)	2000<br>(840-3400)

Location	Year	Sex	Age	Prevalence of current drinkers	Prevalence of abstainers	Population average of standard drinks daily	Percent of total attributable deaths	Percent of total attributable DALYs	Attributable deaths	Attributable DALYs
Serbia	2005	Female	35 to 39	71% (55-85%)	29% (15-45%)	2.8 (1.3-4.9)	4.8% (1.8-8.8%)	10% (2.1-20%)	28 (5.9-55)	2200 (780-4000)
Serbia	2005	Female	40 to 44	70% (53-84%)	30% (16-47%)	3.1 (1.5-5.2)	5.8% (1.9-11%)	11% (3-20%)	57 (16-110)	3400 (1100-6200)
Serbia	2005	Female	45 to 49	69% (47-83%)	31% (17-53%)	3.5 (1.7-6.1)	6.9% (2.3-13%)	12% (3.9-22%)	110 (37-200)	5300 (1800-9700)
Serbia	2005	Female	50 to 54	66% (47-82%)	34% (18-53%)	3.6 (1.9-5.8)	7.5% (2.5-14%)	12% (4.4-21%)	200 (75-370)	8400 (3000-15000)
Serbia	2005	Female	55 to 59	63% (43-80%)	37% (20-57%)	3.5 (1.8-5.9)	7.8% (2.5-15%)	12% (4.1-22%)	260 (95-480)	9300 (3100-17000)
Serbia	2005	Female	60 to 64	60% (39-78%)	40% (22-61%)	3.2 (1.6-5.8)	7.7% (1.5-15%)	11% (2.5-21%)	280 (65-560)	8300 (1600-17000)
Serbia	2005	Female	65 to 69	55% (35-75%)	45% (25-65%)	2.8 (1.4-4.6)	7.5% (1.1-16%)	9.8% (1.6-21%)	500 (84-1000)	12000 (1800-25000)
Serbia	2005	Female	70 to 74	49% (31-67%)	51% (33-69%)	2.3 (1-4.2)	6.4% (-0.36-15%)	8.1% (-0.29-19%)	710 (-25-1600)	13000 (-770-32000)
Serbia	2005	Female	75 to 79	42% (26-62%)	58% (38-74%)	1.8 (0.74-3.4)	5.5% (-0.61-14%)	6.6% (-0.65-16%)	790 (-78-2000)	11000 (-1200-28000)
Serbia	2005	Female	80 to 84	37% (22-55%)	63% (45-78%)	1.6 (0.68-2.9)	5.4% (-0.061-13%)	6.4% (0.01-15%)	660 (0.99-1600)	7100 (-82-17000)
Serbia	2005	Female	85 to 89	36% (22-52%)	64% (48-78%)	1.6 (0.66-3.2)	5.5% (0.45-14%)	6.6% (0.51-17%)	380 (31-980)	2800 (230-7200)
Serbia	2005	Female	90 to 94	37% (24-55%)	63% (45-76%)	1.8 (0.65-3.9)	5.5% (0.36-15%)	6.5% (0.48-18%)	150 (11-400)	750 (47-2000)
Serbia	2005	Female	95 plus	41% (24-62%)	59% (38-76%)	2 (0.55-4.6)	5.5% (-0.19-16%)	6.5% (-0.17-19%)	66 (-1.7-200)	220 (-7.2-650)
Serbia	2005	Male	All Ages	68% (58-79%)	32% (21-42%)	3.5 (2.8-4.3)	11% (5.1-17%)	13% (4.9-21%)	7100 (2700-12000)	190000 (89000-300000)
Serbia	2005	Male	15 to 19	60% (37-81%)	40% (19-63%)	1.6 (0.74-2.7)	4.8% (1.5-8.9%)	8.2% (0.92-18%)	17 (1.9-37)	1800 (570-3300)
Serbia	2005	Male	20 to 24	70% (49-87%)	30% (13-51%)	2.8 (1.4-4.6)	9.8% (3.9-17%)	15% (3-28%)	48 (10-94)	4400 (1800-7600)
Serbia	2005	Male	25 to 29	74% (54-90%)	26% (10-46%)	3.5 (1.9-5.9)	11% (5-18%)	17% (5-32%)	64 (18-120)	5400 (2400-9100)
Serbia	2005	Male	30 to 34	76% (55-91%)	24% (9.3-45%)	4 (2.2-6.4)	11% (4.9-18%)	19% (6.2-33%)	77 (26-140)	5900 (2600-9600)
Serbia	2005	Male	35 to 39	78% (59-91%)	22% (9-41%)	4.3 (2.3-6.8)	12% (5.5-20%)	19% (7.1-33%)	100 (38-180)	6800 (3100-11000)
Serbia	2005	Male	40 to 44	79% (59-91%)	21% (8.5-41%)	4.8 (2.8-7.5)	13% (5.8-22%)	20% (7.8-32%)	180 (72-300)	9900 (4300-16000)
Serbia	2005	Male	45 to 49	79% (57-92%)	21% (8.3-43%)	5.6 (3.2-8.5)	15% (7.4-23%)	20% (9.8-31%)	350 (160-550)	16000 (7800-25000)
Serbia	2005	Male	50 to 54	78% (58-91%)	22% (9-42%)	5.9 (3.7-8.7)	15% (8.1-23%)	19% (10-29%)	630 (340-960)	25000 (14000-38000)

Location	Year	Sex	Age	Prevalence of current drinkers	Prevalence of abstainers	Population average of standard drinks daily	Percent of total attributable deaths	Percent of total attributable DALYs	Attributable deaths	Attributable DALYs
Serbia	2005	Male	55 to 59	76% (56-91%)	24% (8.8-44%)	6.1 (3.6-9.1)	14% (7.5-22%)	18% (9.3-28%)	740 (390-1200)	26000 (13000-40000)
Serbia	2005	Male	60 to 64	75% (53-91%)	25% (9.1-47%)	5.8 (3.2-9.3)	13% (5.7-22%)	16% (6.8-26%)	700 (300-1100)	20000 (8600-33000)
Serbia	2005	Male	65 to 69	74% (50-90%)	26% (10-50%)	5.3 (3.2-7.9)	13% (5.4-21%)	15% (6.2-24%)	1100 (440-1800)	25000 (11000-42000)
Serbia	2005	Male	70 to 74	72% (51-87%)	28% (13-49%)	4.5 (2.6-7.5)	11% (3.1-20%)	12% (3.6-22%)	1200 (370-2200)	24000 (7000-42000)
Serbia	2005	Male	75 to 79	69% (47-86%)	31% (14-53%)	3.8 (2-6.5)	8.9% (1.5-17%)	10% (1.7-19%)	1000 (170-2000)	15000 (2400-30000)
Serbia	2005	Male	80 to 84	67% (42-84%)	33% (16-58%)	3.4 (1.8-5.6)	7.7% (0.89-15%)	8.7% (1.1-18%)	610 (76-1200)	6700 (770-13000)
Serbia	2005	Male	85 to 89	65% (43-83%)	35% (17-57%)	3.1 (1.6-5.2)	6.4% (0.26-14%)	7.2% (0.16-16%)	230 (5.1-490)	1800 (74-3800)
Serbia	2005	Male	90 to 94	65% (44-84%)	35% (16-56%)	2.9 (1.4-5.1)	5.1% (-0.39-13%)	5.6% (-0.59-15%)	59 (-6.3-150)	320 (-25-800)
Serbia	2005	Male	95 plus	65% (39-88%)	35% (12-61%)	2.6 (1-5.3)	4.3% (-1.2-12%)	4.7% (-1.6-14%)	19 (-6.5-56)	68 (-19-200)
Serbia	2010	Female	All Ages	59% (51-67%)	41% (33-49%)	2.5 (2-3.1)	5.9% (1.6-11%)	7.7% (1.3-16%)	4100 (730-8600)	87000 (25000-160000)
Serbia	2010	Female	15 to 19	59% (39-76%)	41% (24-61%)	1.7 (0.74-3.2)	2.8% (1-5%)	7.4% (0.63-16%)	5.1 (0.36-11)	710 (260-1200)
Serbia	2010	Female	20 to 24	68% (50-83%)	32% (17-50%)	2.5 (1-4.6)	4.7% (2.1-7.8%)	9.4% (1.1-20%)	9 (1.1-19)	1400 (650-2300)
Serbia	2010	Female	25 to 29	70% (50-84%)	30% (16-50%)	2.6 (1.1-4.7)	4.9% (2.3-7.9%)	9.5% (1.4-20%)	12 (1.7-24)	1600 (740-2600)
Serbia	2010	Female	30 to 34	71% (50-84%)	29% (16-50%)	2.8 (1.3-4.8)	4.7% (2.2-7.9%)	9.8% (2-19%)	18 (3.8-35)	1900 (870-3200)
Serbia	2010	Female	35 to 39	71% (54-85%)	29% (15-46%)	3.1 (1.4-5.4)	4.9% (2-8.7%)	10% (2.6-20%)	28 (6.9-55)	2400 (970-4100)
Serbia	2010	Female	40 to 44	70% (53-84%)	30% (16-47%)	3.4 (1.7-5.8)	5.7% (2.1-10%)	11% (3.8-20%)	51 (17-94)	3200 (1200-5700)
Serbia	2010	Female	45 to 49	69% (47-83%)	31% (17-53%)	3.8 (1.9-6.8)	6.7% (2.5-12%)	12% (4.6-22%)	93 (35-170)	4700 (1700-8500)
Serbia	2010	Female	50 to 54	66% (47-81%)	34% (19-53%)	3.9 (2.1-6.6)	7.4% (2.8-13%)	12% (4.8-21%)	150 (63-260)	6500 (2500-11000)
Serbia	2010	Female	55 to 59	63% (44-79%)	37% (21-56%)	3.8 (1.9-6.3)	7.7% (2.6-14%)	12% (4.5-21%)	270 (100-490)	10000 (3500-18000)
Serbia	2010	Female	60 to 64	59% (38-78%)	41% (22-62%)	3.5 (1.7-6.2)	7.2% (1.9-14%)	11% (3.2-20%)	320 (95-620)	10000 (2600-19000)
Serbia	2010	Female	65 to 69	55% (36-75%)	45% (25-64%)	3 (1.6-4.9)	7.3% (1.5-15%)	9.9% (2-20%)	380 (75-770)	9300 (1700-19000)
Serbia	2010	Female	70 to 74	49% (31-67%)	51% (33-69%)	2.5 (1.1-4.6)	6.3% (0.034-15%)	8.1% (0.21-19%)	570 (15-1300)	11000 (62-26000)

Location	Year	Sex	Age	Prevalence of current drinkers	Prevalence of abstainers	Population average of standard drinks daily	Percent of total attributable deaths	Percent of total attributable DALYs	Attributable deaths	Attributable DALYs
Serbia	2010	Female	75 to 79	42% (26-60%)	58% (40-74%)	2 (0.79-3.8)	5.5% (-0.45-14%)	6.7% (-0.52-16%)	740 (-54-1800)	11000 (-910-27000)
Serbia	2010	Female	80 to 84	37% (22-55%)	63% (45-78%)	1.8 (0.75-3.2)	5.4% (0.023-13%)	6.4% (0.0082-16%)	700 (0.91-1700)	7600 (36-18000)
Serbia	2010	Female	85 to 89	35% (22-51%)	65% (49-78%)	1.8 (0.73-3.5)	5.5% (0.42-14%)	6.5% (0.52-17%)	540 (44-1400)	4100 (300-11000)
Serbia	2010	Female	90 to 94	37% (23-54%)	63% (46-77%)	1.9 (0.72-4)	5.4% (0.39-15%)	6.4% (0.47-18%)	200 (14-550)	1000 (73-2800)
Serbia	2010	Female	95 plus	40% (23-61%)	60% (39-77%)	2.1 (0.56-4.9)	5.4% (-0.15-15%)	6.4% (-0.15-18%)	52 (-1.2-150)	180 (-4.7-500)
Serbia	2010	Male	All Ages	69% (59-78%)	31% (22-41%)	4 (3.2-4.9)	11% (5.3-18%)	13% (5-21%)	7200 (2900-12000)	190000 (90000-300000)
Serbia	2010	Male	15 to 19	59% (38-80%)	41% (20-62%)	1.7 (0.81-3)	4.9% (1.7-9%)	8.9% (1.1-19%)	14 (1.8-30)	1500 (550-2800)
Serbia	2010	Male	20 to 24	70% (49-87%)	30% (13-51%)	3.1 (1.6-5.2)	10% (4.3-17%)	16% (3.6-30%)	41 (9.4-81)	4000 (1700-6800)
Serbia	2010	Male	25 to 29	74% (54-89%)	26% (11-46%)	3.9 (2-6.4)	12% (5.3-19%)	19% (6-35%)	60 (18-110)	5100 (2300-8400)
Serbia	2010	Male	30 to 34	76% (56-90%)	24% (9.5-44%)	4.4 (2.4-7)	12% (5.5-19%)	20% (7-35%)	81 (27-140)	6100 (2800-10000)
Serbia	2010	Male	35 to 39	79% (61-91%)	21% (8.7-39%)	4.7 (2.6-7.4)	12% (5.6-20%)	20% (8.2-35%)	100 (42-180)	7000 (3400-11000)
Serbia	2010	Male	40 to 44	79% (59-92%)	21% (8.3-41%)	5.3 (3.1-8.1)	13% (6.3-21%)	21% (9.1-34%)	170 (73-270)	9500 (4500-15000)
Serbia	2010	Male	45 to 49	79% (55-92%)	21% (8.4-45%)	6.1 (3.5-9.6)	15% (8.2-23%)	21% (11-32%)	300 (160-470)	14000 (7600-22000)
Serbia	2010	Male	50 to 54	78% (57-91%)	22% (9.3-43%)	6.4 (3.9-9.5)	15% (8.2-23%)	19% (10-29%)	500 (270-750)	20000 (11000-31000)
Serbia	2010	Male	55 to 59	76% (54-91%)	24% (9.3-46%)	6.6 (3.9-9.9)	15% (7.5-23%)	18% (9.3-27%)	810 (440-1300)	28000 (15000-44000)
Serbia	2010	Male	60 to 64	75% (51-91%)	25% (9.2-49%)	6.3 (3.5-10)	13% (6.1-21%)	16% (7.5-25%)	860 (410-1400)	26000 (12000-41000)
Serbia	2010	Male	65 to 69	74% (50-90%)	26% (10-50%)	5.7 (3.5-8.3)	13% (5.5-21%)	15% (6.6-24%)	830 (360-1400)	20000 (8700-34000)
Serbia	2010	Male	70 to 74	71% (49-87%)	29% (13-51%)	4.9 (2.8-8.2)	11% (3.9-20%)	13% (4.4-23%)	1100 (370-1900)	21000 (7100-37000)
Serbia	2010	Male	75 to 79	69% (46-86%)	31% (14-54%)	4.2 (2.2-7.4)	9.2% (2-18%)	10% (2.4-20%)	1100 (250-2100)	16000 (3600-31000)
Serbia	2010	Male	80 to 84	67% (42-85%)	33% (15-58%)	3.8 (2-6.1)	8.2% (1.4-16%)	9.2% (1.5-19%)	750 (130-1500)	8400 (1500-17000)
Serbia	2010	Male	85 to 89	65% (43-83%)	35% (17-57%)	3.4 (1.7-5.7)	6.9% (0.92-15%)	7.7% (0.88-17%)	380 (44-840)	3000 (400-6500)
Serbia	2010	Male	90 to 94	65% (44-85%)	35% (15-56%)	3.2 (1.5-5.6)	5.6% (-0.29-14%)	6.2% (-0.51-16%)	94 (-7.6-240)	510 (-27-1300)

Location	Year	Sex	Age	Prevalence of current drinkers	Prevalence of abstainers	Population average of standard drinks daily	Percent of total attributable deaths	Percent of total attributable DALYs	Attributable deaths	Attributable DALYs
Serbia	2010	Male	95 plus	66% (40-88%)	34% (12-60%)	2.9 (1.1-5.6)	4.8% (-0.79-13%)	5.2% (-1-15%)	17 (-3.3-46)	61 (-9.8-160)
Serbia	2016	Female	All Ages	58% (50-67%)	42% (33-50%)	2.6 (1.9-3.4)	5.6% (1.5-11%)	7.1% (1.1-16%)	3700 (550-8100)	78000 (21000-150000)
Serbia	2016	Female	15 to 19	58% (39-76%)	42% (24-61%)	1.8 (0.72-3.4)	3% (1.1-5.4%)	7.7% (0.8-16%)	5.5 (0.59-12)	720 (260-1300)
Serbia	2016	Female	20 to 24	68% (50-82%)	32% (18-50%)	2.6 (1-4.9)	4.9% (2.2-8.4%)	9.7% (1.1-20%)	9.1 (1.1-19)	1300 (620-2200)
Serbia	2016	Female	25 to 29	70% (50-83%)	30% (17-50%)	2.7 (1.2-5)	5% (2.5-8.1%)	9.8% (1.8-20%)	12 (1.9-24)	1600 (770-2600)
Serbia	2016	Female	30 to 34	70% (50-84%)	30% (16-50%)	2.9 (1.3-5.1)	4.9% (2.4-8.2%)	10% (2.3-20%)	17 (3.7-33)	1800 (870-3000)
Serbia	2016	Female	35 to 39	71% (54-84%)	29% (16-46%)	3.2 (1.5-5.8)	5.1% (2.2-9.3%)	11% (3.1-21%)	29 (7.4-57)	2400 (990-4200)
Serbia	2016	Female	40 to 44	70% (53-84%)	30% (16-47%)	3.6 (1.7-6.1)	5.9% (2.2-11%)	12% (3.7-22%)	54 (17-100)	3500 (1300-6200)
Serbia	2016	Female	45 to 49	68% (46-83%)	32% (17-54%)	3.9 (1.9-7.2)	6.5% (2.6-12%)	12% (4.7-22%)	87 (34-160)	4600 (1800-8200)
Serbia	2016	Female	50 to 54	65% (45-81%)	35% (19-55%)	4 (2-7)	7.1% (2.7-13%)	12% (4.8-21%)	130 (53-240)	5800 (2200-11000)
Serbia	2016	Female	55 to 59	62% (43-80%)	38% (20-57%)	3.9 (1.9-6.6)	7.3% (2.4-13%)	11% (4.2-21%)	190 (70-340)	7100 (2400-13000)
Serbia	2016	Female	60 to 64	59% (38-78%)	41% (22-62%)	3.6 (1.7-6.6)	6.9% (1.7-13%)	10% (2.9-20%)	310 (88-590)	9800 (2500-19000)
Serbia	2016	Female	65 to 69	55% (35-74%)	45% (26-65%)	3.2 (1.6-5.2)	6.8% (1.3-14%)	9.5% (2.1-20%)	440 (96-910)	11000 (2100-23000)
Serbia	2016	Female	70 to 74	48% (30-67%)	52% (33-70%)	2.6 (1.1-4.7)	6% (0.1-14%)	7.9% (0.28-19%)	420 (15-960)	8400 (160-19000)
Serbia	2016	Female	75 to 79	41% (26-60%)	59% (40-74%)	2.1 (0.79-4)	5.1% (-0.26-13%)	6.4% (-0.26-17%)	510 (-21-1300)	7800 (-370-20000)
Serbia	2016	Female	80 to 84	36% (21-55%)	64% (45-79%)	1.9 (0.75-3.4)	4.8% (0.0076-12%)	5.7% (-0.069-15%)	580 (-7.2-1500)	6600 (12-17000)
Serbia	2016	Female	85 to 89	35% (22-51%)	65% (49-78%)	1.8 (0.75-3.6)	4.6% (-0.044-12%)	5.5% (-0.034-15%)	510 (-3.1-1400)	4000 (-38-11000)
Serbia	2016	Female	90 to 94	37% (23-53%)	63% (47-77%)	2 (0.75-4.3)	4.6% (-0.21-14%)	5.5% (-0.3-17%)	280 (-15-850)	1500 (-67-4400)
Serbia	2016	Female	95 plus	40% (23-61%)	60% (39-77%)	2.2 (0.6-5.2)	4.8% (-0.34-14%)	5.7% (-0.44-17%)	82 (-6.3-250)	280 (-20-860)
Serbia	2016	Male	All Ages	69% (59-79%)	31% (21-41%)	4.3 (3.1-5.7)	11% (5.5-18%)	13% (4.9-22%)	6800 (2700-12000)	180000 (88000-290000)
Serbia	2016	Male	15 to 19	59% (37-80%)	41% (20-63%)	1.8 (0.81-3.3)	5.4% (1.7-10%)	9.1% (0.97-19%)	17 (1.9-37)	1700 (550-3200)
Serbia	2016	Male	20 to 24	70% (49-87%)	30% (13-51%)	3.2 (1.5-5.5)	11% (4.3-20%)	16% (3.4-32%)	48 (10-95)	4400 (1700-7700)

Location	Year	Sex	Age	Prevalence of current drinkers	Prevalence of abstainers	Population average of standard drinks daily	Percent of total attributable deaths	Percent of total attributable DALYs	Attributable deaths	Attributable DALYs
Serbia	2016	Male	25 to 29	73% (54-89%)	27% (11-46%)	4.1 (2.1-6.9)	12% (5.6-21%)	19% (6.1-36%)	69 (21-130)	5700 (2600-9500)
Serbia	2016	Male	30 to 34	76% (55-91%)	24% (9.3-45%)	4.7 (2.4-7.7)	13% (6.1-21%)	21% (7.5-36%)	84 (30-150)	6300 (2900-10000)
Serbia	2016	Male	35 to 39	78% (59-91%)	22% (9.4-41%)	4.9 (2.6-8.2)	13% (6-21%)	21% (8-35%)	120 (46-200)	7800 (3600-12000)
Serbia	2016	Male	40 to 44	79% (60-92%)	21% (8.3-40%)	5.6 (3.2-9)	14% (6.8-22%)	22% (9.8-35%)	190 (85-300)	11000 (5000-16000)
Serbia	2016	Male	45 to 49	79% (58-91%)	21% (8.7-42%)	6.4 (3.6-11)	15% (8.4-23%)	21% (11-33%)	300 (160-460)	14000 (7800-22000)
Serbia	2016	Male	50 to 54	78% (57-91%)	22% (9.3-43%)	6.9 (4.1-11)	15% (8.5-23%)	20% (11-30%)	430 (240-650)	18000 (9700-27000)
Serbia	2016	Male	55 to 59	76% (55-91%)	24% (9.3-45%)	7 (4.1-11)	15% (7.7-23%)	19% (9.7-28%)	590 (310-900)	21000 (11000-32000)
Serbia	2016	Male	60 to 64	75% (51-90%)	25% (9.5-49%)	6.8 (3.7-11)	13% (6-21%)	16% (7.5-26%)	860 (410-1400)	26000 (12000-42000)
Serbia	2016	Male	65 to 69	73% (49-90%)	27% (10-51%)	6.1 (3.6-9.3)	12% (5.8-20%)	15% (6.9-24%)	1100 (490-1700)	26000 (12000-43000)
Serbia	2016	Male	70 to 74	71% (49-87%)	29% (13-51%)	5.2 (2.9-8.4)	11% (4-19%)	13% (4.9-23%)	880 (330-1500)	17000 (6400-30000)
Serbia	2016	Male	75 to 79	69% (46-85%)	31% (15-54%)	4.4 (2.2-8)	9.1% (1.9-18%)	10% (2.1-20%)	830 (170-1600)	13000 (2700-24000)
Serbia	2016	Male	80 to 84	67% (43-85%)	33% (15-57%)	3.9 (1.9-6.7)	7.9% (1-16%)	8.9% (1-19%)	730 (86-1600)	8300 (1000-17000)
Serbia	2016	Male	85 to 89	65% (43-84%)	35% (16-57%)	3.6 (1.8-6.2)	6.7% (0.33-16%)	7.4% (0.055-18%)	450 (3.4-1100)	3700 (190-8400)
Serbia	2016	Male	90 to 94	65% (44-85%)	35% (15-56%)	3.3 (1.6-6.1)	5.4% (-0.64-15%)	6% (-1-16%)	160 (-28-430)	870 (-100-2300)
Serbia	2016	Male	95 plus	65% (40-88%)	35% (12-60%)	3 (1.1-6.1)	4.9% (-0.69-13%)	5.4% (-0.96-15%)	30 (-5.5-87)	110 (-15-310)
Slovakia	1990	Female	All Ages	87% (84-89%)	13% (11-16%)	1.9 (1.6-2.2)	2.6% (0.82-4.6%)	1.9% (-1.7-5.7%)	460 (-410-1400)	20000 (6400-35000)
Slovakia	1990	Female	15 to 19	88% (81-93%)	12% (6.9-19%)	2.3 (1.6-3.1)	3.8% (2.4-5.5%)	9.2% (4.9-14%)	7 (3.6-11)	780 (490-1100)
Slovakia	1990	Female	20 to 24	92% (87-95%)	8.3% (5.1-13%)	2.9 (2-4)	5.3% (3.7-7.1%)	10% (6.1-15%)	7.1 (4.1-11)	990 (670-1400)
Slovakia	1990	Female	25 to 29	93% (89-96%)	6.9% (3.9-11%)	2.4 (1.7-3.3)	4.8% (3.4-6.3%)	8.6% (5.2-12%)	8.1 (4.9-11)	1100 (740-1500)
Slovakia	1990	Female	30 to 34	93% (89-96%)	6.6% (3.9-11%)	2.3 (1.7-3.1)	4.6% (3.3-6.1%)	8.9% (6-12%)	13 (8.6-17)	1300 (940-1800)
Slovakia	1990	Female	35 to 39	92% (88-96%)	7.6% (4.5-12%)	2.7 (1.9-3.6)	5.4% (3.8-7.2%)	11% (7.3-14%)	27 (19-36)	1900 (1400-2600)
Slovakia	1990	Female	40 to 44	92% (87-95%)	8.1% (4.9-13%)	2.9 (2.1-3.8)	6.1% (4.4-8.3%)	11% (8.1-15%)	39 (27-52)	2300 (1600-3000)

Location	Year	Sex	Age	Prevalence of current drinkers	Prevalence of abstainers	Population average of standard drinks daily	Percent of total attributable deaths	Percent of total attributable DALYs	Attributable deaths	Attributable DALYs
Slovakia	1990	Female	45 to 49	92% (87-95%)	8% (4.7-13%)	3 (2.1-4.1)	6.6% (4.5-9.4%)	11% (7.7-16%)	52 (36-73)	2500 (1800-3500)
Slovakia	1990	Female	50 to 54	91% (87-95%)	8.6% (5.2-13%)	2.8 (2-3.9)	6.1% (3.7-9.3%)	9.6% (5.8-14%)	64 (38-93)	2600 (1600-3900)
Slovakia	1990	Female	55 to 59	90% (85-94%)	9.8% (6-15%)	2.5 (1.8-3.4)	4.3% (1.8-7.5%)	6.3% (2.6-11%)	68 (28-120)	2400 (970-4100)
Slovakia	1990	Female	60 to 64	89% (82-93%)	11% (6.8-18%)	2.4 (1.6-3.3)	2.4% (-0.6-6%)	3.2% (-1-8.2%)	54 (-17-140)	1600 (-400-4100)
Slovakia	1990	Female	65 to 69	86% (79-92%)	14% (7.8-21%)	2.4 (1.7-3.2)	1.4% (-2.1-4.9%)	1.7% (-2.9-6.3%)	47 (-81-170)	1200 (-1800-4000)
Slovakia	1990	Female	70 to 74	81% (73-88%)	19% (12-27%)	2.1 (1.4-3)	0.88% (-3-4.7%)	0.96% (-4-5.9%)	21 (-88-130)	480 (-1600-2600)
Slovakia	1990	Female	75 to 79	73% (61-83%)	27% (17-39%)	1.8 (1.2-2.8)	0.52% (-2.9-4.3%)	0.45% (-3.8-5.1%)	19 (-160-220)	400 (-2300-3300)
Slovakia	1990	Female	80 to 84	69% (54-80%)	31% (20-46%)	1.8 (1.2-2.6)	0.46% (-2.9-4.3%)	0.34% (-3.7-5%)	17 (-180-240)	280 (-1800-2700)
Slovakia	1990	Female	85 to 89	68% (54-79%)	32% (21-46%)	2 (1.2-2.9)	0.45% (-3.5-4.9%)	0.29% (-4.5-5.7%)	9.5 (-150-190)	130 (-1000-1400)
Slovakia	1990	Female	90 to 94	70% (56-80%)	30% (20-44%)	2.2 (1.3-3.3)	0.67% (-4.1-6.7%)	0.51% (-5.2-7.6%)	6.9 (-70-100)	55 (-340-540)
Slovakia	1990	Female	95 plus	72% (55-85%)	28% (15-45%)	2.3 (1.2-3.7)	1.1% (-4.5-8.3%)	0.96% (-5.6-9.5%)	2.5 (-15-25)	12 (-46-87)
Slovakia	1990	Male	All Ages	84% (80-88%)	16% (12-20%)	5.3 (4.6-6.2)	17% (14-20%)	18% (14-23%)	5500 (4200-7100)	170000 (140000-200000)
Slovakia	1990	Male	15 to 19	78% (66-87%)	22% (13-34%)	3 (1.9-4.1)	8.4% (5.8-12%)	14% (8.7-21%)	25 (15-37)	2300 (1600-3200)
Slovakia	1990	Male	20 to 24	89% (82-94%)	11% (6-18%)	5.5 (4.1-7.2)	17% (13-22%)	25% (18-33%)	60 (41-80)	5100 (3700-6600)
Slovakia	1990	Male	25 to 29	92% (87-96%)	7.6% (4.3-13%)	7.2 (5.2-9.3)	20% (15-24%)	30% (22-38%)	88 (66-110)	6800 (5200-8300)
Slovakia	1990	Male	30 to 34	94% (89-97%)	6% (2.9-11%)	8 (6-10)	21% (16-25%)	31% (24-37%)	140 (110-170)	9500 (7600-11000)
Slovakia	1990	Male	35 to 39	94% (90-97%)	5.8% (3-10%)	7.9 (5.8-10)	21% (17-26%)	30% (23-36%)	220 (170-260)	13000 (10000-15000)
Slovakia	1990	Male	40 to 44	94% (90-97%)	6.1% (3.4-10%)	8.2 (6.1-10)	23% (18-27%)	30% (24-35%)	300 (240-360)	15000 (12000-18000)
Slovakia	1990	Male	45 to 49	93% (87-96%)	7% (3.6-13%)	8.7 (6.4-12)	23% (19-28%)	29% (24-34%)	370 (300-450)	16000 (13000-20000)
Slovakia	1990	Male	50 to 54	92% (86-96%)	8% (4.3-14%)	9.3 (6.8-12)	22% (18-27%)	26% (21-32%)	460 (360-560)	18000 (14000-21000)
Slovakia	1990	Male	55 to 59	91% (84-95%)	8.9% (4.5-16%)	9.8 (7.3-13)	21% (16-26%)	24% (18-30%)	600 (450-760)	20000 (15000-25000)
Slovakia	1990	Male	60 to 64	90% (82-95%)	10% (5.1-18%)	9.2 (6.7-12)	19% (13-24%)	21% (15-28%)	740 (520-990)	21000 (15000-27000)

Location	Year	Sex	Age	Prevalence of current drinkers	Prevalence of abstainers	Population average of standard drinks daily	Percent of total attributable deaths	Percent of total attributable DALYs	Attributable deaths	Attributable DALYs
Slovakia	1990	Male	65 to 69	88% (80-95%)	12% (5.4-20%)	8 (6.1-10)	17% (12-22%)	19% (14-25%)	820 (570-1100)	19000 (14000-26000)
Slovakia	1990	Male	70 to 74	87% (78-92%)	13% (7.6-22%)	6.9 (4.9-9.2)	15% (9.2-20%)	17% (10-23%)	460 (290-640)	8800 (5600-12000)
Slovakia	1990	Male	75 to 79	85% (74-92%)	15% (8.2-26%)	5.9 (3.9-8.5)	12% (5.2-19%)	14% (5.5-21%)	580 (230-900)	8500 (3600-13000)
Slovakia	1990	Male	80 to 84	83% (72-91%)	17% (8.7-28%)	5.3 (3.6-7.4)	11% (4.2-17%)	12% (4.2-19%)	430 (150-690)	4700 (1800-7500)
Slovakia	1990	Male	85 to 89	83% (72-90%)	17% (10-28%)	4.9 (3.4-6.7)	8.8% (2.1-15%)	9.7% (1.7-17%)	180 (31-310)	1400 (330-2400)
Slovakia	1990	Male	90 to 94	83% (73-90%)	17% (9.6-27%)	4.5 (2.9-6.4)	6.6% (-0.4-14%)	7.1% (-1.2-16%)	41 (-7-90)	220 (-14-470)
Slovakia	1990	Male	95 plus	83% (71-92%)	17% (8.1-29%)	4 (2.2-6.3)	4.6% (-2.7-13%)	4.7% (-3.9-14%)	4.6 (-3.8-14)	18 (-11-50)
Slovakia	1995	Female	All Ages	89% (86-91%)	11% (9.3-14%)	1.9 (1.6-2.2)	2.2% (0.25-4.1%)	1.1% (-2.4-4.6%)	280 (-570-1100)	16000 (1800-30000)
Slovakia	1995	Female	15 to 19	89% (83-94%)	11% (6.2-17%)	2.2 (1.5-3)	3.4% (2.2-4.8%)	8.8% (4.6-13%)	6.3 (3.3-9.6)	740 (460-1100)
Slovakia	1995	Female	20 to 24	93% (89-95%)	7.3% (4.6-11%)	2.7 (1.9-3.7)	4.8% (3.3-6.4%)	9.7% (5.6-14%)	5.9 (3.4-8.6)	960 (650-1400)
Slovakia	1995	Female	25 to 29	94% (91-96%)	5.8% (3.5-9%)	2.3 (1.6-3.1)	4.4% (3.3-5.8%)	8.3% (5.1-12%)	5.7 (3.5-8.2)	880 (610-1200)
Slovakia	1995	Female	30 to 34	94% (91-96%)	5.7% (3.5-8.7%)	2.3 (1.6-3.1)	4.3% (3.1-5.7%)	8.7% (6-12%)	10 (7.2-14)	1100 (800-1500)
Slovakia	1995	Female	35 to 39	93% (90-96%)	6.7% (4-10%)	2.6 (1.8-3.5)	4.8% (3.5-6.3%)	10% (7.1-13%)	21 (15-28)	1600 (1100-2100)
Slovakia	1995	Female	40 to 44	93% (89-95%)	7.1% (4.6-11%)	2.8 (2-3.7)	5.3% (3.8-7.1%)	11% (7.7-14%)	37 (26-49)	2200 (1600-2900)
Slovakia	1995	Female	45 to 49	93% (89-96%)	7% (4.3-11%)	2.9 (2.1-3.8)	5.8% (3.9-8%)	11% (7.3-15%)	49 (34-68)	2400 (1700-3300)
Slovakia	1995	Female	50 to 54	92% (89-95%)	7.5% (4.9-11%)	2.7 (1.9-3.7)	5.5% (3.2-8.1%)	8.9% (5.2-13%)	57 (33-84)	2400 (1400-3400)
Slovakia	1995	Female	55 to 59	91% (87-95%)	8.6% (5.4-13%)	2.4 (1.8-3.2)	3.8% (1.4-6.6%)	5.8% (2.1-10%)	53 (19-90)	1800 (700-3200)
Slovakia	1995	Female	60 to 64	90% (85-94%)	9.9% (6.2-15%)	2.3 (1.6-3.2)	1.9% (-0.96-5.2%)	2.7% (-1.4-7.3%)	41 (-20-110)	1200 (-590-3300)
Slovakia	1995	Female	65 to 69	88% (82-93%)	12% (7.2-18%)	2.3 (1.7-3)	0.88% (-2.5-4.1%)	1.1% (-3.5-5.5%)	27 (-83-130)	690 (-2000-3200)
Slovakia	1995	Female	70 to 74	83% (76-88%)	17% (12-24%)	2.1 (1.4-2.8)	0.29% (-3.4-3.9%)	0.25% (-4.4-4.9%)	9.2 (-170-180)	270 (-3000-3600)
Slovakia	1995	Female	75 to 79	74% (63-83%)	26% (17-37%)	1.7 (1.1-2.5)	0.025% (-3.2-3.4%)	-0.15% (-4.1-4%)	-4.1 (-120-110)	14 (-1700-1800)
Slovakia	1995	Female	80 to 84	68% (53-80%)	32% (20-47%)	1.6 (1.1-2.4)	-0.093% (-3.2-3.1%)	-0.3% (-4.2-3.7%)	-14 (-200-180)	-55 (-1900-2000)

Location	Year	Sex	Age	Prevalence of current drinkers	Prevalence of abstainers	Population average of standard drinks daily	Percent of total attributable deaths	Percent of total attributable DALYs	Attributable deaths	Attributable DALYs
Slovakia	1995	Female	85 to 89	67% (53-78%)	33% (22-47%)	1.8 (1.1-2.7)	-0.23% (-3.8-3.9%)	-0.52% (-5-4.5%)	-20 (-190-170)	-78 (-1300-1300)
Slovakia	1995	Female	90 to 94	69% (55-80%)	31% (20-45%)	2 (1.2-3)	-0.11% (-4.5-5.1%)	-0.42% (-5.5-5.7%)	-6.8 (-89-94)	-11 (-430-500)
Slovakia	1995	Female	95 plus	72% (54-85%)	28% (15-46%)	2.1 (1.1-3.4)	0.19% (-5-6.3%)	-0.11% (-6.3-7.2%)	-0.39 (-23-27)	2.8 (-75-97)
Slovakia	1995	Male	All Ages	87% (83-90%)	13% (10-17%)	5.2 (4.5-5.9)	16% (13-19%)	17% (13-22%)	4900 (3700-6200)	150000 (130000-180000)
Slovakia	1995	Male	15 to 19	81% (72-88%)	19% (12-28%)	2.9 (2-3.9)	7.9% (5.4-11%)	13% (8.2-19%)	24 (15-34)	2300 (1500-3200)
Slovakia	1995	Male	20 to 24	91% (85-95%)	9.1% (5.5-15%)	5.3 (3.9-6.8)	16% (12-20%)	24% (16-31%)	57 (39-76)	5000 (3700-6400)
Slovakia	1995	Male	25 to 29	93% (89-96%)	6.7% (4-11%)	6.8 (4.9-8.8)	18% (14-23%)	29% (21-37%)	70 (50-90)	5600 (4200-6900)
Slovakia	1995	Male	30 to 34	95% (91-97%)	5.4% (2.8-9.4%)	7.5 (5.7-9.6)	19% (15-23%)	30% (23-36%)	100 (78-120)	7100 (5700-8500)
Slovakia	1995	Male	35 to 39	95% (91-97%)	5.1% (2.8-8.6%)	7.4 (5.5-9.6)	20% (16-24%)	29% (22-35%)	170 (130-200)	10000 (8100-12000)
Slovakia	1995	Male	40 to 44	95% (91-97%)	5.3% (3-8.7%)	7.7 (5.7-9.8)	21% (17-26%)	29% (23-34%)	300 (230-350)	15000 (12000-18000)
Slovakia	1995	Male	45 to 49	94% (90-97%)	5.9% (3.3-10%)	8.3 (6.1-11)	22% (18-26%)	28% (23-33%)	370 (300-440)	16000 (13000-19000)
Slovakia	1995	Male	50 to 54	93% (89-96%)	7% (4-11%)	8.8 (6.4-11)	22% (18-26%)	26% (22-32%)	420 (340-510)	16000 (13000-20000)
Slovakia	1995	Male	55 to 59	92% (86-96%)	8.1% (4.2-14%)	9.2 (6.9-12)	20% (16-25%)	24% (19-30%)	490 (380-610)	16000 (13000-21000)
Slovakia	1995	Male	60 to 64	91% (84-95%)	9.1% (4.8-16%)	8.7 (6.5-11)	18% (14-23%)	21% (16-27%)	630 (460-810)	18000 (13000-23000)
Slovakia	1995	Male	65 to 69	90% (83-95%)	10% (5.2-17%)	7.6 (5.8-9.7)	16% (12-21%)	19% (13-24%)	690 (490-910)	16000 (12000-21000)
Slovakia	1995	Male	70 to 74	88% (80-93%)	12% (7.3-20%)	6.5 (4.6-8.6)	14% (8.2-19%)	16% (9-22%)	690 (400-970)	13000 (7800-18000)
Slovakia	1995	Male	75 to 79	85% (75-92%)	15% (8.5-25%)	5.5 (3.7-7.7)	11% (4.1-17%)	12% (4.3-20%)	320 (110-510)	4800 (1700-7500)
Slovakia	1995	Male	80 to 84	83% (71-91%)	17% (9-29%)	4.9 (3.4-6.7)	9.2% (3-16%)	10% (3-18%)	340 (100-590)	3800 (1300-6300)
Slovakia	1995	Male	85 to 89	82% (71-90%)	18% (10-29%)	4.4 (3.1-6.1)	7% (0.92-14%)	7.6% (0.41-16%)	160 (8.4-320)	1200 (160-2500)
Slovakia	1995	Male	90 to 94	83% (72-90%)	17% (9.9-28%)	4.1 (2.7-5.8)	4.9% (-1.3-12%)	5.1% (-2.3-14%)	34 (-16-92)	200 (-54-480)
Slovakia	1995	Male	95 plus	83% (70-92%)	17% (8.1-30%)	3.6 (2-5.9)	3.1% (-3.5-11%)	2.9% (-4.9-13%)	3.2 (-5.3-14)	14 (-15-51)
Slovakia	2000	Female	All Ages	89% (87-91%)	11% (9.4-13%)	1.9 (1.7-2.1)	2.1% (0.41-3.7%)	0.92% (-2.2-4%)	220 (-550-980)	15000 (3100-28000)

Location	Year	Sex	Age	Prevalence of current drinkers	Prevalence of abstainers	Population average of standard drinks daily	Percent of total attributable deaths	Percent of total attributable DALYs	Attributable deaths	Attributable DALYs
Slovakia	2000	Female	15 to 19	89%<br>(83-94%)	11%<br>(6.3-17%)	2.1<br>(1.5-2.9)	3.3%<br>(2.1-4.7%)	8.6%<br>(4.5-13%)	5.7<br>(3.1-8.7)	680<br>(430-970)
Slovakia	2000	Female	20 to 24	93%<br>(88-95%)	7.2%<br>(4.6-12%)	2.6<br>(1.9-3.6)	4.6%<br>(3.2-6.2%)	9.3%<br>(5.4-13%)	6.1<br>(3.5-8.9)	1000<br>(680-1500)
Slovakia	2000	Female	25 to 29	94%<br>(91-96%)	5.9%<br>(3.6-9%)	2.2<br>(1.6-2.9)	4.3%<br>(3.1-5.8%)	7.9%<br>(4.8-11%)	6.2<br>(3.8-9)	980<br>(680-1300)
Slovakia	2000	Female	30 to 34	94%<br>(91-96%)	5.8%<br>(3.5-9.1%)	2.1<br>(1.6-2.9)	3.9%<br>(2.9-5.1%)	8.3%<br>(5.7-11%)	7<br>(4.8-9.5)	890<br>(640-1200)
Slovakia	2000	Female	35 to 39	93%<br>(90-96%)	6.8%<br>(4.3-10%)	2.5<br>(1.8-3.3)	4.5%<br>(3.2-5.8%)	9.8%<br>(6.8-13%)	17<br>(12-22)	1300<br>(950-1700)
Slovakia	2000	Female	40 to 44	93%<br>(89-95%)	7.2%<br>(4.7-11%)	2.7<br>(2-3.5)	5.1%<br>(3.6-6.7%)	11%<br>(7.7-14%)	33<br>(24-42)	1900<br>(1400-2500)
Slovakia	2000	Female	45 to 49	93%<br>(89-95%)	7.2%<br>(4.5-11%)	2.7<br>(1.9-3.6)	5.6%<br>(4-7.6%)	10%<br>(7.6-14%)	56<br>(41-73)	2700<br>(2000-3600)
Slovakia	2000	Female	50 to 54	92%<br>(89-95%)	7.8%<br>(5.1-11%)	2.6<br>(1.8-3.4)	5.2%<br>(3.3-7.4%)	9%<br>(5.7-12%)	62<br>(39-85)	2600<br>(1600-3500)
Slovakia	2000	Female	55 to 59	91%<br>(87-94%)	9%<br>(5.7-13%)	2.3<br>(1.7-3.1)	3.8%<br>(1.7-6.2%)	6.2%<br>(2.8-10%)	55<br>(25-87)	1900<br>(850-3000)
Slovakia	2000	Female	60 to 64	90%<br>(85-94%)	10%<br>(6.3-15%)	2.2<br>(1.6-3)	2%<br>(-0.56-4.9%)	3%<br>(-0.66-7.2%)	38<br>(-8.3-93)	1100<br>(-300-2800)
Slovakia	2000	Female	65 to 69	88%<br>(82-93%)	12%<br>(7.2-18%)	2.2<br>(1.6-2.9)	0.92%<br>(-2-3.8%)	1.2%<br>(-2.8-5.3%)	26<br>(-58-110)	640<br>(-1400-2800)
Slovakia	2000	Female	70 to 74	82%<br>(75-88%)	18%<br>(12-25%)	1.9<br>(1.3-2.7)	0.15%<br>(-3-3.3%)	0.084%<br>(-4.1-4.2%)	2.7<br>(-130-140)	130<br>(-2500-2700)
Slovakia	2000	Female	75 to 79	72%<br>(61-82%)	28%<br>(18-39%)	1.6<br>(1-2.2)	-0.15%<br>(-3.1-2.8%)	-0.35%<br>(-4-3.3%)	-17<br>(-190-160)	-140<br>(-2700-2500)
Slovakia	2000	Female	80 to 84	65%<br>(50-78%)	35%<br>(22-50%)	1.5<br>(0.97-2.1)	-0.32%<br>(-3.1-2.4%)	-0.58%<br>(-4.1-2.7%)	-19<br>(-140-88)	-140<br>(-1400-1000)
Slovakia	2000	Female	85 to 89	64%<br>(50-77%)	36%<br>(23-50%)	1.6<br>(0.98-2.4)	-0.53%<br>(-3.8-3%)	-0.88%<br>(-4.8-3.3%)	-34<br>(-190-130)	-190<br>(-1300-1000)
Slovakia	2000	Female	90 to 94	67%<br>(53-79%)	33%<br>(21-47%)	1.8<br>(1-2.7)	-0.47%<br>(-4.4-4.1%)	-0.84%<br>(-5.4-4.5%)	-17<br>(-110-89)	-57<br>(-530-490)
Slovakia	2000	Female	95 plus	70%<br>(52-84%)	30%<br>(16-48%)	1.9<br>(1-3.1)	-0.23%<br>(-5-5.7%)	-0.6%<br>(-6.2-6.3%)	-3<br>(-31-31)	-4.5<br>(-100-110)
Slovakia	2000	Male	All Ages	87%<br>(84-90%)	13%<br>(9.6-16%)	5.3<br>(4.7-5.8)	16%<br>(13-19%)	17%<br>(13-21%)	4800<br>(3700-6000)	150000<br>(130000-180000)
Slovakia	2000	Male	15 to 19	80%<br>(71-88%)	20%<br>(12-29%)	2.7<br>(1.9-3.8)	7.2%<br>(5-9.8%)	13%<br>(7.7-19%)	17<br>(11-25)	1800<br>(1200-2400)
Slovakia	2000	Male	20 to 24	91%<br>(85-95%)	9.2%<br>(5.4-15%)	5.1<br>(3.8-6.6)	15%<br>(11-19%)	23%<br>(16-30%)	58<br>(41-78)	5200<br>(3900-6700)
Slovakia	2000	Male	25 to 29	93%<br>(89-96%)	6.6%<br>(3.8-11%)	6.5<br>(4.8-8.4)	17%<br>(13-22%)	28%<br>(20-35%)	74<br>(54-95)	6000<br>(4600-7400)
Slovakia	2000	Male	30 to 34	95%<br>(91-97%)	5.3%<br>(2.8-9.2%)	7.2<br>(5.5-9.1)	19%<br>(15-23%)	29%<br>(23-36%)	90<br>(70-110)	6400<br>(5000-7600)

Location	Year	Sex	Age	Prevalence of current drinkers	Prevalence of abstainers	Population average of standard drinks daily	Percent of total attributable deaths	Percent of total attributable DALYs	Attributable deaths	Attributable DALYs
Slovakia	2000	Male	35 to 39	95% (91-97%)	5.1% (2.8-8.6%)	7.2 (5.4-9.3)	19% (15-23%)	28% (22-34%)	140 (110-160)	8400 (6700-10000)
Slovakia	2000	Male	40 to 44	95% (91-97%)	5.3% (3.1-9%)	7.4 (5.6-9.4)	21% (17-25%)	29% (23-34%)	260 (210-310)	14000 (11000-16000)
Slovakia	2000	Male	45 to 49	94% (89-97%)	6% (3.2-11%)	7.9 (6-10)	22% (18-26%)	28% (23-33%)	420 (340-500)	19000 (16000-22000)
Slovakia	2000	Male	50 to 54	93% (89-96%)	6.8% (3.9-11%)	8.5 (6.3-11)	22% (18-25%)	27% (22-32%)	510 (410-600)	20000 (16000-23000)
Slovakia	2000	Male	55 to 59	92% (87-96%)	7.7% (4-13%)	8.9 (6.9-11)	20% (16-25%)	24% (19-29%)	510 (400-620)	17000 (14000-21000)
Slovakia	2000	Male	60 to 64	91% (85-95%)	9.2% (4.7-15%)	8.4 (6.3-11)	18% (14-23%)	21% (16-27%)	570 (430-710)	16000 (12000-20000)
Slovakia	2000	Male	65 to 69	89% (82-94%)	11% (5.5-18%)	7.3 (5.6-9.1)	16% (12-20%)	18% (13-24%)	630 (450-820)	15000 (11000-19000)
Slovakia	2000	Male	70 to 74	87% (81-92%)	13% (7.5-19%)	6.3 (4.6-8.3)	13% (7.7-19%)	15% (8.6-21%)	620 (350-870)	12000 (6900-16000)
Slovakia	2000	Male	75 to 79	86% (77-92%)	14% (7.8-23%)	5.5 (3.8-7.6)	11% (4.1-17%)	12% (4.4-19%)	500 (180-800)	7500 (2900-12000)
Slovakia	2000	Male	80 to 84	84% (73-92%)	16% (8-27%)	4.9 (3.5-6.5)	8.7% (2.7-14%)	9.7% (2.7-17%)	220 (61-380)	2400 (760-4100)
Slovakia	2000	Male	85 to 89	84% (73-91%)	16% (9.5-27%)	4.4 (3.1-5.9)	6.5% (0.36-13%)	7% (-0.32-14%)	150 (-6.9-310)	1200 (68-2400)
Slovakia	2000	Male	90 to 94	84% (74-91%)	16% (9.1-26%)	4 (2.7-5.7)	4.4% (-1.7-11%)	4.5% (-2.8-13%)	37 (-23-110)	220 (-85-570)
Slovakia	2000	Male	95 plus	84% (71-93%)	16% (7.4-29%)	3.6 (2-5.7)	2.7% (-3.6-11%)	2.5% (-5-12%)	3.6 (-7.5-18)	16 (-22-67)
Slovakia	2005	Female	All Ages	88% (85-90%)	12% (10-15%)	1.8 (1.6-2)	1.9% (0.48-3.3%)	0.61% (-2.3-3.3%)	150 (-550-840)	14000 (3500-25000)
Slovakia	2005	Female	15 to 19	88% (82-93%)	12% (7-18%)	1.9 (1.4-2.6)	2.9% (1.9-4.1%)	7.7% (4.1-12%)	4 (2.1-6.1)	530 (350-750)
Slovakia	2005	Female	20 to 24	92% (88-95%)	7.8% (4.9-12%)	2.4 (1.7-3.3)	4.5% (3.1-5.9%)	8.4% (5.1-12%)	4.8 (2.9-7)	910 (610-1300)
Slovakia	2005	Female	25 to 29	94% (90-96%)	6.5% (4-10%)	2 (1.5-2.7)	4.1% (3-5.3%)	7.1% (4.4-9.9%)	5.2 (3.2-7.3)	970 (670-1300)
Slovakia	2005	Female	30 to 34	94% (90-96%)	6.4% (3.8-10%)	2 (1.4-2.6)	3.7% (2.8-4.8%)	7.5% (5.2-9.9%)	7.1 (4.9-9.6)	960 (690-1300)
Slovakia	2005	Female	35 to 39	93% (89-96%)	7.4% (4.4-11%)	2.3 (1.6-3.1)	4.2% (3-5.4%)	9.2% (6.3-12%)	13 (9.2-18)	1100 (810-1500)
Slovakia	2005	Female	40 to 44	92% (88-95%)	7.8% (4.8-12%)	2.5 (1.8-3.2)	4.8% (3.4-6.2%)	10% (7.3-13%)	28 (20-36)	1700 (1200-2200)
Slovakia	2005	Female	45 to 49	92% (88-95%)	7.7% (4.7-12%)	2.5 (1.8-3.2)	5.3% (3.8-7.1%)	10% (7.3-13%)	51 (37-67)	2500 (1800-3300)
Slovakia	2005	Female	50 to 54	91% (87-95%)	8.5% (5.5-13%)	2.3 (1.7-3.1)	4.9% (3.1-6.8%)	8.6% (5.7-11%)	68 (45-91)	2800 (1800-3800)

Location	Year	Sex	Age	Prevalence of current drinkers	Prevalence of abstainers	Population average of standard drinks daily	Percent of total attributable deaths	Percent of total attributable DALYs	Attributable deaths	Attributable DALYs
Slovakia	2005	Female	55 to 59	90% (84-94%)	10% (6.2-16%)	2 (1.5-2.7)	3.6% (1.8-5.6%)	6% (3.1-9%)	61 (32-92)	2100 (1100-3200)
Slovakia	2005	Female	60 to 64	88% (82-93%)	12% (7.4-18%)	1.9 (1.4-2.7)	2% (-0.089-4.3%)	3.2% (0.12-6.6%)	40 (1.5-82)	1100 (-52-2400)
Slovakia	2005	Female	65 to 69	85% (78-91%)	15% (8.8-22%)	1.9 (1.4-2.5)	0.88% (-1.5-3.2%)	1.3% (-2.2-4.6%)	23 (-40-83)	560 (-950-2000)
Slovakia	2005	Female	70 to 74	78% (70-85%)	22% (15-30%)	1.6 (1.1-2.3)	0.068% (-2.6-2.6%)	0.016% (-3.5-3.4%)	0.49 (-100-100)	53 (-1900-2000)
Slovakia	2005	Female	75 to 79	69% (56-80%)	31% (20-44%)	1.4 (0.89-2)	-0.29% (-2.7-2.2%)	-0.5% (-3.6-2.7%)	-22 (-160-120)	-240 (-2200-1800)
Slovakia	2005	Female	80 to 84	63% (49-76%)	37% (24-51%)	1.3 (0.88-1.9)	-0.59% (-3.1-1.8%)	-0.88% (-4-2%)	-51 (-230-120)	-440 (-2400-1300)
Slovakia	2005	Female	85 to 89	65% (51-76%)	35% (24-49%)	1.5 (0.94-2.2)	-0.94% (-4-2.2%)	-1.3% (-5.1-2.4%)	-39 (-150-70)	-240 (-1100-560)
Slovakia	2005	Female	90 to 94	67% (54-78%)	33% (22-46%)	1.7 (0.99-2.5)	-0.99% (-4.7-3.2%)	-1.4% (-5.8-3.6%)	-31 (-130-77)	-130 (-610-420)
Slovakia	2005	Female	95 plus	70% (52-84%)	30% (16-48%)	1.8 (0.94-2.9)	-0.86% (-5.2-4.6%)	-1.3% (-6.5-5.3%)	-8.4 (-42-33)	-22 (-140-120)
Slovakia	2005	Male	All Ages	89% (86-91%)	11% (8.8-14%)	5.3 (4.8-5.8)	16% (13-18%)	16% (12-21%)	4600 (3400-5800)	150000 (120000-170000)
Slovakia	2005	Male	15 to 19	81% (71-88%)	19% (12-29%)	2.6 (1.8-3.6)	6.9% (4.8-9.5%)	12% (7.5-18%)	14 (8.6-21)	1500 (1100-2100)
Slovakia	2005	Male	20 to 24	91% (86-95%)	8.8% (5.3-14%)	4.9 (3.7-6.3)	14% (11-18%)	22% (15-29%)	46 (33-61)	4400 (3300-5600)
Slovakia	2005	Male	25 to 29	94% (90-96%)	6.2% (3.7-9.7%)	6.3 (4.8-7.9)	16% (13-20%)	26% (19-33%)	64 (47-81)	5500 (4300-6800)
Slovakia	2005	Male	30 to 34	95% (92-97%)	5% (2.6-8.3%)	6.9 (5.3-8.7)	17% (14-20%)	28% (22-34%)	83 (65-100)	6200 (4900-7500)
Slovakia	2005	Male	35 to 39	95% (92-97%)	4.8% (2.6-7.9%)	6.9 (5.1-9.1)	18% (14-22%)	27% (21-33%)	110 (88-140)	7200 (5600-8600)
Slovakia	2005	Male	40 to 44	95% (92-97%)	5% (2.9-8.2%)	7.1 (5.5-9.1)	19% (15-23%)	28% (22-33%)	210 (160-250)	11000 (8700-13000)
Slovakia	2005	Male	45 to 49	94% (90-97%)	5.7% (3.1-9.6%)	7.6 (5.8-9.6)	21% (17-25%)	28% (23-33%)	380 (310-450)	17000 (14000-20000)
Slovakia	2005	Male	50 to 54	94% (89-96%)	6.4% (3.7-11%)	8.1 (6.2-10)	21% (17-25%)	27% (22-31%)	570 (470-670)	22000 (18000-26000)
Slovakia	2005	Male	55 to 59	93% (87-96%)	7.4% (4-13%)	8.6 (6.8-11)	20% (16-24%)	25% (20-29%)	600 (490-730)	20000 (17000-24000)
Slovakia	2005	Male	60 to 64	91% (85-95%)	8.9% (4.6-15%)	8 (6.1-10)	18% (14-22%)	22% (17-26%)	570 (430-700)	16000 (13000-20000)
Slovakia	2005	Male	65 to 69	90% (83-95%)	10% (5.2-17%)	7 (5.4-8.6)	16% (11-20%)	18% (13-23%)	540 (390-690)	13000 (9500-16000)
Slovakia	2005	Male	70 to 74	88% (81-93%)	12% (7-19%)	6.1 (4.5-7.9)	13% (7.1-18%)	15% (8-21%)	530 (290-770)	10000 (5900-15000)

Location	Year	Sex	Age	Prevalence of current drinkers	Prevalence of abstainers	Population average of standard drinks daily	Percent of total attributable deaths	Percent of total attributable DALYs	Attributable deaths	Attributable DALYs
Slovakia	2005	Male	75 to 79	86% (78-93%)	14% (7.3-22%)	5.2 (3.6-7.2)	9.7% (3.2-16%)	11% (3.3-18%)	440 (130-730)	6600 (2200-11000)
Slovakia	2005	Male	80 to 84	85% (75-92%)	15% (8.1-25%)	4.6 (3.4-6.2)	7.7% (1.9-14%)	8.5% (1.7-16%)	320 (61-580)	3500 (880-6300)
Slovakia	2005	Male	85 to 89	84% (75-90%)	16% (9.6-25%)	4.2 (3-5.6)	5.4% (-0.14-12%)	5.8% (-0.83-13%)	88 (-13-200)	720 (-18-1500)
Slovakia	2005	Male	90 to 94	84% (75-91%)	16% (9-25%)	3.9 (2.6-5.4)	3.3% (-2.4-10%)	3.3% (-3.6-12%)	31 (-34-110)	190 (-140-590)
Slovakia	2005	Male	95 plus	84% (72-93%)	16% (7.2-28%)	3.4 (1.9-5.4)	1.8% (-4.3-9.7%)	1.4% (-5.7-11%)	2.8 (-12-23)	15 (-35-83)
Slovakia	2010	Female	All Ages	87% (85-89%)	13% (11-15%)	1.7 (1.5-1.9)	1.8% (0.58-3.1%)	0.41% (-2.3-3%)	100 (-570-780)	13000 (4600-23000)
Slovakia	2010	Female	15 to 19	87% (80-92%)	13% (8-20%)	1.7 (1.2-2.4)	2.7% (1.8-3.8%)	7.1% (3.8-11%)	2.6 (1.4-4)	410 (260-580)
Slovakia	2010	Female	20 to 24	91% (87-95%)	8.5% (5.4-13%)	2.3 (1.6-3.1)	4.3% (3-5.8%)	7.7% (4.6-11%)	3.8 (2.2-5.5)	810 (540-1200)
Slovakia	2010	Female	25 to 29	93% (89-96%)	7% (4.2-11%)	1.9 (1.4-2.5)	3.9% (2.9-5.2%)	6.7% (4.3-9.1%)	4 (2.5-5.4)	890 (610-1200)
Slovakia	2010	Female	30 to 34	93% (90-96%)	6.7% (3.9-10%)	1.9 (1.3-2.5)	3.7% (2.7-4.9%)	7.4% (5.3-9.8%)	6.7 (4.7-8.9)	1000 (710-1400)
Slovakia	2010	Female	35 to 39	93% (89-95%)	7.4% (4.5-11%)	2.2 (1.5-2.9)	4.1% (3-5.2%)	9% (6.4-12%)	14 (9.7-18)	1200 (870-1600)
Slovakia	2010	Female	40 to 44	92% (88-95%)	8.1% (5.3-12%)	2.3 (1.7-3)	4.6% (3.4-5.9%)	10% (7.5-13%)	23 (17-30)	1500 (1100-1900)
Slovakia	2010	Female	45 to 49	92% (88-95%)	7.7% (4.9-12%)	2.4 (1.7-3.2)	4.9% (3.5-6.5%)	10% (7.5-13%)	40 (30-52)	2000 (1500-2600)
Slovakia	2010	Female	50 to 54	92% (88-94%)	8.4% (5.5-12%)	2.2 (1.6-3)	4.7% (3.1-6.5%)	8.5% (5.8-11%)	63 (43-83)	2600 (1700-3500)
Slovakia	2010	Female	55 to 59	90% (85-94%)	10% (6.4-15%)	2 (1.4-2.6)	3.5% (1.9-5.2%)	6.1% (3.5-8.9%)	67 (38-97)	2300 (1300-3400)
Slovakia	2010	Female	60 to 64	87% (81-92%)	13% (8-19%)	1.8 (1.3-2.5)	2.1% (0.35-4.2%)	3.5% (0.79-6.5%)	49 (11-89)	1400 (240-2700)
Slovakia	2010	Female	65 to 69	83% (75-90%)	17% (10-25%)	1.7 (1.2-2.3)	1% (-0.87-2.9%)	1.7% (-1.3-4.4%)	28 (-22-73)	660 (-560-1800)
Slovakia	2010	Female	70 to 74	75% (67-83%)	25% (17-33%)	1.4 (0.97-2)	0.21% (-1.8-2.2%)	0.25% (-2.6-3%)	6.3 (-65-75)	140 (-1200-1500)
Slovakia	2010	Female	75 to 79	67% (56-78%)	33% (22-44%)	1.3 (0.82-1.8)	-0.28% (-2.3-1.9%)	-0.48% (-3.2-2.3%)	-19 (-130-89)	-210 (-1800-1400)
Slovakia	2010	Female	80 to 84	65% (51-77%)	35% (23-49%)	1.3 (0.9-1.9)	-0.74% (-3.2-1.6%)	-1.1% (-4.2-1.9%)	-59 (-230-100)	-540 (-2300-1200)
Slovakia	2010	Female	85 to 89	66% (52-78%)	34% (22-48%)	1.5 (0.95-2.2)	-1.1% (-4.2-1.9%)	-1.6% (-5.4-2.1%)	-82 (-270-110)	-530 (-1900-890)
Slovakia	2010	Female	90 to 94	68% (55-79%)	32% (21-45%)	1.7 (0.98-2.5)	-1.3% (-4.8-2.8%)	-1.8% (-6.1-3%)	-31 (-100-51)	-130 (-490-290)

Location	Year	Sex	Age	Prevalence of current drinkers	Prevalence of abstainers	Population average of standard drinks daily	Percent of total attributable deaths	Percent of total attributable DALYs	Attributable deaths	Attributable DALYs
Slovakia	2010	Female	95 plus	70% (53-84%)	30% (16-47%)	1.8 (0.94-2.9)	-1.3% (-5.6-3.9%)	-1.8% (-7-4.3%)	-13 (-50-30)	-36 (-160-110)
Slovakia	2010	Male	All Ages	89% (86-91%)	11% (9-14%)	5.2 (4.6-5.8)	15% (12-17%)	16% (12-20%)	4300 (3200-5500)	140000 (110000-160000)
Slovakia	2010	Male	15 to 19	80% (71-88%)	20% (12-29%)	2.5 (1.7-3.4)	6.5% (4.6-8.8%)	12% (7-17%)	11 (6.3-15)	1200 (820-1600)
Slovakia	2010	Male	20 to 24	91% (86-95%)	9.1% (5.3-14%)	4.6 (3.4-6)	14% (10-18%)	21% (14-28%)	38 (26-51)	3700 (2800-4800)
Slovakia	2010	Male	25 to 29	94% (90-96%)	6.5% (3.7-10%)	5.9 (4.4-7.6)	15% (12-19%)	25% (18-32%)	54 (40-69)	4800 (3700-6000)
Slovakia	2010	Male	30 to 34	95% (91-97%)	5.4% (2.8-9.2%)	6.5 (5-8.1)	15% (13-19%)	26% (21-32%)	75 (58-91)	5800 (4600-7100)
Slovakia	2010	Male	35 to 39	95% (92-97%)	5.2% (2.8-8.4%)	6.5 (4.8-8.4)	17% (13-20%)	26% (20-32%)	110 (82-130)	7000 (5400-8400)
Slovakia	2010	Male	40 to 44	95% (92-97%)	5.2% (3.1-8.4%)	6.8 (5.1-8.6)	18% (14-22%)	27% (21-32%)	160 (130-190)	8700 (6900-10000)
Slovakia	2010	Male	45 to 49	94% (90-97%)	5.9% (3.3-9.9%)	7.2 (5.4-9.2)	20% (16-24%)	27% (22-32%)	290 (230-350)	14000 (11000-16000)
Slovakia	2010	Male	50 to 54	93% (89-96%)	6.7% (3.9-11%)	7.7 (5.8-9.8)	21% (17-24%)	27% (22-31%)	500 (400-580)	20000 (16000-23000)
Slovakia	2010	Male	55 to 59	92% (86-96%)	8.1% (4.3-14%)	8 (6.3-10)	20% (16-23%)	25% (20-29%)	660 (530-800)	23000 (18000-27000)
Slovakia	2010	Male	60 to 64	90% (84-95%)	9.5% (5.1-16%)	7.5 (5.7-9.8)	17% (14-22%)	21% (16-26%)	650 (500-800)	19000 (15000-23000)
Slovakia	2010	Male	65 to 69	90% (83-94%)	10% (5.6-17%)	6.6 (5.2-8.5)	15% (11-19%)	18% (13-23%)	530 (390-680)	13000 (9500-16000)
Slovakia	2010	Male	70 to 74	88% (82-93%)	12% (7.3-18%)	5.8 (4.2-7.7)	12% (6.3-17%)	14% (7.2-20%)	440 (230-630)	8600 (4700-12000)
Slovakia	2010	Male	75 to 79	86% (78-92%)	14% (8.1-22%)	4.9 (3.4-6.9)	8.8% (2.9-15%)	10% (3-17%)	370 (110-630)	5600 (1900-9500)
Slovakia	2010	Male	80 to 84	83% (72-91%)	17% (9.3-28%)	4.3 (3-5.9)	6.7% (1.2-12%)	7.3% (0.8-14%)	260 (28-500)	2900 (520-5400)
Slovakia	2010	Male	85 to 89	82% (69-89%)	18% (11-31%)	3.8 (2.6-5.1)	4.5% (-0.39-10%)	4.7% (-1.1-11%)	110 (-28-280)	970 (-84-2200)
Slovakia	2010	Male	90 to 94	82% (72-90%)	18% (10-28%)	3.5 (2.3-5)	2.5% (-2.6-8.9%)	2.3% (-3.7-9.9%)	16 (-26-68)	110 (-110-380)
Slovakia	2010	Male	95 plus	82% (69-92%)	18% (8.3-31%)	3.1 (1.7-5)	1.1% (-4.1-8.5%)	0.57% (-5.8-9.4%)	1.2 (-12-19)	9.4 (-35-71)
Slovakia	2016	Female	All Ages	87% (85-90%)	13% (10-15%)	1.7 (1.4-2.1)	1.8% (0.44-3.3%)	0.53% (-2.4-3.6%)	140 (-630-940)	14000 (3200-25000)
Slovakia	2016	Female	15 to 19	88% (81-93%)	12% (7.1-19%)	1.8 (1.2-2.6)	2.7% (1.8-3.8%)	7.4% (3.8-11%)	2.1 (1.1-3.4)	320 (200-470)
Slovakia	2016	Female	20 to 24	92% (87-95%)	8.3% (5.1-13%)	2.3 (1.5-3.3)	4.3% (3-5.9%)	8% (4.6-12%)	3.1 (1.7-4.6)	690 (460-990)

Location	Year	Sex	Age	Prevalence of current drinkers	Prevalence of abstainers	Population average of standard drinks daily	Percent of total attributable deaths	Percent of total attributable DALYs	Attributable deaths	Attributable DALYs
Slovakia	2016	Female	25 to 29	93% (89-96%)	6.9% (4-11%)	1.9 (1.3-2.7)	4% (3-5.3%)	6.9% (4.4-9.7%)	3.3 (2-4.9)	810 (550-1200)
Slovakia	2016	Female	30 to 34	93% (89-96%)	6.7% (3.9-11%)	1.9 (1.3-2.6)	3.9% (2.8-5.1%)	7.8% (5.5-10%)	6.3 (4.1-8.7)	970 (690-1400)
Slovakia	2016	Female	35 to 39	92% (88-95%)	7.7% (4.5-12%)	2.2 (1.4-3.1)	4.1% (3-5.4%)	9.4% (6.5-12%)	15 (9.8-21)	1400 (970-1800)
Slovakia	2016	Female	40 to 44	92% (87-95%)	8.2% (5-13%)	2.4 (1.6-3.3)	4.4% (3.2-5.9%)	10% (7.5-14%)	23 (16-32)	1500 (1100-2100)
Slovakia	2016	Female	45 to 49	92% (86-95%)	8.1% (4.8-14%)	2.4 (1.6-3.4)	4.8% (3.4-6.5%)	10% (7.5-13%)	34 (24-47)	1800 (1200-2400)
Slovakia	2016	Female	50 to 54	91% (86-95%)	8.8% (5.3-14%)	2.2 (1.5-3.1)	4.6% (3.1-6.4%)	8.9% (6.2-12%)	51 (33-71)	2200 (1400-3100)
Slovakia	2016	Female	55 to 59	90% (84-94%)	10% (6.2-16%)	2 (1.4-2.7)	3.7% (2.1-5.5%)	6.5% (4-9.4%)	64 (38-96)	2200 (1300-3400)
Slovakia	2016	Female	60 to 64	88% (82-93%)	12% (7-18%)	1.9 (1.3-2.7)	2.3% (0.5-4.3%)	4% (1.2-6.9%)	63 (19-110)	1800 (390-3400)
Slovakia	2016	Female	65 to 69	86% (77-92%)	14% (7.8-23%)	1.9 (1.3-2.6)	1.3% (-0.85-3.2%)	2.1% (-1-5.1%)	40 (-20-96)	920 (-630-2500)
Slovakia	2016	Female	70 to 74	80% (71-87%)	20% (13-29%)	1.7 (1.1-2.5)	0.41% (-1.8-2.9%)	0.59% (-2.7-4%)	15 (-64-99)	290 (-1300-2000)
Slovakia	2016	Female	75 to 79	72% (59-83%)	28% (17-41%)	1.4 (0.89-2.2)	-0.12% (-2.4-2.4%)	-0.3% (-3.4-2.9%)	-10 (-120-100)	-85 (-1700-1600)
Slovakia	2016	Female	80 to 84	67% (52-79%)	33% (21-48%)	1.4 (0.9-2.1)	-0.51% (-3.1-2.1%)	-0.82% (-4.1-2.6%)	-43 (-210-140)	-360 (-2200-1500)
Slovakia	2016	Female	85 to 89	66% (52-78%)	34% (22-48%)	1.5 (0.92-2.3)	-0.82% (-3.9-2.4%)	-1.2% (-5-2.7%)	-62 (-250-130)	-380 (-1800-1100)
Slovakia	2016	Female	90 to 94	68% (55-80%)	32% (20-45%)	1.7 (0.95-2.6)	-0.99% (-4.4-3.2%)	-1.5% (-5.8-3.5%)	-56 (-220-130)	-230 (-1000-730)
Slovakia	2016	Female	95 plus	71% (54-85%)	29% (15-46%)	1.8 (0.92-3)	-0.98% (-5.3-4.5%)	-1.5% (-6.8-5.1%)	-8.5 (-38-28)	-22 (-120-100)
Slovakia	2016	Male	All Ages	87% (84-90%)	13% (10-16%)	5.1 (4.1-6.3)	14% (11-17%)	15% (10-20%)	4100 (2700-5600)	130000 (100000-160000)
Slovakia	2016	Male	15 to 19	77% (66-87%)	23% (13-34%)	2.3 (1.4-3.4)	6.3% (4.3-8.6%)	12% (6.6-17%)	7.3 (4.2-11)	880 (590-1200)
Slovakia	2016	Male	20 to 24	89% (82-94%)	11% (6.2-18%)	4.4 (3.1-6)	13% (9.8-17%)	21% (13-28%)	27 (17-38)	2900 (2100-3800)
Slovakia	2016	Male	25 to 29	92% (86-96%)	8% (4.3-14%)	5.7 (3.9-7.6)	15% (11-18%)	25% (17-32%)	42 (29-57)	4000 (3000-5200)
Slovakia	2016	Male	30 to 34	94% (88-97%)	6.3% (3-12%)	6.4 (4.5-8.3)	15% (12-18%)	26% (20-33%)	61 (44-80)	5000 (3800-6400)
Slovakia	2016	Male	35 to 39	94% (89-97%)	6% (3.1-11%)	6.4 (4.5-8.7)	16% (12-20%)	27% (20-33%)	110 (76-140)	7200 (5400-9100)
Slovakia	2016	Male	40 to 44	94% (89-96%)	6.3% (3.6-11%)	6.5 (4.6-9)	17% (13-21%)	27% (21-33%)	160 (110-200)	8800 (6500-11000)

Location	Year	Sex	Age	Prevalence of current drinkers	Prevalence of abstainers	Population average of standard drinks daily	Percent of total attributable deaths	Percent of total attributable DALYs	Attributable deaths	Attributable DALYs
Slovakia	2016	Male	45 to 49	93% (87-96%)	7.2% (3.6-13%)	7 (4.9-9.5)	19% (15-23%)	27% (21-33%)	230 (170-290)	11000 (8200-14000)
Slovakia	2016	Male	50 to 54	92% (85-96%)	8.3% (4.4-15%)	7.4 (5.2-10)	20% (15-24%)	26% (21-31%)	390 (290-490)	16000 (12000-19000)
Slovakia	2016	Male	55 to 59	91% (83-95%)	9.2% (4.6-17%)	7.8 (5.5-10)	19% (15-23%)	24% (20-29%)	580 (440-730)	20000 (16000-25000)
Slovakia	2016	Male	60 to 64	90% (82-95%)	10% (5.1-18%)	7.3 (5-10)	17% (13-21%)	21% (16-26%)	730 (550-950)	21000 (16000-27000)
Slovakia	2016	Male	65 to 69	88% (80-94%)	12% (5.8-20%)	6.4 (4.7-8.5)	14% (10-18%)	17% (12-22%)	600 (410-800)	15000 (10000-19000)
Slovakia	2016	Male	70 to 74	86% (78-92%)	14% (8.1-22%)	5.5 (3.7-7.6)	11% (5.6-16%)	13% (6.4-19%)	450 (210-670)	8900 (4400-13000)
Slovakia	2016	Male	75 to 79	84% (74-91%)	16% (8.9-26%)	4.7 (3-6.8)	8.5% (2.5-14%)	9.8% (2.6-17%)	320 (79-560)	4900 (1400-8400)
Slovakia	2016	Male	80 to 84	82% (71-91%)	18% (9.3-29%)	4.2 (2.7-6)	6.6% (1.1-13%)	7.4% (0.84-14%)	250 (28-510)	2900 (500-5600)
Slovakia	2016	Male	85 to 89	82% (71-90%)	18% (10-29%)	3.8 (2.5-5.5)	4.7% (-0.44-11%)	5% (-1.1-12%)	120 (-27-300)	1000 (-95-2300)
Slovakia	2016	Male	90 to 94	82% (71-90%)	18% (10-29%)	3.5 (2.2-5.3)	2.8% (-2.4-9.4%)	2.6% (-3.5-11%)	39 (-53-150)	250 (-210-840)
Slovakia	2016	Male	95 plus	82% (69-92%)	18% (8.2-31%)	3.2 (1.7-5.2)	1.4% (-3.8-8.9%)	0.9% (-5.4-10%)	1.6 (-9.3-18)	10 (-26-64)
Slovenia	1990	Female	All Ages	67% (62-71%)	33% (29-38%)	2.5 (2.1-2.9)	7.3% (5.6-9.2%)	9.8% (6.8-14%)	920 (630-1300)	21000 (16000-27000)
Slovenia	1990	Female	15 to 19	68% (53-80%)	32% (20-47%)	2.3 (1.5-3.3)	4.6% (2.9-6.7%)	13% (7-19%)	3.1 (1.7-4.9)	320 (190-460)
Slovenia	1990	Female	20 to 24	77% (64-86%)	23% (14-36%)	3 (1.9-4.2)	6.4% (4.4-8.9%)	15% (8.6-21%)	4.9 (2.9-7.3)	540 (370-740)
Slovenia	1990	Female	25 to 29	79% (68-87%)	21% (13-32%)	2.5 (1.7-3.5)	5.3% (3.8-6.9%)	12% (7.3-17%)	4.1 (2.5-5.9)	490 (330-650)
Slovenia	1990	Female	30 to 34	80% (68-88%)	20% (12-32%)	2.3 (1.6-3.2)	4.9% (3.6-6.5%)	12% (7.7-16%)	5.5 (3.5-7.8)	540 (370-710)
Slovenia	1990	Female	35 to 39	79% (67-87%)	21% (13-33%)	2.5 (1.6-3.7)	5.9% (4.2-7.9%)	14% (9.4-18%)	11 (7.3-15)	790 (560-1000)
Slovenia	1990	Female	40 to 44	76% (64-85%)	24% (15-36%)	3.1 (2.2-4.3)	7.4% (5.5-9.7%)	16% (12-20%)	17 (12-22)	980 (720-1200)
Slovenia	1990	Female	45 to 49	72% (61-81%)	28% (19-39%)	3.9 (2.7-5.3)	9.5% (7.1-12%)	18% (14-23%)	30 (23-38)	1500 (1100-1900)
Slovenia	1990	Female	50 to 54	68% (59-76%)	32% (24-41%)	4 (2.8-5.3)	10% (8-14%)	18% (14-23%)	45 (34-57)	1900 (1400-2400)
Slovenia	1990	Female	55 to 59	66% (56-74%)	34% (26-44%)	3.8 (2.6-5)	10% (7.4-13%)	16% (12-21%)	62 (47-81)	2200 (1700-2900)
Slovenia	1990	Female	60 to 64	65% (54-73%)	35% (27-46%)	3.6 (2.5-5)	9.1% (6.4-12%)	14% (9.8-18%)	79 (56-110)	2400 (1700-3200)

Location	Year	Sex	Age	Prevalence of current drinkers	Prevalence of abstainers	Population average of standard drinks daily	Percent of total attributable deaths	Percent of total attributable DALYs	Attributable deaths	Attributable DALYs
Slovenia	1990	Female	65 to 69	64% (54-72%)	36% (28-46%)	3.6 (2.6-4.7)	8.8% (6.1-12%)	13% (8.7-17%)	100 (71-140)	2600 (1800-3500)
Slovenia	1990	Female	70 to 74	61% (50-70%)	39% (30-50%)	3.4 (2.3-4.7)	8.6% (5.1-12%)	11% (6.8-16%)	81 (48-120)	1600 (960-2300)
Slovenia	1990	Female	75 to 79	56% (42-67%)	44% (33-58%)	2.9 (1.9-4.2)	7.4% (3.9-12%)	9.2% (4.8-15%)	160 (81-250)	2400 (1200-3800)
Slovenia	1990	Female	80 to 84	51% (37-64%)	49% (36-63%)	2.5 (1.6-3.7)	6.4% (3.5-11%)	7.8% (4.1-13%)	150 (80-250)	1700 (910-2700)
Slovenia	1990	Female	85 to 89	48% (35-61%)	52% (39-65%)	2.4 (1.5-3.6)	5.9% (2.7-10%)	6.9% (3.1-12%)	100 (45-180)	790 (360-1400)
Slovenia	1990	Female	90 to 94	48% (35-61%)	52% (39-65%)	2.4 (1.4-3.7)	6% (2.3-11%)	7.1% (2.6-13%)	50 (18-94)	260 (100-490)
Slovenia	1990	Female	95 plus	50% (33-66%)	50% (34-67%)	2.5 (1.3-4.2)	6.2% (1.6-13%)	7.3% (1.7-15%)	13 (3.1-26)	44 (12-89)
Slovenia	1990	Male	All Ages	72% (66-78%)	28% (22-34%)	5.2 (4.5-6)	17% (14-20%)	20% (16-24%)	2000 (1500-2400)	59000 (49000-70000)
Slovenia	1990	Male	15 to 19	62% (46-77%)	38% (23-54%)	3 (2-4.3)	9.4% (5.9-14%)	16% (9.3-24%)	11 (6.2-16)	900 (560-1300)
Slovenia	1990	Male	20 to 24	77% (63-87%)	23% (13-37%)	5.4 (3.8-7.1)	19% (13-25%)	29% (19-39%)	34 (22-46)	2500 (1800-3400)
Slovenia	1990	Male	25 to 29	82% (70-90%)	18% (10-30%)	5.5 (4.1-7.3)	19% (14-24%)	30% (21-38%)	35 (24-46)	2500 (1900-3300)
Slovenia	1990	Male	30 to 34	85% (73-92%)	15% (8-27%)	5.7 (4.2-7.5)	18% (14-23%)	29% (22-37%)	43 (31-56)	2900 (2200-3700)
Slovenia	1990	Male	35 to 39	86% (76-92%)	14% (7.8-24%)	5.8 (4.3-7.5)	19% (14-23%)	29% (22-35%)	65 (48-81)	3900 (2900-4700)
Slovenia	1990	Male	40 to 44	85% (76-92%)	15% (8.3-24%)	6.4 (4.8-8.2)	20% (16-24%)	30% (24-35%)	81 (64-99)	4200 (3300-5100)
Slovenia	1990	Male	45 to 49	83% (74-90%)	17% (9.9-26%)	7.5 (5.5-9.7)	22% (19-26%)	30% (25-35%)	120 (100-150)	5500 (4600-6600)
Slovenia	1990	Male	50 to 54	80% (72-86%)	20% (14-28%)	8.4 (6.4-11)	22% (18-25%)	28% (24-33%)	170 (140-210)	6700 (5600-8100)
Slovenia	1990	Male	55 to 59	77% (69-83%)	23% (17-31%)	9.4 (7.2-12)	21% (17-25%)	25% (21-30%)	250 (200-300)	8300 (6700-10000)
Slovenia	1990	Male	60 to 64	77% (69-82%)	23% (18-31%)	10 (7.6-13)	18% (14-22%)	22% (17-27%)	240 (180-300)	6900 (5300-8500)
Slovenia	1990	Male	65 to 69	79% (72-85%)	21% (15-28%)	10 (7.8-13)	17% (13-21%)	20% (15-25%)	220 (160-280)	5200 (3800-6700)
Slovenia	1990	Male	70 to 74	77% (69-83%)	23% (17-31%)	9.4 (7.1-12)	16% (11-20%)	19% (13-24%)	140 (94-180)	2700 (1900-3400)
Slovenia	1990	Male	75 to 79	71% (61-79%)	29% (21-39%)	8 (5.8-10)	14% (9.6-19%)	16% (11-21%)	250 (160-330)	3700 (2500-4800)
Slovenia	1990	Male	80 to 84	68% (56-77%)	32% (23-44%)	6.9 (5-9)	13% (8.5-17%)	14% (9.5-20%)	190 (120-270)	2100 (1400-2900)

Location	Year	Sex	Age	Prevalence of current drinkers	Prevalence of abstainers	Population average of standard drinks daily	Percent of total attributable deaths	Percent of total attributable DALYs	Attributable deaths	Attributable DALYs
Slovenia	1990	Male	85 to 89	69% (57-77%)	31% (23-43%)	6.3 (4.5-8.7)	12% (7.5-17%)	13% (8.4-19%)	90 (57-130)	710 (470-1000)
Slovenia	1990	Male	90 to 94	70% (59-79%)	30% (21-41%)	5.9 (3.9-8.6)	11% (7.3-16%)	13% (8-19%)	30 (19-43)	160 (100-230)
Slovenia	1990	Male	95 plus	71% (56-83%)	29% (17-44%)	5.3 (3-8.1)	11% (5.2-16%)	12% (5.5-18%)	5.4 (2.5-8.1)	19 (9.4-29)
Slovenia	1995	Female	All Ages	67% (62-71%)	33% (29-38%)	2.2 (1.9-2.6)	6.5% (5-8.2%)	8.4% (5.4-12%)	790 (520-1100)	18000 (14000-23000)
Slovenia	1995	Female	15 to 19	68% (53-81%)	32% (19-47%)	2.1 (1.3-2.9)	4.2% (2.6-6.1%)	11% (6-17%)	2.9 (1.6-4.5)	300 (180-440)
Slovenia	1995	Female	20 to 24	77% (64-86%)	23% (14-36%)	2.6 (1.7-3.7)	5.7% (3.9-7.9%)	13% (7.1-19%)	3.5 (2-5.3)	420 (280-580)
Slovenia	1995	Female	25 to 29	79% (68-87%)	21% (13-32%)	2.2 (1.5-3)	4.9% (3.5-6.4%)	11% (6.3-15%)	3.4 (2-4.9)	430 (290-570)
Slovenia	1995	Female	30 to 34	80% (67-88%)	20% (12-33%)	2 (1.3-2.9)	4.6% (3.3-6%)	10% (6.7-15%)	4.6 (2.9-6.6)	470 (330-630)
Slovenia	1995	Female	35 to 39	79% (66-88%)	21% (12-34%)	2.2 (1.4-3.2)	5.4% (3.9-7.2%)	12% (8.6-16%)	9.5 (6.6-13)	680 (500-900)
Slovenia	1995	Female	40 to 44	76% (65-85%)	24% (15-35%)	2.7 (1.9-3.7)	6.7% (4.9-8.6%)	14% (11-18%)	18 (14-24)	1100 (800-1400)
Slovenia	1995	Female	45 to 49	72% (62-81%)	28% (19-38%)	3.4 (2.3-4.6)	8.7% (6.4-11%)	16% (13-21%)	28 (21-36)	1400 (1000-1700)
Slovenia	1995	Female	50 to 54	68% (59-76%)	32% (24-41%)	3.5 (2.5-4.7)	9.4% (7.1-12%)	16% (13-20%)	40 (31-51)	1700 (1300-2100)
Slovenia	1995	Female	55 to 59	66% (56-74%)	34% (26-44%)	3.3 (2.3-4.4)	8.7% (6.5-11%)	15% (11-18%)	47 (36-61)	1700 (1300-2200)
Slovenia	1995	Female	60 to 64	65% (55-74%)	35% (26-45%)	3.2 (2.2-4.4)	8% (5.6-11%)	12% (8.9-16%)	69 (48-92)	2100 (1500-2800)
Slovenia	1995	Female	65 to 69	65% (55-73%)	35% (27-45%)	3.2 (2.3-4.1)	7.5% (5.2-10%)	11% (7.6-14%)	90 (63-120)	2300 (1600-3100)
Slovenia	1995	Female	70 to 74	62% (51-70%)	38% (30-49%)	3 (2-4.1)	7.2% (4.1-11%)	9.7% (5.6-14%)	120 (69-180)	2400 (1400-3600)
Slovenia	1995	Female	75 to 79	56% (43-68%)	44% (32-57%)	2.6 (1.7-3.7)	6.1% (2.9-9.9%)	7.7% (3.7-13%)	70 (33-120)	1100 (510-1800)
Slovenia	1995	Female	80 to 84	52% (37-64%)	48% (36-63%)	2.2 (1.4-3.2)	5.3% (2.6-8.8%)	6.4% (3-11%)	100 (50-170)	1200 (570-1900)
Slovenia	1995	Female	85 to 89	49% (36-62%)	51% (38-64%)	2.1 (1.3-3.1)	4.8% (2-8.6%)	5.7% (2.2-10%)	110 (43-200)	880 (360-1500)
Slovenia	1995	Female	90 to 94	49% (36-62%)	51% (38-64%)	2.2 (1.3-3.3)	4.8% (1.5-9.5%)	5.7% (1.6-11%)	53 (15-100)	280 (84-540)
Slovenia	1995	Female	95 plus	50% (35-66%)	50% (34-65%)	2.2 (1.1-3.6)	5% (0.66-11%)	5.9% (0.6-13%)	15 (1.6-34)	54 (7.2-120)
Slovenia	1995	Male	All Ages	73% (67-78%)	27% (22-33%)	4.8 (4.2-5.4)	16% (14-19%)	19% (16-23%)	1900 (1500-2300)	56000 (47000-66000)

Location	Year	Sex	Age	Prevalence of current drinkers	Prevalence of abstainers	Population average of standard drinks daily	Percent of total attributable deaths	Percent of total attributable DALYs	Attributable deaths	Attributable DALYs
Slovenia	1995	Male	15 to 19	63% (47-78%)	37% (22-53%)	2.7 (1.8-3.8)	8.8% (5.5-13%)	15% (8.5-22%)	11 (6.2-17)	930 (560-1300)
Slovenia	1995	Male	20 to 24	77% (63-87%)	23% (13-37%)	4.8 (3.4-6.3)	17% (12-23%)	27% (18-36%)	28 (18-37)	2100 (1500-2800)
Slovenia	1995	Male	25 to 29	82% (71-90%)	18% (10-29%)	4.9 (3.6-6.3)	17% (12-22%)	27% (19-35%)	28 (19-38)	2100 (1500-2800)
Slovenia	1995	Male	30 to 34	85% (75-92%)	15% (7.9-25%)	5 (3.7-6.5)	17% (13-21%)	27% (20-34%)	35 (26-46)	2400 (1800-3100)
Slovenia	1995	Male	35 to 39	86% (76-92%)	14% (7.8-24%)	5.1 (3.7-6.6)	17% (13-21%)	26% (20-33%)	50 (37-63)	3000 (2300-3800)
Slovenia	1995	Male	40 to 44	85% (77-91%)	15% (8.6-23%)	5.6 (4.3-7.2)	19% (15-23%)	27% (22-32%)	90 (70-110)	4700 (3700-5700)
Slovenia	1995	Male	45 to 49	83% (74-90%)	17% (9.7-26%)	6.6 (4.9-8.4)	21% (17-24%)	28% (24-33%)	110 (91-140)	5100 (4200-6100)
Slovenia	1995	Male	50 to 54	81% (73-86%)	19% (14-27%)	7.4 (5.6-9.2)	21% (18-24%)	27% (23-31%)	150 (130-190)	6100 (5100-7300)
Slovenia	1995	Male	55 to 59	77% (70-83%)	23% (17-30%)	8.2 (6.4-10)	20% (17-24%)	25% (21-29%)	200 (160-240)	6800 (5600-8200)
Slovenia	1995	Male	60 to 64	77% (69-83%)	23% (17-31%)	8.7 (6.7-11)	18% (15-22%)	22% (18-26%)	260 (210-320)	7500 (6000-9200)
Slovenia	1995	Male	65 to 69	79% (72-85%)	21% (15-28%)	8.8 (6.9-11)	17% (13-21%)	20% (15-24%)	260 (200-330)	6300 (4800-7800)
Slovenia	1995	Male	70 to 74	77% (69-83%)	23% (17-31%)	8.2 (6.3-10)	15% (12-19%)	18% (13-22%)	230 (160-290)	4400 (3200-5600)
Slovenia	1995	Male	75 to 79	71% (61-79%)	29% (21-39%)	6.9 (5.1-8.9)	14% (10-18%)	16% (12-20%)	120 (85-150)	1800 (1300-2300)
Slovenia	1995	Male	80 to 84	69% (57-77%)	31% (23-43%)	6 (4.4-7.8)	12% (8.8-16%)	14% (10-19%)	150 (100-200)	1600 (1200-2200)
Slovenia	1995	Male	85 to 89	69% (58-77%)	31% (23-42%)	5.5 (3.9-7.4)	11% (7.8-16%)	13% (8.6-18%)	130 (84-180)	1000 (680-1400)
Slovenia	1995	Male	90 to 94	70% (59-79%)	30% (21-41%)	5.1 (3.4-7.2)	11% (6.6-15%)	12% (7.3-18%)	41 (25-59)	220 (130-310)
Slovenia	1995	Male	95 plus	71% (56-83%)	29% (17-44%)	4.7 (2.6-7.1)	9.7% (3.9-15%)	11% (4.1-17%)	7.8 (2.9-12)	28 (11-43)
Slovenia	2000	Female	All Ages	66% (61-70%)	34% (30-39%)	2 (1.8-2.3)	5.6% (4.4-7%)	7.1% (4.8-9.6%)	680 (450-940)	16000 (12000-20000)
Slovenia	2000	Female	15 to 19	67% (51-81%)	33% (19-49%)	1.8 (1.2-2.6)	3.6% (2.2-5.3%)	9.9% (5.1-15%)	2.1 (1.1-3.3)	230 (140-340)
Slovenia	2000	Female	20 to 24	76% (62-85%)	24% (15-38%)	2.3 (1.5-3.2)	4.9% (3.3-6.7%)	11% (5.9-17%)	2.6 (1.3-3.9)	360 (240-500)
Slovenia	2000	Female	25 to 29	77% (65-86%)	23% (14-35%)	1.9 (1.3-2.6)	4.3% (3.1-5.5%)	9.4% (5.5-14%)	2.2 (1.2-3.2)	330 (220-440)
Slovenia	2000	Female	30 to 34	78% (64-87%)	22% (13-36%)	1.7 (1.1-2.4)	4% (2.9-5.1%)	9.1% (5.9-13%)	3.3 (2.1-4.5)	380 (270-520)

Location	Year	Sex	Age	Prevalence of current drinkers	Prevalence of abstainers	Population average of standard drinks daily	Percent of total attributable deaths	Percent of total attributable DALYs	Attributable deaths	Attributable DALYs
Slovenia	2000	Female	35 to 39	77% (65-87%)	23% (13-35%)	2 (1.3-2.9)	4.8% (3.5-6.2%)	12% (8.2-15%)	7 (5-9.4)	550 (410-710)
Slovenia	2000	Female	40 to 44	76% (64-85%)	24% (15-36%)	2.5 (1.8-3.5)	5.9% (4.4-7.6%)	13% (10-16%)	14 (11-18)	850 (650-1100)
Slovenia	2000	Female	45 to 49	72% (62-80%)	28% (20-38%)	3.1 (2.2-4.3)	7.7% (5.7-9.9%)	15% (12-19%)	29 (22-37)	1400 (1100-1800)
Slovenia	2000	Female	50 to 54	67% (59-76%)	33% (24-41%)	3.1 (2.2-4.1)	8.4% (6.4-11%)	15% (12-19%)	35 (27-44)	1500 (1100-1800)
Slovenia	2000	Female	55 to 59	64% (54-72%)	36% (28-46%)	2.9 (2.1-3.8)	7.7% (5.8-9.8%)	13% (10-17%)	40 (31-51)	1400 (1100-1800)
Slovenia	2000	Female	60 to 64	62% (52-71%)	38% (29-48%)	2.7 (1.9-3.9)	6.9% (4.8-9.4%)	11% (8.2-15%)	48 (35-65)	1500 (1100-2000)
Slovenia	2000	Female	65 to 69	62% (51-71%)	38% (29-49%)	2.7 (1.9-3.6)	6.4% (4.5-8.6%)	9.6% (6.9-13%)	70 (50-95)	1800 (1300-2500)
Slovenia	2000	Female	70 to 74	60% (50-69%)	40% (31-50%)	2.7 (1.9-3.7)	6% (3.7-8.8%)	8.3% (5.2-12%)	95 (58-140)	1900 (1200-2900)
Slovenia	2000	Female	75 to 79	56% (43-68%)	44% (32-57%)	2.3 (1.6-3.4)	5% (2.5-8.1%)	6.5% (3.3-11%)	110 (52-180)	1700 (830-2800)
Slovenia	2000	Female	80 to 84	51% (37-64%)	49% (36-63%)	2 (1.3-2.9)	4.4% (2.2-7%)	5.4% (2.6-8.9%)	86 (42-140)	970 (480-1600)
Slovenia	2000	Female	85 to 89	49% (36-61%)	51% (39-64%)	1.9 (1.2-2.8)	4% (1.6-6.9%)	4.7% (1.8-8.5%)	68 (25-120)	540 (210-940)
Slovenia	2000	Female	90 to 94	49% (37-61%)	51% (39-63%)	1.9 (1.2-3)	3.9% (1-7.7%)	4.6% (1.1-9.3%)	53 (13-110)	290 (79-570)
Slovenia	2000	Female	95 plus	50% (35-65%)	50% (35-65%)	2 (1.1-3.3)	4.1% (0.57-9.2%)	4.7% (0.5-11%)	16 (1.7-38)	57 (8-130)
Slovenia	2000	Male	All Ages	76% (71-80%)	24% (20-29%)	4.7 (4.1-5.2)	16% (13-18%)	19% (16-22%)	1900 (1500-2200)	53000 (45000-62000)
Slovenia	2000	Male	15 to 19	66% (51-80%)	34% (20-49%)	2.6 (1.8-3.6)	7.9% (4.9-11%)	14% (8-21%)	7.9 (4.3-12)	690 (420-980)
Slovenia	2000	Male	20 to 24	79% (66-87%)	21% (13-34%)	4.5 (3.3-5.9)	16% (11-21%)	25% (16-33%)	24 (15-32)	1900 (1300-2500)
Slovenia	2000	Male	25 to 29	83% (74-90%)	17% (9.7-26%)	4.6 (3.4-5.8)	15% (11-19%)	25% (18-33%)	21 (14-28)	1700 (1200-2200)
Slovenia	2000	Male	30 to 34	86% (77-93%)	14% (7.4-23%)	4.7 (3.5-6)	15% (11-19%)	25% (18-33%)	27 (19-35)	2000 (1400-2500)
Slovenia	2000	Male	35 to 39	87% (78-92%)	13% (7.8-22%)	4.7 (3.5-6.1)	15% (12-19%)	25% (19-31%)	40 (30-51)	2500 (1900-3100)
Slovenia	2000	Male	40 to 44	86% (78-92%)	14% (8.3-22%)	5.2 (4-6.6)	17% (14-21%)	26% (21-31%)	69 (52-84)	3700 (2900-4500)
Slovenia	2000	Male	45 to 49	84% (76-90%)	16% (9.8-24%)	6.1 (4.7-7.7)	19% (16-23%)	27% (23-32%)	130 (100-150)	5800 (4700-6900)
Slovenia	2000	Male	50 to 54	82% (75-87%)	18% (13-25%)	6.9 (5.4-8.5)	20% (17-23%)	27% (23-30%)	140 (120-160)	5500 (4600-6500)

Location	Year	Sex	Age	Prevalence of current drinkers	Prevalence of abstainers	Population average of standard drinks daily	Percent of total attributable deaths	Percent of total attributable DALYs	Attributable deaths	Attributable DALYs
Slovenia	2000	Male	55 to 59	79% (72-83%)	21% (17-28%)	7.7 (6.1-9.3)	19% (16-22%)	25% (21-28%)	180 (140-210)	6100 (5000-7300)
Slovenia	2000	Male	60 to 64	78% (72-83%)	22% (17-28%)	8.2 (6.4-10)	18% (15-21%)	22% (18-26%)	220 (170-260)	6400 (5100-7700)
Slovenia	2000	Male	65 to 69	81% (75-86%)	19% (14-25%)	8.3 (6.7-10)	17% (14-20%)	20% (17-24%)	270 (220-330)	6600 (5200-7900)
Slovenia	2000	Male	70 to 74	79% (73-84%)	21% (16-27%)	7.8 (6.2-9.4)	15% (12-18%)	18% (14-22%)	260 (200-320)	5100 (3900-6200)
Slovenia	2000	Male	75 to 79	73% (65-80%)	27% (20-35%)	6.6 (5.1-8.3)	13% (10-16%)	15% (12-19%)	200 (150-250)	3000 (2300-3700)
Slovenia	2000	Male	80 to 84	70% (59-78%)	30% (22-41%)	5.6 (4.2-7.2)	12% (8.9-15%)	14% (10-17%)	130 (97-170)	1500 (1100-1900)
Slovenia	2000	Male	85 to 89	70% (60-78%)	30% (22-40%)	5.1 (3.6-6.7)	11% (7.5-15%)	12% (8.4-17%)	85 (59-120)	670 (470-900)
Slovenia	2000	Male	90 to 94	71% (61-80%)	29% (20-39%)	4.8 (3.2-6.5)	9.8% (5.6-14%)	11% (6.2-16%)	50 (28-73)	270 (150-390)
Slovenia	2000	Male	95 plus	72% (59-83%)	28% (17-41%)	4.3 (2.4-6.5)	8.8% (3.2-14%)	9.9% (3.2-16%)	9 (2.9-14)	33 (12-51)
Slovenia	2005	Female	All Ages	68% (64-71%)	32% (29-36%)	2 (1.7-2.2)	4.7% (3.6-5.9%)	5.8% (3.8-8.2%)	540 (350-780)	13000 (9900-17000)
Slovenia	2005	Female	15 to 19	71% (58-83%)	29% (17-42%)	1.8 (1.2-2.6)	2.9% (1.9-4.2%)	9.2% (4.8-14%)	1.2 (0.61-1.9)	160 (100-230)
Slovenia	2005	Female	20 to 24	79% (67-87%)	21% (13-33%)	2.2 (1.5-3.1)	4.6% (3.2-6.1%)	10% (5.4-15%)	2.2 (1.2-3.4)	320 (210-450)
Slovenia	2005	Female	25 to 29	80% (68-87%)	20% (13-32%)	1.8 (1.3-2.5)	3.9% (2.9-5.1%)	8.4% (4.9-12%)	1.7 (0.94-2.5)	310 (210-420)
Slovenia	2005	Female	30 to 34	80% (70-88%)	20% (12-30%)	1.7 (1.2-2.4)	3.8% (2.8-4.9%)	8.5% (5.5-12%)	2.7 (1.7-3.8)	340 (240-450)
Slovenia	2005	Female	35 to 39	79% (69-88%)	21% (12-31%)	1.8 (1.2-2.6)	4.1% (3.1-5.2%)	10% (7.5-13%)	5.1 (3.6-6.8)	450 (330-580)
Slovenia	2005	Female	40 to 44	75% (65-84%)	25% (16-35%)	2.2 (1.6-3)	4.9% (3.7-6.4%)	12% (8.9-15%)	10 (7.6-13)	660 (500-840)
Slovenia	2005	Female	45 to 49	71% (63-80%)	29% (20-37%)	2.8 (2-3.7)	6.3% (4.8-8%)	13% (10-16%)	20 (16-26)	1000 (800-1300)
Slovenia	2005	Female	50 to 54	70% (63-76%)	30% (24-37%)	3 (2.2-3.9)	7.1% (5.4-9%)	13% (11-16%)	34 (26-42)	1500 (1100-1800)
Slovenia	2005	Female	55 to 59	66% (60-73%)	34% (27-40%)	2.7 (2-3.6)	6.7% (5-8.4%)	12% (9.3-15%)	33 (25-42)	1200 (930-1600)
Slovenia	2005	Female	60 to 64	64% (57-71%)	36% (29-43%)	2.6 (1.9-3.5)	5.8% (4.2-7.8%)	10% (7.5-13%)	38 (28-50)	1200 (860-1600)
Slovenia	2005	Female	65 to 69	63% (57-70%)	37% (30-43%)	2.5 (2-3.2)	5.3% (3.7-6.9%)	8.5% (6.2-11%)	47 (35-62)	1200 (890-1700)
Slovenia	2005	Female	70 to 74	61% (53-67%)	39% (33-47%)	2.4 (1.8-3.2)	4.8% (2.8-7.1%)	7% (4.4-10%)	70 (43-100)	1500 (850-2100)

Location	Year	Sex	Age	Prevalence of current drinkers	Prevalence of abstainers	Population average of standard drinks daily	Percent of total attributable deaths	Percent of total attributable DALYs	Attributable deaths	Attributable DALYs
Slovenia	2005	Female	75 to 79	59% (49-68%)	41% (32-51%)	2.3 (1.6-3)	4.2% (2-6.8%)	5.6% (2.7-8.9%)	85 (40-140)	1400 (630-2200)
Slovenia	2005	Female	80 to 84	54% (41-66%)	46% (34-59%)	1.9 (1.3-2.7)	3.6% (1.6-5.9%)	4.5% (1.9-7.6%)	90 (39-150)	1000 (480-1800)
Slovenia	2005	Female	85 to 89	49% (37-61%)	51% (39-63%)	1.8 (1.1-2.6)	3% (0.93-5.5%)	3.6% (1-6.7%)	56 (16-100)	460 (140-830)
Slovenia	2005	Female	90 to 94	50% (37-60%)	50% (40-63%)	1.8 (1.1-2.6)	3% (0.52-6.1%)	3.5% (0.45-7.4%)	31 (4.1-65)	170 (29-350)
Slovenia	2005	Female	95 plus	51% (36-66%)	49% (34-64%)	1.8 (0.96-2.9)	3.1% (-0.022-7.5%)	3.6% (-0.26-9.1%)	18 (-1.3-46)	64 (-0.44-160)
Slovenia	2005	Male	All Ages	78% (74-82%)	22% (18-26%)	4.6 (4-5.1)	14% (12-16%)	17% (15-20%)	1700 (1400-2000)	47000 (40000-56000)
Slovenia	2005	Male	15 to 19	71% (58-83%)	29% (17-42%)	2.6 (1.8-3.5)	7% (4.3-9.7%)	13% (7.6-20%)	5.3 (3-7.9)	500 (310-700)
Slovenia	2005	Male	20 to 24	81% (69-89%)	19% (11-31%)	4.3 (3.2-5.6)	14% (10-19%)	23% (15-31%)	18 (12-25)	1500 (1100-2000)
Slovenia	2005	Male	25 to 29	86% (78-91%)	14% (8.8-22%)	4.3 (3.3-5.5)	14% (9.9-18%)	23% (15-30%)	21 (14-29)	1700 (1200-2200)
Slovenia	2005	Male	30 to 34	88% (81-93%)	12% (6.8-19%)	4.4 (3.4-5.6)	13% (9.7-16%)	23% (17-30%)	19 (14-25)	1500 (1100-1900)
Slovenia	2005	Male	35 to 39	88% (81-93%)	12% (7.2-19%)	4.4 (3.3-5.6)	13% (10-17%)	23% (17-29%)	28 (20-36)	1900 (1400-2300)
Slovenia	2005	Male	40 to 44	87% (81-92%)	13% (7.8-19%)	4.9 (3.8-6.1)	15% (12-19%)	24% (19-29%)	53 (40-67)	2900 (2300-3600)
Slovenia	2005	Male	45 to 49	86% (80-91%)	14% (9.2-20%)	5.7 (4.5-7.1)	17% (14-20%)	26% (21-30%)	95 (75-120)	4500 (3600-5400)
Slovenia	2005	Male	50 to 54	83% (78-87%)	17% (13-22%)	6.4 (5.1-7.8)	18% (15-21%)	25% (21-28%)	150 (120-170)	6000 (4900-7100)
Slovenia	2005	Male	55 to 59	80% (75-83%)	20% (17-25%)	7.2 (5.8-8.6)	17% (15-20%)	23% (20-26%)	150 (120-180)	5300 (4400-6300)
Slovenia	2005	Male	60 to 64	80% (76-84%)	20% (16-24%)	7.7 (6.1-9.4)	17% (14-20%)	21% (18-25%)	180 (150-220)	5500 (4500-6600)
Slovenia	2005	Male	65 to 69	83% (78-86%)	17% (14-22%)	7.8 (6.4-9.3)	16% (13-18%)	19% (16-22%)	210 (170-260)	5300 (4400-6300)
Slovenia	2005	Male	70 to 74	81% (76-84%)	19% (16-24%)	7.3 (5.9-8.9)	14% (12-17%)	17% (14-20%)	250 (200-300)	4900 (4000-5900)
Slovenia	2005	Male	75 to 79	76% (69-81%)	24% (19-31%)	6.3 (4.8-7.8)	12% (9.7-15%)	14% (11-17%)	210 (160-260)	3300 (2600-4100)
Slovenia	2005	Male	80 to 84	73% (65-79%)	27% (21-35%)	5.4 (4.2-6.7)	11% (8.3-14%)	13% (9.5-16%)	160 (120-200)	1800 (1300-2300)
Slovenia	2005	Male	85 to 89	73% (65-79%)	27% (21-35%)	5 (3.7-6.4)	9.9% (6.8-13%)	11% (7.5-15%)	74 (48-100)	590 (400-810)
Slovenia	2005	Male	90 to 94	74% (65-81%)	26% (19-35%)	4.6 (3.2-6.3)	8.9% (4.6-13%)	10% (4.8-15%)	31 (15-46)	170 (85-240)

Location	Year	Sex	Age	Prevalence of current drinkers	Prevalence of abstainers	Population average of standard drinks daily	Percent of total attributable deaths	Percent of total attributable DALYs	Attributable deaths	Attributable DALYs
Slovenia	2005	Male	95 plus	74% (60-85%)	26% (15-40%)	4.1 (2.3-6.2)	7.8% (2.3-13%)	8.7% (2.2-15%)	11 (2.6-18)	40 (12-66)
Slovenia	2010	Female	All Ages	67% (63-71%)	33% (29-37%)	1.8 (1.5-2.1)	3.9% (2.9-5%)	4.7% (2.8-7%)	450 (270-680)	11000 (7900-14000)
Slovenia	2010	Female	15 to 19	71% (57-82%)	29% (18-43%)	1.7 (1.1-2.4)	2.5% (1.7-3.5%)	8% (4.3-12%)	0.7 (0.37-1.1)	110 (71-160)
Slovenia	2010	Female	20 to 24	79% (67-87%)	21% (13-33%)	2.1 (1.4-2.9)	3.9% (2.8-5.2%)	8.6% (4.7-13%)	1 (0.54-1.5)	230 (150-320)
Slovenia	2010	Female	25 to 29	80% (69-87%)	20% (13-31%)	1.7 (1.2-2.4)	3.6% (2.7-4.7%)	7.4% (4.4-10%)	0.92 (0.54-1.3)	250 (170-360)
Slovenia	2010	Female	30 to 34	81% (71-88%)	19% (12-29%)	1.6 (1.1-2.3)	3.4% (2.5-4.4%)	7.8% (5.2-11%)	1.8 (1.2-2.5)	300 (220-420)
Slovenia	2010	Female	35 to 39	80% (70-88%)	20% (12-30%)	1.7 (1.2-2.6)	3.8% (2.8-4.9%)	10% (7.3-13%)	3.7 (2.7-4.9)	370 (270-480)
Slovenia	2010	Female	40 to 44	76% (66-85%)	24% (15-34%)	2.1 (1.5-2.9)	4.4% (3.3-5.6%)	11% (8.6-14%)	7.8 (5.9-10)	540 (410-700)
Slovenia	2010	Female	45 to 49	71% (63-80%)	29% (20-37%)	2.6 (1.8-3.5)	5.4% (4-6.9%)	12% (9.6-15%)	15 (12-19)	830 (630-1100)
Slovenia	2010	Female	50 to 54	69% (62-76%)	31% (24-38%)	2.7 (2-3.6)	5.9% (4.5-7.5%)	12% (9.4-14%)	24 (19-30)	1100 (860-1400)
Slovenia	2010	Female	55 to 59	65% (58-72%)	35% (28-42%)	2.5 (1.8-3.3)	5.7% (4.2-7.2%)	11% (8.5-13%)	33 (25-41)	1300 (960-1600)
Slovenia	2010	Female	60 to 64	63% (55-70%)	37% (30-45%)	2.4 (1.7-3.2)	5% (3.6-6.7%)	9.1% (7-12%)	30 (23-39)	980 (720-1300)
Slovenia	2010	Female	65 to 69	62% (55-70%)	38% (30-45%)	2.3 (1.8-3)	4.6% (3.3-6.1%)	7.8% (5.9-10%)	38 (28-50)	1000 (740-1400)
Slovenia	2010	Female	70 to 74	60% (52-67%)	40% (33-48%)	2.2 (1.6-2.9)	4.1% (2.5-6%)	6.3% (4.1-8.9%)	47 (29-67)	1000 (630-1500)
Slovenia	2010	Female	75 to 79	57% (47-66%)	43% (34-53%)	2 (1.4-2.8)	3.5% (1.6-5.6%)	4.8% (2.4-7.9%)	67 (32-110)	1100 (520-1800)
Slovenia	2010	Female	80 to 84	52% (40-64%)	48% (36-60%)	1.7 (1.2-2.4)	2.8% (1.2-5%)	3.7% (1.6-6.5%)	71 (30-130)	840 (360-1500)
Slovenia	2010	Female	85 to 89	48% (36-59%)	52% (41-64%)	1.6 (0.96-2.3)	2.4% (0.64-4.7%)	2.9% (0.63-5.9%)	67 (15-140)	560 (140-1100)
Slovenia	2010	Female	90 to 94	48% (36-59%)	52% (41-64%)	1.6 (0.94-2.4)	2.3% (0.11-5.2%)	2.7% (-0.053-6.3%)	33 (-0.65-78)	190 (9.3-420)
Slovenia	2010	Female	95 plus	50% (34-66%)	50% (34-66%)	1.6 (0.84-2.7)	2.4% (-0.44-6.3%)	2.8% (-0.75-7.6%)	12 (-3.4-34)	46 (-8.3-120)
Slovenia	2010	Male	All Ages	78% (74-82%)	22% (18-26%)	4.3 (3.7-5)	13% (11-15%)	16% (14-19%)	1500 (1200-1800)	42000 (36000-50000)
Slovenia	2010	Male	15 to 19	69% (55-81%)	31% (19-45%)	2.3 (1.6-3.2)	5.6% (3.7-7.8%)	12% (6.6-18%)	2.7 (1.4-4.1)	290 (190-420)
Slovenia	2010	Male	20 to 24	81% (70-89%)	19% (11-30%)	4 (2.9-5.3)	12% (8.5-16%)	21% (14-29%)	12 (7.4-16)	1100 (740-1400)

Location	Year	Sex	Age	Prevalence of current drinkers	Prevalence of abstainers	Population average of standard drinks daily	Percent of total attributable deaths	Percent of total attributable DALYs	Attributable deaths	Attributable DALYs
Slovenia	2010	Male	25 to 29	86%<br>(78-91%)	14%<br>(8.7-22%)	4<br>(3-5.2)	11%<br>(8.3-14%)	21%<br>(14-28%)	12<br>(7.7-17)	1100<br>(830-1500)
Slovenia	2010	Male	30 to 34	88%<br>(81-93%)	12%<br>(6.9-19%)	4.1<br>(3-5.3)	11%<br>(8.5-14%)	21%<br>(15-28%)	17<br>(12-22)	1400<br>(1000-1800)
Slovenia	2010	Male	35 to 39	88%<br>(81-93%)	12%<br>(7-19%)	4.1<br>(3.1-5.3)	11%<br>(8.7-15%)	22%<br>(16-28%)	20<br>(14-26)	1500<br>(1100-1900)
Slovenia	2010	Male	40 to 44	87%<br>(80-93%)	13%<br>(7.3-20%)	4.5<br>(3.4-5.7)	14%<br>(10-17%)	24%<br>(18-29%)	41<br>(31-52)	2400<br>(1800-2900)
Slovenia	2010	Male	45 to 49	86%<br>(80-91%)	14%<br>(8.9-20%)	5.3<br>(4.1-6.7)	16%<br>(13-19%)	25%<br>(20-30%)	69<br>(54-86)	3400<br>(2700-4200)
Slovenia	2010	Male	50 to 54	83%<br>(77-87%)	17%<br>(13-23%)	5.8<br>(4.6-7.3)	17%<br>(14-20%)	24%<br>(20-28%)	110<br>(92-140)	4800<br>(3900-5800)
Slovenia	2010	Male	55 to 59	79%<br>(75-83%)	21%<br>(17-25%)	6.5<br>(5.2-8.1)	16%<br>(14-19%)	23%<br>(19-26%)	170<br>(140-210)	6200<br>(5100-7300)
Slovenia	2010	Male	60 to 64	79%<br>(75-83%)	21%<br>(17-25%)	7<br>(5.5-8.6)	16%<br>(13-18%)	21%<br>(17-24%)	160<br>(130-190)	4900<br>(4100-5900)
Slovenia	2010	Male	65 to 69	81%<br>(77-85%)	19%<br>(15-23%)	7<br>(5.7-8.5)	14%<br>(12-17%)	19%<br>(16-22%)	180<br>(150-210)	4600<br>(3800-5400)
Slovenia	2010	Male	70 to 74	80%<br>(75-84%)	20%<br>(16-25%)	6.7<br>(5.3-8.3)	13%<br>(11-16%)	16%<br>(13-19%)	200<br>(170-250)	4200<br>(3400-5100)
Slovenia	2010	Male	75 to 79	75%<br>(68-81%)	25%<br>(19-32%)	5.8<br>(4.4-7.3)	11%<br>(9-14%)	14%<br>(11-17%)	200<br>(160-260)	3300<br>(2600-4100)
Slovenia	2010	Male	80 to 84	72%<br>(64-79%)	28%<br>(21-36%)	5<br>(3.8-6.3)	9.9%<br>(7.3-13%)	12%<br>(8.5-15%)	170<br>(120-220)	1900<br>(1400-2500)
Slovenia	2010	Male	85 to 89	73%<br>(66-79%)	27%<br>(21-34%)	4.6<br>(3.4-6.1)	8.9%<br>(5.6-12%)	10%<br>(6.3-14%)	99<br>(60-140)	810<br>(500-1100)
Slovenia	2010	Male	90 to 94	74%<br>(66-81%)	26%<br>(19-34%)	4.3<br>(2.9-5.9)	7.8%<br>(3.7-12%)	8.9%<br>(3.9-14%)	32<br>(14-50)	180<br>(84-270)
Slovenia	2010	Male	95 plus	74%<br>(62-85%)	26%<br>(15-38%)	3.8<br>(2.2-5.6)	6.7%<br>(1.7-12%)	7.5%<br>(1.4-14%)	7.5<br>(1.4-13)	28<br>(7-48)
Slovenia	2016	Female	All Ages	64%<br>(59-68%)	36%<br>(32-41%)	1.7<br>(1.3-2.2)	3.6%<br>(2.5-5%)	4.3%<br>(2.1-7%)	460<br>(220-760)	10000<br>(7000-14000)
Slovenia	2016	Female	15 to 19	67%<br>(53-80%)	33%<br>(20-47%)	1.6<br>(0.96-2.4)	2.4%<br>(1.6-3.5%)	7.6%<br>(3.8-12%)	0.53<br>(0.27-0.84)	90<br>(58-130)
Slovenia	2016	Female	20 to 24	77%<br>(64-86%)	23%<br>(14-36%)	2<br>(1.2-3.1)	3.9%<br>(2.8-5.3%)	8.5%<br>(4.5-13%)	0.78<br>(0.41-1.2)	180<br>(120-260)
Slovenia	2016	Female	25 to 29	79%<br>(68-87%)	21%<br>(13-32%)	1.7<br>(1.1-2.6)	3.6%<br>(2.7-4.7%)	7.7%<br>(4.6-11%)	0.79<br>(0.46-1.2)	220<br>(150-310)
Slovenia	2016	Female	30 to 34	80%<br>(68-88%)	20%<br>(12-32%)	1.6<br>(0.97-2.4)	3.3%<br>(2.4-4.4%)	7.8%<br>(5.1-11%)	1.4<br>(0.88-2.1)	270<br>(180-380)
Slovenia	2016	Female	35 to 39	79%<br>(67-88%)	21%<br>(12-33%)	1.7<br>(1.1-2.7)	3.6%<br>(2.7-4.7%)	10%<br>(7.3-13%)	3.4<br>(2.3-4.8)	360<br>(270-490)
Slovenia	2016	Female	40 to 44	75%<br>(63-85%)	25%<br>(15-37%)	2.1<br>(1.4-3.1)	4.2%<br>(3.1-5.5%)	11%<br>(8.8-14%)	6.2<br>(4.4-8.5)	460<br>(340-620)

Location	Year	Sex	Age	Prevalence of current drinkers	Prevalence of abstainers	Population average of standard drinks daily	Percent of total attributable deaths	Percent of total attributable DALYs	Attributable deaths	Attributable DALYs
Slovenia	2016	Female	45 to 49	70% (58-79%)	30% (21-42%)	2.6 (1.7-3.7)	5.1% (3.7-6.9%)	12% (9.4-16%)	13 (9-17)	730 (540-980)
Slovenia	2016	Female	50 to 54	65% (56-74%)	35% (26-44%)	2.5 (1.7-3.6)	5.4% (4-7.1%)	11% (8.9-14%)	20 (14-26)	940 (700-1200)
Slovenia	2016	Female	55 to 59	61% (53-71%)	39% (29-47%)	2.3 (1.6-3.3)	5.2% (3.7-6.7%)	10% (7.7-13%)	27 (20-36)	1100 (790-1400)
Slovenia	2016	Female	60 to 64	59% (50-70%)	41% (30-50%)	2.2 (1.5-3.2)	4.6% (3.2-6.3%)	8.6% (6.4-12%)	34 (25-47)	1100 (800-1600)
Slovenia	2016	Female	65 to 69	59% (50-69%)	41% (31-50%)	2.2 (1.5-3.1)	4.2% (2.9-5.8%)	7.4% (5.4-9.9%)	33 (23-46)	920 (620-1300)
Slovenia	2016	Female	70 to 74	57% (47-67%)	43% (33-53%)	2.1 (1.4-3.1)	3.8% (2.2-5.9%)	6.2% (3.8-9%)	43 (25-65)	950 (540-1500)
Slovenia	2016	Female	75 to 79	52% (41-64%)	48% (36-59%)	1.8 (1.2-2.8)	3.2% (1.5-5.5%)	4.6% (2.3-8%)	53 (24-93)	890 (410-1600)
Slovenia	2016	Female	80 to 84	48% (35-60%)	52% (40-65%)	1.6 (0.96-2.4)	2.7% (1-5%)	3.6% (1.3-6.6%)	66 (24-120)	790 (300-1500)
Slovenia	2016	Female	85 to 89	46% (33-57%)	54% (43-67%)	1.5 (0.87-2.4)	2.4% (0.6-4.8%)	3% (0.63-6.1%)	75 (15-160)	620 (160-1300)
Slovenia	2016	Female	90 to 94	46% (34-59%)	54% (41-66%)	1.6 (0.87-2.5)	2.3% (0.098-5.3%)	2.8% (-0.081-6.6%)	56 (-1.7-130)	310 (13-720)
Slovenia	2016	Female	95 plus	48% (33-64%)	52% (36-67%)	1.6 (0.79-2.8)	2.4% (-0.48-6.4%)	2.8% (-0.77-7.8%)	25 (-6.8-69)	90 (-18-240)
Slovenia	2016	Male	All Ages	74% (70-79%)	26% (21-30%)	4.2 (3.3-5.4)	12% (10-15%)	15% (12-18%)	1500 (1200-2000)	40000 (32000-49000)
Slovenia	2016	Male	15 to 19	62% (45-77%)	38% (23-55%)	2.1 (1.2-3.1)	5% (3.3-7.1%)	11% (5.7-17%)	1.9 (0.97-3)	220 (140-310)
Slovenia	2016	Male	20 to 24	76% (62-87%)	24% (13-38%)	3.7 (2.4-5.3)	11% (8.1-15%)	21% (13-30%)	6.9 (4-10)	690 (460-930)
Slovenia	2016	Male	25 to 29	82% (71-90%)	18% (10-29%)	3.8 (2.6-5.4)	11% (8-14%)	21% (13-29%)	8.8 (5.4-13)	880 (620-1200)
Slovenia	2016	Male	30 to 34	85% (74-92%)	15% (7.8-26%)	3.9 (2.6-5.5)	11% (7.9-14%)	21% (14-29%)	13 (8.4-18)	1100 (820-1500)
Slovenia	2016	Male	35 to 39	86% (76-92%)	14% (8-24%)	3.9 (2.7-5.6)	11% (8.6-15%)	23% (16-30%)	20 (14-28)	1500 (1100-2000)
Slovenia	2016	Male	40 to 44	85% (76-91%)	15% (8.6-24%)	4.4 (3-5.9)	13% (9.8-16%)	25% (19-31%)	32 (22-43)	1900 (1400-2500)
Slovenia	2016	Male	45 to 49	84% (75-90%)	16% (9.9-25%)	5.1 (3.6-7.1)	15% (12-19%)	26% (21-32%)	57 (42-77)	2900 (2200-3700)
Slovenia	2016	Male	50 to 54	81% (75-86%)	19% (14-25%)	5.8 (4.2-7.9)	16% (13-20%)	26% (21-30%)	97 (74-130)	4100 (3200-5200)
Slovenia	2016	Male	55 to 59	78% (73-83%)	22% (17-27%)	6.5 (4.8-8.7)	16% (14-19%)	24% (20-27%)	140 (110-180)	5200 (4100-6400)
Slovenia	2016	Male	60 to 64	77% (72-83%)	23% (17-28%)	6.9 (5-9)	15% (13-18%)	21% (17-24%)	200 (160-250)	6100 (4900-7600)

Location	Year	Sex	Age	Prevalence of current drinkers	Prevalence of abstainers	Population average of standard drinks daily	Percent of total attributable deaths	Percent of total attributable DALYs	Attributable deaths	Attributable DALYs
Slovenia	2016	Male	65 to 69	80% (74-84%)	20% (16-26%)	6.9 (5.1-9.1)	14% (11-17%)	19% (15-22%)	180 (140-230)	4600 (3600-5800)
Slovenia	2016	Male	70 to 74	77% (71-82%)	23% (18-29%)	6.5 (4.6-8.7)	12% (10-15%)	16% (13-19%)	190 (140-250)	4000 (3000-5000)
Slovenia	2016	Male	75 to 79	71% (62-78%)	29% (22-38%)	5.4 (3.7-7.4)	10% (7.7-13%)	13% (9.5-16%)	180 (130-250)	3000 (2200-3900)
Slovenia	2016	Male	80 to 84	67% (57-76%)	33% (24-43%)	4.6 (3.1-6.3)	9% (6.1-12%)	11% (7.1-15%)	170 (110-250)	2000 (1400-2800)
Slovenia	2016	Male	85 to 89	69% (58-77%)	31% (23-42%)	4.3 (2.8-6.2)	8.1% (4.7-12%)	9.5% (5.4-14%)	130 (70-200)	1100 (590-1600)
Slovenia	2016	Male	90 to 94	70% (59-79%)	30% (21-41%)	4 (2.5-6)	7.3% (3.3-11%)	8.4% (3.4-13%)	59 (24-98)	320 (150-520)
Slovenia	2016	Male	95 plus	71% (57-83%)	29% (17-43%)	3.7 (2-5.7)	6.5% (1.7-11%)	7.4% (1.5-13%)	13 (2.6-24)	48 (12-85)
Belarus	1990	Female	All Ages	69% (63-75%)	31% (25-37%)	2.8 (2.3-3.3)	6.5% (4.2-9%)	5.9% (1.8-11%)	3300 (1000-6000)	110000 (72000-160000)
Belarus	1990	Female	15 to 19	63% (48-77%)	37% (23-52%)	2.2 (1.2-3.7)	5.2% (3.5-7.5%)	9.9% (5.5-16%)	18 (10-30)	2100 (1400-3100)
Belarus	1990	Female	20 to 24	75% (60-86%)	25% (14-40%)	3.4 (1.8-5.5)	8.5% (6.1-11%)	13% (7.6-19%)	27 (16-39)	3700 (2600-5000)
Belarus	1990	Female	25 to 29	80% (67-88%)	20% (12-33%)	3.8 (2.2-5.9)	9.2% (7.1-12%)	15% (9.7-21%)	40 (26-57)	5200 (3700-6800)
Belarus	1990	Female	30 to 34	83% (69-91%)	17% (9.1-31%)	4.2 (2.6-6.4)	9.8% (7.6-12%)	17% (12-23%)	66 (47-89)	6800 (5000-8800)
Belarus	1990	Female	35 to 39	84% (73-92%)	16% (8.5-27%)	4.4 (2.5-6.7)	11% (8.3-13%)	18% (13-24%)	92 (65-120)	7500 (5700-9500)
Belarus	1990	Female	40 to 44	84% (73-92%)	16% (8.5-27%)	4.4 (2.5-6.8)	12% (8.6-15%)	19% (13-25%)	130 (87-170)	8300 (6100-11000)
Belarus	1990	Female	45 to 49	83% (69-91%)	17% (8.6-31%)	4.3 (2.6-6.8)	12% (8.3-16%)	18% (12-25%)	150 (100-210)	8300 (5900-11000)
Belarus	1990	Female	50 to 54	80% (66-89%)	20% (11-34%)	4.1 (2.4-6.5)	11% (7.4-15%)	16% (10-23%)	300 (180-420)	14000 (8900-19000)
Belarus	1990	Female	55 to 59	75% (59-87%)	25% (13-41%)	3.8 (2.2-5.8)	10% (5.8-15%)	14% (7.7-21%)	370 (200-540)	14000 (7900-20000)
Belarus	1990	Female	60 to 64	71% (53-85%)	29% (15-47%)	3.4 (1.9-5.5)	8.4% (3.4-14%)	11% (3.9-19%)	470 (170-790)	15000 (5800-25000)
Belarus	1990	Female	65 to 69	66% (47-82%)	34% (18-53%)	2.9 (1.7-4.4)	6.6% (1.8-12%)	8.2% (1.8-15%)	450 (97-840)	12000 (3100-21000)
Belarus	1990	Female	70 to 74	59% (41-75%)	41% (25-59%)	2.3 (1.2-4.1)	4.5% (-0.45-11%)	5.3% (-0.93-13%)	270 (-49-670)	5600 (-580-13000)
Belarus	1990	Female	75 to 79	49% (32-68%)	51% (32-68%)	1.8 (0.88-3.2)	3% (-1.5-8.8%)	3.4% (-2.1-11%)	310 (-190-970)	5000 (-2400-15000)
Belarus	1990	Female	80 to 84	43% (26-61%)	57% (39-74%)	1.6 (0.81-2.7)	2.5% (-1.1-7.8%)	2.7% (-1.7-9.1%)	270 (-170-910)	3200 (-1400-9800)

Location	Year	Sex	Age	Prevalence of current drinkers	Prevalence of abstainers	Population average of standard drinks daily	Percent of total attributable deaths	Percent of total attributable DALYs	Attributable deaths	Attributable DALYs
Belarus	1990	Female	85 to 89	40% (24-57%)	60% (43-76%)	1.6 (0.79-2.8)	2.3% (-1.2-7.9%)	2.5% (-1.8-9.2%)	190 (-130-700)	1600 (-850-5300)
Belarus	1990	Female	90 to 94	41% (27-58%)	59% (42-73%)	1.7 (0.8-3.1)	2.3% (-1.5-8.4%)	2.5% (-2.2-9.9%)	84 (-73-320)	490 (-320-1700)
Belarus	1990	Female	95 plus	45% (26-65%)	55% (35-74%)	1.8 (0.7-3.7)	2.5% (-2.1-9.8%)	2.7% (-2.9-12%)	62 (-67-270)	230 (-180-900)
Belarus	1990	Male	All Ages	79% (73-85%)	21% (15-27%)	4.8 (4-5.7)	15% (12-18%)	15% (11-19%)	8000 (5700-10000)	310000 (250000-370000)
Belarus	1990	Male	15 to 19	69% (53-83%)	31% (17-47%)	2.8 (1.5-4.4)	9% (5.7-13%)	14% (8-21%)	61 (36-93)	5000 (3100-7400)
Belarus	1990	Male	20 to 24	82% (69-91%)	18% (9.4-31%)	5.1 (3.1-7.5)	18% (12-25%)	23% (15-33%)	180 (120-250)	14000 (9300-19000)
Belarus	1990	Male	25 to 29	87% (79-93%)	13% (6.7-21%)	6.3 (4-9)	21% (16-27%)	28% (20-36%)	300 (210-390)	22000 (16000-27000)
Belarus	1990	Male	30 to 34	90% (82-96%)	9.7% (4.3-18%)	7.1 (4.9-9.9)	23% (18-28%)	30% (22-36%)	420 (310-530)	28000 (21000-34000)
Belarus	1990	Male	35 to 39	91% (85-96%)	8.7% (4-15%)	7.5 (4.9-11)	23% (18-29%)	30% (22-37%)	500 (370-620)	29000 (22000-35000)
Belarus	1990	Male	40 to 44	92% (85-96%)	8.5% (3.9-15%)	8 (5.1-11)	24% (18-29%)	30% (22-36%)	600 (450-740)	30000 (23000-37000)
Belarus	1990	Male	45 to 49	91% (82-96%)	8.9% (4-18%)	8.5 (5.5-12)	24% (18-28%)	28% (22-34%)	650 (500-790)	29000 (22000-35000)
Belarus	1990	Male	50 to 54	90% (81-95%)	10% (5-19%)	8.2 (5.3-12)	21% (16-25%)	25% (19-30%)	1100 (850-1400)	43000 (33000-53000)
Belarus	1990	Male	55 to 59	88% (78-94%)	12% (6-22%)	7.2 (4.8-10)	18% (13-22%)	21% (15-26%)	1100 (810-1400)	37000 (28000-47000)
Belarus	1990	Male	60 to 64	86% (74-93%)	14% (6.6-26%)	6.3 (3.9-9.4)	14% (8.8-19%)	16% (9.8-22%)	1200 (700-1600)	34000 (21000-46000)
Belarus	1990	Male	65 to 69	85% (72-93%)	15% (7.2-28%)	5.3 (3.5-7.7)	12% (6.4-17%)	13% (6.9-19%)	750 (390-1100)	18000 (9700-26000)
Belarus	1990	Male	70 to 74	82% (69-90%)	18% (9.9-31%)	4.3 (2.5-6.6)	8.5% (2.5-14%)	9.4% (2.4-16%)	370 (99-640)	7300 (2100-12000)
Belarus	1990	Male	75 to 79	78% (63-89%)	22% (11-37%)	3.6 (2.1-5.8)	6% (0.049-12%)	6.4% (-0.42-14%)	350 (-24-760)	5500 (45-11000)
Belarus	1990	Male	80 to 84	74% (57-85%)	26% (15-43%)	3.3 (1.9-4.9)	4.7% (-0.73-11%)	4.9% (-1.3-12%)	250 (-69-620)	2900 (-460-6800)
Belarus	1990	Male	85 to 89	70% (54-82%)	30% (18-46%)	3.1 (1.8-4.9)	3.6% (-1.1-9.9%)	3.7% (-1.8-11%)	110 (-57-340)	990 (-290-2700)
Belarus	1990	Male	90 to 94	69% (54-81%)	31% (19-46%)	2.9 (1.6-4.7)	2.7% (-1.9-8.5%)	2.6% (-2.9-9.5%)	30 (-34-110)	190 (-140-600)
Belarus	1990	Male	95 plus	67% (48-83%)	33% (17-52%)	2.7 (1.3-4.8)	2.1% (-2.8-8.3%)	1.8% (-4-9.1%)	8.7 (-19-44)	41 (-54-160)
Belarus	1995	Female	All Ages	70% (63-76%)	30% (24-37%)	2.7 (2.3-3.2)	6.9% (4.3-9.8%)	6% (1.9-11%)	3900 (1200-7200)	130000 (82000-180000)

Location	Year	Sex	Age	Prevalence of current drinkers	Prevalence of abstainers	Population average of standard drinks daily	Percent of total attributable deaths	Percent of total attributable DALYs	Attributable deaths	Attributable DALYs
Belarus	1995	Female	15 to 19	64% (48-79%)	36% (21-52%)	2.2 (1.2-3.5)	5.5% (3.6-7.9%)	10% (5.9-16%)	21 (12-33)	2300 (1500-3300)
Belarus	1995	Female	20 to 24	76% (61-87%)	24% (13-39%)	3.3 (1.8-5.3)	8.9% (6.3-12%)	14% (8-20%)	32 (19-47)	4100 (2800-5600)
Belarus	1995	Female	25 to 29	80% (68-89%)	20% (11-32%)	3.7 (2.2-5.6)	9.9% (7.4-13%)	15% (9.8-22%)	44 (27-62)	5000 (3600-6600)
Belarus	1995	Female	30 to 34	83% (70-91%)	17% (8.8-30%)	4 (2.5-6.1)	10% (7.9-13%)	17% (12-24%)	79 (53-110)	7400 (5400-9600)
Belarus	1995	Female	35 to 39	84% (73-92%)	16% (8.2-27%)	4.2 (2.5-6.4)	11% (8.6-14%)	19% (13-24%)	130 (92-180)	10000 (7500-13000)
Belarus	1995	Female	40 to 44	84% (74-92%)	16% (7.8-26%)	4.3 (2.6-6.5)	12% (9-16%)	19% (14-25%)	190 (130-250)	11000 (8500-15000)
Belarus	1995	Female	45 to 49	83% (68-92%)	17% (8.4-32%)	4.2 (2.5-6.7)	13% (8.8-17%)	19% (13-26%)	240 (160-330)	12000 (8500-16000)
Belarus	1995	Female	50 to 54	80% (67-89%)	20% (11-33%)	4 (2.3-6.4)	12% (7.8-17%)	17% (11-24%)	260 (160-380)	11000 (7400-16000)
Belarus	1995	Female	55 to 59	76% (61-88%)	24% (12-39%)	3.7 (2.1-5.6)	11% (6.1-16%)	15% (7.8-22%)	490 (260-750)	18000 (9900-27000)
Belarus	1995	Female	60 to 64	71% (54-85%)	29% (15-46%)	3.4 (1.9-5.5)	8.8% (3.3-15%)	11% (3.7-19%)	500 (160-870)	15000 (5600-26000)
Belarus	1995	Female	65 to 69	67% (48-82%)	33% (18-52%)	2.9 (1.7-4.4)	6.5% (1.4-12%)	8% (1.4-15%)	580 (98-1100)	15000 (3300-27000)
Belarus	1995	Female	70 to 74	60% (41-76%)	40% (24-59%)	2.3 (1.2-4)	4.4% (-0.85-11%)	5.1% (-1.5-13%)	450 (-130-1200)	9200 (-1800-23000)
Belarus	1995	Female	75 to 79	50% (33-70%)	50% (30-67%)	1.8 (0.87-3.2)	2.9% (-1.8-8.8%)	3.2% (-2.5-10%)	240 (-190-790)	3800 (-2300-11000)
Belarus	1995	Female	80 to 84	44% (26-62%)	56% (38-74%)	1.6 (0.8-2.7)	2.3% (-1.4-7.4%)	2.5% (-2-8.4%)	290 (-230-980)	3400 (-2100-11000)
Belarus	1995	Female	85 to 89	41% (26-58%)	59% (42-74%)	1.6 (0.8-2.7)	2.2% (-1.5-7.8%)	2.4% (-2-8.8%)	220 (-180-810)	1800 (-1200-6200)
Belarus	1995	Female	90 to 94	42% (27-59%)	58% (41-73%)	1.7 (0.8-3.1)	2.3% (-2-8.8%)	2.4% (-2.6-10%)	110 (-120-460)	610 (-520-2400)
Belarus	1995	Female	95 plus	46% (26-66%)	54% (34-74%)	1.9 (0.71-3.6)	2.5% (-2.6-10%)	2.6% (-3.4-12%)	55 (-72-250)	210 (-210-850)
Belarus	1995	Male	All Ages	80% (74-85%)	20% (15-26%)	4.7 (4.1-5.5)	16% (13-19%)	16% (11-20%)	11000 (7900-14000)	400000 (330000-480000)
Belarus	1995	Male	15 to 19	69% (54-82%)	31% (18-46%)	2.7 (1.6-4.3)	9.4% (5.7-14%)	14% (8-21%)	71 (41-110)	5800 (3600-8400)
Belarus	1995	Male	20 to 24	82% (70-91%)	18% (9.4-30%)	5 (3-7.3)	19% (13-25%)	23% (15-32%)	220 (140-300)	16000 (11000-22000)
Belarus	1995	Male	25 to 29	87% (79-93%)	13% (6.9-21%)	6.2 (3.9-8.7)	23% (16-28%)	28% (20-36%)	330 (230-430)	23000 (16000-29000)
Belarus	1995	Male	30 to 34	90% (82-95%)	9.6% (4.5-18%)	7 (4.6-9.6)	24% (19-29%)	30% (22-36%)	540 (400-670)	34000 (26000-41000)

Location	Year	Sex	Age	Prevalence of current drinkers	Prevalence of abstainers	Population average of standard drinks daily	Percent of total attributable deaths	Percent of total attributable DALYs	Attributable deaths	Attributable DALYs
Belarus	1995	Male	35 to 39	91% (85-96%)	8.6% (4.2-15%)	7.3 (4.9-10)	24% (19-29%)	29% (22-36%)	790 (580-980)	44000 (33000-53000)
Belarus	1995	Male	40 to 44	92% (85-96%)	8.4% (4.1-15%)	7.7 (5.2-11)	25% (19-30%)	30% (23-35%)	970 (730-1200)	47000 (37000-57000)
Belarus	1995	Male	45 to 49	91% (82-96%)	9% (4-18%)	8.2 (5.4-12)	24% (19-29%)	28% (22-34%)	1100 (870-1400)	48000 (38000-58000)
Belarus	1995	Male	50 to 54	90% (81-95%)	10% (4.6-19%)	7.9 (5.1-11)	22% (17-27%)	25% (19-31%)	1100 (790-1300)	40000 (30000-49000)
Belarus	1995	Male	55 to 59	88% (79-94%)	12% (5.6-21%)	7 (4.6-9.9)	19% (13-24%)	21% (15-27%)	1600 (1100-2000)	52000 (37000-67000)
Belarus	1995	Male	60 to 64	87% (76-94%)	13% (6.4-24%)	6.1 (3.9-9.2)	15% (8.6-20%)	16% (9.3-23%)	1400 (800-1900)	39000 (23000-54000)
Belarus	1995	Male	65 to 69	85% (73-93%)	15% (7-27%)	5.2 (3.4-7.5)	12% (5.9-17%)	13% (6.1-19%)	1300 (630-2000)	32000 (15000-46000)
Belarus	1995	Male	70 to 74	82% (70-90%)	18% (9.7-30%)	4.2 (2.6-6.5)	8.4% (2-15%)	9.1% (1.7-16%)	670 (130-1200)	13000 (3100-23000)
Belarus	1995	Male	75 to 79	79% (64-89%)	21% (11-36%)	3.6 (2-5.6)	5.9% (-0.32-12%)	6.3% (-0.82-14%)	280 (-37-600)	4300 (-220-8900)
Belarus	1995	Male	80 to 84	75% (59-86%)	25% (14-41%)	3.3 (1.9-4.9)	4.5% (-1-11%)	4.6% (-1.7-12%)	240 (-87-620)	2800 (-630-6800)
Belarus	1995	Male	85 to 89	71% (55-82%)	29% (18-45%)	3 (1.8-4.7)	3.4% (-1.4-10%)	3.4% (-2.2-11%)	120 (-79-390)	1000 (-430-3000)
Belarus	1995	Male	90 to 94	69% (54-82%)	31% (18-46%)	2.9 (1.6-4.6)	2.4% (-2.2-8.6%)	2.2% (-3.2-9.6%)	31 (-45-130)	200 (-190-710)
Belarus	1995	Male	95 plus	68% (50-84%)	32% (16-50%)	2.7 (1.2-4.7)	1.7% (-3.3-8.3%)	1.4% (-4.5-9.2%)	6 (-19-40)	30 (-57-140)
Belarus	2000	Female	All Ages	71% (64-77%)	29% (23-36%)	2.9 (2.4-3.4)	7.2% (4.5-10%)	6.1% (1.7-11%)	4000 (1100-7600)	130000 (83000-190000)
Belarus	2000	Female	15 to 19	64% (48-79%)	36% (21-52%)	2.2 (1.2-3.6)	5.4% (3.6-7.7%)	11% (5.9-17%)	21 (12-33)	2300 (1600-3400)
Belarus	2000	Female	20 to 24	76% (61-86%)	24% (14-39%)	3.3 (1.8-5.2)	9.1% (6.4-12%)	14% (8.2-21%)	34 (20-50)	4200 (2900-5700)
Belarus	2000	Female	25 to 29	80% (68-89%)	20% (11-32%)	3.8 (2.1-5.8)	10% (7.7-13%)	16% (9.9-23%)	50 (31-72)	5500 (3800-7200)
Belarus	2000	Female	30 to 34	83% (70-91%)	17% (8.7-30%)	4.1 (2.5-6.3)	11% (8.4-14%)	18% (12-25%)	75 (51-100)	6800 (5000-8700)
Belarus	2000	Female	35 to 39	84% (74-92%)	16% (8.2-26%)	4.3 (2.6-6.5)	12% (9-15%)	20% (14-26%)	130 (91-170)	9800 (7200-12000)
Belarus	2000	Female	40 to 44	84% (73-92%)	16% (7.9-27%)	4.4 (2.7-6.4)	13% (9.6-16%)	20% (14-27%)	210 (150-290)	13000 (9900-17000)
Belarus	2000	Female	45 to 49	83% (69-92%)	17% (8.2-31%)	4.3 (2.6-6.4)	13% (9.1-17%)	19% (13-26%)	280 (190-380)	14000 (10000-19000)
Belarus	2000	Female	50 to 54	80% (66-89%)	20% (11-34%)	4 (2.3-6.3)	13% (8.3-18%)	18% (11-25%)	340 (210-480)	14000 (9700-20000)

Location	Year	Sex	Age	Prevalence of current drinkers	Prevalence of abstainers	Population average of standard drinks daily	Percent of total attributable deaths	Percent of total attributable DALYs	Attributable deaths	Attributable DALYs
Belarus	2000	Female	55 to 59	76% (60-88%)	24% (12-40%)	3.7 (2.1-5.8)	12% (6.6-17%)	16% (8.1-23%)	350 (190-510)	12000 (7000-18000)
Belarus	2000	Female	60 to 64	72% (55-85%)	28% (15-45%)	3.4 (1.9-5.6)	9.1% (3.4-15%)	12% (3.9-20%)	550 (180-950)	17000 (6300-28000)
Belarus	2000	Female	65 to 69	67% (49-83%)	33% (17-51%)	2.9 (1.7-4.4)	6.6% (1.3-12%)	8.1% (1.1-16%)	510 (76-1000)	13000 (2500-25000)
Belarus	2000	Female	70 to 74	60% (43-76%)	40% (24-57%)	2.3 (1.3-4)	4.3% (-0.92-11%)	5.1% (-1.6-13%)	530 (-160-1400)	11000 (-2300-26000)
Belarus	2000	Female	75 to 79	51% (33-69%)	49% (31-67%)	1.9 (0.9-3.2)	2.8% (-1.9-8.9%)	3.2% (-2.6-11%)	370 (-310-1200)	5900 (-4000-18000)
Belarus	2000	Female	80 to 84	44% (27-62%)	56% (38-73%)	1.6 (0.79-2.7)	2.3% (-1.6-7.4%)	2.5% (-2.2-8.4%)	200 (-180-690)	2400 (-1600-7600)
Belarus	2000	Female	85 to 89	41% (26-58%)	59% (42-74%)	1.6 (0.78-2.7)	2.3% (-1.5-7.9%)	2.4% (-2-9.1%)	220 (-180-820)	1800 (-1200-6200)
Belarus	2000	Female	90 to 94	43% (27-60%)	57% (40-73%)	1.7 (0.81-3)	2.3% (-2-9.1%)	2.5% (-2.7-10%)	110 (-120-470)	630 (-540-2400)
Belarus	2000	Female	95 plus	47% (26-68%)	53% (32-74%)	1.9 (0.7-3.7)	2.6% (-2.9-11%)	2.7% (-3.8-12%)	52 (-74-240)	190 (-220-810)
Belarus	2000	Male	All Ages	81% (75-86%)	19% (14-25%)	5 (4.3-5.7)	17% (14-20%)	16% (12-21%)	12000 (8500-15000)	430000 (350000-520000)
Belarus	2000	Male	15 to 19	70% (54-83%)	30% (17-46%)	2.7 (1.5-4.4)	9.1% (5.6-14%)	14% (8.1-22%)	68 (39-100)	5500 (3400-8100)
Belarus	2000	Male	20 to 24	82% (70-91%)	18% (9.3-30%)	5 (3.1-7.4)	19% (13-26%)	24% (15-33%)	240 (160-340)	18000 (12000-24000)
Belarus	2000	Male	25 to 29	87% (78-93%)	13% (6.8-22%)	6.2 (3.9-9)	23% (17-29%)	28% (20-36%)	370 (250-480)	25000 (18000-32000)
Belarus	2000	Male	30 to 34	90% (82-96%)	9.5% (4.5-18%)	7 (4.6-9.8)	25% (19-30%)	30% (23-37%)	510 (370-630)	32000 (24000-38000)
Belarus	2000	Male	35 to 39	92% (85-96%)	8.4% (4.2-15%)	7.4 (4.9-10)	25% (19-30%)	30% (23-37%)	780 (580-960)	43000 (33000-52000)
Belarus	2000	Male	40 to 44	92% (85-96%)	8.2% (3.9-15%)	7.8 (5.3-11)	26% (20-31%)	30% (24-36%)	1100 (870-1400)	55000 (43000-66000)
Belarus	2000	Male	45 to 49	91% (82-96%)	8.7% (3.9-18%)	8.3 (5.6-12)	25% (20-30%)	29% (23-35%)	1300 (1000-1600)	57000 (45000-69000)
Belarus	2000	Male	50 to 54	90% (82-95%)	9.9% (4.6-18%)	8 (5.2-11)	23% (17-28%)	26% (20-32%)	1400 (1000-1700)	51000 (39000-63000)
Belarus	2000	Male	55 to 59	88% (78-95%)	12% (5.4-22%)	7.1 (4.7-10)	19% (14-25%)	22% (16-28%)	1100 (810-1500)	37000 (27000-48000)
Belarus	2000	Male	60 to 64	87% (75-94%)	13% (6.3-25%)	6.2 (3.7-9.4)	15% (8.7-21%)	17% (9.4-24%)	1600 (890-2300)	45000 (25000-63000)
Belarus	2000	Male	65 to 69	85% (72-93%)	15% (7-28%)	5.3 (3.4-7.7)	12% (6-18%)	13% (6.2-20%)	1300 (590-2000)	30000 (15000-45000)
Belarus	2000	Male	70 to 74	82% (70-90%)	18% (9.7-30%)	4.3 (2.5-6.6)	8.5% (1.8-15%)	9.3% (1.6-17%)	1000 (180-1900)	20000 (4200-35000)

Location	Year	Sex	Age	Prevalence of current drinkers	Prevalence of abstainers	Population average of standard drinks daily	Percent of total attributable deaths	Percent of total attributable DALYs	Attributable deaths	Attributable DALYs
Belarus	2000	Male	75 to 79	79% (65-89%)	21% (11-35%)	3.6 (2-5.8)	5.8% (-0.64-13%)	6.2% (-1.1-14%)	440 (-82-990)	6600 (-690-14000)
Belarus	2000	Male	80 to 84	75% (60-86%)	25% (14-40%)	3.3 (2-4.9)	4.6% (-1-11%)	4.7% (-1.7-12%)	170 (-60-440)	2000 (-460-4800)
Belarus	2000	Male	85 to 89	71% (56-83%)	29% (17-44%)	3.1 (1.8-4.8)	3.5% (-1.7-10%)	3.5% (-2.5-11%)	100 (-72-320)	850 (-420-2500)
Belarus	2000	Male	90 to 94	69% (56-82%)	31% (18-44%)	2.9 (1.6-4.7)	2.4% (-2.5-8.6%)	2.2% (-3.5-9.5%)	28 (-45-120)	180 (-190-650)
Belarus	2000	Male	95 plus	68% (50-83%)	32% (17-50%)	2.7 (1.3-4.6)	1.7% (-3.5-8.5%)	1.3% (-4.7-9.3%)	5.7 (-21-41)	29 (-60-150)
Belarus	2005	Female	All Ages	71% (65-77%)	29% (23-35%)	3.1 (2.6-3.6)	7.7% (4.8-11%)	6.6% (2-12%)	4300 (1300-8200)	140000 (88000-200000)
Belarus	2005	Female	15 to 19	64% (48-78%)	36% (22-52%)	2.3 (1.2-3.7)	5.1% (3.4-7.2%)	11% (6.4-17%)	16 (9.4-26)	2000 (1400-2900)
Belarus	2005	Female	20 to 24	75% (61-86%)	25% (14-39%)	3.5 (1.9-5.5)	9.2% (6.6-12%)	15% (8.7-22%)	35 (20-51)	4400 (3100-5900)
Belarus	2005	Female	25 to 29	80% (68-89%)	20% (11-32%)	3.9 (2.2-6.2)	11% (8-14%)	17% (11-24%)	55 (34-77)	5800 (4100-7700)
Belarus	2005	Female	30 to 34	83% (71-91%)	17% (8.6-29%)	4.3 (2.6-6.4)	12% (9-15%)	19% (13-25%)	86 (59-120)	7400 (5500-9600)
Belarus	2005	Female	35 to 39	84% (74-92%)	16% (7.9-26%)	4.5 (2.6-6.6)	13% (9.7-16%)	21% (15-27%)	130 (91-170)	9200 (6800-12000)
Belarus	2005	Female	40 to 44	84% (74-92%)	16% (8-26%)	4.5 (2.7-6.8)	13% (9.9-17%)	21% (15-28%)	210 (150-280)	13000 (9800-17000)
Belarus	2005	Female	45 to 49	83% (69-91%)	17% (8.5-31%)	4.4 (2.8-6.7)	13% (9.6-17%)	20% (14-27%)	320 (220-430)	17000 (12000-22000)
Belarus	2005	Female	50 to 54	80% (67-89%)	20% (11-33%)	4.2 (2.5-6.4)	13% (9.1-18%)	19% (13-26%)	400 (270-550)	17000 (12000-23000)
Belarus	2005	Female	55 to 59	76% (60-87%)	24% (13-40%)	3.9 (2.2-6)	13% (7.4-18%)	17% (9.6-24%)	480 (280-700)	17000 (10000-25000)
Belarus	2005	Female	60 to 64	71% (54-85%)	29% (15-46%)	3.6 (2-5.8)	9.9% (4.2-16%)	13% (5-21%)	390 (150-650)	12000 (5000-19000)
Belarus	2005	Female	65 to 69	67% (49-82%)	33% (18-51%)	3 (1.8-4.6)	7% (1.7-13%)	8.7% (1.7-17%)	550 (110-1100)	14000 (3400-26000)
Belarus	2005	Female	70 to 74	61% (43-76%)	39% (24-57%)	2.4 (1.3-4.1)	4.6% (-1-11%)	5.4% (-1.8-14%)	460 (-150-1200)	9500 (-2100-23000)
Belarus	2005	Female	75 to 79	51% (34-69%)	49% (31-66%)	1.9 (0.92-3.3)	3% (-2-9.4%)	3.4% (-2.8-11%)	450 (-370-1500)	7200 (-4700-22000)
Belarus	2005	Female	80 to 84	44% (27-62%)	56% (38-73%)	1.7 (0.86-2.8)	2.7% (-1.4-8.5%)	2.9% (-1.9-9.6%)	380 (-250-1300)	4400 (-2200-14000)
Belarus	2005	Female	85 to 89	42% (25-58%)	58% (42-75%)	1.7 (0.85-2.9)	2.7% (-1.4-8.7%)	2.9% (-2-10%)	190 (-130-650)	1500 (-810-4800)
Belarus	2005	Female	90 to 94	43% (28-60%)	57% (40-72%)	1.8 (0.84-3.2)	2.9% (-1.9-10%)	3.1% (-2.5-12%)	130 (-110-500)	740 (-500-2600)

Location	Year	Sex	Age	Prevalence of current drinkers	Prevalence of abstainers	Population average of standard drinks daily	Percent of total attributable deaths	Percent of total attributable DALYs	Attributable deaths	Attributable DALYs
Belarus	2005	Female	95 plus	47% (27-67%)	53% (33-73%)	2 (0.77-3.9)	3.1% (-2.5-12%)	3.4% (-3.2-14%)	55 (-51-220)	200 (-150-740)
Belarus	2005	Male	All Ages	82% (76-87%)	18% (13-24%)	5.4 (4.6-6.2)	18% (14-21%)	17% (12-22%)	12000 (8900-16000)	450000 (370000-550000)
Belarus	2005	Male	15 to 19	70% (54-83%)	30% (17-46%)	2.8 (1.6-4.5)	9.2% (5.6-13%)	15% (8.3-22%)	62 (35-96)	5200 (3100-7600)
Belarus	2005	Male	20 to 24	82% (70-90%)	18% (9.5-30%)	5.2 (3.2-7.6)	19% (13-25%)	25% (16-34%)	230 (150-310)	17000 (11000-22000)
Belarus	2005	Male	25 to 29	87% (79-93%)	13% (6.7-21%)	6.4 (4.1-9.3)	23% (17-29%)	29% (21-37%)	360 (260-460)	25000 (18000-31000)
Belarus	2005	Male	30 to 34	90% (82-95%)	9.5% (4.6-18%)	7.3 (4.8-10)	25% (19-30%)	31% (23-37%)	530 (400-650)	32000 (25000-39000)
Belarus	2005	Male	35 to 39	92% (85-96%)	8.5% (4.2-15%)	7.7 (5-11)	26% (20-31%)	31% (23-37%)	690 (520-840)	38000 (29000-46000)
Belarus	2005	Male	40 to 44	92% (85-96%)	8.2% (3.9-15%)	8.1 (5.5-11)	27% (21-31%)	31% (25-37%)	1100 (870-1400)	55000 (43000-66000)
Belarus	2005	Male	45 to 49	91% (82-96%)	8.8% (3.9-18%)	8.6 (5.8-12)	26% (20-31%)	30% (24-36%)	1600 (1300-2000)	68000 (55000-83000)
Belarus	2005	Male	50 to 54	90% (81-95%)	9.9% (4.5-19%)	8.3 (5.4-12)	24% (18-28%)	27% (21-33%)	1600 (1200-2000)	62000 (48000-76000)
Belarus	2005	Male	55 to 59	88% (78-95%)	12% (5.3-22%)	7.4 (4.8-11)	20% (15-26%)	23% (16-29%)	1500 (1100-2000)	50000 (36000-64000)
Belarus	2005	Male	60 to 64	87% (76-94%)	13% (6.3-24%)	6.5 (3.9-9.9)	16% (9.4-22%)	18% (10-25%)	1200 (660-1600)	33000 (19000-45000)
Belarus	2005	Male	65 to 69	85% (72-93%)	15% (6.9-28%)	5.5 (3.6-8)	13% (6.5-19%)	14% (6.9-22%)	1500 (720-2200)	35000 (18000-51000)
Belarus	2005	Male	70 to 74	82% (70-90%)	18% (9.6-30%)	4.5 (2.7-6.7)	9.1% (2.3-16%)	9.9% (2.2-18%)	970 (210-1700)	19000 (4600-32000)
Belarus	2005	Male	75 to 79	79% (66-89%)	21% (11-34%)	3.8 (2.1-6)	6.3% (-0.47-13%)	6.7% (-0.99-15%)	670 (-100-1500)	10000 (-780-21000)
Belarus	2005	Male	80 to 84	75% (61-86%)	25% (14-39%)	3.5 (2-5.1)	5.1% (-0.92-12%)	5.3% (-1.6-13%)	280 (-85-720)	3200 (-600-7800)
Belarus	2005	Male	85 to 89	72% (57-83%)	28% (17-43%)	3.2 (1.9-4.8)	3.9% (-1.7-11%)	4% (-2.5-12%)	83 (-53-250)	700 (-310-1900)
Belarus	2005	Male	90 to 94	70% (56-82%)	30% (18-44%)	3 (1.7-4.8)	2.7% (-2.6-9.4%)	2.6% (-3.7-10%)	28 (-40-110)	170 (-170-600)
Belarus	2005	Male	95 plus	68% (50-83%)	32% (17-50%)	2.8 (1.3-4.8)	1.9% (-3.5-8.9%)	1.6% (-4.8-9.7%)	5.4 (-17-33)	26 (-48-120)
Belarus	2010	Female	All Ages	71% (65-77%)	29% (23-35%)	3.3 (2.8-3.9)	8.1% (5.3-11%)	7.1% (2.3-13%)	4600 (1500-8800)	140000 (91000-200000)
Belarus	2010	Female	15 to 19	64% (49-78%)	36% (22-51%)	2.4 (1.3-4)	5.2% (3.5-7.2%)	12% (6.7-18%)	12 (6.9-19)	1500 (1000-2100)
Belarus	2010	Female	20 to 24	76% (62-86%)	24% (14-38%)	3.8 (2.1-6)	8.9% (6.6-12%)	15% (9.5-22%)	26 (16-38)	3900 (2700-5200)

Location	Year	Sex	Age	Prevalence of current drinkers	Prevalence of abstainers	Population average of standard drinks daily	Percent of total attributable deaths	Percent of total attributable DALYs	Attributable deaths	Attributable DALYs
Belarus	2010	Female	25 to 29	80% (68-89%)	20% (11-32%)	4.2 (2.4-6.5)	10% (7.9-13%)	17% (11-24%)	48 (31-67)	5600 (4000-7400)
Belarus	2010	Female	30 to 34	83% (71-91%)	17% (8.8-29%)	4.5 (2.8-7)	12% (9.1-15%)	19% (14-25%)	82 (59-110)	7200 (5400-9200)
Belarus	2010	Female	35 to 39	85% (74-92%)	15% (8.2-26%)	4.8 (2.8-7.4)	13% (9.8-16%)	21% (15-27%)	120 (87-160)	8900 (6700-11000)
Belarus	2010	Female	40 to 44	85% (74-92%)	15% (7.9-26%)	4.9 (2.9-7.3)	14% (10-17%)	23% (16-29%)	180 (130-240)	11000 (8200-14000)
Belarus	2010	Female	45 to 49	83% (70-92%)	17% (8.4-30%)	4.7 (2.9-7.3)	14% (10-18%)	22% (16-28%)	280 (200-370)	15000 (11000-19000)
Belarus	2010	Female	50 to 54	80% (67-90%)	20% (10-33%)	4.5 (2.7-6.9)	14% (9.8-18%)	20% (14-27%)	430 (290-570)	19000 (13000-25000)
Belarus	2010	Female	55 to 59	76% (60-88%)	24% (12-40%)	4.2 (2.4-6.4)	13% (8.3-18%)	18% (11-26%)	540 (340-760)	20000 (12000-27000)
Belarus	2010	Female	60 to 64	72% (55-85%)	28% (15-45%)	3.8 (2.1-6.1)	11% (5.1-16%)	14% (6.5-22%)	480 (220-770)	15000 (7100-23000)
Belarus	2010	Female	65 to 69	67% (49-82%)	33% (18-51%)	3.3 (1.9-4.9)	7.7% (2.3-14%)	9.8% (2.4-19%)	360 (90-690)	9300 (2800-17000)
Belarus	2010	Female	70 to 74	60% (43-76%)	40% (24-57%)	2.6 (1.3-4.4)	5.2% (-0.44-12%)	6.3% (-1.1-16%)	510 (-93-1300)	11000 (-870-25000)
Belarus	2010	Female	75 to 79	51% (34-69%)	49% (31-66%)	2.1 (1-3.7)	3.6% (-1.6-11%)	4.1% (-2.4-13%)	440 (-260-1400)	7100 (-3000-21000)
Belarus	2010	Female	80 to 84	44% (27-62%)	56% (38-73%)	1.8 (0.93-3)	3.2% (-1-9.4%)	3.6% (-1.5-11%)	520 (-220-1600)	6100 (-1900-18000)
Belarus	2010	Female	85 to 89	42% (26-58%)	58% (42-74%)	1.8 (0.9-3.1)	3.2% (-1.3-9.9%)	3.5% (-1.8-12%)	370 (-190-1200)	3000 (-1200-9100)
Belarus	2010	Female	90 to 94	43% (28-60%)	57% (40-72%)	1.9 (0.9-3.5)	3.3% (-1.4-11%)	3.6% (-2.1-12%)	120 (-71-430)	680 (-300-2200)
Belarus	2010	Female	95 plus	47% (28-67%)	53% (33-72%)	2.1 (0.81-4.1)	3.6% (-2.3-12%)	3.9% (-3-15%)	57 (-45-220)	210 (-140-740)
Belarus	2010	Male	All Ages	82% (77-87%)	18% (13-23%)	5.8 (4.8-6.9)	18% (15-22%)	17% (12-22%)	12000 (8700-16000)	430000 (350000-520000)
Belarus	2010	Male	15 to 19	70% (54-83%)	30% (17-46%)	3 (1.7-4.7)	9.1% (5.6-13%)	15% (8.6-23%)	44 (25-67)	3700 (2300-5500)
Belarus	2010	Male	20 to 24	82% (70-91%)	18% (9.5-30%)	5.5 (3.4-8.3)	19% (13-24%)	26% (17-35%)	190 (120-260)	15000 (10000-19000)
Belarus	2010	Male	25 to 29	87% (79-94%)	13% (6.5-21%)	6.9 (4.4-10)	23% (17-28%)	29% (22-37%)	320 (230-400)	22000 (17000-28000)
Belarus	2010	Male	30 to 34	90% (82-96%)	9.5% (4.5-18%)	7.8 (5.2-11)	25% (20-30%)	31% (24-37%)	470 (360-560)	29000 (23000-35000)
Belarus	2010	Male	35 to 39	91% (85-96%)	8.5% (4-15%)	8.2 (5.1-12)	26% (20-31%)	32% (24-38%)	630 (480-750)	35000 (27000-42000)
Belarus	2010	Male	40 to 44	92% (85-96%)	8.2% (4.1-15%)	8.6 (5.8-12)	27% (22-32%)	32% (26-38%)	880 (700-1100)	43000 (35000-52000)

Location	Year	Sex	Age	Prevalence of current drinkers	Prevalence of abstainers	Population average of standard drinks daily	Percent of total attributable deaths	Percent of total attributable DALYs	Attributable deaths	Attributable DALYs
Belarus	2010	Male	45 to 49	91%<br>(83-96%)	8.7%<br>(4-17%)	9.1<br>(6.2-13)	26%<br>(21-31%)	31%<br>(25-37%)	1400<br>(1100-1600)	59000<br>(47000-70000)
Belarus	2010	Male	50 to 54	90%<br>(82-95%)	9.9%<br>(4.8-18%)	8.8<br>(5.8-12)	24%<br>(19-29%)	28%<br>(22-34%)	1800<br>(1400-2200)	67000<br>(52000-81000)
Belarus	2010	Male	55 to 59	88%<br>(79-94%)	12%<br>(5.8-21%)	7.8<br>(5.2-11)	21%<br>(16-26%)	24%<br>(18-30%)	1700<br>(1300-2100)	56000<br>(42000-70000)
Belarus	2010	Male	60 to 64	87%<br>(76-94%)	13%<br>(6.4-24%)	6.9<br>(4.3-10)	17%<br>(11-24%)	19%<br>(12-27%)	1500<br>(930-2100)	43000<br>(27000-59000)
Belarus	2010	Male	65 to 69	85%<br>(73-93%)	15%<br>(6.7-27%)	5.9<br>(3.8-8.6)	14%<br>(7.5-20%)	16%<br>(8.1-23%)	970<br>(510-1400)	23000<br>(13000-33000)
Belarus	2010	Male	70 to 74	82%<br>(70-91%)	18%<br>(9.1-30%)	4.8<br>(2.8-7.2)	10%<br>(3.1-17%)	11%<br>(2.9-19%)	1100<br>(280-1900)	21000<br>(6100-35000)
Belarus	2010	Male	75 to 79	79%<br>(65-89%)	21%<br>(11-35%)	4<br>(2.3-6.4)	7%<br>(-0.21-14%)	7.6%<br>(-0.84-15%)	640<br>(-71-1300)	9700<br>(-290-19000)
Belarus	2010	Male	80 to 84	75%<br>(60-86%)	25%<br>(14-40%)	3.7<br>(2.2-5.5)	5.9%<br>(-0.59-13%)	6.3%<br>(-1.4-15%)	470<br>(-100-1100)	5300<br>(-570-12000)
Belarus	2010	Male	85 to 89	72%<br>(56-83%)	28%<br>(17-44%)	3.4<br>(2.1-5.3)	4.7%<br>(-1.1-12%)	4.9%<br>(-2-13%)	160<br>(-65-430)	1300<br>(-320-3200)
Belarus	2010	Male	90 to 94	70%<br>(55-82%)	30%<br>(18-45%)	3.2<br>(1.8-5.2)	3.5%<br>(-2.3-11%)	3.5%<br>(-3.5-12%)	31<br>(-31-100)	190<br>(-130-560)
Belarus	2010	Male	95 plus	68%<br>(50-83%)	32%<br>(17-50%)	3<br>(1.5-5.2)	2.6%<br>(-3.4-10%)	2.3%<br>(-4.8-11%)	6.7<br>(-14-32)	30<br>(-39-110)
Belarus	2016	Female	All Ages	71%<br>(64-77%)	29%<br>(23-36%)	3.4<br>(2.6-4.4)	7.7%<br>(4.8-12%)	6.9%<br>(1.8-14%)	4200<br>(1100-8600)	130000<br>(75000-190000)
Belarus	2016	Female	15 to 19	64%<br>(48-78%)	36%<br>(22-52%)	2.5<br>(1.3-4.3)	4.8%<br>(3.3-6.9%)	12%<br>(6.8-19%)	6.4<br>(3.4-11)	950<br>(630-1400)
Belarus	2016	Female	20 to 24	75%<br>(61-86%)	25%<br>(14-39%)	3.8<br>(1.9-6.5)	8.7%<br>(6.5-11%)	15%<br>(9.2-22%)	16<br>(9-25)	2700<br>(1900-3800)
Belarus	2016	Female	25 to 29	80%<br>(67-88%)	20%<br>(12-33%)	4.3<br>(2.4-7.1)	9.7%<br>(7.5-12%)	17%<br>(11-23%)	34<br>(21-52)	4800<br>(3400-6600)
Belarus	2016	Female	30 to 34	83%<br>(70-91%)	17%<br>(8.9-30%)	4.7<br>(2.7-7.4)	11%<br>(8.2-13%)	19%<br>(13-25%)	64<br>(41-95)	6500<br>(4700-8700)
Belarus	2016	Female	35 to 39	84%<br>(73-92%)	16%<br>(8.4-27%)	4.9<br>(2.7-7.8)	12%<br>(8.8-15%)	21%<br>(15-27%)	93<br>(58-140)	7500<br>(5300-10000)
Belarus	2016	Female	40 to 44	84%<br>(73-92%)	16%<br>(8-27%)	5<br>(2.8-8.1)	13%<br>(9.2-16%)	22%<br>(16-29%)	140<br>(88-200)	9300<br>(6400-13000)
Belarus	2016	Female	45 to 49	83%<br>(69-91%)	17%<br>(8.7-31%)	4.9<br>(2.7-7.9)	13%<br>(9.1-17%)	22%<br>(15-28%)	180<br>(120-270)	10000<br>(7100-14000)
Belarus	2016	Female	50 to 54	80%<br>(67-89%)	20%<br>(11-33%)	4.6<br>(2.6-7.5)	13%<br>(8.7-17%)	20%<br>(14-27%)	320<br>(200-470)	15000<br>(9900-21000)
Belarus	2016	Female	55 to 59	76%<br>(60-87%)	24%<br>(13-40%)	4.3<br>(2.4-6.6)	12%<br>(7.7-17%)	18%<br>(11-25%)	490<br>(280-730)	19000<br>(12000-27000)
Belarus	2016	Female	60 to 64	71%<br>(55-85%)	29%<br>(15-45%)	3.9<br>(2-6.5)	10%<br>(4.9-16%)	14%<br>(6.5-23%)	470<br>(210-800)	15000<br>(7300-24000)

Location	Year	Sex	Age	Prevalence of current drinkers	Prevalence of abstainers	Population average of standard drinks daily	Percent of total attributable deaths	Percent of total attributable DALYs	Attributable deaths	Attributable DALYs
Belarus	2016	Female	65 to 69	67% (48-83%)	33% (17-52%)	3.3 (1.8-5.3)	7.6% (2.4-14%)	10% (2.6-19%)	410 (98-820)	11000 (3200-21000)
Belarus	2016	Female	70 to 74	60% (42-76%)	40% (24-58%)	2.6 (1.3-4.7)	5.2% (-0.31-12%)	6.6% (-0.97-17%)	270 (-40-720)	5800 (-350-14000)
Belarus	2016	Female	75 to 79	50% (33-69%)	50% (31-67%)	2.1 (0.98-3.9)	3.7% (-1.3-11%)	4.4% (-2.1-14%)	470 (-230-1500)	7700 (-2600-23000)
Belarus	2016	Female	80 to 84	43% (26-62%)	57% (38-74%)	1.9 (0.92-3.3)	3.4% (-0.84-10%)	3.9% (-1.4-12%)	440 (-160-1400)	5100 (-1300-15000)
Belarus	2016	Female	85 to 89	41% (25-58%)	59% (42-75%)	1.8 (0.86-3.3)	3.4% (-0.92-10%)	3.9% (-1.4-12%)	490 (-180-1500)	4000 (-1100-12000)
Belarus	2016	Female	90 to 94	42% (27-60%)	58% (40-73%)	2 (0.9-3.8)	3.6% (-1.4-11%)	4% (-2-13%)	250 (-130-830)	1400 (-550-4300)
Belarus	2016	Female	95 plus	46% (27-68%)	54% (32-73%)	2.2 (0.81-4.3)	3.9% (-2.2-13%)	4.3% (-2.9-15%)	53 (-35-190)	190 (-100-630)
Belarus	2016	Male	All Ages	82% (77-87%)	18% (13-23%)	6 (4.6-7.8)	17% (13-21%)	17% (11-22%)	10000 (6300-14000)	350000 (260000-460000)
Belarus	2016	Male	15 to 19	70% (53-83%)	30% (17-47%)	3.1 (1.7-5.1)	8.4% (5.3-13%)	16% (9-24%)	23 (12-37)	2100 (1300-3200)
Belarus	2016	Male	20 to 24	82% (70-91%)	18% (9.5-30%)	5.7 (3.3-9)	18% (12-23%)	26% (17-36%)	110 (65-150)	8600 (5700-12000)
Belarus	2016	Male	25 to 29	87% (79-93%)	13% (6.8-21%)	7.1 (4.3-11)	21% (16-26%)	30% (21-37%)	240 (160-330)	18000 (13000-24000)
Belarus	2016	Male	30 to 34	90% (82-96%)	9.6% (4.5-18%)	8 (5.2-12)	24% (19-28%)	31% (24-38%)	380 (270-510)	25000 (19000-33000)
Belarus	2016	Male	35 to 39	91% (85-96%)	8.5% (3.9-15%)	8.5 (5.3-13)	25% (19-30%)	32% (24-38%)	480 (340-640)	27000 (20000-36000)
Belarus	2016	Male	40 to 44	92% (85-96%)	8.2% (4-15%)	8.9 (5.5-13)	26% (20-31%)	33% (25-39%)	640 (440-860)	32000 (23000-42000)
Belarus	2016	Male	45 to 49	91% (82-96%)	8.7% (4-18%)	9.4 (6-14)	25% (20-30%)	32% (25-38%)	810 (580-1100)	36000 (26000-47000)
Belarus	2016	Male	50 to 54	90% (82-95%)	10% (4.6-18%)	9.1 (5.7-13)	23% (18-28%)	28% (22-35%)	1300 (920-1700)	50000 (36000-66000)
Belarus	2016	Male	55 to 59	88% (78-94%)	12% (5.7-22%)	8.1 (5.1-12)	20% (15-26%)	24% (18-31%)	1600 (1100-2200)	53000 (37000-71000)
Belarus	2016	Male	60 to 64	86% (76-93%)	14% (6.5-24%)	7.1 (4.3-11)	17% (11-23%)	19% (12-27%)	1500 (870-2100)	42000 (26000-59000)
Belarus	2016	Male	65 to 69	85% (71-93%)	15% (7-29%)	6 (3.7-9.1)	14% (7.4-20%)	16% (8.2-23%)	1100 (580-1700)	27000 (15000-40000)
Belarus	2016	Male	70 to 74	82% (69-90%)	18% (9.7-31%)	4.9 (2.9-7.7)	10% (3.1-16%)	11% (2.9-19%)	560 (140-980)	11000 (3300-18000)
Belarus	2016	Male	75 to 79	78% (64-89%)	22% (11-36%)	4.1 (2.2-6.8)	7.1% (0.26-14%)	7.8% (-0.31-16%)	660 (-27-1400)	10000 (340-21000)
Belarus	2016	Male	80 to 84	74% (58-86%)	26% (14-42%)	3.8 (2.2-5.9)	6% (-0.37-13%)	6.5% (-0.96-15%)	370 (-59-850)	4200 (-260-9300)

Location	Year	Sex	Age	Prevalence of current drinkers	Prevalence of abstainers	Population average of standard drinks daily	Percent of total attributable deaths	Percent of total attributable DALYs	Attributable deaths	Attributable DALYs
Belarus	2016	Male	85 to 89	71%<br>(55-83%)	29%<br>(17-45%)	3.5<br>(2-5.6)	4.9%<br>(-1.3-12%)	5.2%<br>(-2-14%)	250<br>(-93-640)	2100<br>(-530-5000)
Belarus	2016	Male	90 to 94	69%<br>(55-82%)	31%<br>(18-45%)	3.3<br>(1.8-5.5)	3.7%<br>(-2.2-11%)	3.8%<br>(-3.3-12%)	53<br>(-46-170)	320<br>(-190-910)
Belarus	2016	Male	95 plus	68%<br>(49-84%)	32%<br>(16-51%)	3.1<br>(1.4-5.6)	2.8%<br>(-3.2-10%)	2.6%<br>(-4.7-11%)	6.2<br>(-11-27)	27<br>(-31-99)
Estonia	1990	Female	All Ages	80%<br>(77-83%)	20%<br>(17-23%)	1<br>(0.79-1.3)	1.5%<br>(0.16-3%)	-1.8%<br>(-4-0.69%)	-180<br>(-390-66)	4200<br>(440-8600)
Estonia	1990	Female	15 to 19	78%<br>(66-87%)	22%<br>(13-34%)	0.89<br>(0.55-1.3)	3.9%<br>(2.6-5.2%)	6.3%<br>(3.3-9.5%)	1.9<br>(0.98-2.8)	250<br>(160-340)
Estonia	1990	Female	20 to 24	87%<br>(80-92%)	13%<br>(7.9-20%)	1.2<br>(0.75-1.8)	6.1%<br>(4.4-8%)	6.6%<br>(3.5-10%)	2.1<br>(1.1-3.2)	410<br>(290-590)
Estonia	1990	Female	25 to 29	89%<br>(82-93%)	11%<br>(7.1-18%)	1.2<br>(0.79-1.8)	6.7%<br>(5.3-8.3%)	7.3%<br>(4.4-11%)	3<br>(1.8-4.4)	560<br>(400-760)
Estonia	1990	Female	30 to 34	89%<br>(83-93%)	11%<br>(6.5-17%)	1.3<br>(0.9-1.9)	7.4%<br>(6-9.1%)	10%<br>(7.2-14%)	5.4<br>(3.8-7.2)	730<br>(550-950)
Estonia	1990	Female	35 to 39	90%<br>(85-94%)	10%<br>(6.5-15%)	1.5<br>(0.98-2.1)	8.9%<br>(7.3-11%)	13%<br>(10-17%)	11<br>(8.2-14)	1000<br>(810-1300)
Estonia	1990	Female	40 to 44	90%<br>(85-94%)	9.9%<br>(6.1-15%)	1.6<br>(1.1-2.3)	8.8%<br>(7.2-11%)	12%<br>(9.1-16%)	15<br>(11-19)	1100<br>(870-1400)
Estonia	1990	Female	45 to 49	90%<br>(86-93%)	9.8%<br>(7-14%)	1.6<br>(1.1-2.3)	7.9%<br>(6.1-9.9%)	11%<br>(7.9-15%)	17<br>(12-23)	1100<br>(800-1300)
Estonia	1990	Female	50 to 54	88%<br>(84-91%)	12%<br>(8.8-16%)	1.5<br>(1-2.2)	5.9%<br>(4.2-7.9%)	7.6%<br>(4.7-11%)	21<br>(13-31)	1100<br>(790-1500)
Estonia	1990	Female	55 to 59	85%<br>(80-88%)	15%<br>(12-20%)	1.5<br>(1-2.1)	3.7%<br>(1.9-5.7%)	4.4%<br>(1.7-7.4%)	17<br>(6.4-29)	760<br>(380-1200)
Estonia	1990	Female	60 to 64	82%<br>(76-86%)	18%<br>(14-24%)	1.4<br>(0.94-2)	0.91%<br>(-1.1-3.3%)	0.64%<br>(-2.1-4%)	4.2<br>(-14-27)	250<br>(-290-900)
Estonia	1990	Female	65 to 69	79%<br>(72-84%)	21%<br>(16-28%)	1.2<br>(0.8-1.6)	-1.3%<br>(-3.4-1.1%)	-2.1%<br>(-5.1-0.99%)	-19<br>(-45-8.9)	-370<br>(-970-310)
Estonia	1990	Female	70 to 74	71%<br>(63-78%)	29%<br>(22-37%)	0.96<br>(0.59-1.4)	-2.5%<br>(-4.9--0.16%)	-3.6%<br>(-6.5--0.42%)	-32<br>(-59--3.7)	-560<br>(-1100--35)
Estonia	1990	Female	75 to 79	59%<br>(48-70%)	41%<br>(30-52%)	0.78<br>(0.46-1.2)	-2.9%<br>(-5.1--0.67%)	-3.8%<br>(-6.6--0.99%)	-62<br>(-110--17)	-850<br>(-1500--200)
Estonia	1990	Female	80 to 84	51%<br>(40-62%)	49%<br>(38-60%)	0.69<br>(0.41-1.1)	-3%<br>(-5.4--0.92%)	-3.8%<br>(-6.6--1.2%)	-73<br>(-130--23)	-750<br>(-1300--220)
Estonia	1990	Female	85 to 89	50%<br>(38-62%)	50%<br>(38-62%)	0.69<br>(0.4-1.1)	-3%<br>(-5.5--0.89%)	-3.7%<br>(-6.6--1.2%)	-55<br>(-100--17)	-400<br>(-720--120)
Estonia	1990	Female	90 to 94	52%<br>(40-64%)	48%<br>(36-60%)	0.76<br>(0.41-1.2)	-3%<br>(-5.4--0.68%)	-3.8%<br>(-6.7--0.99%)	-26<br>(-45--6.8)	-120<br>(-220--28)
Estonia	1990	Female	95 plus	56%<br>(41-70%)	44%<br>(30-59%)	0.84<br>(0.41-1.4)	-3.2%<br>(-6--0.51%)	-4%<br>(-7.5--0.87%)	-7.9<br>(-15--1.7)	-25<br>(-47--3.8)
Estonia	1990	Male	All Ages	87%<br>(83-90%)	13%<br>(9.8-17%)	2.4<br>(1.9-3)	9%<br>(6.4-12%)	6.7%<br>(3.2-10%)	620<br>(300-960)	31000<br>(22000-41000)

Location	Year	Sex	Age	Prevalence of current drinkers	Prevalence of abstainers	Population average of standard drinks daily	Percent of total attributable deaths	Percent of total attributable DALYs	Attributable deaths	Attributable DALYs
Estonia	1990	Male	15 to 19	78% (66-88%)	22% (12-34%)	1.5 (0.88-2.2)	7.1% (4.5-10%)	9.5% (5.5-14%)	8.5 (4.9-13)	720 (450-1000)
Estonia	1990	Male	20 to 24	88% (80-93%)	12% (6.7-20%)	2.6 (1.6-3.8)	13% (9.1-18%)	16% (10-22%)	21 (14-30)	1700 (1200-2300)
Estonia	1990	Male	25 to 29	91% (85-95%)	8.8% (4.8-15%)	2.9 (1.9-4.2)	15% (11-19%)	18% (12-24%)	29 (19-40)	2300 (1600-3000)
Estonia	1990	Male	30 to 34	93% (87-97%)	6.7% (3.4-13%)	3.2 (2.2-4.6)	16% (12-20%)	19% (14-25%)	42 (31-56)	2900 (2200-3700)
Estonia	1990	Male	35 to 39	94% (89-97%)	6% (2.9-11%)	3.5 (2.3-5)	17% (13-22%)	21% (15-27%)	58 (42-77)	3500 (2700-4500)
Estonia	1990	Male	40 to 44	95% (90-98%)	5.3% (2.4-9.9%)	3.7 (2.5-5.3)	17% (12-22%)	20% (14-26%)	72 (50-96)	3800 (2800-4900)
Estonia	1990	Male	45 to 49	95% (91-98%)	4.8% (2.5-9.1%)	4.1 (2.7-5.8)	16% (11-21%)	19% (13-25%)	83 (57-110)	3800 (2700-5000)
Estonia	1990	Male	50 to 54	95% (91-97%)	4.9% (2.8-8.8%)	4.3 (2.8-6)	14% (9.2-19%)	16% (10-22%)	120 (72-160)	4600 (3000-6400)
Estonia	1990	Male	55 to 59	94% (91-97%)	5.6% (3.4-8.9%)	4.1 (2.8-5.6)	11% (6.1-15%)	12% (6.5-17%)	110 (58-160)	3700 (2200-5400)
Estonia	1990	Male	60 to 64	93% (90-96%)	6.5% (4.1-10%)	3.7 (2.4-5.2)	7.4% (2.8-13%)	7.9% (2.7-14%)	93 (31-160)	2800 (1100-4800)
Estonia	1990	Male	65 to 69	92% (88-95%)	7.9% (4.9-12%)	3.1 (2.1-4.2)	3.9% (0.043-8.3%)	3.9% (-0.62-9.2%)	38 (-6-89)	1000 (11-2200)
Estonia	1990	Male	70 to 74	90% (86-93%)	9.9% (6.8-14%)	2.5 (1.6-3.6)	1.3% (-2.4-5.7%)	0.98% (-3.3-6%)	8.2 (-28-51)	230 (-440-1000)
Estonia	1990	Male	75 to 79	87% (80-92%)	13% (7.6-20%)	2.1 (1.3-3.1)	-0.43% (-4-3.9%)	-0.97% (-5.1-4%)	-10 (-53-42)	-77 (-690-680)
Estonia	1990	Male	80 to 84	84% (75-90%)	16% (9.6-25%)	1.8 (1.2-2.7)	-1.7% (-5-2.1%)	-2.4% (-6.4-1.9%)	-21 (-58-17)	-180 (-560-230)
Estonia	1990	Male	85 to 89	82% (73-88%)	18% (12-27%)	1.7 (1.1-2.5)	-2.3% (-5.6-1.2%)	-3.1% (-7-0.89%)	-16 (-37-4.8)	-110 (-260-55)
Estonia	1990	Male	90 to 94	80% (71-88%)	20% (12-29%)	1.6 (0.93-2.5)	-2.7% (-5.9-0.55%)	-3.6% (-7.4-0.19%)	-5.6 (-11-0.29)	-25 (-54-5.1)
Estonia	1990	Male	95 plus	79% (67-89%)	21% (11-33%)	1.5 (0.73-2.6)	-2.7% (-5.8-0.6%)	-3.7% (-7.5-0.16%)	-1.3 (-2.6-0.055)	-3.7 (-8-0.84)
Estonia	1995	Female	All Ages	80% (77-83%)	20% (17-23%)	1.2 (0.99-1.4)	2.3% (0.87-3.9%)	-0.93% (-3.2-1.7%)	-94 (-330-170)	6500 (2300-11000)
Estonia	1995	Female	15 to 19	78% (66-88%)	22% (12-34%)	1 (0.65-1.4)	4.3% (3-5.7%)	7.4% (4.2-11%)	2.1 (1.2-3.1)	260 (170-350)
Estonia	1995	Female	20 to 24	87% (79-92%)	13% (7.8-21%)	1.4 (0.89-2)	6.6% (4.9-8.4%)	7.6% (4.3-11%)	2.9 (1.7-4.4)	460 (320-620)
Estonia	1995	Female	25 to 29	89% (82-93%)	11% (7.1-18%)	1.4 (0.94-2)	7.4% (5.8-9.2%)	8.7% (5.6-12%)	3.5 (2.2-4.9)	530 (390-700)
Estonia	1995	Female	30 to 34	90% (84-93%)	10% (6.5-16%)	1.5 (1.1-2.1)	8.5% (6.9-10%)	12% (9.4-16%)	6.9 (5.3-9)	760 (600-960)

Location	Year	Sex	Age	Prevalence of current drinkers	Prevalence of abstainers	Population average of standard drinks daily	Percent of total attributable deaths	Percent of total attributable DALYs	Attributable deaths	Attributable DALYs
Estonia	1995	Female	35 to 39	90% (85-94%)	10% (6.4-15%)	1.7 (1.2-2.3)	11% (8.6-13%)	15% (12-19%)	17 (13-21)	1300 (1100-1600)
Estonia	1995	Female	40 to 44	90% (85-94%)	9.8% (6.2-15%)	1.8 (1.3-2.5)	10% (8.3-12%)	14% (11-18%)	23 (17-28)	1500 (1200-1800)
Estonia	1995	Female	45 to 49	90% (86-93%)	9.7% (6.9-14%)	1.8 (1.3-2.5)	9.2% (7.2-11%)	13% (9.4-16%)	28 (21-36)	1500 (1200-1900)
Estonia	1995	Female	50 to 54	89% (84-91%)	11% (8.6-16%)	1.8 (1.2-2.3)	7.2% (5.2-9.5%)	9.4% (6.4-13%)	26 (18-35)	1200 (880-1600)
Estonia	1995	Female	55 to 59	85% (80-89%)	15% (11-20%)	1.7 (1.2-2.3)	4.8% (2.7-7%)	5.9% (2.8-9.1%)	28 (13-44)	1100 (630-1700)
Estonia	1995	Female	60 to 64	82% (76-86%)	18% (14-24%)	1.6 (1.1-2.2)	1.8% (-0.42-4.4%)	1.9% (-1.2-5.5%)	12 (-7.5-35)	460 (-110-1100)
Estonia	1995	Female	65 to 69	79% (72-84%)	21% (16-28%)	1.4 (0.96-1.8)	-0.66% (-3.1-1.9%)	-1.3% (-4.3-2.1%)	-13 (-44-21)	-210 (-940-610)
Estonia	1995	Female	70 to 74	72% (64-78%)	28% (22-36%)	1.1 (0.71-1.6)	-2.1% (-4.4-0.5%)	-2.9% (-5.9-0.36%)	-38 (-77-4.9)	-650 (-1400-160)
Estonia	1995	Female	75 to 79	60% (49-70%)	40% (30-51%)	0.9 (0.55-1.4)	-2.5% (-4.8--0.22%)	-3.3% (-6.1--0.44%)	-39 (-73--5.2)	-530 (-1000--49)
Estonia	1995	Female	80 to 84	52% (41-63%)	48% (37-59%)	0.8 (0.5-1.2)	-2.7% (-5--0.37%)	-3.4% (-6.2--0.63%)	-64 (-120--12)	-650 (-1200--87)
Estonia	1995	Female	85 to 89	51% (39-63%)	49% (37-61%)	0.8 (0.48-1.2)	-2.7% (-5.1--0.41%)	-3.4% (-6.3--0.66%)	-56 (-100--11)	-390 (-730--61)
Estonia	1995	Female	90 to 94	53% (41-65%)	47% (35-59%)	0.89 (0.49-1.4)	-2.8% (-5.3--0.31%)	-3.5% (-6.5--0.53%)	-27 (-50--4.1)	-130 (-250--14)
Estonia	1995	Female	95 plus	57% (41-70%)	43% (30-59%)	1 (0.5-1.7)	-2.9% (-6-0.14%)	-3.7% (-7.3--0.071%)	-7.3 (-14--0.14)	-23 (-47-1.1)
Estonia	1995	Male	All Ages	87% (84-91%)	13% (9.4-16%)	2.8 (2.3-3.3)	12% (9-15%)	9.6% (6-13%)	1100 (660-1500)	46000 (35000-57000)
Estonia	1995	Male	15 to 19	79% (67-88%)	21% (12-33%)	1.7 (1.1-2.5)	8.1% (5.4-12%)	11% (6.6-16%)	9.7 (5.8-14)	800 (510-1100)
Estonia	1995	Male	20 to 24	88% (80-93%)	12% (6.6-20%)	3 (2-4.2)	15% (11-20%)	18% (12-24%)	29 (19-39)	2200 (1500-2900)
Estonia	1995	Male	25 to 29	91% (85-95%)	8.8% (4.7-15%)	3.3 (2.2-4.6)	17% (13-22%)	20% (14-27%)	36 (25-48)	2600 (1900-3300)
Estonia	1995	Male	30 to 34	93% (87-97%)	6.7% (3.2-13%)	3.6 (2.5-5)	19% (14-23%)	22% (16-28%)	56 (42-73)	3600 (2700-4600)
Estonia	1995	Male	35 to 39	94% (90-97%)	5.8% (2.9-10%)	4 (2.7-5.6)	21% (16-26%)	24% (18-31%)	96 (73-120)	5400 (4200-6700)
Estonia	1995	Male	40 to 44	95% (90-98%)	5.2% (2.5-9.8%)	4.3 (3-5.8)	20% (16-26%)	23% (17-29%)	130 (93-160)	6300 (4700-7800)
Estonia	1995	Male	45 to 49	95% (91-98%)	4.8% (2.4-9.2%)	4.7 (3.3-6.5)	20% (15-25%)	22% (16-28%)	150 (110-190)	6500 (4900-8300)
Estonia	1995	Male	50 to 54	95% (91-97%)	4.9% (2.8-8.6%)	4.9 (3.5-6.6)	18% (12-23%)	20% (13-26%)	160 (110-210)	6000 (4200-7800)

Location	Year	Sex	Age	Prevalence of current drinkers	Prevalence of abstainers	Population average of standard drinks daily	Percent of total attributable deaths	Percent of total attributable DALYs	Attributable deaths	Attributable DALYs
Estonia	1995	Male	55 to 59	95% (91-97%)	5.5% (3.4-8.6%)	4.7 (3.4-6.1)	13% (8.5-19%)	15% (9.1-21%)	180 (110-250)	5900 (3700-8200)
Estonia	1995	Male	60 to 64	94% (90-96%)	6.4% (4-10%)	4.2 (2.9-5.7)	9.5% (4.4-15%)	10% (4.4-17%)	140 (60-230)	4000 (1800-6500)
Estonia	1995	Male	65 to 69	92% (88-95%)	7.7% (4.9-12%)	3.5 (2.6-4.6)	5.6% (1.3-10%)	5.8% (0.92-11%)	87 (14-170)	2200 (500-4000)
Estonia	1995	Male	70 to 74	90% (86-93%)	9.8% (6.9-14%)	2.9 (2-4)	2.6% (-1.2-7.6%)	2.5% (-1.8-8.1%)	29 (-21-92)	630 (-280-1800)
Estonia	1995	Male	75 to 79	88% (81-92%)	12% (7.6-19%)	2.4 (1.5-3.5)	0.73% (-3-5.6%)	0.39% (-3.9-5.9%)	3.3 (-33-50)	100 (-420-780)
Estonia	1995	Male	80 to 84	85% (76-90%)	15% (9.5-24%)	2.1 (1.4-3.1)	-0.73% (-4.2-3.3%)	-1.3% (-5.5-3.4%)	-12 (-49-31)	-80 (-470-360)
Estonia	1995	Male	85 to 89	82% (73-89%)	18% (11-27%)	1.9 (1.3-2.8)	-1.7% (-5.2-2.1%)	-2.4% (-6.5-2%)	-14 (-37-11)	-82 (-250-100)
Estonia	1995	Male	90 to 94	81% (71-88%)	19% (12-29%)	1.8 (1.1-2.7)	-2.2% (-5.7-1.4%)	-3% (-7.1-1.1%)	-6 (-14-2.2)	-25 (-66-16)
Estonia	1995	Male	95 plus	80% (67-89%)	20% (11-33%)	1.7 (0.86-2.8)	-2.2% (-5.6-1.5%)	-3.2% (-7.3-1.3%)	-1 (-2.3-0.4)	-2.8 (-7-2)
Estonia	2000	Female	All Ages	79% (76-82%)	21% (18-24%)	1.5 (1.3-1.8)	3.6% (2.3-5.1%)	0.87% (-1.5-3.3%)	82 (-140-310)	9400 (5900-14000)
Estonia	2000	Female	15 to 19	79% (68-87%)	21% (13-32%)	1.3 (0.89-1.9)	4.3% (3.1-5.7%)	8.4% (5.2-12%)	1.8 (1.1-2.6)	230 (160-310)
Estonia	2000	Female	20 to 24	87% (81-92%)	13% (7.9-19%)	1.8 (1.2-2.6)	7% (5.2-8.9%)	8.9% (5.4-13%)	2.3 (1.4-3.4)	420 (290-570)
Estonia	2000	Female	25 to 29	89% (82-93%)	11% (7.2-18%)	1.8 (1.2-2.5)	7.8% (6.1-9.6%)	10% (6.6-13%)	3 (2-4.1)	520 (390-680)
Estonia	2000	Female	30 to 34	89% (83-93%)	11% (6.8-17%)	1.9 (1.4-2.6)	9.1% (7.4-11%)	14% (11-17%)	6.6 (5-8.3)	720 (560-900)
Estonia	2000	Female	35 to 39	90% (85-93%)	10% (6.9-15%)	2.2 (1.6-2.9)	11% (9-13%)	17% (14-20%)	14 (11-17)	1100 (890-1300)
Estonia	2000	Female	40 to 44	90% (86-94%)	9.7% (6.2-14%)	2.4 (1.8-3.2)	11% (9.1-13%)	17% (14-20%)	21 (17-26)	1400 (1100-1700)
Estonia	2000	Female	45 to 49	91% (87-93%)	9.5% (7-13%)	2.4 (1.7-3.2)	11% (8.4-13%)	15% (12-18%)	32 (25-39)	1700 (1400-2100)
Estonia	2000	Female	50 to 54	88% (84-91%)	12% (9.2-16%)	2.2 (1.6-3)	9% (7-11%)	12% (9.5-16%)	35 (26-44)	1600 (1200-2000)
Estonia	2000	Female	55 to 59	83% (80-87%)	17% (13-20%)	2.1 (1.5-2.8)	7% (4.9-9.2%)	9.3% (6.3-12%)	34 (22-45)	1300 (900-1700)
Estonia	2000	Female	60 to 64	79% (72-84%)	21% (16-28%)	1.9 (1.3-2.7)	3.9% (1.7-6.6%)	5.1% (1.9-8.6%)	28 (11-48)	930 (400-1500)
Estonia	2000	Female	65 to 69	74% (66-81%)	26% (19-34%)	1.5 (1.1-2.1)	1.4% (-0.77-3.7%)	1.4% (-1.5-4.6%)	11 (-11-35)	350 (-200-970)
Estonia	2000	Female	70 to 74	64% (54-73%)	36% (27-46%)	1.2 (0.73-1.7)	-0.2% (-2.4-2.1%)	-0.56% (-3.3-2.4%)	-7 (-43-30)	-61 (-730-660)

Location	Year	Sex	Age	Prevalence of current drinkers	Prevalence of abstainers	Population average of standard drinks daily	Percent of total attributable deaths	Percent of total attributable DALYs	Attributable deaths	Attributable DALYs
Estonia	2000	Female	75 to 79	53% (43-64%)	47% (36-57%)	0.96 (0.59-1.5)	-0.88% (-2.9-1.3%)	-1.3% (-4-1.4%)	-21 (-63-22)	-250 (-860-390)
Estonia	2000	Female	80 to 84	49% (37-59%)	51% (41-63%)	0.94 (0.6-1.4)	-1.3% (-3.4-0.83%)	-1.8% (-4.5-0.84%)	-23 (-57-11)	-220 (-570-150)
Estonia	2000	Female	85 to 89	49% (37-61%)	51% (39-63%)	1 (0.6-1.5)	-1.5% (-4-1%)	-2.1% (-5.1-0.93%)	-32 (-79-14)	-210 (-550-140)
Estonia	2000	Female	90 to 94	51% (40-63%)	49% (37-60%)	1.1 (0.62-1.7)	-1.6% (-4.2-1.2%)	-2.1% (-5.4-1.2%)	-18 (-46-11)	-81 (-220-63)
Estonia	2000	Female	95 plus	55% (40-69%)	45% (31-60%)	1.2 (0.63-2.1)	-1.6% (-4.8-1.9%)	-2.2% (-6-1.9%)	-5.4 (-15-4.7)	-15 (-46-18)
Estonia	2000	Male	All Ages	89% (86-92%)	11% (8.3-14%)	3.9 (3.4-4.6)	15% (12-18%)	13% (9.5-17%)	1200 (880-1600)	48000 (39000-57000)
Estonia	2000	Male	15 to 19	80% (70-89%)	20% (11-30%)	2.3 (1.5-3.3)	8.6% (5.8-12%)	13% (7.8-19%)	7.9 (4.8-11)	680 (440-920)
Estonia	2000	Male	20 to 24	89% (82-94%)	11% (5.9-18%)	4 (2.7-5.6)	17% (13-22%)	21% (15-28%)	25 (18-34)	2000 (1500-2600)
Estonia	2000	Male	25 to 29	92% (88-96%)	7.7% (4.2-12%)	4.4 (3-5.9)	19% (14-23%)	24% (17-31%)	31 (23-40)	2300 (1800-2900)
Estonia	2000	Male	30 to 34	94% (90-97%)	5.8% (3-10%)	4.9 (3.4-6.7)	21% (17-26%)	27% (21-34%)	43 (33-54)	2900 (2300-3500)
Estonia	2000	Male	35 to 39	95% (91-97%)	5% (2.7-8.6%)	5.3 (3.7-7.4)	24% (19-29%)	29% (23-36%)	73 (56-89)	4200 (3400-5100)
Estonia	2000	Male	40 to 44	95% (91-98%)	4.7% (2.3-8.5%)	5.7 (4.1-7.7)	25% (20-29%)	29% (23-35%)	120 (96-150)	6100 (4900-7200)
Estonia	2000	Male	45 to 49	96% (92-98%)	4.4% (2.3-8.1%)	6.2 (4.5-8.2)	24% (19-28%)	28% (22-33%)	160 (120-190)	6900 (5500-8200)
Estonia	2000	Male	50 to 54	95% (93-97%)	4.5% (2.6-7.4%)	6.5 (4.6-8.7)	22% (17-26%)	25% (19-30%)	170 (130-200)	6600 (5100-7800)
Estonia	2000	Male	55 to 59	95% (93-97%)	4.9% (3.1-7.4%)	6.3 (4.6-8.2)	18% (14-23%)	21% (15-26%)	160 (110-200)	5400 (3900-6600)
Estonia	2000	Male	60 to 64	94% (92-96%)	5.7% (3.9-8.4%)	5.7 (4-7.7)	14% (8.6-20%)	16% (9.3-22%)	180 (110-250)	5300 (3100-7100)
Estonia	2000	Male	65 to 69	93% (89-95%)	7.3% (4.9-11%)	4.7 (3.5-6.1)	9.9% (4.9-15%)	11% (4.9-17%)	130 (60-210)	3200 (1600-4900)
Estonia	2000	Male	70 to 74	91% (87-93%)	9.5% (6.8-13%)	3.8 (2.6-5.3)	6.2% (1.2-12%)	6.6% (0.8-13%)	89 (11-180)	1800 (360-3400)
Estonia	2000	Male	75 to 79	88% (81-92%)	12% (7.7-19%)	3.1 (2-4.6)	3.6% (-0.88-9.5%)	3.7% (-1.6-11%)	34 (-15-97)	570 (-130-1500)
Estonia	2000	Male	80 to 84	84% (75-91%)	16% (9.5-25%)	2.7 (1.8-3.9)	1.9% (-2.1-6.9%)	1.6% (-3.1-7.4%)	9.7 (-18-45)	140 (-160-520)
Estonia	2000	Male	85 to 89	81% (72-88%)	19% (12-28%)	2.5 (1.6-3.5)	0.56% (-3.2-5.4%)	0.087% (-4.4-5.7%)	0.46 (-23-31)	26 (-150-250)
Estonia	2000	Male	90 to 94	80% (70-88%)	20% (12-30%)	2.3 (1.4-3.5)	-0.29% (-4-4.4%)	-0.94% (-5.5-4.4%)	-2 (-12-9.6)	-3.7 (-52-57)
[truncated: 477,595 more chars]
