# Supplementary material for: Alcohol use and burden for 195 countries and territories, 1990–2016: a systematic analysis for the Global Burden of Disease Study 2016
Source: Lancet. 2018 Sep 22;392(10152):1015–35. doi: 10.1016/S0140-6736(18)31310-2 (PMC6148333; doi:10.1016/S0140-6736(18)31310-2)
Supplement: Supplementary appendix 1 [file mmc1.pdf]

THE LANCET

Supplementary appendix 1

This appendix formed part of the original submission and has been peer reviewed. We post it as supplied by the authors.

Supplement to: GBD 2016 Alcohol Collaborators. Alcohol use and burden for 195 countries and territories, 1990–2016: a systematic analysis for the Global Burden of Disease Study 2016. *Lancet* 2018; published online Aug 23. http://dx.doi.org/10.1016/S0140-6736(18)31310-2.

Methods Appendix to Alcohol use and attributable disease burden in 195 countries and territories, 1990-2016: a systematic analysis of the Global Burden of Disease Study 2016

Contents

I. GATHER Statement 4

II. Location units and time periods of the analysis 7

Methods Appendix Table 2: Location hierarchy and SDI quintile 8

III. Age-standardisation 13

IV. Data sources 14

a. Inclusion criteria 14

V. Consumption estimation 15

a. Definitions 15

b. Data extraction and preparation 15

c. Modeling Strategy 16

Methods Appendix Figure 1: Alcohol Use Flowchart 17

1. Population consumption in liters per capita 18

2. Tourism adjustment 18

Methods Appendix Table 3: Percent change in alcohol consumption due to tourism, by location and 5-year interval 19

3. Unrecorded adjustment 45

Methods Appendix Table 4: Maximum unrecorded estimates by location (incorporated in the final estimates of alcohol consumption were 1000 draws from a uniform distribution between zero and the values reported) 46

4. Individual consumption in grams per day 50

VI. Relative risk estimation 51

a. Motivation for meta-analysis 51

b. Included outcomes 51

c. Search Strategy and Inclusion criteria 51

d. Data preparation 52
e. Modeling Strategy 52
1. Atrial fibrillation and flutter 53
2. Breast cancer 56
3. Cirrhosis 64
4. Colon and rectum cancer 67
5. Diabetes mellitus 72
6. Epilepsy 77
7. Esophageal cancer 80
8. Hemorrhagic stroke 86
9. Hypertensive heart disease 90
10. Interpersonal violence 93
11. Ischaemic heart disease 96
12. Ischaemic stroke 104
13. Larynx cancer 108
14. Lip and oral cavity cancer 112
15. Liver cancer 116
16. Lower respiratory infections 120
17. Pharynx and nasopharynx cancer 123
18. Pancreatitis 126
19. Self-harm 129
20. Transport injuries 132
21. Tuberculosis 135
22. Unintentional injuries 138
VIII. Attributable burden estimation 141
a. TMREL 141
b. Population Attributable Fraction calculations 141
1. Motor vehicle adjustment 141
Methods Appendix Figure 2: Average number of deaths in crash given driver's age, sex, & alcohol's involvement 142
Methods Appendix Figure 3: Percentage of total victims by age & sex, given the male drunk driver's age 143
Methods Appendix Figure 4: Percentage of total victims by age & sex, given the female drunk driver's age 144

c. Attributable burden calculation 145
IX. References 146

I. GATHER Statement

This study complies with the Guidelines for Accurate and Transparent Health Estimates Reporting (GATHER) recommendations. We have documented the steps involved in our analytical procedures and detailed the data sources used in compliance with GATHER. For additional GATHER reporting, please refer to Methods Appendix Table 1 below.

Methods Appendix Table 1: GATHER Checklist

#	GATHER checklist item	Description of compliance	Reference
**Objectives and funding**			
1	Define the indicators, populations, and time periods for which estimates were made.	Narrative provided in paper and methods appendix describing indicators, definitions, and populations	Main text (Methods—Overview, Geographic units and time periods) and methods appendix
2	List the funding sources for the work.	Funding sources listed in paper	Summary (Funding)
**Data Inputs**			
For all data inputs from multiple sources that are synthesized as part of the study:			
3	Describe how the data were identified and how the data were accessed.	Narrative description of data seeking methods provided	Main text (Methods) and methods appendix
4	Specify the inclusion and exclusion criteria. Identify all ad-hoc exclusions.	Narrative about inclusion and exclusion criteria by data type provided; Adhoc exclusions in cause specific write ups	Main text (Methods) and methods appendix
5	Provide information on all included data sources and their main characteristics. For each data source used, report reference information or contact name/institution, population represented, data collection method, year(s) of data collection, sex and age range, diagnostic criteria or measurement method, and sample size, as relevant.	An interactive, online data source tool that provides metadata for data sources by component, geography, cause, risk, or impairment has been developed	Online data citation tools
6	Identify and describe any categories of input data that have potentially important biases (e.g., based on characteristics listed in item 5).	Summary of known biases by cause included in methods appendix	Methods appendix
For data inputs that contribute to the analysis but were not synthesized as part of the study:			
7	Describe and give sources for any other data inputs.	Included in online data source tool	http://ghdx.healthdata.org/gbd-2016/datainput-sourcesrestricted
For all data inputs:			
8	Provide all data inputs in a file format from which data can be efficiently extracted (e.g., a spreadsheet as opposed to a PDF), including all relevant meta-data listed in item 5. For any data inputs that cannot be	Downloads of input data available through online	Online data visualization tools, data query tools, and the Global Health Data

	shared due to ethical or legal reasons, such as third-party ownership, provide a contact name or the name of the institution that retains the right to the data.	tools, including data visualization tools and data query tools; input data not available in tools will be made available upon request	Exchange
**Data analysis**			
9	Provide a conceptual overview of the data analysis method. A diagram may be helpful.	Flow diagrams of the overall methodological processes, as well as cause-specific modelling processes, have been provided	Main text (Methods) and methods appendix
10	Provide a detailed description of all steps of the analysis, including mathematical formulae. This description should cover, as relevant, data cleaning, data pre-processing, data adjustments and weighting of data sources, and mathematical or statistical model(s).	Flow diagrams and Corresponding methodological writeups for each cause, as well as the demographics and causes of death databases and modelling processes, have been provided	Main text (Methods) and methods appendix
11	Describe how candidate models were evaluated and how the final model(s) were selected.	Appendix	Methods appendix
12	Provide the results of an evaluation of model performance, if done, as well as the results of any relevant sensitivity analysis.	Appendix	Methods appendix
13	Describe methods for calculating uncertainty of the estimates. State which sources of uncertainty were, and were not, accounted for in the uncertainty analysis.	Appendix	Methods appendix
14	State how analytic or statistical source code used to generate estimates can be accessed.	Appendix	Methods appendix
**Results and Discussion**			
15	Provide published estimates in a file format from which data can be efficiently extracted.	GBD 2016 results are available through online data visualization tools, the Global Health Data Exchange, and the online data query tool	Main text, supplementary results, and online data tools (data visualization tools, data query tools, and the Global Health Data Exchange)
16	Report a quantitative measure of the uncertainty of the estimates (e.g. uncertainty intervals).	Uncertainty intervals are provided with all results	Main text, methods appendix, and online data tools (data visualization tools, data query tools, and the Global Health Data Exchange)
17	Interpret results in light of existing evidence. If updating a previous set of estimates, describe the reasons for changes in estimates.	Discussion of methodological changes between GBD rounds	Main text (Methods and Discussion) and methods appendix

		provided in the narrative of the Article and methods appendix	
18	Discuss limitations of the estimates. Include a discussion of any modelling assumptions or data limitations that affect interpretation of the estimates.	Discussion of limitations provided in the narrative of the main paper, as well as in the methodological writeups in the methods appendix	Main text (Limitations) and methods appendix

II. Location units and time periods of the analysis

Consistent with the design of the Global Burden of Disease Study 2016, we estimated alcohol consumption and current drinker prevalence from 1990-2016 for 195 locations, which have been arranged into a set of hierarchical categories composed of seven super-regions and a further nested set of 21 regions containing 195 countries and territories, (Table 2), both sexes, and five-year age groups (age group 15-19 through age group 95+). We estimated alcohol-use attributable burden for the same 195 locations from 1990-2016, both sexes, and five-year age groups. Additionally, we calculated aggregate estimates by location and sociodemographic index (SDI). Location-level aggregates (regions, super regions, and global) are consistent with the previously published GBD location hierarchy.

Methods Appendix Table 2: Location hierarchy and SDI quintile

Super region	Region	Location	SDI Quintile
Southeast Asia, East Asia, and Oceania	East Asia	China	Middle SDI
Southeast Asia, East Asia, and Oceania	East Asia	North Korea	Low-middle SDI
Southeast Asia, East Asia, and Oceania	East Asia	Taiwan	High SDI
Southeast Asia, East Asia, and Oceania	Southeast Asia	Cambodia	Low-middle SDI
Southeast Asia, East Asia, and Oceania	Southeast Asia	Indonesia	Middle SDI
Southeast Asia, East Asia, and Oceania	Southeast Asia	Laos	Low-middle SDI
Southeast Asia, East Asia, and Oceania	Southeast Asia	Malaysia	High-middle SDI
Southeast Asia, East Asia, and Oceania	Southeast Asia	Maldives	Middle SDI
Southeast Asia, East Asia, and Oceania	Southeast Asia	Myanmar	Low-middle SDI
Southeast Asia, East Asia, and Oceania	Southeast Asia	Philippines	Middle SDI
Southeast Asia, East Asia, and Oceania	Southeast Asia	Sri Lanka	Middle SDI
Southeast Asia, East Asia, and Oceania	Southeast Asia	Thailand	Middle SDI
Southeast Asia, East Asia, and Oceania	Southeast Asia	Timor-Leste	Low-middle SDI
Southeast Asia, East Asia, and Oceania	Southeast Asia	Vietnam	Middle SDI
Southeast Asia, East Asia, and Oceania	Oceania	Fiji	Middle SDI
Southeast Asia, East Asia, and Oceania	Oceania	Kiribati	Low SDI
Southeast Asia, East Asia, and Oceania	Oceania	Marshall Islands	Low-middle SDI
Southeast Asia, East Asia, and Oceania	Oceania	Federated States of Micronesia	Low-middle SDI
Southeast Asia, East Asia, and Oceania	Oceania	Papua New Guinea	Low SDI
Southeast Asia, East Asia, and Oceania	Oceania	Samoa	Low-middle SDI
Southeast Asia, East Asia, and Oceania	Oceania	Solomon Islands	Low SDI
Southeast Asia, East Asia, and Oceania	Oceania	Tonga	Low-middle SDI
Southeast Asia, East Asia, and Oceania	Oceania	Vanuatu	Low-middle SDI
Central Europe, Eastern Europe, and Central Asia	Central Asia	Armenia	High-middle SDI
Central Europe, Eastern Europe, and Central Asia	Central Asia	Azerbaijan	High-middle SDI
Central Europe, Eastern Europe, and Central Asia	Central Asia	Georgia	High-middle SDI
Central Europe, Eastern Europe, and Central Asia	Central Asia	Kazakhstan	High-middle SDI
Central Europe, Eastern Europe, and Central Asia	Central Asia	Kyrgyzstan	Low-middle SDI
Central Europe, Eastern Europe, and Central Asia	Central Asia	Mongolia	Middle SDI
Central Europe, Eastern Europe, and Central Asia	Central Asia	Tajikistan	Low-middle SDI
Central Europe, Eastern Europe, and Central Asia	Central Asia	Turkmenistan	High-middle SDI
Central Europe, Eastern Europe, and Central Asia	Central Asia	Uzbekistan	Middle SDI
Central Europe, Eastern Europe, and Central Asia	Central Europe	Albania	Middle SDI
Central Europe, Eastern Europe, and Central Asia	Central Europe	Bosnia and Herzegovina	Middle SDI
Central Europe, Eastern Europe, and Central Asia	Central Europe	Bulgaria	High-middle SDI
Central Europe, Eastern Europe, and Central Asia	Central Europe	Croatia	High SDI

Super region	Region	Location	SDI Quintile
Central Europe, Eastern Europe, and Central Asia	Central Europe	Czech Republic	High SDI
Central Europe, Eastern Europe, and Central Asia	Central Europe	Hungary	High-middle SDI
Central Europe, Eastern Europe, and Central Asia	Central Europe	Macedonia	High-middle SDI
Central Europe, Eastern Europe, and Central Asia	Central Europe	Montenegro	High-middle SDI
Central Europe, Eastern Europe, and Central Asia	Central Europe	Poland	High SDI
Central Europe, Eastern Europe, and Central Asia	Central Europe	Romania	High-middle SDI
Central Europe, Eastern Europe, and Central Asia	Central Europe	Serbia	High-middle SDI
Central Europe, Eastern Europe, and Central Asia	Central Europe	Slovakia	High SDI
Central Europe, Eastern Europe, and Central Asia	Central Europe	Slovenia	High SDI
Central Europe, Eastern Europe, and Central Asia	Eastern Europe	Belarus	High-middle SDI
Central Europe, Eastern Europe, and Central Asia	Eastern Europe	Estonia	High SDI
Central Europe, Eastern Europe, and Central Asia	Eastern Europe	Latvia	High SDI
Central Europe, Eastern Europe, and Central Asia	Eastern Europe	Lithuania	High SDI
Central Europe, Eastern Europe, and Central Asia	Eastern Europe	Moldova	Middle SDI
Central Europe, Eastern Europe, and Central Asia	Eastern Europe	Russia	High-middle SDI
Central Europe, Eastern Europe, and Central Asia	Eastern Europe	Ukraine	High-middle SDI
High-income	High-income Asia Pacific	Brunei	High SDI
High-income	High-income Asia Pacific	Japan	High SDI
High-income	High-income Asia Pacific	South Korea	High SDI
High-income	High-income Asia Pacific	Singapore	High SDI
High-income	Australasia	Australia	High SDI
High-income	Australasia	New Zealand	High SDI
High-income	Western Europe	Andorra	High SDI
High-income	Western Europe	Austria	High SDI
High-income	Western Europe	Belgium	High SDI
High-income	Western Europe	Cyprus	High SDI
High-income	Western Europe	Denmark	High SDI
High-income	Western Europe	Finland	High SDI
High-income	Western Europe	France	High SDI
High-income	Western Europe	Germany	High SDI
High-income	Western Europe	Greece	High SDI
High-income	Western Europe	Iceland	High SDI
High-income	Western Europe	Ireland	High SDI

Super region	Region	Location	SDI Quintile
High-income	Western Europe	Israel	High-middle SDI
High-income	Western Europe	Italy	High SDI
High-income	Western Europe	Luxembourg	High SDI
High-income	Western Europe	Malta	High SDI
High-income	Western Europe	Netherlands	High SDI
High-income	Western Europe	Norway	High SDI
High-income	Western Europe	Portugal	High-middle SDI
High-income	Western Europe	Spain	High-middle SDI
High-income	Western Europe	Sweden	High SDI
High-income	Western Europe	Switzerland	High SDI
High-income	Western Europe	United Kingdom	High SDI
High-income	Southern Latin America	Argentina	High-middle SDI
High-income	Southern Latin America	Chile	High-middle SDI
High-income	Southern Latin America	Uruguay	Middle SDI
High-income	High-income North America	Canada	High SDI
High-income	High-income North America	United States	High SDI
Latin America and Caribbean	Caribbean	Antigua and Barbuda	High-middle SDI
Latin America and Caribbean	Caribbean	The Bahamas	High-middle SDI
Latin America and Caribbean	Caribbean	Barbados	High-middle SDI
Latin America and Caribbean	Caribbean	Belize	Low-middle SDI
Latin America and Caribbean	Caribbean	Cuba	High-middle SDI
Latin America and Caribbean	Caribbean	Dominica	Middle SDI
Latin America and Caribbean	Caribbean	Dominican Republic	Middle SDI
Latin America and Caribbean	Caribbean	Grenada	Middle SDI
Latin America and Caribbean	Caribbean	Guyana	Middle SDI
Latin America and Caribbean	Caribbean	Haiti	Low SDI
Latin America and Caribbean	Caribbean	Jamaica	Middle SDI
Latin America and Caribbean	Caribbean	Saint Lucia	Middle SDI
Latin America and Caribbean	Caribbean	Saint Vincent and the Grenadines	Middle SDI
Latin America and Caribbean	Caribbean	Suriname	Middle SDI
Latin America and Caribbean	Caribbean	Trinidad and Tobago	High-middle SDI
Latin America and Caribbean	Andean Latin America	Bolivia	Low-middle SDI
Latin America and Caribbean	Andean Latin America	Ecuador	Middle SDI
Latin America and Caribbean	Andean Latin America	Peru	Middle SDI
Latin America and Caribbean	Central Latin America	Colombia	Middle SDI
Latin America and Caribbean	Central Latin America	Costa Rica	Middle SDI
Latin America and Caribbean	Central Latin America	El Salvador	Middle SDI
Latin America and Caribbean	Central Latin America	Guatemala	Low-middle SDI
Latin America and Caribbean	Central Latin America	Honduras	Low-middle SDI
Latin America and Caribbean	Central Latin America	Mexico	Middle SDI
Latin America and Caribbean	Central Latin America	Nicaragua	Low-middle SDI
Latin America and Caribbean	Central Latin America	Panama	High-middle SDI
Latin America and Caribbean	Central Latin America	Venezuela	Middle SDI
Latin America and Caribbean	Tropical Latin America	Brazil	Middle SDI
Latin America and Caribbean	Tropical Latin America	Paraguay	Middle SDI
North Africa and Middle East	North Africa and Middle East	Algeria	Middle SDI
North Africa and Middle East	North Africa and Middle East	Bahrain	Middle SDI
North Africa and Middle East	North Africa and Middle East	Egypt	Middle SDI
North Africa and Middle East	North Africa and Middle East	Iran	High-middle SDI

Super region	Region	Location	SDI Quintile
North Africa and Middle East	North Africa and Middle East	Iraq	Low-middle SDI
North Africa and Middle East	North Africa and Middle East	Jordan	Middle SDI
North Africa and Middle East	North Africa and Middle East	Kuwait	High-middle SDI
North Africa and Middle East	North Africa and Middle East	Lebanon	High-middle SDI
North Africa and Middle East	North Africa and Middle East	Libya	High-middle SDI
North Africa and Middle East	North Africa and Middle East	Morocco	Low-middle SDI
North Africa and Middle East	North Africa and Middle East	Palestine	Low SDI
North Africa and Middle East	North Africa and Middle East	Oman	Middle SDI
North Africa and Middle East	North Africa and Middle East	Qatar	High-middle SDI
North Africa and Middle East	North Africa and Middle East	Saudi Arabia	High-middle SDI
North Africa and Middle East	North Africa and Middle East	Syria	Low-middle SDI
North Africa and Middle East	North Africa and Middle East	Tunisia	Middle SDI
North Africa and Middle East	North Africa and Middle East	Turkey	High-middle SDI
North Africa and Middle East	North Africa and Middle East	United Arab Emirates	High-middle SDI
North Africa and Middle East	North Africa and Middle East	Yemen	Low SDI
North Africa and Middle East	North Africa and Middle East	Afghanistan	Low SDI
South Asia	South Asia	Bangladesh	Low-middle SDI
South Asia	South Asia	Bhutan	Low-middle SDI
South Asia	South Asia	India	Low-middle SDI
South Asia	South Asia	Nepal	Low-middle SDI
South Asia	South Asia	Pakistan	Low-middle SDI
Sub-Saharan Africa	Central Sub-Saharan Africa	Angola	Low SDI
Sub-Saharan Africa	Central Sub-Saharan Africa	Central African Republic	Low SDI
Sub-Saharan Africa	Central Sub-Saharan Africa	Congo	Low-middle SDI
Sub-Saharan Africa	Central Sub-Saharan Africa	Democratic Republic of the Congo	Low SDI
Sub-Saharan Africa	Central Sub-Saharan Africa	Equatorial Guinea	Middle SDI
Sub-Saharan Africa	Central Sub-Saharan Africa	Gabon	Low-middle SDI
Sub-Saharan Africa	Eastern Sub-Saharan Africa	Burundi	Low SDI
Sub-Saharan Africa	Eastern Sub-Saharan Africa	Comoros	Low SDI
Sub-Saharan Africa	Eastern Sub-Saharan Africa	Djibouti	Low SDI
Sub-Saharan Africa	Eastern Sub-Saharan Africa	Eritrea	Low SDI
Sub-Saharan Africa	Eastern Sub-Saharan Africa	Ethiopia	Low SDI
Sub-Saharan Africa	Eastern Sub-Saharan Africa	Kenya	Low-middle SDI
Sub-Saharan Africa	Eastern Sub-Saharan Africa	Madagascar	Low SDI
Sub-Saharan Africa	Eastern Sub-Saharan Africa	Malawi	Low SDI
Southeast Asia, East Asia, and Oceania	Southeast Asia	Mauritius	High-middle SDI
Sub-Saharan Africa	Eastern Sub-Saharan Africa	Mozambique	Low SDI
Sub-Saharan Africa	Eastern Sub-Saharan Africa	Rwanda	Low SDI
Southeast Asia, East Asia, and Oceania	Southeast Asia	Seychelles	Middle SDI
Sub-Saharan Africa	Eastern Sub-Saharan Africa	Somalia	Low SDI
Sub-Saharan Africa	Eastern Sub-Saharan Africa	Tanzania	Low SDI
Sub-Saharan Africa	Eastern Sub-Saharan Africa	Uganda	Low SDI
Sub-Saharan Africa	Eastern Sub-Saharan Africa	Zambia	Low-middle SDI
Sub-Saharan Africa	Southern Sub-Saharan Africa	Botswana	Middle SDI
Sub-Saharan Africa	Southern Sub-Saharan Africa	Lesotho	Low-middle SDI
Sub-Saharan Africa	Southern Sub-Saharan Africa	Namibia	Low-middle SDI
Sub-Saharan Africa	Southern Sub-Saharan Africa	South Africa	Middle SDI
Sub-Saharan Africa	Southern Sub-Saharan Africa	Swaziland	Low-middle SDI
Sub-Saharan Africa	Southern Sub-Saharan Africa	Zimbabwe	Low-middle SDI

Super region	Region	Location	SDI Quintile
Sub-Saharan Africa	Western Sub-Saharan Africa	Benin	Low SDI
Sub-Saharan Africa	Western Sub-Saharan Africa	Burkina Faso	Low SDI
Sub-Saharan Africa	Western Sub-Saharan Africa	Cameroon	Low-middle SDI
Sub-Saharan Africa	Western Sub-Saharan Africa	Cape Verde	Low-middle SDI
Sub-Saharan Africa	Western Sub-Saharan Africa	Chad	Low SDI
Sub-Saharan Africa	Western Sub-Saharan Africa	Cote d'Ivoire	Low SDI
Sub-Saharan Africa	Western Sub-Saharan Africa	The Gambia	Low SDI
Sub-Saharan Africa	Western Sub-Saharan Africa	Ghana	Low-middle SDI
Sub-Saharan Africa	Western Sub-Saharan Africa	Guinea	Low SDI
Sub-Saharan Africa	Western Sub-Saharan Africa	Guinea-Bissau	Low SDI
Sub-Saharan Africa	Western Sub-Saharan Africa	Liberia	Low SDI
Sub-Saharan Africa	Western Sub-Saharan Africa	Mali	Low SDI
Sub-Saharan Africa	Western Sub-Saharan Africa	Mauritania	Low-middle SDI
Sub-Saharan Africa	Western Sub-Saharan Africa	Niger	Low SDI
Sub-Saharan Africa	Western Sub-Saharan Africa	Nigeria	Low-middle SDI
Sub-Saharan Africa	Western Sub-Saharan Africa	Sao Tome and Principe	Low SDI
Sub-Saharan Africa	Western Sub-Saharan Africa	Senegal	Low SDI
Sub-Saharan Africa	Western Sub-Saharan Africa	Sierra Leone	Low SDI
Sub-Saharan Africa	Western Sub-Saharan Africa	Togo	Low SDI
Southeast Asia, East Asia, and Oceania	Oceania	American Samoa	Middle SDI
Latin America and Caribbean	Caribbean	Bermuda	High-middle SDI
High-income	High-income North America	Greenland	High-middle SDI
Southeast Asia, East Asia, and Oceania	Oceania	Guam	High-middle SDI
Southeast Asia, East Asia, and Oceania	Oceania	Northern Mariana Islands	High-middle SDI
Latin America and Caribbean	Caribbean	Puerto Rico	High SDI
Latin America and Caribbean	Caribbean	Virgin Islands, U.S.	High SDI
High-income	Western Europe	Scotland	High SDI
Sub-Saharan Africa	Eastern Sub-Saharan Africa	South Sudan	Low SDI
North Africa and Middle East	North Africa and Middle East	Sudan	Low-middle SDI
Central Europe, Eastern Europe, and Central Asia	Central Asia	Georgia	High SDI
High-income	Western Europe	Wales	High SDI
High-income	Western Europe	England	High SDI
High-income	Western Europe	Sweden except Stockholm	High SDI
High-income	Western Europe	Stockholm	High SDI

III. Age-standardisation

We used the GBD population standard rates as our age-standardised rates. [1]

IV. Data sources

A systematic review of the literature was performed to extract data on our primary consumption indicators. The Global Health Exchange (GHDx), IHME's online database of health-related data, and Pubmed were searched for population survey data containing participant-level information from which we could formulate the required alcohol use indicators on current drinkers, lifetime abstainers, and levels of alcohol consumption.[2-3] We documented relevant survey variables from each data source in a spreadsheet and extracted using STATA 13.1 and R 3.3.

To generate estimates of population consumption in liters per capita (LPC), we obtained data from FAOSTAT and the WHO GISAH database.[4-5] We obtained data on the number of tourists and their duration of stay from the UNWTO.[6] For unrecorded alcohol stock, we extracted estimates from published papers, consisting of 166 locations.[7-12] A complete list of sources can be found in the GBD data source tool: http://ghdx.healthdata.org/gbd-2015/data-input-sources.

We found studies used in our meta-analysis by searching Pubmed and the GHDx. The meta-analysis below lists all included studies.

For calculating attributable burden, we used estimates from GBD 2016 of deaths and DALYs for the 22 included outcomes. These estimates can be found in the GBD results tool: http://ghdx.healthdata.org/gbd-results-tool.

a. Inclusion criteria

We included nationally representative survey data sources that captured information on alcohol use among individuals age 15 and above. We included only self-reported drinking data and excluded data from questions asking about others' drinking behaviors. We included data that was collected between 1 January 1990 and 31 December 2016 in any of the 195 locations included in this study. For population consumption estimation, we included nationally representative sales data on alcohol availability from sources covering multiple countries. Data were included if they were collected between 1 January 1990 and 31 December 2016 and covered one of the 195 locations included in this study.

For our meta-analysis, we included all cohort and case-control studies reporting a relative risk, hazard ratio, or odds ratio for any risk-outcome pairs we included. Studies were included if they reported a categorical or continuous dose for alcohol consumption, as well as uncertainty measures for their outcomes, and the population under study was representative.

V. Consumption estimation

a. Definitions

We used four indicators to construct alcohol-use consumption, defined as follows:

1. Current drinkers, defined as the proportion of individuals who have consumed at least one alcoholic beverage (or some approximation) in a 12-month period.
2. Lifetime abstainers, defined as the proportion of individuals who have never consumed an alcoholic beverage.
3. Alcohol consumption (in grams per day), defined as grams of alcohol consumed by current drinkers, per day, over a 12-month period.
4. Alcohol liters per capita stock, defined in liters per capita of pure alcohol, over a 12-month period.

We also used three additional indicators to adjust alcohol exposure estimates to account for different types of bias:

1. Number of tourists within a location, defined as the total amount of visitors to a location within a 12 month period.
2. Tourists' duration of stay, defined as the number of days resided in a hosting country.
3. Unrecorded alcohol stock, defined as a percentage of the total alcohol stock produced outside established markets.

We used these indicators, as outlined in the modeling strategy below, to calculate a consumption estimate defined as the grams per day of pure alcohol consumed amongst drinkers.

b. Data extraction and preparation

For data in the current drinkers, lifetime abstainers, and individual-level alcohol consumption models, we extracted primary data from individual-level microdata and survey report tabulations. For microdata, we extracted relevant demographic information, including age, sex, location, and year, as well as survey metadata, including survey weights, primary sampling units, and strata. This information allowed us to tabulate individual-level data in five-year age-sex groups and produce accurate estimates of uncertainty. For survey report tabulations, we extracted data at the most granular age-sex group provided.

For data in the liters per capita mode, to provide more stable time trends in the population consumption model, we transformed FAO sales data (which calculates stock based on primary inputs) to a lagged 5-year average. Given the WHO uses FAO data in locations where the WHO could not find data using their own methods, we did not use FAO data in the locations where the WHO used FAO data to construct their estimate. To correct for bias in the underlying data generating processes between series, we adjusted the input data by running a mixed effect model on the log average of the data with dummy variables for the data series, as well as random effects on super region, region, country, and time. We adjusted the data points using the estimated parameters from the following equation:

$$Log\ Average\ Data = \beta_0 + \beta_1 D + \alpha_s + \alpha_r + \alpha_y$$

$$Transformed\ data = data * e^{\widehat{\beta_1} + \widehat{\alpha_s}}$$

Where D is a dummy variable for a data source, α_s *is a random effect for super-region,* α_r *is a random effect for region, and* α_y *is a random effect for year.*

None of the data sources on liters per capita provided estimates of uncertainty, which is a component required for our eventual modeling strategy. To generate uncertainty, we ran a Loess model on the adjusted data points and the standard deviation between the difference of the Loess smoothed model and the adjusted data points across a five-year span was used as the standard deviation of the data. (i.e. If the total stock changes more variably in a narrow time frame, we believed the data to be more uncertain).

c. Modeling Strategy

In the following paragraphs and shown in the flowchart on the next page (Methods Appendix Figure 1), we outline how we estimated each primary input in the alcohol exposure model, as well as how we combined these inputs to arrive at our final estimate of grams per day of pure alcohol. We estimated all models below using 1000 draws. For all steps, R and associated packages were used. [17-20]

Methods Appendix Figure 1: Alcohol Use Flowchart

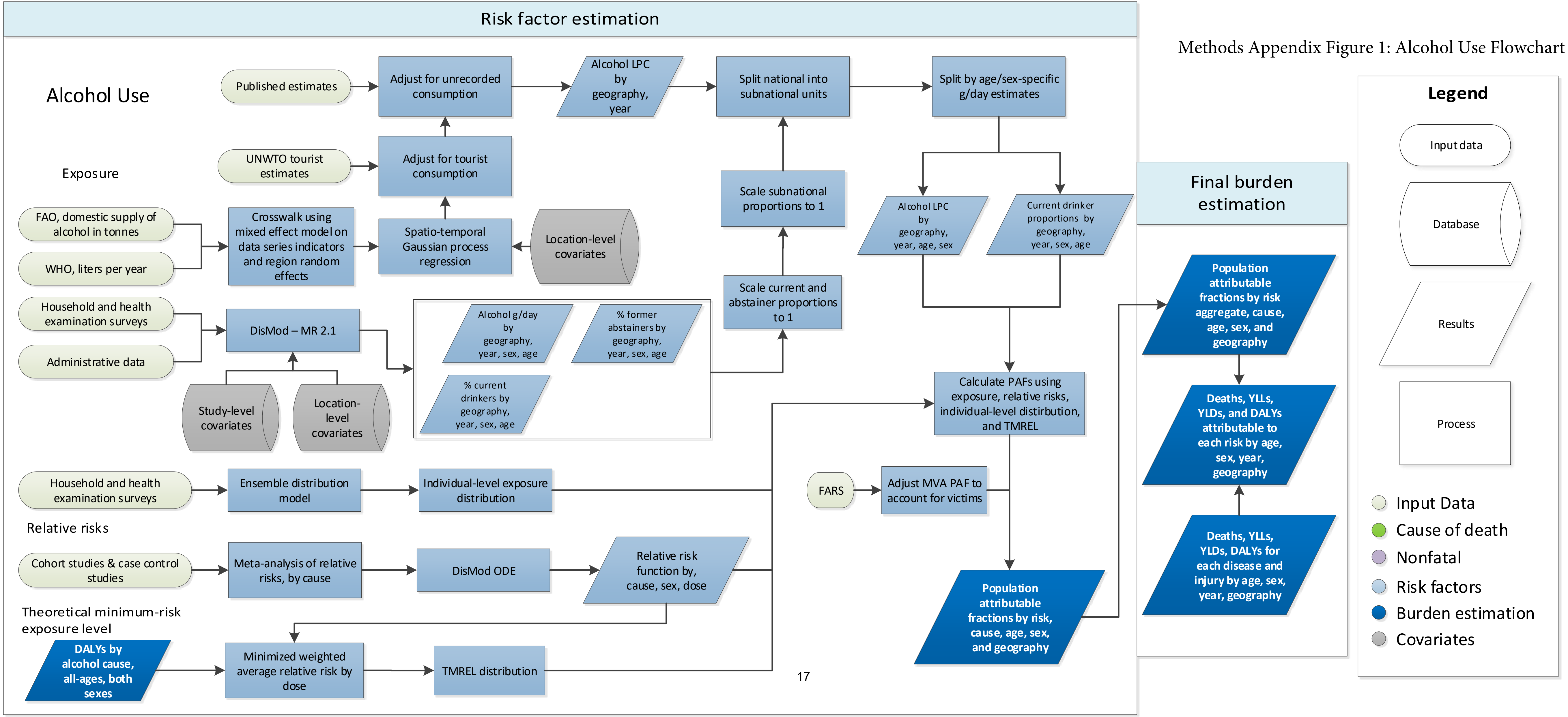

1. Population consumption in liters per capita

We modeled the alcohol liters per capita data, using a spatio-temporal Gaussian process regression (ST-GPR). The model is defined as:

$$Alcohol\ LPC = GP(m_{l,y,a,s}, cov(Alcohol\ LPC)) + \epsilon_{l,y,a,s}$$

$$m_{l,y,a,s} = \beta_0 + \beta_1(\%\ of\ population\ muslim)_{l,y} + \beta_2(SDI)_{l,y} + \alpha_s + \alpha_r + h(r_{l,y})$$

$$\epsilon_{l,y,a,s} = Normal(0, \sigma^2)$$

Where l is a location, y is a year period, a is an age group, and s is a sex, α_s *is a random effect for super region,* α_r *is a random effect for region, and SDI is a location's socio-demographic index. M is the mean Gaussian process function for alcohol lpc, COV is the Matern covariance function of alcohol lpc in time and space, and* $h(r_{l,y})$ *is a smoothing function of the residuals in time and space, derived from hyper-parameters for time and space.*

More details on the spatio-temporal Gaussian process regression and the definition of hyper-parameters within the functions above can be found in the GBD 2016 appendix (pg 18-22). [1] Briefly, the spatio-temporal Gaussian process regression interpolates non-linear trends through time, which is particularly useful for noisy data, and does not require assumptions of a functional form for the underlying data generating process.

Hyper-parameters for the smoothing function and GPR were chosen by performing a grid search over the domains of hyper-parameter combinations. We chose the hyper-parameters and subsequent model, by minimizing the out-of-sample 10-fold cross-validated root mean squared error. Folds were chosen by holding out particular GBD regions.

2. Tourism adjustment

We adjusted the estimates for alcohol LPC for tourist consumption by adding in the per capita rate of consumption abroad and subtracting the per capita rate of tourist consumption domestically. These adjustments can be found in Methods Appendix Table 3.

$$Alcohol\ LPC_d = Unadjusted\ Alcohol\ LPC_d + Alcohol\ LPC_{Domestic\ consumption\ abroad} - Alcohol\ LPC_{Tourist\ consumption\ domestically}$$

$$Alcohol\ LPC_i = \frac{\sum_l Tourist\ Population_l * Proportion\ of\ tourists_{i,l} * Unadjusted\ Alcohol\ LPC_l * \frac{Average\ length\ of\ stay_{i,l}}{365} *}{Population_d}$$

Where l is the set of all locations, d is the domestic location, and i is either domestic consumption abroad or tourist consumption domestically.

Methods Appendix Table 3: Percent change in alcohol consumption due to tourism, by location and 5-year interval

Location	Year	Mean	2.5 percentile	97.5 percentile
Afghanistan	1990	0%	0%	0%
Afghanistan	1995	0%	0%	0%
Afghanistan	2000	0%	0%	0%
Afghanistan	2005	0%	0%	0%
Afghanistan	2010	0%	0%	0%
Afghanistan	2016	0%	0%	0%
Albania	1990	-0.49%	-3.90%	1.33%
Albania	1995	-0.11%	-2.92%	1.47%
Albania	2000	-0.83%	-4.12%	0.67%
Albania	2005	-0.04%	-1.67%	0.96%
Albania	2010	-0.60%	-3.16%	0.51%
Albania	2016	-0.47%	-2.56%	0.57%
Algeria	1990	-0.97%	-1.42%	-0.52%
Algeria	1995	-1.03%	-1.58%	-0.53%
Algeria	2000	-0.93%	-1.39%	-0.50%
Algeria	2005	-0.79%	-1.21%	-0.41%
Algeria	2010	-0.76%	-1.18%	-0.39%
Algeria	2016	-0.79%	-1.33%	-0.38%
American Samoa	1990	-3.12%	-6.79%	-0.40%
American Samoa	1995	-2.80%	-7.28%	0.92%
American Samoa	2000	-2.76%	-6.82%	0.48%
American Samoa	2005	-2.96%	-7.25%	0.22%
American Samoa	2010	-2.64%	-6.36%	0.77%
American Samoa	2016	-2.68%	-5.85%	-0.34%
Andorra	1990	-75.08%	-84.98%	-61.90%
Andorra	1995	-74.36%	-84.33%	-61.62%
Andorra	2000	-75.05%	-83.80%	-63.34%
Andorra	2005	-77.24%	-84.54%	-67.66%
Andorra	2010	-79.30%	-86.50%	-69.68%
Andorra	2016	-80.11%	-87.76%	-70.03%
Angola	1990	-0.03%	-0.18%	0.03%
Angola	1995	-0.03%	-0.18%	0.03%
Angola	2000	-0.02%	-0.15%	0.03%
Angola	2005	-0.01%	-0.08%	0.02%
Angola	2010	-0.01%	-0.08%	0.02%
Angola	2016	-0.01%	-0.06%	0.01%
Antigua and Barbuda	1990	-69.28%	-94.59%	-46.98%
Antigua and Barbuda	1995	-59.31%	-84.05%	-38.72%
Antigua and Barbuda	2000	-48.71%	-68.17%	-31.70%
Antigua and Barbuda	2005	-41.78%	-57.89%	-27.26%
Antigua and Barbuda	2010	-38.97%	-55.37%	-25.03%
Antigua and Barbuda	2016	-38.35%	-56.43%	-23.04%
Argentina	1990	0.35%	0.20%	0.53%
Argentina	1995	0.41%	0.23%	0.65%
Argentina	2000	0.47%	0.25%	0.76%
Argentina	2005	0.52%	0.28%	0.82%

Percent change in alcohol consumption due to tourism, by location and 5-year interval

Location	Year	Mean	2.5 percentile	97.5 percentile
Argentina	2010	0.52%	0.27%	0.85%
Argentina	2016	0.52%	0.26%	0.86%
Armenia	1990	1.35%	0.14%	6.12%
Armenia	1995	1.39%	0.12%	6.40%
Armenia	2000	1.46%	0.09%	6.76%
Armenia	2005	1.36%	0.10%	6.29%
Armenia	2010	1.32%	0.10%	6.10%
Armenia	2016	1.21%	0.12%	5.55%
Australia	1990	1.02%	0.59%	1.75%
Australia	1995	1.14%	0.66%	1.99%
Australia	2000	1.24%	0.67%	2.29%
Australia	2005	1.23%	0.67%	2.22%
Australia	2010	1.17%	0.65%	2.15%
Australia	2016	1.15%	0.62%	2.07%
Austria	1990	3.72%	2.56%	5.00%
Austria	1995	3.85%	2.09%	5.66%
Austria	2000	4.01%	2.45%	5.84%
Austria	2005	4.15%	2.41%	6.07%
Austria	2010	4.33%	2.54%	6.80%
Austria	2016	4.36%	2.63%	6.26%
Azerbaijan	1990	0.84%	0.11%	3.03%
Azerbaijan	1995	0.80%	0.11%	2.93%
Azerbaijan	2000	0.76%	0.10%	2.88%
Azerbaijan	2005	0.72%	0.09%	2.77%
Azerbaijan	2010	0.64%	0.08%	2.31%
Azerbaijan	2016	0.65%	0.09%	2.36%
Bahrain	1990	-5.37%	-8.90%	-2.20%
Bahrain	1995	-6.10%	-10.38%	-2.32%
Bahrain	2000	-7.48%	-13.39%	-2.54%
Bahrain	2005	-8.40%	-14.32%	-3.16%
Bahrain	2010	-9.52%	-16.45%	-3.65%
Bahrain	2016	-10.02%	-19.42%	-3.70%
Bangladesh	1990	-0.21%	-0.31%	-0.07%
Bangladesh	1995	-0.17%	-0.26%	-0.05%
Bangladesh	2000	-0.13%	-0.20%	-0.04%
Bangladesh	2005	-0.11%	-0.17%	-0.04%
Bangladesh	2010	-0.11%	-0.17%	-0.03%
Bangladesh	2016	-0.10%	-0.17%	-0.03%
Barbados	1990	-10.39%	-12.82%	-7.72%
Barbados	1995	-11.55%	-14.82%	-8.34%
Barbados	2000	-12.19%	-15.86%	-8.52%
Barbados	2005	-11.83%	-15.01%	-8.65%
Barbados	2010	-11.60%	-15.23%	-8.39%
Barbados	2016	-11.17%	-14.74%	-7.81%
Belarus	1990	1.04%	0.57%	1.91%
Belarus	1995	1.06%	0.54%	2.03%

Percent change in alcohol consumption due to tourism, by location and 5-year interval

Location	Year	Mean	2.5 percentile	97.5 percentile
Belarus	2000	1.03%	0.51%	1.92%
Belarus	2005	0.99%	0.50%	1.87%
Belarus	2010	0.95%	0.49%	1.78%
Belarus	2016	0.91%	0.49%	1.66%
Belgium	1990	6.25%	3.95%	9.37%
Belgium	1995	6.64%	3.92%	10.32%
Belgium	2000	6.76%	3.92%	10.89%
Belgium	2005	6.01%	3.56%	9.27%
Belgium	2010	5.62%	3.33%	9.21%
Belgium	2016	5.71%	3.23%	9.22%
Belize	1990	-10.79%	-18.22%	-2.94%
Belize	1995	-11.40%	-19.73%	-3.13%
Belize	2000	-11.58%	-19.40%	-3.01%
Belize	2005	-10.29%	-17.62%	-2.70%
Belize	2010	-9.45%	-16.44%	-2.44%
Belize	2016	-8.76%	-15.41%	-2.34%
Benin	1990	-0.85%	-1.59%	-0.33%
Benin	1995	-0.86%	-1.67%	-0.30%
Benin	2000	-0.80%	-1.56%	-0.29%
Benin	2005	-0.79%	-1.56%	-0.29%
Benin	2010	-0.79%	-1.59%	-0.29%
Benin	2016	-0.78%	-1.70%	-0.25%
Bermuda	1990	-12.00%	-16.03%	-3.70%
Bermuda	1995	-11.31%	-15.61%	-3.22%
Bermuda	2000	-11.43%	-15.24%	-3.43%
Bermuda	2005	-12.24%	-16.53%	-3.38%
Bermuda	2010	-13.74%	-18.68%	-3.57%
Bermuda	2016	-15.15%	-20.81%	-4.67%
Bhutan	1990	-0.50%	-1.38%	-0.05%
Bhutan	1995	-0.42%	-1.21%	-0.02%
Bhutan	2000	-0.46%	-1.46%	-0.01%
Bhutan	2005	-0.65%	-2.06%	-0.03%
Bhutan	2010	-0.75%	-2.40%	-0.05%
Bhutan	2016	-0.73%	-2.40%	-0.05%
Bolivia	1990	-0.14%	-0.25%	0.29%
Bolivia	1995	-0.16%	-0.29%	0.32%
Bolivia	2000	-0.16%	-0.29%	0.31%
Bolivia	2005	-0.16%	-0.28%	0.32%
Bolivia	2010	-0.15%	-0.27%	0.34%
Bolivia	2016	-0.14%	-0.25%	0.28%
Bosnia and Herzegovina	1990	1.85%	1.23%	2.62%
Bosnia and Herzegovina	1995	1.93%	1.25%	2.69%
Bosnia and Herzegovina	2000	1.86%	1.26%	2.62%
Bosnia and Herzegovina	2005	1.82%	1.22%	2.56%
Bosnia and Herzegovina	2010	1.75%	1.12%	2.61%
Bosnia and Herzegovina	2016	1.74%	1.12%	2.56%

Percent change in alcohol consumption due to tourism, by location and 5-year interval

Location	Year	Mean	2.5 percentile	97.5 percentile
Botswana	1990	-1.68%	-3.93%	-0.65%
Botswana	1995	-1.61%	-4.10%	-0.51%
Botswana	2000	-1.49%	-4.00%	-0.45%
Botswana	2005	-1.23%	-3.21%	-0.40%
Botswana	2010	-1.12%	-2.95%	-0.34%
Botswana	2016	-1.18%	-2.93%	-0.38%
Brazil	1990	0.05%	-0.03%	0.18%
Brazil	1995	0.05%	-0.03%	0.16%
Brazil	2000	0.04%	-0.02%	0.14%
Brazil	2005	0.04%	-0.02%	0.13%
Brazil	2010	0.04%	-0.02%	0.14%
Brazil	2016	0.04%	-0.02%	0.14%
Brunei	1990	-19.07%	-25.40%	-13.79%
Brunei	1995	-32.59%	-46.08%	-21.16%
Brunei	2000	-59.82%	-85.71%	-38.66%
Brunei	2005	-66.73%	-93.58%	-45.01%
Brunei	2010	-69.24%	-97.21%	-46.16%
Brunei	2016	-66.22%	-93.00%	-42.99%
Bulgaria	1990	-0.30%	-0.46%	-0.08%
Bulgaria	1995	-0.33%	-0.57%	-0.02%
Bulgaria	2000	-0.49%	-0.76%	-0.12%
Bulgaria	2005	-0.38%	-0.68%	-0.03%
Bulgaria	2010	-0.49%	-0.80%	-0.15%
Bulgaria	2016	-0.42%	-0.65%	-0.14%
Burkina Faso	1990	0%	0%	0%
Burkina Faso	1995	0%	0%	0%
Burkina Faso	2000	0%	0%	0%
Burkina Faso	2005	0%	0%	0%
Burkina Faso	2010	0%	0%	0%
Burkina Faso	2016	0%	0%	0%
Burundi	1990	0%	0%	0%
Burundi	1995	0%	0%	0%
Burundi	2000	0%	0%	0%
Burundi	2005	0%	0%	0%
Burundi	2010	0%	0%	0%
Burundi	2016	0%	0%	0%
Cambodia	1990	-1.63%	-3.35%	-0.42%
Cambodia	1995	-0.92%	-1.95%	-0.20%
Cambodia	2000	-0.48%	-1.08%	-0.11%
Cambodia	2005	-0.39%	-0.91%	-0.08%
Cambodia	2010	-0.34%	-0.75%	-0.07%
Cambodia	2016	-0.34%	-0.80%	-0.08%
Cameroon	1990	-0.10%	-0.14%	-0.07%
Cameroon	1995	-0.12%	-0.18%	-0.06%
Cameroon	2000	-0.11%	-0.17%	-0.07%
Cameroon	2005	-0.10%	-0.15%	-0.05%

Percent change in alcohol consumption due to tourism, by location and 5-year interval

Location	Year	Mean	2.5 percentile	97.5 percentile
Cameroon	2010	-0.09%	-0.14%	-0.05%
Cameroon	2016	-0.09%	-0.17%	-0.05%
Canada	1990	-0.95%	-3.88%	3.36%
Canada	1995	-1.03%	-4.62%	3.61%
Canada	2000	-0.93%	-4.55%	3.52%
Canada	2005	-0.98%	-4.26%	3.59%
Canada	2010	-0.98%	-4.33%	3.45%
Canada	2016	-1.02%	-4.17%	3.34%
Cape Verde	1990	0%	0%	0%
Cape Verde	1995	0%	0%	0%
Cape Verde	2000	0%	0%	0%
Cape Verde	2005	0%	0%	0%
Cape Verde	2010	0%	0%	0%
Cape Verde	2016	0%	0%	0%
Central African Republic	1990	-0.02%	-0.04%	-0.01%
Central African Republic	1995	-0.02%	-0.06%	-0.01%
Central African Republic	2000	-0.03%	-0.06%	-0.01%
Central African Republic	2005	-0.03%	-0.07%	-0.01%
Central African Republic	2010	-0.03%	-0.07%	-0.01%
Central African Republic	2016	-0.03%	-0.07%	-0.01%
Chad	1990	-0.09%	-0.12%	-0.06%
Chad	1995	-0.09%	-0.15%	-0.05%
Chad	2000	-0.08%	-0.12%	-0.05%
Chad	2005	-0.08%	-0.12%	-0.04%
Chad	2010	-0.07%	-0.11%	-0.04%
Chad	2016	-0.08%	-0.12%	-0.05%
Chile	1990	-0.09%	-0.26%	0.09%
Chile	1995	-0.11%	-0.37%	0.14%
Chile	2000	-0.13%	-0.49%	0.15%
Chile	2005	-0.12%	-0.41%	0.15%
Chile	2010	-0.11%	-0.38%	0.12%
Chile	2016	-0.10%	-0.29%	0.06%
China	1990	0.05%	0.00%	0.23%
China	1995	0.05%	0.00%	0.20%
China	2000	0.04%	0%	0.18%
China	2005	0.04%	0.00%	0.19%
China	2010	0.03%	0%	0.18%
China	2016	0.03%	0.00%	0.17%
Colombia	1990	-0.03%	-0.10%	0.03%
Colombia	1995	-0.03%	-0.11%	0.05%
Colombia	2000	-0.02%	-0.11%	0.05%
Colombia	2005	-0.03%	-0.11%	0.05%
Colombia	2010	-0.03%	-0.12%	0.05%
Colombia	2016	-0.03%	-0.10%	0.03%
Comoros	1990	-3.58%	-6.49%	-2.09%
Comoros	1995	-4.29%	-7.56%	-2.41%

Percent change in alcohol consumption due to tourism, by location and 5-year interval

Location	Year	Mean	2.5 percentile	97.5 percentile
Comoros	2000	-4.20%	-7.53%	-2.14%
Comoros	2005	-3.65%	-6.42%	-1.92%
Comoros	2010	-3.38%	-6.31%	-1.72%
Comoros	2016	-3.13%	-6.55%	-1.33%
Congo	1990	-0.14%	-0.25%	-0.03%
Congo	1995	-0.19%	-0.42%	-0.03%
Congo	2000	-0.19%	-0.38%	-0.03%
Congo	2005	-0.20%	-0.40%	-0.02%
Congo	2010	-0.17%	-0.36%	-0.01%
Congo	2016	-0.16%	-0.31%	-0.04%
Costa Rica	1990	-1.21%	-1.92%	-0.35%
Costa Rica	1995	-1.18%	-1.91%	-0.33%
Costa Rica	2000	-1.25%	-2.03%	-0.36%
Costa Rica	2005	-1.33%	-2.20%	-0.38%
Costa Rica	2010	-1.35%	-2.29%	-0.39%
Costa Rica	2016	-1.32%	-2.25%	-0.37%
Cote d'Ivoire	1990	-0.04%	-0.10%	-0.01%
Cote d'Ivoire	1995	-0.03%	-0.09%	0.00%
Cote d'Ivoire	2000	-0.03%	-0.08%	0.00%
Cote d'Ivoire	2005	-0.03%	-0.09%	0%
Cote d'Ivoire	2010	-0.03%	-0.10%	0.00%
Cote d'Ivoire	2016	-0.04%	-0.11%	-0.01%
Croatia	1990	-17.79%	-26.24%	-5.98%
Croatia	1995	-17.72%	-26.67%	-5.84%
Croatia	2000	-16.78%	-25.13%	-5.41%
Croatia	2005	-17.64%	-27.23%	-5.37%
Croatia	2010	-19.39%	-29.52%	-6.11%
Croatia	2016	-20.63%	-31.17%	-7.03%
Cuba	1990	-0.88%	-1.19%	-0.54%
Cuba	1995	-0.91%	-1.26%	-0.56%
Cuba	2000	-0.91%	-1.25%	-0.55%
Cuba	2005	-0.85%	-1.15%	-0.52%
Cuba	2010	-0.81%	-1.11%	-0.48%
Cuba	2016	-0.76%	-1.13%	-0.42%
Cyprus	1990	-5.54%	-7.59%	-3.89%
Cyprus	1995	-5.33%	-7.95%	-3.40%
Cyprus	2000	-5.39%	-8.29%	-3.27%
Cyprus	2005	-5.61%	-8.15%	-3.32%
Cyprus	2010	-6.11%	-9.27%	-3.69%
Cyprus	2016	-6.12%	-8.70%	-4.10%
Czech Republic	1990	-0.50%	-2.57%	0.67%
Czech Republic	1995	-0.54%	-3.06%	0.85%
Czech Republic	2000	-0.27%	-2.79%	0.97%
Czech Republic	2005	-0.53%	-2.88%	0.88%
Czech Republic	2010	-0.32%	-2.84%	1.05%
Czech Republic	2016	-0.44%	-2.25%	0.74%

Percent change in alcohol consumption due to tourism, by location and 5-year interval

Location	Year	Mean	2.5 percentile	97.5 percentile
Democratic Republic of the Congo	1990	0.00%	-0.01%	0.01%
Democratic Republic of the Congo	1995	0.00%	-0.02%	0.02%
Democratic Republic of the Congo	2000	0%	-0.02%	0.02%
Democratic Republic of the Congo	2005	0.00%	-0.02%	0.02%
Democratic Republic of the Congo	2010	0.00%	-0.02%	0.02%
Democratic Republic of the Congo	2016	0.00%	-0.01%	0.01%
Denmark	1990	-1.92%	-3.10%	-0.39%
Denmark	1995	-1.89%	-3.57%	0.01%
Denmark	2000	-1.72%	-3.44%	0.22%
Denmark	2005	-1.81%	-3.36%	-0.15%
Denmark	2010	-1.80%	-3.58%	0.07%
Denmark	2016	-2.00%	-3.54%	-0.61%
Djibouti	1990	0%	0%	0%
Djibouti	1995	0%	0%	0%
Djibouti	2000	0%	0%	0%
Djibouti	2005	0%	0%	0%
Djibouti	2010	0%	0%	0%
Djibouti	2016	0%	0%	0%
Dominica	1990	-7.84%	-11.30%	-3.41%
Dominica	1995	-8.15%	-12.47%	-3.36%
Dominica	2000	-8.73%	-13.25%	-3.62%
Dominica	2005	-9.37%	-14.80%	-3.92%
Dominica	2010	-9.09%	-13.98%	-3.85%
Dominica	2016	-8.84%	-14.30%	-3.87%
Dominican Republic	1990	-2.26%	-3.03%	-1.21%
Dominican Republic	1995	-1.97%	-2.64%	-1.06%
Dominican Republic	2000	-1.79%	-2.37%	-0.95%
Dominican Republic	2005	-1.78%	-2.40%	-0.94%
Dominican Republic	2010	-1.77%	-2.40%	-0.93%
Dominican Republic	2016	-1.74%	-2.52%	-0.92%
Ecuador	1990	-0.18%	-0.25%	-0.07%
Ecuador	1995	-0.16%	-0.24%	-0.07%
Ecuador	2000	-0.13%	-0.19%	-0.05%
Ecuador	2005	-0.11%	-0.17%	-0.05%
Ecuador	2010	-0.11%	-0.17%	-0.05%
Ecuador	2016	-0.11%	-0.17%	-0.04%
Egypt	1990	-3.82%	-5.87%	-1.77%
Egypt	1995	-3.82%	-6.18%	-1.68%
Egypt	2000	-3.92%	-6.26%	-1.66%
Egypt	2005	-3.81%	-6.24%	-1.66%
Egypt	2010	-3.85%	-6.28%	-1.65%
Egypt	2016	-3.92%	-7.48%	-1.56%
El Salvador	1990	-0.52%	-1.02%	-0.23%
El Salvador	1995	-0.51%	-1.01%	-0.20%
El Salvador	2000	-0.45%	-0.87%	-0.18%
El Salvador	2005	-0.40%	-0.79%	-0.15%

Percent change in alcohol consumption due to tourism, by location and 5-year interval

Location	Year	Mean	2.5 percentile	97.5 percentile
El Salvador	2010	-0.38%	-0.74%	-0.14%
El Salvador	2016	-0.37%	-0.73%	-0.15%
Equatorial Guinea	1990	0%	0%	0%
Equatorial Guinea	1995	0%	0%	0%
Equatorial Guinea	2000	0%	0%	0%
Equatorial Guinea	2005	0%	0%	0%
Equatorial Guinea	2010	0%	0%	0%
Equatorial Guinea	2016	0%	0%	0%
Eritrea	1990	-0.37%	-0.69%	-0.16%
Eritrea	1995	-0.27%	-0.51%	-0.11%
Eritrea	2000	-0.25%	-0.47%	-0.10%
Eritrea	2005	-0.30%	-0.56%	-0.13%
Eritrea	2010	-0.36%	-0.70%	-0.14%
Eritrea	2016	-0.33%	-0.64%	-0.12%
Estonia	1990	-2.43%	-5.53%	2.01%
Estonia	1995	-2.11%	-5.49%	2.40%
Estonia	2000	-1.73%	-4.14%	1.39%
Estonia	2005	-1.23%	-2.92%	1.41%
Estonia	2010	-1.10%	-2.81%	1.20%
Estonia	2016	-1.13%	-2.47%	0.91%
Ethiopia	1990	-0.02%	-0.04%	0.00%
Ethiopia	1995	-0.02%	-0.04%	0.00%
Ethiopia	2000	-0.02%	-0.04%	0%
Ethiopia	2005	-0.01%	-0.04%	0%
Ethiopia	2010	-0.02%	-0.04%	0.00%
Ethiopia	2016	-0.02%	-0.04%	0.00%
Federated States of Micronesia	1990	-1.95%	-4.13%	-0.55%
Federated States of Micronesia	1995	-1.86%	-3.88%	-0.50%
Federated States of Micronesia	2000	-1.99%	-3.90%	-0.58%
Federated States of Micronesia	2005	-2.26%	-4.51%	-0.66%
Federated States of Micronesia	2010	-2.51%	-5.14%	-0.72%
Federated States of Micronesia	2016	-2.57%	-5.51%	-0.73%
Fiji	1990	-8.31%	-11.15%	-6.61%
Fiji	1995	-8.45%	-11.62%	-6.60%
Fiji	2000	-8.51%	-11.78%	-6.54%
Fiji	2005	-7.92%	-10.98%	-6.20%
Fiji	2010	-6.97%	-9.87%	-5.25%
Fiji	2016	-6.43%	-9.84%	-4.29%
Finland	1990	3.22%	2.38%	4.03%
Finland	1995	3.16%	2.26%	4.21%
Finland	2000	3.13%	2.23%	4.09%
Finland	2005	2.92%	2.07%	3.85%
Finland	2010	2.99%	2.11%	3.92%
Finland	2016	3.25%	2.26%	4.34%
France	1990	-2.78%	-3.52%	-2.11%
France	1995	-3.11%	-4.01%	-2.33%

Percent change in alcohol consumption due to tourism, by location and 5-year interval

Location	Year	Mean	2.5 percentile	97.5 percentile
France	2000	-3.47%	-4.51%	-2.48%
France	2005	-3.67%	-4.78%	-2.66%
France	2010	-4.03%	-5.27%	-2.95%
France	2016	-4.14%	-5.67%	-2.93%
Gabon	1990	0%	0%	0%
Gabon	1995	0%	0%	0%
Gabon	2000	0%	0%	0%
Gabon	2005	0%	0%	0%
Gabon	2010	0%	0%	0%
Gabon	2016	0%	0%	0%
Georgia	1990	0.20%	-0.39%	0.71%
Georgia	1995	0.21%	-0.50%	0.77%
Georgia	2000	0.26%	-0.54%	0.85%
Georgia	2005	0.23%	-0.51%	0.76%
Georgia	2010	0.20%	-0.44%	0.67%
Georgia	2016	0.18%	-0.25%	0.61%
Germany	1990	4.38%	3.31%	5.48%
Germany	1995	4.63%	3.42%	5.93%
Germany	2000	4.93%	3.57%	6.28%
Germany	2005	5.11%	3.64%	6.60%
Germany	2010	5.39%	3.90%	7.14%
Germany	2016	5.68%	3.99%	7.88%
Ghana	1990	-0.15%	-0.22%	-0.09%
Ghana	1995	-0.16%	-0.24%	-0.09%
Ghana	2000	-0.18%	-0.26%	-0.10%
Ghana	2005	-0.17%	-0.27%	-0.09%
Ghana	2010	-0.15%	-0.22%	-0.08%
Ghana	2016	-0.13%	-0.23%	-0.06%
Greece	1990	-1.97%	-2.91%	-1.28%
Greece	1995	-2.12%	-3.24%	-1.36%
Greece	2000	-2.21%	-3.37%	-1.40%
Greece	2005	-2.30%	-3.69%	-1.41%
Greece	2010	-2.26%	-3.59%	-1.40%
Greece	2016	-2.35%	-3.75%	-1.35%
Greenland	1990	0%	0%	0%
Greenland	1995	0%	0%	0%
Greenland	2000	0%	0%	0%
Greenland	2005	0%	0%	0%
Greenland	2010	0%	0%	0%
Greenland	2016	0%	0%	0%
Grenada	1990	-8.65%	-12.63%	-6.34%
Grenada	1995	-8.59%	-13.26%	-5.92%
Grenada	2000	-9.10%	-13.74%	-6.19%
Grenada	2005	-9.46%	-14.54%	-6.17%
Grenada	2010	-10.00%	-15.40%	-6.54%
Grenada	2016	-9.85%	-14.95%	-6.58%

Percent change in alcohol consumption due to tourism, by location and 5-year interval

Location	Year	Mean	2.5 percentile	97.5 percentile
Guam	1990	-34.93%	-60.41%	-17.62%
Guam	1995	-34.44%	-61.83%	-16.97%
Guam	2000	-34.15%	-61.38%	-17.41%
Guam	2005	-34.07%	-62.77%	-17.33%
Guam	2010	-33.57%	-61.27%	-16.60%
Guam	2016	-32.81%	-59.11%	-15.80%
Guatemala	1990	-0.25%	-0.42%	-0.05%
Guatemala	1995	-0.29%	-0.50%	-0.05%
Guatemala	2000	-0.33%	-0.56%	-0.07%
Guatemala	2005	-0.34%	-0.57%	-0.07%
Guatemala	2010	-0.33%	-0.58%	-0.07%
Guatemala	2016	-0.31%	-0.54%	-0.07%
Guinea	1990	-0.10%	-0.17%	-0.06%
Guinea	1995	-0.09%	-0.17%	-0.05%
Guinea	2000	-0.11%	-0.18%	-0.05%
Guinea	2005	-0.12%	-0.21%	-0.05%
Guinea	2010	-0.12%	-0.22%	-0.06%
Guinea	2016	-0.12%	-0.20%	-0.06%
Guinea-Bissau	1990	-0.06%	-0.10%	-0.03%
Guinea-Bissau	1995	-0.06%	-0.13%	-0.02%
Guinea-Bissau	2000	-0.07%	-0.13%	-0.02%
Guinea-Bissau	2005	-0.07%	-0.13%	-0.02%
Guinea-Bissau	2010	-0.07%	-0.13%	-0.02%
Guinea-Bissau	2016	-0.07%	-0.14%	-0.03%
Guyana	1990	-0.04%	-0.31%	0.31%
Guyana	1995	-0.03%	-0.31%	0.30%
Guyana	2000	-0.03%	-0.32%	0.32%
Guyana	2005	-0.05%	-0.42%	0.40%
Guyana	2010	-0.06%	-0.49%	0.45%
Guyana	2016	-0.06%	-0.47%	0.44%
Haiti	1990	-0.35%	-0.61%	-0.13%
Haiti	1995	-0.36%	-0.66%	-0.13%
Haiti	2000	-0.36%	-0.65%	-0.13%
Haiti	2005	-0.36%	-0.66%	-0.13%
Haiti	2010	-0.34%	-0.62%	-0.12%
Haiti	2016	-0.33%	-0.61%	-0.11%
Honduras	1990	-0.78%	-1.56%	-0.18%
Honduras	1995	-0.73%	-1.47%	-0.17%
Honduras	2000	-0.65%	-1.30%	-0.15%
Honduras	2005	-0.59%	-1.24%	-0.13%
Honduras	2010	-0.59%	-1.23%	-0.15%
Honduras	2016	-0.58%	-1.20%	-0.14%
Hungary	1990	-5.45%	-7.38%	-3.40%
Hungary	1995	-5.89%	-8.10%	-3.57%
Hungary	2000	-6.23%	-8.59%	-3.77%
Hungary	2005	-6.20%	-8.47%	-3.82%

Percent change in alcohol consumption due to tourism, by location and 5-year interval

Location	Year	Mean	2.5 percentile	97.5 percentile
Hungary	2010	-7.12%	-9.93%	-4.27%
Hungary	2016	-7.72%	-11.27%	-4.51%
Iceland	1990	-0.71%	-4.33%	2.71%
Iceland	1995	-0.70%	-4.80%	3.12%
Iceland	2000	-0.76%	-4.48%	3.00%
Iceland	2005	-0.60%	-3.96%	2.51%
Iceland	2010	-0.64%	-4.05%	2.11%
Iceland	2016	-0.50%	-3.06%	1.94%
India	1990	-0.01%	-0.01%	0.01%
India	1995	-0.01%	-0.01%	0.01%
India	2000	0.00%	-0.01%	0.01%
India	2005	0.00%	-0.01%	0.01%
India	2010	0.00%	-0.01%	0.01%
India	2016	0.00%	-0.01%	0.01%
Indonesia	1990	-0.86%	-1.23%	-0.64%
Indonesia	1995	-0.80%	-1.16%	-0.59%
Indonesia	2000	-0.74%	-1.07%	-0.55%
Indonesia	2005	-0.71%	-1.04%	-0.52%
Indonesia	2010	-0.68%	-0.99%	-0.50%
Indonesia	2016	-0.61%	-0.92%	-0.42%
Iran	1990	-24.61%	-57.62%	-8.32%
Iran	1995	-19.02%	-44.29%	-6.37%
Iran	2000	-16.58%	-36.04%	-5.79%
Iran	2005	-14.29%	-28.91%	-5.27%
Iran	2010	-12.82%	-26.04%	-4.81%
Iran	2016	-13.01%	-26.37%	-4.82%
Iraq	1990	0.03%	-0.02%	0.10%
Iraq	1995	0.05%	-0.03%	0.15%
Iraq	2000	0.07%	-0.06%	0.21%
Iraq	2005	0.08%	-0.07%	0.26%
Iraq	2010	0.09%	-0.06%	0.30%
Iraq	2016	0.07%	-0.06%	0.24%
Ireland	1990	2.52%	-0.16%	5.53%
Ireland	1995	2.17%	-0.44%	5.26%
Ireland	2000	1.84%	-0.45%	4.40%
Ireland	2005	1.74%	-0.30%	4.16%
Ireland	2010	1.85%	-0.42%	4.53%
Ireland	2016	1.97%	-0.22%	4.59%
Israel	1990	1.51%	-1.71%	5.42%
Israel	1995	1.63%	-2.08%	6.05%
Israel	2000	1.52%	-1.92%	5.38%
Israel	2005	1.28%	-1.67%	4.86%
Israel	2010	1.25%	-1.47%	4.60%
Israel	2016	1.14%	-1.34%	4.19%
Italy	1990	-0.66%	-1.39%	0.56%
Italy	1995	-0.73%	-1.62%	0.78%

Percent change in alcohol consumption due to tourism, by location and 5-year interval

Location	Year	Mean	2.5 percentile	97.5 percentile
Italy	2000	-0.86%	-1.88%	0.86%
Italy	2005	-0.98%	-2.16%	0.95%
Italy	2010	-1.03%	-2.21%	1.06%
Italy	2016	-1.08%	-2.39%	0.88%
Jamaica	1990	-6.32%	-8.92%	-2.97%
Jamaica	1995	-6.67%	-9.67%	-3.16%
Jamaica	2000	-7.06%	-10.12%	-3.38%
Jamaica	2005	-6.72%	-9.63%	-3.12%
Jamaica	2010	-6.10%	-8.93%	-2.81%
Jamaica	2016	-5.75%	-8.94%	-2.51%
Japan	1990	0.61%	0.33%	0.95%
Japan	1995	0.60%	0.32%	0.96%
Japan	2000	0.62%	0.34%	0.97%
Japan	2005	0.66%	0.35%	1.05%
Japan	2010	0.68%	0.36%	1.07%
Japan	2016	0.68%	0.36%	1.13%
Jordan	1990	-18.92%	-26.67%	-12.52%
Jordan	1995	-19.11%	-28.11%	-12.33%
Jordan	2000	-13.19%	-19.46%	-8.58%
Jordan	2005	-9.86%	-14.30%	-6.37%
Jordan	2010	-9.61%	-14%	-6.02%
Jordan	2016	-9.95%	-16.87%	-5.56%
Kazakhstan	1990	0.18%	-0.08%	0.52%
Kazakhstan	1995	0.20%	-0.10%	0.60%
Kazakhstan	2000	0.19%	-0.09%	0.59%
Kazakhstan	2005	0.17%	-0.11%	0.58%
Kazakhstan	2010	0.19%	-0.12%	0.57%
Kazakhstan	2016	0.17%	-0.08%	0.50%
Kenya	1990	-0.19%	-0.22%	-0.15%
Kenya	1995	-0.21%	-0.26%	-0.16%
Kenya	2000	-0.23%	-0.29%	-0.17%
Kenya	2005	-0.24%	-0.31%	-0.18%
Kenya	2010	-0.24%	-0.32%	-0.18%
Kenya	2016	-0.23%	-0.35%	-0.14%
Kiribati	1990	-5.61%	-8.78%	-2.82%
Kiribati	1995	-5.53%	-10.40%	-2.53%
Kiribati	2000	-5.48%	-9.22%	-2.42%
Kiribati	2005	-5.36%	-10.00%	-2.48%
Kiribati	2010	-5.67%	-10.11%	-2.68%
Kiribati	2016	-5.41%	-8.44%	-2.82%
Kuwait	1990	-62.51%	-96.34%	-31.93%
Kuwait	1995	-60.86%	-96.01%	-35.18%
Kuwait	2000	-60.41%	-96.80%	-38.95%
Kuwait	2005	-62.48%	-98.80%	-33.89%
Kuwait	2010	-59.63%	-92.73%	-27.23%
Kuwait	2016	-51.28%	-91.33%	-20.90%

Percent change in alcohol consumption due to tourism, by location and 5-year interval

Location	Year	Mean	2.5 percentile	97.5 percentile
Kyrgyzstan	1990	0.20%	-0.20%	0.66%
Kyrgyzstan	1995	0.24%	-0.29%	0.76%
Kyrgyzstan	2000	0.23%	-0.41%	0.78%
Kyrgyzstan	2005	0.24%	-0.41%	0.78%
Kyrgyzstan	2010	0.21%	-0.57%	0.77%
Kyrgyzstan	2016	0.24%	-0.24%	0.79%
Laos	1990	-0.77%	-1.71%	-0.18%
Laos	1995	-0.78%	-1.91%	-0.18%
Laos	2000	-0.78%	-1.85%	-0.17%
Laos	2005	-0.84%	-1.96%	-0.19%
Laos	2010	-0.83%	-2.05%	-0.19%
Laos	2016	-0.78%	-1.80%	-0.18%
Latvia	1990	-1.42%	-3.40%	0.13%
Latvia	1995	-1.56%	-4.16%	0.30%
Latvia	2000	-1.43%	-4.20%	0.28%
Latvia	2005	-1.30%	-3.49%	0.23%
Latvia	2010	-1.16%	-3.34%	0.15%
Latvia	2016	-1.15%	-2.99%	0.11%
Lebanon	1990	-0.18%	-0.47%	0.28%
Lebanon	1995	-0.21%	-0.64%	0.31%
Lebanon	2000	-0.26%	-0.81%	0.39%
Lebanon	2005	-0.26%	-0.78%	0.41%
Lebanon	2010	-0.27%	-0.81%	0.36%
Lebanon	2016	-0.25%	-0.74%	0.31%
Lesotho	1990	1.78%	0.77%	3.11%
Lesotho	1995	1.77%	0.70%	3.40%
Lesotho	2000	1.70%	0.74%	3.34%
Lesotho	2005	1.54%	0.62%	2.83%
Lesotho	2010	1.51%	0.61%	2.78%
Lesotho	2016	1.48%	0.58%	2.88%
Liberia	1990	0%	0%	0%
Liberia	1995	0%	0%	0%
Liberia	2000	0%	0%	0%
Liberia	2005	0%	0%	0%
Liberia	2010	0%	0%	0%
Liberia	2016	0%	0%	0%
Libya	1990	-15.38%	-47.41%	-6.81%
Libya	1995	-15.53%	-47.06%	-7.42%
Libya	2000	-13.05%	-42.05%	-6.68%
Libya	2005	-9.71%	-29.63%	-4.90%
Libya	2010	-8.70%	-27.71%	-4.17%
Libya	2016	-8.11%	-25.34%	-3.78%
Lithuania	1990	-0.17%	-1.87%	1.98%
Lithuania	1995	-0.21%	-2.36%	2.19%
Lithuania	2000	-0.17%	-1.88%	1.91%
Lithuania	2005	-0.16%	-1.55%	1.54%

Percent change in alcohol consumption due to tourism, by location and 5-year interval

Location	Year	Mean	2.5 percentile	97.5 percentile
Lithuania	2010	-0.16%	-1.48%	1.39%
Lithuania	2016	-0.13%	-1.26%	1.33%
Luxembourg	1990	8.53%	1.77%	16.76%
Luxembourg	1995	8.75%	1.68%	18.64%
Luxembourg	2000	7.42%	1.50%	15.73%
Luxembourg	2005	5.96%	1.05%	13.04%
Luxembourg	2010	5.38%	1.01%	11.55%
Luxembourg	2016	5.52%	1.02%	11.95%
Macedonia	1990	-4.82%	-10.44%	-0.95%
Macedonia	1995	-4.67%	-9.85%	-0.78%
Macedonia	2000	-5.61%	-11.84%	-1.30%
Macedonia	2005	-5.81%	-12.57%	-0.82%
Macedonia	2010	-7.29%	-15.83%	-1.78%
Macedonia	2016	-6.84%	-15.15%	-1.71%
Madagascar	1990	-0.11%	-0.14%	-0.08%
Madagascar	1995	-0.12%	-0.17%	-0.09%
Madagascar	2000	-0.16%	-0.22%	-0.11%
Madagascar	2005	-0.20%	-0.27%	-0.14%
Madagascar	2010	-0.19%	-0.26%	-0.13%
Madagascar	2016	-0.16%	-0.25%	-0.09%
Malawi	1990	-0.08%	-0.19%	-0.02%
Malawi	1995	-0.08%	-0.20%	-0.01%
Malawi	2000	-0.09%	-0.21%	-0.01%
Malawi	2005	-0.08%	-0.20%	-0.01%
Malawi	2010	-0.07%	-0.19%	0%
Malawi	2016	-0.08%	-0.20%	-0.01%
Malaysia	1990	-4.02%	-5.72%	-2.47%
Malaysia	1995	-4.98%	-7.39%	-2.98%
Malaysia	2000	-7.36%	-10.89%	-4.28%
Malaysia	2005	-8.43%	-12.51%	-4.97%
Malaysia	2010	-7.46%	-11.86%	-4.37%
Malaysia	2016	-6.21%	-9.48%	-3.46%
Maldives	1990	-38.86%	-61.33%	-26.39%
Maldives	1995	-30.61%	-49.32%	-21.41%
Maldives	2000	-24.75%	-38.95%	-17.47%
Maldives	2005	-22.91%	-36.29%	-16.03%
Maldives	2010	-22.94%	-37.22%	-15.10%
Maldives	2016	-23.15%	-40.14%	-13.65%
Mali	1990	-0.18%	-0.24%	-0.12%
Mali	1995	-0.18%	-0.25%	-0.12%
Mali	2000	-0.19%	-0.26%	-0.12%
Mali	2005	-0.19%	-0.26%	-0.12%
Mali	2010	-0.18%	-0.26%	-0.11%
Mali	2016	-0.18%	-0.31%	-0.09%
Malta	1990	-13.06%	-16.93%	-10.45%
Malta	1995	-12.48%	-16.19%	-9.47%

Percent change in alcohol consumption due to tourism, by location and 5-year interval

Location	Year	Mean	2.5 percentile	97.5 percentile
Malta	2000	-12.62%	-16.91%	-9.40%
Malta	2005	-12.57%	-15.95%	-9.79%
Malta	2010	-11.78%	-15.81%	-8.86%
Malta	2016	-10.92%	-14.97%	-7.73%
Marshall Islands	1990	-0.56%	-1.25%	0.05%
Marshall Islands	1995	-0.58%	-1.35%	0.12%
Marshall Islands	2000	-0.56%	-1.34%	0.14%
Marshall Islands	2005	-0.56%	-1.34%	0.09%
Marshall Islands	2010	-0.51%	-1.24%	0.14%
Marshall Islands	2016	-0.50%	-1.07%	0.02%
Mauritania	1990	0%	0%	0%
Mauritania	1995	0%	0%	0%
Mauritania	2000	0%	0%	0%
Mauritania	2005	0%	0%	0%
Mauritania	2010	0%	0%	0%
Mauritania	2016	0%	0%	0%
Mauritius	1990	-3.09%	-4.05%	-2.21%
Mauritius	1995	-2.94%	-3.96%	-2.07%
Mauritius	2000	-2.92%	-3.89%	-2.05%
Mauritius	2005	-3.04%	-4.06%	-2.11%
Mauritius	2010	-3.22%	-4.47%	-2.18%
Mauritius	2016	-3.28%	-4.93%	-2.04%
Mexico	1990	-4.14%	-5.48%	-1.62%
Mexico	1995	-3.86%	-5.46%	-1.43%
Mexico	2000	-3.76%	-5.16%	-1.34%
Mexico	2005	-3.89%	-5.25%	-1.39%
Mexico	2010	-4.21%	-5.93%	-1.50%
Mexico	2016	-4.34%	-6.19%	-1.58%
Moldova	1990	2.66%	1.43%	4.71%
Moldova	1995	2.70%	1.44%	4.94%
Moldova	2000	3.03%	1.71%	5.36%
Moldova	2005	3.54%	1.88%	6.54%
Moldova	2010	3.72%	2.08%	6.75%
Moldova	2016	3.66%	2.09%	6.28%
Mongolia	1990	0.47%	0.15%	1.26%
Mongolia	1995	0.49%	0.13%	1.41%
Mongolia	2000	0.43%	0.10%	1.33%
Mongolia	2005	0.38%	0.09%	1.07%
Mongolia	2010	0.31%	0.08%	0.94%
Mongolia	2016	0.27%	0.09%	0.75%
Montenegro	1990	-0.94%	-5.74%	1.58%
Montenegro	1995	-0.95%	-6.10%	1.82%
Montenegro	2000	-0.98%	-5.84%	1.42%
Montenegro	2005	-0.91%	-5.25%	1.45%
Montenegro	2010	-0.90%	-5.06%	1.39%
Montenegro	2016	-0.76%	-4.58%	1.28%

Percent change in alcohol consumption due to tourism, by location and 5-year interval

Location	Year	Mean	2.5 percentile	97.5 percentile
Morocco	1990	-4.71%	-7.03%	-2.66%
Morocco	1995	-4.62%	-6.96%	-2.58%
Morocco	2000	-4.55%	-6.92%	-2.54%
Morocco	2005	-4.76%	-7.21%	-2.63%
Morocco	2010	-6.11%	-9.49%	-3.36%
Morocco	2016	-7.11%	-12.02%	-3.50%
Mozambique	1990	-0.94%	-1.83%	-0.39%
Mozambique	1995	-0.71%	-1.44%	-0.27%
Mozambique	2000	-0.48%	-0.99%	-0.17%
Mozambique	2005	-0.41%	-0.81%	-0.15%
Mozambique	2010	-0.42%	-0.88%	-0.13%
Mozambique	2016	-0.42%	-0.92%	-0.14%
Myanmar	1990	-0.65%	-1.34%	-0.37%
Myanmar	1995	-0.59%	-1.24%	-0.32%
Myanmar	2000	-0.49%	-1.04%	-0.27%
Myanmar	2005	-0.45%	-0.97%	-0.24%
Myanmar	2010	-0.40%	-0.87%	-0.23%
Myanmar	2016	-0.34%	-0.71%	-0.18%
Namibia	1990	-7.58%	-11.67%	-4.51%
Namibia	1995	-3.74%	-5.77%	-2.19%
Namibia	2000	-1.60%	-2.37%	-1.03%
Namibia	2005	-0.93%	-1.39%	-0.56%
Namibia	2010	-0.76%	-1.21%	-0.42%
Namibia	2016	-0.76%	-1.32%	-0.41%
Nepal	1990	-0.75%	-1.06%	-0.49%
Nepal	1995	-0.38%	-0.57%	-0.24%
Nepal	2000	-0.20%	-0.29%	-0.13%
Nepal	2005	-0.15%	-0.22%	-0.10%
Nepal	2010	-0.15%	-0.23%	-0.09%
Nepal	2016	-0.16%	-0.26%	-0.09%
Netherlands	1990	5.07%	3.99%	6.15%
Netherlands	1995	5.16%	3.93%	6.51%
Netherlands	2000	5.22%	3.99%	6.39%
Netherlands	2005	5.27%	3.98%	6.59%
Netherlands	2010	5.39%	3.99%	6.72%
Netherlands	2016	5.41%	3.86%	7.13%
New Zealand	1990	2.22%	1.51%	3.17%
New Zealand	1995	2.43%	1.63%	3.65%
New Zealand	2000	2.67%	1.71%	4.12%
New Zealand	2005	2.52%	1.63%	3.94%
New Zealand	2010	2.45%	1.55%	3.85%
New Zealand	2016	2.47%	1.51%	3.83%
Nicaragua	1990	-0.44%	-0.80%	-0.18%
Nicaragua	1995	-0.46%	-0.88%	-0.17%
Nicaragua	2000	-0.44%	-0.80%	-0.16%
Nicaragua	2005	-0.41%	-0.77%	-0.16%

Percent change in alcohol consumption due to tourism, by location and 5-year interval

Location	Year	Mean	2.5 percentile	97.5 percentile
Nicaragua	2010	-0.43%	-0.81%	-0.16%
Nicaragua	2016	-0.43%	-0.82%	-0.16%
Niger	1990	-0.48%	-0.73%	-0.30%
Niger	1995	-0.50%	-0.79%	-0.29%
Niger	2000	-0.40%	-0.67%	-0.21%
Niger	2005	-0.36%	-0.57%	-0.20%
Niger	2010	-0.39%	-0.64%	-0.21%
Niger	2016	-0.41%	-0.72%	-0.21%
Nigeria	1990	-0.01%	-0.05%	0.00%
Nigeria	1995	-0.01%	-0.04%	0.00%
Nigeria	2000	-0.01%	-0.04%	0.00%
Nigeria	2005	-0.01%	-0.04%	0.00%
Nigeria	2010	-0.01%	-0.05%	0.00%
Nigeria	2016	-0.02%	-0.06%	0.00%
North Korea	1990	0%	0%	0%
North Korea	1995	0%	0%	0%
North Korea	2000	0%	0%	0%
North Korea	2005	0%	0%	0%
North Korea	2010	0%	0%	0%
North Korea	2016	0%	0%	0%
Northern Mariana Islands	1990	-30.47%	-63.29%	-12.53%
Northern Mariana Islands	1995	-29.86%	-65.22%	-11.67%
Northern Mariana Islands	2000	-29.06%	-64.00%	-11.60%
Northern Mariana Islands	2005	-28.42%	-60.77%	-11.49%
Northern Mariana Islands	2010	-28.46%	-60.71%	-11.28%
Northern Mariana Islands	2016	-28.25%	-60.19%	-11.71%
Norway	1990	4.80%	0.52%	9.38%
Norway	1995	4.70%	0.23%	9.68%
Norway	2000	4.33%	0.11%	9.13%
Norway	2005	4.01%	0.08%	8.30%
Norway	2010	3.81%	0.07%	7.98%
Norway	2016	4.02%	0.37%	8.08%
Oman	1990	0%	0%	0%
Oman	1995	0%	0%	0%
Oman	2000	0%	0%	0%
Oman	2005	0%	0%	0%
Oman	2010	0%	0%	0%
Oman	2016	0%	0%	0%
Pakistan	1990	-1.04%	-1.68%	-0.53%
Pakistan	1995	-0.96%	-1.66%	-0.46%
Pakistan	2000	-0.94%	-1.51%	-0.45%
Pakistan	2005	-1.10%	-1.80%	-0.53%
Pakistan	2010	-1.43%	-2.50%	-0.66%
Pakistan	2016	-1.51%	-3.09%	-0.60%
Palestine	1990	-43.78%	-82.89%	-16.32%
Palestine	1995	-36.75%	-69.13%	-14.28%

Percent change in alcohol consumption due to tourism, by location and 5-year interval

Location	Year	Mean	2.5 percentile	97.5 percentile
Palestine	2000	-20.86%	-36.36%	-9.02%
Palestine	2005	-15.61%	-27.44%	-6.90%
Palestine	2010	-18.34%	-32.00%	-7.60%
Palestine	2016	-20.57%	-38.38%	-8.51%
Panama	1990	-1.04%	-1.89%	-0.29%
Panama	1995	-1%	-1.95%	-0.25%
Panama	2000	-0.98%	-1.87%	-0.25%
Panama	2005	-0.91%	-1.78%	-0.23%
Panama	2010	-0.87%	-1.62%	-0.22%
Panama	2016	-0.80%	-1.54%	-0.22%
Papua New Guinea	1990	-0.11%	-0.16%	-0.05%
Papua New Guinea	1995	-0.10%	-0.15%	-0.04%
Papua New Guinea	2000	-0.11%	-0.16%	-0.04%
Papua New Guinea	2005	-0.12%	-0.19%	-0.04%
Papua New Guinea	2010	-0.13%	-0.20%	-0.05%
Papua New Guinea	2016	-0.13%	-0.22%	-0.06%
Paraguay	1990	-1.87%	-4.43%	-0.76%
Paraguay	1995	-1.73%	-4.29%	-0.65%
Paraguay	2000	-1.87%	-4.59%	-0.69%
Paraguay	2005	-2.22%	-5.51%	-0.82%
Paraguay	2010	-2.37%	-5.75%	-0.86%
Paraguay	2016	-2.31%	-5.65%	-0.92%
Peru	1990	-0.15%	-0.27%	-0.04%
Peru	1995	-0.17%	-0.30%	-0.03%
Peru	2000	-0.17%	-0.30%	-0.03%
Peru	2005	-0.18%	-0.32%	-0.04%
Peru	2010	-0.18%	-0.33%	-0.04%
Peru	2016	-0.19%	-0.35%	-0.05%
Philippines	1990	-0.02%	-0.06%	0.03%
Philippines	1995	-0.02%	-0.07%	0.03%
Philippines	2000	-0.02%	-0.06%	0.03%
Philippines	2005	-0.02%	-0.06%	0.03%
Philippines	2010	-0.02%	-0.06%	0.03%
Philippines	2016	-0.02%	-0.06%	0.03%
Poland	1990	-4.89%	-7.61%	-3.05%
Poland	1995	-4.84%	-7.97%	-2.58%
Poland	2000	-4.65%	-7.54%	-2.64%
Poland	2005	-4.27%	-7.03%	-2.31%
Poland	2010	-4.21%	-6.62%	-2.45%
Poland	2016	-4.17%	-6.65%	-2.49%
Portugal	1990	-1.90%	-3.11%	-0.66%
Portugal	1995	-2.07%	-3.43%	-0.63%
Portugal	2000	-2.26%	-3.72%	-0.66%
Portugal	2005	-2.50%	-4.03%	-0.63%
Portugal	2010	-2.54%	-4.44%	-0.62%
Portugal	2016	-2.60%	-4.76%	-0.87%

Percent change in alcohol consumption due to tourism, by location and 5-year interval

Location	Year	Mean	2.5 percentile	97.5 percentile
Puerto Rico	1990	-5.80%	-9.33%	-2.67%
Puerto Rico	1995	-5.66%	-9.38%	-2.54%
Puerto Rico	2000	-5.55%	-9.04%	-2.47%
Puerto Rico	2005	-5.48%	-8.90%	-2.31%
Puerto Rico	2010	-5.35%	-8.72%	-2.29%
Puerto Rico	2016	-5.32%	-8.69%	-2.41%
Qatar	1990	0.75%	-0.16%	2.43%
Qatar	1995	0.99%	-0.33%	3.36%
Qatar	2000	1.21%	-0.23%	3.67%
Qatar	2005	0.87%	-0.20%	2.93%
Qatar	2010	0.72%	-0.19%	2.38%
Qatar	2016	0.69%	-0.07%	2.10%
Romania	1990	0.38%	-0.01%	0.98%
Romania	1995	0.42%	-0.02%	1.10%
Romania	2000	0.46%	-0.02%	1.24%
Romania	2005	0.48%	-0.02%	1.29%
Romania	2010	0.44%	-0.03%	1.18%
Romania	2016	0.43%	-0.01%	1.24%
Russia	1990	0.41%	-0.06%	1.36%
Russia	1995	0.38%	-0.08%	1.31%
Russia	2000	0.34%	-0.07%	1.16%
Russia	2005	0.32%	-0.05%	1.07%
Russia	2010	0.27%	-0.05%	0.95%
Russia	2016	0.28%	-0.05%	0.97%
Rwanda	1990	-0.07%	-0.10%	-0.04%
Rwanda	1995	-0.07%	-0.13%	-0.03%
Rwanda	2000	-0.08%	-0.13%	-0.03%
Rwanda	2005	-0.08%	-0.14%	-0.04%
Rwanda	2010	-0.08%	-0.15%	-0.03%
Rwanda	2016	-0.09%	-0.16%	-0.04%
Saint Lucia	1990	-10.44%	-15.58%	-5.16%
Saint Lucia	1995	-9.39%	-14.01%	-4.42%
Saint Lucia	2000	-9.34%	-14.03%	-4.43%
Saint Lucia	2005	-9.74%	-14.37%	-4.66%
Saint Lucia	2010	-11.09%	-16.45%	-5.44%
Saint Lucia	2016	-11.67%	-17.84%	-5.44%
Saint Vincent and the Grenadines	1990	-7.89%	-9.81%	-5.81%
Saint Vincent and the Grenadines	1995	-8.20%	-10.90%	-5.55%
Saint Vincent and the Grenadines	2000	-8.12%	-10.51%	-5.43%
Saint Vincent and the Grenadines	2005	-7.79%	-10.23%	-5.12%
Saint Vincent and the Grenadines	2010	-7.28%	-9.90%	-4.63%
Saint Vincent and the Grenadines	2016	-7.17%	-10.04%	-4.83%
Samoa	1990	-6.41%	-9.10%	-3.29%
Samoa	1995	-6.09%	-8.72%	-2.34%
Samoa	2000	-5.95%	-8.75%	-2.42%
Samoa	2005	-5.57%	-8.19%	-2.45%

Percent change in alcohol consumption due to tourism, by location and 5-year interval

Location	Year	Mean	2.5 percentile	97.5 percentile
Samoa	2010	-5.65%	-8.37%	-2.67%
Samoa	2016	-5.67%	-9.38%	-2.63%
Sao Tome and Principe	1990	-0.35%	-0.60%	-0.19%
Sao Tome and Principe	1995	-0.26%	-0.47%	-0.12%
Sao Tome and Principe	2000	-0.21%	-0.39%	-0.10%
Sao Tome and Principe	2005	-0.20%	-0.36%	-0.10%
Sao Tome and Principe	2010	-0.22%	-0.39%	-0.11%
Sao Tome and Principe	2016	-0.23%	-0.47%	-0.11%
Saudi Arabia	1990	-5.21%	-8.68%	-2.49%
Saudi Arabia	1995	-6.64%	-11.16%	-2.76%
Saudi Arabia	2000	-8.32%	-14.34%	-3.76%
Saudi Arabia	2005	-8.28%	-14.57%	-3.49%
Saudi Arabia	2010	-7.84%	-14.17%	-3.13%
Saudi Arabia	2016	-6.76%	-13.73%	-2.52%
Senegal	1990	-4.66%	-5.83%	-3.27%
Senegal	1995	-5.64%	-7.44%	-3.74%
Senegal	2000	-6.33%	-8.31%	-4.19%
Senegal	2005	-6.88%	-9.13%	-4.71%
Senegal	2010	-7.70%	-10.31%	-4.81%
Senegal	2016	-7.63%	-11.84%	-4.53%
Serbia	1990	1.68%	0.85%	3.19%
Serbia	1995	1.68%	0.88%	3.03%
Serbia	2000	1.56%	0.66%	3.33%
Serbia	2005	1.43%	0.77%	2.55%
Serbia	2010	1.31%	0.57%	2.68%
Serbia	2016	1.26%	0.61%	2.44%
Seychelles	1990	-10.96%	-14.85%	-8.57%
Seychelles	1995	-9.08%	-12.64%	-6.75%
Seychelles	2000	-7.23%	-9.97%	-5.60%
Seychelles	2005	-6.68%	-9.16%	-5.25%
Seychelles	2010	-7.39%	-10.51%	-5.31%
Seychelles	2016	-7.88%	-12.36%	-4.99%
Sierra Leone	1990	-0.03%	-0.08%	-0.01%
Sierra Leone	1995	-0.03%	-0.08%	0.01%
Sierra Leone	2000	-0.03%	-0.08%	0.00%
Sierra Leone	2005	-0.03%	-0.08%	0%
Sierra Leone	2010	-0.03%	-0.08%	0.00%
Sierra Leone	2016	-0.04%	-0.10%	-0.01%
Singapore	1990	-5.02%	-10.31%	-0.47%
Singapore	1995	-4.22%	-10.20%	0.46%
Singapore	2000	-4.37%	-9.09%	-0.07%
Singapore	2005	-4.15%	-11.11%	0.60%
Singapore	2010	-5.24%	-11.11%	-0.39%
Singapore	2016	-4.97%	-10.54%	-0.77%
Slovakia	1990	-3.67%	-5.00%	-1.98%
Slovakia	1995	-3.91%	-5.92%	-1.59%

Percent change in alcohol consumption due to tourism, by location and 5-year interval

Location	Year	Mean	2.5 percentile	97.5 percentile
Slovakia	2000	-4.07%	-6.10%	-1.76%
Slovakia	2005	-4.07%	-6.07%	-1.03%
Slovakia	2010	-4.42%	-6.44%	-1.63%
Slovakia	2016	-4.48%	-6.48%	-2.37%
Slovenia	1990	-54.66%	-80.83%	-36.15%
Slovenia	1995	-61.54%	-92.74%	-33.91%
Slovenia	2000	-64.58%	-94.66%	-36.12%
Slovenia	2005	-67.28%	-97.42%	-42.75%
Slovenia	2010	-68.27%	-94.22%	-43.82%
Slovenia	2016	-69.98%	-95.83%	-48.99%
Solomon Islands	1990	-0.76%	-1.28%	-0.45%
Solomon Islands	1995	-0.85%	-1.44%	-0.51%
Solomon Islands	2000	-0.77%	-1.38%	-0.43%
Solomon Islands	2005	-0.62%	-1.03%	-0.36%
Solomon Islands	2010	-0.50%	-0.86%	-0.29%
Solomon Islands	2016	-0.45%	-0.75%	-0.24%
Somalia	1990	0%	0%	0%
Somalia	1995	0%	0%	0%
Somalia	2000	0%	0%	0%
Somalia	2005	0%	0%	0%
Somalia	2010	0%	0%	0%
Somalia	2016	0%	0%	0%
South Africa	1990	0.02%	-0.04%	0.07%
South Africa	1995	0.02%	-0.05%	0.08%
South Africa	2000	0.02%	-0.06%	0.08%
South Africa	2005	0.02%	-0.05%	0.08%
South Africa	2010	0.02%	-0.05%	0.07%
South Africa	2016	0.02%	-0.04%	0.08%
South Korea	1990	0.05%	-0.16%	0.26%
South Korea	1995	-0.07%	-0.29%	0.07%
South Korea	2000	0.26%	0.06%	0.56%
South Korea	2005	-0.07%	-0.28%	0.06%
South Korea	2010	0.28%	0.06%	0.61%
South Korea	2016	0.17%	-0.03%	0.45%
South Sudan	1990	0%	0%	0%
South Sudan	1995	0%	0%	0%
South Sudan	2000	0%	0%	0%
South Sudan	2005	0%	0%	0%
South Sudan	2010	0%	0%	0%
South Sudan	2016	0%	0%	0%
Spain	1990	-1.14%	-1.71%	-0.57%
Spain	1995	-1.28%	-2.04%	-0.55%
Spain	2000	-1.38%	-2.20%	-0.57%
Spain	2005	-1.36%	-2.26%	-0.54%
Spain	2010	-1.43%	-2.51%	-0.57%
Spain	2016	-1.48%	-2.39%	-0.67%

Percent change in alcohol consumption due to tourism, by location and 5-year interval

Location	Year	Mean	2.5 percentile	97.5 percentile
Sri Lanka	1990	-0.62%	-1.09%	-0.44%
Sri Lanka	1995	-0.62%	-1.21%	-0.40%
Sri Lanka	2000	-0.46%	-0.91%	-0.29%
Sri Lanka	2005	-0.30%	-0.56%	-0.18%
Sri Lanka	2010	-0.21%	-0.38%	-0.13%
Sri Lanka	2016	-0.18%	-0.33%	-0.11%
Sudan	1990	-0.01%	-0.09%	0.04%
Sudan	1995	-0.01%	-0.08%	0.03%
Sudan	2000	-0.01%	-0.08%	0.03%
Sudan	2005	-0.01%	-0.08%	0.03%
Sudan	2010	-0.01%	-0.07%	0.03%
Sudan	2016	-0.01%	-0.07%	0.03%
Suriname	1990	-0.58%	-1.02%	-0.29%
Suriname	1995	-0.62%	-1.14%	-0.28%
Suriname	2000	-0.55%	-1.03%	-0.24%
Suriname	2005	-0.47%	-0.90%	-0.22%
Suriname	2010	-0.43%	-0.82%	-0.18%
Suriname	2016	-0.41%	-0.78%	-0.19%
Swaziland	1990	-2.88%	-4.46%	-1.82%
Swaziland	1995	-2.84%	-4.53%	-1.56%
Swaziland	2000	-2.86%	-4.64%	-1.61%
Swaziland	2005	-3.21%	-4.95%	-1.79%
Swaziland	2010	-3.92%	-6.27%	-2.11%
Swaziland	2016	-4.06%	-7.42%	-2.07%
Sweden	1990	0.01%	-3.39%	2.38%
Sweden	1995	0.05%	-3.68%	2.74%
Sweden	2000	-0.08%	-3.32%	2.49%
Sweden	2005	0.04%	-3.45%	2.28%
Sweden	2010	0.05%	-3.28%	2.95%
Sweden	2016	-0.12%	-3.34%	1.84%
Switzerland	1990	5.60%	4.55%	7.23%
Switzerland	1995	6.15%	4.77%	8.19%
Switzerland	2000	6.62%	5.27%	8.94%
Switzerland	2005	6.74%	5.24%	9.34%
Switzerland	2010	7.04%	5.42%	9.56%
Switzerland	2016	7.29%	5.19%	10.35%
Syria	1990	-2.74%	-5.72%	-1.58%
Syria	1995	-2.53%	-5.42%	-1.43%
Syria	2000	-2.30%	-5.14%	-1.26%
Syria	2005	-2.20%	-4.82%	-1.20%
Syria	2010	-2.24%	-4.74%	-1.16%
Syria	2016	-2.46%	-5.67%	-1.08%
Taiwan	1990	1.07%	0.45%	1.93%
Taiwan	1995	1.03%	0.21%	2.49%
Taiwan	2000	0.81%	0.12%	1.94%
Taiwan	2005	0.84%	0.18%	1.98%

Percent change in alcohol consumption due to tourism, by location and 5-year interval

Location	Year	Mean	2.5 percentile	97.5 percentile
Taiwan	2010	0.90%	0.25%	2.19%
Taiwan	2016	0.84%	0.33%	1.73%
Tajikistan	1990	0.24%	0.10%	0.40%
Tajikistan	1995	0.24%	0.09%	0.40%
Tajikistan	2000	0.29%	0.11%	0.47%
Tajikistan	2005	0.32%	0.12%	0.52%
Tajikistan	2010	0.35%	0.14%	0.58%
Tajikistan	2016	0.37%	0.14%	0.61%
Tanzania	1990	-0.02%	-0.03%	-0.01%
Tanzania	1995	-0.03%	-0.04%	-0.01%
Tanzania	2000	-0.02%	-0.04%	-0.01%
Tanzania	2005	-0.03%	-0.04%	-0.01%
Tanzania	2010	-0.03%	-0.05%	-0.01%
Tanzania	2016	-0.03%	-0.05%	-0.01%
Thailand	1990	-0.34%	-0.56%	-0.16%
Thailand	1995	-0.27%	-0.59%	-0.03%
Thailand	2000	-0.30%	-0.67%	-0.09%
Thailand	2005	-0.22%	-0.40%	-0.06%
Thailand	2010	-0.28%	-0.65%	-0.04%
Thailand	2016	-0.25%	-0.42%	-0.12%
The Bahamas	1990	-33.28%	-46.46%	-14.05%
The Bahamas	1995	-37.51%	-53.28%	-15.78%
The Bahamas	2000	-41.59%	-56.96%	-18.12%
The Bahamas	2005	-44.31%	-63.00%	-18.26%
The Bahamas	2010	-45.85%	-65.64%	-18.77%
The Bahamas	2016	-46.71%	-67.71%	-18.99%
The Gambia	1990	-4.42%	-6.37%	-2.97%
The Gambia	1995	-4.25%	-6.42%	-2.47%
The Gambia	2000	-3.98%	-6.36%	-2.05%
The Gambia	2005	-3.52%	-5.51%	-1.98%
The Gambia	2010	-3.76%	-6.49%	-2.04%
The Gambia	2016	-4.22%	-7.69%	-2.13%
Timor-Leste	1990	-2.23%	-3.97%	-1.17%
Timor-Leste	1995	-1.85%	-3.44%	-0.83%
Timor-Leste	2000	-1.61%	-3.04%	-0.74%
Timor-Leste	2005	-1.47%	-2.91%	-0.61%
Timor-Leste	2010	-1.46%	-2.96%	-0.56%
Timor-Leste	2016	-1.42%	-3.09%	-0.59%
Togo	1990	-0.10%	-0.18%	-0.04%
Togo	1995	-0.13%	-0.27%	-0.05%
Togo	2000	-0.16%	-0.34%	-0.06%
Togo	2005	-0.16%	-0.33%	-0.05%
Togo	2010	-0.16%	-0.32%	-0.06%
Togo	2016	-0.15%	-0.31%	-0.06%
Tonga	1990	-21.61%	-32.78%	-11.21%
Tonga	1995	-18.07%	-27.04%	-9.50%

Percent change in alcohol consumption due to tourism, by location and 5-year interval

Location	Year	Mean	2.5 percentile	97.5 percentile
Tonga	2000	-14.04%	-21.72%	-6.99%
Tonga	2005	-11.88%	-18.08%	-6.27%
Tonga	2010	-11.83%	-18.91%	-5.69%
Tonga	2016	-11.97%	-20.47%	-5.46%
Trinidad and Tobago	1990	-0.85%	-1.51%	0.40%
Trinidad and Tobago	1995	-0.97%	-1.84%	0.60%
Trinidad and Tobago	2000	-1.10%	-2%	0.60%
Trinidad and Tobago	2005	-1.01%	-1.83%	0.54%
Trinidad and Tobago	2010	-0.93%	-1.76%	0.70%
Trinidad and Tobago	2016	-0.92%	-1.71%	0.41%
Tunisia	1990	-14.06%	-17.04%	-11.22%
Tunisia	1995	-13.80%	-17.06%	-10.82%
Tunisia	2000	-13.51%	-16.89%	-10.46%
Tunisia	2005	-13.30%	-16.65%	-10.37%
Tunisia	2010	-13.03%	-16.58%	-9.89%
Tunisia	2016	-12.70%	-18.96%	-7.99%
Turkey	1990	-3.19%	-5.37%	-1.33%
Turkey	1995	-2.54%	-4.31%	-1.04%
Turkey	2000	-2.15%	-3.73%	-0.90%
Turkey	2005	-2.31%	-4.00%	-0.93%
Turkey	2010	-3.10%	-5.42%	-1.25%
Turkey	2016	-3.66%	-7.35%	-1.34%
Turkmenistan	1990	-0.51%	-1.16%	-0.14%
Turkmenistan	1995	-0.43%	-1.44%	-0.07%
Turkmenistan	2000	-0.35%	-0.91%	-0.08%
Turkmenistan	2005	-0.35%	-1.12%	-0.05%
Turkmenistan	2010	-0.33%	-0.99%	-0.04%
Turkmenistan	2016	-0.38%	-0.86%	-0.11%
Uganda	1990	-0.01%	-0.04%	0.01%
Uganda	1995	-0.01%	-0.04%	0.02%
Uganda	2000	-0.01%	-0.04%	0.02%
Uganda	2005	-0.01%	-0.04%	0.02%
Uganda	2010	-0.01%	-0.05%	0.02%
Uganda	2016	-0.01%	-0.05%	0.02%
Ukraine	1990	0.10%	-0.36%	0.66%
Ukraine	1995	0.10%	-0.44%	0.68%
Ukraine	2000	0.11%	-0.35%	0.69%
Ukraine	2005	0.08%	-0.31%	0.54%
Ukraine	2010	0.07%	-0.27%	0.52%
Ukraine	2016	0.08%	-0.25%	0.53%
United Arab Emirates	1990	-1.59%	-2.84%	-0.73%
United Arab Emirates	1995	-2.49%	-4.53%	-0.89%
United Arab Emirates	2000	-3.68%	-6.79%	-1.22%
United Arab Emirates	2005	-3.94%	-7.15%	-1.33%
United Arab Emirates	2010	-3.98%	-7.00%	-1.63%
United Arab Emirates	2016	-3.80%	-7.44%	-1.62%

Percent change in alcohol consumption due to tourism, by location and 5-year interval

Location	Year	Mean	2.5 percentile	97.5 percentile
United Kingdom	1990	4.87%	2.45%	8.07%
United Kingdom	1995	4.85%	2.32%	8.80%
United Kingdom	2000	4.69%	2.15%	8.73%
United Kingdom	2005	4.69%	2.19%	8.48%
United Kingdom	2010	4.94%	2.21%	9.03%
United Kingdom	2016	4.68%	2.36%	7.74%
United States	1990	1.78%	0.26%	2.53%
United States	1995	1.86%	0.27%	2.79%
United States	2000	1.84%	0.20%	2.73%
United States	2005	1.82%	0.18%	2.66%
United States	2010	1.76%	0.21%	2.61%
United States	2016	1.83%	0.24%	2.85%
Uruguay	1990	-1.29%	-2.27%	-0.74%
Uruguay	1995	-1.24%	-2.41%	-0.60%
Uruguay	2000	-1.24%	-2.40%	-0.58%
Uruguay	2005	-1.36%	-2.48%	-0.63%
Uruguay	2010	-1.44%	-2.79%	-0.65%
Uruguay	2016	-1.45%	-2.66%	-0.77%
Uzbekistan	1990	0.20%	-0.04%	0.62%
Uzbekistan	1995	0.18%	-0.05%	0.60%
Uzbekistan	2000	0.17%	-0.05%	0.53%
Uzbekistan	2005	0.16%	-0.03%	0.52%
Uzbekistan	2010	0.13%	-0.03%	0.44%
Uzbekistan	2016	0.12%	-0.02%	0.38%
Vanuatu	1990	-10.85%	-19.31%	-5.97%
Vanuatu	1995	-11.94%	-21.35%	-6.39%
Vanuatu	2000	-14.24%	-26.81%	-7.51%
Vanuatu	2005	-16.86%	-32.22%	-8.63%
Vanuatu	2010	-18.55%	-35.22%	-9.39%
Vanuatu	2016	-18.06%	-35.31%	-8.55%
Venezuela	1990	0.13%	0.05%	0.29%
Venezuela	1995	0.13%	0.05%	0.32%
Venezuela	2000	0.14%	0.05%	0.32%
Venezuela	2005	0.15%	0.05%	0.35%
Venezuela	2010	0.14%	0.05%	0.34%
Venezuela	2016	0.14%	0.05%	0.34%
Vietnam	1990	-0.57%	-0.86%	-0.33%
Vietnam	1995	-0.39%	-0.62%	-0.22%
Vietnam	2000	-0.22%	-0.36%	-0.12%
Vietnam	2005	-0.14%	-0.22%	-0.08%
Vietnam	2010	-0.11%	-0.17%	-0.05%
Vietnam	2016	-0.10%	-0.16%	-0.05%
Virgin Islands, U.S.	1990	-65.33%	-98.24%	-39.53%
Virgin Islands, U.S.	1995	-65.36%	-97.83%	-38.92%
Virgin Islands, U.S.	2000	-64.07%	-97.41%	-39.79%
Virgin Islands, U.S.	2005	-64.04%	-98.01%	-39.76%

Percent change in alcohol consumption due to tourism, by location and 5-year interval

Location	Year	Mean	2.5 percentile	97.5 percentile
Virgin Islands, U.S.	2010	-64.70%	-97.97%	-38.11%
Virgin Islands, U.S.	2016	-65.41%	-98.29%	-39.43%
Yemen	1990	-0.30%	-0.53%	-0.15%
Yemen	1995	-0.38%	-0.80%	-0.16%
Yemen	2000	-0.70%	-1.67%	-0.30%
Yemen	2005	-1.13%	-2.47%	-0.45%
Yemen	2010	-1.39%	-2.96%	-0.54%
Yemen	2016	-1.24%	-2.53%	-0.50%
Zambia	1990	-0.09%	-0.20%	0.08%
Zambia	1995	-0.09%	-0.22%	0.08%
Zambia	2000	-0.10%	-0.24%	0.09%
Zambia	2005	-0.11%	-0.27%	0.10%
Zambia	2010	-0.11%	-0.28%	0.10%
Zambia	2016	-0.11%	-0.29%	0.10%
Zimbabwe	1990	-0.16%	-0.41%	0.36%
Zimbabwe	1995	-0.19%	-0.51%	0.51%
Zimbabwe	2000	-0.19%	-0.51%	0.56%
Zimbabwe	2005	-0.19%	-0.50%	0.45%
Zimbabwe	2010	-0.19%	-0.50%	0.44%
Zimbabwe	2016	-0.19%	-0.54%	0.43%

3. Unrecorded adjustment

Given the heterogeneous nature of the estimates on unrecorded consumption, as well as the wide variation across countries and time-periods, we took 1000 draws from the uniform distribution of the lowest and highest estimates available for a given country. We did this to incorporate the diffuse uncertainty within the unrecorded estimates reported.

Methods Appendix Table 4 reports the maximum value estimated for the percentage of alcohol stock that is unrecorded. We used these 1000 draws in the above equation. We adjusted LPC only for countries where estimates were available.

We adjusted the alcohol LPC for unrecorded consumption using the following equation:

$$Alcohol\ LPC = \frac{Alcoh\quad LPC}{(1 - \%\ Unrecorded)}$$

Methods Appendix Table 4: Maximum unrecorded estimates by location (*incorporated in the final estimates of alcohol consumption were 1000 draws from a uniform distribution between zero and the values reported here*

Location	Maximum percentage of total alcohol stock estimated to be unrecorded
Albania	37%
Algeria	47%
Andorra	16%
Angola	23%
Antigua and Barbuda	7%
Argentina	15%
Armenia	33%
Australia	20%
Austria	7%
Azerbaijan	41%
Bahrain	5%
Barbados	8%
Belarus	24%
Belgium	5%
Belize	26%
Benin	51%
Bhutan	59%
Bolivia	43%
Bosnia and Herzegovina	44%
Botswana	42%
Brazil	23%
Brunei	31%
Bulgaria	13%
Burkina Faso	44%
Burundi	51%
Cambodia	68%
Cameroon	38%
Canada	26%
Central African Republic	64%
Chile	27%
China	29%
Colombia	40%
Comoros	40%
Congo	48%
Costa Rica	28%
Cote d'Ivoire	48%
Croatia	19%
Cuba	27%
Cyprus	11%
Czech Republic	12%
Democratic Republic of the Congo	51%
Denmark	12%

Maximum unrecorded estimates by location (*incorporated in the final estimates of alcohol consumption were 1000 draws from a uniform distribution between zero and the values reported here)*

Location	Maximum percentage of total alcohol stock estimated to be unrecorded
Djibouti	41%
Dominica	9%
Dominican Republic	14%
Ecuador	52%
Egypt	58%
El Salvador	37%
Equatorial Guinea	11%
Eritrea	60%
Estonia	7%
Fiji	38%
Finland	25%
France	5%
Gabon	25%
Georgia	31%
Germany	6%
Ghana	71%
Greece	24%
Grenada	10%
Guatemala	52%
Guinea-Bissau	41%
Guyana	16%
Haiti	12%
Honduras	31%
Hungary	21%
Iceland	8%
India	53%
Iraq	68%
Ireland	6%
Israel	13%
Italy	4%
Jamaica	38%
Japan	4%
Jordan	37%
Kazakhstan	43%
Kenya	68%
Kyrgyzstan	50%
Laos	20%
Latvia	21%
Lebanon	31%
Lesotho	66%
Liberia	39%
Lithuania	22%

Maximum unrecorded estimates by location (*incorporated in the final estimates of alcohol consumption were 1000 draws from a uniform distribution between zero and the values reported here)*

Location	Maximum percentage of total alcohol stock estimated to be unrecorded
Luxembourg	6%
Madagascar	58%
Malawi	56%
Malaysia	74%
Maldives	28%
Mali	54%
Malta	7%
Mauritius	32%
Mexico	33%
Moldova	65%
Mongolia	33%
Montenegro	46%
Morocco	52%
Mozambique	59%
Myanmar	73%
Namibia	43%
Netherlands	7%
New Zealand	19%
Nicaragua	38%
Niger	71%
Nigeria	14%
North Korea	18%
Norway	18%
Oman	35%
Panama	15%
Papua New Guinea	74%
Paraguay	28%
Peru	38%
Philippines	20%
Poland	18%
Portugal	18%
Qatar	44%
Romania	39%
Russia	32%
Rwanda	35%
Saint Lucia	3%
Saint Vincent and the Grenadines	7%
Samoa	32%
Sao Tome and Principe	43%
Saudi Arabia	69%
Senegal	59%
Serbia	30%

Maximum unrecorded estimates by location (*incorporated in the final estimates of alcohol consumption were 1000 draws from a uniform distribution between zero and the values reported here)*

Location	Maximum percentage of total alcohol stock estimated to be unrecorded
Seychelles	25%
Sierra Leone	43%
Singapore	28%
Slovakia	19%
Slovenia	12%
Solomon Islands	39%
South Africa	36%
South Korea	28%
Spain	15%
Sri Lanka	49%
Sudan	44%
Suriname	22%
Swaziland	22%
Sweden	29%
Switzerland	7%
Syria	34%
Tanzania	41%
Thailand	14%
The Bahamas	8%
The Gambia	29%
Togo	54%
Tonga	36%
Trinidad and Tobago	7%
Tunisia	19%
Turkey	48%
Turkmenistan	56%
Uganda	18%
Ukraine	46%
United Arab Emirates	57%
United Kingdom	14%
United States	8%
Uruguay	18%
Uzbekistan	53%
Vanuatu	44%
Venezuela	20%
Vietnam	63%
Zambia	47%
Zimbabwe	21%

4. Individual consumption in grams per day

We used DisMod-MR 2.1 to construct estimates for each country/year/age/sex on the prevalence of current drinking, abstention, and on individual-level consumption. We chose to use DisMod due to its ability to leverage information across the heterogeneous age groups reported in the surveys, through age-integration, as well as the model's ability to leverage information available from data in nearby locations or time-periods.

After generating complete time series for prevalence of current drinking, abstention, and individual consumption, we made sure the sum of percent current drinkers and percent abstainers summed to one for a given location/year/age/sex. We then calculated the proportion of total consumption for a given location/year by age and sex, using the estimates of individual consumption, the population size, and the percentage of current drinkers. Lastly, we multiplied this proportion of total stock for a given location/year/sex/age by the total stock for a given location/year to calculate the consumption in terms of liter per capita for a given location/year/sex/age. We then converted these estimates to be in terms of grams/per day. The following equations describe these calculations:

$$\%\ Current\ drinkers_{l,y,s,a} = \frac{\%\ Current\ drinkers_{l,y,s,a}}{\%\ Current\ drinkers_{l,y,s,a} + \%\ Abstainers_{l,y,s,a}}$$

$$Proportion\ of\ total\ consumption_{l,y,s,a} = \frac{Alcohol\ \ g/day_{l,y,s,a} * Population_{l,y,s,a} * \%\ Current\ drinkers_{l,y,s,a}}{\sum_{s,a} Alcohol\ \ g/day_{l,y,s,a} * Population_{l,y,s,a} * \%\ Current\ drinkers_{l,y,s,a}}$$

$$Alcohol\ LPC_{l,y,s,a} = \frac{Alcohol\ LPC_{l,y} * Population_{l,y} * Proportion\ of\ total\ consumption_{l,y,s,a}}{\%\ Current\ drinkers_{l,y,s,a} * Population_{l,y,s,a}}$$

$$Alcohol\ g/day_{l,y,s,a} = Alcohol\ LPC_{l,y,s,a} * \frac{1000}{365}$$

where l is a location, y a year, s is a sex, and a is a 5-year age group.

We then used the gamma distribution to estimate individual level variation within location, year, sex, age drinking populations, following the recommendations of other published alcohol studies [15]. We chose parameters of the gamma distribution based on the mean and standard deviation of the 1000 draws of alcohol g/day exposure for a given population.

VI. Relative risk estimation

a. Motivation for meta-analysis

After assessing available evidence on the risk of alcohol use, we decided to conduct a new meta-analysis to improve upon existing approaches and ensure compatibility between our estimates of consumption, relative risk estimates, and aggregate measures of risk. Previous meta-analyses of alcohol use and associated outcomes have not systematically controlled for reference categories and tend to use the midpoint of consumption doses from included studies. For each included outcome, we conducted a new meta-analysis in which we have additionally collected data on the reference category within studies, as well as the width of consumption doses. This allowed us to test the significance of including within our models a confounding variable for reference category choice. It also allowed us to estimate doses continuously despite the reported heterogeneous doses of alcohol consumption, typically estimated categorically within studies.

In the following sections, we report which outcomes we included, our search strategy, the inclusion criteria, and our estimation methods. We also show, for each outcome, the PRISMA flow diagram, the data and estimates for each dose-response curve, and the references for included studies.

b. Included outcomes

Upon assessing Bradford-Hill's criteria for causation and identified studies, we calculated dose-response relative risk curves for the following outcomes: atrial fibrillation, breast cancer, cirrhosis, colo-rectal cancer, diabetes, epilepsy, esophageal cancer, hemorrhagic stroke, hypertension, ischaemic heart disease, ischaemic stroke, intentional injuries, self-harm, unintentional injuries (transport and non-transport), larynx cancer, lip & oral cancer, liver cancer, lower respiratory infection, pharynx cancer, pancreatitis, and tuberculosis.

c. Search Strategy and Inclusion criteria

For each of the above outcomes, we performed a systematic review of literature published between January 1st, 1950 and Dec 31st 2016 using Pubmed and the GHDx. Studies were included if the following conditions were met. Studies were excluded if any of the following conditions were met:

1. The study did not report on the association between alcohol use and one of the included outcomes.

2. The study design was not either a cohort, case-control, or case-crossover.

3. The study did not report a relative measure of risk (either relative risk, risk ratio, odds-ratio, or hazard ratio) and did not report cases and non-cases among those exposed and un-exposed.

4. The study did not report dose-response amounts on alcohol use.

5. The study endpoint did not meet the case definition used in GBD 2016.

For each endpoint, the search strings used, PRISMA flow diagrams, extracted data, and references can be found in the following sections.

d. Data preparation

Risk ratios (relative risk, odds-ratio, or hazard ratio) were extracted from each included study, along with the reference category used, the characteristics of the study population, and all cofounders controlled for in the study. If a study reported results in terms of standard drinkers, drinks were converted to grams per day based on the location of the study, using WHO standard drink measurements. For studies not reporting confidence intervals, we calculated uncertainty using cases, non-cases, and controls. When studies used atypical reference categories (e.g. drinkers who consume 5-10 g/day), we recalculated the relative risk using abstainers as the reference category, if possible.

e. Modeling Strategy

We used these studies to calculate a dose-response, modeled using DisMod ODE 13. We chose DisMod ODE rather than a conventional mixed effect meta-regression because of its ability to estimate nonparametric splines over doses (i.e. for most alcohol causes, there is a non-linear relationship with different doses) and incorporate heterogeneous doses through dose-integration (i.e. most studies report doses categorically in wide ranges. Our model estimates relative risks for specific doses when categories overlap across studies, through an integration step.) Model covariates for reference category choice, sex, average age, type of risk measurement, and publication year were tested. We chose the model which had the best out-of-sample coverage (using a leave-one-out strategy), given the potential covariates and spline points. When potential models had small differences in out-of-sample coverage (less than 0.1% difference), we chose the model with less covariates and spline points. We tested the possibility of estimating each curve by age and sex. If we found no significant differences in results by age or sex (e.g. the uncertainty of the first difference between models overlapped with 0), we estimated the curve for both sexes and all-ages. The majority of causes were estimated for all-ages, both-sexes, with the exception of ischemic heart disease, ischemic stroke, hemorrhagic stroke, and diabetes, which we estimated by sex.

1. Atrial fibrillation and flutter

Summary of the meta-analysis conducted for GBD 2016

Search String:

(((“”ethanol“”[MeSH Terms] OR “”alcohols“”[MeSH Terms]) AND “”atrial fibrillation“”[MeSH Terms]) AND (“”1966/01/01“”[PDAT] : “”2016/12/31“”[PDAT])) AND “”humans“”[MeSH Terms]

PRISMA flow diagram

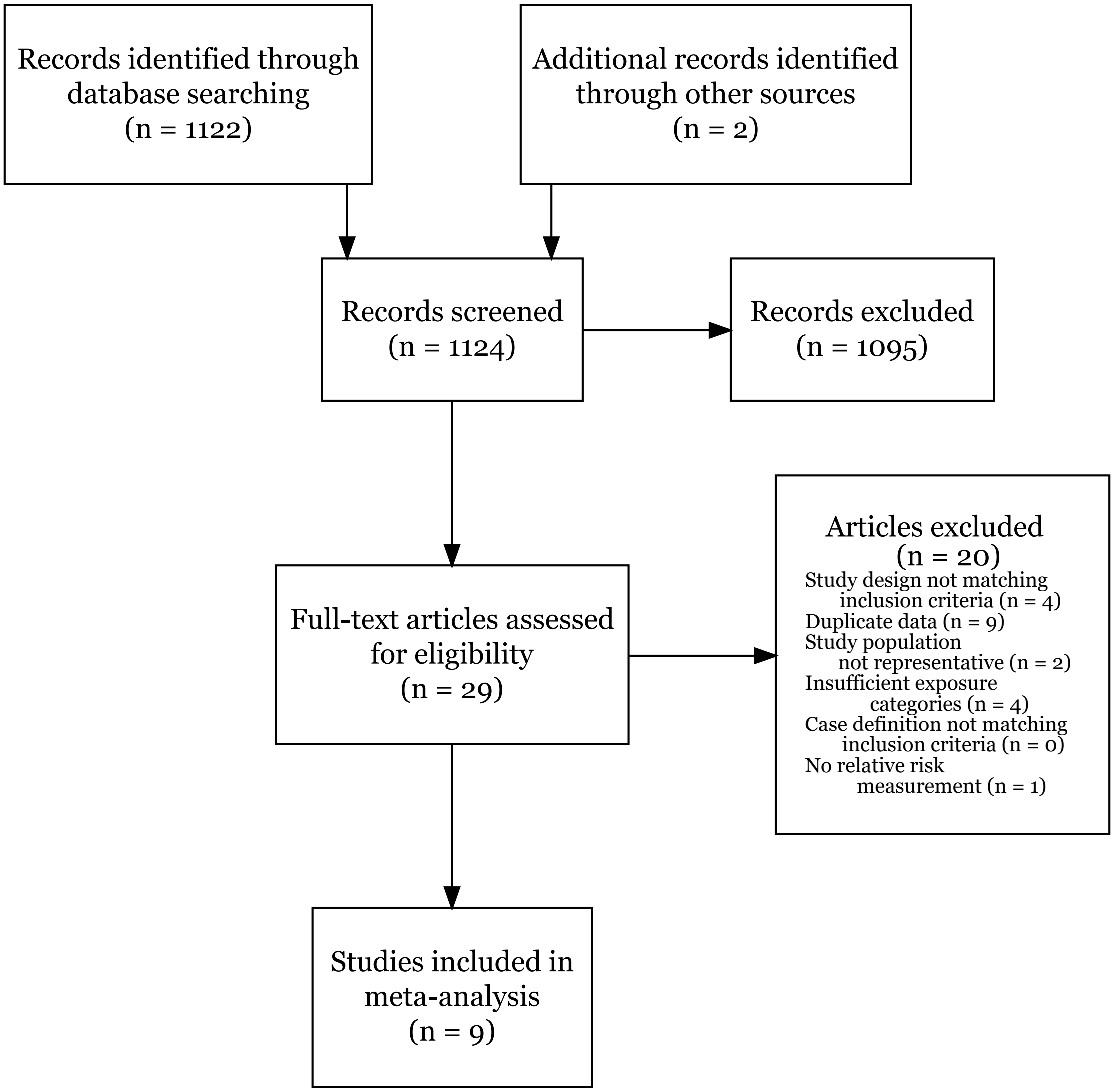

Relative risk (RR) curves for Atrial fibrillation and flutter by number of standard drinks consumed daily. Points are relative risk estimates from studies.
The vertical bars capture the uncertainty in each study, related to the sample size and the horizontal bars capture the range of drinks consumed by individuals in the study.
The black line represents the estimated RR for Atrial fibrillation and flutter at each level of consumption. The shaded green areas represent the 95% uncertainty interval associated with the estimated RR.
Dotted line is a reference for a relative risk of 1.

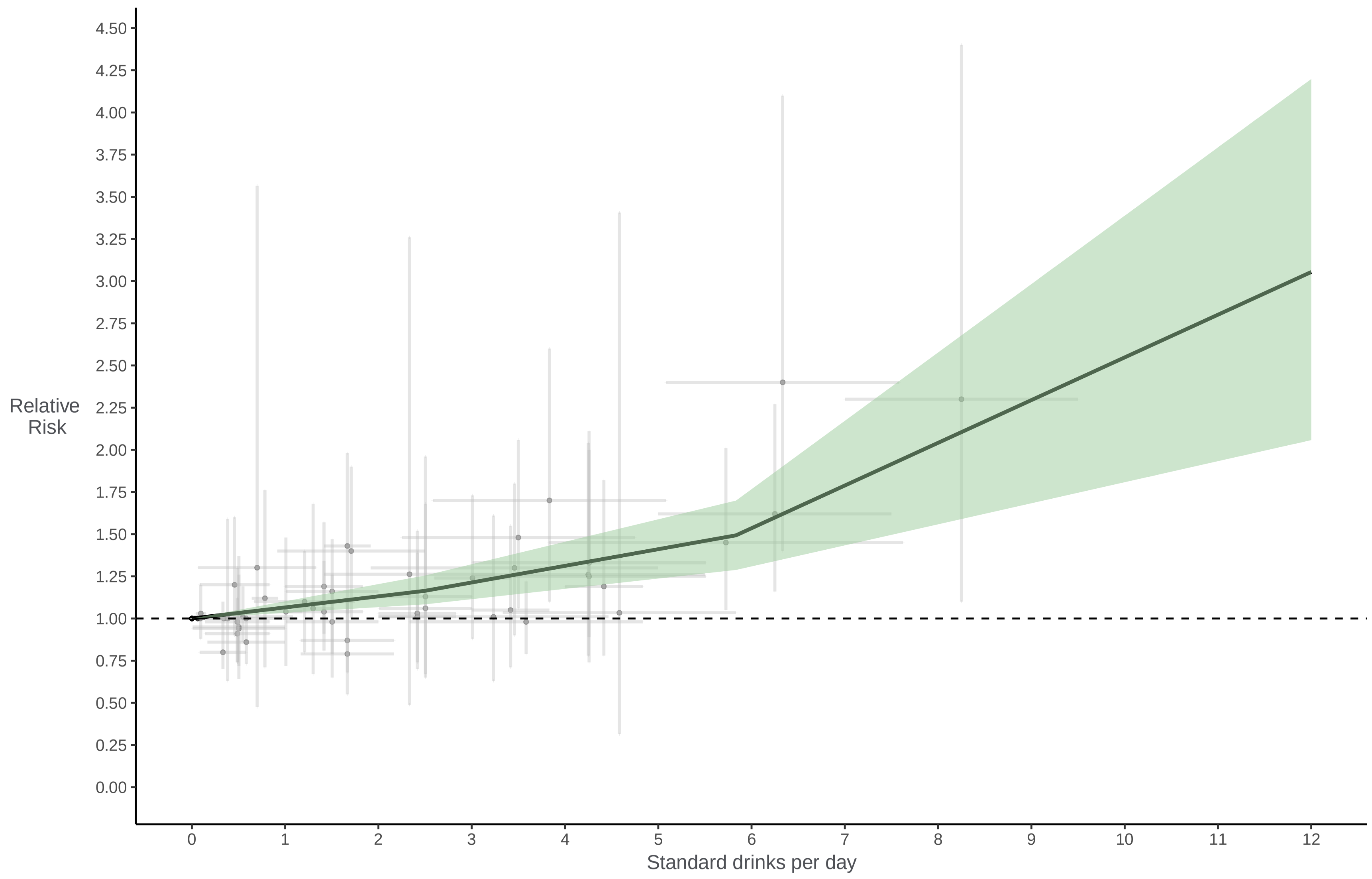

References for Atrial fibrillation and flutter

Cohen EJ, Klatsky AL, Armstrong MA. Alcohol use and supraventricular arrhythmia. Am J Cardiol 1988; 62: 971–3.

Conen D, Tedrow UB, Cook NR, Moorthy MV, Buring JE, Albert CM. Alcohol consumption and risk of incident atrial fibrillation in women. JAMA 2008; 300: 2489–96.

Djousse L, Levy D, Benjamin EJ, et al. Long-term alcohol consumption and the risk of atrial fibrillation in the Framingham Study. Am J Cardiol 2004; 93: 710–3.

Frost L, Vestergaard P. Alcohol and risk of atrial fibrillation or flutter: a cohort study. Arch Intern Med 2004; 164: 1993–8.

Marcus GM, Smith LM, Whiteman D, et al. Alcohol intake is significantly associated with atrial flutter in patients under 60 years of age and a shorter right atrial effective refractory period. Pacing Clin Electrophysiol 2008; 31: 266–72.

Mukamal KJ, Psaty BM, Rautaharju PM, et al. Alcohol consumption and risk and prognosis of atrial fibrillation among older adults: the Cardiovascular Health Study. Am Heart J 2007; 153: 260–6.

Ruigomez A, Johansson S, Wallander MA, Garcia Rodriguez LA. Predictors and prognosis of paroxysmal atrial fibrillation in general practice in the UK. BMC Cardiovasc Disord 2005; 5: 20.

Ruigomez A, Johansson S, Wallander MA, Rodriguez LA. Incidence of chronic atrial fibrillation in general practice and its treatment pattern. J Clin Epidemiol 2002; 55: 358–63.

Mukamal KJ, Tolstrup JS, Friberg J, Jensen G, Gronbaek M. Alcohol Consumption and Risk of Atrial Fibrillation in Men and Women: The Copenhagen City Heart Study. Circulation. 2005; 112(12): 1736–42.

2. Breast cancer

Summary of the meta-analysis conducted for GBD 2016

Search String:

((“”ethanol“”[MeSH Terms] OR “”alcohols“”[MeSH Terms]) AND “”breast neoplasms“”[MeSH Terms] AND (“”0001/01/01“”[PDAT] : “”2016/12/31“”[PDAT])) AND “”humans“”[MeSH Terms] AND “”female“”[MeSH Terms]

PRISMA flow diagram

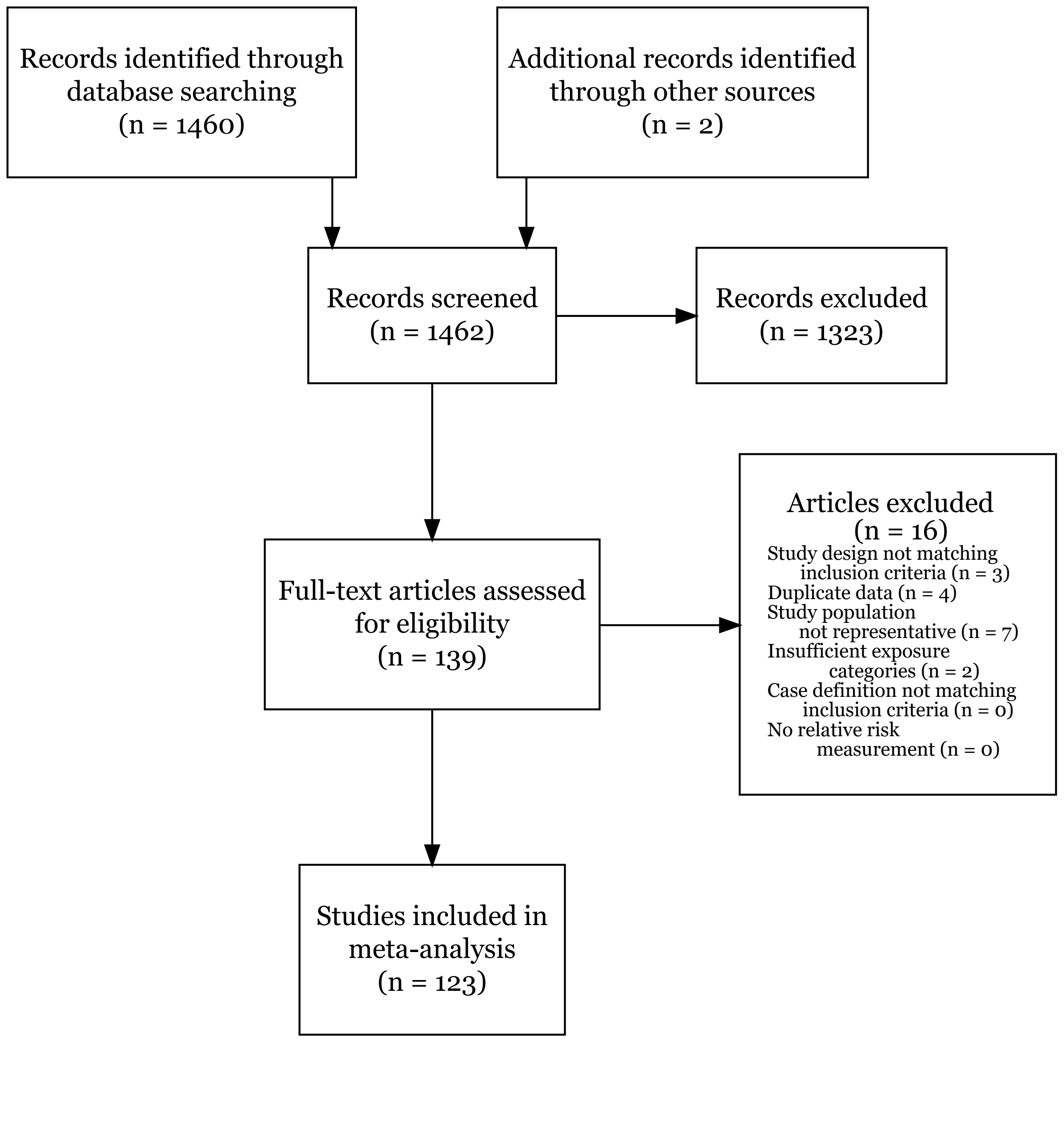

Relative risk (RR) curves for Breast cancer by number of standard drinks consumed daily. Points are relative risk estimates from studies.
The vertical bars capture the uncertainty in each study, related to the sample size and the horizontal bars capture the range of drinks consumed by individuals in the study.
The black line represents the estimated RR for Breast cancer at each level of consumption. The shaded green areas represent the 95% uncertainty interval associated with the estimated RR.
Dotted line is a reference for a relative risk of 1.

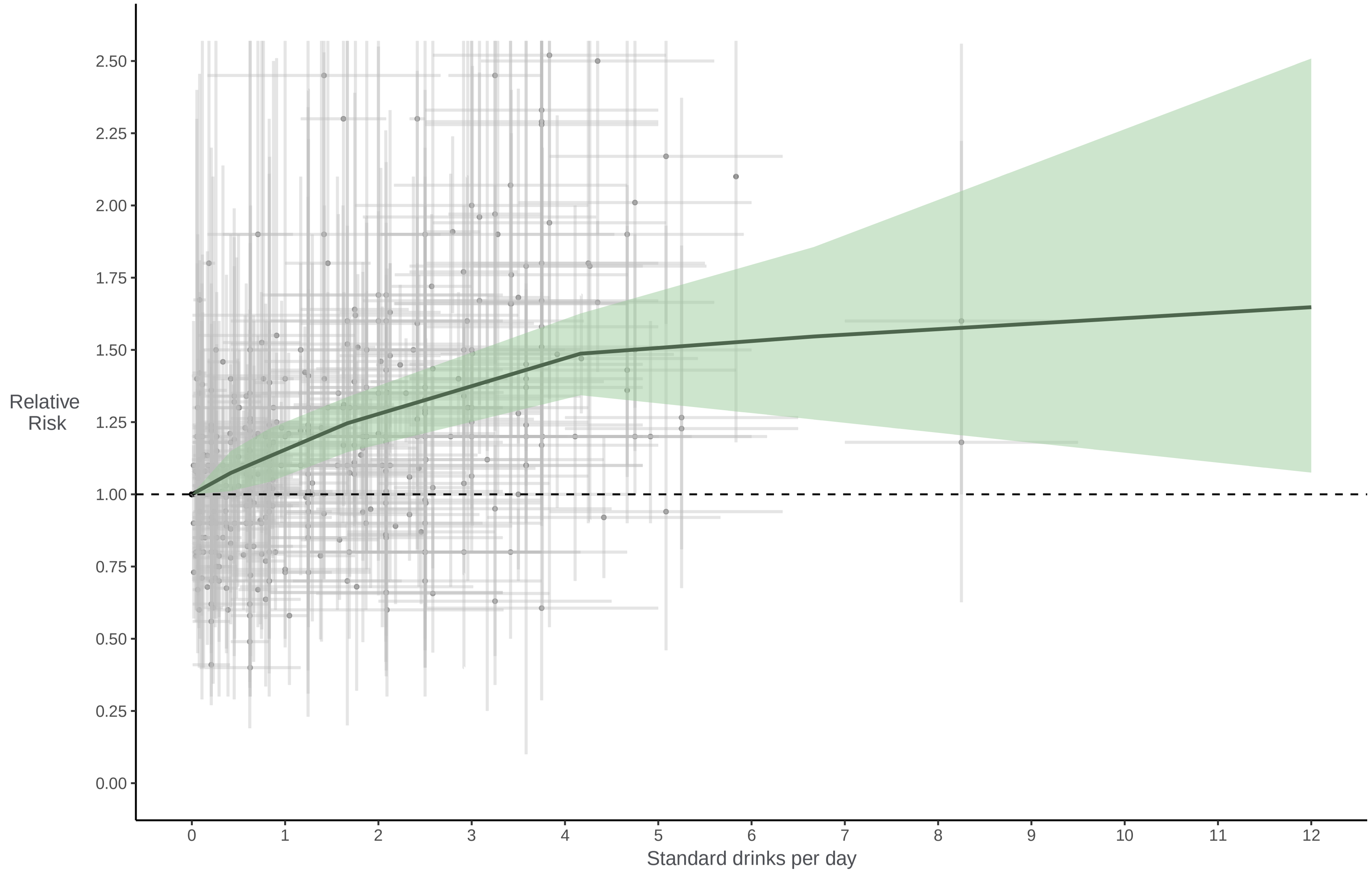

References for Breast cancer

Adami HO, Lund E, Bergstrom R, Meirik O. Cigarette smoking, alcohol consumption and risk of breast cancer in young women. Br J Cancer 1988; 58: 832–7.

Allen NE, Beral V, Casabonne D, et al. Moderate alcohol intake and cancer incidence in women. J Natl Cancer Inst 2009; 101: 296–305.

Baglietto L, English DR, Gertig DM, Hopper JL, Giles GG. Does dietary folate intake modify effect of alcohol consumption on breast cancer risk? Prospective cohort study. BMJ 2005; 331: 80.

Barnes BB, Steindorf K, Hein R, Flesch-Janys D, Chang-Claude J. Population attributable risk of invasive postmenopausal breast cancer and breast cancer subtypes for modifiable and non-modifiable risk factors. Cancer Epidemiol 2011; 35: 345–52.

Baumgartner KB, Annegers JF, McPherson RS, Frankowski RF, Gilliland FD, Samet JM. Is alcohol intake associated with breast cancer in Hispanic women? The New Mexico Women's Health Study. Ethn Dis 2002; 12: 460–9.

Beasley JM, Coronado GD, Livaudais J, et al. Alcohol and risk of breast cancer in Mexican women. Cancer Causes Control 2010; 21: 863–70.

Benzon Larsen S, Vogel U, Christensen J, et al. Interaction between ADH1C Arg(272)Gln and alcohol intake in relation to breast cancer risk suggests that ethanol is the causal factor in alcohol related breast cancer. Cancer Lett 2010; 295: 191–7.

Berstad P, Ma H, Bernstein L, Ursin G. Alcohol intake and breast cancer risk among young women. Breast Cancer Res Treat 2008; 108: 113–20.

Bessaoud F, Daures JP. Patterns of alcohol (especially wine) consumption and breast cancer risk: a case-control study among a population in Southern France. Ann Epidemiol 2008; 18: 467–75.

Bissonauth V, Shatenstein B, Fafard E, et al. Risk of breast cancer among French-Canadian women, noncarriers of more frequent BRCA1/2 mutations and consumption of total energy, coffee, and alcohol. Breast J 2009; 15 Suppl 1: S63-71.

Bowlin SJ, Leske MC, Varma A, Nasca P, Weinstein A, Caplan L. Breast cancer risk and alcohol consumption: results from a large case-control study. Int J Epidemiol 1997; 26: 915–23.

Breslow RA, Chen CM, Graubard BI, Mukamal KJ. Prospective study of alcohol consumption quantity and frequency and cancer-specific mortality in the US population. Am J Epidemiol 2011; 174: 1044–53.

Brown LM, Gridley G, Wu AH, et al. Low level alcohol intake, cigarette smoking and risk of breast cancer in Asian-American women. Breast Cancer Res Treat 2010; 120: 203–10.

Chen WY, Colditz GA, Rosner B, et al. Use of postmenopausal hormones, alcohol, and risk for invasive breast cancer. Ann Intern Med 2002; 137: 798–804.

Chen WY, Rosner B, Hankinson SE, Colditz GA, Willett WC. Moderate alcohol consumption during adult life, drinking patterns, and breast cancer risk. JAMA 2011; 306: 1884–90.

Chu SY, Lee NC, Wingo PA, Webster LA. Alcohol consumption and the risk of breast cancer. Am J Epidemiol 1989; 130: 867–77.

Cotterchio M, Kreiger N, Theis B, Sloan M, Bahl S. Hormonal factors and the risk of breast cancer according to estrogen- and progesterone-receptor subgroup. Cancer Epidemiol Biomarkers Prev 2003; 12: 1053–60.

Croghan IT, Pruthi S, Hays JT, et al. The role of smoking in breast cancer development: an analysis of a Mayo Clinic cohort. Breast J 2009; 15: 489–95.

Dumeaux V, Lund E, Hjartaker A. Use of oral contraceptives, alcohol, and risk for invasive breast cancer. Cancer Epidemiol Biomarkers Prev 2004; 13: 1302–7.

Ericson U, Sonestedt E, Gullberg B, Olsson H, Wirfalt E. High folate intake is associated with lower breast cancer incidence in postmenopausal women in the Malmö Diet and Cancer cohort. Am J Clin Nutr 2007; 86: 434–43.

Ewertz M. Alcohol consumption and breast cancer risk in Denmark. Cancer Causes Control 1991; 2: 247–52.

Feigelson HS, Calle EE, Robertson AS, Wingo PA, Thun MJ. Alcohol consumption increases the risk of fatal breast cancer (United States). Cancer Causes Control 2001; 12: 895–902.

Feigelson HS, Jonas CR, Robertson AS, McCullough ML, Thun MJ, Calle EE. Alcohol, folate, methionine, and risk of incident breast cancer in the American Cancer Society Cancer Prevention Study II Nutrition Cohort. Cancer Epidemiol Biomarkers Prev 2003; 12: 161–4.

Ferraroni M, Decarli A, Willett WC, Marubini E. Alcohol and breast cancer risk: a case-control study from northern Italy. Int J Epidemiol 1991; 20: 859–64.

Franceschi S, Serraino D, Talamini R, Barra S, Bidoli E. Alcohol and breast cancer in an area with high alcohol consumption. Rev Epidemiol Sante Publique 1991; 39: 143–8.

Freudenheim JL, Marshall JR, Graham S, et al. Lifetime alcohol consumption and risk of breast cancer. Nutr Cancer 1995; 23: 11-Jan.

Fuchs CS, Stampfer MJ, Colditz GA, et al. Alcohol consumption and mortality among women. N Engl J Med 1995; 332: 1245–50.

Gapstur SM, Potter JD, Sellers TA, Folsom AR. Increased risk of breast cancer with alcohol consumption in postmenopausal women. Am J Epidemiol 1992; 136: 1221–31.

Garland M, Hunter DJ, Colditz GA, et al. Alcohol consumption in relation to breast cancer risk in a cohort of United States women 25-42 years of age. Cancer Epidemiol Biomarkers Prev 1999; 8: 1017–21.

Goodman MT, Cologne JB, Moriwaki H, Vaeth M, Mabuchi K. Risk factors for primary breast cancer in Japan: 8-year follow-up of atomic bomb survivors. Prev Med 1997; 26: 144–53.

Harris HR, Bergkvist L, Wolk A. Alcohol intake and mortality among women with invasive breast cancer. Br J Cancer 2012; 106: 592–5.

Harris RE, Namboodiri KK, Wynder EL. Breast cancer risk: effects of estrogen replacement therapy and body mass. J Natl Cancer Inst 1992; 84: 1575–82.

Harvey EB, Schairer C, Brinton LA, Hoover RN, Fraumeni JF. Alcohol consumption and breast cancer. J Natl Cancer Inst 1987; 78: 657–61.

Herrinton LJ, Saftlas AF, Stanford JL, Brinton LA, Wolfe JN. Do alcohol intake and mammographic densities interact in regard to the risk of breast cancer? Cancer 1993; 71: 3029–35.

Hiatt RA, Bawol RD. Alcoholic beverage consumption and breast cancer incidence. Am J Epidemiol 1984; 120: 676–83.

Hines LM, Risendal B, Slattery ML, et al. Comparative analysis of breast cancer risk factors among Hispanic and non-Hispanic white women. Cancer 2010; 116: 3215–23.

Hirose K, Hamajima N, Takezaki T, Miura S, Tajima K. Physical exercise reduces risk of breast cancer in Japanese women. Cancer Sci 2003; 94: 193–9.

Horn-Ross PL, Hoggatt KJ, West DW, et al. Recent diet and breast cancer risk: the California Teachers Study (USA). Cancer Causes Control 2002; 13: 407–15.

Hoyer AP, Engholm G. Serum lipids and breast cancer risk: a cohort study of 5,207 Danish women. Cancer Causes Control 1992; 3: 403–8.

Jain MG, Ferrenc RG, Rehm JT, et al. Alcohol and breast cancer mortality in a cohort study. Breast Cancer Res Treat 2000; 64: 201–9.

Kabat GC, Chang CJ, Sparano JA, et al. Urinary estrogen metabolites and breast cancer: a case-control study. Cancer Epidemiol Biomarkers Prev 1997; 6: 505–9.

Kabat GC, Miller AB, Jain M, Rohan TE. Dietary intake of selected B vitamins in relation to risk of major cancers in women. Br J Cancer 2008; 99: 816–21.

Kabat GC, Miller AB, Jain M, Rohan TE. Dietary intake of selected B vitamins in relation to risk of major cancers in women. Br J Cancer 2008; 99: 816–21.

Katsouyanni K, Trichopoulou A, Stuver S, et al. Ethanol and breast cancer: an association that may be both confounded and causal. Int J Cancer 1994; 58: 356–61.

Kawai M, Minami Y, Kakizaki M, et al. Alcohol consumption and breast cancer risk in Japanese women: the Miyagi Cohort study. Breast Cancer Res Treat 2011; 128: 817–25.

Kawase T, Matsuo K, Hiraki A, et al. Interaction of the effects of alcohol drinking and polymorphisms in alcohol-metabolizing enzymes on the risk of female breast cancer in Japan. J Epidemiol 2009; 19: 244–50.

Kim MK, Ko MJ, Han JT. Alcohol consumption and mortality from all-cause and cancers among 1.34 million Koreans: the results from the Korea national health insurance corporation's health examinee cohort in 2000. Cancer Causes Control 2010; 21: 2295–302.

Kinney AY, Millikan RC, Lin YH, Moorman PG, Newman B. Alcohol consumption and breast cancer among black and white women in North Carolina (United States). Cancer Causes Control 2000; 11: 345–57.

Kropp S, Becher H, Nieters A, Chang-Claude J. Low-to-moderate alcohol consumption and breast cancer risk by age 50 years among women in Germany. Am J Epidemiol 2001; 154: 624–34.

Kruk J. Association of lifestyle and other risk factors with breast cancer according to menopausal status: a case-control study in the Region of Western Pomerania (Poland). Asian Pac J Cancer Prev 2007; 8: 513–24.

La Vecchia C, Negri E, Parazzini F, et al. Alcohol and breast cancer: update from an Italian case-control study. Eur J Cancer Clin Oncol 1989; 25: 1711–7.

Le MG, Hill C, Kramar A, Flamanti R. Alcoholic beverage consumption and breast cancer in a French case-control study. Am J Epidemiol 1984; 120: 350–7.

Levi F, Pasche C, Lucchini F, La Vecchia C. Alcohol and breast cancer in the Swiss Canton of Vaud. Eur J Cancer 1996; 32A: 2108–13.

Lew JQ, Freedman ND, Leitzmann MF, et al. Alcohol and risk of breast cancer by histologic type and hormone receptor status in postmenopausal women: the NIH-AARP Diet and Health Study. Am J Epidemiol 2009; 170: 308–17.

Li CI, Chlebowski RT, Freiberg M, et al. Alcohol consumption and risk of postmenopausal breast cancer by subtype: the women's health initiative observational study. J Natl Cancer Inst 2010; 102: 1422–31.

Li CI, Malone KE, Porter PL, Weiss NS, Tang MT, Daling JR. The relationship between alcohol use and risk of breast cancer by histology and hormone receptor status among women 65-79 years of age. Cancer Epidemiol Biomarkers Prev 2003; 12: 1061–6.

Li Y, Baer D, Friedman GD, Udaltsova N, Shim V, Klatsky AL. Wine, liquor, beer and risk of breast cancer in a large population. Eur J Cancer 2009; 45: 843–50.

Lin Y, Kikuchi S, Tamakoshi K, et al. Prospective study of alcohol consumption and breast cancer risk in Japanese women. Int J Cancer 2005; 116: 779–83.

Longnecker MP, Newcomb PA, Mittendorf R, et al. Risk of breast cancer in relation to lifetime alcohol consumption. J Natl Cancer Inst 1995; 87: 923–9.

Longnecker MP, Paganini-Hill A, Ross RK. Lifetime alcohol consumption and breast cancer risk among postmenopausal women in Los Angeles. Cancer Epidemiol Biomarkers Prev 1995; 4: 721–5.

Lucas FL, Cauley JA, Stone RA, et al. Bone mineral density and risk of breast cancer: differences by family history of breast cancer. Study of Osteoporotic Fractures Research Group. Am J Epidemiol 1998; 148: 22–9.

Mannisto S, Virtanen M, Kataja V, Uusitupa M, Pietinen P. Lifetime alcohol consumption and breast cancer: a case-control study in Finland. Public Health Nutr 2000; 3: 8-Nov.

Martin-Moreno JM, Boyle P, Gorgojo L, et al. Alcoholic beverage consumption and risk of breast cancer in Spain. Cancer Causes Control 1993; 4: 345–53.

Maruti SS, Ulrich CM, White E. Folate and one-carbon metabolism nutrients from supplements and diet in relation to breast cancer risk. Am J Clin Nutr 2009; 89: 624–33.

McCarty CA, Reding DJ, Commins J, et al. Alcohol, genetics and risk of breast cancer in the Prostate, Lung, Colorectal and Ovarian (PLCO) Cancer Screening Trial. Breast Cancer Res Treat 2012; 133: 785–92.

McDonald JA, Mandel MG, Marchbanks PA, et al. Alcohol exposure and breast cancer: results of the women's contraceptive and reproductive experiences study. Cancer Epidemiol Biomarkers Prev 2004; 13: 2106–16.

Meara J, McPherson K, Roberts M, Jones L, Vessey M. Alcohol, cigarette smoking and breast cancer. Br J Cancer 1989; 60: 70–3.

Meulepas JM, Newcomb PA, Burnett-Hartman AN, Hampton JM, Trentham-Dietz A. Multivitamin supplement use and risk of invasive breast cancer. Public Health Nutr 2010; 13: 1540–5.

Mezzetti M, La Vecchia C, Decarli A, Boyle P, Talamini R, Franceschi S. Population attributable risk for breast cancer: diet, nutrition, and physical exercise. J Natl Cancer Inst 1998; 90: 389–94.

Morabia A, Bernstein M, Heritier S, Khatchatrian N. Relation of breast cancer with passive and active exposure to tobacco smoke. Am J Epidemiol 1996; 143: 918–28.

Morch LS, Johansen D, Thygesen LC, et al. Alcohol drinking, consumption patterns and breast cancer among Danish nurses: a cohort study. Eur J Public Health 2007; 17: 624–9.

Nasca PC, Baptiste MS, Field NA, et al. An epidemiological case-control study of breast cancer and alcohol consumption. Int J Epidemiol 1990; 19: 532–8.

Newcomb PA, Egan KM, Titus-Ernstoff L, et al. Lactation in relation to postmenopausal breast cancer. Am J Epidemiol 1999; 150: 174–82.

Newcomb PA, Nichols HB, Beasley JM, et al. No difference between red wine or white wine consumption and breast cancer risk. Cancer Epidemiol Biomarkers Prev 2009; 18: 1007–10.

Nielsen NR, Gronbaek M. Interactions between intakes of alcohol and postmenopausal hormones on risk of breast cancer. Int J Cancer 2008; 122: 1109–13.

Ozasa K. Alcohol use and mortality in the Japan Collaborative Cohort Study for Evaluation of Cancer (JACC). Asian Pac J Cancer Prev 2007; 8 Suppl: 81–8.

Petri AL, Tjonneland A, Gamborg M, et al. Alcohol intake, type of beverage, and risk of breast cancer in pre- and postmenopausal women. Alcohol Clin Exp Res 2004; 28: 1084–90.

Richardson S, de Vincenzi I, Pujol H, Gerber M. Alcohol consumption in a case-control study of breast cancer in southern France. Int J Cancer 1989; 44: 84–9.

Rohan TE, McMichael AJ. Alcohol consumption and risk of breast cancer. Int J Cancer 1988; 41: 695–9.

Ronco AL, De Stefani E, Correa P, et al. Dietary benzo[a]pyrene, alcohol drinking, and risk of breast cancer: a case-control study in Uruguay. Asian Pac J Cancer Prev 2011; 12: 1463–7.

Rosenberg L, Palmer JR, Miller DR, Clarke EA, Shapiro S. A case-control study of alcoholic beverage consumption and breast cancer. Am J Epidemiol 1990; 131: 14-Jun.

Rosenberg LU, Einarsdottir K, Friman EI, et al. Risk factors for hormone receptor-defined breast cancer in postmenopausal women. Cancer Epidemiol Biomarkers Prev 2006; 15: 2482–8.

Rossing MA, Stanford JL, Weiss NS, Habel LA. Oral contraceptive use and risk of breast cancer in middle-aged women. Am J Epidemiol 1996; 144: 161–4.

Schatzkin A, Jones DY, Hoover RN, et al. Alcohol consumption and breast cancer in the epidemiologic follow-up study of the first National Health and Nutrition Examination Survey. N Engl J Med 1987; 316: 1169–73.

Simon MS, Carman W, Wolfe R, Schottenfeld D. Alcohol consumption and the risk of breast cancer: a report from the Tecumseh Community Health Study. J Clin Epidemiol 1991; 44: 755–61.

Smith SJ, Deacon JM, Chilvers CE. Alcohol, smoking, passive smoking and caffeine in relation to breast cancer risk in young women. UK National Case-Control Study Group. Br J Cancer 1994; 70: 112–9.

Sneyd MJ, Paul C, Spears GF, Skegg DC. Alcohol consumption and risk of breast cancer. Int J Cancer 1991; 48: 812–5.

Suzuki R, Iwasaki M, Inoue M, et al. Alcohol consumption-associated breast cancer incidence and potential effect modifiers: the Japan Public Health Center-based Prospective Study. Int J Cancer 2010; 127: 685–95.

Suzuki R, Ye W, Rylander-Rudqvist T, Saji S, Colditz GA, Wolk A. Alcohol and postmenopausal breast cancer risk defined by estrogen and progesterone receptor status: a prospective cohort study. J Natl Cancer Inst 2005; 97: 1601–8.

Swanson CA, Coates RJ, Malone KE, et al. Alcohol consumption and breast cancer risk among women under age 45 years. Epidemiology 1997; 8: 231–7.

Terry MB, Zhang FF, Kabat G, et al. Lifetime alcohol intake and breast cancer risk. Ann Epidemiol 2006; 16: 230–40.

Tjonneland A, Christensen J, Olsen A, et al. Alcohol intake and breast cancer risk: the European Prospective Investigation into Cancer and Nutrition (EPIC). Cancer Causes Control 2007; 18: 361–73.

Toniolo P, Riboli E, Protta F, Charrel M, Cappa AP. Breast cancer and alcohol consumption: a case-control study in northern Italy. Cancer Res 1989; 49: 5203–6.

Trentham-Dietz A, Newcomb PA, Storer BE, Remington PL. Risk factors for carcinoma in situ of the breast. Cancer Epidemiol Biomarkers Prev 2000; 9: 697–703.

Viladiu P, Izquierdo A, de Sanjose S, Bosch FX. A breast cancer case-control study in Girona, Spain. Endocrine, familial and lifestyle factors. Eur J Cancer Prev 1996; 5: 329–35.

Wang H, Rothenbacher D, Low M, Stegmaier C, Brenner H, Diepgen TL. Atopic diseases, immunoglobulin E and risk of cancer of the prostate, breast, lung and colorectum. Int J Cancer 2006; 119: 695–701.

Wrensch M, Chew T, Farren G, et al. Risk factors for breast cancer in a population with high incidence rates. Breast Cancer Res 2003; 5(4): R88-102.

Young TB. A case-control study of breast cancer and alcohol consumption habits. Cancer 1989; 64: 552–8.

Zhang M, Holman CD. Low-to-moderate alcohol intake and breast cancer risk in Chinese women. Br J Cancer 2011; 105: 1089–95.

Zhang Y, Kreger BE, Dorgan JF, Splansky GL, Cupples LA, Ellison RC. Alcohol consumption and risk of breast cancer: the Framingham Study revisited. Am J Epidemiol 1999; 149: 93–101.

van den Brandt PA, Goldbohm RA, van, t Veer P. Alcohol and breast cancer: results from The Netherlands Cohort Study. Am J Epidemiol 1995; 141: 907–15.

van't Veer P, Kok FJ, Hermus RJ, Sturmans F. Alcohol dose, frequency and age at first exposure in relation to the risk of breast cancer. Int J Epidemiol 1989; 18: 511–7.

3. Cirrhosis and other chronic liver diseases

Summary of the meta-analysis conducted for GBD 2016

Search String:

(((“”ethanol“”[MeSH Terms] OR “”alcohols“”[MeSH Terms]) AND (“”liver cirrhosis“”[MeSH Terms] OR “”fibrosis“”[MeSH Terms])) AND (“”0001/01/01“”[PDAT] : “”2016/12/31“”[PDAT])) AND “”humans“”[MeSH Terms]

PRISMA flow diagram

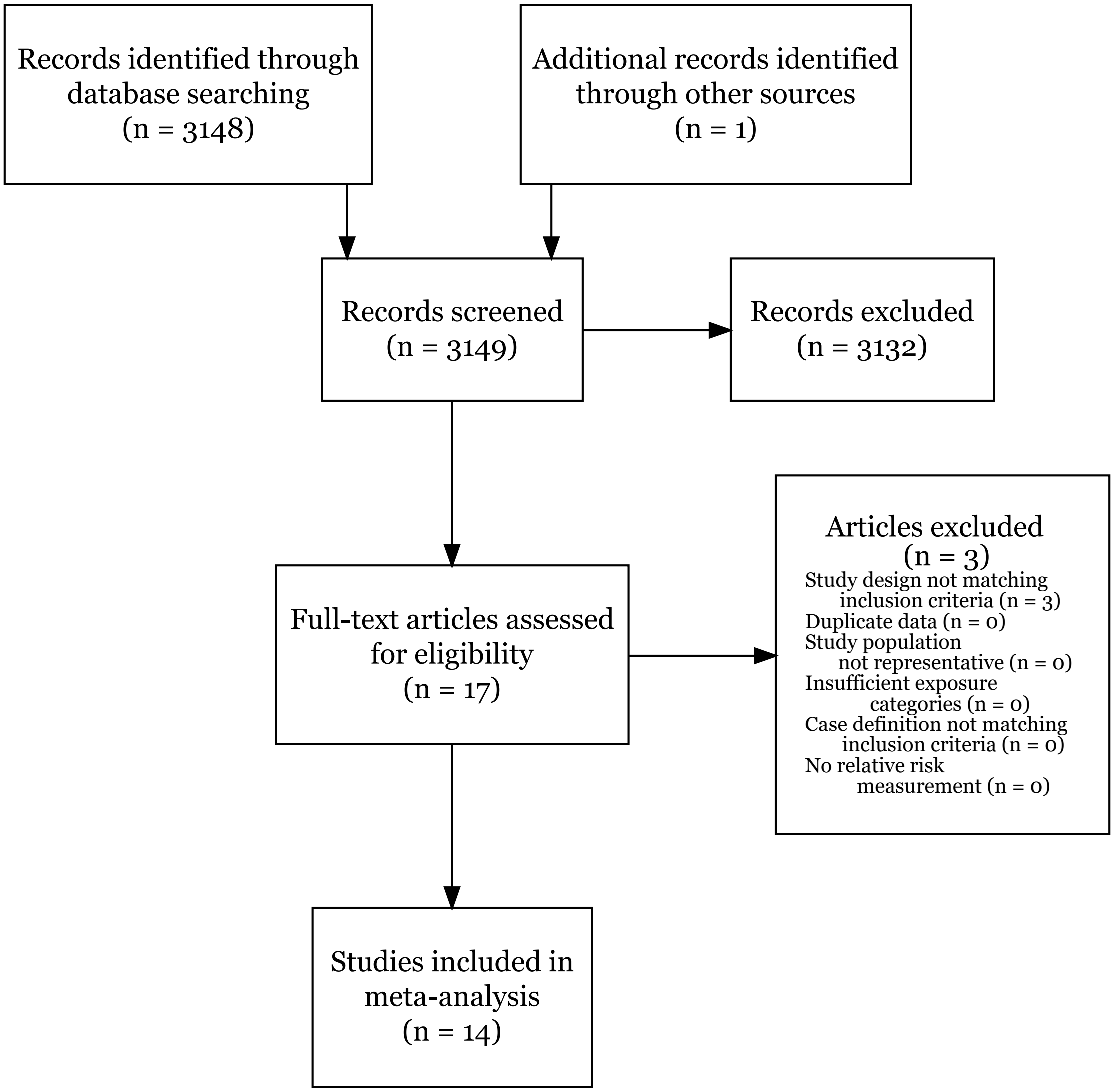

Relative risk (RR) curves for Cirrhosis and other chronic liver diseases by number of standard drinks consumed daily. Points are relative risk estimates from studies.
The vertical bars capture the uncertainty in each study, related to the sample size and the horizontal bars capture the range of drinks consumed by individuals in the study.
The black line represents the estimated RR for Cirrhosis and other chronic liver diseases at each level of consumption. The shaded green areas represent the 95% uncertainty interval associated with the estimated RR.
Dotted line is a reference for a relative risk of 1.

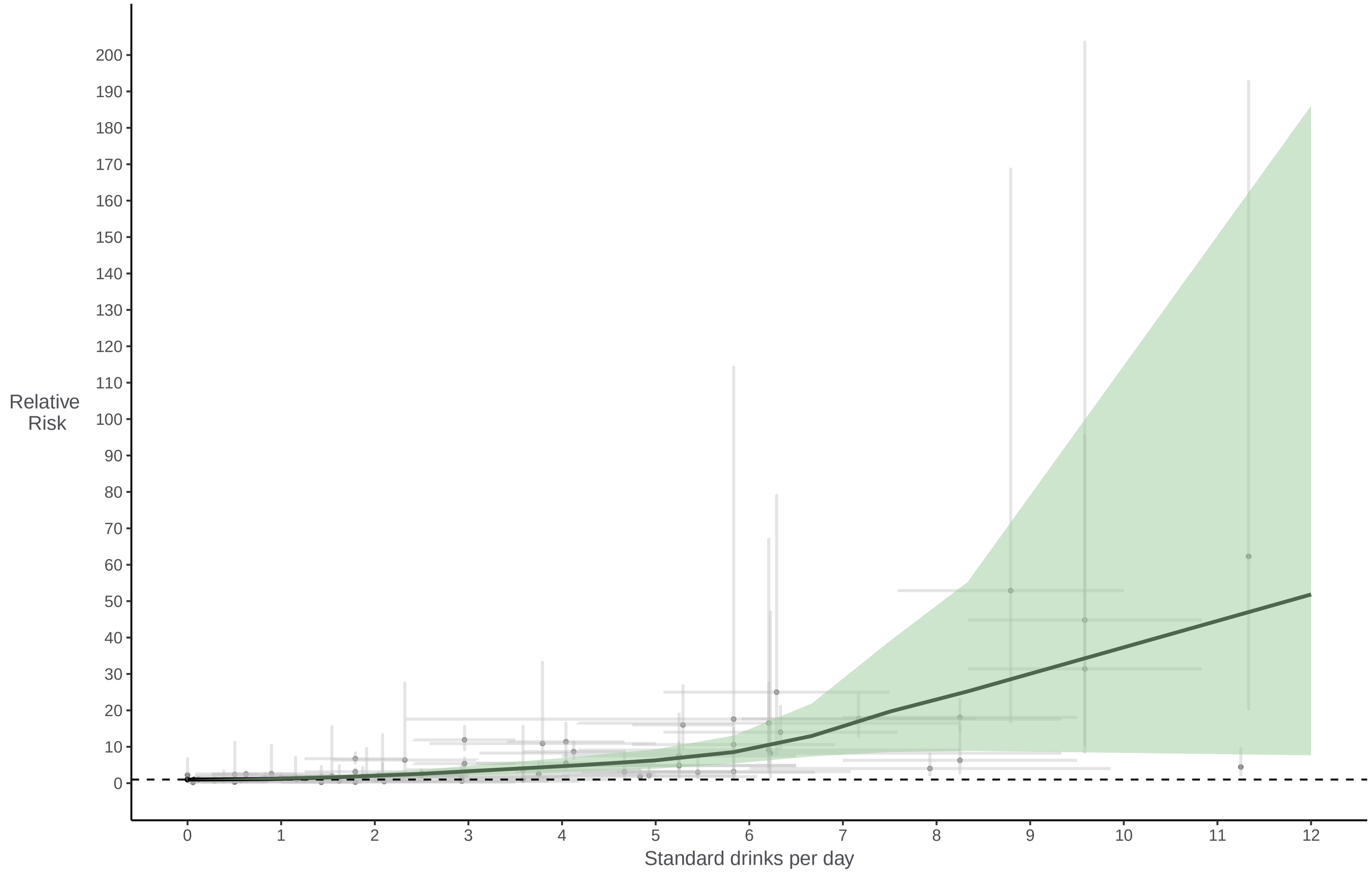

References for Cirrhosis and other chronic liver diseases

Becker U, Deis A, Sorensen TI, et al. Prediction of risk of liver disease by alcohol intake, sex, and age: a prospective population study. Hepatology 1996; 23: 1025–9.

Becker U, Gronbaek M, Johansen D, Sorensen TI. Lower risk for alcohol-induced cirrhosis in wine drinkers. Hepatology 2002; 35: 868–75.

Bellentani S, Saccoccio G, Costa G, et al. Drinking habits as cofactors of risk for alcohol induced liver damage. The Dionysos Study Group. Gut 1997; 41: 845–50.

Blackwelder WC, Yano K, Rhoads GG, Kagan A, Gordon T, Palesch Y. Alcohol and mortality: the Honolulu Heart Study. Am J Med 1980; 68: 164–9.

Boffetta P, Garfinkel L. Alcohol drinking and mortality among men enrolled in an American Cancer Society prospective study. Epidemiology 1990; 1: 342–8.

Corrao G, Arico S, Zambon A, Torchio P, Di Orio F. Female sex and the risk of liver cirrhosis. Collaborative Groups for the Study of Liver Diseases in Italy. Scand J Gastroenterol 1997; 32: 1174–80.

Fuchs CS, Stampfer MJ, Colditz GA, et al. Alcohol consumption and mortality among women. N Engl J Med 1995; 332: 1245–50.

Gordon T, Doyle JT. Drinking and mortality. The Albany Study. Am J Epidemiol 1987; 125: 263–70.

Gordon T, Kannel WB. Drinking and mortality. The Framingham Study. Am J Epidemiol 1984; 120: 97–107.

Kabat GC, Miller AB, Jain M, Rohan TE. Dietary intake of selected B vitamins in relation to risk of major cancers in women. Br J Cancer 2008; 99: 816–21.

Klatsky AL, Friedman GD, Siegelaub AB. Alcohol and mortality. A ten-year Kaiser-Permanente experience. Ann Intern Med 1981; 95: 139–45.

Kono S, Ikeda M, Tokudome S, Nishizumi M, Kuratsune M. Alcohol and mortality: a cohort study of male Japanese physicians. Int J Epidemiol 1986; 15: 527–32.

Thun MJ, Peto R, Lopez AD, et al. Alcohol consumption and mortality among middle-aged and elderly U.S. adults. N Engl J Med 1997; 337: 1705–14.

Yuan JM, Ross RK, Gao YT, Henderson BE, Yu MC. Follow up study of moderate alcohol intake and mortality among middle aged men in Shanghai, China. BMJ 1997; 314: 18–23.

4. Colon and rectum cancer

Summary of the meta-analysis conducted for GBD 2016

Search String:

((“”ethanol“”[MeSH Terms] OR “”alcohols“”[MeSH Terms]) AND colorectal neoplasms“”[MeSH Terms] AND (“”0001/01/01“”[PDAT] : “”2016/12/31“”[PDAT])) AND “”humans“”[MeSH Terms]

PRISMA flow diagram

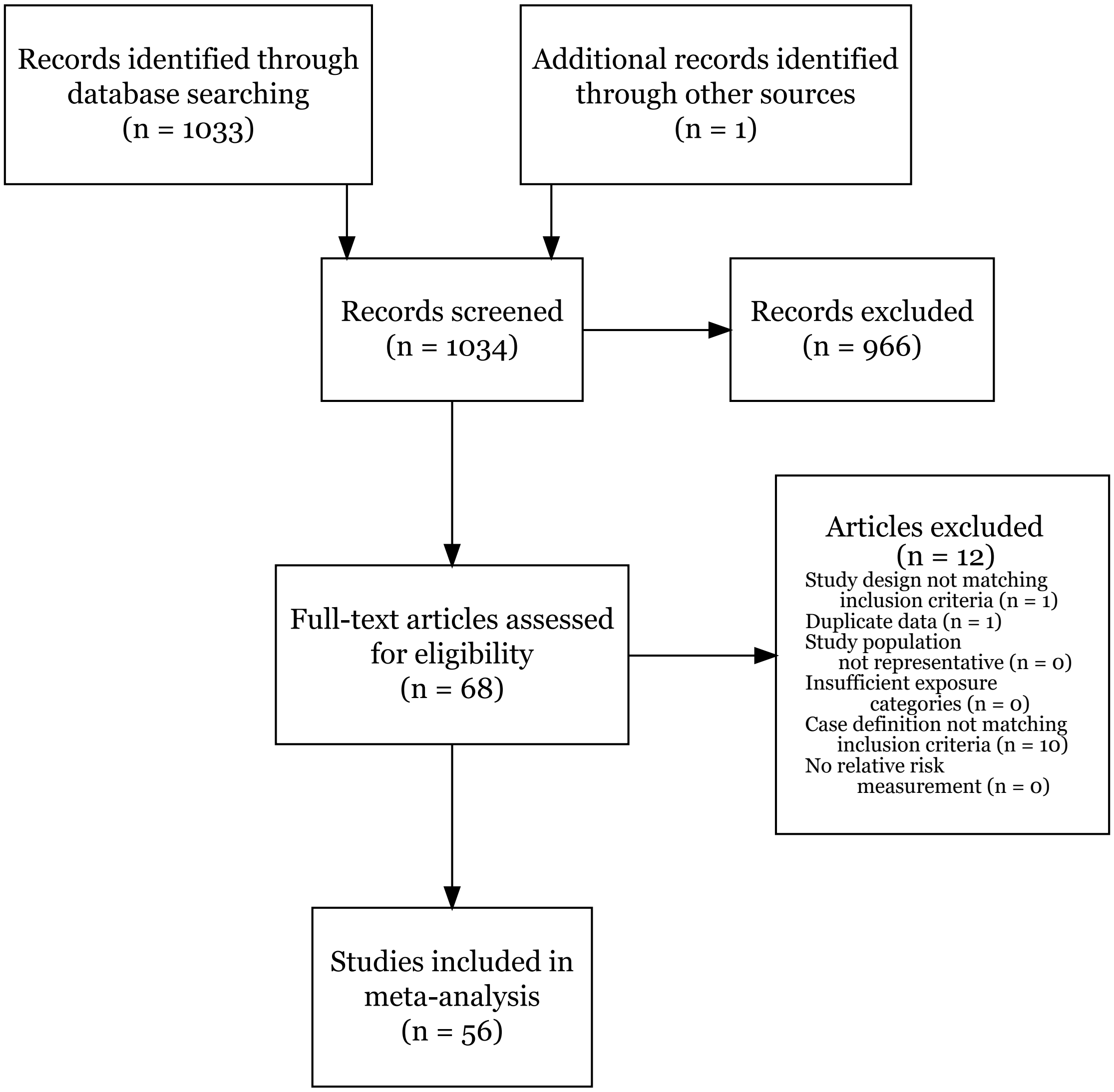

Relative risk (RR) curves for Colon and rectum cancer by number of standard drinks consumed daily. Points are relative risk estimates from studies.
The vertical bars capture the uncertainty in each study, related to the sample size and the horizontal bars capture the range of drinks consumed by individuals in the study.
The black line represents the estimated RR for Colon and rectum cancer at each level of consumption. The shaded green areas represent the 95% uncertainty interval associated with the estimated RR.
Dotted line is a reference for a relative risk of 1.

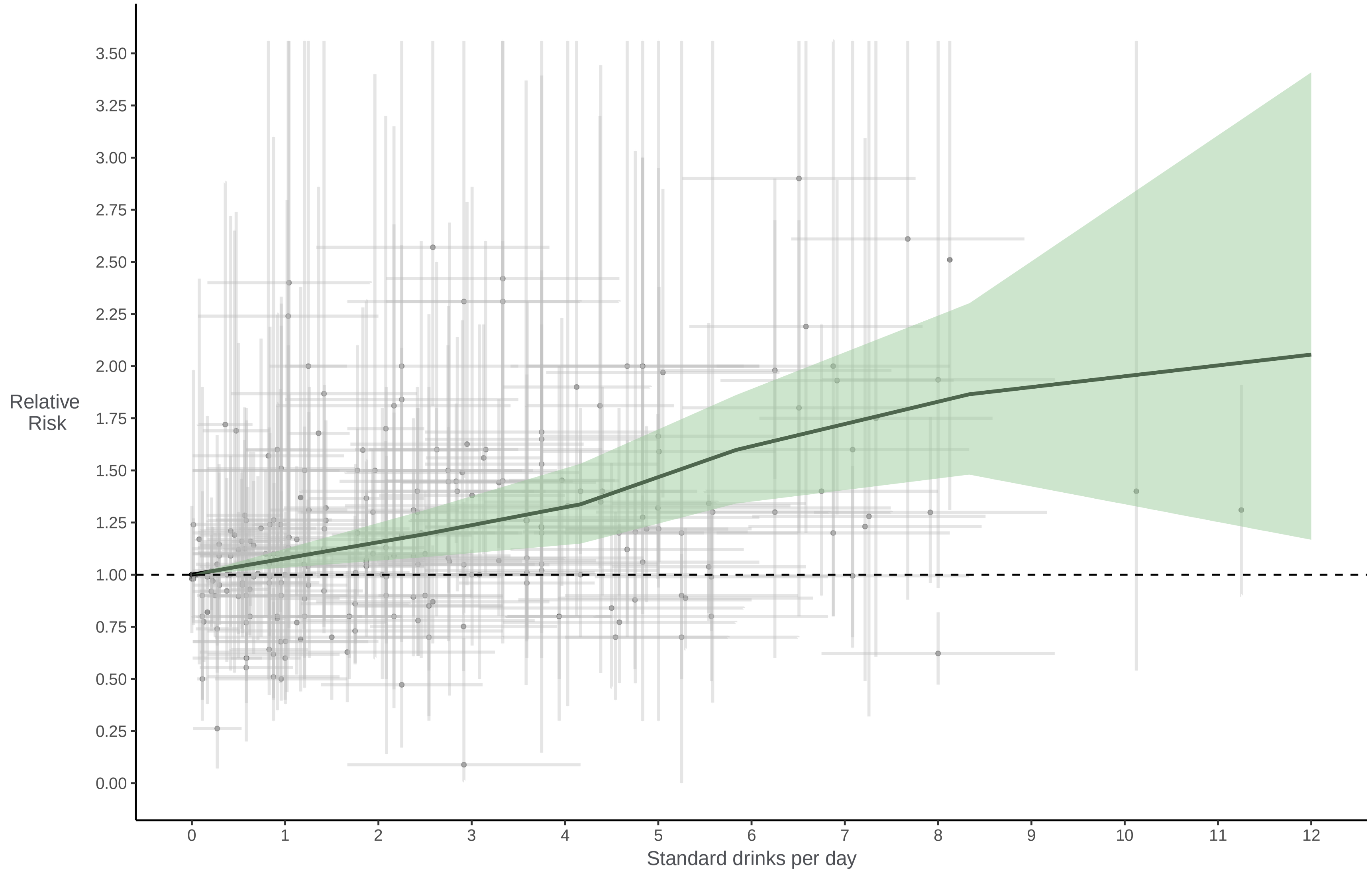

References for Colon and rectum cancer

Akhter M, Kuriyama S, Nakaya N, et al. Alcohol consumption is associated with an increased risk of distal colon and rectal cancer in Japanese men: the Miyagi Cohort Study. Eur J Cancer 2007; 43: 383–90.

Allen NE, Beral V, Casabonne D, et al. Moderate alcohol intake and cancer incidence in women. J Natl Cancer Inst 2009; 101: 296–305.

Barra S, Negri E, Franceschi S, Guarneri S, La Vecchia C. Alcohol and colorectal cancer: a case-control study from northern Italy. Cancer Causes Control 1992; 30: 153–9.

Benedetti A, Parent ME, Siemiatycki J. Lifetime consumption of alcoholic beverages and risk of 13 types of cancer in men: results from a case-control study in Montreal. Cancer Detect Prev 2009; 32: 352–62.

Bongaerts BW, van den Brandt PA, Goldbohm RA, de Goeij AF, Weijenberg MP. Alcohol consumption, type of alcoholic beverage and risk of colorectal cancer at specific subsites. Int J Cancer 2008; 123: 2411–7.

Boutron MC, Faivre J, Dop MC, Quipourt V, Senesse P. Tobacco, alcohol, and colorectal tumors: a multistep process. Am J Epidemiol 1995; 1410: 1038–46.

Breslow RA, Chen CM, Graubard BI, Mukamal KJ. Prospective study of alcohol consumption quantity and frequency and cancer-specific mortality in the US population. Am J Epidemiol 2011; 174: 1044–53.

Chen K, Jiang Q, Ma X, et al. Alcohol drinking and colorectal cancer: a population-based prospective cohort study in China. Eur J Epidemiol 2005; 20: 149–54.

Choi SY, Kahyo H. Effect of cigarette smoking and alcohol consumption in the etiology of cancers of the digestive tract. Int J Cancer 1991; 490: 381–6.

Chyou PH, Nomura AM, Stemmermann GN. A prospective study of colon and rectal cancer among Hawaii Japanese men. Ann Epidemiol 1996; 6: 276–82.

Crockett SD, Long MD, Dellon ES, Martin CF, Galanko JA, Sandler RS. Inverse relationship between moderate alcohol intake and rectal cancer: analysis of the North Carolina Colon Cancer Study. Dis Colon Rectum 2011; 54: 887–94.

Ferrari P, Jenab M, Norat T, et al. Lifetime and baseline alcohol intake and risk of colon and rectal cancers in the European prospective investigation into cancer and nutrition (EPIC). Int J Cancer 2007; 121: 2065–72.

Flood A, Caprario L, Chaterjee N, Lacey JV, Schairer C, Schatzkin A. Folate, methionine, alcohol, and colorectal cancer in a prospective study of women in the United States. Cancer Causes Control 2002; 130: 551–61.

Gao CM, Takezaki T, Wu JZ, et al. Polymorphisms of alcohol dehydrogenase 2 and aldehyde dehydrogenase 2 and colorectal cancer risk in Chinese males. World J Gastroenterol 2008; 14: 5078–83.

Gaziano JM, Gaziano TA, Glynn RJ, et al. Light-to-moderate alcohol consumption and mortality in the Physicians' Health Study enrollment cohort. J Am Coll Cardiol 2000; 350: 96–105.

Gerhardsson de Verdier M, Romelsjo A, Lundberg M. Alcohol and cancer of the colon and rectum. Eur J Cancer Prev 1993; 20: 401–8.

Goldbohm RA, Van den Brandt PA, Van, et al. Prospective study on alcohol consumption and the risk of cancer of the colon and rectum in the Netherlands. Cancer Causes Control 1994; 50: 95–104.

Hassan MM, Phan A, Li D, Dagohoy CG, Leary C, Yao JC. Risk factors associated with neuroendocrine tumors: A U.S.-based case-control study. Int J Cancer 2008; 123: 867–73.

Ho JW, Lam TH, Tse CW, et al. Smoking, drinking and colorectal cancer in Hong Kong Chinese: a case-control study. Int J Cancer 2004; 1090: 587–97.

Hoshiyama Y, Sekine T, Sasaba T. A case-control study of colorectal cancer and its relation to diet, cigarettes, and alcohol consumption in Saitama Prefecture, Japan. Tohoku J Exp Med 1993; 1710: 153–65.

Hu J, Morrison H, Mery L, DesMeules M, Macleod M. Diet and vitamin or mineral supplementation and risk of colon cancer by subsite in Canada. Eur J Cancer Prev 2007; 16: 275–91.

Ji BT, Dai Q, Gao YT, et al. Cigarette and alcohol consumption and the risk of colorectal cancer in Shanghai, China. Eur J Cancer Prev 2002; 110: 237–44.

Kabat GC, Miller AB, Jain M, Rohan TE. Dietary intake of selected B vitamins in relation to risk of major cancers in women. Br J Cancer 2008; 99: 816–21.

Kim J, Kim DH, Lee BH, et al. Folate intake and the risk of colorectal cancer in a Korean population. Eur J Clin Nutr 2009; 630: 1057–64.

Kim MK, Ko MJ, Han JT. Alcohol consumption and mortality from all-cause and cancers among 1.34 million Koreans: the results from the Korea national health insurance corporation's health examinee cohort in 2000. Cancer Causes Control 2010; 21: 2295–302.

Klatsky AL, Armstrong MA, Friedman GD, Hiatt RA. The relations of alcoholic beverage use to colon and rectal cancer. Am J Epidemiol 1988; 1280: 1007–15.

Kono S, Ikeda M, Tokudome S, Nishizumi M, Kuratsune M. Cigarette smoking, alcohol and cancer mortality: a cohort study of male Japanese physicians. Jpn J Cancer Res 1987; 78: 1323–8.

Kune S, Kune GA, Watson LF. Case-control study of alcoholic beverages as etiological factors: the Melbourne Colorectal Cancer Study. Nutr Cancer 1987; 90: 43–56.

Lightfoot TJ, Barrett JH, Bishop T, et al. Methylene tetrahydrofolate reductase genotype modifies the chemopreventive effect of folate in colorectal adenoma, but not colorectal cancer. Cancer Epidemiol Biomarkers Prev 2008; 17: 2421–30.

Lim HJ, Park BJ. [Cohort study on the association between alcohol consumption and the risk of colorectal cancer in the Korean elderly]. J Prev Med Public Health 2008; 41: 23–9.

Morita M, Le Marchand L, Kono S, et al. Genetic polymorphisms of CYP2E1 and risk of colorectal cancer: the Fukuoka Colorectal Cancer Study. Cancer Epidemiol Biomarkers Prev 2009; 18: 235–41.

Murata M, Tagawa M, Watanabe S, Kimura H, Takeshita T, Morimoto K. Genotype difference of aldehyde dehydrogenase 2 gene in alcohol drinkers influences the incidence of Japanese colorectal cancer patients. Jpn J Cancer Res 1999; 900: 711–9.

Murata M, Takayama K, Choi BC, Pak AW. A nested case-control study on alcohol drinking, tobacco smoking, and cancer. Cancer Detect Prev 1996; 20: 557–65.

Nakaya N, Tsubono Y, Kuriyama S, et al. Alcohol consumption and the risk of cancer in Japanese men: the Miyagi cohort study. Eur J Cancer Prev 2005; 14: 169–74.

Newcomb PA, Storer BE, Marcus PM. Cancer of the large bowel in women in relation to alcohol consumption: a case-control study in Wisconsin (United States). Cancer Causes Control 1993; 40: 405–11.

Otani T, Iwasaki M, Yamamoto S, et al. Alcohol consumption, smoking, and subsequent risk of colorectal cancer in middle-aged and elderly Japanese men and women: Japan Public Health Center-based prospective study. Cancer Epidemiol Biomarkers Prev 2003; 120: 1492–500.

Ozasa K. Alcohol use and mortality in the Japan Collaborative Cohort Study for Evaluation of Cancer (JACC). Asian Pac J Cancer Prev 2007; 8 Suppl: 81–8.

Pedersen A, Johansen C, Gronbaek M. Relations between amount and type of alcohol and colon and rectal cancer in a Danish population based cohort study. Gut 2003; 520: 861–7.

Peters RK, Garabrant DH, Yu MC, Mack TM. A case-control study of occupational and dietary factors in colorectal cancer in young men by subsite. Cancer Res 1989; 490: 5459–68.

Riboli E, Cornee J, Macquart-Moulin G, Kaaks R, Casagrande C, Guyader M. Cancer and polyps of the colorectum and lifetime consumption of beer and other alcoholic beverages. Am J Epidemiol 1991; 1340: 157–66.

Sanjoaquin MA, Appleby PN, Thorogood M, Mann JI, Key TJ. Nutrition, lifestyle and colorectal cancer incidence: a prospective investigation of 10998 vegetarians and non-vegetarians in the United Kingdom. Br J Cancer 2004; 90: 118–21.

Sharpe CR, Siemiatycki J, Rachet B. Effects of alcohol consumption on the risk of colorectal cancer among men by anatomical subsite (Canada). Cancer Causes Control 2002; 130: 483–91.

Stern MC, Conti DV, Siegmund KD, et al. DNA repair single-nucleotide polymorphisms in colorectal cancer and their role as modifiers of the effect of cigarette smoking and alcohol in the Singapore Chinese Health Study. Cancer Epidemiol Biomarkers Prev 2007; 16: 2363–72.

Tavani A, Ferraroni M, Mezzetti M, Franceschi S, Lo Re A, La Vecchia C. Alcohol intake and risk of cancers of the colon and rectum. Nutr Cancer 1998; 300: 213–9.

Thun MJ, Peto R, Lopez AD, et al. Alcohol consumption and mortality among middle-aged and elderly U.S. adults. N Engl J Med 1997; 337: 1705–14.

Thygesen LC, Wu K, Gronbaek M, Fuchs CS, Willett WC, Giovannucci E. Alcohol intake and colorectal cancer: a comparison of approaches for including repeated measures of alcohol consumption. Epidemiology 2008; 19: 258–64.

Tsong WH, Koh WP, Yuan JM, Wang R, Sun CL, Yu MC. Cigarettes and alcohol in relation to colorectal cancer: the Singapore Chinese Health Study. Br J Cancer 2007; 96: 821–7.

Wakai K, Kojima M, Tamakoshi K, et al. Alcohol consumption and colorectal cancer risk: findings from the JACC Study. J Epidemiol 2005; 15 Suppl 2(Suppl 2): S173-9.

Wang H, Rothenbacher D, Low M, Stegmaier C, Brenner H, Diepgen TL. Atopic diseases, immunoglobulin E and risk of cancer of the prostate, breast, lung and colorectum. Int J Cancer 2006; 119: 695–701.

Wernli KJ, Wang Y, Zheng Y, Potter JD, Newcomb PA. The relationship between gravidity and parity and colorectal cancer risk. J Womens Health (Larchmt) 2009; 18: 995–1001.

Wu AH, Paganini-Hill A, Ross RK, Henderson BE. Alcohol, physical activity and other risk factors for colorectal cancer: a prospective study. Br J Cancer 1987; 550: 687–94.

Yamada K, Araki S, Tamura M, et al. Case-control study of colorectal carcinoma in situ and cancer in relation to cigarette smoking and alcohol use (Japan). Cancer Causes Control 1997; 80: 780–5.

Yamamoto S, Nakagawa T, Matsushita Y, et al. Visceral fat area and markers of insulin resistance in relation to colorectal neoplasia. Diabetes Care 2010; 33: 184–9.

Yi SW, Sull JW, Linton JA, Nam CM, Ohrr H. Alcohol consumption and digestive cancer mortality in Koreans: the Kangwha Cohort Study. J Epidemiol 2010; 20: 204–11.

Yuan JM, Ross RK, Gao YT, Henderson BE, Yu MC. Follow up study of moderate alcohol intake and mortality among middle aged men in Shanghai, China. BMJ 1997; 314: 18–23.

Zhao J, Zhu Y, Wang PP, et al. Interaction between alcohol drinking and obesity in relation to colorectal cancer risk: a case-control study in Newfoundland and Labrador, Canada. BMC Public Health 2012; 12: 94.

5. Diabetes mellitus

Summary of the meta-analysis conducted for GBD 2016

Search String:

(((“”ethanol“”[MeSH Terms] OR “”alcohols“”[MeSH Terms]) AND (“”diabetes mellitus“”[MeSH Terms] OR (“”diabetes“”[All Fields] AND “”mellitus“”[All Fields]) OR “”diabetes mellitus“”[All Fields] OR “”diabetes“”[All Fields] OR “”diabetes insipidus“”[MeSH Terms] OR (“”diabetes“”[All Fields] AND “”insipidus“”[All Fields]) OR “”diabetes insipidus“”[All Fields])) AND (“”0001/01/01“”[PDAT] : “”2016/12/31“”[PDAT])) AND “”humans“”[MeSH Terms]

PRISMA flow diagram

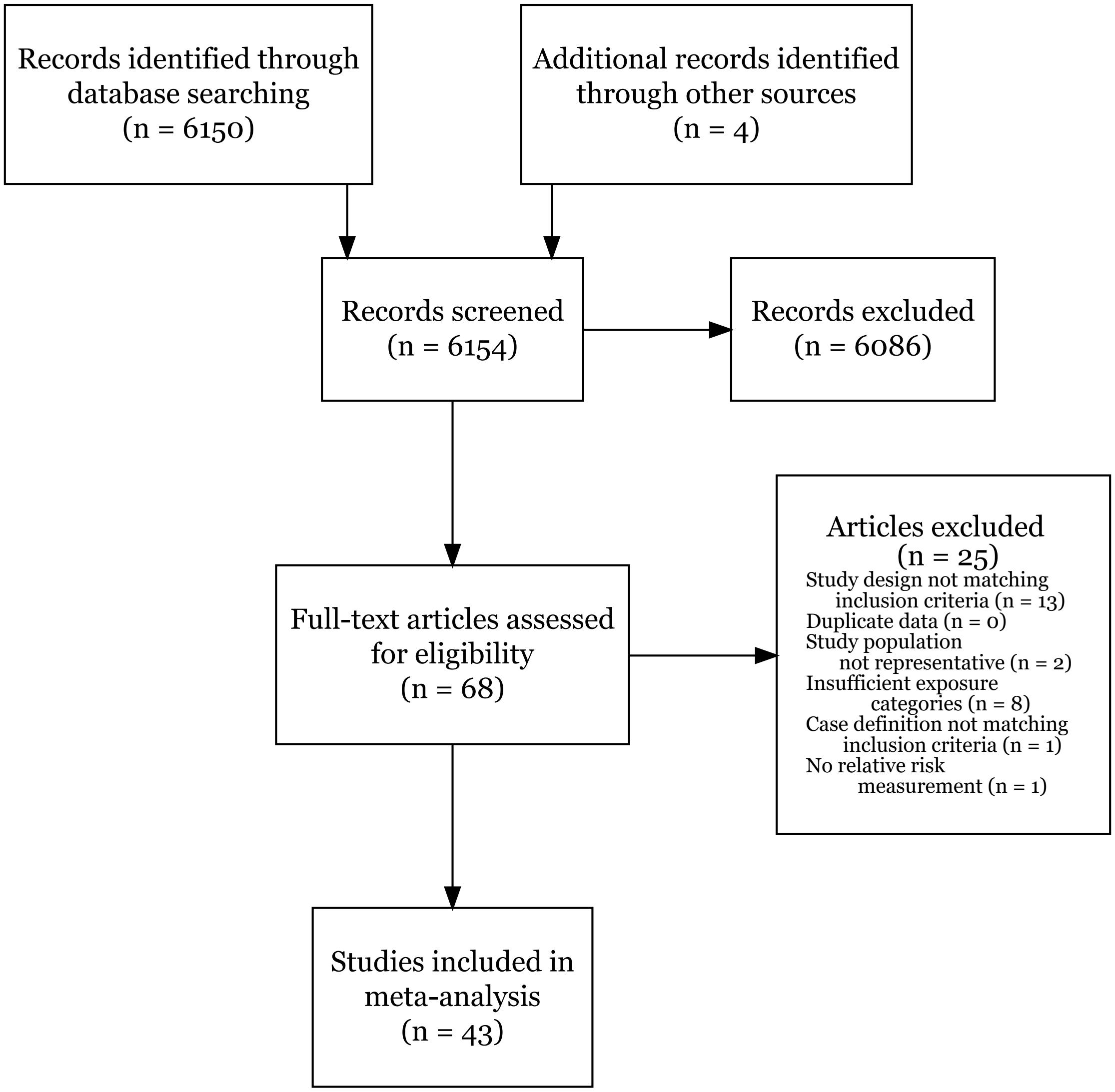

Relative risk (RR) curves for Diabetes mellitus by number of standard drinks consumed daily, by sex. Points are relative risk estimates from studies.
The vertical bars capture the uncertainty in each study, related to the sample size and the horizontal bars capture the range of drinks consumed by individuals in the study.
The black line represents the estimated RR for Diabetes mellitus at each level of consumption. The shaded green areas represent the 95% uncertainty interval associated with the estimated RR.
Dotted line is a reference for a relative risk of 1.

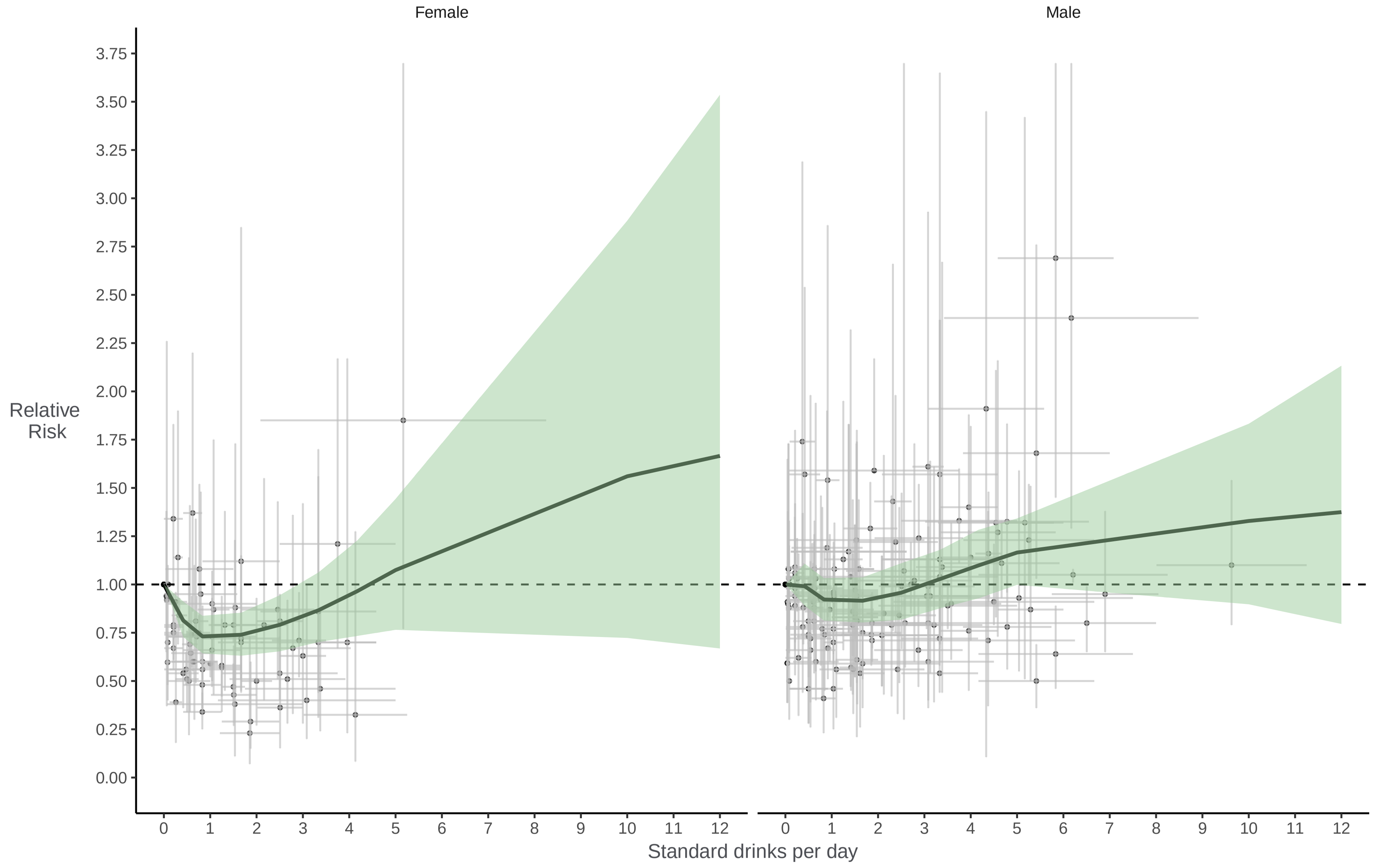

References for Diabetes mellitus

Abbasi A, Corpeleijn E, Gansevoort RT, et al. Role of HDL cholesterol and estimates of HDL particle composition in future development of type 2 diabetes in the general population: the PREVEND study. J Clin Endocrinol Metab 2013; 98(8): E1352-9.

Ajani UA, Hennekens CH, Spelsberg A, Manson JE. Alcohol consumption and risk of type 2 diabetes mellitus among US male physicians. Arch Intern Med 2000; 160: 1025–30.

Balkau B, Soulimane S, Lange C, Gautier A, Tichet J, Vol S. Are the same clinical risk factors relevant for incident diabetes defined by treatment, fasting plasma glucose, and HbA1c? Diabetes Care 2011; 34: 957–9.

Beulens JW, van der Schouw YT, Bergmann MM, et al. Alcohol consumption and risk of type 2 diabetes in European men and women: influence of beverage type and body size The EPIC-InterAct study. J Intern Med 2012; 272: 358–70.

Boggs DA, Rosenberg L, Ruiz-Narvaez EA, Palmer JR. Coffee, tea, and alcohol intake in relation to risk of type 2 diabetes in African American women. Am J Clin Nutr 2010; 92: 960–6.

Carlsson S, Hammar N, Grill V, Kaprio J. Alcohol consumption and the incidence of type 2 diabetes: a 20-year follow-up of the Finnish twin cohort study. Diabetes Care 2003; 26: 2785–90.

Conigrave KM, Hu BF, Camargo CA, Stampfer MJ, Willett WC, Rimm EB. A prospective study of drinking patterns in relation to risk of type 2 diabetes among men. Diabetes 2001; 50: 2390–5.

Cullmann M, Hilding A, Ostenson CG. Alcohol consumption and risk of pre-diabetes and type 2 diabetes development in a Swedish population. Diabet Med 2012; 29: 441–52.

Djousse L, Biggs ML, Mukamal KJ, Siscovick DS. Alcohol consumption and type 2 diabetes among older adults: the Cardiovascular Health Study. Obesity (Silver Spring) 2007; 15: 1758–65.

Heianza Y, Arase Y, Saito K, et al. Role of alcohol drinking pattern in type 2 diabetes in Japanese men: the Toranomon Hospital Health Management Center Study 11 (TOPICS 11). Am J Clin Nutr 2013; 97: 561–8.

Hodge AM, English DR, O, Dea K, Giles GG. Alcohol intake, consumption pattern and beverage type, and the risk of Type 2 diabetes. Diabet Med 2006; 23: 690–7.

Holbrook TL, Barrett-Connor E, Wingard DL. A prospective population-based study of alcohol use and non-insulin-dependent diabetes mellitus. Am J Epidemiol 1990; 132: 902–9.

Hu FB, Manson JE, Stampfer MJ, et al. Diet, lifestyle, and the risk of type 2 diabetes mellitus in women. N Engl J Med 2001; 345: 790–7.

Hu G, Jousilahti P, Peltonen M, Bidel S, Tuomilehto J. Joint association of coffee consumption and other factors to the risk of type 2 diabetes: a prospective study in Finland. Int J Obes (Lond) 2006; 30: 1742–9.

Jee SH, Foong AW, Hur NW, Samet JM. Smoking and risk for diabetes incidence and mortality in Korean men and women. Diabetes Care 2010; 33: 2567–72.

Joosten MM, Chiuve SE, Mukamal KJ, Hu FB, Hendriks HF, Rimm EB. Changes in alcohol consumption and subsequent risk of type 2 diabetes in men. Diabetes 2011; 60: 74–9.

Joosten MM, Grobbee DE, van der A DL, Verschuren WM, Hendriks HF, Beulens JW. Combined effect of alcohol consumption and lifestyle behaviors on risk of type 2 diabetes. Am J Clin Nutr 2010; 91: 1777–83.

Kao WH, Puddey IB, Boland LL, Watson RL, Brancati FL. Alcohol consumption and the risk of type 2 diabetes mellitus: Atherosclerosis Risk in Communities study. Am J Epidemiol 2001; 154: 748–57.

Kawakami N, Takatsuka N, Shimizu H, Ishibashi H. Effects of smoking on the incidence of non-insulin-dependent diabetes mellitus. Replication and extension in a Japanese cohort of male employees. Am J Epidemiol 1997; 145: 103–9.

Koloverou E, Panagiotakos DB, Pitsavos C, et al. Effects of alcohol consumption and the metabolic syndrome on 10-year incidence of diabetes: the ATTICA study. Diabetes Metab 2015; 41: 152–9.

Lee DH, Folsom AR, Jacobs DR. Dietary iron intake and Type 2 diabetes incidence in postmenopausal women: the Iowa Women's Health Study. Diabetologia 2004; 47: 185–94.

Lee DH, Ha MH, Kim JH, et al. Gamma-glutamyltransferase and diabetes–a 4 year follow-up study. Diabetologia 2003; 46: 359–64.

Marques-Vidal P, Vollenweider P, Waeber G, consumption A, incidence of type 2. Alcohol consumption and incidence of type 2 diabetes. Results from the CoLaus study. Nutr Metab Cardiovasc Dis 2015; 25: 75–84.

Maty SC, Lynch JW, Raghunathan TE, Kaplan GA. Childhood socioeconomic position, gender, adult body mass index, and incidence of type 2 diabetes mellitus over 34 years in the Alameda County Study. Am J Public Health 2008; 98: 1486–94.

Meisinger C, Thorand B, Schneider A, Stieber J, Doring A, Lowel H. Sex differences in risk factors for incident type 2 diabetes mellitus: the MONICA Augsburg cohort study. Arch Intern Med 2002; 162: 82–9.

Nagaya T, Yoshida H, Takahashi H, Kawai M. Resting heart rate and blood pressure, independent of each other, proportionally raise the risk for type-2 diabetes mellitus. Int J Epidemiol 2010; 39: 215–22.

Nakanishi N, Suzuki K, Tatara K. Alcohol consumption and risk for development of impaired fasting glucose or type 2 diabetes in middle-aged Japanese men. Diabetes Care 2003; 26: 48–54.

Onat A, Hergenc G, Kucukdurmaz Z, et al. Moderate and heavy alcohol consumption among Turks: long-term impact on mortality and cardiometabolic risk. Turk Kardiyol Dern Ars 2009; 37: 83–90.

Persson LG, Lingfors H, Nilsson M, Molstad S. The possibility of lifestyle and biological risk markers to predict morbidity and mortality in a cohort of young men after 26 years follow-up. BMJ Open 2015; 5: 6798.

Rasouli B, Ahlbom A, Andersson T, et al. Alcohol consumption is associated with reduced risk of Type 2 diabetes and autoimmune diabetes in adults: results from the Nord-Trondelag health study. Diabet Med 2013; 30: 56–64.

Roh WG, Shin HC, Choi JH, Lee YJ, Kim K. Alcohol consumption and higher incidence of impaired fasting glucose or type 2 diabetes in obese Korean men. Alcohol 2009; 43: 643–8.

Sato KK, Hayashi T, Harita N, et al. Relationship between drinking patterns and the risk of type 2 diabetes: the Kansai Healthcare Study. J Epidemiol Community Health 2012; 66: 507–11.

Sawada SS, Lee IM, Muto T, Matuszaki K, Blair SN. Cardiorespiratory fitness and the incidence of type 2 diabetes: prospective study of Japanese men. Diabetes Care 2003; 26: 2918–22.

Shi L, Shu XO, Li H, et al. Physical activity, smoking, and alcohol consumption in association with incidence of type 2 diabetes among middle-aged and elderly Chinese men. PLoS One 2013; 8: 77919.

Stringhini S, Tabak A, Akbaraly TN, et al. Contribution of modifiable risk factors to social inequalities in type 2 diabetes: prospective Whitehall II cohort study. BMJ 2012; 345: e5452.

Strodl E, Kenardy J. Psychosocial and non-psychosocial risk factors for the new diagnosis of diabetes in elderly women. Diabetes Res Clin Pract 2006; 74: 57–65.

Tsai AC, Lee SH. Determinants of new-onset diabetes in older adults–Results of a national cohort study. Clin Nutr 2015; 34: 937–42.

Tsumura K, Hayashi T, Suematsu C, Endo G, Fujii S, Okada K. Daily alcohol consumption and the risk of type 2 diabetes in Japanese men: the Osaka Health Survey. Diabetes Care 1999; 22: 1432–7.

Waki K, Noda M, Sasaki S, et al. Alcohol consumption and other risk factors for self-reported diabetes among middle-aged Japanese: a population-based prospective study in the JPHC study cohort I. Diabet Med 2005; 22: 323–31.

Wannamethee SG, Camargo CA, Manson JE, Willett WC, Rimm EB. Alcohol drinking patterns and risk of type 2 diabetes mellitus among younger women. Arch Intern Med 2003; 163: 1329–36.

Wannamethee SG, Shaper AG, Perry IJ, Alberti KG. Alcohol consumption and the incidence of type II diabetes. J Epidemiol Community Health 2002; 56: 542–8.

Wei M, Gibbons LW, Mitchell TL, Kampert JB, Blair SN. Alcohol intake and incidence of type 2 diabetes in men. Diabetes Care 2000; 23: 18–22.

de Vegt F, Dekker JM, Groeneveld WJ, et al. Moderate alcohol consumption is associated with lower risk for incident diabetes and mortality: the Hoorn Study. Diabetes Res Clin Pract 2002; 57: 53–60.

6. Epilepsy

Summary of the meta-analysis conducted for GBD 2016

Search String:

((("”ethanol“”[MeSH Terms] OR “”alcohols“”[MeSH Terms]) AND (“”epilepsy“”[MeSH Terms] OR “”epilepsy“”[All Fields])) AND (“”0001/01/01“”[PDAT] : “”2016/12/31“”[PDAT])) AND “”humans“”[MeSH Terms]

PRISMA flow diagram

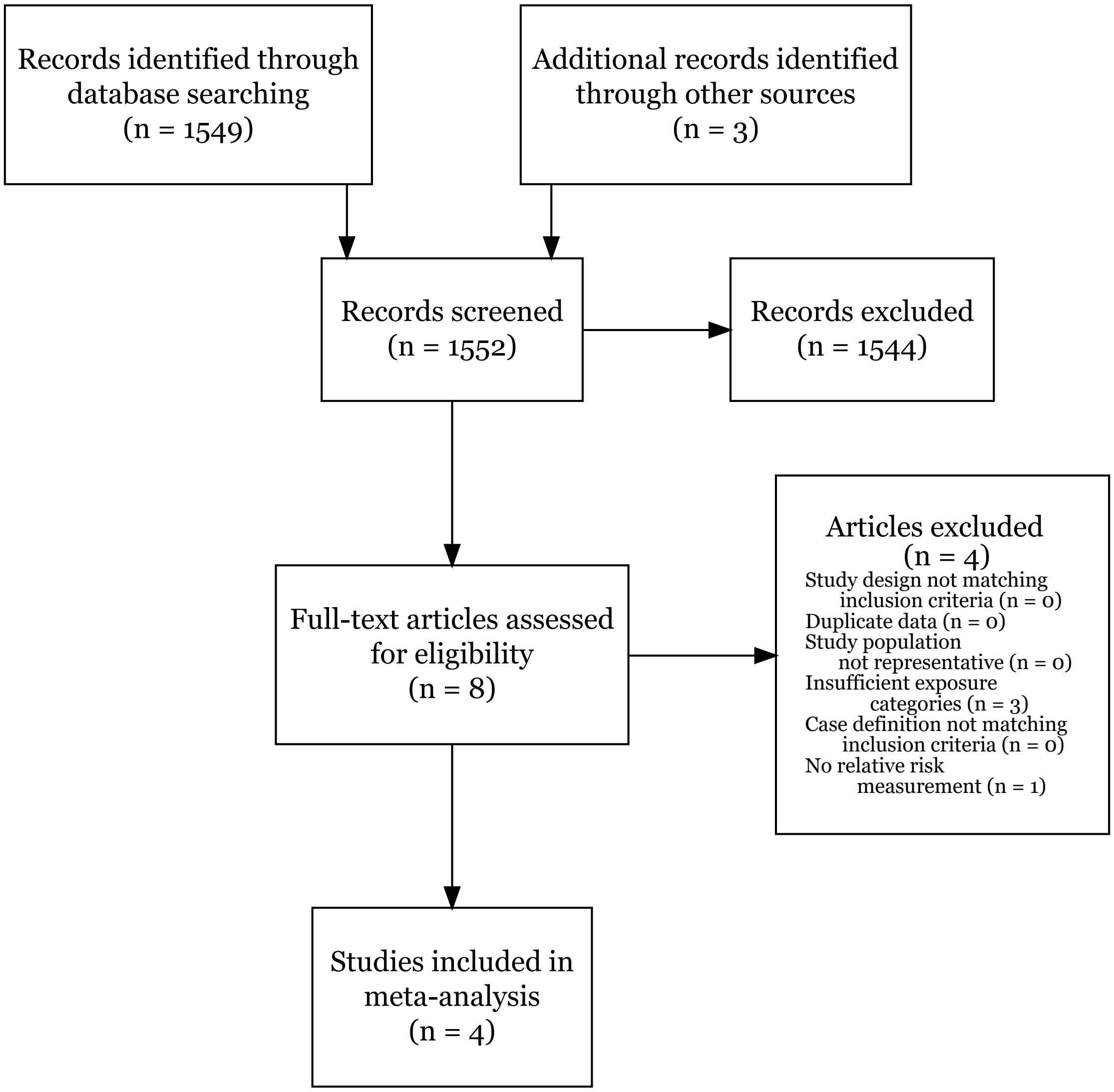

Relative risk (RR) curves for Epilepsy by number of standard drinks consumed daily. Points are relative risk estimates from studies.
The vertical bars capture the uncertainty in each study, related to the sample size and the horizontal bars capture the range of drinks consumed by individuals in the study.
The black line represents the estimated RR for Epilepsy at each level of consumption. The shaded green areas represent the 95% uncertainty interval associated with the estimated RR.
Dotted line is a reference for a relative risk of 1.

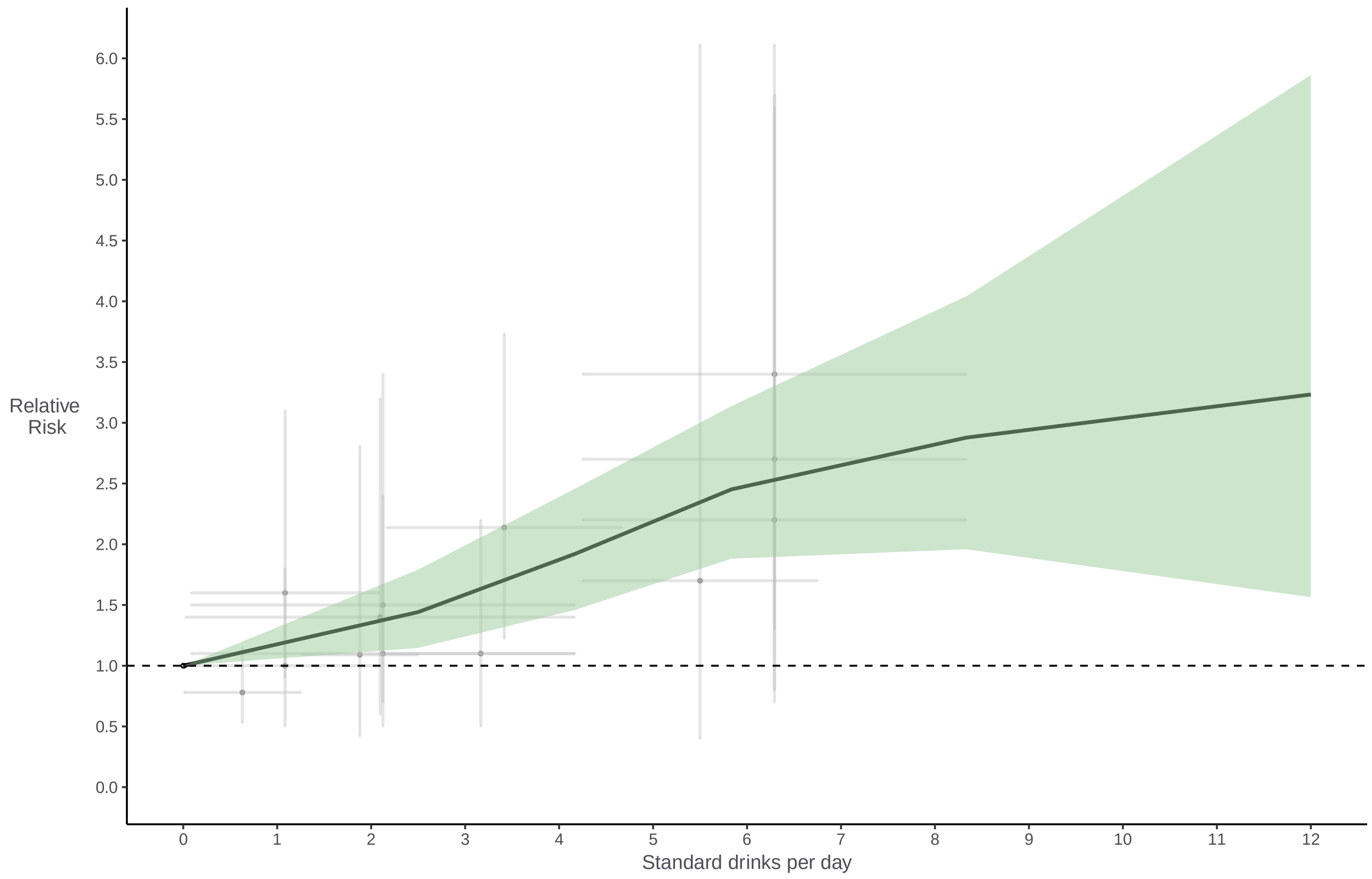

References for Epilepsy

Dworetzky BA, Bromfield EB, Townsend MK, Kang JH. A prospective study of smoking, caffeine, and alcohol as risk factors for seizures or epilepsy in young adult women: data from the Nurses' Health Study II. Epilepsia 2010; 51: 198–205.

Leone M, Bottacchi E, Beghi E, et al. Alcohol use is a risk factor for a first generalized tonic-clonic seizure. The ALC. E. (Alcohol and Epilepsy) Study Group. Neurology 1997; 48: 614–20.

Leone M, Bottacchi E, Beghi E, et al. Risk factors for a first generalized tonic-clonic seizure in adult life. Neurol Sci 2002; 23: 99–106.

Ng SK, Hauser WA, Brust JC, Susser M. Alcohol consumption and withdrawal in new-onset seizures. N Engl J Med 1988; 319: 666–73.

7. Esophageal cancer

Summary of the meta-analysis conducted for GBD 2016

Search String:

((“”ethanol“”[MeSH Terms] OR “”alcohols“”[MeSH Terms]) AND esophageal neoplasms“”[MeSH Terms] AND (“”0001/01/01“”[PDAT] : “”2016/12/31“”[PDAT])) AND “”humans“”[MeSH Terms]

PRISMA flow diagram

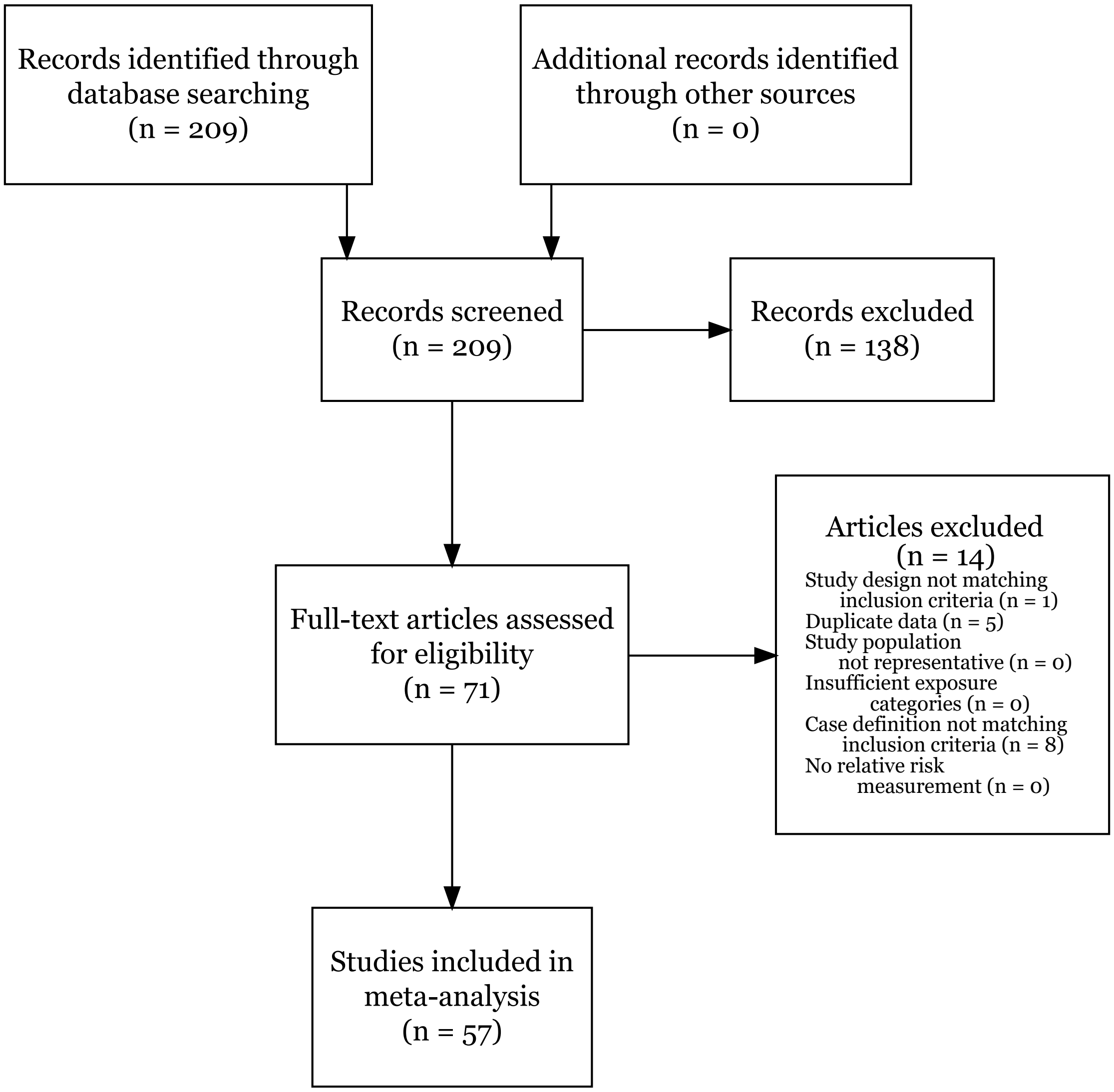

Relative risk (RR) curves for Esophageal cancer by number of standard drinks consumed daily. Points are relative risk estimates from studies.
The vertical bars capture the uncertainty in each study, related to the sample size and the horizontal bars capture the range of drinks consumed by individuals in the study.
The black line represents the estimated RR for Esophageal cancer at each level of consumption. The shaded green areas represent the 95% uncertainty interval associated with the estimated RR.
Dotted line is a reference for a relative risk of 1.

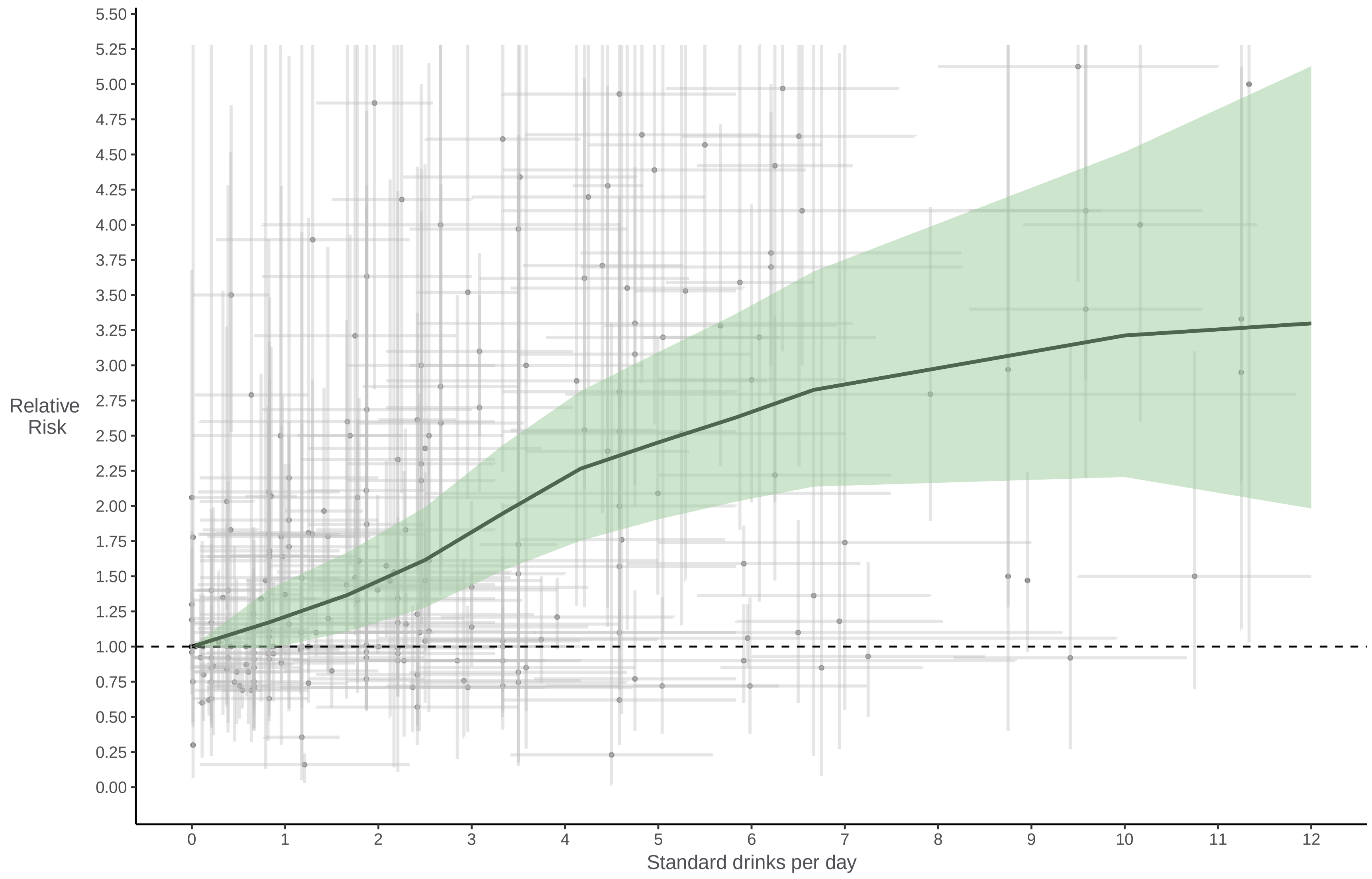

References for Esophageal cancer

Adelhardt M, Moller Jensen O, Sand Hansen H. Cancer of the larynx, pharynx, and oesophagus in relation to alcohol and tobacco consumption among Danish brewery workers. Dan Med Bull 1985; 32: 119–23.

Allen NE, Beral V, Casabonne D, et al. Moderate alcohol intake and cancer incidence in women. J Natl Cancer Inst 2009; 101: 296–305.

Anderson LA, Cantwell MM, Watson RG, et al. The association between alcohol and reflux esophagitis, Barrett's esophagus, and esophageal adenocarcinoma. Gastroenterology 2009; 136: 799–805.

Anderson LA, Cantwell MM, Watson RG, et al. The association between alcohol and reflux esophagitis, Barrett's esophagus, and esophageal adenocarcinoma. Gastroenterology 2009; 136: 799–805.

Benedetti A, Parent ME, Siemiatycki J. Lifetime consumption of alcoholic beverages and risk of 13 types of cancer in men: results from a case-control study in Montreal. Cancer Detect Prev 2009; 32: 352–62.

Boffetta P, Garfinkel L. Alcohol drinking and mortality among men enrolled in an American Cancer Society prospective study. Epidemiology 1990; 1: 342–8.

Boonyaphiphat P, Thongsuksai P, Sriplung H, Puttawibul P. Lifestyle habits and genetic susceptibility and the risk of esophageal cancer in the Thai population. Cancer Lett 2002; 1860: 193–9.

Bosetti C, La Vecchia C, Negri E, Franceschi S. Wine and other types of alcoholic beverages and the risk of esophageal cancer. Eur J Clin Nutr 2000; 540: 918–20.

Brown LM, Hoover RN, Greenberg RS, et al. Are racial differences in squamous cell esophageal cancer explained by alcohol and tobacco use? J Natl Cancer Inst 1994; 860: 1340–5.

Castellsague X, Munoz N, De Stefani E, et al. Independent and joint effects of tobacco smoking and alcohol drinking on the risk of esophageal cancer in men and women. Int J Cancer 1999; 820: 657–64.

Chen J, Zhang N, Ling Y, et al. Alcohol consumption as a risk factor for esophageal adenocarcinoma in North China. Tohoku J Exp Med 2011; 224: 21–7.

Cheng KK, Duffy SW, Day NE, Lam TH, Chung SF, Badrinath P. Stopping drinking and risk of oesophageal cancer. BMJ 1995; 3100: 1094–7.

Choi SY, Kahyo H. Effect of cigarette smoking and alcohol consumption in the etiology of cancers of the digestive tract. Int J Cancer 1991; 490: 381–6.

Corley DA, Kubo A, Zhao W. Abdominal obesity and the risk of esophageal and gastric cardia carcinomas. Cancer Epidemiol Biomarkers Prev 2008; 17: 352–8.

De Stefani E, Ronco AL, Boffetta P, et al. Nutrient intake and risk of squamous cell carcinoma of the esophagus: a case-control study in Uruguay. Nutr Cancer 2006; 56: 149–57.

Fan Y, Yuan JM, Wang R, Gao YT, Yu MC. Alcohol, tobacco, and diet in relation to esophageal cancer: the Shanghai Cohort Study. Nutr Cancer 2008; 60: 354–63.

Freedman ND, Abnet CC, Leitzmann MF, et al. A prospective study of tobacco, alcohol, and the risk of esophageal and gastric cancer subtypes. Am J Epidemiol 2007; 165: 1424–33.

Gammon MD, Schoenberg JB, Ahsan H, et al. Tobacco, alcohol, and socioeconomic status and adenocarcinomas of the esophagus and gastric cardia. J Natl Cancer Inst 1997; 890: 1277–84.

Gao YT, McLaughlin JK, Blot WJ, Ji BT, Benichou J, Dai Q. Risk factors for esophageal cancer in Shanghai, China. I. Role of cigarette smoking and alcohol drinking. Int J Cancer 1994; 580: 192–6.

Garidou A, Tzonou A, Lipworth L, Signorello LB, Kalapothaki V, Trichopoulos D. Life-style factors and medical conditions in relation to esophageal cancer by histologic type in a low-risk population. Int J Cancer 1996; 680: 295–9.

Guo YM, Wang Q, Liu YZ, Chen HM, Qi Z, Guo QH. Genetic polymorphisms in cytochrome P4502E1, alcohol and aldehyde dehydrogenases and the risk of esophageal squamous cell carcinoma in Gansu Chinese males. World J Gastroenterol 2008; 14: 1444–9.

Hanaoka T, Tsugane S, Ando N, et al. Alcohol consumption and risk of esophageal cancer in Japan: a case-control study in seven hospitals. Jpn J Clin Oncol 1994; 240: 241–6.

Hashibe M, Boffetta P, Janout V, et al. Esophageal cancer in Central and Eastern Europe: tobacco and alcohol. Int J Cancer 2007; 120: 1518–22.

Ishiguro S, Sasazuki S, Inoue M, Kurahashi N, Iwasaki M, Tsugane S. Effect of alcohol consumption, cigarette smoking and flushing response on esophageal cancer risk: a population-based cohort study (JPHC study). Cancer Lett 2009; 275: 240–6.

Jayaprakash V, Menezes RJ, Javle MM, et al. Regular aspirin use and esophageal cancer risk. Int J Cancer 2006; 119: 202–7.

Kabat GC, Ng SK, Wynder EL. Tobacco, alcohol intake, and diet in relation to adenocarcinoma of the esophagus and gastric cardia. Cancer Causes Control 1993; 40: 123–32.

Kim MK, Ko MJ, Han JT. Alcohol consumption and mortality from all-cause and cancers among 1.34 million Koreans: the results from the Korea national health insurance corporation's health examinee cohort in 2000. Cancer Causes Control 2010; 21: 2295–302.

Kimm H, Kim S, Jee SH. The independent effects of cigarette smoking, alcohol consumption, and serum aspartate aminotransferase on the alanine aminotransferase ratio in korean men for the risk for esophageal cancer. Yonsei Med J 2010; 51: 310–7.

Kono S, Ikeda M, Tokudome S, Nishizumi M, Kuratsune M. Cigarette smoking, alcohol and cancer mortality: a cohort study of male Japanese physicians. Jpn J Cancer Res 1987; 78: 1323–8.

Lagergren J, Bergstrom R, Lindgren A, Nyren O. The role of tobacco, snuff and alcohol use in the aetiology of cancer of the oesophagus and gastric cardia. Int J Cancer 2000; 850: 340–6.

Launoy G, Milan C, Day NE, Faivre J, Pienkowski P, Gignoux M. Oesophageal cancer in France: potential importance of hot alcoholic drinks. Int J Cancer 1997; 710: 917–23.

Lee CH, Wu DC, Lee JM, et al. Carcinogenetic impact of alcohol intake on squamous cell carcinoma risk of the oesophagus in relation to tobacco smoking. Eur J Cancer 2007; 43: 1188–99.

Lindblad M, Rodríguez LAG, Lagergren J. Body mass, tobacco and alcohol and risk of esophageal, gastric cardia, and gastric non-cardia adenocarcinoma among men and women in a nested case-control study. Cancer Causes Control 2005; 16: 285–94.

Martinez I. Factors associated with ccer of the esophagus, mouth, and pharynx in Puerto Rico. J Natl Cancer Inst 1969; 42: 1069–94.

Mettlin C, Graham S, Priore R, Marshall J, Swanson M. Diet and cancer of the esophagus. Nutr Cancer 1981; 20: 143–7.

Nakaya N, Tsubono Y, Kuriyama S, et al. Alcohol consumption and the risk of cancer in Japanese men: the Miyagi cohort study. Eur J Cancer Prev 2005; 14: 169–74.

Ozasa K. Alcohol use and mortality in the Japan Collaborative Cohort Study for Evaluation of Cancer (JACC). Asian Pac J Cancer Prev 2007; 8 Suppl: 81–8.

Pandeya N, Williams G, Green AC, Webb PM, Whiteman DC. Alcohol consumption and the risks of adenocarcinoma and squamous cell carcinoma of the esophagus. Gastroenterology 2009; 136: 1215–24.

Pottern LM, Morris LE, Blot WJ, Ziegler RG, Fraumeni JF. Esophageal cancer among black men in Washington, D.C. I. Alcohol, tobacco, and other risk factors. J Natl Cancer Inst 1981; 670: 777–83.

Sewram V, De Stefani E, Brennan P, Boffetta P. Mate consumption and the risk of squamous cell esophageal cancer in Uruguay. Cancer Epidemiol Biomarkers Prev 2003; 120: 508–13.

Sharp L, Chilvers CE, Cheng KK, et al. Risk factors for squamous cell carcinoma of the oesophagus in women: a case-control study. Br J Cancer 2001; 850: 1667–70.

Smith M, Zhou M, Whitlock G, et al. Esophageal cancer and body mass index: results from a prospective study of 220,000 men in China and a meta-analysis of published studies. Int J Cancer 2008; 122: 1604–10.

Steevens J, Schouten LJ, Goldbohm RA, van den Brandt PA. Alcohol consumption, cigarette smoking and risk of subtypes of oesophageal and gastric cancer: a prospective cohort study. Gut 2010; 59: 39–48.

Takezaki T, Shinoda M, Hatooka S, et al. Subsite-specific risk factors for hypopharyngeal and esophageal cancer (Japan). Cancer Causes Control 2000; 11: 597–608.

Tuyns AJ, Pequignot G, Abbatucci JS. Oesophageal cancer and alcohol consumption; importance of type of beverage. Int J Cancer 1979; 230: 443–7.

Vassallo A, Correa P, De Stefani E, et al. Esophageal cancer in Uruguay: a case-control study. J Natl Cancer Inst 1985; 750: 1005–9.

Vaughan TL, Davis S, Kristal A, Thomas DB. Obesity, alcohol, and tobacco as risk factors for cancers of the esophagus and gastric cardia: adenocarcinoma versus squamous cell carcinoma. Cancer Epidemiol Biomarkers Prev 1995; 40: 85–92.

Vioque J, Barber X, Bolumar F, et al. Esophageal cancer risk by type of alcohol drinking and smoking: a case-control study in Spain. BMC Cancer 2008; 8: 221.

Wang Y, Ji R, Wei X, et al. Esophageal squamous cell carcinoma and ALDH2 and ADH1B polymorphisms in Chinese females. Asian Pac J Cancer Prev 2011; 12: 2065–8.

Wu AH, Wan P, Bernstein L. A multiethnic population-based study of smoking, alcohol and body size and risk of adenocarcinomas of the stomach and esophagus (United States). Cancer Causes Control 2001; 120: 721–32.

Wu M, Zhang ZF, Kampman E, et al. Does family history of cancer modify the effects of lifestyle risk factors on esophageal cancer? A population-based case-control study in China. Int J Cancer 2011; 128: 2147–57.

Wynder EL, Bross IJ. A study of etiological factors in cancer of the esophagus. Cancer 1961; 140: 389–413.

Yang CX, Wang HY, Wang ZM, et al. Risk factors for esophageal cancer: a case-control study in South-western China. Asian Pac J Cancer Prev 2005; 60: 48–53.

Yokoyama A, Kato H, Yokoyama T, et al. Esophageal squamous cell carcinoma and aldehyde dehydrogenase-2 genotypes in Japanese females. Alcohol Clin Exp Res 2006; 30: 491–500.

Yokoyama T, Yokoyama A, Kato H, et al. Alcohol flushing, alcohol and aldehyde dehydrogenase genotypes, and risk for esophageal squamous cell carcinoma in Japanese men. Cancer Epidemiol Biomarkers Prev 2003; 120: 1227–33.

Yu MC, Garabrant DH, Peters JM, Mack TM. Tobacco, alcohol, diet, occupation, and carcinoma of the esophagus. Cancer Res 1988; 480: 3843–8.

Znaor A, Brennan P, Gajalakshmi V, et al. Independent and combined effects of tobacco smoking, chewing and alcohol drinking on the risk of oral, pharyngeal and esophageal cancers in Indian men. Int J Cancer 2003; 105: 681–6.

Duell EJ, Travier N, Lujan-Barroso L, Clavel-Chapelon F, Boutron-Ruault MC, Morois S, Palli D, Krogh V, Panico S, Tumino R, Sacerdote C, Quiros JR, Sanchez-Cantalejo E, Navarro C, Gurrea AB, Dorronsoro M, Khaw KT, Allen NE, Key TJ, Bueno-de-Mesquita HB, Ros MM, Numans ME, Peeters PH, Trichopoulou A,

Naska A, Dilis V, Teucher B, Kaaks R, Boeing H, Schutze M, Regner S, Lindkvist B, Johansson I, Hallmans G, Overvad K, Egeberg R, Tjonneland A, Lund E, Weiderpass E, Braaten T, Romieu I, Ferrari P, Jenab M, Stenling R, Aune D, Norat T, Riboli E, Gonzalez CA. Alcohol consumption and gastric cancer risk in the European Prospective Investigation into Cancer and Nutrition (EPIC) cohort. Am J Clin Nutr. 2011; 94(5): 1266–75.

Freedman ND, Murray LJ, Kamangar F, Abnet CC, Cook MB, Nyren O, Ye W, Wu AH, Bernstein L, Brown LM, Ward MH, Pandeya N, Green AC, Casson AG, Giffen C, Risch HA, Gammon MD, Chow WH, Vaughan TL, Corley DA, Whiteman DC. Alcohol intake and risk of oesophageal adenocarcinoma: a pooled analysis from the BEACON Consortium. Gut. 2011; 60(8): 1029–37.

Freedman ND, Murray LJ, Kamangar F, Abnet CC, Cook MB, Nyren O, Ye W, Wu AH, Bernstein L, Brown LM, Ward MH, Pandeya N, Green AC, Casson AG, Giffen C, Risch HA, Gammon MD, Chow WH, Vaughan TL, Corley DA, Whiteman DC. Alcohol intake and risk of oesophageal adenocarcinoma: a pooled analysis from the BEACON Consortium. Gut. 2011; 60(8): 1029–37.

8. Hemorrhagic stroke

Summary of the meta-analysis conducted for GBD 2016

Search String:

((“”ethanol“”[MeSH Terms] OR “”alcohols“”[MeSH Terms]) AND “”intracranial hemorrhages“”[MeSH Terms] AND (“”1950/01/01“”[PDAT] : “”2016/12/31“”[PDAT])) AND “”humans“”[MeSH Terms]

PRISMA flow diagram

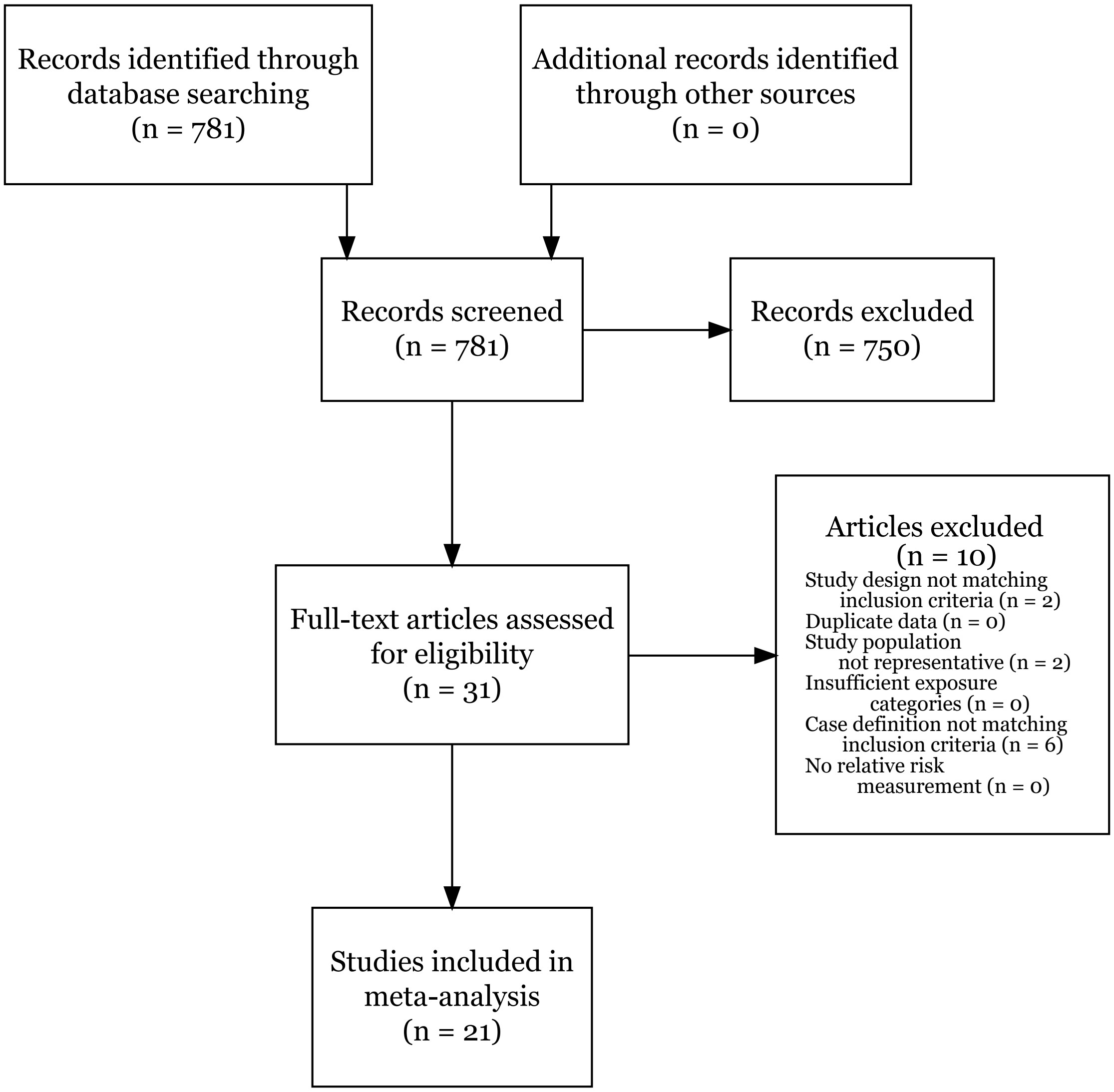

Relative risk (RR) curves for Hemorrhagic stroke by number of standard drinks consumed daily, by sex. Points are relative risk estimates from studies.
The vertical bars capture the uncertainty in each study, related to the sample size and the horizontal bars capture the range of drinks consumed by individuals in the study.
The black line represents the estimated RR for Hemorrhagic stroke at each level of consumption. The shaded green areas represent the 95% uncertainty interval associated with the estimated RR.
Dotted line is a reference for a relative risk of 1.

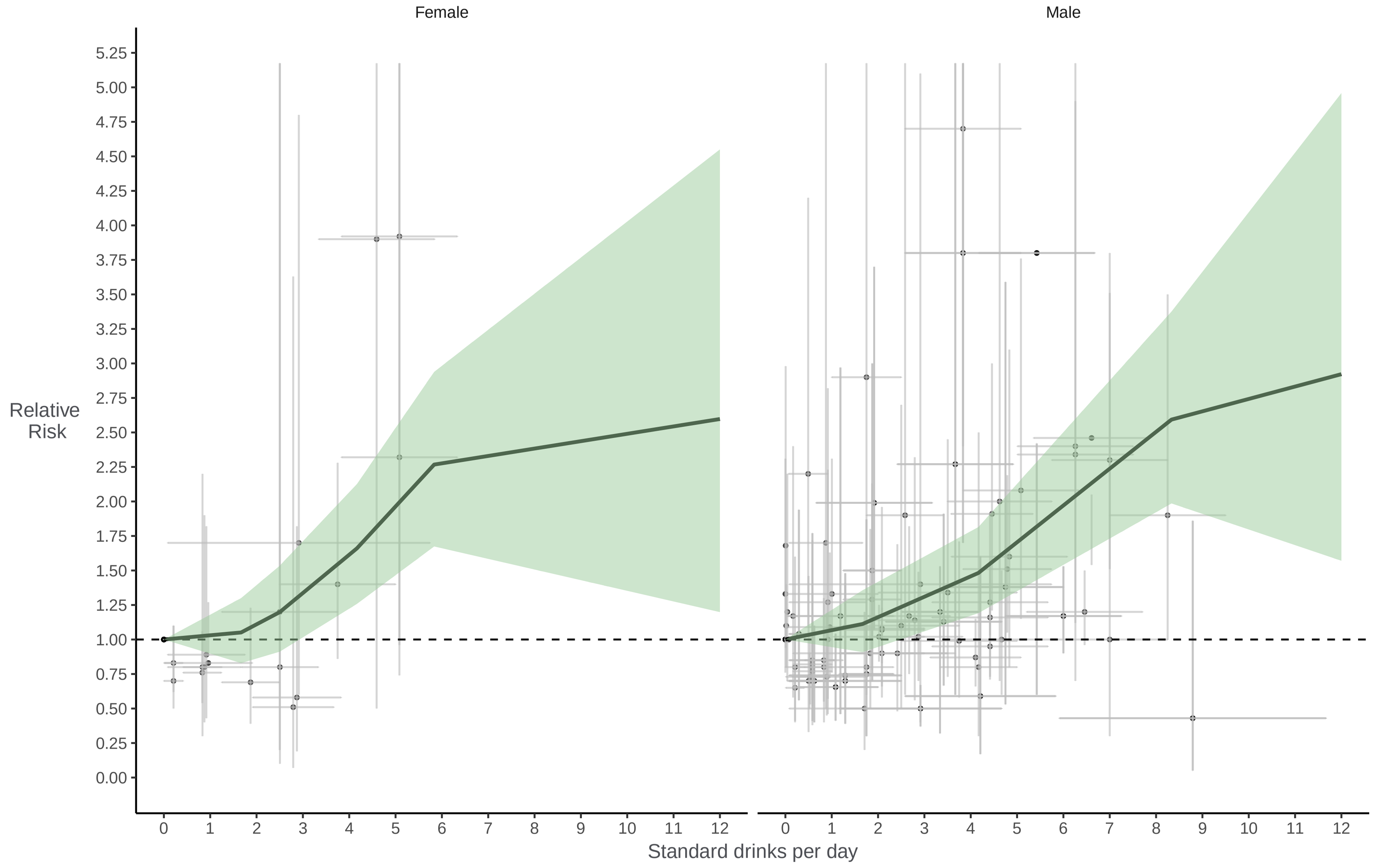

References for Hemorrhagic stroke

Bazzano LA, Gu D, Reynolds K, et al. Alcohol consumption and risk of coronary heart disease among Chinese men. Int J Cardiol 2009; 135: 78–85.

Berger K, Ajani UA, Kase CS, et al. Light-to-moderate alcohol consumption and the risk of stroke among U.S. male physicians. N Engl J Med 1999; 341: 1557–64.

Caicoya M, Rodriguez T, Corrales C, Cuello R, Lasheras C. Alcohol and stroke: a community case-control study in Asturias, Spain. J Clin Epidemiol 1999; 52: 677–84.

Donahue RP, Abbott RD, Reed DM, Yano K. Alcohol and hemorrhagic stroke. The Honolulu Heart Program. JAMA 1986; 255: 2311–4.

Gill JS, Shipley MJ, Tsementzis SA, et al. Alcohol consumption–a risk factor for hemorrhagic and non-hemorrhagic stroke. Am J Med 1991; 90: 489–97.

Ikehara S, Iso H, Toyoshima H, et al. Alcohol consumption and mortality from stroke and coronary heart disease among Japanese men and women: the Japan collaborative cohort study. Stroke 2008; 39: 2936–42.

Ikehara S, Iso H, Yamagishi K, Yamamoto S, Inoue M, Tsugane S. Alcohol consumption, social support, and risk of stroke and coronary heart disease among Japanese men: the JPHC Study. Alcohol Clin Exp Res 2009; 33: 1025–32.

Iso H, Kitamura A, Shimamoto T, et al. Alcohol intake and the risk of cardiovascular disease in middle-aged Japanese men. Stroke 1995; 26: 767–73.

Klatsky AL, Armstrong MA, Friedman GD. Alcohol use and subsequent cerebrovascular disease hospitalizations. Stroke 1989; 20: 741–6.

Kono S, Ikeda M, Tokudome S, Nishizumi M, Kuratsune M. Alcohol and mortality: a cohort study of male Japanese physicians. Int J Epidemiol 1986; 15: 527–32.

Longstreth WT, Nelson LM, Koepsell TD, van Belle G. Cigarette smoking, alcohol use, and subarachnoid hemorrhage. Stroke 1992; 23: 1242–9.

Sankai T, Iso H, Shimamoto T, et al. Prospective study on alcohol intake and risk of subarachnoid hemorrhage among Japanese men and women. Alcohol Clin Exp Res 2000; 24: 386–9.

Stampfer MJ, Colditz GA, Willett WC, Speizer FE, Hennekens CH. A prospective study of moderate alcohol consumption and the risk of coronary disease and stroke in women. N Engl J Med 1988; 319: 267–73.

Suh I, Jee SH, Kim HC, Nam CM, Kim IS, Appel LJ. Low serum cholesterol and haemorrhagic stroke in men: Korea Medical Insurance Corporation Study. Lancet 2001; 357: 922–5.

Thrift AG, Donnan GA, McNeil JJ. Heavy drinking, but not moderate or intermediate drinking, increases the risk of intracerebral hemorrhage. Epidemiology 1999; 10: 307–12.

Chiuve SE, Rexrode KM, Spiegelman D, Logroscino G, Manson JE, Rimm EB. Primary prevention of stroke by healthy lifestyle. Circulation. 2008; 118(9): 947–954.

Chiuve SE, Rexrode KM, Spiegelman D, Logroscino G, Manson JE, Rimm EB. Primary prevention of stroke by healthy lifestyle. Circulation. 2008; 118(9): 947–954.

Hansagi H, Romelsj A, Gerhardsson de Verdier M, Andreasson S, Leifman A. Alcohol consumption and stroke mortality. 20-year follow-up of 15,077 men and women. Stroke. 1995; 26(10): 1768–73.

Jones SB, Loehr L, Avery CL, Gottesman RF, Wruck L, Shahar E, Rosamond WD. Midlife alcohol consumption and the risk of stroke in the Atherosclerosis Risk in Communities Study. Stroke. 2015; 46(11): 3124–30.

Klatsky AL, Armstrong MA, Friedman GD, Sidney S. Alcohol drinking and risk of hemorrhagic stroke. Neuroepidemiology. 2002; 21(3): 115-22.

Nielsen NR, Truelsen T, Barefoot JC, Johnsen SP, Overvad K, Boysen G, Schnohr P, Gronbaek M. Is the effect of alcohol on risk of stroke confined to highly stressed persons?. Neuroepidemiology. 2005; 25(3): 105–13.

Yamada S, Koizumi A, Iso H, Wada Y, Watanabe Y, Date C, Yamamoto A, Kikuchi S, Inaba Y, Toyoshima H, Kondo T, Tamakoshi A, Japan Collaborative Cohort Study Group. Risk factors for fatal subarachnoid hemorrhage: the Japan Collaborative Cohort Study. Stroke. 2003; 34(12): 2781–7.

9. Hypertensive heart disease

Summary of the meta-analysis conducted for GBD 2016

Search String:

((“”ethanol“”[MeSH Terms] OR “”alcohols“”[MeSH Terms]) AND (“”hypertension“”[MeSH Terms] OR “”blood pressure“”[MeSH Terms]) AND (“”1980/01/01“”[PDAT] : “”2016/12/31“”[PDAT])) AND “”humans“”[MeSH Terms]

PRISMA flow diagram

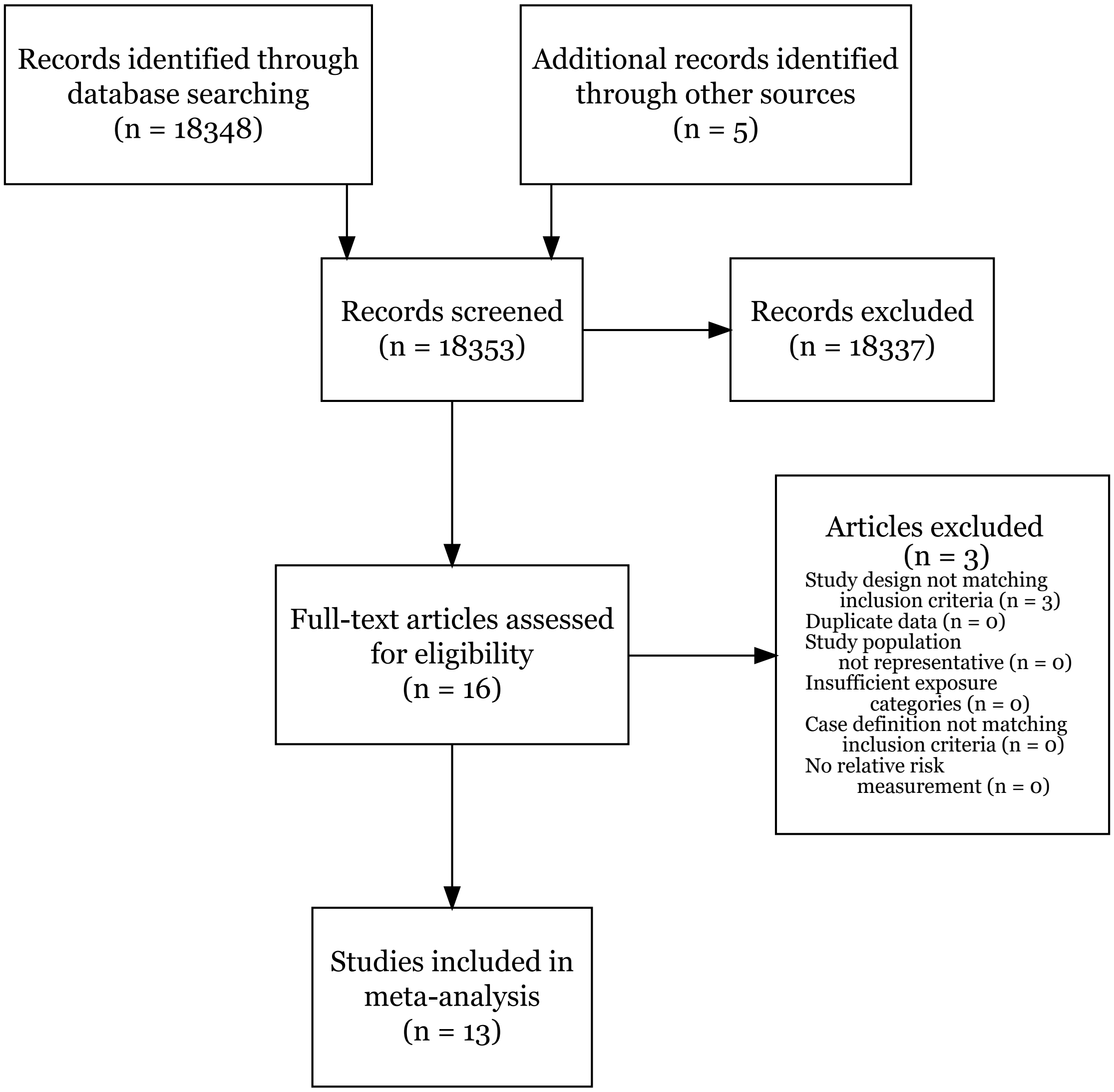

Relative risk (RR) curves for Hypertensive heart disease by number of standard drinks consumed daily. Points are relative risk estimates from studies.
The vertical bars capture the uncertainty in each study, related to the sample size and the horizontal bars capture the range of drinks consumed by individuals in the study.
The black line represents the estimated RR for Hypertensive heart disease at each level of consumption. The shaded green areas represent the 95% uncertainty interval associated with the estimated RR.
Dotted line is a reference for a relative risk of 1.

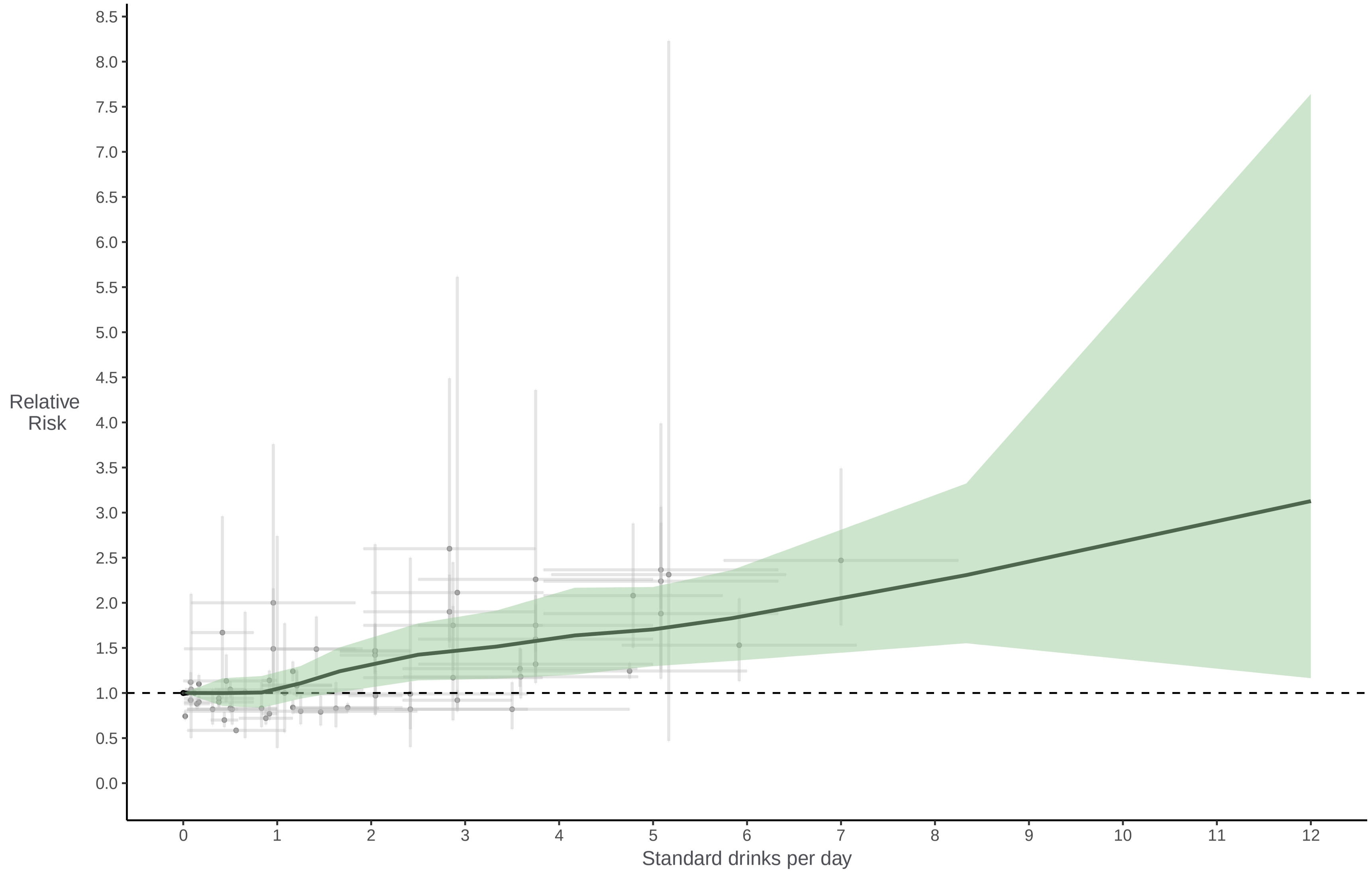

References for Hypertensive heart disease

Ascherio A, Hennekens C, Willett WC, et al. Prospective study of nutritional factors, blood pressure, and hypertension among US women. Hypertension 1996; 27: 1065–72.

Curtis AB, James SA, Strogatz DS, Raghunathan TE, Harlow S. Alcohol consumption and changes in blood pressure among African Americans. The Pitt County Study. Am J Epidemiol 1997; 146: 727–33.

Fuchs FD, Chambless LE, Whelton PK, Nieto FJ, Heiss G. Alcohol consumption and the incidence of hypertension: The Atherosclerosis Risk in Communities Study. Hypertension 2001; 37: 1242–50.

Halanych JH, Safford MM, Kertesz SG, et al. Alcohol consumption in young adults and incident hypertension: 20-year follow-up from the Coronary Artery Risk Development in Young Adults Study. Am J Epidemiol 2010; 171: 532–9.

Klatsky AL, Koplik S, Gunderson E, Kipp H, Friedman GD. Sequelae of systemic hypertension in alcohol abstainers, light drinkers, and heavy drinkers. Am J Cardiol 2006; 98: 1063–8.

Lee SH, Kim YS, Sunwoo S, Huh BY. A retrospective cohort study on obesity and hypertension risk among Korean adults. J Korean Med Sci 2005; 20: 188–95.

Nakanishi N, Makino K, Nishina K, Suzuki K, Tatara K. Relationship of light to moderate alcohol consumption and risk of hypertension in Japanese male office workers. Alcohol Clin Exp Res 2002; 26: 988–94.

Nakanishi N, Yoshida H, Nakamura K, Suzuki K, Tatara K. Alcohol consumption and risk for hypertension in middle-aged Japanese men. J Hypertens 2001; 19: 851–5.

Ohmori S, Kiyohara Y, Kato I, et al. Alcohol intake and future incidence of hypertension in a general Japanese population: the Hisayama study. Alcohol Clin Exp Res 2002; 26: 1010–6.

Sesso HD, Cook NR, Buring JE, Manson JE, Gaziano JM. Alcohol consumption and the risk of hypertension in women and men. Hypertension 2008; 51: 1080–7.

Sesso HD, Cook NR, Buring JE, Manson JE, Gaziano JM. Alcohol consumption and the risk of hypertension in women and men. Hypertension 2008; 51: 1080–7.

Thadhani R, Camargo CA, Stampfer MJ, Curhan GC, Willett WC, Rimm EB. Prospective study of moderate alcohol consumption and risk of hypertension in young women. Arch Intern Med 2002; 162: 569–74.

Tsuruta M, Adachi H, Hirai Y, Fujiura Y, Imaizumi T. Association between alcohol intake and development of hypertension in Japanese normotensive men: 12-year follow-up study. Am J Hypertens 2000; 13: 482–7.

Witteman JC, Willett WC, Stampfer MJ, et al. A prospective study of nutritional factors and hypertension among US women. Circulation 1989; 80: 1320–7.

Yamada Y, Ishizaki M, Kido T, et al. Alcohol, high blood pressure, and serum gamma-glutamyl transpeptidase level. Hypertension 1991; 18: 819–26.

10. Interpersonal violence

Summary of the meta-analysis conducted for GBD 2016

Search String:

((“”ethanol“”[MeSH Terms] OR “”alcohols“”[MeSH Terms]) AND “”violence“”[MeSH Terms] AND (“”1950/01/01“”[PDAT] : “”2016/12/31“”[PDAT])) AND “”humans“”[MeSH Terms]

PRISMA flow diagram

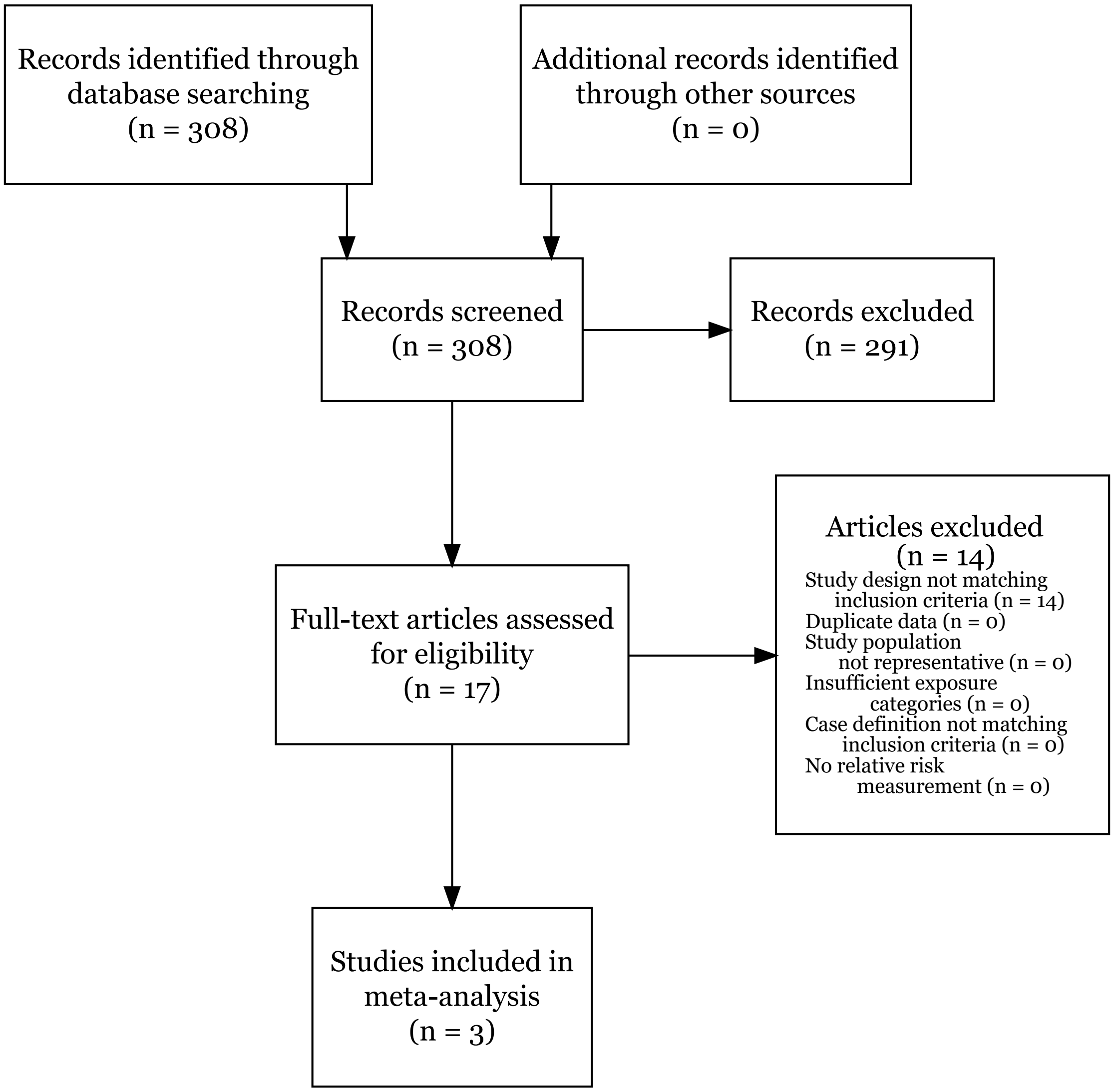

Relative risk (RR) curves for Interpersonal violence by number of standard drinks consumed daily. Points are relative risk estimates from studies.
The vertical bars capture the uncertainty in each study, related to the sample size and the horizontal bars capture the range of drinks consumed by individuals in the study.
The black line represents the estimated RR for Interpersonal violence at each level of consumption. The shaded green areas represent the 95% uncertainty interval associated with the estimated RR.
Dotted line is a reference for a relative risk of 1.

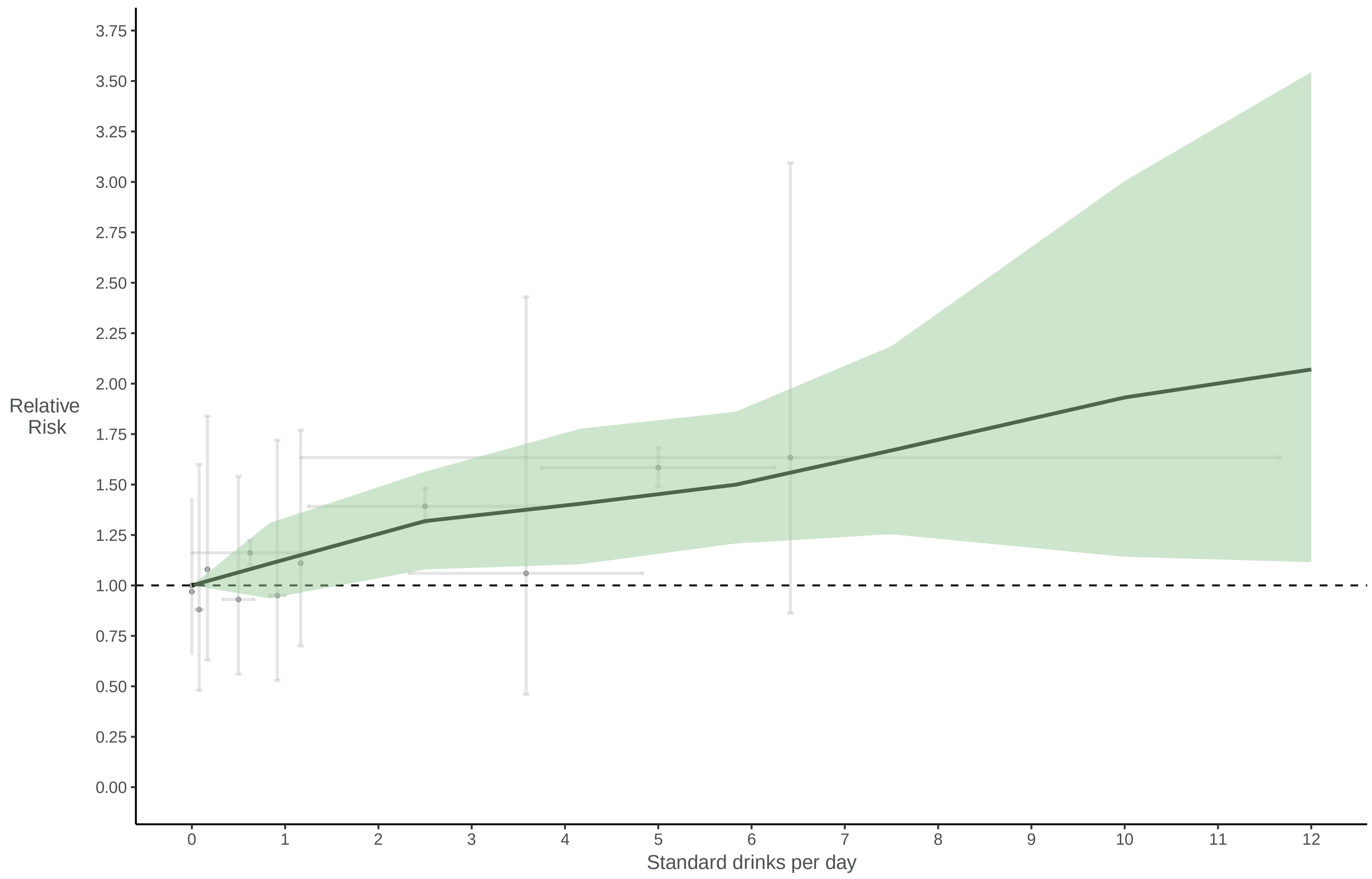

References for Interpersonal violence

Blackwelder WC, Yano K, Rhoads GG, Kagan A, Gordon T, Palesch Y. Alcohol and mortality: the Honolulu Heart Study. Am J Med 1980; 68: 164–9.

Gaziano JM, Gaziano TA, Glynn RJ, Sesso HD, Ajani UA, Stampfer MJ, Manson JE, Hennekens CH, Buring JE. Light-to-moderate alcohol consumption and mortality in the Physicians' Health Study enrollment cohort. J Am Coll Cardiol. 2000; 350(1): 96–105.

Kuendig H, Hasselberg M, Laflamme L, Daeppen JB, Gmel G. Alcohol and nonlethal injuries: a Swiss emergency department study on the risk relationship between acute alcohol consumption and type of injury. J Trauma. 2008; 65(1): 203–11.

11. Ischaemic heart disease

Summary of the meta-analysis conducted for GBD 2016

Search String:

((“”ethanol“”[MeSH Terms] OR “”alcohols“”[MeSH Terms]) AND ((“”myocardial ischemia“”[MeSH Terms] OR “”coronary artery disease“”[MeSH Terms]) OR “”ischemic heart disease“”[All Fields] OR (“”myocardial ischemia“”[MeSH Terms] OR “”coronary artery disease“”[MeSH Terms]) OR “”myocardial ischemia“”[All Fields] OR “”coronary artery disease“”[MeSH Terms] OR “”coronary artery disease“”[All Fields] OR “”atherosclerosis“”[MeSH Terms] OR “”atherosclerosis“”[Title/Abstract] OR “”myocardial infarction“”[Title/Abstract] OR “”heart infarction“”[Title/Abstract]) AND (“”1950/01/01“”[PDAT] : “”2016/12/31“”[PDAT])) AND “”humans“”[MeSH Terms]

PRISMA flow diagram

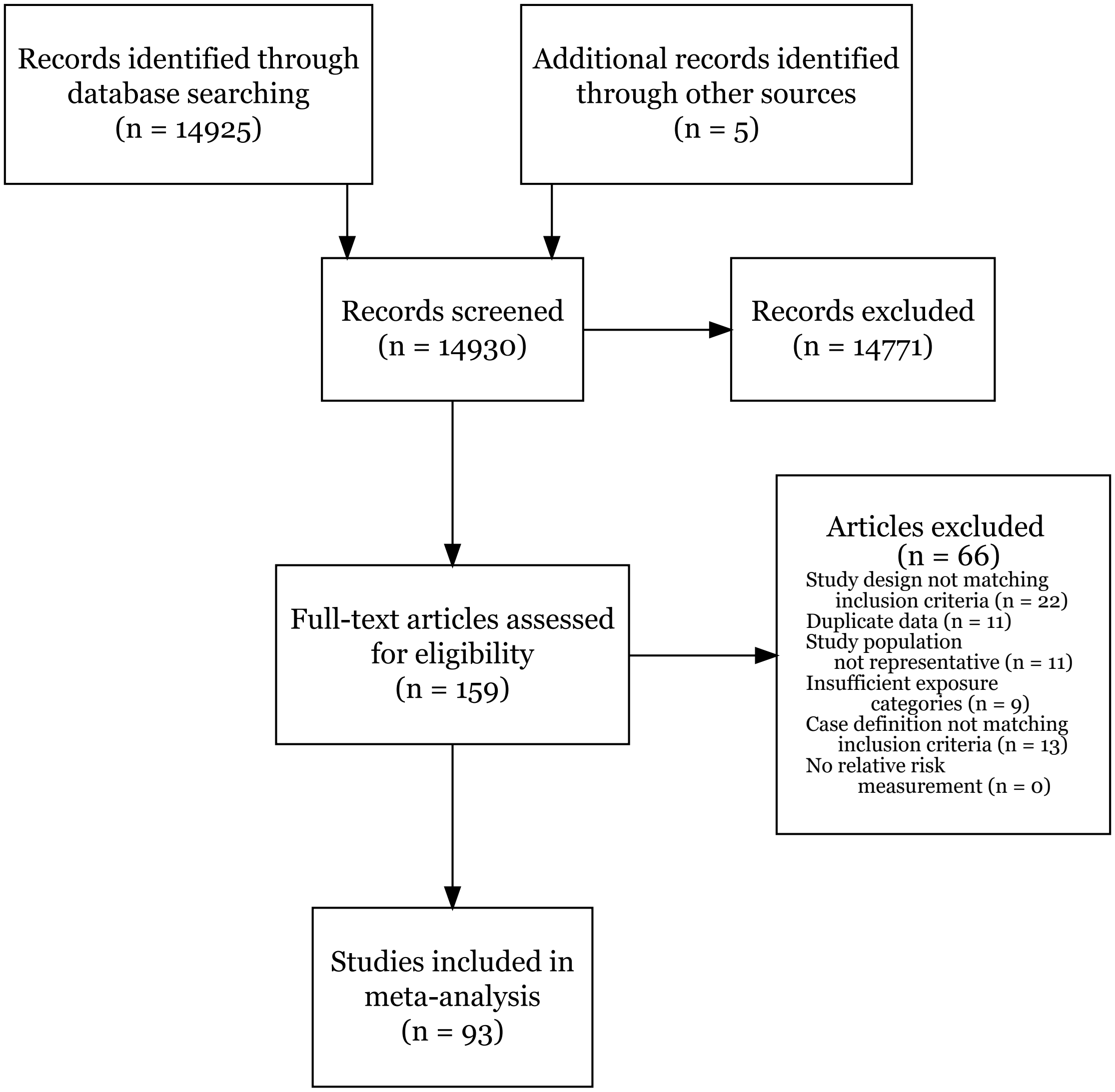

Relative risk (RR) curves for Ischaemic heart disease by number of standard drinks consumed daily, by sex. Points are relative risk estimates from studies.
The vertical bars capture the uncertainty in each study, related to the sample size and the horizontal bars capture the range of drinks consumed by individuals in the study.
The black line represents the estimated RR for Ischaemic heart disease at each level of consumption. The shaded green areas represent the 95% uncertainty interval associated with the estimated RR.
Dotted line is a reference for a relative risk of 1.

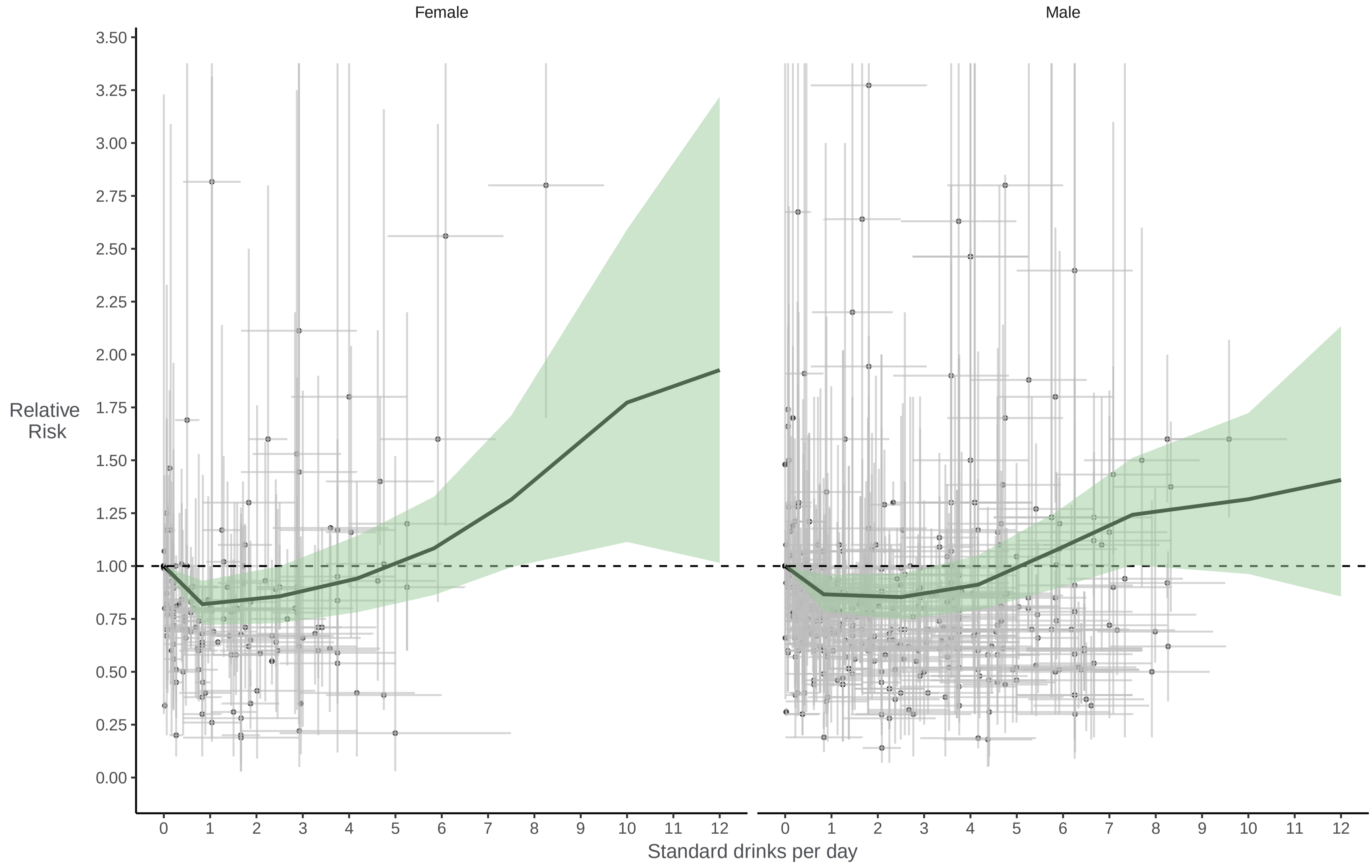

References for Ischaemic heart disease

Albert CM, Manson JE, Cook NR, Ajani UA, Gaziano JM, Hennekens CH. Moderate alcohol consumption and the risk of sudden cardiac death among US male physicians. Circulation 1999; 100: 944–50.

Arriola L, Martinez-Camblor P, Larranaga N, et al. Alcohol intake and the risk of coronary heart disease in the Spanish EPIC cohort study. Heart 2010; 96: 124–30.

Bazzano LA, Gu D, Reynolds K, et al. Alcohol consumption and risk for stroke among Chinese men. Ann Neurol 2007; 62: 569–78.

Bergmann MM, Rehm J, Klipstein-Grobusch K, et al. The association of pattern of lifetime alcohol use and cause of death in the European prospective investigation into cancer and nutrition (EPIC) study. Int J Epidemiol 2013; 42: 1772–90.

Bianchi C, Negri E, La Vecchia C, Franceschi S. Alcohol consumption and the risk of acute myocardial infarction in women. J Epidemiol Community Health 1993; 47: 308–11.

Bobak M, Malyutina S, Horvat P, et al. Alcohol, drinking pattern and all-cause, cardiovascular and alcohol-related mortality in Eastern Europe. Eur J Epidemiol 2016; 31: 21–30.

Boffetta P, Garfinkel L. Alcohol drinking and mortality among men enrolled in an American Cancer Society prospective study. Epidemiology 1990; 1: 342–8.

Chiuve SE, Rimm EB, Mukamal KJ, et al. Light-to-moderate alcohol consumption and risk of sudden cardiac death in women. Heart Rhythm 2010; 7: 1374–80.

Chiuve SE, Rimm EB, Mukamal KJ, et al. Light-to-moderate alcohol consumption and risk of sudden cardiac death in women. Heart Rhythm 2010; 7: 1374–80.

Colditz GA, Branch LG, Lipnick RJ, et al. Moderate alcohol and decreased cardiovascular mortality in an elderly cohort. Am Heart J 1985; 109: 886–9.

Cullen KJ, Knuiman MW, Ward NJ. Alcohol and mortality in Busselton, Western Australia. Am J Epidemiol 1993; 137: 242–8.

Dai J, Mukamal KJ, Krasnow RE, Swan GE, Reed T. Higher usual alcohol consumption was associated with a lower 41-y mortality risk from coronary artery disease in men independent of genetic and common environmental factors: the prospective NHLBI Twin Study. Am J Clin Nutr 2015; 102: 31–9.

Djousse L, Lee IM, Buring JE, Gaziano JM. Alcohol consumption and risk of cardiovascular disease and death in women: potential mediating mechanisms. Circulation 2009; 120: 237–44.

Doll R, Peto R, Boreham J, Sutherland I. Mortality in relation to alcohol consumption: a prospective study among male British doctors. Int J Epidemiol 2005; 34: 199–204.

Dorn JM, Hovey K, Williams BA, et al. Alcohol drinking pattern and non-fatal myocardial infarction in women. Addiction 2007; 102: 730–9.

Ebbert JO, Janney CA, Sellers TA, Folsom AR, Cerhan JR. The association of alcohol consumption with coronary heart disease mortality and cancer incidence varies by smoking history. J Gen Intern Med 2005; 20: 14–20.

Ebrahim S, Lawlor DA, Shlomo YB, et al. Alcohol dehydrogenase type 1C (ADH1C) variants, alcohol consumption traits, HDL-cholesterol and risk of coronary heart disease in women and men: British Women's Heart and Health Study and Caerphilly cohorts. Atherosclerosis 2008; 196: 871–8.

Ebrahim S, Lawlor DA, Shlomo YB, et al. Alcohol dehydrogenase type 1C (ADH1C) variants, alcohol consumption traits, HDL-cholesterol and risk of coronary heart disease in women and men: British Women's Heart and Health Study and Caerphilly cohorts. Atherosclerosis 2008; 196: 871–8.

Fernández-Jarne E, Martínez-Losa E, Serrano-Martínez M, Prado-Santamaría M, Brugarolas-Brufau C, Martínez-González MA. Type of alcoholic beverage and first acute myocardial infarction: a case-control study in a Mediterranean country. Clin Cardiol 2003; 26: 313–8.

Friedman LA, Kimball AW. Coronary heart disease mortality and alcohol consumption in Framingham. Am J Epidemiol 1986; 124: 481–9.

Fuchs CS, Stampfer MJ, Colditz GA, et al. Alcohol consumption and mortality among women. N Engl J Med 1995; 332: 1245–50.

Fuchs FD, Chambless LE, Folsom AR, et al. Association between alcoholic beverage consumption and incidence of coronary heart disease in whites and blacks: the Atherosclerosis Risk in Communities Study. Am J Epidemiol 2004; 160: 466–74.

Garg R, Wagener DK, Madans JH. Alcohol consumption and risk of ischemic heart disease in women. Arch Intern Med 1993; 153: 1211–6.

Gaziano JM, Gaziano TA, Glynn RJ, et al. Light-to-moderate alcohol consumption and mortality in the Physicians' Health Study enrollment cohort. J Am Coll Cardiol 2000; 350: 96–105.

Genchev GD, Georgieva LM, Weijenberg MP, Powles JW. Does alcohol protect against ischaemic heart disease in Bulgaria? A case-control study of non-fatal myocardial infarction in Sofia. Cent Eur J Public Health 2001; 9: 83–6.

Goldberg RJ, Burchfiel CM, Reed DM, Wergowske G, Chiu D. A prospective study of the health effects of alcohol consumption in middle-aged and elderly men. The Honolulu Heart Program. Circulation 1994; 89: 651–9.

Goncalves A, Claggett B, Jhund PS, et al. Alcohol consumption and risk of heart failure: the Atherosclerosis Risk in Communities Study. Eur Heart J 2015; 36: 939–45.

Gordon T, Doyle JT. Drinking and coronary heart disease: the Albany Study. Am Heart J 1985; 110: 331–4.

Gronbaek M, Becker U, Johansen D, et al. Type of alcohol consumed and mortality from all causes, coronary heart disease, and cancer. Ann Intern Med 2000; 133: 411–9.

Gun RT, Pratt N, Ryan P, Gordon I, Roder D. Tobacco and alcohol-related mortality in men: estimates from the Australian cohort of petroleum industry workers. Aust N Z J Public Health 2006; 30: 318–24.

Hammar N, Romelsjo A, Alfredsson L. Alcohol consumption, drinking pattern and acute myocardial infarction. A case referent study based on the Swedish Twin Register. J Intern Med 1997; 241: 125–31.

Harriss LR, English DR, Hopper JL, et al. Alcohol consumption and cardiovascular mortality accounting for possible misclassification of intake: 11-year follow-up of the Melbourne Collaborative Cohort Study. Addiction 2007; 102: 1574–85.

Hart CL, Smith GD. Alcohol consumption and mortality and hospital admissions in men from the Midspan collaborative cohort study. Addiction 2008; 103: 1979–86.

Hein HO, Sorensen H, Suadicani P, Gyntelberg F. Alcohol consumption, Lewis phenotypes, and risk of ischaemic heart disease. Lancet 1993; 341: 392–6.

Henderson SO, Haiman CA, Wilkens LR, Kolonel LN, Wan P, Pike MC. Established risk factors account for most of the racial differences in cardiovascular disease mortality. PLoS One 2007; 2: 377.

Hines LM, Stampfer MJ, Ma J, et al. Genetic variation in alcohol dehydrogenase and the beneficial effect of moderate alcohol consumption on myocardial infarction. N Engl J Med 2001; 344: 549–55.

Hippe M, Vestbo J, Hein HO, Borch-Johnsen K, Jensen G, Sorensen TI. Familial predisposition and susceptibility to the effect of other risk factors for myocardial infarction. J Epidemiol Community Health 1999; 53: 269–76.

Ikehara S, Iso H, Toyoshima H, et al. Alcohol consumption and mortality from stroke and coronary heart disease among Japanese men and women: the Japan collaborative cohort study. Stroke 2008; 39: 2936–42.

Ikehara S, Iso H, Yamagishi K, Yamamoto S, Inoue M, Tsugane S. Alcohol consumption, social support, and risk of stroke and coronary heart disease among Japanese men: the JPHC Study. Alcohol Clin Exp Res 2009; 33: 1025–32.

Iso H, Kitamura A, Shimamoto T, et al. Alcohol intake and the risk of cardiovascular disease in middle-aged Japanese men. Stroke 1995; 26: 767–73.

Jackson R, Scragg R, Beaglehole R. Alcohol consumption and risk of coronary heart disease. BMJ 1991; 303: 211–6.

Jakovljevic B, Stojanov V, Paunovic K, Belojevic G, Milic N. Alcohol consumption and mortality in Serbia: twenty-year follow-up study. Croat Med J 2004; 45: 764–8.

Kabagambe EK, Baylin A, Ruiz-Narvaez E, Rimm EB, Campos H. Alcohol intake, drinking patterns, and risk of nonfatal acute myocardial infarction in Costa Rica. Am J Clin Nutr 2005; 82: 1336–45.

Kagan A, Yano K, Rhoads GG, McGee DL. Alcohol and cardiovascular disease: the Hawaiian experience. Circulation 1981; 64(3 Pt 2): III 27-31.

Kalandidi A, Tzonou A, Toupadaki N, et al. A case-control study of coronary heart disease in Athens, Greece. Int J Epidemiol 1992; 21: 1074–80.

Kaufman DW, Rosenberg L, Helmrich SP, Shapiro S. Alcoholic beverages and myocardial infarction in young men. Am J Epidemiol 1985; 121: 548–54.

Key TJ, Appleby PN, Spencer EA, Travis RC, Roddam AW, Allen NE. Mortality in British vegetarians: results from the European Prospective Investigation into Cancer and Nutrition (EPIC-Oxford). Am J Clin Nutr 2009; 89: 1613–9.

Kitamura A, Iso H, Sankai T, et al. Alcohol intake and premature coronary heart disease in urban Japanese men. Am J Epidemiol 1998; 147: 59–65.

Kivela SL, Nissinen A, Ketola A, Punsar S, Puska P, Karvonen M. Alcohol consumption and mortality in aging or aged Finnish men. J Clin Epidemiol 1989; 42: 61–8.

Kono S, Handa K, Kawano T, Hiroki T, Ishihara Y, Arakawa K. Alcohol intake and nonfatal acute myocardial infarction in Japan. Am J Cardiol 1991; 68: 1011–4.

Kono S, Ikeda M, Tokudome S, Nishizumi M, Kuratsune M. Alcohol and mortality: a cohort study of male Japanese physicians. Int J Epidemiol 1986; 15: 527–32.

Lazarus NB, Kaplan GA, Cohen RD, Leu DJ. Change in alcohol consumption and risk of death from all causes and from ischaemic heart disease. BMJ 1991; 303: 553–6.

Liao Y, McGee DL, Cao G, Cooper RS. Alcohol intake and mortality: findings from the National Health Interview Surveys (1988 and 1990). Am J Epidemiol 2000; 151: 651–9.

Lindschou Hansen J, Tolstrup JS, Jensen MK, et al. Alcohol intake and risk of acute coronary syndrome and mortality in men and women with and without hypertension. Eur J Epidemiol 2011; 26: 439–47.

Makela P, Paljarvi T, Poikolainen K. Heavy and nonheavy drinking occasions, all-cause and cardiovascular mortality and hospitalizations: a follow-up study in a population with a low consumption level. J Stud Alcohol 2005; 66: 722–8.

Malyutina S, Bobak M, Kurilovitch S, et al. Relation between heavy and binge drinking and all-cause and cardiovascular mortality in Novosibirsk, Russia: a prospective cohort study. Lancet 2002; 360: 1448–54.

Marques-Vidal P, Montaye M, Arveiler D, et al. Alcohol consumption and cardiovascular disease: differential effects in France and Northern Ireland. The PRIME study. Eur J Cardiovasc Prev Rehabil 2004; 11: 336–43.

Marques-Vidal P, Montaye M, Arveiler D, et al. Alcohol consumption and cardiovascular disease: differential effects in France and Northern Ireland. The PRIME study. Eur J Cardiovasc Prev Rehabil 2004; 11: 336–43.

Miller GJ, Beckles GL, Maude GH, Carson DC. Alcohol consumption: protection against coronary heart disease and risks to health. Int J Epidemiol 1990; 19: 923–30.

Mukamal KJ, Conigrave KM, Mittleman MA, et al. Roles of drinking pattern and type of alcohol consumed in coronary heart disease in men. N Engl J Med 2003; 348: 109–18.

Murray RP, Connett JE, Tyas SL, et al. Alcohol volume, drinking pattern, and cardiovascular disease morbidity and mortality: is there a U-shaped function? Am J Epidemiol 2002; 155: 242–8.

Pedersen J, Heitmann BL, Schnohr P, Grønbaek M. The combined influence of leisure-time physical activity and weekly alcohol intake on fatal ischaemic heart disease and all-cause mortality. Eur Heart J 2008; 29: 204–12.

Prineas RJ, Folsom AR, Kaye SA. Central adiposity and increased risk of coronary artery disease mortality in older women. Ann Epidemiol 1993; 3: 35–41.

Rehm JT, Bondy SJ, Sempos CT, Vuong CV. Alcohol consumption and coronary heart disease morbidity and mortality. Am J Epidemiol 1997; 146: 495–501.

Renaud SC, Gueguen R, Schenker J, Houtaud A. Alcohol and mortality in middle-aged men from eastern France. Epidemiology 1998; 9: 184–8.

Rimm EB, Giovannucci EL, Willett WC, et al. Prospective study of alcohol consumption and risk of coronary disease in men. Lancet 1991; 338: 464–8.

Roerecke M, Greenfield TK, Kerr WC, Bondy S, Cohen J, Rehm J. Heavy drinking occasions in relation to ischaemic heart disease mortality– an 11-22 year follow-up of the 1984 and 1995 US National Alcohol Surveys. Int J Epidemiol 2011; 40: 1401–10.

Romelsjo A, Allebeck P, Andreasson S, Leifman A. Alcohol, mortality and cardiovascular events in a 35 year follow-up of a nationwide representative cohort of 50,000 Swedish conscripts up to age 55. Alcohol Alcohol 2012; 47: 322–7.

Romelsjo A, Branting M, Hallqvist J, et al. Abstention, alcohol use and risk of myocardial infarction in men and women taking account of social support and working conditions: the SHEEP case-control study. Addiction 2003; 98: 1453–62.

Romelsjo A, Leifman A. Association between alcohol consumption and mortality, myocardial infarction, and stroke in 25 year follow up of 49 618 young Swedish men. BMJ 1999; 319: 821–2.

Rostron B. Alcohol consumption and mortality risks in the USA. Alcohol Alcohol 2012; 47: 334–9.

Schroder H, Masabeu A, Marti MJ, et al. Myocardial infarction and alcohol consumption: a population-based case-control study. Nutr Metab Cardiovasc Dis 2007; 17: 609–15.

Scragg R, Stewart A, Jackson R, Beaglehole R. Alcohol and exercise in myocardial infarction and sudden coronary death in men and women. Am J Epidemiol 1987; 126: 77–85.

Sempos CT, Rehm J, Crespo C, Trevisan M. No Protective Effect of Alcohol Consumption on Coronary Heart Disease (CHD) in African Americans: Average Volume of Drinking over the Life Course and CHD Morbidity and Mortality in a U.S. National Cohort. Contemp Drug Probs 2002; 29: 805–20.

Serdula MK, Koong SL, Williamson DF, et al. Alcohol intake and subsequent mortality: findings from the NHANES I Follow-up Study. J Stud Alcohol 1995; 56: 233–9.

Shaper AG, Wannamethee G, Walker M. Alcohol and coronary heart disease: a perspective from the British Regional Heart Study. Int J Epidemiol 1994; 23: 482–94.

Shaper AG, Wannamethee G, Walker M. Alcohol and coronary heart disease: a perspective from the British Regional Heart Study. Int J Epidemiol 1994; 23: 482–94.

Simons LA, McCallum J, Friedlander Y, Simons J. Alcohol intake and survival in the elderly: a 77 month follow-up in the Dubbo study. Aust N Z J Med 1996; 26: 662–70.

Stampfer MJ, Colditz GA, Willett WC, Speizer FE, Hennekens CH. A prospective study of moderate alcohol consumption and the risk of coronary disease and stroke in women. N Engl J Med 1988; 319: 267–73.

Streppel MT, Ocke MC, Boshuizen HC, Kok FJ, Kromhout D. Long-term wine consumption is related to cardiovascular mortality and life expectancy independently of moderate alcohol intake: the Zutphen Study. J Epidemiol Community Health 2009; 63: 534–40.

Suh I, Shaten BJ, Cutler JA, Kuller LH. Alcohol use and mortality from coronary heart disease: the role of high-density lipoprotein cholesterol. The Multiple Risk Factor Intervention Trial Research Group. Ann Intern Med 1992; 116: 881–7.

Suhonen O, Aromaa A, Reunanen A, Knekt P. Alcohol consumption and sudden coronary death in middle-aged Finnish men. Acta Med Scand 1987; 221: 335–41.

Tavani A, Bertuzzi M, Gallus S, Negri E, La Vecchia C. Risk factors for non-fatal acute myocardial infarction in Italian women. Prev Med 2004; 39: 128–34.

Thun MJ, Peto R, Lopez AD, et al. Alcohol consumption and mortality among middle-aged and elderly U.S. adults. N Engl J Med 1997; 337: 1705–14.

Wannamethee G, Shaper AG. Alcohol and sudden cardiac death. Br Heart J 1992; 68: 443–8.

Wells S, Broad J, Jackson R. Alcohol consumption and its contribution to the burden of coronary heart disease in middle-aged and older New Zealanders: a population-based case-control study. N Z Med J 2004; 117: 793.

Wilkins K. Moderate alcohol consumption and heart disease. Health Rep 2002; 14: 24-Sep.

Xu WH, Zhang XL, Gao YT, et al. Joint effect of cigarette smoking and alcohol consumption on mortality. Prev Med 2007; 45: 313–9.

Yang L, Zhou M, Sherliker P, et al. Alcohol drinking and overall and cause-specific mortality in China: nationally representative prospective study of 220,000 men with 15 years of follow-up. Int J Epidemiol 2012; 41: 1101–13.

Yi SW, Yoo SH, Sull JW, Ohrr H. Association between Alcohol Drinking and Cardiovascular Disease Mortality and All-cause Mortality: Kangwha Cohort Study. J Prev Med Public Health 2004; 37: 120–6.

Younis J, Cooper JA, Miller GJ, Humphries SE, Talmud PJ. Genetic variation in alcohol dehydrogenase 1C and the beneficial effect of alcohol intake on coronary heart disease risk in the Second Northwick Park Heart Study. Atherosclerosis 2005; 180: 225–32.

Yuan JM, Ross RK, Gao YT, Henderson BE, Yu MC. Follow up study of moderate alcohol intake and mortality among middle aged men in Shanghai, China. BMJ 1997; 314: 18–23.

de Labry LO, Glynn RJ, Levenson MR, Hermos JA, LoCastro JS, Vokonas PS. Alcohol consumption and mortality in an American male population: recovering the U-shaped curve–findings from the normative Aging Study. J Stud Alcohol 1992; 53: 25–32.

Drogan D, Sheldrick AJ, Schutze M, Knuppel S, Andersohn F, di Giuseppe R, Herrmann B, Willich SN, Garbe E, Bergmann MM, Boeing H, Weikert C. Alcohol consumption, genetic variants in alcohol deydrogenases, and risk of cardiovascular diseases: a prospective study and meta-analysis. PLoS One. 2012; 7(2): e32176.

Klatsky AL, Armstrong MA, Friedman GD. Alcohol and mortality. Ann Intern Med. 1992; 117(8): 646–54.

Mukamal KJ, Conigrave KM, Mittleman MA, Camargo CA, Stampfer MJ, Willett WC, Rimm EB. Roles of drinking pattern and type of alcohol consumed in coronary heart disease in men. N Engl J Med. 2003; 348(2): 109–18.

12. Ischaemic stroke

Summary of the meta-analysis conducted for GBD 2016

Search String:

((“”ethanol“”[MeSH Terms] OR “”alcohols“”[MeSH Terms]) AND “”brain infarction“”[MeSH Terms] AND (“”1950/01/01“”[PDAT] : “”2016/12/31“”[PDAT])) AND “”humans“”[MeSH Terms]

PRISMA flow diagram

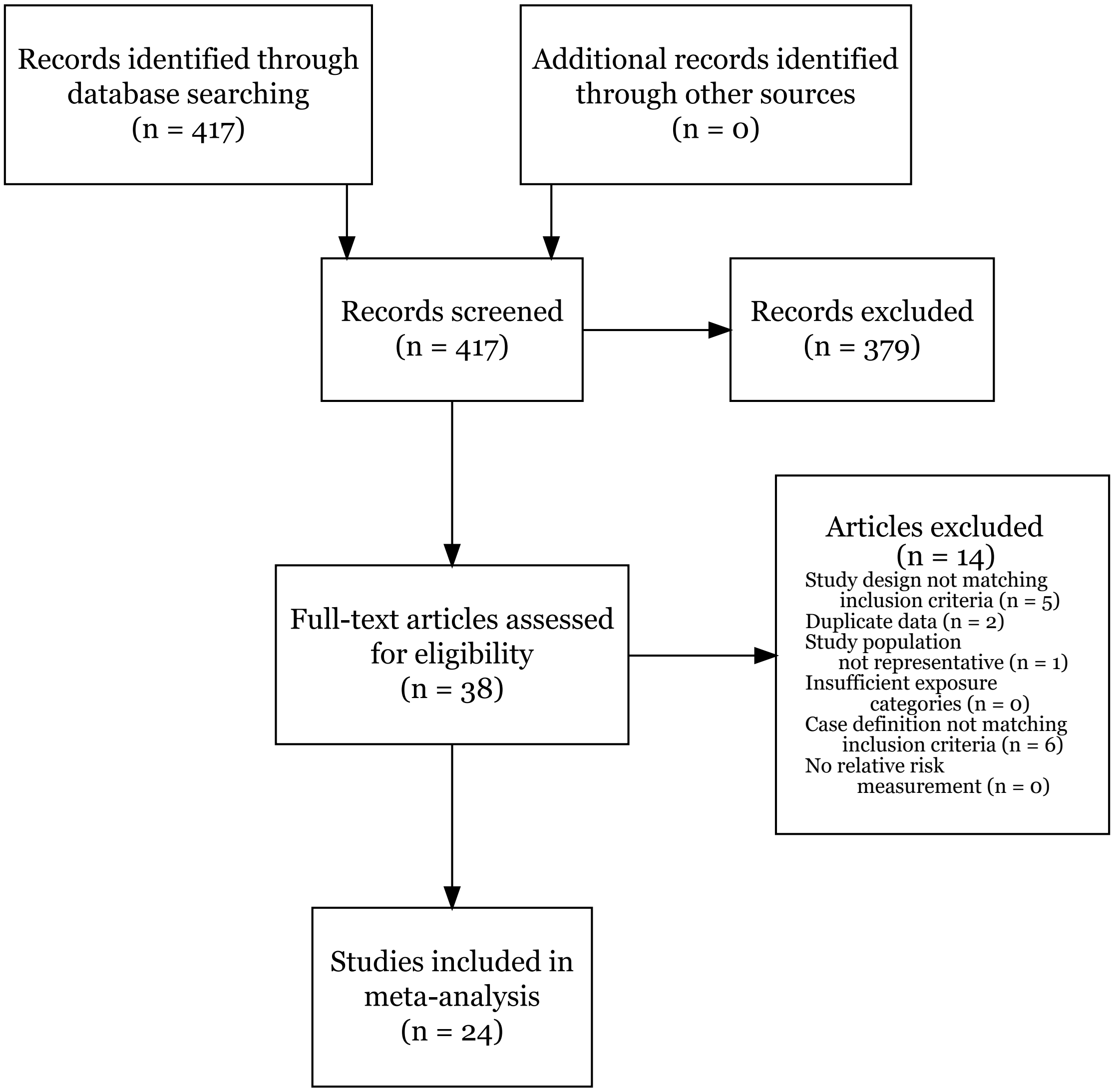

Relative risk (RR) curves for Ischaemic stroke by number of standard drinks consumed daily, by sex. Points are relative risk estimates from studies.
The vertical bars capture the uncertainty in each study, related to the sample size and the horizontal bars capture the range of drinks consumed by individuals in the study.
The black line represents the estimated RR for Ischaemic stroke at each level of consumption. The shaded green areas represent the 95% uncertainty interval associated with the estimated RR.
Dotted line is a reference for a relative risk of 1.

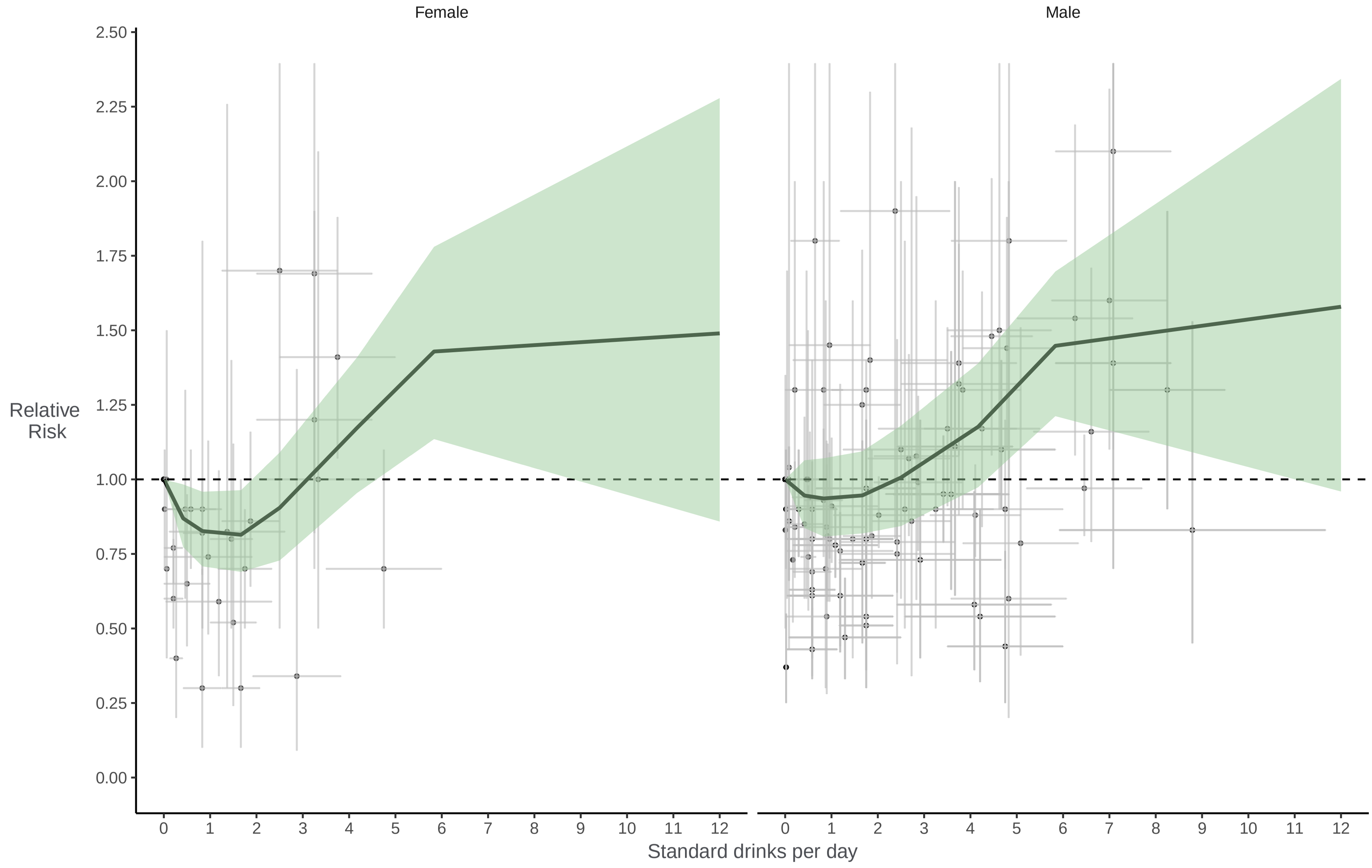

References for Ischaemic stroke

Bazzano LA, Gu D, Reynolds K, et al. Alcohol consumption and risk of coronary heart disease among Chinese men. Int J Cardiol 2009; 135: 78–85.

Berger K, Ajani UA, Kase CS, et al. Light-to-moderate alcohol consumption and the risk of stroke among U.S. male physicians. N Engl J Med 1999; 341: 1557–64.

Caicoya M, Rodriguez T, Corrales C, Cuello R, Lasheras C. Alcohol and stroke: a community case-control study in Asturias, Spain. J Clin Epidemiol 1999; 52: 677–84.

Djousse L, Ellison RC, Beiser A, et al. Alcohol consumption and risk of ischemic stroke: The Framingham Study. Stroke 2002; 33: 907–12.

Gill JS, Shipley MJ, Tsementzis SA, et al. Alcohol consumption–a risk factor for hemorrhagic and non-hemorrhagic stroke. Am J Med 1991; 90: 489–97.

Ikehara S, Iso H, Toyoshima H, et al. Alcohol consumption and mortality from stroke and coronary heart disease among Japanese men and women: the Japan collaborative cohort study. Stroke 2008; 39: 2936–42.

Ikehara S, Iso H, Yamagishi K, Yamamoto S, Inoue M, Tsugane S. Alcohol consumption, social support, and risk of stroke and coronary heart disease among Japanese men: the JPHC Study. Alcohol Clin Exp Res 2009; 33: 1025–32.

Iso H, Kitamura A, Shimamoto T, et al. Alcohol intake and the risk of cardiovascular disease in middle-aged Japanese men. Stroke 1995; 26: 767–73.

Jackson VA, Sesso HD, Buring JE, Gaziano JM. Alcohol consumption and mortality in men with preexisting cerebrovascular disease. Arch Intern Med 2003; 163: 1189–93.

Klatsky AL, Armstrong MA, Friedman GD, Sidney S. Alcohol drinking and risk of hospitalization for ischemic stroke. Am J Cardiol 2001; 88: 703–6.

Klatsky AL, Armstrong MA, Friedman GD. Alcohol use and subsequent cerebrovascular disease hospitalizations. Stroke 1989; 20: 741–6.

Kono S, Ikeda M, Tokudome S, Nishizumi M, Kuratsune M. Alcohol and mortality: a cohort study of male Japanese physicians. Int J Epidemiol 1986; 15: 527–32.

Leppala JM, Paunio M, Virtamo J, et al. Alcohol consumption and stroke incidence in male smokers. Circulation 1999; 100: 1209–14.

Malarcher AM, Giles WH, Croft JB, et al. Alcohol intake, type of beverage, and the risk of cerebral infarction in young women. Stroke 2001; 32: 77–83.

Mukamal KJ, Ascherio A, Mittleman MA, et al. Alcohol and risk for ischemic stroke in men: the role of drinking patterns and usual beverage. Ann Intern Med 2005; 142: 9-Nov.

Mukamal KJ, Chung H, Jenny NS, et al. Alcohol use and risk of ischemic stroke among older adults: the cardiovascular health study. Stroke 2005; 36: 1830–4.

Palomaki H, Kaste M. Regular light-to-moderate intake of alcohol and the risk of ischemic stroke Is there a beneficial effect? Stroke 1993; 24: 1828–32.

Sacco RL, Elkind M, Boden-Albala B, et al. The protective effect of moderate alcohol consumption on ischemic stroke. JAMA 1999; 281: 53–60.

Stampfer MJ, Colditz GA, Willett WC, Speizer FE, Hennekens CH. A prospective study of moderate alcohol consumption and the risk of coronary disease and stroke in women. N Engl J Med 1988; 319: 267–73.

Chiuve SE, Rexrode KM, Spiegelman D, Logroscino G, Manson JE, Rimm EB. Primary prevention of stroke by healthy lifestyle. Circulation. 2008; 118(9): 947–954.

Chiuve SE, Rexrode KM, Spiegelman D, Logroscino G, Manson JE, Rimm EB. Primary prevention of stroke by healthy lifestyle. Circulation. 2008; 118(9): 947–954.

Elkind MSV, Sciacca R, Boden-Albala B, Rundek T, Paik MC, Sacco RL. Moderate alcohol consumption reduces risk of ischemic stroke: the Northern Manhattan Study. Stroke. 2006; 37(1): 13–9.

Hansagi H, Romelsj A, Gerhardsson de Verdier M, Andreasson S, Leifman A. Alcohol consumption and stroke mortality. 20-year follow-up of 15,077 men and women. Stroke. 1995; 26(10): 1768–73.

Henrich JB, Horwitz RI. Evidence against the association between alcohol use and ischemic stroke risk. Arch Intern Med. 1989; 149(6): 1413–6.

Higashiyama A, Wakabayashi I, Ono Y, Watanabe M, Kokubo Y, Okayama A, Miyamoto Y, Okamura T. Association with serum gamma-glutamyltransferase levels and alcohol consumption on stroke and coronary artery disease: the Suita Study. Stroke. 2011; 42(6): 1764–7.

Jones SB, Loehr L, Avery CL, Gottesman RF, Wruck L, Shahar E, Rosamond WD. Midlife alcohol consumption and the risk of stroke in the Atherosclerosis Risk in Communities Study. Stroke. 2015; 46(11): 3124–30.

Nielsen NR, Truelsen T, Barefoot JC, Johnsen SP, Overvad K, Boysen G, Schnohr P, Gronbaek M. Is the effect of alcohol on risk of stroke confined to highly stressed persons?. Neuroepidemiology. 2005; 25(3): 105–13.

13. Larynx cancer

Summary of the meta-analysis conducted for GBD 2016

Search String:

((“”ethanol“”[MeSH Terms] OR “”alcohols“”[MeSH Terms]) AND “”laryngeal neoplasms“”[MeSH Terms] AND (“”0001/01/01“”[PDAT] : “”2016/12/31“”[PDAT])) AND “”humans“”[MeSH Terms]

PRISMA flow diagram

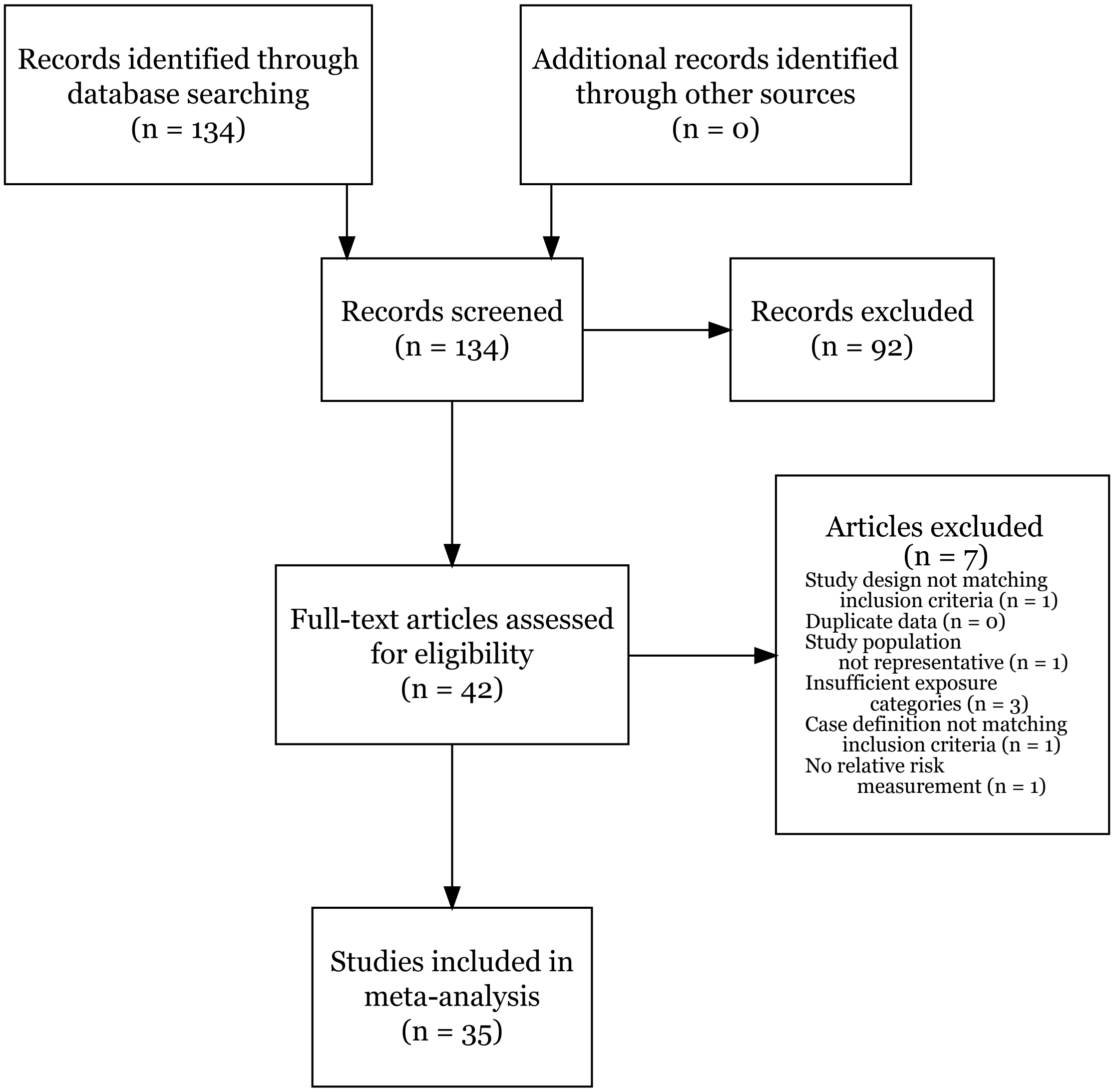

Relative risk (RR) curves for Larynx cancer by number of standard drinks consumed daily. Points are relative risk estimates from studies.
The vertical bars capture the uncertainty in each study, related to the sample size and the horizontal bars capture the range of drinks consumed by individuals in the study.
The black line represents the estimated RR for Larynx cancer at each level of consumption. The shaded green areas represent the 95% uncertainty interval associated with the estimated RR.
Dotted line is a reference for a relative risk of 1.

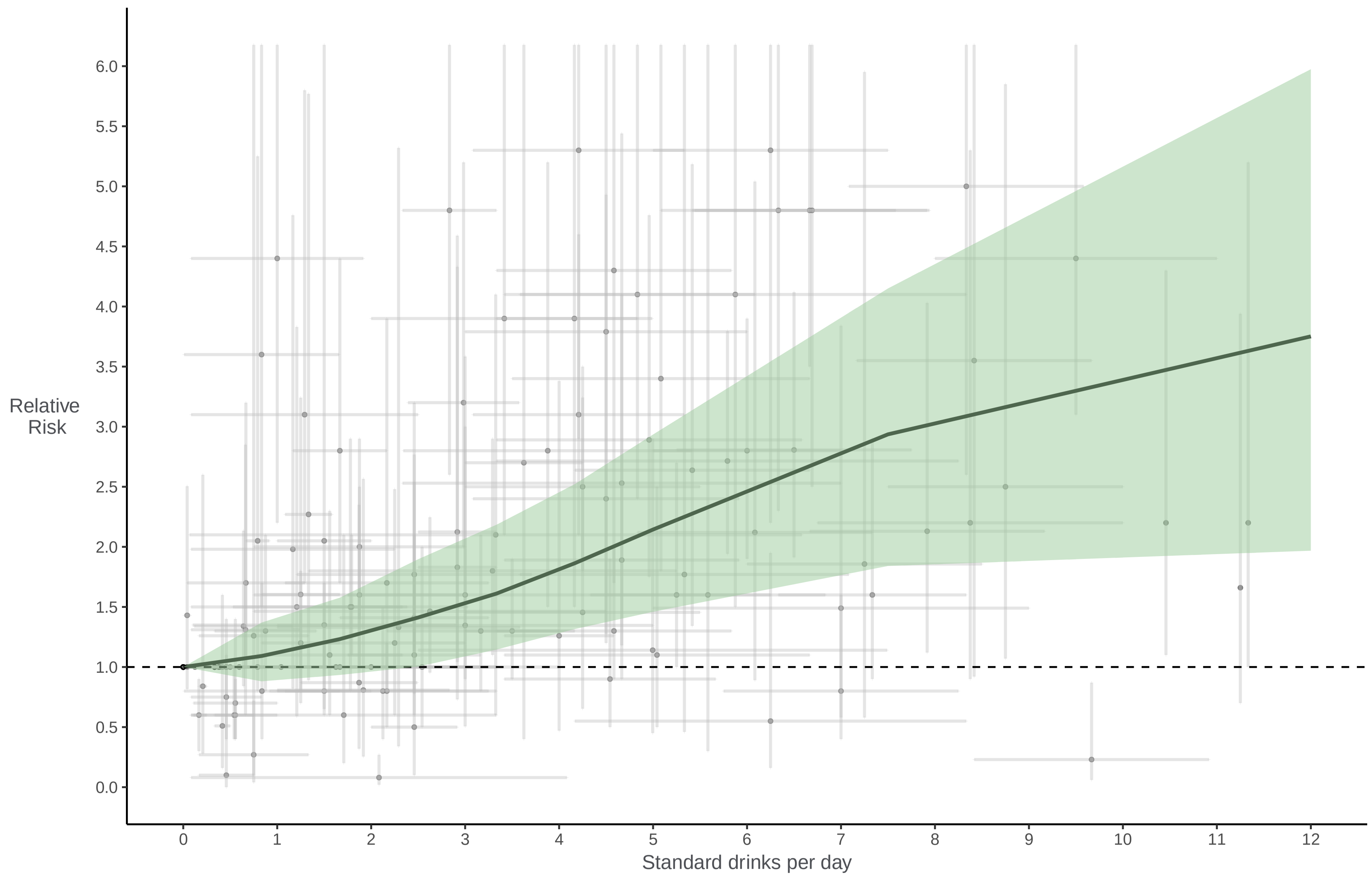

References for Larynx cancer

Adelhardt M, Moller Jensen O, Sand Hansen H. Cancer of the larynx, pharynx, and oesophagus in relation to alcohol and tobacco consumption among Danish brewery workers. Dan Med Bull 1985; 32: 119–23.

Allen NE, Beral V, Casabonne D, et al. Moderate alcohol intake and cancer incidence in women. J Natl Cancer Inst 2009; 101: 296–305.

Andre K, Schraub S, Mercier M, Bontemps P. Role of alcohol and tobacco in the aetiology of head and neck cancer: a case-control study in the Doubs region of France. Eur J Cancer B Oral Oncol 1995; 31B: 301–9.

Bouchardy C, Hirvonen A, Coutelle C, Ward PJ, Dayer P, Benhamou S. Role of alcohol dehydrogenase 3 and cytochrome P-4502E1 genotypes in susceptibility to cancers of the upper aerodigestive tract. Int J Cancer 2000; 87: 734–40.

Brownson RC, Chang JC. Exposure to alcohol and tobacco and the risk of laryngeal cancer. Arch Environ Health 1987; 42: 192–6.

Burch JD, Howe GR, Miller AB, Semenciw R. Tobacco, alcohol, asbestos, and nickel in the etiology of cancer of the larynx: a case-control study. J Natl Cancer Inst 1981; 670: 1219–24.

Cammarota G, Galli J, Cianci R, et al. Association of laryngeal cancer with previous gastric resection. Ann Surg 2004; 240: 817–24.

Choi SY, Kahyo H. Effect of cigarette smoking and alcohol consumption in the aetiology of cancer of the oral cavity, pharynx and larynx. Int J Epidemiol 1991; 20: 878–85.

De Stefani E, Correa P, Oreggia F, et al. Risk factors for laryngeal cancer. Cancer 1987; 60: 3087–91.

Dosemeci M, Gokmen I, Unsal M, Hayes RB, Blair A. Tobacco, alcohol use, and risks of laryngeal and lung cancer by subsite and histologic type in Turkey. Cancer Causes Control 1997; 8: 729–37.

Elwood JM, Pearson JC, Skippen DH, Jackson SM. Alcohol, smoking, social and occupational factors in the aetiology of cancer of the oral cavity, pharynx and larynx. Int J Cancer 1984; 34: 603–12.

Falk RT, Pickle LW, Brown LM, Mason TJ, Buffler PA, Fraumeni JF. Effect of smoking and alcohol consumption on laryngeal cancer risk in coastal Texas. Cancer Res 1989; 49: 4024–9.

Flanders WD, Cann CI, Rothman KJ, Fried MP. Work-related risk factors for laryngeal cancer. Am J Epidemiol 1984; 1190: 23–32.

Freedman ND, Schatzkin A, Leitzmann MF, Hollenbeck AR, Abnet CC. Alcohol and head and neck cancer risk in a prospective study. Br J Cancer 2007; 96: 1469–74.

Garavello W, Bosetti C, Gallus S, et al. Type of alcoholic beverage and the risk of laryngeal cancer. Eur J Cancer Prev 2006; 15: 69–73.

Graham S, Mettlin C, Marshall J, Priore R, Rzepka T, Shedd D. Dietary factors in the epidemiology of cancer of the larynx. Am J Epidemiol 1981; 1130: 675–80.

Guenel P, Chastang JF, Luce D, Leclerc A, Brugere J. A study of the interaction of alcohol drinking and tobacco smoking among French cases of laryngeal cancer. J Epidemiol Community Health 1988; 420: 350–4.

Hashibe M, Boffetta P, Zaridze D, et al. Contribution of tobacco and alcohol to the high rates of squamous cell carcinoma of the supraglottis and glottis in Central Europe. Am J Epidemiol 2007; 165: 814–20.

Hedberg K, Vaughan TL, White E, Davis S, Thomas DB. Alcoholism and cancer of the larynx: a case-control study in western Washington (United States). Cancer Causes Control 1994; 5: 8-Mar.

Hinds MW, Thomas DB, O, Reilly HP. Asbestos, dental X-rays, tobacco, and alcohol in the epidemiology of laryngeal cancer. Cancer 1979; 440: 1114–20.

Kim MK, Ko MJ, Han JT. Alcohol consumption and mortality from all-cause and cancers among 1.34 million Koreans: the results from the Korea national health insurance corporation's health examinee cohort in 2000. Cancer Causes Control 2010; 21: 2295–302.

Lee KW, Kuo WR, Tsai SM, et al. Different impact from betel quid, alcohol and cigarette: risk factors for pharyngeal and laryngeal cancer. Int J Cancer 2005; 117: 831–6.

Lopez-Abente G, Pollan M, Monge V, Martinez-Vidal A. Tobacco smoking, alcohol consumption, and laryngeal cancer in Madrid. Cancer Detect Prev 1992; 16: 265–71.

Matthias C, Bockmuhl U, Jahnke V, et al. Polymorphism in cytochrome P450 CYP2D6, CYP1A1, CYP2E1 and glutathione S-transferase, GSTM1, GSTM3, GSTT1 and susceptibility to tobacco-related cancers: studies in upper aerodigestive tract cancers. Pharmacogenetics 1998; 8: 91–100.

Menvielle G, Luce D, Goldberg P, Bugel I, Leclerc A. Smoking, alcohol drinking and cancer risk for various sites of the larynx and hypopharynx. A case-control study in France. Eur J Cancer Prev 2004; 13: 165–72.

Muscat JE, Wynder EL. Tobacco, alcohol, asbestos, and occupational risk factors for laryngeal cancer. Cancer 1992; 69: 2244–51.

Newhouse ML, Gregory MM, Shannon H. Etiology of carcinoma of the larynx. IARC Sci Publ 1980; nan: 687–95.

Olsen J, Sabreo S, Fasting U. Interaction of alcohol and tobacco as risk factors in cancer of the laryngeal region. J Epidemiol Community Health 1985; 39: 165–8.

Peters ES, McClean MD, Marsit CJ, Luckett B, Kelsey KT. Glutathione S-transferase polymorphisms and the synergy of alcohol and tobacco in oral, pharyngeal, and laryngeal carcinoma. Cancer Epidemiol Biomarkers Prev 2006; 15: 2196–202.

Pisa FE, Barbone F. Diet and the risk of cancers of the lung, oral cavity and pharynx, and larynx: a population-based case-control study in north-east Italy. IARC Sci Publ 2002; 156: 141–3.

Ramroth H, Dietz A, Becher H. Interaction effects and population-attributable risks for smoking and alcohol on laryngeal cancer and its subsites. A case-control study from Germany. Methods Inf Med 2004; 43: 499–504.

Werbrouck J, De Ruyck K, Duprez F, et al. Single-nucleotide polymorphisms in DNA double-strand break repair genes: association with head and neck cancer and interaction with tobacco use and alcohol consumption. Mutat Res 2008; 656: 74–81.

Wynder EL, Covey LS, Mabuchi K, Mushinski M. Environmental factors in cancer of the larynx: a second look. Cancer 1976; 380: 1591–601.

Zang EA, Wynder EL. Reevaluation of the confounding effect of cigarette smoking on the relationship between alcohol use and lung cancer risk, with larynx cancer used as a positive control. Prev Med 2001; 32: 359–70.

Zheng W, Blot WJ, Shu XO, et al. Diet and other risk factors for laryngeal cancer in Shanghai, China. Am J Epidemiol 1992; 136: 178–91.

14. Lip and oral cavity cancer

Summary of the meta-analysis conducted for GBD 2016

Search String:

((“”ethanol“”[MeSH Terms] OR “”alcohols“”[MeSH Terms]) AND (“”lip neoplasms“”[MeSH Terms] OR “”tongue neoplasms“”[MeSH Terms] OR “”salivary gland neoplasms“”[MeSH Terms] OR “”gingival neoplasms“”[MeSH Terms] OR “”mouth neoplasms“”[MeSH Terms]) AND (“”0001/01/01“”[PDAT] : “”2016/12/31“”[PDAT])) AND “”humans“”[MeSH Terms]

PRISMA flow diagram

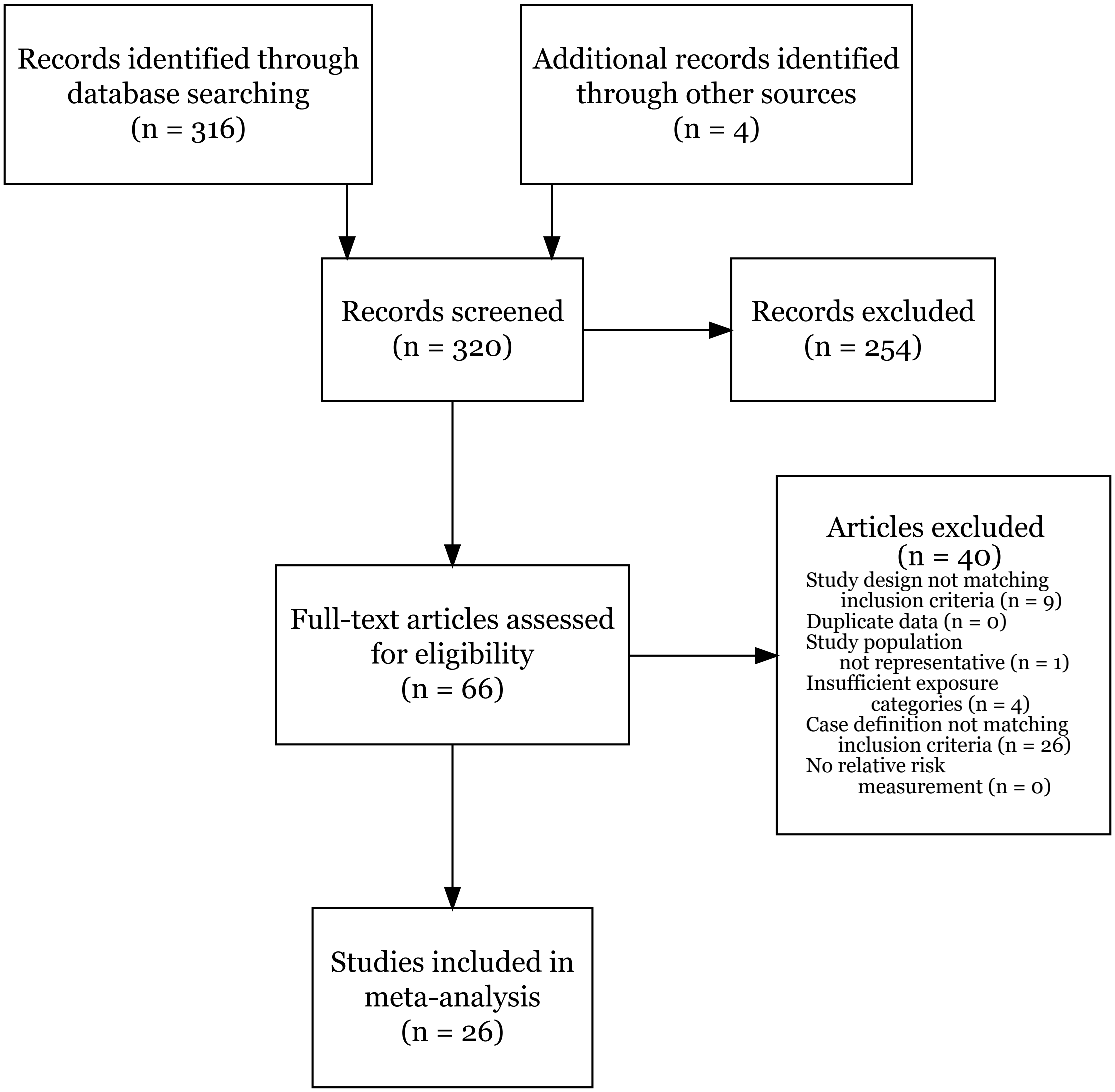

Relative risk (RR) curves for Lip and oral cavity cancer by number of standard drinks consumed daily. Points are relative risk estimates from studies.
The vertical bars capture the uncertainty in each study, related to the sample size and the horizontal bars capture the range of drinks consumed by individuals in the study.
The black line represents the estimated RR for Lip and oral cavity cancer at each level of consumption. The shaded green areas represent the 95% uncertainty interval associated with the estimated RR.
Dotted line is a reference for a relative risk of 1.

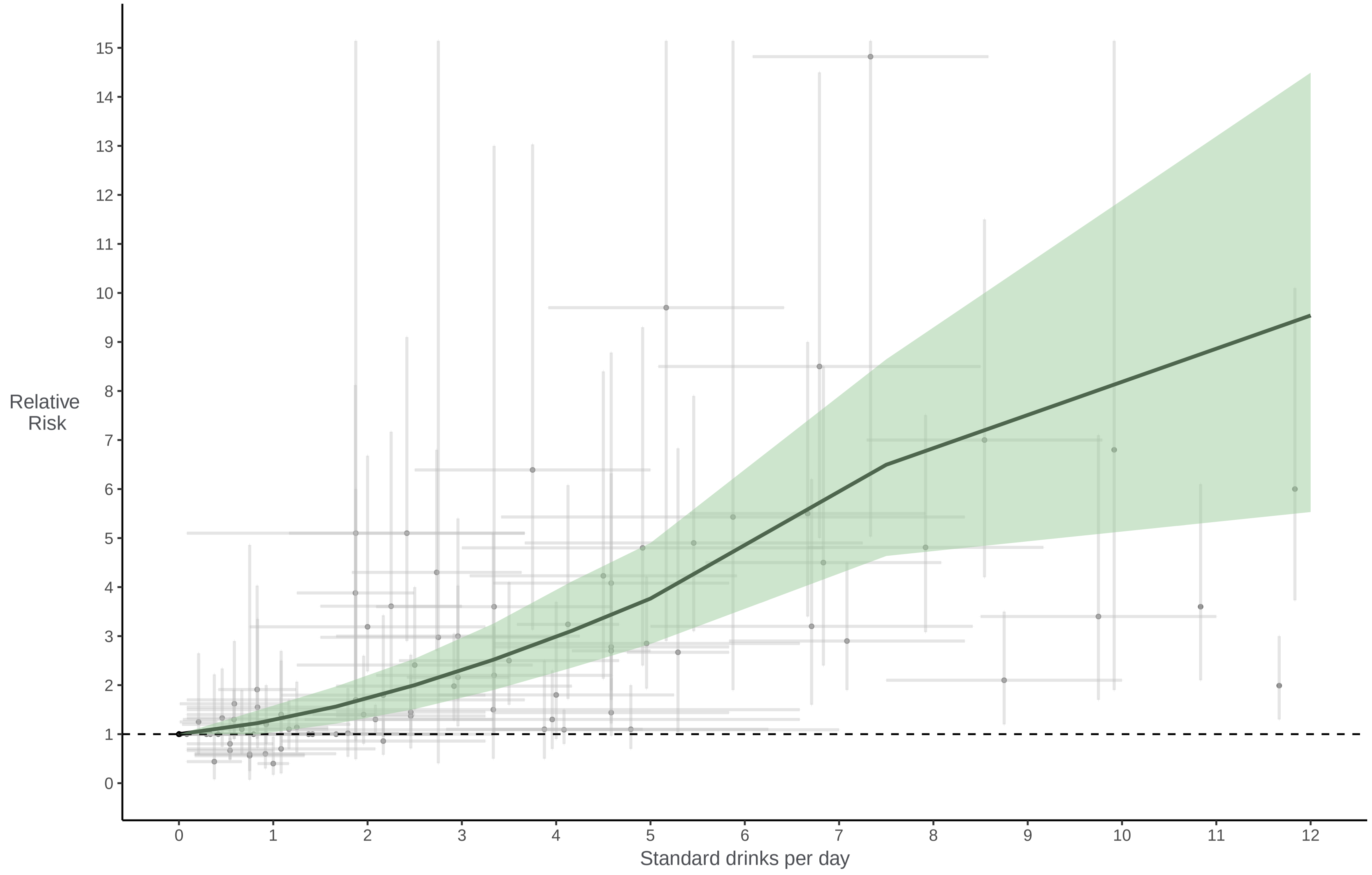

References for Lip and oral cavity cancer

Andre K, Schraub S, Mercier M, Bontemps P. Role of alcohol and tobacco in the aetiology of head and neck cancer: a case-control study in the Doubs region of France. Eur J Cancer B Oral Oncol 1995; 31B: 301–9.

Boffetta P, Garfinkel L. Alcohol drinking and mortality among men enrolled in an American Cancer Society prospective study. Epidemiology 1990; 1: 342–8.

Boffetta P, Mashberg A, Winkelmann R, Garfinkel L. Carcinogenic effect of tobacco smoking and alcohol drinking on anatomic sites of the oral cavity and oropharynx. Int J Cancer 1992; 52: 530–3.

Bross ID, Coombs J. Early onset of oral cancer among women who drink and smoke. Oncology 1976; 33: 136–9.

Bundgaard T, Wildt J, Frydenberg M, Elbrond O, Nielsen JE. Case-control study of squamous cell cancer of the oral cavity in Denmark. Cancer Causes Control 1995; 6: 57–67.

Choi SY, Kahyo H. Effect of cigarette smoking and alcohol consumption in the aetiology of cancer of the oral cavity, pharynx and larynx. Int J Epidemiol 1991; 20: 878–85.

De Stefani E, Boffetta P, Deneo-Pellegrini H, et al. The effect of smoking and drinking in oral and pharyngeal cancers: a case-control study in Uruguay. Cancer Lett 2007; 246: 282–9.

Elwood JM, Pearson JC, Skippen DH, Jackson SM. Alcohol, smoking, social and occupational factors in the aetiology of cancer of the oral cavity, pharynx and larynx. Int J Cancer 1984; 34: 603–12.

Franceschi S, Levi F, La Vecchia C, et al. Comparison of the effect of smoking and alcohol drinking between oral and pharyngeal cancer. Int J Cancer 1999; 83: 4-Jan.

Franceschi S, Talamini R, Barra S, et al. Smoking and drinking in relation to cancers of the oral cavity, pharynx, larynx, and esophagus in northern Italy. Cancer Res 1990; 50: 6502–7.

Freedman ND, Schatzkin A, Leitzmann MF, Hollenbeck AR, Abnet CC. Alcohol and head and neck cancer risk in a prospective study. Br J Cancer 2007; 96: 1469–74.

Graham S, Dayal H, Rohrer T, et al. Dentition, diet, tobacco, and alcohol in the epidemiology of oral cancer. J Natl Cancer Inst 1977; 59: 1611–8.

Herrero R, Castellsague X, Pawlita M, et al. Human papillomavirus and oral cancer: the International Agency for Research on Cancer multicenter study. J Natl Cancer Inst 2003; 95: 1772–83.

Maasland DH, van den Brandt PA, Kremer B, Goldbohm RA, Schouten LJ. Alcohol consumption, cigarette smoking and the risk of subtypes of head-neck cancer: results from the Netherlands Cohort Study. BMC Cancer 2014; 14: 187.

Martinez I. Factors associated with ccer of the esophagus, mouth, and pharynx in Puerto Rico. J Natl Cancer Inst 1969; 42: 1069–94.

Oreggia F, De Stefani E, Correa P, Fierro L. Risk factors for cancer of the tongue in Uruguay. Cancer 1991; 67: 180–3.

Peters ES, McClean MD, Marsit CJ, Luckett B, Kelsey KT. Glutathione S-transferase polymorphisms and the synergy of alcohol and tobacco in oral, pharyngeal, and laryngeal carcinoma. Cancer Epidemiol Biomarkers Prev 2006; 15: 2196–202.

Sankaranarayanan R, Duffy SW, Day NE, Nair MK, Padmakumary G. A case-control investigation of cancer of the oral tongue and the floor of the mouth in southern India. Int J Cancer 1989; 44: 617–21.

Subapriya R, Thangavelu A, Mathavan B, Ramachandran CR, Nagini S. Assessment of risk factors for oral squamous cell carcinoma in Chidambaram, Southern India: a case-control study. Eur J Cancer Prev 2007; 16: 251–6.

Takacs D, Koppany F, Mihalyi S, Suba Z. Decreased oral cancer risk by moderate alcohol consumption in non-smoker postmenopausal women. Oral Oncol 2011; 47: 537–40.

VINCENT RG, MARCHETTA F. The relationship of the use of tobacco and alcohol to cancer of the oral cavity, pharynx or larynx. Am J Surg 1963; 106: 501–5.

Vlajinac HD, Marinkovic JM, Sipetic SB, Andrejic DM, Adanja BJ, Stosic-Divjak SL. Case-control study of oropharyngeal cancer. Cancer Detect Prev 2006; 30: 152–7.

WYNDER EL, BROSS IJ. Aetiological factors in mouth cancer; an approach to its prevention. BMJ 1957; 1: 1137–43.

Zheng TZ, Boyle P, Hu HF, et al. Tobacco smoking, alcohol consumption, and risk of oral cancer: a case-control study in Beijing, People's Republic of China. Cancer Causes Control 1990; 1: 173–9.

15. Liver cancer

Summary of the meta-analysis conducted for GBD 2016

Search String:

((“”ethanol“”[MeSH Terms] OR “”alcohols“”[MeSH Terms]) AND “”liver neoplasms“”[MeSH Terms] AND (“”0001/01/01“”[PDAT] : “”2016/12/31“”[PDAT])) AND “”humans“”[MeSH Terms]

PRISMA flow diagram

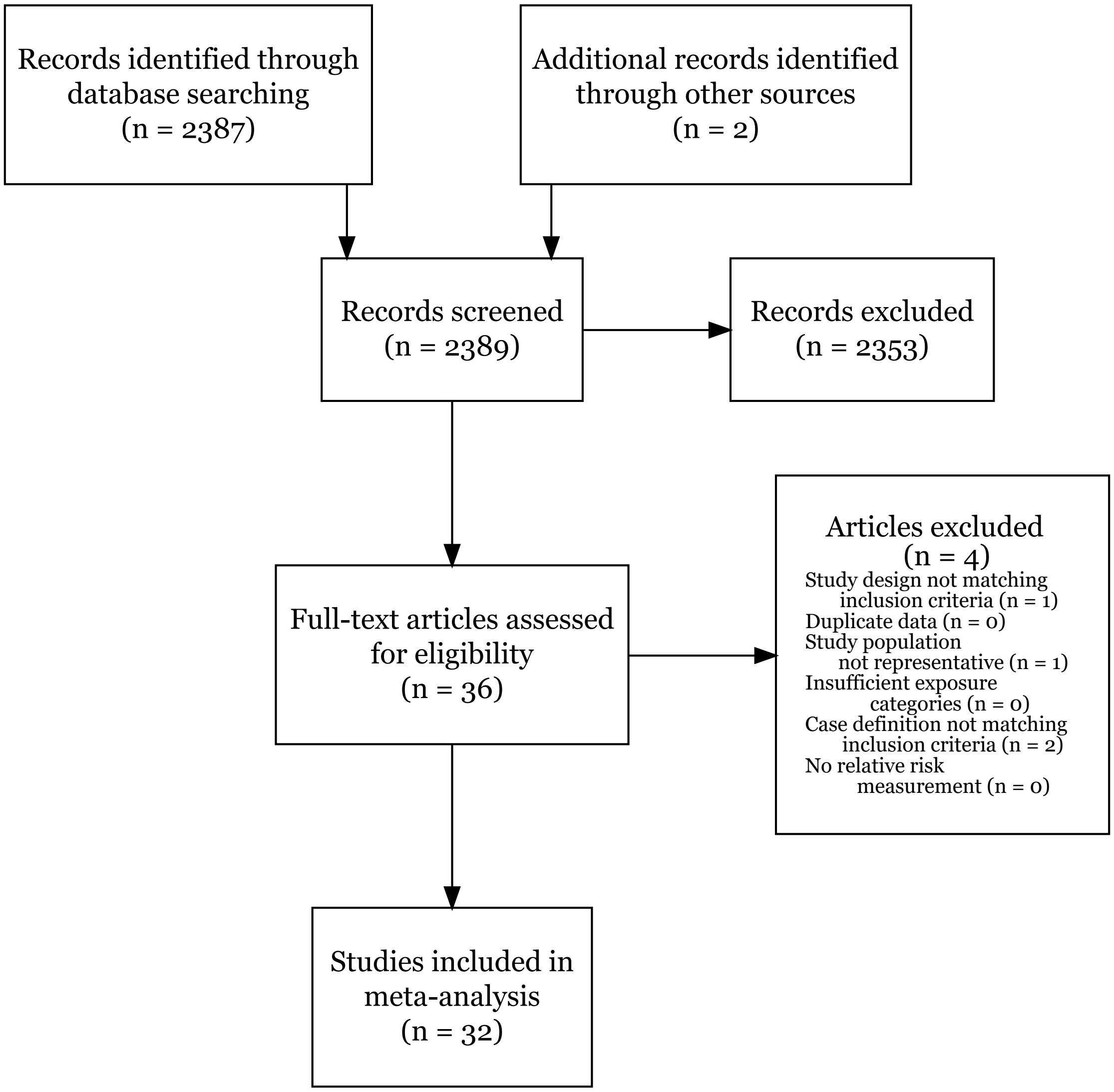

Relative risk (RR) curves for Liver cancer by number of standard drinks consumed daily. Points are relative risk estimates from studies.
The vertical bars capture the uncertainty in each study, related to the sample size and the horizontal bars capture the range of drinks consumed by individuals in the study.
The black line represents the estimated RR for Liver cancer at each level of consumption. The shaded green areas represent the 95% uncertainty interval associated with the estimated RR.
Dotted line is a reference for a relative risk of 1.

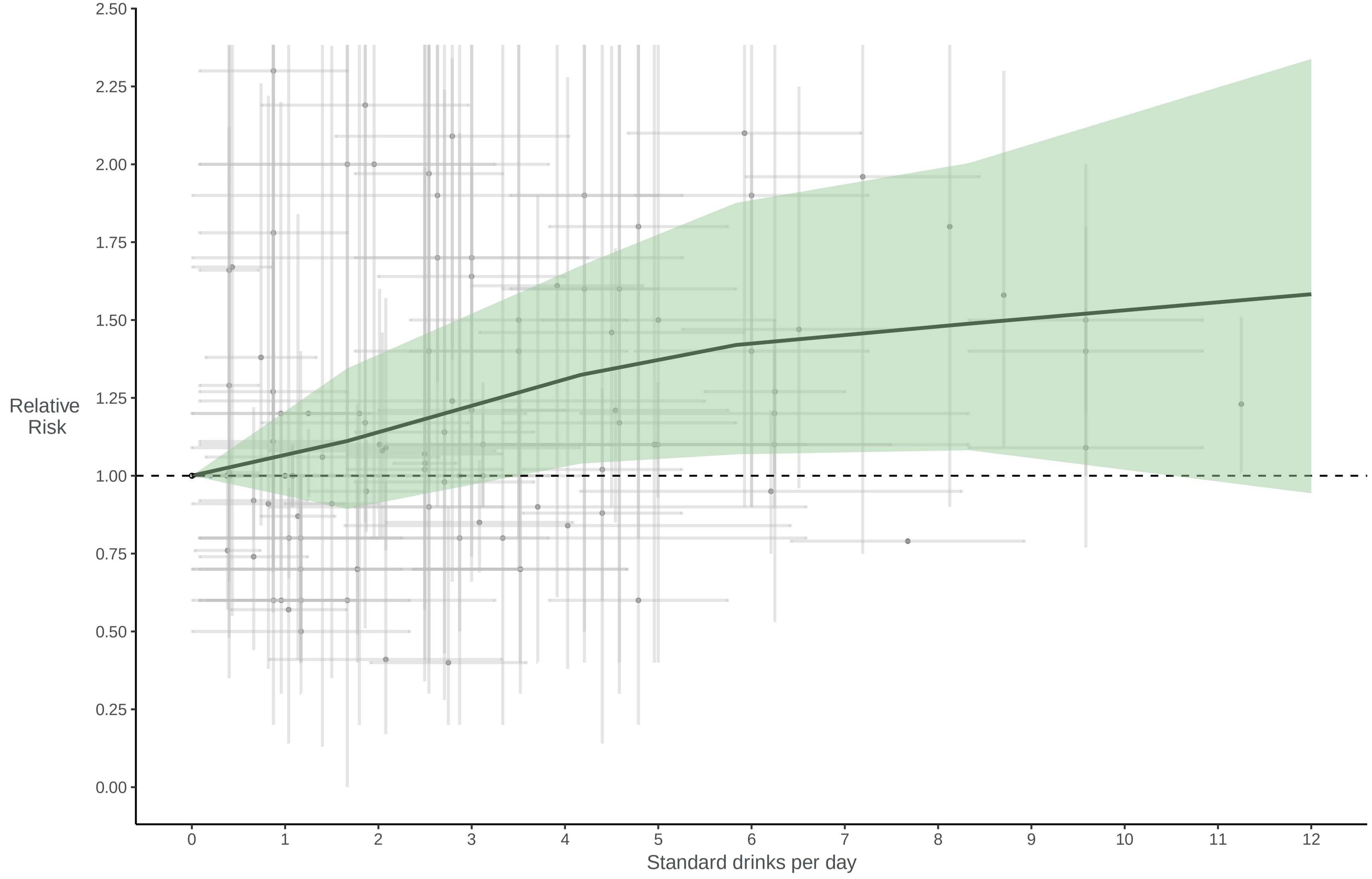

References for Liver cancer

Allen NE, Beral V, Casabonne D, et al. Moderate alcohol intake and cancer incidence in women. J Natl Cancer Inst 2009; 101: 296–305.

Benedetti A, Parent ME, Siemiatycki J. Lifetime consumption of alcoholic beverages and risk of 13 types of cancer in men: results from a case-control study in Montreal. Cancer Detect Prev 2009; 32: 352–62.

Chen CJ, Wang LY, Lu SN, et al. Elevated aflatoxin exposure and increased risk of hepatocellular carcinoma. Hepatology 1996; 24: 38–42.

Choi SY, Kahyo H. Effect of cigarette smoking and alcohol consumption in the aetiology of cancer of the oral cavity, pharynx and larynx. Int J Epidemiol 1991; 20: 878–85.

Cordier S, Le TB, Verger P, et al. Viral infections and chemical exposures as risk factors for hepatocellular carcinoma in Vietnam. Int J Cancer 1993; 55: 196–201.

Donato F, Tagger A, Gelatti U, et al. Alcohol and hepatocellular carcinoma: the effect of lifetime intake and hepatitis virus infections in men and women. Am J Epidemiol 2002; 155: 323–31.

Franceschi S, Montella M, Polesel J, et al. Hepatitis viruses, alcohol, and tobacco in the etiology of hepatocellular carcinoma in Italy. Cancer Epidemiol Biomarkers Prev 2006; 15: 683–9.

Hassan MM, Hwang LY, Hatten CJ, et al. Risk factors for hepatocellular carcinoma: synergism of alcohol with viral hepatitis and diabetes mellitus. Hepatology 2002; 36: 1206–13.

Hassan MM, Kaseb A, Li D, et al. Association between hypothyroidism and hepatocellular carcinoma: a case-control study in the United States. Hepatology 2009; 49: 1563–70.

Jee SH, Ohrr H, Sull JW, Samet JM. Cigarette smoking, alcohol drinking, hepatitis B, and risk for hepatocellular carcinoma in Korea. J Natl Cancer Inst 2004; 96: 1851–6.

Joshi S, Song YM, Kim TH, Cho SI. Socio-economic status and the risk of liver cancer mortality: a prospective study in Korean men. Public Health 2008; 122: 1144–51.

Kim MK, Ko MJ, Han JT. Alcohol consumption and mortality from all-cause and cancers among 1.34 million Koreans: the results from the Korea national health insurance corporation's health examinee cohort in 2000. Cancer Causes Control 2010; 21: 2295–302.

Koh WP, Robien K, Wang R, Govindarajan S, Yuan JM, Yu MC. Smoking as an independent risk factor for hepatocellular carcinoma: the Singapore Chinese Health Study. Br J Cancer 2011; 105: 1430–5.

Kono S, Ikeda M, Tokudome S, Nishizumi M, Kuratsune M. Cigarette smoking, alcohol and cancer mortality: a cohort study of male Japanese physicians. Jpn J Cancer Res 1987; 78: 1323–8.

Kuper H, Tzonou A, Kaklamani E, et al. Tobacco smoking, alcohol consumption and their interaction in the causation of hepatocellular carcinoma. Int J Cancer 2000; 85: 498–502.

Mohamed AE, Kew MC, Groeneveld HT. Alcohol consumption as a risk factor for hepatocellular carcinoma in urban southern African blacks. Int J Cancer 1992; 51: 537–41.

Murata M, Takayama K, Choi BC, Pak AW. A nested case-control study on alcohol drinking, tobacco smoking, and cancer. Cancer Detect Prev 1996; 20: 557–65.

Nakaya N, Tsubono Y, Kuriyama S, et al. Alcohol consumption and the risk of cancer in Japanese men: the Miyagi cohort study. Eur J Cancer Prev 2005; 14: 169–74.

Ohishi W, Fujiwara S, Cologne JB, et al. Risk factors for hepatocellular carcinoma in a Japanese population: a nested case-control study. Cancer Epidemiol Biomarkers Prev 2008; 17: 846–54.

Ozasa K. Alcohol use and mortality in the Japan Collaborative Cohort Study for Evaluation of Cancer (JACC). Asian Pac J Cancer Prev 2007; 8 Suppl: 81–8.

Pyong SJ, Tsukuma H, Hiyama T. Case-control study of hepatocellular carcinoma among Koreans living in Osaka, Japan. Jpn J Cancer Res 1994; 85: 674–9.

Sakamoto T, Hara M, Higaki Y, et al. Influence of alcohol consumption and gene polymorphisms of ADH2 and ALDH2 on hepatocellular carcinoma in a Japanese population. Int J Cancer 2006; 118: 1501–7.

Stemhagen A, Slade J, Altman R, Bill J, factors O risk, liver. Occupational risk factors and liver cancer. A retrospective case-control study of primary liver cancer in New Jersey. Am J Epidemiol 1983; 117: 443–54.

Trichopoulos D, Bamia C, Lagiou P, et al. Hepatocellular carcinoma risk factors and disease burden in a European cohort: a nested case-control study. J Natl Cancer Inst 2011; 103: 1686–95.

Trichopoulos D, Day NE, Kaklamani E, et al. Hepatitis B virus, tobacco smoking and ethanol consumption in the etiology of hepatocellular carcinoma. Int J Cancer 1987; 39: 45–9.

VallMayans M, Calvet X, Bruix J, et al. Risk factors for hepatocellular carcinoma in Catalonia, Spain. Int J Cancer 1990; 46: 378–81.

Yi SW, Sull JW, Linton JA, Nam CM, Ohrr H. Alcohol consumption and digestive cancer mortality in Koreans: the Kangwha Cohort Study. J Epidemiol 2010; 20: 204–11.

Yu MC, Mack T, Hanisch R, Peters RL, Henderson BE, Pike MC. Hepatitis, alcohol consumption, cigarette smoking, and hepatocellular carcinoma in Los Angeles. Cancer Res 1983; 43: 6077–9.

Yuan JM, Gao YT, Ong CN, Ross RK, Yu MC. Prediagnostic level of serum retinol in relation to reduced risk of hepatocellular carcinoma. J Natl Cancer Inst 2006; 98: 482–90.

Yuan JM, Govindarajan S, Arakawa K, Yu MC. Synergism of alcohol, diabetes, and viral hepatitis on the risk of hepatocellular carcinoma in blacks and whites in the U.S. Cancer 2004; 101: 1009–17.

Yun EH, Lim MK, Oh J, et al. Combined effect of socioeconomic status, viral hepatitis, and lifestyles on hepatocelluar carcinoma risk in Korea. Br J Cancer 2010; 103: 741–6.

Zhang JY, Wang X, Han SG, Zhuang H. A case-control study of risk factors for hepatocellular carcinoma in Henan, China. Am J Trop Med Hyg 1998; 59: 947–51.

16. Lower respiratory infections

Summary of the meta-analysis conducted for GBD 2016

Search String:

((“”ethanol“”[MeSH Terms] OR “”alcohols“”[MeSH Terms]) AND (“”pneumonia“”[MeSH Terms] OR “”respiratory tract infections“”[MeSH Terms]) AND (“”1980/01/01“”[PDAT] : “”2016/12/31“”[PDAT])) AND “”humans“”[MeSH Terms]

PRISMA flow diagram

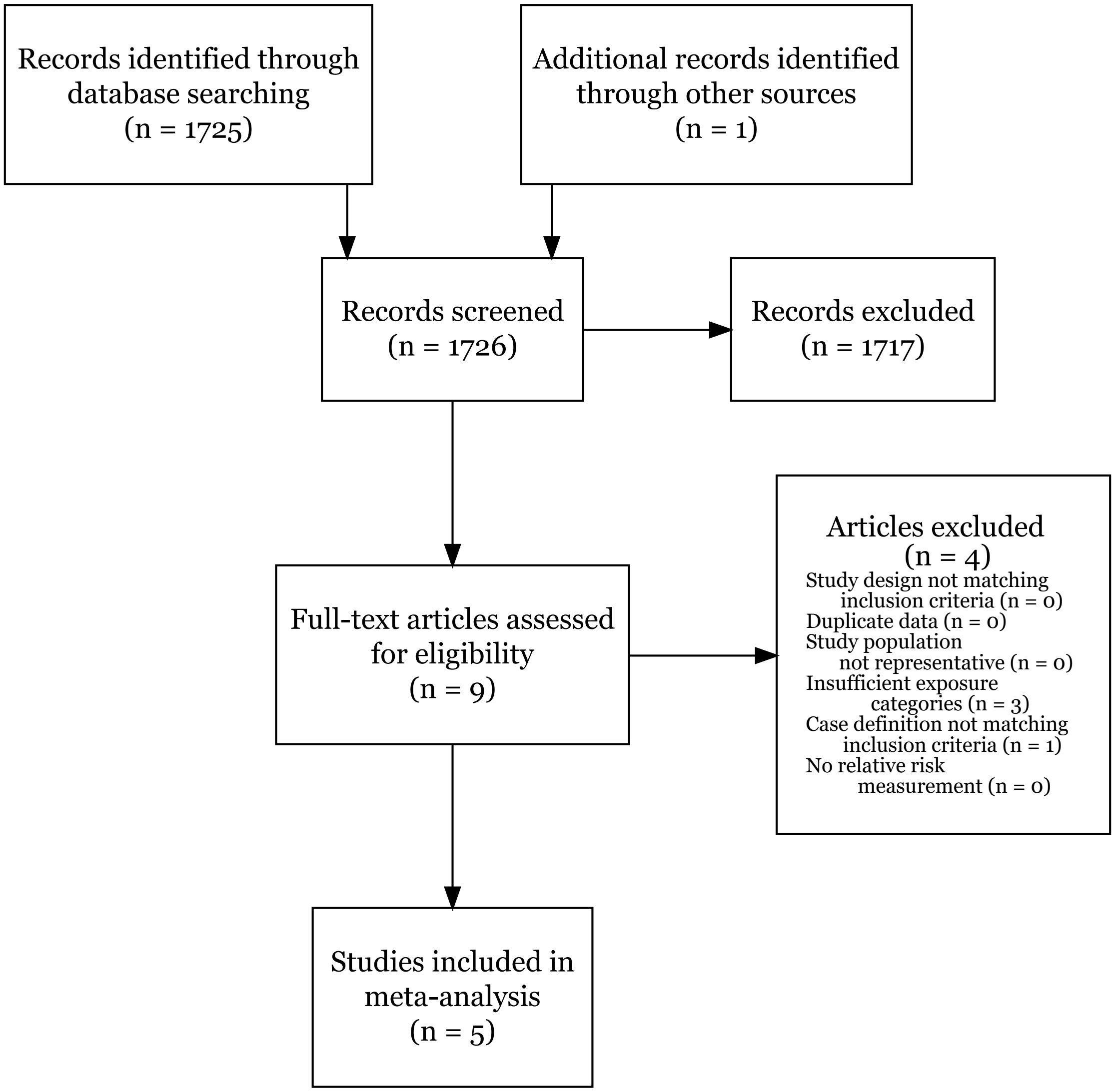

Relative risk (RR) curves for Lower respiratory infections by number of standard drinks consumed daily. Points are relative risk estimates from studies.
The vertical bars capture the uncertainty in each study, related to the sample size and the horizontal bars capture the range of drinks consumed by individuals in the study.
The black line represents the estimated RR for Lower respiratory infections at each level of consumption. The shaded green areas represent the 95% uncertainty interval associated with the estimated RR.
Dotted line is a reference for a relative risk of 1.

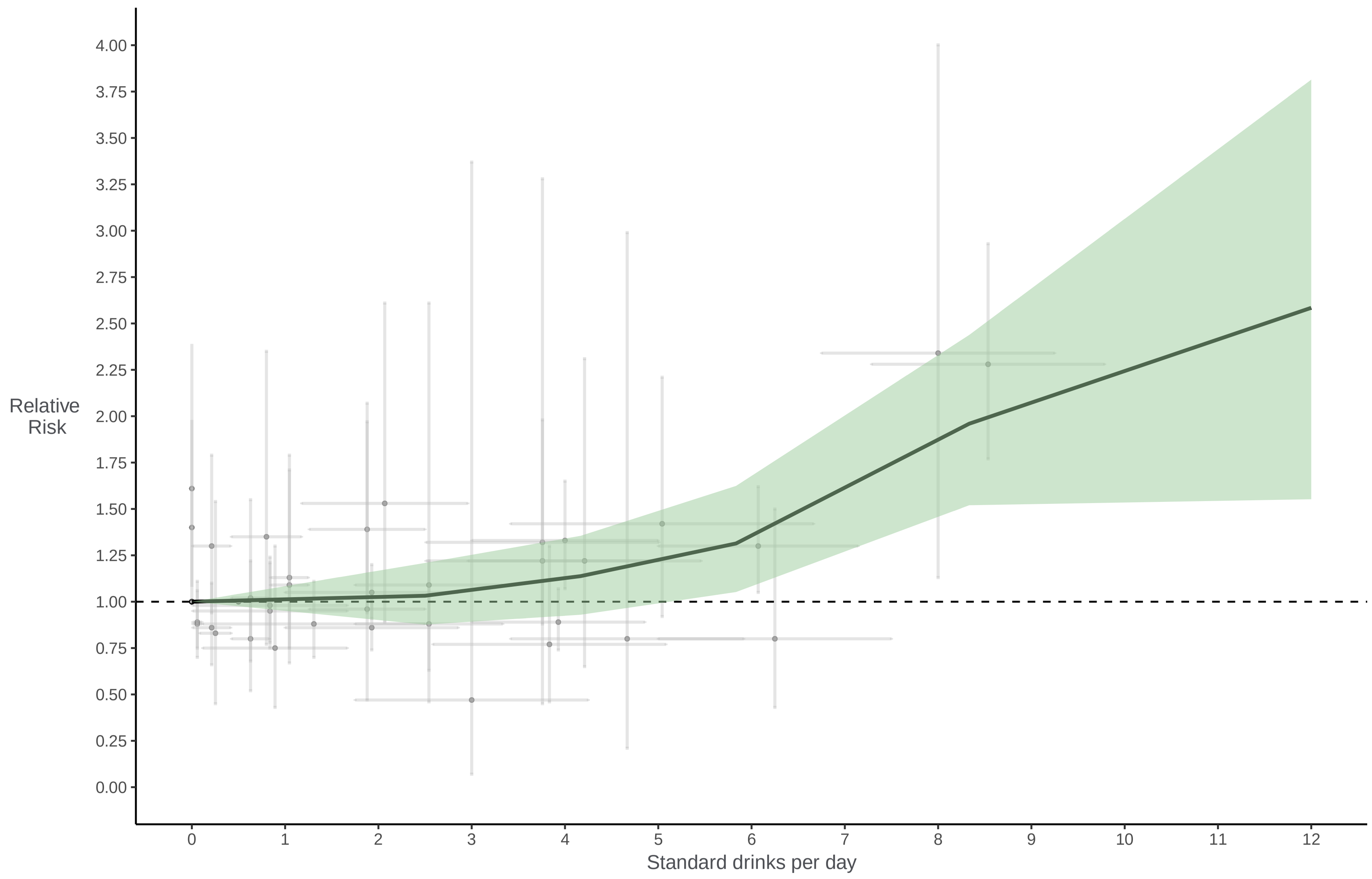

References for Lower respiratory infections

Almirall J, Bolibar I, Balanzo X, Gonzalez CA. Risk factors for community-acquired pneumonia in adults: a population-based case-control study. Eur Respir J 1999; 13: 349–55.

Almirall J, Bolibar I, Serra-Prat M, et al. New evidence of risk factors for community-acquired pneumonia: a population-based study. Eur Respir J 2008; 31: 1274–84.

Baik I, Curhan GC, Rimm EB, Bendich A, Willett WC, Fawzi WW. A prospective study of age and lifestyle factors in relation to community-acquired pneumonia in US men and women. Arch Intern Med 2000; 160: 3082–8.

Baik I, Curhan GC, Rimm EB, Bendich A, Willett WC, Fawzi WW. A prospective study of age and lifestyle factors in relation to community-acquired pneumonia in US men and women. Arch Intern Med 2000; 160: 3082–8.

Kornum JB, Due KM, Norgaard M, et al. Alcohol drinking and risk of subsequent hospitalisation with pneumonia. Eur Respir J 2012; 39: 149–55.

Shen C, Ni MY, Schooling CM, Chan WM, Lee SY, Lam TH. Alcohol use and death from respiratory disease in a prospective Chinese elderly cohort study in Hong Kong. Prev Med 2013; 57: 819–23.

17. Pharynx and nasopharynx cancer

Summary of the meta-analysis conducted for GBD 2016

Search String:

((“”ethanol“”[MeSH Terms] OR “”alcohols“”[MeSH Terms]) AND “”pharyngeal neoplasms“”[MeSH Terms] AND (“”0001/01/01“”[PDAT] : “”2016/12/31“”[PDAT])) AND “”humans“”[MeSH Terms]

PRISMA flow diagram

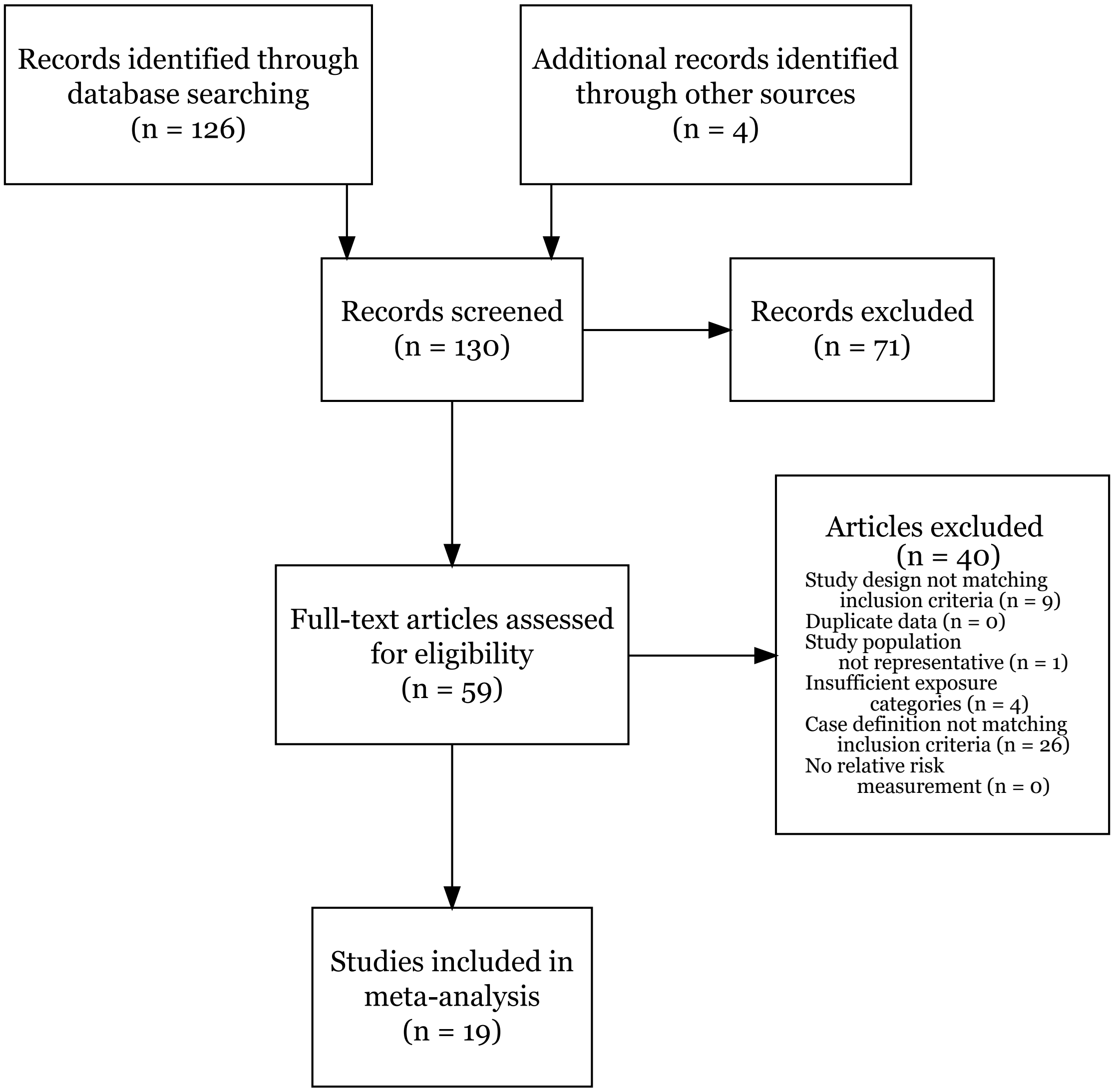

Relative risk (RR) curves for Pharynx and nasopharynx cancer by number of standard drinks consumed daily. Points are relative risk estimates from studies.
The vertical bars capture the uncertainty in each study, related to the sample size and the horizontal bars capture the range of drinks consumed by individuals in the study.
The black line represents the estimated RR for Pharynx and nasopharynx cancer at each level of consumption. The shaded green areas represent the 95% uncertainty interval associated with the estimated RR.
Dotted line is a reference for a relative risk of 1.

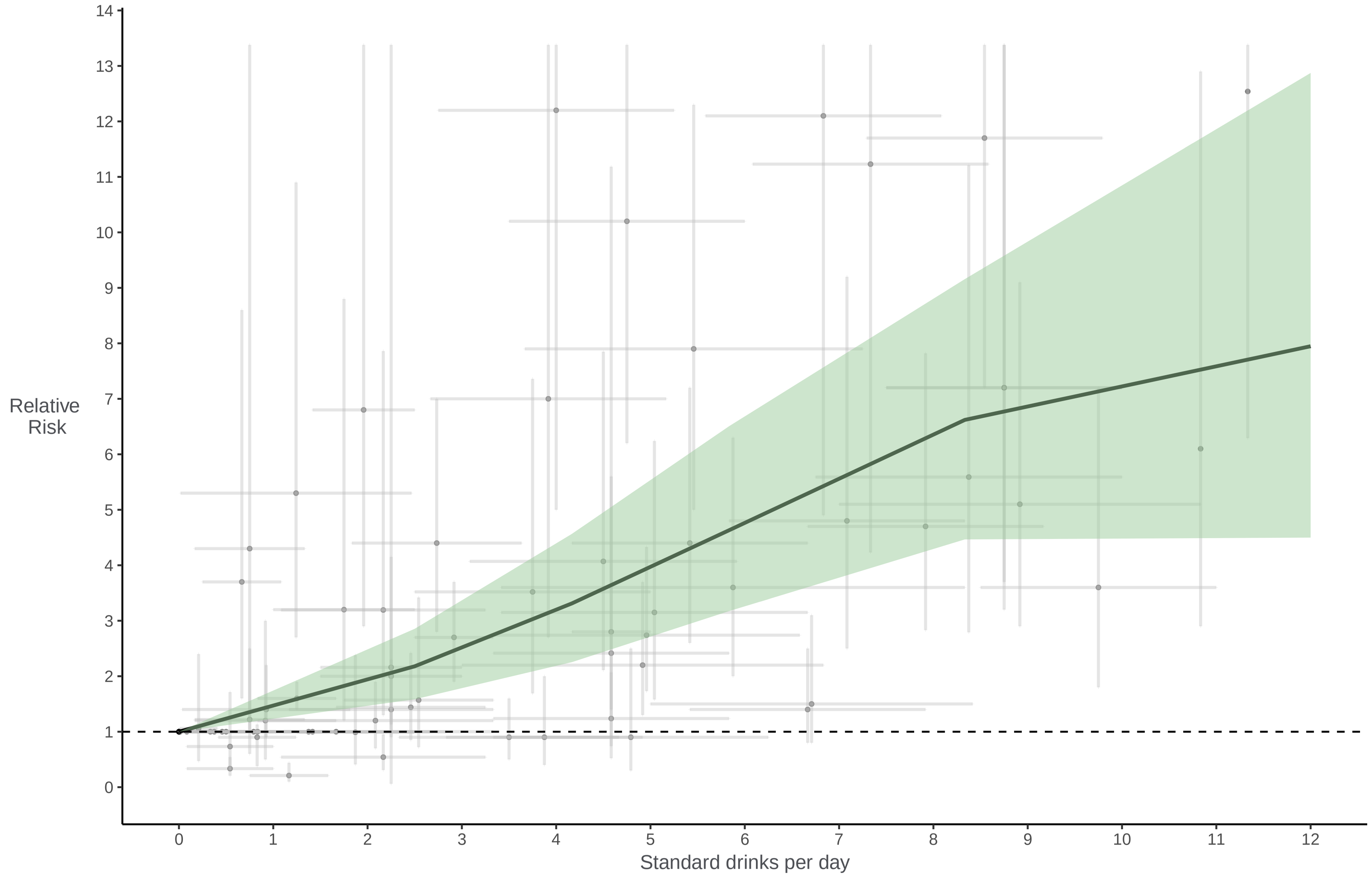

References for Pharynx and nasopharynx cancer

Andre K, Schraub S, Mercier M, Bontemps P. Role of alcohol and tobacco in the aetiology of head and neck cancer: a case-control study in the Doubs region of France. Eur J Cancer B Oral Oncol 1995; 31B: 301–9.

Choi SY, Kahyo H. Effect of cigarette smoking and alcohol consumption in the aetiology of cancer of the oral cavity, pharynx and larynx. Int J Epidemiol 1991; 20: 878–85.

De Stefani E, Boffetta P, Deneo-Pellegrini H, et al. The effect of smoking and drinking in oral and pharyngeal cancers: a case-control study in Uruguay. Cancer Lett 2007; 246: 282–9.

Elwood JM, Pearson JC, Skippen DH, Jackson SM. Alcohol, smoking, social and occupational factors in the aetiology of cancer of the oral cavity, pharynx and larynx. Int J Cancer 1984; 34: 603–12.

Franceschi S, Levi F, La Vecchia C, et al. Comparison of the effect of smoking and alcohol drinking between oral and pharyngeal cancer. Int J Cancer 1999; 83: 4-Jan.

Franceschi S, Talamini R, Barra S, et al. Smoking and drinking in relation to cancers of the oral cavity, pharynx, larynx, and esophagus in northern Italy. Cancer Res 1990; 50: 6502–7.

Freedman ND, Schatzkin A, Leitzmann MF, Hollenbeck AR, Abnet CC. Alcohol and head and neck cancer risk in a prospective study. Br J Cancer 2007; 96: 1469–74.

Herrero R, Castellsague X, Pawlita M, et al. Human papillomavirus and oral cancer: the International Agency for Research on Cancer multicenter study. J Natl Cancer Inst 2003; 95: 1772–83.

Lee KW, Kuo WR, Tsai SM, et al. Different impact from betel quid, alcohol and cigarette: risk factors for pharyngeal and laryngeal cancer. Int J Cancer 2005; 117: 831–6.

Maasland DH, van den Brandt PA, Kremer B, Goldbohm RA, Schouten LJ. Alcohol consumption, cigarette smoking and the risk of subtypes of head-neck cancer: results from the Netherlands Cohort Study. BMC Cancer 2014; 14: 187.

Menvielle G, Luce D, Goldberg P, Bugel I, Leclerc A. Smoking, alcohol drinking and cancer risk for various sites of the larynx and hypopharynx. A case-control study in France. Eur J Cancer Prev 2004; 13: 165–72.

Peters ES, McClean MD, Marsit CJ, Luckett B, Kelsey KT. Glutathione S-transferase polymorphisms and the synergy of alcohol and tobacco in oral, pharyngeal, and laryngeal carcinoma. Cancer Epidemiol Biomarkers Prev 2006; 15: 2196–202.

Rossing MA, Vaughan TL, McKnight B. Diet and pharyngeal cancer. Int J Cancer 1989; 44: 593–7.

Takezaki T, Shinoda M, Hatooka S, et al. Subsite-specific risk factors for hypopharyngeal and esophageal cancer (Japan). Cancer Causes Control 2000; 11: 597–608.

Tuyns AJ, Esteve J, Raymond L, et al. Cancer of the larynx/hypopharynx, tobacco and alcohol: IARC international case-control study in Turin and Varese (Italy), Zaragoza and Navarra (Spain), Geneva (Switzerland) and Calvados (France). Int J Cancer 1988; 41: 483–91.

WYNDER EL, BROSS IJ. Aetiological factors in mouth cancer; an approach to its prevention. BMJ 1957; 1: 1137–43.

18. Pancreatitis

Summary of the meta-analysis conducted for GBD 2016

Search String:

((“”ethanol“”[MeSH Terms] OR “”alcohols“”[MeSH Terms]) AND “”pancreatitis“”[MeSH Terms] AND (“”1980/01/01“”[PDAT] : “”2016/12/31“”[PDAT])) AND “”humans“”[MeSH Terms]

PRISMA flow diagram

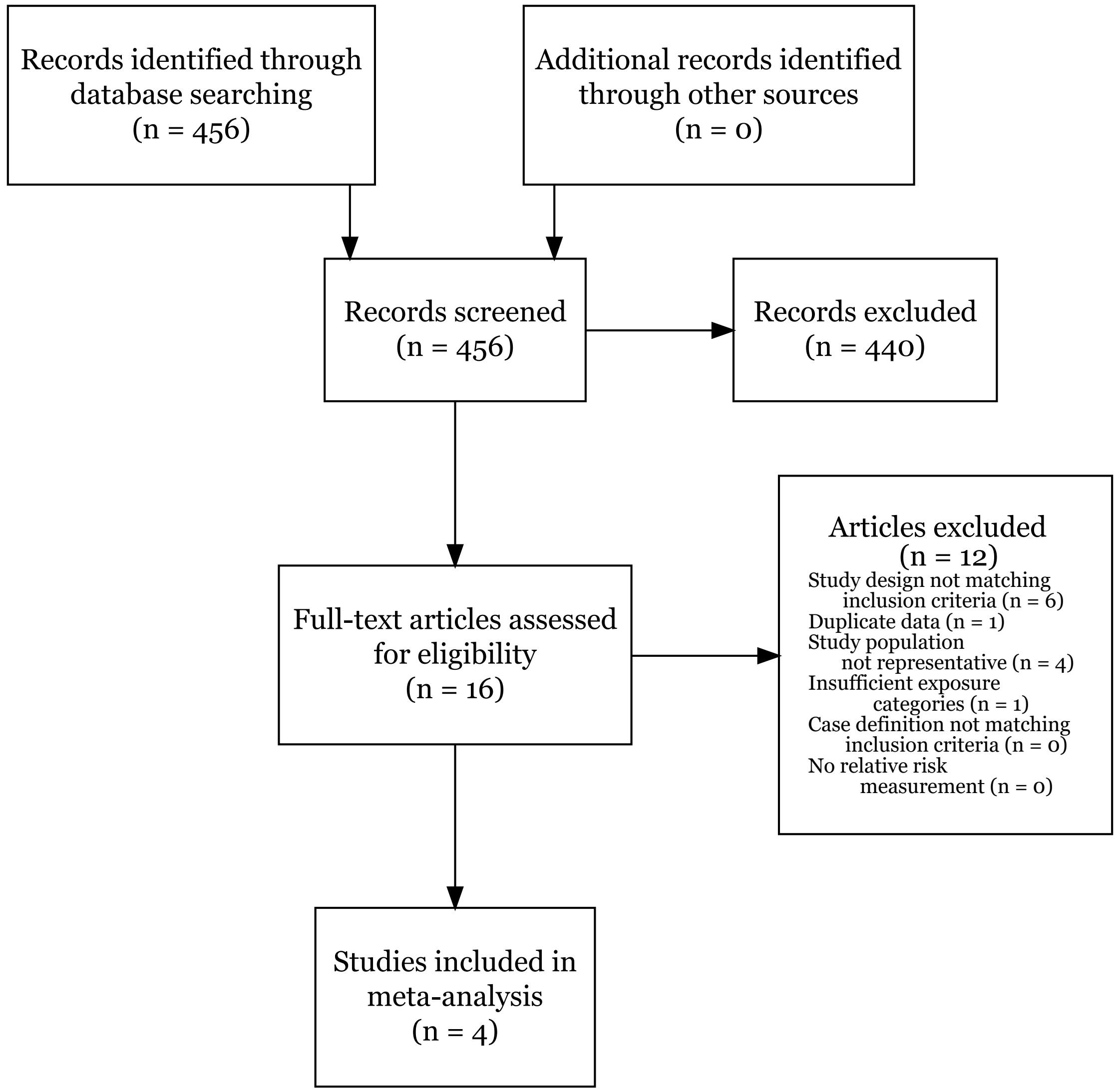

Relative risk (RR) curves for Pancreatitis by number of standard drinks consumed daily. Points are relative risk estimates from studies.
The vertical bars capture the uncertainty in each study, related to the sample size and the horizontal bars capture the range of drinks consumed by individuals in the study.
The black line represents the estimated RR for Pancreatitis at each level of consumption. The shaded green areas represent the 95% uncertainty interval associated with the estimated RR.
Dotted line is a reference for a relative risk of 1.

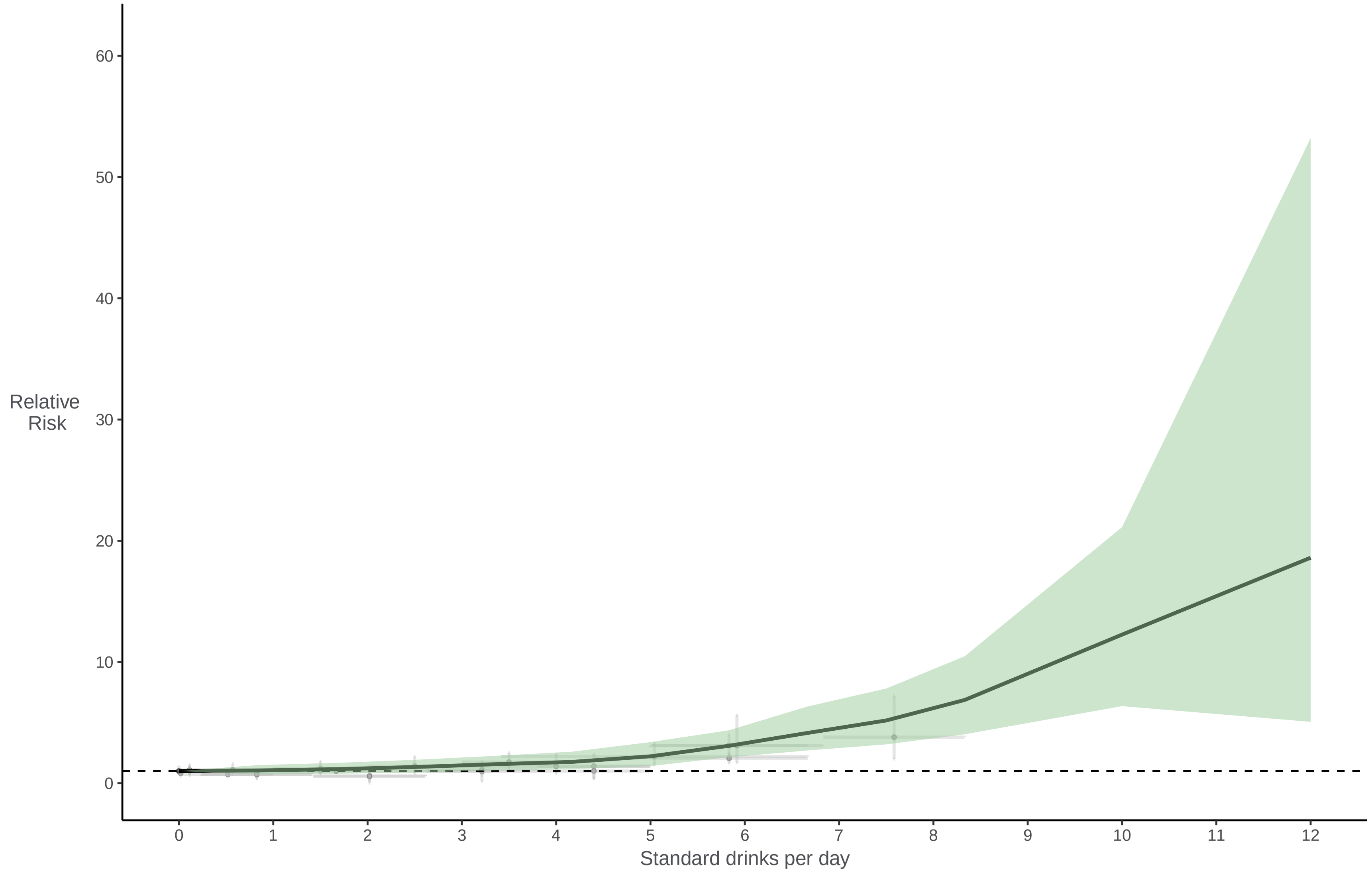

References for Pancreatitis

Blomgren KB, Sundstrom A, Steineck G, Genell S, Sjostedt S, Wiholm BE. A Swedish case-control network for studies of drug-induced morbidity–acute pancreatitis. Eur J Clin Pharmacol 2002; 58: 275–83.

Kristiansen L, Gronbaek M, Becker U, Tolstrup JS. Risk of pancreatitis according to alcohol drinking habits: a population-based cohort study. Am J Epidemiol 2008; 168: 932–7.

Morton C, Klatsky AL, Udaltsova N. Smoking, coffee, and pancreatitis. Am J Gastroenterol 2004; 99: 731–8.

Talamini G, Bassi C, Falconi M, et al. Alcohol and smoking as risk factors in chronic pancreatitis and pancreatic cancer. Dig Dis Sci 1999; 44: 1303–11.

19. Self-harm

Summary of the meta-analysis conducted for GBD 2016

Search String:

((“”ethanol“”[MeSH Terms] OR “”alcohols“”[MeSH Terms]) AND “”self-injurious behavior“”[MeSH Terms] AND (“”1950/01/01“”[PDAT] : “”2016/12/31“”[PDAT])) AND “”humans“”[MeSH Terms]

PRISMA flow diagram

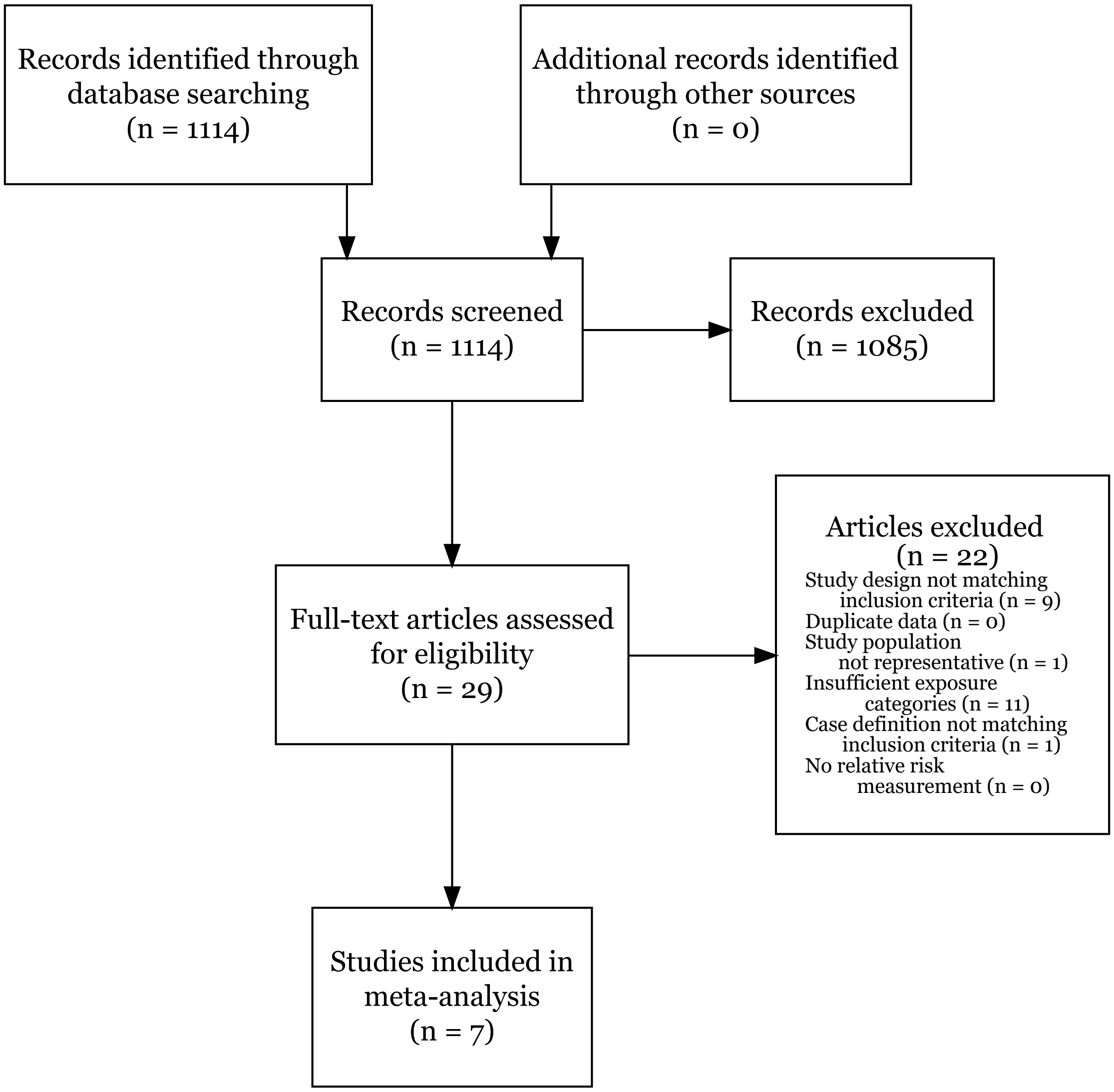

Relative risk (RR) curves for Self−harm by number of standard drinks consumed daily. Points are relative risk estimates from studies.
The vertical bars capture the uncertainty in each study, related to the sample size and the horizontal bars capture the range of drinks consumed by individuals in the study.
The black line represents the estimated RR for Self−harm at each level of consumption. The shaded green areas represent the 95% uncertainty interval associated with the estimated RR.
Dotted line is a reference for a relative risk of 1.

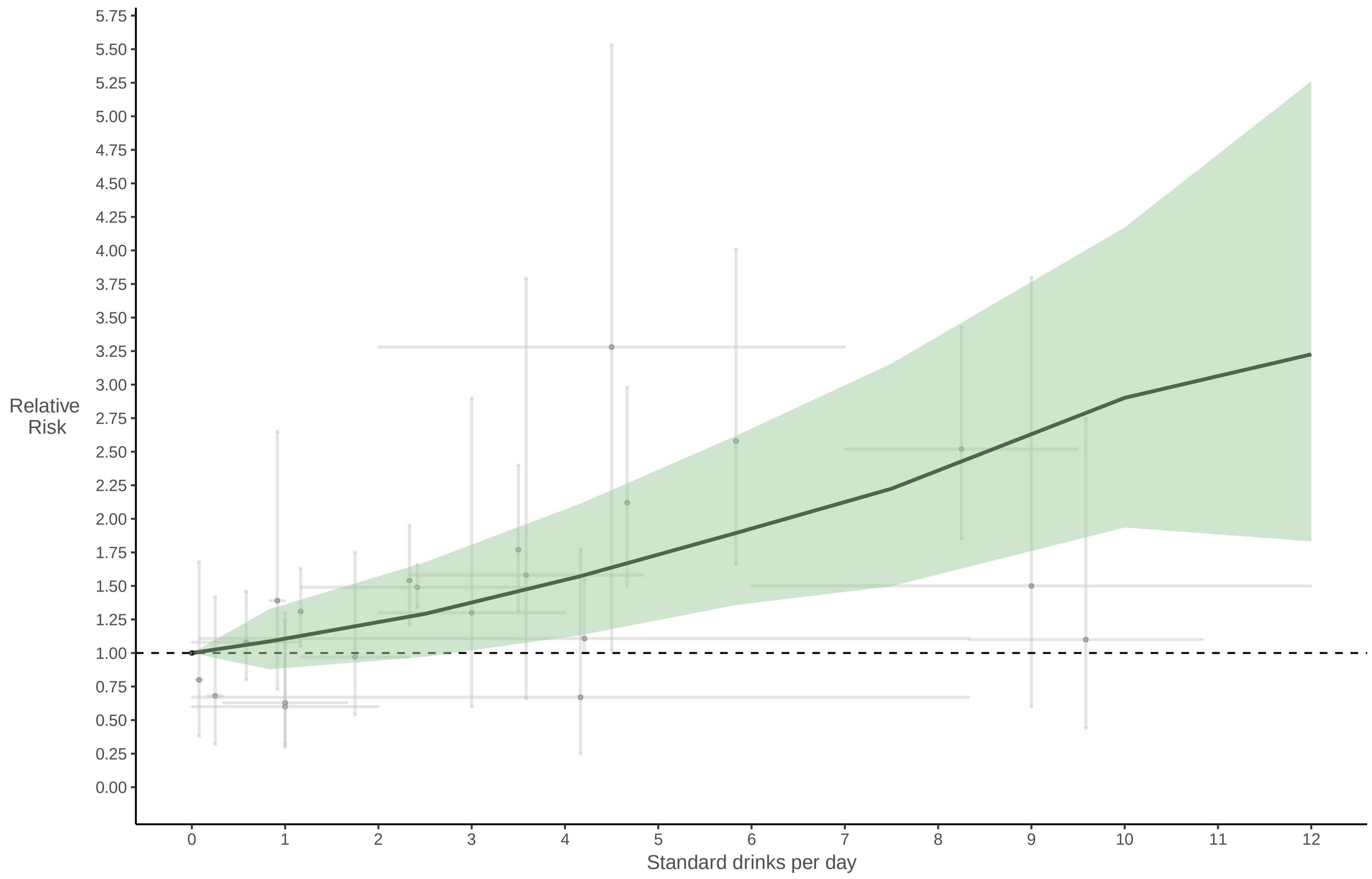

References for Self-harm

Boffetta P, Garfinkel L. Alcohol drinking and mortality among men enrolled in an American Cancer Society prospective study. Epidemiology 1990; 1: 342–8.

Borges G, Rosovsky H. Suicide attempts and alcohol consumption in an emergency room sample. J Stud Alcohol 1996; 57: 543–8.

Andreasson S, Allebeck P, Romelsjö A. Alcohol and mortality among young men: longitudinal study of Swedish conscripts. Br Med J (Clin Res Ed). 1988; 296(6628): 1021–5.

Bagge CL, Lee HJ, Schumacher JA, Gratz KL, Krull JL, Holloman G. Alcohol as an acute risk factor for recent suicide attempts: a case-crossover analysis. J Stud Alcohol Drugs. 2013; 74(4): 552–8.

Chen LH, Baker SP, Li G. Drinking history and risk of fatal injury: comparison among specific injury causes. Accid Anal Prev. 2005; 37(2): 245–51.

Gaziano JM, Gaziano TA, Glynn RJ, Sesso HD, Ajani UA, Stampfer MJ, Manson JE, Hennekens CH, Buring JE. Light-to-moderate alcohol consumption and mortality in the Physicians' Health Study enrollment cohort. J Am Coll Cardiol. 2000; 350(1): 96–105.

Kapur N, Cooper J, King-Hele S, Webb R, Lawlor M, Rodway C, Appleby L. The repetition of suicidal behavior: a multicenter cohort study. J Clin Psychiatry. 2006; 67(10): 1599–609.

Powell KE, Kresnow MJ, Mercy JA, Potter LB, Swann AC, Frankowski RF, Lee RK, Bayer TL. Alcohol Consumption and Nearly Lethal Suicide Attempts. Suicide Life Threat Behav. 2001; 32(1 Suppl): 30–41.

20. Transport injuries

Summary of the meta-analysis conducted for GBD 2016

Search String:

((“”ethanol“”[MeSH Terms] OR “”alcohols“”[MeSH Terms]) AND “”accidents, traffic“”[MeSH Terms] AND (“”1950/01/01“”[PDAT] : “”2016/12/31“”[PDAT])) AND “”humans“”[MeSH Terms]

PRISMA flow diagram

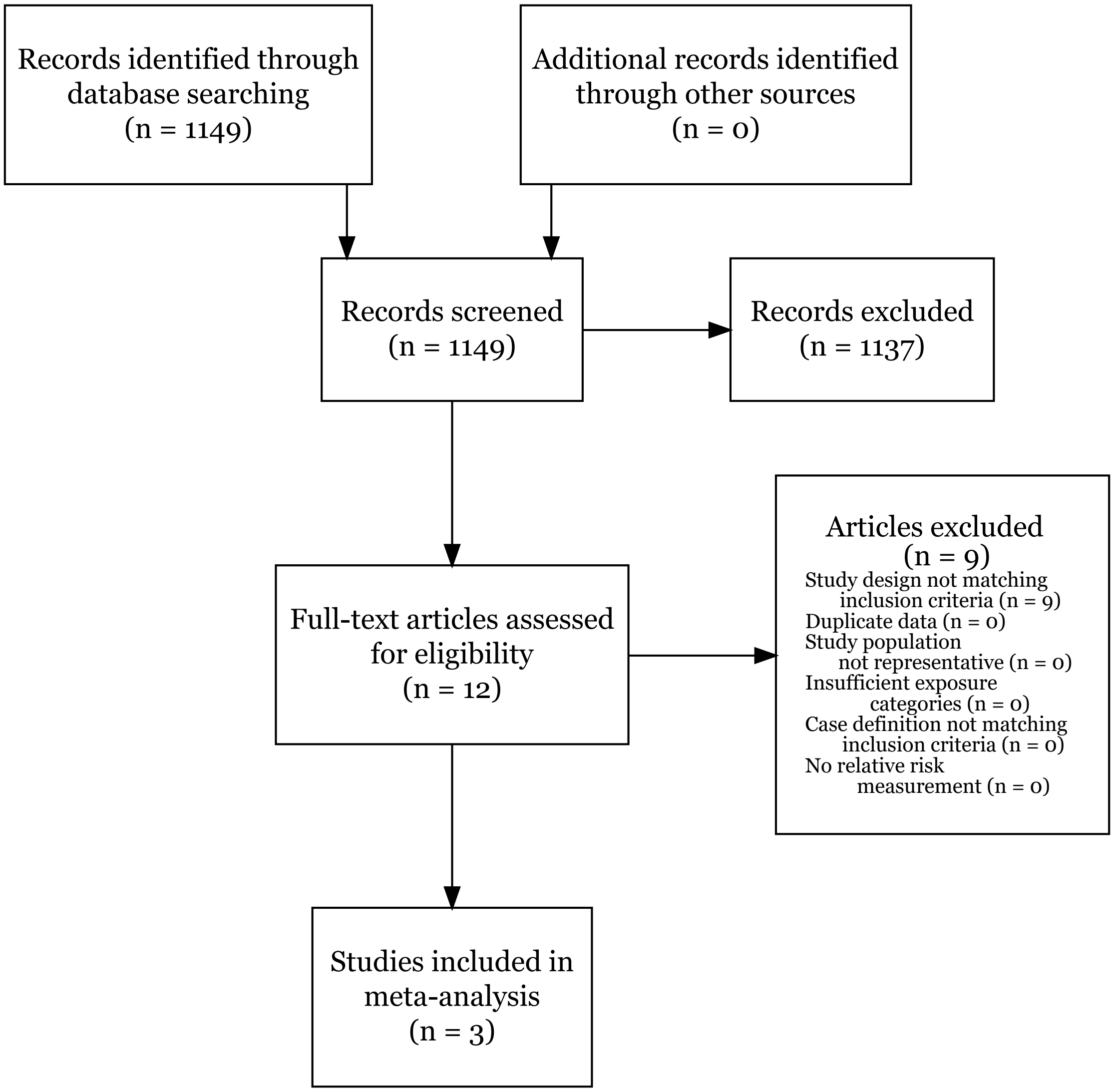

Relative risk (RR) curves for Transport injuries by number of standard drinks consumed daily. Points are relative risk estimates from studies.
The vertical bars capture the uncertainty in each study, related to the sample size and the horizontal bars capture the range of drinks consumed by individuals in the study.
The black line represents the estimated RR for Transport injuries at each level of consumption. The shaded green areas represent the 95% uncertainty interval associated with the estimated RR.
Dotted line is a reference for a relative risk of 1.

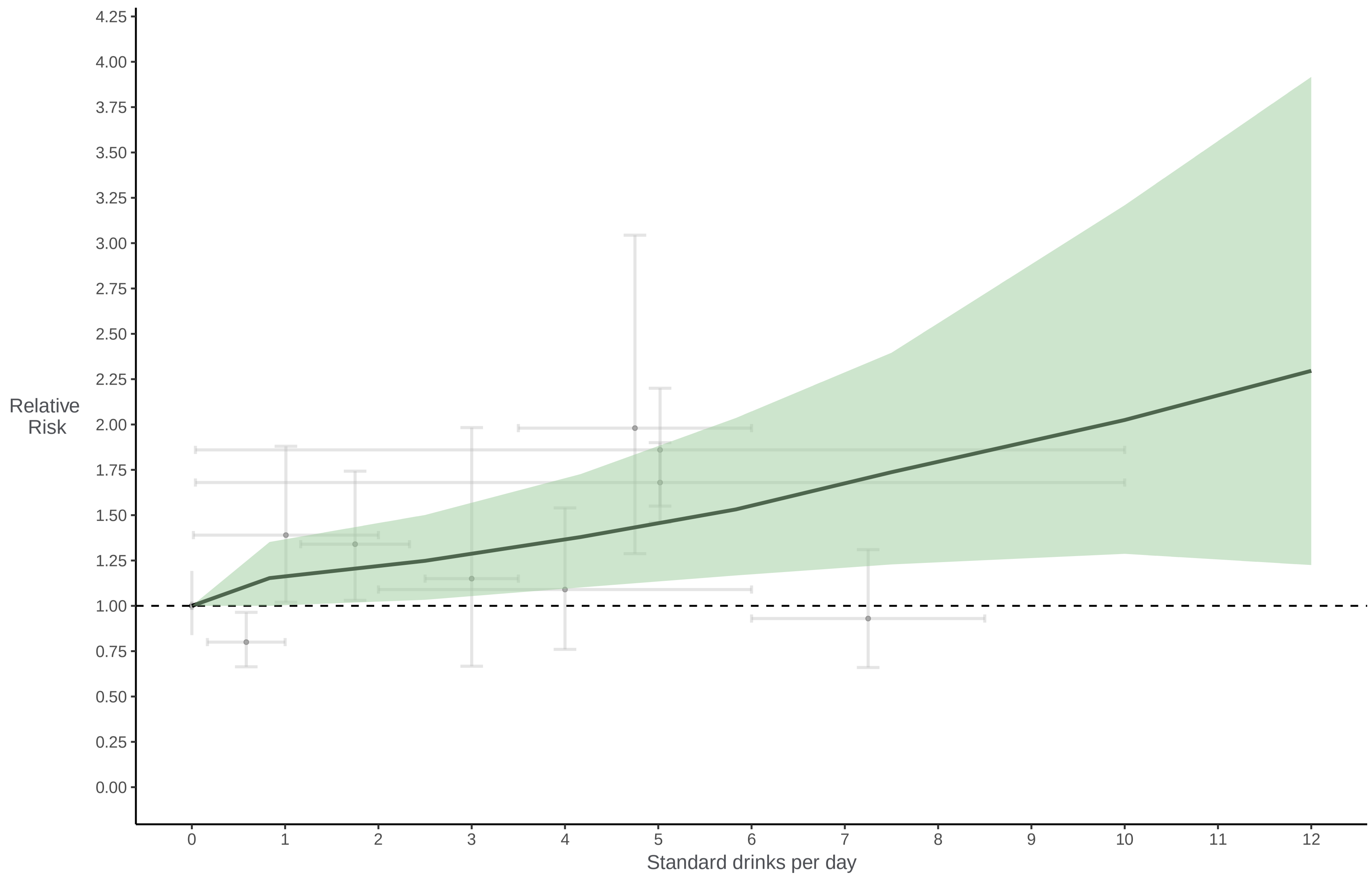

References for Transport injuries

Chen LH, Baker SP, Li G. Drinking history and risk of fatal injury: comparison among specific injury causes. Accid Anal Prev. 2005; 37(2): 245–51.

Bell NS, Amoroso PJ, Yore MM, Smith GS, Jones BH. Self-reported risk-taking behaviors and hospitalization for motor vehicle injury among active duty army personnel. Am J Prev Med. 2000; 18(3 Suppl): 85–95.

Margolis KL, Kerani RP, McGovern P, Songer T, Cauley JA, Ensrud KE, Study Of Osteoporotic Fractures Research Group. Risk factors for motor vehicle crashes in older women. J Gerontol A Biol Sci Med Sci. 2002; 57(3): M186–91.

21. Tuberculosis

Summary of the meta-analysis conducted for GBD 2016

Search String:

((“”ethanol“”[MeSH Terms] OR “”alcohols“”[MeSH Terms]) AND “”tuberculosis“”[MeSH Terms] AND (“”0001/01/01“”[PDAT] : “”2016/12/31“”[PDAT])) AND “”humans“”[MeSH Terms]

PRISMA flow diagram

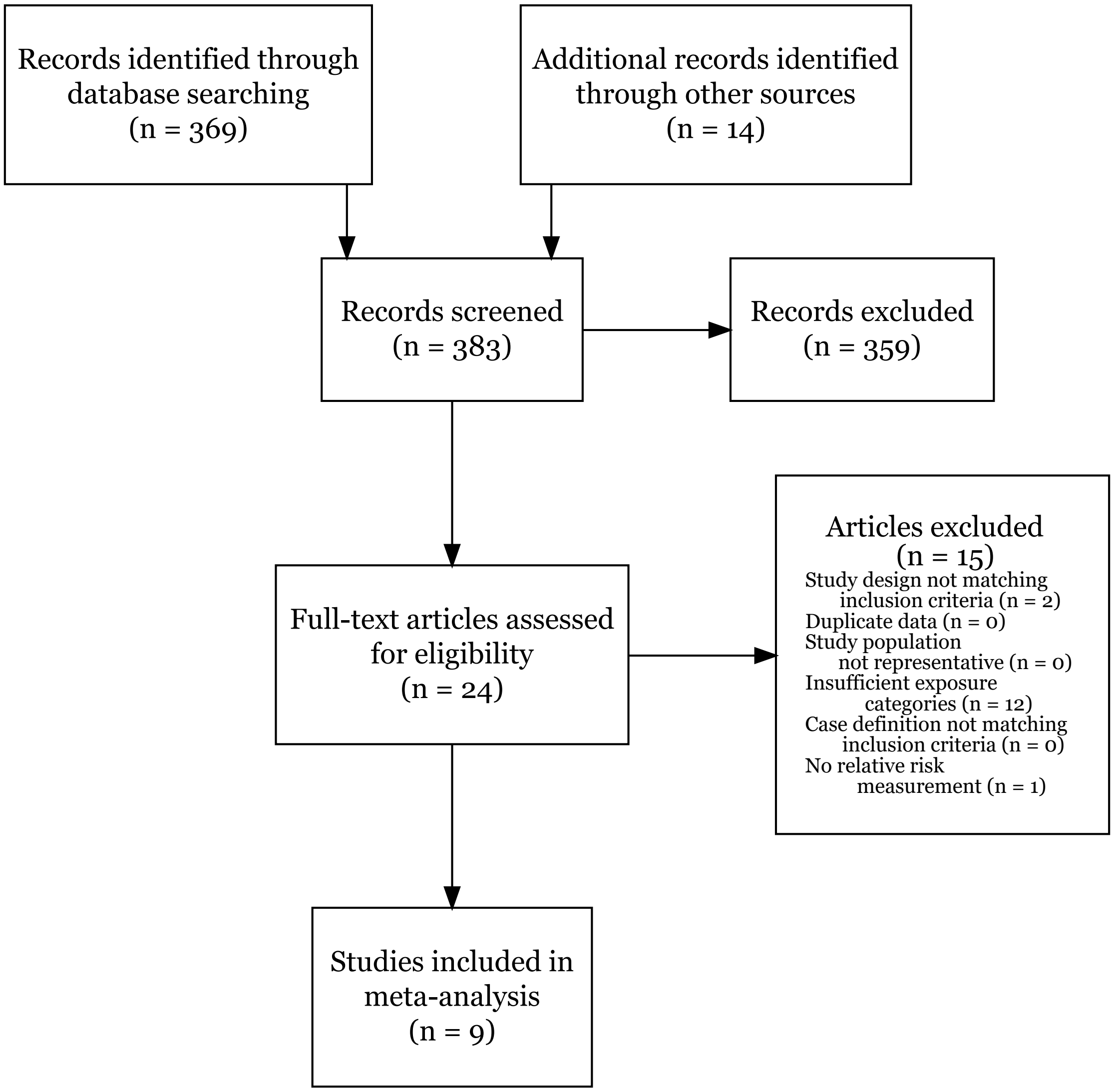

Relative risk (RR) curves for Tuberculosis by number of standard drinks consumed daily. Points are relative risk estimates from studies.
The vertical bars capture the uncertainty in each study, related to the sample size and the horizontal bars capture the range of drinks consumed by individuals in the study.
The black line represents the estimated RR for Tuberculosis at each level of consumption. The shaded green areas represent the 95% uncertainty interval associated with the estimated RR.
Dotted line is a reference for a relative risk of 1.

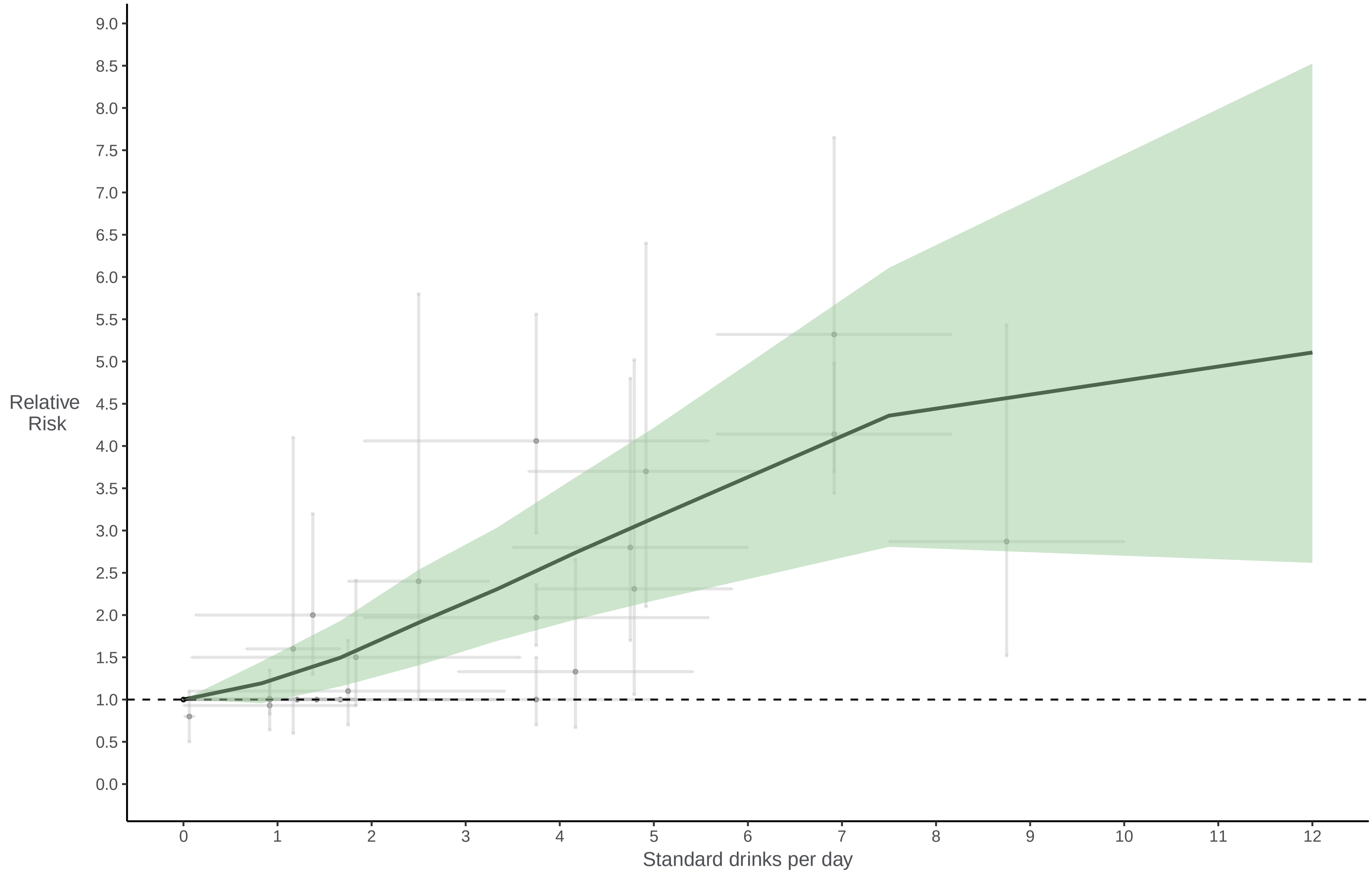

References for Tuberculosis

Buskin SE, Gale JL, Weiss NS, Nolan CM. Tuberculosis risk factors in adults in King County, Washington, 1988 through 1990. Am J Public Health 1994; 84: 1750–6.

Crampin AC, Glynn JR, Floyd S, et al. Tuberculosis and gender: exploring the patterns in a case control study in Malawi. Int J Tuberc Lung Dis 2004; 8: 194–203.

Hemila H, Kaprio J, Pietinen P, Albanes D, Heinonen OP. Vitamin C and other compounds in vitamin C rich food in relation to risk of tuberculosis in male smokers. Am J Epidemiol 1999; 150: 632–41.

Lewis JG, Chamberlain DA. Alcohol consumption and smoking habits in male patients with Pulmonary Tuberculosis. Br J Prev Soc Med 1963; 17: 149–52.

Rosenman KD, Hall N. Occupational risk factors for developing tuberculosis. Am J Ind Med 1996; 30: 148–54.

Tekkel M, Rahu M, Loit HM, Baburin A. Risk factors for pulmonary tuberculosis in Estonia. Int J Tuberc Lung Dis 2002; 6: 887–94.

Tocque K, Bellis MA, Beeching NJ, Syed Q, Remmington T, Davies PD. A case-control study of lifestyle risk factors associated with tuberculosis in Liverpool, North-West England. Eur Respir J 2001; 18: 959–64.

Zaridze D, Brennan P, Boreham J, et al. Alcohol and cause-specific mortality in Russia: a retrospective case-control study of 48,557 adult deaths. Lancet 2009; 373: 2201–14.

22. Unintentional injuries

Summary of the meta-analysis conducted for GBD 2016

Search String:

((“”ethanol“”[MeSH Terms] OR “”alcohols“”[MeSH Terms]) AND ((“”accidents“”[MeSH Terms] NOT “”accidents, traffic“”[MeSH Terms]) OR (“”wounds and injuries“”[MeSH Terms] NOT “”self mutilation“”[MeSH Terms] NOT “”war-related injuries“”[MeSH Terms])) AND (“”1950/01/01“”[PDAT] : “”2016/12/31“”[PDAT])) AND “”humans“”[MeSH Terms]

PRISMA flow diagram

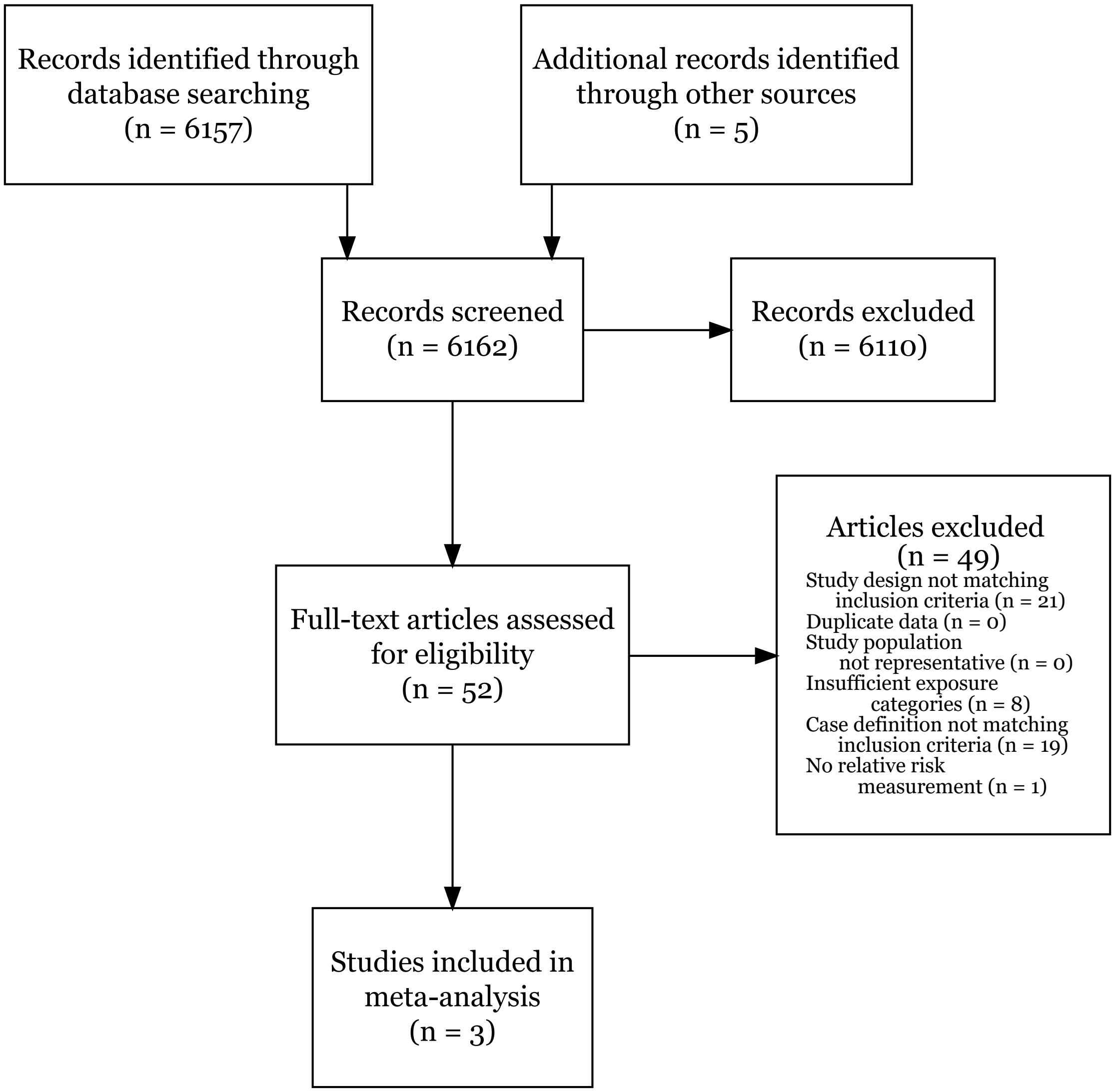

Relative risk (RR) curves for Unintentional injuries by number of standard drinks consumed daily. Points are relative risk estimates from studies.
The vertical bars capture the uncertainty in each study, related to the sample size and the horizontal bars capture the range of drinks consumed by individuals in the study.
The black line represents the estimated RR for Unintentional injuries at each level of consumption. The shaded green areas represent the 95% uncertainty interval associated with the estimated RR.
Dotted line is a reference for a relative risk of 1.

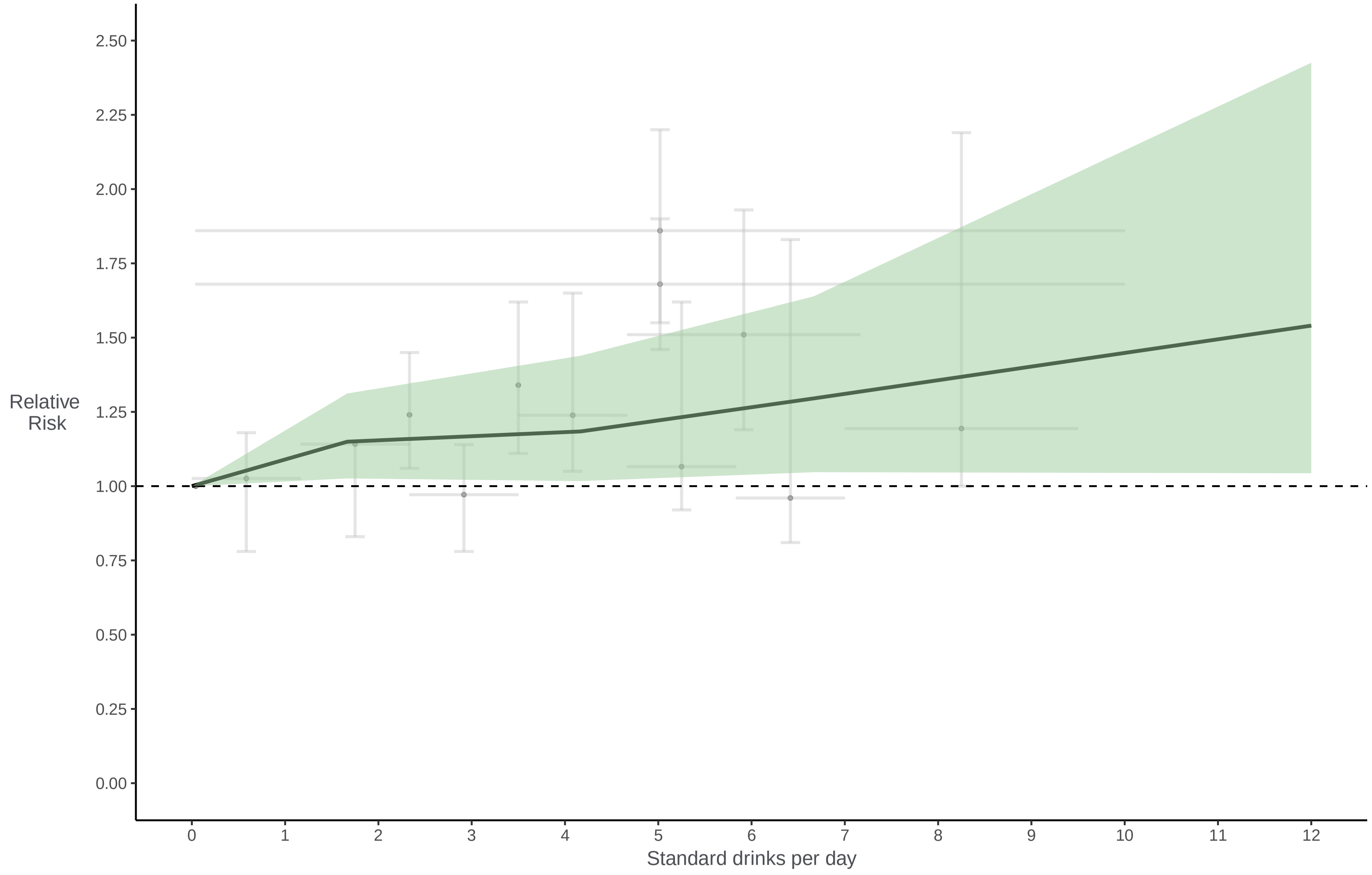

References for Unintentional injuries

Boffetta P, Garfinkel L. Alcohol drinking and mortality among men enrolled in an American Cancer Society prospective study. Epidemiology 1990; 1: 342–8.

Chen LH, Baker SP, Li G. Drinking history and risk of fatal injury: comparison among specific injury causes. Accid Anal Prev. 2005; 37(2): 245–51.

Kanis JA, Johansson H, Johnell O, Oden A, De Laet C, Eisman JA, Pols H, Tenenhouse A. Alcohol intake as a risk factor for fracture. Osteoporos Int. 2005; 16(7): 737–42.

VIII. Attributable burden estimation

a. TMREL

We calculated TMREL by first calculating the overall risk attributable to alcohol. We did this by weighting each relative risk curve by the share of overall DALYs for a given cause. We then took the minimum of this all-cause risk curve as the TMREL of alcohol-use. More formally,

$$TMREL = argmin\ average\ overall\ risk_{\omega}(g/day)$$

$$All - cause\ risk_{\omega}(g/day) = \sum_{\omega} RR_i(g/day) * \frac{DALY_i}{\sum_{\omega} DALY_i}$$

Where ω is the set of all causes associated with alcohol, i is a given cause from that set, DALY is the global DALY rate in 2010 and RR is the dose-response curve for a given cause and exposure level in grams per day.

In other words, we chose TMREL as being the exposure that minimizes your risk of incurring burden from any given cause related to alcohol. We weight the risk for a particular cause in our aggregation by the proportion of DALYs due to that cause. (e.g. since more observed people die from IHD, we weight the risk for IHD more in the above calculation of average risk compared to, say, diabetes, even if both have the same relative risk for a given level of consumption).

b. Population Attributable Fraction calculations

For all causes, we defined PAF as:

$$PAF(x) = \frac{P_A + \int_0^{150} P(x) * RR_C(x)\,dx - 1}{P_A + \int_0^{150} P(x) * RR_C(x)\,dx} \qquad P(x) = P_C * \Gamma(\boldsymbol{p})$$

Where P_c is the prevalence of current drinkers, P_a is the prevalence of abstainers, $RR_c(x)$ is the relative risk function for current drinkers by dose, and ***p*** *are parameters for the gamma distribution determined by the mean and standard deviation of exposure.*

1. Motor vehicle adjustment

In the case of motor vehicle accidents, we adjusted the PAF to account for victims. Using data from the Fatality Analysis Reporting System in the US, we calculated the average number of fatalities in a car crash involving alcohol, as well as the percentage of those fatalities distributed by age and sex (shown in the pages that follow).[14] We aggregated FARS data across the years 1985-2015, given there was little variation in the data temporally and the number of cases in old age groups had too much variance when constructing estimates by year. To adjust PAFs, we multiplied attributable deaths by the average number of fatalities from FARS and redistributed the PAF amongst each population, based on the probability of being a victim to a certain drunk driver by age and sex, based on the FARS data. The following equation describes this process:

$$Adjusted\ PAF_i = \frac{\sum_d PAF_d * DALY_d * Avg\ Fatalities_d * P(i\ is\ a\ victim)_d}{DALY_i}$$

Where i is a population by location year, age, sex and d is the set of all age and sex groups within that location and year.

Methods Appendix Figure 2: Average number of deaths in crash
given driver's age, sex, & alcohol's involvment

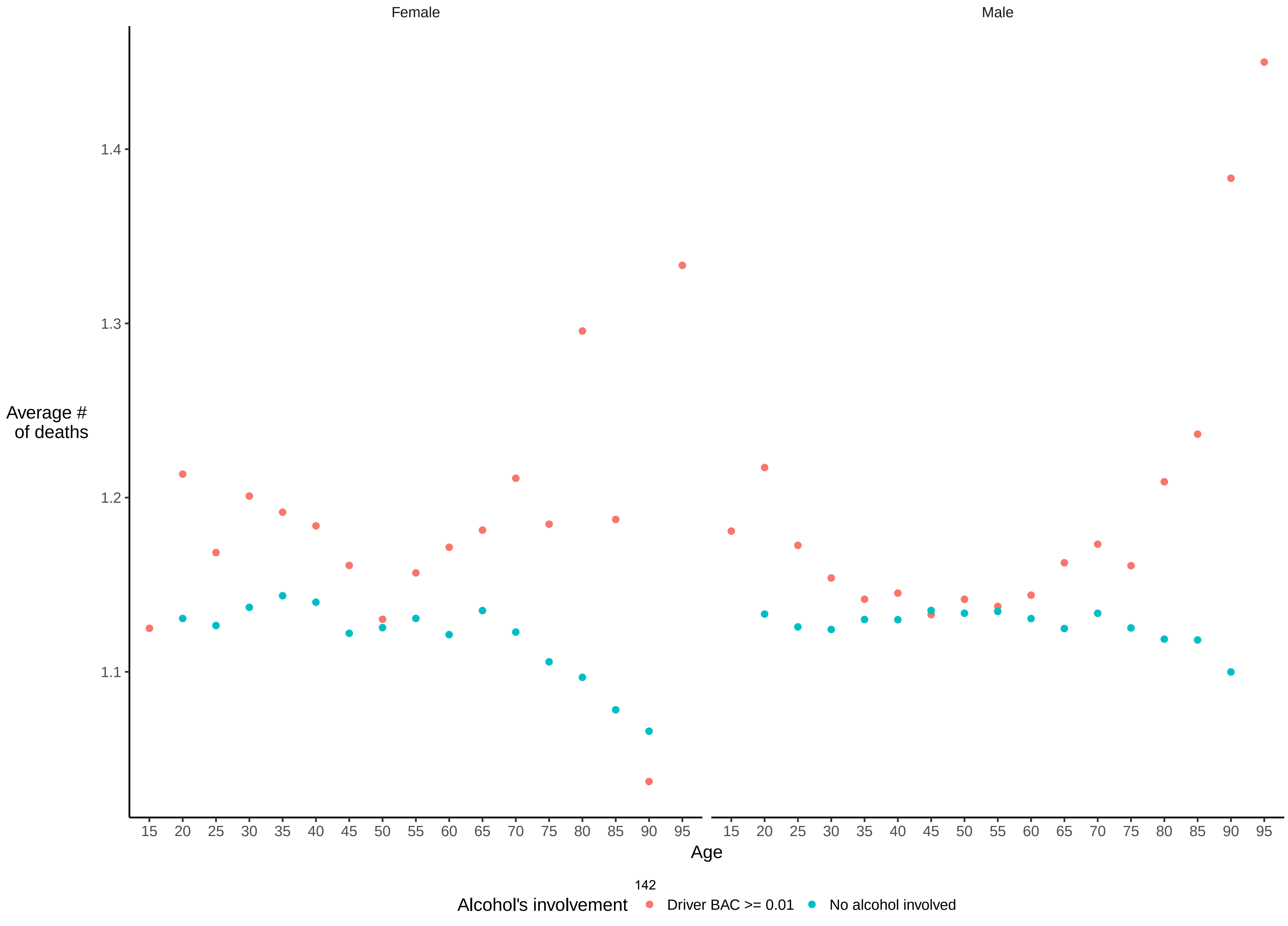

Methods Appendix Figure 3: Percentage of total victims by age & sex, given the male drunk driver's age

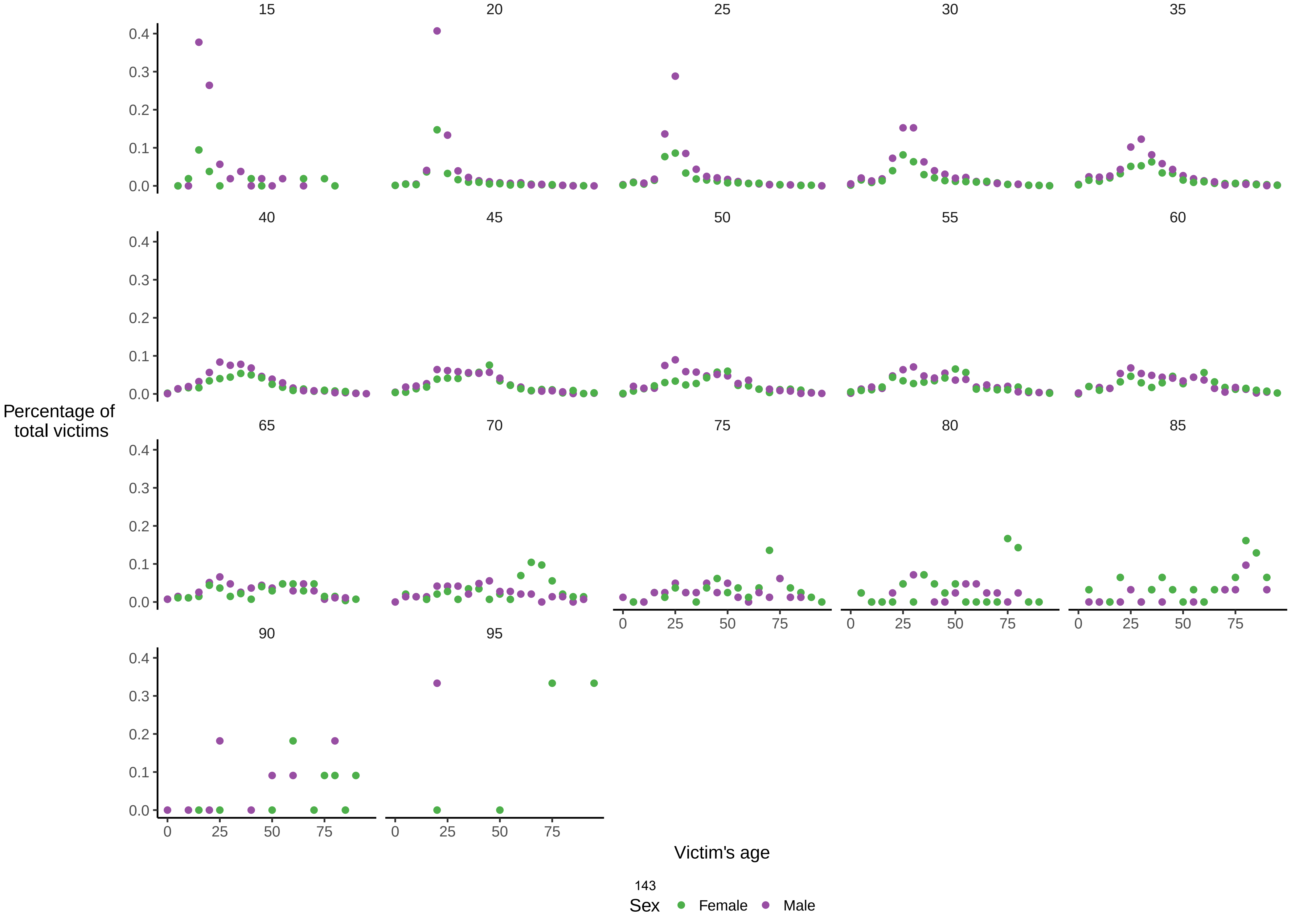

Methods Appendix Figure 4: Percentage of total victims by age & sex, given the female drunk driver's age

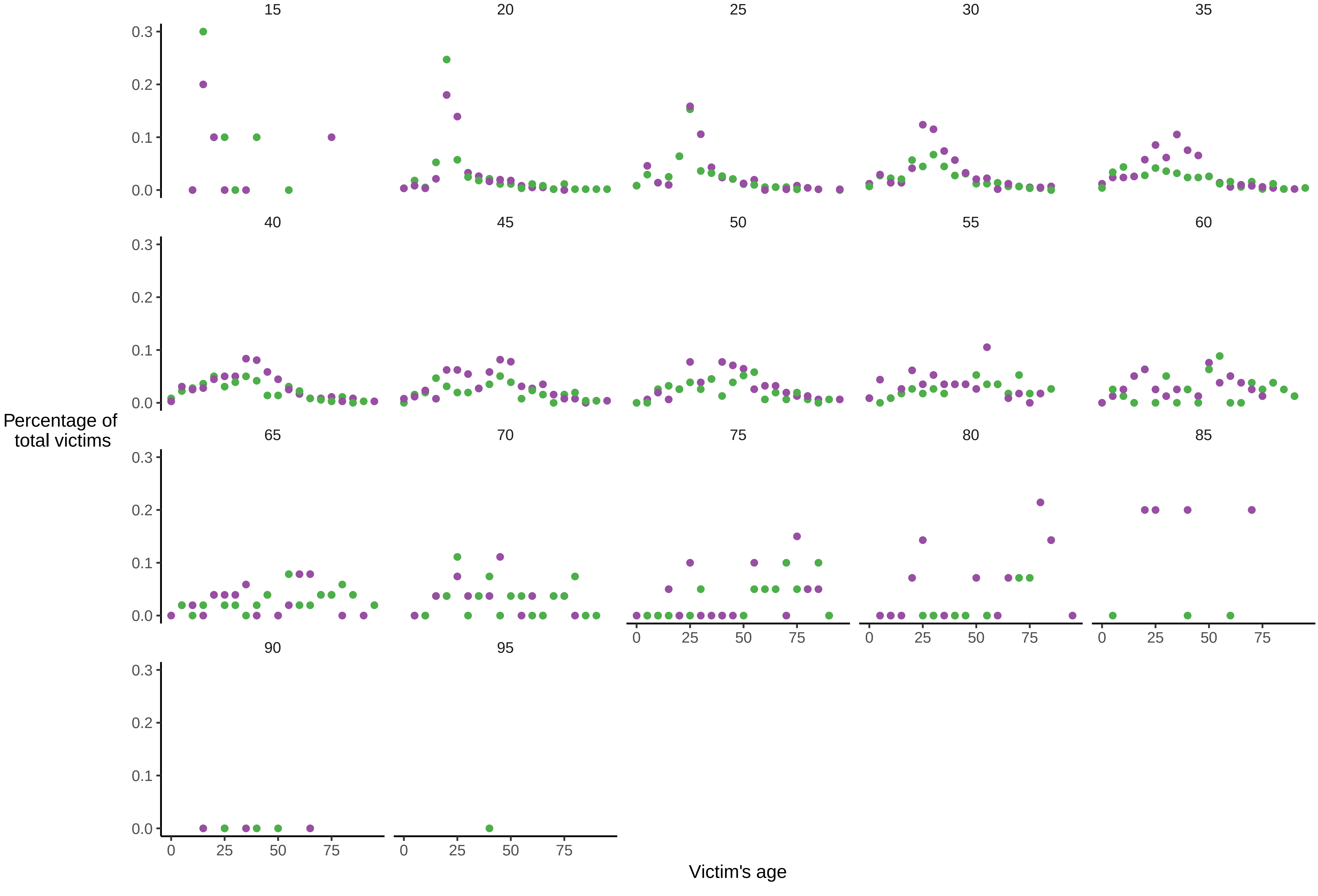

c. Attributable burden calculation

We calculated 1000 draws of the exposure and relative risk models. We then used the estimated PAF draws to calculate YLL, YLDs, and DALYs, following GBD 2016 methods. [1]

IX. References

1. Global, regional, and national comparative risk assessment of 84 behavioural, environmental and occupational, and metabolic risks or clusters of risks, 1990–2016: a systematic analysis for the Global Burden of Disease Study 2016 - The Lancet. http://www.thelancet.com/journals/lancet/article/PIIS0140-6736(17)32366-8/abstract (accessed Feb 2, 2018).
2. Database resources of the National Center for Biotechnology Information. *Nucleic Acids Res* 2016; **44**: D7–19.[2]
3. Global Health Data Exchange | GHDx. http://ghdx.healthdata.org/
4. Food and Agriculture Organization of the United Nations (FAO). FAOSTAT Food Balance Sheets, October 2014. Rome, Italy: Food and Agriculture Organization of the United Nations (FAO).
5. World Health Organization (WHO). WHO Global Health Observatory - Recorded adult per capita alcohol consumption, Total per country. Geneva, Switzerland: World Health Organization (WHO).
6. UN World Tourism Organization (UNWTO). UN World Tourism Organization Compendium of Tourism Statistics 2015 [Electronic]. Madrid, Spain: UN World Tourism Organization (UNWTO), 2016.
7. Norstrom, Thor. "Estimating changes in unrecorded alcohol consumption in Norway using indicators of harm." *Addiction* 93.10 (1998): 1531-1538.
8. Macdonald, Scott. "Unrecorded alcohol consumption in Ontario, Canada: estimation procedures and research implications." *Drug and Alcohol Review* 18.1 (1999): 21-29.
9. Meier, Petra Sylvia, et al. "Adjusting for unrecorded consumption in survey and per capita sales data: quantification of impact on gender-and age-specific alcohol-attributable fractions for oral and pharyngeal cancers in Great Britain." *Alcohol and Alcoholism* 48.2 (2013): 241-249.
10. Hao, Wei, Hanhui Chen, and Zhonghua Su. "China: alcohol today." *Addiction* 100.6 (2005): 737-741.
11. Rehm, Jürgen, and Vladimir Poznyak. "On monitoring unrecorded alcohol consumption." *Alcoholism and Drug Addiction* 28.2 (2015): 79-89.
12. Probst et al. "Unrecorded Alcohol Use: A global modeling study based on Delphi assessments and survey data". Toronto, Canada: CAMH.
13. An Integrative Metaregression Framework for Descriptive Epidemiology. Abraham D. Flaxman, Theo Vos, Christopher J. L. Murray. Seattle: University of Washington Press, [2015]
14. Fatal Accident Reporting System (FARS). National Highway Traffic Safety Administration, National Center for Statistics and Analysis Data Reporting and Information Division (NVS-424); 1985, 1990, 1995, 2000, 2005, 2010, 2015
15. Kehoe T, Gmel G, Shield KD, Gmel G, Rehm J. Determining the best population-level alcohol consumption model and its impact on estimates of alcohol-attributable harms. *Popul Health Metr* 2012; **10**: 6.
16. GHO | By category | Standard drink measures, in grams per unit - Data by country. WHO. http://apps.who.int/gho/data/view.main.54180 (accessed Feb 2, 2017).
17. R Core Team (2013). R: A language and environment for statistical computing. R Foundation for Statistical Computing, Vienna, Austria
18. H. Wickham. ggplot2: Elegant Graphics for Data Analysis. Springer-Verlag New York, 2009.

19. Douglas Bates, Martin Maechler, Ben Bolker, Steve Walker (2015). Fitting Linear Mixed-Effects Models Using lme4. Journal of Statistical Software, 67(1), 1-48.doi:10.18637/jss.v067.i01
